# Transcriptome analysis of immune genes in peripheral blood mononuclear cells of young foals and adult horses

**DOI:** 10.1371/journal.pone.0202646

**Published:** 2018-09-05

**Authors:** Rebecca L. Tallmadge, Minghui Wang, Qi Sun, Maria Julia B. Felippe

**Affiliations:** 1 Equine Immunology Laboratory, Department of Clinical Sciences, College of Veterinary Medicine, Cornell University, Ithaca, NY, United States of America; 2 Cornell Bioinformatics Facility, Cornell University, Ithaca, NY, United States of America; University of Queensland, AUSTRALIA

## Abstract

During the neonatal period, the ability to generate immune effector and memory responses to vaccines or pathogens is often questioned. This study was undertaken to obtain a global view of the natural differences in the expression of immune genes early in life. Our hypothesis was that transcriptome analyses of peripheral blood mononuclear cells (PBMCs) of foals (on day 1 and day 42 after birth) and adult horses would show differential gene expression profiles that characterize natural immune processes. Gene ontology enrichment analysis provided assessment of biological processes affected by age, and a list of 897 genes with ≥2 fold higher (p<0.01) expression in day 42 when compared to day 1 foal samples. Up-regulated genes included B cell and T cell receptor diversity genes; DNA replication enzymes; natural killer cell receptors; granzyme B and perforin; complement receptors; immunomodulatory receptors; cell adhesion molecules; and cytokines/chemokines and their receptors. The list of 1,383 genes that had higher (p<0.01) expression on day 1 when compared to day 42 foal samples was populated by genes with roles in innate immunity such as antimicrobial proteins; pathogen recognition receptors; cytokines/chemokines and their receptors; cell adhesion molecules; co-stimulatory molecules; and T cell receptor delta chain. Within the 742 genes with increased expression between day 42 foal and adult samples, B cell immunity was the main biological process (p = 2.4E-04). Novel data on markedly low (p<0.0001) *TLR3* gene expression, and high (p≤0.01) expression of *IL27*, *IL13RA1*, *IREM-1*, *SIRL-1*, and *SIRP*α on day 1 compared to day 42 foal samples point out potential mechanisms of increased susceptibility to pathogens in early life. The results portray a progression from innate immune gene expression predominance early in life to adaptive immune gene expression increasing with age with a putative overlay of immune suppressing genes in the neonatal phase. These results provide insight to the unique attributes of the equine neonatal and young immune system, and offer many avenues of future investigation.

## Introduction

The naïve neonatal immune system encounters numerous environmental and pathogenic antigens upon leaving the uterus, and faces the task of developing protective immune responses; not responding timely to pathogenic antigens can result in severe infection or death. Research findings over the last decade have better defined specific ways in which the neonatal immune system differs from the adult immune system, although knowledge gaps still challenge the design and development of long-lasting prophylactic measures for the young age [1–3].

The composition of the neonatal immune system differs from the adult in the relative frequency of immune cell populations and expression of selected immune molecules. This difference has been described best in human and mouse neonates and to a lesser extent in other species. Specifically, human neonates have fewer myeloid dendritic cells (DCs), plasmacytoid DCs, and memory-effector T and B cells than adults [4,5]. On the other hand, regulatory T cells are present at higher frequencies in human neonatal peripheral leukocytes than adults [6,7]. Described in humans and mice, a distinct subset of B cells, generally known as B1 cells are prevalent in early life and then decline with age [7]. B1 cells exhibit more innate-like immune functions than conventional B cells, including the unique ability to produce “natural” antibodies without T cell help [8]. Recently, an enriched population of CD71^+^ nucleated red blood cells with immunosuppressive properties has been described in the neonatal humans and mice [9].

Functional distinctions have also been described for neonatal immune cell populations including T cells, B cells, antigen presenting cells (APCs), and natural killer (NK) cells [2]. Neonatal immune responses are reported to have a tendency to polarize toward Th2 and Th17 responses, whereas cytotoxic responses and antigen presentation may be less efficient [10,11]. Th2 responses in the neonatal mouse are heightened by the epigenetic accessibility of the Th2 cytokine gene locus that promotes rapid expression of IL4, IL13, and IL5 cytokines [12]. Th1 responses are generated in neonatal mice after initial antigen exposure but undergo apoptosis upon re-exposure to antigen when mediated through the IL4Rα/IL13Rα1 heteroreceptor [11,13]. Yet, the increasing IL12 production from the expanding DC population with age prevents IL13Rα1-mediated apoptosis and actively promotes Th1 responses [14,15]. It is not yet clear whether this mechanism is shared across species. Further, human neonatal T cells are deficient in the co-stimulatory CD40 ligand molecule, which curtails both T and B cell immune responses [16]. Although neonatal NK cells are present in equivalent or greater numbers than adult NK cells, they are characterized by reduced cytolytic activity and increased use of the inhibitory CD94/NKG2A receptor [17]. Regulatory T cells in the neonatal phase secrete higher levels of IL10 and TGFβ than adult regulatory T cells, with the net result of suppressing APC and effector T cell functions [18,19].

Human neonatal APCs, and DCs in particular, show impaired antigen presentation and T cell stimulation owing to low expression of MHC class II, and co-stimulatory molecules CD80, CD86, and ICAM1 [5,20,21]. Although ample toll-like receptor (TLR) expression is found on neonatal APCs, induction of IL12, interferon α/β, and other effector molecules is limited [10,20,22–26]. Interleukin 27 (IL27) is a cytokine produced by DCs and macrophages at higher levels in neonates and infants than adults, and it exerts immunosuppressive effects on macrophages, CD4+ T cells, and other immune cells [27]. Despite these distinctions, robust and protective neonatal immune responses can be generated. Vaccination can induce long-lived immune memory responses to *Mycobacterium bovis bacillus Calmette-Guérin* (BCG) and hepatitis B in human neonates [28–30]. Also, infectious challenge studies have revealed protective immune responses mounted against BCG and *Listeria monocytogenes* by neonatal mice [31,32].

Studies of the foal immune system have revealed many parallels with the findings in human and mice. Immune cell populations undergo marked expansion in early life before settling to levels found in adult horses [33]. Similar to human neonates, foal peripheral blood mononuclear cells (PBMCs) are comprised of fewer DCs (CD14^-^CD1b^+^CD86^+^), more regulatory T cells (CD4^+^CD25^high^FoxP3^+^), and more B1-like CD5^hi^ cells than adult PBMCs [34–37]. Toll-like receptors are expressed by foal APCs, and IL12p40 and IL12p35 expression is inducible when foal DCs are infected by *Rhodococcus equi*; yet, TLR stimulation may not modulate cytokine expression [37–42]. Equine B cells undergo active development during fetal life: signature B cell molecules and most immunoglobulin (Ig) isotypes are expressed by the time of birth, along with detectable, albeit markedly low levels of serum IgM and IgG [43]. Detailed analysis of Ig sequence diversity during equine developmental stages showed no age-dependent limitations in Ig heavy chain gene usage, and revealed increasing Ig sequence diversity between fetal and neonatal ages, as well as between foals and adult horses [44]. Perhaps a limiting factor for protective immunity, both MHC class II expression and interferon gamma (IFNγ) production have been demonstrated to increase with age in foals [37,40,45,46].

Like humans and mice, neonatal and young foals can mount robust responses to some vaccines, including production of antigen-specific isotype-switched antibodies and cytotoxic T lymphocytes [47–50]. Some foals recover from experimental infectious challenge with *R*. *equi*, depending on bacterial dose and foal age, with adult-like immune responses [51]. Thus, as we better understand the ontogeny and unique attributes of the neonatal immune system, more effective vaccine and therapeutic strategies can be developed. Formulation of vaccines that stimulate multiple TLRs to overcome limitations specific to the neonatal immune system and induce robust immune responses is a promising example [52,53].

The goal of the present study was to identify unique attributes of the equine neonatal and young foal immune systems through transcriptome sequencing of blood leukocytes. Immune gene expression was profiled from a group of foals on days 1 and 42 of age and contrasted with gene expression of their respective dams. To gain a comprehensive view of the immune system in early life, PBMC samples were sampled to allow collective and simultaneous analysis of genes expressed by T lymphocyte, B lymphocyte, monocyte, and dendritic cell populations.

## Materials and methods

### Sample collection and processing

This study was approved by the Cornell University Center for Animal Resources and Education and Institutional Animal Care and Use Committee (protocol #2002–0106) and carried out in strict accordance with the committee's recommendations for the use of vertebrates in research. Healthy Warmblood broodmares (age range from 24 to 27 years) carrying pregnancies from Warmblood stallions were managed at Cornell University Equine Park, Ithaca, NY on grass pasture during the day, with grass hay and grain supplementation, and observed in a stall overnight in the last month of pregnancy. They received standard vaccinations (tetanus toxoid, Eastern and Western encephalitis, rabies, equine herpesvirus-1) and regular herd deworming based on fecal floatation results. Foals were allowed to suckle colostrum naturally after birth under observation, and physical examination was performed daily during the study period. Foals with abnormal physical examination, abnormal complete blood cell count, blood IgG concentration values below 800 mg/dL (SNAP® Foal IgG Test, Idexx, Westbrook, MN), or requiring treatment during the 42-day study period were excluded from the study.

Blood samples were collected by jugular venipuncture into vacutainers containing heparin sulfate (BD Biosciences, San Diego, CA) from four foals on day 1 of life (two female and two male), from the same four foals on day 42 of life, and from four mares (representing adult horses). The blood samples from mares were collected 3 days after foaling; these mares were considered clinically healthy and immunologic sound, with the advantage of sharing the same genetic background, environment and natural immunogenic challenges with the foals in the study. The initial 6 weeks of age were chosen as the sampling period based on the facts that this time would reflect an active naïve immune system responding to environmental pathogens/antigens (e.g. primary exposure and subsequent exposure), and previous data obtained from our and other laboratory’s studies describing marked age-dependent changes in the immune system of foals within the first 3 months of age [33–37,39,40,42–49]. All efforts were made to minimize suffering during blood collection of foals. Enrichment of peripheral blood mononuclear cells (PBMC) was obtained using a previously described protocol of Ficoll-Paque density centrifugation and cell count [37,40,46]. Based on flow cytometric distribution according to cell granularity and size of isolated PBMC, platelet distribution was < 4%, and neutrophil distribution < 20% of total isolated cells. Approximately 5x10^6^ isolated cells per sample were used for RNA extraction.

### Transcriptome sequencing

RNA was isolated from PBMC samples with the RNeasy kit including on-column DNase I digestion, as directed (Qiagen, Valencia CA). RNA quantity was determined with Qubit high sensitivity RNA assay (Thermo Fisher Scientific, Waltham, MA) and RNA integrity was determined with an AATI Fragment Analyzer at the Cornell Genomics Facility (Cornell University, Ithaca NY), with acceptable scores ≥ 7.7. These RNA samples were used to generate transcriptome (RNA-Seq) libraries with TruSeq Stranded mRNA Library Prep Kit (Illumina, San Diego, CA). Libraries were assessed for insert size (260 bp) and DNA concentration (Qubit high sensitivity DNA assay, Thermo Fisher Scientific). After normalization, libraries were multiplexed and sequenced on an Illumina NextSeq500 at the Cornell Genomics Facility. The number of samples multiplexed per run was 12. The RNA-Seq dataset is available in the NCBI Gene Expression Omnibus repository as series GSE101117.

### Data analyses

De-multiplexed sequence data were sent to the Cornell Bioinformatics Facility for quality analysis with FASTQC, and trimmed reads were aligned to the equine genome sequence (NCBI reference assembly Equ Cab 2, INSDC Assembly GCA_000002305.1) with TopHat version 2.1.1, using the Ensembl version 92.2 gene model as the annotation reference for alignment and all downstream analysis [54,55]. Cufflinks version 2.2.1 software was used to quantify transcripts according to the gene model and conduct reference-guided transcript assembly [56]. Differential expression (DE) analysis was carried out using the Bioconductor edgeR package (version 3.3.0), which is based on a generalized linear model of the Negative Binomial family with logarithmic link to capture the quadratic mean-variance relationship. All samples were included in analyses, however pairwise differential expression analyses were performed to identify changes over time (day 1 versus day 42 foals; day 1 versus adult horses; and day 42 foals versus adult horses). The genes with at least 1 read per million in at least 2 samples were kept. Normalization factors and effective library size were applied and tag-wise dispersion (Tgw) was estimated. To minimize false positive results, a cutoff of False Discovery Rate (FDR) <0.05 was used for filtering DE genes. Last, an exact test was performed to detect DE genes between the groups and adjusted p(Tgw) values < 0,05 were considered significant. The distribution of p-values from the transcriptome dataset was also plotted for each age group using Microsoft Excel 2010 to visually inspect the distribution. Differential gene expression was plotted with GraphPad Prism version 6.07 for Windows, GraphPad Software, La Jolla California USA, www.graphpad.com. A multidimensional scaling plot of transcriptome profiles and their relationship calculations were performed using plotMDS function in edgeR package [57].

Enrichment analysis was performed with statistical overrepresentation tests using the PANTHER classification system tools (Gene Ontology Consortium at http://www.geneontology.org) and significance thresholds were p value < 0.01 and log2 fold-change >1 [58,59].

## Results and discussion

The transcriptome of PBMCs was sequenced from foals on 1 day of life, from the same foals again on 42 days of life, and from adult horses (n = 4 per age group). The analyses provided a global overview of immune gene expression at birth, reflective of development in fetal life, and enabled contrasts of lineage-specific immune gene expression during early life and adulthood. Six weeks (day 42) of age represents a transitional phase in the foal’s immune system, including exposure to environmental microorganisms and other antigens, and leukocyte population expansion [33]. Our strategy to obtain paired data from the same foals on days 1 and 42 empowered robust kinetic analysis of gene expression over time. Studies in other species have shown that maternal leukocytes absorbed through colostrum are detected at their peak in the neonate’s blood between 12 and 24hrs of age, and almost no information in this regard is available for the equine species, we assume that most of the cells harvested from foals on day 1 (between 24 and 30 hrs of age) would contain minimal to negligible number of maternal cells. Nevertheless, our data analysis is insufficient to determine the effect of maternal leukocytes in the immune system of the equine neonate.

### Overall outcomes of the transcriptome sequencing

Approximately 34,000,000 to 44,000,000 reads were sequenced from each transcriptome library. The number of annotated genes identified herein was lower than that observed in some other recent equine transcriptome studies [60–64]. This discrepancy is likely due to the considerable efforts put forth in those studies to improve transcript annotation in the horse and thus generation of large transcriptome datasets sampled from multiple tissues (up to 43) and horses (up to 85), with and without *in vitro* stimulation of cultured cells, compilation and re-analysis of multiple transcriptome datasets, as well as differences in filtering strategies [60–64]. The data reported here only considers transcripts annotated by Ensembl release 92.2, similar to our transcriptome analysis of horses with common variable immunodeficiency [65].

**Fig 1:**
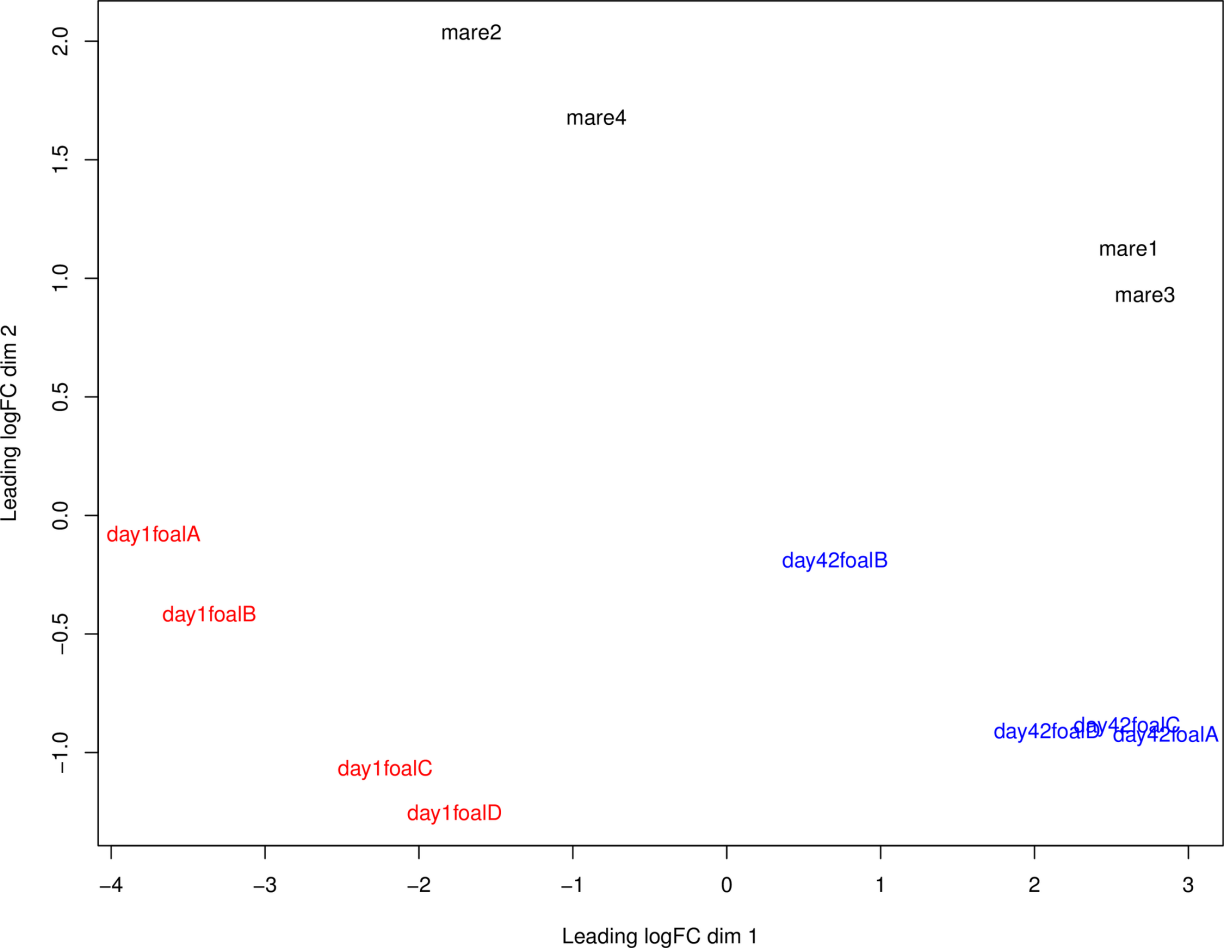
Multidimensional scaling plot of peripheral blood mononuclear cell transcriptome profiles. The transcriptome profiles of day 1 samples are shown in red font, those of day 42 foal samples are shown in blue font, and adult sample profiles are displayed in black font. The correlation among samples within age groups was ≥0.72.

To visually appreciate the relationship of the transcriptome profiles among samples, multidimensional scaling was performed (Fig 1). The samples from day 1 clustered together tightly in contrast to day 42 foal samples. The day 42 foal profiles were distinct from those of day 1 and adult samples. The adult samples formed two groups that were distinct from the foal samples. Variation between the immune cell transcriptome of individuals within an older age group, such as that observed in the adult group, was not surprising because they have encountered different pathogen challenges and environments over their lifetime. The correlation among samples within age groups was ≥0.72.

To identify the dynamic changes occurring in the immune system over time, differential gene expression tests were performed between the transcriptomes of day 1 and day 42 foal samples, and adult samples (S1, S2, and S3 Tables). The distribution of p-values was assessed for each pairwise comparison and values p>0.05 were distributed uniformly (S1 Fig). The p-value distribution pattern was similar for each comparison. The comparison of gene expression with p-values of p<0.05 between day 1 and day 42 foal samples revealed 3,377 genes with ≥2-fold difference in expression levels (Table 1). Between day 42 and adult samples, 2,056 genes were expressed statistically different with the same cut-offs; and 1,750 genes differed in expression levels between day 1 foal and adult samples.

**Table 1:** Differential gene expression of foal and adult horse samples.

	Day 1 vs day 42	Day 42 vs adult	Day 1 vs adult
# transcripts p<0.05	3,592	2,216	1,916
# transcripts p<0.05, FC≥2	3,377	2,056	1,750
# transcripts p<0.01, FC≥2	2,280	1,125	919
# genes with lower expression in younger age group	897	742	575
# genes with higher expression in younger age group	1,383	383	344

‘FC’ abbreviation stands for ‘fold change’

Using a more conservative p<0.01 value of significance, the number of differentially expressed genes between each age group ranged from 919 (day 1 vs adult) to 2,280 (day 1 vs day 42). Between days 1 and 42 foal samples, nearly 40% of the differentially-expressed genes were expressed at lower levels on day 1, and the other 60% had higher expression levels in day 1 than day 42 foal samples. When either day 1 or day 42 foal gene expression levels were compared to adult levels, over 60% of genes were expressed at higher levels in adult samples than in foal samples, and a balance of less than 40% of genes were expressed at higher levels in foal samples.

**Fig 2:**
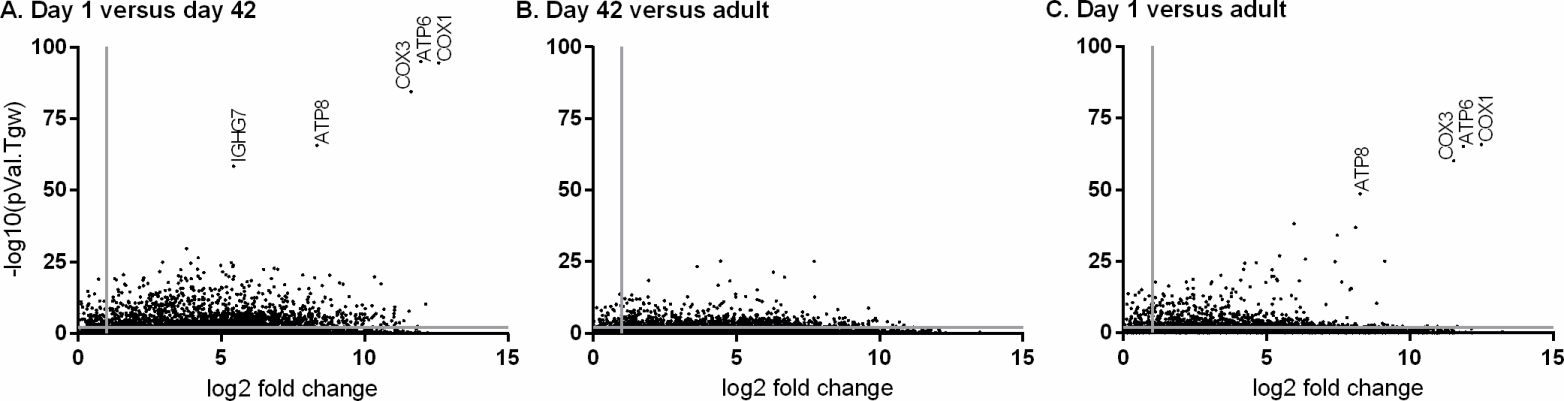
Differential gene expression between day 1 foal, day 42 foal, and adult horse samples. Gene expression levels were compared between groups and plotted with log2 fold expression on the x-axis and–log10 p-value on the y-axis. Gray lines show threshold for levels of differential expression (fold change ≥2) and significance (p-value <0.01). A) Comparison of gene expression from day 1 versus day 42 foal samples; B) comparison of gene expression from day 42 foal versus adult samples; and C) comparison of gene expression from day 1 foal versus adult samples.

To visually appreciate the distribution of the magnitude of fold change in gene expression between age groups and p-value < 0.01 significance levels, plots were generated for each comparison (Fig 2). Five genes showed a striking magnitude of fold change in expression and p-values of ≤5.13x10^-49^ when comparing day 1 and day 42 foal samples (Fig 2 panel A): *COX1*, *COX3*, *ATP6*, *ATP8*, and *IGHG7*. The *COX1*, *COX3*, *ATP6*, *ATP8* genes also had the lowest p-values (≤8.4x10^-54^) when comparing day 1 and adult samples (Fig 2 panel C). The expression of *COX1*, *COX3*, *ATP6* and *ATP8* genes ranged from 269 to 14,951 reads (normalized to counts per million) per foal on day 1 but decreased to zero in day 42 foal and in adult samples. *IGHG7* gene expression was minimal on day 1 (less than 1 read (counts per million) per foal) and reached a range of 63 to 138 normalized reads per foal in day 42 foal samples.

Genes with the statistically most significant (p≤2.5x10^-20^) differential expression between day 42 foal and adult samples included *IGF2BP3*, *IGHE*, *NR3C2* and 2 novel genes that had identity to granzyme B-like transcripts (Fig 2 panel B). *IGF2BP3* gene expression was higher in day 42 foal than in adult samples, while *IGHE*, *NR3C2* and granzyme B-like transcripts were higher in adult than in day 42 foal samples. Expression of granzyme B-like transcripts was also higher (p = 6.0x10^-39^) in adult than day 1 foal samples.

High gene expression of *COX1*, *COX3*, *ATP6* and *ATP8* genes is a novel finding and has not yet been explored in the equine neonate or other species. Similarly, enriched expression of *IGF2BP3* and *NR3C2* during early life has not been investigated. The expression profiles of *IGHG7* and *IGHE* are consistent with previous studies that detect low mRNA and protein amounts at birth and document robust production over the following months [43,66,67]. Expression of granzyme B has been positively correlated with age in human infant lung samples, generally in agreement with the findings described above [68]. Progressive granzyme B expression may reflect the development and proliferation of adaptive immune effector cells with age.

### Enrichment analysis of differentially-expressed genes

Due to the large number of differentially-expressed genes identified between age groups, gene ontology (GO) enrichment analysis was performed to determine which biological processes were predominantly affected. First, the list of 897 genes that had statistically significant (p<0.01) higher expression in day 42 when compared to day 1 foal samples was investigated. The immune response was enriched in this list (p = 6.4x10^-12^) with 74 up-regulated genes representing Igs, T cell receptors, granzyme B, perforin, NK receptors, complement components, immunomodulatory receptors, cell adhesion molecules, cytokines/chemokines and their receptors, and others (Table 2). Thirty-two genes involved in DNA replication were enriched 5-fold in this list, including helicases, primase, polymerases, and mismatch repair and recombination proteins, among others (p = 5.9x10^-11^). A third process, mitosis, was identified based on 39 genes such as cytoskeletal proteins, microtubule and actin binding motor proteins, kinases, and transcription cofactors (p = 3.0x10^-5^). Overall, these results reveal the expansion of the adaptive immune response, which includes proliferating lymphocyte populations that utilize DNA replication machinery for recombination of B cell receptor (BCR Ig) and TCR genes, mismatch repair, and affinity maturation mechanisms.

**Table 2:** Enrichment analysis of 897 genes with statistically significant increased expression in foal samples between days 1 and 42.

Biological process	fold enrichment	p-value	# genes	Representative genes
Immune response	2.73	6.43E-12	74	*ADORA2B*, *BLNK*, *CCL5*, *CD40LG*, *CLNK*, *CRTAM*, *FASLG*, *FCGR3A*, *FCRL1*, *GBP2*, *GBP5*, *GZMB*, *IGHA*, *IGHE*, *IGHG*, *IGHM*, *IGHV*, *IGJ*, *IGKC*, *IGKV*, *IGLC*, *IL2RB*, *IL12RB2*, *KLRK1*, *LY9*, *MASP2*, *PRF1*, *SH2B2*, *SLAMF6*, *STAT1*, *TNFRSF4/OX40*, *TRAC*
DNA replication	5.06	5.92E-11	32	*CDC7*, *DNA2*, *EXO1*, *GEN1*, *LIG1*, *MCM2*, *MCM3*, *MCM4*, *MGME1*, *ORC1*, *ORC6*, *PARP3*, *PCNA*, *POLA1*, *POLA2*, *POLE2*, *POLQ*, *PRIM1*, *RFC5*, *SASS6*, *SMC2*, *TOP2A*, *ZGRF1*
Mitosis	2.60	3.02E-05	39	*BRIP1*, *CCNA2*, *CCNB1*, *CDC16*, *CDK1*, *KIF18A*, *MYO5C*, *NCAPD2*, *NDC80*, *PRC1*, *RASGRP3*, *SEPT11*, *TUBB*, *TUBGCP4*, *ZW10*

Second, the list of 1,383 genes that had lower (p<0.01) expression in day 42 when compared to day 1 foal samples was examined. Again, the immune system process was enriched (p = 2.7x10^-5^) based on 122 genes that were down-regulated over the first 42 days. This list was populated by genes with roles in innate immunity such as antimicrobial proteins, pathogen recognition receptors, complement components, as well as cytokines/chemokines and their receptors, and others (Table 3) rather than the adaptive immune genes that were up-regulated between day 1 and day 42, listed in Table 2. One novel finding was the down-regulation of *IGHD*, which encodes the IgD molecule. The process of cell adhesion was also enriched (p = 1.1x10^-4^) in the list of genes down-regulated over the first 42 days, signified by genes encoding cell adhesion proteins, integrins, cell-collagen interacting proteins, and extracellular matrix proteins among others. An intriguing paradox was identified in this down-regulated gene list: apoptotic (p = 5.5x10^-4^) and cell proliferation (p = 1.98x10^-3^) processes were simultaneously identified. Rapid expansion of immune cell populations has been documented in this time frame, which likely accounts for markers of cell proliferation. The apoptotic processes identified could be the signature of also essential contracting antigen-specific populations or other short-lived immune cells. Finally, an enrichment (p = 8.1x10^-3^) of down-regulated MAPK signaling-associated genes was identified in day 42 foal samples. MAPK are critical regulators of the innate and adaptive arms of the immune system, including cell proliferation, cell differentiation, activation through TLRs, production of inflammatory and anti-inflammatory cytokines, and function of antigen-presenting cells [69]. There are different MAPK modules that are activated by respective stimuli, and the corresponding MAPK signaling leads to gene regulation and immune response. In addition, downregulation of MAPK is important to prevent overt inflammatory responses and tissue damage. It is possible that low MAPK signaling could be an intrinsic condition of day 1 responsible for some of the immunologic differences observed in young foals; concomitantly, this regulatory pathway in the naïve immune system of a young foal presents a greater threshold for activation in order to prevent excessive immune responses, hence inflammation, when environmental stimuli are abundant. This gene ontology analysis emphasized the remarkable immune system dynamics underway in foals during the first 6 weeks of life.

**Table 3:** Gene enrichment analysis of 1,383 genes with statistically significant decreased expression in foal samples between days 1 and 42.

Biological process	fold enrichment	p-value	# genes	Representative genes
Immune system process	1.64	2.75E-05	122	*ADGRE1*, *BPI*, *C1QA*, *C1S*, *C3*, *C4BPA*, *C5AR1*, *CCL3*, *CCL4*, *CCL14*, *CCL23*, *CD18/LTBR*, *CD21/CR2*, *CD163L1*, *CD209*, *CLEC1B*, *CSF2RA*, *CSF3R*, *CXCL3*, *FCER2*, *FCGRT*, *GLIPR2*, *HCST*, *IFIT1*, *IGHD*, *IL13RA1*, *IL27*, *JAK2*, *LTA*, *MAP3K11*, *NFKBIA*, *RELA*, *RUNX1*, *S100A8*, *S100P*, *STAT6*, *TNFRSF14*, *TNFSF14*, *TNS1*
Cell adhesion	2.05	1.06E-04	58	*ADAMTSL4*, *ADAP1*, *AGER*, *ARAP3*, *CUBN*, *ICAM3*, *ITGA2B*, *ITGA7*, *ITGAX*, *ITGB3*, *MEGF9*, *NKIRAS2*, *PCDH17*, *PLXNC1*, *PTK2B*, *RALB*, *ROCK2*, *SIPA1L1*, *TGFBI*, *THY1*, *TSG-6*, *VCAN*
Apoptotic process	2.05	5.47E-04	51	*BCL2L1*, *BNIP3L*, *CFLAR*, *DDIT3*, *DEDD*, *DEDD2*, *DUSP1*, *G0S2*, *GADD45B*, *ING2*, *NOD2*, *NRADD*, *PCBP4*, *RAF1*, *RASSF1*, *UBE2D2*
Cell proliferation	2.71	1.98E-03	26	*BCL6*, *CXCL2*, *HBEGF*, *HGF*, *JAK2*, *JUN*, *LYN*, *PROK2*, *TNFRSF1A*
MAPK cascade	2.05	8.12E-03	39	*GAB2*, *IRAK3*, *MAP3K6*, *MAPK3*, *NUMB*, *PPM1D*, *RAP2C*, *RASGRP4*

Within the 742 genes that were expressed at lower levels in day 42 foal PBMC samples than adult samples, B cell mediated immunity was the only biological process enriched (p = 2.4E-04) (Table 4). This was based on increased expression of several Ig heavy chain constant regions, cell adhesion molecules, cytokine receptors, and signaling molecules. In the complementary list of 383 genes with decreased expression between day 42 foal and adult samples, the process of mitosis and cell cycle was enriched (p = 8.2E-14). This difference may reflect the robust population expansion occurring in the young foal in contrast to the more stable population in the adult [33]. Further, another subset of immune response genes were found to be enriched (p = 7.1E-6), including Ig variable region genes and a member of the Ig isotype switch recombination complex, T cell receptor delta chain, antimicrobial proteins, and others. Decreased expression of the T cell receptor delta chain may indicate that the population of T cells with gamma-delta receptors declines with age, although this has not been determined for horses.

**Table 4:** Gene enrichment analysis of differentially-expressed genes between day 42 foal and adult horse samples.

Biological process	fold enrichment	p-value	# genes	Representative genes
**742 genes with increased expression between day 42 and adult PBMC**				
B cell mediated immunity	3.70	2.36E-04	20	*ADGRE1*, *CNTFR*, *CSF3R*, *FCER1A*, *GAB2*, *IGHE*, *IGHG*, *IL12RB2*, *IL23R*, *KLRG1*, *KLRK1*, *PHLDB3*, *PRLR*, *SIGLEC11*, *TIAM2*
**383 genes with decreased expression between day 42 and adult PBMC**				
Mitosis and cell cycle	3.60	8.23E-14	55	*CCNA2*, *CDC20*, *CDC25B*, *CDK1*, *DNA2*, *E2F8*, *EXO1*, *GEN1*, *HDAC2*, *KIF18A*, *MCM3*, *MYBL2*, *MYO1E*, *NCAPD2*, *NDC80*, *ORC1*, *PRC1*, *RAD51*, *RCC1*, *SMC2*, *TOP2A*, *TUBB*, *UBE2C*	
Immune response	2.96	7.06E-06	34	*ADGRF1*, *ADGRL1*, *CD79B*, *CXCL10*, *FCRL1*, *GBP2*, *IFI35*, *IGHV*, *IGKV*, *STAT1*, *SWAP70*, *TRDC*

Last, the list of genes with differential expression between day 1 foals and adults were analyzed for enriched biological processes, and of the 575 genes that increased (p≤9.87E-03) expression between day 1 and adult samples, 4 processes were identified: B cell mediated immunity, complement activation, cellular component movement, and mitosis (Table 5). Increased expression levels (p<0.01) of various Ig heavy and light chain constant region genes overlapped between the processes of B cell mediated immunity and complement activation, along with additional genes found in each process. On the other hand, 344 genes exhibited lower (p≤4.30E-03) expression levels in adult samples when compared to day 1; these involved immune system processes and endocytosis, and included *IGHD*, *CD1* variants, *OX40L/TNFSF4*, several complement components, antimicrobial proteins, pathogen recognition receptor and others.

**Table 5:** Gene enrichment analysis of differentially-expressed genes between day 1 foal and adult horse samples.

Biological process	fold enrichment	p-value	# genes	Representative genes
**575 genes with increased expression between day 1 and adult PBMC**				
B cell mediated immunity	5.42	3.29E-08	23	*CD2*, *FCER1A*, *FCGR3A*, *IGHA*, *IGHE*, *IGHG constant region isotype genes*, *IGKC*, *IGLC*, *IL12RB2*, *IL23R*
Complement activation	6.74	9.12E-05	12	*C1QTNF2*, *CFB*, *Ig heavy and light constant region genes*, *Perforin 1*
Cellular component movement	2.49	4.93E-03	27	*ABI3*, *CCL5*, *DNAH10*, *DOCK10*, *Kinesin proteins*, *MYO5C*, *PTK6*, *SLA2*, *SRGAP3*
Mitosis	2.55	9.87E-03	24	*CCNA2*, *CCNB1*, *CCNF*, *CDK1*, *NDC80*, *NUF2*, *RAPGEF3*, *SEPT3*, *SEPT8*, *TPX2*, *UBE2C*
**344 genes with decreased expression between day 1 and adult PBMC**				
Immune system process	2.55	1.23E-06	47	*ADGRL1*, *BPI*, *C1QA*, *C3*, *C5AR1*, *CCL3*, *CD1 variants*, *CD209*, *CLEC1B*, *FCGRT*, *ICK*, *IGHD*, *IL13RA1*, *IL27*, *LTA*, *MAP3K9*, *MR1*, *OX40L/TNFSF4*, *PF4*
Endocytosis	3.23	4.30E-03	18	*ARRB1*, *DOCK4*, *SNX30*, *SORL1*, *RHOBTB3*

Maximal expression of the following immune genes was found in day 1 in comparison to either day 42 foal or adult samples: *IGHD*, *FCGRT* (IgG Fc receptor; also known as *FCRN*), *OX40L*, complement components *C1QA*, *C3*, and *C5AR1*, the innate pathogen recognition and cell adhesion receptor *CD209/DC-SIGN*, the C-type lectin-like receptor *CLEC1B*, antimicrobial *BPI*, and cytokines/cytokine receptors *IL18*, *IL27*, *LTA*, *CCL3*, and *IL13RA1*.

OX40L is a molecule that provides co-stimulation when T cells interact with APCs. Maximal expression of *OX40L* in day 1 foal sample is a novel result. It was also surprising that *OX40L* expression levels were below detection level after sequence data filtering in day 42 samples, especially as expression of *OX40*, the gene that encodes the receptor for OX40L, was increased (p = 1.3x10^-9^) between days 1 and 42. Additional analysis of *OX40L* expression by purified cell populations over time is warranted, including post-transcriptional regulatory mechanisms.

Peak expression of *IL27* of day 1 foal samples corresponds to peak *IL27* levels in human and mouse neonates [27]. The IL27 cytokine impairs the ability of neonatal macrophages to control bacterial replication and limits IFNγ expression from CD4^+^ T cells [27]. IL27 may exert similar effects on foal immune responses, applicable to relevant pathogens, such as *Rhodococcus equi*, and could be further investigated.

The result of maximal *IL13RA1* gene expression levels by day 1 foal sample is intriguing and should be further verified in the foal. In a mouse model, neonatal Th1 cells that are generated after primary exposure to antigen express a heterodimeric IL4Rα/IL13Rα1 receptor; upon secondary exposure, the IL4 generated by antigen-specific Th2 cells binds the heterodimeric receptor on the antigen-specific Th1 cells and causes apoptosis [11,13]. *IL13RA1* expression appears to follow a developmentally-regulated program and can be down-regulated by IL12 cytokine expression [14]. It is known that IL12 expression can be induced from foal APC as early as the day of birth [40], but whether sufficient IL12 is produced *in vivo* to regulate *IL13RA1* expression in young foals has not been determined.

### Expression of B lymphocyte genes

As adaptive immunity was a consistent feature of the gene ontology analysis above, we narrowed our focus on the expression of individual genes relevant to the humoral immune system, including Igs and B cell-specific genes. For individual gene expression comparisons between age groups, statistical significance was considered when p<0.05.

**Fig 3:**
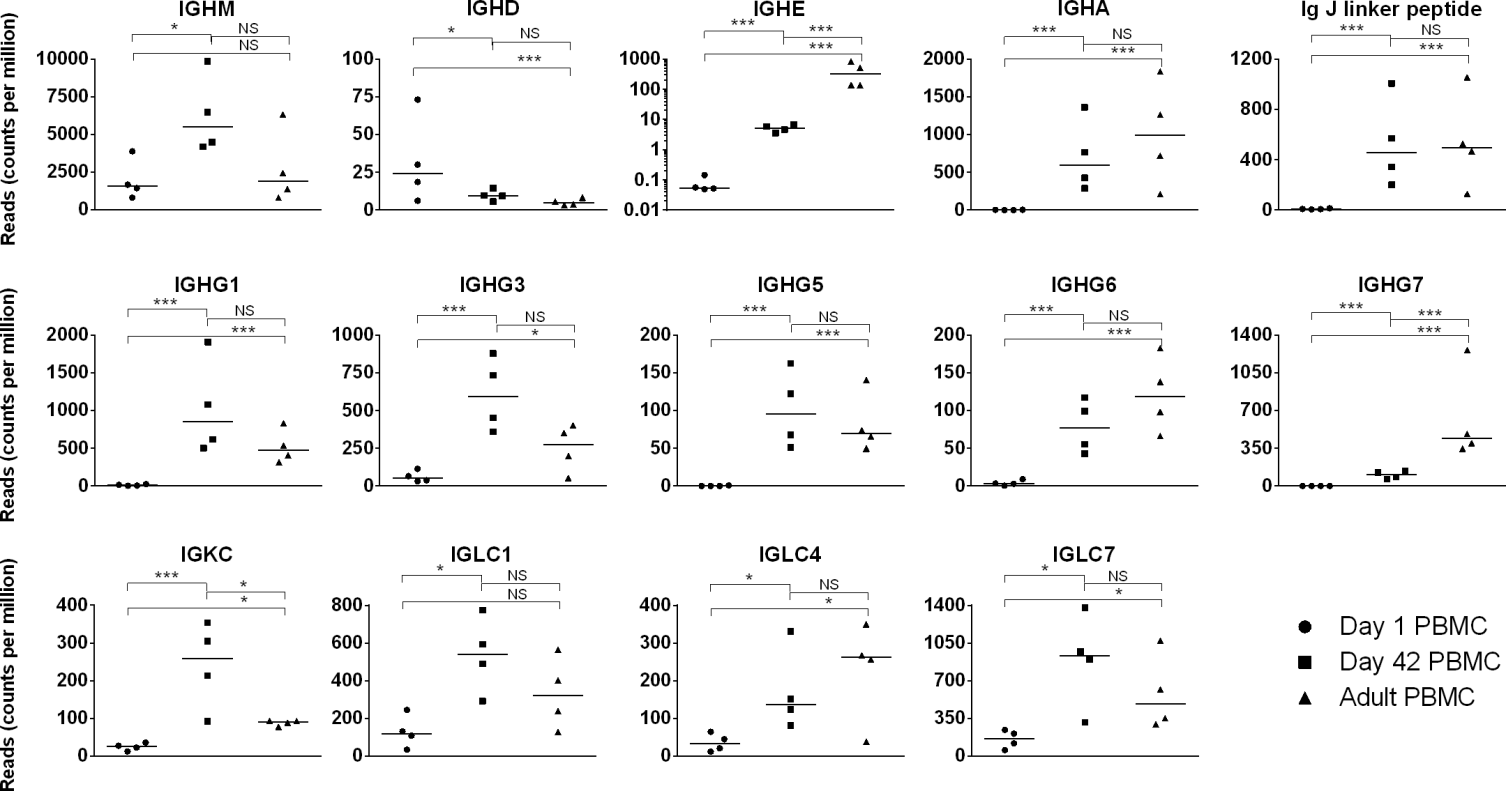
Immunoglobulin gene expression in day 1 foal, day 42 foal, and adult horse samples. Expression (quantified by number of reads on y-axis) of Ig heavy and light chain constant region genes and Ig J linker peptide in day 1 foal (circle), day 42 foal (square), and adult (triangle) samples are plotted on the x-axis. The significance level of comparisons is indicated by asterisks (* p<0.05; ** p≤0.001; *** p≤0.0001); comparisons that did not differ statistically are labeled “NS” to denote not significant.

Expression of Ig heavy chain constant region genes *IGHM*, *IGHD*, *IGHE*, *IGHA*, the associated *Ig J linker peptide*, and 5 of the 7 *IGHG* isotypes was detected in samples of all ages (Fig 3). A three-fold increase (p = 0.019) in *IGHM* expression was found in day 42 versus day 1 foal samples. Detection of *IGHM* transcripts on day 1 is consistent with the presence of serum IgM in pre-suckle neonatal samples reported previously [43]. The increase in *IGHM* transcripts in day 42 foal samples matches the rising trajectory of serum IgM, and the expansion of IgM^+^ cells in germinal centers of foals at 1 and 2 months of age [33,43].

IgD surface expression, encoded by the *IGHD* gene, characterizes two B cell subpopulations in peripheral blood: naïve mature B cells (IgM^+^IgD^+^), also known as recent bone marrow emigrants (CD19^+^IgM^+^IgD^+^CD10^+^CD38^bright^CD27^−^), and non-class-switched memory B cells (IgM^+^IgD^+^CD27^+^) [70]. More recently, IgD has been proposed to induce antimicrobial, opsonizing, proinflammatory, and B cell–stimulating cascades, in addition to binding antigen [71], which could in part underlie the importance of maximal expression early in life. Limited knowledge is available about the IgD isotype in the horse beyond its genome location, and the fact that *IGHD* mRNA is expressed in neonatal, foal, and adult blood and primary lymphoid tissues [43,72]. Maximal *IGHD* expression was measured in day 1 foal samples compared to day 42 foal (p = 0.002) or adult (p = 0.0001) samples; *IGHD* expression in the latter samples was comparable (p>0.05). Although testing IgD protein expression needs to be confirmed and defined using sorted cells, this profile agrees with the profile of human naïve CD27^-^IgD^+^ B lymphocyte populations in peripheral blood, which decreases over the first months of life as cells encounter and respond to antigen [72–74]. In addition, the expression of IgD indicates the stage of mature B cells (IgM+IgD+), which are ready for activation upon antigen encounter and T cell co-stimulation; therefore, the robust expression of IGHD on day 1 suggests that the foal is born with a repertoire of mature B cells in preparation for antigen encounter after birth. Although non-class-switched memory B cells increase in frequency over time, they account for only a small percentage of circulating B cells, and are not numerous enough to alter the overall downward trend [73,75].

The abundance of *IGHG* isotype transcripts in young foal PBMC reported herein is consistent with our previous RT-PCR studies of fetal, neonatal, and foal lymphoid tissues, as well as the presence of negligible IgG1 (IgGa) and IgG4/7 (IgGb) in the serum of newborn foals before they ingest colostrum [43]. *IGHG6* encodes an IgG isotype originally designated IgGc that has not been well characterized in the horse to date [76]. The *IGHG6* expression increases steadily over time and achieves levels similar to *IGHG5* (IgGT).

The reported IgE^+^ cells detected in the first days after birth are the result of maternal IgE proteins bound to the foal’s peripheral blood leukocytes, and endogenous IgE proteins are not produced until 9 months of age [66]. The transcriptome dataset in this study showed low but detectable *IGHE* transcripts on day 1, followed by a 100-fold increase (p<0.0001) in mRNA expression in day 42 foal samples, and another 100-fold increase (p<0.0001) in mRNA expression in adult samples, indicating that *IGHE* transcription occurs at low levels early in life.

Although IgA proteins have not been detected in foal serum prior to colostrum ingestion, *IGHA* transcripts encoding IgA proteins can be detected by RT-PCR in neonatal peripheral blood at birth [43,67]. The transcriptome dataset herein detected low to absent levels of *IGHA* on day 1 (range 0–4 reads per foal, counts per million), followed by a robust increase (p<0.0001) in day 42 foal samples comparable to adult levels. Endogenous IgA protein production has been reported to be in place by 8 weeks of age [43,77]. Expression of the *Ig J polypeptide*, the linker protein for oligomeric IgA and IgM, paralleled that of *IGHA*.

In the horse, B cells utilize the Ig lambda light chain more frequently (92%) than the Ig kappa light chain (8%) [77–79]. Multiple equine Ig lambda constant region genes (*IGLC1*, *IGLC4*, *IGLC5*, *IGLC7*) contribute to Ig lambda molecules, whereas only one Ig kappa constant region gene (*IGKC*) encodes all Ig kappa molecules [79–82]. The transcriptome dataset herein detected expression of *IGLC1*, *IGLC4*, *IGLC5*, and *IGLC7*, as well as *IGKC*. Expression of *IGLC1*, *IGLC4*, *IGLC5*, and *IGLC7* genes increased 4- to 6-fold between days 1 and 42, although *IGLC5* did not reach statistical significance presumably due to large variation between individuals within an age group (day 1 range 1–200 normalized reads; day 42 range 4–945 normalized reads). *IGLC* gene expression was equivalent between day 42 and adult samples. *IGKC* expression increased (p<0.0001) approximately 10 fold between days 1 and 42 of age, and then decreased (p = 0.04) by approximately 3-fold in adult samples. To compare relative expression of the Ig light chains, the number of reads for *IGLC1*, *IGLC4*, *IGLC5*, and *IGLC7* were summed and compared with the number of *IGKC* reads. This calculation revealed that, on day 1, Ig lambda constant region genes contribute to 92% of Ig light chain transcripts; 89% of Ig light chain transcripts on day 42; and 92% of Ig light chain transcripts in adults. The steady ratio of Ig lambda/kappa transcripts throughout equine life contrasts with the Ig light chain ratio of calves and adult cows; IGKC accounts for approximately 20% of Ig light chain transcripts in young calves but only 7% in adult cows [83]. The steady usage of Ig light chains is another instance in which the young foal immune system is equivalent to the adult horse’s immune system.

The wealth of Ig gene expression data provided by the PBMC transcriptome strategy revealed that the only Ig genes that did not reach adult expression levels by day 42 were *IGHG6*, *IGHG7*, and *IGHE*. In addition, expression of *IGKC* was maximal in day 42 foals and reduced (p = 0.041) in adult samples. These data highlight the regulated activity of the humoral immune system in young foals.

**Fig 4:**
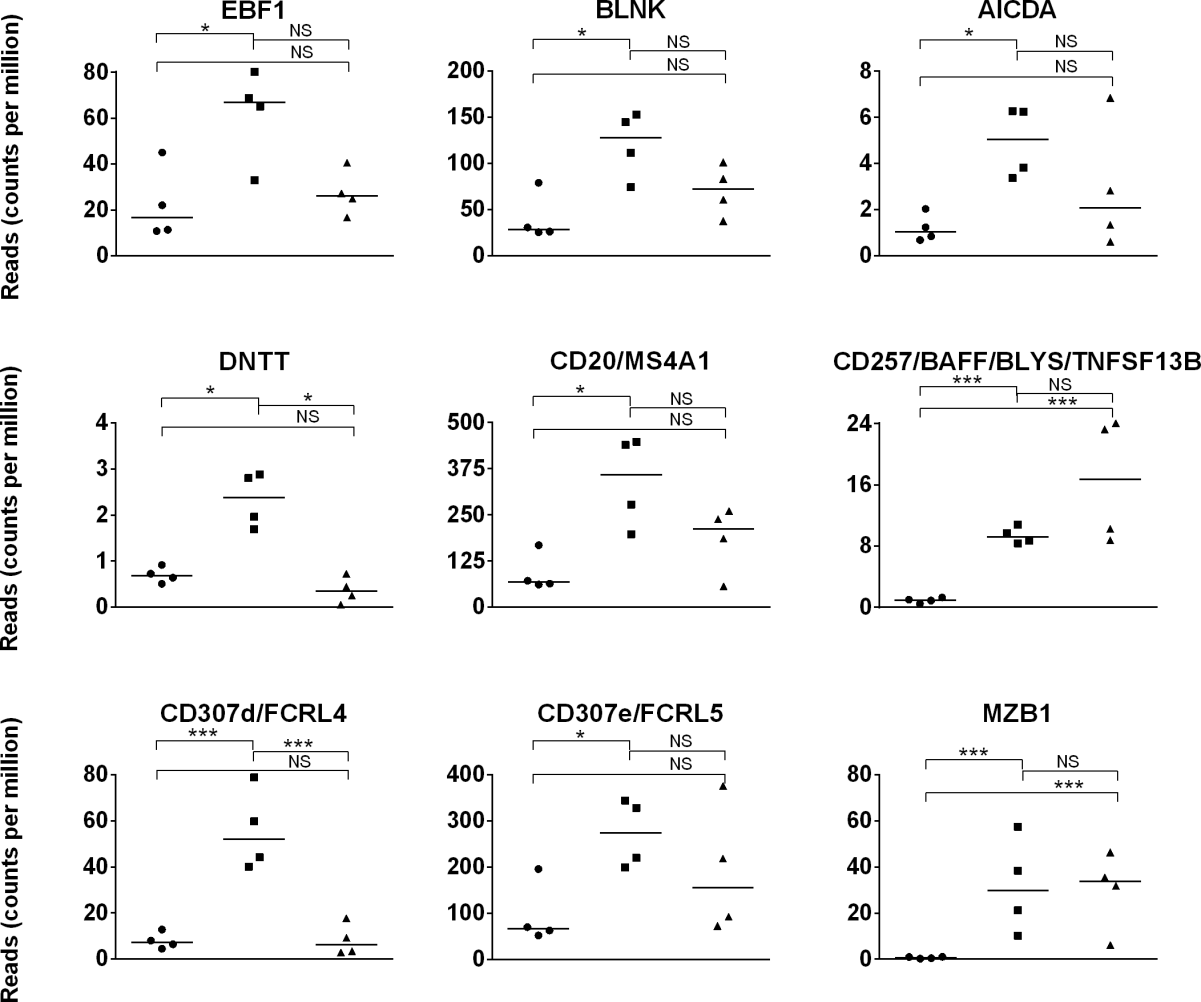
B cell gene expression in day 1 foal, day 42 foal, and adult horse samples. Expression (quantified by number of reads on y-axis) of B lineage genes on day 1 foal (circle), day 42 foal (square), and adult (triangle) samples are plotted on the x-axis. The significance level of comparisons is indicated by asterisks (* p<0.05; ** p≤0.001; *** p≤0.0001); comparisons that did not differ statistically are labeled “NS” to denote not significant.

Expression of essential B cell genes that support B cell development and survival, increase in Ig sequence diversity, modulate intracellular calcium levels, and promote Ig secretion was also analyzed [84–91]. Steady gene expression of transcription factor *PAX5*, cell surface proteins *CD19*, *CD22*, *CD40*, *CD79A*, *CD79B*, *BAFFR/CD268*, *TACI*, and *FCRL1/CD307a*, and signaling molecules *BLK* and *BTK* was detected over time (p>0.05 between all groups). Gene expression was greater (p≤0.05) for *EBF1*, *CD20*, *BLNK*, *BAFF*, *FCRL4*, *FCRL5*, MZB1, *AICDA*, and *DNTT* in day 42 than day 1 foal samples (Fig 4). These increases in gene expression parallel the increase in absolute numbers of B cells and enrichment for adaptive immune gene expression described above in the first 42 days [33].

### Expression of T lymphocyte genes

**Fig 5:**
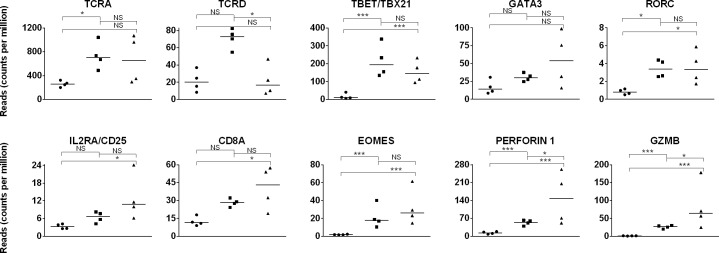
T cell gene expression in day 1 foal, day 42 foal, and adult horse samples. Expression (quantified by number of reads on y-axis) of T lineage genes on day 1 foal (circle), day 42 foal (square), and adult (triangle) samples are plotted on the x-axis. The significance level of comparisons is indicated by asterisks (* p<0.05; ** p≤0.001; *** p≤0.0001); comparisons that did not differ statistically are labeled “NS” to denote not significant.

To examine the kinetics of cellular immunity in young foals, the expression of T cell-specific genes was assessed. T cell receptor constant region genes were examined first; to date, only TCR alpha (*TCRA*) and delta (*TCRD*) are annotated in the equine genome, and these genes were used as representatives of the alpha/beta (αβ) and gamma/delta (γδ) T cell populations, respectively. *TCRA* gene expression increased (p = 0.035) approximately 3-fold over from day 1 to day 42 in foals, when it reached adult-like levels (day 42 foal vs adult p>0.05) (Fig 5). *TCRD* expression was not statistically different (p = 0.11) despite what seemed to be a 3-fold increase from day 1 to day 42 in foals; however, a decline (p = 0.026) in *TCRD* expression ensued when comparing day 42 and adult levels. Knowledge about γδ T cell populations in the horse is scarce. Studies in other species have established that γδ T cells are innate immune cells with antigen-presenting and cytolytic functions [92]. A transient increase in the γδ T cell population occurs in calves over the first 2 months, perhaps similar to that suggested by the equine transcriptome dataset presented here; overall γδ T cells comprise approximately 40% of calf PBMCs at birth and then decline to adult cow levels (approximately 15%) by 5 months of age [93]. The γδ T cell population accounts for a smaller proportion of human peripheral lymphocytes and, in contrast to the bovine system, increases from 1–4% in human neonatal cord blood to 3–12% in adult blood [6,94].

The T cell receptor forms a membrane complex on the cell surface with the four CD3 chains: CD3G, CD3D, CD3E, and CD3Z/CD247 [95]. No changes (p≥0.05) in gene expression were found for any of the CD3 chains over time.

*CD4* gene expression did not change over time, despite the fact that there is an age-dependent increase in peripheral blood subpopulation distributions, and lymphocyte population expansion [46]. In the recent years, different sub-populations of CD4 T cells have been described to change with age. In this study, master transcriptional regulators were utilized as surrogates of CD4 effector lineages (for Th1: *TBET/TBX21*; Th2: *GATA3*; and Th17: *RORC*) because gene expression of archetypal cytokines (*IFN*γ, *IL4*, *IL17F*, respectively) were not detected in the foal transcriptome datasets [96]. The Th1 lineage signature transcription factor *TBET* gene expression was reduced (p≤2.29x10^-8^) in day 1 compared to day 42 foal and adult samples, consistent with the expression profile of human neonatal CD4 T cells and the paradigm of limited Th1 lineage in the neonatal period [11,97]. Th2 lineage transcription factor *GATA3* gene expression was equivalent (p≥0.05) between foal samples on days 1 and 42. Th17 lineage signature gene *RORC* expression paralleled that of TBET in that minimal expression was detected in day 1 foal samples, and adult-like levels were measured in day 42 foal samples (p≤0.018).

The developmental trajectory of regulatory T cells in the horse has been described recently. Published data have shown that the CD4^+^CD25^high^ T cell population in foal PBMCs (age 1–5 months) accounts for 2% of the circulating CD4^+^ T cells, whereas a higher proportion of the foal CD4^+^CD25^high^ T cells also express FoxP3 than the equivalent cells in the adult horse (47% versus 10%) [35]. CD25 is encoded by the interleukin 2 receptor subunit alpha gene (*IL2RA*), and *IL2RA* gene expression did not change by day 42; however *CD25/IL2RA* mRNA expression was approximately 4 times greater (p = 0.03) in adult samples when compared to foal day 1 samples. No difference in *FOXP3* gene expression was detected in the transcriptome dataset, perhaps due to the discrete regulatory T cells population size in peripheral blood.

The CD8 T cells are defined by the co-expression of the T cell receptor and the CD8 alpha/beta heterodimer, although CD8 alpha/alpha homodimers have been described [98]. *CD8A* (CD8 alpha) gene expression differed between day 1 and adult samples (p = 0.045); yet, *CD8B* (CD8 beta) gene expression did not change (p≥0.05) over time. Foals experience age-dependent limitations of IFNγ production, with *IFN*γ gene expression absent or minimal at birth but adult-like levels are reached at approximately 3 months of age [42]. Although *IFN*γ gene expression was not detected in the transcriptome datasets reported here, the cytotoxic transcription factor *EOMES*, and genes encoding the cytolytic proteins perforin (*PRF1*) and granzyme B (*GZMB*) shared by CTLs and NK cells were assessed [99]; the expression of all 3 genes increased (p<0.0001) continually from day 1 to 42 in foals, and through to adulthood. Functional cytotoxic T lymphocytes (CTL) have been detected in foals at 3 weeks of age when inoculated with virulent *Rhodococcus equi* [49]. Functional CTL can also be generated by human fetal and infant immune systems [100,101].

### Expression of natural killer cell genes

**Fig 6:**
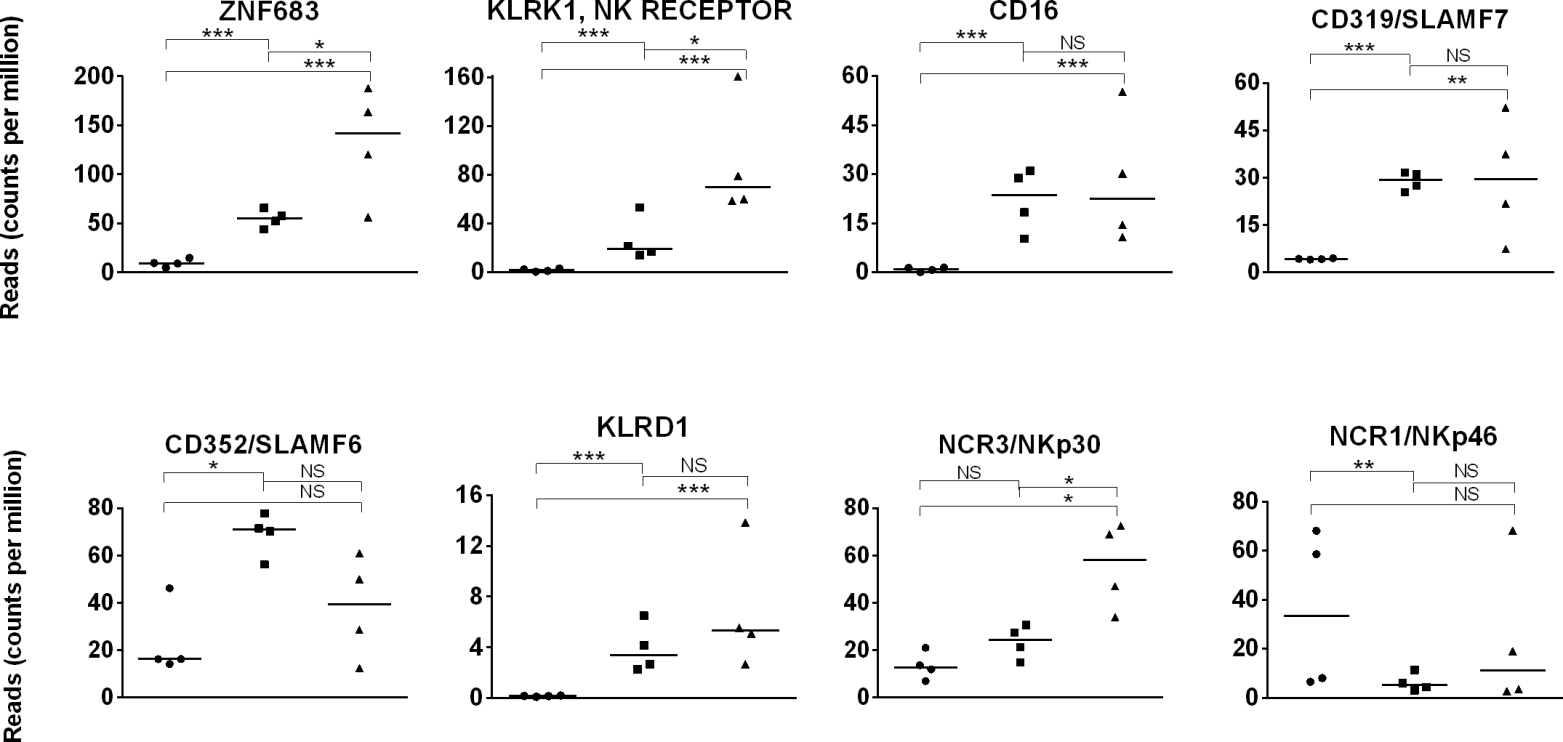
NK cell gene expression in day 1 foal, day 42 foal, and adult horse samples. Expression (quantified by number of reads on y-axis) of NK lineage genes in day 1 foal (circle), day 42 foal (square), and adult (triangle) samples are plotted on the x-axis. The significance level of comparisons is indicated by asterisks (* p<0.05; ** p≤0.001; *** p≤0.0001); comparisons that did not differ statistically are labeled “NS” to denote not significant.

The NK cell population is very heterogeneous and defined by a variety of phenotypic receptors and transcriptional factors, with described age-dependent expression [102]. In this study, the transcription factor *ZNF683/HOBIT* gene expression increased (p = 1.8x10^-6^) over time by approximately 5-fold between days 1 and 42 foals, and another 3-fold (p = 0.01) between day 42 and adults (Fig 6) [103]. These values were paralleled by *KLRK1/NKG2D* expression, which encodes killer cell lectin like receptor K1; the inhibitory marker *KLRD1/CD94/NKG2A*; and the cytotoxicity-activating receptor *NKp30/NCR3* (p≤0.007). CD16 and CD56 are markers often used to phenotype human NK cells and, in combination, CD16+CD56^dim^ NK cells are considered cytolytic cells [102]. The *CD16* gene expression increased (p = 2.6x10^-8^) from day 1 to day 42 in foals, when adult levels were reached. No expression data were found for *CD56* in these PBMC datasets. A similar profile was found for activating markers *CD319* and *CD352*. In contrast, gene expression of another cytotoxicity-activating receptor, *NKp46/NCR1* was equivalent (p>0.05) between day 1 foal and adult samples but lower (p = 0.0005) in day 42 foal samples.

Due to the lack of reagents, few studies have investigated the frequency or phenotype of equine NK cells. In adult equine peripheral blood leukocytes, 0.8 to 2.1% cells are positive for NKp46 and approximately 10% are positive for the NK-5C6 marker [104,105]. Bovine NK cells have been characterized in the neonatal phase and, although in low numbers at birth, they reach the same frequency of the adult NKp46+ cells by 8 days of age, with greater proliferative and cytotoxic activities upon stimulation [106]. In contrast, NK cell numbers in the human neonate are comparable to the adult but with lower cytotoxicity [107,108]. Additional work is needed to comprehend the distribution and cytolytic activity of NK subpopulations in equine blood, but the overall trend was an increasing gene expression from day 1 to day 42 in foals, when mRNA levels of multiple markers reached those found in adult samples.

### Expression of genes involved in antigen presentation and co-stimulation

Presentation of antigen can be mediated by several immune cell populations, including dendritic cells, B cells and macrophages. Antigenic peptides are displayed by MHC class I or class II molecules on the APC and bind cognate T cell receptor molecules. Interactions of additional ligand and receptor pairs on the APC and T cell mediate activating or inhibitory signals, and thereby direct the subsequent T cell functions and fate.

**Fig 7:**
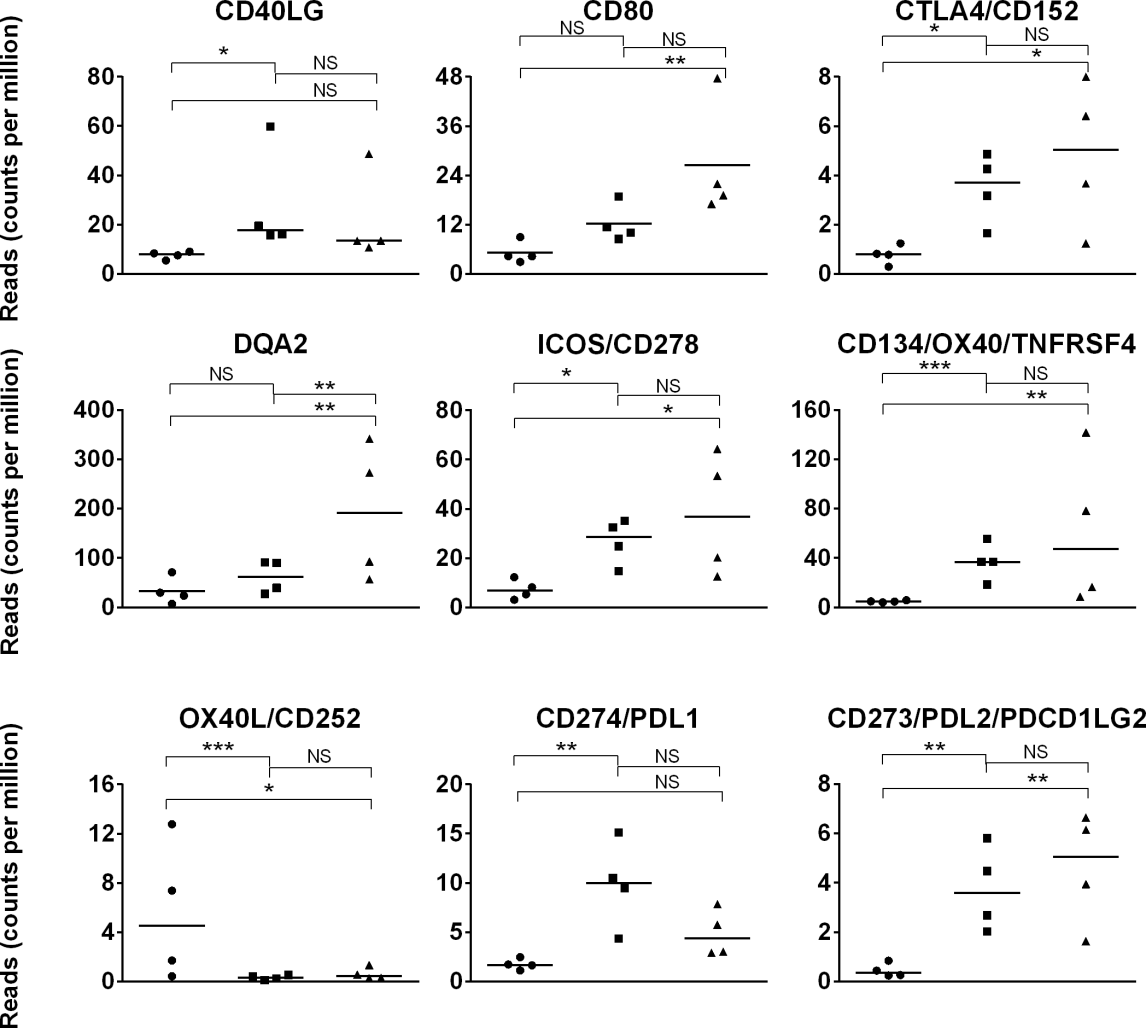
Expression of genes involved in antigen presentation and co-stimulation in day 1 foal, day 42 foal, and adult horse samples. Expression (quantified by number of reads on y-axis) of genes involved in antigen presentation and co-stimulation in day 1 foal (circle), day 42 foal (square), and adult (triangle) samples are plotted on the x-axis. The significance level of comparisons is indicated by asterisks (* p<0.05; ** p≤0.001; *** p≤0.0001); comparisons that did not differ statistically are labeled “NS” to denote not significant.

*OX40L*, a co-stimulatory moleculethat mediates activating signals to T cells, was expressed at higher (p = 4.5x10^-5^) levels in day 1 than day 42 foal and adult horse samples (Fig 7) [109,110]. However, the functional consequences are questionable since the cognate receptor *OX40* exhibited minimal expression in day 1 foal samples (p = 0.0002).

Delayed expression of MHC class II molecules is well-established in the developing foal at the protein level [37,40,45,46] but the contribution of individual MHC class II genes has not been analyzed. The transcriptome dataset showed equivalent (p>0.05) expression over time of *DRA*, *DRB1*, *DRB2*, and *DQA3* genes but revealed increased (p≤0.039) *DQA2* expression in adult in contrast to day 1 or 42 foal samples.

The interaction between CD40L expressed in CD4^+^ T cells and CD40 expressed in B cells and APCs is essential to inducing antibody class switching, germinal center formation, and cytokine production [111]. CD40L expression is low in human cord blood and neonatal CD4^+^ T cells, and increases over the first months of life [112]. Insufficient CD40L levels are a putative limiting factor of Ig class switching in human neonates [113]. In this study, the *CD40* expression did not change (p>0.05) over time in foal and adult samples; yet, the *CD40L* gene expression pattern was consistent with human neonates, as it increased (p = 0.024) between day 1 and day 42 foal samples, when expression was comparable (p = 1) to that of adult samples.

Expression increased (p≤0.03) between day 1 and day 42 foal samples for*PDL1*, *PDL2*, *CTLA-4*, and *ICOS* genes. Expression of *CD80* was greater (p = 0.0004) in adult than day 1 samples. The signals mediated by the encoded protein molecules are activating (ICOS, CD80), inhibitory (CTLA-4), or are dependent on the ligand (PDL1, PDL2) [109].

Altogether, assessment of co-stimulatory gene expression did not define the young foal immune cells as activated or inhibited. Rather, a highly dynamic and complex modulatory system was observed in the young. Since many of these molecules are expressed and/or induced in a variety of cell types under diverse conditions, additional investigations are necessary to better define their role in early life.

### Expression of toll-like receptor genes

A key feature of the innate immune system is the recognition of conserved constituents of pathogens, also known as pathogen-associated molecular patterns (PAMPs), including single- or double-stranded RNA, bacterial CpG DNA, bacterial lipoproteins, and flagellin [114]. A family of 10 Toll-like receptors (TLRs, TLR1 through 10) have been described in a variety of human immune cells [115,116]. Given the importance of innate immunity in early life, the expression of TLR genes was analyzed from the transcriptome dataset. TLR5 is not annotated in the equine genome and thus not reported here.

**Fig 8:**
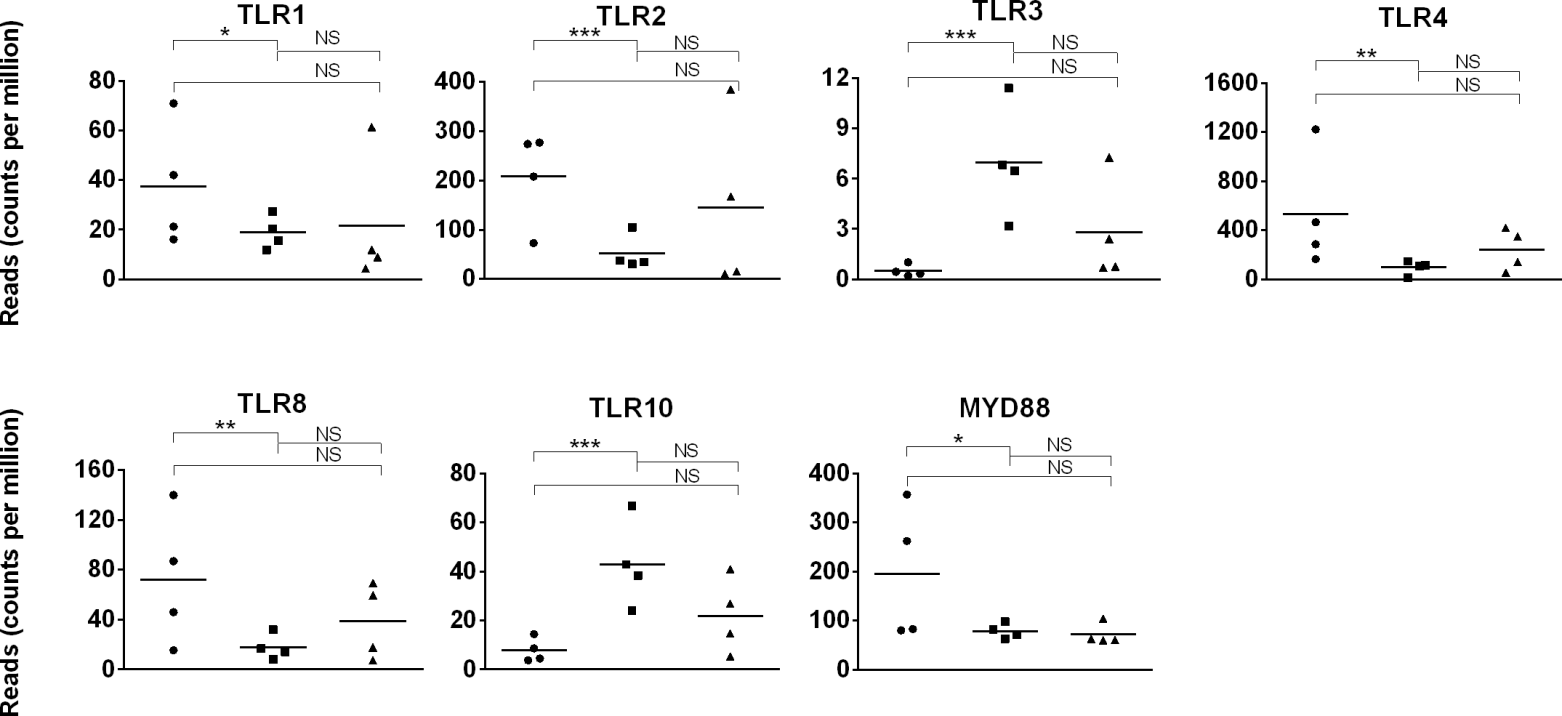
Toll-like receptor gene expression in day 1 foal, day 42 foal, and adult horse samples. Expression (quantified by number of reads on y-axis) of TLR genes in day 1 foal (circle), day 42 foal (square), and adult (triangle) samples are plotted on the x-axis. The significance level of comparisons is indicated by asterisks (* p<0.05; ** p≤0.001; *** p≤0.0001); comparisons that did not differ statistically are labeled “NS” to denote not significant.

*TLR1*, *TLR2*, *TLR4*, *TLR8*, and signaling protein *MYD88* gene expression levels were higher (p≤0.02) in day 1 than day 42 foal samples (Fig 8). No changes (p≥0.05) in *TLR6*, *TLR7*, or *TLR9* expression were detected between day 1 foal, day 42 foal, and adult samples. Comparable expression levels of TLR1, 2, 4, 8, and 9 are found between human neonatal and adult leukocytes, however MYD88 is expressed at lower levels in human neonates than adults [117,118].

The *TLR3* and *TLR10* genes showed the lowest (p≤5.7x10^-5^) expression levels in day 1 foal samples, and both achieved adult levels by day 42 (p>0.05). *TLR3* expression is limited by epigenetic mechanisms in human newborns and, because TLR3 binds viral double-strand RNAs (dsRNAs), lack of TLR3 contributes to susceptibility to viral infections in the neonatal phase [119,120]. Given the similar expression profile, epigenetic regulation of *TLR3* could be involved in young foals. The function of TLR10 is not known, although it is expressed in lymphoid cells and tissues of the horse [121].

### Expression of complement receptor genes

**Fig 9:**
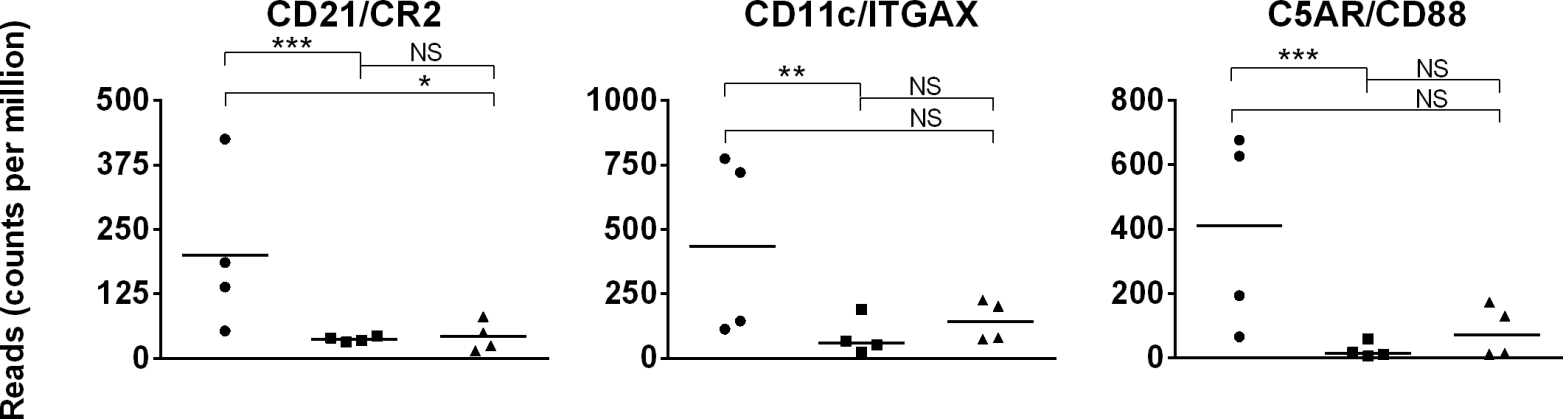
Complement receptor gene expression in day 1 foal, day 42 foal, and adult horse samples. Expression (quantified by number of reads on y-axis) of complement receptor genes in day 1 foal (circle), day 42 foal (square), and adult (triangle) samples are plotted on the x-axis. The significance level of comparisons is indicated by asterisks (* p<0.05; ** p≤0.001; *** p≤0.0001); comparisons that did not differ statistically are labeled “NS” to denote not significant.

We focused our analysis on the expression of complement receptors in PBMC because complement activation proteins are mainly produced by the liver. Eight complement receptors (CR) that are expressed by many cell types have been characterized: CR1 (CD35), CR2 (CD21), CR3 (CD11b/CD18), CR4 (CD11c/CD18), CRIg, C5a receptor (C5AR, CD88), C5L2, and C3a receptor (C3AR1) [122]. CR1 (CD35) is not yet defined in the equine genome and thus not annotated in this dataset. No expression data were detected for *CRIg*, *C5L2*, and *C3AR1* at one or more ages, and so not reported here. *CR3* (*CD11b/CD18*, also known as *ITGAM* or *Mac-1*) expression was equivalent (p>0.05) across age groups. For the remaining receptors, *CR2*, *CR4*, and *C5AR* gene expressions were maximal (p≤0.008) in day 1 foal samples; *CR2*, *CR4*, and *C5AR* expressions were comparable (p≥0.05) between day 42 foal and adult samples (Fig 9). The *CR2* expression profile in equine PBMC is intriguing because it contrasts with the decreased CR2 expression reported for human neonatal B cells [123]. Low CR2 expression has been implicated as the reason why human neonatal B cells do not respond to T cell-independent antigens, such as pneumococcal polysaccharides [124].

### Expression of inhibitory immune receptor genes

**Fig 10:**
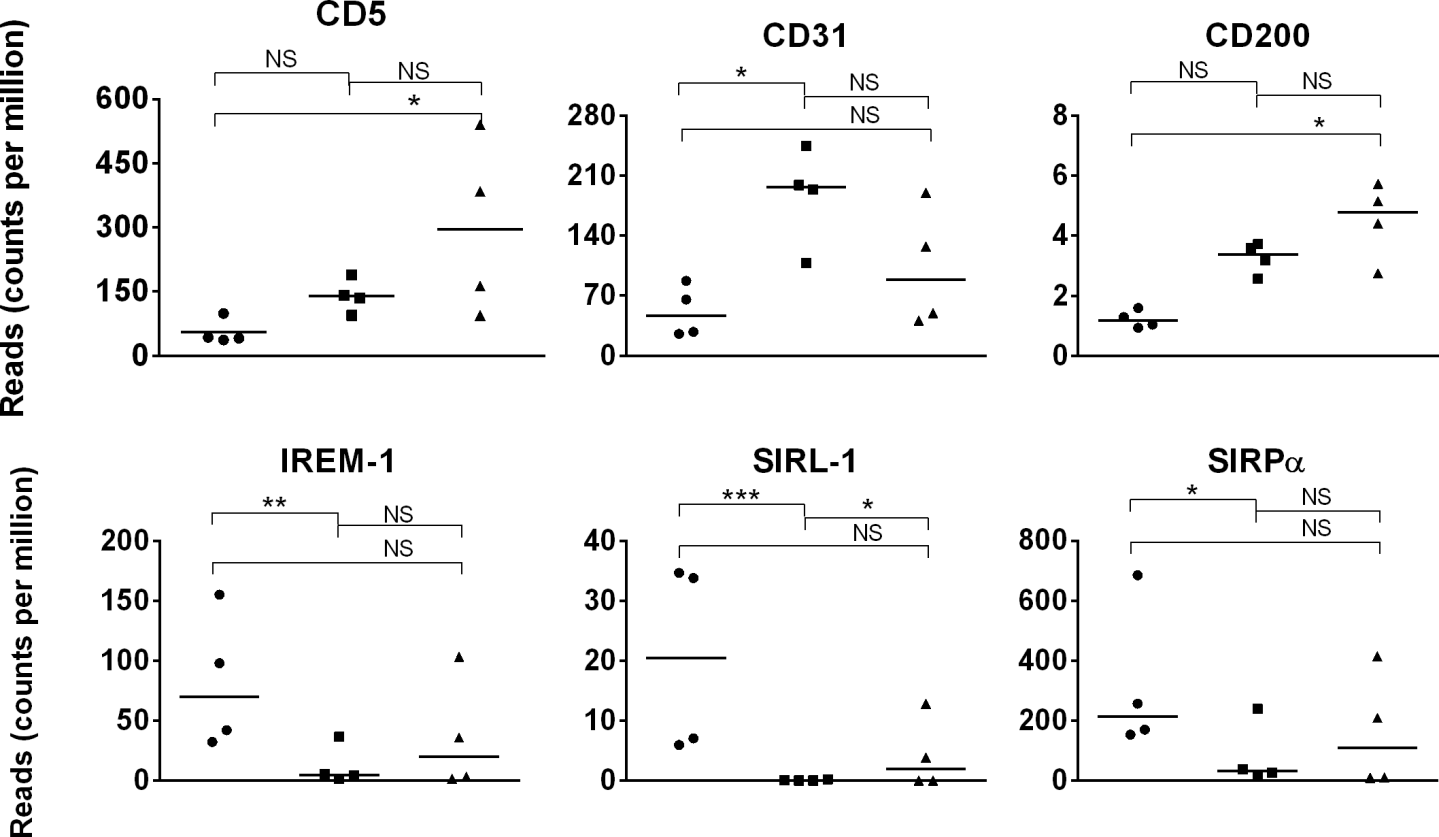
Inhibitory immune receptor gene expression in day 1 foal, day 42 foal, and adult horse samples. Expression (quantified by number of reads on y-axis) of inhibitory immune receptor genes in day 1 foal (circle), day 42 foal (square), and adult (triangle) samples are plotted on the x-axis. The significance level of comparisons is indicated by asterisks (* p<0.05; ** p≤0.001; *** p≤0.0001); comparisons that did not differ statistically are labeled “NS” to denote not significant.

One mechanism that may contribute to dampened immune responses in early life is elevated levels of inhibitory receptors, which can set a higher threshold for immune activation. Indeed, immune inhibitory receptors CD31, CD200, and LAIR-1 are highly expressed in human neonatal T cells [125]. Therefore, expression of 7 immune inhibitory receptors was investigated in this transcriptome dataset. Three immune inhibitory receptor genes showed higher (p≤0.01) expression in day 1 than in day 42 foal samples: immune receptor expressed on myeloid cells-1 (*IREM-1*); signal inhibitory receptor on leukocytes-1 (*SIRL-1*); and signal-regulatory protein alpha (*SIRP*α) (Fig 10). Gene expression of *CD31/PECAM* increased (p = 0.017) between day 1 and day 42 foal samples. *CD5* and *CD200* gene expression increased (p = ≤0.02) between day 1and adult samples.

Immune inhibitory receptors IREM-1, SIRL-1, and SIRPα may be fostering a suppressive environment during the neonatal phase of foals. Expression of these immune inhibitory receptors may differ across immune cell subsets, as well as over time, and further detailed analysis may provide insights to their contributions [125,126].

## Conclusions

The analyses of PBMC transcriptome datasets provided extensive information describing equine neonatal immune system development. More than 2,000 genes exhibited differential expression over the first few weeks of life, and analyses of these genes revealed a list of molecules that may contribute to the particular susceptibilities and putative suppressive environment unique to the neonatal immune system, but also areas of competence. Due to the overlapping, inducible, and complex expression patterns of immune proteins, follow-up experiments with purified cell subsets are necessary to define the mechanisms that are modulating the neonatal immune system. The transcriptional signatures detailed herein may help to clarify the unique susceptibilities and attributes of the neonatal and foal immune system and thus aid the development of more effective vaccination and treatment strategies. Further investigation of genes involved in pathogen detection (e.g. TLRs), cell signaling activation (e.g. MAPK cascade), and antigen presentation (e.g. MHC class II, OX40) would provide applicable insights on how the equine neonate and young foal elaborate immune responses. In addition, since epigenetic regulation has also been shown to be involved in neonatal immune system development, this study provides a platform for epigenetic analysis of the equine immune system, both during early life and in adulthood.

## Supporting information

S1 Table Day 1 versus day 42 PBMC RNA-Seq data. (PDF)

S2 Table Day 42 versus adult PBMC RNA-Seq data. (PDF)

S3 Table Day 1 versus adult PBMC RNA-Seq data. (PDF)

S1 Fig Distribution of p-values for each age comparison. Histograms were constructed to assess the distribution of p-values after differential gene expression analysis. For each comparison between age groups, the distribution of p-values greater than 0.05 was uniform. Each comparison results in more than 1,500 differentially expressed genes with p-values < 0.05.
(PDF)
