# Supplementary material for: Transcriptome analysis of immune genes in peripheral blood mononuclear cells of young foals and adult horses
Source: PLoS One. 2018 Sep 5;13(9):e0202646. doi: 10.1371/journal.pone.0202646 (PMC6124769; doi:10.1371/journal.pone.0202646)
Supplement: S1 Table — (PDF) [file pone.0202646.s001.pdf]

S1 Table. Day 1 versus day 42 PBMC RNA-Seq data

				Raw data (number of reads)							
Gene (Ensembl ID)	log fold change	p value (tag-wise dispersion)	adjusted p value (tag-wise dispersion)	day1foalA	day1foalB	day1foalC	day1foalD	day42foalA	day42foalB	day42foalC	day42foalD
ENSECAG00000027671	11.96291117	1.12E-95	1.27E-91	137518	84132	158360	138085	0	0	0	0
ENSECAG00000027689	12.58707151	3.31E-95	1.88E-91	198914	110243	286985	212133	0	0	0	0
ENSECAG00000027672	11.62158018	4.08E-85	1.54E-81	124700	64151	118245	90510	0	0	0	0
ENSECAG00000027679	8.348753087	2.06E-66	5.86E-63	11461	5470	15373	9851	0	0	0	0
ENSECAG00000007258	5.435459301	4.38E-59	9.94E-56	1	1	6	7	1532	2234	2196	1238
ENSECAG00000020718	3.783778561	2.19E-30	4.15E-27	311	672	513	274	0	8	0	3
ENSECAG00000008161	4.198212091	3.59E-27	5.83E-24	727	668	544	282	0	9	0	2
ENSECAG00000006195	2.95141471	1.43E-25	2.02E-22	330	257	188	155	0	2	0	5
ENSECAG00000018476	5.352462915	1.98E-25	2.50E-22	65	66	58	135	1896	875	1497	2409
ENSECAG00000008322	3.936898396	2.81E-25	3.19E-22	24	10	25	13	582	609	744	536
ENSECAG00000009556	5.420848262	1.62E-24	1.61E-21	0	1	19	2	2244	1091	939	3174
ENSECAG00000023330	4.975995777	1.70E-24	1.61E-21	61	26	62	38	1525	812	1363	1436
ENSECAG00000010523	5.394268773	3.97E-24	3.47E-21	1948	2088	355	447	2	8	3	3
ENSECAG00000021580	6.84203478	1.56E-23	1.26E-20	5900	414	3655	4153	8	20	6	18
ENSECAG00000024357	4.178820257	3.77E-23	2.71E-20	31	47	49	69	866	439	651	903
ENSECAG00000024888	6.964061979	3.82E-23	2.71E-20	113	59	242	123	3838	3684	6935	6335
ENSECAG00000010778	6.480439642	4.26E-23	2.85E-20	1893	926	3851	6010	31	87	18	27
ENSECAG00000001910	3.590621641	5.35E-23	3.38E-20	24	14	24	31	565	416	524	395
ENSECAG00000018883	2.853773077	2.59E-22	1.55E-19	208	277	224	206	2	11	0	2
ENSECAG00000014278	5.049629145	2.90E-22	1.65E-19	759	2559	543	204	1	6	2	1
ENSECAG00000001249	4.386209681	5.15E-22	2.79E-19	326	553	1233	601	34	61	53	43
ENSECAG00000015104	1.589876978	2.68E-21	1.38E-18	58	39	213	97	0	0	0	0
ENSECAG00000018167	5.158421999	3.04E-21	1.50E-18	1661.01	1743	324	382	0	1	9	4
ENSECAG00000020041	7.845555919	3.42E-21	1.62E-18	137	164	97	295	6361	3294	10437	19896
ENSECAG00000003774	8.788304891	4.25E-21	1.93E-18	85	120	332	524	11447	8192	19727	37710
ENSECAG00000014332	3.500888389	7.73E-21	3.38E-18	193	209	322	876	8	21	6	17
ENSECAG00000015806	4.188393415	8.67E-21	3.65E-18	44	38	43	74	804	403	694	982
ENSECAG00000000744	5.447552898	1.13E-20	4.59E-18	1247	1857	1304	742	93	111	147	95
ENSECAG00000006607	5.261648807	1.28E-20	5.03E-18	101	80	83	170	1768	818	1519	1961
ENSECAG00000010847	10.34929947	1.54E-20	5.84E-18	58657	63483	14238	15125	16	193	50	86
ENSECAG00000019659	3.591945715	1.62E-20	5.93E-18	445	769	108	96	0	1	1	1
ENSECAG00000000288	8.259514864	3.44E-20	1.22E-17	23947	2973	2360	2497	3	8	8	1
ENSECAG00000024743	2.297516429	4.57E-20	1.57E-17	4	1	0	4	216	200	272	100
ENSECAG00000015699	3.753919558	4.70E-20	1.57E-17	599	703	114	146	0	0	1	4
ENSECAG00000014517	4.261836763	6.22E-20	2.02E-17	31	44	55	110	835	463	751	952
ENSECAG00000016421	6.753531756	6.78E-20	2.14E-17	7836	2473	249	724	1	1	1	0
ENSECAG00000009575	1.286218259	7.58E-20	2.33E-17	1	1	1	3	67	97	85	138
ENSECAG00000017642	3.951256107	7.94E-20	2.37E-17	25	21	21	73	660	380	634	806
ENSECAG00000008785	5.410633073	1.03E-19	3.01E-17	96	69	95	211	1818	950	1464	2562
ENSECAG00000015583	0.72355415	1.09E-19	3.10E-17	59	56	48	38	0	0	0	0
ENSECAG00000003385	1.329226721	1.75E-19	4.84E-17	27	37	171	116	0	0	0	1
ENSECAG00000012112	2.271753861	2.20E-19	5.95E-17	3	5	9	10	174	127	164	318
ENSECAG00000007545	3.603179668	3.12E-19	8.24E-17	195	111	551	895	4	17	5	8
ENSECAG00000020503	3.808077816	3.47E-19	8.95E-17	4	9	20	20	392	166	702	1136
ENSECAG00000013053	6.048913358	3.55E-19	8.95E-17	180	100	96	218	3039	1505	2505	3622
ENSECAG00000021499	5.900257741	7.95E-19	1.96E-16	2931	463	2220	1675	34	12	3	3
ENSECAG00000026891	3.152432136	1.36E-18	3.29E-16	9	16	20	36	393	185	390	450
ENSECAG00000010117	9.132052065	1.90E-18	4.51E-16	32705	13766	5341	12317	2	66	10	13
ENSECAG00000014384	3.503280202	3.09E-18	7.16E-16	18	30	17	53	398	286	485	625
ENSECAG00000020842	2.441005883	3.49E-18	7.92E-16	78	81	236	376	3	9	1	3
ENSECAG00000021476	10.57851873	4.47E-18	9.96E-16	126308	13991	3793	10668	1	16	16	10
ENSECAG00000022569	1.633229834	6.02E-18	1.31E-15	47	45	127	218	0	4	0	2
ENSECAG00000013081	9.252979971	6.33E-18	1.36E-15	28660	21346	9769	12101	83	363	50	61
ENSECAG00000018808	7.208575245	8.30E-18	1.75E-15	9059	4634	2051	272	16	10	21	13
ENSECAG00000020535	6.330692554	1.15E-17	2.38E-15	1332	3322	2919	2228	423	333	253	239
ENSECAG00000018973	5.723090167	1.19E-17	2.42E-15	2511	2695	401	387	1	12	4	15
ENSECAG00000010654	1.670000022	1.35E-17	2.69E-15	124	24	116	143	0	2	0	2
ENSECAG00000016509	2.285920708	1.91E-17	3.74E-15	89	71	231	280	2	10	1	4
ENSECAG00000009271	8.525861733	2.09E-17	4.02E-15	23277	7723	3295	6889	2	33	5	1
ENSECAG00000021710	5.966767552	2.46E-17	4.67E-15	222	278	296	315	1740	2271	2283	2957
ENSECAG00000024127	5.183312721	4.94E-17	9.20E-15	84	105	114	238	1368	779	1257	2246
ENSECAG00000012506	6.845902762	5.11E-17	9.36E-15	344	263	326	530	4011	3622	6047	4019
ENSECAG00000000281	3.957009989	5.99E-17	1.08E-14	41	43	27	69	674	315	600	853
ENSECAG00000010821	4.013773503	6.43E-17	1.14E-14	42	51	63	92	694	349	609	811
ENSECAG00000010997	2.141703303	7.20E-17	1.26E-14	98	26	180	316	0	3	2	2
ENSECAG00000024185	5.007815385	8.13E-17	1.40E-14	672	440	1437	1932	3	46	8	42
ENSECAG00000025078	5.79693682	8.46E-17	1.44E-14	75	94	88	241	1981	2922	2843	851
ENSECAG00000014452	5.04067146	1.40E-16	2.35E-14	101	75	133	190	1824	898	1254	979
ENSECAG00000010191	3.653109052	1.64E-16	2.71E-14	561	666	82	139	6	0	2	4
ENSECAG00000007625	8.052826971	1.82E-16	2.95E-14	11580	12771	3476	3212	0	42	51	22
ENSECAG00000014953	3.962256696	1.86E-16	2.98E-14	40	47	57	101	621	403	495	853
ENSECAG00000009485	4.614814801	1.91E-16	3.02E-14	1208	1037	298	190	23	19	20	20
ENSECAG00000011788	3.587301681	2.02E-16	3.14E-14	26	37	30	60	453	246	464	715
ENSECAG00000013528	4.390935344	2.06E-16	3.17E-14	288	749	747	913	40	108	35	86
ENSECAG00000014619	3.652840709	2.82E-16	4.27E-14	25	34	43	84	437	327	515	645
ENSECAG00000009647	7.723355671	3.15E-16	4.65E-14	14954	5480	419	1317	6	16	3	4
ENSECAG00000008385	4.786567398	3.15E-16	4.65E-14	71	30	64	143	1177	596	988	1633
ENSECAG00000019369	3.189742536	4.17E-16	6.02E-14	21	21	20	47	367	215	340	499
ENSECAG00000000505	5.369999406	4.19E-16	6.02E-14	3116	363	561	294	2	10	9	6
ENSECAG00000015060	1.400555026	4.59E-16	6.53E-14	4	4	2	6	101	79	82	149
ENSECAG00000024636	4.936423432	6.98E-16	9.79E-14	964	2170	382	109	1	8	2	11

ENSECAG00000007470	4.339839232	7.89E-16	1.09E-13	47	70	64	138	831	462	635	1177
ENSECAG00000021452	4.047692894	7.96E-16	1.09E-13	43	36	48	112	669	410	550	920
ENSECAG00000009966	6.654826514	8.61E-16	1.16E-13	6039	3465	337	1195	18	45	25	27
ENSECAG00000019243	4.663318354	8.97E-16	1.20E-13	1159	1133	359	274	0	19	3	6
ENSECAG00000011068	3.048070843	9.63E-16	1.27E-13	472	330	43	71	1	0	0	2
ENSECAG00000017103	4.222034665	1.22E-15	1.60E-13	62	67	54	124	743	447	665	962
ENSECAG00000017906	3.453288588	1.47E-15	1.90E-13	19	30	23	66	455	236	424	594
ENSECAG00000012742	1.413927824	1.93E-15	2.46E-13	156	69	51	23	0	0	0	0
ENSECAG00000009668	0.348776486	2.05E-15	2.59E-13	44	59	26	21	0	0	0	1
ENSECAG00000008764	5.807319913	2.08E-15	2.60E-13	2161	3353	731	280	1	21	2	11
ENSECAG00000012615	1.809882747	2.92E-15	3.61E-13	53	39	152	254	0	6	0	1
ENSECAG00000006856	2.863916235	3.51E-15	4.29E-13	448	123	127	115	0	6	4	2
ENSECAG00000022085	6.308074885	4.83E-15	5.84E-13	6517	379	478	734	3	10	1	5
ENSECAG00000025146	5.205117567	4.90E-15	5.86E-13	1279	2697	146	179	3	6	4	14
ENSECAG00000021405	1.529991211	5.33E-15	6.31E-13	151	120	22	35	0	0	0	0
ENSECAG00000012930	6.710000494	5.53E-15	6.48E-13	5164	5143	578	958	2	33	4	16
ENSECAG00000021479	2.721003026	6.37E-15	7.39E-13	256	390	75	44	3	1	1	4
ENSECAG00000024691	3.614914191	7.55E-15	8.66E-13	36	17	37	34	465	507	643	252
ENSECAG00000011086	7.314190453	8.89E-15	1.01E-12	8716	6510	1454	771	68	89	78	42
ENSECAG00000019660	8.115117543	9.70E-15	1.09E-12	15169	8827	3719	2886	231	391	161	182
ENSECAG00000021413	4.415555312	1.09E-14	1.21E-12	77	84	81	166	581	864	762	890
ENSECAG00000007499	2.793674843	1.25E-14	1.37E-12	397	226	35	123	0	3	3	0
ENSECAG00000001949	6.44860847	1.26E-14	1.37E-12	5508	2302	1466	154	1	19	11	12
ENSECAG00000016046	2.537431245	1.29E-14	1.39E-12	34	67	663	49	0	0	0	0
ENSECAG00000005838	2.529524174	1.30E-14	1.39E-12	357	179	49	48	0	2	0	0
ENSECAG00000005719	0.580433895	1.32E-14	1.40E-12	0	0	0	3	27	50	88	64
ENSECAG00000025135	3.650845049	1.38E-14	1.46E-12	312	1009	65	135	1	5	2	7
ENSECAG00000003002	5.678199178	1.54E-14	1.61E-12	2228	2560	423	551	90	44	65	59
ENSECAG00000002739	5.538545654	1.87E-14	1.91E-12	125	133	85	277	1918	784	1629	3010
ENSECAG00000022257	2.72554946	1.87E-14	1.91E-12	68	54	284	573	3	10	4	3
ENSECAG00000017890	4.890581051	2.24E-14	2.27E-12	1631	1328	145	167	1	9	0	5
ENSECAG00000016017	5.567987793	2.52E-14	2.53E-12	1913	2816	352	438	0	21	2	7
ENSECAG00000024694	2.625816238	3.06E-14	3.05E-12	11	10	13	34	246	134	239	346
ENSECAG00000010053	2.498556517	3.42E-14	3.37E-12	13	12	15	30	205	148	193	322
ENSECAG00000009704	1.4218249	3.88E-14	3.80E-12	1	4	10	3	78	81	139	115
ENSECAG00000000134	5.821148331	5.18E-14	5.03E-12	2645	2652	384	639	55	89	44	62
ENSECAG00000014536	3.678927027	5.25E-14	5.06E-12	41	23	57	84	518	339	470	600
ENSECAG00000009589	3.229592411	5.46E-14	5.18E-12	30	16	32	13	366	357	439	274
ENSECAG00000010859	3.51582686	5.47E-14	5.18E-12	37	41	46	70	440	263	377	636
ENSECAG00000008193	7.013400383	6.42E-14	6.03E-12	10698	1301	399	580	3	13	10	3
ENSECAG00000006678	0.388104226	7.38E-14	6.87E-12	20	26	44	81	2	2	1	1
ENSECAG00000025028	4.686194572	8.21E-14	7.59E-12	538	682	548	1221	134	185	175	158
ENSECAG00000009163	2.806704616	8.71E-14	7.98E-12	22	18	25	35	296	169	244	348
ENSECAG00000020956	2.587437185	9.06E-14	8.24E-12	170	375	103	73	4	8	0	7
ENSECAG00000002513	2.781253058	9.23E-14	8.32E-12	6	27	12	18	286	117	308	382
ENSECAG00000008821	7.22900715	9.65E-14	8.63E-12	7494	7695	991	558	3	46	11	21
ENSECAG00000016495	4.07367556	1.10E-13	9.75E-12	73	59	57	103	727	363	639	776
ENSECAG00000011895	9.462139993	1.25E-13	1.10E-11	33149	38482	4781	3238	7	164	31	82
ENSECAG00000002971	4.024341762	1.40E-13	1.22E-11	60	89	76	111	483	626	506	711
ENSECAG00000022666	2.182696361	1.63E-13	1.41E-11	7	13	12	19	127	244	146	159
ENSECAG00000010334	3.316250101	1.80E-13	1.55E-11	218	900	41	46	2	0	0	0
ENSECAG00000024847	8.623873228	1.84E-13	1.57E-11	23899	14614	2752	1071	66	148	137	80
ENSECAG00000024287	3.042050614	1.90E-13	1.61E-11	179	328	286	183	22	46	19	30
ENSECAG00000012371	8.034571179	1.98E-13	1.67E-11	20546	2840	1548	1923	53	86	93	44
ENSECAG00000010517	1.226860279	2.12E-13	1.77E-11	20	28	110	177	1	4	0	0
ENSECAG00000014251	4.355936004	2.17E-13	1.80E-11	923	1001	158	242	23	20	3	22
ENSECAG00000006234	7.279788996	2.20E-13	1.81E-11	6683	9116	1065	881	3	53	50	39
ENSECAG00000009716	3.104924355	2.35E-13	1.92E-11	26	21	22	46	351	177	286	514
ENSECAG00000019780	3.350498337	2.77E-13	2.25E-11	18	23	29	75	425	219	357	575
ENSECAG00000017357	4.963664721	3.05E-13	2.46E-11	137	99	207	249	1176	888	1264	1112
ENSECAG00000016075	8.61826443	3.19E-13	2.55E-11	12539	24848	7114	1394	322	312	379	438
ENSECAG00000006595	5.293468994	3.21E-13	2.55E-11	1339	2865	202	158	2	10	15	19
ENSECAG00000019333	6.240645434	3.58E-13	2.82E-11	2673	2426	2074	1174	426	290	297	228
ENSECAG00000021609	6.164098968	3.61E-13	2.83E-11	1506	6479	345	364	6	29	5	24
ENSECAG00000016534	6.063392573	4.30E-13	3.34E-11	2546	3929	781	331	98	61	71	89
ENSECAG00000023828	3.844220457	5.22E-13	4.04E-11	715	697	97	89	2	11	7	10
ENSECAG00000019038	3.458501806	5.28E-13	4.05E-11	21	13	33	74	419	241	381	683
ENSECAG00000023154	2.481987111	5.58E-13	4.25E-11	96	107	210	303	14.0001	31.0001	14	19
ENSECAG00000010783	5.797654651	5.75E-13	4.36E-11	1410	3597	544	1097	58	180	61	98
ENSECAG00000016860	2.930990474	7.28E-13	5.48E-11	25	30	32	34	299	202	223	415
ENSECAG00000008811	7.115826451	7.89E-13	5.90E-11	7009	6667	1044	785	6	64	6	41
ENSECAG00000019868	4.414549652	8.09E-13	5.98E-11	528	810	413	779	69	154	59	50
ENSECAG00000009568	2.538795258	8.11E-13	5.98E-11	10	14	17	36	222	137	180	355
ENSECAG00000020419	4.074060633	8.39E-13	6.15E-11	614	860	229	192	30	38	34	55
ENSECAG00000024845	3.26987985	9.19E-13	6.69E-11	282	721	42	105	8	4	1	2
ENSECAG00000021303	4.402108445	1.14E-12	8.25E-11	1028	980	158	166	32	17	17	32
ENSECAG00000004390	4.224972699	1.23E-12	8.81E-11	55	101	70	166	680	536	862	606
ENSECAG00000020325	3.138361159	1.23E-12	8.81E-11	255	320	129	361	23	41	24	9
ENSECAG00000023216	8.822201579	1.25E-12	8.91E-11	19330	26513	2546	3272	58	306	62	248
ENSECAG00000020645	4.344678307	1.32E-12	9.29E-11	42	27	34	158	810	453	753	1184
ENSECAG00000021765	8.003563559	1.32E-12	9.29E-11	8642	14928	3159	4042	33	349	42	84
ENSECAG00000008506	3.107058685	1.39E-12	9.67E-11	25	14	38	39	360	172	275	524
ENSECAG00000009472	4.074558527	1.43E-12	9.88E-11	68	45	70	127	647	357	608	897
ENSECAG00000008634	4.564235558	1.54E-12	1.06E-10	82	75	144	188	1053	492	759	1165
ENSECAG00000003193	3.413208521	1.77E-12	1.21E-10	150	273	466	466	60	56	24	20

ENSECAG00000020573	4.53533981	2.00E-12	1.36E-10	122	102	125	132	897	515	830	1109
ENSECAG00000012791	6.547421242	2.03E-12	1.38E-10	239	597	283	525	2489	4466	3210	3523
ENSECAG00000009474	5.148895619	2.09E-12	1.41E-10	10	70	56	189	782	1901	1776	1093
ENSECAG00000024563	4.893455599	2.22E-12	1.49E-10	1821	589	405	511	2	26	0	3
ENSECAG00000021044	4.525952266	2.24E-12	1.49E-10	664	980	261	875	46	118	47	28
ENSECAG00000016573	2.532514694	2.40E-12	1.59E-10	96	45	203	480	2	13	3	11
ENSECAG00000019705	3.977435225	2.52E-12	1.65E-10	45	42	60	146	540	366	619	827
ENSECAG00000013057	3.609795345	2.64E-12	1.72E-10	37	45	41	84	540	454	533	245
ENSECAG00000023019	3.109535905	2.73E-12	1.77E-10	15	20	17	66	357	195	293	490
ENSECAG00000009370	4.065322241	2.78E-12	1.79E-10	37	60	64	149	616	355	551	1000
ENSECAG00000019404	1.249909158	3.03E-12	1.94E-10	173	39	21	22	0	0	0	0
ENSECAG00000002575	7.23021456	3.11E-12	1.98E-10	512	502	298	921	5566	3491	5363	8802
ENSECAG00000011776	5.639129583	3.71E-12	2.36E-10	106	221	215	222	1631	2705	2233	702
ENSECAG00000016457	3.836636924	3.84E-12	2.41E-10	38	37	59	114	483	260	598	825
ENSECAG00000004716	0.761498376	3.85E-12	2.41E-10	26	29	47	128	1	0	6	0
ENSECAG00000008787	1.202634319	4.14E-12	2.58E-10	5	2	7	4	102	42	85	123
ENSECAG00000000237	3.401812073	4.33E-12	2.69E-10	300	793	65	93	2	11	8	7
ENSECAG00000024887	6.153650557	4.66E-12	2.87E-10	2887	2695	1436	933	92	328	138	158
ENSECAG00000012913	3.900983226	4.73E-12	2.91E-10	75	83	83	94	466	447	574	612
ENSECAG00000016880	0.914077156	5.27E-12	3.22E-10	3	3	2	4	49	37	76	128
ENSECAG00000012621	1.761162357	5.63E-12	3.42E-10	7	9	14	11	89	141	91	184
ENSECAG00000005925	5.707493807	5.72E-12	3.46E-10	113	187	252	548	2047	1157	1818	2718
ENSECAG00000023587	3.406995565	5.93E-12	3.56E-10	40	43	51	38	395	198	410	592
ENSECAG00000023347	7.507616508	6.00E-12	3.59E-10	7830	9551	1407	1553	201	329	200	230
ENSECAG00000022511	7.301300863	6.31E-12	3.76E-10	5172	10353	1249	1402	163	291	188	219
ENSECAG00000000649	0.985283567	6.42E-12	3.80E-10	0	1	2	9	67	63	118	51
ENSECAG00000019870	4.889180271	6.75E-12	3.97E-10	507	338	1164	1877	74	174	65	154
ENSECAG00000008827	3.081913991	7.18E-12	4.19E-10	27	23	16	43	390	139	293	490
ENSECAG00000009282	2.403816259	7.20E-12	4.19E-10	11	8	21	28	219	222	225	116
ENSECAG00000001069	8.540961798	7.37E-12	4.27E-10	11894	20822	8418	2281	769	547	552	197
ENSECAG00000017089	0.115294175	7.83E-12	4.52E-10	1	0	0	2	26	25	69	39
ENSECAG00000015544	7.168436094	8.84E-12	5.07E-10	4237	10115	1463	1054	62	247	99	178
ENSECAG00000008721	2.720349892	1.01E-11	5.76E-10	5	3	32	23	190	313	284	226
ENSECAG00000019758	2.228408431	1.28E-11	7.27E-10	16	10	25	21	161	137	197	192
ENSECAG00000023860	8.032761041	1.37E-11	7.71E-10	22797	1632	819	484	12	30	17	4
ENSECAG00000008194	4.830471456	1.37E-11	7.71E-10	433	1326	881	581	152	196	170	158
ENSECAG00000009625	8.236022347	1.43E-11	8.03E-10	63	55	3	0	7882	4695	13966	26871
ENSECAG00000023002	1.333819478	1.48E-11	8.22E-10	53	28	90	157	6	10	3	4
ENSECAG00000021052	3.374895914	1.51E-11	8.39E-10	29	37	45	37	195	278	343	786
ENSECAG00000017746	4.664030931	1.70E-11	9.37E-10	97	122	150	270	786	871	1031	814
ENSECAG00000013992	4.818420906	1.71E-11	9.37E-10	113	92	154	263	810	968	1428	783
ENSECAG00000017486	1.782090396	1.78E-11	9.72E-10	55	176	121	61	7	14	7	11
ENSECAG00000024406	3.543090853	1.93E-11	1.05E-09	27	16	36	52	358	590	649	150
ENSECAG00000019352	3.221401133	1.94E-11	1.05E-09	761	23	101	80	0	1	3	0
ENSECAG00000004228	5.839260512	2.13E-11	1.15E-09	3115	2335	417	462	4	62	7	15
ENSECAG00000008466	2.128775001	2.23E-11	1.20E-09	12	10	11	30	154	103	173	225
ENSECAG00000008916	4.879563566	2.25E-11	1.20E-09	77	108	77	283	941	1190	1460	683
ENSECAG00000007176	1.488328604	2.26E-11	1.20E-09	138	111	32	38	0	5	0	0
ENSECAG00000016029	1.993056723	2.40E-11	1.27E-09	11	9	7	21	133	78	157	241
ENSECAG00000014813	3.532576501	2.50E-11	1.31E-09	44	55	39	86	499	229	418	549
ENSECAG00000010078	4.686354326	2.53E-11	1.32E-09	101	93	151	260	901	679	729	1381
ENSECAG00000005083	6.116777823	2.58E-11	1.34E-09	5025	1322	435	337	37	69	38	49
ENSECAG00000008655	2.602412759	2.87E-11	1.49E-09	14	22	10	41	228	139	212	344
ENSECAG00000003626	4.049157907	2.91E-11	1.50E-09	218	1704	58	58	2	7	1	4
ENSECAG00000000494	3.302570363	3.05E-11	1.57E-09	11	27	17	64	158	488	518	333
ENSECAG00000005671	4.400054558	3.89E-11	1.99E-09	54	72	34	204	722	733	984	687
ENSECAG00000008545	2.907519665	4.01E-11	2.04E-09	174	81	279	448	7	36	10	25
ENSECAG00000017525	0.375609524	4.07E-11	2.06E-09	3	1	0	0	60	27	71	34
ENSECAG00000006746	0.124163141	4.19E-11	2.12E-09	0	2	1	4	49	29	35	43
ENSECAG00000013193	5.271235016	4.44E-11	2.23E-09	92	158	146	433	1329	845	1453	2073
ENSECAG00000023776	2.62019259	4.70E-11	2.35E-09	15	17	17	44	245	115	212	362
ENSECAG00000000927	4.353070086	4.77E-11	2.38E-09	1148	488	533	47	21	21	22	37
ENSECAG00000016173	3.898999479	4.87E-11	2.41E-09	26	68	64	149	588	340	599	660
ENSECAG00000013004	2.955511135	4.94E-11	2.44E-09	360	351	105	50	6	16	3	7
ENSECAG00000007464	5.9181665	5.03E-11	2.47E-09	264	114	219	471	2751	1092	2506	2740
ENSECAG00000006711	5.061567254	5.06E-11	2.48E-09	1506	2011	108	119	3	7	10	31
ENSECAG00000006853	6.099408202	5.19E-11	2.52E-09	342	357	435	435	2811	1376	3197	2326
ENSECAG00000020136	7.532644884	5.20E-11	2.52E-09	4467	9938	3660	2913	630	766	780	893
ENSECAG00000023733	12.13369901	5.45E-11	2.63E-09	412179	7274	7069	7230	5	97	95	14
ENSECAG00000024790	2.748847292	5.45E-11	2.63E-09	234	149	138	334	5	21	0	1
ENSECAG00000000583	3.100817178	5.57E-11	2.67E-09	397	445	83	45	2	9	0	0
ENSECAG00000011292	3.031906777	5.60E-11	2.67E-09	142	250	167	493	13	55	16	24
ENSECAG00000022135	6.69686323	5.73E-11	2.73E-09	1829	9175	1564	190	1	47	7	14
ENSECAG00000006663	3.33302226	6.57E-11	3.11E-09	24	15	1	29	190	506	335	570
ENSECAG00000017134	5.131727578	7.15E-11	3.37E-09	1316	1503	577	434	67	173	126	119
ENSECAG00000019129	3.117961462	7.39E-11	3.47E-09	24	51	36	64	316	244	241	457
ENSECAG00000022583	3.698264067	7.60E-11	3.55E-09	671	559	56	141	9	24	10	11
ENSECAG00000019861	2.387604843	8.26E-11	3.85E-09	14	10	34	20	157	134	257	230
ENSECAG00000010928	6.794954795	8.89E-11	4.12E-09	4537	6039	898	1232	46	251	42	57
ENSECAG00000017388	3.040281343	9.05E-11	4.18E-09	24	23	19	72	325	196	282	429
ENSECAG00000027676	9.919756633	1.07E-10	4.93E-09	31656	14115	36720	29727	6250	4473	2751	3567
ENSECAG00000003029	1.561078966	1.10E-10	5.06E-09	6	7	3	12	109	41	111	197
ENSECAG00000007620	3.190041241	1.14E-10	5.20E-09	35	42	26	60	387	174	318	472
ENSECAG00000017647	2.967600532	1.31E-10	5.95E-09	30	36	27	60	292	192	257	394
ENSECAG00000001041	2.694840575	1.38E-10	6.24E-09	21	24	23	49	267	135	217	334

ENSECAG00000012179	8.842511113	1.39E-10	6.24E-09	27184	16257	4046	1430	564	352	347	185
ENSECAG00000018519	4.317691606	1.39E-10	6.24E-09	68	108	182	80	626	549	898	758
ENSECAG00000001621	2.821006015	1.48E-10	6.61E-09	15	25	17	23	254	87	208	576
ENSECAG00000014399	4.606895927	1.49E-10	6.65E-09	442	1495	830	183	17	96	44	32
ENSECAG00000003473	5.165877786	1.67E-10	7.42E-09	1108	2414	284	266	74	81	69	69
ENSECAG00000015456	5.526688812	1.83E-10	8.10E-09	171	214	312	524	1577	981	1658	2263
ENSECAG00000013457	10.26543337	1.85E-10	8.13E-09	50994	69657	13628	7795	12	766	75	274
ENSECAG00000018631	2.890089968	1.87E-10	8.16E-09	109	624	86	77	13	13	8	13
ENSECAG00000005090	7.850859402	1.87E-10	8.16E-09	12665	10089	1299	781	212	249	164	144
ENSECAG00000001688	0.333705459	2.05E-10	8.92E-09	21	22	33	83	1	4	4	3
ENSECAG00000010860	7.042024168	2.07E-10	8.99E-09	12105	53	73	575	1	3	1	0
ENSECAG00000009121	1.682557106	2.21E-10	9.56E-09	12	9	2	12	152	81	115	132
ENSECAG00000020920	3.476779203	2.40E-10	1.03E-08	20	39	14	102	365	376	357	587
ENSECAG00000008085	3.112813569	2.56E-10	1.10E-08	228	295	417	98	8	43	15	8
ENSECAG00000003192	9.733262598	2.56E-10	1.10E-08	28942	41261	13142	11728	2026	3889	2052	2743
ENSECAG00000024139	5.772128708	2.76E-10	1.18E-08	828	4897	640	290	18	103	35	60
ENSECAG00000024558	2.475191391	3.24E-10	1.37E-08	14	9	20	46	186	142	184	322
ENSECAG00000005550	4.257109858	3.25E-10	1.37E-08	465	962	504	238	44	118	44	94
ENSECAG00000018160	6.658786987	3.30E-10	1.39E-08	163	243	113	835	2485	5506	4262	3050
ENSECAG00000020341	4.202455263	3.47E-10	1.45E-08	555	934	434	220	13	90	31	18
ENSECAG00000000400	7.912316173	3.54E-10	1.48E-08	6538	17629	2535	1389	191	530	196	410
ENSECAG00000014493	1.823879482	3.65E-10	1.52E-08	76	287	33	27	0	3	3	5
ENSECAG00000014755	3.364894293	3.66E-10	1.52E-08	53	38	63	79	434	296	304	409
ENSECAG00000022776	5.864992246	3.90E-10	1.61E-08	1064	2572	2075	1088	229	481	188	130
ENSECAG00000009881	6.676516602	3.93E-10	1.62E-08	3120	6722	1482	655	24	240	49	108
ENSECAG00000019877	6.435152419	3.94E-10	1.62E-08	3969	4265	571	592	136	190	169	150
ENSECAG00000012899	3.279369316	4.09E-10	1.67E-08	29	37	42	99	412	275	251	460
ENSECAG00000015261	6.159999763	4.10E-10	1.67E-08	5294	1281	325	445	18	84	12	30
ENSECAG00000014601	9.339584114	4.22E-10	1.71E-08	25682	28302	16562	5300	157	1251	132	92
ENSECAG00000016449	7.271103259	4.28E-10	1.73E-08	7960	6817	805	1407	28	255	108	163
ENSECAG00000018438	2.547538783	4.38E-10	1.76E-08	18	18	28	39	247	109	177	333
ENSECAG00000000639	1.855515501	4.58E-10	1.84E-08	43	49	151	243	8	19	6	8
ENSECAG00000010496	6.412692383	4.92E-10	1.97E-08	3366	5506	412	494	2	79	13	24
ENSECAG00000000360	7.183169167	5.03E-10	2.01E-08	8078	3707	1918	1567	489	379	377	244
ENSECAG00000023992	11.05197584	5.06E-10	2.01E-08	95104	91230	34728	25780	37	2106	148	282
ENSECAG00000013070	7.840998478	5.38E-10	2.13E-08	9070	12031	2092	2148	346	663	415	667
ENSECAG00000023566	4.04430212	5.41E-10	2.14E-08	558	1037	138	132	25	44	15	41
ENSECAG00000018724	0.159549995	5.63E-10	2.21E-08	0	3	1	2	21	65	31	41
ENSECAG00000015967	2.329500722	5.70E-10	2.23E-08	14	21	18	40	156	113	200	264
ENSECAG00000019433	0.769187083	5.85E-10	2.29E-08	1	6	2	10	68	40	53	88
ENSECAG00000018918	2.697797888	6.01E-10	2.34E-08	29	22	20	45	240	148	211	353
ENSECAG00000006322	1.400386999	6.40E-10	2.48E-08	5	11	11	15	87	57	103	135
ENSECAG00000003382	4.625976745	6.82E-10	2.64E-08	859	1227	299	292	85	77	84	140
ENSECAG00000017185	5.802223389	7.15E-10	2.75E-08	268	358	383	610	2220	1900	1745	1552
ENSECAG00000020111	2.057080695	7.58E-10	2.91E-08	20.0106	12.0001	8.00789	23.0001	138.001	125	166.001	177
ENSECAG00000001923	6.866593244	7.67E-10	2.93E-08	399	247	689	562	3957	1504	5563	6979
ENSECAG00000011780	6.562922887	7.73E-10	2.95E-08	279	468	526	914	2866	1891	3060	6037
ENSECAG00000000354	5.753102207	8.55E-10	3.25E-08	632	1536	2160	2296	227	544	144	255
ENSECAG00000023430	8.192004978	8.70E-10	3.30E-08	13231	14013	3453	2454	45	556	50	139
ENSECAG00000024519	1.89015018	8.76E-10	3.31E-08	7	15	12	24	141	68	119	224
ENSECAG00000000197	1.75861455	9.24E-10	3.47E-08	6	9	4	21	59	186	118	135
ENSECAG00000006095	8.194036444	1.01E-09	3.79E-08	558	1328	726	2346	6630	14348	13349	8881
ENSECAG00000003240	2.343717063	1.16E-09	4.34E-08	16	3	18	10	199	82	144	381
ENSECAG00000021167	5.956976638	1.17E-09	4.36E-08	2028	2166	1098	1150	391	373	337	533
ENSECAG00000022185	3.797799465	1.24E-09	4.60E-08	490	635	180	195	44	66	57	65
ENSECAG00000001165	0.790643463	1.25E-09	4.61E-08	49.0106	77.0001	41.0079	29.0001	2.0007	6.00045	2.00104	9.00033
ENSECAG00000019578	1.992365828	1.27E-09	4.68E-08	13	5	14	29	168	89	135	197
ENSECAG00000019948	4.240559253	1.27E-09	4.68E-08	102	97	79	101	1028	302	673	729
ENSECAG00000015381	3.76146015	1.32E-09	4.83E-08	33	40	75	143	397	642	508	363
ENSECAG00000000106	6.0501958	1.33E-09	4.87E-08	222	279	274	471	1513	892	2743	4979
ENSECAG00000008937	4.102461261	1.37E-09	4.98E-08	486	1204	162	139	8	51	28	20
ENSECAG00000015676	7.094135594	1.41E-09	5.10E-08	3685	8104	2272	1441	253	690	373	368
ENSECAG00000010182	0.498812365	1.42E-09	5.13E-08	0	5	3	8	47	45	54	54
ENSECAG00000009285	5.42413824	1.52E-09	5.48E-08	2159	2030	281	148	46	81	63	52
ENSECAG00000004442	2.800174514	1.54E-09	5.55E-08	6	26	3	20	83	273	192	573
ENSECAG00000002113	2.656207735	1.55E-09	5.55E-08	25	31	37	50	206	162	228	287
ENSECAG00000020385	6.041059833	1.60E-09	5.72E-08	1665	3415	1409	927	164	494	197	198
ENSECAG00000019765	5.427602195	1.65E-09	5.87E-08	826	3059	922	331	50	181	61	31
ENSECAG00000007329	5.233132533	1.66E-09	5.91E-08	1186	2596	255	211	87	44	70	95
ENSECAG00000001010	1.124128613	1.75E-09	6.21E-08	1	1	3	12	62	42	167	59
ENSECAG00000015865	10.02841274	1.78E-09	6.27E-08	81289	4019	16577	4391	758	1008	465	584
ENSECAG00000009690	1.19264763	1.80E-09	6.33E-08	100	46	84	30	2	10	2	1
ENSECAG00000022804	1.887698517	1.94E-09	6.81E-08	50	44	167	251	0	5	17	2
ENSECAG00000018094	3.042720311	1.99E-09	6.94E-08	176	476	170	123	14	43	24	41
ENSECAG00000015010	8.408207723	2.05E-09	7.16E-08	12797	18698	3425	3846	197	1144	196	439
ENSECAG00000020164	7.759342655	2.08E-09	7.22E-08	6358	13061	4071	1240	282	841	419	416
ENSECAG00000024209	7.214407987	2.09E-09	7.24E-08	6442	7314	1064	1304	314	428	256	402
ENSECAG00000017267	3.628624937	2.13E-09	7.34E-08	44	10	22	64	311	353	255	1051
ENSECAG00000024181	7.144508025	2.15E-09	7.42E-08	7697	5038	1570	1232	42	332	38	44
ENSECAG00000017109	4.065589819	2.23E-09	7.66E-08	405	936	400	220	33	95	10	36
ENSECAG00000011613	4.388856264	2.29E-09	7.83E-08	570	1529	169	155	11	54	27	50
ENSECAG00000007487	2.648336266	2.30E-09	7.86E-08	20	19	24	52	196	127	207	400
ENSECAG00000008637	5.379894165	2.41E-09	8.19E-08	224	331	222	411	1575	976	1262	1826
ENSECAG00000020698	2.892172382	2.62E-09	8.87E-08	34	19	49	37	313	143	289	334
ENSECAG00000005409	0.309468801	2.69E-09	9.10E-08	0	5	4	1	25	58	42	50

ENSECAG00000018322	2.826933514	2.74E-09	9.20E-08	33	22	21	54	295	141	250	353
ENSECAG00000015494	1.119652595	2.74E-09	9.20E-08	98	80	37	23	1	5	5	7
ENSECAG00000013680	5.160127424	2.75E-09	9.20E-08	167	181	195	414	1251	1116	1651	831
ENSECAG00000009958	10.6775747	2.75E-09	9.20E-08	37530	98951	30451	24899	2876	8951	4177	2212
ENSECAG00000023589	3.603277179	2.79E-09	9.28E-08	8	26	18	103	349	704	510	258
ENSECAG00000020940	1.217590443	2.89E-09	9.60E-08	30	26	94	159	1	9	6	9
ENSECAG00000009570	8.083422395	2.91E-09	9.64E-08	12657	14091	1199	1555	383	403	373	518
ENSECAG00000021949	0.939493436	2.99E-09	9.87E-08	4	7	5	10	81	31	81	83
ENSECAG00000022078	5.992454223	3.18E-09	1.05E-07	2098	3624	893	456	131	299	164	111
ENSECAG00000008363	3.383242422	3.20E-09	1.05E-07	1	3	5	58	270	445	622	330
ENSECAG00000000248	4.589099749	3.23E-09	1.06E-07	540	970	486	647	99	217	194	94
ENSECAG00000019319	7.944592935	3.34E-09	1.09E-07	9071	13314	2734	1865	862	638	587	724
ENSECAG00000010702	4.338665893	3.61E-09	1.18E-07	452	1693	143	133	2	25	4	34
ENSECAG00000015007	2.47600741	3.63E-09	1.18E-07	29	22	23	32	249	122	181	244
ENSECAG00000001159	1.934262192	3.86E-09	1.25E-07	6	13	11	38	110	144	145	149
ENSECAG00000023509	1.335606953	4.56E-09	1.47E-07	8	10	9	12	106	44	82	133
ENSECAG00000008683	11.00867448	4.89E-09	1.57E-07	60114	77680	49485	36233	12094	13685	10602	8506
ENSECAG00000017963	3.295734859	4.90E-09	1.57E-07	38	27	64	63	594	175	308	346
ENSECAG00000016133	2.022201336	4.92E-09	1.58E-07	97	286	48	44	2	11	4	12
ENSECAG00000000339	3.012982502	5.14E-09	1.64E-07	659	22	95	48	4	5	0	4
ENSECAG00000025099	5.376787157	5.40E-09	1.72E-07	579	447	2378	2565	1	87	3	35
ENSECAG00000015547	7.83248502	5.47E-09	1.74E-07	8529	13721	1522	1457	406	579	425	492
ENSECAG00000015853	5.361073785	5.52E-09	1.75E-07	1813	2045	229	339	92	130	71	109
ENSECAG00000016226	2.403603381	5.53E-09	1.75E-07	151	380	58	25	3	2	14	6
ENSECAG00000006830	1.130346304	5.66E-09	1.78E-07	6	9	7	12	63	51	73	127
ENSECAG00000021220	4.173921405	6.01E-09	1.89E-07	35	79	106	129	632	1150	315	450
ENSECAG00000001064	2.946646282	6.73E-09	2.11E-07	3	6	5	46	99	392	524	184
ENSECAG00000016721	9.136679115	6.93E-09	2.16E-07	33411	15217	7738	2714	1543	794	1025	377
ENSECAG00000009742	11.04882912	7.15E-09	2.22E-07	118348	44066	27664	56731	4	1142	25	122
ENSECAG00000018574	5.233278075	7.16E-09	2.22E-07	74	225	110	421	1147	1102	2082	1060
ENSECAG00000019161	2.76051315	7.17E-09	2.22E-07	17	29	38	71	244	179	203	335
ENSECAG00000004666	0.649632083	7.36E-09	2.27E-07	6	2	2	5	67	24	57	81
ENSECAG00000018228	6.88008315	7.37E-09	2.27E-07	2521	8909	1637	880	46	390	140	221
ENSECAG00000024081	4.135549139	8.05E-09	2.47E-07	1023	616	63	139	34	23	39	30
ENSECAG00000004817	3.354842407	8.40E-09	2.57E-07	43	66	58	95	370	212	340	498
ENSECAG00000011878	2.769901831	8.78E-09	2.68E-07	17	29	13	69	261	177	206	358
ENSECAG00000014377	4.604272097	9.52E-09	2.90E-07	101	145	180	301	864	501	875	1050
ENSECAG00000015147	4.870682757	9.56E-09	2.90E-07	164	198	97	309	974	942	801	1294
ENSECAG00000000771	1.918846684	9.61E-09	2.91E-07	13	11	16	28	70	117	183	171
ENSECAG00000009860	3.020632739	9.82E-09	2.97E-07	162	258	207	275	29	80	49	62
ENSECAG00000013981	6.033849652	9.90E-09	2.98E-07	3090	3439	239	561	6	121	17	21
ENSECAG00000007518	5.512816428	1.07E-08	3.21E-07	1852	2819	269	153	20	109	26	48
ENSECAG00000011196	6.887197193	1.09E-08	3.28E-07	3849	7882	961	1394	1	134	6	48
ENSECAG00000024055	8.369576622	1.15E-08	3.43E-07	3449	15078	9023	13976	1353	2544	987	577
ENSECAG00000011101	5.544398893	1.23E-08	3.66E-07	127	253	311	592	1248	1862	2009	1208
ENSECAG00000008096	2.807987987	1.23E-08	3.67E-07	22	51	29	34	231	132	242	417
ENSECAG00000005480	6.661122918	1.25E-08	3.71E-07	246	470	472	1156	2935	2329	2919	6708
ENSECAG00000022251	3.849455564	1.26E-08	3.71E-07	375	884	130	209	58	48	73	56
ENSECAG00000007874	1.372177257	1.29E-08	3.79E-07	33	88	100	90	5	16	4	20
ENSECAG00000010932	6.313464184	1.30E-08	3.82E-07	2611	3690	1198	892	467	410	403	344
ENSECAG00000009402	2.660624813	1.30E-08	3.82E-07	60	331	170	184	35	38	23	37
ENSECAG00000019726	3.708472995	1.35E-08	3.94E-07	244	936	235	137	19	63	22	8
ENSECAG00000001308	5.918433826	1.40E-08	4.10E-07	4187	1119	345	435	87	88	121	174
ENSECAG00000006474	6.730652496	1.48E-08	4.31E-07	3010	6009	1748	1194	187	661	334	176
ENSECAG00000013978	3.393525984	1.59E-08	4.63E-07	46	14	23	86	368	336	582	256
ENSECAG00000023979	2.13882007	1.65E-08	4.79E-07	10	2	12	3	227	93	322	40
ENSECAG00000023348	2.981472732	1.67E-08	4.83E-07	24	65	46	58	241	286	280	270
ENSECAG00000009192	11.54218181	1.76E-08	5.07E-07	142427	128476	31200	16061	8178	8358	6485	4334
ENSECAG00000024473	2.676500495	1.79E-08	5.15E-07	22	18	52	40	280	125	196	319
ENSECAG00000011090	7.494688626	1.84E-08	5.27E-07	3815	10801	2952	2589	618	1078	902	761
ENSECAG00000019711	9.106759726	1.90E-08	5.44E-07	23685	27427	3759	4673	1139	1872	1085	1503
ENSECAG00000013077	5.090877745	1.91E-08	5.44E-07	119	178	179	481	1055	1166	1499	887
ENSECAG00000022423	4.598841517	1.91E-08	5.44E-07	75	42	76	281	844	757	1368	549
ENSECAG00000014923	6.645276004	1.92E-08	5.44E-07	350	548	810	1098	3107	2115	3865	4812
ENSECAG00000021518	3.40446782	1.94E-08	5.49E-07	280	290	283	249	75	104	79	65
ENSECAG00000019124	7.421616363	1.96E-08	5.53E-07	5305	9461	2071	1980	380	667	540	1086
ENSECAG00000003702	2.378859159	2.00E-08	5.63E-07	250	40	98	213	8	28	7	11
ENSECAG00000024992	2.375995685	2.03E-08	5.70E-07	20	16	24	31	244	63	232	221
ENSECAG00000011467	8.291503806	2.05E-08	5.75E-07	11471	19521	1540	2573	565	479	980	238
ENSECAG00000020362	1.234599682	2.08E-08	5.82E-07	13	7	4	10	94	59	90	91
ENSECAG00000015871	3.089944542	2.12E-08	5.93E-07	34	22	47	92	353	196	267	393
ENSECAG00000003772	5.612397712	2.38E-08	6.62E-07	986	2737	705	840	282	313	329	312
ENSECAG00000015233	3.181956697	2.51E-08	6.96E-07	157	123	347	521	94	25	25	24
ENSECAG00000024536	6.601478271	2.52E-08	7.00E-07	2169	7229	1266	1280	1	133	5	74
ENSECAG00000001141	7.122697221	2.65E-08	7.32E-07	8130	4899	714	1227	41	352	33	86
ENSECAG00000015994	3.100918023	2.73E-08	7.54E-07	298	406	161	90	11	44	8	38
ENSECAG00000008106	3.842616346	2.87E-08	7.88E-07	93	53	101	108	521	272	553	649
ENSECAG00000016085	2.853736738	2.99E-08	8.21E-07	345	339	77	22	19	13	7	7
ENSECAG00000014952	6.399653348	3.10E-08	8.48E-07	2526	5296	884	638	182	435	196	250
ENSECAG00000014059	3.062562631	3.17E-08	8.65E-07	28	27	39	101	367	186	278	352
ENSECAG00000016362	4.861779171	3.41E-08	9.28E-07	1292	1255	261	247	120	132	63	82
ENSECAG00000015692	1.883634747	3.51E-08	9.55E-07	7	0	13	11	314	74	99	78
ENSECAG00000022873	4.758876114	3.53E-08	9.58E-07	183	192	189	276	884.996	575	1022	1076
ENSECAG00000017827	6.838776449	3.64E-08	9.85E-07	3481	5443	1762	1455	572	732	518	622
ENSECAG00000003535	5.890270586	3.74E-08	1.01E-06	906	3996	2153	470	1	109	9	6

ENSECAG00000006071	3.340175287	3.85E-08	1.04E-06	53	73	49	101	285	317	380	374
ENSECAG00000008906	5.37435784	3.88E-08	1.04E-06	303	327	204	381	1327	1335	1266	1527
ENSECAG00000015759	5.662872167	3.88E-08	1.04E-06	1289	2283	851	800	338	335	323	486
ENSECAG00000002675	1.248605757	3.93E-08	1.05E-06	3	10	6	24	96	66	78	94
ENSECAG00000002490	4.652459578	4.13E-08	1.10E-06	791	1509	218	223	71	124	72	93
ENSECAG00000002540	6.46284593	4.15E-08	1.10E-06	306	552	434	1074	2951	1725	2659	5156
ENSECAG00000006153	8.07624496	4.18E-08	1.11E-06	12009	12729	3720	1363	99	961	310	211
ENSECAG00000024812	1.251075223	4.41E-08	1.17E-06	4	5	3	20	129	45	51	127
ENSECAG00000018395	9.036687056	4.48E-08	1.18E-06	15692	28570	11246	5581	341	2763	381	648
ENSECAG00000019130	5.417771964	4.55E-08	1.20E-06	1413	1903	955	490	63	333	106	60
ENSECAG00000006485	4.906729899	4.68E-08	1.23E-06	1572	907	314	321	81	171	40	89
ENSECAG00000013223	1.463332739	4.74E-08	1.24E-06	7	10	2	23	114	55	104	129
ENSECAG00000019725	0.653170839	4.75E-08	1.24E-06	7.01056	2.00009	3.00789	9.00007	74.0007	45.0005	41.001	59.0003
ENSECAG00000007351	4.021323306	5.18E-08	1.35E-06	418	1181	124	221	3	46	1	13
ENSECAG00000012148	7.755593146	5.24E-08	1.36E-06	10202	10662	1624	914	119	680	266	301
ENSECAG00000001863	4.212727626	5.32E-08	1.38E-06	78	95	187	214	509	643	682	594
ENSECAG00000001514	6.197633314	5.58E-08	1.45E-06	1702	4020	1081	1036	448	434	353	545
ENSECAG00000016970	5.827081908	5.66E-08	1.46E-06	87	159	340	439	620	1055	3320	3723
ENSECAG00000002412	5.020684805	5.70E-08	1.47E-06	978	1801	448.004	247.004	183	146.002	131.001	126.003
ENSECAG00000010765	3.929954861	5.74E-08	1.48E-06	82	86	112	181	526	428	626	417
ENSECAG00000014699	9.250732694	5.77E-08	1.48E-06	41182	5947	9561	5409	589	1228	2085	1494
ENSECAG00000013126	6.325321975	6.27E-08	1.61E-06	2124	4186	2399	918	21	374	24	73
ENSECAG00000003599	7.086007814	6.41E-08	1.64E-06	5418	7270	923	1008	287	525	392	380
ENSECAG00000010613	7.480706716	6.45E-08	1.65E-06	7648	3814	6011	2730	125	1176	244	222
ENSECAG00000006407	5.384089958	6.70E-08	1.71E-06	509	1206	2009	2010	5	191	10	28
ENSECAG00000008959	6.389908452	6.72E-08	1.71E-06	621	415	614	707	2790	1890	2767	3924
ENSECAG00000024196	5.163503694	6.74E-08	1.71E-06	129	213	159	543	1231	853	1172	1665
ENSECAG00000015839	6.754552363	6.77E-08	1.71E-06	3702	4878	1607	1108	478	578	473	720
ENSECAG00000000003	4.289606591	6.92E-08	1.75E-06	479	979	295	304	131	138	104	134
ENSECAG00000019904	5.283967183	6.95E-08	1.75E-06	909	1456	950	639	312	385	319	276
ENSECAG00000016889	2.791245206	7.13E-08	1.79E-06	31	39	50	72	228	202	245	247
ENSECAG00000009707	1.68154733	7.30E-08	1.83E-06	55	28	109	218	11	22	9	10
ENSECAG00000024959	1.488739315	7.56E-08	1.89E-06	8	10	11	24	97	135	58	96
ENSECAG00000012873	1.838603178	7.76E-08	1.94E-06	71	288	24	48	0	10	0	3
ENSECAG00000003025	3.432881357	7.88E-08	1.96E-06	221	477	375	206	26	88	24	7
ENSECAG00000011465	5.975540246	8.05E-08	2.00E-06	2097	2011	1555	691	485	438	416	411
ENSECAG00000009649	5.683506655	8.21E-08	2.04E-06	2770	1875	469	352	27	222	37	74
ENSECAG00000008300	2.79237079	8.40E-08	2.08E-06	27	60	22	29	238	232	305	193
ENSECAG00000018714	9.244870218	8.72E-08	2.15E-06	18609	33074	10682	7095	684	3731	617	795
ENSECAG00000022964	2.160267123	8.84E-08	2.18E-06	19	22	27	32	167	77	166	215
ENSECAG00000003853	4.779806225	9.10E-08	2.24E-06	750	1291	449	368	156	222	169	154
ENSECAG00000011874	0.205090297	9.17E-08	2.25E-06	1	0	1	9	35	25	71	31
ENSECAG00000015849	8.404258614	9.20E-08	2.25E-06	826	983	1315	4448	12914	10112	14544	11437
ENSECAG00000000650	6.725183685	9.41E-08	2.30E-06	2249	8823	1157	525	9	215	10	34
ENSECAG00000025141	5.080904604	9.48E-08	2.31E-06	886	1777	685	347	70	253	100	217
ENSECAG00000011067	5.710991275	9.52E-08	2.32E-06	2927	2191	187	457	0	44	0	22
ENSECAG00000016962	7.530672098	9.57E-08	2.32E-06	7802	8223	2132	1501	344	1009	423	685
ENSECAG00000024961	3.978804072	9.63E-08	2.33E-06	48	97	113	156	505	839	347	418
ENSECAG00000023266	5.037629637	9.68E-08	2.34E-06	148	111	163	481	1301	840	1433	919
ENSECAG00000016938	2.937479559	9.98E-08	2.41E-06	25	36	27	57	389	136	437	137
ENSECAG00000015657	-0.268809164	1.02E-07	2.46E-06	0	3	0	5	24	23	43	20
ENSECAG00000014338	11.02845321	1.02E-07	2.46E-06	91382	53565	35910	47179	3808	16865	5513	4051
ENSECAG00000013188	2.796460952	1.03E-07	2.47E-06	28	40	29	79	254	169	212	324
ENSECAG00000008461	1.493075669	1.06E-07	2.54E-06	7	7	9	29	109	56	85	154
ENSECAG00000019083	10.39246605	1.13E-07	2.70E-06	56105	58760	14064	11972	5840	5265	4746	5408
ENSECAG00000021722	4.355820569	1.14E-07	2.71E-06	451	959	512	374	41	214	55	95
ENSECAG00000014524	7.489153859	1.14E-07	2.71E-06	7165	5343	3553	2223	1144	1067	994	1022
ENSECAG00000023383	0.012593905	1.14E-07	2.71E-06	0	6	3	2	40	19	47	31
ENSECAG00000008745	4.450897288	1.16E-07	2.76E-06	735	1321	133	322	8	104	19	21
ENSECAG00000008899	4.855361787	1.19E-07	2.81E-06	134	239	169	390	843	1087	911	895
ENSECAG00000024723	9.516028751	1.21E-07	2.85E-06	27829	28068	11885	8359	3022	3891	3513	4663
ENSECAG00000019830	7.201386463	1.26E-07	2.95E-06	2767	11009	1413	1780	189	721	495	513
ENSECAG00000007872	4.381542304	1.27E-07	2.99E-06	79	160	153	272	832	458	610	862
ENSECAG00000021453	2.916887696	1.29E-07	3.01E-06	18	29	17	93	224	204	234	440
ENSECAG00000011219	5.647242268	1.29E-07	3.01E-06	1275	2255	807	802	299	374	464	345
ENSECAG00000015807	6.902614986	1.30E-07	3.02E-06	2495	6658	1748	2078	534	772	619	980
ENSECAG00000009996	1.198176167	1.30E-07	3.03E-06	7	7	10	23	64	63	86	102
ENSECAG00000016018	3.461023224	1.32E-07	3.07E-06	53	56	111	94	272	413	412	354
ENSECAG00000018828	5.28951682	1.33E-07	3.08E-06	1311	2855	168	193	0	54	6	33
ENSECAG00000016161	7.704805711	1.37E-07	3.17E-06	14792	3547	1503	2275	1	198	7	19
ENSECAG00000016508	9.850674589	1.39E-07	3.20E-06	40652	32535	13124	16396	713	5843	1082	1087
ENSECAG00000019423	6.479858739	1.39E-07	3.21E-06	2449	5098	1029	986	337	480	365	629
ENSECAG00000026920	4.255379163	1.40E-07	3.22E-06	595	582	396	316	155	133	174	165
ENSECAG00000023457	5.885100449	1.40E-07	3.22E-06	346	338	550	263	2293	928	2613	2374
ENSECAG00000009543	5.407990508	1.43E-07	3.29E-06	310	185	401	378	1774	1153	1398	1236
ENSECAG00000023888	4.768548879	1.44E-07	3.29E-06	950	1333	470	401	6	138	9	41
ENSECAG00000014660	8.167406359	1.50E-07	3.42E-06	9081	16625	2580	2806	1307	1143	1170	968
ENSECAG00000002017	6.07491956	1.51E-07	3.43E-06	1697	3355	823	1207	518	481	341	438
ENSECAG00000007084	1.931073236	1.52E-07	3.46E-06	31	70	155	261	6	28	5	4
ENSECAG00000018133	9.197632591	1.54E-07	3.49E-06	19343	26476	9659.01	6545.02	2182.01	3529.01	2738	3624
ENSECAG00000018618	7.015248936	1.55E-07	3.50E-06	3027	10568	751	949	28	379	43	149
ENSECAG00000001989	5.261010773	1.56E-07	3.52E-06	2008	951	545	321	124	209	174	190
ENSECAG00000009941	5.247920838	1.58E-07	3.57E-06	1395	1820	264	509	156	225	165	164
ENSECAG00000016330	6.386361395	1.58E-07	3.57E-06	2190	3593	1999	1015	341	720	366	731
ENSECAG00000016507	4.313866647	1.61E-07	3.61E-06	321	1443	230	263	47	93	65	142

ENSECAG00000001784	9.064415128	1.66E-07	3.70E-06	29422	7703	16380	4346	521	2574	960	322
ENSECAG00000020957	6.308861239	1.66E-07	3.70E-06	6378	296	547	561	103	80	141	159
ENSECAG00000010546	2.905648615	1.66E-07	3.70E-06	336	129	171	156	52	36	41	12
ENSECAG00000016702	7.494945483	1.68E-07	3.75E-06	7917	8282	1767	1551	208	983	190	373
ENSECAG00000017168	1.550031534	1.69E-07	3.76E-06	39	93	151	80	3	24	2	5
ENSECAG00000014701	5.456569222	1.72E-07	3.81E-06	1351	1896	705	531	265	357	254	275
ENSECAG00000022399	2.160871272	1.76E-07	3.89E-06	16	2	6	2	287	172	189	34
ENSECAG00000018752	-0.350301922	1.78E-07	3.94E-06	2	3	1	2	29	18	31	24
ENSECAG00000015819	2.031241035	1.80E-07	3.98E-06	19	2	20	23	96	128	186	185
ENSECAG00000007222	7.762430282	1.83E-07	4.03E-06	6127	9060	4856	2997	864	1945	1093	1228
ENSECAG00000016251	2.295330762	1.88E-07	4.13E-06	30	19	28	36	136	171	206	150
ENSECAG00000021134	5.23709452	1.90E-07	4.17E-06	254	205	417	276	1096	870	1294	1734
ENSECAG00000011009	4.612584275	1.91E-07	4.17E-06	143	123	264	262	760	870	782	721
ENSECAG00000020103	3.695021592	1.91E-07	4.17E-06	196	148	555	598	114	98	75	120
ENSECAG00000019932	9.938268031	1.91E-07	4.17E-06	45084	48783	4997	6027	1982	3533	1885	1387
ENSECAG00000018199	3.935090872	1.95E-07	4.25E-06	21	25	60	196	405	663	757	387
ENSECAG00000012629	1.251786538	2.01E-07	4.36E-06	3	8	8	24	114	50	57	118
ENSECAG00000016394	4.231367602	2.05E-07	4.44E-06	1305	139	278	188	18	82	32	28
ENSECAG00000016815	3.56984583	2.06E-07	4.45E-06	384	621	247	53	25	70	33	23
ENSECAG00000016581	1.99806489	2.07E-07	4.47E-06	163	148	26	107	3	18	8	21
ENSECAG00000005547	2.221620082	2.12E-07	4.56E-06	12	25	19	40	133	71	178	294
ENSECAG00000021993	5.1834601	2.13E-07	4.57E-06	2280	648	327	325	141	114	115	165
ENSECAG00000009535	10.01065827	2.13E-07	4.57E-06	43898	46060	11540	11816	647	5722	1188	2119
ENSECAG00000023006	3.587968603	2.16E-07	4.63E-06	400	507	312	107	20	90	43	15
ENSECAG00000007843	3.437632547	2.22E-07	4.75E-06	301	526	195	117	55	70	68	56
ENSECAG00000007123	6.817053898	2.27E-07	4.84E-06	3699	4884	1684	1351	742	709	632	623
ENSECAG00000022278	5.074098348	2.29E-07	4.88E-06	155	138	170	533	1219	789	1011	1585
ENSECAG00000011834	6.791304207	2.33E-07	4.95E-06	2935	5439	2029	1692	367	1080	399	338
ENSECAG00000000426	1.251443171	2.33E-07	4.95E-06	4	15	13	17	78	53	72	127
ENSECAG00000007837	9.696376056	2.34E-07	4.97E-06	33497	39600	7686	9329	1094	5041	1523	1954
ENSECAG00000014292	7.214869079	2.39E-07	5.05E-06	7526	3772	1024	2864	733	712	646	626
ENSECAG00000020270	2.86069937	2.48E-07	5.25E-06	31	35	43	71	317	108	257	331
ENSECAG00000019002	5.790796085	2.50E-07	5.26E-06	3278	884	884	456	237	304	160	163
ENSECAG00000014979	0.453907148	2.51E-07	5.29E-06	2	2	10	7	32	37	45	75
ENSECAG00000011257	8.045034845	2.52E-07	5.29E-06	12241	9660	4432	1263	703	1286	993	576
ENSECAG00000012441	4.916685505	2.53E-07	5.30E-06	140	220	343	331	722	839	1099	1222
ENSECAG00000003925	7.054089253	2.63E-07	5.50E-06	2379	6025	2590	3844	654	1453	784	480
ENSECAG00000022842	6.255451666	2.67E-07	5.57E-06	1772	4136	1531	1165	149	749	167	224
ENSECAG00000014259	7.272998383	2.67E-07	5.57E-06	5109	1543	7283	5108	114	1078	107	177
ENSECAG00000019977	3.974154495	2.75E-07	5.71E-06	658	573	291	262	3	92	10	15
ENSECAG00000019154	6.034455754	2.76E-07	5.74E-06	1529	3920	685	925	241	431	325	454
ENSECAG00000006067	5.54602895	2.78E-07	5.76E-06	1710	1794	691	499	279	328	295	272
ENSECAG00000019821	5.196749309	2.82E-07	5.83E-06	1422	1469	431	412	184	269	185	163
ENSECAG00000009393	0.850945082	3.03E-07	6.26E-06	26	54	86	45	4	17	2	6
ENSECAG00000017469	1.341063537	3.13E-07	6.46E-06	6	14	17	22	96	65	75	105
ENSECAG00000017405	5.363263101	3.14E-07	6.47E-06	929	2074	713	563	322	256	197	409
ENSECAG00000016596	6.958654823	3.16E-07	6.50E-06	3616	5842	1760	2178	349	706	367	1303
ENSECAG00000013486	9.349820144	3.18E-07	6.52E-06	36249	22990	4327	5884	223	2626	490	881
ENSECAG00000008679	6.857481729	3.19E-07	6.53E-06	4166	5736	1151	1075	579	597	474	519
ENSECAG00000016382	1.913719027	3.19E-07	6.53E-06	25	8	19	22	148	108	103	166
ENSECAG00000016594	3.21560686	3.24E-07	6.60E-06	26	50	74	103	331	370	332	204
ENSECAG00000014949	4.438305401	3.30E-07	6.71E-06	355	520	588	710	253	238	223	246
ENSECAG00000010918	8.170660597	3.34E-07	6.80E-06	14408	13909	1585	1844	15	640	48	156
ENSECAG00000016155	2.511618074	3.40E-07	6.90E-06	28	32	32	59	212	130	162	269
ENSECAG00000015835	6.03075128	3.42E-07	6.92E-06	1769	1835	1244	1614	518	485	540	958
ENSECAG00000006881	6.014484151	3.44E-07	6.95E-06	2241	2532	784	979	501	401	293	528
ENSECAG00000016578	4.871449297	3.46E-07	6.98E-06	1316	919	567	409	46	213	16	31
ENSECAG00000014422	5.912125466	3.46E-07	6.98E-06	192	322	352	836	2452	2847	1330	1416
ENSECAG00000013477	2.259644027	3.49E-07	7.02E-06	77	343	104	25	12	19	14	18
ENSECAG00000000199	6.532807127	3.54E-07	7.10E-06	1890	7115	628	821	207	383	251	463
ENSECAG00000012607	8.370650836	3.59E-07	7.19E-06	8104	21437	3141	3683	1060	1620	1569	1544
ENSECAG00000012839	3.563383201	3.68E-07	7.37E-06	284	899	130	57	7	53	8	14
ENSECAG00000017796	6.514655354	3.77E-07	7.53E-06	517	625	825	1101	2986	2187	2676	4065
ENSECAG00000000763	9.033548659	3.91E-07	7.78E-06	17639	6198	15500	19614	3621	4212	2587	1165
ENSECAG00000024810	5.951867409	3.91E-07	7.78E-06	2331	3363	358	657	76	364	121	207
ENSECAG00000023478	6.468741657	4.01E-07	7.97E-06	2791	4600	1581	1253	35	561	49	137
ENSECAG00000015981	5.185763269	4.02E-07	7.97E-06	876	1454	707	589	286	309	271	407
ENSECAG00000013692	7.453181919	4.05E-07	8.02E-06	454	750	1236	2189	4159	4121	7400	9227
ENSECAG00000005624	0.777871645	4.09E-07	8.07E-06	0	6	1	16	60	35	76	76
ENSECAG00000012045	4.825481459	4.09E-07	8.07E-06	660	1503	834	395	8	184	15	38
ENSECAG00000020003	2.337046983	4.18E-07	8.23E-06	76	26	333	224	14	27	18	4
ENSECAG00000023690	5.389311233	4.33E-07	8.52E-06	1341	1727	771	397	309	301	260	242
ENSECAG00000015095	5.737208246	4.35E-07	8.54E-06	2194	2677	261	471	150	270	177	183
ENSECAG00000015641	0.589343157	4.36E-07	8.54E-06	5	7	3	4	55	17	50	93
ENSECAG00000013655	5.206732982	4.37E-07	8.54E-06	232	287	219	290	819	784	1071	2415
ENSECAG00000015483	3.214122771	4.37E-07	8.54E-06	166	765	113	68	0	32	6	12
ENSECAG00000017840	7.551817344	4.47E-07	8.71E-06	6200	9745	2489	2329	349	1421	218	475
ENSECAG00000023555	1.687909647	4.55E-07	8.84E-06	13	22	16	26	122	75	89	150
ENSECAG00000017970	6.849831456	5.09E-07	9.89E-06	4031	6294	1895	821	32	595	88	191
ENSECAG00000022739	0.367460678	5.12E-07	9.91E-06	33	33	53	32	0	11	0	0
ENSECAG00000009572	3.396454976	5.12E-07	9.91E-06	267	427	167	211	85	80	72	95
ENSECAG00000000026	4.671234269	5.25E-07	1.01E-05	755	1106	323	428	238	93	158	222
ENSECAG00000003573	1.757283595	5.26E-07	1.01E-05	66	239	42	43	13	8	15	1
ENSECAG00000011797	8.603960862	5.28E-07	1.02E-05	22249	12480	3225	1556	843	1344	941	1148
ENSECAG00000003418	5.747482075	5.33E-07	1.02E-05	723	4330	513	520	217	198	249	275

ENSECAG00000019453	5.14346518	5.44E-07	1.04E-05	1146	1271	484	590	272	277	227	337
ENSECAG00000019365	5.696762436	5.54E-07	1.06E-05	1116	3248	807	459	186	403	173	302
ENSECAG00000014689	4.265532162	5.56E-07	1.06E-05	391	895	285	401	139	149	167	186
ENSECAG00000024620	3.993812309	5.56E-07	1.06E-05	192	253	1217	537	0	50	0	13
ENSECAG00000016033	5.047564789	5.59E-07	1.07E-05	198	272	248	428	879	777	1024	1577
ENSECAG00000021968	10.32112048	5.64E-07	1.07E-05	71917	33181	15931	8867	5436	5153	4138	1870
ENSECAG00000017386	1.807582557	5.65E-07	1.07E-05	19	7	27	11	96	89	124	188
ENSECAG00000006725	6.827042737	5.77E-07	1.09E-05	3264	5302	1651	1523	818	842	587	672
ENSECAG00000019432	6.044432828	5.92E-07	1.12E-05	1609	3468	897	1004	299	641	348	341
ENSECAG00000000735	6.846522024	6.00E-07	1.13E-05	2508	8089	1103	1021	353	629	397	573
ENSECAG00000013228	4.023014073	6.00E-07	1.13E-05	570	554	315	209	70	155	110	81
ENSECAG00000005863	9.678903054	6.10E-07	1.15E-05	20163	28308	20953	15556	5699	7911	5619	6258
ENSECAG00000022397	2.353982857	6.17E-07	1.16E-05	96	360	56	59	21	22	23	18
ENSECAG00000008183	2.575003654	6.19E-07	1.16E-05	103	523	43	53	0	16	0	3
ENSECAG00000016158	6.265186656	6.27E-07	1.18E-05	1640	4180	1311	987	406	553	351	753
ENSECAG00000024980	4.865216046	6.48E-07	1.21E-05	380	1777	846	240	122	221	157	158
ENSECAG00000002234	0.652553365	6.52E-07	1.22E-05	4	5	4	13	28	56	39	94
ENSECAG00000022571	3.982746528	6.56E-07	1.22E-05	886	539	103	137	6	74	12	31
ENSECAG00000008622	7.199432337	6.57E-07	1.22E-05	5272.01	6680	1732.01	1700	484.001	1108	444.001	850
ENSECAG00000014364	5.874062784	7.03E-07	1.31E-05	2263	1289	1206	995	378	555	524	323
ENSECAG00000017538	5.007842186	7.12E-07	1.32E-05	837	1700	605	226	174	217	199	167
ENSECAG00000010185	8.177352677	7.15E-07	1.32E-05	5051	25485	1891	1268	465	1055	364	700
ENSECAG00000009020	3.946639244	7.16E-07	1.32E-05	50	132	164	138	387	559	563	488
ENSECAG00000010196	7.745699007	7.17E-07	1.32E-05	3928	13860	2900	3359	656	1801	707	977
ENSECAG00000016582	3.529077315	7.30E-07	1.35E-05	347	436	222	181	46	50	88	140
ENSECAG00000020009	5.916446118	7.31E-07	1.35E-05	2346	2535	643	657	196	468	341	248
ENSECAG00000019531	2.855763431	7.34E-07	1.35E-05	172	518	69	48	22	17	17	43
ENSECAG00000004969	2.858176717	7.41E-07	1.36E-05	14	22	35	107	220	240	265	288
ENSECAG00000023722	5.034320097	7.50E-07	1.37E-05	628	2328	382	306	70	229	129	142
ENSECAG00000018585	3.775774361	7.50E-07	1.37E-05	659	595	89	79	59	43	46	21
ENSECAG00000012475	1.918384999	7.56E-07	1.38E-05	15	18	21	41	147	71	129	167
ENSECAG00000018638	2.090665543	7.61E-07	1.39E-05	204	137	47	50	11	25	15	18
ENSECAG00000013276	4.809090282	7.63E-07	1.39E-05	503	573	781	926	286	354	321	300
ENSECAG00000013090	4.785781994	7.77E-07	1.41E-05	204	164	266	327	932	635	941	980
ENSECAG00000018650	5.814608008	7.78E-07	1.41E-05	345	348	228	785	2120	1117	1830	2507
ENSECAG00000021014	6.032834804	7.79E-07	1.41E-05	184	315	241	970	2301	3078	2305	1233
ENSECAG00000020892	4.507786689	7.86E-07	1.42E-05	67	175	86	298	444	625	783	1318
ENSECAG00000022363	2.361740156	7.97E-07	1.44E-05	157	320	59	48	1	27	8	6
ENSECAG00000014711	3.882790203	8.01E-07	1.44E-05	399	340	341	405	96	194	97	141
ENSECAG00000000633	2.849106351	8.09E-07	1.46E-05	29	36	43	94	263	143	260	311
ENSECAG00000009700	1.702145672	8.20E-07	1.47E-05	14	12	23	30	87	133	119	90
ENSECAG00000009012	6.151388987	8.54E-07	1.53E-05	956	2799	1451	2425.01	480	845	737	443
ENSECAG00000019847	5.216858837	8.77E-07	1.57E-05	1614	1197	394	457	239	177	235	280
ENSECAG00000019060	1.529963781	8.78E-07	1.57E-05	9	15	21	12	74	92	166	61
ENSECAG00000014074	6.367960657	9.17E-07	1.64E-05	238	486	138	1099	2658	3509	3165	2223
ENSECAG00000012440	0.542665227	9.29E-07	1.66E-05	5	4	6	8	76	21	64	37
ENSECAG00000022127	8.135330167	9.38E-07	1.67E-05	17755	2093	8492	1885	123	1007	152	47
ENSECAG00000009079	3.417881083	9.39E-07	1.67E-05	341	775	67	47	1	32	2	5
ENSECAG00000014092	2.310872127	9.52E-07	1.69E-05	142	223	90	67	32	38	23	18
ENSECAG00000015006	4.563410784	9.55E-07	1.69E-05	849	1440	206	217	5	84	1	21
ENSECAG00000011728	7.357385439	9.61E-07	1.70E-05	7091	5088	1864	2820	539	1493	598	711
ENSECAG00000005199	7.38107398	9.65E-07	1.70E-05	6205	5302	2320	2991	1031	1358	1219	823
ENSECAG00000003121	10.5974512	9.72E-07	1.71E-05	47415	64165	25609	22395	8301	12242	9144	10964
ENSECAG00000017237	7.047754645	9.94E-07	1.75E-05	10540	1683	328	659	11	234	15	56
ENSECAG00000000214	6.226963403	1.01E-06	1.78E-05	3413	3636	430	315	254	121	279	312
ENSECAG00000011922	5.453894899	1.02E-06	1.80E-05	1201	2426	590	460	137	400	124	230
ENSECAG00000005292	7.914697038	1.03E-06	1.80E-05	7965	12435	2681	2111	1196	1294	1171	1252
ENSECAG00000009167	1.416910952	1.04E-06	1.82E-05	74	103	74	34	12	22	14	6
ENSECAG00000021110	6.809493752	1.05E-06	1.84E-05	3978	7015	878	678	55	567	90	105
ENSECAG00000011792	1.950941216	1.10E-06	1.92E-05	52	105	106	161	32	24	43	29
ENSECAG00000001372	1.920495443	1.10E-06	1.92E-05	50	45	157	204	14	30	18	33
ENSECAG00000022699	-0.00842522	1.11E-06	1.93E-05	3	2	2	7	19	47	30	33
ENSECAG00000012804	8.412299016	1.11E-06	1.93E-05	9783	13164	7521	4709	2170	3047	1522	2595
ENSECAG00000017470	4.203095759	1.13E-06	1.96E-05	401	816	313	323	184	136	152	161
ENSECAG00000012789	6.084401635	1.13E-06	1.97E-05	2982	3776	427	435	4	170	4	25
ENSECAG00000012954	6.760966216	1.17E-06	2.03E-05	2575	6316	1236	1392	485	587	543	993
ENSECAG00000013814	3.011657159	1.18E-06	2.03E-05	203	263	198	148	79	68	47	77
ENSECAG00000016328	2.660993557	1.19E-06	2.05E-05	37	55	689	68	7	25	3	13
ENSECAG00000012222	5.812105265	1.20E-06	2.06E-05	1306	3722	566	540	217	433	206	151
ENSECAG00000012768	4.831793757	1.20E-06	2.06E-05	1553	1289	41	101	35	48	21	92
ENSECAG00000024047	8.05225554	1.22E-06	2.09E-05	10068	12545	2915	1952	941	1141	1160	1821
ENSECAG00000021009	1.256800992	1.22E-06	2.09E-05	9	7	18	10	39	125	71	89
ENSECAG00000008024	3.368749765	1.26E-06	2.16E-05	65	51	41	100	367	385	451	178
ENSECAG00000013368	2.925457876	1.28E-06	2.18E-05	161	522	92	67	26	50	23	23
ENSECAG00000016469	6.916459793	1.29E-06	2.21E-05	324	500	428	1887	3870	4311	5379	3230
ENSECAG00000011540	1.826817636	1.30E-06	2.22E-05	88	131	88	70	18	39	15	15
ENSECAG00000003398	5.834711818	1.30E-06	2.22E-05	3787	1052	506	321	153	242	151	42
ENSECAG00000002892	5.222366339	1.33E-06	2.25E-05	212	334	259	527	1114	826	1220	1601
ENSECAG00000014490	2.932935306	1.33E-06	2.25E-05	207	255	100	239	45	47	39	101
ENSECAG00000010881	8.589625425	1.34E-06	2.27E-05	22783	11213	3944	1981	554	1869	254	275
ENSECAG00000004906	6.198578823	1.36E-06	2.30E-05	222	511	321	1187	2054	2660	2816	2265
ENSECAG00000013543	0.927378758	1.37E-06	2.31E-05	3	10	9	17	53	61	97	42
ENSECAG00000019309	6.605576711	1.41E-06	2.38E-05	542	894	630	932	2511	4450	2309	3308
ENSECAG00000021133	5.371255427	1.44E-06	2.43E-05	344	331	238	447	1313	942	1321	1749
ENSECAG00000019874	6.488326587	1.44E-06	2.43E-05	672	736	673	981	2517	2611	2747	3522

ENSECAG00000019790	4.328341738	1.45E-06	2.43E-05	62	33	136	208	687	628	1262	257
ENSECAG00000013595	6.632752454	1.49E-06	2.50E-05	2384	5401	1348	1310	470	882	453	684
ENSECAG00000009170	6.585697585	1.51E-06	2.52E-05	1844	5570	1259	1462	665	715	581	703
ENSECAG00000022453	0.927188454	1.51E-06	2.52E-05	9	7.99979	8	17	54	46	71	81
ENSECAG00000024360	5.988131742	1.53E-06	2.55E-05	2404	1917	1045	796	491	512	480	423
ENSECAG00000013745	6.15755953	1.54E-06	2.57E-05	2752	4083	309	399	281	234	274	198
ENSECAG00000012880	5.63772108	1.55E-06	2.58E-05	1007	2113	970	833	429	475	487	486
ENSECAG00000019000	4.980486445	1.57E-06	2.61E-05	848	1557	552	368	134	345	98	156
ENSECAG00000007562	0.743827638	1.57E-06	2.61E-05	3	10	4	18	60	49	55	58
ENSECAG00000021173	5.720593195	1.66E-06	2.75E-05	1062	2756	834	776	356	513	358	393
ENSECAG00000023683	-0.284478884	1.67E-06	2.76E-05	2	3	3	4	27	18	22	38
ENSECAG00000009896	9.201052513	1.68E-06	2.77E-05	42452	8187	3003	4021	976	2155	1366	968
ENSECAG00000016700	4.448262222	1.72E-06	2.83E-05	328	893	556	427	168	223	210	252
ENSECAG00000023203	5.739990783	1.72E-06	2.84E-05	355	419	507	668	1794	1030	1742	2177
ENSECAG00000023475	5.468697406	1.73E-06	2.85E-05	1934	1294	1125	270	96	362	195	68
ENSECAG00000010208	0.901646119	1.73E-06	2.85E-05	35	60	47	52	13	14	16	13
ENSECAG00000021269	8.688167717	1.79E-06	2.93E-05	25229	3726	10248	2616	1755	1739	1167	628
ENSECAG00000017157	6.844109078	1.82E-06	2.98E-05	1269	5742	3811	3732	46	899	84	384
ENSECAG00000010089	6.32979746	1.84E-06	3.01E-05	3572	2222	1153	1550	106	769	130	390
ENSECAG00000009430	6.466353736	1.88E-06	3.07E-05	1963	2937	1581	2211	1063	824	867	812
ENSECAG00000006670	4.502694256	1.89E-06	3.08E-05	350	924	465	543	179	225	254	249
ENSECAG00000009073	2.344463089	1.89E-06	3.08E-05	9	15	41	64	173	150	155	222
ENSECAG00000023420	11.02233279	1.92E-06	3.12E-05	32385	70707	89722	62178	5612	22057	3248	2416
ENSECAG00000015406	5.536097529	1.97E-06	3.20E-05	857	678	2095	1188	601	456	383	377
ENSECAG00000003761	4.742431817	2.02E-06	3.27E-05	538	1317	480	462	142	328	136	181
ENSECAG00000019025	5.922749714	2.05E-06	3.31E-05	2404	3148	409	468	78	415	165	173
ENSECAG00000009865	5.911032678	2.06E-06	3.32E-05	1917	3318	905	561	51	490	78	84
ENSECAG00000008215	9.828728568	2.07E-06	3.34E-05	17449	35036	23629	19181	10180	5390	8602	6028
ENSECAG00000012502	6.953318155	2.09E-06	3.37E-05	4250	5730	1391	1332	596	965	544	724
ENSECAG00000027699	10.66553664	2.16E-06	3.47E-05	64868	17016	44770	42763	16649	9291	8272	8780
ENSECAG00000017042	6.666447404	2.20E-06	3.54E-05	5159	3641	1114	918	79	631	40	154
ENSECAG00000019173	2.102501817	2.24E-06	3.59E-05	113	98	231	47	9	41	7	12
ENSECAG00000014957	1.288624982	2.25E-06	3.60E-05	95	92	24	48	2	16	12	11
ENSECAG00000004025	8.140458898	2.27E-06	3.63E-05	10885	8916	4908	3429	2056	2078	1841	1403
ENSECAG00000003758	4.782486347	2.29E-06	3.65E-05	884	1295	470	341	49	289	49	64
ENSECAG00000023351	0.643182978	2.32E-06	3.70E-05	9	2	8	4	36	49	38	93
ENSECAG00000020932	7.160641039	2.34E-06	3.73E-05	2533	8288	1822	2627	1068	1011	939	971
ENSECAG00000008527	5.038396249	2.40E-06	3.82E-05	226	195	265	441	1103	840	1435	772
ENSECAG00000016862	5.714432372	2.41E-06	3.83E-05	1789	1758	992	602	601	358	302	355
ENSECAG00000024798	2.726334123	2.44E-06	3.87E-05	23	42	37	86	236	260	214	156
ENSECAG00000004420	6.524299556	2.46E-06	3.90E-05	3292	2143	2247	1288	608	940	566	871
ENSECAG00000000608	6.103610136	2.48E-06	3.93E-05	1471	3759	642	1318	454	438	419	696
ENSECAG00000000715	5.694364315	2.57E-06	4.05E-05	363	296	510	690	2084	1114	1657	1666
ENSECAG00000010215	0.431744492	2.57E-06	4.06E-05	1	5	7	12	35	28	40	80
ENSECAG00000024046	8.789407348	2.58E-06	4.06E-05	13893	18192	7041	5847	2605	3600	2659	3128
ENSECAG00000021081	2.75803564	2.58E-06	4.06E-05	7	28	25	93	146	282	288	234
ENSECAG00000011155	6.987679031	2.59E-06	4.07E-05	3550	4543	2475	2102	833	1237	1199	876
ENSECAG00000013153	7.779872853	2.62E-06	4.11E-05	7675	3587	9175	3325	1104	2348	557	648
ENSECAG00000005316	-0.205845279	2.62E-06	4.11E-05	0	3	0	9	25	23	24	42
ENSECAG00000024361	5.077699872	2.63E-06	4.11E-05	1072	932	564	674	249	400	271	319
ENSECAG00000018870	10.08349253	2.64E-06	4.13E-05	41787	56068	9397	5766	5159	3747	4189	5008
ENSECAG00000007242	7.164448892	2.69E-06	4.19E-05	3288	5137	4621	2430	1146	1399	893	287
ENSECAG00000011486	5.780462435	2.73E-06	4.26E-05	708	1456	2345	1826	527	552	374	89
ENSECAG00000007480	3.624346477	2.75E-06	4.28E-05	71	119	84	142	366	413	395	372
ENSECAG00000003315	6.967722343	2.76E-06	4.29E-05	3597	2133	3311	5200	1338	499	995	156
ENSECAG00000019571	5.871497987	2.81E-06	4.36E-05	1008	3002	1037	988	380	545	361	671
ENSECAG00000021631	5.828906714	3.06E-06	4.74E-05	1277	1935	1453	901	569	703	426	476
ENSECAG00000023192	8.892226187	3.06E-06	4.74E-05	33614	9446	1004	2554	1012	1064	1127	1031
ENSECAG00000021011	5.738107783	3.08E-06	4.76E-05	793	1551	1828	1455	281	847	389	447
ENSECAG00000013677	5.695551924	3.09E-06	4.78E-05	847	3155	1050	709	90	556	202	264
ENSECAG00000018702	7.698944673	3.11E-06	4.80E-05	7904	9640	1890	1747	1235	979	1140	996
ENSECAG00000017295	8.548030849	3.19E-06	4.91E-05	24811	10017	774	1579	1006	711	721	910
ENSECAG00000016126	5.086887792	3.31E-06	5.09E-05	745	898	733	924	514	342	344	262
ENSECAG00000001471	10.25635511	3.44E-06	5.29E-05	49898	42751	38510	13047	14	3076	115	119
ENSECAG00000022970	5.342006436	3.45E-06	5.29E-05	924	860	1068	1009	437	374	392	673
ENSECAG00000021841	6.498223335	3.51E-06	5.37E-05	3351	3757	1097	980	725	650	437	238
ENSECAG00000008851	9.255625503	3.51E-06	5.37E-05	15418	14300	20179	14397	4772	7724	3200	3335
ENSECAG00000023355	2.864087621	3.52E-06	5.38E-05	24	28	71	89	218	168	227	372
ENSECAG00000020037	3.121623161	3.53E-06	5.39E-05	64	38	55	88	357	169	344	284
ENSECAG00000008270	6.167812338	3.62E-06	5.51E-05	588	340	629	596	3372	1765	2582	1638
ENSECAG00000022506	6.346481578	3.77E-06	5.73E-05	1961	2280	2223	1348	864	776	688	1047
ENSECAG00000018028	7.265593882	3.79E-06	5.75E-05	4912	5564	3992	1515	568	1716	632	743
ENSECAG00000013747	6.195958589	3.83E-06	5.80E-05	2232	2423	1686	1477	247	1024	209	236
ENSECAG00000014691	9.98346326	3.91E-06	5.93E-05	31104	50564	10074	12724	6068	5692	6825	5965
ENSECAG00000000638	7.53767715	3.98E-06	6.02E-05	975	1099	1532	2561	6055	4452	7011	6777
ENSECAG00000015468	5.990327965	3.98E-06	6.02E-05	2377	2303	1011	544	455	401	548	341
ENSECAG00000024205	9.785279057	4.01E-06	6.05E-05	14921	20389	25035	32790	9614	9014	7203	8851
ENSECAG00000016070	4.060596966	4.03E-06	6.07E-05	66	140	110	232	307	609	649	584
ENSECAG00000016341	5.482145162	4.03E-06	6.07E-05	736	1591	1010	1236	277	404	284	832
ENSECAG00000011602	4.483357873	4.20E-06	6.31E-05	16	8	46	102	262	68	318	3276
ENSECAG00000021455	6.072015144	4.20E-06	6.31E-05	1352	1921	1385	1828	657	854	714	796
ENSECAG00000000362	6.60434876	4.28E-06	6.42E-05	3633	3653	1104	1167	689	512	643	909
ENSECAG00000023970	4.639044742	4.30E-06	6.43E-05	497	532	793	1034	82	310	39	246
ENSECAG00000016422	2.701480484	4.30E-06	6.43E-05	24	39	60	82	184	162	247	254
ENSECAG00000019430	4.445144448	4.34E-06	6.47E-05	125	253	102	212	551	560	718	1010

ENSECAG00000002437	7.83851954	4.36E-06	6.51E-05	11149	5420	4478	2214	1585	891	1469	282
ENSECAG00000016219	0.747980379	4.46E-06	6.64E-05	7	6	8	16	64	28	60	70
ENSECAG00000017321	2.475690913	4.49E-06	6.67E-05	64	47	268	310	18	42	25	51
ENSECAG00000008760	2.254329542	4.49E-06	6.67E-05	21	27	16	53	139	79	163	284
ENSECAG00000015375	6.513556561	4.64E-06	6.88E-05	3658	3793	1001	630	518	518	453	640
ENSECAG00000020162	1.384162546	4.69E-06	6.95E-05	19	5	6	16	61	59	132	112
ENSECAG00000017436	8.522082719	4.70E-06	6.96E-05	13218	15045	12599	2638	6	1142	42	101
ENSECAG00000000107	3.075521335	4.73E-06	7.00E-05	65	33	55	66	411	276	230	196
ENSECAG00000019115	6.803518582	4.77E-06	7.04E-05	4288	4723	2034	1128	103	1002	123	191
ENSECAG00000005501	2.895809898	4.82E-06	7.10E-05	43	29	35	78	146	156	245	498
ENSECAG00000008606	2.408254731	4.90E-06	7.21E-05	37	9	30	14	105	123	292	246
ENSECAG00000021048	7.184955345	5.15E-06	7.57E-05	3679	5762	3455	3046	306	1925	445	594
ENSECAG00000017482	3.277838781	5.19E-06	7.61E-05	360	525	62	60	44	35	44	46
ENSECAG00000017561	2.437794261	5.28E-06	7.74E-05	37	198	170	217	44	50	38	29
ENSECAG00000014153	7.351417097	5.31E-06	7.77E-05	5619	3544	4072	2857	1454	1752	1092	1019
ENSECAG00000010014	2.435442989	5.36E-06	7.84E-05	26	9	31	3	258	100	334	108
ENSECAG00000015719	5.194046951	5.37E-06	7.84E-05	631	2161	395	646	300	300	217	280
ENSECAG00000024290	7.215583726	5.38E-06	7.85E-05	3359	5955	2948	2708	982	1635	1323	1195
ENSECAG00000000697	4.807594009	5.50E-06	8.01E-05	283	1039	886	852	159	428	170	200
ENSECAG00000017205	7.297446903	5.54E-06	8.04E-05	6125	6623	1523	1464	882	1021	845	937
ENSECAG00000023766	2.726433104	5.54E-06	8.04E-05	46	135	665	47	0	22	0	2
ENSECAG00000018281	3.000489092	5.54E-06	8.05E-05	201	367	141.002	104.004	54.0016	51.0021	79.0018	51.0024
ENSECAG00000026816	7.179721967	5.61E-06	8.13E-05	5471	5767	1867	1337	782	1137	830	858
ENSECAG00000013319	5.69420843	5.76E-06	8.34E-05	720	2555	1049	994	405	591	330	555
ENSECAG00000024870	0.820568506	5.95E-06	8.59E-05	9	2	15	8	46	57	49	85
ENSECAG00000018032	5.079728295	6.02E-06	8.69E-05	670.007	926	660	953	388	406	417	381
ENSECAG00000018665	4.072383944	6.05E-06	8.72E-05	32	112	83	271	488	444	446	940
ENSECAG00000000454	7.567889684	6.09E-06	8.78E-05	671	976	869	2914	5179	3927	6114	11436
ENSECAG00000011590	4.379259769	6.10E-06	8.78E-05	577	1102	303	141	126	136	111	162
ENSECAG00000011545	5.049738525	6.13E-06	8.80E-05	843	1330	442	595	308	282	274	381
ENSECAG00000016312	2.422648291	6.24E-06	8.95E-05	29	23	33	51	280	71	173	208
ENSECAG00000018503	4.060219436	6.30E-06	9.03E-05	106	126	136	241	416	436	529	708
ENSECAG00000020259	5.528196791	6.37E-06	9.11E-05	251	163	486	686	1593	1290	1814	1208
ENSECAG00000012280	6.240305245	6.43E-06	9.18E-05	965	2964	1886	1933	522	815	620	1129
ENSECAG00000011686	5.117446974	6.48E-06	9.25E-05	1300	1308	358	424	199	310	234	234
ENSECAG00000004556	7.751363668	6.50E-06	9.26E-05	5278	12965	2549	2178	1235	1799	841	726
ENSECAG00000020506	6.994136323	6.51E-06	9.27E-05	915	904	1043	1309	4632	2461	4172	5342
ENSECAG00000021509	2.03513939	6.52E-06	9.27E-05	57	173	94	106	36	39	29	32
ENSECAG00000013462	1.202255336	6.90E-06	9.78E-05	3	13	7	20	63	147	58	39
ENSECAG00000014175	6.782617723	6.90E-06	9.78E-05	3568	4572	1399	1344	655	791	798	1036
ENSECAG00000012516	9.701246917	6.90E-06	9.78E-05	19207	21005	23330	21978	9561	8383	7461	4441
ENSECAG00000026935	5.778333017	6.97E-06	9.87E-05	2144	1811	650	650	466	379	422	403
ENSECAG00000024318	3.744699134	7.11E-06	0.000100454	365	936	73	95	56	74	38	16
ENSECAG00000003516	7.31812685	7.17E-06	0.000101189	3186	3862	5425	3566	2174	1384	1515	1346
ENSECAG00000017969	5.110509066	7.50E-06	0.000105662	1446	1239	951	338	1	151	4	12
ENSECAG00000007337	7.168470436	7.52E-06	0.000105838	3203	6304	2977	1960	1195	1267	1018	1418
ENSECAG00000010789	6.283649909	7.58E-06	0.000106541	2599	1608	2045	1277	587	1007	493	667
ENSECAG00000023417	8.290997052	7.63E-06	0.000107122	14274	9648	3741	3094	2157	1665	2232	1272
ENSECAG00000019111	8.636744423	7.64E-06	0.000107122	11338	2901	29883	7749	4	803	3	140
ENSECAG00000022304	6.169140626	7.72E-06	0.000108111	1637	1611	2133	1503	600	913	714	950
ENSECAG00000010242	5.775979526	7.93E-06	0.000110957	1968	3317	517	304	12	315	24	72
ENSECAG00000014113	7.506343076	8.08E-06	0.000112952	8834	4283	2424	2248	1644	1038	1013	575
ENSECAG00000006159	6.037068707	8.09E-06	0.000112969	1388	2370	1465	1072	650	703	649	738
ENSECAG00000013622	4.992068413	8.20E-06	0.000114157	269	283	261	366	902	706	1097	1214
ENSECAG00000002083	4.007038761	8.20E-06	0.000114157	608	706	129	179	56	130	64	119
ENSECAG00000016722	6.125551746	8.25E-06	0.000114699	1798	4617	700	286	314	333	323	408
ENSECAG00000013988	4.505375475	8.30E-06	0.00011514	920	172	1261	346	3	131	5	17
ENSECAG00000008154	2.128466223	8.30E-06	0.00011514	297	37	39	65	26	10	13	6
ENSECAG00000001640	2.878665476	8.44E-06	0.000116955	141	427	133	104	27.0001	75.0001	13	42
ENSECAG00000022554	5.951129844	8.45E-06	0.000117006	1083	2439	1093	1462	499	726	589	789
ENSECAG00000023723	5.741307266	8.53E-06	0.00011786	442	349	536	696	1729	1202	1814	1827
ENSECAG00000013130	1.381587367	8.71E-06	0.000120281	115	54	32	64	6	21	13	19
ENSECAG00000009903	3.003284842	8.80E-06	0.000121309	22	39	51	69	120	271	590	125
ENSECAG00000012104	4.140372395	8.99E-06	0.000123772	385	446	961	410	4	143	8	10
ENSECAG00000009100	1.945178944	9.03E-06	0.000124161	301	23	27	29	4	15	3	8
ENSECAG00000017926	2.662901305	9.04E-06	0.000124161	13	42	5	60	171	140	157	475
ENSECAG00000023625	5.657425806	9.12E-06	0.000125175	1250	2235	843	602	485	462	363	459
ENSECAG00000000757	2.3143856	9.27E-06	0.000127077	31	32	42	49	181	115	143	198
ENSECAG00000015208	0.76864027	9.36E-06	0.000128108	11	4	5	15	59	51	69	43
ENSECAG00000004463	6.389609663	9.37E-06	0.000128108	3393	2411	1113	1078	752	612	471	772
ENSECAG00000009337	1.287956437	9.50E-06	0.000129664	34	47	90	95	31	10	18	20
ENSECAG00000026955	5.721770609	9.51E-06	0.000129664	1800	1007	1392	779	615	499	484	396
ENSECAG00000020720	2.867520574	9.56E-06	0.000130292	42	41	36	107	196	201	242	314
ENSECAG00000017406	3.586927607	9.82E-06	0.00013361	531	357	103	176	97	57	79	89
ENSECAG00000003181	5.08437516	9.85E-06	0.000133842	221	385	177	346	1241	594	1210	1322
ENSECAG00000024194	4.299119898	9.98E-06	0.000135456	126	140	250	203	599	402	604	865
ENSECAG00000019016	1.679518008	1.01E-05	0.000136335	14	9	20	32	75	46	139	184
ENSECAG00000003836	2.061594374	1.01E-05	0.000136949	65	173	67	132	31	39	37	34
ENSECAG00000023706	5.309512234	1.01E-05	0.000136949	1033	1367	552	816	361	415	421	413
ENSECAG00000006442	5.949443255	1.03E-05	0.000139104	1841	1704	977	1332	549	781	582	536
ENSECAG00000020010	6.71279104	1.06E-05	0.00014345	1518	2175	3657	3488	991	1483	983	998
ENSECAG00000014615	3.141037326	1.06E-05	0.000143474	461	170	143	106	20	88	29	32
ENSECAG00000020448	1.420695847	1.07E-05	0.000144275	17	15	12	24	90	54	80	129
ENSECAG00000020366	5.983341088	1.07E-05	0.000144353	2338	2306	888	577	578	403	499	395
ENSECAG00000009884	0.236317709	1.11E-05	0.000149295	7	1	7	7	35	31	47	38

ENSECAG00000011051	0.424233361	1.11E-05	0.000149295	1	7	7	15	35	36	52	50
ENSECAG00000022677	5.129750456	1.13E-05	0.000151417	1563	880	294	620	268	296	246	201
ENSECAG00000013625	3.858073332	1.15E-05	0.000153635	73	115	114	236	460	394	517	426
ENSECAG00000008446	7.956683066	1.15E-05	0.000153646	11033	8829	2629	2035	1063	1918	1380	1144
ENSECAG00000000701	6.82564945	1.15E-05	0.000153646	866	461	13511	529	242	429	49	59
ENSECAG00000015196	2.669172779	1.19E-05	0.000158261	101	456	102	39	33	37	31	22
ENSECAG00000022349	0.614942146	1.19E-05	0.000158422	5	6	12	9	51	19	68	65
ENSECAG00000006162	8.141137242	1.20E-05	0.000159024	9130	14741	2465	2612	1289	2126	1538	1450
ENSECAG00000005250	5.731957698	1.20E-05	0.000159095	1500	1510	1018	893	595	521	559	589
ENSECAG00000001129	6.821982622	1.20E-05	0.000159427	3375	2736	2579	2333	1143	1224	1195	596
ENSECAG00000023667	7.835508925	1.21E-05	0.000160432	9306	6123	4580	2266	1875	1770	1636	1037
ENSECAG00000015218	0.156902397	1.21E-05	0.000160432	3	6	3	9	21	50	24	47
ENSECAG00000023358	8.666574479	1.22E-05	0.000160955	11097	19404	7517	3635	2640	3566	2578	1941
ENSECAG00000017126	4.486879923	1.22E-05	0.000160955	83	171	173	399	649	802	689	662
ENSECAG00000004433	6.935245637	1.22E-05	0.000161266	3860	7913	739	465	445	609	434	481
ENSECAG00000020728	1.039291545	1.22E-05	0.000161266	27	36	52	118	8	14	17	22
ENSECAG00000004372	0.986808503	1.23E-05	0.000162008	7	3	5	25	66	64	97	38
ENSECAG00000000284	2.168735995	1.23E-05	0.0001621	25	30	28	38	161	209	108	98
ENSECAG00000020763	5.387946939	1.24E-05	0.000163411	2024	1295	869	538	0	175	5	23
ENSECAG00000024868	1.446004717	1.26E-05	0.00016566	21	7	8	21	81	122	84	73
ENSECAG00000014207	3.818563767	1.26E-05	0.00016566	200	474	446	356	118	214	102	75
ENSECAG00000014770	2.357880572	1.31E-05	0.000171936	33	20	49	43	183	93	186	215
ENSECAG00000010104	2.768375627	1.34E-05	0.000174841	27	37	67	74	203	153	150	407
ENSECAG00000007804	6.717640818	1.35E-05	0.000176399	3345	3562	1626	1629	745	1130	698	1052
ENSECAG00000003863	0.469584559	1.38E-05	0.000179709	8	5	2	9	28	67	51	33
ENSECAG00000015410	1.420742824	1.41E-05	0.000183089	44	46	160	70	2	30	4	8
ENSECAG00000013762	4.699761896	1.41E-05	0.000183581	1023	1365	154	135	57	98	80	224
ENSECAG00000012172	7.593805764	1.42E-05	0.000185172	9638	2654	4954	1715	1628	1052	1217	615
ENSECAG00000023036	6.186578126	1.44E-05	0.000186478	1510	3054	1309	1424	504	1034	429	650
ENSECAG00000020494	2.91717919	1.44E-05	0.000187046	11	76	37	65	200	122	362	375
ENSECAG00000019048	1.601179807	1.45E-05	0.000187954	10	7	18	27	40	61	92	243
ENSECAG00000017338	4.957380582	1.46E-05	0.000189345	1052	1081	426	400	245	326	214	264
ENSECAG00000008601	10.13876393	1.46E-05	0.000189345	30188	51159	14554	17441	8745	8278	7966	10303
ENSECAG00000014087	4.674463782	1.47E-05	0.00019003	110	123	142	474	744	803	1165	591
ENSECAG00000024647	0.068349904	1.48E-05	0.000190991	2	7	3	10	35	26	38	32
ENSECAG00000004240	4.717597461	1.50E-05	0.000193004	656	1143	508	270	156	247	229	274
ENSECAG00000000216	7.114104575	1.54E-05	0.000198562	3051	4486	2969	2897	1400	1492	1454	1419
ENSECAG00000024971	2.690696336	1.55E-05	0.000198668	145	354	93	75	38	54	45	35
ENSECAG00000008274	5.329908454	1.55E-05	0.000198668	1226	2996	216	224	1	125	2	21
ENSECAG00000009180	5.792932299	1.56E-05	0.000199742	2483	1762	881	318	150	519	256	332
ENSECAG00000001757	5.861754207	1.60E-05	0.000204989	1259	1247	1694	1466	884	591	690	348
ENSECAG00000005023	3.145559932	1.62E-05	0.000206807	62	25	39	33	441	58	475	313
ENSECAG00000024168	6.261118752	1.62E-05	0.000206824	3121	2902	639	762	543	528	467	532
ENSECAG00000017976	3.778242942	1.63E-05	0.000207702	53	84	116	238	375	453	503	396
ENSECAG00000014867	10.14217466	1.64E-05	0.000208832	27695	50715	38420	14336	1047	11257	765	1658
ENSECAG00000007779	4.950543176	1.65E-05	0.000209567	198	279	402	320	777	767	919	1319
ENSECAG00000006647	2.566926987	1.65E-05	0.000209567	23	58	59	48	188	136	199	238
ENSECAG00000009621	5.470912609	1.66E-05	0.000210711	272	316	608	522	1307	967	1252	1966
ENSECAG00000017361	6.018304444	1.67E-05	0.000211774	247	318	570	1179	2207	1663	2737	1704
ENSECAG00000023808	5.574759817	1.68E-05	0.00021344	284	269	404	856	1596	1011	1390	2010
ENSECAG00000012397	7.87657607	1.69E-05	0.000213997	13279	6498	4368	1683	18	625	10	4
ENSECAG00000023070	6.539046729	1.70E-05	0.000214487	2789	3200	1468	1365	930	814	791	954
ENSECAG00000009921	7.952604418	1.73E-05	0.000219058	16275	2988	2660	1940	1796	831	817	470
ENSECAG00000022716	6.687804908	1.76E-05	0.000222605	3920	5310	1198	1345	6	538	17	72
ENSECAG00000016134	7.72537077	1.77E-05	0.00022326	4906	10182	3139	3158	1653	1893	1898	1296
ENSECAG00000009210	0.803849827	1.78E-05	0.000223695	5	7	9	17	60	48	94	25
ENSECAG00000000404	1.749778604	1.80E-05	0.000227057	38	267	61	18	21	12	3	12
ENSECAG00000023628	3.067640673	1.82E-05	0.000228802	304	294	77	148	60	57	48	85
ENSECAG00000019300	3.745748959	1.87E-05	0.000235375	251	697	304	299	14	166	17	30
ENSECAG00000007320	3.29016282	1.90E-05	0.000237888	136	350	306	204	78	112	62	131
ENSECAG00000000458	4.611369277	1.93E-05	0.000241245	609	891	690	510	47	365	48	67
ENSECAG00000006995	7.610917094	1.97E-05	0.000246129	6513	7261	2971	2259	1293	1551	1418	1945
ENSECAG00000019483	2.503552532	1.97E-05	0.000246324	40	34	42	65	188	123	198	213
ENSECAG00000010277	8.637662721	1.99E-05	0.000248195	6901	7145	17559	12070	4710	3933	3150	1414
ENSECAG00000003816	8.865809567	1.99E-05	0.000248214	2756	26193	13022	12161	1972	4985	3086	1852
ENSECAG00000000713	5.431225187	2.00E-05	0.000249413	1366	2223	312	409	244	316	290	292
ENSECAG00000008295	5.084971582	2.02E-05	0.000250911	552	253	1825	2121	1	124	1	96
ENSECAG00000011348	6.940629493	2.02E-05	0.000251429	720	699	785	1798	4041	2818	6196	2914
ENSECAG00000012411	6.50736911	2.04E-05	0.000252794	1835	4199	1880	1191	616.001	1130	696.001	702.001
ENSECAG00000002429	3.469566398	2.05E-05	0.000254106	244	305	202	342.999	143	73.9996	157	82
ENSECAG00000017005	5.641583249	2.07E-05	0.000255859	1847	2440	803	315	27	455	56	87
ENSECAG00000020295	2.174603536	2.07E-05	0.000255859	25	16	23	64	141	105	126	230
ENSECAG00000008718	5.502593914	2.07E-05	0.000256465	366	366	404	607	1591	825	1516	1628
ENSECAG00000009907	6.231201717	2.09E-05	0.000258553	326	403	370	1356	1802	2761	3472	1713
ENSECAG00000004655	7.361577153	2.11E-05	0.000260289	1962	4163	5299	6129	2451	1512	1521	1160
ENSECAG00000021525	9.883473288	2.12E-05	0.000260838	24912	32147	15035	20694	9562	6981	7924	11114
ENSECAG00000020578	1.015242695	2.13E-05	0.000262331	15	9	5	5	83	33	106	53
ENSECAG00000023787	1.075743569	2.14E-05	0.000263245	68	73	26	41	14	16	14	16
ENSECAG00000026927	4.765608667	2.16E-05	0.000265104	675	1130	396	403	263	266	215	297
ENSECAG00000010836	5.509671045	2.18E-05	0.000267024	266	514	349	649	1370	921	1599	1710
ENSECAG00000011930	1.805945058	2.18E-05	0.000267593	84	42	105	147	12	27	21	51
ENSECAG00000024327	6.702316324	2.20E-05	0.000268877	3414	4589	1040	1180	701	829	618	936
ENSECAG00000016071	6.434219616	2.28E-05	0.000278266	2908	1598	2058	1491	958	783	689	1086
ENSECAG00000013569	5.027011861	2.28E-05	0.000278266	1006	1039	540	456	374	317	277	296
ENSECAG00000004107	4.633820035	2.35E-05	0.000286543	298	684	1007	515	308	201	348	224

ENSECAG00000016271	0.66245274	2.46E-05	0.00029973	8	6	7	18	62	36	40	64
ENSECAG00000015500	5.790047504	2.47E-05	0.000300723	1087	4086	703	518	16	77	507	60
ENSECAG00000010914	6.662065965	2.47E-05	0.000300754	3828	3359	1153	1295	888	662	862	855
ENSECAG00000015172	9.377912695	2.49E-05	0.000303078	19411	15757	18778	10776	7370	5334	6747	5331
ENSECAG00000014234	4.868320162	2.51E-05	0.000304985	212	365	185	283	907	636	936	1159
ENSECAG00000013504	4.933777108	2.53E-05	0.000307157	302	213	239	252	1264	533	1024	1058
ENSECAG00000021093	2.361127831	2.54E-05	0.000307792	77	311	78	74	34	35	36	41
ENSECAG00000017598	2.828654449	2.54E-05	0.000307826	174	339	113	99	37	54	82	30
ENSECAG00000010300	1.962812028	2.57E-05	0.000310072	19	28	16	54	107	122	142	127
ENSECAG00000006896	1.914555869	2.58E-05	0.000311085	17	122	157	138	21	28	27	37
ENSECAG00000022899	3.657573429	2.62E-05	0.00031581	183	561	295	223	153	108	91	144
ENSECAG00000017203	9.237117738	2.63E-05	0.000316569	20426	16306	13614	10274	4988	3951	7347	1714
ENSECAG00000014359	1.189174475	2.64E-05	0.000317803	93	35	50	54	11	27	9	7
ENSECAG00000024242	6.365948963	2.64E-05	0.000317803	461	588	908	1240	2257	2686	3046	2050
ENSECAG00000017075	4.778342804	2.67E-05	0.000320447	1203	1024	195	220	172	172	155	199
ENSECAG00000024925	2.16108002	2.68E-05	0.000321963	84	61	291	69	19	50	22	14
ENSECAG00000021072	3.85904593	2.72E-05	0.000326098	95	171	75	135	467	311	400	688
ENSECAG00000024955	0.841949397	2.75E-05	0.000329346	29	84	23	55	4	16	12	15
ENSECAG00000024918	2.56892975	2.75E-05	0.000329346	126	141	193	120	54	70	52	63
ENSECAG00000014775	1.873542563	2.79E-05	0.000333901	16	16	36	11	149	156	40	152
ENSECAG00000019918	5.735793674	2.81E-05	0.000334925	1285	1465	1292	869	524	671	611	616
ENSECAG00000018671	1.615458603	2.82E-05	0.000335748	49	163	55	52	13	25	23	28
ENSECAG00000021529	5.701794482	2.86E-05	0.000340481	368	447	602	722	1775	1097	1524	1842
ENSECAG00000017396	5.176194812	2.95E-05	0.000351358	266	288	382	511	941	1003	870	1560
ENSECAG00000013078	-0.184267692	2.99E-05	0.000355035	5	2	5	5	28	21	23	36
ENSECAG00000016664	3.630451748	3.00E-05	0.000356291	542	233	319	187	12	151	39	51
ENSECAG00000008285	5.499604777	3.01E-05	0.000357406	606	2063	724	1188	321	614	368	511
ENSECAG00000006998	0.372614505	3.07E-05	0.000363597	19	22	86	21	0	13	4	4
ENSECAG00000019230	1.866273088	3.07E-05	0.000363843	47	248	120	11	8	26	5	2
ENSECAG00000020795	2.374478445	3.08E-05	0.000364418	110	365	50	94	0	31	0	5
ENSECAG00000019620	5.215497303	3.12E-05	0.000369073	509	920	1418	890	483	536	322	263
ENSECAG00000016866	5.858367183	3.18E-05	0.000374968	1548	901	1958	1073	730	673	559	564
ENSECAG00000016336	1.860911061	3.21E-05	0.000378455	11	28	22	51	110	89	103	168
ENSECAG00000021545	6.741878359	3.23E-05	0.000379674	494	599	824	1884	3696	3135	4338	2430
ENSECAG00000017374	2.419565656	3.23E-05	0.000379674	225	162	38	102	41	22	32	49
ENSECAG00000011152	3.793317057	3.24E-05	0.000381014	75	147.999	101	106	795	256	386	354
ENSECAG00000008705	9.972277475	3.27E-05	0.000383688	33868	42296	11479	11150	6973	7933	7371	6783
ENSECAG00000011413	1.63794616	3.28E-05	0.000384762	14	26	20	31	87	55	113	148
ENSECAG00000010339	8.627312063	3.29E-05	0.000385214	21585	5794	8940	3412	2721	1797	2073	269
ENSECAG00000020463	6.50630524	3.30E-05	0.00038577	1163	3492	2310	2830	414	1501	432	796
ENSECAG00000008472	3.816195624	3.30E-05	0.00038577	252	442	360	314	100	207	129	172
ENSECAG00000019691	2.424480314	3.33E-05	0.000388364	123	91	197	167	61	33	59	10
ENSECAG00000008730	7.734174159	3.34E-05	0.000389334	6242	7016	3734	3359	2092	1996	1729	2175
ENSECAG00000015342	10.94550849	3.35E-05	0.000389984	69808	86020	37796	46183	22	5182	72	28
ENSECAG00000000090	6.742375474	3.35E-05	0.000389984	3023	2004	4143	1565	915	1445	473	826
ENSECAG00000007522	5.735055167	3.40E-05	0.000394658	1076	2308	979	733	517	487	625	547
ENSECAG00000022741	4.822661548	3.40E-05	0.000394658	506	1890	310	234	230	141	199	215
ENSECAG00000026945	2.945074378	3.41E-05	0.000396274	87	86	213	560	81	63	52	24
ENSECAG00000008113	4.362583739	3.44E-05	0.000398441	149	144	201	265	449	809	724	506
ENSECAG00000003343	3.230780101	3.47E-05	0.000401469	415	277	193	111	7	101	9	20
ENSECAG00000023398	4.035703277	3.47E-05	0.000401923	835	667	50	75	75	45	83	69
ENSECAG00000017628	3.30131271	3.56E-05	0.000411322	401	346	79	197	7	83	40	75
ENSECAG00000016684	5.728638507	3.57E-05	0.00041167	1583	1967	701	737	475	566	421	534
ENSECAG00000020098	4.429116515	3.59E-05	0.000414031	172	157	200	326	662	496	711	725
ENSECAG00000007417	5.833954879	3.61E-05	0.000416191	1706	3141	616	869	27	597	99	86
ENSECAG00000018345	7.269534913	3.62E-05	0.00041709	3137	3838	5095	3161	1218	2225	1465	1428
ENSECAG00000024354	6.754635202	3.63E-05	0.000417188	2829	2433	2451	2503	1630	1092	948	1037
ENSECAG00000008109	7.308585811	3.63E-05	0.000417531	828	1103	1148	2402	4350	5436	6771	3501
ENSECAG00000019631	6.931125562	3.70E-05	0.000424371	987	800	1152	1355	4274	2442	4084	4647
ENSECAG00000020840	7.091138094	3.73E-05	0.000427612	4036	7015	1142	1421	884	902	969	1101
ENSECAG00000012750	5.629704317	3.73E-05	0.000427612	1280	2551	728	352	322	455	401	317
ENSECAG00000010204	1.851186763	3.74E-05	0.000428065	16	19	25	50	118	63	124	164
ENSECAG00000023190	3.955795037	3.78E-05	0.000431742	338	662	237	261	130	118	154	213
ENSECAG00000004216	11.36841271	3.82E-05	0.00043617	73502	91206	70409	29759	15188	32990	14441	20400
ENSECAG00000002390	2.556510103	3.84E-05	0.000437492	73	151	165	219	68	69	56	50
ENSECAG00000003313	1.663373906	4.01E-05	0.000457168	80	21	73	180	30	20	16	27
ENSECAG00000004339	2.553672077	4.09E-05	0.000465516	29	21	65	73	180	132	184	266
ENSECAG00000015319	3.077730016	4.11E-05	0.000467162	54	82	70	85	224	193	246	401
ENSECAG00000021685	1.515233734	4.24E-05	0.000481376	11	16	28	32	91	56	89	130
ENSECAG00000023076	9.102109489	4.25E-05	0.000482713	18766	24019	5672	5750	4994	2925	4285	3252
ENSECAG00000018864	2.694108853	4.26E-05	0.000482713	130	326	96	99	41	57	52	63
ENSECAG00000020734	1.678631751	4.31E-05	0.000488649	131	148	41	20	4	27	1	7
ENSECAG00000007974	4.010184858	4.33E-05	0.00048989	498	710	225	175	64	205	67	111
ENSECAG00000026830	0.574105191	4.35E-05	0.000492321	5	9	10	15	51	28	49	59
ENSECAG00000010188	4.1567953	4.44E-05	0.000501993	381	694	349	274	151	221	187	171
ENSECAG00000020213	6.430888614	4.52E-05	0.000509605	2674	2376	1179	1749	873	786	914	1058
ENSECAG00000022468	7.556588934	4.53E-05	0.000510905	6166	2953	4426	4372	2255	1625	1781	1926
ENSECAG00000024499	6.124583412	4.57E-05	0.000514381	2277	1484	1134	1548	741	851	723	608
ENSECAG00000002905	3.900738543	4.57E-05	0.000514385	180	633	399	311	122	115	96	270
ENSECAG00000010425	5.708194668	4.69E-05	0.000527373	1283	1926	858	796	665	486	541	536
ENSECAG00000002920	3.512792581	4.73E-05	0.00053104	59	111	139	121	315	324	416	329
ENSECAG00000001697	4.463380578	4.76E-05	0.000533998	471	792	462	374	187	318	231	180
ENSECAG00000016179	3.707444638	4.77E-05	0.000533998	86	96	130	200	419	341	431	382
ENSECAG00000005194	4.30281502	4.81E-05	0.000538768	112	118	210	295	473	461	476	1097
ENSECAG00000015109	8.847568712	4.84E-05	0.000541287	2123	1155	4702	4314	18008	5152	25127	16148

ENSECAG00000011160	1.931627322	4.89E-05	0.000546053	117	181	54	39	31	27	23	6
ENSECAG00000020809	0.831409675	4.95E-05	0.000551955	16	47	44	86	19	11	14	15
ENSECAG00000007961	5.599030487	4.96E-05	0.000553238	207	364	543	739	1010	2194	1299	1408
ENSECAG00000000141	2.173622885	4.97E-05	0.000553238	42	176	109	185	21	66	22	19
ENSECAG00000008859	2.718820337	5.07E-05	0.000563893	44	183	260	233	66	47	65	72
ENSECAG00000024082	8.338666227	5.11E-05	0.000568447	13553	14496	2156	2974	449	3058	935	1291
ENSECAG00000008358	5.635984003	5.16E-05	0.000572632	271	538	488	778	1380	1136	1426	2049
ENSECAG00000017082	5.822894023	5.16E-05	0.000572632	1762	1770	863	796	589	537	543	647
ENSECAG00000021224	1.479725949	5.22E-05	0.000578702	124	86	61	38	0	22	0	1
ENSECAG00000007415	4.269305235	5.24E-05	0.000580102	74	139	204	299	499	778	689	398
ENSECAG00000020874	4.987881825	5.33E-05	0.000589289	630	746	1366	920	50	518	69	77
ENSECAG00000016054	2.456319059	5.36E-05	0.0005926	32	53	25	52	121	232	194	147
ENSECAG00000017747	6.174618371	5.37E-05	0.0005926	1198	2003	1753	1830	805	893	1075	824
ENSECAG00000008722	4.571366268	5.42E-05	0.000597509	551	927	520	336	205	359	144	137
ENSECAG00000022695	4.027278349	5.43E-05	0.000598432	195.002	290	690	695	69	273	55	120.001
ENSECAG00000022252	5.787644706	5.44E-05	0.000598572	436	365	532	876	1908	1310	1956	1462
ENSECAG00000018837	5.745381655	5.44E-05	0.000598572	1088	1351	1377	1083	735	697	633	609
ENSECAG00000024613	5.222717138	5.49E-05	0.000602947	1604	1131	464	279	310	264	242	335
ENSECAG00000019530	6.311826683	5.50E-05	0.000603387	2419	2095	1437	1291	756	841	890	943
ENSECAG00000004701	5.242299679	5.50E-05	0.000603387	360	262	380	419	1188	729	1473	1228
ENSECAG00000003773	1.807508052	5.57E-05	0.000609822	76	169	63	44	16	28	31	32
ENSECAG00000015424	5.829275039	5.58E-05	0.000610694	236	453	619	1033	1655	1436	1792	2062
ENSECAG00000013239	5.173128237	5.59E-05	0.000611776	1395	337	948	839	390	435	194	205
ENSECAG00000000591	5.387269087	5.62E-05	0.000613764	1693	996	669	486	380	423	385	285
ENSECAG00000014869	5.907739861	5.85E-05	0.000637715	713	1390	1871	1928	909	764	608	695
ENSECAG00000007708	2.720938288	5.85E-05	0.000637715	39	58	60	46	235	101	198	326
ENSECAG00000015791	3.846170468	5.88E-05	0.000640281	113	101	153	196	453	337	447	495
ENSECAG00000013913	5.347522891	5.91E-05	0.000643348	257	334	346	746	1254	1089	1389	1129
ENSECAG00000018683	8.61173955	5.96E-05	0.000648242	6099	16757	7678	8873	3853	4196	3022	4462
ENSECAG00000010999	2.293277207	6.00E-05	0.00065228	90	71	199	184	43	68	14	21
ENSECAG00000009137	3.750744197	6.06E-05	0.000657809	429	544	186	153	91	128	141	51
ENSECAG00000020343	7.560589124	6.10E-05	0.000661083	9437	5948	1326	1380	872	1210	1422	777
ENSECAG00000024310	0.859233335	6.10E-05	0.000661083	7	12	6	18	98	45	59	30
ENSECAG00000016418	1.626995368	6.15E-05	0.000666006	8	14	34	3	58	104	65	210
ENSECAG00000017059	4.492043445	6.22E-05	0.000672368	1011	531	255	278	209	151	135	246
ENSECAG00000024286	7.817612581	6.23E-05	0.000672368	8037	8363	2682	2491	1736	1713	1513	2289
ENSECAG00000016646	0.294382051	6.33E-05	0.00068303	4	1	10	14	44	35	37	37
ENSECAG00000019137	4.240949607	6.35E-05	0.000683989	282	454	759	358	289	212	162	186
ENSECAG00000011361	5.040597343	6.37E-05	0.000686391	478	1027	785	839	365	515	355	362
ENSECAG00000006931	7.52902232	6.39E-05	0.000687943	1629	4464	5987	8088	1793	2395	1807	1504
ENSECAG00000024552	2.818720165	6.47E-05	0.000695304	51	37	88	60	214	182	216	273
ENSECAG00000012903	4.242063973	6.51E-05	0.000698521	119	143	161	337	613	412	541	741
ENSECAG00000019324	1.985948563	6.51E-05	0.000698521	254	92	5	44	10	7	25	7
ENSECAG00000000196	3.39646792	6.53E-05	0.000700267	7	38	20	183	262	494	386	340
ENSECAG00000024358	2.646149424	6.59E-05	0.000706149	78	28	476	161	35	42	36	46
ENSECAG00000003916	4.55544404	6.66E-05	0.000712022	182	213	304	256	720	561	742	757
ENSECAG00000022360	1.518751812	6.66E-05	0.000712022	34	209	24	47	8	23	14	16
ENSECAG00000015971	5.09750579	6.67E-05	0.000712488	299	299	350	459	1137	780	961	1168
ENSECAG00000023981	8.176901465	6.70E-05	0.000714371	6727	12175	4639	4672	2733	2343	2240	3692
ENSECAG00000015134	1.031837089	6.70E-05	0.00071459	32	81	38	55	15	19	22	14
ENSECAG00000012451	4.88890572	6.76E-05	0.000719108	306	451	1105	1263	362	398	285	286
ENSECAG00000024882	2.84432577	6.76E-05	0.000719108	286	148	59	381	1	51	0	6
ENSECAG00000006453	4.803111381	6.89E-05	0.000732078	1275	511	382	369	235	220	239	280
ENSECAG00000014104	2.000824964	7.00E-05	0.000743565	59	236	63	71	13	49	20	20
ENSECAG00000009306	1.141808979	7.01E-05	0.000743565	107	38	43	30	9	25	6	7
ENSECAG00000012336	6.297161717	7.02E-05	0.000743565	2620	3439	885	682	457	764	721	385
ENSECAG00000011320	2.030526751	7.04E-05	0.000744944	18	38	24	39	147	54	184	148
ENSECAG00000020428	6.171124333	7.09E-05	0.000749854	1202	2500	1644	1396	881	791	816	988
ENSECAG00000019229	5.178323847	7.15E-05	0.000755633	225	365	395	548	1090	805	1299	1080
ENSECAG00000023469	0.864116054	7.21E-05	0.000761208	1	8	3	25	27	88	79	48
ENSECAG00000011970	5.246427677	7.35E-05	0.000775436	133	167	294	774	1069	1466	1405	859
ENSECAG00000004042	4.421167407	7.49E-05	0.00078907	110	612	357	1512	174	237	177	111
ENSECAG00000016658	0.53185551	7.52E-05	0.00079181	4	4	19	8	52	42	51	36
ENSECAG00000024000	7.025745837	7.53E-05	0.00079181	584	1027	899	2252	3852	4141	5230	3114
ENSECAG00000010656	3.992758943	7.53E-05	0.000791961	602	91	872	123	115	120	84	34
ENSECAG00000024982	5.686604177	7.69E-05	0.000807537	1444	1719	900	644	568	466	518	647
ENSECAG00000003569	5.745510834	7.75E-05	0.000812362	215	581	418	909	1671	1228	2224	1459
ENSECAG00000020437	6.389823991	7.75E-05	0.000812362	1770	904	3036	2795	1242	800	961	556
ENSECAG00000007818	9.088189423	7.80E-05	0.000816504	19453	21025	5524	6368	4488	3745	4702	3949
ENSECAG00000021869	4.959676299	7.80E-05	0.000816504	620	730	800	708	498	340	425	296
ENSECAG00000018398	2.455249827	7.92E-05	0.000827379	139	323	62	35	41	28	29	41
ENSECAG00000012386	5.104558354	7.94E-05	0.000828865	532	1331	944	484	320	398	321	499
ENSECAG00000007726	5.996883204	8.08E-05	0.000842521	432	499	697	1052	1946	1361	2091	2245
ENSECAG00000002611	4.301656411	8.10E-05	0.000844493	163	139	263	216	581	477	610	689
ENSECAG00000008158	0.914403737	8.55E-05	0.00089063	7	12	12	26	65	49	54	64
ENSECAG00000016395	2.820641957	8.57E-05	0.000891301	55	64	22	54	193	178	176	392
ENSECAG00000022883	8.088728528	8.57E-05	0.000891301	8755	10802	2772	3915	2050	2346	2644	1993
ENSECAG00000011496	8.247191062	8.61E-05	0.00089393	5253	6706	13749	7086	2317	4888	1286	1406
ENSECAG00000011939	6.237572682	8.65E-05	0.000898001	1313	2767	1612	1512	810	867	1118	575
ENSECAG00000010888	2.135971017	8.69E-05	0.000900957	30	35	26	37	71	120	148	231
ENSECAG00000023263	7.221992589	8.72E-05	0.000903349	4205	4889	2612	2365	1438	1533	1753	1171
ENSECAG00000020431	8.515419715	8.74E-05	0.000903857	8340	10150	10653	9795	5183	3159	2439	609
ENSECAG00000023264	2.457798856	8.74E-05	0.000903857	19	51	19	79	207	117	230	160
ENSECAG00000004770	3.674684254	8.77E-05	0.000906081	346	735	109	106	41	60	92	147
ENSECAG00000011623	4.543672933	8.81E-05	0.000909598	200	213	268	275	691	559	682	813

ENSECAG00000018544	2.643804869	8.86E-05	0.000913572	32	55	35	92	210	126	191	266
ENSECAG00000016417	5.415919577	8.94E-05	0.000921027	383	235	516	457	1493	781	1178	1847
ENSECAG00000014763	4.375349607	8.97E-05	0.000923283	160	214	178	294	634	471	567	801
ENSECAG00000016258	3.882295473	9.00E-05	0.000925538	682	364	212	115	75	141	83	136
ENSECAG00000014016	6.875692577	9.08E-05	0.000932817	439	589	669	2238	3456	4805	4533	2321
ENSECAG00000020522	4.762224653	9.09E-05	0.000932817	678	1110	497	287	242	332	185	279
ENSECAG00000024465	5.83915031	9.11E-05	0.000934686	627	297	430	668	1846	1252	1752	2294
ENSECAG00000012854	8.022886768	9.22E-05	0.000944415	5781	11452	3937	4319	1886	2288	2023	3658
ENSECAG00000025116	1.553390827	9.27E-05	0.000949198	53	187	41	30	16	30	11	8
ENSECAG00000017186	5.022826656	9.31E-05	0.000951808	245	108	485	252	1368	724	1044	1001
ENSECAG00000011902	6.356763514	9.34E-05	0.000954459	3742	2056	1066	656	772	579	589	656
ENSECAG00000018086	5.188456437	9.49E-05	0.000969162	595	1780	593	690	172	482	235	484
ENSECAG00000018785	0.556227202	9.61E-05	0.000979493	19	37	67	27	12	14	10	11
ENSECAG00000019873	6.36322442	9.61E-05	0.000979493	4926	979	1702	970	20	680	23	143
ENSECAG00000002984	2.00652606	9.62E-05	0.000979493	23	16	43	20	164	148	46	173
ENSECAG00000009027	1.490362928	9.67E-05	0.000983647	8	11	24	41	94	86	117	58
ENSECAG00000019596	9.524813009	9.73E-05	0.000988886	44781	9103	13124	10862	101	5161	147	996
ENSECAG00000012655	6.428211	9.78E-05	0.000993158	4867	2127	456	622	488	590	533	290
ENSECAG00000022213	6.826036016	0.000100103	0.001015513	1880	4510	2027	2513	1144	1295	1248	1371
ENSECAG00000024457	8.813394538	0.000100344	0.001017043	13595	20715	5082	5283	2771	4677	3726	1816
ENSECAG00000012389	7.098671315	0.000100721	0.001019954	3036	4557	2566	2742	1473	1167	1507	2022
ENSECAG00000023399	1.89715235	0.00010099	0.001021771	13	56	366	51	6	29	2	2
ENSECAG00000005590	3.784541506	0.000101549	0.001026512	67	155	125	193	476	275	484	441
ENSECAG00000022702	4.414404554	0.000102572	0.001035934	376	272	581	1000	324	253	206	119
ENSECAG00000020066	5.823301253	0.0001036	0.001045385	828	1743	1347	1333	771	614	812	737
ENSECAG00000021457	0.991586815	0.00010479	0.001056244	11	15	1	11	78	23	74	97
ENSECAG00000022374	1.900105918	0.000104862	0.001056244	52	246	52	42	25	29	16	30
ENSECAG00000000307	5.869845155	0.00010498	0.00105649	1979	2271	601	679	645	570	402	344
ENSECAG00000015687	8.230387136	0.000105821	0.00106402	8856	14252	3151	3035	2391	1968	2280	2934
ENSECAG00000010011	3.417548049	0.000105991	0.001064782	231	232	395	163	146	104	116	93
ENSECAG00000020945	1.637860976	0.000107096	0.001074935	15	7	36	31	137	65	79	123
ENSECAG00000026846	7.082429224	0.000108405	0.001087107	2668	5845	2529	2155	1203	1502	1617	993
ENSECAG00000008155	3.757598867	0.000109544	0.001097568	69	119	120	223	410	421	489	303
ENSECAG00000018134	0.865739794	0.000109864	0.001099805	10	0	9	6	116	17	91	31
ENSECAG00000015121	5.07915549	0.000110375	0.001103941	249	402	298	436	860	818	1291	1019
ENSECAG00000020620	6.225255772	0.000111017	0.001109332	471	692	808	1225	2325	2103	2047	2329
ENSECAG00000012215	4.560269789	0.000111109	0.001109332	1019	668	205	283	214	200	146	213
ENSECAG00000017631	3.959995873	0.00011144	0.001111664	125	125	143	237	511	382	474	477
ENSECAG00000011811	0.281790931	0.000111666	0.001112937	6	0	9	10	29	53	43	27
ENSECAG00000010420	7.382535442	0.000112108	0.001116365	814	911	1402	2912	5457	4989	6951	3629
ENSECAG00000007665	6.518543465	0.000112605	0.001120336	2703	3557	1136	1226	908	744	620	1231
ENSECAG00000020193	8.741150995	0.000113201	0.001125279	6615	25527	10374	5586	1013	5742	568	1357
ENSECAG00000011688	5.969775604	0.000113518	0.001127438	1129	3468.01	803.003	763.998	516	518.993	561.009	724.004
ENSECAG00000001653	6.095867585	0.000113868	0.001129926	432	531	849	1080	1977	1627	1844	2741
ENSECAG00000000466	6.809514444	0.00011461	0.001136233	2841	5301	1472	1221	866	1099	997	992
ENSECAG00000021542	4.328053421	0.000114703	0.001136233	482	638	439	337	137	335	177	179
ENSECAG00000000431	1.325533472	0.000115703	0.001145141	36	64	125	52	4	26	5	31
ENSECAG00000015859	1.765397858	0.000116129	0.001148351	19	18	33	42	98	71	102	156
ENSECAG00000022574	7.427789372	0.000116415	0.001150183	6200	5879	2384	1685	1529	1442	1236	1677
ENSECAG00000009363	5.860399013	0.000117943	0.001163374	1144	4439	468	311	82	579	97	236
ENSECAG00000005126	6.525983801	0.000117955	0.001163374	600	1196	787	1065	2803	2425	2554	3316
ENSECAG00000000968	4.283263867	0.00011807	0.001163494	174	1158	341	371	152	101	135	310
ENSECAG00000021378	1.838700155	0.000118738	0.001169063	23.0106	34.0001	29.0079	16.0001	128.001	71.0005	145.001	111
ENSECAG00000012584	6.776853936	0.000120081	0.001181265	2736	4125	1761	1667	1249	804	1151	1385
ENSECAG00000006192	1.405321592	0.000120323	0.001182621	13	7	15	33	65	35	87	172
ENSECAG00000018264	7.635032501	0.000120605	0.001184372	1270	1254	2056	2857	7052	4953	7586	5031
ENSECAG00000000972	1.544414107	0.000122023	0.001197257	29	58	93	130	24	25	27	39
ENSECAG00000006934	6.133410236	0.000122135	0.001197324	1493	3287	1065	952	445	712	485	1074
ENSECAG00000021876	5.738815617	0.00012226	0.001197519	986	3286	521	676	241	674	284	409
ENSECAG00000017398	5.646045491	0.000122475	0.001198585	271	553	217	858	1139	1537	1731	1789
ENSECAG00000011679	9.32733378	0.000123032	0.001203001	15406	29441	9602	8147	5625	5501	6253	5675
ENSECAG00000006964	4.571241915	0.000123612	0.001207639	445	997	323	482	259	200	288	316
ENSECAG00000015412	4.114097421	0.00012513	0.001221413	130	138	186	243	540	376	406	769
ENSECAG00000007789	2.496663045	0.000125953	0.001228396	40	18	40	75	196	154	251	117
ENSECAG00000003634	3.731272415	0.000126358	0.001231287	100	148	115	137	402	274	361	587
ENSECAG00000004928	1.467843098	0.000127348	0.001239868	14	19	24	30	55	61	117	113
ENSECAG00000007807	4.477637836	0.00012789	0.001244076	197	1165	433	490	287	216	207	162
ENSECAG00000012668	2.675736261	0.00013025	0.001265956	97	119	223	195	81	52	62	97
ENSECAG00000020238	6.479223308	0.000131258	0.001274662	1464	6035	1189	511	554	657	609	766
ENSECAG00000016204	2.904107614	0.000134981	0.001309699	333	239	95	46	29	51	39	82
ENSECAG00000013653	4.017792302	0.000135217	0.001310864	320	410	479	328	193	266	134	132
ENSECAG00000021672	10.67216413	0.000135722	0.001314639	29471	46756	62975	28635	10087	26440	9504	12984
ENSECAG00000015182	3.602213427	0.000136311	0.001319221	94	57	102	142	453	263	617	201
ENSECAG00000012235	4.278421843	0.000136698	0.001321841	572	779	296	233	108	286	79	165
ENSECAG00000024702	8.269773807	0.000136896	0.001322629	15396	7614	2660	3169	2151	2707	2047	1442
ENSECAG00000005945	5.723110957	0.00013728	0.001325211	943	2329	915	796	476	623	548	675
ENSECAG00000018367	2.758130191	0.000138428	0.001335156	32	38	87	83	213	123	239	278
ENSECAG00000009611	7.614348144	0.000140117	0.001349868	8803	4347	2827	2368	2112	1364	1813	748
ENSECAG00000005489	6.298389518	0.00014019	0.001349868	3224	3616	1321	523	7	575	14	59
ENSECAG00000024727	5.983475828	0.000141982	0.001365957	1635	2407	785	1209	438	987	464	562
ENSECAG00000024553	1.729839754	0.000142268	0.001367554	15	24	33	41	103	65	122	120
ENSECAG00000020495	3.114390057	0.000144506	0.001387737	28	36	36	179	213	209	312	421
ENSECAG00000008137	1.411421279	0.000144623	0.001387737	17	12	25	30	105	58	76	90
ENSECAG00000010890	4.226455588	0.000144734	0.001387737	470	962	457	189	9	227	15	59
ENSECAG00000026958	5.885938174	0.000145027	0.001389371	1645	2408	712	813	611	436	809	390

ENSECAG00000010267	9.693193688	0.000145365	0.001391434	18776	28341	22170	10448	7942	9302	7149	8589
ENSECAG00000005881	1.873223627	0.000145674	0.001393226	95	82	54	124	33	33	30	53
ENSECAG00000011632	4.36591171	0.00014589	0.001394115	351	532	402	673	283	327	192	208
ENSECAG00000003104	5.821840379	0.000146433	0.001398124	1368	1385	898.004	1601	473	648.002	499.001	1116
ENSECAG00000019890	4.568576394	0.000146642	0.001398952	237	213	236	188	771	460	811	843
ENSECAG00000018272	7.04134001	0.000148427	0.001414784	986	739	1743	1450	4389	3925	4191	3613
ENSECAG00000017892	5.871838282	0.000149827	0.001426934	1147	1593	1373	1242	617	645	569	1163
ENSECAG00000024198	2.91131797	0.000150138	0.001428705	45	47	82	103	255	143	212	326
ENSECAG00000020441	3.476955417	0.00015117	0.00143615	80	83	48	175	349	195	382	495
ENSECAG00000018755	3.948264985	0.000151174	0.00143615	132	106	133	235	508	326	548	473
ENSECAG00000014260	11.21766394	0.000151691	0.001439858	35356	123923	74946	37507	4924	34289	5716	3241
ENSECAG00000007942	9.267617329	0.000152653	0.001447779	28421	19700	5943	3962	4786	2951	3913	5284
ENSECAG00000017773	3.25055981	0.000153925	0.001458628	299	373	92	136	77	80	81	100
ENSECAG00000024232	1.804281473	0.000154712	0.001464864	44	51	153	132	11	39	34	39
ENSECAG00000011864	7.283417673	0.000156818	0.001483566	3531	7865	1675	2186	1162	1218	1302	1928
ENSECAG00000002438	4.119472255	0.000158398	0.001497265	1020	456	237	138	0	87	0	14
ENSECAG00000008108	5.496315327	0.00016207	0.001530705	1135	1396	746	799	534	594	542	421
ENSECAG00000009096	6.887012368	0.000162835	0.001536652	2723	5432	1773	1581	780	1004	760	1902
ENSECAG00000005770	6.741459071	0.000163316	0.001539913	3942	3315	1705	907	1116	824	986	890
ENSECAG00000017629	6.351451902	0.000163679	0.001541719	1626	2969	1470	1474	1012	686	957	1231
ENSECAG00000001900	1.086979425	0.000163786	0.001541719	84	29	67	41	4	30	3	7
ENSECAG00000011694	3.7229601	0.000163914	0.001541719	143	233	450	547	171	83	136	225
ENSECAG00000003789	0.436142031	0.000165166	0.001551915	21	49	37	28	13	12	14	3
ENSECAG00000018564	3.114070232	0.000165272	0.001551915	60	79	69	49	260	410	277	132
ENSECAG00000024219	1.934802789	0.000165806	0.001554651	20	11	30	36	125	151	186	33
ENSECAG00000002814	6.472239767	0.000165887	0.001554651	1486	1838	3082	1967	1240	1037	1251	894
ENSECAG00000021389	1.627144636	0.000165974	0.001554651	39	98	54	133	38	36	21	19
ENSECAG00000017777	4.590103005	0.000166192	0.001555415	130	292	251	322	648	587	580	1077
ENSECAG00000021241	8.000691357	0.000168812	0.001578633	12387	7612	1903	1970	1964	1615	1614	1841
ENSECAG00000009328	4.215468221	0.000169085	0.001578972	228	195	1028	510	176	153	239	213
ENSECAG00000011954	7.763204682	0.00016928	0.001578972	4602	9203	3782	3262	1955	2467	1973	2350
ENSECAG00000019617	4.671065758	0.000169312	0.001578972	1049	716	194	397	156	266	178	275
ENSECAG00000012496	5.179791184	0.000169404	0.001578972	748	1420	584	605	337	446	327	537
ENSECAG00000013722	6.200982194	0.000170015	0.001583365	712	2113	3166	1562	523	1149	579	885
ENSECAG00000023126	4.130018803	0.000170181	0.001583621	575	1026	53	122	81	57	92	171
ENSECAG00000023826	7.261578861	0.00017064	0.001586592	1412	6546	3494	4561	2123	1184	1880	1048
ENSECAG00000024389	5.643035671	0.000172582	0.001603337	1668	1468	540	880	620	459	576	381
ENSECAG00000023847	6.325760246	0.000175982	0.001633588	693	571	957	982	2823	1579	2105	3194
ENSECAG00000014984	9.752464856	0.000176719	0.001639083	32151	29099	8769	11021	7732	7191	7247	6862
ENSECAG00000017245	4.841623632	0.000177899	0.001648684	504	1120	413	649	318	349	257	428
ENSECAG00000009229	3.206060555	0.00017874	0.00165513	73	83	88	110	315	255	274	251
ENSECAG00000000483	7.471502899	0.000181785	0.001681953	517	598	2034	2237	4000	2007	9662	8944
ENSECAG00000012142	5.45403753	0.000183158	0.001693276	967	1819	828	486	379	564	459	416
ENSECAG00000017048	4.537147066	0.000183423	0.001694348	1245	449	218	199	137	185	108	242
ENSECAG00000009105	3.35609195	0.000184103	0.001699253	56	59	137	147	264	327	274	375
ENSECAG00000016385	2.021574929	0.000184666	0.001703061	4	16	12	41	83	376	55	34
ENSECAG00000001499	2.749816364	0.000186429	0.001717927	357	69	111	120	12	81	29	36
ENSECAG00000019264	7.967944877	0.000186613	0.001718231	7927	10324	3110	2412	2100	2306	2111	2001
ENSECAG00000010727	7.579078902	0.00018746	0.001724631	6544	6448	2480	2328	2131	1301	1872	1719
ENSECAG00000019506	7.168548291	0.000188855	0.001735331	1996	10265	1576	1174	833	1100	1123	1096
ENSECAG00000004471	3.37803759	0.000188928	0.001735331	441	347	97	83	81	56	85	98
ENSECAG00000023459	8.478466845	0.000190827	0.001751358	11175	4905	10559	7311	3388	4463	2929	3695
ENSECAG00000021301	5.209910717	0.000194316	0.001781936	300	241	387	638	907	968	1094	1411
ENSECAG00000012725	8.747808816	0.000194689	0.001783914	12896	13587	5887	8035	3919	4274	3370	5636
ENSECAG00000019187	5.415955974	0.000195509	0.001789105	272	296	578	627	1313	1369	1467	858
ENSECAG00000016629	2.276537931	0.000195634	0.001789105	63	109	167	150	47	71	36	52
ENSECAG00000017419	6.788026512	0.000195728	0.001789105	2839	2187	2724	2405	1625	1223	1333	1029
ENSECAG00000004611	2.011615928	0.000197813	0.001806709	107	252	33	27	8	30	11	37
ENSECAG00000017994	4.251406949	0.000198564	0.001812119	162	176	213	240	544	397	622	674
ENSECAG00000018996	9.102010733	0.000198727	0.001812152	34251	7643	6848	2636	2656	4004	3232	1025
ENSECAG00000013954	0.477145821	0.00019982	0.001820653	9	8	9	7	47	17	50	62
ENSECAG00000008014	4.459524551	0.000207831	0.001892126	160	962	377	796	162	257	243	272
ENSECAG00000014859	3.860080791	0.00020857	0.001897332	135	123	132	173	487	277	510	464
ENSECAG00000013151	7.116220412	0.000210074	0.001909485	3964	1034	7967	1531	1599	531	1298	911
ENSECAG00000021917	3.565249646	0.000210736	0.001913976	102	97	144	110	313	278	409	418
ENSECAG00000011117	3.744352305	0.000211419	0.001918649	399	894	103	79	6	124	5	20
ENSECAG00000011636	4.927394607	0.000212275	0.001924875	113	32	176	4	1629	359	2531	157
ENSECAG00000006177	3.367002739	0.000213662	0.001935906	48	85	71	111	107	595	233	381
ENSECAG00000020227	8.091403548	0.000214154	0.001938823	6602	11130	4786	3669	2028	3595	2043	2694
ENSECAG00000011136	4.358268434	0.000216928	0.001962372	461	1125	197	212	179	175	200	154
ENSECAG00000002235	4.191582632	0.000218644	0.001976315	146	89	167	318	592	388	659	579
ENSECAG00000013476	3.910053966	0.000220515	0.001990137	145	96	129	220	431	364	472	531
ENSECAG00000002764	4.700688465	0.000220523	0.001990137	26	116	52	350	167	751	402	2672
ENSECAG00000002522	1.256084855	0.000221364	0.001995821	54	46	66	59	26	22	24	29
ENSECAG00000016948	11.09791637	0.000221504	0.001995821	88921	37891	48762	29461	19725	23708	20675	11375
ENSECAG00000003954	4.208999066	0.000222562	0.002003765	643	380	406	224	198	175	222	190
ENSECAG00000000918	1.039595705	0.000228088	0.002051889	9	13	19	24	47	43	76	87
ENSECAG00000009794	7.114476996	0.00022963	0.002064126	3424	5214	1811	2557	1520	1410	1139	1793
ENSECAG00000019543	1.030123808	0.000230547	0.002070735	73	94	23	27	0	22	1	7
ENSECAG00000014096	4.647868207	0.000236082	0.002118774	642	695	575	330	332	229	321	304
ENSECAG00000015898	1.4619095	0.000238469	0.002138307	81	23	126	53	21	29	23	8
ENSECAG00000002472	4.918488806	0.000238635	0.002138307	843	1825	302	163	32	322	110	194
ENSECAG00000011364	3.437656478	0.000239086	0.002140659	69	104	67	163	391	168	380	406
ENSECAG00000020930	0.363486461	0.000239763	0.002145032	34	38	23	26	7	8	14	15
ENSECAG00000008826	7.31090744	0.000241698	0.002160636	4370	5257	2898	2194	1540	1753	1202	2136

ENSECAG00000002456	2.212507689	0.000243846	0.002178125	29	33	39	54	147	66	143	233
ENSECAG00000015245	7.530543195	0.000245654	0.002192554	11233	1920	1436	2242	1213	1046	1119	1540
ENSECAG00000022239	1.607478086	0.000245962	0.002193583	109	8	207	23	4	27	5	2
ENSECAG00000019559	2.758250882	0.000247547	0.002205987	116	365	87	116	47	88	51	37
ENSECAG00000024031	6.260327748	0.000249121	0.002218266	3175	1972	998	740	839	625	627	731
ENSECAG00000000895	3.845791504	0.000249556	0.002220403	100	166	145	170	499	286	378	551
ENSECAG00000015370	5.869908604	0.000252149	0.00224172	1125	1652	929	1814	517	1044	576	665
ENSECAG00000011988	4.083156988	0.000255044	0.002265683	77	206	90	205	231	395	625	947
ENSECAG00000005331	3.936518266	0.00025758	0.002286424	76	131	151	291	414	425	507	466
ENSECAG00000021198	0.811018951	0.000259303	0.002299924	9	8	20	13	37	35	61	86
ENSECAG00000007767	7.674495255	0.000259734	0.002300705	7033	5530	3486	2598	1997	1842	2343	1941
ENSECAG00000009520	4.698930401	0.000259796	0.002300705	182	292	303	346	748	534	737	1017
ENSECAG00000017026	4.996788526	0.000262014	0.002317923	297	292	336	431	905	651	950	1225
ENSECAG00000011353	3.793214048	0.000262148	0.002317923	152	478	395	362	92	238	129	146
ENSECAG00000007197	5.320240029	0.000264608	0.002337855	211	356	526	686	1197	1121	1190	1120
ENSECAG00000006038	2.294642699	0.000265357	0.00234265	21	66	16	29	181	75	144	258
ENSECAG00000021486	5.834897402	0.000266613	0.002351907	1236	932	1682	1381	695	934	555	705
ENSECAG00000024974	6.129804884	0.00026868	0.002368301	2923	1125	1114	1058	771	530	752	797
ENSECAG00000010050	6.476559881	0.000271193	0.00238652	1083	4741	1203	1848	715	892	773	1295
ENSECAG00000006023	5.177939713	0.00027127	0.00238652	782	1263	623	625	398	436	543	325
ENSECAG00000008662	6.693162777	0.000271377	0.00238652	724	561	1642	1028	3142	3033	3625	2763
ENSECAG00000017587	6.990658803	0.000272263	0.002392458	2333	3737	2703	3083	1983	1345	1398	1553
ENSECAG00000000015	3.878874398	0.000273935	0.002405294	78	43	78	267	431	464	798.999	207
ENSECAG00000010961	6.06232566	0.00027489	0.002411817	1556	2218	1443	831	881	612	732	904
ENSECAG00000014868	7.002153603	0.00027994	0.002454227	2314	6149	1871	2091	1034	1603	1213	1296
ENSECAG00000022290	4.539539666	0.000280752	0.002459448	1257	513	174	167	226	123	107	201
ENSECAG00000005800	1.008473048	0.0002813	0.002462353	6	15	6	30	64	50	37	107
ENSECAG00000022786	6.31837911	0.000282032	0.00246686	3072	1800	1084	1140	708	874	807	878
ENSECAG00000020605	3.660502426	0.000283054	0.002473895	72	70	68	256	397	324	551	300
ENSECAG00000018769	4.331085265	0.000286	0.002496477	138	212	227	306	591	433	601	709
ENSECAG00000022498	7.949600884	0.000286077	0.002496477	2271	1307	2252	2809	8072	6277	10534	5874
ENSECAG00000000064	5.62784935	0.000286975	0.002502385	1504	1516	791	592	594	533	456	596
ENSECAG00000023010	5.581036365	0.000290673	0.002532691	1290	844	1178	894	677	521	601	652
ENSECAG00000013962	7.759665153	0.000291691	0.002539609	10052	7161	1378	1614	1617	1463	1740	1213
ENSECAG00000009872	5.488132931	0.000291948	0.002539901	1111	1588	518	855	483	512	447	635
ENSECAG00000009119	7.186569984	0.000293553	0.00255191	4291	6248	1355	1978	1288	1705	1045	1086
ENSECAG00000015249	3.702838502	0.000294951	0.002560493	67	122	166	190	348	279	460	461
ENSECAG00000012263	8.336016503	0.000295001	0.002560493	5958	10565	7799	6468	3688	4513	3137	3198
ENSECAG00000015262	6.000949983	0.000295216	0.002560493	910	2487	1163	1435	650	684	867	987
ENSECAG00000019671	8.857566782	0.000298617	0.002588012	16169	18622	3849	5143	3820	3581	4006	4090
ENSECAG00000003363	5.332100873	0.000300539	0.002600115	500	1003	995	1293	494	393	711	593
ENSECAG00000018578	1.530257704	0.000300668	0.002600115	12	25	16	42	71	55	114	121
ENSECAG00000011733	8.504649517	0.0003007	0.002600115	8427	21174	5277	3846	588	5186	783	1417
ENSECAG00000008020	2.332952187	0.000301007	0.002600792	142	162	81	84	36	48	46	72
ENSECAG00000021024	-0.162133506	0.000301646	0.002604332	1	1	3	15	19	21	21	50
ENSECAG00000012675	3.092099507	0.000302868	0.002612901	59	65	78	138	230	263	245	272
ENSECAG00000000436	9.944564387	0.000303785	0.00261882	23203	20780	58930	22433	18	6218	45	545
ENSECAG00000020122	10.7057102	0.000304615	0.002623981	62405	55666	15126	22839	14147	10517	12646	21491
ENSECAG00000012073	3.880539778	0.000305163	0.002626714	289	562	249	254	162	183	169	168
ENSECAG00000025069	0.426292505	0.000306162	0.002633317	31.0002	41.0006	46.0004	16.0007	4.00023	20.0003	7.00076	5.00044
ENSECAG00000019841	6.45073692	0.000308627	0.002652511	2398	2556	1404	1452	944	1038	1078	1023
ENSECAG00000016117	2.30953076	0.00030905	0.002654137	102	185	68	116	53	45	48	64
ENSECAG00000000521	6.547084345	0.000311051	0.002669308	641	734	708	1545	3401	3109	3200	1518
ENSECAG00000024627	5.866060789	0.000319844	0.002742689	1228	1843	1066	1144	648	572	552	1166
ENSECAG00000004845	4.010282515	0.000320132	0.002743094	80	75	219	273	459	501	371	632
ENSECAG00000022118	7.131537241	0.000322529	0.00276155	660	1814	168	379	3821	2888	4550	9030
ENSECAG00000023904	1.603221711	0.000325334	0.002783466	99	183	21	16	0	21	10	18
ENSECAG00000001845	2.824722334	0.000327139	0.002796803	121	46	308	284	68	78	54	73
ENSECAG00000023941	4.624457551	0.000328395	0.002805434	817	933	235	313	220	323	127	212
ENSECAG00000020076	6.765654882	0.000330498	0.002821279	3292	4365	1344	1104	876	1119	840	1285
ENSECAG00000017955	6.674682746	0.000331728	0.002829651	2804	2752	1649	2054	1106	1422	847	1330
ENSECAG00000017707	7.449539324	0.000334989	0.002855325	4929	8638	1494	2026	857	2055	1584	982
ENSECAG00000002370	2.121239777	0.000336383	0.002865056	57	385	15	29	11	23	3	32
ENSECAG00000005116	3.4089573	0.000339822	0.002891312	260	425	147	151	118	102	109	115
ENSECAG00000011333	3.300588203	0.000339975	0.002891312	155	320	230	224	107	141	79	141
ENSECAG00000023892	4.764735865	0.000340749	0.002895729	142	277	331	412	617	951	950	631
ENSECAG00000000733	3.866394114	0.000341583	0.002900643	129	136	146	195	422	317	479	484
ENSECAG00000022754	1.321450629	0.000344969	0.002927213	27	75	73	73	21	31	21	31
ENSECAG00000016674	6.151087819	0.00034547	0.002929272	1516	2233	1448	1161	832	948	805	955
ENSECAG00000017770	1.036354815	0.00034628	0.002933958	13	12	15	25	77	37	61	75
ENSECAG00000004044	2.454200658	0.000347457	0.002941738	35	34	37	92	194	108	148	231
ENSECAG00000008726	1.281353661	0.000347751	0.002942028	3	8	37	21	49	89	79	94
ENSECAG00000011535	3.934867304	0.000353025	0.0029826	125	175	140	183	527	301	442	533
ENSECAG00000018634	5.289807365	0.000353174	0.0029826	251	146	542	613	1029	1038	1649	1033
ENSECAG00000007601	4.822002597	0.00035354	0.0029826	775	1034	354	386	289	280	332	343
ENSECAG00000021177	3.656697228	0.000353596	0.0029826	63	131	157	174	394	257	354	501
ENSECAG00000022089	2.161227864	0.000354788	0.002987818	29	41	26	63	171	95	138	141
ENSECAG00000014135	3.880240325	0.000354967	0.002987818	107	107	176	190	543	201	489	561
ENSECAG00000009918	3.341839776	0.000355004	0.002987818	77	58	141	114	356	216	343	305
ENSECAG00000002072	4.485140391	0.000357715	0.003008411	145	241	178	386	648	414	694	915
ENSECAG00000009276	0.765375828	0.000358056	0.003009049	13	32	47	104	5	25	5	12
ENSECAG00000018529	7.598822169	0.000360961	0.003031223	1072	1428	1207	3416	7990	4457	8009	3821
ENSECAG00000012020	8.001331594	0.000365639	0.003068239	1184	2604	1942	4233	7875	8472	8681	6233
ENSECAG00000013287	0.816540323	0.000366526	0.003073412	26	70	59	32	6	29	3	6
ENSECAG00000012390	4.955252184	0.000369745	0.003098118	672	842	524	700	370	425	420	419

ENSECAG00000004303	0.792267576	0.000370297	0.003098579	2	14	11	16	38	28	32	133
ENSECAG00000015690	6.338026795	0.000370756	0.003098579	717	697	801	1189	2331	1832	3063	2231
ENSECAG00000018046	8.829092014	0.00037081	0.003098579	13531	13655	7936	8023	2969	7211	3860	3197
ENSECAG00000021238	3.675582458	0.000370891	0.003098579	255	586	268	129	96	190	101	84
ENSECAG00000018545	3.35945499	0.000371309	0.003099791	74	116	102	119	331	211	307	368
ENSECAG00000019276	6.616878363	0.000372995	0.003109723	849	871	1073	1135	3329	1706	2752	4079
ENSECAG00000015469	4.184180839	0.000373192	0.003109723	155	201	1046	606	197	177	233	98
ENSECAG00000008973	2.858126003	0.00037332	0.003109723	120	76	365	188	46	77	97	71
ENSECAG00000017644	6.952592826	0.000376814	0.003136525	2723	4580	2982	2866	323	2371	295	568
ENSECAG00000023476	6.296873376	0.000378887	0.003151475	1569	2886	1233	1549	765	1151	981	791
ENSECAG00000020958	1.001417474	0.000379563	0.003154789	12	8	16	27	74	37	73	60
ENSECAG00000014199	5.862676706	0.000382725	0.00317874	1401	1929	860	1008	629	663	616	920
ENSECAG00000010200	6.977734791	0.000388477	0.003224162	2497	6601	1497	1570	1096	1119	1185	1467
ENSECAG00000001555	1.866633213	0.0003889	0.003225315	13	12	33	7	140	90	263	12
ENSECAG00000010482	5.655077185	0.000391539	0.003244834	1206	1797	713	793	680	499	532	693
ENSECAG00000016537	2.46007416	0.000394617	0.003267959	32	48	70	48	166	109	147	257
ENSECAG00000008769	7.451893525	0.000395131	0.003269832	5273	4527	3519	2505	2018	1982	1722	1967
ENSECAG00000004400	3.896527982	0.000401308	0.003318533	558	626	80	144	94	122	154	82
ENSECAG00000016329	1.626395493	0.000406812	0.003361596	56	163	55	31	20	34	17	30
ENSECAG00000013515	2.753440768	0.000408637	0.003371501	125	359	99	86	50	89	32	58
ENSECAG00000016974	2.878273115	0.000408676	0.003371501	34	113	28	40	208	148	263	346
ENSECAG00000006445	3.052788202	0.000409133	0.003371501	45	55	100	121	191	286	314	199
ENSECAG00000001774	7.347840162	0.000409197	0.003371501	6335	5329	1542	1719	1178	1606	1503	1417
ENSECAG00000018810	3.67808822	0.00041035	0.003378548	67	108	119	213	423	232	269	663
ENSECAG00000004312	7.521443772	0.000411466	0.003385286	7956	6249	3418	1854	95	2233	126	127
ENSECAG00000017243	2.066059944	0.000415086	0.003410928	25	27	28	74	131	113	130	132
ENSECAG00000004523	6.482319236	0.000415183	0.003410928	779	712	856	1392	2871	1722	2599	3460
ENSECAG00000004960	6.555228879	0.00041646	0.003418625	216	1315	406	934	1505	5393	2259	2994
ENSECAG00000014314	2.902566496	0.000416722	0.003418625	21	68	30	131	247	118	225	395
ENSECAG00000011128	5.414832197	0.000419628	0.003439985	648	1067	1238	923	641	649	443	558
ENSECAG00000010657	5.100276833	0.000420037	0.003440854	283	259	382	600	910	809	997	1254
ENSECAG00000020711	4.663530458	0.000420852	0.003445046	163	197	348	387	699	476	997	810
ENSECAG00000021419	3.426299439	0.00042205	0.003452359	162	183	314	385	101	163	148	117
ENSECAG00000021171	5.525138427	0.000424245	0.00346782	608	1677	845	1115	562	546	631	680
ENSECAG00000021505	5.574457599	0.000424751	0.003469459	1238	1270	557	1212	629	603	438	674
ENSECAG00000021780	4.781377659	0.000427069	0.003485806	211	262	368	346	858	471	873	1013
ENSECAG00000011935	5.909524756	0.000427613	0.003485806	921	1638	1204	1670	862	744	753	991
ENSECAG00000008852	6.562490001	0.000427914	0.003485806	1428	1653	2904	3091	795	1434	1332	1293
ENSECAG00000007886	0.499379871	0.000428083	0.003485806	7	42	52	50	11	18	7	8
ENSECAG00000011807	8.476326436	0.000428286	0.003485806	9881	13216	5244	4715	3560	3835	3392	3602
ENSECAG00000017804	3.119232205	0.000432848	0.003520416	197	388	165	107	62	106	99	23
ENSECAG00000002320	5.006609317	0.000435889	0.003541613	432	380	1147	1199	366	400	420	477
ENSECAG00000013700	1.91116908	0.000436078	0.003541613	125	121	37	72	21	30	26	58
ENSECAG00000014514	8.737317689	0.000436919	0.003545906	7838	16696	8001	7938	5100	3914	4491	5751
ENSECAG00000017083	1.723475143	0.000443694	0.003598324	52	74	97	93	36	37	26	53
ENSECAG00000000632	2.746058681	0.000444616	0.003603225	149	276	151	66	60	89	47	54
ENSECAG00000024967	8.132988619	0.000445799	0.003608112	3340	7743	10171	8778	1380	5437	1559	2171
ENSECAG00000002831	3.023266996	0.000445854	0.003608112	254	229.998	137	136.998	49.9998	133	62	65
ENSECAG00000020304	7.50539883	0.000449574	0.003635626	4828	6565	2565	2748	2057	1812	2073	1823
ENSECAG00000017370	6.267452745	0.000453482	0.003664623	1593	1972	1966	1492	775	1388	770	843
ENSECAG00000021614	3.799366981	0.000457228	0.003692272	122	137	163	142	431	278	435	494
ENSECAG00000009524	7.564085387	0.000460345	0.003714798	4085	8469	2502	2844	2049	1511	1781	2585
ENSECAG00000016641	5.248439308	0.000460911	0.003716728	284	366	469	617	955	967	1174	1221
ENSECAG00000018530	1.424258495	0.0004652	0.00374712	18	17	20	37	73	55	79	120
ENSECAG00000024413	8.199490302	0.000465339	0.00374712	6413	8361	6714	5543	4117	2963	3503	3503
ENSECAG00000004860	3.395616952	0.000466087	0.003750483	123	291	357	280	85	192	102	83
ENSECAG00000013186	2.726982194	0.000468083	0.003763876	142	117	219	145	61	60	112	73
ENSECAG00000017019	0.972986296	0.000469698	0.003774195	11	9	15	29	56	52	43	87
ENSECAG00000003495	3.706195287	0.000474404	0.003809317	612	372	132	100	68	177	46	68
ENSECAG00000015317	2.185463733	0.00047578	0.003817663	50	99	121	200	44	68	32	52
ENSECAG00000014340	2.951974949	0.000479421	0.003843337	64.0034	57	77	108	218	191	248	257.994
ENSECAG00000006840	4.962858159	0.000479656	0.003843337	158	204	257	541	1015	1116	1355	321
ENSECAG00000017241	9.678625499	0.000483394	0.003870555	58747	2953	12128	2845	5824	2196	4210	775
ENSECAG00000008015	1.283479537	0.000485162	0.003880405	39	41	94	66	22	35	18	24
ENSECAG00000007393	0.593240273	0.000485307	0.003880405	25	33	36	58	6	17	23	7
ENSECAG00000014368	6.65763203	0.000486453	0.003886836	591	922	1206	1683	3258	2260	2954	3445
ENSECAG00000004162	8.700461481	0.000489471	0.0039056	20368	14169	2339	1882	2655	2145	2768	3245
ENSECAG00000008866	6.977142257	0.000489489	0.0039056	672	1292	1258	1239	4430	5636	3626	1642
ENSECAG00000009456	4.28196607	0.000497779	0.003968956	825	602	148	195	183	96	128	268
ENSECAG00000002482	4.283005189	0.00050178	0.003998057	440	928	174	291	194	167	245	178
ENSECAG00000014974	6.464470758	0.000502575	0.00400158	2557	3333	1042	1124	772	1141	845	858
ENSECAG00000009444	2.937152878	0.000503082	0.004002058	90	91	304	372	41	134	54	45
ENSECAG00000012393	6.986943891	0.000503339	0.004002058	3927	5511	1451	984	1480	1029	1019	956
ENSECAG00000008187	4.026948302	0.000505393	0.00401558	65	87	265	226	641	280	463	639
ENSECAG00000006337	4.920424614	0.000506907	0.00402479	554	966	840	380	441	365	315	379
ENSECAG00000020179	4.266053042	0.000514597	0.004083	390	608	407	312	224	279	212	248
ENSECAG00000000543	1.885341276	0.000522084	0.004138883	132	59	96	68	15	60	18	30
ENSECAG00000022369	7.653099442	0.000522369	0.004138883	5244	5748	4484	2890	2503	2349	2386	1759
ENSECAG00000014780	6.764339985	0.000522925	0.004140398	1820	2021	3449	2802	1656	1197	1324	1602
ENSECAG00000007419	3.857013388	0.000525299	0.0041563	93	120	123	265	368	443	568	319
ENSECAG00000024511	5.080290351	0.000528005	0.004174805	219	363	242	662	881	983	931	1141
ENSECAG00000017371	1.16376377	0.000528794	0.004178137	7	26	95	141	10	19	16	20
ENSECAG00000023364	6.094021244	0.000531211	0.004194318	955	2144	1706	1474	704	1068	861	938
ENSECAG00000024489	2.195108238	0.000534336	0.00421606	30	31	61	39	174	150	119	105
ENSECAG00000021043	3.821039816	0.00053754	0.004238394	372	455	304	142	178	135	156	158

ENSECAG00000023865	6.508093771	0.000540853	0.004261564	2000	2115	1785	2173	1378	1120	1078	1279
ENSECAG00000018516	0.717067714	0.000541278	0.004261719	6.00006	10	14.0001	18.0006	23	73.0001	59.0001	42.0001
ENSECAG00000014883	5.405260951	0.000541623	0.004261719	461	336	375	577	1356	831	1227	1570
ENSECAG00000017582	2.899608458	0.000545885	0.004292281	66	71	44	90	184	163	259	304
ENSECAG00000022638	2.95476056	0.000550736	0.004327286	209	367	86	90	41	106	32	74
ENSECAG00000021098	5.407184344	0.000551099	0.004327286	252	331	313	956	1042	1147	1422	1412
ENSECAG00000015379	4.5933737	0.000553602	0.004343942	1057	733	326	204	47	378	67	127
ENSECAG00000010080	5.018698876	0.000554791	0.004350268	765	984	460	656	507	330	360	450
ENSECAG00000009453	2.986839074	0.000559546	0.004384528	60	56	106	92	227	183	255	275
ENSECAG00000017223	5.434747026	0.00056021	0.004386701	2791	262	397	399	362	209	230	366
ENSECAG00000020102	6.142901834	0.000561367	0.004390921	1395	1717	1359	1823	828	1073	1007	907
ENSECAG00000009631	4.442402702	0.000561522	0.004390921	395	792	469	329	199	345	235	259
ENSECAG00000015394	1.731967967	0.000563626	0.004404349	9	28	17	61	108	83	89	134
ENSECAG00000003759	7.324892332	0.000570937	0.00445841	4326	6761	1998	1782	1681	1421	1699	1594
ENSECAG00000018533	1.846000007	0.000575606	0.004491781	10	22	34	54	58	153	143	88
ENSECAG00000021595	4.013523695	0.000576129	0.004492776	110	138	175	255	479	250	493	716
ENSECAG00000019222	1.201468615	0.00057725	0.004498436	39	59	41	98	16	38	19	8
ENSECAG00000016973	1.675369116	0.000578668	0.004503118	10	24	8	11	52	27	344	28
ENSECAG00000023853	5.927599514	0.000579025	0.004503118	370	557	765	1112	1670	1596	1645	2073
ENSECAG00000004973	8.185511295	0.00057904	0.004503118	621	2669	2097	5080	5427	7810	10843	13929
ENSECAG00000014936	2.930945609	0.000580878	0.004514322	51	69	79	109	256	155	255	235
ENSECAG00000017584	6.043853106	0.000585675	0.004548486	357	635	851	1159	1731	1691	2088	2109
ENSECAG00000010731	4.862365335	0.000591934	0.004593957	1313	732	211	343	244	182	204	424
ENSECAG00000013162	6.213805026	0.000593379	0.004602025	453	521	1145	1090	2562	1358	2398	2486
ENSECAG00000022992	9.375252759	0.000594375	0.004606606	11509	32368	17041	14712	1042	10336	1848	371
ENSECAG00000015563	7.113121519	0.000595126	0.004609285	1181	1146	1260	2131	4116	3470	3971	4896
ENSECAG00000010930	6.683329406	0.000601432	0.004654952	1876	2298	2017	3271	1251	1532	1198	1480
ENSECAG00000009653	3.686954908	0.000604005	0.004671686	208	351	370	324	78	257	131	102
ENSECAG00000020262	5.776875077	0.000608279	0.004701544	1152	1963	929	837	617	691	752	652
ENSECAG00000000158	2.102100833	0.000610233	0.004713438	16	30	51	42	127	153	196	52
ENSECAG00000014279	5.372182997	0.000612361	0.004726659	333	326	471	733	1040	979	1085	1690
ENSECAG00000008401	7.337239895	0.000613416	0.004731588	2096	4857	4400	4083	1812	2391	1941	1894
ENSECAG00000016139	2.312988069	0.000615233	0.004742383	36	28	37	83	153	100	165	188
ENSECAG00000005295	1.833635788	0.00061639	0.004748087	75	186	44	45	21	47	26	21
ENSECAG00000019862	5.423132495	0.000617725	0.004753292	373	255	485	709	1178	764	1353	1821
ENSECAG00000019165	2.033500345	0.000617903	0.004753292	33	24	34	53	125	77	109	193
ENSECAG00000023901	4.631564951	0.00061987	0.0047652	225	288	272	287	689	594	805	733
ENSECAG00000017012	7.269575149	0.000620627	0.004767259	4619	3844	2163	3219	1918	1686	1626	1923
ENSECAG00000009618	3.311990638	0.000620977	0.004767259	43	57	167	95	194	391	220	422
ENSECAG00000016587	4.382253035	0.000623335	0.004782129	130	260	163	338	479	441	705	839
ENSECAG00000010966	4.115021514	0.000624889	0.004790815	101	143	167	347	492	341	572	644
ENSECAG00000024946	2.70263647	0.000630153	0.004827915	137	329	109	51	48	68	56	62
ENSECAG00000008809	2.997637584	0.000633666	0.004851559	20	111	45	37	174	523	173	152
ENSECAG00000009347	7.508417928	0.000634928	0.004857949	6816	4206	3827	1675	1977	1868	2012	1056
ENSECAG00000023113	2.239346765	0.000637606	0.004875152	32	32	49	69	161	102	126	175
ENSECAG00000000007	5.440365561	0.000641295	0.004900063	1398	1645	514	354	468	430	387	439
ENSECAG00000014674	3.520476022	0.000642119	0.004903059	96	43	111	180	337	198	408	481
ENSECAG00000024062	4.735853875	0.000643646	0.004911423	172	180	197	572	758	769.002	516	1166
ENSECAG00000020165	2.489491797	0.000644357	0.004913549	10	19	69	87	190	109	282	145
ENSECAG00000021363	1.797346482	0.000645829	0.004921471	43	51	203	65	21	41	26	42
ENSECAG00000023827	-0.086040271	0.000649831	0.004948647	3	7	11	4	27	29	25	27
ENSECAG00000020819	2.205798443	0.00065453	0.004978061	53	270	78	66	29	28	34	74
ENSECAG00000019746	3.518289552	0.00065457	0.004978061	57	119	93	157	487	458	158	279
ENSECAG00000007003	4.252751	0.000655043	0.00497833	135	177	250	288	674	432	560	509
ENSECAG00000008178	3.327755348	0.000659107	0.005005863	171	196	186	417	162	127	110	96
ENSECAG00000014428	5.025047103	0.000665984	0.005054714	241	395	320	498	772	702	1138	1126
ENSECAG00000022051	10.77382857	0.000666459	0.005054948	68350	29198	26631	33616	16245	18692	17164	20162
ENSECAG00000020022	3.443515305	0.000671377	0.00508885	81	89	129	147	420	229	364	260
ENSECAG00000007534	6.387823056	0.000672822	0.005096404	1310	3425	1233	1850	766	1223	936	1106
ENSECAG00000017618	8.827523388	0.000679678	0.005144904	10392	13460	10044	8524	3982	7355	4066	4845
ENSECAG00000012220	6.431431886	0.000681921	0.005158449	1692	3144	1551	1455	918	1331	816	1097
ENSECAG00000025003	1.289624543	0.000682895	0.00516238	23	45	41	168	12	34	20	16
ENSECAG00000006182	3.209010529	0.000687018	0.005190095	74	85	84	132	251	185	282	380
ENSECAG00000005196	0.765516014	0.000689527	0.005205586	7	43	110	34	1	20	8	11
ENSECAG00000014186	4.913530079	0.000691647	0.005218125	73	89	559	321	718	1056	1402	664
ENSECAG00000024392	1.452449156	0.000692615	0.005221958	18	20	28	29	99	45	93	92
ENSECAG00000018970	6.197441278	0.000693586	0.005225814	343	601	754	1409	2280	2075	3010	1328
ENSECAG00000013712	5.535495329	0.000699219	0.005264759	1402	2561	369	1177	1	298	0	33
ENSECAG00000016067	5.177573098	0.000701466	0.005278183	388	256	412	440	1143	802	1331	919
ENSECAG00000014460	8.003862436	0.000704204	0.005294355	7901	9172	3128	3247	2133	2421	2368	3574
ENSECAG00000009355	2.993728886	0.000704547	0.005294355	50	69	114	85	231	159	272	286
ENSECAG00000022075	7.763834927	0.000708942	0.005323856	6105	7841	2595	3294	2400	2140	2202	2562
ENSECAG00000023974	5.643576796	0.00070999	0.00532821	764	1157	1220	1277	676	726	708	790
ENSECAG00000020935	6.835614608	0.000714478	0.005358352	941	1042	1400	1442	2883	2788	3690	4038
ENSECAG00000011924	4.464214608	0.000718236	0.005382978	201	180	306	278	677	509	690	639
ENSECAG00000017522	2.08966425	0.000723093	0.005415809	190	153	38	23	22	11	20	61
ENSECAG00000024077	2.409542197	0.000735641	0.005504067	74	173	205	82	45	90	36	29
ENSECAG00000026827	3.581119297	0.000735846	0.005504067	78	132	95	180	497	196	332	407
ENSECAG00000024930	4.34382112	0.000738577	0.005519453	158	226	236	293	501	514	558	733
ENSECAG00000013038	7.413222361	0.000738874	0.005519453	3096	6912	3064	2957	1576	2489	1896	1648
ENSECAG00000021678	6.647866611	0.000740298	0.005526459	2555	2886	1598	1813	1280	1130	1233	1338
ENSECAG00000017676	1.333256617	0.000742221	0.005537176	4	9	27	33	27	58	128	115
ENSECAG00000008177	1.561095853	0.000750061	0.005591753	52	214	69	0	8	20	1	3
ENSECAG00000026880	2.970007294	0.000750521	0.005591753	159	317	119	128	92	77	84	97
ENSECAG00000011419	4.997041575	0.000752904	0.005605829	284	230	372	527	989	595	951	1167

ENSECAG00000014764	1.350171245	0.000758044	0.005639191	21	15	22	26	80	50	108	68
ENSECAG00000018443	3.523302122	0.000758377	0.005639191	84	80	106	218	316	308	296	431
ENSECAG00000017893	5.455033294	0.000761898	0.005661666	879	1300	683	987	538	513	671	662
ENSECAG00000019488	3.407623958	0.000762922	0.00566557	157	177	311	463	59	216	83	82
ENSECAG00000022594	1.412399315	0.000765287	0.005678591	9	13	35	34	49	87	107	79
ENSECAG00000006895	2.543641821	0.000765675	0.005678591	45	120	234	243	51	16	52	113
ENSECAG00000007763	5.772670443	0.000768822	0.005698207	366	399	704	1047	1376	1417	1765	1707
ENSECAG00000023331	4.874847547	0.000770653	0.005705827	260	243	349	475	875	673	728	1069
ENSECAG00000019619	6.075084597	0.000770854	0.005705827	1189	2354	1262	1227	809	923	814	952
ENSECAG00000014658	1.053255372	0.00077186	0.005708821	11	15	24	17	61	53	36	102
ENSECAG00000023101	1.461485717	0.000772263	0.005708821	53	79	48	76	37	30	26	31
ENSECAG00000008799	2.847143212	0.000778143	0.005748547	74	35	60	87	265	144	240	228
ENSECAG00000009768	1.783345137	0.000779221	0.005752766	43	124	129	116	0	51	3	6
ENSECAG00000014855	5.426327516	0.000788691	0.005815483	379	2229	791	850	404	630	461	356
ENSECAG00000017141	5.178465293	0.00078874	0.005815483	231	307	359	731	1313	972	1056	816
ENSECAG00000011464	6.040424208	0.000789672	0.00581858	619	536	621	933	2594	1483	2321	1283
ENSECAG00000021076	4.286350276	0.0007928	0.005837843	138	156	220	363	502	581	629	507
ENSECAG00000006306	4.379013291	0.000794422	0.005846	120	163	179	464	668	514	572	682
ENSECAG00000011528	6.51519246	0.000797309	0.005860011	3217	2870	1181	841	926	831	1068	923
ENSECAG00000024475	6.175134093	0.000797358	0.005860011	502	480	880	1345	1980	2167	2393	1793
ENSECAG00000022424	1.349366875	0.000803268	0.00589963	112	28	45	56	12	33	24	10
ENSECAG00000006244	5.337776529	0.00080941	0.0059409	929	800	1044	742	658	450	584	497
ENSECAG00000008759	0.414494197	0.000812947	0.005963013	12	48	52	22	8	19	5	11
ENSECAG00000017880	4.598829653	0.000816475	0.005985024	228	172	347	288	788	503	717	796
ENSECAG00000007597	0.432586919	0.000818762	0.005997923	2	11	11	17	38	31	60	31
ENSECAG00000009654	4.043462024	0.000819624	0.005998087	152	164	185	205	509	339	548	495
ENSECAG00000017017	5.575359415	0.00082021	0.005998087	208	378	373	1024	1370	1567	1960	784
ENSECAG00000000268	2.304720398	0.000820369	0.005998087	46	36	38	61	160	128	114	189
ENSECAG00000024482	3.221448536	0.000820914	0.005998215	199	254	300	325	4	157	8	27
ENSECAG00000016019	6.493844989	0.000822507	0.006005994	2530	2625	1477	1260	1172	970	951	1246
ENSECAG00000024215	1.374704016	0.000828038	0.006042494	20	9	21	35	106	47	69	96
ENSECAG00000016922	6.917311569	0.000836074	0.00609722	6254	1063	2347	1096	1220	958	818	1126
ENSECAG00000017930	4.944870611	0.000841472	0.006132654	552	939	641	570	367	446	410	449
ENSECAG00000014229	11.04661066	0.000858573	0.006253271	9236	10704	36396	19358	55829	85323	75227	46456
ENSECAG00000019606	4.047360795	0.000871293	0.00634185	258	251	417	634	231	175	279	209
ENSECAG00000026832	0.851589046	0.000873694	0.006355254	15	3	19	14	62	32	60	69
ENSECAG00000014934	2.79019986	0.000878429	0.006385611	45	65	79	99	202	179	212	208
ENSECAG00000005344	4.570191062	0.000880077	0.006393504	97	151	287	478	722	371	864	941
ENSECAG00000020412	1.198628948	0.000883115	0.006411472	13	13	8	40	91	41	83	67
ENSECAG00000007179	5.581242564	0.000886576	0.006432491	545	397	505	508	1201	939	1481	2018
ENSECAG00000020583	5.393465442	0.000890449	0.006456465	1013	440	1581	1116	636	557	416	139
ENSECAG00000004544	1.324895455	0.000915539	0.006634154	48	165	29	11	8	23	16	18
ENSECAG00000017383	5.930824669	0.000923172	0.006683076	2181	649	1618	1017	925	515	779	481
ENSECAG00000008121	2.425592378	0.000923467	0.006683076	34	46	57	66	130	91	237	191
ENSECAG00000012539	3.427786	0.000925556	0.006693935	70	76	44	215	358	268	446	244
ENSECAG00000020919	0.80963513	0.000927319	0.006699636	11	14	16	16	58	29	57	64
ENSECAG00000011638	7.694625417	0.000927766	0.006699636	4335	10750	2418	2183	1727	2002	2326	2053
ENSECAG00000008273	6.554584924	0.000928114	0.006699636	2360	2579	1605	1657	1121	1198	1161	1329
ENSECAG00000016217	8.906642165	0.000940447	0.006784353	3461	3609	4503	7901	15214	15956	13510	12440
ENSECAG00000012233	2.413827048	0.000943506	0.006796599	26	40	32	101	145	99	159	261
ENSECAG00000022504	3.613910828	0.000943922	0.006796599	228	697	155	198	24	217	32	67
ENSECAG00000022190	6.527579951	0.000943939	0.006796599	2775	2397	1279	1579	1157	1024	1114	1179
ENSECAG00000011890	10.57250688	0.000950381	0.006838648	34640	68048	20879	18634	15131	15024	16077	17158
ENSECAG00000012169	5.210013601	0.000961744	0.006916036	433	1114	725	1161	396	572	483	578
ENSECAG00000008283	1.358253904	0.000963057	0.006921095	8	20	31	31	73	103	67	59
ENSECAG00000014055	4.386068098	0.000963923	0.006922939	207	192	208	303	604	407	655	736
ENSECAG00000026820	3.955482694	0.000970866	0.0069684	276	111	994	286	46	237	102	146
ENSECAG00000015612	1.254660314	0.000974971	0.006993447	21	114	30	86	8	27	13	34
ENSECAG00000019444	0.526988091	0.000990433	0.007099871	5	12	7	16	21	35	31	93
ENSECAG00000024277	5.541088414	0.000995113	0.007128921	459	464	435	706	1499	929	1234	1669
ENSECAG00000019738	7.771463843	0.001004069	0.007188179	4510	7820	3705	3911	2570	2256	3099	2548
ENSECAG00000019133	3.633029689	0.00100465	0.007188179	184	435	309	213	133	197	129	151
ENSECAG00000018481	0.318238833	0.001007008	0.007200519	6	9	9	16	34	32	37	40
ENSECAG00000009612	4.330915798	0.001008091	0.007203733	395	389	567	428	343	228	304	224
ENSECAG00000011651	1.370888267	0.001016346	0.007258156	68	106	43	24	20	28	28	16
ENSECAG00000020081	3.698240839	0.001021561	0.007290811	202	247	319	419	205	156	190	167
ENSECAG00000008221	9.010854142	0.001025056	0.007311165	6149	14739	18108	13008	3723	9384	5228	4182
ENSECAG00000021398	8.096594018	0.001028822	0.007333425	3720	11079	5390	5886	2885	3821	2865	3361
ENSECAG00000013451	3.617883738	0.001029548	0.007333998	221	177	439	367	249	114	133	68
ENSECAG00000015040	6.18565283	0.001036477	0.007374884	554	631	1015	1128	2172	1555	2025	2583
ENSECAG00000012738	8.588343876	0.001036586	0.007374884	9666	12967	5827	6471	4185	4487	4048	4950
ENSECAG00000015341	1.468117819	0.001037682	0.007377212	84	99	30	48	40	8	30	20
ENSECAG00000021212	1.797145101	0.001038212	0.007377212	55	54	85	154	28	53	42	29
ENSECAG00000017662	4.036439072	0.001046364	0.007428098	325	412	1171	107	1	142	0	19
ENSECAG00000009356	6.547897372	0.001046681	0.007428098	1446	3331	1970	1795	990	1405	916	1486
ENSECAG00000009646	3.747723224	0.001048176	0.007434065	112	323	431	541	207	156	173	55
ENSECAG00000019885	7.389869353	0.001052694	0.007461454	2926	7947	2750	2195	1649	2112	1441	2059
ENSECAG00000017077	8.56751682	0.001058616	0.007498753	9655	15263	4637	5398	3588	4203	4421	3819
ENSECAG00000015723	4.49269061	0.001068349	0.007561164	344	616	336	775	360	280	230	360
ENSECAG00000022063	6.016173763	0.001068758	0.007561164	1397	2348	1498	583	920	664	662	797
ENSECAG00000001119	8.895427614	0.001069687	0.007563026	6542	5538	13534	24296	6466	5247	5536	4979
ENSECAG00000004286	3.850262208	0.001072919	0.007581159	106	102	207	182	484	233	356	656
ENSECAG00000016820	3.149103714	0.001076617	0.007602564	73	161	240	476	69	69	97	174
ENSECAG00000016002	2.86389325	0.00108335	0.007645356	59	40	90	93	247	183	268	161
ENSECAG00000007182	2.61133569	0.001093966	0.007713118	29	41	86	91	214	129	175	211

ENSECAG00000010990	7.850789375	0.00109431	0.007713118	4541	13843	3254	3165	249	3891	467	730
ENSECAG00000011552	4.644768358	0.001098837	0.00774023	249	217	290	345	750	494	827	779
ENSECAG00000022492	6.509930364	0.001110296	0.007816096	1034	815	850	1016	2675	1974	2773	3255
ENSECAG00000022664	7.923903236	0.001112583	0.007827349	8144	7151	3260	3083	2494	2945	2305	2499
ENSECAG00000010872	3.873188694	0.001114894	0.007835452	277	420	293	297	185	204	172	229
ENSECAG00000012182	5.57362246	0.001115801	0.007835452	1644	1050	610	774	494	512	648	627
ENSECAG00000002387	4.997423577	0.001115804	0.007835452	746	1440	407	333	221	334	284	540
ENSECAG00000006253	3.989244963	0.001116907	0.007838358	146	64	236	111	652	241	454	643
ENSECAG00000014582	4.323741189	0.001122167	0.007867945	176	146	286	272	714	451	575	542
ENSECAG00000019855	4.110213567	0.001122508	0.007867945	749	332	195	221	203	121	160	229
ENSECAG00000012080	6.298163374	0.001135395	0.007950801	817	799	822	959	2195	1748	2364	2721
ENSECAG00000022971	7.149407542	0.001135729	0.007950801	3820	5171	1441	2527	1344	1731	1799	1286
ENSECAG00000019232	3.987430237	0.001139306	0.00797093	169	137	133	120	500	196	491	794
ENSECAG00000025055	7.458075884	0.001142542	0.007988656	2831	5416	4027	3825	1959	2277	2175	2758
ENSECAG00000022924	6.925626894	0.001148026	0.008022063	797	872	1536	2112	3814	2679	3870	3961
ENSECAG00000018562	4.99669681	0.001148943	0.008023531	332	171	311	404	1439	614	1206	560
ENSECAG00000017865	3.868461334	0.001157537	0.008078585	124	94	218	115	424	206	483	685
ENSECAG00000017625	3.443677004	0.001159988	0.008090718	247	118	199	508	145	128	72	165
ENSECAG00000010611	3.232611858	0.001168014	0.008137128	299	137	260	144	92	133	88	110
ENSECAG00000007681	7.224768885	0.001168074	0.008137128	1397	1413	1782	1268	4245	2966	4752	6057
ENSECAG00000002238	1.326466884	0.001174396	0.008169433	21	17	16	31	101	43	78	78
ENSECAG00000017459	5.112710256	0.001174787	0.008169433	756	783	864	680	293	688	364	419
ENSECAG00000002652	3.410318766	0.001174868	0.008169433	61	96	147	159	242	257	370	363
ENSECAG00000018111	5.304032246	0.00117906	0.008193562	566	1699	537	887	327	627	481	469
ENSECAG00000009055	4.617127115	0.001180586	0.008199156	219	211	291	407	757	567	678	756
ENSECAG00000014752	5.721106417	0.001182041	0.008204246	813	1595	1018	1213	669	692	802	841
ENSECAG00000006723	1.552336981	0.001185072	0.00822026	13	26	25	28	68	168	41	76
ENSECAG00000026969	2.94299616	0.0011865	0.00822028	55	101	203	507	61	87	90	83
ENSECAG00000017217	3.214690041	0.001186522	0.00822028	671	127	15	29	66	46	17	36
ENSECAG00000016494	6.690479019	0.001189964	0.008239108	3466	3539	1048	1118	1102	1040	1055	1098
ENSECAG00000012784	2.507344167	0.00119185	0.00824714	49	29	51	87	185	110	204	183
ENSECAG00000021270	4.808730712	0.001193048	0.008250404	495	648	702	601	410	422	422	325
ENSECAG00000013414	1.840556136	0.001199464	0.008289727	19	37	42	40	88	83	118	132
ENSECAG00000014116	3.942107145	0.001206184	0.008331102	118	106	171	293	411	383	471	499
ENSECAG00000015037	1.413411866	0.001209632	0.008349841	27	193	24	32	13	28	9	25
ENSECAG00000013524	0.876448422	0.001214619	0.008376547	21	24	88	55	10	22	11	27
ENSECAG00000023916	5.08494257	0.001214975	0.008376547	593	757	713	904	477	552	434	494
ENSECAG00000007863	2.209357283	0.001231615	0.008486116	36	36	48	52	185	83	169	112
ENSECAG00000009926	6.805024145	0.001244116	0.008567059	3335	2404	2338	1665	1398	1458	1125	1468
ENSECAG00000009799	4.971348952	0.001245008	0.008568007	938	154	1302	668	346	139	476	323
ENSECAG00000000859	6.882635442	0.001248524	0.008587007	1199	2842	3772	3547	1614	1204	1852	1596
ENSECAG00000014216	7.351503236	0.001253927	0.00861895	3746	6009	2679	2452	1377	2384	1486	2206
ENSECAG00000019946	6.061287304	0.001256307	0.008627067	1537	1812	1071	1350	849	879	927	943
ENSECAG00000025023	6.738763174	0.001256627	0.008627067	2814	4192	1395	1245	986	1452	1138	1027
ENSECAG00000015151	4.872897967	0.001289932	0.00885037	433	796	595	774	413	303	519	456
ENSECAG00000013944	5.877889371	0.001294032	0.008873141	819	2318	981	1210	715	662	870	861
ENSECAG00000026842	1.392878273	0.001309083	0.008970929	54	53	113	50	6	51	10	15
ENSECAG00000013998	4.499110471	0.001311214	0.00898012	166	225	184	421	869	455	472	842
ENSECAG00000015745	6.213091217	0.001312789	0.00898549	462	517	588	1633	2065	2363	2794	1463
ENSECAG00000016959	6.834815055	0.001319839	0.009028306	2338	3803	2290	1757	1666	1057	1511	1545
ENSECAG00000013398	5.638037197	0.001335895	0.009132634	541	1073	1565	1386	752	672	640	813
ENSECAG00000007486	3.53168777	0.001336808	0.009133378	103	121	127	141	299	247	341	447
ENSECAG00000015355	7.753272438	0.001340455	0.009152793	2985	4801	6063	6454	3410	2605	2756	2748
ENSECAG00000026017	1.325373805	0.001341479	0.009154285	34	106	41	76	11	48	11	14
ENSECAG00000020462	7.47752752	0.001343094	0.009159802	3751	6834	3067	2612	1872	2206	2466	1688
ENSECAG00000021756	2.523835712	0.001353455	0.00922018	56	34	34	88	139	165	158	230
ENSECAG00000018569	5.961782375	0.00135357	0.00922018	423	568	857	1108	1621	1631	1704	2058
ENSECAG00000005317	0.627967799	0.001373145	0.009347915	10	12	10	16	38	25	43	80
ENSECAG00000021704	5.290796412	0.001376279	0.009363639	502	1000	752	1305	474	588	574	614
ENSECAG00000015074	3.525913676	0.001403211	0.009541166	224	421	193	198	105	162	123	174
ENSECAG00000018369	3.484270961	0.001406529	0.009558006	72	162	439	545	100	173	118	93
ENSECAG00000022599	3.298836798	0.001408868	0.009563619	73	93	127	121	290	175	279	411
ENSECAG00000024254	0.763587453	0.001409039	0.009563619	28	11	87	67	22	15	3	0
ENSECAG00000026027	0.962363817	0.001410545	0.009568128	16	35	58	95	19	21	9	29
ENSECAG00000015678	6.722871771	0.001414575	0.009589741	2386	4363	1326	1459	1127	994	1359	1427
ENSECAG00000012681	2.914709576	0.001422625	0.00963856	100	152	277	205	113	45	65	148
ENSECAG00000007444	6.190361491	0.001424625	0.009646359	1241	3101	1171	1091	1041	683	864	1048
ENSECAG00000018806	5.321293871	0.00142646	0.009653033	318	308	486	765	1111	935	1270	1188
ENSECAG00000011978	2.150632703	0.001432654	0.009689177	26	35	58	55	150	80	117	182
ENSECAG00000010180	3.70548489	0.001433705	0.009690513	295	301	253	301	149	116	157	280
ENSECAG00000011392	4.027814676	0.001434666	0.009691248	97	183	160	156	298	255	356	1182
ENSECAG00000016912	7.779222308	0.001436674	0.009699045	9498	4147	2642	2843	2492	1790	2093	2620
ENSECAG00000020608	2.27987817	0.001442806	0.009733941	35	54	45	54	138	94	139	205
ENSECAG00000008981	4.525275719	0.001443837	0.009733941	147	265	269	360	514	651	890	542
ENSECAG00000011735	1.833478273	0.001444413	0.009733941	33	100	68	197	13	63	10	35
ENSECAG00000016978	3.824819832	0.001458657	0.009824104	12	31	177	112	53	189	1117	702
ENSECAG00000019037	1.297162046	0.001464369	0.009856729	109	74	18	21	27	13	18	20
ENSECAG00000010948	7.142519453	0.001465513	0.009858588	4335	3422	2451	2177	2260	1333	1539	1567
ENSECAG00000012089	9.905079674	0.001467772	0.009867942	13423	25056	21797	30704	12852	12963	11043	13086
ENSECAG00000021451	6.037560933	0.001470098	0.009877735	1187	2821	794	1149	684	755	815	973
ENSECAG00000011122	5.077733177	0.00147489	0.009904076	406	261	332	428	1109	720	999	1044
ENSECAG00000015198	5.053986115	0.001479824	0.00993134	224	297	443	567	1126	815	1128	670
ENSECAG00000011870	2.532515239	0.001487838	0.00997923	33	65	46	86	159	100	210	222
ENSECAG00000009473	6.927758467	0.001509359	0.010117606	2565	3765	1822	2799	1290	1810	1600	1447
ENSECAG00000005419	0.872892065	0.001521908	0.010194492	27.0071	115	11	34	18	3	22	9

ENSECAG00000020861	2.990351789	0.001522624	0.010194492	62	87	62	68	270	118	410	172
ENSECAG00000020708	2.746549955	0.001529184	0.010232383	45	52	72	105	204	118	272	197
ENSECAG00000012474	5.472994186	0.001530132	0.010232704	199	231	702	801	1067	786	1714	1801
ENSECAG00000010748	3.695108657	0.001543296	0.010314664	225	504	278	168	148	100	164	225
ENSECAG00000011157	4.185480563	0.00157117	0.010494788	152	152	201	329	517	384	585	574
ENSECAG00000016585	6.364835522	0.001578045	0.010534519	1612	2531	1274	1803	1192	1009	1211	1067
ENSECAG00000019685	5.667600714	0.001585656	0.01057911	755	1008	1271	1430	767	664	703	937
ENSECAG00000012830	3.199240807	0.001595048	0.010635523	91	59	98	107	177	239	400	267
ENSECAG00000008643	2.715117776	0.001597141	0.010643237	20	46	75	85	92	119	159	494
ENSECAG00000018383	5.512982365	0.001620513	0.010782249	424	436	527	680	1206	828	1226	1981
ENSECAG00000010036	6.295590821	0.001620575	0.010782249	2473	2181	939	1371	569	1389	711	827
ENSECAG00000019220	2.53105551	0.001620849	0.010782249	23	18	43	122	206	183	236	90
ENSECAG00000001584	2.963050196	0.001631573	0.010847238	34	113	62	51	169	126	200	515
ENSECAG00000023814	5.443557947	0.001638732	0.010888466	469	285	503	667	1350	915	1285	1419
ENSECAG00000013142	3.832812319	0.001654978	0.010989982	134	116	168	202	393	299	529	403
ENSECAG00000014323	3.146184344	0.001657873	0.010996545	52	41	103	173	258	235	205	360
ENSECAG00000010371	0.463610457	0.001657902	0.010996545	4	11	18	11	52	35	45	27
ENSECAG00000000264	0.949788523	0.001659509	0.011000785	8	8	34	12	60	38	72	63
ENSECAG00000018736	2.851414527	0.001665409	0.011029172	204	126	155	149	75	91	95	95
ENSECAG00000016911	6.030228326	0.001665733	0.011029172	1805	2403	1184	564	670	640	1082	291
ENSECAG00000016847	6.151056806	0.00166988	0.011050188	610	708	667	1295	1926	1702	2331	2050
ENSECAG00000022900	0.658283405	0.001671816	0.011054477	7	13	11	18	20	73	39	53
ENSECAG00000003448	6.629652714	0.001672474	0.011054477	2445	3043	1592	1528	1115	1148	1303	1488
ENSECAG00000017336	5.934298901	0.001673622	0.011055635	1492	1759	1017	956	790	807	791	852
ENSECAG00000007476	3.40715599	0.001680486	0.011094529	505	169	101	157	124	127	66	75
ENSECAG00000012416	1.630752303	0.00168962	0.011148353	34	19	15	19	161	71	46	110
ENSECAG00000023172	8.109319506	0.001699809	0.011203723	5521	7964	5527	6358	2752	4949	2566	3257
ENSECAG00000010009	4.965647019	0.001700006	0.011203723	310	322	349	449	783	653	1030	1033
ENSECAG00000006714	2.932729898	0.00170097	0.011203723	61	52	529	223	95	53	87	29
ENSECAG00000001926	5.439906673	0.001710972	0.011257615	317	354	638	606	1647	956	1588	767
ENSECAG00000010840	0.557458225	0.001711134	0.011257615	10	18	9	5	47	44	38	44
ENSECAG00000015796	3.669673118	0.001722532	0.011326047	125	107	106	200	451	248	394	377
ENSECAG00000017805	6.021489635	0.001728451	0.01135839	647	609	754	957	2002	1515	1860	1893
ENSECAG00000015318	1.940102233	0.001733451	0.011384666	40	9	39	30	170	88	123	93
ENSECAG00000005266	5.710132272	0.001735085	0.011388812	2352	957	536	601	582	510	575	521
ENSECAG00000007993	6.708832866	0.001756875	0.01152518	2212	4263	1534	1384	1225	1187	1417	1126
ENSECAG00000016852	6.424739659	0.001763598	0.01156261	1836	1131	3381	1454	1278	911	905	1376
ENSECAG00000024886	3.942425179	0.001785203	0.011697506	231	552	523	680	0	151	0	7
ENSECAG00000021605	0.516562377	0.001792945	0.011741468	8	11	13	17	39	28	45	52
ENSECAG00000015034	8.687127279	0.001802186	0.011795183	4708	2779	2473	4975	11230	14149	13352	11032
ENSECAG00000020336	5.969102459	0.001815946	0.0118784	1352	2844	756	730	773	644	882	502
ENSECAG00000009576	3.727667372	0.001824356	0.011919782	70	90	222	207	394	319	339	488
ENSECAG00000024350	4.861893958	0.001824371	0.011919782	291	237	274	519	849	666	727	1055
ENSECAG00000022579	6.465430554	0.001830073	0.011950167	845	823	986	1348	2740	1918	2600	2744
ENSECAG00000005816	1.199281306	0.001832569	0.01195959	3	19	29	21	39	76	120	45
ENSECAG00000011705	5.659758567	0.001833939	0.011961664	993	1435	751	1176	691	592	793	802
ENSECAG00000020782	4.972517159	0.001838999	0.011987783	429	518	805	1121	564	359	402	515
ENSECAG00000021538	3.960409333	0.001842995	0.012006941	130	70	252	176	595	270	506	484
ENSECAG00000016346	5.318541395	0.001849696	0.012041886	976	1203	624	623	596	529	515	426
ENSECAG00000008926	5.287069146	0.001851504	0.012041886	752	694	863	1164	786	468	546	394
ENSECAG00000023907	6.051111528	0.001851538	0.012041886	310	1000	533	1017	1403	1414	2055	3040
ENSECAG00000018888	5.08781387	0.001862124	0.012103809	638	1137	596	617	329	569	479	411
ENSECAG00000012542	4.489876836	0.001869004	0.01214158	160	238	246	381	884	449	695	512
ENSECAG00000013793	4.865909848	0.001871784	0.012152691	212	247	345	583	690	842	884	834
ENSECAG00000010402	2.702496779	0.001887152	0.012245472	40	79	62	82	150	197	251	153
ENSECAG00000013742	6.963451848	0.001900945	0.01232793	2939	6148	1078	1359	1180	1186	1243	1559
ENSECAG00000020234	8.604402849	0.00192407	0.012470785	2810	4072	3499	6073	11239	8017	11531	15653
ENSECAG00000015059	0.426139129	0.001925233	0.012471205	3	6	19	16	33	33	32	60
ENSECAG00000006991	6.784301963	0.001926644	0.012473239	1165	517	534	1804	3586	3243	4457	2139
ENSECAG00000015822	1.447047731	0.001929003	0.012481394	20	6	254	52	4	34	8	7
ENSECAG00000018699	2.197260824	0.001936982	0.012525888	17	49	39.9705	80	117	136	116.982	170
ENSECAG00000008892	2.756835146	0.001939152	0.012529173	48	82	46	95	177	134	210	280
ENSECAG00000022400	5.275832077	0.001939695	0.012529173	350	361	391	694	1167	809	1355	1010
ENSECAG00000003996	1.06187667	0.001942353	0.012539215	22	6	15	17	79	27	58	96
ENSECAG00000013238	1.673881808	0.001944212	0.012544088	12	16	49	40	65	68	100	157
ENSECAG00000006573	3.191792596	0.001949646	0.012572005	64	100	71	118	266	101	369	379
ENSECAG00000009624	6.709099821	0.001957753	0.012617126	2023	2391	2640	2101	1583	1421	1455	1345
ENSECAG00000010694	6.236921473	0.001976592	0.012731314	1031	2892	1489	1418	814	1063	780	1338
ENSECAG00000016069	3.057552911	0.001984783	0.012769911	44	75	114	103	204	273	347	143
ENSECAG00000002520	4.752455876	0.001984832	0.012769911	472	1213	371	373	288	258	320	460
ENSECAG00000011037	9.444816729	0.001991317	0.012804384	6423	6966	5082	9579	22859	16445	18486	25451
ENSECAG00000009297	2.239026649	0.001994959	0.012820545	134	58	180	65	75	43	38	45
ENSECAG00000010521	2.961803259	0.002001788	0.012857157	287	226	202	117	3	122	2	14
ENSECAG00000012449	2.466350134	0.002008584	0.01289352	18.0014	55	43.0011	104.002	140.001	134.001	237.002	150.002
ENSECAG00000007059	3.116564599	0.002020313	0.012961491	28	232	489	240	18	126	46	98
ENSECAG00000008613	5.711271002	0.00202902	0.01301	1344	1848	576	778	581	637	649	725
ENSECAG00000011966	7.577135158	0.002042068	0.01308628	4988	6372	2224	3512	2395	1560	1929	3259
ENSECAG00000014479	3.789063564	0.00204847	0.013119908	384	587	232	94	194	166	93	40
ENSECAG00000010108	6.288434049	0.00205082	0.013127559	925	3487	1139	1693	916	909	1020	1147
ENSECAG00000010414	0.459466212	0.002058042	0.013166372	24	45	40	19	12	22	8	9
ENSECAG00000008416	4.359916476	0.002065089	0.013200875	149	206	212	390	498	544	464	820
ENSECAG00000011493	5.80747113	0.002065759	0.013200875	1924	332	2764	386	664	198	630	567
ENSECAG00000019499	5.369072806	0.002072063	0.013222791	929	2590	253	196	386	305	418	292
ENSECAG00000020201	1.380191289	0.002072365	0.013222791	23	14	23	27	103	91	70	40
ENSECAG00000007072	4.369513713	0.002073318	0.013222791	192	204	281	269	571	427	619	697

ENSECAG00000009862	5.706754693	0.002075345	0.013222791	1607	1719	639	630	405	796	336	790
ENSECAG00000022545	6.315880634	0.002076017	0.013222791	1212	3180	1263	1502	900	1147	936	1180
ENSECAG00000012514	2.709367237	0.002076171	0.013222791	29.0071	84	47	101	156	188	142	297
ENSECAG00000008141	2.683532894	0.002088813	0.013295853	38	50	84	106	177	159	194	209
ENSECAG00000007355	5.383977146	0.002093786	0.013320046	575	469	1502	1464	464	431	480	917
ENSECAG00000006213	5.224372822	0.002097774	0.013337945	387	1377	842	855	435	649	476	398
ENSECAG00000009168	6.899167729	0.002116862	0.013451779	860	1522	6658	4496	915	2242	763	409
ENSECAG00000013000	2.553820261	0.002121922	0.013464678	51	56	41	70	183	103	123	307
ENSECAG00000015714	6.059983523	0.002122	0.013464678	1522.01	2466	1106.01	748	783.001	900	710.001	887
ENSECAG00000020548	3.555631244	0.002122447	0.013464678	59	148	92	212	300	266	299	520
ENSECAG00000008715	4.346645213	0.002131729	0.013516021	413	678	378	289	278	256	243	304
ENSECAG00000011029	4.750219695	0.002161217	0.013695343	210	255	273	536	892	609	806	695
ENSECAG00000016921	5.270733244	0.0021665	0.013713715	1135	1038	576	532	481	529	527	386
ENSECAG00000011310	3.938764361	0.00216653	0.013713715	146	130	181	235	407	359	506	447
ENSECAG00000008017	4.50124004	0.002175844	0.013757598	201	175	287	392	643	566	722	587
ENSECAG00000013872	0.780492058	0.002175885	0.013757598	10	3	26	8	73	23	42	78
ENSECAG00000021069	7.174926912	0.002178251	0.013764898	4980	6194	813.996	1178	1386	1249	1555	1034
ENSECAG00000015093	0.634174269	0.002188464	0.013821748	10	11	3	22	39	22	47	83
ENSECAG00000025034	4.930916385	0.002192267	0.013838074	249	244	412	544	851	690	1016	837
ENSECAG00000024430	2.074832811	0.002196307	0.013855877	14	39	31	82	102	150	134	110
ENSECAG00000015031	3.8160913	0.002207862	0.01391754	268	553	183	274	129	210	208	125
ENSECAG00000000949	2.749550595	0.002208531	0.01391754	184	206	157	227	2	105	2	4
ENSECAG00000007737	1.74526479	0.002220009	0.01398212	11	14	30	64	50	65	143	163
ENSECAG00000008773	4.101803008	0.002222579	0.013990552	137	166	243	206	359	494	683	399
ENSECAG00000010980	4.883634501	0.002227199	0.014008942	235	284	373	521	729	604	911	1055
ENSECAG00000015579	2.937292881	0.002227967	0.014008942	157	218	160	145	123	57	104	110
ENSECAG00000015385	2.704330981	0.002230321	0.014015988	66	84	161	402	60	90	89	33
ENSECAG00000023468	5.966493012	0.002231684	0.014016802	1693	1580	941	1066	900	691	826	941
ENSECAG00000011892	2.486724185	0.002234602	0.014027373	35	72	30	69	200	73	197	215
ENSECAG00000008390	5.849529462	0.002245018	0.014084977	951	1739	864	1552	739	892	616	994
ENSECAG00000009400	3.446385397	0.002270174	0.014234941	109	657	175	160	48	159	43	155
ENSECAG00000017666	1.483134074	0.00227199	0.014238474	29	67	112	59	35	31	27	37
ENSECAG00000023177	4.395750242	0.002278659	0.014272397	157	186	249	404	733	462	580	578
ENSECAG00000000690	4.32943402	0.002279949	0.014272608	173	174	233	370	551	472	570	645
ENSECAG00000022769	4.19568842	0.002290494	0.014330723	135	125	168	371	815	360	557	397
ENSECAG00000005675	6.294611352	0.002292403	0.014334773	1648	3053	1692	747	1094	1224	621	581
ENSECAG00000008101	5.98159643	0.002294308	0.014338796	588	412	821	1075	1877	1286	1962	2063
ENSECAG00000024245	1.530010618	0.002301003	0.01437273	94	96	21	50	33	26	31	22
ENSECAG00000016273	5.541798254	0.002305415	0.014392377	880	971	1256	863	745	582.002	718	686
ENSECAG00000009984	5.64022634	0.00230761	0.014398172	785	1452	885	1209	621	775	711	794
ENSECAG00000010153	6.819883216	0.002330592	0.01453358	1149	9665	601	385	691	707	709	1063
ENSECAG00000025151	3.559913912	0.002359059	0.014698882	102	107	123	192	323	220	368	439
ENSECAG00000007629	3.791842088	0.0023605	0.014698882	176	382	436	263	149	194	150	261
ENSECAG00000016810	1.400379804	0.00236098	0.014698882	9	28	17	25	130	70	107	17
ENSECAG00000009733	2.321527912	0.002365189	0.014717017	49	70	220	165	58	49	78	48
ENSECAG00000002193	4.987610515	0.002368603	0.014730199	462	684	767	858	575	372	420	553
ENSECAG00000019023	6.324198867	0.002375304	0.014763786	2186	2609	1045	1094	1145	1065	831	849
ENSECAG00000000095	5.138577964	0.00237789	0.014771782	315	360	434	566	1002	687	1041	1184
ENSECAG00000019147	5.188136267	0.002393536	0.014855352	916	807	804	654	358	742	292	464
ENSECAG00000018342	7.336708161	0.002393958	0.014855352	3777	4489	3117	2728	2397	1671	1921	2487
ENSECAG00000013298	3.422664433	0.002408284	0.014936091	95	81	153	137	344	230	332	314
ENSECAG00000007907	3.286177771	0.002416167	0.01497681	140	295	311	298	24	222	22	59
ENSECAG00000006577	4.991768935	0.002439587	0.015113731	311	294	351	539	999	654	937	941
ENSECAG00000018648	5.599808464	0.002447338	0.015153493	1287	1284	713	793	596	660	623	730
ENSECAG00000006554	1.0721455	0.002448817	0.015154392	15	3	15	24	146	35	51	32
ENSECAG00000023765	0.847692498	0.002451602	0.015156336	22	93	25	34	6	19	16	26
ENSECAG00000026917	5.401379895	0.002451799	0.015156336	741	822	1278	888	661	604	716	378
ENSECAG00000014462	1.275268539	0.002461415	0.015207502	52	72	50	51	15.9982	47	15	21
ENSECAG00000010333	6.84272285	0.002467229	0.015235139	1376	4915	2282	2116	1218	1651	1526	1290
ENSECAG00000017331	6.595941293	0.002500596	0.015432794	2914	2496	1443	1374	1281	1108	1307	1155
ENSECAG00000018327	1.753312638	0.002519185	0.015533288	47	87	76	103	39	53	39	35
ENSECAG00000014929	2.669763464	0.002519614	0.015533288	23	63	76	99	147	186	269	142
ENSECAG00000017235	5.071989645	0.002522578	0.015543129	555	976	679	721	363	608	415	509
ENSECAG00000005764	3.722450693	0.002528912	0.015573711	357	374	192	206	175	135	186	177
ENSECAG00000013554	8.213960562	0.002530607	0.01557571	9484	8338	4673	3382	3178	3574	3535	3229
ENSECAG00000012334	1.833341844	0.002534779	0.015589745	95	141	50	60	12	72	16	15
ENSECAG00000010437	5.607542658	0.002535632	0.015589745	412	393	778	611	1582	1125	1807	966
ENSECAG00000012707	5.132558594	0.002542149	0.015621363	475	713	854	1062	512	562	474	558
ENSECAG00000007313	8.665204463	0.002567047	0.015761692	10380	10435	7933	7057	5814	4629	5653	4130
ENSECAG00000011711	6.257314768	0.00256776	0.015761692	1509	1092	2069	2155	1205	1120	1158	783
ENSECAG00000020587	7.790846812	0.002571301	0.015770394	2925	7265	9518	5091	463	4718	269	691
ENSECAG00000007635	4.056321393	0.002571954	0.015770394	125	141	212	302	398	367	494	619
ENSECAG00000019351	1.934326194	0.002573573	0.015771807	29	31	54	35	110	77	118	140
ENSECAG00000012550	1.255109644	0.002576979	0.01578417	13	70	25	156	21	27	17	23
ENSECAG00000023189	2.565341385	0.002580087	0.015794693	40	280	104	172	37	56	70	96
ENSECAG00000021161	3.788937526	0.002582121	0.015798634	153	129	142	165	434	281	443	412
ENSECAG00000016782	8.543708803	0.002593806	0.015861586	8684	15362	5813	3943	3573	4589	3369	5204
ENSECAG00000017663	0.357989695	0.002597967	0.015878485	8	1	14	14	29	19	47	60
ENSECAG00000013889	5.205274935	0.002616332	0.015982129	301	435	441	593	866	889	956	1374
ENSECAG00000019270	4.913714438	0.002620319	0.015997886	550	1211	419	471	392	399	406	355
ENSECAG00000015061	7.889826353	0.002637457	0.01609387	5924	7273	3806	3828	3520	2783	2555	2933
ENSECAG00000019848	4.883493519	0.002641238	0.016108292	294	277	294	511	713	650	938	992
ENSECAG00000017148	6.081955621	0.002657654	0.016193478	2345	2205	700	627	932	705	758	511
ENSECAG00000018924	8.242550301	0.002658056	0.016193478	9364	5876	5948	5126	3007	3928	3443	4566
ENSECAG00000012199	6.063972198	0.002659659	0.016194556	779	4553	638	619	326	778	610	815

ENSECAG00000025138	4.009892453	0.002661115	0.016194744	139	362	395	657	206	190	197	303
ENSECAG00000013659	4.227797984	0.002667804	0.016226758	476	418	391	297	291	174	262	302
ENSECAG00000024408	1.832841821	0.002675558	0.016265215	20	77	95	185	44	34	46	32
ENSECAG00000019329	4.590210848	0.002677032	0.016265477	43	171	264	520	549	997	788	566
ENSECAG00000003387	4.145926538	0.002680492	0.016277794	177	196	223	198	529	410	487	544
ENSECAG00000021567	7.481891404	0.002685711	0.016300774	3486	6115	3082	3196	2139	1807	2467	2936
ENSECAG00000015306	4.66215081	0.002697686	0.016364714	396	921	346	597	247	336	411	371
ENSECAG00000023958	2.472607408	0.00270521	0.016401596	26	46	63	103	128	133	177	209
ENSECAG00000020696	2.087870349	0.002711282	0.016422463	59	141	111	78	56	57	42	53
ENSECAG00000013763	7.448268837	0.002711542	0.016422463	2737	5441	3707	3796	2159	2267	2152	2946
ENSECAG00000012207	1.414136984	0.002719059	0.016449132	23	17	26	33	66	50	78	119
ENSECAG00000003597	2.199145146	0.002719667	0.016449132	25	47	42	72	130	75	130	211
ENSECAG00000009639	1.089920179	0.002720289	0.016449132	16	16	13	27	36	37	71	113
ENSECAG00000022507	6.49634897	0.002733351	0.016519328	1337	4325	1239	1454	661	1030	1025	1634
ENSECAG00000019475	6.626942144	0.002762453	0.016686334	1162	1321	4181	2997	1617	1460	1042	950
ENSECAG00000016957	2.619179912	0.002776371	0.016761492	96	166	168	144	78	115	54	45
ENSECAG00000021570	3.662789671	0.002778157	0.016763368	47	78	144	297	348	311	416	419
ENSECAG00000018929	5.084873232	0.002789751	0.016824393	679	932	672	581	513	459	472	501
ENSECAG00000013574	3.674146482	0.002795877	0.016847654	216	469	220	220	167	160	183	161
ENSECAG00000016984	1.889033017	0.002796574	0.016847654	131	70	51	86	28	66	17	35
ENSECAG00000016367	2.360101988	0.002802812	0.016876284	86	131	192	65	72	36	60	79
ENSECAG00000018195	7.508655176	0.002809049	0.016904883	3288	6491	3437	3105	2206	2756	1996	2312
ENSECAG00000011650	4.486677458	0.002815932	0.016937331	548	683	390	315	191	392	269	277
ENSECAG00000020138	5.12247797	0.002829266	0.01700853	492	720	707	1201	483	356	539	723
ENSECAG00000004636	5.229834488	0.002832599	0.017019563	650	635	1028	1028	721	545	508	350
ENSECAG00000012682	3.437535328	0.002856162	0.017152067	98	85	146	121	283	198	477	296
ENSECAG00000022082	3.797982073	0.00285862	0.017157759	114	183	169	155	337	351	431	436
ENSECAG00000008884	6.072686872	0.002864988	0.017186901	750	673	672	644	1643	1140	1818	3403
ENSECAG00000012978	4.987104213	0.002886925	0.017309361	563	661	895	594	466	490	475	420
ENSECAG00000010547	4.886483353	0.002892743	0.017335096	184	478	313	352	614	817	1067	831
ENSECAG00000000512	5.572411081	0.002905949	0.017405058	356	453	674	808	1280	901	1226	1951
ENSECAG00000008103	5.196036556	0.002940287	0.017599493	409	467	308	359	984	707	1101	1428
ENSECAG00000024201	3.461658828	0.00294151	0.017599493	46	122	90	188	274	420	444	149
ENSECAG00000015330	2.721433755	0.002943505	0.017602161	61	28	67	107	208	141	227	201
ENSECAG00000024438	5.71535166	0.002946647	0.01761168	370	459	628	1066	2000	1007	1373	1532
ENSECAG00000019492	5.513129691	0.00294971	0.017620715	1252	1481	692	475	641	389	655	595
ENSECAG00000013046	6.823835863	0.002961956	0.017684575	1233	912	4995	5551	1614	1380	1178	263
ENSECAG00000017817	2.327522338	0.002966478	0.017702273	95	93	254	61	30	100	20	33
ENSECAG00000012670	8.999853548	0.002970121	0.017714707	20097	10458	8154	5468	4656	6704	4844	5843
ENSECAG00000022955	9.034098686	0.002974372	0.017726416	11159	21782	6484	8083	5961	5528	6823	6269
ENSECAG00000019797	6.24182085	0.002975205	0.017726416	778	543	789	1256	2315	1266	2383	2771
ENSECAG00000024869	6.24465279	0.002982224	0.017752309	909	2159	1604	2175	905	974	1400	1093
ENSECAG00000014498	7.262158537	0.002982675	0.017752309	3301	4743	2302	3358	2164	1450	1341	3071
ENSECAG00000014753	6.609969532	0.002984978	0.01775671	695	574	754	2273	3215	2428	3616	2177
ENSECAG00000010395	0.310222764	0.002991362	0.017785375	9	38	38	37	4	20	11	11
ENSECAG00000014181	7.175886453	0.003050573	0.018116049	1428	1324	1616	1889	3633	3090	4702	5304
ENSECAG00000015344	0.872656576	0.003051245	0.018116049	10	16	16	28	48	43	53	63
ENSECAG00000017922	9.01410179	0.003051762	0.018116049	11643	24456	5495	5788	5569	3959	6363	6632
ENSECAG00000006249	5.125530769	0.003061806	0.018163121	229	247	510	694	886	1037	1042	918
ENSECAG00000023656	3.853079658	0.003062889	0.018163121	99	150	119	283	413	233	480	549
ENSECAG00000022880	3.982262919	0.003074149	0.018220388	365	403	501	249	84	376	58	107
ENSECAG00000009791	5.872650057	0.003082645	0.018261216	422	686	735	1027	1793	1469	1535	1619
ENSECAG00000011202	1.499244328	0.003088034	0.018283605	15	22	34	32	75	36	76	157
ENSECAG00000011354	4.197751436	0.003096526	0.018324336	251	524	369	488	207	282	323	228
ENSECAG00000015398	4.210314606	0.003098546	0.018326748	163	214	133	326	528	359	563	655
ENSECAG00000011844	5.343109177	0.003100374	0.018328017	796	687	1020	1001	622	701	544	480
ENSECAG00000024116	5.560910396	0.003106752	0.018346234	650.004	1259	1159.01	1087	618	857.003	481.005	779
ENSECAG00000008688	5.444041914	0.0031079	0.018346234	555	893	1440	1349	477	1015	271	360
ENSECAG00000004861	-0.169248867	0.00310861	0.018346234	4.00006	3.00004	10.0001	11.0006	23	24.0001	27.0001	23.0001
ENSECAG00000022151	5.581392156	0.003109914	0.018346234	326	1134	1174	2202	436	890	499	644
ENSECAG00000003235	4.962771062	0.003116392	0.018374905	202	380	373	572	821	683	806	1154
ENSECAG00000013881	4.860969332	0.003119937	0.018386266	225	237	384	565	887	751	812	746
ENSECAG00000015083	2.960991541	0.003124394	0.018402986	39	100	33	114	260	91	216	415
ENSECAG00000014258	5.378328908	0.003131248	0.018425856	346	470	607	583	1106	891	1111	1476
ENSECAG00000010412	-0.092042898	0.00313152	0.018425856	2	3	11	13	20	22	20	45
ENSECAG00000013478	3.289320827	0.003152961	0.018539781	75	89	116	163	325	202	276	300
ENSECAG00000015753	2.543766019	0.003154481	0.018539781	63	18	72	43	118	182	244	162
ENSECAG00000006103	1.386430159	0.003155777	0.018539781	18	28	26	25	56	50	85	115
ENSECAG00000011173	6.191791983	0.003158997	0.018549109	1400	1905	1314	1623	1215	848	1094	1106
ENSECAG00000008242	2.882670041	0.003164693	0.018572956	94	356	306	108	1	108	1	15
ENSECAG00000015492	3.748861667	0.003167222	0.018578199	151	81	172	157	413	254	418	470
ENSECAG00000009892	5.35187396	0.003186157	0.018679628	920	1306	570	675	614	420	600	595
ENSECAG00000013839	7.427752534	0.003188758	0.018685235	2454	3983	3647	5721	2546	2085	2220	2915
ENSECAG00000008833	8.947971795	0.003203947	0.018764561	8925	15264	8641	11750	5489	6808	5655	8136
ENSECAG00000011422	4.390414031	0.003219183	0.01884408	390	829	511	165	259	161	366	208
ENSECAG00000018173	5.588281532	0.003227967	0.018877407	953	1278	877	934	619	679	749	774
ENSECAG00000013970	5.415152745	0.003228199	0.018877407	336	431	626	690	1090	879	1305	1433
ENSECAG00000017191	3.232419832	0.003243883	0.018959364	242	289	242	77	104	160	54	84
ENSECAG00000008441	2.397842084	0.003248367	0.018975805	55	43	34	28	177	60	298	125
ENSECAG00000005949	5.456829763	0.003252538	0.01899041	320	484	547	729	1140	739	1143	1983
ENSECAG00000002942	5.951444663	0.00329115	0.019205982	1139	1244	1414	1449	979	837	899	1039
ENSECAG00000001996	1.802299743	0.003306406	0.019282534	21	245	55	39	15	50	17	28
ENSECAG00000014404	4.785444098	0.003307662	0.019282534	141	281.999	376.998	540	637	770	674	1011
ENSECAG00000017783	2.106085852	0.003310863	0.019291298	46	5	43	23	173	58	116	219
ENSECAG00000019509	8.608646123	0.003317818	0.019321909	9169	10945	7563	6667	4722	3934	4284	7354

ENSECAG00000020235	3.931757045	0.003322582	0.019339743	221	982	109	161	180	83	200	132
ENSECAG00000012252	5.331133174	0.003325286	0.019345568	188	269	351	1052	988	1265	1396	1006
ENSECAG00000017984	7.401889404	0.003337658	0.019403102	7031	6112	1416	707	1997	1519	1444	562
ENSECAG00000010199	4.8768451	0.00333859	0.019403102	314	386	288	328	790	733	839	893
ENSECAG00000016491	8.932841641	0.003341223	0.019408473	7379	22484	7835	7747	4538	6852	5475	6091
ENSECAG00000023740	5.019137657	0.003345069	0.019413176	301	313	416	547	859	818	874	978
ENSECAG00000001117	3.450481895	0.00334545	0.019413176	165	187	303	326	162	119	179	170
ENSECAG00000006886	5.604251653	0.003349553	0.019427066	2138	1319	272	440	431	484	604	394
ENSECAG00000021359	5.767666592	0.003366264	0.019514026	1278	1518	979	793	729	667	669	979
ENSECAG00000013180	2.06130159	0.003370548	0.019528899	35	66	217	100	49	42	44	57
ENSECAG00000019249	2.404407107	0.003380217	0.019574939	26	60	57	58	99	264	105	148
ENSECAG00000000794	6.462375477	0.003386608	0.019601953	2429	3022	1212	976	1393	671	1225	960
ENSECAG00000016586	4.86865948	0.003396158	0.019646674	290	247	354	415	991	528	565	1271
ENSECAG00000024171	5.243287877	0.003397792	0.019646674	950	716	708	775	558	488	571	575
ENSECAG00000016323	0.902660329	0.003402339	0.019662955	63	28	60	17	17	16	28	10
ENSECAG00000009873	3.727457584	0.003446694	0.019909167	137	838	189	137	156	106	193	59
ENSECAG00000019191	0.999227464	0.003450621	0.019921726	0	3	32	5	17	141	31	75
ENSECAG00000019700	1.883325662	0.003452539	0.019922675	34	14	54	26	108	66	135	137
ENSECAG00000024138	3.783998553	0.003455578	0.019930093	213	659	461	211	1	239	29	32
ENSECAG00000017937	1.60437468	0.003468122	0.019992292	39	23	124	132	23	19	23	65
ENSECAG00000009296	3.339671428	0.003471453	0.019994081	80	206	425	336	57	76	83	264
ENSECAG00000014406	2.012922554	0.003471952	0.019994081	108	210	40	17	35	34	36	36
ENSECAG00000021080	3.534698212	0.00348471	0.020057385	72	106	136	230	320	323	333	317
ENSECAG00000012302	1.191252725	0.003492451	0.020089054	19	161	24	26	12	19	31	10
ENSECAG00000011549	3.173991923	0.003493749	0.020089054	139	240	208	213	128	80	149	149
ENSECAG00000020471	2.582828605	0.003506924	0.020154614	44	68	63	79	148	118	183	241
ENSECAG00000021447	4.765842315	0.003512889	0.020178689	906	626	344	423	383	179	324	488
ENSECAG00000002225	1.150082943	0.003528607	0.020258731	10	77	34	115	13	17	22	36
ENSECAG00000007133	4.648894657	0.003538032	0.020302585	72	299	373	138	676	1007	1149	226
ENSECAG00000008160	5.179162885	0.003546191	0.02033913	400	432	312	487	1107	749	1084	1101
ENSECAG00000014262	4.461136505	0.00355384	0.020367761	208	191	312	311	798	515	570	547
ENSECAG00000022080	6.518532597	0.003554768	0.020367761	1461	2351	2185	1971	1265	1486	1292	1261
ENSECAG00000013728	2.012327983	0.003561206	0.020394367	103	71	92	120	59	68	25	12
ENSECAG00000014046	3.072022873	0.003597302	0.020590702	52	122	66	123	211	208	275	267
ENSECAG00000019201	3.343266022	0.003620404	0.020712504	107	93	88	148	256	227	283	393
ENSECAG00000014741	5.235150299	0.003630834	0.020742357	1080	664	885	611	789	230	417	536
ENSECAG00000002410	0.54669813	0.003631771	0.020742357	11	12	17	9	39	35	37	55
ENSECAG00000005486	5.629544091	0.003632188	0.020742357	666	2021	912	781	567	705	696	709
ENSECAG00000016378	4.345595663	0.003632925	0.020742357	229	679	371	546	281	261	258	366
ENSECAG00000002855	5.738465597	0.00363831	0.02076267	669	1323	1587	1425	452	1201	388	754
ENSECAG00000010048	0.785103185	0.00364077	0.020764686	9	5	6	38	36	47	57	66
ENSECAG00000010666	2.907802545	0.003642318	0.020764686	49	50	109	118	217	141	203	314
ENSECAG00000001533	1.763752325	0.003656323	0.020834071	24	40	26	28	102	35	120	158
ENSECAG00000013869	0.626433139	0.003678911	0.020948781	4	10	17	26	51	32	50	43
ENSECAG00000006826	1.66167374	0.003680141	0.020948781	31	122	68	80	26	51	29	39
ENSECAG00000009039	8.262739579	0.00369757	0.021037449	3497	3063	1423	3366	11054	5851	9499	11013
ENSECAG00000008975	3.259922911	0.003699618	0.021038568	187	345	204	109	118	131	117	107
ENSECAG00000023751	0.699805286	0.003701993	0.021041543	12	35	71	49	17	27	10	4
ENSECAG00000000548	11.85550368	0.003706151	0.021054641	14121	28875	31904	80016	77439	73133	118408	194438
ENSECAG00000014185	3.651847695	0.003711524	0.021074632	82	126	170	211	364	232	374	448
ENSECAG00000018662	6.037307717	0.003714338	0.021080077	395	834	551	1346	1836	1805	1525	2235
ENSECAG00000013244	6.509707701	0.003734747	0.021185322	1834	2745	1659	1537	1324	1054	1397	1335
ENSECAG00000000660	1.219847103	0.003742504	0.021218729	82	71	33	18	29	23	19	19
ENSECAG00000011044	1.339300939	0.003746325	0.021229798	21	152	47	34	18	42	11	14
ENSECAG00000024224	1.614558389	0.003750434	0.021239215	20	32	22	48	95	64	113	78
ENSECAG00000011174	6.341617217	0.003751725	0.021239215	349	447	796	2135	2044	2377	2642	2464
ENSECAG00000013419	3.214527452	0.003764989	0.021303687	108	381	126	248	109	108	134	121
ENSECAG00000014197	4.908616755	0.003769249	0.021317174	329	1394	402	665	320	465	269	463
ENSECAG00000021987	5.175049463	0.003773137	0.021328547	1505	709	371	425	441	394	449	354
ENSECAG00000018849	6.704960138	0.003778514	0.021348325	1726	1678	2937	3364	1852	1513	1468	749
ENSECAG00000023657	0.891147245	0.003783257	0.021364498	49	27	27	79	10	37	12	8
ENSECAG00000024698	4.315593885	0.003789653	0.021384793	142	171	277	369	628	413	461	727
ENSECAG00000018135	0.828813324	0.003790616	0.021384793	2	11	10	30	79	28	96	16
ENSECAG00000020847	5.043934318	0.003809953	0.021474669	395	697	821	1058	463	589	482	404
ENSECAG00000001796	1.005820631	0.003810327	0.021474669	0	3	25	7	19	12	220	29
ENSECAG00000009562	6.495384505	0.003818119	0.021507918	847	1221	4875	2775	895	1889	470	342
ENSECAG00000017924	0.854138769	0.003822248	0.021520505	5	97	28	65	11	27	11	5
ENSECAG00000004925	6.212018975	0.003828994	0.021547812	639	841	887	1204	2060	1549	2147	2435
ENSECAG00000017304	1.184270202	0.003840319	0.021600843	11	19	25	35	55	48	84	72
ENSECAG00000020317	6.672399166	0.003849233	0.021640271	2553	3592	1310	1365	1435	1091	1396	1172
ENSECAG00000013688	4.576395665	0.003853514	0.021653625	283	220	267	269	760	447	788	691
ENSECAG00000006800	5.99933494	0.003860622	0.021674643	1058	2264	1138	1059	878	832	895	984
ENSECAG00000020856	6.442214286	0.00386107	0.021674643	2227	1941	1439	1734	1025	1114	1559	1110
ENSECAG00000005377	4.298434005	0.003865034	0.021686178	202	161	208	316	593	317	543	793
ENSECAG00000023208	3.964907232	0.003897641	0.021858339	136	166	170	256	520	292	424	506
ENSECAG00000000321	-0.077296961	0.003900361	0.021862804	7	3	10	9	25	26	19	35
ENSECAG00000009665	4.906412357	0.003903491	0.021869557	236	267	502	445	828	660	973	833
ENSECAG00000008796	4.004939894	0.003906487	0.021873645	575	500	124	182	117	181	189	218
ENSECAG00000014572	5.048102828	0.003909124	0.021873645	572	691	660	915	547	542	382	521
ENSECAG00000022568	3.969890789	0.003909996	0.021873645	480	588	154	141	136	131	186	252
ENSECAG00000019741	3.020000496	0.00392235	0.021931958	72	79	104	100	217	213	260	215
ENSECAG00000021290	6.454504427	0.003925816	0.021940539	1169	3802	1191	1922	685	1186	931	1696
ENSECAG00000007552	0.701724848	0.003933322	0.021961762	12	13	17	17	37	39	48	59
ENSECAG00000020085	3.948303897	0.003933479	0.021961762	189	612	349	245	187	254	166	192
ENSECAG00000019247	5.818833481	0.00394285	0.02200327	1304	1602	865	949	748.997	708	805	928

ENSECAG00000014213	5.6170091	0.003977917	0.022188067	439	463	685	828	1345	1104	1242	1675
ENSECAG00000015360	2.322134257	0.003982382	0.022202074	19	39	45	109	112	158	156	156
ENSECAG00000012987	2.991213494	0.003994352	0.022256118	35	51	100	150	122	211	233	387
ENSECAG00000024038	8.037439908	0.003995994	0.022256118	4637	11266	4203	3596	2718	3006	3439	3767
ENSECAG00000020551	6.846290137	0.004008843	0.022316744	2851	4147	1480	1730	2306	816	1202	1192
ENSECAG00000012847	4.36720229	0.004027722	0.022390831	219	203	202	315	510	405	747	644
ENSECAG00000013263	3.513598784	0.004028019	0.022390831	67	53	168	202	395	228	476	237
ENSECAG00000016899	4.005972915	0.004028064	0.022390831	147	103	217	278	418	386	473	521
ENSECAG00000020886	0.358025155	0.004031358	0.022398185	5	11	12	16	39	17	48	42
ENSECAG00000015340	0.605076253	0.004036218	0.022414228	9	13	14	19	52	32	31	57
ENSECAG00000016972	4.557387525	0.004040352	0.022426221	355	710	497	458	359	273	383	370
ENSECAG00000006957	6.957301485	0.004052327	0.022481711	372	1026	825	3164	3029	3951	3424	4666
ENSECAG00000021208	7.036721981	0.004057431	0.02249904	3278	4196	1911	1931	1802	1682	1552	1664
ENSECAG00000021627	4.687542722	0.004060773	0.022506585	250	231	346	344	919	685	756	480
ENSECAG00000014941	6.661456721	0.004070161	0.022547621	683	1133	993	1997	2440	2737	2963	3243
ENSECAG00000001360	5.002920277	0.004083611	0.022611104	270	402	391	508	824	703	904	1062
ENSECAG00000000749	5.585674218	0.004093347	0.022653974	515	411	596	758	1378	991	1446	1469
ENSECAG00000010559	3.767333358	0.004102679	0.022690913	92	110	158	275	319	327	320	589
ENSECAG00000007911	3.729939043	0.004104016	0.022690913	177	271	288	463	147	183	213	233
ENSECAG00000021291	5.945447281	0.004117216	0.022752825	717	559	723	820	1790	1446	1853	1715
ENSECAG00000016298	4.946854109	0.004129744	0.022810962	230	408	337	502	731	562	1031	1115
ENSECAG00000015053	6.992239506	0.004142531	0.022870474	5045	3237	1109	1422	1362	1201	1319	1758
ENSECAG00000021174	1.213937189	0.00414705	0.022884302	8	32	20	28	53	66	84	62
ENSECAG00000022962	3.830515939	0.004154586	0.022914759	186	247	323	585	194	142	141	332
ENSECAG00000015186	4.768744807	0.004166338	0.022968429	733	866	326	376	395	262	373	388
ENSECAG00000022357	3.809236304	0.004176137	0.023011284	267	511	205	297	237	228	68	136
ENSECAG00000017025	3.262033197	0.004198895	0.023125472	298	146	183	190	106	133	105	150
ENSECAG00000022137	2.75104142	0.004201559	0.023128931	49	57	63	120	171	123	181	313
ENSECAG00000015905	6.280530401	0.004222071	0.023214803	1071	1191	2509	2240	1341	897	1158	1205
ENSECAG00000013123	5.734173832	0.00422267	0.023214803	896	1539	1132	940	735	717	691	1019
ENSECAG00000014648	4.621479652	0.004223288	0.023214803	206	331	305	341	671	584	714	706
ENSECAG00000023805	5.532947021	0.004237968	0.023284233	874	1353	753	928	568	696	689	747
ENSECAG00000008957	6.659092296	0.004249953	0.023338792	2268	3275	1467	1762	1251	1466	1570	944
ENSECAG00000003982	3.633267693	0.004290103	0.023547897	78	146	46	223	294	398	549	221
ENSECAG00000000967	2.709092066	0.004297045	0.023574614	57	206	146	213	74	60	90	121
ENSECAG00000013050	9.256655384	0.004304824	0.023605891	18403	21799	8150	5291	6619	5666	5704	9576
ENSECAG00000007964	5.26255692	0.004307645	0.023609965	202	216	415	921	1195	1116	1459	645
ENSECAG00000005573	1.846517064	0.004321318	0.023664387	86	80	87	66	35	67	33	34
ENSECAG00000007165	5.883022069	0.004322555	0.023664387	603	490	702	981	1936	1250	1873	1459
ENSECAG00000010260	6.246338324	0.004323822	0.023664387	1742	2588	990	1155	971	860	937	1305
ENSECAG00000018137	2.711777498	0.004329237	0.023682614	45	63	89	95	205	162	194	171
ENSECAG00000024750	3.370407999	0.004331487	0.02368352	111	54	156	89	331	192	328	365
ENSECAG00000004249	4.900340017	0.004356121	0.023803636	685	807	532	458	471	370	421	434
ENSECAG00000003049	1.635113696	0.004357645	0.023803636	37	56	159	53	30	47	38	16
ENSECAG00000016361	0.296899254	0.004371599	0.023868384	35	16	34	24	21	5	14	10
ENSECAG00000012108	5.568626091	0.004388079	0.023946854	379	721	510	664	1389	1054	1364	1386
ENSECAG00000009901	10.84395634	0.004390411	0.02394808	54983	35362	39835	30508	26535	23704	25253	13695
ENSECAG00000008173	6.60408906	0.004404474	0.024013259	2049	3053	1836	1388	1152	1503	1355	1228
ENSECAG00000013149	8.62186913	0.004422747	0.024101318	9259	10648	6680	7648	5327	4297	5102	6338
ENSECAG00000007835	4.723825785	0.004430461	0.024131784	169	313	344	473	582	622	914	784
ENSECAG00000016445	5.244406508	0.00443972	0.02416731	909	1073	649	528	600	437	459	618
ENSECAG00000023946	1.782450584	0.004441238	0.02416731	24	31	14	59	87	146	90	75
ENSECAG00000026911	6.125558334	0.004444031	0.024170935	1795	1718	1109	1191	964	985	829	1119
ENSECAG00000024218	2.777691815	0.00445245	0.024203156	65	127	226	246	84	60	82	140
ENSECAG00000018670	3.608050479	0.004454216	0.024203156	80	96	221	164	330	310	301	433
ENSECAG00000014696	6.288193154	0.004465805	0.024239815	760	723	1036	1075	1945	1495	2008	3432
ENSECAG00000012479	4.788875456	0.004465814	0.024239815	234	254	372	494	837	617	868	687
ENSECAG00000018826	1.361265154	0.004467362	0.024239815	49	102	32	47	30	27	23	38
ENSECAG00000012772	0.283295617	0.004482687	0.024311357	3	5	21	10	24	24	50	43
ENSECAG00000022965	8.366809469	0.004485511	0.024314524	5830	11567	7419	4701	4134	4860	4086	3719
ENSECAG00000016557	4.033235592	0.004487551	0.024314524	165	143	197	210	480	256	399	759
ENSECAG00000006763	0.501594202	0.004493564	0.024335499	13	48	22	65	4	28	3	11
ENSECAG00000008227	3.686626971	0.004501585	0.02436732	220	521	209	179	157	151	169	194
ENSECAG00000024640	9.397994963	0.004525552	0.024485392	15888	29484	26529	6519	50	8290	101	97
ENSECAG00000019662	5.580089105	0.004554708	0.024631409	841	1909	719	688	748	515	750	537
ENSECAG00000011541	4.683778373	0.004558665	0.024641078	211	199	277	494	807	359	659	1157
ENSECAG00000014306	5.438477065	0.004575603	0.024720874	482	451	503	605	1185	924	1195	1458
ENSECAG00000006913	10.2758499	0.004579662	0.024731044	23402	25832	36357	26166	17744	18572	17614	11521
ENSECAG00000016811	1.108736866	0.004586848	0.024758086	7	14	18	45	58	39	68	88
ENSECAG00000023494	5.083946706	0.004696727	0.025339131	347	546	1281	976	521	519	493	401
ENSECAG00000026912	0.726133515	0.004701452	0.025352588	17	10	7	17	37	25	47	90
ENSECAG00000019186	6.265470615	0.004712868	0.02540209	695	716	1007	1310	1921.99	1915	2006	2626
ENSECAG00000019160	2.907974745	0.004727281	0.025467696	65	35	112	105	227	182	245	207
ENSECAG00000018147	4.028958099	0.004748873	0.025570824	174	143	139	289	478	429	396	512
ENSECAG00000009327	9.281342575	0.004750925	0.025570824	13766	14239	12627	13637	8147	8736	8047	9072
ENSECAG00000008088	2.178481609	0.004756646	0.025589496	42	38	55	38	157	76	181	112
ENSECAG00000019875	6.197338565	0.004765976	0.025627554	1563	2915	919	1082	790	1226	978	566
ENSECAG00000016638	4.470176634	0.004781439	0.025698536	223	1327	359	188	186	240	286	269
ENSECAG00000022446	6.933454015	0.004800471	0.025788628	433	1137	1346	2542	2950	4459	4288	2481
ENSECAG00000017998	5.817203389	0.004809613	0.025825532	385	751	494	1078	1605	1108	1399	2246
ENSECAG00000006938	0.931267143	0.004838611	0.025960788	16	68	42	53	16	22	24	26
ENSECAG00000024065	9.366015335	0.004839372	0.025960788	37214	12831	5382	2579	7513	2063	8254	1656
ENSECAG00000007853	2.136115404	0.004847245	0.025990747	25	51	48	67	130	94	136	136
ENSECAG00000000680	3.797193812	0.004889049	0.026202534	138	118	183	178	450	284	511	308
ENSECAG00000009288	2.627997	0.004903537	0.026267791	24	73	35	122	163	222	204	129

ENSECAG00000024935	6.046987524	0.004911613	0.026294185	1531	1534	1332	1115	1130	895	929	825
ENSECAG00000018423	7.076311249	0.004913092	0.026294185	1917	4660	2919	2532	1854	1594	1469	2561
ENSECAG00000017800	5.131285753	0.004916354	0.026299255	184	261	351	900	885	814	1271	1018
ENSECAG00000020125	4.462079391	0.004922283	0.026318578	293	435	823	474	203	469	273	229
ENSECAG00000014076	4.579286391	0.004931908	0.026340717	164	231	207	541	781	675	674	521
ENSECAG00000019344	6.56034842	0.004931954	0.026340717	1343	3609	1654	1754	1038	1473	1091	1554
ENSECAG00000002988	8.739849416	0.004933378	0.026340717	8506	14327	8144	6672	5202	5892	5129	6008
ENSECAG00000005768	4.420234269	0.004954422	0.026440647	199	192	280	353	525	510	759	543
ENSECAG00000003734	0.642709758	0.004961023	0.026463447	11	14	13	18	37	26	47	68
ENSECAG00000003887	7.327719091	0.004966561	0.026480558	2684	5688	2925	2852	1956	1918	2178	2706
ENSECAG00000013757	6.378601015	0.004978738	0.026533029	2439	2307	1117	1090	1261	904	1136	1020
ENSECAG00000007024	2.849907994	0.00499406	0.02660221	45.0011	58	111.001	111	207.001	209	198	192
ENSECAG00000022483	7.362315372	0.004997643	0.026608818	5798	2165	3457	2814	2574	2079	2002	1131
ENSECAG00000007238	0.93231871	0.00500641	0.026643013	9	22	10	26	37	28	69	93
ENSECAG00000007656	4.294674303	0.005013	0.026652084	159	213	274	300	548	360	531	733
ENSECAG00000012955	1.288286553	0.005013818	0.026652084	22	50	194	23	1	38	0	3
ENSECAG00000005359	3.738621193	0.005015152	0.026652084	286	333	219	291	192	136	224	215
ENSECAG00000020654	2.763169409	0.00505188	0.02682889	43	58	104	98	174	138	217	243
ENSECAG00000013865	4.425843127	0.005053144	0.02682889	211	178	240	388	665	462	713	524
ENSECAG00000022390	3.152238558	0.005064342	0.026872256	8	152	46	26	94	606	226	224
ENSECAG00000023446	3.838999941	0.005066042	0.026872256	129	132	198	211	428	306	469	371
ENSECAG00000008432	5.179342044	0.005086372	0.026967504	326	141.001	337	776	1255	716	1429	825
ENSECAG00000024316	5.968257991	0.005092238	0.026986011	576	411	907	1111	1673	1493	1689	2102
ENSECAG00000023236	7.483719325	0.005108412	0.027059104	2768	7230	2744	3468	1664	2895	2117	2461
ENSECAG00000021375	4.386428725	0.005124039	0.02711758	200	216	224	373	557	482	562	681
ENSECAG00000025956	3.047100699	0.005124225	0.02711758	200	215	152	134	107	87	111	140
ENSECAG00000007069	4.280513353	0.005141727	0.027197531	156.002	242	219	328	531	387	573	639.001
ENSECAG00000012304	4.046537889	0.005157341	0.027267429	187	164	155	240	542	364	456	468
ENSECAG00000017056	4.608892021	0.005174322	0.027329247	508	856	348	355	340	308	327	358
ENSECAG00000010560	8.538868113	0.005174641	0.027329247	8875	8950	21663	6424	20	3364	1	35
ENSECAG00000015405	7.392185533	0.005176249	0.027329247	914	1265	1339	4016	4074	5318	6233	3928
ENSECAG00000009418	4.239864021	0.005192497	0.027402298	255.001	555	287.001	576.001	225.001	242	288.001	356.001
ENSECAG00000021791	3.677486004	0.005199396	0.027425972	114	157	142	173	326	253	358	494
ENSECAG00000023882	1.668250487	0.00520408	0.027437936	77	57	68	77	35	45	24	56
ENSECAG00000022305	1.065367418	0.00520919	0.027452144	12	7	2	45	59	74	75	46
ENSECAG00000005000	4.292501815	0.005219268	0.027481533	100	235	236	363	411	393	551	882
ENSECAG00000022302	3.946133776	0.005219605	0.027481533	170	194	123	164	436	292	577	444
ENSECAG00000011180	5.701475809	0.005234458	0.02754697	361	468	827	834	1158	934	1363	2480
ENSECAG00000008873	1.690320175	0.005259092	0.027663795	33	29	27	31	83	56	87	153
ENSECAG00000024278	3.29313452	0.005271193	0.02771462	82	62	136	168	257	262	271	307
ENSECAG00000011304	7.218060446	0.005295414	0.027829089	3692	1000	5581	3689	1952	936	2138	2353
ENSECAG00000009131	3.189421968	0.005323755	0.027965094	64	107	96	153	291	198	284	242
ENSECAG00000014095	4.23097065	0.005333791	0.028004869	135	145	197	416	503	339	501	804
ENSECAG00000008486	3.431467463	0.005366492	0.028163551	117	464	210	197	132	133	177	107
ENSECAG00000006698	0.668836228	0.005374537	0.028187303	7	12	16	22	56	17	66	44
ENSECAG00000018205	2.487408327	0.00537598	0.028187303	55	36	70	68	197	96	183	170
ENSECAG00000024659	3.852704253	0.00538785	0.028236507	134	127	206	212	361	311	414	513
ENSECAG00000025695	0.934049146	0.00540787	0.028322283	19	31	40	98	21	22	22	24
ENSECAG00000015326	4.374046477	0.005409202	0.028322283	154	155	359	327	658	498	620	506
ENSECAG00000015966	6.834372859	0.005417677	0.028353589	1173	1071	1554	1096	3408	2114	2872	4854
ENSECAG00000018002	7.881881069	0.005437949	0.028446585	6997.01	1117	8328	8581	1365	4701	1097	296
ENSECAG00000016773	5.548752932	0.005468523	0.028593354	1024	1652	601	754	917	338	597	644
ENSECAG00000005467	7.349232936	0.005488168	0.028675543	2241	2595	4985	5340	3102	1754	2080	2299
ENSECAG00000014441	3.080998708	0.005489289	0.028675543	63	91	93	134	257	135	268	301
ENSECAG00000007582	6.741511204	0.005504306	0.028740773	1511	565	973	458	4714	1617	3266	3753
ENSECAG00000017181	7.160433127	0.00553236	0.028873988	6805	3336	1959	1716	75	2619	109	674
ENSECAG00000012951	4.579191566	0.00553907	0.028895735	310	1015	364	438	278	364	259	384
ENSECAG00000006917	0.654924603	0.005562467	0.028987893	20	34	76	20	6	10	20	29
ENSECAG00000018141	7.076744394	0.005563113	0.028987893	2593	4819	2015	2328	1532	1787	1799	2132
ENSECAG00000017462	3.115566188	0.00556439	0.028987893	89	249	190	264	107	100	131	152
ENSECAG00000015287	3.329801906	0.005590437	0.029110241	190	292	200	177	135	109	171	148
ENSECAG00000018307	6.090343213	0.005615092	0.029225231	594	493	1108	986	1784	1276	1726	2974
ENSECAG00000023622	4.551513339	0.005619889	0.029236804	181	287	298	389	584	523	582	877
ENSECAG00000000085	5.52635373	0.005624686	0.029248368	570	874	1219	1373	744	709	655	759
ENSECAG00000017987	3.07266425	0.005642237	0.029326213	56	116	94	113	225	178	239	305
ENSECAG00000023703	2.375608114	0.005652909	0.029368243	42	46	66	54	117	166	83	236
ENSECAG00000016177	-0.097795138	0.005659716	0.029390169	4	13	4	8	22	24	31	25
ENSECAG00000017034	4.881814361	0.005688414	0.029525703	565	800	583	504	412	460	397	475
ENSECAG00000009549	7.899566747	0.005693788	0.0295401	4244	10585	3271	3334	2949	2312	2545	4126
ENSECAG00000007453	3.729331028	0.005713297	0.029627789	149	300	442	310	166	197	131	264
ENSECAG00000020673	0.398505417	0.005736858	0.029736396	34	25	29	25	18	9	13	20
ENSECAG00000022985	4.295093441	0.005750031	0.029791088	228	180	238	241	609	357	569	651
ENSECAG00000023648	5.416713591	0.0057605	0.029825549	411	479	515	674	1278	777	1178	1441
ENSECAG00000018834	2.264019716	0.005761933	0.029825549	32	47	26	74	247	53	172	106
ENSECAG00000024365	5.23715197	0.005764985	0.029827757	273	371	486	781	903	966	1046	1180
ENSECAG00000021673	3.206604567	0.005780342	0.029893605	48	64	88	179	251	273	453	103
ENSECAG00000013435	5.269223641	0.005783351	0.029895559	391	589	297	281	1559	1081	972	779
ENSECAG00000024797	7.197118455	0.005807077	0.030004551	2321	3426	4205	2987	2378	1663	1646	2793
ENSECAG00000013255	8.114836914	0.005814598	0.030029756	5216	6559	5999	6518	3948	4266	2963	4449
ENSECAG00000021199	4.260871322	0.005828663	0.030088716	256	438	518	444	335	250	239	346
ENSECAG00000009014	2.32774663	0.005862152	0.030241054	27	42	80	64	149	99	205	121
ENSECAG00000024189	7.159300493	0.005863496	0.030241054	724	809	1371	3502	3909	4240	5613	2966
ENSECAG00000023071	1.957012923	0.005871752	0.030269893	25	45	44	50	128	67	121	124
ENSECAG00000007134	6.804626232	0.005883906	0.030318794	1913	3513	2053	2198	1297	1730	1488	1808
ENSECAG00000015633	3.066929377	0.005901189	0.030385126	39	88	47	179	242	357	182	185

ENSECAG00000017949	1.902570671	0.005902128	0.030385126	165	88	24	43	32	38	42	35
ENSECAG00000017294	4.789028197	0.005907606	0.030399558	559	681	438	626	430	360	351	527
ENSECAG00000007007	6.386365846	0.00591745	0.030436428	693	926	1115	1309	2547	1455	2348	2946
ENSECAG00000005263	4.842760264	0.005941818	0.030547934	448	1259	373	446	362	413	280	456
ENSECAG00000018532	2.793464039	0.005944768	0.030549276	65	18	86	47	402	77	291	104
ENSECAG00000021651	6.356621861	0.005954559	0.030574755	536	640	1177	1540	2073	3022	2367	1588
ENSECAG00000024512	4.198758019	0.005955108	0.030574755	157	112	291	289	624	486	549	376
ENSECAG00000004900	5.236334598	0.005966242	0.030618084	345	341	509	638	1217	725	1251	923
ENSECAG00000011823	2.65295765	0.006013768	0.03084805	55	37	89	91	187	131	178	221
ENSECAG00000009302	1.938231271	0.006032342	0.030929364	24	21	71	36	140	96	112	93
ENSECAG00000016316	6.493693309	0.00603929	0.030951022	1520	2610	1767	1758	1239	1171	1309	1639
ENSECAG00000023009	11.05073182	0.00604432	0.030962836	94630	49602	16885	17913	23458	18484	22875	23104
ENSECAG00000007773	4.229968494	0.00605139	0.03098508	194	217	152	294	597	334	602	558
ENSECAG00000018952	3.447091127	0.006058319	0.031006588	90	131	129	163	306	281	310	291
ENSECAG00000013749	5.125922815	0.00608461	0.031122924	981	662	663	555	497	438	382	673
ENSECAG00000006364	5.302267469	0.006086529	0.031122924	831	1252	569	647	514	507	657	563
ENSECAG00000012264	1.11253006	0.006105853	0.031207694	43	49	61	37	16	29	34	25
ENSECAG00000020998	5.858097221	0.006135578	0.031341852	1151	1804	1006	886	861	682	810	1014
ENSECAG00000016519	3.706057004	0.006139099	0.031341852	247	314	256	292	224	148	144	253
ENSECAG00000012926	0.857521578	0.006140377	0.031341852	8	17	20	23	31	49	38	90
ENSECAG00000000125	6.509438413	0.006187725	0.031569343	1904	2018	1331	2509	1308	1358	1269	1426
ENSECAG00000018560	5.274532613	0.006249995	0.03187222	628	943	1057	938	266	927	227	567
ENSECAG00000015813	4.315510227	0.0062527	0.03187222	241	452	674	381	266	294	261	359
ENSECAG00000007008	2.717871525	0.006277543	0.031984503	87	202	147	156	82	111	59	100
ENSECAG00000018964	0.741658332	0.006292939	0.032048576	21	8	12	4	75	23	51	53
ENSECAG00000023441	4.463613661	0.006311675	0.032129595	969	324	285	232	302	262	193	259
ENSECAG00000016313	4.838726442	0.006327525	0.032195854	288	231	405	468	810	603	770	941
ENSECAG00000018034	6.590364711	0.006350508	0.032298329	2505	2120	1856	1486	1428	1092	1423	1529
ENSECAG00000020056	1.928264811	0.006368191	0.032373774	34	36	27	60	93	84	100	158
ENSECAG00000011250	2.721026469	0.006385188	0.032433733	50	72	80	97	189	154	205	181
ENSECAG00000013592	2.15308661	0.006385695	0.032433733	38	28	66	55	144	90	126	145
ENSECAG00000003563	1.880217746	0.006392504	0.032453814	26	42	51	35	101	92	107	111
ENSECAG00000005987	4.281824119	0.006438501	0.032672731	178.007	494	522	561	243	245	345	328
ENSECAG00000006721	6.758558161	0.006456024	0.032740952	3323	2343	1899	1426	1519	1429	1471	1258
ENSECAG00000023886	6.003044355	0.006457708	0.032740952	812	709	645	626	2004	1349	1745	2094
ENSECAG00000022092	6.621452115	0.006500229	0.03294184	1660	2829	1789	2120	1381	1281	1594	1579
ENSECAG00000008917	7.554354088	0.006522514	0.033040039	3696	4784	4364	3306	2693	2577	2884	2419
ENSECAG00000020012	5.349939039	0.006528415	0.03305519	338	424	611	639	1062	1320	928	1060
ENSECAG00000013713	4.967647163	0.006558375	0.033192098	568	791	661	576	470	465	387	585
ENSECAG00000018646	3.802408307	0.006603839	0.033407312	100	104	224	247	378	333	391	438
ENSECAG00000019263	8.170473171	0.006608011	0.033413538	6284	10939	3458	4502	3642	3446	3662	3933
ENSECAG00000018498	3.401028973	0.006615833	0.033425456	69	110	138	190	276	225	284	389
ENSECAG00000016882	0.674014208	0.006616252	0.033425456	5	11	16	29	27	50	42	61
ENSECAG00000015086	9.713071055	0.006633251	0.033496441	16751	37207	8685	13187	7186	10534	7987	14556
ENSECAG00000009982	7.111138006	0.006648117	0.0335566	2730	3204	3083	2762	2048	1888	2050	2040
ENSECAG00000003539	3.95255798	0.006654794	0.033575388	145	151	162	287	412	335	452	498
ENSECAG00000001234	4.58687687	0.006663646	0.033605123	204	274	325	396	611	561	668	745
ENSECAG00000004617	6.619739792	0.006667245	0.033608358	482	1024	1390	1841	2169	3483	2719	2559
ENSECAG00000013304	5.801678422	0.006704131	0.033777674	1532	1625	707	751	880	699	734	678
ENSECAG00000007609	2.613516965	0.006707485	0.033777674	48	49	89	48	122	76	232	312
ENSECAG00000011248	6.11317368	0.006710344	0.033777674	1402	1674	1308	1351	962	998	1003	1214
ENSECAG00000026984	7.090052446	0.006712726	0.033777674	2223	6397	1969	1618	1340	1434	1873	2159
ENSECAG00000010055	2.354069857	0.006770453	0.034053072	43	36	82	40	199	78	145	177
ENSECAG00000024002	3.310887623	0.006773507	0.034053355	40	116	86	204	167	343	297	329
ENSECAG00000013606	4.4024614	0.006814644	0.03424502	202	201	268	365	659	429	544	667
ENSECAG00000018324	7.7579839	0.006898236	0.034649759	6842	5418	2812	3188	2144	3173	2583	2951
ENSECAG00000012484	6.398447258	0.006923503	0.034761309	3216	1246	1896	782	1319	1271	679	580
ENSECAG00000021210	4.011133151	0.006937926	0.034818337	149	146	248	229	403	360	459	542
ENSECAG00000021865	1.930978212	0.006969382	0.034960759	198	50	24	67	11	63	17	36
ENSECAG00000010939	1.431049485	0.007033009	0.035264364	17	4	21	41	66	131	111	17
ENSECAG00000006884	3.760061391	0.007039205	0.035279861	90	144	196	233	353	294	433	403
ENSECAG00000025029	3.601660706	0.007047673	0.035306729	96	372	291	360	160	141	188	203
ENSECAG00000022780	1.493669062	0.007052956	0.035317624	14	27	10	46	33	157	57	85
ENSECAG00000023084	6.247897167	0.007062473	0.035349699	694	528	1170	1276	2347	1592	2104	2373
ENSECAG00000004830	2.335475059	0.007114203	0.035579199	38	52	64	66	186	99	148	131
ENSECAG00000022601	6.450661389	0.007116094	0.035579199	738	1056	1037	1481	2609	2061	2361	2462
ENSECAG00000013492	6.206862548	0.007117719	0.035579199	1702	1943	1110	1368	903	1137	1052.01	1173
ENSECAG00000019879	5.566967009	0.00714171	0.035664048	635	1185	1121	1081	823	712	697	723
ENSECAG00000019051	5.489613498	0.007141719	0.035664048	469	420	621	656	1259	1027	1529	1034
ENSECAG00000014283	6.38440198	0.00714411	0.035664048	711	624	851	1841	2488	2073	2793	1902
ENSECAG00000009992	4.036030868	0.007147872	0.035667157	304	456	296.001	323	180	236	267	281
ENSECAG00000007364	4.440730981	0.007171287	0.035768288	370	527	405	515	303	345	320	367
ENSECAG00000021999	9.109839255	0.00717694	0.035780778	11983	21609	6673	8251	6947	6448	7650	6648
ENSECAG00000000186	7.595655797	0.007183779	0.035799164	4411	2753	5556	4687	3642	2412	2756	1282
ENSECAG00000002257	3.472926325	0.007191337	0.03582112	24	156	125	170	317	196	495	305
ENSECAG00000016549	2.338913903	0.007195883	0.035828055	40	24	95	31	136	92	183	194
ENSECAG00000020432	8.239642253	0.00721956	0.035930197	8212	9154	5349	3320	4841	1975	3643	4766
ENSECAG00000022172	7.315571213	0.007241974	0.036025967	2851	7235	2642	1611	2251	2437	1168	1679
ENSECAG00000019341	0.308505307	0.007259335	0.036096525	20	15	27	57	6	20	17	7
ENSECAG00000011226	1.918518425	0.007316634	0.036365525	33	29	40	60	123	81	90	130
ENSECAG00000007922	4.020654362	0.007322276	0.036377659	370	727	508	217	1	262	2	19
ENSECAG00000006248	3.172145619	0.007335732	0.036423615	99	223	140	411	106	135	64	173
ENSECAG00000018188	4.746900475	0.007337938	0.036423615	983	847	139	310	371	310	269	241
ENSECAG00000011700	3.706237579	0.007352746	0.03646616	114	438	461	347	44	336	47	81
ENSECAG00000023059	2.935058208	0.007352929	0.03646616	83	86	46	83	283	136	294	173

ENSECAG00000019484	7.044974391	0.007363034	0.036500348	2757	3972	2093	2419	1753	1476	1849	2394
ENSECAG00000019599	4.999115088	0.007404118	0.036688001	612	1186	381	532	404	423	433	527
ENSECAG00000020630	5.268044176	0.007414277	0.036722326	259	380	435	894	1015	918	1019	1257
ENSECAG00000002936	3.532817738	0.007423747	0.036753209	80	145	143	182	245	262	328	449
ENSECAG00000021300	6.95556283	0.007428738	0.0367619	2331	3970	2161	2210	2029	1766	1569	1523
ENSECAG00000012069	7.443487335	0.007449728	0.036849719	3350	5670	3313	2638	2758	2180	2236	2390
ENSECAG00000007734	0.26790446	0.007453143	0.036850571	7	11	3	17	62	32	20	22
ENSECAG00000007741	5.647828455	0.007466537	0.036900738	453	679	642	693	1262	1002	1435	1734
ENSECAG00000013580	5.112313651	0.007480156	0.036951969	854	571	764	643	519	522	515	494
ENSECAG00000013139	3.896914106	0.0074888	0.036978594	347	398	222	269	217	196	238	193
ENSECAG00000010367	2.00601	0.007498391	0.037009869	43	24	38	52	129	61	107	173
ENSECAG00000024559	5.652760646	0.007506622	0.037034407	1042	1103	1049	1003	929	789	746	495
ENSECAG00000015297	3.627247733	0.007511072	0.037040278	106	114	179	177	352	242	268	523
ENSECAG00000020000	0.347516726	0.007531856	0.037126657	5	10	19	10	22	31	31	60
ENSECAG00000017687	8.616824157	0.007538531	0.037133452	9530	11619	6091	6339	4665	5373	5263	5502
ENSECAG00000012941	9.300347101	0.007542357	0.037133452	18058	20394	7848	7894	8421	8989	6698	6228
ENSECAG00000000669	8.065854572	0.007543039	0.037133452	5213	7036	5380	5431	3642	4061	3568	3900
ENSECAG00000006955	5.3150235	0.007553876	0.037170699	400	494	422	636	1091	860	1057	1292
ENSECAG00000021804	7.699490895	0.007593546	0.037349725	8596	1960	5711	1778	3399	2427	1677	1267
ENSECAG00000007680	4.997285182	0.007616882	0.037448298	341	334	341	509	982	523	1002	1000
ENSECAG00000014898	5.344545714	0.00763987	0.03754507	345	446	621	618	1091	881	944	1490
ENSECAG00000024048	8.198686661	0.007646378	0.037560806	9390	4341	5994	5837	5131	3615	3902	1745
ENSECAG00000008318	3.615343085	0.007664668	0.037634383	125	94	150	207	314	287	361	384
ENSECAG00000009325	4.736456864	0.007672035	0.03765428	208	292	347	498	670	514	763	955
ENSECAG00000001856	3.900124581	0.007676547	0.037660159	111	178	160	266	357	260	437	617
ENSECAG00000018995	6.141174965	0.007685467	0.037687644	1489	2372	995	1067	1115	981	819	1029
ENSECAG00000009648	7.302828367	0.007713942	0.037810964	5146	4766	1828	1538	1898	1819	2081	1797
ENSECAG00000008126	7.060949037	0.007778195	0.038109465	1231	1346	1785	1868	4130	2802	4171	3756
ENSECAG00000018997	5.44518448	0.007809069	0.038244241	235	328	619	1022	1110	1015	1393	1315
ENSECAG00000016238	8.942444159	0.007874488	0.038543555	11410	17170	6851	7246	4942	6030	5536	8920
ENSECAG00000016501	2.408529546	0.00787697	0.038543555	44	44	58	91	140	130	122	203
ENSECAG00000025208	1.312979058	0.007937762	0.038824302	24	36	77	106	43	31	23	22
ENSECAG00000022784	7.643036217	0.007942302	0.038829792	2421	1458	2392	2216	6561	4206	5935	6217
ENSECAG00000002871	4.949308827	0.007956737	0.03888363	579	1456	318	347	351	322	351	545
ENSECAG00000022242	6.769411725	0.008012894	0.03914123	2794	2988	1785	1508	1385	1565	1527	1485
ENSECAG00000012085	5.663345138	0.00802022	0.039160182	1173	1536	626	847	763	645	665	835
ENSECAG00000008724	4.7378548	0.008058064	0.039328062	260	639	843	631	331	459	400	401
ENSECAG00000016839	0.621335902	0.008068686	0.039360273	37	49	10	48	8	31	7	11
ENSECAG00000022154	5.45393458	0.008071593	0.039360273	851	1178	707	892	521	593	772	773
ENSECAG00000020519	7.965776209	0.008118399	0.039571517	8505	7263	2578	2721	3710	2610	2392	3213
ENSECAG00000019241	2.121709171	0.008122065	0.039571517	34	19	59	49	132	159	170	43
ENSECAG00000019285	5.033774161	0.008125361	0.039571517	314	385	432	492	908	885	708	994
ENSECAG00000001080	3.056975416	0.008147608	0.039662861	62	64	143	107	170	197	273	296
ENSECAG00000021034	2.794879325	0.008187207	0.039838564	33	137	141	431	77	59	97	103
ENSECAG00000024689	4.825315347	0.008196467	0.039861397	276	303	416	409	709	619	861	849
ENSECAG00000006717	5.042561551	0.008198916	0.039861397	248	1167	1071	708	499	668	164	263
ENSECAG00000014739	0.245298457	0.008214258	0.039918904	10	9	13	7	27	20	34	52
ENSECAG00000024158	2.708405227	0.00823936	0.040008153	60	110	377	140	37	150	39	24
ENSECAG00000001072	4.316757379	0.008246886	0.040008153	302	558	321	533	243	364	276	299
ENSECAG00000001317	7.301386268	0.008248875	0.040008153	2435	8206	2639	1085	1623	1986	1933	1753
ENSECAG00000001439	5.344754504	0.008249365	0.040008153	756	888	630	1187	859	447	597	565
ENSECAG00000021336	1.236664076	0.008250229	0.040008153	37	66	71	50	11	56	17	12
ENSECAG00000013808	2.984309693	0.008264941	0.040062398	56	71	87	160	228	202	227	215
ENSECAG00000009044	5.808561931	0.008313738	0.040281747	785	1158	2502	580	829	755	633	864
ENSECAG00000008693	7.675816039	0.00836093	0.040493133	5553	6137	2843	2776	2636	3061	2481	2291
ENSECAG00000016553	2.660950215	0.008389365	0.040613532	50	64	81	93	155	128	188	234
ENSECAG00000010646	5.331642279	0.008393865	0.040618014	380	492	440	705	1055	881	1115	1273
ENSECAG00000001042	7.183903399	0.008399689	0.040628893	1359	4991	3528	3384	1849	1996	1836	2539
ENSECAG00000016933	6.135840355	0.008417735	0.040698852	1073	2952	889	1203	982	746	1120	1039
ENSECAG00000001066	5.138007468	0.008448649	0.040830946	532	1676	557	371	556	429	507	255
ENSECAG00000006704	3.60976804	0.008499112	0.041057358	122	111	150	196	362	241	336	399
ENSECAG00000023935	6.378879254	0.008555558	0.041312475	877	826	824	1496	2383	1777	2229	2736
ENSECAG00000013698	4.132116511	0.00857877	0.041406961	201	313	621	456	237	167	205	434
ENSECAG00000011827	5.769099136	0.008583679	0.041413063	1413	1730	589	789	742	671	862	681
ENSECAG00000023456	2.620756438	0.008587451	0.041413674	36	49	74	117	212	152	214	111
ENSECAG00000000419	8.862771069	0.008591533	0.041415783	4384	4078	6201	5652	12434	7957	13441	20551
ENSECAG00000004389	7.078038243	0.00862615	0.041565019	2035	2379	3347	4229	2043	2002	1928	2120
ENSECAG00000016244	5.035081979	0.008656262	0.041692432	208	339	540	582	739	780	844	1208
ENSECAG00000010891	5.501233796	0.008762114	0.042184381	517	1576	984	849	566	767	621	710
ENSECAG00000017313	2.91157376	0.008785546	0.042270701	141	151	111	281	66	145	96	93
ENSECAG00000014580	1.804085698	0.008788557	0.042270701	32	65	60	193	33	38	38	66
ENSECAG00000023717	7.062328757	0.008791205	0.042270701	3005	4977	1530	2003	1841	1784	1880	1371
ENSECAG00000007385	6.465078326	0.008814491	0.042364741	5588	735	482	415	672	699	868	829
ENSECAG00000000369	4.076786866	0.008839584	0.042467379	162	770	348	244	183	203	199	316
ENSECAG00000008739	0.772970798	0.008851637	0.04250731	11	44	63	43	21	19	13	27
ENSECAG00000012523	2.401558424	0.00888399	0.042644654	42	45	68	85	132	119	146	190
ENSECAG00000010672	1.489830714	0.008902262	0.042699849	13	18	40	50	65	78	92	78
ENSECAG00000005595	4.580904363	0.008903005	0.042699849	262	215	359	280	736	460	781	641
ENSECAG00000009897	3.895187741	0.008912497	0.042727339	99	150	212	273	433	350	504	326
ENSECAG00000019762	7.345819461	0.008923919	0.042764051	2840	6116	2167	3362	1833	2698	2509	1367
ENSECAG00000018153	3.679582352	0.008969166	0.04296276	185	414	225	278	173	179	152	247
ENSECAG00000004193	1.395640722	0.008973201	0.042963976	23	10	28	38	56	51	55	150
ENSECAG00000016061	5.606862417	0.008995105	0.043035992	780	1441	826	1032	683	819	721	739
ENSECAG00000006851	0.910871709	0.008995818	0.043035992	6	19	24	18	25	97	42	48
ENSECAG00000009608	5.133829824	0.009003445	0.043054354	832	1194	381	526	477	528	469	425

ENSECAG00000012706	5.556870965	0.009012373	0.043078914	539	973	1245	1315	764	630	738	873
ENSECAG00000012862	9.210720044	0.009045241	0.043217844	19256	19986	5276	6480	5953	7284	9104	4315
ENSECAG00000018467	7.049907451	0.0090512	0.043228138	3624	4281	1786	1457	1697	1804	1694	1562
ENSECAG00000011093	6.111953228	0.009076042	0.043328569	1742	776	1897	1544	1267	1035	927	583
ENSECAG00000022845	1.363062463	0.009111103	0.04347768	29	69	93	61	25	21	57	5
ENSECAG00000017507	0.255692412	0.009123485	0.043518489	6	4	7	26	32	22	37	43
ENSECAG00000015948	1.434106972	0.009166929	0.043707362	19	32	27	35	74	59	64	104
ENSECAG00000011276	5.318376562	0.009200716	0.043835453	932	903	690	712	649	592	554	596
ENSECAG00000012257	6.315808623	0.009204276	0.043835453	734	721	1149	1373	2047	1749	2156	2705
ENSECAG00000017886	4.440529958	0.009205368	0.043835453	160	221	319	411	582	498	549	706
ENSECAG00000024686	6.196534404	0.009214714	0.043861131	1224	1713	1017	2376	1069	1067	1029	1318
ENSECAG00000012134	3.976717602	0.009218481	0.043861131	271	380	271	407	255	171	228	315
ENSECAG00000017221	8.931731796	0.009248137	0.043977219	8127	20741	6659	7799	5745	6104	6597	6934
ENSECAG00000011223	3.420302418	0.009250621	0.043977219	131	440	393	110	27	260	47	62
ENSECAG00000017445	0.991191717	0.009309095	0.044236695	24	23	45	100	29	19	21	28
ENSECAG00000009533	9.607949604	0.00932866	0.044311136	23270	16122	13403	12635	11807	8127	10561	11209
ENSECAG00000019894	6.290102487	0.009344178	0.044366298	1916	1917	1655	931	1270	923	1164	1115
ENSECAG00000005330	5.05089036	0.009379141	0.044513702	412	1023	610	806	644	389	446	551
ENSECAG00000007115	4.559884327	0.009386663	0.044530798	380	903	358	364	313	329	360	332
ENSECAG00000015543	6.411680687	0.009399715	0.044574108	836	817	1064	1472	2366	2007	1932	2973
ENSECAG00000003428	10.44488498	0.009408972	0.044599391	33930	25074	31522	28480	20076	17686	22039	18199
ENSECAG00000017160	5.05685597	0.009418295	0.044614538	378	960	1456	695	94	891	68	171
ENSECAG00000021289	5.687856652	0.009420021	0.044614538	342	204	1050	589	2032	1255	2033	670
ENSECAG00000012316	3.550567185	0.009443785	0.044708454	237	257	250	239	167	190	115	215
ENSECAG00000018363	4.998134465	0.009466235	0.044796067	799	449	701	708	396	600	350	522
ENSECAG00000001985	2.903272737	0.009477841	0.044832321	193	268	96	80	84	62	105	123
ENSECAG00000025051	2.632011884	0.009497643	0.044907292	44	72	47	99	154	81	282	202
ENSECAG00000009953	4.650003629	0.009506548	0.044924073	183	291	304	509	668	621	719	674
ENSECAG00000012776	6.948168568	0.009511849	0.044924073	2088	2524	3126	2907	2185	1850	1470	1861
ENSECAG00000013847	6.15703214	0.009513054	0.044924073	637	871	852	1208	1886	1592	1715	2532
ENSECAG00000004618	1.306203554	0.009523504	0.044954736	34	31	89	73	35	35	26	30
ENSECAG00000025031	3.069287762	0.009534051	0.044985832	193	238	133	141	134	91	90	149
ENSECAG00000023838	0.708073734	0.009623925	0.045391049	17	8	13	20	70	27	42	45
ENSECAG00000005856	7.274792262	0.009628692	0.045394689	995	1562	1875	3040	3447	3246	4604	6294
ENSECAG00000016570	0.56889548	0.009644118	0.045448557	24	30	39	36	21	17	11	25
ENSECAG00000001037	5.649744248	0.009663509	0.045521055	814	1737	749	1089	453	485	555	1405
ENSECAG00000010107	10.07839282	0.009686903	0.045612345	18924	41052	18160	16535	15327	12900	15704	13746
ENSECAG00000008327	4.928365532	0.00973484	0.045802741	263	391	394	486	824	766	907	724
ENSECAG00000016649	0.226617013	0.0097354	0.045802741	13	21	24	50	12	18	10	13
ENSECAG00000004314	4.618441494	0.009766553	0.045930288	263	285	279	361	648	466	696	856
ENSECAG00000023262	5.918168179	0.009774869	0.045950375	560.011	724	657.008	1109	1714	1320	1686	1761
ENSECAG00000016711	3.466899197	0.0098139	0.046114776	83	75	201	150	406	200	341	291
ENSECAG00000009519	2.195970979	0.009834518	0.04619003	76	97	131	94	48	69	56	82
ENSECAG00000017795	8.790259628	0.009838045	0.04619003	4762	6744	17312	14882	6661	4516	8707	2887
ENSECAG00000020223	0.015254874	0.009865525	0.046299914	11	22	29	30	9	20	6	7
ENSECAG00000009781	3.93185296	0.009878205	0.046340283	150	176	162	242	401	271	470	530
ENSECAG00000025129	3.899861109	0.009901101	0.046428421	183	644	421	187	76	372	66	119
ENSECAG00000009114	5.458324934	0.009905166	0.046428421	435	435	543	800	1293	824	1271	1343
ENSECAG00000003462	4.62124413	0.009969925	0.046698868	375	373	922	504	306	488	338	273
ENSECAG00000016390	5.154114328	0.009971084	0.046698868	336	340	592	457	922	651	913	1435
ENSECAG00000007556	8.375906207	0.009989002	0.046763511	7887	9265	4819	6247	4090	4751	4955	4356
ENSECAG00000021033	5.184169549	0.010005725	0.046822507	593	922	737	766	582	632	487	550
ENSECAG00000009046	9.681070716	0.010018039	0.046860832	18978	29287	11821	10453	9995	9248	9317	14307
ENSECAG00000015940	5.369504237	0.010024168	0.046864641	392	398	540	778	1163	872	1072	1321
ENSECAG00000023564	4.310258735	0.010027103	0.046864641	172	200	155	430	637	331	559	685
ENSECAG00000021147	8.890510259	0.010072041	0.047055318	5113	3189	5400	6862	15024	7896	13283	19213
ENSECAG00000013225	2.678161906	0.010106188	0.047176515	40	192	348	110	10	139	17	55
ENSECAG00000018187	6.682100929	0.010106287	0.047176515	1231	2598	2043	3285	1603	1757	1309	1458
ENSECAG00000001853	4.24467859	0.010118956	0.047201864	143	240	293	253	621	384	573	466
ENSECAG00000021501	6.177990638	0.010120026	0.047201864	737	825	860	1145	1764	1565	2050	2454
ENSECAG00000007736	2.55508888	0.010158363	0.047342539	66	134	187	145	59	93	65	110
ENSECAG00000013300	4.379649761	0.010158521	0.047342539	3	26	245	84	182	2546	25	79
ENSECAG00000009845	4.350148114	0.010174555	0.047388329	196	277	224	290	566	406	654	572
ENSECAG00000002716	6.12180256	0.010179898	0.047388329	1860	2032	910	934	1118	890	1041	735
ENSECAG00000006915	8.175543782	0.010180858	0.047388329	8789	10948	2010	3134	4023	2553	2660	4283
ENSECAG00000022206	5.571632855	0.010248154	0.047655814	374	589	655	835	1075	1073	1439	1480
ENSECAG00000017425	7.753169153	0.010250299	0.047655814	3716	7747	4154	3015	2592	2909	2954	3443
ENSECAG00000016396	6.376506454	0.010250907	0.047655814	697	824	1278	1394	2072	1887	2199	2837
ENSECAG00000016465	5.990395293	0.0102613	0.047684617	1613	1097	1105	1382	1160	808	781	1075
ENSECAG00000017917	8.20219961	0.010345872	0.048057972	1851	2768	3417	5672	7622	10321	9570	5422
ENSECAG00000003859	1.826895881	0.010370036	0.04815053	37	39	21	25	58	61	95	218
ENSECAG00000017943	4.685308486	0.010381905	0.048185948	142	427	159	364	697	959	958	299
ENSECAG00000005244	6.225615544	0.010389979	0.048203732	666.001	848	1100	1136	1831	1671	2276	2261
ENSECAG00000024122	6.175266979	0.010404103	0.048249558	1563	2191	906	1327	1073	893	1150	1091
ENSECAG00000021462	5.498445495	0.010417217	0.048290666	282	239	402	1277	1497	1170	1398	1038
ENSECAG00000024622	4.880969208	0.010444608	0.048378852	395	439	715	1074	500	307	401	640
ENSECAG00000014994	7.733897778	0.010445726	0.048378852	5026	4658	3857	4336	2821	3100	2569	3774
ENSECAG00000000612	1.145574375	0.010449015	0.048378852	12	19	29	31	67	33	57	93
ENSECAG00000023275	1.380365084	0.010492874	0.048562132	12	27	29	40	68	34	80	117
ENSECAG00000012111	0.651734395	0.010518797	0.048662284	24	43	60	18	10	17	32	5
ENSECAG00000014430	1.45669067	0.010571902	0.048888056	14	13	42	40	45	73	54	150
ENSECAG00000012321	6.234426869	0.010629626	0.04913499	618	957	938	917	1858	1947	3382	1123
ENSECAG00000007747	6.44720396	0.010658025	0.049231759	812	900	1209	1413	2596	1714	2309	2862
ENSECAG00000013632	4.747375941	0.010659226	0.049231759	801	599	307	468	344	330	352	495
ENSECAG00000019651	3.799240089	0.010699047	0.049395602	173	550	232	263	214	143	243	183

ENSECAG00000009760	4.164571227	0.010776064	0.049730967	135	170	339	211	578	303	585	513
ENSECAG00000018756	4.820616727	0.010787286	0.049762542	203	335	373	480	657	1107	567	687
ENSECAG00000025091	8.623127631	0.010824465	0.049913788	8662	11704	6609	6602	4553	6045	4988	6074
ENSECAG00000023130	0.611613304	0.010844717	0.049986886	10	4	24	17	39	21	54	61
ENSECAG00000000982	4.738270146	0.010882584	0.050138044	133	222.999	426	495.999	580	354	824	1344
ENSECAG00000023479	4.786062234	0.010886337	0.050138044	260	300	264	538	839	539	944	659
ENSECAG00000014211	5.542933048	0.010957166	0.050443811	554	410	641	615	1182	862	1385	1672
ENSECAG00000024263	1.717803818	0.010968562	0.050468979	28	111	62	114	22	32	30	81
ENSECAG00000016424	8.148492724	0.010971517	0.050468979	7366	7024	5021	4300	3667	4323	3273	4289
ENSECAG00000016614	5.104855843	0.010978797	0.050482029	379	254	182	641	1333	673	1439	509
ENSECAG00000023748	4.794339198	0.010986269	0.05049595	238	243	414	528	682	665	707	917
ENSECAG00000008711	4.729088611	0.010996111	0.050518984	233	282	372	459	545	615	762	920
ENSECAG00000014831	7.643247072	0.011000173	0.050518984	4809	5827	2770	3432	2259	2263	2845	3765
ENSECAG00000007250	4.775200672	0.011009962	0.050541911	258	428	308	324	752	639	786	759
ENSECAG00000024781	3.531205217	0.011014062	0.050541911	115	123	128	188	335	251	332	332
ENSECAG00000001652	3.743393952	0.011025334	0.050573214	176	168	246	684	189	157	175	252
ENSECAG00000024764	3.096758272	0.011060579	0.05069991	151	148	200	244	127	156	91	121
ENSECAG00000013217	5.004135407	0.01106188	0.05069991	686	881	563	497	379	562	502	435
ENSECAG00000023795	1.768386751	0.011081683	0.050770193	61	53	93	95	42	57	51	30
ENSECAG00000016645	4.969053409	0.011099209	0.050829992	316	670	897	890	388	591	403	539
ENSECAG00000024981	2.91326676	0.011126018	0.050932241	80	46	89	109	286	168	243	146
ENSECAG00000022197	4.951278913	0.011135034	0.050952984	793	1450	271	167	345	230	449	406
ENSECAG00000020858	3.780374301	0.011141247	0.050960887	199	596	263	188	105	282	187	83
ENSECAG00000014499	1.684353367	0.011148419	0.050967492	29	24	36	41	122	54	124	61
ENSECAG00000022470	1.902265699	0.011151662	0.050967492	52	51	121	122	30	60	40	68
ENSECAG00000016309	4.161082567	0.01116295	0.05099857	289	306	582	348	335	257	207	283
ENSECAG00000024933	4.1490035	0.011175556	0.051034189	337	470	343	313	319	261	251	229
ENSECAG00000019815	9.103288275	0.01117973	0.051034189	9012	18922	9327	10774	7677	6178	9232	7678
ENSECAG00000019940	2.098076711	0.011213988	0.051170013	30	83	72	259	47	44	77	38
ENSECAG00000023015	1.458293654	0.011227385	0.051197005	27	49	79	106	54	31	32	22
ENSECAG00000010151	6.342758587	0.011228915	0.051197005	1521	2726	1082	1511	1140	1089	1469	960.001
ENSECAG00000004180	2.633567046	0.011246928	0.051258562	58	168	159	196	55	108	107	57
ENSECAG00000002408	5.214453088	0.011263112	0.051311741	481	522	1129	1131	673	480	517	660
ENSECAG00000014375	2.586135956	0.011270486	0.051324754	66	144	113	227	75	68	86	115
ENSECAG00000017874	6.824271392	0.011303684	0.051447828	1824	6602	777	1229	800	1498	843	1949
ENSECAG00000009610	6.392496639	0.011306568	0.051447828	476	484	1126	1919	2293	2319	3483	1368
ENSECAG00000006109	0.816380247	0.011322958	0.05150178	80	19	34	23	5	38	8	8
ENSECAG00000000265	4.935399938	0.011337014	0.051545078	403	337	273	360	914	561	1035	861
ENSECAG00000009494	4.62486728	0.011358319	0.05162129	173	208	509	318	639	548	600	933
ENSECAG00000023784	5.744373439	0.011397305	0.051742269	479	489	747	1050	1328	1404	1504	1447
ENSECAG00000009830	3.234456047	0.011397323	0.051742269	120	224	247	243	99	122	102	232
ENSECAG00000021275	5.897646889	0.0113986	0.051742269	673	565	828	845	1735	1262	1753	1650
ENSECAG00000011125	8.24563133	0.011413339	0.051788479	6845	9300	4566	4933	4412	4007	3726	4642
ENSECAG00000016526	1.40859227	0.011434538	0.051863958	17	19	38	41	84	65	79	62
ENSECAG00000015901	4.415778784	0.011457707	0.051943604	196	191	250	444	624	472	551	646
ENSECAG00000000396	5.632032978	0.011461241	0.051943604	564	578	725	398	1238	1023	1535	1663
ENSECAG00000021751	3.209777842	0.011474501	0.051982968	95	82	100	154	204	219	274	319
ENSECAG00000015003	3.097634714	0.011495576	0.052057687	76	91	83	153	230	196	199	318
ENSECAG00000006239	5.101000692	0.011508502	0.052095457	338	250	406	721	1044	703	863	1129
ENSECAG00000021973	3.19200802	0.011523598	0.052132921	70	85	99	179	329	159	279	245
ENSECAG00000017839	2.588500167	0.011528298	0.052132921	222	141	65	69	63	78	55	91
ENSECAG00000018823	9.094905338	0.011530543	0.052132921	12094	17205	7994	9263	8149	6374	6544	9085
ENSECAG00000020631	3.759140467	0.011564022	0.052263491	185	396	257	330	179	176	149	313
ENSECAG00000015899	6.730725299	0.011598877	0.052400175	1809	2986	1929	2202	1430	1480	1707	1818
ENSECAG00000022704	4.555830415	0.011631441	0.052514005	143	387	258	368	566	488	612	922
ENSECAG00000007432	4.358029336	0.011633317	0.052514005	149	183	371	319	623	358	611	647
ENSECAG00000010001	5.981328222	0.011646861	0.052554265	995	1621	1246	1371	1063	919	958	1052
ENSECAG00000021899	7.210304384	0.011661514	0.052599492	3529	2814	2944	3026	2355	1796	2056	2551
ENSECAG00000004384	0.226401672	0.011682834	0.052674746	3	8	3	27	36	14	39	45
ENSECAG00000010758	1.799226682	0.011688573	0.052679716	22	61	19	27	103	48	113	148
ENSECAG00000024780	5.149358585	0.011694601	0.05268019	299	567	432	451	865	844	944	1152
ENSECAG00000010174	3.442495554	0.011697951	0.05268019	211	368	185	154	124	187	142	141
ENSECAG00000004790	2.803569815	0.011720305	0.052759949	70	73	92	62	228	110	237	212
ENSECAG00000006022	4.089280435	0.011733214	0.052797138	301	410	355	341	245	290	214	295
ENSECAG00000020442	3.445705092	0.011751588	0.052858885	74	130	122	210	233	290	310	350
ENSECAG00000010476	5.908293969	0.011818266	0.053137768	583	556	822	1094	1512	1375	1520	2032
ENSECAG00000019856	1.627462445	0.011927116	0.053605968	20	23	33	59	105	63	106	68
ENSECAG00000019131	8.81137965	0.011944673	0.05366365	8988	16783	4932	8876	4788	7904	5538	4784
ENSECAG00000020299	0.573687898	0.011988205	0.053837939	46	12	11	69	12	18	19	12
ENSECAG00000022288	2.227078755	0.012041675	0.054056702	46	58	167	186	42	83	61	52
ENSECAG00000018308	1.927211111	0.012071644	0.054169835	25	24	57	48	79	64	77	232
ENSECAG00000019323	5.767384443	0.012099308	0.054272537	471	605	874	856	1476	1224	1396	1679
ENSECAG00000011031	0.197082219	0.01212067	0.054346904	2	6	16	19	30	20	40	36
ENSECAG00000008584	5.671194564	0.012132257	0.054377397	707	1615	691	1330	792	647	731	978
ENSECAG00000001756	4.149391236	0.012154675	0.054456394	163	191	245	295	429	398	481	586
ENSECAG00000014958	5.150891306	0.012180205	0.054549268	429	1232	516	898	457	437	546	717
ENSECAG00000014214	3.50061718	0.012242062	0.05480469	76	130	170	178	264	253	275	449
ENSECAG00000008340	4.213050577	0.012256313	0.054846882	256	395	546	409	245	385	182	290
ENSECAG00000000537	8.097946708	0.012278346	0.054890189	5637	11699	4241	2318	2708	4207	3509	3041
ENSECAG00000017792	5.312042896	0.012279614	0.054890189	437	343	574	595	1194	748	1226	1110
ENSECAG00000010628	5.30515941	0.012280484	0.054890189	465	830	787	1364	585	529	710	703
ENSECAG00000011848	0.327972863	0.012294886	0.054932952	4	13	7	23	30	32	24	53
ENSECAG00000022330	5.243932065	0.012300621	0.05493697	286	423	464	803	966	1032	1031	979
ENSECAG00000022164	7.009434581	0.012334889	0.055066613	1073	1047	2109	1839	4128	2311	2874	5300
ENSECAG00000024762	5.710821876	0.012339341	0.055066613	510	659	591	862	1340	1017	1274	2031

ENSECAG00000008727	2.330443387	0.012375008	0.0552041	73	141	234	131	0	99	1	12
ENSECAG00000014410	3.186934112	0.012393712	0.055265838	54	35	163	155	335	282	212	203
ENSECAG00000002917	0.589519548	0.012407045	0.05530359	8	56	20	64	13	22	19	8
ENSECAG00000013947	4.420587529	0.012428452	0.055357723	102	235	276	477	436	619	535	756
ENSECAG00000015350	0.561714416	0.012428934	0.055357723	2	5	10	36	22	54	64	29
ENSECAG00000008912	6.157506145	0.012441182	0.055390559	3063	914	1044	801	933	1058	933	422
ENSECAG00000013357	0.930448551	0.012447497	0.055396969	20	11	4	25	107	22	50	48
ENSECAG00000006985	1.459162117	0.012458267	0.055407313	4	81	135	81	9	37	6	46
ENSECAG00000018548	4.063526443	0.012460365	0.055407313	207	366	564	317	235	278	280	196
ENSECAG00000018172	3.125875005	0.012464451	0.055407313	73	107	83	147	189	180	290	303
ENSECAG00000009882	3.77036294	0.012479227	0.055451303	147	153	153	195	458	307	335	369
ENSECAG00000012260	4.680792219	0.012510579	0.055568882	290	1259	278	477	291	431	280	339
ENSECAG00000017202	6.187168802	0.012537611	0.055667189	579	761	898	1504	2204	1443	1898	2340
ENSECAG00000022676	5.385064593	0.012549117	0.055685353	1651	166	1315	735	877	411	261	78
ENSECAG00000019054	1.426897602	0.012551504	0.055685353	17	18	29	51	56	77	105	58
ENSECAG00000014030	1.823499527	0.012562572	0.055712703	8	22	37	82	57	144	89	119
ENSECAG00000008225	3.099842832	0.012567672	0.055713575	60	73	112	165	232	166	326	221
ENSECAG00000013644	4.178710742	0.012584869	0.055764704	323	598	304	276	276	246	278	264
ENSECAG00000023753	4.895112831	0.012589022	0.055764704	360	400	1114	837	481	581	339	312
ENSECAG00000007145	2.959787598	0.01261455	0.055843982	84	85	514	213	12	184	8	12
ENSECAG00000019386	5.103080903	0.012616749	0.055843982	416	1424	521	610	439	454	443	659
ENSECAG00000019637	5.104895893	0.01262562	0.055855826	205	322	244	930	916	1151	964	760
ENSECAG00000020378	4.605952941	0.012629257	0.055855826	201	271	365	381	567	411	781	890
ENSECAG00000019915	5.033852567	0.012712258	0.056188237	594	755	565	776	572	513	407	576
ENSECAG00000000357	8.536290768	0.012714307	0.056188237	3036	2858	5137	6664	10716	7797	10691	12613
ENSECAG00000006803	4.033533285	0.012723932	0.05620891	150	119	192	334	412	515	499	339
ENSECAG00000013391	4.002539002	0.012739692	0.056249496	142	89	267	272	442	306	494	535
ENSECAG00000016279	2.210724584	0.012743021	0.056249496	108	105	109	76	81	73	52	29
ENSECAG00000023820	8.405823509	0.012760332	0.056304037	4956	5574	12670	7968	3554	6986	4085	3735
ENSECAG00000013796	5.878074831	0.012791813	0.05642103	908	1597	1057	1323	814	923	1003	955
ENSECAG00000023601	1.469075406	0.012800534	0.056431564	11	14	12	72	55	114	100	50
ENSECAG00000011211	3.199174042	0.012804134	0.056431564	55	61	153	174	227	242	288	255
ENSECAG00000013789	0.499141282	0.012829156	0.056519918	12	23	38	62	15	20	21	8
ENSECAG00000008261	5.291496242	0.012850753	0.056572417	404	403	632	507	1090	917	1117	1028
ENSECAG00000010678	4.073878327	0.01285103	0.056572417	212	643	311	292	181	231	196	366
ENSECAG00000000906	7.21074252	0.012897523	0.056755096	1464	5293	2688	4000	1669	2268	1750	2778
ENSECAG00000010755	3.280443252	0.012907292	0.056767064	128	210	242	266	149	128	187	118
ENSECAG00000021023	4.203772967	0.012910235	0.056767064	149	190	238	368	505	410	558	488
ENSECAG00000006640	5.518812663	0.012952444	0.056930628	371	498	639	863	971	1372	1049	1467
ENSECAG00000023894	4.46813148	0.012966504	0.056950554	324	311	825	476	273	245	315	518
ENSECAG00000023099	5.532970325	0.012967003	0.056950554	554	2029	591	862	566	642	706	710
ENSECAG00000012717	-0.030842279	0.013027305	0.057178846	10	4	8	10	20	18	26	43
ENSECAG00000022350	3.598018783	0.013029047	0.057178846	108	145	124	202	273	214	410	435
ENSECAG00000019742	2.324746623	0.013093421	0.05743917	70	192	62	120	63	50	63	94
ENSECAG00000024704	4.684948865	0.013127304	0.057565585	306	277	278	374	684	477	745	881
ENSECAG00000022513	1.300229939	0.01315207	0.057651937	9	92	125	58	1	51	2	2
ENSECAG00000022087	2.594070679	0.013166871	0.057694559	26	71	37	133	100	188	218	183
ENSECAG00000010962	2.839615248	0.013176239	0.057713347	60	69	103	105	186	145	211	242
ENSECAG00000003056	4.67675827	0.013228961	0.057921946	380	881	520	488	176	626	289	259
ENSECAG00000023697	5.737987605	0.013250349	0.057993247	437	356	866	1070	1438	1443	1356	1501
ENSECAG00000014555	2.449116209	0.013281117	0.058105525	39	66	64	67	112	103	120	294
ENSECAG00000001414	4.657535315	0.013315207	0.058232251	124	270	345.002	574	695	587	667.001	793
ENSECAG00000011752	2.733108878	0.013327861	0.058265161	45	66	89	119	169	149	212	195
ENSECAG00000011550	5.328801548	0.01333928	0.058292652	657	1433	591	673	700	499	591	597
ENSECAG00000013816	4.628929056	0.013344484	0.058292977	259	356	183	346	500	558	652	1034
ENSECAG00000014222	4.260609269	0.013361797	0.058325284	182	887	297	352	230	322	257	220
ENSECAG00000026999	4.506111071	0.013362672	0.058325284	278	860	376	437	274	364	323	373
ENSECAG00000023086	5.264735679	0.013368155	0.058325284	456	362	481	596	1151	852	1030	1058
ENSECAG00000009828	8.678830792	0.013372414	0.058325284	8335	17698	6397	4206	8904	3608	4516	2297
ENSECAG00000015551	5.630280059	0.013411426	0.058472993	611	1417	1046	1097	802	798	794	717
ENSECAG00000022585	5.018506379	0.013436824	0.058561254	325	234	494	559	901	596	1060	952
ENSECAG00000016195	4.17652845	0.013540892	0.058977641	372	674	197	274	259	210	304	225
ENSECAG00000000025	1.812992624	0.013542745	0.058977641	39	46	138	102	46	43	48	52
ENSECAG00000023227	8.133233247	0.013566943	0.059060384	3990	9611	5517	5274	3685	4079	3729	4678
ENSECAG00000020198	1.522791538	0.013599245	0.059163573	38	53	93	73	40	40	21	54
ENSECAG00000002186	6.454123823	0.013601061	0.059163573	1239	2206	2477	2430	446	2493	617	695
ENSECAG00000019825	5.438152265	0.013639321	0.059274406	649	495	156	310	1368	883	1269	1596
ENSECAG00000017653	2.712976064	0.013639357	0.059274406	49	78	73	105	226	110	192	195
ENSECAG00000011366	7.230160626	0.01364668	0.059274406	2079	2999	3937	4363	2910	2465	1757	1689
ENSECAG00000004679	4.620007006	0.013647408	0.059274406	919	59	1455	482	0	251	1	0
ENSECAG00000020513	2.283403632	0.01365402	0.059280466	40	73	36	62	116	126	148	148
ENSECAG00000021434	0.497736265	0.013663087	0.05929717	22	12	75	27	6	29	6	13
ENSECAG00000011249	4.174560444	0.013730831	0.059568423	330	408	404	387	186	409	189	269
ENSECAG00000013037	4.643920678	0.013792055	0.059811193	273	300	369	174	746	454	820	751
ENSECAG00000019760	4.738602789	0.013809455	0.059863801	1163	317	405	241	366	270	341	348
ENSECAG00000019585	0.697441658	0.013816238	0.059870365	11	19	11	23	49	39	51	36
ENSECAG00000016283	6.581576818	0.013861424	0.060043272	1083	2727	1665	2981	1135	1512	1343	1864
ENSECAG00000008755	7.701959923	0.01394942	0.060401415	6368	6737	2164	2050	2467	2826	2703	2386
ENSECAG00000021265	6.229910715	0.014042176	0.060779886	899	1455	2958	1532	737	1765	697	944
ENSECAG00000020904	3.815950239	0.014069858	0.060876515	400	345	168	236	220	119	204	250
ENSECAG00000011145	7.019274697	0.01413295	0.061126221	1266	4155	3575	2698	2446	1960	1572	1353
ENSECAG00000011492	5.425816811	0.014171422	0.06126929	401	394	524	859	1272	668	1268	1467
ENSECAG00000022325	7.211374633	0.014189093	0.061322355	1681	1208	1693	2249	4321	3014	5337	3776
ENSECAG00000006850	4.293916369	0.014226927	0.061442039	175	256	225	323	550	338	627	592
ENSECAG00000003034	1.861541646	0.014227601	0.061442039	37	37	21	52	93	53	119	152

ENSECAG00000001964	5.500270664	0.014240738	0.061475405	493	509	570	707	1389	913	1208	1282
ENSECAG00000008485	8.329498438	0.014252183	0.061501442	3118	3457	4377	3774	7401	9004	7369	12192
ENSECAG00000024648	7.49971863	0.014302285	0.061694216	2913	3938	4115	4843	2710	3487	1758	2674
ENSECAG00000022922	4.268466711	0.014320011	0.061747236	192	149	312	305	479	508	534	531
ENSECAG00000006854	4.124733104	0.014332294	0.061776754	158	354	517	546	269	299	277	198
ENSECAG00000006857	4.581580439	0.014399455	0.06199666	355	660	423	553	382	381	327	415
ENSECAG00000012760	6.362367558	0.014399674	0.06199666	657	934	1336	1177	1772	2125	1753	3298
ENSECAG00000015820	5.151222808	0.014399682	0.06199666	506	470	1162	981	786	428	505	458
ENSECAG00000018515	6.010052469	0.014413765	0.062033789	569	765	999	964	1740	1601	1648	1814
ENSECAG00000019169	5.189687656	0.01442459	0.062056867	483	1142	673	779	735	416	546	568
ENSECAG00000013268	0.816763026	0.014453777	0.062158902	20	6	23	15	56	36	47	58
ENSECAG00000017605	1.370679366	0.014466478	0.062189984	18	62	39	137	35	39	26	25
ENSECAG00000010682	5.210270166	0.014482406	0.062215366	324	301.996	560	736	923	903	958	1156
ENSECAG00000023209	3.678900546	0.014483334	0.062215366	86	108	200	252	302	292	340	461
ENSECAG00000012724	6.855505359	0.014498225	0.062255797	2117	3078	2133	2361	1830	1270	1655	2320
ENSECAG00000011862	1.032442617	0.014558346	0.062490339	18	18	24	23	49	51	74	47
ENSECAG00000023688	2.823418874	0.014575105	0.06253865	51	65	89	137	164	159	199	254
ENSECAG00000010800	4.658686695	0.014581645	0.062543093	221	341	252	469	688	531	648	833
ENSECAG00000010790	4.555451126	0.014628396	0.062719937	277	237	306	324	625	468	662	767
ENSECAG00000011365	3.1000509	0.014654573	0.062808472	34	37	101	222	283	234	299	166
ENSECAG00000012132	4.839186129	0.01472568	0.063089432	292	1803	186	368	274	290	306	493
ENSECAG00000021350	4.882187342	0.014734728	0.063104401	244	494	319	403	693	673	793	1004
ENSECAG00000005306	1.856490333	0.014744201	0.063111808	41	37	31	26	133	134	54	86
ENSECAG00000017568	0.511808279	0.014751728	0.063111808	8	5	12	30	42	48	39	26
ENSECAG00000017682	3.224776844	0.014753121	0.063111808	138	395	140	200	45	233	49	100
ENSECAG00000022948	4.396598776	0.014825181	0.063391018	298	487	465	503	403	290	300	359
ENSECAG00000020675	3.863580512	0.014829548	0.063391018	310	383	276	223	248	226	213	160
ENSECAG00000024044	4.365491814	0.014837935	0.063394486	238	201	273	293	507	461	662	565
ENSECAG00000009940	1.369998873	0.014841518	0.063394486	47	37	89	56	32	50	24	24
ENSECAG00000014402	4.444717383	0.014869849	0.063491627	195	206	328	371	510	414	565	870
ENSECAG00000010919	9.533332362	0.014914624	0.063658888	15991	25035	11674	10209	8296	11448	9297	11101
ENSECAG00000017242	5.702385669	0.014950082	0.063786269	889	1003	1034	1353	952	659	918	865
ENSECAG00000000836	7.401893358	0.01497693	0.06384254	2879	5932	2163	3800	1828	3104	2481	1848
ENSECAG00000016707	5.278283116	0.01497768	0.06384254	401	447	526	604	950	856	1007	1288
ENSECAG00000018788	7.100683154	0.014980128	0.06384254	1758	4221	3041	2840	2141	1746	1756	2646
ENSECAG00000022897	1.459637161	0.015024395	0.064007192	22.0071	37	19	35	80	48	104	73
ENSECAG00000021724	3.078535678	0.015042109	0.064058636	21	74	75	223	180	211	305	282
ENSECAG00000014948	3.92393357	0.015064116	0.064128318	120	136	209	297	427	272	359	605
ENSECAG00000011021	2.304727791	0.015121336	0.064347799	46	45	59	77	130	112	155	139
ENSECAG00000018600	11.06773991	0.015144653	0.064422894	79734	30425	33813	31464	28145	24751	25712	33332
ENSECAG00000009564	5.009716414	0.01515266	0.064432828	272	334	419	661	921	684	736	1087
ENSECAG00000014705	3.276292465	0.015166282	0.064466628	90	110	122	142	272	185	315	275
ENSECAG00000017440	5.467298037	0.015176926	0.064487745	695	1187	1016	724	697	546	707	863
ENSECAG00000007829	5.562144697	0.015195487	0.064517521	384	381	898	736	986	1386	1216	1462
ENSECAG00000021399	2.674868899	0.015199648	0.064517521	59	208	105	213	75	106	85	86
ENSECAG00000021617	6.715675747	0.015200968	0.064517521	530	757	2087	1699	2446	3424	3612	2160
ENSECAG00000019459	5.253890648	0.015310327	0.064957405	369	463	486	638	985	749	1008	1312
ENSECAG00000000147	1.312299631	0.015365511	0.065150052	54	72	49	32	25	34	30	40
ENSECAG00000011746	7.003847802	0.015367201	0.065150052	4311	1201	3956	1482	2183	1739	1526	866
ENSECAG00000000703	3.08483472	0.015383251	0.065193772	56	136	70	121	194	147	347	264
ENSECAG00000014561	7.762304429	0.015394175	0.065205971	1779	1878	3011	3863	5772	4826	7981	5369
ENSECAG00000011647	8.372602264	0.015397608	0.065205971	6522	14416	3612	4169	3449	4548	3844	5300
ENSECAG00000019086	4.24250407	0.015411622	0.065232837	165	192	191	400	545	301	543	682
ENSECAG00000012773	3.604048681	0.015415435	0.065232837	88	137	194	160	257	466	247	338
ENSECAG00000021686	7.766089603	0.015488468	0.065517487	2728	2385	1874	2565	6436	4444	6690	6896
ENSECAG00000017563	6.562034071	0.015535738	0.065692985	829	932	1127	1915	2700	2123	2988	2329
ENSECAG00000006079	7.036229008	0.015639756	0.066108227	894	1007	1772	2842	4115	3098	4358	2897
ENSECAG00000024441	1.838158084	0.015653163	0.06614029	28	38	39	52	97	51	114	137
ENSECAG00000020278	6.186003172	0.015697096	0.066301264	1191	2314	724	2078	896	1320	980	1026
ENSECAG00000018726	5.517914753	0.015717124	0.066361191	280	516	437	1137	956	1423	1326	1224
ENSECAG00000024376	5.564807793	0.015749267	0.066441142	356	449	550	1080	1378	1224	1577	870
ENSECAG00000012498	6.515002723	0.015752554	0.066441142	758	741	1900	843	2632	1541	2466	3728
ENSECAG00000007794	6.157141528	0.015753603	0.066441142	1140	1721	1369	1649	1168	996	1182	1241
ENSECAG00000000817	6.271931393	0.015780851	0.066517923	817	897	891	1231	2258	1425	2199	2433
ENSECAG00000017695	4.599538289	0.015789178	0.066517923	198	262	430	344	677	474	680	756
ENSECAG00000007700	4.230236262	0.015789371	0.066517923	213	552	303	560	323	223	326	288
ENSECAG00000021388	3.093752516	0.015852594	0.066759516	70	285	238	191	60	159	117	124
ENSECAG00000023790	3.441142466	0.01586134	0.066771597	116	93	168	132	318	224	330	306
ENSECAG00000008479	4.134773394	0.015889677	0.066866114	190	142	209	275	452	246	777	473
ENSECAG00000024074	4.7460717	0.015946982	0.067082418	162	336	330	535	576	502	638	1256
ENSECAG00000022727	4.337830583	0.0159847	0.067216196	232	190	276	296	534	417	595	611
ENSECAG00000023813	3.339852611	0.016022357	0.067349619	180	214	441	251	13	261	5	36
ENSECAG00000010909	4.02535873	0.016064453	0.067501596	163	199	618	598	319	198	266	92
ENSECAG00000006680	3.961299178	0.016070464	0.06750189	165	427	361	391	217	254	199	295
ENSECAG00000005421	3.045858845	0.016107088	0.067630722	60	112	93	126	274	146	264	218
ENSECAG00000011604	3.097261558	0.016121449	0.067666016	86	177	243	259	117	143	113	136
ENSECAG00000013327	1.913468823	0.016156893	0.067789742	14	33	35	91	99	112	88	121
ENSECAG00000009928	7.410301691	0.01616883	0.067814784	2956	5723	2909	2775	2682	2096	2232	2919
ENSECAG00000016399	6.384830567	0.016228215	0.068018033	765	750	1250	1529	2271	1908	2104	2649
ENSECAG00000002281	5.416788965	0.016229263	0.068018033	430	574	516	650	1045	905	1249	1309
ENSECAG00000007694	8.107097104	0.016264739	0.068130118	4868	8402	4841	5200	3869	3629	4423	4311
ENSECAG00000014783	4.375360671	0.016267999	0.068130118	40	107	154	566	554	397	1373	214
ENSECAG00000018425	5.634238264	0.016276962	0.068142536	647	1558	914	1092	535	953	641	915
ENSECAG00000018117	3.245020497	0.01630918	0.068252268	149	257	176	216	118	117	147	196
ENSECAG00000024202	1.341722584	0.016320827	0.068275862	19	89	70	60	12	49	22	37

ENSECAG00000019917	2.910440608	0.016336818	0.068317601	109	101	247	205	112	106	109	117
ENSECAG00000025184	1.332812623	0.016346328	0.068332222	25	40	80	91	18	45	32	34
ENSECAG00000009294	2.235727123	0.016353093	0.068335361	56	62	169	142	77	56	61	72
ENSECAG00000013691	6.045437234	0.016390801	0.068450908	654	704	686	1301	2013	1230	1763	2107
ENSECAG00000001598	5.654476738	0.016395756	0.068450908	534	787	449	671	1281	1334	1387	1350
ENSECAG00000020381	3.468704267	0.016398818	0.068450908	229	343	144	202	162	147	117	216
ENSECAG00000022855	5.749514604	0.016428774	0.068550763	1220	1122	1110	841	890.002	845	813	771
ENSECAG00000024822	5.852516217	0.016463699	0.068671274	956	1460	1145	1144	986	833	857	1031
ENSECAG00000010844	4.967553091	0.016479875	0.068713518	260	700	848	984	449	530	533	423
ENSECAG00000022388	8.460850856	0.016507573	0.068803759	6757	9294	7348	6185	5114	5036	4976	5652
ENSECAG00000009239	4.799772629	0.016549374	0.068933319	202	279	359	625	704	624	627	1036
ENSECAG00000005510	2.129894515	0.016550792	0.068933319	18	74	158	226	73	72	22	15
ENSECAG00000008840	7.931308736	0.016562689	0.068957593	2408	2339	3174	3694	7300	4727	6339	8772
ENSECAG00000011197	1.055348318	0.016601481	0.069093784	18	21	16	20	61	14	71	97
ENSECAG00000022070	6.049181233	0.016626058	0.069170731	899	2792	1082	908	1120	576	1023	1083
ENSECAG00000020638	3.355447892	0.01669558	0.069434547	61	204	131	685	99	177	120	112
ENSECAG00000022018	0.158125802	0.016751781	0.069642785	30	12	31	23	11	20	12	4
ENSECAG00000020810	3.774574561	0.016776428	0.069719744	150	123	121	172	722	141	453	261
ENSECAG00000018413	3.208146894	0.016784132	0.069726254	60	90	150	155	245	176	248	344
ENSECAG00000023323	1.213499534	0.016800909	0.069770441	2	5	41	35	34	55	49	150
ENSECAG00000014144	7.701308631	0.01684667	0.069934916	1746	1887	2428	4059	5510	4094	4897	8909
ENSECAG00000012023	6.167904492	0.016869263	0.070003127	1054	1536	1565	1844	1295	1081	1173	1084
ENSECAG00000007212	1.807658503	0.01691375	0.070162114	38	68	145	89	24	89	20	24
ENSECAG00000008378	3.506439614	0.016954559	0.070305731	125	110	126	185	284	304	346	276
ENSECAG00000008251	3.938918187	0.01696843	0.070337579	146	164	193	271	425	296	436	478
ENSECAG00000021754	7.315047068	0.017095744	0.070839476	2506	5657	2693	2767	1755	2089	2109	3327
ENSECAG00000006686	5.409209399	0.017111618	0.070879402	231	272	407	1246	1398	741	1148	1526
ENSECAG00000020269	2.186680779	0.017150685	0.071015336	134	104	69	65	72	45	67	58
ENSECAG00000012701	7.910709563	0.01718765	0.07114247	5038	3167	6178	6285	4078	3591	3044	3549
ENSECAG00000026853	3.319132098	0.0172081	0.071196583	68	87	152	192	262	231	227	366
ENSECAG00000003345	10.60303712	0.017215315	0.071196583	38544	24609	34397	32493	21402	21384	21869	26329
ENSECAG00000015123	7.394661868	0.017219522	0.071196583	2863	3503	3538	4389	2871	1942	2721	2925
ENSECAG00000014150	5.003616073	0.017254861	0.071316743	427	435	850	1120	682	479	409	426
ENSECAG00000018142	6.523916164	0.017361624	0.071731918	480	864	899	2425	2449	2664	3069	1917
ENSECAG00000011078	5.866697041	0.017377254	0.071770397	688	942	1738	1750	1101	932	911	700
ENSECAG00000007912	3.140543521	0.017444278	0.072021035	67	66	142	157	238	192	283	243
ENSECAG00000014276	5.561450953	0.017452776	0.072029945	475	475	616	900	1301	1134	1147	1362
ENSECAG00000010906	-0.078839536	0.017496541	0.072168558	2	6	4	22	15	17	29	43
ENSECAG00000024637	6.182272761	0.017499065	0.072168558	878	281	838	1252	3021	1254	1915	2068
ENSECAG00000017794	8.70804458	0.017544393	0.072329244	3497	10227	15033	11801	6837	8502	2653	3491
ENSECAG00000012481	6.705289861	0.017583279	0.072463265	2918	2236	2865	850	1381	1700	1614	509
ENSECAG00000008150	4.73303766	0.017633022	0.072641912	241	204	432	495	769	583	751	718
ENSECAG00000013630	4.104036691	0.017673707	0.072783133	142	147	172	351	209	607	369	734
ENSECAG00000018298	4.244723203	0.017712382	0.072915973	162	233	236	295	681	402	631	313
ENSECAG00000009381	5.254198292	0.017722053	0.07292936	905	670	717	751	640	478	683	598
ENSECAG00000015126	5.217746904	0.017746899	0.073005167	466	628	697	1508	690	430	524	734
ENSECAG00000006600	3.211342709	0.017754887	0.073011592	94	188	221	329.008	151	124	110	178
ENSECAG00000009805	4.267728585	0.017792004	0.073131044	200	363	501	636	239	334	343	285
ENSECAG00000022064	3.628692566	0.017796808	0.073131044	137	111	168	177	371	231	308	431
ENSECAG00000011712	3.096635837	0.017822127	0.073208605	83	77	115	131	265	143	234	294
ENSECAG00000005320	7.092524291	0.01783282	0.073226059	2786	2977	2680	2832	2346	1588	2244	2203
ENSECAG00000007875	3.466837295	0.017855489	0.073292654	143	106	126	127	293	229	334	348
ENSECAG00000026878	1.754556842	0.017955731	0.073677508	29	46	34	37	103	65	103	97
ENSECAG00000025004	4.519189043	0.017986082	0.073775402	202	248	369	364	707	488	578	640
ENSECAG00000012793	3.056098344	0.018024334	0.073905624	41	48	142	166	207	295	184	233
ENSECAG00000008165	4.796572485	0.018141505	0.074359229	236	320	451	444	641	609	832	834
ENSECAG00000018687	5.151906781	0.018156278	0.074392941	337	337	614	502	992	605	1180	1030
ENSECAG00000008751	6.894967641	0.018165319	0.074403154	2245	4635	1455	1774	1566	1124	1469	2558
ENSECAG00000007598	4.73480107	0.018284496	0.074841852	208	272	495	296	978	333	637	1028
ENSECAG00000022422	5.228807342	0.01829035	0.074841852	371	505	370	666	938	827	944	1268
ENSECAG00000008738	7.537672156	0.018292187	0.074841852	5687	3122	4540	2267	2860	3638	1310	1662
ENSECAG00000023448	5.019267398	0.018344697	0.075029677	184	180	685	344	1302	225	1456	873
ENSECAG00000011753	2.798703109	0.018357051	0.075053188	111	271	158	87	87	102	121	21
ENSECAG00000012282	3.479263169	0.018389391	0.075120106	97	103	175	170	367	234	354	239
ENSECAG00000010845	5.309857445	0.018392164	0.075120106	828	1084	582	727	617	403	510	933
ENSECAG00000010244	4.135978282	0.018393252	0.075120106	223	344	424	502	242	265	257	374
ENSECAG00000004387	5.274355562	0.018410458	0.075163358	902	1007	605	561	547	522	607	691
ENSECAG00000018230	1.912217785	0.018438322	0.075250076	25	50	47	45	129	58	112	116
ENSECAG00000024346	4.466242193	0.018462356	0.075321109	155	174	251	572	518	592	552	724
ENSECAG00000017579	6.944372753	0.018471126	0.075326755	3077	2914	2083	1915	2120	1420	1826	1893
ENSECAG00000009005	6.136341006	0.018476999	0.075326755	1521	1095	2032	1150	1413	1041	961	818
ENSECAG00000009840	3.874287223	0.018504038	0.075409927	144	183	163	247	376	342	378	454
ENSECAG00000016216	5.995746955	0.018511315	0.075412534	1515	1632	1178	768	1050	789	973	1025
ENSECAG00000023460	7.755931095	0.018546411	0.075528429	3152	5802	4394	5111	3139	2914	3640	3571
ENSECAG00000022099	4.556639521	0.018565444	0.075578852	233	203	372	385	695	431	702	679
ENSECAG00000018414	0.647834291	0.018585681	0.075634136	8	2	16	31	39	75	38	22
ENSECAG00000019395	3.21191781	0.018605618	0.07568816	78	134	98	130	303	165	239	302
ENSECAG00000022516	5.063101626	0.018627923	0.075751776	432	204	497	428	891	669	1179	904
ENSECAG00000017127	1.851248483	0.018673632	0.075910486	32	22	34	71	71	87	89	157
ENSECAG00000021281	7.663318687	0.018680865	0.075912728	4098	5778	3097	3895	3206	2888	3017	2831
ENSECAG00000024775	6.223247187	0.018754191	0.076183453	997	2473	1072	1802	1071	1090	949	1511
ENSECAG00000008080	3.838465444	0.018767736	0.076211227	100	189	147	291	337	285	427	496
ENSECAG00000011456	10.42204271	0.018790439	0.076276159	39216	36349	18469	18411	19327	17084	19134	19445
ENSECAG00000022710	7.546966596	0.018803967	0.076303811	3085	6269	3144	3294	2760	2487	3069	2675
ENSECAG00000014039	3.953567847	0.018812711	0.076312037	171	166	188	239	412	309	473	454

ENSECAG00000019151	1.783173567	0.018915743	0.076675362	47	144	44	68	50	34	27	69
ENSECAG00000019795	7.242442538	0.018915775	0.076675362	3099	2845	2487	4561	2079	2569	2698	1547
ENSECAG00000021826	6.764222451	0.018999069	0.076985527	872	958	1517	2238	3096	2597	2632	3410
ENSECAG00000012553	3.328511815	0.019027772	0.07707435	74	90	176	160	270	269	271	261
ENSECAG00000019949	4.111869582	0.019070015	0.077217928	329	459	343	305	177	224	176	473
ENSECAG00000021985	0.421604834	0.019155587	0.077536793	6	13	15	21	27	28	38	50
ENSECAG00000011036	5.246408676	0.019172872	0.077579121	739	737	1019	588	783	467	588	534
ENSECAG00000023994	5.063979987	0.019183441	0.077594252	330	404	380	613	830	708	797	1209
ENSECAG00000023977	2.268667713	0.019193755	0.077608345	45	37	47	95	123	103	140	161
ENSECAG00000022581	3.557439357	0.019208361	0.07763977	128	108	155	185	284	274	346	350
ENSECAG00000026860	5.499454247	0.01927709	0.077847568	453	510	622	780	1126	945	1229	1439
ENSECAG00000010577	3.196036879	0.019279052	0.077847568	112	509	107	180	9	206	39	97
ENSECAG00000021200	5.255101868	0.019280325	0.077847568	813	740	769	748	610	688	608	473
ENSECAG00000020910	2.704379752	0.019366188	0.078166477	33	81	80	122	152	132	209	223
ENSECAG00000012759	0.896756828	0.019417503	0.078345762	30	73	22	33	21	23	23	27
ENSECAG00000024834	6.627757201	0.019486797	0.078597441	1702	2281	1945	2218	1594	1619	1451	1586
ENSECAG00000020677	1.026369494	0.019504054	0.078639126	1	11	24	38	24	55	126	35
ENSECAG00000019256	7.061788844	0.019515545	0.078657545	2001	5847	1915	1719	1812	1785	1889	1900
ENSECAG00000019612	5.656496662	0.019544968	0.078725798	504	516	612	1006	1449	1026	1483	1337
ENSECAG00000009343	4.707720124	0.019546337	0.078725798	242	217	392	495	664	807	570	702
ENSECAG00000020481	6.587647473	0.019637526	0.079052662	2130	2719	1412	1568	1791	1255	1512	1130
ENSECAG00000022291	7.651808428	0.019642819	0.079052662	3234	5413	4494	3777	3279	2366	3330	3326
ENSECAG00000010500	8.338333489	0.019658048	0.079052662	2953	4353	3026	4760	9585	5752	8005	13137
ENSECAG00000024908	4.525452194	0.019659847	0.079052662	354	466	567	520	352	415	401	290
ENSECAG00000019911	5.021412892	0.019672172	0.079052662	363	397	426	442	801	748	752	1129
ENSECAG00000014995	3.323349561	0.01967468	0.079052662	92	102	109	194	265	208	307	292
ENSECAG00000010255	3.374212624	0.019676195	0.079052662	67	118	118	224	233	250	294	339
ENSECAG00000021441	2.953733561	0.019693087	0.07907569	72	70	109	118	184	162	280	211
ENSECAG00000019889	6.349389993	0.019695846	0.07907569	2220	1730	1540	991	1206	1262	1221	1081
ENSECAG00000014822	6.926492615	0.019723837	0.079160099	978	1277	1446	2453	3248	3302	3623	2885
ENSECAG00000022341	8.654755912	0.019748545	0.079204334	9827	9184	6693	7571	5432	6090	5046	6898
ENSECAG00000022115	3.340091818	0.019753767	0.079204334	133	104	90	130	274	211	287	334
ENSECAG00000006904	10.02801251	0.019755772	0.079204334	23712	23188	16277	24273	18204	12550	15982	14721
ENSECAG00000014969	3.71588426	0.019782576	0.079283818	294	324	247	206	178	257	131	199
ENSECAG00000011027	4.444539738	0.019825322	0.079406553	148	343	267	348	476	472	524	875
ENSECAG00000012968	4.390939954	0.019827178	0.079406553	150	278	259	410	530	406	552	750
ENSECAG00000017727	3.659145861	0.019852384	0.079479489	86	148	188	213	271	291	277	533
ENSECAG00000000395	4.833626534	0.019860569	0.079484251	460	362	878	724	585	299	411	519
ENSECAG00000010357	1.460464016	0.019875562	0.079516243	52	64	61	75	7	68	21	31
ENSECAG00000009277	1.011061429	0.019936551	0.079732167	1	19	16	35	108	10	74	52
ENSECAG00000018344	3.926826275	0.020007637	0.079988308	142	126	249	242	338	311	398	598
ENSECAG00000018113	9.679958712	0.020024654	0.080028181	10831	28770	37938	16474	102	17318	157	1865
ENSECAG00000013362	0.662507242	0.020092069	0.080269371	11	13	17	17	21	83	22	46
ENSECAG00000003015	1.433593862	0.020130885	0.080396173	125	7	113	22	5	53	4	0
ENSECAG00000003965	4.713648222	0.020144725	0.080422536	277	324	367	331	704	451	691	968
ENSECAG00000017925	1.149267154	0.020151642	0.080422536	22	19	20	33	59	42	68	71
ENSECAG00000023793	5.331967684	0.020195262	0.080558636	403	424	511	772	989	873	1144	1216
ENSECAG00000023975	3.694216408	0.020201495	0.080558636	110	179	137	221	310	281	321	476
ENSECAG00000024244	6.359657913	0.020207166	0.080558636	754	501	1454	921	3826	822	2896	1893
ENSECAG00000016233	3.473775207	0.020214106	0.080558636	339	254	91	230	191	129	96	191
ENSECAG00000011280	3.68095774	0.020237454	0.080623405	77	410	250	454	172	193	157	221
ENSECAG00000018541	6.139870959	0.02025811	0.080677407	910	1764	1665	1572	969	1197	889	1473
ENSECAG00000009243	1.420186844	0.020276334	0.080721692	9	20	31	55	37	116	78	64
ENSECAG00000022508	6.481686217	0.020289987	0.080747751	1172	2140	1935	2249	1540	1242	1404	1608
ENSECAG00000012659	5.240845786	0.020339946	0.080825545	510	1133	815	661	537	648	579	618
ENSECAG00000008331	5.270841924	0.020348179	0.080825545	733	695	783	895	570	635	615	703
ENSECAG00000012139	3.579149624	0.020349438	0.080825545	88	93	178	244	346	208	396	347
ENSECAG00000014136	3.487401684	0.020353783	0.080825545	188	260	209	267	177	170	186	169
ENSECAG00000014912	4.348083617	0.020354675	0.080825545	211	182	276	370	567	417	630	535
ENSECAG00000012168	5.367716599	0.020355514	0.080825545	1368	475	654	785	611	611	573	594
ENSECAG00000018029	2.164809663	0.02035933	0.080825545	61	62	121	172	40	99	48	52
ENSECAG00000020074	1.288664516	0.020408482	0.080969229	27	16	2	40	73	57	53	106
ENSECAG00000015930	5.393853868	0.020410251	0.080969229	446	452	595	668	1292	879	1157	1067
ENSECAG00000018159	2.146290641	0.020416902	0.080969229	40	69	115	192	52	59	72	66
ENSECAG00000008140	4.150655708	0.020523014	0.081361647	201	178	255	228	596	403	493	387
ENSECAG00000010596	6.100771513	0.020572771	0.081530458	605	1488	2194	1752	924	1066	975	1367
ENSECAG00000006921	4.324490567	0.020614055	0.081665585	177	200	284	360	624	297	627	602
ENSECAG00000020416	8.891953015	0.020659958	0.081818906	12005	12522	6485	8114	7509	5800	7146	6605
ENSECAG00000015587	6.63793848	0.020673135	0.081842565	1300	2092	2397	2704	1768	1438	1709	1427
ENSECAG00000016242	9.463599516	0.020782635	0.082247403	13435	17643	13653	14772	11321	11542	8312	10658
ENSECAG00000000692	7.603693752	0.020818153	0.082359279	1507	2076	2668	3583	5164	4862	5067	5962
ENSECAG00000015957	4.86839597	0.020831891	0.082360591	251	207	464	579	793	631	1039	649
ENSECAG00000012265	4.657289847	0.020839356	0.082360591	243	281	383	402	738	549	617	733
ENSECAG00000024673	4.490759785	0.02084023	0.082360591	225	210	392	312	593	481	666	631
ENSECAG00000010929	3.075273911	0.020879574	0.082487387	67	77	117	158	248	167	213	282
ENSECAG00000009093	6.725219461	0.02089005	0.082500086	1287	1077	1317	1398	2983	2277	2946	3204
ENSECAG00000026922	3.260589221	0.020915964	0.082573726	99	306	176	259	128	95	171	183
ENSECAG00000006712	4.594214328	0.02092577	0.082580371	204	305	334	422	588	482	684	788
ENSECAG00000016726	8.115852828	0.020932184	0.082580371	6075	8867	3317	4852	3960	3835	3493	4566
ENSECAG00000022042	5.045188637	0.020955257	0.082642704	234	463	220	654	575	1479	644	891
ENSECAG00000005374	7.869761483	0.021043521	0.082962001	4430	4968	4528	5632	3758	3473	3270	3913
ENSECAG00000013194	0.8248981	0.021054997	0.082966287	7	21	14	34	39	45	51	56
ENSECAG00000021042	4.526545745	0.021059213	0.082966287	128	181	263	588	657	754	745	341
ENSECAG00000014269	1.475973173	0.021130267	0.083217363	30	17	109	117	37	22	26	55
ENSECAG00000003554	4.51560854	0.021140289	0.083227984	160	208	456	336	542	760	484	641

ENSECAG00000023870	5.681237846	0.021168595	0.083310555	450	462	742	1066	1383	950	1557	1542
ENSECAG00000010633	5.118908251	0.02120159	0.083411519	691	1029	412	697	687	402	500	543
ENSECAG00000021942	4.102309615	0.021239651	0.083511133	143	227	200	309	345	369	534	578
ENSECAG00000013220	4.438087531	0.02124161	0.083511133	332	947	218	342	325	254	342	306
ENSECAG00000011261	2.350839608	0.021253403	0.083528595	32	354	51	53	61	67	58	21
ENSECAG00000020244	0.575411303	0.021280078	0.083593347	9	33	67	33	5	28	19	12
ENSECAG00000021951	5.973118092	0.021284594	0.083593347	768	3449	480	967	437	1169	691	835
ENSECAG00000008375	3.365054278	0.021313936	0.08367966	71	102	107	239	224	325	272	282
ENSECAG00000021430	7.667780844	0.021329237	0.08371081	1837	2017	1892	4121	5317	6753	5623	4347
ENSECAG00000011689	5.921057461	0.021359851	0.083802013	609	481	1083	866	2054	1205	1675	1534
ENSECAG00000005912	1.46062125	0.021376658	0.083839002	7	35	37	44	68	80	61	93
ENSECAG00000016770	5.97680154	0.021392821	0.083859771	704	1843	1534	1256	797	1184	857	1095
ENSECAG00000018311	3.208242508	0.021396715	0.083859771	64	109	120	168	196	192	317	291
ENSECAG00000016435	3.680962058	0.021429733	0.083960199	103	551	197	299	156	215	145	203
ENSECAG00000011555	4.784960983	0.021437118	0.083960199	256	293	416	477	694	514	836	856
ENSECAG00000011779	4.720079341	0.021448665	0.083976474	535	857	345	391	366	354	445	420
ENSECAG00000014908	4.692330476	0.021499815	0.084147744	257	207	442	378	903	406	718	763
ENSECAG00000020454	0.687897673	0.021524344	0.084214738	20	32	28	64	15	13	24	33
ENSECAG00000012271	3.48307956	0.021541439	0.084252611	127	425	158	270	120	140	131	263
ENSECAG00000016150	8.284841352	0.021584091	0.08439038	6146	6599	6083	7180	4658	4424	4548	5525
ENSECAG00000018579	1.271161342	0.021609589	0.084461008	47	24	70	69	34	36	23	37
ENSECAG00000001441	6.994862185	0.02165471	0.08460826	834	1042	2027	2565	3416	3614	4011	2757
ENSECAG00000024527	3.612046017	0.021663023	0.084611641	96	101	236	179	364	269	332	343
ENSECAG00000023397	0.434505916	0.021683087	0.084660905	8	12	18	15	27	43	48	24
ENSECAG00000022617	5.25624087	0.02170452	0.084715477	699	1239	623	537	712	422	556	657
ENSECAG00000023990	5.035711236	0.021726335	0.084771503	690	760	653	512	601	347	600	518
ENSECAG00000014920	1.300167077	0.02173951	0.084793793	43	53	81	39	30	32	50	10
ENSECAG00000024701	3.205832183	0.021784053	0.084938368	48	78	89	230	188	346	158	333
ENSECAG00000006979	5.401155652	0.021854862	0.085185231	353	824	685	2114	694	465	671	766
ENSECAG00000023758	3.257678538	0.021902599	0.085342021	208	404	85	121	178	64	82	172
ENSECAG00000010343	7.398934202	0.021927144	0.085408368	1883	6671	2761	3619	1680	3103	2107	2674
ENSECAG00000007542	0.422036213	0.021943386	0.085436884	19	28	37	36	3	28	17	13
ENSECAG00000019684	2.731322115	0.021949504	0.085436884	72	76	84	66	193	123	214	194
ENSECAG00000024314	3.28507561	0.021958877	0.085444095	109	173	382	195	149	100	147	191
ENSECAG00000015465	5.796701471	0.02200481	0.085593511	496	831	617	895	1805	950	1492	1662
ENSECAG00000019913	7.083875842	0.022016148	0.085608308	2214	5162	2032	2041	2220	1841	2004	1730
ENSECAG00000019776	5.383013129	0.022057041	0.085721923	486	632	461	379	1123	805	1241	1337
ENSECAG00000017474	3.747485014	0.022060456	0.085721923	222	414	224	259	148	220	144	299
ENSECAG00000007441	6.502094686	0.022086311	0.085793048	786	540	1318	1979	2321	2063	2891	2558
ENSECAG00000004675	5.668549598	0.022097286	0.085806344	1037	1169	905	925	919	755	825	750
ENSECAG00000011648	6.818321128	0.02214009	0.085943185	1805	2636	2550	2356	1964	1589	1639	1977
ENSECAG00000019995	1.797245556	0.022166151	0.086014962	24	37	50	37	117	62	149	55
ENSECAG00000013725	5.541384272	0.022233258	0.08624261	460	420	631	952	1220	1116	1196	1332
ENSECAG00000014532	6.561930338	0.022240725	0.08624261	892	1131	1213	1633	2406	2065	2773	2742
ENSECAG00000022913	7.144870804	0.022248547	0.08624261	2711	2253	4233	2741	2499	1718	2623	1711
ENSECAG00000019555	3.829855397	0.022255178	0.08624261	232	363	289	278	181	238	220	255
ENSECAG00000015264	1.821366941	0.022270591	0.086272914	163	33	63	26	47	39	38	36
ENSECAG00000021773	6.217527873	0.022305448	0.086378492	2538	1850	642	879	901	1128	921	1050
ENSECAG00000008576	0.403463543	0.022354698	0.086539721	18	22	3	98	28	3	4	12
ENSECAG00000018825	4.629869948	0.022383711	0.086622522	212	319	394	347	703	701	581	584
ENSECAG00000005699	4.284748589	0.022450663	0.086836349	409	836	159	242	168	326	300	224
ENSECAG00000016443	2.769673715	0.02245425	0.086836349	61	13	124	22	347	101	323	72
ENSECAG00000015805	7.112128002	0.022494427	0.086962124	3257	4991	1430	1788	1832	1887	1443	2474
ENSECAG00000022058	1.880677473	0.022521818	0.087008855	120	61	62	56	39	58	34	62
ENSECAG00000001268	0.1603675	0.022521831	0.087008855	3	19	6	14	26	28	28	37
ENSECAG00000009031	2.416624226	0.02256665	0.087152372	50	48	61	93	106	133	171	165
ENSECAG00000023153	4.903596519	0.022574574	0.087153352	237	387	359	614	726	646	793	975
ENSECAG00000018131	2.650845304	0.022584075	0.087160413	53	56	81	95	84	237	145	222
ENSECAG00000001496	5.510864137	0.022612235	0.08723946	854	810	902	1122	827	728	782	624
ENSECAG00000019396	2.496558651	0.022685877	0.087493869	34	70	85	84	141	115	178	175
ENSECAG00000004944	5.570383484	0.022780626	0.087829477	397	362	827	888	1173	892	1169	1900
ENSECAG00000024703	5.944745144	0.022825089	0.087971054	1180	1999	802	1053	752	973	710	1302
ENSECAG00000023166	4.419145438	0.02306814	0.088864857	220	316	281	234	529	453	637	645
ENSECAG00000010464	5.207485871	0.023080179	0.088864857	721	1054	556	632	669	422	631	596
ENSECAG00000010024	2.094979104	0.02308046	0.088864857	106	65	120	84	39	86	67	16
ENSECAG00000013617	3.076765701	0.023111339	0.0889536	57	80	126	160	224	162	282	241
ENSECAG00000021223	9.216142808	0.023156313	0.08909652	8916	12844	11159	18884	8982	8547	8972	10099
ENSECAG00000023644	4.420748716	0.023199567	0.089212018	198	150	303	460	536	446	597	710
ENSECAG00000010479	8.229485248	0.023202034	0.089212018	5669	8476	5539	5223	4268	4621	4017	5074
ENSECAG00000024195	3.088971106	0.023217345	0.089240688	93	114	66	108	217	136	306	282
ENSECAG00000023248	4.614146544	0.023246749	0.08932349	214	403	601	1014	361	364	320	498
ENSECAG00000007379	2.139787516	0.023272114	0.08937454	45	172	111	109	12	122	13	31
ENSECAG00000010069	1.205540785	0.023275767	0.08937454	16	26	21	32	93	23	54	89
ENSECAG00000018348	11.80709005	0.02328579	0.089382821	66794	72773	81664	80855	61569	43431	57176	56981
ENSECAG00000004256	4.818126554	0.023392045	0.089760357	584	847	401	420	452	344	432	515
ENSECAG00000007347	1.468089515	0.023420963	0.089840981	12	34	35	46	63	57	88	92
ENSECAG00000016500	7.798956551	0.02345206	0.089929902	4328	5830	4180	4044	3127	3689	3214	3388
ENSECAG00000003013	3.130757271	0.023512407	0.090130892	57	132	115	123	263	153	276	254
ENSECAG00000014365	2.968622216	0.023526133	0.090153093	82	79	104	108	241	158	220	219
ENSECAG00000010349	2.579335384	0.023621726	0.09048889	86	110	162	189	147	90	41	42
ENSECAG00000005942	0.577273797	0.023700223	0.090758992	12	8	20	21	31	30	44	55
ENSECAG00000017200	4.183208946	0.023712218	0.090774334	103	206	159	471	391	377	613	603
ENSECAG00000000887	5.575435282	0.023730058	0.090812032	844	691	1405	1060	963	352	847	873
ENSECAG00000015671	3.357218384	0.023755851	0.090880127	120	146	105	67	315	192	333	296
ENSECAG00000023493	5.784906636	0.023836059	0.091156279	1198	1278	768	1145	801	815	773	1131

ENSECAG00000022918	7.304221147	0.023862373	0.091214357	2529	5089	2824	2736	2323	2145	2358	2783
ENSECAG00000011177	5.395224663	0.023867302	0.091214357	2301	353	255	386	519	286	492	621
ENSECAG00000023169	1.634218172	0.023891539	0.091276282	21	33	49	32	51	115	76	93
ENSECAG00000012033	1.950754411	0.02394206	0.091416824	35	45	35	53	88	55	107	185
ENSECAG00000005166	6.642061032	0.023944417	0.091416824	1262	1514	3104	2780	1679	1426	1473	1794
ENSECAG00000016769	4.226642972	0.023987176	0.091549311	166	153	229	424	567	348	570	510
ENSECAG00000012794	3.68749599	0.024046899	0.091746431	108	187	151	204	374	255	319	420
ENSECAG00000003474	5.508278015	0.024062073	0.091754226	1328	603	716	1138	906	403	293	1059
ENSECAG00000010483	4.964757845	0.024065094	0.091754226	820	762	420	444	460	450	449	534
ENSECAG00000022968	7.569127925	0.024087475	0.091808752	5242	5660	2292	2205	2737	1983	3053	2790
ENSECAG00000013481	7.025050172	0.024125086	0.091921269	3862	4206	1240	1295	1918	1847	1191	1918
ENSECAG00000012095	2.249424172	0.024162532	0.092033085	27	52	34	112	112	150	113	144
ENSECAG00000010526	3.283025496	0.024192347	0.092115765	311	144	175	155	208	108	119	104
ENSECAG00000023502	5.692313615	0.024223089	0.092187541	516	540	738	948	1212	1279	1251	1627
ENSECAG00000003770	3.920090584	0.024232533	0.092187541	86	290	87	225	303	264	433	739
ENSECAG00000014594	0.412975227	0.024235538	0.092187541	23	35	31	21	15	16	25	11
ENSECAG00000011218	5.381512021	0.024249974	0.09221158	516	448	517	609	1248	825	1037	1282
ENSECAG00000015857	2.401007311	0.024294584	0.092325531	63.0011	42	55.001	41.0005	221.001	212	55.0005	106
ENSECAG00000015087	3.045929688	0.024296192	0.092325531	66	67	132	144	272	194	207	209
ENSECAG00000020406	7.459053153	0.024315029	0.092363889	4270	5577	2048	2488	2617	2011	2691	2721
ENSECAG00000018293	4.058838884	0.024322545	0.092363889	133	117	277	340	435	386	454	498
ENSECAG00000011697	3.053269974	0.024346418	0.092423656	74	33	122	140	176	324	280	134
ENSECAG00000014371	7.046492756	0.024396923	0.092584447	1620	4025	2379	3296	1964	1921	1899	2439
ENSECAG00000024928	6.16515651	0.024458301	0.092781762	801.011	1867	1687.01	1758	866.001	1390	965.001	1332
ENSECAG00000020879	5.998662687	0.024465249	0.092781762	336	754	915	1422	1438	1525	1991	1858
ENSECAG00000014508	4.917841387	0.024501089	0.092886677	154	316	423	734	705	834	746	930
ENSECAG00000018613	4.956487086	0.024534415	0.092981995	286	298	418	612	1062	525	967	728
ENSECAG00000007826	3.321138584	0.024555865	0.093032259	84	125	127	155	282	163	243	398
ENSECAG00000023989	1.699012196	0.024615811	0.093228282	19	36	47	49	67	91	73	118
ENSECAG00000008185	1.769595588	0.024924062	0.094364274	33	38	14	53	98	48	77	171
ENSECAG00000024262	5.368675079	0.02494476	0.094390647	552	1469	652	745	549	627	617	789
ENSECAG00000016898	6.682803371	0.024947642	0.094390647	847	1447	982	1913	2714	2291	3534	2449
ENSECAG00000001058	7.288114413	0.024959779	0.094405129	3123	3762.01	2734.01	3309.01	1785.01	2984.01	2102.01	2579.01
ENSECAG00000022912	0.69458443	0.02498468	0.094451214	12	74	43	31	1	38	3	15
ENSECAG00000022914	2.596238351	0.024988589	0.094451214	20	67	52	152	161	122	204	191
ENSECAG00000003158	3.122788735	0.025026811	0.094564225	57	70	134	182	213	223	255	240
ENSECAG00000017024	5.076969013	0.025037513	0.094573212	357	354	508	508	769	605	992	1214
ENSECAG00000006470	1.174572662	0.025087653	0.094731112	15	100	15	90	9	33	26	33
ENSECAG00000018816	4.734779003	0.02511294	0.094795092	190	244	215	699	632	948	803	467
ENSECAG00000017282	4.066523985	0.025133918	0.09484277	150	167	255	247	390	420	700	271
ENSECAG00000020475	6.603049923	0.025243657	0.095214327	2027	2555	1714	1563	1998	1312	1035	1619
ENSECAG00000003511	5.688877718	0.025249143	0.095214327	1043	1415	890	784	912	467	754	1127
ENSECAG00000005228	3.618119445	0.025275587	0.095262744	64	107	120	341	374	243	344	388
ENSECAG00000008603	6.258246156	0.025287005	0.095262744	486.007	854	1012	1762	2029	1792	2252	2022
ENSECAG00000015392	7.344811552	0.025295159	0.095262744	1089	1358	2827	3043	3953	3821	4409	5565
ENSECAG00000017696	2.265546838	0.025302015	0.095262744	71	70	134	149	53	93	50	81
ENSECAG00000020451	2.709047053	0.025303904	0.095262744	76	82	209	227	122	95	82	82
ENSECAG00000014267	2.691279619	0.025353697	0.095413431	79	451.003	77.0018	62.0025	9.00295	156.002	6	5.00318
ENSECAG00000007589	3.020837031	0.025360725	0.095413431	115	203	236	216	18	218	59	92
ENSECAG00000000877	3.442984052	0.025403726	0.095543574	92	161	111	153	293	177	445	264
ENSECAG00000000473	4.366592885	0.025412501	0.095544948	212	258	201	390	539	458	526	642
ENSECAG00000021736	4.212607055	0.02542245	0.095550735	196	380	487	515	303	297	296	308
ENSECAG00000017630	5.503306057	0.025455707	0.095609652	1172	1208	621	570	665	766	605	652
ENSECAG00000010563	2.496814307	0.025459735	0.095609652	48	70	78	74	144	119	151	190
ENSECAG00000005038	6.57301876	0.02546337	0.095609652	1919	2766	1390	1603	1455	1532	1310	1545
ENSECAG00000024817	0.718053604	0.025478992	0.095636707	14	19	21	12	36	41	44	54
ENSECAG00000021855	7.24271787	0.025498572	0.09567859	3511	3610	2369	2769	2124	2474	2051	2488
ENSECAG00000008923	8.441825354	0.025517496	0.095717989	4125	3183	32640	551	198	7213	309	341
ENSECAG00000023998	6.348048457	0.02553651	0.095757699	1013	759	1197	1014	2411	1675	2285	2303
ENSECAG00000024428	0.948094916	0.025552112	0.095784592	32	25	60	51	12	25	29	37
ENSECAG00000022379	5.700819139	0.025597123	0.095921673	1769	927	765	566	735	620	808	837
ENSECAG00000018844	5.46203788	0.025662688	0.096135661	431	513	590	817	1062	970	1221	1309
ENSECAG00000006943	5.774172276	0.025691294	0.096211102	691	546	678	852	1427	1251	1353	1706
ENSECAG00000011302	0.242863621	0.025718236	0.096280264	5	7	2	29	25	54	28	21
ENSECAG00000007945	7.739173629	0.025732534	0.096302058	2172	2149	2010	4147	5838	5861	6247	5089
ENSECAG00000010394	5.872539213	0.0257645	0.096372799	1798	1850	594	450	844	458	878	1000
ENSECAG00000009215	5.672220767	0.025771222	0.096372799	1684	59	2185	994	995	473	447	84
ENSECAG00000025068	8.283379102	0.025780221	0.096372799	6756	11542	3063	4658	4026	3844	4356	5191
ENSECAG00000015346	4.684976352	0.025785364	0.096372799	117	7	217	442	853	21	1317	1403
ENSECAG00000023527	4.925416664	0.025814921	0.096444655	201	235	474	702	1012	510	800	955
ENSECAG00000024991	9.639989824	0.025821567	0.096444655	15162	27671	10977	13121	10060	11602	12489	11629
ENSECAG00000019188	4.508643534	0.025830487	0.096446268	234	163	345	433	629	483	584	714
ENSECAG00000011953	5.00244061	0.025839441	0.096448005	365	335	322	619	903	590	865	1035
ENSECAG00000016003	3.724173854	0.025934971	0.096772789	143	142	160	211	418	236	378	370
ENSECAG00000000721	1.082343492	0.026029254	0.097092706	21	38	57	73	22	22	33	41
ENSECAG00000016672	4.432684134	0.026194962	0.097678753	164	236	310	440	596	606	554	478
ENSECAG00000009787	3.546413632	0.02623843	0.09780874	67	123	772	353	94	250	63	4
ENSECAG00000012767	1.00768141	0.026298179	0.097984147	10	24	24	31	34	56	57	67
ENSECAG00000012055	6.690902023	0.026309113	0.097984147	2148	2970	1371	1868	1612	1400	1613	1708
ENSECAG00000023433	0.197126099	0.026311356	0.097984147	8	6	11	21	32	25	38	24
ENSECAG00000011996	4.470602922	0.026412826	0.098329794	226	610	548	525	280	314	227	586
ENSECAG00000014130	6.324531381	0.026480208	0.098533389	1468	2377	1259	1353	1111	1280	1184	1449
ENSECAG00000007359	8.038501578	0.026484859	0.098533389	6517	6390	4290	4179	4890	2839	4297	3398
ENSECAG00000017715	1.854302854	0.026517995	0.0986042	21	25	63	63	91	85	94	121
ENSECAG00000008443	3.243871278	0.026521249	0.0986042	119	270	135	294	127	155	119	176

ENSECAG00000009949	3.801743597	0.026578723	0.098785557	102	148	188	278	286	255	374	599
ENSECAG00000023914	4.426365553	0.026625429	0.098926789	184	242	302	419	558	427	621	635
ENSECAG00000012637	4.670330247	0.026638161	0.098941741	319	749	502	534	425	310	491	417
ENSECAG00000008723	6.350206948	0.026692817	0.099100575	758	839	1022	1686	2008	1646	2271	2713
ENSECAG00000024716	7.076931651	0.026698368	0.099100575	1446	2307	2028	7063	2088	1961	2207	1698
ENSECAG00000018694	2.742513996	0.02672119	0.099152894	44	60	65	159	160	126	174	282
ENSECAG00000005458	1.368259977	0.026743153	0.099201994	23	30	139	51	32	33	43	19
ENSECAG00000009518	1.223135169	0.026762549	0.099241543	58	54	45	32	29	19	40	39
ENSECAG00000016628	3.852340917	0.026795005	0.099329477	236	315	298	320	227	203	246	266
ENSECAG00000005451	3.599117409	0.026805324	0.099335321	156	237	398	233	189	203	151	213
ENSECAG00000010569	1.010726026	0.026822209	0.099365484	35	9	7	6	70	28	75	67
ENSECAG00000020196	1.610787546	0.026868257	0.099503629	12	35	14	71	70	66	146	61
ENSECAG00000000928	3.265574652	0.02698876	0.099894037	115	73	120	149	282	201	212	346
ENSECAG00000012048	8.129371422	0.02699126	0.099894037	4841	9535	3573	5599	3881	4105	4121	4436
ENSECAG00000006011	7.239519435	0.027016918	0.09995644	1120	1269	1215	3927	4546	4351	4714	2931
ENSECAG00000000511	3.388916296	0.027052525	0.100053439	159	224	227	252	172	153	169	178
ENSECAG00000006847	9.440364465	0.027060748	0.100053439	12950	22354	11436	11389	9401	8526	8310	14939
ENSECAG00000015474	6.344839396	0.027086289	0.100115295	836	699	1045	1660	2217	1719	2178	2486
ENSECAG00000024730	2.865546704	0.027120643	0.100209675	47	97	83	134	215	126	227	218
ENSECAG00000016636	5.542879987	0.027132421	0.100220599	384	120	703	362	2770	931	2009	79
ENSECAG00000005213	6.304493495	0.027159808	0.100283669	1725	1597	1395	1579	1264	1401	1229	1094
ENSECAG00000016438	5.219806863	0.027167148	0.100283669	420	986	663	1074	507	645	592	682
ENSECAG00000021106	4.982315224	0.027209688	0.100408077	515	838	547	631	483	508	511	563
ENSECAG00000007498	4.118851404	0.027223838	0.100427676	125	165	224	408	493	337	552	458
ENSECAG00000011247	3.564958453	0.02723432	0.100433737	88	105	137	273	291	187	353	464
ENSECAG00000020313	0.600771595	0.027293392	0.100614625	13	3	9	31	42	15	60	56
ENSECAG00000005312	2.000974994	0.027301081	0.100614625	42	34	32	61	180	43	98	131
ENSECAG00000013072	1.460994681	0.027322791	0.100661981	26	26	44	22	87	56	73	81
ENSECAG00000004262	5.090633938	0.027333945	0.100670434	472	982	550	791	453	542	546	675
ENSECAG00000008072	5.596790087	0.027352172	0.100704917	683	660	1259	1488	734	864	704	877
ENSECAG00000026909	7.355457095	0.027394057	0.100826457	1744	1334	2710	2042	4651	3065	4020	6360
ENSECAG00000014665	4.17405871	0.027412045	0.100859993	170	219	227	306	482	275	519	651
ENSECAG00000007446	5.591094905	0.02753356	0.101274299	611	581	542	685	1341	1074	1303	1306
ENSECAG00000007377	5.968995509	0.02754417	0.101280538	768	2486	947	1052	1046	801	1032	933
ENSECAG00000023335	0.971307459	0.027582307	0.101387957	8	7	46	15	63	52	74	29
ENSECAG00000019560	5.007334979	0.027612244	0.10145407	309	339	565	465	931	609	813	1016
ENSECAG00000006312	3.436278667	0.027618152	0.10145407	135	324	212	239	141	182	190	152
ENSECAG00000000544	1.616314778	0.02763257	0.101474227	37	136	55	39	30	55	42	27
ENSECAG00000016083	7.087246481	0.027708752	0.101721112	2160	3670	2485	2964	2106	1963	1836	2693
ENSECAG00000000437	2.248353025	0.027742982	0.101781975	35	58	54.0001	81	147	80	145	139
ENSECAG00000022905	6.299719553	0.027747352	0.101781975	2192	2143	980	892	1298	914	1130	1266
ENSECAG00000012612	7.861325966	0.027752205	0.101781975	6028	7012	2574	3325	3455	2935	3930	2739
ENSECAG00000014595	0.710832696	0.027786892	0.101876306	14	8	20	24	46	21	36	78
ENSECAG00000020669	8.431372591	0.027814631	0.10194511	9457	6724	6292	5296	5345	4592	5419	4880
ENSECAG00000014565	0.574732607	0.027855079	0.102060435	7	14	9	23	26	87	39	9
ENSECAG00000007095	5.752978355	0.027914131	0.102243827	414	260	578	1392	1643	1668	1923	677
ENSECAG00000017900	6.621113389	0.027957548	0.102362436	1191	2985	1765	2395	1210	1436	1440	2181
ENSECAG00000016654	5.741998496	0.027964531	0.102362436	347	863	508	1068	1163	1028	1460	2129
ENSECAG00000021142	4.11336327	0.027977055	0.102375298	200	199	204	262	404	331	546	546
ENSECAG00000020740	7.974345366	0.028012843	0.102473253	7691	4739	4316	3975	5480	2137	4307	1731
ENSECAG00000015637	3.434147862	0.028056841	0.102601167	175	146	281	334	211	79	174	208
ENSECAG00000022494	7.14598048	0.028089913	0.102689059	3863	4046	1761	1676	2175	1888	2021	1958
ENSECAG00000017860	5.797360251	0.028127058	0.102791776	464.002	995.002	2042	1460.04	856.001	1084	761.001	721.004
ENSECAG00000006819	4.878194572	0.028157568	0.102848779	328	271	419	513	754	590	787	945
ENSECAG00000012866	3.168385632	0.02816076	0.102848779	164	237	148	182	131	137	147	138
ENSECAG00000010007	8.293623012	0.028183898	0.102900209	7531	7776	4558	5747	5131	4599	4965	3694
ENSECAG00000014975	8.445630133	0.028208786	0.10295799	8287	13031	3743	3823	4981	4881	5048	3840
ENSECAG00000002798	3.771370636	0.028228306	0.102978427	112	166	211	217	334	265	385	456
ENSECAG00000019793	5.439753874	0.028232512	0.102978427	251	242	531	1236	1138	1019	1665	921
ENSECAG00000020055	6.94862716	0.028244171	0.102982351	900	1696	1684	2080	3145	3218	3483	3246
ENSECAG00000006614	0.862728757	0.028251715	0.102982351	13	26	16	21	64	48	35	45
ENSECAG00000021853	2.432348566	0.028274284	0.103031564	61	39	76	72	139	125	178	137
ENSECAG00000023164	3.652013635	0.028292059	0.103063282	97	137	153	261	370	197	397	381
ENSECAG00000011060	2.329109706	0.028383939	0.103340778	31	59	67	88	119	94	138	194
ENSECAG00000006004	4.485875941	0.028386426	0.103340778	299	570	467	492	363	375	360	370
ENSECAG00000008940	1.570172942	0.028453082	0.103550261	22	41	133	90	33	49	49	15
ENSECAG00000021603	8.614500717	0.028522127	0.103748749	6632	15820	5659	5032	4484	5154	5379	7245
ENSECAG00000014069	3.864699147	0.028527781	0.103748749	169	344	242	514	271	256	162	218
ENSECAG00000021598	2.38589666	0.028535015	0.103748749	98	57	225	86	126	39	63	49
ENSECAG00000015390	6.384207671	0.028584188	0.103894289	948	728	1281	1304	2291	1825	2203	2485
ENSECAG00000009898	2.267021103	0.028600151	0.103919065	36	39	86	70	108	129	131	146
ENSECAG00000016754	5.589570561	0.028637793	0.10402257	881	1334	758	871	797	722	899	646
ENSECAG00000003445	0.526117093	0.028734685	0.10434116	13	9	13	21	60	29	17	50
ENSECAG00000000096	2.51184866	0.028779771	0.104450768	67	221	117	94	38	113	80	76
ENSECAG00000011042	2.187112741	0.028783256	0.104450768	72	98	179	50	71	84	30	54
ENSECAG00000016016	2.874383501	0.028814196	0.104529659	48	87	97	138	176	153	191	267
ENSECAG00000024214	4.517105983	0.028828849	0.104549435	148	230	357	486	506	556	477	903
ENSECAG00000016532	4.673411188	0.028840346	0.104557757	275	212	393	419	864	434	698	706
ENSECAG00000011831	0.988507551	0.028897567	0.104731788	14	17	12	45	59	44	54	55
ENSECAG00000017935	7.655803523	0.028916709	0.104767743	4932	6268	2232	2955	2957	2847	2828	2925
ENSECAG00000021531	-0.054458558	0.029074677	0.105306497	1	4	5	25	36	8	24	39
ENSECAG00000015185	3.497574725	0.029154223	0.105560958	112	112	149	191	330	178	378	318
ENSECAG00000022195	3.694068155	0.029189236	0.10565406	149	268	286	402	258	176	193	207
ENSECAG00000020252	9.624666776	0.029208372	0.105689658	18708	15684	14524	15469	11462	10682	10917	14989
ENSECAG00000000622	1.877358449	0.029232647	0.105743818	12	47	50	38	25	180	73	141

ENSECAG00000008277	0.8104235	0.029304063	0.105968417	16	20	22	12	44	41	64	37
ENSECAG00000010232	6.555303433	0.029318894	0.105988316	1490	2987	1689	1578	1128	1812	1154	1681
ENSECAG00000018128	1.208279485	0.029441047	0.10639041	9	26	29	43	52	50	64	82
ENSECAG00000009157	6.553900506	0.029454044	0.10639041	1861	2905	1289.01	1502	1342	1409.06	1505	1519
ENSECAG00000009626	8.647469603	0.029460245	0.10639041	3895	1764	27938	9112	4957	7909	2711	957
ENSECAG00000008083	2.423581063	0.029469679	0.10639041	70.0016	120.002	83	200.038	71.0006	76.0019	59.0011	115.004
ENSECAG00000017367	1.550336003	0.029476942	0.10639041	20	26	22	69	76	78	84	75
ENSECAG00000007730	0.91429204	0.029595744	0.106785279	27	72	27	37	9	37	19	28
ENSECAG00000018893	3.496235625	0.029617317	0.106829191	105	128	170	169	284	224	284	402
ENSECAG00000021923	4.886453155	0.029642743	0.106857198	341	301	408	471	843	552	902	786
ENSECAG00000011230	5.508533937	0.029643891	0.106857198	858	959	972	849	1033	671	603	627
ENSECAG00000010537	0.542091293	0.029669625	0.106875899	16	40	43	27	18	19	28	7
ENSECAG00000022286	4.563765756	0.029674337	0.106875899	463	868	324	255	334	330	327	407
ENSECAG00000002732	4.759708029	0.029677298	0.106875899	236	388	408	395	714	540	730	827
ENSECAG00000022245	1.544889229	0.029751568	0.107109415	14	20	51	49	89	40	97	95
ENSECAG00000009193	5.91912797	0.02976439	0.107121635	989.011	1729	860.008	1316	884.001	914	1029	1087
ENSECAG00000011806	7.308239078	0.02978932	0.107177406	1591	1488	2271	2635	4244	3705	4743	4208
ENSECAG00000010623	2.988148333	0.02980473	0.107198905	65	99	105	115	143	263	170	269
ENSECAG00000023923	2.020495963	0.029817169	0.107209709	40	111	113	98	29	86	46	62
ENSECAG00000007762	4.612169355	0.02986386	0.107343619	341	280	926	564	427	425	302	362
ENSECAG00000011804	1.488192882	0.029876283	0.107354309	7	34	14	69	58	74	107	74
ENSECAG00000014827	2.878368269	0.030017465	0.107827516	69	172	163	245	120	86	132	114
ENSECAG00000023411	3.680866231	0.030046966	0.107899378	176.996	469.997	234.988	182.98	171.994	150.989	182.999	273.999
ENSECAG00000024800	1.030096865	0.030128026	0.108153868	22	23	22	13	53	34	51	87
ENSECAG00000024598	4.3242115	0.030136873	0.108153868	194	271	272	295	460	402	659	566
ENSECAG00000000314	5.796013774	0.030152023	0.108174073	444	701	859	999	1351	1243	1281	1905
ENSECAG00000015048	0.653561171	0.030255381	0.108510617	32	35	35	25	23	21	23	17
ENSECAG00000020563	8.61724127	0.03027256	0.108537969	5837	5337	13428	11007	7410	6307	5945	2643
ENSECAG00000015274	2.771650483	0.03028671	0.108554447	61	104	52	104	192	169	141	235
ENSECAG00000000555	6.171013819	0.03030277	0.10857776	750	790	887	1355	1849	1467	1868	2379
ENSECAG00000021458	4.035125583	0.030453706	0.10908158	161	211	184	291	379	341	458	540
ENSECAG00000024968	4.017159516	0.030462582	0.10908158	149	480	220	606	218	230	167	377
ENSECAG00000021792	4.867413916	0.030628166	0.109639956	329	339	397	425	856	510	918	764
ENSECAG00000002211	3.031881962	0.030672603	0.109764444	52	87	82	186	187	186	156	372
ENSECAG00000015817	6.246523047	0.030697798	0.109808872	1910	1702	1043	1296	1122	1197	1120	1279
ENSECAG00000013964	4.125769683	0.030704347	0.109808872	166	150	219	377	509	303	507	530
ENSECAG00000020322	4.58447066	0.030884525	0.110418493	249	795	430	547	302	338	420	470
ENSECAG00000020695	4.237279248	0.030908961	0.110471096	203	188	226	315	744	267	567	440
ENSECAG00000016725	4.564522995	0.030947202	0.110561189	205	282	375	394.001	680	430	656	697
ENSECAG00000019451	7.355450092	0.03095363	0.110561189	4399	4423	1984	2280	2512	1822	2564	2676
ENSECAG00000020508	5.183146419	0.030992591	0.110661474	985	980	442	506	574	505	723	250
ENSECAG00000008310	5.78522084	0.031001186	0.110661474	490	705	691	996	2129	976	1417	1260
ENSECAG00000000828	4.867118213	0.031087251	0.110933839	240	217	510	585	792	775	821	650
ENSECAG00000000343	2.021445047	0.031114518	0.110996282	73	119	155	78	1	102	1	18
ENSECAG00000005604	1.69244098	0.031127483	0.111007679	19	46	46	38	78	67	112	88
ENSECAG00000009464	1.412040744	0.0311592	0.11108592	49	92	40	37	32	38	35	41
ENSECAG00000009935	1.842097315	0.031189341	0.111158498	18.0001	51	34.0001	68.0006	110	75.0001	104	95.0001
ENSECAG00000013822	2.065549067	0.031287053	0.111471779	29	35	40	89	141	47	100	184
ENSECAG00000011001	0.981966608	0.031337807	0.111617607	7	31	16	32	35	44	60	75
ENSECAG00000017170	1.772807667	0.031486735	0.112099625	39	27	50	32	107	80	103	76
ENSECAG00000007965	6.652569258	0.031492871	0.112099625	1373	2653	2088	2211	1357	1605	1556	2001
ENSECAG00000021961	4.489154414	0.031565477	0.112322878	114	252	292	546	450	543	538	884
ENSECAG00000000545	6.053835533	0.031678274	0.112653696	684	759	818	1232	1755	1268	2003	1915
ENSECAG00000026890	3.128653525	0.031678275	0.112653696	48	336	132	309	85	91	96	219
ENSECAG00000009511	5.058998446	0.031689415	0.112658051	261	393	500	659	862	648	1020	924
ENSECAG00000026994	5.478912673	0.031847075	0.113145434	675	1054	854	998	702	764	794	707
ENSECAG00000024022	2.78961998	0.031855307	0.113145434	69	85	104	64	178	172	212	173
ENSECAG00000007392	6.99839232	0.031856385	0.113145434	3009	3968	1526	1803	1817	1655	1609	2485
ENSECAG00000016867	4.845222443	0.031896672	0.113250526	344	1086	412	838	135	177	211	1075
ENSECAG00000024811	4.716365671	0.031905909	0.113250526	343	286	1066	673	608	433	247	244
ENSECAG00000010621	4.538766497	0.031996647	0.113537133	244	291	309	369	553	502	584	773
ENSECAG00000023195	4.7159098	0.032045958	0.113616927	223	348	299	505	619	904	525	663
ENSECAG00000018231	5.453156771	0.032046112	0.113616927	672	865	931	1069	884	669	705	681
ENSECAG00000011130	1.844435638	0.032049133	0.113616927	29	24	57	59	107	67	86	128
ENSECAG00000024033	2.771188619	0.032185732	0.114052577	32	71	103	129	108	235	214	177
ENSECAG00000015301	4.995147776	0.032192098	0.114052577	449	143	1554	921	695	377	414	189
ENSECAG00000017066	6.111258071	0.032210876	0.114083531	788	660	938	1210	1887	1462	1783	2092
ENSECAG00000009527	4.791647526	0.032249005	0.114182985	266	245	426	535	871	507	734	780
ENSECAG00000013303	2.44782693	0.032343135	0.114480592	30	106	138	257	61	105	65	65
ENSECAG00000012730	4.06838495	0.032405371	0.114665159	106	217	254	307	612	331	376	447
ENSECAG00000010015	5.927409014	0.032455141	0.114800939	628	565	690	1269	2004	932	1932	1602
ENSECAG00000018566	5.677167082	0.032463952	0.114800939	388	592	738	1089	1192	1323	1119	1663
ENSECAG00000000444	1.771630386	0.032574877	0.11515736	31	26	63	31	85	63	99	126
ENSECAG00000007801	6.969648938	0.032619998	0.115249892	1454	4839	2031	2378	1711	1791	1551	2542
ENSECAG00000014505	5.957163727	0.032634896	0.115249892	649	1812	1284	1463	915	1022	845	1272
ENSECAG00000017044	5.877485463	0.032637448	0.115249892	593	475	1023	819	1935	1178	2203	939
ENSECAG00000022323	3.748464849	0.032641626	0.115249892	169	413	283	249	195	185	167	306
ENSECAG00000009286	7.352355646	0.032737643	0.115552999	2944	4077	3276	3007	2418	2142	2927	2861
ENSECAG00000024602	6.292314323	0.032881812	0.116025822	1097	2107	1244	2041	989	1349	1174	1587
ENSECAG00000011662	4.177744887	0.03295072	0.11623287	158	275	178	297	582	351	628	346
ENSECAG00000008748	3.091282945	0.033013135	0.116416897	58	84	71	207	322	179	271	151
ENSECAG00000015458	3.076548314	0.033036715	0.116463903	171	247	116	149	150	96	105	159
ENSECAG00000017597	0.520225104	0.033047909	0.116467228	7	12	23	20	40	28	37	45
ENSECAG00000026921	5.218095518	0.033148166	0.116750826	333	549	449	642	810	764	1075	1224
ENSECAG00000011523	2.280286571	0.033148932	0.116750826	61	102	83	178	73	63	88	69

ENSECAG00000014862	4.545715115	0.033177758	0.11681614	277	1054	518	227	395	228	498	89
ENSECAG00000017058	4.005505286	0.033190063	0.116823262	179	168	201	267	514	315	472	375
ENSECAG00000024193	6.947040852	0.033203873	0.11683568	1815	2681	2517	3276	2113	1865	1844	2190
ENSECAG00000015619	1.710222003	0.033231831	0.116897853	57	117	54	47	31	66	45	30
ENSECAG00000014990	6.644359548	0.033245643	0.116910243	1136	3026	1968	2377	1566	1980	1230	1447
ENSECAG00000015008	4.095572786	0.033262245	0.116914551	116	167	208	412	469	258	460	664
ENSECAG00000010127	2.68632944	0.033269356	0.116914551	111	59	310	101	52	134	56	95
ENSECAG00000018794	5.369600424	0.033282112	0.116914551	438	399	745	533	1233	725	999	1415
ENSECAG00000012998	6.51591728	0.033293579	0.116914551	1537	3082	1314	1496	1319	1453	1440	1456
ENSECAG00000018288	2.101604744	0.033298318	0.116914551	39	21	63	76	84	91	140	155
ENSECAG00000019491	3.44082441	0.033334593	0.116970289	104	141	125	184	257	254	295	327
ENSECAG00000013605	3.662693258	0.033334782	0.116970289	93	105	169	300	330	378	292	328
ENSECAG00000008596	5.064291644	0.033399675	0.117161811	225	2138	272	410	286	468	453	487
ENSECAG00000017657	6.765311238	0.033419571	0.117195421	4101	2011	2495	635	2241	705	1743	170
ENSECAG00000024564	6.185816609	0.033442074	0.117210523	620	841	944	1500	1826	1743	2297	1674
ENSECAG00000013261	4.290220641	0.033444509	0.117210523	160	365	209	246	448	525	505	573
ENSECAG00000015430	1.468035203	0.033464496	0.117213078	31	23	16	51	82	70	75	69
ENSECAG00000011894	3.940257681	0.033465871	0.117213078	122	164	222	315	362	356	421	455
ENSECAG00000011822	2.849265963	0.033491427	0.11726644	25	189	397	130	26	186	52	40
ENSECAG00000008338	5.433360863	0.033563823	0.11748372	376	584	607	758	986	918	1049	1532
ENSECAG00000012939	3.031421973	0.033658164	0.11777766	80	399	99	137	98	84	131	142
ENSECAG00000007343	3.1673854	0.033684886	0.117834875	60	254	212	281	107	124	159	153
ENSECAG00000013308	5.47438711	0.033703183	0.117862594	560	1270	776	1031	710	731	623	876
ENSECAG00000018824	4.593270063	0.033717092	0.117874954	517	813	326	268	348	395	345	341
ENSECAG00000008541	1.662780999	0.033740819	0.117911486	38	23	24	55	85	74	73	110
ENSECAG00000017516	4.13338017	0.03375271	0.117911486	159	161	231	374	472	355	598	408
ENSECAG00000020277	2.09644699	0.033758675	0.117911486	8	39	51	102	103	135	154	81
ENSECAG00000017929	7.373653992	0.033860097	0.118229385	3252	5163	2904	2147	2755	1890	2664	2738
ENSECAG00000018492	6.023248136	0.033881893	0.118269147	566	913	713	1287	1588	1440	1724	2017
ENSECAG00000022826	4.175449327	0.033899064	0.118290857	159	215	241	350	455	356	496	571
ENSECAG00000019019	6.842037852	0.033908935	0.118290857	1274	1451	1291	1723	3062	2488	3129	3491
ENSECAG00000004470	5.723859159	0.033949384	0.118395611	509	656	755	920	1466	1025	1295	1676
ENSECAG00000019355	7.849520398	0.033963099	0.118407097	1915	1831	3039	4121	4376	10372	6919	3265
ENSECAG00000018377	4.848021182	0.034084165	0.118792725	387	255	404	384	803	584	840	790
ENSECAG00000011667	6.413567083	0.034211153	0.119198751	1906	3332	958	731	1205	1205	1374	964
ENSECAG00000022649	2.736784165	0.034269458	0.119355572	167	90	159	126	115	96	62	122
ENSECAG00000016660	5.89496064	0.034277172	0.119355572	1099	1577	1179	844	950	963	863	1054
ENSECAG00000009287	2.093577453	0.034338841	0.119533672	28	42	67	68	80	79	136	168
ENSECAG00000007524	6.36422917	0.034395563	0.119694451	1154	1524	1791	2415	1623	1019	1611	1196
ENSECAG00000009855	3.771369789	0.03441871	0.119738329	71	162	214	278	313	279	378	486
ENSECAG00000008786	5.718997217	0.034572358	0.120231483	482	676	656	1035	1406	998	1358	1700
ENSECAG00000010373	5.465837563	0.034585198	0.120231483	486	385	608	841	1498	841	1119	1149
ENSECAG00000020684	3.981920466	0.034597129	0.120231483	184	298	430	422	314	197	288	231
ENSECAG00000023092	2.404715656	0.034602794	0.120231483	75	17.0004	408	78	21	124.001	16	2
ENSECAG00000014083	6.488442368	0.034616484	0.120242277	1524	2349	1572	1776	1320	1235	1800	1475
ENSECAG00000017684	6.004253928	0.034676484	0.120413879	670	1559	1148	2109	988	1082	1045	1091
ENSECAG00000013051	3.605458596	0.034693387	0.120435768	133	127	138	219	336	222	361	362
ENSECAG00000013764	6.036100222	0.034764445	0.120645578	462.001	764	1060	1166	1320	1747	2501	1288
ENSECAG00000016696	5.701541497	0.034778004	0.120655781	613	447	808	683	1635	653	1279	2108
ENSECAG00000004897	5.113036602	0.034864912	0.120920371	1136	423	1119	327	118	1006	218	218
ENSECAG00000004911	6.287953561	0.034876566	0.120923876	895	1131	886	1020	2034	1794	2072	2262
ENSECAG00000007021	4.864151609	0.035083292	0.121603526	238	516	286	405	774	884	908	473
ENSECAG00000022265	6.23838695	0.035146605	0.121763071	770	687	1035	1505	2362	1637	1900	1969
ENSECAG00000010075	5.482395748	0.035153881	0.121763071	363	479	727	891	1179	923	1147	1365
ENSECAG00000002061	0.69652807	0.035172373	0.121763071	9	19	5	34	34	28	58	57
ENSECAG00000017061	7.670434106	0.035178042	0.121763071	1452	1907	3236	4027	5093	5305	6202	5110
ENSECAG00000024722	4.507772724	0.035188456	0.121763071	463	662	388	291	324	419	259	397
ENSECAG00000010493	4.90760623	0.035193621	0.121763071	297	260	474	582	769	726	788	804
ENSECAG00000008538	3.270343861	0.035236295	0.121873602	79	109	157	143	229	304	216	255
ENSECAG00000018394	4.879511039	0.035293573	0.122034563	289	494	901	838	537	404	460	551
ENSECAG00000008567	6.516313382	0.035308415	0.122048741	1070	1007	1181	1343	2808	1512	2407	3018
ENSECAG00000000367	4.960214332	0.035341386	0.122125555	293	406	360	617	845	616	865	882
ENSECAG00000007644	9.459789645	0.035409255	0.122322882	16011	15541	11807	13723	9979	10552	9876	12832
ENSECAG00000010533	1.172071562	0.035457019	0.122450654	16	53	57	86	35	16	10	58
ENSECAG00000016917	6.870660298	0.035487777	0.122518579	3454	3522	1104	1204	1752	1204	1750	1884
ENSECAG00000011543	4.944641851	0.035498254	0.122518579	417	485	1027	639	577	371	620	439
ENSECAG00000005704	7.554421053	0.035696369	0.123137391	1160	1756	2768	4244	4595	4953	5362	5234
ENSECAG00000000891	5.792762828	0.035699222	0.123137391	718	1441	1019	1350	1103	772	781	1058
ENSECAG00000016542	3.760877598	0.035790133	0.123413503	86	143	192	287	387	182	432	462
ENSECAG00000020717	3.820972899	0.035811459	0.123449573	85	130	223	305	271	358	302	589
ENSECAG00000026919	1.065424908	0.035834658	0.123479656	20	36	90	38	24	35	27	26
ENSECAG00000007788	5.743959169	0.035841921	0.123479656	748	1318	1107	1138	752	894	890	1068
ENSECAG00000017003	2.438648171	0.035860407	0.123505895	86	99	131	145	85	90	41	111
ENSECAG00000015231	2.304367616	0.035886118	0.123556993	54	252	74	58	56	69	93	27
ENSECAG00000000934	6.852979044	0.03593017	0.123671188	1919	2769	2424	2274	1968	1736	1634	2144
ENSECAG00000016053	2.28395698	0.03596773	0.123762975	38	147	99	150	51	61	73	103
ENSECAG00000015689	5.639999145	0.036009459	0.123869051	785	819	1011	1446	929	731	919	792
ENSECAG00000009706	9.196230153	0.036065588	0.124024579	10431	16600	11117	10176	9318	7882	10428	8293
ENSECAG00000012796	0.237497236	0.036190811	0.124417548	8	7	13	21	21	30	40	30
ENSECAG00000013248	0.869388282	0.036206689	0.124434483	25	27	55	49	36	22	24	19
ENSECAG00000018521	6.676714851	0.036294312	0.124697905	3204	1436	1531	1823	1557	1428	1464	1730
ENSECAG00000000529	6.427834562	0.036383024	0.124932862	900	2671	1406	2357	1090	1512	1443	1432
ENSECAG00000004349	3.773240896	0.036384689	0.124932862	51	251	135	114	198	920	240	166
ENSECAG00000025131	6.091897578	0.036404428	0.124936804	1139	2132	1019	1199	877	1075	971	1441
ENSECAG00000010670	2.581937595	0.036407829	0.124936804	48	55	102	93	158	141	135	200

ENSECAG00000025164	3.007249977	0.036491459	0.125163072	72	96	98	143	224	193	209	211
ENSECAG00000011099	6.81605905	0.036495798	0.125163072	2181	3260	1516	2163	2139	1515	1812	1521
ENSECAG00000016384	4.784981099	0.036556528	0.125333517	358	182	450	370	803	663	589	860
ENSECAG00000011585	7.355429796	0.036694037	0.125767013	1694	3339	4764	4577	2258	3249	2243	2440
ENSECAG00000023375	5.235587928	0.036902325	0.126429781	383	402	557	694	977	865	953	1056
ENSECAG00000016881	5.670435389	0.036927665	0.126429781	435	458	997	699	1258	809	1021	2488
ENSECAG00000022071	1.69798477	0.036931108	0.126429781	51	126	89	26	21	87	14	21
ENSECAG00000009848	1.878829038	0.036931917	0.126429781	25	32	38	85	90	87	119	94
ENSECAG00000016829	5.969172453	0.036946807	0.126442658	1971	609	1026	1364	997	607	995	1229
ENSECAG00000018358	5.068007749	0.036964895	0.126466467	425	663	890	762	593	435	638	606
ENSECAG00000020279	0.397902613	0.037000994	0.126551865	13	43	19	38	11	11	20	27
ENSECAG00000023484	1.178037857	0.037113739	0.126899278	36	14	17	11	108	38	52	57
ENSECAG00000007882	5.638435471	0.037130503	0.126918404	494	604	694	910	1313	1036	1227	1530
ENSECAG00000001065	1.950685926	0.03717638	0.12702822	31	141	82	85	51	45	77	45
ENSECAG00000007526	5.49884682	0.03718499	0.12702822	497	973	1077	1216	627	837	724	843
ENSECAG00000020925	5.002306626	0.037204821	0.127057763	179	409	413	761	677	850	891	921
ENSECAG00000001099	1.976675981	0.037233655	0.127118026	38	30	53	52	99	52	77	217
ENSECAG00000018757	2.691364504	0.037290712	0.127274579	54	71	97	96	208	109	151	223
ENSECAG00000014480	3.992705175	0.037414509	0.127658756	60	224	119	421	389	455	421	461
ENSECAG00000024510	4.709035665	0.037488995	0.1278745	154	70	571	484	625	488	892	960
ENSECAG00000023016	8.334452325	0.037510405	0.127909128	5110	6686	6929	8312	6091	4452	4794	5290
ENSECAG00000009111	5.842769523	0.0375342	0.127951868	1001	1175	1009	1387	927	1021	846	1049
ENSECAG00000014890	3.904161657	0.037572588	0.128044316	117	181	174	311	337	266	352	651
ENSECAG00000006321	7.279587416	0.037597313	0.128090156	3354	3433	3394	2278	3121	1971	2287	2258
ENSECAG00000023909	5.880322177	0.037660835	0.128231	539	459	982	1190	1649	1140	1647	1729
ENSECAG00000013562	5.97043616	0.037661226	0.128231	653	854	782	1007	1817	1194	1696	1784
ENSECAG00000015695	0.580987085	0.037709513	0.128356945	18	14	7	7	18	95	19	32
ENSECAG00000021907	6.772761028	0.037745505	0.128420937	2854	2723	1644	1358	1872	1217	1728	1821
ENSECAG00000011077	3.591088314	0.037760963	0.128420937	132	143	138	193	380	209	324	354
ENSECAG00000019363	7.250418331	0.037762221	0.128420937	1738	1635	1852	2418	4106	3533	4001	4491
ENSECAG00000014836	3.590618958	0.037851379	0.128680556	103	186	158	138	223	201	411	469
ENSECAG00000011085	3.93897702	0.037861213	0.128680556	98	262	171	250	292	324	382	653
ENSECAG00000011569	6.05435116	0.037904332	0.128788583	554.011	1067	718.008	1010	1189	2038	1171	2697
ENSECAG00000018378	6.550960354	0.037937264	0.128861942	1830	2381	1775	1418	1522	1290	1441	1804
ENSECAG00000024705	4.965013581	0.037979604	0.128967203	500	365	2186	389	12	821	10	53
ENSECAG00000023356	4.351162576	0.038115451	0.129366123	249	248	224	319	495	542	522	539
ENSECAG00000022426	6.911494001	0.038119854	0.129366123	1047	1229	1321	2668	3653	2714	3826	2559
ENSECAG00000003775	7.127236509	0.038188503	0.129560396	1215	1793	1950	2309	3615	3342	3858	3870
ENSECAG00000013124	4.93482046	0.038207407	0.129585839	705	702	697	413	624	519	451	132
ENSECAG00000014833	4.625048405	0.038266068	0.129746066	241	315	409	331	568	578	612	784
ENSECAG00000005741	3.969436433	0.038313961	0.129869698	125	132	194	385	472	296	468	415
ENSECAG00000005667	4.046931774	0.038328577	0.129874187	151	215	190	288	558	228	535	429
ENSECAG00000017450	1.176965598	0.038342274	0.129874187	7	25	6	59	63	36	58	101
ENSECAG00000000816	5.549487226	0.038358437	0.129874187	320	313	404	1458	1142	1495	1139	1243
ENSECAG00000015058	6.66067111	0.038361008	0.129874187	1102	522	1941	1381	2811	2497	3018	2566
ENSECAG00000016966	3.415054593	0.038448851	0.130119286	82	82	43	289	344	373	322	138
ENSECAG00000015460	9.418589201	0.038462406	0.130119286	20615	16657	7855	9095	9921	8115	11030	10189
ENSECAG00000004590	3.314976304	0.038467759	0.130119286	98	84	134	190	268	194	341	244
ENSECAG00000012618	3.885262871	0.038631256	0.1305847	158.001	524	273.001	287.001	182.001	287	187.001	253.001
ENSECAG00000019148	3.286723114	0.038649305	0.1305847	53	106	180	164	257	217	196	377
ENSECAG00000006359	1.625822611	0.038651011	0.1305847	25	36	40	40	93	38	96	106
ENSECAG00000000386	2.598768437	0.038651324	0.1305847	70	264	80	103	97	83	86	76
ENSECAG00000009231	5.299201976	0.038693427	0.130688085	327	439	633	766	966	939	881	1258
ENSECAG00000013071	5.344693117	0.038758342	0.130868434	570	1069	765	896	541	467	706	1017
ENSECAG00000000173	6.867471746	0.038907605	0.131277295	2674	4828	1059	1086	2184	1509	1622	910
ENSECAG00000014905	0.822591755	0.038913114	0.131277295	15	15	23	24	59	33	58	34
ENSECAG00000024610	1.30845696	0.038914094	0.131277295	14	4	36	52	47	71	108	48
ENSECAG00000017106	0.524133309	0.038958422	0.131387829	32	13	42	32	20	16	26	13
ENSECAG00000009436	4.187197518	0.039013129	0.131533285	154	201	291	339	507	408	470	483
ENSECAG00000013382	2.685237451	0.039094632	0.13173221	24	38	90	148	125	332	150	95
ENSECAG00000017348	4.927453913	0.039095319	0.13173221	680	731	492	451	520	471	456	505
ENSECAG00000000168	6.885164621	0.03917082	0.1319365	1849	2209	6721	2792	34	2848	28	17
ENSECAG00000022113	4.943372063	0.039179172	0.1319365	288	307	439	640	814	586	752	1041
ENSECAG00000021688	2.675456241	0.039203256	0.131952189	70	53	105	71	221	148	165	144
ENSECAG00000018675	0.918062848	0.039207058	0.131952189	12	68	47	41	10	28	24	36
ENSECAG00000003051	5.290237137	0.039229179	0.131987542	1368	475	647	512	571	585	643	491
ENSECAG00000005548	4.803824622	0.039283821	0.132132262	238	346	478	442	603	597	723	964
ENSECAG00000003494	2.466387187	0.039335687	0.132225578	77	64	200	147	79	105	80	53
ENSECAG00000011661	6.966390907	0.039336056	0.132225578	1035	1131	1882	2544	3601	2419	3186	4162
ENSECAG00000020091	6.619398537	0.039355596	0.132225578	1103	1071	1410	1458	2785	1922	2378	3269
ENSECAG00000008212	7.285419609	0.039358115	0.132225578	2539	3331	3129	3856	2808	1833	2698	2846
ENSECAG00000021733	0.365670236	0.039408108	0.132353385	18	33	37	19	14	7	16	31
ENSECAG00000024614	2.997032065	0.039419456	0.132353385	70	50	151	126	199	179	228	246
ENSECAG00000013685	4.428550014	0.039447844	0.132409572	240	373	629	577	288	336	302	508
ENSECAG00000006658	2.254338458	0.039549036	0.132710027	29	66	51	79	81	115	90	240
ENSECAG00000013289	4.375221849	0.039602878	0.132848336	113	231	275	510	415	638	530	585
ENSECAG00000010539	3.503494898	0.039613639	0.132848336	78	241	318	387	135	165	144	264
ENSECAG00000020949	4.88549712	0.039639176	0.132894754	397	357	1034	737	489	292	433	724
ENSECAG00000002830	6.412691644	0.039769293	0.133281	1074	2179	1879	1850	1220	1485	1477	1436
ENSECAG00000018424	5.69061104	0.039780114	0.133281	587	552	675	912	1681	953	1232	1479
ENSECAG00000024706	6.628426926	0.039793866	0.133281	1258	1967	2551	2537	2016	1666	1406	1332
ENSECAG00000016965	0.717922431	0.039801305	0.133281	11	17	18	28	38	40	39	53
ENSECAG00000015830	5.509153919	0.039835498	0.133356196	331	476	603	1127	1168	977	1131	1459
ENSECAG00000013578	5.684594134	0.039866861	0.133413447	423	504	817	1100	1320	1061	1234	1714
ENSECAG00000009447	2.886634266	0.039876084	0.133413447	54	65	101	163	181	161	188	255

ENSECAG00000015574	0.556021584	0.039910062	0.133487821	14	6	19	23	43	32	42	36
ENSECAG00000014360	6.031807245	0.039952163	0.13358931	352	431	1174	1482	2022	1518	2423	1060
ENSECAG00000008668	2.725161387	0.039997935	0.133677693	45	49	468	97	63	127	45	79
ENSECAG00000006366	7.659194729	0.040002126	0.133677693	2224	7188	3380	4460	2786	2627	3260	3903
ENSECAG00000013965	1.531909602	0.040115603	0.13401749	13	54	150	56	39	30	52	27
ENSECAG00000015241	0.534306367	0.040176392	0.13418112	16	14	18	7	54	24	36	40
ENSECAG00000012630	6.752539586	0.0402343	0.13429913	1697	2739	2151	2127	1938	1499	1818	1795
ENSECAG00000025567	1.679445611	0.040244928	0.13429913	41	51	108	78	45	61	46	29
ENSECAG00000014679	2.943344721	0.040247187	0.13429913	73	109	73	122	194.001	154	183.001	290.001
ENSECAG00000018811	6.739986307	0.040276067	0.13435604	2372	3153	1469	1509	1899	1060	1900	1712
ENSECAG00000022298	2.13584393	0.040350004	0.134563178	27	30	43	118	97	110	114	155
ENSECAG00000009290	7.861024598	0.040440298	0.134824725	5732	8725	2017	2455	3406	3285	3541	2492
ENSECAG00000024442	3.45105823	0.040490665	0.134953045	124	173	457	301	80	338	46	68
ENSECAG00000018627	4.853338267	0.040522152	0.135018384	514	763	488	506	549	369	454	540
ENSECAG00000021812	3.061991864	0.040535578	0.135023522	84	282	193	151	86	137	119	164
ENSECAG00000023388	1.844551494	0.04055779	0.135057917	36	60	191	51	45	84	15	27
ENSECAG00000023413	5.980439512	0.040721222	0.135562416	677	584	1013	1065	1864	1174	1781	1735
ENSECAG00000004023	3.704480754	0.040734511	0.135566935	144	158	145	219	355	244	366	395
ENSECAG00000010316	6.348614304	0.040748896	0.135575096	1366	1503	1856	1798	1530	1256	1192	1502
ENSECAG00000021079	3.342828913	0.040808746	0.135734477	148	283	210	180	164	133	182	170
ENSECAG00000008767	6.490547508	0.040827663	0.135741066	1686	1716	2132	1611	1361	1556	1248	1743
ENSECAG00000018419	3.975723827	0.040834621	0.135741066	253	141	708	274	312	234	218	150
ENSECAG00000007962	4.667288828	0.040885204	0.135847643	303	398	271	268	758	579	708	604
ENSECAG00000018401	7.795141639	0.040890595	0.135847643	2046	2414	3227	3784	6115	5275	6153	5886
ENSECAG00000008202	5.286587552	0.040932848	0.135948266	597	611	1140	844	860	571	625	514
ENSECAG00000000901	5.173408955	0.041012797	0.136173992	441	367	1480	867	575	521	406	787
ENSECAG00000022709	3.159080593	0.041029702	0.136190322	114	92	122	95	241	207	259	233
ENSECAG00000017373	2.843881664	0.041165208	0.136600202	73	141	170	239	114	89	111	143
ENSECAG00000015700	5.575447083	0.041251937	0.136848032	410	607	677	944	1230	1016	1239	1378
ENSECAG00000020046	5.484227193	0.041400834	0.137263202	530	550	557	714	1282	891	1142	1282
ENSECAG00000004050	2.072981172	0.041401249	0.137263202	33	27	45	75	45	244	81	87
ENSECAG00000016185	1.354880538	0.041444028	0.137364949	10	29	34	41	71	26	54	137
ENSECAG00000022060	3.442332823	0.041516245	0.137564181	99	91	168	208	331	199	281	332
ENSECAG00000020598	8.058445364	0.041537102	0.137593164	4599	8965	3676	4671	3972	3669	3483	5253
ENSECAG00000007830	4.241482338	0.041582788	0.137704354	201	174	158	446	553	439	373	637
ENSECAG00000023732	4.749917735	0.041623702	0.137799681	195	427	428	379	782	499	634	889
ENSECAG00000006471	3.198630312	0.041687863	0.137971892	198.001	360	75.001	117	161.001	84.0005	134	138
ENSECAG00000023055	5.698952393	0.041869143	0.138531509	504	800	618	828	1156	1000	1496	1732
ENSECAG00000011785	2.779685412	0.041895378	0.138577959	60	53	118	107	152	157	141	290
ENSECAG00000007173	4.17932553	0.041909166	0.138583221	184	123	358	204	583	339	270	805
ENSECAG00000013945	4.051103214	0.041940002	0.138644835	157	257	170	277	408	349	426	541
ENSECAG00000023959	3.466111242	0.042110748	0.139168795	131	105	146	171	292	239	235	397
ENSECAG00000002313	1.069249475	0.042144194	0.13923883	16	24	23	31	43	50	38	92
ENSECAG00000022648	0.135655053	0.042177385	0.139307978	10	21	43	22	14	24	5	8
ENSECAG00000007346	6.959257111	0.042194639	0.139324466	2576	2904	2252	2157	2106	1877	1624	2341
ENSECAG00000018969	0.692525054	0.042210246	0.139335507	19	14	15	17	49	23	58	41
ENSECAG00000004121	4.414204449	0.042272202	0.139488118	215	214	391	299	606	417	561	622
ENSECAG00000009432	3.48736301	0.042281031	0.139488118	99	361	179	331	149	193	157	198
ENSECAG00000008387	6.639222802	0.042302817	0.139519479	1123	3527	1886	1777	1298	1500	1439	2084
ENSECAG00000001532	2.6142847	0.042329701	0.139567632	32	80	51	136	101	131	157	290
ENSECAG00000020007	2.028967083	0.042412949	0.139801545	37	21	14	83	111	59	27	309
ENSECAG00000024238	7.126627151	0.042463623	0.139927983	2618	4134	2420	2121	2845	2057	1900	1788
ENSECAG00000021764	6.214073535	0.042495651	0.139992921	344	832	1038	1863	1744	2158	1687	2250
ENSECAG00000005400	3.11972718	0.042525943	0.140052107	75	92	141	78	243	400	88	207
ENSECAG00000021294	5.998385035	0.042559338	0.140121472	1542	1361	814	1260	1130	761	1129	1106
ENSECAG00000012359	4.966880933	0.042581249	0.140153	1271	208	588	350	495	357	414	471
ENSECAG00000000113	2.898650269	0.042595474	0.140159216	69	60	85	162	182	213	227	158
ENSECAG00000018570	6.137008514	0.042613342	0.140177415	661	653	1270	1196	1999	1436	1810	2052
ENSECAG00000023067	4.008713576	0.042678202	0.140350138	159	145	242	304	520	320	418	409
ENSECAG00000024248	5.607834712	0.042758792	0.140574477	530	427	694	984	1252	1047	1325	1388
ENSECAG00000007009	3.827431981	0.042778478	0.140589618	152	290	355	394	213	223	261	241
ENSECAG00000015739	6.887413524	0.042788145	0.140589618	1192	875	1845	2205	3300	2369	2850	4157
ENSECAG00000021428	5.762983535	0.042835908	0.140705864	1478	1469	536	720	679	761	849	1015
ENSECAG00000021817	4.888283385	0.043057536	0.141392985	336	745	587	771	468	346	564	633
ENSECAG00000000258	5.539766367	0.043148645	0.141651228	516	2592	328	610	906	530	619	461
ENSECAG00000016191	3.454031337	0.043221579	0.141827592	107	161	100	183	297	190	317	354
ENSECAG00000011726	2.938672178	0.043227332	0.141827592	87	109	66	99	226	215	185	179
ENSECAG00000019900	10.18578109	0.043259507	0.141892181	32008	22875	16695	20822	18333	14571	18944	18770
ENSECAG00000008326	3.780316099	0.043420233	0.142378265	98	173	169.004	304	368	312	363	377
ENSECAG00000016058	4.671954153	0.043465069	0.142462126	231	275	286	594	610	705	647	647
ENSECAG00000012077	5.390087467	0.043470884	0.142462126	606	1771	488	573	576	511	590	894
ENSECAG00000021810	2.175431859	0.043502287	0.14251885	42	48	52	76	109	70	153	150
ENSECAG00000008493	4.67865524	0.043530318	0.14251885	285	200	1489	1202	1	521	0	75
ENSECAG00000018603	2.989868587	0.043534913	0.14251885	95	242	258	181	6	227	41	73
ENSECAG00000008074	4.816843365	0.043538367	0.14251885	367	283	393	401	758	549	743	869
ENSECAG00000007383	1.893406774	0.043574911	0.142586117	16	39	48	78	119	47	113	126
ENSECAG00000009988	7.149372632	0.043584015	0.142586117	2514	3232	2433	3340	2416	2226	1975	2604
ENSECAG00000000614	4.219931596	0.043655707	0.142779545	188	844	238	315	197	238	279	405
ENSECAG00000024777	0.243471262	0.043683979	0.142830896	10	7	14	18	25	28	24	45
ENSECAG00000011962	2.058845564	0.043711632	0.142848174	93	145	57	55	30	108	31	40
ENSECAG00000015585	4.004002616	0.043714408	0.142848174	124	210	247	271	443	289	537	393
ENSECAG00000018649	9.588260686	0.043860455	0.143254671	16574	30099	10509	5903	9434	10938	12019	10375
ENSECAG00000013359	4.848964394	0.043871695	0.143254671	301	408	1038	717	528	417	417	534
ENSECAG00000007170	5.267497728	0.043876629	0.143254671	317	380	571	832	645	1083	1149	1093
ENSECAG00000011396	1.296661441	0.043894687	0.143272459	16	15	51	28	66	41	54	107

ENSECAG00000008005	7.157609273	0.04393687	0.143368958	1421	2127	1538	2039	3527	3767	4551	3152
ENSECAG00000019828	0.61609837	0.04403812	0.143658088	22	73	16.9999	17	22	21	7	24
ENSECAG00000017156	5.686882257	0.044053934	0.143661787	1159	955	889	992	716	929	954	764
ENSECAG00000009579	4.455696493	0.044064542	0.143661787	221	218	332	414	623	458	538	629
ENSECAG00000005815	5.316605883	0.044169852	0.143963814	628	873	890	815	876	494	784	475
ENSECAG00000000399	5.8189846	0.044233966	0.144131437	910	1479	816	1333	1212	714	974	829
ENSECAG00000021895	2.526778413	0.044320814	0.144373018	48	63	115	24	119	174	164	175
ENSECAG00000015149	8.003598828	0.044338899	0.144390532	2215	3040	3429	4578	7810	4536	6715	8443
ENSECAG00000021155	1.953102687	0.044368384	0.14444515	17	90	18	36	82	81	124	151
ENSECAG00000010122	2.507364885	0.044515734	0.144883347	30	64	79	113	86	158	168	195
ENSECAG00000010047	6.145797801	0.044589797	0.145082837	638	942	1005	1272	1816	1549	1876	2001
ENSECAG00000018370	6.013353775	0.04465344	0.14524832	611	709	1032	1137	1690	1204	1577	2239
ENSECAG00000002142	3.443655098	0.044699212	0.145355594	206	241	185	237	174	138	138	259
ENSECAG00000000227	6.580795682	0.044821521	0.145711623	799	1257	1384	1711	2138	2707	2316	2679
ENSECAG00000017638	2.063096714	0.044839218	0.145727458	48	36	61	39	143	68	153	86
ENSECAG00000012712	6.325476854	0.044944886	0.146029108	644	1688	2676	2051	992	1675	858	1530
ENSECAG00000018643	3.665045136	0.044969283	0.14605458	117	130	136	285	305	227	397	405
ENSECAG00000000428	5.223474572	0.044978435	0.14605458	776	472	772	968	788	482	590	622
ENSECAG00000012952	6.249975516	0.0450195	0.146146159	1101	1602	1706	1704	1258	1135	1250	1549
ENSECAG00000003600	4.397648644	0.045068206	0.146262484	191	239	316	408	511	469	538	632
ENSECAG00000019750	6.391719252	0.045098273	0.146318269	1718	1120	2189	1700	1573	1017	1154	1805
ENSECAG00000017382	5.822766665	0.04514761	0.146436524	682	1709	776	1527	839	849	782	1273
ENSECAG00000010715	4.315912446	0.045203055	0.146567937	367	434	327	438	370	288	353	315
ENSECAG00000007365	6.019504814	0.045213925	0.146567937	708	836	939	827	1526	2176	1295	1657
ENSECAG00000005616	4.228476941	0.045352345	0.146960416	157	313	210	308	475	370	479	623
ENSECAG00000010650	4.069155953	0.045360868	0.146960416	283	194	475	428	293	219	294	301
ENSECAG00000016299	4.067383501	0.045442118	0.147181682	151	340	303	691	266	293	211	308
ENSECAG00000011882	5.52872082	0.045695705	0.147960842	358	569	650	1002	1181	1080	1287	1143
ENSECAG00000015914	6.955694785	0.04571564	0.147983221	1230	1495	1641	2118	3142	3077	3157	3559
ENSECAG00000013860	2.611100241	0.045882234	0.148454623	63	87	56	87	197	129	130	190
ENSECAG00000017120	4.694358822	0.0458874	0.148454623	375	584	681	436	452	475	323	459
ENSECAG00000019590	1.217638624	0.045984575	0.148726657	26	48	49	85	25	58	16	25
ENSECAG00000000462	3.134865792	0.046172288	0.149272787	153	437	120	94	49	263	19	36
ENSECAG00000017397	2.01378086	0.046179708	0.149272787	21	68	30	72	75	87	110	168
ENSECAG00000009789	6.565492937	0.046208055	0.149321935	1270	2866	1562	2062	1381	1444	1206	2182
ENSECAG00000011466	6.237934248	0.046243549	0.149394142	783	2512	1456	1592	962	1637	955	1189
ENSECAG00000000834	6.564782389	0.046281665	0.14947478	1101	1255	1222	1215	2916	1845	2697	2407
ENSECAG00000024148	0.943344834	0.046323248	0.149566566	9	17	23	35	23	92	47	39
ENSECAG00000026885	6.081610455	0.046336446	0.149566675	627	765	699	1546	1804	1944	1233	2041
ENSECAG00000019104	3.830094954	0.046382722	0.149673528	100	131	244	294	439	352	331	347
ENSECAG00000008366	6.308809449	0.046401756	0.149692434	1100	2489	1456	1329	1168	1268	1224	1528
ENSECAG00000006245	2.372459813	0.046457987	0.149831293	68	77	261	131	6	155	5	28
ENSECAG00000011134	5.26672782	0.04647831	0.149854301	480	463	549	498	934	932	957	1113
ENSECAG00000010984	3.053138419	0.046506614	0.149885434	70	123	241	312	122	121	135	140
ENSECAG00000021610	6.884449931	0.04651435	0.149885434	2449	2733	1808	2373	1985	1564	1844	2278
ENSECAG00000012216	3.859370686	0.046580275	0.150046049	181	386	230	403	191	212	238	318
ENSECAG00000001445	8.033064074	0.046590606	0.150046049	6074	5386	4352	4982	4480	3621	3943	4522
ENSECAG00000024522	4.444031817	0.046648317	0.150189338	193	276	253	448	692	364	649	546
ENSECAG00000022275	5.115999474	0.046850922	0.150798915	344	296	462	733	881	493	864	1473
ENSECAG00000010842	6.271671428	0.046939835	0.151025292	706	2773	1334	1691	1147	1040	1291	1504
ENSECAG00000009150	4.750567987	0.046947838	0.151025292	259	246	373	571	881	515	814	564
ENSECAG00000017248	4.746696063	0.046990916	0.151110897	215	199	377	655	753	761	807	456
ENSECAG00000020597	6.672129127	0.047005783	0.151110897	1156	1251	1307	1526	2944	1923	2794	3006
ENSECAG00000011374	7.403905615	0.047014348	0.151110897	3270	4251	2905	3094	2829	2469	2466	3115
ENSECAG00000025109	4.258419533	0.047132108	0.151446554	125	390	757	476.001	179	179	241	585
ENSECAG00000016144	3.367379109	0.047162598	0.15150168	85	139	143	173	296	201	233	345
ENSECAG00000008434	1.384736754	0.047189046	0.151505604	15	21	43	46	90	52	80	51
ENSECAG00000018596	1.524292375	0.047190489	0.151505604	18	34	35	54	84	54	80	82
ENSECAG00000008938	5.011090888	0.047237883	0.151614923	307	738	862	774	501	567	516	581
ENSECAG00000022563	2.768154749	0.047389152	0.152057484	36	43	64.0016	176	277	185	241.001	50
ENSECAG00000007231	4.267956162	0.047464945	0.152257682	206	199	224	401	449	391	517	635
ENSECAG00000015420	5.21759136	0.047511815	0.152365013	948	707	678	514	586	400	684	742
ENSECAG00000009702	8.051958489	0.047547766	0.152437279	2556	3150.01	3398	4622	7308.03	6104	7151.04	7374
ENSECAG00000014125	5.471347079	0.047564432	0.152447694	189	740	325	1100	1204	928	1455	1175
ENSECAG00000017661	1.010381971	0.047636792	0.152636556	15	49	52	62	13	32	26	41
ENSECAG00000004975	7.204153485	0.047807744	0.153132141	2013	3135	3094	4034	2480	2332	2111	2820
ENSECAG00000010572	6.780448294	0.047818415	0.153132141	1020	1389	1482	1947	3066	2209	2852	3338
ENSECAG00000015959	2.947965223	0.047848809	0.153186296	105	188	143	232	69	56	133	222
ENSECAG00000011777	4.634808928	0.047892499	0.15328298	305	1110	323	337	458	242	479	299
ENSECAG00000019289	2.754586526	0.047951786	0.153429511	41	77	104	129	153	150	165	244
ENSECAG00000011262	5.536400146	0.048014618	0.153574204	607	566	1315	1428	849	582	693	1019
ENSECAG00000022694	5.228341404	0.048033739	0.153574204	944	1062	354	546	600	477	649	588
ENSECAG00000005203	5.741673432	0.048037557	0.153574204	881	1770	665	976	781	1076	743	770
ENSECAG00000019661	5.55342805	0.048100725	0.153709472	462	476	790	844	1263	929	1178	1434
ENSECAG00000016830	6.789382942	0.048126964	0.153709472	1977	3596	1624	1657	1775	1291	1953	1976
ENSECAG00000018017	4.121356012	0.048129463	0.153709472	166	133	328	295	482	343	460	526
ENSECAG00000009904	6.508267834	0.048133982	0.153709472	1129	1025	1318	1150	2470	1865	2540	2599
ENSECAG00000015230	4.205998167	0.04825733	0.154060069	378	621	226	227	307	235	256	345
ENSECAG00000014189	4.505845236	0.048352716	0.154321225	291	428	639	504	384	373	459	288
ENSECAG00000017905	0.441305506	0.048382508	0.154372946	8	13	26	8	20	32	40	51
ENSECAG00000022438	9.169930381	0.048414712	0.15443233	5927	19918	13866	10252	6191	12523	7492	7671
ENSECAG00000000489	10.13582551	0.048530917	0.15475955	21325	33797	18637	17176	16590	17149	16915	18765
ENSECAG00000008471	4.835253372	0.048593559	0.154915829	301	302	369	518	737	387	690	1228
ENSECAG00000010315	4.794126386	0.048657777	0.155077045	265	301	408	502	634	523	540	1229
ENSECAG00000002944	8.949195715	0.048681031	0.155107649	7359	14630	9299	9453	6981	7365	8296	8644

ENSECAG00000007977	1.168052171	0.048726142	0.155207858	23	80	56	29	25	34	30	36
ENSECAG00000000700	7.760726848	0.04890723	0.155741017	2329	2129	2920	3749	6144	4263	5771	6843
ENSECAG00000010529	4.340169155	0.048927716	0.155762597	315	431	422	434	380	319	314	361
ENSECAG00000009547	5.868746203	0.048993519	0.155885452	504	570	1076	1055	1721	1214	1560	1493
ENSECAG00000022368	7.630500607	0.0490064	0.155885452	5077	4372	2594	3552	3601	2690	2812	3171
ENSECAG00000014796	5.546417813	0.049007467	0.155885452	364	930	1246	1521	635	819	682	967
ENSECAG00000011837	8.897047454	0.049115003	0.156183786	5683	11352	10222	13395	7788	6960	8184	7780
ENSECAG00000018538	6.298937319	0.049131314	0.156191939	928	793	1214	1166	2472	1623	2016	2012
ENSECAG00000021995	4.816046244	0.049183673	0.156314656	256	383	458	428	691	638	612	934
ENSECAG00000011657	6.299960399	0.049247922	0.156473092	794	2132	2269	1435	1065	1742	1026	1151
ENSECAG00000013032	3.856895252	0.049261068	0.156473092	137	243	144	218	402	287	343	480
ENSECAG00000022958	5.98548346	0.049297383	0.156534444	784	754	884	821	2002	1149	1678	1736
ENSECAG00000020629	3.616216815	0.049307937	0.156534444	86	105	178	296	289	311	364	307
ENSECAG00000016626	3.276870639	0.049321934	0.156535145	81	92	175	144	242	245	344	176
ENSECAG00000007966	6.14582497	0.049397633	0.156731613	1446	1552	1240	1252	1352	919	1133	1323
ENSECAG00000013735	3.927240335	0.049501784	0.157018221	182	239	126	195	368	302	398	541
ENSECAG00000024151	6.237979719	0.049557208	0.15710833	1269	1726	1484	1458	1376	1092	1293	1340
ENSECAG00000020678	7.452379977	0.049557847	0.15710833	3425	3982	3393	3141	3127	2611	2591	2969
ENSECAG00000022654	2.734331076	0.04962836	0.157287986	179	154	87	100	78	85	123	111
ENSECAG00000012780	6.06490289	0.049668355	0.157370846	822	751	925	956	2048	1191	1733	1980
ENSECAG00000010325	8.768239323	0.049697909	0.157389096	9160	13462	6882	5454	6550	5226	7398	7374
ENSECAG00000000455	2.195598572	0.04970182	0.157389096	82	179	61	52	59	50	53	101
ENSECAG00000021247	4.997471887	0.049750401	0.157499041	253	402	313	798	786	751	906	838
ENSECAG00000008028	2.544693939	0.049873017	0.157843238	41	174	182	112	73	65	99	115
ENSECAG00000023224	6.852094546	0.049923917	0.157960329	1077	1016	1702	2366	2924	2774	2593	3846
ENSECAG00000026939	1.981747696	0.049959653	0.158029391	30	59	32	71	107	78	105	127
ENSECAG00000016746	6.67175421	0.050020443	0.158177644	754	2803	2277	3228	1043	2017	1275	2065
ENSECAG00000012876	5.735216826	0.050070265	0.158247817	704	821	489	513	1037	1011	1711	1945
ENSECAG00000000562	4.215618857	0.05007049	0.158247817	106	203	242	475	431	497	568	424
ENSECAG00000012240	1.699201127	0.050085991	0.158252789	21	51	92	134	49	39	50	52
ENSECAG00000018065	1.449060031	0.050143275	0.158389738	29	29	25	39	66	36	76	117
ENSECAG00000014516	5.803625099	0.050161198	0.158402316	498	714	711	1166	1214	1183	1513	1813
ENSECAG00000024816	0.827206707	0.050191156	0.15845288	23	14	21	14	40	29	52	68
ENSECAG00000019591	6.380251439	0.050216021	0.158487343	1121	1147	2225	2553	1150	954	1859	1617
ENSECAG00000007083	4.673849498	0.050505153	0.159355608	145	315	321	638	525	694	644	783
ENSECAG00000013447	5.923200535	0.050528485	0.159384966	899	1439	1147	1319	1024	936	1079	1115
ENSECAG00000020479	5.242460004	0.05067543	0.159785729	342	376	490	888	866	729	1110	1204
ENSECAG00000017447	6.485445555	0.050683662	0.159785729	1310	1614	2282	2066	1400	1699	1335	1575
ENSECAG00000012286	3.868410691	0.050719371	0.159834275	131	141	209	297	442	303	255	528
ENSECAG00000013334	7.461680808	0.050727402	0.159834275	3986	582	8458	3004	3760	1677	2562	1171
ENSECAG00000018802	5.903251587	0.050744772	0.159834275	780	409	1127	509	1942	882	1557	2151
ENSECAG00000005661	5.376257638	0.050755331	0.159834275	807	1273	664	485	601	631	686	743
ENSECAG00000000496	7.206427857	0.050795108	0.159915218	1757	3636	3297	3650	2158	2407	2144	3013
ENSECAG00000016467	6.346529497	0.05083073	0.159983035	1061	2036	1622	1844	1314	1248	1446	1516
ENSECAG00000020315	3.997876058	0.050857891	0.160024193	150	182	221	309	413	293	435	505
ENSECAG00000016601	5.267204601	0.050903287	0.160116134	461	438	547	605	994	885	961	1063
ENSECAG00000018474	5.619932846	0.050915296	0.160116134	290	459	565	1420	1375	1321	1453	967
ENSECAG00000000566	2.990011259	0.050949512	0.1601794	73	62	122	148	179	154	289	214
ENSECAG00000018354	3.961477559	0.050968131	0.16019361	252	519	300	178	249	194	180	359
ENSECAG00000006117	6.166982735	0.050989521	0.160198399	807	3555	715	1053	846	1471	898	850
ENSECAG00000005015	2.423830368	0.050997853	0.160198399	45	148	128	274	5	160	2	17
ENSECAG00000007757	2.42149039	0.051095937	0.160457079	44	57	77	95	155	98	148	158
ENSECAG00000010867	6.325090559	0.051120325	0.160457079	795	969	976	1582	1918	1560	1916	2955
ENSECAG00000010166	5.293085412	0.051129333	0.160457079	339	404	579	802	869	760	1567	851
ENSECAG00000013733	7.967982426	0.051141425	0.160457079	3005	2374	3384	3787	7164	5574	6740	6992
ENSECAG00000017346	5.174219507	0.051150813	0.160457079	331	465	499	688	985	859	1014	780
ENSECAG00000018525	3.773320358	0.051168805	0.160469215	126	135	226	248	349	293	351	411
ENSECAG00000018858	5.594852819	0.051195663	0.160509141	538	927	1297	1232	798	919	615	945
ENSECAG00000022935	4.783233103	0.051231382	0.160576818	488	833	393	454	348	492	376	567
ENSECAG00000020670	3.103289741	0.051304891	0.160762871	77	91	113	171	196	217	216	257
ENSECAG00000009693	5.126249757	0.051341041	0.160831793	285	513	516	605	873	651	948	1102
ENSECAG00000023082	2.179053058	0.05140143	0.160976586	54	34	77	34	110	79	173	129
ENSECAG00000009902	7.327586185	0.051438212	0.161047386	1916	7056	1789	3079	1476	3457	2013	2235
ENSECAG00000015438	2.717794299	0.051476568	0.161123076	33	62	123	124	176	131	175	217
ENSECAG00000011809	6.447156405	0.051648615	0.161617065	871	1283	2629	2783	1736	1160	1475	1403
ENSECAG00000015278	5.036249648	0.051681569	0.16165136	282	440	446	646	982	765	786	779
ENSECAG00000017729	5.505392207	0.051688029	0.16165136	455	515	656	870	1348	893	1232	1133
ENSECAG00000013216	6.396377095	0.051782855	0.161903356	861	1080	1030	1546	2087	1573	2343	2716
ENSECAG00000011530	6.946365407	0.05190107	0.162196664	1105	1700	1732	1922	2808	3446	2804	3755
ENSECAG00000002612	0.591501506	0.051914398	0.162196664	46	23	51	7	35	14	14	2
ENSECAG00000011676	2.872897486	0.051919492	0.162196664	53	93	93	145	168	168	191	235
ENSECAG00000018758	4.800518719	0.05204113	0.162531974	251	273	344	676	660	656	623	928
ENSECAG00000016512	3.221629667	0.052139595	0.162794745	161	144	209	249	141	97	200	172
ENSECAG00000006497	0.964956022	0.052159643	0.162812601	42	51	41	24	28	34	31	9
ENSECAG00000009270	4.377847998	0.052214696	0.162939681	186	220	323	414	514	428	491	700
ENSECAG00000008070	5.035024769	0.052229053	0.162939732	453	899	536	728	553	444	521	717
ENSECAG00000009730	6.039274187	0.052290746	0.163087415	1379	807	1631	1424	1338	959	1131	882
ENSECAG00000023858	2.404179726	0.052334223	0.163178221	70	176	86	103	76	64	84	103
ENSECAG00000012582	4.716191455	0.052451264	0.163468583	535	630	343	564	365	455	280	618
ENSECAG00000012438	3.959822966	0.052456122	0.163468583	144	165	228	308	352	334	440	467
ENSECAG00000024618	4.5811915	0.052528384	0.163648889	111	215	326	677	498	658	598	775
ENSECAG00000017857	6.965382511	0.052562169	0.163709255	1880	2016	3589	2860	2466	1994	1695	2021
ENSECAG00000008774	4.664600603	0.052615182	0.163829459	257	207	433	474	655	509	639	814
ENSECAG00000009128	2.008410533	0.052826192	0.164441423	49	18	42	77	124	91	117	97
ENSECAG00000017954	7.022533807	0.052851245	0.164474349	3675	3222	1784	1374	2138	2013	1820	1769

ENSECAG00000011163	6.153522269	0.052952482	0.164744279	986	1104	1353	2517	1354	1138	1143	1167
ENSECAG00000019505	0.778362995	0.05306495	0.165048991	14	16	25	22	60	29	37	51
ENSECAG00000024383	5.594142449	0.053091618	0.165086745	537	579	558	944	1241	1053	1063	1578
ENSECAG00000008943	4.468938586	0.053120305	0.165130754	506	628	264	311	377	261	375	395
ENSECAG00000012277	1.524292306	0.053139004	0.165139045	46	41	69	83	39	47	33	56
ENSECAG00000012107	6.321317804	0.053152041	0.165139045	1086	2060	1286	2055	1216	1157	1256	1805
ENSECAG00000021782	3.879653507	0.053215446	0.16529084	262	306	281	314	305	184	275	213
ENSECAG00000015993	3.220723876	0.053311154	0.165542863	95	127	88	156	270	143	235	343
ENSECAG00000008632	6.476479359	0.05339877	0.165769623	1290	2156	1936	1733	1772	1365	1378	1466
ENSECAG00000022551	0.799904832	0.05369586	0.16664637	25	18	50	59	32	8	21	36
ENSECAG00000021117	2.246690085	0.053782632	0.166870089	43.0106	100	133.008	141	57.0007	94.0005	42.001	92.0003
ENSECAG00000016168	4.477410593	0.053870927	0.16709841	205	218	253	495	894	391	669	372
ENSECAG00000019325	3.910648612	0.053930481	0.16716983	119	164	193	340	368	260	365	587
ENSECAG00000008784	5.560313137	0.053931577	0.16716983	468	447	845	826	1324	994	1245	1232
ENSECAG00000000329	8.176783037	0.053938092	0.16716983	4985	7281	3839	7743	5035	4466	3753	5277
ENSECAG00000008355	0.900481811	0.054051643	0.167447011	15	11	31	28	39	46	65	42
ENSECAG00000016120	5.518510415	0.054057	0.167447011	616	1105	799	1156	792	799	819	711
ENSECAG00000007879	0.956418671	0.054207625	0.167867821	28	9	23	18	63	32	51	62
ENSECAG00000018122	1.771025357	0.054266575	0.167979687	16	44	37	73	68	66	113	115
ENSECAG00000020171	2.866159175	0.054273317	0.167979687	101	145	269	205	16	233	10	57
ENSECAG00000011835	6.167476499	0.054387374	0.16828686	897	989	798	1003	1910	1440	2001	2072
ENSECAG00000021522	2.187029262	0.054414229	0.168320101	34	117	121	123	81	64	65	68
ENSECAG00000015380	4.124978988	0.054427746	0.168320101	160	185	275	337	462	404	430	472
ENSECAG00000016319	5.60052332	0.054466372	0.168393718	424	505	739	1064	1152	1111	1225	1434
ENSECAG00000015128	8.623757481	0.054509197	0.168480275	8115	9862	5894	7788	6093	5933	7203	5537
ENSECAG00000018880	7.198879437	0.054719909	0.169085562	1993	2605	3842	3767	2604	2085	2392	2721
ENSECAG00000009999	5.032190315	0.054735842	0.169088807	539	1058	500	482	497	610	432	568
ENSECAG00000010159	5.365213097	0.054844756	0.169379213	718	832	756	911	800	619	745	659
ENSECAG00000009478	5.978851908	0.054900476	0.16947261	638	565	881	1353	1905	1556	1626	1337
ENSECAG00000022606	3.670008398	0.054913635	0.16947261	117	139	187	244	287	311	323	377
ENSECAG00000023596	7.584257796	0.054919746	0.16947261	4607	5500	2338	2642	3266	2794	2899	2714
ENSECAG00000014791	3.40630939	0.05493563	0.169475598	148	60	392	376	228	152	138	90
ENSECAG00000021028	3.314591721	0.055095709	0.169923301	139	316	195	158	131	197	119	173
ENSECAG00000010675	0.65898336	0.055166216	0.17009245	11	24	12	15	28	77	41	17
ENSECAG00000009312	4.449366472	0.055180494	0.17009245	205	330	289	351	619	455	614	524
ENSECAG00000008647	5.045529412	0.055387486	0.170684191	372	509	359	464	854	535	888	1165
ENSECAG00000023752	4.788003749	0.055476244	0.170908199	315	611	703	608	501	482	419	480
ENSECAG00000010087	6.341404697	0.055490261	0.170908199	2212	2074	986	920	1375	1059	1360	1206
ENSECAG00000015088	3.843288172	0.055513051	0.170932055	130	197	211	224	396	308	433	323
ENSECAG00000009670	3.992387807	0.055568622	0.171048825	113	239	176	313	431	206	494	558
ENSECAG00000024801	6.185516402	0.055591512	0.171048825	1592	1557	1259	1183	1213	1160	1282	1139
ENSECAG00000022145	3.74758202	0.055596137	0.171048825	149	175	165	207	375	239	373	398
ENSECAG00000007580	4.626201171	0.055655624	0.17118549	480	928	231	322	334	289	395	505
ENSECAG00000015522	4.975132138	0.0557477	0.171422209	438	853	530	668	518	469	563	597
ENSECAG00000004844	4.143355741	0.05576432	0.171422209	205	510	310	418	235	317	259	361
ENSECAG00000013191	7.771039326	0.055777848	0.171422209	3745	4614	4398	4907	3505	3247	4132	3437
ENSECAG00000020459	5.912616288	0.055964826	0.17195034	683	833	775	901	1391	1193	1717	1867
ENSECAG00000015858	7.852415662	0.055983862	0.171962301	6824	3931	5198	2649	5241	2842	3685	1184
ENSECAG00000013485	5.082971561	0.055998989	0.171962301	402	504	423	414	836	655	844	1169
ENSECAG00000013274	3.110546161	0.056032862	0.172019825	61	83	144	162	224	165	162	371
ENSECAG00000025054	6.932878581	0.056048459	0.17202123	2424	3144	2047	2007	2018	1891	2165	1766
ENSECAG00000011786	3.808410784	0.056170944	0.172350598	89	202	205	242	245	286	325	636
ENSECAG00000018297	4.96236087	0.056196356	0.17237853	263	400	452	633	863	628	779	883
ENSECAG00000006613	1.39329748	0.05621039	0.17237853	24	42	68	95	43	34	37	44
ENSECAG00000011438	4.964598587	0.056293517	0.172586869	510	577	876	521	577	287	598	653
ENSECAG00000015887	4.047302069	0.056393024	0.172845302	150	269	400	619	251	238	273	340
ENSECAG00000019391	3.937181667	0.056418646	0.172877199	57	393	64	119	233	291	311	1004
ENSECAG00000004434	6.095845658	0.056475812	0.17300571	790	734	1042	1082	1705	1563	1955	1727.99
ENSECAG00000014383	5.856781587	0.056625263	0.173416777	693	551	395	957	600	3360	1005	1252
ENSECAG00000025118	4.554825008	0.056990901	0.174473509	230	272	291	494	677	419	641	666
ENSECAG00000009379	6.667389827	0.057001027	0.174473509	2806	2561	878	1692	1542	1566	1834	1146
ENSECAG00000020816	2.232510866	0.057017196	0.174475998	54	45	117	212	85	68	66	60
ENSECAG00000009015	3.748606692	0.05704155	0.174503523	125.014	308	317	385	162	228	231	268
ENSECAG00000023115	3.274105418	0.057084328	0.174587384	75	82	509	255	74	216	130	107
ENSECAG00000014737	6.432414982	0.057145647	0.17472789	841	966	976	1919	2426	1557	2432	2535
ENSECAG00000011300	0.865292086	0.057410256	0.175443883	23	15	21	13	44	29	32	94
ENSECAG00000013212	4.023076102	0.057410698	0.175443883	385	320	216	337	286	184	259	339
ENSECAG00000023831	3.502342516	0.057453262	0.17550901	68	178	147	214	293	225	275	389
ENSECAG00000023641	4.601551966	0.057462904	0.17550901	135	271	330	597	796	520	795	409
ENSECAG00000005649	2.121770093	0.057484975	0.175529235	36	111	100	131	44	64	62	98
ENSECAG00000011113	3.162812502	0.057628818	0.175921178	81	125	223	378	133	90	168	180
ENSECAG00000013250	6.629531712	0.057687611	0.176053354	2034	2114	1775	1780	1488	1314	1691	2126
ENSECAG00000007508	7.148330326	0.057741641	0.176170926	3358	4474	1750	1533	2076	1760	2322	2478
ENSECAG00000011034	0.282756308	0.057790853	0.176273737	9	16	5	20	19	31	27	49
ENSECAG00000010145	2.874942734	0.057889342	0.176526758	57	48	90	184	212	189	233	135
ENSECAG00000024092	6.813626179	0.057906363	0.176531285	3296	2346	1393	1558	1663	1464	1650	2220
ENSECAG00000015600	5.272613174	0.057952308	0.17662396	281	553	529	775	860	688	954	1518
ENSECAG00000018928	1.400802938	0.058107918	0.177050727	29	55	48	94	38	43	26	52
ENSECAG00000007717	5.892482898	0.058131702	0.177075711	864	1177	1270	1432	1237	716	1093	1069
ENSECAG00000021408	5.255353321	0.058209596	0.177265461	301	396	589	847	975	853	1264	788
ENSECAG00000024410	6.028367133	0.058290361	0.177463849	1036	1245	1607	1254	1228	1013	1131	1092
ENSECAG00000002449	1.33929321	0.058339642	0.177566303	25	21	44	24	73	49	87	55
ENSECAG00000009751	6.357513696	0.058495216	0.177992139	786	900	1192	1639	2133	1723	1880	2675
ENSECAG00000014727	3.328974761	0.058535055	0.178065675	119	73	149	167	309	233	281	216
ENSECAG00000002016	5.025907369	0.058567724	0.178072793	375	409	483	477	822	674	833	972

ENSECAG00000017938	6.212728951	0.05856874	0.178072793	670	943	665	1624	1854	2334	2319	1119
ENSECAG00000017351	2.305855138	0.058606377	0.178139555	31	84	154	184	57	94	66	75
ENSECAG00000014885	1.062234755	0.05863554	0.178178204	18	28	20	29	55	32	61	70
ENSECAG00000003033	2.017226925	0.058650456	0.178178204	27	22	186	168	37	25	94	57
ENSECAG00000011488	3.124375603	0.058677331	0.178212198	219	52	620	75	4	189	0	0
ENSECAG00000007739	3.093955558	0.058699162	0.178230859	83	102	122	127	218	130	225	328
ENSECAG00000000020	2.937736246	0.058783915	0.178440514	88	220	142	186	125	85	113	173
ENSECAG00000016164	2.612965196	0.058804794	0.178456215	58	45	74	121	212	83	241	119
ENSECAG00000019275	0.638312263	0.058885679	0.178653963	39	9	49	32	20	13	12	38
ENSECAG00000017046	3.926537697	0.058991143	0.178926151	121	237	166	298	344	343	444	423
ENSECAG00000024086	6.343358757	0.059120499	0.179270646	1183	868	891	1090	2489	1546	2329	2090
ENSECAG00000000021	3.521931377	0.059159406	0.179340761	132	62	161	231	316	267	346	271
ENSECAG00000000741	3.317375824	0.059196643	0.179405776	78	133	201	80	221	225	325	279
ENSECAG00000022429	3.6701375	0.059213377	0.179408638	336	263	214	163	256	155	223	156
ENSECAG00000021163	4.78708807	0.059229991	0.179411132	173	253	619	479	731	577	784	760
ENSECAG00000016914	2.037415005	0.05935473	0.179741057	47	97	73	127	60	61	64	74
ENSECAG00000023930	5.693932297	0.059441621	0.17995622	558	427	893	911	1672	1049	1417	1147
ENSECAG00000013874	3.719542137	0.059522473	0.180152993	27	359	796	295	14	383	5	36
ENSECAG00000005469	4.795769018	0.059609268	0.180367645	473	652	529	504	589	352	459	492
ENSECAG00000000861	5.511125158	0.059674389	0.180516615	553	501	530	861	1315	855	1302	1173
ENSECAG00000018435	9.720008239	0.05970625	0.180564921	17596	20519	15021	13713	13871	12550	13176	13968
ENSECAG00000020767	1.945994724	0.059733432	0.180586511	12	28	37	113	166	79	103	71
ENSECAG00000010624	1.155228424	0.059745176	0.180586511	12	27	30	33	76	27	89	43
ENSECAG00000000226	4.354294279	0.059783267	0.180653585	197	519	448	536	284	337	273	487
ENSECAG00000012047	2.423762295	0.059848923	0.180792368	41	76	64	93	164	109	138	144
ENSECAG00000019290	4.781872778	0.059861018	0.180792368	283	185	480	536	777	591	807	648
ENSECAG00000012623	2.31147153	0.059989759	0.181117971	29	40	92	94	100	106	154	164
ENSECAG00000003621	3.745230924	0.060000708	0.181117971	142	155	212	188	400	231	399	350
ENSECAG00000010405	6.488963868	0.060086478	0.181328702	733	494	1282	2375	2731	2072	2514	2141
ENSECAG00000019366	7.061591359	0.060190924	0.181595667	1312	1156	1904	2747	3593	3250	3973	3037
ENSECAG00000012944	3.556533745	0.060310147	0.181907058	231	352	166	168	182	181	195	187
ENSECAG00000019869	0.524261177	0.060365718	0.182026351	27	7	70	19	21	22	14	14
ENSECAG00000018860	8.281914123	0.060452742	0.182240398	4944	5954	7408	7613	4478	6897	4429	3830
ENSECAG00000014466	6.034877504	0.0604895	0.182302839	1232	1275	1173	1446	833	1083	1063	1499
ENSECAG00000000313	2.708649769	0.060539006	0.182379634	56	65	74	129	216	132	80	278
ENSECAG00000017290	7.239329672	0.060570064	0.182379634	2135	3180	3076	4040	2301	2728	2529	2458
ENSECAG00000003357	6.14636641	0.060577263	0.182379634	752	1496	1599	2025	1096	1341	1146	1232
ENSECAG00000017207	5.188125026	0.060579189	0.182379634	790	634	825	552	768	524	596	530
ENSECAG00000021302	4.16199399	0.06062427	0.182421046	194	168	289	292	592	286	405	576
ENSECAG00000005530	5.254199432	0.060625054	0.182421046	368	452	542	772	926	828	941	1155
ENSECAG00000019628	4.133765775	0.060671374	0.182512087	159	197	298	305	514	330	457	485
ENSECAG00000015368	2.027899352	0.060755333	0.182583225	38	39	54	48	125	24	179	126
ENSECAG00000009788	4.395021075	0.060759482	0.182583225	174	245	303	445	385	505	557	701
ENSECAG00000005538	5.948271187	0.060761257	0.182583225	527	822	698	1357	1719	1693	1107	1775
ENSECAG00000023254	6.460733601	0.060769172	0.182583225	1081	886	1407	1214	2443	1943	2417	2226
ENSECAG00000010956	0.463959245	0.060781095	0.182583225	8	17	22	14	32	35	31	41
ENSECAG00000013521	3.321971546	0.06079144	0.182583225	73	54	210	175	336	148	264	331
ENSECAG00000013544	4.023110294	0.060814814	0.182605156	199	340	338	560	157	475	193	131
ENSECAG00000022762	3.570651038	0.060872702	0.182730684	110	130	182	215	277	218	343	386
ENSECAG00000017092	2.908193188	0.060897859	0.182757918	237	106	67	220	69	207	46	69
ENSECAG00000008690	1.245017247	0.061040749	0.183138365	26	43	47	87	23	32	40	48
ENSECAG00000016250	1.288575205	0.061097928	0.183217213	10	29	33	47	45	62	86	60
ENSECAG00000011272	4.699622386	0.06109928	0.183217213	251	438	698	810	431	418	358	565
ENSECAG00000010953	1.300874693	0.061168667	0.183376886	13	20	53	29	87	43	48	84
ENSECAG00000020273	3.706278041	0.061277583	0.183654946	109	205	156	225	339	236	385	383
ENSECAG00000011405	4.789950082	0.061424129	0.184045611	274	356	294	590	771	488	698	880
ENSECAG00000008162	3.973016409	0.061462234	0.184111231	88	132	118	495	467	447	513	242
ENSECAG00000019410	6.887616473	0.061482533	0.184123494	1267	2653	2941	3101	1640	2327	1916	1894
ENSECAG00000014268	7.406625707	0.061533624	0.18422794	2885	3449	3678	3577	2870	2596	2905	2915
ENSECAG00000011500	4.68191726	0.061601198	0.184381669	239	335	342	506	629	496	699	779
ENSECAG00000012634	4.115882285	0.061735994	0.184716312	198	599	297	312	338	208	270	320
ENSECAG00000018150	4.516803387	0.061745516	0.184716312	260	203	380	377	637	463	687	544
ENSECAG00000008013	0.799663662	0.061779622	0.184767015	19	43	40	42	20	28	16	37
ENSECAG00000016031	5.594819567	0.061794988	0.184767015	961	1270	715	773	994	557	730	952
ENSECAG00000012923	3.211961263	0.061921121	0.185095443	94	91	145	128	242	363	158	189
ENSECAG00000013266	1.22469027	0.061984958	0.185237532	35	38	42	89	55	34	11	31
ENSECAG00000014320	2.454938345	0.062170505	0.18574317	36	50	115	58	124	146	238	74
ENSECAG00000009686	5.298079599	0.062211929	0.185776691	476	694	274	488	1113	811	858	1367
ENSECAG00000008051	5.002019108	0.062214426	0.185776691	209	400	441	771	791	608	903	986
ENSECAG00000016370	3.864952506	0.062268651	0.185889758	95	204	206	268	378	324	567	232
ENSECAG00000023546	5.417789959	0.06233088	0.18602665	1361	986	451	432	540	850	571	557
ENSECAG00000004274	7.528147072	0.062446445	0.186322613	1866	1908	2765	3228	4769	4369	4297	5784
ENSECAG00000018790	0.797586258	0.062561186	0.186615962	30	65	20	24	24	13	31	28
ENSECAG00000020576	3.524527646	0.062622281	0.186749175	114	119	171	199	364	173	264	402
ENSECAG00000015514	9.273938344	0.062638868	0.186749625	10677	17265	12901	9016	8389	11094	9058	10492
ENSECAG00000013246	2.806214098	0.062674184	0.186805896	55	67	133	108	170	135	201	223
ENSECAG00000013955	7.042328271	0.062693837	0.186815466	1511	4526	1769	3267	1611	2177	1831	2816
ENSECAG00000001581	6.317385357	0.062800367	0.18708384	935	2910	1259	1369	1103	1364	1233	1475
ENSECAG00000013410	1.369243733	0.062842069	0.187159001	36	84	35	55	35	41	33	44
ENSECAG00000009773	5.057697799	0.062873474	0.187203462	169	253	871	437	595	1272	809	828
ENSECAG00000008235	1.442751611	0.063076795	0.187759639	19	14	55	36	96	96	42	51
ENSECAG00000010922	5.912018779	0.063285097	0.188258298	578	733	824	1207	1522	1250	1299	2072
ENSECAG00000017188	5.007049679	0.063309604	0.188258298	388	436	401	489	781	726	837	896
ENSECAG00000009407	1.900339109	0.063311423	0.188258298	22	217	121	42	0	94	0	6
ENSECAG00000015498	6.47984608	0.063340574	0.188258298	1822	2101	1381	1591	1686	1142	1379	1776

ENSECAG00000019698	5.585236039	0.063342806	0.188258298	271	508	827	1135	1038	1087	1291	1529
ENSECAG00000011669	1.831720256	0.063343731	0.188258298	51	18	27	58	106	46	94	147
ENSECAG00000008754	4.853199778	0.063403129	0.188326821	254	253	396	717	832	570	817	734
ENSECAG00000009109	4.689221583	0.063404921	0.188326821	314	238	753	934	493	342	421	450
ENSECAG00000020127	3.267189139	0.063416513	0.188326821	111	147	115	74	325	265	244	172
ENSECAG00000018841	2.569395864	0.063542184	0.188650717	66	2	82	14	346	32	359	21
ENSECAG00000011982	1.818047069	0.0636021	0.188692943	27	44	42	65	83	75	106	98
ENSECAG00000006784	7.881755894	0.063602967	0.188692943	2704	9150	3585	4266	3108	3323	3247	5152.01
ENSECAG00000004119	2.957094073	0.063613841	0.188692943	87	71	114	118	243	133	239	193
ENSECAG00000016464	11.06179621	0.063622836	0.188692943	50064	58716	28139	31346	29018	34807	31423	36477
ENSECAG00000007856	4.151110871	0.063664859	0.188768301	256	643	236	294	344	163	281	357
ENSECAG00000020430	3.693969364	0.063757347	0.188976435	213	257	297	250	200	173	242	263
ENSECAG00000009746	1.333736907	0.06376832	0.188976435	22	33	29	37	68	64	56	68
ENSECAG00000022527	10.06422653	0.063793731	0.189002444	18220	39472	16418	12820	15718	18984	14344	15356
ENSECAG00000014236	2.619406783	0.063865767	0.18916654	59	88	57	106	160	128	151	200
ENSECAG00000008540	0.900806448	0.063904817	0.189232873	17	20	20	30	49	32	59	50
ENSECAG00000017558	4.664973105	0.06395101	0.189284477	169	263	285	712	532	584	764	744
ENSECAG00000003194	0.436162563	0.063979424	0.189284477	34	23	21	27	12	13	19	32
ENSECAG00000016742	1.204383531	0.063983111	0.189284477	25	17	29	30	43	99	37	59
ENSECAG00000020146	3.017891741	0.063991403	0.189284477	69	79	92	189	222	138	217	272
ENSECAG00000012420	5.848604135	0.064005541	0.189284477	505	702	873	1170	1372	1297	1511	1621
ENSECAG00000019226	2.418829805	0.06404109	0.18930441	42	47	81	107	147	92	163	156
ENSECAG00000017435	4.649846417	0.064045604	0.18930441	316	652	481	603	310	516	258	584
ENSECAG00000021234	5.250209274	0.064090172	0.189347029	345	583	439	725	793	1026	946	1068
ENSECAG00000022722	5.465279904	0.06409784	0.189347029	443	2378	432	484	548	519	493	1039
ENSECAG00000007934	1.282925315	0.06411539	0.189347029	51	43	53	46	36	48	32	26
ENSECAG00000015165	5.496741696	0.064130504	0.189347029	431	943	1077	1333	577	670	696	1174
ENSECAG00000021354	4.019986951	0.064143348	0.189347029	143	191	239	317	454	279	449	476
ENSECAG00000009149	7.533970513	0.064251139	0.18961596	842	2459	1754	4913	3975	5226	5910	4650
ENSECAG00000022043	5.658036265	0.064328174	0.189747316	944	719	1268	1028	1001	917	706	791
ENSECAG00000019065	5.35793667	0.06432905	0.189747316	614	562	1340	834	939	638	638	493
ENSECAG00000025072	7.798656635	0.064368487	0.189776411	3180	4313	4753	6216	3345	4194	3434	3707
ENSECAG00000020649	5.065129296	0.064372319	0.189776411	306	406	575	573	862	657	789	1085
ENSECAG00000007936	0.908819024	0.064414408	0.189851057	29	66	24	32	22	26	36	21
ENSECAG00000026901	1.627522624	0.064436713	0.189851057	26	22	54	50	93	60	81	86
ENSECAG00000012842	0.623039619	0.064447767	0.189851057	13	20	42	60	26	25	17	17
ENSECAG00000010029	4.77343192	0.064469904	0.189867041	479	536	581	511	465	444	468	511
ENSECAG00000009776	2.600640277	0.06450289	0.189890979	44	76	89	108	134	163	132	195
ENSECAG00000015602	4.990925814	0.064511457	0.189890979	278	1045	598	731	385	661	446	578
ENSECAG00000017739	5.473721461	0.064549396	0.189953441	549	916	687	1619	447	842	571	1110
ENSECAG00000015542	6.815000938	0.064642277	0.190177511	2643	2783	1469	1781	1847	1637	1832	1926
ENSECAG00000014712	4.591741789	0.064703343	0.19030789	184	247	363	568	559	628	545	715
ENSECAG00000017215	3.581152965	0.064732413	0.190335991	99	137	136	280	319	215	318	387
ENSECAG00000015215	5.2327312	0.064746401	0.190335991	360	428	636	668	1072	783	944	980
ENSECAG00000022743	4.864960382	0.064811843	0.190450437	253	314	434	622	655	810	534	960
ENSECAG00000026976	2.920015162	0.064818856	0.190450437	123	261	90	134	131	109	120	107
ENSECAG00000011232	3.308660389	0.065156207	0.191392145	81	138	132	175	232	195	228	373
ENSECAG00000009120	0.951770543	0.065237324	0.191580894	20	19	28	20	51	33	47	69
ENSECAG00000020928	1.794567301	0.065256181	0.19158675	40	27	35	66	77	73	93	119
ENSECAG00000024142	4.526773155	0.065304986	0.19168051	252	403	623	658	451	195	409	520
ENSECAG00000000711	4.742244851	0.065394871	0.191871186	244	256	531	439	728	498	671	840
ENSECAG00000023743	2.857419041	0.065403723	0.191871186	76	87	94	115	173	147	194	233
ENSECAG00000022653	2.659453614	0.065436601	0.191918084	43	69	118	97	145	170	127	214
ENSECAG00000021600	2.779508396	0.065465685	0.191953835	71	50	108	120	191	129	157	245
ENSECAG00000015831	6.730952242	0.065528563	0.19208863	1262	1367	1590	1187	3078	2218	2877	2819
ENSECAG00000019730	1.174747169	0.065588075	0.192213493	21	36	26	128	37	40	22	23
ENSECAG00000013642	1.250743883	0.065653352	0.192355178	15	44	15	32	56	31	70	100
ENSECAG00000010965	8.183993229	0.065762714	0.192608532	3880	8568	4831	6924	3948	5591	5252	3584
ENSECAG00000023365	4.015547801	0.065773729	0.192608532	108	47	279	364	462	134	676	564
ENSECAG00000014795	3.366267077	0.065794702	0.192620304	88	107	134	203	319	111	247	434
ENSECAG00000008732	5.061413752	0.065901792	0.192754449	462	785	670	751	411	724	461	662
ENSECAG00000017041	2.344514012	0.065903446	0.192754449	58	74	47	54	111	100	102	235
ENSECAG00000012705	3.176934817	0.065907853	0.192754449	107	95	397	187	188	97	127	149
ENSECAG00000008857	4.588235196	0.065908382	0.192754449	314	324	279	319	653	479	681	641
ENSECAG00000017258	6.884617784	0.065943965	0.192808887	2054	3569	1844	1858	1820	1954	1857	2022
ENSECAG00000010554	4.115006115	0.065993261	0.192903377	292	176	137	173	593	245	493	541
ENSECAG00000014942	4.528683887	0.066118613	0.19321305	218	272	335	460	610	453	635	629
ENSECAG00000016324	3.634064488	0.066133212	0.19321305	589	122	161	60	158	204	169	99
ENSECAG00000017476	0.560851621	0.066209997	0.193387658	8	4	23	29	24	20	41	75
ENSECAG00000013516	3.985091859	0.066237616	0.193418604	89	158	200	443	439	377	399	422
ENSECAG00000016531	5.761409631	0.066279635	0.193478108	484	684	832	1000	1316	892	1309	2079
ENSECAG00000016212	5.627098992	0.06629205	0.193478108	588	378	769	928	1514	928	1318	1296
ENSECAG00000003436	5.590620512	0.066340087	0.193568584	314	613	798	995	1603	963	1266	1061
ENSECAG00000006855	5.129105119	0.066447792	0.19383307	614	718	725	654	798	495	528	575
ENSECAG00000015063	1.280184217	0.066588354	0.194190776	28	25	22	18	58	126	14	63
ENSECAG00000023741	4.280061935	0.0666197	0.194190776	899	87	299	284	513	106	270	49
ENSECAG00000013660	2.146582971	0.06662169	0.194190776	145	108	7	130	98	67	13	21
ENSECAG00000012174	4.182712176	0.066670711	0.19428382	177	227	272	316	550	359	433	492
ENSECAG00000024523	1.909202614	0.066714889	0.194362709	62	41	19	21	111	51	136	122
ENSECAG00000024852	5.277345629	0.066778652	0.194498601	226	566	294	1045	1008	1098	1106	798
ENSECAG00000005915	2.883597282	0.066822621	0.194576785	41.0071	117	109	118	154	157	237	220
ENSECAG00000009550	11.14334302	0.066936763	0.194859211	42744	66350	38077	33086	29980	36994	34686	41063
ENSECAG00000000609	5.976636799	0.066975066	0.194892191	1183	1199	1238	1235	1248	951	1071	1054
ENSECAG00000019527	7.226902842	0.066982398	0.194892191	3317	5026	2159	1246	2301	1927	2216	2683
ENSECAG00000004289	6.557139141	0.067055818	0.195055863	1015	1384	900	1600	2877	2424	2593	1729

ENSECAG00000006410	1.703353002	0.067081337	0.195080151	20	27	45	73	87	48	99	109
ENSECAG00000011778	6.036156081	0.067160207	0.195259537	878	690	891	954	1820	1274	1944	1671
ENSECAG00000016958	4.818799864	0.067221834	0.195388713	154	419	302	688	474	858	712	870
ENSECAG00000012961	0.674890159	0.067313197	0.195570458	9	18	16	28	43	24	74	24
ENSECAG00000020435	1.260993391	0.067322885	0.195570458	41	32	63	75	79	14	20	16
ENSECAG00000020144	5.351415934	0.067336	0.195570458	563	418	463	375	2152	619	1217	439
ENSECAG00000020161	9.496621231	0.067376345	0.19563103	13632	16520	12848	14682	12769	9876	12057	12475
ENSECAG00000010190	2.029124382	0.067391291	0.19563103	33	77	37	39	133	65	145	91
ENSECAG00000013815	7.212851317	0.067465041	0.195795096	2153	2177	3682	4351	2886	2362	2127	2518
ENSECAG00000002155	6.1128204	0.067570182	0.196039945	649	819	1102	1284	1633	1562	1941	1835
ENSECAG00000005231	1.547950004	0.067583917	0.196039945	50	3	14	11	259	19	55	26
ENSECAG00000014431	6.358400811	0.067703542	0.196336816	870	1050	1116	1441	1852	1803	2275	2382
ENSECAG00000014575	5.788115225	0.067834012	0.196664977	1118	1427	765	907	880	895	916	1008
ENSECAG00000024907	6.137603526	0.067885709	0.196764648	635	872	1256	1127	1755	1604	1605	2169
ENSECAG00000008915	6.289841372	0.068005419	0.197012326	541	751	1089	2056	1738	1827	1856	2691
ENSECAG00000015382	5.182560242	0.068005839	0.197012326	405	619	2585	335	16	1059	6	71
ENSECAG00000023673	5.840171118	0.068067995	0.197142124	716	1179	1148	1582	986	799	891	1358
ENSECAG00000011046	0.387261573	0.068107095	0.197205099	12	11	20	9	29	12	42	56
ENSECAG00000017714	2.979098884	0.068163942	0.197319418	66	76	165	95	227	186	225	176
ENSECAG00000020425	8.366518195	0.068260972	0.197549965	6224	7835	5139	7120	5471	4978	5658	5433
ENSECAG00000011917	10.24976693	0.068326396	0.197688072	25395	42676	14671	14152	15879	18576	20756	18120
ENSECAG00000024824	4.158226268	0.068343491	0.197688072	286	330	420	393	287	384	173	342
ENSECAG00000008412	4.085561629	0.068401156	0.197784281	163	226	224	315	410	318	458	540
ENSECAG00000008445	3.125486602	0.06841261	0.197784281	83	118	208	378	125	112	92	229
ENSECAG00000018279	3.1028737	0.068428974	0.197784281	60	207	252	249	84	220	100	104
ENSECAG00000004870	4.070705317	0.06846423	0.197835855	119	294	208	288	370	333	452	576
ENSECAG00000009278	6.302764755	0.068488987	0.197857075	779	542	1475	1464	2425	1342	2229	2223
ENSECAG00000008144	2.248098524	0.068601045	0.198094976	32	53	63	101	105	115	147	120
ENSECAG00000019618	5.823833207	0.068606207	0.198094976	619	852	663	916	1404	1161	1296	1908
ENSECAG00000014808	7.582977318	0.0687694	0.198515733	1937	1983	2894	3384	5555	4043	4703	5623
ENSECAG00000018388	0.768686348	0.068809183	0.198516483	4	18	14	45	34	46	45	52
ENSECAG00000024766	9.040583778	0.068820173	0.198516483	9475	10033	10876	12230	9096	8428	9483	7316
ENSECAG00000013197	7.28074241	0.068822076	0.198516483	3861	2925	2320	2902	2508	2243	2378	2992
ENSECAG00000008011	4.910984012	0.068858255	0.198570431	248	286	373	802	726	698	728	917
ENSECAG00000022166	0.806771393	0.068983711	0.198881736	8	3	35	21	13	56	16	117
ENSECAG00000011097	3.855751279	0.069029078	0.198962047	102.011	206	727.008	304	140.001	361	208.001	102
ENSECAG00000023761	5.528185574	0.06906655	0.199019564	397	461	744	1029	1191	990	1166	1307
ENSECAG00000016539	2.418796434	0.069203529	0.199363717	37	68	84	90	143	111	123	175
ENSECAG00000014645	4.004625884	0.069387266	0.199842363	86	440	21	58	356	506	530	490
ENSECAG00000002428	4.528137272	0.069418152	0.199880649	327	187	369	241	839	455	662	444
ENSECAG00000008519	5.336656161	0.069444046	0.199904547	405	823	867	1252	590	687	692	835
ENSECAG00000005833	2.630940954	0.069668404	0.200499596	80	95	218	142	49	158	66	93
ENSECAG00000008226	6.47659055	0.069798701	0.200784761	1090	611	1560	1294	3347	1531	2106	2365
ENSECAG00000016693	6.298929399	0.069802834	0.200784761	1305	2529	1133	1208	1344	1100	1568	1137
ENSECAG00000024711	6.749082285	0.069976622	0.201233706	1368	1019	1524	1632	3075	1960	3422	2698
ENSECAG00000009762	4.938389249	0.070045657	0.201348565	66.8056	443.991	152.822	888.985	374.992	852	819	1443.07
ENSECAG00000017560	5.739873594	0.070060016	0.201348565	1084	1122	886	973	995	877	872	899
ENSECAG00000016230	2.890917371	0.070069726	0.201348565	67	113	69	133	188	152	221	204
ENSECAG00000011337	5.635141893	0.070105894	0.201401561	483	711	757	806	1197	1032	1227	1537
ENSECAG00000014475	4.961775162	0.070160107	0.201499329	398	309	557	305	964	467	871	954
ENSECAG00000011503	4.981296858	0.070175396	0.201499329	355	386	440	546	779	575	906	928
ENSECAG00000009583	8.467939281	0.070196545	0.201501616	5705	6322	8689	8171	5628	4752	5341	7987
ENSECAG00000021001	6.299833967	0.070211662	0.201501616	1158	1241	1711	2268	1298	1226	1139	1826
ENSECAG00000012956	5.427674274	0.070441989	0.202111586	363	619	668	708	895	1199	1373	839
ENSECAG00000011522	4.516575423	0.070501295	0.202230677	190	313	321	452	513	491	507	818
ENSECAG00000021622	3.60810745	0.070522159	0.202235401	82	123	215	251	394	244	329	281
ENSECAG00000010917	2.918811206	0.07053854	0.202235401	46	92	126	91	164	411	96	121
ENSECAG00000015712	3.685165168	0.070586899	0.202279502	117	245	104	181	293	273	282	508
ENSECAG00000016326	6.426874752	0.070589529	0.202279502	918	1110	1361	1196	2245	1719	1844	3049
ENSECAG00000024367	4.417539749	0.070625355	0.202331137	293	760	387	438	79	706	147	210
ENSECAG00000019699	7.940058688	0.070662229	0.202341453	4293	6174	4889	4121	4362	3707	4249	3725
ENSECAG00000024844	8.451482411	0.070669645	0.202341453	6585	8340	5721	7169	5661	5594	5432	6222
ENSECAG00000010336	7.205894013	0.070682382	0.202341453	1554	1884	2100	2190	4400	2683	4124	4124
ENSECAG00000000101	2.152651745	0.07076787	0.202535149	28	52	69	80	94	143	110	106
ENSECAG00000012452	4.109739846	0.070847087	0.202710803	207	318	363	532	344	194	316	339
ENSECAG00000011102	5.672804613	0.070888008	0.202776824	541	721	744	790	1294	1015	1220	1611
ENSECAG00000024674	1.731046225	0.071020912	0.203105865	32	48	94	119	80	28	42	51
ENSECAG00000017501	2.985059796	0.071184758	0.203523207	89	339	84	150	85	102	120	174
ENSECAG00000021066	5.731862573	0.071262422	0.203601166	484	676	885	895	1307	961	1153	2026
ENSECAG00000000387	8.24613153	0.071268011	0.203601166	6720	11150	4969	1574	3250	5897	4287	3613
ENSECAG00000021084	4.766371538	0.071283834	0.203601166	185	297	407	669	632	655	793	668
ENSECAG00000021271	2.923176478	0.07130307	0.203601166	78	86	108	114	238	126	245	174
ENSECAG00000018457	3.158006445	0.071313527	0.203601166	76	115	127	160	214	191	271	231
ENSECAG00000026937	4.336258672	0.071319542	0.203601166	255	478	455	425	294	368	260	462
ENSECAG00000020948	4.883879163	0.071418256	0.203831758	526	626	489	635	580	445	533	478
ENSECAG00000007894	6.599775024	0.071631378	0.20438868	2051	2265	1660	1515	1978	1671	1472	1173
ENSECAG00000021403	6.85999346	0.071663137	0.204427959	1834	2661	2328	2432	1746	2252	1843	1908
ENSECAG00000010744	7.207924086	0.07174987	0.204624003	3410	4017	2139	1853	2819	1883	2212	2475
ENSECAG00000003934	4.191855842	0.071774437	0.204630625	150	276	315	232	384	340	464	705
ENSECAG00000020617	1.056597071	0.071788213	0.204630625	16	22	33	27	63	31	68	51
ENSECAG00000016088	2.644684974	0.071846937	0.204746652	72	39	87	116	186	121	181	164
ENSECAG00000011478	2.784320442	0.071939743	0.20495972	57	75	63	167	149	140	226	203
ENSECAG00000014802	1.363415577	0.072081655	0.205312551	13	32	32	50	54	48	53	116
ENSECAG00000020032	4.529844008	0.072101291	0.205317009	200	213	315	565	687	610	541	491
ENSECAG00000024911	2.618015157	0.072199624	0.205545508	63	61	74	117	210	109	156	159

ENSECAG00000020929	7.368184116	0.072257744	0.205646877	2552	3668	4646	2534	3550	2652	2515	1887
ENSECAG00000011200	5.811346198	0.072279222	0.205646877	540	586	927	1108	1485	1126	1363	1694
ENSECAG00000022420	0.973621926	0.072295171	0.205646877	12	38	44	83	25	39	28	16
ENSECAG00000012877	6.453823962	0.072307628	0.205646877	1165	4146	856	1081	1013	1230	1981	944
ENSECAG00000019774	1.505171838	0.072437442	0.20596452	52	77	39	58	49	27	32	66
ENSECAG00000011738	5.781872901	0.072473327	0.205987613	970	1176	1029	1037	940	844	1001	1048
ENSECAG00000010144	3.611391938	0.072481823	0.205987613	81	132	223	233	238	285	274	469
ENSECAG00000020504	6.149873897	0.07251397	0.206027439	520	820	991	1729	1679	1630	1759	2159
ENSECAG00000011854	2.161008348	0.072669332	0.206417238	55	20	84	40	104	77	178	125
ENSECAG00000014543	3.855194789	0.072689288	0.206422316	104	186	215	301	346	268	418	446
ENSECAG00000013656	0.72124145	0.072713959	0.206440779	15	17	17	24	49	20	31	73
ENSECAG00000012566	2.77162255	0.072973176	0.207124964	70	87	108	78	204	157	177	160
ENSECAG00000021148	3.45524612	0.073037179	0.207254852	78	152	143	210	212	200	240	509
ENSECAG00000002091	3.806674557	0.073269044	0.207841158	116	193	192	266	416	309	377	306
ENSECAG00000008989	4.805811851	0.073293729	0.207841158	486	709	570	390	433	379	625	445
ENSECAG00000003091	6.23081253	0.073298673	0.207841158	1514	1940	1004	1298	1167	1071	1299	1514
ENSECAG00000011721	5.596079256	0.073395742	0.208042407	479	547	786	873	1266	1257	1340	943
ENSECAG00000008570	3.843312254	0.073406267	0.208042407	157	262	264	574	235	343	179	142
ENSECAG00000006920	6.635562315	0.073522036	0.208318547	1132	1008	1546	1654	2482	2424	2346	2874
ENSECAG00000021898	6.967611441	0.073576216	0.208402491	1474	1616	1572	1834	3343	2529	3126	3978
ENSECAG00000012409	4.500988246	0.073588346	0.208402491	294	288	293	286	759	403	613	536
ENSECAG00000022787	6.385951459	0.073654534	0.208537956	1307	1692	1641	1929	1455	1155	1549	1692
ENSECAG00000017694	6.218443067	0.073689719	0.208585596	872	773	982	1429	2000	1868	1793	1829
ENSECAG00000004868	5.934836764	0.07375216	0.208710347	895	1562	1074	1255	993	1084	928	1217
ENSECAG00000022442	8.224763392	0.073880004	0.209020072	2486.01	3200	3810	6711	8362	6620	9211	6918
ENSECAG00000022353	4.05135122	0.073957739	0.20918791	143	424	284	557	258	226	238	397
ENSECAG00000019097	5.119540582	0.073987777	0.209220787	896	639	543	526	686	465	625	519
ENSECAG00000011144	5.340709535	0.074034961	0.209302121	632	556	791	1351	941	411	656	784
ENSECAG00000024898	6.285550595	0.07417418	0.20964354	728	2584	1253	1903	1031	1436	1285	1402
ENSECAG00000021647	2.059121286	0.074223617	0.209731095	142	71	8	135	79	47	15	69
ENSECAG00000014742	3.432146403	0.074262708	0.209789382	71	89	212	220	265	251	259	339
ENSECAG00000006906	2.256320211	0.074295456	0.209829722	75	229	39	48	64	57	58	91
ENSECAG00000021794	1.094086439	0.074349579	0.209930396	27	45	53	48	25	36	36	32
ENSECAG00000012319	2.163503684	0.074437292	0.210096278	42	105	123	105	69	48	70	101
ENSECAG00000010152	2.80118977	0.07444531	0.210096278	50	82	73	160	210	100	249	172
ENSECAG00000008626	5.296208389	0.074532589	0.21029036	552	483	990	1189	792	590	660	676
ENSECAG00000000051	5.906304115	0.074595929	0.210404582	900	753	3982	833	47	1813	29	12
ENSECAG00000014535	3.857099707	0.074610109	0.210404582	137	478	208.004	383	226	167	281	283
ENSECAG00000006769	5.26528261	0.074743577	0.210622444	436	295	805	500	1055	809	1066	1004
ENSECAG00000023598	4.516589438	0.074750739	0.210622444	138	266	345	557	477	530	567	770
ENSECAG00000021548	5.975622794	0.074754835	0.210622444	581	684	1157	1101	1447	1406	1647	1838
ENSECAG00000020759	0.468493704	0.074761514	0.210622444	10	13	19	20	43	25	21	52
ENSECAG00000009655	4.127070108	0.07479935	0.2106768	171	208	224	351	512	259	499	519
ENSECAG00000020841	5.190296234	0.074823694	0.210693139	683	636	642	851	638	539	682	693
ENSECAG00000017758	8.261252839	0.075030938	0.211224359	7228	6822	4253	5544	4874	4016	4749	6281
ENSECAG00000011605	4.557424774	0.075059913	0.211253588	217	283	333	491	555	466	677	670
ENSECAG00000014914	5.96737385	0.075186196	0.2115258	769	437	635	1337	1719	834	2849	1251
ENSECAG00000010634	3.706780976	0.075193866	0.2115258	108	356	231	418	168	205	179	313
ENSECAG00000010689	6.006523513	0.075260455	0.211660716	452	708	912	1591	1262	1473	1574	2308
ENSECAG00000021680	2.691916658	0.075309876	0.211747293	113	150	113	139	117	113	60	124
ENSECAG00000018957	6.257998345	0.075331157	0.211754726	788	623	1350	1451	2200	1700	1808	2079
ENSECAG00000023883	4.446899912	0.075411198	0.21192729	234	210	453	256	579	518	579	534
ENSECAG00000020049	2.835419431	0.075650645	0.212547633	47	97	113	118	119	186	185	248
ENSECAG00000022211	4.621809437	0.075688479	0.212601359	233	254	443	420	629	493	845	516
ENSECAG00000000361	4.533908156	0.075768953	0.212774802	256	291	364	340	564	476	519	787
ENSECAG00000023373	5.941866255	0.075914285	0.213130247	919	1325	1122	1452	1125	876.001	1210	1098
ENSECAG00000019542	4.391357282	0.075951872	0.213183096	279	231	582	662	348	405	298	358
ENSECAG00000012864	2.78122109	0.076164585	0.213727343	76	70	80	115	221	78	180	253
ENSECAG00000018625	2.427841215	0.076196359	0.213763713	84	199	73	74	73	61	87	111
ENSECAG00000011798	1.679983995	0.076239747	0.213832635	22	51	100	110	43	36	53	65
ENSECAG00000014425	4.300965309	0.076382151	0.21417917	219	238	301	316	495	451	453	574
ENSECAG00000015502	6.397522015	0.076449398	0.214314844	1741	2088	1374	1222	1470	1034	1562	1601
ENSECAG00000019399	5.082597513	0.076589663	0.214655093	392	425	364	664	835	631	823	1163
ENSECAG00000008862	1.563741104	0.076615944	0.214675797	14	19	29	86	55	106	84	63
ENSECAG00000008451	5.252363831	0.076637098	0.214682128	490	713	913	930	747	525	795	597
ENSECAG00000024774	6.796961666	0.076712812	0.214841256	739	1514	1083	2833	2589	2425	3443	3111
ENSECAG00000018679	0.829122348	0.076748152	0.214887261	24	17	71	39	30	34	19	14
ENSECAG00000009909	6.941855885	0.076771192	0.214898812	1330	2734	2444	3789	2035	1999	1795	2507
ENSECAG00000025018	6.216919968	0.076856129	0.215083581	709	2087	1530	1797	1038	1509	1162	1287
ENSECAG00000014369	2.67806966	0.076962095	0.215327092	46	105	64	108	168	89	152	275
ENSECAG00000022111	2.358651255	0.07698207	0.215329614	38	76	69	81	93	127	154	151
ENSECAG00000012208	4.408422655	0.0770009	0.215329614	248	493	385	588	393	266	414	407
ENSECAG00000015033	4.626108887	0.077050574	0.215388816	251	386	654	785	467	315	339	591
ENSECAG00000015496	2.094262386	0.077059984	0.215388816	40	40	53	77	88	62	97	212
ENSECAG00000005179	0.466841146	0.0772148	0.215747222	4	28	23	80	9	34	13	7
ENSECAG00000017166	5.871312979	0.077226188	0.215747222	1052	1000	1048	1442	1055	866	1001	1192
ENSECAG00000009633	5.078555044	0.07726581	0.21576954	366	309	488	725	903	688	870	942
ENSECAG00000013540	6.079144661	0.077272158	0.21576954	688	796	2070	2283	1008	1294	1003	1213
ENSECAG00000008598	2.037580967	0.077321659	0.215854715	24	49	67	71	130	65	92	142
ENSECAG00000008186	7.28284894	0.077362	0.215914283	1752	1672	2552	2169	4293	3213	4238	4349
ENSECAG00000013629	8.865222392	0.077579836	0.216469081	6939	4278	4792	6479	13303	9065	13853	13398
ENSECAG00000009181	6.037603528	0.077709777	0.216778417	739	926	664	1204	1627	1220	1565	2338
ENSECAG00000016373	2.056247095	0.077864023	0.217155383	22	56	37	99	79	82	124	154
ENSECAG00000019985	1.766151976	0.077887183	0.217166667	31	49	41	44	80	52	93	130
ENSECAG00000000031	5.895823008	0.077994646	0.217412947	947	1838	792	1060	1005	1005	890	1099

ENSECAG00000010205	5.684123172	0.078094245	0.217578407	727	1254	1019	1003	771	965	835	1006
ENSECAG00000009651	6.580983861	0.07809535	0.217578407	1071	830	1275	2070	2466	2095	2357	2874
ENSECAG00000023213	5.915731357	0.078111452	0.217578407	665	1619	1376	1230	826	1270	716	1264
ENSECAG00000018741	7.151614536	0.078134836	0.217590198	1341	2099	1756	2335	4300	2374	4252	3836
ENSECAG00000006339	5.234816594	0.078171912	0.217640104	322	565	518	704	943	645	988	1243
ENSECAG00000010934	3.215022661	0.07825021	0.217804724	83	53	210	84.0001	294	66	321	362.001
ENSECAG00000003137	3.024676278	0.078367781	0.217996219	65	91	125	160	181	169	232	249
ENSECAG00000022539	3.889950211	0.078371057	0.217996219	122	195	214	290	301	291	402	517
ENSECAG00000007378	4.994732651	0.07839334	0.217996219	391	698	620	826	526	514	618	587
ENSECAG00000017333	6.651328598	0.07841481	0.217996219	1305	1034	1591	1265	2683	2194	2592	2850
ENSECAG00000017655	4.44755744	0.07841494	0.217996219	337	154	131	455	603	514	551	626
ENSECAG00000009680	9.070600126	0.078610099	0.21848531	9963	16665	7655	8357	7285	8626	9443	8787
ENSECAG00000010819	7.886127014	0.078641224	0.218518363	3513	6655	4012	4792	3646	3711	3816	4546
ENSECAG00000011139	2.549126306	0.078697116	0.218620204	37	71	86	107	124	77	157	270
ENSECAG00000001583	1.382958594	0.078723866	0.218641057	24	16	30	58	61	75	77	52
ENSECAG00000015790	2.387802933	0.078751981	0.218665692	50	65	57	95	101	101	146	196
ENSECAG00000024384	0.904318097	0.078853265	0.218893427	10	19	23	39	31	65	41	51
ENSECAG00000018303	7.541893263	0.078887558	0.218935132	2785	4943	3221	3964	3132	2806	2780	3811
ENSECAG00000006558	6.367936721	0.078918498	0.218967514	803	851	1354	1602	2437	1893	1976	1997
ENSECAG00000014560	7.450387512	0.078975629	0.219072534	1527	2630	2122	3052	5572	3406	3714	5523
ENSECAG00000010123	1.458012323	0.079001635	0.219091183	22	25	40	44	110	27	71	82
ENSECAG00000010574	4.356681781	0.079221531	0.219647398	274	428	440	475	347	350	297	440
ENSECAG00000011098	4.384426798	0.079251221	0.21967611	382	443	402	378	268	370	307	487
ENSECAG00000021219	7.787111756	0.079329713	0.219839263	5042	5359	3505	3139	3802	3253	4015	3079
ENSECAG00000025085	3.680905939	0.079359913	0.219839263	122	120	182	274	322	205	433	357
ENSECAG00000001256	0.855449321	0.079368127	0.219839263	34	53	34	21	16	39	19	27
ENSECAG00000016042	5.623507699	0.079717086	0.22075202	625	659	694	631	1385	927	1322	1343
ENSECAG00000008059	5.754977513	0.079799418	0.220926166	299	514	556	1687	1419	1663	1516	959
ENSECAG00000023466	5.714626744	0.079852796	0.22102009	856	1266	865	1051	967	845	831	1022
ENSECAG00000023565	5.753918942	0.079941815	0.221212592	1092	829	1134	1073	1006	744	1081	907
ENSECAG00000009246	3.011927005	0.08001977	0.221374391	243	192	80	96	153	85	135	112
ENSECAG00000017624	2.443869862	0.080108149	0.221564943	43	55	88	103	124	138	130	161
ENSECAG00000022698	3.537238121	0.080232486	0.221854834	79	123	178	268	287	245	250	412
ENSECAG00000024958	6.386534166	0.080315226	0.222029585	645	2248	1838	2381	939	1657	1247	1730
ENSECAG00000010893	2.901885599	0.080528729	0.222565659	97	89	209	240	135	77	159	115
ENSECAG00000021916	6.559576676	0.080554246	0.22258204	1680	1884	1723	2025	1534	1511	1590	1903
ENSECAG00000017989	8.169840894	0.08070654	0.222948628	4511	8503	4027	6200	4781	3160	4718	6410
ENSECAG00000009632	5.738277765	0.080777403	0.223090145	744	764	596	619	1359	1050	1344	1717
ENSECAG00000024809	4.180321196	0.08098545	0.223610373	146	209	307	355	429	316	545	550
ENSECAG00000008317	3.945478773	0.081133149	0.22396376	294	203.979	318	393	255	264	236.99	296
ENSECAG00000003847	2.663760927	0.081157372	0.22397621	80	52	41	138	210	156	133	164
ENSECAG00000022480	6.699998831	0.081238061	0.224104599	748	1557	1431	2045	2146	2734	2877	2805
ENSECAG00000008876	7.401691317	0.081243341	0.224104599	3672	5647	2285	1579	2160	2995	2718	2451
ENSECAG00000014806	4.268603711	0.08128354	0.224151236	124	336	216	395	449	491	430	585
ENSECAG00000000295	5.142513103	0.081299704	0.224151236	310	339	444	926	832	917	810	999
ENSECAG00000009465	1.898385651	0.081470876	0.224568677	25	98	64	142	38	51	59	81
ENSECAG00000022218	1.004165016	0.081634472	0.224957279	18	9	29	39	57	52	32	65
ENSECAG00000024211	2.839808518	0.081651454	0.224957279	70	92	115	92	190	185	174	175
ENSECAG00000022888	3.379721568	0.081677335	0.224957512	98	87	170	211	279	229	295	252
ENSECAG00000003310	7.643716329	0.081691137	0.224957512	1414	2473	3163	4003	4763	4821	4742	6353
ENSECAG00000019813	6.656666696	0.081789783	0.225174584	802	972	1568	2315	2553	2135	3324	2303
ENSECAG00000014504	0.578286755	0.081881482	0.225372431	14	7	14	27	80	32	32	11
ENSECAG00000015463	6.125759893	0.081904476	0.225381122	382	603	1261	1651	1524	2692	1348	1579
ENSECAG00000007474	5.089540441	0.081983492	0.225543931	481	1064	458	670	615	432	645	627
ENSECAG00000023403	6.120164425	0.082089207	0.225746576	611	586	1137	1611	1980	1611	1722	1718
ENSECAG00000015576	3.5347866	0.082096889	0.225746576	51	202	126	200	182	154	250	718
ENSECAG00000008488	4.670779609	0.082145174	0.225824695	239	265	361	575	590	663	688	594
ENSECAG00000011597	2.59066209	0.082189038	0.225890625	24	105	83	94	130	94	164	260
ENSECAG00000019381	4.342148045	0.082216476	0.225911389	274	505	367	449	280	347	354	425
ENSECAG00000023824	3.628561909	0.082258628	0.225972566	126	153	197	171	262	343	204	458
ENSECAG00000022444	3.668994489	0.08232156	0.22604138	97	157	176	282	297	311	284	393
ENSECAG00000017703	3.613582899	0.082323467	0.22604138	169	301	269	222	204	207	181	240
ENSECAG00000007845	6.546673316	0.082465017	0.226375337	1260	1163	1054	1371	2523	1797	2256	3043
ENSECAG00000007274	3.949142672	0.082492965	0.226397359	267	156	559	286	354	139	211	305
ENSECAG00000010392	5.255724084	0.082548036	0.22649379	452	549	471	600	992	769	964	1105
ENSECAG00000020408	5.701751426	0.082761398	0.227024387	653	742	594	762	1408	838	1344	1733
ENSECAG00000012589	4.004676795	0.082901838	0.227319049	300	355	307	265	337	234	234	285
ENSECAG00000006513	4.406010738	0.082913378	0.227319049	95	371	302	379	481	279	672	819
ENSECAG00000012679	5.764730311	0.082928838	0.227319049	506	641	613	1317	1470	984	1258	1832
ENSECAG00000020688	4.68871169	0.083020645	0.227515816	951	297	321	327	396	324	451	419
ENSECAG00000006526	3.643291713	0.083143421	0.227797336	106	197	177	194	346	226	292	410
ENSECAG00000019178	6.348810784	0.083337522	0.228274089	985	1002	1045	1370	2275	1539	2203	2226
ENSECAG00000016246	7.700140286	0.083375245	0.228322374	4011	5170	3533	3474	3463	3529	3371	3322
ENSECAG00000021132	2.602229287	0.083447855	0.22846615	45	89	225	176	93	111	75	99
ENSECAG00000026844	4.48413433	0.083511814	0.228586179	333	325	739	421	530	244	452	259
ENSECAG00000022670	4.960050017	0.083598639	0.228768724	299	465	431	552	770	682	795	840
ENSECAG00000017735	1.277286923	0.083695559	0.228965471	15	40	96	99	1	78	7	12
ENSECAG00000015022	3.061536276	0.08371084	0.228965471	104	123	80	107	220	172	246	219
ENSECAG00000016289	5.8233923	0.083778686	0.229095891	814	1083	1072	1488	1072	938	1006	984
ENSECAG00000017576	3.251839889	0.083863126	0.229271617	67	131	135	189	261	173	263	275
ENSECAG00000009392	2.040132881	0.083920258	0.229313919	44	85	78	138	53	71	61	79
ENSECAG00000007908	4.386628692	0.083936743	0.229313919	226	263	225	435	636	417	548	499
ENSECAG00000012086	2.858944987	0.083939147	0.229313919	72	182	187	170	102	169	64	119
ENSECAG00000017540	4.177391975	0.083989871	0.229378225	160	685	257	408	212	253	231	486
ENSECAG00000024364	6.393262544	0.084003062	0.229378225	634	1215	1346	1552	2122	1688	2243	2444

ENSECAG00000023936	0.863102863	0.084101244	0.229591142	2	6	44	19	11	34	135	25
ENSECAG00000015768	5.980689127	0.084150062	0.229669229	1718	600	1256	1267	1148	1068	1008	892
ENSECAG00000013489	5.3374359	0.084214532	0.229761321	395	600	545	714	1095	728	1102	1126
ENSECAG00000020240	6.287578133	0.084224248	0.229761321	902	527	1399	1333	2452	1699	2280	1553
ENSECAG00000020581	1.812929582	0.084363816	0.230086816	44	40	38	44	81	73	127	79
ENSECAG00000010400	3.484604545	0.08445268	0.230273903	231	364	109	162	192	138	168	214
ENSECAG00000015694	3.592560571	0.084570641	0.230529248	98	210	133	205	245	247	350	392
ENSECAG00000017080	2.057320122	0.084586907	0.230529248	25	51	68	76	97	97	106	123
ENSECAG00000024696	6.848413866	0.084662173	0.230679042	1152	1264	1410	2500	3456	2315	2696	3364
ENSECAG00000013331	6.894273363	0.084731686	0.230813095	1345	3626	2287	2492	1653	2620	1635	1841
ENSECAG00000003471	7.101288338	0.08485729	0.231099839	2278	3730	2521	2215	2484	1864	2265	2613
ENSECAG00000011265	0.813951653	0.084885219	0.231120502	8	9	25	42	36	21	65	63
ENSECAG00000000421	1.846225864	0.084912248	0.231138707	32	47	49	53	86	61	108	113
ENSECAG00000008409	3.836675703	0.085055409	0.231457231	114	242	607	295	137	355	134	248
ENSECAG00000009617	3.614520872	0.085087714	0.231457231	116	152	164	236	402	216	267	359
ENSECAG00000003075	1.322800782	0.085090376	0.231457231	6	56	17	34	41	54	69	107
ENSECAG00000023145	6.157319926	0.085198374	0.23169553	720	1398	2045	1736	977	1557	1015	1290
ENSECAG00000025048	6.340135632	0.085230897	0.231728511	794	834	919	2004	2285	1524	2080	2382
ENSECAG00000020174	1.571764211	0.085367613	0.232044694	26	19	55	48	89	56	78	82
ENSECAG00000025083	5.068505905	0.085418279	0.232126881	249	661	667	1333	603	429	621	643
ENSECAG00000019292	3.866672469	0.085494593	0.232278709	193	446	259	256	242	202	182	360
ENSECAG00000017967	3.874823591	0.085539284	0.232344571	130	140	209	345	411	277	405	391
ENSECAG00000021900	0.265315872	0.085582032	0.232405126	5	46	26	23	20	13	18	13
ENSECAG00000009967	4.727705162	0.085624896	0.232441217	229	367	504	346	668	453	631	958
ENSECAG00000012331	5.075642941	0.085636238	0.232441217	628	338	160	375	984	619	995	1030
ENSECAG00000016733	3.695684446	0.085661733	0.232454888	190	305	257	265	186	261	199	230
ENSECAG00000010693	4.853891168	0.085774493	0.23269128	273	265	578	503	710	585	815	798
ENSECAG00000018200	4.412865912	0.085789806	0.23269128	258	184	308	415	521	518	488	610
ENSECAG00000016694	4.501280605	0.085817386	0.232710534	350	876	199	371	345	300	337	479
ENSECAG00000010463	6.003778552	0.08590853	0.232839153	828	1601	1226	1401	1261	888	1053	1308
ENSECAG00000027114	0.428176231	0.085923866	0.232839153	3	2	5	48	69	29	22	25
ENSECAG00000009427	0.698296813	0.085932266	0.232839153	23	59	27	19	23	14	34	21
ENSECAG00000009479	3.064666666	0.085949048	0.232839153	228	289	60	71	166	124	99	85
ENSECAG00000005070	0.665953477	0.085967281	0.232839153	8	7	22	38	43	45	51	21
ENSECAG00000001870	4.763402967	0.08600019	0.232872773	336	175	463	442	806	379	963	673
ENSECAG00000018415	2.465822987	0.086064968	0.232992653	59	73	246	100	64	120	81	63
ENSECAG00000011663	1.79680199	0.086120735	0.233045055	45	45	123	82	50	25	89	48
ENSECAG00000018720	0.160697049	0.086125346	0.233045055	6	12	20	11	25	28	35	21
ENSECAG00000024020	6.60311636	0.086294838	0.233448084	1415	1623	2199	2612	2209	1094	1752	1692
ENSECAG00000020533	5.172336892	0.086339219	0.233483725	446	1061	502	867	474	593	687	721
ENSECAG00000007200	6.440985805	0.086349112	0.233483725	953	994	1328	1487	2328	1563	2077	2879
ENSECAG00000018273	2.729848359	0.086377147	0.233501271	76	156	129	182	106	115	88	130
ENSECAG00000007796	6.01193108	0.086396703	0.233501271	638	781	852	1374	1757	1268	1536	1932
ENSECAG00000009944	1.92533502	0.086486896	0.233637877	14	38	48	100	70	111	103	110
ENSECAG00000025092	7.560023399	0.086488374	0.233637877	1660	2080	3194	3277	5230	3223	4369	6941
ENSECAG00000000500	4.403528845	0.0865901	0.233791013	78	404	251	375	510	834	530	304
ENSECAG00000010957	3.187774471	0.086591004	0.233791013	74	82	170	172	220	217	237	249
ENSECAG00000011946	2.493277758	0.086606792	0.233791013	52	53	87	98	163	77	137	213
ENSECAG00000010231	4.407451241	0.086828872	0.234334833	431	362	430	432	215	473	238	487
ENSECAG00000016140	6.244624775	0.087029338	0.234820074	971	1491	1135	2705	1613	1032	1481	1044
ENSECAG00000024429	7.310693775	0.087069622	0.234872992	2003	5940	1899	3186	2760	3002	2154	2085
ENSECAG00000017933	5.690565434	0.087100247	0.234899836	613	1736	940	817	717	1096	683	931
ENSECAG00000015927	2.909076937	0.087165145	0.235019074	59	163	110	347	161	88	136	81
ENSECAG00000012571	6.186305162	0.087264557	0.235205384	615	2348	1409	1610	1112	1113	1413	1280
ENSECAG00000018468	5.05734473	0.08728206	0.235205384	399.996	478.998	1007.99	814.996	572	562.997	482.995	709
ENSECAG00000018890	5.291039948	0.087296348	0.235205384	412	871	753	1161	735	642	488	880
ENSECAG00000005773	4.653792766	0.087320742	0.235215332	139	1156	427	437	377	203	417	586
ENSECAG00000010683	4.416478597	0.087407871	0.235387234	234	560	334	598	338	326	371	448
ENSECAG00000017724	7.685893341	0.087434413	0.235387234	6888	2217	3854	3139	5166	2368	3294	1158
ENSECAG00000011234	2.908224882	0.08744671	0.235387234	70	149	268	149	121	153	70	129
ENSECAG00000024503	6.617345458	0.087522865	0.235536426	1631	2926	1582	1466	1691	1384	1483	2073
ENSECAG00000013601	4.38883481	0.087602597	0.235695172	221	234	332	372	566	357	605	577
ENSECAG00000011905	2.640662647	0.087772203	0.23609559	37	47	56	190	148	194	199	108
ENSECAG00000009911	6.101410647	0.087806044	0.236130716	310	570	1008	2046	1552	2242	1543	1759
ENSECAG00000017271	4.59080763	0.087845365	0.236180558	224	150	298	669	683	568	710	503
ENSECAG00000013423	8.916973396	0.087954525	0.236328079	5512	7349	13534	15072	7828	8698	5954	9499
ENSECAG00000008964	5.982585225	0.08795749	0.236328079	1061	1453	1064	1322	961	1336	846	1193
ENSECAG00000003388	6.809729193	0.087962634	0.236328079	1690	2523	2121	2526	2047	1620	2077	2002
ENSECAG00000015055	4.971340973	0.088024423	0.236425255	366	445	344	531	851	765	515	1038
ENSECAG00000014965	5.446253003	0.08804042	0.236425255	337	440	742	997	1025	920	954	1521
ENSECAG00000016944	2.80680172	0.088079736	0.236474945	75	169	142	191	81	105	122	157
ENSECAG00000001488	3.75848483	0.088137593	0.236574374	140	51	258	106	833	49.0001	513	176
ENSECAG00000016935	5.023584481	0.088183432	0.236641511	354	374	447	632	921	546	986	811
ENSECAG00000011453	6.500928169	0.088218874	0.236678931	1045	2845	1835	1489	1486	1499	1383	1762
ENSECAG00000020312	2.851206689	0.088239038	0.236678931	63	122	129	337	108	147	114	75
ENSECAG00000010655	4.142079463	0.088276165	0.236722631	224	407	405	357	261	351	280	322
ENSECAG00000011639	6.964957412	0.088299897	0.2367304	1171	1178	2221	2279	2930	3076	2789	4020
ENSECAG00000021891	5.819715437	0.08841649	0.236958489	534	593	932	1171	1334	1258	1329	1725
ENSECAG00000006391	3.138381264	0.088426685	0.236958489	89	169	144	383	94	231	98	115
ENSECAG00000015051	5.114550882	0.088686638	0.237599053	224	393	576	826	893	727	863	1007
ENSECAG00000007088	1.743335486	0.088739416	0.237684405	26	28	52	61	90	35	110	118
ENSECAG00000021662	7.984443751	0.088791744	0.237724892	3749	2757	2822	2727	8217	3734	5941	9373
ENSECAG00000018037	5.452300624	0.088796377	0.237724892	540	365	537	928	1569	739	1115	1042
ENSECAG00000008396	8.067549059	0.088958546	0.238102946	3570	4014	2993	2033	7823	5941	8758	5789
ENSECAG00000017325	6.670880531	0.088992475	0.238137658	1946	3811	1136	1038	1533	1332	1653	1927

ENSECAG00000003961	1.044288965	0.089069795	0.238288442	19	14	21	45	44	31	71	67
ENSECAG00000023534	7.765731062	0.089112097	0.23834549	925	2204	2101	6657	5083	7489	6744	3659
ENSECAG00000019053	2.515272158	0.089177604	0.238414932	44	73	102	79	109	134	152	190
ENSECAG00000009450	2.149428574	0.089180026	0.238414932	84	28	225	48	49	28	118	50
ENSECAG00000000103	4.301520466	0.089425157	0.239014027	137	209	352	435	448	437	552	544
ENSECAG00000020079	5.242311786	0.089454884	0.23903725	491	1320	455	749	471	490	704	888
ENSECAG00000003314	0.905556318	0.089574118	0.239299583	17	36	71	33	18	17	45	29
ENSECAG00000010877	4.99937859	0.089598593	0.2393087	212	376	587	674	743	855	732	858
ENSECAG00000014231	5.880437987	0.089793319	0.239667599	385	604	1011	1416	1226	1494	1364	1885
ENSECAG00000024595	0.413394028	0.089795742	0.239667599	12	9	13	27	42	25	38	27
ENSECAG00000015122	6.875019596	0.089796248	0.239667599	2593	2959	1396	2036	1983	1707	1903	2170
ENSECAG00000023593	6.914697827	0.089870414	0.23978035	1060	1529	1804	2284	2565	2876	2987	3879
ENSECAG00000005775	0.597441906	0.0898807	0.23978035	15	33	43	33	13	29	26	18
ENSECAG00000010178	1.831320596	0.089946524	0.239899626	39	90	81	77	57	56	54	62
ENSECAG00000009943	6.319528179	0.090004187	0.23992085	1865	2096	1100	925	1342	1275	1283	1335
ENSECAG00000010233	2.650291713	0.09003285	0.23992085	43	87	100	104	150	120	205	165
ENSECAG00000024699	7.584285428	0.090038528	0.23992085	3686	4917	3457	2774	3236	2773	3216	3533
ENSECAG00000005917	3.401252187	0.090038946	0.23992085	73	123	172	224	297	239	260	267
ENSECAG00000018403	0.822938068	0.090079139	0.23997167	17	45	22	65	20	32	24	27
ENSECAG00000009552	8.712020416	0.090204267	0.240248684	6688	9351	7946	9636	7552	5841	7889	6912
ENSECAG00000011460	2.942938551	0.09025459	0.240296137	67	78	88	185	172	164	218	232
ENSECAG00000014530	2.044817315	0.090264383	0.240296137	15	70	52	61	68	137	175	51
ENSECAG00000008976	5.753089732	0.090386872	0.240565856	1115	1231	796	910	1067	814	836	992
ENSECAG00000021699	2.645986043	0.090470825	0.240685998	69	76	187	202	106	88	116	96
ENSECAG00000023141	3.388764567	0.090474379	0.240685998	199	130	215	295	231	157	119	192
ENSECAG00000005248	4.773494837	0.090523755	0.240760979	294	335	351	540	770	433	697	863
ENSECAG00000011371	4.854655502	0.09054716	0.24076687	339	341	439	467	751	602	865	654
ENSECAG00000022076	4.984359503	0.090597798	0.240845151	243	1067	499	827	494	414	468	772
ENSECAG00000016942	0.399340835	0.090692353	0.241040119	8	32	36	34	15	24	12	23
ENSECAG00000000259	1.103278228	0.090758118	0.241117052	22	60	46	46	23	32	39	37
ENSECAG00000018846	3.580756656	0.090763742	0.241117052	113	184	181	160	299	216	336	363
ENSECAG00000023764	7.784057485	0.090839412	0.241261663	1930	2541	3271	4300	6244	4360	7040	4987
ENSECAG00000023680	6.189823646	0.090866893	0.241278251	1505	2600	696	810	1237	1001	1136	1272
ENSECAG00000020500	6.679192842	0.090901104	0.241312698	1810	2447	1551	2130	1837	1539	1752	1906
ENSECAG00000009169	4.881826185	0.090943969	0.241370096	253	326	503	605	652	701	618	981
ENSECAG00000014794	4.630630681	0.091029521	0.241455715	245	734	472	540	342	419	440	502
ENSECAG00000021487	2.602059743	0.091034678	0.241455715	57	54	72	141	163	120	159	180
ENSECAG00000022714	1.395733038	0.091057372	0.241455715	16	22	86	129	37	41	51	11
ENSECAG00000012881	3.358150767	0.091061234	0.241455715	86	146	120	206	240	184	335	284
ENSECAG00000009253	1.075548714	0.091181937	0.241719359	13	31	24	33	42	36	46	95
ENSECAG00000010351	5.303858549	0.091310439	0.242003546	786	1512	504	255	618	607	670	531
ENSECAG00000024883	6.722938325	0.091434198	0.242275038	1433	2744	1916	2263	1783	1762	1806	1917
ENSECAG00000017416	1.597623953	0.091531735	0.242441655	37	27	26	54	67	45	82	124
ENSECAG00000010837	4.192295167	0.091539755	0.242441655	95	150	271	531	404	452	379	666
ENSECAG00000008559	4.572656989	0.091572099	0.242470796	385	433	541	490	455	476	362	342
ENSECAG00000001830	2.031315555	0.091855967	0.243149697	23	116	91	127	62	57	83	47
ENSECAG00000011938	5.913205318	0.091871295	0.243149697	866	1107	1299	1460	1025	773	1093	1431
ENSECAG00000018099	1.486654641	0.091964275	0.2433391	75	12	128	30	73	28	28	4
ENSECAG00000019687	3.699101012	0.092040927	0.243485217	91	100	257	268	365	409	273	265
ENSECAG00000018963	1.722232406	0.092065647	0.243493921	28	75	163	80	2	105	0	9
ENSECAG00000020642	5.72710821	0.092117817	0.2435752	716	1220	961	1232	929	930	904	989
ENSECAG00000010051	9.285006823	0.092254453	0.243879734	5293	5521	11460	11020	14377	11740	13585	26904
ENSECAG00000022681	1.988862958	0.092280158	0.243890941	66	91	63	98	48	86	42	73
ENSECAG00000019925	4.471638298	0.092319236	0.243937479	241	405	561	593	450	262	422	430
ENSECAG00000009496	5.594788411	0.092384604	0.244021562	868	1236	779	775	912	812	816	781
ENSECAG00000021085	6.207240741	0.092399604	0.244021562	797	996	1098	1160	1691	1740	2258	1665
ENSECAG00000021716	5.87339958	0.092415489	0.244021562	591	1569	1105	1454	1037	939	912	1220
ENSECAG00000010303	3.434962591	0.092511788	0.244219084	82	130	151	245	276	233	273	307
ENSECAG00000005487	7.113898841	0.092612657	0.244428575	102	28	276	4183	986	68	1425	18372
ENSECAG00000020436	3.977542416	0.092714636	0.244640895	122	191	235	345	344	406	330	501
ENSECAG00000014470	2.232543963	0.092758161	0.244685487	61	73	161	88	86	60	76	82
ENSECAG00000016295	4.709730832	0.092794458	0.244685487	221	492	231	479	664	486	639	890
ENSECAG00000015564	3.342003485	0.092813323	0.244685487	132	257	184	233	142	158	209	178
ENSECAG00000006892	5.373668256	0.092836774	0.244685487	291	980	1152	1112	367	766	661	995
ENSECAG00000023200	3.070217094	0.092839213	0.244685487	97	142	270	185	137	82	132	214
ENSECAG00000000276	0.134161138	0.092898825	0.244761491	11	7	20	8	26	28	23	31
ENSECAG00000019557	5.442601544	0.092931497	0.244761491	1058	491	1333	509	575	980	407	789
ENSECAG00000000040	3.694169951	0.092932677	0.244761491	154	124	188	225	335	221	302	468
ENSECAG00000016300	5.338210656	0.09297937	0.244827718	406	1090	630	1197	733	527	536	1025
ENSECAG00000016493	7.443146688	0.093018834	0.244874883	2122	3658	3734	4801	2383	3140	2602	3753
ENSECAG00000005178	1.271260641	0.093133678	0.245039566	14	41	35	28	69	58	53	63
ENSECAG00000013760	4.335679707	0.093139146	0.245039566	186	202	220	527	447	353	532	746
ENSECAG00000002919	6.83922264	0.093146091	0.245039566	2239	3327	2028	1233	1563	2125	1558	2170
ENSECAG00000016806	3.909902895	0.093188873	0.245095366	219	307	378	278	227	158	284	378
ENSECAG00000008118	1.975288897	0.093215721	0.24510924	23	44	44	95	108	130	80	80
ENSECAG00000012325	0.577583313	0.093408137	0.245523666	7	11	23	31	26	39	31	54
ENSECAG00000009540	5.70897736	0.093416547	0.245523666	443	523	835	1238	1369	1044	1373	1449
ENSECAG00000024671	1.826020162	0.093452932	0.245545136	28	48	95	143	59	61	53	44
ENSECAG00000009322	0.446888752	0.093467938	0.245545136	14	11	11	23	23	29	63	22
ENSECAG00000012986	5.304106128	0.093494662	0.245558564	605	376	944	1355	712	784	606	558
ENSECAG00000025107	5.523466303	0.093532322	0.245600703	365	416	738	1124	1336	1203	1191	858
ENSECAG00000024025	4.298087275	0.093570729	0.245644782	207	300	268	299	508	364	497	604
ENSECAG00000016040	4.946785182	0.093600086	0.245665091	276	403	412	672	778	543	765	1015
ENSECAG00000010757	3.773882866	0.093636966	0.245705129	126	149	190	302	291	297	350	440
ENSECAG00000014766	4.949858336	0.093765273	0.245985	212	371	550	659	687	746	691	969

ENSECAG00000008497	0.278274607	0.093828285	0.246093484	4	48	20	36	3	13	7	38
ENSECAG00000013583	4.149742137	0.09397005	0.246408425	176	368	304	637	305	223	286	421
ENSECAG00000020387	2.093634602	0.094023071	0.246436948	56	55	33	55	121	56	132	141
ENSECAG00000000644	6.148450841	0.094028647	0.246436948	687	941	1090	1282	1658	1774	1588	2026
ENSECAG00000018735	0.235987949	0.094045996	0.246436948	8	14	12	20	23	28	26	38
ENSECAG00000000879	2.090802488	0.094143195	0.246634766	30	62	47	83	97	75	108	160
ENSECAG00000008356	2.710628244	0.09429137	0.246966008	36	111	360	91	50	62	174	90
ENSECAG00000023488	8.72282104	0.094349083	0.247060216	10041	9691	6539	6206	6855	6863	7824	5449
ENSECAG00000007776	2.275775746	0.094501806	0.247368401	39	76	45	87	100	77	166	160
ENSECAG00000000308	6.25616447	0.094510318	0.247368401	1626	1677	1204	1250	1247	1442	1191	1297
ENSECAG00000001342	3.569796388	0.094609284	0.247570403	66	295	265	412	157	198	218	202
ENSECAG00000018531	2.766295809	0.094716649	0.247794282	78	53	340	160	167	111	69	19
ENSECAG00000020260	7.838612377	0.094776771	0.247894492	2843	7780	3613	4276	3173	4098	3587	4136
ENSECAG00000002889	7.420098415	0.094834492	0.247985147	1702	1565	2638	3508	4297	3648	4224	5459
ENSECAG00000023529	0.283125187	0.094855083	0.247985147	15	24	11	52	12	21	13	21
ENSECAG00000011582	5.899608154	0.094904035	0.247995101	501	836	822	1276	1403	1456	1443	1617
ENSECAG00000014879	5.044996763	0.094922006	0.247995101	401	531	402	401	889	577	979	892
ENSECAG00000016353	5.136579591	0.09492437	0.247995101	389	389	576	621	921	751	843	971
ENSECAG00000003998	4.201884984	0.094963154	0.248039392	127	197	248	477	576	481	444	344
ENSECAG00000015243	4.144981511	0.095001029	0.248081289	150	158	350	341	462	404	449	455
ENSECAG00000013718	4.998865323	0.095069319	0.248202574	295	267	635	618	804	702	860	831
ENSECAG00000007813	6.81330251	0.095111935	0.24825679	1427	3179	1986	2594	1429	2824	1564	1468
ENSECAG00000017449	5.347323644	0.095144905	0.248285809	279	363	864	820	1066	999	745	1326
ENSECAG00000015358	5.917509434	0.095223208	0.248433085	1421	1276	935	869	1085	1031	1075	857
ENSECAG00000025102	5.528227645	0.095287102	0.248542712	904	1032	802	726	755	799	880	744
ENSECAG00000015091	4.997752522	0.095333497	0.248606654	297	763	685	843	537	504	632	571
ENSECAG00000023178	6.293453467	0.095390423	0.248698024	2118	1608	1005	1066	1263	1131	1387	1408
ENSECAG00000022408	2.325473282	0.095567827	0.249103383	28	73	46	102	58	191	77	205
ENSECAG00000012266	5.667995228	0.095794423	0.24963675	535	491	949	892	1376	1124	1210	1339
ENSECAG00000000294	6.129651232	0.095832979	0.24967996	613	841	1083	1467	1463	1594	1605	2379
ENSECAG00000012136	7.626182993	0.0958737	0.249728789	2045	2552	2755	3313	4722	4356	5256	5849
ENSECAG00000015333	2.744716253	0.095903847	0.24975006	155	187	62	136	54	192	57	79
ENSECAG00000012872	2.625965685	0.096018745	0.249957987	87	95	169	154	119	59	81	153
ENSECAG00000002199	3.673696803	0.09602769	0.249957987	93	148	257	210	273	320	286	409
ENSECAG00000017444	4.870712605	0.096089429	0.250032925	507	521	547	663	557	468	545	499
ENSECAG00000012377	0.818510183	0.096100491	0.250032925	12	38	42	58	25	36	22	19
ENSECAG00000010409	4.405301864	0.096372751	0.250683882	185	383	510	727	349	478	320	263
ENSECAG00000018895	5.424531784	0.096474024	0.250889874	176	511	255	1468	1055	1208	1290	965
ENSECAG00000018949	5.984611432	0.096503285	0.250908541	546	902	916	1258	1438	1320	1739	1814
ENSECAG00000020502	3.519181336	0.096562742	0.251005691	117	103	206	197	293	218	265	391
ENSECAG00000019284	4.410344225	0.096624042	0.251107587	157	595	480	529	293	302	370	498
ENSECAG00000016844	0.319435835	0.096650675	0.251119362	8	8	17	25	21	38	23	41
ENSECAG00000008388	2.038082637	0.09692114	0.251764517	32	29	80	71	76	112	128	102
ENSECAG00000026502	1.92145681	0.096974506	0.25183361	3	61	91	250	81	61	14	25
ENSECAG00000023061	9.997645297	0.096992068	0.25183361	24586	28447	14229	10955	14041	15193	18038	17276
ENSECAG00000021263	2.849065601	0.097225082	0.252380941	47	77	77	189	230	101	171	256
ENSECAG00000022083	7.366852295	0.0972682	0.252435195	3308	3481	2803	3123	2640	2270	2971	3371
ENSECAG00000026882	3.019588347	0.097303355	0.252468764	80	267	111	210	123	105	148	156
ENSECAG00000023344	5.190252022	0.097409346	0.25268607	290	511	496	793	860	778	709	1340
ENSECAG00000015912	5.70994264	0.097448739	0.252730556	708	1706	710	1009	721	1169	833	746
ENSECAG00000000035	5.729903062	0.097549446	0.252934004	529	756	604	1116	1250	1082	1264	1717
ENSECAG00000020182	7.193925836	0.097605352	0.25302122	1242	1449	1946	3463	4048	3456	4854	2592
ENSECAG00000008489	6.321643094	0.097650406	0.253043004	798	830	1191	1725	1950	1626	1902	2581
ENSECAG00000015826	4.28567166	0.097690218	0.253043004	231	501	495	311	365	301	249	432
ENSECAG00000013041	6.984578162	0.097699906	0.253043004	3780	1410	2776	1697	3077	1597	1711	1567
ENSECAG00000009822	6.996027775	0.097730493	0.253043004	1516	1733	3829	3642	2626	1984	2216	1766
ENSECAG00000019708	6.95353569	0.097765252	0.253043004	2846	2979	1078	2720	2223	1573	1886	2466
ENSECAG00000017116	6.737302297	0.097767219	0.253043004	799	1101	1683	2470	2523	3202	2724	2275
ENSECAG00000021708	3.624074507	0.097769652	0.253043004	101	198	107	179	239	95	224	852
ENSECAG00000012126	6.502544441	0.097820197	0.253116165	754	610	1237	2506	2403	2010	3096	1797
ENSECAG00000008170	3.825205047	0.097874191	0.253198216	97	70	253	356	304	319	259	627
ENSECAG00000022974	1.203786595	0.097998375	0.253449179	20	56	58	55	28	33	31	52
ENSECAG00000023980	4.827252656	0.098015815	0.253449179	329	686	605	631	359	525	436	674
ENSECAG00000008835	7.183136275	0.098121936	0.253620272	1607	2110	1822	2179	3691	2903	4220	4028
ENSECAG00000004093	2.331555837	0.098126625	0.253620272	61	54	52	84	157	70	152	141
ENSECAG00000007780	3.152052571	0.098357023	0.254112603	284	141	150	95	139	180	139	66
ENSECAG00000005098	4.543270515	0.098376134	0.254112603	463	676	256	381	478	275	333	481
ENSECAG00000015005	7.507196924	0.098384205	0.254112603	2951	4197	3668	3500	2850	3528	3119	2714
ENSECAG00000020173	4.305559569	0.098427449	0.254166518	202	419	725	411	107	666	115	239
ENSECAG00000000017	0.682698493	0.098636526	0.254648536	10	15	17	37	38	33	41	48
ENSECAG00000012666	7.223090835	0.098743132	0.254865848	1657	1984	2299	1848	4150	2372	3695	5366
ENSECAG00000018606	4.409108537	0.098910229	0.255239161	186	262	354	409	521	486	432	675
ENSECAG00000021260	3.688718886	0.099042167	0.255521595	74	223	102	307	327	229	340	448
ENSECAG00000005393	4.791671809	0.099221206	0.255925391	493	537	395	706	537	430	430	556
ENSECAG00000020238	4.848650238	0.099270321	0.255993961	306	335	559	387	759	612	766	726
ENSECAG00000015223	0.7270306	0.099600189	0.256786329	0	74	40	46	3	35	20	13
ENSECAG00000013509	5.238235465	0.099698453	0.256884767	765	592	682	823	705	631	704	594
ENSECAG00000020692	6.496888208	0.099715689	0.256884767	863	991	1215	2053	2386	1621	2279	2882
ENSECAG00000008904	2.041194988	0.099719611	0.256884767	31	37	43	108	81	100	103	138
ENSECAG00000007623	3.270817718	0.099728807	0.256884767	115	69	159	152	338	131	228	311
ENSECAG00000008003	4.720980522	0.09976311	0.256914882	267	330	426	469	608	532	694	780
ENSECAG00000024049	0.894466405	0.099808821	0.256931537	15	37	55	46	33	21	27	33
ENSECAG00000007640	7.189991546	0.099820684	0.256931537	2317	3720	3677	1870	1901	2762	3077	1702
ENSECAG00000002581	4.794323477	0.099843172	0.256931537	183	508	261	605	648	534	761	887
ENSECAG00000014209	3.552108542	0.099860031	0.256931537	91	128	164	280	297	284	236	365

ENSECAG00000023139	2.954615896	0.099901173	0.256979199	48	11	101	220	236	64	293	293
ENSECAG00000012963	1.860885744	0.099979539	0.257122573	29	36	54	72	85	78	77	129
ENSECAG00000021007	6.593506369	0.100074164	0.257307684	1062	2677	2093	1872	1707	1712	1582	1630
ENSECAG00000024666	4.421214853	0.100522204	0.258401196	317	620	358	356	394	401	302	362
ENSECAG00000010270	2.16016704	0.100602088	0.258548049	10	43	72	108	65	95	160	159
ENSECAG00000012929	5.868435534	0.100746397	0.258860372	1094	664	1462	1366	1286	700	855	1242
ENSECAG00000007311	5.983499489	0.10085514	0.259055134	612	1008	1698	1931	1067	1150	1037	1169
ENSECAG00000013768	6.412771805	0.100867797	0.259055134	2062	1781	1234	1250	1451	1231	1428	1666
ENSECAG00000010440	5.260805654	0.100924647	0.259127145	565	1064	582	741	625	519	713	840
ENSECAG00000005185	2.304899987	0.100941449	0.259127145	10	112	24	81	176	43	139	202
ENSECAG00000022023	6.416183189	0.100997605	0.259212736	1134	2397	1671	1465	1517	1112	1705	1599
ENSECAG00000021391	6.819711263	0.101039062	0.259260574	2017	2194	2303	2199	1938	1873	1939	2069
ENSECAG00000012473	2.460898269	0.101205516	0.259629052	24	139	83	266	58	71	72	136
ENSECAG00000012315	7.756631578	0.101249689	0.259683738	2222	2028	4087	3233	5468	4210	5060	7750
ENSECAG00000010019	1.374885785	0.10133509	0.259844118	24	40	29	34	71	59	55	75
ENSECAG00000020652	4.031131169	0.101659821	0.26061798	176	163	237	337	421	308	440	468
ENSECAG00000000110	3.190604083	0.101683158	0.260619004	92	152	67	142	218	220	119	410
ENSECAG00000016569	2.676283613	0.101850877	0.260930222	42	60	134	103	130	117	154	268
ENSECAG00000017129	3.934101214	0.101864019	0.260930222	177	174	215	275	379	341	349	452
ENSECAG00000025030	5.973143724	0.101888338	0.260930222	671	1015	1458	2020	1080	1039	994	1342
ENSECAG00000010027	2.660763183	0.101896444	0.260930222	105	109	160	127	101	56	116	152
ENSECAG00000023250	3.373659347	0.101925341	0.26094541	71	116	183	208	254	177	246	387
ENSECAG00000020524	3.238697427	0.10202951	0.261136596	106	179	263	215	142	146	191	164
ENSECAG00000008772	6.3871444	0.102045985	0.261136596	1550	1933	1344	1513	1473	1256	1468	1646
ENSECAG00000019205	5.686811487	0.10230034	0.261728545	953	1459	834	627	911	737	821	1062
ENSECAG00000008887	6.242424968	0.102337196	0.261763896	650	784	1243	1693	2038	1511	1967	2072
ENSECAG00000020042	1.813374746	0.102389497	0.261838726	35	25	60	54	85	95	121	54
ENSECAG00000018238	6.278048186	0.10249645	0.262053255	1072	1737	1700	1520	1462	1165	1428	1434
ENSECAG00000019405	2.981164699	0.102525305	0.262068058	135	151	156	171	141	132	138	122
ENSECAG00000000482	5.474285939	0.102778791	0.262622322	397	1025	957	1254	734	737	941	697
ENSECAG00000010771	4.832462813	0.102788371	0.262622322	309	457	331	478	649	657	730	782
ENSECAG00000013437	4.776008923	0.10286445	0.262757617	328	208	418	556	824	502	790	639
ENSECAG00000008653	4.885949504	0.102909891	0.262779548	427	747	494	605	510	414	539	639
ENSECAG00000021039	6.739487822	0.102932361	0.262779548	1554	2296	1992	2609	1677	2127	1722	1857
ENSECAG00000013165	2.213980547	0.102956708	0.262779548	77	85	107	94	69	86	64	86
ENSECAG00000001389	6.127912037	0.102965547	0.262779548	927.011	1669	1167.01	1760	1005	1104	1128	1723
ENSECAG00000018542	6.893073757	0.103002873	0.262815775	1280	1852	1585	1620	3139	2574	2861	3500
ENSECAG00000023238	7.145749736	0.103050992	0.262879518	1918	3568	2621	3180	2270	2599	1975	2856
ENSECAG00000019092	3.048181018	0.103129862	0.263021659	95	107	107	124	190	152	231	267
ENSECAG00000021071	5.253342692	0.103280848	0.26334762	409	738	910	995	717	557	663	800
ENSECAG00000000962	0.750583227	0.103311713	0.263367217	14	35	39	51	18	30	23	30
ENSECAG00000000129	7.377197891	0.103381194	0.263445514	1503	1695	2727	3266	3544	4605	3349	5486
ENSECAG00000009689	0.660161683	0.103406831	0.263445514	13	50	36	32	19	39	18	10
ENSECAG00000015525	1.196478938	0.103411987	0.263445514	14	41	20	31	38	24	79	103
ENSECAG00000021091	4.912011367	0.103499233	0.263561661	428	530	624	769	574	547	506	514
ENSECAG00000024767	4.337825999	0.103503972	0.263561661	248	584	300	467	284	379	369	354
ENSECAG00000024419	4.817591282	0.103541744	0.263595424	347	420	682	814	451	450	536	577
ENSECAG00000012875	6.602870332	0.103563631	0.263595424	1193	2456	1884	2154	1674	1687	1696	1723
ENSECAG00000022859	5.883424475	0.10373321	0.263967912	444	1090	669	1096	1266	1225	1333	2185
ENSECAG00000008658	1.1996227	0.103861174	0.264234361	12	24	25	57	39	51	63	81
ENSECAG00000018313	2.836495137	0.103901936	0.26427889	64	87	124	103	180	129	241	173
ENSECAG00000008844	3.786144949	0.104032306	0.264551266	353	333	198	152	185	320	166	191
ENSECAG00000025223	0.941943299	0.104241095	0.265022896	18	42	33	67	41	17	27	33
ENSECAG00000021990	4.144191734	0.104326533	0.265180775	141	195	276	409	488	359	446	468
ENSECAG00000010772	5.370262293	0.104393142	0.265282653	372	488	628	908	1150	779	926	1267
ENSECAG00000021619	4.340266193	0.104417063	0.265282653	141	277	323	427	466	499	432	620
ENSECAG00000011072	4.581565889	0.104436658	0.265282653	189	412	530	917	368	368	407	519
ENSECAG00000021986	6.026661637	0.104511399	0.26541317	693	911	946	1103	1586	1309	1673	1915
ENSECAG00000024113	6.929787132	0.104555393	0.26546556	1448	911	2110	1891	3930	1690	2683	4586
ENSECAG00000010995	3.752981741	0.104586492	0.265485194	123	115	258	214	630	236	294	226
ENSECAG00000008052	5.586512718	0.104612991	0.265493144	445	671	858	607	1848	617	1388	1053
ENSECAG00000021997	6.482718055	0.104643262	0.265500171	967	1248	1290	1382	2526	1420	2333	2791
ENSECAG00000013636	8.957550867	0.104662495	0.265500171	3795	8522	5216	8861	12722	14029	15196	9022
ENSECAG00000006219	4.438542628	0.104810362	0.265815923	246	287	281	387	565	465	621	487
ENSECAG00000006080	3.684954342	0.105091015	0.266468225	163	180	169	148	383	200	353	376
ENSECAG00000010600	0.423446004	0.105213225	0.266718578	10	34	23	44	16	19	13	30
ENSECAG00000015666	6.306807582	0.105262539	0.26673109	952	1037	2232	2288	1392	1009	1273	1929
ENSECAG00000022449	4.070537447	0.105273965	0.26673109	184	247	260	215	407	325	459	490
ENSECAG00000013393	5.865391254	0.105288588	0.26673109	745	1458	1283	1172	972	1494	705	706
ENSECAG00000002400	3.247183355	0.105341348	0.26680526	91	74	180	172	218	219	271	249
ENSECAG00000015009	3.635136212	0.105438834	0.266992395	122	160	163	242	311	233	268	443
ENSECAG00000018012	1.359799078	0.105465383	0.266992395	4	53	159	36	5	70	13	27
ENSECAG00000012328	7.254157624	0.10548573	0.266992395	1606	1798	2026	2964	4121	3072	4150	4153
ENSECAG00000013439	1.418128267	0.105638455	0.267319405	17	104	52	61	18	63	10	56
ENSECAG00000017677	8.184656589	0.105763786	0.267576961	1800	2991	4618	7077	6266	6808	7554	9555
ENSECAG00000024920	6.364035298	0.105928162	0.267818242	616	1170	1070	1889	2240	1739	1666	2667
ENSECAG00000023498	2.209441403	0.105936417	0.267818242	36	62	60	88	145	87	107	128
ENSECAG00000000753	6.436102916	0.105949335	0.267818242	898	1102	1108	1771	2310	1702	2412	2221
ENSECAG00000022644	0.634385162	0.105953441	0.267818242	33	22	23	45	13	38	19	18
ENSECAG00000020149	4.250893229	0.105997196	0.267869249	193	198	287	405	448	455	470	507
ENSECAG00000021887	1.199905537	0.106055068	0.2679559	14	26	41	34	41	73	57	57
ENSECAG00000000759	4.430717274	0.106362183	0.268672104	314	431	521	393	362	384	392	380
ENSECAG00000017672	2.102333866	0.106417752	0.268730126	39	23	93	61	143	84	88	131
ENSECAG00000018353	6.029130264	0.106432456	0.268730126	491	697	987	1632	1620	1220	2052	1677
ENSECAG00000019157	6.245199395	0.106599208	0.269091358	1362	1735	1093	1599	1148	1266	1424	1461

ENSECAG00000014762	5.948544669	0.106678458	0.269231595	654	734	760	1353	1661	1071	1754	1695
ENSECAG00000017850	4.887233795	0.106872011	0.269654169	418	403	612	968	567	558	335	609
ENSECAG00000022013	3.412685205	0.106893362	0.269654169	118	168	345	242	185	175	126	238
ENSECAG00000008628	0.895747451	0.106929633	0.269685792	30	28	51	37	38	17	31	29
ENSECAG00000017870	4.496415025	0.106957831	0.269689969	166	359	228	513	481	376	549	923
ENSECAG00000008661	6.283437287	0.106978762	0.269689969	1021	1505	1734	1902	1601	1292	1428	1162
ENSECAG00000018885	3.749523888	0.10710769	0.269913757	181	172	337	400	209	195	250	286
ENSECAG00000000855	5.102953811	0.107125352	0.269913757	537	803	587	692	591	626	582	629
ENSECAG00000019327	2.433306849	0.107142006	0.269913757	28	19	140	29	250	61	301	13
ENSECAG00000011271	0.592936031	0.107162556	0.269913757	16	79	21	7	32	20	14	10
ENSECAG00000008697	3.795156529	0.107215865	0.269988177	96	207	157	325	326	298	436	333
ENSECAG00000008211	4.231889549	0.107407067	0.270386795	156	225	316	305	349	568	200	836
ENSECAG00000011680	0.705507342	0.107421756	0.270386795	7	21	24	27	51	37	46	27
ENSECAG00000008134	1.107232271	0.107522724	0.270580994	20	52	51	51	32	27	43	33
ENSECAG00000021118	3.130784899	0.107634442	0.27075724	74	26	135	211	241	80	331	309
ENSECAG00000016953	0.522395805	0.107652883	0.27075724	15	6	22	22	40	20	43	42
ENSECAG00000019014	2.698183162	0.107684863	0.27075724	51	94	102	101	146	150	146	213
ENSECAG00000015324	5.751502585	0.107706159	0.27075724	995	1035	1043	991	1135	666	908	1130
ENSECAG00000014019	4.261394927	0.10771191	0.27075724	360	428	302	352	364	286	315	376
ENSECAG00000017197	6.000624428	0.107811385	0.270947348	565	919	964	1221	1725	1243	1579	1816
ENSECAG00000003905	3.657761703	0.1080356	0.271450792	172	346	159	330	172	191	179	322
ENSECAG00000019360	5.286051264	0.108171031	0.271730986	444	876	854	894	648	615	646	898
ENSECAG00000012922	4.988758285	0.108270065	0.271919646	241	173	507	905	858	744	916	714
ENSECAG00000018942	5.127340221	0.108300392	0.271935702	329	328	556	816	906	742	857	964
ENSECAG00000024267	5.221016152	0.108368319	0.272046142	385	292	612	836	1090	854	862	906
ENSECAG00000005790	0.758983928	0.108418494	0.272078601	24	78	19	12	29	23	18	23
ENSECAG00000009317	5.38846272	0.108429142	0.272078601	543	835	976	947	697	949	661	603
ENSECAG00000014446	10.08992566	0.108483603	0.272155154	11265	7164	19662	20183	30112	20365	31618	31912
ENSECAG00000017720	5.629492446	0.108585889	0.272351626	491	893	1353	1269	791	730	832	1200
ENSECAG00000012684	2.073601873	0.108664173	0.272487824	17	62	45	97	79	72	164	124
ENSECAG00000013120	7.621245874	0.108697222	0.272510555	2782	4684	4352	3733	3302	3402	3187	3598
ENSECAG00000013456	4.555612976	0.108744071	0.272567866	355	668	393	379	424	349	449	408
ENSECAG00000007532	6.152518626	0.10879387	0.272632542	791	727	1085	1416	1810	1390	1971	1916
ENSECAG00000011983	4.124412993	0.108837946	0.272682853	163	232	257	335	380	345	434	581
ENSECAG00000001211	3.781928006	0.108912922	0.272810543	140	621	123	244	157	236	260	203
ENSECAG00000013780	2.792637331	0.108951003	0.272845778	89	62	81	121	192	96	182	250.001
ENSECAG00000007722	2.048692609	0.10897888	0.27285545	50	17	89	27	123	44	143	142
ENSECAG00000006638	5.163185669	0.1090457	0.272955842	251	502	402	874	766	559	758	1659
ENSECAG00000013738	2.63801389	0.109067023	0.272955842	61	54	98	120	197	106	158	171
ENSECAG00000006958	4.785987638	0.109127211	0.273046326	337	754	524	525	446	475	485	536
ENSECAG00000006093	6.648977381	0.109360072	0.273516085	739	1098	1658	2278	2231	2444	3038	2357
ENSECAG00000022752	6.547383847	0.109363103	0.273516085	939	1020	1565	1761	2514	1856	2221	2790
ENSECAG00000022901	4.457966938	0.109432233	0.273588196	230	400	226	333	513	456	580	652
ENSECAG00000012720	4.131672616	0.109440094	0.273588196	135	490	381	432	273	246	277	420
ENSECAG00000002541	7.370266425	0.109662939	0.274084979	2012	2650	1786	2043	4605	3633	4759	3899
ENSECAG00000000409	1.859926707	0.109694666	0.274103981	60	73	80	69	52	65	45	75
ENSECAG00000007533	7.179093407	0.109767851	0.274226543	2351	332	2265	264	7411	998	6548	2242
ENSECAG00000010986	7.51972039	0.109920021	0.274546334	4622	3391	2623	3180	3392	2586	3070	3209
ENSECAG00000017297	2.028335125	0.109946855	0.274553004	30	58	81	21	94	139	123	64
ENSECAG00000009602	6.45306797	0.110127201	0.274942926	866	1002	1556	1530	2177	2115	2088	2305
ENSECAG00000009024	5.3270886	0.110208395	0.275052072	295	401	540	1120	853	1016	1176	960
ENSECAG00000019602	3.304140638	0.110219335	0.275052072	92	62	217	143	182	273	167	410
ENSECAG00000022217	5.025066501	0.110303737	0.275202253	369	339	559	567	784	673	782	988
ENSECAG00000011690	1.795091327	0.110432485	0.275445468	43	44	37	46	94	86	88	79
ENSECAG00000002267	6.747909883	0.110449705	0.275445468	1806	3010	1478	1983	1749	1645	1850	2136
ENSECAG00000016667	6.65136455	0.110506634	0.275526964	2568	3229	455	1535	1088	1963	1594	1416
ENSECAG00000020973	2.975781933	0.110557073	0.275547835	141	279	108	80	106	147	104	136
ENSECAG00000020561	0.76923929	0.110563508	0.275547835	15	17	28	20	42	26	34	71
ENSECAG00000000547	5.92582319	0.110656271	0.275718541	620	594	977	1306	1404	1445	1448	1721
ENSECAG00000009435	2.962947166	0.110887668	0.276189885	59	84	128	161	230	149	167	244
ENSECAG00000014967	3.670376804	0.110894055	0.276189885	166	119	163	231	389	214	293	396
ENSECAG00000016315	4.052836282	0.111082588	0.27656282	219	197	174	303	448	397	405	397
ENSECAG00000015738	1.552784819	0.111092476	0.27656282	32	20	24	70	55	55	85	109
ENSECAG00000023142	0.387596864	0.111144688	0.276632189	10	13	16	21	13	40	30	46
ENSECAG00000022980	0.566810767	0.111176713	0.276651296	17	21	55	26	31	15	16	26
ENSECAG00000009254	0.924489513	0.111244444	0.276728688	17	72	27	37	23	33	25	31
ENSECAG00000015452	2.204147734	0.111270671	0.276728688	31	48	57	111	136	67	99	177
ENSECAG00000015339	2.642547685	0.111280882	0.276728688	64	168	113	202	43	52	53	248
ENSECAG00000024839	4.29499614	0.111310273	0.276741209	171	231	290	415	606	247	559	581
ENSECAG00000020937	4.994569129	0.111389581	0.2768778	312	243	622	595	976	405	858	1036
ENSECAG00000023300	6.74021124	0.111508172	0.277085359	1694	2756	1482	2441	1640	1587	1503	2707
ENSECAG00000017978	3.365346978	0.111525093	0.277085359	133	277	191	208	190	163	131	223
ENSECAG00000012611	6.324718409	0.111546245	0.277085359	894	1330	1001	1094	1906	1554	2518	2063
ENSECAG00000017883	6.232679036	0.111580872	0.277110791	2094	1980	511	1016	1353	911	1210	1355
ENSECAG00000015728	2.106837922	0.111646979	0.277196702	40	52	56	74	87	78	92	188
ENSECAG00000006166	3.776344582	0.111665112	0.277196702	143	218	170	210	393	262	389	321
ENSECAG00000015099	7.44718302	0.111688655	0.277196702	2087	2195	2584	2345	3763	3573	4967	5567
ENSECAG00000004461	1.245318582	0.111768344	0.277237306	45	53	33	53	40	38	19	51
ENSECAG00000012520	0.319560714	0.111775318	0.277237306	6	16	20	15	23	19	33	50
ENSECAG00000021657	11.33840187	0.111778217	0.277237306	36231	63692	47747	61489	40798	50634	31871	50769
ENSECAG00000008992	6.264147663	0.111896273	0.277447157	1024	1857	1277	1872	1370	1094	1620	1353
ENSECAG00000022378	4.020138133	0.111911663	0.277447157	137	79	306	277	751	301	594	90
ENSECAG00000007066	2.66565507	0.111966868	0.277523463	37	102	89	96	98	260	182	101
ENSECAG00000013937	2.358007435	0.112012109	0.277575046	69	48	66	58	89	180	176	76
ENSECAG00000017040	5.454415383	0.112041172	0.277586522	554	901	851	1121	828	760	729	840

ENSECAG00000024555	5.558214803	0.112085902	0.277636803	606	614	576	794	1195	866	1277	1348
ENSECAG00000000751	3.073279856	0.112124344	0.277671489	48	75	156	171	117	329	257	152
ENSECAG00000010826	4.711212059	0.112605949	0.278803398	175	386	325	636	683	454	698	803
ENSECAG00000012383	4.838763909	0.112631185	0.278805124	322	297	443	562	679	696	726	703
ENSECAG00000021929	5.498775025	0.112784253	0.279120761	483	474	795	827	1239	1014	993	1203
ENSECAG00000019876	1.69904325	0.112807827	0.279120761	22	34	51	66	95	76	73	79
ENSECAG00000017554	6.161770633	0.112838289	0.279135344	1110	1556	1356	1459	1289	1019	1378	1433
ENSECAG00000003368	7.662250462	0.112930628	0.27930296	2542	2273	2798	3105	5934	4089	5601	4983
ENSECAG00000020781	5.722633104	0.112978252	0.279359934	524	715	953	779	1092	1242	1527	1335
ENSECAG00000014401	2.807847371	0.11301931	0.279400654	66	67	143	82	248	105	225	142
ENSECAG00000012470	2.735878865	0.113070386	0.279466113	119	159	161	79	133	108	118	64
ENSECAG00000019914	7.262220998	0.113307348	0.279990885	1293	1517	7292	4609.04	2665	3991	1314	342.004
ENSECAG00000012884	7.100398825	0.113436332	0.280225567	1902	3150	2708	3069	2237	2216	2526	2586
ENSECAG00000009627	6.091320285	0.113451646	0.280225567	1380	1677	1142	892	1440	904	1115	1231
ENSECAG00000021435	4.144313055	0.113546842	0.280399742	212	366	468	348	373	244	352	267
ENSECAG00000010871	1.203021424	0.113619358	0.280517849	19	30	26	41	59	44	46	82
ENSECAG00000003845	8.730594553	0.113866099	0.281029751	3298	15722	11701	5531	4127	9372	8276	3800
ENSECAG00000015812	7.591268376	0.113876164	0.281029751	4063	3694	3147	3944	3171	2739	2481	4846
ENSECAG00000009390	7.998663122	0.113927457	0.281095282	2594	3300	3548	4558	6128	5309	6528	8124
ENSECAG00000018887	8.060740751	0.113989838	0.281178148	2182	3456	4221	4950	7166	5009	8512	6543
ENSECAG00000013668	6.967910583	0.114027405	0.281178148	1066	2850	2488	4222	1748	2279	1761	2768
ENSECAG00000015042	7.079278312	0.114035285	0.281178148	1403	1860	1789	2382	3538	2616	3612	3919
ENSECAG00000012075	0.848715807	0.114247883	0.281641233	33	56	24	23	29	21	27	32
ENSECAG00000012945	6.170943885	0.114369354	0.281879522	679	1264	877	1151	1806	1159	2146	2169
ENSECAG00000010671	3.446004666	0.114429526	0.281966661	95	158	124	228	307	185	275	332
ENSECAG00000024018	4.514464794	0.114498119	0.282074506	382	150	924	470	398	518	309	185
ENSECAG00000021005	5.789285344	0.114547448	0.282105493	576	682	671	1213	1501	835	1457	1775
ENSECAG00000010663	5.863151086	0.114560354	0.282105493	1143	532	1729	1308	1598	821	888	479
ENSECAG00000013094	0.601095412	0.114702534	0.282394408	12	13	19	23	42	73	13	22
ENSECAG00000012993	3.766807771	0.114770444	0.282398232	193	92	736	202	100	437	90	110
ENSECAG00000022012	0.741073391	0.114776479	0.282398232	20	10	31	8	54	16	64	41
ENSECAG00000006824	2.426259193	0.114778651	0.282398232	60	125	88	169	76	83	67	133
ENSECAG00000022461	4.462589851	0.114972782	0.282733172	164	291	330	500	544	410	632	605
ENSECAG00000019749	3.650349315	0.114973101	0.282733172	156	167	161	178	348	191	355	377
ENSECAG00000017542	2.791702319	0.114995471	0.282733172	64	64	116	99	72	331	156	146
ENSECAG00000009536	6.993389421	0.115014321	0.282733172	1359	1729	1675	2244	3328	2944	2908	3629
ENSECAG00000010099	2.619962315	0.115095265	0.28287095	34	193	160	135	81	101	75	136
ENSECAG00000015527	1.138391856	0.115371232	0.283487876	14	96	42	34	8	36	28	52
ENSECAG00000016697	3.604370363	0.115403197	0.283505109	116	158	207	191	355	229	316	309
ENSECAG00000007198	8.628896935	0.115494319	0.283620113	2535	4422	4631	10612	11616	9550	12391	7210
ENSECAG00000024175	0.729025108	0.115499935	0.283620113	17	26	50	40	24	20	26	33
ENSECAG00000014556	5.498973252	0.115557848	0.28370101	460	706	597	803	1212	907	1134	1194
ENSECAG00000012567	3.010681125	0.115697561	0.283912358	90	43	113	182	253	175	215	179
ENSECAG00000013994	3.922854258	0.115697985	0.283912358	158	136	268	286	399	345	420	336
ENSECAG00000004913	3.531145072	0.115718899	0.283912358	77	237	172	121	220	284	335	338
ENSECAG00000003124	7.638325632	0.115786554	0.284017018	2626	4826	3766	4694	3188	2948	4136	3491
ENSECAG00000019455	1.653047661	0.115820307	0.284026337	23	46	46	48	94	55	78	88
ENSECAG00000004670	4.421810416	0.115845575	0.284026337	541	405	106	736	607	271	82	321
ENSECAG00000009910	5.253552695	0.115884653	0.284026337	202	361	812	829	1151	1076	895	695
ENSECAG00000013523	4.216532509	0.115890345	0.284026337	170	298	252	323	420	353	452	632
ENSECAG00000014343	1.691708016	0.11597539	0.284173471	20	73	70	116	35	56	63	47
ENSECAG00000009504	7.227613587	0.116000983	0.284174896	1423	1649	2488	2800	4308	2737	3791	4398
ENSECAG00000011340	4.035834382	0.116031227	0.284187713	124.011	244	155.008	405	370.001	301	409.001	593
ENSECAG00000012206	1.867435119	0.116156359	0.284432876	30	50	27	81	93	59	124	97
ENSECAG00000019621	6.023992708	0.116181879	0.284434068	701	835	918	1249	1558	1372	1634	1863
ENSECAG00000010849	6.787161869	0.116252863	0.284533817	1141	1107	2052	1806	3151	1959	2992	3085
ENSECAG00000016918	6.734602087	0.116272708	0.284533817	1762	2130	2158	2142	1895	1543	2008	2078
ENSECAG00000008231	2.668637612	0.116311038	0.284566326	38	220	148	129	71	83	135	117
ENSECAG00000008082	6.700199289	0.116398333	0.284718591	1593	3865	1215	1459	1886	1125	2142	1703
ENSECAG00000025090	5.530477008	0.116445679	0.284773096	566	650	961	1595	1004	696	700	895
ENSECAG00000022139	1.766070868	0.116529008	0.284915557	28	79	132	54	21	95	38	39
ENSECAG00000009961	4.006936486	0.116601424	0.285031277	188	453	250	377	285	214	294	333
ENSECAG00000019123	2.379363994	0.11671597	0.285249914	52	52	81	88	141	71	151	169
ENSECAG00000012815	6.974777548	0.116917571	0.28568117	2726	3042	1704	2030	1993	2347	1964	2106
ENSECAG00000010902	4.85912108	0.116949003	0.285696533	472	798	415	514	441	473	577	534
ENSECAG00000014906	5.130642654	0.116989827	0.285734826	408	878	663	811.008	461	747	593	654
ENSECAG00000007479	5.66457116	0.117039208	0.285736606	522	706	804	847	1406	956	1413	1208
ENSECAG00000010431	6.350117376	0.117063768	0.285736606	967	983	1091	1519	1909	1718	1968	2531
ENSECAG00000012013	5.955178193	0.117066001	0.285736606	752	524	890	1321	1645	1265	1711	1557
ENSECAG00000022333	4.691947003	0.117148755	0.28587718	329	329	862	585	521	232	420	640
ENSECAG00000023135	1.07818449	0.117214139	0.285923304	15	28	36	25	44	38	68	61
ENSECAG00000008025	2.871348225	0.117217986	0.285923304	49	64	80	217	187	172	156	235
ENSECAG00000010415	2.480210705	0.117275675	0.286002623	55	151	128	118	68	111	89	97
ENSECAG00000003955	6.651217985	0.117356826	0.28613911	961	1170	1155	2433	2334	2521	2179	3070
ENSECAG00000009146	4.252461496	0.117389363	0.286157035	125	317	199	449	494	353	488	590
ENSECAG00000014926	3.884241295	0.117446704	0.286235403	150	188	206	287	377	272	414	400
ENSECAG00000018522	3.360530768	0.117508647	0.286246301	83	103	204	175	339	201	291	199
ENSECAG00000026932	5.610309474	0.117509123	0.286246301	592	393	916	728	1418	840	1763	904
ENSECAG00000000869	4.496834839	0.117526755	0.286246301	247	264	391	351	465	400	679	713
ENSECAG00000024530	5.752919476	0.117562151	0.286271145	546	714	1733	1451	760	857	1021	1169
ENSECAG00000013938	3.813836622	0.117720104	0.286594348	164	185	159	263	308	294	386	412
ENSECAG00000012601	5.297258994	0.117951352	0.287017378	616	391	1048	1110	770	666	755	526
ENSECAG00000016296	5.438995187	0.117956586	0.287017378	681	1160	668	784	634	793	796	820
ENSECAG00000007248	5.656898168	0.117969649	0.287017378	408	584	797	1195	1214	1123	1133	1515
ENSECAG00000022228	6.051228827	0.118002071	0.287034796	960	2004	1113	1018	973	1314	1091	1166

ENSECAG00000010941	4.642243248	0.118089507	0.287185997	224	189	412	594	694	795	487	499
ENSECAG00000020147	4.071932709	0.118129862	0.287187728	186	172	233	357	445	314	430	484
ENSECAG00000006923	5.498057543	0.118140771	0.287187728	544	1159	930	876	832	568	780	1073
ENSECAG00000023726	5.747048405	0.118297879	0.287508129	411	692	742	1307	1399	1490	1104	1284
ENSECAG00000012154	5.870719226	0.118531465	0.288014222	694	1409	1137	1326	1021	912	1104	1168
ENSECAG00000011263	4.392693484	0.118762337	0.288480958	263	255	287	347	596	375	589	521
ENSECAG00000024998	3.1141512	0.118774329	0.288480958	73	140	116	114	377	107	243	167
ENSECAG00000019817	5.335277417	0.118939366	0.288820064	516	448	588	681	1191	720	1005	1091
ENSECAG00000009153	7.384059673	0.119001005	0.288907996	2567	4763	2099	3752	2910	2071	2728	3762
ENSECAG00000001174	3.625786	0.119166672	0.289248393	89	86	155	133	30	1185	22	140
ENSECAG00000010665	3.64201997	0.119354481	0.289622967	80	160	211	268	288	264	332	362
ENSECAG00000019849	7.118196626	0.119371973	0.289622967	1575	2113	1632	2130	3480	2828	3281	4551
ENSECAG00000009749	0.512814558	0.119398217	0.289624795	8	38	21	57	19	34	10	14
ENSECAG00000022179	8.031946956	0.1195124	0.28983989	4710	7173	3902	4368	4708	3718	4467	4841
ENSECAG00000010668	6.081304713	0.119543427	0.289853269	592	852	1063	1425	1519	1438	1615	2158
ENSECAG00000010508	5.936753733	0.11970568	0.290184753	591	889	778	1187	1914	1053	1915	1211
ENSECAG00000015518	6.011555638	0.119784789	0.290314585	577	761	1015	1412	1499	1488	1557	1818
ENSECAG00000021306	5.348964158	0.120028299	0.290797159	481	494	614	707	1124	869	924	1083
ENSECAG00000022797	6.078597288	0.120089333	0.290797159	920	1320	1590	1403	1232	1008	1211	1423
ENSECAG00000015869	4.42999491	0.120100605	0.290797159	469	594	309	222	378	298	374	400
ENSECAG00000000685	11.51510706	0.120114144	0.290797159	48674	89548	48312	41327	48750	43130	51299	51365
ENSECAG00000023898	1.083400597	0.120135876	0.290797159	39	61	27	55	7	74	16	6
ENSECAG00000020137	1.212560592	0.120137464	0.290797159	22	42	74	52	24	28	54	39
ENSECAG00000018420	3.057513708	0.120304305	0.291138981	42	74	194	145	200	227	138	293
ENSECAG00000005100	4.189647617	0.120457701	0.291401926	216	316	464	447	312	302	340	347
ENSECAG00000012228	5.743611728	0.120464253	0.291401926	664	1553	819	1121	892	852	924	1134
ENSECAG00000002992	4.841442709	0.120575448	0.291608821	83	1114	386	1057	190	453	328	797
ENSECAG00000012469	5.980964706	0.120698924	0.291845324	964	1382	1197	1269	1174	908	1249	1208
ENSECAG00000010158	1.497724296	0.120755085	0.291918994	22	95	64	50	33	57	30	53
ENSECAG00000007916	6.457697302	0.120797209	0.291958708	983	2923	1435	1629	1064	1738	1468	1710
ENSECAG00000022953	2.177891385	0.12100137	0.292389954	49	47	67	75	116	98	119	117
ENSECAG00000001649	9.111914155	0.121073345	0.292501669	3506	8054	5601	13409	13900	15532	17555	9682
ENSECAG00000015206	4.962655759	0.12112052	0.292553432	323	893	594	658	406	662	543	550
ENSECAG00000010387	5.640700993	0.121155267	0.292575163	450	508	959	968	1030	1196	1037	1697
ENSECAG00000015867	2.054495824	0.121269227	0.292788133	45	86	76	133	81	50	70	76
ENSECAG00000019802	5.975817229	0.121486843	0.293242999	764	1814	1799	669	1449	1196	870	547
ENSECAG00000022619	2.656205999	0.121509245	0.293242999	52	142	120	222	69	110	134	99
ENSECAG00000016681	6.95658562	0.121596625	0.293391559	1132	1717	1959	2122	2879	2715	2744	4225
ENSECAG00000017845	1.388680863	0.121625106	0.293397974	24	20	38	54	60	63	59	78
ENSECAG00000008058	2.146140954	0.121818581	0.293802317	46	41	62	86	107	94	122	119
ENSECAG00000008456	4.017872304	0.122020264	0.294226281	190	259	154	265	444	280	388	523
ENSECAG00000011702	3.767826695	0.122055235	0.294248161	106.984	429	180.985	419.976	179.983	209.988	220.971	309.976
ENSECAG00000015983	4.701476231	0.122296786	0.294767943	292	266	459	451	653	521	724	665
ENSECAG00000005161	6.256109405	0.122542244	0.295296919	945	1815	1046	2380	887	1558	1343	1511
ENSECAG00000013436	6.268596529	0.122631773	0.295450001	1102	910	1024	1059	2148	1471	2115	1984
ENSECAG00000000306	5.936805494	0.122678222	0.29549925	674	821	952	1017	1572	1251	1442	1771
ENSECAG00000012966	5.012403963	0.122730442	0.295553501	241	490	390	752	663	764	659	1141
ENSECAG00000018009	4.244929726	0.12275277	0.295553501	177	280	260	358	412	341	523	611
ENSECAG00000001101	5.654118237	0.122814162	0.295638667	486	884	1059	1765	704	679	757	1471
ENSECAG00000007885	3.458113773	0.123085651	0.296229435	107	177	133	182	285	206	239	375
ENSECAG00000011347	3.109725203	0.123153047	0.296328869	74	110	166	118	201	153	259	257
ENSECAG00000000519	2.42419499	0.123211186	0.296405991	42	50	65	131	84	128	106	239
ENSECAG00000018835	1.565325119	0.123423184	0.296853136	5	72	31	24	64	29	92	144
ENSECAG00000013922	9.209624415	0.123489765	0.296903801	8754	14566	11690	11632	9778	8416	10037	12659
ENSECAG00000016963	3.330712393	0.12350205	0.296903801	100	136	167	145	256	193	296	255
ENSECAG00000023837	6.06539485	0.123522643	0.296903801	759	1356	1700	1514	1095	1461	1070	1056
ENSECAG00000021913	5.462990809	0.123564483	0.296941552	441	1126	1003	982	492	1096	627	805
ENSECAG00000024363	5.392667919	0.123632065	0.297041133	603	778	734	1166	576	794	704	948
ENSECAG00000022499	0.819615675	0.123658833	0.297042635	8	11	37	32	28	46	44	59
ENSECAG00000010514	1.974777016	0.123691557	0.297058439	61	111	64	68	51	74	60	69
ENSECAG00000012128	3.887786418	0.123779192	0.297206082	248	268	411	246	180	429	153	190
ENSECAG00000010947	4.490368624	0.123831073	0.297267832	675	424	454	230	689	329	194	36
ENSECAG00000020633	7.779073835	0.12391681	0.297369081	2411.01	2865	2866.01	3854	5721	4328	5752	6475
ENSECAG00000015559	6.49574765	0.123947621	0.297369081	1163	2565	1104	2321	1759	1388	1376	1767
ENSECAG00000016013	5.437360927	0.123951766	0.297369081	633	782	819	1107	847	661	816	824
ENSECAG00000014022	0.533144271	0.124098752	0.297658861	13	36	40	25	19	18	29	21
ENSECAG00000011074	2.43482473	0.124153064	0.29772628	88	37	192	141	93	37	62	154
ENSECAG00000011384	6.488903233	0.124402774	0.298262149	1626	3036	1042	1112	1614	1185	1605	1618
ENSECAG00000012755	4.205199896	0.124492768	0.298414944	84.0007	252	381.001	312.001	345.001	649	517.001	331.001
ENSECAG00000004412	0.548193496	0.124585753	0.298529446	14	38	36	29	16	34	19	15
ENSECAG00000000397	7.473575813	0.124593085	0.298529446	1866	2200	2713	3037	4269	3974	4724	4895
ENSECAG00000008612	3.853862921	0.124680023	0.298674767	145	173	288	188	410	284	359	385
ENSECAG00000011819	7.104579819	0.124761238	0.298806321	2435	2826	2570	2726	2384	2327	2206	2689
ENSECAG00000010712	5.040711893	0.124830988	0.298910366	574	750	616	516	644	535	560	592
ENSECAG00000007975	4.915388186	0.125095225	0.299479972	298	691	562	871	446	497	597	619
ENSECAG00000022295	7.146818777	0.125258793	0.299775807	1401	2596	3026	4853	2449	2515	2203	2656
ENSECAG00000012825	4.315508708	0.125292267	0.299775807	258	259	213	244	376	543	924	190
ENSECAG00000000014	4.080458204	0.12529795	0.299775807	105	273	231	965	368	252	266	197
ENSECAG00000000578	6.923031741	0.125371291	0.299834295	2441	2746	1842	2172	2176	1736	2553	1839
ENSECAG00000007660	6.550990052	0.125375175	0.299834295	1117	883	1528	1708	2217	2037	2467	2622
ENSECAG00000009110	4.498189343	0.12545492	0.29996187	278	251	359	342	742	398	644	458
ENSECAG00000009191	2.50319036	0.125567095	0.300166911	95	97	128	120	82	105	78	118
ENSECAG00000014668	4.105102986	0.125647256	0.30023345	218	182	237	251	681	189	568	328
ENSECAG00000010335	6.038208375	0.125647778	0.30023345	1407	1215	1122	1119	1221	972	1188	1256
ENSECAG00000017252	4.646062962	0.125694889	0.300264304	189	437	236	557	582	421	697	837

ENSECAG00000020969	7.14238707	0.125713545	0.300264304	2313	1919	3315	3785	2908	2684	2194	1836
ENSECAG00000009497	5.303123399	0.125769058	0.300282822	413	424	1440	1044	918	637	663	446
ENSECAG00000010218	0.780618228	0.125792723	0.300282822	7	15	21	43	35	69	26	39
ENSECAG00000023567	8.00457611	0.125800584	0.300282822	3228	4146	6205	7454	4474	4535	4374	4371
ENSECAG00000020570	5.965682001	0.126073654	0.300871425	599	502	1243	1226	1635	1051	1572	2058
ENSECAG00000023651	2.618480595	0.126555958	0.301925212	71	122	229	90	97	163	53	58
ENSECAG00000013756	2.787103009	0.126568367	0.301925212	84	113	185	175	140	90	137	102
ENSECAG00000005643	3.412635261	0.126753047	0.302302293	105	92	171	209	210	402	173	276
ENSECAG00000020859	1.569486572	0.126936001	0.302675098	32	35	96	83	52	49	35	54
ENSECAG00000017372	6.15669623	0.127030855	0.302837721	850	1422	1852	1493	1584	1128	1248	1100
ENSECAG00000008287	3.432025946	0.127064483	0.302854343	112	87	209	178	264	163	341	330
ENSECAG00000020627	4.236425021	0.12719135	0.303031142	203	472	327	498	333	224	336	455
ENSECAG00000005360	1.299342134	0.1272262	0.303031142	17	35	120	64	8	89	5	11
ENSECAG00000010746	4.097841057	0.127233528	0.303031142	179	141	340	297	483	322	384	527
ENSECAG00000025328	2.454349871	0.127245342	0.303031142	17	85	284	118	59	122	74	60
ENSECAG00000023445	2.380725083	0.127344846	0.303204556	66	52	66	87	128	115	132	145
ENSECAG00000007978	7.417020383	0.127464523	0.3033591	1573	1835	2703	3574	3921	4212	4246	4781
ENSECAG00000020319	2.020957178	0.12746807	0.3033591	47	26	83	45	118	67	109	123
ENSECAG00000020771	2.503635839	0.127489852	0.3033591	38	72	63	138	111	105	141	226
ENSECAG00000013409	6.222988952	0.127605244	0.303564325	791	1309	712	1262	1987	1165	1991	2438
ENSECAG00000008048	5.191191333	0.127672096	0.303564325	343	501	529	749	927	729	794	1150
ENSECAG00000017064	7.263790012	0.127696769	0.303564325	1910	3360	3466	3545	2201	3134	2527	2772
ENSECAG00000018042	5.988779009	0.127703891	0.303564325	883	916	901	589	1395	1353	1790	1856
ENSECAG00000020482	0.787707008	0.127724542	0.303564325	4	6	23	55	47	43	37	49
ENSECAG00000017196	1.568749476	0.127736405	0.303564325	34	75	58	70	45	41	31	77
ENSECAG00000003532	4.599735681	0.127837542	0.30365321	309	715	380	467	368	377	478	484
ENSECAG00000014495	6.788902393	0.12785284	0.30365321	1273	2249	2272	3106	1827	1864	1870	2283
ENSECAG00000013402	4.627370568	0.127853983	0.30365321	278	210	461	406	596	329	637	975.001
ENSECAG00000006063	2.20389596	0.127953938	0.30376816	55	45	61	76	94	76	138	161
ENSECAG00000023064	5.945267483	0.127961975	0.30376816	521	2338	805	1318	968	994	997	1234
ENSECAG00000010745	3.020720011	0.12798259	0.30376816	56	50	136	214	219	195	232	175
ENSECAG00000020181	7.429177037	0.128267921	0.304381812	1618	1444	2566	4150	4341	4043	4756	4270
ENSECAG00000012290	0.136295488	0.128495615	0.304770179	2	12	9	30	24	21	33	29
ENSECAG00000017566	2.339695363	0.128503944	0.304770179	40	210	107	59	60	109	54	84
ENSECAG00000012358	1.756433921	0.128519805	0.304770179	24	42	50	63	54	94	86	102
ENSECAG00000011246	0.419428962	0.128541153	0.304770179	17	42	24	24	5	30	8	31
ENSECAG00000005457	5.781657425	0.128565699	0.304770179	616	880	1338	1561	992	859	1022	1119
ENSECAG00000008293	4.500805897	0.128622599	0.304841462	181	68	520	265	930	255	1261	84
ENSECAG00000019979	7.233518904	0.128760512	0.305101727	1446	1591	2642	2770	3936	3200	3275	4804
ENSECAG00000023170	6.224173325	0.128786119	0.305101727	926	839	1249	981	2022	1086	1505	3091
ENSECAG00000001194	3.749366222	0.128880545	0.305261779	202	305	270	256	262	268	204	193
ENSECAG00000018305	7.10408515	0.128912541	0.305273924	1164	1667	2179	2851	3557	2697	3580	4024
ENSECAG00000002462	2.270463578	0.128949504	0.305297825	53	130	62	156	50	93	105	58
ENSECAG00000007847	7.337569425	0.128977851	0.305301323	3748	3913	2316	2081	2910	2246	2582	3150
ENSECAG00000024909	0.532063885	0.129090829	0.305455098	6	14	15	38	35	31	43	32
ENSECAG00000005795	4.703393421	0.129121553	0.305455098	199	243	473	614	541	664	756	606
ENSECAG00000023991	5.464434653	0.129123467	0.305455098	913	879	842	727	557	1258	552	539
ENSECAG00000024124	6.3799714	0.129163628	0.3054865	673	1371	1203	1441	2525	1190	2566	2102
ENSECAG00000008473	3.217429018	0.129380141	0.305934893	130.001	235	120.001	251.001	121.001	131	194.001	193.001
ENSECAG00000018186	6.039805316	0.129483275	0.306087551	996	1460	1210	1350	1089	963	1142	1568
ENSECAG00000009090	6.5237035	0.129498579	0.306087551	1931	2541	1248	1128	1305	1615	1445	1868
ENSECAG00000018114	5.473643422	0.129537165	0.306091385	353	445	767	1069	986	970	1002	1457
ENSECAG00000016543	8.159996342	0.129603235	0.306091385	3637	3322	3950	4317	7421	6295	7691	7522
ENSECAG00000006129	3.918656717	0.129607897	0.306091385	81	286	182	303	296	355	348	533
ENSECAG00000010236	5.302623733	0.129607961	0.306091385	398	765	952	1051	555	720	612	953
ENSECAG00000010993	2.770663906	0.129654979	0.306138792	69	87	92	122	159	123	194	207
ENSECAG00000018635	3.087564627	0.129731417	0.306255633	88	71	136	172	224	141	228	266
ENSECAG00000011773	3.474039824	0.129820165	0.306401478	174	122	115	144	327	155	329	329
ENSECAG00000006184	6.207786059	0.129856691	0.306424033	1828	1405	1041	1126	1209	1139	1308	1458
ENSECAG00000017861	7.013697688	0.129900586	0.306463966	2084	2198	2777	3028	2073	2240	2438	2330
ENSECAG00000010206	3.343779248	0.129977611	0.306559786	81	170	112	202	214	247	246	304
ENSECAG00000024362	4.831000978	0.129995163	0.306559786	251	354	428	641	613	645	704	825
ENSECAG00000016183	1.693409515	0.130200473	0.306980241	24	47	42	51	58	53	65	160
ENSECAG00000017550	7.360242621	0.130269592	0.307079483	2227	3162	2729	5161	2737	2506	2910	3403
ENSECAG00000000273	5.909258236	0.130456181	0.307455534	446	550	1342	1172	1483	1417	1458	1608
ENSECAG00000017091	2.604526904	0.130507496	0.307512685	42	47	106	145	129	139	171	174
ENSECAG00000018577	2.056225791	0.130571231	0.307599073	37	52	38	91	115	52	121	139
ENSECAG00000004253	5.6964774	0.130696837	0.307831149	768	1467	781	934	1206	724	841	882
ENSECAG00000017300	3.977144625	0.13092364	0.308238524	113	326	165	240	343	213	432	650
ENSECAG00000012573	6.992760791	0.130924055	0.308238524	1439	1151	2367	6486	2528	1754	2406	1717
ENSECAG00000018583	5.141652643	0.131153284	0.308699443	552	693	597	844	643	547	620	759
ENSECAG00000023551	3.381646552	0.131174169	0.308699443	79	113	177	218	231	297	313	186
ENSECAG00000013272	4.231168323	0.131275454	0.308873828	210	213	296	343	477	445	384	534
ENSECAG00000023731	4.478689461	0.131483983	0.309251457	229	234	335	493	521	527	498	639
ENSECAG00000024417	4.798223038	0.131490388	0.309251457	276	471	326	496	634	734	702	639
ENSECAG00000020623	5.820943757	0.131644908	0.309550795	567	646.999	939	1130	1295	1292	1206	1762
ENSECAG00000019833	4.641637401	0.131755618	0.309625935	299	420	227	394	592	527	821	538
ENSECAG00000008684	7.381320587	0.131756333	0.309625935	1829	1564	2189	3707	3494	4479	3795	5054
ENSECAG00000015443	1.83542931	0.131758616	0.309625935	27	34	67	63	98	66	97	95
ENSECAG00000006587	4.312710045	0.131859139	0.309741716	221	217	302	392	552	397	451	553
ENSECAG00000014874	4.676878713	0.131862408	0.309741716	216	240	363	683	683	528	752	569
ENSECAG00000008635	4.781520814	0.131964346	0.309900482	227	318	394	684	598	582	622	928
ENSECAG00000016130	1.439156206	0.132010017	0.309900482	8	53	28	43	52	33	64	142
ENSECAG00000013284	0.728763258	0.132011823	0.309900482	19	16	15	29	38	46	40	37
ENSECAG00000022243	0.837788534	0.132078547	0.309993071	10	24	27	28	50	23	51	54

ENSECAG00000019803	3.754534629	0.132191471	0.31019403	110	378	229	387	265	194	225	250
ENSECAG00000017635	4.835272508	0.132263077	0.310261671	266	333	512	553	760	567	676	793
ENSECAG00000025155	1.126459266	0.132274911	0.310261671	13	12	30	60	60	49	51	58
ENSECAG00000009413	7.415608727	0.132414619	0.310525263	1672	4805	3532	3803	2609	2798	3401	2948
ENSECAG00000026822	3.363421628	0.132530578	0.310733064	126	108	140	187	264	226	283	243
ENSECAG00000013080	6.532855911	0.132578994	0.310735757	966	1022	1463	1765	1945	1609	2173	3682
ENSECAG00000019989	6.083146671	0.132586424	0.310735757	850	893	922	1072	1830	1140	1848	1930
ENSECAG00000009622	3.379428994	0.132821883	0.311223392	100	107	187	177	392	183	233	233
ENSECAG00000015586	0.374788924	0.132886789	0.311311278	23	31	19	24	27	15	14	22
ENSECAG00000010862	8.016951306	0.133015421	0.311548384	3902	7506	4484	4242	4345	4797	4192	4195
ENSECAG00000016785	6.360624124	0.133080487	0.31163654	980	948	1252	1451	1926	1844	2003	2320
ENSECAG00000021648	4.177334364	0.133228127	0.311879595	157	210	530	648	361	252	354	278
ENSECAG00000022212	5.856500537	0.133239179	0.311879595	997	1171	1162	1001	1064	890	1039	1184
ENSECAG00000007751	8.321662055	0.133412817	0.312184807	5844	7548	4854	6405	4961	5790	5029	6119
ENSECAG00000006378	1.794214453	0.133431413	0.312184807	21	44	47	75	65	89	69	125
ENSECAG00000015533	4.024995456	0.133451999	0.312184807	225	512	203	319	229	281	256	357
ENSECAG00000004651	8.534906719	0.133525764	0.312279119	10746	8036	3879	4728	7162	4704	6110	6045
ENSECAG00000012342	4.322441634	0.133547284	0.312279119	367	503	317	303	385	241	367	416
ENSECAG00000023879	10.55358919	0.133751365	0.312691978	39016	38479	18340	15976	25015	22677	25696	23750
ENSECAG00000016193	6.26926848	0.133808642	0.312761528	1120	2472	809	1576	1403	1081	1315	1548
ENSECAG00000024265	5.100713851	0.133885885	0.312831793	402	725	658	901	595	489	658	764
ENSECAG00000020745	3.277360621	0.133893769	0.312831793	58	197	249	331	119	184	162	182
ENSECAG00000019367	1.141906222	0.133948869	0.312896186	14	17	41	42	53	61	34	71
ENSECAG00000025039	3.476123948	0.134112183	0.313213284	239	162	284	150	195	145	212	210
ENSECAG00000024391	4.239900192	0.134191009	0.313247771	464	484	211	236	410	292	328	205
ENSECAG00000021615	6.453156252	0.134208288	0.313247771	1080	1273	997	1477	2130	1817	2258	2494
ENSECAG00000014421	5.378653002	0.134209659	0.313247771	403	566	589	865	919	955	1159	1012
ENSECAG00000023066	3.15723172	0.134286725	0.313303939	103	69	121	196	204	195	210	288
ENSECAG00000015070	5.043516343	0.134288874	0.313303939	326	469	459	655	804	641	909	869
ENSECAG00000024905	6.406498367	0.134647201	0.314075445	919	934.001	1389	1611	2052	1900	2065	2334.01
ENSECAG00000000939	1.737434168	0.134695956	0.314091184	22	45	44	68	70	94	73	92
ENSECAG00000003822	1.321134305	0.134709236	0.314091184	15	57	17	29	57	37	69	97
ENSECAG00000006082	1.182491601	0.134738371	0.314094659	31	32	68	48	36	38	31	40
ENSECAG00000014740	7.061624039	0.134849096	0.314270926	1395	1641	1846	2581	3968	2319	3661	3491
ENSECAG00000018318	9.477838263	0.134869304	0.314270926	14320	13011	13573	13793	12060	10775	13570	12580
ENSECAG00000020875	3.894771566	0.135007363	0.314528124	180	570	223	193	165	310	213	289
ENSECAG00000023501	7.779271466	0.135112933	0.314709542	2053	3784	2357	3751	5921	3797	6721	5955
ENSECAG00000015168	5.055034093	0.135166707	0.314770265	283	511	400	741	713	642	966	962
ENSECAG00000019195	1.444666527	0.135269636	0.314945411	41	51	73	53	26	72	21	47
ENSECAG00000013040	4.889633552	0.135403474	0.315192434	414	323	425	411	839	397	1023	726
ENSECAG00000003619	6.381968764	0.135472444	0.315288388	1220	1257	2170	1842	1475	1390	1655	1444
ENSECAG00000014669	4.045472628	0.135653458	0.31564501	142	196	262	358	435	301	463	429
ENSECAG00000014940	6.107157593	0.135691164	0.315668102	859	855	2057	1794	1388	1248	1171	1031
ENSECAG00000017369	5.902350083	0.135787919	0.315809356	873	668	850	807	1743	1039	1616	1568
ENSECAG00000006612	3.560785582	0.135807473	0.315809356	81	233	146	184	199	255	316	427
ENSECAG00000019563	6.316452789	0.135844491	0.315830797	828	1646	1629	2306	1254	1515	1216	1723
ENSECAG00000019268	1.649300159	0.135950476	0.315959071	26	53	42	37	59	56	92	110
ENSECAG00000017771	4.202625846	0.135955281	0.315959071	347	147	693	280	439	198	307	279
ENSECAG00000004945	7.025118085	0.135988924	0.315969557	1279	1720	2066	1863	3779	1310	3036	5635
ENSECAG00000011505	6.707706637	0.136015411	0.315969557	998	1004	1724	2240	2410	2944	2848	2117
ENSECAG00000003783	0.958837456	0.136120154	0.316148239	26	26	12	26	44	38	52	60
ENSECAG00000010991	5.902548693	0.136225468	0.316328176	616	634	1849	1920	1210	1057	1012	862
ENSECAG00000022396	7.1367727	0.136416943	0.316704572	1574	1500	2162	2597	3657	2771	3311	4449
ENSECAG00000022192	3.980051758	0.136470454	0.316704572	209	224	337	490	321	233	286	284
ENSECAG00000022247	2.167248216	0.13649848	0.316704572	64	118	126	125	7	160	0	26
ENSECAG00000018163	0.376070166	0.136499057	0.316704572	24	8	12	6	43	20	32	37
ENSECAG00000012586	4.303369117	0.136641129	0.316969478	193	265	345	321	446	382	551	560
ENSECAG00000026973	4.474323396	0.136734853	0.317122147	231	266	753	573	387	297	375	516
ENSECAG00000015572	5.351160066	0.136873218	0.317315698	350	539	673	833	1020	749	1188	1045
ENSECAG00000012664	4.304215345	0.136874163	0.317315698	199	265	290	382	454	428	457	594
ENSECAG00000020574	1.74646746	0.137047081	0.317651761	38	29	57	46	90	59	111	75
ENSECAG00000002021	4.030595857	0.137096582	0.317701686	192	217	210	287	396	275	446	507
ENSECAG00000023875	1.069954463	0.137489779	0.318547893	20	15	30	40	65	28	43	76
ENSECAG00000013973	5.985811582	0.137594461	0.318725436	599	567	946	1545	1931	1607	1673	1030
ENSECAG00000014996	6.635987686	0.13762444	0.318729901	3470	1321	1411	938	2066	1213	1700	1387
ENSECAG00000008410	2.743553802	0.137659835	0.318746901	75	129	132	222	71	130	66	181
ENSECAG00000024569	6.604643912	0.137815468	0.318956828	970	1203	1547	1872	2416	2097	2276	2825
ENSECAG00000019986	5.297007326	0.137817313	0.318956828	424	493	612	738	1025	746	1033	1035
ENSECAG00000018233	8.361215925	0.137844711	0.318956828	6204	6837	5508	6665	6220	4925	5416	6250
ENSECAG00000020013	1.09804777	0.13793602	0.318956828	17	42	48	71	29	22	29	57
ENSECAG00000016480	4.653726772	0.137952771	0.318956828	372	402	625	535	514	401	478	414
ENSECAG00000014600	5.095623742	0.137962592	0.318956828	255	292	682	786	775	906	925	756
ENSECAG00000015795	3.713371565	0.137964174	0.318956828	163	72	253	213	395	280	352	293
ENSECAG00000020971	1.374799671	0.13798899	0.318956828	18	14	24	80	75	41	84	65
ENSECAG00000007187	5.756745923	0.138003148	0.318956828	972	1179	851	1022	989	938	956	986
ENSECAG00000003550	3.739888918	0.138031845	0.318958272	62	212	151	332	220	466	331	321
ENSECAG00000019024	6.201933051	0.138089671	0.31902701	1204	1825	1189	1340	1390	1127	1521	1168
ENSECAG00000019217	6.769367487	0.138126696	0.319037809	1548	2012	2473	2474	1765	1678	1906	2494
ENSECAG00000018207	3.390105709	0.138177389	0.319037809	62	231	102	151	177	182	238	513
ENSECAG00000001262	2.568384804	0.138178583	0.319037809	56	82	155	212	81	134	81	91
ENSECAG00000013828	7.71370031	0.138341954	0.319316741	2423	4305	3353	7270	3438	3690	3194	4280
ENSECAG00000020261	7.527228103	0.138355599	0.319316741	2521	3746	3682	4674	3669	2959	3307	3013
ENSECAG00000006191	1.670852271	0.138502562	0.319591005	28	29	52	52	79	78	125	35
ENSECAG00000000048	6.786923722	0.13854033	0.319613246	1328	895	1964	1898	3275	2189	2780	2850
ENSECAG00000010084	5.307797818	0.138603012	0.31969294	551	487	427	702	1006	672	1057	1214

ENSECAG00000025088	5.653639951	0.138828888	0.32014894	415	943	1141	1681	623	1026	879	1029
ENSECAG00000011592	2.713463948	0.139049984	0.320593734	87	139	236	187	1	237	5	35
ENSECAG00000021313	2.529217619	0.13914534	0.3207485	67	65	95	72	143	90	179	171
ENSECAG00000016807	6.560629621	0.139243153	0.320851489	1222	1918	1987	2235	1517	1503	1722	2089
ENSECAG00000016497	5.890598529	0.139246497	0.320851489	575	1149	1208	1875	974	1090	1074	1120
ENSECAG00000014342	3.491409063	0.139389585	0.321116072	89	147	141	263	203	244	256	430
ENSECAG00000017734	4.530162856	0.139558108	0.321439129	275	326	452	837	363	305	418	578
ENSECAG00000024987	3.686313773	0.139672488	0.321592215	92	204	151	275	267	341	447	217
ENSECAG00000008757	3.202491089	0.139681181	0.321592215	79	131	108	195	210	154	227	340
ENSECAG00000027210	1.307211136	0.139817634	0.321754403	7	14	118	104	76	19	21	13
ENSECAG00000016252	5.963878006	0.139823136	0.321754403	1014	1585	1023	1055	1131	1003	1141	1175
ENSECAG00000026873	9.414342773	0.139836582	0.321754403	14850	11584	13795	11749	13958	10727	11714	9763
ENSECAG00000017182	7.837650098	0.139883976	0.321798286	2247	2121	3020	5572	5912	6016	5675	5338
ENSECAG00000000645	3.68670524	0.13997707	0.321947262	103	158	209	269	261	237	326	466
ENSECAG00000023120	0.949667134	0.140061096	0.322075324	26	54	24	47	40	14	31	37
ENSECAG00000008462	4.347107282	0.140160615	0.322238952	248	260	566	555	398	287	371	403
ENSECAG00000020139	7.879360863	0.140212414	0.322292827	4828	4277	4475	4272	4611	3766	3981	3889
ENSECAG00000021308	2.508766672	0.140249371	0.322312571	30	148	108	212	61	118	69	115
ENSECAG00000008563	6.736406678	0.140360048	0.322501691	1394	2910	1801	2182	1693	1980	1732	2086
ENSECAG00000016063	5.380983214	0.140551254	0.322875728	312	512	722	934	868	966	965	1298
ENSECAG00000020885	9.50994397	0.140617269	0.322896851	13800	17641	12603	11547	13818	10950	11609	13473
ENSECAG00000024708	4.082442528	0.140618089	0.322896851	147	218	230	389	321	409	462	470
ENSECAG00000021880	8.320417496	0.140680996	0.322896851	2609	4934	4153	6232	8170	6565	7232	10550
ENSECAG00000022684	7.385212283	0.1406856	0.322896851	1560	2746	1838	3157	4287	3443	4247	4867
ENSECAG00000019394	4.452534041	0.140702544	0.322896851	243	243	441	288	576	402	625	560
ENSECAG00000012953	5.252899222	0.140805712	0.323068355	178	436	411	1229	813	1297	890	822
ENSECAG00000007492	1.717396289	0.140985367	0.323412545	27	52	34	60	79	82	65	99
ENSECAG00000015944	4.819962476	0.141012652	0.323412545	169	364	385	772	595	700	768	725
ENSECAG00000018459	6.387288075	0.141094353	0.323534619	1025	784	1344	1564	2181	1750	2255	2073
ENSECAG00000008252	5.683463782	0.141130724	0.323552721	449	515	1132	886	1443	998	1113	1537
ENSECAG00000026898	3.698286333	0.141184535	0.32361079	255	256	215	242	220	162	257	279
ENSECAG00000002414	2.358190048	0.141328342	0.323777976	45	54	87	93	105	151	130	117
ENSECAG00000019538	2.675899409	0.141334894	0.323777976	78	131	122	177	103	114	116	105
ENSECAG00000019720	2.040766078	0.141342964	0.323777976	30	55	25	107	97	58	116	157
ENSECAG00000008495	5.96140281	0.141375592	0.323787436	632	1037	1273	2159	961	1066	1090	1372
ENSECAG00000024635	5.187251298	0.141723267	0.324518291	363	675	728	1181	715	592	695	618
ENSECAG00000021818	8.041764977	0.141868922	0.324786357	3407	4032	3023	3231	6675	5227	6840	8153
ENSECAG00000009095	8.046738285	0.141983602	0.324983419	2841	6624	5011	6910	4301	4314	4441	5436
ENSECAG00000013959	2.400251504	0.142017193	0.324994833	51	77	61	94	131	94	157	144
ENSECAG00000022320	5.373771744	0.142240149	0.325439503	416	855	1045	955	751	698	831	709
ENSECAG00000012495	5.856520521	0.142423496	0.325750499	418	812	1055	1130	1462	1243	1609	1354
ENSECAG00000006876	5.928416265	0.142473451	0.325750499	695	694	748	1374	1618	1202	1471	1704
ENSECAG00000022219	5.124166257	0.142475886	0.325750499	529	942	584	571	639	609	469	764
ENSECAG00000023677	0.98979307	0.142490757	0.325750499	47	32	32	49	12	68	10	17
ENSECAG00000011440	1.764905664	0.142541534	0.325801028	26	34	34	84	43	59	97	154
ENSECAG00000022940	5.849890739	0.142591804	0.32584453	680	1283	1012	1550	983	826	1005	1403
ENSECAG00000009148	1.951099905	0.142617924	0.32584453	15	48	55	95	82	70	111	131
ENSECAG00000010874	7.866306546	0.142686621	0.325935945	3579	5716	4307	4495	3357	4372	3629	4854
ENSECAG00000019190	2.050431764	0.1428145	0.326162483	26	55	50	94	66	114	94	142
ENSECAG00000024378	4.987516309	0.143144568	0.326814668	264	296	1049	1162	779	403	570	391
ENSECAG00000023599	1.183853958	0.143157596	0.326814668	27	13	12	59	43	57	67	63
ENSECAG00000007864	8.12514897	0.143409196	0.327179252	4078	7128.97	5015.97	5657.99	3576	4864	4096.93	6885.97
ENSECAG00000009757	5.21145224	0.143419567	0.327179252	372	413	1352	991	829	543	788	332
ENSECAG00000018007	0.630721881	0.143475732	0.327179252	4	26	19	27	24	31	49	50
ENSECAG00000012042	5.824287469	0.143479506	0.327179252	452	760	909	1213	1186	1518	1077	1781
ENSECAG00000020116	7.119561476	0.143489933	0.327179252	1846	2894	2483	3887	1881	2447	3065	2338
ENSECAG00000016916	8.160219121	0.143509323	0.327179252	2939	4539	6044	10773	5490	4739	5212	4180
ENSECAG00000006965	5.393401063	0.143518869	0.327179252	333	869	859	1409	692	675	661	992
ENSECAG00000015288	6.314386018	0.143588623	0.327272605	1608	2025	1029	1219	1530	1138	1353	1554
ENSECAG00000022310	3.73378754	0.143619396	0.327277092	88	131	733	218	136	192	233	291
ENSECAG00000019269	3.712897563	0.143772665	0.327560663	117	151	231	232	318	155	390	472
ENSECAG00000006633	1.848417424	0.143954822	0.32790992	18	72	92	150	12	52	33	120
ENSECAG00000002972	4.577531966	0.144034033	0.327920984	272	283	333	454	453	575	442	905
ENSECAG00000017545	6.356626241	0.144037589	0.327920984	1008	1344	2140	1963	1530	1223	1338	1869
ENSECAG00000019997	5.573092911	0.144046262	0.327920984	598	471	732	895	1346	817	1177	1360
ENSECAG00000017234	7.273355406	0.144253277	0.32832647	1002	2292	1623	4002	3619	4251	3923	3751
ENSECAG00000024871	1.526406259	0.144333251	0.328360256	30	62	61	83	22	37	46	83
ENSECAG00000003510	5.429209201	0.144365246	0.328360256	814	543	1040	877	1035	614	746	663
ENSECAG00000013985	6.841007421	0.144374417	0.328360256	1488	3188	1707	2618	1822	2181	1664	2334
ENSECAG00000011342	8.302610284	0.144383721	0.328360256	5059	6350	5515	7811	5146	5568	5065	6315
ENSECAG00000007906	4.394127281	0.144488546	0.328532891	219	244	351	401	506	435	523	586
ENSECAG00000015422	4.976942337	0.144583643	0.328683344	364	365	434	652	761	613	761	950
ENSECAG00000000223	5.746727812	0.144703936	0.328844578	702	1208	1033	1327	833	1434	854	511
ENSECAG00000013579	4.869877613	0.144712453	0.328844578	313	294	513	562	715	742	779	596
ENSECAG00000008523	5.950559454	0.14489407	0.329130817	254	615	831	1992	1242	1980	1503	1515
ENSECAG00000024506	3.091098128	0.144896352	0.329130817	70	125	89	195	202	180	249	214
ENSECAG00000006787	6.941922994	0.14494195	0.329137296	1871	2605	2249	2777	2169	1955	2113	2529
ENSECAG00000021507	7.958503982	0.144957141	0.329137296	2905	5933	4834	6230	4073	3963	4273	5212
ENSECAG00000002178	0.802525258	0.144998284	0.329164936	29	32	36	38	13	41	24	28
ENSECAG00000009512	8.404085416	0.145051595	0.329220181	5470	5082	8554	7622	6286	5400	6410	5435
ENSECAG00000017437	1.60396641	0.145100986	0.329229181	34	35	35	52	83	50	95	73
ENSECAG00000006451	3.914740825	0.145113513	0.329229181	169	192	497	373	323	160	324	244
ENSECAG00000005084	5.049093033	0.145178043	0.329309828	477	362	888	900	732	512	568	547
ENSECAG00000021641	0.631106964	0.145215979	0.32933013	6	40	34	50	22	24	20	26
ENSECAG00000023251	1.861544115	0.145273648	0.329395168	27	70	35	51	92	46	90	148

ENSECAG00000004807	7.829076059	0.145311682	0.329401174	5345	6223	2562	2844	4305	3975	3523	3060
ENSECAG00000017287	1.849443642	0.145343127	0.329401174	25	31	56	84	113	58	69	125
ENSECAG00000009844	6.058434894	0.145363271	0.329401174	958	945	827	800	1633	1369	1502	2161
ENSECAG00000011593	6.802207785	0.145547582	0.329734393	2388	2074	1445	2563	1956	2081	2129	1477
ENSECAG00000011898	1.614468012	0.145568361	0.329734393	53	12	30	21	229	41	30	36
ENSECAG00000001580	4.095854678	0.145816312	0.330230206	171	132	322	355	405	330	445	521
ENSECAG00000022346	4.746970157	0.145911156	0.330379146	235	87	517	539	1396	349	894	224
ENSECAG00000020641	3.74318168	0.145981582	0.330411427	28	126	92	513	260	394	476	283
ENSECAG00000019207	8.697982798	0.145983574	0.330411427	4450	3943	6032	8669	11255	7570	11686	11738
ENSECAG00000018412	4.269505283	0.146239795	0.330925424	246	245	322	230	570	386	505	435
ENSECAG00000017586	3.84618235	0.146294853	0.330984095	206	195	390	331	254	255	240	272
ENSECAG00000011055	6.053471766	0.146440377	0.331247375	844	736	2057	1767	1612	863	1222	961
ENSECAG00000020833	2.327648264	0.146632658	0.331616293	50	60	66	91	89	81	154	185
ENSECAG00000013534	8.202798215	0.146668038	0.331630297	6235	9790	2908	3348	5754	3583	5009	4933
ENSECAG00000017052	5.446407985	0.146709628	0.331658335	459	408	677	1012	1032	1050	1015	1157
ENSECAG00000016883	8.276255435	0.146822055	0.331846467	5478	8083	5202	4870	4610	5569	5209	5858
ENSECAG00000003466	4.281773129	0.146920444	0.332002801	262	165	219	355	843	292	637	215
ENSECAG00000011664	4.414544304	0.147004441	0.332070799	218	188	333	462	839	445	298	551
ENSECAG00000008716	3.830087903	0.147008988	0.332070799	143	144	282	229	437	252	313	411
ENSECAG00000021954	4.528111257	0.147075072	0.332154039	217	305	375	448	584	513	503	643
ENSECAG00000002758	5.469063388	0.147227357	0.332431882	323	577	778	970	1167	889	1045	1237
ENSECAG00000015080	2.917415612	0.147424525	0.33281094	54	77	107	191	158	188	139	276
ENSECAG00000006437	4.698929365	0.147503732	0.3329236	259	329	434	483	654	482	723	674
ENSECAG00000019260	4.351997329	0.14755876	0.332981655	158	223	352	478	561	398	473	578
ENSECAG00000022756	5.25973428	0.147631367	0.333079346	745	1319	452	340	620	585	789	542
ENSECAG00000017685	3.113833386	0.147716936	0.33320624	102	171	197	212	163	130	165	150
ENSECAG00000013633	0.737722976	0.147766983	0.33325297	12	22	69	50	2	56	11	12
ENSECAG00000011553	10.69479299	0.147845455	0.333363774	34077	44522	23042	23748	30272	18634	29974	33751
ENSECAG00000012531	0.783175872	0.148121871	0.333920773	15	16	33	20	38	45	51	32
ENSECAG00000022923	5.120972623	0.148207098	0.334046626	334	309	634	710	1406	647	675	729
ENSECAG00000002378	1.686689542	0.148281435	0.334147891	30	43	29	67	77	51	87	108
ENSECAG00000010177	3.559897003	0.148315475	0.334158323	83	161	146	289	214	232	302	441
ENSECAG00000012900	10.05306501	0.14847784	0.334457815	23798	29054	12630	14148	16964	15361	18761	19872
ENSECAG00000019554	2.168751404	0.148550987	0.334556256	44	55	49	96	102	93	114	137
ENSECAG00000000382	4.300607426	0.148683346	0.334787987	531	373	301	226	367	424	269	235
ENSECAG00000002531	2.787601754	0.148847023	0.335025619	53	80	137	110	183	106	217	187
ENSECAG00000016979	4.291626046	0.148847855	0.335025619	131	252	384	384	429	409	461	638
ENSECAG00000018426	5.866336525	0.148896701	0.335069185	468	1019	2085	1233	1415	851	951	839
ENSECAG00000006575	6.011123783	0.149049704	0.335347076	660	683	843	1574	1551	1123	1577	2186
ENSECAG00000014592	5.942832906	0.149450393	0.33606907	1007	1010	1338	1295	1124	1082	1162	1089
ENSECAG00000022718	6.319927901	0.149479805	0.33606907	760	894	1384	1627	2088	1445	1922	2447
ENSECAG00000013321	3.377379549	0.149491819	0.33606907	100	119	156	217	228	219	247	332
ENSECAG00000014769	6.903927079	0.149502591	0.33606907	1489	2013	3158	2853	2212	1968	2133	2215
ENSECAG00000011402	7.195566122	0.149546285	0.33606907	1234	1419	2198	3675	4256	3303	3422	3681
ENSECAG00000013468	2.5704786	0.149548074	0.33606907	86	126	93	160	107	103	75	123
ENSECAG00000021202	7.55626698	0.149665759	0.336233848	1204	2170	2193	5284	4566	5035	6242	3132
ENSECAG00000010361	2.660808742	0.149680585	0.336233848	79	109	150	169	84	105	78	170
ENSECAG00000026914	5.215772846	0.149771841	0.336372338	794	925	582	412	583	466	706	848
ENSECAG00000018901	4.10965102	0.149808128	0.336387343	127	236	253	400	345	369	495	488
ENSECAG00000005827	5.367208585	0.149959383	0.336604403	506	431	664	759	959	854	1045	1172
ENSECAG00000017508	3.647830681	0.149998191	0.336604403	103	136	218	269	303	215	347	379
ENSECAG00000018657	5.630511169	0.149999186	0.336604403	601	870	1188	1195	945	689	842	1185
ENSECAG00000018900	5.072890371	0.150023297	0.336604403	393	481	432	610	861	721	872	809
ENSECAG00000017463	5.975979226	0.150100259	0.336710591	779	540	1191	1009	1430	1358	1361	2115
ENSECAG00000021492	6.486019399	0.150341805	0.337185865	1204	982	1249	1468	2822	1383	2303	2461
ENSECAG00000025161	3.47949266	0.150461403	0.337387501	58	104	140	348	268	254	189	435
ENSECAG00000010404	6.922229138	0.150510131	0.337427453	2151	2014	2200	2963	2120	2120	2055	2310
ENSECAG00000020507	7.124105638	0.150538616	0.337427453	1351	1688	2445	2426	3386	3148	3191	4166
ENSECAG00000007901	0.948848372	0.150597986	0.337493949	24	27	61	41	30	14	35	45
ENSECAG00000007581	3.355675109	0.150630075	0.337499293	101	115	154	209	295	173	265	280
ENSECAG00000019162	7.480098805	0.150877827	0.337987749	1686	2709	2495	3124	4417	3507	5164	4761
ENSECAG00000024623	8.424059901	0.150964517	0.338115285	4898	8165	7928	5837	6691	6455	5142	5285
ENSECAG00000009886	3.581519289	0.151017772	0.338167899	135	75	238	208	355	202	326	322
ENSECAG00000000446	5.972841044	0.151049348	0.338171958	587	581	935	1615	1559	1611	1413	1565
ENSECAG00000005865	3.355334148	0.15111286	0.3382475	75	223	99	145	189	183	264	419
ENSECAG00000024331	4.304100896	0.151311677	0.338625817	149	249	303	463	454	323	504	689
ENSECAG00000020792	2.552418905	0.151569942	0.339136999	36	53	58	159	324	65	167	61
ENSECAG00000007960	8.617065315	0.151707892	0.339378828	5389	12408	5552	7436	6180	5358	7109	8401
ENSECAG00000022500	9.909801514	0.151749716	0.339405566	10008	11398	15702	16149	24771	19276	26259	26658
ENSECAG00000010304	4.94124738	0.151811899	0.339477819	447	279	440	522	871	570	775	816
ENSECAG00000011994	9.661581562	0.151955557	0.339732199	9414	12533	9395	11715	20468	18451	24247	18393
ENSECAG00000018487	3.917049301	0.152044513	0.339864205	172	222	183	257	440	238	448	367
ENSECAG00000011457	7.065287139	0.152166398	0.34000325	1710	1594	2121	1822	3510	2657	3333	3925
ENSECAG00000024309	3.801357604	0.152166566	0.34000325	77	295	355	490	150	367	166	226
ENSECAG00000012138	3.895513332	0.152232231	0.34002147	90	235	194	353	337	313	360	468
ENSECAG00000006539	4.775498725	0.152234573	0.34002147	356	333	419	421	727	534	698	713
ENSECAG00000017169	4.804367159	0.152987762	0.341636586	330	251	449	576	669	648	583	835
ENSECAG00000014310	5.118743763	0.153020311	0.341642124	349	593	407	617	927	672	640	1207
ENSECAG00000001164	3.225336009	0.15309371	0.341738846	80	83	165	192	199	343	153	227
ENSECAG00000014719	2.070921286	0.153305375	0.342035842	49	27	73	64	89	57	102	189
ENSECAG00000007373	1.907401016	0.153316629	0.342035842	44	43	51	51	76	134	59	101
ENSECAG00000020976	6.551643308	0.153333525	0.342035842	679	1236	1249	2287	2048	2231	3433	1578
ENSECAG00000024197	5.784080437	0.153347173	0.342035842	618	597	834	1160	1437	1038	1428	1477
ENSECAG00000015717	0.887383635	0.153566171	0.342457081	13	39	20	15	37	39	41	68
ENSECAG00000005362	0.974622239	0.153648145	0.34257265	8	47	95	21	11	53	19	22

ENSECAG00000009171	8.60492889	0.153684468	0.34258641	2757	3963	6273	9630	9193	8381	10384	11554
ENSECAG00000021599	0.714793993	0.153742605	0.342614662	13	12	18	41	31	41	42	45
ENSECAG00000018866	7.364909632	0.153781086	0.342614662	1061	1613	2782	4244	3538	4232	4060	4761
ENSECAG00000006400	7.146543678	0.153818002	0.342614662	4162	2095	2204	1820	2763	1871	2152	2769
ENSECAG00000016389	5.634087749	0.153829392	0.342614662	688	649	682	719	1209	1142	1280	1197
ENSECAG00000017667	2.426305571	0.153847914	0.342614662	54	46	102	90	143	118	133	137
ENSECAG00000008991	6.152650684	0.154018279	0.342893555	787	2059	1035	1698	1313	1171	1407	1150
ENSECAG00000014133	6.050649444	0.15405547	0.342893555	898	1454	1418	1286	974	1151	1207	1488
ENSECAG00000009197	4.887293569	0.154063686	0.342893555	429	418	261	465	977	660	684	597
ENSECAG00000011017	4.893765613	0.15422097	0.343176393	212	370	461	740	664	552	845	869
ENSECAG00000008742	6.202513499	0.154259959	0.343195937	692	366	1703	1414	1863	1637	1899	2040
ENSECAG00000024988	5.47320469	0.15435701	0.343344626	448	820	350	863	1224	858	933	1424
ENSECAG00000015792	5.632658921	0.154519538	0.34363887	623	510	628	900	2367	614	1201	846
ENSECAG00000011860	4.2885859	0.154713574	0.343964302	211	611	321	384	325	426	245	356
ENSECAG00000002782	0.69078476	0.154735236	0.343964302	15	11	35	14	19	47	40	54
ENSECAG00000026811	1.92644911	0.15475669	0.343964302	41	45	40	56	199	29	102	66
ENSECAG00000004168	2.200591481	0.154809443	0.344014255	43	77	62	56	87	130	139	96
ENSECAG00000024919	3.307471266	0.155057825	0.344432618	123	82	147	192	285	211	247	234
ENSECAG00000010189	2.933468381	0.155072322	0.344432618	91	45	160	105	217	142	180	238
ENSECAG00000007426	7.060684704	0.155088653	0.344432618	2556	2559	2035	3010	2475	2586	2042	2208
ENSECAG00000013421	4.960061063	0.155151837	0.344505603	198	843	490	1070	515	461	599	613
ENSECAG00000018050	5.323506913	0.155226832	0.34457329	786	656	922	605	908	648	688	588
ENSECAG00000007374	8.61471355	0.155242974	0.34457329	4466	7884	9898	9328	6558	5616	6707	8861
ENSECAG00000014810	7.745527179	0.155342128	0.344726026	3489	4720	3640	4704	3615	3836	3041	4637
ENSECAG00000019027	2.456301975	0.155455228	0.344909647	51	122	128	158	37	142	86	86
ENSECAG00000006916	2.749996741	0.155571542	0.345100323	59	30	581	28	3	232	11	6
ENSECAG00000012022	7.61563879	0.155738664	0.34528632	1598	2598	2831	4301	4559	4410	5454	5116
ENSECAG00000023223	5.518452895	0.155741739	0.34528632	504	879	1048	1153	720	847	828	961
ENSECAG00000021409	5.067335589	0.155766573	0.34528632	330	417	535	689	953	689	756	851
ENSECAG00000005990	7.032980242	0.155776947	0.34528632	2246	4376	1429	1957	1517	2407	2390	2478
ENSECAG00000021404	0.355543375	0.156049307	0.345822552	57	11	7	17	19	19	3	27
ENSECAG00000022530	0.221482203	0.156163436	0.346007987	8	15	27	48	6	13	19	30
ENSECAG00000016076	4.768939762	0.156198947	0.346019192	209	254	571	555	942	447	752	562
ENSECAG00000008719	4.033093366	0.156316803	0.34605535	174	154	271	345	408	321	414	464
ENSECAG00000020784	4.037050695	0.156323373	0.34605535	138	310	210	245	471	216	373	606
ENSECAG00000021489	4.266906281	0.156327162	0.34605535	251	569	318	327	311	373	338	328
ENSECAG00000023237	8.34338297	0.156337099	0.34605535	4193	3261	5375	4541	9644	6058	8314	8880
ENSECAG00000015106	8.278167553	0.156434359	0.346165239	2516	4929	6923	12567	5793	4232	4854	6664
ENSECAG00000018688	6.344789279	0.156481001	0.346165239	734	1105	1388	1518	1797	2004	1808	2319
ENSECAG00000022016	2.316088949	0.156491562	0.346165239	51	68	55	87	85	127	84	208
ENSECAG00000006468	4.804898746	0.156534518	0.346165239	259	375	517	482	681	580	691	758
ENSECAG00000002306	6.023929125	0.156560449	0.346165239	1113	1502	1209	1004	1071	1149	1056	1399
ENSECAG00000002176	5.412146391	0.156569544	0.346165239	306	1085	395	326	1472	361	1533	1161
ENSECAG00000011156	3.108410614	0.156707923	0.346403779	37	145	134	169	159	184	208	327
ENSECAG00000024298	6.037271238	0.156798757	0.346537149	1036	1057	460	756	1582	1482	1456	2148
ENSECAG00000009292	4.341775178	0.156831261	0.346541577	331	549	340	289	422	287	331	397
ENSECAG00000011158	4.904355523	0.156899407	0.346624745	425	491	606	779	567	533	563	522
ENSECAG00000010268	7.727925472	0.156934411	0.346634677	3403	5052	3544	4245	3613	3362	3847	4137
ENSECAG00000018575	3.180919095	0.157064387	0.346816814	70	131	163	139	187	282	165	258
ENSECAG00000022081	5.581309151	0.15707792	0.346816814	510	644	643	1007	1200	1039	1149	1241
ENSECAG00000022849	7.311762934	0.157221621	0.346990809	3566	3732	2076	2370	2669	2570	2621	3060
ENSECAG00000017942	5.843492669	0.157238471	0.346990809	912	1207	1138	1027	1104	950	1018	1108
ENSECAG00000008098	7.47864884	0.157248343	0.346990809	1889	1492	3523	2941	4585	2744	4332	6691
ENSECAG00000000331	6.234290325	0.157289539	0.346994773	1287	1744	1113	1519	1320	1582	1144	1277
ENSECAG00000009395	3.708196401	0.15731122	0.346994773	137	154	174	281	316	260	396	306
ENSECAG00000012562	4.247387971	0.157458688	0.347196685	214	311	460	516	375	329	335	331
ENSECAG00000012289	4.156615874	0.157492242	0.347196685	124.002	247	213	465	340	359	425	663.001
ENSECAG00000012464	6.436921992	0.15749443	0.347196685	892	1001	1380	1733	2565	1426	2070	2520
ENSECAG00000009514	6.662141261	0.157634593	0.347407161	1411	2663	1533	2188	1557	1648	2004	2009
ENSECAG00000009232	6.402731864	0.157651058	0.347407161	822	1734	1831	2421	1287	1630	1293	1882
ENSECAG00000009893	3.45122835	0.1577648	0.347590394	136	83	130	259	283	236	294	271
ENSECAG00000025110	8.481061353	0.157910967	0.347844981	4205	4784	3886	6593	7723	7382	8601	12648
ENSECAG00000021356	3.05376298	0.158006268	0.347987443	58	165	94	130	132	185	193	336
ENSECAG00000019669	6.714373944	0.158051283	0.348019122	1005	1193	1973	1882	2716	2185	2587	2873
ENSECAG00000017457	8.476915406	0.158167691	0.348207965	11470	7579	3447	3231	6912	3812	6466	5692
ENSECAG00000018417	5.409543961	0.158310595	0.348435279	703	1102	611	746	758	584	867	854
ENSECAG00000020050	4.986007304	0.158353722	0.348435279	366	465	466	508	788	696	726	856
ENSECAG00000025265	6.790900538	0.158362945	0.348435279	203	491	5409	5452	3567	1080	548	926
ENSECAG00000016220	4.492343638	0.158393874	0.348435857	243	373	330	314	529	442	622	605
ENSECAG00000004218	3.962098431	0.158445012	0.348480879	251	353	320	248	332	215	331	219
ENSECAG00000002401	3.957367287	0.158499773	0.348533853	169	285	366	408	293	297	247	273
ENSECAG00000009000	4.393183807	0.15861203	0.348713212	371	543	347	293	471	282	348	385
ENSECAG00000017465	4.236471759	0.158865504	0.349202914	187	258	349	265	580	321	529	419
ENSECAG00000017356	2.138745247	0.159004342	0.349389511	38	163	65	100	23	60	54	144
ENSECAG00000014977	4.712652035	0.159011896	0.349389511	161	517	612	920	402	455	470	524
ENSECAG00000000348	6.610133854	0.159086925	0.349486782	929	949	1646	2204	2275	2182	2286	2906
ENSECAG00000012176	5.178464193	0.159160809	0.349556621	724	1047	403	486	573	524	601	834
ENSECAG00000018843	5.217060015	0.159180246	0.349556621	776	667	519	775	700	584	642	754
ENSECAG00000020888	8.630786717	0.159253361	0.349649601	5708	7574	9943	8014	9062	6441	5316	6770
ENSECAG00000001299	3.467190437	0.159315093	0.349717559	114	171	144	171	336	129	304	347
ENSECAG00000016872	2.588997267	0.159346294	0.349718484	68	230	86	87	96	66	111	126
ENSECAG00000016546	2.667982227	0.159400021	0.34974856	73	105	198	129	98	112	94	133
ENSECAG00000020898	5.26262853	0.159447144	0.34974856	215	515	499	1055	1118	1013	1034	606
ENSECAG00000011269	3.680236416	0.159452345	0.34974856	89	171	236	251	268	283	353	353
ENSECAG00000008062	5.958332825	0.159576571	0.349953484	789	931	1350	1858	1264	1050	841	1379

ENSECAG00000007791	2.539493007	0.159694714	0.35014499	65	76	83	79	191	73	177	144
ENSECAG00000020289	7.566988701	0.159742586	0.350182377	1857	1946	2904	4189	4615	4386	4417	5488
ENSECAG00000017296	3.422929808	0.159825314	0.350296144	157	155	99	143	238	228	261	347
ENSECAG00000007306	1.214464238	0.160077953	0.350776558	22	21	50	19	61	28	60	88
ENSECAG00000013443	7.98208041	0.160106251	0.350776558	2792	7843	3785	5750	3740	4679	4050	4985
ENSECAG00000026941	4.035088131	0.160157688	0.350821602	158	278	208	283	374	306	483	445
ENSECAG00000021368	3.011292384	0.160355534	0.351187275	121	85	224	220	208	97	128	116
ENSECAG00000012960	4.593685204	0.160411611	0.351242384	324	392	317	221	617	558	758	451
ENSECAG00000010631	5.814668596	0.16046014	0.351244294	492	530	767	1579	1460	988	1435	1711
ENSECAG00000008963	6.59488897	0.16049253	0.351244294	1121	1003	1574	1806	2380	1758	2409	3048
ENSECAG00000011960	5.058973454	0.16053054	0.351244294	212	366	495	936	770	748	1071	687
ENSECAG00000022421	3.655607581	0.160560754	0.351244294	102	162	194	279	297	277	289	370
ENSECAG00000011164	3.558992556	0.160590846	0.351244294	107	191	134	235	252	244	357	304
ENSECAG00000022524	5.55686661	0.160625599	0.351244294	492	1060	764.004	1415	810	761	980	870
ENSECAG00000016701	5.431440178	0.160628881	0.351244294	361	622	590	997	985	813	1167	1255
ENSECAG00000017656	5.071671455	0.160940906	0.351858876	372	723	622	916	587	524	742	599
ENSECAG00000008184	6.73976399	0.161055835	0.352042399	1048	2944	1831	2735	1511	1512	1984	2636
ENSECAG00000006422	2.606226979	0.161094263	0.352058669	35	97	76	129	93	139	142	244
ENSECAG00000009834	3.182138404	0.161208643	0.352240884	75	205	107	393	114	181	123	189
ENSECAG00000013484	0.484071044	0.161334075	0.352447176	9	24	60	43	0	49	0	7
ENSECAG00000010332	4.233138351	0.161420907	0.352569079	241	216	281	313	483	341	538	476
ENSECAG00000006540	5.554206289	0.161514281	0.352705219	467	473	776	1071	1309	936	1110	1219
ENSECAG00000013324	6.594338234	0.161649704	0.352933117	1496	1234	1067	1288	2759	2104	2453	2269
ENSECAG00000020051	7.157379014	0.161752348	0.353089372	2738	4257	1007	3039	2173	2318	3091	1874
ENSECAG00000016210	5.683760093	0.161950776	0.353407087	339	597	1086	954	916	1187	802	2324
ENSECAG00000013631	4.004674793	0.161965865	0.353407087	303	264	264	374	322	268	300	252
ENSECAG00000024306	8.14325998	0.161991208	0.353407087	2527	2697	3605	7475	7721	7211	7333	5973
ENSECAG00000008758	4.785644651	0.162145749	0.35367633	296	435	798	677	521	275	490	728
ENSECAG00000013497	2.940887831	0.162269259	0.353877796	46	79	400	153	170	57	140	122
ENSECAG00000023338	4.912580632	0.162366378	0.354021645	311	298	539	621	742	614	722	855
ENSECAG00000017941	5.870906715	0.162454279	0.354058754	721	1903	934	911	922	835	983	1438
ENSECAG00000011108	5.800080533	0.162454984	0.354058754	929	1162	988	1072	1097	758	907	1335
ENSECAG00000009727	7.734683254	0.162476883	0.354058754	4209	4702	3825	3231	3598	3657	4338	3078
ENSECAG00000008929	4.566363412	0.162563238	0.354179006	257	230	378	512	592	483	688	541
ENSECAG00000023202	5.53976108	0.162990294	0.355032259	863	1030	728	795	820	794	714	1039
ENSECAG00000005876	3.441774465	0.163017598	0.355032259	140	151	250	322	212	165	197	200
ENSECAG00000010293	7.278711002	0.163048612	0.355032259	2573	3886	2798	2494	3126	2598	2443	2708
ENSECAG00000014072	4.998726849	0.163396344	0.35572126	262	321	618	711	734	637	853	903
ENSECAG00000000643	5.580410018	0.163446384	0.355727318	446	659	713	1026	1096	1093	1079	1356
ENSECAG00000008807	4.207736812	0.163461743	0.355727318	175	216	292	402	397	418	411	575
ENSECAG00000016486	5.460037866	0.163589718	0.355937643	377	1301	560	1318	777	670	861	814
ENSECAG00000015180	0.636365091	0.163702332	0.356114474	22	48	26	22	23	35	19	15
ENSECAG00000014973	4.672608935	0.163734657	0.356116609	643	290	525	456	704	230	505	292
ENSECAG00000022937	6.405388079	0.163840871	0.356221488	1400	1905	1115	2019	1466	1560	1247	1812
ENSECAG00000001130	5.480895749	0.163845581	0.356221488	443	619	648	927	1057	969	1006	1288
ENSECAG00000012306	7.422345686	0.16390059	0.356259962	2008	3626	3725	4238	2977	2704	3205	3387
ENSECAG00000026930	5.105454305	0.163944854	0.356259962	304	710	848	880	526	624	612	739
ENSECAG00000017894	2.470079467	0.163979309	0.356259962	14	46	121	130	144	110	128	191
ENSECAG00000016291	6.690821742	0.163999653	0.356259962	1054	1209	1739	1932	2571	2247	2053	3360
ENSECAG00000005273	1.497245251	0.164020055	0.356259962	37	45	59	82	45	52	52	37
ENSECAG00000003330	0.331950732	0.164064171	0.356287673	7	14	11	31	33	26	29	32
ENSECAG00000016434	6.039325754	0.164163681	0.356346198	1355	1385	1154	895	1232	997	1157	1310
ENSECAG00000018407	5.273415652	0.164194065	0.356346198	446	456	597	740	1082	708	956	1012
ENSECAG00000011341	2.737091659	0.164216388	0.356346198	46	122	66	129	149	100	175	257
ENSECAG00000022831	3.230259363	0.164217956	0.356346198	108	104	110	198	254	145	282	252
ENSECAG00000023123	5.647552884	0.164298319	0.356346198	544	872	633	799	1235	1071	1113	1462
ENSECAG00000017484	3.872324985	0.164308322	0.356346198	222	264	341	294	251	333	175	271
ENSECAG00000018434	3.711314454	0.164351336	0.356346198	100	198	201	258	262	358	281	376
ENSECAG00000005394	4.885723547	0.164354319	0.356346198	223	494	524	1280	463	513	499	618
ENSECAG00000009112	3.852378183	0.164373387	0.356346198	202	337	294	258	264	215	238	326
ENSECAG00000015158	2.647815565	0.16456355	0.356690395	83	67	101	69	168	93	146	231
ENSECAG00000014140	3.316209417	0.164709996	0.356939725	99	133	130	202	190	214	268	307
ENSECAG00000014515	1.31542666	0.164816241	0.357101856	53	37	50	55	11	69	25	45
ENSECAG00000022482	5.741999201	0.165038183	0.357469531	990	1426	807	707	957	735	819	1320
ENSECAG00000014999	2.304653224	0.165048861	0.357469531	57	51	49	109	121	89	151	130
ENSECAG00000019339	1.517643497	0.165204763	0.357714917	25	41	41	43	71	48	67	97
ENSECAG00000014981	5.426702764	0.165289951	0.357714917	420	381	798	948	1109	860	1084	1148
ENSECAG00000000971	6.374813695	0.165292237	0.357714917	1801	1024	1888	1475	1748	1525	1308	1235
ENSECAG00000004942	7.547631303	0.165318246	0.357714917	3224	1603	5043	6349	4298	4064	2489	534
ENSECAG00000019978	3.012879559	0.165319576	0.357714917	86	91	225	277	188	69	148	148
ENSECAG00000021732	4.802172609	0.165436122	0.357898938	523	511	496	524	544	508	483	477
ENSECAG00000021984	3.755516808	0.165644783	0.35828213	146	173	158	292	305	235	376	417
ENSECAG00000014301	4.993847585	0.165700098	0.358333558	412	487	796	779	602	518	697	500
ENSECAG00000006511	0.99143938	0.165793491	0.358445485	17	4	56	13	56	32	42	79
ENSECAG00000019132	6.400880393	0.165814951	0.358445485	1825	1486	1200	1724	1744	1269	1544	1469
ENSECAG00000011439	6.665342407	0.165927181	0.358619865	1143	4209	945	1723	1620	1860	1787	1460
ENSECAG00000021932	3.312959811	0.165963181	0.358629452	136	263	144	228	196	111	133	263
ENSECAG00000014955	3.667461121	0.166239013	0.35915719	70	135	188	355	192	312	414	352
ENSECAG00000009594	2.58567239	0.16638168	0.359397081	57	59	86	135	132	122	162	177
ENSECAG00000000588	1.16142122	0.16650381	0.359592527	17	71	61	28	31	39	28	42
ENSECAG00000021937	3.780246371	0.166564612	0.359655478	106	250	153	272	274	263	350	483
ENSECAG00000001409	0.85748527	0.166767072	0.35999992	15	26	18	33	37	39	39	61
ENSECAG00000013610	6.288291451	0.1667875	0.35999992	1006	923	1148	1311	1944	1426	1774	2586
ENSECAG00000021154	2.028164139	0.167052558	0.360503545	45	32	47	95	122	69	88	130
ENSECAG00000011448	5.072076685	0.167236316	0.360831565	562	604	711	624	697	466	625	683

ENSECAG00000008379	3.057837086	0.167371727	0.361034566	65	167	109	111	213	152	207	260
ENSECAG00000009525	3.81830757	0.167420747	0.361034566	133	160	241	287	336	304	377	352
ENSECAG00000021297	9.856093668	0.167425728	0.361034566	12054	10403	14723	12307	23380	17965	23842	28727
ENSECAG00000015348	5.89479835	0.167547318	0.361144481	610	890	691	1272	1239	1309	1406	1864
ENSECAG00000014325	3.294413841	0.167588472	0.361144481	105	95	176	175	246	192	222	309
ENSECAG00000000507	6.313467442	0.167598293	0.361144481	752	829	1190	1961	1902	1751	1806	2337
ENSECAG00000009054	4.566191017	0.167615314	0.361144481	361	550	503	530	315	845	130	158
ENSECAG00000006701	2.158311969	0.167635627	0.361144481	29	54	87	62	105	155	37	155
ENSECAG00000010803	1.063504623	0.167691819	0.361197051	13	18	21	53	59	24	105	23
ENSECAG00000017074	1.79501712	0.167748354	0.361250341	24	33	60	75	98	62	73	111
ENSECAG00000011103	3.940380847	0.167816648	0.361267177	258	346	219	325	275	249	272	306
ENSECAG00000014809	6.483887499	0.167888077	0.361267177	1013	1882	1750	2378	1532	1465	1725	1811
ENSECAG00000019458	7.802402469	0.167905454	0.361267177	2066	2459	4537	3574	5360	4928	6101	5847
ENSECAG00000006649	7.569281395	0.167907698	0.361267177	3584	4862	2602	3189	3319	3135	3348	3413
ENSECAG00000018292	0.409036402	0.167915152	0.361267177	12	46	23	22	35	12	11	21
ENSECAG00000018686	6.194662484	0.168337641	0.362045042	871	737	1483	968	2305	1184	1996	1787
ENSECAG00000011270	4.031470092	0.168340429	0.362045042	159	227	238	326	363	352	433	436
ENSECAG00000014491	7.844980876	0.168492458	0.36223239	1875	3265	3772	4297	5351	4952	5489	7168
ENSECAG00000014903	4.197323167	0.168518963	0.36223239	247	219	331	170	592	382	466	371
ENSECAG00000015229	3.639213344	0.168523184	0.36223239	84	255	440	295	94	382	102	209
ENSECAG00000009798	3.467277307	0.168796567	0.36275139	189	382	96	179	95	204	215	217
ENSECAG00000004567	4.677228385	0.168845898	0.362788785	282	301	742	742	526	425	415	470
ENSECAG00000001426	8.673223169	0.168978883	0.363005875	5482	8096	8791	9978	6633	8053	5860	8155
ENSECAG00000006801	5.978543612	0.169087043	0.363169561	1132	1893	813	815	1002	1086	994	1342
ENSECAG00000020592	6.533827694	0.169149159	0.363234312	605	1513	993	2263	1800	3225	2197	1874
ENSECAG00000025154	2.759945553	0.169245815	0.363316432	39	76	110	167	145	142	180	207
ENSECAG00000022811	4.505895423	0.169289177	0.363316432	233	249	328	530	598	465	531	612
ENSECAG00000022180	1.93319814	0.169310059	0.363316432	24	108	75	120	20	53	48	125
ENSECAG00000006081	6.121104333	0.169334804	0.363316432	935	1332	1639	1398	1286	1219	1200	1390
ENSECAG00000002856	6.146920491	0.169347283	0.363316432	824	710	1236	1297	1821	1192	1897	2073
ENSECAG00000012273	5.405834361	0.169407185	0.363357652	534	549	528	814	1065	995	950	1082
ENSECAG00000000217	1.337997061	0.169430456	0.363357652	27	30	69	83	30	56	44	29
ENSECAG00000017895	7.583164343	0.169533158	0.363452554	3147	3516	3008	5242	3624	2991	3372	3687
ENSECAG00000016428	5.986830388	0.169538685	0.363452554	541	419	1586	1095	2076	1222	1311	1754
ENSECAG00000002165	2.830659384	0.169684693	0.363675156	89	283	75	116	146	99	153	53
ENSECAG00000004931	3.047989249	0.169706537	0.363675156	119	69	257	242	172	61	204	116
ENSECAG00000007228	6.592560158	0.169762857	0.363727245	815	575	2059	2117	2130	2619	3002	1843
ENSECAG00000024924	3.794283885	0.169823431	0.363788429	142	272	126	214	401	243	317	423
ENSECAG00000013759	8.510047371	0.169857702	0.363793254	5209.02	10954	5433.02	6730	4669	7272	5712.97	7392
ENSECAG00000000516	2.944080499	0.170304284	0.364680979	22	76	132	214	187	134	255	214
ENSECAG00000004843	5.656685974	0.17041851	0.364856813	866	928	776	1202	846	923	852	1080
ENSECAG00000021851	4.973112173	0.170491194	0.364943661	193	491	372	826	668	852	786	756
ENSECAG00000017349	5.62735116	0.170561744	0.365025906	897	1275	675	785	922	884	922	780
ENSECAG00000012693	3.948296687	0.170596987	0.365032575	332	360	195	269	454	199	233	153
ENSECAG00000010810	7.015716664	0.170639165	0.365054075	1553	1354	1758	2516	4887	2437	2845	2729
ENSECAG00000014963	4.449132205	0.170696764	0.365108552	171	341	322	457	505	440	482	709
ENSECAG00000016612	7.327110086	0.170776581	0.365198834	1659	1866	2313	3368	4185	3092	4273	4401
ENSECAG00000017149	5.689860166	0.170803257	0.365198834	525	434	1028	1047	1371	1045	1159	1480
ENSECAG00000009629	5.096293036	0.170936437	0.365376762	318	438	1145	975	522	332	390	1200
ENSECAG00000003995	3.130458124	0.170950789	0.365376762	133	146	187	204	160	124	164	179
ENSECAG00000011267	7.084951037	0.17109226	0.365610355	1582	1684	2204	2089	3377	3408	2843	3757
ENSECAG00000021994	5.917943503	0.171477908	0.36636555	580	1312	1367	1525	1147	1016	1297	907
ENSECAG00000006429	6.925433713	0.171540164	0.366397569	3311	1738	2553	1443	1351	3441	1328	1682
ENSECAG00000018384	3.09000588	0.171577482	0.366397569	101	80	150	125	222	104	266	269
ENSECAG00000008438	0.382152914	0.171589638	0.366397569	13	15	11	23	24	20	31	52
ENSECAG00000023081	5.58773437	0.171693006	0.366486813	316	489	1046	1032	1518	955	1147	1098
ENSECAG00000022269	7.037458254	0.171695943	0.366486813	1320	3225	2893	3002	2007	2208	2179	3012
ENSECAG00000015043	5.233899498	0.171844578	0.366735179	423	507	496	756	923	667	873	1219
ENSECAG00000019348	1.597163927	0.172066691	0.367076348	26	51	26	55	67	52	73	110
ENSECAG00000024418	4.859230251	0.172079167	0.367076348	392	304	459	459	933	495	738	675
ENSECAG00000012559	7.504811205	0.172101365	0.367076348	4063	4784	2246	2262	3156	2879	3264	3065
ENSECAG00000004260	4.264883299	0.172214279	0.367248243	157	274	230	479	411	422	453	595
ENSECAG00000016136	4.300668131	0.172263492	0.367284255	428	360	280	371	427	286	351	340
ENSECAG00000024732	5.536150893	0.172543113	0.367811416	507	476	917	792	1298	967	1047	1189
ENSECAG00000008761	1.009749913	0.172672673	0.368018555	16	64	39	39	43	20	31	35
ENSECAG00000011885	1.351079524	0.172711349	0.368031948	20	28	76	91	34	35	39	58
ENSECAG00000009216	0.447588019	0.172763222	0.368050036	16	16	17	14	39	19	36	38
ENSECAG00000020540	3.889718742	0.172784623	0.368050036	141	247	221	227	296	280	375	511
ENSECAG00000011899	7.038215273	0.172934603	0.368273287	1351	1320	2282	2545	2657	3363	2610	4463
ENSECAG00000022028	4.81004943	0.172954256	0.368273287	722	585	345	364	649	473	438	348
ENSECAG00000013048	3.647896528	0.173057973	0.368276441	125	424	199	227	230	194	201	250
ENSECAG00000009488	9.395227078	0.173064529	0.368276441	12729	17877	6846	14543	10943	11187	15098	7929
ENSECAG00000000253	5.023530926	0.173081234	0.368276441	506	780	507	628	530	496	640	715
ENSECAG00000024179	4.831167929	0.173098542	0.368276441	274	427	391	578	735	489	769	773
ENSECAG00000016439	7.2715947	0.173117803	0.368276441	3890	4199	1614	1552	2872	1918	2630	2955
ENSECAG00000002756	3.195114154	0.173244452	0.368428646	91	93	183	145	252	156	235	263
ENSECAG00000017675	3.419421658	0.173254203	0.368428646	111	188	210	356	176	139	220	232
ENSECAG00000022692	9.118009989	0.173338274	0.368538448	7631	7815	14015	15419	12407	8824	9336	8234
ENSECAG00000004316	1.256932428	0.173468227	0.36871657	27	25	25	46	69	36	59	71
ENSECAG00000009644	5.900248838	0.173490363	0.36871657	425	1212	1285	2036	886	1176	930	1277
ENSECAG00000016420	4.920479844	0.173519407	0.36871657	358	368	446	581	759	561	785	830
ENSECAG00000014271	7.143010552	0.173586845	0.368790899	1349	1836	2102	2866	3128	2816	4024	4037
ENSECAG00000013918	5.889966198	0.173731683	0.36902961	684	1353	986	1599	1192	1065	1091	952
ENSECAG00000018765	5.281888608	0.173848299	0.369208295	414	562	657	615	1002	873	833	1041
ENSECAG00000009932	2.957675779	0.173994309	0.369449325	45	90	145	170	177	196	169	224

ENSECAG00000018981	2.10394191	0.174097141	0.369598601	22.6574	63	27.04	123.175	72.5927	103.658	113.403	147.608
ENSECAG00000013776	3.429564819	0.17458557	0.370566271	107	132	166	206	321	158	301	282
ENSECAG00000000551	5.519664968	0.174756763	0.370860355	274	344	612	1514	1296	861	1732	728
ENSECAG00000022950	4.94145079	0.175211631	0.371756219	403	349	850	824	634	421	509	683
ENSECAG00000016819	4.524669818	0.175535165	0.372373142	219	247	414	483	551	472	553	658
ENSECAG00000015951	4.425585085	0.175658888	0.372566042	168	310	338	457	410	591	524	545
ENSECAG00000008478	4.250600698	0.175737342	0.372662874	296	469	267	397	302	262	359	461
ENSECAG00000008427	2.850863448	0.17597963	0.373107026	49	96	62	204	160	146	157	267
ENSECAG00000020981	3.557817009	0.176050661	0.373187987	131	212	237	353	185	187	226	253
ENSECAG00000010331	3.622053806	0.176118271	0.373235736	160	203	347	239	231	207	203	242
ENSECAG00000020691	4.276779792	0.176138885	0.373235736	213	490	330	484	273	271	301	569
ENSECAG00000002156	0.997460707	0.176214319	0.373325954	22	31	46	59	32	24	27	49
ENSECAG00000014563	3.925671568	0.176456558	0.373769465	144	116	319	295	490	313	305	389
ENSECAG00000016141	3.604854949	0.176878876	0.374576649	156	102	209	171	383	103	310	465
ENSECAG00000013506	4.291621874	0.176903564	0.374576649	225	409	359	538	310	246	393	494
ENSECAG00000013791	6.004635597	0.176959769	0.374625841	907	1898	924	1102	1096	910	1173	1458
ENSECAG00000019093	6.117546002	0.17699863	0.374638308	727	1872	1430	1351	928	1240	1311	1541
ENSECAG00000017334	4.792252594	0.177369471	0.375296579	331	376	779	709	685	458	431	415
ENSECAG00000008782	1.776627484	0.177375693	0.375296579	44	50	107	68	72	38	61	60
ENSECAG00000008516	4.550303854	0.177446237	0.375375935	260	356	659	568	482	412	368	439
ENSECAG00000000572	2.595663955	0.177608669	0.375649608	31	50	95	173	167	120	193	124
ENSECAG00000017366	0.489881835	0.177676744	0.375723649	12	8	28	18	17	62	26	29
ENSECAG00000014962	2.99427429	0.177728358	0.37576286	51	86	183	118	155	151	131	386
ENSECAG00000014363	0.752123265	0.177784717	0.375812085	14	53	27	37	27	24	29	26
ENSECAG00000014291	6.985855744	0.177847255	0.375874351	1890	4024	1732	1984	2552	1671	2160	2506
ENSECAG00000010635	5.599559156	0.177990162	0.376106419	577	1039	1073	1007	845	847	796	1103
ENSECAG00000014225	1.00626875	0.178066802	0.376198402	14	29	49	77	29	40	18	39
ENSECAG00000024024	2.469886795	0.178515022	0.377075234	58	67	168	161	117	72	105	84
ENSECAG00000004755	7.795365747	0.178585196	0.377153345	2486	6998	3626	4308	3380	3872	3786	4511
ENSECAG00000015490	2.751168687	0.178686782	0.377297754	46	74	105	165	177	160	148	175
ENSECAG00000015673	3.788620992	0.178786337	0.377437823	107	148	207	363	338	293	343	377
ENSECAG00000014481	5.833005211	0.178828806	0.377457345	470	764	901	1298	1244	1299	1329	1648
ENSECAG00000013804	0.227425089	0.179055862	0.377866401	4	11	21	21	17	46	21	26
ENSECAG00000015818	6.137728672	0.179098155	0.377885467	998	1377	1466	1476	1346	1139	1311	1399
ENSECAG00000000738	3.494046426	0.179189529	0.378008063	154	108	139	222	287	221	293	305
ENSECAG00000009682	3.409442415	0.179227532	0.378018047	117	176	323	221	155	170	194	239
ENSECAG00000006424	3.249620967	0.179314445	0.378131167	114	132	281	233	178	207	118	154
ENSECAG00000000364	3.300689577	0.179366864	0.378168169	108	151	107	181	264	171	259	276
ENSECAG00000021573	3.526328211	0.179398559	0.378168169	126	139	145	250	284	248	256	335
ENSECAG00000017231	5.71776345	0.179588397	0.378498121	468	1079	1163	1501	698	1039	999	1089
ENSECAG00000006350	3.508899802	0.179829606	0.378936199	373	305	25	131	169	155	214	155
ENSECAG00000017704	5.75284532	0.180073288	0.379349917	661	568	914	946	1680	930	1189	1469
ENSECAG00000008627	4.623456072	0.180121548	0.379349917	437	523	385	467	436	372	476	518
ENSECAG00000024412	5.074335757	0.180157634	0.379349917	412	1291	511	357	532	601	696	457
ENSECAG00000000580	7.420553995	0.180159492	0.379349917	2492	3694	3058	3960	2814	2834	3126	3559
ENSECAG00000024657	5.932520699	0.180198307	0.379361343	653	928	962	973	1564	1074	1535	1779
ENSECAG00000001013	4.006503955	0.180425099	0.379768427	237	330	271	379	320	243	246	367
ENSECAG00000009782	2.48507372	0.18084639	0.38058468	92	131	100	96	98	94	102	92
ENSECAG00000012171	7.050185992	0.180919555	0.380668145	1815	2778	2714	2901	2385	2162	2383	2661
ENSECAG00000023197	5.420728421	0.180953477	0.380669025	394	872	793	1371	748	708	811	875
ENSECAG00000017531	7.534869778	0.181036895	0.38077401	3226	2942	3986	4042	3630	3188	3536	2787
ENSECAG00000004777	0.340600418	0.181073204	0.380779891	5	14	49	42	23	3	11	36
ENSECAG00000011499	3.690396927	0.181202514	0.380981303	107	228	177	229	293	270	345	348
ENSECAG00000007910	3.299546322	0.181310973	0.381138811	150	109	89	170	290	166	268	257
ENSECAG00000022750	5.246654048	0.181422711	0.381195639	550	891	538	885	617	609	712	855
ENSECAG00000011322	5.982069655	0.181442713	0.381195639	1434	1047	1091	995	1237	997	944	1376
ENSECAG00000002977	1.871874273	0.18147057	0.381195639	15	68	51	55	61	45	82	198
ENSECAG00000004662	1.00875268	0.181472207	0.381195639	15	29	19	40	36	44	78	37
ENSECAG00000024501	6.812516597	0.181561015	0.381311693	1468	2506	2101	2594	1970	1919	2118	2133
ENSECAG00000023705	5.024612825	0.181907248	0.38196824	340	429	498	648	924	602	765	851
ENSECAG00000012166	6.220186971	0.181971	0.382031505	713	972	4240	1790	13	2802	11	273
ENSECAG00000019842	5.431630108	0.182559877	0.383196993	472	435	670	982	1031	878	1018	1260
ENSECAG00000016198	1.726013923	0.182628993	0.383204698	20	25	62	70	40	52	84	167
ENSECAG00000016403	6.181162431	0.182631002	0.383204698	797	1374	1374	2195	1179	1329	1256	1560
ENSECAG00000006901	4.8037544	0.18268933	0.383256309	323	461	350	441	642	457	754	887
ENSECAG00000010971	2.193558595	0.182724701	0.383259747	39	52	101	205	73	75	100	50
ENSECAG00000023171	4.329883447	0.182766222	0.383276083	217	358	541	447	372	334	363	403
ENSECAG00000010501	4.447723897	0.183048882	0.383735831	250	462	482	477	418	339	378	477
ENSECAG00000017673	8.134954329	0.183081615	0.383735831	4282	5767	6287	5732	3551	6476	6124	2892
ENSECAG00000017953	6.915548858	0.183086775	0.383735831	1176	1565	2145	2039	3345	2333	2720	3530
ENSECAG00000008524	1.899977572	0.183292613	0.384096398	38	49	50	62	114	53	108	92
ENSECAG00000021771	6.51680694	0.183408083	0.384267498	2346	1609	1137	1496	1538	1630	1612	1624
ENSECAG00000001075	6.467142344	0.183945363	0.385285436	1214	1772	1908	1822	1591	1529	1530	1851
ENSECAG00000010522	6.111001451	0.183961757	0.385285436	941	568	1812	2336	1403	1236	1239	980
ENSECAG00000019415	6.204577651	0.184399065	0.386130146	707	937	1194	1545	1788	1639	1838	1847
ENSECAG00000010834	2.033836719	0.184453237	0.386172412	35	42	79	56	98	178	58	65
ENSECAG00000016302	2.57142123	0.184507516	0.386214885	57	82	86	105	127	112	169	175
ENSECAG00000024760	5.898991535	0.184678641	0.386501881	602	822	1764	1618	1267	737	1258	1082
ENSECAG00000009951	2.14508067	0.184822374	0.386731457	34	60	95	190	62	66	71	99
ENSECAG00000000478	2.838909702	0.18491173	0.386847188	70	59	148	111	148	253	176	120
ENSECAG00000009804	0.747123957	0.185849848	0.388682405	17	28	14	21	32	26	42	66
ENSECAG00000009028	6.452274271	0.185857376	0.388682405	1687	1675	1521	1540	1483	1476	1494	1918
ENSECAG00000022758	0.960263828	0.185984113	0.388875872	13	16	35	40	43	46	42	56
ENSECAG00000023132	2.440624926	0.186102225	0.389051238	60	185	87	96	84	37	93	155
ENSECAG00000010851	6.676139811	0.186143856	0.389066684	1456	2004	1885	2492	1985	1700	1715	2079

ENSECAG00000012221	6.546938784	0.186391428	0.389512488	1106	1585	2172	2461	1726	1560	1754	1820
ENSECAG00000021193	6.328440764	0.186498626	0.389664838	1061	1376	2059	1662	1656	1404	1288	1538
ENSECAG00000015336	8.117045253	0.186597387	0.389757687	4601	6218	4751	5492	5016	4911	4737	5044
ENSECAG00000020866	4.612658883	0.186623384	0.389757687	284	280	457	383	633	478	594	663
ENSECAG00000021731	3.610385494	0.186645976	0.389757687	132	101	224	243	324	219	332	322
ENSECAG00000018321	2.977415237	0.18679249	0.38993928	98	79	137	114	213	148	179	237
ENSECAG00000027594	1.655478947	0.186801575	0.38993928	8	35	161	89	89	34	10	48
ENSECAG00000016138	2.959518781	0.18690232	0.389979811	144	197	118	120	94	154	162	118
ENSECAG00000021172	4.112280209	0.186913952	0.389979811	142	199	229	466	410	312	421	559
ENSECAG00000007336	2.649218986	0.186923962	0.389979811	60	50	108	133	212	142	120	142
ENSECAG00000016767	4.587561933	0.187262485	0.390614349	466	589	369	318	458	404	404	447
ENSECAG00000020774	3.602480398	0.187331956	0.390635201	89	97	257	258	351	164	249	471
ENSECAG00000010068	7.994622159	0.187341244	0.390635201	4356	4549	4939	5616	5138	4127	4512	4445
ENSECAG00000011075	4.382354935	0.187492102	0.390822095	272	424	449	432	377	387	387	375
ENSECAG00000015634	6.697697943	0.187499669	0.390822095	978	1085	1757	2316	2393	2590	2353	2785
ENSECAG00000006403	5.575237942	0.187833616	0.391446359	640	829	984	1176	857	814	785	1108
ENSECAG00000008214	4.077922454	0.1879105	0.39153477	170	167	351	289	392	366	360	530
ENSECAG00000022169	1.631294688	0.187968414	0.391539741	31	41	39	55	75	53	82	92
ENSECAG00000022891	3.786132486	0.187981807	0.391539741	136	136	250	267	285	204	333	563
ENSECAG00000012378	4.061414193	0.188053125	0.391616497	159	310	216	260	370	327	395	544
ENSECAG00000010197	3.346840395	0.188116468	0.391676618	83	105	242	140	302	145	285	284
ENSECAG00000027060	1.035534749	0.188442991	0.392284586	7	27	99	46	20	35	13	54
ENSECAG00000024596	3.802548791	0.188510198	0.392352605	126	192	240	256	334	284	329	405
ENSECAG00000024719	8.472900925	0.188845898	0.392979321	8808	11705	3952	2681	9512	2006	8426	1754
ENSECAG00000003636	7.390946121	0.188947	0.39311771	2116	1365	2297	3690	4490	3165	4209	4925
ENSECAG00000015437	0.698571561	0.1892516	0.393679363	18	19	22	19	22	28	46	63
ENSECAG00000021689	0.174381452	0.18958104	0.394292473	5	9	10	33	19	23	23	43
ENSECAG00000010726	1.366273279	0.189641393	0.394300063	38	38	39	90	30	53	52	32
ENSECAG00000020347	4.307652543	0.189688094	0.394300063	170	275	324	411	487	390	490	543
ENSECAG00000021642	2.922169028	0.1896888	0.394300063	67	32	124	185	357	72	215	143
ENSECAG00000024842	8.15720858	0.190036319	0.394886926	2175	3329	3303	7689	6159	7904	10022	4298
ENSECAG00000024352	5.503147892	0.190040636	0.394886926	393	588	368	1294	1125	891	1757	712
ENSECAG00000012512	7.265585797	0.190085926	0.394908811	1884	3139	2999	4069	2707	2315	3089	3053
ENSECAG00000011332	2.745386409	0.190244165	0.395165303	44	57	115	174	154	181	137	188
ENSECAG00000014336	4.999404144	0.190434164	0.395484456	268	305	729	555	546	527	801	1349
ENSECAG00000015175	7.320933666	0.19046743	0.395484456	2275	5103	1614	3367	2382	2523	2708	3583
ENSECAG00000008863	4.852415183	0.190867173	0.396242065	255	470	494	478	748	607	664	758
ENSECAG00000017766	4.294245053	0.190925066	0.396289844	204	207	350	393	465	422	523	476
ENSECAG00000023608	2.292826463	0.191011584	0.396397008	22	108	48	271	45	81	81	103
ENSECAG00000000291	8.882728492	0.191104131	0.396516643	4566	6699	4363	10984	11357	11569	15095	8920
ENSECAG00000018551	5.407543687	0.191296144	0.396784681	416	557	682	874	1080	827	896	1301
ENSECAG00000013991	0.46990166	0.191303158	0.396784681	2	16	11	43	25	33	44	32
ENSECAG00000015363	3.888943125	0.1915571	0.39723887	133	148	321	256	331	245	400	487
ENSECAG00000023256	7.802371798	0.191717044	0.397498004	1837	3387	3756	3612	4874	3546	4742	9679
ENSECAG00000018054	2.285410587	0.191891255	0.397786616	43	58	59	113	132	108	145	88
ENSECAG00000012094	3.77245787	0.191943459	0.397822252	88	220	235	261	313	277	328	418
ENSECAG00000008094	5.608270392	0.192034438	0.397938224	262	522	470	1686	988	1573	1472	785
ENSECAG00000018921	1.860791777	0.192225316	0.39826113	33	21	76	67	97	71	102	88
ENSECAG00000017146	7.055972368	0.192340254	0.398426612	989	1115	3321	2289	2817	3436	3249	3744
ENSECAG00000018174	0.396232814	0.19254222	0.398772275	16	7	25	13	38	27	30	31
ENSECAG00000015662	2.147927542	0.192589255	0.398796995	65	43	66	50	140	75	120	104
ENSECAG00000007113	5.215329195	0.192659309	0.398869364	464	424	604	652	929	728	848	1088
ENSECAG00000026944	3.345358223	0.192865276	0.399223041	129	180	115	112	226	204	287	286
ENSECAG00000015236	4.9326599	0.193225887	0.399896635	227	182	837	518	894	738	819	567
ENSECAG00000016405	1.432950913	0.193278362	0.399932389	17	29	46	56	73	40	82	69
ENSECAG00000008029	8.103997849	0.193314463	0.399934256	4772	6116	4116	5852	5020	5061	4926	4411
ENSECAG00000013055	5.317510267	0.193350478	0.399935943	461.992	1054.99	698	831.027	566.996	840.991	647.994	837.975
ENSECAG00000015742	7.789833105	0.193542364	0.400259981	3191	5586	4215	3865	3655	3164	3853	5264
ENSECAG00000010756	4.919596255	0.193658372	0.400390767	431	373	453	423	759	577	833	764
ENSECAG00000005293	5.211170677	0.193695491	0.400390767	566	700	615	895	655	590	727	786
ENSECAG00000015409	5.371506831	0.193737591	0.400390767	366	797	1042	1080	597	798	815	816
ENSECAG00000019321	6.235161389	0.193746562	0.400390767	637	869	1525	1457	1916	1375	1768	2317
ENSECAG00000011091	3.718909915	0.193825221	0.400480479	279	289	201	184	253	186	188	310
ENSECAG00000021257	0.264543166	0.193871913	0.400504122	9	12	7	30	13	45	31	24
ENSECAG00000011652	7.75991158	0.193918676	0.400527903	2698	4206	4324	5861	3763	3677	3630	4616
ENSECAG00000020069	4.281897079	0.194078828	0.40078583	201	399	528	378	366	325	278	454
ENSECAG00000001393	4.89868317	0.194157609	0.40087566	579	470	543	597	672	500	454	551
ENSECAG00000018925	5.240511597	0.194248936	0.400991355	459	1106	535	778	601	713	543	883
ENSECAG00000009324	5.528606107	0.194312263	0.401049215	967	1138	665	541	908	652	785	961
ENSECAG00000005870	4.338948226	0.194445782	0.401218942	133	338	247	493	457	468	457	589
ENSECAG00000017473	4.847757459	0.194465122	0.401218942	300	449	332	595	854	663	535	721
ENSECAG00000004386	0.313483983	0.194553804	0.401329034	4	15	13	31	24	31	42	20
ENSECAG00000014112	5.909114404	0.194652074	0.401418178	484	648	1123	1383	1638	1443	1342	1363
ENSECAG00000017640	7.02751509	0.19467855	0.401418178	1051	1315	2398	2994	3383	2765	2979	3763
ENSECAG00000015797	2.544863772	0.194703008	0.401418178	57	71	102	273	115	122	71	77
ENSECAG00000019556	4.698263819	0.194814381	0.401574928	243	453	229	582	591	515	628	813
ENSECAG00000016476	3.973600222	0.195049233	0.401986103	146	294	393	414	298	193	300	361
ENSECAG00000000755	6.475833248	0.195274269	0.402376904	750	874	1171	2552	2392	1424	1956	3145
ENSECAG00000017183	1.036269942	0.195457206	0.402597258	21	24	17	44	43	34	49	75
ENSECAG00000018547	5.007870185	0.195497733	0.402597258	511	859	454	545	594	493	541	723
ENSECAG00000025094	3.547913245	0.195506887	0.402597258	89	131	160	312	300	217	218	428
ENSECAG00000009295	6.858393733	0.195522942	0.402597258	1505	1964	2473	3136	2193	1889	2020	2379
ENSECAG00000016199	4.002084237	0.195844455	0.403071805	178	274	444	349	335	223	283	332
ENSECAG00000008042	4.684068816	0.195869624	0.403071805	228	286	421	612	638	437	687	739
ENSECAG00000008093	6.539685811	0.195873246	0.403071805	852.99	1258	1316	2118	2129	2011	2248	2654

ENSECAG00000012787	7.197813379	0.19589531	0.403071805	3361	3516	1806	1975	2578	2417	2627	2569
ENSECAG00000000303	4.388751189	0.195999834	0.403160969	163	146	892	607	426	365	352	266
ENSECAG00000020637	9.490016218	0.196043216	0.403160969	8813	15694	15172	16836	12814	11287	12813	14706
ENSECAG00000009899	3.162652835	0.196047154	0.403160969	65	124	144	188	207	151	265	256
ENSECAG00000011696	9.592785297	0.196109639	0.403160969	8109	7829	11246	17153	18463	17432	20584	20116
ENSECAG00000014989	5.665380353	0.19611606	0.403160969	797	1047	917	999	886	904	740	1240
ENSECAG00000022850	1.637782145	0.196299394	0.403464854	30	55	21	53	62	51	66	136
ENSECAG00000013025	0.586889798	0.196527915	0.403861489	9	13	44	62	18	23	30	19
ENSECAG00000013909	5.489727189	0.196747675	0.404239979	451	908	813	1377	970	674	712	978
ENSECAG00000024786	6.787074438	0.196785185	0.404243947	1171	1671	1629	1840	3041	2009	2744	3033
ENSECAG00000009480	2.363122422	0.19722947	0.405083376	36	68	92	95	134	87	147	136
ENSECAG00000013810	2.234319589	0.197452928	0.405373985	13	61	135	32	71	175	126	106
ENSECAG00000017483	0.633238409	0.197456903	0.405373985	15	4	27	31	33	47	24	46
ENSECAG00000021652	6.247312681	0.197477998	0.405373985	620	686	1235	2021	2169	1232	2485	1601
ENSECAG00000021160	6.465991149	0.197571456	0.405492572	990	1433	1271	1317	2168	1534	2326	2635
ENSECAG00000009293	4.451608604	0.197712851	0.405694893	205	276	330	492	470	453	679	500
ENSECAG00000019642	4.288377167	0.197741447	0.405694893	128	407	523	538	410	300	396	285
ENSECAG00000004932	5.242800364	0.198010508	0.406173567	541	722	857	712	846	618	572	768
ENSECAG00000014215	6.35179586	0.198074824	0.406216669	1366	992	967	979	2109	1591	2303	2050
ENSECAG00000009159	4.611351442	0.198103025	0.406216669	130	316	490	427	158	1015	637	636
ENSECAG00000024534	5.552548571	0.198228883	0.406401402	455	639	717	997	1056	887	1118	1478
ENSECAG00000013643	0.147778892	0.198373499	0.406624517	19	10	13	2	24	19	27	39
ENSECAG00000013093	2.520657406	0.198499247	0.406782975	71	74	171	138	99	86	79	139
ENSECAG00000020872	6.322863027	0.198522408	0.406782975	1074	942	1406	1041	1994	1699	2055	2031
ENSECAG00000019470	1.095544146	0.198604699	0.406878217	17	33	18	43	32	61	55	58
ENSECAG00000007703	6.083096404	0.198747724	0.407097825	691	743	1217	1371	1485	1644	1485	1924
ENSECAG00000019536	4.860266575	0.198851086	0.407236127	404	579	605	590	568	426	652	502
ENSECAG00000007147	8.246281848	0.198936535	0.407297808	3285	4649	4143	4969	7580	6708	7320	8353
ENSECAG00000014240	3.617308268	0.198970262	0.407297808	208	96	503	160	156	180	288	205
ENSECAG00000021528	6.096221206	0.198988746	0.407297808	698	806	1056	1505	1384	1815	1651	1712
ENSECAG00000010739	-0.006173798	0.199078855	0.407408854	14	8	20	35	19	7	11	23
ENSECAG00000020118	5.885939909	0.199174605	0.407531399	518	902	637	1434	914	1696	1467	1668
ENSECAG00000000576	1.962593006	0.199241706	0.407595294	26	100	91	97	63	70	61	69
ENSECAG00000010095	1.884774109	0.19946164	0.407971764	26	46	69	60	83	72	141	64
ENSECAG00000009336	7.646114591	0.199543556	0.408065853	1396	1665	2829	6136	5209	4397	5845	4560
ENSECAG00000007244	3.746805542	0.199630274	0.408169727	147	106	178	350	307	355	338	301
ENSECAG00000010505	2.928605279	0.199713861	0.408267163	98	102	209	196	183	114	141	89
ENSECAG00000008334	7.766588621	0.199793627	0.408356754	1641	2630	4001	4599	5055	4907	5679	5819
ENSECAG00000009251	5.127994815	0.199998512	0.408576063	678	802	551	502	562	621	791	524
ENSECAG00000001410	0.759499672	0.199999225	0.408576063	23	49	35	21	13	12	44	38
ENSECAG00000019052	6.41279809	0.200012285	0.408576063	1021	466	660	2555	3126	1226	1068	3569
ENSECAG00000004503	2.783547054	0.200044766	0.408576063	71	89	174	232	122	146	112	90
ENSECAG00000001114	2.999501899	0.20008614	0.408587118	90	77	143	132	156	235	224	157
ENSECAG00000008819	7.664777412	0.200148247	0.4086405	2647	4332	3277	5654	3592	3315	3439	4347
ENSECAG00000011843	3.670532273	0.20029719	0.408779232	167	150	198	195	320	243	314	360
ENSECAG00000021859	2.020510379	0.200310429	0.408779232	96	57	63	98	24	126	40	62
ENSECAG00000016535	3.975973341	0.200378902	0.408779232	108	143	761	339	259	294	318	167
ENSECAG00000007814	4.464445538	0.200391905	0.408779232	171	379	272	490	501	565	524	528
ENSECAG00000023030	4.56144316	0.200396086	0.408779232	316	326	318	369	623	470	555	630
ENSECAG00000007848	1.860113103	0.200487943	0.40884041	21	36	61	87	92	55	84	131
ENSECAG00000013901	7.700970527	0.200503269	0.40884041	3837	4563	2862	4387	3416	2928	3283	5379
ENSECAG00000015946	3.963699373	0.200534026	0.40884041	255	249	358	307	335	294	258	235
ENSECAG00000008142	3.496622519	0.200584016	0.408868962	122	111	182	236	343	210	250	295
ENSECAG00000009399	3.766930837	0.200673994	0.408978999	141	210	344	384	265	231	236	256
ENSECAG00000004669	2.293487704	0.200924769	0.409416647	107	45	8	24	124	64	269	78
ENSECAG00000006983	6.765437412	0.201049663	0.409597682	2998	1509	1514	1802	2039	2145	1537	1798
ENSECAG00000011565	5.560319811	0.201088228	0.409602805	415	553	845	1040	990	1118	989	1451
ENSECAG00000003722	2.651280156	0.201206515	0.409703189	54	104	57	128	138	74	197	228
ENSECAG00000015553	2.510855398	0.201209628	0.409703189	47	78	128	242	54	47	170	111
ENSECAG00000008091	8.259705362	0.201311461	0.409820388	5825	4930	6186	6153	6120	5407	5032	5228
ENSECAG00000006748	5.819364902	0.201339325	0.409820388	1106	1036	912	1065	900	1156	1098	931
ENSECAG00000019326	6.738635151	0.201465719	0.41000421	1439	2903	1542	2250	2007	1976	1866	1805
ENSECAG00000026845	4.838553627	0.201732022	0.410472642	170	739	585	843	411	481	541	627
ENSECAG00000003981	7.226751084	0.201822406	0.410583023	1540	1017	3549	2150	4091	1723	4381	5265
ENSECAG00000010439	-0.150832857	0.201918809	0.410705606	3	2	21	16	19	17	37	11
ENSECAG00000011181	6.196714828	0.201972243	0.410740761	472	754	1262	2001	1516	1874	1468	2352
ENSECAG00000025153	6.094959746	0.202011447	0.410746969	916	1357	1480	1609	1987	1040	1211	555
ENSECAG00000012948	7.742744279	0.202186058	0.411028448	2997	5780	3586	3956	3385	4022	3586	4264
ENSECAG00000016920	4.233309035	0.202294157	0.411174635	155	274	263	435	439	366	439	579
ENSECAG00000003503	5.161396157	0.202554238	0.411629629	386	439	565	734	871	656	905	1014
ENSECAG00000022717	6.664459033	0.202631492	0.411712986	1129	1244	1409	2083	2323	1635	2532	3572
ENSECAG00000020376	1.358221199	0.202737016	0.411853742	32	17	40	43	71	59	74	43
ENSECAG00000026929	1.810742212	0.202841433	0.4119922	22	58	41	66	32	86	126	105
ENSECAG00000002691	5.954945912	0.202961783	0.412162964	856	1357	979	1487	1167	1019	1148	1275
ENSECAG00000012399	7.598485243	0.203212142	0.412597634	2605	1649	3651	2589	6276	3226	5246	4663
ENSECAG00000005354	3.515116122	0.203383239	0.412871245	124	65	215	245	318	198	354	261
ENSECAG00000012249	1.994590786	0.203439106	0.412910884	79	70	32	132	99	70	38	52
ENSECAG00000016099	5.654796872	0.203495035	0.412950632	751	992	564	1646	1179	706	1121	589
ENSECAG00000007177	3.261600678	0.203725672	0.413344838	113	67	190	164	265	171	272	236
ENSECAG00000008262	7.526153559	0.203954782	0.413735804	2687	4342	3853	3056	3236	2884	3110	4108
ENSECAG00000018471	7.78103922	0.204092587	0.413941444	3010	5055	4766	3995	3373	3832	3586	5086
ENSECAG00000012851	6.256012608	0.204177074	0.41398769	984	731	1182	1516	2076	1559	1843	1929
ENSECAG00000010374	5.27591823	0.204215747	0.41398769	454	622	911	1012	676	679	620	920
ENSECAG00000009544	0.668356675	0.204224696	0.41398769	7	34	28	64	28	30	16	23
ENSECAG00000017791	7.057865639	0.20433711	0.414141678	1832	3349	1821	3255	2515	1907	2509	2717

ENSECAG00000009016	4.337914737	0.20437848	0.414151647	252	337	385	600	440	320	400	322
ENSECAG00000012657	6.653493566	0.204809802	0.414951671	1123	1503	1428	1731	2441	2213	2437	2665
ENSECAG00000009985	5.018060379	0.204954971	0.415171757	299	301	604	740	704	925	751	717
ENSECAG00000008405	3.672524102	0.205065986	0.415283146	87	79	298	252	224	220	179	720
ENSECAG00000021051	7.431495337	0.205083061	0.415283146	2254	3856	2693	4669	3383	2428	3354	3356
ENSECAG00000001155	7.028260508	0.205134159	0.415312601	800	1583	1866	3609	2246	3320	3088	4360
ENSECAG00000007905	4.919011313	0.205500571	0.41598031	330	604	793	590	652	427	556	608
ENSECAG00000008288	4.71172857	0.205697713	0.416296571	322	234	481	470	814	563	582	565
ENSECAG00000024126	6.351706362	0.205739535	0.416296571	666	877	1241	2185	2143	1496	1785	2603
ENSECAG00000014571	1.953439643	0.205766726	0.416296571	31	50	52	83	87	57	106	132
ENSECAG00000024078	5.083164426	0.205940829	0.416486568	591	804	534	534	680	501	606	702
ENSECAG00000019990	5.194941107	0.205949209	0.416486568	305	580.997	753.998	1372	735.997	510.998	706	732.997
ENSECAG00000010033	7.049222393	0.205970606	0.416486568	1813	2195	3153	3130	2754	2267	2584	1928
ENSECAG00000015618	5.709875134	0.206080294	0.416634217	681	779	811	644	1469	885	1446	1283
ENSECAG00000012455	5.863047003	0.2061347	0.416670069	893	1632	816	981	836	853	959	1617
ENSECAG00000011296	5.222050477	0.206588663	0.417513409	718	700	648	639	569	654	633	895
ENSECAG00000025867	5.121993954	0.206699418	0.417662953	52	402	744	2548	1305	420	52	94
ENSECAG00000012526	3.767612365	0.206781152	0.417753813	257	206	335	211	267	216	305	175
ENSECAG00000018988	6.859800591	0.206868163	0.41779885	1382	1124	2049	2093	3142	2617	2682	2889
ENSECAG00000024751	3.917144792	0.206876988	0.41779885	96	177	225	422	381	298	409	384
ENSECAG00000013850	3.999077375	0.206945958	0.41784929	115	316	193	300	307	355	343	568
ENSECAG00000019675	9.472807022	0.206975515	0.41784929	8932	9141	9954	11222	17539	15025	18867	18901
ENSECAG00000000286	6.721053071	0.207111411	0.418049362	1318	2258	1980	2558	1854	1656	1952	2331
ENSECAG00000015512	3.679904108	0.207197794	0.418149437	144	181	213	185	332	267	351	282
ENSECAG00000024040	5.897654128	0.207275623	0.41823222	700	1142	667	788	1488	986	1808	1565
ENSECAG00000004504	1.024553738	0.20751474	0.418640355	11	36	44	78	29	30	33	40
ENSECAG00000011671	7.471585191	0.207776451	0.419021251	2115	5681	2701	3187	3040	3447	3116	2821
ENSECAG00000011243	7.696641896	0.207809182	0.419021251	2894	4131	3735	5297	3400	3636	3541	4509
ENSECAG00000013366	10.22083026	0.207814183	0.419021251	10294	12815	17765	29389	25683	25556	32469	35052
ENSECAG00000010573	1.498196933	0.207928233	0.419176825	13	18	125	94	41	29	50	58
ENSECAG00000013930	7.443639015	0.208041509	0.419330783	3198	4118	2540	3042	3347	2752	2806	3579
ENSECAG00000024369	2.603314935	0.20812418	0.419405657	55	58	109	119	218	85	133	166
ENSECAG00000011287	5.12718652	0.208152482	0.419405657	182	414	312	1220	845	968	1030	598
ENSECAG00000024257	8.557093255	0.208254125	0.419536058	4743	4026	10994	11222	8975	5503	6977	4787
ENSECAG00000007521	5.68166476	0.20837219	0.41969949	419	610	927	1170	1053	1365	1212	1269
ENSECAG00000000450	5.420983018	0.208457435	0.41979677	649	1035	697	783	612	730	886	934
ENSECAG00000025132	0.536637781	0.20862821	0.420066153	21	45	13	29	23	27	13	27
ENSECAG00000000102	7.096956658	0.208697423	0.420066153	1963	1522	1723	2343	3796	2504	3327	3918
ENSECAG00000007592	2.650600696	0.208702115	0.420066153	56	127	143	175	99	117	100	126
ENSECAG00000010786	7.911392287	0.208909983	0.420340855	4202	4384	4564	4976	4187	4140	4513	4518
ENSECAG00000023483	3.767842391	0.208912586	0.420340855	142	126	289	219	350	199	328	474
ENSECAG00000018555	6.887850657	0.208968113	0.420368604	1596	1912	2656	3035	2344	1908	1864	2583
ENSECAG00000000188	4.562690932	0.209000374	0.420368604	272	275	356	482	704	477	510	575
ENSECAG00000013903	5.116837556	0.20916143	0.420607523	303	468	893	1168	712	525	553	761
ENSECAG00000018093	9.285539787	0.209193198	0.420607523	9952	11705	13658	12210	11401	10299	10908	12288
ENSECAG00000024656	2.036353347	0.209364613	0.420877696	47	32	61	81	131	52	122	101
ENSECAG00000011942	5.913241059	0.209411954	0.420898394	518	921	960	1217	1528	1328	1551	1347
ENSECAG00000017872	5.12166384	0.209483373	0.420967471	349	866	600	875	571	588	669	750
ENSECAG00000025168	5.284283484	0.209702768	0.421333837	336	536	588	904	798.994	862	904.999	1182
ENSECAG00000001869	5.874183673	0.209902019	0.421659608	393	848	952	1374	1224	1561	1584	1252
ENSECAG00000021930	7.619133309	0.210073549	0.421829137	3244	4368	3056	4164	3482	2868	3260	4693
ENSECAG00000014232	7.937171965	0.210080577	0.421829137	1832	710	5562	4163	11571	3458	10891	632
ENSECAG00000017546	5.490075439	0.21009779	0.421829137	232	568	825	1136	975	1228	1198	935
ENSECAG00000015850	4.225533653	0.210312701	0.42210787	171	293	223	420	480	336	428	572
ENSECAG00000012485	6.074108696	0.210313679	0.42210787	833	2040	1073	1126	1384	909	1266	1350
ENSECAG00000010385	1.233477922	0.210348069	0.42210787	14	21	19	59	13	158	15	49
ENSECAG00000001674	1.071079953	0.210473823	0.422285639	16	25	24	47	44	27	60	75
ENSECAG00000022110	7.966113078	0.210636518	0.422537451	5142	4695	4121	4508	4466	4287	3619	5533
ENSECAG00000006338	1.20188792	0.210990738	0.423138261	17	22	37	49	41	72	46	60
ENSECAG00000010701	5.555784083	0.211010507	0.423138261	469	759	679	875	1161	886	1277	1180
ENSECAG00000016127	5.826725331	0.211402279	0.423849073	668	761	740	1155	1601	1010	1357	1505
ENSECAG00000017572	3.873835999	0.211482332	0.423870478	239	211	317	338	255	299	200	312
ENSECAG00000024574	4.509959393	0.211487567	0.423870478	201	174	591	356	607	449	453	744
ENSECAG00000015829	2.903867118	0.211687844	0.424197052	41	82	100	221	140	153	220	230
ENSECAG00000010983	4.128414188	0.211772813	0.424292488	161	237	278	368	406	362	444	462
ENSECAG00000014400	4.712618792	0.21187601	0.424424406	274	182	580	495	714	463	615	784
ENSECAG00000016337	6.661871675	0.212012474	0.424531299	1029	1487	2570	3112	2095	1138	1891	2323
ENSECAG00000015016	2.134073847	0.212027639	0.424531299	6	40	69	118	20	73	141	247
ENSECAG00000024644	2.836735203	0.212041464	0.424531299	102	64	121	89	232	123	144	210
ENSECAG00000018796	8.007278617	0.212137289	0.424648322	2802	6176	5010	6239	4128	4542	4350	5574
ENSECAG00000024161	6.832588278	0.212209291	0.424717627	703	1151	1859	3117	2570	3848	2892	1796
ENSECAG00000011969	6.323137417	0.212263165	0.424750631	1452	1374	1615	1466	1729	1373	1592	1114
ENSECAG00000019359	3.957751896	0.21230176	0.424753054	90	335	240	657	260	261	261	313
ENSECAG00000017011	0.651294119	0.21236981	0.424814399	12	27	14	25	35	49	43	20
ENSECAG00000018678	2.83269282	0.212440088	0.424834593	65	111	93	126	193	102	259	146
ENSECAG00000008895	5.417305462	0.212455041	0.424834593	770	613	1157	621	759	563	668	1166
ENSECAG00000020651	7.761361545	0.212492078	0.424834593	1925	3284	2993	4455	4855	4608	5069	6873
ENSECAG00000022987	4.055608302	0.212698924	0.424997839	114	403	172	215	248	340	366	741
ENSECAG00000001100	3.081000554	0.212705777	0.424997839	127	169	185	153	151	111	142	210
ENSECAG00000024243	10.22801848	0.212718159	0.424997839	16146	25144	20857	31544	21466	19679	21073	24364
ENSECAG00000016981	2.830340693	0.212723351	0.424997839	69	75	120	143	148	135	215	194
ENSECAG00000018209	3.227983975	0.212761447	0.42499922	96	104	162	177	238	210	182	277
ENSECAG00000016699	6.429096276	0.212954587	0.425310251	648	1223	1453	1885	1890	2145	1876	2403
ENSECAG00000011830	4.476882383	0.212996116	0.42531843	203	230	424	482	589	408	551	600
ENSECAG00000016668	1.388708428	0.213035105	0.425321537	11	21	57	57	59	50	68	80

ENSECAG00000015132	6.322405702	0.213108906	0.425329659	1215	1602	1593	1558	1464	1176	1238	2105
ENSECAG00000018624	7.347995426	0.213114042	0.425329659	2626	3992	2342	3272	2863	2628	2896	3392
ENSECAG00000010338	2.405916027	0.213418629	0.425862744	68	62	93	49.0001	117	79	123	217.001
ENSECAG00000023017	0.483883468	0.213730756	0.426325748	9	18	14	29	41	26	11	58
ENSECAG00000016850	4.469606056	0.213751646	0.426325748	274	271	286	451	585	396	446	727
ENSECAG00000019125	7.860200904	0.213763227	0.426325748	1625	3397	3485	5297	5585	4691	5346	7439
ENSECAG00000013618	3.312010678	0.213827707	0.426379502	237	183	130	163	177	149	150	227
ENSECAG00000014127	4.941373828	0.214004676	0.426657506	252	396	621	578	734	553	718	971
ENSECAG00000001903	4.954961022	0.214053437	0.426661909	295	362	564	639	707	806	577	872
ENSECAG00000004867	5.657381718	0.214081988	0.426661909	547	623	774	1055	1486	809	1174	1406
ENSECAG00000008620	4.593359831	0.214310303	0.426957511	197	435	508	816	379	373	508	514
ENSECAG00000006116	2.357816114	0.214343543	0.426957511	34	123	125	134	73	79	118	81
ENSECAG00000015413	5.391735236	0.214370312	0.426957511	382	290	573	1294	1332	895	1046	828
ENSECAG00000022401	4.121562439	0.21438062	0.426957511	127	290	244	381	447	337	417	480
ENSECAG00000000594	4.065575988	0.214427935	0.426976902	194	369	305	414	268	329	308	321
ENSECAG00000017761	2.780745002	0.214526183	0.427040671	52	113	145	264	98	121	104	158
ENSECAG00000004219	6.198300627	0.21453513	0.427040671	648	887	1385	3375	1502	1001	1702	994
ENSECAG00000010897	9.297562606	0.214693196	0.427280451	7643	5126	10625	11822	17340	9290	17618	19258
ENSECAG00000019910	7.068550342	0.215010721	0.427837445	1277	4043	2173	3056	2382	1935	2304	3080
ENSECAG00000013545	4.764165819	0.215157146	0.428053843	381	392	639	650	571	383	535	542
ENSECAG00000006749	7.099955492	0.215260541	0.428184571	4021	2010	1594	2248	2928	1124	2279	3254
ENSECAG00000010659	4.92192085	0.215392141	0.428320999	337	539	673	789	591	439	562	688
ENSECAG00000013333	4.599573784	0.215404523	0.428320999	267	278	414	471	560	446	577	758
ENSECAG00000010105	6.161437802	0.215564243	0.428523607	541	998	1219	1586	1449	1503	2009	1954.99
ENSECAG00000003157	7.335888162	0.215581847	0.428523607	1841	2102	3677	5556	2987	2834	3077	2694
ENSECAG00000009573	11.49382592	0.215724243	0.428731543	36206	41282	78266	74385	55615	39512	52806	60336
ENSECAG00000013885	5.554433744	0.215810181	0.428731543	458	841	1086	1296	777	797	879	1065
ENSECAG00000020653	5.247658061	0.215822552	0.428731543	407	466	1098	1055	878	599	562	751
ENSECAG00000000392	6.741315382	0.21583739	0.428731543	1100	1070	1737	2447	2709	2275	2656	2752
ENSECAG00000014330	6.811123087	0.215982679	0.428945149	1098	625	2678	1377	5309	1373	4371	668
ENSECAG00000012518	5.987890561	0.216448944	0.429754164	529	650	877	1837	1388	1502	1747	1490
ENSECAG00000022392	4.858343584	0.21649059	0.429754164	187	338	546	694	538	781	521	987
ENSECAG00000018673	9.87538305	0.216503506	0.429754164	18054	20913	17294	13221	18400	12129	15980	20601
ENSECAG00000015011	5.687440213	0.216644588	0.429959093	666	656	784	872	1280	959	1168	1559
ENSECAG00000023074	2.22520263	0.216833429	0.430258719	50	50	83	76	105	107	115	123
ENSECAG00000017574	4.478689361	0.21695235	0.430419522	333	327	209	350.999	482	561	540.999	567
ENSECAG00000016949	1.134708368	0.21700115	0.430441178	22	37	18	36	46	51	59	54
ENSECAG00000023862	7.200937067	0.217050896	0.430464702	2053	2850	2882	3581	2147	1870	3594	3168
ENSECAG00000015924	7.344546001	0.21715249	0.430547442	1739	2385	2417	2776	3746	3376	4014	4775
ENSECAG00000003681	0.771402739	0.217168402	0.430547442	20	24	44	43	21	29	29	33
ENSECAG00000013971	4.669372786	0.217259699	0.430653298	329	394	331	380	617	446	646	757
ENSECAG00000026371	0.50220676	0.217306423	0.430670779	13	21	33	44	28	18	14	31
ENSECAG00000015161	6.369541245	0.217374498	0.430730563	924	1032	1347	1558	1965	2044	1931	1950
ENSECAG00000000606	2.94378226	0.217544297	0.430922252	143	78	71	73	187	84	304	216
ENSECAG00000023461	6.632160225	0.21754709	0.430922252	1130	1450	1261	1893	2487	1879	2243	3071
ENSECAG00000010079	5.286226797	0.21760079	0.430949769	460	469	599	776	1043	750	919	1021
ENSECAG00000005817	4.11135644	0.217673879	0.430949769	163	395	198	167	328	272	443	708
ENSECAG00000020616	2.564196928	0.217709474	0.430949769	45	57	84	159	115	133	127	207
ENSECAG00000016265	8.744977094	0.217712698	0.430949769	6965	14008	5377	5826	7197	6367	8192	8157
ENSECAG00000006436	5.143677697	0.217869024	0.431184088	683	375	810	830	934	519	712	326
ENSECAG00000006301	1.766475017	0.21834573	0.432051312	23	64	29	68	86	57	64	132
ENSECAG00000018144	5.328599395	0.218383267	0.432051312	626	781	740	815	813	620	800	771
ENSECAG00000020123	3.495898999	0.218508886	0.432224575	89	151	211	214	245	209	316	323
ENSECAG00000025056	6.578906079	0.218817386	0.432759466	1072	973	1845	1692	2287	1918	2283	2805
ENSECAG00000014313	0.019957365	0.218888157	0.432824094	4	18	28	31	19	11	22	7
ENSECAG00000021555	4.694853554	0.218975783	0.432922019	211	262	484	613	604	728	475	675
ENSECAG00000018106	5.345382374	0.219097756	0.433087806	311	668	541	943	950	837	1144	969
ENSECAG00000000190	1.63420284	0.219223929	0.433261833	19	27	57	71	49	96	78	76
ENSECAG00000008948	4.408664701	0.219408792	0.433489042	208	333	327	332	394	298	427	1025
ENSECAG00000014983	4.960701724	0.219445214	0.433489042	262	551	482	549	709	532	835	935
ENSECAG00000023140	6.644617347	0.219457729	0.433489042	1397	2952	1401	1792	1988	1859	1912	1335
ENSECAG00000026992	4.407561873	0.219491503	0.433489042	229	328	601	500	289	365	356	561
ENSECAG00000024026	1.905489885	0.219658214	0.433742477	28	40	73	70	72	83	92	116
ENSECAG00000021998	1.79570234	0.219696176	0.433742477	24	70	117	67	58	60	60	55
ENSECAG00000006315	5.825716045	0.219737601	0.433748891	687	940	1071	1752	1218	868	1304	772
ENSECAG00000012844	4.685484117	0.219955702	0.434103993	312	371	375	430	621	517	651	669
ENSECAG00000000485	6.474603956	0.220047928	0.434210586	1199	2209	1671	1568	1468	1419	1579	2122
ENSECAG00000015209	4.810575226	0.22014296	0.434322679	307	416	479	436	646	563	704	768
ENSECAG00000017911	5.624535752	0.220331426	0.434585301	674	732	594	791	1177	915	1400	1259
ENSECAG00000022793	5.773214542	0.220352572	0.434585301	495	724	927	1156	1020	1289	1222	1722
ENSECAG00000006833	1.574549215	0.220486457	0.434773884	47	74	55	43	46	44	59	54
ENSECAG00000025866	0.453305005	0.220579062	0.434881018	5	27	34	47	14	32	14	22
ENSECAG00000017115	2.112867006	0.220821112	0.435274129	82	31	117	105	90	51	67	88
ENSECAG00000011518	5.438088653	0.220855074	0.435274129	643	896	954	709	1002	688	800	708
ENSECAG00000007536	6.156678975	0.220909281	0.435305454	900	1090	962	952	2328	1977	1652	908
ENSECAG00000022389	9.430564816	0.22097441	0.435358288	9844	14459	14537	13987	13036	11220	12528	13061
ENSECAG00000000557	4.285800935	0.221229281	0.435784863	137	238	342	478	452	404	490	532
ENSECAG00000010201	1.341584467	0.221590736	0.436421207	14	40	125	56	7	101	2	15
ENSECAG00000019428	4.154632088	0.221690635	0.436489885	168	221	324	348	449	331	463	464
ENSECAG00000006601	3.799972138	0.22170244	0.436489885	136	172	200	317	320	255	322	455
ENSECAG00000017402	0.735432208	0.221814202	0.436634263	11	28.0004	25	20	34	35.0011	25	67
ENSECAG00000013322	3.330947227	0.221990147	0.436904912	131	293	208	192	35	387	67	120
ENSECAG00000023522	3.433520009	0.22207058	0.436987518	57	372	213	240	118	213	135	276
ENSECAG00000016166	1.399190195	0.222150755	0.43706959	33	3	56	8	106	12	169	6
ENSECAG00000002810	0.174563825	0.222486219	0.437653812	1	37	28	25	19	17	13	16

ENSECAG00000016932	5.095253214	0.222657393	0.437859318	188	412	668	846	787	804	880	825
ENSECAG00000023027	7.972732511	0.222667764	0.437859318	5911	1032	7343	6330	8757	2491	3399	1167
ENSECAG00000021846	3.727642807	0.222881739	0.438151891	177	116	180	279	340	212	338	409
ENSECAG00000021185	3.084006495	0.222893675	0.438151891	104	80	134	161	202	219	187	206
ENSECAG00000016675	7.698995957	0.222976058	0.438208128	5165	3852	3477	2415	3886	2483	3365	5018
ENSECAG00000018815	4.676857973	0.222999419	0.438208128	203	332	341	686	636	509	737	576
ENSECAG00000017144	5.867591813	0.223358848	0.438838532	594	712	1134	1038	1358	1196	1423	1617
ENSECAG00000024875	2.154517451	0.223661431	0.439290567	39	94	18	60	27	204	64	163
ENSECAG00000017921	2.883423557	0.22366625	0.439290567	66	57	107	201	153	139	238	194
ENSECAG00000023155	4.706965523	0.223753794	0.439386555	329	274	321	610	611	509	657	741
ENSECAG00000021956	9.508825273	0.224033794	0.439742348	6797	13676	18323	20288	12985	10685	15492	13240
ENSECAG00000012974	2.463554857	0.224038658	0.439742348	61	63	89	93.0001	102	103	149	185.001
ENSECAG00000016779	3.952364834	0.224051087	0.439742348	146	314	146	269	299	303	401	517
ENSECAG00000024249	4.911287864	0.224174372	0.439851973	210	798	504	935	511	333	459	909
ENSECAG00000007221	3.364392719	0.224184367	0.439851973	106	165	139	162	266	191	378	163
ENSECAG00000015069	6.558686683	0.224249753	0.439904298	951	489	5051	1914	1016	3319	765	465
ENSECAG00000007062	1.712106454	0.224382128	0.440087992	28	83	72	77	49	36	35	106
ENSECAG00000017903	2.325447039	0.224473887	0.440177656	54	42	50	129	129	49	138	194
ENSECAG00000021036	4.652677565	0.224533163	0.440177656	312	302	414	428	669	463	675	600
ENSECAG00000006963	0.86393703	0.224569797	0.440177656	8	36	78	44	2	72	3	12
ENSECAG00000014876	4.736032256	0.224641272	0.440177656	352	336	306	548	579	587	683	701
ENSECAG00000013736	6.690974211	0.224645634	0.440177656	1183	1857	2437	2552	2021	1655	2064	1886
ENSECAG00000022705	7.902050253	0.224660291	0.440177656	2498	6518	5298	4241	3833	4127	4023	5250
ENSECAG00000006230	2.074748463	0.224950523	0.440670318	39	62	102	137	58	67	72	98
ENSECAG00000008319	6.979934262	0.22504773	0.440722549	1469	1129	2612	1954	3285	2192	2814	4265
ENSECAG00000014661	0.652748422	0.225054764	0.440722549	18	14	23	22	20	25	46	62
ENSECAG00000016022	7.113532015	0.225207029	0.440944729	1255	1645	2448	2869	3281	2725	3273	4293
ENSECAG00000008733	5.872260308	0.225397912	0.441242432	622	1215	1106	1585	956	1007	1115	1313
ENSECAG00000015505	6.46796577	0.225466155	0.441299992	611	985	1801	1977	2580	2194	1550	2256
ENSECAG00000017831	5.864902275	0.225699519	0.441680664	545	1033	817	1034	1639	979	1499	1503
ENSECAG00000020790	3.781096213	0.225790475	0.441782569	180	155	239	183	435	214	412	277
ENSECAG00000013746	1.927264884	0.225883812	0.441889097	40	25	168	116	28	142	26	4
ENSECAG00000006414	3.731377309	0.226116866	0.442210989	169	339	241	245	237	212	274	245
ENSECAG00000000412	4.235516014	0.226126197	0.442210989	382	300	349	339	449	287	367	257
ENSECAG00000026934	3.190632936	0.226283151	0.442435125	49	241	184	280	126	179	135	196
ENSECAG00000019583	3.9707156	0.226341486	0.442435125	156	185	249	345	329	358	381	430
ENSECAG00000016599	4.417433491	0.226357629	0.442435125	298	307	411	623	459	362	369	395
ENSECAG00000003146	3.276403493	0.226471677	0.442581904	141	242	164	172	152	163	180	205
ENSECAG00000019388	4.884214422	0.226548674	0.442656239	392	408	390	498	771	493	706	883
ENSECAG00000022972	2.419611975	0.226885358	0.443132818	24	77	65	145	86	133	125	186
ENSECAG00000008927	4.772710495	0.22690003	0.443132818	526	228	729	571	461	465	520	536
ENSECAG00000018813	4.111116453	0.226909588	0.443132818	73	194	297	487	258	394	620	435
ENSECAG00000009102	8.093939963	0.227155033	0.443486804	4490	5049	5610	5405	4777	4639	4660	5843
ENSECAG00000024710	0.431068003	0.227168914	0.443486804	5	9	25	32	21	34	38	34
ENSECAG00000023654	2.191284095	0.227216725	0.443503938	39	100	118	102	71	75	66	109
ENSECAG00000023913	4.429523953	0.227332259	0.443653234	157	273	282	611	452	451	485	708
ENSECAG00000013312	3.755128097	0.227410303	0.443729326	179	163	189	228	380	204	354	375
ENSECAG00000018697	5.420405463	0.227641861	0.444092361	386	778	599.967	764.994	859.995	818.829	1083	1377
ENSECAG00000007743	8.453207064	0.227804716	0.444092361	2533	3461	6391	8676	8238	7297	8838	10394
ENSECAG00000015004	2.418570177	0.227835239	0.444092361	77	88	120	121	116	82	94	87
ENSECAG00000015891	6.439199364	0.227847265	0.444092361	895.011	1571	1956.01	2391	1917	1167	1653	1730
ENSECAG00000000261	5.901172134	0.227871051	0.444092361	603	755	1032	1206	1425	1233	1478	1563
ENSECAG00000014875	7.694806542	0.227897278	0.444092361	1093	2652	2862	6003	4647	4544	6363	4969
ENSECAG00000023602	7.461047883	0.227901919	0.444092361	1263	1930	2721	4788	3915	3969	4190	5288
ENSECAG00000021194	4.882580213	0.227909044	0.444092361	358	1081	412	361	473	575	591	422
ENSECAG00000009136	4.229671139	0.227950625	0.444097223	159	253	326	380	486	268	574	490
ENSECAG00000006377	7.856061772	0.228057482	0.444156147	2095	2636	4004	4962	5283	4695	5238	7652
ENSECAG00000011229	1.818932986	0.228085777	0.444156147	41	45	35	183	67	29	37	102
ENSECAG00000016122	3.775156088	0.228113113	0.444156147	128	137	260	289	273	337	341	360
ENSECAG00000016036	2.120686379	0.228152248	0.444156147	40	30	94	78	103	105	126	86
ENSECAG00000017368	4.78819536	0.228176327	0.444156147	281	371	439	561	698	464	730	756
ENSECAG00000012116	2.868732899	0.228265169	0.444217635	26	42	80	290	185	172	227	153
ENSECAG00000024850	0.396347843	0.228286109	0.444217635	19	19	27	36	7	34	17	22
ENSECAG00000024826	5.347706306	0.228465702	0.44446997	321	644	631	883	1006	865	999	1010
ENSECAG00000022626	4.48971757	0.228494024	0.44446997	267	264	338	452	499	531	455	660
ENSECAG00000001333	0.380729281	0.2285958	0.444477808	15	8	19	22	32	19	37	35
ENSECAG00000015455	4.421109466	0.228622495	0.444477808	263	303	248	392	679	246	682	494
ENSECAG00000019437	7.106174831	0.228647773	0.444477808	1239	1263	3605	1699	3568	2197	3232	4914
ENSECAG00000008002	3.386540508	0.228685547	0.444477808	179	367	89	144	274	150	149	133
ENSECAG00000009516	0.435163672	0.228693651	0.444477808	8	52	15.0036	30	11	20	22	30
ENSECAG00000022885	2.977196304	0.22875276	0.444516651	74	85	126	169	233	116	161	266
ENSECAG00000015195	5.736439268	0.229142897	0.445176926	478	479	706	1555	1618	1040	1721	795
ENSECAG00000009595	6.234609688	0.229170906	0.445176926	955	1254	1091	901	1692	1516	1828	2283
ENSECAG00000005071	3.348794434	0.229347027	0.445397967	58	265	125	97	194	172	309	358
ENSECAG00000022687	6.863953605	0.229363097	0.445397967	1253	1619	1606	2290	2635	2038	2753	4043
ENSECAG00000020517	3.433049367	0.2294337	0.445458937	128	263	212	219	184	168	142	296
ENSECAG00000017007	4.110597051	0.229501392	0.445492402	155	203	338	329	362	340	347	633
ENSECAG00000003668	5.088568214	0.229529354	0.445492402	276	681	323	695	759	922	1016	566
ENSECAG00000020821	5.40746092	0.229611331	0.445575397	527	659	581	659	1221	673	1123	1074
ENSECAG00000013084	4.78124974	0.229946846	0.446150284	430	461	582	556	600	429	480	552
ENSECAG00000016595	1.919600597	0.230155268	0.446478429	35	68	86	111	53	55	68	90
ENSECAG00000004996	1.28866506	0.230226032	0.446539462	19	97	16	63	26	30	46	55
ENSECAG00000024216	0.449883121	0.230321434	0.446648257	9	11	33	61	20	22	16	26
ENSECAG00000024642	4.635313295	0.230425415	0.446773645	244	216	443	597	648	438	658	657
ENSECAG00000019658	0.971978578	0.230484256	0.446811485	10	3	33	54	33	12	83	77

ENSECAG00000020753	3.756846229	0.230627073	0.447012078	146	108	547	316	125	294	171	341
ENSECAG00000022815	6.765670281	0.23073652	0.447102352	953	1100	2121	2357	2228	2709	2481	3139
ENSECAG00000017256	9.078839957	0.230752349	0.447102352	3900	9369	13593	18589	9382	8209	11359	9536
ENSECAG00000013112	2.541023267	0.230985255	0.447477317	87	196	58	95	103	67	100	133
ENSECAG00000016510	2.467077837	0.231091288	0.447606413	96	30	42	95	295	102	116	50
ENSECAG00000013770	6.38314389	0.231387356	0.448103484	1195	2097	1209	1713	1471	1361	1466	1898
ENSECAG00000025016	5.307087168	0.231597249	0.448433529	356	550	522	983	839	842	879	1248
ENSECAG00000016202	1.083905855	0.231663069	0.448478383	13	18	100	46	48	20	31	35
ENSECAG00000023869	3.80209188	0.231699358	0.448478383	120	291	183	548	262	242	231	266
ENSECAG00000009438	3.402831016	0.231789682	0.448576796	178	226	178	199	247	127	220	170
ENSECAG00000018130	6.096262461	0.231966621	0.448842771	1408	1572	963	934	1290	1135	1205	1344
ENSECAG00000015558	6.826918419	0.232061946	0.448883038	1317	1339	2110	1709	3431	2185	3026	2370
ENSECAG00000012800	6.348355556	0.232075419	0.448883038	953	1095	1330	1380	1950	1759	1778	2328
ENSECAG00000011132	11.05516692	0.232105954	0.448883038	45757	40575	30783	39068	40119	34136	37242	40414
ENSECAG00000011150	5.355402851	0.232154198	0.448899931	302	576	668	971	914	1057	960	954
ENSECAG00000024653	7.299924987	0.232218393	0.448947658	2365	3716	3202	2494	3138	2979	2539	2694
ENSECAG00000020905	5.841699382	0.232507724	0.449430548	650	1252	1172	1277	1051	970	1242	1017
ENSECAG00000000269	3.397245017	0.232791675	0.449810744	136	171	267	230	229	140	199	204
ENSECAG00000008790	4.487815604	0.232859656	0.449810744	130	293	200	735	523	637	693	329
ENSECAG00000015691	3.861468238	0.232911996	0.449810744	179	143	394	442	278	281	247	238
ENSECAG00000009267	4.856886636	0.23294377	0.449810744	323	365	468	563	642	607	586	943
ENSECAG00000011367	1.861733666	0.232957569	0.449810744	32	35	55	84	97	75	70	109
ENSECAG00000016196	2.8532015	0.232979461	0.449810744	58	92	195	256	139	112	153	99
ENSECAG00000012598	5.013055176	0.233001235	0.449810744	328	395	528	688	853	553	944	734
ENSECAG00000023122	2.264845871	0.233022704	0.449810744	4	73	38	98	6	240.998	4	303.998
ENSECAG00000019984	1.024382043	0.233097225	0.449810744	16	18	29	48	40	51	55	46
ENSECAG00000009725	3.805326634	0.233100305	0.449810744	110	291	187	210	293	267	318	493
ENSECAG00000022821	2.759378585	0.233197292	0.449888166	80.0001	95	90	104	197.001	82	171	220
ENSECAG00000022893	6.2633398	0.233219618	0.449888166	718	1015	1293	1561	1852	1119	1971	2572
ENSECAG00000020614	2.125911653	0.233414056	0.45018681	43	66	80	168	55	89	62	95
ENSECAG00000024970	2.573867014	0.233507086	0.450289802	38	99	383	63	0	237	7	19
ENSECAG00000016369	3.568091323	0.233555987	0.450307674	96	138	219	258	320	251	262	304
ENSECAG00000019200	5.256434386	0.233731941	0.450570464	412	382	660	842	1098	706	931	923
ENSECAG00000014700	10.37155214	0.233778117	0.450583031	22059	26540	26783	24205	28260	22117	19641	25532
ENSECAG00000018326	1.627383895	0.233846826	0.450639017	30	43	27	67	88	44	103	65
ENSECAG00000019317	4.578929995	0.234130893	0.451109921	294	342	665	534	518	310	474	479
ENSECAG00000017744	6.921370337	0.234333222	0.451406847	1388	2385	2267	3484	1863	2503	2017	2465
ENSECAG00000007136	3.082922927	0.234378491	0.451406847	109	240	124	165	153	110	171	175
ENSECAG00000004338	0.403170832	0.234424034	0.451406847	18	42	21	15	28	10	15	31
ENSECAG00000022933	5.337491372	0.234443918	0.451406847	1152	685	599	387	1089	547	634	532
ENSECAG00000019570	5.425540042	0.234608608	0.451647408	490	1603	497	647	804	637	878	748
ENSECAG00000012769	5.813866906	0.234725553	0.451795991	703	684	779	1143	1469	993	1388	1551
ENSECAG00000022657	3.824751519	0.235201103	0.452634644	227	251	319	255	342	180	295	216
ENSECAG00000012627	0.731142936	0.235309138	0.452765865	3	30	23	31	29	56	37	35
ENSECAG00000006341	2.941922539	0.235531612	0.453117198	32	93	153	179	160	192	201	197
ENSECAG00000013192	5.541611915	0.235776009	0.453510582	412	731	714	968	1149	982	1035	1265
ENSECAG00000008116	1.789234134	0.235908024	0.453687706	44	47	57	30	82	74	79	100
ENSECAG00000023520	4.702168641	0.236103664	0.453923923	219	371	381	620	606	559	630	683
ENSECAG00000018661	5.30740172	0.236139666	0.453923923	525	592	849	1047	809	619	774	782
ENSECAG00000024965	8.031877924	0.236150705	0.453923923	4121	4555	5231	5957	5130	4458	4692	4818
ENSECAG00000015897	6.381019658	0.236210576	0.453962207	854	1333	2125	2240	1369	1662	1565	1556
ENSECAG00000007626	7.131405701	0.236282158	0.454022979	1340	6144	1703	1840	1372	2190	1515	4587
ENSECAG00000019547	3.711261946	0.236558375	0.454418881	221	229	256	259	251	200	296	211
ENSECAG00000014502	3.689210215	0.236568182	0.454418881	173	87	225	246	330	313	299	301
ENSECAG00000022447	1.857556018	0.236759304	0.454709129	26	33	39	109	67	64	137	87
ENSECAG00000016855	7.183870638	0.236947679	0.454994005	1563	2125	1946	2807	3778	3506	3360	3371
ENSECAG00000017417	5.007427612	0.237054315	0.455061652	306	570	908	736	484	646	408	841
ENSECAG00000011928	6.819416718	0.23706301	0.455061652	1384	881	1845	2370	3537	1448	2990	3271
ENSECAG00000024604	8.982699552	0.237173945	0.455125358	10819	5525	12115	10024	14098	7133	8250	5610
ENSECAG00000009658	7.003952025	0.237176311	0.455125358	1434	3256	2453	2737	1975	2629	2200	2516
ENSECAG00000023716	5.316349094	0.237249188	0.455155186	449	491	581	855	1058	690	1103	956
ENSECAG00000022576	6.640038162	0.237284258	0.455155186	1030	1237	1393	2289	2390	1897	2198	3224
ENSECAG00000016824	4.160753303	0.237312033	0.455155186	137	68	329	510	374	386	776	250
ENSECAG00000008574	2.448208798	0.237428371	0.45530146	72	101	111	135	94	104	59	131
ENSECAG00000012628	0.476688625	0.237790184	0.455918339	10	17	45	40	10	32	20	23
ENSECAG00000017599	2.737353764	0.237897333	0.455967685	70	63	79	173	172	115	163	202
ENSECAG00000007465	5.834455061	0.237909872	0.455967685	616	1354	983	1405	1067	949	931	1340
ENSECAG00000011693	3.053929733	0.237958034	0.455967685	38	192	141	339	123	119	167	167
ENSECAG00000021560	2.804613771	0.23798817	0.455967685	71	66	142	115	208	144	195	126
ENSECAG00000022491	4.827783283	0.238016576	0.455967685	317	342	471	563	731	501	720	758
ENSECAG00000021318	9.562182328	0.238076814	0.456006198	9541	8850	11483	12662	17317	16484	18599	21917
ENSECAG00000015427	4.426498437	0.238335764	0.456298401	153	426	553	591	299	406	389	483
ENSECAG00000027628	1.693780615	0.23835051	0.456298401	33	25	66	161	86	67	26	20
ENSECAG00000010247	4.938925074	0.238353971	0.456298401	316	862	503	654	579	433	488	789
ENSECAG00000008913	1.133800986	0.238390011	0.456298401	28	22	70	58	18	71	24	20
ENSECAG00000021075	2.317267295	0.238452199	0.456328711	48	60	90	220	105	64	98	76
ENSECAG00000000607	0.873723366	0.238486172	0.456328711	47	12	36	41	52	15	28	21
ENSECAG00000021114	6.356557591	0.238570305	0.456412831	668	1210	1292	1810	1624	1974	2061	2175
ENSECAG00000020984	7.928145476	0.238692244	0.456569238	2397	3844	2215	5577	5599	6303	7062	4766
ENSECAG00000020621	3.366061668	0.238758156	0.456618443	265	90	255	143	133	251	140	175
ENSECAG00000022077	6.782666123	0.238872874	0.456760955	1475	1813	2597	2585	1965	2458	1550	2049
ENSECAG00000007742	4.994383318	0.238983298	0.45689521	334	603	521	1060	510	502	477	904
ENSECAG00000012822	9.682623787	0.23906695	0.456912893	9043	12657	21963	22096	15387	12645	16310	15234
ENSECAG00000024954	2.88241327	0.239072976	0.456912893	54	100	118	154	119	139	158	317
ENSECAG00000022048	6.491537946	0.239117744	0.456921596	841	1180	1356	2073	2018	1896	2310	2409

ENSECAG00000019336	7.622284953	0.239278286	0.457151486	2835	4488	3567	3983	4051	2704	3827	3879
ENSECAG00000020512	3.237471466	0.239339146	0.457190883	111	100	125	208	230	167	222	297
ENSECAG00000021650	4.322178027	0.239430954	0.457289376	224	293	254	404	490	389	403	641
ENSECAG00000007797	5.816260858	0.239579386	0.457495963	670	657	876	1143	1578	1066	1290	1445
ENSECAG00000018057	2.251668222	0.239671829	0.457506433	67	52	50	85	105	69	145	150
ENSECAG00000021484	5.346002435	0.23970394	0.457506433	707	1027	690	546	1083	564	806	455
ENSECAG00000012583	4.029173935	0.239705668	0.457506433	126	257	315	270	445	300	386	438
ENSECAG00000013418	4.150307679	0.239833853	0.457674208	187	281	254	319	465	340	392	500
ENSECAG00000023794	2.628041399	0.239883895	0.457692832	96	203	66	94.9998	122.999	64	130	111
ENSECAG00000000477	4.607324362	0.240015834	0.457867681	259	397	413	876	566	365	464	400
ENSECAG00000008611	4.967446592	0.240298838	0.458330602	446	322	503	507	924	602	835	629
ENSECAG00000007043	2.437730528	0.240454557	0.458550634	66	121	86	142	95	85	101	106
ENSECAG00000017054	0.525246857	0.240536132	0.458561868	9	40	26	38	16	30	11	33
ENSECAG00000004299	6.484248396	0.240596758	0.458561868	931	1900	1623	2512	1590	1609	1591	1877
ENSECAG00000019354	4.132926545	0.240640267	0.458561868	159	214	323	355	339	366	385	602
ENSECAG00000011381	6.442966551	0.2406623	0.458561868	776	1842	2393	1826	1250	2010	1194	1864
ENSECAG00000009719	6.118553824	0.240740852	0.458561868	828	1511	1265	1661	1318	1137	1384	1374
ENSECAG00000016809	4.725603146	0.240773696	0.458561868	176	315	425	728	706	466	717	664
ENSECAG00000002212	6.677609095	0.240786967	0.458561868	1211	1026	2211	3990	2590	1489	1643	1653
ENSECAG00000000933	5.31744335	0.240823481	0.458561868	586	638	832	919	870	580	754	809
ENSECAG00000012843	6.929542364	0.240823681	0.458561868	2804	2329	1660	2074	2804	1003	2609	2332
ENSECAG00000006561	10.44687651	0.240958536	0.458614891	15614	15812	26849	19839	34091	19841	35714	51084
ENSECAG00000008200	1.127489166	0.240979629	0.458614891	26	15	48	17	64	78	30	38
ENSECAG00000007086	3.621724852	0.240983244	0.458614891	120	253	246	349	203	140	231	340
ENSECAG00000018739	1.027595373	0.241012983	0.458614891	19	35	43	72	14	13	21	86
ENSECAG00000019198	5.858023017	0.241098969	0.458701689	916	1203	895	1248	1117	941	1064	1237
ENSECAG00000011768	8.853100723	0.241144003	0.458710557	12088	6455	8065	6793	11426	7059	7125	6568
ENSECAG00000018637	3.815564927	0.241195279	0.458731295	251	159	400	239	235	249	291	239
ENSECAG00000000464	6.130632851	0.241282643	0.458789042	716	862	1354	1243	1732	1241	1813	1948
ENSECAG00000001391	0.001744934	0.2413064	0.458789042	6	6	10.0036	29	23	23	25	20
ENSECAG00000012292	8.240312207	0.241375242	0.458809872	4876	5857	5854	6131	5571	5336	4790	6396
ENSECAG00000017536	5.201460104	0.241398118	0.458809872	358	529	438	904	942	708	840	1035
ENSECAG00000015453	3.587368454	0.241544822	0.458914089	122	194	357	267	188	190	292	201
ENSECAG00000013247	3.443651996	0.241585628	0.458914089	105	196	291	297	343	203	95	99
ENSECAG00000002931	4.368448963	0.241621713	0.458914089	189	252	366	444	511	386	548	524
ENSECAG00000018149	6.476205598	0.241644594	0.458914089	1082	1332	1378	1320	2073	1903	1954	2641
ENSECAG00000005662	4.563206497	0.241654902	0.458914089	220	406	374	395	558	422	640	643
ENSECAG00000017135	5.124429153	0.2418242	0.459151449	331	541	477	757	828	670	790	1048
ENSECAG00000015276	4.423675474	0.241860713	0.459151449	217	227	353	493	505	356	479	743
ENSECAG00000010640	6.501657402	0.242022497	0.459343174	1483	1948	1626	1577	1666	1489	1922	1621
ENSECAG00000021406	3.724009009	0.242080467	0.459343174	114	207	192	252	197	235	311	567
ENSECAG00000019373	7.059605689	0.242082989	0.459343174	1545	1645	1648	2938	3377	2611	2785	4294
ENSECAG00000025139	2.489833122	0.242272942	0.459626843	43	106	89	228	87	83	121	101
ENSECAG00000025150	4.480969241	0.242366633	0.459727828	269	478	451	486	341	384	350	604
ENSECAG00000021103	7.916475345	0.242451557	0.459793579	2016	1491	3648	7115	6551	3171	4591	10720
ENSECAG00000022847	5.358600791	0.242501546	0.459793579	579	624	516	591	1044	803	905	1193
ENSECAG00000014226	11.29156442	0.242555043	0.459793579	11269	24824	121029	65204	44432	54682	34919	31002
ENSECAG00000015973	4.755524949	0.242563168	0.459793579	227	398	358	668	684	485	684	735
ENSECAG00000023846	5.164889634	0.242630187	0.459843901	356	452	592	775	827	877	735	955
ENSECAG00000025162	7.866607363	0.242716789	0.459926905	2634	4112	5057	6628	4276	3827	4110	4957
ENSECAG00000015266	8.760654876	0.242754942	0.459926905	6652	14395	4116	7595	7277	6697	8526	7935
ENSECAG00000012161	3.728648039	0.242798049	0.459931882	117	256	343	351	200	197	120	449
ENSECAG00000016468	2.107787125	0.242963134	0.46009691	38	47	57	106	104	82	98	133
ENSECAG00000018641	0.749307871	0.242966156	0.46009691	14	20	13	42	31	28	44	58
ENSECAG00000011079	3.680898153	0.243056212	0.460190749	113	169	232	250	293	251	300	387
ENSECAG00000001415	6.765347154	0.243259158	0.460498259	1054	1069	2028	2340	2575	1858	2469	3803
ENSECAG00000019349	0.90701899	0.24337967	0.460649643	39	35	28	32	32	25	22	47
ENSECAG00000019481	6.150595452	0.2434396	0.460686332	835	881	1120	1370	1720	1352	1685	2052
ENSECAG00000021551	4.593160794	0.243719254	0.461098716	417	299	582	490	515	420	420	427
ENSECAG00000012688	3.513904844	0.243738681	0.461098716	180	359	123	180	167	158	212	281
ENSECAG00000015036	8.102236877	0.24389505	0.461317722	3201	3493	4044	5074	6914	5780	7266	6737
ENSECAG00000010368	3.8133578	0.244132114	0.461645578	135	207	187	285	276	414	187	484
ENSECAG00000011932	3.233062912	0.244149646	0.461645578	75	109	149	224	158	185	214	367
ENSECAG00000006379	5.331019096	0.244292275	0.461802805	620	668	798	891	833	735	725	724
ENSECAG00000009563	3.732391826	0.244355542	0.461802805	125	175	196	282	497	265	284	227
ENSECAG00000012788	3.986446437	0.244417291	0.461802805	189	156	242	338	432	263	485	344
ENSECAG00000013042	3.384251628	0.244433073	0.461802805	85	136	170	230	207	252	307	229
ENSECAG00000021844	3.019101796	0.244436021	0.461802805	74	155	189	227	100	156	116	211
ENSECAG00000002377	3.269954654	0.244780186	0.462376138	127	251	141	205	207	147	154	192
ENSECAG00000015471	4.079178515	0.244944023	0.462608709	220	158	303	290	488	363	378	385
ENSECAG00000015601	4.444943228	0.245096215	0.462819212	273	246	325	438	531	471	512	552
ENSECAG00000020796	2.389051906	0.245401721	0.463319102	39	53	76	141	110	119	125	152
ENSECAG00000022947	3.743140028	0.245563313	0.463547161	133	128	232	307	351	278	352	297
ENSECAG00000014082	5.213520443	0.245677103	0.463581177	458	522	664	424	729	1246	879	661
ENSECAG00000006910	3.662159863	0.245701104	0.463581177	105	139	236	277	305	191	397	334
ENSECAG00000018222	7.648371886	0.245703736	0.463581177	2314	2969	3119	2997	4879	3932	4865	5928
ENSECAG00000008487	2.882642322	0.245996978	0.46405739	63	78	144	137	223	143	239	106
ENSECAG00000012431	6.294730915	0.246142515	0.464254856	933	1112	1696	2407	1592	1370	1181	1728
ENSECAG00000013059	3.475443199	0.246209906	0.464304888	123	240	222	268	159	205	171	284
ENSECAG00000019950	7.382925686	0.246348515	0.464489185	3416	3388	2592	2730	3263	2067	2861	4029
ENSECAG00000018573	2.894272296	0.246506334	0.464709635	74	258	133	100	117	117	124	164
ENSECAG00000011390	6.56764422	0.246899107	0.465346893	987	1781	2357	2219	1774	1806	1492	1978
ENSECAG00000000648	4.896862217	0.246926282	0.465346893	318	360	512	587	543	623	658	1042
ENSECAG00000026866	3.335572738	0.247015791	0.465438379	119	165	168	345	209	156	209	157
ENSECAG00000013086	1.410965827	0.247234653	0.465773525	19	51	28	43	46	43	58	116

ENSECAG00000023594	5.67601392	0.247307947	0.465834366	881	1544	644	607	911	755	976	1080
ENSECAG00000020062	7.245300358	0.247411175	0.465951561	1525	1929	2634	2850	3644	3592	3237	4179
ENSECAG00000011007	3.595619258	0.247482331	0.465967739	189	303	173	224	205	150	234	302
ENSECAG00000000074	4.602621841	0.247501787	0.465967739	259	171	574	207	1112	487	841	60
ENSECAG00000008477	4.542154616	0.247607401	0.466036949	232	353	313	492	523	634	530	505
ENSECAG00000008311	7.369610918	0.247633455	0.466036949	2713	3278	2948	3363	3027	2789	2964	3403
ENSECAG00000009841	5.804019972	0.2476616	0.466036949	626	779	1126	1873	1118	887	1053	1138
ENSECAG00000013820	3.013454147	0.24786372	0.466340054	54	154	245	204	118	185	99	161
ENSECAG00000016552	5.831885976	0.247952174	0.466386455	490	706	1078	1181	1145	1399	1002	1935
ENSECAG00000013285	6.502880395	0.247984636	0.466386455	810	961	1702	2038	2141	2340	1812	2386
ENSECAG00000015402	3.248528551	0.248016565	0.466386455	90	186	176	301	144	186	166	192
ENSECAG00000013158	2.30241053	0.248052574	0.466386455	72	124	99	76	141	77	66	52
ENSECAG00000018018	7.498929289	0.248435304	0.466981691	1926	1803	3861	2851	4995	3116	4275	5430
ENSECAG00000017172	5.710243209	0.248451357	0.466981691	77	1202	679	691	404	3782	630	479
ENSECAG00000019100	2.669536868	0.248494806	0.466986103	41	130	37	114	28	349	112	149
ENSECAG00000021473	4.664613452	0.248575306	0.467011448	183	518	306	486	530	433	555	978
ENSECAG00000017216	3.607273854	0.248615736	0.467011448	97	161	225	221	235	469	264	185
ENSECAG00000020011	3.019745588	0.248631601	0.467011448	57	99	161	158	182	217	238	136
ENSECAG00000020878	8.183732166	0.248676019	0.467017673	2372	4401	4665	5682	5544	8310	7218	7040
ENSECAG00000020368	2.100313734	0.248891741	0.467345557	48	43	56	90	135	50	100	137
ENSECAG00000004707	6.310759092	0.248962244	0.467400696	1127	804	1976	2295	1865	1020	1415	1593
ENSECAG00000006077	4.74040668	0.24902566	0.467442515	165	354	360	791	602	536	663	785
ENSECAG00000013260	8.870058578	0.249067346	0.467443539	6941	10095	8917	9369	8213	8026	8133	9820
ENSECAG00000009934	2.231326773	0.249112157	0.467450425	37	52	162	137	71	72	56	127
ENSECAG00000021030	3.131321657	0.249293388	0.467713256	104	94	155	147	214	188	220	215
ENSECAG00000019791	3.647506553	0.249404398	0.467844274	176	248	180	347	216	165	281	266
ENSECAG00000000925	3.883227919	0.24966737	0.468260261	98	218	183	402	304	286	366	478
ENSECAG00000025037	5.085099268	0.24983576	0.468498747	304	996	547	763	516	604	578	815
ENSECAG00000012044	0.319988167	0.24993317	0.468604072	7	15	8	35	25	32	37	21
ENSECAG00000008999	4.372360019	0.250082864	0.468807376	202	309	239	485	443	516	359	670
ENSECAG00000012483	3.369988151	0.250216764	0.468981009	148	255	173	190	159	140	241	216
ENSECAG00000014866	4.60330924	0.250306392	0.469045867	531	692	239	257	392	410	395	527
ENSECAG00000010564	2.924750543	0.250401307	0.469045867	92	83	132	119	159	169	151	256
ENSECAG00000017190	2.583233085	0.250454618	0.469045867	84	77	169	128	128	86	93	123
ENSECAG00000015656	4.504340866	0.250456236	0.469045867	175	346	372	477	421	421	508	857
ENSECAG00000022864	5.06680853	0.250457778	0.469045867	392	452	497	644	782	610	734	1086
ENSECAG00000013510	10.05023539	0.250540469	0.469069464	10639	20357	23692	30338	20317	14345	15974	27359
ENSECAG00000019408	7.405034047	0.250552946	0.469069464	1315	2591	3103	2966	2982	4760	4526	4097
ENSECAG00000012453	3.349481937	0.250607547	0.46909439	110	124	130	235	246	221	246	260
ENSECAG00000021498	5.420726355	0.250761065	0.469304434	987	891	495	649	910	788	737	644
ENSECAG00000009279	3.106021533	0.250886474	0.469435641	54	100	149	217	176	213	190	251
ENSECAG00000011861	3.010048267	0.250913805	0.469435641	74	77	127	192	248	99	231	212
ENSECAG00000014676	9.156054955	0.251288377	0.470039466	9743	16990	7316	8139	8894	10283	11363	9656
ENSECAG00000007013	2.629151828	0.251319288	0.470039466	49	80	75	159	138	99	161	207
ENSECAG00000000565	6.333955932	0.251471085	0.470245962	822	880	947	2260	2113	1565	1852	2271
ENSECAG00000013999	6.852180714	0.251721674	0.470637101	1730	2064	2365	2519	2094	2178	2166	2088
ENSECAG00000004819	3.493126367	0.251767699	0.470645706	127	154	132	245	275	245	259	294
ENSECAG00000019465	2.648392372	0.251892532	0.470801603	64	105	196	128	117	53	125	155
ENSECAG00000007104	7.870946261	0.252088696	0.471022404	2592	5685	5128	4637	3444	4541	3872	5215
ENSECAG00000019202	0.227484478	0.252104693	0.471022404	12	5	13	28	28	41	21	17
ENSECAG00000017121	3.521661102	0.252135035	0.471022404	109	130	217	226	304	214	284	296
ENSECAG00000017225	2.968693676	0.252259776	0.471177968	73	84	131	173	173	189	184	201
ENSECAG00000023672	4.291281216	0.252556033	0.47156238	181	214	271	526	451	492	537	374
ENSECAG00000020499	2.992356726	0.252556892	0.47156238	92	88	199	257	165	111	161	134
ENSECAG00000012040	3.089230124	0.252590093	0.47156238	56	192	202	245	116	223	129	116
ENSECAG00000024259	8.601956211	0.25280254	0.471881463	4551	3503	5900	7769	15864	8302	9648	4462
ENSECAG00000014040	3.142338098	0.252887639	0.471962771	77	88	176	180	178	233	224	206
ENSECAG00000007816	5.03413677	0.252966302	0.472022115	476	602	871	513	645	865	370	451
ENSECAG00000017713	6.772533329	0.253002524	0.472022115	1176	1907	1465	1756	2445	2589	2587	2886
ENSECAG00000008068	5.895951054	0.253327383	0.472475889	673	639	1189	1032	1611	1278	1338	1433
ENSECAG00000013383	5.896457369	0.253328913	0.472475889	709	877	838	1081	1352	1140	1377	1832
ENSECAG00000017887	7.552283687	0.253498511	0.472714604	3964	2110	4288	3501	3488	2836	3748	3540
ENSECAG00000018283	4.244248749	0.253587099	0.472802203	73	322	290	463	308	403	438	733
ENSECAG00000022440	7.40687962	0.253705423	0.472945204	1131	1605	3277	4366	3594	3438	4010	5761
ENSECAG00000023088	2.804562053	0.253833064	0.472978252	63	114	177	204	159	90	141	110
ENSECAG00000011658	1.043344356	0.253849554	0.472978252	25	9	47	19	64	71	21	43
ENSECAG00000011591	7.545513313	0.253889199	0.472978252	1562	2278	3173	4226	3833	4887	4079	5287
ENSECAG00000008775	1.293333652	0.253935684	0.472978252	25	84	39	35	51	33	36	45
ENSECAG00000019099	2.66934716	0.253939023	0.472978252	37	49	158	117	95	111	120	326
ENSECAG00000002987	5.735043604	0.253980325	0.472978252	806	1313	938	827	1102	693	828	1385
ENSECAG00000023912	1.230614869	0.254035159	0.472978252	18	37	76	55	43	23	35	59
ENSECAG00000017167	6.410639821	0.254065009	0.472978252	849	1293	1523	1365	1895	1735	2314	2181
ENSECAG00000007105	7.979628981	0.254097804	0.472978252	2393	2531	4761	5311	6370	4585	6482	7314
ENSECAG00000021278	2.374128062	0.25435279	0.473339959	49	11	138	17	199	17	358	7
ENSECAG00000009156	4.629949868	0.254375443	0.473339959	282	267	420	502	702	380	611	686
ENSECAG00000008164	9.267916669	0.254750347	0.473959955	6581	6391	9834	13046	19198	10327	17649	13343
ENSECAG00000023517	7.701649393	0.25484346	0.473996526	5383	4237	2512	2546	3817	3344	4015	3473
ENSECAG00000008542	11.19844253	0.254902786	0.473996526	22570	37648	64332	65109	44787	32167	45373	49436
ENSECAG00000010226	5.052785564	0.254956261	0.473996526	313	490	459	762	774	663	790	926
ENSECAG00000018402	4.828745366	0.254962494	0.473996526	207	466	336	730	515	729	677	795
ENSECAG00000007963	6.416742653	0.255017783	0.473996526	1554	901	1176	832	2471	1661	2223	2010
ENSECAG00000011914	6.943890997	0.25502031	0.473996526	1798	2090	1214	1520	2995	2330	3120	3687
ENSECAG00000008105	3.894736196	0.25513854	0.474066931	179	307	233	418	290	288	239	281
ENSECAG00000017863	6.966713424	0.255203355	0.474066931	1136	2078	2759	4135	2413	1986	2319	2501
ENSECAG00000014124	5.61807916	0.255224776	0.474066931	568	549	691	1141	1392	814	1328	1158

ENSECAG00000021981	4.572518459	0.255256407	0.474066931	313	424	491	552	404	427	475	487
ENSECAG00000000626	4.792101057	0.255266809	0.474066931	429	638	434	516	553	394	585	545
ENSECAG00000009739	1.757697489	0.255493859	0.474411051	11	20	107	46	93	38	118	94
ENSECAG00000013122	2.734244648	0.25556034	0.474456958	71	103	157	191	120	128	103	127
ENSECAG00000010103	8.409673387	0.255673729	0.474589921	6909	4687	6194	7484	5296	5988	5771	7715
ENSECAG00000003776	4.712605134	0.255774379	0.474699199	265	154	691	373	549	412	455	1259
ENSECAG00000011526	3.368369617	0.255857878	0.474776614	107	102	196	201	262	186	262	283
ENSECAG00000001003	3.131823233	0.255978248	0.474922412	102	113	186	296	179	152	117	187
ENSECAG00000018247	0.748901206	0.256032594	0.474945686	26	9	24	21	64	18	32	50
ENSECAG00000000696	0.200381513	0.256209034	0.475195403	12	18	23	33	19	15	25	14
ENSECAG00000017541	6.70816488	0.256330259	0.475233962	1083	2173	1995	2922	1599	1983	1659	2538
ENSECAG00000012764	3.010531801	0.256340703	0.475233962	79	192	153	203	102	121	138	227
ENSECAG00000007723	3.06949078	0.256355303	0.475233962	83	139	108	150	206	127	229	252
ENSECAG00000010711	6.978534562	0.256512374	0.475447569	1841	2460	2310	2879	2336	2239	2072	2721
ENSECAG00000000878	2.059703651	0.256767054	0.475841994	87	77	68	69	57	97	65	67
ENSECAG00000010092	6.406238783	0.256879452	0.475870455	904	903	1228	2111	2008	1770	1943	2418
ENSECAG00000006543	3.57840611	0.256900805	0.475870455	146	141	170	236	290	243	337	265
ENSECAG00000007090	0.355734234	0.25690806	0.475870455	9	8	21	29	26	19	34	41
ENSECAG00000020761	4.1701165	0.256970356	0.475908262	131	248	274	454	433	324	413	565
ENSECAG00000021661	5.035248016	0.257074167	0.476003709	280	364	379	1012	711	681	768	1007
ENSECAG00000022510	4.173154026	0.257105682	0.476003709	201	359	92	312	501	595	172	517
ENSECAG00000013689	4.410827829	0.257183265	0.476069771	193	283	381	430	568	406	418	639
ENSECAG00000014928	10.32045189	0.257297899	0.476135744	14177	18781	33722	35370	24391	18811	23842	26668
ENSECAG00000009463	3.520637025	0.257368935	0.476135744	106	202	147	212	224	322	244	300
ENSECAG00000009313	2.647626575	0.257379811	0.476135744	59	58	119	121	174	85	115	248
ENSECAG00000015176	2.923084201	0.257386529	0.476135744	74	112	122	125	156	126	190	266
ENSECAG00000026862	3.235684251	0.257446433	0.476169033	89	85	177	202	254	195	289	163
ENSECAG00000005265	6.667508886	0.25757908	0.476336835	1118	1745	790	2218	2279	2478	2279	2828
ENSECAG00000024292	6.328032261	0.25764726	0.476385381	1097	1244	1697	2043	1432	1422	1614	1579
ENSECAG00000025052	2.610009386	0.257702999	0.476410914	71	83	83	107	149	133	153	146
ENSECAG00000010601	3.546185372	0.257973274	0.476832981	127	202	266	306	193	181	217	280
ENSECAG00000011008	2.891515039	0.258053459	0.47685073	45	73	135	191	147	165	134	284
ENSECAG00000016006	0.316587751	0.258076771	0.47685073	22	23	16	28	25	16	13	27
ENSECAG00000007050	5.862711187	0.258136402	0.47685073	803	541	773	1275	1540	1210	1671	1131
ENSECAG00000024655	4.378874054	0.258150752	0.47685073	237	283	848	290	364	609	192	237
ENSECAG00000026814	3.396981008	0.258240775	0.476939481	95	105	215	211	239	197	268	311
ENSECAG00000015881	5.244091008	0.258429091	0.477105111	464	584	891	936	727	604	745	802
ENSECAG00000014846	5.741811774	0.258472334	0.477105111	427	611	1277	938	1291	1086	1434	1297
ENSECAG00000016489	10.75459279	0.25847589	0.477105111	22402	21909	47883	44298	36769	22458	32587	34494
ENSECAG00000005115	1.683738481	0.258498421	0.477105111	43	45	45	122	49	61	35	76
ENSECAG00000023363	2.733939311	0.258622795	0.477257137	87	144	101	175	134	77	127	146
ENSECAG00000012014	1.546682301	0.258897264	0.477624992	29	51	25	52	68	48	86	79
ENSECAG00000024341	6.909614228	0.258906207	0.477624992	1165	3051	2375	2713	2094	2092	2023	2677
ENSECAG00000011107	8.182590244	0.258990276	0.477702519	3325.01	6885	5524	6683.01	4988	5287	5744	5185
ENSECAG00000015445	5.486027726	0.259161937	0.477941557	433	436	693	1209	1130	890	1065	1195
ENSECAG00000006392	7.021309703	0.259342713	0.478197322	1948	1768	3539	2617	2698	2008	1955	2959
ENSECAG00000016089	3.661193365	0.259396727	0.47821931	185	195	136	165	380	179	383	291
ENSECAG00000017882	0.899094963	0.2594483	0.478236791	10	22	33	38	38	40	56	40
ENSECAG00000019306	3.488998333	0.259647781	0.47852686	66	130	195	299	255	264	295	261
ENSECAG00000007431	5.151556148	0.259816981	0.478761035	369	479	568	730	928	750	911	755
ENSECAG00000020735	4.369419056	0.259861732	0.47876585	253	453	362	480	404	381	354	409
ENSECAG00000015580	7.314359538	0.259921912	0.478799087	1863	2117	2470	2763	3963	3238	4244	3904
ENSECAG00000015730	7.970486187	0.260525595	0.47983333	2712	3348	6748	7456	4906	4701	5222	3109
ENSECAG00000009502	4.109117235	0.26059508	0.479883517	129	193	256	490	349	411	364	534
ENSECAG00000016971	5.542236203	0.260760443	0.480110218	417	362	1214	794	1174	983	1300	1023
ENSECAG00000016333	5.761945645	0.260994946	0.480464124	587	754	902	1006	1316	1373	1164	1238
ENSECAG00000024941	3.587593757	0.26167395	0.481636063	124	152	216	214	244	240	274	396
ENSECAG00000015476	6.715024811	0.261721407	0.481645388	857	1222	1994	2332	2267	2672	2140	3011
ENSECAG00000018310	2.058246408	0.261803496	0.481671018	29	43	77	94	91	78	110	122
ENSECAG00000004757	5.325039265	0.261820121	0.481671018	625	717	702	942	785	444	634	1196
ENSECAG00000018095	0.196425611	0.261910124	0.481758594	12	6	17	22	21	33	19	32
ENSECAG00000019714	5.232276628	0.262064797	0.481965073	467	682	361	630	841	632	961	1213
ENSECAG00000026972	0.305916005	0.262161176	0.482064295	6.00713	15	18	26	26	33	21	33
ENSECAG00000010774	8.17709716	0.262241346	0.482133684	3486	6673	5243	6832	5016	4925	5369	6012
ENSECAG00000014411	4.082341965	0.262373719	0.482299013	200	269	254	240	400	261	369	620
ENSECAG00000011852	4.489475785	0.262598995	0.482635035	177	501	296	330	455	537	582	573
ENSECAG00000016518	7.174122367	0.262766413	0.482834133	2049	2504	3185	3177	2740	2523	2468	3005
ENSECAG00000012354	0.935404312	0.262792314	0.482834133	18	12	42	29	35	60	38	46
ENSECAG00000007600	6.714028737	0.262987482	0.483114594	1208	2585	1973	2190	1706	2059	1690	2333
ENSECAG00000021884	4.161960849	0.263136556	0.483310305	143	278	248	424	413	361	443	487
ENSECAG00000008834	4.703116308	0.263273488	0.48346588	114	819	988	426	31	1114	75	222
ENSECAG00000004762	5.571028264	0.26330636	0.48346588	601	445	955	1933	994	1258	578	468
ENSECAG00000006398	0.571062162	0.263373357	0.483510758	7	14	33	24	16	38	40	46
ENSECAG00000016884	6.281375976	0.263473899	0.483617196	1570	1431	1268	1276	1362	1417	1399	1591
ENSECAG00000013623	7.958026762	0.263597103	0.483743719	5708	4422	2411	5735	3842	4473	5477	3823
ENSECAG00000021626	3.113125701	0.263653919	0.483743719	71	78	179	188	224	171	195	244
ENSECAG00000024439	4.297189906	0.263705608	0.483743719	288	306	421	448	389	304	308	489
ENSECAG00000009513	7.70024445	0.263735406	0.483743719	1994	2714	3169	4531	4464	4362	4928	6464
ENSECAG00000019020	2.506176503	0.263755707	0.483743719	39	62	121	110	108	101	156	188
ENSECAG00000013118	6.878165936	0.26381699	0.483778025	1233	2153	2698	3095	1936	1983	2246	2615
ENSECAG00000005614	0.697759453	0.26388041	0.483816236	8	11	6	61	22	42	19	79
ENSECAG00000011920	2.252420564	0.263981193	0.483922928	48	85	40	88	83	97	110	177
ENSECAG00000021752	6.588426277	0.264134381	0.484125639	1566	875	2984	1995	2571	1649	1376	1295
ENSECAG00000026942	3.2816524	0.264223204	0.484143729	84	144	162	185	197	218	240	271
ENSECAG00000023118	0.416718299	0.264270679	0.484143729	8	49	14	33	10	29	26	15

ENSECAG00000014482	4.124911148	0.264277946	0.484143729	161	398	346	442	256	333	298	412
ENSECAG00000026993	1.126476956	0.264314694	0.484143729	17	26	21	56	52	26	74	58
ENSECAG00000005905	5.016668272	0.264459297	0.484258159	296	220	1242	1030	1078	413	496	192
ENSECAG00000018798	2.412392025	0.264462408	0.484258159	84	68	128	130	54	105	141	67
ENSECAG00000011363	7.261898726	0.26470893	0.484631464	1577	3235	2997	4234	2369	3043	2472	3433
ENSECAG00000006899	5.186227594	0.264901445	0.484884128	223	491	699.99	840	650	1011	932	863
ENSECAG00000007051	6.030472692	0.264937805	0.484884128	614	1548	1289	1577	1069	1064	1164	1640
ENSECAG00000024841	0.055931812	0.264978561	0.484884128	4	8	19	22	35	22	26	11
ENSECAG00000008665	5.875708944	0.265017641	0.484884128	801	827	1168	1681	950	1130	1060	1291
ENSECAG00000018190	7.297116667	0.265061017	0.484885409	2517	1383	2590	1963	4377	2118	4117	5124
ENSECAG00000011192	8.423726409	0.265164935	0.484997423	9468	6802	3311	4624	7010	3901	6351	6977
ENSECAG00000014091	2.508533308	0.26529742	0.485136996	42	80	65	148	136	109	114	194
ENSECAG00000019106	3.113498158	0.265326641	0.485136996	67	129	107	214	198	183	186	266
ENSECAG00000000194	4.550604206	0.265544888	0.485457928	216	451	500	669	395	450	303	605
ENSECAG00000006203	0.460400965	0.266176339	0.486534035	23	17	29	31	26	21	20	23
ENSECAG00000022843	6.443656589	0.266251429	0.48656353	789	1361	1268	1848	2113	1557	2005	2701
ENSECAG00000026979	4.13953157	0.266278123	0.48656353	170	313	214	347	466	381	404	417
ENSECAG00000013320	6.892045161	0.266336836	0.486592559	1417	1168	1609	2812	3187	2300	3456	2520
ENSECAG00000019719	9.699811213	0.266390335	0.486612055	9628	12500	21143	22575	16729	12110	16627	15526
ENSECAG00000015725	6.46751726	0.266440484	0.486625426	1056	1347	1260	1505	2116	1784	2360	2167
ENSECAG00000013890	6.765690971	0.266570636	0.486759264	805	1606	1480	2780	2372	2483	2283	3394
ENSECAG00000023044	5.902380199	0.266599445	0.486759264	662	785	960	1183	1634	1070	1296	1701
ENSECAG00000021785	8.661595678	0.26668077	0.486829516	397	9391	17047	10944	875	9247	7735	6819
ENSECAG00000009492	4.567213376	0.266875845	0.487107366	199	225	404	640	604	515	638	495
ENSECAG00000015181	5.596625101	0.266972532	0.487205575	981	584	1243	876	1653	790	610	253
ENSECAG00000021086	2.430254485	0.267128558	0.487412025	32	54	38	196	133	92	135	178
ENSECAG00000002086	3.974445496	0.267382236	0.487770455	213	178	238	278	397	303	384	412
ENSECAG00000015977	6.469690407	0.267410858	0.487770455	1101	903	2822	2103	1799	1540	1575	1609
ENSECAG00000022049	4.604054293	0.26754792	0.48794213	240	410	332	451	547	354	558	908
ENSECAG00000020722	3.83550188	0.26769839	0.488138198	196	255	294	319	236	225	316	293
ENSECAG00000007682	6.752735243	0.267823339	0.488287673	1916	2358	1591	1991	2271	1687	2245	1725
ENSECAG00000009970	7.311025321	0.267930514	0.488323362	1832	3505	3450	3390	2582	2861	2248	4100
ENSECAG00000010156	5.415050503	0.267979878	0.488323362	454	574	689	847	1026	752	1317	946
ENSECAG00000023230	3.82773224	0.267988748	0.488323362	132	306	314	343	208	247	261	342
ENSECAG00000020058	4.087548717	0.268014829	0.488323362	205	227	195	371	355	374	341	559
ENSECAG00000021649	2.608284386	0.268078919	0.488361821	73	62	99	109	181	90	145	175
ENSECAG00000000383	7.000626423	0.268351534	0.488696845	1942	1613	1826	1664	3212	2355	3154	3783
ENSECAG00000017376	6.353260662	0.268360469	0.488696845	649	1080	1099	2241	1691	1650	1886	2676
ENSECAG00000021734	2.60697584	0.26839186	0.488696845	47	107	124	216	93	103	102	139
ENSECAG00000016028	3.749122888	0.268569079	0.488941175	195	242	269	289	229	249	269	253
ENSECAG00000010028	1.787454615	0.268770939	0.48917171	30	54	88	109	50	36	31	125
ENSECAG00000021305	6.197054743	0.268781816	0.48917171	1317	991	1307	1799	1269	1305	1362	1577
ENSECAG00000016653	9.950404521	0.268936032	0.48937399	13133	14698	25771	23202	20912	13459	18959	19529
ENSECAG00000020054	0.981015267	0.269099933	0.489593826	23	26	40	64	41	37	23	31
ENSECAG00000021339	0.369323786	0.269326986	0.489928469	14	13	8	30	23	41	22	33
ENSECAG00000005163	2.968842459	0.26938418	0.489954066	130	130	171	146	131	170	121	141
ENSECAG00000014244	6.681917004	0.269483928	0.49005704	1291	1266	3584	1844	2005	1076	1851	2685
ENSECAG00000007352	2.804479084	0.269571431	0.490137718	79	85	106	128	113	164	169	225
ENSECAG00000021789	5.388154617	0.269797376	0.490470046	608	772	778	922	737	726	866	855
ENSECAG00000003182	0.075994776	0.269923563	0.490500333	4	11.0028	5.00342	34.0025	15	20.0017	38.0092	25.0042
ENSECAG00000014564	3.418274146	0.269926985	0.490500333	106	138	176	214	268	212	282	251
ENSECAG00000018511	0.949388373	0.269943547	0.490500333	18	20	34.9999	30	46	30	38	69.9999
ENSECAG00000024895	0.240833359	0.270199186	0.490880941	11	10	15	24	33	16	31	29
ENSECAG00000014063	6.213836134	0.27023942	0.490880941	914	1631	1424	1584	1375	1030	1588	1643
ENSECAG00000008882	7.125242372	0.270629832	0.491511533	1737	2586	2371	4137	2333	2535	2445	3071
ENSECAG00000015517	2.375097128	0.2709861	0.492036415	20	68	60	160	79	154	109	166
ENSECAG00000002904	5.498133888	0.271005447	0.492036415	522	831	1068	982	1004	631	885	923
ENSECAG00000008284	2.333540723	0.271090888	0.492112905	61	101	112	107	75	94	73	121
ENSECAG00000013823	6.994638587	0.271174487	0.492186026	1062	2449	2651	4122	1909	2461	2247	2768
ENSECAG00000022005	8.884933117	0.271440114	0.492589454	4767	7831	6179	8614	12040	9785	10189	14120
ENSECAG00000017669	4.831695113	0.271609811	0.492818695	321	328	400	675	619	598	594	901
ENSECAG00000008949	4.943717895	0.271718374	0.492936958	338	469	511	513	738	634	716	812
ENSECAG00000012337	6.788378208	0.271855807	0.493107547	1188	3090	1683	2482	1756	1776	1992	2692
ENSECAG00000014904	7.321170388	0.271942538	0.493186131	2331	3249	2816	3622	2848	3179	2593	3191
ENSECAG00000014334	11.27092601	0.272174341	0.493527747	28970	36906	63924	65120	49812	34935	45128	52471
ENSECAG00000017543	5.059129512	0.272364209	0.493793226	347	391	673	595	817	585	862	906
ENSECAG00000016034	5.483720387	0.272459121	0.493878639	828	1107	636	611	1038	567	806	914
ENSECAG00000004167	1.026478849	0.272537995	0.493878639	13	34	17	46	27	37	54	79
ENSECAG00000020881	6.718229528	0.272541724	0.493878639	1153	1441	1549	2082	1955	2204	2337	3702
ENSECAG00000023053	2.598116584	0.272717021	0.494062289	64	74	101	106	158	87	169	170
ENSECAG00000000463	3.362070642	0.272730036	0.494062289	56	170	129	259	172	217	195	434
ENSECAG00000020060	2.773192329	0.272826959	0.494159079	49	103	219	181	113	134	121	115
ENSECAG00000007206	5.701591256	0.273037062	0.494460806	480	627	846	1276	1217	1004	1245	1476
ENSECAG00000000438	6.217550858	0.273090423	0.494478628	1764	981	1259	1273	1303	919	1578	1737
ENSECAG00000022701	5.407541716	0.273236488	0.494643061	445	621	599	910	999	828	1101	1077
ENSECAG00000019935	0.315013833	0.273268306	0.494643061	4	30	35	28	15	16	25	22
ENSECAG00000022022	5.985799356	0.273395827	0.494789409	594	1259	1592	1451	939	1139	1007	1703
ENSECAG00000005717	4.682730263	0.273436253	0.494789409	390	364	527	632	562	459	423	489
ENSECAG00000018080	5.858644422	0.273496432	0.494819499	513	658	1046	1370	1291	1376	1119	1733
ENSECAG00000000686	10.46795986	0.273603355	0.494934138	22791	31527	24727	26104	26636	23005	26678	27482
ENSECAG00000018485	8.475717494	0.273741151	0.495087207	4247	5468	5517	4843	8676	6977	8402	10732
ENSECAG00000024036	3.314317326	0.273782997	0.495087207	92	93	176	235	247	159	269	285
ENSECAG00000015307	5.408553454	0.273818694	0.495087207	373	507	772	979	1097	960	959	980
ENSECAG00000000963	4.123056498	0.27414421	0.4955969	199	277	473	377	369	308	333	284
ENSECAG00000008848	7.074080647	0.274784134	0.496674727	1469	1940	1849	2588	2635	2304	2955	5405

ENSECAG00000008511	10.78676829	0.274829393	0.49667752	24841	23509	40246	49667	41013	25019	33737	29491
ENSECAG00000024554	4.268306726	0.275107075	0.496993035	151	295	311	399	308	580	318	636
ENSECAG00000008241	2.390395651	0.275115111	0.496993035	39	71	230	82	79	70	85	132
ENSECAG00000004212	4.393133751	0.275135204	0.496993035	247	315	436	619	395	290	441	469
ENSECAG00000013348	1.179466392	0.275516475	0.497602637	28	41	24	15	51	90	45	28
ENSECAG00000002263	7.267223786	0.275675427	0.497810584	2288	4404	2204	2455	2559	2576	2720	3441
ENSECAG00000026810	3.222305112	0.275768846	0.497900147	97	126	109	216	225	196	169	308
ENSECAG00000011640	5.293615027	0.27581599	0.497906145	194	645	691	1913	651	646	1059	375
ENSECAG00000015658	4.191107158	0.275876741	0.4979367	182	197	283	442	484	294	583	382
ENSECAG00000000602	1.323474365	0.276180375	0.498265234	33	22	43	33	72	37	59	72
ENSECAG00000013898	2.943326599	0.27618349	0.498265234	69	124	120	134	192	138	199	206
ENSECAG00000018075	5.835466451	0.276190322	0.498265234	724	975	1100	1507	1168	927	1070	1193
ENSECAG00000020143	4.241230758	0.276290358	0.498366574	216	226	305	379	515	409	478	374
ENSECAG00000019597	5.586859551	0.276364857	0.498421827	870	644	810	1207	1035	669	954	984
ENSECAG00000010540	8.652408797	0.276583984	0.498728508	3555	5340	6433	8753	9159	8574	11039	10188
ENSECAG00000010776	5.725446705	0.276622694	0.498728508	636	582	1045	843	1516	846	1524	1161
ENSECAG00000024846	4.997319375	0.276684301	0.498760435	281	614	716	866	570	569	597	679
ENSECAG00000012041	4.621441044	0.276730242	0.498764119	304	368	788	396	518	389	479	443
ENSECAG00000006597	4.788188601	0.276878902	0.49887506	329	398	392	519	699	499	746	665
ENSECAG00000000897	5.209312449	0.27687961	0.49887506	444	383.999	894	1188	752	581	753	681
ENSECAG00000020354	3.987896028	0.276927246	0.498881778	341	220	262	314	243	254	275	409
ENSECAG00000020889	6.903090969	0.2771941	0.499149352	1306	3171	1899	2769	2267	1770	2077	2819
ENSECAG00000003837	9.757419656	0.277266385	0.499149352	17593	13683	25404	11023	19093	4543	30080	3045
ENSECAG00000000760	3.449441118	0.277266919	0.499149352	217	128	251	214	169	117	147	374
ENSECAG00000015530	6.206985572	0.277277342	0.499149352	1566	1075	1313	1312	1498	1335	1285	1367
ENSECAG00000012445	8.186685179	0.277295433	0.499149352	3891	6000	4817	7594	5794	4979	4801	5993
ENSECAG00000023532	7.977213239	0.277366922	0.49919895	2632	5525	5145	6323	4096	4597	3911	6069
ENSECAG00000003869	7.521946464	0.277432948	0.499238701	2663	3897	3304	3919	3262	3742	2647	3912
ENSECAG00000016662	4.331789225	0.277791781	0.499763928	164	241	356	413	236	623	226	911
ENSECAG00000003276	6.421827737	0.277815147	0.499763928	2717	1648	675	802	1593	873	1863	1677
ENSECAG00000009688	6.582279017	0.277883341	0.499763928	1152	2078	1989	1962	1692	1705	1780	2045
ENSECAG00000004257	4.151405222	0.277917451	0.499763928	153	215	286	437	429	318	365	595
ENSECAG00000016734	6.845992071	0.277944751	0.499763928	1731	3085	1852	1753	1841	2023	2156	2460
ENSECAG00000005757	9.511309486	0.278072611	0.499847985	26149	10568	6416	6297	11956	11465	12376	13835
ENSECAG00000000592	3.771838507	0.2781099	0.499847985	224	292	237	235	247	182	292	300
ENSECAG00000020532	6.833113959	0.278123479	0.499847985	1251	1448	2036	2006	2791	2301	2797	3032
ENSECAG00000008257	2.924548758	0.278226757	0.499954518	73	86	135	150	219	113	202	196
ENSECAG00000015528	5.411708339	0.278291328	0.499991473	674	948	696	754	791	643	755	1049
ENSECAG00000002911	2.582648611	0.278459776	0.500215016	55	231	81	97	62	57	94	208
ENSECAG00000010581	5.492975412	0.278521337	0.500246512	891	1008	605	674	981	671	830	870
ENSECAG00000017479	6.392936813	0.278691675	0.500346125	584	997	1225	2461	1577	2150	1782	2580
ENSECAG00000016955	7.040804268	0.278697282	0.500346125	1892	2384	2398	3255	2468	2336	2395	2635
ENSECAG00000022473	4.351837068	0.278708909	0.500346125	145	202	442	469	413	412	408	748
ENSECAG00000014142	4.530982584	0.278885412	0.500540415	284	258	444	855	482	320	546	360
ENSECAG00000001897	6.331541217	0.278905243	0.500540415	936	1119	1231	1501	2110	1510	2070	1948
ENSECAG00000018014	2.850817516	0.278990524	0.500614392	148	107	141	122	132	116	129	146
ENSECAG00000021798	3.576490719	0.279145116	0.500812697	135	146	201	207	345	147	277	392
ENSECAG00000001892	7.062774578	0.279336196	0.501076391	1326	2840	2562	3760	2358	2344	1895	3376
ENSECAG00000010644	8.573812227	0.279428611	0.501163044	4701	6142	5029	5581	8574	8362	9124	10992
ENSECAG00000019969	3.910154065	0.279543282	0.501289579	113	336	291	465	161	330	228	376
ENSECAG00000016078	5.615810962	0.279592536	0.501298784	996	1089	773	624	1023	624	1300	622
ENSECAG00000022088	7.323806284	0.279817095	0.501555126	1524	2861	2251	2710	3095	3327	3889	5271
ENSECAG00000007459	2.272467576	0.279823794	0.501555126	120	102	57	66	45	135	77	62
ENSECAG00000013920	4.849664506	0.279884228	0.501584322	264	374	525	603	688	578	560	915
ENSECAG00000007150	6.776124007	0.279961681	0.501644002	1020	1316	1912	2408	2227	2708	2453	3083
ENSECAG00000021742	6.538196851	0.280168183	0.501934861	1491	1644	1494	2289	1807	1817	1919	1332
ENSECAG00000010795	6.830271874	0.280268394	0.502035234	937	2088	2282	3911	1834	2378	2184	1916
ENSECAG00000017211	4.307254936	0.280382264	0.502160038	324	325	438	353	388	263	449	392
ENSECAG00000024783	3.657439321	0.280538315	0.502360337	177	130	214	190	381	211	335	281
ENSECAG00000023395	0.626331406	0.280713637	0.502574856	5	13	26	41	34	50	37	21
ENSECAG00000000420	4.092265362	0.280746577	0.502574856	173	474	233	418	283	273	331	382
ENSECAG00000002702	3.39239693	0.280897191	0.502765263	76	108	171	562	162	157	161	266
ENSECAG00000010162	2.486634934	0.280953958	0.502787663	26	97	93	277	45	119	70	145
ENSECAG00000021188	6.493143656	0.281008985	0.502806944	655	1144	1855	1891	1695	2012	2107	2846
ENSECAG00000022163	4.87466754	0.281081221	0.502857004	484	629	400	609	554	524	530	600
ENSECAG00000017421	2.040017094	0.281307484	0.503182563	18	67	63	93	92	97	89	116
ENSECAG00000006773	5.75626519	0.281618489	0.503659573	990	1001	953	908	1098	910	1033	1031
ENSECAG00000017209	5.203359356	0.282033753	0.504183464	325	813	443	564	925	765	1077	735
ENSECAG00000016890	5.264349107	0.282036994	0.504183464	580	780	613	849	760	489	666	1032
ENSECAG00000014678	5.592287528	0.282044543	0.504183464	611	770	733	680	1173	856	1192	1371
ENSECAG00000019392	6.237649281	0.282172621	0.504259556	856	1558	1296	2048	1428	1217	1676	1372
ENSECAG00000001347	4.820613937	0.282175872	0.504259556	197	338	364	1601	584	560	267	577
ENSECAG00000015035	4.070673234	0.282246651	0.504306723	244	196	259	256	435	302	427	437
ENSECAG00000024474	4.965634059	0.282412244	0.504523257	347	477	512	521	824	556	667	917
ENSECAG00000023969	1.063096723	0.282762664	0.505069862	18	32	45	70	37	20	52	33
ENSECAG00000019035	2.106998768	0.283073739	0.505529832	53	74	100	101	78	42	91	101
ENSECAG00000022359	3.896463777	0.283119117	0.505529832	131	192	231	357	356	265	349	451
ENSECAG00000026317	0.497668044	0.283171687	0.505529832	18	29	35	21	32	21	12	27
ENSECAG00000000060	4.573436518	0.28319815	0.505529832	240	303	497	383	512	518	509	720
ENSECAG00000015268	3.882592197	0.283268722	0.505576378	157	209	200	313	299	283	334	494
ENSECAG00000019607	3.745980904	0.283336894	0.505618626	124	141	201	351	363	231	391	294
ENSECAG00000020982	4.191368427	0.283510815	0.505849541	246	269	241	292	411	363	510	445
ENSECAG00000023539	5.980486239	0.283742704	0.506183797	685	693	1056	1422	1387	1380	1470	1735
ENSECAG00000002063	0.675543528	0.283842077	0.506281582	18	23	12	29	44	25	44	36
ENSECAG00000022690	8.000550569	0.28406593	0.506601334	3655	1597	4516	4192	10238	4862	7142	3012

ENSECAG00000020775	0.806245774	0.284256137	0.506860989	7	39	15	31	49	29	29	61
ENSECAG00000000371	5.909916668	0.28449739	0.507142719	1312	898	1065	985	1436	919	1162	932
ENSECAG00000021839	3.333677988	0.284529351	0.507142719	111	115	141	231	219	247	206	283
ENSECAG00000014886	0.31613751	0.284583229	0.507142719	7	38	29	22	6	24	7	40
ENSECAG00000022930	1.799862249	0.284626727	0.507142719	10	47	43	105	63	70	113	93
ENSECAG00000020987	3.340949748	0.28463731	0.507142719	167	83	322	203	243	103	179	208
ENSECAG00000023334	0.738072699	0.284808127	0.507367504	3	17	41	30	42	27	45	45
ENSECAG00000017711	8.117511339	0.284877925	0.507412288	2643	6789	5764	6496	3825	5827	4369	6315
ENSECAG00000020421	4.553369646	0.285113457	0.50775221	284	282	452	361	581	445	515	691
ENSECAG00000025149	6.57088816	0.285181419	0.50779365	1693	2700	1279	1129	1421	1900	1623	1963
ENSECAG00000009429	0.627049373	0.285237685	0.507814255	12	15	38	14	39	41	23	41
ENSECAG00000016805	5.029515205	0.285504475	0.508209595	144	519	516	833	533	1106	869	597
ENSECAG00000000424	0.864793378	0.28595738	0.508936051	16	17	22	45	41	34	41	55
ENSECAG00000017033	6.980841029	0.286028349	0.508982631	1359	2361	2835	3225	2216	2490	2026	2682
ENSECAG00000009471	5.344590689	0.286099234	0.509029047	252	1077	558	1441	485	638	631	1246
ENSECAG00000006356	2.222575399	0.286469755	0.50960848	90	87	69	90	87	76	82	89
ENSECAG00000011805	6.156201126	0.286832149	0.510117222	622	964	1115	1724	1701	1492	1716	1838
ENSECAG00000024178	4.657973999	0.28686648	0.510117222	248	324	616	821	414	385	653	426
ENSECAG00000023269	7.578414727	0.286890429	0.510117222	1981	2199	3500	3693	5189	4026	4575	4541
ENSECAG00000015212	0.807081294	0.28717301	0.510539782	24	24	74	11	21	49	15	22
ENSECAG00000024379	4.084503132	0.287387843	0.510797525	172	232	435	487	332	330	276	324
ENSECAG00000013894	3.572811407	0.287407901	0.510797525	137	174	279	326	247	178	249	212
ENSECAG00000000263	3.38333515	0.28788186	0.511559853	129	206	217	229	206	182	159	231
ENSECAG00000019853	3.185183255	0.288194686	0.512035657	136	23	120	192	372	141	337	68
ENSECAG00000019622	3.023344991	0.288421464	0.51226718	83	80	237	256	169	165	122	120
ENSECAG00000007094	5.174568547	0.288469198	0.51226718	303	643	945	925	605	628	602	904
ENSECAG00000001627	4.066506364	0.288475868	0.51226718	158	442	292	379	298	285	307	363
ENSECAG00000025597	1.094771234	0.288505341	0.51226718	8	13	29	163	38	63	1	9
ENSECAG00000017769	5.861079789	0.288596658	0.512349255	570	774	733	1502	1322	1031	1616	1568
ENSECAG00000018645	7.002182244	0.288749243	0.512540057	1587	2915	2521	2602	2403	2281	2334	2553
ENSECAG00000015251	4.848568983	0.288921012	0.512764844	386	599	615	529	541	692	477	412
ENSECAG00000007704	4.397053592	0.289017241	0.51285552	374	287	295	628	442	207	381	561
ENSECAG00000025009	2.893353377	0.289252724	0.513193231	63	168	152	195	139	99	145	163
ENSECAG00000008968	7.495172929	0.28933773	0.513210305	2036	2118	3073	3430	4722	3385	4521	4740
ENSECAG00000020764	2.225974707	0.289352686	0.513210305	49	92	59	170	74	64	97	100
ENSECAG00000011889	4.743216724	0.289813876	0.513948066	362	673	370	574	533	375	385	740
ENSECAG00000008481	3.113847625	0.289921861	0.514059329	92	107	118	191	229	166	146	292
ENSECAG00000013420	6.05852929	0.290012458	0.514139732	745	1812	1112	1302	1105	1228	1352	1305
ENSECAG00000015076	4.414375929	0.290272515	0.514520486	208	186	430	473	513	476	620	402
ENSECAG00000022485	4.649644633	0.290378449	0.514627973	227	406	409	479	520	453	664	746
ENSECAG00000012660	11.52436403	0.290720335	0.514960733	31029	32120	50613	56743	85075	74527	86252	37614
ENSECAG00000013256	6.259786117	0.290755207	0.514960733	641	1428	1480	2574	1332	1109	1237	2135
ENSECAG00000008220	2.364248908	0.290768343	0.514960733	38	106	109	157	72	108	95	89
ENSECAG00000021437	2.054520541	0.290773255	0.514960733	40	124	69	81	85	57.0001	77	75
ENSECAG00000024184	3.71934891	0.290792824	0.514960733	115	189	387	357	334	196	250	179
ENSECAG00000002018	5.312683694	0.291063455	0.515320804	557	854	825	659	926	576	670	849
ENSECAG00000018696	1.145791969	0.291086862	0.515320804	28	37	44	56	39	34	41	40
ENSECAG00000019312	6.507898128	0.291332235	0.515674848	1321	2028	1436	1905	1874	1421	1709	1879
ENSECAG00000001399	8.192523539	0.291432516	0.515727014	4255	12413	2120	3004	5265	3364	4208	7700
ENSECAG00000022471	5.355255757	0.291500742	0.515727014	706	661	763	830	894	606	657	973
ENSECAG00000010916	3.11599195	0.29157265	0.515727014	64	122	150	186	233	143	215	238
ENSECAG00000022150	2.438351307	0.291585603	0.515727014	63	72	157	133	90	40	103	161
ENSECAG00000023917	4.200140343	0.291588658	0.515727014	88	242	371	448	376	403	455	530
ENSECAG00000014542	5.291272675	0.2918001	0.516020659	171	266	378	1581	494	488	638	2588
ENSECAG00000023674	6.680082568	0.2919288	0.516167915	1181	1887	2096	2676	1912	1714	2059	2061
ENSECAG00000011279	4.16079077	0.29206466	0.516263471	177	222	221	471	441	291	405	582
ENSECAG00000014388	1.633437043	0.292073719	0.516263471	21	35	37	89	73	66	82	72
ENSECAG00000018567	5.32648373	0.292185072	0.516339558	420	629	666	697	821	901	949	1098
ENSECAG00000016153	4.632729358	0.29221556	0.516339558	315	309	396	437	701	384	658	613
ENSECAG00000020001	6.926550027	0.292305899	0.516339558	1726	2607	2046	2682	2314	2143	2368	2256
ENSECAG00000013018	0.846551843	0.292315499	0.516339558	25	19	21	26	49	37	25	59
ENSECAG00000011633	5.084234496	0.292395688	0.516339558	411	479	482	653	759	694	908	822
ENSECAG00000012938	1.421313225	0.292427402	0.516339558	14	25	48	68	83	36	67	71
ENSECAG00000002578	6.202688065	0.292444602	0.516339558	765	852	1148	1742	1807	1250	2088	1868
ENSECAG00000009756	3.026390123	0.29248032	0.516339558	93	101	133	143	174	144	255	203
ENSECAG00000020567	5.735523893	0.292606453	0.516481981	552	1046	920	1579	858	1005	993	1196
ENSECAG00000024577	6.108334376	0.292781402	0.516710513	960	2067	764	1302	987	1316	1009	1799
ENSECAG00000013226	4.236995927	0.29291144	0.516850909	187	320	257	359	452	340	370	642
ENSECAG00000008614	4.967094716	0.292951932	0.516850909	289	839.002	571	686.002	376.004	633.005	512	820
ENSECAG00000018629	4.88601114	0.293185531	0.517182736	351	408	369	642	711	469	787	848
ENSECAG00000009022	6.019623986	0.293263109	0.51723928	781	938	944	1191	1696	1207	1652	1572
ENSECAG00000024416	2.647649957	0.293483558	0.517547755	66	99	90	102	140	109	167	183
ENSECAG00000021921	1.413778859	0.293576438	0.517631206	36	34	75	57	41	64	41	34
ENSECAG00000024029	6.866988377	0.293698905	0.51776679	1162	1402	1863	2661.99	2510	2616	2729	3300.99
ENSECAG00000003667	1.546700575	0.29382947	0.517846344	34	50	61	76	48	51	47	61
ENSECAG00000020426	3.399254266	0.293835186	0.517846344	83	188	174	180	243	191	284	287
ENSECAG00000014026	3.684876797	0.294001391	0.518058902	79	256	135	298	268	191	347	455
ENSECAG00000009053	4.001654737	0.294157997	0.518254483	205	114	412	553	328	281	309	241
ENSECAG00000022534	7.250509155	0.294432711	0.518658056	2025	1684	2646	2385	4137	2980	3940	3566
ENSECAG00000014832	3.883267024	0.294571798	0.518761302	202	602	134	146	328	154	283	276
ENSECAG00000021065	11.51461485	0.294622804	0.518761302	36759	38680	78322	75129	60334	44213	50989	61090
ENSECAG00000011435	4.865359685	0.294628294	0.518761302	304	236	548	680	887	463	854	579
ENSECAG00000019340	5.034764766	0.294699036	0.518805461	437	450	716	868	660	609	528	697
ENSECAG00000023724	3.477939659	0.294952053	0.519082607	102	199	237	337	147	173	221	292
ENSECAG00000011865	5.416118381	0.294955893	0.519082607	520	385	844	809	1000	982	991	1049

ENSECAG00000014347	5.724364247	0.294993522	0.519082607	527	1106	1041	1356	912	1094	874	1126
ENSECAG00000024658	9.654439387	0.295141162	0.519201152	23992	18208	5978	7321	14482	10861	15167	15466
ENSECAG00000005951	0.810297436	0.295152283	0.519201152	31	46	22	25	30	25	21	42
ENSECAG00000004450	4.203003386	0.295446909	0.519621316	242	167	403	207	588	309	630	250
ENSECAG00000016647	7.939696569	0.295539688	0.519621316	3421	5099	4704	5158	4523	4214	4706	4855
ENSECAG00000014405	2.000006142	0.29555123	0.519621316	38	47	145	89	51	105	46	69
ENSECAG00000023304	6.365919227	0.295574068	0.519621316	869	939	1320	1931	2009	1732	1871	2193
ENSECAG00000005750	3.149937494	0.295725901	0.519807811	94	99	161	162	259	126	175	301
ENSECAG00000019715	3.566651408	0.295945761	0.519978021	121	133	231	209	344	187	389	209
ENSECAG00000007689	5.911991315	0.2959518	0.519978021	835	1434	1103	1006	996	947	1212	1430
ENSECAG00000006205	4.848423141	0.29596003	0.519978021	234	252	768	497	637	578	648	910
ENSECAG00000015393	5.588176541	0.296192257	0.520243201	950	867	786	791	1065	730	898	939
ENSECAG00000014132	1.658800111	0.296202541	0.520243201	25	34	53	71	80	70	80	65
ENSECAG00000023400	2.025516937	0.29630893	0.520349624	28	88	91	120	36	100	64	80
ENSECAG00000009108	1.55251777	0.296760144	0.521061203	33	72	46	69	44	39	49	77
ENSECAG00000021004	0.359245702	0.296805853	0.521061203	9	18	20	19	44	21	20	33
ENSECAG00000017710	2.919790451	0.296933637	0.521205003	100	108	229	135	155	96	174	125
ENSECAG00000023859	3.980675596	0.29704789	0.521325012	140	136	715	313	442	264	264	105
ENSECAG00000002455	1.169336627	0.297109834	0.521353194	15	41	18	50	35	46	58	75
ENSECAG00000012155	4.010224707	0.297161089	0.521362616	231	412	339	196	371	325	278	182
ENSECAG00000020036	5.841067302	0.2972423	0.521424581	833	741	1395	1336	1273	730	1352	982
ENSECAG00000014982	2.850659203	0.297364125	0.521557763	85	230	97	124	151	99	123	148
ENSECAG00000016305	7.037018037	0.297552262	0.521722778	1245	1603	2290	2839	3159	2724	2904	3804
ENSECAG00000017464	6.628039503	0.297572733	0.521722778	1165	1490	1338	1822	2104	1968	2001	3453
ENSECAG00000020284	5.115043261	0.29763916	0.521722778	365	611	896	747	628	666	649	684
ENSECAG00000020293	6.626548197	0.297641881	0.521722778	995	2532	1852	2123	1506	1977	1938	1944
ENSECAG00000009846	2.160585386	0.298093187	0.522433255	33	68	57	91	93	51	59	252
ENSECAG00000015459	5.89453786	0.298372472	0.522827551	644	772	1544	1657	1235	994	1208	1054
ENSECAG00000012118	6.417310711	0.298410198	0.522827551	1450	1890	1749	1021	1582	1444	1461	1908
ENSECAG00000017154	11.87107438	0.29847093	0.522853331	40249	50993	97036	110807	71640	52607	72501	81109
ENSECAG00000013442	6.036419917	0.298585744	0.522895762	720	1259	1337	1694	925	1362	1054	1634
ENSECAG00000013825	3.463839528	0.298587194	0.522895762	106	173	369	252	112	411	108	94
ENSECAG00000002668	5.992753594	0.298730049	0.523013562	1072	1635	891	1100	1585	1203	1302	410
ENSECAG00000012538	2.100899888	0.298746525	0.523013562	25	97	84	140	59	62	80	104
ENSECAG00000013676	8.220792416	0.298859178	0.523130177	5484	6866	5308	3859	5591	5750	5412	4859
ENSECAG00000011096	2.104770107	0.298936098	0.523184218	43	65	53	82	107	55	123	129
ENSECAG00000024094	2.272389247	0.299574961	0.524221577	51	45	54	131	132	78	138	115
ENSECAG00000012224	4.684094294	0.299765369	0.524420111	374	299	459	323	766	432	577	682
ENSECAG00000008897	3.822623667	0.299780727	0.524420111	69	148	167	496	268	506	352	225
ENSECAG00000009189	1.617706103	0.300044248	0.524793031	22	50	38	65	60	60	71	101
ENSECAG00000004417	3.652272538	0.300120055	0.524793031	85	193	198	286	288	154	373	408
ENSECAG00000005765	3.405565601	0.300132469	0.524793031	126	204	186	286	214	173	173	235
ENSECAG00000020291	4.585356238	0.300348287	0.525089589	206	462	713	460	437	401	475	490
ENSECAG00000011559	3.588053303	0.300735878	0.525686314	105	309	235	286	113	280	178	302
ENSECAG00000007683	5.564238166	0.300804799	0.525725908	361	602	593	1441	1003	1031	1175	1286
ENSECAG00000024460	4.516818504	0.300899298	0.525732181	302	585	409	371	441	314	430	552
ENSECAG00000022025	2.655347194	0.300900931	0.525732181	66	65	63	176	176	101	132	202
ENSECAG00000023359	3.18279564	0.300956496	0.525748418	75	177	112	166	168	183	234	286
ENSECAG00000020724	8.260668748	0.301022108	0.52576077	3648	4237	4936	4908	7646	5973	6654	9438
ENSECAG00000019667	5.625139346	0.301056114	0.52576077	482	547	1035	972	1174	1000	1142	1335
ENSECAG00000014896	8.320270012	0.301157545	0.525820489	6130	7548	4180	5184	5632	5980	5700	5965
ENSECAG00000003160	1.480084044	0.301182868	0.525820489	32	46	39	28	44	47	107	69
ENSECAG00000019460	1.808102553	0.301838457	0.52688409	96	57	53	42	120	39	19	56
ENSECAG00000012231	6.876053353	0.302092489	0.527246523	1503	1609	1861	1813	3241	2151	2714	3157
ENSECAG00000016482	2.440326888	0.30220697	0.527365318	46	20	327	86	141	132	33	11
ENSECAG00000014178	5.037159414	0.302307626	0.527447259	538	790	469	546	586	563	633	696
ENSECAG00000018278	1.533156464	0.302387262	0.527447259	22	56	29	53	63	55	53	107
ENSECAG00000021466	8.230045621	0.302393192	0.527447259	2220	3542	5326	7422	6950	7169	6963	7800
ENSECAG00000020582	1.984238579	0.302525371	0.527525923	26	77	159	51	53	78	85	51
ENSECAG00000022059	7.335904217	0.302531149	0.527525923	2015	1503	3225	6440	3810	2309	2326	3436
ENSECAG00000014256	1.422157842	0.302717588	0.527770022	14	46	84	71	39	52	53	37
ENSECAG00000022861	5.243605477	0.303024324	0.528223746	536	1062	587	543	702	661	693	784
ENSECAG00000018379	7.108730405	0.303148216	0.528289984	1717	2616	2794	3377	2383	2645	1994	3354
ENSECAG00000016383	1.516275574	0.303155315	0.528289984	42	59	71	35	56	39	43	64
ENSECAG00000024916	5.35087947	0.303288069	0.528362769	564	624	820	1027	754	734	730	914
ENSECAG00000009713	4.802544797	0.303290088	0.528362769	187	193	740	500	976	628	964	159
ENSECAG00000014459	1.794320105	0.303368121	0.52841769	30	37	72	59	95	74	86	70
ENSECAG00000013694	3.869411002	0.303466764	0.528508487	102	332	291	432	208	233	264	388
ENSECAG00000016278	7.453867595	0.303531916	0.528540939	4574	3723	1818	2032	2960	2802	3554	3232
ENSECAG00000020333	4.660808016	0.303686835	0.528729669	286	423	631	570	539	412	549	410
ENSECAG00000007800	6.363813896	0.303749991	0.528756291	851	928	1299	1999	1943	1575	1982	2321
ENSECAG00000000319	6.83310496	0.3037952	0.528756291	1721	1307	1926	1409	2928	1980	2753	3394
ENSECAG00000006779	4.879503561	0.304148088	0.529256241	372	464	649	725	685	496	576	473
ENSECAG00000018523	4.46670663	0.304175608	0.529256241	288	243	325	459	601	382	545	562
ENSECAG00000015267	5.827348893	0.304456785	0.529664369	466	604	2116	1404	1014	1378	840	857
ENSECAG00000010532	8.42562317	0.305065578	0.530612808	4705	8227	5557	7252	5993	5432	6933	7254
ENSECAG00000018373	2.421248005	0.30509536	0.530612808	48	98	85	66	136	126	168	76
ENSECAG00000006865	4.72359166	0.305220213	0.530646427	228	558	306	472	509	440	728	867
ENSECAG00000016946	6.223718325	0.305234498	0.530646427	912	1160	1648	1952	1558	1091	1608	1450
ENSECAG00000017721	1.874068212	0.305254801	0.530646427	47	55	106	67	73	40	66	85
ENSECAG00000015596	2.752618515	0.305360882	0.530743316	59	96	94	151	139	123	178	203
ENSECAG00000021195	6.996756251	0.30540396	0.530743316	1541	1493	1873	2673	3343	2291	2906	3745
ENSECAG00000012977	4.331275237	0.305555791	0.530925967	182	204	367	495	559	350	475	525
ENSECAG00000012505	2.869606173	0.305652997	0.531013663	77	170	138	175	89	113	105	233
ENSECAG00000015821	6.958410385	0.305849922	0.531274547	1735	2659	2400	2402	2248	2249	2232	2615

ENSECAG00000023505	8.307277952	0.306128329	0.531669926	5188	5958	5229	7114	6124	5565	5561	6328
ENSECAG00000025022	9.240622315	0.306171125	0.531669926	7693	7830	15442	16256	12596	8846	11036	12551
ENSECAG00000014044	4.935509569	0.306532002	0.532215252	238	453	466	755	808	515	761	815
ENSECAG00000005707	2.773650076	0.306691288	0.532410453	67	117	79	130	117	201	168	157
ENSECAG00000010761	3.487474489	0.306865997	0.532631194	70	172	146	303	224	228	244	384
ENSECAG00000020166	3.536485067	0.306912201	0.532631194	168	220	222	241	248	206	180	235
ENSECAG00000021329	0.4691768	0.307053738	0.532795444	13	13	27	19	33	22	28	45
ENSECAG00000005944	5.628316431	0.307128342	0.532843521	484	563	956	1058	1296	920	1148	1293
ENSECAG00000015638	7.244978241	0.307219167	0.532919722	1284	1759	2578	3732	3301	3231	3388	4683
ENSECAG00000007549	5.600474426	0.307282416	0.532948071	636	727	655	844	1142	1012	1213	1177
ENSECAG00000000053	3.243062415	0.307424297	0.533112769	102	126	145	186	223	161	217	302
ENSECAG00000017776	7.237846816	0.307727237	0.533556671	1507	3523	3137	3427	2087	2557	3162	3510
ENSECAG00000007716	1.110013918	0.30805043	0.534035548	15	30	38	36	34	36	77	56
ENSECAG00000016568	6.916874479	0.308199847	0.534213068	1182	1722	2059	2303	2775	2855	2439	3430
ENSECAG00000019028	3.289865941	0.30826105	0.534237653	91	106	170	222	179	208	211	338
ENSECAG00000014688	6.900373665	0.308319432	0.534257342	1111	2107	2758	3439	1843	1920	2026	3249
ENSECAG00000007803	4.772770355	0.308891039	0.535166207	311	308	473	567	707	461	612	809
ENSECAG00000019121	5.504287482	0.309045649	0.535320216	423	622	697	1087	968	961	1125	1200
ENSECAG00000020726	7.270363184	0.309074161	0.535320216	1770	1616	2830	2907	4502	2913	4292	3050
ENSECAG00000017418	9.994135457	0.309193726	0.53544568	21763	31914	9339	9967	17597	24196	16266	11523
ENSECAG00000015384	3.240102658	0.309384484	0.535694378	63	117	211	174	174	262	288	166
ENSECAG00000012867	4.014856338	0.309624188	0.536027735	161	260	393	431	321	286	244	371
ENSECAG00000009722	3.662635942	0.309743255	0.536152173	213	116	422	193	272	206	225	217
ENSECAG00000010815	0.128158045	0.309907369	0.536214731	1	20	23	8	24	17	9	56
ENSECAG00000017251	6.270422823	0.309913272	0.536214731	996	1096	1321	1070	1964	1373	1952	2047
ENSECAG00000022014	3.806849232	0.309920977	0.536214731	143	245	205	213	321	443	334	205
ENSECAG00000010292	1.43751313	0.310220588	0.536644552	26	38	46	38	71	31	81	75
ENSECAG00000016433	8.038619201	0.310277776	0.536644552	5456	4321	4493	4606	5368	3919	5050	5172
ENSECAG00000012312	7.568391123	0.310311099	0.536644552	4012	4503	2272	2648	3360	3289	3829	3318
ENSECAG00000016406	0.1491182	0.310408928	0.53673204	19	9	39	13	20	23	9	16
ENSECAG00000007672	3.456695335	0.310757499	0.537252997	172	107	150	188	292	195	234	332
ENSECAG00000011514	-0.174501162	0.310819078	0.537277707	5	4	22	13	23	14	21	21
ENSECAG00000004306	2.84439683	0.310948668	0.537419952	89	134	99	234	132	102	92	205
ENSECAG00000016401	6.40817043	0.311129047	0.537510943	1032.01	1140	1504.01	1347	2075	1697	1854	2420
ENSECAG00000010118	2.491062358	0.31113495	0.537510943	27	37	197	58	142	124	138	149
ENSECAG00000004444	6.23516359	0.311143238	0.537510943	1205	1125	1557	1695	2003	1334	1271	1005
ENSECAG00000008099	4.064098893	0.311250901	0.537615192	147	272	252	349	370	334	389	484
ENSECAG00000009859	4.723976084	0.311317398	0.537648317	254	543	557	654	502	483	414	618
ENSECAG00000006875	6.033675693	0.311584496	0.538027819	674	1602	1204	1448	1112	1295	1047	1514
ENSECAG00000024200	0.871815343	0.311664191	0.538075765	12	39	50	38	35	23	32	35
ENSECAG00000018059	3.560180187	0.311706978	0.538075765	158	243	96	132	434	176	256	284
ENSECAG00000016565	4.433683637	0.311815604	0.538181512	226	358	252	464	524	369	490	672
ENSECAG00000008237	1.811366015	0.311897662	0.538241379	70	73	62	60	35	139	18	25
ENSECAG00000013892	2.304210861	0.311950416	0.538250665	42	50	96	98	114	153	64	137
ENSECAG00000024434	4.601834068	0.312052922	0.538345779	294	588	417	493	366	512	422	530
ENSECAG00000023579	3.575110485	0.312237392	0.538582245	144	115	250	184	335	207	281	312
ENSECAG00000018978	6.407246713	0.312440257	0.538850364	790	2380	1464	1793	1406	1474	1451	2081
ENSECAG00000008815	4.564960268	0.312699963	0.539216419	498	466	402	285	484	276	524	485
ENSECAG00000018875	1.480800612	0.312807482	0.539235998	17	47	96	61	47	28	69	49
ENSECAG00000000030	4.17117184	0.312839218	0.539235998	173	250	265	419	407	344	445	500
ENSECAG00000024984	5.839005222	0.312853697	0.539235998	639	1510	725	1443	876	1154	931	1349
ENSECAG00000010321	2.934559278	0.313014937	0.539432081	150	93	174	143	154	187	93	106
ENSECAG00000014759	5.60015463	0.313108424	0.539511361	513	363	967	1135	1205	834	1417	1162
ENSECAG00000021040	4.192827176	0.31344013	0.540001024	165	328	501	440	353	434	361	164
ENSECAG00000002521	6.701982707	0.313513749	0.54004597	1360	1527	1363	1816	2408	1944	2754	2819
ENSECAG00000018098	9.944712612	0.313634552	0.540172166	14024	16767	19184	24806	19908	15817	17433	20643
ENSECAG00000016517	5.287008443	0.313690782	0.540187126	526	635	858	860	703	738	754	796
ENSECAG00000000716	5.327786766	0.313902827	0.540470362	538	873	766	748	724	783	809	731
ENSECAG00000011509	5.843335773	0.314055871	0.540651941	800	1006	642	824	1441	1098	1304	1619
ENSECAG00000009676	5.978799141	0.314133225	0.540703182	777	747	1724	1702	1954	885	1159	607
ENSECAG00000020242	3.99714133	0.314658582	0.541525418	119	219	255	403	362	327	423	391
ENSECAG00000015199	1.952490746	0.314778778	0.541650231	30	71	26	87	104	134	38	94
ENSECAG00000001300	2.316147339	0.315210086	0.542310266	29	71	51	145	104	165	72	133
ENSECAG00000011611	4.856342216	0.315823629	0.543283585	282	411	359	740	623	545	695	879
ENSECAG00000024483	6.326027429	0.315946951	0.543413451	758	1092	1379	1723	2028	1622	1714	2200
ENSECAG00000021679	5.73731747	0.316059131	0.543524117	868	949	716	1367	1003	944	968	1162
ENSECAG00000008268	2.335727708	0.316413809	0.544018591	55	50	94	89	134	75	98	181
ENSECAG00000008354	0.1523334	0.316442428	0.544018591	11	12	30	30	19	8	14	32
ENSECAG00000010198	4.823311247	0.316693305	0.544367524	339	550	325	428	692	556	665	762
ENSECAG00000026897	4.106310032	0.317082328	0.544953775	190	204	358	272	467	310	440	405
ENSECAG00000017934	4.804215711	0.317225564	0.544986089	284	455	361	599	635	705	656	597
ENSECAG00000024820	5.188479541	0.317237329	0.544986089	479	431	945	911	588	818	587	751
ENSECAG00000015331	1.645534929	0.317245027	0.544986089	36	36	109	57	62	41	57	61
ENSECAG00000017838	5.688016687	0.317419937	0.545204131	494	1881	723	734	802	773	985	1278
ENSECAG00000008209	6.508939114	0.317616218	0.545458807	1353	654	2918	2170	2480	1539	1275	1317
ENSECAG00000008163	6.731343695	0.317785916	0.545667761	1039	1286	1710	2507	2399	2422	2405	2825
ENSECAG00000010754	5.680321888	0.318301407	0.546470321	502	1296	908	1138	1031	892	891	1101
ENSECAG00000011660	2.899642437	0.318428856	0.546606537	83	113	42	157	312	267	104	40
ENSECAG00000024770	3.611250233	0.318580724	0.546615662	95	197	216	235	270	278	281	317
ENSECAG00000022120	8.64099804	0.318616422	0.546615662	4007	5495	6249	8132	9178	8427	8932	11882
ENSECAG00000005957	5.17566008	0.318681659	0.546615662	366	692	718	1007	628	472	534	1161
ENSECAG00000015428	5.229561045	0.318705637	0.546615662	867	750	386	606	870	580	729	586
ENSECAG00000012570	4.184002308	0.318773293	0.546615662	164	499	323.011	383.002	325.002	289.057	358	399
ENSECAG00000011215	1.544147958	0.31888822	0.546615662	99	24	39	39	24	53	82	31
ENSECAG00000022803	2.681833848	0.318943464	0.546615662	70	43	126	128	182	68	218	158

ENSECAG00000000508	2.396506139	0.318968662	0.546615662	78	47	125	154	88	89	103	100
ENSECAG00000023954	6.98438352	0.318974464	0.546615662	923	1865	2038	2980	2515	2839	2414	4462
ENSECAG00000000002	4.870897899	0.318980173	0.546615662	285	498	455	537	564	638	716	823
ENSECAG00000014611	2.231777737	0.31899654	0.546615662	61	53	81	57	179	70	134	67
ENSECAG00000016590	4.835906812	0.319011482	0.546615662	263	556	526	860	608	506	495	563
ENSECAG00000011358	0.337145837	0.31917477	0.546812987	8	38	22	25	20	18	16	29
ENSECAG00000024896	6.436794908	0.319268517	0.546891133	1134	2194	1278	1760	1584	1322	1756	1933
ENSECAG00000012511	4.580703846	0.319327145	0.546909108	238	259	485	486	542	529	484	696
ENSECAG00000013800	3.217862545	0.319695255	0.547391623	96	124	140	195	235	225	206	202
ENSECAG00000000370	11.77409685	0.319705229	0.547391623	38714	49217	91714	96084	69713	50881	68060	72825
ENSECAG00000009086	4.339662269	0.319763152	0.547408308	233	331	565	390	416	298	311	522
ENSECAG00000021620	3.410753357	0.319973403	0.547632297	142	153	144	169	275	184	276	272
ENSECAG00000022006	5.889999623	0.319990391	0.547632297	1072	1505	883	793	1869	669	1089	703
ENSECAG00000022464	4.457026566	0.320189287	0.547870458	260	301	359	397	534	467	476	573
ENSECAG00000010799	3.883152088	0.320225991	0.547870458	121	125	249	427	322	315	439	320
ENSECAG00000011370	7.153125843	0.320317315	0.547875784	1354	3021	1693	2190	3013	2766	3699	4271
ENSECAG00000019235	4.38090181	0.320325544	0.547875784	224.001	542	357.001	422.001	405.001	326	444.001	402.001
ENSECAG00000009354	4.712298767	0.320526577	0.548137112	322	212	859	648	545	477	382	544
ENSECAG00000024311	7.514112367	0.320936236	0.54869977	2076	2934	2063	3689	4456	3286	5017	4795
ENSECAG00000015628	1.621990912	0.32095218	0.54869977	40	29	48	52	92	55	76	66
ENSECAG00000018335	2.975686324	0.321106452	0.548792135	66	95	142	171	145	164	181	259
ENSECAG00000000078	5.705784448	0.321132916	0.548792135	708	684	933	682	1442	1034	1514	911
ENSECAG00000014721	1.152481494	0.321172888	0.548792135	14	35	34	43	48	44	57	57
ENSECAG00000000728	2.992764453	0.32119941	0.548792135	78	103	73	219	206	131	156	279
ENSECAG00000020589	5.942769731	0.321255536	0.548805504	616	965	998	1178	1339	1235	1499	1701
ENSECAG00000022646	4.807458079	0.32140588	0.548976159	213	402	516	616	753	461	749	676
ENSECAG00000009091	5.423519031	0.321452067	0.548976159	437	580	1111	1193	988	732	746	795
ENSECAG00000002847	3.553690835	0.321564608	0.549043448	119	153	221	209	284	199	279	351
ENSECAG00000012027	0.233110108	0.321588114	0.549043448	14	8	15	21	49	17	10	33
ENSECAG00000014372	6.206405686	0.321758274	0.549233691	1682	2385	535	545	1058	962	1170	2111
ENSECAG00000021475	4.358636477	0.321811307	0.549233691	453	475	220	274	368	425	324	380
ENSECAG00000023563	6.267540115	0.321844562	0.549233691	740	1947	1482	1635	1294	1310.99	1488	1775
ENSECAG00000004022	0.007670471	0.3220636	0.549524947	6	10	20	44	21	17	17	5
ENSECAG00000020283	7.575351051	0.322258003	0.549774089	2093	3582	4375	4576	3411	3137	4258	3514
ENSECAG00000018265	6.80870201	0.322363297	0.549871157	1147	2946	1893	2526	1678	2116	1800	2844
ENSECAG00000015531	1.839455359	0.322511996	0.550042225	35	49	44	78	97	63	70	108
ENSECAG00000019411	6.19652818	0.322656539	0.550206152	406	4333	593	398	1157	1869	807	905
ENSECAG00000025087	6.269950941	0.322761954	0.550303319	1272	851	1181	1023	1894	1373	1867	2264
ENSECAG00000015906	2.910947959	0.322987887	0.550605907	57	102	109	186	128	132	188	278
ENSECAG00000020168	6.768151148	0.323178707	0.550848555	985	1280	1586	2943	2441	2281	2549	3117
ENSECAG00000023121	2.378952116	0.323330161	0.551024042	70	66	60	91	185	66	122	127
ENSECAG00000018653	4.575107524	0.323413365	0.551083181	402	367	434	523	554	346	407	518
ENSECAG00000020151	0.403241658	0.323465262	0.551088964	7	21	46	27	13	23	23	27
ENSECAG00000008877	4.847417682	0.323882448	0.551716997	300	680	515	682	337	704	505	603
ENSECAG00000009365	3.057200512	0.324038961	0.551900865	95	95	139	161	211	147	182	250
ENSECAG00000017910	7.481660122	0.324117149	0.551951296	2219	2537	1936	3806	4847	3361	4515	4396
ENSECAG00000019179	2.57505749	0.324745927	0.55292008	87	128	87	136	126	76	108	131
ENSECAG00000010360	6.182797026	0.324783367	0.55292008	843	267	2856	2164	1968	1108	1469	392
ENSECAG00000017018	5.495929662	0.324870205	0.552985058	568	843	809	1130	878	669	1004	937
ENSECAG00000017555	5.044938706	0.324962137	0.553009383	335	370	624	704	731	527	830	1048
ENSECAG00000022015	0.756558079	0.325029263	0.553009383	6	15	11	61	22	29	17	101
ENSECAG00000020071	4.99903786	0.32503051	0.553009383	540	737	432	567	726	442	629	632
ENSECAG00000021105	0.798019446	0.325231092	0.553267805	9	30	18	39	32	29	55	46
ENSECAG00000016800	9.052092347	0.325286565	0.553279334	5498	7003	7643	11735	13151	10137	12971	14829
ENSECAG00000022848	5.351130645	0.325534499	0.553618168	431	583	575	916	929	752	852	1328
ENSECAG00000021325	2.588884687	0.325835134	0.554046511	50	68	219	153	50	169	74	120
ENSECAG00000011330	1.861097355	0.326192984	0.554393772	21	34	62	89.0001	40	40	66	227.001
ENSECAG00000019070	3.49774313	0.326202891	0.554393772	110	177	186	202	234	270	249	303
ENSECAG00000012818	4.122337222	0.326246502	0.554393772	204	320	234	249	449	272	458	475
ENSECAG00000016753	3.793413081	0.326273573	0.554393772	120	199	189	337	313	402	204	384
ENSECAG00000021926	5.502977036	0.326283326	0.554393772	575	712	575	828	1204	758	1110	1202
ENSECAG00000018392	3.717929147	0.326503166	0.554684355	129	133	324	170	367	165	517	222
ENSECAG00000022608	5.463077619	0.326600105	0.554766093	420	461	876	997	981	1001	946	1204
ENSECAG00000020655	5.017629009	0.326754665	0.554945667	480	707	526	618	635	539	688	612
ENSECAG00000019034	5.153326425	0.326980975	0.555247024	255	504	548	949	886	682	741	1061
ENSECAG00000007760	7.712505855	0.327068975	0.555313463	2804	2364	3277	3778	5604	4026	5238	5180
ENSECAG00000009049	7.597368277	0.327269966	0.555571695	2344	1528	4539	2731	5100	2813	4407	6793
ENSECAG00000006603	6.001536073	0.327422177	0.555602655	404	840	793	2020	1619	1403	2059	996
ENSECAG00000009003	6.702996019	0.327501627	0.555602655	1555	2332	1439	2320	1903	1702	1762	2563
ENSECAG00000016412	7.503989451	0.327502231	0.555602655	2821	3115	2858	4766	3629	3001	3385	3676
ENSECAG00000013905	2.002059471	0.327546746	0.555602655	32	73	95	117	57	108	45	68
ENSECAG00000012752	0.84135898	0.327553917	0.555602655	38	15	16	68	15	55	16	27
ENSECAG00000006302	4.622161672	0.327581604	0.555602655	199	600	485	618	351	624	426	414
ENSECAG00000013531	7.047116706	0.327663754	0.55565904	1351	2251	3216	3402	2400	2286	2523	2794
ENSECAG00000006492	7.441369577	0.327845534	0.555884339	1724	1745	2866	3809	6322	5449	3410	1400
ENSECAG00000012779	7.533439408	0.32793831	0.555905187	2541	2689	2955	2279	4579	3657	4105	5496
ENSECAG00000012515	4.993224029	0.327955683	0.555905187	267	530	533	640	724	578	803	887
ENSECAG00000021497	2.567499313	0.328125942	0.556110822	57	91	342	34	6	276	7	19
ENSECAG00000006710	5.564185017	0.328188306	0.556133562	1004	711	908	682	1092	830	829	796
ENSECAG00000022061	6.273445467	0.328262785	0.556176821	822	793	1640	1434	2276	1269	1980	1813
ENSECAG00000007423	4.948631367	0.328368732	0.556245285	306	546	892	587	472	525	679	678
ENSECAG00000021909	2.795116855	0.328401107	0.556245285	23	120	80	201	161	147	184	178
ENSECAG00000005666	6.012960053	0.328513523	0.556352757	917	985	1268	1631	1355	1008	990	1638
ENSECAG00000020760	6.436380307	0.328621373	0.556452464	1017	1190	1269	1752	2007	1820	1802	2539
ENSECAG00000020320	1.239029901	0.328702148	0.556506304	33	51	56	36	24	81	24	23

ENSECAG00000008289	3.681078266	0.328832085	0.556643347	121	166	230	263	249	278	275	408
ENSECAG00000022231	1.566975576	0.329090102	0.556997131	24	29	66	50	77	46	67	91
ENSECAG00000017760	5.668414977	0.329226646	0.557145242	601	464	1011	1024	1180	1116	1232	1230
ENSECAG00000020031	5.20219995	0.329434308	0.557352979	371	580	572	734	882	691	867	1006
ENSECAG00000020822	8.613967317	0.32944751	0.557352979	6921	9444	6329	5442	7249	6433	7483	7818
ENSECAG00000011622	3.525126508	0.329527885	0.557405959	74	235	130	250	215	182	294	429
ENSECAG00000000341	1.422239396	0.329661857	0.557549572	18	39	72	84	33	55	42	56
ENSECAG00000012083	9.811022389	0.329730487	0.557582645	6736	13413	12483	22662	21745	18167	22892	23699
ENSECAG00000020897	4.17795252	0.329856275	0.557710279	182	300	477	419	317	283	442	328
ENSECAG00000015974	4.370142155	0.329904135	0.557710279	171	406	490	528	356	355	322	546
ENSECAG00000019722	4.120285183	0.330019045	0.557789702	216	157	512	460	342	247	289	436
ENSECAG00000007745	6.086131795	0.330049302	0.557789702	685	740	1206	1594	1610	1372	1516	1902
ENSECAG00000019350	4.910645835	0.330254927	0.557861142	310	433	517	577	719	659	796	612
ENSECAG00000017726	5.098807582	0.330275521	0.557861142	436	732	649	683	686	605	643	698
ENSECAG00000010846	5.439692896	0.330313394	0.557861142	484	540	585	1071	980	924	912	1257
ENSECAG00000008513	2.84385844	0.330388283	0.557861142	85	111	83	135	170	132	192	184
ENSECAG00000019307	6.166872774	0.330422693	0.557861142	980	1503	1048	1777	1375	1127	1546	1456
ENSECAG00000017319	6.806953968	0.330458373	0.557861142	1202	1393	2285	1802	2661	1916	2761	3386
ENSECAG00000024058	0.872743811	0.330480333	0.557861142	12	47	41	40	44	20	40	18
ENSECAG00000015349	3.794081241	0.330484364	0.557861142	202	388	209	196	232	199	298	309
ENSECAG00000016906	2.272020862	0.330631345	0.558005964	45	71	59	108	124	94	130	105
ENSECAG00000020064	6.202220086	0.330668382	0.558005964	1094	1236	1566	1470	1458	1409	1306	1450
ENSECAG00000017830	5.99253427	0.330726505	0.558021169	498	714	1278	1500	1174	1868	1531	1366
ENSECAG00000002386	2.732618037	0.330903991	0.558237735	73	76	97	142	202	102	200	125
ENSECAG00000021401	6.766629123	0.331538383	0.559224926	1765	1634	2353	2210	2167	1870	2049	2182
ENSECAG00000004558	5.901349781	0.331599151	0.559244404	792	1014	1142	1485	1184	867	1157	1435
ENSECAG00000009007	5.405505501	0.331662793	0.559268723	486	482	730	898	987	770	932	1308
ENSECAG00000012317	6.942089239	0.331745963	0.559325958	1689	2581	2282	2527	2358	1931	2614	2421
ENSECAG00000005540	8.234726795	0.332066197	0.559782809	3396	5191	4801	10434	5183	6475	5538	4985
ENSECAG00000024966	4.739912024	0.332584391	0.560562726	217	406	387	664	504	580	647	779
ENSECAG00000015449	6.43345811	0.332671404	0.560562726	719	1118	1262	2291	2216	2220	2296	1345
ENSECAG00000022134	5.520689278	0.332709258	0.560562726	485	636	888	1575	833	890	711	1073
ENSECAG00000018754	5.915837958	0.332748231	0.560562726	720	766	1010	1161	1557	1088	1525	1487
ENSECAG00000010768	6.428086259	0.332775531	0.560562726	1030	724	1574	1905	2212	1621	2076	2262
ENSECAG00000005769	4.47687741	0.333272638	0.561316886	224	382	497	603	363	532	349	429
ENSECAG00000023542	6.261710604	0.333409214	0.561382196	668	1211	1740	2435	1196	1588	1268	1759
ENSECAG00000012979	7.233758638	0.333437136	0.561382196	1970	2308	3645	3433	2797	2810	2683	3074
ENSECAG00000011040	0.499615662	0.333459641	0.561382196	10	30	26	41	16	15	24	41
ENSECAG00000010251	4.725977846	0.333559876	0.561467749	248	521	297	491	462	932	631	431
ENSECAG00000014960	4.074287749	0.333929318	0.562004793	192	220	311	284	432	349	417	370
ENSECAG00000009643	-0.012364492	0.334025165	0.562004793	6	10	14	22	25	12	28	23
ENSECAG00000018608	5.227772948	0.334027316	0.562004793	439	474	675	684	907	797	793	997
ENSECAG00000011655	6.492474117	0.33411948	0.562076626	846	1283	1413	2021	2202	1796	1746	2787
ENSECAG00000011331	8.374565612	0.334210471	0.562146465	3027	5034	5694	6127	7168	8453	9037	6745
ENSECAG00000008399	7.129752703	0.334413756	0.562351589	2340	3998	1957	1713	2483	2180	2364	3391
ENSECAG00000023878	3.806463083	0.33443141	0.562351589	124	206	365	395	259	239	249	310
ENSECAG00000000767	6.895910985	0.334533134	0.5624394	1063	975	1801	3635	3057	2347	2971	3056
ENSECAG00000020977	3.275180498	0.334714381	0.562660866	67	119	342	253	173	156	126	256
ENSECAG00000018889	4.847904858	0.334802052	0.562724988	409	830	345	492	478	424	531	755
ENSECAG00000024333	6.274698416	0.334942105	0.562832323	966	897	1111	1717	1703	1648	1681	2267
ENSECAG00000019474	8.573736733	0.334964986	0.562832323	20698	135	130	8213	13529	191	146	6076
ENSECAG00000021515	7.93598496	0.335158521	0.56304134	1335	2888	2336	8951	6095	6878	5627	5060
ENSECAG00000015214	4.963234542	0.33518849	0.56304134	402	355	574	528	788	563	863	704
ENSECAG00000017604	6.762416381	0.33539635	0.563223837	1430	1648	2055	3120	2180	2073	1719	2266
ENSECAG00000022603	5.909673141	0.335415985	0.563223837	1032	1319	905	1008	968	1059	1015	1558
ENSECAG00000009761	8.924890212	0.335445846	0.563223837	7176.99	6353	6970.98	6804	11911	9796	12549	12466
ENSECAG00000012663	5.688385853	0.335543327	0.563304267	820	535	761	856	1497	839	1267	1282
ENSECAG00000007006	7.664223485	0.335670605	0.563379014	1824	2270	3297	5167	5190	3802	4658	5816
ENSECAG00000010593	5.454905091	0.33568702	0.563379014	355	909	870	1257.01	814	710	876	968
ENSECAG00000017001	5.02615712	0.335780712	0.563427882	362	367	542	741	700	672	739	940
ENSECAG00000026975	2.554247637	0.335830852	0.563427882	89	113	99	127	90	101	106	139
ENSECAG00000009052	3.205081053	0.335864905	0.563427882	93	86	190	182	218	151	201	314
ENSECAG00000003592	5.3515935	0.336594082	0.564567753	538	568	604	717	1110	755	895	1065
ENSECAG00000000037	5.543005633	0.336714707	0.564612935	495	777	1041	1211	867	787	927	1026
ENSECAG00000024914	5.425445458	0.336720405	0.564612935	447	359	1070	718	1314	699	1080	992
ENSECAG00000020275	3.873240495	0.336849268	0.564745666	185	286	316	289	310	237	306	258
ENSECAG00000012036	6.667997022	0.336983391	0.564887178	1759	2128	1687	1719	2288	1171	2194	2066
ENSECAG00000024435	5.109632648	0.337278497	0.565298464	349	573	555	1216	557	652	652	773
ENSECAG00000003414	5.71641911	0.337497364	0.565581866	520	526	1386	1760	1190	664	1166	957
ENSECAG00000012708	6.287925054	0.337646748	0.565748762	1061	943	1368	1226	1905	1523	1980	1941
ENSECAG00000014058	5.770939925	0.337849946	0.566005763	474	853	1207	1744	996	1041	1024	1103
ENSECAG00000016147	3.561759499	0.338322607	0.566564223	176	172	349	169	282	209	165	218
ENSECAG00000003616	7.18320719	0.338324893	0.566564223	1776	2407	1964	2281	3828	2441	3958	3664
ENSECAG00000019246	7.022332489	0.338375911	0.566564223	1134	1363	2705	2803	2704	3571	2944	3040
ENSECAG00000018364	3.705566419	0.338382751	0.566564223	117	191	181	313	268	252	348	359
ENSECAG00000010135	3.176349268	0.338544253	0.566751113	94	196	170	221	149	142	182	208
ENSECAG00000022255	8.503218593	0.338634813	0.566819203	8503	6516	5946	4427	7156	4229	6772	8774
ENSECAG00000003393	3.159956281	0.33871809	0.566844219	166	183	121	172	160	179	204	102
ENSECAG00000009162	8.654612161	0.338749538	0.566844219	7408	7415	7373	6883	8453	6135	6952	8685
ENSECAG00000010638	1.36931089	0.338895378	0.566916933	27	42	31	43	56	68	47	67
ENSECAG00000010433	4.363384791	0.338952466	0.566916933	329	245	289	302	567	480	344	547
ENSECAG00000022204	5.546456097	0.339000614	0.566916933	820	762	794	962	1012	680	939	973
ENSECAG00000021659	2.495536497	0.339069788	0.566916933	37	111	144	152	83	108	94	125
ENSECAG00000019039	5.114563879	0.33911448	0.566916933	388	443	485	815	787	665	1003	776
ENSECAG00000022270	6.632400851	0.339170612	0.566916933	1010	1332	1535	2209	1990	2384	2049	2932

ENSECAG00000027691	8.09472627	0.339180824	0.566916933	3776	2663	4630	4594	4900	7387	4500	9660
ENSECAG00000012459	6.788817485	0.339225347	0.566916933	1041	1460	1958	2318	2493	3004	2194	2671
ENSECAG00000009741	5.980130144	0.339281163	0.566916933	533	564	1370	1506	1395	1433	1308	1833
ENSECAG00000011876	2.705265546	0.339324968	0.566916933	58	107	97	118	85	151	174	210
ENSECAG00000026034	0.541879166	0.339341846	0.566916933	14	12	54	35	28	40	5	16
ENSECAG00000011751	8.124988118	0.339515966	0.567124435	2731	2950	5425	5827	5673	7621	5714	7527
ENSECAG00000021821	4.874833764	0.339596143	0.567174978	412	496	590	640	584	502	594	583
ENSECAG00000017269	6.447041568	0.339661598	0.567200922	712	622	1292	2898	2100	1858	2840	1526
ENSECAG00000016716	0.395049164	0.339748463	0.567262606	4	20	13	35	24	46	22	26
ENSECAG00000016039	4.09043042	0.339846496	0.567342917	114	337	455	430	252	271	300	468
ENSECAG00000016157	1.916623162	0.339958069	0.567445803	24	57	71	67	86	48	124	102
ENSECAG00000024204	2.611530504	0.340122742	0.567637279	82	75	151	155	97	82	105	172
ENSECAG00000000470	6.65443577	0.340254694	0.567774097	538	1808	1512	4796	1624	1599	1061	3146
ENSECAG00000009685	3.575470611	0.34074702	0.568426029	103	188	143	297	296	226	258	346
ENSECAG00000023499	5.062608921	0.340783175	0.568426029	301	604	762	900	562	528.002	578	919
ENSECAG00000023253	6.890222689	0.340795468	0.568426029	1514	1124	1441	3045	3242	1914	2906	3361
ENSECAG00000023312	4.634850441	0.340902281	0.568446557	327	520	232	309	617	378	673	720
ENSECAG00000009774	4.981145431	0.340907837	0.568446557	286	394	462	846	690	566	681	1058
ENSECAG00000012255	3.516676518	0.341098246	0.568680597	84	325	256	211	178	170	161	347
ENSECAG00000008640	3.221972846	0.341241905	0.568836638	76	144	180	160	221	209	244	196
ENSECAG00000007044	0.802894339	0.341345985	0.568926666	15	24	4	108	17	37	13	41
ENSECAG00000006641	0.181537484	0.341695183	0.569425149	15	10	26	32	19	21	19	14
ENSECAG00000011568	5.37891555	0.34185706	0.569598512	320	474	1423	1135	1101	740	715	493
ENSECAG00000018084	3.638591479	0.341899477	0.569598512	107	142	234	287	284	249	249	395
ENSECAG00000021774	2.89377741	0.34211533	0.569874561	38	92	122	210	145	145	221	198
ENSECAG00000006582	2.862877285	0.342257212	0.569954801	48	121	113.002	159	170	171	181.001	158
ENSECAG00000019587	1.622247576	0.342355831	0.569954801	37	48	35	50	64	58	71	96
ENSECAG00000006745	8.015699355	0.34240832	0.569954801	2098	5808	6284	6419	3153	7778	2835.01	4716
ENSECAG00000000413	6.366091897	0.34241335	0.569954801	1055	1078	1226	1573	1916	1420	1862	2632
ENSECAG00000018229	4.462070796	0.342414317	0.569954801	159	309	383	978	538	287	482	322
ENSECAG00000023311	5.904082562	0.342887484	0.570601299	448	537	850	2018	1403	1014	1442	1908
ENSECAG00000011537	4.094397941	0.342951466	0.570601299	155	388	209	239	387	260	407	592
ENSECAG00000000737	7.241063784	0.342975962	0.570601299	2699	2105	1624	1600	4674	2420	4187	3410
ENSECAG00000016027	3.533335414	0.343003598	0.570601299	71	169	199	283	214	260	285	334
ENSECAG00000026970	1.692895754	0.343197448	0.570771369	48	35	52	37	105	55	77	67
ENSECAG00000017940	1.793561648	0.343219209	0.570771369	40	71	81	67	62	83	61	35
ENSECAG00000011855	3.924925014	0.343256536	0.570771369	121	234	228	357	311	315	295	517
ENSECAG00000019449	4.433845539	0.343524584	0.570997234	371	497	395	257	429	273	420	522
ENSECAG00000006233	5.013146484	0.34353224	0.570997234	335	280	789	556	856	506	852	854
ENSECAG00000010410	7.449562877	0.343543134	0.570997234	2213	1434	2881	3929	4094	3419	4405	4836
ENSECAG00000002133	2.858491099	0.343698131	0.571055854	67	107	101	160	173	139	192	176
ENSECAG00000014620	7.450588586	0.343793416	0.571055854	1636	2938	3551	5723	2730	3134	3613	3695
ENSECAG00000010766	6.405324719	0.343832682	0.571055854	653	1315	1230	2105	1490	1825	1845	2919
ENSECAG00000006141	3.712546534	0.34390749	0.571055854	130	255	161	228	300	224	307	414
ENSECAG00000020358	8.770427066	0.343920947	0.571055854	2396	5830	8807	9651	7839	11514	16021	6474
ENSECAG00000024709	5.23091252	0.343926017	0.571055854	441	459	703	669	952	805	677	1080
ENSECAG00000012070	6.951750564	0.34397442	0.571055854	1588	3007	2233	2287	2154	2290	2615	2250
ENSECAG00000020689	0.48499916	0.344003382	0.571055854	16	18	23	14	42	19	36	31
ENSECAG00000020572	4.823203414	0.344083581	0.571055854	291	461	502	456	553	620	764	694
ENSECAG00000005219	8.247210785	0.344106553	0.571055854	4220	4731	6642	7429	6328	5342	6680	4256
ENSECAG00000022613	7.010512255	0.344131265	0.571055854	1754	2518	2428	2856	2811	2079	2332	2594
ENSECAG00000011564	5.535579175	0.344207427	0.57109883	993	791	726	698	975	782	800	965
ENSECAG00000024180	5.099807164	0.344344909	0.571243518	424	403	566	683	991	652	802	748
ENSECAG00000016281	7.50008065	0.344656785	0.571677429	3246	3449	3006	3314	3618	2578	3468	4073
ENSECAG00000000465	9.452979856	0.344767415	0.571777458	9663	26513	6053	9474	8777	15056	13286	12598
ENSECAG00000020924	4.320053921	0.344940087	0.571962955	123	333	347	432	319	594	351	619
ENSECAG00000000734	3.82591464	0.344979945	0.571962955	105	149	314	317	316	306	315	399
ENSECAG00000001468	4.368085457	0.345193717	0.572233879	325	305	224	316	520	390	517	519
ENSECAG00000022335	4.668968806	0.345332548	0.572380513	210	282	502	601	493	470	555	905
ENSECAG00000001815	2.316917353	0.345507088	0.572586279	56	69	110	148	92	92	99	79
ENSECAG00000022682	3.250262606	0.345572435	0.572611056	101	104	130	242	203	187	207	305
ENSECAG00000011281	4.000465573	0.345748127	0.572818638	185	261	486	270	290	361	178	362
ENSECAG00000003570	3.676502205	0.346026522	0.573196289	110	180	267	219	253	307	308	324
ENSECAG00000024672	5.512493551	0.346577234	0.574024859	435	457	844	1144	1018	894	1125	1245
ENSECAG00000011981	1.019361477	0.346720189	0.574177932	28	15	28	35	79	22	53	37
ENSECAG00000009809	2.261618133	0.346887113	0.574322354	34	113	76	158	52	93	69	131
ENSECAG00000013346	5.12108518	0.346908494	0.574322354	525	824	464	682	672	637	599	752
ENSECAG00000015073	2.438928814	0.347081539	0.574525123	54	69	178	117	112	66	102	118
ENSECAG00000011080	7.809873189	0.347148527	0.574544275	4130	4725	3500	3675	4130	4504	3894	4154
ENSECAG00000004586	5.661782953	0.347194243	0.574544275	159	459	633	2048	1444	971	1662	853
ENSECAG00000010227	6.788180959	0.347355937	0.574625874	774	1697	2195	2184	2426	2787	2401	2793
ENSECAG00000010798	5.438764484	0.347391442	0.574625874	497	1029	778	881	699	752	847	1053
ENSECAG00000017123	5.738142867	0.347478087	0.574625874	504	1141	1086	1293	925	910	1120	1166
ENSECAG00000019390	1.487290936	0.347490854	0.574625874	42	23	55	22	80	24	83	87
ENSECAG00000022072	8.941580695	0.347496425	0.574625874	6797	5311	7788	7654	18851	11069	11659	5500
ENSECAG00000000655	3.311489688	0.347597529	0.574709419	129	174	118	142	234	199	269	231
ENSECAG00000005718	5.999572774	0.34774821	0.574860232	619	472	1144	1774	1713	1685	1337	1261
ENSECAG00000016227	4.481286302	0.347789934	0.574860232	143	338	343	587	415	645	482	539
ENSECAG00000007400	4.567125899	0.347955102	0.575049581	290	461	563	419	486	369	429	544
ENSECAG00000012698	5.534716133	0.348016183	0.57506688	748	732	796	1079	922	859	733	1067
ENSECAG00000020265	2.930298411	0.348353683	0.57550286	82	139	168	192	186	73	149	163
ENSECAG00000022235	4.534134912	0.34838133	0.57550286	225	293	355	571	612	405	613	535
ENSECAG00000014746	6.747267599	0.348655305	0.575871723	764	1558	1580	5169	1841	2092	2261	1647
ENSECAG00000011828	0.744159522	0.34871353	0.575884175	18	26	51	30	13	44	18	36
ENSECAG00000025860	0.564106163	0.349072936	0.576330156	27	14	43	24	26	46	9	11

ENSECAG00000022604	7.063563464	0.349127993	0.576330156	1631	2617	2696	3108	2252	2603	2017	3311
ENSECAG00000024440	4.690562016	0.349135756	0.576330156	322	325	437	470	602	471	667	671
ENSECAG00000024753	3.425569272	0.349269074	0.576453368	107	341	224	129	151	200	108	329
ENSECAG00000015423	5.278483177	0.349386822	0.576453368	371	823	659	1099	832	616	711	834
ENSECAG00000021787	1.847140066	0.349411829	0.576453368	45	98	62	55	64	44	69	85
ENSECAG00000020600	5.295824361	0.349525793	0.576453368	565	411	749	566	1031	864	975	791
ENSECAG00000005422	5.693558845	0.349587219	0.576453368	493	593	781	1405	1099	1103	1061	1604
ENSECAG00000017698	7.097875903	0.34959491	0.576453368	1339	3166	2780	3138	2309	2650	2240	3173
ENSECAG00000005326	3.330493458	0.349618933	0.576453368	107	117	185	194	253	157	254	287
ENSECAG00000008344	4.467464974	0.34962719	0.576453368	211	268	345	562	501	461	448	663
ENSECAG00000000077	1.3303154	0.349676801	0.576453368	21	72	43	52	48	32	57	39
ENSECAG00000015334	3.562393432	0.349733308	0.576453368	127	240	234	286	202	169	207	328
ENSECAG00000022240	0.710401358	0.349768484	0.576453368	13	21.0001	34.0001	59.0001	17.0001	29.0001	31.0001	34.0001
ENSECAG00000015546	5.704990565	0.349977833	0.576714741	580	528	1144	844	1950	869	1387	720
ENSECAG00000007495	5.188809257	0.350120972	0.576840077	725	513	647	683	723	577	738	758
ENSECAG00000020480	6.015783658	0.350155431	0.576840077	790	1190	823	1000	1368	1364	1612	1728
ENSECAG00000015177	4.953316057	0.350317271	0.577023026	359	363	494	692	806	578	718	785
ENSECAG00000010885	6.171418699	0.350410706	0.577093267	784	1301	857	1395	1643	1374	1935	1818
ENSECAG00000022314	4.621550097	0.350468509	0.577104811	311	272	509	362	702	442	644	517
ENSECAG00000007727	3.047258074	0.35064467	0.577239132	94	63	159	176	194	153	223	212
ENSECAG00000009691	5.537749012	0.350701453	0.577239132	675	644	929	1178	915	931	854	864
ENSECAG00000010970	5.632077406	0.350702493	0.577239132	440	936	1221	1177	932	765	856	1299
ENSECAG00000018257	5.309694842	0.35081772	0.577345153	551	818	644	864	727	748	693	894
ENSECAG00000019346	7.499313395	0.350901057	0.577398669	2147	2108	3322	3203	4773	3618	4061	4721
ENSECAG00000007445	4.120663598	0.351054444	0.577546519	161	226	353	330	385	413	364	454
ENSECAG00000010322	2.040945717	0.351148422	0.577546519	6	37	55	148	27	75	166	147
ENSECAG00000012246	6.160852369	0.351172691	0.577546519	932	1335	1376	1620	1240	1353	1399	1524
ENSECAG00000017375	2.241792146	0.35120965	0.577546519	28	55	82	123	97	94	94	166
ENSECAG00000017246	5.518434897	0.351245067	0.577546519	714	615	633	675	991	826	1127	1389
ENSECAG00000020292	6.050819467	0.351351085	0.57763725	417	905	1125	1732	1102	2173	1056	1919
ENSECAG00000016109	3.70516341	0.35140284	0.577638753	138	275	134	205	314	215	269	454
ENSECAG00000026130	2.958663625	0.351642225	0.577816122	52	32	167	460	237	151	60	62
ENSECAG00000000442	2.326008196	0.351677696	0.577816122	82	108	82	88	92	59	75	147
ENSECAG00000000909	4.395956821	0.351704853	0.577816122	274	485	318	456	383	385	376	470
ENSECAG00000000773	4.628276431	0.351757635	0.577816122	168	281	454	681	495	537	590	706
ENSECAG00000024815	1.475672613	0.351765016	0.577816122	39	39	59	69	67	26	48	60
ENSECAG00000001648	4.869105238	0.351950116	0.578036602	394	441	403	455	1039	400	647	683
ENSECAG00000023965	5.188016067	0.352187457	0.578204751	467	927	627	612	601	545	785	878
ENSECAG00000000946	1.458903151	0.352215375	0.578204751	38	39	44	100	5	82	27	65
ENSECAG00000001214	1.906215903	0.35227063	0.578204751	38	69	47	54	60	115	77	97
ENSECAG00000000176	3.932971409	0.352338994	0.578204751	157	281	364	345	241	284	325	312
ENSECAG00000024693	5.378586949	0.352346886	0.578204751	558	559	759	567	1041	797	1067	980
ENSECAG00000008822	4.636582436	0.352357833	0.578204751	227	271	463	604	619	529	517	656
ENSECAG00000020895	4.701915227	0.352418231	0.578220353	249	310	414	660	612	578	487	756
ENSECAG00000000036	6.437644641	0.352534224	0.578288645	1192	1857	1512	1739	1572	1511	1673	1919
ENSECAG00000008069	3.610046749	0.352561648	0.578288645	140	190	196	194	241	227	311	370
ENSECAG00000004259	0.760206339	0.352640735	0.578334878	32	6	19	25	67	20	35	39
ENSECAG00000014807	3.388238442	0.352809325	0.578527861	81	181	281	274	150	218	207	201
ENSECAG00000023922	4.835311407	0.353011744	0.578776253	502	807	239	479	460	396	498	801
ENSECAG00000011344	2.651178497	0.353101746	0.578818412	12	111	151	63	22	238	288	74
ENSECAG00000020493	6.450228742	0.353149829	0.578818412	914	1088	854	2507	2240	2294	1127	2675
ENSECAG00000023516	4.090104059	0.353190288	0.578818412	172	145	382	338	499	280	465	362
ENSECAG00000015556	0.507776338	0.353332214	0.578967496	9	19	24	61	15	31	27	19
ENSECAG00000011076	7.560881963	0.353427933	0.579040833	2053	2357	2915	4157	4447	3821	4709	4914
ENSECAG00000010259	6.185255778	0.353568329	0.579187335	635	838	1348	1757	1512	1351	1574	2482
ENSECAG00000015950	2.677558075	0.353797872	0.579420393	57	55	136	133	198	85	135	197
ENSECAG00000023376	4.31729447	0.353837777	0.579420393	255	435	311	460	439	347	320	422
ENSECAG00000010524	6.900869077	0.35386359	0.579420393	1874	1362	2810	2873	2266	1932	2223	2686
ENSECAG00000003967	6.675827288	0.354040543	0.579607448	1566	2045	1858	1911	1920	1703	1948	2273
ENSECAG00000022522	3.786462233	0.354079854	0.579607448	170	144	425	314	324	196	294	216
ENSECAG00000017309	1.751856365	0.35421463	0.579730662	37	28	47	84	84	65	78	88
ENSECAG00000015929	9.330937946	0.354257172	0.579730662	6089	9524	10487	12896	15240	13511	15560	16992
ENSECAG00000023953	7.530250518	0.354391886	0.57984553	3731	3285	2909	3158	3364	3194	3460	3898
ENSECAG00000010114	8.407874491	0.354429432	0.57984553	3653	3007	5293	8657	9984	4497	8095	10581
ENSECAG00000019520	6.473254289	0.354725273	0.580245977	1315	1400	1983	1780	1860	1415	1627	1957
ENSECAG00000002187	3.706420978	0.354815491	0.580310006	185	77	270	220	372	275	331	256
ENSECAG00000011360	3.694730799	0.354877889	0.580328522	109	189	192	313	244	295	317	353
ENSECAG00000017701	4.609383699	0.355096215	0.580552947	181	243	511	611	480	528	745	533
ENSECAG00000016215	6.308444078	0.35516669	0.580552947	920	1253	1808	1999	1394	1666	1183	1837
ENSECAG00000007504	3.312983669	0.355168415	0.580552947	150	114	149	141	246	122	220	378
ENSECAG00000022984	3.137662666	0.355321022	0.58071885	35	66	529	132	61	306	71	121
ENSECAG00000014032	7.981792235	0.355609196	0.580913787	4137	5323	3821	5091	4593	4812	4867	4691
ENSECAG00000023951	6.788574574	0.355735092	0.580913787	1059	1343	1807	2661	2498	2370	2230	3372
ENSECAG00000010977	6.930087783	0.355740499	0.580913787	996	3410	2155	2784	1987	2159	2136	2925
ENSECAG00000002759	2.97908268	0.355745936	0.580913787	95	138	154	204	128	138	132	197
ENSECAG00000013215	0.849809014	0.355791873	0.580913787	27	22	33	50	20	32	35	38
ENSECAG00000008582	5.223051229	0.355814287	0.580913787	648	762	697	514	704	749	705	670
ENSECAG00000007349	5.986084752	0.355815712	0.580913787	801	980	809	1210	1369	1382	1417	1752
ENSECAG00000022735	7.106842521	0.355849319	0.580913787	1922	3375	1974	2901	2534	2941	2856	1864
ENSECAG00000002362	1.200850724	0.355946772	0.580989402	10	49	37	27	79	41	79	17
ENSECAG00000002087	5.971169215	0.356176835	0.581281413	697	887	968	1305	1507	1219	1518	1599
ENSECAG00000021471	7.034876147	0.356448055	0.5816405	1518	3253	1902	3194	2091	2296	2102	3495
ENSECAG00000012859	5.60951945	0.356764901	0.581979396	743	1156	812	747	939	814	1005	988
ENSECAG00000023583	6.443502101	0.356768117	0.581979396	1404	1640	707	973	2063	1625	2359	2317
ENSECAG00000009261	7.039486005	0.35681939	0.581979396	4185	1992	808	1997	2924	2819	1167	2318

ENSECAG00000017286	5.847881378	0.356860628	0.581979396	651	922	859	1041	1578	857	1532	1448
ENSECAG00000016895	4.173514174	0.357157136	0.58237936	158.007	302	408	528	277	313	399	388
ENSECAG00000017829	3.602593664	0.357477512	0.582818123	103	201	198	235	323	305	274	220
ENSECAG00000008710	2.123198474	0.357597031	0.582867757	60	67	105	108	59	164	30	32
ENSECAG00000015840	0.745385216	0.357756223	0.582867757	4	18	29	44	26	33	22	79
ENSECAG00000009182	3.937307991	0.35779304	0.582867757	219	164	379	401	421	250	285	177
ENSECAG00000021822	4.651393476	0.35779455	0.582867757	428	518	407	420	541	418	388	581
ENSECAG00000008529	4.685313701	0.357801481	0.582867757	327	316	661	623	467	495	477	531
ENSECAG00000016357	6.201442766	0.357815755	0.582867757	798	1014	1236	1468	1639	1510	1525	2226
ENSECAG00000018623	5.636404293	0.358144497	0.583319636	451	479	838	1387	1044	889	1011	1805
ENSECAG00000023217	5.501216494	0.35827561	0.583335545	307	506	966	1096	798	1267	793	1406
ENSECAG00000012967	-0.134628844	0.358301742	0.583335545	18	6	10	4	23	7	23	32
ENSECAG00000003001	1.440921204	0.358308288	0.583335545	19	19	57	64	44	56	86	68
ENSECAG00000015282	4.101904027	0.358449704	0.583482168	143	187	286	467	341	320	381	588
ENSECAG00000023271	5.453954387	0.358818102	0.583998177	422	678	749	866	858	867	1053	1317
ENSECAG00000023762	7.551146345	0.358869629	0.583998384	1858	2558	3204	3826	4107	4065	4701	4833
ENSECAG00000022122	4.960652223	0.35914007	0.584354787	341	428	546	597	622	697	761	800
ENSECAG00000008526	2.437424379	0.359325868	0.584573384	75	86	140	96	93	99	77	132
ENSECAG00000011335	6.438566881	0.359484482	0.5847477	1601	863	2352	1542	2079	749	1696	2072
ENSECAG00000018269	7.113173772	0.359597571	0.584771103	918	1831	2983	2889	2766	3889	2829	3586
ENSECAG00000019652	3.897517364	0.359601804	0.584771103	145	126	418	194	423	335	356	289
ENSECAG00000001700	5.851399749	0.359763286	0.584949979	747	538	1214	918	1941	913	1369	1234
ENSECAG00000019845	6.270538697	0.359885769	0.585065405	1150	1619	1341	1444	1279	1448	1318	1905
ENSECAG00000008974	7.82705737	0.359992461	0.585140978	2982	2559	5945	6015	4397	4895	3930	3629
ENSECAG00000005731	6.99783787	0.360035256	0.585140978	1604	1613	2035	2349	3346	2139	3072	3601
ENSECAG00000008691	0.18312917	0.360199606	0.58532436	6	15	14	26	19	33	24	24
ENSECAG00000012064	0.049553684	0.360301138	0.585405625	6	22	20	28	15	14	21	17
ENSECAG00000014416	1.776269811	0.360478381	0.585532701	26	53	52	67	48	67	76	135
ENSECAG00000006138	5.489677555	0.360482419	0.585532701	511	610	841	776	1164	871	965	1179
ENSECAG00000008063	1.272001318	0.360834867	0.586021406	19.0027	12	51.0001	52.0029	79	19	29	112
ENSECAG00000023584	7.338491049	0.36099985	0.586054524	2586	2895	2235	4257	3089	2876	3272	2990
ENSECAG00000003632	6.85039521	0.361023048	0.586054524	1845	1028	1855	1882	3068	2168	2743	3020
ENSECAG00000022610	7.437684987	0.361053806	0.586054524	1359	3184	2862	3140	3970	3534	4434	4507
ENSECAG00000009836	8.081708023	0.361094638	0.586054524	2416	2265	5201	6978	7249	4759	6782	7253
ENSECAG00000017991	8.788149985	0.36111316	0.586054524	5771	6887	9515	11414	7586	7892	8072	9973
ENSECAG00000012781	5.403852551	0.361603728	0.586698149	353	884	717.002	1331	639	788	747.001	1081
ENSECAG00000017006	5.80203641	0.36161302	0.586698149	747	1397	795	1090	1156	877	1041	1230
ENSECAG00000016149	5.252515299	0.3618125	0.586937981	362	599	708	1301	632	766	670	853
ENSECAG00000011135	3.807418763	0.361881415	0.586965973	106.011	204	184.008	379	361.001	186	409.001	376
ENSECAG00000012578	2.007241803	0.362132185	0.587181128	49	78	39	49	67	71	76	177
ENSECAG00000009597	3.360622502	0.362160852	0.587181128	87	304	137	253	154	195	176	238
ENSECAG00000015256	6.041485714	0.362215405	0.587181128	716	1665	1238	1244	1352	1361	1281	970
ENSECAG00000015920	6.282429262	0.362220782	0.587181128	576	907	1126	2400	2064	1430	1830	2045
ENSECAG00000023537	3.209583567	0.362323547	0.587263929	103	120	164	338	194	176	166	148
ENSECAG00000009084	0.256394396	0.362554829	0.58755498	10	26	22	30	8	12	22	38
ENSECAG00000020263	4.614508986	0.362918473	0.587844583	230	306	835	513	453	368	527	516
ENSECAG00000012279	6.911543318	0.362943564	0.587844583	1652	1858	1712	1690	2290	3249	2925	2834
ENSECAG00000010808	4.482713975	0.362962199	0.587844583	297	171	826	427	616	242	460	333
ENSECAG00000001265	2.780348869	0.362964424	0.587844583	79	155	131	144	93	101	157	164
ENSECAG00000022117	7.394270717	0.362992219	0.587844583	2062.02	3973	3106	3320	2819.98	2781.98	2898	4365
ENSECAG00000019673	6.70959725	0.363143811	0.588006268	1058	1419	1408	2601	2242	2176	2253	3229
ENSECAG00000008196	3.617471384	0.363293127	0.588164222	291	269	155	144	121	419	122	183
ENSECAG00000024191	6.580058012	0.363617004	0.588604701	1826	1309	1919	1756.99	1892	1820	1871	1665
ENSECAG00000015107	5.273284118	0.363806236	0.58882713	596	741	607	829	747	695	689	866
ENSECAG00000018554	9.417060264	0.363911778	0.588914062	7534	10506	15306	19847	13802	10531	14477	12796
ENSECAG00000024665	5.265809664	0.364072884	0.589090873	294	459	691	1023	805	892	870	1017
ENSECAG00000001294	3.046078135	0.364307926	0.58938725	71	134	217	217	167	114	182	155
ENSECAG00000011722	8.310613354	0.36445434	0.589540179	3400	5518	7753	7651	6107	5338	6544	6031
ENSECAG00000014218	4.037268912	0.364587455	0.589671554	188	280	636	411	3	794	2	37
ENSECAG00000008522	6.137496792	0.364670218	0.589721466	665	1006	1188	1537	1516	1546	1475	2033
ENSECAG00000024979	6.602215614	0.36475925	0.589730652	817	2033	2614	1982	1372	1887	1417	2736
ENSECAG00000011450	5.485698288	0.364779706	0.589730652	524	521	737	953	1478	648	1105	974
ENSECAG00000015734	4.631761047	0.364924348	0.589880558	157	309	418	708	519	649	459	695
ENSECAG00000022273	2.562027237	0.365005636	0.589907973	76	66	110	84	117	103	159	180
ENSECAG00000024529	1.555810382	0.365068724	0.589907973	16	26	61	72	70	67	43	97
ENSECAG00000000012	6.407245507	0.365097066	0.589907973	882	449	1642	2262	2403	1152	2555	2090
ENSECAG00000006981	8.680657996	0.365377199	0.590276658	8419	7982	4225	8836	6844	7492	8073	8040
ENSECAG00000015148	6.198737727	0.365457707	0.590322784	691.999	884.996	1246	1802.99	1535	1537	1610	2212.99
ENSECAG00000019018	6.046005948	0.365559483	0.590403248	498	849	1371	1465	1608	1179	1702	1719
ENSECAG00000023920	2.86485769	0.365742345	0.590614628	64	128	108	134	157	142	178	206
ENSECAG00000001899	7.299940964	0.366115822	0.591081345	2986	3955	1940	2196	2831	2493	3074	3418
ENSECAG00000016340	10.73830491	0.366172991	0.591081345	15718	25370	40462	54317	33184	27119	35025	33247
ENSECAG00000013335	3.592552928	0.366187431	0.591081345	188	166	275	258	216	136	187	390
ENSECAG00000024492	2.162693115	0.366419891	0.591372556	37	61	82	82	97	56	104	173
ENSECAG00000003238	5.750570057	0.366653293	0.591665206	634	634	938	1109	1270	1035	1386	1307
ENSECAG00000017249	6.079317305	0.36685547	0.591907392	696	938	1896	1638	1472	1310	1220	1137
ENSECAG00000023687	5.696929444	0.36699647	0.592001976	527	887	1026	1508	1107	806	1141	959
ENSECAG00000010491	6.025518433	0.3670183	0.592001976	452	821	1202	1706	1547	1327	1356	1903
ENSECAG00000001043	6.242137168	0.36729462	0.592245877	637	896	1590	1646	1764	1449	1927	1955
ENSECAG00000005869	5.681385183	0.367312525	0.592245877	452	635	1002	1158	1320	1037	1061	1357
ENSECAG00000019299	5.967950627	0.367325884	0.592245877	535	1174	1199	1940	1137	1136	1260	1277
ENSECAG00000024929	1.329504327	0.367432511	0.592333739	20	30	33	64	43	48	69	73
ENSECAG00000017265	6.976016803	0.367593317	0.592508905	1192	1139	1493	4063	3274	2605	3855	2228
ENSECAG00000024684	7.295849547	0.36776428	0.592573598	1860	2143	2770	2571	3876	2934	4026	4023
ENSECAG00000016798	3.558898214	0.36778296	0.592573598	107	216	294	281	161	242	245	238

ENSECAG00000024080	2.855660952	0.367789915	0.592573598	64	98	123	154	151	164	149	212
ENSECAG00000022437	6.486787053	0.3680832	0.592962047	1541	1227	1046	1191	2422	1649	2100	2341
ENSECAG00000018902	0.95877166	0.368170581	0.593018732	19	23	69	35	39	37	22	35
ENSECAG00000000039	3.33318438	0.368430056	0.593334052	0	100	76	339	0	366	128	672
ENSECAG00000016084	3.407945626	0.368470786	0.593334052	85	114	197	266	224	210	219	351
ENSECAG00000015767	5.714100065	0.368578991	0.593424189	808	794	1166	1012	1193	915	935	1013
ENSECAG00000014773	3.620112077	0.368775267	0.593656076	105	188	193	500	243	214	245	215
ENSECAG00000023259	1.539412847	0.36926229	0.594317716	22	86	43	68	39	49	54	65
ENSECAG00000010422	3.402704695	0.369290889	0.594317716	79	82	172	335	202	223	222	365
ENSECAG00000011062	5.686656479	0.369494699	0.594561503	710	938	1091	983	945	761	1213	1094
ENSECAG00000002783	3.907605844	0.36976006	0.594904248	133	169	286	350	404	297	296	405
ENSECAG00000013265	5.072271748	0.369932679	0.595097706	463	544	739	699	744	549	626	695
ENSECAG00000022620	5.975025853	0.370080363	0.59517965	762	863	1101	1082	1462	1206	1483	1713
ENSECAG00000027056	2.149340249	0.370095502	0.59517965	23	46	232	60	61	104	78	55
ENSECAG00000024759	2.237173928	0.370140768	0.59517965	37	86	140	99	78	101	67	95
ENSECAG00000019150	1.245959549	0.37029377	0.595304725	9	26	27	79	49	37	93	44
ENSECAG00000019502	7.235674	0.37032334	0.595304725	1811	2271	2040	2868	3585	3228	3848	3495
ENSECAG00000003367	1.875365201	0.370469351	0.595393178	33	40	51	93	91	49	81	128
ENSECAG00000011409	4.835303755	0.370483169	0.595393178	285	365	506	631	684	552	675	735
ENSECAG00000017901	6.516228242	0.370723908	0.595695805	1607	2080	1222	1549	1865	1662	1726	1689
ENSECAG00000020316	11.3237904	0.370938186	0.595764409	29105	38592	65944	64274	57194	34424	57801	43724
ENSECAG00000011707	4.523723949	0.370942892	0.595764409	181	405	457	762	475	416	483	367
ENSECAG00000018775	7.146803333	0.37097283	0.595764409	2413	2241	2379	3294	2795	2590	2837	2521
ENSECAG00000019800	0.340293945	0.370976342	0.595764409	13	14	25	10	13	56	24	20
ENSECAG00000005437	5.694058252	0.371113173	0.595899925	800	1056	873	950	1065	974	951	1002
ENSECAG00000020350	4.9448936	0.371308881	0.596129928	302	500	516	585	734	646	677	790
ENSECAG00000010549	5.353914733	0.371451148	0.596186015	364	777	450	937	864	993	860	1091
ENSECAG00000011747	1.838039683	0.371510519	0.596186015	40	33	90	116	85	42	52	80
ENSECAG00000010820	4.128453929	0.371537566	0.596186015	108	313	295	707	282	263	391	382
ENSECAG00000017104	2.595447844	0.37158316	0.596186015	57	52	274	93	145	59	113	116
ENSECAG00000019452	6.163624752	0.371606175	0.596186015	1026	1231	1442	1505	1288	1392	1297	1569
ENSECAG00000004370	3.034734334	0.371771803	0.596367532	147	136	99	219	169	173	80	181
ENSECAG00000019955	3.735796237	0.37205321	0.596703089	98	232	255	224	249	395	278	311
ENSECAG00000011630	3.28276141	0.372086022	0.596703089	67	60	142	345	263	133	209	347
ENSECAG00000008534	3.47377245	0.372145204	0.596713775	119	138	228	180	307	204	237	291
ENSECAG00000022306	4.227916428	0.372339808	0.596820868	259	296	363	456	330	384	368	353
ENSECAG00000006252	2.888332604	0.372357424	0.596820868	75	143	102	108	175	119	201	204
ENSECAG00000001057	1.905606668	0.372369577	0.596820868	18	65	49	91	68	78	65	147
ENSECAG00000024773	5.965885855	0.373029264	0.597793865	939	1390	1013	1111	1173	1080	1344	1215
ENSECAG00000012735	3.439227391	0.373129286	0.597869828	70	166	116	328	221	194	245	379
ENSECAG00000009318	7.256769902	0.373284474	0.59803415	2337	2197	4252	2461	3056	1941	2491	4243
ENSECAG00000009695	7.099723785	0.373447794	0.598211453	1359	2860	2643	3615	2994	2248	2696	2505
ENSECAG00000023911	4.030439785	0.373508819	0.598224866	202	302	302	398	327	253	318	367
ENSECAG00000014409	5.586966532	0.373758928	0.598472949	719	557	1086	1175	949	876	943	942
ENSECAG00000010327	6.051000512	0.373858339	0.598472949	1076	731	1926	1102	1554	907	1220	1394
ENSECAG00000002317	3.228440704	0.373866058	0.598472949	84	496	43	81	95	95	217	244
ENSECAG00000015116	4.191558684	0.373874405	0.598472949	153	304	479	467	324	344	306	425
ENSECAG00000007822	6.617239163	0.374151096	0.59883149	1306	1992	1738	2145	1894	1652	1999	2035
ENSECAG00000008987	5.309980306	0.374251718	0.598908172	339	599	640	930	888	871	845	1075
ENSECAG00000016731	6.330030413	0.37454768	0.599119234	1290	1461	1350	1662	1641	1368	1533	1683
ENSECAG00000014344	2.590356725	0.374555898	0.599119234	56	72	94	141	123	97	140	212
ENSECAG00000006065	7.416671932	0.374561869	0.599119234	1283	2877	3636	5917	3110	2661	3343	3862
ENSECAG00000024355	6.254137742	0.374613625	0.599119234	923	891.045	2215	1766	1550	1577	1526	1092
ENSECAG00000009759	7.388342025	0.374689902	0.599119234	2287	4016	3217	2657	3218	3070	3507	2814
ENSECAG00000013844	3.852040295	0.374699989	0.599119234	103	215	203	392	312	271	352	423
ENSECAG00000022573	6.056115521	0.37476685	0.599141826	751	965	863	1507	1918	1311	1554	1399
ENSECAG00000020813	7.570907381	0.375015794	0.59934875	2474	2592	2961	3158	5011	3741	4001	5261
ENSECAG00000014119	9.751984281	0.375020491	0.59934875	13178	15110	17324	17923	14904	14460	13746	22741
ENSECAG00000014716	1.914364544	0.375054534	0.59934875	33	45	60	87	90	66	89	106
ENSECAG00000000285	3.28189903	0.375288924	0.599621193	104	231	162	226	151	157	221	207
ENSECAG00000016188	6.695266643	0.375330569	0.599621193	1235	1656	2359	2537	1912	2066	1694	2283
ENSECAG00000008902	5.123178741	0.375419797	0.599623809	470	571	508	498	811	698	805	931
ENSECAG00000018681	6.275080625	0.37545581	0.599623809	336	1454	1074	2056	1402	2015	1918	1991
ENSECAG00000019332	5.383578992	0.37549053	0.599623809	572	687	737	1064	851	753	818	819
ENSECAG00000016392	7.701419991	0.375688171	0.599855115	2858	3376	3024	2574	5638	3433	5374	5448
ENSECAG00000023406	5.427946037	0.375905944	0.600031989	393	574	736	1015	1027	834	1208	914
ENSECAG00000012196	2.506137651	0.375943748	0.600031989	74	45	124	76	186	116	80	158
ENSECAG00000020407	1.258850357	0.375994001	0.600031989	11	21	29	79	77	87	37	19
ENSECAG00000007746	0.758409424	0.376010189	0.600031989	6	10	16	68	32	25	51	51
ENSECAG00000020996	4.315330516	0.376074908	0.60005099	137	501	314.004	597	290	367	428	420
ENSECAG00000020694	3.9794604	0.376223129	0.600203201	145	137	336	364	344	374	282	481
ENSECAG00000020610	6.166228512	0.376326923	0.600245614	961	1483	1389	1358	1433	1301	1346	1473
ENSECAG00000004159	5.501232987	0.376445914	0.600245614	598	516	1010	1296	1101	765	860	745
ENSECAG00000012050	4.299010191	0.376450897	0.600245614	221	214	401	347	615	407	523	279
ENSECAG00000011754	5.330782063	0.376484744	0.600245614	415	320	861	903	1199	645	1092	848
ENSECAG00000025005	5.094925145	0.376513861	0.600245614	341	518	860	879	809	598	648	565
ENSECAG00000004006	4.020671303	0.37658495	0.60027472	301	613	114	155	461	180	408	39
ENSECAG00000000008	7.965746213	0.377115723	0.600983284	2001	4019	5459	8540	4775	3823	5182	5156
ENSECAG00000021364	5.130338656	0.37713526	0.600983284	275	190	897	836	931	702	1026	658
ENSECAG00000021905	2.296653144	0.377230115	0.601050142	38	107	78	167	60	117	46	129
ENSECAG00000018845	7.620356324	0.377429687	0.601283806	1892	2784	3079	4373	4768	3991	4471	5354
ENSECAG00000024452	2.243915116	0.377548742	0.60138915	49	71	71	82	95	59	131	167
ENSECAG00000009486	4.400836198	0.377693813	0.601535899	190	294	348	495	526	422	407	612
ENSECAG00000013755	6.320377453	0.377882845	0.601752611	1300	1158	1574	1758	1796	1277	1758	1305
ENSECAG00000024528	3.743659411	0.378112545	0.602034016	110	512	147	235	193	182	251	371

ENSECAG00000017730	3.117224826	0.378352926	0.602332345	69	122	136	211	199	168	211	232
ENSECAG00000000300	1.629955889	0.378415998	0.602348357	17	85	31	37	88	71	58	74
ENSECAG00000007477	8.175052914	0.37853186	0.602448381	3813	5668	5518	6482	5725	4027	5872	6569
ENSECAG00000022834	6.033910542	0.378720136	0.602609772	1158	1213	1020	1233	1180	1249	1111	1515
ENSECAG00000005483	4.85602524	0.37873934	0.602609772	266	595	583	738	541	515	624	554
ENSECAG00000016611	5.648231323	0.378945979	0.602792981	688	1315	781	792	947	675	1055	1212
ENSECAG00000016077	7.936044897	0.378960594	0.602792981	7501	2562	2759	3807	4439	3308	4648	5661
ENSECAG00000018267	5.624738893	0.379118796	0.602960213	582	934	571	848	1455	779	1150	1247
ENSECAG00000013933	0.117367812	0.379291018	0.603149692	5	36	20	19	7	20	9	33
ENSECAG00000018048	0.971960662	0.379651525	0.603576144	8	21	58	74	19	23	26	67
ENSECAG00000009758	4.164246055	0.379665438	0.603576144	146	333	292	317	421	323	613	313
ENSECAG00000014613	5.582089726	0.379719519	0.603577669	511	842	1314	887	906	971	722	1095
ENSECAG00000024469	5.693162633	0.3797916	0.6036078	432	895	1088	1587	952	952	932	1169
ENSECAG00000008781	4.877938474	0.379960027	0.603791025	404	520	311	477	569	557	708	938
ENSECAG00000007895	4.654500025	0.380186411	0.604066285	186	487	275	622	521	567	493	789
ENSECAG00000020825	5.427438446	0.38057605	0.604441492	459	622	766	805	861	915	895	1333
ENSECAG00000024334	2.711441726	0.380601913	0.604441492	41	65	188	98	131	157	124	210.999
ENSECAG00000012891	7.916410866	0.380665401	0.604441492	4424	5219	3487	3993	4801	3681	4810	5017
ENSECAG00000019476	8.640437156	0.380709542	0.604441492	10147	5848	6411	5018	7904	5646	7492	8706
ENSECAG00000021207	8.969692569	0.380718303	0.604441492	6236	6526	7273	9795	12206	9677	12880	12793
ENSECAG00000013487	1.480245661	0.38074175	0.604441492	20	39	69	90	43	46	51	60
ENSECAG00000020530	5.10937676	0.380992354	0.604754838	257	557	839	1048	605	568	628	870
ENSECAG00000015816	11.92207107	0.381146516	0.604844454	44746	50779	102547	102818	84481	50349	82276	77109
ENSECAG00000016334	6.477540888	0.38115528	0.604844454	894	1607	1366	1547	1902	1994	1827	2609
ENSECAG00000016583	0.792584622	0.381261353	0.604886074	35	40	15	29	21	35	39	22
ENSECAG00000000642	7.755749623	0.381287983	0.604886074	2482	2998	3910	3497	5789	4533	4412	5656
ENSECAG00000017951	6.05812687	0.381367987	0.604928531	1098	1276	1202	1135	1389	1150	1312	1286
ENSECAG00000019245	6.497859005	0.381429532	0.604941701	840	1291	1689	1803	2134	1686	2032	2623
ENSECAG00000014617	7.923888474	0.381738714	0.60534756	2688	3219	4432	4264	6424	4535	6150	5820
ENSECAG00000012620	5.702927019	0.381916938	0.605545667	677	759	701	999	1409	796	1319	1348
ENSECAG00000008046	8.692131871	0.382270882	0.606022291	4061	5120	7407	8778	9788	8338	9475	11678
ENSECAG00000013110	8.438742221	0.382529789	0.606348139	3448	5298	4080	8410	8045	7852	7871	9058
ENSECAG00000000402	4.436458649	0.382726913	0.606575978	150	413	450	689	292	428	458	467
ENSECAG00000024795	6.759786825	0.383175946	0.607180652	1419	1120	2442	1055	3480	3836	1025	1918
ENSECAG00000024788	10.46679922	0.383297893	0.607180652	28843	36696	13738	20064	25014	20241	27743	32417
ENSECAG00000026989	2.530160663	0.38330623	0.607180652	32	156	53	87	163	78	133	187
ENSECAG00000005386	2.825507822	0.383322198	0.607180652	92	156	132	134	150	103	122	161
ENSECAG00000016947	5.591914379	0.383387202	0.607198967	727	975	899	828	916	903	711	1219
ENSECAG00000017565	6.304563694	0.383564204	0.607381169	833	1205	1475	2569	1488	1295	1712	1619
ENSECAG00000011931	4.575670193	0.38360916	0.607381169	274	512	577	391	328	637	340	482
ENSECAG00000005169	6.289604623	0.383780854	0.607568352	495	1040	1633	3287	1322	1553	1290	1731
ENSECAG00000013378	2.919553596	0.383858811	0.607607106	77	112	110	152	141	121	187	269
ENSECAG00000000160	5.829507098	0.384316881	0.608247444	583	757	981	1242	1328	1144	1331	1460
ENSECAG00000019980	3.519845224	0.384383394	0.608267983	127	108	192	278	261	229	246	338
ENSECAG00000004311	10.16944072	0.384583321	0.608475728	10552	17955	27103	35231	23261	17168	24019	22901
ENSECAG00000019553	3.69201944	0.384621781	0.608475728	117	197	267	424	218	260	253	246
ENSECAG00000001958	3.547821914	0.38487617	0.608793407	110	482	154	111	151	251	223	213
ENSECAG00000016736	4.670997288	0.384989436	0.608869097	292	257	459	572	638	515	544	668
ENSECAG00000002413	7.528927563	0.385031197	0.608869097	1311	3306	2758	7798	3424	3313	3935	3058
ENSECAG00000012028	4.744115351	0.385252379	0.609113582	326	407	536	727	549	523	486	513
ENSECAG00000020980	4.155066573	0.385357845	0.609113582	281	440	261	270	363	300	324	373
ENSECAG00000015395	4.257656759	0.385379571	0.609113582	58	678	138	162	335	832	400	273
ENSECAG00000014558	4.156223494	0.38540945	0.609113582	139	212	321	463	377	439	438	391
ENSECAG00000018986	8.025090108	0.385453851	0.609113582	3076	6056	5622	4471	4895	4788	4616	5485
ENSECAG00000009561	3.249751934	0.385850506	0.609655605	114	122	105	230	193	181	220	302
ENSECAG00000013416	4.35482218	0.385931716	0.609699131	173	356	434	612	372	352	416	437
ENSECAG00000022383	1.898782191	0.386189458	0.610021495	49	25	99	21	138	92	80	44
ENSECAG00000020400	1.913814805	0.386359431	0.610205151	44	56	56	133	56	72	86	58
ENSECAG00000009994	4.145987712	0.386596717	0.610495052	170	408	365	376	230	355	395	369
ENSECAG00000014435	8.741617752	0.386675236	0.61053419	6040	8144	4513	5092	8770	10922	10416	10612
ENSECAG00000013857	2.927688381	0.386841556	0.610711929	72	125	109	152	170	149	169	222
ENSECAG00000005495	2.58494627	0.386966607	0.610745174	115	82	123	104	128	107	93	117
ENSECAG00000012914	3.497392323	0.38697012	0.610745174	124	117	170	284	310	206	279	256
ENSECAG00000003481	5.886605191	0.387113257	0.610818346	727	1822	1067	626	1099	757	1444	1191
ENSECAG00000012372	6.268689626	0.387151499	0.610818346	751	1349	1619	2186	1190	1589	1142	2034
ENSECAG00000021962	4.96576695	0.387208197	0.610818346	426	381	887	626	673	337	490	939
ENSECAG00000009142	5.121581616	0.387231521	0.610818346	228	387	333	1325	779	769	945	795
ENSECAG00000023757	0.929177849	0.387372606	0.610956073	17	34	33	60	25	23	46	40
ENSECAG00000026861	3.739041176	0.387472609	0.610990601	158	217	280	338	274	203	287	264
ENSECAG00000012297	2.300912509	0.387575672	0.610990601	38	54	111	195	91	87	87	93
ENSECAG00000022570	9.179046844	0.38762811	0.610990601	11215	10227	9085	10806	9492	8882	11223	14577
ENSECAG00000000746	2.81215093	0.38770249	0.610990601	66	79	160	109	212	80	171	208
ENSECAG00000017534	5.162505191	0.38771523	0.610990601	479	437	532	726	833	831	773	861
ENSECAG00000000564	2.399264751	0.387717148	0.610990601	121	77	63	109	124	52	104	109
ENSECAG00000019775	7.132540931	0.387908606	0.611207541	1320.01	2035	2386.01	2932	3097	3052	3005	4039
ENSECAG00000022742	7.606647823	0.38816083	0.611520154	2684	3698	2390	2181	4845	3825	4568	5355
ENSECAG00000013377	5.94464741	0.388248237	0.611573058	333	1070	726	1796	876	1813	1582	1510
ENSECAG00000007371	7.626929113	0.388519663	0.611915777	1858	2316	4089	3912	4743	4690	4943	4109
ENSECAG00000005934	0.020068744	0.388710101	0.612130862	21	33	7	7	19	30	7	4
ENSECAG00000022010	2.086898091	0.388811496	0.612205685	58	40	71	69	146	54	77	127
ENSECAG00000009030	6.252323785	0.388966213	0.612292134	1514	1555	1067	1132	1626	1212	1520	1467
ENSECAG00000014391	5.688469692	0.389052414	0.612292134	597	696	761	1136	1364	813	1218	1417
ENSECAG00000022936	5.024184702	0.389083629	0.612292134	347	430	953	726	595	470	584	890
ENSECAG00000007020	2.121928875	0.389158973	0.612292134	39	33	159	115	112	71	65	60
ENSECAG00000000084	5.687167734	0.389188147	0.612292134	607	865	1092	1240	954	995	977	1092

ENSECAG00000015096	4.749579867	0.389260799	0.612292134	229	375	370	742	623	472	659	760
ENSECAG00000006911	4.390828158	0.389263852	0.612292134	243	160	446	440	521	405	502	532
ENSECAG00000006522	5.291710739	0.389297516	0.612292134	491	394	95	1260	1532	1322	285	676
ENSECAG00000020210	2.587859654	0.389415768	0.612380018	12	62	220	33	31	225	259	77
ENSECAG00000021950	8.275216373	0.389461187	0.612380018	3716	2991	5613	6353	9167	5167	7575	7564
ENSECAG00000008404	7.131645266	0.389961146	0.613081297	2255	2737	2307	2792	2655	2178	2733	3243
ENSECAG00000015847	2.431541969	0.390288593	0.613429086	104	40	73	66	143	86	94	200
ENSECAG00000005156	3.669645295	0.390290342	0.613429086	170	151	215	436	289	215	185	279
ENSECAG00000014387	9.472866445	0.39041879	0.613546099	7846	7601	14239	13265	16247	15063	17712	18249
ENSECAG00000001463	1.510827387	0.390726073	0.61394408	35	62	72	36	38	36	70	62
ENSECAG00000020556	0.235011364	0.390784241	0.613950573	6	28	14	12	22	32	21	30
ENSECAG00000011057	7.551285497	0.391123826	0.614229057	1802	2149	2991	4796	3939	4057	4298	5426
ENSECAG00000008829	2.228175387	0.391153092	0.614229057	32	59	211	68	49	110	59	111
ENSECAG00000013557	5.830248939	0.39116886	0.614229057	1140	1052	790	959	1283	854	1188	1042
ENSECAG00000020643	5.663674264	0.391177738	0.614229057	540	802	974	1530	1185	780	979	1005
ENSECAG00000000359	3.767263228	0.391503291	0.614655299	112.014	224	396	308	262	230	253	294
ENSECAG00000022236	3.562830933	0.391816472	0.615029506	104	176	224	210	238	263	162	458
ENSECAG00000023181	6.044464361	0.391898229	0.615029506	736	1440	1340	1332	1047	1191	1196	1711
ENSECAG00000009636	7.369131961	0.391951934	0.615029506	2782	2618	3464	3059	3824	2569	3322	2882
ENSECAG00000005086	4.325684171	0.391966994	0.615029506	309	284	549	320	223	555	393	307
ENSECAG00000001807	2.764316876	0.392032572	0.615029506	66	70	85	195	166	102	200	173
ENSECAG00000013724	4.344856946	0.392066424	0.615029506	358	660	217	180	455	194	386	483
ENSECAG00000009913	4.123538535	0.392135907	0.615053585	205	235	302	303	398	223	428	606
ENSECAG00000010284	3.321162583	0.392221563	0.615103023	142	92	196	149	266	186	243	240
ENSECAG00000011529	1.332919479	0.392285181	0.615117889	19	50	19	52	36	71	30	101
ENSECAG00000014311	10.15692521	0.392570495	0.615409494	14164	15434	28051	29522	24805	17691	21118	23404
ENSECAG00000010283	7.24759066	0.392591273	0.615409494	1495	1800	2644	3526	3645	2909	3773	3976
ENSECAG00000010855	6.460116264	0.39263364	0.615409494	1074	1920	1440	2030	1621	1518	1602	2111
ENSECAG00000006072	5.926894643	0.392963863	0.615842125	628	1335	1165	1377	1044	1212	1144	1316
ENSECAG00000000193	6.37529659	0.393153573	0.616054462	833	1494	1781	2156	1762	1341	1504	1863
ENSECAG00000008521	1.998949522	0.393351047	0.616278902	47	57	109	83	65	78	66	85
ENSECAG00000019639	5.201088476	0.39363362	0.616615803	429	404	667	776	969	619	945	883
ENSECAG00000021705	10.46348808	0.393732925	0.616615803	16430	19768	35720	36354	29906	22274	26293	29121
ENSECAG00000011995	5.999895198	0.393803959	0.616615803	623	976	1116	1272	1322	1288	1647	1674
ENSECAG00000002481	8.531526434	0.393853458	0.616615803	12828	5299	2926	3092	5977	6617	6302	7567
ENSECAG00000006457	1.212532272	0.393857749	0.616615803	13	20	46	59	41	43	58	73
ENSECAG00000021365	4.511194531	0.3938917	0.616615803	344	216	413	367	596	409	507	623
ENSECAG00000014738	5.759778124	0.394191862	0.61700068	466	855	981	1954	828	1038	1092	1217
ENSECAG00000021946	7.414823328	0.394462242	0.617338842	1640	3068	1924	3725	3793	4470	4468	3178
ENSECAG00000014664	4.657932827	0.394552339	0.617394805	230	473	714	477	360	423	604	556
ENSECAG00000010213	4.984572941	0.394812112	0.617716223	338	374	715	518	707	717	757	736
ENSECAG00000016960	5.456094094	0.39492204	0.617803142	472	819	754	1249	797	735	1029	853
ENSECAG00000000271	0.590422411	0.394989494	0.6178236	9	8	43	21	11	50	34	43
ENSECAG00000010913	7.820033453	0.395494877	0.618366288	1674	2247	5576	4653	5200	4303	6022	5981
ENSECAG00000021698	2.120626797	0.395506497	0.618366288	32	31	111	78	55	109	47	214
ENSECAG00000014208	4.170561182	0.395588794	0.618366288	119	322	343.002	651	276	332	304.001	462
ENSECAG00000008533	4.34704935	0.395602928	0.618366288	211	368	480	486	215	381	265	709
ENSECAG00000007323	5.223620983	0.395608568	0.618366288	375	386	631	984	887	703	887	999
ENSECAG00000022109	7.040322528	0.395706461	0.618376289	1630	2607	1567	1809	3226	2561	3151	3511
ENSECAG00000017111	5.303305974	0.395723816	0.618376289	448	814	786	848	672	608	728	1101
ENSECAG00000002890	3.897705226	0.39594064	0.618630027	145	231	248	292	406	246	345	389
ENSECAG00000025015	6.517348278	0.396027038	0.61867994	922	1350	1251	2193	2068	1556	2368	2610
ENSECAG00000024860	7.080656098	0.396183975	0.618793966	1990	3178	2693	1840	2427	1881	2336	3794
ENSECAG00000008630	3.773700283	0.396208951	0.618793966	107	215	245	524	224	270	190	352
ENSECAG00000014880	2.737851571	0.396294591	0.618842652	96	160	131	98	138	159	128	47
ENSECAG00000006087	6.178654723	0.396511759	0.619044738	781	1277	1663	1673	1275	1375	1420	1559
ENSECAG00000023934	4.362693993	0.396580984	0.619044738	148	228	674	623	549	245	535	176
ENSECAG00000014089	6.755776395	0.39665988	0.619044738	1082	1145	1820	2752	2703	1962	2676	2813
ENSECAG00000015203	6.254215809	0.396702775	0.619044738	719	1173	1444	1395	1704	1336	2089	1990
ENSECAG00000000826	1.131928986	0.396723757	0.619044738	23	20	37	45	56	46	57	39
ENSECAG00000019482	3.458362654	0.396790465	0.619044738	106	183	248	302	162	202	166	312
ENSECAG00000021778	0.953562015	0.396805389	0.619044738	43	18	46	33	7	71	25	20
ENSECAG00000019843	6.088592299	0.396940399	0.619170348	878	947	1105	1177	1629	1445	1573	1641
ENSECAG00000014129	1.327440849	0.397026572	0.619219754	29	52	35	68	42	38	49	53
ENSECAG00000010696	2.225377535	0.397249894	0.619483021	66	29	95	73	118	63	122	143
ENSECAG00000010222	7.67336644	0.397357692	0.61956609	3596	2508	5196	3509	4962	3112	4032	3302
ENSECAG00000011253	5.246834681	0.397514969	0.619726274	319	476	607	1031	724	719	852	1273
ENSECAG00000007124	5.559924696	0.39780518	0.620093629	538	442	881	1087	1060	910	980	1461
ENSECAG00000019218	4.439944852	0.398049771	0.62038978	200	284	427	449	414	429	565	605
ENSECAG00000012019	8.568227792	0.398339926	0.620756857	4218	4563	5919	8482	10023	7153	7822	11045
ENSECAG00000009080	1.76227691	0.3985207	0.620799159	24	55	51	71	96	48	81	91
ENSECAG00000018884	5.837333521	0.398550415	0.620799159	578	977	779	1217	1427	1198	1193	1467
ENSECAG00000018362	3.609365753	0.398550827	0.620799159	139	143	195	271	266	249	263	356
ENSECAG00000003882	2.912615474	0.398591816	0.620799159	98	75	132	138	164	159	206	169
ENSECAG00000010933	4.028483183	0.398640263	0.620799159	135	210	203	492	359	354	323	491
ENSECAG00000013269	5.515074669	0.398872811	0.621009196	566	622	800	789	1112	747	1006	1409
ENSECAG00000015124	4.342651829	0.398930939	0.621009196	208	296	326	427	450	378	412	652
ENSECAG00000019895	0.180920456	0.398939106	0.621009196	15	3	45	23	33	12	20	4
ENSECAG00000011727	3.370365856	0.399151128	0.621246321	91	265	344	140	17	463	38	131
ENSECAG00000016580	6.198014883	0.399222747	0.621246321	554	827	1284	2080	1439	1877	1612	1898
ENSECAG00000020988	3.322834026	0.399255469	0.621246321	116	169	152	152	207	216	214	294
ENSECAG00000022300	1.893013152	0.39933525	0.621285377	39	52	54	145	52	52	82	86
ENSECAG00000012605	4.717206357	0.399422555	0.621334295	492	254	356	373	630	455	672	725
ENSECAG00000017443	2.381879975	0.399476063	0.621334295	49	98	76	80	97	114	110	166
ENSECAG00000010098	3.443658795	0.399537964	0.621345518	92	228	240	269	163	273	167	208

ENSECAG00000016863	3.531348952	0.399694001	0.621503112	146	109	228	204	316	157	294	325
ENSECAG00000023095	4.721764482	0.399992346	0.621881915	285	643	403	609	462	444	472	678
ENSECAG00000005256	2.406397426	0.400080429	0.621898467	53	81	58	124	134	89	117	157
ENSECAG00000022326	7.244539759	0.400119644	0.621898467	2384	2789	1474	1707	3464	3782	3700	3369
ENSECAG00000025152	3.130658255	0.400167197	0.621898467	79	239	199	143	112	261	79	177
ENSECAG00000017173	2.527997083	0.400372358	0.621973563	55	67	99	129	149	103	136	148
ENSECAG00000005653	8.212034871	0.4005426	0.621973563	4626	5083	5543	6395	6381	4173	5434	6878
ENSECAG00000014786	6.778385635	0.400562243	0.621973563	854	1775	1776	2509	2665	2117	2716	2780
ENSECAG00000005594	2.064290649	0.400603732	0.621973563	39	162	53	52	62	43	75	124
ENSECAG00000003055	4.03656431	0.400626857	0.621973563	183	272	375	387	336	294	313	324
ENSECAG00000016857	3.563287811	0.400647814	0.621973563	69	152	181	354	236	213	386	276
ENSECAG00000024566	7.11678563	0.400654117	0.621973563	1346	1912	3754	3743	2918	1973	2508	3307
ENSECAG00000022386	8.148030502	0.40068266	0.621973563	3223	6347	5375	6214	4670	5124	4996	6989
ENSECAG00000014782	4.684790391	0.400760689	0.621973563	361	455	539	489	558	442	460	543
ENSECAG00000024664	3.378884401	0.400762934	0.621973563	90	309	249	146	112	388	115	97
ENSECAG00000006611	6.053599689	0.400955906	0.622188064	779	1044	955	1280	1576	1443	1348	1780
ENSECAG00000022285	5.503324771	0.401047479	0.62224518	626	734	874	1058	876	890	853	918
ENSECAG00000008100	7.333536795	0.401180889	0.622367185	2493	2533	2716	4125	3268	2649	3153	3344
ENSECAG00000024125	4.791030415	0.401416429	0.622647573	382	315	614	747	628	476	456	587
ENSECAG00000019763	6.478108068	0.401621695	0.622880931	769	1313	1455	2108	2000	1654	2212	2459
ENSECAG00000002544	8.717598023	0.401868731	0.623042314	8335	8230	6368	6643	7268	6546	8252	9967
ENSECAG00000005968	2.356430921	0.4018713	0.623042314	37	80	118	168	89	101	75	112
ENSECAG00000004993	4.096011206	0.401890259	0.623042314	175	165	346	379	366	446	355	407
ENSECAG00000023897	2.741064484	0.402185389	0.623293439	59	122	128	206	105	108	109	184
ENSECAG00000022266	2.093508638	0.402214538	0.623293439	35	83	55	76	100	67	93	142
ENSECAG00000000373	2.686887219	0.402271448	0.623293439	52	63	56	227	184	189	133	93
ENSECAG00000008147	4.783405605	0.402271676	0.623293439	348	372	662	658	682	398	544	515
ENSECAG00000017324	7.226940545	0.402407268	0.623418513	3625	1183	2725	3090	3110	2045	3174	2937
ENSECAG00000019293	10.70118982	0.402480217	0.62344652	18758	23375	39259	46979	35188	25971	30651	35549
ENSECAG00000007356	5.414240193	0.402557007	0.623480468	616	829	854	731	912	684	868	863
ENSECAG00000021139	1.048204973	0.402695303	0.623609654	11	36	39	32	41	52	37	58
ENSECAG00000016206	5.094481037	0.402787022	0.623666686	361	517	651	1067	662	552	693	763
ENSECAG00000013074	3.572980871	0.402935091	0.623810942	101	165	181	299	214	289	277	321
ENSECAG00000013771	6.741260359	0.403126166	0.62402173	1784	463	4578	1471	3660	1256	1875	725
ENSECAG00000005014	8.190076577	0.403204552	0.624058047	3032	4157	5060	5555	6395	6543	6380	8133
ENSECAG00000017913	1.233755345	0.403837494	0.624952548	15	50	44	74	15	39	48	64
ENSECAG00000017289	1.810652399	0.403968056	0.625069461	20	64.0004	80	115	43	31.0011	80	103
ENSECAG00000016706	4.138134495	0.404138812	0.625144858	275	107	570	399	481	190	456	155
ENSECAG00000017079	2.670972469	0.404186973	0.625144858	44	74	133	142	153	107	124	218
ENSECAG00000026813	10.60794351	0.404207662	0.625144858	18232	20257	39137	42439	32611	23234	31832	31542
ENSECAG00000010788	5.404520199	0.404236866	0.625144858	317	848	946	1134	754	810	747	971
ENSECAG00000017461	6.588482445	0.40431841	0.62518587	759	2263	1679	2688	1611	1933	1853	1969
ENSECAG00000016843	8.060792052	0.404406448	0.625236911	3083	4465	5366	7400	4481	5022	4577	6440
ENSECAG00000010435	3.603270379	0.40452752	0.625333916	78	178	194	319	195	272	262	415
ENSECAG00000007668	6.519862261	0.404579265	0.625333916	545	1170	1912	2145	1492	1466	1471	4613
ENSECAG00000024231	3.107627608	0.404866579	0.625692882	85	108	125	213	227	158	174	245
ENSECAG00000018208	5.833358337	0.405433616	0.626483985	695	1441	998	958	1130	906	1165	1235
ENSECAG00000013910	3.904353255	0.405531686	0.626550315	250	352	236	204	255	248	315	327
ENSECAG00000012159	2.993262759	0.405631264	0.626618956	30	117	129	228	139	191	169	256
ENSECAG00000010288	7.237187262	0.405730551	0.62668713	2478	3198	1928	3304	3161	3046	3050	2064
ENSECAG00000021328	4.911223686	0.40583558	0.626764152	314	425	491	651	627	740	654	732
ENSECAG00000013171	5.541765013	0.405975605	0.626895193	664	682	1017	1006	929	829	849	1044
ENSECAG00000008240	4.217703415	0.406102038	0.627005212	274	159	453	508	388	330	379	318
ENSECAG00000009972	2.772254369	0.406244164	0.627068677	65	98	104	147	162	106	175	196
ENSECAG00000010769	6.055865332	0.406304904	0.627068677	1014	1238	1150	1355	1596	1092	1170	1317
ENSECAG00000004091	3.307583247	0.406308713	0.627068677	66	73	213	272	269	143	326	200
ENSECAG00000011929	5.500696916	0.406517968	0.627306418	468	11	1250	475	3112	675	1027	14
ENSECAG00000013355	4.171333616	0.406870118	0.627704139	229	294	375	419	376	301	352	373
ENSECAG00000008131	7.5827109	0.406886198	0.627704139	3083	2607	3871	4506	4285	2694	3799	3995
ENSECAG00000016106	2.891864504	0.407660108	0.628812672	93	74	101	177	192	130	206	162
ENSECAG00000006197	6.296767876	0.407938603	0.629156835	1135	1240	1654	1654	1543	1296	1560	1756
ENSECAG00000019988	3.115495393	0.408087335	0.629300801	52	167	129	183	182	162	156	324
ENSECAG00000026902	2.034107781	0.408330847	0.629540494	50	42	136	80	57	93	54	92
ENSECAG00000027692	11.06061557	0.408375766	0.629540494	36050	18905	37317	27460	52012	48781	43487	59497
ENSECAG00000019231	3.429165837	0.408408993	0.629540494	90	85	171	329	295	205	365	143
ENSECAG00000017834	0.483784749	0.408559817	0.629687553	6	14	29	30	35	19	31	42
ENSECAG00000024284	7.228136852	0.408986156	0.630170313	2013	1586	1999	3463	3955	2868	3927	3314
ENSECAG00000023851	8.015747262	0.409036887	0.630170313	3953	4787	3948	6263	5518	4496	4577	5241
ENSECAG00000009352	1.308539975	0.409050073	0.630170313	21	46	62	56	36	29	44	72
ENSECAG00000010340	2.262997351	0.409251069	0.630170313	43.0071	83	77	80	117	79	114	137
ENSECAG00000009628	8.030782932	0.409287941	0.630170313	3647	4865	4653	6090	5318	4516	4462	5850
ENSECAG00000017176	4.625543045	0.409291744	0.630170313	321	318	424	428	468	523	569	725
ENSECAG00000001215	2.515640789	0.409302385	0.630170313	42	74	126	101	146	64	132	205
ENSECAG00000020978	10.39240756	0.409367998	0.630170313	16264	17724	35165	33273	28231	19850	25826	29108
ENSECAG00000010661	10.10778745	0.409372213	0.630170313	13062	15390	26945	28857	23855	16874	21051	22733
ENSECAG00000020590	6.320148778	0.409704335	0.630596133	677	948	1640	1791	1495	1388	1736	2964
ENSECAG00000022566	6.552215383	0.409972298	0.630720134	1210	2013	1577	2033	1760	1382	2219	1946
ENSECAG00000024652	6.245199904	0.410032258	0.630720134	1185	1835	1246	1078	956	1424	1579	1881
ENSECAG00000013254	2.791892705	0.410034031	0.630720134	57	101	84	186	131	135	173	209
ENSECAG00000003931	6.826490803	0.410081784	0.630720134	1802	1629	1530	1502	3026	1988	2927	2752
ENSECAG00000011380	7.167706219	0.410138175	0.630720134	3012	3382	1906	1566	2884	1900	2797	3320
ENSECAG00000021116	10.50810725	0.410144879	0.630720134	26022	30796	20565	26213	26799	23018	26485	35037
ENSECAG00000011907	2.751174565	0.41020828	0.630720134	42	123	58	187	143	214	92	181
ENSECAG00000021890	0.311421798	0.41022899	0.630720134	7	14	36	38	23	27	19	11
ENSECAG00000005996	0.355525188	0.410435365	0.630952052	13	22	26	31	12	30	21	23

ENSECAG00000004908	6.323906354	0.410561084	0.630995335	1897	1496	1132	867	1752	1078	1642	1621
ENSECAG00000023506	3.956329512	0.410574592	0.630995335	126	243	421	400	304	258.002	294	339
ENSECAG00000021727	6.972268998	0.410893234	0.631399638	1680	641	3461	4405	4265	1509	2231	1059
ENSECAG00000008855	8.194788092	0.41098931	0.631408066	3503	3980	4561	5639	7328	6227	6819	7053
ENSECAG00000000212	4.889757436	0.411009862	0.631408066	278	431	541	608	579	568	617	1001
ENSECAG00000009188	10.37241096	0.411089218	0.631444598	17650	18775	33644	29221	30309	20817	25073	24960
ENSECAG00000001394	6.587179302	0.411213381	0.631484043	934	773	2956	799	1483	4747	1622	1128
ENSECAG00000017124	4.479557559	0.411226055	0.631484043	177	286	386	583	491	396	522	672
ENSECAG00000013624	3.119789305	0.411442476	0.631731002	96	107	137	188	237	149	223	196
ENSECAG00000021311	7.430361415	0.411749323	0.632116715	1802	2955	2687	2971	3877	3018	4121	5305
ENSECAG00000019630	5.06611273	0.41188905	0.632210942	498	801	523	537	709	455	716	729
ENSECAG00000017626	5.208286901	0.411985672	0.632210942	377	723	507	639	858	554	911	1147
ENSECAG00000007646	6.020348393	0.412001935	0.632210942	881	702	1728	1628	2234	989	915	722
ENSECAG00000010086	6.600793635	0.412060097	0.632210942	1384	1780	1796	2050	1953	1552	2050	2014
ENSECAG00000008222	1.272500191	0.412118073	0.632210942	24	35	20	60	55	54	57	54
ENSECAG00000012227	4.275269058	0.412187847	0.632210942	174	307	305	425	448	321	575	448
ENSECAG00000008021	2.819267406	0.412200199	0.632210942	85.0017	76	125	129	197	84	219	164.001
ENSECAG00000015089	11.2280167	0.412272292	0.63223617	26270	32978	57187	69332	53409	33622	48249	48296
ENSECAG00000021488	5.967313044	0.412382718	0.632320167	360	1150	1026	1443	1059	1743	1487	1505
ENSECAG00000019839	3.856862888	0.41264857	0.632626793	192	206	204	264	318	286	373	360
ENSECAG00000019004	3.325163148	0.412694049	0.632626793	161	160	104	135	231	183	251	276
ENSECAG00000017285	3.528321931	0.412893447	0.632830691	112	158	136	314	259	240	247	327
ENSECAG00000014250	1.679616545	0.412947122	0.632830691	50	58	47	82	25	109	39	47
ENSECAG00000012744	3.859258668	0.413004558	0.632830691	102	179	311	307	276	162	301	677
ENSECAG00000011003	8.246575485	0.413049851	0.632830691	5865	3495	6352	6856	9614	4348	6183	1900
ENSECAG00000012879	6.951792099	0.413107062	0.632833011	1468	2781	1820	3161	1943	2113	2076	3471
ENSECAG00000019676	5.155143073	0.413305219	0.633051214	337	510	715	669	813	645	840	1003
ENSECAG00000008129	7.707924032	0.413363448	0.633055061	2586	3941	4669	4250	3463	4254	3884	4423
ENSECAG00000018090	6.632532901	0.41347234	0.633136486	1080	1315	1856	1858	2216	2261	1947	2798
ENSECAG00000012792	5.608412218	0.413768876	0.633473758	268	1147	1069	1321	869	1006	1020	802
ENSECAG00000020564	7.220660268	0.413804104	0.633473758	2127	1220	5192	2724	2883	2460	2112	3862
ENSECAG00000013154	3.292347274	0.414108366	0.633854136	70	160	142	232	208	181	139	409
ENSECAG00000015726	3.736804893	0.414227925	0.633868801	135	292	201	454	48	539	78	239
ENSECAG00000012543	6.658497998	0.414317497	0.633868801	1308	2353	1610	2064	1877	2022	2100	1734
ENSECAG00000005515	4.439386911	0.414350808	0.633868801	298	250	416	261	927	170	620	366
ENSECAG00000016577	3.765404977	0.41439213	0.633868801	125	265	241	402	302	220	267	255
ENSECAG00000016812	4.654086466	0.414432384	0.633868801	301	390	654	503	453	412	425	685
ENSECAG00000011454	4.932579184	0.414452677	0.633868801	397	332	537	580	1085	488	621	646
ENSECAG00000017909	3.360231768	0.414580234	0.633956908	98	215	213	239	173	168	207	242
ENSECAG00000018287	5.57808898	0.414621878	0.633956908	574	602	804	985	1134	926	1126	1210
ENSECAG00000016931	8.584586992	0.414691254	0.633977667	5736	6979	6677	8765	8163	6651	8125	6278
ENSECAG00000003138	1.512120316	0.414937356	0.634268564	14	48	51	55	37	88	61	75
ENSECAG00000020668	0.915849882	0.415053125	0.634360183	18	14	56	56	37	29	27	39
ENSECAG00000020253	11.20969	0.415599256	0.634986149	27191	33582	59105	61223	50117	33871	50724	46760
ENSECAG00000009766	5.755036082	0.415605224	0.634986149	537	398	1152	1339	1229	923	1271	1668
ENSECAG00000013096	0.728252494	0.41566794	0.634986149	19	14	15	44	37	18	43	55
ENSECAG00000014438	3.318332792	0.415686233	0.634986149	70	122	182	259	237	193	203	299
ENSECAG00000006415	0.683193548	0.41577291	0.635033176	9	23	13	47	19	37	41	49
ENSECAG00000019253	2.504918648	0.416147037	0.63545968	19	83	120	129	101	95	178	166
ENSECAG00000017478	2.470391891	0.41616401	0.63545968	61	75	98	91	161	80	118	160
ENSECAG00000016214	6.102767465	0.416282781	0.635555624	551	965	1250	1635	1445	1510	1483	1931
ENSECAG00000000302	6.615570264	0.416426154	0.635689099	740	1408	2653	2910	1721	2005	1585	2205
ENSECAG00000009265	3.104284945	0.416786806	0.636154177	69.0071	117	181	160	184	152	164	311
ENSECAG00000020310	3.436648358	0.416883287	0.636215972	202	193	232	126	250	201	210	145
ENSECAG00000011634	4.648767598	0.416988372	0.636290878	267	423	513	670	461	489	521	475
ENSECAG00000009708	6.223104579	0.417392169	0.636821515	819	634	1499	1681	2513	1096	1996	1398
ENSECAG00000019751	5.573135075	0.41771979	0.637235803	771	736	692	1244	1112	771	1048	753
ENSECAG00000004593	3.852174071	0.417855553	0.637357335	117	410	307	242	240	209	191	472
ENSECAG00000015269	7.276034267	0.417987134	0.637404166	1265	1560	3525	3439	3599	2833	4189	3998
ENSECAG00000005659	2.973224522	0.417998454	0.637404166	102	131	187	164	98	238	84	152
ENSECAG00000019712	3.726762924	0.418091485	0.637460474	141	292	295	237	261	219	264	276
ENSECAG00000009259	7.421576859	0.418189178	0.637494198	1599	2447	2576	4100	3470	4294	3549	4688
ENSECAG00000000603	3.613632818	0.418225819	0.637494198	108	156	170	339	215	257	339	325
ENSECAG00000000356	2.325822854	0.41830782	0.637533663	61	71	48	114	128	107	122	105
ENSECAG00000019736	6.203280971	0.418443039	0.637654213	1203	1808	974	1174	1283	1375	1220	1818
ENSECAG00000010456	4.62059198	0.418558376	0.637744437	239	497	342	418	455	506	573	754
ENSECAG00000016621	6.003392845	0.418617881	0.637749579	738	1199	1236	1550	1255	1147	1290	1329
ENSECAG00000023887	1.166709852	0.418809742	0.637956333	7	57	52	59	26	45	33	52
ENSECAG00000014943	4.380843344	0.419081941	0.638207693	254	464	373	414	378	407	470	347
ENSECAG00000007599	6.81730296	0.419087097	0.638207693	1438	3036	1732	1917	1847	1923	2285	2640
ENSECAG00000010383	3.239479076	0.419334132	0.63842759	81	116	164	230	196	159	246	281
ENSECAG00000006707	2.896843469	0.419343874	0.63842759	122	188	101	112	170	85	161	144
ENSECAG00000014473	7.470387577	0.419738348	0.638829487	2708	4815	2585	2469	3879	2560	3813	3224
ENSECAG00000012834	5.082141463	0.419896648	0.638829487	252.011	1063	435.008	809	506.001	546	598.001	962
ENSECAG00000008041	4.021355321	0.419969762	0.638829487	135	472	268	330	313	294	349	276
ENSECAG00000011491	6.496272603	0.420025074	0.638829487	1130	1702	1343	2526	1616	1806	1567	2020
ENSECAG00000020379	3.216726639	0.420044108	0.638829487	114	344	133	75	137	255	127	141
ENSECAG00000018926	6.091865697	0.420120647	0.638829487	835	891	1131	1331	1765	1219	1784	1531
ENSECAG00000024649	4.455127785	0.420133076	0.638829487	244	298	445	347	604	319	562	556
ENSECAG00000014882	6.892196546	0.420150468	0.638829487	732	2016	2133	2559	1702	3489	3438	2421
ENSECAG00000012886	5.272561248	0.420169948	0.638829487	366	715	761	497	720	699	1093	1096
ENSECAG00000016381	2.823031469	0.420206996	0.638829487	77	50	161	135	192	154	186	124
ENSECAG00000004592	0.77921956	0.420226332	0.638829487	20	3	39	26	85	32	35	8
ENSECAG00000011148	4.641092834	0.420295277	0.638848821	159	491	457	861	399	523	470	511
ENSECAG00000022160	6.747917086	0.420613437	0.639246907	1729	2355	1412	2179	1935	2065	2485	1701

ENSECAG00000018466	2.866733045	0.420747469	0.639310235	68	112	174	202	163	105	126	161
ENSECAG00000021243	7.318979479	0.420767641	0.639310235	3403	3226	1783	2634	3252	2681	3267	2876
ENSECAG00000024729	8.257977441	0.420891795	0.639413368	3269	3475	6470	5443	8060	5308	8107	7382
ENSECAG00000019716	7.008699161	0.420988973	0.639475496	871	1347	2532	3586	3238	2667	2985	3209
ENSECAG00000012556	4.978964212	0.421214018	0.63973181	353	788	470	685	523	632	677	603
ENSECAG00000004150	0.243491962	0.421560241	0.640172073	10	21	33	20	17	16	27	20
ENSECAG00000006365	7.066515675	0.421859678	0.64053149	1722	2428	2277	3527	2462	2252	2428	3295
ENSECAG00000001192	5.335422821	0.421941843	0.64053149	324	612	966	1222	869	640	801	833
ENSECAG00000001444	1.913997539	0.421966045	0.64053149	32	52	61	80	61	108	95	78
ENSECAG00000006802	0.201249694	0.422077203	0.640608656	8	12	32	34	10	14	24	29
ENSECAG00000013471	3.002728067	0.422185405	0.640608656	81	73	161	177	156	169	197	223
ENSECAG00000014686	5.398400083	0.422186025	0.640608656	468	591	721	843	1119	831	793	1145
ENSECAG00000024173	5.643818025	0.422289639	0.640680315	596	595	1483	1041	1073	748	927	1165
ENSECAG00000022296	5.388399638	0.422420995	0.640794038	414	846	857	991	720	764	703	1108
ENSECAG00000017799	9.878948043	0.422832684	0.641332927	9090	29240	13308	18664	14704	16325	18464	21948
ENSECAG00000021482	3.5072896	0.423253819	0.641885998	74	222	185	224	222	236	266	335
ENSECAG00000024532	6.765473019	0.423430879	0.641931411	1223	2000	2052	2857	1923	1849	2267	2444
ENSECAG00000010318	4.62092735	0.423511548	0.641931411	196	365	449	548	582	688	412	567
ENSECAG00000021989	5.25994676	0.423514815	0.641931411	378	1079	304	420	1033	885	862	821
ENSECAG00000004362	6.212169662	0.423546969	0.641931411	1222	1581	1111	1286	1651	1205	1503	1388
ENSECAG00000022162	8.342256078	0.423566255	0.641931411	3575	5437	4569	11746	6346	5742	6808	5753
ENSECAG00000021143	0.433721236	0.423739065	0.642040315	24	12	22	6	41	17	26	41
ENSECAG00000014477	8.037522653	0.423786315	0.642040315	3055	3272	4647	5023	6098	5578	5624	7335
ENSECAG00000015062	1.933099151	0.423807636	0.642040315	26	57	61	88	84	59	97	115
ENSECAG00000007207	3.982435055	0.424250265	0.642545308	147	181	313	352	379	364	266	452
ENSECAG00000005017	0.70021153	0.424254084	0.642545308	5	14	23	55	29	43	38	36
ENSECAG00000013505	3.464328458	0.424443198	0.642705016	153	121	165	217	291	189	321	220
ENSECAG00000009920	3.77347842	0.424472667	0.642705016	138	178	265	259	284	267	239	491
ENSECAG00000005210	6.901363783	0.424539348	0.64272033	1357	1775	2231	1813	3414	1986	3330	2437
ENSECAG00000008615	3.824432	0.424637846	0.642771646	145	290	302	308	271	290	218	312
ENSECAG00000013433	5.253276091	0.424686388	0.642771646	239	485	1013	1326	674	754	816	652
ENSECAG00000026905	10.89291507	0.424815277	0.642876736	18506	28206	48037	52621	38852	29971	40145	36528
ENSECAG00000020565	3.211200356	0.424935025	0.642876736	88	110	201	166	198	144	262	262
ENSECAG00000009173	3.281554602	0.424975019	0.642876736	59	37	242	265	389	158	272	111
ENSECAG00000006181	4.226882075	0.424982148	0.642876736	375	389	249	264	380	346	334	370
ENSECAG00000007696	3.017213541	0.425193671	0.643069508	87	194	52	116	100	150	169	373
ENSECAG00000011186	0.719960976	0.425294272	0.643069508	11	29	24	28	41	22	34	53
ENSECAG00000000586	0.174070577	0.425329363	0.643069508	4	19	30	32	14	23	11	26
ENSECAG00000011567	6.677618027	0.425335975	0.643069508	1128	2193	2050	2163	1987	1951	1959	2030
ENSECAG00000000398	5.029760681	0.425581582	0.643288007	366	449	848	765	572	574	699	710
ENSECAG00000008346	6.285947014	0.425638921	0.643288007	641.003	1121	1586	1555	1614	1175	1666	2960
ENSECAG00000007319	3.749319274	0.425650346	0.643288007	100	210	200	345	301	279	325	336
ENSECAG00000019747	6.51519112	0.426034417	0.643746476	1554	455	3266	1650	2553	1403	1889	804
ENSECAG00000018611	5.371271969	0.426067021	0.643746476	471	596	1066	927	725	753	842	924
ENSECAG00000023440	4.080494789	0.426168381	0.643814007	137	237	218	488	335	315	346	595
ENSECAG00000016817	2.757695416	0.426502699	0.644233405	79	124	109	69	160	119	234	117
ENSECAG00000012006	7.147794394	0.426751033	0.644522828	1191	1677	4080	4289	2315	2153	2356	4141
ENSECAG00000013777	3.457289639	0.427026341	0.644797113	239	102	130	357	236	94	51	442
ENSECAG00000001599	11.44335264	0.427046143	0.644797113	27966	37557	66637	85380	57526	40491	60067	55260
ENSECAG00000002621	6.201098355	0.427161009	0.64488485	1085	1159	1016	1046	1674	1455	1893	1799
ENSECAG00000023327	1.20403246	0.427324815	0.64504644	19	52	19	36	45	22	70	81
ENSECAG00000022434	5.876934498	0.427538553	0.645204498	1252	903	783	1112	1338	895	1134	1186
ENSECAG00000021593	5.469152121	0.427543097	0.645204498	448	755	765	776	951	970	896	1259
ENSECAG00000016287	3.512834548	0.42760025	0.645205052	102	124	256	229	258	245	227	328
ENSECAG00000013222	4.242567363	0.427970968	0.645627838	189	230	319	459	407	373	501	459
ENSECAG00000013054	5.502122456	0.427994092	0.645627838	1266	520	554	683	951	557	891	1069
ENSECAG00000024271	1.869591986	0.428207151	0.645835902	41	24	62	89	62	92	111	70
ENSECAG00000016402	3.453573914	0.428245704	0.645835902	91	152	394	199	196	185	171	281
ENSECAG00000011198	5.284977065	0.428370861	0.645899453	469	314	1241	951	1020	661	585	709
ENSECAG00000009721	9.017121785	0.428458729	0.645899453	6942	8858	9053	13830	9626	8055	10191	12250
ENSECAG00000020596	4.466736041	0.428497073	0.645899453	184	140	649	398	759	342	480	512
ENSECAG00000014503	5.431003864	0.428562804	0.645899453	633	767	509	601	1021	785	1037	1172
ENSECAG00000007022	2.851121901	0.42857208	0.645899453	83	77	97	181	211	125	133	205
ENSECAG00000000497	4.585669708	0.428641165	0.645917893	328	302	412	394	719	372	576	559
ENSECAG00000011531	5.837444092	0.428738858	0.645958154	432	1320	1083	1508	989	1157	1078	1202
ENSECAG00000021016	4.910009494	0.428794755	0.645958154	311	334	688	1000	603	324	625	807
ENSECAG00000010618	2.612806748	0.42883844	0.645958154	53	85	99	136	108	130	109	226
ENSECAG00000019139	4.953310797	0.428944574	0.646032376	263	387	447	911	688	636	657	890
ENSECAG00000014151	6.285800481	0.429573151	0.646893326	770	1269	1183	1666	1638	1684	1915	1945
ENSECAG00000010494	1.595724869	0.429870127	0.647214801	56	37	36	25	56	38	84	111
ENSECAG00000020839	4.590068664	0.429941732	0.647214801	265	424	260	526	551	434	462	808
ENSECAG00000017045	1.038310098	0.429975379	0.647214801	22	12	56	21	34	27	77	54
ENSECAG00000013963	3.560332862	0.430014481	0.647214801	77	213	252	386	167	203	201	334
ENSECAG00000014065	5.486124677	0.430298209	0.647556059	560	884	526	686	1163	679	1045	1311
ENSECAG00000016068	5.239756313	0.430557133	0.647785239	423	539	666	717	936	719	754	1075
ENSECAG00000024683	0.11712558	0.430564525	0.647785239	6	20	23	30	14	18	23	17
ENSECAG00000020268	3.089426957	0.431788331	0.649443153	114	93	238	191.628	205	75	135	238
ENSECAG00000014300	7.002560335	0.431844024	0.649443153	1341	1328	2184	3212	3212	2198	3368	3241
ENSECAG00000023595	5.974265394	0.431851539	0.649443153	928	686	874	1264	2179	1286	1329	1006
ENSECAG00000013853	3.354469775	0.431928492	0.649443153	126	175	116	189	261	207	184	298
ENSECAG00000020491	4.169048291	0.431952289	0.649443153	173	307	340	273	303	396	350	630
ENSECAG00000012394	1.814574343	0.432356602	0.649929724	29	29	125	92	44	76	60	72
ENSECAG00000022668	5.843284534	0.432390317	0.649929724	312	575	740	2182	840	1733	1413	1385
ENSECAG00000014662	5.672889428	0.432512206	0.650026943	506	981	695	973	1038	992	1118	1586
ENSECAG00000019654	3.122710991	0.432586052	0.650051941	49	122	115	273	200	189	190	233

ENSECAG00000017292	5.326342025	0.432942685	0.650428537	538	775	639	956	901	504	926	826
ENSECAG00000016453	3.036919666	0.432974701	0.650428537	47	178	131	296	142	147	121	207
ENSECAG00000009099	6.777999419	0.433008401	0.650428537	1334	1730	1859	1622	3075	1597	2504	3168
ENSECAG00000015081	0.791486845	0.433076586	0.650444966	14	20	33	67	14	36	36	33
ENSECAG00000007941	3.905132628	0.43354373	0.651049983	170	168	422	370	241	251	239	433
ENSECAG00000019294	1.025316823	0.433594017	0.651049983	9	24	34	54	68	23	40	57
ENSECAG00000002580	5.6726018	0.433769916	0.651228037	453	1330	937	1039	830	934	1159	1040
ENSECAG00000019069	6.982133007	0.433833612	0.651237615	2036	2492	2433	2058	1529	3192	2485	2336
ENSECAG00000009936	7.049054304	0.434206333	0.651640977	1201	1468	2277	3494	2925	2967	2599	3904
ENSECAG00000003565	4.656961256	0.434217025	0.651640977	235	357	246	768	589	410	568	807
ENSECAG00000016692	7.62772676	0.434333387	0.651664573	2170	2844	3336	3731	5291	3257	5128	4860
ENSECAG00000011227	3.035905661	0.434347457	0.651664573	49	30	363	289	253	100	173	4
ENSECAG00000021402	1.385858802	0.434919264	0.652436319	22	47	20	62	52	45	50	97
ENSECAG00000002250	2.674261593	0.434991265	0.652458186	136	53	137	133	216	87	65	99
ENSECAG00000017904	6.986102089	0.435312554	0.652853912	1318	1896	2752	3722	2506	2100	2598	2634
ENSECAG00000021317	2.687734771	0.435476825	0.653002457	47	81	165	98	164	121	134	182
ENSECAG00000016207	0.441486679	0.43553752	0.653002457	14	14	29	15	33	25	42	20
ENSECAG00000020622	4.829476735	0.435584019	0.653002457	338	442	447	519	631	541	652	791
ENSECAG00000014546	5.52092192	0.435797283	0.65323598	741	555	1011	1166	427	1753	303	808
ENSECAG00000019916	8.276052083	0.435878089	0.65327092	4086	4553	6025	8605	6555	5022	5541	6895
ENSECAG00000012137	4.438974412	0.436197842	0.653663925	239	222	444	452	476	446	516	556
ENSECAG00000026858	3.582347873	0.436610334	0.654140525	96	147	277	230	264	259	314	263
ENSECAG00000027002	4.83363759	0.436636567	0.654140525	332	382	455	604	608	468	713	853
ENSECAG00000018610	1.437369272	0.436688601	0.654140525	31	29	55	90	40	52	58	45
ENSECAG00000012197	0.899729858	0.436780206	0.654191497	6	35	52	52	48	18	31	33
ENSECAG00000000350	4.111859797	0.43699734	0.654400418	98	220	343	464	357	360	442	444
ENSECAG00000018727	1.965701293	0.437034886	0.654400418	35	56	61	82	113	69	96	79
ENSECAG00000014357	0.724461881	0.437095868	0.654405489	17	2	36	33	62	32	50	8
ENSECAG00000015352	4.880816211	0.437329777	0.654669424	496	584	426	549	517	532	549	709
ENSECAG00000014653	7.343031984	0.437387736	0.654669932	2003	2216	2617	2999	3984	2849	3810	4579
ENSECAG00000001582	6.961102002	0.437455136	0.65468457	1740	1667	1750	2224	3527	2332	3577	2138
ENSECAG00000000780	5.256318393	0.437618477	0.654811852	397	430	852	1263	828	622	671	867
ENSECAG00000014265	4.933592779	0.437655448	0.654811852	539	742	373	450	646	454	550	725
ENSECAG00000013245	5.873196867	0.437731558	0.654839495	608	1044	711	1275	1151	1151	1214	1950
ENSECAG00000008744	5.988860948	0.437879275	0.654974239	1200	1540	591	1062	1509	978	1300	1064
ENSECAG00000015018	5.998260556	0.438139495	0.655156838	676	1137	900	1240	1269	1462	1504	1633
ENSECAG00000017514	10.67492942	0.43820716	0.655156838	21648	20397	40592	40441	38081	23155	31544	33175
ENSECAG00000021777	7.178803994	0.438237843	0.655156838	2569	2758	2511	2315	2537	2760	2767	3094
ENSECAG00000021082	1.207005662	0.438302268	0.655156838	16.0001	33	35.0001	51.0006	48	76.0001	31.0001	52.0001
ENSECAG00000007184	0.585847	0.438396676	0.655156838	13	34	32	28	25	17	28	36
ENSECAG00000012492	5.600558854	0.438438404	0.655156838	588	400	1164	822	1376	839	1146	1150
ENSECAG00000016986	6.259173083	0.438449235	0.655156838	959	1382	1144	1050	2182	1205	2065	1671
ENSECAG00000014710	0.250923129	0.438492314	0.655156838	11	11	18	25	22	34	20	27
ENSECAG00000012143	4.960756061	0.438520309	0.655156838	285	372	663	526	321	1649	329	556
ENSECAG00000017105	6.168923055	0.438678714	0.655307329	1071	740	1203	1276	2219	1024	1639	1859
ENSECAG00000021191	6.996486935	0.438795883	0.65539619	1728	1631	1860	2479	3064	2590	2649	3616
ENSECAG00000018490	5.948646476	0.438925129	0.655503065	888	1297	767	1497	1193	1067	1423	1119
ENSECAG00000021671	1.650297893	0.43901198	0.655546605	24	94	90	30	20	83	90	14
ENSECAG00000023818	5.985305429	0.439127341	0.655632701	743	1421	870	1629	1315	1030	1545	1020
ENSECAG00000023450	2.468054933	0.439461502	0.656045406	68	66	88	105	131	128	145	100
ENSECAG00000000940	4.306176674	0.439541011	0.656068654	182	659	340	263	511	301	445	219
ENSECAG00000013639	2.374629753	0.439613361	0.656068654	22	54	112	133	41	134	170	144
ENSECAG00000012038	6.410402031	0.439693408	0.656068654	795	1124	1445	2066	1738	1774	2111	2228
ENSECAG00000026875	2.658447123	0.439708044	0.656068654	50	69	81	194	103	116	119	264
ENSECAG00000020538	7.163438902	0.439784223	0.656096158	1794	2400	2683	3787	2783	2387	3005	2978
ENSECAG00000011670	10.29685213	0.440467232	0.656969861	10931	19964	29373	38029	24656	19620	26771	25654
ENSECAG00000008008	6.857171115	0.440485513	0.656969861	1681	2679	2121	1736	2388	1903	2163	2534
ENSECAG00000021875	4.998293273	0.440567102	0.657005305	291	578	773	752	839	518	516	620
ENSECAG00000026888	2.740147892	0.441135596	0.657766751	60	112	92	143	148	139	126	206
ENSECAG00000004264	0.491695757	0.441230259	0.657821573	4	5	63	43	19	37	21	10
ENSECAG00000021941	7.363598023	0.441315963	0.657863025	3141	3201	2628	2465	3361	2318	3371	3688
ENSECAG00000015338	5.603119741	0.441477643	0.657866784	551	949	940	1102	898	966	952	1000
ENSECAG00000011742	4.729255714	0.441543039	0.657866784	364	536	485	499	574	473	538	485
ENSECAG00000023114	0.448418382	0.441548839	0.657866784	36	28	8	16	20	27	21	24
ENSECAG00000006287	4.251927488	0.441550088	0.657866784	276	282	266	300	458	332	384	600
ENSECAG00000023756	3.328177252	0.44166286	0.657948527	98	167	141	211	211	217	199	300
ENSECAG00000022168	6.322697669	0.441820682	0.657979281	836	960	1000	2304	1485	1780	1694	2499
ENSECAG00000021022	2.252319382	0.44182639	0.657979281	33	48	139	65	100	83	133	130
ENSECAG00000015228	3.271946673	0.441901904	0.657979281	83	142	138	241	170	197	290	234
ENSECAG00000014003	3.370461798	0.441945087	0.657979281	130	219	212	180	228	174	182	212
ENSECAG00000000429	1.887188752	0.441973057	0.657979281	23	48	38	121	80	75	103	83
ENSECAG00000013288	3.730313209	0.442160436	0.658044867	109	213	218	507	235	227	172	389
ENSECAG00000021766	2.625732342	0.442237605	0.658044867	28	47	110	214	140	158	125	153
ENSECAG00000011268	1.529851328	0.442244241	0.658044867	31	35	78	76	42	85	50	22
ENSECAG00000024339	5.517080192	0.442248777	0.658044867	412	450	945	1145	1047	866	941	1404
ENSECAG00000017552	0.070949194	0.442463187	0.658079787	6	7	42	24	21	25	8	12
ENSECAG00000012351	5.912394629	0.442504727	0.658079787	577	750	1031	1502	1167	1337	1505	1518
ENSECAG00000007577	4.258961176	0.442569608	0.658079787	248.011	435	298.008	387	349.001	336	347.001	467
ENSECAG00000020871	6.524140891	0.442618932	0.658079787	1110	1677	1799	2158	1824	1605	1909	1875
ENSECAG00000021493	6.195377393	0.442634329	0.658079787	1064	1327	825	1049	1729	1286	1989	1822
ENSECAG00000017272	5.916193247	0.442673375	0.658079787	750	746	1322	1688	1322	1125	1100	1168
ENSECAG00000013253	6.787874447	0.442677681	0.658079787	1248	1497	2817	2748	2342	1740	2303	2217
ENSECAG00000017322	2.455891662	0.442847463	0.658246059	35	109	111	172	86	90	81	157
ENSECAG00000015953	7.490793781	0.442961172	0.658288257	4056	2464	3670	2191	4724	2355	3496	3072
ENSECAG00000024336	10.25566387	0.443045799	0.658288257	13588	16504	33404	29548	25679	18541	25722	24149

ENSECAG00000009975	4.842636839	0.443049666	0.658288257	198	513	441	676	611	492	948	602
ENSECAG00000024165	3.369792732	0.443176548	0.658390682	62	121	234	241	194	269	263	224
ENSECAG00000023926	6.204240146	0.443376223	0.658525065	1149	1564	1117	1363	1395	1119	1382	1919
ENSECAG00000012391	5.621909337	0.443409181	0.658525065	381	859	1059	1510	851	735	1384	851
ENSECAG00000013408	3.413659471	0.443535425	0.658525065	127	169	120	228	273	186	269	256
ENSECAG00000012487	2.922281846	0.44359598	0.658525065	90	171	120	170	137	120	132	194
ENSECAG00000008518	1.666968995	0.443598321	0.658525065	15	43	33	104	69	33	71	131
ENSECAG00000018906	6.77994635	0.443621315	0.658525065	1265	1765	1961	3304	2071	1867	1897	2775
ENSECAG00000007411	3.664508196	0.443672714	0.658525065	171	197	187	193	318	213	282	364
ENSECAG00000009635	4.930427216	0.443826305	0.658596636	363	460	409	658	760	563	648	836
ENSECAG00000017875	3.069158521	0.443891829	0.658596636	91	78	175	169	191	159	227	198
ENSECAG00000021502	2.840573593	0.443894828	0.658596636	88	109	181	146	131	102	130	189
ENSECAG00000011743	6.045666339	0.444004	0.658649475	649	1350	1298	1608	1149	1194	1194	1667
ENSECAG00000016644	1.03028968	0.444055829	0.658649475	13	38	63	41	16	63	29	30
ENSECAG00000017659	6.022358172	0.444146282	0.658649475	761	721	1332	1218	1600	1436	1236	1711
ENSECAG00000014355	2.657457214	0.44416232	0.658649475	64	53	135	132	164	112	115	199
ENSECAG00000017891	2.191935381	0.444372402	0.658875014	50	47	114	51	78	68	122	165
ENSECAG00000024285	4.203398984	0.444481874	0.6589041	152	235	325	470	343	342	465	559
ENSECAG00000012054	2.453101796	0.444554713	0.6589041	39	65	169	174	37	198	40	102
ENSECAG00000018242	8.004786457	0.444565994	0.6589041	2793	3584	4696	4631	6918	4362	6070	6734
ENSECAG00000021768	5.134491619	0.444733577	0.659066507	563	548	584	833	688	915	587	478
ENSECAG00000012436	3.249000954	0.445036549	0.659346434	74	128	111	293	208	206	194	275
ENSECAG00000015112	5.204300677	0.445038532	0.659346434	336	499	634	883	905	521	856	1161
ENSECAG00000020525	1.526576811	0.445259717	0.659588123	32	33	69	82	54	66	37	51
ENSECAG00000017081	0.38831354	0.445793995	0.660226082	19	7	24.9998	17	37	22	23	34.9998
ENSECAG00000003230	0.743946185	0.445806592	0.660226082	19	24	19	29	27	27	30	70
ENSECAG00000008151	2.498280656	0.445948379	0.660306204	51	98	71	122	106	103	152	162
ENSECAG00000017868	4.570232451	0.445976924	0.660306204	263	477	455	550	437	354	354	738
ENSECAG00000020667	5.806690561	0.446196844	0.660545738	699	1025	1101	1219	1061	1043	1156	1147
ENSECAG00000022455	6.073944649	0.446509874	0.660923032	532	776	1394	1660	1504	1258	1554	1946
ENSECAG00000009442	5.998127954	0.446594488	0.66096217	767	1416	971	1493	1072	1053	1259	1667
ENSECAG00000015135	8.767117729	0.446867094	0.66127949	6104	6395	10197	9389	9304	7982	7918	8284
ENSECAG00000015415	5.791631524	0.447022624	0.661423499	753	1098	689	690	1094	1259	1207	1573
ENSECAG00000021675	5.795315601	0.447121606	0.661483813	438	730	1660	1514	721	1333	654	1554
ENSECAG00000012241	3.125300779	0.447274809	0.661624318	68	105	170	208	178	190	191	246
ENSECAG00000021472	5.308733828	0.447683975	0.662143364	414	611	528	946	924	645	986	1113
ENSECAG00000000279	4.563433869	0.447769096	0.662183062	303	306	529	604	513	471	417	436
ENSECAG00000017389	5.634122618	0.447951281	0.662321892	529	994	980	1128	973	852	949	1158
ENSECAG00000010008	4.828864774	0.447979558	0.662321892	221	841	500	544	349	563	561	720
ENSECAG00000010407	4.788298392	0.448057268	0.662350596	306	395	532	479	576	568	650	736
ENSECAG00000024240	4.232068876	0.448280606	0.662594542	191	211	400	375	486	311	400	545
ENSECAG00000020851	6.577433288	0.448392359	0.662673515	867	1040	2011	2182	2121	1900	2070	2790
ENSECAG00000000682	5.224380608	0.448602436	0.662790588	332	534	658	870	823	651	933	1042
ENSECAG00000008919	2.99445594	0.44860756	0.662790588	109	181	130.002	148	110	135	179.001	186
ENSECAG00000019159	6.283108768	0.448646578	0.662790588	729	1292	1290	1588	1625	1639	1652	2275
ENSECAG00000010489	5.963203352	0.448800164	0.662870871	831	1377	1174	1039	1181	1092	1224	1407
ENSECAG00000011998	2.550485822	0.448817604	0.662870871	38	165	75	173	76	84	117	163
ENSECAG00000014633	6.183395731	0.449346715	0.663506034	459	869	1066	2421	1495	1658	1507	2123
ENSECAG00000000619	4.977195091	0.449364454	0.663506034	411	407	501	617	836	558	712	785
ENSECAG00000005884	4.132309853	0.449479973	0.663590366	223	239	347	240	417	418	399	364
ENSECAG00000020450	2.648864698	0.44956309	0.663626846	66	67	83	171	127	123	140	192
ENSECAG00000010580	3.950390008	0.449786901	0.663870976	154	198	295	321	401	258	347	421
ENSECAG00000016105	4.264116887	0.449897909	0.663939178	239	218	551	412	467	328	313	380
ENSECAG00000021272	1.200816721	0.44994998	0.663939178	38	20	40	24	69	45	42	54
ENSECAG00000008935	4.888585235	0.450173866	0.664183283	486	717	419	423	652	462	623	561
ENSECAG00000024547	5.537491715	0.450310588	0.664224422	456	853	1148	976	1102	629	991	936
ENSECAG00000020672	7.740746294	0.450319573	0.664224422	2519	4713	3540	5005	3504	3806	4164	5165
ENSECAG00000016343	6.402392484	0.45045623	0.664224422	763	1493	1688	2490	1518	1588	1612	1905
ENSECAG00000019384	3.294742322	0.450463985	0.664224422	89	161	149	365	142	171	177	263
ENSECAG00000016190	11.29744423	0.450547873	0.664224422	29885	34515	56277	70841	55596	37874	47695	53661
ENSECAG00000018193	6.637137262	0.45055251	0.664224422	859	1475	1726	2274	2548	1865	2469	2314
ENSECAG00000022112	5.064936552	0.450670352	0.664311954	266	471	665	739	606	672	668	1171
ENSECAG00000009900	2.797126508	0.450830827	0.664353204	83	67	160	94	146	71	199	249
ENSECAG00000018250	5.86330106	0.450858713	0.664353204	722	643	1102	1137	1480	915	1423	1578
ENSECAG00000007410	6.715969477	0.450873751	0.664353204	1162	2089	893	2208	2741	1719	2929	2464
ENSECAG00000017856	3.718939374	0.45102301	0.664486961	119	89	301	324	279	237	381	331
ENSECAG00000009207	2.581884272	0.451211522	0.664626068	82	54	94	125	160	105	141	147
ENSECAG00000015552	2.07169068	0.451234421	0.664626068	50	51	80	64	91	77	92	128
ENSECAG00000024721	5.12520632	0.451356777	0.664720116	517	482	587	477	929	705	768	800
ENSECAG00000013895	4.661696985	0.451625379	0.664993942	243	300	632	763	447	445	525	558
ENSECAG00000021095	3.079621516	0.451659765	0.664993942	68	116	130	220	197	156	199	229
ENSECAG00000013673	6.37509352	0.451962054	0.665291718	658	1219	1416	2055	1759	1779	1615	2543
ENSECAG00000007315	4.337137047	0.452023876	0.665291718	167	310	452	623	353	374	360	491
ENSECAG00000012018	0.67676321	0.452094515	0.665291718	12	9	35	34	28	25	21	75
ENSECAG00000016066	2.288750757	0.452096229	0.665291718	41	85	65	197	58	82	52	173
ENSECAG00000010719	10.18106919	0.452186786	0.665338806	13410	15139	27828	32392	24398	15892	27444	21806
ENSECAG00000004595	4.334343403	0.452408588	0.665529282	212	367	461	446	331	300	541	406
ENSECAG00000023393	5.350751961	0.45243339	0.665529282	399	559	714	929	927	737	1041	1040
ENSECAG00000021326	1.579397097	0.452906271	0.665876814	43	64	67	39	32	17	70	103
ENSECAG00000021279	6.189834279	0.452908597	0.665876814	1289	853	1473	1581	1481	1279	1588	1334
ENSECAG00000006696	4.215434191	0.452913755	0.665876814	132	346	325	660	228	462	264	459
ENSECAG00000002233	5.682414308	0.452940969	0.665876814	552	592	885	1955	901	960	1090	1042
ENSECAG00000017818	3.916333733	0.452991351	0.665876814	107	312	222	298	309	234	414	454
ENSECAG00000020674	4.093474177	0.453021279	0.665876814	240	254	221	303	398	270	432	483
ENSECAG00000009087	4.396032111	0.453186493	0.665948783	194	340	312	470	356	435	351	833

ENSECAG00000007952	4.650760088	0.453232755	0.665948783	395	404	467	509	508	382	487	610
ENSECAG00000007420	1.2699101	0.453288584	0.665948783	16	20	55	48	71	96	19	31
ENSECAG00000020077	4.641923899	0.45330469	0.665948783	296	312	409	527	528	329	744	727
ENSECAG00000006859	7.360115188	0.453380622	0.665974225	3040	3136	2502	2790	3390	2800	3594	2839
ENSECAG00000005471	4.388417173	0.453627572	0.666250836	263	305	423	260	536	386	480	528
ENSECAG00000000782	9.310821386	0.453734426	0.666321642	9471	9438	7988	9996	16812	11633	14603	16724
ENSECAG00000006405	6.310752462	0.454252294	0.666995938	723	1131	1479	1725	1677	1707	1719	2200
ENSECAG00000024054	0.309030404	0.454402502	0.667105955	19	16	26	25	10	18	39	17
ENSECAG00000019938	4.442090623	0.454444648	0.667105955	210	295	391	480	423	386	524	677
ENSECAG00000007654	3.242711783	0.454562904	0.667154325	108	146	130	191	221	186	187	279
ENSECAG00000011972	5.272705559	0.454648006	0.667154325	641	394	429	841	967	700	978	935
ENSECAG00000015778	3.06440205	0.454676959	0.667154325	119	94	216	193	252	138	151	78
ENSECAG00000010187	3.993366259	0.454712471	0.667154325	154	319	177	323	273	301	363	553
ENSECAG00000009185	4.710410318	0.454946834	0.667359641	282	544	500	583	463	438	469	704
ENSECAG00000007175	3.710678364	0.45496988	0.667359641	164	128	266	237	349	213	350	297
ENSECAG00000023798	5.595804727	0.455073136	0.667424934	677	999	644	1147	1013	800	1087	891
ENSECAG00000015020	3.889751737	0.455220196	0.667554448	135	155	271	386	400	235	369	367
ENSECAG00000002746	4.481179444	0.455509901	0.6678096	316	432	367	472	439	433	402	476
ENSECAG00000009738	11.52294734	0.455511741	0.6678096	32178	41350	75122	75398	61697	43543	61814	60609
ENSECAG00000007467	3.839075697	0.455615687	0.667811126	259	269	253	213	240	395	208	229
ENSECAG00000019114	6.503712199	0.455630333	0.667811126	1083	1000	1414	2204	2082	1909	1935	2451
ENSECAG00000011351	1.126771814	0.455805535	0.667981747	12	92	28	28	22	32	24	76
ENSECAG00000023057	6.468789625	0.455961109	0.668055702	930	1187	1312	2192	2057	1829	2136	2114
ENSECAG00000000881	0.698956393	0.455973594	0.668055702	28	21	22	9	46	39	35	26
ENSECAG00000005528	7.110507476	0.45615265	0.668231874	1708	1680	3538	3432	2957	2167	2511	3148
ENSECAG00000005351	3.743593458	0.456237918	0.668270623	56	89	353	364	272	425	376	167
ENSECAG00000007500	9.700392967	0.456465706	0.668449052	10196	11418	19782	21266	17986	12539	16585	17385
ENSECAG00000014345	4.964908046	0.456477398	0.668449052	249	595	758	749	655	595	657	515
ENSECAG00000023451	4.935040335	0.457050545	0.669202099	376	371	661	868	638	570	573	615
ENSECAG00000027675	11.52677625	0.457245177	0.669400812	44036	27333	58963	41455	59653	74157	57280	86762
ENSECAG00000000365	2.608533553	0.457305189	0.669402416	52	61	126	140	159	109	132	164
ENSECAG00000000352	7.419274512	0.45746203	0.669458042	1322	2404	3527	3569	3198	3437	3530	5988
ENSECAG00000012770	5.035242819	0.457470618	0.669458042	319	440	569	766	792	683	624	905
ENSECAG00000017593	5.042757778	0.457543401	0.669458042	309	492	581	717	742	646	734	897
ENSECAG00000000893	1.826485786	0.457626952	0.669458042	29	70	39	145	25	112	37	73
ENSECAG00000021445	1.556661697	0.457729141	0.669458042	27	83	43	61	63	31	40	85
ENSECAG00000015444	4.045444494	0.457734914	0.669458042	138	201	345	368	333	316	472	396
ENSECAG00000017136	4.351374121	0.457755636	0.669458042	219	388	351	553	402	279	404	532
ENSECAG00000012726	4.60692598	0.457907688	0.669543185	217	459	370	475	471	437	705	632
ENSECAG00000002434	3.111146686	0.45793171	0.669543185	54	162	155	310	149	176	137	191
ENSECAG00000011011	2.610747403	0.458002599	0.669557761	33	107	118	232	74	109	111	163
ENSECAG00000021517	3.880231128	0.458093297	0.669557761	166	201	247	298	342	262	385	360
ENSECAG00000012010	4.190223043	0.458118468	0.669557761	212	428	135	283	503	348	272	602
ENSECAG00000007927	4.743118524	0.458375383	0.669783502	257	374	489	584	605	561	567	713
ENSECAG00000019888	4.359666862	0.458390821	0.669783502	205	312	360	408	443	342	480	633
ENSECAG00000010502	7.083954202	0.45854701	0.669857017	1215	2703	3217	3086	2271	2871	2244	3113
ENSECAG00000013283	5.300337275	0.458559046	0.669857017	250	351	907	1065	814	675	1422	759
ENSECAG00000017699	0.355132903	0.4589111	0.670285116	24	15	13	9	26	39	16	33
ENSECAG00000020395	0.760394165	0.459041244	0.670330052	7	51	26	43	28	35	32	20
ENSECAG00000012200	1.175330462	0.459059861	0.670330052	11	31	16	77	47	89	29	38
ENSECAG00000016657	3.647816651	0.459202014	0.670382711	138	99	260	284	323	291	265	269
ENSECAG00000024495	3.086327705	0.459213927	0.670382711	96	86	164	169	276	114	222	176
ENSECAG00000025059	6.539648909	0.459311519	0.670439039	973	1345	1923	2812	1828	1592	1868	2015
ENSECAG00000015120	2.239157706	0.459848944	0.670701199	49	39	80	120	127	66	127	118
ENSECAG00000014603	2.834432675	0.459877494	0.670701199	35	91	142	186	133	201	126	199
ENSECAG00000023620	3.928912743	0.459893089	0.670701199	155	217	256	327	315	326	377	370
ENSECAG00000014071	3.591195898	0.459949797	0.670701199	171	177	170	188	291	156	292	392
ENSECAG00000015765	0.89641947	0.4600051	0.670701199	19	37	26	16	58	13	50	52
ENSECAG00000011959	2.100832364	0.460038547	0.670701199	23	73	65	187	45	83	86	99
ENSECAG00000018773	10.02520369	0.460067786	0.670701199	13437	13023	25442	26443	23894	15698	19816	21245
ENSECAG00000009398	2.705055791	0.460078068	0.670701199	93	45	69	180	188	92	119	221
ENSECAG00000014585	6.663533339	0.460119379	0.670701199	2426	735	576	1526	1756	3077	3054	1678
ENSECAG00000017787	3.920288027	0.460126301	0.670701199	189	425	182	288	395	256	274	228
ENSECAG00000009660	2.57994541	0.460206888	0.670701199	36	53	82	342	125	116	121	62
ENSECAG00000008537	6.472208369	0.46022783	0.670701199	1010	1872	1392	2258	1435	1639	1659	2259
ENSECAG00000016758	9.572407235	0.460258515	0.670701199	9699	10182	18776	18346	16958	12737	14596	14425
ENSECAG00000020206	4.203205539	0.460520322	0.670968151	156	243	359	419	360	449	425	443
ENSECAG00000011336	3.855732108	0.460559814	0.670968151	203	164	254	248	387	289	331	317
ENSECAG00000009021	3.244473208	0.460833976	0.671281491	79	169	260	208	132	176	131	291
ENSECAG00000017193	5.547678897	0.461084321	0.671560063	439	586	799	1198	1100	897	1148	1137
ENSECAG00000001385	7.371598202	0.461378552	0.671902474	2465	2425	3218	4071	4631	2448	3359	2279
ENSECAG00000006101	3.585733295	0.461607293	0.672008117	165	116	187	262	273	221	320	291
ENSECAG00000020999	5.20354378	0.461608784	0.672008117	499	1005	487	610	589	641	566	1096
ENSECAG00000007904	1.894157899	0.46162853	0.672008117	52	55	84	75	68	63	57	92
ENSECAG00000015889	6.01451702	0.462089563	0.672593085	509	964	1987	1474	1390	1177	1350	1080
ENSECAG00000012905	8.206034648	0.46218289	0.672642756	5374	6677	4310	4110	6174	4671	5766	6109
ENSECAG00000010973	7.172647915	0.462398922	0.672825535	2072	969	3150	2074	6498	973	2979	3630
ENSECAG00000018617	7.166048106	0.462426914	0.672825535	1735	2064	2984	3982	2787	2499	2527	3432
ENSECAG00000016355	2.515765113	0.462591889	0.672959843	59	75	102	111	113	124	140	145
ENSECAG00000011126	5.753658039	0.46274169	0.672959843	534	564	1532	645	1196	612	1345	2021
ENSECAG00000021422	6.895331489	0.462785703	0.672959843	1851	2910	1481	2156	2335	1990	2088	2850
ENSECAG00000006476	7.000067906	0.462864477	0.672959843	1842	3093	1841	2333	2380	1889	2346	3423
ENSECAG00000010752	0.733714609	0.462872351	0.672959843	11	16	31	38	24	32	26	70
ENSECAG00000000416	2.140043594	0.462874597	0.672959843	25	62	65	209	72	93	69	86
ENSECAG00000011547	10.81005702	0.462969848	0.673012208	21171	23257	46979	44292	39234	27519	37562	34397

ENSECAG00000024128	1.273565306	0.463082661	0.673090085	18	34	44	45	27	51	49	96
ENSECAG00000018839	6.662037192	0.463229393	0.673215106	1041	2371	1801	2224	1944	1771	1979	2237
ENSECAG00000000631	7.545633778	0.463287178	0.673215106	2021	2010	3908	3641	4094	4572	3264	5462
ENSECAG00000001336	1.026576154	0.463442132	0.673326029	16	14	35	55	44	57	59	21
ENSECAG00000017665	4.849050947	0.463482034	0.673326029	453	318	704	616	859	422	556	380
ENSECAG00000021790	6.766487392	0.463612073	0.673428839	2875	1790	1167	1385	2338	1404	2195	2401
ENSECAG00000011451	7.960991549	0.463676501	0.673436329	2937	3112	4254	5036	5394	4911	5693	7269
ENSECAG00000022394	3.027161262	0.464477138	0.674512939	46	114	121	241	156	192	258	142
ENSECAG00000021974	2.657916306	0.464600559	0.674533415	50	69	68	206	128	93	81	310
ENSECAG00000011239	6.023014815	0.464609972	0.674533415	552	515	1898	2214	1383	1042	1489	1065
ENSECAG00000016020	3.148453442	0.464766151	0.67467395	94	79	290	213	209	155	167	135
ENSECAG00000020739	4.642347598	0.464858974	0.674698337	209	541	519	590	437	521	469	518
ENSECAG00000010652	0.572570307	0.464945759	0.674698337	7	11	26	44	28	27	36	42
ENSECAG00000008210	8.833904955	0.464961096	0.674698337	4671	9830	5997	6637	12030	9897	9072	11713
ENSECAG00000011376	6.889823486	0.465198096	0.674903609	1735	2413	2017	2283	2256	2247	2141	2610
ENSECAG00000004246	3.812284592	0.465221358	0.674903609	165	182	259	256	290	266	388	342
ENSECAG00000021833	4.91836984	0.465421017	0.675107059	325	486	603	853	601	314	598	902
ENSECAG00000017390	4.101633061	0.465493681	0.67512627	210	254	474	306	361	288	297	398
ENSECAG00000007129	2.559721564	0.465648316	0.675157264	76	66	173	127	92	187	56	89
ENSECAG00000013060	5.352074303	0.465663687	0.675157264	492	467	446	1162	875	848	1322	700
ENSECAG00000014118	3.214287845	0.465693318	0.675157264	65	173	116	222	131	187	193	365
ENSECAG00000019370	7.151544027	0.465897867	0.67536764	1893	2476	2822	3206	2209	2709	2222	4013
ENSECAG00000009659	5.578455359	0.466309345	0.67587789	562	710	770	935	950	1097	1051	1251
ENSECAG00000025080	10.87974091	0.466885532	0.676563368	21349	24973	47202	49606	41015	27659	38284	39301
ENSECAG00000016757	4.433849643	0.466934083	0.676563368	124	629	163	387	540	492	564	418
ENSECAG00000019095	4.809494279	0.467038687	0.676563368	110	607	609	930	276	454	704	711
ENSECAG00000021393	5.833069013	0.467146367	0.676563368	633	562	955	1424	1938	618	1704	1068
ENSECAG00000010295	5.020747197	0.467163649	0.676563368	331	627	528	928	573	575	621	799
ENSECAG00000007103	5.838436178	0.46718578	0.676563368	592	979	930	1063	1289	1008	1671	1273
ENSECAG00000012180	3.916701548	0.467253947	0.676563368	101	203	281	392	257	380	336	413
ENSECAG00000011487	6.646053902	0.467258647	0.676563368	1182	2174	1932	1954	1800	1880	1895	2281
ENSECAG00000023789	5.993948028	0.467593898	0.67694687	647	948	1298	1922	1101	1310	1197	1392
ENSECAG00000008032	0.871531909	0.467642667	0.67694687	23	16	34	29	53	28	39	44
ENSECAG00000023105	1.535967502	0.467794067	0.67707977	28	26	50	70	74	77	42	68
ENSECAG00000025119	7.516669114	0.468108533	0.677448625	1922	2697	2923	3860	3826	3822	3900	5496
ENSECAG00000013529	6.162307854	0.468363526	0.677731327	968	571	1831	913	1631	1328	1722	2020
ENSECAG00000010818	5.884349487	0.468561957	0.677932123	1549	678	943	915	1688	683	1691	243
ENSECAG00000013916	5.946341335	0.468741177	0.678037236	795	918	894	1184	1438	1115	1415	1704
ENSECAG00000012210	8.301213109	0.468753959	0.678037236	3834	6586	5820	6883	5402	6134	5664	7316
ENSECAG00000018873	3.14466889	0.468851034	0.678091324	96	83	178	190	233	157	199	226
ENSECAG00000017352	1.385459609	0.468942743	0.678137641	21	20	62	52	43	56	92	45
ENSECAG00000020298	8.700386131	0.469097347	0.678274886	6101	9198	6660	7808	8048	6572	7993	9775
ENSECAG00000015144	3.488571492	0.470148697	0.679695472	185	127	215	86	343	367	123	204
ENSECAG00000005253	2.199598644	0.470199473	0.679695472	25	82	69	107	71	84	122	151
ENSECAG00000005853	3.630023508	0.470324817	0.679719194	118	274	203	325	189	217	209	353
ENSECAG00000020995	1.60294354	0.470398447	0.679719194	23	69	80	51	49	64	43	66
ENSECAG00000007236	5.362474869	0.470411016	0.679719194	327	903	735	1145	617	947	728	905
ENSECAG00000005608	8.271886821	0.470455179	0.679719194	4061	4707	4429	5302	7347	5879	6586	9121
ENSECAG00000006646	0.149329536	0.470596546	0.679836994	18	14	32	11	25	8	9	34
ENSECAG00000016297	5.053037393	0.470725554	0.679936911	310	403	576	847	957	767	560	740
ENSECAG00000013915	10.4787036	0.470957767	0.679950589	15488	21432	31288	40359	29034	21915	28591	31633
ENSECAG00000010391	5.167546479	0.470959379	0.679950589	265	414	638	1049	741	915	700	934
ENSECAG00000010873	3.258667441	0.470962479	0.679950589	115	204	180	188	199	178	174	187
ENSECAG00000013845	1.881719947	0.4709744	0.679950589	64	33	51	56	79	64	83	116
ENSECAG00000019180	7.373864433	0.471155208	0.680125203	3925	3073	2262	1838	3449	2495	3229	3536
ENSECAG00000021934	6.0413786	0.471278583	0.68021192	493	781	1342	1686	1337	1301	1472	1995
ENSECAG00000011681	5.914283134	0.471335016	0.68021192	656	921	996	1212	1413	1053	1518	1546
ENSECAG00000005781	5.151421123	0.471498253	0.680271283	340	537	588	774	639	1158	692	712
ENSECAG00000020016	1.90588988	0.471557443	0.680271283	41	86	84	60	55	100	37	81
ENSECAG00000015608	5.638347103	0.471604453	0.680271283	671	512	995	1555	1144	729	1190	828
ENSECAG00000003502	3.003892824	0.471615639	0.680271283	67	153	122	146	180	117	173	282
ENSECAG00000014680	2.009123739	0.471935411	0.680646121	49	31	83	73	126	60	109	75
ENSECAG00000005690	2.077883487	0.472149372	0.680648218	44	50	54	109	79	98	84	125
ENSECAG00000003122	4.930465749	0.472159124	0.680648218	392	629	382	396	710	625	631	846
ENSECAG00000013136	8.515902728	0.472176414	0.680648218	5153	4112	6653	5873	9784	6462	8669	9350
ENSECAG00000010807	2.99011614	0.472176488	0.680648218	40	278	133	153	102	126	93	279
ENSECAG00000013604	7.094504648	0.472383488	0.68086023	2180	2900	1857	2758	2796	2592	2950	2180
ENSECAG00000007778	4.780937145	0.47254474	0.681006258	165	610	404	563	619	518	710	691
ENSECAG00000010461	3.723986416	0.472769956	0.681244419	168	289	243	239	259	197	276	304
ENSECAG00000021331	0.054775096	0.472927284	0.681384708	8	14	27	25	15	19	9	27
ENSECAG00000020192	3.869791618	0.473115079	0.681568852	264	214	189	368	304	250	322	254
ENSECAG00000016598	0.230871652	0.473513396	0.682008093	13	16	19	14	26	24	30	21
ENSECAG00000024079	10.85914374	0.473542437	0.682008093	20441	23601	47511	50156	37918	28677	40805	36472
ENSECAG00000013128	5.092292904	0.473600058	0.682008093	377	599	628	944	426	946	484	771
ENSECAG00000022472	5.302832048	0.473723207	0.682098983	387	570	1010	985	723	837	775	748
ENSECAG00000015197	3.390930247	0.473877989	0.682235392	83	217	187	310	179	228	201	192
ENSECAG00000026954	5.789883622	0.474034871	0.682374788	569	796	1250	1532	1277	897	1161	1023
ENSECAG00000001898	4.896462564	0.474141912	0.682442412	437	719	582	324	643	531	575	560
ENSECAG00000021706	5.146660903	0.47439257	0.682716705	368	607	729	913	688	704	628	775
ENSECAG00000019532	5.004626726	0.474475076	0.682748964	337	707	652	622	580	593	673	684
ENSECAG00000005682	4.314726341	0.474657939	0.682811734	63	442	236	928	297	291	272	653
ENSECAG00000006626	4.954006294	0.474687219	0.682811734	291	461	399	845	667	617	671	893
ENSECAG00000023719	5.680358379	0.474698986	0.682811734	503	795	894	1067	1192	1085	1243	1130
ENSECAG00000008638	7.173839579	0.474869408	0.682970407	1648	1435	3117	2759	3099	3137	3183	3983
ENSECAG00000019343	1.726656872	0.475067915	0.683169428	28	74	110	44	18	150	12	33

ENSECAG00000020604	1.620708617	0.475468425	0.683658851	24	65	44	100	44	54	46	84
ENSECAG00000019146	3.067785754	0.475571959	0.683721194	62	114	135	224	160	173	208	230
ENSECAG00000024130	6.343690959	0.475885453	0.683956073	986	914	2106	2039	1793	1384	1458	1749
ENSECAG00000017775	2.806313649	0.475905491	0.683956073	65	63	123	189	129	137	152	232
ENSECAG00000008889	2.179500307	0.475990934	0.683956073	38	47	108	82	115	67	135	100
ENSECAG00000000042	8.315467248	0.476012971	0.683956073	3453	6790	5883	7537	5840	5432	5056	8636
ENSECAG00000016744	5.186686648	0.476036316	0.683956073	349	519	674	771	705	578	947	1142
ENSECAG00000021690	4.391206994	0.476421832	0.684365054	204	373	358	642	352	352	493	452
ENSECAG00000023523	7.852344235	0.476472593	0.684365054	3474	5184	3810	3890	5137	3482	4692	4671
ENSECAG00000021781	7.2153572	0.476501667	0.684365054	1848	2470	2745	3935	2776	2300	3226	3391
ENSECAG00000022404	0.878091375	0.476636412	0.684472056	15	20	64	37	34	18	27	54
ENSECAG00000000076	0.617650907	0.476736103	0.6845287	14	19	26	57	44	30	17	13
ENSECAG00000013141	5.31200656	0.477169541	0.685064483	316	600	532	1146	875	792	908	1077
ENSECAG00000015928	3.918678402	0.477300571	0.685166024	129	250	293	483	235	270	336	338
ENSECAG00000023720	0.761074038	0.477884743	0.685917944	20	18	38	15	45	48	25	32
ENSECAG00000020914	4.881917187	0.478006388	0.686005884	248	518	643	812	523	509	630	666
ENSECAG00000019857	5.200688084	0.478207412	0.686097952	318	548	883	1041	670	741	704	768
ENSECAG00000013908	6.085797998	0.4783105	0.686097952	777	1236	1548	1369	1476	1241	1411	1204
ENSECAG00000014273	5.396640721	0.478336305	0.686097952	177	527	981	1081	622	1269	804	1211
ENSECAG00000000140	7.072422345	0.47833885	0.686097952	2718	2198	2320	1995	2779	2273	2391	3029
ENSECAG00000024095	10.25744638	0.478372467	0.686097952	13756	17133	30963	30504	26883	18760	25206	24255
ENSECAG00000004464	0.868966003	0.478469578	0.686118198	25.0001	19	40.0001	47.0006	43	26.0001	20.0001	43.0001
ENSECAG00000009712	2.386692724	0.478554319	0.686118198	43	94	122	135	76	124	79	113
ENSECAG00000008644	3.960969513	0.478567745	0.686118198	131	182	366	309	307	383	317	416
ENSECAG00000014324	3.204854424	0.478633861	0.686126411	71	149	137	225	212	141	222	281
ENSECAG00000010468	4.360499339	0.478775709	0.68618224	268	328	464	412	439	373	393	410
ENSECAG00000007554	3.41930257	0.478793592	0.68618224	118	121	189	235	208	217	187	381
ENSECAG00000014670	4.825913095	0.478951479	0.686321946	527	640	346	416	654	438	586	527
ENSECAG00000021258	2.850092887	0.479091139	0.686435501	48	86	155	168	164	160	195	138
ENSECAG00000015520	5.094987406	0.479179942	0.68647617	322	674	875	637	504	686	592	910
ENSECAG00000022488	5.526370323	0.479538586	0.686903355	629	788	1091	766	793	962	698	1183
ENSECAG00000006462	5.785110685	0.479958228	0.687292238	814	803	1370	921	1274	846	1171	1056
ENSECAG00000006592	4.081184896	0.479968518	0.687292238	118	331	277	345	400	295	442	419
ENSECAG00000015145	3.46610603	0.479991543	0.687292238	183	192	99	146	325	235	215	245
ENSECAG00000014950	2.697888873	0.480089112	0.687293331	43	135	107	217	113	120	136	120
ENSECAG00000002606	3.562328538	0.480116596	0.687293331	142	177	131	274	325	219	304	231
ENSECAG00000000584	5.532748209	0.480173778	0.687293331	569	536	901	876	1278	808	1091	1055
ENSECAG00000024053	2.281818418	0.480312215	0.687324804	95	52	123	69	91	56	70	154
ENSECAG00000017723	3.598421531	0.480324818	0.687324804	138	99	254	264	281	249	298	281
ENSECAG00000015589	4.913075006	0.480377246	0.687324804	337	443	642	430	601	609	737	811
ENSECAG00000015682	4.68014339	0.480598449	0.687484104	218	367	418	650	488	533	496	849
ENSECAG00000006525	4.421198606	0.480718187	0.687484104	209	513	351	495	394	288	490	523
ENSECAG00000013411	5.854598396	0.480768416	0.687484104	384	805	1188	1401	1070	1366	1161	1734
ENSECAG00000004031	4.902978772	0.480773084	0.687484104	619	636.007	580	181	737	358	723.003	451
ENSECAG00000016977	5.274654677	0.480791119	0.687484104	554	739	434	534	1037	648	1132	747
ENSECAG00000018599	6.746065443	0.480863326	0.687500832	1525	1581	1521	1792	2719	1818	2916	2470
ENSECAG00000018251	5.199644623	0.481529224	0.688306745	407	608	616	649	668	758	841	1101
ENSECAG00000021058	1.626247939	0.48154817	0.688306745	33	43	51	54	89	49	59	84
ENSECAG00000012680	5.437683627	0.481779714	0.688551083	477	344	1009	903	1069	906	1184	802
ENSECAG00000022641	4.367108362	0.482092457	0.688911394	219	341	311	688	344	285	424	584
ENSECAG00000019865	4.087335667	0.482235721	0.688983295	231	162	325	322	422	341	387	403
ENSECAG00000011761	6.43323755	0.482264051	0.688983295	742	766	1978	2119	2035	1705	2018	2231
ENSECAG00000023666	2.777279403	0.48245321	0.689109809	94	67	106	144	147	116	167	204
ENSECAG00000006431	4.111412285	0.48250092	0.689109809	126	409	317	453	238	284	321	515
ENSECAG00000011019	1.969712123	0.48264121	0.689109809	26	30	86	102	91	82	54	137
ENSECAG00000016358	6.776297703	0.482653001	0.689109809	1693	1632	2197	2324	2151	2242	2143	1997
ENSECAG00000015911	5.157343703	0.482655858	0.689109809	422	650	490	627	719	737	850	954
ENSECAG00000000870	2.439467107	0.482835905	0.689280256	69	65	102	83	137	78	145	140
ENSECAG00000024299	4.345913207	0.483044494	0.6894914	208	236	402	447	421	416	468	552
ENSECAG00000016203	3.047450836	0.48339211	0.689882197	85	91	127	214	179	139	262	179
ENSECAG00000024491	4.063951116	0.483468021	0.689882197	151	402	292	385	338	296	261	417
ENSECAG00000008677	0.574992303	0.483500435	0.689882197	7	27	36	42	44	12	29	18
ENSECAG00000009133	2.273370187	0.483647911	0.690005972	38	111	93	152	12	235	17	46
ENSECAG00000018343	8.260949036	0.483740432	0.690025781	4793	6896	4791	5229	4695	7130	4698	7061
ENSECAG00000020469	4.96036066	0.483793857	0.690025781	309	610	588	782	590	564	591	725
ENSECAG00000015578	4.193753367	0.483843988	0.690025781	216	185	351	395	444	333	455	441
ENSECAG00000020917	7.041280641	0.483922284	0.690050828	2249	2923	1816	2171	2862	1932	2724	2762
ENSECAG00000020787	1.13803158	0.484539714	0.690844551	22	24	49	72	44	13	35	70
ENSECAG00000024456	5.561961248	0.48494366	0.691333735	530	774	903	1268	1030	833	916	968
ENSECAG00000000536	5.905832809	0.485079003	0.691439923	610	1183	777	1150	1345	1013	1269	1937
ENSECAG00000015289	5.943718772	0.485224663	0.691516493	723	866	1325	1661	1054	1304	807	1694
ENSECAG00000006043	5.679217553	0.485295553	0.691516493	485	451	866	1558	1092	1137	1267	1192
ENSECAG00000017977	3.866398222	0.48533874	0.691516493	159.542	112.631	228.968	430.401	356.516	308.126	278.36	401.495
ENSECAG00000013864	6.051117537	0.485376169	0.691516493	1768	946	813	821	1464	982	1294	1375
ENSECAG00000021249	6.279788222	0.485499132	0.691604958	1278	1616	953	1589	1651	1345	1431	1681
ENSECAG00000019032	6.746189604	0.48580787	0.691958007	1616	1446	1976	2755	2264	1738	2132	2329
ENSECAG00000017606	1.80082089	0.485918996	0.692029535	41	36	36	95	118	60	70	70
ENSECAG00000000854	0.391239019	0.486164423	0.692250159	6	16	18	36	26	33	17	39
ENSECAG00000010954	5.816155139	0.486230315	0.692250159	515	902	1005	1172	1261	1160	1476	1218
ENSECAG00000000910	6.459006153	0.486315823	0.692250159	955	1271	1474	1814	2557	1298	2142	2121
ENSECAG00000000792	6.171410926	0.486339093	0.692250159	752	1355	1764	1390	1342	1488	1159	1685
ENSECAG00000007896	5.603518333	0.486412363	0.692250159	475	505	894	1281	995	952	1041	1491
ENSECAG00000014042	1.944813755	0.486439471	0.692250159	67	24	131	54	81	73	52	75
ENSECAG00000012486	6.45213957	0.486759254	0.692533387	936	1450	2112	1965	1862	1122	1745	2255
ENSECAG00000009032	3.932672084	0.486760396	0.692533387	105	320	188	343	227	236	439	535

ENSECAG00000020276	6.326417282	0.486912098	0.692602052	1308	1418	1470	1397	1701	1335	1663	1635
ENSECAG00000020154	4.434745828	0.486930575	0.692602052	267	325	403	323	500	450	453	568
ENSECAG00000006124	4.685773706	0.487097453	0.692752692	291	236	443	672	666	412	615	680
ENSECAG00000006455	5.100577947	0.487180243	0.69278372	220	452	723	848	1067	524	687	902
ENSECAG00000010780	5.326911151	0.487370036	0.692949926	423	903	684	907	883	564	897	840
ENSECAG00000022066	5.821376056	0.487452741	0.692949926	1136	985	728	1011	1366	869	890	1329
ENSECAG00000021612	7.276767588	0.487556132	0.692949926	2492	2625	2396	3568	3240	2543	2819	3588
ENSECAG00000021592	5.105223269	0.487642693	0.692949926	372	602	612	961	592	602	579	973
ENSECAG00000007078	3.466712517	0.48765135	0.692949926	192	155	117	166	298	191	263	269
ENSECAG00000014389	2.860055507	0.487663053	0.692949926	89	58	135	163	166	153	168	177
ENSECAG00000024105	7.846398553	0.487801903	0.693060551	1170	2822	2728	8606	5455	6347	5424	4154
ENSECAG00000016322	0.765482973	0.488052096	0.693329321	29	43	7	42	34	24	3	57
ENSECAG00000003736	4.095255497	0.488441947	0.6937964	168	178	507	448	376	256	422	265
ENSECAG00000014028	3.578429393	0.488750488	0.694057792	137	203	138	254	274	202	297	326
ENSECAG00000024063	6.865380419	0.488782327	0.694057792	1451	1190	2172	2424	2688	2351	3167	2513
ENSECAG00000012658	3.903419194	0.488870485	0.694057792	88	294	242	555	237	257	326	336
ENSECAG00000002460	3.513662876	0.488893516	0.694057792	367	74	181	121	291	139	213	209
ENSECAG00000010792	7.202216252	0.4889314	0.694057792	1875	2938	2498	3392	2657	2991	2625	3241
ENSECAG00000013942	5.429802254	0.489057625	0.694150247	788	599	453	615	1226	652	1213	904
ENSECAG00000022445	3.503720951	0.489446323	0.694615178	132	134	278	301	275	182	233	186
ENSECAG00000016276	6.632950364	0.489569913	0.694703803	1189	2427	1687	1804	2239	1455	1739	2412
ENSECAG00000013399	2.03834106	0.489689448	0.694786652	36	77	103	86	85	60	72	95
ENSECAG00000008585	8.638146324	0.489807276	0.694867058	4457	4840	10504	10539	7112	6234	6001	11940
ENSECAG00000018405	6.946897641	0.489960005	0.694996951	1077	1661	2500	2630	2592	2583	2765	3461
ENSECAG00000008435	4.976232492	0.490206091	0.695186675	181	458	426	1021	529	930	640	780
ENSECAG00000024190	3.993964791	0.490216127	0.695186675	191	271	247	260	386	292	356	423
ENSECAG00000020072	8.281743856	0.490527131	0.695330721	3125	6500	5637	8017	5799	5953	5796	6693
ENSECAG00000010942	8.007443902	0.490532612	0.695330721	5809	4612	3454	3492	5387	4206	5477	4679
ENSECAG00000024681	7.839266556	0.490544617	0.695330721	2957	4669	4412	4478	4417	4184	4615	4670
ENSECAG00000021948	2.889943099	0.490575255	0.695330721	49	124	86	210	142	108	225	217
ENSECAG00000016864	5.917825587	0.490651625	0.695330721	467	613	1011	1928	1340	1384	1347	1470
ENSECAG00000005335	1.360846801	0.49073397	0.695330721	18	52	39	41	61	45	55	72
ENSECAG00000013119	4.183884491	0.490746088	0.695330721	186	207	435	550	387	295	310	438
ENSECAG00000022321	5.122351007	0.490890439	0.695378194	491	351	514	805	838	608	760	1002
ENSECAG00000014139	4.180624082	0.490901997	0.695378194	183	208	328	436	432	264	514	459
ENSECAG00000015910	7.169370873	0.491500102	0.695985322	2476	2015	2979	2695	2985	2700	2540	3012
ENSECAG00000017095	5.649300455	0.491501751	0.695985322	494	750	922	1033	1251	969	999	1359
ENSECAG00000021426	3.604360543	0.491584435	0.695985322	137	153	166	308	293	218	303	298
ENSECAG00000016841	6.833261663	0.491593683	0.695985322	1182	2586	2200	2388	1776	2362	1900	2901
ENSECAG00000004043	5.156168273	0.491636877	0.695985322	452	397	821	963	752	655	690	711
ENSECAG00000018064	5.12164774	0.491771978	0.696089849	336	698	566	556	803	733	674	974
ENSECAG00000013577	5.782169488	0.492039419	0.69637546	704	787	996	896	1372	1066	1256	1315
ENSECAG00000018100	2.324136087	0.492096336	0.69637546	80	0	0	119	293	0	0	311
ENSECAG00000007272	7.275736933	0.492158118	0.696376157	1131	2037	4152	4877	2479	3171	2987	3331
ENSECAG00000024136	3.860826503	0.4922974	0.696481179	110	156	291	390	318	274	360	382
ENSECAG00000017063	3.384128172	0.492384391	0.696481179	122	141	156	227	227	214	221	291
ENSECAG00000024758	5.00773191	0.49244082	0.696481179	591	336	407	539	842	579	743	801
ENSECAG00000019283	5.632116119	0.492499016	0.696481179	511	1026	1054	1001	847	959	1012	1113
ENSECAG00000012756	0.766140863	0.492538838	0.696481179	25	73	13	3	23	27	26	40
ENSECAG00000021168	0.478185649	0.492635169	0.69653071	15	19	39	25	23	10	26	41
ENSECAG00000017743	5.012884965	0.492755669	0.696614397	288	350	730	708	650	533	987	799
ENSECAG00000004727	2.472988491	0.493176236	0.697072597	84	21	145	49	267	52	158	54
ENSECAG00000015548	5.919467352	0.493202482	0.697072597	438	831	1294	1380	1187	1448	1400	1478
ENSECAG00000008984	2.195429461	0.493335857	0.697174378	24	82	77	101	72	97	100	154
ENSECAG00000006066	4.699324374	0.493593816	0.69736751	211	372	538	547	677	396	614	704
ENSECAG00000019077	7.550226704	0.493595275	0.69736751	2696	3778	2228	1997	4485	3271	4286	5610
ENSECAG00000016589	5.284793105	0.493677179	0.697396507	266	1052	651	952	657	828	730	816
ENSECAG00000018240	6.71641009	0.494034953	0.697815159	1763	1597	1785	2291	1973	1783	1992	2575
ENSECAG00000004620	8.204629436	0.494343161	0.698163704	3329	7246	4447	6550	5652	5284	6135	5863
ENSECAG00000017004	2.698264714	0.494587551	0.698390012	63	72	100	171	198	110	118	171
ENSECAG00000021564	5.341373811	0.494626336	0.698390012	490	483	741	836	991	829	914	944
ENSECAG00000021835	6.706045679	0.494814426	0.698568776	1231	1901	2103	2373	2082	1682	2136	2380
ENSECAG00000001462	4.339475039	0.494891076	0.698590186	139	351	436	358	393	395	596	468
ENSECAG00000008893	1.452892467	0.495013469	0.698596303	23	24	66	99	41	55	56	46
ENSECAG00000023659	4.033962758	0.49501838	0.698596303	190	242	363	404	358	274	299	362
ENSECAG00000021629	9.048638424	0.495246366	0.698706108	6233	7010	13552	12874	11730	8517	10454	10560
ENSECAG00000010265	6.22844725	0.495261428	0.698706108	901	1229	1131	1366	1664	1695	1887	1549
ENSECAG00000017807	0.43752455	0.495280672	0.698706108	6	14	36	21	43	17	26	34
ENSECAG00000012758	3.544167655	0.495527681	0.698967785	90	125	250	287	251	207	278	340
ENSECAG00000007234	4.936498978	0.49610283	0.699627697	324	253	1114	648	878	463	659	319
ENSECAG00000026850	8.480030299	0.496118672	0.699627697	3002	4925	9084	10742	7377	5927	7087	7363
ENSECAG00000024996	5.309181065	0.496184714	0.699633994	239	418	1039	864	581	1240	1171	613
ENSECAG00000017228	8.382814878	0.496525194	0.699948427	5748	5042	5755	7219	7879	5610	6080	6413
ENSECAG00000010486	1.564588606	0.49653092	0.699948427	18	37	33	96	50	60	101	57
ENSECAG00000018221	3.310358935	0.496704054	0.700105628	113	378	137	76	211	280	130	87
ENSECAG00000016050	5.619599759	0.496773513	0.700116677	642	857	923	1094	939	964	870	1141
ENSECAG00000008403	10.51298229	0.497149837	0.700554838	11973	22059	34492	45300	29257	23486	32135	28519
ENSECAG00000005234	2.228976305	0.497207729	0.700554838	53	60	123	109	118	58	83	99
ENSECAG00000007770	5.051327695	0.497294723	0.700590533	592	453	427	879	751	539	714	598
ENSECAG00000016816	5.151584857	0.497397388	0.700648293	451	436	646	680	875	753	710	889
ENSECAG00000006883	5.539733464	0.497599694	0.700846377	412	637	728	1254	1143	749	1227	1145
ENSECAG00000008568	1.60303206	0.497780288	0.701013837	33	38	57	51	67	56	73	78
ENSECAG00000022003	7.754315367	0.497903418	0.70102452	2894	3311	2474	4418	5020	4241	5142	5598
ENSECAG00000003319	3.64720369	0.497911273	0.70102452	131	180	192	277	334	156	262	416
ENSECAG00000016673	10.39130391	0.498178016	0.701172093	14889	18430	20673	29573	35143	21746	35209	33004

ENSECAG00000007061	5.879882815	0.498195166	0.701172093	389	973	706	1791	1152	1570	1623	1008
ENSECAG00000019837	7.170201549	0.498201224	0.701172093	2946	1154	3227	2902	3785	2255	2841	2146
ENSECAG00000013950	7.985643253	0.498433202	0.701411696	3113	3181	4299	5036	5103	6059	5440	6750
ENSECAG00000017179	6.360601364	0.498662793	0.701647883	930	1519	1581	1958	1726	1421	1606	1776
ENSECAG00000011881	2.846669463	0.498751522	0.701680494	90	143	151	128	163	107	142	144
ENSECAG00000013849	2.849607659	0.498809484	0.701680494	78	71	105	198	174	132	167	188
ENSECAG00000000498	1.837427661	0.498878323	0.701680787	32	29	52	110	81	64	99	81
ENSECAG00000011599	6.474015242	0.498933205	0.701680787	816	1411	1652	1774	1863	1727	2060	2511
ENSECAG00000019840	6.538724367	0.499503315	0.702395627	845	1176	1708	2280	1938	2184	1799	2592
ENSECAG00000024006	5.537413259	0.499712042	0.70260218	465	696	821	1528	976	750	993	963
ENSECAG00000000309	0.97970269	0.500005907	0.702928373	31	34	19	20	34	40	45	59
ENSECAG00000008939	7.009089561	0.500237887	0.703100631	1856	2070	2605	2714	2814	2114	2649	2559
ENSECAG00000022267	5.453579104	0.5002522	0.703100631	392	586	871	987	1021	909	923	1131
ENSECAG00000003968	3.702571613	0.500331637	0.703125301	159	160	214	275	324	230	301	333
ENSECAG00000013493	5.576648609	0.500934543	0.703820485	407	479	856	1409	930	981	1060	1420
ENSECAG00000024742	1.206299826	0.500962083	0.703820485	27	16	48	42	75	31	61	41
ENSECAG00000018509	6.323035472	0.501012153	0.703820485	825	1039	1592	1639	1823	1619	1824	2038
ENSECAG00000004476	2.698712131	0.501395953	0.704272569	46	75	119	167	162	56	246	144
ENSECAG00000020803	4.077125798	0.50150532	0.704339116	202	182	320	353	375	337	413	409
ENSECAG00000004919	6.585029843	0.501614329	0.704405143	1276	1181	1846	1559	2583	1639	2309	2294
ENSECAG00000010848	5.208936718	0.501751685	0.704446332	300	494	532	1111	899	814	827	821
ENSECAG00000022215	6.340244474	0.501767661	0.704446332	1070	1278	1316	1258	2013	1520	1848	2034
ENSECAG00000017363	6.596842514	0.502012114	0.704702451	755	1510	1516	2512	1918	2324	2205	2391
ENSECAG00000000540	6.000178612	0.502496896	0.705295829	1185	1220	1134	802	1426	1103	1194	1299
ENSECAG00000018454	4.034462959	0.502918356	0.705800193	172	290	310	225	325	455	317	384
ENSECAG00000009946	6.769700101	0.502980481	0.7058002	1454	1935	2042	2426	2158	1865	2002	2637
ENSECAG00000000506	1.028535028	0.503056956	0.705820343	12	27	31	53	31	33	68	51
ENSECAG00000021136	10.74407346	0.503532456	0.706364355	18581	25169	41374	43923	35261	26892	36087	36058
ENSECAG00000001316	3.897686924	0.503569026	0.706364355	134	240	238	530	257	271	248	394
ENSECAG00000016892	5.193892505	0.503794087	0.706592818	348	363	1054	505	807	453	855	1351
ENSECAG00000016426	5.029704864	0.504007272	0.70680457	645	546	505	514	666	521	664	731
ENSECAG00000011975	6.194080231	0.504101442	0.706849387	730	1174	1588	1958	1435	1322	1479	1570
ENSECAG00000020194	4.58932452	0.504169681	0.706857837	367	352	473	510	448	453	542	453
ENSECAG00000022100	4.852519851	0.504242133	0.706872191	320	412	489	608	641	580	684	711
ENSECAG00000018500	4.870579945	0.504757647	0.707415881	305	484	610	755	586	565	464	710
ENSECAG00000007541	6.63352494	0.504789007	0.707415881	1470	1882	1135	2694	2205	1657	2248	1637
ENSECAG00000015605	4.007633466	0.504816755	0.707415881	146	316	200	346	311	349	347	464
ENSECAG00000022748	1.25215931	0.505237749	0.707918523	17	50	24	46	57	18	64	83
ENSECAG00000019789	2.985171051	0.505441663	0.707978581	65	125	133	290	114	165	152	174
ENSECAG00000024605	7.644266922	0.505451208	0.707978581	3203	3436	3923	3545	4379	3311	4044	3940
ENSECAG00000021178	6.093558253	0.505467545	0.707978581	1031	1065	1554	1147	1460	1188	1355	1403
ENSECAG00000015740	5.987159411	0.505910766	0.708512033	667	565	1356	1485	1718	1192	1452	1441
ENSECAG00000011172	7.969360679	0.506401015	0.709106272	2962	3445	6031	6231	5467	4523	3948	5719
ENSECAG00000021579	6.691856148	0.506521485	0.709106272	1192	1522	2259	2701	1695	2337	1623	2459
ENSECAG00000022725	2.35221618	0.506522312	0.709106272	50	97	101	127	86	98	76	132
ENSECAG00000023326	4.315356857	0.506588519	0.709111587	208	305	387	345	426	440	469	463
ENSECAG00000022502	6.564412998	0.506921427	0.709482296	897	1278	1415	2525	2496	1442	2153	2671
ENSECAG00000008951	1.212515792	0.507028689	0.709482296	23	79	27	36	17	74	37	32
ENSECAG00000008514	6.938847842	0.507040683	0.709482296	1271	1659	2032	2831	2884	2303	2949	3152
ENSECAG00000019881	6.49835973	0.507364186	0.70984754	1102	2770	876	1689	1617	1353	1968	2144
ENSECAG00000015752	5.003298329	0.507527289	0.709988311	380	489	570	549	779	504	841	805
ENSECAG00000009959	6.439040542	0.507898457	0.710339366	1109	1952	1426	1692	2015	1173	2017	1663
ENSECAG00000014887	4.307976429	0.507906294	0.710339366	159	283	343	503	423	460	465	441
ENSECAG00000024917	3.457126821	0.508006038	0.710339366	80	158	163	310	193	233	250	333
ENSECAG00000024819	4.716590062	0.508028313	0.710339366	205	451	315	735	527	489	543	874
ENSECAG00000023244	3.990987733	0.508319139	0.710658553	211	156	313	298	422	304	327	398
ENSECAG00000005146	4.962031936	0.508566837	0.710800753	327	446	450	778	712	596	741	781
ENSECAG00000020078	1.112698768	0.508585405	0.710800753	17	28	36	41	29	117	13	31
ENSECAG00000023713	5.192708544	0.508608531	0.710800753	383	749	497	631	718	751	802	1076
ENSECAG00000022331	1.924197079	0.508759636	0.710840054	58	50	75	86	57	72	69	90
ENSECAG00000007382	4.506824563	0.508811737	0.710840054	261	226	467	479	582	426	623	432
ENSECAG00000016416	7.001309564	0.508837281	0.710840054	1278	1707	2317	2843	2644	2716	2679	3770
ENSECAG00000015376	3.128422382	0.509089077	0.710840054	57	129	180	191	171	202	166	262
ENSECAG00000017458	3.698400839	0.509116415	0.710840054	147	179	305	324	310	224	229	253
ENSECAG00000000022	3.718360455	0.509182918	0.710840054	109	110	186	454	205	439	260	293
ENSECAG00000001851	5.392677206	0.509247942	0.710840054	295	814	497	1110	847	668	1322	1042
ENSECAG00000007112	4.122129906	0.50925524	0.710840054	140	134	332	539	385	259	413	571
ENSECAG00000014461	4.67013307	0.509261151	0.710840054	155	452	333	722	455	533	459	924
ENSECAG00000014261	6.210938744	0.509262281	0.710840054	660	816	1439	1926	1527	1537	1604	2128
ENSECAG00000022866	1.7829454	0.509463858	0.711034069	25.0008	79.0015	56	107.004	35.002	136.002	36	28.0055
ENSECAG00000023078	4.697669469	0.509623378	0.711086876	241	444	532	720	413	408	551	701
ENSECAG00000011063	0.642279097	0.509626864	0.711086876	14	27	45	34	7	77	7	3
ENSECAG00000018249	5.823780833	0.509846172	0.711305526	421	768	1056	1489	1225	1229	1184	1530
ENSECAG00000015327	4.545945131	0.510044341	0.711494635	288	386	294	476	589	415	598	521
ENSECAG00000026833	6.511864781	0.510435297	0.711952596	881	1687	1189	1917	1314	2126	1383	3740
ENSECAG00000006335	0.821841262	0.510530437	0.711980182	4	34	46	52	34	25	35	30
ENSECAG00000022174	3.566043299	0.510580401	0.711980182	123	134	220	274	264	232	263	320
ENSECAG00000014057	3.609607116	0.510715033	0.7120708	100	123	213	358	319	197	354	248
ENSECAG00000017230	3.65549793	0.510890323	0.7120708	129	148	346	322	271	258	253	186
ENSECAG00000013495	6.686139894	0.510895252	0.7120708	1447	1101	2035	3053	2199	1524	1893	2554
ENSECAG00000011570	0.997728174	0.510896071	0.7120708	19	26	26	46	37	54	46	38
ENSECAG00000010616	4.701873434	0.510995957	0.712122662	191	370	864	548	409	549	455	617
ENSECAG00000017393	7.274397876	0.511121634	0.71221045	1795	2207	2125	3534	3984	2338	4367	3645
ENSECAG00000019545	8.598903279	0.511282411	0.712347119	4254	5446	4436	10298	8319	7162	9452	11230
ENSECAG00000013514	6.675510133	0.511401439	0.712425594	1841	413	3533	1834	3279	1774	1788	625

ENSECAG00000024304	4.29559265	0.51170407	0.712759795	271	474	281	345	359	292	436	469
ENSECAG00000004587	1.220341621	0.511860964	0.712890938	13	21	26	83	23	64	93	29
ENSECAG00000012163	6.822888	0.51194945	0.712926787	1829	2305	1492	2332	2427	1900	2627	1890
ENSECAG00000007276	4.823996704	0.512023772	0.712942904	363	280	857	596	739	279	384	848
ENSECAG00000015025	0.373791443	0.512174249	0.712965857	11	24	47	9	18	32	24	12
ENSECAG00000026808	5.100430917	0.512236464	0.712965857	264	733	540	1061	654	616	773	652
ENSECAG00000011769	1.158568561	0.512269021	0.712965857	13	31	30	63	41	41	57	61
ENSECAG00000018299	6.171161131	0.512291257	0.712965857	926	1039	1238	1241	1676	1331	1620	1963
ENSECAG00000008294	4.187403226	0.512425082	0.713064763	158	269	346	395	389	421	354	487
ENSECAG00000009190	10.30093797	0.512547569	0.713147867	17910	31989	15660	24235	22011	23062	28436	24145
ENSECAG00000009291	1.236290922	0.512704568	0.713278965	9	24	59	53	51	45	51	66
ENSECAG00000022763	6.70208021	0.512807784	0.713335216	1147	1782	1457	2106	2216	2138	2069	3164
ENSECAG00000010959	5.360278158	0.512968563	0.713471515	277	470	822	1164	766	882	891	1237
ENSECAG00000010568	5.097793217	0.513134883	0.713615489	430	531	439	732	825	574	740	1000
ENSECAG00000012141	5.754155437	0.513277771	0.713726843	479	1309	772.999	1469	752	1228	699.999	1561
ENSECAG00000009499	3.136946724	0.513690441	0.714213263	91	114	238	209	154	161	172	196
ENSECAG00000006942	6.404325034	0.513898748	0.714415462	829	1471	1153	1934	1763	1595	1893	2537
ENSECAG00000020880	2.386412347	0.514231374	0.714790417	46	93	76	105	118	82	136	142
ENSECAG00000000204	6.718046713	0.514704602	0.715304149	906	1921	2034	3132	1716	2307	2195	1992
ENSECAG00000013772	4.149756569	0.514726872	0.715304149	286	211	501	249	340	274	445	325
ENSECAG00000017496	5.749099099	0.515013981	0.715520838	593	670	920	1266	1138	969	1312	1489
ENSECAG00000020658	3.64292553	0.515071908	0.715520838	183	162	196	208	267	230	278	369
ENSECAG00000023042	3.126582178	0.515126889	0.715520838	74	76	188	222	189	169	197	247
ENSECAG00000022272	8.736829357	0.5151347	0.715520838	6026	7381	6781	10860	8514	7366	8175	9459
ENSECAG00000012124	5.612358042	0.515221833	0.71553545	900	1183	603	607	1081	682	1087	992
ENSECAG00000011432	4.611255319	0.515271172	0.71553545	297	459	459	545	437	627	383	456
ENSECAG00000022567	10.57413782	0.515519505	0.715756508	16954	20733	37671	39168	33022	24074	31008	31421
ENSECAG00000018565	3.617867939	0.515609922	0.715756508	89	248	116	323	204	209	288	449
ENSECAG00000014602	2.277502191	0.515667738	0.715756508	52	39	122	76	76	90	85	204
ENSECAG00000003236	4.264225335	0.515682343	0.715756508	266	431	279	371	402	279	419	424
ENSECAG00000023379	6.642851286	0.515835141	0.715881138	1690	1683	1407	2247	2227	1713	2021	1910
ENSECAG00000019529	4.191910688	0.516091626	0.716149616	184	199	344	444	527	389	405	328
ENSECAG00000009412	3.787054005	0.516294631	0.716263547	198	266	247	255	286	226	267	312
ENSECAG00000023096	2.691144353	0.516299811	0.716263547	81	78	179	130	124	108	120	147
ENSECAG00000013560	2.955377994	0.516511114	0.716469208	73	132	112	163	181	122	167	245
ENSECAG00000016772	5.41419643	0.516715226	0.716570727	430	785	577	907	870	920	975	1102
ENSECAG00000011900	3.658914874	0.516768142	0.716570727	128	156	217	470	313	210	254	199
ENSECAG00000023470	4.671179724	0.516773502	0.716570727	277	350	648	598	523	452	400	653
ENSECAG00000007363	5.256237232	0.517019474	0.716816221	426	542	887	954	771	831	738	663
ENSECAG00000016814	7.209598865	0.517076725	0.716816221	2013	3194	2691	2591	3187	2400	2591	3530
ENSECAG00000008572	3.076908709	0.517185545	0.71687961	53	203	153	230	126	170	174	176
ENSECAG00000008680	8.005785542	0.517345937	0.716958683	1939	3178	4975	6651	5169	6129	5212	7230
ENSECAG00000020997	4.059285848	0.517373788	0.716958683	203	206	286	346	394	293	450	376
ENSECAG00000014202	2.636169337	0.517431896	0.716958683	87	86	93	99.0001	143	93	141	197.001
ENSECAG00000010779	5.109003063	0.517810643	0.717320204	431	472	585	670	758	653	852	866
ENSECAG00000012821	5.616454418	0.517819074	0.717320204	609	942	835	1125	951	822	1150	991
ENSECAG00000023134	1.52425272	0.518130115	0.717581511	18	44	51	63	57	47	61	97
ENSECAG00000016351	1.079745708	0.518141609	0.717581511	46	34	38	21	27	53	35	36
ENSECAG00000013883	3.26680257	0.518197175	0.717581511	49	38	655	64	87	445	33	43
ENSECAG00000016632	5.274477474	0.518472312	0.71787502	527	412	701	769	960	705	876	984
ENSECAG00000021494	3.640570521	0.518579742	0.717936277	88	188.992	295	390	193.971	274.997	193.964	304
ENSECAG00000010329	3.772583078	0.518743603	0.718075635	105	420	230	240	210	237	235	386
ENSECAG00000006078	5.476107964	0.518828989	0.718106343	565.001	875.001	812.001	905.003	996.001	680	923.002	965
ENSECAG00000017341	7.085967243	0.518918053	0.718142134	1804	2213	2672	3155	2796	2651	2691	2525
ENSECAG00000007201	10.09002791	0.519108632	0.718318387	10654	15041	27652	29604	21374	17830	23854	22233
ENSECAG00000021312	3.613598779	0.519441538	0.718691519	76	212	312	341	163	237	219	335
ENSECAG00000010355	5.39244833	0.519696725	0.718888305	489	380	689	1139	1101	942	1026	725
ENSECAG00000003427	3.740195975	0.51971031	0.718888305	83	177	255	359	244	204	380	411
ENSECAG00000004005	3.544499788	0.519855275	0.718927075	68	163	289	226	251	212	376	228
ENSECAG00000015098	6.850254515	0.519892769	0.718927075	1955	2167	1868	2046	2375	2651	1591	2359
ENSECAG00000024411	6.446440458	0.519928162	0.718927075	1104	1915	1790	1338	1581	1706	1828	1759
ENSECAG00000008583	6.690464529	0.520092433	0.71893031	1150	1614	2050	2848	2186	1905	2122	1929
ENSECAG00000015597	8.822414088	0.520170059	0.71893031	6675	3236	14209	9974	11823	10085	7923	3780
ENSECAG00000005021	1.674969887	0.520173365	0.71893031	27	31	92	94	43	45	100	47
ENSECAG00000021543	4.415247369	0.520183602	0.71893031	151	96	510	540	523	517	973	18
ENSECAG00000021514	3.661316773	0.520348061	0.719070138	131	119	208	356	283	283	299	281
ENSECAG00000013415	2.278214545	0.520759592	0.719385356	0	71	15	196	8	230	5	256
ENSECAG00000008955	4.315214009	0.520772351	0.719385356	232	320	269	424	515	360	441	491
ENSECAG00000007267	3.931873316	0.520773254	0.719385356	265	222	312	254	325	295	263	313
ENSECAG00000000091	0.827014395	0.520829426	0.719385356	16	46	27	36	21	39	32	35
ENSECAG00000016724	6.73960338	0.520942913	0.719399544	1576	1622	1681	1475	2642	1899	2546	2752
ENSECAG00000014000	4.04138273	0.520966331	0.719399544	154	319	286	464	313	282	261	452
ENSECAG00000022623	5.850011032	0.521468747	0.720005822	532	699	945	1601	1435	1065	1554	1187
ENSECAG00000013045	4.450034132	0.521538724	0.720014943	494	375	268	310	387	381	439	505
ENSECAG00000017076	3.618007786	0.521607335	0.720022177	152	298	181	236	232	210	258	263
ENSECAG00000004183	1.630621863	0.521771416	0.72016118	40	64	57	55	54	50	63	67
ENSECAG00000016677	5.139435986	0.5219385	0.720202669	221	524	889	1104	629	738	708	675
ENSECAG00000011357	6.37936343	0.521984425	0.720202669	1151	1449	1662	1633	1627	1620	1684	1669
ENSECAG00000020392	5.349726543	0.521991637	0.720202669	618	419	735	711	1088	737	858	1042
ENSECAG00000008148	6.395900229	0.522083882	0.720242481	1116	1203	1263	1627	1862	1593	2121	2099
ENSECAG00000013190	4.635756705	0.522265451	0.720405494	205	367	580	734	401	415	518	644
ENSECAG00000009977	1.282235669	0.522412932	0.720475892	22	52	34	69	46	33	42	61
ENSECAG00000015108	3.081158277	0.522527919	0.720475892	100	110	160	147	188	175	161	247
ENSECAG00000026908	4.625956758	0.522563109	0.720475892	299	584	330	545	520	421	490	525
ENSECAG00000018857	-0.11919653	0.522570131	0.720475892	5	13	25	22	19	5	19	19

ENSECAG00000022430	5.340553205	0.522774447	0.720670137	576	262	1037	571	945	636	979	1221
ENSECAG00000004355	2.588608536	0.523024902	0.720927931	42	120	88	121	95	99	162	201
ENSECAG00000013627	3.789972024	0.523192139	0.721070971	239	260	242	203	227	257	287	314
ENSECAG00000019823	4.962266472	0.523329623	0.72115119	289	375	571	800	700	701	600	822
ENSECAG00000020403	4.758610903	0.523377285	0.72115119	188	386	494	720	653	576	563	664
ENSECAG00000026938	9.72235434	0.523586354	0.721284675	7575	11954	20811	24511	16625	14998	17193	17157
ENSECAG00000011238	2.236544729	0.523601126	0.721284675	42	58	136	117	74	79	112	93
ENSECAG00000023902	6.268093297	0.524247706	0.722087821	873	2160	941	1524	1430	1366	1461	1833
ENSECAG00000011763	6.643780463	0.524346916	0.722136929	881	1958	1512	1964	2229	1882	2167	2927
ENSECAG00000009585	7.651375249	0.524526756	0.722238026	1743	2606	5149	6004	3598	4193	3474	4418
ENSECAG00000023617	0.200723857	0.524547455	0.722238026	10	12	25	16	25	16	32	26
ENSECAG00000012365	6.355873512	0.524773832	0.722462169	1071	970	1752	1215	2533	1390	1701	1882
ENSECAG00000014537	5.170813433	0.525051445	0.722756787	392	369	764	782	804	688	908	871
ENSECAG00000011016	3.183915348	0.525364394	0.723099969	56	39	491	137	130	209	157	165
ENSECAG00000014577	1.770370868	0.525432882	0.723106639	59	86	34	63	14	143	32	44
ENSECAG00000018445	4.454928975	0.525933067	0.723629725	290	319	354	385	547	372	500	577
ENSECAG00000010090	8.24992424	0.525940351	0.723629725	1835	3998	4499	9929	6162	6247	6968	9045
ENSECAG00000013326	3.876917414	0.526150557	0.723831291	158	202	322	405	317	251	308	281
ENSECAG00000020246	8.906034384	0.526368178	0.724043008	5475	6648	9562	7837	11910	8421	11776	12300
ENSECAG00000016488	3.289607545	0.526497823	0.724133672	121	78	225	176	258	205	210	216
ENSECAG00000002024	0.292135633	0.526660647	0.724151352	9	12	21	28	27	15	36	28
ENSECAG00000018501	1.497597791	0.526741976	0.724151352	21	64	54	73	31	101	26	40
ENSECAG00000005087	5.804384897	0.526764604	0.724151352	712	899	1191	1197	1063	1027	956	1440
ENSECAG00000024737	6.235188729	0.526765615	0.724151352	864	1075	1482	1292	1565	1482	1750	2072
ENSECAG00000014852	3.986186616	0.52709116	0.724511222	185	217	240	350	479	234	352	382
ENSECAG00000019056	5.320089358	0.527337078	0.724640163	345	624	617	1018	990	823	802	1006
ENSECAG00000012736	7.14963042	0.527344235	0.724640163	1256	824	3269	3860	2915	2887	2429	5182
ENSECAG00000015110	1.157026441	0.527438198	0.724640163	23	35	43	26	40	59	35	64
ENSECAG00000006519	6.523426279	0.527459527	0.724640163	1008	2024	1611	2002	1825	1365	1920	2253
ENSECAG00000000301	4.847058638	0.527503854	0.724640163	273	353	461	793	534	695	638	734
ENSECAG00000006090	5.299298327	0.527694554	0.724814497	427	524	535	1057	875	755	886	1067
ENSECAG00000009145	8.525059665	0.527850009	0.724940385	8572	7009	4666	4212	6644	6464	7900	7387
ENSECAG00000009143	5.496699721	0.528004234	0.725064553	834	895	593	738	902	781	1034	844
ENSECAG00000007461	5.317595399	0.528138759	0.725161641	628	622	854	668	736	655	973	808
ENSECAG00000003790	6.658616185	0.528261028	0.72524188	867	2334	1636	2695	1724	1769	1996	2525
ENSECAG00000018350	5.753979295	0.528480426	0.725380247	620	864	899	1006	1077	1117	1242	1454
ENSECAG00000024754	5.079492447	0.528570709	0.725380247	410	524	506	674	818	533	862	867
ENSECAG00000015861	4.815049636	0.528579738	0.725380247	360	360	486	554	650	478	693	738
ENSECAG00000014970	2.109564896	0.52871085	0.725380247	54	82	54	59	129	78	79	106
ENSECAG00000012982	4.233837719	0.528733459	0.725380247	185	171	403	452	497	292	365	583
ENSECAG00000012674	5.290821268	0.528747655	0.725380247	455	619	654	725	959	697	960	929
ENSECAG00000015493	7.173114552	0.528808712	0.725380247	896	1399	3582	3530	2631	4720	3664	2094
ENSECAG00000013115	6.255830517	0.529045285	0.725617157	1059	1265	1486	1618	1497	1539	1423	1613
ENSECAG00000005685	3.230036129	0.529184771	0.725720866	112	175	186	200	171	201	140	220
ENSECAG00000010097	4.015292433	0.529280002	0.725763865	164	209	283	573	305	288	348	329
ENSECAG00000003601	6.903375243	0.529352179	0.725775245	1720	1483	2230	1783	2875	2496	2839	2730
ENSECAG00000009141	3.609444853	0.529466333	0.725814064	88	184	209	312	308	252	271	273
ENSECAG00000021385	9.281270685	0.529566868	0.725814064	6215	9209	9903	14073	13622	12334	14523	16975
ENSECAG00000010605	4.85607454	0.529572134	0.725814064	283	839	367	593	509	596	587	573
ENSECAG00000020991	5.666492623	0.529776643	0.72600678	1299	617	338	1317	2060	445	265	1082
ENSECAG00000021056	6.18287369	0.530019385	0.72613961	857	1040	1872	1518	1377	1383	1038	2001
ENSECAG00000016436	5.200873109	0.53003123	0.72613961	366	545	540	1353	868	567	716	765
ENSECAG00000014814	4.827810657	0.53007429	0.72613961	341	521	456	731	516	503	613	637
ENSECAG00000006796	1.803376724	0.530129208	0.72613961	50	39	59	53	62	50	95	113
ENSECAG00000015902	3.379209746	0.530367786	0.726378832	130	132	198	175	195	248	194	308
ENSECAG00000013325	5.149990887	0.530517563	0.72649639	327	473	572	950	709	672	908	940
ENSECAG00000015119	6.048970776	0.530656832	0.726588682	805	315	1503	2755	2477	518	1552	363
ENSECAG00000026824	2.174772248	0.530712856	0.726588682	54	65	90	119	103	84	71	87
ENSECAG00000016834	4.163954104	0.530813919	0.726639487	336	482	186	203	232	228	327	626
ENSECAG00000017992	4.125443166	0.531199054	0.727079105	174	234	518	363	238	370	386	370
ENSECAG00000008348	4.974131171	0.531277973	0.727099534	257	518	549	708	583	552	693	1063
ENSECAG00000006959	6.337594162	0.531597961	0.727449841	1160	1652	1512	1311	1579	1629	1635	1541
ENSECAG00000006228	3.070533923	0.531711422	0.727517482	93	134	178	98	217	146	189	216
ENSECAG00000016459	11.02548183	0.531976107	0.727791996	23025	26428	53380	53967	44773	32528	45203	41037
ENSECAG00000022184	6.301059518	0.532234684	0.728058088	739	1058	930	2428	1325	1838	1613	2473
ENSECAG00000022294	0.962472221	0.532403668	0.728130931	7	61	32	45	13	33	32	62
ENSECAG00000019406	4.267015924	0.53243365	0.728130931	235	262	415	269	447	410	413	472
ENSECAG00000011168	7.688006973	0.532480189	0.728130931	1616.08	2534.59	3420.8	5955.13	3937.99	5231.62	3744.92	6096.39
ENSECAG00000009275	6.436376526	0.532728137	0.72832227	927	1171	1656	1755	1887	1566	2218	2225
ENSECAG00000004691	6.772087156	0.532748318	0.72832227	1547	1373	1756	2011	2362	2596	2194	2800
ENSECAG00000017250	2.443661886	0.532825986	0.728340815	47	88	84	119	96	109	126	164
ENSECAG00000023575	7.138421058	0.533620065	0.729239146	3760	1735	2294	1442	3508	2076	2995	2226
ENSECAG00000024104	0.489904166	0.533643533	0.729239146	5	14	46	15	27	31	44	20
ENSECAG00000014761	5.221301515	0.533771615	0.729239146	262	590	672	930	765	833	911	862
ENSECAG00000011476	2.447032265	0.533826618	0.729239146	42	87	88	126	118	112	139	122
ENSECAG00000016651	5.350825258	0.533833033	0.729239146	483	398	818	902	1023	771	813	1112
ENSECAG00000012915	4.582328584	0.533937053	0.729239146	234	376	432	486	505	474	575	612
ENSECAG00000024443	4.374458061	0.533986075	0.729239146	157	409	271	763	334	431	325	536
ENSECAG00000010782	4.521036978	0.533996628	0.729239146	150	483	513	581	421	463	459	458
ENSECAG00000012229	8.879685718	0.53410762	0.729303061	5017	2371	12976	8699	16445	8993	12860	5562
ENSECAG00000020020	7.580494929	0.534189596	0.729327348	2593	3976	3121	3954	3567	3521	3755	4279
ENSECAG00000008406	1.793585058	0.534312176	0.729407058	36	48	46	81	79	56	83	95
ENSECAG00000020595	4.22599126	0.534416392	0.729461683	252	401	307	351	351	271	380	497
ENSECAG00000012600	6.506806757	0.534664105	0.729712139	915	1276	1777	1837	2276	1641	2051	2326
ENSECAG00000015202	3.253833266	0.534807366	0.729819997	135	173	182	179	186	192	184	181

ENSECAG00000010203	0.35690604	0.535069896	0.730090568	16	8	18	28	24	11	32	48
ENSECAG00000021896	2.279438616	0.535368742	0.73038758	45	93	73	79	61	91	139	155
ENSECAG00000014148	2.99207127	0.535416137	0.73038758	45	124	195	139	169	163	153	246
ENSECAG00000000018	4.604236259	0.535526222	0.730450053	209	582	442	542	424	483	511	501
ENSECAG00000014191	6.461586883	0.53579943	0.730734981	973	1485	1235	1869	1942	1803	1901	2376
ENSECAG00000005916	3.391785795	0.536228606	0.73117418	125	204	132	172	243	197	228	289
ENSECAG00000013460	1.86432532	0.536250171	0.73117418	28	48	90	110	56	53	92	74
ENSECAG00000014420	3.556121409	0.536436055	0.731339867	104	191	186	269	260	252	226	328
ENSECAG00000018237	6.500048318	0.536708523	0.731551172	1174	1504	1851	1949	1845	1604	1894	1871
ENSECAG00000011329	4.461730253	0.536719818	0.731551172	181	363	278	606	340	481	429	780
ENSECAG00000000880	0.348641959	0.536942856	0.73176739	22	19	21	22	23	18	30	19
ENSECAG00000008468	5.094928692	0.537097794	0.731826724	394	785	442	844	493	592	669	982
ENSECAG00000019242	5.141149581	0.537115213	0.731826724	273	366	456	1269	928	790	937	544
ENSECAG00000016116	6.012233475	0.5373138	0.731972214	519	799	1124	1822	1965	1037	1511	1383
ENSECAG00000006951	3.733561237	0.537350839	0.731972214	98	188	261	313	369	252	352	228
ENSECAG00000012467	4.210489139	0.537438897	0.732004405	143	177	452	438	454	324	493	417
ENSECAG00000020160	2.880863834	0.537577987	0.732031112	91	72	132	158	212	122	192	142
ENSECAG00000024424	6.643018627	0.537587361	0.732031112	1304	1645	1480	1684	2460	1890	2106	2686
ENSECAG00000026867	2.858718293	0.53778272	0.732205311	47	105	127	189	158	176	136	186
ENSECAG00000021938	0.508699493	0.537844176	0.732205311	14	23	34	28	23	33	16	29
ENSECAG00000011764	7.300789797	0.537981373	0.732244234	2487	3038	2164	3430	3071	2455	3103	3911
ENSECAG00000009997	6.276207829	0.538133912	0.732244234	1210	1420	1669	1054	1605	1476	1685	1329
ENSECAG00000018894	5.568627165	0.538158269	0.732244234	455	784	880	1422	1052	683	1052	1016
ENSECAG00000008558	4.727239725	0.538187883	0.732244234	336	467	326	500	573	445	558	856
ENSECAG00000012927	6.215576622	0.538195001	0.732244234	510	1139	1230	2032	1367	1804	1399	2223
ENSECAG00000011141	3.022402807	0.538536582	0.732621245	67	127	122	312	116	154	177	180
ENSECAG00000016437	2.066207803	0.538654913	0.732694495	28	51	77	110	90	83	84	123
ENSECAG00000008649	5.241357121	0.53884122	0.732860179	523	830	556	733	803	668	794	743
ENSECAG00000010129	3.229698333	0.539028669	0.733021846	73	146	172	206	169	191	190	309
ENSECAG00000009754	2.784308967	0.539152574	0.733021846	52	108	90	191	147	129	172	179
ENSECAG00000011579	2.887809447	0.539199848	0.733021846	61	99	136	175	123	167	175	206
ENSECAG00000017731	4.819985621	0.539309085	0.733021846	282	752	404	590	454	465	590	745
ENSECAG00000008867	5.214925966	0.539324818	0.733021846	407	684	607	1011	722	700	759	781
ENSECAG00000000640	3.327665159	0.539347177	0.733021846	98	158	164	205	190	141	207	398
ENSECAG00000013798	6.623367512	0.53980336	0.733554094	1044	1338	2484	2455	2405	1387	1979	2084
ENSECAG00000016458	0.087777523	0.539867981	0.733554173	19	6	28	18	22	21	11	18
ENSECAG00000010458	5.307887416	0.540140292	0.733708817	721	635	487	896	717	704	783	954
ENSECAG00000016025	4.856416249	0.540144785	0.733708817	390	430	519	429	736	486	684	727
ENSECAG00000010136	7.173910184	0.540222602	0.733708817	2622	3919	1519	1748	3235	2021	3794	2064
ENSECAG00000016010	7.356126378	0.540240095	0.733708817	2375	2761	3064	3477	3868	2839	2943	3304
ENSECAG00000017772	4.714075999	0.540382006	0.733813835	279	374	499	513	598	562	689	503
ENSECAG00000000235	2.667074873	0.540512166	0.733859315	39	91	75	207	136	99	135	220
ENSECAG00000017997	2.147953674	0.540695104	0.733859315	22	66	80	114	150	78	89	84
ENSECAG00000015497	4.968767991	0.54082488	0.733859315	337	501	624	833	601	619	488	798
ENSECAG00000010148	2.733740244	0.540849025	0.733859315	55	184	111	132	99	119	136	160
ENSECAG00000008977	5.974694758	0.540897047	0.733859315	1587	568	1080	949	1566	994	1259	1073
ENSECAG00000010545	5.531299788	0.540915504	0.733859315	498	473	980	1025	1161	755	1047	1262
ENSECAG00000023285	1.788348063	0.540928358	0.733859315	16	40	84	78	74	84	79	72
ENSECAG00000020908	1.973505143	0.54093221	0.733859315	32	60	76	73	85	61	99	111
ENSECAG00000012427	2.264216048	0.54107384	0.733963821	52	69	71	100	96	68	95	185
ENSECAG00000019625	3.8166864	0.541218627	0.734072585	157	261	307	288	237	330	251	284
ENSECAG00000019067	6.210929757	0.541513895	0.734251983	475	1095	1203	2161	1272	1790	1555	2157
ENSECAG00000014294	3.713808726	0.541562352	0.734251983	138	249	272	293	302	247	285	188
ENSECAG00000013671	7.855919474	0.541590254	0.734251983	2858	2923	3843	4829	6229	4256	5308	5453
ENSECAG00000026223	0.138776512	0.541609388	0.734251983	4	13	21	51	35	28	0	3
ENSECAG00000020274	4.884748316	0.542119111	0.734855325	368	547	327	566	911	563	728	465
ENSECAG00000022561	3.531436694	0.542187747	0.734860693	131	164	215	208	242	225	242	342
ENSECAG00000020665	11.85441927	0.542302644	0.73492875	40119	47777	92804	98086	81783	55226	78876	75699
ENSECAG00000015000	2.494986685	0.542473466	0.735072572	44	59	194	140	93	134	146	35
ENSECAG00000007688	0.355986522	0.542861052	0.73551005	8	19	26	40	26	17	23	24
ENSECAG00000014873	6.777177483	0.543111128	0.735761135	2298	2367	1243	1396	2271	1812	2304	2181
ENSECAG00000008642	8.113628055	0.543583962	0.736313898	2973	3306	4553	6880	6576	5144	6794	6946
ENSECAG00000010266	5.910306567	0.543802608	0.736522259	888	1529	791	956	801	1504	918	1484
ENSECAG00000000377	4.776997699	0.543995335	0.73669547	286	282	754	726	600	466	503	608
ENSECAG00000009763	3.36612816	0.544069377	0.736707933	117	162	212	260	203	176	243	186
ENSECAG00000008201	7.312544011	0.544215579	0.73681809	2144	2404	2481	2648	3553	3118	3789	4102
ENSECAG00000013588	5.37142745	0.544336724	0.736874489	402	944	746	896	875	654	870	918
ENSECAG00000023744	1.869225957	0.544444556	0.736874489	63	44	87	60	48	74	100	49
ENSECAG00000019012	1.41441662	0.54447374	0.736874489	24	39	47	50	62	43	65	68
ENSECAG00000018189	3.457466901	0.544539743	0.736874489	124	231	144	150	307	140	394	166
ENSECAG00000000845	0.380029262	0.544581507	0.736874489	22	20	25	19	15	29	15	33
ENSECAG00000003862	5.534618829	0.544879539	0.737189964	473	805	702	977	1137	963	948	1130
ENSECAG00000013251	5.329262764	0.545020139	0.737246829	291.999	513	730.998	1146	815	921	915	985
ENSECAG00000000568	3.226655699	0.545051343	0.737246829	188	131	122	201	210	124	176	226
ENSECAG00000012398	3.144241543	0.545141412	0.737280886	67	142	180	171	184	184	176	260
ENSECAG00000011923	5.963109518	0.545565209	0.737766235	715	700	1700	1485	1569	1016	1127	1244
ENSECAG00000009339	0.185516893	0.545648086	0.737790498	17	17	6	19	21	20	22	36
ENSECAG00000018851	6.225068422	0.545818719	0.737933399	681	1354	1373	2173	1432	1398	1516	1614
ENSECAG00000020785	4.339618086	0.546253744	0.738433675	117	443	374	634	215	460	263	644
ENSECAG00000022177	4.10955898	0.546494479	0.738671219	255	335	265	348	350	253	377	399
ENSECAG00000016891	0.933620709	0.546585167	0.738705919	19	27	45	47	39	12	38	53
ENSECAG00000016661	8.293168094	0.546733896	0.738819045	1978	1458	9553	6618	14328	5063	9318	1046
ENSECAG00000008458	8.209539861	0.546977425	0.739060233	3401	4743	3956	6389	6843	5517	6511	8430
ENSECAG00000011178	7.885614905	0.54717746	0.739174343	2688.01	4927	5024.01	4402	4686	3813	4885	5389
ENSECAG00000023676	2.860688424	0.547191991	0.739174343	84	94	197	157	103	92	127	252

ENSECAG00000000423	5.918498313	0.547310698	0.739204546	684	722	1054	1430	1578	1166	1222	1512
ENSECAG00000017347	2.855474166	0.547344468	0.739204546	125	91	122	191	46	265	55	162
ENSECAG00000014704	4.183196742	0.547527037	0.739265553	171	247	368	374	396	342	298	631
ENSECAG00000017337	3.550146653	0.547559969	0.739265553	108	112	296	227	266	180	240	398
ENSECAG00000013843	1.520307912	0.547584835	0.739265553	25	29	53	70	75	48	50	85
ENSECAG00000010817	0.590186596	0.54801258	0.73975513	11	24	40	33	16	40	19	31
ENSECAG00000009177	5.756486184	0.548095817	0.739779599	617	1079	999	1170	980	991	1201	1158
ENSECAG00000020083	5.139569831	0.548344577	0.739963805	417	718	475	548	695	697	799	1019
ENSECAG00000000902	0.427973749	0.548362545	0.739963805	8	10	31	30	26	30	27	33
ENSECAG00000001012	1.712640904	0.549246643	0.74105183	25	54	68	98	50	54	89	53
ENSECAG00000012314	3.072825288	0.549311236	0.74105183	61	95	152	242	224	161	175	204
ENSECAG00000000242	7.855006835	0.549524093	0.74105183	3208	4788	3756	4678	4188	4563	4318	5221
ENSECAG00000017101	2.648855798	0.549628168	0.74105183	92	77	145	131	119	99	108	165
ENSECAG00000019473	1.566117006	0.54973729	0.74105183	15	40	35	99	48	60	75	84
ENSECAG00000010243	5.455332116	0.5497472	0.74105183	512	657	668	952	920	831	1025	1195
ENSECAG00000010467	0.974045489	0.54975199	0.74105183	18	36	36	52	30	30	20	66
ENSECAG00000018274	4.756948893	0.549786496	0.74105183	488	262	416	429	739	452	653	624
ENSECAG00000014312	4.619157365	0.549817572	0.74105183	202	337	433	641	616	372	635	616
ENSECAG00000015565	1.265287227	0.549863751	0.74105183	52	38	23	47	44	40	48	46
ENSECAG00000021972	2.810106197	0.549970503	0.74105183	71	71	153	144	149	152	193	136
ENSECAG00000009428	1.836028465	0.549971505	0.74105183	42	42	70	58	89	45	78	114
ENSECAG00000006267	1.381230429	0.550072278	0.74105183	9	33	72	92	25	51	34	81
ENSECAG00000002027	0.113400423	0.550138986	0.74105183	11	21	18	23	24	16	22	13
ENSECAG00000021602	3.231422135	0.550186568	0.74105183	100	174	158	281	119	376	87	95
ENSECAG00000004668	2.768373969	0.550212396	0.74105183	55	145	107	206	78	127	107	218
ENSECAG00000015620	3.663102412	0.550299408	0.741061701	134	124	221	336	264	213	322	358
ENSECAG00000010141	4.410822294	0.55035017	0.741061701	233	277	279	583	556	385	441	548
ENSECAG00000015868	6.794363703	0.550458604	0.741119879	1257	1720	2380	2784	2302	2078	2074	2371
ENSECAG00000019897	4.206536153	0.550739042	0.741409596	140	260	300	517	352	413	362	551
ENSECAG00000009490	5.841770341	0.551114075	0.741826575	661	1066	1004	1415	955	1162	936	1552
ENSECAG00000011391	5.632175488	0.551375256	0.742050408	476	480	1066	1213	1136	1085	1396	842
ENSECAG00000012194	4.996084109	0.551410983	0.742050408	262	472	564	799	698	601	766	823
ENSECAG00000021443	6.536906037	0.5514802	0.742055664	1005	1277	1223	2483	2225	1543	2176	2565
ENSECAG00000000965	7.331376796	0.551639512	0.742182135	2268	3020	3496	2608	2609	3985	2187	3815
ENSECAG00000026947	0.862903769	0.551742125	0.742232303	19	7	67	42	39	11	41	40
ENSECAG00000026852	2.79047411	0.551897557	0.742287443	79	49	175	121	197	112	171	152
ENSECAG00000013073	6.203608284	0.551925076	0.742287443	869	231	1816	1649	2803	1761	1572	652
ENSECAG00000012871	0.636965103	0.552042541	0.742287443	23	29	28	25	28	42	21	18
ENSECAG00000015895	4.738513203	0.552080037	0.742287443	403	593	347	499	505	469	520	637
ENSECAG00000015238	0.648969806	0.552109768	0.742287443	7	25	20	42	26	41	30	39
ENSECAG00000023631	5.597115391	0.552302095	0.742391671	657	530	1301	1002	1031	610	862	1409
ENSECAG00000022781	0.738794785	0.552317972	0.742391671	26	5	18	40	51	5	16	88
ENSECAG00000014692	7.842924688	0.552441466	0.742404579	3271	4108	4861	3999	5058	3236	4994	4968
ENSECAG00000024863	4.687208673	0.552458257	0.742404579	145	315	471	806	431	584	575	768
ENSECAG00000016627	0.990947243	0.552884674	0.742889743	16	53	43	27	38	19	45	45
ENSECAG00000023185	7.292244411	0.553396203	0.74348914	2070	3004	2965	3118	2932	2633	3228	3710
ENSECAG00000023341	3.253636242	0.553635897	0.743651895	112	184	136	141	253	143	204	274
ENSECAG00000002924	2.834331976	0.553648247	0.743651895	97	57	111	283	233	124	106	69
ENSECAG00000009051	2.880491262	0.55381891	0.743730472	58	114	178	205	100	136	154	183
ENSECAG00000010518	5.884992543	0.553837663	0.743730472	652	765	1217	1130	1373	1040	1458	1486
ENSECAG00000024152	5.334893258	0.553988594	0.743762579	365	308	1041	911	700	709	981	1347
ENSECAG00000008249	5.073521548	0.553992493	0.743762579	565	430	517	488	844	680	830	685
ENSECAG00000012878	4.986873186	0.554138581	0.74386059	356	310	773	560	1032	550	676	618
ENSECAG00000012970	6.203222602	0.554224669	0.74386059	1091	1192	1373	1522	1536	1325	1218	1842
ENSECAG00000019234	6.879495228	0.554261904	0.74386059	1546.99	2424.99	1876.99	2537	2201	2482.98	2031.99	2601.99
ENSECAG00000008175	1.146534646	0.554401059	0.743903407	24	55	36	39	27	48	38	50
ENSECAG00000020158	6.306596627	0.554424754	0.743903407	840	867	1791	1554	1806	1196	1718	2590
ENSECAG00000019601	7.481913048	0.554989162	0.744552115	2850	3573	3115	2835	3662	3022	3659	3816
ENSECAG00000018452	2.580865916	0.55503929	0.744552115	49	109	110	102	130	116	144	150
ENSECAG00000019081	3.434195155	0.555438613	0.744999825	127	194	123	220	187	222	236	341
ENSECAG00000007001	8.823288562	0.555663007	0.745212828	5511	10180	7859	8986	7155	9558	7690	11293
ENSECAG00000016097	5.855233804	0.555894805	0.745410423	681	1060	760	1116	1263	1082	1298	1619
ENSECAG00000018421	2.656975778	0.555941553	0.745410423	100	130	137	72	246	78	94	53
ENSECAG00000008985	1.650862673	0.556017012	0.745423633	29	42	70	91	74	48	61	54
ENSECAG00000006544	7.619958821	0.556126707	0.745482733	2629	2642	3679	5480	3833	3683	3996	4046
ENSECAG00000009827	5.36050496	0.556653803	0.746056115	551	980	663	649	781	676	931	896
ENSECAG00000014440	2.93764985	0.55670404	0.746056115	102	95	122	244	127	169	134	163
ENSECAG00000012976	5.947970875	0.556788406	0.746056115	528	1185	1150.01	1732	1154	1005	1451	1333
ENSECAG00000024607	6.798709531	0.556817097	0.746056115	1129	1373	2241	2359	3278	2177	2876	1744
ENSECAG00000006020	3.281242049	0.556948707	0.746113949	121	136	138	207	204	178	212	290
ENSECAG00000019122	5.650479688	0.556991596	0.746113949	748	542	1500	797	1466	840	1046	542
ENSECAG00000010401	5.85456117	0.557311399	0.746279461	693	872	872	1227	1337	1235	1332	1292
ENSECAG00000009858	3.57510232	0.557335589	0.746279461	141	131	182	303	294	243	278	257
ENSECAG00000007369	7.135319031	0.557397984	0.746279461	1595	3697	2097	2631	2580	2854	2623	3012
ENSECAG00000018805	5.850674502	0.557422282	0.746279461	645	910	881	1236	1207	1270	1109	1631
ENSECAG00000001110	1.024816108	0.557485269	0.746279461	29	17	75	21	60	26	32	29
ENSECAG00000018940	4.78005082	0.557538584	0.746279461	248	575	538	651	470	691	529	458
ENSECAG00000021449	6.634072085	0.557574929	0.746279461	1511	1654	1748	2061	2100	1762	2012	2046
ENSECAG00000015883	3.135038708	0.557700072	0.746359036	101	87	198	158	219	203	208	157
ENSECAG00000023952	5.979228413	0.557831783	0.746446579	939	1417	914	1079	1157	1030	1296	1583
ENSECAG00000001035	3.618108025	0.55789688	0.746446579	87	237	237	367	241	160	261	314
ENSECAG00000011118	3.471191546	0.558074356	0.746551571	135	188	218	253	184	183	251	263
ENSECAG00000010753	4.98509489	0.558106763	0.746551571	333	571	431	675	609	670	644	947
ENSECAG00000023048	5.873988575	0.558232523	0.746576834	728	1036	1035	1397	1235	959	1112	1431
ENSECAG00000015162	7.420649028	0.558257066	0.746576834	1649	2327	3882	5119	3172	3282	3236	3870

ENSECAG00000024102	3.16344963	0.558328662	0.746584708	61	119	139	270	182	170	243	218
ENSECAG00000023874	1.811938772	0.558439808	0.746645458	26	45	63	136	64	51	70	82
ENSECAG00000023179	12.20767297	0.558574723	0.746737969	56103	58372	111559	126852	107160	71820	94930	100303
ENSECAG00000001651	6.052681852	0.558821723	0.74692241	1011	986	1220	1450	1316	1136	1438	1436
ENSECAG00000019814	6.938339805	0.558844166	0.74692241	1118	1422	2681	2695	2411	3182	2116	3472
ENSECAG00000020660	10.03146459	0.558910554	0.746923279	9234	14433	27410	28645	18828	19442	19983	24274
ENSECAG00000007295	0.404119798	0.559061373	0.747035839	14	4	23	35	19	25	29	43
ENSECAG00000013159	5.335617366	0.559126278	0.747035839	311	527	783	1060	925	835	821	1075
ENSECAG00000023549	4.951876368	0.559202547	0.747049893	467	535	654	480	634	555	703	569
ENSECAG00000015523	0.881053486	0.55937397	0.747191047	12	23	43	31	31	40	51	39
ENSECAG00000013449	4.394866789	0.559530007	0.747278584	156	535	263	356	442	338	485	678
ENSECAG00000027000	2.697871227	0.559571043	0.747278584	53	85	125	237	99	151	131	112
ENSECAG00000008891	6.500048254	0.559768805	0.747297781	704	1082	1876	2317	1975	1515	1890	2977
ENSECAG00000010059	2.37760209	0.559769446	0.747297781	37	55	101	141	85	95	155	137
ENSECAG00000014787	1.441947405	0.559782733	0.747297781	24	84	38	46	29	54	37	83
ENSECAG00000004874	2.794943909	0.559921763	0.747316914	77	69	119	171	149	113	212	155
ENSECAG00000022878	5.715556673	0.559928612	0.747316914	529	405	1548	837	1002	869	991	2063
ENSECAG00000022895	4.029738392	0.56007136	0.747364238	158	377	217.004	441	296	294	346	357
ENSECAG00000017326	6.461422857	0.560122898	0.747364238	1041	2155	1449	1571	1729	1657	1928	1673
ENSECAG00000014586	4.948577139	0.560161402	0.747364238	483	595	494	553	720	466	680	603
ENSECAG00000020931	4.783500532	0.560640665	0.747801313	223	413	491	980	525	469	542	663
ENSECAG00000009065	11.60955763	0.56068418	0.747801313	35679	43384	67754	86310	69003	49809	62511	66112
ENSECAG00000010446	4.044836899	0.560686445	0.747801313	201	219	367	410	360	242	296	427
ENSECAG00000020374	1.988659467	0.560907801	0.747901513	29	16	156	26	201	80	68	19
ENSECAG00000009917	1.935670244	0.560939351	0.747901513	35	86	97	56	37	94	73	82
ENSECAG00000000057	4.390740742	0.560959047	0.747901513	249	239	387	456	559	306	537	503
ENSECAG00000005752	4.930764114	0.561138253	0.748052662	259	419	811	793	533	561	644	695
ENSECAG00000019963	4.491222511	0.561305531	0.748187874	278	287	491	575	415	427	403	556
ENSECAG00000021222	7.409476827	0.56155496	0.748432546	1684	2735	3672	4610	2953	3547	2692	4292
ENSECAG00000023660	5.290873736	0.562003912	0.748889161	293	423	783	1125	882	772	951	942
ENSECAG00000000469	4.488107648	0.562054298	0.748889161	258	298	380	494	541	414	578	483
ENSECAG00000013754	1.327826949	0.562095297	0.748889161	12	62	28	48	50	47	55	74
ENSECAG00000011678	6.51280536	0.562367879	0.749164479	1015	804	1700	2414	2205	1784	2013	2317
ENSECAG00000014554	1.721795777	0.562461332	0.749201131	29	84	42	34	65	53	84	99
ENSECAG00000016107	3.099397923	0.56265124	0.749366239	82	168	102	176	144	259	218	142
ENSECAG00000011429	3.087785438	0.562727319	0.749379723	124	83	83	360	205	158	194	80
ENSECAG00000021819	4.30907291	0.562829515	0.74942798	268	344	431	344	481	335	361	403
ENSECAG00000011195	3.281878347	0.562933114	0.749478092	85	169	167	308	191	151	181	247
ENSECAG00000010736	10.10287794	0.56327031	0.749775837	14444	39280	9268	14734	16441	8454	15874	46602
ENSECAG00000002876	4.910112898	0.563288729	0.749775837	300	387	500	776	747	524	662	789
ENSECAG00000022600	2.746509386	0.563509564	0.749922672	72	118	168	123	162	103	118	141
ENSECAG00000023165	1.249831777	0.563531048	0.749922672	10	63	19	93	14	57	36	63
ENSECAG00000011497	5.728690612	0.564153354	0.750662888	411	1104	726	1182	973	1113	1200	1536
ENSECAG00000015610	5.843639685	0.564617356	0.751190845	715	719	979	1232	1374	1142	1460	1180
ENSECAG00000003118	3.405695262	0.564759623	0.751190845	111	172	199	299	185	129	258	273
ENSECAG00000006052	2.568803295	0.564780567	0.751190845	48	78	112	138	100	87	165	192
ENSECAG00000018799	7.143607877	0.564814591	0.751190845	1641	2169	2386	2654	3354	2623	3156	3783
ENSECAG00000004270	4.043430511	0.565089719	0.751468795	261	241	259	205	503	200	421	390
ENSECAG00000006268	4.924585332	0.565333689	0.751705252	324	223	695	724	796	649	687	608
ENSECAG00000012595	3.604983974	0.565931412	0.752409081	110	196	143	337	254	264	286	292
ENSECAG00000015244	5.143518471	0.56599546	0.752409081	408	306	683	882	935	509	960	819
ENSECAG00000023738	3.997757098	0.566138122	0.752510686	150	216	398	424	370	300	313	274
ENSECAG00000010557	2.816308874	0.566249775	0.752528489	60	74	136	181	210	146	171	104
ENSECAG00000011306	1.336096348	0.5663485	0.752528489	19	32	48	56	52	39	57	78
ENSECAG00000023131	4.945847499	0.566350212	0.752528489	304	603	514	830	638	533	668	629
ENSECAG00000010969	7.932366104	0.566596309	0.752767453	3278	3418	5545	5401	4963	4381	4424	5671
ENSECAG00000024677	5.507413671	0.566663302	0.752768436	475	451	1041	954	1008	754	739	1711
ENSECAG00000021784	2.892635511	0.566998583	0.752952157	87	109	132	218	165	152	162	90
ENSECAG00000012575	3.371751022	0.567039006	0.752952157	77	199	164	220	206	198	238	298
ENSECAG00000020966	4.615879503	0.567085894	0.752952157	173	370	454	905	407	534	380	615
ENSECAG00000023180	7.262042233	0.567098652	0.752952157	1708	2213	3720	3641	3278	2503	3153	3301
ENSECAG00000012156	3.25732739	0.567132948	0.752952157	46	213	113	388	157	266	123	170
ENSECAG00000014798	2.190353512	0.567506401	0.75335994	62	61	48	100	146	44	114	115
ENSECAG00000023410	4.791816699	0.567843986	0.7536612	335	276	498	670	594	632	575	688
ENSECAG00000023646	3.388140152	0.567903241	0.7536612	138	355	110	115	241	192	223	140
ENSECAG00000007205	5.032424974	0.567932335	0.7536612	441	557	633	672	686	474	731	754
ENSECAG00000025158	2.793673478	0.568043428	0.753720592	66	157	120	159	154	81	163	146
ENSECAG00000013635	6.912784984	0.568147884	0.753771165	1126	1595	2125	2997	3016	2256	2785	2911
ENSECAG00000010571	4.695565998	0.568344569	0.753944074	343	291	472	521	596	601	579	539
ENSECAG00000012031	4.254719716	0.56842494	0.753962663	221	296	337	339	372	363	373	630
ENSECAG00000023011	4.660703051	0.568788841	0.754228089	304	377	464	707	345	707	425	499
ENSECAG00000011841	1.807859536	0.568801235	0.754228089	47	51	55	51	91	55	88	80
ENSECAG00000010010	1.837632135	0.568824195	0.754228089	46	44	68	52	73	81	107	55
ENSECAG00000001722	5.131848832	0.569047789	0.754361857	352	459	826	599	798	834	706	804
ENSECAG00000019655	3.756690535	0.569057866	0.754361857	124	281	279	295	249	235	275	317
ENSECAG00000014732	2.060998617	0.569203322	0.754368625	29	60	128	97	70	118	68	51
ENSECAG00000013452	1.14857572	0.569247783	0.754368625	17	19	60	37	46	33	51	69
ENSECAG00000012700	3.656729545	0.569315031	0.754368625	70	206	222	500	185	223	358	207
ENSECAG00000024150	1.703720878	0.569328548	0.754368625	49	50	49	34	89	51	47	111
ENSECAG00000016982	7.312659131	0.569398161	0.75437289	1450	2323	2929	3553	4097	3236	2974	4195
ENSECAG00000008950	5.006300534	0.569677366	0.754579236	336	968	431	526	548	478	721	822
ENSECAG00000012669	3.90837052	0.569686735	0.754579236	142	133	308	392	497	261	343	256
ENSECAG00000008746	8.688948897	0.569780198	0.754615062	5243	4992	8020	7267	11512	7955	8969	9289
ENSECAG00000012731	4.047767355	0.570342099	0.754975485	148	317	259	507.008	277	277	315	455
ENSECAG00000020900	5.085583078	0.570358149	0.754975485	348	435	652	766	764	675	676	951

ENSECAG00000015733	5.987948505	0.570360065	0.754975485	631	1379	730	1247	1402	1083	1518	1796
ENSECAG00000020134	6.153401873	0.57046809	0.754975485	1171	1094	1535	1134	1545	720	1279	2241
ENSECAG00000004829	5.184779741	0.570491726	0.754975485	472	1045	418	582	650	714	593	931
ENSECAG00000020913	6.544038775	0.570550366	0.754975485	1045	919	1524	2583	2608	1793	1965	2101
ENSECAG00000009310	2.940801852	0.570606062	0.754975485	156	89	140	137	150	100	181	171
ENSECAG00000014275	4.314789842	0.570751419	0.754975485	207	442	447	322	278	339	320	664
ENSECAG00000012988	4.554767185	0.570783054	0.754975485	355	363	358	335	591	434	447	663
ENSECAG00000013105	2.713744817	0.57083615	0.754975485	88	85	85	227	139	81	107	189
ENSECAG00000011714	1.458397027	0.570837988	0.754975485	1	40.9997	86	33.999	8.99956	70	97	80.9951
ENSECAG00000014115	0.359431071	0.570868311	0.754975485	7	17	37	32	24	28	13	24
ENSECAG00000015515	4.579149364	0.570916156	0.754975485	234	308	421	592	553	414	459	752
ENSECAG00000017757	5.424807024	0.571032421	0.755023253	389	471	855	1127	935	830	979	1144
ENSECAG00000002222	3.613070471	0.571101645	0.755023253	185	108	209	248	299	243	289	272
ENSECAG00000014128	2.450525168	0.571151633	0.755023253	45	68	88	150	115	131	106	137
ENSECAG00000019212	4.062469299	0.571248746	0.75506378	148	216	282	445	409	224	464	427
ENSECAG00000024349	8.935474484	0.571438379	0.755226575	8302	6847	9011	10314	10407	7282	9722	11621
ENSECAG00000013242	5.954367	0.571579886	0.755285274	434	770	1400	1518	1223	1559	1385	1408
ENSECAG00000006452	5.337873853	0.571615743	0.755285274	419	764	788	950	900	702	831	814
ENSECAG00000002508	6.615985279	0.571809454	0.755453375	1122	2065	1631	2283	1444	1314	1696	3530
ENSECAG00000012002	5.471729008	0.572249627	0.755947014	409	340	827	1380	1115	681	1290	966
ENSECAG00000009630	0.291535213	0.572466553	0.756145661	6	15	23	47	14	13	21	39
ENSECAG00000009481	5.532898782	0.572699204	0.756174918	384	444	985	1269	1088	865	1321	908
ENSECAG00000002115	7.175403935	0.572706599	0.756174918	1719	2153	2676	2445	3157	3196	2843	3957
ENSECAG00000019899	5.653296549	0.572713538	0.756174918	824	689	518	1018	1277	761	1144	1429
ENSECAG00000004437	6.599511768	0.572754915	0.756174918	1155	2081	1551	2160	1249	2055	2310	2077
ENSECAG00000019992	2.776190496	0.572915866	0.756299531	102	85	78	245	152	114	194	54
ENSECAG00000005413	2.634506017	0.573217519	0.756577636	57	123	119	156	118	70	174	123
ENSECAG00000013499	6.985927922	0.573275012	0.756577636	1620	2726	2136	2487	2425	2356	2444	2884
ENSECAG00000020779	2.348897596	0.573326303	0.756577636	34	79	196	71	58	116	62	150
ENSECAG00000020867	8.646061889	0.573437899	0.756637023	9971	6854	4277	5111	7932	6760	7943	8612
ENSECAG00000000460	1.933263274	0.573577971	0.756733966	50	76	42	52	94	61	91	99
ENSECAG00000004992	5.048180714	0.57366749	0.756764196	311	493	733	912	661	607	682	701
ENSECAG00000006675	6.250835455	0.573945393	0.757042902	1075	1080	1265	2045	1671	1253	1541	1658
ENSECAG00000024402	6.606265857	0.574107748	0.75716915	1474	2365	1165	1719	1973	1683	1558	2579
ENSECAG00000008428	4.759895261	0.574691081	0.757850518	292	401	575	708	581	530	511	543
ENSECAG00000015049	3.411980886	0.575033457	0.758112322	100	150	201	229	242	182	261	277
ENSECAG00000019905	0.62182582	0.575065962	0.758112322	8	17	19	50	21	37	50	24
ENSECAG00000017832	6.990793788	0.575089782	0.758112322	1778	1717	2351	1903	3145	2497	3031	2879
ENSECAG00000005459	6.666199319	0.575185572	0.758150634	1203	1860	1628	2674	2200	1863	1995	2047
ENSECAG00000020424	7.509214362	0.575709871	0.758753689	1301	2605	3369	4747	3745	3889	3481	5621
ENSECAG00000014176	3.500591627	0.575925183	0.758949424	147	109	161	297	260	202	321	239
ENSECAG00000019227	4.86932916	0.576140475	0.759070718	213	334	362	1068	742	590	833	475
ENSECAG00000014049	5.542294374	0.576150842	0.759070718	540	676	741	1023	1121	783	1168	1131
ENSECAG00000007262	5.128055796	0.576288821	0.759164474	512	722	368	864	642	796	745	561
ENSECAG00000007497	2.898326554	0.576379401	0.759195775	184	35	83	79	320	35	109	262
ENSECAG00000007702	6.944711479	0.576481506	0.759242248	1048	2536	2172	3453	1965	2599	2207	3008
ENSECAG00000015275	2.974348305	0.576724294	0.759473972	55	92	237	86	77	431	121	66
ENSECAG00000009669	4.798267422	0.576841097	0.759539754	403	403	422	478	693	434	673	722
ENSECAG00000019549	3.655076444	0.577032685	0.759611531	193	95	264	198	398	107	342	326
ENSECAG00000017022	6.675629191	0.577070688	0.759611531	1118	1629	1838	2954	2092	1793	2072	2231
ENSECAG00000019772	6.607525894	0.577096175	0.759611531	1933	1648	1314	1680	2021	2011	1856	1802
ENSECAG00000006300	2.851010439	0.577326086	0.759826131	49	127	129	152	127	95	188	257
ENSECAG00000009530	7.319992028	0.577593699	0.760090296	1970	1148	4167	2630	4889	2323	4048	3509
ENSECAG00000026818	-0.021383138	0.577704915	0.76014861	8	14	7	26	27	23	20	11
ENSECAG00000009060	2.152710613	0.578268052	0.760676649	44	56	52	128	88	104	74	134
ENSECAG00000013896	8.34368547	0.57827972	0.760676649	2518	4075	5340	9558	7065	8054	8542	5737
ENSECAG00000022627	9.424024674	0.578307067	0.760676649	8512	9693	11272	13297	15889	13921	15710	17179
ENSECAG00000007194	0.996029416	0.578409371	0.760723147	8	18	56	39	69	30	56	21
ENSECAG00000016479	6.097105519	0.578516577	0.760776082	974	875	1210	1160	1869	1102	1719	1510
ENSECAG00000020559	2.68012159	0.578750116	0.760926538	69	70	142	115	172	141	160	98
ENSECAG00000026883	2.087792984	0.57876493	0.760926538	40	92	59	63	92	70	107	115
ENSECAG00000009750	4.607806832	0.578857977	0.760960816	191	296	405.002	740	531	552	566.001	539
ENSECAG00000016440	5.834656033	0.579120428	0.761217758	481	1031	828	1384	1118	1256	1434	1318
ENSECAG00000003947	1.81070626	0.579372712	0.761461278	26	39	65	93	69	83	66	94
ENSECAG00000020351	4.488075777	0.579524472	0.761572641	227	310	403	511	459	405	491	678
ENSECAG00000012400	7.087771752	0.579803574	0.761851302	136	2309	3619	2469	417	5274	5295	1758
ENSECAG00000000539	7.25612555	0.579884275	0.761869233	2581	2199	2342	1778	4379	2528	3466	3677
ENSECAG00000000684	3.950642248	0.58018718	0.762113711	159	118	274	463	373	262	355	420
ENSECAG00000023306	6.586522074	0.580204506	0.762113711	1558	1888	1047	1065	2194	1668	2473	2491
ENSECAG00000004924	4.865081208	0.580276514	0.762120189	256	441	602	884	512	475	537	833
ENSECAG00000017253	1.413850314	0.580417524	0.76221728	18	27	56	105	51	67	35	41
ENSECAG00000011175	3.556803046	0.580490335	0.762224799	126	215	236	270	220	170	267	285
ENSECAG00000011412	4.164234319	0.580594054	0.762265414	82	170	574	620	155	306	462	471
ENSECAG00000020453	5.651737091	0.580655444	0.762265414	602	711	1042	1277	1077	976	952	1033
ENSECAG00000006358	3.00624274	0.580980029	0.762603406	82	102	114	213	162	151	127	297
ENSECAG00000021253	3.392219577	0.581139882	0.762725117	96	171	200	197	277	186	266	213
ENSECAG00000011563	2.341238926	0.581270415	0.762808322	48	59	105	106	121	88	113	134
ENSECAG00000013281	1.693312454	0.581423535	0.762921146	30	16	76	78	105	57	81	46
ENSECAG00000010209	5.377366775	0.581860839	0.763364526	664	551	640	662	1021	712	1001	1027
ENSECAG00000021836	5.978458816	0.581895807	0.763364526	538	1266	904	1351	1176	1508	1346	1647
ENSECAG00000017261	6.969831364	0.582114369	0.763556392	2501	2208	1591	2279	4483	1621	1583	2138
ENSECAG00000016559	0.374544655	0.582176468	0.763556392	8	7	26	35	52	10	31	20
ENSECAG00000026456	1.432730256	0.582260019	0.763561052	7	21	22	195	153	11	7	8
ENSECAG00000002280	6.229107594	0.582314427	0.763561052	894	915	1817	1836	1580	1378	1419	1641
ENSECAG00000007427	3.670064012	0.582725454	0.76401184	169	172	196	254	292	259	273	318

ENSECAG00000012217	5.21561915	0.582827232	0.764057114	187	2638	11	35	712	1486	9	22
ENSECAG00000012554	1.005276954	0.583770001	0.765045167	14	33	30	43	34	23	56	67
ENSECAG00000010550	6.997624461	0.583773818	0.765045167	895.922	1724.41	2105.2	3803.87	2399.01	3319.38	2273.09	3648.61
ENSECAG00000020438	3.655773046	0.583782926	0.765045167	209	116	203	180	238	707	85	85
ENSECAG00000000641	4.726143345	0.584090255	0.765315636	578	274	370	574	438	539	464	660
ENSECAG00000019257	5.458276381	0.584124027	0.765315636	743	646	587	633	1036	975	1121	806
ENSECAG00000010728	2.177090808	0.584356468	0.765506128	84	94	25	131	11	228	17	45
ENSECAG00000019898	0.977288935	0.584404168	0.765506128	27	32	38	38	31	52	23	38
ENSECAG00000001098	0.97095928	0.584539748	0.76559546	12	11	39	60	29	57	42	43
ENSECAG00000024015	4.084017226	0.584757963	0.765764179	206	238	291	331	332	315	468	409
ENSECAG00000009236	6.714877106	0.58480336	0.765764179	898	1673	1807	2457	2029	1988	2046	3583
ENSECAG00000017195	5.80598533	0.58544914	0.766463993	524	875	1099	1658	1015	1103	1136	1237
ENSECAG00000015258	10.25794063	0.585472716	0.766463993	9357	17920	29046	37942	23897	21689	24919	26365
ENSECAG00000013501	3.895951536	0.585591202	0.766486929	105	360	296	330	275	290	301	310
ENSECAG00000011713	6.255258892	0.585625157	0.766486929	799	1122	1720	1966	1757	1251	1690	1424
ENSECAG00000008550	7.321993029	0.585927157	0.766535418	2056	1640	4249	3781	3605	3040	2875	3132
ENSECAG00000024606	6.004461127	0.585983854	0.766535418	642	1040	1189	1244	1480	1145	1291	1913
ENSECAG00000004232	4.165624754	0.585988761	0.766535418	189	182	297	507	399	345	404	472
ENSECAG00000011355	8.138736166	0.585998268	0.766535418	2842	5982	3284	5355	4776	4955	5700	10929
ENSECAG00000009344	8.798571517	0.586054129	0.766535418	5846	6933	7055	7438	11647	8184	10094	10755
ENSECAG00000002607	1.342166187	0.586066994	0.766535418	25	23	42	65	76	35	38	79
ENSECAG00000000558	7.177945876	0.586329509	0.766764383	1837	2367	3039	3144	3129	2898	2481	3017
ENSECAG00000009195	4.7602724	0.586396603	0.766764383	636	64	455	264	1444	416	534	155
ENSECAG00000024268	10.29687873	0.586444507	0.766764383	13049	16474	31677	32840	26476	19481	26529	27708
ENSECAG00000011826	5.755660516	0.587050804	0.767468788	596	826	904	1141	1118	1001	1344	1401
ENSECAG00000023996	2.039685692	0.587155472	0.76750771	45	129	71	37	86	55	68	107
ENSECAG00000022045	6.469465325	0.587257535	0.76750771	1313	1171	1912	1856	1982	1516	1800	1813
ENSECAG00000020746	6.65369746	0.587324755	0.76750771	924	1815	1477	2253	1952	2114	1765	3386
ENSECAG00000003332	4.012613041	0.587350778	0.76750771	190	246	386	333	235	329	381	336
ENSECAG00000008307	6.049620621	0.587473544	0.767579854	718	1004	1034	1510	1487	1213	1384	1918
ENSECAG00000009309	6.183351042	0.588034469	0.768224403	763	1310	1497	1681	1425	1420	1398	1599
ENSECAG00000019108	0.481672088	0.588134826	0.76823806	14	19	27	39	35	30	16	17
ENSECAG00000017928	6.064322405	0.588180151	0.76823806	602	1085	1421	1871	1224	1031	1769	1349
ENSECAG00000011444	7.132139228	0.588343039	0.768358726	1482	2077	2412	3000	3080	2749	3110	3815
ENSECAG00000018429	5.398915336	0.588464056	0.768358726	501	604	661	934	1022	839	956	958
ENSECAG00000000282	3.26002458	0.588475412	0.768358726	78	129	153	264	168	165	213	331
ENSECAG00000013214	5.375566664	0.588980201	0.768929454	446	661	677	884	878	841	891	1119
ENSECAG00000011783	7.733212154	0.589050955	0.768933473	2881	3061	3981	5467	4622	3502	3863	5081
ENSECAG00000020944	4.47156363	0.589384814	0.769255237	198	282	500	462	437	394	485	701
ENSECAG00000024957	4.401793036	0.589432854	0.769255237	129	488	373	604	350	545	366	385
ENSECAG00000024838	3.498974549	0.589557077	0.76932899	107	205.003	208.003	308.002	203	185.002	248.009	266.004
ENSECAG00000017650	6.426088736	0.589741967	0.769481882	891	1902	1430	1960	1622	1537	1846	1906
ENSECAG00000021745	4.410822501	0.590071001	0.769822794	241	294	515	488	482	289	498	442
ENSECAG00000013765	4.868497424	0.590206003	0.769910517	354	368	490	941	690	410	608	646
ENSECAG00000023234	1.075452394	0.590523192	0.770187078	16	30	41	39	33	28	43	86
ENSECAG00000015431	2.770821174	0.590579989	0.770187078	82	95	112	132	144	162	133	170
ENSECAG00000007948	1.49903904	0.59062137	0.770187078	17	53	42	62	55	87	48	57
ENSECAG00000011896	4.190119641	0.59082934	0.770369861	280	296	318	372	433	306	382	336
ENSECAG00000024728	5.02162694	0.591323297	0.770925451	354	465	763	771	729	555	615	721
ENSECAG00000015832	11.3360444	0.591750472	0.771286218	23209	36595	63900	71843	55415	39220	54889	56369
ENSECAG00000022024	6.544484022	0.591816885	0.771286218	1244	1769	1943	1561	1923	1602	1951	2029
ENSECAG00000008125	3.457798296	0.591884828	0.771286218	154	174	141	198	254	169	235	344
ENSECAG00000008470	4.170599784	0.591885742	0.771286218	204	252	379	473	322	333	409	376
ENSECAG00000000393	7.308264017	0.59196441	0.771286218	1327	2141	3544	3275	3095	3405	3093	4902
ENSECAG00000002556	2.707068739	0.592007314	0.771286218	103	47	146	84	236	74	150	140
ENSECAG00000008561	7.21252206	0.592141552	0.771372657	1331	1717	2859	3839	2819	3357	2915	4437
ENSECAG00000014002	8.758038061	0.59242152	0.771648895	6455	10434	6381	6637	7451	8387	8957	9419
ENSECAG00000023993	1.997939118	0.59258955	0.771779283	35	26	148	92	92	55	89	65
ENSECAG00000024638	7.105442959	0.592726419	0.771836836	1530	1642	2537	3115	3059	2988	2224	4307
ENSECAG00000011004	6.517478272	0.59279684	0.771836836	724	1459	1240	2659	1915	2133	2233	1953
ENSECAG00000021107	7.055384814	0.592837534	0.771836836	1467	3522	1958	2490	2555	2686	2320	3000
ENSECAG00000024056	4.938033947	0.593500646	0.772611634	363	375	615	601	717	558	791	682
ENSECAG00000006430	10.64843649	0.593592269	0.772642383	15202	24091	36723	44579	34090	23759	37202	32838
ENSECAG00000009115	5.740532189	0.593707452	0.772703788	658	1156	872	1062	1092	870	1073	1300
ENSECAG00000020716	11.32969366	0.594257927	0.773331642	42093	49342	47185	38558	51780	41434	52056	59962
ENSECAG00000017404	7.442874358	0.594650822	0.77375431	1628	2669	4411	4202	3643	3008	3762	3445
ENSECAG00000008557	0.346278291	0.594728667	0.773766989	22	20	23	17	17	22	19	34
ENSECAG00000011724	0.609831994	0.595217062	0.774313747	18	36	27	21	23	18	27	45
ENSECAG00000008411	3.891293833	0.595580008	0.774539347	71	328	401	328	140	443	243	307
ENSECAG00000010390	1.836712905	0.595614501	0.774539347	17	75	54	127	58	62	73	78
ENSECAG00000017355	1.778560432	0.595698387	0.774539347	33	57	52	109	64	49	74	78
ENSECAG00000021477	5.856125758	0.595750089	0.774539347	494	753	1037	1570	1212	985	1477	1578
ENSECAG00000014036	4.391926965	0.59579285	0.774539347	354	616	208	217	447	378	427	390
ENSECAG00000022837	3.094714816	0.595799498	0.774539347	164	113	137	166	157	155	183	177
ENSECAG00000002700	2.147460983	0.596043208	0.77472933	38	33	136	63	140	65	145	50
ENSECAG00000013654	4.198869688	0.596082011	0.77472933	172	317.001	395.002	455	361	316	405	391
ENSECAG00000019829	5.776348155	0.596158513	0.774740138	882	1139	786	921	1222	838	972	1407
ENSECAG00000017836	7.662215585	0.596418633	0.774989537	2551	3538	3119	3214	5203	3058	4854	5470
ENSECAG00000021031	9.29927469	0.596998102	0.775653795	7206	7712	17781	13959	10577	9018	8781	22509
ENSECAG00000012588	4.770441438	0.597396362	0.776082491	357	659	459	399	533	492	571	590
ENSECAG00000014616	5.641661737	0.597476292	0.776097591	520	632	754	1362	972	946	830	1822
ENSECAG00000016530	4.333160004	0.597754665	0.77633755	311	247	369	293	451	495	367	490
ENSECAG00000003791	5.520820377	0.597797679	0.77633755	726	969	648	780	938	768	1015	973
ENSECAG00000022853	6.008162831	0.598272918	0.776839132	1086	172	2460	974	1795	784	1478	879
ENSECAG00000020797	5.406197418	0.598320652	0.776839132	491	744	689	746	884	886	833	1212

ENSECAG00000004024	6.771437834	0.598926711	0.777486333	1342	1989	1942	2554	1852	2321	1873	2715
ENSECAG00000016908	1.03687359	0.598955982	0.777486333	11	9	49	59	70	43	49	17
ENSECAG00000005075	4.217636863	0.599204394	0.777557142	214	319	384	410	364	261	448	431
ENSECAG00000024342	5.267901032	0.599205957	0.777557142	312	593	597	1061	858	697	861	1066
ENSECAG00000015649	4.070068516	0.599277378	0.777557142	162	321	328	407	279	352	276	438
ENSECAG00000001568	5.031383133	0.599284272	0.777557142	351	497	795	719	530	726	541	829
ENSECAG00000024404	4.93850933	0.5994907	0.777736166	363	337	750	480	841	624	689	590
ENSECAG00000006505	6.653185345	0.599559452	0.777736556	1151	1882	1842	2357	1871	1847	2154	2223
ENSECAG00000019041	4.797740367	0.599691834	0.777819477	445	458	357	676	578	444	622	596
ENSECAG00000000723	2.713781705	0.599817937	0.777894235	61	78	129	150	168	95	151	180
ENSECAG00000013539	4.656317456	0.599931775	0.777953073	378	342	476	572	533	465	511	516
ENSECAG00000019646	3.692762459	0.600137095	0.778130512	97	142	375	369	263	233	263	260
ENSECAG00000010350	2.543734074	0.600591572	0.778630926	38	63	151.001	123	144	107	159	113
ENSECAG00000013277	2.510805057	0.600754056	0.778752719	44	122	87	99	110	115	96	197
ENSECAG00000011203	4.615174026	0.601002754	0.778970296	232	548	480	492	459	391	515	617
ENSECAG00000001628	3.2221323	0.60105902	0.778970296	91	144	162	196	212	196	236	186
ENSECAG00000013553	5.417935405	0.601289602	0.77912312	418	793	699	1223	709	763	897	1093
ENSECAG00000004549	6.156072113	0.601332125	0.77912312	952	763	1495	1272	1682	1361	1677	1704
ENSECAG00000007920	7.326940194	0.601382659	0.77912312	2218	2558	2915	3759	3384	2464	3338	3752
ENSECAG00000009178	5.812304627	0.601947422	0.779765889	818	1075	888	1107	1234	876	1144	1304
ENSECAG00000016854	6.879883629	0.603262448	0.781046388	3982	1550	811	860	2668	1770	2250	2389
ENSECAG00000024479	2.823377893	0.603272895	0.781046388	93	98	109	211	147	110	147	155
ENSECAG00000000722	2.862265549	0.603324267	0.781046388	113	64	189	149	193	113	120	142
ENSECAG00000003551	2.377726604	0.603450785	0.781046388	25	60	159	87	82	122	137	127
ENSECAG00000016937	6.77865208	0.603473587	0.781046388	1027	1724	2238	3196	2309	1746	2381	2394
ENSECAG00000010434	4.687820583	0.603486685	0.781046388	293	253	581	790	533	338	492	727
ENSECAG00000011621	6.833155146	0.603539203	0.781046388	977	1375	1832	3423	2185	2467	2434	3284
ENSECAG00000019441	5.018431704	0.603628373	0.781046388	405	380	953	581	610	416	631	981
ENSECAG00000001048	2.520654648	0.603647106	0.781046388	72	55	149	139	113	96	108	132
ENSECAG00000000513	4.595512775	0.603683726	0.781046388	270	395	534	530	546	405	497	500
ENSECAG00000011411	6.620080937	0.603692077	0.781046388	1078	1736	1888	2418	2057	1980	1662	2202
ENSECAG00000011254	5.556853301	0.603928946	0.781263883	576	865	645	1291	852	834	950	1180
ENSECAG00000006997	5.081485678	0.604333905	0.781698752	622	502	613	547	805	571	719	613
ENSECAG00000008386	1.422121368	0.604605899	0.781872504	34	42	67	46	50	69	61	13
ENSECAG00000016359	4.551181406	0.60469683	0.781872504	226	394	522	561	479	369	535	509
ENSECAG00000020820	6.10177131	0.604711417	0.781872504	937	1285	1174	1407	1628	1244	1139	1531
ENSECAG00000021500	2.795794486	0.604743493	0.781872504	31	113	199	191	122	147	150	112
ENSECAG00000009417	4.184240582	0.604913622	0.78191453	162	306	259	458	395	304	461	480
ENSECAG00000009389	10.6985423	0.605011675	0.78191453	16398	23311	38194	46709	35924	25560	38919	31935
ENSECAG00000023836	4.413313973	0.60504939	0.78191453	196	319	363	723	400	414	311	587
ENSECAG00000011589	3.160630168	0.605051272	0.78191453	54	152	114	267	141	186	166	327
ENSECAG00000022700	5.715055501	0.605273002	0.782023797	576	694	833	1327	1267	998	1165	1279
ENSECAG00000014227	4.84877741	0.605273479	0.782023797	285	324	681	582	672	582	578	757
ENSECAG00000026952	2.087181408	0.605425459	0.782131219	28	64	67	170	68	65	54	143
ENSECAG00000015133	5.481629505	0.605561054	0.78221745	485	646	673	1072	1563	579	1029	870
ENSECAG00000006589	6.644977686	0.606054974	0.782766467	1319	2213	1408	2091	1884	1561	1797	2881
ENSECAG00000013344	1.734685778	0.606156862	0.782809077	23	58	27	103	86	65	69	76
ENSECAG00000005626	3.021762062	0.606237743	0.782824552	89	136	177	179	162	142	175	163
ENSECAG00000016998	6.133567266	0.606511811	0.783089454	622	1372	1096	2157	1895	1520	1202	890
ENSECAG00000025074	4.765135296	0.607045998	0.783661978	298	395	577	704	592	369	574	678
ENSECAG00000011445	4.114262427	0.607093182	0.783661978	242	298	345	316	334	334	296	429
ENSECAG00000000796	4.66813235	0.607446039	0.78393311	293	382	484	452	577	480	575	646
ENSECAG00000015809	1.800447166	0.607483528	0.78393311	23	45	132	58	48	96	58	55
ENSECAG00000010876	1.481374634	0.607510213	0.78393311	14	37	58	107	33	58	35	84
ENSECAG00000025036	6.321749518	0.607672129	0.784053001	812	1007	1749	1618	1939	1483	1856	1920
ENSECAG00000012882	9.052836711	0.607991416	0.784257198	7185	6211	10128	9396	13564	9637	12260	12951
ENSECAG00000024591	3.353806305	0.608122695	0.784257198	169	258	102	160	178	149	216	269
ENSECAG00000020301	2.079424898	0.608130871	0.784257198	32	54	84	99	91	60	97	134
ENSECAG00000015499	7.368142883	0.608175443	0.784257198	1498	1850	3428	4084	4255	3582	3411	3664
ENSECAG00000009247	5.825891168	0.608175512	0.784257198	653	529	1360	1703	1785	1016	694	994
ENSECAG00000002048	5.301764217	0.608398791	0.784433133	347	851	529	792	748	623	905	1334
ENSECAG00000015963	2.735145948	0.608450027	0.784433133	44	85	120	189	135	131	146	188
ENSECAG00000022535	5.760366539	0.608590979	0.784525834	559	968	895	1529	1091	919	1189	1189
ENSECAG00000020188	4.209437043	0.608700975	0.784578614	178	467	214	482	444	359	391	257
ENSECAG00000011856	0.594212307	0.608802044	0.784619876	7	23	21	41	33	28	28	41
ENSECAG00000011220	6.212245428	0.609119962	0.78494057	2362	363	1447	647	2748	669	1830	264
ENSECAG00000012353	4.372202407	0.609496357	0.78533654	139	323	313	609	328	366	503	696
ENSECAG00000019322	7.247304294	0.609625635	0.785414045	1026	1896	2443	4895	3801	2068	3299	4933
ENSECAG00000021041	3.745323531	0.609902106	0.785681148	192	212	247	287	300	186	323	266
ENSECAG00000023152	8.120195803	0.610014002	0.785736208	4030	6457	4195	4564	4723	5713	5986	5622
ENSECAG00000001187	6.194602523	0.610164659	0.785841176	637.011	1413	1098.01	1602	1504	1496	1579	2027
ENSECAG00000016910	0.38989593	0.610245539	0.785856263	10	16	25	25	25	26	23	38
ENSECAG00000011014	3.698334882	0.610889273	0.786516988	104	213	203	330	305	251	290	317
ENSECAG00000020528	0.453675314	0.61091751	0.786516988	24	6	30	12	40	19	26	35
ENSECAG00000014090	11.37020645	0.610966286	0.786516988	26853	34139	63345	74023	57393	41177	58875	53758
ENSECAG00000005065	2.520536998	0.611485222	0.787047668	45	62	119	144	118	82	120	205
ENSECAG00000023146	3.627076125	0.611568078	0.787047668	87	185	244	429	280	168	298	234
ENSECAG00000010835	2.692855219	0.611586328	0.787047668	58	95	158	164	124	106	105	177
ENSECAG00000018693	1.362350466	0.611720538	0.787131229	37	18	30	69	47	45	54	84
ENSECAG00000010473	4.59070208	0.612324904	0.78777585	329	277	436	472	662	453	531	507
ENSECAG00000013049	5.471796377	0.612427954	0.78777585	465	524	913	995	1013	777	1090	1107
ENSECAG00000021867	4.900793136	0.612429509	0.78777585	433	400	444	578	717	445	865	663
ENSECAG00000009767	3.214356306	0.612648471	0.787843243	77	129	165	352	62	327	115	192
ENSECAG00000022785	4.876532489	0.612727631	0.787843243	427	358	560	465	745	571	774	529
ENSECAG00000021957	5.636150561	0.612745843	0.787843243	493	641	960	1174	1086	1036	1186	1126

ENSECAG00000012113	7.791008901	0.612759262	0.787843243	1688	2922	3750	6443	4689	4264	5698	5495
ENSECAG00000023094	7.676464255	0.612906526	0.78794342	2865	3146	3817	4734	3999	3418	4569	4454
ENSECAG00000021459	5.91728456	0.613027198	0.787976862	741	1178	1175	1142	1219	950	1257	1496
ENSECAG00000001546	8.062974535	0.613071243	0.787976862	2490	3169	6063	5623	5168	5217	5776	8328
ENSECAG00000002379	5.930223569	0.613309472	0.788075821	688	571	1115	1631	1351	1208	1299	1640
ENSECAG00000012858	1.79390158	0.613313175	0.788075821	64	11	122	51	92	43	104	10
ENSECAG00000024373	6.380930545	0.613356318	0.788075821	906	1317	1640	1422	2422	1347	1878	1866
ENSECAG00000021530	6.107368673	0.61346105	0.788121264	786	1022	1114	1515	1582	1349	1453	1807
ENSECAG00000011967	5.450836606	0.613610888	0.788224636	536	508	917	829	988	941	995	974
ENSECAG00000020326	3.278351718	0.613766342	0.7883352	144	94	160	188	288	117	110	391
ENSECAG00000007798	4.609623258	0.61394735	0.788478556	226	274	492	619	438	485	708	556
ENSECAG00000021307	2.8553808	0.61419509	0.788707573	87	139	109	98	136	190	102	223
ENSECAG00000014987	4.59547209	0.614388011	0.788866153	254	323	450	542	527	542	524	556
ENSECAG00000024938	4.606208166	0.614582031	0.788973364	229	356	467	769	348	539	347	722
ENSECAG00000003229	8.810543484	0.614610389	0.788973364	5310	7852	6713	8090	11156	8355	10092	11233
ENSECAG00000016813	4.780724189	0.614715039	0.789018558	355	398	421	563	683	449	569	778
ENSECAG00000007811	3.291520446	0.614907526	0.789156405	64	121	204	372	178	160	206	229
ENSECAG00000010823	4.148347203	0.614961346	0.789156405	213	157	344	429	461	308	360	471
ENSECAG00000021411	2.3166703	0.615559287	0.789834513	24	129	98	119	82	115	91	91
ENSECAG00000012790	0.683276566	0.615699298	0.789924957	13	17	11	57	32	32	39	36
ENSECAG00000013929	6.414196218	0.615948299	0.790155196	676	1041	1857	2112	1832	1739	2094	2002
ENSECAG00000007655	4.657356196	0.616608754	0.790827426	239	267	384	799	579	451	586	661
ENSECAG00000022194	6.705146657	0.616611527	0.790827426	1987	1548	1646	1879	2245	1731	2134	2278
ENSECAG00000014168	7.148363377	0.61668871	0.790837147	1732	2730	2466	3195	3187	2241	3169	2802
ENSECAG00000024390	7.23189728	0.616859621	0.790901338	1532	2917	2793	3703	2541	3000	3327	3151
ENSECAG00000012046	10.53052215	0.616940773	0.790901338	11506	22170	38891	39509	27061	24483	36365	29775
ENSECAG00000012924	1.082963949	0.616947593	0.790901338	15	26	42	75	30	38	28	63
ENSECAG00000014743	4.678807052	0.61721729	0.791157812	341	554	391	486	487	479	473	630
ENSECAG00000017219	5.225850501	0.617457029	0.791331559	324	444	920	763	730	743	664	1265
ENSECAG00000024976	5.44593322	0.617492132	0.791331559	579	792	625	696	958	852	854	1264
ENSECAG00000000595	4.605937451	0.617599303	0.791356172	314	337	378	515	549	426	578	632
ENSECAG00000021815	3.218693329	0.617650637	0.791356172	75	146	205	268	150	137	201	257
ENSECAG00000008349	2.510984911	0.617934213	0.791630232	63	73	116	99	140	65	179	131
ENSECAG00000023054	5.096391781	0.618350126	0.792073747	287	567	713	967	602	637	782	735
ENSECAG00000020068	6.674255306	0.619200748	0.792887405	611	1297	2160	2843	1909	1987	2298	3136
ENSECAG00000013345	7.820139567	0.61921754	0.792887405	3108	5821	3284	3355	4210	4563	4323	4857
ENSECAG00000014110	1.473422952	0.619261737	0.792887405	40	44	51	55	62	43	61	46
ENSECAG00000007288	3.867117513	0.619264463	0.792887405	122	146	275	321	14	1089	24	185
ENSECAG00000014270	3.501459239	0.6193794	0.79294521	157	209	201	217	233	202	210	262
ENSECAG00000014412	5.721905985	0.61945337	0.792950562	839	832	742	770	1336	797	1303	1342
ENSECAG00000009404	2.777071885	0.619802627	0.793308263	80	75	170	92	145	79	221	181
ENSECAG00000011433	6.00588704	0.620044188	0.793528054	912	846	1117	1130	1290	1454	1447	1548
ENSECAG00000000381	3.785604237	0.620150135	0.793574258	156	242	346	269	83	607	182	133
ENSECAG00000020478	6.588511711	0.62052451	0.793963905	879	1766	1444	3120	2017	2028	1132	2539
ENSECAG00000010240	5.886216563	0.620642457	0.794025402	516	922	1219	1213	1272	1112	1293	1647
ENSECAG00000000718	6.774910653	0.620817073	0.794159376	1268	1402	1825	2571	2524	2176	2473	2683
ENSECAG00000000081	0.216047995	0.620967499	0.79426238	12	25	9	16	7	30	20	43
ENSECAG00000010106	1.954235208	0.621094573	0.794335495	24	24	105	96	76	43	112	124
ENSECAG00000023124	4.429464312	0.621481639	0.794669832	340	385	251	305	529	306	634	481
ENSECAG00000015383	1.727615583	0.62155013	0.794669832	17	70	53	64	53	63	42	145
ENSECAG00000020148	2.658081532	0.621565816	0.794669832	80	21	165	122	190	108	140	136
ENSECAG00000010378	5.474141207	0.621720572	0.794778255	547	776	781	1047	995	683	1032	898
ENSECAG00000012157	3.553893024	0.621939395	0.794887956	89	209	236	222	306	224	218	306
ENSECAG00000005597	2.423010726	0.621946306	0.794887956	65	94	99	121	145	69	94	115
ENSECAG00000015743	6.993047276	0.622241589	0.795175901	1132	1419	2490	3417	3414	2490	2645	2957
ENSECAG00000002816	4.952489776	0.622868102	0.795887019	287	397	626	733	714	610	628	821
ENSECAG00000009764	5.365340615	0.62313103	0.79613345	616	670	628	598	913	805	987	986
ENSECAG00000006591	3.246169771	0.623295034	0.796230073	57	165	277	212	119	152	173	313
ENSECAG00000023577	4.901063822	0.623465155	0.796230073	377	370	551	609	662	556	688	768
ENSECAG00000024675	2.661953582	0.623564642	0.796230073	44	103	149	178	74	110	146	168
ENSECAG00000020443	3.148247245	0.623623197	0.796230073	101	104	216	127	238	118	187	265
ENSECAG00000020404	5.031034897	0.623626798	0.796230073	451.999	333	778.999	772.997	729.999	658.999	646.999	573.999
ENSECAG00000011434	6.108293012	0.623627127	0.796230073	565	1273	1141	1538	1350	1333	1593	1945
ENSECAG00000013127	5.762544793	0.623718593	0.796257377	839	762	965	731	1391	955	1138	1411
ENSECAG00000005271	4.892713021	0.623852536	0.796338896	364	483	638	612	606	472	517	834
ENSECAG00000009590	4.614610797	0.624054915	0.796507745	296	358	358	554	595	579	510	482
ENSECAG00000006588	5.744348957	0.62431085	0.796744903	729	657	780	1256	1329	983	1222	1266
ENSECAG00000002452	1.177378366	0.624381058	0.796745011	12	55	25	44	51	29	43	80
ENSECAG00000004773	4.945927639	0.624469217	0.796768022	431	382	605	503	677	675	694	696
ENSECAG00000024771	7.432060851	0.624649144	0.796908104	1239	2055	3165	5187	3637	4459	3735	3675
ENSECAG00000023151	5.022942571	0.624815017	0.796984297	326	597	565	593	717	635	719	836
ENSECAG00000018761	0.499460364	0.624849156	0.796984297	14	20	25	21	28	15	40	40
ENSECAG00000012499	6.717307411	0.624940764	0.797011669	1973	1748	1482	1903	2332	1689	2335	2093
ENSECAG00000006486	4.662436056	0.625237836	0.797301043	254	267	509	636	687	410	626	549
ENSECAG00000007189	1.42975151	0.62538423	0.79736428	18	35	31	88	55	55	77	49
ENSECAG00000022770	6.079509011	0.625429855	0.79736428	897	1494	1102	1177	1250	1274	1474	1456
ENSECAG00000003211	1.791827305	0.62549796	0.79736428	48	98	23	67	76	46	62	83
ENSECAG00000006885	7.668336109	0.625813338	0.797647743	2589	3220	3489	5399	4610	3038	3974	4832
ENSECAG00000016617	3.668215704	0.625982524	0.797647743	115	202	211	420	210	211	255	347
ENSECAG00000022301	4.365038349	0.625999402	0.797647743	209	329	445	509	433	372	482	365
ENSECAG00000014145	0.719401279	0.626028673	0.797647743	18	17	42	38	31	14	31	48
ENSECAG00000022114	5.40387507	0.626116656	0.797647743	452	679	507	1122	957	727	953	1182
ENSECAG00000021707	5.783527997	0.626218478	0.797647743	339.999	751.999	1077	1560	850.999	1152	1072	1954
ENSECAG00000000375	7.115707785	0.62632132	0.797647743	1805	1617	2895	2341	3649	2469	3918	2453
ENSECAG00000013047	1.212473175	0.626337604	0.797647743	16	23	58	76	42	46	38	47

ENSECAG00000001625	4.046419852	0.626352153	0.797647743	172	201	432	401	319	377	288	327
ENSECAG00000018659	7.547924217	0.627323016	0.798794588	2119	5440	2497	3041	3099	3519	3273	5104
ENSECAG00000020854	6.205951253	0.627472671	0.798895617	1344	764	981	1364	1719	1256	1627	2111
ENSECAG00000026849	5.762763087	0.627567243	0.7989265	824	1242	910	680	1003	880	1136	1383
ENSECAG00000014589	0.602995482	0.627775753	0.799102409	15	9	34	31	15	24	53	40
ENSECAG00000007820	1.823579862	0.627915694	0.799175942	31	55	50	128	65	79	74	49
ENSECAG00000009383	4.45662013	0.627974195	0.799175942	234	317	504	530	456	361	488	471
ENSECAG00000022474	4.799527162	0.628336176	0.799249424	348	263	675	475	718	548	446	802
ENSECAG00000002171	4.468703406	0.628339598	0.799249424	289	219	797	293	456	726	144	351
ENSECAG00000019318	3.92108309	0.628343993	0.799249424	221	526	121	162	512	358	136	130
ENSECAG00000018276	6.058353403	0.628374672	0.799249424	936	999	987	1238	1489	1286	1478	1727
ENSECAG00000019416	6.505949759	0.628383657	0.799249424	1035	1084	1592	2200	1964	2070	1704	2410
ENSECAG00000019103	3.508593888	0.62856659	0.799392612	245	148	129	241	206	189	200	315
ENSECAG00000017344	4.624091573	0.628784061	0.799421968	219	442	526	609	434	470	442	653
ENSECAG00000001626	5.34203246	0.628824449	0.799421968	451	509	815	1198	744	782	743	1020
ENSECAG00000021927	10.55182534	0.628837942	0.799421968	14950	21311	36879	38089	32873	23027	32632	31995
ENSECAG00000006861	4.764417331	0.62887111	0.799421968	462	441	350	358	616	507	691	626
ENSECAG00000000124	8.212012227	0.628993159	0.799473903	3668	4343	5556	4953	5863	6353	6436	8212
ENSECAG00000008056	5.116627183	0.629052692	0.799473903	252	606	596	829	648	880	1057	481
ENSECAG00000021521	4.331907989	0.6291873	0.799555542	196	322	346	440	368	524	444	443
ENSECAG00000025140	4.594404618	0.629485311	0.799844789	350	559	301	443	385	426	497	646
ENSECAG00000007671	6.917473872	0.629686642	0.80001114	1492	1816	2061	2243	3106	2025	2721	3089
ENSECAG00000008097	5.013607461	0.629856744	0.800103999	597	531	461	577	681	578	620	730
ENSECAG00000010085	5.041400999	0.629987791	0.800103999	352	455	629	697	766	491	802	910
ENSECAG00000016826	5.521240741	0.630069419	0.800103999	542	648	796	965	959	1154	928	1020
ENSECAG00000017946	8.170082801	0.630112815	0.800103999	2632	4760	5284	5917	5353	6037	6852	7849
ENSECAG00000008616	3.45766173	0.630167121	0.800103999	171	90	280	241	299	187	244	125
ENSECAG00000007827	7.632331731	0.630182247	0.800103999	2071	3904	3745	4629	3708	3935	3750	4544
ENSECAG00000022405	6.991916906	0.630511896	0.800337354	1672	2576	2232	2426	2649	2557	2237	2776
ENSECAG00000023718	2.497069262	0.630543435	0.800337354	53	72	77	161	110	79	136	188
ENSECAG00000018112	0.719701774	0.630577362	0.800337354	18	9	51	40	25	28	47	18
ENSECAG00000008341	5.42228012	0.631530304	0.801457312	601	454	790	869	1104	738	966	1039
ENSECAG00000016939	10.29398522	0.631693794	0.801575261	15315	15958	28997	31342	28473	19574	24867	28098
ENSECAG00000023204	4.269871771	0.631775395	0.801589284	199	510	317	320	402	320	417	411
ENSECAG00000015391	5.652939615	0.632116352	0.801624376	497	667	1185	1349	975	743	945	1463
ENSECAG00000011151	7.431795509	0.632145285	0.801624376	2094	1885	3171	3933	4635	3018	3839	4135
ENSECAG00000023093	3.516322592	0.63215622	0.801624376	94	296	157	286	139	292	185	282
ENSECAG00000023369	7.161064946	0.632187256	0.801624376	2490	2403	2732	2077	3536	2176	2909	2868
ENSECAG00000014382	2.598444906	0.632216732	0.801624376	57	84	92	156	139	112	120	171
ENSECAG00000014591	1.484258447	0.632226371	0.801624376	33	38	32	67	58	54	47	89
ENSECAG00000007897	5.178717957	0.632332044	0.801668901	372	512	656	826	878	647	718	1008
ENSECAG00000015369	1.125522387	0.632431517	0.801702287	10	19	97	37	52	13	51	46
ENSECAG00000016393	4.921763412	0.632539305	0.801702287	260	707	584	623	544	510	552	863
ENSECAG00000000809	9.314212427	0.632589545	0.801702287	6710	9583	15762	14471	12901	11613	12150	14426
ENSECAG00000009202	4.040741819	0.632640618	0.801702287	144	279	267	534	293	321	356	346
ENSECAG00000000351	2.853183543	0.632938501	0.801990326	133	83	82	125	187	133	157	172
ENSECAG00000023961	4.524830751	0.633065936	0.802062352	268	196	724	486	495	382	483	483
ENSECAG00000010324	5.667176278	0.633540308	0.802573863	789	603	1454	664	1446	798	903	920
ENSECAG00000012943	4.546226526	0.633646871	0.80261937	227	407	451	609	504	400	439	553
ENSECAG00000023573	5.870309724	0.633956907	0.802867227	735	878	886	1720	1139	1083	1257	1257
ENSECAG00000018655	4.489419986	0.633983872	0.802867227	484	430	253	291	439	393	532	421
ENSECAG00000018217	8.008287625	0.6342255	0.803083711	3241	3444	4116	5532	6398	4664	5815	6427
ENSECAG00000010802	2.398417531	0.634325458	0.803120778	55	38	90	157	101	120	126	122
ENSECAG00000007888	2.851393615	0.634412638	0.80314166	70	112	113	166	158	136	170	179
ENSECAG00000018867	2.587585471	0.634526909	0.803196829	57	149	69	87	111	92	137	209
ENSECAG00000006741	4.734207661	0.634668232	0.803272931	348	505	437	336	539	489	687	674
ENSECAG00000020989	6.159820539	0.634728426	0.803272931	1034	1061	1212	1718	1473	1268	1558	1494
ENSECAG00000021823	6.862610288	0.634995194	0.803521037	1124	1820	2221	2366	2449	2459	2360	3217
ENSECAG00000024420	6.904522117	0.635142742	0.803618244	1084	1743	2505	3632	2278	2247	2305	2826
ENSECAG00000010212	7.505030884	0.635584562	0.804087718	3249	3692	2201	3156	4816	2992	3468	3188
ENSECAG00000020401	7.170888175	0.636119524	0.804624364	1619	2183	2141	3312	2932	3494	2615	3912
ENSECAG00000015935	11.41078826	0.636150384	0.804624364	82408	40237	22880	23573	50202	47104	57943	57300
ENSECAG00000000781	3.015505087	0.636531646	0.804994146	38	113	108	288	110	167	182	279
ENSECAG00000002088	5.974588508	0.636616354	0.804994146	783	1119	934	1643	1262	989	1441	1430
ENSECAG00000013413	5.070144028	0.636700352	0.804994146	281	803	599	733	585	634	773	715
ENSECAG00000017490	5.568486783	0.636780212	0.804994146	529	815	825	857	946	832	1165	1318
ENSECAG00000000252	4.835946792	0.636796988	0.804994146	223	491	572	587	606	664	684	573
ENSECAG00000018943	6.137959584	0.636878672	0.80500784	1080	2022	856	736	1373	1210	1439	1617
ENSECAG00000026834	2.35335593	0.637268975	0.805411579	74	101	77	97	101	80	104	119
ENSECAG00000019831	5.365309365	0.637623169	0.805769597	552	741	706	872	920	680	856	900
ENSECAG00000024700	7.053831244	0.637818383	0.805926653	1614	1185	2760	2972	4062	1982	3148	2864
ENSECAG00000010381	5.089431171	0.637948858	0.806001882	307	664	547	683	679	762	640	963
ENSECAG00000010175	5.732219569	0.6382665	0.806249318	393	1178	945	1392	903	1117	1081	1177
ENSECAG00000019987	4.384484698	0.638294315	0.806249318	334	415	357	299	360	460	403	447
ENSECAG00000023664	0.68801788	0.638357583	0.806249318	24	26	26	31	37	17	29	38
ENSECAG00000013542	6.913818808	0.638544173	0.806395342	1780	2753	1993	1691	2476	2299	2751	2076
ENSECAG00000022038	6.994932028	0.639125799	0.806931189	1800	1762	2778	2661	2768	1982	2655	2934
ENSECAG00000012848	5.416090983	0.639164328	0.806931189	581	640	506	977	941	703	945	1268
ENSECAG00000023962	8.243678955	0.639181544	0.806931189	4000	5884	5821	5542	5497	6001	5598	7286
ENSECAG00000013819	6.833969885	0.639298186	0.806936344	1726	2043	1476	2741	2176	1859	2182	3057
ENSECAG00000022469	10.54558682	0.639327669	0.806936344	15529	21776	32797	40350	34390	23588	30726	31493
ENSECAG00000019508	0.858090878	0.639584859	0.807171295	8	14	35	84	44	26	28	34
ENSECAG00000016887	3.258517752	0.639724094	0.807257348	152	219	138	132	185	154	203	221
ENSECAG00000022460	6.802065594	0.63985535	0.807333313	1350	1919	1732	2021	2405	2307	2308	3019
ENSECAG00000017803	4.852823883	0.64000536	0.807378632	345	384	424	721	710	487	647	745

ENSECAG00000021288	5.194603373	0.640104172	0.807378632	528	513	852	646	700	781	597	881
ENSECAG00000012032	1.467666016	0.640104446	0.807378632	48	21	59	64	44	71	24	69
ENSECAG00000017956	4.85341141	0.640244321	0.80746542	227	327	605	788	535	621	742	681
ENSECAG00000005195	6.415201329	0.640396	0.807567075	795	1211	1371	2291	1823	1770	2000	2036
ENSECAG00000016554	8.15544993	0.640738474	0.807749242	3353	5651	5028	6316	5038	5680	5365	6873
ENSECAG00000013783	1.370788127	0.640891531	0.807749242	90	19	39	11	124	25	25	11
ENSECAG00000017899	8.429562312	0.640904944	0.807749242	4611	5868	5577	8558	6332	6306	6578	8626
ENSECAG00000016635	4.618717763	0.640926238	0.807749242	256	528	387	577	485	448	548	502
ENSECAG00000011210	4.399689638	0.640942435	0.807749242	217	345	421	540	387	364	457	507
ENSECAG00000008073	8.070954403	0.64096701	0.807749242	3658	4752	4053	6667	6167	4827	4702	5948
ENSECAG00000023225	5.545718951	0.641072713	0.807792853	505	751	1166	903	1067	698	1066	967
ENSECAG00000021816	6.337200826	0.641218535	0.807887003	946	963	2048	1888	2009	1267	1578	1697
ENSECAG00000014998	3.851232522	0.641296308	0.807895404	213	145	369	289	319	202	372	261
ENSECAG00000017165	7.179163806	0.641390065	0.807923938	2250	2198	2761	2881	3358	2076	3580	2656
ENSECAG00000013687	3.221531135	0.641478759	0.807946088	127	156	127	249	159	175	183	229
ENSECAG00000019403	4.334479566	0.642140391	0.808460432	251	205	463	542	466	350	407	399
ENSECAG00000017137	2.641179363	0.642182637	0.808460432	36	118	126	118	94	159	188	110
ENSECAG00000002265	3.78247466	0.642190798	0.808460432	160	224	207	281	275	245	320	394
ENSECAG00000017035	1.50557535	0.642202933	0.808460432	16	55	70	69	31	25	42	124
ENSECAG00000002635	5.0171436	0.642242902	0.808460432	240	350	729	866	571	587	759	1017
ENSECAG00000017453	5.689059037	0.642525774	0.808552755	505	577	791	1590	1036	1148	962	1483
ENSECAG00000014051	3.823153893	0.642541902	0.808552755	156	239	234	268	313	245	379	325
ENSECAG00000019517	2.912267299	0.642561887	0.808552755	87	233	77	121	120	154	138	178
ENSECAG00000022196	4.360650807	0.642600895	0.808552755	214	255	358	523	458	386	481	507
ENSECAG00000023467	3.054389013	0.642863052	0.808710471	89	121	107	214	155	118	228	251
ENSECAG00000000725	7.20913246	0.64289634	0.808710471	1753	1550	2999	3234	3467	2852	3354	3667
ENSECAG00000010685	6.714073174	0.642939772	0.808710471	1830	1422	2154	1757	2454	2076	2053	1801
ENSECAG00000011469	2.49492023	0.643183823	0.808927894	41.0007	144	92.0005	130.001	95.0006	112	96.0011	139.001
ENSECAG00000010625	4.294115712	0.643399491	0.809109575	242	356	395	375	388	341	425	436
ENSECAG00000027681	11.45018015	0.643545656	0.809203822	46268	33269	72027	42721	55063	61863	44338	60816
ENSECAG00000013773	1.664929518	0.643978806	0.809658868	45	45	50	82	56	37	46	111
ENSECAG00000025013	5.300725703	0.644354869	0.809968686	399	601	895	953	805	653	834	923
ENSECAG00000017833	0.906818653	0.644367801	0.809968686	27	26	44	29	26	24	31	62
ENSECAG00000023931	8.498844501	0.644511735	0.810059993	3449	6206	5517	8222	6842	8743	7171	9855
ENSECAG00000024789	5.71055372	0.64478891	0.810318725	771	1048	617	726	1104	925	1330	1358
ENSECAG00000013066	3.367559398	0.644877209	0.810340063	70	70	218	339	208	321	232	149
ENSECAG00000018079	6.855000347	0.644958577	0.810352687	1159	2023	1789	2504	2673	2127	2516	3128
ENSECAG00000011033	4.389999843	0.645180146	0.810541444	204	219	491	460	430	359	439	668
ENSECAG00000012497	6.023325111	0.645284564	0.810582998	443	789	1415	1755	1405	1577	1675	1110
ENSECAG00000009566	5.474143945	0.645565809	0.810846642	505	642	690	1055	943	773	1041	1228
ENSECAG00000003617	2.838822087	0.645706102	0.810933208	103	62	130	145	203	86	191	166
ENSECAG00000019274	5.836135634	0.646048444	0.811273477	859	1029	736	852	1265	1063	1277	1530
ENSECAG00000021267	4.318628244	0.646125154	0.811280142	189	343	350	575	409	341	429	436
ENSECAG00000022176	6.158602037	0.646249364	0.811346439	856	916	1165	1697	1786	1383	1573	1629
ENSECAG00000017962	5.385069066	0.646391826	0.811413683	599	810	630	836	912	676	819	1005
ENSECAG00000025122	2.388551047	0.646445755	0.811413683	71	72	105	64	130	123	126	81
ENSECAG00000008944	4.229498604	0.646588272	0.811502921	124	518	420	286	348	475	250	404
ENSECAG00000020212	1.322155309	0.646770101	0.811551033	26	46	46	57	50	54	49	35
ENSECAG00000015293	3.949910007	0.64682242	0.811551033	165	323	268	337	345	302	272	322
ENSECAG00000000889	3.477250837	0.646840887	0.811551033	135	245	117	182	227	185	255	341
ENSECAG00000019650	5.041395914	0.646934466	0.811578823	420	586	464	593	819	518	880	728
ENSECAG00000023627	2.561784075	0.647089977	0.811684292	28	113	128	178	98	116	129	116
ENSECAG00000007528	0.647696759	0.647317944	0.811822852	13	13	46	19	25	17	33	64
ENSECAG00000009113	7.413062939	0.647403991	0.811822852	3156	2734	2825	2771	3754	3159	2953	3837
ENSECAG00000000104	3.367119254	0.647468118	0.811822852	111	105	239	306	181	157	210	283
ENSECAG00000011043	4.977527552	0.647486243	0.811822852	257	340	582	958	562	640	738	890
ENSECAG00000024983	6.198252211	0.6478758	0.812136454	582	1225	1069	2035	1626	1301	1815	1860
ENSECAG00000008454	7.080945156	0.647879319	0.812136454	1320	2054	3335	3190	2897	2134	2873	3066
ENSECAG00000001767	4.050807608	0.647977208	0.812169558	132	271	400	261	395	202	438	471
ENSECAG00000007605	7.427556805	0.64816209	0.812231041	1575	3752	3067	4125	2933	3546	2989	4409
ENSECAG00000013990	4.604788986	0.648169235	0.812231041	236	494	448	563	470	429	443	641
ENSECAG00000014339	3.42399943	0.648292718	0.812285906	74	242	162	325	150	308	122	256
ENSECAG00000011362	6.872440104	0.648356001	0.812285906	1079	1530	2210	2926	2826	2466	2035	3226
ENSECAG00000021201	3.331359458	0.648451168	0.812315564	174	39	184	385	539	99	84	7
ENSECAG00000016704	4.674529195	0.648584969	0.812351619	306	614	413	429	530	403	574	563
ENSECAG00000024738	3.164700032	0.648693292	0.812351619	53	305	85	214	107	139	152	315
ENSECAG00000010261	4.703819545	0.648721423	0.812351619	257	426	457	765	517	441	449	722
ENSECAG00000023592	8.607840943	0.648815405	0.812351619	4703	6007	7442	10111	9026	6267	7962	8214
ENSECAG00000019956	0.027314688	0.648888339	0.812351619	0	9	20	33	27	17	17	24
ENSECAG00000006542	5.69691376	0.649023068	0.812351619	561	743	879	1193	890	1109	913	1763
ENSECAG00000023985	3.857521905	0.649081966	0.812351619	160	198	296	272	351	241	302	406
ENSECAG00000021432	4.541682373	0.649126537	0.812351619	498	307	287	454	432	431	494	514
ENSECAG00000014655	1.975239569	0.649163724	0.812351619	34	85	56	109	63	51	81	115
ENSECAG00000011133	3.526463664	0.649194922	0.812351619	244	211	149	148	171	303	252	166
ENSECAG00000024549	5.853342014	0.649399202	0.812517755	566	504	1167	2177	1114	959	1139	1471
ENSECAG00000012836	2.779692425	0.649945417	0.813111631	98	67	121	138	153	119	160	183
ENSECAG00000017767	1.355173324	0.650056014	0.813160457	31	35	28	90	49	41	61	43
ENSECAG00000010723	5.201568579	0.650307788	0.813295427	397	504	709	785	832	738	710	1003
ENSECAG00000000621	6.584313093	0.65047949	0.813295427	889	1889	1611	2622	1674	2129	1842	2074
ENSECAG00000010445	6.471887279	0.65048417	0.813295427	1035	1192	1157	2415	2029	1761	1709	2484
ENSECAG00000014531	5.051672867	0.650501691	0.813295427	338	546	653	599	688	592	737	957
ENSECAG00000015075	1.738097127	0.650521813	0.813295427	35	65	63	72	31	58	91	78
ENSECAG00000010128	7.773708205	0.650595243	0.813297739	3466	2964	3012	6141	5103	2816	3999	5898
ENSECAG00000021568	0.627720824	0.650917053	0.81353388	32	28	10	6	34	20	59	23
ENSECAG00000007748	4.243903706	0.650927346	0.81353388	212	248	341	426	356	394	384	559

ENSECAG00000023052	3.73379862	0.651113798	0.813573656	90	271	263	362	223	211	230	415
ENSECAG00000020110	6.326872197	0.651188017	0.813573656	940	1519	1127	1491	1662	1866	1691	1921
ENSECAG00000010710	5.674075585	0.651222647	0.813573656	579	679	958	1500	1065	1035	862	1182
ENSECAG00000016808	6.473226494	0.65124559	0.813573656	893	648	2648	2525	2773	1276	1319	1737
ENSECAG00000019850	5.949908778	0.651354387	0.813620112	716	654	1147	1536	1542	1114	1381	1493
ENSECAG00000020612	0.707775392	0.652193212	0.814520844	3	12	21	69	78	32	13	18
ENSECAG00000007142	6.187219915	0.652218856	0.814520844	701	1214	992	1895	1657	1471	1579	1803
ENSECAG00000010784	4.659835265	0.65236779	0.814617302	190	344	389	792	548	508	481	734
ENSECAG00000012186	2.906396149	0.652442336	0.81462086	99	110	155	163	215	116	141	121
ENSECAG00000015999	7.061494119	0.652566554	0.81463983	1349	2425	2599	3317	2624	2205	2875	3145
ENSECAG00000023144	6.266210041	0.652600927	0.81463983	799	1696	1164	1841	1479	1462	1259	2069
ENSECAG00000013923	8.484231565	0.652773752	0.814709969	4419	7604	4696	8810	6848	6526	7203	8271
ENSECAG00000021753	2.021822768	0.652800524	0.814709969	65	82	60	64	80	65	79	95
ENSECAG00000012599	2.193163604	0.653125853	0.815026462	36	88	59	156	98	61	88	113
ENSECAG00000015193	7.428772894	0.653363598	0.815233604	1816	2567	3142	3593	3894	3265	3379	5049
ENSECAG00000019296	3.573920009	0.653491701	0.81530391	71	138	239	361	246	252	246	323
ENSECAG00000027677	8.778076043	0.653677173	0.815436852	6236	4806	9831	6033	10368	9241	6562	13936
ENSECAG00000018517	4.760408969	0.653741796	0.815436852	400	607	408	415	569	546	465	606
ENSECAG00000022721	3.923749755	0.653890119	0.815477943	100	172	266	653	290	218	331	379.001
ENSECAG00000022375	8.486674759	0.653918284	0.815477943	4205	4965	6624	7069	8027	7524	7462	9300
ENSECAG00000015595	5.720428158	0.654036876	0.815502925	651	989	815	1260	1014	844	1144	1321
ENSECAG00000023201	8.71270832	0.654115489	0.815502925	5617	8829	6652	8233	7765	7582	9191	9243
ENSECAG00000020891	2.608463578	0.65415364	0.815502925	62	124	106	141	123	127	105	125
ENSECAG00000009175	5.145537813	0.654298421	0.815529242	187	351	721	1204	720	875	670	909
ENSECAG00000011673	4.592756939	0.654318304	0.815529242	1148	91	68	98	1422	103	106	112
ENSECAG00000024843	7.251335316	0.654571807	0.815755717	1008	1606	2553	5344	3489	3168	3388	3729
ENSECAG00000015584	4.302019897	0.65473396	0.815868311	178	370	247	490	388	279	556	560
ENSECAG00000018731	8.179788679	0.654908313	0.81599608	4490	6664	4002	4602	5148	6594	4638	6766
ENSECAG00000010751	3.334480052	0.655046024	0.816078171	42	123	139	380	103	365	79	372
ENSECAG00000013445	4.959937657	0.655268443	0.816179989	300	527	699	711	650	551	625	709
ENSECAG00000014972	4.460373526	0.655324622	0.816179989	384	322	303	303	569	419	439	543
ENSECAG00000020357	4.123060905	0.655343253	0.816179989	143	249	271	502	347	281	415	529
ENSECAG00000023477	4.077635373	0.655579132	0.816332086	225	187	456	322	385	318	298	355
ENSECAG00000000266	5.579757072	0.655609072	0.816332086	634	645	900	807	1665	1034	938	587
ENSECAG00000017353	8.720528018	0.655698	0.81635335	5148	6114	10773	8117	8584.97	6207	8971	10467
ENSECAG00000015054	1.214078224	0.65584116	0.816442124	12	40	29	66	46	50	36	71
ENSECAG00000021235	1.537088558	0.65626498	0.816570239	44	40	52	62	53	43	58	72
ENSECAG00000024784	4.780617486	0.656305273	0.816570239	332	624	394	564	456	444	559	792
ENSECAG00000021813	3.113245282	0.656344715	0.816570239	200	152	79	127	173	148	162	202
ENSECAG00000017293	4.678237118	0.656351427	0.816570239	325	427	403	657	575	398	496	623
ENSECAG00000017556	5.214742467	0.656418334	0.816570239	708	552	438	473	825	688	971	849
ENSECAG00000018428	5.705755057	0.656429546	0.816570239	460	827	1186	1356	1016	1015	741	1489
ENSECAG00000018965	5.795227882	0.656467895	0.816570239	468	889	1053	1272	1086	1141	1276	1448
ENSECAG00000000789	4.306009814	0.656581745	0.816570239	216	509	264	393	421	273	390	528
ENSECAG00000010645	4.949968599	0.656590891	0.816570239	336	541	513	830	583	495	751	692
ENSECAG00000009002	0.434189382	0.656892078	0.816800553	2	32	27	38	16	24	11	46
ENSECAG00000018723	5.501581985	0.656970744	0.816800553	466	846	665	1309	774	852	802	1276
ENSECAG00000008102	7.026436725	0.657037368	0.816800553	905	2550	2350	2753	2451	3101	2911	3217
ENSECAG00000007305	4.353940452	0.657104433	0.816800553	296	127	614	197	737	309	366	452
ENSECAG00000017430	4.226422484	0.657135527	0.816800553	179	279	320	448	495	283	501	390
ENSECAG00000014803	7.334519811	0.657350179	0.816977984	1932	2546	3527	3517	3550	2840	3059	3627
ENSECAG00000000743	4.353349731	0.657474394	0.817042991	218	414	298	358	387	307	462	700
ENSECAG00000011070	3.342642641	0.657548413	0.817045611	86	222	177	149	229	127	301	260
ENSECAG00000011562	4.760241107	0.657630967	0.817058835	238	267	449	887	523	472	637	816
ENSECAG00000010882	5.66526394	0.657904678	0.817309529	404	765	977	1663	965	762	1131	1292
ENSECAG00000007164	6.056347925	0.658572377	0.817937606	761	1625	1211	1006	1443	956	1515	1506
ENSECAG00000021912	1.139783072	0.658587483	0.817937606	39	37	29	40	31	33	39	67
ENSECAG00000008447	0.635594233	0.658626224	0.817937606	15	14	34	49	22	15	13	67
ENSECAG00000010426	3.883937834	0.658774975	0.817998085	138	230	327	379	255	254	264	429
ENSECAG00000007880	1.141468571	0.658818912	0.817998085	24	23	24	92	22	47	33	64
ENSECAG00000001608	1.099421921	0.659373375	0.818597059	8	47	37	66	35	41	40	44
ENSECAG00000012548	3.148616791	0.659518277	0.818687498	100	84	175	298	181	157	176	191
ENSECAG00000021261	6.715221918	0.659641891	0.81873424	1085	1133	2021	2709	2476	2547	2031	2286
ENSECAG00000008605	5.51646693	0.659769154	0.81873424	489	980	651	1137	901	798	885	1158
ENSECAG00000005889	3.014803919	0.659772109	0.81873424	45	132	93	273	148	155	163	266
ENSECAG00000016354	5.989904911	0.659886382	0.818786619	706	924	1358	1582	1288	1075	1315	1521
ENSECAG00000022436	6.241611558	0.660052971	0.818903894	819	1072	1218	1850	1535	1477	1932	1801
ENSECAG00000015919	5.39124754	0.660342503	0.819173656	521	962	544	892	981	679	884	865
ENSECAG00000007943	0.196483106	0.660650575	0.819314438	18	12	23	7	21	20	29	27
ENSECAG00000017281	5.030364004	0.660659314	0.819314438	246	317	699	968	790	611	660	874
ENSECAG00000015242	9.490823341	0.660719233	0.819314438	8931	8739	17463	16943	16460	11062	14406	16266
ENSECAG00000024012	6.065645883	0.66074877	0.819314438	251	999	1109	2269	809	2111	1127	2000
ENSECAG00000004865	2.910944634	0.660816539	0.819314438	56	99	125	217	96	179	157	242
ENSECAG00000017879	1.556961665	0.660938004	0.819375624	28	45	38	104	51	44	68	64
ENSECAG00000008555	7.039654748	0.661081752	0.819464419	1277	3186	1373	2381	2870	1534	2839	4994
ENSECAG00000023524	6.947153936	0.661354335	0.819642236	1700	1854	2271	2894	2764	2008	2469	2793
ENSECAG00000020187	5.354121733	0.661454674	0.819642236	456	521	848	833	914	722	970	1044
ENSECAG00000015965	7.100821359	0.661462206	0.819642236	1948	1382	2369	2974	3945	2910	1995	3509
ENSECAG00000002645	2.020805206	0.661513757	0.819642236	47	68	44	90	77	120	59	99
ENSECAG00000000526	6.271324151	0.661591845	0.819649607	770	1124	1561	1589	1672	1298	1864	2104
ENSECAG00000002676	8.36603928	0.661934938	0.819920862	6059	3635	7666	5288	9799	5722	6085	4482
ENSECAG00000015773	6.107214337	0.661965229	0.819920862	646	1202	1447	1702	1527	1079	1453	1575
ENSECAG00000010884	3.03272571	0.662027283	0.819920862	112	137	147	95	189	125	209	212
ENSECAG00000024663	6.603153324	0.662264432	0.820125174	1637	1074	1860	1305	2070	1584	2242	2940
ENSECAG00000007483	6.272648325	0.6625132	0.820264002	752	1306	1546	1393	1735	1823	1464	1838

ENSECAG00000018946	4.373195297	0.662520925	0.820264002	146.007	280	363	616	422	384	603	437
ENSECAG00000012748	3.600417255	0.662762547	0.820473748	122	248	240	249	202	261	279	224
ENSECAG00000006122	3.882764868	0.663168787	0.820750476	105	352	190	297	201	236	368	545
ENSECAG00000005243	4.675416751	0.663200688	0.820750476	248	433	454	527	563	538	510	657
ENSECAG00000024526	-0.044234513	0.663202792	0.820750476	2	27	20	17	11	11	19	27
ENSECAG00000005798	5.813278761	0.663435385	0.820757146	811	765	937	1011	1535	994	1217	1264
ENSECAG00000017053	4.716608599	0.663472529	0.820757146	183	319	624	643	483	653	457	754
ENSECAG00000010894	2.253370523	0.663492539	0.820757146	39	90	84	87	106	101	105	107
ENSECAG00000019424	2.916759454	0.663497129	0.820757146	89	79	184	120	169	167	167	165
ENSECAG00000009714	6.910976219	0.664356948	0.821731292	1472	3775	1455	1523	1957	2878	2599	2035
ENSECAG00000009877	1.038955652	0.664534127	0.821833161	28	16	28	51	53	34	39	53
ENSECAG00000018168	1.001208464	0.664610928	0.821833161	16	25	34	71	32	40	46	31
ENSECAG00000000327	5.682401731	0.664656303	0.821833161	686	1130	839	855	931	834	1143	1298
ENSECAG00000019947	7.590371735	0.665012045	0.822038079	2446	2862	3541	3265	4490	3884	4253	4654
ENSECAG00000018076	6.716231829	0.665026111	0.822038079	1060	1918	2013	2612	1904	1963	1744	2981
ENSECAG00000015757	10.45377033	0.66509658	0.822038079	16293	18871	32219	34548	32298	23032	29284	28518
ENSECAG00000016192	7.409785345	0.665111429	0.822038079	1309	3744	2162	3798	3365	5085	3719	2876
ENSECAG00000019694	3.808622152	0.665216353	0.822078333	123	238	296	363	266	222	269	384
ENSECAG00000013003	3.527866793	0.665455376	0.82224566	108	192	280	143	270	162	344	260
ENSECAG00000018628	7.608986099	0.665500091	0.82224566	2845	3783	2778	2302	4256	3762	4163	5517
ENSECAG00000023640	6.487280931	0.665568855	0.82224566	989	1679	1485	2271	1696	1410	2156	2069
ENSECAG00000000323	0.377279525	0.666121977	0.822686195	21	15	11	22	9	64	5	31
ENSECAG00000018341	0.331671574	0.666125593	0.822686195	3	4	27	45	33	13	41	20
ENSECAG00000002284	3.991141836	0.666142668	0.822686195	145	259	238	402	343	256	460	359
ENSECAG00000022559	6.370075956	0.666550914	0.823016583	885	1308	1503	2304	1612	1303	1951	1897
ENSECAG00000020190	6.978686707	0.666555061	0.823016583	1341	1635	2269	3013	2665	2721	2578	3332
ENSECAG00000000224	3.257765829	0.666816912	0.823184571	102	167	99	244	175	173	224	287
ENSECAG00000018483	5.161652172	0.666836014	0.823184571	254	793	612	975	637	814	592	840
ENSECAG00000014830	7.5827173	0.667116489	0.823441341	2079	2454	4467	5038	3909	3888	3312	4354
ENSECAG00000024076	7.271109965	0.667249652	0.823467034	1682	2750	2244	3223	3284	2872	3785	3946
ENSECAG00000009890	6.403872102	0.667282255	0.823467034	880	1311	1650	1653	1844	1733	1879	2081
ENSECAG00000009813	6.718161183	0.667417184	0.823544097	1534	336	2208	2409	4481	2308	2397	347
ENSECAG00000019810	6.2810803	0.667508504	0.823567338	921	996	1464	1653	1985	1480	1492	1988
ENSECAG00000000985	4.323325394	0.667671698	0.823667641	239	266	422	343	378	282	496	657
ENSECAG00000023737	0.539778844	0.667835175	0.823667641	19	11	32	38	26	24	35	21
ENSECAG00000011829	0.736902475	0.667862866	0.823667641	12	19	51	15	25	62	19	35
ENSECAG00000000770	3.785522809	0.667879773	0.823667641	109	287	316	281	219	233	358	300
ENSECAG00000020415	4.38752479	0.668538652	0.824390727	200	309	397	468	467	377	439	585
ENSECAG00000010062	5.433352532	0.66876982	0.824531714	492	442	995	1229	1011	828	736	925
ENSECAG00000021725	5.646411771	0.668836183	0.824531714	731	389	1333	1109	1284	706	932	1152
ENSECAG00000026829	2.342008187	0.668870693	0.824531714	63	66	105	125	93	67	122	122
ENSECAG00000014724	8.297849467	0.669121986	0.824695418	3230	6143	5339.01	8111	5697	5851	6040	7937
ENSECAG00000007190	7.561453332	0.669148658	0.824695418	2426	3126	3219	3015	4303	3721	4046	4909
ENSECAG00000006890	9.111282895	0.669250307	0.824731235	6816	7718	10294	15318	10575	10304	11536	12244
ENSECAG00000013840	3.217690037	0.669380033	0.824746389	109	197	131	216	161	172	176	239
ENSECAG00000012727	3.358203555	0.66940778	0.824746389	90	148	269	239	230	151	205	241
ENSECAG00000017581	2.996992695	0.669588951	0.824880154	90	132	181	159	148	123	163	210
ENSECAG00000014964	6.164327374	0.66966167	0.824880301	919	857	1270	1577	1877	1279	1471	1781
ENSECAG00000012173	3.039392169	0.669900059	0.825002159	50	195	120	161	132	213	164	222
ENSECAG00000020805	5.385827917	0.669905819	0.825002159	409	760	694	854	888	692	1012	1151
ENSECAG00000021100	6.500418441	0.670217339	0.825296348	1028	1666	1487	1569	1899	1871	1990	2316
ENSECAG00000024964	5.517201718	0.670452892	0.825481534	1498	422	405	462	1057	690	992	882
ENSECAG00000017553	6.566169571	0.670513033	0.825481534	1561	1762	1166	1138	2218	1880	2134	2270
ENSECAG00000010020	3.373947929	0.670701409	0.825623988	36	8	782	10	299	133	40	289
ENSECAG00000012024	5.248833143	0.671202974	0.8261519	313	954	920	530	407	1299	448	806
ENSECAG00000020473	1.896900758	0.671330913	0.826219869	35	32	111	94	64	75	76	72
ENSECAG00000014198	6.08102936	0.671447421	0.826273757	869	1074	844	1547	1526	1393	1448	1657
ENSECAG00000009606	6.962520304	0.671686048	0.826450209	1314	1674	2383	2770	2778	2437	2727	3238
ENSECAG00000009335	5.656936657	0.671772369	0.826450209	885	876	811	793	988	834	911	1409
ENSECAG00000019713	3.84027097	0.671812104	0.826450209	158	213	163	397	251	222	377	443
ENSECAG00000004152	4.38863107	0.671899962	0.826450209	191	180	383	665	483	348	550	495
ENSECAG00000018847	2.904811074	0.6719545	0.826450209	87	197	113	117	149	91	191	168
ENSECAG00000009638	3.643480585	0.672200784	0.826663633	156	150	244	241	348	166	236	378
ENSECAG00000012454	4.406220525	0.672663287	0.826981575	155	308	389	579	439	434	474	538
ENSECAG00000008525	5.304186806	0.672663773	0.826981575	287	527	797	1067	723	725	877	1232
ENSECAG00000022578	2.997474077	0.672677672	0.826981575	59	153	88	222	148	169	220	171
ENSECAG00000009068	6.301384029	0.672790068	0.827021745	1074	1399	1173	1876	1475	1375	2029	1527
ENSECAG00000011472	3.160944577	0.672855924	0.827021745	103	159	141	156	218	171	213	188
ENSECAG00000008117	3.739260655	0.673273139	0.827445041	139	173	281	382	351	258	238	218
ENSECAG00000019512	0.306772923	0.673354431	0.827455445	24	10	32	13	17	7	32	35
ENSECAG00000012384	5.603493137	0.673892717	0.827987826	380	732	929	1604	938	814	1058	1159
ENSECAG00000020681	6.828148207	0.673933411	0.827987826	1205	2008	2317	2613	2331	1952	2419	2553
ENSECAG00000013475	7.073822472	0.674069913	0.828027053	1249	1935	3040	2541	2893	2264	2611	4490
ENSECAG00000006216	4.089869032	0.674123234	0.828027053	271	306	243	336	404	289	306	382
ENSECAG00000024995	5.021910884	0.67418397	0.828027053	243	463	604	884	574	763	603	958
ENSECAG00000013982	1.83952278	0.674259055	0.828029765	35	42	86	95	66	68	82	61
ENSECAG00000019387	3.336740373	0.674626755	0.828391785	73	187	221	260	186	150	228	252
ENSECAG00000005081	7.614751871	0.674741317	0.828442927	2767	2321	4067	2994	4665	2520	4407	6386
ENSECAG00000024587	6.454807973	0.674989166	0.828657688	905	1332	1411	2115	2222	1863	1705	1994
ENSECAG00000022911	0.914172286	0.67541456	0.829040663	10	21	43	44	34	39	42	47
ENSECAG00000006949	7.994405413	0.675497488	0.829040663	3292	3557	4834	4370	6170	5011	5127	6612
ENSECAG00000018146	5.474384609	0.675520019	0.829040663	568	644	700	943	1037	805	1068	1035
ENSECAG00000021027	7.886725351	0.675747988	0.829230872	3954	4014	3662	4528	4751	4205	4528	5731
ENSECAG00000012545	4.523515783	0.675912787	0.82934353	146	397	336	679	406	456	556	642
ENSECAG00000007576	5.057451564	0.67606711	0.829373392	394	238	625	948	846	527	703	927

ENSECAG00000019613	3.650281685	0.676158357	0.829373392	91	291	190	229	189	204	251	505
ENSECAG00000019497	8.488810615	0.676179421	0.829373392	4357	5579	4642	8395	6834	9319	8384	7385
ENSECAG00000003883	4.090712444	0.676284986	0.829373392	183	382	246	389	287	327	355	412
ENSECAG00000018097	4.470442069	0.676302101	0.829373392	196	388	321	737	432	435	329	605
ENSECAG00000011758	6.433015261	0.676540453	0.829576153	1151	2009	1319	1430	1720	1887	1647	1706
ENSECAG00000021355	4.517872316	0.676722074	0.829638966	293	377	309	475	477	513	410	634
ENSECAG00000012029	6.08471417	0.676807859	0.829638966	607	1602	798	1338	1093	1189	1619	2261
ENSECAG00000014138	4.738130435	0.676810736	0.829638966	342	290	429	675	657	493	625	592
ENSECAG00000019908	5.990265876	0.677190409	0.829906699	616	1115	1088	1802	1237	1133	1366	1458
ENSECAG00000009326	7.24991915	0.677230844	0.829906699	1789	1961	2455	3688	3683	2745	3099	4177
ENSECAG00000021244	1.194770782	0.677248276	0.829906699	20	36	53	52	45	33	46	52
ENSECAG00000023543	3.331506993	0.677326072	0.829912523	91	92	244	236	229	202	222	241
ENSECAG00000007596	4.752181284	0.677771537	0.830368795	250	230	609	715	864	517	514	498
ENSECAG00000024902	9.703413674	0.677990189	0.830505226	7799	11996	18532	23574	17936	12910	18832	17851
ENSECAG00000008676	9.808373895	0.678029086	0.830505226	10128	9747	16915	21677	24699	15302	20210	20614
ENSECAG00000017504	6.629889899	0.67819186	0.830538198	1654	1373	1750	2008	2196	1863	2033	1924
ENSECAG00000023968	6.167951975	0.678202457	0.830538198	880	789	1537	1996	1492	1352	1415	1591
ENSECAG00000006936	6.634997111	0.678275298	0.830538198	794	1117	2083	2736	2585	2121	1946	2204
ENSECAG00000014968	4.629350565	0.678364274	0.830557638	322	150	705	628	628	469	510	355
ENSECAG00000012382	2.152468321	0.678476487	0.830605522	38	66	79	152	31	60	41	224
ENSECAG00000023354	0.463650527	0.678914647	0.830840565	6	20	18	41	23	27	37	29
ENSECAG00000017291	4.056532767	0.67899742	0.830840565	215	365.999	265	292.999	306	300.999	356.999	386
ENSECAG00000001854	2.467060673	0.679001692	0.830840565	44	138	102	107	115	102	105	114
ENSECAG00000021480	0.08478623	0.679114592	0.830840565	18	3	14	23	29	15	18	27
ENSECAG00000016043	5.887480548	0.67914448	0.830840565	511	841	1385	1623	1241	1058	1265	1256
ENSECAG00000023967	3.85903278	0.679160952	0.830840565	82	183	324	397	276	314	260	452
ENSECAG00000007350	4.385456082	0.679180352	0.830840565	151	365	447	582	413	366	512	396
ENSECAG00000004251	1.956523044	0.679426713	0.830927847	38	66	92	29	107	27	191	25
ENSECAG00000019957	3.340916612	0.679460813	0.830927847	74	141	188	272	259	151	276	217
ENSECAG00000005272	1.117837192	0.679514024	0.830927847	32	31	30	32	34	35	65	55
ENSECAG00000021485	1.060910995	0.679560787	0.830927847	5	28	27	76	16	39	60	69
ENSECAG00000024660	2.725345738	0.679649498	0.830927847	57	65	92	226	173	147	168	93
ENSECAG00000023809	3.53624977	0.679690496	0.830927847	141	208	196	269	261	221	201	252
ENSECAG00000016930	0.703698183	0.679947338	0.830964948	23	27	23	35	27	21	31	45
ENSECAG00000021796	8.105365362	0.680043236	0.830964948	2034	3867	4302	8420	5904	7262	6963	4256
ENSECAG00000013474	0.781720436	0.680079272	0.830964948	10	28	35	32	34	27	44	42
ENSECAG00000007758	4.857936937	0.680165677	0.830964948	196	969	339	576	390	825	448	618
ENSECAG00000009116	6.179682771	0.680214262	0.830964948	891	1149	1177	1423	1679	1403	1639	1716
ENSECAG00000011717	6.026777247	0.680256453	0.830964948	666	1092	1369	1551	1333	972	1444	1616
ENSECAG00000011619	5.108023823	0.680299986	0.830964948	276	321	714	1045	928	581	856	712
ENSECAG00000022790	6.466534743	0.680321648	0.830964948	1322	774	2246	1906	2160	1259	1949	1818
ENSECAG00000009981	4.432994641	0.680380845	0.830964948	250	327	399	410	499	390	535	488
ENSECAG00000023942	0.211365207	0.680452198	0.830964948	4	25	26	26	13	33	19	15
ENSECAG00000011346	5.625278289	0.680614523	0.831073853	567	761	1037	1136	895	837	1098	1229
ENSECAG00000006443	4.764474386	0.680699786	0.831088649	433	538	352	287	709	471	682	565
ENSECAG00000026848	6.197210614	0.680909561	0.831255445	996	946	1656	1554	1763	1218	1518	1481
ENSECAG00000022189	3.536610538	0.681088011	0.83135145	160	131	194	248	286	202	196	356
ENSECAG00000018244	3.99307072	0.681134541	0.83135145	141	199	355	356	310	371	403	316
ENSECAG00000021924	2.04167424	0.681355586	0.83153192	29	56	60	125	75	64	103	125
ENSECAG00000014319	3.753518887	0.681493176	0.83161051	177	160	289	330	324	251	272	235
ENSECAG00000024169	4.277476689	0.681656887	0.831720956	209	146	433	488	616	346	458	300
ENSECAG00000013803	6.449648388	0.682521655	0.832686679	1159	1570	1759	1551	1791	1556	2014	1753
ENSECAG00000013213	7.682470983	0.683038796	0.833111442	2604	3757	4374	3605	4335	3640	3487	5289
ENSECAG00000021637	6.904760275	0.683107311	0.833111442	1664	1681	2579	2470	2657	2168	2439	2460
ENSECAG00000021996	6.92564931	0.68310838	0.833111442	1682	1575	2766	2532	2439	1866	2425	3239
ENSECAG00000005517	7.399931987	0.683163114	0.833111442	2684	2843	1950	2777	3758	3073	3444	5016
ENSECAG00000023976	7.111507356	0.68394424	0.833942904	1372	1776	2561	3443	3158	2779	3312	3077
ENSECAG00000002353	1.81879151	0.68399172	0.833942904	14	84	56	110	32	75	66	99
ENSECAG00000024353	5.574823188	0.684178958	0.834081687	513	739	779	1424	890	815	1069	1134
ENSECAG00000015986	0.345685575	0.684321771	0.83409372	14	10	33	15	28	15	33	32
ENSECAG00000008439	8.570515469	0.684401328	0.83409372	5142	6381	6437	5471	10581	6262	8518	8910
ENSECAG00000024149	8.881644071	0.684409061	0.83409372	6386	9171	8156	9109	9118	8217	10515	10400
ENSECAG00000016968	4.729378961	0.684615719	0.834100986	310	318	430	681	669	378	684	639
ENSECAG00000000260	5.391305241	0.684631472	0.834100986	709	584	819	731	906	856	996	611
ENSECAG00000001358	6.893384977	0.684686068	0.834100986	1727	1443	2007	2305	2714	2064	2950	2933
ENSECAG00000013968	5.536732303	0.684766731	0.834100986	475.011	790	839.008	1240	893.001	719	1186	1006
ENSECAG00000015489	4.532453488	0.684858084	0.834100986	221	336	497	460	525	544	457	509
ENSECAG00000011179	3.679503259	0.68488698	0.834100986	176	151	220	264	301	242	274	319
ENSECAG00000007917	7.269778904	0.684928903	0.834100986	2191	1919	4137	2519	3610	2811	2709	3354
ENSECAG00000014322	6.216819229	0.685167686	0.834282085	823	1754	1325	1258	1387	1231	1575	1907
ENSECAG00000002866	5.524926805	0.685242737	0.834282085	774	667	767	927	1273	661	999	813
ENSECAG00000014117	3.886684656	0.685297897	0.834282085	354	341	97	137	285	181	352	366
ENSECAG00000019216	5.427849958	0.685410237	0.834304702	352	606	824	1119	845	908.002	905	1169
ENSECAG00000006660	2.919996474	0.685463333	0.834304702	74	103	128	186	164	138	209	157
ENSECAG00000021379	5.942842692	0.685613156	0.834397673	797	775	1258	1540	1360	1141	1038	1497
ENSECAG00000009350	3.865700394	0.685933467	0.834698089	157	133	336	449	328	280	241	324
ENSECAG00000012072	1.111978888	0.68605501	0.834756588	8	47	16	60	7	124	9	45
ENSECAG00000017164	9.212267408	0.686256155	0.834824166	6118	8311	15225	13505	12078	8976	13138	14113
ENSECAG00000012816	4.101385206	0.686324506	0.834824166	175	215	375	356	395	301	431	395
ENSECAG00000024013	6.177097292	0.686330974	0.834824166	859	1033	1329	1454	1573	1503	1602	1732
ENSECAG00000009577	1.087965595	0.686519429	0.834964007	18	29	41	64	49	25	44	45
ENSECAG00000000004	3.530638907	0.686744979	0.83514893	124	156	202	269	250	213	238	327
ENSECAG00000022645	4.621855568	0.686883713	0.835228248	245	384	525	623	413	608	507	446
ENSECAG00000015504	5.970729385	0.687090998	0.835271938	1000	1090	958	1207	1355	1212	1233	1304
ENSECAG00000001455	4.780501096	0.687093315	0.835271938	307	374	691	583	677	398	594	577

ENSECAG00000010542	5.742503814	0.687196025	0.835271938	445	914	1082	1482	974	1095	1086	1205
ENSECAG00000019598	5.736116944	0.687213702	0.835271938	741	731.989	1072.98	1170.98	1256	922.994	1096	1079.99
ENSECAG00000013603	4.787756281	0.687535163	0.835573273	246	334	666	586	616	564	511	768
ENSECAG00000006019	6.797586006	0.687751543	0.835746849	1239	2334	1594	1843	2451	2039	2818	2642
ENSECAG00000010359	6.09384904	0.688381401	0.836422786	784	1143	1398	1503	1374	1042	1500	1713
ENSECAG00000015824	0.687508583	0.688639377	0.836646771	10	21	33	35	26	29	49	32
ENSECAG00000020814	5.394236089	0.689032702	0.836877169	491	628	675	942	910	851	987	965
ENSECAG00000014826	4.307950673	0.689048523	0.836877169	168	322	446	505	366	334	449	467
ENSECAG00000010263	7.71580735	0.689049983	0.836877169	2504	4793	3458	3916	3486	3571	4150	6005
ENSECAG00000006944	2.660700857	0.689474251	0.837055777	132	30	127	71	166	134	141	123
ENSECAG00000011617	2.408460834	0.689560235	0.837055777	47	67	127	148	102	58	101	167
ENSECAG00000019522	3.965270391	0.689777175	0.837055777	300	302	239	175	416	216	326	300
ENSECAG00000022098	1.000838684	0.689870296	0.837055777	15	35	25	48	37	27	46	65
ENSECAG00000020197	5.088035124	0.689872752	0.837055777	408	901	422	603	595	514	777	902
ENSECAG00000017712	5.649093686	0.689894536	0.837055777	557	796	1169	1031	1209	620	1149	1157
ENSECAG00000016442	8.051372187	0.689950129	0.837055777	2772	2019	7062	5488	7901	4286	5257	6597
ENSECAG00000011808	1.455718515	0.689962602	0.837055777	20.0007	34	54.0005	67.001	70.0006	43.0005	62.0011	64.001
ENSECAG00000019029	7.219113262	0.689990609	0.837055777	1548	1860	2812	3599	3734	2518	3311	3819
ENSECAG00000022486	3.928828472	0.690005608	0.837055777	172	215	245	358	329	284	361	374
ENSECAG00000013740	8.313695963	0.690070375	0.837055777	3322.01	4577	8276.01	6819	6158	5422	6621	7705
ENSECAG00000016264	1.1511142	0.690081101	0.837055777	17	21	49	78	42	16	26	89
ENSECAG00000009878	5.256173523	0.690342353	0.837283285	293	560	946	737	936	734	937	775
ENSECAG00000018411	4.869524772	0.690486986	0.8372861	455	582.999	401	304	618	589.999	693	703
ENSECAG00000024832	6.903586959	0.690544168	0.8372861	1565	1275	2367	2475	3138	2409	2315	2818
ENSECAG00000018001	1.763283425	0.690616949	0.8372861	46	57	43	89	47	57	77	86
ENSECAG00000019546	7.030261017	0.690644217	0.8372861	1344	1844	2587	2759	2663	2430	2707	3970
ENSECAG00000023986	7.398454887	0.690808448	0.8372861	3487	6072	492	1087	6407	4943	432	363
ENSECAG00000019952	4.508047872	0.690826513	0.8372861	207	203	693	359	410	934	220	425
ENSECAG00000012016	3.654195059	0.690926374	0.8372861	120	308	203	255	171	226	276	349
ENSECAG00000022130	5.803682552	0.69099181	0.8372861	906	832	1028	602	1245	1112	1353	1258
ENSECAG00000001312	3.51881965	0.6910079	0.8372861	93	87	362	204	359	131	337	205
ENSECAG00000019261	2.166425152	0.69120357	0.837433884	85	76	42	50	93	100	103	100
ENSECAG00000010801	7.221087735	0.691584919	0.837806573	1804	3345	2559	2650	2608	2865	2900	3780
ENSECAG00000008254	2.112914219	0.69192836	0.838133265	15	40	107	129	48	90	97	154
ENSECAG00000000797	8.906175973	0.692028818	0.838133768	5165	8448	10031	10778	9150	7661	9798	12654
ENSECAG00000005198	4.939603557	0.692088869	0.838133768	558	546	435	489	639	542	616	705
ENSECAG00000016211	2.233008297	0.692150075	0.838133768	46	62	38	159	98	63	144	115
ENSECAG00000020657	2.278204707	0.692333346	0.838266355	45	56	98	114	110	68	118	135
ENSECAG00000016637	3.266824542	0.692425376	0.838288452	67	107	188	290	203	224	228	191
ENSECAG00000008264	7.708179962	0.692577027	0.838332511	2591	4137	3750	4156	4256	3577	5006	4131
ENSECAG00000024134	4.43388263	0.692609336	0.838332511	292	380	429	381	511	343	466	443
ENSECAG00000021059	4.259239787	0.693229885	0.838910097	129	268	388	511	309	473	482	423
ENSECAG00000022584	0.756681683	0.693261034	0.838910097	10	15	10	74	47	24	44	30
ENSECAG00000016926	5.082084722	0.693370571	0.838910097	483	430	528	715	860	625	789	720
ENSECAG00000000471	3.393205795	0.693381863	0.838910097	90	193	146	258	214	194	226	302
ENSECAG00000015731	4.089968285	0.693496799	0.838959821	255	225	236	353	387	333	359	430
ENSECAG00000022969	2.218018823	0.693634134	0.839036627	86	7	196	41	240	27	60	15
ENSECAG00000012841	1.322108153	0.69405301	0.839426866	12	41	39	69	50	54	54	58
ENSECAG00000024208	6.119552969	0.694104507	0.839426866	714	1054	1401	1865	1270	1375	1162	1899
ENSECAG00000001999	4.85174992	0.694307983	0.839583578	370	359	557	570	661	571	662	654
ENSECAG00000006648	3.860726758	0.694449188	0.839588914	153	141	241	434	271	280	363	378
ENSECAG00000025403	-0.022631702	0.694460184	0.839588914	8	14	21	23	20	22	6	21
ENSECAG00000020255	4.512759636	0.694596536	0.839664416	338	420	375	244	532	371	564	575
ENSECAG00000013161	2.888492487	0.69468784	0.83968545	98	63	136	173	219	128	168	139
ENSECAG00000020939	3.709771821	0.694877697	0.839825592	90	180	338	370	217	330	207	287
ENSECAG00000010812	7.144675605	0.694987478	0.839868935	1321	2515	2502	2880	2712	2928	2924	4126
ENSECAG00000004972	1.380756116	0.69507866	0.839889794	26	45	43	69	48	52	40	61
ENSECAG00000010938	5.941088541	0.695263449	0.840023746	592	688	1180	1667	1760	1040	1423	1233
ENSECAG00000016666	4.351012985	0.695454629	0.840093011	397	647.007	88	178	298	279	409.003	656
ENSECAG00000024629	6.624994149	0.695468656	0.840093011	1022	2362	1475	2099	1922	1676	1752	2759
ENSECAG00000019120	5.114237578	0.695795213	0.84039813	163	461	763	987	307	877	702	1255
ENSECAG00000003561	3.267066401	0.695891412	0.840424981	74	244	152	219	176	138	219	246
ENSECAG00000009778	2.311444286	0.696076364	0.840559001	30	95	79	119	92	88	130	129
ENSECAG00000011782	9.922209869	0.69624818	0.840589444	9035	16312	19608	26258	20965	17417	18707	21687
ENSECAG00000021026	5.339071352	0.696249539	0.840589444	462	294	1188	1024	1154	617	858	625
ENSECAG00000004465	6.841887823	0.696775376	0.841043858	1707	1635	1642	2194	2537	2249	2477	2987
ENSECAG00000017498	4.649225536	0.697010523	0.841043858	346	296	451	508	495	454	643	636
ENSECAG00000015508	5.935374921	0.697103671	0.841043858	651	1349	960	1351	963	1285	1119	1641
ENSECAG00000010224	7.418247091	0.697164745	0.841043858	2376	2297	2854	3276	3717	3771	3844	3864
ENSECAG00000007663	6.888201339	0.697188007	0.841043858	1368	1556	2748	1861	3726	1627	1991	3402
ENSECAG00000010639	8.256961013	0.697215618	0.841043858	2947	5270	4424	7411	6134	7013	6070	8128
ENSECAG00000014248	6.250847084	0.697287419	0.841043858	556	1161	1174	2304	1450	1506	1506	2382
ENSECAG00000017741	7.570031168	0.697384195	0.841043858	2752	3390	2542	3004	4139	3950	3931	4998
ENSECAG00000018817	5.336870735	0.697728646	0.841043858	289	606	756	1096	780	908	855	1037
ENSECAG00000017588	5.13881223	0.697738225	0.841043858	318	452	636	961	858	436	929	945
ENSECAG00000016999	2.873698499	0.697742322	0.841043858	50	163	99	161	146	115	159	239
ENSECAG00000006454	5.674105009	0.697789195	0.841043858	348	1433	782	1119	676	1309	999	1105
ENSECAG00000000287	7.490109172	0.697815072	0.841043858	1515	3069	3150	4160	3754	3398	3851	5158
ENSECAG00000016874	6.151428843	0.697821308	0.841043858	776	999	1390	1483	1506	1402	1658	1733
ENSECAG00000021856	3.082083249	0.697843019	0.841043858	82	102	133	233	133	337	170	87
ENSECAG00000015057	6.386669795	0.697894237	0.841043858	690	1644	1624	2153	1236	1242	1550	2919
ENSECAG00000022546	9.082912837	0.697967312	0.841043858	4817	10808	11839	11757	10054	10973	10004	12918
ENSECAG00000009847	8.383616036	0.697981718	0.841043858	3330	5846	5918	6482	7026	6715	6894	9357
ENSECAG00000016254	6.210957321	0.69807427	0.841043858	968	1325	1102	1268	1677	1294	1822	1802
ENSECAG00000007795	7.646938024	0.698241261	0.841043858	2349	3998	4163	3493	4285	3405	3940	4729

ENSECAG00000009160	6.000087108	0.698253331	0.841043858	1210	1156	900	937	1450	1212	1117	1425
ENSECAG00000014326	4.44969489	0.698254419	0.841043858	197	251	414	611	526	467	490	439
ENSECAG00000006013	4.23587326	0.698406261	0.841137581	203	259	490	386	393	308	365	477
ENSECAG00000012110	8.404645943	0.698668971	0.841364796	4206	5345	6354	8269	6391	5110	6866	9501
ENSECAG00000008907	3.257017787	0.698769324	0.841396467	101	128	158	235	167	175	237	271
ENSECAG00000000520	7.652189856	0.698885398	0.841447059	3281	5066	2630	2465	6224	1856	4900	3213
ENSECAG00000007718	0.172879096	0.699236932	0.8417811	11	7	16	33	25	18	17	34
ENSECAG00000004492	3.339834118	0.699632852	0.842123734	101	157	140	264	206	179	255	258
ENSECAG00000003766	3.593920217	0.699669781	0.842123734	77	144	223	375	335	168	420	150
ENSECAG00000021868	9.550635251	0.699795186	0.842178945	10168	9157	13463	14750	20403	13470	15642	17782
ENSECAG00000007391	6.687045554	0.699863897	0.842178945	1299	2068	1287	1788	2216	1524	2223	3389
ENSECAG00000011087	6.55356728	0.700224275	0.842479691	934	1176	1655	2513	2563	1599	1922	2302
ENSECAG00000003312	5.111300106	0.700497105	0.842479691	406	642	592	780	683	620	699	839
ENSECAG00000006545	5.658527252	0.700501387	0.842479691	859	815	799	915	1005	879	1046	1219
ENSECAG00000011206	4.120034087	0.700538929	0.842479691	104	466	155	407	285	395	364	516
ENSECAG00000010503	3.497846521	0.700539779	0.842479691	111	157	195	365	222	203	245	242
ENSECAG00000005903	2.066796577	0.700558715	0.842479691	61	36	82	77	108	73	107	79
ENSECAG00000017585	4.806008315	0.700836779	0.842651121	240	497	428	692	578	412	712	805
ENSECAG00000018553	7.691632349	0.700929436	0.842651121	2771	3384	4041	4297	4114	3000	3360	6626
ENSECAG00000021882	8.857379265	0.700952576	0.842651121	9548	6026	9005	6086	11723	7280	10563	7663
ENSECAG00000022851	1.055749118	0.700997923	0.842651121	21	28	29	49	30	40	69	39
ENSECAG00000022755	3.359640112	0.701465493	0.843093122	158	174	126	163	313	161	229	208
ENSECAG00000019192	7.744481248	0.701514027	0.843093122	2562	3065	3544	4675	5143	3803	4739	5532
ENSECAG00000007968	6.464335074	0.701788751	0.843334086	961	1563	1820	1905	1884	1495	2002	1848
ENSECAG00000026959	4.763269035	0.702198395	0.843737115	272	430	540	710	523	483	548	677
ENSECAG00000021283	5.749926705	0.702448233	0.843948062	683	986	737	1007	1053	1069	1456	1175
ENSECAG00000016200	5.66455586	0.702757537	0.844230401	441	839	1067	1344	867	1022	1179	1067
ENSECAG00000022999	1.775294609	0.702970412	0.844396852	23	95	24	68	56	54	78	119
ENSECAG00000016556	2.046100444	0.70327713	0.844663346	46	40	136	70	70	60	125	67
ENSECAG00000000577	6.608453799	0.703340954	0.844663346	1225	1714	1795	2088	2039	1503	2047	2457
ENSECAG00000024960	5.529871682	0.703498535	0.844710434	545	437	928	1473	882	937	953	982
ENSECAG00000021436	8.238028893	0.703528854	0.844710434	2674	4037	7924	7649	6408	5093	6336	6718
ENSECAG00000013170	6.849928703	0.703655747	0.84477352	1017	1501	2826	3196	2114	1762	2573	3022
ENSECAG00000010238	3.544342654	0.703952312	0.845040271	91	117	166	535	252	236	254	178
ENSECAG00000016749	2.208585914	0.704148885	0.845186946	46	57	75	120	110	64	97	140
ENSECAG00000023978	1.833843262	0.704295109	0.845273163	39.9996	37	86	89.9991	75.9984	66	73	62.9995
ENSECAG00000014843	6.067091988	0.704621661	0.845575762	567	880	1521	1526	1487	1334	1470	1659
ENSECAG00000010641	3.566348462	0.704749062	0.845639333	119	280	211	214	257	214	258	225
ENSECAG00000000453	3.600817256	0.705067486	0.845932077	136	112	266	278	303	221	252	300
ENSECAG00000007525	4.210017633	0.705205824	0.845961888	174	240	390	554	314	353	340	506
ENSECAG00000020743	4.838562915	0.705241243	0.845961888	363	380	680	373	552	441	589	1007
ENSECAG00000023318	6.266906172	0.706068995	0.846865398	748	1886	1165	1030	1380	2205	1642	1538
ENSECAG00000024426	5.892369815	0.706281131	0.846972715	793	1037	1133	1127	1359	947	1242	1341
ENSECAG00000017960	4.488214143	0.706307558	0.846972715	329	384	374	445	414	496	389	527
ENSECAG00000013231	7.055206375	0.706460369	0.846992966	1597	2578	2230	2945	3035	2140	2484	3255
ENSECAG00000024944	5.73814759	0.706508296	0.846992966	474	873	997	1559	1095	864	1234	1191
ENSECAG00000010211	6.047583917	0.706548084	0.846992966	1031	1401	1042	939	1174	1206	1373	1667
ENSECAG00000021237	2.103597209	0.706696291	0.847081259	43	54	61	119	108	68	75	130
ENSECAG00000010272	5.232539924	0.706882418	0.847214983	364	399	745	1027	818	717	799	1008
ENSECAG00000023277	2.849733959	0.70714464	0.847439869	82	119	124	128	118	136	168	218
ENSECAG00000017451	1.451409146	0.707284031	0.847517523	6	52	58	60	41	45	87	67
ENSECAG00000006867	0.998953765	0.707393021	0.847558738	12	19	65	27	35	52	34	50
ENSECAG00000015801	6.263970284	0.707741749	0.847789174	707	955	1366	2169	1668	1435	1666	2083
ENSECAG00000014120	3.529616753	0.707762401	0.847789174	144	141	207	334	217	226	227	264
ENSECAG00000022067	3.901418196	0.707873228	0.847789174	145	290	262	368	240	269	315	398
ENSECAG00000006518	5.2746066	0.707883814	0.847789174	344	543	553	1186	839	655	845	1123
ENSECAG00000000127	5.452317642	0.708090975	0.847947898	472	527	833	1075	1043	783	946	1112
ENSECAG00000016794	5.353715543	0.70824556	0.847973142	427	394	616	1337	1049	773	926	875
ENSECAG00000013518	5.29766447	0.708273301	0.847973142	404	624	769	1028	748	760	652	1079
ENSECAG00000024143	1.119485881	0.708349503	0.847973142	46	21	29	21	26	84	36	39
ENSECAG00000004756	4.153354394	0.708410585	0.847973142	232	244	358	412	339	288	390	444
ENSECAG00000017544	0.615714668	0.708558724	0.848061121	12	31	19	45	18	31	22	43
ENSECAG00000008139	4.486251991	0.708838259	0.848248448	232	233	423	612	533	420	495	535
ENSECAG00000017520	0.847608826	0.708895216	0.848248448	14	22	59	36	3	86	11	23
ENSECAG00000023186	4.202639157	0.708939207	0.848248448	241	169	433	333	489	328	427	385
ENSECAG00000018489	7.290998503	0.709087258	0.848336256	3189	1290	2605	3599	3127	2248	2998	4469
ENSECAG00000016441	0.609262985	0.709278929	0.848421435	9	24	19	58	30	25	29	29
ENSECAG00000015884	4.607922653	0.709307799	0.848421435	199	324	603	672	528	384	613	458
ENSECAG00000020780	6.149155749	0.709541396	0.848585819	636	1079	1216	1828	1395	1571	1475	1840
ENSECAG00000024393	5.081138526	0.709594602	0.848585819	268	461	549	1035	744	570	699	1019
ENSECAG00000007976	8.638202898	0.709681521	0.848600447	3919	5788	8906	10547	8670	6593	8943	8201
ENSECAG00000010622	10.45518333	0.709969978	0.848791224	15274	17243	32211	37967	32606	21230	30541	30027
ENSECAG00000000009	0.871525274	0.709990476	0.848791224	18	23	30	38	37	9	38	80
ENSECAG00000013591	4.265451227	0.710156368	0.848900226	225	223	407	526	439	316	370	449
ENSECAG00000000366	2.904143778	0.710236839	0.848907108	57	68	215	152	164	192	154	145
ENSECAG00000012646	4.233112597	0.710332871	0.848932584	197	218	347	488	398	376	430	455
ENSECAG00000022712	6.134527494	0.710876847	0.849375496	593	1035	1141	1985	1281	1249	1588	2184
ENSECAG00000024217	5.176666621	0.710961344	0.849375496	293	480	727	949	939	657	860	737
ENSECAG00000011911	1.665892117	0.711035632	0.849375496	25	16	69	96	113	52	86	25
ENSECAG00000016280	4.575394686	0.711041576	0.849375496	130	545	382	529	480	423	638	584
ENSECAG00000013314	5.603141455	0.71107725	0.849375496	593	739	1257	786	1165	739	1069	1015
ENSECAG00000017226	1.843211678	0.711172896	0.849400446	35	42	81	67	75	104	77	51
ENSECAG00000022362	5.404287634	0.711407112	0.849590878	422	882	805	873	865	924	685	982
ENSECAG00000024329	4.247497753	0.711604248	0.849593883	148	254	521	472	335	319	478	413
ENSECAG00000007397	10.26313554	0.711629579	0.849593883	13067	15945	27662	33172	28246	19133	26613	26166

ENSECAG00000024129	5.874121467	0.711633954	0.849593883	892	978	1053	1054	1118	1135	1170	1390
ENSECAG00000013711	6.234496917	0.712305688	0.850306496	797	1004	1028.01	2214	1642	1599	1717	1694
ENSECAG00000009069	9.710944969	0.712404087	0.850334619	7820	20394	14006	17536	12920	14946	20973	19275
ENSECAG00000023553	3.579322764	0.712605338	0.850485489	86	142	251	330	223	256	317	256
ENSECAG00000016996	8.428409359	0.712991555	0.850857057	3644	4221	6530	8332	7219	6843	7187	9638
ENSECAG00000004869	1.338468731	0.713178028	0.85096857	16	36	50	83	48	54	37	55
ENSECAG00000011048	5.999576572	0.713234791	0.85096857	1126	428	2057	824	2451	743	1419	416
ENSECAG00000021433	4.128221854	0.714048771	0.851840551	145	292	313	533	317	318	390	400
ENSECAG00000012547	2.257057706	0.714150376	0.851840551	34	54	163	99	106	102	55	111
ENSECAG00000011275	5.560437617	0.714190555	0.851840551	654	802	665	855	1033	1026	1018	1074
ENSECAG00000002867	1.168706631	0.714300183	0.85188188	43	6	79	22	64	29	55	19
ENSECAG00000016755	7.104016404	0.714427451	0.851936744	1333	2461	2922	3179	2333	3140	2382	3325
ENSECAG00000019219	4.192579109	0.71454431	0.851936744	187	161	477	375	558	391	279	386
ENSECAG00000022237	3.871917418	0.714583677	0.851936744	134	265	298	353	190	294.002	315	394
ENSECAG00000012525	5.750546169	0.71464611	0.851936744	402	760	1111	1387	1072	988	1226	1511
ENSECAG00000000712	4.134149682	0.714841041	0.851987399	245	241	399	325	414	311	403	293
ENSECAG00000016123	2.861226967	0.714872504	0.851987399	97	85	191	131	126	125	165	168
ENSECAG00000013600	7.188863877	0.715004098	0.851987399	1180	2452	3142	3980	2735	3281	2621	3174
ENSECAG00000010039	6.063172038	0.715066034	0.851987399	678	997	1173	1578	1415	1108	1802	1616
ENSECAG00000000916	4.967395506	0.715082022	0.851987399	212	583	590	713	628	572	776	801
ENSECAG00000008878	5.861866658	0.715228476	0.851987399	857	613	986	1264	1421	906	1413	1443
ENSECAG00000013206	5.71440603	0.715260847	0.851987399	498	750	695	1610	1122	1029	1095	1419
ENSECAG00000003460	4.610105532	0.715288488	0.851987399	319	381	341	521	527	375	548	733
ENSECAG00000024388	2.188395575	0.715466362	0.851991546	67	3	171	5	204	4	204	19
ENSECAG00000024533	4.754280447	0.715526715	0.851991546	153	430	562	696	469	629	531	766
ENSECAG00000008167	5.831688421	0.715553742	0.851991546	995	878	805	1155	1443	925	1110	1188
ENSECAG00000023374	5.561637492	0.715591914	0.851991546	532	700	676	1208	1136	803	1163	1077
ENSECAG00000011056	2.273042493	0.715863502	0.852225598	36	88	87	99	90	60	116	167
ENSECAG00000008301	6.590070921	0.716017596	0.85231974	1031	1556	1409	2341	2052	2074	1945	2462
ENSECAG00000007744	1.439679216	0.716177255	0.852415496	19	68	45	60	32	20	40	125
ENSECAG00000003259	2.883015937	0.716305544	0.852415496	73	141	149	156	120	157	148	166
ENSECAG00000013375	7.194235204	0.716323108	0.852415496	1761	3635	2287	2380	2698	2391	2890	4060
ENSECAG00000018572	6.279197677	0.716665365	0.852691253	763	1500	1667	1562	1486	1417	1570	1916
ENSECAG00000018958	6.431910606	0.716704935	0.852691253	1152	1462	1573	1208	2184	1403	2003	2106
ENSECAG00000001893	2.543879189	0.716882635	0.852784832	82	124	54	140	96	101	138	130
ENSECAG00000021067	7.244422912	0.716934258	0.852784832	2409	2429	2154	3456	3687	2088	2844	3870
ENSECAG00000010852	11.44862278	0.717021056	0.852784832	31963	38616	60608	70496	64839	44770	58101	60429
ENSECAG00000008941	7.09891215	0.717083813	0.852784832	2468	2518	2151	1926	3204	2314	2719	2928
ENSECAG00000021864	6.786908254	0.718060336	0.853856781	900	1776	2649	2778	1865	2519	1646	2967
ENSECAG00000019507	4.145071204	0.718233092	0.853904221	128	160	309	632	412	366	476	304
ENSECAG00000004882	6.723488931	0.718250541	0.853904221	1495	2283	1758	1606	2250	1776	2162	2477
ENSECAG00000006472	5.098792866	0.71843773	0.854037402	470	433	667	831	864	550	687	711
ENSECAG00000016432	6.94468472	0.718568698	0.854103729	1128	2376	2357	3029	2839	2203	2755	2211
ENSECAG00000019779	1.021773867	0.718856704	0.854334113	32	10	41	35	42	28	80	25
ENSECAG00000009219	8.6226964	0.719015976	0.854334113	9828	6071	3655	5523	8494	6420	8230	8681
ENSECAG00000020539	5.399345503	0.719047993	0.854334113	416	823	775	979	741	787	925	1019
ENSECAG00000023260	4.315559334	0.719063292	0.854334113	218	228	372	487	481	229	297	815
ENSECAG00000018203	6.11536042	0.719211725	0.854421123	670	1476	1070	1262	1407	1298	1528	1943
ENSECAG00000018243	8.671417876	0.720056118	0.855334826	5385	7552	6646	8889	7706	7735	8386	9432
ENSECAG00000016827	5.841509373	0.720317366	0.855555709	614	1149	1007	1264	982	1168	957	1620
ENSECAG00000012267	4.611195898	0.720644815	0.855855168	341	668	287	332	562	369	553	505
ENSECAG00000013294	6.403491002	0.72073635	0.855874415	1130	1738	1494	1406	1864	1672	1678	1690
ENSECAG00000021889	7.127212296	0.721192202	0.856237204	1189	3271	2255	2254	2797	3292	3002	3307
ENSECAG00000017145	8.326353503	0.721193674	0.856237204	3859	4854	5228	6730	7518	5612	8208	7292
ENSECAG00000015836	5.067568825	0.721267935	0.856237204	398	457	660	663	756	598	775	835
ENSECAG00000020755	9.036556262	0.721749586	0.856713671	6422	5242	12614	9468	14595	9316	12069	10930
ENSECAG00000019518	4.157597059	0.7218201	0.856713671	130	258	292	533	238	339	416	609
ENSECAG00000016499	5.08830971	0.721945551	0.856773067	436	728	510	660	700	647	868	544
ENSECAG00000021159	6.569808928	0.722103514	0.856871031	1135	1241	2249	2108	1744	1743	2090	2223
ENSECAG00000012703	5.489338171	0.722408997	0.857103212	520	673	947	1027	930	781	1018	967
ENSECAG00000018450	1.708226712	0.722464066	0.857103212	12	46	53	108	47	72	56	113
ENSECAG00000006331	4.062883732	0.722525486	0.857103212	208	189	301	389	406	302	417	345
ENSECAG00000004792	5.817554928	0.722653472	0.857165544	683	1259	732	1234	1264	852	1157	1384
ENSECAG00000017213	1.31700137	0.722884	0.857329016	16	23	78	65	46	64	34	44
ENSECAG00000015485	4.104129376	0.723070564	0.857329016	155	281	314.004	500	371	249	370	424
ENSECAG00000018030	4.376767131	0.723091025	0.857329016	195	345	330	645	334	498	379	471
ENSECAG00000012622	6.490239512	0.723145754	0.857329016	1128	1133	1745	1814	2106	1532	2104	2250
ENSECAG00000012743	4.922613216	0.72324243	0.857329016	418	702	482	431	679	565	560	674
ENSECAG00000020547	11.25509743	0.723325464	0.857329016	19932	34739	60630	66170	52773	41987.1	55701	47956
ENSECAG00000001554	2.0477552	0.723345488	0.857329016	103	100	24	25.0001	65.0001	63.0001	75	123
ENSECAG00000023572	3.036586541	0.7234363	0.857329016	93	207	72	129	179	122	215	218
ENSECAG00000007301	8.541707302	0.723586684	0.857329016	3123	8240	6414	9278	6025	7620	6142	10677
ENSECAG00000010985	5.092879826	0.723598497	0.857329016	468	500	664	518	729	738	738	795
ENSECAG00000001481	6.693688765	0.723648632	0.857329016	863	4210	285	635	2667	1936	2117	2818
ENSECAG00000013983	7.638080562	0.723705147	0.857329016	2356	3920	2991	3309	4117	3664	4211	5893
ENSECAG00000005207	3.620417032	0.723805047	0.857329016	162	180	206	315	331	186	262	218
ENSECAG00000010706	4.02731766	0.723847672	0.857329016	72	290	277	617.008	283	339	339	347
ENSECAG00000012624	5.167360122	0.724553008	0.858074972	567	579	554	506	901	675	821	773
ENSECAG00000008659	5.152918174	0.724637613	0.858085728	424	737	515	798	690	808	644	754
ENSECAG00000011111	3.312491972	0.724832654	0.858227242	130	115	259	190	184	262	140	204
ENSECAG00000011974	7.629012546	0.724923815	0.858245742	1824	3250	4213	5124	3729	3913	3530	5028
ENSECAG00000018513	1.134358728	0.725138963	0.858411013	15	32	21	73	27	41	58	64
ENSECAG00000011687	4.846881585	0.725884908	0.859175585	301	360	433	837	665	570	753	536
ENSECAG00000012230	6.137401375	0.725990857	0.859175585	755	711	1421	1824	1783	1249	1653	1547
ENSECAG00000007750	8.425920084	0.726011687	0.859175585	3622	5318	5539	8068	7625	7151	6040	9953

ENSECAG00000024187	8.557754272	0.726189331	0.859246653	4856	4235	9874	7772	7784	6614	8298	7993
ENSECAG00000016338	3.707777633	0.726230041	0.859246653	209	289	176	191	304	193	333	226
ENSECAG00000012364	1.674755878	0.726298615	0.859246653	20	48	67	99	55	39	71	88
ENSECAG00000016082	8.085844897	0.726415982	0.859258939	4738	4807	4222	4380	6053	4310	5596	6286
ENSECAG00000014246	0.640881103	0.726460251	0.859258939	9	44	29	23	19	35	26	36
ENSECAG00000016897	4.014735219	0.726957505	0.85975759	119	249	264	465	304	338	374	410
ENSECAG00000007404	5.400667563	0.727202353	0.859913617	336	610	680	1264	840	882	925	1087
ENSECAG00000014636	0.724132469	0.727346631	0.859913617	14	21	34	31	21	46	46	24
ENSECAG00000020447	7.865119719	0.727350143	0.859913617	1884	5621	3177	6284	4247	3976	4107	6838
ENSECAG00000000467	2.600623439	0.727392164	0.859913617	41	124	103	177	84	123	71	210
ENSECAG00000012298	4.916788738	0.727519841	0.859975077	297	373	565	988	553	664	515	733
ENSECAG00000005447	1.462407616	0.727666725	0.860012057	13	35	73	55	24	113	37	60
ENSECAG00000008692	6.50018904	0.727757561	0.860012057	827	2030	1440	2157	1829	1815	1864	1878
ENSECAG00000015321	7.136502612	0.72781244	0.860012057	2062	2196	2700	2716	3157	2677	2718	2932
ENSECAG00000009993	5.394910735	0.727911482	0.860012057	411.011	549	847.008	1266	816.001	831	924.001	868
ENSECAG00000021187	6.241375809	0.727929585	0.860012057	1002	1326	1240	1674	1496	1281	1577	1901
ENSECAG00000010516	5.337678739	0.728101478	0.860125701	474	625	920	807	925	724	846	823
ENSECAG00000020848	5.306050232	0.728475808	0.860407449	559	243	1479	489	764	872	617	928
ENSECAG00000014559	3.619617712	0.728491433	0.860407449	149	250	175	191	226	182	371	315
ENSECAG00000015226	7.307576956	0.729073728	0.860922817	1789	3329	2811	3144	3089	2884	2959	4090
ENSECAG00000014992	0.927785864	0.729099629	0.860922817	25	29	20	60	10	82	27	15
ENSECAG00000000122	3.779300832	0.729155102	0.860922817	66	91	592	157	225	401	263	332
ENSECAG00000011614	6.216411033	0.729248829	0.860944014	895	1298	996	1614	1339	1391	1705	2190
ENSECAG00000020575	5.657641326	0.729519856	0.861174504	531	813	1171	1048	937	958	926	1352
ENSECAG00000017686	0.818266466	0.729596739	0.86117579	19	18	42	25	32	68	32	13
ENSECAG00000024260	1.689180218	0.729675658	0.861179478	18	62	58	98	35	63	80	73
ENSECAG00000008588	3.795476572	0.730129059	0.861593782	126	299	201	270	256	330	293	339
ENSECAG00000022322	6.682605177	0.730219905	0.861593782	1271	1659	1718	2613	2373	1772	2245	2022
ENSECAG00000009315	0.445420043	0.730254192	0.861593782	5	11	17	55	39	20	26	29
ENSECAG00000014053	8.747778889	0.730440505	0.861724121	5350	6338	6211	9871	9335	7821	9278	12103
ENSECAG00000026871	3.06178708	0.730598308	0.861771852	141	165	125	118	200	137	147	191
ENSECAG00000018622	7.286064735	0.730632657	0.861771852	1974	2957	2766	3162	3568	2690	3854	2574
ENSECAG00000002734	5.519650142	0.730731642	0.86179914	526	701	957	1047	857	847	934	1153
ENSECAG00000005303	2.934880375	0.73114664	0.862199079	100	126	134	169	175	164	102	173
ENSECAG00000015755	7.923782029	0.731320352	0.862203595	2874	3515	3403	6089	5394	4600	5259	6416
ENSECAG00000009409	3.9726409	0.731374728	0.862203595	296	224	211	199	378	344	332	326
ENSECAG00000009033	6.574868013	0.731378124	0.862203595	1844	1568	1317	1527	1900	1520	2019	2413
ENSECAG00000018783	2.319411644	0.731489743	0.862245716	46	66	103	107	96	84	127	132
ENSECAG00000003253	5.137359862	0.732213087	0.863008827	351	473	605	927	681	869	572	977
ENSECAG00000018868	3.208963569	0.732415201	0.863118168	104	81	214	203	231	178	216	186
ENSECAG00000006450	1.426453647	0.732457787	0.863118168	32	26	48	62	59	51	59	62
ENSECAG00000023777	6.476990184	0.732683419	0.86324052	1118	872	2207	2163	1982	1625	1730	1960
ENSECAG00000023915	4.104534264	0.732713569	0.86324052	187	190	330	433	426	299	446	341
ENSECAG00000005805	6.706105877	0.733189381	0.863467635	1468	1599	1882	1576	3137	1704	2456	1952
ENSECAG00000018725	2.571536989	0.733266826	0.863467635	67	81	127	145	110	98	161	106
ENSECAG00000007939	0.556755399	0.733277865	0.863467635	14	15	46	26	15	50	21	19
ENSECAG00000016447	6.351376481	0.733542751	0.863467635	1428	1996	792	1115	1604	1576	1706	1751
ENSECAG00000014429	9.196823566	0.733657091	0.863467635	5722	7770	12291	13007	12462	13772	10227	15740
ENSECAG00000017916	7.540432272	0.733707487	0.863467635	2137	3927	2943	4043	3341	3944	3559	4349
ENSECAG00000003030	7.58536303	0.733729955	0.863467635	2230	3681	3553	4005	3675	3100	3931	5158
ENSECAG00000013582	4.801348055	0.733897757	0.863467635	210	685	488	407	395	775	534	753
ENSECAG00000008865	3.756853094	0.733916702	0.863467635	113	279	225	355	207	241	301	357
ENSECAG00000011836	4.57521675	0.733965218	0.863467635	320	313	300	616	542	421	532	615
ENSECAG00000016833	3.684429012	0.733971412	0.863467635	92	195	198	384	153	250	258	498
ENSECAG00000009755	3.937901637	0.734027428	0.863467635	128	43	312	559	383	405	510	51
ENSECAG00000015926	1.117452454	0.734098459	0.863467635	21	33	38	41	61	29	50	46
ENSECAG00000011403	6.742053829	0.734108889	0.863467635	1075	1597	2316	2748	1698	2613	2191	2170
ENSECAG00000000116	5.928060843	0.734135846	0.863467635	561	1078	874	1547	1087	1336	1191	1782
ENSECAG00000005886	3.447850919	0.734152237	0.863467635	94	187	181	335	134	222	261	266
ENSECAG00000021561	9.766244955	0.734198277	0.863467635	10044	11371	17008	18240	20603	16100	19470	21500
ENSECAG00000021299	7.876199905	0.734412582	0.863630279	2530	3116	5719	5554	4918	4107	4130	6173
ENSECAG00000014690	6.483947449	0.734731273	0.863915629	1082	1199	1347	2252	2027	1315	1975	2712
ENSECAG00000009968	0.614641881	0.73493012	0.864044723	34	12	25	31	3	86	2	11
ENSECAG00000018804	4.06306648	0.734993157	0.864044723	155	143	402	423	448	296	282	461
ENSECAG00000004120	3.462625415	0.73514398	0.864128008	139	127	260	251	255	210	137	295
ENSECAG00000020356	6.25141817	0.73528981	0.864128008	697.021	1359	1723.02	1671	1771	1520	1708.98	1182
ENSECAG00000010590	4.947294807	0.735324692	0.864128008	284	607	577	725	578	538	654	778
ENSECAG00000008645	2.523639217	0.735368219	0.864128008	60	87	114	104	127	99	102	181
ENSECAG00000023607	5.650172378	0.735597181	0.86430767	885	1484	399	454	958	990	925	1188
ENSECAG00000023299	5.402641837	0.735674849	0.864309547	394	662	738	1037	898	808	1032	985
ENSECAG00000009307	7.167732951	0.735756232	0.864315788	1416	2471	2470	3073	3597	2318	2926	4032
ENSECAG00000024590	4.267998746	0.735885879	0.864345012	171	316	429	467	372	335	441	432
ENSECAG00000002357	3.84049069	0.735933255	0.864345012	110	361	254	288	293	240	333	299
ENSECAG00000007490	3.4716907	0.736241628	0.864617816	94	142	212	375	180	250	200	265
ENSECAG00000023910	3.373415898	0.73668287	0.865046587	49	248	176	297	136	179	165	366
ENSECAG00000007883	4.794819136	0.736871762	0.865178977	286	555	622	476	345	758	430	715
ENSECAG00000020882	5.589156562	0.737005231	0.865246273	436	893	812	1047	1076	934	1147	1074
ENSECAG00000012635	4.953857507	0.737126984	0.865299803	241	522	500	1032	634	590	562	757
ENSECAG00000019794	2.938416971	0.737403045	0.865534441	94	72	160	167	148	169	148	209
ENSECAG00000021840	7.284633061	0.737577046	0.865546024	1773	1882	2946	3673	3398	3486	3110	3792
ENSECAG00000018110	4.904872389	0.737629288	0.865546024	271	382	578	796	685	585	692	670
ENSECAG00000006021	5.58417099	0.737677206	0.865546024	517.001	525.001	1174	1248	1230	707	1065	940
ENSECAG00000016520	4.176643275	0.737751933	0.865546024	157	163	390	523	422	319	367	499
ENSECAG00000022650	4.18132818	0.737822436	0.865546024	270	334	326	286	397	317	386	384
ENSECAG00000017908	4.47431176	0.737869987	0.865546024	207	380	402	478	458	511	429	549

ENSECAG00000011715	6.182242452	0.73808422	0.865707949	930	738	1231	1885	1896	1208	1859	1455
ENSECAG00000010150	6.150903265	0.738517529	0.866060807	895.003	829	1727	1556	1605	1433	1326	1446
ENSECAG00000001775	1.507393982	0.73857546	0.866060807	42	26	61	39	61	109	20	51
ENSECAG00000013541	5.240049824	0.738613732	0.866060807	185	784	438	1187	518	1128	784	894
ENSECAG00000012333	5.78069785	0.738763318	0.866146818	776	679	1125	911	1268	959	1443	1178
ENSECAG00000013502	7.705394871	0.738876672	0.866190337	2764	5194	2749	3524	3776	3145	3234	7180
ENSECAG00000010012	5.424146642	0.738962713	0.86620183	474	631	781	1189	804	617	878	1285
ENSECAG00000002380	5.401416519	0.739115348	0.866291375	577	933	760	561	966	712	791	1013
ENSECAG00000021748	1.794695862	0.739362417	0.866460974	27	52	87	82	59	54	99	60
ENSECAG00000019128	6.882526511	0.739422308	0.866460974	2717	2364	1273	1046	2387	2038	2751	2360
ENSECAG00000012896	6.636003606	0.739488828	0.866460974	1166	1838	1788	2164	2115	1671	2219	2187
ENSECAG00000017595	3.266238703	0.739777876	0.866710273	64	190	163	219	150	226	185	288
ENSECAG00000019677	4.27196388	0.739862465	0.866720005	123	251	276	694	486	483	470	241
ENSECAG00000007386	1.169244232	0.739981242	0.866769781	19	41	69	24	35	44	47	46
ENSECAG00000011424	4.535912484	0.740245959	0.866978408	217	341	518	594	476	409	455	571
ENSECAG00000022103	8.959328914	0.74032003	0.866978408	4017	7530	9245	12748	10398	10251	11286	12342
ENSECAG00000024525	0.559458132	0.740388267	0.866978408	20	12	35	31	35	15	27	35
ENSECAG00000020963	6.404977558	0.740887999	0.86747418	1007	1382	1557	2014	1634	1523	1821	2021
ENSECAG00000023292	6.081540927	0.741037765	0.867560133	596	1250	1039	1626	1225	1449	1326	2007
ENSECAG00000006984	5.613178848	0.741135814	0.867585527	621	773	1076	908	922	1020	854	1238
ENSECAG00000016548	6.104280151	0.741257721	0.867598649	964	1074	1178	1534	1518	1040	1508	1636
ENSECAG00000012037	3.98207957	0.741299743	0.867598649	157	341	240	273	299	372	315	400
ENSECAG00000015373	3.346902683	0.741468301	0.867706544	143	102	271	191	287	113	244	180
ENSECAG00000011716	5.49144518	0.741581703	0.867749878	328	750	802	1178	777	848	1030	1346
ENSECAG00000007579	5.268949014	0.74166524	0.867750091	252	498	862	1042	675	910	723	1102
ENSECAG00000015154	4.801677821	0.741734631	0.867750091	138	507	539	726	538	648	467	820
ENSECAG00000008450	7.488810356	0.742221719	0.868215356	2521	3358	2937	3529	4749	3011	3958	2895
ENSECAG00000007777	4.928179734	0.742359297	0.868215356	329	554	575	677	595	551	562	813
ENSECAG00000019777	5.061639856	0.742361572	0.868215356	389	657	755	504	538	1093	500	514
ENSECAG00000019974	4.7909551	0.743184224	0.869088015	318	437	491	554	613	575	663	566
ENSECAG00000022801	4.826413692	0.743424135	0.8691443	464	366	497	418	878	391	744	495
ENSECAG00000000135	7.142670779	0.743455489	0.8691443	1730	1936	3051	2341	3218	2446	3044	3908
ENSECAG00000012824	3.568262069	0.743461842	0.8691443	77	298	168	319	166	179	231	400
ENSECAG00000008292	4.456367438	0.743582049	0.869195396	231	320	325	586	485	447	490	502
ENSECAG00000004313	7.321630758	0.743671661	0.869210721	2430	2906	2323	3245	3168	3073	2998	3893
ENSECAG00000009214	5.791183129	0.743899845	0.869319299	745	1380	732	860	1251	959	949	1408
ENSECAG00000013870	1.305378965	0.74391758	0.869319299	17	38	94	23	49	65	32	40
ENSECAG00000007380	0.294101526	0.744355171	0.869741203	12	13	21	26	34	12	35	21
ENSECAG00000017692	5.811914987	0.744440678	0.869751669	646	624	1215	1225	1240	1071	1199	1452
ENSECAG00000010480	7.362821263	0.744661902	0.86992068	2289	2673	3155	3234	3799	2740	3512	3465
ENSECAG00000022413	9.269520233	0.744841095	0.870040559	6588	7934	16048	13839	13789	11501	12687	12306
ENSECAG00000009106	4.448887722	0.745159796	0.870323356	215	401	362	442	784	293	435	419
ENSECAG00000000844	0.318992867	0.745409328	0.870455211	0	18	22	39	16	25	39	23
ENSECAG00000011023	4.198287358	0.745425911	0.870455211	143	286	343	465	286	421	416	491
ENSECAG00000012021	3.387821545	0.745654502	0.87058704	86	143	174	303	208	210	207	299
ENSECAG00000009529	6.557880974	0.74569205	0.87058704	918	1644	1666	2549	1926	1705	2079	2040
ENSECAG00000014825	6.094397799	0.745852748	0.870685187	766	1319	1066	1252	1589	1299	1337	1820
ENSECAG00000000518	7.502522593	0.746232497	0.870973811	1833	1717	4061	5877	3739	3664	3943	3354
ENSECAG00000027244	3.615258444	0.746340684	0.870973811	31	44	142	861	594	188	94	46
ENSECAG00000011732	1.428004987	0.746380003	0.870973811	22	86	30	44	31	44	68	67
ENSECAG00000014217	5.604967838	0.746455713	0.870973811	548	713	753	1210	1044	834	1182	1240
ENSECAG00000014298	2.489453875	0.746546185	0.870973811	41	95	107	125	120	122	111	136
ENSECAG00000000942	1.803303105	0.746559932	0.870973811	45	21	108	37	112	39	86	71
ENSECAG00000015938	2.96196436	0.746659294	0.871000298	54	131	155	232	160	150	167	148
ENSECAG00000016131	4.531182799	0.746779205	0.871050747	90	191	538	819	344	911	281	478
ENSECAG00000009675	5.300041554	0.746863572	0.871059731	558	394	797	780	1105	555	744	1115
ENSECAG00000007559	4.82131865	0.7472679	0.871441844	343	385	617	636	594	568	575	580
ENSECAG00000022869	5.280122712	0.747436493	0.871548997	413	365	1132	618	940	429	740	1431
ENSECAG00000023512	3.615511555	0.747600808	0.871651142	169	273	141	250	251	214	346	175
ENSECAG00000011952	5.251710036	0.747825113	0.871735725	417	635	661	987	829	618	746	969
ENSECAG00000007560	6.946933669	0.747849205	0.871735725	1412	1534	2159	3054	2707	2863	2308	2995
ENSECAG00000019668	2.151191361	0.747903524	0.871735725	30.0007	40	146.001	112.001	96.0006	62.0005	114.001	78.001
ENSECAG00000000556	4.76164687	0.748089944	0.87178076	280	555	395	526	655	413	594	747
ENSECAG00000011603	7.542281442	0.748095618	0.87178076	1790	2470	4041	4090	4652	3281	4167	4463
ENSECAG00000015561	5.17963673	0.748403568	0.871972672	565	629	495	549	750	744	785	914
ENSECAG00000026974	3.352221611	0.748486484	0.871972672	84	199	176	207	201	149	237	324
ENSECAG00000012777	5.782939017	0.74850223	0.871972672	586	1127	855	980	1347	820	1123	1623
ENSECAG00000007859	0.262908705	0.748567281	0.871972672	14	16	22	27	22	20	25	21
ENSECAG00000010110	8.343599442	0.748727272	0.872069633	3560	5106	5708	6822	6852	6664	7237	8042
ENSECAG00000019382	7.951565807	0.748821077	0.872089491	2565	4908	5572	4313	4505	4510	4711	6680
ENSECAG00000020923	0.370736548	0.749094972	0.872319061	11	25	16	24	16	28	28	35
ENSECAG00000019645	3.848376337	0.749251902	0.872412391	199	166	363	271	333	170	446	221
ENSECAG00000013667	5.219569786	0.749379154	0.872431751	333	798	596	925	748	653	826	844
ENSECAG00000000550	4.780642209	0.749422099	0.872431751	268	385	598	570	502	645	532	732
ENSECAG00000021397	4.068152539	0.749739459	0.872600926	228	258	335	328	369	300	364	340
ENSECAG00000024994	7.409091825	0.749783271	0.872600926	1872	3348	2729	4065	2976	3588	3393	3957
ENSECAG00000017915	4.311012695	0.749797821	0.872600926	195	101	458	587	743	327	449	232
ENSECAG00000012463	11.35641042	0.750011067	0.872748133	24331	32764	57026	60819	59401	43560	78186	51990
ENSECAG00000013338	2.035760358	0.750125402	0.872748133	31	57	70	145	103	80	78	59
ENSECAG00000019233	7.742724252	0.750230576	0.872748133	2926	4149	2860	3413	5136	4192	5384	4207
ENSECAG00000017119	3.330603678	0.750231563	0.872748133	180	128	144	225	196	137	227	266
ENSECAG00000010157	10.1748606	0.750357927	0.872805771	11285	14456	25750	33488	25274	18750	24920	25937
ENSECAG00000003277	7.945556675	0.750653944	0.873014713	3002	2395	5233	5222	10786	5779	3312	1763
ENSECAG00000015874	0.317650332	0.750691229	0.873014713	18	14	28	20	25	16	35	16
ENSECAG00000022582	4.618268114	0.750792681	0.873016129	341	487	385	447	541	441	502	535

ENSECAG00000011326	5.248508456	0.750891787	0.873016129	475	539	759	679	885	743	871	832
ENSECAG00000020965	3.658950601	0.750938995	0.873016129	144	161	289	221	285	231	280	315
ENSECAG00000017645	8.318396304	0.750999793	0.873016129	5432	5602	5113	5171	7515	4999	6980	6677
ENSECAG00000013178	5.168085936	0.751161429	0.873114697	331	512	715	856	809	662	907	777
ENSECAG00000021020	6.868460213	0.751566935	0.873496677	911	2165	1934	2828	2323	2507	2308	3226
ENSECAG00000011129	7.555033889	0.751754962	0.873625844	2557	4734	1956	2099	3980	3099	4281	5606
ENSECAG00000000823	4.797633136	0.751847264	0.873632708	266	441	390	937	522	578	643	521
ENSECAG00000023944	2.430695552	0.7519486	0.873632708	63	60	142	112	120	81	107	127
ENSECAG00000018393	4.979962527	0.752005856	0.873632708	420	596	518	620	683	636	691	569
ENSECAG00000007211	1.018269057	0.752068431	0.873632708	9	28	71	36	23	50	34	46
ENSECAG00000000236	8.459928753	0.752252632	0.873746637	3449	4336	7308	8426	7702	7256	7320	8982
ENSECAG00000012801	0.645983206	0.752320309	0.873746637	24	24	24	29	28	22	18	53
ENSECAG00000010312	2.958706493	0.752429314	0.873783919	50	117	104	326	205	208	57	137
ENSECAG00000007275	3.153875108	0.752853318	0.874186959	100	66	230	250	218	108	195	203
ENSECAG00000019266	4.148814922	0.753484395	0.874558884	170	256	251	518	379	381	392	399
ENSECAG00000023474	4.883576324	0.753527074	0.874558884	245	506	624	585	801	469	706	623
ENSECAG00000018966	0.172858745	0.753540087	0.874558884	5	22	21	19	19	23	18	32
ENSECAG00000003017	2.217384987	0.753587917	0.874558884	36	89	69	142	66	89	85	133
ENSECAG00000013307	5.133216011	0.753608392	0.874558884	375	544	839	706	666	723	685	813
ENSECAG00000005797	7.38247875	0.75367772	0.874558884	2622	2803	2361	2478	3674	3389	3671	4051
ENSECAG00000005906	6.169711035	0.753712426	0.874558884	670	1059	1381	1720	1673	1316	1377	2014
ENSECAG00000020497	3.330981536	0.75391909	0.874680554	96	168	197	188	286	101	177	343
ENSECAG00000014242	5.165381065	0.754074201	0.874680554	363	423	747	875	746	748	867	779
ENSECAG00000007970	1.854675279	0.754236026	0.874680554	38.0001	52	77	86.0003	76.0008	44	87	82.0002
ENSECAG00000016348	2.774679297	0.754297264	0.874680554	54	100	117	185	120	142	172	164
ENSECAG00000023235	6.507408114	0.754321158	0.874680554	1242	2047	1262	1601	2047	1512	1781	2175
ENSECAG00000025043	0.622895405	0.754407999	0.874680554	9	4	36	50	45	24	25	36
ENSECAG00000008836	2.377853281	0.75449009	0.874680554	47	41	33	234	131	60	133	141
ENSECAG00000019674	6.836358277	0.754507455	0.874680554	975	1679	2442	3298	2090	2078	2713	2501
ENSECAG00000009074	5.646378968	0.754510131	0.874680554	460	368	1153	1458	1003	1100	1236	1062
ENSECAG00000015480	3.492308712	0.754885193	0.875019105	88	113	215	356	221	171	312	294
ENSECAG00000015828	5.213218237	0.754956195	0.875019105	399	571	680	993	817	587	707	974
ENSECAG00000023727	3.855328897	0.755193398	0.875120322	68	19	968	138	1	923	2	2
ENSECAG00000018202	6.636570991	0.755197567	0.875120322	619	1364	1671	3298	1628	2486	2261	2394
ENSECAG00000024210	4.566109844	0.755677007	0.875586596	215	459	381	641	463	415	545	526
ENSECAG00000010610	8.855821733	0.755824421	0.875666652	5699	6576	8396	9197	8904	7509	9246	16201
ENSECAG00000017318	2.609543228	0.75590024	0.875666652	41	155	103	86	105	154	180	88
ENSECAG00000013778	2.75109334	0.756613828	0.876403947	83	105	83	159	161	122	136	171
ENSECAG00000023633	3.021815586	0.756792541	0.876521596	63	158	123	190	141	138	196	244
ENSECAG00000019902	5.456291071	0.757228901	0.876937598	586	707	886	830	844	955	693	1129
ENSECAG00000004287	5.538321481	0.757443298	0.877062916	407	955	641	1081	880	906	955	1377
ENSECAG00000027669	11.81223653	0.757543264	0.877062916	56739	33983	85160	46671	75302	87285	73422	82353
ENSECAG00000011430	6.152634612	0.75756869	0.877062916	959	760	1473	1829	1674	1244	1516	1416
ENSECAG00000012908	4.165772995	0.757712546	0.877140086	179	251	302	468	407	308	375	495
ENSECAG00000021189	0.72707061	0.757849649	0.877205421	12	19	38	47	31	23	39	33
ENSECAG00000008260	5.966528644	0.757994671	0.877205421	621	803	1365	1390	1487	1140	1397	1483
ENSECAG00000016738	4.054631091	0.758000601	0.877205421	188	160	501	336	539	312	272	208
ENSECAG00000012745	3.792436865	0.758252948	0.877408086	109	184	263	386	279	286	268	387
ENSECAG00000010584	5.444488809	0.758490753	0.877593883	559	790	659	770	964	873	858	1136
ENSECAG00000000019	5.925222378	0.758840799	0.877909496	608	861	1162	1431	1479	1001	1471	1406
ENSECAG00000001359	6.390930644	0.75910085	0.878059609	1032	1845	1212	1675	1383	1700	1611	2239
ENSECAG00000018035	8.87554962	0.759125113	0.878059609	8228	9311	5210	8584	9910	10835	10017	6947
ENSECAG00000012597	7.546728267	0.759230856	0.878092527	3658	3128	2774	2630	4964	2832	3714	3812
ENSECAG00000024397	9.279235538	0.759516793	0.878333822	7004	12463	8078	12367	12338	12560	14238	16079
ENSECAG00000024679	6.303762072	0.759852953	0.878584165	1067	1407	1223	1299	1813	1440	1664	2068
ENSECAG00000015782	8.472835707	0.759916648	0.878584165	8192	5469	3269	6113	13864	4776	3955	6128
ENSECAG00000024091	9.464884532	0.760010602	0.878584165	7897	8982	17542	16169	16204	11130	14585	16282
ENSECAG00000026924	3.853498656	0.760121921	0.878584165	168	256	279	298	247	286	273	383
ENSECAG00000025124	3.720279256	0.760263261	0.878584165	129	176	224	432	290	213	295	280
ENSECAG00000008394	6.775825379	0.760376415	0.878584165	1644	2690	949	1277	2561	2009	2991	2164
ENSECAG00000024744	6.739290805	0.760399671	0.878584165	1219	1641	1989	2120	2205	2113	2265	2863
ENSECAG00000027377	9.867122501	0.760432178	0.878584165	250	1900	908	87595	58188	7094	1835	1288
ENSECAG00000021362	4.355945447	0.760437554	0.878584165	274	328	325	376	392	343	444	639
ENSECAG00000024227	3.730307849	0.760506536	0.878584165	116	301	237	193	369	218	350	226
ENSECAG00000018528	2.178039042	0.760613993	0.87861897	43	60	123	93	124	62	77	100
ENSECAG00000010512	1.417067251	0.760961795	0.878853431	22	42	43	61	41	24	54	120
ENSECAG00000011095	3.3102919	0.760971665	0.878853431	116	105	192	237	214	178	230	249
ENSECAG00000023353	5.437214188	0.761229595	0.879061963	681	786	817	587	1064	803	869	814
ENSECAG00000015988	6.178926144	0.761778759	0.879606733	887	1092	1236	1502	1840	1308	1592	1629
ENSECAG00000007666	0.940014685	0.762031403	0.879631106	17	28	25	50	39	26	45	54
ENSECAG00000013948	4.351900403	0.762059414	0.879631106	227	277	415	421	510	355	443	485
ENSECAG00000015843	8.051470338	0.762087017	0.879631106	2808	5442	3986	4845	5252	5710	5872	6632
ENSECAG00000022520	6.170374017	0.762109541	0.879631106	667	1314	1546	1586	1489	1251	1628	1583
ENSECAG00000000689	6.027952207	0.762317437	0.879781689	962	1305	978	1149	1923	817	1241	1425
ENSECAG00000011765	7.194085269	0.762553183	0.879895802	2128	2006	2565	3472	3471	2367	3099	3132
ENSECAG00000010566	3.997863077	0.762606217	0.879895802	86	238	202	596	262	354	360	436
ENSECAG00000019867	6.171729831	0.762662797	0.879895802	1175	1724	1002	836	1190	2497	896	1099
ENSECAG00000015015	4.064539292	0.762726083	0.879895802	195	274	263	354	320	313	410	426
ENSECAG00000013572	0.54735193	0.762979081	0.880037616	9	16	36	29	21	30	43	27
ENSECAG00000021229	3.042894677	0.763078832	0.880037616	64	151	150	231	156	135	154	229
ENSECAG00000003103	6.84310561	0.763114175	0.880037616	1830	1991	1827	2069	2520	1803	2505	2665
ENSECAG00000019472	3.673460877	0.763221416	0.880037616	132	173	245	366	250	268	236	286
ENSECAG00000001650	6.981116572	0.763309609	0.880037616	1665	2802	2101	1983	2747	2231	2572	2849
ENSECAG00000023408	4.40953998	0.76331737	0.880037616	347	219	481	261	460	440	588	369
ENSECAG00000018201	3.404918733	0.763391193	0.880037616	96	201	197	258	177	172	253	267

ENSECAG00000016788	4.495370432	0.763634902	0.880229254	192	451	570	369	413	407	447	595
ENSECAG00000016045	1.542583488	0.763791478	0.880320427	18	46	53	73	75	51	68	55
ENSECAG00000007657	7.145397657	0.763960759	0.880426224	1413	2624	2696	3338	2617	2904	2531	3613
ENSECAG00000008931	3.82640568	0.764378739	0.880757471	138	222	254	405	283	220	280	391
ENSECAG00000013936	7.965857197	0.764403223	0.880757471	2255	3221	5025	6365	4961	6192	4205	6724
ENSECAG00000004436	4.598393896	0.764590806	0.880853021	320	361	403	473	487	508	484	646
ENSECAG00000019769	2.040845511	0.764641203	0.880853021	25	36	78	140	45	146	71	90
ENSECAG00000014760	6.034143891	0.764741002	0.880878677	553	1138	1493	1127	845	1445	1165	2408
ENSECAG00000024069	4.251315602	0.764877817	0.880901843	290	406	282	284	428	306	504	315
ENSECAG00000005290	2.200288934	0.765025189	0.880901843	51	70	72	100	110	81	100	108
ENSECAG00000000898	2.003111796	0.765050703	0.880901843	43	67	85	52	97	65	57	135
ENSECAG00000012339	7.420487989	0.765147848	0.880901843	1880	2009	3951	3440	3770	3449	3488	4467
ENSECAG00000012056	7.53579144	0.765148767	0.880901843	1648	2138	5596	4148	3013	4351	3634	4090
ENSECAG00000012310	7.659005626	0.765229218	0.880905205	2238	3237	3984	3657	4988	3681	4271	4964
ENSECAG00000018336	7.366505603	0.765551129	0.881186499	1936	1871	2606	4603	3780	3293	3427	4099
ENSECAG00000016372	4.499889987	0.765652186	0.881190291	332	218	463	424	454	243	462	898
ENSECAG00000006493	1.864454594	0.765709536	0.881190291	44	36	81	94	76	58	92	62
ENSECAG00000016387	4.051273457	0.766031261	0.881340574	157	228	258	477	368	320	350	417
ENSECAG00000003116	3.053093514	0.766041709	0.881340574	65	101	144	254	180	127	212	213
ENSECAG00000008536	4.947681303	0.766120296	0.881340574	267	392	674	933	494	648	520	887
ENSECAG00000026807	3.435897224	0.766150401	0.881340574	115	189	188	207	209	206	240	295
ENSECAG00000000984	5.667393573	0.766547933	0.88167998	769	547	1107	1078	1084	744	829	1600
ENSECAG00000016804	0.502330719	0.76666767	0.88167998	10	43	20	20	26	17	26	38
ENSECAG00000014838	3.596777252	0.766728615	0.88167998	86	212	218	371	228	234	226	301
ENSECAG00000002550	1.703485705	0.766755842	0.88167998	22	57	56	76	68	56	72	86
ENSECAG00000011958	3.886250053	0.766891894	0.881747187	129	199	299	368	341	315	271	366
ENSECAG00000009006	2.886554587	0.767236236	0.882053841	78	74	177	153	157	197	110	177
ENSECAG00000009835	3.863082746	0.767517827	0.882288299	225	103	410	268	386	145	265	407
ENSECAG00000019569	6.58596681	0.767666017	0.882347111	1007	1332	2011	2549	2111	1769	2289	1709
ENSECAG00000009667	4.99156343	0.767724304	0.882347111	532	524	518	352	770	510	865	660
ENSECAG00000014272	6.03547253	0.768463825	0.883107716	773	1012	1271	1207	1364	1169	1546	1707
ENSECAG00000012898	0.649897054	0.768591593	0.883133452	16	14	49	27	19	27	23	51
ENSECAG00000007384	3.864064763	0.768747084	0.883133452	129	208	264	379	365	254	316	342
ENSECAG00000024746	1.961173263	0.768841671	0.883133452	40	34	60	122	94	50	118	77
ENSECAG00000009811	2.09371233	0.768854093	0.883133452	90	29	105	7	174	40	133	34
ENSECAG00000019540	3.221070641	0.768874856	0.883133452	73	115	222	275	168	151	179	267
ENSECAG00000014744	0.108881223	0.769092071	0.883194419	11	11	15	36	16	19	21	22
ENSECAG00000001822	0.416066181	0.769117184	0.883194419	6	22	28	26	31	31	29	17
ENSECAG00000011251	2.957414707	0.769228861	0.883194419	80	130	159	177	125	137	117	260
ENSECAG00000011059	4.426050175	0.769238863	0.883194419	228	475	249	559	376	375	373	661
ENSECAG00000001645	5.324760175	0.769465602	0.88330975	418	361	1122	1002	1199	607	746	734
ENSECAG00000019681	5.315755152	0.769523069	0.88330975	615	320	829	775	1263	536	901	829
ENSECAG00000021963	5.261768164	0.769674249	0.88330975	381	546	883	927	849	767	812	719
ENSECAG00000000325	8.888430596	0.769757794	0.88330975	2886	6979	8586	18296	4573	6446	5421	23502
ENSECAG00000007669	4.50140766	0.769867506	0.88330975	197	394	387	659	450	386	540	490
ENSECAG00000021369	7.105148951	0.76990891	0.88330975	1656	2348	2505	3124	2954	2467	2807	3140
ENSECAG00000007505	6.90916835	0.769937361	0.88330975	1035	1667	2422	3010	2491	2206	2320	3696
ENSECAG00000022868	4.262507752	0.769961254	0.88330975	195	236	439	401	402	325	351	628
ENSECAG00000024159	5.055199131	0.770193325	0.883403322	568	617	373	455	669	684	719	858
ENSECAG00000012933	6.236288043	0.77019832	0.883403322	296	1534	1354	1977	1024	2500	2201	785
ENSECAG00000000883	6.071019682	0.770380349	0.883522916	998	850	1194	1598	1448	1233	1388	1486
ENSECAG00000010684	6.930432678	0.770642295	0.883690403	1612	1970	1874	3066	2393	2416	2356	2889
ENSECAG00000024368	7.25215729	0.77068194	0.883690403	1601	2414	3395	2479	3591	3941	2874	2881
ENSECAG00000016259	5.364858357	0.770761279	0.883692195	433	269	876	1185	1473	799	1141	190
ENSECAG00000023150	8.05126661	0.771130618	0.884021223	2436	3430	6716	6736	5262	5113	5806	5554
ENSECAG00000003985	1.867989981	0.771203869	0.884021223	47	88	60	46	87	51	103	46
ENSECAG00000021834	5.443558459	0.771510915	0.884147114	371	1070	686	934	903	969	788	900
ENSECAG00000000675	6.056741147	0.771664055	0.884147114	823	974	1252	1614	1259	1375	1322	1540
ENSECAG00000023239	6.82807256	0.771685893	0.884147114	1379	1633	2462	2493	2455	1886	2212	2881
ENSECAG00000011756	6.311404549	0.771812751	0.884147114	1263	1253	1148	1725	1911	1267	2044	1299
ENSECAG00000012837	5.140553491	0.771841625	0.884147114	402	666	549	843	746	614	774	786
ENSECAG00000020679	7.773546315	0.771851225	0.884147114	2452	3815	3903	3976	4255	4836	3994	6297
ENSECAG00000014281	6.188545909	0.771931717	0.884147114	918	955	1555	1683	1689	1037	1568	1783
ENSECAG00000008095	0.846432534	0.771936224	0.884147114	10	20	25	62	36	39	46	29
ENSECAG00000019574	3.783360758	0.772261688	0.884430733	170	256	219	304	279	184	328	350
ENSECAG00000014253	7.03834385	0.772468138	0.884578007	1703	1907	2777	2758	2815	2055	2905	3125
ENSECAG00000011022	3.545761969	0.772705859	0.884761057	103	184	154	346	262	240	265	251
ENSECAG00000002706	2.855361828	0.772932951	0.884931901	58	148	133	129	131	134	180	189
ENSECAG00000016603	8.437520067	0.773040257	0.884965582	3688	6440	7236	7021	6034	7428	6295	8751
ENSECAG00000009396	7.201335123	0.773243359	0.88510891	1513	1901	2974	4306	3069	2643	2839	3612
ENSECAG00000007172	1.268664856	0.773485009	0.885272134	7	18	94	61	50	49	45	38
ENSECAG00000010046	4.922773779	0.773589778	0.885272134	359	629	498	435	771	545	659	687
ENSECAG00000000029	4.409434822	0.773619699	0.885272134	334	381	326	377	546	297	499	409
ENSECAG00000012270	5.865426771	0.77377763	0.885363689	917	691	1039	1042	1266	1214	1309	1314
ENSECAG00000024593	0.851548733	0.773958057	0.885480963	17	27	32	48	31	41	40	24
ENSECAG00000019009	6.769423457	0.774047199	0.885493785	1858	1979	1730	1627	2490	1859	2211	2442
ENSECAG00000016012	3.491853221	0.774247981	0.885595543	51	196	196	333	169	260	256	303
ENSECAG00000014894	2.614979234	0.774292037	0.885595543	52	85	91	182	149	89	133	168
ENSECAG00000005564	2.856339887	0.774462761	0.88570165	97	52	133	188	169	124	129	218
ENSECAG00000018640	4.244424016	0.774607519	0.885734951	228	200	385	433	293	530	425	391
ENSECAG00000018855	0.281077206	0.774647792	0.885734951	0	21	19	37	25	24	30	20
ENSECAG00000024301	5.766183306	0.775124623	0.886097673	470	647	1430	1119	1160	1068	965	1626
ENSECAG00000006293	6.385890105	0.775246349	0.886097673	903	1423	1530	2048	1588	1547	1732	2075
ENSECAG00000006343	5.289156608	0.775260468	0.886097673	496	751	553	695	814	849	938	804
ENSECAG00000008342	5.490855302	0.775276973	0.886097673	369	842	994	1008	789	826	720	1419

ENSECAG00000024831	5.850961003	0.775643432	0.886362644	844	613	1149	1087	1868	920	1256	1024
ENSECAG00000018676	5.565212799	0.775819979	0.886362644	566	607	1040	1117	975	810	852	1314
ENSECAG00000009510	6.193930204	0.775856382	0.886362644	918	883	1813	1501	1667	1546	1379	1407
ENSECAG00000007422	8.179255846	0.775865394	0.886362644	2565	5414	4484	8689	4365	6673	5063	7679
ENSECAG00000021351	6.237566376	0.775974257	0.886362644	414	1330	1282	2198	1555	2076	1791	1110
ENSECAG00000005412	4.638058097	0.775976872	0.886362644	296	392	355	591	569	424	585	609
ENSECAG00000024276	4.648407058	0.776162687	0.886408526	499	322	378	439	524	480	527	526
ENSECAG00000026837	5.417504871	0.776192418	0.886408526	438	651	843	1111	843	835	904	950
ENSECAG00000003831	1.815220125	0.776299434	0.886408526	21	45	78	89	88	40	66	116
ENSECAG00000002173	6.450467304	0.776370985	0.886408526	910	1318	1713	2315	1573	2022	1481	2121
ENSECAG00000011683	6.590946335	0.776407116	0.886408526	1131	1817	1504	1763	2088	1998	2221	2137
ENSECAG00000005729	6.319117877	0.776567004	0.886413816	1068	1208	1545	1268	1523	1315	2001	2242
ENSECAG00000018277	4.825704837	0.776567781	0.886413816	303	531	511	635	557	494	711	579
ENSECAG00000019852	6.648433087	0.776844435	0.886429602	1350	1333	1565	2278	2178	2065	2210	2353
ENSECAG00000021721	0.326713085	0.776878377	0.886429602	8	27	9	31	19	43	20	19
ENSECAG00000016189	4.956540121	0.77692373	0.886429602	270	569	445	804	588	566	635	961
ENSECAG00000017014	0.582595068	0.776947245	0.886429602	5	34	43	23	7	67	15	15
ENSECAG00000027684	11.57573114	0.776981851	0.886429602	56662	35315	68320	42316	60623	61878	53590	74026
ENSECAG00000017013	2.951334658	0.777077275	0.886429602	50	108	163	203	139	156	186	195
ENSECAG00000023207	0.148992546	0.77712773	0.886429602	18	10	18	25	18	22	5	37
ENSECAG00000017131	4.787719343	0.777255325	0.886486148	421	339	518	469	595	601	613	597
ENSECAG00000000872	6.28230887	0.777651088	0.886848506	1276	1515	1102	1249	1744	1344	1657	1684
ENSECAG00000005600	2.875001569	0.777825862	0.886958796	49	171	97	215	114	92	152	246
ENSECAG00000020467	4.904519072	0.778007283	0.887076643	353	341	564	734	599	615	689	715
ENSECAG00000025086	6.367863741	0.778143116	0.887142493	931	1675	1283	1855	1754	1551	1666	1861
ENSECAG00000000795	1.656267551	0.778421887	0.887371273	16	40	78	74	41	70	63	99
ENSECAG00000015984	8.251171465	0.779058107	0.887793414	3806	5997	3658	5992	6603	5240	7293	7954
ENSECAG00000010289	3.999156286	0.77909343	0.887793414	89	283	394	310	326	241	443	405
ENSECAG00000023362	3.829581974	0.779159247	0.887793414	153	267	208	295	352	248	338	304
ENSECAG00000019901	3.225236555	0.779181226	0.887793414	60	109	271	252	143	211	167	235
ENSECAG00000018284	2.242873539	0.779182883	0.887793414	39	75	89	106	81	101	115	112
ENSECAG00000019462	0.388936348	0.779268031	0.8878014	18	12	20	26	33	24	17	35
ENSECAG00000014853	5.297561116	0.77957411	0.887966064	311	566	821	982	772	711	1141	821
ENSECAG00000016263	5.667923845	0.779575143	0.887966064	537	652	1092	1110	1182	957	1151	1160
ENSECAG00000015760	3.577893407	0.779704081	0.887966064	113.971	141	273	336.006	266	158	209	359
ENSECAG00000022981	2.384009103	0.779725174	0.887966064	45	47	127	126	70	121	86	182
ENSECAG00000009061	4.016453179	0.779826809	0.887992805	145	228	205	528	353	331	385	343
ENSECAG00000022106	5.188809021	0.779949247	0.888005889	373	354	1048	845	848	706	671	771
ENSECAG00000015041	3.617030531	0.779994611	0.888005889	129	124	268	367	286	209	217	295
ENSECAG00000013403	3.312624989	0.780157852	0.888102747	114	109	207	221	243	169	202	258
ENSECAG00000012274	5.664499728	0.780448658	0.888276615	507	896	955	985	1151	815	1154	1356
ENSECAG00000019670	5.773643892	0.780466946	0.888276615	876	1419	551	717	976	1267	950	1277
ENSECAG00000012184	4.122712816	0.780573356	0.888308742	175	244	369	366	403	321	409	388
ENSECAG00000000624	5.599096496	0.780664104	0.88832304	680	721	832	866	1049	955	1123	1110
ENSECAG00000009333	5.662781116	0.781076386	0.888703174	629	951	663	1029	1088	1000	1076	1279
ENSECAG00000021667	2.458748629	0.781419542	0.88900459	67	58	113	149	126	86	110	123
ENSECAG00000020676	7.328960367	0.781678034	0.889188629	1770	3539	2512	3365	3199	2266	3625	4315
ENSECAG00000000010	3.792466514	0.781799488	0.889188629	175	137	181	514	340	248	272	268
ENSECAG00000014123	4.541823937	0.781816089	0.889188629	217	268	572	506	484	553	531	451
ENSECAG00000010430	6.443949189	0.782017202	0.889322436	1115	1548	1538	1784	2126	1505	1691	1891
ENSECAG00000014635	8.258272483	0.782091178	0.889322436	4109	3679	6991	4705	7622	3681	6742	9574
ENSECAG00000017521	4.666262999	0.782168554	0.889322436	478	438	440	277	620	360	589	523
ENSECAG00000018235	4.51636539	0.782730659	0.889872498	333	413	333	469	605	322	418	557
ENSECAG00000023080	3.933542543	0.783048854	0.890103074	124	214	312	468	258	293	305	403
ENSECAG00000000384	6.073694278	0.78315519	0.890103074	673	981	1122	1766	1437	1450	1430	1569
ENSECAG00000023194	0.572628876	0.783168494	0.890103074	16	18	32	22	20	31	25	49
ENSECAG00000011012	7.768605334	0.783406351	0.890284353	2416	3350	4879	4756	4559	3269	4105	6282
ENSECAG00000025040	4.629090983	0.784134082	0.890957891	308	381	384	540	532	439	594	603
ENSECAG00000000596	3.729847826	0.784155863	0.890957891	173	169	282	297	259	346	211	256
ENSECAG00000023340	4.416427671	0.784395023	0.891140512	231	312	445	415	450	433	451	531
ENSECAG00000021636	7.203779162	0.784633556	0.891322382	1586	2749	2178	3132	3094	3218	2951	3699
ENSECAG00000009368	8.578798771	0.785173348	0.891717153	11242	3244	42	11637	14909	3597	36	11343
ENSECAG00000024821	0.288803784	0.785215172	0.891717153	15	20.9995	19.9991	21.9997	20	19.9967	18.997	32.9923
ENSECAG00000016885	4.352185399	0.785216522	0.891717153	154	273	459	621	471	446	452	281
ENSECAG00000016187	4.472921888	0.78546922	0.891914978	302	423	388	383	520	364	385	577
ENSECAG00000009744	5.793342356	0.785761946	0.891997892	535.001	1649	273.001	1041	974.001	1700	1283	857.001
ENSECAG00000010839	0.793058618	0.78578266	0.891997892	23	27	34	29	43	18	38	35
ENSECAG00000012175	7.098719356	0.785868656	0.891997892	1726	2980	1584	3178	3069	2427	2714	3106
ENSECAG00000013227	4.690230934	0.785960861	0.891997892	258	325	521	632	660	438	603	558
ENSECAG00000005329	4.55791774	0.785993058	0.891997892	201	367	416	731	489	426	533	492
ENSECAG00000010775	2.0232196	0.786013281	0.891997892	36	61	85	80	76	98	83	91
ENSECAG00000013737	4.668072874	0.786145828	0.892059213	271	374	382	675	607	445	578	597
ENSECAG00000012699	5.05092331	0.7862563	0.892095475	255	539	809	615	578	767	609	957
ENSECAG00000000311	5.833639446	0.786756795	0.892574209	554	926	825	1542	1105	1029	1208	1702
ENSECAG00000015786	3.195864838	0.787111939	0.892849062	58	196	138	219	160	166	253	224
ENSECAG00000016783	2.01001452	0.787197967	0.892849062	28	64	85	86	68	58	76	154
ENSECAG00000017636	3.726728624	0.787234809	0.892849062	145	214	203	307	263	235	287	379
ENSECAG00000013727	4.500305396	0.787563864	0.893133109	235	353	422	488	487	405	469	628
ENSECAG00000013626	1.697878802	0.787709094	0.893208656	42	27	34	106	92	41	80	68
ENSECAG00000000852	1.996113578	0.787865925	0.89329734	42	46	71	98	99	50	46	160
ENSECAG00000012025	1.799549311	0.788272028	0.893668607	50	29	73	87	66	68	71	71
ENSECAG00000013834	2.968831526	0.788402906	0.893687793	32	102	267	111	35	444	59	124
ENSECAG00000023316	6.772207019	0.788446263	0.893687793	1236	1803	1612	2552	2506	1767	2768	2607
ENSECAG00000010421	4.747669968	0.788596165	0.893768541	349	445	256	703	521	571	562	701
ENSECAG00000017854	0.646227938	0.788851446	0.893968694	7	8	55	29	26	28	41	36

ENSECAG00000009331	4.187537658	0.789033205	0.894085496	214	290	272	506	382	311	386	429
ENSECAG00000026946	0.711329292	0.789435391	0.894452025	17	27	26.0036	40	22	33	31	39
ENSECAG00000017489	3.135278036	0.790034495	0.894979885	79	130	139	240	178	147	178	272
ENSECAG00000016304	5.175213364	0.790078761	0.894979885	476	494	680	690	844	587	848	896
ENSECAG00000021124	4.053976949	0.790137584	0.894979885	209	215	287	374	445	268	383	356
ENSECAG00000012813	3.122833358	0.790556547	0.89536518	101	95	125	258	231	149	199	180
ENSECAG00000021483	7.722108459	0.790689166	0.895426124	2231	4211	3201	4089	4247	4223	4338	5870
ENSECAG00000010181	2.994087891	0.790877007	0.895549587	98	77	159	186	211	141	171	170
ENSECAG00000008498	4.615034266	0.79121614	0.895810799	275	396	499	539	501	595	420	487
ENSECAG00000008036	5.72225877	0.791302615	0.895810799	458	1057	930.001	1319	818	1322	892	1309
ENSECAG00000008176	5.55568539	0.791344216	0.895810799	581	716	814	962	1000	947	988	1176
ENSECAG00000013925	6.985932974	0.791688534	0.89611129	1702	1662	2579	2880	3026	2295	2308	2858
ENSECAG00000019466	5.652931499	0.791893805	0.896227857	697	1017	736	939	1112	770	1185	1120
ENSECAG00000006243	4.523371135	0.791949277	0.896227857	325	292	455	395	469	328	642	591
ENSECAG00000018215	6.574742549	0.792112699	0.896320229	1226	2166	1302	1765	1744	1821	2181	2132
ENSECAG00000023322	5.597596305	0.792200076	0.896320229	204	940	786	1455	926	922	856	1616
ENSECAG00000012574	4.729342733	0.792267564	0.896320229	299	376	437	653	567	477	597	684
ENSECAG00000012910	0.175297184	0.792431075	0.896323024	6	8	53	9	8	27	22	24
ENSECAG00000007148	3.027454146	0.792485268	0.896323024	184	113	115	101	179	124	201	158
ENSECAG00000012606	3.999994691	0.792506698	0.896323024	159	227	353	319	340	321	307	432
ENSECAG00000023167	4.404973993	0.792663794	0.896363234	227	299	382	502	396	414	435	615
ENSECAG00000021478	9.247106731	0.792700033	0.896363234	7989	7305	15889	11081	14939	10926	12779	11177
ENSECAG00000010824	5.879412733	0.793251917	0.896898028	672	748	1106	1399	1414	1208	1292	1209
ENSECAG00000015219	2.243044284	0.793404021	0.896980745	40	90	63	145	109	67	87	121
ENSECAG00000015150	9.057697779	0.793530255	0.897034202	5409	6955	12753	13233	11848	9611	10616	11905
ENSECAG00000010597	5.082393463	0.793974029	0.89744657	326	440	653	878	608	737	709	910
ENSECAG00000012555	3.712188595	0.79410528	0.897452993	104	252	255	325	204	226	340	309
ENSECAG00000017575	4.442349891	0.794137686	0.897452993	114	258	297	1047	729	268	540	212
ENSECAG00000021396	6.024042683	0.794364559	0.897620101	716	1345	992	1159	1357	1297	1519	1520
ENSECAG00000011309	3.017868168	0.794509674	0.897694801	101	64	221	196	103	366	44	101
ENSECAG00000019250	2.424532259	0.794709423	0.89778095	38	60	140	155	76	121	89	146
ENSECAG00000023366	4.567648267	0.794743953	0.89778095	340	469	323	344	556	390	563	575
ENSECAG00000016365	3.872182895	0.795070668	0.898060734	158	304	265	203	344	241	425	267
ENSECAG00000023698	4.53149803	0.795323956	0.898196242	269	321	518	394	492	510	614	382
ENSECAG00000025100	7.256901166	0.79534874	0.898196242	2160	2234	2498	2890	3672	2924	3424	3409
ENSECAG00000011635	1.725820158	0.795658328	0.898402395	40	29	98	60	53	43	69	102
ENSECAG00000020115	8.488723059	0.79568943	0.898402395	2415	5657	5011	11959	7774	7531	6698	9835
ENSECAG00000011944	7.140263346	0.795890894	0.89845881	1495	1967	2451	3531	3398	2692	3324	2948
ENSECAG00000006127	6.651320266	0.79591255	0.89845881	1422	1626	1223	2185	2310	1893	2034	2620
ENSECAG00000008651	4.752177826	0.795976622	0.89845881	377.999	444	446.999	439.998	555.999	563.999	614.999	609.999
ENSECAG00000000974	7.729002643	0.796071186	0.89847629	3282	4241	3397	3170	4623	3961	3934	5017
ENSECAG00000015703	5.672548028	0.796680425	0.899074592	518	918	926	1227	970	859	1104	1332
ENSECAG00000003793	1.988694832	0.796849005	0.899158552	50	44	125	19	69	56	108	118
ENSECAG00000020611	0.46091546	0.796913098	0.899158552	12	22	27	21	26	43	20	22
ENSECAG00000007913	4.851530726	0.797594415	0.899837925	242	424	601	693	582	592	690	650
ENSECAG00000023658	5.327309266	0.797777261	0.899873299	501	688	809	564	815	882	925	869
ENSECAG00000021693	6.956175886	0.79779927	0.899873299	2385	2281	1177	1504	2705	2469	2809	2991
ENSECAG00000020569	7.496028592	0.79786337	0.899873299	1717	3895	2740	4449	3019	4101	3097	4667
ENSECAG00000016223	6.196984756	0.798049233	0.899979704	633	1113	1280	1985	1630	1320	1562	1947
ENSECAG00000001749	2.753973646	0.798116133	0.899979704	102	84	118	117	158	149	139	135
ENSECAG00000011343	4.269302481	0.798555779	0.900386103	267	323	330	387	385	361	435	411
ENSECAG00000009329	3.079960543	0.798746783	0.9005121	43	150	111	281	178	159	176	229
ENSECAG00000023133	0.824632703	0.798977829	0.900677971	19	41	15	30	38	35	40	35
ENSECAG00000018069	5.209126243	0.799088048	0.900677971	470	276	1104	752	847	628	682	907
ENSECAG00000000546	3.060599143	0.799131722	0.900677971	55	83	199	237	177	166	218	163
ENSECAG00000027694	10.78271015	0.799695671	0.901224183	23482	18403	44548	26415	35952	36480	30889	53320
ENSECAG00000013517	4.691952569	0.79987419	0.901321869	464	302	429	531	554	653	445	447
ENSECAG00000009314	4.039026679	0.799941008	0.901321869	155	196	435	306	338	399	317	371
ENSECAG00000006289	8.962210987	0.800115616	0.901429214	7091	8867	8741	9176	9662	9258	10385	11999
ENSECAG00000016301	5.60423153	0.800273516	0.901517717	493	695	1225	1013	1089	830	1074	1047
ENSECAG00000011385	5.668558225	0.800839434	0.901845392	426.007	730	1150	1377	1074	909	1179	1051
ENSECAG00000016162	4.57815145	0.800883721	0.901845392	484	327	321	415	581	428	463	495
ENSECAG00000005102	0.457941249	0.80092324	0.901845392	12	24	4	44	25	19	34	37
ENSECAG00000017524	3.562977398	0.800967318	0.901845392	65	244	165	403	200	225	305	230
ENSECAG00000014393	5.143848604	0.800985119	0.901845392	380	566	660	886	664	794	547	924
ENSECAG00000000187	5.347109024	0.801087643	0.901845392	793	539	627	693	872	715	855	933
ENSECAG00000006699	2.959556911	0.801120009	0.901845392	73	95	150	202	213	131	169	163
ENSECAG00000018433	3.732686656	0.801260066	0.901913698	81	194	385	316	271	180	470	154
ENSECAG00000010544	7.302572056	0.801537449	0.902085335	1982	2118	3861	2119	3612	2080	3054	5471
ENSECAG00000015777	3.884288374	0.801571338	0.902085335	111	282	278	314	411	195	387	302
ENSECAG00000017201	6.266469599	0.801944853	0.902274115	740	960	1820	1654	1682	1990	1351	1651
ENSECAG00000024986	5.091474604	0.801983163	0.902274115	661	505	625	416	926	384	1082	395
ENSECAG00000009640	4.038391945	0.802003839	0.902274115	182	336	339	262	361	290	289	422
ENSECAG00000026826	1.730136366	0.802056729	0.902274115	33	51	57	70	58	45	112	71
ENSECAG00000023182	2.817787424	0.802843112	0.903025519	84	78	167	122	136	229	95	142
ENSECAG00000015855	2.654552045	0.802883629	0.903025519	46	155	92	113	109	122	131	189
ENSECAG00000012973	7.845233313	0.803241063	0.903338113	2010	4632	3290	5406	4586	5809	4698	4948
ENSECAG00000010306	2.77472311	0.803578954	0.903568019	61	82	132	184	137	109	185	166
ENSECAG00000023112	3.7914568	0.803604544	0.903568019	136	138	346.999	378	308	282	281	258
ENSECAG00000020832	5.217333901	0.804512047	0.904498899	190	778	570	1033	747	669	807	1066
ENSECAG00000010759	7.400370615	0.804609193	0.904518616	1823	3260	2641	4159	3399	3084	3375	4178
ENSECAG00000022254	6.60062738	0.805016848	0.904819963	1178	1061	2063	2122	2088	1851	2594	1941
ENSECAG00000026520	0.77007074	0.805114459	0.904819963	3	7	23	103	72	14	17	23
ENSECAG00000007648	3.498709718	0.805120172	0.904819963	141	226	194	204	270	210	255	186
ENSECAG00000021248	5.85655468	0.805195797	0.904819963	1081	745	1340	608	1333	463	1399	1679

ENSECAG00000006724	3.529302941	0.805421628	0.90491552	120	161	221	319	224	199	208	326
ENSECAG00000007152	7.324933031	0.805440121	0.90491552	1591	4085	2697	2649	2839	2913	3289	4286
ENSECAG00000000479	6.16494115	0.805585605	0.904944203	768	1171	1272	1547	1586	1280	1695	1724
ENSECAG00000014919	2.801630418	0.805624944	0.904944203	65	103	116	178	109	100	196	209
ENSECAG00000019073	7.028762688	0.805960413	0.905013515	1438	1804	2859	2505	3246	2376	2892	2946
ENSECAG00000008250	5.978306913	0.806016097	0.905013515	765	1027	1107	1478	1280	1140	1458	1362
ENSECAG00000007787	7.203868769	0.806027747	0.905013515	2008	2990	2394	1781	3536	2428	3614	3439
ENSECAG00000025008	5.573439375	0.806071395	0.905013515	375	842	1058	1134	1040	811	1028	1083
ENSECAG00000007914	6.138101069	0.806118089	0.905013515	569	1043	1144	2107	1265	1323	1345	2313
ENSECAG00000014093	4.289317642	0.806164565	0.905013515	192	342	357	402	427	334	472	476
ENSECAG00000000811	3.768311854	0.806324619	0.905103766	133	241	241	282	302	205	343	345
ENSECAG00000024945	4.569250498	0.806412966	0.905113516	190	389	588	415	417	579	527	535
ENSECAG00000016600	1.416175293	0.80690502	0.905439735	19	57	54	54	47	34	78	52
ENSECAG00000014528	4.636736957	0.80692746	0.905439735	467	530	274	316	590	410	539	511
ENSECAG00000022303	4.724496055	0.806942683	0.905439735	324	329	570	506	756	492	518	533
ENSECAG00000012999	5.022657475	0.807044466	0.905464522	548	581	403	461	699	697	653	789
ENSECAG00000014614	4.943448418	0.80728703	0.905647239	376	441	454	767	696	593	592	810
ENSECAG00000017332	3.065596008	0.807480897	0.905775294	42	164	152	260	133	127	186	243
ENSECAG00000023232	4.642914162	0.807855686	0.90610625	221	324	513	646	597	456	526	604
ENSECAG00000015439	2.426204792	0.80807722	0.906265263	104	105	63	68	100	103	119.999	110
ENSECAG00000014304	6.017238126	0.808242824	0.906359	826	726	1464	1212	1625	1009	1028	2100
ENSECAG00000012921	6.431198355	0.808339731	0.906359	844	942	2412	2006	1681	1140	1571	2897
ENSECAG00000011124	6.541022595	0.808400115	0.906359	997	2081	1186	2222	1987	1854	1862	1993
ENSECAG00000026980	6.453534984	0.808744602	0.906587301	874	1073	1970	1982	1796	2068	1384	2425
ENSECAG00000000047	4.432907694	0.808793603	0.906587301	207	410	374	435	477	366	527	518
ENSECAG00000023932	8.503218481	0.80885462	0.906587301	3509	6021	6534	10029	7795	6301	7294	8752
ENSECAG00000019040	4.391128158	0.808922906	0.906587301	239	296	337	523	594	323	430	490
ENSECAG00000009340	4.13472886	0.809223331	0.906834549	167	337	279	457	389	327	357	377
ENSECAG00000005267	5.508424171	0.809518267	0.907075597	388	571	1004	1148	800	759	809	1696
ENSECAG00000018129	4.309351204	0.809978814	0.907502148	336	301	308	274	444	387	356	552
ENSECAG00000002745	5.12672252	0.810163944	0.907620068	607	837	376	421	766	627	904	562
ENSECAG00000016103	3.954959469	0.810415166	0.907714778	123	250	327	344	274	367	306	400
ENSECAG00000022459	5.33898812	0.81046247	0.907714778	422	696	822	887	764	709	819	1097
ENSECAG00000024008	1.775546976	0.810488155	0.907714778	44	33	58	104	62	73	50	88
ENSECAG00000006975	7.323887267	0.810649389	0.90774911	2033	3291	1944	2936	4486	2091	4265	3324
ENSECAG00000004706	1.326024813	0.810678597	0.90774911	15	34	30	87	88	10	36	87
ENSECAG00000023111	6.185876847	0.81083242	0.907753954	858	835	1611	1850	1655	1018	1514	1923
ENSECAG00000012244	1.561183406	0.810842711	0.907753954	22	38	57	96	28	73	62	68
ENSECAG00000010252	6.710449913	0.810949958	0.907784573	1189	2022	1823	2216	2015	2109	1969	2611
ENSECAG00000022587	6.554298912	0.811129423	0.907803829	1121	1650	1684	2046	1912	1846	1996	2040
ENSECAG00000001222	2.706417103	0.811134209	0.907803829	34	141	106	156	121	139	138	169
ENSECAG00000021415	4.293916952	0.811206855	0.907803829	187	461	237	389	325	499	552	311
ENSECAG00000019493	2.842924645	0.811458856	0.90795525	62	129	136	180	149	112	163	164
ENSECAG00000014597	3.280418967	0.811501986	0.90795525	144	142	202	163	226	212	121	235
ENSECAG00000023585	7.850753367	0.811732597	0.908123845	3453	2112	6383	3906	5859	2758	4625	6053
ENSECAG00000014547	6.344374662	0.811908903	0.908231659	995	1196	1463	1676	1720	1622	1462	2334
ENSECAG00000022484	6.866576357	0.812106452	0.908262152	1060	2236	1816	2683	2446	2165	2148	3572
ENSECAG00000016771	0.844985383	0.812135391	0.908262152	18	30	37	35	23	27	50	39
ENSECAG00000020509	0.139297133	0.812175978	0.908262152	8	21	12	32	12	19	20	31
ENSECAG00000004224	6.528487169	0.812383673	0.908372951	981	1486	1791	1850	2095	1801	1964	2210
ENSECAG00000014694	5.08791797	0.812434952	0.908372951	375	567	593	709	688	612	757	923
ENSECAG00000024999	6.035401957	0.812620331	0.90849082	622	847	1304	1695	1337	1435	1204	1759
ENSECAG00000023803	6.283833022	0.813117969	0.908957729	1318	1427	1151	1210	1552	1209	1555	2223
ENSECAG00000022720	2.454590707	0.813354031	0.909132169	68	94	96	114	136	74	102	138
ENSECAG00000016728	4.295618818	0.813923742	0.909679476	207	374	264	535	403	355	423	446
ENSECAG00000008623	0.529786613	0.814033609	0.909712783	10	16	23	54	9	58	11	26
ENSECAG00000021885	6.990264747	0.814133552	0.909734994	1298	1692	2804	2705	2880	2522	2635	3108
ENSECAG00000005938	3.840805881	0.814330077	0.90975562	137.011	284	236.008	347	292.001	261	295.001	337
ENSECAG00000014366	5.317095438	0.814379183	0.90975562	615	643	595	652	1081	596	1018	792
ENSECAG00000018213	5.683619894	0.814392221	0.90975562	359	878	1067	1212	875	1059	1060	1523
ENSECAG00000025006	4.919609171	0.814484041	0.909768745	425	502	553	564	827	437	705	540
ENSECAG00000009183	7.743986088	0.814752976	0.909934545	3811	3263	2717	3375	4610	4395	4801	5011
ENSECAG00000026857	3.962311231	0.814812676	0.909934545	152	208	374	300	293	294	293	491
ENSECAG00000018339	8.144053877	0.814874714	0.909934545	3598	4417	5571	6003	5843	4459	6955	6278
ENSECAG00000018375	5.461473435	0.814952819	0.909934545	555	668	850	965	850	988	921	876
ENSECAG00000020501	7.782624004	0.815092154	0.909968211	2841	3478	4031	3827	5091	4239	4799	5188
ENSECAG00000010946	3.456832679	0.815143148	0.909968211	76	197	283	227	155	253	201	290
ENSECAG00000000628	0.696308838	0.815295084	0.910048408	25	22	21	37	36	14	19	59
ENSECAG00000014373	5.635469891	0.815846994	0.910575004	545	584	1104	1092	1246	795	1293	1006
ENSECAG00000010996	4.799613583	0.815933975	0.910582636	232	451	572	745	593	489	634	595
ENSECAG00000008664	7.226010021	0.816169065	0.910664274	1160	2263	2800	4077	3382	2827	3134	3829
ENSECAG00000013424	9.922967151	0.816176576	0.910664274	10387	10832	25385	22836	21124	16720	19780	22967
ENSECAG00000011301	3.754665682	0.816247577	0.910664274	99	221	263	401	247	290	376	178
ENSECAG00000000133	1.502294997	0.816924657	0.911247117	28	34	63	72	66	34	39	90
ENSECAG00000018027	6.404093187	0.816958893	0.911247117	971	1214	1776	1617	1996	1506	1766	2160
ENSECAG00000021655	4.568341909	0.817010595	0.911247117	237	195	655	510	419	413	456	820
ENSECAG00000009059	7.668467766	0.817150844	0.911266891	2390	2841	3992	4167	4606	4158	4394	4633
ENSECAG00000010356	6.990208361	0.81718873	0.911266891	1001	1734	2840	3766	2302	2170	2780	3355
ENSECAG00000022178	4.837107685	0.817454718	0.911474043	305	526	484	562	713	446	610	738
ENSECAG00000000484	1.87551214	0.817696931	0.911654649	49	47	59	76	65	74	76	100
ENSECAG00000014723	2.329442129	0.818017408	0.911812592	81	60	87	80	145	119	81	84
ENSECAG00000014850	6.125633573	0.818058185	0.911812592	1024	739	1414	1329	1887	1029	1727	1484
ENSECAG00000019975	8.619959693	0.818110237	0.911812592	5395	3684	11366	6900	8781	4493	6161	13816
ENSECAG00000006950	7.003557056	0.8181596	0.911812592	1526	3053	1904	2223	2021	2841	2639	3102
ENSECAG00000011140	3.734967169	0.818299966	0.911879581	126	176	185	423	305	203	291	372

ENSECAG00000015560	4.377115338	0.818761262	0.912205271	319	254	491	328	501	419	405	381
ENSECAG00000007621	7.522520267	0.818814453	0.912205271	1565	3506	1831	6683	2726	2755	3062	6977
ENSECAG00000006692	7.494807614	0.818834233	0.912205271	2390	3110	2665	3432	4187	3332	4284	3998
ENSECAG00000021192	1.896682316	0.818957252	0.912205271	22.5565	49.0001	94.0079	81.0001	59.9866	58.0005	65.001	143.985
ENSECAG00000021720	4.961514595	0.819033262	0.912205271	332	533	487	844	593	531	602	888
ENSECAG00000019812	5.342241532	0.819073946	0.912205271	392	458	691	1259	881	924	724	1001
ENSECAG00000022108	3.900044735	0.819675184	0.9127854	117	187	325	395	362	328	284	317
ENSECAG00000022541	4.314803919	0.81983626	0.912822932	249	239	433	463	375	421	360	492
ENSECAG00000007926	-0.047889267	0.819869568	0.912822932	19	3	22	15	21	7	14	30
ENSECAG00000009013	3.04861942	0.819991666	0.912832178	56	114	180	214	178	166	217	152
ENSECAG00000009240	7.533604963	0.820093758	0.912832178	2319	3272	3564	3554	3430	3423	3923	4664
ENSECAG00000016128	11.09276303	0.820250104	0.912832178	25563	27593	47311	54380	53006	35133	46682	46498
ENSECAG00000011527	4.736446768	0.820273962	0.912832178	263	285	392	907	682	506	512	627
ENSECAG00000010134	4.121423394	0.820279576	0.912832178	142	213	386	452	376	367	334	437
ENSECAG00000023715	7.49509218	0.820409037	0.912886835	1940	2657	3492	3901	4049	3227	4538	3993
ENSECAG00000025106	5.938886659	0.820523805	0.912925135	847	1102	897	1319	1174	1286	1303	1321
ENSECAG00000020650	5.896289104	0.820639102	0.91294601	572	856	962	1678	1306	1165	1063	1692
ENSECAG00000009235	3.561554989	0.820703269	0.91294601	97	163	193	357	241	196	270	328
ENSECAG00000017788	4.147265542	0.820937611	0.913117292	230	210	412	369	408	299	366	397
ENSECAG00000005097	3.636407196	0.821058408	0.913162258	84	217	222	385	218	258	237	310
ENSECAG00000008911	6.768718886	0.821425091	0.913480659	1384	2002	1656	1982	2320	2244	2404	2550
ENSECAG00000010077	2.482546385	0.821858642	0.913867447	69	58	140	88	142	73	103	174
ENSECAG00000023108	6.498945189	0.821933765	0.913867447	1069	1588	1694	1890	1835	1636	1605	2506
ENSECAG00000017222	4.585873922	0.822319191	0.914206521	194	335	470.002	652	475	429	516.001	688
ENSECAG00000023280	2.670571775	0.822433442	0.914244083	86	97	68	156	180	125	82	166
ENSECAG00000005751	0.39933625	0.823042106	0.914638367	8	20	17	47	22	5	24	51
ENSECAG00000012404	3.836673557	0.823141997	0.914638367	130	224	257	350	321	224	295	415
ENSECAG00000011767	5.98332061	0.823248596	0.914638367	830	923	1270	1319	1110	1233	1160	1798
ENSECAG00000010441	-0.016688772	0.823259994	0.914638367	4	15	20	21	19	14	27	18
ENSECAG00000020104	5.673982875	0.823263218	0.914638367	392	972	947	1145	940	1144	1316	1017
ENSECAG00000008453	4.941371449	0.82327113	0.914638367	364	378	610	691	688	610	627	748
ENSECAG00000010589	6.209619255	0.823436015	0.914732108	664	1197	1185	2000	1600	1191	1476	2280
ENSECAG00000015187	1.552204447	0.823562574	0.914735248	30	19	92	64	64	45	67	56
ENSECAG00000012380	6.135314023	0.823599858	0.914735248	724.458	857.369	1422.03	1762.6	1525.48	1461.87	1567.64	1554.5
ENSECAG00000019377	4.98039132	0.823992431	0.915081811	341	489	682	690.001	769	652	632	547
ENSECAG00000010246	6.142441557	0.824285249	0.915266137	529	878	1313	2238	1532	1259	1514	1908
ENSECAG00000020824	8.091123146	0.82431952	0.915266137	4608	4680	2917	4672	6827	4998	6056	6073
ENSECAG00000001552	0.002696581	0.824727736	0.915629914	9	11	23	22	12	26	19	15
ENSECAG00000004226	1.492363577	0.824853564	0.915680136	19	48	44	73	61	35	70	76
ENSECAG00000015479	4.347709319	0.825119813	0.915886217	187	336	391	450	416	496	441	397
ENSECAG00000012569	5.188015841	0.825500589	0.916190116	444	652	603	804	780	612	800	855
ENSECAG00000014779	6.548050615	0.825591088	0.916190116	1088	1698	1540	1782	1837	1769	2113	2483
ENSECAG00000000138	1.790700051	0.825688905	0.916190116	35	40	73	74	57	141	52	35
ENSECAG00000014757	5.128351957	0.82571614	0.916190116	364	584	589	930	681	721	628	883
ENSECAG00000013454	5.437451153	0.825805739	0.916200059	450	775	877	908	920	814	875	1004
ENSECAG00000026983	2.626984803	0.826021229	0.916291949	48	125	88	147	118	121	158	136
ENSECAG00000022282	6.167917316	0.826059488	0.916291949	688	1391	1195	1506	1407	1346	1395	2182
ENSECAG00000016919	7.090925876	0.826130498	0.916291949	1658	1979	2642	3258	2396	2369	2353	4360
ENSECAG00000000864	0.111248187	0.826372391	0.916470777	3	19	22	30	12	12	35	20
ENSECAG00000022872	0.812047119	0.826523125	0.916487018	14	23	38	46	39	21	9	70
ENSECAG00000026981	7.812056836	0.826560073	0.916487018	2468	3331	4979	5024	4743	4603	4262	5008
ENSECAG00000000629	3.869642303	0.826629024	0.916487018	177	150	315	325	325	274	332	339
ENSECAG00000017455	2.26412706	0.826718244	0.916496505	88	95	60	62	137	76	91	83
ENSECAG00000017412	3.487675497	0.827045653	0.916686689	84	178	186	320	212	234	231	298
ENSECAG00000008958	6.807721791	0.827085672	0.916686689	1132	1865	1844	2665	2447	2313	2703	2273
ENSECAG00000009937	3.536948662	0.827131837	0.916686689	132	189	169	323	252	244	240	213
ENSECAG00000013295	4.171755357	0.827256285	0.916735191	191	169	457	471	431	345	303	410
ENSECAG00000008444	6.026100752	0.827423565	0.916756616	940	794	1217	1268	1547	1232	1466	1428
ENSECAG00000015652	6.470342295	0.827436992	0.916756616	744	1189	1883	2666	1702	1767	1207	2750
ENSECAG00000017510	3.798086575	0.827755447	0.917020026	177	242	215	318	256	245	247	414
ENSECAG00000020150	0.785811059	0.828080045	0.917290189	14	28	31	44	24	30	27	54
ENSECAG00000016822	5.300864113	0.828376073	0.917445513	493	630	591	874	914	753	700	1079
ENSECAG00000007738	2.063633499	0.828381757	0.917445513	42	58	51	125	93	78	73	117
ENSECAG00000009500	3.038338797	0.828540149	0.917531499	59	132	105	269	156	168	205	181
ENSECAG00000013563	5.688392928	0.828846167	0.917740767	404	845	1103	1362	972	975	1186	1159
ENSECAG00000023774	6.40830262	0.82896816	0.917740767	755	1247	1626	2175	1787	1609	1766	2281
ENSECAG00000013585	4.154639759	0.82897144	0.917740767	265	321	258	344	323	334	394	428
ENSECAG00000017646	6.470347075	0.829159986	0.91781603	709	1845	1441	1971	1706	1749	2081	2218
ENSECAG00000018527	7.726747817	0.829200981	0.91781603	3210.01	3369	3961.01	3672	4154	4028	4578	4840
ENSECAG00000010680	4.809852118	0.829285988	0.917820709	293	414	575	588	561	516	639	731
ENSECAG00000018948	9.805079917	0.829459833	0.9179237	13350	10309	16558	17200	26530	13338	19720	19227
ENSECAG00000000023	5.027730809	0.829835978	0.918076214	332	383	803	801	743	596	741	621
ENSECAG00000023807	4.708878517	0.829958563	0.918076214	278	362	584	612	505	476	551	651
ENSECAG00000003760	0.913737829	0.830037584	0.918076214	26	47	23	22	37	35	35	40
ENSECAG00000022667	2.238209679	0.830080199	0.918076214	36	80	98	95	91	59	92	174
ENSECAG00000018245	2.73557318	0.830104781	0.918076214	42	89	134	193	138	134	136	168
ENSECAG00000019632	4.916947812	0.830147898	0.918076214	246	495	604	829	540	722	512	722
ENSECAG00000003271	8.077257501	0.830163265	0.918076214	4302	4933	3808	4932	5924	5058	5554	5861
ENSECAG00000004604	7.007891709	0.830518652	0.918379846	1944	2290	1894	2509	2998	2182	2624	2923
ENSECAG00000022956	9.025980637	0.830602134	0.91838278	4465	8111	9960	12927	10537	10513	11437	13273
ENSECAG00000020868	4.129736949	0.830741726	0.918447746	151	186	421	519	323	295	259	592
ENSECAG00000006211	4.732006254	0.830868477	0.918498505	248	717	442	399	566	339	617	708
ENSECAG00000008107	8.142238068	0.830953671	0.918503318	2995	5034	4836	7054	4730	5910	5881	6945
ENSECAG00000019439	5.272323247	0.831140917	0.918620924	333	848	562	831	694	841	704	1143
ENSECAG00000014842	6.450935052	0.831237041	0.918637804	1314	1345	1436	1808	1919	1552	1862	1966

ENSECAG00000012127	3.717008565	0.831439403	0.918772077	145	338	178	224	277	222	295	293
ENSECAG00000008577	5.942090242	0.831917391	0.919210872	953	1114	859	1162	1333	1039	1285	1488
ENSECAG00000016625	2.211637888	0.832131669	0.919212728	0	33	162	183	0	74	106	184
ENSECAG00000006602	3.980410188	0.832189152	0.919212728	271	352	160	155	331	262	453	335
ENSECAG00000006527	2.379010699	0.832266155	0.919212728	49	71	115	131	88	78	120	141
ENSECAG00000022361	5.971270871	0.832375969	0.919212728	635	923	1016	1686	1241	1212	1194	1859
ENSECAG00000003209	1.832452281	0.832390643	0.919212728	33	74	34	108	72	37	99	80
ENSECAG00000023503	2.679123834	0.832422552	0.919212728	85	94	92	139	123	162	126	136
ENSECAG00000008587	3.5873632	0.832485388	0.919212728	121	159	259	312	265	183	226	329
ENSECAG00000009876	4.718684638	0.832583128	0.919231319	315	330	558	536	569	422	650	661
ENSECAG00000011858	6.104570365	0.832751781	0.919239779	1060	1197	881	1206	1613	1207	1482	1724
ENSECAG00000008800	5.645904501	0.8327526	0.919239779	435	947	769	1236	1287	747	995	1378
ENSECAG00000023868	8.363288363	0.832900776	0.919314029	4610	4884	5287	6438	7860	5523	7531	8052
ENSECAG00000022640	7.471467123	0.833376244	0.919749479	1957	1909	4078	3959	4158	3181	4088	4130
ENSECAG00000005708	4.207954072	0.833889661	0.920156511	250	157	400	406	338	470	476	299
ENSECAG00000017968	4.534842059	0.83394566	0.920156511	254	343	444	498	566	350	483	634
ENSECAG00000014848	6.967433834	0.833988009	0.920156511	1662	2311	2042	2492	2461	2590	2334	3022
ENSECAG00000007412	0.986647018	0.834238703	0.920194187	38	17	29	44	49	42	42	18
ENSECAG00000011873	6.214451955	0.834241404	0.920194187	948	1387	1169	1561	1538	1598	1657	1337
ENSECAG00000013168	5.824629384	0.834265122	0.920194187	477	1201	1043	1270	828	1241	1130	1524
ENSECAG00000001400	4.129788524	0.834452173	0.920241067	153	314	268	448	350	341	399	431
ENSECAG00000009071	4.880440986	0.834540067	0.920241067	414	502	492	574	706	479	591	687
ENSECAG00000000449	3.669378623	0.834571176	0.920241067	99	280	210	253	192	288	265	362
ENSECAG00000014497	5.161169269	0.834631596	0.920241067	381	498	626	1042	748	550	768	941
ENSECAG00000015844	0.84995414	0.834792344	0.920328995	11	14	53	38	31	51	29	37
ENSECAG00000024890	2.571785991	0.834914926	0.920331916	28	103	102	176	114	118	136	146
ENSECAG00000008861	4.834201674	0.835007688	0.920331916	354	310	488	762	563	562	631	728
ENSECAG00000020723	2.381299824	0.835037997	0.920331916	50	78	120	115	89	125	115	88
ENSECAG00000023221	7.539516542	0.835365377	0.920603434	1712	3011	3522	5114	4073	3375	4163	3835
ENSECAG00000013761	4.168812261	0.835453631	0.920611401	113	252	413	548	327	354	321	492
ENSECAG00000020699	10.86739189	0.835565105	0.92064495	21200	26851	38882	44678	41484	38076	34166	41089
ENSECAG00000022411	4.166006776	0.836001966	0.921036977	230	222	353	371	398	403	320	431
ENSECAG00000015789	2.701509306	0.836319041	0.921265609	107	94	90	109	155	70	202	141
ENSECAG00000014621	5.54267487	0.836398016	0.921265609	519	635	994	1121	921	747	1100	1144
ENSECAG00000025082	2.305291382	0.836522073	0.921265609	22	87	85	172	78	99	90	133
ENSECAG00000020838	7.292335321	0.836533822	0.921265609	1981	1789	3091	3519	3320	3337	3011	4036
ENSECAG00000019982	2.317733459	0.836926744	0.921477979	44	41	125	124	127	85	133	82
ENSECAG00000009987	6.426765337	0.837035109	0.921477979	1324	1401	1514	1489	2005	1449	1870	1856
ENSECAG00000007116	7.150730024	0.837045391	0.921477979	1241	1666	3120	3756	2629	2775	3171	3912
ENSECAG00000010249	6.15472213	0.837051067	0.921477979	912	1325	981	1668	1542	1009	1623	1824
ENSECAG00000019610	4.490821139	0.837170232	0.921519878	272	248	498	468	596	368	605	378
ENSECAG00000003585	1.729310883	0.837365337	0.921645351	22	46	71	81	61	86	66	66
ENSECAG00000000162	8.421419294	0.83764328	0.921861967	5494	6138	5481	5578	6752	6723	6509	8555
ENSECAG00000023695	0.574964271	0.837764948	0.92190657	11	32	16	32	27	30	18	49
ENSECAG00000020342	0.03662708	0.837966151	0.92203868	7	18	21	21	7	28	16	23
ENSECAG00000014767	6.197262964	0.838252351	0.92226428	545	1062	1808	1913	1223	1473	1297	2153
ENSECAG00000016566	0.753839912	0.838682615	0.922648322	28	21	23	26	28	55	29	25
ENSECAG00000005828	4.848410722	0.83902014	0.922857782	317	423	578	594	694	408	717	702
ENSECAG00000003697	1.246511629	0.83903546	0.922857782	6	40	42	72	39	66	46	46
ENSECAG00000006619	2.97419043	0.839280032	0.922981111	45	145	191	183	123	141	173	210
ENSECAG00000010911	6.083489073	0.839310054	0.922981111	714	1095	1366	1571	1478	1213	1344	1644
ENSECAG00000001560	2.365671488	0.839639622	0.923254175	34	100	82	128	72	96	99	183
ENSECAG00000017394	7.736696574	0.839806777	0.923288488	2646	3627	4021	4379	4715	3851	3481	5829
ENSECAG00000010964	6.433143547	0.839867217	0.923288488	742	1950	1482	1885	1338	1847	1554	2485
ENSECAG00000019228	5.489946091	0.839914611	0.923288488	820	593	628	748	1223	740	904	1063
ENSECAG00000020052	4.517405563	0.840326832	0.923652265	140	278	740	390	242	1040	397	238
ENSECAG00000020815	2.204025836	0.840414502	0.923659274	44	50	85	129	85	83	116	112
ENSECAG00000013019	2.416635453	0.840865608	0.92400122	89	46	85	114	150	60	130	126
ENSECAG00000018312	2.896093974	0.840888278	0.92400122	94	131	124	158	232	156	69	147
ENSECAG00000013877	7.85573392	0.841068444	0.924109821	2891	3593	4109	5504	5530	3609	5413	4764
ENSECAG00000009507	3.375951076	0.841205292	0.924170811	117	194	212	139	204	244	206	240
ENSECAG00000013232	4.129105813	0.84139238	0.924286979	143	210	376	548	332	362	348	405
ENSECAG00000013570	3.76128407	0.84152779	0.92434636	182	295	205	218	247	292	197	389
ENSECAG00000000647	0.934045907	0.841723235	0.924462822	29	29	26	29	48	25	40	48
ENSECAG00000013422	4.950288779	0.841837408	0.924462822	432	277	622	826	720	523	701	626
ENSECAG00000000517	6.141841875	0.841878889	0.924462822	769	1232	1216	1461	1369	1404	1660	1709
ENSECAG00000013649	4.976498429	0.842008784	0.924462822	404	386	560	734	865	439	635	824
ENSECAG00000008465	5.989857519	0.84204064	0.924462822	649	899	891	1915	1562	1120	1341	1525
ENSECAG00000012603	4.337396776	0.842265143	0.9245068	186	384	282	507	369	437	488	453
ENSECAG00000021181	2.581542937	0.842384205	0.9245068	70	90	84	173	112	117	117	143
ENSECAG00000013508	6.479404545	0.842398538	0.9245068	1192	1168	1908	1520	2123	1679	2202	1739
ENSECAG00000021097	8.981366997	0.84240617	0.9245068	6370	9212	9218	9771	9046	10943	8634	13471
ENSECAG00000000001	5.40200955	0.842563434	0.924590085	322	424	1004	1211	852	1043	735	1034
ENSECAG00000024913	4.388716733	0.842901656	0.924804866	303	278	358	490	553	270	385	554
ENSECAG00000012357	6.96807656	0.84292195	0.924804866	1224	2420	1901	2802	2583	2331	2637	3439
ENSECAG00000016288	3.511112013	0.843175757	0.924994009	110	206	219	215	250	231	235	269
ENSECAG00000006583	0.686661752	0.843432369	0.925186192	18	22	26	41	25	21	23	58
ENSECAG00000013026	3.930291768	0.843632086	0.925315934	115	186	341	409	242	269	255	586
ENSECAG00000012035	6.151624826	0.843819478	0.925432134	520	735	1597	2456	1445	1278	1405	1812
ENSECAG00000014736	6.456147621	0.844087292	0.925583841	1389	918	1161	2647	2315	1289	885	2931
ENSECAG00000019486	4.678840257	0.844120733	0.925583841	369	318	537	513	638	455	536	497
ENSECAG00000004371	2.890916443	0.844402346	0.92568567	53	118	185	139	125	155	158	204
ENSECAG00000012193	4.432539352	0.844445506	0.92568567	250	511	242	378	480	271	537	619
ENSECAG00000017072	2.686896347	0.844458015	0.92568567	33	102	144	194	90	86	157	200
ENSECAG00000014290	2.304428643	0.844820399	0.925957463	81	40	104	81	144	72	81	133

ENSECAG00000007949	2.765641306	0.844868951	0.925957463	83	170	112	84	85	150	224	85
ENSECAG00000017477	4.966468444	0.84519293	0.926223193	623	519	365	358	702	650	694	668
ENSECAG00000005570	5.739248003	0.845321683	0.926247216	795	890	1089	758	1147	922	1124	1281
ENSECAG00000011295	0.10964386	0.845384025	0.926247216	12	6	22	24	22	29	11	24
ENSECAG00000018820	6.17734985	0.845538858	0.926247216	568	1559	1323	1669	1320	1613	1234	1860
ENSECAG00000025123	5.768222476	0.845565017	0.926247216	747	872	755	1173	1318	1170	1161	1050
ENSECAG00000021820	7.468652328	0.845622458	0.926247216	2306	2637	3245	4052	5051	2738	3249	3750
ENSECAG00000024972	2.504626707	0.84608827	0.926592373	70	122	109	73	119	88	148	108
ENSECAG00000013441	3.098196187	0.846100675	0.926592373	130	160	97	145	176	150	204	214
ENSECAG00000000691	7.070998665	0.846285612	0.92661986	1866	2459	2106	2116	3149	2528	3020	3032
ENSECAG00000011789	2.20295899	0.846322439	0.92661986	51	110	71	74	69	94	87	125
ENSECAG00000017649	2.3021366	0.846370437	0.92661986	38	93	77	119	84	95	144	98
ENSECAG00000001515	0.556362561	0.846569465	0.926703141	16	15	27	32	36	27	33	24
ENSECAG00000021803	3.723706445	0.846651208	0.926703141	206	267	125	220	210	304	254	382
ENSECAG00000016091	5.808627261	0.846739644	0.926703141	547	771	1107	1391	1282	1057	1126	1417
ENSECAG00000001235	4.937178912	0.846772752	0.926703141	310	520	588	724	646	503	699	719
ENSECAG00000015401	4.379800854	0.847021027	0.926885573	92	322	611	409	273	694	402	409
ENSECAG00000011050	4.50290501	0.847104509	0.926887657	189	329.003	517.003	507.002	479	510.002	484.009	480.004
ENSECAG00000022495	3.681487932	0.847488344	0.92721835	142	170	260	328	315	164	241	357
ENSECAG00000022039	4.012114838	0.847967525	0.927653285	229	130	339	428	485	207	393	246
ENSECAG00000016991	4.496514494	0.848169713	0.927785143	175	296	493	595	461	336	469	726
ENSECAG00000018848	4.441292858	0.848660993	0.928142119	195	189	453	767	405	480	491	408
ENSECAG00000015139	5.52673924	0.848701804	0.928142119	332	804	756	1304	828	889	1032	1281
ENSECAG00000010370	5.000300323	0.848741121	0.928142119	276	392	717	819	707	635	711	720
ENSECAG00000023415	6.688313126	0.849239096	0.928555014	1338	1542	1985	1840	2364	1901	2433	2277
ENSECAG00000025125	5.706912387	0.849287056	0.928555014	764	686	1118	948	1177	979	1042	1164
ENSECAG00000010777	6.583188364	0.849363867	0.928555014	2193	1388	872	1138	2590	1392	2434	2018
ENSECAG00000021407	4.593955943	0.849635715	0.928762844	183	316	546	625	483	580	436	587
ENSECAG00000021970	3.861069352	0.84994741	0.929014187	163	286	228	319	235	248	339	393
ENSECAG00000017527	2.452095872	0.850227735	0.929141274	50	97	115	116	99	96	118	136
ENSECAG00000011770	4.613361686	0.850330058	0.929141274	500	450	228	262	526	418	474	742
ENSECAG00000016038	2.419691489	0.850484763	0.929141274	50	58	129	118	110	129	83	136
ENSECAG00000006481	6.819117758	0.850491881	0.929141274	1635	2449	1717	1641	2435	1687	2605	2750
ENSECAG00000013490	3.871599064	0.850532094	0.929141274	182	311	209	284	396	231	327	256
ENSECAG00000012932	8.361730224	0.850554338	0.929141274	3524	4945	5145	8567	7257	5989	7511	8044
ENSECAG00000008104	6.578242106	0.850685627	0.929195356	946	1732	1814	1861	1794	1821	1943	2822
ENSECAG00000024661	6.069536747	0.850878273	0.929316442	653	1410	1200	1390	1296	1469	1188	1645
ENSECAG00000005508	4.900260805	0.851026763	0.929360297	367	328	650	742	677	639	507	655
ENSECAG00000023319	0.671286302	0.851117713	0.929360297	3	17	24	64	25	40	32	33
ENSECAG00000019280	5.638664869	0.851193581	0.929360297	497	871	966	1148	1183	815	1160	1007
ENSECAG00000023709	6.298591573	0.851245609	0.929360297	1154	620	1896	1765	1632	1648	1361	1911
ENSECAG00000011120	4.681931498	0.851529508	0.929526941	254	343	599	616	577	434	515	623
ENSECAG00000020647	7.79255458	0.851622833	0.929526941	2270	3334	4701	4458	3890	5282	2825	7524
ENSECAG00000026825	2.142249055	0.851643677	0.929526941	21	124	84	54	88	106	107	73
ENSECAG00000008278	5.707442491	0.851871682	0.929647784	672	583	1136	1236	1068	1023	1119	1148
ENSECAG00000024646	3.588697979	0.851933649	0.929647784	118	217	213	246	224	186	374	260
ENSECAG00000026906	1.702870808	0.852103745	0.929647784	31	49	58	88	66	45	87	64
ENSECAG00000010257	3.124465649	0.852106684	0.929647784	58	167	148	218	146	145	235	233
ENSECAG00000023261	5.450498235	0.852224083	0.929647784	351	789	888	1084	831	712	970	1169
ENSECAG00000020933	7.220600524	0.852278025	0.929647784	1003	1748	2634	5933	3817	2531	3516	2448
ENSECAG00000000407	5.248394609	0.852485677	0.929647784	519	624	555	768	746	730	925	897
ENSECAG00000012874	5.603980147	0.852528796	0.929647784	768	790	746	923	916	865	997	1314
ENSECAG00000026910	1.25396218	0.852561595	0.929647784	3	3	79	103	8	10	78	93
ENSECAG00000017750	4.954630279	0.852664966	0.929647784	620	403	492	352	851	555	693	592
ENSECAG00000018698	5.929746928	0.852719846	0.929647784	610	1123	810	1777	1353	1217	1007	1518
ENSECAG00000012102	4.902711739	0.852736244	0.929647784	261	591	406	873	705	603	519	658
ENSECAG00000020184	5.116586138	0.852971395	0.929814927	375	296	811	882	869	533	871	752
ENSECAG00000010629	5.926320421	0.853189133	0.929889313	764	887	675	1737	1413	1583	653	1628
ENSECAG00000019062	5.126119662	0.853203317	0.929889313	530	490	800	494	871	614	693	732
ENSECAG00000002157	0.864549396	0.85343444	0.92999073	15	29	41	28	38	30	46	37
ENSECAG00000006256	2.261365423	0.853489718	0.92999073	48	91	88	80	102	72	132	106
ENSECAG00000007999	4.335630206	0.853541924	0.92999073	232	286	470	406	558	239	378	526
ENSECAG00000015322	7.275856302	0.853739123	0.930116398	2170	2596	2724	2977	3394	2868	3322	3337
ENSECAG00000008076	6.401656623	0.854089448	0.930401623	887	1482	1586	1938	1484	1794	1696	2090
ENSECAG00000015762	4.958664507	0.8541647	0.930401623	253	554	577	836	531	673	675	705
ENSECAG00000015925	7.180251126	0.854340405	0.930503804	1918	1785	3001	2790	3343	2606	3416	3286
ENSECAG00000023937	0.184792963	0.854463462	0.93054863	8	23	23	19	13	17	13	44
ENSECAG00000020484	4.10186632	0.855228236	0.93122431	147	310	425	315	304	241	494	396
ENSECAG00000010813	6.948093612	0.855247815	0.93122431	1636	2632	1522	2052	2481	2989	3382	1747
ENSECAG00000000346	5.818460895	0.855381507	0.931280632	541	1191	913	1284	1065	997	939	1771
ENSECAG00000011237	5.819605736	0.855551369	0.931376321	531	761	1100	1481	1078	987	1225	1661
ENSECAG00000019577	5.743854121	0.856114647	0.931860072	393	831	884	1640	832	1245	1036	1556
ENSECAG00000017143	4.484512884	0.856159769	0.931860072	224	8	264	1091	850	15	439	775
ENSECAG00000001800	0.113953968	0.856516365	0.932158902	10	15	22	23	22	15	4	41
ENSECAG00000022772	0.922232819	0.856744564	0.932294192	39	21	18	30	74	15	30	42
ENSECAG00000020850	8.197074625	0.856804784	0.932294192	4146	4153	5361	6394	6752	5326	6374	5987
ENSECAG00000000987	6.499997702	0.85689614	0.932304313	1399	1814	1155	1269	1932	1522	2233	2235
ENSECAG00000006134	7.374664227	0.857112931	0.932450892	1809	2806	2394	4103	3383	3186	3677	4255
ENSECAG00000019788	4.005214595	0.857329022	0.932566927	116	283	267	497	295	338	352	341
ENSECAG00000003080	4.70635515	0.857383746	0.932566927	282	287	585	697	610	543	440	575
ENSECAG00000022050	6.648580396	0.857601193	0.932714153	766	1729	1100	3415	2933	1624	2671	1483
ENSECAG00000021601	5.642425339	0.857785057	0.932824829	512	811	716	1353	1123	892	1071	1264
ENSECAG00000023940	3.878005642	0.85811234	0.933091436	131	225	254	392	283	298.002	303	390
ENSECAG00000022660	8.071276929	0.85824273	0.933097123	3197	6037	3817	5235	5059	4898	5225	7404
ENSECAG00000014812	1.109194358	0.858281819	0.933097123	21	20	45	53	47	35	55	43

ENSECAG00000023647	6.801832811	0.858443628	0.9331018	774	2325	1601	3408	1721	2323	2045	3255
ENSECAG00000012920	6.888381369	0.858450371	0.9331018	1700	2241	1443	2608	2515	2171	2629	2568
ENSECAG00000000860	7.21220394	0.858560567	0.933121674	964	2710	4937	2043	506	7614	1132	2213
ENSECAG00000017288	6.733781353	0.858686451	0.933121674	1134	1795	2148	2331	1858	2352	2094	2569
ENSECAG00000007563	6.565338243	0.858789092	0.933121674	722	1333.99	1695	2882.99	1856.99	2127.99	1875	2364
ENSECAG00000023455	8.937882764	0.858797161	0.933121674	5617	8412	9615	10295	9295	9214	10396	12137
ENSECAG00000010673	3.104636902	0.859133833	0.933199168	102	113	159	192	168	177	178	218
ENSECAG00000018478	5.919812289	0.859267613	0.933199168	676	596	1449	1367	1581	1198	1283	1169
ENSECAG00000022011	1.429361512	0.85934113	0.933199168	32	22	66	51	66	50	52	59
ENSECAG00000004055	6.046087402	0.859391615	0.933199168	1319	268	1761	1033	2360	765	1582	718
ENSECAG00000018169	5.712823519	0.859425601	0.933199168	610	961	937	917	1120	1155	1318	908
ENSECAG00000013871	6.020922092	0.859514378	0.933199168	702	1282	968	1337	1313	1104	1455	1813
ENSECAG00000016332	6.599697005	0.859555017	0.933199168	773	1229	2161	2921	1907	1932	1878	2388
ENSECAG00000019838	1.846912903	0.859606869	0.933199168	14	72	31	126	14	65	12	236
ENSECAG00000016379	6.363927543	0.859607682	0.933199168	743	1366	1760	1955	1601	1687	1601	1999
ENSECAG00000012406	5.107144965	0.859764934	0.933214053	344	718	509	822	710	635	678	867
ENSECAG00000021807	4.651636286	0.8598874	0.933214053	244	319	561	582	594	475	567	536
ENSECAG00000006428	3.444086648	0.85989766	0.933214053	88	250	161	262	176	221	227	277
ENSECAG00000024001	5.858630121	0.859949933	0.933214053	629	1116	928	1157	1364	1056	993	1661
ENSECAG00000014009	1.268712413	0.860107042	0.933295407	11	33	56	73	31	61	54	41
ENSECAG00000012829	8.688821343	0.860207505	0.933315286	5231	8410	7348	6817	8267	7758	8040	10478
ENSECAG00000013291	7.341385964	0.860294183	0.933320205	1725	842	6658	2328	5316	3787	2955	909
ENSECAG00000019816	4.335565768	0.860396316	0.933341888	340	347	220	427	476	457	469	243
ENSECAG00000012732	7.722063347	0.860857257	0.933693202	1568	3347	3152	6705	3976	5410	3565	5381
ENSECAG00000006549	6.149116852	0.860884526	0.933693202	701	1140	1527	1607	1256	1498	1415	1768
ENSECAG00000006989	6.590326871	0.861428322	0.934193814	794	1725	1695	2696	1714	1824	2090	2453
ENSECAG00000021719	0.355665832	0.861651869	0.934347063	14	20	11	32	26	14	35	30
ENSECAG00000011795	2.586023291	0.861760736	0.934375621	56	63	136	149	108	127	137	142
ENSECAG00000024023	6.590237529	0.861910871	0.934375621	1340	1769	1870	1089	2030	1797	2310	2257
ENSECAG00000009976	5.489310543	0.861924915	0.934375621	639	867	673	807	933	810	1046	967
ENSECAG00000008909	6.719040235	0.862231679	0.934618997	1609	1432	2136	1877	2459	1693	2402	2271
ENSECAG00000012890	5.924211277	0.862505158	0.934826252	536	1026	1020	1763	1365	1129	1288	1290
ENSECAG00000012845	5.895966569	0.862801376	0.935058111	1018	1062	743	863	1424	1085	1288	1384
ENSECAG00000001039	4.27372371	0.863000649	0.935184871	265	326	351	353	440	358	381	434
ENSECAG00000004794	6.038620175	0.86309861	0.935201832	746	1165	1198	1417	1326	1128	1235	1866
ENSECAG00000018506	3.399184488	0.863402186	0.935388871	113	79	174	361	243	301	219	128
ENSECAG00000026943	3.597933281	0.863435881	0.935388871	112	216	229	249	280	248	238	276
ENSECAG00000002323	3.08955786	0.863765348	0.935537197	101	80	190	193	166	142	196	236
ENSECAG00000020852	5.824766158	0.863786393	0.935537197	909	580	952	1418	1363	895	1287	1203
ENSECAG00000000220	3.195223663	0.863895363	0.935537197	77	139	188	247	174	204	186	187
ENSECAG00000019214	3.924005699	0.863927541	0.935537197	230	206	357	210	422	253	283	301
ENSECAG00000022819	8.084970996	0.863984493	0.935537197	2779	4419	4192	6965	6436	4668	5620	7072
ENSECAG00000013788	10.29918271	0.864095879	0.935568647	25135	22906	14815	17178	24330	20473	29262	31489
ENSECAG00000016705	5.720493886	0.86443255	0.935843986	796	1219	632	788	1075	992	886	1479
ENSECAG00000024121	3.018540126	0.864527276	0.935857366	172	128	114	96	50	370	81	130
ENSECAG00000004011	1.147742974	0.864641137	0.935891454	28	35	44	27	48	48	30	60
ENSECAG00000023903	6.336513686	0.865091919	0.936290187	986	1340	1386	1847	1535	1627	1480	2147
ENSECAG00000015450	2.197655373	0.865413363	0.936547078	49	42	103	109	82	66	100	151
ENSECAG00000010781	3.706110809	0.865494131	0.936547078	177	250	211	231	235	331	244	260
ENSECAG00000009067	5.649794705	0.865713508	0.936695256	1201	644	515	545	1459	792	1550	546
ENSECAG00000000678	6.164833224	0.866036429	0.936955428	779	1469	1199	1471	1376	1470	1452	1704
ENSECAG00000009129	1.653872966	0.866217913	0.93698113	34	28	132	20	19	151	55	6
ENSECAG00000019644	1.786636329	0.866225118	0.93698113	25	84	60	66	47	85	45	101
ENSECAG00000006839	7.013699444	0.866329389	0.937004714	1400	1823	2945	2431	2752	2169	3067	3320
ENSECAG00000007322	7.866025339	0.866528623	0.937130993	2465	3778	4115	6089	5063	4165	4981	5322
ENSECAG00000014013	2.331017138	0.866666752	0.937162435	61	83	110	82	110	70	118	116
ENSECAG00000024436	1.192082356	0.866722661	0.937162435	35	38	23	53	49	22	17	99
ENSECAG00000003113	6.331666016	0.867013596	0.937387809	1008	1043	1682	1837	1967	1226	1681	1911
ENSECAG00000020120	5.357413004	0.867105567	0.937398045	604	741	626	750	852	761	759	1075
ENSECAG00000000332	5.918260221	0.867398446	0.937625454	820	941	1242	1066	1289	1117	1279	1385
ENSECAG00000018381	8.25217511	0.867717506	0.937661071	3798	4339	5766	6188	6310	5354	6850	8206
ENSECAG00000000372	4.970970235	0.8677501	0.937661071	394	413	620	646	706	518	700	806
ENSECAG00000015720	5.700773744	0.867798352	0.937661071	554	1048	831	996	1047	1150	939	1370
ENSECAG00000023693	4.800702325	0.867811995	0.937661071	284	425	589	569	579	499	642	699
ENSECAG00000006877	4.819043837	0.867912567	0.937661071	310	261	776	528	328	1256	346	431
ENSECAG00000016504	6.357639879	0.867944333	0.937661071	1384	1426	1317	1198	1750	1346	1729	2080
ENSECAG00000018406	5.879798006	0.8680572	0.937661071	594	750	1731	1053	2151	857	1287	545
ENSECAG00000015555	4.215769236	0.86810389	0.937661071	158	290	388	485	346	384	401	413
ENSECAG00000010037	5.218213724	0.86817413	0.937661071	314	508	757	995	763	656	706	1135
ENSECAG00000013309	7.950498052	0.868494017	0.937917405	2906	4931	4439	4564	4870	4785	4955	6108
ENSECAG00000022381	3.838589336	0.868963481	0.938223367	118	182	262	480	291	255	270	379
ENSECAG00000001086	1.042851687	0.869001607	0.938223367	9	47	18	61	19	44	34	77
ENSECAG00000016427	4.765256761	0.869180625	0.938223367	267	445	454	673	706	404	458	818
ENSECAG00000013117	4.765911751	0.869457935	0.938223367	140	488	423	976	456	599	545	658
ENSECAG00000001472	0.605245854	0.869534307	0.938223367	25	14	22	28	46	21	51	6
ENSECAG00000026951	3.133447807	0.869558609	0.938223367	54	115	148	302	158	200	206	187
ENSECAG00000007321	6.242167392	0.869610148	0.938223367	868	1167	1152	2124	1581	1354	1620	1803
ENSECAG00000010042	7.307376867	0.869675848	0.938223367	1550	3127	3347	2928	2867	3843	3135	3261
ENSECAG00000000708	6.357773086	0.869805364	0.938223367	1068	1317	1796	1345	1926	1372	1772	1813
ENSECAG00000022458	4.948153614	0.869934898	0.938223367	337	592	511	682	562	643	721	644
ENSECAG00000020472	3.653499035	0.869947818	0.938223367	140	239	189	296	266	238	225	320
ENSECAG00000014548	4.864506753	0.870075597	0.938223367	325	305	617	728	660	619	741	475
ENSECAG00000008022	3.359725315	0.870077585	0.938223367	83	127	197	303	201	202	218	266
ENSECAG00000006729	1.027396006	0.870128571	0.938223367	28	19	41	47	47	25	41	49
ENSECAG00000024544	8.148846005	0.870339497	0.938223367	3108	4566	5517	5653	6416	6496	5502	6114

ENSECAG00000022246	5.398191215	0.870426882	0.938223367	641	659	698	810	958	690	914	984
ENSECAG00000018984	6.592654042	0.870509244	0.938223367	1378	1569	1527	2036	2050	1650	2097	2321
ENSECAG00000013340	3.495300676	0.870535629	0.938223367	172	136	241	203	240	211	271	208
ENSECAG00000003872	4.391564145	0.870546986	0.938223367	191	401	384	491	464	272	522	510
ENSECAG00000013455	3.989819132	0.87064455	0.938223367	182	186	298	453	304	286	350	387
ENSECAG00000020393	6.128232651	0.870703302	0.938223367	520	962	787	2736	1614	1315	1341	1842
ENSECAG00000001596	7.111053412	0.870732039	0.938223367	1417	1910	3205	2746	3147	2492	3215	3178
ENSECAG00000006771	5.576009747	0.87083416	0.938223367	706	406	1165	819	1311	683	956	1231
ENSECAG00000021665	0.801175603	0.870836254	0.938223367	31	38	21	16	86	17	22	9
ENSECAG00000010538	3.335363833	0.870987719	0.938223367	122	128	185	266	243	185	208	197
ENSECAG00000022249	4.948706994	0.871006297	0.938223367	649	514	398	373	1032	517	597	396
ENSECAG00000022293	2.292893746	0.871006871	0.938223367	38	73	82	138	69	89	128	136
ENSECAG00000012308	3.123455033	0.871419609	0.938578975	93	150	155	203	192	127	235	167
ENSECAG00000011539	3.368961233	0.871695354	0.938786978	86	168	155	297	178	279	177	249
ENSECAG00000023513	5.238979299	0.872002902	0.939029189	459	664	578	783	781	727	728	1048
ENSECAG00000019199	4.510116219	0.872256675	0.939213451	244	303	545	493	487	456	460	495
ENSECAG00000006687	5.423032667	0.872486866	0.939254696	399	842	761	991	853	737	1090	915
ENSECAG00000018458	1.166167601	0.872529043	0.939254696	17	43	47	45	24	62	40	49
ENSECAG00000022765	1.927786708	0.872542978	0.939254696	34	45	76	99	96	68	62	98
ENSECAG00000000665	6.417575073	0.872633069	0.939256324	662	1379	1830	2273	1634	1903	1712	1866
ENSECAG00000004780	4.483389596	0.872729623	0.939256324	180	295	499	640	409	442	454	565
ENSECAG00000019206	0.843998082	0.872828532	0.939256324	20	18	39	35	37	34	27	51
ENSECAG00000020498	3.285837369	0.872875157	0.939256324	84	113	250	213	212	127	200	318
ENSECAG00000024654	7.644234492	0.873015801	0.939269948	1984	5364	2318	4019	4025	4221	3926	4469
ENSECAG00000013696	6.359837023	0.873083651	0.939269948	1001	1943	1334	1179	1851	1354	1809	1883
ENSECAG00000018803	4.663564341	0.873135821	0.939269948	323	419	489	409	634	429	628	497
ENSECAG00000023964	0.395272584	0.873495313	0.939567713	5.01056	18.0001	20.0079	42.0001	20.0007	22.0005	21.001	45.0003
ENSECAG00000023612	2.91060431	0.8736469	0.93964181	68	97	114	264	158	138	155	168
ENSECAG00000024114	7.986605913	0.874068969	0.939987089	3589	3835	4567	5191	5564	4701	5307	5653
ENSECAG00000024380	0.794608146	0.874185109	0.939987089	14	49	4	39	36	53	25	27
ENSECAG00000020523	10.3874669	0.874216121	0.939987089	12610	16470	31721	35377	26093	24953	29639	31417
ENSECAG00000014047	7.620596446	0.874404943	0.940101151	2361	4519	2422	3358	4067	3671	4386	5071
ENSECAG00000008879	4.993683083	0.87460925	0.940150435	378	581	444	699	664	562	601	956
ENSECAG00000014015	1.462841295	0.874616273	0.940150435	55	35	46	36	72	80	36	26
ENSECAG00000020360	4.905997099	0.874734462	0.940188531	287	339	563	891	625	593	637	741
ENSECAG00000000475	4.708643907	0.874950088	0.940331338	265	328	684	473	575	564	402	727
ENSECAG00000011258	1.976122285	0.87504931	0.940349026	17	67	66	121	84	72	59	123
ENSECAG00000009397	0.629466412	0.875503302	0.940714385	26	11	37	15	34	16	37	43
ENSECAG00000002955	4.914350144	0.875657687	0.940714385	362	257	758	740	662	479	676	715
ENSECAG00000019236	0.469981242	0.875682573	0.940714385	14	10	39	29	23	22	37	23
ENSECAG00000023692	8.139390245	0.875720476	0.940714385	3984	3533	6127	5546	7419	4972	5529	5616
ENSECAG00000019593	8.271335093	0.875937013	0.940858039	2604	4323	7879	7352	8134	7106	6025	4062
ENSECAG00000005758	6.039605201	0.876058187	0.940899246	704	1201	1066	1598	1657	1053	1368	1453
ENSECAG00000008678	8.368230256	0.876553981	0.941341196	3816	4665	5182	8608	7769	5795	8075	7105
ENSECAG00000008712	5.184141691	0.876635381	0.941341196	483	553	594	754	756	724	821	837
ENSECAG00000010342	3.06734898	0.876824123	0.94136568	84	132	172	166	143	191	158	228
ENSECAG00000007287	0.081028353	0.876932019	0.94136568	4	19	12	31	11	23	19	31
ENSECAG00000007653	4.105176066	0.876941899	0.94136568	147	384	259	355	312	266	335	603
ENSECAG00000022822	5.456191851	0.87698959	0.94136568	514.003	671	953	869.003	911	760	876	1159
ENSECAG00000009063	5.568332561	0.877288471	0.94145437	611	848	907	818	1044	861	1028	1047
ENSECAG00000012639	5.911985109	0.877358874	0.94145437	637	767	1394	1430	1207	1075	1128	1677
ENSECAG00000013036	0.882923778	0.877394214	0.94145437	18	13	58	26	56	20	31	48
ENSECAG00000001318	6.233940661	0.877403655	0.94145437	1121	831	1591	1330	1883	1465	1875	1252
ENSECAG00000005387	5.876535598	0.877510541	0.941480147	637	849	1165	1460	1385	885	1339	1344
ENSECAG00000011406	6.534459159	0.877807117	0.941578843	1209	1232	1442	2290	2270	1672	2427	1628
ENSECAG00000005229	6.472296037	0.878042781	0.941578843	1317	1775	1293	1461	2027	1458	2080	1884
ENSECAG00000009221	3.91751728	0.878076028	0.941578843	146	185	260	449	411	251	262	388
ENSECAG00000013939	2.521257882	0.878077898	0.941578843	38	173	81	100	64	132	164	103
ENSECAG00000009871	2.670180383	0.878113634	0.941578843	68	81	99	179	130	106	144	169
ENSECAG00000019778	4.259492127	0.878207472	0.941578843	235	277	359	445	422	407	412	344
ENSECAG00000002116	2.520851564	0.878255513	0.941578843	51	94	79	184	163	82	126	98
ENSECAG00000012840	4.958230464	0.878294957	0.941578843	180.007	681	460	933	548	717	638	673
ENSECAG00000019558	4.726471024	0.878483047	0.941578843	300	446	529	537	632	411	528	661
ENSECAG00000013174	8.309434303	0.878492587	0.941578843	3067	4372	6294	7936	6146	5929	6510	9217
ENSECAG00000005632	0.768228543	0.8785681	0.941578843	8	22	32	58	27	40	31	33
ENSECAG00000008997	6.298488558	0.87859698	0.941578843	979	1344	1466	1560	1735	1300	1744	1853
ENSECAG00000018145	3.127548884	0.879009069	0.941931627	134	98	162	194	215	105	172	241
ENSECAG00000026940	3.061211769	0.879119048	0.941960639	76	163	128	187	176	122	190	238
ENSECAG00000026874	6.315867403	0.879247118	0.94200903	939	984	1731	1671	2010	1156	1895	1888
ENSECAG00000019259	1.956565802	0.87984675	0.942488483	43	24	99	106	82	64	92	76
ENSECAG00000007302	6.250925852	0.87986053	0.942488483	769	1028	1221	2232	1612	1549	1601	1832
ENSECAG00000022412	4.299586149	0.880265771	0.942770577	177	357	294	567	456	334	360	504
ENSECAG00000010978	5.675274238	0.88028983	0.942770577	993	666	777	698	1409	851	1180	985
ENSECAG00000000411	2.757188529	0.880484792	0.942890501	78	157	75	120	158	106	150	170
ENSECAG00000007289	3.859852874	0.880570085	0.94289297	115	194	241	465	260	294	377	318
ENSECAG00000015794	8.652286047	0.881034374	0.942927768	8053	6554	6802	3938	8018	10883	7357	6645
ENSECAG00000008402	1.38554951	0.881161066	0.942927768	35	35	62	38	64	37	54	55
ENSECAG00000022536	6.175616561	0.881171811	0.942927768	1111	1226	1075	1381	1706	1321	1486	1544
ENSECAG00000024486	3.870651709	0.881209123	0.942927768	153	209	235	394	313	221	370	366
ENSECAG00000020730	5.614636625	0.881238849	0.942927768	462	984	827	1005	940	1168	1119	968
ENSECAG00000010132	9.11695481	0.881245599	0.942927768	10235	11202	8158	5526	14273	9130	12313	10540
ENSECAG00000010309	3.885535034	0.881254919	0.942927768	119	204	269	480	349	195	266	439
ENSECAG00000007781	6.472247726	0.881266499	0.942927768	594	1444	1595	2605	1466	1967	1908	2366
ENSECAG00000018607	0.831808328	0.881553883	0.943146442	25	42	10	27	32	41	55	17
ENSECAG00000022976	5.020008508	0.882036971	0.943523234	342	412	637	818	748	544	806	709

ENSECAG00000025060	2.622176488	0.882072152	0.943523234	53	102	173	94	77	121	150	159
ENSECAG00000011846	6.680845545	0.882168548	0.943537517	1078	883	2234	2893	2776	2110	2236	1703
ENSECAG00000026879	3.309579045	0.882425069	0.943678859	119	122	146	309	185	138	230	280
ENSECAG00000015012	4.219499688	0.882466808	0.943678859	107	376	172	729	259	457	419	396
ENSECAG00000007555	4.730889697	0.882932126	0.944087598	203	470	539	677	466	513	556	695
ENSECAG00000008503	13.15347473	0.883169951	0.944153217	64769	118060	173852	287520	202205	233679	217551	125249
ENSECAG00000014847	3.628904834	0.883184436	0.944153217	95	206	266	311	200	237	240	359
ENSECAG00000024437	4.650106108	0.883254453	0.944153217	332	383	412	518	617	453	485	618
ENSECAG00000007192	11.11943982	0.883325885	0.944153217	42562	34926	35060	29771	48045	34077	48800	56606
ENSECAG00000009724	4.41254	0.883585746	0.944342135	346	312	293	463	527	338	446	475
ENSECAG00000012242	1.671204065	0.883874713	0.944562123	57	62	36	44	78	56	58	65
ENSECAG00000014784	8.380245857	0.883982721	0.944588703	3338	4655	5103	10957	8183	5085	8577	5997
ENSECAG00000006891	3.66833165	0.884104571	0.944630067	125	302	162	240	258	208	208	442
ENSECAG00000015408	3.209990367	0.884700726	0.9449714	86	211	154	181	132	156	171	320
ENSECAG00000017756	7.986292131	0.884745541	0.9449714	2850	3930	5375	5419	5070	5958	4222	5877
ENSECAG00000020152	8.242544111	0.884784156	0.9449714	3746	3490	7502	6236	7846	4063	7204	6367
ENSECAG00000015810	2.915044101	0.88479249	0.9449714	88	92	130	192	160	143	203	137
ENSECAG00000019768	5.618495945	0.884839881	0.9449714	665	548	1134	1012	1087	536	1117	1455
ENSECAG00000019528	4.25484768	0.88518295	0.945163418	194	281	361	454	372	301	452	538
ENSECAG00000022744	3.130617209	0.885227155	0.945163418	38	126	169	290	139	145	178	305
ENSECAG00000015506	4.736882347	0.885269239	0.945163418	355	464	562	391	669	463	622	467
ENSECAG00000018789	6.502292471	0.88542936	0.945232993	1278	1998	1067	1388	1964	1467	2418	2040
ENSECAG00000005171	4.638762481	0.885512512	0.945232993	279	240	733	482	524	631	314	594
ENSECAG00000022262	6.867627671	0.885621495	0.945232993	1534	1727	1948	2812	2442	2415	2252	2664
ENSECAG00000011147	8.26517806	0.885667175	0.945232993	5444	4478	4422	4859	6550	5967	7172	7040
ENSECAG00000023632	6.565937968	0.885795049	0.945280675	1133	1216	2538	1598	1951	1657	1671	2740
ENSECAG00000020873	2.46222279	0.885889207	0.945292371	59	127	54	114	102	102	119	151
ENSECAG00000010248	2.885112997	0.886497757	0.945852898	53	131	90	234	123	93	151	285
ENSECAG00000022158	5.881502138	0.887337865	0.946660359	1072	1233	609	831	1346	1017	1335	1237
ENSECAG00000000775	5.41587366	0.887435574	0.946675711	462	932	746	748	777	1028	713	1044
ENSECAG00000006290	1.484163749	0.887547425	0.946706144	31	70	33	37	72	41	79	43
ENSECAG00000018058	0.942269437	0.88800426	0.947092637	27	42	34	16	18	45	22	68
ENSECAG00000023315	8.207247334	0.888115846	0.947092637	4280	3923	6120	4719	7067	5145	6528	7000
ENSECAG00000021111	6.388584767	0.888188208	0.947092637	1201	1338	1453	1620	1992	1693	1682	1626
ENSECAG00000011190	2.05514182	0.888259486	0.947092637	27	58	78.0016	137	63	106	101.001	61
ENSECAG00000005577	0.19173323	0.888326547	0.947092637	8	25	10	32	32	26	11	15
ENSECAG00000025163	4.941857194	0.888459618	0.947122133	416	461	592	526	757	593	709	579
ENSECAG00000009619	4.17143171	0.88852093	0.947122133	169	252	448	392	343	333	439	389
ENSECAG00000011315	0.673895196	0.888681527	0.947155196	15	18	34	32	21	30	8	76
ENSECAG00000012059	4.540508155	0.888718671	0.947155196	97	386	404	784	358	568	371	725
ENSECAG00000000800	3.619422178	0.888857355	0.947194682	136	217	245	204	274	221	267	298
ENSECAG00000009459	5.460509916	0.888922451	0.947194682	364	661	947	1173	778	918	900	1099
ENSECAG00000025174	1.213782476	0.889032857	0.947223492	35	21	45	51	68	29	40	49
ENSECAG00000010892	6.308800406	0.8891379	0.947246584	744	1153	1273	2529	1495	1444	1755	1985
ENSECAG00000019626	5.790578187	0.889431475	0.947413446	260	310	1110	2468	1332	816	1326	1385
ENSECAG00000013993	4.643336666	0.889592796	0.947413446	235	428	407	693	466	447	613	570
ENSECAG00000006495	5.10694122	0.889631519	0.947413446	382	501	590	856	791	722	734	710
ENSECAG00000000406	6.850796277	0.889661734	0.947413446	1795	2082	1517	2246	2627	2163	2578	2277
ENSECAG00000005633	3.49557293	0.889711447	0.947413446	100	247	220	204	227	251	169	290
ENSECAG00000018063	3.649280286	0.889935274	0.947562982	71	197	264	386	206	312	186	334
ENSECAG00000011637	5.634112321	0.89027473	0.947750322	322	532	1779	947	1302	892	1059	876
ENSECAG00000018829	7.633703348	0.890278048	0.947750322	2044	4673	2565	3740	4623	3347	5832	3378
ENSECAG00000009401	1.631039042	0.891026181	0.948365752	57	48	46	43	79	43	68	61
ENSECAG00000019064	6.98646537	0.891086303	0.948365752	1769	2374	2185	2098	2765	2104	2895	2912
ENSECAG00000018052	1.033221606	0.891106729	0.948365752	19	31	39	49	33	27	32	73
ENSECAG00000015900	3.338262127	0.891190031	0.948365752	105	145	215	209	201	203	216	248
ENSECAG00000013751	6.721425163	0.891768304	0.948812205	1620	1541	1828	2051	2349	1846	1800	2936
ENSECAG00000023125	6.935277614	0.891776583	0.948812205	1155	1304	2566	3491	2712	2121	2955	2851
ENSECAG00000024639	4.106662029	0.89210341	0.949038732	220	255	298	360	429	307	317	436
ENSECAG00000012838	5.306005909	0.892156548	0.949038732	715	733	230	910	779	989	643	862
ENSECAG00000010237	4.761135625	0.892320659	0.949124446	160	467	384	917	474	587	567	718
ENSECAG00000009870	1.784705747	0.892588328	0.949320283	20	49	75	102	48	70	82	78
ENSECAG00000006808	6.118721239	0.892784347	0.949439887	883	1218	1328	1248	1311	1374	1401	1767
ENSECAG00000022844	4.272511374	0.893156201	0.949600521	214	268	373	443	353	361	370	597
ENSECAG00000007118	4.66446051	0.893168261	0.949600521	231	520	416	596	524	385	590	643
ENSECAG00000010167	2.713883509	0.89323457	0.949600521	73	127	97	128	151	136	139	132
ENSECAG00000023381	4.059278276	0.893269703	0.949600521	130	297	361	386	357	258	368	420
ENSECAG00000008480	5.187134862	0.893998366	0.950286223	535	428	682	736	873	696	736	838
ENSECAG00000008704	5.360978468	0.894713594	0.950947687	293	700	599	1300	812	818	871	1058
ENSECAG00000009199	0.751366848	0.894854765	0.950947687	18	10	33	47	30	40	30	37
ENSECAG00000023324	5.648741941	0.894871737	0.950947687	611	504	1181	1073	1084	875	1030	1377
ENSECAG00000023089	4.00945089	0.895132961	0.951136323	187	191	372	364	391	225	345	396
ENSECAG00000007070	3.954221222	0.895289268	0.951177873	171	239	291	332	317	295	326	399
ENSECAG00000009424	3.534934402	0.895339496	0.951177873	144	126	221	314	230	217	267	250
ENSECAG00000023220	4.250465437	0.895455285	0.951211944	102	238	257	793	418	472	400	332
ENSECAG00000000762	4.850237834	0.895762438	0.951449268	460	301	406	798	707	454	672	586
ENSECAG00000016102	8.30770696	0.896137292	0.951758451	3836	4817	6317	5943	6381	7024	6253	7747
ENSECAG00000020625	7.53275375	0.89622124	0.951758643	2075	4949	1887	2972	3766	4196	4604	3349
ENSECAG00000014448	2.38841952	0.896384657	0.951820885	58	71	71	149	117	80	115	138
ENSECAG00000010570	1.34242936	0.896461492	0.951820885	18	29	62	59	70	42	53	46
ENSECAG00000015487	8.79992651	0.896649729	0.951820885	5906	10131	6833	6577	8751	8491	10515	9550
ENSECAG00000020843	2.455101421	0.896697702	0.951820885	50	104	71	140	84	97	126	165
ENSECAG00000012500	5.548014675	0.896698711	0.951820885	366	808	902	1150	964	1054	1022	969
ENSECAG00000008548	11.04566943	0.896850495	0.951893071	20628	29220	48364	51367	48464	34923	48190	46199
ENSECAG00000013091	7.8060378	0.897009111	0.951970283	1884	2331	3026	9570	4760	4670	5200	3923

ENSECAG00000015440	6.668049026	0.897090813	0.951970283	2178	1612	1296	877	2396	1436	4316	610
ENSECAG00000018441	5.377624355	0.897604912	0.95237684	387	608	912	1023	972	775	749	1004
ENSECAG00000011612	10.81487076	0.897641575	0.95237684	20640	29187	31567	38636	33784	38303	31266	53754
ENSECAG00000021012	3.291498297	0.897794688	0.95245035	53	166	122	388	121	272.002	204	200
ENSECAG00000022749	2.298193323	0.898019789	0.952481087	96	57	89	51	151	47	113	115
ENSECAG00000015693	4.780285101	0.898055652	0.952481087	317	427	597	534	514	435	644	734
ENSECAG00000012870	1.350646075	0.898075153	0.952481087	21	31	71	52	42	83	21	53
ENSECAG00000022642	2.586066315	0.898275118	0.952542582	33	96	154	146	99	135	129	127
ENSECAG00000010814	4.654503854	0.898300807	0.952542582	335	332	501	548	363	685	354	696
ENSECAG00000000066	8.749967582	0.89846733	0.952630255	4870	4865	9194	10262	10782	7710	9117	9768
ENSECAG00000012403	4.467081494	0.898830232	0.95288846	184	421	308	603	333	443	399	756
ENSECAG00000001911	7.219905059	0.898880661	0.95288846	2272	2195	2763	2685	3329	2589	3014	3644
ENSECAG00000012348	-0.020180902	0.899024937	0.95288846	8	6	25	26	20	24	13	14
ENSECAG00000011084	1.687070455	0.899114925	0.95288846	35	33	57	88	64	55	71	82
ENSECAG00000026923	2.258410913	0.899130187	0.95288846	20	76	111	141	63	83	134	111
ENSECAG00000019204	1.904073524	0.899270579	0.95294836	40	29	141	27	73	49	129	68
ENSECAG00000021146	6.875388468	0.899499228	0.953101765	2401	1610	1994	1392	2868	1987	2459	2541
ENSECAG00000013581	9.242409794	0.899904701	0.953442486	7080	15596	7393	10431	12322	11084	12334	15252
ENSECAG00000006280	6.850537645	0.90012186	0.953521439	1445	2080	2025	2258	2643	1893	2423	2792
ENSECAG00000017980	6.890764649	0.900147066	0.953521439	1449	2148	1963	2520	2480	2560	2306	2580
ENSECAG00000018000	6.420729123	0.900668686	0.953985048	1023	1333	1235	2370	1839	1270	2149	1989
ENSECAG00000016487	6.638759158	0.900957142	0.954117346	1989	1703	1163	1149	2259	1727	2528	2128
ENSECAG00000013200	4.951275987	0.900961539	0.954117346	331	405	588	852	692	530	706	673
ENSECAG00000014476	4.772135283	0.901128051	0.954204746	257	423	569	668	654	404	632	615
ENSECAG00000013103	0.622781943	0.901807668	0.954835404	13	22	20	43	35	27	34	29
ENSECAG00000004513	6.271105127	0.901983642	0.954932738	844	1351	1123	2060	1440	1320	1592	2207
ENSECAG00000024608	8.300873531	0.902394765	0.955190631	5307	5296	3436	5894	7515	5785	7051	6993
ENSECAG00000013147	3.178897294	0.902452103	0.955190631	153	147	124	163	136	151	196	279
ENSECAG00000003755	3.365017354	0.902479442	0.955190631	132	152	170	247	246	167	214	234
ENSECAG00000013002	4.737625346	0.902767827	0.95540686	323	498	395	595	529	508	611	590
ENSECAG00000019783	4.988719146	0.903288153	0.955868492	268	512	558	858	578	661	702	795
ENSECAG00000006469	5.759412252	0.904645058	0.957004407	726	606	1189	1184	1468	886	1200	990
ENSECAG00000022979	4.777949855	0.904679833	0.957004407	190	550	415	746	507	440	743	691
ENSECAG00000009962	5.822723885	0.904692852	0.957004407	603	808	878	1715	1144	1206	1074	1326
ENSECAG00000018882	4.510186645	0.904698499	0.957004407	222	343	438	548	536	395	429	614
ENSECAG00000009482	6.883291627	0.904902282	0.957130862	925	1785	2009	3873	2263	2293	2168	3245
ENSECAG00000017247	3.62092426	0.905416487	0.957529803	141	169	246	263	304	227	241	284
ENSECAG00000003257	3.452030273	0.905576127	0.957529803	114	107	333	205	234	127	252	312
ENSECAG00000023040	5.186350863	0.905621204	0.957529803	462	626	615	683	747	681	901	805
ENSECAG00000018499	4.76178885	0.905628403	0.957529803	314	469	523	537	633	408	613	639
ENSECAG00000024371	1.590505177	0.905700827	0.957529803	46	75	17	40	62	17	91	92
ENSECAG00000020380	1.550578632	0.905857622	0.957599126	27	43	73	54	77	50	48	62
ENSECAG00000020865	5.495946207	0.905934959	0.957599126	517	572	800	1183	1058	802	869	1181
ENSECAG00000011182	4.844752533	0.906027012	0.95760734	353	579	368	643	564	487	582	802
ENSECAG00000004124	3.477395254	0.906331872	0.957802487	98	161	215	288	225	150	288	304
ENSECAG00000008928	10.40974081	0.906380245	0.957802487	14718	16602	32084	32096	31219	22670	29207	31657
ENSECAG00000024651	5.726290924	0.906826334	0.958100135	672	594	1334	1015	1099	990	1102	1271
ENSECAG00000006659	4.883266358	0.906830563	0.958100135	369	519	566	465	584	661	601	684
ENSECAG00000015615	6.726443104	0.907009401	0.958199983	1086	1666	2222	2397	2186	1808	1996	3006
ENSECAG00000016591	2.203136504	0.907351227	0.958417366	48	68	95	104	148	37	120	74
ENSECAG00000018612	1.34033402	0.907383876	0.958417366	31	49	40	36	58	42	64	47
ENSECAG00000019044	5.087511912	0.907697821	0.958659847	228	448	790	1012	730	739	691	672
ENSECAG00000017299	0.883159954	0.907917958	0.958803219	3	33	36	60	28	47	48	17
ENSECAG00000006568	4.709414753	0.90811266	0.958919707	251	403	492	696	745	555	444	426
ENSECAG00000021345	2.503427115	0.908335931	0.959065826	90	91	88	83	138	90	154	100
ENSECAG00000003512	4.601599796	0.908445661	0.959065826	217	492	450	520	385	516	537	598
ENSECAG00000014573	0.257748457	0.908504268	0.959065826	16	14	18	23	23	24	14	35
ENSECAG00000014598	2.393545055	0.908791352	0.95927976	48	83	100	119	121	90	103	135
ENSECAG00000019923	6.838805605	0.909099399	0.95951578	1500	2031	1423	2580	2926	2385	2647	1831
ENSECAG00000017571	5.591062609	0.909221019	0.959555008	884	729	691	688	1015	1102	999	1012
ENSECAG00000019258	4.563373945	0.909458341	0.959716325	270	318	485	514	507	439	534	555
ENSECAG00000020477	5.121874436	0.909779505	0.959936704	331	480	682	905	733	784	754	703
ENSECAG00000007125	8.007823808	0.909906729	0.959936704	3220	3743	4817	5904	5319	4956	5408	5983
ENSECAG00000020465	6.168860669	0.909920639	0.959936704	882	1065	1200	1666	1593	990	1666	2041
ENSECAG00000025104	9.749751715	0.910383719	0.960336071	14517	12667	13389	13822	21476	15745	18529	18778
ENSECAG00000018910	5.016133497	0.910641056	0.960498848	279	411	684	948	795	621	682	606
ENSECAG00000024140	0.64414597	0.910707102	0.960498848	15	21	21	42	19	49	25	32
ENSECAG00000006305	1.499394739	0.910963695	0.960680295	18	39	60	73	54	82	55	40
ENSECAG00000024884	4.695623885	0.911224116	0.960787935	223	469	345	810	460	453	509	776
ENSECAG00000011992	6.317967542	0.911419977	0.960787935	1035	1496	1294	1521	1641	1489	1764	1832
ENSECAG00000005556	6.020106146	0.911465857	0.960787935	400	1118	1240	1813	1133	1115	1164	2292
ENSECAG00000022334	7.133141451	0.911484022	0.960787935	1851	2573	2614	2325	2888	2549	2873	3556
ENSECAG00000019087	5.011918555	0.91149248	0.960787935	324	504	688	726	542	891	500	748
ENSECAG00000022675	7.028004705	0.911575545	0.960787935	1196	1757	2969	3050	2704	2464	2624	3545
ENSECAG00000013775	5.199129228	0.911657695	0.960787935	335	605	643	939	665	728	813	966
ENSECAG00000020954	4.483403961	0.912006231	0.961020299	249	370	422	448	448	421	507	550
ENSECAG00000011291	8.292560412	0.912079763	0.961020299	4099	4675	5121	6860	8082	4934	7692	6451
ENSECAG00000006458	2.60423656	0.912131922	0.961020299	79	111	128	79	142	88	133	143
ENSECAG00000024447	3.646061032	0.912342238	0.961040195	98	266	159	362	211	182	190	487
ENSECAG00000017400	5.638808726	0.912407924	0.961040195	516	457	1124	1353	1171	1224	1122	705
ENSECAG00000000298	6.545289883	0.912427184	0.961040195	1168	1463	1377	2423	1888	1795	1825	2384
ENSECAG00000021380	7.833933493	0.912550904	0.961040195	2527	3287	4100	6071	4789	4260	4536	5681
ENSECAG00000022307	5.852858791	0.912573725	0.961040195	1053	1036	742	719	1329	1002	1410	1252
ENSECAG00000021184	6.878886868	0.912688833	0.961072337	1106	1571	3115	2475	2282	1584	2139	4114
ENSECAG00000013897	6.998327107	0.912908527	0.96119761	1640	2051	2261	2739	3015	2228	3029	2468

ENSECAG00000020230	5.178861453	0.913139352	0.96119761	347	732	562	868	647	781	733	882
ENSECAG00000011951	5.741405702	0.913144558	0.96119761	879	569	1148	944	1640	859	1012	979
ENSECAG00000015783	3.895020945	0.91314619	0.96119761	164	192	274	378	352	318	306	291
ENSECAG00000014187	0.821393561	0.913282583	0.961252127	3	29	8	91	28	29	35	46
ENSECAG00000008886	0.666750961	0.913443081	0.961332001	9	24	20	51	20	27	32	52
ENSECAG00000019782	8.539899828	0.913669993	0.961407727	4796	5801	7240	7428	7069	8305	6252	9684
ENSECAG00000022238	0.61490157	0.913684267	0.961407727	5	25	23	49	36	24	39	24
ENSECAG00000007266	2.95740762	0.914356065	0.961911069	72	93	127	265	196	166	164	108
ENSECAG00000022057	2.612063395	0.914421697	0.961911069	69	61	155	135	111	108	167	118
ENSECAG00000006736	3.111170519	0.914445197	0.961911069	93	114	173	199	155	178	195	210
ENSECAG00000014872	3.212904283	0.914501264	0.961911069	77	179	130	244	181	161	204	253
ENSECAG00000013813	3.3861007	0.914832176	0.962139177	84	193	170	290	240	150	238	250
ENSECAG00000025095	3.370031943	0.91488749	0.962139177	94	157	234	211	212	165	236	280
ENSECAG00000020281	7.052356198	0.915099273	0.962266231	2444	2416	1590	1685	3045	2130	3410	2932
ENSECAG00000011235	5.848155996	0.915333348	0.962266231	816	957	844	1243	1318	1144	1179	1195
ENSECAG00000021824	4.555401658	0.915336536	0.962266231	383	286	445	436	472	459	503	540
ENSECAG00000018674	0.317574038	0.915347071	0.962266231	14	10	22	31	19	22	38	20
ENSECAG00000002321	5.936359731	0.915551879	0.962392492	1144	689	1005	1013	1610	989	1231	1480
ENSECAG00000009487	6.304288922	0.915644286	0.96240059	987	1422	1251	1691	1686	1288	1802	1923
ENSECAG00000009537	3.600567388	0.915985423	0.962670093	140	151	216	311	286	194	333	225
ENSECAG00000010552	6.677696623	0.916227336	0.962777079	872	795	1677	4069	2362	2271	2570	1535
ENSECAG00000022209	8.3467191	0.916286687	0.962777079	3088	4800	6354	8863	6320	7013	6091	7945
ENSECAG00000021606	5.270649796	0.91634143	0.962777079	294	672	607	1124	635	925	767	987
ENSECAG00000021450	0.280339322	0.91644412	0.96279594	9	10	22	41	18	32	18	23
ENSECAG00000011061	4.141611848	0.91664314	0.962854828	289	260	279	290	303	434	306	469
ENSECAG00000011558	2.199949101	0.916744151	0.962854828	24	49	88	165	119	66	116	89
ENSECAG00000008391	5.035435654	0.916886535	0.962854828	427	410	422	1028	705	606	631	826
ENSECAG00000004462	2.90011026	0.916894403	0.962854828	57	130	190	119	239	60	247	94
ENSECAG00000008960	6.381361341	0.91692389	0.962854828	894	1769	1030	1868	1657	1604	1758	2198
ENSECAG00000001304	1.513601368	0.917101156	0.962951976	32	45	34.0016	82	62	43	53.0009	75
ENSECAG00000011706	4.890108112	0.917347151	0.963121265	359	474	519	670	686	529	658	621
ENSECAG00000014533	5.752553222	0.917440868	0.963130661	620	1298	807	748	951	1323	1006	1346
ENSECAG00000005988	5.104597075	0.917670496	0.963282721	421	673	552	600	625	618	775	964
ENSECAG00000015448	6.659977887	0.917939454	0.963476035	1049	2499	1402	1878	1925	2012	2275	2292
ENSECAG00000022614	4.090971209	0.91821465	0.963675859	200	302	312	337	372	303	395	359
ENSECAG00000017691	6.689124295	0.918816542	0.964146044	1105	1489	1872	2769	2261	1749	2488	2208
ENSECAG00000013794	1.302847874	0.918832368	0.964146044	10	61	27	66	38	48	54	66
ENSECAG00000018660	3.074100599	0.919207999	0.96445113	64	115	176	247	193	175	165	162
ENSECAG00000010149	7.16754659	0.919377607	0.964478808	1680	3012	1893	2877	2991	3277	2375	3758
ENSECAG00000016571	4.234284604	0.919404152	0.964478808	145	394	335	403	322	383	377	543
ENSECAG00000014800	7.215880054	0.919638284	0.964635357	1595	3396	1862	2957	3542	2840	3967	2387
ENSECAG00000009826	4.226297467	0.919851328	0.96472532	168	331	369	434	350	345	405	478
ENSECAG00000025049	3.720153951	0.919893867	0.96472532	161	289	212	208	318	197	296	297
ENSECAG00000024881	2.260575607	0.920226348	0.964954958	21	52	65	225	97	96	82	117
ENSECAG00000019921	3.088266958	0.92028269	0.964954958	90	147	115	217	202	213	117	188
ENSECAG00000012713	5.883968626	0.920381487	0.964969499	523	1131	1027	1447	1124	1152	1196	1522
ENSECAG00000017442	4.384481132	0.920582015	0.964993908	162	387	452	420	361	447	425	564
ENSECAG00000018023	0.441407401	0.920637117	0.964993908	17	16	30	23	37	13	23	33
ENSECAG00000000623	6.838151598	0.920659564	0.964993908	655	1858	1694	4311	2585	1952	2641	2452
ENSECAG00000016462	3.416071465	0.921028378	0.965222332	86	181	164	305	232	161	221	311
ENSECAG00000013900	2.195658131	0.921070608	0.965222332	25	96	63	142	69	78	108	123
ENSECAG00000019884	3.339656623	0.921199249	0.965222332	57	86	205	388	314	193	195	158
ENSECAG00000019447	3.684106379	0.921274557	0.965222332	128	215	240	280	197	319	279	298
ENSECAG00000013966	5.161292287	0.92138977	0.965222332	331	600	695	811	623	640	602	1265
ENSECAG00000021658	7.283438482	0.921574744	0.965222332	1676	2552	2596	4034	3400	2492	3835	3447
ENSECAG00000006357	4.17274804	0.921613576	0.965222332	164	294	424	332	278	352	469	452
ENSECAG00000008071	2.365460758	0.921626471	0.965222332	118	35	78	92	121	109	110	79
ENSECAG00000020337	8.720134381	0.92164206	0.965222332	7014	7169	6163	7117	10297	7294	9024	8907
ENSECAG00000009947	4.121198495	0.921985753	0.965410025	284	241	304	274	530	245	426	292
ENSECAG00000023021	8.205744708	0.922056151	0.965410025	2761	3957	6781	7411	6416	5443	6148	6910
ENSECAG00000022920	6.27824569	0.922144279	0.965410025	928	745	1931	1792	1949	1226	1383	2037
ENSECAG00000018651	2.309047085	0.922161151	0.965410025	30	69	84	164	99	94	110	117
ENSECAG00000023073	5.173110877	0.922418141	0.965544874	416	588	599	816	884	711	668	835
ENSECAG00000002656	0.343163728	0.92245992	0.965544874	5	28	23	28	20	26	35	14
ENSECAG00000021225	7.416058039	0.922742832	0.965723993	1304	1542	4021	5344	3629	3363	3014	4840
ENSECAG00000013328	1.343383527	0.922801038	0.965723993	29	21	30	97	46	40	82	34
ENSECAG00000024212	6.567475305	0.922964984	0.965806608	1188	1663	1421	2003	2101	2174	1652	2205
ENSECAG00000016471	4.792566632	0.923132217	0.965888688	227	493	470	774	468	678	528	641
ENSECAG00000007955	9.276443712	0.923213444	0.965888688	9425	11089	10546	9556	13620	11982	11637	15093
ENSECAG00000020445	5.192565878	0.923471757	0.966069985	484	694	598	589	869	655	912	698
ENSECAG00000016703	0.286189444	0.923776568	0.966299886	23	12	22	16	11	17	17	51
ENSECAG00000012593	3.405280516	0.923891511	0.966322386	100	134	268	237	162	242	163	320
ENSECAG00000009711	4.996453492	0.923968175	0.966322386	281	461	588	889	555	535	668	1025
ENSECAG00000018271	6.229221282	0.924093457	0.96636446	541	1173	1401	2175	1630	1420	1616	1806
ENSECAG00000019624	7.721143475	0.924246595	0.966435653	3504	3753	3421	2926	4715	3832	5071	4106
ENSECAG00000009673	7.353667859	0.924627926	0.966699059	1549	2370	3279	4385	4314	2299	3052	4268
ENSECAG00000005033	2.863960955	0.924677183	0.966699059	111	144	56	148	118	92	123	309
ENSECAG00000020369	5.737921201	0.92479504	0.966699059	193	790	1132	1907	878	1191	1352	1022
ENSECAG00000021925	3.175016715	0.924966408	0.966699059	78	129	166	248	170	192	190	218
ENSECAG00000011277	4.726023258	0.924978806	0.966699059	381	434	515	410	688	456	553	527
ENSECAG00000007191	3.174610957	0.925008993	0.966699059	71	169	124	256	193	150	164	273
ENSECAG00000014299	2.79654691	0.925272837	0.966885861	62	79	124	216	115	125	126	234
ENSECAG00000024085	2.314224804	0.9254606	0.96694871	49	51	103	134	110	102	106	101
ENSECAG00000019479	6.307195127	0.925503188	0.96694871	1053	1332	1128	1847	2011	1403	1627	1634
ENSECAG00000024338	1.21910117	0.925694653	0.967059824	11	47	31	67	31	39	62	62

ENSECAG00000021750	5.90133364	0.925962239	0.967248343	424	958	317	2817	818	1795	300	2085
ENSECAG00000017511	4.12083892	0.926045368	0.967248343	148	291	223	584	392	286	310	485
ENSECAG00000017573	5.097954698	0.926260121	0.96734185	434	569	558	700	673	465	995	837
ENSECAG00000013380	2.646053795	0.926341344	0.96734185	63	83	149	135	113	142	98	165
ENSECAG00000003959	1.878312105	0.926493406	0.96734185	23	29	71	137	81	65	101	60
ENSECAG00000012609	5.069858031	0.926531228	0.96734185	422	458	741	600	724	638	760	762
ENSECAG00000014263	0.449028693	0.926632119	0.96734185	5	13	31	42	25	26	33	24
ENSECAG00000018469	1.479430401	0.926645722	0.96734185	31	49	58	44	65	48	53	60
ENSECAG00000008695	5.861324694	0.92733215	0.967951736	1107	802	990	794	1217	997	1226	1490
ENSECAG00000007634	3.836787154	0.927400334	0.967951736	168	295	184	289	285	213	307	437
ENSECAG00000004879	4.305538475	0.927911466	0.96839626	233	353	435	299	485	324	455	398
ENSECAG00000008993	9.335103699	0.928040526	0.968441996	7016	15047	7559	14391	12850	15736	14144	10893
ENSECAG00000010536	0.864590607	0.928233709	0.968490132	14	10	51	45	31	42	24	52
ENSECAG00000019110	7.170981099	0.928257132	0.968490132	2117	2567	1959	2927	3016	2621	2996	3568
ENSECAG00000019135	0.54552726	0.928444422	0.968596596	23	12	32	24	23	27	20	45
ENSECAG00000027695	9.625766747	0.92853123	0.968598222	14342	8813	17615	10193	17626	15499	16577	16656
ENSECAG00000019174	2.955846139	0.928747833	0.968735231	83	123	147	177	160	158	156	170
ENSECAG00000009694	6.304203849	0.929181363	0.969098462	943	815	1424	2390	1423	1334	1507	2494
ENSECAG00000010520	3.837234009	0.929274429	0.969106569	129	320	218	286	296	244	315	377
ENSECAG00000021588	5.267025063	0.929495043	0.969247676	310	644	572	1172	657	966	664	1011
ENSECAG00000015019	5.835418181	0.929713594	0.969322097	844	1031	808	1083	1585	915	1181	1138
ENSECAG00000022391	5.230477939	0.929737037	0.969322097	395	434	797	1016	785	781	725	868
ENSECAG00000004034	4.452620086	0.929863708	0.969365212	541	282	259	240	636	169	704	335
ENSECAG00000007715	6.634660728	0.93003072	0.969374544	1162	1963	1491	1952	2004	1873	1962	2770
ENSECAG00000018881	0.024164693	0.930087982	0.969374544	11	7	22	25	18	19	14	25
ENSECAG00000008502	8.721938068	0.930191904	0.969374544	4735	6113	8653	8940	9386	8290	8318	10481
ENSECAG00000021209	5.507912104	0.93029291	0.969374544	589	571	776	1124	1312	757	995	837
ENSECAG00000013640	3.469047517	0.930299245	0.969374544	76	155	176	379	175	205	216	363
ENSECAG00000014734	2.385277241	0.930485631	0.969479848	44	80	109	130	74	97	99	166
ENSECAG00000012718	4.615858221	0.931078743	0.970008865	318	452	375	455	506	609	482	482
ENSECAG00000014315	1.889485578	0.93128905	0.970033942	73	90	20	28	147	97	13	52
ENSECAG00000022402	2.406901199	0.931341868	0.970033942	62	59	101	143	117	124	109	81
ENSECAG00000022552	4.467569128	0.93135894	0.970033942	248	315	479	440	456	498	468	461
ENSECAG00000017754	4.424224958	0.931507668	0.970078957	250	512	267	400	367	384	420	654
ENSECAG00000015464	9.266811944	0.931572918	0.970078957	6742	7697	13352	15327	15320	9754	13771	13275
ENSECAG00000009477	7.926104846	0.931745636	0.970133722	2295	4184	4464	6103	4794	4986	4604	6172
ENSECAG00000009179	0.552374409	0.931907994	0.970133722	14	6	64	5	42	25	23	29
ENSECAG00000013062	10.48775288	0.931936648	0.970133722	16240	20005	23510	41045	34668	25534	24111	37565
ENSECAG00000004574	1.853353902	0.931999119	0.970133722	38	50	61	90	79	89	68	63
ENSECAG00000023800	5.40292506	0.93207559	0.970133722	605	824	534	764	783	843	910	1110
ENSECAG00000019640	6.651192574	0.932137814	0.970133722	1071	1115	2443	2238	2475	1725	2529	1906
ENSECAG00000022532	6.660562066	0.932411582	0.970245408	996	2014	1607	2376	2141	2208	2427	1663
ENSECAG00000019967	4.445327792	0.932425368	0.970245408	177	389	364	611	400	411.001	415	620
ENSECAG00000020568	6.108542291	0.932526316	0.970245408	769	1262	1166	1411	1290	1505	1433	1694
ENSECAG00000012417	4.357490969	0.932586701	0.970245408	208	242	315	702	461	384	508	359
ENSECAG00000008395	6.317614741	0.932759196	0.970247951	948	968	1401	2130	1804	1402	1564	2135
ENSECAG00000023023	0.505049352	0.932759934	0.970247951	16	17	29	30	32	47	12	15
ENSECAG00000008321	2.402832655	0.933031307	0.970265835	45	55	133	139	110	119	106	97
ENSECAG00000010450	4.412325391	0.933046073	0.970265835	260	243	582	358	605	363	462	350
ENSECAG00000005217	5.860692617	0.933060689	0.970265835	516	736	1286	1601	1126	998	1278	1538
ENSECAG00000024939	3.143806289	0.93311871	0.970265835	63	120	260	182	110	413	40	135
ENSECAG00000019055	2.149837637	0.933279262	0.970327618	33	51	72	154	104	57	87	132
ENSECAG00000018791	3.421234443	0.93334893	0.970327618	100	197	206	233	202	191	227	281
ENSECAG00000018119	6.300871657	0.933503861	0.970399896	1055	1401	1116	1726	1610	1363	1507	2242
ENSECAG00000010363	9.301084309	0.933768973	0.970490572	7897	9542	11103	14629	12640	12199	13503	14972
ENSECAG00000011242	5.377677899	0.93396507	0.970490572	511	590	685	1015	940	770	978	875
ENSECAG00000021714	3.821060981	0.933991748	0.970490572	116	138	343	421	292	205	258	449
ENSECAG00000000637	3.949671032	0.934069987	0.970490572	127	188	391	391	210	308	277	516
ENSECAG00000008291	8.028200816	0.934102799	0.970490572	3067	3492	3806	7987	5505	4414	5674	6573
ENSECAG00000024650	2.357306838	0.934172237	0.970490572	65	80	70	119	112	111	78	133
ENSECAG00000023471	1.175676354	0.934231309	0.970490572	14	53	41	43	36	40	51	54
ENSECAG00000002701	7.056571574	0.934327518	0.970490572	2467	1525	1925	2787	3030	2477	3456	2183
ENSECAG00000020907	4.741380978	0.934421213	0.970490572	327	455	446	582	542	480	597	643
ENSECAG00000011382	6.146949984	0.934445244	0.970490572	956	1777	1013	825	1489	1082	1807	1631
ENSECAG00000000376	1.936709195	0.934633599	0.970494254	52	52	72	76	94	59	88	73
ENSECAG00000022628	5.715046222	0.934636093	0.970494254	661	1039	816	991	1052	890	1017	1524
ENSECAG00000010579	0.719694734	0.934705036	0.970494254	18	38	16	33	41	24	37	27
ENSECAG00000025134	5.708857971	0.935402676	0.971082556	542	961	834	1198	902	1114	1117	1363
ENSECAG00000020636	5.059115111	0.935514699	0.971082556	246	629	523	927	747	564	732	834
ENSECAG00000009117	7.939507623	0.935749382	0.971082556	3169	3717	4056	5395	6193	3979	5265	5807
ENSECAG00000025075	5.177262345	0.93576188	0.971082556	487	482	772	638	794	582	890	854
ENSECAG00000000590	5.327891517	0.935765474	0.971082556	432	524	847	935	930	728	873	920
ENSECAG00000021003	3.106918147	0.935784449	0.971082556	57	263	79	171	144	151	163	289
ENSECAG00000004824	6.30949783	0.935872319	0.971085049	1231	1139	1452	1406	2510	1262	1489	1421
ENSECAG00000003748	1.490758841	0.936386486	0.97135676	18	53	63	50	39	69	40	87
ENSECAG00000010974	2.6660574	0.936399835	0.97135676	73	35	140	201	106	187	121	99
ENSECAG00000021295	7.261699839	0.936460084	0.97135676	1862	2244	1810	4828	3185	2280	3403	4237
ENSECAG00000020128	4.768541022	0.936476144	0.97135676	263	531	607	449	561	673	476	563
ENSECAG00000019385	6.142194879	0.936784401	0.971413606	937	1328	1143	1286	1690	1200	1515	1576
ENSECAG00000003090	4.598608996	0.936796386	0.971413606	234	147	775	515	890	317	572	294
ENSECAG00000020461	3.610980015	0.937106489	0.971413606	178	128	150	362	197	224	266	368
ENSECAG00000021982	5.735664399	0.937157209	0.971413606	729	654	983	1259	1443	853	1148	1058
ENSECAG00000024978	2.114261642	0.937202534	0.971413606	58	54	55	117	67	81	76	146
ENSECAG00000017914	0.815598728	0.937205251	0.971413606	16	17	45	35	38	37	25	43
ENSECAG00000008421	3.778736591	0.937211905	0.971413606	146	174	281	354	292	300	328	217

ENSECAG00000011704	1.485145925	0.937214923	0.971413606	16	78	43	42	20	161	10	29
ENSECAG00000023288	7.084889211	0.93745963	0.971489435	1451	2083	2541	3147	3221	2336	3086	3062
ENSECAG00000020309	6.584592932	0.937579999	0.971489435	1137	792	2379	2248	2499	2017	1517	2207
ENSECAG00000024246	6.846424381	0.937615701	0.971489435	1492.99	1972	1910.99	2370.99	2528	2054.99	2562	2591.99
ENSECAG00000022271	2.374251438	0.937630096	0.971489435	37	115	53	158	72	108	99	151
ENSECAG00000024859	0.801170643	0.93774473	0.971519615	29	27	21	31	41	27	45	24
ENSECAG00000002570	2.953502166	0.938154665	0.971833596	24	136	74	354	92	232	100	211
ENSECAG00000012723	6.200731865	0.938218862	0.971833596	901	1108	1264	1762	1626	1526	1423	1623
ENSECAG00000018586	4.311358269	0.938316269	0.971845893	260	276	337	447	359	376	510	455
ENSECAG00000022027	3.711787002	0.938692301	0.972146743	126	277	183	312	297	243	241	321
ENSECAG00000000698	2.084886741	0.939130092	0.972511493	35	71	67	113	59	137	72	82
ENSECAG00000018980	8.623261613	0.939268278	0.972565951	3565	6693	7062	9830	7428	8149	7588	10935
ENSECAG00000020449	6.601064721	0.939421878	0.972636357	1612	1549	1193	1861	1788	2237	1900	2400
ENSECAG00000001166	5.81767005	0.939529354	0.972658999	720	687	1102	1281	1380	777	1148	1605
ENSECAG00000024560	5.985973271	0.939735121	0.972690385	821	721	1356	1349	1469	1115	1415	1455
ENSECAG00000020618	4.277716591	0.939744827	0.972690385	164	363	285	524	314	418	549	367
ENSECAG00000020025	2.948795527	0.939816498	0.972690385	85	99	201	138	183	83	130	263
ENSECAG00000018333	9.129339454	0.940089481	0.972884296	9913	11652	7065	6685	14190	8687	14057	10279
ENSECAG00000018768	4.649979988	0.940254368	0.972949329	220	408	347	778	555	341	551	738
ENSECAG00000000390	6.017856878	0.940323586	0.972949329	610	838	1350	1796	1287	1571	1006	1583
ENSECAG00000014550	5.963543001	0.940419626	0.972960098	838.001	968	1184	1186	1176	1399	1281	1393
ENSECAG00000013270	4.260356214	0.940629694	0.973088827	221	471	241	305	506	225	463	467
ENSECAG00000007602	5.105362607	0.94130562	0.973699422	432	480	585	861	944	519	767	680
ENSECAG00000007619	2.779921921	0.941462285	0.973758993	47	103	155	181	168	116	169	115
ENSECAG00000022315	4.283625806	0.941534615	0.973758993	325	394	201	296	446	290	472	473
ENSECAG00000014020	0.385229669	0.941829203	0.973922143	16	21	24	20	23	18	28	33
ENSECAG00000015079	8.070346846	0.941863801	0.973922143	3929	4926	3700	5222	5891	4417	6457	6024
ENSECAG00000014380	5.288429342	0.942330115	0.974188821	292	482	737	1285	788	766	771	1042
ENSECAG00000003669	7.829207978	0.942350227	0.974188821	1941	3306	4272	6395	5125	5459	5619	3026
ENSECAG00000016797	5.244155687	0.942383315	0.974188821	449	643	700	725	832	692	727	1017
ENSECAG00000003179	3.667880396	0.942464665	0.974188821	116	197	180	388	230	205	300	362
ENSECAG00000010551	7.093261728	0.94257338	0.974212567	2221	2814	1661	1851	3049	2370	3194	3173
ENSECAG00000012309	4.704489812	0.942764861	0.974254521	274	356	487	704	541	560	455	641
ENSECAG00000013658	3.624956392	0.942785465	0.974254521	89	322	135	309	201	244	248	346
ENSECAG00000007824	6.652197574	0.943481254	0.974801789	1053.01	1438	1709	2870	2350	1585	2386	2213
ENSECAG00000010375	6.963260262	0.943548798	0.974801789	1865	2259	2071	1956	2781	1747	2782	3364
ENSECAG00000000605	2.449934969	0.943583814	0.974801789	61	91	95	112	118	85	133	128
ENSECAG00000023041	4.899587094	0.943658236	0.974801789	221	638	478	758	531	534	585	894
ENSECAG00000011968	9.224464176	0.943814627	0.974874709	6858	6909	15197	11343	11724	9947	9816	20988
ENSECAG00000019089	6.263339572	0.943973968	0.974950662	610	1093	1453	2269	1399	1568	1692	1948
ENSECAG00000014652	8.618412338	0.944422331	0.975216409	3694	5603	7333	10662	8046	10122	8690	6410
ENSECAG00000015838	6.989727266	0.944487514	0.975216409	1567	2238	2075	2720	2738	2375	2617	3041
ENSECAG00000017858	6.026396028	0.944540833	0.975216409	806	1076	1029	1441	1477	1006	1480	1677
ENSECAG00000005572	7.662076203	0.944593607	0.975216409	2654	3686	3285	3629	4123	3908	4296	5142
ENSECAG00000006737	7.067276099	0.944660429	0.975216409	1649	2401	2374	2606	3079	2222	2866	3233
ENSECAG00000007294	6.265485145	0.944849299	0.975303056	835	1145	1428	1927	1470	1607	1570	1857
ENSECAG00000023437	6.268427757	0.944932914	0.975303056	682	1571	1286	1810	1490	1537	1818	1657
ENSECAG00000016472	5.454490404	0.945028006	0.975303056	402	994	699	844	778	871	856	1282
ENSECAG00000013884	0.478377704	0.945087718	0.975303056	13	18	27	29	31	19	25	38
ENSECAG00000017819	6.304176489	0.94564108	0.975785483	1025	1181	1455	1680	1473	1504	1867	1849
ENSECAG00000016802	1.651179814	0.945824435	0.975886054	28	54	51	74	58	97	73	26
ENSECAG00000012677	8.839544126	0.945982631	0.975960652	5667	6865	9222	9376	9622	9315	9003	10785
ENSECAG00000000184	4.788312069	0.946269384	0.976080575	353	417	524	535	559	508	604	704
ENSECAG00000000541	1.759292035	0.946270686	0.976080575	26	66	45	89	56	69	43	119
ENSECAG00000020237	6.191538922	0.946410698	0.976123045	797	1090	1389	1780	1499	1404	1648	1629
ENSECAG00000012444	3.131319166	0.946483682	0.976123045	61	143	148	274	170	164	191	208
ENSECAG00000011387	5.606068566	0.946586735	0.976140723	605	692	948	1067	1034	868	1059	1173
ENSECAG00000024877	5.867285069	0.946766945	0.976237955	642	970	1205	1091	1251	1066	1254	1453
ENSECAG00000001495	3.550104431	0.946856782	0.976239232	86	268	133	316	207	248	284	257
ENSECAG00000007611	6.533316911	0.946960401	0.976239232	747	1167	2156	2442	1719	2071	1640	2537
ENSECAG00000018197	6.531605348	0.947025948	0.976239232	1053	1413	2058	1774	2010	1963	1795	2030
ENSECAG00000013187	7.124240124	0.947185623	0.976315254	2860	2702	788	2113	4232	2502	3100	2093
ENSECAG00000007156	8.002269141	0.947646602	0.976701804	2625	4026	4515	6757	5225	4439	5727	6418
ENSECAG00000016142	4.992496417	0.948092334	0.977072571	272	389	678	898	645	655	613	818
ENSECAG00000009140	2.256858841	0.948382476	0.977282939	22	81	65	171	119	84	87	114
ENSECAG00000001181	4.380967249	0.948682713	0.977435864	145	233	596	520	374	431	510	431
ENSECAG00000005760	6.152238844	0.948702931	0.977435864	853	1401	1056	1393	1284	1383	1464	2009
ENSECAG00000012925	2.815617957	0.949087169	0.977743078	80	89	136	167	171	120	140	168
ENSECAG00000010878	4.654323483	0.949739019	0.978325905	293	412	399	618	617	474	465	570
ENSECAG00000009889	9.202131059	0.950083742	0.978592283	7936	10432	8907	11358	12966	11128	12387	14265
ENSECAG00000019425	3.704374769	0.950171734	0.978594203	142	176	212	378	265	280	256	290
ENSECAG00000010254	4.715151145	0.95049282	0.978775928	196	394	554	697	416	677	411	740
ENSECAG00000022663	4.671430792	0.950566461	0.978775928	279	425	438	605	554	426	560	623
ENSECAG00000012338	6.711065716	0.950651604	0.978775928	985	1214	1741	3488	2288	1845	2445	2443
ENSECAG00000017099	6.373312359	0.95069276	0.978775928	747	1311	1549	2140	1666	1716	1734	1991
ENSECAG00000009449	4.259357568	0.950903342	0.978872632	232	168	409	500	536	327	346	432
ENSECAG00000021423	5.761424382	0.950958996	0.978872632	537	439	1060	1821	1246	902	1418	1088
ENSECAG00000004664	1.714351047	0.951185393	0.979016979	44	57	39	74	66	45	83	77
ENSECAG00000012435	3.344937585	0.95184288	0.979604964	143	123	200	213	259	136	254	206
ENSECAG00000001440	1.604750022	0.952125836	0.979694544	16	47	52	95	56	41	76	83
ENSECAG00000018148	5.286115033	0.952128595	0.979694544	390	597	755	971	792	670	857	1002
ENSECAG00000018994	5.172158365	0.952204046	0.979694544	375	574	494	1085	787	831	471	955
ENSECAG00000022635	7.752402914	0.952274823	0.979694544	2247	3756	3457	5493	4362	3926	4689	5350
ENSECAG00000020682	1.223124187	0.952441504	0.979777309	21	26	32	83	54	35	50	49
ENSECAG00000009174	2.411033069	0.952614292	0.979806663	60	94	69	128	109	81	108	157

ENSECAG00000012105	0.496219423	0.952663567	0.979806663	7	12	44	29	23	27	34	28
ENSECAG00000005829	2.545034861	0.952728746	0.979806663	70	67	125	124	162	59	134	146
ENSECAG00000008452	6.034191125	0.953331628	0.980337945	1212	1013	868	1087	1468	1134	1565	1380
ENSECAG00000012894	6.409061152	0.953444039	0.980364812	1308	1640	1324	1210	1987	1458	1744	2034
ENSECAG00000023832	7.479184644	0.953590783	0.980426972	2114	3356	2998	3355	3448	3645	3710	4542
ENSECAG00000021330	6.798948449	0.953994741	0.980652587	1903	1528	2155	1595	3106	2202	2020	2032
ENSECAG00000016995	7.342365675	0.954053778	0.980652587	4181	2598	1341	1423	3420	2759	3867	3700
ENSECAG00000021232	2.592779963	0.954069151	0.980652587	77	79	77	179	142	126	76	158
ENSECAG00000018536	5.154856986	0.954279541	0.980773568	183	504	930	929	546	626	894	1015
ENSECAG00000019636	5.873404065	0.954359494	0.980773568	643	955	1045	1327	1190	1052	1373	1424
ENSECAG00000026889	4.961976022	0.954809003	0.980903289	342	465	631	700	588	629	662	758
ENSECAG00000020656	5.127885628	0.954895415	0.980903289	532	311	843	646	946	628	672	703
ENSECAG00000017806	4.845443798	0.954911393	0.980903289	351	547	451	546	667	451	502	873
ENSECAG00000014289	4.752032666	0.954912663	0.980903289	248	322	690	630	657	581	582	428
ENSECAG00000013565	5.868573719	0.95491738	0.980903289	552	927	1169	1366	1336	1119	1031	1541
ENSECAG00000013079	1.279897445	0.955455129	0.981051913	15	29	71	47	47	52	57	42
ENSECAG00000002350	8.025925765	0.955458915	0.981051913	2486	4159	5732	5723	5164	5377	5090	6458
ENSECAG00000000732	1.461984469	0.955608822	0.981051913	3	36	63	103	2	96	42	77
ENSECAG00000012810	6.714540031	0.955765838	0.981051913	1634	1241	1706	2336	2612	1676	2429	2323
ENSECAG00000015304	1.809205647	0.955908996	0.981051913	50	66	55	46	64	61	101	67
ENSECAG00000010193	4.342796226	0.955914099	0.981051913	183	324	416	490	347	393	480	495
ENSECAG00000009554	1.805118549	0.955967706	0.981051913	41	82	41	62	53	75	68	91
ENSECAG00000020855	7.861900556	0.955973912	0.981051913	2405	6854	2554	3551	4353	5883	4652	4550
ENSECAG00000010081	6.518112818	0.955988386	0.981051913	1162	1979	1292	1632	1983	1754	1678	2378
ENSECAG00000022877	2.122598047	0.956038543	0.981051913	53	103	56	69	87	55	99	124
ENSECAG00000012942	0.898901325	0.956097976	0.981051913	40	4.00004	60	1.00012	88	2	54	11.0006
ENSECAG00000000474	6.900029107	0.956098208	0.981051913	1876	1622	2577	1571	3142	1770	2761	2628
ENSECAG00000018212	8.112153526	0.956503269	0.98128445	2920	4060	4746	7319	5248	6108	5227	7175
ENSECAG00000016924	3.720457442	0.956577084	0.98128445	133	182	306	287	187	365	183	367
ENSECAG00000012746	0.717070749	0.956676475	0.98128445	13	22	24	52	29	39	19	42
ENSECAG00000013395	7.536061127	0.95669599	0.98128445	2726	2335	3547	3813	3671	3569	3443	5134
ENSECAG00000010729	8.920091809	0.956823953	0.98128445	3311	6298	9797	15416	8047	8968	9791	15323
ENSECAG00000017621	4.182274223	0.956843023	0.98128445	200	204	426	405	333	387	451	370
ENSECAG00000014356	7.037144429	0.957097942	0.981384257	1450	1839	2707	2936	2776	2443	2596	3515
ENSECAG00000018309	6.423435994	0.957113092	0.981384257	1896	1181	1115	1089	1650	1336	1978	2379
ENSECAG00000017559	2.309036726	0.957255415	0.981441619	51	86	87	103	115	71	130	103
ENSECAG00000022225	5.898816381	0.958400045	0.982460287	459	901	1315	1503	1112	1050	966	2070
ENSECAG00000018194	6.662082223	0.958421917	0.982460287	1208	1337	2319	2039	2085	2180	1628	2701
ENSECAG00000022961	3.855982593	0.958642749	0.982485335	107	302	221	367	160	309	328	447
ENSECAG00000019928	7.337274855	0.958651866	0.982485335	397	6333	40	4884	882	6041	54	6511
ENSECAG00000018495	6.248884225	0.958705765	0.982485335	862	916	1885	1559	1814	1294	1361	2022
ENSECAG00000000410	3.681587691	0.958816834	0.98251054	144	170	252	302	247	231	232	395
ENSECAG00000007838	6.916859285	0.959070516	0.982681865	1237	1604	2397	3111	2718	2263	3061	2277
ENSECAG00000012352	3.679615024	0.95916566	0.982690733	155	245	187	270	281	232	197	378
ENSECAG00000011461	6.284744158	0.959254456	0.982693096	865	884	1649	1969	1913	1281	1657	1862
ENSECAG00000016376	2.637584103	0.959570325	0.982928059	40	114	151	117	74	130	100	230
ENSECAG00000022942	8.497797125	0.959681652	0.982953478	4423	6782	5755	6974	7421	7421	8034	8078
ENSECAG00000015237	3.374935825	0.959899452	0.983002804	116	138	158.999	306.999	217.999	177	241	236.999
ENSECAG00000012082	7.70244442	0.959902844	0.983002804	2146	2123	4678	5564	3902	3452	4403	6353
ENSECAG00000007107	3.553377563	0.960077934	0.983013454	109	187	131	406	214	244	243	284
ENSECAG00000003079	8.539099299	0.960086279	0.983013454	4735	5281	7292	7931	8725	7004	7152	8687
ENSECAG00000017751	5.852759034	0.960173234	0.983013902	487	887	1154	1559	1054	1268	1025	1555
ENSECAG00000021276	6.289451815	0.960278346	0.983032937	790	1297	1594	1632	1806	1462	1742	1683
ENSECAG00000023588	0.569482224	0.96049657	0.98316775	11	14	39	32	26	59	14	15
ENSECAG00000013323	4.2480279	0.96102531	0.983620356	149	179	419	634	534	306	340	427
ENSECAG00000020521	7.659021719	0.961763173	0.984194209	2538	3887	3327	3524	4433	3623	4116	5261
ENSECAG00000017180	0.507418669	0.962037721	0.984194209	9	5	24	62	16	15	20	63
ENSECAG00000014570	4.703325513	0.962077619	0.984194209	323	452	507	430	547	491	543	651
ENSECAG00000023401	4.026998549	0.962098686	0.984194209	56	349	213	583	231	327	336	487
ENSECAG00000026949	1.511279805	0.962103201	0.984194209	26	55	35	76	31	78	51	70
ENSECAG00000000487	1.233263153	0.962240754	0.984194209	32	57	29	23	92	25	20	60
ENSECAG00000011626	7.412060144	0.962272217	0.984194209	4438	2059	1777	1848	3277	2073	3442	5975
ENSECAG00000007328	7.85979581	0.962327253	0.984194209	1451	2737	4446	8615	4873	4640	4516	5666
ENSECAG00000011964	7.034468343	0.962365575	0.984194209	885	2222	2454	3867.02	2518	2346	2679	3656
ENSECAG00000020591	4.493757802	0.962455958	0.984198056	227.001	348	446.001	535.001	470.001	463	557.001	398.001
ENSECAG00000004547	4.052399074	0.962997556	0.984638861	266	243	279	290	415	275	361	350
ENSECAG00000022783	0.989222794	0.963060347	0.984638861	28	24	37	34	43	24	30	69
ENSECAG00000005927	4.224976985	0.963435346	0.984782038	217	216	482	339	570	309	295	427
ENSECAG00000023949	3.554421126	0.963458192	0.984782038	111	118	450	130	9	854	4	11
ENSECAG00000015030	7.716992957	0.963460406	0.984782038	3175	1277	7247	2227	7068	3408	5287	1775
ENSECAG00000000246	6.136532871	0.963576012	0.984811609	576	1387	1355	1507	1387	1524	1434	1665
ENSECAG00000024878	2.672928299	0.963740773	0.984884913	62	53	230	89	104	173	85	165
ENSECAG00000014518	6.851709348	0.963896591	0.984884913	533	4270	1423	1432	1669	4368	1734	1828
ENSECAG00000018672	2.123624525	0.964033938	0.984884913	16	61	116	120	47	55	124	140
ENSECAG00000020384	6.63538769	0.964135022	0.984884913	1163	1265	1757	2629	1764	1346	3146	2336
ENSECAG00000013766	1.146503971	0.964139883	0.984884913	26	26	37	54	58	36	41	47
ENSECAG00000024626	8.537166178	0.96420174	0.984884913	3041	5677	7901	9327	6561	10316	5559	9152
ENSECAG00000005108	3.131277672	0.964254512	0.984884913	86	168	120	217	174	141	207	227
ENSECAG00000019709	4.859026248	0.96526204	0.985825376	415	472	508	491	604	452	634	817
ENSECAG00000022001	4.13713858	0.965546645	0.985899324	207	277	289	410	437	245	419	410
ENSECAG00000015670	7.233488513	0.965586366	0.985899324	1306	2103	2755	4587	2925	2746	3062	4118
ENSECAG00000000326	2.702145256	0.965665017	0.985899324	45	100	146	166	168	93	193	85
ENSECAG00000004439	1.737605338	0.965780197	0.985899324	34	64	38	83	51	50	69	113
ENSECAG00000010013	7.481207886	0.965909197	0.985899324	1791	2295	3731	4733	3891	3045	3828	4479
ENSECAG00000023747	4.433254507	0.965942217	0.985899324	183	208	438	714	350	335	359	847

ENSECAG00000000016	5.043676605	0.965962177	0.985899324	502	492	495	689	631	628	701	843
ENSECAG00000016925	7.542002397	0.966028619	0.985899324	2726	3013	3044	3529	4362	3106	3948	4471
ENSECAG00000012412	3.471506383	0.966284937	0.986072342	115	128	256	268	231	192	252	261
ENSECAG00000023029	4.732847702	0.966521845	0.986225523	243	499	477	616	449	553	452	812
ENSECAG00000011695	6.361253465	0.966732166	0.986239178	647	1088	1724	2372	1604	1834	1448	2153
ENSECAG00000021762	5.319733837	0.966796274	0.986239178	466	579	1007	632	927	818	884	729
ENSECAG00000024720	3.953802508	0.966806658	0.986239178	93	259	318	425	275	335	308	403
ENSECAG00000020397	8.103545376	0.966882433	0.986239178	5650	4713	3161	3476	6391	4570	6089	6358
ENSECAG00000018290	5.222612412	0.966971882	0.986241878	620	1015	291	388	816	678	1023	621
ENSECAG00000015188	5.344895205	0.967321446	0.986509853	925	642	312	561	1007	638	846	1014
ENSECAG00000013390	2.288866416	0.968129781	0.987115166	47	49	92	155	114	109	94	83
ENSECAG00000008043	5.286532268	0.968166269	0.987115166	271	598	742	1167	679	753	777	1155
ENSECAG00000012442	4.609868187	0.968234423	0.987115166	204	475	410	610	509	459	385	731
ENSECAG00000018062	8.239148573	0.968413036	0.987115166	3612	6233	4184	5994	6324	5924	6668	6978
ENSECAG00000023160	2.786475209	0.968473016	0.987115166	43	96	91	266	151	169	137	118
ENSECAG00000024592	4.800618681	0.968508633	0.987115166	321	451	491	613	614	468	550	768
ENSECAG00000022693	7.993590476	0.968616285	0.987115166	4467	4033	3884	3927	6179	4140	5653	5729
ENSECAG00000024543	4.146647008	0.96869067	0.987115166	171	291	356	401	396	256	465	386
ENSECAG00000022309	4.866168715	0.968696893	0.987115166	216	425	607	835	560	553	545	828
ENSECAG00000021513	4.56206226	0.968964983	0.987299806	190	441	342	694	394	339	513	790
ENSECAG00000011560	6.194393348	0.969152276	0.987366523	1094	1325	662	1783	2065	1488	1052	1584
ENSECAG00000017952	3.203937131	0.969204263	0.987366523	69	275	110	153	172	189	280	132
ENSECAG00000021465	5.672398073	0.969459458	0.987537956	585	857	841	1194	1072	857	1229	1184
ENSECAG00000020890	1.408724631	0.969591515	0.987583935	29	54	44	44	48	42	60	68
ENSECAG00000017428	2.718208875	0.969708894	0.987584663	88	72	150	128	110	96	123	232
ENSECAG00000013904	5.508137445	0.969766069	0.987584663	656	816	668	849	998	851	953	1062
ENSECAG00000007436	2.074429854	0.969923397	0.987656358	41	62	70	115	99	66	87	98
ENSECAG00000012902	5.098950972	0.970195219	0.987843393	350	442	728	867	784	624	789	702
ENSECAG00000012950	3.128616869	0.97028096	0.987843393	137	108	143	187	162	181	234	150
ENSECAG00000016094	2.465833181	0.970696844	0.988178259	54	122	64	132	117	85	112	150
ENSECAG00000024236	4.477952029	0.970787768	0.988182281	206	326	330	719	397	362	357	806
ENSECAG00000014169	5.024294812	0.971352058	0.988648967	416	277	837	668	851	556	708	664
ENSECAG00000012980	3.935865568	0.971422709	0.988648967	142	309	275	316	263	269	283	494
ENSECAG00000000328	1.95943056	0.971565963	0.988648967	37	37	93	101	105	64	96	53
ENSECAG00000019809	4.079199928	0.971594294	0.988648967	123	105	471	519	528	377	340	167
ENSECAG00000017413	5.572362505	0.972049423	0.98902351	355	793	835	1411	871	967	1005	1198
ENSECAG00000025062	4.248570726	0.972376092	0.989182704	154	277	290	642	448	377	399	374
ENSECAG00000024188	3.712512172	0.972380005	0.989182704	213	123	268	248	280	238	339	255
ENSECAG00000008831	5.786841571	0.972597905	0.989268764	833	722	1084	967	1226	999	1306	1187
ENSECAG00000024034	5.01231131	0.972723458	0.989268764	364	283	685	936	613	733	637	738
ENSECAG00000013258	4.377785514	0.972776812	0.989268764	258	235	419	497	491	348	442	499
ENSECAG00000010534	6.915241784	0.972812876	0.989268764	1439	2023	2281	2318	2420	2364	2592	2971
ENSECAG00000016419	5.821246233	0.972986745	0.989357025	823	1182	863	774	1680	689	1364	1085
ENSECAG00000016838	5.470745609	0.973477233	0.989725578	517	607	908	972	1136	751	940	967
ENSECAG00000018179	6.522861289	0.973523417	0.989725578	1048	1471	1645	2062	1893	1683	2253	2039
ENSECAG00000014541	1.816148188	0.973681347	0.989797572	20.0071	119	27	67	82	43	79	89
ENSECAG00000015936	5.880246051	0.973882511	0.989913499	392	946	1229	1651	1223	1236	1154	1376
ENSECAG00000010070	3.195417061	0.974140028	0.990030246	96	73	277	176	210	182	185	198
ENSECAG00000024825	0.502674277	0.974238495	0.990030246	12	14	32	33	30	24	37	21
ENSECAG00000011508	6.493911346	0.974258773	0.990030246	960	1420	1694	2131	1957	1748	1619	2360
ENSECAG00000015581	7.848206695	0.974408424	0.990093768	3100	4175	3446	4444	4978	3873	5386	5590
ENSECAG00000023652	4.555318179	0.974527054	0.990125761	280	476	352	444	551	387	499	579
ENSECAG00000001324	4.530050251	0.97465035	0.990162489	193	778	268	251	650	628	478	158
ENSECAG00000005549	8.24965779	0.974850592	0.990248754	3419	5483	5814	5647	5864	6119	7797	6170
ENSECAG00000022703	7.416183125	0.974909573	0.990248754	3000	2234	2197	3607	4173	2660	3512	4401
ENSECAG00000011947	4.849030278	0.975173473	0.990428265	321	617	451	518	704	527	619	606
ENSECAG00000019657	3.841503038	0.975456313	0.990626978	157	202	324	291	276	282	324	327
ENSECAG00000024977	5.339266714	0.975625865	0.990685607	506	563	944	699	1029	814	680	904
ENSECAG00000008594	6.705768786	0.97568843	0.990685607	555	3210	1226	2239	1328	2696	2732	1979
ENSECAG00000012093	5.987856048	0.975889273	0.990800994	509	1031	1146	1778	1124	1399	1060	1865
ENSECAG00000019832	3.577092201	0.976257852	0.990916694	117	77	345	301	396	147	268	194
ENSECAG00000014264	4.050220294	0.976348066	0.990916694	153	255	301	447	358	277	332	444
ENSECAG00000019822	7.513266704	0.976352051	0.990916694	2687	3461	2670	3092	4719	2314	3960	4719
ENSECAG00000024050	1.789731124	0.976352085	0.990916694	46	39	68	75	80	74	54	75
ENSECAG00000015945	3.27756615	0.976545059	0.990989404	85	166	175	245	177	187	176	281
ENSECAG00000002715	6.421498306	0.97664068	0.990989404	1053	1430	1464	1834	1798	1620	1767	2117
ENSECAG00000024461	8.903682485	0.976823344	0.990989404	6799	8234	8433	7999	9999	8915	10445	11431
ENSECAG00000007233	6.133603093	0.976904926	0.990989404	773	1061	1368	1576	1814	1274	1561	1330
ENSECAG00000020044	3.835002196	0.976905923	0.990989404	200	140	346	266	326	275	312	287
ENSECAG00000018636	3.770549657	0.977021767	0.990989404	181	215	230	282	266	323	250	305
ENSECAG00000009743	3.181515099	0.977034264	0.990989404	70	111	222	230	202	148	249	168
ENSECAG00000024956	8.636064596	0.977586233	0.99146075	3575	8136	7929	7571	5697	9950	9111	8757
ENSECAG00000001258	0.805945265	0.977787455	0.991568187	20	24	29	40	39	25	45	30
ENSECAG00000019702	5.776547675	0.977900386	0.991568187	642	874	1118	1065	1047	1050	905	1700
ENSECAG00000006878	4.203314767	0.977953979	0.991568187	157	275	340	527	386	300	367	521
ENSECAG00000014160	5.710988502	0.978221568	0.99168879	475	825	1027	1333	1070	878	1038	1509
ENSECAG00000023825	5.503317007	0.978247488	0.99168879	698	931	539	728	937	825	969	1161
ENSECAG00000014173	5.297847969	0.978430641	0.99178597	549	631	671	752	715	775	707	1167
ENSECAG00000007573	6.284181075	0.978664522	0.991888944	1027	1102	1441	1639	1878	1270	1637	1914
ENSECAG00000008168	5.048735504	0.978825566	0.991888944	483	426	610	682	760	671	608	765
ENSECAG00000006987	4.962402651	0.97884766	0.991888944	462	480	535	569	768	668	619	565
ENSECAG00000016415	5.825257213	0.978881423	0.991888944	495	953	871	1684	1042	1152	1309	1306
ENSECAG00000014918	4.550407532	0.978985491	0.991905935	250	295	363	726	557	355	545	552
ENSECAG00000009323	5.299858586	0.979326542	0.992163012	381	483	842	1061	855	627	867	1021
ENSECAG00000016209	5.409870378	0.97942791	0.99217724	364	652	749	1223	916	854	826	1035

ENSECAG00000015084	6.385998008	0.979777152	0.992442542	842	1238	1608	2087	1813	1573	1749	2027
ENSECAG00000010347	7.122977796	0.979905878	0.992484452	1558	785	3997	3347	4456	3111	3719	305
ENSECAG00000000232	4.138970867	0.980297644	0.992792747	184	457	241	268	362	326	515	286
ENSECAG00000001218	8.506716805	0.980411553	0.992819613	3679	4380	9134	8083	8150	6727	7444	8673
ENSECAG00000020218	3.967633555	0.980688449	0.993011511	220	214	332	264	338	314	316	352
ENSECAG00000017466	0.906050825	0.980801143	0.993037122	33	16	28	38	53	26	35	39
ENSECAG00000023972	2.044283465	0.980896255	0.99304493	54	34	80	107	102	73	85	84
ENSECAG00000015982	6.104747909	0.981247515	0.993312034	1209	1163	933	1073	1552	1140	1567	1613
ENSECAG00000015913	0.857955575	0.981350528	0.993327813	15	37	38	24	52	13	40	44
ENSECAG00000011273	5.245142774	0.981517116	0.993357016	361	636	604	1027	791	625	880	961
ENSECAG00000006668	2.571876397	0.981554235	0.993357016	24	119	123	155	115	98	133	153
ENSECAG00000008007	4.121039916	0.9819053	0.993582582	161	281	364	385	388	326	445	313
ENSECAG00000015142	6.324796624	0.981952017	0.993582582	947	1128	1555	1843	1865	1445	1612	1911
ENSECAG00000018832	4.611089665	0.982100237	0.993644069	135	393	636	577	346	462	760	496
ENSECAG00000021304	4.518750233	0.98219518	0.993651646	223	222	595	555	499	485	459	502
ENSECAG00000007513	7.874876756	0.982350213	0.993709535	3194	2187	5841	4648	7040	4022	4897	3895
ENSECAG00000005814	7.368979032	0.982427319	0.993709535	2972	2364	2722	2386	5296	2910	4376	1332
ENSECAG00000006930	5.129848146	0.982685791	0.993882496	390	384	782	854	736	717	821	696
ENSECAG00000011933	4.951655925	0.982903828	0.994014534	550	441	393	610	609	589	641	796
ENSECAG00000007334	4.622506134	0.983166432	0.994136625	191	348	572	628	490	427	533	652
ENSECAG00000002197	0.833939052	0.983199548	0.994136625	8	59	27	19	21	27	45	51
ENSECAG00000009258	4.797768685	0.983498577	0.994350492	282	237	474	1008	713	490	620	528
ENSECAG00000009891	3.649221339	0.984421837	0.995117606	106	208	206	353	234	266	281	280
ENSECAG00000026987	5.765925775	0.984572372	0.995117606	632	751	966	1374	1223	906	1219	1319
ENSECAG00000018442	6.995489087	0.984605516	0.995117606	1003	1863	2139	4173	2703	2481	2994	2641
ENSECAG00000021928	6.107822284	0.984607651	0.995117606	862	1255	926	1599	1606	1256	1224	1809
ENSECAG00000009415	5.502365053	0.984806476	0.995230025	637	603	924	846	1012	759	993	1107
ENSECAG00000016730	4.865894006	0.984994871	0.995331886	663	343	723	1	844	889.008	619.008	3
ENSECAG00000006210	6.464040435	0.985637085	0.99581617	1075	1529	1513	1785	1859	1499	2005	2219
ENSECAG00000011818	5.577548616	0.985812037	0.99581617	439.441	792.609	936.259	1127.48	857.642	925	1027	1283.36
ENSECAG00000019456	2.406580321	0.985862139	0.99581617	73	126	75	59	101	79	118	150
ENSECAG00000012489	6.996251984	0.985886349	0.99581617	1718	1730	2608	2441	2855	1993	3123	2997
ENSECAG00000010140	4.001769501	0.985912348	0.99581617	103	288	316	419	288	326	380	365
ENSECAG00000021352	5.917815968	0.986031319	0.995847809	592	1055	1226	1246	1110	1359	1312	1353
ENSECAG00000010511	5.242672806	0.986205167	0.99593486	408	530	807	853	640	829	770	977
ENSECAG00000018861	3.213021756	0.986441417	0.996084907	124	87	234	170	242	151	201	191
ENSECAG00000022311	3.986686672	0.98657632	0.9961326	149	217	303	440	288	245	489	321
ENSECAG00000007149	8.112911433	0.986981997	0.996334594	3191	4136	5630	5886	5579	5407	4984	7733
ENSECAG00000009458	7.581126463	0.98701423	0.996334594	3151	2958	2924	3296	4414	4757	3320	3675
ENSECAG00000022136	3.479016484	0.987099854	0.996334594	131	145	233	246	279	215	201	244
ENSECAG00000014821	6.651049999	0.987127136	0.996334594	1132	1921	1810	1851	1869	1981	2292	2451
ENSECAG00000008535	1.34171708	0.987359066	0.996449447	15	26	75	57	37	72	65	28
ENSECAG00000006925	3.429004346	0.987426596	0.996449447	98	207	232	185	229	153	259	281
ENSECAG00000020975	4.477378505	0.987550962	0.996449447	303	341	296	557	480	369	335	734
ENSECAG00000021002	7.696547615	0.987591729	0.996449447	3307	3982	2524	3545	4904	3802	4074	4913
ENSECAG00000017912	3.110639443	0.987821691	0.996592973	80	69	204	254	187	138	298	100
ENSECAG00000011574	3.587115481	0.987931027	0.996614785	111	205	243	264	263	244	269	232
ENSECAG00000004791	3.659276292	0.988128827	0.996725828	66	132	422	286	355	282	286	120
ENSECAG00000000229	0.923477001	0.988484479	0.996962291	12	20	63	30	4	102	23	15
ENSECAG00000020558	3.487006069	0.988619631	0.996962291	114	185	143	330	190	224	167	381
ENSECAG00000008092	1.658557483	0.988626486	0.996962291	47	29	59	71	65	52	101	39
ENSECAG00000022544	8.345008187	0.98883361	0.997082666	3751	5054	6021	7339	6831	6398	6807	7658
ENSECAG00000000099	1.929655461	0.989137358	0.99730044	44	49	58	104	88	58	72	102
ENSECAG00000022019	8.226504378	0.989546108	0.997501246	3642	3388	7041	6366	7030	5404	6091	7038
ENSECAG00000012654	4.010420417	0.989598644	0.997501246	100	237	324	499	305	330	416	303
ENSECAG00000023670	5.737698597	0.989661807	0.997501246	774	870	825	1045	1111	1056	969	1419
ENSECAG00000021055	5.909560076	0.989734131	0.997501246	478	1460	849	1322	1004	1028	1127	2056
ENSECAG00000007707	7.309957936	0.989810559	0.997501246	1942	1901	3103	3947	3461	2528	3598	4023
ENSECAG00000023721	9.679506968	0.989863276	0.997501246	11942	17643	10398	12726	12262	23588	11706	21987
ENSECAG00000024396	3.888865219	0.990648918	0.998204417	184	149	315	364	385	216	324	335
ENSECAG00000007240	7.233669117	0.990937282	0.998245597	2082	2966	2092	2690	3207	2991	3027	3635
ENSECAG00000016143	2.052834446	0.990950729	0.998245597	28	55	64	146	71	84	78	114
ENSECAG00000019248	4.714113262	0.990953361	0.998245597	257	254	615	723	594	505	545	581
ENSECAG00000010787	5.655549901	0.991220252	0.998368415	661	707	826	1215	1078	953	931	1347
ENSECAG00000013264	5.605546786	0.99125102	0.998368415	479	894	820	1150	865	980	823	1509
ENSECAG00000017343	7.446505158	0.991621271	0.998485505	1528	2920	4628	2861	3301	3671	4713	3032
ENSECAG00000022373	4.629730122	0.991625158	0.998485505	268	463	412	530	586	425	549	544
ENSECAG00000011236	4.095763265	0.991630914	0.998485505	206	225	364	360	409	266	370	412
ENSECAG00000022385	5.395187537	0.991888513	0.998652349	327	725	777	1126	763	875	1021	915
ENSECAG00000009158	2.37855786	0.992028659	0.998652349	42	71	126	116	99	103	95	141
ENSECAG00000003698	3.319707379	0.992060294	0.998652349	109	253	100	192	181	142	221	316
ENSECAG00000009950	3.770759858	0.992223959	0.998728616	155	218	275	269	307	239	269	345
ENSECAG00000007120	1.892302825	0.992314949	0.998731726	36	85	83	32	40	157	54	45
ENSECAG00000019688	2.394444772	0.992671632	0.998949865	31	73	137	125	103	97	94	150
ENSECAG00000016335	2.554365562	0.992707527	0.998949865	55	82	101	168	133	94	135	131
ENSECAG00000026960	5.021899489	0.992849562	0.999004314	268	531	682	780	736	425	885	743
ENSECAG00000013024	2.62812541	0.993587726	0.999558747	47	82	106	201	96	145	138	137
ENSECAG00000008798	5.324665083	0.993686664	0.999558747	362.011	589	810.008	1042	807.001	824	718.001	1068
ENSECAG00000006288	7.328783277	0.993707135	0.999558747	4196	236	3974	1246	4914	2520	5690	258
ENSECAG00000022963	5.869632907	0.993834488	0.999558747	591	940	981	1530	1260	972	1474	1291
ENSECAG00000011608	5.786096749	0.993840447	0.999558747	524	855	1088	1364	1064	971	1321	1360
ENSECAG00000000391	2.758843142	0.994220603	0.999708614	67	56	156	194	175	135	115	140
ENSECAG00000008531	6.106410004	0.994313202	0.999708614	890	1022	1255	1460	1390	1344	1537	1594
ENSECAG00000019611	3.652781138	0.994400091	0.999708614	133	121	347	259	355	201	270	239
ENSECAG00000025173	4.400628451	0.994401083	0.999708614	262	304	358	506	433	372	407	595

ENSECAG00000005107	3.830130397	0.994429392	0.999708614	170	278	194	306	269	277	279	384
ENSECAG00000018072	5.839684601	0.994797373	0.999867184	707	964	1166	961	1285	830	1216	1613
ENSECAG00000009454	2.792122161	0.994896581	0.999867184	62	171	122	99	111	218	78	168
ENSECAG00000019013	5.493882973	0.994917967	0.999867184	343	716	1048	1046	885	899	1000	1054
ENSECAG00000018903	5.991758021	0.99497588	0.999867184	1121	1256	797	807	1267	1025	1602	1556
ENSECAG00000022557	5.161713281	0.995027129	0.999867184	554	457	700	626	962	635	711	737
ENSECAG00000019748	3.979863446	0.995390419	1	159	166	419	342	354	280	280	435
ENSECAG00000003237	6.113760505	0.995426155	1	871	1290	1131	1298	1655	1179	1545	1537
ENSECAG00000009780	6.205069697	0.995595364	1	1163	1395	1013	1163	1557	1361	1643	1732
ENSECAG00000005995	6.662523176	0.99563091	1	728	1445	2393	2638	2202	2443	1616	2309
ENSECAG00000009349	4.064534082	0.996067325	1	143	315	308	383	319	294	369	448
ENSECAG00000018626	7.887974826	0.996072151	1	3036	4774	3737	4020	4649	4544	5625	5397
ENSECAG00000007881	2.635214828	0.99626118	1	190	99	21	28	99	200	81	133
ENSECAG00000016473	6.972784131	0.996920461	1	1162	2343	2068	3104	2514	2564	2175	3511
ENSECAG00000019277	4.991077089	0.997198784	1	292	527	396	1028	655	524	763	776
ENSECAG00000025021	5.457208842	0.997266577	1	410	767	663	1220	850	847	930	1123
ENSECAG00000013404	7.427811164	0.997605849	1	1973	1397	4902	3592	4309	3137	3147	4120
ENSECAG00000013824	5.291247405	0.997614613	1	453	804	676	661	918	755	738	922
ENSECAG00000010094	5.537169677	0.997866729	1	567	636	749	1227	1246	767	918	1031
ENSECAG00000017780	6.664154677	0.997888126	1	1068	1281	2111	2583	2131	2188	1752	2569
ENSECAG00000018745	7.51206188	0.998078632	1	2025	2989	3298	4132	3878	3518	3708	4489
ENSECAG00000016030	5.170958464	0.998359039	1	438	413	683	939	695	737	791	840
ENSECAG00000016695	4.496283783	0.998476275	1	174	388	389	639	387	413	484	648
ENSECAG00000017002	8.060578446	0.998941351	1	2539	2712	6130	7531	6071	4892	5419	6446
ENSECAG00000010981	6.879337214	0.99903574	1	1447	1830	2495	2097	2717	2037	2756	2533
ENSECAG00000019609	4.134714243	0.999426338	1	218	184	380	414	373	296	371	460
ENSECAG00000023559	5.653383371	0.999459958	1	598	761	926	1126	1056	878	1176	1189
ENSECAG00000014284	1.805792303	1	1	31.0071	49	78	77	68	54	97	71
ENSECAG00000009796	1.205291235	1	1	25	16	67	44	41	62	33	49
ENSECAG00000011656	3.360748868	1	1	306	75	95	112	225	99	276	285
ENSECAG00000018010	0.118653338	1	1	3	13	20	37	28	8	28	19
ENSECAG00000018831	0.011053065	1	1	7	14	22	19	19	18	14	27
ENSECAG00000016386	1.765903672	1	1	51	30	70	68	86	59	62	76
ENSECAG00000019096	0.378689732	1	1	5	7	30	48	17	26	43	13
ENSECAG00000013597	0.649951814	1	1	14	24	21	44	10	65	7	39
ENSECAG00000006208	0.599689696	1	1	13	21	24	40	14	22	47	39
ENSECAG00000025017	1.958545204	1	1	30	42	81	119	80	63	71	111
ENSECAG00000015521	1.657208647	1	1	28	31	55	105	62	45	72	83
ENSECAG00000021809	1.017425737	1	1	45	32	12	29	39	31	43	53
ENSECAG00000004395	1.009371448	1	1	21	48	37	18	29	27	29	83
ENSECAG00000000785	1.936871447	1	1	57	25	49	127	102	52	74	92
ENSECAG00000013350	1.842864313	1	1	49	43	40	106	76	63	89	69
ENSECAG00000018792	1.847952801	1	1	33	49	72	89	41	65	95	100
ENSECAG00000014107	1.665561636	1	1	22	38	95	60	81	56	61	63
ENSECAG00000020584	3.4729502	1	1	83	175	234	285	171	236	276	252
ENSECAG00000001088	1.314651218	1	1	17	28	57	68	57	31	59	58
ENSECAG00000017122	0.865096149	1	1	29	18	29	38	40	51	21	32
ENSECAG00000016502	2.702116674	1	1	64	57	104	236	121	96	140	197
ENSECAG00000013488	1.54082588	1	1	42	71	33	32	80	26	78	58
ENSECAG00000005101	2.880497781	1	1	71	195	99	117	156	100	178	192
ENSECAG00000012893	1.039650118	1	1	19	32	20	66	39	32	53	43
ENSECAG00000016375	2.540809998	1	1	57	148	67	110	120	82	148	142
ENSECAG00000005128	4.105549756	1	1	218	225	267	461	371	290	325	489
ENSECAG00000009308	1.641849647	1	1	28	47	61	73	54	55	58	93
ENSECAG00000019221	2.684747002	1	1	70	65	129	178	135	101	133	176
ENSECAG00000012218	3.243500457	1	1	94	135	187	233	161	180	216	246
ENSECAG00000003274	1.084589843	1	1	42	24	31	31	58	36	35	43
ENSECAG00000002833	5.296260128	1	1	330	837	618	920	655	821	768	1115
ENSECAG00000023035	4.453405206	1	1	237	342	397	519	523	363	457	527
ENSECAG00000015467	7.164345523	1	1	2299	2124	2068	2877	3170	2576	3366	3123
ENSECAG00000014569	5.002703976	1	1	310	578	469	859	660	569	618	903
