# Supplementary material for: Transcriptome analysis of immune genes in peripheral blood mononuclear cells of young foals and adult horses
Source: PLoS One. 2018 Sep 5;13(9):e0202646. doi: 10.1371/journal.pone.0202646 (PMC6124769; doi:10.1371/journal.pone.0202646)
Supplement: S2 Table — (PDF) [file pone.0202646.s002.pdf]

				Raw data (number of reads)							
Gene (Ensembl ID)	log fold change	p value (tag-wise dispersion)	adjusted p value (tag-wise dispersion)	day42foalA	day42foalB	day42foalC	day42foalD	mare1	mare2	mare3	mare4
ENSECAG00000016797	4.444915056	6.41E-26	4.40E-22	832	692	727	1017	13	33	28	48
ENSECAG00000009575	7.713069998	7.75E-26	4.40E-22	67	97	85	138	9479	2821	16138	2703
ENSECAG00000016605	3.628848515	4.99E-24	1.89E-20	9	14	5	5	564	392	582	197
ENSECAG00000009282	6.283673925	4.43E-22	1.26E-18	219	222	225	116	3532	2414	2662	1741
ENSECAG00000008721	6.677406539	2.50E-20	5.68E-17	190	313	284	226	4905	2148	4946	1929
ENSECAG00000011113	1.925933341	3.79E-19	7.17E-16	133	90	168	180	1	0	3	6
ENSECAG00000016614	4.768442624	5.41E-19	8.78E-16	1333	673	1439	509	57	30	77	54
ENSECAG00000006338	4.358458768	1.50E-17	2.13E-14	41	72	46	60	952	373	987	464
ENSECAG00000023062	5.716855594	6.23E-16	7.86E-13	43	26	4	8	428	987	1702	4508
ENSECAG00000009700	4.970746743	1.84E-14	2.01E-11	87	133	119	90	1242	570	1849	549
ENSECAG00000025096	0.933297895	1.95E-14	2.01E-11	0	4	0	0	86	41	95	38
ENSECAG00000024524	1.347833332	3.67E-14	3.47E-11	0	5	1	2	136	56	109	45
ENSECAG00000023860	5.321251722	1.31E-13	1.14E-10	12	30	17	4	209	3488	580	1008
ENSECAG00000008322	7.728802154	1.91E-13	1.44E-10	582	609	744	536	8415	3934	14370	2470
ENSECAG00000023537	4.977622226	2.00E-13	1.44E-10	194	176	166	148	1221	643	1259	794
ENSECAG00000024394	1.920106195	2.06E-13	1.44E-10	5	2	0	2	213	83	202	30
ENSECAG00000002389	3.289329162	2.15E-13	1.44E-10	10	11	12	3	94	484	131	609
ENSECAG00000000649	1.103944106	7.83E-13	4.94E-10	67	63	118	51	3	4	3	4
ENSECAG00000019048	1.516538375	1.15E-12	6.78E-10	40	61	92	243	0	2	5	3
ENSECAG00000019436	1.632743852	1.19E-12	6.78E-10	7	1	4	2	165	84	36	124
ENSECAG00000024812	1.268981652	1.56E-12	8.43E-10	129	45	51	127	2	0	6	4
ENSECAG00000008606	5.722298179	1.63E-12	8.44E-10	105	123	292	246	2398	1106	2813	686
ENSECAG00000016243	1.373343337	2.38E-12	1.17E-09	7	6	3	4	142	58	82	56
ENSECAG00000022666	5.795913111	2.99E-12	1.41E-09	127	244	146	159	1914	1396	3774	477
ENSECAG00000006176	2.480266838	3.98E-12	1.81E-09	1	9	5	1	424	138	135	59
ENSECAG00000000521	6.39061001	5.54E-12	2.42E-09	3401	3109	3200	1518	214	234	431	611
ENSECAG00000019333	5.904108961	6.90E-12	2.90E-09	426	290	297	228	1896	2111	961	2271
ENSECAG00000015055	4.555735001	9.82E-12	3.98E-09	851	765	515	1038	83	68	201	123
ENSECAG00000015927	1.75542106	2.46E-11	9.65E-09	161	88	136	81	5	7	9	15
ENSECAG00000019204	1.176526985	2.62E-11	9.91E-09	73	49	129	68	3	3	9	0
ENSECAG00000020640	4.493238514	3.18E-11	1.17E-08	7	47	29	8	1060	114	1630	489
ENSECAG00000025070	1.956936899	3.68E-11	1.30E-08	5	16	4	10	115	118	224	63
ENSECAG00000011440	4.076949484	5.25E-11	1.81E-08	43	59	97	154	567	387	689	474
ENSECAG00000017881	1.766551104	6.44E-11	2.12E-08	8	9	14	14	72	65	133	172
ENSECAG00000016361	2.692171343	6.52E-11	2.12E-08	21	5	14	10	310	293	186	54
ENSECAG00000015834	0.806369188	7.74E-11	2.39E-08	2	4	2	3	28	43	44	113
ENSECAG00000020859	3.179260503	7.78E-11	2.39E-08	52	49	35	54	342	248	255	258
ENSECAG00000024870	3.451940024	9.14E-11	2.73E-08	46	57	49	85	444	225	331	343
ENSECAG00000009450	0.801822427	1.01E-10	2.93E-08	49	28	118	50	0	2	2	5
ENSECAG00000024974	6.554636531	1.03E-10	2.93E-08	771	530	752	797	3682	2191	2683	2268
ENSECAG00000007329	4.419868255	1.59E-10	4.41E-08	87	44	70	95	345	1376	334	594
ENSECAG00000008660	2.181123407	1.86E-10	5.02E-08	21	11	7	5	166	69	280	102
ENSECAG00000010486	0.960229974	2.05E-10	5.42E-08	50	60	101	57	1	8	2	3
ENSECAG00000013088	1.047485996	3.39E-10	8.75E-08	9	4	7	7	93	49	71	45
ENSECAG00000026892	0.615793607	3.96E-10	9.98E-08	2	7	3	5	40	42	65	45
ENSECAG00000004180	4.209270947	4.04E-10	9.98E-08	55	108	107	57	503	288	509	1063
ENSECAG00000016994	3.392126263	5.78E-10	1.40E-07	10	27	36	26	313	85	890	207
ENSECAG00000016715	0.42089379	8.17E-10	1.93E-07	2	4	4	3	35	52	26	50
ENSECAG00000016098	0.11872539	1.06E-09	2.45E-07	0	3	3	4	37	29	28	38
ENSECAG00000015637	2.252884613	1.11E-09	2.53E-07	211	79	174	208	10	8	10	31
ENSECAG00000008174	2.487728195	1.33E-09	2.97E-07	17	25	18	16	295	68	255	116
ENSECAG00000024888	9.615525167	1.58E-09	3.45E-07	3838	3684	6935	6335	29386	19316	28624	16603
ENSECAG00000015959	4.329630986	1.68E-09	3.59E-07	69	56	133	222	521	580	634	710
ENSECAG00000011770	6.300827781	1.90E-09	3.99E-07	526	418	474	742	3659	1926	2559	1146
ENSECAG00000022741	4.82592656	2.01E-09	4.14E-07	230	141	199	215	1113	727	1007	490
ENSECAG00000013327	1.646731465	2.49E-09	5.05E-07	99	112	88	121	5	15	8	17
ENSECAG00000009297	3.091743169	3.90E-09	7.77E-07	75	43	38	45	227	261	285	249
ENSECAG00000015853	4.021059147	4.05E-09	7.94E-07	92	130	71	109	484	634	383	396
ENSECAG00000008808	0.692486463	4.42E-09	8.51E-07	4	7	3	2	76	36	67	25
ENSECAG00000022400	5.131327677	5.13E-09	9.71E-07	1167	809	1355	1010	176	240	274	332
ENSECAG00000006595	2.542901678	5.29E-09	9.82E-07	2	10	15	19	45	298	105	313
ENSECAG00000007258	8.53720612	5.36E-09	9.82E-07	1532	2234	2196	1238	22410	7892	9135	6466
ENSECAG00000022555	1.427388781	6.84E-09	1.23E-06	8	2	12	12	128	71.0001	109	34
ENSECAG00000000022	5.523694419	7.28E-09	1.29E-06	205	439	260	293	1042	1231	902	2251
ENSECAG00000017842	2.306432141	8.46E-09	1.48E-06	30	13	13	32	122	73	248	194
ENSECAG00000013193	5.475564882	9.83E-09	1.69E-06	1329	845	1453	2073	369	264	438	171
ENSECAG00000023601	4.006647133	1.10E-08	1.85E-06	55	114	100	50	221	448	545	796
ENSECAG00000023912	0.27672551	1.11E-08	1.85E-06	43	23	35	59	1	2	4	6
ENSECAG00000010886	1.892277292	1.18E-08	1.93E-06	8	23	2	7	113	97	83	185
ENSECAG00000019460	0.737187922	1.34E-08	2.18E-06	120	39	19	56	0	1	7	0
ENSECAG00000026965	2.145201144	1.39E-08	2.22E-06	14	6	9	2	262	198	33	70
ENSECAG00000000354	6.442773829	1.42E-08	2.25E-06	227	544	144	255	1091	3511	1001	5489
ENSECAG00000010510	1.198413783	1.50E-08	2.32E-06	10	13	7	6	60	56	125	47

ENSECAG00000008726	3.833703626	1.51E-08	2.32E-06	49	89	79	94	614	333	640	176
ENSECAG00000013130	1.776751117	1.63E-08	2.46E-06	6	21	13	19	122	97	72	127
ENSECAG00000006636	1.578674066	1.98E-08	2.96E-06	13	1	5	9	84	31	68	212
ENSECAG00000008973	3.636047512	2.01E-08	2.96E-06	46	77	97	71	537	394	334	210
ENSECAG00000011221	1.287677451	2.47E-08	3.60E-06	4	11	1	5	51	153	64	37
ENSECAG00000019884	2.75151012	2.95E-08	4.23E-06	314	193	195	158	37	30	43	41
ENSECAG00000008445	2.014118648	3.29E-08	4.67E-06	125	112	92	229	7	5	23	27
ENSECAG00000016075	7.303649837	3.51E-08	4.93E-06	322	312	379	438	851	12868	1091	5004
ENSECAG00000013169	1.372395931	3.73E-08	5.16E-06	11	9	16	17	68	91	93	56
ENSECAG00000019124	6.732449032	4.25E-08	5.82E-06	380	667	540	1086	1825	4402	1842	4425
ENSECAG00000006474	6.12235789	4.38E-08	5.92E-06	187	661	334	176	1643	3398	1502	1848
ENSECAG00000003002	3.664307126	5.34E-08	7.14E-06	90	44	65	59	507	582	181	230
ENSECAG00000020238	6.37990144	5.84E-08	7.71E-06	554	657	609	766	1440	3802	2074	1987
ENSECAG00000004258	1.339639258	6.21E-08	8.11E-06	2	7	11	13	153	34	67	65
ENSECAG00000009125	0.503239668	7.55E-08	9.75E-06	5	4	7	4	42	64	37	24
ENSECAG00000005083	3.868707927	9.05E-08	1.14E-05	37	69	38	49	177	1127	196	278
ENSECAG00000012681	1.442196834	9.06E-08	1.14E-05	113	45	65	148	12	3	19	8
ENSECAG00000019154	6.072273778	1.27E-07	1.59E-05	241	431	325	454	1065	4367	1176	1248
ENSECAG00000020592	6.218708195	1.33E-07	1.64E-05	1800	3225	2197	1874	368	292	907	635
ENSECAG00000016804	2.178243414	1.38E-07	1.68E-05	26	17	26	38	84	113	134	211
ENSECAG00000015233	3.064691045	1.45E-07	1.76E-05	94	25	25	24	285	234	276	234
ENSECAG00000011261	3.334857531	1.50E-07	1.79E-05	61	67	58	21	468	227	385	162
ENSECAG00000011559	2.749156254	1.51E-07	1.79E-05	113	280	178	302	30	32	58	28
ENSECAG00000019016	1.640506601	1.58E-07	1.85E-05	75	46	139	184	3	1	14	18
ENSECAG00000005671	4.693954091	1.71E-07	1.98E-05	722	733	984	687	121	185	260	246
ENSECAG00000021710	8.143971737	2.09E-07	2.37E-05	1740	2271	2283	2957	9986	7353	10154	4645
ENSECAG00000006060	2.533087884	2.09E-07	2.37E-05	16	18	18	45	307	83	290	65
ENSECAG00000000248	4.941406511	2.13E-07	2.40E-05	99	217	194	94	551	1654	371	1077
ENSECAG00000010836	7.480255517	2.96E-07	3.28E-05	1370	921	1599	1710	6195	1979	6354	6439
ENSECAG00000002764	4.642319989	2.97E-07	3.28E-05	167	751	402	2672	4	71	12	2
ENSECAG00000006973	0.527906685	3.12E-07	3.41E-05	1	4	10	0	47	61	25	41
ENSECAG00000013992	4.919229294	3.22E-07	3.47E-05	810	968	1428	783	62	58	180	338
ENSECAG00000021174	0.990298211	3.23E-07	3.47E-05	53	66	84	62	0	4	18	3
ENSECAG00000006722	1.563076588	3.48E-07	3.69E-05	11	2	4	22	61	28	149	153
ENSECAG00000018476	5.720886542	3.77E-07	3.97E-05	1896	875	1497	2409	439	233	690	225
ENSECAG00000011874	2.868790986	3.95E-07	4.11E-05	35	25	71	31	303	175	115	278
ENSECAG00000021413	6.637145371	4.29E-07	4.42E-05	581	864	762	890	3228	2076	5176	1222
ENSECAG00000009589	5.679004745	4.32E-07	4.42E-05	366	357	439	274	2609	1101	1728	547
ENSECAG00000011101	5.736600545	4.37E-07	4.42E-05	1248	1862	2009	1208	341	413	338	614
ENSECAG00000022278	5.231477455	4.40E-07	4.42E-05	1219	789	1011	1585	256	190	555	244
ENSECAG00000015819	4.116565462	4.48E-07	4.46E-05	96	128	186	185	523	495	463	433
ENSECAG00000009020	4.044371413	4.70E-07	4.64E-05	387	559	563	488	74	112	177	144
ENSECAG00000020204	1.459498774	4.91E-07	4.80E-05	9	10	23	20	57	63	85	122
ENSECAG00000012924	0.322901238	5.15E-07	5.00E-05	30	38	28	63	4	2	5	8
ENSECAG00000014225	2.298874844	5.29E-07	5.09E-05	29	40	18	39	167	130	179	101
ENSECAG00000015574	2.835636595	5.36E-07	5.12E-05	43	32	42	36	401	123	265	84
ENSECAG00000022305	3.603214939	6.66E-07	6.30E-05	59	74	75	46	100	306	345	757
ENSECAG00000005857	1.243462297	7.08E-07	6.65E-05	12	12	11	3	41	118	43	79
ENSECAG00000018258	1.436643113	8.10E-07	7.55E-05	22	11	10	21	47	79	85	106
ENSECAG00000008710	4.136347517	8.51E-07	7.86E-05	59	164	30	32	190	443	715	930
ENSECAG00000021075	1.447048339	8.86E-07	8.10E-05	105	64	98	76	17	12	8	21
ENSECAG00000009485	4.693456526	8.98E-07	8.10E-05	23	19	20	20	20	2651	31	585
ENSECAG00000011051	2.759142403	8.98E-07	8.10E-05	35	36	52	50	240	197	287	78
ENSECAG00000017903	1.933926126	9.43E-07	8.37E-05	129	49	138	194	17	22	28	8
ENSECAG00000016534	4.5642748	9.43E-07	8.37E-05	98	61	71	89	301	1956	121	465
ENSECAG00000004705	1.10685087	1.07E-06	9.45E-05	12	10	8	7	83	32	128	26
ENSECAG00000025092	7.299817616	1.10E-06	9.58E-05	5230	3223	4369	6941	1213	1085	1951	545
ENSECAG00000004883	0.441104883	1.11E-06	9.58E-05	8	3	7	4	70	30	23	35
ENSECAG00000016066	1.387093756	1.14E-06	9.73E-05	58	82	52	173	2	11	1	16
ENSECAG00000023141	2.444660525	1.14E-06	9.73E-05	231	157	119	192	17	18	32	57
ENSECAG00000024158	3.692266656	1.21E-06	0.0001026	37	150	39	24	381	380	460	378
ENSECAG00000007453	4.370182124	1.29E-06	0.0001084	166	197	131	264	604	585	528	521
ENSECAG00000021072	3.925684874	1.58E-06	0.0001317	467	311	400	688	88	111	181	107
ENSECAG00000011086	5.707260905	1.59E-06	0.0001319	68	89	78	42	106	5656	170	563
ENSECAG00000012910	1.985008718	1.70E-06	0.0001403	8	27	22	24	97	203	92	71
ENSECAG00000006267	2.756246701	1.81E-06	0.0001478	25	51	34	81	227	167	246	146
ENSECAG00000021484	7.02276245	1.85E-06	0.0001498	1083	564	806	455	8965	2078	3305	1415
ENSECAG00000011492	5.283653977	1.88E-06	0.0001515	1272	668	1268	1467	453	229	423	241
ENSECAG00000019779	2.828865845	2.01E-06	0.0001602	42	28	80	25	122	167	242	323
ENSECAG00000021438	1.127289723	2.02E-06	0.0001602	11	15	3	11	47	40	51	126
ENSECAG00000000852	1.366646753	2.09E-06	0.0001649	99	50	46	160	7	20	3	4
ENSECAG00000020645	4.665360769	2.14E-06	0.000168	810	453	753	1184	161	101	383	132
ENSECAG00000016953	3.043801198	2.35E-06	0.0001829	40	20	43	42	558	72	347	75
ENSECAG00000012249	1.061637916	2.44E-06	0.0001883	99	70	38	52	10	5	16	12
ENSECAG00000024127	5.48785118	2.77E-06	0.0002102	1368	779	1257	2246	248	244	724	198
ENSECAG00000024200	2.026073969	2.77E-06	0.0002102	35	23	32	35	114	96	151	99
ENSECAG00000004544	1.499306124	2.78E-06	0.0002102	8	23	16	18	43	110	103	74
ENSECAG00000010876	2.925950105	2.97E-06	0.0002232	33	58	35	84	242	224	315	108

ENSECAG00000018101	2.181224556	2.99E-06	0.0002235	12	9	45	21	108	220	161	58
ENSECAG00000010078	4.905661887	3.02E-06	0.0002245	901	679	729	1381	271	187	395	115
ENSECAG00000013053	6.412045657	3.07E-06	0.0002265	3039	1505	2505	3622	691	370	1290	307
ENSECAG00000022890	1.320322003	3.16E-06	0.0002315	12	4	19	7	96	34	156	31
ENSECAG00000008015	2.067418594	3.34E-06	0.0002427	22	35	18	24	188	138	70	85
ENSECAG00000014020	1.932181336	3.36E-06	0.0002427	23	18	28	33	145	55	173	78
ENSECAG00000012930	4.636443022	3.46E-06	0.0002475	2	33	4	16	8	2566	75	534
ENSECAG00000021043	4.306378881	3.47E-06	0.0002475	178	135	156	158	973	377	632	264
ENSECAG00000008432	5.157256339	3.61E-06	0.000256	1255	716	1429	825	224	183	434	395
ENSECAG00000023417	7.856749302	3.69E-06	0.0002601	2157	1665	2232	1272	3098	9926	7206	5798
ENSECAG00000023779	1.238375665	3.75E-06	0.0002627	14	13	5	24	83	69	82	39
ENSECAG00000024055	8.78682514	3.81E-06	0.0002651	1353	2544	987	577	1557	26033	5317	22816
ENSECAG00000023608	1.304313889	3.83E-06	0.0002651	45	81	81	103	8	21	12	12
ENSECAG00000024523	4.150268258	3.91E-06	0.0002692	111	51	136	122	868	170	987	202
ENSECAG00000024847	6.32972616	3.99E-06	0.0002728	66	148	137	80	110	8124	130	1718
ENSECAG00000002814	6.777052731	4.01E-06	0.0002728	1240	1037	1251	894	3159	2857	3188	2368
ENSECAG00000023733	8.59765713	4.48E-06	0.0003032	5	97	95	14	47	41309	16	8621
ENSECAG00000000214	5.272210676	4.61E-06	0.0003099	254	121	279	312	415	2503	708	790
ENSECAG00000021555	4.424158324	4.86E-06	0.0003248	604	728	475	675	162	182	176	243
ENSECAG00000000404	1.919310966	4.89E-06	0.0003248	21	12	3	12	38	293	63	62
ENSECAG00000012938	1.065110718	4.92E-06	0.0003249	83	36	67	71	11	14	11	13
ENSECAG00000013685	5.138979409	4.95E-06	0.0003249	288	336	302	508	828	873	1391	689
ENSECAG00000021765	6.470867039	5.07E-06	0.0003299	33	349	42	84	370	8345	314	2141
ENSECAG00000015865	7.694947265	5.08E-06	0.0003299	758	1008	465	584	1587	16764	981	5671
ENSECAG00000006647	4.552769771	5.50E-06	0.0003553	188	136	199	238	849	441	1179	257
ENSECAG00000020975	6.12560247	5.54E-06	0.0003553	480	369	335	734	4592	622	1823	1232
ENSECAG00000009483	1.331586008	5.58E-06	0.0003562	9	16	19	15	96	34	121	49
ENSECAG00000019243	3.322169704	5.91E-06	0.0003752	0	19	3	6	8	564	28	740
ENSECAG00000008355	2.96485467	6.14E-06	0.0003876	39	46	65	42	341	124	407	80
ENSECAG00000000763	8.717632649	6.33E-06	0.0003974	3621	4212	2587	1165	13263	12868	6598	15553
ENSECAG00000008215	9.857935675	6.65E-06	0.000415	10180	5390	8602	6028	15359	29775	18099	40626
ENSECAG00000017301	2.331925213	6.70E-06	0.0004156	16	18	37	31	288	175	83	48
ENSECAG00000003776	4.407677046	6.98E-06	0.0004311	549	412	455	1259	116	155	281	111
ENSECAG00000017267	5.883073338	7.09E-06	0.0004351	311	353	255	1051	1445	1186	3134	1162
ENSECAG00000014203	2.025406917	7.12E-06	0.0004351	2	2	28	4	35	45	130	343
ENSECAG00000016513	1.054643824	7.26E-06	0.0004409	22	9	12	6	77	38	79	47
ENSECAG00000013872	0.734242956	7.58E-06	0.0004577	73	23	42	78	8	1	5	14
ENSECAG00000013869	2.892952707	7.87E-06	0.0004726	51	32	50	43	257	346	208	52
ENSECAG00000007625	6.503576167	7.95E-06	0.0004726	0	42	51	22	28	9955	64	1555
ENSECAG00000020404	5.962636402	7.95E-06	0.0004726	729.999	658.999	646.999	573.999	2158	1471	1569	1261
ENSECAG00000016974	2.955001513	8.07E-06	0.000476	208	148	263	346	35	43	100	54
ENSECAG00000017827	6.123042479	8.09E-06	0.000476	572	732	518	622	1229	3042	1085	2090
ENSECAG00000016573	0.8789547	8.18E-06	0.000479	2	13	3	11	34	44	27	117
ENSECAG00000013266	2.34444286	8.60E-06	0.0004991	55	34	11	31	186	94	197	124
ENSECAG00000004107	5.190727502	8.61E-06	0.0004991	308	201	348	224	2154	755	849	403
ENSECAG00000010847	8.406072515	8.92E-06	0.0005141	16	193	50	86	43.0004	35047	18	8693
ENSECAG00000011486	6.67095788	9.08E-06	0.0005211	527	552	374	89	462	3761	1435	7293
ENSECAG00000013524	1.383990215	9.16E-06	0.0005227	10	22	11	27	65	92	65	70
ENSECAG00000015456	5.801756166	9.36E-06	0.0005317	1577	981	1658	2263	421	554	786	395
ENSECAG00000011788	3.926050401	9.42E-06	0.0005323	453	246	464	715	106	117	191	71
ENSECAG00000014780	7.117345931	9.63E-06	0.0005416	1656	1197	1324	1602	3188	4330	4005	2960
ENSECAG00000000927	2.966295202	9.79E-06	0.0005478	21	21	22	37	49	671	44	173
ENSECAG00000012652	1.898645479	1.02E-05	0.0005699	18	19	29	21	224	41	90	85
ENSECAG00000000596	4.849959401	1.04E-05	0.0005764	259	346	211	256	1034	402	834	886
ENSECAG00000022510	3.839103911	1.06E-05	0.0005822	501	595	172	517	73	42	117	158
ENSECAG00000006234	5.533284048	1.13E-05	0.0006191	3	53	50	39	30	4685	34	1149
ENSECAG00000014422	6.147908584	1.17E-05	0.0006365	2452	2847	1330	1416	565	517	900	623
ENSECAG00000019318	5.356174838	1.17E-05	0.0006365	512	358	136	130	1711	1260	742	954
ENSECAG00000002437	7.370536355	1.18E-05	0.0006405	1585	891	1469	282	5795	7071	3128	3044
ENSECAG00000009192	9.830412011	1.23E-05	0.0006628	8178	8358	6485	4334	17569	41828	13733	27810
ENSECAG00000001481	6.342695042	1.25E-05	0.0006692	2667	1936	2117	2818	330	498	1105	1058
ENSECAG00000009966	4.863807501	1.26E-05	0.0006692	18	45	25	27	8	3236	65	332
ENSECAG00000021264	0.260155252	1.26E-05	0.0006692	3	6	2	14	28	31	52	26
ENSECAG00000010932	5.564005174	1.29E-05	0.0006819	467	410	403	344	898	2361	809	971
ENSECAG00000017297	3.868438343	1.33E-05	0.0006971	94	139	123	64	528	269	759	174
ENSECAG00000008506	3.399980966	1.40E-05	0.0007307	360	172	275	524	73	44	147	52
ENSECAG00000016457	4.144999231	1.40E-05	0.0007307	483	260	598	825	141	125	232	92
ENSECAG00000014187	0.213580202	1.45E-05	0.0007497	28	29	35	46	3	5	12	5
ENSECAG00000024925	2.242078751	1.45E-05	0.0007511	19	50	22	14	152	212	81	99
ENSECAG00000015663	1.866276412	1.46E-05	0.0007517	20	18	10	34	135	49	226	42
ENSECAG00000015469	4.3618732	1.52E-05	0.000779	197	177	233	98	680	676	543	337
ENSECAG00000020066	6.060067914	1.54E-05	0.0007858	771	614	812	737	2003	1851	1380	1509
ENSECAG00000018134	0.924541285	1.57E-05	0.0007944	116	17	91	31	4	2	16	0
ENSECAG00000009494	4.553306598	1.59E-05	0.0008025	639	548	600	933	154	181	239	314
ENSECAG00000014332	2.063276975	1.62E-05	0.0008128	8	21	6	17	13	131	53	337
ENSECAG00000015350	0.472409442	1.63E-05	0.0008135	22	54	64	29	4	11	6	3
ENSECAG00000019940	0.730054728	1.65E-05	0.0008231	47	44	77	38	2	11	3	14
ENSECAG00000000134	3.253777373	1.68E-05	0.0008357	55	89	44	62	142	524	161	246

ENSECAG00000017486	1.469266756	1.77E-05	0.0008747	7	14	7	11	19	201	24	83
ENSECAG00000004372	3.327165454	1.79E-05	0.0008768	66	64	97	38	184	128	256	632
ENSECAG00000011848	2.291228415	1.79E-05	0.0008768	30	32	24	53	217	102	185	61
ENSECAG00000016140	5.445636325	1.92E-05	0.0009383	1613	1032	1481	1044	226	158	471	643
ENSECAG00000018564	4.916894644	1.94E-05	0.0009434	260	410	277	132	801	639	940	938
ENSECAG00000020535	5.823852017	1.96E-05	0.0009474	423	333	253	239	513	3783	418	1801
ENSECAG00000017567	0.285201304	1.98E-05	0.0009531	7	7	7	9	21	40	30	41
ENSECAG00000018979	1.146993239	2.11E-05	0.0010104	10	20	7	12	63	39	33	122
ENSECAG00000008385	5.154077356	2.13E-05	0.0010153	1177	596	988	1633	322	186	546	123
ENSECAG00000008785	5.805776506	2.15E-05	0.0010161	1818	950	1464	2562	516	407	821	194
ENSECAG00000002157	0.344924105	2.16E-05	0.0010161	38	30	46	37	2	6	14	6
ENSECAG00000019505	2.44065775	2.16E-05	0.0010161	60	29	37	51	190	104	210	107
ENSECAG00000013783	3.226064588	2.16E-05	0.0010161	124	25	25	11	283	264	464	161
ENSECAG00000018574	5.569132272	2.18E-05	0.0010162	1147	1102	2082	1060	521	287	456	554
ENSECAG00000012791	6.849884856	2.18E-05	0.0010162	2489	4466	3210	3523	872	328	1320	1399
ENSECAG00000019677	3.73732626	2.28E-05	0.001056	486	483	470	241	28	49	55	189
ENSECAG00000006607	5.674282888	2.29E-05	0.001056	1768	818	1519	1961	554	265	782	271
ENSECAG00000017103	4.601514248	2.36E-05	0.0010835	743	447	665	962	220	209	335	164
ENSECAG00000011905	2.461685775	2.47E-05	0.0011285	148	194	199	108	35	40	39	51
ENSECAG00000008300	3.011717822	2.47E-05	0.0011285	238	232	305	193	27	79	63	71
ENSECAG00000008471	4.648729533	2.52E-05	0.0011471	737	387	690	1228	130	153	364	241
ENSECAG00000022399	2.442431802	2.62E-05	0.0011866	287	172	189	34	33	30	26	27
ENSECAG00000020111	2.364471084	2.74E-05	0.0012348	138.001	125	166.001	177	49.0006	38.0007	39.0001	41.0239
ENSECAG00000016611	6.459480669	3.00E-05	0.0013479	947	675	1055	1212	1793	2771	2895	1583
ENSECAG00000009044	6.256101748	3.20E-05	0.0014242	829	755	633	864	1634	2821	2579	995
ENSECAG00000027377	8.904976158	3.20E-05	0.0014242	58188	7094	1835	1288	447	1381	1248	1319
ENSECAG00000013081	7.093580049	3.21E-05	0.0014242	83	363	50	61	58	11094	127	6421
ENSECAG00000001249	3.598642969	3.38E-05	0.0014905	34	61	53	43	180	1048	65	120
ENSECAG00000016123	3.86582933	3.39E-05	0.0014905	126	125	165	168	454	300	542	264
ENSECAG00000012179	6.661209568	3.47E-05	0.0015199	564	352	347	185	654	9033	386	1929
ENSECAG00000018613	4.847047534	3.50E-05	0.0015282	1062	525	967	728	194	233	346	337
ENSECAG00000023216	6.35393318	3.51E-05	0.0015285	58	306	62	248	97	4742	178	5583
ENSECAG00000020393	5.766838976	3.58E-05	0.0015502	1614	1315	1341	1842	408	324	716.999	805
ENSECAG00000004420	6.093039116	3.65E-05	0.0015774	608	940	566	871	1817	2002	1642	1460
ENSECAG00000000483	7.561754394	3.67E-05	0.0015774	4000	2007	9662	8944	832	608	2902	1037
ENSECAG00000013477	1.925834781	3.80E-05	0.0016272	12	19	14	18	26	303	48	65
ENSECAG00000024139	4.029984166	3.87E-05	0.001653	18	103	35	60	42	1107	135	728
ENSECAG00000021452	4.432335408	3.93E-05	0.001673	669	410	550	920	178	148	351	124
ENSECAG00000015060	1.721964963	3.97E-05	0.001684	101	79	82	149	20	16	44	7
ENSECAG00000012010	5.343767473	3.99E-05	0.0016846	503	348	272	602	1254	948	1407	656
ENSECAG00000008538	4.667063006	4.05E-05	0.001705	229	304	216	255	955	527	840	385
ENSECAG00000018716	2.185744235	4.08E-05	0.0017115	22	21	34	4	266	86	34	163
ENSECAG00000006931	7.741986879	4.13E-05	0.0017235	1793	2395	1807	1504	5896	8813	2820	5207
ENSECAG00000012516	9.761210443	4.17E-05	0.0017366	9561	8383	7461	4441	15977	32677	15328	30086
ENSECAG00000008821	5.171533955	4.22E-05	0.0017478	3	46	11	21	9	3418	3	1221
ENSECAG00000014384	3.89612876	4.31E-05	0.0017773	398	286	485	625	98	130	216	62
ENSECAG00000024205	9.898412575	4.32E-05	0.0017773	9614	9014	7203	8851	17140	30815	14423	40388
ENSECAG00000021434	1.294379712	4.41E-05	0.0018066	6	29	6	13	90	77	36	77
ENSECAG00000017232	1.873070729	4.42E-05	0.0018071	21	14	45	17	84	105	54	173
ENSECAG00000017239	1.401566754	4.63E-05	0.0018839	27	13	21	25	81	55	63	87
ENSECAG00000000400	6.133523295	4.69E-05	0.0018984	191	530	196	410	398	4307	398	3249
ENSECAG00000023251	3.399242642	4.71E-05	0.0018984	92	46	90	148	149	228	432	381
ENSECAG00000021042	4.450023028	4.73E-05	0.0018984	657	754	745	341	125	174	198	273
ENSECAG00000014953	4.361865605	4.74E-05	0.0018984	621	403	495	853	175	152	310	169
ENSECAG00000017388	3.372725624	4.75E-05	0.0018984	325	196	282	429	94	102	104	53
ENSECAG00000008085	2.406000993	4.76E-05	0.0018984	8	43	15	8	21	245	85	309
ENSECAG00000002892	5.419460608	4.95E-05	0.0019612	1114	826	1220	1601	403	431	618	370
ENSECAG00000022648	1.20282136	4.96E-05	0.0019612	14	24	5	8	36	44	65	126
ENSECAG00000004945	6.811985187	5.01E-05	0.001971	3779	1310	3036	5635	1336	547	1278	743
ENSECAG00000004587	0.798876788	5.01E-05	0.001971	23	64	93	29	7	11	10	9
ENSECAG00000008655	2.918201811	5.16E-05	0.0020193	228	139	212	344	31	53	106	53
ENSECAG00000009012	6.22254811	5.23E-05	0.0020364	480	845	737	443	1370.01	3445	937	2245
ENSECAG00000023632	6.167761106	5.23E-05	0.0020364	1951	1657	1671	2740	508	814	741	930
ENSECAG00000015172	9.60467842	5.28E-05	0.0020458	7370	5334	6747	5331	28014	34725	8052	13526
ENSECAG00000007470	4.748759341	5.32E-05	0.0020563	831	462	635	1177	213	234	378	237
ENSECAG00000019309	6.797380776	5.53E-05	0.0021308	2511	4450	2309	3308	729	781	1201	1554
ENSECAG00000022137	2.719769685	5.58E-05	0.0021414	171	123	181	313	50	41	64	68
ENSECAG00000017905	2.405706255	5.62E-05	0.0021487	20	32	40	51	235	156	183	37
ENSECAG00000019571	5.715774295	5.68E-05	0.0021641	380	545	361	671	868	2581	1198	881
ENSECAG00000015147	6.583726482	5.72E-05	0.0021641	974	942	801	1294	2974	2414	3663	1086
ENSECAG00000016700	4.293129403	5.73E-05	0.0021641	168	223	210	252	444	528	463	563
ENSECAG00000023280	2.209764189	5.73E-05	0.0021641	180	125	82	166	41	21	31	48
ENSECAG00000013979	2.891295242	5.98E-05	0.0022474	37	65	67	12	91	136	337	342
ENSECAG00000020385	5.272972233	6.01E-05	0.002254	164	494	197	198	502	2009	482	1341
ENSECAG00000015340	0.49458296	6.04E-05	0.0022575	52	32	31	57	1	9	5	14
ENSECAG00000011878	3.064170935	6.13E-05	0.0022767	261	177	206	358	72	64	108	36
ENSECAG00000006337	5.068618909	6.13E-05	0.0022767	441	365	315	379	755	976	967	697
ENSECAG00000016085	2.122359024	6.21E-05	0.0022986	19	13	7	7	8	356	23	142

ENSECAG00000017126	4.673083984	6.34E-05	0.0023397	649	802	689	662	229	187	324	315
ENSECAG00000014536	4.039241156	6.37E-05	0.0023412	518	339	470	600	135	139	252	73
ENSECAG00000001227	0.812149295	6.39E-05	0.002343	2	10	17	7	68	26	95	21
ENSECAG00000024694	3.012058948	6.48E-05	0.0023618	246	134	239	346	63	57	107	49
ENSECAG00000005090	6.126874938	6.49E-05	0.0023618	212	249	164	144	227	7107	140	893
ENSECAG00000000942	3.313133508	6.64E-05	0.0024102	112	39	86	71	465	174	389	113
ENSECAG00000013094	2.620911141	6.72E-05	0.0024293	42	73	13	22	298	115	209	105
ENSECAG00000014501	0.709153601	6.80E-05	0.0024536	10	11	4	9	44	92	21	23
ENSECAG00000022653	4.192374648	6.83E-05	0.0024559	145	170	127	214	598	184	956	352
ENSECAG00000004810	0.760836865	6.96E-05	0.0024956	8	13	11	19	50	27	59	49
ENSECAG00000001268	0.033184577	7.35E-05	0.0026184	26	28	28	37	2	7	11	2
ENSECAG00000000362	5.917680653	7.35E-05	0.0026184	689	512	643	909	1509	2013	1425	1089
ENSECAG00000019423	5.448366819	7.47E-05	0.0026508	337	480	365	629	731	1746	966	1037
ENSECAG00000017647	3.276447817	7.51E-05	0.0026587	292	192	257	394	83	62	139	70
ENSECAG00000009472	4.393187788	7.64E-05	0.0026965	647	357	608	897	265	132	259	88
ENSECAG00000010552	6.318349106	7.76E-05	0.002729	2362	2271	2570	1535	899	470	977	976
ENSECAG00000017483	2.242234049	7.78E-05	0.002729	33	47	24	46	170	58	208	104
ENSECAG00000023561	2.028698769	7.87E-05	0.0027503	15	7	13	32	372	34	67	30
ENSECAG00000000454	7.779184612	7.90E-05	0.002754	5179	3927	6114	11436	1209	1265	3826	1712
ENSECAG00000014730	0.240806842	7.99E-05	0.0027767	4	9	11	7	36	26	27	37
ENSECAG00000019660	6.460220411	8.29E-05	0.0028657	231	391	161	182	156	8173	186	2159
ENSECAG00000001910	5.822008761	8.30E-05	0.0028657	565	416	524	395	3250	1152	1366	479
ENSECAG00000019161	3.051220995	8.37E-05	0.0028821	244	179	203	335	64	71	102	67
ENSECAG00000012705	3.847559147	8.46E-05	0.0029032	188	97	127	149	554	302	463	221
ENSECAG00000016866	5.81569834	8.84E-05	0.0030233	730	673	559	564	1262	1829	1148	1364
ENSECAG00000009958	10.26309607	8.89E-05	0.0030266	2876	8951	4177	2212	5961	95054	5538	40781
ENSECAG00000005925	6.056073962	8.91E-05	0.0030266	2047	1157	1818	2718	780	654	834	270
ENSECAG00000010914	6.109430946	8.93E-05	0.0030266	888	662	862	855	1806	1960	1441	1571
ENSECAG00000008465	5.691214924	9.07E-05	0.0030655	1562	1120	1341	1525	521	437	704	675
ENSECAG00000022447	1.50892878	9.14E-05	0.0030798	67	64	137	87	14	2	36	18
ENSECAG00000012939	3.443387495	9.17E-05	0.0030803	98	84	131	142	218	271	378	275
ENSECAG00000016328	1.344622477	9.24E-05	0.0030803	7	25	3	13	39	69	29	165
ENSECAG00000005194	4.183094068	9.24E-05	0.0030803	473	461	476	1097	42	9	288	39
ENSECAG00000024185	3.663634705	9.25E-05	0.0030803	3	46	8	42	19	586	27	1029
ENSECAG00000003925	7.155077625	9.64E-05	0.0031938	654	1453	784	480	1823	9748	1188	3303
ENSECAG00000008634	4.893307988	9.64E-05	0.0031938	1053	492	759	1165	308	214	459	152
ENSECAG00000011262	6.542000152	9.92E-05	0.0032712	849	582	693	1019	6042	1503	1670	1081
ENSECAG00000020125	4.878815658	9.94E-05	0.0032712	203	469	273	229	935	647	742	763
ENSECAG00000016460	2.045594813	0.0001008	0.0033078	28	14	14	38	25	244	70	135
ENSECAG00000025109	3.198257912	0.0001019	0.0033356	179	179	241	585	117	44.0005	64.0006	24.0004
ENSECAG00000021453	3.221656833	0.0001023	0.0033384	224	204	234	440	94	85	96	53
ENSECAG00000016653	10.69120737	0.0001034	0.0033668	20912	13459	18959	19529	49267	41169	40853	37822
ENSECAG00000009163	3.166857734	0.0001066	0.0034591	296	169	244	348	77	68	127	47
ENSECAG00000014517	4.70483233	0.0001073	0.0034737	835	463	751	952	242	293	334	131
ENSECAG00000013398	6.031253006	0.0001077	0.0034759	752	672	640	813	2724	1092	1814	1100
ENSECAG00000016962	6.013653478	0.0001089	0.0035058	344	1009	423	685	1064	2455	1601	1705
ENSECAG00000016394	3.453587383	0.0001105	0.0035415	18	82	32	28	84	904	43	264
ENSECAG00000019083	9.072113647	0.0001107	0.0035415	5840	5265	4746	5408	11330	22290	9253	12730
ENSECAG00000014601	8.448007005	0.0001112	0.0035485	157	1251	132	92	445	34880	251	8392
ENSECAG00000015728	1.890113188	0.0001116	0.0035515	87	78	92	188	26	17	37	38
ENSECAG00000007518	4.566324776	0.0001135	0.0035911	20	109	26	48	46	2337	33	479
ENSECAG00000020137	2.03594558	0.0001135	0.0035911	24	28	54	39	100	73	148	128
ENSECAG00000019668	1.591024004	0.0001149	0.0036258	96.0006	62.0005	114.001	78.001	13.0009	23.001	32.0011	27
ENSECAG00000020164	6.284287078	0.0001167	0.0036706	282	841	419	416	864	5217	647	1982
ENSECAG00000025265	5.629329012	0.000117	0.0036721	3567	1080	548	926	206	505	246	396
ENSECAG00000011895	7.081426163	0.0001174	0.0036729	7	164	31	82	8	13230	9	4216
ENSECAG00000008760	2.418446397	0.0001209	0.0037726	139	79	163	284	25	35	76	26
ENSECAG00000019705	4.326089483	0.0001223	0.0038054	540	366	619	827	176	199	283	84
ENSECAG00000018681	6.054949598	0.0001246	0.0038657	1402	2015	1918	1991	394	573	763	1054
ENSECAG00000003773	1.620887333	0.0001279	0.0039573	16	28	31	32	74	62	128	71
ENSECAG00000013344	1.270810808	0.0001294	0.0039905	86	65	69	76	2	8	18	31
ENSECAG00000021220	4.470019581	0.0001296	0.0039905	632	1150	315	450	117	215	197	205
ENSECAG00000005550	3.752403445	0.00013	0.0039909	44	118	44	94	129	1008	144	246
ENSECAG00000003816	8.76579714	0.0001316	0.004029	1972	4985	3086	1852	5556	22150	4263	16872
ENSECAG00000005927	5.189745607	0.0001324	0.0040418	570	309	295	427	1304	809	977	644
ENSECAG00000016384	6.527000528	0.0001331	0.0040527	803	663	589	860	6195	1249	2278	720
ENSECAG00000006803	5.416988539	0.0001342	0.0040755	412	515	499	339	1885	661	1031	878
ENSECAG00000023203	5.928726597	0.000138	0.0041734	1794	1030	1742	2177	622	568	991	469
ENSECAG00000018069	5.988599276	0.0001381	0.0041734	847	628	682	907	1918	1212	2017	1195
ENSECAG00000013722	6.298978073	0.0001385	0.0041743	523	1149	579	885	1437	3117	1412	2150
ENSECAG00000000281	4.367253942	0.0001417	0.0042568	674	315	600	853	214	146	310	88
ENSECAG00000015782	9.613265572	0.0001421	0.0042574	13864	4776	3955	6128	13223	24772	28657	19522
ENSECAG00000015807	6.049420265	0.0001431	0.0042763	534	772	619	980	909	2372	2049	1442
ENSECAG00000023776	2.965361637	0.000144	0.004292	245	115	212	362	71	46	102	53
ENSECAG00000011651	1.726758462	0.0001456	0.0043288	20	28	28	16	76	164	87	36
ENSECAG00000015544	5.587838825	0.0001462	0.0043349	62	247	99	178	57	4406	193	1142
ENSECAG00000019150	0.871894684	0.0001479	0.0043685	49	37	93	44	6	5	5	25
ENSECAG00000023736	-0.043903477	0.0001483	0.0043685	8	0	10	4	31	27	25	21

ENSECAG00000017373	3.541441485	0.0001484	0.0043685	114	89	111	143	358	387	329	146
ENSECAG00000009570	6.744249223	0.0001503	0.0044111	383	403	373	518	315	10096	383	1757
ENSECAG00000001042	7.513072821	0.0001525	0.0044634	1849	1996	1836	2539	3959	6049	4713	3660
ENSECAG00000015839	5.895415643	0.0001562	0.0045605	478	578	473	720	928	3153	1086	1001
ENSECAG00000023383	0.263840256	0.0001572	0.0045796	40	19	47	31	8	5	11	7
ENSECAG00000000271	2.15290214	0.0001592	0.0046219	11	50	34	43	109	71	209	122
ENSECAG00000017625	3.829338834	0.0001595	0.0046219	145	128	72	165	653	147	491	291
ENSECAG00000021813	4.12251833	0.0001605	0.0046375	173	148	162	202	618	512	536	172
ENSECAG00000011250	2.676222882	0.0001613	0.0046517	189	154	205	181	44	39	94	59
ENSECAG00000013965	2.045222001	0.0001618	0.0046522	39	30	52	27	139	83	122	98
ENSECAG00000004338	1.357498781	0.0001626	0.0046632	28	10	15	31	87	55	95	43
ENSECAG00000009568	2.921574942	0.000164	0.0046818	222	137	180	355	65	48	103	57
ENSECAG00000009543	5.627874279	0.000164	0.0046818	1774	1153	1398	1236	669	239	772	310
ENSECAG00000009941	4.058292302	0.0001657	0.0047167	156	225	165	164	381	499	373	437
ENSECAG00000023826	7.637401971	0.0001678	0.0047574	2123	1184	1880	1048	1190	9042	4367	7637
ENSECAG00000018002	9.124651258	0.0001679	0.0047574	1365	4701	1097	296	2706	12823	4899	52237
ENSECAG00000022511	6.039581312	0.0001684	0.0047578	163	291	188	219	127	6569	202	884
ENSECAG00000014399	3.691393906	0.0001688	0.0047586	17	96	44	32	46	1160	132	188
ENSECAG00000009479	3.615033518	0.0001709	0.004805	166	124	99	85	370	206	514	240
ENSECAG00000026891	3.59482167	0.0001717	0.0048051	393	185	390	450	152	83	139	62
ENSECAG00000019242	4.845229779	0.0001717	0.0048051	928	790	937	544	170	211	337	422
ENSECAG00000017696	1.275218461	0.0001725	0.0048134	53	93	50	81	20	17	16	21
ENSECAG00000019869	1.082117115	0.0001773	0.0049255	21	22	14	14	57	47	54	65
ENSECAG00000000356	1.994373392	0.0001774	0.0049255	128	107	122	105	28	14	38	51
ENSECAG00000016842	0.467841959	0.0001829	0.0050668	13	9.00189	8.00006	9.00006	57.0021	37	21	30
ENSECAG00000020362	1.484833162	0.0001846	0.0051011	94	59	90	91	7	25	37	12
ENSECAG00000017666	2.093054217	0.0001861	0.0051321	35	31	27	37	198	104	141	33
ENSECAG00000010800	4.585821095	0.000189	0.0051916	688	531	648	833	179	155	241	403
ENSECAG00000018650	6.060377497	0.0001892	0.0051916	2120	1117	1830	2507	697	308	1174	622
ENSECAG00000010277	8.662524128	0.0001922	0.0052604	4710	3933	3150	1414	4995	13874	7318	18734
ENSECAG00000010020	5.104921758	0.0001971	0.0053831	299	133	40	289	178	2264	396	1166
ENSECAG00000010014	2.585269565	0.0001984	0.0054056	258	100	334	108	23	63	5	5
ENSECAG00000012759	1.449570762	0.0002035	0.0055236	21	23	23	27	75	99	63	48
ENSECAG00000007487	2.954971742	0.0002037	0.0055236	196	127	207	400	52	55	113	52
ENSECAG00000018665	4.355007637	0.000207	0.0055867	488	444	446	940	181	237	236	174
ENSECAG00000014680	3.340609816	0.0002071	0.0055867	126	60	109	75	449	175	321	157
ENSECAG00000009648	7.372573156	0.0002085	0.0056126	1898	1819	2081	1797	5102	4086	4514	2935
ENSECAG00000018420	4.374354901	0.000211	0.0056662	200	227	138	293	575	501	866	262
ENSECAG00000013350	3.628441558	0.0002142	0.0057392	76	63	89	69	1044	74	165	178
ENSECAG00000011973	0.582922358	0.0002161	0.0057756	0	15	2	0	19	49	22	92
ENSECAG00000021609	3.133262155	0.0002191	0.0058427	6	29	5	24	5	637	7	465
ENSECAG00000012441	6.65784747	0.0002213	0.005884	722	839	1099	1222	4720	1411	3977	982
ENSECAG00000024558	2.83192181	0.0002217	0.005884	186	142	184	322	60	46	108	46
ENSECAG00000016581	0.884830092	0.0002233	0.005912	3	18	8	21	31	76	42	52
ENSECAG00000023752	5.308041836	0.0002265	0.0059831	501	482	419	480	977	1148	849	893
ENSECAG00000025076	1.997865069	0.0002285	0.0060118	12	19	10	77	127	104	138	88
ENSECAG00000014240	4.196868387	0.0002286	0.0060118	156	180	288	205	477	395	509	477
ENSECAG00000010174	3.796931984	0.0002294	0.0060171	124	187	142	141	313	413	371	317
ENSECAG00000023398	2.847963926	0.0002327	0.0060904	75	45	83	69	140	281	209	127
ENSECAG00000000039	2.925311285	0.0002337	0.0061033	0	366	128	672	0	8	1	23
ENSECAG00000019000	4.409800338	0.0002353	0.0061305	134	345	98	156	344	810	440	696
ENSECAG00000006670	4.47710077	0.0002368	0.0061562	179	225	254	249	488	952	355	461
ENSECAG00000019877	4.98670929	0.0002391	0.0062004	136	190	169	150	126	2641	196	655
ENSECAG00000023019	3.501319274	0.0002413	0.006243	357	195	293	490	119	103	138	46
ENSECAG00000016817	3.881078363	0.0002449	0.0063213	160	119	234	117	408	302	412	400
ENSECAG00000000859	7.038276093	0.0002479	0.0063847	1614	1204	1852	1596	3923	2583	3151	3416
ENSECAG00000008811	5.055951305	0.0002572	0.0066089	6	64	6	41	4	2964	3	1312
ENSECAG00000019821	4.343524262	0.0002589	0.0066375	184	269	185	163	387	769	325	604
ENSECAG00000011636	5.155604834	0.0002639	0.0067522	1629	359	2531	157	160	145	401	59
ENSECAG00000005328	1.484440701	0.0002675	0.006827	8	7	28	40	57	72	143	49
ENSECAG00000007620	3.520818748	0.0002717	0.0069134	387	174	318	472	111	56	184	75
ENSECAG00000014137	1.21015765	0.0002721	0.0069134	22	6	14	33	74	36	95	51
ENSECAG00000024196	5.490697021	0.0002755	0.0069859	1231	853	1172	1665	477	517	674	312
ENSECAG00000016405	2.892114199	0.0002768	0.0069956	73	40	82	69	273	265	159	90
ENSECAG00000009370	4.442614524	0.0002771	0.0069956	616	355	551	1000	252	169	318	114
ENSECAG00000017839	3.005335713	0.0002791	0.0070304	63	78	55	91	79	185	424	217
ENSECAG00000016495	4.40968127	0.00028	0.0070359	727	363	639	776	244	121	341	74
ENSECAG00000014784	7.937407382	0.0002868	0.0071904	8183	5085	8577	5997	2037	1010	4012	2795
ENSECAG00000015261	3.490640319	0.0002925	0.0073166	18	84	12	30	28	863	37	433
ENSECAG00000008827	3.447423497	0.0002934	0.0073166	390	139	293	490	97	43	168	78
ENSECAG00000015806	4.628220065	0.0002937	0.0073166	804	403	694	982	214	257	370	94
ENSECAG00000015014	1.376003491	0.0002954	0.0073306	6	38	12	9	40	50	110	103
ENSECAG00000017324	7.890534027	0.0002956	0.0073306	3110	2045	3174	2937	5516	6106	6008	5948
ENSECAG00000018322	3.149678396	0.0002966	0.0073396	295	141	250	353	84	84	109	42
ENSECAG00000019765	4.323492571	0.0003003	0.0074162	50	181	61	31	155	1730	87	357
ENSECAG00000009443	0.621283449	0.0003039	0.0074791	19	9	1	3	72	18	60	29
ENSECAG00000007804	6.4289538	0.0003045	0.0074791	745	1130	698	1052	1158	3675	1865	2042
ENSECAG00000009337	1.172124827	0.0003049	0.0074791	31	10	18	20	56	61	63	56

ENSECAG00000014470	2.892431214	0.000306	0.0074913	86	60	76	82	245	107	221	208
ENSECAG00000013180	2.367005829	0.0003074	0.0075086	49	42	44	57	197	63	188	112
ENSECAG00000026955	5.480371714	0.0003093	0.0075382	615	499	484	396	1191	1557	827	830
ENSECAG00000014674	3.480744602	0.0003181	0.007736	337	198	408	481	25	64	206	21
ENSECAG00000003473	3.535422201	0.0003195	0.0077556	74	81	69	69	95	850	94	241
ENSECAG00000000660	1.474522078	0.0003233	0.0078262	29	23	19	19	78	79	118	28
ENSECAG00000014594	1.079909886	0.0003244	0.0078262	15	16	25	11	83	59	48	33
ENSECAG00000007459	3.126848141	0.0003245	0.0078262	45	135	77	62	236	151	311	255
ENSECAG00000008194	4.513650359	0.0003272	0.0078737	152	196	170	158	532	1481	225	213
ENSECAG00000005905	6.8122837	0.0003281	0.0078804	1078	413	496	192	10017	1453	1925	335
ENSECAG00000001757	5.872006356	0.0003299	0.0079064	884	591	690	348	1289	2066	1169	1365
ENSECAG00000024723	8.589577786	0.0003319	0.0079366	3022	3891	3513	4663	6836	18761	6473	7546
ENSECAG00000019825	5.437905985	0.0003329	0.0079453	1368	883	1269	1596	403	142	890	199
ENSECAG00000024357	4.647012019	0.0003339	0.0079515	866	439	651	903	260	184	437	127
ENSECAG00000019319	6.304185462	0.0003365	0.0079969	862	638	587	724	1139	4558	846	1680
ENSECAG00000015547	6.134534145	0.0003386	0.0080306	406	579	425	492	490	5147	551	1507
ENSECAG00000011090	6.781953879	0.0003398	0.0080419	618	1078	902	761	1265	7026	951	2583
ENSECAG00000021133	5.61439467	0.0003415	0.0080552	1313	942	1321	1749	592	290	738	604
ENSECAG00000010859	3.904119895	0.0003418	0.0080552	440	263	377	636	146	117	240	94
ENSECAG00000020946	0.373152305	0.0003466	0.0081518	18	8	4	9	32	28	37	39
ENSECAG00000000650	5.388198907	0.00035	0.008215	9	215	10	34	50	3797	55	1355
ENSECAG00000019038	3.876626767	0.0003516	0.008234	419	241	381	683	140	109	237	71
ENSECAG00000018253	2.227160164	0.0003589	0.008388	38	4	10	2	375	28	202	10
ENSECAG00000009862	5.776218835	0.0003624	0.0084539	405	796	336	790	1413	1876	1594	734
ENSECAG00000006854	4.594027809	0.0003658	0.0085146	269	299	277	198	416	584	500	967
ENSECAG00000023190	3.795503534	0.0003834	0.0089053	130	118	154	213	270	362	557	249
ENSECAG00000020787	2.243984096	0.0003872	0.0089764	44	13	35	70	150	61	227	100
ENSECAG00000002513	3.149300462	0.000389	0.0090001	286	117	308	382	57	30	164	38
ENSECAG00000020944	4.182734449	0.0003924	0.0090584	437	394	485	701	133	219	257	152
ENSECAG00000009393	0.334162515	0.0003965	0.009135	4	17	2	6	44	13	39	45
ENSECAG00000010496	5.099647657	0.0004012	0.0092259	2	79	13	24	3	3512	5	822
ENSECAG00000019828	1.257702538	0.0004125	0.0094658	22	21	7	24	23	69	92	80
ENSECAG00000022585	4.927479726	0.0004166	0.0095409	901	596	1060	952	142	121	536	400
ENSECAG00000016531	5.647948784	0.00042	0.0095994	1316	892	1309	2079	368	296	782	752
ENSECAG00000020971	2.865165817	0.0004252	0.009697	75	41	84	65	188	79	171	362
ENSECAG00000012200	0.786973102	0.0004262	0.009697	47	89	29	38	3	10	4	22
ENSECAG00000018054	2.068054104	0.0004275	0.009697	132	108	145	88	38	36	24	46
ENSECAG00000013079	2.312541937	0.0004277	0.009697	47	52	57	42	113	88	151	169
ENSECAG00000020664	0.47861576	0.0004362	0.0098554	6	16	8	11	22	27	40	60
ENSECAG00000004471	2.954654138	0.0004364	0.0098554	81	56	85	98	198	242	246	115
ENSECAG00000005775	1.318238807	0.000438	0.0098718	13	29	26	18	55	81	73	52
ENSECAG00000024408	1.989955365	0.00044	0.009898	44	34	46	32	79	90	110	134
ENSECAG00000010821	4.426603857	0.0004429	0.0099427	694	349	609	811	196	174	387	105
ENSECAG00000021993	4.081096452	0.0004456	0.009983	141	114	115	165	157	1116	231	304
ENSECAG00000024722	4.882846063	0.000448	0.010002	324	419	259	397	885	547	801	702
ENSECAG00000024209	5.818582647	0.0004482	0.010002	314	428	256	402	291	4437	392	1138
ENSECAG00000018928	1.998458758	0.0004496	0.0100134	38	43	26	52	116	87	117	94
ENSECAG00000013239	4.917480221	0.0004622	0.0102737	390	435	194	205	624	711	566	1245
ENSECAG00000018870	9.407926928	0.0004638	0.0102862	5159	3747	4189	5008	5494	50494	4163	14537
ENSECAG00000008662	6.869243842	0.0004645	0.0102862	3142	3033	3625	2763	1195	723	1733	1577
ENSECAG00000024358	2.1603147	0.0004688	0.0103525	35	42	36	46	184	126	114	49
ENSECAG00000015809	2.758072438	0.0004694	0.0103525	48	96	58	55	106	202	189	216
ENSECAG00000003029	1.921678706	0.0004803	0.0105702	109	41	111	197	26	21	52	24
ENSECAG00000022707	1.364016939	0.0004811	0.0105702	15	21	26	26	50	54	139	40
ENSECAG00000014813	3.88195572	0.0004832	0.0105964	499	229	418	549	168	147	182	67
ENSECAG00000014104	1.970816322	0.0004902	0.0107291	13	49	20	20	69	218	41	101
ENSECAG00000023564	4.309452438	0.0004931	0.0107716	637	331	559	685	261	225	174	146
ENSECAG00000020868	3.707195242	0.0004989	0.0108765	323	295	259	592	150	119	123	138
ENSECAG00000020573	4.909211424	0.0005058	0.0109856	897	515	830	1109	291	256	551	192
ENSECAG00000016721	7.740423711	0.0005068	0.0109856	1543	794	1025	377	878	19020	655	4188
ENSECAG00000004666	0.967807502	0.0005068	0.0109856	67	24	57	81	13	18	20	7
ENSECAG00000017538	4.29957678	0.0005112	0.0110528	174	217	199	167	248	837	278	673
ENSECAG00000016973	1.786290842	0.0005118	0.0110528	52	27	344	28	14	4	22	23
ENSECAG00000019060	3.518767132	0.0005189	0.0111664	74	92	166	61	611	142	337	178
ENSECAG00000020720	3.066059644	0.000519	0.0111664	196	201	242	314	62	45	148	74
ENSECAG00000019151	2.269652315	0.0005241	0.0112545	50	34	27	69	128	64	236	105
ENSECAG00000005614	0.344885962	0.0005252	0.011257	22	42	19	79	5	0	0	15
ENSECAG00000007305	5.537470107	0.0005287	0.01131	737	309	366	452	2272	785	1378	498
ENSECAG00000004655	7.229305226	0.0005391	0.0115114	2451	1512	1521	1160	2969	4644	3012	4446
ENSECAG00000010772	5.277492979	0.0005418	0.0115458	1150	779	926	1267	527	359	537	376
ENSECAG00000014359	0.920923465	0.0005432	0.0115557	11	27	9	7	76	51	35	40
ENSECAG00000006686	5.489793693	0.0005453	0.0115739	1398	741	1148	1526	546	418	697	425
ENSECAG00000016362	3.41593609	0.0005464	0.0115739	120	132	63	82	290	432	166	215
ENSECAG00000002581	4.716376257	0.0005475	0.0115739	648	534	761	887	351	213	368	257
ENSECAG00000025141	4.152963406	0.0005482	0.0115739	70	253	100	217	273	745	317	553
ENSECAG00000017352	2.580479618	0.0005559	0.0117148	43	56	92	45	113	151	178	185
ENSECAG00000021378	3.616710771	0.0005674	0.0118957	128.001	71.0005	145.001	111	555.001	151.001	506	143.024
ENSECAG00000013070	6.727841422	0.0005677	0.0118957	346	663	415	667	316	9366	440	1973

ENSECAG00000008080	3.812820907	0.0005677	0.0118957	337	285	427	496	123	105	203	175
ENSECAG00000010928	5.435379529	0.0005687	0.0118957	46	251	42	57	39	4449	48	699
ENSECAG00000023744	2.76319175	0.0005703	0.0119077	48	74	100	49	214	130	197	169
ENSECAG00000000546	3.949164177	0.0005718	0.0119184	177	166	218	163	368	348	393	422
ENSECAG00000023015	0.334489921	0.0005729	0.0119191	54	31	32	22	11	5	12	8
ENSECAG00000010327	6.853176398	0.0005947	0.0123486	1554	907	1220	1394	4466	2035	4222	1335
ENSECAG00000008851	9.154810811	0.0005971	0.0123771	4772	7724	3200	3335	6696	27558	7289	19571
ENSECAG00000023607	6.336423738	0.0006009	0.0124335	958	990	925	1188	2323	1327	2864	1468
ENSECAG00000009921	6.977539025	0.0006092	0.012571	1796	831	817	470	1916	8034	1929	1652
ENSECAG00000003193	2.221930876	0.0006098	0.012571	60	56	24	20	145	84	117	159
ENSECAG00000019726	2.746444055	0.000615	0.0126554	19	63	22	8	44	539	33	161
ENSECAG00000005624	2.571470421	0.0006168	0.0126703	60	35	76	76	164	152	194	105
ENSECAG00000012371	4.405028147	0.0006197	0.0127069	53	86	93	44	16	1955	38	514
ENSECAG00000016029	2.38342943	0.0006267	0.0128271	133	78	157	241	64	35	44	43
ENSECAG00000023440	3.830518429	0.0006309	0.0128825	335	315	346	595	94	157	139	193
ENSECAG00000009430	6.149196398	0.0006317	0.0128825	1063	824	867	812	1455	1875	1455	2007
ENSECAG00000008221	9.264871502	0.0006369	0.0129642	3723	9384	5228	4182	9951	22006	8462	24773
ENSECAG00000002540	6.718843874	0.0006399	0.0130035	2951	1725	2659	5156	653	901	2100	406
ENSECAG00000011074	1.549161278	0.000649	0.0131633	93	37	62	154	19	18	32	28
ENSECAG00000009285	3.407649586	0.0006514	0.0131885	46	81	63	52	41	795	79	278
ENSECAG00000008866	7.085306468	0.0006772	0.0136884	4430	5636	3626	1642	984	1901	711	1083
ENSECAG00000003770	3.91601862	0.000679	0.0136996	303	264	433	739	100	97	229	185
ENSECAG00000014952	5.166702354	0.0006809	0.0137124	182	435	196	250	234	2120	354	1211
ENSECAG00000000591	5.105076209	0.0006822	0.0137158	380	423	385	285	558	1388	546	939
ENSECAG00000008461	1.784236889	0.0006844	0.0137348	109	56	85	154	12	32	45	27
ENSECAG00000007123	6.046971564	0.0006891	0.0138047	742	709	632	623	901	3101	829	1826
ENSECAG00000008985	2.670013729	0.0006904	0.0138069	74	48	61	54	89	83	221	307
ENSECAG00000009707	0.78495144	0.0006947	0.0138693	11	22	9	10	48	31	26	78
ENSECAG00000021260	3.615928179	0.0006974	0.0138968	327	229	340	448	124	99	163	144
ENSECAG00000020234	8.735131327	0.0007065	0.0140541	11239	8017	11531	15653	4680	3909	6147	4474
ENSECAG00000012693	4.638174291	0.0007109	0.0140745	454	199	233	153	666	688	880	360
ENSECAG00000006746	0.408057707	0.0007115	0.0140745	49	29	35	43	1	3	7	21
ENSECAG00000020188	3.703863817	0.0007122	0.0140745	444	359	391	257	121	49	202	146
ENSECAG00000006714	2.986778785	0.0007128	0.0140745	95	53	87	29	305	277	84	177
ENSECAG00000013661	2.339869977	0.0007137	0.0140745	66	9	50	26	255	71	180	73
ENSECAG00000020957	3.672891879	0.0007213	0.0141991	103	80	141	159	155	653	226	287
ENSECAG00000010752	0.408821668	0.0007235	0.014219	24	32	26	70	7	6	9	16
ENSECAG00000022453	1.156392195	0.0007354	0.0144281	54	46	71	81	22	12	32	10
ENSECAG00000008693	7.7987151	0.000748	0.0146495	2636	3061	2481	2291	6438	5686	6134	3775
ENSECAG00000010840	2.061486529	0.000751	0.0146828	47	44	38	44	123	82	135	88
ENSECAG00000003105	2.631146824	0.0007551	0.0147367	37	9	74	41	288	50	372	49
ENSECAG00000013457	8.664270635	0.0007568	0.0147451	12	766	75	274	10	43113	23	8341
ENSECAG00000009247	7.018125061	0.0007593	0.0147681	1785	1016	694	994	5957	678	6176	2135
ENSECAG00000015849	8.808256679	0.0007641	0.0148364	12914	10112	14544	11437	4053	2530	6631	6407
ENSECAG00000011970	5.514788609	0.0007662	0.0148512	1069	1466	1405	859	359	510	503	669
ENSECAG00000013323	3.847103983	0.0007732	0.0149613	534	306	340	427	76	110	264	144
ENSECAG00000007805	1.352937012	0.0007781	0.0150319	27	15	17	5	32	17	166	95
ENSECAG00000020270	3.077659353	0.0007814	0.0150688	317	108	257	331	72	47	145	27
ENSECAG00000017428	2.197342064	0.0007828	0.0150699	110	96	123	232	14	21	91	20
ENSECAG00000023430	6.841344234	0.0007852	0.0150918	45	556	50	139	53	11420	48	2675
ENSECAG00000020612	2.280878067	0.0007866	0.015092	78	32	13	18	67	69	229	207
ENSECAG00000008594	6.35958779	0.0008026	0.015374	1328	2696	2732	1979	887	840	1325	609
ENSECAG00000010158	2.131529729	0.0008123	0.0155335	33	57	30	53	88	132	106	124
ENSECAG00000000744	4.491573466	0.0008158	0.0155736	93	111	147	95	58	1884	71	556
ENSECAG00000023992	9.59232824	0.0008212	0.015651	37	2106	148	282	94	80904	57	16594
ENSECAG00000016546	3.248226393	0.0008336	0.015861	98	112	94	133	192	231	284	244
ENSECAG00000019433	1.147040854	0.000836	0.0158806	68	40	53	88	19	17	28	13
ENSECAG00000017892	5.989833893	0.0008447	0.0160191	617	645	569	1163	1480	2335	1582	850
ENSECAG00000013622	5.187823604	0.0008543	0.0161729	902	706	1097	1214	400	238	677	379
ENSECAG00000015994	2.175078998	0.0008582	0.0162204	11	44	8	38	15	259	83	164
ENSECAG00000023347	5.746862009	0.0008636	0.0162767	201	329	200	230	132	4934	108	990
ENSECAG00000012421	1.455184366	0.0008641	0.0162767	9	11	30	36	54	99	28	113
ENSECAG00000021968	9.060192528	0.0008655	0.0162774	5436	5153	4138	1870	4723	30598	5942	16424
ENSECAG00000011465	5.453493507	0.0008772	0.0164704	485	438	416	411	606	2223	517	1075
ENSECAG00000020784	3.875822026	0.0008812	0.0165165	471	216	373	606	146	123	242	91
ENSECAG00000011602	4.694633372	0.0008906	0.0166593	262	68	318	3276	59	18	135	234
ENSECAG00000012922	4.862273159	0.0008917	0.0166593	858	744	916	714	123	189	353	527
ENSECAG00000019802	6.650680596	0.0008954	0.0167002	1449	1196	870	547	4013	2258	3278	1008
ENSECAG00000015905	6.482363725	0.000911	0.0169419	1341	897	1158	1205	2763	1654	1883	2253
ENSECAG00000004913	3.317517979	0.0009113	0.0169419	220	284	335	338	28	121	41	134
ENSECAG00000021750	7.036859065	0.0009128	0.0169419	818	1795	300	2085	1944	3923	4582	3877
ENSECAG00000016155	2.788140776	0.0009154	0.0169632	212	130	162	269	62	55	107	56
ENSECAG00000000609	6.355199522	0.0009213	0.0170443	1248	951	1071	1054	1673	1858	2325	1915
ENSECAG00000017342	1.80704731	0.0009346	0.0172616	36	13	18	24	192	33	175	15
ENSECAG00000007522	5.382703411	0.0009446	0.0174175	517	487	625	547	994	976	971	1003
ENSECAG00000014251	2.446578724	0.0009479	0.0174503	23	20	3	22	5	449	9	191
ENSECAG00000013626	1.339952043	0.0009654	0.0177314	92	41	80	68	22	19	24	25
ENSECAG00000017926	3.001011159	0.0009677	0.0177314	171	140	157	475	55	76	88	79

ENSECAG00000027676	9.624730832	0.0009678	0.0177314	6250	4473	2751	3567	1994	31195	2830	58741
ENSECAG00000012186	3.815549252	0.0009738	0.0178125	215	116	141	121	555	217	489	213
ENSECAG00000023894	4.818956563	0.0009806	0.0179074	273	245	315	518	1025	494	821	487
ENSECAG00000014619	4.063859609	0.0009848	0.0179562	437	327	515	645	151	214	218	51
ENSECAG00000017906	3.922727229	0.0009928	0.0180724	455	236	424	594	161	142	239	83
ENSECAG00000005228	3.626010725	0.0009948	0.0180805	374	243	344	388	158	120	164	82
ENSECAG00000001784	7.83306824	0.0010045	0.0182267	521	2574	960	322	1007	14730	1094	9931
ENSECAG00000010028	0.968166733	0.0010151	0.0183897	50	36	31	125	16	16	16	1
ENSECAG00000011776	6.042292084	0.0010219	0.0184571	1631	2705	2233	702	480	365	1247	517
ENSECAG00000018438	2.899591507	0.0010221	0.0184571	247	109	177	333	45	62	123	54
ENSECAG00000006934	6.209306173	0.0010328	0.0186223	445	712	485	1074	965	4529	914	1227
ENSECAG00000018163	0.258993404	0.0010392	0.0187071	43	20	32	37	11	4	15	6
ENSECAG00000010232	6.870185285	0.0010432	0.0187489	1128	1812	1154	1681	3164	2690	3102	2438
ENSECAG00000016549	3.877481148	0.0010518	0.0188748	136	92	183	194	400	238	803	164
ENSECAG00000018395	8.253350444	0.001061	0.0190089	341	2763	381	648	650	26060	609	9162
ENSECAG00000016173	4.326039255	0.0010668	0.0190834	588	340	599	660	272	239	192	156
ENSECAG00000015967	2.663980931	0.0010779	0.0192508	156	113	200	264	72	57	80	23
ENSECAG00000017252	4.548425952	0.001091	0.019454	582	421	697	837	172	203	309	371
ENSECAG00000005078	0.818776112	0.0010961	0.0195159	7	15	25	9	40	33	91	28
ENSECAG00000017003	1.518432877	0.0011028	0.0196045	85	90	41	111	18	18	16	43
ENSECAG00000014246	1.652416918	0.0011214	0.0199038	19	35	26	36	90	87	121	33
ENSECAG00000020003	1.32097491	0.0011233	0.0199063	14	27	18	4	137	69	18	50
ENSECAG00000010546	2.072316472	0.0011277	0.0199524	52	36	41	12	193	60	112	92
ENSECAG00000006522	6.842794382	0.0011309	0.0199783	1532	1322	285	676	1617	956	6004	4974
ENSECAG00000008844	4.246163013	0.0011464	0.020221	185	320	166	191	513	283	623	513
ENSECAG00000000713	5.261110825	0.0011545	0.0203321	244	316	290	292	212	2849	366	677
ENSECAG00000016537	3.908131936	0.0011567	0.0203384	166	109	147	257	597	270	422	239
ENSECAG00000001989	4.51293123	0.0011692	0.0204735	124	209	174	190	173	1667	252	336
ENSECAG00000009881	5.06810783	0.0011695	0.0204735	24	240	49	108	18	2737	75	1239
ENSECAG00000009121	2.082563951	0.0011698	0.0204735	152	81	115	132	30	21	77	31
ENSECAG00000001166	5.516752826	0.001182	0.0206554	1380	777	1148	1605	674	508	589	319
ENSECAG00000013300	4.438800626	0.0011876	0.0207213	182	2546	25	79	22	18	20	171
ENSECAG00000013108	0.723736191	0.0011904	0.0207391	21	13	9	13	63	22	57	31
ENSECAG00000007646	6.867468671	0.0011983	0.0208437	2234	989	915	722	4716	1853	3848	1893
ENSECAG00000000090	6.340642686	0.0012003	0.0208466	915	1445	473	826	1697	2409	1581	2347
ENSECAG00000017017	5.638892236	0.0012061	0.020905	1370	1567	1960	784	177	277	337	1026
ENSECAG00000016382	2.239630975	0.0012073	0.020905	148	108	103	166	26	50	57	50
ENSECAG00000007385	5.960382012	0.0012122	0.0209586	672	699	868	829	2075	1192	2079	797
ENSECAG00000023617	1.473396811	0.0012198	0.0209713	25	16	32	26	71	20	106	106
ENSECAG00000024058	1.824898267	0.0012198	0.0209713	44	20	40	18	159	80	100	39
ENSECAG00000000360	5.310640651	0.0012201	0.0209713	489	379	377	244	337	2069	624	1072
ENSECAG00000008185	1.652031644	0.0012218	0.0209713	98	48	77	171	16	4	60	9
ENSECAG00000022164	7.069004434	0.0012248	0.0209713	4128	2311	2874	5300	1360	1064	2335	1440
ENSECAG00000008363	3.823403887	0.0012265	0.0209713	270	445	622	330	60	42	87	283
ENSECAG00000011753	3.797210819	0.0012275	0.0209713	87	102	121	21	88	1090	158	208
ENSECAG00000008683	10.45536437	0.0012277	0.0209713	12094	13685	10602	8506	11575	88967	12244	36538
ENSECAG00000013390	3.153796128	0.001248	0.0212851	114	109	94	83	180	170	290	263
ENSECAG00000012183	0.940602538	0.001251	0.021305	11	18	8	24	73	69	41	17
ENSECAG00000017253	2.296177191	0.0012618	0.0214559	51	67	35	41	110	79	127	198
ENSECAG00000018421	3.771601694	0.0012695	0.0215543	246	78	94	53	728	188	237	337
ENSECAG00000002211	4.31212629	0.0012726	0.0215755	187	186	156	372	350	344	428	917
ENSECAG00000021662	7.894384583	0.0012757	0.0215949	8217	3734	5941	9373	3390	2083	3127	923
ENSECAG00000018354	4.316033293	0.0012833	0.0216922	249	194	180	359	325	508	564	561
ENSECAG00000007880	2.164844709	0.0012878	0.0217358	22	47	33	64	81	47	204	165
ENSECAG00000014338	10.8499427	0.0013044	0.0219827	3808	16865	5513	4051	1298	52633	11954	167738
ENSECAG00000020007	1.769242863	0.0013122	0.0220583	111	59	27	309	0	0	0	31
ENSECAG00000005458	1.785717815	0.0013127	0.0220583	32	33	43	19	41	112	125	85
ENSECAG00000012290	0.001584814	0.0013243	0.0222203	24	21	33	29	6	10	5	8
ENSECAG00000021167	5.364427212	0.001338	0.0224169	391	373	337	533	475	2138	422	1144
ENSECAG00000011579	3.835184132	0.0013559	0.0226831	123	167	175	206	476	323	267	332
ENSECAG00000017134	4.065104587	0.0013799	0.0230497	67	173	126	119	126	1157	122	396
ENSECAG00000012172	7.387550822	0.0013879	0.0231396	1628	1052	1217	615	1242	13265	1119	2527
ENSECAG00000011422	4.392197975	0.0013893	0.0231396	259	161	366	208	632	540	493	394
ENSECAG00000015196	2.072754369	0.0013928	0.0231632	33	37	31	22	32	251	46	121
ENSECAG00000001796	1.137163051	0.0013951	0.0231682	19	12	220	29	4	9	20	10
ENSECAG00000008317	4.382514634	0.0013993	0.0232039	255	264	236.99	296	413.99	375	624	624
ENSECAG00000021303	2.672822102	0.0014114	0.0233448	32	17	17	32	12	640	11	70
ENSECAG00000010242	4.643517703	0.0014119	0.0233448	12	315	24	72	135	1656	197	991
ENSECAG00000013777	4.672432234	0.0014158	0.0233746	236	94	51	442	1542	259	282	797
ENSECAG00000004645	0.985660528	0.0014184	0.0233835	14	23	19	14	27	32	70	80
ENSECAG00000014696	6.342280811	0.0014212	0.0233959	1945	1495	2008	3432	511	621	1628	1000
ENSECAG00000014113	6.570794336	0.0014269	0.0234553	1644	1038	1013	575	1754	3378	1762	2486
ENSECAG00000024302	0.860223281	0.0014321	0.0235076	10	15	11	31	52	42	65	28
ENSECAG00000015010	7.349423949	0.0014528	0.0237805	197	1144	196	439	120	16051	213	3036
ENSECAG00000018398	2.135251454	0.0014529	0.0237805	41	28	29	41	26	230	62	152
ENSECAG00000017672	2.010885124	0.0014664	0.0239672	143	84	88	131	27	32	64	35
ENSECAG00000023907	6.18304525	0.0014779	0.0241206	1403	1414	2055	3040	539	505	1392	954
ENSECAG00000023145	6.5967234	0.0014803	0.0241244	977	1557	1015	1290	2933	2407	2282	1646

ENSECAG00000009595	6.149088849	0.0014898	0.0242449	1692	1516	1828	2283	906	739	1201	675
ENSECAG00000024959	1.800349057	0.0014973	0.0243199	97	135	58	96	18	24	55	30
ENSECAG00000015671	3.251125392	0.0014987	0.0243199	315	192	333	296	60	60	189	15
ENSECAG00000018696	1.964648548	0.0015081	0.0244371	39	34	41	40	144	110	100	44
ENSECAG00000022078	4.853018138	0.0015235	0.024651	131	299	164	111	160	2164	117	758
ENSECAG00000017059	3.912046021	0.0015332	0.0247728	209	151	135	246	298	359	432	386
ENSECAG00000006930	5.909285369	0.0015372	0.0248024	736	717	821	696	2196	1367	957	1221
ENSECAG00000011780	6.879031753	0.0015491	0.0249586	2866	1891	3060	6037	687	1041	2475	485
ENSECAG00000017026	5.132865353	0.0015525	0.0249784	905	651	950	1225	457	235	605	371
ENSECAG00000005945	5.471141909	0.001558	0.0250319	476	623	548	675	841	1207	1254	896
ENSECAG00000009039	8.414577104	0.0015627	0.0250717	11054	5851	9499	11013	4454	1995	5853	2964
ENSECAG00000024763	2.751071253	0.0015665	0.0250979	36	62	103	13	87	149	181	339
ENSECAG00000012481	7.18827573	0.001569	0.0251012	1381	1700	1614	509	8100	2755	3698	1232
ENSECAG00000007807	4.250114278	0.0015801	0.0252432	287	216	207	162	510	625	329	387
ENSECAG00000024519	2.268414942	0.0016018	0.0255543	141	68	119	224	36	41	72	35
ENSECAG00000017243	2.171158426	0.0016114	0.0256401	131	113	130	132	15	29	62	63
ENSECAG00000010672	2.82331417	0.0016117	0.0256401	65	78	92	78	180	184	243	107
ENSECAG00000008899	5.155351806	0.0016384	0.026029	843	1087	911	895	270	323	391	652
ENSECAG00000012756	1.462853306	0.0016427	0.0260604	23	27	26	40	56	72	67	79
ENSECAG00000015676	5.914023207	0.0016741	0.0265214	253	690	373	368	337	4230	340	1642
ENSECAG00000017075	4.111559476	0.0016765	0.0265224	172	172	155	199	304	906	221	296
ENSECAG00000010053	2.909237458	0.0016808	0.0265531	205	148	193	322	73	59	128	24
ENSECAG00000009716	3.537609656	0.0016974	0.0267555	351	177	286	514	86	112	210	60
ENSECAG00000009860	2.794274953	0.0016983	0.0267555	29	80	49	62	39	356	109	244
ENSECAG00000022185	3.213310205	0.0017127	0.0269459	44	66	57	65	55	769	51	135
ENSECAG00000018671	1.605809864	0.0017258	0.0271137	13	25	23	28	13	126	42	152
ENSECAG00000012804	7.722684974	0.0017817	0.0278448	2170	3047	1522	2595	3949	8959	3575	4339
ENSECAG00000005166	6.953982049	0.0017819	0.0278448	1679	1426	1473	1794	3735	3068	2611	2339
ENSECAG00000014660	6.981305046	0.0017827	0.0278448	1307	1143	1170	968	1830	6653	697	3881
ENSECAG00000020304	7.261140305	0.0017831	0.0278448	2057	1812	2073	1823	4707	3576	3365	3003
ENSECAG00000010151	6.534767042	0.0017846	0.0278448	1140	1089	1469	960.001	1702	2720	1683	2707
ENSECAG00000022127	7.14654506	0.0017892	0.0278784	123	1007	152	47	196	15007	113	1699
ENSECAG00000005480	6.96428472	0.0018102	0.0281672	2935	2329	2919	6708	486	1131	2686	509
ENSECAG00000022424	1.28987755	0.0018163	0.0282238	12	33	24	10	67	101	33	51
ENSECAG00000007718	1.530495866	0.0018457	0.0286007	25	18	17	34	185	30	65	33
ENSECAG00000022325	7.28005949	0.0018465	0.0286007	4321	3014	5337	3776	2237	1070	2112	1813
ENSECAG00000024623	8.901281687	0.0018481	0.0286007	6691	6455	5142	5285	12604	11574	10086	11915
ENSECAG00000008787	1.593984057	0.0018615	0.0287352	102	42	85	123	33	10	48	12
ENSECAG00000006614	0.765569919	0.0018619	0.0287352	64	48	35	45	3	19	7	20
ENSECAG00000024301	5.539059979	0.0018705	0.028829	1160	1068	965	1626	459	579	503	707
ENSECAG00000013528	3.268973561	0.0018763	0.0288791	40	108	35	86	34	563	121	349
ENSECAG00000011108	6.311048822	0.0018866	0.0289873	1097	758	907	1335	2151	1591	2938	1078
ENSECAG00000021932	3.919871555	0.0018884	0.0289873	196	111	133	263	203	361	647	347
ENSECAG00000007934	1.955119059	0.0019063	0.0292217	36	48	32	26	174	43	131	66
ENSECAG00000010145	2.68668213	0.0019199	0.0293579	212	189	233	135	17	10	46	124
ENSECAG00000021586	1.845625584	0.0019203	0.0293579	48	12	32	18	89	44	258	32
ENSECAG00000024352	5.399125561	0.0019312	0.0294844	1125	891	1757	712	457	264	497	677
ENSECAG00000018918	3.10196636	0.0019354	0.0295083	240	148	211	353	99	87	114	58
ENSECAG00000017582	3.034503027	0.0019731	0.0300416	184	163	259	304	75	40	140	90
ENSECAG00000000632	2.585707598	0.0019768	0.0300416	60	89	47	54	91	162	157	202
ENSECAG00000022444	3.590488306	0.0019783	0.0300416	297	311	284	393	88	103	193	161
ENSECAG00000019507	3.862532288	0.0019877	0.0301439	412	366	476	304	119	82	249	194
ENSECAG00000019780	3.798819039	0.0020175	0.030555	425	219	357	575	134	89	281	78
ENSECAG00000001041	3.070150101	0.0020296	0.0306987	267	135	217	334	93	76	128	35
ENSECAG00000012601	5.7350187	0.0020383	0.0307886	770	666	755	526	1734	1049	1409	900
ENSECAG00000017316	0.735575555	0.0020476	0.030888	7	20	18	13	36	52	24	55
ENSECAG00000000199	5.147042672	0.0020617	0.0310592	207	383	251	463	289	2124	424	816
ENSECAG00000008202	5.646105917	0.0020666	0.0310927	860	571	625	514	926	1454	1172	1180
ENSECAG00000013514	7.526704313	0.0020719	0.0311312	3279	1774	1788	625	6455	4474	5787	2634
ENSECAG00000003192	8.628929476	0.002081	0.0312266	2026	3889	2052	2743	1861	28792	1841	10809
ENSECAG00000023667	7.349554035	0.0021037	0.0314849	1875	1770	1636	1037	3966	8502	1715	2251
ENSECAG00000021312	4.254585485	0.0021038	0.0314849	163	237	219	335	422	515	581	333
ENSECAG00000017237	5.338876621	0.002112	0.0315658	11	234	15	56	30	4187	7	716
ENSECAG00000023722	4.236642748	0.0021174	0.0316026	70	229	129	142	127	933	112	889
ENSECAG00000024054	1.158643969	0.00212	0.0316026	10	18	39	17	51	62	69	46
ENSECAG00000018585	2.459410713	0.002127	0.0316656	59	43	46	21	86	337	42	113
ENSECAG00000020136	6.64421999	0.0021395	0.031791	630	766	780	893	624	7802	725	1418
ENSECAG00000020074	2.73119081	0.002141	0.031791	73	57	53	106	177	192	227	79
ENSECAG00000012451	4.789034506	0.0021545	0.0319487	362	398	285	286	643	739	430	848
ENSECAG00000001923	7.319143123	0.002164	0.0320484	3957	1504	5563	6979	1613	1879	1815	1489
ENSECAG00000008059	5.741733532	0.0021736	0.0321488	1419	1663	1516	959	440	509	515	987
ENSECAG00000017796	6.816175795	0.0021918	0.0323656	2986	2187	2676	4065	1299	1221	1956	926
ENSECAG00000022353	3.384637371	0.0021962	0.0323656	258	226	238	397	126	97	137	109
ENSECAG00000000734	3.66638765	0.0021968	0.0323656	316	306	315	399	150	151	147	129
ENSECAG00000011660	2.578563249	0.0022009	0.0323831	312	267	104	40	4	37	36	72
ENSECAG00000001069	6.496372322	0.0022308	0.0327806	769	547	552	197	153	5807	367	4012
ENSECAG00000023737	1.403153256	0.0022507	0.0330301	26	24	35	21	39	49	102	83
ENSECAG00000006290	1.074204478	0.002255	0.0330513	72	41	79	43	8	31	16	12

ENSECAG00000000416	1.541407995	0.0023207	0.0339705	72	93	69	86	16	15	25	51
ENSECAG00000000794	6.409615877	0.0023339	0.0341198	1393	671	1225	960	1191	2603	1902	2489
ENSECAG00000008937	2.87842262	0.0023569	0.0344108	8	51	28	20	5	600	11	253
ENSECAG00000019341	0.75973148	0.0023734	0.0345701	6	20	17	7	13	24	88	64
ENSECAG00000021082	2.468392437	0.0023739	0.0345701	48	76.0001	31.0001	52.0001	287	92.0005	118.001	83.0004
ENSECAG00000012336	5.664918798	0.0024019	0.0349338	457	764	721	385	992	1988	882	1044
ENSECAG00000009352	0.707919661	0.0024208	0.0351632	36	29	44	72	10	10	28	9
ENSECAG00000011885	2.177546745	0.0024299	0.0352511	34	35	39	58	128	75	268	32
ENSECAG00000008679	5.818113051	0.0024884	0.0360533	579	597	474	519	616	3394	529	1143
ENSECAG00000017231	6.140819078	0.0025025	0.0361649	698	1039	999	1089	1511	1651	1701	1696
ENSECAG00000014153	6.92405217	0.0025025	0.0361649	1454	1752	1092	1019	1432	4813	2038	3776
ENSECAG00000016557	4.083769435	0.0025542	0.0368652	480	256	399	759	158	84	315	190
ENSECAG00000014968	5.830793343	0.0025606	0.0369117	628	469	510	355	4363	510	851	480
ENSECAG00000001088	2.386576864	0.0025644	0.0369192	57	31	59	58	218	166	97	52
ENSECAG00000008829	2.968484233	0.002571	0.0369668	49	110	59	111	103	160	227	326
ENSECAG00000017522	1.595480678	0.0025833	0.0370966	22	11	20	61	130	34	89	69
ENSECAG00000011613	2.600300779	0.0025885	0.0371248	11	54	27	50	27	470	31	143
ENSECAG00000000224	2.964394925	0.0025993	0.0372324	175	173	224	287	58	52	78	138
ENSECAG00000008722	4.322596037	0.0026134	0.0373876	205	359	144	137	352	674	341	640
ENSECAG00000013515	2.724833988	0.002647	0.0377837	50	89	32	58	71	369	96	149
ENSECAG00000014516	5.80195262	0.0026478	0.0377837	1214	1183	1513	1813	595	480	763	963
ENSECAG00000001653	6.316003794	0.0026576	0.0378761	1977	1627	1844	2741	1183	724	1272	828
ENSECAG00000023853	6.099693546	0.002665	0.037923	1670	1596	1645	2073	593	682	1093	1104
ENSECAG00000010783	3.97564195	0.0026675	0.037923	58	180	61	98	61	1075	69	540
ENSECAG00000013291	8.47192811	0.0026916	0.0382178	5316	3787	2955	909	14621	11158	8774	3180
ENSECAG00000005800	1.211529328	0.0027356	0.0387936	64	50	37	107	17	11	34	24
ENSECAG00000006002	1.117272458	0.0027571	0.0390504	9	20	7	45	57	49	71	49
ENSECAG00000021869	4.940888719	0.0027957	0.0395066	498	340	425	296	613	833	648	817
ENSECAG00000001845	2.602418704	0.0027963	0.0395066	68	78	54	73	126	134	122	220
ENSECAG00000023105	1.192977652	0.0028108	0.0396617	74	77	42	68	3	30	10	25
ENSECAG00000012416	3.337114611	0.0028182	0.0397176	161	71	46	110	497	108	364	127
ENSECAG00000003554	4.547695495	0.0028405	0.0399819	542	760	484	641	180	300	374	238
ENSECAG00000018841	4.784986825	0.0028545	0.0400903	346	32	359	21	1420	714	726	326
ENSECAG00000001114	4.022011949	0.0028553	0.0400903	156	235	224	157	247	498	454	387
ENSECAG00000001072	4.548638827	0.0028616	0.0401297	243	364	276	299	485	686	522	520
ENSECAG00000015375	5.360609832	0.0028697	0.0401479	518	518	453	640	796	1380	794	862
ENSECAG00000004556	6.989552792	0.00287	0.0401479	1235	1799	841	726	2083	7728	1481	1667
ENSECAG00000008759	0.583096835	0.002882	0.0402662	8	19	5	11	21.9999	20.9999	19	98.9998
ENSECAG00000012215	4.015557001	0.002904	0.0405242	214	200	146	213	490	494	329	243
ENSECAG00000014923	6.968479645	0.0029293	0.040827	3107	2115	3865	4812	1337	1371	2265	492
ENSECAG00000010357	2.077804254	0.002942	0.0409539	7	68	21	31	97	156	39	164
ENSECAG00000004636	5.422231134	0.0029717	0.0413166	721	545	508	350	753	1225	841	1275
ENSECAG00000000003	3.608482133	0.002982	0.0413796	131	138	104	134	207	614	174	210
ENSECAG00000011257	6.872587286	0.0029835	0.0413796	703	1286	993	576	633	8570	927	2300
ENSECAG00000008118	3.360054328	0.0030123	0.0417271	108	130	80	80	522	70	283	222
ENSECAG00000020103	3.079567378	0.0030201	0.0417826	114	98	75	120	179	162	295	202
ENSECAG00000020085	4.141324519	0.0030236	0.0417826	187	254	166	192	256	738	403	327
ENSECAG00000010267	9.431544535	0.003037	0.0419161	7942	9302	7149	8589	13844	25109	10953	16456
ENSECAG00000020428	6.05604947	0.0030772	0.0424196	881	791	816	988	1257	2253	1129	1529
ENSECAG00000019531	1.376971153	0.003085	0.0424754	22	17	17	43	39	119	42	60
ENSECAG00000009676	6.558739301	0.003111	0.042743	1954	885	1159	607	2437	1728	3066	2097
ENSECAG00000015468	5.351973819	0.0031119	0.042743	455	401	548	341	430	2069	613	919
ENSECAG00000019837	7.850950448	0.0031446	0.0431399	3785	2255	2841	2146	8576	4978	6122	3067
ENSECAG00000018150	4.497409517	0.0031791	0.0434757	637	463	687	544	253	174	403	272
ENSECAG00000003516	6.941031091	0.0031834	0.0434757	2174	1384	1515	1346	2953	2579	2709	3391
ENSECAG00000020920	3.969315828	0.0031842	0.0434757	365	376	357	587	100	120	296	204
ENSECAG00000024287	1.821264659	0.0031844	0.0434757	22	46	19	30	34	157	36	143
ENSECAG00000014125	5.496596948	0.0032238	0.0439609	1204	928	1455	1175	291	303	659	884
ENSECAG00000015871	3.471178833	0.0032282	0.0439682	353	196	267	393	134	121	164	60
ENSECAG00000014074	6.761397101	0.0032338	0.0439825	2658	3509	3165	2223	683	524	1898	1816
ENSECAG00000004592	2.426061191	0.0032382	0.0439825	85	32	35	8	254	80	242	41
ENSECAG00000022426	6.940399501	0.0032409	0.0439825	3653	2714	3826	2559	1227	828	2274	1633
ENSECAG00000008773	5.254099904	0.0032453	0.0439895	359	494	683	399	934	510	1178	1112
ENSECAG00000010918	6.611309482	0.0032721	0.0443	15	640	48	156	25	8230	32	3868
ENSECAG00000018229	3.916754842	0.0032771	0.0443156	538	287	482	322	134	76	290	172
ENSECAG00000001870	5.983467438	0.0032904	0.0443971	806	379	963	673	3328	828	1525	775
ENSECAG00000009742	9.245493361	0.003291	0.0443971	4	1142	25	122	35	57581	0	20860
ENSECAG00000015095	4.31083919	0.0033041	0.0445212	150	270	177	183	206	1035	232	527
ENSECAG00000008141	3.875777855	0.0033157	0.0445781	177	159	194	209	395	193	458	394
ENSECAG00000018895	5.381186814	0.0033162	0.0445781	1055	1208	1290	965	185	224	513	888
ENSECAG00000012876	5.731155096	0.0033458	0.0449233	1037	1011	1711	1945	328	354	907	925
ENSECAG00000014198	5.880599379	0.0033515	0.0449462	1526	1393	1448	1657	508	578	706	1124
ENSECAG00000022583	0.948131367	0.0033586	0.044988	9	24	10	11	10	97	21	76
ENSECAG00000017203	8.713620269	0.003379	0.0452085	4988	3951	7347	1714	5906	11139	10436	15802
ENSECAG00000007464	6.350184247	0.0034006	0.0454236	2751	1092	2506	2740	1403	625	1139	304
ENSECAG00000025062	3.899687259	0.0034036	0.0454236	448	377	399	374	68	146	188	253
ENSECAG00000023468	5.915637439	0.0034071	0.0454236	900	691	826	941	1193	1047	1971	1457
ENSECAG00000004770	2.882568605	0.0034188	0.0455262	41	60	92	147	163	199	211	164

ENSECAG00000020032	4.503309893	0.0034255	0.0455617	687	610	541	491	198	202	302	382
ENSECAG00000020987	3.857610635	0.003439	0.0456565	243	103	179	208	340	327	419	316
ENSECAG00000011785	2.764012991	0.0034406	0.0456565	152	157	141	290	74	65	103	50
ENSECAG00000014973	5.342287549	0.0034505	0.0456886	704	230	505	292	1781	1062	929	352
ENSECAG00000016674	6.006142944	0.0034511	0.0456886	832	948	805	955	1408	1190	1503	1856
ENSECAG00000023070	6.101362978	0.0034599	0.0457526	930	814	791	954	1174	2653	1248	1340
ENSECAG00000019904	5.019600266	0.0034724	0.0458319	312	385	319	276	415	1910	402	499
ENSECAG00000014832	4.329901126	0.003474	0.0458319	328	154	283	276	389	443	652	456
ENSECAG00000015245	6.769600078	0.0034817	0.0458559	1213	1046	1119	1540	1416	5559	1796	1848
ENSECAG00000022304	5.925552101	0.0034839	0.0458559	600	913	714	950	1093	1998	1344	1232
ENSECAG00000015121	5.292352967	0.0035195	0.0462703	860	818	1291	1019	399	359	653	564
ENSECAG00000021841	5.784716608	0.0035242	0.0462789	725	650	437	238	906	3279	564	821
ENSECAG00000007090	0.175828307	0.0035549	0.0465756	26	19	34	41	14	8	9	6
ENSECAG00000018752	-0.041782966	0.003555	0.0465756	29	18	31	24	6	2	16	7
ENSECAG00000010999	2.275358031	0.0035602	0.0465814	43	68	14	21	81	115	37	301
ENSECAG00000013126	5.376851062	0.0035636	0.0465814	21	374	24	73	26	3588	40	1364
ENSECAG00000019475	6.634924041	0.00357	0.0466116	1617	1460	1042	950	2769	2535	1891	2148
ENSECAG00000018683	8.366775898	0.0036041	0.0470022	3853	4196	3022	4462	4491	12307	5261	10035
ENSECAG00000015760	4.347661585	0.0036182	0.0471324	266	158	209	359	730	346.005	700	256.002
ENSECAG00000007337	6.746432498	0.0036237	0.0471498	1195	1267	1018	1418	1533	5100	1415	2322
ENSECAG00000008466	2.557286916	0.0036281	0.0471527	154	103	173	225	57	61	94	26
ENSECAG00000015859	1.936640433	0.0036472	0.0473464	98	71	102	156	18	22	68	45
ENSECAG00000021363	1.638215576	0.0036702	0.0475913	21	41	26	42	75	43	78	120
ENSECAG00000006612	3.481469281	0.0036838	0.0476933	199	255	316	427	80	134	150	137
ENSECAG00000008926	5.396637728	0.0036865	0.0476933	786	468	546	394	902	697	1286	1168
ENSECAG00000013981	4.178357354	0.0036911	0.0476985	6	121	17	21	4	1780	5	407
ENSECAG00000003546	0.574057473	0.0036957	0.0477033	5	16	23	10	40	42	33	32
ENSECAG00000000697	4.654640727	0.0037001	0.0477069	159	428	170	200	340	1170	228	833
ENSECAG00000003539	4.031146712	0.0037192	0.0478987	412	335	452	498	160	139	267	227
ENSECAG00000019533	0.771062887	0.0037244	0.0479105	23	10	8	12	100	23	46	12
ENSECAG00000014430	2.902294496	0.0037439	0.0480861	45	73	54	150	178	94	370	158
ENSECAG00000016862	5.198822528	0.0037465	0.0480861	601	358	302	355	697	1786	494	593
ENSECAG00000011637	6.416082719	0.003753	0.0481158	1302	892	1059	876	3839	1220	2182	1167
ENSECAG00000024081	1.823066241	0.0037719	0.0483032	34	23	39	30	22	182	63	97
ENSECAG00000017205	6.152352127	0.0037899	0.0484588	882	1021	845	937	985	2352	1514	1807
ENSECAG00000007242	6.510994954	0.0037926	0.0484588	1146	1399	893	287	1448	3414	1376	2996
ENSECAG00000022933	5.962398482	0.0038359	0.0489569	1089	547	634	532	2766	1463	1473	530
ENSECAG00000020118	5.775983456	0.0038613	0.0492266	914	1696	1467	1668	329	569	603	1051
ENSECAG00000013529	7.082824568	0.0038784	0.049389	1631	1328	1722	2020	4950	2486	4549	1423
ENSECAG00000021518	2.889285546	0.0038988	0.0495934	75	104	79	65	87	226	185	244
ENSECAG00000009024	5.270383432	0.0039036	0.0495978	853	1016	1176	960	248	311	580	715
ENSECAG00000010048	0.896956822	0.0039129	0.0496609	36	47	57	66	7	5	22	31
ENSECAG00000006023	5.097919161	0.0039347	0.0498557	398	436	543	325	614	1182	633	802
ENSECAG00000000608	5.422203714	0.003937	0.0498557	454	438	419	696	582	1989	582	966
ENSECAG00000016018	3.710341808	0.0039748	0.050278	272	413	412	354	49	56	202	230
ENSECAG00000006913	10.45833528	0.0039883	0.0503285	17744	18572	17614	11521	29635	48099	19025	39484
ENSECAG00000014315	3.248556221	0.003992	0.0503285	147	97	13	52	442	251	281	72
ENSECAG00000003772	4.704697411	0.0039921	0.0503285	282	313	329	312	498	1100	374	506
ENSECAG00000009896	6.985457249	0.0040479	0.0509397	976	2155	1366	968	1721	3225	1847	6071
ENSECAG00000024364	6.407614121	0.0040534	0.0509397	2122	1688	2243	2444	1087	937	1375	1024
ENSECAG00000017294	4.92961455	0.004054	0.0509397	430	360	351	527	775	560	720	784
ENSECAG00000016852	6.333681984	0.0040782	0.0511762	1278	911	905	1376	2088	1227	2443	1826
ENSECAG00000006162	7.396342729	0.0040831	0.0511762	1289	2126	1538	1450	1418	10317	1907	3408
ENSECAG00000000545	6.11197959	0.0040883	0.0511762	1755	1268	2003	1915	975	742	1032	824
ENSECAG00000021364	6.07232286	0.0040941	0.0511762	931	702	1026	658	2930	1046	1549	1008
ENSECAG00000008488	4.642702634	0.0040954	0.0511762	590	663	688	594	259	312	278	361
ENSECAG00000017295	6.234922416	0.0041062	0.051201	1006	711	721	910	906	3853	906	1653
ENSECAG00000013046	6.718905636	0.0041064	0.051201	1614	1380	1178	263	2157	3137	2178	3247
ENSECAG00000013577	5.656819227	0.0041311	0.0514248	1372	1066	1256	1315	615	554	787	680
ENSECAG00000026820	4.246347847	0.0041334	0.0514248	46	237	102	146	63	415	161	1568
ENSECAG00000026816	6.21770199	0.0041403	0.0514552	782	1137	830	858	1390	3045	1168	1418
ENSECAG00000023805	5.700685726	0.0041758	0.0518389	568	696	689	747	1474	1607	780	946
ENSECAG00000019559	2.953204999	0.0041806	0.0518416	47	88	51	37	86	557	32	143
ENSECAG00000017337	4.402170463	0.0042018	0.0520482	266	180	240	398	510	561	691	279
ENSECAG00000024360	5.238743519	0.0042199	0.0522158	491	512	480	423	832	1339	622	723
ENSECAG00000024031	5.769265476	0.0042391	0.0523954	839	625	627	731	1074	1998	1107	886
ENSECAG00000024181	5.17223125	0.00425	0.0524739	42	332	38	44	26	3166	17	1103
ENSECAG00000004055	7.272426901	0.0042681	0.0526173	2360	765	1582	718	9642	1990	4778	708
ENSECAG00000021028	3.643111166	0.0042709	0.0526173	131	197	119	173	353	286	305	252
ENSECAG00000022992	9.071635101	0.0042835	0.0527149	1042	10336	1848	371	6919	12187	15318	29898
ENSECAG00000018515	6.102814567	0.0042893	0.0527293	1740	1601	1648	1814	893	820	911	949
ENSECAG00000016701	5.392551049	0.0043043	0.0528339	985	813	1167	1255	517	473	595	579
ENSECAG00000001598	5.756106884	0.0043089	0.0528339	1281	1334	1387	1350	658	731	768	626
ENSECAG00000024047	6.926524015	0.0043183	0.0528339	941	1141	1160	1821	1115	6807	1393	2806
ENSECAG00000021269	7.523452404	0.0043205	0.0528339	1755	1739	1167	628	961	14392	1067	3185
ENSECAG00000015520	5.612466293	0.004321	0.0528339	504	686	592	910	1341	902	1407	922
ENSECAG00000011102	5.682360786	0.0043288	0.0528721	1294	1015	1220	1611	652	515	847	681
ENSECAG00000003428	10.56788826	0.0043419	0.0529646	20076	17686	22039	18199	39181	40232	27658	36044

ENSECAG00000005292	6.708766481	0.0043457	0.0529646	1196	1294	1171	1252	1409	4525	1385	2705
ENSECAG00000019125	7.808426723	0.0043722	0.0532298	5585	4691	5346	7439	2834	2137.99	3432	2836
ENSECAG00000007505	6.704359961	0.0044229	0.05379	2491	2206	2320	3696	1023	1035	1974	1282
ENSECAG00000017804	2.969294649	0.0044439	0.053987	62	106	99	23	108	308	123	271
ENSECAG00000011746	7.153847503	0.0044499	0.054003	2183	1739	1526	866	5196	3453	4546	1231
ENSECAG00000020875	4.250385234	0.0044548	0.0540046	165	310	213	289	438	485	452	423
ENSECAG00000004463	5.63774554	0.0044648	0.0540676	752	612	471	772	1144	1644	832	980
ENSECAG00000017122	1.820883619	0.0044707	0.0540706	40	51	21	32	73	42	113	138
ENSECAG00000006896	1.684801036	0.0044745	0.0540706	21	28	27	37	43	169	22	92
ENSECAG00000013186	2.702168941	0.0045031	0.0543487	61	60	112	73	204	138	160	133
ENSECAG00000001141	5.202260609	0.0045071	0.0543487	41	352	33	86	29	3175	23	1145
ENSECAG00000011413	3.098157491	0.0045351	0.0546285	87	55	113	148	125	250	320	163
ENSECAG00000011348	7.231599512	0.0045804	0.0551153	4041	2818	6196	2914	2185	1053	2584	1090
ENSECAG00000010758	1.901402867	0.0045868	0.0551343	103	48	113	148	43	40	40	25
ENSECAG00000020856	6.533927488	0.0046126	0.0553386	1025	1114	1559	1110	1630	2678	1446	2909
ENSECAG00000021509	1.6157225	0.0046136	0.0553386	36	39	29	32	66	72	62	97
ENSECAG00000010748	3.63015443	0.0046603	0.0558406	148	100	164	225	270	327	344	241
ENSECAG00000012607	7.446945321	0.0046678	0.0558712	1060	1620	1569	1544	1003	12655	1103	3377
ENSECAG00000015521	1.283057358	0.0046772	0.0559247	62	45	72	83	14	15	33	35
ENSECAG00000009905	1.672996851	0.004732	0.0564641	7	38	27	33	79	105	16	135
ENSECAG00000008535	2.289069918	0.0047323	0.0564641	37	72	65	28	111	71	135	186
ENSECAG00000011185	1.014650847	0.0047497	0.0566121	17	25	19	12	101	34	45	25
ENSECAG00000017217	2.476284695	0.0047612	0.0566854	66	46	17	36	47	368	30	144
ENSECAG00000015195	5.660201437	0.00477	0.0566854	1618	1040	1721	795	601	361	871	645
ENSECAG00000024750	3.466991682	0.0047739	0.0566854	331	192	328	365	158	54	202	64
ENSECAG00000014460	7.791690876	0.0047758	0.0566854	2133	2421	2368	3574	3951	10106	3704	3489
ENSECAG00000006725	6.011871449	0.0048246	0.0572047	818	842	587	672	780	3030	805	1615
ENSECAG00000001308	3.883933872	0.0048305	0.0572152	87	88	121	174	65	1152	166	167
ENSECAG00000016970	6.1290078	0.0048496	0.0573818	620	1055	3320	3723	1361	168	569	171
ENSECAG00000015563	7.318217936	0.0048943	0.0578503	4116	3470	3971	4896	1995	1244	3083	1697
ENSECAG00000021679	6.211512195	0.0049021	0.0578819	1003	944	968	1162	2285	1244	2106	1282
ENSECAG00000013762	4.074415291	0.0049107	0.0579236	57	98	80	224	49	1349	67	365
ENSECAG00000011180	6.883119953	0.0049276	0.0580632	1158	934	1363	2480	3608	2176	4672	1352
ENSECAG00000012584	6.486971246	0.004935	0.0580898	1249	804	1151	1385	1840	3548	1506	1458
ENSECAG00000018202	6.429819896	0.0049418	0.0581095	1628	2486	2261	2394	633	1039	1102	1483
ENSECAG00000005126	6.758808845	0.0049829	0.0584985	2803	2425	2554	3316	998	889	2260	1378
ENSECAG00000010596	6.267893778	0.0049852	0.0584985	924	1066	975	1367	1744	1918	1803	1549
ENSECAG00000014269	0.383873075	0.0050003	0.058507	37	22	26	55	9	15	7	13
ENSECAG00000022516	5.14915624	0.0050011	0.058507	891	669	1179	904	477	267	674	354
ENSECAG00000014862	4.875004363	0.0050013	0.058507	395	228	498	89	586	1101	393	880
ENSECAG00000021501	6.269463649	0.0050205	0.0586708	1764	1565	2050	2454	770	632	1262	1323
ENSECAG00000007562	1.097583676	0.0050466	0.0589149	60	49	55	58	20	17	30	21
ENSECAG00000009646	3.9142394	0.0050647	0.0590658	207	156	173	55	472	95	768	330
ENSECAG00000004942	8.324691947	0.0050754	0.0591295	4298	4064	2489	534	7948	6644	3069	16963
ENSECAG00000020810	5.379005577	0.0050842	0.0591613	722	141	453	261	2570	497	1094	326
ENSECAG00000024644	3.801262856	0.0050885	0.0591613	232	123	144	210	421	251	424	253
ENSECAG00000019848	4.97983566	0.0051121	0.059364	713	650	938	992	260	208	644	448
ENSECAG00000012830	4.539708081	0.0051164	0.059364	177	239	400	267	896	272	899	309
ENSECAG00000023192	6.736148885	0.0051463	0.0596503	1012	1064	1127	1031	974	6811	1216	1603
ENSECAG00000019911	5.082461359	0.005156	0.0596941	801	748	752	1129	367	328	569	479
ENSECAG00000024327	5.944486983	0.0051606	0.0596941	701	829	618	936	656	2236	1176	1782
ENSECAG00000008951	2.057391111	0.0051861	0.0598842	17	74	37	32	66	123	174	73
ENSECAG00000024577	5.658681372	0.0051876	0.0598842	987	1316	1009	1799	518	678	712	653
ENSECAG00000014524	6.47149058	0.0052135	0.0601217	1144	1067	994	1022	1207	3982	1168	2062
ENSECAG00000024473	3.066606738	0.0052211	0.0601486	280	125	196	319	105	60	145	53
ENSECAG00000010251	4.518914286	0.0052271	0.0601564	462	932	631	431	90	253	195	411
ENSECAG00000002866	6.242609153	0.0052399	0.0602432	1273	661	999	813	2841	1006	2737	942
ENSECAG00000022853	6.874928816	0.0052518	0.060319	1795	784	1478	879	5834	2058	3624	726
ENSECAG00000020431	8.152621846	0.0052787	0.0605665	5183	3159	2439	609	5493	6785	5828	11758
ENSECAG00000017213	2.139618138	0.0053139	0.0609091	46	64	34	44	162	54	147	86
ENSECAG00000023690	4.546009653	0.0053227	0.0609477	309	301	260	242	394	940	321	561
ENSECAG00000020847	5.212858919	0.0053313	0.0609848	463	589	482	404	1100	709	645	1009
ENSECAG00000011797	6.764020527	0.0053456	0.0610874	843	1344	941	1148	1154	6693	899	2082
ENSECAG00000015496	2.022356065	0.005362	0.0611565	88	62	97	212	21	28	57	56
ENSECAG00000020782	4.148367848	0.0053631	0.0611565	564	359	402	515	132	193	228	295
ENSECAG00000019483	2.759593768	0.0053678	0.0611565	188	123	198	213	80	85	84	46
ENSECAG00000023808	5.851457651	0.0054157	0.0616053	1596	1011	1390	2010	633	447	1305	533
ENSECAG00000022374	1.69842994	0.0054181	0.0616053	25	29	16	30	28	232	24	50
ENSECAG00000014699	7.646716386	0.005428	0.0616568	589	1228	2085	1494	456	15913	774	4687
ENSECAG00000003382	3.437223992	0.0054396	0.0617265	85	77	84	140	76	655	67	319
ENSECAG00000016154	0.757062199	0.0054583	0.0618767	21	14	10	20	63	17	64	28
ENSECAG00000015450	1.864601404	0.0054643	0.0618832	82	66	100	151	24	21	68	39
ENSECAG00000018714	8.084857514	0.0055157	0.0624036	684	3731	617	795	512	21188	963	8023
ENSECAG00000020395	0.130732151	0.0055291	0.0624931	28	35	32	20	17	5	2	9
ENSECAG00000017157	6.303502401	0.0055425	0.0625815	46	899	84	384	142	2491	130	7031
ENSECAG00000019658	0.839174311	0.0056028	0.0632002	33	12	83	77	18	9	9	21
ENSECAG00000020698	3.295109784	0.0056095	0.0632122	313	143	289	334	140	63	168	52
ENSECAG00000022581	3.595323037	0.0056266	0.0632481	284	274	346	350	116	96	234	137

ENSECAG00000022911	0.649424071	0.0056314	0.0632481	34	39	42	47	13	11	18	19
ENSECAG00000026027	0.939566963	0.0056332	0.0632481	19	21	9	29	53	62	33	37
ENSECAG00000019369	3.717559951	0.0056349	0.0632481	367	215	340	499	192	118	207	76
ENSECAG00000015149	8.055564582	0.0056749	0.0635234	7810	4536	6715	8443	3798	2545	3700	3155
ENSECAG00000016860	3.398459563	0.0056758	0.0635234	299	202	223	415	90	144	166	67
ENSECAG00000003853	4.119329745	0.0056762	0.0635234	156	222	169	154	215	972	167	363
ENSECAG00000007964	5.436424535	0.0056886	0.063551	1195	1116	1459	645	450	333	528	774
ENSECAG00000011922	4.401733852	0.0056899	0.063551	137	400	124	230	351	861	271	616
ENSECAG00000024152	6.06860613	0.0057081	0.0636918	700	709	981	1347	2013	1096	1848	1317
ENSECAG00000011540	1.400399824	0.0057251	0.0638183	18	39	15	15	42	135	19	72
ENSECAG00000016978	3.931809372	0.0057431	0.0638224	53	189	1117	702	67	5	267	17
ENSECAG00000003315	6.248047382	0.0057463	0.0638224	1338	499	995	156	790	2834	1600	2713
ENSECAG00000014974	6.125631403	0.0057514	0.0638224	772	1141	845	858	1050	1926	1224	2343
ENSECAG00000011631	2.971610774	0.0057538	0.0638224	66	23	146	14	289	103	74	407
ENSECAG00000018702	6.68900349	0.0057596	0.0638224	1235	979	1140	996	851	5671	1153	2502
ENSECAG00000006698	0.76154008	0.0057608	0.0638224	56	17	66	44	12	10	26	14
ENSECAG00000018288	3.258565641	0.0057648	0.0638224	84	91	140	155	200	164	412	182
ENSECAG00000001312	4.686513234	0.0057911	0.0640517	359	131	337	205	1476	346	749	157
ENSECAG00000021809	1.961971257	0.0058114	0.0642133	39	31	43	53	151	92	115	33
ENSECAG00000011454	5.850901931	0.0058234	0.0642837	1085	488	621	646	2432	708	1725	839
ENSECAG00000019410	7.209489476	0.0058475	0.0643956	1640	2327	1916	1894	3529	4028	2669	3506
ENSECAG00000023683	-0.00519917	0.0058487	0.0643956	27	18	22	38	15	8	4	4
ENSECAG00000003599	5.643071934	0.0058505	0.0643956	287	525	392	380	201	3637	242	1160
ENSECAG00000011851	1.574962036	0.0058647	0.0644893	21	2	28	54	132	86	60	36
ENSECAG00000013223	1.886733514	0.005882	0.0645637	114	55	104	129	42	23	63	26
ENSECAG00000010613	6.343036948	0.0058843	0.0645637	125	1176	244	222	216	5242	187	3651
ENSECAG00000006721	6.748589905	0.0058885	0.0645637	1519	1429	1471	1258	2410	3448	2188	1866
ENSECAG00000021173	5.136397825	0.0058963	0.0645802	356	513	358	393	470	1536	426	943
ENSECAG00000010729	8.661255541	0.0059014	0.0645802	8047	8968	9791	15323	4526	4842	6364	4212
ENSECAG00000015406	5.200475904	0.005941	0.0649122	601	456	383	377	663	1313	490	989
ENSECAG00000017361	6.334970504	0.0059432	0.0649122	2207	1663	2737	1704	707	500	1208	1563
ENSECAG00000015759	4.982870021	0.0060467	0.06597	338	335	323	486	369	1509	474	678
ENSECAG00000018032	4.940860007	0.0060517	0.06597	388	406	417	381	423	928	607	918
ENSECAG00000023595	6.872777705	0.0060901	0.0663258	2179	1286	1329	1006	4589	1832	2294	2611
ENSECAG00000000633	3.195833873	0.0061069	0.0664446	263	143	260	311	120	99	128	66
ENSECAG00000008113	4.552103217	0.0061549	0.0669025	449	809	724	506	118	160	276	486
ENSECAG00000014770	3.963260262	0.0061779	0.0669891	183	93	186	215	790	206	501	122
ENSECAG00000018292	0.975848516	0.006179	0.0669891	35	12	11	21	59	37	74	28
ENSECAG00000023263	6.791981484	0.0061805	0.0669891	1438	1533	1753	1171	2570	3104	2244	2300
ENSECAG00000008927	5.147542846	0.006192	0.0670497	461	465	520	536	752	871	794	804
ENSECAG00000009901	10.78402353	0.0062112	0.0671297	26535	23704	25253	13695	37739	39552	41391	50351
ENSECAG00000019025	4.623853785	0.0062112	0.0671297	78	415	165	173	104	1411	439	657
ENSECAG00000012260	4.715925995	0.0062481	0.0674285	291	431	280	339	323	705	733	751
ENSECAG00000000216	6.741025246	0.0062578	0.0674285	1400	1492	1454	1419	2173	3232	1963	2416
ENSECAG00000013486	7.618185609	0.0062622	0.0674285	223	2626	490	881	340	14504	641	6888
ENSECAG00000019276	6.816676532	0.0062626	0.0674285	3329	1706	2752	4079	1699	985	1938	890
ENSECAG00000018519	4.753033127	0.0062984	0.0677498	626	549	898	758	188	311	590	201
ENSECAG00000018864	2.204396379	0.0063319	0.0680456	41	57	52	63	88	151	130	77
ENSECAG00000022071	2.045571872	0.0063388	0.0680557	21	87	14	21	75	151	86	119
ENSECAG00000017561	2.085643116	0.0063516	0.0681283	44	50	38	29	71	207	36	108
ENSECAG00000027244	2.981040088	0.0063803	0.0683716	594	188	94	46	37	83	40	80
ENSECAG00000006745	8.661504801	0.0064187	0.0687186	3153	7778	2835.01	4716	9855	10813	5188.01	13981
ENSECAG00000014273	6.155663928	0.006437	0.0687855	622	1269	804	1211	1552	1523	1851	1689
ENSECAG00000002520	4.663682707	0.0064392	0.0687855	288	258	320	460	764	743	474	378
ENSECAG00000012148	5.527120234	0.0064459	0.0687855	119	680	266	301	123	3022	383	1447
ENSECAG00000000085	5.682654045	0.0064492	0.0687855	744	709	655	759	912	1127	1041	1613
ENSECAG00000024286	7.419679393	0.0064696	0.0689387	1736	1713	1513	2289	2082	10290	2307	2115
ENSECAG00000000934	7.065582398	0.0065191	0.0694002	1968	1736	1634	2144	3090	2947	3177	3080
ENSECAG00000021921	2.073696557	0.0065391	0.0695478	41	64	41	34	111	46	178	99
ENSECAG00000004025	7.232766086	0.0065573	0.0696762	2056	2078	1841	1403	2198	5535	2500	4019
ENSECAG00000016587	4.558704646	0.0065795	0.0697275	479	441	705	839	187	184	439	354
ENSECAG00000021647	0.919090763	0.0065798	0.0697275	79	47	15	69	10	9	35	12
ENSECAG00000014490	2.413857975	0.0065805	0.0697275	45	47	39	101	77	181	74	197
ENSECAG00000006704	3.677868922	0.006607	0.0698971	362	241	336	399	149	78	225	164
ENSECAG00000010091	0.841795959	0.0066088	0.0698971	11	12	29	17	74	45	23	33
ENSECAG00000021278	2.195417491	0.0066513	0.0702808	199	17	358	7	44	21	11	22
ENSECAG00000020295	2.492301561	0.0066769	0.0704855	141	105	126	230	48	49	75	79
ENSECAG00000014428	5.180952895	0.0066975	0.0706374	772	702	1138	1126	336	249	591	648
ENSECAG00000000971	6.774746825	0.0067482	0.0710432	1748	1525	1308	1235	3667	2091	2404	2054
ENSECAG00000000795	2.498375034	0.0067485	0.0710432	41	70	63	99	126	110	193	126
ENSECAG00000009996	1.562823164	0.006768	0.0711826	64	63	86	102	32	42	25	19
ENSECAG00000019578	2.430987676	0.0067902	0.0713509	168	89	135	197	57	68	73	25
ENSECAG00000009746	2.442514555	0.006827	0.0716713	68	64	56	68	176	141	117	87
ENSECAG00000022702	4.348305183	0.0068512	0.0718584	324	253	206	119	299	341	368	1064
ENSECAG00000009935	2.989607116	0.0068687	0.071976	110	75.0001	104	95.0001	180	167	328.001	112
ENSECAG00000009704	1.961437839	0.0068975	0.0722106	78	81	139	115	34	52	34	42
ENSECAG00000012506	7.372491414	0.0069144	0.0723214	4011	3622	6047	4019	1952	548	3161	2081
ENSECAG00000000735	5.572602027	0.0069733	0.0728502	353	629	397	573	300	2732	604	1095

ENSECAG00000024168	5.383132833	0.0069778	0.0728502	543	528	467	532	523	1765	686	939
ENSECAG00000012498	7.784663243	0.0069923	0.0729345	2632	1541	2466	3728	9852	3291	7495	1818
ENSECAG00000013205	0.658891754	0.0070075	0.0730261	21	13	17	16	42	36	31	40
ENSECAG00000005344	4.772777597	0.0070933	0.0738526	722	371	864	941	406	290	422	173
ENSECAG00000018756	4.844427209	0.0071217	0.0740802	657	1107	567	687	191	376	180	504
ENSECAG00000010881	6.664753714	0.0071316	0.0741156	554	1869	254	275	1742	5401	318	3512
ENSECAG00000019129	3.537983603	0.0071584	0.0743254	316	244	241	457	108	166	181	54
ENSECAG00000013865	4.544555993	0.0071862	0.0745461	665	462	713	524	274	174	402	327
ENSECAG00000000962	1.250673586	0.0071986	0.0746069	18	30	23	30	35	91	54	49
ENSECAG00000011621	6.689708227	0.0072211	0.0747718	2185	2467	2434	3284	1124	955	1530	1760
ENSECAG00000017482	1.971056335	0.0072522	0.074922	44	35	44	46	83	169	83	45
ENSECAG00000007321	5.945710843	0.0072664	0.074922	1581	1354	1620	1803	377	585	738	1330
ENSECAG00000017587	6.836582332	0.0072668	0.074922	1983	1345	1398	1553	3101	2407	2350	2656
ENSECAG00000024576	1.236360951	0.0072681	0.074922	43	25	16	11	44	63	55	71
ENSECAG00000024511	5.227331883	0.0072686	0.074922	881	983	931	1141	212	265	438	856
ENSECAG00000009811	3.397788845	0.0072999	0.0751766	174	40	133	34	497	141	452	75
ENSECAG00000007020	2.697755245	0.00732	0.0753067	112	71	65	60	156	105	244	139
ENSECAG00000016779	3.880406725	0.0073258	0.0753067	299	303	401	517	197	122	209	195
ENSECAG00000021073	0.324182601	0.0073459	0.0754454	16	11	8	15	26	26	48	21
ENSECAG00000020366	5.274620925	0.007365	0.0755723	578	403	499	395	639	1758	662	583
ENSECAG00000020688	4.864146622	0.0073721	0.0755772	396	324	451	419	837	509	883	499
ENSECAG00000010789	5.83104757	0.0073826	0.0756168	587	1007	493	667	691	1989	887	1869
ENSECAG00000011196	4.673957034	0.0074394	0.0760397	1	134	6	48	3	2545	1	592
ENSECAG00000023814	5.608893896	0.0074482	0.0760397	1350	915	1285	1419	652	292	968	607
ENSECAG00000014260	11.18235534	0.0074505	0.0760397	4924	34289	5716	3241	4681	138257	6181	121223
ENSECAG00000024811	4.939799137	0.0074507	0.0760397	608	433	247	244	938	607	764	636
ENSECAG00000019095	5.491341873	0.0075087	0.0765631	276	454	704	711	550	512	2627	1071
ENSECAG00000020949	4.218437952	0.0075433	0.0768473	489	292	433	724	265	212	219	208
ENSECAG00000008715	4.326055926	0.0075996	0.0772713	278	256	243	304	395	585	424	436
ENSECAG00000011467	6.794782939	0.007605	0.0772713	565	479	980	238	93	10030	102	2284
ENSECAG00000020506	7.283132409	0.0076117	0.0772713	4632	2461	4172	5342	2533	1199	2750	917
ENSECAG00000005968	2.969315465	0.0076122	0.0772713	89	101	75	112	266	132	262	110
ENSECAG00000018345	7.103039319	0.0076327	0.0774102	1218	2225	1465	1428	1498	6078	2246	3443
ENSECAG00000015182	3.870442441	0.0076495	0.0775117	453	263	617	201	151	127	169	212
ENSECAG00000004574	2.679249472	0.0076685	0.0776346	79	89	68	63	161	163	91	199
ENSECAG00000010736	9.559780041	0.0076915	0.0777038	16441	8454	15874	46602	5537	4446	15592	7535
ENSECAG00000016019	6.278923342	0.0076961	0.0777038	1172	970	951	1246	2173	1893	1849	1155
ENSECAG00000023494	5.202631328	0.0076991	0.0777038	521	519	493	401	1021	1098	645	614
ENSECAG00000013050	8.996061013	0.0077027	0.0777038	6619	5666	5704	9576	9788	14941	13107	9884
ENSECAG00000016449	4.600483615	0.0077388	0.0779987	28	255	108	163	20	1523	70	1165
ENSECAG00000009149	7.451532391	0.0077592	0.0781312	3975	5226	5910	4650	444	1016	1015	4376
ENSECAG00000004906	6.585847266	0.0077657	0.0781312	2054	2660	2816	2265	560	845	1561	1814
ENSECAG00000011718	0.454442772	0.0077768	0.0781736	13	16	12	15	44	23	40	24
ENSECAG00000014404	4.94552254	0.0077937	0.078273	637	770	674	1011	362	412	456.998	342
ENSECAG00000006641	0.812058398	0.0078004	0.078273	19	21	19	14	47	48	26	45
ENSECAG00000017795	8.896866077	0.007861	0.0787431	6661	4516	8707	2887	13878	14679	9674	7789
ENSECAG00000024961	4.382891209	0.0078611	0.0787431	505	839	347	418	222	241	292	232
ENSECAG00000012095	2.28562109	0.0078746	0.078765	112	150	113	144	42	34	34	94
ENSECAG00000008840	8.039890531	0.0078831	0.078765	7300	4727	6339	8772	3980	2660	4108	2413
ENSECAG00000020548	3.704902986	0.0078841	0.078765	300	266	299	520	111	87	273	154
ENSECAG00000012595	3.432267198	0.0079329	0.0791824	254	264	286	292	114	136	135	136
ENSECAG00000007680	5.091575229	0.0079801	0.079584	982	523	1002	1000	507	275	626	306
ENSECAG00000006153	5.876990129	0.0079902	0.0796149	99	961	310	211	215	3498	270	2511
ENSECAG00000004044	2.658234659	0.008	0.0796427	194	108	148	231	54	32	118	79
ENSECAG00000012838	5.013431726	0.0080492	0.079997	779	989	643	862	263	546	396	352
ENSECAG00000012789	4.085445265	0.0080497	0.079997	4	170	4	25	5	1316	25	719
ENSECAG00000016662	4.186547333	0.0080785	0.0802131	236	623	226	911	156	225	181	218
ENSECAG00000014698	1.393659351	0.0081091	0.0804461	37	2	35	8	41	22	107	128
ENSECAG00000014532	6.653282766	0.0081271	0.0805547	2406	2065	2773	2742	1459	1006	1775	1089
ENSECAG00000026921	5.274801209	0.0081693	0.0809023	810	764	1075	1224	505	360	686	490
ENSECAG00000009258	4.529958137	0.0081854	0.0809916	713	490	620	528	232	150	416	362
ENSECAG00000009572	2.79219786	0.008203	0.0810944	85	80	72	95	108	246	127	177
ENSECAG00000018981	2.057987396	0.008257	0.0815576	72.5927	103.658	113.403	147.608	34.3279	42.5809	46.804	59
ENSECAG00000014364	5.200046761	0.0082947	0.0818592	378	555	524	323	522	1502	584	855
ENSECAG00000015342	9.730904515	0.0083137	0.0819748	22	5182	72	28	169	60293	249	48737
ENSECAG00000011128	5.401231355	0.0083289	0.0820535	641	649	443	558	1006	1202	986	669
ENSECAG00000005590	4.006383047	0.008351	0.0821998	476	275	484	441	260	91	236	182
ENSECAG00000021994	6.302526888	0.0083655	0.0822713	1147	1016	1297	907	1960	2087	1453	1626
ENSECAG00000010646	5.449304599	0.008381	0.0823523	1055	881	1115	1273	503	425	796	614
ENSECAG00000019264	7.321392045	0.0084182	0.0826209	2100	2306	2111	2001	2731	4981	3190	3846
ENSECAG00000008094	5.56479214	0.0084228	0.0826209	988	1573	1472	785	422	291	635	938
ENSECAG00000011016	3.786292922	0.0084815	0.0831245	130	209	157	165	509	122	396	345
ENSECAG00000016217	9.129764323	0.0084899	0.0831354	15214	15956	13510	12440	7375	3725	10496	6286
ENSECAG00000006249	5.296193027	0.0085049	0.0832108	886	1037	1042	918	378	398	575	691
ENSECAG00000009621	5.734521353	0.0085319	0.0834027	1307	967	1252	1966	644	478	1195	413
ENSECAG00000012475	2.247690138	0.0085443	0.0834524	147	71	129	167	48	58	71	23
ENSECAG00000009417	4.008782149	0.008587	0.0837974	395	304	461	480	153	195	203	245
ENSECAG00000012222	5.162447428	0.0085945	0.083798	217	433	206	151	89	2882	151	689

ENSECAG00000016117	2.211804695	0.0086333	0.0841042	53	45	48	64	38	129	162	134
ENSECAG00000009611	6.990617487	0.0087565	0.0852314	2112	1364	1813	748	1745	4822	2226	3428
ENSECAG00000013977	0.708620169	0.0087678	0.0852684	23	20	12	5	20	35	38	71
ENSECAG00000024674	2.533278749	0.0087963	0.0854721	80	28	42	51	60	18	423	183
ENSECAG00000013724	4.823324989	0.0088061	0.085494	455	194	386	483	754	552	969	430
ENSECAG00000018057	3.30531187	0.0088193	0.0855493	105	69	145	150	431	194	226	117
ENSECAG00000015973	4.693889061	0.008845	0.0857257	684	485	684	735	335	244	407	360
ENSECAG00000007222	6.906584973	0.0088737	0.0859304	864	1945	1093	1228	1197	5594	1150	3882
ENSECAG00000006205	5.590342732	0.008918	0.0862855	637	578	648	910	1002	957	1620	853
ENSECAG00000013816	4.697238618	0.0089272	0.0863009	500	558	652	1034	157	187	445	471
ENSECAG00000013595	5.744544297	0.0089614	0.086558	470	882	453	684	610	2639	937	938
ENSECAG00000025078	6.391684	0.0090031	0.0868871	1981	2922	2843	851	759	715	895	1514
ENSECAG00000024053	3.014932123	0.0090622	0.0873176	91	56	70	154	308	102	329	92
ENSECAG00000008529	5.148426024	0.0090631	0.0873176	467	495	477	531	768	504	867	1158
ENSECAG00000016921	5.124935306	0.0090876	0.0874797	481	529	527	386	849	805	773	747
ENSECAG00000022014	3.614539507	0.0091124	0.0876443	321	443	334	205	93	103	60	252
ENSECAG00000019099	2.538628992	0.0091386	0.0878214	95	111	120	326	61	53	97	31
ENSECAG00000002490	3.174534942	0.0091686	0.0880353	71	124	72	93	91	483	83	238
ENSECAG00000016507	3.093772436	0.0092283	0.0885336	47	93	65	142	84	484	116	164
ENSECAG00000015543	6.556282991	0.0092416	0.0885866	2366	2007	1932	2973	1156	980	1661	1286
ENSECAG00000022090	1.30278432	0.0092886	0.0889276	37	36	15	7	63	40	65	83
ENSECAG00000026017	1.569772336	0.0092928	0.0889276	11	48	11	14	16	198	24	73
ENSECAG00000023355	3.227370093	0.0093348	0.089254	218	168	227	372	102	115	141	82
ENSECAG00000008454	7.610574676	0.0093531	0.0893539	2897	2134	2873	3066	4481	4695	4218	4425
ENSECAG00000013188	3.18954203	0.0093632	0.0893678	254	169	212	324	92	115	152	66
ENSECAG00000011219	5.086886536	0.0093703	0.0893678	299	374	464	345	452	1945	352	520
ENSECAG00000017249	6.511772743	0.0093843	0.0894266	1472	1310	1220	1137	2574	1770	1849	2059
ENSECAG00000016158	5.615493637	0.0094281	0.0897686	406	553	351	753	484	3107	552	645
ENSECAG00000020677	1.029606442	0.0094372	0.0897801	24	55	126	35	5	7	1	40
ENSECAG00000002675	1.690475296	0.009472	0.0900355	96	66	78	94	29	29	57	29
ENSECAG00000024310	2.584879114	0.0095074	0.0902964	98	45	59	30	282	119	195	33
ENSECAG00000010624	2.647562166	0.009528	0.0904162	76	27	89	43	389	124	96	42
ENSECAG00000017046	3.940800795	0.0095412	0.090466	344	343	444	423	152	221	197	184
ENSECAG00000008003	4.715435005	0.0096049	0.0909941	608	532	694	780	322	296	442	316
ENSECAG00000006166	3.729953569	0.0096257	0.0910678	393	262	389	321	149	87	264	151
ENSECAG00000011543	5.215513082	0.0096287	0.0910678	577	371	620	439	1287	700	874	587
ENSECAG00000010409	4.774594342	0.0096678	0.0913611	349	478	320	263	698	771	470	591
ENSECAG00000020620	6.492941851	0.0096964	0.0915551	2325	2103	2047	2329	888	1022	1606	1342
ENSECAG00000011555	4.849695125	0.0097148	0.0916534	694	514	836	856	351	268	527	357
ENSECAG00000022249	5.842714461	0.0097356	0.091773	1032	517	597	396	3175	770	1388	480
ENSECAG00000013789	0.736396118	0.0097816	0.0921305	15	20	21	8	14	36	66	52
ENSECAG00000017141	5.400735674	0.0098624	0.0928145	1313	972	1056	816	506	430	538	716
ENSECAG00000022402	3.300320498	0.009885	0.0929101	117	124	109	81	535	148	167	130
ENSECAG00000017383	5.612437415	0.0098948	0.0929101	925	515	779	481	934	1207	1185	1155
ENSECAG00000010961	5.867780383	0.0098971	0.0929101	881	612	732	904	2099	1376	1311	632
ENSECAG00000016062	0.365197628	0.0099897	0.0937017	4	20	14	9	32	12	44	41
ENSECAG00000014701	4.510795295	0.0100494	0.094184	265	357	254	275	352	805	341	623
ENSECAG00000010844	5.121657392	0.0100986	0.094487	449	530	533	423	727	825	710	891
ENSECAG00000008758	4.213636499	0.0101099	0.094487	521	275	490	728	248	244	266	68
ENSECAG00000009828	8.702104454	0.0101169	0.094487	8904	3608	4516	2297	9887	13762	12502	5019
ENSECAG00000019186	6.426970785	0.0101216	0.094487	1921.99	1915	2006	2626	1120	876	1647	1012
ENSECAG00000023644	4.480197069	0.0101233	0.094487	536	446	597	710	205	141	436	354
ENSECAG00000000269	3.839026489	0.0101481	0.0946403	229	140	199	204	382	253	431	278
ENSECAG00000008154	0.722463582	0.0101611	0.0946839	26	10	13	6	32	92	27	12
ENSECAG00000005987	4.413419615	0.0102968	0.0958699	243	245	345	328	690	438	453	379
ENSECAG00000023420	10.17808717	0.0103277	0.0960256	5612	22057	3248	2416	10213	45892	5116	67697
ENSECAG00000017386	3.320871514	0.0103304	0.0960256	96	89	124	188	301	146	413	135
ENSECAG00000021631	5.400127058	0.010351	0.096138	569	703	426	476	729	1525	653	977
ENSECAG00000006103	1.496453523	0.0103891	0.0964133	56	50	85	115	10	20	45	41
ENSECAG00000017241	8.335644876	0.0104396	0.0968025	5824	2196	4210	775	5093	16282	6688	4633
ENSECAG00000013217	5.176897877	0.0104604	0.0969168	379	562	502	435	572	1332	634	788
ENSECAG00000011930	2.159269678	0.0104934	0.0971428	12	27	21	51	19	13	22	483
ENSECAG00000008356	3.220032979	0.0105036	0.0971585	50	62	174	90	63	405	392	115
ENSECAG00000017657	6.999124711	0.0105231	0.097259	2241	705	1743	170	5566	2903	4192	1068
ENSECAG00000004807	8.267169036	0.0105486	0.0974161	4305	3975	3523	3060	13692	6416	6881	2906
ENSECAG00000009865	4.850285251	0.0106205	0.0979572	51	490	78	84	101	2024	83	985
ENSECAG00000014914	5.954517365	0.0106245	0.0979572	1719	834	2849	1251	488	244	1318	855
ENSECAG00000012359	4.081969524	0.0106529	0.0981042	495	357	414	471	118	275	180	234
ENSECAG00000008596	4.023925016	0.0106621	0.0981042	286	468	453	487	95	230	148	274
ENSECAG00000012768	2.923912925	0.0106663	0.0981042	35	48	21	92	2	563	67	207
ENSECAG00000023322	5.408178062	0.0107053	0.098383	926	922	856	1616	784	383	555	439
ENSECAG00000013283	6.060956254	0.010743	0.0986502	814	675	1422	759	2012	1087	1868	1256
ENSECAG00000021157	1.367358074	0.0107563	0.0986919	46	80	135	17	14	19	26	33
ENSECAG00000010182	0.890611478	0.0107762	0.0987946	47	45	54	54	21	11	34	2
ENSECAG00000020621	3.833728582	0.0107958	0.0988947	133	251	140	175	403	337	483	160
ENSECAG00000019642	4.714700484	0.0108249	0.0990812	410	300	396	285	864	578	652	351
ENSECAG00000009632	5.791284305	0.0108339	0.0990837	1359	1050	1344	1717	715	475	1028	786
ENSECAG00000022252	6.073758979	0.010852	0.0991499	1908	1310	1956	1462	846	543	1133	1067

ENSECAG00000020419	2.271880144	0.0108586	0.0991499	30	38	34	55	15	339	34	111
ENSECAG00000000268	3.467073003	0.0108779	0.0992465	160	128	114	189	263	211	364	207
ENSECAG00000012397	5.782798795	0.0109178	0.0994413	18	625	10	4	85	4293	98	2222
ENSECAG00000014869	5.700330207	0.0109194	0.0994413	909	764	608	695	1187	1078	1106	1295
ENSECAG00000024875	2.023289076	0.0109255	0.0994413	27	204	64	163	17	19	43	66
ENSECAG00000021457	1.261113336	0.0109424	0.0995152	78	23	74	97	8	7	55	19
ENSECAG00000011145	7.124003456	0.0110416	0.1003374	2446	1960	1572	1353	3427	4093	3372	2055
ENSECAG00000009655	4.113269717	0.0111264	0.100954	512	259	499	519	219	104	262	272
ENSECAG00000006901	4.762152316	0.0111272	0.100954	642	457	754	887	278	260	481	397
ENSECAG00000020273	3.729075207	0.0111734	0.1011804	339	236	385	383	156	160	199	147
ENSECAG00000014920	1.530797511	0.0111784	0.1011804	30	32	50	10	58	85	76	64
ENSECAG00000009347	7.259874001	0.0111789	0.1011804	1977	1868	2012	1056	2545	7883	1881	2318
ENSECAG00000018849	6.719227208	0.0112197	0.1014684	1852	1513	1468	749	2276	2631	2440	2433
ENSECAG00000014092	1.529117237	0.0112592	0.1017444	32	38	23	18	22	126	41	97
ENSECAG00000024219	3.529163737	0.0112764	0.1018197	125	151	186	33	295	202	189	482
ENSECAG00000001714	0.54710068	0.0112918	0.1018774	6	20	10	21	33	12	44	57
ENSECAG00000024510	4.839396927	0.0113367	0.1022017	625	488	892	960	417	151	436	427
ENSECAG00000011728	6.20874232	0.0113982	0.1026315	539	1493	598	711	678	2867	1181	2502
ENSECAG00000013970	5.567666665	0.0114025	0.1026315	1090	879	1305	1433	447	470	929	707
ENSECAG00000021201	4.601379063	0.0114132	0.1026462	539	99	84	7	791	276	1379	454
ENSECAG00000017436	7.133790586	0.0114513	0.1028433	6	1142	42	101	31	10690	12	6817
ENSECAG00000024391	4.53403529	0.0114532	0.1028433	410	292	328	205	521	343	704	627
ENSECAG00000023936	0.866888869	0.0114914	0.1031045	11	34	135	25	17	9	8	21
ENSECAG00000008603	6.384287041	0.0115112	0.1032012	2029	1792	2252	2022	1241	933	1270	1080
ENSECAG00000006182	3.378288523	0.0115823	0.1037284	251	185	282	380	110	51	170	165
ENSECAG00000012502	5.821722321	0.0115883	0.1037284	596	965	544	724	751	2271	792	1449
ENSECAG00000010178	2.24757399	0.0116064	0.1038091	57	56	54	62	132	123	142	57
ENSECAG00000020009	4.620331998	0.0116514	0.1040823	196	468	341	248	377	614	590	749
ENSECAG00000022964	2.532118523	0.0116553	0.1040823	167	77	166	215	54	34	122	50
ENSECAG00000011658	2.162745186	0.0117141	0.1045253	64	71	21	43	119	58	147	126
ENSECAG00000001214	2.860465158	0.0118532	0.1056832	60	115	77	97	131	99	193	293
ENSECAG00000025028	3.863568007	0.0118897	0.1059258	134	185	175	158	154	625	139	491
ENSECAG00000021895	3.590524768	0.0119241	0.1061491	119	174	164	175	314	278	367	173
ENSECAG00000022483	7.202008268	0.0120091	0.1068219	2574	2079	2002	1131	4013	3191	3852	2718
ENSECAG00000018793	0.820691004	0.0120209	0.1068432	9	16	29	11	11	28	59	85
ENSECAG00000004869	0.921478243	0.0120337	0.1068732	48	54	37	55	11	11	29	28
ENSECAG00000024257	8.955785402	0.0120807	0.1071872	8975	5503	6977	4787	14364	12026	9110	10583
ENSECAG00000002321	6.608181051	0.0120949	0.1071872	1610	989	1231	1480	3659	1267	2853	1431
ENSECAG00000023706	4.912169625	0.0121235	0.1071872	361	415	421	413	589	1060	497	584
ENSECAG00000018312	3.636008794	0.0121282	0.1071872	232	156	69	147	227	189	552	284
ENSECAG00000020786	1.440607927	0.012132	0.1071872	33	19	37	9	124	27	119	20
ENSECAG00000016467	6.556219863	0.0121349	0.1071872	1314	1248	1446	1516	1820	2261	2046	2222
ENSECAG00000024314	3.64725021	0.0121351	0.1071872	149	100	147	191	436	407	302	75
ENSECAG00000019348	1.516273452	0.012154	0.1072707	67	52	73	110	15	22	43	44
ENSECAG00000012069	7.424083737	0.0121639	0.1072743	2758	2180	2236	2390	3543	4759	4364	2990
ENSECAG00000000309	0.718372341	0.0121875	0.1073991	34	40	45	59	21	3	31	5
ENSECAG00000023555	2.055260167	0.0122224	0.1076237	122	75	89	150	53	49	53	29
ENSECAG00000016702	5.861023774	0.0122321	0.1076256	208	983	190	373	248	3895	198	1837
ENSECAG00000022776	4.861159407	0.0122771	0.1078865	229	481	188	130	202	1068	140	1663
ENSECAG00000022579	6.665105463	0.0122808	0.1078865	2740	1918	2600	2744	1558	895	1707	1292
ENSECAG00000008048	5.160544169	0.0123467	0.1082991	927	729	794	1150	321	292	574	684
ENSECAG00000018099	2.060611484	0.0123468	0.1082991	73	28	28	4	243	72	115	25
ENSECAG00000009314	4.69038245	0.0124078	0.1087498	338	399	317	371	691	648	555	444
ENSECAG00000023723	6.002416068	0.0124383	0.1087893	1729	1202	1814	1827	554	333	1481	950
ENSECAG00000016134	7.098006334	0.0124401	0.1087893	1653	1893	1898	1296	1832	4501	1592	5061
ENSECAG00000000382	4.643027135	0.0124467	0.1087893	367	424	269	235	466	785	649	423
ENSECAG00000012280	5.846587845	0.0124506	0.1087893	522	815	620	1129	1020	2156	960	1111
ENSECAG00000018533	2.078742969	0.0125083	0.1091563	58	153	143	88	34	50	29	60
ENSECAG00000015206	5.255155391	0.0125258	0.1091563	406	662	543	550	940	734	930	857
ENSECAG00000024746	1.68214474	0.0125282	0.1091563	94	50	118	77	37	13	57	31
ENSECAG00000016959	6.670771595	0.0125355	0.1091563	1666	1057	1511	1545	2398	1971	3581	1498
ENSECAG00000023657	0.904791566	0.012547	0.1091563	10	37	12	8	50	75	25	33
ENSECAG00000024887	4.528613618	0.0125547	0.1091563	92	328	138	158	85	1665	80	588
ENSECAG00000000477	4.16547025	0.0125686	0.1091563	566	365	464	400	201	165	229	313
ENSECAG00000007133	4.707329095	0.0125695	0.1091563	676	1007	1149	226	39	174	35	565
ENSECAG00000004316	1.104502451	0.0126118	0.1094405	69	36	59	71	6	7	23	45
ENSECAG00000012059	5.186478288	0.0126897	0.1100322	358	568	371	725	770	899	1130	570
ENSECAG00000011807	8.123569058	0.0127325	0.110319	3560	3835	3392	3602	4907	11163	4675	4951
ENSECAG00000018846	3.580095703	0.0127471	0.1103612	299	216	336	363	106	130	208	158
ENSECAG00000020858	3.871082757	0.0127664	0.1103963	105	282	187	83	440	482	287	209
ENSECAG00000022506	6.031827922	0.0127758	0.1103963	864	776	688	1047	964	2951	1120	981
ENSECAG00000003104	5.667866196	0.0127803	0.1103963	473	648.002	499.001	1116	1012	1870	1060	726.001
ENSECAG00000023787	0.474783198	0.012828	0.1107243	14	16	14	16	26	37	26	39
ENSECAG00000011405	4.831438205	0.0128463	0.110798	771	488	698	880	403	326	382	375
ENSECAG00000012954	5.663002631	0.0128645	0.1108712	485	587	543	993	679	2207	713	1086
ENSECAG00000014314	3.195861411	0.0128836	0.1109512	247	118	225	395	90	70	185	85
ENSECAG00000015319	3.345023134	0.0129785	0.1116838	224	193	246	401	66	107	186	132
ENSECAG00000010118	3.426345787	0.0130216	0.1119699	142	124	138	149	309	130	424	185

ENSECAG00000011343	4.860316667	0.0131609	0.1130821	385	361	435	411	700	883	622	426
ENSECAG00000018810	3.897076842	0.0132365	0.1136458	423	232	269	663	103	126	257	232
ENSECAG00000016488	4.022434634	0.0133439	0.1144818	258	205	210	216	461	261	475	314
ENSECAG00000017271	4.609052194	0.0133608	0.1145402	683	568	710	503	262	130	446	400
ENSECAG00000017777	4.836520452	0.0133839	0.1146327	648	587	580	1077	275	420	450	318
ENSECAG00000002676	8.993167534	0.0133918	0.1146327	9799	5722	6085	4482	18056	8698	14784	7466
ENSECAG00000014496	0.445279468	0.0134041	0.1146519	14	15	13	17	19	26	51	33
ENSECAG00000017188	5.048365566	0.0134283	0.1147724	781	726	837	896	325	433	504	499
ENSECAG00000020519	8.035817841	0.0134657	0.1150053	3710	2610	2392	3213	2297	13902	2948	6082
ENSECAG00000005250	5.340590862	0.0134844	0.1150503	595	521	559	589	649	1246	724	1018
ENSECAG00000016129	0.405688707	0.0134912	0.1150503	11	10	14	22	37	31	41	15
ENSECAG00000005244	6.363665612	0.0135056	0.1150866	1831	1671	2276	2261	1246	859	1474	900.001
ENSECAG00000009456	3.741679261	0.0135429	0.1153177	183	96	128	268	180	487	421	198
ENSECAG00000014421	5.372231569	0.0135835	0.115577	919	955	1159	1012	547	336	685	663
ENSECAG00000017630	5.580114802	0.0136339	0.1158757	665	766	605	652	1337	1198	1004	747
ENSECAG00000008897	4.758762216	0.0136443	0.1158757	268	506	352	225	354	768	540	903
ENSECAG00000016957	2.658172723	0.0136492	0.1158757	78	115	54	45	204	137	121	145
ENSECAG00000009170	5.697672069	0.0136621	0.1158979	665	715	581	703	530	2000	753	1528
ENSECAG00000023782	0.472718806	0.0137002	0.1160836	6	9	9	33	16	35	41	43
ENSECAG00000019758	3.723798868	0.0137044	0.1160836	161	137	197	192	439	207	454	169
ENSECAG00000024536	4.313078984	0.0137261	0.1161806	1	133	5	74	0	1110	11	1400
ENSECAG00000008103	5.373424694	0.0137617	0.1163956	984	707	1101	1428	475	247	847	611
ENSECAG00000007826	3.388975922	0.0137804	0.1164666	282	163	243	398	141	110	163	97
ENSECAG00000000368	0.903401134	0.0138421	0.1169015	10	30	21	2	30	85	43	30
ENSECAG00000013545	5.120888993	0.013888	0.1171909	571	383	535	542	794	702	860	763
ENSECAG00000014015	2.357259279	0.013897	0.1171909	72	80	36	26	147	151	173	44
ENSECAG00000008938	5.23055045	0.0139428	0.1174902	501	567	516	581	851	931	781	770
ENSECAG00000012675	3.329728836	0.014023	0.1180776	230	263	245	272	92	101	139	167
ENSECAG00000016177	0.004137834	0.0141258	0.1188559	22	24	31	25	2	7	5	20
ENSECAG00000018544	2.879783485	0.0141941	0.1193417	210	126	191	266	33	44	125	131
ENSECAG00000001533	1.922424279	0.0142307	0.1195613	102	35	120	158	24	25	71	42
ENSECAG00000018094	1.861331092	0.0142575	0.1196976	14	43	24	41	18	250	28	72
ENSECAG00000021300	6.931965884	0.0142854	0.1197555	2029	1766	1569	1523	3359	2538	2766	2383
ENSECAG00000025124	4.287319244	0.0142855	0.1197555	290	213	295	280	519	398	625	268
ENSECAG00000018327	1.799870227	0.0143059	0.1198387	39	53	39	35	53	78.3333	110	92
ENSECAG00000019365	4.541545836	0.0143569	0.1201766	186	403	173	302	254	1142	327	503
ENSECAG00000011828	1.337590449	0.0143783	0.1202278	13	44	18	36	74	51	45	73
ENSECAG00000019437	8.024295481	0.0143842	0.1202278	3568	2197	3232	4914	8260	5279	8743	2648
ENSECAG00000012002	5.34383016	0.0144215	0.1202709	1115	681	1290	966	485	280	742	639
ENSECAG00000011234	3.254650199	0.0144312	0.1202709	121	153	70	129	136	313	208	244
ENSECAG00000003550	3.691795879	0.0144315	0.1202709	220	466	331	321	92	133	98	262
ENSECAG00000012854	7.61269891	0.0144317	0.1202709	1886	2288	2023	3658	3087	9043	3041	3070
ENSECAG00000008072	5.763148505	0.0145209	0.1209255	734	864	704	877	1247	880	1666	1122
ENSECAG00000001471	8.461421422	0.0145466	0.1209819	14	3076	115	119	47	27418	53	16340
ENSECAG00000013630	4.19938346	0.0145574	0.1209819	209	607	369	734	183	132	345	229
ENSECAG00000008559	4.937546559	0.0145615	0.1209819	455	476	362	342	847	910	578	451
ENSECAG00000020745	3.583084779	0.0145702	0.1209819	119	184	162	182	297	271	315	222
ENSECAG00000009251	5.491247496	0.0145953	0.1211013	562	621	791	524	1205	1099	1209	581
ENSECAG00000021257	1.362717366	0.0146666	0.1216046	13	45	31	24	69	53	83	45
ENSECAG00000011160	1.25982048	0.0147333	0.1220685	31	27	23	6	66	113	39	18
ENSECAG00000010569	2.370214247	0.0147523	0.1221364	70	28	75	67	101	73	278	80
ENSECAG00000003445	0.5505221	0.0147893	0.1222819	60	29	17	50	8	3	22	21
ENSECAG00000019899	5.536860158	0.0147914	0.1222819	1277	761	1144	1429	574	353	796	780
ENSECAG00000026502	3.218647731	0.0148461	0.1226454	81	61	14	25	3	399	4	708
ENSECAG00000003619	6.664722755	0.01487	0.1227031	1475	1390	1655	1444	2273	1873	2582	2351
ENSECAG00000017566	2.666499145	0.0148747	0.1227031	60	109	54	84	166	95	255	110
ENSECAG00000022712	5.997417415	0.0148892	0.1227051	1281	1249	1588	2184	617	890	1010	920
ENSECAG00000019932	8.122550874	0.0148966	0.1227051	1982	3533	1885	1387	830	21542	1037	5540
ENSECAG00000022913	7.269381841	0.0149084	0.1227107	2499	1718	2623	1711	4307	3546	3593	2638
ENSECAG00000020091	6.696385398	0.0149189	0.1227107	2785	1922	2378	3269	1785	869	1711	1131
ENSECAG00000015971	5.3671332	0.0149574	0.1228395	1137	780	961	1168	621	416	743	452
ENSECAG00000008545	1.517299945	0.0149625	0.1228395	7	36	10	25	7	102	8	199
ENSECAG00000011099	6.935422109	0.014967	0.1228395	2139	1515	1812	1521	2186	2732	3203	2989
ENSECAG00000003259	3.472151045	0.0149855	0.1229026	120	157	148	166	345	232	269	181
ENSECAG00000013090	5.141557449	0.015003	0.1229576	932	635	941	980	470	279	647	503
ENSECAG00000020441	3.732370184	0.0150593	0.1233301	349	195	382	495	146	53	285	152
ENSECAG00000022063	5.748176317	0.0150953	0.1235359	920	664	662	797	1620	1307	1149	747
ENSECAG00000016839	0.711947979	0.0151165	0.1236205	8	31	7	11	13	70	27	52
ENSECAG00000001098	0.777672177	0.0151298	0.1236401	29	57	42	43	15	17	17	22
ENSECAG00000016330	5.52512024	0.0151944	0.1240788	341	720	366	731	565	2349	500	938
ENSECAG00000024946	2.490409921	0.0152102	0.1241181	48	68	56	62	66	323	68	85
ENSECAG00000009473	6.768573746	0.0152281	0.1241755	1290	1810	1600	1447	2673	2273	2009	2868
ENSECAG00000024797	7.210809459	0.0153173	0.1248128	2378	1663	1646	2793	3690	3479	2828	3352
ENSECAG00000024971	2.163800827	0.0153849	0.1251623	38	54	45	35	27	239	43	136
ENSECAG00000019100	2.546280977	0.0153915	0.1251623	28	349	112	149	34	35	72	78
ENSECAG00000000265	5.069248023	0.0153932	0.1251623	914	561	1035	861	529	246	638	338
ENSECAG00000010199	5.058625757	0.0154418	0.1254675	790	733	839	893	378	322	517	579
ENSECAG00000019776	5.490028502	0.0154614	0.125537	1123	805	1241	1337	683	409	940	339

ENSECAG00000012776	7.027032812	0.0155077	0.1256634	2185	1850	1470	1861	2809	3329	2436	3140
ENSECAG00000003462	4.696077631	0.0155083	0.1256634	306	488	338	273	634	611	572	546
ENSECAG00000021048	6.645150616	0.0155102	0.1256634	306	1925	445	594	558	5905	421	3626
ENSECAG00000016279	2.385878787	0.0155549	0.1259362	81	73	52	29	59	158	133	161
ENSECAG00000009215	5.594636092	0.015574	0.1259836	995	473	447	84	2208	685	1635	539
ENSECAG00000007941	3.495798664	0.0155919	0.1259836	241	251	239	433	182	73	172	128
ENSECAG00000010011	3.316963869	0.0155941	0.1259836	146	104	116	93	251	448	126	115
ENSECAG00000026958	5.389123696	0.0156402	0.1262664	611	436	809	390	627	1318	754	1137
ENSECAG00000013913	5.659027832	0.0156748	0.1264428	1254	1089	1389	1129	724	637	820	567
ENSECAG00000019004	3.216621889	0.0156843	0.1264428	231	183	251	276	95	93	115	157
ENSECAG00000017581	3.574641791	0.0157243	0.1266309	148	123	163	210	357	266	317	164
ENSECAG00000017994	4.464219978	0.0157299	0.1266309	544	397	622	674	203	151	450	346
ENSECAG00000013362	0.739798189	0.0158142	0.1272193	21	83	22	46	11	18	20	13
ENSECAG00000025086	6.120583272	0.0159164	0.1279511	1754	1551	1666	1861	557	1187	996	913
ENSECAG00000003514	-0.024727516	0.016038	0.1288136	12	9	8	12	25	23	20	20
ENSECAG00000025085	3.703074444	0.016049	0.1288136	322	205	433	357	159	115	218	169
ENSECAG00000010339	7.493927224	0.0160688	0.1288136	2721	1797	2073	269	993	7013	2790	8087
ENSECAG00000014995	3.418561762	0.0160691	0.1288136	265	208	307	292	125	97	157	163
ENSECAG00000021110	4.942568097	0.0162357	0.1299943	55	567	90	105	62	1872	126	1330
ENSECAG00000018813	3.976737438	0.0162393	0.1299943	258	394	620	435	42	124	163	352
ENSECAG00000017970	5.167025568	0.0163186	0.1305372	32	595	88	191	53	2470	138	1318
ENSECAG00000009535	8.402953555	0.0163756	0.130901	647	5722	1188	2119	675	22878	1568	11482
ENSECAG00000000025	1.914759877	0.0163878	0.1309065	46	43	48	52	118	70	100	65
ENSECAG00000023922	4.383617573	0.0164077	0.1309737	460	396	498	801	137	361	256	263
ENSECAG00000019626	6.450524167	0.0164785	0.1313869	1332	816	1326	1385	1283	1124	2261	3603
ENSECAG00000017840	6.412352282	0.0164826	0.1313869	349	1421	218	475	281	6024	179	2612
ENSECAG00000009940	1.584529809	0.0165049	0.1314719	32	50	24	24	48	124	53	59
ENSECAG00000009073	2.77929087	0.0165176	0.1314809	173	150	155	222	74	95	104	56
ENSECAG00000006867	1.954392212	0.0165343	0.1315222	35	52	34	50	116	156	50	45
ENSECAG00000018064	5.763763379	0.0165704	0.1317168	803	733	674	974	1206	697	1480	1566
ENSECAG00000016847	6.365427213	0.0165904	0.1317837	1926	1702	2331	2050	870	822	1274	1535
ENSECAG00000007467	4.344511829	0.0166282	0.1319582	240	395	208	229	322	554	595	421
ENSECAG00000024189	7.39408828	0.0166415	0.1319582	3909	4240	5613	2966	1468	1736	2233	3143
ENSECAG00000021716	6.093801357	0.0166472	0.1319582	1037	939	912	1220	1551	1273	1604	1601
ENSECAG00000017463	6.00172704	0.0166909	0.1322123	1430	1358	1361	2115	742	721	1294	774
ENSECAG00000016863	4.306275979	0.0167332	0.1324547	316	157	294	325	633	333	566	310
ENSECAG00000010405	6.617495805	0.0167805	0.1327367	2731	2072	2514	2141	1488	1137	1550	1171
ENSECAG00000009649	3.573649306	0.0168067	0.1328515	27	222	37	74	38	720	80	433
ENSECAG00000019455	2.614573442	0.016826	0.1329114	94	55	78	88	137	114	238	99
ENSECAG00000022270	6.582897907	0.0168804	0.1332486	1990	2384	2049	2932	855	1153	1643	1565
ENSECAG00000015930	5.482181269	0.0168924	0.1332502	1292	879	1157	1067	805	409	755	403
ENSECAG00000022369	7.422746294	0.016944	0.1335652	2503	2349	2386	1759	3045	6617	3453	2599
ENSECAG00000023478	5.027320499	0.0169696	0.1336737	35	561	49	137	58	2388	99	1071
ENSECAG00000021615	6.498531352	0.0170029	0.1338433	2130	1817	2258	2494	1177	1012	1413	1419
ENSECAG00000003025	2.343745144	0.017021	0.1338596	26	88	24	7	20	306	35	180
ENSECAG00000008567	6.60018417	0.0170285	0.1338596	2808	1512	2407	3018	1767	832	1632	871
ENSECAG00000021277	0.844374839	0.0171154	0.1344494	17	7	2	34	12	27	25	128
ENSECAG00000014836	3.653872749	0.0171794	0.1348355	223	201	411	469	119	101	210	197
ENSECAG00000017984	6.804124401	0.0171883	0.1348355	1997	1519	1444	562	3029	3066	3443	1206
ENSECAG00000011686	4.36663202	0.0172868	0.1355145	199	310	234	234	232	937	220	553
ENSECAG00000000757	2.632435637	0.0173629	0.1360177	181	115	143	198	89	49	105	55
ENSECAG00000010315	4.82713046	0.0174543	0.1366389	634	523	540	1229	248	247	588	389
ENSECAG00000007349	5.946762991	0.0175515	0.1373055	1369	1382	1417	1752	920	674	983	846
ENSECAG00000003418	4.283965275	0.0176054	0.1376232	217	198	249	275	157	638	219	863
ENSECAG00000019530	5.980393748	0.0176163	0.1376232	756	841	890	943	863	2510	1089	1221
ENSECAG00000012089	9.82571315	0.0176478	0.1377739	12852	12963	11043	13086	14837	23644	16632	27928
ENSECAG00000022397	1.211895394	0.0177129	0.1381876	21	22	23	18	15	131	13	68
ENSECAG00000016629	2.109920841	0.0177271	0.1382032	47	71	36	52	59	123	91	136
ENSECAG00000011067	3.479284825	0.0177984	0.1386638	0	44	0	22	0	1141	0	239
ENSECAG00000009356	6.431607073	0.0178349	0.1387328	990	1405	916	1486	1392	2972	1579	1797
ENSECAG00000015301	5.056673024	0.0178612	0.1387328	695	377	414	189	1004	632	1165	423
ENSECAG00000007238	1.036608771	0.0178729	0.1387328	37	28	69	93	4	8	31	35
ENSECAG00000000465	8.907349564	0.0178776	0.1387328	8777	15056	13286	12598	1942	6176	6033	8617
ENSECAG00000008637	5.822296816	0.0178799	0.1387328	1575	976	1262	1826	1008	429	982	604
ENSECAG00000026827	3.812784702	0.0178827	0.1387328	497	196	332	407	190	139	239	137
ENSECAG00000022971	6.811499257	0.0178927	0.1387328	1344	1731	1799	1286	1511	2651	2378	3796
ENSECAG00000006995	6.760809151	0.0179283	0.1387689	1293	1551	1418	1945	1583	3023	2308	2833
ENSECAG00000020076	6.129873273	0.0179303	0.1387689	876	1119	840	1285	1257	1753	1543	1644
ENSECAG00000022150	1.862402172	0.017934	0.1387689	90	40	103	161	37	23	74	25
ENSECAG00000015858	8.064807459	0.0179764	0.1388921	5241	2842	3685	1184	9686	4988	8076	3542
ENSECAG00000020102	6.043526169	0.0179903	0.1388921	828	1073	1007	907	1425	1722	1222	1440
ENSECAG00000023625	5.008065822	0.0179951	0.1388921	485	462	363	459	504	1063	549	777
ENSECAG00000022855	5.811772194	0.0179989	0.1388921	890.002	845	813	771	1148	1307	1247	1234
ENSECAG00000023935	6.542080186	0.0180278	0.1389631	2383	1777	2229	2736	1219	722	1801	1399
ENSECAG00000001360	5.168042356	0.0180325	0.1389631	824	703	904	1062	417	418	629	509
ENSECAG00000022075	7.565138017	0.0181053	0.1394294	2400	2140	2202	2562	2776	9621	2402	2772
ENSECAG00000021923	4.970322207	0.0181724	0.1398511	843	552	902	786	308	310	577	492
ENSECAG00000020369	6.26902508	0.0182004	0.1399715	878	1191	1352	1022	1585	2013	1478	1748

ENSECAG00000023344	5.216600213	0.0182292	0.1400988	860	778	709	1340	428	322	640	620
ENSECAG00000019637	5.295761871	0.018378	0.1411463	916	1151	964	760	512	334	594	661
ENSECAG00000014710	1.132043196	0.0184247	0.1414096	22	34	20	27	40	40	79	49
ENSECAG00000009402	1.48059566	0.0184612	0.1415205	35	38	23	37	57	78	42	81
ENSECAG00000005116	3.12535151	0.0184641	0.1415205	118	102	109	115	180	327	159	136
ENSECAG00000003736	4.628802975	0.0184843	0.1415802	376	256	422	265	915	451	635	307
ENSECAG00000003758	4.105308158	0.0185244	0.1417917	49	289	49	64	48	1378	32	387
ENSECAG00000003748	2.295518417	0.0186491	0.1426359	39	69	40	87	69	163	82	151
ENSECAG00000023974	5.645179756	0.0186598	0.1426359	676	726	708	790	1025	1479	923	968
ENSECAG00000006067	4.662329508	0.0186895	0.1427663	279	328	295	272	218	1374	327	470
ENSECAG00000009180	4.918958709	0.0187076	0.1428091	150	519	256	332	136	1397	344	1148
ENSECAG00000024878	3.351890545	0.0187609	0.1430324	104	173	85	165	275	149	204	338
ENSECAG00000007069	4.418278346	0.0187621	0.1430324	531	387	573	639.001	269.001	164.001	434	272
ENSECAG00000013151	6.305917974	0.0187798	0.1430718	1599	531	1298	911	2010	1873	1133	2206
ENSECAG00000015040	6.419592991	0.0188641	0.1436177	2172	1555	2025	2583	1266	840	1584	1047
ENSECAG00000019023	6.11742512	0.0189017	0.1437168	1145	1065	831	849	1227	2254	1288	1404
ENSECAG00000021486	5.697300238	0.0189149	0.1437168	695	934	555	705	1375	1476	935	838
ENSECAG00000017405	4.514304137	0.0189151	0.1437168	322	256	197	409	448	965	313	363
ENSECAG00000018392	3.615123657	0.018937	0.1437867	367	165	517	222	130	88	138	221
ENSECAG00000019160	3.976685688	0.0189542	0.1438215	227	182	245	207	525	273	403	250
ENSECAG00000006038	2.617498118	0.0190128	0.1441014	181	75	144	258	56	44	133	52
ENSECAG00000002412	3.732650872	0.0190286	0.1441014	183	146.002	131.001	126.003	108.001	700	125	343.001
ENSECAG00000016493	7.703727957	0.0190291	0.1441014	2383	3140	2602	3753	3744	5604	4175	5226
ENSECAG00000010930	6.541907251	0.0190846	0.1443587	1251	1532	1198	1480	2251	1846	1696	2447
ENSECAG00000005331	4.164233946	0.0190886	0.1443587	414	425	507	466	108	203	209	370
ENSECAG00000016922	6.179245682	0.0191245	0.1445341	1220	958	818	1126	1029	2297	1447	1692
ENSECAG00000020933	6.957067055	0.0191447	0.1445908	3817	2531	3516	2448	1577	801	2204	1985
ENSECAG00000014822	7.064328795	0.019185	0.1447797	3248	3302	3623	2885	1504	1166	2129	2383
ENSECAG00000023656	4.017134875	0.0192196	0.1447797	413	233	480	549	145	97	301	275
ENSECAG00000024648	7.58665365	0.0192198	0.1447797	2710	3487	1758	2674	4131	4972	3904	4423
ENSECAG00000010666	3.098967912	0.0192323	0.1447797	217	141	203	314	91	95	141	100
ENSECAG00000000373	2.556544634	0.0192334	0.1447797	184	189	133	93	59	71	40	90
ENSECAG00000018802	6.930514288	0.0192998	0.1450924	1942	882	1557	2151	3967	1504	4985	1445
ENSECAG00000011229	1.160425652	0.0193005	0.1450924	67	29	37	102	22	19	34	21
ENSECAG00000019620	4.953761826	0.0193451	0.1453316	483	536	322	263	554	1110	539	630
ENSECAG00000012233	2.662603702	0.0193863	0.1455443	145	99	159	261	73	90	83	47
ENSECAG00000021525	9.362426587	0.0194036	0.1455781	9562	6981	7924	11114	17587	22026	12119	8340
ENSECAG00000016389	5.658245885	0.0194236	0.1456319	1209	1142	1280	1197	761	477	800	761
ENSECAG00000017082	5.484830825	0.0194454	0.1456608	589	537	543	647	523	2104	701	759
ENSECAG00000012020	8.312015998	0.0194531	0.1456608	7875	8472	8681	6233	2806	3532	4773	5482
ENSECAG00000006575	6.022537635	0.0194908	0.1458473	1551	1123	1577	2186	913	489	955	1167
ENSECAG00000006090	5.215838728	0.0195313	0.1460542	875	755	886	1067	519	413	613	508
ENSECAG00000020150	0.413355592	0.0195513	0.1461077	24	30	27	54	9	12	24	9
ENSECAG00000014662	5.605393957	0.019596	0.1462772	1038	992	1118	1586	510	563	940	688
ENSECAG00000025099	3.286864903	0.0195998	0.1462772	1	87	3	35	0	585	5	613
ENSECAG00000009619	4.711373075	0.0196189	0.1463234	343	333	439	389	759	514	625	454
ENSECAG00000016204	2.088897518	0.0196406	0.1463897	29	51	39	82	69	170	67	94
ENSECAG00000026935	4.994252655	0.0197412	0.1470425	466	379	422	403	442	1330	444	678
ENSECAG00000013725	5.654980075	0.0197625	0.1471049	1220	1116	1196	1332	553	448	899	895
ENSECAG00000011811	0.556888067	0.0198066	0.1473364	29	53	43	27	6	12	4	30
ENSECAG00000007542	0.812414808	0.0198394	0.1474148	3	28	17	13	12	33	29	107
ENSECAG00000009530	8.182798771	0.0198431	0.1474148	4889	2323	4048	3509	12138	5499	8026	2343
ENSECAG00000007894	6.8242459	0.0199669	0.1482381	1978	1671	1472	1173	3004	2886	2487	1847
ENSECAG00000022089	2.405138237	0.0200246	0.1485691	171	95	138	141	35	40	89	87
ENSECAG00000020583	5.285491638	0.0200385	0.1485753	636	557	416	139	2117	491	737	531
ENSECAG00000017933	5.944208041	0.020093	0.1488521	717	1096	683	931	1061	2071	1400	989
ENSECAG00000023588	1.450281822	0.020102	0.1488521	26	59	14	15	41	57	100	75
ENSECAG00000020325	1.349319843	0.0201715	0.1492694	23	41	24	9	85	105	39	19
ENSECAG00000018207	3.33207559	0.0202299	0.1496036	177	182	238	513	28	103	156	161
ENSECAG00000011827	5.760036414	0.0202839	0.1499059	742	671	862	681	1557	1789	850	656
ENSECAG00000023484	1.278340022	0.0203625	0.1503884	108	38	52	57	42	24	20	10
ENSECAG00000000294	6.157370017	0.0203918	0.1505075	1463	1594	1605	2379	633	632	1216	1404
ENSECAG00000014416	2.66085688	0.0204903	0.151136	48	67	76	135	101	112	259	144
ENSECAG00000024574	5.445176584	0.020526	0.1513008	607	449	453	744	1799	645	1478	304
ENSECAG00000003363	5.219256268	0.0205648	0.1514887	494	393	711	593	940	817	807	736
ENSECAG00000024868	1.784511542	0.0205898	0.1515749	81	122	84	73	63	17	51	12
ENSECAG00000021752	6.992135703	0.0206733	0.152091	2571	1649	1376	1295	4450	2057	3361	1992
ENSECAG00000000500	4.442898639	0.0207755	0.152744	510	834	530	304	138	282	222	388
ENSECAG00000021864	7.310043697	0.0208039	0.1528532	1865	2519	1646	2967	3502	4181	4284	2437
ENSECAG00000024355	6.736632473	0.0208315	0.1528592	1550	1577	1526	1092	4307	1916	1909	1645
ENSECAG00000016578	3.553745651	0.0208316	0.1528592	46	213	16	31	41	614	21	628
ENSECAG00000024774	6.851296314	0.0208699	0.1530276	2589	2425	3443	3111	859	925	1911	2379
ENSECAG00000014145	1.376288895	0.0208815	0.1530276	31	14	31	48	85	44	86	34
ENSECAG00000021998	2.188971721	0.0209078	0.1531218	58	60	60	55	100	127	117	76
ENSECAG00000009243	1.511394691	0.0209373	0.1532387	37	116	78	64	10	32	28	46
ENSECAG00000010702	1.939165601	0.0209559	0.153276	2	25	4	34	0	194	1	264
ENSECAG00000000015	5.303607041	0.0209963	0.153335	431	464	798.999	207	940	306	2008	811
ENSECAG00000019191	1.158741312	0.0209966	0.153335	17	141	31	75	2	0	53	6

ENSECAG00000019646	4.189584347	0.0210044	0.153335	263	233	263	260	601	317.999	454	290
ENSECAG00000021016	5.353815997	0.0210433	0.1533786	603	324	625	807	1374	674	973	699
ENSECAG00000010402	2.83859666	0.0210502	0.1533786	150	197	251	153	35	44	191	60
ENSECAG00000019747	7.200674766	0.0210509	0.1533786	2553	1403	1889	804	7833	1602	4428	1076
ENSECAG00000023461	6.621476186	0.0210691	0.1534126	2487	1879	2243	3071	1062	773	1859	1731
ENSECAG00000018940	5.257257648	0.0211153	0.1536491	470	691	529	458	659	1052	753	951
ENSECAG00000014530	3.220377971	0.0211392	0.1536491	68	137	175	51	127	126	363	331
ENSECAG00000015690	6.601299545	0.0211421	0.1536491	2331	1832	3063	2231	1072	896	1405	1900
ENSECAG00000019323	5.912827713	0.0211658	0.1536548	1476	1224	1396	1679	833	709	805	990
ENSECAG00000015791	4.135331524	0.02117	0.1536548	453	337	447	495	257	143	294	241
ENSECAG00000025048	6.391003865	0.0212382	0.1539829	2285	1524	2080	2382	822	631	1800	1362
ENSECAG00000024215	1.594951654	0.0212423	0.1539829	106	47	69	96	45	38	32	11
ENSECAG00000011634	5.105221942	0.0212799	0.1541569	461	489	521	475	1001	879	717	473
ENSECAG00000017655	4.557489829	0.0212945	0.1541647	603	514	551	626	257	217	416	387
ENSECAG00000019529	4.91858051	0.0213498	0.1543787	527	389	405	328	994	335	966	550
ENSECAG00000021295	7.055270247	0.0213513	0.1543787	3185	2280	3403	4237	1931	1417	2262	1616
ENSECAG00000026951	3.752722978	0.0214091	0.1546984	158	200	206	187	310	307	361	245
ENSECAG00000024909	1.50555202	0.0214279	0.1547362	35	31	43	32	75	44	58	86
ENSECAG00000015884	5.112219368	0.0214903	0.1550877	528	384	613	458	855	437	1156	747
ENSECAG00000009951	2.479368953	0.0216122	0.1556534	62	66	71	99	133	114	168	102
ENSECAG00000006668	3.234451948	0.0216227	0.1556534	115	98	133	153	280	270	180	130
ENSECAG00000004503	3.180613658	0.0216275	0.1556534	122	146	112	90	207	237	269	131
ENSECAG00000010246	6.012197704	0.0216373	0.1556534	1532	1259	1514	1908	825	732	1267	799
ENSECAG00000016264	0.693249544	0.0216652	0.1556534	42	16	26	89	17	9	20	18
ENSECAG00000023628	2.341325844	0.0216767	0.1556534	60	57	48	85	100	201	89	78
ENSECAG00000012386	4.763786708	0.0216768	0.1556534	320	398	321	499	404	702	518	825
ENSECAG00000019236	0.113395835	0.0216783	0.1556534	23	22	37	23	6	9	13	15
ENSECAG00000003474	5.582294846	0.0217077	0.1557167	906	403	293	1059	1044	857	1855	815
ENSECAG00000022638	2.433149598	0.0217196	0.1557167	41	106	32	74	70	127	147	183
ENSECAG00000023130	0.780825718	0.0217282	0.1557167	39	21	54	61	18	17	23	15
ENSECAG00000012929	6.2351342	0.0217802	0.1559909	1286	700	855	1242	3096	1654	1420	761
ENSECAG00000020000	0.503406944	0.0219304	0.1569675	22	31	31	60	20	13	14	10
ENSECAG00000002611	4.591451736	0.0220423	0.1576694	581	477	610	689	270	261	488	292
ENSECAG00000018828	2.830250114	0.0220611	0.1577042	0	54	6	33	2	404	0	471
ENSECAG00000001394	6.439481957	0.0221005	0.1578869	1483	4747	1622	1128	1062	259	912	1607
ENSECAG00000013580	5.169111936	0.0221441	0.1580988	519	522	515	494	1176	735	750	553
ENSECAG00000018058	1.664858408	0.0221723	0.1582002	18	45	22	68	70	85	84	61
ENSECAG00000002193	5.017900347	0.0222222	0.1584572	575	372	420	553	738	642	880	630
ENSECAG00000005000	4.435671181	0.0222585	0.1586162	411	393	551	882	174	136	384	415
ENSECAG00000023942	0.887166211	0.0222791	0.1586634	13	33	19	15	25	64	45	39
ENSECAG00000010415	2.799472854	0.0223014	0.1587226	68	111	89	97	108	186	172	177
ENSECAG00000015723	3.571918428	0.0223341	0.1588563	360	280	230	360	49	192	138	177
ENSECAG00000007767	7.162211686	0.0223665	0.158987	1997	1842	2343	1941	3644	4012	2313	2744
ENSECAG00000023358	7.821780839	0.0223923	0.1590706	2640	3566	2578	1941	3512	11422	2343	3795
ENSECAG00000016815	2.427038636	0.0224101	0.1590979	25	70	33	23	27	374	3	165
ENSECAG00000009093	6.840401151	0.022432	0.1591536	2983	2277	2946	3204	1883	802	2333	1103
ENSECAG00000001129	6.323430596	0.0225291	0.1597432	1143	1224	1195	596	1425	3239	1333	1275
ENSECAG00000017004	2.521142826	0.0225664	0.1597734	198	110	118	171	48	52	42	119
ENSECAG00000002113	3.117072155	0.022573	0.1597734	206	162	228	287	100	58	175	106
ENSECAG00000023076	8.381502406	0.0225756	0.1597734	4994	2925	4285	3252	3246	18314	3099	6863
ENSECAG00000003494	2.756146028	0.0226816	0.1603307	79	105	80	53	111	257	78	187
ENSECAG00000009287	2.157453926	0.0226826	0.1603307	80	79	136	168	53	25	66	66
ENSECAG00000013908	6.513355681	0.0227219	0.1605086	1476	1241	1411	1204	2286	2305	2115	1355
ENSECAG00000015946	4.310926493	0.0228578	0.1613505	335	294	258	235	478	450	495	359
ENSECAG00000011852	4.471549012	0.0228695	0.1613505	455	537	582	573	248	327	312	294
ENSECAG00000015034	8.990312942	0.0228993	0.1614605	11230	14149	13352	11032	5053	5020	9142	7902
ENSECAG00000011457	7.101700921	0.0230047	0.1621034	3510	2657	3333	3925	2242	1395	2286	1547
ENSECAG00000004339	2.896912701	0.0230404	0.1622543	180	132	184	266	120	63	117	66
ENSECAG00000020478	7.1564201	0.0231336	0.1626264	2017	2028	1132	2539	3104	4977	1800	3007
ENSECAG00000021685	1.823729936	0.0231449	0.1626264	91	56	89	130	56	26	54	27
ENSECAG00000009105	3.652959184	0.0231576	0.1626264	264	327	274	375	115	152	200	187
ENSECAG00000016773	5.442963271	0.0231586	0.1626264	917	338	597	644	1114	750	1419	720
ENSECAG00000014030	3.023725488	0.0231649	0.1626264	57	144	89	119	102	152	286	250
ENSECAG00000023791	1.348300274	0.0231941	0.1627311	24	16	1	36	1	77	85	122
ENSECAG00000016028	4.12306137	0.0232143	0.1627721	229	249	269	253	331	325	419	495
ENSECAG00000013847	6.32098598	0.0232536	0.1629473	1886	1592	1715	2532	938	821	1575	1166
ENSECAG00000010019	1.381040263	0.0232788	0.1630235	71	59	55	75	18	25	40	38
ENSECAG00000009112	4.163951829	0.0233241	0.1631508	264	215	238	326	472	320	452	366
ENSECAG00000022025	2.577232564	0.0233262	0.1631508	176	101	132	202	81	41	105	65
ENSECAG00000010675	1.931574429	0.0233401	0.1631508	28	77	41	17	99	104	47	118
ENSECAG00000015379	4.134157589	0.0234489	0.1636739	47	378	67	127	85	1090	208	414
ENSECAG00000017081	0.228556822	0.0234635	0.1636739	37	22	23	34.9998	11	8	5	21
ENSECAG00000012629	1.663421975	0.0234678	0.1636739	114	50	57	118	39	20	66	11
ENSECAG00000014151	6.234222921	0.0234726	0.1636739	1638	1684	1915	1945	857	1010	1054	1250
ENSECAG00000020605	3.860300929	0.0234913	0.1637043	397	324	551	300	56	57	110	401
ENSECAG00000020473	2.492975393	0.0236613	0.1647283	64	75	76	72	196	123	128	75
ENSECAG00000018545	3.609638332	0.0236673	0.1647283	331	211	307	368	177	97	198	164
ENSECAG00000023264	2.759469433	0.0236831	0.1647373	207	117	230	160	54	26	174	63

ENSECAG00000018422	0.593525542	0.023777	0.1651935	1	2	22	26	17	34	100	13
ENSECAG00000017642	4.620229942	0.0237777	0.1651935	660	380	634	806	220	179	510	398
ENSECAG00000009399	4.089330569	0.0237968	0.1652246	265	231	236	256	372	341	436	372
ENSECAG00000002541	7.448116552	0.0238301	0.1653551	4605	3633	4759	3899	1980	2107	2811	2603
ENSECAG00000023509	2.879147843	0.0238492	0.1653863	106	44	82	133	242	81	320	92
ENSECAG00000024065	8.761728605	0.0239225	0.1656852	7513	2063	8254	1656	9656	19472	8670	4569
ENSECAG00000019226	2.452856189	0.0239316	0.1656852	147	92	163	156	84	48	90	41
ENSECAG00000015381	4.199224711	0.023936	0.1656852	397	642	508	363	118	99	129	489
ENSECAG00000001399	7.66455724	0.0240025	0.1660439	5265	3364	4208	7700	1605	2342	3852	2970
ENSECAG00000020052	5.371630661	0.0240317	0.1661449	242	1040	397	238	787	817	695	1761
ENSECAG00000017390	4.560943246	0.0240532	0.1661921	361	288	297	398	774	488	518	340
ENSECAG00000009091	5.773292874	0.0241238	0.1665786	988	732	746	795	1250	1213	1000	1306
ENSECAG00000006407	3.658626926	0.024182	0.166879	5	191	10	28	12	680	6	796
ENSECAG00000023200	3.358401516	0.0242307	0.1671138	137	82	132	214	282	184	331	162
ENSECAG00000012136	7.69309223	0.0242581	0.167194	4722	4356	5256	5849	2336	2069	3771	3197
ENSECAG00000022842	5.190199275	0.0242795	0.167194	149	749	167	224	178	2357	203	968
ENSECAG00000002856	6.159262303	0.0242939	0.167194	1821	1192	1897	2073	1108	529	1353	968
ENSECAG00000018949	6.031568579	0.0243012	0.167194	1438	1320	1739	1814	764	917	731	1145
ENSECAG00000015633	3.257151751	0.0243245	0.1672534	242	357	182	185	61	120	84	177
ENSECAG00000002235	4.471513298	0.0243444	0.1672884	592	388	659	579	363	133	433	236
ENSECAG00000016684	5.172078345	0.0243901	0.167501	475	566	421	534	548	1258	560	846
ENSECAG00000012993	4.063940782	0.0244668	0.1678231	100	437	90	110	381	611	306	324
ENSECAG00000025118	4.606014622	0.0244739	0.1678231	677	419	641	666	340	174	377	412
ENSECAG00000022791	0.836372959	0.0244813	0.1678231	14	22	35	139	8	3	13	35
ENSECAG00000020928	1.833625502	0.02456	0.1682611	77	73	93	119	44	50	45	25
ENSECAG00000022914	2.763077266	0.0245909	0.1682804	161	122	204	191	89	63	105	85
ENSECAG00000011055	6.316479253	0.0246051	0.1682804	1612	863	1222	961	1958	1130	2369	1778
ENSECAG00000021200	5.355862836	0.0246073	0.1682804	610	688	608	473	984	779	835	1028
ENSECAG00000008140	5.224189056	0.0246265	0.1683107	596	403	493	387	1805	438	1076	305
ENSECAG00000021071	5.490669084	0.0246787	0.1685658	717	557	663	800	855	1042	1105	920
ENSECAG00000005770	6.037729359	0.0247065	0.1685953	1116	824	986	890	1205	2059	1334	1165
ENSECAG00000021722	3.389410932	0.0247127	0.1685953	41	214	55	95	50	525	137	346
ENSECAG00000010434	5.122192011	0.0247595	0.1688132	533	338	492	727	882	669	852	690
ENSECAG00000006470	1.152400787	0.0248019	0.1689178	9	33	26	33	27	50	52	83
ENSECAG00000021155	2.078692682	0.024812	0.1689178	82	81	124	151	33	69	61	28
ENSECAG00000007526	5.669192019	0.0248194	0.1689178	627	837	724	843	1007	1275	1278	902
ENSECAG00000005070	1.831614954	0.024883	0.1692492	43	45	51	21	151	58	101	36
ENSECAG00000015006	2.655193197	0.0249364	0.169511	5	84	1	21	7	475	2	242
ENSECAG00000021949	1.425319653	0.0250558	0.1702208	81	31	81	83	38	16	38	29
ENSECAG00000007486	3.719752652	0.0251663	0.170869	299	247	341	447	144	79	237	222
ENSECAG00000016508	8.3254783	0.0251817	0.1708713	713	5843	1082	1087	477	22214	698	11407
ENSECAG00000013677	4.76885278	0.0252016	0.1709043	90	556	202	264	165	1299	275	980
ENSECAG00000009702	8.160530988	0.025305	0.1714615	7308.03	6104	7151.04	7374	3508	2520	5442	4196.02
ENSECAG00000007936	1.178047094	0.0253139	0.1714615	22	26	36	21	51	84	38	33
ENSECAG00000012240	0.938795227	0.0253325	0.1714724	49	39	50	52	13	22	33	18
ENSECAG00000024263	1.731758776	0.0253457	0.1714724	22	32	30	81	49	70	88	110
ENSECAG00000018884	5.792222338	0.0254337	0.1719651	1427	1198	1193	1467	724	500	991	916
ENSECAG00000021132	1.890216201	0.0254496	0.1719705	93	111	75	99	32	32	24	77
ENSECAG00000027114	0.529619216	0.0254722	0.1720206	69	29	22	25	10	12	26	10
ENSECAG00000021529	5.998636148	0.0254888	0.1720308	1775	1097	1524	1842	1073	792	1062	543
ENSECAG00000017629	5.996160163	0.0255116	0.172082	1012	686	957	1231	1680	1245	1474	1194
ENSECAG00000012730	4.942959104	0.0256931	0.1732033	612	331	376	447	780	638	936	437
ENSECAG00000017556	5.109370768	0.0257446	0.173352	825	688	971	849	411	309	550	630
ENSECAG00000013510	10.40799846	0.0257536	0.173352	20317	14345	15974	27359	28965	21709	27813	46316
ENSECAG00000019928	8.739987923	0.0257832	0.173352	882	6041	54	6511	1833	17061	18875	11544
ENSECAG00000024378	5.218124546	0.0257903	0.173352	779	403	570	391	823	869	821	802
ENSECAG00000023323	1.424050484	0.0257914	0.173352	34	55	49	150	16	27	46	26
ENSECAG00000024590	4.783818455	0.0258156	0.1734121	372	335	441	432	834	606	659	368
ENSECAG00000016948	10.47580283	0.0258937	0.1738335	19725	23708	20675	11375	29241	46226	26123	28220
ENSECAG00000015517	2.333980762	0.0259298	0.1739729	79	154	109	166	47	49	66	80
ENSECAG00000022451	0.746230915	0.0259818	0.174219	15	26	17	17	62	37	24	30
ENSECAG00000016316	6.448278462	0.0260233	0.1743944	1239	1171	1309	1639	1821	1921	1982	1857
ENSECAG00000006095	8.683536162	0.0261424	0.1750148	6630	14348	13349	8881	912	3999	6783	7265
ENSECAG00000003398	4.101848688	0.0261705	0.1750148	153	242	151	42	98	1269	64	297
ENSECAG00000017146	7.099743257	0.0261791	0.1750148	2817	3436	3249	3744	1987	1702	2354	1448
ENSECAG00000015297	3.763153265	0.0261888	0.1750148	352	242	268	523	149	165	275	103
ENSECAG00000000466	6.247792876	0.0262249	0.1750148	866	1099	997	992	1276	3565	1080	935
ENSECAG00000002307	0.553130276	0.0262261	0.1750148	15	9	17	29	31	26	39	38
ENSECAG00000009137	3.477781587	0.0262335	0.1750148	91	128	141	51	40	733	64	276
ENSECAG00000011835	6.285164065	0.0262391	0.1750148	1910	1440	2001	2072	1097	944	1336	1027
ENSECAG00000015773	6.542056201	0.0262893	0.1750648	1527	1079	1453	1575	2360	1299	2562	2088
ENSECAG00000015459	6.240666471	0.0262931	0.1750648	1235	994	1208	1054	2008	1602	1284	1703
ENSECAG00000018028	6.794305089	0.0263047	0.1750648	568	1716	632	743	199	7754	314	3226
ENSECAG00000023892	5.793242006	0.026309	0.1750648	617	951	950	631	984	715	1699	1723
ENSECAG00000018528	2.750850488	0.0263237	0.1750648	124	62	77	100	192	127	175	126
ENSECAG00000005870	5.062557745	0.0263679	0.1752561	457	468	457	589	723	677	1100	506
ENSECAG00000009686	5.408378933	0.0263931	0.1753212	1113	811	858	1367	550	529	863	388
ENSECAG00000024663	6.509741153	0.0264583	0.1756513	2070	1584	2242	2940	1208	902	1831	1192

ENSECAG00000018228	4.812243252	0.0264938	0.1757848	46	390	140	221	14	1166	74	1906
ENSECAG00000008622	5.734825107	0.0265251	0.1758894	484.001	1108	444.001	850	847.001	1804	998	1167.02
ENSECAG00000013435	5.48279964	0.0265682	0.1759477	1559	1081	972	779	440	737	809	313
ENSECAG00000001192	5.743108743	0.0265725	0.1759477	869	640	801	833	1135	591	1395	1758
ENSECAG00000008784	6.492770849	0.0265942	0.1759477	1324	994	1245	1232	4254	990	2319	955
ENSECAG00000025018	6.389980228	0.026599	0.1759477	1038	1509	1162	1287	1775	1410	2031	2216
ENSECAG00000000519	3.360439348	0.0266113	0.1759477	84	128	106	239	148	276	309	226
ENSECAG00000019479	6.130328778	0.0266394	0.1760308	2011	1403	1627	1634	723	933	1327	941
ENSECAG00000007380	1.104332983	0.0266868	0.1762323	34	12	35	21	63	39	69	31
ENSECAG00000014323	3.416563664	0.0267009	0.1762323	258	235	205	360	124	138	148	141
ENSECAG00000000564	2.890967254	0.0267432	0.1764089	124	52	104	109	257	112	218	114
ENSECAG00000010939	1.65315123	0.0268329	0.1768264	66	131	111	17	16	22	47	42
ENSECAG00000023086	5.421674193	0.0268376	0.1768264	1151	852	1030	1058	665	462	776	493
ENSECAG00000018133	8.112035999	0.0269605	0.1775327	2182.01	3529.01	2738	3624	1037	15886	2722.01	7235.01
ENSECAG00000010727	6.97266804	0.0270065	0.177733	2131	1301	1872	1719	1794	4554	2312	2566
ENSECAG00000011351	1.777399091	0.0270651	0.1779325	22	32	24	76	36	170	49	70
ENSECAG00000020448	1.790322802	0.0270682	0.1779325	90	54	80	129	40	34	61	29
ENSECAG00000023977	2.376591983	0.0271041	0.1780308	123	103	140	161	62	27	89	77
ENSECAG00000020182	7.259987423	0.0271145	0.1780308	4048	3456	4854	2592	1914	1167	2852	2307
ENSECAG00000008358	5.960160823	0.0271696	0.17829	1380	1136	1426	2049	758	871	976	873
ENSECAG00000000494	3.835897724	0.0271959	0.178359	158	488	518	333	68	126	110	333
ENSECAG00000022492	6.746101228	0.027459	0.1798912	2675	1974	2773	3255	1659	699	2403	1068
ENSECAG00000008904	2.029203002	0.0274668	0.1798912	81	100	103	138	20	35	44	87
ENSECAG00000000138	2.73521205	0.0274785	0.1798912	57	141	52	35	340	109	93	112
ENSECAG00000005949	5.649558339	0.0274928	0.1798912	1140	739	1143	1983	594	412	1008	750
ENSECAG00000000733	4.126022497	0.0275827	0.180335	422	317	479	484	218	203	323	200
ENSECAG00000015231	2.474595632	0.0276043	0.180335	56	69	93	27	100	263	111	56
ENSECAG00000010573	1.95174144	0.0276083	0.180335	41	29	50	58	181	39	132	32
ENSECAG00000013328	2.00740224	0.0276331	0.1803933	46	40	82	34	75	71	110	123
ENSECAG00000018929	5.087001058	0.0276643	0.1804934	513	459	472	501	506	1126	662	705
ENSECAG00000015551	5.703167698	0.027686	0.1805309	802	798	794	717	1213	1239	1234	854
ENSECAG00000007597	0.703435489	0.0278053	0.1812051	38	31	60	31	12	14	24	21
ENSECAG00000019901	3.730317282	0.0278302	0.1812153	143	211	167	235	274	281	328	312
ENSECAG00000013942	5.356096581	0.0278388	0.1812153	1226	652	1213	904	680	359	751	432
ENSECAG00000019465	3.293216837	0.0278907	0.1813768	117	53	125	155	535	236	66	109
ENSECAG00000002370	0.884783357	0.0279097	0.1813768	11	23	3	32	9	42	30	107
ENSECAG00000002920	4.60819323	0.0279202	0.1813768	315	324	416	329	535	268	869	602
ENSECAG00000011590	3.430717719	0.0279275	0.1813768	126	136	111	162	105	490	154	249
ENSECAG00000011662	4.293119348	0.0279436	0.1813779	582	351	628	346	262	200	309	269
ENSECAG00000021055	5.73110338	0.0280415	0.1819095	1004	1028	1127	2056	542	579	861	952
ENSECAG00000015355	7.656356252	0.0280779	0.1820418	3410	2605	2756	2748	3234	4773	3550	6569
ENSECAG00000016212	5.714299424	0.0280989	0.1820738	1514	928	1318	1296	690	412	1014	830
ENSECAG00000012389	6.762333467	0.0281634	0.1823875	1473	1167	1507	2022	1965	4056	1910	1683
ENSECAG00000005515	4.281754427	0.0282532	0.182865	927	170	620	366	314	205	276	66
ENSECAG00000024046	8.11332494	0.0283137	0.1829571	2605	3600	2659	3128	1605	17967	2182	4742
ENSECAG00000013170	7.322053511	0.0283229	0.1829571	2114	1762	2573	3022	3659	2588	5463	2897
ENSECAG00000017245	4.615794135	0.0283332	0.1829571	318	349	257	428	317	920	495	470
ENSECAG00000012235	3.942420246	0.0283363	0.1829571	108	286	79	165	88	784	128	511
ENSECAG00000018112	1.319205023	0.028348	0.1829571	25	28	47	18	66	70	56	38
ENSECAG00000007917	7.770476056	0.0283929	0.1831433	3610	2811	2709	3354	7248	3472	5974	3226
ENSECAG00000021900	0.606038323	0.0284133	0.1831709	20	13	18	13	29	64	38	10
ENSECAG00000020475	6.665123401	0.0285108	0.1835871	1998	1312	1035	1619	2871	2159	2154	1793
ENSECAG00000018279	3.305201286	0.028524	0.1835871	84	220	100	104	207	219	258	240
ENSECAG00000013378	2.823741602	0.0285264	0.1835871	141	121	187	269	74	64	108	111
ENSECAG00000011545	4.534187919	0.028602	0.1838694	308	282	274	381	192	756	416	768
ENSECAG00000021177	3.90813133	0.0286042	0.1838694	394	257	354	501	196	153	315	123
ENSECAG00000011552	4.883449715	0.0286188	0.1838694	750	494	827	779	406	367	444	398
ENSECAG00000009562	6.252901386	0.0287118	0.1843625	895	1889	470	342	1597	2463	1227	1962
ENSECAG00000020463	6.054851588	0.0287599	0.1844064	414	1501	432	796	785	2785	733	1982
ENSECAG00000013516	4.065893772	0.0287669	0.1844064	439	377	399	422	141	225	204	306
ENSECAG00000010090	8.182078893	0.0287854	0.1844064	6162	6247	6968	9045	2807	3744	5080	4206
ENSECAG00000017309	1.67898796	0.0287898	0.1844064	84	65	78	88	43	32	41	39
ENSECAG00000016271	1.020341174	0.0288146	0.1844064	62	36	40	64	25	24	28	14
ENSECAG00000024784	4.50983853	0.028824	0.1844064	456	444	559	792	200	239	370	419
ENSECAG00000024419	5.056296811	0.0288323	0.1844064	451	450	536	577	727	609	881	714
ENSECAG00000014312	5.226261454	0.0288612	0.1844875	616	372	635	616	948	694	1071	620
ENSECAG00000020301	2.873963654	0.0289065	0.1846732	91	60	97	134	189	117	326	80
ENSECAG00000016744	5.109080991	0.0289291	0.1846761	705	578	947	1142	470	392	655	386
ENSECAG00000014372	5.733754921	0.0289395	0.1846761	1058	962	1170	2111	749	640	1079	413
ENSECAG00000015037	0.964654669	0.0290356	0.1851858	13	28	9	25	12	119	29	26
ENSECAG00000000622	1.990812227	0.0290973	0.1854747	25	180	73	141	23	38	67	40
ENSECAG00000013742	6.539539525	0.0291185	0.1855061	1180	1186	1243	1559	1690	3768	1768	1051
ENSECAG00000019722	4.459389836	0.0292336	0.1861346	342	247	289	436	445	371	613	542
ENSECAG00000000607	1.532640011	0.0293165	0.1865578	52	15	28	21	159	60	59	10
ENSECAG00000019809	4.703586575	0.0293425	0.1866188	528	377	340	167	561	468	631	747
ENSECAG00000015134	0.653355059	0.0293875	0.1868008	15	19	22	14	61	32	19	31
ENSECAG00000003836	1.483955812	0.0294609	0.1871624	31	39	37	34	54	76	48	74
ENSECAG00000013988	2.785581385	0.0295545	0.1876523	3	131	5	17	23	366	13	380

ENSECAG00000022962	3.827667304	0.0295844	0.1877371	194	142	141	332	403	267	396	234
ENSECAG00000010953	1.369827572	0.0296069	0.1877748	87	43	48	84	36	13	39	31
ENSECAG00000021199	4.319855942	0.029683	0.1881522	335	250	239	346	470	488	427	374
ENSECAG00000013763	7.337078477	0.0297681	0.1885867	2159	2267	2152	2946	3698	4335	3465	2741
ENSECAG00000006942	6.35398636	0.0298112	0.1887544	1763	1595	1893	2537	1094	966	1384	1215
ENSECAG00000022786	5.910756419	0.0298911	0.1891391	708	874	807	878	733	2467	786	1386
ENSECAG00000011988	4.316894069	0.0299053	0.1891391	231	395	625	947	59	138	197	496
ENSECAG00000021528	6.117822754	0.0299259	0.1891646	1384	1815	1651	1712	992	733	1353	854
ENSECAG00000017398	5.844746953	0.0299579	0.1892615	1139	1537	1731	1789	130	244	647	1594
ENSECAG00000021693	6.819762014	0.0299747	0.1892624	2705	2469	2809	2991	1379	813	2057	2003
ENSECAG00000006921	4.453088226	0.0300117	0.1893903	624	297	627	602	311	231	309	323
ENSECAG00000002691	6.226323556	0.0300979	0.189829	1167	1019	1148	1275	1663	1280	1925	1675
ENSECAG00000020115	8.354367628	0.0303006	0.1910011	7774	7531	6698	9835	4033	4397	5564	3833
ENSECAG00000023483	4.604185026	0.0303244	0.1910451	350	199	328	474	749	685	544	232
ENSECAG00000000816	5.607506081	0.0303756	0.191262	1142	1495	1139	1243	233	224	590	1335
ENSECAG00000000295	5.20295667	0.030431	0.1913769	832	917	810	999	396	272	712	665
ENSECAG00000011478	2.844172146	0.030441	0.1913769	149	140	226	203	63	64	107	129
ENSECAG00000026846	6.575330982	0.0304444	0.1913769	1203	1502	1617	993	1675	3310	1426	2007
ENSECAG00000003698	3.088709404	0.0304956	0.1915928	181	142	221	316	78	99	114	139
ENSECAG00000010595	0.909364118	0.0305516	0.1917169	25	26	24	13	25	43	59	47
ENSECAG00000008638	7.09912389	0.0305588	0.1917169	3099	3137	3183	3983	1969	1914	2450	1036
ENSECAG00000009005	6.195933006	0.0305796	0.1917169	1413	1041	961	818	2204	1545	1668	1091
ENSECAG00000022301	4.844811453	0.0305829	0.1917169	433	372	482	365	899	557	477	612
ENSECAG00000001012	2.312748886	0.0306415	0.1918934	50	54	89	53	185	128	71	74
ENSECAG00000016436	4.916821173	0.0306448	0.1918934	868	567	716	765	274	363	547	480
ENSECAG00000006917	0.571690417	0.0306862	0.1920464	6	10	20	29	19	63	23	31
ENSECAG00000016512	3.490764812	0.030785	0.192559	141	97	200	172	380	220	312	129
ENSECAG00000007993	6.380313829	0.0308478	0.1928452	1225	1187	1417	1126	1420	2349	1913	1579
ENSECAG00000023476	6.066664292	0.0308889	0.1929963	765	1151	981	791	917	2404	987	1654
ENSECAG00000021011	5.178159942	0.0309216	0.1930739	281	847	389	447	801	676	744	1103
ENSECAG00000024465	6.151629923	0.0309438	0.1930739	1846	1252	1752	2294	1246	318	1465	778
ENSECAG00000020313	0.745430652	0.0309523	0.1930739	42	15	60	56	16	6	20	28
ENSECAG00000014431	6.452307203	0.0310179	0.1933277	1852	1803	2275	2382	1160	871	1601	1372
ENSECAG00000016882	1.792172914	0.0310271	0.1933277	27	50	42	61	73	57	96	95
ENSECAG00000006779	5.245839056	0.0310768	0.1935231	685	496	576	473	1139	596	868	765
ENSECAG00000024173	6.103970948	0.0310925	0.1935231	1073	748	927	1165	2538	1521	1450	670
ENSECAG00000009725	4.519437712	0.0311256	0.193623	293	267	318	493	523	534	470	486
ENSECAG00000000685	11.686127	0.0313691	0.1950312	48750	43130	51299	51365	79302	62832	81139	71031
ENSECAG00000006414	4.057216445	0.0314033	0.1951371	237	212	274	245	353	422	396	299
ENSECAG00000017200	4.338541287	0.0315045	0.1955887	391	377	613	603	204	200	325	364
ENSECAG00000003982	3.837226239	0.0315105	0.1955887	294	398	549	221	79	69	309	240
ENSECAG00000021403	7.048696618	0.0315889	0.195886	1746	2252	1843	1908	2862	3417	3095	2290
ENSECAG00000018806	5.553015583	0.0315929	0.195886	1111	935	1270	1188	453	420	965	814
ENSECAG00000017304	2.309624378	0.0316605	0.1961982	55	48	84	72	166	99	127	66
ENSECAG00000017754	4.218442784	0.0317101	0.1963987	367	384	420	654	191	203	305	311
ENSECAG00000012842	0.842963906	0.031744	0.1964447	26	25	17	17	38	47	30	46
ENSECAG00000009682	3.719648183	0.0317521	0.1964447	155	170	194	239	213	336	324	305
ENSECAG00000014427	0.663376419	0.03177	0.1964485	16	22	13	18	47	38	55	9
ENSECAG00000011496	7.694628408	0.0318438	0.1966091	2317	4888	1286	1406	2549	7137	3430	6610
ENSECAG00000017469	1.755462465	0.0318765	0.1966091	96	65	75	105	61	37	32	25
ENSECAG00000017628	2.452454251	0.0318775	0.1966091	7	83	40	75	41	330	40	135
ENSECAG00000023262	6.102696882	0.0318929	0.1966091	1714	1320	1686	1761	952.001	946.001	1001	987.024
ENSECAG00000019391	4.068142642	0.0318985	0.1966091	233	291	311	1004	78	134	397	196
ENSECAG00000014211	5.718954656	0.0318999	0.1966091	1182	862	1385	1672	813	568	1032	538
ENSECAG00000015874	0.971988447	0.0319582	0.1966808	25	16	35	16	72	29	49	32
ENSECAG00000012899	3.75749356	0.0319784	0.1966808	412	275	251	460	85	66	301	231
ENSECAG00000021234	5.291470488	0.0319913	0.1966808	793	1026	946	1068	387	286	502	924
ENSECAG00000025029	3.606957379	0.0319961	0.1966808	160	141	188	203	285	345	238	211
ENSECAG00000016076	5.542293845	0.0319981	0.1966808	942	447	752	562	1261	765	1543	674
ENSECAG00000017817	2.021192967	0.032031	0.196777	30	100	20	33	85	102	101	103
ENSECAG00000012263	8.003936146	0.0320634	0.196869	3688	4513	3137	3198	5718	7219	4558	5255
ENSECAG00000011902	5.482570354	0.0322422	0.19786	772	579	589	656	897	1481	743	766
ENSECAG00000019165	2.27908595	0.0322788	0.197978	125	77	109	193	45	31	114	46
ENSECAG00000017190	2.951204584	0.0323242	0.1980758	128	86	93	123	144	154	170	234
ENSECAG00000002083	2.895492983	0.0323296	0.1980758	56	130	64	119	109	288	110	181
ENSECAG00000014118	3.055501059	0.0323596	0.1981523	131	187	193	365	33	77	133	153
ENSECAG00000015384	3.169425015	0.0324738	0.1987448	174	262	288	166	64	86	176	128
ENSECAG00000019662	5.42072064	0.0325433	0.1990625	748	515	750	537	929	1077	849	867
ENSECAG00000012664	4.331126268	0.0326102	0.1990636	454	428	457	594	255	249	320	284
ENSECAG00000015318	3.224634717	0.0326106	0.1990636	170	88	123	93	392	106	278	121
ENSECAG00000024764	3.168756976	0.0326208	0.1990636	127	156	91	121	206	183	220	206
ENSECAG00000016480	4.927738022	0.0326277	0.1990636	514	401	478	414	479	627	780	812
ENSECAG00000002668	6.491761364	0.0326311	0.1990636	1585	1203	1302	410	697	1511	3399	3321
ENSECAG00000015695	0.663322463	0.0326496	0.1990694	18	95	19	32	12	26	4	10
ENSECAG00000016143	2.661807241	0.0327047	0.1992987	71	84	78	114	115	126	131	204
ENSECAG00000018122	1.849987141	0.0327348	0.1993751	68	66	113	115	33	34	43	63
ENSECAG00000011638	7.139618675	0.0327637	0.199444	1727	2002	2326	2053	2678	4591	2450	2664
ENSECAG00000026830	0.820789986	0.032859	0.199894	51	28	49	59	1	10	38	22

ENSECAG00000019751	5.2700334	0.0328728	0.199894	1112	771	1048	753	683	381	578	475
ENSECAG00000021804	7.438325503	0.0329302	0.2000643	3399	2427	1677	1267	5959	4874	4153	1316
ENSECAG00000001554	1.666204523	0.032936	0.2000643	65.0001	63.0001	75	123	24.0007	15	51.0001	59
ENSECAG00000016826	5.404653951	0.0329831	0.2002431	959	1154	928	1020	458	314	764	816
ENSECAG00000015390	6.519428978	0.0331234	0.2009874	2291	1825	2203	2485	1640	709	1556	1242
ENSECAG00000017910	7.474018215	0.0331522	0.201055	4847	3361	4515	4396	2494	2451	2297	2452
ENSECAG00000006244	5.245098604	0.033315	0.2019347	658	450	584	497	1250	761	899	472
ENSECAG00000001065	2.071417424	0.0333635	0.2021204	51	45	77	45	89	124	84	83
ENSECAG00000007528	1.530268389	0.0333899	0.2021724	25	17	33	64	103	50	103	23
ENSECAG00000003436	6.513554843	0.0334892	0.2026658	1603	963	1266	1061	2722	932	4390	888
ENSECAG00000009536	7.051194495	0.0335571	0.2029684	3328	2944	2908	3629	1230	1440	2134	2533
ENSECAG00000000035	5.800727058	0.033603	0.2030795	1250	1082	1264	1717	675	577	911	1014
ENSECAG00000008677	1.146539156	0.0336112	0.2030795	44	12	29	18	28	34	73	78
ENSECAG00000014446	10.168745	0.0336364	0.2030929	30112	20365	31618	31912	19906	8750	20580	12702
ENSECAG00000009774	4.952807477	0.0336589	0.2030929	690	566	681	1058	449	365	479	425
ENSECAG00000004462	3.871895999	0.033667	0.2030929	239	60	247	94	942	182	243	102
ENSECAG00000007313	8.484173697	0.0336999	0.2031232	5814	4629	5653	4130	9553	10080	7061	5200
ENSECAG00000012045	3.414436471	0.0337498	0.2031232	8	184	15	38	5	648	16	535
ENSECAG00000006492	8.38732787	0.033751	0.2031232	6322	5449	3410	1400	8305	3259	12241	9464
ENSECAG00000008852	6.504967439	0.0337557	0.2031232	795	1434	1332	1293	1286	739	2429	4325
ENSECAG00000009381	5.324755417	0.0337615	0.2031232	640	478	683	598	1128	844	877	651
ENSECAG00000015500	4.605466811	0.0338169	0.203349	16	77	507	60	2	555	1114	1293
ENSECAG00000012390	4.797992976	0.0338818	0.203562	370	425	420	419	582	758	588	501
ENSECAG00000018699	2.433934282	0.0338882	0.203562	117	136	116.982	170	76	61	93	36
ENSECAG00000014207	3.360599456	0.0339406	0.2036747	118	214	102	75	168	201	184	421
ENSECAG00000016910	0.214471467	0.0339428	0.2036747	25	26	23	38	8	7	23	12
ENSECAG00000018675	1.289314563	0.0340016	0.2039198	10	28	24	36	12	83	18	128
ENSECAG00000008723	6.504569145	0.0340678	0.2041432	2008	1646	2271	2713	1241	1122	1305	1477
ENSECAG00000005973	0.307650962	0.0340748	0.2041432	21	16	27	70	13	0	24	8
ENSECAG00000004870	4.149450362	0.0341361	0.2044026	370	333	452	576	229	162	308	273
ENSECAG00000019714	5.22946974	0.0343242	0.2054207	841	632	961	1213	354	399	645	692
ENSECAG00000012794	3.812534483	0.034364	0.2055507	374	255	319	420	196	145	225	193
ENSECAG00000014636	1.501123028	0.0343837	0.2055602	21	46	46	24	55	42	110	63
ENSECAG00000012319	2.490337913	0.0344084	0.2055998	69	48	70	101	222	61	184	72
ENSECAG00000024410	6.1835015	0.0344503	0.2056757	1228	1013	1131	1092	1616	1566	1678	1408
ENSECAG00000005580	0.491312772	0.0344573	0.2056757	21	12	18	15	22	21	37	48
ENSECAG00000016971	6.236724085	0.0345027	0.2058382	1174	983	1300	1023	2126	1288	2256	1055
ENSECAG00000005023	5.047707016	0.0345497	0.2060104	441	58	475	313	1501	60	2006	195
ENSECAG00000018307	7.075008696	0.0345775	0.2060684	1784	1276	1726	2974	3896	1952	5162	1623
ENSECAG00000004264	0.943778667	0.0346088	0.2061462	19	37	21	10	63	32	40	43
ENSECAG00000013712	5.155329903	0.0346695	0.2064	1	298	0	33	2	2485	1	1990
ENSECAG00000018406	6.561656514	0.0347269	0.2066155	2151	857	1287	545	3856	1368	2859	990
ENSECAG00000005865	3.387755372	0.0347421	0.2066155	189	183	264	419	105	100	182	159
ENSECAG00000014691	8.901348344	0.0347988	0.2068444	6068	5692	6825	5965	4881	23273	6552	8938
ENSECAG00000023118	0.778362048	0.0348742	0.2070851	10	29	26	15	39	32	54	33
ENSECAG00000019711	6.916066459	0.0348758	0.2070851	1139	1872	1085	1503	732	6098	867	3753
ENSECAG00000005199	6.565163278	0.034902	0.2071329	1031	1358	1219	823	591	3566	618	4316
ENSECAG00000019713	3.711009515	0.0349361	0.2072265	251	222	377	443	159	148	219	172
ENSECAG00000008892	2.983388719	0.0350289	0.2076645	177	134	210	280	66	60	129	148
ENSECAG00000009231	5.417243828	0.0350691	0.2076645	966	939	881	1258	568	452	751	671
ENSECAG00000013492	6.157142878	0.0350827	0.2076645	903	1137	1052.01	1173	1341	2109	1320.01	1384
ENSECAG00000024699	7.759984376	0.0351004	0.2076645	3236	2773	3216	3533	6787	3857	5424	3295
ENSECAG00000014244	7.150813507	0.0351013	0.2076645	2005	1076	1851	2685	6292	2092	4064	1078
ENSECAG00000023798	5.310427065	0.0353452	0.2089987	1013	800	1087	891	330	548	661	656
ENSECAG00000012108	5.782925451	0.0353739	0.2090596	1389	1054	1364	1386	815	543	1046	761
ENSECAG00000019459	5.398618711	0.0354947	0.2096643	985	749	1008	1312	554	379	850	618
ENSECAG00000006884	3.931629226	0.0355135	0.2096665	353	294	433	403	211	148	194	264
ENSECAG00000018832	5.135842054	0.0355899	0.2099066	346	462	760	496	972	585	808	766
ENSECAG00000023459	8.045051302	0.0355912	0.2099066	3388	4463	2929	3695	4797	10008	3916	4782
ENSECAG00000008126	7.249756017	0.0356889	0.2103428	4130	2802	4171	3756	2658	1319	2907	1421
ENSECAG00000000300	2.507519823	0.0357273	0.2103428	88	71	58	74	55	157	116	201
ENSECAG00000007974	3.137584206	0.0357285	0.2103428	64	205	67	111	200	170	209	249
ENSECAG00000021610	6.982235034	0.0357392	0.2103428	1985	1564	1844	2278	2591	2899	2396	3112
ENSECAG00000012706	5.600613846	0.0357905	0.2103556	764	630	738	873	1053	1075	982	1050
ENSECAG00000006840	5.244116966	0.035791	0.2103556	1015	1116	1355	321	167	499	737	443
ENSECAG00000003121	9.676204577	0.0358075	0.2103556	8301	12242	9144	10964	7306	39561	8399	20345
ENSECAG00000014150	5.107128846	0.0358155	0.2103556	682	479	409	426	997	423	896	799
ENSECAG00000014360	7.114172318	0.035841	0.2103969	2022	1518	2423	1060	6531	1751	3952	1123
ENSECAG00000014169	5.521107139	0.0358783	0.2104805	851	556	708	664	1237	769	1264	773
ENSECAG00000016733	3.936584078	0.0358923	0.2104805	186	261	199	230	241	296	427	421
ENSECAG00000024982	5.273714955	0.035928	0.2105813	568	466	518	647	514	1477	608	802
ENSECAG00000013216	6.5070968	0.0359855	0.2108091	2087	1573	2343	2716	1217	798	1685	1494
ENSECAG00000004031	5.341308978	0.0361148	0.211458	737	358	723.003	451	856.003	434	1838	745
ENSECAG00000000306	6.003378623	0.0361407	0.2115002	1572	1251	1442	1771	905	736	1097	955
ENSECAG00000021780	5.881169158	0.0361717	0.211573	858	471	873	1013	2696	624	1647	596
ENSECAG00000009822	7.21012758	0.0361935	0.2115912	2626	1984	2216	1766	4749	2561	3320	2582
ENSECAG00000017805	6.252172306	0.0362554	0.2117598	2002	1515	1860	1893	990	583	1462	1304
ENSECAG00000006081	6.366197946	0.0362596	0.2117598	1286	1219	1200	1390	1901	1760	1929	1512

ENSECAG00000014689	3.563279531	0.0362934	0.2118481	139	149	167	186	174	465	204	213
ENSECAG00000013194	0.98104145	0.0363337	0.2119748	39	45	51	56	25	19	21	27
ENSECAG00000012470	3.028384994	0.0363647	0.2120465	133	108	118	64	189	270	164	118
ENSECAG00000024169	4.9101726	0.0364537	0.2124038	616	346	458	300	824	412	913	596
ENSECAG00000024614	3.115816603	0.036472	0.2124038	199	179	228	246	84	83	127	159
ENSECAG00000021689	0.125774213	0.0364873	0.2124038	19	23	23	43	16	3	18	7
ENSECAG00000017724	7.855059254	0.0365147	0.2124038	5166	2368	3294	1158	7421	5161	6439	2851
ENSECAG00000007113	5.234184114	0.0365195	0.2124038	929	728	848	1088	468	345	740	593
ENSECAG00000025023	6.330588854	0.0365745	0.212615	986	1452	1138	1027	1632	2687	1421	1282
ENSECAG00000007058	0.695350556	0.0366646	0.212985	17	19	18	23	52	23	23	48
ENSECAG00000004911	6.43508449	0.0366756	0.212985	2034	1794	2072	2262	1183	836	1681	1292
ENSECAG00000018775	7.500261471	0.0367011	0.2130045	2795	2590	2837	2521	4738	2943	5190	3314
ENSECAG00000016722	4.900354079	0.0367165	0.2130045	314	333	323	408	160	1730	208	713
ENSECAG00000011008	3.67219874	0.0369277	0.2140221	147	165	134	284	327	161	343	339
ENSECAG00000010995	4.695774959	0.0369593	0.2140221	630	236	294	226	811	404	924	319
ENSECAG00000010728	1.46271894	0.0369666	0.2140221	11	228	17	45	19	14	5	46
ENSECAG00000015641	0.99680983	0.03697	0.2140221	55	17	50	93	6	19	47	10
ENSECAG00000009413	7.653020174	0.0369861	0.2140221	2609	2798	3401	2948	4010	6077	4011	3539
ENSECAG00000023126	2.953572959	0.0370222	0.2141187	81	57	92	171	107	336	165	106
ENSECAG00000012968	4.512731008	0.0370405	0.2141187	530	406	552	750	205	207	460	388
ENSECAG00000008570	3.162449075	0.0370797	0.2141778	235	343	179	142	48	56	200	132
ENSECAG00000019300	2.965081099	0.0370942	0.2141778	14	166	17	30	23	451	20	340
ENSECAG00000005547	2.64809723	0.0371073	0.2141778	133	71	178	294	115	66	74	24
ENSECAG00000002831	2.796395434	0.0371358	0.2141785	49.9998	133	62	65	85	332	52.9993	184
ENSECAG00000021040	4.589925334	0.0371625	0.2141785	353	434	361	164	402	475	547	783
ENSECAG00000004791	4.332653456	0.0371749	0.2141785	355	282	286	120	792	270	572	272
ENSECAG00000013793	5.090967424	0.0371828	0.2141785	690	842	884	834	321	370	541	661
ENSECAG00000010050	5.950655474	0.0372107	0.2142302	715	892	773	1295	951	1833	1012	1596
ENSECAG00000014189	4.749084697	0.0373206	0.214678	384	373	459	288	500	924	597	365
ENSECAG00000013247	3.885553832	0.0373263	0.214678	343	203	95	99	521	350	310	206
ENSECAG00000010778	3.153810284	0.0373677	0.2148073	31	87	18	27	0	490	0	565
ENSECAG00000022162	8.002264535	0.0374894	0.2153497	6346	5742	6808	5753	2555	2643	5233	3859
ENSECAG00000019685	5.624085892	0.0374999	0.2153497	767	664	703	937	886	928	1218	1246
ENSECAG00000023888	3.008985541	0.0375283	0.2154039	6	138	9	41	8	525	8	352
ENSECAG00000020179	4.074458141	0.0376316	0.2156685	224	279	212	248	391	469	285	331
ENSECAG00000021494	4.079623691	0.0376362	0.2156685	193.971	274.997	193.964	304	467	242.997	304	506.982
ENSECAG00000010761	3.452494591	0.0376438	0.2156685	224	228	244	384	102	139	152	182
ENSECAG00000014802	1.424132444	0.0376504	0.2156685	54	48	53	116	30	22	46	30
ENSECAG00000009474	6.682269956	0.037725	0.2159413	782	1901	1776	1093	1755	2746	1292	3521
ENSECAG00000023512	3.257056869	0.0377427	0.2159413	251	214	346	175	24	116	123	180
ENSECAG00000007726	6.325536404	0.037755	0.2159413	1946	1361	2091	2245	948	1115	1576	918
ENSECAG00000023036	5.531805591	0.0378618	0.216317	504	1034	429	650	797	1109	852	1369
ENSECAG00000022251	2.348027655	0.0378646	0.216317	58	48	73	56	45	265	50	113
ENSECAG00000002072	4.74365163	0.0378779	0.216317	648	414	694	915	250	185	474	542
ENSECAG00000009835	4.521311922	0.0379109	0.2163971	386	145	265	407	1036	308	690	172
ENSECAG00000019432	5.080335394	0.0379791	0.2166775	299	641	348	341	304	1616	308	908
ENSECAG00000016984	1.868832033	0.0380448	0.2169432	28	66	17	35	38	220	26	64
ENSECAG00000008446	6.921616488	0.0380704	0.2169801	1063	1918	1380	1144	541	6013	960	4083
ENSECAG00000022574	6.7056412	0.0380918	0.2169933	1529	1442	1236	1677	1592	4184	1636	1762
ENSECAG00000019492	5.260242711	0.0381763	0.2172958	641	389	655	595	1015	790	1114	487
ENSECAG00000009990	0.810192145	0.0381832	0.2172958	9	39	14	9	19	56	26	65
ENSECAG00000015430	1.583158784	0.0382148	0.217367	82	70	75	69	58	17	35	31
ENSECAG00000018615	0.651607211	0.0382427	0.2174165	12	21	20	21	28	56	29	26
ENSECAG00000020212	1.85956931	0.0382671	0.2174469	50	54	49	35	85	83	107	56
ENSECAG00000020481	6.553654138	0.0383854	0.2179621	1791	1255	1512	1130	2206	1958	2203	1794
ENSECAG00000019797	6.447083974	0.0384093	0.2179621	2315	1266	2383	2771	1605	441	1750	915
ENSECAG00000019874	6.877155286	0.0384154	0.2179621	2517	2611	2747	3522	1281	920	2553	1863
ENSECAG00000022859	5.968730587	0.03851	0.2183442	1266	1225	1333	2185	786	607	1182	1011
ENSECAG00000005530	5.347797242	0.0385212	0.2183442	926	828	941	1155	620	448	750	510
ENSECAG00000019111	6.188621999	0.0386649	0.2190496	4	803	3	140	13	4465	2	4527
ENSECAG00000000438	5.856786183	0.0387712	0.2194265	1303	919	1578	1737	753	646	1159	761
ENSECAG00000012029	5.988529098	0.0387839	0.2194265	1093	1189	1619	2261	601	861	1280	822
ENSECAG00000019122	6.136075633	0.0388414	0.2194265	1466	840	1046	542	2182	1419	2100	748
ENSECAG00000023297	0.532466411	0.038851	0.2194265	17	13	11	25	16	58	20	35
ENSECAG00000021686	7.955085228	0.0388613	0.2194265	6436	4444	6690	6896	4226	1647	4919	2747
ENSECAG00000005616	4.327075535	0.0388665	0.2194265	475	370	479	623	310	163	351	285
ENSECAG00000019474	9.177803429	0.0388946	0.2194265	13529	191	146	6076	22661	10471	16576	15670
ENSECAG00000018662	6.270667367	0.0388981	0.2194265	1836	1805	1525	2235	630	951	1138	1580
ENSECAG00000020976	6.592606955	0.0389053	0.2194265	2048	2231	3433	1578	898	793	2049	1589
ENSECAG00000015360	3.670572941	0.0390211	0.2199708	112	158	156	156	114	113	140	926
ENSECAG00000013860	2.713978628	0.0391531	0.2205343	197	129	130	190	66	63	121	91
ENSECAG00000006701	2.106234351	0.0391599	0.2205343	105	155	37	155	30	23	34	100
ENSECAG00000009168	6.559381136	0.0392444	0.2209005	915	2242	763	409	1923	2504	640	4015
ENSECAG00000023831	3.567238326	0.0393199	0.2212157	293	225	275	389	182	176	170	75
ENSECAG00000000341	2.234562	0.0393925	0.2215146	33	55	42	56	60	334	34	22
ENSECAG00000000426	1.720971932	0.0394479	0.2217162	78	53	72	127	37	43	43	36
ENSECAG00000021708	3.645099218	0.039496	0.2218769	239	95	224	852	128	110	267	69
ENSECAG00000004162	7.487331882	0.0396655	0.2226858	2655	2145	2768	3245	2617	3505	4747	5139

ENSECAG00000019069	7.403694714	0.0396885	0.2226858	1529	3192	2485	2336	3534	4789	2451	4233
ENSECAG00000012726	4.521077032	0.0396988	0.2226858	471	437	705	632	193	271	276	487
ENSECAG00000017353	9.167400543	0.0397774	0.2230019	8584.97	6207	8971	10467	13975	9702	18848	9574
ENSECAG00000019839	3.798423581	0.0398135	0.2230019	318	286	373	360	167	197	212	175
ENSECAG00000002250	3.29839246	0.0398141	0.2230019	216	87	65	99	103	461	102	254
ENSECAG00000000487	2.132422788	0.0399105	0.2233561	92	25	20	60	102	204	81	30
ENSECAG00000024622	4.22014738	0.0399166	0.2233561	500	307	401	640	146	312	321	205
ENSECAG00000011309	3.745232807	0.0400354	0.2238901	103	366	44	101	375	223	485	256
ENSECAG00000012220	6.159729318	0.0400615	0.2238901	918	1331	816	1097	957	1633	1326	2426
ENSECAG00000017425	7.688942756	0.0400829	0.2238901	2592	2909	2954	3443	4087	7039	4255	2781
ENSECAG00000024184	4.142299898	0.0400964	0.2238901	334	196	250	179	699	186	454	303
ENSECAG00000000982	4.839939238	0.0401106	0.2238901	580	354	824	1344	555	176.999	538.999	88.9999
ENSECAG00000026902	1.568004485	0.0402095	0.224332	57	93	54	92	23	24	26	64
ENSECAG00000020623	5.883802098	0.0403623	0.2250738	1295	1292	1206	1762	723.999	713	999	965
ENSECAG00000021745	4.861820543	0.0403881	0.2251074	482	289	498	442	729	797	552	459
ENSECAG00000020984	7.876161771	0.0404469	0.2253236	5599	6303	7062	4766	1015	2095	2646	5987
ENSECAG00000013655	5.594835168	0.0404702	0.2253236	819	784	1071	2415	225	483	1304	491
ENSECAG00000018353	6.778002729	0.0405169	0.2253236	1620	1220	2052	1677	2988	1421	3373	2098
ENSECAG00000020633	7.855815025	0.0405229	0.2253236	5721	4328	5752	6475	3729	2608	3332	3193.02
ENSECAG00000017770	2.278428144	0.0405261	0.2253236	77	37	61	75	160	97	157	42
ENSECAG00000013319	5.1973028	0.0405661	0.2254354	405	591	330	555	328	1815	468	719
ENSECAG00000008231	2.937170646	0.0406318	0.2256903	71	83	135	117	188	271	129	101
ENSECAG00000009354	5.085254237	0.0406794	0.2258259	545	477	382	544	613	1230	590	529
ENSECAG00000020454	0.774842565	0.040696	0.2258259	15	13	24	33	38	28	29	59
ENSECAG00000021505	5.300929373	0.0408626	0.2266399	629	603	438	674	1095	520	746	1130
ENSECAG00000017558	4.765372901	0.0409961	0.2272659	532	584	764	744	308	208	568	438
ENSECAG00000016252	6.159598416	0.0410155	0.2272659	1131	1003	1141	1175	1667	1360	1885	1258
ENSECAG00000025567	1.869088677	0.0411343	0.227813	45	61	46	29	70	127	64	69
ENSECAG00000012548	3.624514467	0.0412254	0.2282065	181	157	176	191	172	255	263	419
ENSECAG00000012331	5.237299613	0.0412622	0.2282285	984	619	995	1030	518	341	847	432
ENSECAG00000022699	1.588888372	0.0412696	0.2282285	19	47	30	33	57	26	212	22
ENSECAG00000005419	0.398860087	0.041366	0.2286504	18	3	22	9	6.00043	63.0001	22	32
ENSECAG00000003257	3.968151683	0.0414418	0.2289576	234	127	252	312	287	275	406	441
ENSECAG00000015957	5.025325367	0.0414802	0.2290587	793	631	1039	649	318	323	595	578
ENSECAG00000017631	4.262187991	0.0415756	0.2294741	511	382	474	477	191	193	284	386
ENSECAG00000016336	2.245495044	0.0416225	0.2296209	110	89	103	168	55	40	84	64
ENSECAG00000022111	2.406992378	0.0416446	0.2296314	93	127	154	151	58	36	81	93
ENSECAG00000007926	0.733748083	0.0416819	0.2297258	21	7	14	30	76	21	56	8
ENSECAG00000007829	5.738132954	0.0417966	0.2302462	986	1386	1216	1462	840	427	1024	755
ENSECAG00000011870	2.786473834	0.041871	0.2305441	159	100	210	222	102	49	124	80
ENSECAG00000010476	6.081570932	0.0420205	0.231255	1512	1375	1520	2032	1101	615	1380	796
ENSECAG00000015794	8.421957053	0.0420423	0.231263	8018	10883	7357	6645	3453	3688	4560	6739
ENSECAG00000019777	5.539314273	0.0420684	0.2312949	538	1093	500	514	901	1150	1018	1050
ENSECAG00000018826	1.319934292	0.0422368	0.2321083	30	27	23	38	31	120	32	42
ENSECAG00000022114	5.336511458	0.0423379	0.2325512	957	727	953	1182	565	476	659	613
ENSECAG00000011172	8.34932855	0.0424573	0.2329137	5467	4523	3948	5719	8040	5760	8420	6662
ENSECAG00000016570	0.640353506	0.0424669	0.2329137	21	17	11	25	48	37	15	38
ENSECAG00000024666	4.70695341	0.0424838	0.2329137	394	401	302	362	264	890	489	686
ENSECAG00000014859	4.143663309	0.0424859	0.2329137	487	277	510	464	216	125	415	209
ENSECAG00000023847	6.656093173	0.0425128	0.2329486	2823	1579	2105	3194	1637	1090	1771	1204
ENSECAG00000008907	3.092806603	0.0427059	0.2337994	167	175	237	271	64	78	125	173
ENSECAG00000009359	0.369895992	0.0427202	0.2337994	11	23	15	9	29	34	19	32
ENSECAG00000016626	4.120941297	0.0427591	0.2337994	242	245	344	176	348	355	444	401
ENSECAG00000004590	3.431462082	0.0427601	0.2337994	268	194	341	244	172	123	131	140
ENSECAG00000010501	4.777979465	0.0427709	0.2337994	418	339	378	477	922	351	763	435
ENSECAG00000015753	2.818606132	0.0428051	0.2338735	118	182	244	162	35	69	114	131
ENSECAG00000017216	3.562630899	0.0428609	0.2340656	235	469	264	185	106	91	157	240
ENSECAG00000022118	7.598552768	0.042907	0.23417	3821	2888	4550	9030	2758	1146	4852	1362
ENSECAG00000022930	2.637135385	0.0429312	0.23417	63	70	113	93	214	102	125	123
ENSECAG00000018743	0.517691827	0.0429418	0.23417	11	31	3	13	47	30	44	15
ENSECAG00000002578	6.213128426	0.0430369	0.2345756	1807	1250	2088	1868	1220	806	1076	1115
ENSECAG00000000284	2.553142222	0.0430659	0.2346216	161	209	108	98	61	50	129	51
ENSECAG00000000781	2.873535999	0.0431646	0.2350462	110	167	182	279	70	106	68	117
ENSECAG00000011340	4.10655758	0.043228	0.2352788	370.001	301	409.001	593	229.001	183.001	248	286.024
ENSECAG00000014259	5.37894637	0.0432614	0.2353476	114	1078	107	177	220	2302	264	1465
ENSECAG00000017987	3.274028861	0.0432951	0.2354185	225	178	239	305	117	87	188	129
ENSECAG00000005501	3.255246815	0.0433899	0.2358211	146	156	245	498	42	129	229	37
ENSECAG00000007875	3.625331116	0.0434747	0.2361686	293	229	334	348	150	102	272	149
ENSECAG00000002176	5.478348482	0.0435067	0.2362125	1472	361	1533	1161	349	363	1047	601
ENSECAG00000011673	5.924624602	0.0435243	0.2362125	1422	103	106	112	3692	2722	59	105
ENSECAG00000010980	5.08911806	0.0437276	0.2370762	729	604	911	1055	422	249	735	518
ENSECAG00000009444	2.668857894	0.043766	0.2370762	41	134	54	45	36	133	102	367
ENSECAG00000011541	4.893853061	0.0437665	0.2370762	807	359	659	1157	420	407	523	230
ENSECAG00000011938	6.109491966	0.0437669	0.2370762	1025	773	1093	1431	1589	1563	1612	1166
ENSECAG00000002017	5.31701467	0.0438189	0.2371411	518	481	341	438	192	2724	349	476
ENSECAG00000021985	1.497110853	0.0438207	0.2371411	27	28	38	50	104	27	91	43
ENSECAG00000012720	4.331275535	0.0438796	0.237347	273	246	277	420	459	469	502	330
ENSECAG00000016501	2.591012724	0.0439717	0.2377317	140	130	122	203	72	59	124	58

ENSECAG00000022891	4.565895072	0.0441372	0.2384214	285	204	333	563	663	244	823	470
ENSECAG00000011860	4.584670144	0.044162	0.2384214	325	426	245	356	583	768	445	315
ENSECAG00000012743	5.372201335	0.0441686	0.2384214	679	565	560	674	1110	468	1412	751
ENSECAG00000011333	3.085514339	0.0441832	0.2384214	107	141	79	141	166	152	157	296
ENSECAG00000016067	5.456933521	0.04421	0.2384524	1143	802	1331	919	613	368	1001	503
ENSECAG00000015714	5.813389592	0.0442575	0.2385958	783.001	900	710.001	887	836.001	2035	1023	976.024
ENSECAG00000018657	5.893196276	0.0443079	0.2387541	945	689	842	1185	1266	1624	1033	1150
ENSECAG00000003585	2.41250842	0.0443346	0.2387849	61	86	66	66	179	131	81	87
ENSECAG00000009934	1.708540716	0.0443901	0.2389184	71	72	56	127	50	38	44	25
ENSECAG00000010196	6.550471099	0.0444015	0.2389184	656	1801	707	977	438	5308	625	2562
ENSECAG00000026890	3.103313533	0.0445079	0.2392757	85	91	96	219	217	135	221	212
ENSECAG00000018205	2.699198896	0.0445212	0.2392757	197	96	183	170	81	41	125	88
ENSECAG00000021247	5.124124891	0.0445311	0.2392757	786	751	906	838	470	377	482	643
ENSECAG00000011337	5.727488728	0.0446376	0.2397344	1197	1032	1227	1537	730	614	1116	613
ENSECAG00000008487	2.850886779	0.0447218	0.2400355	223	143	239	106	126	70	69	89
ENSECAG00000000507	6.375618595	0.0447359	0.2400355	1902	1751	1806	2337	953	903	1573	1403
ENSECAG00000023941	4.026025873	0.0447701	0.240038	220	323	127	212	195	431	334	526
ENSECAG00000022070	5.968778921	0.0447786	0.240038	1120	576	1023	1083	1577	1407	1716	797
ENSECAG00000020471	2.80198046	0.0448184	0.2401127	148	118	183	241	76	62	115	113
ENSECAG00000016435	3.632266067	0.0448348	0.2401127	156	215	145	203	211	261	347	288
ENSECAG00000013228	3.244675609	0.0449783	0.2407293	70	155	110	81	78	429	42	359
ENSECAG00000019862	5.694919687	0.0449924	0.2407293	1178	764	1353	1821	864	298	1075	644
ENSECAG00000014615	2.051741069	0.0450355	0.2407838	20	88	29	32	26	183	36	158
ENSECAG00000016628	3.983230711	0.0450615	0.2407838	227	203	246	266	357	374	311	332
ENSECAG00000009436	4.304007978	0.0450662	0.2407838	507	408	470	483	284	209	349	269
ENSECAG00000010663	6.171760124	0.0451054	0.2408803	1598	821	888	479	3303	835	1757	893
ENSECAG00000019120	4.907083652	0.0451464	0.240937	307	877	702	1255	203	154	307	798
ENSECAG00000022754	1.018987494	0.0451585	0.240937	21	31	21	31	40	33	54	55
ENSECAG00000005409	0.764195766	0.0452453	0.2412485	25	58	42	50	8	1	39	22
ENSECAG00000019343	2.816728742	0.0452593	0.2412485	18	150	12	33	20	158	18	586
ENSECAG00000024435	4.784303628	0.0453274	0.2414261	557	652	652	773	318	313	328	559
ENSECAG00000020992	0.533514421	0.0453351	0.2414261	18	14	21	19	36	27	35	29
ENSECAG00000012304	4.251816293	0.0453713	0.2415055	542	364	456	468	203	159	392	310
ENSECAG00000017545	6.598201209	0.0454262	0.2416845	1530	1223	1338	1869	2830	2078	1877	1548
ENSECAG00000018503	4.447497786	0.0454496	0.2416959	416	436	529	708	263	266	358	335
ENSECAG00000024024	2.764868889	0.0454852	0.2417349	117	72	105	84	184	116	205	113
ENSECAG00000012961	0.678682594	0.045501	0.2417349	43	24	74	24	8	2	16	37
ENSECAG00000024964	5.225508513	0.0455208	0.2417349	1057	690	992	882	440	216	836	606
ENSECAG00000018618	4.378712826	0.0456382	0.2422449	28	379	43	149	15	1255	32	951
ENSECAG00000003314	1.099159851	0.0457817	0.2428931	18	17	45	29	57	37	66	35
ENSECAG00000017776	7.545624774	0.0458472	0.2431272	2087	2557	3162	3510	3912	4473	4854	3147
ENSECAG00000020031	5.178739428	0.0458831	0.2431287	882	691	867	1006	626	386	665	370
ENSECAG00000002942	5.917810444	0.0458903	0.2431287	979	837	899	1039	1068	1379	1143	1576
ENSECAG00000014290	3.014435519	0.0459552	0.2433588	144	72	81	133	347	100	196	111
ENSECAG00000008711	4.894825076	0.0460146	0.2434173	545	615	762	920	304	416	470	474
ENSECAG00000025015	6.513188369	0.0460189	0.2434173	2068	1556	2368	2610	1320	1224	1392	1308
ENSECAG00000020341	2.34528936	0.0460305	0.2434173	13	90	31	18	3	343	18	166
ENSECAG00000008643	4.025667295	0.0462604	0.2445192	92	119	159	494	248	693	239	322
ENSECAG00000020151	0.77973701	0.0463082	0.2445379	13	23	23	27	25	46	42	39
ENSECAG00000007165	6.118742902	0.0463097	0.2445379	1936	1250	1873	1459	863	780	1437	934
ENSECAG00000017930	4.812888531	0.0463285	0.2445379	367	446	410	449	639	767	572	452
ENSECAG00000013261	5.126156908	0.046416	0.2448859	448	525	505	573	1047	653	1053	369
ENSECAG00000021354	4.08049461	0.046545	0.2453907	454	279	449	476	355	165	183	188
ENSECAG00000009685	4.209145181	0.0465655	0.2453907	296	226	258	346	385	310	526	410
ENSECAG00000007696	2.916282166	0.0465936	0.2453907	100	150	169	373	44	64	101	157
ENSECAG00000016515	0.625252911	0.0465981	0.2453907	23	10	20	25	29	28	42	38
ENSECAG00000015813	4.308109502	0.0466927	0.2457324	266	294	261	359	373	556	384	401
ENSECAG00000006306	4.686564352	0.0467098	0.2457324	668	514	572	682	346	377	342	368
ENSECAG00000021399	2.694545808	0.0467561	0.2457324	75	106	85	86	134	198	110	131
ENSECAG00000019978	3.314759149	0.0467699	0.2457324	188	69	148	148	354	172	216	162
ENSECAG00000023916	5.021363378	0.0467776	0.2457324	477	552	434	494	747	718	576	755
ENSECAG00000012987	4.016803856	0.0468248	0.2457324	122	211	233	387	403	285	410	356
ENSECAG00000008601	9.296335462	0.0468339	0.2457324	8745	8278	7966	10303	7985	24664	9580	13473
ENSECAG00000010739	0.383615254	0.0468361	0.2457324	19	7	11	23	56	14	23	25
ENSECAG00000011077	3.703822652	0.0469052	0.2458686	380	209	324	354	200	147	230	125
ENSECAG00000007382	5.091153557	0.0469369	0.2458686	582	426	623	432	739	582	735	920
ENSECAG00000018837	5.519108511	0.0469404	0.2458686	735	697	633	609	554	1098	758	1638
ENSECAG00000024362	4.882705582	0.0469486	0.2458686	613	645	704	825	426	312	554	391
ENSECAG00000006065	7.731659307	0.047009	0.2459857	3110	2661	3343	3862	4633	5255	4126	4344
ENSECAG00000003634	4.033395308	0.0470143	0.2459857	402	274	361	587	204	225	329	115
ENSECAG00000003761	3.974297107	0.0470415	0.2460151	142	328	136	181	164	662	203	411
ENSECAG00000024254	0.173549825	0.0470856	0.2461324	22	15	3	0	19	33	13	42
ENSECAG00000016126	4.663976562	0.0471262	0.2462313	514	342	344	262	695	610	420	492
ENSECAG00000011528	5.92688059	0.0472222	0.2466191	926	831	1068	923	1486	1386	1417	929
ENSECAG00000024555	5.642521029	0.0472903	0.2468615	1195	866	1277	1348	771	541	870	714
ENSECAG00000023904	0.326145881	0.0473729	0.2471789	0	21	10	18	14	54	26	23
ENSECAG00000017005	4.336378059	0.0474493	0.2474641	27	455	56	87	37	1331	68	680
ENSECAG00000023824	4.409648767	0.0475428	0.2477869	262	343	204	458	419	372	695	424

ENSECAG00000009055	4.859170555	0.0475548	0.2477869	757	567	678	756	436	260	574	379
ENSECAG00000014825	6.586367123	0.0476011	0.2479145	1589	1299	1337	1820	2344	1843	2190	1848
ENSECAG00000019870	3.403655882	0.0476896	0.2481007	74	174	65	154	63	455	54	454
ENSECAG00000014650	1.104059434	0.0476965	0.2481007	15.0007	14.0005	51.001	4.00033	105.001	27.0007	73.0001	12.0239
ENSECAG00000021001	6.444808326	0.0477024	0.2481007	1298	1226	1139	1826	1536	1753	2067	2140
ENSECAG00000017083	1.533089376	0.047904	0.2489945	36	37	26	53	59	67	39	93
ENSECAG00000023859	4.308842195	0.0479181	0.2489945	442	264	264	105	344	488	515	467
ENSECAG00000022924	7.22467316	0.0479418	0.2490035	3814	2679	3870	3961	2686	1404	2351	1948
ENSECAG00000000939	1.771336436	0.0479698	0.2490354	70	94	73	92	32	34	61	46
ENSECAG00000010671	3.503401216	0.0480434	0.2492408	307	185	275	332	176	122	175	140
ENSECAG00000013162	6.51340113	0.0480544	0.2492408	2562	1358	2398	2486	1562	1051	1736	758
ENSECAG00000021435	4.363872091	0.04809	0.2492408	373	244	352	267	471	376	501	456
ENSECAG00000020982	4.189145356	0.0480972	0.2492408	411	363	510	445	261	191	303	266
ENSECAG00000009013	3.61120866	0.0481315	0.2493049	178	166	217	152	294	242	300	242
ENSECAG00000009778	2.168978434	0.048225	0.2496756	92	88	130	129	57	38	79	58
ENSECAG00000017331	6.297653656	0.0483208	0.2500573	1281	1108	1307	1155	1837	1890	1695	1291
ENSECAG00000006883	5.480326747	0.0484513	0.2506187	1143	749	1227	1145	454	371	899	831
ENSECAG00000024924	3.82488812	0.048592	0.2512319	401	243	317	423	146	117	231	277
ENSECAG00000013574	3.546538212	0.0487738	0.2518895	167	160	183	161	155	324	293	264
ENSECAG00000016162	5.0276671	0.0487827	0.2518895	581	428	463	495	984	465	835	589
ENSECAG00000002257	3.581789408	0.0487885	0.2518895	317	196	495	305	21	38	98	356
ENSECAG00000024482	2.910653889	0.0488079	0.2518895	4	157	8	27	11	230	19	596
ENSECAG00000019027	1.800336391	0.0488352	0.2519158	37	142	86	86	15	29	41	72
ENSECAG00000015657	1.180499111	0.0488979	0.2521251	24	23	43	20	36	59	26	84
ENSECAG00000022006	6.245003431	0.0491227	0.2531693	1869	669	1089	703	1874	1324	2772	1017
ENSECAG00000010689	6.127003683	0.0491488	0.2531887	1262	1473	1574	2308	742	873	1290	1132
ENSECAG00000003481	6.249189253	0.0492145	0.2534122	1099	757	1444	1191	2519	996	2448	934
ENSECAG00000005146	4.906547186	0.0493943	0.2542227	712	596	741	781	375	315	461	555
ENSECAG00000021819	4.68969102	0.0495366	0.2548399	481	335	361	403	561	496	596	582
ENSECAG00000022265	6.387201988	0.0496063	0.2549843	2362	1637	1900	1969	1303	728	1718	1100
ENSECAG00000016911	5.625879438	0.0496096	0.2549843	670	640	1082	291	783	1678	722	1232
ENSECAG00000009791	6.10676353	0.049727	0.2554722	1793	1469	1535	1619	862	906	1431	804
ENSECAG00000023641	4.730969202	0.0497717	0.2555211	796	520	795	409	405	313	336	390
ENSECAG00000005330	5.051271268	0.0497987	0.2555211	644	389	446	551	572	605	764	957
ENSECAG00000002924	3.358218931	0.049804	0.2555211	233	124	106	69	333	109	349	188
ENSECAG00000000548	12.07402665	0.049858	0.2556826	77439	73133	118408	194438	14524	49102	123508	26191
ENSECAG00000006177	3.713670368	0.0499581	0.2560315	107	595	233	381	130	109	273	138
ENSECAG00000015889	6.365166044	0.0500205	0.2560315	1390	1177	1350	1080	2198	2000	1562	1296
ENSECAG00000006462	6.153833601	0.0500206	0.2560315	1274	846	1171	1056	2068	1149	2010	1022
ENSECAG00000000715	6.073847086	0.0500381	0.2560315	2084	1114	1657	1666	1089	393	1499	809
ENSECAG00000016058	4.800376566	0.0500387	0.2560315	610	705	647	647	330	307	522	429
ENSECAG00000006071	3.798955713	0.0500761	0.2561074	285	317	380	374	117	101	281	259
ENSECAG00000012637	4.767349667	0.050179	0.2565184	425	310	491	417	627	706	550	464
ENSECAG00000013026	3.763672433	0.0502296	0.2566615	242	269	255	586	133	172	254	168
ENSECAG00000013815	7.351115659	0.0502579	0.2566631	2886	2362	2127	2518	3073	3581	3106	4416
ENSECAG00000000367	5.095712282	0.0502751	0.2566631	845	616	865	882	455	420	574	515
ENSECAG00000026850	8.864786677	0.0503402	0.2568312	7377	5927	7087	7363	11930	12260	8241	8059
ENSECAG00000019051	5.681345906	0.050395	0.2568312	1259	1027	1529	1034	773	362	822	954
ENSECAG00000007212	2.07160936	0.0503963	0.2568312	24	89	20	24	22	163	17	221
ENSECAG00000008344	5.109582068	0.0504084	0.2568312	501	461	448	663	1023	700	923	393
ENSECAG00000020878	8.161260088	0.0504484	0.2568312	5544	8310	7218	7040	2038	1778	5732	5965
ENSECAG00000022290	3.618667359	0.0504613	0.2568312	226	123	107	201	188	540	178	191
ENSECAG00000024309	4.063951259	0.0504663	0.2568312	150	367	166	226	315	546	257	370
ENSECAG00000011355	8.049971879	0.0505923	0.2572659	4776	4955	5700	10929	2083	3220	5190	4074
ENSECAG00000007274	4.120086008	0.0505971	0.2572659	354	139	211	305	511	359	475	217
ENSECAG00000008401	7.159024471	0.0507962	0.2581107	1812	2391	1941	1894	2163	3898	1562	5092
ENSECAG00000007837	7.842704451	0.0508087	0.2581107	1094	5041	1523	1954	749	11337	1836	8682
ENSECAG00000024743	4.122893845	0.0508955	0.2581972	216	200	272	100	134	277	110	1223
ENSECAG00000013850	4.003012013	0.0508956	0.2581972	307	355	343	568	141	136	270	326
ENSECAG00000017265	6.928655701	0.0509163	0.2581972	3274	2605	3855	2228	861	730	2220	2761
ENSECAG00000012393	6.232851406	0.0509166	0.2581972	1480	1029	1019	956	1498	2264	1218	1466
ENSECAG00000008542	11.48486203	0.0510533	0.258775	44787	32167	45373	49436	64274	77507	51656	55759
ENSECAG00000007640	7.339167769	0.0510935	0.2588633	1901	2762	3077	1702	2925	4273	3726	3320
ENSECAG00000011366	7.234302615	0.0511574	0.2590557	2910	2465	1757	1689	3808	3576	3061	2725
ENSECAG00000019673	6.702967047	0.051197	0.2590557	2242	2176	2253	3229	1037	1162	2026	1779
ENSECAG00000001898	5.299430504	0.0511999	0.2590557	643	531	575	560	704	1365	850	495
ENSECAG00000015007	3.004434716	0.0512702	0.2592145	249	122	181	244	92	56	172	105
ENSECAG00000021595	4.288276889	0.0512834	0.2592145	479	250	493	716	332	145	363	225
ENSECAG00000008274	3.150554728	0.0512998	0.2592145	1	125	2	21	4	574	0	470
ENSECAG00000018358	5.211894044	0.0513456	0.2593309	593	435	638	606	1121	606	852	640
ENSECAG00000025153	6.391944945	0.0514567	0.2597764	1987	1040	1211	555	2212	1898	1752	1598
ENSECAG00000024167	0.321413628	0.0515314	0.2600379	16	62	22	21	10	3	29	9
ENSECAG00000017917	8.389328208	0.0516507	0.2605241	7622	10321	9570	5422	2309	2649	3995	8425
ENSECAG00000018656	1.130473494	0.0517054	0.2605503	28	39	15	24	58	18	79	51
ENSECAG00000024194	4.674525865	0.0517097	0.2605503	599	402	604	865	343	385	429	254
ENSECAG00000013747	5.400618911	0.0517288	0.2605503	247	1024	209	236	308	1753	155	1981
ENSECAG00000024860	7.403728043	0.0517476	0.2605503	2427	1881	2336	3794	4201	3484	4449	2711
ENSECAG00000005071	3.380621546	0.0518209	0.2608033	194	172	309	358	173	72	177	131

ENSECAG00000021267	4.059992082	0.0518667	0.2609182	409	341	429	436	124	275	190	295
ENSECAG00000000987	6.407144431	0.0520131	0.261539	1932	1522	2233	2235	1296	1086	1415	1128
ENSECAG00000022349	1.96826238	0.0520837	0.2617784	51	19	68	65	133	69	124	40
ENSECAG00000021289	6.920860054	0.052298	0.2626865	2032	1255	2033	670	4968	1871	4559	516
ENSECAG00000000383	7.043030816	0.0523107	0.2626865	3212	2355	3154	3783	1948	1561	2388	1662
ENSECAG00000006857	4.624303059	0.0523401	0.2627183	382	381	327	415	490	528	494	612
ENSECAG00000012738	8.239605339	0.0526809	0.2643121	4185	4487	4048	4950	6938	9692	4463	5093
ENSECAG00000011439	6.156464472	0.0527119	0.2643509	1620	1860	1787	1460	526	1162	942	1302
ENSECAG00000016586	5.098553663	0.0527696	0.2644407	991	528	565	1271	542	311	669	388
ENSECAG00000009432	3.600442776	0.0527764	0.2644407	149	193	157	198	201	340	197	324
ENSECAG00000018128	1.292712113	0.0528169	0.2644619	52	50	64	82	7	16	38	52
ENSECAG00000005573	1.80072195	0.0528389	0.2644619	35	67	33	34	52	146	54	62
ENSECAG00000018686	6.991353276	0.0528505	0.2644619	2305	1184	1996	1787	5474	1811	2470	1718
ENSECAG00000024082	6.897143048	0.0528997	0.2645016	449	3058	935	1291	1350	4517	1539	3833
ENSECAG00000005752	5.29161086	0.0529141	0.2645016	533	561	644	695	970	776	1008	619
ENSECAG00000013051	3.725943245	0.0529283	0.2645016	336	222	361	362	170	113	282	166
ENSECAG00000000828	5.02016172	0.0530602	0.2650446	792	775	821	650	417	314	470	633
ENSECAG00000019128	6.666674347	0.0534085	0.2666668	2387	2038	2751	2360	1833	975	1789	1237
ENSECAG00000013060	5.217996615	0.0534368	0.266691	875	848	1322	700	157	254	428	1044
ENSECAG00000023981	7.534287781	0.0534779	0.2667305	2733	2343	2240	3692	2686	6287	3475	3688
ENSECAG00000016383	1.90023254	0.0534964	0.2667305	56	39	43	64	106	45	129	64
ENSECAG00000015161	6.41445381	0.0535152	0.2667305	1965	2044	1931	1950	1043	1280	1212	1359
ENSECAG00000014906	5.355620563	0.0535843	0.2667873	461	747	593	654	648.007	1194	913	778
ENSECAG00000010611	2.903292667	0.0536081	0.2667873	92	133	88	110	158	174	190	142
ENSECAG00000010916	3.052564587	0.053611	0.2667873	233	143	215	238	79	71	223	62
ENSECAG00000014258	5.611070615	0.0536359	0.2667873	1106	891	1111	1476	695	506	916	746
ENSECAG00000020437	5.935490774	0.0536475	0.2667873	1242	800	961	556	1850	1490	1001	982
ENSECAG00000016987	0.078883512	0.0536675	0.2667873	30.0007	27.0005	29.001	13.0003	8.00055	8.00066	6.00012	21.0239
ENSECAG00000006819	5.008996908	0.0537597	0.2671288	754	590	787	945	557	247	601	426
ENSECAG00000017136	4.068580998	0.053797	0.2671972	402	279	404	532	178	244	221	276
ENSECAG00000009780	6.088842378	0.05388	0.2674923	1557	1361	1643	1732	912	909	1085	1073
ENSECAG00000002575	7.830251888	0.0540158	0.2680495	5566	3491	5363	8802	1936	1028	5187	4149
ENSECAG00000005316	0.208600592	0.0541242	0.2684701	25	23	24	42	4	21	17	4
ENSECAG00000003954	3.872696091	0.0541614	0.2685375	198	175	222	190	277	654	198	166
ENSECAG00000010848	5.176416244	0.054427	0.2697367	899	814	827	821	397	527	604	542
ENSECAG00000000482	5.677030763	0.0544973	0.2699674	734	737	941	697	1018	1404	797	1128
ENSECAG00000015867	2.315743547	0.0545728	0.2702233	81	50	70	76	78	81	128	165
ENSECAG00000022302	4.144891863	0.0547813	0.2710832	436	292	577	444	293	114	412	131
ENSECAG00000022617	5.228767058	0.0547942	0.2710832	712	422	556	657	830	722	979	701
ENSECAG00000019847	4.007832487	0.0548407	0.271195	239	177	235	280	204	543	279	382
ENSECAG00000020820	6.474655679	0.0548796	0.2712526	1628	1244	1139	1531	2423	1720	1944	1523
ENSECAG00000024972	3.020009861	0.0549058	0.2712526	119	88	148	108	235	148	154	181
ENSECAG00000003075	1.443772745	0.0549239	0.2712526	41	54	69	107	29	16	37	50
ENSECAG00000010392	5.360367457	0.0550822	0.2719159	992	769	964	1105	616	450	706	622
ENSECAG00000021419	2.427377863	0.0552299	0.2723252	101	163	148	117	37	44	62	123
ENSECAG00000012978	4.93623253	0.0552354	0.2723252	466	490	475	420	655	767	738	477
ENSECAG00000014746	6.375828088	0.055237	0.2723252	1841	2092	2261	1647	722	755	1318	1891
ENSECAG00000005171	5.094816433	0.0553273	0.2726144	524	631	314	594	678	793	794	699
ENSECAG00000026920	3.595699862	0.0553447	0.2726144	155	133	174	165	100	517	119	359
ENSECAG00000016033	6.169169866	0.0553677	0.2726144	879	777	1024	1577	2481	755	2493	878
ENSECAG00000010324	6.047288478	0.0555198	0.2732451	1446	798	903	920	1679	989	1864	1260
ENSECAG00000011004	6.47345147	0.0555508	0.2732793	1915	2133	2233	1953	1051	1438	1189	1376
ENSECAG00000024684	7.300067322	0.0555965	0.2733854	3876	2934	4026	4023	2120	1990	2295	2571
ENSECAG00000016585	6.245906842	0.055721	0.273879	1192	1009	1211	1067	989	2387	1049	2137
ENSECAG00000000040	3.760121204	0.0557582	0.2739437	335	221	302	468	162	120	304	163
ENSECAG00000011670	10.65757873	0.0558053	0.2740562	24656	19620	26771	25654	41720	41209	27142	29888
ENSECAG00000015018	5.986991409	0.0558439	0.2741273	1269	1462	1504	1633	740	930	1016	995
ENSECAG00000024543	3.974829314	0.0559598	0.2745776	396	256	465	386	254	171	227	213
ENSECAG00000024977	5.784073465	0.0559944	0.2746287	1029	814	680	904	1442	932	1448	936
ENSECAG00000020991	6.242936098	0.0560419	0.2746766	2060	445	265	1082	2980	1126	692	2424
ENSECAG00000009730	6.118142426	0.0560525	0.2746766	1338	959	1131	882	1584	1407	1623	1329
ENSECAG00000015579	2.772606152	0.0560833	0.2746841	123	57	104	110	152	125	157	174
ENSECAG00000022508	6.541839741	0.0561439	0.2746841	1540	1242	1404	1608	1887	2458	1535	1994
ENSECAG00000012659	5.2961932	0.0561634	0.2746841	537	648	579	618	546	1114	857	861
ENSECAG00000021275	6.10442879	0.0561646	0.2746841	1735	1262	1753	1650	1207	843	1064	861
ENSECAG00000018012	1.653950387	0.0561985	0.2746841	5	70	13	27	21	139	14	140
ENSECAG00000010948	6.855050523	0.0562207	0.2746841	2260	1333	1539	1567	2248	4214	1723	1844
ENSECAG00000009612	4.238966539	0.0562233	0.2746841	343	228	304	224	534	448	446	231
ENSECAG00000001342	3.7712567	0.0562611	0.2747505	157	198	218	202	332	432	177	246
ENSECAG00000006134	7.268135365	0.0562998	0.2748216	3383	3186	3677	4255	2059	1796	2640	2392
ENSECAG00000024318	2.295057667	0.0563361	0.2748805	56	74	38	16	20	331	18	107
ENSECAG00000007417	4.773744257	0.0563791	0.274972	27	597	99	86	29	2072	68	690
ENSECAG00000000551	5.588142349	0.0564227	0.2750669	1296	861	1732	728	503	339	838	983
ENSECAG00000010970	5.947449381	0.0565318	0.2753632	932	765	856	1299	1425	1597	1367	874
ENSECAG00000019207	8.787847728	0.056532	0.2753632	11255	7570	11686	11738	6753	4754	7727	5766
ENSECAG00000006486	4.570258448	0.0565695	0.2754278	687	410	626	549	341	268	514	204
ENSECAG00000010731	4.336757306	0.0566317	0.2755512	244	182	204	424	110	950	255	536
ENSECAG00000015317	2.010295514	0.0566434	0.2755512	44	68	32	52	36	169	50	112

ENSECAG00000013982	2.325611118	0.0567146	0.2757796	66	68	82	61	74	136	130	106
ENSECAG00000013276	4.514900171	0.0567588	0.2758765	286	354	321	300	284	819	237	680
ENSECAG00000023589	4.234273769	0.0568013	0.2759314	349	704	510	258	128	173	245	422
ENSECAG00000011120	5.143606865	0.0568187	0.2759314	577	434	515	623	898	540	1246	470
ENSECAG00000023253	6.884868285	0.0568758	0.2760908	3242	1914	2906	3361	2232	893	2197	1315
ENSECAG00000017978	3.568791905	0.0569543	0.2763537	190	163	131	223	210	212	301	327
ENSECAG00000000895	4.143762537	0.0570722	0.2768073	499	286	378	551	316	184	309	161
ENSECAG00000014406	1.715057034	0.0571581	0.2771057	35	34	36	36	5	151	39	120
ENSECAG00000015518	6.109148079	0.0572373	0.2773716	1499	1488	1557	1818	987	910	1150	1008
ENSECAG00000020432	7.992142554	0.0574373	0.278222	4841	1975	3643	4766	6710	5014	7887	3274
ENSECAG00000007474	5.22925385	0.0575049	0.2783889	615	432	645	627	671	1037	773	713
ENSECAG00000021091	5.115499225	0.0575567	0.2783889	574	547	506	514	864	658	729	713
ENSECAG00000007008	2.658546806	0.0575584	0.2783889	82	111	59	100	108	123	143	193
ENSECAG00000010127	1.728519093	0.0575698	0.2783889	52	134	56	95	25	26	12	81
ENSECAG00000013141	5.285823755	0.0576601	0.2787066	875	792	908	1077	481	663	551	517
ENSECAG00000022955	8.691044679	0.0577289	0.2789209	5961	5528	6823	6269	7680	13046	8232	6842
ENSECAG00000006305	1.288949298	0.057765	0.2789764	54	82	55	40	24	26	32	36
ENSECAG00000018135	1.134264942	0.0578591	0.2790963	79	28	96	16	27	27	24	16
ENSECAG00000000773	5.208020906	0.0578806	0.2790963	495	537	590	706	706	624	1039	828
ENSECAG00000012770	5.573878502	0.0579054	0.2790963	792	683	624	905	1213	883	929	1018
ENSECAG00000016958	4.919121164	0.0579075	0.2790963	474	858	712	870	354	195	463	659
ENSECAG00000020840	6.047633887	0.0579127	0.2790963	884	902	969	1101	604	2473	1191	1498
ENSECAG00000009547	6.014339359	0.0579479	0.2791477	1721	1214	1560	1493	928	882	873	1048
ENSECAG00000016931	8.915069145	0.0579971	0.2791479	8163	6651	8125	6278	9969	10471	10902	10685
ENSECAG00000022787	6.524225619	0.058021	0.2791479	1455	1155	1549	1692	2131	1918	2084	1672
ENSECAG00000011220	6.8874144	0.0580267	0.2791479	2748	669	1830	264	5042	1579	4585	806
ENSECAG00000005195	6.352949947	0.0580462	0.2791479	1823	1770	2000	2036	877	872	1252	1737
ENSECAG00000020763	3.33489403	0.0583417	0.2804501	0	175	5	23	6	514	7	652
ENSECAG00000005084	5.265405386	0.0586068	0.2816053	732	512	568	547	1031	709	1011	582
ENSECAG00000009845	4.547071352	0.0588026	0.2823963	566	406	654	572	347	278	408	299
ENSECAG00000007105	8.026914	0.0588212	0.2823963	6370	4585	6482	7314	3628	3117	4069	4057
ENSECAG00000015287	3.263590713	0.0589177	0.2826541	135	109	171	148	205	213	171	249
ENSECAG00000020309	7.092945767	0.0589246	0.2826541	2499	2017	1517	2207	2550	3923	3077	2244
ENSECAG00000019894	6.171649821	0.0590428	0.2831014	1270	923	1164	1115	1702	1470	2107	963
ENSECAG00000006680	4.055850271	0.0590848	0.283183	217	254	199	295	217	501	265	471
ENSECAG00000011558	1.996483713	0.0591452	0.2833531	119	66	116	89	72	39	35	50
ENSECAG00000009069	10.12651943	0.0593362	0.2841485	12920	14946	20973	19275	27389	22842	26870	20592
ENSECAG00000020010	6.200604435	0.0594217	0.2844378	991	1483	983	998	1888	1012	1836	1699
ENSECAG00000015083	3.164857132	0.0594984	0.2846851	260	91	216	415	96	66	238	15
ENSECAG00000013225	2.529787862	0.0595321	0.2847263	10	139	17	55	31	149	60	357
ENSECAG00000010845	5.296426385	0.0596231	0.2850416	617	403	510	933	1104	653	907	743
ENSECAG00000008108	5.120305959	0.0596614	0.2851048	534	594	542	421	583	952	738	712
ENSECAG00000007847	7.502366683	0.0598054	0.2855553	2910	2246	2582	3150	5593	4144	3898	2171
ENSECAG00000021114	6.397751577	0.0598128	0.2855553	1624	1974	2061	2175	902	932	1561	1547
ENSECAG00000009128	2.14658223	0.0598311	0.2855553	124	91	117	97	65	30	89	45
ENSECAG00000012701	7.877634854	0.059994	0.2860699	4078	3591	3044	3549	4243	5589	4428	6065
ENSECAG00000019491	3.496553559	0.060036	0.2860699	257	254	295	327	137	32	174	239
ENSECAG00000019855	3.658776306	0.0600606	0.2860699	203	121	160	229	160	495	206	249
ENSECAG00000020653	5.471003549	0.060063	0.2860699	878	599	562	751	904	857	991	1033
ENSECAG00000012102	4.736490649	0.0600649	0.2860699	705	603	519	658	343	373	403	396
ENSECAG00000020193	7.800600407	0.0600997	0.2860859	1013	5742	568	1357	1330	9752	920	10490
ENSECAG00000020171	3.21544981	0.0601216	0.2860859	16	233	10	57	37	246	65	650
ENSECAG00000007762	4.648857394	0.0601438	0.2860859	427	425	302	362	549	567	632	422
ENSECAG00000012112	2.952910781	0.0602423	0.2864348	174	127	164	318	87	108	155	45
ENSECAG00000008099	4.06351594	0.060332	0.2866602	370	334	389	484	242	191	269	245
ENSECAG00000011689	6.788439577	0.0603402	0.2866602	2054	1205	1675	1534	4029	1692	3045	1163
ENSECAG00000016206	4.82996609	0.0604131	0.2868865	662	552	693	763	431	417	386	371
ENSECAG00000011141	2.699471554	0.0605136	0.2872439	116	154	177	180	72	80	69	117
ENSECAG00000003632	7.614755224	0.0606394	0.2875844	3068	2168	2743	3020	9427	2706	3602	2068
ENSECAG00000017042	4.828724689	0.0606531	0.2875844	79	631	40	154	50	1610	37	1300
ENSECAG00000023142	0.386104531	0.0606761	0.2875844	13	40	30	46	2	14	17	21
ENSECAG00000006895	2.123193005	0.0606867	0.2875844	51	16	52	113	62	143	120	77
ENSECAG00000008668	2.674424997	0.0607344	0.2876904	63	127	45	79	121	296	80	79
ENSECAG00000021304	4.972239851	0.0607832	0.2877813	499	485	459	502	796	606	675	604
ENSECAG00000016191	3.595084621	0.0608042	0.2877813	297	190	317	354	159	140	206	165
ENSECAG00000017685	3.365859791	0.0609381	0.2882951	163	130	165	150	233	254	211	195
ENSECAG00000017127	2.045684794	0.0609988	0.2884617	71	87	89	157	58	47	59	47
ENSECAG00000010394	5.710116061	0.061053	0.288598	844	458	878	1000	1827	812	1330	650
ENSECAG00000018648	5.417969522	0.0610926	0.288665	596	660	623	730	584	1220	768	1081
ENSECAG00000005816	1.515646316	0.0614307	0.2900978	39	76	120	45	33	26	21	52
ENSECAG00000010482	5.291924517	0.0614469	0.2900978	680	499	532	693	534	1117	764	938
ENSECAG00000017618	8.490671886	0.0615287	0.2903634	3982	7355	4066	4845	5092	10898	5215	10669
ENSECAG00000013796	5.881642452	0.0615787	0.2904786	814	923	1003	955	1216	1438	1294	1035
ENSECAG00000023809	3.963460535	0.0616379	0.290531	261	221	201	252	377	229	497	284
ENSECAG00000018634	5.661807387	0.061641	0.290531	1029	1038	1649	1033	675	409	816	1003
ENSECAG00000020449	6.496384848	0.0618694	0.2914085	1788	2237	1900	2400	1168	1328	1556	1218
ENSECAG00000018264	8.018302233	0.0619153	0.2914085	7052	4953	7586	5031	3986	2077	4841	3780
ENSECAG00000014330	7.904552451	0.0619167	0.2914085	5309	1373	4371	668	5061	2686	13578	3361

ENSECAG00000021336	1.166915385	0.0619298	0.2914085	11	56	17	12	40	100	39	28
ENSECAG00000021622	3.712000713	0.0620799	0.2919457	394	244	329	281	172	112	202	237
ENSECAG00000020278	6.087426198	0.0620953	0.2919457	896	1320	980	1026	1324	1491	1560	1416
ENSECAG00000010890	3.342129646	0.0622652	0.2926233	9	227	15	59	20	484	20	565
ENSECAG00000023967	4.508866977	0.0623123	0.2927237	276	314	260	452	211	594	348	888
ENSECAG00000022184	6.258211725	0.0623654	0.2928519	1325	1838	1613	2473	648	912	1554	1311
ENSECAG00000001985	2.680949474	0.0624791	0.2931575	84	62	105	123	127	146	130	159
ENSECAG00000014402	4.615920265	0.0625017	0.2931575	510	414	565	870	329	222	515	337
ENSECAG00000014796	5.622708505	0.0625079	0.2931575	635	819	682	967	981	1140	974	1068
ENSECAG00000023082	3.161638856	0.0625349	0.293163	110	79	173	129	329	118	290	94
ENSECAG00000014653	7.328027044	0.06258	0.2931896	3984	2849	3810	4579	2415	1677	3153	2039
ENSECAG00000007375	0.696357838	0.0625922	0.2931896	28	23	15	15	39	24	48	33
ENSECAG00000015505	7.122077157	0.0627742	0.2938248	2580	2194	1550	2256	3114	2935	3079	2862
ENSECAG00000013964	4.277093583	0.0627795	0.2938248	509	303	507	530	214	179	381	330
ENSECAG00000022436	6.18434132	0.0629098	0.2939595	1535	1477	1932	1801	867	740	1371	1314
ENSECAG00000009183	7.649217512	0.0629184	0.2939595	4610	4395	4801	5011	2844	2151	4001	2640
ENSECAG00000021098	5.699872588	0.0629196	0.2939595	1042	1147	1422	1412	523	253	976	1175
ENSECAG00000017148	5.574538405	0.0629245	0.2939595	932	705	758	511	914	1130	1227	831
ENSECAG00000008617	1.342944168	0.0629416	0.2939595	33	39	39	13	36	50	44	102
ENSECAG00000005469	4.927505951	0.0629636	0.2939595	589	352	459	492	753	575	740	549
ENSECAG00000013810	2.047950675	0.063079	0.2943775	71	175	126	106	18	96	3	0
ENSECAG00000009294	2.269505093	0.0632128	0.2948627	77	56	61	72	179	71	90	90
ENSECAG00000019121	5.504343678	0.0632497	0.2948627	968	961	1125	1200	403	521	727	970
ENSECAG00000006122	3.71110027	0.0632838	0.2948627	201	236	368	545	45	148	184	281
ENSECAG00000010888	3.307657819	0.0632868	0.2948627	71	120	148	231	239	174	349	145
ENSECAG00000014435	8.736078056	0.06338	0.2951757	8770	10922	10416	10612	3494	4541	6864	8950
ENSECAG00000016131	5.189806557	0.0634154	0.2952194	344	911	281	478	521	590	868	1382
ENSECAG00000010537	0.682931904	0.0634466	0.2952438	18	19	28	7	22	45	14	64
ENSECAG00000014135	5.049408417	0.0634878	0.2953142	543	201	489	561	1710	356	775	265
ENSECAG00000001215	2.452638974	0.0636218	0.2958166	146	64	132	205	47	57	96	83
ENSECAG00000012755	5.030398568	0.0636691	0.2959154	345.001	649	517.001	331.001	507.001	595.001	508.001	1324
ENSECAG00000007320	2.783186995	0.0637301	0.2960779	78	112	62	131	87	181	133	215
ENSECAG00000020880	2.304240092	0.0637672	0.2961292	118	82	136	142	48	52	77	83
ENSECAG00000020532	6.863410008	0.0639427	0.2968226	2791	2301	2797	3032	1672	1866	1540	1511
ENSECAG00000022554	5.398482941	0.0642102	0.2978613	499	726	589	789	900	1059	684	929
ENSECAG00000017938	6.368279249	0.0642189	0.2978613	1854	2334	2319	1119	942	1007	1423	1325
ENSECAG00000021388	3.070432275	0.0642503	0.2978781	60	159	117	124	115	245	165	227
ENSECAG00000011900	4.043277937	0.0642833	0.2978781	313	210	254	199	265	239	522	455
ENSECAG00000023658	5.244736737	0.0643222	0.2978781	815	882	925	869	455	496	597	656
ENSECAG00000007419	4.175548152	0.0643274	0.2978781	368	443	568	319	202	180	300	326
ENSECAG00000014326	4.347923365	0.0643752	0.2979779	526	467	490	439	218	225	241	444
ENSECAG00000003343	2.170868271	0.0644665	0.298279	7	101	9	20	8	337	12	102
ENSECAG00000009573	11.72424195	0.0646051	0.2987986	55615	39512	52806	60336	74312	75665	63678	78292
ENSECAG00000020656	5.556708573	0.0648192	0.2992828	946	628	672	703	988	935	1346	776
ENSECAG00000017336	5.704512094	0.0648387	0.2992828	790	807	791	852	847	1491	982	1075
ENSECAG00000017226	2.535329112	0.0648673	0.2992828	75	104	77	51	67	90	195	190
ENSECAG00000027594	0.851617184	0.0648948	0.2992828	89	34	10	48	17	26	22	11
ENSECAG00000013745	4.31539899	0.0649069	0.2992828	281	234	274	198	96	901	156	684
ENSECAG00000016880	2.427749039	0.0649099	0.2992828	49	37	76	128	93	54	270	107
ENSECAG00000018569	6.249498947	0.0649174	0.2992828	1621	1631	1704	2058	1194	953	1332	1006
ENSECAG00000025208	1.286635027	0.0649206	0.2992828	43	31	23	22	30	92	27	68
ENSECAG00000011353	3.536006159	0.0650496	0.2997557	92	238	129	146	117	399	146	390
ENSECAG00000017135	5.746770482	0.0653024	0.3006838	828	670	790	1048	1700	1021	1206	687
ENSECAG00000011966	7.183405332	0.0653039	0.3006838	2395	1560	1929	3259	2532	2709	3613	3806
ENSECAG00000016351	1.631790728	0.0653708	0.3007149	27	53	35	36	22	47	72	153
ENSECAG00000000308	6.36122725	0.0653775	0.3007149	1247	1442	1191	1297	1548	1841	1951	1621
ENSECAG00000018789	6.386063997	0.0654337	0.3007149	1964	1467	2418	2040	712	930	1399	1777
ENSECAG00000010559	3.987784778	0.0654634	0.3007149	319	327	320	589	118	142	353	267
ENSECAG00000005915	2.991149039	0.0654688	0.3007149	154	157	237	220	75.0004	115	94	135
ENSECAG00000011093	5.991517247	0.0654695	0.3007149	1267	1035	927	583	1714	1096	1672	1081
ENSECAG00000011809	6.538647134	0.0655973	0.3011801	1736	1160	1475	1403	2776	1805	1898	1455
ENSECAG00000005295	1.257622034	0.0656334	0.3012242	21	47	26	21	29	91	32	61
ENSECAG00000022770	6.420908782	0.0656741	0.301289	1250	1274	1474	1456	1792	1761	2166	1546
ENSECAG00000024000	7.416551055	0.0658831	0.3020063	3852	4141	5230	3114	1222	1876	2347	3926
ENSECAG00000015218	0.577671794	0.0659023	0.3020063	21	50	24	47	13	18	27	10
ENSECAG00000016625	3.5137072	0.0659313	0.3020063	0	74	106	184	198	2	541	634
ENSECAG00000019381	4.516210603	0.0659368	0.3020063	280	347	354	425	394	600	561	409
ENSECAG00000003563	2.832001353	0.0660328	0.3023242	101	92	107	111	156	111	263	114
ENSECAG00000006476	6.728645717	0.0660825	0.3024297	2380	1889	2346	3423	1218	1065	2135	1797
ENSECAG00000014868	6.510386912	0.0661364	0.3025546	1034	1603	1213	1296	885	3587	1207	2291
ENSECAG00000020037	3.550422591	0.0662439	0.3029245	357	169	344	284	123	61	273	178
ENSECAG00000000436	8.725235612	0.0663404	0.3032438	18	6218	45	545	36	15411	20	37094
ENSECAG00000015241	1.519676967	0.0664001	0.3033944	54	24	36	40	72	53	98	36
ENSECAG00000026842	1.058158913	0.0664402	0.3034557	6	51	10	15	23	35	18	129
ENSECAG00000010024	2.120865313	0.0666905	0.3044086	39	86	67	16	133	128	57	80
ENSECAG00000012023	6.232294752	0.0667024	0.3044086	1295	1081	1173	1084	1731	2033	1563	1045
ENSECAG00000021614	4.09127938	0.0668582	0.3049967	431	278	435	494	258	243	305	132
ENSECAG00000022106	5.557455575	0.0669291	0.3051976	848	706	671	771	934	969	1130	946

ENSECAG00000014573	0.883967894	0.067001	0.3054019	23	24	14	35	57	23	49	35
ENSECAG00000000516	3.735506059	0.0670276	0.3054019	187	134	255	214	349	263	393	175
ENSECAG00000009021	3.635461286	0.0670926	0.3055752	132	176	131	291	203	355	359	185
ENSECAG00000004860	3.290498738	0.0671527	0.3057263	85	192	102	83	69	402	67	375
ENSECAG00000005510	1.984967888	0.0672202	0.3059111	73	72	22	15	52	131	50	137
ENSECAG00000015330	3.01470232	0.0673364	0.3063171	208	141	227	201	111	60	151	119
ENSECAG00000021817	4.989280143	0.0673663	0.3063304	468	346	564	633	716	587	786	634
ENSECAG00000023934	4.722729267	0.0675492	0.3070395	549	245	535	176	513	734	592	502
ENSECAG00000000325	9.447090637	0.0676196	0.3072362	4573	6446	5421	23502	13437	10391	13528	28249
ENSECAG00000012086	3.043379837	0.0677205	0.3075718	102	169	64	119	202	208	218	111
ENSECAG00000010333	6.640295106	0.0677624	0.3076393	1218	1651	1526	1290	1248	4014	1247	2117
ENSECAG00000024346	4.664680133	0.067805	0.307648	518	592	552	724	294	221	475	474
ENSECAG00000010343	7.331991106	0.067843	0.307648	1680	3103	2107	2674	2275	4648	3092	3987
ENSECAG00000006230	2.37084632	0.0678456	0.307648	58	67	72	98	162	78	120	99
ENSECAG00000012750	4.691014021	0.0679143	0.3078368	322	455	401	317	346	848	405	636
ENSECAG00000011896	4.558040357	0.0680747	0.3084404	433	306	382	336	529	453	599	445
ENSECAG00000017400	5.526951624	0.0681809	0.3087985	1171	1224	1122	705	562	588	893	625
ENSECAG00000015565	1.673718756	0.0682741	0.3090974	44	40	48	46	77	72	60	67
ENSECAG00000024788	10.11529184	0.0683502	0.3092561	25014	20241	27743	32417	15180	14687	20013	13447
ENSECAG00000021648	4.396523344	0.0683889	0.3092561	361	252	354	278	719	423	424	268
ENSECAG00000000669	7.974512902	0.0684448	0.3092561	3642	4061	3568	3900	4438	7316	4180	5607
ENSECAG00000015398	4.45623936	0.0684949	0.3092561	528	359	563	655	298	162	511	287
ENSECAG00000008293	5.770748949	0.068504	0.3092561	930	255	1261	84	2469	604	2018	455
ENSECAG00000012006	6.821860705	0.0685063	0.3092561	2315	2153	2356	4141	1725	1074	2661	998
ENSECAG00000005486	5.427346307	0.0685204	0.3092561	567	705	696	709	651	1067	861	1074
ENSECAG00000014827	2.938542187	0.0685269	0.3092561	120	86	132	114	172	165	171	159
ENSECAG00000012880	4.964961077	0.0687752	0.3101639	429	475	487	486	735	947	467	500
ENSECAG00000013713	4.936225151	0.0687827	0.3101639	470	465	387	585	590	431	986	683
ENSECAG00000020691	4.519814381	0.0688883	0.3104939	273	271	301	569	308	582	621	493
ENSECAG00000020051	7.269436889	0.0689106	0.3104939	2173	2318	3091	1874	3444	2892	3739	3288
ENSECAG00000022677	4.107441213	0.0689711	0.3106436	268	296	246	201	234	394	353	528
ENSECAG00000011688	5.332935396	0.0690444	0.3107913	516	518.993	561.009	724.004	554.001	1769.99	543.008	605.004
ENSECAG00000025132	0.788868786	0.0690755	0.3107913	23	27	13	27	39	26	50	37
ENSECAG00000014757	4.968512724	0.0691015	0.3107913	681	721	628	883	512	434	447	398
ENSECAG00000014462	1.087868971	0.0691134	0.3107913	15.9982	47	15	21	28.9998	91	37	32.999
ENSECAG00000014882	6.83830435	0.069173	0.3109364	1702	3489	3438	2421	678	1618	1255	2507
ENSECAG00000014215	6.432468482	0.069216	0.3110066	2109	1591	2303	2050	1544	787	1710	1015
ENSECAG00000011193	0.442073804	0.0694218	0.3114071	14	20	12	21	21	14	48	39
ENSECAG00000010766	6.415648937	0.0694288	0.3114071	1490	1825	1845	2919	814	982	1477	1683
ENSECAG00000016565	4.440680985	0.0694333	0.3114071	524	369	490	672	320	242	430	257
ENSECAG00000011939	5.837423652	0.069435	0.3114071	810	867	1118	575	756	1814	957	1396
ENSECAG00000018172	3.327267708	0.0694428	0.3114071	189	180	290	303	121	108	155	169
ENSECAG00000012611	6.439130123	0.0694696	0.3114071	1906	1554	2518	2063	1329	1035	1711	1032
ENSECAG00000018367	3.127483449	0.0695582	0.3116812	213	123	239	278	111	67	171	128
ENSECAG00000021066	5.841032348	0.0696141	0.3116944	1307	961	1153	2026	563	650	1263	888
ENSECAG00000026873	9.569097845	0.0696161	0.3116944	13958	10727	11714	9763	15233	18302	17804	14383
ENSECAG00000011518	5.667004875	0.0696828	0.3118195	1002	688	800	708	944	1163	1216	981
ENSECAG00000006602	3.839392997	0.0696989	0.3118195	331	262	453	335	286	128	249	112
ENSECAG00000022892	1.227848648	0.0698309	0.3121663	14	32	38	26	16	24	58	127
ENSECAG00000013735	4.062414254	0.0698314	0.3121663	368	302	398	541	149	147	377	276
ENSECAG00000011438	5.089147622	0.0699591	0.3126142	577	287	598	653	673	810	965	505
ENSECAG00000018848	4.854804856	0.0700458	0.3128551	405	480	491	408	614	449	757	689
ENSECAG00000004219	6.397915569	0.0700681	0.3128551	1502	1001	1702	994	2218	1459	2319	1322
ENSECAG00000020459	6.054408225	0.0701698	0.313186	1391	1193	1717	1867	1073	719	1291	856
ENSECAG00000016568	6.948007137	0.0703307	0.3137333	2775	2855	2439	3430	1527	1509	1882	2249
ENSECAG00000017769	5.905587392	0.0703476	0.3137333	1322	1031	1616	1568	875	728	964	971
ENSECAG00000012411	5.851112593	0.0703826	0.3137659	616.001	1130	696.001	702.001	535.001	2441	662.001	1450
ENSECAG00000019885	6.891952309	0.0705198	0.3142545	1649	2112	1441	2059	2285	3037	1924	2881
ENSECAG00000002988	8.542637855	0.0705949	0.3144655	5202	5892	5129	6008	5422	12198	6622	7894
ENSECAG00000006350	2.799155706	0.0706469	0.314574	169	155	214	155	24	61	97	167
ENSECAG00000013540	6.161300765	0.0707741	0.314958	1008	1294	1003	1213	1739	1438	1453	1399
ENSECAG00000021443	6.503736595	0.0707886	0.314958	2225	1543	2176	2565	1015	902	1520	1887
ENSECAG00000010243	5.412921424	0.0708561	0.3151204	920	831	1025	1195	555	400	692	852
ENSECAG00000022168	6.303175441	0.0708806	0.3151204	1485	1780	1694	2499	685	767	1689	1485
ENSECAG00000000576	2.217539949	0.070909	0.3151236	63	70	61	69	93	127	109	76
ENSECAG00000016312	2.866028884	0.0710846	0.3155936	280	71	173	208	105	61	153	56
ENSECAG00000004727	3.611524991	0.071093	0.3155936	267	52	158	54	885	69	207	70
ENSECAG00000009758	4.901876611	0.0711054	0.3155936	421	323	613	313	1238	390	857	259
ENSECAG00000024957	4.79357527	0.0711259	0.3155936	350	545	366	385	606	777	445	541
ENSECAG00000007601	4.352813158	0.0711979	0.3157894	289	280	332	343	380	599	439	325
ENSECAG00000023113	2.529153723	0.0714259	0.3165919	161	102	126	175	88	32	107	79
ENSECAG00000011954	7.217109239	0.071437	0.3165919	1955	2467	1973	2350	1915	4869	2070	4014
ENSECAG00000002700	2.923382479	0.0714859	0.3165919	140	65	145	50	284	121	216	83
ENSECAG00000015336	8.333884115	0.0714933	0.3165919	5016	4911	4737	5044	9566	6311	6534	5405
ENSECAG00000011679	8.679901728	0.0715182	0.3165919	5625	5501	6253	5675	4766	16895	4639	9585
ENSECAG00000022695	3.423765628	0.0716063	0.3168584	69	273	55	120.001	123.001	350.001	108	424
ENSECAG00000006072	6.218535446	0.0717734	0.3174742	1044	1212	1144	1316	1525	1806	1613	1315
ENSECAG00000001496	5.560710301	0.0719477	0.317901	827	728	782	624	872	1103	1038	968

ENSECAG00000016223	6.126257863	0.0719568	0.317901	1630	1320	1562	1947	785	898	1161	1287
ENSECAG00000008472	3.498336807	0.0719787	0.317901	100	207	129	172	86	374	182	379
ENSECAG00000021433	4.524399894	0.0719818	0.317901	317	318	390	400	548	556	440	405
ENSECAG00000018893	3.642800036	0.0720481	0.3180498	284	224	284	402	125	182	231	158
ENSECAG00000003616	7.199380917	0.0720715	0.3180498	3828	2441	3958	3664	2528	2020	2335	1434
ENSECAG00000021672	10.09502084	0.0721969	0.3184796	10087	26440	9504	12984	14361	30235	15195	39421
ENSECAG00000022455	6.08634137	0.0722388	0.3184974	1504	1258	1554	1946	1047	827	1143	1021
ENSECAG00000020932	6.14601902	0.072257	0.3184974	1068	1011	939	971	600	3069	916	1587
ENSECAG00000026860	5.660629103	0.072346	0.3187069	1126	945	1229	1439	763	382	1095	752
ENSECAG00000017894	2.556832011	0.0723607	0.3187069	144	110	128	191	49	88	117	56
ENSECAG00000017593	5.02569895	0.0724451	0.318936	742	646	734	897	426	467	481	515
ENSECAG00000021876	5.007084449	0.0724689	0.318936	241	674	284	409	221	1676	317	698
ENSECAG00000010715	4.424718469	0.0725244	0.3190566	370	288	353	315	543	313	543	461
ENSECAG00000018281	2.105827931	0.0726033	0.3192597	54.0016	51.0021	79.0018	51.0024	73.0019	144.002	67.0022	91.0022
ENSECAG00000015652	6.293615351	0.0726267	0.3192597	1702	1767	1207	2750	1288	1204	1152	798
ENSECAG00000006436	5.4017721	0.0727062	0.3194853	934	519	712	326	825	844	1459	652
ENSECAG00000019602	4.113273308	0.0728419	0.3197672	182	273	167	410	435	293	593	235
ENSECAG00000022139	1.966922814	0.072865	0.3197672	21	95	38	39	49	103	75	133
ENSECAG00000011304	6.909909737	0.0728745	0.3197672	1952	936	2138	2353	2502	2891	1654	3319
ENSECAG00000021014	6.55903558	0.0729215	0.3197672	2301	3078	2305	1233	519	1157	1469	1984
ENSECAG00000017234	7.36686815	0.0729689	0.3197672	3619	4251	3923	3751	1454	1510	2968	3519
ENSECAG00000015587	6.653944938	0.0730064	0.3197672	1768	1438	1709	1427	2288	2245	2022	1924
ENSECAG00000012264	0.998863291	0.0730113	0.3197672	16	29	34	25	24	40	50	63
ENSECAG00000013232	4.531363008	0.0730272	0.3197672	332	362	348	405	438	452	516	566
ENSECAG00000006116	2.610887892	0.073081	0.3197672	73	79	118	81	148	145	122	116
ENSECAG00000019499	4.639285801	0.0731173	0.3197672	386	305	418	292	316	1059	313	473
ENSECAG00000019430	4.865755667	0.0731192	0.3197672	551	560	718	1010	255	197	758	455
ENSECAG00000024710	1.325777435	0.0731319	0.3197672	21	34	38	34	18	36	80	100
ENSECAG00000019280	6.06693511	0.0731363	0.3197672	1183	815	1160	1007	2067	1320	1368	956
ENSECAG00000019979	7.339166214	0.0732449	0.3199667	3936	3200	3275	4804	2547	1956	2944	1941
ENSECAG00000022218	1.993236599	0.0732734	0.3199667	57	52	32	65	23	86	96	166
ENSECAG00000025174	1.853174392	0.0732756	0.3199667	68	29	40	49	163	49	90	31
ENSECAG00000024704	4.855645724	0.0733096	0.3199667	684	477	745	881	315	186	614	544
ENSECAG00000024438	6.570787636	0.0733228	0.3199667	2000	1007	1373	1532	2151	1220	3358	1704
ENSECAG00000023784	5.958430486	0.0734981	0.3206085	1328	1404	1504	1447	717	762	1200	1027
ENSECAG00000010751	4.115550577	0.0735686	0.3207929	103	365	79	372	284	266	303	783
ENSECAG00000000732	2.24916247	0.0737249	0.3212659	2	96	42	77	111	115	163	80
ENSECAG00000020753	3.23346366	0.0737395	0.3212659	125	294	171	341	141	64	214	76
ENSECAG00000006004	4.570473413	0.0737966	0.3212659	363	375	360	370	564	542	473	434
ENSECAG00000011854	3.093393848	0.0738012	0.3212659	104	77	178	125	252	227	171	102
ENSECAG00000009292	4.515942409	0.0738363	0.3212659	422	287	331	397	494	411	595	463
ENSECAG00000014140	3.348625377	0.0738663	0.3212659	190	214	268	307	81	106	186	189
ENSECAG00000016757	4.439328774	0.073875	0.3212659	540	492	564	418	315	349	308	241
ENSECAG00000012847	4.599613612	0.0740041	0.3217041	510	405	747	644	337	173	463	416
ENSECAG00000022772	1.599485455	0.0741303	0.3221294	74	15	30	42	82	60	52	77
ENSECAG00000016071	5.888142059	0.0743535	0.3228827	958	783	689	1086	676	2353	1019	1030
ENSECAG00000014479	3.243867963	0.0743867	0.3228827	194	166	93	40	226	207	148	283
ENSECAG00000011509	5.885962404	0.0744002	0.3228827	1441	1098	1304	1619	823	602	1194	918
ENSECAG00000013760	4.434380517	0.0744198	0.3228827	447	353	532	746	290	168	342	428
ENSECAG00000024760	6.186995503	0.0744684	0.3228827	1267	737	1258	1082	2777	961	1988	766
ENSECAG00000002408	5.214261477	0.0744772	0.3228827	673	480	517	660	1077	620	883	598
ENSECAG00000020774	3.65701957	0.0745027	0.3228827	351	164	249	471	151	113	251	185
ENSECAG00000010152	2.917074678	0.0746103	0.3232257	210	100	249	172	97	64	114	129
ENSECAG00000011001	1.158567256	0.0748752	0.3242498	35	44	60	75	32	16	39	25
ENSECAG00000017338	4.29867443	0.0749463	0.3243503	245	326	214	264	141	862	215	535
ENSECAG00000011648	6.832604757	0.0749555	0.3243503	1964	1589	1639	1977	3035	2124	2565	1999
ENSECAG00000011037	9.754673545	0.0750501	0.324636	22859	16445	18486	25451	16348	9974	15153	6591
ENSECAG00000013206	5.667533302	0.0751223	0.3246929	1122	1029	1095	1419	689	598	967	789
ENSECAG00000008007	4.581177503	0.075143	0.3246929	388	326	445	313	594	481	454	510
ENSECAG00000014537	5.700684729	0.075149	0.3246929	804	688	908	871	1409	643	1570	902
ENSECAG00000024105	7.811841896	0.0752156	0.3247996	5455	6347	5424	4154	1567	2248	3146	5425
ENSECAG00000009099	6.772235756	0.0752309	0.3247996	3075	1597	2504	3168	1623	1092	2294	1376
ENSECAG00000016056	0.850418516	0.0753139	0.3249617	13	17	31	35	44	33	51	30
ENSECAG00000009884	1.471722647	0.0753257	0.3249617	35	31	47	38	36	60	54	94
ENSECAG00000023331	5.156398939	0.0754694	0.3254583	875	673	728	1069	424	319	743	616
ENSECAG00000017542	2.776094637	0.0755011	0.3254712	72	331	156	146	19	35	49	200
ENSECAG00000000064	5.239345509	0.0756117	0.325794	594	533	456	596	467	1609	495	672
ENSECAG00000005400	3.207855575	0.0756333	0.325794	243	400	88	207	85	42	45	254
ENSECAG00000020892	4.961869781	0.0757014	0.3258981	444	625	783	1318	269	108	799	518
ENSECAG00000007446	5.769854827	0.0757318	0.3258981	1341	1074	1303	1306	833	491	1143	792
ENSECAG00000019719	9.946824435	0.0757436	0.3258981	16729	12110	16627	15526	22445	22651	17794	21501
ENSECAG00000024780	5.342131483	0.0758811	0.3263665	865	844	944	1152	621	346	970	449
ENSECAG00000006619	3.440730899	0.0759386	0.3263939	123	141	173	210	194	324	237	182
ENSECAG00000009119	6.437111978	0.075945	0.3263939	1288	1705	1045	1086	1050	2785	1568	2046
ENSECAG00000002378	1.752768893	0.0762417	0.3275452	77	51	87	108	41	38	52	45
ENSECAG00000007896	5.600166722	0.0762706	0.3275453	995	952	1041	1491	666	591	833	794
ENSECAG00000021291	6.18840306	0.0763251	0.3276556	1790	1446	1853	1715	1273	522	1547	944
ENSECAG00000017393	7.232205307	0.0764666	0.3280343	3984	2338	4367	3645	2522	1068	3056	1987

ENSECAG00000017256	9.312812785	0.0765203	0.3280343	9382	8209	11359	9536	14739	18007	9654	11898
ENSECAG00000017346	5.921421863	0.0765243	0.3280343	985	859	1014	780	2200	644	1619	901
ENSECAG00000014669	4.115421884	0.0765288	0.3280343	435	301	463	429	178	233	256	314
ENSECAG00000004928	1.816392936	0.076617	0.3282885	55	61	117	113	36	19	69	57
ENSECAG00000016105	4.585032684	0.0767355	0.3284566	467	328	313	380	490	570	558	426
ENSECAG00000010909	3.955880124	0.0767539	0.3284566	319	198	266	92	352	291	284	457
ENSECAG00000015005	7.736563507	0.0767737	0.3284566	2850	3528	3119	2714	3033	8176	2501	4711
ENSECAG00000021093	1.737643247	0.0767879	0.3284566	34	35	36	41	13	230	34	29
ENSECAG00000000197	2.263625118	0.0768008	0.3284566	59	186	118	135	14	14	47	140
ENSECAG00000023959	3.603830647	0.0768761	0.3285418	292	239	235	397	201	94	209	177
ENSECAG00000018313	2.928240235	0.0768899	0.3285418	180	129	241	173	93	90	133	100
ENSECAG00000019619	5.931642963	0.0769174	0.3285418	809	923	814	952	655	2714	810	1074
ENSECAG00000012872	2.809323264	0.0769365	0.3285418	119	59	81	153	177	105	223	125
ENSECAG00000013410	1.493303712	0.0769688	0.3285564	35	41	33	44	34	69	56	88
ENSECAG00000007532	6.254339734	0.0770546	0.3287992	1810	1390	1971	1916	1205	633	1574	1125
ENSECAG00000000291	8.966358512	0.0771101	0.3289009	11357	11569	15095	8920	4820	5790	8922	8912
ENSECAG00000007359	7.979497262	0.0771966	0.3289009	4890	2839	4297	3398	4825	7001	5146	4647
ENSECAG00000002021	4.096578566	0.0772229	0.3289009	396	275	446	507	208	187	384	204
ENSECAG00000013077	5.632436323	0.0772269	0.3289009	1055	1166	1499	887	464	471	863	1068
ENSECAG00000000449	3.514386663	0.0772441	0.3289009	192	288	265	362	82	122	148	263
ENSECAG00000017525	1.859694354	0.0772525	0.3289009	60	27	71	34	101	68	128	36
ENSECAG00000008227	3.503328126	0.0772915	0.3289009	157	151	169	194	197	369	222	185
ENSECAG00000015703	5.520102206	0.0773101	0.3289009	970	859	1104	1332	510	493	770	939
ENSECAG00000021791	3.886787288	0.0773851	0.3290966	326	253	358	494	201	108	356	172
ENSECAG00000023475	3.981626245	0.0774558	0.3291643	96	362	195	68	111	740	127	503
ENSECAG00000013895	4.991800478	0.0774589	0.3291643	447	445	525	558	1020	518	635	549
ENSECAG00000017077	8.047545157	0.0774973	0.3292042	3588	4203	4421	3819	4423	8049	5141	4996
ENSECAG00000018050	5.502154504	0.0775428	0.3292742	908	648	688	588	1200	864	1033	751
ENSECAG00000010769	6.345295772	0.0776029	0.3293717	1596	1092	1170	1317	2160	1487	1689	1519
ENSECAG00000011661	7.154755904	0.0776237	0.3293717	3601	2419	3186	4162	2234	1818	2493	1741
ENSECAG00000019200	5.308046596	0.0776699	0.3294446	1098	706	931	923	670	521	606	504
ENSECAG00000014514	8.257309087	0.0778516	0.3300169	5100	3914	4491	5751	6938	6622	6176	6322
ENSECAG00000017908	4.386832287	0.077863	0.3300169	458	511	429	549	204	311	322	354
ENSECAG00000020597	6.838907582	0.0780001	0.3304409	2944	1923	2794	3006	1741	1286	1951	1732
ENSECAG00000008340	4.257491735	0.0780224	0.3304409	245	385	182	290	286	508	259	617
ENSECAG00000006339	5.342431074	0.0780503	0.3304409	943	645	988	1243	725	356	750	541
ENSECAG00000021487	2.708787594	0.078213	0.3309203	163	120	159	180	66	70	109	113
ENSECAG00000024954	2.895112377	0.0782459	0.3309203	119	139	158	317	76	72	136	117
ENSECAG00000024798	3.906904194	0.0782509	0.3309203	236	260	214	156	215	201	433	508
ENSECAG00000002522	0.893861403	0.0783655	0.3311358	26	22	24	29	33	37	38	51
ENSECAG00000018342	7.069338683	0.0783704	0.3311358	2397	1671	1921	2487	2907	3166	2443	2827
ENSECAG00000014375	2.585386307	0.0783893	0.3311358	75	68	86	115	98	74	146	225
ENSECAG00000009229	3.531596833	0.0785798	0.3317538	315	255	274	251	153	74	220	200
ENSECAG00000012377	1.024359335	0.078594	0.3317538	25	36	22	19	31	62	24	60
ENSECAG00000006485	3.064294662	0.0786721	0.3319602	81	171	40	89	34	264	68	442
ENSECAG00000002472	3.73059003	0.0787113	0.3320021	32	322	110	194	120	491	309	328
ENSECAG00000010009	5.222598788	0.0787965	0.3322382	783	653	1030	1033	512	311	832	551
ENSECAG00000024834	6.637505116	0.078829	0.3322518	1594	1619	1451	1586	1522	2533	1992	2352
ENSECAG00000007343	3.229005915	0.0788819	0.3323516	107	124	159	153	166	313	144	184
ENSECAG00000017850	5.058201251	0.0789479	0.3323735	567	558	335	609	562	678	694	922
ENSECAG00000019690	0.270611981	0.0789497	0.3323735	18	17	12	12	40	23	19	20
ENSECAG00000017370	5.984012638	0.0789749	0.3323735	775	1388	770	843	1061	1226	1028	2181
ENSECAG00000012670	8.506615615	0.0790649	0.3326288	4656	6704	4844	5843	7596	8492	5676	9450
ENSECAG00000000227	6.745843381	0.0791999	0.3330734	2138	2707	2316	2679	1364	1440	1981	1549
ENSECAG00000007683	5.604007971	0.079389	0.333641	1003	1031	1175	1286	663	415	870	940
ENSECAG00000018457	3.228808446	0.0793936	0.333641	214	191	271	231	53	104	144	201
ENSECAG00000003495	2.806091267	0.0795003	0.3339661	68	177	46	68	136	244	114	133
ENSECAG00000011856	1.230810529	0.0796165	0.3342763	33	28	28	41	47	55	63	38
ENSECAG00000011096	2.093665832	0.079633	0.3342763	107	55	123	129	49	39	84	54
ENSECAG00000009296	2.214044434	0.0797183	0.3344492	57	76	83	264	31	59	71	67
ENSECAG00000009959	6.816508949	0.0797601	0.3344492	2015	1173	2017	1663	3826	1484	2950	1663
ENSECAG00000014202	3.311129009	0.0798094	0.3344492	143	93	141	197.001	297	180	335	87
ENSECAG00000007816	5.305354309	0.0798143	0.3344492	645	865	370	451	1164	693	987	619
ENSECAG00000001468	4.361416619	0.0798214	0.3344492	520	390	517	519	193	166	376	441
ENSECAG00000021389	1.1467409	0.0799151	0.3347182	38	36	21	19	32	57	40	65
ENSECAG00000006302	4.912385803	0.0800029	0.3349624	351	624	426	414	624	752	516	672
ENSECAG00000024179	4.888379383	0.0801735	0.3355528	735	489	769	773	516	355	386	441
ENSECAG00000025168	5.34759091	0.0802391	0.335704	798.994	862	904.999	1182	616	545	711	533
ENSECAG00000019990	4.824512434	0.0805241	0.3367722	735.997	510.998	706	732.997	527	337	523.998	208
ENSECAG00000015745	6.552068412	0.0806732	0.3372714	2065	2363	2794	1463	1105	752	1624	1948
ENSECAG00000009773	5.204785513	0.0807637	0.3374691	595	1272	809	828	216	435	483	875
ENSECAG00000021493	6.215091918	0.0808041	0.3374691	1729	1286	1989	1822	1139	849	1360	1082
ENSECAG00000023546	5.359816841	0.0808647	0.3374691	540	850	571	557	875	899	722	984
ENSECAG00000014831	7.609925887	0.0808684	0.3374691	2259	2263	2845	3765	1385	7547	2650	5710
ENSECAG00000017900	6.632795897	0.080869	0.3374691	1210	1436	1440	2181	1967	2814	1875	1685
ENSECAG00000024290	6.501649369	0.0809131	0.3375291	982	1635	1323	1195	945	3407	954	2576
ENSECAG00000023800	5.300158527	0.0809511	0.3375638	783	843	910	1110	414	529	609	754
ENSECAG00000014136	3.542859848	0.081012	0.3376935	177	170	186	169	228	248	206	318

ENSECAG00000020491	4.772634869	0.0811328	0.3380732	303	396	350	630	488	298	910	761
ENSECAG00000008981	4.790682328	0.0812135	0.3382852	514	651	890	542	230	355	388	577
ENSECAG00000012966	5.108004298	0.0813887	0.338891	663	764	659	1141	386	566	523	506
ENSECAG00000006203	0.765192596	0.081471	0.3391093	26	21	20	23	52	25	35	34
ENSECAG00000014481	5.915064788	0.0815496	0.3393064	1244	1299	1329	1648	698	851	1081	969
ENSECAG00000014727	3.466228003	0.0815957	0.3393064	309	233	281	216	190	95	205	126
ENSECAG00000000384	5.995366381	0.081608	0.3393064	1437	1450	1430	1569	688	1019	738	1224
ENSECAG00000023134	2.249586491	0.0817455	0.3397539	57	47	61	97	152	60	185	44
ENSECAG00000019285	5.803072154	0.0819385	0.3404314	908	885	708	994	1626	710	1632	881
ENSECAG00000004262	5.153025033	0.0819818	0.3404867	453	542	546	675	569	1049	632	743
ENSECAG00000019351	2.220542209	0.0820218	0.3405284	110	77	118	140	66	56	76	51
ENSECAG00000003774	9.549470895	0.0821457	0.3405794	11447	8192	19727	37710	7373	10489	16197	6017
ENSECAG00000015743	7.572236729	0.0821584	0.3405794	3414	2490	2645	2957	7006	2975	3998	2619
ENSECAG00000006513	5.260816677	0.0821626	0.3405794	481	279	672	819	845	379	1969	451
ENSECAG00000020925	5.188694758	0.0821815	0.3405794	677	850	891	921	547	509	555	523
ENSECAG00000014185	3.897536538	0.082184	0.3405794	364	232	374	448	177	164	330	181
ENSECAG00000012348	0.635526811	0.082257	0.3407576	20	24	13	14	46	10	80	12
ENSECAG00000018469	1.970288234	0.0824836	0.3415718	65	48	53	60	87	83	103	67
ENSECAG00000009744	6.355589832	0.0826194	0.3418105	974.001	1700	1283	857.001	1395	2297	1055	2273
ENSECAG00000022211	4.730678305	0.0826267	0.3418105	629	493	845	516	384	462	322	303
ENSECAG00000006424	3.480699741	0.0826315	0.3418105	178	207	118	154	284	252	242	187
ENSECAG00000021731	3.673119295	0.0827607	0.3421823	324	219	332	322	175	137	240	176
ENSECAG00000020865	5.941594718	0.0827817	0.3421823	1058	802	869	1181	1204	699	1915	1552
ENSECAG00000024020	6.736373629	0.082847	0.3423277	2209	1094	1752	1692	1721	2086	2693	2662
ENSECAG00000000817	6.477821498	0.0829958	0.3426929	2258	1425	2199	2433	1438	774	1910	1182
ENSECAG00000008963	6.689588698	0.0830161	0.3426929	2380	1758	2409	3048	1459	1372	1996	1282
ENSECAG00000012515	5.017551239	0.0830259	0.3426929	724	578	803	887	487	418	531	467
ENSECAG00000000470	6.186877218	0.083116	0.3429403	1624	1599	1061	3146	431	508	579	2265
ENSECAG00000006588	5.712768365	0.0831586	0.3429915	1329	983	1222	1266	645	560	997	948
ENSECAG00000014732	2.509136525	0.0832094	0.3430462	70	118	68	51	104	68	162	189
ENSECAG00000019890	4.932292815	0.083242	0.3430462	771	460	811	843	459	345	682	282
ENSECAG00000011503	5.105933252	0.0832625	0.3430462	779	575	906	928	543	404	604	478
ENSECAG00000012542	5.401928067	0.0834741	0.3437935	884	449	695	512	1347	467	1353	612
ENSECAG00000009897	4.118953617	0.0835839	0.3441209	433	350	504	326	280	171	320	224
ENSECAG00000009953	4.838873554	0.0836301	0.3441841	668	621	719	674	196	390	514	535
ENSECAG00000020630	5.49633924	0.0836599	0.3441841	1015	918	1019	1257	473	364	1018	835
ENSECAG00000012780	6.231247066	0.083744	0.3444054	2048	1191	1733	1980	1164	766	1462	1090
ENSECAG00000024022	2.946501781	0.0838373	0.3446644	178	172	212	173	104	112	146	50
ENSECAG00000026034	0.947811584	0.0839741	0.3451019	28	40	5	16	29	78	31	34
ENSECAG00000022422	5.429812706	0.084034	0.345223	938	827	944	1268	493	519	822	741
ENSECAG00000023703	3.400869434	0.0841666	0.3456429	117	166	83	236	238	141	475	134
ENSECAG00000020289	7.648676778	0.0842754	0.3459353	4615	4386	4417	5488	1912	1781	3972	4047
ENSECAG00000021599	0.760465143	0.084306	0.3459353	31	41	42	45	7	20	27	28
ENSECAG00000007914	6.070541205	0.0843387	0.3459353	1265	1323	1345	2313	972	843	1054	1106
ENSECAG00000023493	5.772892966	0.0843596	0.3459353	801	815	773	1131	1452	962	1318	884
ENSECAG00000017372	6.329033754	0.0843929	0.345947	1584	1128	1248	1100	1709	2034	1477	1499
ENSECAG00000017048	3.562266487	0.0846106	0.3465858	137	185	108	242	98	347	221	387
ENSECAG00000019856	1.819279522	0.08463	0.3465858	105	63	106	68	16	31	71	62
ENSECAG00000007623	4.158023237	0.0846505	0.3465858	338	131	228	311	696	146	645	186
ENSECAG00000008906	5.8632907	0.0847024	0.3465858	1327	1335	1266	1527	826	355	1472	791
ENSECAG00000021907	6.708970748	0.0847292	0.3465858	1872	1217	1728	1821	2666	1982	2781	1514
ENSECAG00000014306	5.663225643	0.0847318	0.3465858	1185	924	1195	1458	986	371	1038	574
ENSECAG00000012221	6.758986936	0.0847891	0.3466954	1726	1560	1754	1820	2783	2361	2227	1740
ENSECAG00000013377	5.850298224	0.0848334	0.3467517	876	1813	1582	1510	204	407	482	1840
ENSECAG00000011935	5.701567177	0.084867	0.3467642	862	744	753	991	787	1053	1121	1425
ENSECAG00000024311	7.558776636	0.0849421	0.3468988	4456	3286	5017	4795	2805	2125	3153	2962
ENSECAG00000024236	4.316206504	0.084961	0.3468988	397	362	357	806	205	205	332	389
ENSECAG00000013554	7.774298072	0.0850241	0.3470315	3178	3574	3535	3229	3794	5404	3796	5612
ENSECAG00000020522	4.275723807	0.0851447	0.347399	242	332	185	279	141	883	199	491
ENSECAG00000023391	0.157381499	0.0852253	0.3475386	19	8	10	18	12	32	30	21
ENSECAG00000006955	5.519994106	0.0852574	0.3475386	1091	860	1057	1292	938	319	811	610
ENSECAG00000015796	3.94476179	0.0852707	0.3475386	451	248	394	377	261	94	249	258
ENSECAG00000000023	5.442614606	0.0853259	0.3476389	743	596	741	621	1290	945	858	583
ENSECAG00000016898	6.899018007	0.0856753	0.348937	2714	2291	3534	2449	1669	1526	2008	1789
ENSECAG00000015175	6.904738537	0.0857201	0.3489946	2382	2523	2708	3583	1123	1872	2124	1849
ENSECAG00000018467	6.742278806	0.085809	0.3492314	1697	1804	1694	1562	2248	2462	2226	2052
ENSECAG00000024077	1.996872347	0.085937	0.3496268	45	90	36	29	172	55	61	75
ENSECAG00000017164	9.59510344	0.0863536	0.351043	12078	8976	13138	14113	20022	17796	15175	12920
ENSECAG00000000226	4.455627698	0.0863655	0.351043	284	337	273	487	524	467	445	422
ENSECAG00000011834	5.6064938	0.0863778	0.351043	367	1080	399	338	167	2310	196	1961
ENSECAG00000017792	5.529563743	0.0865021	0.3514226	1194	748	1226	1110	641	384	899	827
ENSECAG00000010047	6.314941528	0.0865439	0.3514667	1816	1549	1876	2001	1097	1053	1464	1178
ENSECAG00000022368	7.611219687	0.086829	0.3523773	3601	2690	2812	3171	4625	4498	4025	3400
ENSECAG00000007059	2.890685316	0.0868302	0.3523773	18	126	46	98	13	472	13	229
ENSECAG00000024238	7.124186382	0.0869825	0.3528695	2845	2057	1900	1788	3915	2497	3457	2160
ENSECAG00000019577	5.642917596	0.0871108	0.3530069	832	1245	1036	1556	416	683	787	1007
ENSECAG00000012655	5.191225511	0.0871148	0.3530069	488	590	533	290	303	1868	284	775
ENSECAG00000023250	3.454493356	0.0871396	0.3530069	254	177	246	387	190	162	155	82

ENSECAG00000013246	2.931031432	0.0871407	0.3530069	170	135	201	223	69	73	155	127
ENSECAG00000012080	6.608792596	0.0872388	0.3532784	2195	1748	2364	2721	1432	903	2106	1433
ENSECAG00000016116	6.703612471	0.0872732	0.3532918	1965	1037	1511	1383	4949	911	2767	954
ENSECAG00000019925	4.632667112	0.0874126	0.3535567	450	262	422	430	637	403	631	457
ENSECAG00000011704	1.10778306	0.0874404	0.3535567	20	161	10	29	3	14	12	50
ENSECAG00000024935	5.931961937	0.0875383	0.3535567	1130	895	929	825	909	1896	1158	1164
ENSECAG00000007287	0.666020219	0.087554	0.3535567	11	23	19	31	40	29	35	32
ENSECAG00000019270	4.715242427	0.0875561	0.3535567	392	399	406	355	353	978	398	504
ENSECAG00000026927	4.147160917	0.0875632	0.3535567	263	266	215	297	296	643	211	357
ENSECAG00000008175	1.532084262	0.0875671	0.3535567	27	48	38	50	63	56	76	56
ENSECAG00000008826	6.748917171	0.0875877	0.3535567	1540	1753	1202	2136	1443	2884	1714	3139
ENSECAG00000012064	0.377789676	0.0876813	0.3538088	15	14	21	17	22	36	30	21
ENSECAG00000021451	5.656646122	0.0879992	0.3549653	684	755	815	973	757	1289	1012	1161
ENSECAG00000017225	2.992065345	0.0880852	0.3551863	173	189	184	201	105	90	167	82
ENSECAG00000010349	2.629871726	0.088127	0.3552286	147	90	41	42	130	229	87	105
ENSECAG00000008295	3.152416197	0.0882716	0.355681	1	124	1	96	3	427	3	579
ENSECAG00000004216	10.59178089	0.08832	0.355681	15188	32990	14441	20400	14882	51497	14458	58233
ENSECAG00000002652	3.676681004	0.088375	0.355681	242	257	370	363	104	100	193	309
ENSECAG00000023165	0.798034409	0.0883946	0.355681	14	57	36	63	7	17	16	38
ENSECAG00000009626	8.333621935	0.0883958	0.355681	4957	7909	2711	957	10434	4905	5705	9060
ENSECAG00000006717	4.984017418	0.0884424	0.3557428	499	668	164	263	1246	1080	331	207
ENSECAG00000018599	6.748770673	0.088522	0.3559369	2719	1818	2916	2470	1834	1300	1543	1617
ENSECAG00000021989	5.305885419	0.0886029	0.3561199	1033	885	862	821	536	411	711	696
ENSECAG00000020874	4.190794365	0.0886302	0.3561199	50	518	69	77	77	645	77	1047
ENSECAG00000026849	6.104328465	0.0887378	0.356426	1003	880	1136	1383	1409	1911	1344	1064
ENSECAG00000018299	6.169091944	0.0888522	0.3566051	1676	1331	1620	1963	841	961	1237	1272
ENSECAG00000007147	8.33593398	0.0888766	0.3566051	7580	6708	7320	8353	4271	3896	5726	5097
ENSECAG00000016428	6.67437522	0.0888766	0.3566051	2076	1222	1311	1754	1702	1285	4062	2094
ENSECAG00000020479	5.401691447	0.0892059	0.3578003	866	729	1110	1204	666	423	687	734
ENSECAG00000026909	7.534998742	0.0892474	0.3578402	4651	3065	4020	6360	3572	1703	3964	1154
ENSECAG00000024559	5.589203553	0.0893327	0.3580127	929	789	746	495	1208	832	1402	719
ENSECAG00000005827	5.460725543	0.0893535	0.3580127	959	854	1045	1172	536	566	899	645
ENSECAG00000010166	5.388484661	0.0895747	0.3587727	869	760	1567	851	268	463	405	1130
ENSECAG00000007311	6.112139592	0.0896264	0.3588266	1067	1150	1037	1169	1849	1249	1620	1100
ENSECAG00000022868	4.803054571	0.0896514	0.3588266	402	325	351	628	891	284	917	409
ENSECAG00000009665	5.69474868	0.0899215	0.3597202	828	660	973	833	1354	617	1500	998
ENSECAG00000023135	1.166851312	0.0899568	0.3597202	44	38	68	61	19	23	44	30
ENSECAG00000022275	5.278319149	0.0899696	0.3597202	881	493	864	1473	519	402	833	519
ENSECAG00000006865	4.751585164	0.0900289	0.359825	509	440	728	867	289	330	396	537
ENSECAG00000013269	5.534255321	0.0900592	0.359825	1112	747	1006	1409	714	586	837	632
ENSECAG00000000077	1.668075187	0.0901836	0.3601953	48	32	57	39	42	77	57	101
ENSECAG00000018529	7.974758846	0.0902354	0.3602755	7990	4457	8009	3821	3313	1122	4563	4948
ENSECAG00000011177	5.0072601	0.090362	0.360654	519	286	492	621	609	1168	735	271
ENSECAG00000019386	4.984910977	0.0904141	0.3607355	439	454	443	659	698	785	794	410
ENSECAG00000000555	6.349099926	0.0905138	0.3609871	1849	1467	1868	2379	1131	705	1885	1199
ENSECAG00000019812	5.778672374	0.0905407	0.3609871	881	924	724	1001	1301	823.997	1507	1032
ENSECAG00000021545	7.171329325	0.0906306	0.3610594	3696	3135	4338	2430	1052	1219	3046	2946
ENSECAG00000011361	4.792163404	0.0906389	0.3610594	365	515	355	362	329	903	326	838
ENSECAG00000014987	5.072481798	0.0906542	0.3610594	527	542	524	556	609	692	808	723
ENSECAG00000021024	1.057207056	0.0907527	0.3613247	19	21	21	50	34	32	99	26
ENSECAG00000008101	6.273333427	0.0909405	0.3618731	1877	1286	1962	2063	1375	678	1617	934
ENSECAG00000015229	4.003738641	0.0909541	0.3618731	94	382	102	209	97	162	360	966
ENSECAG00000016912	7.148731318	0.0910834	0.362238	2492	1790	2093	2620	2689	3802	2833	2600
ENSECAG00000013268	1.808286374	0.0911096	0.362238	56	36	47	58	119	52	92	46
ENSECAG00000019841	6.055138358	0.0911513	0.3622769	944	1038	1078	1023	970	1885	821	1932
ENSECAG00000014528	5.02521852	0.0913541	0.362956	590	410	539	511	854	450	950	559
ENSECAG00000012724	6.833842849	0.0914587	0.3631648	1830	1270	1655	2320	1363	3721	2289	2347
ENSECAG00000023209	3.897080612	0.0914888	0.3631648	302	292	340	461	174	192	276	217
ENSECAG00000018348	11.78731764	0.0915026	0.3631648	61569	43431	57176	56981	68533	91886	57934	81754
ENSECAG00000004670	3.615814277	0.0917336	0.3639547	607	271	82	321	83	25	370	127
ENSECAG00000013357	1.225862186	0.0919928	0.3648556	107	22	50	48	27	14	34	39
ENSECAG00000016401	6.463614181	0.0920897	0.3651125	2075	1697	1854	2420	1217	1263	1507	1307.02
ENSECAG00000013133	0.459924285	0.0922381	0.3655537	5	27	23	11	28	29	40	24
ENSECAG00000020764	1.826942542	0.0922964	0.3655537	74	64	97	100	58	38	55	37
ENSECAG00000019875	5.918519035	0.0923311	0.3655537	790	1226	978	566	789	1887	978	1525
ENSECAG00000016309	4.143562311	0.0923466	0.3655537	335	257	207	283	463	418	364	257
ENSECAG00000012473	2.578168727	0.0923619	0.3655537	58	71	72	136	99	225	73	120
ENSECAG00000009116	6.149229345	0.0924121	0.3656248	1679	1403	1639	1716	890	924	1218	1244
ENSECAG00000017367	1.7154873	0.0925774	0.3661512	76	78	84	75	17	28	76	51
ENSECAG00000009907	6.661372052	0.0926564	0.3662638	1802	2761	3472	1713	615	602	1595	2736
ENSECAG00000014760	6.54311631	0.0927212	0.3662638	845	1445	1165	2408	1455	2714	1931	1829
ENSECAG00000018653	4.857701155	0.0927305	0.3662638	554	346	407	518	842	409	647	586
ENSECAG00000016489	10.97663472	0.0927892	0.3662638	36769	22458	32587	34494	48963	42814	36079	43647
ENSECAG00000019439	5.169036314	0.09281	0.3662638	694	841	704	1143	294	411	566	809
ENSECAG00000009844	6.163078807	0.0928146	0.3662638	1633	1369	1502	2161	1076	937	1602	630
ENSECAG00000022769	4.48926166	0.0928315	0.3662638	815	360	557	397	461	159	374	259
ENSECAG00000022195	3.799640882	0.0929397	0.3665634	258	176	193	207	437	320	243	191
ENSECAG00000019760	3.832702975	0.0932054	0.3673815	366	270	341	348	190	174	189	258

ENSECAG00000008536	5.313534042	0.0932118	0.3673815	494	648	520	887	856	753	1111	667
ENSECAG00000009053	4.25894851	0.0934747	0.3682896	328	281	309	241	614	251	506	299
ENSECAG00000017416	1.695372289	0.0935808	0.3685362	67	45	82	124	38	18	44	66
ENSECAG00000023978	2.274015233	0.0936022	0.3685362	75.9984	66	73	62.9995	82	71.9993	181.999	98
ENSECAG00000021325	2.032367021	0.0937163	0.3688577	50	169	74	120	28	52	9	92
ENSECAG00000018378	6.530644832	0.0938024	0.3689583	1522	1290	1441	1804	2018	1737	2176	1784
ENSECAG00000012282	4.368153304	0.0938314	0.3689583	367	234	354	239	866	199	520	271
ENSECAG00000018029	2.137692696	0.0938738	0.3689583	40	99	48	52	59	90	128	119
ENSECAG00000009418	4.174975213	0.0939166	0.3689583	225.001	242	288.001	356.001	303.001	561.001	301.001	358
ENSECAG00000018303	7.609968689	0.0939346	0.3689583	3132	2806	2780	3811	3727	3945	4456	4413
ENSECAG00000022797	6.240915717	0.0939367	0.3689583	1232	1008	1211	1423	1657	1878	1619	1139
ENSECAG00000006442	5.399820208	0.0941141	0.369527	549	781	582	536	398	1188	539	1559
ENSECAG00000007013	2.667282195	0.0943451	0.3703063	138	99	161	207	80	89	95	83
ENSECAG00000007432	5.334231976	0.094381	0.3703189	623	358	611	647	1575	393	1696	178
ENSECAG00000004042	2.894779779	0.0945099	0.3706967	174	237	177	111	70	64	106	158
ENSECAG00000018423	6.872758585	0.0945974	0.3709121	1854	1594	1469	2561	2120	2985	2157	2584
ENSECAG00000012680	5.389972246	0.0948202	0.3716574	1069	906	1184	802	315	623	348	1011
ENSECAG00000015687	7.243200153	0.0949159	0.3718273	2391	1968	2280	2934	2236	4203	2959	3385
ENSECAG00000014940	6.263722283	0.0949291	0.3718273	1388	1248	1171	1031	2076	1491	1813	1109
ENSECAG00000017191	2.911654064	0.0950189	0.3720511	104	160	54	84	214	111	79	275
ENSECAG00000018363	4.919826419	0.0950536	0.3720584	396	600	350	522	463	514	699	943
ENSECAG00000022619	2.836719703	0.0951698	0.3723525	69	110	134	99	152	200	100	161
ENSECAG00000023732	4.916359987	0.0951943	0.3723525	782	499	634	889	391	472	500	398
ENSECAG00000022134	5.75850633	0.0952352	0.3723587	833	890	711	1073	932	1155	1110	1316
ENSECAG00000014676	9.342851401	0.0952614	0.3723587	8894	10283	11363	9656	13186	17228	12968	11654
ENSECAG00000005538	6.10994415	0.0953032	0.3723939	1719	1693	1107	1775	864	771	1358	1157
ENSECAG00000024801	6.245492886	0.0953652	0.3724823	1213	1160	1282	1139	2024	1744	1540	1037
ENSECAG00000024651	6.129608323	0.0954393	0.3724823	1099	990	1102	1271	2039	918	1987	1070
ENSECAG00000011273	5.1419735	0.0954669	0.3724823	791	625	880	961	408	517	498	646
ENSECAG00000011268	1.930725121	0.0954687	0.3724823	42	85	50	22	95	97	92	55
ENSECAG00000000007	4.855096237	0.0954898	0.3724823	468	430	387	439	343	1052	415	649
ENSECAG00000013688	4.805168529	0.0955359	0.3725154	760	447	788	691	521	143	642	280
ENSECAG00000013153	6.45238107	0.0955639	0.3725154	1104	2348	557	648	3138	2306	916	1424
ENSECAG00000014117	3.623976404	0.0956404	0.3726858	285	181	352	366	128	90	296	189
ENSECAG00000024095	10.56337447	0.0957092	0.3728259	26883	18760	25206	24255	34448	32862	28335	32682
ENSECAG00000011111	3.028258308	0.0959857	0.373775	184	262	140	204	90	51	66	215
ENSECAG00000011429	3.490086949	0.0960228	0.3737914	205	158	194	80	363	134	332	185
ENSECAG00000014577	1.27135225	0.0960757	0.3738692	14	143	32	44	18	17	37	37
ENSECAG00000022446	7.238927599	0.0961222	0.3739104	2950	4459	4288	2481	1286	1575	1739	3789
ENSECAG00000017963	3.814897414	0.0961521	0.3739104	594	175	308	346	345	123	185	45
ENSECAG00000018429	5.379392864	0.0963248	0.3741712	1022	839	956	958	570	472	779	688
ENSECAG00000012321	6.486745719	0.0963268	0.3741712	1858	1947	3382	1123	899	1204	1306	1676
ENSECAG00000000399	5.91458614	0.0963277	0.3741712	1212	714	974	829	2118	698	1382	1027
ENSECAG00000017272	6.183894539	0.096351	0.3741712	1322	1125	1100	1168	1451	1208	1884	1577
ENSECAG00000024187	8.949277942	0.0964692	0.3745022	7784	6614	8298	7993	14833	8407	12553	6954
ENSECAG00000010001	5.959758347	0.0965739	0.3747807	1063	919	958	1052	1118	1470	1024	1552
ENSECAG00000016406	0.488814379	0.0966569	0.3749745	20	23	9	16	23	33	11	54
ENSECAG00000021326	1.9875934	0.0966919	0.3749824	32	17	70	103	121	55	115	71
ENSECAG00000010784	4.616527949	0.0967927	0.3752453	548	508	481	734	268	325	407	438
ENSECAG00000004249	4.824754578	0.0969204	0.3754868	471	370	421	434	504	1069	479	345
ENSECAG00000004932	5.439843388	0.0969211	0.3754868	846	618	572	768	754	832	1015	1048
ENSECAG00000014999	2.372446435	0.0970411	0.3758013	121	89	151	130	55	54	67	103
ENSECAG00000012227	4.272336181	0.0970685	0.3758013	448	321	575	448	268	285	245	305
ENSECAG00000013124	5.06365999	0.0971526	0.3759988	624	519	451	132	1067	1293	397	229
ENSECAG00000024512	5.116754316	0.0972376	0.3761996	624	486	549	376	1308	400	1041	357
ENSECAG00000012822	9.896130011	0.0972872	0.3762631	15387	12645	16310	15234	20493	23824	16346	19766
ENSECAG00000000307	5.020961851	0.0973956	0.3763946	645	570	402	344	571	885	541	754
ENSECAG00000014400	5.364381508	0.097399	0.3763946	714	463	615	784	1309	606	1194	471
ENSECAG00000024930	4.614351335	0.0974206	0.3763946	501	514	558	733	298	180	635	332
ENSECAG00000011475	0.481349526	0.0974811	0.3765003	9	25	22	7	68	10	29	19
ENSECAG00000011117	2.37083584	0.0976077	0.3768613	6	124	5	20	12	351	2	169
ENSECAG00000019274	5.820204848	0.0976634	0.3769482	1265	1063	1277	1530	853	657	985	926
ENSECAG00000014737	6.590793537	0.0977772	0.3772592	2426	1557	2432	2535	996	914	2233	1663
ENSECAG00000012012	0.749508432	0.097858	0.3774352	27	9	21	32	45	18	62	25
ENSECAG00000005451	3.622736229	0.0978893	0.3774352	189	203	151	213	253	302	272	218
ENSECAG00000015596	2.767841281	0.0980104	0.3776724	139	123	178	203	92	69	97	120
ENSECAG00000017033	7.216511648	0.0980173	0.3776724	2216	2490	2026	2682	2852	3770	3186	2692
ENSECAG00000024917	3.416232968	0.0980952	0.3778445	193	233	250	333	92	144	169	193
ENSECAG00000014236	2.748355812	0.098302	0.3785128	160	128	151	200	68	53	123	130
ENSECAG00000005115	1.233909182	0.0986202	0.3795799	49	61	35	76	31	30	23	34
ENSECAG00000022013	3.554537284	0.098646	0.3795799	185	175	126	238	234	291	249	224
ENSECAG00000002782	0.732338078	0.0987273	0.3797643	19	47	40	54	10	10	50	10
ENSECAG00000009518	1.19946549	0.098775	0.3798188	29	19	40	39	59	59	54	25
ENSECAG00000016981	2.874659582	0.098932	0.3802941	148	135	215	194	87	65	125	133
ENSECAG00000021052	4.727917221	0.0993067	0.3815026	195	278	343	786	536	299	1190	441
ENSECAG00000013562	6.154159523	0.0993471	0.3815026	1817	1194	1696	1784	902	861	1426	1132
ENSECAG00000011206	4.05656104	0.0993472	0.3815026	285	395	364	516	149	264	243	285
ENSECAG00000006159	5.441465806	0.0994452	0.38175	650	703	649	738	597	1203	787	1045

ENSECAG00000024822	5.882914644	0.0996641	0.3824612	986	833	857	1031	698	1222	1069	2049
ENSECAG00000024380	1.614708737	0.0998529	0.3829432	36	53	25	27	60	28	214	10
ENSECAG00000022656	0.501648896	0.0998805	0.3829432	24	13	4	29	33	48	26	15
ENSECAG00000011230	5.538030006	0.0999827	0.3829432	1033	671	603	627	1110	1043	922	810
ENSECAG00000015739	7.091063529	0.1000909	0.3829432	3300	2369	2850	4157	2156	1521	2614	1820
ENSECAG00000013042	3.428685457	0.1000998	0.3829432	207	252	307	229	157	127	154	170
ENSECAG00000009510	6.543830156	0.1001151	0.3829432	1667	1546	1379	1407	2117	1970	1893	1766
ENSECAG00000019408	7.468086116	0.10015	0.3829432	2982	4760	4526	4097	2182	1669	2872	3622
ENSECAG00000019975	8.370118875	0.1001521	0.3829432	8781	4493	6161	13816	6350	4499	5294	1452
ENSECAG00000020292	6.639056988	0.1001864	0.3829432	1102	2173	1056	1919	2178	1920	2406	1999
ENSECAG00000019700	2.989893011	0.1001951	0.3829432	108	66	135	137	352	65	213	97
ENSECAG00000018724	0.680905176	0.1002167	0.3829432	21	65	31	41	6	4	56	6
ENSECAG00000017954	6.937937297	0.1002226	0.3829432	2138	2013	1820	1769	3035	2067	3392	1986
ENSECAG00000008738	7.326788449	0.100228	0.3829432	2860	3638	1310	1662	2961	3403	3509	4152
ENSECAG00000021598	2.303741503	0.1002654	0.3829573	126	39	63	49	134	78	101	125
ENSECAG00000004253	5.875628616	0.1003412	0.382993	1206	724	841	882	2056	814	1356	823
ENSECAG00000016348	2.678729035	0.1003422	0.382993	120	142	172	164	74	74	97	110
ENSECAG00000016439	7.428017816	0.1004366	0.3831886	2872	1918	2630	2955	7220	2583	3383	1871
ENSECAG00000022764	1.058236651	0.1004609	0.3831886	11	17	57	135	16	15	29	33
ENSECAG00000020319	2.128688728	0.1006856	0.3839168	118	67	109	123	47	32	76	82
ENSECAG00000019192	7.701227564	0.1012223	0.385784	5143	3803	4739	5532	3304	2417	3319	3269
ENSECAG00000009465	1.982134617	0.1012935	0.385784	38	51	59	81	68	81	69	124
ENSECAG00000022320	5.527400218	0.1012972	0.385784	751	698	831	709	1017	923	924	953
ENSECAG00000015974	4.620358748	0.1013112	0.385784	356	355	322	546	602	433	539	501
ENSECAG00000020400	1.542592586	0.1015417	0.3865322	56	72	86	58	45	31	23	49
ENSECAG00000018736	2.587903518	0.1016246	0.3867182	75	91	95	95	89	152	151	126
ENSECAG00000013849	3.447104824	0.1018215	0.3873378	174	132	167	188	273	105	341	250
ENSECAG00000019205	5.75048453	0.1019745	0.3877419	911	737	821	1062	1213	1130	1152	956
ENSECAG00000022534	7.30903345	0.1020004	0.3877419	4137	2980	3940	3566	2855	2031	2589	1788
ENSECAG00000021652	6.960284033	0.1020553	0.3877419	2169	1232	2485	1601	1457	2743	1819	4859
ENSECAG00000005271	5.21390248	0.1020971	0.3877419	606	472	517	834	789	685	927	716
ENSECAG00000020608	2.58878616	0.1021307	0.3877419	138	94	139	205	106	56	97	70
ENSECAG00000007665	5.771644692	0.1021325	0.3877419	908	744	620	1231	643	1290	1302	1405
ENSECAG00000010104	3.17600343	0.1023504	0.3883403	203	153	150	407	82	210	100	56
ENSECAG00000006497	1.0000539	0.1023586	0.3883403	28	34	31	9	64	33	42	36
ENSECAG00000024412	5.231850214	0.1024011	0.3883719	532	601	696	457	649	1234	819	472
ENSECAG00000021736	4.258234611	0.102446	0.3884125	303	297	296	308	339	474	448	347
ENSECAG00000013066	3.943619745	0.1025399	0.3886389	208	321	232	149	406	243	332	355
ENSECAG00000008396	8.245496598	0.1027515	0.389311	7823	5941	8758	5789	5459	2835	6273	3037
ENSECAG00000018640	4.728604149	0.1029128	0.3897149	293	530	425	391	544	551	475	672
ENSECAG00000003955	7.327284494	0.1029662	0.3897149	2334	2521	2179	3070	3476	1915	5726	3050
ENSECAG00000022692	9.279931587	0.1029875	0.3897149	12407	8824	9336	8234	13742	12728	11926	14421
ENSECAG00000022326	7.281733319	0.1029954	0.3897149	3464	3782	3700	3369	1393	2585	2427	2599
ENSECAG00000020937	5.742785433	0.1032216	0.3903279	976	405	858	1036	1861	621	1750	556
ENSECAG00000004830	2.588446778	0.1032261	0.3903279	186	99	148	131	93	62	98	78
ENSECAG00000022582	4.988761022	0.1033053	0.3904478	541	441	502	535	476	840	740	606
ENSECAG00000019748	4.403476908	0.1033286	0.3904478	354	280	280	435	377	310	646	496
ENSECAG00000022390	3.566734209	0.1033609	0.3904478	94	606	226	224	171	117	253	55
ENSECAG00000003872	4.790622753	0.103407	0.3904921	464	272	522	510	667	493	732	467
ENSECAG00000021356	3.132802724	0.1034565	0.3905419	132	185	193	336	72	122	130	155
ENSECAG00000010136	6.905994638	0.1034964	0.3905419	3235	2021	3794	2064	1605	1050	2215	2169
ENSECAG00000007559	5.179104228	0.1035233	0.3905419	594	568	575	580	961	530	828	738
ENSECAG00000007907	2.99835313	0.1037301	0.3911921	24	222	22	59	46	334	30	371
ENSECAG00000015527	1.218681451	0.1037786	0.3912448	8	36	28	52	30	38	66	76
ENSECAG00000000014	3.527340513	0.1038324	0.3913057	368	252	266	197	80	129	192	239
ENSECAG00000002402	1.452708423	0.1038636	0.3913057	73	13	43	162	27	10	69	23
ENSECAG00000021986	6.154825203	0.103963	0.3915501	1586	1309	1673	1915	1269	792	1202	1039
ENSECAG00000022657	4.128240126	0.1040688	0.3918188	342	180	295	216	601	222	555	186
ENSECAG00000019115	5.135532231	0.1041237	0.3918311	103	1002	123	191	120	1565	106	1682
ENSECAG00000014975	8.27858482	0.1041411	0.3918311	4981	4881	5048	3840	8896	7762	6764	3219
ENSECAG00000013368	1.437226304	0.1042274	0.3920263	26	50	23	23	7	124	20	97
ENSECAG00000023411	3.673207152	0.1042732	0.3920685	171.994	150.989	182.999	273.999	290	378.99	181.995	223.992
ENSECAG00000006013	4.614949659	0.1043313	0.3921571	393	308	365	477	556	675	486	334
ENSECAG00000019277	4.88586881	0.104511	0.392647	655	524	763	776	412	412	397	511
ENSECAG00000009189	1.625862026	0.1045307	0.392647	60	60	71	101	29	46	34	51
ENSECAG00000019638	1.317413534	0.1045693	0.3926619	34	28	46	12	129	41	41	15
ENSECAG00000005675	5.848148836	0.1046324	0.3927693	1094	1224	621	581	1171	1240	1177	1307
ENSECAG00000013640	3.331573257	0.1048049	0.3932867	175	205	216	363	118	99	143	205
ENSECAG00000021161	4.086340751	0.104873	0.3934107	434	281	443	412	236	178	336	249
ENSECAG00000012682	3.733718982	0.1049303	0.3934107	283	198	477	296	237	132	215	164
ENSECAG00000014276	5.779548402	0.1049599	0.3934107	1301	1134	1147	1362	759	760	986	829
ENSECAG00000000369	3.91235508	0.1049765	0.3934107	183	203	199	316	149	284	287	612
ENSECAG00000023836	4.756070424	0.1050142	0.3934221	400	414	311	587	404	448	646	827
ENSECAG00000018419	3.95574805	0.1050574	0.3934545	312	234	218	150	536	237	256	315
ENSECAG00000021147	9.150034775	0.1051156	0.3935424	15024	7896	13283	19213	8239	3372	14831	6322
ENSECAG00000011549	3.049010792	0.1051722	0.3936247	128	80	149	149	167	201	175	162
ENSECAG00000020262	5.462269773	0.1052512	0.3937619	617	691	752	652	533	1489	740	934
ENSECAG00000021248	6.327885263	0.1052782	0.3937619	1333	463	1399	1679	3031	824	2407	974

ENSECAG00000013399	2.412881669	0.1053176	0.3937798	85	60	72	95	72	141	88	157
ENSECAG00000024114	8.380074636	0.1054132	0.3939744	5564	4701	5307	5653	7267	5187	7357	8613
ENSECAG00000016422	3.758729161	0.105439	0.3939744	184	162	247	254	310	182	370	310
ENSECAG00000008283	2.429505552	0.1055008	0.3940756	73	103	67	59	154	83	76	158
ENSECAG00000007788	5.829008852	0.1055497	0.3941286	752	894	890	1068	811	1698	940	1272
ENSECAG00000010746	4.207440975	0.1057117	0.3946036	483	322	384	527	302	228	347	200
ENSECAG00000019868	2.710537408	0.1058896	0.3951381	69	154	59	50	154	240	41	144
ENSECAG00000016638	3.991982659	0.1061802	0.396011	186	240	286	269	204	412	370	372
ENSECAG00000020851	6.60914945	0.1062684	0.396011	2121	1900	2070	2790	1396	1468	1509	1483
ENSECAG00000012878	5.530452094	0.1063444	0.396011	1032	550	676	618	1364	510	1401	762
ENSECAG00000005374	7.812263018	0.1063474	0.396011	3758	3473	3270	3913	4762	4341	4763	5048
ENSECAG00000023583	6.495920105	0.1063502	0.396011	2063	1625	2359	2317	1681	770	2032	906
ENSECAG00000008818	2.4517745	0.1063541	0.396011	126	16	53	0	199	15	392	21
ENSECAG00000008147	5.065153447	0.1064011	0.396011	682	398	544	515	707	579	1000	572
ENSECAG00000021554	-0.084512125	0.1064153	0.396011	13	0	0	25	21	26	35	6
ENSECAG00000008319	7.677929032	0.1064373	0.396011	3285	2192	2814	4265	6962	2510	6685	2128
ENSECAG00000010882	5.504666631	0.1067471	0.3970333	965	762	1131	1292	656	692	808	562
ENSECAG00000019245	6.540470973	0.1068773	0.3973877	2134	1686	2032	2623	1234	1423	1624	1326
ENSECAG00000026939	2.1529702	0.106953	0.3975388	107	78	105	127	63	44	79	59
ENSECAG00000008485	9.18148917	0.1070734	0.3978563	7401	9004	7369	12192	15735	7025	20324	8604
ENSECAG00000024992	2.913405314	0.1071591	0.3980445	244	63	232	221	157	50	141	36
ENSECAG00000017661	1.118890109	0.1073457	0.3986074	13	32	26	41	19	48	25	101
ENSECAG00000002452	1.078414574	0.1074822	0.3988599	51	29	43	80	16	14	33	44
ENSECAG00000003503	5.229956498	0.1075406	0.3988599	871	656	905	1014	720	391	647	477
ENSECAG00000020493	6.491133333	0.1075745	0.3988599	2240	2294	1127	2675	1388	1400	1557	864
ENSECAG00000020540	3.968349163	0.1076136	0.3988599	296	280	375	511	219	183	277	233
ENSECAG00000023989	2.540289418	0.1076263	0.3988599	67	91	73	118	146	91	147	120
ENSECAG00000015913	1.438503456	0.1076454	0.3988599	52	13	40	44	59	86	35	52
ENSECAG00000008879	4.890505804	0.1076595	0.3988599	664	562	601	956	280	266	628	584
ENSECAG00000019178	6.486195853	0.1077274	0.3989813	2275	1539	2203	2226	1837	991	1533	950
ENSECAG00000022042	5.291258455	0.1079095	0.3995254	575	1479	644	891	405	470	722	661
ENSECAG00000020165	2.929873372	0.10796	0.399582	190	109	282	145	84	82	87	157
ENSECAG00000011792	1.248513226	0.1081176	0.3999955	32	24	43	29	33	41	33	101
ENSECAG00000009789	6.565183255	0.1081421	0.3999955	1381	1444	1206	2182	1728	2155	2113	1874
ENSECAG00000015165	5.578656982	0.1082367	0.400215	577	670	696	1174	1275	884	897	930
ENSECAG00000019613	4.200182423	0.1083481	0.4004262	189	204	251	505	292	469	575	264
ENSECAG00000018906	7.048867646	0.1083643	0.4004262	2071	1867	1897	2775	2404	2153	3528	3183
ENSECAG00000014175	5.817400079	0.1085195	0.4008691	655	791	798	1036	323	2027	715	1857
ENSECAG00000020110	6.291357765	0.1085569	0.4008772	1662	1866	1691	1921	668	981	1582	1479
ENSECAG00000010547	5.182395586	0.1086661	0.4010522	614	817	1067	831	372	424	592	754
ENSECAG00000019830	5.522820232	0.1086749	0.4010522	189	721	495	513	26	2002	82	2477
ENSECAG00000023751	0.340222373	0.1087516	0.4011324	17	27	10	4	12	41	27	30
ENSECAG00000017419	6.353352907	0.1087673	0.4011324	1625	1223	1333	1029	1668	1924	1430	1752
ENSECAG00000021721	0.933375681	0.108981	0.4017377	19	43	20	19	46	35	45	38
ENSECAG00000000484	2.417917077	0.1090022	0.4017377	65	74	76	100	118	55	161	141
ENSECAG00000020998	5.673973878	0.109049	0.4017801	861	682	810	1014	1011	898	1040	1287
ENSECAG00000019188	4.71694859	0.1092483	0.4023839	629	483	584	714	339	295	501	441
ENSECAG00000022545	6.092514781	0.1093331	0.4025588	900	1147	936	1180	1063	2623	926	1072
ENSECAG00000013994	4.575623686	0.1093667	0.4025588	399	345	420	336	597	298	616	535
ENSECAG00000018426	6.007788282	0.1095374	0.402957	1415	851	951	839	1512	1606	1392	876
ENSECAG00000022005	8.954450795	0.1095459	0.402957	12040	9785	10189	14120	6793	4787	9398	8503
ENSECAG00000006991	7.135694932	0.1097108	0.4034333	3586	3243	4457	2139	908	827	2483	3659
ENSECAG00000007306	1.267117684	0.1098563	0.403777	61	28	60	88	34	18	59	9
ENSECAG00000013623	7.585823026	0.1098754	0.403777	3842	4473	5477	3823	2328	1864	3691	3528
ENSECAG00000006851	1.177309993	0.109988	0.4040008	25	97	42	48	19	20	20	50
ENSECAG00000013165	2.398110623	0.1100074	0.4040008	69	86	64	86	65	104	105	187
ENSECAG00000005876	3.643135591	0.1101188	0.4042791	212	165	197	200	373	202	263	225
ENSECAG00000024569	6.723094063	0.1102915	0.4045984	2416	2097	2276	2825	1590	1215	2028	1573
ENSECAG00000003034	2.085942509	0.1103064	0.4045984	93	53	119	152	45	19	86	76
ENSECAG00000017747	5.919501359	0.110377	0.4045984	805	893	1075	824	634	2220	653	1628
ENSECAG00000022599	3.608756393	0.110389	0.4045984	290	175	279	411	125	156	239	181
ENSECAG00000017484	4.059230333	0.1104572	0.4045984	251	333	175	271	290	309	452	382
ENSECAG00000008769	6.960976038	0.1104619	0.4045984	2018	1982	1722	1967	2043	4246	1866	2236
ENSECAG00000009478	6.141184285	0.1104646	0.4045984	1905	1556	1626	1337	700	700	1279	1544
ENSECAG00000024730	3.063430452	0.1104933	0.4045984	215	126	227	218	121	74	143	135
ENSECAG00000011198	5.54865748	0.1105264	0.4045984	1020	661	585	709	1245	713	1329	709
ENSECAG00000010947	4.718277821	0.110675	0.4050119	689	329	194	36	951	996	345	159
ENSECAG00000021328	4.894502166	0.1108633	0.4055704	627	740	654	732	202	399	496	627
ENSECAG00000012156	2.879493686	0.1110221	0.4060206	157	266	123	170	24	139	62	136
ENSECAG00000018867	2.517958584	0.111099	0.406171	111	92	137	209	77	49	117	75
ENSECAG00000017440	5.427880948	0.1111771	0.4061724	697	546	707	863	873	1038	948	701
ENSECAG00000019556	4.76758733	0.1112513	0.4061724	591	515	628	813	241	266	480	614
ENSECAG00000020174	1.711606549	0.1112553	0.4061724	89	56	78	82	38	51	41	43
ENSECAG00000016465	5.86542172	0.1112769	0.4061724	1160	808	781	1075	1319	1045	1328	1155
ENSECAG00000009882	3.987961854	0.1112782	0.4061724	458	307	335	369	298	140	265	214
ENSECAG00000012923	3.996073071	0.1113233	0.4062067	242	363	158	189	383	288	394	316
ENSECAG00000005303	3.329430171	0.1114433	0.4064588	175	164	102	173	229	165	288	189
ENSECAG00000020462	7.104103822	0.111464	0.4064588	1872	2206	2466	1688	1836	5140	1960	2673

ENSECAG00000020792	3.537314471	0.1117056	0.4072093	324	65	167	61	412	92	429	175
ENSECAG00000022968	7.377249591	0.1118095	0.4074573	2737	1983	3053	2790	4123	3804	3949	2167
ENSECAG00000021482	3.514243242	0.1118859	0.4074911	222	236	266	335	143	127	197	196
ENSECAG00000005021	2.063158833	0.1118905	0.4074911	43	45	100	47	136	52	109	74
ENSECAG00000007066	2.64639104	0.1119576	0.4076046	98	260	182	101	14	35	18	209
ENSECAG00000009368	9.409741764	0.1120253	0.4077203	14909	3597	36	11343	15502	28132	7453	16566
ENSECAG00000025866	0.665102734	0.112199	0.4082218	14	32	14	22	25	49	32	28
ENSECAG00000020813	7.616216493	0.1122921	0.4084296	5011	3741	4001	5261	2624	2579	3486	3023
ENSECAG00000005179	0.464592447	0.1125139	0.4091054	9	34	13	7	10	47	27	37
ENSECAG00000011155	6.109625037	0.1125623	0.4091506	833	1237	1199	876	1019	2478	780	1509
ENSECAG00000018075	6.067241059	0.1125993	0.4091541	1168	927	1070	1193	1630	1545	1184	1153
ENSECAG00000013149	8.351728961	0.1127268	0.4094544	5327	4297	5102	6338	7626	5107	8627	6520
ENSECAG00000007350	4.769052631	0.1127541	0.4094544	413	366	512	396	872	602	448	370
ENSECAG00000024076	7.244537936	0.1127967	0.4094691	3284	2872	3785	3946	1930	2127	2445	2530
ENSECAG00000008582	5.457499949	0.1128882	0.4094691	704	749	705	670	913	987	1029	719
ENSECAG00000014655	2.363335961	0.1129124	0.4094691	63	51	81	115	136	105	113	87
ENSECAG00000021375	4.636746098	0.1129262	0.4094691	557	482	562	681	346	240	486	420
ENSECAG00000000473	4.537746291	0.1129618	0.4094691	539	458	526	642	327	145	505	400
ENSECAG00000023081	6.191397498	0.113002	0.4094691	1518	955	1147	1098	1910	1329	1681	1190
ENSECAG00000017911	5.708836423	0.1130104	0.4094691	1177	915	1400	1259	748	538	835	1041
ENSECAG00000011995	6.027272312	0.1131763	0.4099394	1322	1288	1647	1674	1298	761	1026	815
ENSECAG00000011657	6.35931802	0.1132639	0.4100935	1065	1742	1026	1151	853	1816	1516	2896
ENSECAG00000016120	5.596944156	0.1132911	0.4100935	792	799	819	711	758	906	1082	1306
ENSECAG00000026857	4.412826221	0.113339	0.4101362	293	294	293	491	459	389	615	350
ENSECAG00000024197	5.892186698	0.1135215	0.410666	1437	1038	1428	1477	809	741	908	1131
ENSECAG00000009016	3.989420938	0.1137023	0.4110991	440	320	400	322	315	232	136	188
ENSECAG00000010300	2.426511289	0.1137578	0.4110991	107	122	142	127	68	68	104	59
ENSECAG00000021406	3.757868567	0.1137598	0.4110991	197	235	311	567	138	164	168	282
ENSECAG00000005815	5.414936967	0.113786	0.4110991	876	494	784	475	842	1344	785	601
ENSECAG00000013119	4.476412949	0.1139805	0.4116383	387	295	310	438	502	292	546	556
ENSECAG00000019114	6.533281399	0.1140162	0.4116383	2082	1909	1935	2451	1545	1309	1606	1126
ENSECAG00000013474	1.371921388	0.114044	0.4116383	34	27	44	42	76	39	73	36
ENSECAG00000016313	5.613648589	0.1141334	0.4118027	810	603	770	941	1171	649	1831	625
ENSECAG00000015391	5.980088549	0.1141621	0.4118027	975	743	945	1463	1513	1498	1340	888
ENSECAG00000016582	2.903003313	0.1142106	0.4118471	46	50	88	140	27	589	8	71
ENSECAG00000014059	3.638891751	0.114279	0.4119628	367	186	278	352	224.999	122	258	100
ENSECAG00000006957	7.322958228	0.1143826	0.4120903	3029	3951	3424	4666	1283	1502	2412	4070
ENSECAG00000026905	11.16715138	0.1143869	0.4120903	38852	29971	40145	36528	52124	53670	39595	47580
ENSECAG00000013489	5.454595919	0.1145369	0.4124067	1095	728	1102	1126	554	393	631	1024
ENSECAG00000022716	4.509518481	0.1145473	0.4124067	6	538	17	72	6	1594	10	820
ENSECAG00000020060	3.03710365	0.1146638	0.4126951	113	134	121	115	133	248	113	202
ENSECAG00000017880	4.927505807	0.1148178	0.4131186	788	503	717	796	397	301	721	416
ENSECAG00000001130	5.579357962	0.1148696	0.4131742	1057	969	1006	1288	724	537	981	686
ENSECAG00000016078	5.885170469	0.1149559	0.4132075	1023	624	1300	622	653	832	1310	2452
ENSECAG00000007700	4.277281487	0.1149649	0.4132075	323	223	326	288	718	451	309	179
ENSECAG00000008178	2.396405468	0.114988	0.4132075	162	127	110	96	40	40	123	86
ENSECAG00000000647	1.5402643	0.1151364	0.413525	48	25	40	48	54	68	117	21
ENSECAG00000009610	6.691955718	0.1151868	0.413525	2293	2319	3483	1368	1143	1098	1805	2002
ENSECAG00000008025	3.004596623	0.1152048	0.413525	187	172	156	235	143	101	122	82
ENSECAG00000019557	5.498682123	0.115222	0.413525	575	980	407	789	1296	1128	561	816
ENSECAG00000008298	0.338582236	0.1152614	0.4135358	19	27	4	0	52	23	35	8
ENSECAG00000010092	6.494201211	0.1154625	0.4141264	2008	1770	1943	2418	1356	1030	1755	1391
ENSECAG00000003668	5.175136561	0.1155034	0.4141424	759	922	1016	566	475	376	617	671
ENSECAG00000006582	3.459548861	0.1156801	0.414645	170	171	181.001	158	252	237	235	203
ENSECAG00000014867	8.748960391	0.1157927	0.4147984	1047	11257	765	1658	284	20366	421	23880
ENSECAG00000007717	5.972773048	0.1158549	0.4147984	1237	716	1093	1069	1546	1075	1435	1177
ENSECAG00000023527	5.682612244	0.1158777	0.4147984	1012	510	800	955	1609	1005	1101	613
ENSECAG00000009074	5.615338585	0.1158822	0.4147984	1003	1100	1236	1062	650	546	869	915
ENSECAG00000022361	5.901278549	0.1159055	0.4147984	1241	1212	1194	1859	676	544	1146	1253
ENSECAG00000000869	4.592549724	0.1160553	0.4151654	465	400	679	713	207	273	461	478
ENSECAG00000000193	6.155673682	0.1160811	0.4151654	1762	1341	1504	1863	1262	1067	1099	827
ENSECAG00000017209	5.769311593	0.1161194	0.4151718	925	765	1077	735	1432	728	1431	1031
ENSECAG00000016210	5.764762717	0.1162308	0.415439	916	1187	802	2324	744	773	1234	367
ENSECAG00000015953	7.811922684	0.1163459	0.4157198	4724	2355	3496	3072	8495	3129	5451	2601
ENSECAG00000017695	4.808555004	0.1166332	0.4166152	677	474	680	756	307	353	660	357
ENSECAG00000015576	3.634409886	0.1167402	0.4168626	182	154	250	718	241	44	282	78
ENSECAG00000017109	2.169057515	0.1167759	0.4168626	33	95	10	36	14	214	3	211
ENSECAG00000018530	1.737814001	0.1170117	0.4175734	73	55	79	120	14	20	80	61
ENSECAG00000007716	1.103514206	0.1170569	0.4176035	34	36	77	56	13	16	50	32
ENSECAG00000009098	0.38091646	0.1171105	0.4176453	13	15	19	18	53	28	21	8
ENSECAG00000008443	3.245410343	0.1171422	0.4176453	127	155	119	176	141	274	215	175
ENSECAG00000020244	0.395721202	0.1174698	0.4186581	5	28	19	12	14	27	39	35
ENSECAG00000020055	7.17447159	0.1175	0.4186581	3145	3218	3483	3246	2234	1757	2755	1942
ENSECAG00000000749	5.849883485	0.1176229	0.4189648	1378	991	1446	1469	1114	503	1142	718
ENSECAG00000016376	2.445624648	0.1177152	0.4190825	74	130	100	230	56	105	80	38
ENSECAG00000017404	7.764741664	0.1177584	0.4190825	3643	3008	3762	3445	4650	5521	3765	4138
ENSECAG00000010027	2.78858704	0.1177666	0.4190825	101	56	116	152	174	115	148	157
ENSECAG00000026845	5.010586782	0.1178074	0.4190949	411	481	541	627	629	905	567	562

ENSECAG00000007095	6.02221184	0.1178455	0.4190949	1643	1668	1923	677	861	621	1078	1223
ENSECAG00000009618	3.685372057	0.1178808	0.4190949	194	391	220	422	98	149	345	133
ENSECAG00000007444	5.800254181	0.117924	0.4191173	1041	683	864	1048	1024	1454	971	1123
ENSECAG00000005699	4.107119784	0.1179638	0.4191275	168	326	300	224	262	584	236	376
ENSECAG00000018573	3.109869062	0.1181106	0.4195181	117	117	124	164	133	277	150	168
ENSECAG00000022291	7.581627532	0.1181676	0.4195892	3279	2366	3330	3326	4534	4516	3772	3147
ENSECAG00000024960	5.834735233	0.1187187	0.4214143	882	937	953	982	1207	925	1314	1290
ENSECAG00000020963	6.280605665	0.1188663	0.4218062	1634	1523	1821	2021	1210	941	1359	1243
ENSECAG00000016637	3.801286932	0.1189411	0.4218923	203	224	228	191	176	200	368	480
ENSECAG00000008705	9.016355705	0.1189648	0.4218923	6973	7933	7371	6783	3969	24733	5281	10753
ENSECAG00000014123	5.000583705	0.1190986	0.4219214	484	553	531	451	945	444	740	584
ENSECAG00000010502	7.364033158	0.1191075	0.4219214	2271	2871	2244	3113	2776	4296	4052	2725
ENSECAG00000019019	7.05199712	0.1191177	0.4219214	3062	2488	3129	3491	2264	1274	2622	1841
ENSECAG00000013853	3.328127636	0.1191216	0.4219214	261	207	184	298	101	67	211	196
ENSECAG00000015069	6.750821424	0.1193241	0.4225069	1016	3319	765	465	4248	1720	2840	1184
ENSECAG00000000512	5.837208762	0.1194268	0.4227375	1280	901	1226	1951	937	452	1393	664
ENSECAG00000016742	1.34470006	0.1194789	0.4227375	43	99	37	59	35	19	18	53
ENSECAG00000024702	7.170658854	0.1195442	0.4227375	2151	2707	2047	1442	2008	6024	1947	2238
ENSECAG00000015223	0.774624722	0.1195889	0.4227375	3	35	20	13	42	56	1	58
ENSECAG00000021917	3.915366761	0.1196079	0.4227375	313	278	409	418	211	171	373	118
ENSECAG00000013312	3.80700527	0.1196125	0.4227375	380	204	354	375	214	118	315	169
ENSECAG00000008451	5.394544443	0.1197192	0.4229617	747	525	795	597	896	1190	620	760
ENSECAG00000007747	6.684007191	0.1197504	0.4229617	2596	1714	2309	2862	1337	962	2543	1431
ENSECAG00000015062	1.906954528	0.1198284	0.4230801	84	59	97	115	29	41	55	77
ENSECAG00000004023	3.880935409	0.1198584	0.4230801	355	244	366	395	201	166	294	208
ENSECAG00000021678	6.255397127	0.1200713	0.4236999	1280	1130	1233	1338	1622	1684	1194	1767
ENSECAG00000001393	5.08780015	0.1202534	0.4240878	672	500	454	551	951	589	747	562
ENSECAG00000010837	4.354792818	0.1202559	0.4240878	404	452	379	666	217	201	390	403
ENSECAG00000022527	10.03333412	0.1203453	0.4241238	15718	18984	14344	15356	22614	30780	21870	13266
ENSECAG00000006984	5.956067622	0.1203629	0.4241238	922	1020	854	1238	1099	1679	1275	1056
ENSECAG00000010033	7.237067423	0.1203781	0.4241238	2754	2267	2584	1928	4271	2230	3317	2984
ENSECAG00000010508	6.06380269	0.1205694	0.4246089	1914	1053	1915	1211	987	642	1119	1258
ENSECAG00000012467	4.191481397	0.1205906	0.4246089	454	324	493	417	336	232	326	162
ENSECAG00000016326	6.597821099	0.1208688	0.4254566	2245	1719	1844	3049	1421	1255	1962	1259
ENSECAG00000010560	7.319782127	0.1209886	0.4256969	20	3364	1	35	87	6497	61	12159
ENSECAG00000020708	3.074416094	0.1210146	0.4256969	204	118	272	197	97	85	172	125
ENSECAG00000010206	3.44676878	0.1210495	0.4256969	214	247	246	304	100	142	173	210
ENSECAG00000019256	6.862933932	0.1212172	0.4260365	1812	1785	1889	1900	1533	3370	2471	2356
ENSECAG00000012021	3.309387836	0.1212211	0.4260365	208	210	207	299	175	95	127	166
ENSECAG00000010644	8.657058047	0.1213126	0.4262262	8574	8362	9124	10992	5731	5341	7560	5473
ENSECAG00000014996	6.668829191	0.1214097	0.4262623	2066	1213	1700	1387	2667	2716	2053	1094
ENSECAG00000026917	5.246281065	0.1214318	0.4262623	661	604	716	378	856	968	619	714
ENSECAG00000012403	4.365579935	0.1214354	0.4262623	333	443	399	756	211	282	320	384
ENSECAG00000011302	0.490206053	0.121538	0.4264905	25	54	28	21	9	18	8	29
ENSECAG00000013416	4.614940099	0.1217272	0.4269235	372	352	416	437	452	561	438	578
ENSECAG00000015063	1.411885091	0.1217365	0.4269235	58	126	14	63	21	9	19	72
ENSECAG00000013915	10.76250275	0.1218185	0.4269637	29034	21915	28591	31633	43614	37217	30524	34103
ENSECAG00000017800	6.024798748	0.1218232	0.4269637	885	814	1271	1018	643	1026	1120	2919
ENSECAG00000004861	0.111694251	0.1219979	0.4273609	23	24.0001	27.0001	23.0001	8.0001	8.0005	9.00057	25.0004
ENSECAG00000022742	7.661176969	0.1220837	0.4273609	4845	3825	4568	5355	3417	1919	3692	3129
ENSECAG00000001234	4.82881719	0.1221174	0.4273609	611	561	668	745	341	407	452	494
ENSECAG00000024428	0.979055486	0.1221538	0.4273609	12	25	29	37	13	34	40	89
ENSECAG00000013653	3.632571439	0.1221598	0.4273609	193	266	134	132	165	239	251	429
ENSECAG00000024499	5.511517086	0.1221622	0.4273609	741	851	723	608	1000	1012	851	894
ENSECAG00000019879	5.52930735	0.1224532	0.4280597	823	712	697	723	985	1282	717	796
ENSECAG00000016487	6.58456231	0.1224749	0.4280597	2259	1727	2528	2128	1537	1110	1846	1372
ENSECAG00000019470	1.148686749	0.1225133	0.4280597	32	61	55	58	17	17	31	49
ENSECAG00000021117	2.286590863	0.1225206	0.4280597	57.0007	94.0005	42.001	92.0003	90.0006	125.001	116	88.0239
ENSECAG00000000540	6.277200022	0.1225504	0.4280597	1426	1103	1194	1299	1820	1916	1543	1106
ENSECAG00000020929	7.416265044	0.1227194	0.428518	3550	2652	2515	1887	4079	4033	3985	2365
ENSECAG00000014076	4.834067423	0.1229396	0.4289161	781	675	674	521	183	284	452	718
ENSECAG00000000095	5.420871196	0.12294	0.4289161	1002	687	1041	1184	623	410	906	679
ENSECAG00000007072	4.636018158	0.1229466	0.4289161	571	427	619	697	513	196	461	291
ENSECAG00000022762	3.717248446	0.1230658	0.4291999	277	218	343	386	190	123	271	189
ENSECAG00000021244	0.903757367	0.1232758	0.429681	45	33	46	52	12	18	15	47
ENSECAG00000017357	6.079172608	0.1232794	0.429681	1176	888	1264	1112	1627	956	1615	1454
ENSECAG00000010464	5.173008748	0.1234479	0.4301364	669	422	631	596	437	758	879	978
ENSECAG00000014670	4.578786366	0.1236352	0.4305481	654	438	586	527	304	174	577	375
ENSECAG00000002133	3.448390807	0.1236419	0.4305481	173	139	192	176	297	171	279	185
ENSECAG00000013673	6.406958945	0.1236978	0.4306109	1759	1779	1615	2543	1013	1297	1519	1315
ENSECAG00000019065	5.468265069	0.1237695	0.4307283	939	638	638	493	1436	437	1362	655
ENSECAG00000004240	3.911272707	0.1238738	0.4309582	156	247	229	274	164	469	234	414
ENSECAG00000010089	4.844264641	0.1239422	0.4309582	106	769	130	390	205	754	176	1596
ENSECAG00000013074	3.577878517	0.1239493	0.4309582	214	289	277	321	150	179	137	211
ENSECAG00000008697	3.927531303	0.1239883	0.4309618	326	298	436	333	189	207	214	272
ENSECAG00000009712	2.709658489	0.1242693	0.4315474	76	124	79	113	115	142	196	112
ENSECAG00000012725	8.116990367	0.1242695	0.4315474	3919	4274	3370	5636	3501	9087	3966	6828
ENSECAG00000022242	6.52485498	0.1243332	0.4315474	1385	1565	1527	1485	2415	1887	1919	1388

ENSECAG00000022072	9.698745153	0.1243376	0.4315474	18851	11069	11659	5500	13587	9366	45257	11121
ENSECAG00000007480	4.080136465	0.1243467	0.4315474	366	413	395	372	145	218	382	253
ENSECAG00000008891	6.509822741	0.1243947	0.4315819	1975	1515	1890	2977	1561	1250	1498	1176
ENSECAG00000023445	2.483185158	0.1245167	0.4318732	128	115	132	145	72	44	117	84
ENSECAG00000019327	3.759727123	0.1245933	0.4319047	250	61	301	13	730	220	333	47
ENSECAG00000012618	3.935637752	0.124621	0.4319047	182.001	287	187.001	253.001	158.001	395.001	234.001	534
ENSECAG00000024392	1.801856549	0.1246398	0.4319047	99	45	93	92	47	27	78	38
ENSECAG00000000520	8.035662081	0.124906	0.4326951	6224	1856	4900	3213	5854	4031	8819	4542
ENSECAG00000014943	4.663964913	0.1252637	0.4337237	378	407	470	347	391	601	484	640
ENSECAG00000012018	1.396877339	0.1253126	0.4337237	28	25	21	75	52	41	108	36
ENSECAG00000017007	4.161137081	0.1253175	0.4337237	362	340	347	633	186	163	436	277
ENSECAG00000006826	1.431317711	0.1253574	0.4337297	26	51	29	39	31	55	36	114
ENSECAG00000018113	9.529890442	0.1254533	0.4338464	102	17318	157	1865	147	26853	306	56873
ENSECAG00000017470	3.453179034	0.1254758	0.4338464	184	136	152	161	133	451	111	234
ENSECAG00000017653	2.940559244	0.1255057	0.4338464	226	110	192	195	76	68	152	140
ENSECAG00000000314	5.990006561	0.1257364	0.434503	1351	1243	1281	1905	1111	647	1260	868
ENSECAG00000015413	6.067713578	0.1257721	0.434503	1332	895	1046	828	2627	785	1661	756
ENSECAG00000011597	2.711776488	0.1259209	0.4347463	130	94	164	260	44	36	113	161
ENSECAG00000025059	6.347043179	0.1259751	0.4347463	1828	1592	1868	2015	1032	1157	1322	1455
ENSECAG00000005253	2.172493418	0.1259875	0.4347463	71	84	122	151	47	35	88	79
ENSECAG00000010584	5.401590956	0.1259957	0.4347463	964	873	858	1136	517	433	771	856
ENSECAG00000009766	5.799056866	0.1261103	0.4350098	1229	923	1271	1668	551	737	1167	944
ENSECAG00000010293	7.406375007	0.1262319	0.435297	3126	2598	2443	2708	4608	3048	3801	2786
ENSECAG00000024383	5.761059503	0.1263376	0.4353768	1241	1053	1063	1578	836	471	1086	933
ENSECAG00000016138	2.510647744	0.1263618	0.4353768	94	154	162	118	46	87	91	87
ENSECAG00000009624	6.475110323	0.1264112	0.4353768	1583	1421	1455	1345	1821	1993	1656	1819
ENSECAG00000003755	3.220852963	0.1264131	0.4353768	246	167	214	234	114	138	129	149
ENSECAG00000007895	4.670175567	0.1264467	0.4353768	521	567	493	789	234	301	324	619
ENSECAG00000021350	5.121975356	0.1265415	0.4354737	693	673	793	1004	490	521	636	450
ENSECAG00000017644	6.349030629	0.1265515	0.4354737	323	2371	295	568	432	4105	180	3017
ENSECAG00000012271	3.410026071	0.1266108	0.4355457	120	140	131	263	148	192	198	384
ENSECAG00000018384	3.795797056	0.1266994	0.4356626	222	104	266	269	399	196	425	196
ENSECAG00000011248	5.993177418	0.1267535	0.4356626	962	998	1003	1214	1251	1583	1114	1250
ENSECAG00000016705	5.559815393	0.1267598	0.4356626	1075	992	886	1479	369	369	761	1273
ENSECAG00000018195	7.190888381	0.1268796	0.4359423	2206	2756	1996	2312	3363	3464	2467	2825
ENSECAG00000014096	4.225377122	0.1269531	0.4360631	332	229	321	304	402	209	570	438
ENSECAG00000022190	6.082679677	0.1270791	0.436155	1157	1024	1114	1179	1266	1416	1285	1584
ENSECAG00000019640	7.074634242	0.1270922	0.436155	2475	1725	2529	1906	3760	2376	2739	2421
ENSECAG00000010420	7.808332867	0.1270951	0.436155	5457	4989	6951	3629	1454	1794	4395	5294
ENSECAG00000009322	0.520409956	0.1271978	0.4363755	23	29	63	22	2	6	21	36
ENSECAG00000018208	6.063768149	0.1273312	0.4365369	1130	906	1165	1235	1471	1401	1413	1195
ENSECAG00000015485	3.936747574	0.1274024	0.4365369	371	249	370	424	234	232	225	195
ENSECAG00000015087	3.254014604	0.1274357	0.4365369	272	194	207	209	118	91	230	115
ENSECAG00000010657	5.444522097	0.1274645	0.4365369	910	809	997	1254	697	395	733	819
ENSECAG00000023477	4.400560969	0.1274678	0.4365369	385	318	298	355	431	451	534	349
ENSECAG00000014197	4.621925628	0.1275459	0.4365369	320	465	269	463	431	791	344	484
ENSECAG00000022485	4.695664163	0.1276214	0.4365369	520	453	664	746	271	380	396	485
ENSECAG00000026825	2.683283938	0.1276283	0.4365369	88	106	107	73	121	110	259	83
ENSECAG00000021210	4.262429472	0.1276314	0.4365369	403	360	459	542	298	164	396	293
ENSECAG00000000644	6.292212409	0.1276901	0.4365369	1658	1774	1588	2026	1296	771	1477	1266
ENSECAG00000021567	7.179403993	0.1276954	0.4365369	2139	1807	2467	2936	2400	4231	2916	2486
ENSECAG00000009286	7.314680162	0.127706	0.4365369	2418	2142	2927	2861	3207	3751	3252	2985
ENSECAG00000008091	8.415061462	0.1279431	0.4372158	6120	5407	5032	5228	7860	7555	7332	5799
ENSECAG00000019923	6.73734854	0.1279889	0.4372408	2926	2385	2647	1831	813	1431	1451	2500
ENSECAG00000016926	5.056276774	0.128169	0.4377244	860	625	789	720	510	400	599	505
ENSECAG00000007275	3.528220707	0.1284901	0.4384618	218	108	195	203	295	198	305	188
ENSECAG00000018187	6.568868834	0.1284949	0.4384618	1603	1757	1309	1458	1928	1369	1966	2702
ENSECAG00000019795	7.197386828	0.1285118	0.4384618	2079	2569	2698	1547	1961	4779	2218	3396
ENSECAG00000010204	2.30032941	0.1285646	0.4384618	118	63	124	164	55	52	111	56
ENSECAG00000006191	2.494243781	0.1285779	0.4384618	79	78	125	35	82	88	150	184
ENSECAG00000021724	3.394019045	0.128691	0.4386155	180	211	305	282	96	122	187	200
ENSECAG00000007629	3.574180911	0.1287246	0.4386155	149	194	150	261	221	254	270	258
ENSECAG00000012474	5.851479214	0.1287388	0.4386155	1067	786	1714	1801	873	599	1325	692
ENSECAG00000009512	8.538757743	0.1288417	0.4388345	6286	5400	6410	5435	10456	7471	8118	5293
ENSECAG00000014692	8.138739636	0.1290452	0.4393959	5058	3236	4994	4968	6888	4895	7224	4830
ENSECAG00000005092	0.787460169	0.1291297	0.4395203	13	29	29	17	71	12	32	36
ENSECAG00000021956	9.680206748	0.1291592	0.4395203	12985	10685	15492	13240	18399	19830	14645	15487
ENSECAG00000012484	5.967764039	0.129318	0.4395277	1319	1271	679	580	1482	1391	1180	1211
ENSECAG00000007579	5.229515646	0.1293343	0.4395277	675	910	723	1102	463	656	562	537
ENSECAG00000007194	1.797022174	0.1293376	0.4395277	69	30	56	21	154	33	120	18
ENSECAG00000015559	6.568678241	0.1293538	0.4395277	1759	1388	1376	1767	1555	1836	2251	2220
ENSECAG00000021044	2.279081926	0.1293548	0.4395277	46	118	47	28	110	217	31	68
ENSECAG00000024627	5.505447167	0.1295125	0.439932	648	572	552	1166	716	1328	1182	587
ENSECAG00000018608	5.280097104	0.1297082	0.4401708	907	797	793	997	478	512	742	642
ENSECAG00000008584	5.601230823	0.1297336	0.4401708	792	647	731	978	689	788	925	1698
ENSECAG00000006453	3.960976106	0.1297388	0.4401708	235	220	239	280	262	475	255	300
ENSECAG00000011898	2.679843286	0.1297428	0.4401708	229	41	30	36	133	141	216	108
ENSECAG00000021311	7.46947253	0.1297766	0.4401708	3877	3018	4121	5305	2791	2607	2974	2176

ENSECAG00000020694	4.527649456	0.1298837	0.4404026	344	374	282	481	350	354	713	564
ENSECAG00000016726	7.971427382	0.130146	0.4411605	3960	3835	3493	4566	2644	5852	5506	7406
ENSECAG00000018458	1.647275728	0.1302608	0.4414178	24	62	40	49	31	75	100	69
ENSECAG00000020213	5.825847091	0.1305654	0.442318	873	786	914	1058	939	1559	666.001	1506
ENSECAG00000020988	3.331068734	0.1307954	0.442965	207	216	214	294	112	118	150	200
ENSECAG00000023351	1.127366712	0.130888	0.4431465	36	49	38	93	7	9	72	22
ENSECAG00000009903	3.45271377	0.1309955	0.443255	120	271	590	125	18	73	154	288
ENSECAG00000006525	4.713267856	0.1310108	0.443255	394	288	490	523	420	707	511	536
ENSECAG00000000891	5.811221934	0.1310487	0.443255	1103	772	781	1058	1253	802	1303	1320
ENSECAG00000016503	0.754582062	0.1311362	0.443255	29	18	27	19	37	27	28	49
ENSECAG00000024350	5.13659669	0.1311603	0.443255	849	666	727	1055	449	178	796	679
ENSECAG00000009390	8.142805185	0.1311669	0.443255	6128	5309	6528	8124	4561	4080	5278	2829
ENSECAG00000026944	3.44451237	0.1311932	0.443255	226	204	287	286	126	171	153	174
ENSECAG00000016599	4.612847245	0.131346	0.4436395	459	362	369	395	528	460	540	504
ENSECAG00000006129	4.035536559	0.1314211	0.4437612	296	355	348	533	347	127	240	233
ENSECAG00000021522	2.233348234	0.1315947	0.4441377	81	64	65	68	95	127	66	106
ENSECAG00000026912	1.064233253	0.1316108	0.4441377	37	25	47	90	33	18	35	22
ENSECAG00000007083	4.86944984	0.1317556	0.4444943	525	694	644	783	401	456	503	381
ENSECAG00000011890	10.01721865	0.1318746	0.4444977	15131	15024	16077	17158	14898	39669	15168	16990
ENSECAG00000015899	6.632764787	0.1318914	0.4444977	1430	1480	1707	1818	1639	2880	1893	1733
ENSECAG00000016812	4.915278977	0.1319256	0.4444977	453	412	425	685	605	519	939	495
ENSECAG00000016854	7.149153123	0.131928	0.4444977	2668	1770	2250	2389	2668	4162	2897	2050
ENSECAG00000021223	9.20053486	0.1319523	0.4444977	8982	8547	8972	10099	6992	12366	9332	21798
ENSECAG00000019029	7.193343228	0.1319983	0.4445129	3734	2518	3311	3819	2481	2146	2293	1778
ENSECAG00000002833	5.199540653	0.1320351	0.4445129	655	821	768	1115	341	601	633	613
ENSECAG00000006450	1.953111275	0.1321406	0.4447365	59	51	59	62	101	50	99	82
ENSECAG00000012818	4.146302061	0.1321866	0.4447593	449	272	458	475	246	345	258	149
ENSECAG00000014678	5.669374037	0.1323244	0.445091	1173	856	1192	1371	763	772	912	648
ENSECAG00000009670	4.137869865	0.1324693	0.4454466	431	206	494	558	295	91	388	243
ENSECAG00000013659	4.070682012	0.1325184	0.44548	291	174	262	302	269	632	236	263
ENSECAG00000015580	7.402632954	0.1326541	0.445804	3963	3238	4244	3904	2673	1887	3251	2446
ENSECAG00000019817	5.470790055	0.1327165	0.4458525	1191	720	1005	1091	756	612	732	576
ENSECAG00000010893	2.373744723	0.132754	0.4458525	135	77	159	115	95	57	86	45
ENSECAG00000014480	4.157139534	0.1327862	0.4458525	389	455	421	461	81	92	262	545
ENSECAG00000023874	1.510567975	0.1328496	0.4458566	64	51	70	82	42	21	69	22
ENSECAG00000015951	5.001544755	0.1329413	0.4458566	410	591	524	545	559	539	863	737
ENSECAG00000014304	5.936956601	0.1329645	0.4458566	1625	1009	1028	2100	1236	765	1139	431
ENSECAG00000021530	6.110011864	0.1329791	0.4458566	1582	1349	1453	1807	1015	1077	1060	1045
ENSECAG00000015331	1.2310808	0.1329973	0.4458566	62	41	57	61	7	25	49	41
ENSECAG00000008518	1.549282452	0.133023	0.4458566	69	33	71	131	22	7	107	8
ENSECAG00000011763	6.667483482	0.1331707	0.4461865	2229	1882	2167	2927	1306	1412	1647	1822
ENSECAG00000023437	6.178270512	0.1332	0.4461865	1490	1537	1818	1657	849	1208	1006	1278
ENSECAG00000012569	5.077718797	0.133259	0.4462056	780	612	800	855	424	479	577	565
ENSECAG00000001532	2.81414107	0.1333032	0.4462056	101	131	157	290	65	55	139	135
ENSECAG00000015412	4.495989372	0.1333554	0.4462056	540	376	406	769	376	267	426	273
ENSECAG00000003080	5.064240264	0.1333628	0.4462056	610	543	440	575	548	559	788	915
ENSECAG00000017925	2.005607304	0.1334827	0.4463979	59	42	68	71	94	89	108	51
ENSECAG00000012182	5.180099159	0.1334989	0.4463979	494	512	648	627	477	1220	540	759
ENSECAG00000015492	4.015235239	0.1336448	0.4467543	413	254	418	470	307	140	387	76
ENSECAG00000023040	5.118367482	0.1337461	0.4469615	747	681	901	805	513	582	461	501
ENSECAG00000007798	5.069366386	0.1338445	0.4471587	438	485	708	556	692	613	706	777
ENSECAG00000020068	7.184254195	0.1338924	0.4471871	1909	1987	2298	3136	4495	2703	3381	1714
ENSECAG00000024204	2.881975092	0.1339659	0.447267	97	82	105	172	131	203	173	117
ENSECAG00000025031	2.895701092	0.1340183	0.447267	134	91	90	149	146	139	203	147
ENSECAG00000013423	8.975838604	0.1340453	0.447267	7828	8698	5954	9499	8686	10627	8562	14550
ENSECAG00000006723	1.879379836	0.134108	0.447267	68	168	41	76	44	27	14	91
ENSECAG00000024436	0.918810202	0.1341131	0.447267	49	22	17	99	21	21	12	35
ENSECAG00000023327	1.168926762	0.1342602	0.4475971	45	22	70	81	13	14	35	52
ENSECAG00000007681	8.226422855	0.1343016	0.4475971	4245	2966	4752	6057	10141	2300	12330	2817
ENSECAG00000019162	7.611635738	0.1343538	0.4475971	4417	3507	5164	4761	3228	2441	3737	2353
ENSECAG00000024771	7.92119813	0.1343698	0.4475971	3637	4459	3735	3675	4542	5152	4871	5669
ENSECAG00000023353	5.790530326	0.1344318	0.4476726	1064	803	869	814	1301	1366	1294	630
ENSECAG00000022236	3.489621985	0.1345128	0.4477265	238	263	162	458	253	48	247	50
ENSECAG00000022266	2.078435347	0.1345268	0.4477265	100	67	93	142	27	45	98	63
ENSECAG00000004050	2.253812671	0.1346411	0.4479426	45	244	81	87	35	27	45	128
ENSECAG00000023838	0.976663751	0.1346707	0.4479426	70	27	42	45	15	15	20	49
ENSECAG00000011157	4.503686166	0.1347862	0.4481356	517	384	585	574	287	282	435	366
ENSECAG00000019512	0.793261312	0.1348076	0.4481356	17	7	32	35	28	20	95	17
ENSECAG00000012496	4.705698712	0.1348587	0.4481742	337	446	327	537	318	549	478	868
ENSECAG00000023502	5.906185656	0.1351404	0.4489791	1212	1279	1251	1627	947	800	1108	828
ENSECAG00000021826	7.010426941	0.135198	0.4490393	3096	2597	2632	3410	1799	1222	2753	2117
ENSECAG00000019935	0.517514324	0.1352467	0.4490697	15	16	25	22	19	38	40	22
ENSECAG00000009007	5.452823267	0.135329	0.4492115	987	770	932	1308	859	463	778	549
ENSECAG00000011122	5.393870338	0.1354576	0.449507	1109	720	999	1044	786	307	980	462
ENSECAG00000012050	4.879953208	0.1356184	0.4499092	615	407	523	279	845	404	791	493
ENSECAG00000016216	5.856626091	0.1356787	0.4499779	1050	789	973	1025	995	1361	1276	1112
ENSECAG00000023170	6.911420434	0.1357927	0.4502247	2022	1086	1505	3091	3320	1767	4078	1378
ENSECAG00000020945	2.111337769	0.1359293	0.4504439	137	65	79	123	52	41	75	72

ENSECAG00000020013	0.527257786	0.1359382	0.4504439	29	22	29	57	9	17	7	34
ENSECAG00000014213	5.891351758	0.1360247	0.4505992	1345	1104	1242	1675	864	621	1335	857
ENSECAG00000002458	0.091353462	0.1361749	0.4507507	12	12	10	21	8	25	22	33
ENSECAG00000026940	2.954233447	0.1361854	0.4507507	176	122	190	238	97	88	174	87
ENSECAG00000021362	4.306439683	0.1361895	0.4507507	392	343	444	639	297	253	330	299
ENSECAG00000009219	8.43748943	0.136307	0.4510082	8494	6420	8230	8681	5739	3109	6951	5113
ENSECAG00000001964	5.726277497	0.1363814	0.4510176	1389	913	1208	1282	991	477	1042	725
ENSECAG00000020771	2.627425654	0.1363892	0.4510176	111	105	141	226	70	72	101	105
ENSECAG00000010185	5.66089371	0.136629	0.4516789	465	1055	364	700	132	2497	321	1610
ENSECAG00000006577	5.278798225	0.1368113	0.4521501	999	654	937	941	619	316	738	690
ENSECAG00000020674	4.101849357	0.1369333	0.4524215	398	270	432	483	164	253	300	295
ENSECAG00000012712	6.300936266	0.137074	0.4527546	992	1675	858	1530	1321	1954	1502	1758
ENSECAG00000010755	3.287570207	0.1371142	0.4527558	149	128	187	118	192	275	102	253
ENSECAG00000000096	2.424736437	0.1371816	0.4527652	38	113	80	76	75	153	104	130
ENSECAG00000014441	3.339372653	0.1371967	0.4527652	257	135	268	301	135	63	237	150
ENSECAG00000016838	5.842019076	0.1374957	0.4536199	1136	751	940	967	1210	1077	1528	940
ENSECAG00000012538	2.338338191	0.1375482	0.4536614	59	62	80	104	82	153	98	92
ENSECAG00000003235	5.246094736	0.1376059	0.4537199	821	683	806	1154	372	510	768	660
ENSECAG00000016646	0.765871172	0.1377058	0.4538225	44	35	37	37	17	17	36	19
ENSECAG00000018670	4.404061005	0.1377864	0.4538225	330	310	301	433	416	332	677	376
ENSECAG00000013751	7.08410979	0.137792	0.4538225	2349	1846	1800	2936	3105	1940	3581	2833
ENSECAG00000008291	7.916458919	0.1377968	0.4538225	5505	4414	5674	6573	3558	2220	4569	4292
ENSECAG00000007948	1.39057919	0.13792	0.4540965	55	87	48	57	19	36	10	62
ENSECAG00000022709	3.350978406	0.1380907	0.4545269	241	207	259	233	127	76	213	182
ENSECAG00000008116	2.546524872	0.1385902	0.4558835	82	74	79	100	169	58	260	49
ENSECAG00000024349	9.235383005	0.1386074	0.4558835	10407	7282	9722	11621	15864	8745	16345	10436
ENSECAG00000024122	5.982083604	0.1386483	0.4558835	1073	893	1150	1091	1310	1264	1299	1288
ENSECAG00000024495	3.697032456	0.1386634	0.4558835	276	114	222	176	258	143	544	223
ENSECAG00000020589	6.01210686	0.1387242	0.4559516	1339	1235	1499	1701	872	904	1153	1046
ENSECAG00000022681	2.140992425	0.1389769	0.4565634	48	86	42	73	60	166	87	63
ENSECAG00000004617	6.919968749	0.1389908	0.4565634	2169	3483	2719	2559	1328	1812	2663	1443
ENSECAG00000021473	4.740483812	0.1391745	0.4570115	530	433	555	978	336	243	511	514
ENSECAG00000015278	5.21403497	0.1392077	0.4570115	982	765	786	779	539	443	709	581
ENSECAG00000007184	0.882074092	0.1393116	0.4572205	25	17	28	36	48	33	39	33
ENSECAG00000010042	7.666424851	0.1395387	0.4576951	2867	3843	3135	3261	3878	4016	4721	4363
ENSECAG00000012903	4.639380627	0.1395432	0.4576951	613	412	541	741	306	198	589	416
ENSECAG00000021189	1.11708881	0.1395771	0.4576951	31	23	39	33	52	38	49	42
ENSECAG00000011946	2.640953971	0.1396392	0.4577668	163	77	137	213	102	67	95	85
ENSECAG00000017507	1.201242936	0.1398588	0.4583543	32	22	37	43	49	48	65	32
ENSECAG00000015075	2.140362285	0.1399752	0.4586034	31	58	91	78	93	115	115	56
ENSECAG00000019715	3.601425579	0.1402075	0.45918	344	187	389	209	240	163	122	146
ENSECAG00000017235	4.906722877	0.140232	0.45918	363	608	415	509	734	493	456	828
ENSECAG00000007355	5.181031297	0.1405007	0.4599272	464	431	480	917	1033	1078	494	409
ENSECAG00000009336	8.293924848	0.1406121	0.4600672	5209	4397	5845	4560	6057	3617	8751	8703
ENSECAG00000008799	3.883552672	0.1406535	0.4600672	265	144	240	228	432	141	645	132
ENSECAG00000020779	2.7005948	0.140665	0.4600672	58	116	62	150	145	188	139	83
ENSECAG00000011419	5.316642707	0.1407822	0.460318	989	595	951	1167	765	356	898	368
ENSECAG00000000103	4.927944241	0.1408699	0.460361	448	437	552	544	764	651	641	453
ENSECAG00000014635	8.771822311	0.1408764	0.460361	7622	3681	6742	9574	13552	8170	11705	4347
ENSECAG00000005863	8.833861008	0.1409503	0.4604067	5699	7911	5619	6258	3254	19909	2945	13494
ENSECAG00000010929	3.301713589	0.1410095	0.4604067	248	167	213	282	122	111	161	181
ENSECAG00000015673	3.884978838	0.1410446	0.4604067	338	293	343	377	153	163	265	296
ENSECAG00000013433	5.482572985	0.1410525	0.4604067	674	754	816	652	707	1163	882	911
ENSECAG00000014960	4.113277677	0.1411804	0.460692	432	349	417	370	313	251	235	210
ENSECAG00000013325	5.165878953	0.1412829	0.460894	709	672	908	940	492	462	635	605
ENSECAG00000015168	5.179915544	0.1413732	0.4609435	713	642	966	962	661	469	596	458
ENSECAG00000021851	5.583008649	0.1413793	0.4609435	668	852	786	756	680	686	998	1707
ENSECAG00000008170	3.987127428	0.1414333	0.4609872	304	319	259	627	290	194	306	114
ENSECAG00000023122	2.305263547	0.1415339	0.4611828	6	240.998	4	303.998	32	94	22.9999	31
ENSECAG00000024159	5.01923242	0.1416801	0.461527	669	684	719	858	326	450	654	542
ENSECAG00000020407	2.080706165	0.1417597	0.4616538	77	87	37	19	57	56	221	67
ENSECAG00000011374	7.388465491	0.1418425	0.461791	2829	2469	2466	3115	2828	4482	3183	3317
ENSECAG00000024968	3.965676778	0.1419396	0.4619746	218	230	167	377	341	412	258	287
ENSECAG00000007599	6.59267063	0.1420325	0.4621446	1847	1923	2285	2640	1109	1345	1560	1881
ENSECAG00000008032	0.844343047	0.1422031	0.4625544	53	28	39	44	17	13	33	31
ENSECAG00000000966	0.141441137	0.1423035	0.4625544	20	11	10	13	28	26	37	3
ENSECAG00000014138	4.701970038	0.1423054	0.4625544	657	493	625	592	388	204	576	413
ENSECAG00000007789	2.94727529	0.1423213	0.4625544	196	154	251	117	58	81	113	176
ENSECAG00000016443	3.958053479	0.1423673	0.4625716	347	101	323	72	496	137	724	131
ENSECAG00000007626	6.65006403	0.1424395	0.4626737	1372	2190	1515	4587	770	1385	1369	2226
ENSECAG00000025223	1.052535737	0.1425055	0.4627557	41	17	27	33	34	37	36	68
ENSECAG00000009136	4.829085239	0.1426735	0.4630878	486	268	574	490	916	455	570	451
ENSECAG00000021957	5.640441456	0.1427469	0.4630878	1086	1036	1186	1126	689	597	880	913
ENSECAG00000015135	9.023704762	0.1428933	0.4630878	9304	7982	7918	8284	11213	11571	9335	11064
ENSECAG00000022039	4.403242847	0.142929	0.4630878	485	207	393	246	554	316	535	401
ENSECAG00000019590	1.217097969	0.142943	0.4630878	25	58	16	25	29	56	53	63
ENSECAG00000013481	6.75183111	0.1429461	0.4630878	1918	1847	1191	1918	2228	1523	4090	1533
ENSECAG00000024920	6.517811297	0.1429605	0.4630878	2240	1739	1666	2667	1163	911	1837	1746

ENSECAG00000005666	6.227973145	0.142973	0.4630878	1355	1008	990	1638	1581	993	2072	1657
ENSECAG00000009830	3.151443893	0.1429756	0.4630878	99	122	102	232	225	197	152	174
ENSECAG00000018047	0.813304392	0.1430154	0.4630878	61	13	27	72	28	5	40	12
ENSECAG00000011391	5.621718191	0.1432176	0.4635111	1136	1085	1396	842	1081	379	875	597
ENSECAG00000024023	6.548874796	0.1432277	0.4635111	2030	1797	2310	2257	1436	1231	2022	1083
ENSECAG00000011726	3.132361054	0.1433693	0.4636809	226	215	185	179	96	68	207	140
ENSECAG00000019725	1.233543563	0.1433706	0.4636809	74.0007	45.0005	41.001	59.0003	28.0006	10.0007	62.0001	26.0239
ENSECAG00000012302	0.522349058	0.1434027	0.4636809	12	19	31	10	15	44	13	49
ENSECAG00000019587	1.650198833	0.1434854	0.4638165	64	58	71	96	44	33	42	53
ENSECAG00000006422	2.687331301	0.1435324	0.4638364	93	139	142	244	42	74	87	149
ENSECAG00000007377	5.863011985	0.1436461	0.4638844	1046	801	1032	933	954	1541	1336	943
ENSECAG00000014325	3.378475804	0.1437168	0.4638844	246	192	222	309	138	83	262	130
ENSECAG00000001499	1.592184791	0.1437506	0.4638844	12	81	29	36	45	39	108	84
ENSECAG00000015700	5.764044714	0.1437552	0.4638844	1230	1016	1239	1378	960	586	1012	799
ENSECAG00000012816	4.575385847	0.1437777	0.4638844	395	301	431	395	656	535	507	285
ENSECAG00000019106	3.139222813	0.1437923	0.4638844	198	183	186	266	69	65	254	123
ENSECAG00000021301	6.087743019	0.1438571	0.4639617	907	968	1094	1411	1404	1974	1618	653
ENSECAG00000024195	3.306835143	0.1439663	0.4640834	217	136	306	282	178	50	179	155
ENSECAG00000006322	1.98412429	0.1439998	0.4640834	87	57	103	135	33	17	101	66
ENSECAG00000009926	6.377562711	0.1440174	0.4640834	1398	1458	1125	1468	1461	2081	1542	1696
ENSECAG00000010808	4.141087912	0.144074	0.4641341	616	242	460	333	276	111	427	216
ENSECAG00000020696	1.940587888	0.144155	0.4641501	56	57	42	53	59	177	27	61
ENSECAG00000023388	1.765191486	0.1441607	0.4641501	45	84	15	27	50	69	30	157
ENSECAG00000017563	6.809328873	0.1442418	0.4642796	2700	2123	2988	2329	1220	1233	2145	2235
ENSECAG00000015081	1.124946021	0.1443013	0.4643396	14	36	36	33	46	68	22	46
ENSECAG00000009847	8.362581842	0.1443728	0.4644381	7026	6715	6894	9357	4557	4621	5549	5085
ENSECAG00000010391	5.195232624	0.1444289	0.4644871	741	915	700	934	399	467	628	729
ENSECAG00000016612	7.457141525	0.1444715	0.4644923	4185	3092	4273	4401	2941	2396	2952	2294
ENSECAG00000017143	5.436314424	0.1446112	0.46481	850	15	439	775	1857	81	337	2083
ENSECAG00000009520	5.054352827	0.1447343	0.4650742	748	534	737	1017	494	502	633	372
ENSECAG00000016289	5.910703169	0.1448439	0.4650964	1072	938	1006	984	1258	1007	1449	1239
ENSECAG00000020780	6.138528719	0.1448536	0.4650964	1395	1571	1475	1840	1194	821	1294	1043
ENSECAG00000014248	6.234600092	0.1448641	0.4650964	1450	1506	1506	2382	993	739	1468	1445
ENSECAG00000013746	1.036564804	0.1449568	0.4652586	28	142	26	4	5	11	10	56
ENSECAG00000001621	4.321440689	0.1450526	0.4652586	254	87	208	576	463	84	1284	175
ENSECAG00000015348	5.991219879	0.1451029	0.4652586	1239	1309	1406	1864	567	613	1317	1396
ENSECAG00000008052	5.712562841	0.1451167	0.4652586	1848	617	1388	1053	650	610	1444	394
ENSECAG00000020587	7.250044477	0.1451202	0.4652586	463	4718	269	691	421	6429	302	7921
ENSECAG00000013991	0.56407548	0.1451604	0.4652586	25	33	44	32	10	28	12	21
ENSECAG00000024245	1.10261444	0.1452376	0.4653749	33	26	31	22	31	104	29	16
ENSECAG00000005690	2.056192486	0.1453838	0.4657121	79	98	84	125	30	53	72	75
ENSECAG00000022437	6.571005281	0.1455699	0.4661299	2422	1649	2100	2341	1368	1473	1622	1353
ENSECAG00000017691	6.607899084	0.1456028	0.4661299	2261	1749	2488	2208	1490	1443	1566	1461
ENSECAG00000013892	2.326624566	0.1456374	0.4661299	114	153	64	137	39	67	60	104
ENSECAG00000019861	3.063694265	0.1456951	0.4661436	157	134	257	230	113	83	181	108
ENSECAG00000013334	7.29645624	0.1457237	0.4661436	3760	1677	2562	1171	5660	3830	3039	1137
ENSECAG00000009585	7.916563745	0.1457724	0.4661679	3598	4193	3474	4418	5245	5200	4454	5070
ENSECAG00000007797	5.903227961	0.14586	0.4663169	1578	1066	1290	1445	1009	974	803	802
ENSECAG00000021106	4.979345223	0.1464837	0.4681694	483	508	511	563	565	740	628	650
ENSECAG00000018679	0.839866641	0.1465219	0.4681694	30	34	19	14	46	29	32	43
ENSECAG00000016130	1.554250253	0.1468231	0.4689999	52	33	64	142	21	12	62	59
ENSECAG00000001064	4.487383637	0.1470316	0.4694617	99	392	524	184	88	501	422	1071
ENSECAG00000014320	3.375688684	0.1470503	0.4694617	124	146	238	74	181	167	94	480
ENSECAG00000007671	7.421216573	0.1471877	0.4697164	3106	2025	2721	3089	5966	1959	4009	2719
ENSECAG00000005814	7.89559704	0.1472128	0.4697164	5296	2910	4376	1332	5464	2956	10552	2937
ENSECAG00000009675	5.264675803	0.1472703	0.4697677	1105	555	744	1115	463	466	862	557
ENSECAG00000020198	0.685589768	0.1473755	0.4699716	40	40	21	54	8	43	15	4
ENSECAG00000011550	5.188653864	0.147459	0.470029	700	499	591	597	562	817	704	917
ENSECAG00000017862	0.555576891	0.1474763	0.470029	20	15	23	23	30	42	20	27
ENSECAG00000014232	9.004989848	0.1476228	0.470248	11571	3458	10891	632	10265	3869	27122	10321
ENSECAG00000001409	0.953302849	0.1476679	0.470248	37	39	39	61	21	28	33	20
ENSECAG00000022950	5.079155293	0.1477074	0.470248	634	421	509	683	595	598	871	747
ENSECAG00000009794	6.481129745	0.1477106	0.470248	1520	1410	1139	1793	1124	2049	1842	2383
ENSECAG00000018394	4.892485473	0.1477997	0.4703227	537	404	460	551	669	543	536	695
ENSECAG00000011690	1.935401157	0.1478169	0.4703227	94	86	88	79	50	37	61	66
ENSECAG00000009497	5.393757113	0.1478813	0.470396	918	637	663	446	818	1063	672	910
ENSECAG00000011326	5.224206239	0.1479909	0.4706128	885	743	871	832	533	464	602	688
ENSECAG00000011450	6.030245909	0.1480827	0.4707137	1478	648	1105	974	1865	885	1921	909
ENSECAG00000018243	8.512609435	0.1481123	0.4707137	7706	7735	8386	9432	3939	4402	7139	6679
ENSECAG00000015966	7.139362128	0.148147	0.4707137	3408	2114	2872	4854	2788	1550	2832	1167
ENSECAG00000014958	5.026325171	0.1484652	0.4715063	457	437	546	717	576	708	603	789
ENSECAG00000019644	1.574450834	0.1484794	0.4715063	47	85	45	101	29	32	32	64
ENSECAG00000012570	4.389974057	0.1487066	0.4718062	325.002	289.057	358	399	526	383	439	383.002
ENSECAG00000022899	3.010294568	0.1487275	0.4718062	153	108	91	144	143	197	120	215
ENSECAG00000004817	3.90688826	0.1487444	0.4718062	370	212	340	498	267	192	301	99
ENSECAG00000001892	7.261186505	0.1487712	0.4718062	2358	2344	1895	3376	2051	3583	2994	4183
ENSECAG00000008647	5.213904291	0.1488214	0.4718062	854	535	888	1165	406	348	1008	512
ENSECAG00000016040	5.075929348	0.1488231	0.4718062	778	543	765	1015	365	313	656	718

ENSECAG00000017490	5.56895906	0.1490829	0.4723691	946	832	1165	1318	622	627	936	754
ENSECAG00000012274	6.06132096	0.1490838	0.4723691	1151	815	1154	1356	1408	1489	1343	1191
ENSECAG00000015383	1.655403853	0.149319	0.4728762	53	63	42	145	23	51	53	38
ENSECAG00000014690	6.461209636	0.1493271	0.4728762	2027	1315	1975	2712	1415	959	1876	1189
ENSECAG00000013728	1.775384211	0.1494039	0.4729875	59	68	25	12	30	167	20	85
ENSECAG00000016857	4.108855151	0.1495739	0.4733937	236	213	386	276	407	293	416	330
ENSECAG00000015208	1.298084349	0.1497619	0.4736856	59	51	69	43	22	27	43	41
ENSECAG00000000357	9.319294176	0.1497709	0.4736856	10716	7797	10691	12613	14333	8755	16768	14395
ENSECAG00000011036	5.187190462	0.1497912	0.4736856	783	467	588	534	786	778	911	544
ENSECAG00000000686	10.64024027	0.1499357	0.4737234	26636	23005	26678	27482	39648	26906	37868	29444
ENSECAG00000014199	5.438381258	0.1499427	0.4737234	629	663	616	920	514	1121	826	1122
ENSECAG00000019671	7.931377049	0.1499656	0.4737234	3820	3581	4006	4090	3418	7959	4178	4622
ENSECAG00000018637	4.011700036	0.14997	0.4737234	235	249	291	239	259	323	254	516
ENSECAG00000022383	2.650443911	0.1502025	0.4743258	138	92	80	44	165	81	77	228
ENSECAG00000008992	6.377655034	0.1503864	0.4745381	1370	1094	1620	1353	1666	2220	1241	1621
ENSECAG00000001066	4.854863699	0.1503901	0.4745381	556	429	507	255	1053	483	659	292
ENSECAG00000017543	5.1391981	0.1504241	0.4745381	817	585	862	906	453	468	654	586
ENSECAG00000019948	5.621074935	0.1504368	0.4745381	1028	302	673	729	2586	333	1529	169
ENSECAG00000009173	4.064990135	0.1505193	0.4746665	389	158	272	111	607	146	713	104
ENSECAG00000002281	5.649888878	0.1505789	0.4747226	1045	905	1249	1309	576	598	1080	867
ENSECAG00000002621	6.252311246	0.150675	0.4748581	1674	1455	1893	1799	1121	1321	1261	912
ENSECAG00000000541	1.611043819	0.1507055	0.4748581	56	69	43	119	26	47	51	39
ENSECAG00000001181	4.768561183	0.1508344	0.4751325	374	431	510	431	785	482	650	362
ENSECAG00000022028	4.90298442	0.150908	0.4752325	649	473	438	348	483	653	680	679
ENSECAG00000023886	6.288864504	0.1509556	0.4752509	2004	1349	1745	2094	1699	691	1637	671.999
ENSECAG00000017788	4.485920652	0.1510038	0.4752708	408	299	366	397	521	436	477	409
ENSECAG00000022115	3.581522576	0.1511861	0.4756986	274	211	287	334	226	91	234	154
ENSECAG00000023260	4.241447196	0.1512359	0.4756986	481	229	297	815	243	165	471	232
ENSECAG00000008076	6.295576943	0.1512654	0.4756986	1484	1794	1696	2090	849	1501	1202	1156
ENSECAG00000000703	3.341049291	0.1514313	0.475782	194	147	347	264	134	69	242	145
ENSECAG00000022217	5.151285529	0.1514553	0.475782	784	673	782	988	682	310	717	460
ENSECAG00000005447	1.345446544	0.1514554	0.475782	24	113	37	60	25	24	50	32
ENSECAG00000022117	7.133037079	0.1514959	0.475782	2819.98	2781.98	2898	4365	1591.99	2342	2907	1604.05
ENSECAG00000019260	4.472536364	0.1515339	0.475782	561	398	473	578	421	254	343	313
ENSECAG00000015463	6.318571586	0.1515432	0.475782	1524	2692	1348	1579	706	1142	1352	1549
ENSECAG00000006881	4.831174761	0.1517406	0.4762034	501	401	293	528	313	1200	345	516
ENSECAG00000016998	5.928723758	0.1517613	0.4762034	1895	1520	1202	890	440	801	1032	1325
ENSECAG00000025060	2.426361625	0.1518532	0.4762669	77	121	150	159	82	66	116	31
ENSECAG00000022080	6.375193533	0.1518654	0.4762669	1265	1486	1292	1261	1148	2347	1193	2122
ENSECAG00000006077	4.839767165	0.1519496	0.4762898	602	536	663	785	407	445	507	369
ENSECAG00000020477	5.494429493	0.1519565	0.4762898	733	784	754	703	809	949	916	1003
ENSECAG00000012953	5.34345807	0.1521232	0.4766808	813	1297	890	822	192	290	499	1294
ENSECAG00000019520	6.696987095	0.1522422	0.4769221	1860	1415	1627	1957	2784	1687	2499	1641
ENSECAG00000011676	3.059386895	0.1523738	0.477145	168	168	191	235	107	105	147	128
ENSECAG00000011164	3.648046554	0.152413	0.477145	252	244	357	304	89	209	187	233
ENSECAG00000021009	1.743968143	0.1524394	0.477145	39	125	71	89	19	7	101	47
ENSECAG00000008307	6.061477428	0.1525544	0.4773735	1487	1213	1384	1918	790	891	1186	1259
ENSECAG00000011671	7.602308401	0.1526009	0.4773874	3040	3447	3116	2821	5307	4045	4029	2764
ENSECAG00000008574	2.653907883	0.1527154	0.477614	94	104	59	131	141	114	121	155
ENSECAG00000000021	3.705348062	0.152808	0.477772	316	267	346	271	167	78	300	228
ENSECAG00000020482	1.60632631	0.1529014	0.4779327	47	43	37	49	31	70	115	49
ENSECAG00000008070	5.06415588	0.1536958	0.480246	553	444	521	717	715	630	782	624
ENSECAG00000019322	7.231572504	0.1537623	0.480246	3801	2068	3299	4933	2868	1446	3276	1376
ENSECAG00000012867	4.222738816	0.1537684	0.480246	321	286	244	371	351	356	432	408
ENSECAG00000015266	8.393240951	0.1539137	0.4805678	7277	6697	8526	7935	4295	5078	5148	5641
ENSECAG00000018014	3.076328636	0.1541238	0.4810917	132	116	129	146	203	87	274	170
ENSECAG00000017376	6.417367263	0.1542358	0.481309	1691	1650	1886	2676	642	918	1418	2162
ENSECAG00000013409	6.35209587	0.1543611	0.4815675	1987	1165	1991	2438	1119	530	2182	1195
ENSECAG00000024810	3.919268036	0.1547421	0.4825184	76	364	121	207	39	676	146	510
ENSECAG00000020227	7.408563393	0.154855	0.4825184	2028	3595	2043	2694	2156	6199	2428	3389
ENSECAG00000013307	5.448109273	0.1548766	0.4825184	666	723	685	813	1116	792	991	685
ENSECAG00000020954	4.417314443	0.1548815	0.4825184	448	421	507	550	394	252	338	301
ENSECAG00000019675	9.590452599	0.1548859	0.4825184	17539	15025	18867	18901	14004	7726	15239	9621
ENSECAG00000023035	4.831271482	0.1549207	0.4825184	523	363	457	527	697	445	765	473
ENSECAG00000014533	5.693295632	0.155043	0.4827668	951	1323	1006	1346	583	655	816	1104
ENSECAG00000014928	10.4865957	0.1551104	0.4828443	24391	18811	23842	26668	29946	32874	26249	29420
ENSECAG00000015158	2.713729973	0.1551999	0.4829905	168	93	146	231	120	127	37	51
ENSECAG00000014620	7.18826972	0.1552852	0.4831237	2730	3134	3613	3695	1915	2157	1927	2746
ENSECAG00000000984	5.522276978	0.155492	0.483443	1084	744	829	1600	643	470	1232	473
ENSECAG00000013618	3.455780368	0.1554926	0.483443	177	149	150	227	253	235	251	174
ENSECAG00000016933	5.898714122	0.1555432	0.483443	982	746	1120	1039	1333	1684	1101	745
ENSECAG00000025184	1.215090728	0.1555581	0.483443	18	45	32	34	36	72	29	56
ENSECAG00000022378	4.974808888	0.155658	0.4836211	751	301	594	90	1428	306	729	407
ENSECAG00000022108	4.315914903	0.1557328	0.4837212	362	328	284	317	386	422	441	393
ENSECAG00000017282	4.244676083	0.1558562	0.4838742	390	420	700	271	134	208	154	544
ENSECAG00000006253	5.00990831	0.1558672	0.4838742	652	241	454	643	975	409	1256	244
ENSECAG00000024214	4.741337562	0.1559259	0.4839242	506	556	477	903	361	398	507	344
ENSECAG00000018009	4.351227673	0.1559882	0.4839755	412	341	523	611	343	317	375	159

ENSECAG00000003291	0.697054205	0.1560685	0.4839755	10	15	17	48	33	19	63	27
ENSECAG00000010712	5.150095466	0.1561134	0.4839755	644	535	560	592	736	852	761	549
ENSECAG00000016806	4.012349821	0.1561488	0.4839755	227	158	284	378	365	301	475	227
ENSECAG00000013254	2.795621005	0.156177	0.4839755	131	135	173	209	58	70	105	162
ENSECAG00000020098	4.830753765	0.1562104	0.4839755	662	496	711	725	353	232	654	505
ENSECAG00000023726	5.894984145	0.1562407	0.4839755	1399	1490	1104	1284	1106	848	896	748
ENSECAG00000009129	2.44016133	0.1563333	0.4841305	19	151	55	6	163	232	111	9
ENSECAG00000016548	5.996020825	0.1563794	0.4841412	1518	1040	1508	1636	975	884	1145	941
ENSECAG00000025695	0.762387204	0.1564583	0.4842534	21	22	22	24	21	31	10	83
ENSECAG00000011929	6.760014775	0.1565919	0.4844984	3112	675	1027	14	3964	616	6183	793
ENSECAG00000016233	3.30919656	0.1566736	0.4844984	191	129	96	191	86	260	264	244
ENSECAG00000011019	2.567225484	0.1566763	0.4844984	91	82	54	137	77	147	153	128
ENSECAG00000000352	7.45491909	0.1567081	0.4844984	3198	3437	3530	5988	2121	2430	3880	2228
ENSECAG00000015238	0.609792281	0.1567587	0.484523	26	41	30	39	16	20	14	27
ENSECAG00000023807	5.032996827	0.1568105	0.4845512	505	476	551	651	580	654	787	669
ENSECAG00000024442	3.475574412	0.1568694	0.4846013	80	338	46	68	162	410	33	411
ENSECAG00000017720	5.751000379	0.1569884	0.4848371	791	730	832	1200	1359	1294	1138	623
ENSECAG00000020316	11.55376483	0.1572205	0.4853506	57194	34424	57801	43724	66177	70505	49413	63181
ENSECAG00000012881	3.511826908	0.1572401	0.4853506	240	184	335	284	134	146	186	205
ENSECAG00000008209	6.678099563	0.157342	0.4855331	2480	1539	1275	1317	2281	1963	2608	1703
ENSECAG00000005595	4.840605603	0.157434	0.4856508	736	460	781	641	544	247	618	316
ENSECAG00000024116	5.411845679	0.1574656	0.4856508	618	857.003	481.005	779	798.008	1035.01	600	1057
ENSECAG00000023093	3.287257408	0.1575723	0.4858478	139	292	185	282	107	102	155	198
ENSECAG00000015380	4.303140487	0.1576797	0.4860471	462	404	430	472	245	265	348	339
ENSECAG00000022206	5.828122446	0.1579186	0.4866514	1075	1073	1439	1480	931	715	1089	788
ENSECAG00000010249	6.058679083	0.1580215	0.4868365	1542	1009	1623	1824	871	916	1057	1253
ENSECAG00000024967	7.73411187	0.1582954	0.4874586	1380	5437	1559	2171	698	6232	922	12126
ENSECAG00000005957	4.882094026	0.1583093	0.4874586	628	472	534	1161	545	256	704	243
ENSECAG00000008129	7.93165324	0.1584734	0.4878318	3463	4254	3884	4423	4921	4385	5883	5134
ENSECAG00000024413	7.765484976	0.1585442	0.4879174	4117	2963	3503	3503	3415	6083	4080	4327
ENSECAG00000007582	7.90590518	0.1586416	0.4880851	4714	1617	3266	3753	12237	803	8254	1484
ENSECAG00000000877	3.674133301	0.1587244	0.4882074	293	177	445	264	239	121	224	157
ENSECAG00000012169	5.026407665	0.1589102	0.4886206	396	572	483	578	449	1097	353	788
ENSECAG00000020843	2.337498821	0.1589771	0.4886206	84	97	126	165	62	39	76	108
ENSECAG00000015902	3.871883021	0.1589877	0.4886206	195	248	194	308	333	288	345	252
ENSECAG00000009799	4.353630289	0.1591843	0.4890922	346	139	476	323	415	542	264	485
ENSECAG00000015099	7.616991083	0.1592603	0.4891936	3763	3573	4967	5567	3094	2078	3981	2879
ENSECAG00000010500	8.600386982	0.1594087	0.4895169	9585	5752	8005	13137	6233	2961	9446	4796
ENSECAG00000008409	3.890483259	0.1595041	0.4895936	137	355	134	248	149	282	288	583
ENSECAG00000022076	5.00238903	0.1595198	0.4895936	494	414	468	772	683	526	754	690
ENSECAG00000023954	7.052331842	0.1598077	0.4903446	2515	2839	2414	4462	1260	1561	2261	2926
ENSECAG00000022359	3.965509012	0.1600105	0.490777	356	265	349	451	205	210	276	246
ENSECAG00000017248	4.941168734	0.160035	0.490777	753	761	807	456	270	275	579	710
ENSECAG00000014589	0.527049166	0.1600978	0.4908368	15	24	53	40	12	13	36	12
ENSECAG00000016530	4.306989854	0.1601934	0.4909975	451	495	367	490	207	175	302	492
ENSECAG00000017605	0.49943879	0.1603837	0.491434	35	39	26	25	11	19	13	27
ENSECAG00000012598	5.092757737	0.1604672	0.491434	853	553	944	734	703	420	457	452
ENSECAG00000006428	3.287444662	0.1605106	0.491434	176	221	227	277	105	187	103	152
ENSECAG00000020507	7.267764323	0.1605287	0.491434	3386	3148	3191	4166	1985	2119	3217	2148
ENSECAG00000011048	6.495741229	0.1605521	0.491434	2451	743	1419	416	3706	1772	2284	389
ENSECAG00000010990	7.049155514	0.1606762	0.4916813	249	3891	467	730	193	8326	177	4025
ENSECAG00000011923	6.253897209	0.1608002	0.4919283	1569	1016	1127	1244	2474	1035	1825	1063
ENSECAG00000019349	1.093257766	0.1610165	0.4924546	32	25	22	47	34	38	59	48
ENSECAG00000017534	5.213164175	0.1611057	0.4924546	833	831	773	861	587	406	565	715
ENSECAG00000000638	8.528882358	0.1611539	0.4924546	6055	4452	7011	6777	8135	5392	10152	7524
ENSECAG00000023520	4.793562086	0.1611705	0.4924546	606	559	630	683	426	343	520	411
ENSECAG00000011332	2.835844578	0.161189	0.4924546	154	181	137	188	46	97	105	154
ENSECAG00000021999	8.754261645	0.1613991	0.4928484	6947	6448	7650	6648	7117	12343	7252	8839
ENSECAG00000019986	5.427042968	0.1614047	0.4928484	1025	746	1033	1035	653	464	870	691
ENSECAG00000007853	2.39736328	0.161495	0.492875	130	94	136	136	35	36	91	132
ENSECAG00000007656	5.08563535	0.1615002	0.492875	548	360	531	733	1242	337	889	452
ENSECAG00000018791	3.295151637	0.1618046	0.4936659	202	191	227	281	90	164	174	142
ENSECAG00000023397	1.418986961	0.161867	0.4936659	27	43	48	24	131	26	55	23
ENSECAG00000022606	3.841539866	0.1619375	0.4936659	287	311	323	377	224	129	261	248
ENSECAG00000009389	10.99728499	0.1619498	0.4936659	35924	25560	38919	31935	48583	46827	36933	36577
ENSECAG00000013032	4.03071299	0.1620762	0.4936659	402	287	343	480	231	101	359	291
ENSECAG00000016039	4.302687519	0.1620791	0.4936659	252	271	300	468	515	382	471	273
ENSECAG00000020678	7.42480399	0.1621547	0.4936659	3127	2611	2591	2969	3941	3646	3339	3161
ENSECAG00000012228	5.812675938	0.1621962	0.4936659	892	852	924	1134	1168	996	1332	1087
ENSECAG00000017258	6.968185441	0.1622179	0.4936659	1820	1954	1857	2022	1494	5439	1734	1690
ENSECAG00000006321	7.212411267	0.1622213	0.4936659	3121	1971	2287	2258	4076	2551	3519	2232
ENSECAG00000008403	10.77380877	0.1622374	0.4936659	29257	23486	32135	28519	41547	40542	28573	33549
ENSECAG00000022784	7.952064421	0.1623305	0.4938169	6561	4206	5935	6217	5006	2179	5505	2112
ENSECAG00000018468	5.141014567	0.1624193	0.4939071	572	562.997	482.995	709	877.992	770.995	657	566.997
ENSECAG00000020756	0.423981306	0.1624471	0.4939071	18	9	21	15	26	56	35	0
ENSECAG00000018516	1.117694438	0.1626892	0.4943604	23	73.0001	59.0001	42.0001	23.0001	12.0005	38.0006	41.0004
ENSECAG00000001625	4.351842901	0.1626953	0.4943604	319	377	288	327	534	410	368	366
ENSECAG00000023220	4.654561702	0.1627268	0.4943604	418	472	400	332	426	437	640	602

ENSECAG00000023587	4.773928745	0.1628678	0.4944913	395	198	410	592	1318	153	888	165
ENSECAG00000026882	2.537126311	0.1628989	0.4944913	123	105	148	156	60	88	105	79
ENSECAG00000007722	2.916666622	0.1629005	0.4944913	123	44	143	142	266	72	238	97
ENSECAG00000014473	7.731027341	0.1630729	0.4948825	3879	2560	3813	3224	7772	2498	4870	2986
ENSECAG00000018726	5.694339555	0.1631224	0.4948926	956	1423	1326	1224	214	343	573	1768
ENSECAG00000023688	3.045742622	0.1631634	0.4948926	164	159	199	254	43	97	181	153
ENSECAG00000026844	4.539216897	0.1633222	0.4950716	530	244	452	259	486	496	515	441
ENSECAG00000024547	5.777933093	0.1633564	0.4950716	1102	629	991	936	919	1160	1534	920
ENSECAG00000002905	3.349112412	0.16339	0.4950716	122	115	96	270	94	471	129	168
ENSECAG00000016329	0.868584227	0.1633967	0.4950716	20	34	17	30	16	31	48	61
ENSECAG00000013436	6.431920836	0.1634858	0.4950842	2148	1471	2115	1984	1536	873	1706	1244
ENSECAG00000011663	1.858243112	0.1635249	0.4950842	50	25	89	48	89	70	112	43
ENSECAG00000005266	4.575629592	0.1635316	0.4950842	582	510	575	521	187	401	225	551
ENSECAG00000018305	7.252378951	0.1636425	0.4952877	3557	2697	3580	4024	2937	1599	2964	1784
ENSECAG00000007006	7.743599079	0.1637961	0.4956206	5190	3802	4658	5816	3069	2079	4404	3653
ENSECAG00000010563	2.718676115	0.1639975	0.4959945	144	119	151	190	96	60	130	99
ENSECAG00000011725	0.414189221	0.164007	0.4959945	14	18	24	17	22	16	38	35
ENSECAG00000025122	2.347257236	0.1641042	0.4961565	130	123	126	81	66	62	61	95
ENSECAG00000015832	11.62842751	0.1642627	0.4963806	55415	39220	54889	56369	76874	75005	54638	54299
ENSECAG00000011097	3.937523363	0.1642978	0.4963806	140.001	361	208.001	102	319.001	319.001	48.0001	688.024
ENSECAG00000020790	4.419223399	0.1643378	0.4963806	435	214	412	277	655	283	671	242
ENSECAG00000018924	7.879904339	0.1644089	0.4963806	3007	3928	3443	4566	3532	8194	4338	3381
ENSECAG00000007211	1.407512037	0.1644287	0.4963806	23	50	34	46	76	36	77	38
ENSECAG00000000017	1.422542035	0.1644405	0.4963806	38	33	41	48	33	63	60	67
ENSECAG00000020673	0.163169481	0.1647834	0.4972833	18	9	13	20	31	22	27	11
ENSECAG00000020025	2.769790428	0.1648403	0.4973231	183	83	130	263	109	90	141	35
ENSECAG00000006853	6.710832491	0.1650202	0.4976336	2811	1376	3197	2326	1509	654	2770	1440
ENSECAG00000026941	4.131449479	0.1650309	0.4976336	374	306	483	445	321	148	406	176
ENSECAG00000014787	1.766028372	0.1651673	0.4979127	29	54	37	83	75	42	76	99
ENSECAG00000020056	2.785679984	0.1652449	0.4980144	93	84	100	158	119	89	198	187
ENSECAG00000017710	3.143870324	0.1654993	0.4984715	155	96	174	125	228	196	200	117
ENSECAG00000016972	4.442918854	0.1655253	0.4984715	359	273	383	370	252	684	362	494
ENSECAG00000025022	9.424102043	0.1655282	0.4984715	12596	8846	11036	12551	13828	15091	11835	15859
ENSECAG00000015752	5.0254496	0.1657948	0.4990911	779	504	841	805	559	383	564	485
ENSECAG00000015608	5.439060638	0.1658258	0.4990911	1144	729	1190	828	640	417	1029	598
ENSECAG00000021880	8.477121048	0.1659381	0.4990911	8170	6565	7232	10550	5077	3934	5798	6853
ENSECAG00000004349	4.054449814	0.1659422	0.4990911	198	920	240	166	137	221	341	198
ENSECAG00000001300	2.376189488	0.1659536	0.4990911	104	165	72	133	59	83	70	74
ENSECAG00000008155	4.139230758	0.166092	0.4993749	410	421	489	303	110	148	301	465
ENSECAG00000005086	3.982930862	0.1662968	0.4998586	223	555	393	307	105	143	141	479
ENSECAG00000011139	2.693457533	0.1666282	0.500628	124	77	157	270	40	56	133	134
ENSECAG00000008517	0.712855849	0.166641	0.500628	57	1	6	8	90	7	28	29
ENSECAG00000013933	0.37664236	0.1668364	0.5009882	7	20	9	33	15	43	24	25
ENSECAG00000016770	5.899491524	0.1669696	0.5009882	797	1184	857	1095	1075	1438	1177	1161
ENSECAG00000022576	6.745892034	0.1669791	0.5009882	2390	1897	2198	3224	1333	1213	1994	2103
ENSECAG00000010823	4.624750254	0.1669797	0.5009882	461	308	360	471	683	268	737	416
ENSECAG00000020049	3.543814506	0.1670251	0.5009882	119	186	185	248	213	130	381	289
ENSECAG00000012813	3.060665533	0.1670255	0.5009882	231	149	199	180	108	82	142	157
ENSECAG00000004845	4.44390385	0.1673943	0.5019619	459	501	371	632	235	312	393	376
ENSECAG00000008967	0.453954894	0.1674445	0.5019801	16	18	16	26	26	38	32	15
ENSECAG00000001854	2.242902579	0.1676705	0.5025247	115	102	105	114	45	101	52	57
ENSECAG00000011017	5.012442304	0.1677834	0.5027306	664	552	845	869	633	401	599	314
ENSECAG00000010263	7.561991295	0.1679748	0.5031712	3486	3571	4150	6005	2310	2577	3556	3111
ENSECAG00000014763	4.734832481	0.1680366	0.5031712	634	471	567	801	167	320	743	379
ENSECAG00000023648	5.699604738	0.1680633	0.5031712	1278	777	1178	1441	860	562	1083	732
ENSECAG00000022213	6.323980045	0.1681873	0.5034097	1144	1295	1248	1371	863	2811	988	1911
ENSECAG00000001057	1.910590918	0.1682372	0.5034262	68	78	65	147	28	22	63	93
ENSECAG00000018037	5.62661668	0.1683981	0.5036836	1569	739	1115	1042	794	408	834	979
ENSECAG00000019982	2.797246179	0.1684118	0.5036836	127	85	133	82	158	174	136	110
ENSECAG00000021265	6.054562863	0.1685843	0.5040667	737	1765	697	944	1240	1078	1231	2073
ENSECAG00000024354	6.209655751	0.1686818	0.5042254	1630	1092	948	1037	1404	2442	1152	1033
ENSECAG00000023622	4.822919306	0.1689325	0.5047133	584	523	582	877	435	289	496	512
ENSECAG00000018481	1.311648298	0.1689339	0.5047133	34	32	37	40	84	20	55	53
ENSECAG00000000428	5.218390607	0.1690372	0.5047235	788	482	590	622	776	652	848	778
ENSECAG00000014369	3.436658518	0.1690445	0.5047235	168	89	152	275	229	100	393	237
ENSECAG00000017830	6.069547214	0.1690745	0.5047235	1174	1868	1531	1366	770	922	1024	1374
ENSECAG00000013944	5.531821214	0.16915	0.5047235	715	662	870	861	846	1061	910	917
ENSECAG00000007818	8.073177344	0.1691595	0.5047235	4488	3745	4702	3949	3650	10016	4314	4215
ENSECAG00000016097	5.877089988	0.1695322	0.5057029	1263	1082	1298	1619	884	556	1133	1100
ENSECAG00000018532	3.938327605	0.1699143	0.5067096	402	77	291	104	479	295	515	98
ENSECAG00000015187	1.360527064	0.169971	0.5067456	64	45	67	56	36	27	27	49
ENSECAG00000016220	4.607275423	0.1701463	0.5069368	529	442	622	605	446	226	484	333
ENSECAG00000014903	4.834963543	0.1701508	0.5069368	592	382	466	371	802	476	842	303
ENSECAG00000007206	5.78884585	0.170169	0.5069368	1217	1004	1245	1476	847	545	1268	815
ENSECAG00000012380	6.519193994	0.17024	0.5070153	1525.48	1461.87	1567.64	1554.5	1757.64	1298.91	1931.14	2582.74
ENSECAG00000016595	1.537404086	0.17048	0.5074859	53	55	68	90	25	27	54	56
ENSECAG00000009383	4.745835892	0.1704874	0.5074859	456	361	488	471	559	536	488	604
ENSECAG00000013391	4.766298109	0.1707165	0.5079348	442	306	494	535	702	334	886	400

ENSECAG00000013277	2.482599989	0.1707329	0.5079348	110	115	96	197	57	83	102	76
ENSECAG00000009583	8.486826304	0.1707723	0.5079348	5628	4752	5341	7987	6834	8805	6291	7495
ENSECAG00000013045	4.244101934	0.1708976	0.5081744	387	381	439	505	180	291	306	359
ENSECAG00000021642	3.034831342	0.1710672	0.5084091	357	72	215	143	181	42	106	112
ENSECAG00000021290	6.20582572	0.1710825	0.5084091	685	1186	931	1696	577	3263	916	1414
ENSECAG00000017104	2.88747829	0.1711201	0.5084091	145	59	113	116	303	62	233	65
ENSECAG00000024979	6.333782711	0.1711556	0.5084091	1372	1887	1417	2736	554	1061	1617	1657
ENSECAG00000015008	4.31457759	0.1712712	0.5086194	469	258	460	664	393	217	378	192
ENSECAG00000015256	6.238803856	0.1714005	0.5088648	1352	1361	1281	970	1387	1554	1564	1659
ENSECAG00000023506	4.190021328	0.1714434	0.5088648	304	258.002	294	339	422.002	395	396	283
ENSECAG00000013451	3.278685789	0.1715335	0.5089992	249	114	133	68	155	359	153	153
ENSECAG00000014254	0.028046111	0.1716749	0.5091536	25	9	23	35	21	5	11	11
ENSECAG00000013024	2.424900547	0.1716752	0.5091536	96	145	138	137	17	52	43	167
ENSECAG00000000641	4.551498322	0.1718867	0.509648	438	539	464	660	377	262	419	376
ENSECAG00000016196	2.422580424	0.1719424	0.5096799	139	112	153	99	35	51	45	157
ENSECAG00000013543	1.459029603	0.1722888	0.5103924	53	61	97	42	24	31	18	69
ENSECAG00000018369	3.012596723	0.1722961	0.5103924	100	173	118	93	145	137	134	271
ENSECAG00000013771	7.06991626	0.1723175	0.5103924	3660	1256	1875	725	6191	1831	3584	613
ENSECAG00000014830	7.870169452	0.1723855	0.5104393	3909	3888	3312	4354	4982	3609	5512	5362
ENSECAG00000010441	0.483164643	0.1724232	0.5104393	19	14	27	18	27	23	37	28
ENSECAG00000014261	6.257441488	0.1724952	0.5105192	1527	1537	1604	2128	1100	1071	1489	1116
ENSECAG00000012669	4.412624371	0.1725541	0.5105605	497	261	343	256	471	256	482	597
ENSECAG00000006192	1.87970363	0.172605	0.5105781	65	35	87	172	31	43	93	33
ENSECAG00000021100	6.510647097	0.1727039	0.5106847	1899	1871	1990	2316	1147	1435	1525	1538
ENSECAG00000000517	6.107149488	0.1727546	0.5106847	1369	1404	1660	1709	705	1213	1108	1185
ENSECAG00000018995	5.886810058	0.1728314	0.5106847	1115	981	819	1029	926	1181	1252	1474
ENSECAG00000024277	5.865298543	0.1728564	0.5106847	1499	929	1234	1669	901	386	1468	865
ENSECAG00000019651	3.628086454	0.1728894	0.5106847	214	143	243	183	218	358	254	190
ENSECAG00000021306	5.500576209	0.1729495	0.5106847	1124	869	924	1083	612	575	881	766
ENSECAG00000001075	6.59592299	0.1729928	0.5106847	1591	1529	1530	1851	1939	2212	1859	1787
ENSECAG00000020958	1.411357202	0.1730007	0.5106847	74	37	73	60	46	15	54	31
ENSECAG00000022423	5.229204937	0.1730826	0.5107294	844	757	1368	549	178	283	568	1113
ENSECAG00000013635	6.929399518	0.1731057	0.5107294	3016	2256	2785	2911	1866	1927	1353	2144
ENSECAG00000010631	5.958463346	0.1733176	0.5112218	1460	988	1435	1711	1027	574	1061	1196
ENSECAG00000015139	5.501450925	0.1733935	0.5113127	828	889	1032	1281	687	676	815	627
ENSECAG00000013258	4.77600635	0.1735293	0.5115805	491	348	442	499	744	441	815	310
ENSECAG00000014503	5.498736146	0.1739092	0.5124834	1021	785	1037	1172	616	569	901	748
ENSECAG00000010356	6.851309805	0.1739556	0.5124834	2302	2170	2780	3355	2716	882	1928	1407
ENSECAG00000022379	5.477343564	0.1739709	0.5124834	735	620	808	837	936	939	993	741
ENSECAG00000011310	4.241281931	0.174207	0.5130456	407	359	506	447	318	174	470	185
ENSECAG00000012105	0.959897823	0.1744515	0.5134568	23	27	34	28	36	24	45	58
ENSECAG00000015054	1.169145329	0.174459	0.5134568	46	50	36	71	35	28	28	30
ENSECAG00000010289	4.453747477	0.1744822	0.5134568	326	241	443	405	703	335	535	268
ENSECAG00000000557	4.394740145	0.1745391	0.5134912	452	404	490	532	323	276	356	329
ENSECAG00000000446	6.105906261	0.1746467	0.5135852	1559	1611	1413	1565	625	720	1429	1502
ENSECAG00000002061	0.939300984	0.1746615	0.5135852	34	28	58	57	9	26	25	39
ENSECAG00000000264	1.35459323	0.1748728	0.5140173	60	38	72	63	41	22	54	24
ENSECAG00000016424	7.969254287	0.1748989	0.5140173	3667	4323	3273	4289	2936	9936	3898	3939
ENSECAG00000023296	0.558304747	0.1750675	0.5143723	19	22	17	24	21	19	49	35
ENSECAG00000024918	2.01181414	0.1751165	0.5143723	54	70	52	63	77	131	61	63
ENSECAG00000022883	7.227465594	0.1751556	0.5143723	2050	2346	2644	1993	1277	6010	1551	3685
ENSECAG00000023931	8.498056111	0.1753439	0.5145737	6842	8743	7171	9855	4219	4589	6932	6377
ENSECAG00000018565	3.628957583	0.1753648	0.5145737	204	209	288	449	143	121	221	250
ENSECAG00000006771	5.505625016	0.1754676	0.5145737	1311	683	956	1231	969	614	807	294
ENSECAG00000007911	3.662170688	0.1754704	0.5145737	147	183	213	233	118	322	162	472
ENSECAG00000014908	5.470016433	0.175495	0.5145737	903	406	718	763	1700	396	1274	486
ENSECAG00000011044	0.718021488	0.1755535	0.5145737	18	42	11	14	28	33	21	57
ENSECAG00000025088	5.769242487	0.1755569	0.5145737	623	1026	879	1029	975	1025	1940	677
ENSECAG00000025129	3.5516944	0.175624	0.5145737	76	372	66	119	215	313	109	394
ENSECAG00000012858	2.201940782	0.1756318	0.5145737	92	43	104	10	159	67	111	75
ENSECAG00000020252	9.501779975	0.1759689	0.5154283	11462	10682	10917	14989	12203	15792	15695	16161
ENSECAG00000015719	4.175627967	0.1760294	0.5154726	300	300	217	280	235	641	155	467
ENSECAG00000000048	6.944248908	0.1761922	0.5158162	3275	2189	2780	2850	2335	1150	2521	1571
ENSECAG00000014377	5.127164774	0.1764937	0.5165045	864	501	875	1050	788	322	749	183
ENSECAG00000015738	1.693358395	0.1765182	0.5165045	55	55	85	109	34	16	47	80
ENSECAG00000023029	4.625387695	0.1767942	0.5171789	449	553	452	812	189	321	428	540
ENSECAG00000019905	1.305441823	0.1768422	0.5171859	21	37	50	24	19	64	21	109
ENSECAG00000020684	4.00191829	0.1769514	0.5172182	314	197	288	231	341	397	273	299
ENSECAG00000000129	7.551459825	0.1769796	0.5172182	3544	4605	3349	5486	1830	2836	3840	2915
ENSECAG00000022341	8.511303068	0.177	0.5172182	5432	6090	5046	6898	5689	11649	6308	6264
ENSECAG00000003033	1.133478497	0.1770353	0.5172182	37	25	94	57	12	5	22	69
ENSECAG00000023048	6.132610732	0.1771407	0.5173628	1235	959	1112	1431	1660	1151	1883	1077
ENSECAG00000024299	4.374662417	0.1771759	0.5173628	421	416	468	552	263	331	350	313
ENSECAG00000024434	4.800174638	0.1772369	0.517408	366	512	422	530	446	639	635	566
ENSECAG00000010972	0.130773856	0.1773563	0.5176234	13	17.0001	16.0001	9.00009	31.0001	27.0005	5.00057	25.0004
ENSECAG00000027002	4.866276279	0.1774021	0.517624	608	468	713	853	342	481	434	501
ENSECAG00000008739	0.558203875	0.1774617	0.5176651	21	19	13	27	11	55	23	31
ENSECAG00000012773	3.854535258	0.1778056	0.5185349	257	466	247	338	256	176	294	120

ENSECAG00000018401	8.035610039	0.178008	0.5189821	6115	5275	6153	5886	3971	3435	4564	4130
ENSECAG00000012354	1.595230318	0.1780503	0.5189821	35	60	38	46	42	57	74	81
ENSECAG00000012442	4.969704336	0.1781362	0.5190669	509	459	385	731	780	587	762	455
ENSECAG00000019708	6.498303404	0.1782045	0.5190669	2223	1573	1886	2466	832	1029	2033	1755
ENSECAG00000006707	3.146743861	0.1782195	0.5190669	170	85	161	144	208	116	268	166
ENSECAG00000013581	9.138014144	0.1782622	0.5190669	12322	11084	12334	15252	6655	9380	9726	8302
ENSECAG00000017729	5.692669835	0.1783198	0.5191015	1348	893	1232	1133	738	470	1167	873
ENSECAG00000001830	2.08071309	0.1784838	0.5194459	62	57	83	47	74	121	47	109
ENSECAG00000018773	10.25833129	0.1787647	0.5201302	23894	15698	19816	21245	27490	25220	22455	25536
ENSECAG00000008014	3.939549828	0.1788844	0.5202226	162	257	243	272	158	344	172	636
ENSECAG00000012890	5.834145476	0.178888	0.5202226	1365	1129	1288	1290	1137	867	700	739
ENSECAG00000013541	5.116684507	0.1789613	0.5202359	518	1128	784	894	103	353	274	1149
ENSECAG00000007205	5.300962113	0.1789842	0.5202359	686	474	731	754	737	640	1224	683
ENSECAG00000014116	4.272655846	0.17913	0.5203142	411	383	471	499	236	123	369	443
ENSECAG00000019822	7.424762018	0.1791922	0.5203142	4719	2314	3960	4719	2262	2198	3988	2091
ENSECAG00000018996	7.547585421	0.1792158	0.5203142	2656	4004	3232	1025	2093	5955	2480	5444
ENSECAG00000022468	6.856760909	0.17924	0.5203142	2255	1625	1781	1926	2330	3132	1732	2195
ENSECAG00000020368	2.768213437	0.1792402	0.5203142	135	50	100	137	210	84	197	98
ENSECAG00000017080	2.228006451	0.1794287	0.5205543	97	97	106	123	60	62	74	73
ENSECAG00000015508	5.815739776	0.1794643	0.5205543	963	1285	1119	1641	693	836	1032	934
ENSECAG00000015070	5.162771847	0.1795132	0.5205543	804	641	909	869	338	387	717	771
ENSECAG00000013738	2.792588728	0.1795191	0.5205543	197	106	158	171	120	78	94	108
ENSECAG00000016520	4.142020915	0.179552	0.5205543	422	319	367	499	240	224	438	170
ENSECAG00000013274	3.296384402	0.179603	0.5205692	224	165	162	371	157	158	166	79
ENSECAG00000013658	3.50167633	0.1796964	0.5207069	201	244	248	346	113	172	133	238
ENSECAG00000012800	6.464660324	0.1797481	0.5207241	1950	1759	1778	2328	1267	1294	1390	1534
ENSECAG00000009182	4.167020865	0.1798711	0.5208409	421	250	285	177	406	414	363	305
ENSECAG00000013418	4.242885079	0.1798959	0.5208409	465	340	392	500	304	260	315	270
ENSECAG00000000463	3.451951207	0.179926	0.5208409	172	217	195	434	156	119	178	190
ENSECAG00000017442	4.768668838	0.1800462	0.5210559	361	447	425	564	590	631	627	383
ENSECAG00000011280	3.532012184	0.1801851	0.5213252	172	193	157	221	303	159	241	262
ENSECAG00000007942	8.178677928	0.1803705	0.5217288	4786	2951	3913	5284	1824	15438	2341	4807
ENSECAG00000012999	4.987546088	0.1805058	0.5219104	699	697	653	789	286	416	617	631
ENSECAG00000002732	4.973731422	0.1805755	0.5219104	714	540	730	827	475	438	633	392
ENSECAG00000022668	5.88677732	0.1806115	0.5219104	840	1733	1413	1385	492	679	704	1620
ENSECAG00000014736	6.325381092	0.1806171	0.5219104	2315	1289	885	2931	904	1119	1489	1332
ENSECAG00000019738	7.31166452	0.1807752	0.5220409	2570	2256	3099	2548	2694	3854	2952	3461
ENSECAG00000022864	5.159669333	0.1808793	0.5220409	782	610	734	1086	603	489	644	464
ENSECAG00000003831	2.329496951	0.1809114	0.5220409	88	40	66	116	155	57	137	84
ENSECAG00000001318	6.62965479	0.180943	0.5220409	1883	1465	1875	1252	2558	1252	1961	2446
ENSECAG00000019449	4.644188473	0.1809724	0.5220409	429	273	420	522	487	484	835	304
ENSECAG00000001155	7.14774254	0.181023	0.5220409	2246	3320	3088	4360	1370	1199	2653	3410
ENSECAG00000016808	6.309966152	0.1810574	0.5220409	2773	1276	1319	1737	1168	1147	1277	1239
ENSECAG00000002087	6.050137695	0.1810832	0.5220409	1507	1219	1518	1599	892	970	1142	1134
ENSECAG00000016677	5.391127937	0.1811218	0.5220409	629	738	708	675	679	1151	702	855
ENSECAG00000020948	4.939462152	0.1811218	0.5220409	580	445	533	478	665	487	675	692
ENSECAG00000004992	5.301181162	0.1812149	0.5221765	661	607	682	701	825	691	805	879
ENSECAG00000021650	4.405854604	0.1812697	0.522202	490	389	403	641	265	197	465	380
ENSECAG00000021447	4.391539695	0.1813999	0.5224447	383	179	324	488	412	501	594	251
ENSECAG00000012197	1.17684754	0.1815064	0.5226188	48	18	31	33	82	37	44	26
ENSECAG00000012932	8.765241053	0.1816413	0.5227816	7257	5989	7511	8044	9440	5874	11833	9443
ENSECAG00000003460	4.580677915	0.1817206	0.5227816	527	375	548	733	442	346	412	233
ENSECAG00000013680	5.773990437	0.1817604	0.5227816	1251	1116	1651	831	611	758	824	1132
ENSECAG00000010322	2.013670231	0.1818047	0.5227816	27	75	166	147	4	34	24	127
ENSECAG00000024113	7.082777336	0.1818218	0.5227816	3930	1690	2683	4586	2606	1055	3475	915
ENSECAG00000003837	10.02941741	0.1818391	0.5227816	19093	4543	30080	3045	28743	14335	51543	6274
ENSECAG00000024842	8.281218141	0.1819471	0.5228376	6159	7904	10022	4298	2605	4391	3332	7566
ENSECAG00000023027	8.08847211	0.1819506	0.5228376	8757	2491	3399	1167	5639	4001	11817	3608
ENSECAG00000010015	6.704410791	0.1820082	0.5228707	2004	932	1932	1602	4403	1287	2555	788
ENSECAG00000006410	1.89029306	0.1820869	0.5229645	87	48	99	109	40	29	79	63
ENSECAG00000008719	4.161057604	0.1822047	0.523023	408	321	414	464	248	188	379	286
ENSECAG00000011269	3.809855494	0.1822233	0.523023	268	283	353	353	195	180	250	223
ENSECAG00000022571	1.511159053	0.1822453	0.523023	6	74	12	31	2	127	15	122
ENSECAG00000003511	5.591771231	0.1823759	0.5231941	912	467	754	1127	889	1117	1157	780
ENSECAG00000007635	4.355615177	0.1823971	0.5231941	398	367	494	619	479	172	298	267
ENSECAG00000004492	3.304552306	0.1826401	0.5233525	206	179	255	258	182	140	147	107
ENSECAG00000004400	3.004739917	0.1826849	0.5233525	94	122	154	82	46	360	90	186
ENSECAG00000023498	2.891933377	0.1827046	0.5233525	145	87	107	128	188	81	198	166
ENSECAG00000016517	5.479703174	0.1827073	0.5233525	703	738	754	796	732	985	874	1013
ENSECAG00000022923	5.765929359	0.1827187	0.5233525	1406	647	675	729	741	1098	733	1989
ENSECAG00000023910	3.143480946	0.1827592	0.5233525	136	179	165	366	53	100	120	223
ENSECAG00000020509	0.516725972	0.1827748	0.5233525	12	19	20	31	26	30	25	34
ENSECAG00000022016	2.995963819	0.182943	0.523702	85	127	84	208	173	145	244	119
ENSECAG00000006468	4.930965983	0.1832818	0.5242643	681	580	691	758	530	420	500	422
ENSECAG00000018492	6.231553468	0.1833169	0.5242643	1588	1440	1724	2017	740	732	1407	1787
ENSECAG00000019329	4.988084406	0.1833987	0.5242643	549	997	788	566	170	327	471	875
ENSECAG00000008857	4.772044395	0.1834832	0.5242643	653	479	681	641	419	254	649	372
ENSECAG00000012449	3.394377257	0.1834854	0.5242643	140.001	134.001	237.002	150.002	151.002	257.002	181.002	285

ENSECAG00000003049	1.217047953	0.1834872	0.5242643	30	47	38	16	41	53	42	57
ENSECAG00000001048	2.813371172	0.1834977	0.5242643	113	96	108	132	133	146	136	164
ENSECAG00000018844	5.683804918	0.1835388	0.5242643	1062	970	1221	1309	869	513	1112	743
ENSECAG00000021773	5.507065981	0.1835548	0.5242643	901	1128	921	1050	551	613	734	919
ENSECAG00000010919	9.299062981	0.1836392	0.5243734	8296	11448	9297	11101	10308	20662	12132	8731
ENSECAG00000022309	5.218457838	0.1837092	0.5244414	560	553	545	828	999	611	952	511
ENSECAG00000017045	0.844882158	0.1837772	0.5245037	34	27	77	54	0	0	73	2
ENSECAG00000004437	6.438562898	0.1838662	0.5245075	1249	2055	2310	2077	1289	1379	1344	1284
ENSECAG00000007663	7.40538203	0.1838709	0.5245075	3726	1627	1991	3402	6886	2078	3576	1971
ENSECAG00000011804	1.697525025	0.1840129	0.524781	58	74	107	74	9	12	35	109
ENSECAG00000004312	6.023764706	0.1841056	0.5249135	95	2233	126	127	155	2697	114	3478
ENSECAG00000011647	8.088215254	0.1842184	0.5251035	3449	4548	3844	5300	3649	10571	4243	3890
ENSECAG00000018880	7.19127578	0.1842667	0.5251093	2604	2085	2392	2721	3134	2586	3052	3182
ENSECAG00000000409	1.946878441	0.1844836	0.5255082	52	65	45	75	62	78	107	76
ENSECAG00000022710	7.382421921	0.1844992	0.5255082	2760	2487	3069	2675	3332	4191	2964	3052
ENSECAG00000009796	1.636035114	0.1846829	0.5258081	41	62	33	49	66	46	61	87
ENSECAG00000017703	3.683867797	0.1846971	0.5258081	204	207	181	240	329	197	333	213
ENSECAG00000012289	4.263346646	0.1848218	0.5258996	340	359	425	663.001	147.001	183.001	314	499
ENSECAG00000010259	6.266704873	0.1848235	0.5258996	1512	1351	1574	2482	929	1165	1506	1190
ENSECAG00000007961	6.03693347	0.1849268	0.5258996	1010	2194	1299	1408	481	730	1035	1673
ENSECAG00000023762	7.632903317	0.1849436	0.5258996	4107	4065	4701	4833	3318	2605	3311	2961
ENSECAG00000012736	7.186891827	0.1849607	0.5258996	2915	2887	2429	5182	2726	1999	2700	1329
ENSECAG00000016914	2.056221538	0.1850291	0.5259624	60	61	64	74	74	81	100	90
ENSECAG00000009840	4.109984804	0.1851423	0.5260734	376	342	378	454	242	168	304	342
ENSECAG00000023132	2.542978722	0.1851668	0.5260734	84	37	93	155	106	112	146	131
ENSECAG00000014809	6.596581989	0.1852071	0.5260734	1532	1465	1725	1811	1979	2173	2065	1585
ENSECAG00000017066	6.334838358	0.1853158	0.5262505	1887	1462	1783	2092	1591	691	1628	1125
ENSECAG00000004303	1.225553189	0.1854213	0.5264186	38	28	32	133	23	13	71	16
ENSECAG00000014880	3.000679643	0.1855266	0.5265858	138	159	128	47	187	189	171	131
ENSECAG00000022169	1.737447225	0.1856409	0.5267786	75	53	82	92	52	38	50	48
ENSECAG00000023096	2.976564966	0.1857029	0.526823	124	108	120	147	208	106	229	124
ENSECAG00000000158	3.167824447	0.1858198	0.527023	127	153	196	52	147	106	251	290
ENSECAG00000008493	4.285626165	0.185871	0.5270367	1	521	0	75	26	269	52	1851
ENSECAG00000008104	6.547352688	0.1859338	0.5270832	1794	1821	1943	2822	1133	1083	1634	1986
ENSECAG00000012760	6.622339977	0.186007	0.527159	1772	2125	1753	3298	826	1552	2038	1628
ENSECAG00000008616	3.788493952	0.1861224	0.5273545	299	187	244	125	392	197	405	190
ENSECAG00000022885	3.608483492	0.1861846	0.5273603	233	116	161	266	396	140	345	166
ENSECAG00000007346	6.894188365	0.1862173	0.5273603	2106	1877	1624	2341	1912	2666	2605	2532
ENSECAG00000018596	1.71628355	0.1863869	0.527709	84	54	80	82	26	32	81	48
ENSECAG00000020886	0.659570124	0.186778	0.5286318	39	17	48	42	5	12	23	40
ENSECAG00000021818	8.209272999	0.1868059	0.5286318	6675	5227	6840	8153	5180	2986	6640	3428
ENSECAG00000014263	0.905231148	0.1869349	0.5288652	25	26	33	24	37	48	28	38
ENSECAG00000022831	3.333972415	0.1871975	0.5293718	254	145	282	252	99	70	232	199
ENSECAG00000014762	6.103829724	0.1872297	0.5293718	1661	1071	1754	1695	1436	711	1343	751
ENSECAG00000024604	9.114176298	0.1872748	0.5293718	14098	7133	8250	5610	13241	10826	15097	7808
ENSECAG00000002984	2.475576626	0.1873004	0.5293718	164	148	46	173	91	26	46	133
ENSECAG00000008550	7.593916784	0.1873959	0.5294847	3605	3040	2875	3132	4920	4388	3621	2833
ENSECAG00000012630	6.751581652	0.1874336	0.5294847	1938	1499	1818	1795	1757	3364	1694	1882
ENSECAG00000017285	3.57329877	0.1875005	0.5295202	259	240	247	327	145	153	178	236
ENSECAG00000008412	4.257809941	0.1875394	0.5295202	410	318	458	540	261	202	320	387
ENSECAG00000012431	6.441051683	0.1878089	0.5301495	1592	1370	1181	1728	1500	1586	1737	2242
ENSECAG00000024986	5.493303736	0.1879385	0.5303834	926	384	1082	395	1561	835	1021	418
ENSECAG00000024457	7.838766904	0.1880165	0.5304497	2771	4677	3726	1816	1811	11186	2038	4267
ENSECAG00000012378	4.182939556	0.1880554	0.5304497	370	327	395	544	286	211	309	303
ENSECAG00000014299	2.680938924	0.1881216	0.5305048	115	125	126	234	40	80	123	125
ENSECAG00000017269	6.446140454	0.188199	0.5305913	2100	1858	2840	1526	227	839	805	2935
ENSECAG00000019344	6.372281148	0.1883337	0.530751	1038	1473	1091	1554	949	3556	1014	1244
ENSECAG00000000008	8.166960766	0.1884226	0.530751	4775	3823	5182	5156	6365	7081	4294	5593
ENSECAG00000011136	3.589143121	0.1884373	0.530751	179	175	200	154	109	547	116	227
ENSECAG00000019790	4.963632711	0.1884628	0.530751	687	628	1262	257	418	295	549	564
ENSECAG00000009873	3.191386865	0.188492	0.530751	156	106	193	59	84	438	101	148
ENSECAG00000010601	3.747208639	0.1885735	0.530751	193	181	217	280	203	378	245	271
ENSECAG00000013331	6.907624568	0.1886095	0.530751	1653	2620	1635	1841	2016	2236	2111	3580
ENSECAG00000002931	4.467475866	0.1886295	0.530751	511	386	548	524	358	319	357	315
ENSECAG00000007504	3.348329127	0.188741	0.5309335	246	122	220	378	222	144	117	86
ENSECAG00000008434	1.557182836	0.1889723	0.5312657	90	52	80	51	10	22	58	69
ENSECAG00000024820	5.379033717	0.1890028	0.5312657	588	818	587	751	1162	617	940	704
ENSECAG00000001275	3.360501275	0.189041	0.5312657	169	126	200	515	77	107	309	98
ENSECAG00000015768	5.958669018	0.1890462	0.5312657	1148	1068	1008	892	1556	958	1667	949
ENSECAG00000020333	4.852402178	0.1892179	0.5316167	539	412	549	410	610	534	652	568
ENSECAG00000027699	9.515556911	0.1893571	0.5318763	16649	9291	8272	8780	4512	15632	7451	37036
ENSECAG00000022826	4.37520936	0.1895053	0.5320463	455	356	496	571	234	198	506	348
ENSECAG00000008862	2.373494515	0.1895293	0.5320463	55	106	84	63	71	86	105	181
ENSECAG00000005556	6.390438016	0.1895582	0.5320463	1133	1115	1164	2292	1185	1803	1949	1951
ENSECAG00000006443	4.766197178	0.1896665	0.5321402	709	471	682	565	468	294	571	344
ENSECAG00000010340	2.287569536	0.1897762	0.5321402	117	79	114	137	92.0004	58.0001	83	45
ENSECAG00000020484	4.470843538	0.1897975	0.5321402	304	241	494	396	664	474	443	249
ENSECAG00000006855	5.173400676	0.1898033	0.5321402	798	495	528	575	697	791	868	590

ENSECAG00000013778	2.695778357	0.1898259	0.5321402	161	122	136	171	68	69	94	146
ENSECAG00000009561	3.292475866	0.189896	0.5322055	193	181	220	302	138	102	126	211
ENSECAG00000018267	5.687343117	0.1899479	0.5322195	1455	779	1150	1247	569	546	1367	759
ENSECAG00000022718	6.473609344	0.1901331	0.5326071	2088	1445	1922	2447	1447	1152	1448	1499
ENSECAG00000009576	4.098260532	0.1904976	0.5334179	394	319	339	488	189	233	330	295
ENSECAG00000006405	6.370766898	0.1905165	0.5334179	1677	1707	1719	2200	1075	1082	1691	1377
ENSECAG00000022064	4.336266603	0.1905961	0.5335093	371	231	308	431	387	302	653	359
ENSECAG00000010503	3.825606594	0.1907203	0.5337256	222	203	245	242	396	169	420	216
ENSECAG00000023680	6.094886015	0.1914361	0.5355497	1237	1001	1136	1272	1117	1205	1855	1414
ENSECAG00000016810	1.729409379	0.1914664	0.5355497	130	70	107	17	102	9	35	8
ENSECAG00000019399	5.259549393	0.1916381	0.5357532	835	631	823	1163	512	369	719	788
ENSECAG00000002746	4.722132345	0.1916592	0.5357532	439	433	402	476	404	548	537	665
ENSECAG00000011444	7.160059769	0.1916807	0.5357532	3080	2749	3110	3815	1879	1931	2627	2456
ENSECAG00000026813	10.81503546	0.1918764	0.5360129	32611	23234	31832	31542	40284	38082	31989	36995
ENSECAG00000008261	5.557593921	0.1918826	0.5360129	1090	917	1117	1028	850	630	916	531
ENSECAG00000006248	2.362373449	0.1919152	0.5360129	106	135	64	173	82	89	41	65
ENSECAG00000010984	3.014407173	0.1922221	0.53656	122	121	135	140	159	121	217	180
ENSECAG00000004972	1.171394505	0.1922846	0.53656	48	52	40	61	21	29	30	43
ENSECAG00000019716	7.539851145	0.1922897	0.53656	3238	2667	2985	3209	5846	2600	5069	2166
ENSECAG00000008833	8.620446171	0.1923	0.53656	5489	6808	5655	8136	7580	8621	7418	8574
ENSECAG00000016445	4.974347072	0.1923968	0.5366983	600	437	459	618	574	651	648	676
ENSECAG00000019104	4.042613945	0.1925745	0.5370619	439	352	331	347	169	279	300	242
ENSECAG00000017325	6.180715984	0.1926442	0.5371244	1533	1332	1653	1927	831	1273	1387	1007
ENSECAG00000023820	8.225396125	0.1926962	0.5371378	3554	6986	4085	3735	4078	7214	3583	10308
ENSECAG00000021974	2.669838895	0.1927976	0.5372885	128	93	81	310	90	52	121	99
ENSECAG00000011464	6.889514712	0.1930384	0.5378275	2594	1483	2321	1283	3108	1675	3065	2110
ENSECAG00000023406	5.503416208	0.1933597	0.5385908	1027	834	1208	914	717	696	726	666
ENSECAG00000006288	7.942582667	0.1935557	0.5390044	4914	2520	5690	258	8235	4808	7607	2040
ENSECAG00000009918	4.248685932	0.1937618	0.5394295	356	216	343	305	553	395	458	176
ENSECAG00000007780	3.132604797	0.1938033	0.5394295	139	180	139	66	111	240	200	190
ENSECAG00000024151	6.256380969	0.1939676	0.5395902	1376	1092	1293	1340	1389	2101	1234	1393
ENSECAG00000004567	4.801348057	0.1940196	0.5395902	526	425	415	470	796	461	584	444
ENSECAG00000025110	8.634605799	0.1940965	0.5395902	7723	7382	8601	12648	3840	4567	8899	7238
ENSECAG00000016693	5.864735721	0.1941091	0.5395902	1344	1100	1568	1137	614	786	978	1233
ENSECAG00000018586	4.672012563	0.1941328	0.5395902	359	376	510	455	530	519	538	483
ENSECAG00000022664	7.314536632	0.1941461	0.5395902	2494	2945	2305	2499	2047	4606	2474	3890
ENSECAG00000015600	5.469006363	0.1942023	0.5396006	860	688	954	1518	725	603	926	482
ENSECAG00000016340	10.92749253	0.1942545	0.5396006	33184	27119	35025	33247	43846	43838	35218	36022
ENSECAG00000008611	5.079235753	0.1942923	0.5396006	924	602	835	629	593	382	617	495
ENSECAG00000004006	4.268803251	0.1944755	0.5399773	461	180	408	39	285	707	358	325
ENSECAG00000021673	3.567022253	0.1945627	0.5400874	251	273	453	103	199	185	117	153
ENSECAG00000025083	5.102941878	0.194619	0.5401118	603	429	621	643	980	585	672	561
ENSECAG00000001546	8.529811833	0.1947771	0.5404184	5168	5217	5776	8328	9294	7393	9463	4545
ENSECAG00000013402	4.72118213	0.1949564	0.540657	596	329	637	975.001	599	294	508	97
ENSECAG00000009398	3.271579378	0.1949883	0.540657	188	92	119	221	276	117	247	180
ENSECAG00000010694	5.92235924	0.1950059	0.540657	814	1063	780	1338	962	1907	975	1039
ENSECAG00000017857	6.945915893	0.1950838	0.5407412	2466	1994	1695	2021	2909	2439	2846	1890
ENSECAG00000011882	5.724668329	0.1954527	0.5416315	1181	1080	1287	1143	1198	692	668	644
ENSECAG00000007295	0.388495553	0.1956943	0.5420935	19	25	29	43	9	18	19	21
ENSECAG00000006885	7.51232228	0.1957149	0.5420935	4610	3038	3974	4832	3137	2950	2404	2512
ENSECAG00000019712	3.973469987	0.1959075	0.5424322	261	219	264	276	335	403	352	195
ENSECAG00000007319	3.781570013	0.1960239	0.5424322	301	279	325	336	182	251	138	228
ENSECAG00000022642	2.972391344	0.1960451	0.5424322	99	135	129	127	149	230	153	112
ENSECAG00000006197	6.495449386	0.1960821	0.5424322	1543	1296	1560	1756	1754	1381	1983	2223
ENSECAG00000015479	4.307532739	0.1961267	0.5424322	416	496	441	397	213	281	323	385
ENSECAG00000018111	4.969746115	0.1961589	0.5424322	327	627	481	469	268	1095	324	925
ENSECAG00000010572	7.002716668	0.1961906	0.5424322	3066	2209	2852	3338	2246	1507	2519	1706
ENSECAG00000011322	6.074146832	0.1962192	0.5424322	1237	997	944	1376	1716	911	1748	1170
ENSECAG00000007050	5.978328504	0.1964802	0.5428963	1540	1210	1671	1131	992	613	1395	966
ENSECAG00000021432	4.80387639	0.1964827	0.5428963	432	431	494	514	509	571	641	550
ENSECAG00000010973	7.850749762	0.1965963	0.5430781	6498	973	2979	3630	6893	2202	9423	2529
ENSECAG00000016473	6.909839563	0.1967927	0.5433709	2514	2564	2175	3511	1340	1908	2249	1923
ENSECAG00000024825	0.966708082	0.1967979	0.5433709	30	24	37	21	55	47	37	20
ENSECAG00000023646	3.13190882	0.1968538	0.5433931	241	192	223	140	69	116	103	212
ENSECAG00000010080	4.627286914	0.1971657	0.5439584	507	330	360	450	452	554	526	475
ENSECAG00000008292	4.439953624	0.1971713	0.5439584	485	447	490	502	296	313	297	414
ENSECAG00000009090	6.149362089	0.1972023	0.5439584	1305	1615	1445	1868	992	1023	1282	1157
ENSECAG00000009625	9.7622802	0.197405	0.5443349	7882	4695	13966	26871	13136	25454	35817	4071
ENSECAG00000014178	4.803071878	0.1974346	0.5443349	586	563	633	696	341	403	438	533
ENSECAG00000013487	1.694338263	0.1975537	0.5445311	43	46	51	60	66	72	66	60
ENSECAG00000016254	6.222645667	0.1976348	0.5446225	1677	1294	1822	1802	1311	1069	1239	1025
ENSECAG00000023566	1.333011589	0.1977251	0.5447391	25	44	15	41	4	83	11	127
ENSECAG00000024650	2.25055538	0.1980683	0.5455463	112	111	78	133	96	60	43	65
ENSECAG00000012526	3.908491263	0.1981141	0.5455463	267	216	305	175	251	263	470	282
ENSECAG00000012627	1.474034196	0.1983159	0.5458728	29	56	37	35	35	78	25	93
ENSECAG00000011604	3.031055573	0.1983288	0.5458728	117	143	113	136	108	257	142	164
ENSECAG00000021065	11.67288572	0.1985475	0.5463422	60334	44213	50989	61090	70221	72043	55208	68675
ENSECAG00000024334	3.294351454	0.1987057	0.5466453	131	157	124	210.999	112	162	223	336

ENSECAG00000018221	3.643242371	0.1989597	0.5470575	211	280	130	87	181	506	302	81
ENSECAG00000023716	5.423741285	0.1990037	0.5470575	1058	690	1103	956	822	513	781	546
ENSECAG00000016396	6.664411315	0.1990129	0.5470575	2072	1887	2199	2837	1370	1312	2007	1707
ENSECAG00000014874	5.253304633	0.1990482	0.5470575	683	528	752	569	976	454	900	825
ENSECAG00000001090	0.778273476	0.1990966	0.5470582	22	13	48	81	25	28	22	9
ENSECAG00000020775	0.854535261	0.1992712	0.5471862	49	29	29	61	13	5	33	44
ENSECAG00000007152	7.628720668	0.1992768	0.5471862	2839	2913	3289	4286	3588	3996	5315	3404
ENSECAG00000021187	6.141023198	0.1993435	0.5471862	1496	1281	1577	1901	930	919	1072	1486
ENSECAG00000007845	6.749035115	0.1994043	0.5471862	2523	1797	2256	3043	1688	1217	2314	1544
ENSECAG00000008527	5.533079011	0.1994158	0.5471862	1103	840	1435	772	631	315	815	1082
ENSECAG00000014984	8.81579197	0.1994322	0.5471862	7732	7191	7247	6862	6455	13707	6699	9887
ENSECAG00000021732	4.9198763	0.1995321	0.5472749	544	508	483	477	672	614	636	528
ENSECAG00000013718	5.583938519	0.1995609	0.5472749	804	702	860	831	1125	645	1576	667
ENSECAG00000013255	7.918641686	0.1996631	0.5473994	3948	4266	2963	4449	2742	7075	4172	5905
ENSECAG00000024044	4.62670838	0.1997027	0.5473994	507	461	662	565	385	250	457	439
ENSECAG00000021655	4.988115818	0.2001334	0.5484475	419	413	456	820	950	428	875	410
ENSECAG00000025151	3.89122019	0.2002091	0.5484778	323	220	368	439	215	140	332	222
ENSECAG00000019086	4.511897343	0.2002815	0.5484778	545	301	543	682	320	224	459	406
ENSECAG00000002508	6.44158148	0.2003149	0.5484778	1444	1314	1696	3530	1131	1360	1476	1350
ENSECAG00000022430	5.794595875	0.2003376	0.5484778	945	636	979	1221	1400	924	1396	816
ENSECAG00000024916	5.520150272	0.2003991	0.5485142	754	734	730	914	863	733	1127	1007
ENSECAG00000007365	6.66638419	0.2005567	0.5487132	1526	2176	1295	1657	1368	2075	2135	2784
ENSECAG00000010316	6.341537527	0.2005685	0.5487132	1530	1256	1192	1502	1910	1716	1465	1404
ENSECAG00000009153	7.404587608	0.2008077	0.5492354	2910	2071	2728	3762	2778	3048	4757	3465
ENSECAG00000025074	5.046691667	0.2009433	0.5493929	592	369	574	678	555	478	658	1045
ENSECAG00000018628	7.622349195	0.200962	0.5493929	4256	3762	4163	5517	2766	3058	4082	2242
ENSECAG00000020528	0.441916652	0.2011935	0.5494317	40	19	26	35	18	8	32	14
ENSECAG00000024201	3.80861632	0.2011947	0.5494317	274	420	444	149	73	158	163	388
ENSECAG00000007533	8.314086195	0.2011999	0.5494317	7411	998	6548	2242	15483	2841	11263	720
ENSECAG00000019596	7.360864022	0.2012586	0.5494317	101	5161	147	996	154	5896	215	10426
ENSECAG00000014879	5.206969064	0.2012685	0.5494317	889	577	979	892	730	270	834	450
ENSECAG00000002919	6.803647675	0.2013252	0.5494317	1563	2125	1558	2170	2693	1637	3227	1743
ENSECAG00000012210	8.528550966	0.2014233	0.5494317	5402	6134	5664	7316	7244	9034	7611	6180
ENSECAG00000024492	2.758279586	0.201451	0.5494317	97	56	104	173	120	100	119	234
ENSECAG00000006451	4.05597342	0.2015461	0.5494317	323	160	324	244	487	362	416	132
ENSECAG00000014658	1.443595166	0.2015994	0.5494317	61	53	36	102	36	15	65	36
ENSECAG00000010446	4.308450313	0.2016117	0.5494317	360	242	296	427	317	412	551	354
ENSECAG00000008486	3.133498419	0.2016645	0.5494317	132	133	177	107	246	189	143	143
ENSECAG00000020641	3.969737498	0.2016919	0.5494317	260	394	476	283	358	114	246	204
ENSECAG00000015935	11.20066873	0.20171	0.5494317	50202	47104	57943	57300	40745	25175	54479	24535
ENSECAG00000020078	1.868709629	0.201747	0.5494317	29	117	13	31	65	41	73	151
ENSECAG00000022700	5.731862405	0.2017501	0.5494317	1267	998	1165	1279	618	538	1003	1186
ENSECAG00000015267	5.555483929	0.2020422	0.5500952	1014	1378	840	857	731	685	745	729
ENSECAG00000024353	5.463864905	0.2021291	0.5501999	890	815	1069	1134	645	448	957	744
ENSECAG00000016070	4.568805882	0.2025245	0.5508145	307	609	649	584	199	251	392	583
ENSECAG00000023926	6.033150075	0.202558	0.5508145	1395	1119	1382	1919	970	906	1346	905
ENSECAG00000011547	11.03288228	0.2025651	0.5508145	39234	27519	37562	34397	42694	46936	37897	43371
ENSECAG00000012082	8.092592945	0.2026215	0.5508145	3902	3452	4403	6353	7931	4773	6901	3136
ENSECAG00000015734	5.117896379	0.2026687	0.5508145	519	649	459	695	845	617	749	611
ENSECAG00000023567	8.066551632	0.2027339	0.5508145	4474	4535	4374	4371	6794	4299	5210	5671
ENSECAG00000020424	7.53628255	0.202751	0.5508145	3745	3889	3481	5621	2972	2747	3741	1927
ENSECAG00000010993	2.909025136	0.2027789	0.5508145	159	123	194	207	113	66	131	136
ENSECAG00000019157	5.903912949	0.2028557	0.5508145	1148	1266	1424	1461	545	868	1160	1165
ENSECAG00000016083	6.992252235	0.2028881	0.5508145	2106	1963	1836	2693	3093	1882	2984	2493
ENSECAG00000018017	4.320719785	0.2029134	0.5508145	482	343	460	526	415	176	426	199
ENSECAG00000016675	7.828539896	0.2029725	0.5508145	3886	2483	3365	5018	8156	2744	6147	2346
ENSECAG00000024224	1.962189404	0.2029853	0.5508145	95	64	113	78	54	61	52	52
ENSECAG00000010862	8.054595035	0.2030871	0.5509592	4345	4797	4192	4195	6424	5072	6081	4235
ENSECAG00000018173	5.3955991	0.2031494	0.5509966	619	679	749	774	758	1087	750	772
ENSECAG00000023324	6.00688106	0.2032957	0.5512618	1084	875	1030	1377	1733	985	1569	967
ENSECAG00000024658	9.276588627	0.2036364	0.5520538	14482	10861	15167	15466	10818	7006	13579	6849
ENSECAG00000004121	4.629799293	0.2037281	0.5521708	606	417	561	622	398	254	455	430
ENSECAG00000017767	1.699267877	0.2038066	0.5522516	49	41	61	43	107	34	56	75
ENSECAG00000010506	0.586334123	0.2039058	0.5523042	13	19	26	29	38	27	34	22
ENSECAG00000021785	8.784218327	0.2039627	0.5523042	875	9247	7735	6819	1602	14538	11204	12606
ENSECAG00000020291	4.763141395	0.2039719	0.5523042	437	401	475	490	523	708	467	482
ENSECAG00000005773	4.555391474	0.2040393	0.5523553	377	203	417	586	495	429	635	391
ENSECAG00000016379	6.673622063	0.2042403	0.5526604	1601	1687	1601	1999	2222	2191	2354	1490
ENSECAG00000010069	1.472412039	0.2042517	0.5526604	93	23	54	89	52	24	51	24
ENSECAG00000019092	3.207137148	0.204298	0.5526604	190	152	231	267	157	82	151	161
ENSECAG00000010375	6.883886657	0.2043782	0.5527458	2781	1747	2782	3364	2142	1431	2430	1298
ENSECAG00000016636	6.753161652	0.2045749	0.5531461	2770	931	2009	79	3516	1520	4293	901
ENSECAG00000016596	5.477014249	0.204708	0.5533741	349	706	367	1303	379	1856	368	1134
ENSECAG00000007182	3.000034698	0.2048712	0.5535404	214	129	175	211	124	86	188	78
ENSECAG00000000910	6.47841061	0.2049034	0.5535404	2557	1298	2142	2121	1962	1245	1529	584
ENSECAG00000019263	7.800778512	0.2049157	0.5535404	3642	3446	3662	3933	2998	6444	4457	4246
ENSECAG00000011125	7.989614224	0.2051208	0.5539627	4412	4007	3726	4642	3947	6993	4548	5121
ENSECAG00000022790	6.759665809	0.2052628	0.5540136	2160	1259	1949	1818	3158	2030	2351	1341

ENSECAG00000008134	1.180443133	0.2052833	0.5540136	32	27	43	33	67	33	57	30
ENSECAG00000009761	9.017520566	0.205286	0.5540136	11911	9796	12549	12466	10409	5407	10271	5665
ENSECAG00000001294	3.298092986	0.2053979	0.5541673	167	114	182	155	361	112	217	141
ENSECAG00000010189	3.607662114	0.2054784	0.5541673	217	142	180	238	392	149	371	129
ENSECAG00000023032	0.23499027	0.2054893	0.5541673	5	21	27	7	17	14	28	40
ENSECAG00000022701	5.508925222	0.2057376	0.5547053	999	828	1101	1077	587	756	680	800
ENSECAG00000001580	4.232828915	0.2059	0.5549083	405	330	445	521	374	194	399	178
ENSECAG00000024232	1.246620197	0.2059106	0.5549083	11	39	34	39	9	30	23	152
ENSECAG00000009932	3.092116795	0.2059695	0.5549353	177	196	169	224	87	110	141	169
ENSECAG00000024564	6.429946689	0.2061872	0.5553903	1826	1743	2297	1674	1319	1179	1303	1568
ENSECAG00000021840	7.284904159	0.2062985	0.5555493	3398	3486	3110	3792	2526	1972	3023	2207
ENSECAG00000013320	7.011679586	0.2063441	0.5555493	3187	2300	3456	2520	1990	1266	2205	2550
ENSECAG00000018413	3.472633003	0.2064304	0.5556499	245	176	248	344	210	94	199	164
ENSECAG00000012843	7.10287211	0.2065075	0.5557258	2804	1003	2609	2332	4828	1533	4280	1298
ENSECAG00000024999	6.401208609	0.2066164	0.5558339	1337	1435	1204	1759	1357	1419	1808	2305
ENSECAG00000015831	6.942122594	0.2066455	0.5558339	3078	2218	2877	2819	2333	1436	2626	1213
ENSECAG00000017576	3.434175923	0.2070157	0.5565739	261	173	263	275	142	162	209	137
ENSECAG00000022670	5.125385773	0.2070382	0.5565739	770	682	795	840	584	335	763	516
ENSECAG00000021005	5.947049391	0.2070676	0.5565739	1501	835	1457	1775	603	691	1374	1208
ENSECAG00000021912	1.597428331	0.2073171	0.5570283	31	33	39	67	15	139	25	74
ENSECAG00000017116	6.928903707	0.2073348	0.5570283	2523	3202	2724	2275	1641	1835	1506	2411
ENSECAG00000013830	1.113863912	0.207404	0.5570672	21	50	22	17	6	31	39	118
ENSECAG00000010190	2.258154017	0.2074473	0.5570672	133	65	145	91	55	44	100	79
ENSECAG00000001815	2.538771007	0.2076012	0.5572993	92	92	99	79	141	134	119	83
ENSECAG00000007820	2.162301694	0.2076319	0.5572993	65	79	74	49	56	56	108	165
ENSECAG00000005351	3.800553952	0.207754	0.5574952	272	425	376	167	176	150	176	311
ENSECAG00000014371	6.960156425	0.2078876	0.5576722	1964	1921	1899	2439	2253	3854	2062	1839
ENSECAG00000003636	7.524095443	0.2079181	0.5576722	4490	3165	4209	4925	2742	1149	3130	4276
ENSECAG00000003783	1.123009072	0.2080033	0.5577689	44	38	52	60	34	18	33	36
ENSECAG00000008748	3.892924094	0.2080834	0.557852	322	179	271	151	514	174	401	182
ENSECAG00000012357	6.947719666	0.2081421	0.5578778	2583	2331	2637	3439	1572	1896	1954	2223
ENSECAG00000008627	4.740982402	0.2082802	0.5581161	436	372	476	518	514	382	682	624
ENSECAG00000012273	5.534503045	0.2084286	0.558292	1065	995	950	1082	590	395	1005	946
ENSECAG00000009878	5.641015807	0.2084441	0.558292	936	734	937	775	944	945	1189	954
ENSECAG00000003510	5.514547604	0.2085495	0.5583111	1035	614	746	663	892	738	1389	762
ENSECAG00000005604	1.938729832	0.2085495	0.5583111	78	67	112	88	49	63	67	38
ENSECAG00000024657	6.066858811	0.2087625	0.5586535	1564	1074	1535	1779	1213	734	1378	902
ENSECAG00000024271	2.559981627	0.2087758	0.5586535	62	92	111	70	28	77	112	312
ENSECAG00000020625	7.483176391	0.2089421	0.5589336	3766	4196	4604	3349	1477	2917	3132	3356
ENSECAG00000024210	4.457028888	0.2089789	0.5589336	463	415	545	526	380	328	280	343
ENSECAG00000020438	3.605971288	0.2094941	0.5601264	238	707	85	85	183	113	264	91
ENSECAG00000023364	5.808765347	0.2095235	0.5601264	704	1068	861	938	507	1553	717	1844
ENSECAG00000007965	6.612491836	0.2096171	0.5602449	1357	1605	1556	2001	1322	2872	1606	2061
ENSECAG00000008902	5.20562417	0.2097031	0.5603428	811	698	805	931	568	477	732	548
ENSECAG00000016814	7.443679589	0.2099198	0.5607899	3187	2400	2591	3530	4499	3158	3886	2678
ENSECAG00000016782	8.015589592	0.2100507	0.5609671	3573	4589	3369	5204	3257	8840	4545	4454
ENSECAG00000024598	4.548359747	0.2100849	0.5609671	460	402	659	566	380	219	472	384
ENSECAG00000012479	5.087959747	0.2103021	0.5612854	837	617	868	687	553	352	797	430
ENSECAG00000023061	9.918481923	0.2103774	0.5612854	14041	15193	18038	17276	18380	20986	20012	19508
ENSECAG00000017967	4.050573205	0.2104007	0.5612854	411	277	405	391	243	142	347	292
ENSECAG00000009254	0.964576467	0.2104017	0.5612854	23	33	25	31	61	28	57	17
ENSECAG00000002706	3.28217843	0.2105374	0.5614413	131	134	180	189	218	129	252	213
ENSECAG00000013413	5.330651391	0.2105717	0.5614413	585	634	773	715	963	823	830	623
ENSECAG00000025049	4.068198548	0.2106085	0.5614413	318	197	296	297	444	225	432	291
ENSECAG00000021368	2.571624168	0.2107392	0.5614886	208	97	128	116	64	75	40	148
ENSECAG00000023466	5.745945584	0.2107561	0.5614886	967	845	831	1022	1010	794	1322	1242
ENSECAG00000000412	4.381661225	0.2107908	0.5614886	449	287	367	257	485	444	443	328
ENSECAG00000013733	8.20552651	0.2108239	0.5614886	7164	5574	6740	6992	5830	3370	5855	3104
ENSECAG00000008268	2.952985661	0.2109573	0.5617122	134	75	98	181	183	109	306	77
ENSECAG00000012041	4.819350149	0.2111815	0.5620474	518	389	479	443	861	548	668	258
ENSECAG00000014231	6.085740627	0.2111822	0.5620474	1226	1494	1364	1885	1102	1010	1192	951
ENSECAG00000006712	4.833540928	0.2112603	0.5621235	588	482	684	788	471	372	561	367
ENSECAG00000024804	0.690656376	0.2114269	0.5623575	32	16	18	23	39	8	71	23
ENSECAG00000008024	3.924991907	0.2114472	0.5623575	367	385	451	178	144	100	205	432
ENSECAG00000011129	7.554983184	0.2115714	0.5624087	3980	3099	4281	5606	2313	2397	4442	2625
ENSECAG00000014311	10.35389102	0.2116234	0.5624087	24805	17691	21118	23404	28605	29215	21273	27086
ENSECAG00000013883	2.707868893	0.2116593	0.5624087	87	445	33	43	87	59	39	132
ENSECAG00000015306	4.394452882	0.2116645	0.5624087	247	336	411	371	259	597	349	507
ENSECAG00000017455	2.676095722	0.2118029	0.5624737	137	76	91	83	249	93	140	60
ENSECAG00000000311	5.828110953	0.211805	0.5624737	1105	1029	1208	1702	701	686	1099	1109
ENSECAG00000014955	3.801046171	0.2118375	0.5624737	192	312	414	352	217	103	173	330
ENSECAG00000023164	3.886635263	0.2120287	0.5628497	370	197	397	381	183	128	351	246
ENSECAG00000011769	1.165623228	0.2121373	0.5630064	41	41	57	61	29	23	50	24
ENSECAG00000005768	4.715444133	0.212299	0.563154	525	510	759	543	410	210	580	437
ENSECAG00000012486	6.68784782	0.2123031	0.563154	1862	1122	1745	2255	2441	1626	3081	1409
ENSECAG00000006377	7.980360583	0.2123416	0.563154	5283	4695	5238	7652	4855	2342	5305	3157
ENSECAG00000022803	2.745163586	0.2124307	0.5632588	182	68	218	158	79	53	194	66
ENSECAG00000013629	9.089887385	0.2125764	0.5635135	13303	9065	13853	13398	9035	3856	13791	7136

ENSECAG00000020495	4.116595121	0.2126672	0.5636226	213	209	312	421	364	195	478	423
ENSECAG00000000186	7.315743732	0.2128128	0.5638771	3642	2412	2756	1282	1965	2563	4905	4250
ENSECAG00000004868	5.952794499	0.2128712	0.5639003	993	1084	928	1217	1005	1232	1431	1327
ENSECAG00000010574	4.41741759	0.2131462	0.564497	347	350	297	440	397	389	440	510
ENSECAG00000023030	4.68172916	0.2133754	0.5648497	623	470	555	630	319	274	591	430
ENSECAG00000024262	5.261059411	0.2133788	0.5648497	549	627	617	789	542	728	754	1098
ENSECAG00000002313	1.82977606	0.2136059	0.565319	43	50	38	92	83	57	84	71
ENSECAG00000014779	6.543965302	0.2139316	0.5660204	1837	1769	2113	2483	1502	1396	1592	1370
ENSECAG00000004218	4.074516843	0.2139706	0.5660204	332	215	331	219	358	390	388	241
ENSECAG00000006802	0.47301854	0.2140949	0.5662175	10	14	24	29	8	33	55	21
ENSECAG00000011251	3.282720296	0.2142917	0.5666059	125	137	117	260	162	195	253	199
ENSECAG00000013689	4.905330286	0.214395	0.5667469	568	406	418	639	527	479	838	625
ENSECAG00000024907	6.332855396	0.2144728	0.5667469	1755	1604	1605	2169	1618	874	1587	986
ENSECAG00000010383	3.287783681	0.2144947	0.5667469	196	159	246	281	140	151	138	150
ENSECAG00000016393	4.779413527	0.2146225	0.5668795	544	510	552	863	314	515	447	391
ENSECAG00000024534	5.676842027	0.2146503	0.5668795	1056	887	1118	1478	916	512	1040	772
ENSECAG00000011846	7.098652722	0.2147384	0.5668795	2776	2110	2236	1703	2145	1684	2491	5247
ENSECAG00000021946	7.48142863	0.2147926	0.5668795	3793	4470	4468	3178	1630	2588	2305	4236
ENSECAG00000022471	5.128837645	0.2147944	0.5668795	894	606	657	973	698	396	690	383
ENSECAG00000017300	4.140305321	0.2148999	0.5670263	343	213	432	650	280	143	424	230
ENSECAG00000000572	3.24475824	0.2150051	0.5671721	167	120	193	124	237	121	283	160
ENSECAG00000018646	4.118251135	0.2152101	0.5675811	378	333	391	438	245	192	367	278
ENSECAG00000020387	2.29273477	0.2154948	0.5679489	121	56	132	141	69	58	105	52
ENSECAG00000017144	5.99660343	0.2155836	0.5679489	1358	1196	1423	1617	1093	876	1136	922
ENSECAG00000008745	1.868205448	0.2156383	0.5679489	8	104	19	21	3	219	4	111
ENSECAG00000024029	6.966213402	0.2156985	0.5679489	2510	2616	2729	3300.99	2705	1625.99	1958	1359.99
ENSECAG00000000397	7.652254585	0.2157456	0.5679489	4269	3974	4724	4895	3658	2139	3764	2998
ENSECAG00000023887	1.375884673	0.2157467	0.5679489	26	45	33	52	30	65	54	64
ENSECAG00000016434	6.095958746	0.2157481	0.5679489	1232	997	1157	1310	1313	1286	1358	1519
ENSECAG00000016444	0.785095112	0.2157496	0.5679489	18	22	25	35	13	34	46	50
ENSECAG00000006378	1.935441921	0.2158738	0.5681442	65	89	69	125	31	39	82	69
ENSECAG00000013983	7.645162047	0.2159523	0.5682192	4117	3664	4211	5893	2965	2781	3697	3033
ENSECAG00000008260	5.968072651	0.2161349	0.5684214	1487	1140	1397	1483	922	618	1165	1265
ENSECAG00000012134	3.875337217	0.216196	0.5684214	255	171	228	315	387	226	411	198
ENSECAG00000004780	4.799423611	0.2162202	0.5684214	409	442	454	565	571	593	696	403
ENSECAG00000016660	5.815518523	0.2162593	0.5684214	950	963	863	1054	1235	1174	1077	1005
ENSECAG00000006511	1.210882386	0.2162794	0.5684214	56	32	42	79	29	30	44	26
ENSECAG00000000287	7.502840565	0.2164771	0.5688095	3754	3398	3851	5158	2658	2682	3201	2733
ENSECAG00000018412	4.839554326	0.2167217	0.5693205	570	386	505	435	705	350	824	508
ENSECAG00000015869	4.032058897	0.2168072	0.5694133	378	298	374	400	251	218	255	278
ENSECAG00000021891	6.008007102	0.2169061	0.5695415	1334	1258	1329	1725	923	947	1282	922
ENSECAG00000008088	2.524225024	0.2171153	0.569959	157	76	181	112	114	41	109	68
ENSECAG00000000529	6.385070501	0.217282	0.5702647	1090	1512	1443	1432	1093	2671	1235	1706
ENSECAG00000009564	5.263204049	0.2174597	0.570355	921	684	736	1087	511	381	990	556
ENSECAG00000018086	4.516794614	0.2175292	0.570355	172	482	235	484	128	710	261	858
ENSECAG00000021208	6.635895227	0.217545	0.570355	1802	1682	1552	1664	1627	2361	1933	2051
ENSECAG00000012848	5.433299138	0.2175499	0.570355	941	703	945	1268	515	453	934	836
ENSECAG00000020692	6.659840377	0.2175674	0.570355	2386	1621	2279	2882	1146	698	2657	1898
ENSECAG00000005829	3.065782736	0.2177053	0.5705849	162	59	134	146	248	53	398	68
ENSECAG00000012138	4.054250837	0.2178772	0.5707827	337	313	360	468	260	223	301	239
ENSECAG00000008211	4.370440635	0.2178813	0.5707827	349	568	200	836	97	294	503	314
ENSECAG00000014633	6.272404583	0.2179825	0.570916	1495	1658	1507	2123	1061	992	1420	1427
ENSECAG00000024339	5.963929196	0.2180394	0.5709335	1047	866	941	1404	1596	760	1666	1117
ENSECAG00000010551	7.05134749	0.2181628	0.571125	3049	2370	3194	3173	2320	1280	2782	1962
ENSECAG00000015868	7.032486168	0.2182678	0.5712682	2302	2078	2074	2371	3101	2036	3408	2191
ENSECAG00000026861	3.955450726	0.2185407	0.5718506	274	203	287	264	326	295	275	362
ENSECAG00000015940	5.65296647	0.2186381	0.5719055	1163	872	1072	1321	855	560	981	798
ENSECAG00000019289	2.972219196	0.2186906	0.5719055	153	150	165	244	78	111	115	159
ENSECAG00000008987	5.386471052	0.2187127	0.5719055	888	871	845	1075	586	707	560	726
ENSECAG00000017396	5.999235021	0.2189695	0.5721916	941	1003	870	1560	1243	1181	1864	950
ENSECAG00000022539	4.072120006	0.2190098	0.5721916	301	291	402	517	199	222	336	281
ENSECAG00000017374	1.251306948	0.2190872	0.5721916	41	22	32	49	55	66	43	28
ENSECAG00000010682	5.854098796	0.2191544	0.5721916	923	903	958	1156	1305	681	1458	1301
ENSECAG00000008730	6.953427137	0.2191694	0.5721916	2092	1996	1729	2175	1450	4417	1594	2550
ENSECAG00000010224	7.432988116	0.2192609	0.5721916	3717	3771	3844	3864	2798	2423	2784	2722
ENSECAG00000019506	5.99151311	0.2193456	0.5721916	833	1100	1123	1096	624	1877	976	1688
ENSECAG00000000361	4.717128215	0.2194325	0.5721916	564	476	519	787	438	300	548	359
ENSECAG00000013497	2.34274605	0.2194847	0.5721916	170	57	140	122	138	30	87	13
ENSECAG00000008626	5.329987947	0.219485	0.5721916	792	590	660	676	868	543	1089	803
ENSECAG00000018456	0.523846298	0.2195022	0.5721916	18	29	18	16	28	22	30	36
ENSECAG00000019424	3.360623614	0.2195925	0.5721916	169	167	167	165	214	152	290	198
ENSECAG00000022482	5.431027437	0.2195966	0.5721916	957	735	819	1320	566	679	768	685
ENSECAG00000011843	3.795864214	0.2196127	0.5721916	320	243	314	360	220	166	267	201
ENSECAG00000021588	5.204021266	0.2196149	0.5721916	657	966	664	1011	364	382	543	970
ENSECAG00000007563	6.550577098	0.2196975	0.5721916	1856.99	2127.99	1875	2364	1030	1385.99	1587.99	1877.99
ENSECAG00000009428	2.364341124	0.219698	0.5721916	89	45	78	114	152	96	113	66
ENSECAG00000007498	4.363562505	0.219748	0.5721916	493	337	552	458	314	179	322	449
ENSECAG00000006899	5.686369737	0.2197792	0.5721916	650	1011	932	863	1186	837	1326	848

ENSECAG00000021995	5.008166758	0.220058	0.5727117	691	638	612	934	526	255	668	572
ENSECAG00000021542	3.782466663	0.2201282	0.5727117	137	335	177	179	107	325	236	505
ENSECAG00000012523	2.70504307	0.2201555	0.5727117	132	119	146	190	99	67	131	94
ENSECAG00000000725	7.62448958	0.2202666	0.5727117	3467	2852	3354	3667	4458	2939	4875	3928
ENSECAG00000010303	3.603043933	0.2202941	0.5727117	276	233	273	307	149	174	141	260
ENSECAG00000024595	1.147363334	0.2203223	0.5727117	42	25	38	27	57	31	61	34
ENSECAG00000024208	6.011043467	0.2203319	0.5727117	1270	1375	1162	1899	938	703	1057	1361
ENSECAG00000015091	5.077493184	0.220451	0.5728902	537	504	632	571	662	906	475	648
ENSECAG00000019212	4.073375183	0.2205957	0.5730731	409	224	464	427	354	158	301	207
ENSECAG00000020265	3.1358744	0.2206223	0.5730731	186	73	149	163	204	157	184	181
ENSECAG00000008960	6.358970267	0.2207248	0.5731115	1657	1604	1758	2198	976	1043	1732	1484
ENSECAG00000001599	11.64449606	0.2208116	0.5731115	57526	40491	60067	55260	74282	68034	52019	65341
ENSECAG00000020310	3.659034222	0.2208655	0.5731115	250	201	210	145	288	281	312	160
ENSECAG00000003845	8.686681703	0.2209338	0.5731115	4127	9372	8276	3800	6335	11138	11476	5806
ENSECAG00000014712	4.788602933	0.2209503	0.5731115	559	628	545	715	286	347	515	572
ENSECAG00000025052	3.195150338	0.2209755	0.5731115	149	133	153	146	236	104	322	122
ENSECAG00000012571	6.193332864	0.2209903	0.5731115	1112	1113	1413	1280	1253	1999	1157	1411
ENSECAG00000018875	1.673220038	0.2210413	0.5731131	47	28	69	49	78	46	113	35
ENSECAG00000010873	3.50425066	0.2213132	0.5736871	199	178	174	187	163	280	226	254
ENSECAG00000020581	2.015585782	0.2214023	0.5736905	81	73	127	79	50	61	65	56
ENSECAG00000007660	6.727318899	0.2214155	0.5736905	2217	2037	2467	2622	1409	1153	2288	1892
ENSECAG00000015144	3.498481141	0.2215235	0.5737177	343	367	123	204	239	48	114	233
ENSECAG00000026880	2.543045343	0.2215589	0.5737177	92	77	84	97	61	249	63	103
ENSECAG00000022627	9.462682208	0.2215775	0.5737177	15889	13921	15710	17179	11853	9123	11845	10871
ENSECAG00000014283	6.702450978	0.2218329	0.5742479	2488	2073	2793	1902	1268	848	1779	2575
ENSECAG00000017915	4.80406498	0.2220011	0.5742691	743	327	449	232	485	462	392	1013
ENSECAG00000014291	7.031660299	0.2220147	0.5742691	2552	1671	2160	2506	2060	2688	2907	2971
ENSECAG00000014560	7.665932331	0.2220697	0.5742691	5572	3406	3714	5523	3430	2137	4278	2854
ENSECAG00000010985	5.101620299	0.2220707	0.5742691	729	738	738	795	476	551	575	536
ENSECAG00000007906	4.536370412	0.2220938	0.5742691	506	435	523	586	404	249	426	370
ENSECAG00000009003	6.479894566	0.222171	0.5743377	1903	1702	1762	2563	870	1135	1783	1853
ENSECAG00000009754	2.809393493	0.2222783	0.5743953	147	129	172	179	77	96	136	111
ENSECAG00000005540	8.416429518	0.2223241	0.5743953	5183	6475	5538	4985	4639	5778	6377	11677
ENSECAG00000000513	4.858113237	0.2223882	0.5743953	546	405	497	500	754	444	623	537
ENSECAG00000018222	7.780716753	0.2223955	0.5743953	4879	3932	4865	5928	3329	3021	4630	2774
ENSECAG00000017145	8.333287591	0.22279	0.5752512	7518	5612	8208	7292	5063	4436	5294	5175
ENSECAG00000008318	3.898640339	0.2228282	0.5752512	314	287	361	384	122	144	377	278
ENSECAG00000002456	2.619156774	0.2230602	0.5756145	147	66	143	233	43	28	161	124
ENSECAG00000020649	5.25778652	0.2230703	0.5756145	862	657	789	1085	577	387	754	704
ENSECAG00000014591	1.432043239	0.2232394	0.5759201	58	54	47	89	13	21	49	67
ENSECAG00000017351	2.230941333	0.2233034	0.5759545	57	94	66	75	106	95	88	95
ENSECAG00000020347	4.432116447	0.2234008	0.5759763	487	390	490	543	330	225	432	366
ENSECAG00000007779	5.403470397	0.2234957	0.5759763	777	767	919	1319	602	513	1061	503
ENSECAG00000017101	2.937701778	0.223508	0.5759763	119	99	108	165	211	78	239	124
ENSECAG00000008121	2.808110187	0.2235147	0.5759763	130	91	237	191	133	48	161	68
ENSECAG00000012175	6.997015054	0.2237055	0.5762359	3069	2427	2714	3106	1614	1317	2588	2554
ENSECAG00000017584	6.426976609	0.2238343	0.5762359	1731	1691	2088	2109	1207	687	2086	1517
ENSECAG00000022440	7.547482593	0.2238835	0.5762359	3594	3438	4010	5761	3203	2011	3969	2545
ENSECAG00000012941	8.875510527	0.2239033	0.5762359	8421	8989	6698	6228	7524	13785	8825	8064
ENSECAG00000022113	5.167754351	0.223923	0.5762359	814	586	752	1041	493	442	610	710
ENSECAG00000015086	9.262342286	0.2240155	0.5762359	7186	10534	7987	14556	8369	19453	13220	9298
ENSECAG00000012075	0.887878929	0.2240507	0.5762359	29	21	27	32	51	42	38	18
ENSECAG00000017646	6.842872822	0.2240592	0.5762359	1706	1749	2081	2218	2396	2347	3027	1588
ENSECAG00000012866	3.096876782	0.224072	0.5762359	131	137	147	138	167	190	155	182
ENSECAG00000009325	5.020478716	0.2241501	0.5763061	670	514	763	955	533	310	586	604
ENSECAG00000005317	1.666613774	0.2244541	0.5768627	38	25	43	80	111	17	128	28
ENSECAG00000017574	4.608880695	0.2244681	0.5768627	482	561	540.999	567	402.998	212	509	405
ENSECAG00000017595	3.695735295	0.2246947	0.5772599	150	226	185	288	199	299	235	324
ENSECAG00000009899	3.759961373	0.2247243	0.5772599	207	151	265	256	362	107	370	314
ENSECAG00000011858	6.106380832	0.2248869	0.5774574	1613	1207	1482	1724	1010	1009	1273	1075
ENSECAG00000019632	5.212853324	0.2249142	0.5774574	540	722	512	722	733	822	769	652
ENSECAG00000000321	0.277469101	0.2250359	0.5774574	25	26	19	35	13	10	29	12
ENSECAG00000016963	3.484865976	0.225069	0.5774574	256	193	296	255	177	99	241	170
ENSECAG00000024759	2.460947034	0.2250766	0.5774574	78	101	67	95	76.9997	120	79.9999	178
ENSECAG00000023230	3.996868283	0.2251062	0.5774574	208	247	261	342	234	414	321	324
ENSECAG00000011364	3.851588679	0.2252176	0.5776126	391	168	380	406	207	67	392	208
ENSECAG00000017299	1.28676634	0.2253077	0.5776487	28	47	48	17	32	58	50	62
ENSECAG00000019222	0.676796978	0.2253333	0.5776487	16	38	19	8	10	23	21	84
ENSECAG00000005682	3.9752622	0.2254866	0.5779111	297	291	272	653	95	68	281	472
ENSECAG00000011681	5.979037775	0.2256482	0.5781949	1413	1053	1518	1546	966	1003	1037	951
ENSECAG00000002936	3.823747192	0.2258124	0.5783237	245	262	328	449	197	111	275	286
ENSECAG00000021990	4.319627487	0.2258313	0.5783237	488	359	446	468	326	260	306	340
ENSECAG00000008836	2.318254278	0.2258793	0.5783237	131	60	133	141	68	81	107	23
ENSECAG00000007843	2.087262559	0.2259021	0.5783237	55	70	68	56	41	160	39	109
ENSECAG00000015012	3.954669549	0.2260439	0.5785369	259	457	419	396	17	72	103	613
ENSECAG00000019585	0.957543181	0.2261695	0.5785369	49	39	51	36	5	15	36	48
ENSECAG00000007062	1.283354858	0.2261717	0.5785369	49	36	35	106	16	30	58	31
ENSECAG00000010872	3.626283473	0.2261891	0.5785369	185	204	172	229	142	403	197	260

ENSECAG00000010934	3.42478875	0.2262918	0.5786692	294	66	321	362.001	178	78	306	52
ENSECAG00000013488	1.975196995	0.2263779	0.5786778	80	26	78	58	109	64	103	54
ENSECAG00000021665	0.549585374	0.2264374	0.5786778	86	17	22	9	34	15	13	6
ENSECAG00000017296	3.571913371	0.226448	0.5786778	238	228	261	347	114	115	300	205
ENSECAG00000017451	1.395405973	0.2265933	0.5788398	41	45	87	67	41	13	71	23
ENSECAG00000019736	6.375831464	0.2266172	0.5788398	1283	1375	1220	1818	1380	1872	1694	1691
ENSECAG00000018377	5.067510327	0.2266643	0.5788398	803	584	840	790	735	238	747	351
ENSECAG00000013757	5.988696577	0.2268491	0.5791816	1261	904	1136	1020	1518	1155	1244	1166
ENSECAG00000023705	5.559025616	0.2269226	0.579239	924	602	765	851	1338	452	1453	717
ENSECAG00000016189	4.944504306	0.2270434	0.5793842	588	566	635	961	333	440	544	599
ENSECAG00000014775	2.422938337	0.2270815	0.5793842	149	156	40	152	91	22	107	83
ENSECAG00000018343	8.54294374	0.2272091	0.5795797	4695	7130	4698	7061	4871	14631	7025	4030
ENSECAG00000024700	7.066811612	0.2273145	0.5796653	4062	1982	3148	2864	3102	1274	2153	1647
ENSECAG00000020139	7.925126253	0.2274112	0.5796653	4611	3766	3981	3889	5312	5071	5734	3661
ENSECAG00000024018	4.483807029	0.2274411	0.5796653	398	518	309	185	496	313	612	463
ENSECAG00000001652	3.08923256	0.2274468	0.5796653	189	157	175	252	124	83	97	198
ENSECAG00000020761	4.286036232	0.2276882	0.5798925	433	324	413	565	274	280	375	283
ENSECAG00000020279	0.289142314	0.2277118	0.5798925	11	11	20	27	19	40	23	14
ENSECAG00000006539	4.922608656	0.227732	0.5798925	727	534	698	713	535	394	600	367
ENSECAG00000023180	7.502303175	0.2277496	0.5798925	3278	2503	3153	3301	3795	4194	3273	3292
ENSECAG00000021455	5.538242329	0.2277912	0.5798925	657	854	714	796	572	1404	523	1241
ENSECAG00000013889	5.504837249	0.2278612	0.5799408	866	889	956	1374	433	407	809	1188
ENSECAG00000010212	7.800479949	0.2279261	0.5799644	4816	2992	3468	3188	8184	2789	4344	3224
ENSECAG00000011237	5.810723898	0.2279726	0.5799644	1078	987	1225	1661	776	903	938	909
ENSECAG00000019508	0.590021165	0.2281867	0.580379	44	26	28	34	22	8	18	32
ENSECAG00000002186	6.306072615	0.2284235	0.5808514	446	2493	617	695	432	3104	415	2962
ENSECAG00000014808	7.812587462	0.2284992	0.5808629	5555	4043	4703	5623	3221	2784	4207	3905
ENSECAG00000012143	4.937951256	0.2285303	0.5808629	321	1649	329	556	118	310	236	977
ENSECAG00000020665	12.09656375	0.2291007	0.581856	81783	55226	78876	75699	102738	103807	72406	74900
ENSECAG00000016103	3.921259274	0.2291075	0.581856	274	367	306	400	148	223	259	292
ENSECAG00000019130	3.48670058	0.2291656	0.581856	63	333	106	60	61	678	46	181
ENSECAG00000018799	7.189639122	0.2291851	0.581856	3354	2623	3156	3783	2425	2179	2380	2096
ENSECAG00000018755	4.386488499	0.2292482	0.581856	508	326	548	473	287	227	459	337
ENSECAG00000016494	6.015443794	0.2293349	0.581856	1102	1040	1055	1098	697	1843	1105	1560
ENSECAG00000023227	7.940898348	0.2294	0.581856	3685	4079	3729	4678	2975	7775	4810	4362
ENSECAG00000019367	1.822141672	0.22942	0.581856	53	61	34	71	54	93	73	68
ENSECAG00000023101	1.043964938	0.2294212	0.581856	37	30	26	31	29	35	60	45
ENSECAG00000009312	4.65120831	0.2294427	0.581856	619	455	614	524	437	252	435	442
ENSECAG00000007653	4.058675673	0.2294845	0.581856	312	266	335	603	302	165	315	241
ENSECAG00000010425	5.111457853	0.2295969	0.581941	665	486	541	536	392	1261	452	674
ENSECAG00000021824	4.489268413	0.2296205	0.581941	472	459	503	540	274	328	355	435
ENSECAG00000015634	6.84989412	0.229717	0.5819769	2393	2590	2353	2785	1903	1300	2033	2026
ENSECAG00000010244	4.079041078	0.2298304	0.5819769	242	265	257	374	337	286	351	399
ENSECAG00000012986	5.31658665	0.2298352	0.5819769	712	784	606	558	790	636	920	896
ENSECAG00000014042	2.203298904	0.2298911	0.5819769	81	73	52	75	124	63	166	42
ENSECAG00000002701	6.962057673	0.2299381	0.5819769	3030	2477	3456	2183	953	1357	2147	3202
ENSECAG00000015919	5.280262334	0.2299421	0.5819769	981	679	884	865	458	569	656	751
ENSECAG00000014926	4.040615762	0.2300404	0.5820961	377	272	414	400	319	194	272	223
ENSECAG00000003766	4.117430666	0.2305211	0.5831825	335	168	420	150	622	344	342	149
ENSECAG00000008916	6.063945116	0.2306258	0.5833173	941	1190	1460	683	738	1155	1144	2561
ENSECAG00000004370	3.24337811	0.230949	0.5836756	169	173	80	181	154	262	188	171
ENSECAG00000013569	4.290531417	0.2309752	0.5836756	374	317	277	296	322	635	260	349
ENSECAG00000000124	8.240697929	0.2310452	0.5836756	5863	6353	6436	8212	4794	4034	6164	3929
ENSECAG00000008391	4.970238262	0.2310777	0.5836756	705	606	631	826	352	430	526	650
ENSECAG00000009533	9.273014963	0.2312115	0.5836756	11807	8127	10561	11209	11975	13615	12673	11847
ENSECAG00000018925	5.330012294	0.2312243	0.5836756	601	713	543	883	746	706	788	1004
ENSECAG00000009691	5.717695141	0.2312304	0.5836756	915	931	854	864	1343	905	1098	875
ENSECAG00000011593	6.847713196	0.2312371	0.5836756	1956	2081	2129	1477	2129	2563	2210	2387
ENSECAG00000018890	5.330062479	0.2312616	0.5836756	735	642	488	880	675	807	845	913
ENSECAG00000017458	3.960023562	0.2312924	0.5836756	310	224	229	253	436	208	443	211
ENSECAG00000015385	2.208807848	0.2313629	0.5836756	60	90	89	33	53	60	141	148
ENSECAG00000000364	3.442968698	0.2314287	0.5836756	264	171	259	276	176	133	197	157
ENSECAG00000017369	6.513194732	0.2314687	0.5836756	1743	1039	1616	1568	2511	613	3437	1361
ENSECAG00000000700	8.012501242	0.2315261	0.5836756	6144	4263	5771	6843	4471	2974	4892	3832
ENSECAG00000000002	4.952441015	0.2316222	0.5836756	564	638	716	823	324	391	557	664
ENSECAG00000000398	4.856291604	0.2316264	0.5836756	572	574	699	710	381	440	390	572
ENSECAG00000013018	1.489612017	0.2316409	0.5836756	49	37	25	59	62	51	100	24
ENSECAG00000021235	1.866735026	0.2317451	0.5837844	53	43	58	72	106	49	114	38
ENSECAG00000000303	4.421759755	0.2317869	0.5837844	426	365	352	266	467	388	379	502
ENSECAG00000013945	4.267018992	0.231886	0.5839046	408	349	426	541	221	170	390	421
ENSECAG00000007513	8.235729431	0.2323059	0.5848321	7040	4022	4897	3895	8003	4836	7448	4654
ENSECAG00000006392	7.141445587	0.2323612	0.5848417	2698	2008	1955	2959	3265	2683	3036	2424
ENSECAG00000009892	5.032111971	0.232473	0.5849934	614	420	600	595	554	592	894	625
ENSECAG00000015597	9.064361826	0.2326173	0.5851653	11823	10085	7923	3780	13954	11619	10235	8596
ENSECAG00000022408	3.096772944	0.2326882	0.5851653	58	191	77	205	132	113	253	244
ENSECAG00000015693	4.715040581	0.2326958	0.5851653	514	435	644	734	387	364	485	406
ENSECAG00000011197	1.358967361	0.2327577	0.5851914	61	14	71	97	33	23	67	17
ENSECAG00000011354	4.051828032	0.2328731	0.5853008	207	282	323	228	226	638	222	253

ENSECAG00000015498	6.079353811	0.2329043	0.5853008	1686	1142	1379	1776	735	1210	969	1270
ENSECAG00000024338	1.703724722	0.2330271	0.5854412	31	39	62	62	24	91	42	114
ENSECAG00000011630	3.384502623	0.2330632	0.5854412	263	133	209	347	172	117	185	156
ENSECAG00000015602	4.987521572	0.2332677	0.5857052	385	661	446	578	388	762	500	935
ENSECAG00000015043	5.372967037	0.2332714	0.5857052	923	667	873	1219	809	436	684	670
ENSECAG00000018554	9.592172415	0.233339	0.5857454	13802	10531	14477	12796	16626	16954	12288	16391
ENSECAG00000023748	5.076159045	0.2333992	0.5857669	682	665	707	917	611	394	659	461
ENSECAG00000020935	7.229804166	0.2335651	0.5859588	2883	2788	3690	4038	2185	1301	3400	2626
ENSECAG00000009511	5.287083799	0.2335788	0.5859588	862	648	1020	924	614	486	949	416
ENSECAG00000022965	8.008081923	0.2337407	0.5862356	4134	4860	4086	3719	5682	7134	4452	3474
ENSECAG00000017771	3.732407115	0.2341614	0.5871609	439	198	307	279	371	125	166	98
ENSECAG00000018579	1.126189175	0.2343966	0.5876211	34	36	23	37	55	21	43	61
ENSECAG00000020513	2.589555952	0.2345335	0.587771	116	126	148	148	54	95	93	111
ENSECAG00000014936	3.319825075	0.2345599	0.587771	256	155	255	235	167	75	241	126
ENSECAG00000023300	6.767178864	0.2346527	0.5878737	1640	1587	1503	2707	1736	1989	1983	3147
ENSECAG00000006080	3.855759784	0.2347527	0.5879947	383	200	353	376	229	113	371	181
ENSECAG00000019020	2.596127348	0.2349034	0.5882208	108	101	156	188	79	41	108	130
ENSECAG00000023513	5.218489608	0.2349486	0.5882208	781	727	728	1048	447	470	649	781
ENSECAG00000024125	4.985654801	0.2349983	0.5882208	628	476	456	587	510	518	755	800
ENSECAG00000009106	4.421639574	0.2350806	0.588297	784	293	435	419	461	188	386	261
ENSECAG00000004504	1.140096639	0.2353095	0.5886373	29	30	33	40	69	48	26	33
ENSECAG00000020336	5.4083115	0.2353556	0.5886373	773	644	882	502	624	1090	694	1005
ENSECAG00000021982	6.06170483	0.235372	0.5886373	1443	853	1148	1058	1911	814	1362	1359
ENSECAG00000018076	6.604716218	0.2355172	0.5888706	1904	1963	1744	2981	1199	1551	1768	1613
ENSECAG00000006638	5.34652491	0.2355812	0.5888884	766	559	758	1659	506	395	714	909
ENSECAG00000011983	4.2628808	0.2356279	0.5888884	380	345	434	581	200	137	404	451
ENSECAG00000008334	7.924900815	0.2357588	0.5889792	5055	4907	5679	5819	4208	3610	4173	3082
ENSECAG00000014127	5.066502068	0.235768	0.5889792	734	553	718	971	577	469	661	388
ENSECAG00000023194	1.037420116	0.2360039	0.5894389	20	31	25	49	40	26	58	45
ENSECAG00000015465	6.041895702	0.2360737	0.5894837	1805	950	1492	1662	1260	512	1618	765
ENSECAG00000023006	1.781933566	0.2361993	0.5896434	20	90	43	15	15	166	10	108
ENSECAG00000024231	3.160551698	0.2362415	0.5896434	227	158	174	245	104	125	111	191
ENSECAG00000012306	7.515291182	0.2363338	0.5896784	2977	2704	3205	3387	3536	4987	3129	2972
ENSECAG00000009689	0.747175633	0.2363593	0.5896784	19	39	18	10	16	57	7	59
ENSECAG00000020740	7.73474187	0.2365609	0.5900517	5480	2137	4307	1731	3741	4652	6368	3207
ENSECAG00000008220	2.54114354	0.2366162	0.5900601	72	108	95	89	115	151	79	126
ENSECAG00000007264	0.449503612	0.2366748	0.5900767	15	15	35	12	36	24	26	23
ENSECAG00000023856	0.376647868	0.2367693	0.5901296	15	25	35	38	11	15	29	14
ENSECAG00000010628	5.230876547	0.2367999	0.5901296	585	529	710	703	826	956	495	698
ENSECAG00000024716	6.485002407	0.2371673	0.5909153	2088	1961	2207	1698	954	625	1569	2440
ENSECAG00000021807	5.006842518	0.2372991	0.5910653	594	475	567	536	617	712	553	681
ENSECAG00000011227	3.349598767	0.2373315	0.5910653	253	100	173	4	429	131	335	61
ENSECAG00000018000	6.367759249	0.237428	0.591176	1839	1270	2149	1989	1212	1265	1321	1395
ENSECAG00000016807	6.628710953	0.2374975	0.5912193	1517	1503	1722	2089	1900	1988	2004	2004
ENSECAG00000010665	3.806658065	0.237638	0.5914396	288	264	332	362	172	167	203	313
ENSECAG00000022956	9.00932417	0.2377231	0.5915218	10537	10513	11437	13273	7264	8266	10033	6415
ENSECAG00000021441	3.214057709	0.2379395	0.5919306	184	162	280	211	166	82	221	91
ENSECAG00000020261	7.597077007	0.2381625	0.5922377	3669	2959	3307	3013	5375	3167	4206	3021
ENSECAG00000021107	6.900265783	0.2382187	0.5922377	2555	2686	2320	3000	1194	1588	1949	2719
ENSECAG00000014672	0.429886479	0.2382507	0.5922377	16	31	10	17	20	14	33	44
ENSECAG00000006613	1.377854879	0.2382715	0.5922377	43	34	37	44	78	26	70	42
ENSECAG00000023312	4.722484224	0.238402	0.5922749	617	378	673	720	507	219	650	265
ENSECAG00000011742	4.949766893	0.2384046	0.5922749	574	473	538	485	683	686	707	412
ENSECAG00000012297	1.981707439	0.2384478	0.5922749	91	87	87	93	17	51	59	96
ENSECAG00000019853	3.240500508	0.2385247	0.5922749	372	141	337	68	271	24	172	23
ENSECAG00000022704	4.840851504	0.2385734	0.5922749	566	488	612	922	244	345	550	642
ENSECAG00000013999	6.977763133	0.2386088	0.5922749	2094	2178	2166	2088	2944	2141	2868	2243
ENSECAG00000019206	1.299316383	0.2386619	0.5922749	37	34	27	51	33	23	80	73
ENSECAG00000000450	5.52516236	0.2388114	0.5922749	612	730	886	934	990	653	994	1082
ENSECAG00000012417	4.270578507	0.2388538	0.5922749	461	384	508	359	155	222	360	451
ENSECAG00000022286	4.380157616	0.2388821	0.5922749	334	330	327	407	263	433	551	455
ENSECAG00000012688	3.17170705	0.2388905	0.5922749	167	158	212	281	90	98	144	209
ENSECAG00000011265	1.035789537	0.2389122	0.5922749	36	21	65	63	17	24	46	27
ENSECAG00000010105	6.309613925	0.23909	0.5925865	1449	1503	2009	1954.99	1157	1084	1526	1291
ENSECAG00000016887	3.527555972	0.239292	0.5929084	185	154	203	221	252	136	331	242
ENSECAG00000008470	4.42351472	0.2393351	0.5929084	322	333	409	376	419.001	492.001	371	431
ENSECAG00000016569	2.865667193	0.2394244	0.5929084	130	117	154	268	138	74	144	77
ENSECAG00000010227	7.276667627	0.2394287	0.5929084	2426	2787	2401	2793	2861	3805	2612	3122
ENSECAG00000022999	1.742574227	0.2394956	0.5929448	56	54	78	119	26	30	54	81
ENSECAG00000009015	3.778338537	0.2396396	0.593172	162	228	231	268	167.001	432	228	282
ENSECAG00000011881	3.09741284	0.239883	0.5936449	163	107	142	144	107	170	195	234
ENSECAG00000003274	1.478496018	0.2399352	0.5936449	58	36	35	43	60	35	74	61
ENSECAG00000018318	9.518651591	0.2400895	0.5938975	12060	10775	13570	12580	15901	18490	12557	11960
ENSECAG00000001439	5.189463201	0.2402098	0.5940656	859	447	597	565	810	654	850	639
ENSECAG00000008917	7.300143299	0.24034	0.5941812	2693	2577	2884	2419	2605	3821	3406	2861
ENSECAG00000014476	5.091555267	0.2404232	0.5941812	654	404	632	615	768	367	859	823
ENSECAG00000011238	2.482209593	0.2404485	0.5941812	74	79	112	93	117	121	125	92
ENSECAG00000007179	5.953633316	0.2404658	0.5941812	1201	939	1481	2018	1192	383	1525	799

ENSECAG00000020815	2.622721707	0.2405662	0.5943	85	83	116	112	85	159	138	120
ENSECAG00000008173	6.294759589	0.2406614	0.5943499	1152	1503	1355	1228	1307	884	1921	2399
ENSECAG00000004669	3.129667561	0.2406911	0.5943499	124	64	269	78	281	136	232	101
ENSECAG00000019555	3.786571609	0.2409725	0.5949156	181	238	220	255	182	389	192	352
ENSECAG00000018611	5.584138105	0.2410295	0.5949271	725	753	842	924	834	1343	994	648
ENSECAG00000011967	5.847376137	0.2411013	0.5949749	988	941	995	974	1531	1026	1325	754
ENSECAG00000009928	7.187611937	0.2413945	0.5955692	2682	2096	2232	2919	3430	2987	2951	2339
ENSECAG00000012206	2.050651526	0.2414682	0.5956216	93	59	124	97	63	28	79	73
ENSECAG00000011969	6.433941796	0.2416866	0.5959618	1729	1373	1592	1114	2252	1313	1953	1491
ENSECAG00000011563	2.80538791	0.2417111	0.5959618	121	88	113	134	158	103	130	181
ENSECAG00000021984	3.89452423	0.2420901	0.5967667	305	235	376	417	256	132	259	270
ENSECAG00000026866	3.050750702	0.2425352	0.5974764	209	156	209	157	119	97	160	127
ENSECAG00000014666	0.3697117	0.2425554	0.5974764	15	12	29	16	46	23	23	11
ENSECAG00000007129	2.248004257	0.2425943	0.5974764	92	187	56	89	37	67	56	104
ENSECAG00000016645	4.865533939	0.2426152	0.5974764	388	591	403	539	420	768	450	705
ENSECAG00000013643	0.305901138	0.242647	0.5974764	24	19	27	39	7	15	36	7
ENSECAG00000021085	6.396073646	0.2426936	0.5974764	1691	1740	2258	1665	1009	891	1545	1899
ENSECAG00000001648	5.362214994	0.2431647	0.5984313	1039	400	647	683	946	549	1185	731
ENSECAG00000021606	5.239798232	0.2432357	0.5984313	635	925	767	987	387	623	647	696
ENSECAG00000020146	3.230652517	0.2432395	0.5984313	222	138	217	272	136	75	190	172
ENSECAG00000003996	2.09080043	0.2433134	0.5984835	79	27	58	96	117	72	156	24
ENSECAG00000014723	2.78140575	0.2433741	0.5985032	145	119	81	84	206	102	176	90
ENSECAG00000025034	5.628625068	0.2434862	0.5986493	851	690	1016	837	944	768	1075	1199
ENSECAG00000007441	6.795716005	0.2436003	0.5988002	2321	2063	2891	2558	1676	904	2725	1788
ENSECAG00000012792	5.800804612	0.2436547	0.5988044	869	1006	1020	802	1337	1471	847	766
ENSECAG00000016706	4.399994029	0.2437075	0.5988044	481	190	456	155	702	170	824	186
ENSECAG00000022469	10.80227624	0.2438008	0.5988056	34390	23588	30726	31493	37650	39687	30832	35653
ENSECAG00000024138	2.911194375	0.243866	0.5988056	1	239	29	32	5	198	14	537
ENSECAG00000007061	5.919266168	0.2438783	0.5988056	1152	1570	1623	1008	397	829	876	1579
ENSECAG00000009519	2.069047529	0.2439188	0.5988056	48	69	56	82	73	139	32	95
ENSECAG00000024809	4.373427299	0.2439783	0.5988223	429	316	545	550	334	246	329	378
ENSECAG00000000391	3.124949383	0.2442567	0.5990695	175	135	115	140	226	156	155	172
ENSECAG00000018697	5.553244938	0.2443157	0.5990695	859.995	818.829	1083	1377	769.999	606.999	1008	604.995
ENSECAG00000013038	6.999646513	0.2443978	0.5990695	1576	2489	1896	1648	703	5918	883	3082
ENSECAG00000013804	0.930371309	0.2443986	0.5990695	17	46	21	26	50	36	35	33
ENSECAG00000017559	2.219622224	0.2444103	0.5990695	115	71	130	103	89	54	89	38
ENSECAG00000009594	2.71909197	0.244489	0.5990695	132	122	162	177	52	73	161	112
ENSECAG00000013624	3.17721792	0.2445075	0.5990695	237	149	223	196	150	135	109	142
ENSECAG00000022043	5.656802392	0.2445419	0.5990695	1001	917	706	791	840	1101	1065	1026
ENSECAG00000014847	3.505600907	0.2445537	0.5990695	200	237	240	359	104	177	102	279
ENSECAG00000010180	3.416313843	0.2448509	0.5996082	149	116	157	280	156	333	178	196
ENSECAG00000017854	1.116675814	0.2449583	0.5996082	26	28	41	36	58	26	66	29
ENSECAG00000018402	4.944864899	0.2449686	0.5996082	515	729	677	795	301	492	473	634
ENSECAG00000008285	4.811300507	0.2450253	0.5996082	321	614	368	511	357	820	393	701
ENSECAG00000005320	6.945447516	0.2450869	0.5996082	2346	1588	2244	2203	2741	2771	2443	1906
ENSECAG00000008688	5.191199771	0.2450903	0.5996082	477	1015	271	360	482	1134	178	1284
ENSECAG00000015467	7.122991826	0.2451794	0.5996969	3170	2576	3366	3123	1703	1781	2905	2465
ENSECAG00000007746	0.813836209	0.2454004	0.6001084	32	25	51	51	17	7	21	49
ENSECAG00000007872	4.945426213	0.2454884	0.6001202	832	458	610	862	524	482	621	268
ENSECAG00000018542	7.102453634	0.2455983	0.6001202	3139	2574	2861	3500	1832	2064	2492	2275
ENSECAG00000022749	2.765449803	0.2456098	0.6001202	151	47	113	115	215	91	210	65
ENSECAG00000008632	6.447234572	0.2456166	0.6001202	1772	1365	1378	1466	1885	1653	1880	1547
ENSECAG00000007745	6.19769829	0.24584	0.6005369	1610	1372	1516	1902	1153	898	1328	1313
ENSECAG00000015944	4.993933192	0.2460082	0.6008032	595	700	768	725	384	484	451	657
ENSECAG00000025403	0.297893094	0.2460548	0.6008032	20	22	6	21	19	28	34	17
ENSECAG00000007094	5.333993786	0.2461945	0.6010151	605	628	602	904	809	1088	644	651
ENSECAG00000017437	1.768347259	0.2462851	0.6011072	83	50	95	73	43	46	59	50
ENSECAG00000007589	2.86034507	0.2464715	0.6014329	18	218	59	92	27	215	98	308
ENSECAG00000013244	6.246023668	0.2465721	0.6015491	1324	1054	1397	1335	991	2223	1193	1626
ENSECAG00000018577	2.228577076	0.2468585	0.6020331	115	52	121	139	85	46	90	53
ENSECAG00000024397	9.281143152	0.2468765	0.6020331	12338	12560	14238	16079	8952	6330	13391	10706
ENSECAG00000003869	7.650145928	0.2469975	0.602199	3262	3742	2647	3912	3509	4422	4099	4154
ENSECAG00000023016	8.247869361	0.2471351	0.6024051	6091	4452	4794	5290	5861	5424	7137	6294
ENSECAG00000012046	10.77725295	0.2473202	0.602727	27061	24483	36365	29775	38059	41358	31787	30070
ENSECAG00000018994	5.110909836	0.247393	0.602775	787	831	471	955	586	401	735	450
ENSECAG00000000496	7.171279163	0.2474746	0.60284	2158	2407	2144	3013	2295	4035	2630	2554
ENSECAG00000001495	3.489243987	0.2475258	0.60284	207	248	284	257	241	119	140	173
ENSECAG00000014645	4.855738769	0.2477731	0.603313	356	506	530	490	448	324	1320	426
ENSECAG00000008158	1.365579333	0.2480479	0.6037554	65	49	54	64	30	9	71	38
ENSECAG00000018823	8.809732937	0.2482722	0.6037554	8149	6374	6544	9085	6022	11002	7865	11452
ENSECAG00000009261	7.176077192	0.2482763	0.6037554	2924	2819	1167	2318	1599	3360	1952	5088
ENSECAG00000007802	0.446454866	0.2483076	0.6037554	23	15	20	21	25	17	49	20
ENSECAG00000020617	1.259935423	0.2483123	0.6037554	63	31	68	51	43	30	41	19
ENSECAG00000009277	2.12079499	0.2483396	0.6037554	108	10	74	52	74	18	259	64
ENSECAG00000002173	6.739817267	0.2483668	0.6037554	1573	2022	1481	2121	1356	1712	2558	3175
ENSECAG00000023166	4.681949594	0.2483801	0.6037554	529	453	637	645	376	349	594	298
ENSECAG00000012926	1.198570998	0.2485517	0.6039502	31	49	38	90	22	17	54	37
ENSECAG00000005185	3.158793993	0.2485665	0.6039502	176	43	139	202	331	68	263	117

ENSECAG00000010777	6.57432581	0.2486504	0.6039597	2590	1392	2434	2018	1784	1184	2109	906
ENSECAG00000022819	8.056089111	0.2486768	0.6039597	6436	4668	5620	7072	2494	3116	5571	5576
ENSECAG00000024048	7.803970683	0.2490053	0.6046283	5131	3615	3902	1745	4583	5394	4315	3967
ENSECAG00000019340	5.194149183	0.2491128	0.60476	660	609	528	697	732	464	985	815
ENSECAG00000005097	3.950177494	0.2491982	0.6048381	218	258	237	310	287	430	257	260
ENSECAG00000009706	9.061311449	0.249439	0.604901	9318	7882	10428	8293	12803	8351	12438	10074
ENSECAG00000000458	3.437785976	0.2494759	0.604901	47	365	48	67	43	367	24	555
ENSECAG00000000396	6.354877796	0.2495008	0.604901	1238	1023	1535	1663	2487	1160	2527	666
ENSECAG00000024267	5.412027144	0.2495135	0.604901	1090	854	862	906	769	383	816	738
ENSECAG00000020104	6.023799732	0.2495293	0.604901	940	1144	1316	1017	1121	1383	1192	1486
ENSECAG00000000475	5.057262099	0.2495436	0.604901	575	564	402	727	683	679	797	522
ENSECAG00000025095	3.750238778	0.2499572	0.6057742	212	165	236	280	336	278	336	149
ENSECAG00000011033	4.413087189	0.2500197	0.6057963	430	359	439	668	281	251	425	384
ENSECAG00000016991	4.873065686	0.2500949	0.6058493	461	336	469	726	763	445	843	367
ENSECAG00000013918	6.023236892	0.2502608	0.6060043	1192	1065	1091	952	587	1294	1308	2159
ENSECAG00000012821	5.477766627	0.2502655	0.6060043	951	822	1150	991	514	829	531	860
ENSECAG00000013154	3.319552065	0.2505484	0.6064411	208	181	139	409	91	85	331	98
ENSECAG00000014440	3.181752205	0.2505543	0.6064411	127	169	134	163	165	153	196	228
ENSECAG00000011892	2.883150284	0.2506304	0.6064411	200	73	197	215	103	36	167	133
ENSECAG00000017955	6.150414289	0.250718	0.6064411	1106	1422	847	1330	1056	2125	916	1550
ENSECAG00000009086	4.542571704	0.2507346	0.6064411	416	298	311	522	714	378	629	209
ENSECAG00000025163	5.285321835	0.2507705	0.6064411	757	593	709	579	935	642	992	588
ENSECAG00000013844	3.937713594	0.2508197	0.6064411	312	271	352	423	198	206	262	283
ENSECAG00000013962	6.556767793	0.2508948	0.6064937	1617	1463	1740	1213	875	3742	1388	1558
ENSECAG00000021492	6.663106233	0.2510276	0.6066857	2822	1383	2303	2461	1910	1063	1819	1602
ENSECAG00000018018	8.061263533	0.25115	0.6068524	4995	3116	4275	5430	8743	4001	6501	2860
ENSECAG00000022922	4.547651844	0.2513196	0.6071195	479	508	534	531	454	214	493	309
ENSECAG00000022502	6.625689557	0.2513723	0.6071195	2496	1442	2153	2671	1335	931	2230	1837
ENSECAG00000012942	1.585139168	0.2514378	0.6071195	88	2	54	11.0006	42	41	181	24
ENSECAG00000016459	11.24166086	0.2514844	0.6071195	44773	32528	45203	41037	54808	47759	46459	46496
ENSECAG00000020299	0.156692643	0.2515995	0.6071195	12	18	19	12	38	10	16	24
ENSECAG00000006430	10.89205012	0.2516165	0.6071195	34090	23759	37202	32838	42269	43383	31522	35520
ENSECAG00000013839	6.778526899	0.2516347	0.6071195	2546	2085	2220	2915	977	1976	1476	2307
ENSECAG00000011747	1.506577125	0.2517133	0.6071802	85	42	52	80	66	13	52	28
ENSECAG00000025956	2.795710838	0.2528522	0.6097979	107	87	111	140	134	224	87	110
ENSECAG00000008046	8.793003481	0.253187	0.6104757	9788	8338	9475	11678	7971	5359	7567	6881
ENSECAG00000007234	5.175916482	0.2532445	0.6104847	878	463	659	319	1224	582	775	410
ENSECAG00000007103	5.912266108	0.2533598	0.610633	1289	1008	1671	1273	1044	856	943	951
ENSECAG00000024242	6.850975635	0.2535291	0.6109115	2257	2686	3046	2050	1512	1321	2261	2210
ENSECAG00000019403	4.583120146	0.2536278	0.6110196	466	350	407	399	568	388	547	432
ENSECAG00000019563	6.381194493	0.2537816	0.6111365	1254	1515	1216	1723	1372	2036	1593	1608
ENSECAG00000024034	5.324189996	0.2538434	0.6111365	613	733	637	738	758	505	1167	862
ENSECAG00000015392	7.644101965	0.253857	0.6111365	3953	3821	4409	5565	3052	2411	4804	2440
ENSECAG00000020533	5.183692324	0.2539907	0.6111365	474	593	687	721	747	807	577	750
ENSECAG00000008878	5.874226569	0.2539953	0.6111365	1421	906	1413	1443	1108	475	1262	895
ENSECAG00000024530	5.80315974	0.2539991	0.6111365	760	857	1021	1169	1119	1504	1060	738
ENSECAG00000011864	6.449052574	0.2543263	0.6117941	1162	1218	1302	1928	562	3710	1115	1690
ENSECAG00000023208	4.281693898	0.2547417	0.6124757	520	292	424	506	307	144	496	278
ENSECAG00000013443	8.021750022	0.2547607	0.6124757	3740	4679	4050	4985	5342	6312	5851	3454
ENSECAG00000002377	3.418132026	0.2547713	0.6124757	207	147	154	192	181	128	230	350
ENSECAG00000014724	8.175573693	0.2548314	0.6124905	5697	5851	6040	7937	3842	4479	5209	4604
ENSECAG00000009781	4.23380948	0.2549059	0.61254	401	271	470	530	318	247	339	266
ENSECAG00000008390	5.203010927	0.254984	0.6125981	739	892	616	994	404	429	596	881
ENSECAG00000022245	1.816149507	0.2551326	0.6127064	89	40	97	95	77	39	57	25
ENSECAG00000023551	3.552519054	0.255137	0.6127064	231	297	313	186	174	108	269	173
ENSECAG00000014948	4.215674717	0.2552247	0.6127875	427	272	359	605	310	225	345	277
ENSECAG00000017129	4.105670062	0.2554055	0.613092	379	341	349	452	200	172	380	334
ENSECAG00000017343	7.777131068	0.2556411	0.6135279	3301	3671	4713	3032	4230	4272	4468	4756
ENSECAG00000020854	6.258539603	0.2558131	0.6137183	1719	1256	1627	2111	1343	886	1465	1206
ENSECAG00000019159	6.363122528	0.2558475	0.6137183	1625	1639	1652	2275	980	1314	1419	1509
ENSECAG00000016855	7.327864851	0.2559892	0.6137183	3778	3506	3360	3371	2282	2724	2385	2582
ENSECAG00000013939	2.363656404	0.256051	0.6137183	64	132	164	103	31	65	86	114
ENSECAG00000019293	10.87665112	0.2560756	0.6137183	35188	25971	30651	35549	37869	41597	31759	39682
ENSECAG00000025080	11.07888809	0.2561252	0.6137183	41015	27659	38284	39301	49522	46529	36727	40986
ENSECAG00000014998	3.697258245	0.2562738	0.6137183	319	202	372	261	189	129	328	159
ENSECAG00000014783	4.774066719	0.2562901	0.6137183	554	397	1373	214	332	131	855	309
ENSECAG00000003995	3.239650491	0.2563074	0.6137183	160	124	164	179	218	143	246	167
ENSECAG00000012316	3.413103915	0.2563181	0.6137183	167	190	115	215	102	255	214	307
ENSECAG00000008326	4.019449603	0.2563371	0.6137183	368	312	363	377	268	188	290	267
ENSECAG00000014740	7.245142961	0.2563688	0.6137183	3968	2319	3661	3491	2918	1916	2923	1731
ENSECAG00000017714	3.204668618	0.2564575	0.6138012	227	186	225	176	192	86	155	124
ENSECAG00000026818	0.524736612	0.2566548	0.6141441	27	23	20	11	40	30	14	29
ENSECAG00000008664	7.592742376	0.2567608	0.6142683	3382	2827	3134	3829	3443	2796	4089	5423
ENSECAG00000006429	6.481247203	0.2568718	0.6144045	1351	3441	1328	1682	1062	1250	1057	2018
ENSECAG00000024038	7.630089255	0.2569656	0.6144995	2718	3006	3439	3767	2015	7476	3101	3343
ENSECAG00000010520	3.799957032	0.2571299	0.6147628	296	244	315	377	223	217	226	188
ENSECAG00000011435	4.98272635	0.2572244	0.6148323	887	463	854	579	591	323	606	459
ENSECAG00000020995	1.344850826	0.2572672	0.6148323	49	64	43	66	32	37	50	26

ENSECAG00000018970	6.642406026	0.2575531	0.615282	2280	2075	3010	1328	1149	1311	1723	2059
ENSECAG00000004395	1.445349013	0.2576181	0.615282	29	27	29	83	19	60	78	72
ENSECAG00000023456	2.955912963	0.2576373	0.615282	212	152	214	111	175	75	117	89
ENSECAG00000005790	0.127716783	0.2577215	0.615282	29	23	18	23	25	6	8	16
ENSECAG00000014339	3.705114863	0.2577559	0.615282	150	308	122	256	189	193	319	395
ENSECAG00000020923	0.312208289	0.2577803	0.615282	16	28	28	35	10	8	32	17
ENSECAG00000019366	7.313208305	0.2579432	0.6155414	3593	3250	3973	3037	2035	2109	3085	2839
ENSECAG00000013122	2.873446116	0.2580063	0.6155626	120	128	103	127	144	135	187	132
ENSECAG00000008587	3.492166459	0.2581189	0.615702	265	183	226	329	151	188	188	157
ENSECAG00000011315	0.513245808	0.2583489	0.6161212	21	30	8	76	3	32	12	20
ENSECAG00000018153	3.498669379	0.2585337	0.6162882	173	179	152	247	178	297	229	204
ENSECAG00000011722	8.475961521	0.2585948	0.6162882	6107	5338	6544	6031	7321	8331	5850	6936
ENSECAG00000021733	0.238495438	0.2586114	0.6162882	14	7	16	31	11	25	27	31
ENSECAG00000022793	5.912766151	0.2586371	0.6162882	1020	1289	1222	1722	869	863	1176	939
ENSECAG00000017318	2.582741244	0.2586959	0.6162882	105	154	180	88	70	75	84	122
ENSECAG00000010524	7.05798566	0.2587445	0.6162882	2266	1932	2223	2686	3595	2341	2758	2018
ENSECAG00000000366	3.330553017	0.2591253	0.6170659	164	192	154	145	210	221	196	183
ENSECAG00000014087	5.184033436	0.259241	0.617212	744	803	1165	591	201	243	542	1193
ENSECAG00000004874	3.262337003	0.2595852	0.6177855	149	113	212	155	124	161	279	235
ENSECAG00000024566	6.877959108	0.2595906	0.6177855	2918	1973	2508	3307	2706	2087	1393	728
ENSECAG00000020050	5.136073537	0.2596871	0.6178855	788	696	726	856	416	443	657	722
ENSECAG00000016188	6.864095026	0.2597534	0.6179138	1912	2066	1694	2283	1888	2381	2392	2686
ENSECAG00000025860	0.786577149	0.2600721	0.6184946	26	46	9	11	33	59	35	14
ENSECAG00000026960	5.339819537	0.2601064	0.6184946	736	425	885	743	760	675	1007	836
ENSECAG00000004355	3.067560397	0.2602421	0.618598	95	99	162	201	147	133	229	185
ENSECAG00000019652	3.998741914	0.2602588	0.618598	423	335	356	289	354	166	240	215
ENSECAG00000024079	11.05600219	0.2605896	0.6192546	37918	28677	40805	36472	46857	45737	36132	42121
ENSECAG00000010983	4.261696948	0.2607582	0.6195258	406	362	444	462	281	261	330	332
ENSECAG00000014334	11.39187238	0.2611427	0.6203094	49812	34935	45128	52471	58290	61303	45359	50288
ENSECAG00000023488	8.659688115	0.2613119	0.6205817	6855	6863	7824	5449	7529	9456	8119	7442
ENSECAG00000023998	6.623964822	0.2614402	0.6206923	2411	1675	2285	2303	2226	1011	1786	1192
ENSECAG00000007231	4.479028062	0.2614678	0.6206923	449	391	517	635	259	169	541	447
ENSECAG00000006554	1.505970582	0.2617203	0.6210937	146	35	51	32	54	8	77	14
ENSECAG00000019621	6.192077019	0.2617463	0.6210937	1558	1372	1634	1863	992	605	1843	1294
ENSECAG00000021436	8.499475437	0.2619585	0.6214676	6408	5093	6336	6718	8467	8006	6565	5914
ENSECAG00000006543	3.677229616	0.2620584	0.6214888	290	243	337	265	258	119	285	124
ENSECAG00000011829	1.415450951	0.2621222	0.6214888	25	62	19	35	23	58	5	145
ENSECAG00000019772	6.834988089	0.26214	0.6214888	2021	2011	1856	1802	3118	1981	2368	1722
ENSECAG00000010051	9.514579326	0.2621863	0.6214888	14377	11740	13585	26904	12665	9335	15734	7772
ENSECAG00000016378	4.133415746	0.2622825	0.6215871	281	261	258	366	238	551	273	335
ENSECAG00000009309	6.409180255	0.2624278	0.6218018	1425	1420	1398	1599	1358	1969	1547	1848
ENSECAG00000011027	4.693351608	0.2626508	0.6221875	476	472	524	875	545	164	593	310
ENSECAG00000004434	6.32117486	0.2627406	0.6221875	1705	1563	1955	1727.99	1526	781	1699	1122
ENSECAG00000012315	7.946608532	0.2628468	0.6221875	5468	4210	5060	7750	4625	2844	6216	1572
ENSECAG00000019133	3.241199586	0.2628852	0.6221875	133	197	129	151	250	164	151	201
ENSECAG00000011158	5.003287861	0.2629096	0.6221875	567	533	563	522	667	625	686	579
ENSECAG00000023123	5.807352342	0.2629729	0.6221875	1235	1071	1113	1462	623	721	1151	1107
ENSECAG00000023579	3.662037462	0.2630452	0.6221875	335	207	281	312	252	214	187	97
ENSECAG00000015282	4.188012519	0.2630771	0.6221875	341	320	381	588	181	231	415	318
ENSECAG00000017223	4.168250132	0.263107	0.6221875	362	209	230	366	226	692	326	204
ENSECAG00000003381	0.36361046	0.2631635	0.6221875	21	26	18	50	5	6	20	36
ENSECAG00000012338	6.687744958	0.2632141	0.6221875	2288	1845	2445	2443	1972	1106	1540	1896
ENSECAG00000004464	0.596900835	0.2632587	0.6221875	43	26.0001	20.0001	43.0001	17.0001	24.0005	16.0006	23.0004
ENSECAG00000025155	1.796773374	0.2633027	0.6221875	60	49	51	58	97	67	45	69
ENSECAG00000011270	4.179926265	0.2633624	0.6221992	363	352	433	436	279	260	341	254
ENSECAG00000024329	4.508827853	0.2635062	0.6224095	335	319	478	413	513	418	575	333
ENSECAG00000000701	4.033025039	0.2637836	0.6227824	242	429	49	59	32	275	20	1240
ENSECAG00000020012	5.671678192	0.2638062	0.6227824	1062	1320	928	1060	797	710	948	791
ENSECAG00000009049	8.183136121	0.2638286	0.6227824	5100	2813	4407	6793	9674	3976	9120	1986
ENSECAG00000004701	5.695458804	0.2640649	0.6232107	1188	729	1473	1228	985	445	1340	518
ENSECAG00000024706	6.586590712	0.2642973	0.6235804	2016	1666	1406	1332	2937	1267	2220	1408
ENSECAG00000016298	5.268292289	0.2643313	0.6235804	731	562	1031	1115	396	415	749	880
ENSECAG00000024336	10.44620539	0.2644537	0.6235844	25679	18541	25722	24149	31931	32860	22887	23869
ENSECAG00000023213	5.973205833	0.2644604	0.6235844	826	1270	716	1264	618	2430	1022	969
ENSECAG00000023334	0.852718084	0.2644977	0.6235844	42	27	45	45	37	25	23	12
ENSECAG00000006124	4.747704082	0.2647261	0.6239934	666	412	615	680	521	317	534	314
ENSECAG00000005906	6.181600874	0.2649068	0.624181	1673	1316	1377	2014	818	980	1131	1667
ENSECAG00000006979	4.882563389	0.2649803	0.624181	694	465	671	766	454	307	569	545
ENSECAG00000021026	5.58289688	0.2650258	0.624181	1154	617	858	625	952	887	1165	859
ENSECAG00000013521	3.5847682	0.2650571	0.624181	336	148	264	331	202	168	238	122
ENSECAG00000000925	4.015753992	0.2650805	0.624181	304	286	366	478	192	229	348	244
ENSECAG00000012956	5.644618891	0.2652045	0.6242659	895	1199	1373	839	743	638	790	982
ENSECAG00000009499	3.366055544	0.2652265	0.6242659	154	161	172	196	211	221	269	136
ENSECAG00000018310	2.172032109	0.2654187	0.6245891	91	78	110	122	74	62	87	43
ENSECAG00000013531	7.190073537	0.2655939	0.6248399	2400	2286	2523	2794	2898	3280	2750	2678
ENSECAG00000017912	3.009424368	0.2656353	0.6248399	187	138	298	100	85	79	99	207
ENSECAG00000024766	8.999649256	0.2657508	0.6249821	9096	8428	9483	7316	11329	10782	10948	8211
ENSECAG00000009486	4.838451989	0.2658463	0.6250772	526	422	407	612	473	550	654	621

ENSECAG00000021229	2.914031616	0.2659679	0.6252337	156	135	154	229	138	94	94	124
ENSECAG00000016716	0.474008091	0.2662652	0.6258032	24	46	22	26	8	16	22	28
ENSECAG00000006736	3.06847804	0.2663557	0.6258862	155	178	195	210	107	107	152	148
ENSECAG00000023369	7.394285454	0.2665553	0.6262257	3536	2176	2909	2868	3909	2930	3903	2845
ENSECAG00000024074	5.424106678	0.2667546	0.6265644	576	502	638	1256	943	526	1375	724
ENSECAG00000021339	0.467431234	0.2668551	0.6266709	23	41	22	33	5	19	33	17
ENSECAG00000019466	5.918468018	0.2670619	0.6270155	1112	770	1185	1120	1512	911	1325	1094
ENSECAG00000016673	10.46461345	0.2671122	0.6270155	35143	21746	35209	33004	23794	19300	22769	22260
ENSECAG00000019131	8.494012769	0.2675968	0.6280232	4788	7904	5538	4784	4334	12877	5530	6333
ENSECAG00000009148	2.605300377	0.2676905	0.6281132	82	70	111	131	173	83	179	72
ENSECAG00000005489	3.998558499	0.2678352	0.6282713	7	575	14	59	19	789	16	711
ENSECAG00000000372	5.29834349	0.2679389	0.6282713	706	518	700	806	868	597	987	715
ENSECAG00000012142	4.838368179	0.267975	0.6282713	379	564	459	416	358	1018	252	664
ENSECAG00000022269	7.104923685	0.2679791	0.6282713	2007	2208	2179	3012	2045	3547	2486	2884
ENSECAG00000001304	1.858622874	0.2680852	0.6283903	62	43	53.0009	75	54	76	78	85
ENSECAG00000022405	7.255503739	0.268186	0.628497	2649	2557	2237	2776	4177	3179	3545	1486
ENSECAG00000016020	3.375949802	0.2685033	0.6291107	209	155	167	135	320	178	253	104
ENSECAG00000005731	7.107251151	0.2685991	0.6291191	3346	2139	3072	3601	2570	1759	2467	1909
ENSECAG00000018370	6.250408535	0.2686176	0.6291191	1690	1204	1577	2239	1388	841	1632	1022
ENSECAG00000014487	0.906416364	0.2687121	0.6292038	29	14	31	38	24	26	86	23
ENSECAG00000025008	5.84165766	0.2687646	0.6292038	1040	811	1028	1083	1437	851	1431	894
ENSECAG00000011084	2.091575557	0.2688418	0.6292336	64	55	71	82	59	55	101	139
ENSECAG00000006696	4.407299456	0.2688881	0.6292336	228	462	264	459	368	302	511	568
ENSECAG00000021954	4.67973754	0.2692025	0.6298396	584	513	503	643	326	262	574	477
ENSECAG00000024475	6.59369894	0.2692596	0.6298434	1980	2167	2393	1793	1422	1078	1536	2107
ENSECAG00000007228	7.168821765	0.2694178	0.6300837	2130	2619	3002	1843	3308	2388	2331	3580
ENSECAG00000012116	3.502253694	0.2695854	0.6303459	185	172	227	153	115	269	290	256
ENSECAG00000019897	4.250531352	0.26981	0.6307412	352	413	362	551	398	222	303	257
ENSECAG00000009344	8.853994064	0.2699182	0.6308644	11647	8184	10094	10755	6262	6479	8544	7906
ENSECAG00000014366	5.695964127	0.2699928	0.6309088	1081	596	1018	792	1388	608	1666	648
ENSECAG00000022948	4.314941068	0.2701789	0.6311883	403	290	300	359	380	410	378	418
ENSECAG00000009739	1.895404709	0.2702235	0.6311883	93	38	118	94	65	67	57	14
ENSECAG00000009631	3.989201176	0.2703828	0.6314305	199	345	235	259	270	373	235	397
ENSECAG00000009140	2.17858092	0.2705267	0.6315889	119	84	87	114	44	50	58	113
ENSECAG00000011614	6.240180789	0.2705618	0.6315889	1339	1391	1705	2190	875	1089	1395	1470
ENSECAG00000019831	5.267366127	0.2707619	0.6318486	920	680	856	900	416	641	664	709
ENSECAG00000011469	2.767035367	0.2708223	0.6318486	95.0006	112	96.0011	139.001	79.0009	162.001	134.001	178
ENSECAG00000018830	0.367735673	0.2708902	0.6318486	26	12	49	28	4	6	36	22
ENSECAG00000015327	4.970355985	0.2709019	0.6318486	589	415	598	521	878	384	775	519
ENSECAG00000023875	1.248297009	0.2709512	0.6318486	65	28	43	76	29	32	46	28
ENSECAG00000011951	6.049624732	0.2710396	0.631925	1640	859	1012	979	1684	1352	1327	926
ENSECAG00000011514	0.419541808	0.2710984	0.6319325	23	14	21	21	35	19	31	20
ENSECAG00000021973	3.512245978	0.2712431	0.632039	329	159	279	245	155	115	207	227
ENSECAG00000014806	4.484268573	0.2712554	0.632039	449	491	430	585	276	283	376	475
ENSECAG00000001756	4.429009059	0.2713392	0.6321047	429	398	481	586	373	254	471	265
ENSECAG00000023602	7.62246256	0.2714703	0.6322729	3915	3969	4190	5288	3123	1840	3945	3672
ENSECAG00000008815	4.710191852	0.2715763	0.6322729	484	276	524	485	694	468	671	297
ENSECAG00000007175	3.773377212	0.2716385	0.6322729	349	213	350	297	269	125	240	211
ENSECAG00000015901	4.70786415	0.2716882	0.6322729	624	472	551	646	365	239	555	510
ENSECAG00000012573	6.600006683	0.2717231	0.6322729	2528	1754	2406	1717	1338	1013	1910	1962
ENSECAG00000011694	2.747372737	0.2717454	0.6322729	171	83	136	225	144	83	112	54
ENSECAG00000015585	4.234770452	0.2718412	0.6323661	443	289	537	393	270	191	485	245
ENSECAG00000017934	4.893435163	0.2719613	0.6325161	635	705	656	597	265	479	427	665
ENSECAG00000005217	5.821840413	0.2721682	0.6327199	1126	998	1278	1538	1022	916	940	689
ENSECAG00000010209	5.434332474	0.2722277	0.6327199	1021	712	1001	1027	703	490	914	677
ENSECAG00000015897	6.51013402	0.2722634	0.6327199	1369	1662	1565	1556	1427	2564	1298	1887
ENSECAG00000004232	4.18125378	0.2722717	0.6327199	399	345	404	472	324	113	477	228
ENSECAG00000015948	1.766658687	0.2724927	0.6329888	74	59	64	104	46	45	69	41
ENSECAG00000021538	4.379393309	0.2725386	0.6329888	595	270	506	484	396	174	461	272
ENSECAG00000021297	10.03985097	0.2725547	0.6329888	23380	17965	23842	28727	19142	10657	24567	12273
ENSECAG00000016749	2.67131435	0.2727037	0.6332055	110	64	97	140	163	161	142	53
ENSECAG00000022388	8.257332397	0.2727678	0.6332249	5114	5036	4976	5652	5615	7618	5662	5463
ENSECAG00000013105	2.493147336	0.2729886	0.6336079	139	81	107	189	41	69	72	146
ENSECAG00000013884	0.924889143	0.2732124	0.6337969	31	19	25	38	79	23	27	24
ENSECAG00000016190	11.4827308	0.2733119	0.6337969	55596	37874	47695	53661	64730	59486	48637	56235
ENSECAG00000011962	1.848508006	0.2733235	0.6337969	30	108	31	40	49	95	59	95
ENSECAG00000023408	4.802207983	0.2733442	0.6337969	460	440	588	369	492	306	719	796
ENSECAG00000010226	5.177317743	0.2734466	0.6337969	774	663	790	926	596	380	688	655
ENSECAG00000010218	1.49038115	0.2734834	0.6337969	35	69	26	39	48	70	57	52
ENSECAG00000016082	7.983021088	0.273542	0.6337969	6053	4310	5596	6286	4153	3399	5017	3465
ENSECAG00000007322	7.803474816	0.2735613	0.6337969	5063	4165	4981	5322	3568	3185	3569	3759
ENSECAG00000019394	4.991300051	0.2735722	0.6337969	576	402	625	560	833	391	875	505
ENSECAG00000023474	5.247488154	0.2743227	0.6353004	801	469	706	623	963	571	912	618
ENSECAG00000006920	6.856688688	0.274333	0.6353004	2482	2424	2346	2874	1870	1233	2733	1618
ENSECAG00000021033	5.096652034	0.2745005	0.6355213	582	632	487	550	313	1066	610	757
ENSECAG00000021560	2.942333265	0.2745403	0.6355213	208	144	195	126	111	97	112	143
ENSECAG00000019749	3.833364268	0.2748457	0.6360986	348	191	355	377	248	130	301	211
ENSECAG00000024404	5.364490796	0.2752568	0.636505	841	624	689	590	1340	539	1012	479

ENSECAG00000020872	6.476670551	0.2752632	0.636505	1994	1699	2055	2031	1912	861	1888	1021
ENSECAG00000019395	3.502856104	0.2752909	0.636505	303	165	239	302	190	109	245	161
ENSECAG00000011200	6.007242092	0.2752922	0.636505	1485	1126	1363	1694	749	555	1734	1142
ENSECAG00000015726	3.263221457	0.2753296	0.636505	48	539	78	239	29	116	67	285
ENSECAG00000014602	2.816459493	0.2753575	0.636505	76	90	85	204	160	136	245	55
ENSECAG00000014279	5.768727826	0.2755945	0.6369232	1040	979	1085	1690	827	529	1127	1037
ENSECAG00000014002	8.608925881	0.2757194	0.6370385	7451	8387	8957	9419	4829	6623	6924	6076
ENSECAG00000025813	0.214009466	0.2758582	0.6370385	76	26	4	5	0	22	0	22
ENSECAG00000012654	4.336091837	0.2758738	0.6370385	305	330	416	303	535	317	447	331
ENSECAG00000023846	5.294077669	0.2759073	0.6370385	827	877	735	955	503	492	893	642
ENSECAG00000014833	4.860085425	0.2759248	0.6370385	568	578	612	784	536	303	575	433
ENSECAG00000017875	3.552284035	0.2760437	0.6371836	191	159	227	198	355	136	281	192
ENSECAG00000020500	6.659924918	0.2762169	0.6372786	1837	1539	1752	1906	1845	1786	2517	1938
ENSECAG00000021458	4.267585755	0.2762583	0.6372786	379	341	458	540	286	121	414	395
ENSECAG00000014368	7.055713504	0.2763031	0.6372786	3258	2260	2954	3445	3017	1672	2610	896
ENSECAG00000008712	5.17904966	0.2763539	0.6372786	756	724	821	837	563	391	793	588
ENSECAG00000024846	5.127215868	0.2763795	0.6372786	570	569	597	679	770	580	773	663
ENSECAG00000020283	7.717348599	0.2764215	0.6372786	3411	3137	4258	3514	3703	5154	3904	3975
ENSECAG00000012118	6.55591939	0.2767337	0.6376621	1582	1444	1461	1908	2103	2470	2028	891
ENSECAG00000024905	6.585824157	0.2767658	0.6376621	2052	1900	2065	2334.01	1832	1112	2100	1126
ENSECAG00000023125	6.920998305	0.2767835	0.6376621	2712	2121	2955	2851	2402	1077	2259	1971
ENSECAG00000015293	4.215802176	0.2768124	0.6376621	345	302	272	322	532	353	380	227
ENSECAG00000007668	7.034221291	0.2769046	0.6377307	1492	1466	1471	4613	2777	1777	4214	2268
ENSECAG00000006518	5.283195373	0.2769544	0.6377307	839	655	845	1123	353	494	579	1013
ENSECAG00000013993	4.582477559	0.2773558	0.6385256	466	447	613	570	426	405	341	306
ENSECAG00000023902	6.117442611	0.2775713	0.6385774	1430	1366	1461	1833	716	1469	935	1170
ENSECAG00000024544	8.508981191	0.2776092	0.6385774	6416	6496	5502	6114	9469	6193	8812	5115
ENSECAG00000010467	1.223253885	0.2776186	0.6385774	30	30	20	66	49	36	74	33
ENSECAG00000012646	4.63738408	0.2776409	0.6385774	398	376	430	455	776	298	708	276
ENSECAG00000015552	2.612858693	0.2776817	0.6385774	91	77	92	128	156	52	255	67
ENSECAG00000017929	7.192187065	0.2777157	0.6385774	2755	1890	2664	2738	3070	3241	3180	2179
ENSECAG00000022918	7.131437042	0.2779386	0.6389605	2323	2145	2358	2783	3189	3254	2528	2130
ENSECAG00000010545	5.925873127	0.2780863	0.6391708	1161	755	1047	1262	1344	899	1771	917
ENSECAG00000021087	0.963310769	0.2781535	0.639196	15	24	27	125	12	33	48	6
ENSECAG00000026930	5.187032839	0.2783209	0.6394512	526	624	612	739	669	842	599	759
ENSECAG00000017883	5.801591949	0.278538	0.6398056	1353	911	1210	1355	864	790	1093	842
ENSECAG00000016142	4.971451464	0.2785878	0.6398056	645	655	613	818	388	398	569	644
ENSECAG00000010913	7.927241163	0.2787825	0.6400007	5200	4303	6022	5981	4550	2054	4422	4396
ENSECAG00000017673	8.24032878	0.2788215	0.6400007	3551	6476	6124	2892	3081	6200	4253	11712
ENSECAG00000017819	6.6026276	0.2788418	0.6400007	1473	1504	1867	1849	2509	1237	2590	1549
ENSECAG00000009992	3.847030221	0.2789593	0.6401411	180	236	267	281	291	358	251	243
ENSECAG00000013578	5.934369182	0.2791053	0.6402882	1320	1061	1234	1714	805	768	1280	1106
ENSECAG00000011721	5.813233306	0.2791361	0.6402882	1266	1257	1340	943	980	771	1020	811
ENSECAG00000020658	3.694926098	0.279251	0.6403951	267	230	278	369	196	130	265	223
ENSECAG00000019522	4.242025229	0.2792955	0.6403951	416	216	326	300	474	266	621	214
ENSECAG00000006800	5.711406236	0.2796052	0.6409132	878	832	895	984	784	1479	917	932
ENSECAG00000016542	4.025517425	0.2796343	0.6409132	387	182	432	462	288	144	302	278
ENSECAG00000008807	4.364138862	0.2797078	0.6409524	397	418	411	575	351	252	400	300
ENSECAG00000023990	4.920717785	0.2797648	0.6409537	601	347	600	518	716	609	698.001	404
ENSECAG00000020022	4.281444686	0.27985	0.6409605	420	229	364	260	499	237	706	202
ENSECAG00000024361	4.28187549	0.2799438	0.6409605	249	400	271	319	171	467	219	742
ENSECAG00000005213	6.196585539	0.2799891	0.6409605	1264	1401	1229	1094	1026	1487	1535	1815
ENSECAG00000011623	4.969333214	0.2800344	0.6409605	691	559	682	813	616	340	653	383
ENSECAG00000017074	2.396108488	0.2800499	0.6409605	98	62	73	111	128	92	135	74
ENSECAG00000004433	5.064244514	0.2805056	0.6418741	445	609	434	481	59	1409	277	1077
ENSECAG00000015148	6.305069013	0.2805657	0.6418823	1535	1537	1610	2212.99	1181	789.997	1584	1564
ENSECAG00000011301	3.64014618	0.2807005	0.6420613	247	290	376	178	131	168	166	288
ENSECAG00000010616	4.479705594	0.2808035	0.6421108	409	549	455	617	186	461	108	520
ENSECAG00000017739	5.04763743	0.2808351	0.6421108	447	842	571	1110	749	236	443	600
ENSECAG00000019938	4.499850384	0.2809109	0.6421548	423	386	524	677	236	226	545	438
ENSECAG00000013504	5.411876038	0.2810365	0.6421864	1264	533	1024	1058	925	282	1147	285
ENSECAG00000009275	6.510784106	0.2810378	0.6421864	1887	1566	2218	2225	1513	1232	1735	1390
ENSECAG00000020977	3.413952756	0.2812134	0.6424322	173	156	126	256	181	258	289	139
ENSECAG00000019625	4.039321951	0.2812585	0.6424322	237	330	251	284	373	252	388	317
ENSECAG00000010966	4.493299581	0.2813223	0.6424487	492	341	572	644	174	142	451	633
ENSECAG00000017169	4.974429843	0.2817744	0.643142	669	648	583	835	420	377	646	574
ENSECAG00000020317	6.21103324	0.2817887	0.643142	1435	1091	1396	1172	1145	1641	1395	1682
ENSECAG00000014267	1.98271325	0.2818607	0.643142	9.00295	156.002	6	5.00318	13	228	76.002	40
ENSECAG00000021241	6.689101699	0.28187	0.643142	1964	1615	1614	1841	1716	2835	1617	1946
ENSECAG00000001347	4.477582558	0.281909	0.643142	584	560	267	577	251	278	203	606
ENSECAG00000007078	3.532985529	0.2819933	0.6432052	298	191	263	269	118	166	273	164
ENSECAG00000016418	2.217629609	0.2820713	0.643254	58	104	65	210	51	43	32	133
ENSECAG00000011029	5.107466119	0.2821412	0.6432841	892	609	806	695	362	394	732	718
ENSECAG00000003471	7.065666259	0.2822434	0.6433131	2484	1864	2265	2613	2442	3183	2962	2074
ENSECAG00000014743	4.905434143	0.2822672	0.6433131	487	479	473	630	669	560	648	506
ENSECAG00000008068	6.031826526	0.2825052	0.6435269	1611	1278	1338	1433	1339	664	1141	1039
ENSECAG00000009397	0.562190144	0.2825149	0.6435269	34	16	37	43	20	12	39	10
ENSECAG00000009162	8.79331791	0.2825309	0.6435269	8453	6135	6952	8685	11673	7180	11198	6076

ENSECAG00000014919	2.77200314	0.2826665	0.6437066	109	100	196	209	73	75	146	123
ENSECAG00000005393	4.825892342	0.2827485	0.6437643	537	430	430	556	505	524	603	632
ENSECAG00000021143	1.031068933	0.2829103	0.6440037	41	17	26	41	66	25	51	24
ENSECAG00000010347	7.596181596	0.2830627	0.6441669	4456	3111	3719	305	4304	3544	5930	3117
ENSECAG00000008165	5.058240203	0.2831909	0.6441669	641	609	832	834	576	315	786	464
ENSECAG00000008399	6.888953008	0.2832567	0.6441669	2483	2180	2364	3391	1489	1720	2744	1642
ENSECAG00000015089	11.39673014	0.2832733	0.6441669	53409	33622	48249	48296	62171	57179	44384	51596
ENSECAG00000024653	7.384041729	0.283295	0.6441669	3138	2979	2539	2694	2831	3843	3548	3105
ENSECAG00000010826	4.897068558	0.2833223	0.6441669	683	454	698	803	353	286	541	711
ENSECAG00000015783	4.232126958	0.283563	0.6444864	352	318	306	291	281	378	410	439
ENSECAG00000008264	7.957120639	0.2836262	0.6444864	4256	3577	5006	4131	5036	5471	4667	4605
ENSECAG00000021475	4.466677855	0.2838679	0.6444864	368	425	324	380	358	414	605	410
ENSECAG00000018769	4.738130678	0.2838751	0.6444864	591	433	601	709	383	365	555	399
ENSECAG00000013041	6.882877938	0.2838949	0.6444864	3077	1597	1711	1567	2669	2017	3026	1896
ENSECAG00000017640	7.20378516	0.2839158	0.6444864	3383	2765	2979	3763	2716	1820	2873	1983
ENSECAG00000000125	6.282348946	0.2839651	0.6444864	1308	1358	1269	1426	1145	1702	1409	1900
ENSECAG00000016215	6.459482285	0.2839918	0.6444864	1394	1666	1183	1837	1548	2000	1801	1604
ENSECAG00000010261	4.935267713	0.2839989	0.6444864	517	441	449	722	538	587	746	576
ENSECAG00000023115	3.109180842	0.2840444	0.6444864	74	216	130	107	169	293	84	149
ENSECAG00000004586	5.83362117	0.2840869	0.6444864	1444	971	1662	853	898	766	827	1093
ENSECAG00000008160	5.534523847	0.2843583	0.6449733	1107	749	1084	1101	905	459	1000	617
ENSECAG00000000037	5.702133178	0.2846955	0.6456092	867	787	927	1026	1005	1276	865	922
ENSECAG00000008755	7.308990416	0.2848434	0.6457042	2467	2826	2703	2386	2100	5736	2047	2681
ENSECAG00000000680	4.127465534	0.2848511	0.6457042	450	284	511	308	392	154	383	145
ENSECAG00000000212	4.9684879	0.2850077	0.6458193	579	568	617	1001	377	398	745	488
ENSECAG00000015370	5.494407416	0.2850156	0.6458193	517	1044	576	665	298	1486	378	1536
ENSECAG00000005495	2.761283221	0.2851972	0.6461018	128	107	93	117	152	93	181	129
ENSECAG00000015914	7.218227573	0.2853011	0.6461862	3142	3077	3157	3559	2373	1739	2923	2518
ENSECAG00000012110	8.654668538	0.2853964	0.6461862	6391	5110	6866	9501	9109	5520	8530	9585
ENSECAG00000000381	4.135199189	0.2854051	0.6461862	83	607	182	133	179	223	324	827
ENSECAG00000024026	2.043623043	0.2854914	0.6462528	72	83	92	116	61	37	76	74
ENSECAG00000009552	8.688151996	0.2856326	0.6464437	7552	5841	7889	6912	6418	7678	10580	8811
ENSECAG00000023585	8.222530217	0.2856983	0.6464635	5859	2758	4625	6053	13943	3325	5847	2035
ENSECAG00000019103	3.357898433	0.285858	0.646696	206	189	200	315	118	133	168	213
ENSECAG00000007694	7.902409547	0.2859636	0.6467121	3869	3629	4423	4311	3301	6565	4339	4833
ENSECAG00000009504	7.433730816	0.2859819	0.6467121	4308	2737	3791	4398	3452	2172	3145	2138
ENSECAG00000003388	6.811877137	0.2860359	0.6467121	2047	1620	2077	2002	2523	2329	2051	1977
ENSECAG00000008058	2.329507634	0.286157	0.6468196	107	94	122	119	92	48	102	62
ENSECAG00000004389	6.889492962	0.2861973	0.6468196	2043	2002	1928	2120	1733	2388	2094	3267
ENSECAG00000005758	5.994422607	0.28649	0.6473103	1657	1053	1368	1453	718	1062	1122	1156
ENSECAG00000020676	7.245564474	0.2865567	0.6473103	3199	2266	3625	4315	2273	1884	3309	2257
ENSECAG00000008964	5.981979961	0.2865854	0.6473103	961	1336	846	1193	1086	1241	1287	1408
ENSECAG00000004436	4.941565812	0.2867136	0.6474712	487	508	484	646	536	481	747	708
ENSECAG00000024008	2.110954699	0.2868654	0.6476852	62	73	50	88	66	43	93	159
ENSECAG00000020576	4.161942656	0.2870468	0.6479555	364	173	264	402	408	201	667	237
ENSECAG00000015920	6.740265382	0.2871026	0.6479555	2064	1430	1830	2045	2690	1315	1992	2611
ENSECAG00000016346	4.945660686	0.2871674	0.6479555	596	529	515	426	416	772	541	722
ENSECAG00000013110	8.549303358	0.2872929	0.6479555	8045	7852	7871	9058	5475	3601	8035	6910
ENSECAG00000018277	5.103710663	0.287339	0.6479555	557	494	711	579	862	721	734	420
ENSECAG00000017246	5.638109002	0.2873512	0.6479555	991	826	1127	1389	830	655	992	733
ENSECAG00000015420	5.140936763	0.2873967	0.6479555	586	400	684	742	478	1055	725	542
ENSECAG00000026875	2.726221643	0.2874415	0.6479555	103	116	119	264	71	99	144	84
ENSECAG00000011756	6.23595394	0.2877774	0.648492	1911	1267	2044	1299	846	1034	1452	1484
ENSECAG00000024443	4.578699304	0.287821	0.648492	334	431	325	536	375	382	464	712
ENSECAG00000014222	4.03474178	0.2878508	0.648492	230	322	257	220	166	666	167	310
ENSECAG00000017119	3.213722099	0.2879795	0.6485675	196	137	227	266	148	96	205	127
ENSECAG00000015236	5.604252351	0.2880176	0.6485675	894	738	819	567	2523	421	911	267
ENSECAG00000007379	1.792866395	0.2880786	0.6485675	12	122	13	31	23	97	24	159
ENSECAG00000024602	6.210312957	0.2881788	0.6485675	989	1349	1174	1587	1125	1579	1227	1940
ENSECAG00000026919	0.89791406	0.2881855	0.6485675	24	35	27	26	37	45	32	32
ENSECAG00000020442	3.757176422	0.2882528	0.6485675	233	290	310	350	223	157	283	186
ENSECAG00000021283	5.790506923	0.288329	0.6485675	1053	1069	1456	1175	915	733	1053	864
ENSECAG00000002739	6.289931364	0.288341	0.6485675	1918	784	1629	3010	958	277	2104	1557
ENSECAG00000024664	3.571202663	0.2885235	0.6488495	112	388	115	97	230	334	267	157
ENSECAG00000016918	6.757627961	0.2885866	0.6488628	1895	1543	2008	2078	2125	2195	2437	1832
ENSECAG00000014301	5.09854434	0.2887171	0.6489839	602	518	697	500	928	393	875	597
ENSECAG00000010036	5.792364662	0.2888017	0.6489839	569	1389	711	827	373	1849	738	1558
ENSECAG00000023446	4.171453309	0.2888629	0.6489839	428	306	469	371	261	182	434	271
ENSECAG00000024240	4.673558149	0.288869	0.6489839	486	311	400	545	714	392	658	308
ENSECAG00000022974	0.732658261	0.2889657	0.6489964	28	33	31	52	30	21	20	20
ENSECAG00000023740	5.348087951	0.2889888	0.6489964	859	818	874	978	723	659	757	436
ENSECAG00000007619	3.13745028	0.2891697	0.6492743	168	116	169	115	272	138	105	196
ENSECAG00000020760	6.558483539	0.2893519	0.6494582	2007	1820	1802	2539	1264	1162	1874	1814
ENSECAG00000015073	2.616770865	0.289412	0.6494582	112	66	102	118	180	122	134	62
ENSECAG00000008558	4.779929734	0.2894231	0.6494582	573	445	558	856	321	315	646	480
ENSECAG00000018566	5.932634578	0.2894829	0.6494639	1192	1323	1119	1663	931	785	1460	780
ENSECAG00000006293	6.325355664	0.2897327	0.6498461	1588	1547	1732	2075	994	1384	1355	1380
ENSECAG00000006452	5.233737123	0.2897676	0.6498461	900	702	831	814	598	409	670	740

ENSECAG00000022180	1.968241802	0.2899427	0.6498771	20	53	48	125	49	112	53	106
ENSECAG00000019583	4.104783138	0.289949	0.6498771	329	358	381	430	224	208	396	269
ENSECAG00000007445	4.562810296	0.2899531	0.6498771	385	413	364	454	343	445	628	490
ENSECAG00000023451	5.132628519	0.2900641	0.6499976	638	570	573	615	975	437	800	616
ENSECAG00000013191	7.724044842	0.290146	0.650053	3505	3247	4132	3437	3865	5696	3237	3889
ENSECAG00000020046	5.733490886	0.2902567	0.6501727	1282	891	1142	1282	956	677	1005	790
ENSECAG00000011914	7.084744329	0.2903285	0.6501739	2995	2330	3120	3687	2320	876	3838	1684
ENSECAG00000011296	5.302922509	0.2904068	0.6501739	569	654	633	895	877	611	912	752
ENSECAG00000009293	4.943005094	0.2905063	0.6501739	470	453	679	500	776	476	666	546
ENSECAG00000020512	3.371730832	0.290569	0.6501739	230	167	222	297	145	111	219	173
ENSECAG00000024931	0.361401284	0.2906155	0.6501739	29	14	9	21	28	10	49	19
ENSECAG00000010937	1.214056551	0.2906418	0.6501739	20	14	30	82	38	31	71	54
ENSECAG00000021938	0.756204176	0.2906579	0.6501739	23	33	16	29	22	40	44	28
ENSECAG00000020647	7.774615178	0.2908307	0.6503912	3890	5282	2825	7524	2759	3757	4073	3020
ENSECAG00000011460	3.159241022	0.2908937	0.6503912	172	164	218	232	110	119	197	129
ENSECAG00000005626	3.269265866	0.2909268	0.6503912	162	142	175	163	233	167	234	145
ENSECAG00000011920	2.386255554	0.2909946	0.6504147	83	97	110	177	82	55	104	75
ENSECAG00000003367	1.961343659	0.2911814	0.6506055	91	49	81	128	53	46	54	76
ENSECAG00000002462	2.323952471	0.2912096	0.6506055	50	93	105	58	40	80	108	195
ENSECAG00000016664	2.222569282	0.2912983	0.6506055	12	151	39	51	45	135	31	190
ENSECAG00000012107	6.275631747	0.2913743	0.6506055	1216	1157	1256	1805	1232	1402	1565	1960
ENSECAG00000012840	4.897686038	0.2913773	0.6506055	548	717	638	673	333	485	481	575
ENSECAG00000015474	6.627096545	0.2914236	0.6506055	2217	1719	2178	2486	1613	967	2108	1728
ENSECAG00000008855	8.294871422	0.2916914	0.6506632	7328	6227	6819	7053	3922	4177	4922	6825
ENSECAG00000009232	6.467867842	0.2917433	0.6506632	1287	1630	1293	1882	1417	2371	1631	1538
ENSECAG00000019241	3.066471595	0.2917958	0.6506632	132	159	170	43	79	152	132	347
ENSECAG00000013773	1.446982081	0.2918035	0.6506632	56	37	46	111	56	30	54	13
ENSECAG00000004973	8.697397798	0.2918201	0.6506632	5427	7810	10843	13929	7309	4860	11006	2488
ENSECAG00000024424	6.716460657	0.2918399	0.6506632	2460	1890	2106	2686	1317	1426	2219	1829
ENSECAG00000022472	5.491454748	0.2918922	0.6506632	723	837	775	748	1039	956	829	711
ENSECAG00000024758	5.073765468	0.2919077	0.6506632	842	579	743	801	507	224	824	613
ENSECAG00000006437	4.863499053	0.2919908	0.6507208	654	482	723	674	449	344	640	435
ENSECAG00000010635	5.69148514	0.2926714	0.6520917	845	847	796	1103	1079	1205	977	788
ENSECAG00000024719	8.460968313	0.2927676	0.6520917	9512	2006	8426	1754	5541	4993	17335	3959
ENSECAG00000022346	4.964660126	0.2927782	0.6520917	1396	349	894	224	741	286	612	170
ENSECAG00000018509	6.405949251	0.2929175	0.6522741	1823	1619	1824	2038	1372	1372	1439	1237
ENSECAG00000020148	2.69561053	0.2930318	0.6523214	190	108	140	136	112	60	160	63
ENSECAG00000000603	3.684000431	0.2930718	0.6523214	215	257	339	325	111	189	201	288
ENSECAG00000024365	5.558468395	0.2931686	0.6523214	903	966	1046	1180	496	553	864	1115
ENSECAG00000000606	3.128433218	0.2932509	0.6523214	187	84	304	216	139	98	197	96
ENSECAG00000022568	3.414612794	0.2932795	0.6523214	136	131	186	252	89	249	208	328
ENSECAG00000006603	6.129279662	0.2932834	0.6523214	1619	1403	2059	996	806	614	1364	1677
ENSECAG00000021184	7.202290352	0.2934543	0.6525738	2282	1584	2139	4114	3788	2832	4058	1388
ENSECAG00000019497	8.522865082	0.2938377	0.6532496	6834	9319	8384	7385	4336	4441	7050	7543
ENSECAG00000018333	9.471463256	0.2938732	0.6532496	14190	8687	14057	10279	23552	11308	15639	7561
ENSECAG00000025867	5.254111178	0.294082	0.653586	1305	420	52	94	62	1860	34	1518
ENSECAG00000019873	4.230344495	0.2941442	0.6535962	20	680	23	143	30	829	32	846
ENSECAG00000009288	3.480492024	0.2942633	0.6536269	163	222	204	129	142	242	178	344
ENSECAG00000001358	6.938481448	0.2943102	0.6536269	2714	2064	2950	2933	1708	1726	2310	2122
ENSECAG00000015249	4.13276022	0.2943306	0.6536269	348	279	460	461	278	268	380	170
ENSECAG00000012383	5.022954086	0.294427	0.6537132	679	696	726	703	543	360	745	447
ENSECAG00000015855	2.634441976	0.294525	0.653803	109	122	131	189	66	91	88	128
ENSECAG00000013304	5.443358238	0.2947051	0.6540748	880	699	734	678	688	952	865	932
ENSECAG00000026885	6.343376614	0.2948496	0.6542678	1804	1944	1233	2041	1051	1257	1143	1681
ENSECAG00000022228	6.060764445	0.2949536	0.6543706	973	1314	1091	1166	914	1827	1224	1290
ENSECAG00000019097	5.060493063	0.2950651	0.6544379	686	465	625	519	718	602	684	645
ENSECAG00000004773	5.332271181	0.295159	0.6544379	677	675	694	696	1263	583	742	627
ENSECAG00000018030	4.268907633	0.295287	0.6544379	334	498	379	471	186	235	327	460
ENSECAG00000014962	3.626673297	0.2953303	0.6544379	155	151	131	386	334	289	283	104
ENSECAG00000020797	5.460138926	0.2954113	0.6544379	884	886	833	1212	547	623	797	861
ENSECAG00000020919	1.215109776	0.2954131	0.6544379	58	29	57	64	31	56	24	13
ENSECAG00000013998	4.892878898	0.2954515	0.6544379	869	455	472	842	634	266	505	458
ENSECAG00000005649	2.055210635	0.2954625	0.6544379	44	64	62	98	48	69	112	114
ENSECAG00000001117	3.248916153	0.2955024	0.6544379	162	119	179	170	272	176	207	112
ENSECAG00000011700	3.26900656	0.2956249	0.6545815	44	336	47	81	31	380	61	367
ENSECAG00000008935	5.054880277	0.2957179	0.6546599	652	462	623	561	575	591	718	763
ENSECAG00000012141	5.948968739	0.2959095	0.6549564	752	1228	699.999	1561	1038	1539	1065	1247
ENSECAG00000007963	6.57886475	0.2961961	0.655463	2471	1661	2223	2010	1630	926	2674	948
ENSECAG00000015850	4.362351075	0.2962779	0.6555163	480	336	428	572	417	179	320	375
ENSECAG00000018364	3.809992726	0.2964383	0.6557434	268	252	348	359	217	208	271	181
ENSECAG00000024142	4.512562014	0.296572	0.6559115	451	195	409	520	573	328	495	449
ENSECAG00000002971	5.08726491	0.2966409	0.6559361	483	626	506	711	673	589	1060	443
ENSECAG00000020652	4.225875916	0.2967414	0.6560306	421	308	440	468	334	180	353	321
ENSECAG00000021688	2.958191359	0.2968289	0.6560965	221	148	165	144	139	85	155	98
ENSECAG00000019631	7.386861091	0.2968883	0.6561	4274	2442	4084	4647	4849	1169	3238	781
ENSECAG00000001317	6.730495854	0.2973162	0.6568957	1623	1986	1933	1753	2131	2116	1939	2200
ENSECAG00000024781	3.834040842	0.297364	0.6568957	335	251	332	332	277	128	289	203
ENSECAG00000021500	3.075574035	0.2977103	0.6575329	122	147	150	112	83	191	94	323

ENSECAG00000023627	2.82739491	0.2978637	0.6576792	98	116	129	116	143	211	94	113
ENSECAG00000010661	10.26950562	0.2978924	0.6576792	23855	16874	21051	22733	24871	27864	19985	25246
ENSECAG00000017744	6.660805446	0.2980084	0.6578075	1863	2503	2017	2465	843	1891	1285	2235
ENSECAG00000013383	6.030328212	0.2982515	0.6582157	1352	1140	1377	1832	1264	676	1397	891
ENSECAG00000016199	4.096539879	0.2983092	0.6582157	335	223	283	332	330	284	328	425
ENSECAG00000009524	6.8400076	0.2983839	0.6582527	2049	1511	1781	2585	1842	3111	2228	1870
ENSECAG00000014886	0.41856782	0.2985454	0.6582838	6	24	7	40	15	36	33	23
ENSECAG00000011344	3.401746043	0.2986152	0.6582838	22	238	288	74	203	163	366	200
ENSECAG00000013600	7.442184628	0.2986178	0.6582838	2735	3281	2621	3174	2427	4013	2989	4434
ENSECAG00000001100	3.178593185	0.2986298	0.6582838	151	111	142	210	172	176	204	175
ENSECAG00000005886	3.313794643	0.2989717	0.6589095	134	222	261	266	133	113	111	243
ENSECAG00000013632	4.459940059	0.2992433	0.6591592	344	330	352	495	489	398	370	484
ENSECAG00000010222	7.829405806	0.2992484	0.6591592	4962	3112	4032	3302	6220	4052	4776	3231
ENSECAG00000026223	0.72313796	0.2992591	0.6591592	35	28	0	3	0	45	1	110
ENSECAG00000020651	7.932459556	0.2996176	0.6598209	4855	4608	5069	6873	3508	3047	4913	4216
ENSECAG00000024693	5.492884035	0.2998755	0.6602609	1041	797	1067	980	909	555	852	569
ENSECAG00000008959	6.959894417	0.3001464	0.6606075	2790	1890	2767	3924	2232	646	3889	1143
ENSECAG00000009654	4.42778436	0.3001493	0.6606075	509	339	548	495	450	178	433	300
ENSECAG00000017347	3.22983131	0.3002581	0.6606532	46	265	55	162	31	142	107	560
ENSECAG00000014782	4.857909468	0.3003319	0.6606532	558	442	460	543	568	427	726	611
ENSECAG00000018283	4.400912008	0.3003445	0.6606532	308	403	438	733	223	247	432	435
ENSECAG00000011003	8.465445127	0.3006385	0.6610529	9614	4348	6183	1900	11451	6188	9951	2465
ENSECAG00000010108	5.538322543	0.3006426	0.6610529	916	909	1020	1147	620	674	877	835
ENSECAG00000008620	4.693375233	0.3007058	0.6610639	379	373	508	514	678	522	474	372
ENSECAG00000018570	6.392160271	0.3008385	0.661151	1999	1436	1810	2052	1651	835	1807	1143
ENSECAG00000008427	3.030278362	0.3008618	0.661151	160	146	157	267	103	131	184	81
ENSECAG00000019997	5.7487617	0.3010857	0.6613251	1346	817	1177	1360	979	491	1279	739
ENSECAG00000021548	6.193007151	0.3011833	0.6613251	1447	1406	1647	1838	1442	824	1557	902
ENSECAG00000024647	0.610493047	0.3013804	0.6613251	35	26	38	32	13	9	47	18
ENSECAG00000011617	2.688942112	0.3014349	0.6613251	102	58	101	167	161	112	184	73
ENSECAG00000017040	5.50372057	0.301452	0.6613251	828	760	729	840	931	665	911	1094
ENSECAG00000017160	4.542438539	0.3015105	0.6613251	94	891	68	171	100	614	160	1213
ENSECAG00000008653	4.91231602	0.3015416	0.6613251	510	414	539	639	539	559	689	606
ENSECAG00000010122	2.770354621	0.3016601	0.6613251	86	158	168	195	72	136	125	71
ENSECAG00000001926	6.290711965	0.3016678	0.6613251	1647	956	1588	767	3619	748	1706	522
ENSECAG00000014256	1.535282544	0.3016745	0.6613251	39	52	53	37	48	63	48	71
ENSECAG00000006400	7.112856673	0.3017091	0.6613251	2763	1871	2152	2769	3519	2504	3234	1832
ENSECAG00000009756	3.13517951	0.3017171	0.6613251	174	144	255	203	124	164	153	88
ENSECAG00000005306	2.166968053	0.3017373	0.6613251	133	134	54	86	58	20	25	146
ENSECAG00000016139	3.187356057	0.3017958	0.6613251	153	100	165	188	194	129	324	113
ENSECAG00000014112	6.385081901	0.3018472	0.6613251	1638	1443	1342	1363	1833	1201	2050	1601
ENSECAG00000016417	6.284803405	0.3019283	0.6613251	1493	781	1178	1847	2373	769	2627	778
ENSECAG00000007962	4.934198493	0.3019871	0.6613251	758	579	708	604	382	296	589	693
ENSECAG00000012445	8.288538713	0.301989	0.6613251	5794	4979	4801	5993	6419	4739	6794	7216
ENSECAG00000005219	8.401722942	0.3021903	0.6616384	6328	5342	6680	4256	9215	7172	6121	4535
ENSECAG00000011182	4.769756156	0.3025667	0.6621157	564	487	582	802	202	292	469	749
ENSECAG00000025161	3.688131198	0.3026175	0.6621157	268	254	189	435	166	224	232	170
ENSECAG00000016939	10.52034633	0.302629	0.6621157	28473	19574	24867	28098	30075	31823	24984	29755
ENSECAG00000020230	5.126793678	0.3026415	0.6621157	647	781	733	882	577	539	339	707
ENSECAG00000018910	5.313806569	0.3027271	0.6621753	795	621	682	606	1142	555	970	540
ENSECAG00000009061	3.998820433	0.3027853	0.6621753	353	331	385	343	151	141	229	468
ENSECAG00000007795	7.890715981	0.3028737	0.6622411	4285	3405	3940	4729	5929	3688	6141	3439
ENSECAG00000000110	3.854413791	0.3030376	0.6623667	218	220	119	410	246	157	562	270
ENSECAG00000010463	6.002759581	0.3030478	0.6623667	1261	888	1053	1308	1106	1243	1413	1299
ENSECAG00000007200	6.65663773	0.3031075	0.6623697	2328	1563	2077	2879	1805	971	2194	1570
ENSECAG00000020190	7.027323267	0.3031739	0.6623874	2665	2721	2578	3332	2035	1959	2070	2239
ENSECAG00000026862	3.375292824	0.3033211	0.6625287	254	195	289	163	133	154	156	192
ENSECAG00000018902	1.138250296	0.3034726	0.6625287	39	37	22	35	26	26	39	89
ENSECAG00000008022	3.342462912	0.303616	0.6625287	201	202	218	266	163	108	214	152
ENSECAG00000012485	6.139284146	0.303636	0.6625287	1384	909	1266	1350	1762	1005	1854	1043
ENSECAG00000022311	3.930189091	0.3036838	0.6625287	288	245	489	321	322	200	254	159
ENSECAG00000023238	7.140024204	0.3037314	0.6625287	2270	2599	1975	2856	2727	3320	2786	2314
ENSECAG00000024819	4.780441957	0.3037587	0.6625287	527	489	543	874	292	293	548	622
ENSECAG00000022081	5.752315819	0.3037727	0.6625287	1200	1039	1149	1241	703	639	1170	1009
ENSECAG00000009985	5.180842825	0.3037764	0.6625287	704	925	751	717	535	484	580	711
ENSECAG00000007492	1.856948944	0.3038218	0.6625287	79	82	65	99	14	33	66	98
ENSECAG00000008775	1.382219776	0.3041652	0.6631501	51	33	36	45	59	54	52	41
ENSECAG00000024775	6.058910972	0.3042815	0.6631798	1071	1090	949	1511	1113	1746	1556	854
ENSECAG00000011569	6.308216119	0.3044366	0.6631798	1189	2038	1171	2697	441.001	1272	1670	1559.02
ENSECAG00000013776	3.999644706	0.3044676	0.6631798	321	158	301	282	441	130	537	242
ENSECAG00000023813	2.819592589	0.3044697	0.6631798	13	261	5	36	11	259	9	382
ENSECAG00000022051	10.06880247	0.3044707	0.6631798	16245	18692	17164	20162	15301	31053	14675	24095
ENSECAG00000023968	6.391246453	0.3046781	0.6634868	1492	1352	1415	1591	2135	1389	1596	1495
ENSECAG00000011998	2.731429856	0.3047777	0.6634868	76	84	117	163	118	191	111	107
ENSECAG00000011537	4.208446314	0.3047869	0.6634868	387	260	407	592	238	180	455	310
ENSECAG00000006890	9.002104408	0.3048607	0.6635203	10575	10304	11536	12244	6615	6917	10392	8916
ENSECAG00000022350	3.891817314	0.3049421	0.6635704	273	214	410	435	162	110	328	335
ENSECAG00000010110	8.37081661	0.3050272	0.6636284	6852	6664	7237	8042	5369	5031	5988	4618

ENSECAG00000022434	6.030673009	0.3051019	0.6636639	1338	895	1134	1186	1295	814	1840	1343
ENSECAG00000022335	4.76935084	0.305361	0.6637549	493	470	555	905	249	428	459	575
ENSECAG00000018217	8.058987558	0.305415	0.6637549	6398	4664	5815	6427	4331	2715	5400	4710
ENSECAG00000016594	4.249574324	0.3054706	0.6637549	331	370	332	204	266	228	380	702
ENSECAG00000009904	6.773112392	0.3054861	0.6637549	2470	1865	2540	2599	1832	1642	2047	1480
ENSECAG00000024316	6.331524472	0.3055664	0.6637549	1673	1493	1689	2102	1232	906	1813	1318
ENSECAG00000001869	6.358029337	0.3055738	0.6637549	1224	1561	1584	1252	1378	1778	1377	1921
ENSECAG00000013452	1.621067957	0.3055973	0.6637549	46	33	51	69	74	40	79	56
ENSECAG00000014344	2.665136934	0.3056994	0.6637549	123	97	140	212	59	57	115	155
ENSECAG00000016010	7.562224838	0.3057999	0.6637549	3868	2839	2943	3304	4930	2544	4678	3158
ENSECAG00000001640	1.592751444	0.3058137	0.6637549	27.0001	75.0001	13	42	2.00073	84	16.0001	163
ENSECAG00000010351	4.818786083	0.3058143	0.6637549	618	607	670	531	325	420	497	548
ENSECAG00000013903	5.192798527	0.305845	0.6637549	712	525	553	761	619	711	689	869
ENSECAG00000023011	4.88528816	0.3060222	0.6640127	345	707	425	499	512	468	583	827
ENSECAG00000020161	9.422199194	0.3063835	0.6646696	12769	9876	12057	12475	11751	14751	12667	15439
ENSECAG00000016353	5.340198071	0.3065724	0.6649525	921	751	843	971	578	547	698	786
ENSECAG00000015232	0.024391781	0.3066766	0.6649909	4.99921	12.9995	19.9992	19.9994	14.9995	22	23	17.9998
ENSECAG00000021774	3.015923268	0.3067928	0.6649909	145	145	221	198	122	107	119	147
ENSECAG00000014292	5.398073433	0.3068991	0.6649909	733	712	646	626	274	1639	484	971
ENSECAG00000019228	5.510902462	0.3069055	0.6649909	1223	740	904	1063	745	567	899	742
ENSECAG00000006021	5.835164134	0.3069109	0.6649909	1230	707	1065	940	1589	899.002	1200	856.001
ENSECAG00000025102	5.548434942	0.3069669	0.6649909	755	799	880	744	709	1139	578	1252
ENSECAG00000022601	6.778501816	0.3069999	0.6649909	2609	2061	2361	2462	1910	1307	2460	1423
ENSECAG00000015678	6.130652358	0.3070721	0.6650204	1127	994	1359	1427	1030	1574	1375	1530
ENSECAG00000009972	2.858625744	0.3071822	0.6651319	162	106	175	196	100	101	124	120
ENSECAG00000024738	2.947951892	0.3073192	0.6653019	107	139	152	315	50	69	130	213
ENSECAG00000024202	0.999469851	0.3073891	0.6653263	12	49	22	37	36	45	39	39
ENSECAG00000010177	3.74559445	0.3074973	0.6654338	214	232	302	441	171	189	236	241
ENSECAG00000008968	7.63678124	0.3076043	0.6655386	4722	3385	4521	4740	3946	2221	3480	3041
ENSECAG00000013881	5.219989774	0.3078833	0.6659574	887	751	812	746	610	390	812	606
ENSECAG00000005231	1.918002493	0.3080225	0.6659574	259	19	55	26	13	18	119	56
ENSECAG00000004259	1.407732018	0.3080618	0.6659574	67	20	35	39	49	93	20	47
ENSECAG00000004709	1.429827437	0.3080763	0.6659574	8	182	12	41	11	51	33	37
ENSECAG00000022541	4.585875483	0.3080911	0.6659574	375	421	360	492	456	310	523	649
ENSECAG00000023964	0.786881799	0.3084876	0.6666876	20.0007	22.0005	21.001	45.0003	36.0006	24.0007	38.0001	38.0239
ENSECAG00000024086	6.59955145	0.3086112	0.6667301	2489	1546	2329	2090	2024	1043	2159	991
ENSECAG00000014970	2.570237878	0.3086772	0.6667301	129	78	79	106	129	95	157	101
ENSECAG00000012974	3.088442025	0.3088252	0.6667301	102	103	149	185.001	336	90	258	41
ENSECAG00000008164	9.81301985	0.3088334	0.6667301	19198	10327	17649	13343	26320	12610	24164	10900
ENSECAG00000015830	5.779867824	0.308866	0.6667301	1168	977	1131	1459	905	736	1115	815
ENSECAG00000012666	7.441002123	0.3089083	0.6667301	4150	2372	3695	5366	3348	1673	4329	1724
ENSECAG00000020726	7.406426477	0.3089181	0.6667301	4502	2913	4292	3050	3456	2309	2959	1939
ENSECAG00000009087	4.453307238	0.3090206	0.6668248	356	435	351	833	200	302	292	555
ENSECAG00000024896	6.55264059	0.3091405	0.6669567	1584	1322	1756	1933	1496	1530	1967	2475
ENSECAG00000016704	4.896151007	0.3092717	0.667113	530	403	574	563	563	539	763	511
ENSECAG00000020913	6.620928285	0.309583	0.6676577	2608	1793	1965	2101	1576	1093	2157	1586
ENSECAG00000009590	4.65981135	0.3098145	0.6680301	595	579	510	482	465	340	474	324
ENSECAG00000021437	2.181956041	0.3099967	0.6681182	85	57.0001	77	75	107	111	78	63
ENSECAG00000000643	5.742956283	0.3100121	0.6681182	1096	1093	1079	1356	609	761	845	1219
ENSECAG00000019350	5.01707206	0.3101045	0.6681182	719	659	796	612	629	312	556	570
ENSECAG00000010410	7.583269759	0.3101147	0.6681182	4094	3419	4405	4836	3170	1886	4318	3040
ENSECAG00000014981	5.596393103	0.3101494	0.6681182	1109	860	1084	1148	1051	461	1008	593
ENSECAG00000017281	5.424834074	0.3103696	0.6683522	790	611	660	874	1257	850	940	383
ENSECAG00000017579	6.699644713	0.3103757	0.6683522	2120	1420	1826	1893	1736	1870	2418	2254
ENSECAG00000021714	3.763218036	0.3105575	0.6686169	292	205	258	449	223	184	249	193
ENSECAG00000016577	3.969625939	0.310639	0.6686187	302	220	267	255	527	251	295	186
ENSECAG00000015993	3.475496962	0.3106761	0.6686187	270	143	235	343	157	126	177	230
ENSECAG00000014133	6.116412136	0.3108643	0.668897	974	1151	1207	1488	986	1825	1271	1352
ENSECAG00000013419	2.833261591	0.3113519	0.6694378	109	108	134	121	155	166	129	114
ENSECAG00000018251	5.282396666	0.3113739	0.6694378	668	758	841	1101	562	529	747	675
ENSECAG00000025162	7.960941571	0.31144	0.6694378	4276	3827	4110	4957	3678	4929	4411	6883
ENSECAG00000024456	5.745746528	0.3114554	0.6694378	1030	833	916	968	1255	875	1245	872
ENSECAG00000008519	5.326406004	0.3114586	0.6694378	590	687	692	835	769	767	818	804
ENSECAG00000006663	4.616374747	0.3114692	0.6694378	190	506	335	570	551	218	1078	282
ENSECAG00000001697	3.864092915	0.3118814	0.670197	187	318	231	180	144	507	136	380
ENSECAG00000008201	7.392021319	0.3119413	0.6701988	3553	3118	3789	4102	2905	2253	3153	2464
ENSECAG00000017012	6.71814471	0.3120111	0.6702219	1918	1686	1626	1923	1098	2902	1663	2682
ENSECAG00000009111	5.790456734	0.3121865	0.6703918	927	1021	846	1049	851	1106	1023	1379
ENSECAG00000017879	1.383131998	0.3122082	0.6703918	51	44	68	64	32	29	30	60
ENSECAG00000000690	4.691854653	0.3124244	0.6704991	551	472	570	645	406	311	415	518
ENSECAG00000023577	4.951990249	0.312436	0.6704991	662	556	688	768	491	396	657	455
ENSECAG00000019214	4.236108811	0.3124497	0.6704991	422	253	283	301	522	333	506	172
ENSECAG00000007734	1.195061665	0.3124942	0.6704991	62	32	20	22	30	20	63	79
ENSECAG00000001110	1.24123792	0.3125582	0.6705098	60	26	32	29	56	40	57	36
ENSECAG00000022413	9.520056086	0.3129062	0.6711295	13789	11501	12687	12306	17621	14468	13624	12604
ENSECAG00000001187	6.262309697	0.3130886	0.6713938	1504	1496	1579	2027	1121	1212	1275	1331.02
ENSECAG00000003968	4.10790796	0.3132235	0.6715563	324	230	301	333	392	253	400	336
ENSECAG00000022022	6.095102172	0.3134827	0.6719684	939	1139	1007	1703	906	1564	1595	1359

ENSECAG00000012016	3.53233653	0.313534	0.6719684	171	226	276	349	231	116	201	171
ENSECAG00000013696	6.624747078	0.3136313	0.6720501	1851	1354	1809	1883	1901	2235	2187	1440
ENSECAG00000016829	5.437935135	0.3137562	0.6721911	997	607	995	1229	897	716	697	389
ENSECAG00000011761	6.845108878	0.3138606	0.6722879	2035	1705	2018	2231	2504	1450	3159	2203
ENSECAG00000008692	6.426060435	0.3139199	0.6722881	1829	1815	1864	1878	848	1440	1709	1533
ENSECAG00000010141	4.466103331	0.3141727	0.6727026	556	385	441	548	395	413	281	271
ENSECAG00000019763	6.59166686	0.3143391	0.6729321	2000	1654	2212	2459	1299	1537	1586	1779
ENSECAG00000018798	2.532598617	0.3145369	0.6732069	54	105	141	67	131	89	94	157
ENSECAG00000014110	1.74529342	0.314586	0.6732069	62	43	61	46	52	39	127	62
ENSECAG00000017421	2.56826233	0.3146733	0.6732669	92	97	89	116	129	104	137	105
ENSECAG00000020965	3.660025587	0.314782	0.6733726	285	231	280	315	110	206	183	279
ENSECAG00000021281	7.440104411	0.3149668	0.6736411	3206	2888	3017	2831	2911	3557	3232	4068
ENSECAG00000018383	5.873393755	0.3150897	0.6737346	1206	828	1226	1981	884	502	1740	689
ENSECAG00000009109	4.621830888	0.3151841	0.6737346	493	342	421	450	514	459	522	452
ENSECAG00000005608	8.369234636	0.3152562	0.6737346	7347	5879	6586	9121	4869	4216	6836	5360
ENSECAG00000023951	6.913424495	0.3152629	0.6737346	2498	2370	2230	3372	2087	1510	2289	1881
ENSECAG00000015344	1.256971294	0.3153071	0.6737346	48	43	53	63	25	21	46	49
ENSECAG00000000511	3.317530817	0.3157437	0.6745406	172	153	169	178	215	201	224	154
ENSECAG00000019139	5.054633554	0.3158867	0.6747192	688	636	657	890	445	551	589	539
ENSECAG00000018969	0.964488709	0.3159682	0.6747664	49	23	58	41	49	9	30	21
ENSECAG00000006087	6.333300862	0.3160706	0.6748583	1275	1375	1420	1559	1775	1692	1592	1250
ENSECAG00000017895	7.627002593	0.3161789	0.6749189	3624	2991	3372	3687	3073	2887	4852	5202
ENSECAG00000004752	0.333481842	0.3162178	0.6749189	15	22	23	13	24	30	29	14
ENSECAG00000015493	7.252226848	0.3163616	0.6750225	2631	4720	3664	2094	1738	2233	2018	3445
ENSECAG00000017230	3.858510317	0.3164473	0.6750225	271	258	253	186	237	348	287	279
ENSECAG00000006985	0.952084754	0.3164977	0.6750225	9	37	6	46	2	111	2	47
ENSECAG00000002401	4.035907147	0.3165295	0.6750225	293	297	247	273	235	350	328	392
ENSECAG00000000235	3.129337867	0.3166537	0.6750225	136	99	135	220	107	139	202	269
ENSECAG00000008008	7.012228049	0.3167507	0.6750225	2388	1903	2163	2534	2552	2850	2749	2031
ENSECAG00000023054	5.312357452	0.3167617	0.6750225	602	637	782	735	812	886	825	596
ENSECAG00000019339	1.673408164	0.3168099	0.6750225	71	48	67	97	54	39	74	21
ENSECAG00000010237	4.75431967	0.316892	0.6750225	474	587	567	718	291	493	403	503
ENSECAG00000020169	0.567296165	0.3169033	0.6750225	15	11	23	42	24	43	24	23
ENSECAG00000006398	1.164868472	0.31692	0.6750225	16	38	40	46	37	50	42	48
ENSECAG00000010254	5.032174079	0.3171268	0.6751337	416	677	411	740	486	757	672	682
ENSECAG00000016207	0.996029128	0.3171431	0.6751337	33	25	42	20	21	38	78	27
ENSECAG00000017197	6.204630509	0.317274	0.6751337	1725	1243	1579	1816	1248	1240	1267	959
ENSECAG00000000261	6.047592043	0.3173867	0.6751337	1425	1233	1478	1563	1444	765	1167	877
ENSECAG00000021913	5.494632523	0.3173934	0.6751337	492	1096	627	805	625	1022	687	1257
ENSECAG00000022848	5.466986932	0.3174159	0.6751337	929	752	852	1328	561	478	941	900
ENSECAG00000016711	3.81036096	0.3174274	0.6751337	406	200	341	291	292	137	309	136
ENSECAG00000024171	4.988575489	0.3174477	0.6751337	558	488	571	575	544	793	599	549
ENSECAG00000019985	1.963751537	0.3175511	0.675227	80	52	93	130	47	14	100	73
ENSECAG00000011118	3.687504005	0.3176163	0.6752392	184	183	251	263	246	257	238	280
ENSECAG00000013740	8.545202288	0.3176962	0.6752828	6158	5422	6621	7705	7067	8426	6643	7360.02
ENSECAG00000000047	4.800403308	0.3178128	0.6754042	477	366	527	518	972	363	579	335
ENSECAG00000015662	2.329157484	0.3179873	0.6756487	140	75	120	104	90	67	84	61
ENSECAG00000021551	4.366687204	0.318299	0.6761845	515	420	420	427	290	311	248	437
ENSECAG00000023593	7.147984196	0.3186358	0.6767733	2565	2876	2987	3879	1973	2123	2905	2133
ENSECAG00000012605	4.833394625	0.3187279	0.6768424	630	455	672	725	462	325	666	389
ENSECAG00000004604	7.271753195	0.3188548	0.6769625	2998	2182	2624	2923	3608	2558	3917	2306
ENSECAG00000018042	6.202582477	0.3189037	0.6769625	1395	1353	1790	1856	1053	849	2030	892
ENSECAG00000010965	8.125117158	0.3190634	0.6770405	3948	5591	5252	3584	3440	8194	3528	7059
ENSECAG00000017111	5.152483866	0.3190771	0.6770405	672	608	728	1101	497	386	742	683
ENSECAG00000016230	3.130756103	0.3191192	0.6770405	188	152	221	204	149	82	174	143
ENSECAG00000013403	3.676279536	0.3194263	0.6775654	243	169	202	258	279	209	361	185
ENSECAG00000021603	8.328840479	0.3195688	0.6775883	4484	5154	5379	7245	4214	7186	7978	6449
ENSECAG00000002225	0.016146484	0.3196078	0.6775883	13	17	22	36	4	29	9	6
ENSECAG00000017035	1.765565207	0.319616	0.6775883	31	25	42	124	58	55	115	56
ENSECAG00000017449	5.584556657	0.3197764	0.6777238	1066	999	745	1326	732	741	936	682
ENSECAG00000016453	3.187314458	0.3198506	0.6777238	142	147	121	207	204	162	180	180
ENSECAG00000011650	4.102656197	0.3198875	0.6777238	191	392	269	277	261	475	223	401
ENSECAG00000016063	5.571355624	0.3199528	0.6777238	868	966	965	1298	579	808	905	769
ENSECAG00000015338	5.793141366	0.3200857	0.6777238	898	966	952	1000	1375	1302	979	675
ENSECAG00000019326	6.79603741	0.3200898	0.6777238	2007	1976	1866	1805	2487	2351	2018	1867
ENSECAG00000024826	5.791749668	0.3200978	0.6777238	1006	865	999	1010	1003	1205	1016	1092
ENSECAG00000015572	5.535284554	0.3202152	0.6777238	1020	749	1188	1045	889	762	695	589
ENSECAG00000022987	4.232279585	0.3202169	0.6777238	248	340	366	741	181	224	486	301
ENSECAG00000000887	5.462382267	0.3203372	0.6777613	963	352	847	873	1280	801	933	518
ENSECAG00000021803	3.694634539	0.3203539	0.6777613	210	304	254	382	80	249	209	247
ENSECAG00000010381	5.141102158	0.3205117	0.6779689	679	762	640	963	538	580	665	472
ENSECAG00000012033	2.242738567	0.3207085	0.678156	88	55	107	185	38	31	117	100
ENSECAG00000022083	7.330279629	0.3207208	0.678156	2640	2270	2971	3371	2976	3419	3149	3150
ENSECAG00000010597	5.099348363	0.3209534	0.678156	608	737	709	910	403	457	602	739
ENSECAG00000021515	8.077683132	0.3209797	0.678156	6095	6878	5627	5060	2137	2323	5312	7363
ENSECAG00000009848	2.163972067	0.3209839	0.678156	90	87	119	94	67	47	54	100
ENSECAG00000007172	1.527353532	0.3211027	0.678156	50	49	45	38	33	55	78	66
ENSECAG00000017863	7.065860107	0.3211505	0.678156	2413	1986	2319	2501	4011	2037	2903	1772

ENSECAG00000002267	6.717589938	0.3211655	0.678156	1749	1645	1850	2136	1619	2052	2383	2283
ENSECAG00000011500	4.902140425	0.3211665	0.678156	629	496	699	779	411	310	737	489
ENSECAG00000014810	7.773351801	0.3211972	0.678156	3615	3836	3041	4637	3032	4637	4646	5142
ENSECAG00000011622	3.641688722	0.3212986	0.678244	215	182	294	429	165	97	265	260
ENSECAG00000008150	5.028325458	0.3214164	0.6783347	769	583	751	718	445	327	840	519
ENSECAG00000021402	1.470134337	0.321461	0.6783347	52	45	50	97	27	33	62	43
ENSECAG00000015093	1.062570011	0.3215412	0.6783779	39	22	47	83	16	7	33	61
ENSECAG00000000556	4.789839196	0.321737	0.6786648	655	413	594	747	422	400	456	483
ENSECAG00000004996	1.301177896	0.3218442	0.6787084	26	30	46	55	61	55	42	35
ENSECAG00000002234	1.176379652	0.3218771	0.6787084	28	56	39	94	2	1	71	47
ENSECAG00000000909	4.551240712	0.3219955	0.6788321	383	385	376	470	298	485	478	604
ENSECAG00000020905	5.945659429	0.3221558	0.679044	1051	970	1242	1017	957	1410	1088	1365
ENSECAG00000013668	6.958471665	0.3224578	0.6793599	1748	2279	1761	2768	1840	3489	2206	2240
ENSECAG00000005995	6.636860841	0.3224705	0.6793599	2202	2443	1616	2309	1808	847	2154	1651
ENSECAG00000023676	3.071907114	0.3225177	0.6793599	103	92	127	252	135	154	196	197
ENSECAG00000020379	3.422171733	0.3225557	0.6793599	137	255	127	141	406	281	101	76
ENSECAG00000023717	6.652201229	0.3226732	0.6793599	1841	1784	1880	1371	2038	2459	1827	1549
ENSECAG00000018148	5.554326509	0.3226738	0.6793599	792	670	857	1002	903	942	1016	820
ENSECAG00000011599	6.563825945	0.32276	0.6793599	1863	1727	2060	2511	1803	1083	1828	1439
ENSECAG00000015628	2.166448027	0.3227842	0.6793599	92	55	76	66	73	52	170	81
ENSECAG00000011448	5.14829818	0.3228982	0.6794387	697	466	625	683	638	507	1108	622
ENSECAG00000019769	2.025714479	0.3229469	0.6794387	45	146	71	90	60	46	85	51
ENSECAG00000000442	2.471544572	0.3230011	0.6794387	92	59	75	147	104	118	108	112
ENSECAG00000011370	7.301775911	0.3231981	0.6796692	3013	2766	3699	4271	2085	1958	3311	2873
ENSECAG00000013437	5.325966948	0.3232303	0.6796692	824	502	790	639	1109	442	1017	681
ENSECAG00000007189	1.894038343	0.3233313	0.6797558	55	55	77	49	58	43	78	125
ENSECAG00000021081	3.894142729	0.3234191	0.6797598	146	282	288	234	129	112	473	575
ENSECAG00000011738	5.775424022	0.3234529	0.6797598	940	844	1001	1048	984	1323	1065	892
ENSECAG00000022087	2.943230252	0.323702	0.6801574	100	188	218	183	66	108	51	221
ENSECAG00000011466	6.146346266	0.323971	0.6805968	962	1637	955	1189	958	2102	951	1547
ENSECAG00000023944	2.69635411	0.3243426	0.6810526	120	81	107	127	129	92	172	132
ENSECAG00000023858	2.301367338	0.3244013	0.6810526	76	64	84	103	86	72	101	138
ENSECAG00000018803	4.983718994	0.3244362	0.6810526	634	429	628	497	683	602	699	518
ENSECAG00000013123	5.504296075	0.3244467	0.6810526	735	717	691	1019	700	1248	940	662
ENSECAG00000009798	2.962733582	0.3245245	0.6810526	95	204	215	217	11	131	45	236
ENSECAG00000001608	1.3310577	0.3246249	0.6810526	35	41	40	44	65	46	47	39
ENSECAG00000018626	7.870320749	0.3246843	0.6810526	4649	4544	5625	5397	3580	3558	4217	3617
ENSECAG00000009150	5.343805784	0.324688	0.6810526	881	515	814	564	1067	463	1117	657
ENSECAG00000023248	4.479994641	0.3247276	0.6810526	361	364	320	498	374	554	391	428
ENSECAG00000015981	4.258226886	0.3248192	0.6810611	286	309	271	407	185	706	282	335
ENSECAG00000021706	5.342103767	0.3248516	0.6810611	688	704	628	775	563	603	788	1303
ENSECAG00000011823	3.004764364	0.3251813	0.6815453	187	131	178	221	98	64	260	83
ENSECAG00000016590	4.981714263	0.3252361	0.6815453	608	506	495	563	575	814	499	574
ENSECAG00000011844	5.116429844	0.3252771	0.6815453	622	701	544	480	517	674	590	975
ENSECAG00000026942	3.410617312	0.3253553	0.6815453	197	218	240	271	122	198	180	156
ENSECAG00000015251	4.975933649	0.3254229	0.6815453	541	692	477	412	670	622	715	496
ENSECAG00000016153	4.761073005	0.3254425	0.6815453	701	384	658	613	504	324	529	376
ENSECAG00000012793	3.788843519	0.3255497	0.681644	207	295	184	233	156	322	339	297
ENSECAG00000019591	6.320908041	0.3262241	0.6824442	1150	954	1859	1617	1672	1063	1367	2285
ENSECAG00000010892	6.282281063	0.3262669	0.6824442	1495	1444	1755	1985	1239	975	1486	1381
ENSECAG00000010150	6.378473777	0.3262991	0.6824442	1605	1433	1326	1446	1916	1315	1852	1489
ENSECAG00000023869	3.526204034	0.3263261	0.6824442	262	242	231	266	123	141	186	271
ENSECAG00000011818	5.572659092	0.3263884	0.6824442	857.642	925	1027	1283.36	679.159	694.877	770	926.458
ENSECAG00000002481	8.253145245	0.3264012	0.6824442	5977	6617	6302	7567	4574	3728	7300	4168
ENSECAG00000008548	11.33285909	0.3264099	0.6824442	48464	34923	48190	46199	57467	55878	42975	47381
ENSECAG00000013603	4.825662154	0.3264125	0.6824442	616	564	511	768	545	334	567	372
ENSECAG00000012621	2.927667257	0.326613	0.6826997	89	141	91	184	134	108	265	125
ENSECAG00000005234	2.440635928	0.3267147	0.6826997	118	58	83	99	127	110	76	117
ENSECAG00000018142	6.860746005	0.3267622	0.6826997	2449	2664	3069	1917	1016	1278	1777	3261
ENSECAG00000023917	4.338548076	0.3267751	0.6826997	376	403	455	530	217	426	305	304
ENSECAG00000000107	3.670202268	0.3269414	0.6829215	411	276	230	196	167	82	360	196
ENSECAG00000018189	3.945012448	0.3270894	0.6831051	307	140	394	166	296	129	569	322
ENSECAG00000020866	4.775732157	0.3271947	0.6831993	633	478	594	663	451	416	558	326
ENSECAG00000015096	4.842698715	0.3273171	0.6833293	623	472	659	760	345	360	375	731
ENSECAG00000008955	4.385334827	0.3274032	0.6833836	515	360	441	491	403	243	350	334
ENSECAG00000009669	4.859882655	0.3275403	0.6835441	693	434	673	722	463	375	639	395
ENSECAG00000016168	5.185782281	0.3278096	0.683935	894	391	669	372	1165	285	1477	235
ENSECAG00000020590	6.426021098	0.3278481	0.683935	1495	1388	1736	2964	1234	980	2184	1220
ENSECAG00000018373	2.979984609	0.3280257	0.6840176	136	126	168	76	266	99	147	130
ENSECAG00000014543	4.082943122	0.3281066	0.6840176	346	268	418	446	287	174	357	268
ENSECAG00000019889	6.09940773	0.3281139	0.6840176	1206	1262	1221	1081	1347	1108	1399	1553
ENSECAG00000008742	6.736231572	0.3281285	0.6840176	1863	1637	1899	2040	2636	1588	1806	2387
ENSECAG00000012217	5.582217113	0.3282946	0.6842383	712	1486	9	22	1630	2322	99	236
ENSECAG00000016654	6.044472311	0.3283854	0.6842827	1163	1028	1460	2129	744	977	1261	1283
ENSECAG00000015898	0.699605301	0.3284821	0.6842827	21	29	23	8	0	36	7	97
ENSECAG00000023150	8.301733412	0.3284966	0.6842827	5262	5113	5806	5554	5780	7578	5415	6030
ENSECAG00000015439	2.727790335	0.3286058	0.6843845	100	103	119.999	110	130	52	201	170
ENSECAG00000008588	3.818935115	0.3286948	0.6844445	256	330	293	339	185	159	309	249

ENSECAG00000013485	5.304307016	0.3287798	0.684496	836	655	844	1169	972	280	842	419
ENSECAG00000001996	0.92044215	0.3289503	0.6847254	15	50	17	28	16	60	29	45
ENSECAG00000011933	4.931887833	0.3290285	0.6847627	609	589	641	796	469	316	644	557
ENSECAG00000011132	11.11038817	0.3290935	0.6847725	40119	34136	37242	40414	28698	42164	46070	61003
ENSECAG00000008931	3.738762876	0.3292395	0.6849507	283	220	280	391	128	206	256	249
ENSECAG00000024914	5.898693432	0.3295053	0.6853783	1314	699	1080	992	1491	770	2012	635
ENSECAG00000024190	4.072822751	0.3297344	0.6857293	386	292	356	423	237	211	298	328
ENSECAG00000019852	6.988519827	0.3298513	0.6858468	2178	2065	2210	2353	2297	1480	3353	3181
ENSECAG00000017364	0.914206552	0.3299862	0.6860017	1	31	66	89	0	9	40	43
ENSECAG00000017573	5.079303071	0.3301232	0.6860714	673	465	995	837	666	320	794	391
ENSECAG00000013877	7.800626213	0.3301583	0.6860714	5530	3609	5413	4764	3840	3287	3392	3636
ENSECAG00000015185	3.780855879	0.3302009	0.6860714	330	178	378	318	251	145	247	222
ENSECAG00000018578	1.967143619	0.3303103	0.6860727	71	55	114	121	11	27	55	131
ENSECAG00000022504	2.670852306	0.3303288	0.6860727	24	217	32	67	25	242	23	257
ENSECAG00000020898	5.804901402	0.3303828	0.6860727	1118	1013	1034	606	1632	960	1043	798
ENSECAG00000021766	3.10648466	0.3305959	0.6863898	140	158	125	153	190	178	159	154
ENSECAG00000008481	3.220493527	0.330748	0.686563	229	166	146	292	108	59	217	201
ENSECAG00000016106	2.976768146	0.3308483	0.686563	192	130	206	162	87	67	152	184
ENSECAG00000011508	6.478357034	0.3308606	0.686563	1957	1748	1619	2360	1041	1284	1533	1911
ENSECAG00000013112	2.634945178	0.3311208	0.6868628	103	67	100	133	41	89	157	235
ENSECAG00000017477	4.967297633	0.3311261	0.6868628	702	650	694	668	282	389	464	846
ENSECAG00000008991	5.874347399	0.3312713	0.6869558	1313	1171	1407	1150	641	1043	730	1281
ENSECAG00000023140	6.702140784	0.3312918	0.6869558	1988	1859	1912	1335	1413	2294	1956	2590
ENSECAG00000019317	4.703938705	0.3313841	0.6870217	518	310	474	479	780	408	637	273
ENSECAG00000024919	3.49937353	0.3315002	0.6871369	285	211	247	234	179	125	261	154
ENSECAG00000007742	4.772582828	0.3316066	0.6872321	510	502	477	904	475	354	500	418
ENSECAG00000009913	4.217862851	0.3318279	0.6875077	398	223	428	606	272	352	295	232
ENSECAG00000022500	10.12064305	0.3319646	0.6875077	24771	19276	26259	26658	20107	14059	22390	14855
ENSECAG00000019451	7.106075455	0.3321068	0.6875077	2512	1822	2564	2676	3797	2028	3266	1943
ENSECAG00000004159	5.654083031	0.3321283	0.6875077	1101	765	860	745	1405	704	1091	805
ENSECAG00000019481	6.309709479	0.3321619	0.6875077	1720	1352	1685	2052	1259	1147	1478	1279
ENSECAG00000005708	4.558400424	0.3322089	0.6875077	338	470	476	299	634	313	509	437
ENSECAG00000019247	5.539128992	0.3322124	0.6875077	748.997	708	805	928	416	1141	766	1369
ENSECAG00000017663	0.805071458	0.3322237	0.6875077	29	19	47	60	14	9	57	21
ENSECAG00000017541	6.795373121	0.3325492	0.6880558	1599	1983	1659	2538	1813	2388	2454	2115
ENSECAG00000014767	6.450473141	0.3328585	0.6882682	1223	1473	1297	2153	1403	1810	2511	1262
ENSECAG00000015924	7.518485336	0.3328841	0.6882682	3746	3376	4014	4775	2801	2410	4171	2518
ENSECAG00000019724	0.865443311	0.3328948	0.6882682	2	54	94	17	1	29	32	27
ENSECAG00000012055	6.490504438	0.3329131	0.6882682	1612	1400	1613	1708	1708	1853	1992	1486
ENSECAG00000009143	5.382775273	0.3329548	0.6882682	902	781	1034	844	509	615	818	763
ENSECAG00000007556	8.089525793	0.3330206	0.6882789	4090	4751	4955	4356	3563	9863	3734	4198
ENSECAG00000020888	8.648484061	0.3332438	0.6886149	9062	6441	5316	6770	8068	6697	7902	9418
ENSECAG00000017531	7.582271735	0.3333645	0.6887367	3630	3188	3536	2787	3344	4252	4766	2901
ENSECAG00000017868	4.744613169	0.3334844	0.6887367	437	354	354	738	461	504	759	431
ENSECAG00000019908	5.919439115	0.3334846	0.6887367	1237	1133	1366	1458	872	1128	850	999
ENSECAG00000017794	8.441385878	0.3337422	0.6891434	6837	8502	2653	3491	3681	14216	2723	7296
ENSECAG00000009999	4.996184313	0.3339184	0.6893031	497	610	432	568	231	1153	554	597
ENSECAG00000011982	2.445705975	0.3339549	0.6893031	83	75	106	98	100	112	135	88
ENSECAG00000018269	7.567074021	0.3340016	0.6893031	2766	3889	2829	3586	3891	3652	4328	3206
ENSECAG00000016667	6.077064378	0.3341152	0.6894122	1088	1963	1594	1416	187	758	839	2281
ENSECAG00000020618	4.263925077	0.3347406	0.6902549	314	418	549	367	237	305	322	348
ENSECAG00000011782	10.17797	0.3347469	0.6902549	20965	17417	18707	21687	31214	29244	15915	14666
ENSECAG00000013359	4.801395875	0.3347776	0.6902549	528	417	417	534	968	310	587	385
ENSECAG00000026874	6.325818242	0.3347812	0.6902549	2010	1156	1895	1888	1737	1036	1166	1174
ENSECAG00000019509	8.171715842	0.3349284	0.6902549	4722	3934	4284	7354	5386	6153	5956	5332
ENSECAG00000021704	5.027943855	0.3349295	0.6902549	474	588	574	614	485	818	566	681
ENSECAG00000022348	0.211467181	0.3349489	0.6902549	9	11	34	13	21	14	32	24
ENSECAG00000013348	1.807009179	0.3351951	0.6904659	51	90	45	28	53	60	95	79
ENSECAG00000016738	4.357269942	0.3352198	0.6904659	539	312	272	208	765	283	404	219
ENSECAG00000018276	6.127581893	0.3352337	0.6904659	1489	1286	1478	1727	964	1007	1494	1116
ENSECAG00000004931	2.636921333	0.3353406	0.6905608	172	61	204	116	106	55	145	73
ENSECAG00000008411	4.151635243	0.3355789	0.6909264	140	443	243	307	138	652	280	358
ENSECAG00000012620	5.816714369	0.3358857	0.69142	1409	796	1319	1348	1053	609	1166	842
ENSECAG00000022727	4.637188806	0.3359404	0.69142	534	417	595	611	475	302	528	292
ENSECAG00000024198	3.38942952	0.3360756	0.6915729	255	143	212	326	187	76	264	134
ENSECAG00000009080	1.835597416	0.3364376	0.6917381	96	48	81	91	72	17	57	66
ENSECAG00000018783	2.702857552	0.336465	0.6917381	96	84	127	132	102	133	159	126
ENSECAG00000006710	5.669878998	0.3364709	0.6917381	1092	830	829	796	1134	901	1033	925
ENSECAG00000024126	6.532380451	0.336522	0.6917381	2143	1496	1785	2603	1421	945	1956	1751
ENSECAG00000024402	6.492899305	0.3365935	0.6917381	1973	1683	1558	2579	1049	1256	1638	1903
ENSECAG00000017976	4.313508107	0.3366231	0.6917381	375	453	503	396	162	215	342	537
ENSECAG00000017787	4.090179163	0.336641	0.6917381	395	256	274	228	398	242	443	291
ENSECAG00000005359	3.505997242	0.3366623	0.6917381	192	136	224	215	218	322	160	188
ENSECAG00000013909	5.565419542	0.3367039	0.6917381	970	674	712	978	871	609	1287	1035
ENSECAG00000015801	6.320620336	0.3368712	0.6918762	1668	1435	1666	2083	1249	1081	1430	1452
ENSECAG00000010701	5.718220969	0.336893	0.6918762	1161	886	1277	1180	1012	689	932	777
ENSECAG00000008366	6.216417316	0.3371438	0.691885	1168	1268	1224	1528	1025	1949	1525	1314
ENSECAG00000023066	3.722140391	0.337169	0.691885	204	195	210	288	276	118	413	285

ENSECAG00000023651	2.643543134	0.3372031	0.691885	97	163	53	58	40	106	76	307
ENSECAG00000009636	7.520798279	0.3372068	0.691885	3824	2569	3322	2882	5190	3273	3728	2413
ENSECAG00000011202	2.430634953	0.3372668	0.691885	75	36	76	157	260	53	101	36
ENSECAG00000012464	6.62641264	0.3373071	0.691885	2565	1426	2070	2520	1573	970	1914	1982
ENSECAG00000000612	1.92952124	0.3373258	0.691885	67	33	57	93	109	69	84	43
ENSECAG00000015824	0.689883861	0.3373845	0.691885	26	29	49	32	12	23	36	21
ENSECAG00000006312	3.33032479	0.3376544	0.6923135	141	182	190	152	180	280	131	197
ENSECAG00000019246	7.164106387	0.3379283	0.69275	2704	3571	2944	3040	2125	1898	2263	2912
ENSECAG00000022392	5.024886555	0.3380959	0.6929685	538	781	521	987	369	572	623	505
ENSECAG00000005548	5.045589832	0.3382941	0.6930751	603	597	723	964	276	568	694	573
ENSECAG00000012829	8.637919821	0.3383529	0.6930751	8267	7758	8040	10478	6439	5266	7483	6475
ENSECAG00000016539	2.662104813	0.3383566	0.6930751	143	111	123	175	87	79	140	89
ENSECAG00000008522	6.261006598	0.338392	0.6930751	1516	1546	1475	2033	1274	1173	1465	1064
ENSECAG00000020168	6.901255763	0.3385789	0.6932535	2441	2281	2549	3117	1357	1261	2357	2794
ENSECAG00000005769	4.622769042	0.3387837	0.6932535	363	532	349	429	357	466	453	685
ENSECAG00000005917	3.624500309	0.3388238	0.6932535	297	239	260	267	167	156	190	264
ENSECAG00000019067	6.30058086	0.3388703	0.6932535	1272	1790	1555	2157	976	1195	1317	1598
ENSECAG00000009782	2.547404157	0.3388832	0.6932535	98	94	102	92	91	135	144	96
ENSECAG00000014769	6.945398619	0.3389581	0.6932535	2212	1968	2133	2215	2853	2309	3254	1425
ENSECAG00000005856	7.631862609	0.3389701	0.6932535	3447	3246	4604	6294	2252	1794	4812	4048
ENSECAG00000006658	2.989693942	0.3389764	0.6932535	81	115	90	240	81	243	230	95
ENSECAG00000011640	4.993466388	0.3390737	0.6932535	651	646	1059	375	528	479	445	532
ENSECAG00000020570	6.471997968	0.3391743	0.6932535	1635	1051	1572	2058	1965	1349	2203	1534
ENSECAG00000006824	2.028008582	0.3391971	0.6932535	76	83	67	133	34	70	87	54
ENSECAG00000013905	2.137421409	0.339246	0.6932535	57	108	45	68	50	107	93	104
ENSECAG00000019250	2.703181519	0.3392749	0.6932535	76	121	89	146	108	141	193	85
ENSECAG00000016305	7.186906591	0.3393335	0.6932535	3159	2724	2904	3804	1902	2024	2747	2766
ENSECAG00000021253	3.444749062	0.3397746	0.6939621	277	186	266	213	170	160	235	118
ENSECAG00000009174	2.369471246	0.3398026	0.6939621	109	81	108	157	57	90	105	62
ENSECAG00000023674	6.789297227	0.3399654	0.69417	1912	1714	2059	2061	2321	2302	2147	1880
ENSECAG00000023672	4.414158265	0.3402696	0.6946661	451	492	537	374	245	132	385	590
ENSECAG00000019217	6.801581723	0.3403993	0.6948061	1765	1678	1906	2494	1811	2634	2016	2256
ENSECAG00000016491	8.435721874	0.3405915	0.6950735	4538	6852	5475	6091	3586	11901	4173	7691
ENSECAG00000009327	8.940521571	0.340707	0.6951843	8147	8736	8047	9072	10213	9521	9210	9876
ENSECAG00000021261	7.079169419	0.3409039	0.695461	2476	2547	2031	2286	1987	2194	3414	3271
ENSECAG00000000682	5.320379911	0.341068	0.6956542	823	651	933	1042	547	537	655	842
ENSECAG00000019452	6.024253282	0.3411246	0.6956542	1288	1392	1297	1569	862	1111	1121	1110
ENSECAG00000016038	2.375586949	0.3411823	0.6956542	110	129	83	136	29	95	64	116
ENSECAG00000013298	3.797861478	0.3415553	0.6962897	344	230	332	314	297	235	209	106
ENSECAG00000018811	6.569212863	0.341807	0.6966778	1899	1060	1900	1712	3065	1344	2035	1143
ENSECAG00000004618	1.117467055	0.3419363	0.6967675	35	35	26	30	22	75	5	70
ENSECAG00000023239	7.06204387	0.3420252	0.6967675	2455	1886	2212	2881	2524	2346	2848	2842
ENSECAG00000013468	2.622325194	0.3421017	0.6967675	107	103	75	123	92	124	97	177
ENSECAG00000017001	5.148184264	0.3421661	0.6967675	700	672	739	940	649	391	636	624
ENSECAG00000020672	7.584148319	0.3421926	0.6967675	3504	3806	4164	5165	2737	3356	3660	2510
ENSECAG00000009537	3.586657354	0.3422352	0.6967675	286	194	333	225	232	139	206	177
ENSECAG00000020600	5.720792317	0.3423284	0.6967675	1031	864	975	791	1256	794	1359	786
ENSECAG00000013270	4.238752203	0.3424119	0.6967675	506	225	463	467	225	184	485	324
ENSECAG00000022243	1.45744038	0.3425662	0.6967675	50	23	51	54	60	42	76	42
ENSECAG00000023969	1.168231389	0.3425911	0.6967675	37	20	52	33	52	45	54	26
ENSECAG00000008895	5.476691084	0.3426287	0.6967675	759	563	668	1166	1010	968	738	749
ENSECAG00000024292	6.422181877	0.3426999	0.6967675	1432	1422	1614	1579	1501	1552	1994	1698
ENSECAG00000013040	5.092406197	0.3427	0.6967675	839	397	1023	726	529	327	865	489
ENSECAG00000002847	3.659565655	0.3427098	0.6967675	284	199	279	351	290	113	162	219
ENSECAG00000024522	4.699293422	0.3428532	0.6969343	692	364	649	546	479	262	535	396
ENSECAG00000007794	6.033601783	0.3430893	0.6972189	1168	996	1182	1241	1194	1645	1053	1172
ENSECAG00000004117	0.231606952	0.343146	0.6972189	18	10	18	25	20	23	24	22
ENSECAG00000012539	4.280042742	0.3431773	0.6972189	358	268	446	244	307	239	525	516
ENSECAG00000004270	4.100575762	0.3433779	0.6975017	503	200	421	390	173	192	496	240
ENSECAG00000024460	4.323402084	0.3435137	0.6976527	441	314	430	552	275	282	409	322
ENSECAG00000011211	3.52845317	0.3436148	0.6977333	227	242	288	255	106	84	213	322
ENSECAG00000008021	2.906917243	0.3439389	0.6981685	197	84	219	164.001	124.001	90.0013	180	68.0004
ENSECAG00000022780	1.93229385	0.3439878	0.6981685	33	157	57	85	35	37	58	92
ENSECAG00000019657	4.134822235	0.3440135	0.6981685	276	282	324	327	383	372	390	239
ENSECAG00000014982	2.958809734	0.3441659	0.6983531	151	99	123	148	96	133	150	248
ENSECAG00000023795	1.532447883	0.3443553	0.6984521	42	57	51	30	38	47	51	96
ENSECAG00000005014	8.311682101	0.3443802	0.6984521	6395	6543	6380	8133	5061	4843	6084	4427
ENSECAG00000016872	2.566764725	0.3444451	0.6984521	96	66	111	126	135	125	109	98
ENSECAG00000011836	4.61321121	0.3446068	0.6984521	542	421	532	615	358	292	435	495
ENSECAG00000015836	5.112214109	0.3446093	0.6984521	756	598	775	835	589	452	661	544
ENSECAG00000005422	5.819552534	0.3446431	0.6984521	1099	1103	1061	1604	796	626	1390	918
ENSECAG00000019283	5.805817382	0.3446451	0.6984521	847	959	1012	1113	1273	1128	1122	835
ENSECAG00000023837	6.103970844	0.3448063	0.6986541	1095	1461	1070	1056	1100	2007	1135	1101
ENSECAG00000014389	2.937616741	0.3449231	0.698766	166	153	168	177	78	85	160	159
ENSECAG00000011306	1.372522495	0.3450566	0.6989119	52	39	57	78	45	30	58	20
ENSECAG00000006898	0.636741938	0.3451517	0.6989498	21	40	13	17	38	24	30	30
ENSECAG00000003122	5.03553123	0.3451984	0.6989498	710	625	631	846	485	444	680	527
ENSECAG00000022100	4.933007225	0.3453535	0.6991393	641	580	684	711	478	334	618	562

ENSECAG00000010407	5.198146681	0.3454881	0.6991663	576	568	650	736	1060	397	1073	456
ENSECAG00000014665	4.450728957	0.34549	0.6991663	482	275	519	651	322	210	541	344
ENSECAG00000015605	4.081450822	0.3456162	0.6992171	311	349	347	464	203	203	257	411
ENSECAG00000019793	6.098970767	0.3456382	0.6992171	1138	1019	1665	921	1369	903	1494	1712
ENSECAG00000020637	9.532547755	0.3458067	0.6994336	12814	11287	12813	14706	16488	14547	15695	11857
ENSECAG00000008614	5.079835553	0.3459004	0.6994983	376.004	633.005	512	820	413	845.003	717.004	701.003
ENSECAG00000003981	7.852143659	0.3460791	0.6997352	4091	1723	4381	5265	8850	1438	6976	2371
ENSECAG00000014572	4.855586678	0.3461711	0.6997415	547	542	382	521	374	554	690	693
ENSECAG00000009659	5.674241566	0.3462083	0.6997415	950	1097	1051	1251	649	694	1012	989
ENSECAG00000014289	5.030973644	0.346267	0.6997415	657	581	582	428	634	571	690	688
ENSECAG00000014039	4.250013781	0.346458	0.6999917	412	309	473	454	365	181	388	290
ENSECAG00000009313	3.268495125	0.3465141	0.6999917	174	85	115	248	348	40	364	87
ENSECAG00000015048	0.513618849	0.3466733	0.7001889	23	21	23	17	23	9	60	25
ENSECAG00000001440	1.966081475	0.3468103	0.7002184	56	41	76	83	98	98	52	57
ENSECAG00000000081	0.19666981	0.3468112	0.7002184	7	30	20	43	3	4	20	33
ENSECAG00000016881	5.940532583	0.3470061	0.7004874	1258	809	1021	2488	1121	577	1754	491
ENSECAG00000012333	6.114729232	0.3470771	0.7005062	1268	959	1443	1178	1748	1269	1484	940
ENSECAG00000009340	4.077549254	0.3471661	0.7005613	389	327	357	377	239	287	235	300
ENSECAG00000014498	6.833293343	0.3476673	0.701448	2164	1450	1341	3071	2319	1289	3158	2538
ENSECAG00000026922	3.080925999	0.3477598	0.7015101	128	95	171	183	136	193	191	152
ENSECAG00000008354	0.273540251	0.3480092	0.7018354	19	8	14	32	11	25	33	25
ENSECAG00000011626	7.763289392	0.3480446	0.7018354	3277	2073	3442	5975	7729	2390	6565	1509
ENSECAG00000011819	7.099953861	0.3481611	0.7019458	2384	2327	2206	2689	2143	2584	2940	3181
ENSECAG00000022077	6.861638447	0.3484411	0.7023856	1965	2458	1550	2049	2465	2276	2347	2074
ENSECAG00000012988	4.62778175	0.3485528	0.7024861	591	434	447	663	413	294	509	385
ENSECAG00000009841	5.616293802	0.3488174	0.7027083	1118	887	1053	1138	1041	606	818	704
ENSECAG00000017079	3.132378705	0.3488235	0.7027083	153	107	124	218	143	180	214	163
ENSECAG00000009487	6.285208296	0.3488975	0.7027083	1686	1288	1802	1923	1089	1253	1118	1557
ENSECAG00000022620	6.101154882	0.3489106	0.7027083	1462	1206	1483	1713	1146	873	1246	1229
ENSECAG00000019390	2.104480448	0.3490234	0.7027265	80	24	83	87	151	56	124	27
ENSECAG00000019832	3.89442232	0.3490433	0.7027265	396	147	268	194	295	247	408	256
ENSECAG00000026888	2.823869995	0.3492991	0.7031169	148	139	126	206	83	95	100	158
ENSECAG00000023153	5.190477223	0.3493776	0.7031503	726	646	793	975	558	612	557	612
ENSECAG00000009577	1.343785028	0.3496658	0.7036056	49	25	44	45	62	31	66	44
ENSECAG00000000544	1.351044385	0.3499054	0.7037018	30	55	42	27	16	70	45	73
ENSECAG00000017501	2.83463175	0.3499411	0.7037018	85	102	120	174	112	200	123	125
ENSECAG00000008069	3.723804304	0.349949	0.7037018	241	227	311	370	200	214	228	189
ENSECAG00000014049	5.615378654	0.3499614	0.7037018	1121	783	1168	1131	976	704	808	667
ENSECAG00000024933	3.96637393	0.3500588	0.7037611	319	261	251	229	425	238	316	263
ENSECAG00000010719	10.34042172	0.3501148	0.7037611	24398	15892	27444	21806	31354	26397	19587	24762
ENSECAG00000023913	4.585641272	0.3501968	0.7038015	452	451	485	708	255	304	378	595
ENSECAG00000018190	7.853892206	0.3504328	0.7041511	4377	2118	4117	5124	7432	3020	7059	1690
ENSECAG00000000413	6.499600372	0.3505722	0.7041995	1916	1420	1862	2632	1412	1008	1893	1638
ENSECAG00000011855	4.046267245	0.3505809	0.7041995	311	315	295	517	246	261	264	273
ENSECAG00000011705	5.352914815	0.3508462	0.70453	691	592	793	802	724	935	788	728
ENSECAG00000023277	2.858489763	0.3509947	0.70453	118	136	168	218	55	99	135	159
ENSECAG00000008378	3.805823338	0.3510088	0.70453	284	304	346	276	209	93	357	241
ENSECAG00000023794	2.702082424	0.3510249	0.70453	122.999	64	130	111	48	134	212	144
ENSECAG00000024703	5.753031869	0.3510555	0.70453	752	973	710	1302	581	993	940	1799
ENSECAG00000017901	6.630886392	0.3516277	0.7054502	1865	1662	1726	1689	1622	1426	2619	2255
ENSECAG00000000424	1.392899406	0.3516383	0.7054502	41	34	41	55	63	40	53	50
ENSECAG00000011894	4.206543894	0.3518547	0.7057597	362	356	421	455	242	182	356	412
ENSECAG00000022050	6.65447166	0.3520698	0.705876	2933	1624	2671	1483	1297	761	2242	2243
ENSECAG00000008781	4.999182517	0.3520706	0.705876	569	557	708	938	435	516	483	609
ENSECAG00000006366	7.489318332	0.3520991	0.705876	2786	2627	3260	3903	2538	4434	3316	3812
ENSECAG00000019967	4.414018469	0.3522571	0.7059617	400	411.001	415	620	264	378	362	350
ENSECAG00000018388	1.027602569	0.3523304	0.7059617	34	46	45	52	7	36	20	50
ENSECAG00000015268	4.334058417	0.3523867	0.7059617	299	283	334	494	315	268	502	542
ENSECAG00000007554	3.498895723	0.3523904	0.7059617	208	217	187	381	144	167	254	150
ENSECAG00000019918	5.140867996	0.3527249	0.7064126	524	671	611	616	483	991	613	659
ENSECAG00000005108	3.442509245	0.3528013	0.7064126	174	141	207	227	247	196	243	175
ENSECAG00000008167	6.06358068	0.3528021	0.7064126	1443	925	1110	1188	1538	643	1617	1592
ENSECAG00000017474	3.562555571	0.3529283	0.7065034	148	220	144	299	221	220	261	239
ENSECAG00000019688	2.719496003	0.3529934	0.7065034	103	97	94	150	143	158	123	93
ENSECAG00000004900	5.570531808	0.3530571	0.7065034	1217	725	1251	923	1000	490	1138	439
ENSECAG00000024268	10.49086957	0.3530962	0.7065034	26476	19481	26529	27708	32042	31211	24082	25573
ENSECAG00000018311	3.509190201	0.3532729	0.706664	196	192	317	291	194	95	278	160
ENSECAG00000023524	6.862578871	0.3533009	0.706664	2764	2008	2469	2793	2251	1816	1831	1527
ENSECAG00000009738	11.67607273	0.3535752	0.7070882	61697	43543	61814	60609	69145	71111	55403	61340
ENSECAG00000022837	2.961187977	0.3536485	0.7071103	157	155	183	177	87	95	153	155
ENSECAG00000003597	2.609235311	0.3537666	0.7072218	130	75	130	211	93	77	151	55
ENSECAG00000008137	2.360887301	0.3541009	0.7077656	105	58	76	90	136	43	217	42
ENSECAG00000021489	4.304042996	0.3545693	0.7084899	311	373	338	328	240	361	442	535
ENSECAG00000014139	4.26740449	0.3546637	0.7084899	432	264	514	459	363	266	308	284
ENSECAG00000012435	3.644003341	0.3546779	0.7084899	259	136	254	206	269	154	315	272
ENSECAG00000024839	4.498324549	0.3548019	0.7084899	606	247	559	581	322	184	587	372
ENSECAG00000014621	5.781872524	0.3548749	0.7084899	921	747	1100	1144	1188	1019	1201	889
ENSECAG00000024040	6.088626786	0.3548813	0.7084899	1488	986	1808	1565	1053	774	1512	1140

ENSECAG00000023151	5.088882863	0.3548998	0.7084899	717	635	719	836	521	524	633	527
ENSECAG00000019180	7.199789997	0.3549652	0.7084959	3449	2495	3229	3536	2431	1668	3575	1950
ENSECAG00000019888	4.452253161	0.3550969	0.7085657	443	342	480	633	366	317	397	324
ENSECAG00000009724	4.679778819	0.3551669	0.7085657	527	338	446	475	636	349	485	558
ENSECAG00000011281	4.121274513	0.3552401	0.7085657	290	361	178	362	380	317	365	317
ENSECAG00000015193	7.488943426	0.3552497	0.7085657	3894	3265	3379	5049	3091	2085	3949	2588
ENSECAG00000005938	4.092538315	0.3554657	0.7088721	292.001	261	295.001	337	362.001	348.001	358	269.024
ENSECAG00000024965	8.097312103	0.3558224	0.7093335	5130	4458	4692	4818	6156	4315	6043	5220
ENSECAG00000002088	5.900707081	0.3558258	0.7093335	1262	989	1441	1430	915	911	807	1208
ENSECAG00000002871	4.501637479	0.3559593	0.7093335	351	322	351	545	267	677	465	368
ENSECAG00000017479	6.565297712	0.3559755	0.7093335	1577	2150	1782	2580	1331	1401	1808	1639
ENSECAG00000023793	5.62481933	0.3561162	0.7093335	989	873	1144	1216	723	537	1032	961
ENSECAG00000013421	4.661861549	0.356154	0.7093335	515	461	599	613	361	519	296	398
ENSECAG00000005717	4.795947297	0.3561714	0.7093335	562	459	423	489	528	447	564	654
ENSECAG00000016681	7.181553257	0.3561967	0.7093335	2879	2715	2744	4225	2401	1882	2828	2328
ENSECAG00000012199	5.349486761	0.356704	0.7102192	326	778	610	815	100	1468	232	1570
ENSECAG00000008516	4.618134522	0.3569738	0.7105806	482	412	368	439	613	445	474	393
ENSECAG00000018257	5.442473061	0.3570106	0.7105806	727	748	693	894	643	913	863	977
ENSECAG00000022649	2.570267301	0.3574624	0.7112338	115	96	62	122	170	91	105	106
ENSECAG00000023756	3.423224946	0.3575626	0.7112338	211	217	199	300	176	141	184	177
ENSECAG00000017508	3.830542981	0.3575751	0.7112338	303	215	347	379	231	137	369	180
ENSECAG00000024656	2.193247002	0.3576553	0.7112338	131	52	122	101	93	33	115	36
ENSECAG00000015423	5.408583079	0.3576839	0.7112338	832	616	711	834	780	586	861	1135
ENSECAG00000024945	4.890742426	0.3577712	0.7112338	417	579	527	535	609	582	589	542
ENSECAG00000000160	5.948968274	0.3577771	0.7112338	1328	1144	1331	1460	1100	959	884	1039
ENSECAG00000024246	6.82695346	0.3578678	0.7112897	2528	2054.99	2562	2591.99	1838	1318	2557	1736
ENSECAG00000016621	6.146204485	0.3579972	0.7114224	1255	1147	1290	1329	1311	1325	1535	1353
ENSECAG00000009214	6.010660704	0.3580659	0.7114344	1251	959	949	1408	972	1497	1321	1231
ENSECAG00000023598	4.747104062	0.3582148	0.7116059	477	530	567	770	363	192	668	533
ENSECAG00000015542	6.679361005	0.3585287	0.7119984	1847	1637	1832	1926	2031	1887	2357	1762
ENSECAG00000021193	6.394000222	0.358578	0.7119984	1656	1404	1288	1538	1511	2110	1425	1451
ENSECAG00000020381	3.232199172	0.3586005	0.7119984	162	147	117	216	145	213	188	196
ENSECAG00000016319	5.851091205	0.3587645	0.712044	1152	1111	1225	1434	882	658	1322	960
ENSECAG00000014047	7.641884217	0.3588129	0.712044	4067	3671	4386	5071	3229	2750	3522	3434
ENSECAG00000011275	5.883492299	0.3588162	0.712044	1033	1026	1018	1074	1409	863	1382	999
ENSECAG00000018725	2.824713743	0.3589408	0.712044	110	98	161	106	149	151	130	127
ENSECAG00000019654	3.230711078	0.358949	0.712044	200	189	190	233	182	147	116	132
ENSECAG00000003793	2.458530863	0.3591343	0.712044	69	56	108	118	200	44	192	31
ENSECAG00000009713	5.444289237	0.3591433	0.712044	976	628	964	159	1558	599	1267	259
ENSECAG00000017686	1.330260572	0.3591502	0.712044	32	68	32	13	41	44	17	102
ENSECAG00000024417	4.985696399	0.3591876	0.712044	634	734	702	639	381	372	457	819
ENSECAG00000001463	1.650470101	0.3595788	0.7122572	38	36	70	62	63	70	59	53
ENSECAG00000006457	1.712056696	0.3596231	0.7122572	41	43	58	73	54	88	69	45
ENSECAG00000012836	3.170036409	0.3596892	0.7122572	153	119	160	183	221	117	219	165
ENSECAG00000007191	3.152582461	0.359737	0.7122572	193	150	164	273	126	99	135	196
ENSECAG00000000009	1.341221741	0.3597551	0.7122572	37	9	38	80	30	52	61	62
ENSECAG00000015682	4.766752826	0.3598065	0.7122572	488	533	496	849	342	365	440	600
ENSECAG00000017946	8.233149882	0.3598366	0.7122572	5353	6037	6852	7849	5182	3049	7164	4264
ENSECAG00000014340	3.387282249	0.3598479	0.7122572	218	191	248	257.994	129.003	77.9924	305	161.998
ENSECAG00000022060	3.705686054	0.3599318	0.7122572	331	199	281	332	252	97	309	176
ENSECAG00000014475	5.544547005	0.359953	0.7122572	964	467	871	954	1235	754	1308	454
ENSECAG00000019676	5.531246313	0.3599849	0.7122572	813	645	840	1003	871	883	985	862
ENSECAG00000017228	8.547768485	0.3601058	0.7123723	7879	5610	6080	6413	9415	5590	8359	6469
ENSECAG00000022109	7.179410474	0.3607445	0.7134385	3226	2561	3151	3511	2112	1909	3093	2374
ENSECAG00000009195	5.392622579	0.3607704	0.7134385	1444	416	534	155	1021	274	2308	241
ENSECAG00000021875	5.155072133	0.3609355	0.7136409	839	518	516	620	689	668	842	608
ENSECAG00000024418	5.037667488	0.3610299	0.7137032	933	495	738	675	677	268	706	475
ENSECAG00000023818	6.136977429	0.3613034	0.7140517	1315	1030	1545	1020	1094	1002	1668	1854
ENSECAG00000020816	2.106714062	0.3613319	0.7140517	85	68	66	60	121	47	75	102
ENSECAG00000005719	1.852883514	0.3616767	0.7146088	27	50	88	64	35	102	113	44
ENSECAG00000004595	4.542984789	0.3617768	0.7146574	331	300	541	406	773	391	520	187
ENSECAG00000004523	6.920701553	0.3618272	0.7146574	2871	1722	2599	3460	2570	1315	2771	1156
ENSECAG00000019698	6.118661945	0.3619299	0.7147361	1038	1087	1291	1529	1317	1348	1683	1095
ENSECAG00000020885	9.476135338	0.3622338	0.7152118	13818	10950	11609	13473	14084	13235	15241	13730
ENSECAG00000017922	8.366006204	0.3623026	0.7152218	5569	3959	6363	6632	3897	11910	5433	4596
ENSECAG00000021781	7.085332994	0.3623648	0.7152218	2776	2300	3226	3391	2466	1956	2451	1904
ENSECAG00000020924	4.777975711	0.362571	0.7155045	319	594	351	619	355	648	708	477
ENSECAG00000011336	3.950496644	0.3626866	0.7155586	387	289	331	317	280	174	312	226
ENSECAG00000019768	5.879729125	0.3627693	0.7155586	1087	536	1117	1455	1072	922	1784	961
ENSECAG00000018525	4.027472637	0.3627874	0.7155586	349	293	351	411	302	229	300	209
ENSECAG00000016824	4.743417418	0.3629069	0.71567	374	386	776	250	692	543	351	533
ENSECAG00000000332	6.163900651	0.3629806	0.7156912	1289	1117	1279	1385	1518	1542	1212	1260
ENSECAG00000022676	4.698772044	0.3634265	0.7162869	877	411	261	78	516	613	640	386
ENSECAG00000008923	7.381839448	0.3634499	0.7162869	198	7213	309	341	798	2261	357	12328
ENSECAG00000024673	4.775483403	0.3634731	0.7162869	593	481	666	631	635	297	548	270
ENSECAG00000008831	6.073048514	0.363535	0.7162869	1226	999	1306	1187	1911	877	1447	1085
ENSECAG00000006861	4.834672195	0.3638513	0.7167858	616	507	691	626	342	418	615	481
ENSECAG00000009982	6.8517764	0.3639464	0.7168489	2048	1888	2050	2040	2902	2412	2287	1442

ENSECAG00000014794	4.619026489	0.3641343	0.7170277	342	419	440	502	412	663	436	396
ENSECAG00000003887	6.957643061	0.3642115	0.7170277	1956	1918	2178	2706	1865	3025	2469	2344
ENSECAG00000022442	8.498676775	0.3642266	0.7170277	8362	6620	9211	6918	5003	6087	4953	6871
ENSECAG00000002380	5.647584027	0.3646665	0.717747	966	712	791	1013	1649	677	1117	548
ENSECAG00000023155	5.160983774	0.3647183	0.717747	611	509	657	741	858	410	934	660
ENSECAG00000000738	3.68471438	0.3649392	0.717879	287	221	293	305	176	175	272	202
ENSECAG00000000785	2.286889094	0.3649481	0.717879	102	52	74	92	147	37	132	85
ENSECAG00000024373	6.74586502	0.364975	0.717879	2422	1347	1878	1866	2265	1315	3204	1894
ENSECAG00000016094	2.406366781	0.3650766	0.7179545	117	85	112	150	47	101	97	77
ENSECAG00000023448	5.401198197	0.3651966	0.7180661	1302	225	1456	873	378	460	1490	340
ENSECAG00000023124	4.828401266	0.3652826	0.718077	529	306	634	481	857	368	751	321
ENSECAG00000007170	5.851974069	0.3653286	0.718077	645	1083	1149	1093	531	860	1698	1638
ENSECAG00000016651	5.706013024	0.365605	0.7182713	1023	771	813	1112	1113	752	1224	1020
ENSECAG00000015212	0.903005363	0.3656566	0.7182713	21	49	15	22	25	41	8	75
ENSECAG00000013825	3.59651284	0.3657263	0.7182713	112	411	108	94	91	300	150	464
ENSECAG00000025158	3.002909991	0.3657302	0.7182713	154	81	163	146	171	152	209	109
ENSECAG00000011994	9.891815768	0.3657436	0.7182713	20468	18451	24247	18393	16124	10742	20076	14775
ENSECAG00000016788	4.742774211	0.3658315	0.7183198	413	407	447	595	298	708	494	597
ENSECAG00000011735	1.074735916	0.3659574	0.7184427	13	63	10	35	16	30	35	94
ENSECAG00000010754	5.783736429	0.3660387	0.7184781	1031	892	891	1101	1089	1050	1118	1019
ENSECAG00000016276	6.786156459	0.3661081	0.7184903	2239	1455	1739	2412	2331	1724	2594	2078
ENSECAG00000005881	1.201251319	0.3662873	0.7186221	33	33	30	53	60	32	41	46
ENSECAG00000019087	5.278679086	0.3663018	0.7186221	542	891	500	748	624	866	793	760
ENSECAG00000019249	3.287891811	0.36637	0.7186318	99	264	105	148	355	219	120	87
ENSECAG00000000881	1.231053961	0.3667335	0.7192207	46	39	35	26	54	43	69	21
ENSECAG00000012193	4.452521093	0.3668873	0.7192425	480	271	537	619	347	318	421	319
ENSECAG00000023620	4.331818196	0.366897	0.7192425	315	326	377	370	225	329	489	576
ENSECAG00000019377	5.240380735	0.3669608	0.7192425	769	652	632	547	1023	693	809.001	449
ENSECAG00000010075	5.756400639	0.3670137	0.7192425	1179	923	1147	1365	788	666	1002	1100
ENSECAG00000021270	4.545147121	0.3670612	0.7192425	410	422	422	325	278	488	344	739
ENSECAG00000022522	3.925457926	0.3672142	0.7194181	324	196	294	216	419	304	294	182
ENSECAG00000004969	3.483628491	0.3673377	0.7195359	220	240	265	288	47	47	189	400
ENSECAG00000012976	6.122572317	0.3674301	0.7195929	1154	1005	1451	1333	1503	1324	1517	1087
ENSECAG00000012438	4.209617685	0.3675652	0.7196995	352	334	440	467	292	186	423	305
ENSECAG00000016535	3.562853067	0.3676113	0.7196995	259	294	318	167	75	302	76	226
ENSECAG00000018242	8.122021108	0.3679158	0.7200749	6918	4362	6070	6734	4892	4285	4919	3792
ENSECAG00000024789	5.779362345	0.3679766	0.7200749	1104	925	1330	1358	1090	811	1090	562
ENSECAG00000002482	3.545604746	0.3680152	0.7200749	194	167	245	178	121	316	190	294
ENSECAG00000013197	7.167662053	0.3681005	0.7200749	2508	2243	2378	2992	1947	3104	3087	3178
ENSECAG00000018098	10.0412636	0.36812	0.7200749	19908	15817	17433	20643	24162	14962	25441	19657
ENSECAG00000019553	3.831349332	0.3684407	0.7205781	218	260	253	246	272	305	292	246
ENSECAG00000013171	5.684567053	0.3685845	0.7207259	929	829	849	1044	1119	875	1248	788
ENSECAG00000016195	3.867483843	0.3687279	0.7207259	259	210	304	225	220	231	282	431
ENSECAG00000008691	0.296379124	0.3687556	0.7207259	19	33	24	24	18	15	21	14
ENSECAG00000004558	5.758879505	0.3687701	0.7207259	1184	867	1157	1435	805	950	1049	711
ENSECAG00000013627	3.652319767	0.3689811	0.7210129	227	257	287	314	121	165	246	271
ENSECAG00000012864	3.025289668	0.3691064	0.7210129	221	78	180	253	110	56	234	114
ENSECAG00000014411	4.233479422	0.3691145	0.7210129	400	261	369	620	307	211	321	367
ENSECAG00000018629	5.02102533	0.3691708	0.7210129	711	469	787	848	718	340	561	471
ENSECAG00000011605	4.787956929	0.3692604	0.7210639	555	466	677	670	406	342	555	499
ENSECAG00000024883	6.689578826	0.3693912	0.7211954	1783	1762	1806	1917	1783	2199	1871	2165
ENSECAG00000007534	5.914859843	0.3694562	0.7211984	766	1223	936	1106	556	2204	622	1317
ENSECAG00000024929	1.441310917	0.3696319	0.7213761	43	48	69	73	43	29	59	33
ENSECAG00000023359	3.323405378	0.3698994	0.7213761	168	183	234	286	172	98	167	196
ENSECAG00000017914	1.180481967	0.3699674	0.7213761	38	37	25	43	26	29	44	82
ENSECAG00000009188	10.50958002	0.369979	0.7213761	30309	20817	25073	24960	27852	33453	22447	30232
ENSECAG00000021466	8.39698956	0.3700072	0.7213761	6950	7169	6963	7800	5142	4109	7214	5564
ENSECAG00000011168	7.788441696	0.3700281	0.7213761	3937.99	5231.62	3744.92	6096.39	3350.95	3210.58	4290.05	3461.37
ENSECAG00000009540	6.205296626	0.3701467	0.7213761	1369	1044	1373	1449	1607	1177	1948	1098
ENSECAG00000020598	7.868853518	0.3702126	0.7213761	3972	3669	3483	5253	2821	5455	4792	5389
ENSECAG00000009947	4.445716773	0.3702431	0.7213761	530	245	426	292	511	463	444	298
ENSECAG00000024474	5.378611236	0.370278	0.7213761	824	556	667	917	759	622	1081	835
ENSECAG00000010756	5.09976754	0.3702852	0.7213761	759	577	833	764	565	408	815	463
ENSECAG00000004386	0.500740496	0.3703094	0.7213761	24	31	42	20	12	28	25	13
ENSECAG00000006078	5.679680738	0.3705047	0.7214492	996.001	680	923.002	965	1726	616.002	1098	639.001
ENSECAG00000015288	6.288710315	0.3705129	0.7214492	1530	1138	1353	1554	1531	1238	1787	1575
ENSECAG00000012352	3.637906196	0.3705794	0.7214492	281	232	197	378	118	158	232	283
ENSECAG00000018424	5.964959846	0.3706511	0.7214492	1681	953	1232	1479	1179	761	1157	984
ENSECAG00000015480	3.523214476	0.3706645	0.7214492	221	171	312	294	174	125	220	215
ENSECAG00000018035	8.756301458	0.3708814	0.7217479	9910	10835	10017	6947	2315	10236	5572	8050
ENSECAG00000019312	6.594313805	0.3710014	0.7218287	1874	1421	1709	1879	1870	1536	2332	1858
ENSECAG00000005248	4.982659591	0.3712189	0.7218287	770	433	697	863	378	277	640	764
ENSECAG00000019988	3.226771077	0.3712796	0.7218287	182	162	156	324	143	148	188	106
ENSECAG00000007044	0.85942196	0.3712946	0.7218287	17	37	13	41	11	14	69	59
ENSECAG00000021459	5.849134595	0.3713309	0.7218287	1219	950	1257	1496	921	909	965	957
ENSECAG00000015810	2.897999923	0.3714866	0.7218287	160	143	203	137	97	117	76	163
ENSECAG00000015765	0.990920777	0.3715111	0.7218287	58	13	50	52	29	23	45	18
ENSECAG00000014345	5.132329698	0.3715265	0.7218287	655	595	657	515	900	719	554	548

ENSECAG00000004944	5.878847554	0.371541	0.7218287	1173	892	1169	1900	1158	702	1312	665
ENSECAG00000020660	10.20582037	0.3715584	0.7218287	18828	19442	19983	24274	22694	27777	20755	20994
ENSECAG00000013059	3.220616031	0.371636	0.7218559	159	205	171	284	59	159	182	178
ENSECAG00000011449	0.746766103	0.3721761	0.7225613	14	49	38	51	26	2	62	1
ENSECAG00000012769	6.239866964	0.3722728	0.7225613	1469	993	1388	1551	1358	1230	1768	1583
ENSECAG00000005633	3.769233036	0.3722851	0.7225613	227	251	169	290	313	283	230	239
ENSECAG00000008928	10.67666252	0.3723521	0.7225613	31219	22670	29207	31657	35100	33694	30873	28730
ENSECAG00000011403	6.664857224	0.3723593	0.7225613	1698	2613	2191	2170	1255	994	1601	2720
ENSECAG00000012427	2.313632755	0.3723808	0.7225613	96	68	95	185	46	46	112	104
ENSECAG00000005267	5.520555961	0.372499	0.7226671	800	759	809	1696	575	903	1040	401
ENSECAG00000010623	3.294082319	0.3726099	0.7226843	143	263	170	269	160	114	161	181
ENSECAG00000013460	2.068503227	0.3726351	0.7226843	56	53	92	74	141	55	64	72
ENSECAG00000022811	4.685088921	0.3727729	0.7228282	598	465	531	612	301	299	520	559
ENSECAG00000010605	4.732339785	0.3729561	0.7230599	509	596	587	573	284	400	440	584
ENSECAG00000023305	0.559824379	0.3732982	0.7235145	36	11	26	14	38	35	34	8
ENSECAG00000016069	3.49630608	0.373318	0.7235145	204	273	347	143	89	182	218	212
ENSECAG00000006335	0.972057874	0.3734292	0.7236067	34	25	35	30	52	36	26	36
ENSECAG00000018531	2.57658627	0.3735267	0.7236722	167	111	69	19	110	142	97	136
ENSECAG00000009708	6.354464119	0.3736198	0.7237292	2513	1096	1996	1398	1453	1155	1577	1102
ENSECAG00000003253	5.461285326	0.3736842	0.7237305	681	869	572	977	683	942	997	828
ENSECAG00000019816	4.282874671	0.3739045	0.7239284	476	457	469	243	291	333	287	310
ENSECAG00000016242	9.266592653	0.3739139	0.7239284	11321	11542	8312	10658	8890	19785	8737	10532
ENSECAG00000024852	5.532538514	0.3740218	0.724014	1008	1098	1106	798	255	667	569	1400
ENSECAG00000015581	7.846128596	0.374115	0.7240199	4978	3873	5386	5590	3407	4028	3573	3714
ENSECAG00000001164	3.782074601	0.3741524	0.7240199	199	343	153	227	295	193	332	287
ENSECAG00000011687	4.880357793	0.3742195	0.7240264	665	570	753	536	304	411	402	757
ENSECAG00000010589	6.510356204	0.3744292	0.7240856	1600	1191	1476	2280	1998	1282	2170	1750
ENSECAG00000026932	6.179778907	0.3745795	0.7240856	1418	840	1763	904	2077	1077	2156	590
ENSECAG00000007201	10.25133639	0.3746676	0.7240856	21374	17830	23854	22233	23853	24004	20993	26770
ENSECAG00000022847	5.515206813	0.3746709	0.7240856	1044	803	905	1193	945	396	1103	562
ENSECAG00000019624	7.990958617	0.3747209	0.7240856	4715	3832	5071	4106	6291	4133	5811	3924
ENSECAG00000019762	6.994742738	0.3747389	0.7240856	1833	2698	2509	1367	1078	3816	1793	3436
ENSECAG00000019363	7.553823508	0.374752	0.7240856	4106	3533	4001	4491	3480	2060	3930	2823
ENSECAG00000022524	5.611301577	0.3747927	0.7240856	810	761	980	870	836	1254	691	977
ENSECAG00000019699	7.848509847	0.3748238	0.7240856	4362	3707	4249	3725	5167	4770	4575	3533
ENSECAG00000001822	0.824572057	0.3749489	0.7242041	31	31	29	17	52	35	30	19
ENSECAG00000011046	0.664660803	0.3752341	0.7246318	29	12	42	56	35	9	31	14
ENSECAG00000005293	5.279578555	0.3753467	0.7246625	655	590	727	786	649	629	885	891
ENSECAG00000018727	2.062035815	0.3753833	0.7246625	113	69	96	79	56	58	60	80
ENSECAG00000016504	6.337238902	0.3754414	0.7246625	1750	1346	1729	2080	1366	1308	1500	1096
ENSECAG00000013142	4.240719214	0.3756064	0.7248579	393	299	529	403	331	190	381	322
ENSECAG00000017179	6.518826442	0.3758712	0.7250042	1726	1421	1606	1776	1776	1740	1833	1767
ENSECAG00000012723	6.451229196	0.3758755	0.7250042	1626	1526	1423	1623	1639	1282	2119	1859
ENSECAG00000021058	2.08889023	0.3759097	0.7250042	89	49	59	84	77	37	135	101
ENSECAG00000020978	10.5190018	0.3759376	0.7250042	28231	19850	25826	29108	31414	30502	25334	27548
ENSECAG00000021095	3.167446237	0.3763535	0.7255901	197	156	199	229	117	77	155	220
ENSECAG00000009221	4.245499954	0.3764759	0.7255901	411	251	262	388	597	246	330	328
ENSECAG00000014053	8.795571167	0.3765139	0.7255901	9335	7821	9278	12103	7949	4650	9232	7171
ENSECAG00000011186	0.774172487	0.3765532	0.7255901	41	22	34	53	20	2	48	29
ENSECAG00000017756	8.245656602	0.3765753	0.7255901	5070	5958	4222	5877	5171	6713	6101	5834
ENSECAG00000007744	1.715911345	0.3766246	0.7255901	32	20	40	125	32	75	105	58
ENSECAG00000008531	6.115103668	0.3767321	0.7255945	1390	1344	1537	1594	1109	1135	1155	1115
ENSECAG00000012824	3.466647702	0.3768175	0.7255945	166	179	231	400	166	118	208	210
ENSECAG00000010849	7.022324876	0.3768397	0.7255945	3151	1959	2992	3085	2492	1579	2428	1958
ENSECAG00000020358	8.923838824	0.3768824	0.7255945	7839	11514	16021	6474	8217	8136	4632	8832
ENSECAG00000017554	6.171298688	0.377082	0.7258556	1289	1019	1378	1433	1575	1542	1314	1132
ENSECAG00000007863	2.657266785	0.3772507	0.7260574	185	83	169	112	129	60	136	65
ENSECAG00000009308	1.972270581	0.3774282	0.7262747	54	55	58	93	102	36	108	72
ENSECAG00000012777	6.097242121	0.377504	0.7262747	1347	820	1123	1623	1248	972	1591	1602
ENSECAG00000017465	4.73418791	0.3776052	0.7262747	580	321	529	419	698	371	619	427
ENSECAG00000008346	6.393979437	0.3778379	0.7262747	1614	1175	1666	2960	1199	881.003	2407	1076
ENSECAG00000015373	3.606192743	0.3778381	0.7262747	287	113	244	180	260	121	401	224
ENSECAG00000016334	6.613522212	0.3778567	0.7262747	1902	1994	1827	2609	1507	1421	1892	1613
ENSECAG00000016084	3.528623679	0.3778624	0.7262747	224	210	219	351	163	177	147	235
ENSECAG00000001893	2.779052141	0.3778751	0.7262747	96	101	138	130	148	146	154	92
ENSECAG00000016955	7.12576475	0.3779672	0.7263287	2468	2336	2395	2635	2299	2932	2767	2905
ENSECAG00000006892	5.325687247	0.3780474	0.7263532	367	766	661	995	454	1007	916	797
ENSECAG00000025030	5.9698707	0.3781078	0.7263532	1080	1039	994	1342	1206	1087	1036	1539
ENSECAG00000017712	5.87323062	0.3782341	0.7263541	1209	620	1149	1157	1584	657	1478	970
ENSECAG00000009395	3.896544636	0.3782361	0.7263541	316	260	396	306	247	147	351	220
ENSECAG00000026943	3.607605623	0.378333	0.7264173	280	248	238	276	144	157	233	248
ENSECAG00000018129	4.337633432	0.3784461	0.7265116	444	387	356	552	278	222	436	384
ENSECAG00000020343	6.011435975	0.3785957	0.7266761	872	1210	1422	777	442	1823	887	1982
ENSECAG00000011531	5.977360645	0.3787064	0.7267657	989	1157	1078	1202	1232	1541	1120	958
ENSECAG00000018297	5.206358129	0.3790508	0.7273038	863	628	779	883	455	454	780	743
ENSECAG00000010867	6.591665756	0.3793538	0.7277621	1918	1560	1916	2955	1384	987	2275	1759
ENSECAG00000017568	1.314101996	0.3794805	0.7278824	42	48	39	26	34	40	43	80
ENSECAG00000021159	6.516772482	0.3797155	0.7280951	1744	1743	2090	2223	1312	1309	1349	1987

ENSECAG00000012927	6.304909423	0.3797304	0.7280951	1367	1804	1399	2223	713	1168	1579	1698
ENSECAG00000008583	6.857804405	0.3798379	0.7280951	2186	1905	2122	1929	2112	2185	2195	2572
ENSECAG00000002323	3.422799458	0.3798993	0.7280951	166	142	196	236	233	147	333	154
ENSECAG00000006987	5.243913629	0.379928	0.7280951	768	668	619	565	963	628	953	455
ENSECAG00000021833	5.077338843	0.379976	0.7280951	601	314	598	902	586	698	716	650
ENSECAG00000021136	10.90655081	0.3800638	0.7281404	35261	26892	36087	36058	38804	43171	30925	36775
ENSECAG00000022822	5.434170025	0.3801605	0.7282028	911	760	876	1159	831	575.003	912	507.001
ENSECAG00000000539	7.343994705	0.3803699	0.7283698	4379	2528	3466	3677	2783	1844	4154	1860
ENSECAG00000019325	4.162356486	0.3804154	0.7283698	368	260	365	587	190	179	309	469
ENSECAG00000012351	6.030899234	0.38044	0.7283698	1167	1337	1505	1518	1078	881	1250	1095
ENSECAG00000022972	2.603154163	0.3806349	0.7284506	86	133	125	186	69	83	77	143
ENSECAG00000016399	6.704333018	0.3806446	0.7284506	2271	1908	2104	2649	1587	1104	2505	1726
ENSECAG00000026829	2.580183511	0.3808081	0.7284506	93	67	122	122	170	72	121	114
ENSECAG00000000429	1.993139816	0.380863	0.7284506	80	75	103	83	54	78	51	54
ENSECAG00000012208	4.405078812	0.3808717	0.7284506	393	266	414	407	427	521	381	312
ENSECAG00000022449	4.285131475	0.3808819	0.7284506	407	325	459	490	332	192	494	258
ENSECAG00000003759	6.530377783	0.3809311	0.7284506	1681	1421	1699	1594	1124	2948	1313	1736
ENSECAG00000015790	3.003621305	0.3812915	0.729017	101	101	146	196	85	103	290	189
ENSECAG00000014561	8.125744135	0.3816618	0.7294092	5772	4826	7981	5369	3642	3964	4694	5729
ENSECAG00000010540	8.827600062	0.3816755	0.7294092	9159	8574	11039	10188	7147	5497	9535	7534
ENSECAG00000002890	3.997376427	0.3817907	0.7294092	406	246	345	389	366	141	298	214
ENSECAG00000015015	4.101555187	0.3818021	0.7294092	320	313	410	426	252	279	312	258
ENSECAG00000000640	3.759332431	0.3818177	0.7294092	190	141	207	398	258	160	548	167
ENSECAG00000010710	5.612292952	0.3819074	0.7294579	1065	1035	862	1182	647	654	911	1004
ENSECAG00000009216	0.642381909	0.3822338	0.7298486	39	19	36	38	27	13	36	14
ENSECAG00000012612	7.5607237	0.3822405	0.7298486	3455	2935	3930	2739	2734	4505	3097	4423
ENSECAG00000017874	5.859948354	0.3824732	0.7301703	800	1498	843	1949	394	1143	1079	1049
ENSECAG00000014694	5.382581524	0.382929	0.7308101	688	612	757	923	913	674	946	729
ENSECAG00000005098	4.481298738	0.382937	0.7308101	478	275	333	481	287	417	443	619
ENSECAG00000008913	1.249802044	0.3831681	0.7310254	18	71	24	20	8	45	20	125
ENSECAG00000015515	4.653621633	0.3831785	0.7310254	553	414	459	752	332	312	448	542
ENSECAG00000011608	5.791965407	0.3833283	0.7311883	1064	971	1321	1360	837	1021	909	816
ENSECAG00000008098	7.670321566	0.3835473	0.7313425	4585	2744	4332	6691	4721	2575	4491	958
ENSECAG00000019425	3.664081664	0.3836156	0.7313425	265	280	256	290	170	118	178	338
ENSECAG00000023182	3.184794994	0.383695	0.7313425	136	229	95	142	205	160	174	183
ENSECAG00000000605	2.423044877	0.3837077	0.7313425	118	85	133	128	114	59	98	60
ENSECAG00000004450	4.36729023	0.3838548	0.7313425	588	309	630	250	419	189	437	269
ENSECAG00000019609	4.427242026	0.3839145	0.7313425	373	296	371	460	534	235	667	307
ENSECAG00000019035	2.20518879	0.3839668	0.7313425	78	42	91	101	76	79	101	110
ENSECAG00000009727	7.741294135	0.38403	0.7313425	3598	3657	4338	3078	3038	5482	2857	5311
ENSECAG00000015546	6.193972893	0.3840493	0.7313425	1950	869	1387	720	2235	752	2207	829
ENSECAG00000020724	8.424691362	0.384117	0.7313425	7646	5973	6654	9438	5449	4405	7634	5004
ENSECAG00000011061	4.444986169	0.3841276	0.7313425	303	434	306	469	296	501	427	481
ENSECAG00000007730	0.648544151	0.3842922	0.7313425	9	37	19	28	9	38	29	47
ENSECAG00000016502	2.660571661	0.3843416	0.7313425	121	96	140	197	79	55	137	129
ENSECAG00000019950	7.437150068	0.384357	0.7313425	3263	2067	2861	4029	4057	3179	4732	1863
ENSECAG00000007960	8.279981277	0.3844292	0.7313425	6180	5358	7109	8401	3122	6542	5010	4996
ENSECAG00000009872	4.898306551	0.3844768	0.7313425	483	512	447	635	373	734	457	757
ENSECAG00000011349	0.153599149	0.3845086	0.7313425	10	18	15	23	11	15	32	28
ENSECAG00000017106	0.320057473	0.3845787	0.7313425	20	16	26	13	19	24	20	31
ENSECAG00000010450	4.696144893	0.3846663	0.7313425	605	363	462	350	652	501	595	298
ENSECAG00000005765	3.521890027	0.3847139	0.7313425	214	173	173	235	267	226	223	181
ENSECAG00000012339	7.746438716	0.384778	0.7313425	3770	3449	3488	4467	4778	3526	5210	3456
ENSECAG00000000722	3.068307504	0.3848256	0.7313425	193	113	120	142	208	115	225	126
ENSECAG00000013222	4.3507877	0.3848945	0.7313511	407	373	501	459	276	246	422	388
ENSECAG00000010638	1.496125316	0.3850677	0.7315579	56	68	47	67	30	33	56	53
ENSECAG00000005033	2.816656914	0.3851514	0.7315779	118	92	123	309	50	89	106	184
ENSECAG00000019502	7.37978305	0.3852811	0.7315779	3585	3228	3848	3495	2616	2587	2977	2634
ENSECAG00000010655	4.142877	0.3854404	0.7315779	261	351	280	322	243	357	305	483
ENSECAG00000004819	3.652250993	0.385472	0.7315779	275	245	259	294	167	204	228	203
ENSECAG00000027681	11.64591524	0.3854797	0.7315779	55063	61863	44338	60816	67550	74746	57792	50241
ENSECAG00000018796	8.047563526	0.3855608	0.7315779	4128	4542	4350	5574	5192	6060	5320	4067
ENSECAG00000003393	3.27787652	0.3855688	0.7315779	160	179	204	102	160	129	250	247
ENSECAG00000021887	1.811847821	0.385651	0.7315779	41	73	57	57	76	67	86	48
ENSECAG00000002016	5.269503635	0.3857087	0.7315779	822	674	833	972	748	433	869	485
ENSECAG00000008503	13.48641973	0.3857465	0.7315779	202205	233679	217551	125249	200019	220884	212054	277932
ENSECAG00000019741	3.401710727	0.3858216	0.7315779	217	213	260	215	154	91	256	182
ENSECAG00000016128	11.33847622	0.3858599	0.7315779	53006	35133	46682	46498	61151	47619	44540	49754
ENSECAG00000010571	5.064068015	0.3859471	0.7315779	596	601	579	539	541	345	920	903
ENSECAG00000012591	0.568824178	0.3860184	0.7315779	6	25	34	24	16	33	25	41
ENSECAG00000007400	4.691938596	0.3860443	0.7315779	486	369	429	544	513	425	687	418
ENSECAG00000000273	6.144033022	0.3862275	0.7317905	1483	1417	1458	1608	1136	887	1413	1248
ENSECAG00000016433	8.133109702	0.3862854	0.7317905	5368	3919	5050	5172	8626	3564	6723	3576
ENSECAG00000016375	2.848032602	0.386585	0.7321269	120	82	148	142	165	86	165	160
ENSECAG00000010493	5.190991906	0.3865919	0.7321269	769	726	788	804	629	479	745	547
ENSECAG00000016965	0.962550163	0.3869242	0.7326342	38	40	39	53	2	34	45	29
ENSECAG00000010378	5.664460989	0.3871308	0.732903	995	683	1032	898	1014	760	1107	1092
ENSECAG00000016960	5.591541066	0.3872359	0.7329798	797	735	1029	853	1014	970	856	880

ENSECAG00000024396	4.191307877	0.3875678	0.733486	385	216	324	335	460	162	560	310
ENSECAG00000020882	5.898432701	0.3877029	0.7335541	1076	934	1147	1074	1289	1157	1262	916
ENSECAG00000008270	7.120650021	0.387733	0.7335541	3372	1765	2582	1638	4648	2204	3369	1031
ENSECAG00000018284	2.600397519	0.3879375	0.7338189	81	101	115	112	85	130	134	129
ENSECAG00000002197	0.752890037	0.3880138	0.7338409	21	27	45	51	10	18	27	42
ENSECAG00000013054	5.333160453	0.3882468	0.7341318	951	557	891	1069	750	550	790	537
ENSECAG00000017783	3.111587062	0.3883983	0.7341318	173	58	116	219	307	94	300	32
ENSECAG00000004757	5.121407315	0.3884179	0.7341318	785	444	634	1196	567	359	861	497
ENSECAG00000015264	1.327171701	0.3884261	0.7341318	47	39	38	36	34	74	36	48
ENSECAG00000021513	4.535223068	0.3885584	0.7342597	394	339	513	790	257	279	424	538
ENSECAG00000017741	7.637543854	0.3887712	0.7345397	4139	3950	3931	4998	3156	2603	4111	3200
ENSECAG00000005075	4.41698921	0.388897	0.734655	364	261	448	431	386	413	353.001	512
ENSECAG00000021942	4.349394309	0.3889995	0.7347266	345	369	534	578	84	151	421	633
ENSECAG00000005377	4.670466383	0.3891733	0.7348303	593	317	543	793	472	168	610	409
ENSECAG00000000602	1.895169131	0.3892403	0.7348303	72	37	59	72	91	41	155	24
ENSECAG00000011873	6.184505763	0.3892485	0.7348303	1538	1598	1657	1337	1019	1336	950	1353
ENSECAG00000026954	5.939937306	0.3893814	0.7348815	1277	897	1161	1023	1248	1029	1359	1153
ENSECAG00000010371	0.910521926	0.3894426	0.7348815	52	35	45	27	40	12	46	11
ENSECAG00000016556	2.27502844	0.3894697	0.7348815	70	60	125	67	93	85	113	93
ENSECAG00000020269	1.877843357	0.389719	0.7351108	72	45	67	58	48	58	74	112
ENSECAG00000020218	4.256047768	0.3897207	0.7351108	338	314	316	352	574	240	447	258
ENSECAG00000010439	0.506539522	0.3899749	0.7354681	19	17	37	11	19	25	17	49
ENSECAG00000009046	9.248950935	0.390131	0.7356404	9995	9248	9317	14307	8779	15530	13017	10377
ENSECAG00000009688	6.670235148	0.3903046	0.7358006	1692	1705	1780	2045	1594	2227	1791	2268
ENSECAG00000015215	5.477577087	0.390372	0.7358006	1072	783	944	980	880	606	856	569
ENSECAG00000020578	2.088485186	0.3904606	0.7358006	83	33	106	53	72	33	120	128
ENSECAG00000007104	7.926965154	0.390475	0.7358006	3444	4541	3872	5215	3677	5698	4812	4841
ENSECAG00000018790	0.612914554	0.390628	0.7359023	24	13	31	28	23	33	25	35
ENSECAG00000019783	5.273550045	0.3906586	0.7359023	578	661	702	795	982	591	733	708
ENSECAG00000000655	3.44706431	0.3910952	0.736468	234	199	269	231	218	117	208	153
ENSECAG00000014600	5.584045192	0.3911273	0.736468	775	906	925	756	858	928	816	1114
ENSECAG00000007760	7.864986406	0.3912386	0.736468	5604	4026	5238	5180	4831	2455	4983	2937
ENSECAG00000014766	5.181729697	0.3912832	0.736468	687	746	691	969	441	489	818	648
ENSECAG00000025139	2.193416343	0.3914975	0.736468	87	83	121	101	53	60	66	102
ENSECAG00000010404	6.930307015	0.391518	0.736468	2120	2120	2055	2310	1858	2171	2505	3044
ENSECAG00000015109	9.455382771	0.3915383	0.736468	18008	5152	25127	16148	5016	21268	4493	9187
ENSECAG00000020572	4.954509973	0.3915965	0.736468	553	620	764	694	522	508	568	406
ENSECAG00000017473	5.031942483	0.3916153	0.736468	854	663	535	721	518	386	684	561
ENSECAG00000021941	7.193819259	0.3916359	0.736468	3361	2318	3371	3688	2579	1223	4182	1689
ENSECAG00000014700	10.41133939	0.3917149	0.736468	28260	22117	19641	25532	26947	25075	27670	27451
ENSECAG00000020064	6.308926088	0.3917404	0.736468	1458	1409	1306	1450	1578	1504	1826	1259
ENSECAG00000025055	7.034456785	0.3918428	0.736468	1959	2277	2175	2758	1783	3755	2290	2321
ENSECAG00000024013	6.490592747	0.3918666	0.736468	1573	1503	1602	1732	1942	1563	1829	1639
ENSECAG00000020805	5.452178422	0.3921696	0.7368751	888	692	1012	1151	737	715	653	726
ENSECAG00000023731	4.681120481	0.3922129	0.7368751	521	527	498	639	438	261	516	468
ENSECAG00000011424	4.752494159	0.3928288	0.7378849	476	409	455	571	496	520	640	451
ENSECAG00000021237	2.481908979	0.3929329	0.7378849	108	68	75	130	124	76	113	130
ENSECAG00000019416	6.585202581	0.3929489	0.7378849	1964	2070	1704	2410	1138	1259	2060	1907
ENSECAG00000016471	4.747504876	0.3930154	0.7378849	468	678	528	641	252	642	280	474
ENSECAG00000017772	5.06889613	0.3930752	0.7378849	598	562	689	503	613	613	708	686
ENSECAG00000023911	3.884998061	0.3932732	0.7381345	327	253	318	367	206	236	248	255
ENSECAG00000005203	5.626115532	0.3934638	0.7383703	781	1076	743	770	427	1164	913	1397
ENSECAG00000025043	1.013659523	0.3936	0.7385038	45	24	25	36	37	35	44	40
ENSECAG00000001514	4.905736472	0.3938385	0.7388293	448	434	353	545	23	1896	85	416
ENSECAG00000015219	2.156317828	0.3941758	0.7393399	109	67	87	121	77	57	74	67
ENSECAG00000010745	3.263324778	0.3943625	0.7393968	219	195	232	175	128	82	173	229
ENSECAG00000001863	5.126983107	0.3943989	0.7393968	509	643	682	594	705	425	1169	529
ENSECAG00000015452	2.443268256	0.3944014	0.7393968	136	67	99	177	57	69	90	120
ENSECAG00000012242	1.957982834	0.3946871	0.7398104	78	56	58	65	81	68	114	46
ENSECAG00000007609	3.028518122	0.3948147	0.7399274	122	76	232	312	109	68	252	86
ENSECAG00000002449	2.028214568	0.3951468	0.7403716	73	49	87	55	96	56	150	33
ENSECAG00000001856	4.230342234	0.3951821	0.7403716	357	260	437	617	174	128	372	533
ENSECAG00000005549	8.533220685	0.3953648	0.7405076	5864	6119	7797	6170	7322	5704	9323	7119
ENSECAG00000010775	2.388563401	0.395385	0.7405076	76	98	83	91	141	95	112	64
ENSECAG00000005413	2.830687357	0.3955797	0.7407017	118	70	174	123	126	138	132	164
ENSECAG00000012837	5.372090682	0.3956835	0.7407017	746	614	774	786	1183	603	871	587
ENSECAG00000015274	3.093167398	0.3957703	0.7407017	192	169	141	235	132	75	189	151
ENSECAG00000000536	6.010556517	0.3958624	0.7407017	1345	1013	1269	1937	763	777	1226	1483
ENSECAG00000013025	0.561594596	0.3960599	0.7407017	18	23	30	19	25	16	55	21
ENSECAG00000014000	4.209126379	0.3962307	0.7407017	313	282	261	452	393	257	427	385
ENSECAG00000012266	5.890845408	0.3962711	0.7407017	1376	1124	1210	1339	1427	651	958	815
ENSECAG00000011300	1.171736606	0.3962788	0.7407017	44	29	32	94	34	14	62	25
ENSECAG00000021981	4.676730528	0.3963214	0.7407017	404	427	475	487	647	478	451	406
ENSECAG00000020746	6.73326484	0.3963406	0.7407017	1952	2114	1765	3386	1109	1306	2302	2288
ENSECAG00000026994	5.394244211	0.3963848	0.7407017	702	764	794	707	861	775	824	797
ENSECAG00000020259	6.409192557	0.3963883	0.7407017	1593	1290	1814	1208	2250	931	2460	1198
ENSECAG00000006601	3.961054163	0.3964432	0.7407017	320	255	322	455	252	155	407	203
ENSECAG00000017355	1.981431973	0.3964534	0.7407017	64	49	74	78	77	93	70	65

ENSECAG00000017597	0.83284606	0.3964668	0.7407017	40	28	37	45	21	29	42	12
ENSECAG00000004311	10.28571617	0.3967543	0.7410141	23261	17168	24019	22901	26471	29867	18486	21910
ENSECAG00000015826	4.276131791	0.3967645	0.7410141	365	301	249	432	596	286	415	234
ENSECAG00000017761	2.8343534	0.3968407	0.7410346	98	121	104	158	81	105	139	249
ENSECAG00000013925	7.211325481	0.3971626	0.7414944	3026	2295	2308	2858	2340	2387	3673	3347
ENSECAG00000012684	2.328049049	0.3972175	0.7414944	79	72	164	124	112	37	69	88
ENSECAG00000009711	5.280165373	0.3978338	0.7424057	555	535	668	1025	553	575	963	999
ENSECAG00000019415	6.396537961	0.3978489	0.7424057	1788	1639	1838	1847	1055	1243	1500	1746
ENSECAG00000016387	4.095893889	0.3979018	0.7424057	368	320	350	417	248	231	330	303
ENSECAG00000020187	5.425901504	0.3980089	0.7424836	914	722	970	1044	804	612	766	630
ENSECAG00000025072	7.712392594	0.3981705	0.7426631	3345	4194	3434	3707	4040	3791	3918	4653
ENSECAG00000016798	3.719379335	0.3982494	0.7426884	161	242	245	238	419	298	156	151
ENSECAG00000024869	5.952467796	0.3983585	0.7427087	905	974	1400	1093	943	1491	1131	1213
ENSECAG00000011591	7.731712837	0.3983911	0.7427087	3833	4887	4079	5287	3211	3228	4131	3298
ENSECAG00000009438	3.491376537	0.3985689	0.7429182	247	127	220	170	310	118	324	161
ENSECAG00000015506	5.000550118	0.3986636	0.7429728	669	463	622	467	717	737	667	370
ENSECAG00000011236	4.372438683	0.39878	0.742982	409	266	370	412	418	395	307	486
ENSECAG00000002238	1.782929986	0.3988754	0.742982	101	43	78	78	77	21	81	30
ENSECAG00000000252	4.90493874	0.3989059	0.742982	606	664	684	573	481	516	393	522
ENSECAG00000000879	2.340586512	0.3989301	0.742982	97	75	108	160	75	57	95	90
ENSECAG00000007145	2.102326979	0.3990478	0.7430794	12	184	8	12	24	232	11	92
ENSECAG00000011060	2.647905224	0.3994969	0.7437766	119	94	138	194	95	81	133	86
ENSECAG00000014585	7.164413425	0.3995532	0.7437766	1756	3077	3054	1678	1556	2768	1841	5337
ENSECAG00000010468	4.525966919	0.3996834	0.743897	439	373	393	410	499	474	456	356
ENSECAG00000009939	-0.003608704	0.3998779	0.7440435	7	22	9	20	23	15	13	23
ENSECAG00000009629	4.748401758	0.3998931	0.7440435	522	332	390	1200	445	520	516	180
ENSECAG00000010938	6.267472942	0.4000022	0.7441246	1760	1040	1423	1233	1732	1097	1781	1446
ENSECAG00000019034	5.300986015	0.4002093	0.7441664	886	682	741	1061	438	617	744	781
ENSECAG00000024140	1.014618468	0.4002393	0.7441664	19	49	25	32	23	58	42	33
ENSECAG00000002350	8.287539981	0.4002593	0.7441664	5164	5377	5090	6458	6173	6221	6603	5421
ENSECAG00000018435	9.581498352	0.4002867	0.7441664	13871	12550	13176	13968	14338	19033	12744	13246
ENSECAG00000024691	4.45626854	0.4003906	0.7442377	465	507	643	252	266	278	254	580
ENSECAG00000013080	6.750269459	0.4006413	0.7445819	1945	1609	2173	3682	1825	1244	2658	1372
ENSECAG00000003502	3.09426413	0.4008513	0.7448503	180	117	173	282	141	66	197	142
ENSECAG00000007739	3.732873877	0.4010142	0.7449586	218	130	225	328	412	142	472	82
ENSECAG00000018757	3.331430222	0.4010407	0.7449586	208	109	151	223	243	104	339	139
ENSECAG00000002212	6.781148894	0.4020593	0.7466595	2590	1489	1643	1653	4750	988	2347	874
ENSECAG00000008342	5.711694024	0.4021149	0.7466595	789	826	720	1419	1019	984	1459	671
ENSECAG00000023743	3.114298452	0.4022607	0.7466595	173	147	194	233	180	112	128	123
ENSECAG00000000173	6.5188545	0.4024333	0.7466595	2184	1509	1622	910	1770	2381	951	1989
ENSECAG00000023292	6.143067616	0.4024931	0.7466595	1225	1449	1326	2007	1046	1143	1318	1131
ENSECAG00000012246	6.031744131	0.4026003	0.7466595	1240	1353	1399	1524	979	1092	1022	1173
ENSECAG00000007864	8.102256145	0.4026055	0.7466595	3576	4864	4096.93	6885.97	4380.98	6626	6029.94	4480.97
ENSECAG00000005741	4.2592163	0.4026121	0.7466595	472	296	468	415	176	197	383	488
ENSECAG00000014739	0.649066786	0.4026174	0.7466595	27	20	34	52	25	10	36	21
ENSECAG00000017444	4.875291513	0.4026701	0.7466595	557	468	545	499	590	466	646	595
ENSECAG00000015349	3.88480346	0.4026795	0.7466595	232	199	298	309	269	216	344	340
ENSECAG00000010779	5.203378484	0.4028203	0.7467988	758	653	852	866	710	586	616	473
ENSECAG00000019536	4.93368452	0.4030836	0.7469791	568	426	652	502	687	597	666	430
ENSECAG00000015649	4.258275628	0.4032019	0.7469791	279	352	276	438	339	385	337	425
ENSECAG00000013950	8.384328827	0.4032089	0.7469791	5103	6059	5440	6750	8390	6780	6970	4039
ENSECAG00000018244	4.333302295	0.4032861	0.7469791	310	371	403	316	361	310	558	373
ENSECAG00000001174	3.936301574	0.4032937	0.7469791	30	1185	22	140	83	163	86	469
ENSECAG00000021031	9.150818863	0.4033121	0.7469791	10577	9018	8781	22509	9492	8534	13769	4587
ENSECAG00000012990	0.959844113	0.4034518	0.7470846	11	68	37	52	24	4	26	56
ENSECAG00000009693	5.385537992	0.4035593	0.7470846	873	651	948	1102	824	476	824	627
ENSECAG00000010768	6.850829563	0.4035898	0.7470846	2212	1621	2076	2262	2667	1400	3256	1907
ENSECAG00000021116	10.33296697	0.4036322	0.7470846	26799	23018	26485	35037	20649	23196	22223	16694
ENSECAG00000016122	3.94668775	0.4037446	0.747171	273	337	341	360	272	161	304	264
ENSECAG00000016774	-0.133283178	0.4040737	0.747344	21	18	9	26	20	7	15	6
ENSECAG00000014409	5.456194461	0.404128	0.747344	949	876	943	942	538	506	879	984
ENSECAG00000022231	2.070683653	0.4042083	0.747344	77	46	67	91	59	74	155	53
ENSECAG00000020158	6.697552862	0.4042161	0.747344	1806	1196	1718	2590	2777	1700	3067	804
ENSECAG00000007704	4.498662588	0.4042484	0.747344	442	207	381	561	652	322	583	250
ENSECAG00000021308	2.447858585	0.4042845	0.747344	61	118	69	115	107	136	86	95
ENSECAG00000024525	0.419000809	0.404362	0.747344	35	15	27	35	31	9	20	15
ENSECAG00000023713	5.288462695	0.4044188	0.747344	718	751	802	1076	382	682	662	806
ENSECAG00000021996	7.125081862	0.4044303	0.747344	2439	1866	2425	3239	2616	2334	4113	2046
ENSECAG00000024134	4.655222429	0.404615	0.7475636	511	343	466	443	688	431	530	322
ENSECAG00000009949	4.105811727	0.4047564	0.7477034	286	255	374	599	303	141	419	259
ENSECAG00000016580	6.602466813	0.4048333	0.7477238	1439	1877	1612	1898	1397	1359	1842	3095
ENSECAG00000011357	6.29726471	0.404928	0.7477771	1627	1620	1684	1669	1293	1080	1623	1218
ENSECAG00000005781	5.241186331	0.4050342	0.7478516	639	1158	692	712	411	533	435	1020
ENSECAG00000000196	4.099593927	0.4052354	0.7481016	262	494	386	340	87	232	238	510
ENSECAG00000012161	3.430422943	0.4056941	0.7488267	200	197	120	449	90	118	232	242
ENSECAG00000008341	5.769145405	0.4058941	0.7490741	1104	738	966	1039	1566	718	1391	645
ENSECAG00000012859	5.730953298	0.4061101	0.7492307	939	814	1005	988	1445	810	1326	599
ENSECAG00000016986	6.385296162	0.4061109	0.7492307	2182	1205	2065	1671	1535	809	1894	1303

ENSECAG00000011433	6.350988431	0.4065535	0.7499254	1290	1454	1447	1548	2524	1250	1305	1254
ENSECAG00000022498	8.806728115	0.4067756	0.7502134	8072	6277	10534	5874	13529	5246	11137	6164
ENSECAG00000021705	10.57282956	0.4069441	0.7502884	29906	22274	26293	29121	29147	30076	27729	31909
ENSECAG00000018443	3.981900419	0.406964	0.7502884	316	308	296	431	255	230	322	215
ENSECAG00000016696	5.989541331	0.4070461	0.7502884	1635	653	1279	2108	1412	614	1644	423
ENSECAG00000026911	5.777661776	0.4070805	0.7502884	964	985	829	1119	912	1296	953	1045
ENSECAG00000022057	2.887822782	0.4072275	0.7504109	111	108	167	118	140	106	161	180
ENSECAG00000021751	3.5527393	0.4072798	0.7504109	204	219	274	319	144	101	335	188
ENSECAG00000026934	3.212522381	0.4073629	0.7504109	126	179	135	196	154	203	171	195
ENSECAG00000012735	3.560343567	0.4074429	0.7504109	221	194	245	379	152	83	179	336
ENSECAG00000001262	2.194336402	0.4074773	0.7504109	81	134	81	91	45	64	65	106
ENSECAG00000017165	7.376567848	0.407885	0.7510399	3358	2076	3580	2656	3856	3549	3055	2452
ENSECAG00000017417	5.076960445	0.4079569	0.7510506	484	646	408	841	476	837	701	612
ENSECAG00000000319	7.008020132	0.4081426	0.7512147	2928	1980	2753	3394	2162	2165	2430	1600
ENSECAG00000011924	4.883751166	0.4082113	0.7512147	677	509	690	639	534	259	653	502
ENSECAG00000018137	3.434665364	0.4082444	0.7512147	205	162	194	171	334	112	295	132
ENSECAG00000024608	8.322508935	0.4084124	0.7514021	7515	5785	7051	6993	5694	5499	5405	3985
ENSECAG00000021307	2.901277604	0.4085057	0.7514392	136	190	102	223	48	121	118	173
ENSECAG00000004975	7.101145618	0.4085649	0.7514392	2480	2332	2111	2820	2293	1977	3171	3421
ENSECAG00000021105	0.944309667	0.4086478	0.7514701	32	29	55	46	33	15	43	24
ENSECAG00000010978	5.70525637	0.4087183	0.751478	1409	851	1180	985	1018	677	1176	552
ENSECAG00000017332	3.303186239	0.4088003	0.7515072	133	127	186	243	188	222	167	189
ENSECAG00000024640	7.510484287	0.4088867	0.7515443	50	8290	101	97	405	6099	922	9484
ENSECAG00000009392	2.005745361	0.4091401	0.7518885	53	71	61	79	38	100	59	117
ENSECAG00000016440	5.930943925	0.4095298	0.7524039	1118	1256	1434	1318	972	1012	980	1019
ENSECAG00000014365	3.284602502	0.4095531	0.7524039	241	158	220	219	168	80	236	146
ENSECAG00000007363	5.419664263	0.4097627	0.7525226	771	831	738	663	688	699	861	1099
ENSECAG00000022861	5.316471676	0.4097839	0.7525226	702	661	693	784	707	793	808	770
ENSECAG00000016476	4.027934818	0.4098164	0.7525226	298	193	300	361	393	291	337	252
ENSECAG00000021784	3.08776215	0.4099885	0.7527168	165	152	162	90	161	177	145	182
ENSECAG00000008568	2.028264766	0.4100758	0.7527294	67	56	73	78	63	53	94	114
ENSECAG00000005718	6.426726955	0.4101279	0.7527294	1713	1685	1337	1261	1743	1030	1644	2379
ENSECAG00000019791	3.72742555	0.4102147	0.7527672	216	165	281	266	241	192	286	328
ENSECAG00000017928	6.236410216	0.4102997	0.7528015	1224	1031	1769	1349	1674	1371	1377	1406
ENSECAG00000016432	7.154285175	0.4103677	0.7528046	2839	2203	2755	2211	3125	2987	2589	2327
ENSECAG00000017289	1.933040068	0.4106109	0.753105	43	31.0011	80	103	93	94	60	49
ENSECAG00000000518	7.712096911	0.4106723	0.753105	3739	3664	3943	3354	5234	3050	4514	3698
ENSECAG00000016809	4.9103926	0.4107303	0.753105	706	466	717	664	443	462	531	524
ENSECAG00000016519	3.465587678	0.4110469	0.7535053	224	148	144	253	148	166	277	293
ENSECAG00000014855	4.887488984	0.4110813	0.7535053	404	630	461	356	112	1579	208	415
ENSECAG00000022604	6.899381258	0.4113432	0.7537671	2252	2603	2017	3311	1665	2086	2145	1861
ENSECAG00000017064	7.254038878	0.4114181	0.7537671	2201	3134	2527	2772	2077	3908	2832	3035
ENSECAG00000010819	7.802119569	0.4114818	0.7537671	3646	3711	3816	4546	3819	5912	3955	3531
ENSECAG00000012658	4.057597719	0.4115134	0.7537671	237	257	326	336	370	426	205	275
ENSECAG00000025149	6.615487962	0.4116423	0.7537671	1421	1900	1623	1963	2319	1766	2511	1106
ENSECAG00000007423	5.046558909	0.4116504	0.7537671	472	525	679	678	661	595	695	615
ENSECAG00000016982	7.410693673	0.4116886	0.7537671	4097	3236	2974	4195	2715	2451	2696	3215
ENSECAG00000020654	3.144818177	0.411785	0.753822	174	138	217	243	181	57	204	123
ENSECAG00000016785	6.579147435	0.4119117	0.7539326	1926	1844	2003	2320	1124	1257	2085	1908
ENSECAG00000019179	2.683754817	0.4123106	0.7543582	126	76	108	131	166	91	126	122
ENSECAG00000009804	1.371648994	0.4123962	0.7543582	32	26	42	66	113	15	45	34
ENSECAG00000012452	4.083802546	0.4124282	0.7543582	344	194	316	339	276	227	384	461
ENSECAG00000014144	8.066715795	0.4124928	0.7543582	5510	4094	4897	8909	3990	3026	6450	4244
ENSECAG00000007404	5.469074883	0.4124975	0.7543582	840	882	925	1087	730	645	878	672
ENSECAG00000014265	5.055049154	0.4125428	0.7543582	646	454	550	725	556	582	944	540
ENSECAG00000017375	2.376283867	0.4127335	0.7545642	97	94	94	166	69	80	124	51
ENSECAG00000021404	0.220556764	0.4127883	0.7545642	19	19	3	27	9	49	19	10
ENSECAG00000007240	7.497888186	0.4130823	0.7549438	3207	2991	3027	3635	3198	3190	4335	3463
ENSECAG00000025003	0.46673379	0.4131918	0.7549438	12	34	20	16	17	39	20	28
ENSECAG00000013539	4.844264606	0.4131953	0.7549438	533	465	511	516	548	363	779	593
ENSECAG00000008017	4.894482583	0.4135677	0.7555027	643	566	722	587	487	273	711	502
ENSECAG00000022294	1.114899937	0.4137265	0.7556712	13	33	32	62	40	59	26	40
ENSECAG00000012391	5.765229717	0.4138714	0.7558143	851	735	1384	851	1401	1129	889	774
ENSECAG00000018831	0.343239642	0.4141253	0.7561564	19	18	14	27	16	18	18	44
ENSECAG00000011342	8.295820864	0.4144486	0.756536	5146	5568	5065	6315	7042	7132	5867	4318
ENSECAG00000009210	1.430709337	0.4144664	0.756536	60	48	94	25	28	31	48	54
ENSECAG00000020897	4.292947015	0.4147864	0.7568014	317	283	442	328	364	468	328	349
ENSECAG00000000084	5.821827522	0.4148948	0.7568014	954	995	977	1092	1487	738	1169	1020
ENSECAG00000016107	3.159094136	0.4149492	0.7568014	144	259	218	142	49	159	143	198
ENSECAG00000020277	2.4880323	0.4150542	0.7568014	103	135	154	81	61	86	90	110
ENSECAG00000014216	6.736815508	0.415102	0.7568014	1377	2384	1486	2206	1479	2176	1828	2855
ENSECAG00000008495	5.965275523	0.4151546	0.7568014	961	1066	1090	1372	1048	1190	1274	1316
ENSECAG00000013103	0.963739828	0.415215	0.7568014	35	27	34	29	26	33	48	44
ENSECAG00000010197	3.559572899	0.4152944	0.7568014	302	145	285	284	197	148	249	165
ENSECAG00000021601	5.686345672	0.41533	0.7568014	1123	892	1071	1264	916	808	908	749
ENSECAG00000020184	5.414123099	0.4153575	0.7568014	869	533	871	752	1036	622	1092	608
ENSECAG00000000122	4.154742207	0.4154298	0.7568014	225	401	263	332	192	352	396	473
ENSECAG00000026371	0.53543026	0.4154963	0.7568014	28	18	14	31	26	36	32	15

ENSECAG00000012512	7.300823289	0.4155288	0.7568014	2707	2315	3089	3053	3199	3746	2610	2577
ENSECAG00000019346	7.655778556	0.4155445	0.7568014	4773	3618	4061	4721	3710	2498	3990	3062
ENSECAG00000000260	5.586641413	0.4156616	0.7568844	906	856	996	611	916	813	999	1016
ENSECAG00000000091	1.005767319	0.4157234	0.7568844	21	39	32	35	19	57	44	34
ENSECAG00000025134	5.717820077	0.4157901	0.7568845	902	1114	1117	1363	433	805	911	1282
ENSECAG00000017980	6.877488394	0.415982	0.7571126	2480	2560	2306	2580	1682	1844	1817	2312
ENSECAG00000005437	5.808809571	0.4161655	0.7573214	1065	974	951	1002	1186	1047	1191	903
ENSECAG00000008450	7.706498332	0.4162301	0.7573214	4749	3011	3958	2895	3964	4377	3579	4309
ENSECAG00000006876	6.146652691	0.4165738	0.7578254	1618	1202	1471	1704	1078	815	1416	1414
ENSECAG00000023373	5.92155445	0.4167456	0.7580165	1125	876.001	1210	1098	1148	1475	998	1008
ENSECAG00000006915	7.568773387	0.4173401	0.7586858	4023	2553	2660	4283	4126	3591	4603	2599
ENSECAG00000009054	4.595322475	0.4173501	0.7586858	315	845	130	158	258	709	105	901
ENSECAG00000009721	8.870139158	0.4174109	0.7586858	9626	8055	10191	12250	7183	6938	9013	7474
ENSECAG00000022654	2.545535711	0.4174579	0.7586858	78	85	123	111	95	75	150	147
ENSECAG00000021928	6.117856048	0.4175452	0.7586858	1606	1256	1224	1809	1068	784	1283	1475
ENSECAG00000007736	2.316136828	0.4176141	0.7586858	59	93	65	110	42	164	58	123
ENSECAG00000016179	4.215777749	0.4177929	0.7586858	419	341	431	382	348	191	338	335
ENSECAG00000017838	5.787828196	0.41782	0.7586858	802	773	985	1278	1247	1483	1338	284
ENSECAG00000007801	6.732889833	0.4179136	0.7586858	1711	1791	1551	2542	1748	2570	1839	2023
ENSECAG00000007198	8.907159712	0.4179159	0.7586858	11616	9550	12391	7210	5897	6543	7453	11100
ENSECAG00000019540	3.442538123	0.4179439	0.7586858	168	151	179	267	163	194	253	246
ENSECAG00000020881	7.165035486	0.4180085	0.7586858	1955	2204	2337	3702	2504	1432	4768	3010
ENSECAG00000006640	5.844250519	0.4180212	0.7586858	971	1372	1049	1467	624	989	980	1153
ENSECAG00000019956	0.491096694	0.4180646	0.7586858	27	17	17	24	21	13	12	63
ENSECAG00000013585	4.388717004	0.4181154	0.7586858	323	334	394	428	273	396	535	445
ENSECAG00000010431	6.581061134	0.4182084	0.7587334	1909	1718	1968	2531	1364	1195	2285	1572
ENSECAG00000019453	3.995594217	0.4182988	0.7587761	272	277	227	337	199	377	315	351
ENSECAG00000019518	4.201478845	0.4187237	0.7590492	238	339	416	609	256	162	414	372
ENSECAG00000021934	6.400268488	0.41883	0.7590492	1337	1301	1472	1995	1624	1470	2016	1460
ENSECAG00000015080	3.136090998	0.4188609	0.7590492	158	188	139	276	98	137	220	106
ENSECAG00000010891	5.279294387	0.4188797	0.7590492	566	767	621	710	381	1162	458	1000
ENSECAG00000025150	4.290536352	0.4188831	0.7590492	341	384	350	604	285	230	372	393
ENSECAG00000020315	4.256383404	0.4189206	0.7590492	413	293	435	505	355	166	473	264
ENSECAG00000007373	2.09775289	0.4189596	0.7590492	76	134	59	101	31	31	51	145
ENSECAG00000024871	1.499721202	0.4190022	0.7590492	22	37	46	83	37	59	52	73
ENSECAG00000013671	7.959945739	0.4190769	0.7590492	6229	4256	5308	5453	5101	3090	4654	3402
ENSECAG00000010456	4.743497618	0.4191176	0.7590492	455	506	573	754	500	341	550	364
ENSECAG00000016843	8.170535399	0.4192136	0.7590834	4481	5022	4577	6440	4965	4955	6918	5848
ENSECAG00000022066	5.955354908	0.4193312	0.7590834	1366	869	890	1329	1158	831	1605	1303
ENSECAG00000008479	4.497581004	0.419337	0.7590834	452	246	777	473	361	272	464	368
ENSECAG00000008468	4.989182647	0.4194699	0.7591498	493	592	669	982	370	497	484	703
ENSECAG00000013700	1.057097993	0.4195724	0.7591498	21	30	26	58	20	59	45	36
ENSECAG00000004461	1.19748499	0.4196891	0.7591498	40	38	19	51	26	32	49	74
ENSECAG00000020123	3.660814611	0.4197153	0.7591498	245	209	316	323	309	176	174	132
ENSECAG00000005081	7.986663151	0.4198049	0.7591498	4665	2520	4407	6386	6586	4349	6961	2388
ENSECAG00000010029	4.738066565	0.4199011	0.7591498	465	444	468	511	435	575	502	547
ENSECAG00000011402	7.699947947	0.4199527	0.7591498	4256	3303	3422	3681	3805	2594	4540	5572
ENSECAG00000008348	5.068855158	0.4199826	0.7591498	583	552	693	1063	488	393	651	680
ENSECAG00000008096	3.529242771	0.4200301	0.7591498	231	132	242	417	177	105	302	164
ENSECAG00000018209	3.403638271	0.4202515	0.7591498	238	210	182	277	169	89	230	199
ENSECAG00000021690	4.553586979	0.4202678	0.7591498	352	352	493	452	522	556	333	387
ENSECAG00000021397	4.287091438	0.4203223	0.7591498	369	300	364	340	475	292	424	334
ENSECAG00000014425	4.539952204	0.4203682	0.7591498	495	451	453	574	334	258	532	418
ENSECAG00000016416	7.114434548	0.4204398	0.7591498	2644	2716	2679	3770	1873	2096	2499	2628
ENSECAG00000011695	6.380434873	0.4204522	0.7591498	1604	1834	1448	2153	1305	1539	1298	1266
ENSECAG00000012834	4.919132907	0.4204687	0.7591498	506.001	546	598.001	962	291.001	497.001	614	570.024
ENSECAG00000017154	11.93293234	0.4205098	0.7591498	71640	52607	72501	81109	74991	74717	65918	89008
ENSECAG00000001623	3.873305623	0.4206206	0.7592293	298	285	307	363	137	285	257	254
ENSECAG00000019148	3.917239347	0.4208691	0.7594559	257	217	196	377	376	309	404	112
ENSECAG00000018466	2.695703266	0.4209032	0.7594559	163	105	126	161	89	69	156	102
ENSECAG00000023976	7.189495027	0.4210044	0.7594559	3158	2779	3312	3077	2433	2114	2776	2270
ENSECAG00000002091	4.058010595	0.4210136	0.7594559	416	309	377	306	338	176	298	267
ENSECAG00000000133	1.783551856	0.4212967	0.7598459	66	34	39	90	94	71	81	25
ENSECAG00000023499	5.175570739	0.4214069	0.759924	562	528.002	578	919	660.002	884	566	656
ENSECAG00000013231	7.261368582	0.4215642	0.760087	3035	2140	2484	3255	4074	2193	3553	2261
ENSECAG00000023207	0.403417607	0.4216564	0.7601325	18	22	5	37	29	19	29	24
ENSECAG00000000140	7.19771785	0.4220321	0.7606217	2779	2273	2391	3029	2945	2696	3610	2236
ENSECAG00000006573	3.616545286	0.4220617	0.7606217	266	101	369	379	107	41	327	306
ENSECAG00000004790	3.18334329	0.422517	0.7611177	228	110	237	212	148	112	229	92
ENSECAG00000012038	6.540361871	0.4225217	0.7611177	1738	1774	2111	2228	1206	1302	1898	1790
ENSECAG00000014022	0.505581783	0.4225379	0.7611177	19	18	29	21	11	18	26	55
ENSECAG00000011611	5.005534709	0.4226869	0.7612654	623	545	695	879	477	348	656	648
ENSECAG00000012520	0.570788805	0.422902	0.7615321	23	19	33	50	18	16	26	26
ENSECAG00000019598	5.932733166	0.42298	0.7615517	1256	922.994	1096	1079.99	1472.99	928	1394	961.993
ENSECAG00000011806	7.633878333	0.4232065	0.7616801	4244	3705	4743	4208	4251	2212	3957	2596
ENSECAG00000016583	0.956906106	0.4232343	0.7616801	21	35	39	22	7	68	24	51
ENSECAG00000005988	5.122138906	0.4232525	0.7616801	625	618	775	964	355	556	578	778
ENSECAG00000013238	2.137562642	0.423842	0.7624614	65	68	100	157	117	60	52	31

ENSECAG00000023563	6.355237907	0.4238817	0.7624614	1294	1310.99	1488	1775	1384	1997	1560	1327
ENSECAG00000003931	6.969332332	0.423888	0.7624614	3026	1988	2927	2752	2539	1262	2757	1722
ENSECAG00000007552	1.118544755	0.4239611	0.7624722	37	39	48	59	15	33	50	33
ENSECAG00000020825	5.56271001	0.4240567	0.7625234	861	915	895	1333	686	674	893	877
ENSECAG00000015548	6.271051466	0.4241277	0.7625304	1187	1448	1400	1478	1458	1110	1930	1555
ENSECAG00000015844	0.853428773	0.4242432	0.7626172	31	51	29	37	25	20	22	39
ENSECAG00000008321	2.676875171	0.4244099	0.7627962	110	119	106	97	132	81	129	163
ENSECAG00000019606	3.684873104	0.4247585	0.7633019	231	175	279	209	315	201	294	200
ENSECAG00000019650	5.114012173	0.4248484	0.7633428	819	518	880	728	537	328	721	709
ENSECAG00000016280	4.631409246	0.4251506	0.763765	480	423	638	584	248	265	461	648
ENSECAG00000006141	4.141833227	0.4253684	0.7640353	300	224	307	414	185	446	401	348
ENSECAG00000016091	6.082268425	0.4257154	0.7645377	1282	1057	1126	1417	1383	1039	1639	1216
ENSECAG00000010539	3.340009112	0.4260668	0.7650478	135	165	144	264	191	231	200	164
ENSECAG00000022254	6.910615938	0.4261917	0.7650484	2088	1851	2594	1941	3008	1505	2132	2792
ENSECAG00000014226	10.93437602	0.4262018	0.7650484	44432	54682	34919	31002	30128	37531	24055	31512
ENSECAG00000006280	7.088371255	0.426397	0.7652477	2643	1893	2423	2792	2920	2467	2721	2442
ENSECAG00000013676	8.266621641	0.4264476	0.7652477	5591	5750	5412	4859	6850	4662	6861	5843
ENSECAG00000007666	0.961109891	0.4265299	0.7652746	39	26	45	54	30	16	38	33
ENSECAG00000016899	4.668334033	0.4267114	0.76542	418	386	473	521	567	291	762	412
ENSECAG00000015405	7.819703663	0.4267457	0.76542	4074	5318	6233	3928	1685	1456	4257	7230
ENSECAG00000023958	3.215364952	0.4270554	0.7655621	128	133	177	209	139	133	274	198
ENSECAG00000000823	4.738463014	0.4270886	0.7655621	522	578	643	521	265	309	433	729
ENSECAG00000006589	6.553623425	0.4270978	0.7655621	1884	1561	1797	2881	1022	1161	1349	2603
ENSECAG00000014032	7.813102798	0.4271706	0.7655621	4593	4812	4867	4691	3167	3418	4568	3647
ENSECAG00000001903	5.13576143	0.4271834	0.7655621	707	806	577	872	505	510	789	521
ENSECAG00000014894	2.635283435	0.4272294	0.7655621	149	89	133	168	87	43	131	135
ENSECAG00000011173	5.893385267	0.4274172	0.7656533	1215	848	1094	1106	1168	962	1088	1376
ENSECAG00000008462	4.368822573	0.4275194	0.7656533	398	287	371	403	541	317	334	409
ENSECAG00000015464	9.526056589	0.4275455	0.7656533	15320	9754	13771	13275	15050	15838	11666	14480
ENSECAG00000022298	2.446144833	0.4275499	0.7656533	97	110	114	155	30	60	80	166
ENSECAG00000008911	7.073061949	0.4276186	0.7656557	2320	2244	2404	2550	3278	1704	3785	1948
ENSECAG00000008943	4.307011779	0.4281234	0.7662595	377	261	375	395	379	361	433	365
ENSECAG00000010156	5.571014598	0.428154	0.7662595	1026	752	1317	946	1135	442	775	749
ENSECAG00000012555	3.950260681	0.4281582	0.7662595	204	226	340	309	342	347	332	181
ENSECAG00000022942	8.513300899	0.4282515	0.7663057	7421	7421	8034	8078	5210	5955	6550	6134
ENSECAG00000016591	2.50852214	0.4286397	0.7668796	148	37	120	74	158	107	104	80
ENSECAG00000008865	3.96957579	0.4289383	0.767293	207	241	301	357	240	325	298	354
ENSECAG00000007621	7.776431971	0.4291499	0.7675505	2726	2755	3062	6977	2769	2088	4804	8328
ENSECAG00000008489	6.563911946	0.4294507	0.7679676	1950	1626	1902	2581	1504	917	2231	1706
ENSECAG00000018308	2.306237262	0.4295235	0.7679769	79	64	77	232	85	38	157	26
ENSECAG00000017170	2.103569766	0.4296403	0.7679977	107	80	103	76	60	54	124	31
ENSECAG00000024259	9.148240544	0.4296703	0.7679977	15864	8302	9648	4462	9358	6611	19708	11383
ENSECAG00000019618	6.083772818	0.4298893	0.7681735	1404	1161	1296	1908	919	807	1555	1269
ENSECAG00000023979	3.437470488	0.429904	0.7681735	227	93	322	40	205	50	196	471
ENSECAG00000024447	3.580732832	0.4300001	0.7682245	211	182	190	487	165	73	367	175
ENSECAG00000008502	8.744150339	0.4301132	0.7683056	9386	8290	8318	10481	6295	6510	7745	7525
ENSECAG00000014555	2.818961006	0.4301869	0.7683165	112	103	120	294	66	93	203	87
ENSECAG00000023549	5.147998134	0.4305395	0.7686429	634	555	703	569	958	666	895	274
ENSECAG00000017406	2.260170051	0.430543	0.7686429	97	57	79	89	113	122	88	47
ENSECAG00000011371	5.095525609	0.4307405	0.7686429	751	602	865	654	591	448	635	577
ENSECAG00000010360	6.198340206	0.4307692	0.7686429	1968	1108	1469	392	1853	1357	1538	1088
ENSECAG00000011239	6.134869931	0.4307764	0.7686429	1383	1042	1489	1065	1764	1123	1144	1391
ENSECAG00000012171	7.072596488	0.4307902	0.7686429	2385	2162	2383	2661	2210	3063	2486	2608
ENSECAG00000021927	10.73920015	0.4308914	0.7686429	32873	23027	32632	31995	42939	36773	25821	26478
ENSECAG00000019199	4.746469194	0.430911	0.7686429	487	456	460	495	416	403	589	699
ENSECAG00000023089	4.257145963	0.4311117	0.7688801	391	225	345	396	430	266	515	301
ENSECAG00000008005	7.477632968	0.4315172	0.7694791	3527	3767	4551	3152	2656	3207	2989	2685
ENSECAG00000021036	4.83229823	0.4316238	0.7694791	669	463	675	600	402	460	496	500
ENSECAG00000006052	2.648697718	0.4316507	0.7694791	100	87	165	192	89	68	120	121
ENSECAG00000009142	5.581941664	0.4318747	0.7697575	779	769	945	795	344	588	975	1991
ENSECAG00000021178	6.236172588	0.4320056	0.76987	1460	1188	1355	1403	1460	1505	1564	1267
ENSECAG00000025125	5.94018898	0.4321279	0.7699673	1177	979	1042	1164	1622	1132	1270	722
ENSECAG00000019201	3.750142388	0.4321986	0.7699724	256	227	283	393	183	135	347	221
ENSECAG00000009095	7.765083304	0.4322901	0.7700147	4301	4314	4441	5436	3517	3928	3618	3035
ENSECAG00000005107	3.814247409	0.4326959	0.7705302	269	277	279	384	115	217	240	330
ENSECAG00000019845	6.384054309	0.4327152	0.7705302	1279	1448	1318	1905	1198	2339	1496	1351
ENSECAG00000023133	1.184584767	0.4328424	0.7706359	38	35	40	35	38	27	63	50
ENSECAG00000019299	5.841597614	0.4329247	0.770654	1137	1136	1260	1277	989	983	865	906
ENSECAG00000024773	6.08874335	0.4329894	0.770654	1173	1080	1344	1215	1462	1736	1222	781
ENSECAG00000003697	1.594654619	0.4330883	0.770654	39	66	46	46	64	61	71	38
ENSECAG00000007708	3.620084545	0.433124	0.770654	235	101	198	326	280	143	439	145
ENSECAG00000017865	4.350570132	0.4331978	0.7706646	424	206	483	685	350	189	542	256
ENSECAG00000011712	3.434113394	0.4333199	0.7707612	265	143	234	294	185	89	266	164
ENSECAG00000013493	5.692075953	0.4334531	0.7707904	930	981	1060	1420	622	633	980	1202
ENSECAG00000025138	3.664057378	0.4334898	0.7707904	206	190	197	303	244	195	240	311
ENSECAG00000009651	6.846565302	0.4335399	0.7707904	2466	2095	2357	2874	1306	1445	2644	2282
ENSECAG00000009339	0.667994279	0.4337181	0.7709865	21	20	22	36	14	9	61	45
ENSECAG00000023064	5.63481231	0.4338198	0.7710366	968	994	997	1234	704	611	804	1166

ENSECAG00000015977	6.520916583	0.4341029	0.7710366	1799	1540	1575	1609	2271	1510	2191	1182
ENSECAG00000024908	4.392851815	0.4341247	0.7710366	352	415	401	290	437	380	355	454
ENSECAG00000021570	4.140211901	0.4341859	0.7710366	348	311	416	419	256	228	398	280
ENSECAG00000022011	1.434077357	0.4342086	0.7710366	66	50	52	59	27	23	63	55
ENSECAG00000000782	9.452245808	0.4343053	0.7710366	16812	11633	14603	16724	11710	9952	13156	11095
ENSECAG00000014648	4.984215667	0.4343088	0.7710366	671	584	714	706	351	402	700	650
ENSECAG00000008541	2.004318797	0.434366	0.7710366	85	74	73	110	80	39	82	50
ENSECAG00000014090	11.5439468	0.4345074	0.7710366	57393	41177	58875	53758	62219	64483	47890	56122
ENSECAG00000021926	5.64077887	0.4345454	0.7710366	1204	758	1110	1202	793	335	1237	978
ENSECAG00000018489	7.47504288	0.4345537	0.7710366	3127	2248	2998	4469	3150	2772	3658	4376
ENSECAG00000018405	7.069002624	0.4345657	0.7710366	2592	2583	2765	3461	2505	1881	2647	1808
ENSECAG00000011952	5.221397708	0.4346287	0.7710366	829	618	746	969	661	606	629	542
ENSECAG00000018551	5.595242968	0.4347255	0.7710879	1080	827	896	1301	618	581	1011	1019
ENSECAG00000010181	3.317416898	0.4348728	0.7712287	211	141	171	170	202	149	217	214
ENSECAG00000003236	4.422467239	0.4350859	0.771432	402	279	419	424	398	269	500	525
ENSECAG00000009355	3.462724446	0.4351232	0.771432	231	159	272	286	176	135	279	127
ENSECAG00000014448	2.390875707	0.4352084	0.7714338	117	80	115	138	82	42	108	102
ENSECAG00000000834	6.851182765	0.4352689	0.7714338	2916	1845	2697	2407	2493	1034	2703	1397
ENSECAG00000016995	7.32419157	0.4354143	0.7714338	3420	2759	3867	3700	3531	1495	3827	1710
ENSECAG00000009000	4.39169691	0.4354178	0.7714338	471	282	348	385	464	301	508	375
ENSECAG00000015042	7.318956213	0.4355153	0.7714338	3538	2616	3612	3919	3441	1455	3465	2186
ENSECAG00000018946	4.447468331	0.4356615	0.7714338	422	384	603	437	321	416	305	351
ENSECAG00000007107	3.498671446	0.4356735	0.7714338	214	244	243	284	71	77	209	365
ENSECAG00000015820	4.997591325	0.4357176	0.7714338	786	428	505	458	1085	329	929	252
ENSECAG00000009937	3.777824908	0.4357355	0.7714338	252	244	240	213	323	273	210	250
ENSECAG00000016510	2.715142999	0.4358761	0.7715626	295	102	116	50	111	71	142	81
ENSECAG00000026975	2.657629419	0.4362387	0.7720049	90	101	106	139	98	100	152	147
ENSECAG00000014388	2.117224325	0.436262	0.7720049	73	66	82	72	91	53	106	92
ENSECAG00000010198	4.979697991	0.4363499	0.7720402	692	556	665	762	541	328	768	468
ENSECAG00000019259	2.219601369	0.4364289	0.7720598	82	64	92	76	60	105	81	115
ENSECAG00000013937	3.031221045	0.436514	0.77209	89	180	176	76	87	142	64	367
ENSECAG00000021568	1.090020386	0.4367078	0.7723127	34	20	59	23	48	47	31	36
ENSECAG00000020253	11.32289743	0.4368408	0.7724097	50117	33871	50724	46760	52570	55036	40726	49622
ENSECAG00000022151	5.186375461	0.4368987	0.7724097	436	890	499	644	521	823	311	1185
ENSECAG00000006364	4.982176191	0.4371221	0.7726845	514	507	657	563	530	529	566	829
ENSECAG00000026938	9.881961793	0.4372742	0.7727134	16625	14998	17193	17157	22347	24294	12021	13648
ENSECAG00000007654	3.340899215	0.437325	0.7727134	221	186	187	279	92	101	242	225
ENSECAG00000018816	5.088923618	0.4373426	0.7727134	632	948	803	467	312	389	666	856
ENSECAG00000013820	3.064152528	0.437518	0.7727468	118	185	99	161	111	220	107	210
ENSECAG00000012982	4.600623918	0.4375982	0.7727468	497	292	365	583	562	374	606	363
ENSECAG00000014262	4.852241902	0.4376611	0.7727468	798	515	570	547	437	381	503	572
ENSECAG00000002521	6.866764155	0.4377003	0.7727468	2408	1944	2754	2819	2286	1641	2284	1455
ENSECAG00000013212	3.916232634	0.4377017	0.7727468	286	184	259	339	303	305	382	191
ENSECAG00000022722	4.853676834	0.4378451	0.77288	548	519	493	1039	156	914	228	401
ENSECAG00000024613	4.041847879	0.4381531	0.7733035	310	264	242	335	240	475	286	261
ENSECAG00000004183	1.474607331	0.4383195	0.7734128	54	50	63	67	37	54	46	30
ENSECAG00000016045	1.899552203	0.4383512	0.7734128	75	51	68	55	80	78	85	46
ENSECAG00000000176	3.769078126	0.4384611	0.7734146	241	284	325	312	112	270	230	253
ENSECAG00000002048	5.394047376	0.4384978	0.7734146	748	623	905	1334	570	575	831	809
ENSECAG00000010817	0.803702637	0.4385565	0.7734146	16	40	19	31	11	21	31	76
ENSECAG00000021641	0.543323632	0.4388012	0.7737259	22	24	20	26	26	30	21	31
ENSECAG00000018555	6.920391943	0.4390663	0.7739698	2344	1908	1864	2583	2394	2169	2360	2446
ENSECAG00000023067	4.284917764	0.4391192	0.7739698	520	320	418	409	474	266	341	161
ENSECAG00000010650	3.967355257	0.4391439	0.7739698	293	219	294	301	319	255	347	298
ENSECAG00000010265	6.348463457	0.4394348	0.7743625	1664	1695	1887	1549	1555	1069	1329	1424
ENSECAG00000004371	2.906386803	0.4396572	0.7744947	125	155	158	204	78	156	121	112
ENSECAG00000021756	3.002745918	0.4396949	0.7744947	139	165	158	230	112	117	221	62
ENSECAG00000025039	3.468953177	0.4397145	0.7744947	195	145	212	210	316	248	202	93
ENSECAG00000000359	3.879394802	0.4398254	0.77457	262	230	253	294	268.981	296	285	286
ENSECAG00000003056	4.352317914	0.4399959	0.7747501	176	626	289	259	251	526	392	443
ENSECAG00000023395	0.79487019	0.4403656	0.7750342	34	50	37	21	24	9	21	47
ENSECAG00000004313	7.519275504	0.4404036	0.7750342	3168	3073	2998	3893	3144	3577	4180	3370
ENSECAG00000012365	6.45877659	0.4405202	0.7750342	2533	1390	1701	1882	2029	1370	1508	752
ENSECAG00000008615	3.969003288	0.4405298	0.7750342	271	290	218	312	254	420	289	240
ENSECAG00000020502	3.749416567	0.4406184	0.7750342	293	218	265	391	287	81	284	221
ENSECAG00000008421	3.764364366	0.4406361	0.7750342	292	300	328	217	241	167	236	234
ENSECAG00000021272	1.661225825	0.4407006	0.7750342	69	45	42	54	57	83	57	44
ENSECAG00000015694	3.839304138	0.4407714	0.7750342	245	247	350	392	131	146	372	294
ENSECAG00000022851	1.452377293	0.4407921	0.7750342	30	40	69	39	29	67	48	67
ENSECAG00000010430	6.652620499	0.4408622	0.7750342	2126	1505	1691	1891	2357	1623	2201	1619
ENSECAG00000008909	6.950927781	0.4409627	0.7750342	2459	1693	2402	2271	3166	1653	2674	2190
ENSECAG00000017510	4.032612279	0.440976	0.7750342	256	245	247	414	197	291	396	407
ENSECAG00000017540	4.07760775	0.4411832	0.7751244	212	253	231	486	138	445	281	459
ENSECAG00000012463	11.69807814	0.4411839	0.7751244	59401	43560	78186	51990	63502	71744	44756	77274
ENSECAG00000009673	7.339915019	0.441314	0.7751244	4314	2299	3052	4268	2854	1619	3791	2505
ENSECAG00000015528	5.492877551	0.4413334	0.7751244	791	643	755	1049	544	1059	859	1005
ENSECAG00000020083	5.24691128	0.4413685	0.7751244	695	697	799	1019	613	478	781	657
ENSECAG00000018655	4.407576302	0.4415043	0.7751294	439	393	532	421	380	269	451	296

ENSECAG00000017704	6.222964485	0.4415078	0.7751294	1680	930	1189	1469	1905	549	2401	1220
ENSECAG00000010529	4.276795753	0.441619	0.7751908	380	319	314	361	366	344	436	358
ENSECAG00000024015	4.167379439	0.4416793	0.7751908	332	315	468	409	240	230	297	404
ENSECAG00000007751	8.285620191	0.4418499	0.7753704	4961	5790	5029	6119	3626	6442	5181	9175
ENSECAG00000012028	4.877411877	0.4421119	0.7757103	549	523	486	513	348	553	775	637
ENSECAG00000021352	5.941835109	0.4423417	0.7759937	1110	1359	1312	1353	878	1059	1023	1080
ENSECAG00000013843	1.922219835	0.4426335	0.7760905	75	48	50	85	55	67	78	95
ENSECAG00000012668	2.09437398	0.4427061	0.7760905	81	52	62	97	110	58	95	71
ENSECAG00000010318	5.011066869	0.4427124	0.7760905	582	688	412	567	405	793	778	535
ENSECAG00000006135	0.554188259	0.4428222	0.7760905	20	38	19	11	16	36	26	33
ENSECAG00000007602	5.102135677	0.4428784	0.7760905	944	519	767	680	781	421	540	488
ENSECAG00000009876	4.761252863	0.4429029	0.7760905	569	422	650	661	498	410	509	355
ENSECAG00000013091	8.043250336	0.442948	0.7760905	4760	4670	5200	3923	4681	3722	5994	6434
ENSECAG00000007422	8.121602847	0.442965	0.7760905	4365	6673	5063	7679	2869	4755	4978	5514
ENSECAG00000020284	5.207763509	0.4430118	0.7760905	628	666	649	684	656	852	665	649
ENSECAG00000009858	3.93667613	0.4437445	0.7772543	294	243	278	257	319	193	328	364
ENSECAG00000012841	1.36196272	0.4438776	0.7773676	50	54	54	58	22	18	66	54
ENSECAG00000022880	3.368152531	0.4440662	0.777578	84	376	58	107	67	314	78	378
ENSECAG00000018287	5.709037768	0.4443964	0.7780362	1134	926	1126	1210	621	828	984	1038
ENSECAG00000020559	2.764875479	0.4445933	0.778261	172	141	160	98	83	110	156	82
ENSECAG00000010946	3.683390322	0.4447323	0.7783109	155	253	201	290	197	264	277	263
ENSECAG00000016916	7.864798575	0.4447589	0.7783109	5490	4739	5212	4180	2846	3123	4310	5081
ENSECAG00000015777	4.220640876	0.4450186	0.7783865	411	195	387	302	551	236	507	195
ENSECAG00000019195	1.4182189	0.4450661	0.7783865	26	72	21	47	26	46	27	113
ENSECAG00000023299	5.473330845	0.4450991	0.7783865	898	808	1032	985	765	542	869	787
ENSECAG00000024686	5.973476395	0.4451259	0.7783865	1069	1067	1029	1318	816	829	1386	1932
ENSECAG00000004924	4.786799579	0.4451447	0.7783865	512	475	537	833	403	400	477	530
ENSECAG00000014214	3.852422619	0.445288	0.7785172	264	253	275	449	175	180	341	254
ENSECAG00000009550	10.97776626	0.4455392	0.7787167	29980	36994	34686	41063	45013	32434	53079	28792
ENSECAG00000013736	6.739433169	0.4455923	0.7787167	2021	1655	2064	1886	2468	2082	1993	1663
ENSECAG00000009032	4.032701863	0.4456256	0.7787167	227	236	439	535	115	209	336	397
ENSECAG00000004824	6.310215176	0.4456763	0.7787167	2510	1262	1489	1421	1457	1264	1419	1039
ENSECAG00000014466	5.953191342	0.4460664	0.7792783	833	1083	1063	1499	1073	1234	1206	1247
ENSECAG00000018834	2.71697209	0.446218	0.7793177	247	53	172	106	82	35	221	81
ENSECAG00000010200	6.09388585	0.446259	0.7793177	1096	1119	1185	1467	765	1747	1099	1628
ENSECAG00000007420	1.371343062	0.4462947	0.7793177	71	96	19	31	31	26	15	76
ENSECAG00000010610	9.176629301	0.4464158	0.7793844	8904	7509	9246	16201	9575	10983	14214	10740
ENSECAG00000007803	4.929672593	0.4465114	0.7793844	707	461	612	809	530	414	533	528
ENSECAG00000012703	5.681067802	0.4465388	0.7793844	930	781	1018	967	980	964	943	1039
ENSECAG00000018233	8.318890082	0.4467327	0.7795129	6220	4925	5416	6250	5197	6090	6833	6743
ENSECAG00000023236	7.065082438	0.4467882	0.7795129	1664	2895	2117	2461	1001	4104	1707	3613
ENSECAG00000003182	0.274675355	0.4468183	0.7795129	15	20.0017	38.0092	25.0042	16.0024	9.00166	19.0081	25.0045
ENSECAG00000013178	5.463742099	0.4469786	0.7796728	809	662	907	777	889	898	716	865
ENSECAG00000003882	3.329863206	0.4471433	0.7797076	164	159	206	169	208	147	279	161
ENSECAG00000015180	0.609601908	0.4471851	0.7797076	23	35	19	15	23	27	15	51
ENSECAG00000020453	5.815929353	0.4473901	0.7797076	1077	976	952	1033	1174	983	988	1171
ENSECAG00000018336	7.433356596	0.4474687	0.7797076	3780	3293	3427	4099	2842	2284	3162	3148
ENSECAG00000006519	6.666126421	0.4475134	0.7797076	1825	1365	1920	2253	2143	1657	2365	1717
ENSECAG00000007835	5.09681907	0.4475331	0.7797076	582	622	914	784	400	591	464	757
ENSECAG00000007776	2.540290695	0.4476176	0.7797076	100	77	166	160	107	60	75	121
ENSECAG00000018820	6.162187252	0.4476626	0.7797076	1320	1613	1234	1860	841	1320	1306	1239
ENSECAG00000016692	7.764626962	0.4477165	0.7797076	5291	3257	5128	4860	3926	3843	3623	2676
ENSECAG00000012420	6.113147736	0.4477312	0.7797076	1372	1297	1511	1621	1063	878	1375	1321
ENSECAG00000024376	6.172115702	0.4477536	0.7797076	1378	1224	1577	870	1196	831	1769	1939
ENSECAG00000008974	7.656771258	0.44806	0.7798664	4397	4895	3930	3629	3096	3181	3623	3318
ENSECAG00000007410	6.855896115	0.4480892	0.7798664	2741	1719	2929	2464	1539	1178	2642	2386
ENSECAG00000013000	3.005904198	0.4480938	0.7798664	183	103	123	307	133	76	246	59
ENSECAG00000009658	7.054089509	0.4481194	0.7798664	1975	2629	2200	2516	1793	3890	2247	2248
ENSECAG00000017211	4.39078079	0.4481908	0.7798713	388	263	449	392	335	377	400	516
ENSECAG00000019881	6.381700981	0.448432	0.7799974	1617	1353	1968	2144	891	1586	1455	1530
ENSECAG00000001444	2.018971731	0.4484464	0.7799974	61	108	95	78	39	91	41	70
ENSECAG00000012582	4.595920357	0.4484856	0.7799974	365	455	280	618	494	532	448	388
ENSECAG00000005265	6.863933875	0.4485515	0.7799974	2279	2478	2279	2828	1272	1714	1890	2770
ENSECAG00000013501	4.068119814	0.4486301	0.7799974	275	290	301	310	350	405	271	254
ENSECAG00000007088	1.996797783	0.4488149	0.7799974	90	35	110	118	59	14	122	56
ENSECAG00000014613	5.687891381	0.4489049	0.7799974	906	971	722	1095	889	1100	1185	796
ENSECAG00000024406	4.736974113	0.4489611	0.7799974	358	590	649	150	194	670	660	627
ENSECAG00000024180	5.250459117	0.4490434	0.7799974	991	652	802	748	660	428	749	694
ENSECAG00000014058	5.864090256	0.4490941	0.7799974	996	1041	1024	1103	1433	1059	1042	923
ENSECAG00000017397	2.33326609	0.4491663	0.7799974	75	87	110	168	42	39	107	131
ENSECAG00000016259	5.768425402	0.4491838	0.7799974	1473	799	1141	190	1658	895	1110	711
ENSECAG00000009640	4.002624001	0.4492003	0.7799974	361	290	289	422	248	178	367	266
ENSECAG00000004152	4.735287379	0.4492246	0.7799974	483	348	550	495	825	288	615	376
ENSECAG00000013253	6.909752116	0.4492959	0.7800018	2342	1740	2303	2217	2257	2524	2019	2419
ENSECAG00000021330	7.06291361	0.449405	0.7800721	3106	2202	2020	2032	3771	2341	2691	1607
ENSECAG00000014036	4.278121301	0.4494791	0.7800814	447	378	427	390	193	397	244	397
ENSECAG00000004582	-0.042376255	0.4495546	0.7800933	13	17	22	26	22	8	17	6
ENSECAG00000018845	7.772668369	0.4496704	0.7801424	4768	3991	4471	5354	4408	2704	4562	2769

ENSECAG00000013475	7.401802547	0.4497203	0.7801424	2893	2264	2611	4490	3676	2481	4037	3081
ENSECAG00000024708	4.303272306	0.449994	0.780383	321	409	462	470	341	294	352	301
ENSECAG00000009859	4.818055036	0.4499963	0.780383	502	483	414	618	472	453	729	556
ENSECAG00000003240	3.142007863	0.4500916	0.7804291	199	82	144	381	198	41	244	74
ENSECAG00000015936	6.117019157	0.4502124	0.7805193	1223	1236	1154	1376	1397	1373	1455	1096
ENSECAG00000011174	7.093892632	0.4505203	0.7805788	2044	2377	2642	2464	2210	1360	2136	5206
ENSECAG00000013711	6.303381207	0.4505822	0.7805788	1642	1599	1717	1694	1903	1190	1089	922
ENSECAG00000018966	0.146534553	0.450618	0.7805788	19	23	18	32	3	7	9	41
ENSECAG00000021846	3.912892346	0.4506429	0.7805788	340	212	338	409	211	117	384	285
ENSECAG00000020162	2.438597814	0.4506462	0.7805788	61	59	132	112	100	87	219	39
ENSECAG00000006471	2.600415902	0.4506633	0.7805788	161.001	84.0005	134	138	68.0011	96.0005	119	101.001
ENSECAG00000024884	4.948106857	0.4507277	0.7805788	460	453	509	776	362	368	729	1012
ENSECAG00000000326	2.99740618	0.4509375	0.7807398	168	93	193	85	231	125	157	115
ENSECAG00000015818	6.176257849	0.4509753	0.7807398	1346	1139	1311	1399	1413	1637	1289	1164
ENSECAG00000012194	5.094101258	0.4511463	0.7807398	698	601	766	823	748	455	597	436
ENSECAG00000013200	5.180934068	0.4511636	0.7807398	692	530	706	673	751	504	774	791
ENSECAG00000008092	1.961764366	0.4511644	0.7807398	65	52	101	39	104	67	66	65
ENSECAG00000019411	6.1609321	0.4513398	0.7809244	1157	1869	807	905	496	2247	727	2121
ENSECAG00000018459	6.619656968	0.4515427	0.7811564	2181	1750	2255	2073	1985	1067	1938	1565
ENSECAG00000014875	8.175780798	0.4516847	0.7812831	4647	4544	6363	4969	6038	5530	4793	5957
ENSECAG00000024998	3.364848031	0.4521575	0.7819818	377	107	243	167	223	72	248	113
ENSECAG00000011453	6.431572997	0.4523647	0.7822211	1486	1499	1383	1762	946	2213	1377	2087
ENSECAG00000016938	3.666117185	0.4525952	0.7825006	389	136	437	137	195	78	357	189
ENSECAG00000015725	6.65201745	0.4529044	0.7829161	2116	1784	2360	2167	1714	1330	1999	1670
ENSECAG00000003118	3.576531388	0.4530689	0.7830154	185	129	258	273	276	147	294	226
ENSECAG00000023758	2.490651193	0.4531525	0.7830154	178	64	82	172	37	128	68	103
ENSECAG00000013765	5.036484335	0.4531687	0.7830154	690	410	608	646	713	412	802	649
ENSECAG00000020584	3.470205403	0.4533846	0.7831348	171	236	276	252	190	166	168	185
ENSECAG00000010801	7.163667845	0.4534441	0.7831348	2608	2865	2900	3780	1842	2522	2381	2638
ENSECAG00000022082	4.200647816	0.4535208	0.7831348	337	351	431	436	336	189	434	259
ENSECAG00000013245	5.994085749	0.453552	0.7831348	1151	1151	1214	1950	608	776	1224	1625
ENSECAG00000023584	7.436863087	0.4536968	0.7831348	3089	2876	3272	2990	2175	2272	4178	5193
ENSECAG00000022641	4.520270719	0.4537022	0.7831348	344	285	424	584	375	661	420	299
ENSECAG00000024091	9.669730696	0.4537204	0.7831348	16204	11130	14585	16282	17621	16108	13600	15486
ENSECAG00000015334	3.678512495	0.4538198	0.7831874	202	169	207	328	256	271	335	140
ENSECAG00000016415	6.065670774	0.4540215	0.7833606	1042	1152	1309	1306	1159	853	1582	1655
ENSECAG00000019829	5.932095606	0.4540581	0.7833606	1222	838	972	1407	1192	872	1411	1274
ENSECAG00000010087	5.904920113	0.4541389	0.783381	1375	1059	1360	1206	1007	867	1141	971
ENSECAG00000024216	0.504068596	0.4543215	0.7835564	20	22	16	26	1	19	24	69
ENSECAG00000022970	4.739186483	0.4543786	0.7835564	437	374	392	673	419	782	211	624
ENSECAG00000017445	0.60759141	0.4544574	0.7835734	29	19	21	28	33	40	22	17
ENSECAG00000009157	6.33662727	0.4547316	0.7838202	1342	1409.06	1505	1519	1154	1991	1414	1602
ENSECAG00000018839	6.789545478	0.4547386	0.7838202	1944	1771	1979	2237	1744	2647	2144	1938
ENSECAG00000011023	4.250383177	0.4548602	0.7839109	286	421	416	491	183	351	393	309
ENSECAG00000010422	3.559318522	0.4549889	0.7840137	202	223	222	365	157	136	193	277
ENSECAG00000014705	3.615683733	0.4550806	0.7840529	272	185	315	275	134	124	332	220
ENSECAG00000007276	5.00018366	0.4553547	0.7843658	739	279	384	848	792	394	1210	238
ENSECAG00000022219	5.119030331	0.4554012	0.7843658	639	609	469	764	586	852	644	572
ENSECAG00000010255	3.711272077	0.4554694	0.7843658	233	250	294	339	154	165	199	329
ENSECAG00000017034	4.618174404	0.4556507	0.7845591	412	460	397	475	367	451	606	489
ENSECAG00000005163	3.062549612	0.4557762	0.7846563	131	170	121	141	89	117	146	315
ENSECAG00000020072	8.408962831	0.4561006	0.7850949	5799	5953	5796	6693	5646	5580	7106	8228
ENSECAG00000018474	5.916039066	0.456198	0.7850949	1375	1321	1453	967	395	563	1330	1709
ENSECAG00000014459	1.95072038	0.4562383	0.7850949	95	74	86	70	40	38	66	98
ENSECAG00000005229	6.694240016	0.4563136	0.7851055	2027	1458	2080	1884	1978	1517	2581	2020
ENSECAG00000012745	4.096768651	0.4565099	0.7853242	279	286	268	387	284	290	363	392
ENSECAG00000017585	4.868011699	0.4567826	0.7856068	578	412	712	805	453	255	551	674
ENSECAG00000009246	2.813574327	0.4568125	0.7856068	153	85	135	112	152	107	124	164
ENSECAG00000023986	7.702110261	0.4569082	0.7856525	6407	4943	432	363	7219	7716	1267	768
ENSECAG00000022189	3.604667805	0.4570052	0.7857004	286	202	196	356	161	171	235	224
ENSECAG00000012277	1.394178887	0.4574187	0.7862923	39	47	33	56	52	34	74	45
ENSECAG00000020128	5.011046841	0.457534	0.7863713	561	673	476	563	420	627	762	704
ENSECAG00000011057	7.94613009	0.4578577	0.786632	3939	4057	4298	5426	5100	3763	6048	4408
ENSECAG00000016419	6.080100183	0.4578912	0.786632	1680	689	1364	1085	1692	884	1741	1018
ENSECAG00000011043	5.065428184	0.4578934	0.786632	562	640	738	890	451	454	598	713
ENSECAG00000015911	5.278191931	0.4580041	0.7867033	719	737	850	954	491	597	793	701
ENSECAG00000014495	6.772214265	0.4581763	0.7868639	1827	1864	1870	2283	1777	2544	2047	1994
ENSECAG00000010810	7.240129173	0.4583267	0.7868639	4887	2437	2845	2729	3880	1585	2681	1634
ENSECAG00000005889	3.053879583	0.4584466	0.7868639	148	155	163	266	46	86	97	288
ENSECAG00000003332	4.198036396	0.4584959	0.7868639	235	329	381	336	419	411	404	187
ENSECAG00000010621	4.820329481	0.4585114	0.7868639	553	502	584	773	579	292	685	316
ENSECAG00000014934	3.244012249	0.4585983	0.7868639	202	179	212	208	132	108	150	221
ENSECAG00000014821	6.677604592	0.4586492	0.7868639	1869	1981	2292	2451	1766	1533	2016	1486
ENSECAG00000013808	3.37178159	0.4586518	0.7868639	228	202	227	215	158	106	192	218
ENSECAG00000016724	6.854888681	0.4591277	0.7869212	2642	1899	2546	2752	2601	1354	2293	1381
ENSECAG00000017462	2.820307027	0.4592212	0.7869212	107	100	131	152	108	188	135	111
ENSECAG00000010135	3.280408553	0.4592441	0.7869212	149	142	182	208	219	200	141	184
ENSECAG00000023596	7.368971114	0.4593126	0.7869212	3266	2794	2899	2714	4523	2835	3272	2181

ENSECAG00000017461	6.69251197	0.4593564	0.7869212	1611	1933	1853	1969	1672	2482	1674	2058
ENSECAG00000015395	4.45800528	0.4593876	0.7869212	335	832	400	273	135	267	457	551
ENSECAG00000006011	7.612949735	0.4594238	0.7869212	4546	4351	4714	2931	2037	1604	4070	5255
ENSECAG00000003916	5.035050909	0.4594392	0.7869212	720	561	742	757	754	364	692.001	348
ENSECAG00000024654	7.871220534	0.459466	0.7869212	4025	4221	3926	4469	4654	4706	5761	3105
ENSECAG00000012698	5.63302609	0.4594812	0.7869212	922	859	733	1067	746	898	1113	1079
ENSECAG00000012406	5.32438428	0.4594947	0.7869212	710	635	678	867	598	667	864	979
ENSECAG00000017222	4.871488946	0.459588	0.7869212	475	429	516.001	688	694	464	663	450
ENSECAG00000018500	5.0245103	0.4596214	0.7869212	586	565	464	710	437	630	612	839
ENSECAG00000023553	3.914927843	0.4596551	0.7869212	223	256	317	256	404	242	341	190
ENSECAG00000012364	1.88587976	0.4598263	0.7869706	55	39	71	88	82	79	59	62
ENSECAG00000004439	1.72018837	0.4598828	0.7869706	51	50	69	113	56	39	44	63
ENSECAG00000023363	2.491704721	0.4599763	0.7869706	134	77	127	146	48	55	121	136
ENSECAG00000000562	4.764103022	0.4599972	0.7869706	431	497	568	424	531	397	640	554
ENSECAG00000010368	3.994308757	0.4600662	0.7869706	276	414	187	484	199	305	285	227
ENSECAG00000011522	4.771620872	0.4600996	0.7869706	513	491	507	818	311	375	610	519
ENSECAG00000014876	4.90999259	0.4602418	0.7870953	579	587	683	701	280	303	760	672
ENSECAG00000013698	3.570853722	0.4604411	0.7873176	237	167	205	434	295	97	199	168
ENSECAG00000017181	5.276767137	0.4607545	0.7877348	75	2619	109	674	93	941	105	907
ENSECAG00000013592	2.875844612	0.460896	0.7878581	144	90	126	145	161	93	261	78
ENSECAG00000017766	4.730237334	0.4611179	0.7880427	465	422	523	476	661	340	512	549
ENSECAG00000014130	6.130920764	0.4613143	0.7880427	1111	1280	1184	1449	1035	1649	1164	1490
ENSECAG00000000880	0.527711253	0.4613832	0.7880427	23	18	30	19	32	20	17	39
ENSECAG00000017723	3.982823825	0.4614257	0.7880427	281	249	298	281	419	188	403	237
ENSECAG00000008976	5.682495252	0.4615912	0.7880427	1067	814	836	992	1023	782	1108	1051
ENSECAG00000005169	6.138963226	0.4616139	0.7880427	1322	1553	1290	1731	899	1234	1248	1276
ENSECAG00000018119	6.311725535	0.4616307	0.7880427	1610	1363	1507	2242	998	1131	1718	1482
ENSECAG00000023970	3.422825206	0.4617832	0.7880427	82	310	39	246	91	300	47	428
ENSECAG00000014119	9.845246543	0.4619083	0.7880427	14904	14460	13746	22741	16315	22274	20767	11987
ENSECAG00000023167	4.458348595	0.4620632	0.7880427	396	414	435	615	349	332	367	392
ENSECAG00000015691	3.905260225	0.4621449	0.7880427	278	281	247	238	371	251	328	214
ENSECAG00000014095	4.632583669	0.4621557	0.7880427	503	339	501	804	405	218	647	386
ENSECAG00000008940	1.225352401	0.4621735	0.7880427	33	49	49	15	20	45	46	72
ENSECAG00000024285	4.329860613	0.4622107	0.7880427	343	342	465	559	340	276	314	383
ENSECAG00000016034	5.298866439	0.4622283	0.7880427	1038	567	806	914	691	653	680	555
ENSECAG00000017511	4.09934741	0.4622315	0.7880427	392	286	310	485	178	204	251	479
ENSECAG00000004611	0.597798334	0.4622695	0.7880427	8	30	11	37	2	86	2	25
ENSECAG00000007524	6.258194204	0.4623197	0.7880427	1623	1019	1611	1196	1862	1623	1245	1108
ENSECAG00000022445	3.652205911	0.4623221	0.7880427	275	182	233	186	260	147	332	258
ENSECAG00000002759	3.085378933	0.4624217	0.7880942	128	138	132	197	143	119	191	214
ENSECAG00000016552	6.018080951	0.4625876	0.7881479	1145	1399	1002	1935	852	896	1377	1206
ENSECAG00000024978	2.103445293	0.4626	0.7881479	67	81	76	146	77	36	59	96
ENSECAG00000015586	0.313772681	0.4626614	0.7881479	27	15	14	22	17	20	22	33
ENSECAG00000026947	0.998841232	0.4629211	0.7883773	39	11	41	40	29	31	38	56
ENSECAG00000008225	3.752072962	0.4629567	0.7883773	232	166	326	221	220	231	238	360
ENSECAG00000016238	8.479349544	0.4630794	0.7883773	4942	6030	5536	8920	4396	9706	6142	7257
ENSECAG00000004651	8.395464168	0.4631032	0.7883773	7162	4704	6110	6045	7540	4998	8466	5466
ENSECAG00000013147	3.140963834	0.463143	0.7883773	136	151	196	279	87	183	134	149
ENSECAG00000003905	3.326156966	0.4632248	0.7883984	172	191	179	322	124	99	181	246
ENSECAG00000009787	2.832422403	0.4633227	0.7884468	94	250	63	4	113	231	55	180
ENSECAG00000011287	5.322550996	0.4634437	0.7885346	845	968	1030	598	224	310	470	1545
ENSECAG00000021543	5.023574852	0.4636339	0.78874	523	517	973	18	874	484	957	437
ENSECAG00000016367	1.867532437	0.4638094	0.7889204	72	36	60	79	52	112	69	45
ENSECAG00000018160	7.511749583	0.4640486	0.7892091	2485	5506	4262	3050	1724	2282	3468	4476
ENSECAG00000015203	6.40226598	0.4641562	0.7892739	1704	1336	2089	1990	1365	1458	1543	1220
ENSECAG00000011683	6.659290357	0.4642337	0.7892876	2088	1998	2221	2137	1602	1865	1719	1451
ENSECAG00000020973	2.534676646	0.4643087	0.789297	106	147	104	136	37	87	87	150
ENSECAG00000022714	1.165241384	0.4645622	0.7894718	37	41	51	11	36	30	75	38
ENSECAG00000016295	4.9716378	0.4646819	0.7894718	664	486	639	890	340	377	673	702
ENSECAG00000017341	7.224143305	0.46471	0.7894718	2796	2651	2691	2525	2796	2632	3240	2917
ENSECAG00000024452	2.379309548	0.4647205	0.7894718	95	59	131	167	96	45	107	82
ENSECAG00000016885	4.312062742	0.4647672	0.7894718	471	446	452	281	276	252	362	412
ENSECAG00000012514	3.178445872	0.4648286	0.7894718	156	188	142	297	103	85.0001	279	128
ENSECAG00000007289	3.87212382	0.4650324	0.7894826	260	294	377	318	150	227	142	406
ENSECAG00000019370	7.277865752	0.4650485	0.7894826	2209	2709	2222	4013	2723	3681	3375	2199
ENSECAG00000018110	4.976970701	0.4650787	0.7894826	685	585	692	670	524	448	627	491
ENSECAG00000019902	5.657233289	0.4651129	0.7894826	844	955	693	1129	735	1193	930	995
ENSECAG00000007741	5.99280478	0.4653552	0.7897758	1262	1002	1435	1734	769	708	1951	898
ENSECAG00000018603	2.640157082	0.4657314	0.7901007	6	227	41	73	12	79	24	431
ENSECAG00000017435	4.569491073	0.4658018	0.7901007	310	516	258	584	254	533	521	550
ENSECAG00000022145	4.021192229	0.4658615	0.7901007	375	239	373	398	252	148	437	245
ENSECAG00000008439	8.654238548	0.4658928	0.7901007	10581	6262	8518	8910	8358	5768	7141	5031
ENSECAG00000015805	6.726736579	0.4658944	0.7901007	1832	1887	1443	2474	1532	2163	2296	2194
ENSECAG00000020739	4.774245317	0.466039	0.790228	437	521	469	518	474	482	494	659
ENSECAG00000026937	4.28178251	0.4663617	0.7903704	294	368	260	462	366	376	310	441
ENSECAG00000017638	2.397698895	0.466406	0.7903704	143	68	153	86	59	67	161	49
ENSECAG00000012784	2.985733436	0.4664754	0.7903704	185	110	204	183	143	50	223	100
ENSECAG00000022288	1.885117681	0.466494	0.7903704	42	83	61	52	28	78	47	137

ENSECAG00000021185	3.548536521	0.466512	0.7903704	202	219	187	206	225	150	372	186
ENSECAG00000011765	7.158885766	0.4665405	0.7903704	3471	2367	3099	3132	2842	2255	2081	2130
ENSECAG00000018499	4.990508481	0.4670204	0.7909726	633	408	613	639	548	497	789	644
ENSECAG00000016917	6.501873879	0.4671133	0.7909726	1752	1204	1750	1884	1893	1380	2070	1669
ENSECAG00000014013	2.57945426	0.4672474	0.7909726	110	70	118	116	136	86	160	88
ENSECAG00000020166	3.625256999	0.4673264	0.7909726	248	206	180	235	230	182	255	294
ENSECAG00000012334	1.037018017	0.4673468	0.7909726	12	72	16	15	3	42	25	98
ENSECAG00000024591	3.518730627	0.4673812	0.7909726	178	149	216	269	131	191	311	274
ENSECAG00000020530	4.97852947	0.4674389	0.7909726	605	568	628	870	545	583	472	444
ENSECAG00000018034	6.23968186	0.4674531	0.7909726	1428	1092	1423	1529	1193	1640	1487	1452
ENSECAG00000018309	6.661387578	0.4676348	0.7911153	1650	1336	1978	2379	2292	1545	2721	1361
ENSECAG00000013072	2.140022308	0.4677159	0.7911153	87	56	73	81	121	46	140	46
ENSECAG00000022600	2.919617884	0.4677463	0.7911153	162	103	118	141	206	134	145	100
ENSECAG00000017760	6.053802554	0.468066	0.7914886	1180	1116	1232	1230	1736	763	1576	1113
ENSECAG00000024987	3.917385608	0.4681064	0.7914886	267	341	447	217	147	254	186	369
ENSECAG00000010956	1.104775325	0.4682964	0.791692	32	35	31	41	46	51	57	11
ENSECAG00000009762	5.194061816	0.4684867	0.7918959	374.992	852	819	1443.07	22.999	110	151.997	1841.4
ENSECAG00000020900	5.184732819	0.4685706	0.7919198	764	675	676	951	409	546	780	691
ENSECAG00000005210	7.302900553	0.4686855	0.7919962	3414	1986	3330	2437	3980	2310	4348	1853
ENSECAG00000011409	4.975704121	0.4688164	0.7920012	684	552	675	735	587	530	502	434
ENSECAG00000011582	6.151459359	0.4689383	0.7920012	1403	1456	1443	1617	1178	924	1529	1153
ENSECAG00000017899	8.596220721	0.4689481	0.7920012	6332	6306	6578	8626	6627	5778	9239	8746
ENSECAG00000002265	3.861717003	0.4689709	0.7920012	275	245	320	394	252	184	310	210
ENSECAG00000007328	7.863219763	0.4690371	0.7920012	4873	4640	4516	5666	4288	2536	5515	3239
ENSECAG00000024217	5.495717517	0.4691541	0.7920811	939	657	860	737	1288	650	1131	463
ENSECAG00000010851	6.695662559	0.4692396	0.7921076	1985	1700	1715	2079	2162	1892	2169	1741
ENSECAG00000019916	8.385251253	0.4694391	0.7923267	6555	5022	5541	6895	7339	4669	7896	6299
ENSECAG00000024767	4.303369608	0.4696855	0.7926248	284	379	369	354	259	491	336	426
ENSECAG00000010370	5.269830758	0.4697866	0.7926776	707	635	711	720	867	633	735	723
ENSECAG00000021651	7.047082972	0.4699664	0.7927567	2073	3022	2367	1588	1323	2339	2205	4595
ENSECAG00000005517	7.491471618	0.4700289	0.7927567	3758	3073	3444	5016	2981	2366	3656	2983
ENSECAG00000012497	6.350158125	0.4701106	0.7927567	1405	1577	1675	1110	1350	1394	1417	2137
ENSECAG00000019558	4.955572996	0.4701126	0.7927567	632	411	528	661	636	382	897	536
ENSECAG00000024006	5.460015455	0.4702481	0.7928674	976	750	993	963	706	666	699	839
ENSECAG00000022404	0.656947199	0.4704448	0.7929664	34	18	27	54	24	27	31	9
ENSECAG00000000190	2.154451422	0.4704823	0.7929664	49	96	78	76	62	81	113	93
ENSECAG00000018988	7.075374836	0.4705162	0.7929664	3142	2617	2682	2889	2859	1550	2464	2058
ENSECAG00000014603	2.970056727	0.4707318	0.793212	133	201	126	199	124	127	121	125
ENSECAG00000010068	7.996465678	0.4708653	0.7933187	5138	4127	4512	4445	5360	5046	4953	4295
ENSECAG00000023178	6.169305818	0.4709348	0.7933187	1263	1131	1387	1408	1306	1680	1388	1097
ENSECAG00000016469	7.632242041	0.4710786	0.7934433	3870	4311	5379	3230	2561	1486	3291	5724
ENSECAG00000016227	4.633815928	0.4711484	0.7934433	415	645	482	539	228	404	426	560
ENSECAG00000005335	1.477014059	0.4714416	0.7938194	61	45	55	72	36	30	66	43
ENSECAG00000021508	0.680068658	0.4715664	0.7939117	53	6	18	23	38	15	43	30
ENSECAG00000018980	8.649883499	0.4717617	0.7939399	7428	8149	7588	10935	5087	6052	7498	7913
ENSECAG00000010668	6.314632644	0.4718257	0.7939399	1519	1438	1615	2158	853	1027	1774	1706
ENSECAG00000010387	5.878607546	0.4718887	0.7939399	1030	1196	1037	1697	749	906	1241	1036
ENSECAG00000020803	4.176481156	0.4720049	0.7939399	375	337	413	409	441	151	374	218
ENSECAG00000023055	5.987436502	0.47201	0.7939399	1156	1000	1496	1732	826	681	1568	1222
ENSECAG00000012900	9.710110474	0.4720744	0.7939399	16964	15361	18761	19872	15137	10527	18555	11489
ENSECAG00000017650	6.365638727	0.4721186	0.7939399	1622	1537	1846	1906	982	1567	1409	1476
ENSECAG00000011841	2.223310115	0.4721423	0.7939399	91	55	88	80	61	34	173	116
ENSECAG00000010971	2.152861554	0.4723873	0.794158	73	75	100	50	80	73	110	85
ENSECAG00000005661	5.214801569	0.4724118	0.794158	601	631	686	743	788	425	1062	655
ENSECAG00000012231	7.296307608	0.4726265	0.7944013	3241	2151	2714	3157	4088	1906	3996	2392
ENSECAG00000016304	5.457496889	0.4727876	0.7945546	844	587	848	896	948	535	1168	795
ENSECAG00000008020	1.584237193	0.472947	0.7946038	36	48	46	72	34	76	60	59
ENSECAG00000009513	7.896497119	0.4729897	0.7946038	4464	4362	4928	6464	4022	2880	4838	4152
ENSECAG00000020999	5.063691234	0.4730268	0.7946038	589	641	566	1096	231	547	525	863
ENSECAG00000012382	1.947841712	0.4731338	0.7946661	31	60	41	224	14	71	68	74
ENSECAG00000005685	3.390795551	0.4732484	0.7947411	171	201	140	220	126	219	280	197
ENSECAG00000020767	2.64613156	0.4734587	0.7949767	166	79	103	71	121	46	157	183
ENSECAG00000013250	6.501990641	0.4736302	0.7951148	1488	1314	1691	2126	1942	1574	1810	1616
ENSECAG00000000550	5.074305617	0.4736907	0.7951148	502	645	532	732	488	555	685	888
ENSECAG00000006138	5.640226198	0.4737509	0.7951148	1164	871	965	1179	882	781	712	907
ENSECAG00000014499	2.082764895	0.4741924	0.7956555	122	54	124	61	87	21	98	60
ENSECAG00000016288	3.801521838	0.4743179	0.7956555	250	231	235	269	353	209	283	234
ENSECAG00000023909	6.190864673	0.4745781	0.7956555	1649	1140	1647	1729	1359	719	1777	1077
ENSECAG00000013631	4.017486293	0.4746495	0.7956555	322	268	300	252	313	247	349	349
ENSECAG00000017953	7.138829082	0.4746575	0.7956555	3345	2333	2720	3530	1818	2498	2373	2572
ENSECAG00000017831	6.284626505	0.4746756	0.7956555	1639	979	1499	1503	2113	1072	1702	1159
ENSECAG00000024062	5.229327128	0.4746969	0.7956555	758	769.002	516	1166	411	353	989	774
ENSECAG00000021232	2.862302551	0.4747078	0.7956555	142	126	76	158	122	86	147	220
ENSECAG00000021365	4.65718426	0.4747664	0.7956555	596	409	507	623	424	240	606	422
ENSECAG00000021649	3.085623787	0.4748166	0.7956555	181	90	145	175	204	81	266	132
ENSECAG00000014019	4.230390242	0.4748436	0.7956555	364	286	315	376	286	323	393	452
ENSECAG00000022330	5.812314078	0.4749963	0.7957939	966	1032	1031	979	628	936	1494	1352
ENSECAG00000013759	8.200969842	0.4751797	0.7959838	4669	7272	5712.97	7392	2148.99	5363.97	5137	6446.05

ENSECAG00000023365	4.721687742	0.4753651	0.796177	462	134	676	564	787	145	450	760
ENSECAG00000019077	7.697242146	0.4754448	0.7961931	4485	3271	4286	5610	2883	3185	4656	3103
ENSECAG00000011276	5.06368502	0.4755265	0.7962124	649	592	554	596	639	609	802	532
ENSECAG00000006981	8.75308134	0.4757189	0.7964173	6844	7492	8073	8040	4730	6601	8709	14137
ENSECAG00000023975	4.027512572	0.4760967	0.7969323	310	281	321	476	199	220	300	353
ENSECAG00000013115	6.399365903	0.4762396	0.797054	1497	1539	1423	1613	1357	1897	1564	1604
ENSECAG00000012679	6.01349599	0.4764322	0.797173	1470	984	1258	1832	758	466	1600	1544
ENSECAG00000013954	1.381652122	0.476451	0.797173	47	17	50	62	59	48	57	36
ENSECAG00000000902	0.834517981	0.476624	0.797345	26	30	27	33	38	25	41	31
ENSECAG00000010874	7.834315253	0.4767434	0.7973661	3357	4372	3629	4854	3640	6216	4360	3251
ENSECAG00000010505	2.949063287	0.4768249	0.7973661	183	114	141	89	198	129	153	120
ENSECAG00000019253	2.655553252	0.4768472	0.7973661	101	95	178	166	138	52	92	117
ENSECAG00000007882	5.926451501	0.4770651	0.7976129	1313	1036	1227	1530	1191	724	1153	1003
ENSECAG00000008436	0.077975957	0.4772973	0.7978837	10	10	25	20	20	10	31	18
ENSECAG00000021411	2.185149905	0.4776678	0.7982838	82	115	91	91	47	57	74	109
ENSECAG00000007916	6.152879586	0.4776772	0.7982838	1064	1738	1468	1710	709	1506	1548	897
ENSECAG00000014843	6.358548798	0.4777625	0.7983089	1487	1334	1470	1659	1534	1667	1726	1335
ENSECAG00000017689	-0.017061719	0.4779088	0.7984359	9	1	24	24	3	33	31	8
ENSECAG00000008640	3.645810545	0.4782663	0.7988282	221	209	244	196	333	258	224	145
ENSECAG00000015945	3.542720156	0.478322	0.7988282	177	187	176	281	173	115	316	330
ENSECAG00000017056	4.252899943	0.4784258	0.7988282	340	308	327	358	138	515	404	424
ENSECAG00000012699	5.33662242	0.4784755	0.7988282	578	767	609	957	567	844	954	755
ENSECAG00000006839	7.062729543	0.4784952	0.7988282	2752	2169	3067	3320	1671	2188	2984	2066
ENSECAG00000011181	6.423505522	0.4786892	0.7990295	1516	1874	1468	2352	1066	1232	1776	1679
ENSECAG00000018741	7.434611807	0.478873	0.7990295	4300	2374	4252	3836	3883	1654	3773	2156
ENSECAG00000017063	3.491442493	0.4788936	0.7990295	227	214	221	291	157	165	136	263
ENSECAG00000011432	4.775937876	0.4788972	0.7990295	437	627	383	456	305	456	511	880
ENSECAG00000005944	6.001516546	0.4790773	0.7991376	1296	920	1148	1293	1342	973	1444	1181
ENSECAG00000018403	0.351919471	0.4791027	0.7991376	20	32	24	27	13	33	14	10
ENSECAG00000014617	8.088664764	0.4792072	0.7991947	6424	4535	6150	5820	5025	3805	4785	4365
ENSECAG00000018298	4.601326241	0.4793867	0.7991962	681	402	631	313	434	301	473	385
ENSECAG00000023088	2.571997354	0.4795038	0.7991962	159	90	141	110	107	89	102	78
ENSECAG00000008100	7.428570756	0.4795635	0.7991962	3268	2649	3153	3344	3280	2486	4052	3623
ENSECAG00000006233	5.421841299	0.4795827	0.7991962	856	506	852	854	1156	572	1201	449
ENSECAG00000017115	2.11307593	0.4796408	0.7991962	90	51	67	88	120	51	106	62
ENSECAG00000008036	5.928427714	0.4796627	0.7991962	818	1322	892	1309	873	1540	1130	1101
ENSECAG00000019617	3.649040992	0.4798035	0.7991962	156	266	178	275	150	224	245	363
ENSECAG00000012845	5.942646254	0.4798944	0.7991962	1424	1085	1288	1384	1292	720	1497	601
ENSECAG00000002580	5.779763724	0.4799747	0.7991962	830	934	1159	1040	950	1165	1155	923
ENSECAG00000019473	1.963750997	0.480009	0.7991962	48	60	75	84	91	50	97	66
ENSECAG00000024711	7.045787709	0.4800534	0.7991962	3075	1960	3422	2698	2643	1785	2674	1638
ENSECAG00000021313	2.777673162	0.480075	0.7991962	143	90	179	171	115	80	141	107
ENSECAG00000005757	9.199439412	0.4801867	0.7991962	11956	11465	12376	13835	9713	8357	13503	7559
ENSECAG00000013642	1.572104007	0.4802749	0.7991962	56	31	70	100	42	17	57	69
ENSECAG00000022947	4.170686063	0.4803184	0.7991962	351	278	352	297	360	307	441	289
ENSECAG00000020425	8.235890755	0.4804783	0.7991962	5471	4978	5658	5433	4214	5251	6532	7518
ENSECAG00000018063	3.883080932	0.4806365	0.7991962	206	312	186	334	227	291	261	361
ENSECAG00000013904	5.739984719	0.4806692	0.7991962	998	851	953	1062	953	644	1428	1165
ENSECAG00000010942	7.878584123	0.4807896	0.7991962	5387	4206	5477	4679	3883	3926	3763	3855
ENSECAG00000012779	7.716049019	0.48079	0.7991962	4579	3657	4105	5496	4110	2829	4491	2529
ENSECAG00000011707	4.617607158	0.4808901	0.7991962	475	416	483	367	492	457	504	433
ENSECAG00000017553	6.663535545	0.4808949	0.7991962	2218	1880	2134	2270	2041	978	2187	1612
ENSECAG00000016746	6.488052825	0.4809186	0.7991962	1043	2017	1275	2065	1240	1966	1589	2108
ENSECAG00000023559	5.887355155	0.4809327	0.7991962	1056	878	1176	1189	1272	1047	1139	1059
ENSECAG00000010134	4.404794523	0.4809868	0.7991962	376	367	334	437	497	354	450	330
ENSECAG00000013601	4.638107287	0.4810633	0.7991962	566	357	605	577	439	294	583	344
ENSECAG00000002556	3.136939388	0.4811627	0.7991962	236	74	150	140	219	58	309	138
ENSECAG00000010085	5.351884225	0.4811781	0.7991962	766	491	802	910	824	862	741	680
ENSECAG00000019946	5.658105808	0.4813155	0.7993074	849	879	927	943	606	1332	773	1123
ENSECAG00000018986	8.109763136	0.4816205	0.7994447	4895	4788	4616	5485	5248	6241	5184	4460
ENSECAG00000013093	2.528902709	0.4816286	0.7994447	99	86	79	139	80	103	140	127
ENSECAG00000018498	3.780089968	0.4816461	0.7994447	276	225	284	389	178	121	289	321
ENSECAG00000024374	1.460971338	0.4816797	0.7994447	151	18	66	11	105	9	31	4
ENSECAG00000014595	1.07595751	0.4817891	0.7994571	46	21	36	78	50	13	30	33
ENSECAG00000006101	3.961975191	0.481833	0.7994571	273	221	320	291	385	228	381	221
ENSECAG00000011056	2.324436061	0.4818983	0.7994571	90	60	116	167	61	55	126	80
ENSECAG00000012914	3.901496314	0.4820593	0.7995278	310	206	279	256	403	154	354	266
ENSECAG00000024742	1.324253532	0.4821357	0.7995278	75	31	61	41	29	36	55	34
ENSECAG00000015237	3.365022627	0.482152	0.7995278	217.999	177	241	236.999	98.9998	150.999	167	247.999
ENSECAG00000008534	3.888621157	0.4823016	0.799659	307	204	237	291	500	174	289	194
ENSECAG00000013340	3.732587177	0.4824375	0.7996718	240	211	271	208	330	161	351	205
ENSECAG00000009259	7.570509435	0.4824501	0.7996718	3470	4294	3549	4688	2088	3015	3581	3899
ENSECAG00000022212	5.845448182	0.4826323	0.7998122	1064	890	1039	1184	1186	1100	1148	946
ENSECAG00000016042	5.882360561	0.4826756	0.7998122	1385	927	1322	1343	1195	579	1469	728
ENSECAG00000017550	7.320357554	0.4828184	0.7998288	2737	2506	2910	3403	2859	2368	3435	3768
ENSECAG00000023204	4.435425554	0.4829738	0.7998288	402	320	417	411	467	331	536	346
ENSECAG00000011277	4.962357059	0.4829981	0.7998288	688	456	553	527	798	452	694	472
ENSECAG00000009191	2.178428545	0.4831196	0.7998288	82	105	78	118	53	108	53	60

ENSECAG00000008056	5.198386127	0.4832917	0.7998288	648	880	1057	481	738	373	981	339
ENSECAG00000014558	4.304229868	0.4833401	0.7998288	377	439	438	391	267	289	299	438
ENSECAG00000002867	1.485123236	0.4833718	0.7998288	64	29	55	19	147	23	59	1
ENSECAG00000007978	7.675133056	0.4833747	0.7998288	3921	4212	4246	4781	3471	2400	4539	3306
ENSECAG00000021762	5.57609383	0.4834381	0.7998288	927	818	884	729	1594	605	934	571
ENSECAG00000024727	5.192458011	0.483449	0.7998288	438	987	464	562	265	1406	495	638
ENSECAG00000024902	9.879731362	0.4834602	0.7998288	17936	12910	18832	17851	19426	19949	15276	17498
ENSECAG00000025140	4.763445117	0.4836996	0.8000781	385	426	497	646	450	677	538	406
ENSECAG00000000018	4.752989348	0.4838096	0.8000781	424	483	511	501	333	452	618	701
ENSECAG00000008428	4.944252826	0.4838222	0.8000781	581	530	511	543	330	531	469	1072
ENSECAG00000023505	8.360794152	0.4839835	0.8001555	6124	5565	5561	6328	5134	6005	6950	7403
ENSECAG00000018350	5.863914188	0.4840099	0.8001555	1077	1117	1242	1454	1245	646	1255	756
ENSECAG00000006904	9.509422801	0.4842727	0.8004449	18204	12550	15982	14721	13092	12828	11371	10096
ENSECAG00000021477	5.964593868	0.4843259	0.8004449	1212	985	1477	1578	1011	1071	940	1094
ENSECAG00000012545	4.612610307	0.4844794	0.8005822	406	456	556	642	431	391	395	385
ENSECAG00000000147	0.984731097	0.4848115	0.8009998	25	34	30	40	17	57	35	39
ENSECAG00000000168	5.912678066	0.4848732	0.8009998	34	2848	28	17	126	422	89	5058
ENSECAG00000014569	5.026489037	0.484991	0.8010315	660	569	618	903	519	579	526	513
ENSECAG00000015619	1.435730029	0.4850844	0.8010315	31	66	45	30	32	62	25	88
ENSECAG00000011932	3.693477324	0.485132	0.8010315	158	185	214	367	240	202	414	172
ENSECAG00000010884	3.377384033	0.4851745	0.8010315	189	125	209	212	226	128	295	172
ENSECAG00000021579	6.821996031	0.4854258	0.8011175	1695	2337	1623	2459	2185	1981	2069	2474
ENSECAG00000010236	5.294761665	0.4854312	0.8011175	555	720	612	953	563	704	857	910
ENSECAG00000016834	4.021442215	0.4854382	0.8011175	232	228	327	626	285	208	290	274
ENSECAG00000014380	5.534813082	0.4859121	0.8017347	788	766	771	1042	816	1021	965	725
ENSECAG00000017443	2.524439931	0.4859903	0.8017347	97	114	110	166	92	60	124	96
ENSECAG00000013822	2.749136874	0.4860419	0.8017347	141	47	100	184	147	75	215	104
ENSECAG00000010114	8.58692861	0.4860945	0.8017347	9984	4497	8095	10581	7302	3552	8211	6515
ENSECAG00000017701	4.761735929	0.4862217	0.8017619	480	528	745	533	484	357	730	226
ENSECAG00000013814	2.009140108	0.4863433	0.8017619	79	68	47	77	49	101	51	106
ENSECAG00000017856	4.104724095	0.4864712	0.8017619	279	237	381	331	387	308	385	247
ENSECAG00000019670	5.745752705	0.486516	0.8017619	976	1267	950	1277	653	1053	1019	814
ENSECAG00000015030	8.003547202	0.4865199	0.8017619	7068	3408	5287	1775	6011	4374	7418	2768
ENSECAG00000010473	4.91794233	0.4866004	0.8017619	662	453	531	507	708	474	786	387
ENSECAG00000000099	1.91218351	0.486605	0.8017619	88	58	72	102	71	39	96	32
ENSECAG00000013295	4.379395427	0.486771	0.801919	431	345	303	410	412	350	404	434
ENSECAG00000002544	8.560158279	0.4868559	0.8019427	7268	6546	8252	9967	6657	5821	7564	4910
ENSECAG00000006936	6.946319385	0.4871127	0.8021666	2585	2121	1946	2204	2269	2043	3080	2190
ENSECAG00000008704	5.411134643	0.4871402	0.8021666	812	818	871	1058	526	671	806	836
ENSECAG00000000060	4.739001442	0.4872038	0.8021666	512	518	509	720	554	427	498	263
ENSECAG00000007955	9.508154616	0.487321	0.802177	13620	11982	11637	15093	12899	15750	14587	12734
ENSECAG00000023719	5.810232996	0.4874392	0.802177	1192	1085	1243	1130	910	781	944	1112
ENSECAG00000024384	1.176540941	0.4874862	0.802177	31	65	41	51	15	24	25	71
ENSECAG00000014477	8.189304909	0.4875396	0.802177	6098	5578	5624	7335	4796	3767	6851	4158
ENSECAG00000000968	2.924349565	0.4876936	0.802177	152	101	135	310	26	259	25	112
ENSECAG00000023433	0.926719476	0.4877044	0.802177	32	25	38	24	78	9	42	20
ENSECAG00000009114	5.802998514	0.4877044	0.802177	1293	824	1271	1343	1393	492	975	839
ENSECAG00000018513	1.184631282	0.4878249	0.8022511	27	41	58	64	23	41	48	26
ENSECAG00000023523	7.963074812	0.4879278	0.8022511	5137	3482	4692	4671	4418	4739	5619	4523
ENSECAG00000024191	6.451272207	0.4879613	0.8022511	1892	1820	1871	1665	1569	1445	1653.99	1137
ENSECAG00000011530	7.254008497	0.4881829	0.8024985	2808	3446	2804	3755	2417	2503	3358	1850
ENSECAG00000011499	3.8953479	0.4882531	0.8024985	293	270	345	348	277	131	363	219
ENSECAG00000008563	6.722849613	0.4884337	0.8026791	1693	1980	1732	2086	1673	3245	1579	1469
ENSECAG00000015151	4.578695749	0.4885734	0.8027744	413	303	519	456	383	738	428	265
ENSECAG00000019989	6.320703744	0.488633	0.8027744	1830	1140	1848	1930	1718	1096	1526	961
ENSECAG00000016392	7.866422396	0.4887721	0.8028869	5638	3433	5374	5448	4945	2481	5611	2500
ENSECAG00000009993	5.573534796	0.4889331	0.8030351	816.001	831	924.001	868	976.001	804.001	1055	816.024
ENSECAG00000008162	4.326653522	0.4892392	0.8033979	467	447	513	242	192	250	531	360
ENSECAG00000007347	1.812518803	0.4892954	0.8033979	63	57	88	92	19	28	88	88
ENSECAG00000010129	3.330824495	0.4894492	0.8034576	169	191	190	309	129	209	155	145
ENSECAG00000025106	6.150743267	0.4894733	0.8034576	1174	1286	1303	1321	782	1605	1298	1760
ENSECAG00000004756	4.339312277	0.4896846	0.8036242	339	288	390	444	354	354	420	430
ENSECAG00000021163	5.093715775	0.4897163	0.8036242	731	577	784	760	566	487	607	611
ENSECAG00000016408	1.151188333	0.4898169	0.8036733	18	122	25	16	15	55	25	28
ENSECAG00000006741	5.045612933	0.4901831	0.804158	539	489	687	674	683	499	821	557
ENSECAG00000013978	4.193727579	0.4903142	0.8042505	368	336	582	256	194	194	270	530
ENSECAG00000010097	3.915190225	0.4903811	0.8042505	305	288	348	329	156	191	262	381
ENSECAG00000022136	3.731811679	0.4908332	0.8048757	279	215	201	244	198	283	305	239
ENSECAG00000012948	7.739187284	0.4909394	0.8048889	3385	4022	3586	4264	3334	5477	3963	3521
ENSECAG00000017514	10.77629829	0.4910982	0.8048889	38081	23155	31544	33175	36391	34604	29256	34377
ENSECAG00000002876	5.017561013	0.4911227	0.8048889	747	524	662	789	614	368	616	564
ENSECAG00000017565	6.399247764	0.4912043	0.8048889	1488	1295	1712	1619	1196	1319	1888	2142
ENSECAG00000010079	5.478026995	0.491221	0.8048889	1043	750	919	1021	983	620	735	605
ENSECAG00000004289	6.844006576	0.4913597	0.8048889	2877	2424	2593	1729	1108	1238	2041	3228
ENSECAG00000021023	4.551930185	0.4913659	0.8048889	505	410	558	488	411	413	388	319
ENSECAG00000016164	2.940622535	0.491524	0.8048889	212	83	241	119	143	59	189	105
ENSECAG00000012707	4.884035725	0.4915599	0.8048889	512	562	474	558	510	674	492	565
ENSECAG00000021517	4.006716315	0.4916063	0.8048889	342	262	385	360	226	247	299	285

ENSECAG00000002284	4.077380331	0.4916207	0.8048889	343	256	460	359	273	236	296	304
ENSECAG00000020622	4.958982008	0.4918556	0.8051482	631	541	652	791	546	463	681	380
ENSECAG00000018882	4.548357427	0.4919586	0.8051482	536	395	429	614	285	340	412	511
ENSECAG00000026130	2.605623864	0.4919917	0.8051482	237	151	60	62	93	111	105	64
ENSECAG00000024618	4.915717349	0.4922385	0.805349	498	658	598	775	553	355	676	436
ENSECAG00000005172	0.244981162	0.4922835	0.805349	18	20	27	30	11	10	32	17
ENSECAG00000016758	9.684100171	0.4923271	0.805349	16958	12737	14596	14425	15048	16868	12264	18847
ENSECAG00000018903	6.022607168	0.4925783	0.8053668	1267	1025	1602	1556	1063	838	1492	999
ENSECAG00000007334	4.642771439	0.4926057	0.8053668	490	427	533	652	463	358	416	410
ENSECAG00000008109	7.896037794	0.4927119	0.8053668	4350	5436	6771	3501	2038	2605	4072	6945
ENSECAG00000015098	6.982782084	0.4927193	0.8053668	2375	2651	1591	2359	1591	2926	2786	2463
ENSECAG00000022744	3.158043625	0.4927423	0.8053668	139	145	178	305	120	152	153	147
ENSECAG00000008695	5.879661497	0.4927634	0.8053668	1217	997	1226	1490	965	766	1321	927
ENSECAG00000003116	3.110347611	0.492951	0.8054943	180	127	212	213	144	95	201	126
ENSECAG00000023217	5.891131526	0.4929832	0.8054943	798	1267	793	1406	1001	1344	1346	862
ENSECAG00000016813	4.87311329	0.4932673	0.805747	683	449	569	778	449	328	718	479
ENSECAG00000008999	4.559381119	0.4934718	0.805747	443	516	359	670	377	268	397	518
ENSECAG00000010593	5.539649004	0.4934962	0.805747	814	710	876	968	970.007	1053	701	778
ENSECAG00000017746	5.37985544	0.4934983	0.805747	786	871	1031	814	366	515	595	1261
ENSECAG00000021142	4.434635894	0.4935749	0.805747	404	331	546	546	334	239	508	369
ENSECAG00000016864	6.061697818	0.4936094	0.805747	1340	1384	1347	1470	1096	783	1328	1302
ENSECAG00000012398	3.251712051	0.4936344	0.805747	184	184	176	260	181	110	179	151
ENSECAG00000016946	6.095313326	0.4938523	0.8058141	1558	1091	1608	1450	973	1229	1106	1213
ENSECAG00000005934	0.136744974	0.4938877	0.8058141	19	30	7	4	4	40	4	34
ENSECAG00000015448	6.672802337	0.4939113	0.8058141	1925	2012	2275	2292	1831	1304	2237	1498
ENSECAG00000015011	6.099187625	0.4939593	0.8058141	1280	959	1168	1559	1571	744	2151	967
ENSECAG00000002353	2.022229779	0.494367	0.8059882	32	75	66	99	33	111	51	118
ENSECAG00000010268	7.479071238	0.4943762	0.8059882	3613	3362	3847	4137	2665	2556	3073	3574
ENSECAG00000013985	6.81292537	0.4944231	0.8059882	1822	2181	1664	2334	1132	2101	2120	3412
ENSECAG00000017943	5.42174427	0.4944584	0.8059882	697	959	958	299	758	666	586	1347
ENSECAG00000023692	8.359705998	0.4944651	0.8059882	7419	4972	5529	5616	6738	5854	7141	5674
ENSECAG00000011715	6.266210493	0.4944918	0.8059882	1896	1208	1859	1455	1414	874	1578	1310
ENSECAG00000007112	4.502528695	0.4945857	0.8060257	385	259	413	571	677	445	361	249
ENSECAG00000010247	4.973681644	0.4947516	0.8061804	579	433	488	789	488	625	602	691
ENSECAG00000013705	0.389393454	0.4949429	0.8063765	18	15	24	25	46	9	30	14
ENSECAG00000017058	4.324978631	0.4950809	0.8064855	514	315	472	375	322	227	422	364
ENSECAG00000011565	5.764821072	0.4952148	0.8065881	990	1118	989	1451	751	1019	914	909
ENSECAG00000000558	7.3249774	0.4954212	0.8068085	3129	2898	2481	3017	2796	2788	3409	3387
ENSECAG00000014686	5.737765656	0.4956301	0.807033	1119	831	793	1145	924	870	1311	1014
ENSECAG00000006210	6.501570454	0.4957572	0.8071243	1859	1499	2005	2219	1579	1184	1617	1699
ENSECAG00000017182	8.095180802	0.4958914	0.807227	5912	6016	5675	5338	3469	2344	6472	6178
ENSECAG00000007120	2.220062518	0.4959755	0.8072483	40	157	54	45	91	56	84	141
ENSECAG00000009617	3.875596531	0.4960532	0.8072591	402	216	267	359	184	165	299	323
ENSECAG00000017333	6.935135714	0.496268	0.8074623	2683	2194	2592	2850	2741	1670	2452	1222
ENSECAG00000019037	0.311511657	0.4963217	0.8074623	27	13	18	20	15	37	14	23
ENSECAG00000010696	2.671623797	0.4964077	0.8074623	118	63	122	143	115	111	201	76
ENSECAG00000011175	3.717223269	0.4964624	0.8074623	220	170	267	285	259	248	241	259
ENSECAG00000012801	0.867699432	0.4968173	0.8076633	28	22	18	53	28	20	49	44
ENSECAG00000011493	4.899873577	0.4970349	0.8076633	664	198	630	567	1395	273	515	198
ENSECAG00000000258	4.911850867	0.4971248	0.8076633	906	530	619	461	327	353	574	743
ENSECAG00000022566	6.65586575	0.4971915	0.8076633	1760	1382	2219	1946	1917	2283	1698	1735
ENSECAG00000005942	0.938621015	0.4972984	0.8076633	31	30	44	55	23	13	28	52
ENSECAG00000014094	0.986188688	0.4973064	0.8076633	58	27	30	3	60	22	69	10
ENSECAG00000023259	1.631614481	0.4973072	0.8076633	39	49	54	65	21	90	52	73
ENSECAG00000016354	6.151473638	0.4974547	0.8076633	1288	1075	1315	1521	1367	1254	1437	1362
ENSECAG00000020044	4.088319596	0.4974995	0.8076633	326	275	312	287	462	166	373	331
ENSECAG00000009177	5.698767306	0.4975105	0.8076633	980	991	1201	1158	613	993	822	996
ENSECAG00000016147	3.650577063	0.4975752	0.8076633	282	209	165	218	211	247	136	370
ENSECAG00000017183	1.263085108	0.4975911	0.8076633	43	34	49	75	33	29	38	48
ENSECAG00000014912	4.679109698	0.4977205	0.8076633	567	417	630	535	479	241	578	420
ENSECAG00000023099	5.201756168	0.4977305	0.8076633	566	642	706	710	468	1040	581	692
ENSECAG00000020616	3.053186018	0.4978687	0.8076633	115	133	127	207	78	125	223	234
ENSECAG00000008071	2.620461839	0.4978847	0.8076633	121	109	110	79	95	80	133	175
ENSECAG00000010252	6.91186066	0.4980121	0.8076633	2015	2109	1969	2611	2377	2492	2629	1726
ENSECAG00000017992	4.287013578	0.4980559	0.8076633	238	370	386	370	498	464	384	152
ENSECAG00000022815	6.979470505	0.4980632	0.8076633	2228	2709	2481	3139	1695	1987	2673	2089
ENSECAG00000018441	5.589532699	0.4981284	0.8076633	972	775	749	1004	975	907	935	845
ENSECAG00000003847	2.968107778	0.4981981	0.8076633	210	156	133	164	105	81	77	232
ENSECAG00000011022	3.844476024	0.4982364	0.8076633	262	240	265	251	284	265	237	311
ENSECAG00000010640	6.532873019	0.4982541	0.8076633	1666	1489	1922	1621	2101	1485	1849	1650
ENSECAG00000019296	3.911080593	0.4982925	0.8076633	246	252	246	323	193	260	258	455
ENSECAG00000002455	1.35271539	0.4983749	0.8076816	35	46	58	75	23	38	63	35
ENSECAG00000011960	5.516435449	0.4984719	0.8076907	770	748	1071	687	721	629	1057	1159
ENSECAG00000023316	6.841864661	0.4985227	0.8076907	2506	1767	2768	2607	2265	1659	1946	1719
ENSECAG00000013749	4.778722888	0.4986378	0.8077619	497	438	382	673	446	560	583	515
ENSECAG00000026993	1.622618308	0.4988111	0.8078431	52	26	74	58	60	52	54	69
ENSECAG00000025016	5.50624399	0.4988794	0.8078431	839	842	879	1248	632	716	785	899
ENSECAG00000012154	5.861240213	0.4989012	0.8078431	1021	912	1104	1168	943	1422	898	1116

ENSECAG00000000743	4.434905671	0.4991628	0.8081514	387	307	462	700	249	256	571	377
ENSECAG00000016185	2.053736304	0.4993988	0.8083259	71	26	54	137	79	95	117	35
ENSECAG00000000800	3.648354991	0.4994129	0.8083259	274	221	267	298	233	88	291	221
ENSECAG00000015394	2.284471892	0.499489	0.8083339	108	83	89	134	43	49	137	88
ENSECAG00000008097	4.961036756	0.4996177	0.8084003	681	578	620	730	330	445	615	690
ENSECAG00000012229	9.305599332	0.4996724	0.8084003	16445	8993	12860	5562	12685	7335	12405	17860
ENSECAG00000017997	2.568726172	0.4999708	0.8085485	150	78	89	84	163	67	194	52
ENSECAG00000007465	5.878351606	0.4999893	0.8085485	1067	949	931	1340	838	1105	1161	1401
ENSECAG00000021034	2.274180314	0.5000246	0.8085485	77	59	97	103	96	115	87	69
ENSECAG00000008848	7.267887576	0.5000882	0.8085485	2635	2304	2955	5405	2343	2193	3835	1932
ENSECAG00000016372	4.552121085	0.5001407	0.8085485	454	243	462	898	388	287	638	241
ENSECAG00000022494	6.814542875	0.5002498	0.8085485	2175	1888	2021	1958	1434	2938	2143	2063
ENSECAG00000019284	4.349424193	0.5003818	0.8085485	293	302	370	498	231	457	341	536
ENSECAG00000023400	2.062331222	0.5005207	0.8085485	36	100	64	80	74	69	66	114
ENSECAG00000012024	5.441041396	0.500598	0.8085485	407	1299	448	806	327	788	1028	1337
ENSECAG00000013326	3.787050822	0.5006358	0.8085485	317	251	308	281	146	200	232	325
ENSECAG00000010329	3.901068905	0.500636	0.8085485	210	237	235	386	159	302	319	381
ENSECAG00000016601	5.54637491	0.500646	0.8085485	994	885	961	1063	1126	547	768	656
ENSECAG00000009549	7.369496243	0.5006893	0.8085485	2949	2312	2545	4126	1984	4796	2699	3096
ENSECAG00000005632	1.002374683	0.5010847	0.8088641	27	40	31	33	37	31	37	46
ENSECAG00000010622	10.63790839	0.5010896	0.8088641	32606	21230	30541	30027	31904	35273	23501	30764
ENSECAG00000023195	5.03017009	0.5010984	0.8088641	619	904	525	663	337	408	655	777
ENSECAG00000021867	4.992871426	0.501226	0.8089551	717	445	865	663	786	407	512	372
ENSECAG00000002716	5.731574504	0.5013907	0.8091059	1118	890	1041	735	686	1261	1167	953
ENSECAG00000003146	3.309687268	0.5015642	0.8091662	152	163	180	205	130	178	201	259
ENSECAG00000000584	5.86592153	0.5015705	0.8091662	1278	808	1091	1055	1242	1047	1066	1081
ENSECAG00000010996	5.001743127	0.5017262	0.8093024	593	489	634	595	600	668	597	568
ENSECAG00000021600	3.070107347	0.5020085	0.8095591	191	129	157	245	230	69	138	99
ENSECAG00000013375	7.150336414	0.5020467	0.8095591	2698	2391	2890	4060	2268	2505	2796	1831
ENSECAG00000015176	3.112438969	0.5020991	0.8095591	156	126	190	266	130	101	160	173
ENSECAG00000016291	6.921970991	0.5021801	0.8095748	2571	2247	2053	3360	1514	1618	2617	2415
ENSECAG00000026924	4.056925639	0.5023578	0.8097463	247	286	273	383	199	276	361	461
ENSECAG00000002977	2.483759822	0.502572	0.8098867	61	45	82	198	251	46	99	49
ENSECAG00000016338	3.899924646	0.5026422	0.8098867	304	193	333	226	247	328	269	297
ENSECAG00000012127	3.69222052	0.5026588	0.8098867	277	222	295	293	197	251	241	146
ENSECAG00000015333	2.205782333	0.5027802	0.8099674	54	192	57	79	58	80	43	97
ENSECAG00000015107	5.365054415	0.503039	0.8101706	747	695	689	866	731	726	841	850
ENSECAG00000007301	8.484745871	0.503049	0.8101706	6025	7620	6142	10677	3879	6666	6554	6429
ENSECAG00000006357	4.202363059	0.5034129	0.8104031	278	352	469	452	191	366	320	319
ENSECAG00000018084	4.032654	0.5034381	0.8104031	284	249	249	395	215	320	328	398
ENSECAG00000014963	4.886739182	0.5034614	0.8104031	505	440	482	709	359	422	994	580
ENSECAG00000015525	1.486590814	0.5034787	0.8104031	38	24	79	103	37	18	83	38
ENSECAG00000015262	5.459035317	0.5035946	0.8104748	650	684	867	987	523	961	843	1039
ENSECAG00000020862	0.613964844	0.5036683	0.8104786	40	6	8	45	44	24	19	28
ENSECAG00000018522	3.623974518	0.503893	0.8106221	339	201	291	199	201	136	298	181
ENSECAG00000018095	0.717543065	0.5039002	0.8106221	21	33	19	32	20	13	49	46
ENSECAG00000017833	0.768499167	0.5040278	0.8106664	26	24	31	62	14	21	44	24
ENSECAG00000021051	7.440563332	0.5040705	0.8106664	3383	2428	3354	3356	4395	2112	4283	2799
ENSECAG00000013272	4.463270806	0.5043243	0.8109598	477	445	384	534	389	288	383	403
ENSECAG00000012216	3.741431898	0.5044	0.8109666	191	212	238	318	268	267	261	226
ENSECAG00000011243	7.701184024	0.5052377	0.812114	3400	3636	3541	4509	3838	3721	4449	4005
ENSECAG00000011678	6.640463284	0.5052566	0.812114	2205	1784	2013	2317	1683	1168	2115	1789
ENSECAG00000019833	4.883352732	0.5053585	0.8121629	592	527	821	538	415	212	737	629
ENSECAG00000000708	6.369187139	0.5055176	0.8122294	1926	1372	1772	1813	1342	1442	1470	1244
ENSECAG00000024439	4.36198991	0.5056021	0.8122294	389	304	308	489	416	337	387	436
ENSECAG00000010215	1.435742124	0.5056144	0.8122294	35	28	40	80	36	36	118	28
ENSECAG00000021727	7.038821247	0.5059985	0.8125538	4265	1509	2231	1059	3341	1193	2656	3330
ENSECAG00000010480	7.332392641	0.5060825	0.8125538	3799	2740	3512	3465	3216	2592	2599	2188
ENSECAG00000018901	4.560307369	0.5061846	0.8125538	345	369	495	488	366	309	546	622
ENSECAG00000012898	0.87182598	0.5062172	0.8125538	19	27	23	51	16	57	29	34
ENSECAG00000016332	6.804192482	0.5063964	0.8125538	1907	1932	1878	2388	1624	2203	2319	2427
ENSECAG00000008642	8.239466142	0.5065322	0.8125538	6576	5144	6794	6946	4171	3394	6054	6693
ENSECAG00000018506	3.714774457	0.5065957	0.8125538	243	301	219	128	372	135	321	212
ENSECAG00000010361	2.643780535	0.5066028	0.8125538	84	105	78	170	66	145	157	118
ENSECAG00000017860	5.596915803	0.5066586	0.8125538	856.001	1084	761.001	721.004	828	803.001	752	1331
ENSECAG00000009660	2.659298147	0.5067078	0.8125538	125	116	121	62	118	78	84	214
ENSECAG00000021707	5.891672762	0.5069417	0.8125538	850.999	1152	1072	1954	949.998	895.988	1240	884
ENSECAG00000006943	6.096018299	0.5069707	0.8125538	1427	1251	1353	1706	1286	595	1665	1109
ENSECAG00000000478	3.364098955	0.5069926	0.8125538	148	253	176	120	187	99	342	204
ENSECAG00000012025	2.017275977	0.5070201	0.8125538	66	68	71	71	80	44	133	63
ENSECAG00000021294	5.625355633	0.5070799	0.8125538	1130	761	1129	1106	591	687	1018	1031
ENSECAG00000018052	1.282514034	0.5071504	0.8125538	33	27	32	73	28	30	51	80
ENSECAG00000024193	6.7920058	0.5071808	0.8125538	2113	1865	1844	2190	1742	2483	2037	2160
ENSECAG00000011702	3.685593444	0.5071888	0.8125538	179.983	209.988	220.971	309.976	239.974	269.974	211.969	257
ENSECAG00000005329	4.543095845	0.5072846	0.8125538	489	426	533	492	436	312	377	417
ENSECAG00000004908	6.18450581	0.5073224	0.8125538	1752	1078	1642	1621	1460	997	1520	890
ENSECAG00000011034	0.947970797	0.5073922	0.8125538	19	31	27	49	66	13	45	25
ENSECAG00000021385	9.410598059	0.5074259	0.8125538	13622	12334	14523	16975	11266	7383	15919	11343

ENSECAG00000018490	6.059613259	0.507468	0.8125538	1193	1067	1423	1119	1692	774	1534	1174
ENSECAG00000022690	8.513839331	0.5075535	0.8125538	10238	4862	7142	3012	10674	4315	8714	5367
ENSECAG00000011103	3.952366126	0.5076047	0.8125538	275	249	272	306	230	183	435	376
ENSECAG00000019135	0.505583519	0.50781	0.8126004	23	27	20	45	20	18	22	24
ENSECAG00000013534	8.061821937	0.5079082	0.8126004	5754	3583	5009	4933	5365	3797	7294	4500
ENSECAG00000018428	5.638497909	0.5079459	0.8126004	1016	1015	741	1489	559	456	630	1636
ENSECAG00000007856	3.990607175	0.5079525	0.8126004	344	163	281	357	382	263	330	250
ENSECAG00000012455	5.653323478	0.5079915	0.8126004	836	853	959	1617	762	593	1110	939
ENSECAG00000010213	5.136983215	0.508133	0.8126545	707	717	757	736	533	656	558	568
ENSECAG00000000543	1.00140295	0.5081684	0.8126545	15	60	18	30	16	22	29	91
ENSECAG00000019915	4.850113348	0.5082487	0.8126684	572	513	407	576	547	427	486	759
ENSECAG00000019750	6.263467094	0.5083422	0.8127036	1573	1017	1154	1805	1721	2004	1378	716
ENSECAG00000017368	4.974029163	0.5084478	0.8127581	698	464	730	756	529	295	613	673
ENSECAG00000023224	7.172057051	0.5088985	0.8131489	2924	2774	2593	3846	1985	1670	3336	2777
ENSECAG00000017313	2.633232424	0.5089574	0.8131489	66	145	96	93	59	34	61	355
ENSECAG00000018450	2.054529394	0.5089685	0.8131489	47	72	56	113	85	88	67	75
ENSECAG00000016497	5.880028302	0.5090156	0.8131489	974	1090	1074	1120	1022	1181	984	1273
ENSECAG00000015358	5.841151459	0.5091002	0.8131489	1085	1031	1075	857	607	1421	762	1577
ENSECAG00000012960	5.068620218	0.5091218	0.8131489	617	558	758	451	599	706	543	703
ENSECAG00000018065	1.775809464	0.5093104	0.813253	66	36	76	117	85	33	59	35
ENSECAG00000009447	3.206871858	0.5094373	0.813253	181	161	188	255	108	78	260	170
ENSECAG00000013159	5.454558574	0.5095006	0.813253	925	835	821	1075	872	685	757	594
ENSECAG00000010233	2.932544074	0.5095542	0.813253	150	120	205	165	121	108	153	114
ENSECAG00000021657	11.25398204	0.5095818	0.813253	40798	50634	31871	50769	32340	51596	47936	57002
ENSECAG00000023169	2.016948349	0.5096166	0.813253	51	115	76	93	53	64	70	67
ENSECAG00000016357	6.36722986	0.509731	0.8133214	1639	1510	1525	2226	1398	1142	1659	1368
ENSECAG00000013938	4.051792091	0.5098802	0.8134451	308	294	386	412	144	232	336	386
ENSECAG00000021627	5.099230994	0.5100339	0.8134721	919	685	756	480	632	295	773	601
ENSECAG00000019691	0.998264933	0.5100403	0.8134721	61	33	59	10	37	31	20	28
ENSECAG00000023375	5.537331766	0.510571	0.8141981	977	865	953	1056	755	629	1041	720
ENSECAG00000011451	8.108491859	0.5106389	0.8141981	5394	4911	5693	7269	5061	4335	4606	4243
ENSECAG00000024441	2.217991849	0.5107254	0.8142218	97	51	114	137	66	41	134	61
ENSECAG00000024629	6.795415725	0.5108846	0.8143613	1922	1676	1752	2759	1386	1869	2694	2700
ENSECAG00000022464	4.621182328	0.5109815	0.8144015	534	467	476	573	360	311	516	466
ENSECAG00000011380	7.235073458	0.5111071	0.8144873	2884	1900	2797	3320	3367	3304	3315	1575
ENSECAG00000016013	5.443154361	0.5112891	0.8145763	847	661	816	824	994	834	918	568
ENSECAG00000020058	4.27519268	0.5113064	0.8145763	355	374	341	559	257	270	389	374
ENSECAG00000005833	2.454507564	0.5115775	0.8148915	49	158	66	93	115	103	95	111
ENSECAG00000013078	0.399331179	0.5116804	0.8148915	28	21	23	36	27	6	38	7
ENSECAG00000001211	3.636240974	0.5117194	0.8148915	157	236	260	203	83	240	244	413
ENSECAG00000016518	7.214504513	0.5118074	0.8149173	2740	2523	2468	3005	2359	3332	2975	2660
ENSECAG00000000377	4.90881059	0.5119079	0.8149631	600	466	503	608	527	565	685	524
ENSECAG00000016543	8.42999669	0.5120379	0.8150128	7421	6295	7691	7522	6421	3910	7822	5048
ENSECAG00000003822	1.598011129	0.5121202	0.8150128	57	37	69	97	49	10	69	63
ENSECAG00000016193	6.197066504	0.5122037	0.8150128	1403	1081	1315	1548	966	1552	1428	1647
ENSECAG00000011331	8.560017166	0.5123216	0.8150128	7168	8453	9037	6745	4722	6164	5963	8011
ENSECAG00000012022	7.868017714	0.5123412	0.8150128	4559	4410	5454	5116	3649	4229	4125	3414
ENSECAG00000002387	4.343799914	0.5123853	0.8150128	221	334	284	540	79	181	232	1184
ENSECAG00000008043	5.319056364	0.5124764	0.8150128	679	753	777	1155	740	649	817	442
ENSECAG00000014535	3.742155577	0.5125729	0.8150128	226	167	281	283	188	319	234	278
ENSECAG00000013897	7.000524826	0.5125849	0.8150128	3015	2228	3029	2468	2917	1326	2879	1437
ENSECAG00000017389	5.563226349	0.5126799	0.8150435	973	852	949	1158	779	768	856	758
ENSECAG00000018645	7.050608314	0.5127477	0.8150435	2403	2281	2334	2553	2174	2898	2306	2693
ENSECAG00000022877	2.377079228	0.5129824	0.8150773	87	55	99	124	65	95	112	130
ENSECAG00000001101	5.671394395	0.5130535	0.8150773	704	679	757	1471	213	841	900	2136
ENSECAG00000000057	4.503822292	0.5131543	0.8150773	559	306	537	503	345	279	523	376
ENSECAG00000020054	1.01005998	0.5131907	0.8150773	41	37	23	31	46	31	36	38
ENSECAG00000022321	5.243967597	0.5133378	0.8150773	838	608	760	1002	749	329	1030	460
ENSECAG00000023709	6.492405902	0.5134985	0.8150773	1632	1648	1361	1911	1775	1619	2080	1416
ENSECAG00000011445	4.04971885	0.513513	0.8150773	334	334	296	429	299	317	290	165
ENSECAG00000007576	5.144222789	0.5135385	0.8150773	846	527	703	927	754	287	907	435
ENSECAG00000022130	5.904715444	0.5135879	0.8150773	1245	1112	1353	1258	867	1149	1108	853
ENSECAG00000002063	0.850271505	0.5135982	0.8150773	44	25	44	36	22	18	12	54
ENSECAG00000001426	8.649605589	0.5136629	0.8150773	6633	8053	5860	8155	5871	9635	6888	8256
ENSECAG00000021111	6.598674053	0.5137676	0.8150773	1992	1693	1682	1626	2289	1565	1948	1601
ENSECAG00000020689	0.95347342	0.5138074	0.8150773	42	19	36	31	41	32	24	47
ENSECAG00000006748	5.828821675	0.5138495	0.8150773	900	1156	1098	931	1069	1058	1184	1022
ENSECAG00000008435	5.122063238	0.5138957	0.8150773	529	930	640	780	486	524	617	686
ENSECAG00000016820	2.537928784	0.5139522	0.8150773	69	69	97	174	140	100	156	58
ENSECAG00000011390	6.603452417	0.5139888	0.8150773	1774	1806	1492	1978	1744	2142	1824	1646
ENSECAG00000015900	3.605922062	0.5142065	0.8151748	201	203	216	248	267	231	226	203
ENSECAG00000012207	2.176896849	0.5142385	0.8151748	66	50	78	119	116	43	157	46
ENSECAG00000012518	6.202833731	0.5142727	0.8151748	1388	1502	1747	1490	1077	652	1508	1756
ENSECAG00000006869	0.483903501	0.5144255	0.8151748	4	31	1	50	15	28	34	32
ENSECAG00000001568	5.189814239	0.5144515	0.8151748	530	726	541	829	455	821	948	592
ENSECAG00000015618	5.920931769	0.5147845	0.8151748	1469	885	1446	1283	1383	813	1181	644
ENSECAG00000025013	5.459719623	0.5148098	0.8151748	805	653	834	923	844	830	873	795
ENSECAG00000002379	6.239101142	0.5148477	0.8151748	1351	1208	1299	1640	1478	850	2225	1381

ENSECAG00000022315	4.324844739	0.5148868	0.8151748	446	290	472	473	331	294	355	346
ENSECAG00000018843	5.204032505	0.514954	0.8151748	700	584	642	754	764	496	816	764
ENSECAG00000007827	7.786440328	0.5149719	0.8151748	3708	3935	3750	4544	3423	5003	4405	4022
ENSECAG00000006649	7.515619354	0.5150605	0.8151748	3319	3135	3348	3413	2836	3352	4181	3747
ENSECAG00000009333	5.927083865	0.5150734	0.8151748	1088	1000	1076	1279	1210	965	1522	983
ENSECAG00000021478	9.44468394	0.5150788	0.8151748	14939	10926	12779	11177	16245	13307	12708	11101
ENSECAG00000009160	5.970963439	0.5151268	0.8151748	1450	1212	1117	1425	791	1004	1208	1210
ENSECAG00000015258	10.39709041	0.5152199	0.8151924	23897	21689	24919	26365	26358	32498	20777	22559
ENSECAG00000014410	3.616864495	0.5152814	0.8151924	335	282	212	203	359	130	207	83
ENSECAG00000024049	0.797844479	0.5156732	0.8156986	33	21	27	33	45	41	24	17
ENSECAG00000021794	0.981680064	0.5160229	0.8159657	25	36	36	32	23	51	26	46
ENSECAG00000010590	4.927269438	0.5160682	0.8159657	578	538	654	778	422	582	550	454
ENSECAG00000015861	4.932918728	0.516091	0.8159657	650	478	693	738	557	337	611	548
ENSECAG00000007773	4.631600981	0.5161914	0.8159657	597	334	602	558	431	217	589	433
ENSECAG00000000004	3.605257533	0.5162137	0.8159657	250	213	238	327	120	151	276	262
ENSECAG00000009065	11.74588492	0.5163493	0.8159657	69003	49809	62511	66112	72820	73427	53863	61140
ENSECAG00000000578	6.849604445	0.5163951	0.8159657	2176	1736	2553	1839	1924	1645	2692	2713
ENSECAG00000018610	1.546312299	0.5164167	0.8159657	40	52	58	45	79	57	59	26
ENSECAG00000013294	6.576100258	0.5165818	0.816113	1864	1672	1678	1690	1946	1934	1632	1693
ENSECAG00000021720	4.949782181	0.517072	0.8164358	593	531	602	888	443	335	497	786
ENSECAG00000019268	2.186396718	0.5170885	0.8164358	59	56	92	110	46	84	127	99
ENSECAG00000011235	5.869233347	0.5171147	0.8164358	1318	1144	1179	1195	795	749	1068	1329
ENSECAG00000023139	3.664449751	0.5171372	0.8164358	236	64	293	293	329	86	148	450
ENSECAG00000020036	5.902434492	0.5171455	0.8164358	1273	730	1352	982	1148	1109	1380	949
ENSECAG00000000691	7.340871657	0.5178467	0.8172684	3149	2528	3020	3032	3651	2401	3743	2737
ENSECAG00000015133	5.584367314	0.5178731	0.8172684	1563	579	1029	870	918	554	1209	533
ENSECAG00000000030	4.334190291	0.5179638	0.8172684	407	344	445	500	226	258	364	494
ENSECAG00000021962	5.083269343	0.5180396	0.8172684	673	337	490	939	835	594	1053	205
ENSECAG00000000940	4.412543525	0.5180405	0.8172684	511	301	445	219	666	329	463	201
ENSECAG00000019053	3.050582671	0.5181726	0.8172684	109	134	152	190	132	95	267	165
ENSECAG00000018188	3.834941948	0.5181765	0.8172684	371	310	269	241	118	326	211	253
ENSECAG00000015928	4.036765218	0.5184249	0.8175466	235	270	336	338	289	329	331	303
ENSECAG00000024429	7.176387951	0.5187749	0.8179851	2760	3002	2154	2085	1937	5345	1565	1999
ENSECAG00000024503	6.311960552	0.5190317	0.8181686	1691	1384	1483	2073	1240	1252	1346	1481
ENSECAG00000000963	4.187996373	0.5190838	0.8181686	369	308	333	284	359	406	307	307
ENSECAG00000016932	5.318748917	0.5191074	0.8181686	787	804	880	825	620	556	736	768
ENSECAG00000009602	6.707615979	0.5193191	0.8183886	2177	2115	2088	2305	1915	1159	2409	1607
ENSECAG00000015906	3.085303455	0.5197143	0.8188979	128	132	188	278	105	97	199	158
ENSECAG00000003160	1.672867821	0.5200124	0.8191257	44	47	107	69	43	40	86	33
ENSECAG00000022587	6.558860591	0.5200665	0.8191257	1912	1846	1996	2040	1597	1493	1541	1670
ENSECAG00000020538	7.061995945	0.5200752	0.8191257	2783	2387	3005	2978	2423	2272	2220	1923
ENSECAG00000015835	5.14948512	0.5201848	0.8191846	518	485	540	958	253	1432	308	679
ENSECAG00000001791	0.61483599	0.5202851	0.819229	57	9	62	1	38	7	28	12
ENSECAG00000023666	2.908479035	0.5204963	0.819448	147	116	167	204	139	78	158	117
ENSECAG00000015689	5.539312661	0.5206805	0.8196244	929	731	919	792	1044	733	881	882
ENSECAG00000018407	5.487287805	0.5207713	0.8196537	1082	708	956	1012	1018	396	901	701
ENSECAG00000000985	4.389821549	0.5208991	0.8197414	378	282	496	657	525	217	482	149
ENSECAG00000019913	6.765316608	0.521159	0.8198586	2220	1841	2004	1730	2218	2488	2140	1412
ENSECAG00000022099	4.896509755	0.5211976	0.8198586	695	431	702	679	652	260	571	507
ENSECAG00000016800	9.243650275	0.5212274	0.8198586	13151	10137	12971	14829	10156	6768	14033	10009
ENSECAG00000022396	7.397234375	0.5212968	0.8198586	3657	2771	3311	4449	2739	2190	3730	2719
ENSECAG00000022694	4.796378068	0.5213345	0.8198586	600	477	649	588	472	313	643	448
ENSECAG00000012708	6.462941751	0.5216492	0.8202399	1905	1523	1980	1941	1899	1166	1781	1068
ENSECAG00000009158	2.3754867	0.5218399	0.8203791	99	103	95	141	98	67	129	39
ENSECAG00000018415	2.310360236	0.5218822	0.8203791	64	120	81	63	42	73	89	188
ENSECAG00000016278	7.235769627	0.5221126	0.8206278	2960	2802	3554	3232	2542	2120	3090	2373
ENSECAG00000023901	5.305625341	0.5223711	0.8208202	689	594	805	733	1302	383	944	467
ENSECAG00000000590	5.376947157	0.5223795	0.8208202	930	728	873	920	654	646	622	841
ENSECAG00000022179	7.941954966	0.5226757	0.8211346	4708	3718	4467	4841	4014	6408	4557	3663
ENSECAG00000007600	6.750975187	0.5227582	0.8211346	1706	2059	1690	2333	1772	2637	1839	1882
ENSECAG00000000760	3.199359063	0.5228286	0.8211346	169	117	147	374	93	114	302	97
ENSECAG00000005038	6.1353385	0.5228963	0.8211346	1455	1532	1310	1545	646	1437	1247	1315
ENSECAG00000007905	4.951156444	0.5229411	0.8211346	652	427	556	608	704	492	723	459
ENSECAG00000018731	8.114059455	0.5230683	0.8212208	5148	6594	4638	6766	3389	5107	5429	4434
ENSECAG00000000684	4.068677163	0.5232822	0.8212648	373	262	355	420	352	187	299	273
ENSECAG00000017977	4.220713856	0.5233862	0.8212648	356.516	308.126	278.36	401.495	355.357	268.09	504.858	321.257
ENSECAG00000016355	2.899524146	0.5234064	0.8212648	113	124	140	145	213	97	159	108
ENSECAG00000000672	6.235265672	0.5234554	0.8212648	1287	1571	1006	1583	1163	1459	1425	1697
ENSECAG00000019488	2.754642681	0.5234578	0.8212648	59	216	83	82	108	78	87	263
ENSECAG00000013936	8.043613043	0.5236715	0.8214564	4961	6192	4205	6724	3495	4887	4997	4069
ENSECAG00000019622	3.05196626	0.5237246	0.8214564	169	165	122	120	155	145	181	158
ENSECAG00000006544	7.749993823	0.5243605	0.822231	3833	3683	3996	4046	4199	2833	4753	4922
ENSECAG00000008635	5.02257934	0.5243632	0.822231	598	582	622	928	481	385	707	625
ENSECAG00000015424	6.38199525	0.5246495	0.8225665	1655	1436	1792	2062	1550	1185	1642	1242
ENSECAG00000003137	3.532662056	0.5249507	0.8226422	181	169	232	249	219	176	256	241
ENSECAG00000000928	3.617567454	0.5249951	0.8226422	282	201	212	346	109	165	298	244
ENSECAG00000000285	3.37086661	0.5250061	0.8226422	151	157	221	207	201	216	196	174
ENSECAG00000024659	4.24326503	0.5251149	0.8226422	361	311	414	513	389	184	448	248

ENSECAG00000018324	7.023475034	0.5252033	0.8226422	2144	3173	2583	2951	1185	2592	2578	2240
ENSECAG00000006729	0.96964233	0.5252929	0.8226422	47	25	41	49	21	11	56	35
ENSECAG00000014271	7.384129168	0.5252949	0.8226422	3128	2816	4024	4037	2885	2146	3623	2611
ENSECAG00000021148	3.74141164	0.525364	0.8226422	212	200	240	509	106	177	303	296
ENSECAG00000010332	4.454529145	0.5254016	0.8226422	483	341	538	476	492	208	435	326
ENSECAG00000019234	7.007962762	0.525427	0.8226422	2201	2482.98	2031.99	2601.99	2070	2354	2738.99	2708.99
ENSECAG00000010084	5.540786442	0.5256118	0.8226422	1006	672	1057	1214	722	324	1405	746
ENSECAG00000016996	8.521734504	0.5256296	0.8226422	7219	6843	7187	9638	5958	5027	7835	5894
ENSECAG00000000637	4.175314042	0.5256394	0.8226422	210	308	277	516	298	330	461	311
ENSECAG00000011396	1.655250927	0.5257502	0.8227024	66	41	54	107	45	55	70	26
ENSECAG00000016299	3.693279942	0.5260013	0.8229605	266	293	211	308	139	173	303	247
ENSECAG00000023575	7.251123709	0.52606	0.8229605	3508	2076	2995	2226	3935	2779	3874	1270
ENSECAG00000020069	4.316427754	0.5263483	0.823298	366	325	278	454	232	607	344	321
ENSECAG00000020525	1.346689923	0.5264693	0.823374	54	66	37	51	27	37	27	64
ENSECAG00000009643	0.155947728	0.526653	0.823418	25	12	28	23	10	8	27	21
ENSECAG00000008533	4.178143861	0.5267028	0.823418	215	381	265	709	128	304	376	372
ENSECAG00000010790	4.90646235	0.526715	0.823418	625	468	662	767	507	285	743	502
ENSECAG00000011246	0.276685695	0.5268164	0.8234632	5	30	8	31	9	27	16	38
ENSECAG00000020824	8.163766661	0.5270405	0.8235322	6827	4998	6056	6073	3754	4128	5574	5782
ENSECAG00000018607	0.85362007	0.5271492	0.8235322	32	41	55	17	13	36	30	28
ENSECAG00000013691	6.653396862	0.5271493	0.8235322	2013	1230	1763	2107	3847	527	2670	1046
ENSECAG00000020839	4.734091341	0.5272641	0.8235322	551	434	462	808	350	277	605	567
ENSECAG00000011862	1.712883841	0.527347	0.8235322	49	51	74	47	41	59	51	99
ENSECAG00000025597	1.023813606	0.5274385	0.8235322	38	63	1	9	2	80	4	76
ENSECAG00000014208	4.245788193	0.5274956	0.8235322	276	332	304.001	462	313	386	359	385
ENSECAG00000001455	4.957830851	0.5275068	0.8235322	677	398	594	577	726	620	461	538
ENSECAG00000009891	3.890939239	0.527513	0.8235322	234	266	281	280	274	243	318	304
ENSECAG00000009525	4.042425339	0.527696	0.8236103	336	304	377	352	282	187	336	297
ENSECAG00000014176	3.616625341	0.5277646	0.8236103	260	202	321	239	207	153	275	179
ENSECAG00000008950	5.136798194	0.5278141	0.8236103	548	478	721	822	744	629	898	437
ENSECAG00000012173	3.391792604	0.5278531	0.8236103	132	213	164	222	208	80	384	179
ENSECAG00000001488	4.580042123	0.5280244	0.823667	833	49.0001	513	176	536.001	133	1271	159
ENSECAG00000011009	5.252514126	0.5280344	0.823667	760	870	782	721	746	454	706	652
ENSECAG00000014764	1.864174682	0.528159	0.82367	80	50	108	68	82	17	78	54
ENSECAG00000003055	4.146728198	0.5281814	0.82367	336	294	313	324	365	386	268	315
ENSECAG00000009984	5.346020337	0.5283962	0.8236886	621	775	711	794	445	831	547	1290
ENSECAG00000021084	5.055436863	0.5284113	0.8236886	632	655	793	668	481	540	549	641
ENSECAG00000019137	3.352354998	0.5284798	0.8236886	289	212	162	186	153	199	130	168
ENSECAG00000000066	9.013085017	0.5284834	0.8236886	10782	7710	9117	9768	8799	9555	9467	11800
ENSECAG00000013490	4.085337416	0.528638	0.8238166	396	231	327	256	401	270	353	279
ENSECAG00000017221	8.480087943	0.5289882	0.8242492	5745	6104	6597	6934	3369	10608	5184	7980
ENSECAG00000010633	4.669645571	0.5291852	0.8244431	687	402	500	543	391	517	295	439
ENSECAG00000008718	6.036746407	0.5294128	0.8246846	1591	825	1516	1628	1457	602	1583	789
ENSECAG00000002171	4.656434141	0.5297439	0.8250872	456	726	144	351	417	320	185	1081
ENSECAG00000013506	4.304812296	0.5298414	0.825126	310	246	393	494	327	372	356	452
ENSECAG00000013495	6.606880422	0.5300195	0.8252399	2199	1524	1893	2554	1256	1243	2028	2092
ENSECAG00000010149	7.211660352	0.5300617	0.8252399	2991	3277	2375	3758	1714	2716	2374	2984
ENSECAG00000012252	6.020301889	0.5301325	0.8252399	988	1265	1396	1006	974	1146	1161	1682
ENSECAG00000023197	5.433991815	0.5302495	0.8253061	748	708	811	875	693	989	696	873
ENSECAG00000008767	6.350440435	0.5303204	0.8253061	1361	1556	1248	1743	1134	1705	1224	2123
ENSECAG00000011434	6.220886122	0.5305263	0.8253591	1350	1333	1593	1945	1159	967	1739	1216
ENSECAG00000014979	1.466922639	0.5305621	0.8253591	32	37	45	75	41	28	93	56
ENSECAG00000000770	3.727631328	0.5305724	0.8253591	219	233	358	300	278	204	215	163
ENSECAG00000014401	3.377800165	0.5306542	0.8253732	248	105	225	142	355	136	229	93
ENSECAG00000004549	6.266893409	0.5310507	0.8257835	1682	1361	1677	1704	1837	851	1579	901
ENSECAG00000023415	6.753878295	0.5310634	0.8257835	2364	1901	2433	2277	2110	1813	1561	1638
ENSECAG00000017571	5.831427802	0.531249	0.8258587	1015	1102	999	1012	996	967	1334	1060
ENSECAG00000011529	1.471571405	0.5314314	0.8258587	36	71	30	101	22	27	18	99
ENSECAG00000016966	4.116881263	0.5317587	0.8258587	344	373	322	138	220	227	255	667
ENSECAG00000016979	4.735436803	0.5317743	0.8258587	429	409	461	638	614	476	625	327
ENSECAG00000019545	8.737428149	0.5317948	0.8258587	8319	7162	9452	11230	5783	3840	9655	9683
ENSECAG00000011564	5.590845995	0.5318198	0.8258587	975	782	800	965	950	794	1091	844
ENSECAG00000013084	4.831103716	0.5319537	0.8258587	600	429	480	552	647	389	563	585
ENSECAG00000007682	6.780496352	0.5319717	0.8258587	2271	1687	2245	1725	2663	1706	2380	1690
ENSECAG00000020643	5.760097084	0.5321386	0.8258587	1185	780	979	1005	1206	704	1080	1165
ENSECAG00000000301	4.974247712	0.5321837	0.8258587	534	695	638	734	508	514	603	471
ENSECAG00000007541	6.544196778	0.5322267	0.8258587	2205	1657	2248	1637	1160	1881	1150	1907
ENSECAG00000014461	4.798419113	0.5323477	0.8258587	455	533	459	924	320	317	469	746
ENSECAG00000020071	5.067857125	0.5323568	0.8258587	726	442	629	632	606	372	960	698
ENSECAG00000023374	5.837568545	0.53239	0.8258587	1136	803	1163	1077	984	782	1409	1245
ENSECAG00000003030	7.756590668	0.5324459	0.8258587	3675	3100	3931	5158	4474	3511	4882	3775
ENSECAG00000022480	6.983187185	0.532489	0.8258587	2146	2734	2877	2805	1278	2146	1878	3057
ENSECAG00000024333	6.453723759	0.5325138	0.8258587	1703	1648	1681	2267	1218	1088	1917	1753
ENSECAG00000021855	6.983172323	0.5325621	0.8258587	2124	2474	2051	2488	2377	2712	2111	2364
ENSECAG00000023234	1.453905921	0.5326973	0.8258587	33	28	43	86	75	33	55	46
ENSECAG00000008447	0.79998847	0.5327156	0.8258587	22	15	13	67	47	20	43	23
ENSECAG00000018894	5.704469782	0.5327319	0.8258587	1052	683	1052	1016	742	972	878	1377
ENSECAG00000013775	5.250839958	0.532787	0.8258587	665	728	813	966	591	702	745	495

ENSECAG00000016088	2.96420438	0.5328208	0.8258587	186	121	181	164	156	93	142	117
ENSECAG00000010685	6.867505142	0.5328566	0.8258587	2454	2076	2053	1801	2109	2456	2512	1830
ENSECAG00000012342	4.063568963	0.5331544	0.8258702	385	241	367	416	182	231	286	408
ENSECAG00000000467	2.505206475	0.5331824	0.8258702	84	123	71	210	50	121	73	108
ENSECAG00000005256	2.558774294	0.5332004	0.8258702	134	89	117	157	62	67	117	138
ENSECAG00000011889	4.794270256	0.5333898	0.8258702	533	375	385	740	444	398	624	685
ENSECAG00000020675	3.59915604	0.5334013	0.8258702	248	226	213	160	319	184	264	167
ENSECAG00000021134	5.8576054	0.533512	0.8258702	1096	870	1294	1734	1702	632	1104	343
ENSECAG00000010684	7.11038336	0.5335357	0.8258702	2393	2416	2356	2889	2109	2150	3415	3034
ENSECAG00000016027	3.709367841	0.5336798	0.8258702	214	260	285	334	163	181	284	243
ENSECAG00000019233	7.833811111	0.5336899	0.8258702	5136	4192	5384	4207	3582	3258	4615	3871
ENSECAG00000008661	6.253725182	0.5339758	0.8258702	1601	1292	1428	1162	1484	1657	1022	1570
ENSECAG00000024652	6.117490833	0.5339763	0.8258702	956	1424	1579	1881	724	725	2001	1316
ENSECAG00000023659	4.17044522	0.5339947	0.8258702	358	274	299	362	344	177	445	446
ENSECAG00000025090	5.502371938	0.534056	0.8258702	1004	696	700	895	1048	855	664	844
ENSECAG00000026833	6.623526153	0.5340982	0.8258702	1314	2126	1383	3740	487	1809	1876	2266
ENSECAG00000011472	3.249994068	0.5341035	0.8258702	218	171	213	188	136	139	219	134
ENSECAG00000019110	7.19142239	0.5341154	0.8258702	3016	2621	2996	3568	2652	1917	2790	2475
ENSECAG00000010579	0.966022966	0.5341321	0.8258702	41	24	37	27	24	39	38	45
ENSECAG00000017402	1.251577139	0.5341916	0.8258702	34	35.0011	25	67	50	55	59	16
ENSECAG00000000444	2.121077474	0.5342775	0.8258702	85	63	99	126	124	33	88	28
ENSECAG00000016566	1.104879759	0.5343182	0.8258702	28	55	29	25	19	29	37	81
ENSECAG00000000141	1.059571573	0.5345127	0.8260211	21	66	22	19	11	62	24	61
ENSECAG00000009890	6.694185126	0.5345612	0.8260211	1844	1733	1879	2081	1957	2292	1838	1717
ENSECAG00000003760	1.146838215	0.5347517	0.826203	37	35	35	40	36	19	83	36
ENSECAG00000018046	7.998761295	0.5351634	0.8267266	2969	7211	3860	3197	1923	8611	2526	6518
ENSECAG00000014721	1.332129297	0.5354077	0.8269315	48	44	57	57	37	33	59	30
ENSECAG00000000765	0.03220486	0.5354416	0.8269315	12	17	12	22	8	24	12	29
ENSECAG00000016385	2.736286928	0.5356126	0.8270832	83	376	55	34	42	168	77	102
ENSECAG00000004260	4.492808883	0.5358254	0.8272993	411	422	453	595	280	302	460	472
ENSECAG00000021118	3.776621076	0.5362505	0.8278431	241	80	331	309	421	73	167	430
ENSECAG00000010542	5.905779555	0.5363278	0.8278499	974	1095	1086	1205	944	1337	1025	1207
ENSECAG00000018411	4.97479652	0.5364135	0.8278697	618	589.999	693	703	470	530	574	523.999
ENSECAG00000000242	7.779760362	0.536538	0.8279493	4188	4563	4318	5221	3400	3569	3723	3950
ENSECAG00000005376	0.607199047	0.5368237	0.8282776	54	10	38	23	12	8	50	23
ENSECAG00000016833	3.746307251	0.5369797	0.8283354	153	250	258	498	127	194	194	354
ENSECAG00000014294	3.627610531	0.5371003	0.8283354	302	247	285	188	183	227	145	238
ENSECAG00000022194	6.844826591	0.5372137	0.8283354	2245	1731	2134	2278	2393	1636	2567	2252
ENSECAG00000008977	5.853996466	0.537325	0.8283354	1566	994	1259	1073	1631	1073	486	439
ENSECAG00000012337	6.805510814	0.5373886	0.8283354	1756	1776	1992	2692	1922	2071	2129	2395
ENSECAG00000023985	3.950901506	0.5374002	0.8283354	351	241	302	406	325	192	246	255
ENSECAG00000020173	4.16150697	0.5374602	0.8283354	107	666	115	239	82	491	86	770
ENSECAG00000000751	3.641317049	0.5375388	0.8283354	117	329	257	152	179	211	289	297
ENSECAG00000019054	2.123215146	0.5375518	0.8283354	56	77	105	58	67	53	99	121
ENSECAG00000009302	2.667051282	0.5376805	0.8283354	140	96	112	93	153	80	171	91
ENSECAG00000024995	5.114297948	0.5376875	0.8283354	574	763	603	958	383	687	548	660
ENSECAG00000020194	4.716333343	0.5377362	0.8283354	448	453	542	453	499	482	547	473
ENSECAG00000008744	6.072957869	0.5378909	0.8284525	1509	978	1300	1064	1508	691	1974	1119
ENSECAG00000010550	7.126323207	0.5380992	0.8284525	2399.01	3319.38	2273.09	3648.61	1983.05	2489.42	2660.95	2164.63
ENSECAG00000011076	7.743596021	0.5381328	0.8284525	4447	3821	4709	4914	4085	2627	4587	3164
ENSECAG00000021810	2.507745249	0.5381539	0.8284525	109	70	153	150	87	50	103	129
ENSECAG00000026807	3.505621829	0.5381768	0.8284525	209	206	240	295	153	199	196	195
ENSECAG00000011012	7.96512583	0.5382932	0.8285193	4559	3269	4105	6282	6065	4808	5927	2465
ENSECAG00000013610	6.516181558	0.5386995	0.8290324	1944	1426	1774	2586	2029	780	1997	1396
ENSECAG00000016341	4.678042977	0.5388126	0.829094	277	404	284	832	111	898	222	736
ENSECAG00000013604	7.19406771	0.5389099	0.8290963	2796	2592	2950	2180	2446	3053	2921	2735
ENSECAG00000018231	5.343062863	0.5389601	0.8290963	884	669	705	681	876	681	854	680
ENSECAG00000009608	4.728694227	0.5394491	0.8295045	477	528	469	425	493	406	578	559
ENSECAG00000010855	6.542125221	0.5394549	0.8295045	1621	1518	1602	2111	1449	1698	1847	2094
ENSECAG00000027677	9.103843558	0.5396082	0.8295045	10368	9241	6562	13936	10590	9623	13477	8922
ENSECAG00000013579	5.108250332	0.5396209	0.8295045	715	742	779	596	566	402	622	730
ENSECAG00000005797	7.473923019	0.5397062	0.8295045	3674	3389	3671	4051	3281	2663	3278	2671
ENSECAG00000010753	5.10023889	0.5397907	0.8295045	609	670	644	947	370	554	657	716
ENSECAG00000024025	4.559028356	0.5398056	0.8295045	508	364	497	604	423	274	490	400
ENSECAG00000021768	5.007188194	0.5398096	0.8295045	688	915	587	478	252	800	308	664
ENSECAG00000019493	2.80986172	0.5399653	0.8296316	149	112	163	164	65	129	117	140
ENSECAG00000022375	8.596179889	0.5400544	0.8296317	8027	7524	7462	9300	6356	6437	7090	5887
ENSECAG00000006950	6.995581488	0.5401115	0.8296317	2021	2841	2639	3102	1759	1820	2395	2610
ENSECAG00000010188	3.386147787	0.5406699	0.8302673	151	221	187	171	113	229	208	253
ENSECAG00000013896	8.470268473	0.5406714	0.8302673	7065	8054	8542	5737	3880	6520	4962	7866
ENSECAG00000016771	0.766598954	0.5408934	0.8304806	23	27	50	39	10	15	45	35
ENSECAG00000013990	4.76090404	0.5409566	0.8304806	470	429	443	641	524	469	518	550
ENSECAG00000016183	1.956084323	0.5412207	0.8306695	58	53	65	160	28	64	98	56
ENSECAG00000011907	2.915204614	0.5412259	0.8306695	143	214	92	181	110	178	90	90
ENSECAG00000014896	8.128662592	0.5413173	0.8306976	5632	5980	5700	5965	3195	5567	4782	4937
ENSECAG00000017013	3.258497647	0.5414084	0.8307251	139	156	186	195	268	186	163	109
ENSECAG00000006079	7.446337731	0.5416562	0.8309406	4115	3098	4358	2897	2115	2194	3786	3700
ENSECAG00000024981	3.588576883	0.5416952	0.8309406	286	168	243	146	188	135	298	327

ENSECAG00000009265	3.503097272	0.5418695	0.8310958	184	152	164	311	225	285	274	86
ENSECAG00000023961	4.472657748	0.5420107	0.8312001	495	382	483	483	287	413	417	355
ENSECAG00000020910	3.091501538	0.5422071	0.8312805	152	132	209	223	140	109	176	136
ENSECAG00000006243	4.79946051	0.5422095	0.8312805	469	328	642	591	638	354	648	514
ENSECAG00000023720	0.880139677	0.5424073	0.8314716	45	48	25	32	11	23	11	63
ENSECAG00000017731	4.749770933	0.5425502	0.8315785	454	465	590	745	460	325	500	522
ENSECAG00000000102	7.329680597	0.5428198	0.8318277	3796	2504	3327	3918	3546	1472	3444	2390
ENSECAG00000015458	2.843825348	0.5428602	0.8318277	150	96	105	159	131	88	213	131
ENSECAG00000012036	6.526935306	0.5429325	0.8318277	2288	1171	2194	2066	1129	1694	1804	1538
ENSECAG00000018794	5.699064212	0.5432017	0.8320587	1233	725	999	1415	1027	779	1121	555
ENSECAG00000009638	3.96353364	0.5432298	0.8320587	348	166	236	378	305	212	509	206
ENSECAG00000021012	3.245013637	0.543458	0.832296	121	272.002	204	200	77.0021	217	68	222
ENSECAG00000009944	2.235770295	0.5437002	0.8323949	70	111	103	110	33	44	94	133
ENSECAG00000023952	5.927433232	0.5437239	0.8323949	1157	1030	1296	1583	881	977	1333	931
ENSECAG00000005570	5.938182544	0.5438015	0.8323949	1147	922	1124	1281	741	1537	1198	1154
ENSECAG00000013321	3.609282661	0.5438507	0.8323949	228	219	247	332	119	161	308	230
ENSECAG00000004867	6.055875597	0.5439731	0.8323949	1486	809	1174	1406	1580	728	1629	1225
ENSECAG00000011505	6.971621795	0.5441388	0.8323949	2410	2944	2848	2117	1794	1426	2464	2800
ENSECAG00000018612	1.369053027	0.5441635	0.8323949	58	42	64	47	24	28	56	56
ENSECAG00000015003	3.489954188	0.5441698	0.8323949	230	196	199	318	149	150	243	206
ENSECAG00000013288	3.59505691	0.5442488	0.8323949	235	227	172	389	195	199	258	141
ENSECAG00000018339	8.134620345	0.5443105	0.8323949	5843	4459	6955	6278	5403	3391	6129	4033
ENSECAG00000013478	3.717711761	0.5443287	0.8323949	325	202	276	300	279	117	279	201
ENSECAG00000012492	5.960912726	0.5446225	0.8327322	1376	839	1146	1150	1582	892	1714	648
ENSECAG00000009290	7.245496689	0.5448153	0.8328272	3406	3285	3541	2492	864	3239	1941	3724
ENSECAG00000022473	4.757751714	0.5448313	0.8328272	413	412	408	748	630	445	664	343
ENSECAG00000019542	4.302137685	0.5449269	0.8328612	348	405	298	358	367	316	319	502
ENSECAG00000001265	2.616121708	0.5450691	0.8329665	93	101	157	164	107	97	91	97
ENSECAG00000008466	4.108188466	0.5455531	0.8334857	410	284	359	385	337	170	392	261
ENSECAG00000007573	6.332581433	0.5455556	0.8334857	1878	1270	1637	1914	1454	1249	1599	1127
ENSECAG00000022935	4.420085122	0.5457776	0.8337127	348	492	376	567	239	399	397	380
ENSECAG00000013285	6.903967491	0.5459949	0.83388	2141	2340	1812	2386	1754	1877	2841	2789
ENSECAG00000007411	4.023532318	0.546034	0.83388	318	213	282	364	374	176	435	285
ENSECAG00000020814	5.493441959	0.5461979	0.8339496	910	851	987	965	850	551	929	727
ENSECAG00000005467	7.009746706	0.5462985	0.8339496	3102	1754	2080	2299	3526	1239	3955	1507
ENSECAG00000020996	4.380249824	0.5462998	0.8339496	290	367	428	420	326	400	485	382
ENSECAG00000016043	6.046935535	0.5465231	0.8341058	1241	1058	1265	1256	1594	1104	1475	862
ENSECAG00000018141	6.638937371	0.546549	0.8341058	1532	1787	1799	2132	1177	2091	1820	2499
ENSECAG00000008834	4.651249672	0.5466812	0.8341956	31	1114	75	222	24	538	63	1513
ENSECAG00000009279	3.542504538	0.5470135	0.8345905	176	213	190	251	128	187	281	311
ENSECAG00000016895	4.252728568	0.5471562	0.8346164	277	313	399	388	277	383	344	444
ENSECAG00000000020	2.792446875	0.5471964	0.8346164	125	85	113	173	133	123	111	159
ENSECAG00000004306	2.634826962	0.5472778	0.8346164	132	102	92	205	52	99	188	67
ENSECAG00000011697	3.743909137	0.5473244	0.8346164	176	324	280	134	220	211	165	441
ENSECAG00000021462	6.142398842	0.5474412	0.8346825	1497	1170	1398	1038	1314	876	1589	1679
ENSECAG00000018072	5.880974239	0.5476273	0.8347494	1285	830	1216	1613	1041	1027	1092	773
ENSECAG00000000537	7.572461976	0.5477886	0.8347494	2708	4207	3509	3041	2004	4773	3137	4605
ENSECAG00000011059	4.619988356	0.5478666	0.8347494	376	375	373	661	305	264	574	796
ENSECAG00000007378	4.95367603	0.5478731	0.8347494	526	514	618	587	558	673	475	621
ENSECAG00000015242	9.643231227	0.5479076	0.8347494	16460	11062	14406	16266	13976	15378	12699	18844
ENSECAG00000009464	1.143881241	0.547926	0.8347494	32	38	35	41	21	80	35	26
ENSECAG00000012031	4.573042614	0.5481747	0.8349395	372	363	373	630	286	402	500	649
ENSECAG00000017453	5.803577041	0.5483378	0.8349395	1036	1148	962	1483	948	712	1066	1061
ENSECAG00000020669	8.130782484	0.5484018	0.8349395	5345	4592	5419	4880	6706	5178	4265	5073
ENSECAG00000020722	3.893480193	0.5484136	0.8349395	236	225	316	293	359	212	304	263
ENSECAG00000014209	3.822737172	0.5484227	0.8349395	297	284	236	365	155	214	243	323
ENSECAG00000012487	3.043345154	0.5484918	0.8349395	137	120	132	194	72	117	195	265
ENSECAG00000015984	8.329966528	0.5488516	0.8350337	6603	5240	7293	7954	6771	3123	7277	4591
ENSECAG00000019528	4.301687176	0.5489536	0.8350337	372	301	452	538	218	279	304	506
ENSECAG00000009071	4.887760858	0.5489737	0.8350337	706	479	591	687	570	405	467	530
ENSECAG00000010813	7.224205823	0.5490108	0.8350337	2481	2989	3382	1747	2703	2840	2490	3380
ENSECAG00000016373	2.380968307	0.5490118	0.8350337	79	82	124	154	90	54	110	85
ENSECAG00000006592	4.223796954	0.5490236	0.8350337	400	295	442	419	236	258	349	407
ENSECAG00000020501	7.860348707	0.5490683	0.8350337	5091	4239	4799	5188	3970	3575	4454	3589
ENSECAG00000015983	4.947051607	0.5491682	0.8350739	653	521	724	665	445	465	427	710
ENSECAG00000019396	2.863855452	0.5493231	0.8351975	141	115	178	175	97	75	177	135
ENSECAG00000004212	4.454644993	0.5495424	0.8353421	395	290	441	469	549	272	509	361
ENSECAG00000012862	8.594093705	0.5495814	0.8353421	5953	7284	9104	4315	3355	13482	4033	8354
ENSECAG00000018274	4.890681631	0.5497383	0.8353421	739	452	653	624	479	333	738	468
ENSECAG00000021428	5.306902129	0.5497963	0.8353421	679	761	849	1015	420	858	704	631
ENSECAG00000010007	8.00734498	0.5498938	0.8353421	5131	4599	4965	3694	3877	6116	4236	5257
ENSECAG00000011539	3.414904801	0.5499811	0.8353421	178	279	177	249	135	116	228	231
ENSECAG00000018393	5.161442532	0.5499885	0.8353421	683	636	691	569	724	561	963	512
ENSECAG00000008073	8.217139805	0.5500065	0.8353421	6167	4827	4702	5948	6717	4455	6193	5483
ENSECAG00000011713	6.392912009	0.5501194	0.8354019	1757	1251	1690	1424	2018	1215	1691	1482
ENSECAG00000024430	2.846164678	0.550264	0.8355098	102	150	134	110	90	87	146	240
ENSECAG00000022684	7.655867684	0.5504709	0.8357123	4287	3443	4247	4867	3729	2350	4219	3362
ENSECAG00000011126	6.155896679	0.5506751	0.8358346	1196	612	1345	2021	2049	1253	1948	346

ENSECAG00000017521	4.860763952	0.5506987	0.8358346	620	360	589	523	713	484	775	277
ENSECAG00000016695	4.719247206	0.5508971	0.8358683	387	413	484	648	438	434	627	523
ENSECAG00000010894	2.347445926	0.5509706	0.8358683	106	101	105	107	68	75	107	81
ENSECAG00000025036	6.43808553	0.5509909	0.8358683	1939	1483	1856	1920	2009	1172	1460	1153
ENSECAG00000002531	3.045172653	0.5510152	0.8358683	183	106	217	187	150	103	187	103
ENSECAG00000009910	5.742268039	0.5514895	0.836476	1151	1076	895	695	1318	687	1290	840
ENSECAG00000009396	7.378961069	0.5516161	0.8365563	3069	2643	2839	3612	2753	3680	2882	3256
ENSECAG00000024121	2.932550399	0.5517514	0.8366498	50	370	81	130	93	187	84	95
ENSECAG00000008002	3.338389491	0.5520639	0.8370121	274	150	149	133	139	124	234	298
ENSECAG00000007164	6.211836324	0.5521478	0.8370275	1443	956	1515	1506	1690	1416	1732	814
ENSECAG00000020237	6.399854953	0.5522763	0.8370467	1499	1404	1648	1629	1588	1098	2076	1747
ENSECAG00000013916	6.093612827	0.5523078	0.8370467	1438	1115	1415	1704	1153	826	1393	1283
ENSECAG00000019574	3.746798265	0.5524254	0.8370604	279	184	328	350	145	121	216	406
ENSECAG00000012727	3.517640238	0.5525432	0.8370604	230	151	205	241	241	182	252	201
ENSECAG00000012921	6.399593684	0.5526031	0.8370604	1681	1140	1571	2897	1207	1663	2208	501
ENSECAG00000024607	7.142969335	0.5526117	0.8370604	3278	2177	2876	1744	3435	2634	3125	1648
ENSECAG00000012511	4.759648715	0.5526906	0.8370684	542	529	484	696	449	337	629	422
ENSECAG00000017321	1.049336092	0.5527991	0.837109	18	42	25	51	27	19	28	85
ENSECAG00000010795	6.651292267	0.5528648	0.837109	1834	2378	2184	1916	1069	1617	1219	2730
ENSECAG00000016479	6.404657815	0.552943	0.8371157	1869	1102	1719	1510	1323	1288	2177	1755
ENSECAG00000016359	4.713864214	0.5531007	0.8372429	479	369	535	509	667	283	785	325
ENSECAG00000012657	6.8778131	0.5532828	0.8374031	2441	2213	2437	2665	1456	1719	2470	2327
ENSECAG00000020642	5.684227011	0.553354	0.8374031	929	930	904	989	842	1094	954	976
ENSECAG00000007800	6.745630344	0.5542341	0.8383059	1943	1575	1982	2321	2958	1056	2686	1658
ENSECAG00000017727	4.019106397	0.5543356	0.8383059	271	291	277	533	213	191	403	287
ENSECAG00000016699	6.650142825	0.5544126	0.8383059	1890	2145	1876	2403	1136	1492	1957	2214
ENSECAG00000021398	7.499792869	0.5544212	0.8383059	2885	3821	2865	3361	2176	4937	2868	3680
ENSECAG00000000526	6.379570152	0.5544616	0.8383059	1672	1298	1864	2104	1333	946	1699	1704
ENSECAG00000002944	8.756627074	0.5545135	0.8383059	6981	7365	8296	8644	5429	10393	7747	9297
ENSECAG00000024244	7.062545913	0.5545501	0.8383059	3826	822	2896	1893	4156	1179	4083	1349
ENSECAG00000021789	5.271697276	0.5546109	0.8383059	737	726	866	855	638	551	766	658
ENSECAG00000009767	3.054713393	0.5546224	0.8383059	62	327	115	192	53	80	157	244
ENSECAG00000019123	2.654238534	0.554765	0.8383059	141	71	151	169	119	64	135	94
ENSECAG00000004417	4.066547399	0.5548254	0.8383059	288	154	373	408	381	261	415	237
ENSECAG00000018931	0.583372175	0.5548361	0.8383059	35	15	14	31	66	8	31	9
ENSECAG00000023996	2.174755296	0.5549779	0.8384085	86	55	68	107	75	67	166	48
ENSECAG00000004843	5.659786555	0.5554984	0.8390833	846	923	852	1080	852	726	1041	1248
ENSECAG00000007364	4.228021661	0.5557377	0.8393332	303	345	320	367	273	561	236	325
ENSECAG00000012139	3.952984827	0.555889	0.8394501	346	208	396	347	278	206	362	188
ENSECAG00000023256	8.044544908	0.5561554	0.8397407	4874	3546	4742	9679	4773	3079	7357	2634
ENSECAG00000023348	3.92276596	0.5562675	0.8397984	241	286	280	270	265	178	531	229
ENSECAG00000002889	7.719884195	0.5563662	0.8398357	4297	3648	4224	5459	3346	2665	4870	3473
ENSECAG00000020867	8.551907708	0.5568008	0.8403547	7932	6760	7943	8612	6492	5235	7560	6011
ENSECAG00000017743	5.366730527	0.5569233	0.8403547	650	533	987	799	325	649	808	1443
ENSECAG00000004865	3.237803634	0.5569851	0.8403547	96	179	157	242	193	171	208	151
ENSECAG00000017656	5.085435609	0.5570059	0.8403547	587	524	742	599	667	842	466	556
ENSECAG00000015061	7.348300885	0.557149	0.8403737	3520	2783	2555	2933	2973	3743	2860	2710
ENSECAG00000012286	4.174760398	0.5571956	0.8403737	442	303	255	528	398	140	378	292
ENSECAG00000000592	3.820754209	0.55729	0.8403737	247	182	292	300	336	277	271	186
ENSECAG00000020255	4.617677704	0.5573739	0.8403737	532	371	564	575	413	371	488	374
ENSECAG00000012589	3.921136606	0.557427	0.8403737	337	234	234	285	308	225	380	255
ENSECAG00000000878	2.076600518	0.5574863	0.8403737	57	97	65	67	54	110	54	98
ENSECAG00000017572	3.895777984	0.5575364	0.8403737	255	299	200	312	347.999	227	338.998	227.999
ENSECAG00000022650	4.354638064	0.5577174	0.8405349	397	317	386	384	356	304	472	439
ENSECAG00000024150	2.088589485	0.5580001	0.8408494	89	51	47	111	87	40	140	69
ENSECAG00000006886	4.745557401	0.5581138	0.8409093	431	484	604	394	336	667	475	549
ENSECAG00000022373	4.668725967	0.5586233	0.8414998	586	425	549	544	433	330	454	492
ENSECAG00000008790	4.714747392	0.5586539	0.8414998	523	637	693	329	191	239	404	900
ENSECAG00000002115	7.299076537	0.5589431	0.8418238	3157	3196	2843	3957	2657	3297	2623	1763
ENSECAG00000021139	1.48351478	0.5598011	0.8426494	41	52	37	58	18	82	72	40
ENSECAG00000000263	3.436112633	0.5598772	0.8426494	206	182	159	231	196	191	211	225
ENSECAG00000020020	7.508677095	0.559961	0.8426494	3567	3521	3755	4279	2896	3248	3011	2970
ENSECAG00000011072	4.319026205	0.5601139	0.8426494	368	368	407	519	383	330	297	305
ENSECAG00000000232	4.176004374	0.5602243	0.8426494	362	326	515	286	255	280	291	369
ENSECAG00000017687	8.181150539	0.5602857	0.8426494	4665	5373	5263	5502	3063	8495	4817	5529
ENSECAG00000014016	7.513689026	0.5604987	0.8426494	3456	4805	4533	2321	1328	2520	2782	5424
ENSECAG00000004168	2.459879918	0.5605189	0.8426494	87	130	139	96	51	85	99	120
ENSECAG00000018362	3.761019785	0.5605415	0.8426494	266	249	263	356	163	139	322	292
ENSECAG00000023053	2.792692918	0.5606229	0.8426494	158	87	169	170	147	77	158	71
ENSECAG00000001999	5.125920924	0.5606427	0.8426494	661	571	662	654	666	644	746	589
ENSECAG00000011652	7.748247289	0.5606602	0.8426494	3763	3677	3630	4616	3345	4974	4002	3923
ENSECAG00000023738	4.130988438	0.5607041	0.8426494	370	300	313	274	351	294	379	312
ENSECAG00000020134	6.065923789	0.5608234	0.8426494	1545	720	1279	2241	1495	778	1746	466
ENSECAG00000007500	9.789520662	0.5608431	0.8426494	17986	12539	16585	17385	17290	18425	14813	16427
ENSECAG00000008093	6.765292279	0.560899	0.8426494	2129	2011	2248	2654	1531.99	1221	2387	2301
ENSECAG00000000792	6.27853364	0.5609151	0.8426494	1342	1488	1159	1685	1371	1741	1393	1319
ENSECAG00000004919	6.723855015	0.5609373	0.8426494	2583	1639	2309	2294	2537	1212	2130	1218
ENSECAG00000019842	5.657090455	0.5609487	0.8426494	1031	878	1018	1260	657	637	1019	1128

ENSECAG00000014664	4.741150734	0.5610491	0.8426494	360	423	604	556	635	419	414	558
ENSECAG00000015000	2.311983272	0.5610724	0.8426494	93	134	146	35	88	108	77	30
ENSECAG00000015838	7.021606447	0.5611233	0.8426494	2738	2375	2617	3041	2296	1748	2409	2327
ENSECAG00000021209	5.760055245	0.5612935	0.8427935	1312	757	995	837	1345	1019	1287	495
ENSECAG00000000595	4.897427277	0.5615477	0.8430638	549	426	578	632	501	445	661	677
ENSECAG00000004257	4.346112317	0.5617613	0.843273	429	318	365	595	380	280	386	313
ENSECAG00000023594	5.489713859	0.5619535	0.8433278	911	755	976	1080	615	847	826	723
ENSECAG00000022900	1.191329227	0.5619562	0.8433278	20	73	39	53	41	19	34	47
ENSECAG00000019257	5.590917624	0.5620205	0.8433278	1036	975	1121	806	702	756	1098	713
ENSECAG00000008011	5.205891207	0.5621645	0.8433718	726	698	728	917	377	539	679	893
ENSECAG00000022735	7.163152692	0.5621984	0.8433718	2534	2941	2856	1864	1994	2306	2610	4138
ENSECAG00000008819	7.652138569	0.5624586	0.8435435	3592	3315	3439	4347	2597	3218	4696	5052
ENSECAG00000012372	6.346251806	0.562471	0.8435435	1190	1589	1142	2034	1056	1892	1497	1699
ENSECAG00000022458	4.96360811	0.562595	0.8435435	562	643	721	644	377	571	537	593
ENSECAG00000021249	6.378942231	0.5627027	0.8435435	1651	1345	1431	1681	1740	1112	2157	1398
ENSECAG00000019681	5.610216462	0.5627476	0.8435435	1263	536	901	829	1383	489	1288	658
ENSECAG00000009635	5.077540459	0.5628141	0.8435435	760	563	648	836	429	438	770	665
ENSECAG00000001222	2.772934202	0.5628326	0.8435435	121	139	138	169	87	132	105	116
ENSECAG00000024411	6.389123489	0.5634161	0.8442025	1581	1706	1828	1759	1273	1286	1718	1417
ENSECAG00000024298	6.497874448	0.5634229	0.8442025	1582	1482	1456	2148	1719	1080	2090	2077
ENSECAG00000023994	5.400239699	0.5634954	0.8442025	830	708	797	1209	702	354	895	933
ENSECAG00000018649	9.002090114	0.5636178	0.8442746	9434	10938	12019	10375	5910	11948	5880	9358
ENSECAG00000007743	8.89214832	0.5638542	0.8444574	8238	7297	8838	10394	7852	6718	13313	9190
ENSECAG00000023599	1.74453803	0.5638885	0.8444574	43	57	67	63	52	38	103	68
ENSECAG00000024877	6.098910042	0.5639831	0.8444877	1251	1066	1254	1453	1822	943	1547	927
ENSECAG00000013303	2.13141809	0.5643053	0.8446679	61	105	65	65	69	106	43	110
ENSECAG00000020138	4.85234452	0.5644069	0.8446679	483	356	539	723	241	733	470	753
ENSECAG00000009490	5.795554975	0.5645238	0.8446679	955	1162	936	1552	908	749	1010	1095
ENSECAG00000000074	5.249792255	0.5645274	0.8446679	1112	487	841	60	1298	587	1045	158
ENSECAG00000014181	7.624332346	0.5645717	0.8446679	3633	3090	4702	5304	3441	1509	6060	2568
ENSECAG00000011178	7.812543968	0.5646742	0.8446679	4686	3813	4885	5389	3626	4448	3382	3370.02
ENSECAG00000018240	6.819227098	0.5647496	0.8446679	1973	1783	1992	2575	1989	1858	2644	2152
ENSECAG00000016753	3.974784274	0.5649618	0.8446679	313	402	204	384	166	300	315	254
ENSECAG00000013798	6.559930451	0.5649772	0.8446679	2405	1387	1979	2084	2234	1240	1621	1216
ENSECAG00000010798	5.50567569	0.5651102	0.8446679	699	752	847	1053	749	680	1202	863
ENSECAG00000020582	1.980620346	0.565188	0.8446679	53	78	85	51	51	88	47	111
ENSECAG00000021059	4.357554031	0.5652593	0.8446679	309	473	482	423	366	343	395	255
ENSECAG00000017730	3.287478108	0.5652695	0.8446679	199	168	211	232	153	158	179	153
ENSECAG00000021605	1.00196963	0.565307	0.8446679	39	28	45	52	17	22	51	36
ENSECAG00000024063	7.017356073	0.5653226	0.8446679	2688	2351	3167	2513	2802	1475	2644	1831
ENSECAG00000017588	5.225420023	0.5653424	0.8446679	858	436	929	945	361	395	699	1068
ENSECAG00000016442	8.362229112	0.5653675	0.8446679	7901	4286	5257	6597	5633	5789	6085	7651
ENSECAG00000012586	4.544334523	0.5654766	0.8447197	446	382	551	560	354	391	503	317
ENSECAG00000002607	1.706535863	0.5657767	0.8448725	76	35	38	79	64	48	78	59
ENSECAG00000009027	2.09874265	0.5657793	0.8448725	94	86	117	58	19	76	56	116
ENSECAG00000012951	4.169047883	0.5658071	0.8448725	278	364	259	384	184	334	313	551
ENSECAG00000000392	6.972407705	0.5658953	0.8448725	2709	2275	2656	2752	1912	1501	2852	2324
ENSECAG00000023275	2.091342238	0.5660314	0.8448725	68	34	80	117	49	135	92	46
ENSECAG00000022110	7.941034065	0.5660559	0.8448725	4466	4287	3619	5533	4302	6014	4743	3488
ENSECAG00000003791	5.657869075	0.5660995	0.8448725	938	768	1015	973	985	1001	1228	621
ENSECAG00000012094	4.003973736	0.566316	0.8450525	313	277	328	418	281	248	311	228
ENSECAG00000016942	0.225755442	0.5664159	0.8450525	15	24	12	23	22	28	19	14
ENSECAG00000026959	4.744325664	0.5665095	0.8450525	523	483	548	677	532	420	544	293
ENSECAG00000015440	6.703847833	0.5665605	0.8450525	2396	1436	4316	610	1387	1922	1023	2277
ENSECAG00000010534	6.965920733	0.5665921	0.8450525	2420	2364	2592	2971	1960	1850	2490	2169
ENSECAG00000012952	6.134776542	0.5666917	0.84509	1258	1135	1250	1549	1315	1090	1488	1446
ENSECAG00000007022	3.011057302	0.5668404	0.8451491	211	125	133	205	144	94	174	121
ENSECAG00000009171	8.884168485	0.5668801	0.8451491	9193	8381	10384	11554	9025	5878	10918	6188
ENSECAG00000016273	5.230620426	0.5674304	0.8457596	745	582.002	718	686	509.002	842	613	859
ENSECAG00000022705	7.888632991	0.5674698	0.8457596	3833	4127	4023	5250	4737	5487	5181	2586
ENSECAG00000013947	4.818404434	0.5675442	0.8457596	436	619	535	756	307	451	586	553
ENSECAG00000011095	3.391388393	0.5675874	0.8457596	214	178	230	249	198	132	195	171
ENSECAG00000014798	2.312766531	0.5677295	0.8457913	146	44	114	115	101	48	84	86
ENSECAG00000024816	1.468302954	0.5677576	0.8457913	40	29	52	68	70	23	99	26
ENSECAG00000014791	3.143838995	0.5682134	0.8463594	228	152	138	90	245	98	248	104
ENSECAG00000014420	3.684215201	0.5684656	0.8465947	260	252	226	328	186	189	270	214
ENSECAG00000013322	2.868742924	0.5685205	0.8465947	35	387	67	120	36	142	46	211
ENSECAG00000009749	0.349714416	0.5687612	0.8466785	19	34	10	14	7	34	10	42
ENSECAG00000025005	5.176516824	0.5687837	0.8466785	809	598	648	565	645	718	710	658
ENSECAG00000026901	1.948780052	0.5688004	0.8466785	93	60	81	86	48	30	107	69
ENSECAG00000020160	3.253061301	0.5689778	0.8468316	212	122	192	142	325	106	201	106
ENSECAG00000009834	2.864918324	0.5690727	0.8468619	114	181	123	189	60	151	105	149
ENSECAG00000019306	3.903781905	0.5692757	0.8470171	255	264	295	261	291	283	265	291
ENSECAG00000018858	5.498731651	0.5693261	0.8470171	798	919	615	945	567	1013	728	1100
ENSECAG00000001010	2.263138051	0.5695256	0.8472028	62	42	167	59	117	57	111	89
ENSECAG00000014965	5.730238389	0.5696424	0.8472657	1025	920	954	1521	795	811	1207	803
ENSECAG00000016109	3.884885788	0.5699054	0.8475459	314	215	269	454	200	125	379	299
ENSECAG00000011876	2.881776762	0.5703568	0.8481062	85	151	174	210	63	125	128	161

ENSECAG00000018761	0.602916042	0.5709502	0.8486005	28	15	40	40	17	17	36	23
ENSECAG00000016830	6.562754316	0.5710547	0.8486005	1775	1291	1953	1976	1809	1607	2022	1729
ENSECAG00000008798	5.536202328	0.5711722	0.8486005	807.001	824	718.001	1068	826.001	889.001	1088	714.024
ENSECAG00000008861	5.085700495	0.5712043	0.8486005	563	562	631	728	827	448	791	535
ENSECAG00000008993	9.347395752	0.5712543	0.8486005	12850	15736	14144	10893	7477	12437	9580	13436
ENSECAG00000006239	5.489759066	0.5712673	0.8486005	1044	703	863	1129	765	431	992	892
ENSECAG00000012606	4.078726393	0.5712691	0.8486005	340	321	307	432	272	231	341	292
ENSECAG00000016003	4.062939675	0.5712871	0.8486005	418	236	378	370	220	120	465	337
ENSECAG00000024148	1.292347189	0.5713616	0.8486005	23	92	47	39	18	18	11	99
ENSECAG00000021483	7.808075146	0.5715343	0.8487289	4247	4223	4338	5870	3030	3288	4986	3928
ENSECAG00000010981	7.103319099	0.5716396	0.8487289	2717	2037	2756	2533	3193	2396	2848	2007
ENSECAG00000021276	6.348304992	0.5716722	0.8487289	1806	1462	1742	1683	1374	1332	1654	1156
ENSECAG00000011779	4.239908335	0.5718823	0.8487425	366	354	445	420	104	437	314	369
ENSECAG00000024665	5.608202254	0.5718981	0.8487425	805	892	870	1017	900	849	1060	884
ENSECAG00000010771	5.09523779	0.5719055	0.8487425	649	657	730	782	601	397	729	602
ENSECAG00000002972	4.817325956	0.5720281	0.8488134	453	575	442	905	375	277	583	676
ENSECAG00000003859	2.593173704	0.5721472	0.8488562	58	61	95	218	77	75	253	84
ENSECAG00000025051	3.050762147	0.5722063	0.8488562	154	81	282	202	52	32	185	275
ENSECAG00000020833	2.591713405	0.5723087	0.8488972	89	81	154	185	80	94	129	90
ENSECAG00000020668	0.974890486	0.5725126	0.8489169	37	29	27	39	38	18	61	33
ENSECAG00000008117	3.698170825	0.5725193	0.8489169	351	258	238	218	206	186	200	264
ENSECAG00000012085	5.317332836	0.5726139	0.8489169	763	645	665	835	399	847	882	915
ENSECAG00000012035	6.32871901	0.5726209	0.8489169	1445	1278	1405	1812	1759	1417	1619	1268
ENSECAG00000019373	7.287026624	0.5727929	0.8489755	3377	2611	2785	4294	2762	2122	3445	2270
ENSECAG00000022901	4.72620956	0.5728099	0.8489755	513	456	580	652	475	469	525	293
ENSECAG00000020861	3.521250466	0.5731947	0.8494298	270	118	410	172	150	102	394	127
ENSECAG00000008222	1.436574518	0.573333	0.8494298	55	54	57	54	39	17	76	44
ENSECAG00000013971	4.887442202	0.5733968	0.8494298	617	446	646	757	404	400	672	538
ENSECAG00000023009	10.23531169	0.5734156	0.8494298	23458	18484	22875	23104	25682	22779	24401	18770
ENSECAG00000001099	2.637222354	0.573652	0.8496693	99	52	77	217	208	92	137	45
ENSECAG00000011226	2.346847462	0.5738116	0.8497948	123	81	90	130	77	44	132	84
ENSECAG00000006830	1.88179557	0.5739696	0.849918	63	51	73	127	71	47	100	17
ENSECAG00000019232	4.484015681	0.5742204	0.8501664	500	196	491	794	751	86	467	146
ENSECAG00000010680	4.884362216	0.5742871	0.8501664	561	516	639	731	427	467	526	565
ENSECAG00000007383	2.511379954	0.5744615	0.8503103	119	47	113	126	114	66	182	86
ENSECAG00000006911	4.744327506	0.574534	0.8503103	521	405	502	532	550	373	616	512
ENSECAG00000015828	5.38173772	0.5746296	0.8503411	817	587	707	974	904	619	921	729
ENSECAG00000016666	4.245531364	0.57479	0.8504675	298	279	409.003	656	79.0032	440	293	403
ENSECAG00000011974	7.787898441	0.5749498	0.8505926	3729	3913	3530	5028	3578	4711	4419	4026
ENSECAG00000015733	6.117830391	0.5750242	0.8505926	1402	1083	1518	1796	722	831	1657	1564
ENSECAG00000017413	5.616223909	0.575325	0.8508489	871	967	1005	1198	717	852	987	760
ENSECAG00000006252	3.061780102	0.5754141	0.8508489	175	119	201	204	135	70	187	166
ENSECAG00000010144	3.909834954	0.5755463	0.8508489	238	285	274	469	314	203	327	154
ENSECAG00000011091	3.687040678	0.5756758	0.8508489	253	186	188	310	263	182	252	287
ENSECAG00000000453	3.89118583	0.5757012	0.8508489	303	221	252	300	272	198	377	298
ENSECAG00000009788	4.674533056	0.575772	0.8508489	385	505	557	701	214	312	413	767
ENSECAG00000007885	3.720603593	0.5758063	0.8508489	285	206	239	375	173	163	308	245
ENSECAG00000015126	4.828331684	0.575951	0.8508489	690	430	524	734	603	498	430	336
ENSECAG00000011928	7.265979118	0.5760388	0.8508489	3537	1448	2990	3271	3675	1802	4808	1849
ENSECAG00000005100	4.170360157	0.5760653	0.8508489	312	302	340	347	414	350	375	222
ENSECAG00000004093	2.632494806	0.5760668	0.8508489	157	70	152	141	81	67	171	93
ENSECAG00000013174	8.373259637	0.5760964	0.8508489	6146	5929	6510	9217	6768	3989	6768	4887
ENSECAG00000017464	6.828095798	0.5761715	0.8508492	2104	1968	2001	3453	1711	1704	2328	1953
ENSECAG00000001119	8.075947825	0.5763891	0.8510054	6466	5247	5536	4979	3932	3399	5708	5318
ENSECAG00000024843	7.380543421	0.576471	0.8510054	3489	3168	3388	3729	2714	2821	3300	2380
ENSECAG00000012544	0.52190213	0.5766066	0.8510054	41	8	15	58	4	7	37	38
ENSECAG00000002320	4.533362458	0.5766281	0.8510054	366	400	420	477	199	402	397	788
ENSECAG00000017215	3.874283158	0.5766698	0.8510054	319	215	318	387	169	106	466	265
ENSECAG00000014679	3.279900107	0.5767268	0.8510054	194.001	154	183.001	290.001	139	73	281	165
ENSECAG00000011456	10.015932	0.576893	0.8511401	19327	17084	19134	19445	15685	27840	16962	17187
ENSECAG00000010338	2.64151562	0.5771126	0.8512019	117	79	123	217.001	170	50	126	57
ENSECAG00000017366	1.050908884	0.5771176	0.8512019	17	62	26	29	30	47	46	32
ENSECAG00000000402	4.508833708	0.5772334	0.8512019	292	428	458	467	388	573	363	376
ENSECAG00000023914	4.754944165	0.5772717	0.8512019	558	427	621	635	527	298	556	449
ENSECAG00000014091	2.706804706	0.5773648	0.8512019	136	109	114	194	46	77	98	203
ENSECAG00000003179	3.712105693	0.5774464	0.8512019	230	205	300	362	170	176	258	273
ENSECAG00000021884	4.362777311	0.5776089	0.8512019	413	361	443	487	354	346	285	375
ENSECAG00000021930	7.595500787	0.5777837	0.8512019	3482	2868	3260	4693	3549	2911	4373	4004
ENSECAG00000024637	6.825888526	0.5779847	0.8512019	3021	1254	1915	2068	2158	1156	3474	2173
ENSECAG00000024124	6.844907902	0.578117	0.8512019	2525	1190	2566	2102	2507	1353	3577	1599
ENSECAG00000021948	3.041014622	0.5781467	0.8512019	142	108	225	217	165	119	153	100
ENSECAG00000024501	6.796100143	0.5781601	0.8512019	1970	1919	2118	2133	1975	2135	2464	1850
ENSECAG00000017751	6.058863739	0.5783148	0.8512019	1054	1268	1025	1555	1600	1108	1305	1020
ENSECAG00000014093	4.365597583	0.5783462	0.8512019	427	334	472	476	273	363	332	402
ENSECAG00000011279	4.353417454	0.5783557	0.8512019	441	291	405	582	292	192	485	425
ENSECAG00000016343	6.488738265	0.5783686	0.8512019	1518	1588	1612	1905	1550	1614	1876	1748
ENSECAG00000021181	2.560117092	0.5784585	0.8512019	112	117	117	143	81	49	109	150
ENSECAG00000004442	3.633701801	0.5785004	0.8512019	83	273	192	573	158	12	524	132

ENSECAG00000009239	5.163550461	0.5785124	0.8512019	704	624	627	1036	484	428	818	723
ENSECAG00000020832	5.471505182	0.5785673	0.8512019	747	669	807	1066	974	709	869	799
ENSECAG00000016694	4.117802026	0.5786399	0.8512019	345	300	337	479	133	220	331	479
ENSECAG00000020629	3.946688152	0.5787498	0.8512019	289	311	364	307	165	242	265	353
ENSECAG00000004034	4.71029684	0.5788072	0.8512019	636	169	704	335	614	180	1037	311
ENSECAG00000017942	5.829982093	0.5788079	0.8512019	1104	950	1018	1108	1165	1060	1170	887
ENSECAG00000023094	7.631468733	0.5788083	0.8512019	3999	3418	4569	4454	3030	3264	3326	3682
ENSECAG00000025054	6.75397601	0.5789327	0.8512746	2018	1891	2165	1766	2336	2186	1987	1606
ENSECAG00000017290	7.11314572	0.5794579	0.8519366	2301	2728	2529	2458	2656	3245	2593	1907
ENSECAG00000002018	5.3588936	0.5800345	0.8525641	926	576	670	849	834	706	941	635
ENSECAG00000010533	0.512689476	0.5801046	0.8525641	35	16	10	58	25	23	28	7
ENSECAG00000022459	5.513022084	0.5802751	0.8525641	764	709	819	1097	927	717	929	881
ENSECAG00000009052	3.394975521	0.5803085	0.8525641	218	151	201	314	189	158	203	145
ENSECAG00000016968	5.025730777	0.5803329	0.8525641	669	378	684	639	761	291	1022	494
ENSECAG00000010917	3.278373103	0.5803351	0.8525641	164	411	96	121	186	98	70	256
ENSECAG00000013922	9.119587531	0.5804212	0.8525804	9778	8416	10037	12659	8838	10032	14974	9063
ENSECAG00000021212	0.94562949	0.5807217	0.8529116	28	53	42	29	29	16	32	43
ENSECAG00000007003	4.911526428	0.5808507	0.852944	674	432	560	509	840	364	732	384
ENSECAG00000014554	1.857793646	0.580894	0.852944	65	53	84	99	40	40	81	75
ENSECAG00000015963	2.846491985	0.5810623	0.8530809	135	131	146	188	67	151	121	125
ENSECAG00000000933	5.357207314	0.5812876	0.8533013	870	580	754	809	715	834	695	828
ENSECAG00000014688	6.750099767	0.5813844	0.8533331	1843	1920	2026	3249	1315	1872	1531	2456
ENSECAG00000011007	3.611268889	0.5817182	0.8536676	205	150	234	302	177	292	232	219
ENSECAG00000025094	3.78476438	0.5817737	0.8536676	300	217	218	428	151	162	295	319
ENSECAG00000019814	7.077881431	0.5818377	0.8536676	2411	3182	2116	3472	1624	2049	2813	2673
ENSECAG00000010977	6.970792452	0.5822162	0.8541127	1987	2159	2136	2925	2373	2950	2428	1670
ENSECAG00000014322	6.20007756	0.5824823	0.8541989	1387	1231	1575	1907	1319	1306	1326	984
ENSECAG00000015188	5.402685152	0.582505	0.8541989	1007	638	846	1014	802	619	866	576
ENSECAG00000014929	3.175963162	0.582528	0.8541989	147	186	269	142	212	97	131	148
ENSECAG00000020819	1.28462115	0.5825758	0.8541989	29	28	34	74	12	93	23	51
ENSECAG00000012583	4.24456534	0.582729	0.8543131	445	300	386	438	346	238	348	343
ENSECAG00000024777	0.873649016	0.5829403	0.8545127	25	28	24	45	48	14	64	15
ENSECAG00000023920	3.247576191	0.5831344	0.8546387	157	142	178	206	239	126	235	130
ENSECAG00000010834	2.602321072	0.5831768	0.8546387	98	178	58	65	180	56	37	198
ENSECAG00000003669	8.084875224	0.5832832	0.8546845	5125	5459	5619	3026	3515	4919	4379	8025
ENSECAG00000012128	3.814989095	0.5834939	0.854883	180	429	153	190	166	270	117	532
ENSECAG00000027694	11.05571554	0.5837351	0.855126	35952	36480	30889	53320	40012	37673	49517	35888
ENSECAG00000007282	0.556169765	0.583816	0.8551342	38	22	18	40	16	20	18	34
ENSECAG00000012884	6.849900111	0.5839621	0.8552379	2237	2216	2526	2586	1368	2402	1521	2315
ENSECAG00000005990	6.930023954	0.5842115	0.8554929	1517	2407	2390	2478	1441	3523	1692	2457
ENSECAG00000012469	5.946337096	0.5842942	0.8555037	1174	908	1249	1208	1098	1307	968	1228
ENSECAG00000012871	0.780281045	0.5845198	0.8555744	28	42	21	18	17	61	32	15
ENSECAG00000016884	6.137426165	0.5845329	0.8555744	1362	1417	1399	1591	1091	1181	1267	1223
ENSECAG00000012459	6.981666592	0.5845684	0.8555744	2493	3004	2194	2671	1857	1904	2060	2684
ENSECAG00000006787	6.899963003	0.5848022	0.8557001	2169	1955	2113	2529	1749	2701	2101	2422
ENSECAG00000009051	2.998185058	0.584859	0.8557001	100	136	154	183	145	161	162	136
ENSECAG00000017348	4.742490393	0.5848803	0.8557001	520	471	456	505	456	465	588	523
ENSECAG00000008191	0.434727034	0.5850446	0.8558302	18	24	29	36	11	24	25	21
ENSECAG00000008105	3.930017508	0.5851345	0.8558515	290	288	239	281	271	230	379	290
ENSECAG00000023965	5.080004581	0.585278	0.8559511	601	545	785	878	496	598	633	545
ENSECAG00000024363	5.364356686	0.5854714	0.8560912	576	794	704	948	644	875	741	833
ENSECAG00000009653	3.101101142	0.5855245	0.8560912	78	257	131	102	55	262	65	272
ENSECAG00000014393	5.148221617	0.5856766	0.8562034	664	794	547	924	431	571	620	763
ENSECAG00000017287	2.355267062	0.586455	0.8572311	113	58	69	125	78	73	115	127
ENSECAG00000011947	5.07122125	0.5867808	0.8574946	704	527	619	606	675	511	868	520
ENSECAG00000007426	6.823098879	0.5867863	0.8574946	2475	2586	2042	2208	1150	1825	1813	2801
ENSECAG00000004091	3.499972967	0.5868872	0.8575317	269	143	326	200	209	153	182	201
ENSECAG00000022488	5.627825788	0.5870193	0.8576144	793	962	698	1183	1137	885	892	801
ENSECAG00000022979	4.836473417	0.5872838	0.8578906	507	440	743	691	427	614	450	387
ENSECAG00000021771	6.287848103	0.5873695	0.8579054	1538	1630	1612	1624	958	1530	1684	1103
ENSECAG00000026871	3.231042202	0.5877068	0.8582877	200	137	147	191	149	161	270	144
ENSECAG00000026906	1.908410754	0.5879119	0.8584768	66	45	87	64	80	39	92	77
ENSECAG00000000407	5.319435345	0.5880992	0.8586085	746	730	925	897	522	411	870	938
ENSECAG00000006847	8.926918112	0.5881532	0.8586085	9401	8526	8310	14939	5597	11002	9559	5986
ENSECAG00000017418	9.707592618	0.5886002	0.8589263	17597	24196	16266	11523	4727	19722	6119	22143
ENSECAG00000020604	1.704356879	0.5886236	0.8589263	44	54	46	84	62	47	53	83
ENSECAG00000022163	4.920695297	0.5886357	0.8589263	554	524	530	600	360	492	689	783
ENSECAG00000024211	3.143535584	0.5886734	0.8589263	190	185	174	175	137	120	140	186
ENSECAG00000007592	2.642359372	0.5889083	0.8591587	99	117	100	126	113	141	101	111
ENSECAG00000014347	5.777982904	0.5890125	0.8592003	912	1094	874	1126	667	1151	1007	1314
ENSECAG00000021636	7.465188863	0.589487	0.8595766	3094	3218	2951	3699	2873	2832	4218	3622
ENSECAG00000015088	4.144878599	0.5895249	0.8595766	396	308	433	323	453	205	289	224
ENSECAG00000005817	4.56408075	0.5895418	0.8595766	328	272	443	708	293	327	792	453
ENSECAG00000022391	5.429165354	0.5895731	0.8595766	785	781	725	868	923	837	806	661
ENSECAG00000010922	6.20563294	0.5899518	0.8597453	1522	1250	1299	2072	1008	878	1536	1641
ENSECAG00000017806	4.894277776	0.5899795	0.8597453	667	451	502	873	356	365	698	614
ENSECAG00000009471	5.142153923	0.5900211	0.8597453	485	638	631	1246	332	516	560	951
ENSECAG00000012298	5.078336202	0.5901034	0.8597453	553	664	515	733	465	527	682	908

ENSECAG00000006300	2.96421413	0.5902172	0.8597453	127	95	188	257	65	76	185	194
ENSECAG00000004513	6.467460853	0.5903089	0.8597453	1440	1320	1592	2207	1054	1242	2360	2207
ENSECAG00000011179	3.782576211	0.5903226	0.8597453	301	242	274	319	231	174	261	258
ENSECAG00000020197	5.064381108	0.5903602	0.8597453	595	514	777	902	511	394	808	582
ENSECAG00000017529	0.41560168	0.5904102	0.8597453	13	11	31	33	27	11	23	37
ENSECAG00000011347	3.38302726	0.5905857	0.8597453	201	153	259	257	210	121	153	204
ENSECAG00000018326	1.866125139	0.5906978	0.8597453	88	44	103	65	41	51	58	83
ENSECAG00000010238	3.493035267	0.5907335	0.8597453	252	236	254	178	185	125	180	258
ENSECAG00000019612	6.000879316	0.5907355	0.8597453	1449	1026	1483	1337	1369	548	1437	1036
ENSECAG00000008526	2.494417417	0.590799	0.8597453	93	99	77	132	128	85	114	100
ENSECAG00000002086	4.172121282	0.5908269	0.8597453	397	303	384	412	391	169	421	240
ENSECAG00000008254	2.448889958	0.5908999	0.8597453	48	90	97	154	89	81	159	95
ENSECAG00000017692	5.909740957	0.5911349	0.8598793	1240	1071	1199	1452	662	926	1348	1179
ENSECAG00000014300	7.350100193	0.5911434	0.8598793	3212	2198	3368	3241	3559	2522	3414	2923
ENSECAG00000016754	5.398437709	0.5912269	0.8598866	797	722	899	646	642	803	726	1007
ENSECAG00000002429	2.694685287	0.5914155	0.8598866	143	73.9996	157	82	121.997	111	144	117
ENSECAG00000026946	0.902861093	0.5914327	0.8598866	22	33	31	39	46	49	21	18
ENSECAG00000015057	6.533101552	0.5914695	0.8598866	1236	1242	1550	2919	1591	1310	1860	2332
ENSECAG00000001596	7.356282532	0.5916533	0.8598866	3147	2492	3215	3178	3165	3343	3097	2725
ENSECAG00000012440	1.257375156	0.5916592	0.8598866	76	21	64	37	59	11	64	16
ENSECAG00000022495	3.681293926	0.5916783	0.8598866	315	164	241	357	260	178	190	217
ENSECAG00000011786	4.133142533	0.5920042	0.8600933	245	286	325	636	192	209	402	384
ENSECAG00000009719	5.984519562	0.5920592	0.8600933	1318	1137	1384	1374	693	1102	1075	1400
ENSECAG00000000916	5.235554109	0.5921041	0.8600933	628	572	776	801	759	518	1010	606
ENSECAG00000000775	5.440055969	0.592134	0.8600933	777	1028	713	1044	536	607	867	946
ENSECAG00000007912	3.536803699	0.5921991	0.8600933	238	192	283	243	190	180	218	184
ENSECAG00000010494	1.781865324	0.5923311	0.8601551	56	38	84	111	62	31	82	48
ENSECAG00000014989	5.672869895	0.5923931	0.8601551	886	904	740	1240	704	779	1174	1248
ENSECAG00000003079	8.751106089	0.592592	0.8603339	8725	7004	7152	8687	8834	5836	9178	9210
ENSECAG00000015460	9.066412439	0.5926777	0.8603484	9921	8115	11030	10189	10118	10836	10736	8874
ENSECAG00000011535	4.427616415	0.5929546	0.8606404	527	301	442	533	351	168	636	327
ENSECAG00000025328	2.220869055	0.5931194	0.8607114	59	122	74	60	103	121	56	66
ENSECAG00000012567	3.53235209	0.5932204	0.8607114	253	175	215	179	320	110	302	168
ENSECAG00000007233	6.189683179	0.5932308	0.8607114	1814	1274	1561	1330	1108	1250	1053	1478
ENSECAG00000013834	3.40090467	0.5935644	0.860918	35	444	59	124	37	310	72	415
ENSECAG00000001749	3.033795561	0.5935778	0.860918	158	149	139	135	183	126	194	122
ENSECAG00000022255	8.510036045	0.5936006	0.860918	7156	4229	6772	8774	8062	6831	8670	4300
ENSECAG00000006542	5.794927262	0.5936962	0.8609467	890	1109	913	1763	374	924	1143	1302
ENSECAG00000014083	6.308782259	0.5938139	0.8610074	1320	1235	1800	1475	1569	1612	1309	1428
ENSECAG00000024161	7.298164131	0.5939632	0.8610766	2570	3848	2892	1796	2389	2197	2762	4808
ENSECAG00000020596	4.645964447	0.5940132	0.8610766	759	342	480	512	603	380	473	192
ENSECAG00000016598	0.625459574	0.5943279	0.8614229	26	24	30	21	26	15	50	25
ENSECAG00000015670	7.272983768	0.5945699	0.8614535	2925	2746	3062	4118	2302	2393	2845	2930
ENSECAG00000013404	7.645379128	0.5946194	0.8614535	4309	3137	3147	4120	4350	3347	4314	3208
ENSECAG00000002816	5.230958836	0.5946372	0.8614535	714	610	628	821	635	632	731	838
ENSECAG00000025123	6.007520946	0.5946524	0.8614535	1318	1170	1161	1050	1369	955	1331	1219
ENSECAG00000012600	6.65024777	0.5948758	0.8614805	2276	1641	2051	2326	1576	1925	1722	1513
ENSECAG00000000889	3.588628155	0.5948853	0.8614805	227	185	255	341	166	111	281	258
ENSECAG00000012944	3.381122236	0.5948986	0.8614805	182	181	195	187	121	222	193	252
ENSECAG00000010331	3.424676535	0.5951244	0.8616965	231	207	203	242	172	171	220	152
ENSECAG00000005869	6.013327431	0.5951994	0.8616965	1320	1037	1061	1357	1306	1017	1543	1041
ENSECAG00000026837	5.430342804	0.5953615	0.8618212	843	835	904	950	833	556	863	687
ENSECAG00000020001	6.790270629	0.5956212	0.8619491	2314	2143	2368	2256	1648	1312	2492	2159
ENSECAG00000020595	4.168567638	0.5956982	0.8619491	351	271	380	497	310	299	332	259
ENSECAG00000019292	3.756296022	0.5957262	0.8619491	242	202	182	360	168	271	227	358
ENSECAG00000020517	3.435297036	0.5958044	0.8619491	184	168	142	296	197	202	202	216
ENSECAG00000010933	4.200205857	0.595952	0.8619491	359	354	323	491	201	235	354	452
ENSECAG00000012574	4.811341712	0.5960163	0.8619491	567	477	597	684	472	283	577	585
ENSECAG00000012908	4.251562213	0.5961915	0.8619491	407	308	375	495	439	222	282	326
ENSECAG00000016661	8.791811828	0.5962425	0.8619491	14328	5063	9318	1046	5405	3593	23931	5122
ENSECAG00000022024	6.671254054	0.5962901	0.8619491	1923	1602	1951	2029	2120	2105	1991	1422
ENSECAG00000009760	4.763160015	0.5963436	0.8619491	578	303	585	513	744	408	627	297
ENSECAG00000008416	4.983346503	0.5964837	0.8619491	498	544	464	820	420	375	915	763
ENSECAG00000017292	5.250418844	0.5966409	0.8619491	901	504	926	826	762	661	544	557
ENSECAG00000007596	5.053185561	0.5966815	0.8619491	864	517	514	498	965	468	601	485
ENSECAG00000007115	4.210817558	0.5967899	0.8619491	313	329	360	332	293	505	324	256
ENSECAG00000016891	1.038650402	0.5967956	0.8619491	39	12	38	53	49	34	43	26
ENSECAG00000012168	4.869376188	0.5968316	0.8619491	611	611	573	594	562	373	575	471
ENSECAG00000008630	3.636048377	0.5969058	0.8619491	224	270	190	352	216	152	144	305
ENSECAG00000020350	5.277939825	0.596979	0.8619491	734	646	677	790	970	646	761	544
ENSECAG00000013382	3.123186387	0.5969814	0.8619491	125	332	150	95	98	172	122	163
ENSECAG00000024649	4.618437057	0.5970975	0.8619491	604	319	562	556	474	329	532	324
ENSECAG00000012155	4.031425889	0.5971243	0.8619491	371	325	278	182	376	246	387	245
ENSECAG00000018926	6.257779902	0.5972177	0.8619491	1765	1219	1784	1531	1686	1090	1105	1247
ENSECAG00000000376	2.156691926	0.5972361	0.8619491	94	59	88	73	78	82	98	78
ENSECAG00000023059	3.40448596	0.5973005	0.8619491	283	136	294	173	305	81	203	104
ENSECAG00000009776	2.904513649	0.5973791	0.8619491	134	163	132	195	60	91	114	226
ENSECAG00000014502	4.107957466	0.5974228	0.8619491	330	313	299	301	284	193	410	447

ENSECAG00000014355	2.819289258	0.5976159	0.8620249	164	112	115	199	148	62	137	120
ENSECAG00000006997	5.217880632	0.5977379	0.8620249	805	571	719	613	1016	533	742	529
ENSECAG00000026914	5.13894626	0.597804	0.8620249	583	466	706	848	803	377	1001	554
ENSECAG00000014057	3.949885686	0.597811	0.8620249	319	197	354	248	327	216	306	329
ENSECAG00000008513	3.03701927	0.5978547	0.8620249	170	132	192	184	137	91	157	162
ENSECAG00000020610	6.238502113	0.5979332	0.8620286	1433	1301	1346	1473	1199	1656	1330	1453
ENSECAG00000015816	11.97239463	0.5980584	0.8620996	84481	50349	82276	77109	94524	76549	58433	72856
ENSECAG00000009335	5.794793825	0.5981451	0.8621153	988	834	911	1409	951	808	1358	1122
ENSECAG00000016412	7.543360028	0.5982539	0.8621627	3629	3001	3385	3676	2982	2556	4275	4544
ENSECAG00000001626	5.477041043	0.598501	0.862366	744	782	743	1020	627	754	899	1097
ENSECAG00000001555	2.950638426	0.5985468	0.862366	140	90	263	12	286	172	104	37
ENSECAG00000005457	5.766347498	0.5986401	0.8623909	992	859	1022	1119	1001	1458	803	756
ENSECAG00000021602	3.067787977	0.5989018	0.8626585	119	376	87	95	106	121	79	223
ENSECAG00000024728	5.151038662	0.5990778	0.8627623	729	555	615	721	673	595	737	677
ENSECAG00000024605	7.742769103	0.5992277	0.8627623	4379	3311	4044	3940	4670	3658	5210	2812
ENSECAG00000020670	3.424684408	0.5992328	0.8627623	196	217	216	257	133	133	207	247
ENSECAG00000024660	2.829942129	0.5995136	0.8627623	173	147	168	93	151	68	178	74
ENSECAG00000007975	4.882683715	0.5995193	0.8627623	446	497	597	619	433	641	474	661
ENSECAG00000015368	2.401009831	0.5995199	0.8627623	125	24	179	126	113	46	144	38
ENSECAG00000007479	5.925435365	0.5995217	0.8627623	1406	956	1413	1208	1079	873	1195	992
ENSECAG00000006495	5.351633909	0.5996136	0.8627623	791	722	734	710	406	548	985	1234
ENSECAG00000013890	6.986321261	0.5996574	0.8627623	2372	2483	2283	3394	1513	1667	2427	3036
ENSECAG00000007634	3.885580564	0.6002092	0.8634468	285	213	307	437	269	130	278	319
ENSECAG00000012593	3.623698619	0.6003455	0.8635094	162	242	163	320	265	270	292	110
ENSECAG00000014253	7.203200179	0.6005613	0.8635094	2815	2055	2905	3125	3016	2146	3108	2955
ENSECAG00000022267	5.600266005	0.6006061	0.8635094	1021	909	923	1131	790	926	756	774
ENSECAG00000025075	5.239302814	0.6006087	0.8635094	794	582	890	854	689	641	771	444
ENSECAG00000017131	5.040803648	0.6007409	0.8635094	595	601	613	597	638	526	780	557
ENSECAG00000006213	4.862850897	0.6007644	0.8635094	435	649	476	398	76	1304	139	661
ENSECAG00000015186	4.275008825	0.6007848	0.8635094	395	262	373	388	266	316	508	396
ENSECAG00000007134	6.441525939	0.6009786	0.8635401	1297	1730	1488	1808	1086	2540	1339	1481
ENSECAG00000024389	4.833883508	0.6010938	0.8635401	620	459	576	381	267	793	458	623
ENSECAG00000008898	0.720870239	0.601138	0.8635401	23	13	27	77	44	6	38	12
ENSECAG00000000718	6.903994357	0.6011571	0.8635401	2524	2176	2473	2683	1883	1751	2121	2373
ENSECAG00000018471	7.75269486	0.601212	0.8635401	3373	3832	3586	5086	2854	4736	4511	4227
ENSECAG00000014918	4.774556643	0.6013171	0.8635401	557	355	545	552	495	300	708	617
ENSECAG00000011329	4.588180134	0.6013383	0.8635401	340	481	429	780	229	251	434	709
ENSECAG00000010445	6.75581201	0.6014989	0.863624	2029	1761	1709	2484	1777	1273	3012	2329
ENSECAG00000014548	5.115360149	0.6016488	0.863624	660	619	741	475	931	479	785	451
ENSECAG00000011773	3.747971094	0.6016999	0.863624	327	155	329	329	317	69	371	148
ENSECAG00000017061	8.047706962	0.6017456	0.863624	5093	5305	6202	5110	4436	4352	4811	4218
ENSECAG00000008761	0.696564619	0.6017896	0.863624	43	20	31	35	20	29	30	20
ENSECAG00000011837	8.71728308	0.6018529	0.863624	7788	6960	8184	7780	7319	9853	6745	7576
ENSECAG00000009759	7.434442508	0.6020101	0.8636497	3218	3070	3507	2814	3090	3191	3854	2997
ENSECAG00000010991	5.836808142	0.6020229	0.8636497	1210	1057	1012	862	1476	998	1112	716
ENSECAG00000022412	4.317728398	0.602128	0.8636914	456	334	360	504	453	254	301	321
ENSECAG00000012767	1.399708827	0.6025274	0.8640363	34	56	57	67	29	37	48	54
ENSECAG00000000365	2.75287414	0.6025283	0.8640363	159	109	132	164	62	48	140	199
ENSECAG00000011124	6.547752718	0.6026252	0.8640363	1987	1854	1862	1993	1517	1991	1021	1620
ENSECAG00000005471	4.54807193	0.6026727	0.8640363	536	386	480	528	507	309	439	319
ENSECAG00000021675	5.683749186	0.6030313	0.8642663	721	1333	654	1554	684	966	853	923
ENSECAG00000021409	5.46621051	0.6030354	0.8642663	953	689	756	851	864	965	773	690
ENSECAG00000014804	0.428881973	0.6030615	0.8642663	18	23	31	14	26	15.9997	42	15.9999
ENSECAG00000003790	6.60198146	0.6032849	0.8643153	1724	1769	1996	2525	1441	1640	1996	1538
ENSECAG00000017201	6.521008422	0.603319	0.8643153	1682	1990	1351	1651	2340	1213	1932	1501
ENSECAG00000018643	3.999866272	0.6033651	0.8643153	305	227	397	405	206	173	374	337
ENSECAG00000023237	8.625959164	0.6034	0.8643153	9644	6058	8314	8880	8273	5549	8417	4420
ENSECAG00000001775	1.875261847	0.6036503	0.8645649	61	109	20	51	40	70	35	135
ENSECAG00000016627	0.864970091	0.6039431	0.8648492	38	19	45	45	23	22	38	31
ENSECAG00000007904	1.770796822	0.6040011	0.8648492	68	63	57	92	44	65	59	49
ENSECAG00000003276	6.349399367	0.6041864	0.8650055	1593	873	1863	1677	1825	754	2581	1243
ENSECAG00000014592	5.922343414	0.6045255	0.8650943	1124	1082	1162	1089	972	1159	1262	1169
ENSECAG00000007999	4.562223529	0.6045736	0.8650943	558	239	378	526	873	354	453	131
ENSECAG00000009328	3.215675301	0.6046091	0.8650943	176	153	239	213	214	195	132	45
ENSECAG00000020848	5.439195674	0.6046997	0.8650943	764	872	617	928	832	536	1128	841
ENSECAG00000003209	2.033691678	0.6047126	0.8650943	72	37	99	80	68	51	143	58
ENSECAG00000011733	6.971595368	0.6047326	0.8650943	588	5186	783	1417	317	2632	632	6523
ENSECAG00000008510	0.094889388	0.6048457	0.8650943	5	32	21	5	12	26	6	32
ENSECAG00000011147	8.340218574	0.6048578	0.8650943	6550	5967	7172	7040	4801	4303	6967	6089
ENSECAG00000022470	1.530853206	0.6054065	0.8656593	30	60	40	68	97	44	37	36
ENSECAG00000014838	3.580261758	0.605454	0.8656593	228	234	226	301	140	194	179	279
ENSECAG00000013215	0.885552172	0.6054814	0.8656593	20	32	35	38	31	36	29	38
ENSECAG00000005912	2.100380613	0.6058713	0.8661078	68	80	61	93	89	51	111	77
ENSECAG00000006527	2.571322464	0.606028	0.8662227	88	78	120	141	137	112	113	84
ENSECAG00000010106	2.336828346	0.606115	0.8662381	76	43	112	124	164	28	161	48
ENSECAG00000007267	4.043937455	0.6062698	0.8662857	325	295	263	313	282	330	404	234
ENSECAG00000006906	1.937782091	0.60646	0.8662857	64	57	58	91	46	102	59	76
ENSECAG00000007392	6.678020377	0.6065857	0.8662857	1817	1655	1609	2485	1135	2478	1922	2159

ENSECAG00000011808	1.791626715	0.606661	0.8662857	70.0006	43.0005	62.0011	64.001	134.001	36.001	48.0011	42
ENSECAG00000020406	7.099277575	0.6066981	0.8662857	2617	2011	2691	2721	1307	3320	3292	2517
ENSECAG00000017186	5.629237277	0.6068967	0.8662857	1368	724	1044	1001	779	372	1826	476
ENSECAG00000011150	5.578838667	0.6069066	0.8662857	914	1057	960	954	641	720	929	971
ENSECAG00000008074	5.129662054	0.6069155	0.8662857	758	549	743	869	693	247	959	519
ENSECAG00000013213	7.824787836	0.6071146	0.8662857	4335	3640	3487	5289	3543	4681	5388	3608
ENSECAG00000017261	7.103874311	0.6071374	0.8662857	4483	1621	1583	2138	5045	2091	1917	1417
ENSECAG00000000016	5.242678167	0.6071587	0.8662857	631	628	701	843	590	656	841	778
ENSECAG00000007244	3.994003012	0.6073283	0.8662857	307	355	338	301	344	163	283	280
ENSECAG00000014941	7.10409898	0.6073482	0.8662857	2440	2737	2963	3243	1416	1621	3507	2964
ENSECAG00000017166	5.642862291	0.6073495	0.8662857	1055	866	1001	1192	612	717	931	1151
ENSECAG00000012622	6.599465282	0.6073513	0.8662857	2106	1532	2104	2250	1820	1372	2104	1335
ENSECAG00000020542	0.389536086	0.6073754	0.8662857	34.4073	10.3421	33.5965	25.3916	13.6721	20.4191	25.196	19
ENSECAG00000019147	4.717098225	0.607551	0.8662857	358	742	292	464	379	549	333	726
ENSECAG00000016036	2.577099079	0.6075857	0.8662857	103	105	126	86	131	97	143	82
ENSECAG00000015009	3.904289946	0.6075974	0.8662857	311	233	268	443	219	156	430	221
ENSECAG00000000029	4.581338266	0.6077801	0.8664376	546	297	499	409	500	375	532	412
ENSECAG00000021472	5.471979067	0.6078643	0.8664488	924	645	986	1113	682	689	849	800
ENSECAG00000014452	6.14365581	0.6081885	0.8665924	1824	898	1254	979	2525	391	2488	322
ENSECAG00000027000	2.821777217	0.6084334	0.8665924	99	151	131	112	66	104	135	241
ENSECAG00000004547	4.269979662	0.6084449	0.8665924	415	275	361	350	522	315	401	221
ENSECAG00000021219	7.604198956	0.6084904	0.8665924	3802	3253	4015	3079	4628	3477	4129	2504
ENSECAG00000026811	2.556470436	0.6085226	0.8665924	199	29	102	66	101	22	325	52
ENSECAG00000018478	6.001805322	0.6086085	0.8665924	1581	1198	1283	1169	1278	1096	876	1025
ENSECAG00000010639	8.372231284	0.6086167	0.8665924	6134	7013	6070	8128	5086	4622	6355	6467
ENSECAG00000023181	5.958759405	0.6086293	0.8665924	1047	1191	1196	1711	767	1242	1129	1047
ENSECAG00000000870	2.800286441	0.6086517	0.8665924	137	78	145	140	178	79	162	115
ENSECAG00000008402	1.609792876	0.6088102	0.8666898	64	37	54	55	95	38	78	21
ENSECAG00000017887	7.363040388	0.6089027	0.8666898	3488	2836	3748	3540	2863	3449	2297	2256
ENSECAG00000022429	3.466040891	0.608949	0.8666898	256	155	223	156	229	238	217	146
ENSECAG00000009449	4.500287549	0.6090499	0.866699	536	327	346	432	689	272	509	263
ENSECAG00000018487	4.167182031	0.609108	0.866699	440	238	448	367	371	135	399	315
ENSECAG00000018804	4.343656636	0.6093694	0.8667194	448	296	282	461	527	256	463	306
ENSECAG00000017962	5.373561494	0.6094222	0.8667194	912	676	819	1005	675	574	812	771
ENSECAG00000010132	9.320089227	0.6094784	0.8667194	14273	9130	12313	10540	14624	14722	13461	5546
ENSECAG00000002745	5.294014134	0.6094824	0.8667194	766	627	904	562	814	635	788	726
ENSECAG00000019803	3.500164824	0.6096053	0.8667194	265	194	225	250	148	233	154	203
ENSECAG00000009714	7.040367552	0.6096232	0.8667194	1957	2878	2599	2035	2093	3380	1973	2357
ENSECAG00000011430	6.30940956	0.6097669	0.8667194	1674	1244	1516	1416	1314	1283	1654	1749
ENSECAG00000019599	4.619632629	0.60979	0.8667194	404	423	433	527	208	594	468	588
ENSECAG00000009514	6.613331298	0.6098091	0.8667194	1557	1648	2004	2009	1464	2120	1547	2176
ENSECAG00000000204	6.81383777	0.6100849	0.8668315	1716	2307	2195	1992	1884	1965	2537	2160
ENSECAG00000023121	2.831955858	0.6101403	0.8668315	185	66	122	127	286	45	127	93
ENSECAG00000019849	7.40954483	0.610148	0.8668315	3480	2828	3281	4551	2984	1991	3927	2779
ENSECAG00000016844	0.641727093	0.610246	0.8668315	21	38	23	41	24	25	26	20
ENSECAG00000017025	2.7887845	0.6102695	0.8668315	106	133	105	150	102	136	120	161
ENSECAG00000016725	4.89274687	0.6106704	0.8670229	680	430	656	697	687	336	549	434
ENSECAG00000025037	4.920305357	0.6107495	0.8670229	516	604	578	815	264	412	701	694
ENSECAG00000023338	5.157439108	0.6108644	0.8670229	742	614	722	855	603	516	727	588
ENSECAG00000014518	7.144119551	0.6108933	0.8670229	1669	4368	1734	1828	603	5850	2489	1759
ENSECAG00000016002	3.37774206	0.6109095	0.8670229	247	183	268	161	268	95	246	80
ENSECAG00000008387	6.413596081	0.6110703	0.8670229	1298	1500	1439	2084	1417	1985	1602	1352
ENSECAG00000024479	2.754934963	0.6111091	0.8670229	147	110	147	155	98	51	135	167
ENSECAG00000022120	8.855272408	0.6111092	0.8670229	9178	8427	8932	11882	8239	7080	8829	7181
ENSECAG00000024085	2.365145568	0.6111232	0.8670229	110	102	106	101	83	66	89	100
ENSECAG00000021792	5.200211335	0.6111973	0.8670229	856	510	918	764	804	482	858	345
ENSECAG00000014711	2.934169361	0.6112439	0.8670229	96	194	97	141	185	133	235	42
ENSECAG00000003883	4.055773454	0.6114219	0.8671671	287	327	355	412	119	257	325	415
ENSECAG00000006245	1.750307094	0.6115752	0.8671867	6	155	5	28	12	64	4	193
ENSECAG00000000302	6.673233742	0.6115884	0.8671867	1721	2005	1585	2205	1594	2138	2075	1859
ENSECAG00000015053	6.090126964	0.6116761	0.8672027	1362	1201	1319	1758	635	1069	1411	1540
ENSECAG00000007476	2.521646854	0.6120733	0.8676576	124	127	66	75	115	184	77	48
ENSECAG00000013517	4.868423846	0.6122538	0.8676696	554	653	445	447	623	363	769	496
ENSECAG00000005483	4.935762125	0.6122902	0.8676696	541	515	624	554	518	741	484	527
ENSECAG00000024552	3.410632526	0.6123395	0.8676696	214	182	216	273	227	110	275	104
ENSECAG00000016403	6.177721501	0.6124341	0.8676696	1179	1329	1256	1560	1033	1715	1047	1582
ENSECAG00000001039	4.459619753	0.6124637	0.8676696	440	358	381	434	426	389	500	347
ENSECAG00000023862	7.188756315	0.6127345	0.8677382	2147	1870	3594	3168	1935	2720	2648	3776
ENSECAG00000015363	4.301892894	0.6127666	0.8677382	331	245	400	487	376	299	450	375
ENSECAG00000019405	2.919893553	0.612817	0.8677382	141	132	138	122	94	109	104	271
ENSECAG00000013509	5.17332517	0.6129076	0.8677382	705	631	704	594	788	567	848	534
ENSECAG00000023186	4.480954993	0.6129313	0.8677382	489	328	427	385	556	314	430	388
ENSECAG00000008873	2.158410417	0.6130183	0.8677382	83	56	87	153	40	33	159	68
ENSECAG00000019486	4.861215643	0.6130468	0.8677382	638	455	536	497	568	461	675	503
ENSECAG00000022535	5.888358397	0.6134743	0.8679236	1091	919	1189	1189	1115	1114	1298	924
ENSECAG00000011667	6.030742833	0.6134848	0.8679236	1205	1205	1374	964	1162	1560	962	1160
ENSECAG00000023660	5.586870777	0.6135319	0.8679236	882	772	951	942	831	578	1230	1053
ENSECAG00000007778	5.106882944	0.613542	0.8679236	619	518	710	691	853	447	738	578

ENSECAG00000010355	5.550858333	0.6135599	0.8679236	1101	942	1026	725	839	690	834	815
ENSECAG00000009350	4.00850077	0.6138239	0.868189	328	280	241	324	270	290	332	323
ENSECAG00000022544	8.557675309	0.6139348	0.8682378	6831	6398	6807	7658	6730	4858	9706	7805
ENSECAG00000021742	6.562056487	0.6141032	0.8682949	1807	1817	1919	1332	1512	1597	1862	2176
ENSECAG00000009185	4.804384682	0.614175	0.8682949	463	438	469	704	517	492	584	514
ENSECAG00000012292	8.230442324	0.6142045	0.8682949	5571	5336	4790	6396	5230	6327	5683	5330
ENSECAG00000015275	3.411630193	0.6144434	0.8683298	77	431	121	66	273	195	128	222
ENSECAG00000018567	5.525092551	0.6145025	0.8683298	821	901	949	1098	765	731	989	660
ENSECAG00000013190	4.738965735	0.6145029	0.8683298	401	415	518	644	463	501	511	531
ENSECAG00000015792	5.903316553	0.614535	0.8683298	2367	614	1201	846	690	478	2328	662
ENSECAG00000023698	4.616965347	0.614726	0.8684916	492	510	614	382	314	500	346	460
ENSECAG00000001003	3.140360534	0.6148744	0.8685932	179	152	117	187	195	170	167	129
ENSECAG00000023120	0.845813136	0.6150663	0.8687563	40	14	31	37	20	43	24	43
ENSECAG00000017527	2.651538486	0.6151575	0.868777	99	96	118	136	178	92	121	83
ENSECAG00000018278	1.733924966	0.6155202	0.8690211	63	55	53	107	31	41	31	107
ENSECAG00000017173	2.898724584	0.6155974	0.8690211	149	103	136	148	115	95	230	139
ENSECAG00000024991	9.300621632	0.6156085	0.8690211	10060	11602	12489	11629	8820	17196	9294	11652
ENSECAG00000013260	8.861477687	0.6156363	0.8690211	8213	8026	8133	9820	8102	10310	9096	7444
ENSECAG00000022027	3.913997568	0.6159045	0.8692917	297	243	241	321	258	283	341	257
ENSECAG00000009117	8.003091055	0.616137	0.8693977	6193	3979	5265	5807	5286	3333	4795	3979
ENSECAG00000021195	7.20547637	0.6162315	0.8693977	3343	2291	2906	3745	3006	1382	3373	2383
ENSECAG00000011358	0.103217545	0.6162509	0.8693977	20	18	16	29	9	7	25	24
ENSECAG00000015341	0.29997014	0.6163071	0.8693977	40	8	30	20	17	13	8	33
ENSECAG00000015986	0.436241865	0.6163654	0.8693977	28	15	33	32	22	32	14	10
ENSECAG00000011589	3.282946697	0.6164389	0.8693977	141	186	166	327	90	125	175	257
ENSECAG00000019219	4.29948691	0.6168532	0.8698741	558	391	279	386	445	268	354	247
ENSECAG00000017120	4.553165603	0.6170786	0.8700838	452	475	323	459	404	375	447	550
ENSECAG00000020392	5.65185023	0.617224	0.8701808	1088	737	858	1042	1138	628	1087	966
ENSECAG00000010788	5.470919731	0.6175219	0.8704227	754	810	747	971	867	734	831	896
ENSECAG00000023306	6.733529303	0.6175498	0.8704227	2194	1668	2473	2491	2236	1322	2638	1085
ENSECAG00000015530	6.074607801	0.6176254	0.8704227	1498	1335	1285	1367	1218	910	1437	1061
ENSECAG00000008062	5.933569651	0.6178147	0.8705815	1264	1050	841	1379	1233	985	1308	1085
ENSECAG00000020293	6.648636998	0.6178951	0.8705868	1506	1977	1938	1944	1608	2203	1797	1880
ENSECAG00000018624	7.160763842	0.618084	0.8706903	2863	2628	2896	3392	2200	2127	3238	2242
ENSECAG00000006833	1.548399652	0.6181219	0.8706903	46	44	59	54	48	74	39	52
ENSECAG00000010205	5.60150076	0.6182503	0.8707633	771	965	835	1006	855	957	936	882
ENSECAG00000006287	4.415674181	0.6187008	0.8709955	458	332	384	600	368	232	476	384
ENSECAG00000011693	3.000233673	0.6187343	0.8709955	123	119	167	167	161	175	102	155
ENSECAG00000013462	2.209182227	0.6187617	0.8709955	63	147	58	39	42	82	117	118
ENSECAG00000011050	4.752683498	0.6187623	0.8709955	479	510.002	484.009	480.004	675.002	552.002	497.008	291.004
ENSECAG00000012599	2.124753187	0.6187986	0.8709955	98	61	88	113	81	68	59	72
ENSECAG00000017251	6.476198129	0.6191028	0.8713046	1964	1373	1952	2047	1864	1303	1750	1133
ENSECAG00000016571	4.463222237	0.6191716	0.8713046	322	383	377	543	462	371	552	291
ENSECAG00000012550	0.434795459	0.6192781	0.8713464	21	27	17	23	29	22	25	21
ENSECAG00000013366	10.48318553	0.6194288	0.8713955	25683	25556	32469	35052	21149	16697	35334	25530
ENSECAG00000011363	7.267688595	0.6195291	0.8713955	2369	3043	2472	3433	2391	3606	3220	2358
ENSECAG00000008912	5.377308296	0.6197087	0.8713955	933	1058	933	422	850	677	585	639
ENSECAG00000020614	1.889085143	0.6197171	0.8713955	55	89	62	95	63	52	53	70
ENSECAG00000021450	0.482364055	0.6197295	0.8713955	18	32	18	23	18	23	38	23
ENSECAG00000016296	5.376817066	0.6197732	0.8713955	634	793	796	820	596	1018	863	627
ENSECAG00000008388	2.573497193	0.6198567	0.871405	76	112	128	102	56	157	110	123
ENSECAG00000017546	5.742543408	0.6199697	0.871456	975	1228	1198	935	703	1071	969	858
ENSECAG00000020326	3.367916566	0.6203665	0.8715641	288	117	110	391	312	48	272	42
ENSECAG00000012758	3.700142253	0.6203949	0.8715641	251	207	278	340	171	190	259	256
ENSECAG00000005916	3.526547374	0.6204331	0.8715641	243	197	228	289	90	170	233	280
ENSECAG00000009365	3.241530546	0.6204417	0.8715641	211	147	182	250	94	86	200	258
ENSECAG00000022192	3.956247915	0.6205263	0.8715641	321	233	286	284	429	154	359	250
ENSECAG00000022958	6.311579614	0.6205401	0.8715641	2002	1149	1678	1736	1773	952	1817	885
ENSECAG00000004287	5.640466038	0.6207147	0.8715641	880	906	955	1377	658	663	1098	1019
ENSECAG00000008125	3.768314989	0.6207163	0.8715641	254	169	235	344	209	177	440	236
ENSECAG00000026754	0.24707953	0.6207501	0.8715641	19	14	11	34	17	28	10	28
ENSECAG00000023915	4.208105392	0.6208139	0.8715641	426	299	446	341	339	226	376	313
ENSECAG00000014491	8.278806177	0.6208952	0.8715705	5351	4952	5489	7168	6675	5418	7660	3903
ENSECAG00000014026	3.896060314	0.6210583	0.8716678	268	191	347	455	143	131	298	436
ENSECAG00000021359	5.356214181	0.6212301	0.8716678	729	667	669	979	729	827	688	806
ENSECAG00000024895	0.467658552	0.6213994	0.8716678	33	16	31	29	25	9	28	23
ENSECAG00000006808	6.158137555	0.6214015	0.8716678	1311	1374	1401	1767	978	1145	1367	1391
ENSECAG00000001372	0.551504743	0.6214118	0.8716678	14	30	18	33	6	47	16	36
ENSECAG00000019332	5.459040331	0.6215566	0.8716678	851	753	818	819	764	599	1052	938
ENSECAG00000017829	3.787811176	0.621577	0.8716678	323	305	274	220	164	192	233	336
ENSECAG00000014342	3.748745215	0.6215784	0.8716678	203	244	256	430	170	85	363	304
ENSECAG00000017845	1.685379645	0.6220785	0.872239	60	63	59	78	50	42	62	55
ENSECAG00000014072	5.249643932	0.6221393	0.872239	734	637	853	903	583	464	885	694
ENSECAG00000011523	2.085456217	0.6223548	0.8723409	73	63	88	69	32	60	53	180
ENSECAG00000007149	8.157516193	0.6223656	0.8723409	5579	5407	4984	7733	5032	4581	6323	3450
ENSECAG00000009454	2.803283537	0.6225496	0.8724386	111	218	78	168	47	148	87	162
ENSECAG00000005572	7.726527966	0.6225889	0.8724386	4123	3908	4296	5142	3918	3193	4387	2913
ENSECAG00000021593	5.636096283	0.6227867	0.8726082	951	970	896	1259	718	695	1035	971

ENSECAG00000015004	2.418781219	0.6228957	0.8726532	116	82	94	87	112	76	139	80
ENSECAG00000015502	6.102938937	0.6229899	0.8726775	1470	1034	1562	1601	1111	1302	1415	822
ENSECAG00000024128	1.427956999	0.6233723	0.8729834	27	51	49	96	45	50	38	34
ENSECAG00000007920	7.290982015	0.6234844	0.8729834	3384	2464	3338	3752	2793	2190	3130	2575
ENSECAG00000018942	5.405655509	0.6235836	0.8729834	906	742	857	964	713	555	921	727
ENSECAG00000009870	1.980949536	0.6236048	0.8729834	48	70	82	78	89	73	72	59
ENSECAG00000012037	4.08324371	0.62363	0.8729834	299	372	315	400	224	273	351	298
ENSECAG00000016736	5.001746492	0.6236693	0.8729834	638	515	544	668	721	523	654	511
ENSECAG00000007461	5.27663001	0.6238739	0.8731621	736	655	973	808	548	671	710	697
ENSECAG00000002830	6.272625167	0.6240585	0.8732564	1220	1485	1477	1436	810	2336	1202	1374
ENSECAG00000009981	4.700401981	0.624154	0.8732564	499	390	535	488	574	399	538	450
ENSECAG00000007824	6.694405738	0.6242114	0.8732564	2350	1585	2386	2213	2142	1808	1570	1405
ENSECAG00000025173	4.450761844	0.6242689	0.8732564	433	372	407	595	362	373	379	355
ENSECAG00000020755	9.326086707	0.6243257	0.8732564	14595	9316	12069	10930	15651	12291	11783	8562
ENSECAG00000022866	1.882795956	0.6245609	0.8734779	35.002	136.002	36	28.0055	28.0022	108.004	27.0022	115.003
ENSECAG00000017726	5.006925995	0.6248934	0.8736652	686	605	643	698	442	576	472	666
ENSECAG00000020895	5.045157995	0.6249634	0.8736652	612	578	487	756	504	375	844	827
ENSECAG00000008196	3.378381746	0.6249984	0.8736652	121	419	122	183	95	111	144	330
ENSECAG00000022507	5.886445764	0.6250025	0.8736652	661	1030	1025	1634	469	1739	985	1233
ENSECAG00000013158	2.279023938	0.6251965	0.8738288	141	77	66	52	98	103	95	68
ENSECAG00000003319	3.980719774	0.6257293	0.8743688	334	156	262	416	267	175	388	392
ENSECAG00000022703	7.465230486	0.6257573	0.8743688	4173	2660	3512	4401	2907	1357	5029	3018
ENSECAG00000003967	6.724585982	0.6258769	0.8743688	1920	1703	1948	2273	2259	1967	2104	1594
ENSECAG00000020499	2.987974844	0.6258907	0.8743688	165	111	161	134	128	134	179	160
ENSECAG00000024786	7.033002155	0.626035	0.8744486	3041	2009	2744	3033	2483	2186	2647	1541
ENSECAG00000009459	5.638973119	0.6261976	0.8744486	778	918	900	1099	999	751	1145	873
ENSECAG00000000780	5.333926137	0.6262046	0.8744486	828	622	671	867	754	593	695	996
ENSECAG00000018575	3.644429724	0.6263103	0.8744486	187	282	165	258	293	110	410	170
ENSECAG00000004256	4.56472718	0.6263484	0.8744486	452	344	432	515	454	560	410	331
ENSECAG00000006769	5.586505926	0.6264098	0.8744486	1055	809	1066	1004	1085	579	905	705
ENSECAG00000011085	4.290621016	0.6265629	0.8744494	292	324	382	653	164	230	415	522
ENSECAG00000020699	11.04439611	0.6266453	0.8744494	41484	38076	34166	41089	35731	33408	38583	52756
ENSECAG00000007977	0.654516216	0.6266511	0.8744494	25	34	30	36	14	34	12	33
ENSECAG00000018635	3.37177918	0.6267182	0.8744494	224	141	228	266	185	115	227	172
ENSECAG00000012400	7.21975502	0.6269074	0.8745039	417	5274	5295	1758	343	4805	289	3252
ENSECAG00000018001	1.685627443	0.6269113	0.8745039	47	57	77	86	20	26	43	117
ENSECAG00000019943	0.495427458	0.6269904	0.8745069	13	21	21	38	19	17	59	12
ENSECAG00000009809	2.278075959	0.6273241	0.8746295	52	93	69	131	62	122	74	102
ENSECAG00000020655	5.069543362	0.627437	0.8746295	635	539	688	612	608	529	708	690
ENSECAG00000002280	6.341233078	0.6274528	0.8746295	1580	1378	1419	1641	1577	1267	1774	1495
ENSECAG00000022334	7.167111823	0.6274736	0.8746295	2888	2549	2873	3556	1913	2846	2410	2487
ENSECAG00000000893	1.603363934	0.6274926	0.8746295	25	112	37	73	20	21	71	84
ENSECAG00000008540	1.458264454	0.6275403	0.8746295	49	32	59	50	56	24	93	39
ENSECAG00000000266	5.889998676	0.6279329	0.8750693	1665	1034	938	587	1773	750	1368	663
ENSECAG00000016890	5.142386361	0.6280298	0.8750969	760	489	666	1032	460	400	810	766
ENSECAG00000021823	6.99306423	0.628178	0.8751961	2449	2459	2360	3217	2078	2200	2552	1819
ENSECAG00000002178	0.69437954	0.628372	0.875359	13	41	24	28	22	37	28	30
ENSECAG00000010491	6.372551793	0.6290029	0.8761021	1547	1327	1356	1903	2089	1222	2143	863
ENSECAG00000021748	1.95596687	0.6290596	0.8761021	59	54	99	60	73	84	57	72
ENSECAG00000012973	7.940527528	0.6292065	0.8761991	4586	5809	4698	4948	3409	4780	4681	3663
ENSECAG00000017677	8.507793935	0.6294842	0.8764784	6266	6808	7554	9555	6153	3842	8407	6702
ENSECAG00000015881	5.280828509	0.6297658	0.8767631	727	604	745	802	919	631	776	590
ENSECAG00000010260	5.772448647	0.6300373	0.8770295	971	860	937	1305	866	1235	1186	796
ENSECAG00000021445	1.651573035	0.6301618	0.8770295	63	31	40	85	23	30	52	138
ENSECAG00000001159	2.947420068	0.6302455	0.8770295	110	144	145	149	147	148	234	63
ENSECAG00000022755	3.660522015	0.630266	0.8770295	313	161	229	208	341	124	328	184
ENSECAG00000022247	1.824314141	0.6304584	0.8771235	7	160	0	26	0	63	4	233
ENSECAG00000020914	4.97705997	0.6304979	0.8771235	523	509	630	666	603	636	544	560
ENSECAG00000014546	5.32425855	0.6306558	0.8771235	427	1753	303	808	220	396	677	1362
ENSECAG00000013055	5.310313619	0.6306918	0.8771235	566.996	840.991	647.994	837.975	402.997	987.994	771	812.999
ENSECAG00000009967	5.014547368	0.6307197	0.8771235	668	453	631	958	728	319	811	355
ENSECAG00000017185	6.508607329	0.6308744	0.8772313	2220	1900	1745	1552	2335	1351	1389	1004
ENSECAG00000000479	6.262777058	0.631061	0.8773833	1586	1280	1695	1724	1272	1010	1498	1500
ENSECAG00000018851	6.32907605	0.6311975	0.8774209	1432	1398	1516	1614	1488	1580	1495	1426
ENSECAG00000006568	4.904393946	0.6312628	0.8774209	745	555	444	426	531	467	721	563
ENSECAG00000024744	6.842199433	0.6313197	0.8774209	2205	2113	2265	2863	2174	1591	2439	1648
ENSECAG00000007549	5.802314476	0.6314601	0.8774947	1142	1012	1213	1177	916	747	1092	1082
ENSECAG00000014383	6.245765146	0.6315691	0.8774947	600	3360	1005	1252	229	853	2166	1868
ENSECAG00000019108	0.608410145	0.6316046	0.8774947	35	30	16	17	41	20	41	11
ENSECAG00000006458	2.807378854	0.6317797	0.8775884	142	88	133	143	90	135	186	121
ENSECAG00000011964	7.236240864	0.6318266	0.8775884	2518	2346	2679	3656	3563.01	2691	3254	1864
ENSECAG00000011014	3.990043812	0.6320591	0.8777084	305	251	290	317	331	310	278	265
ENSECAG00000024815	1.513741956	0.6320675	0.8777084	67	26	48	60	62	37	56	58
ENSECAG00000022801	5.104170183	0.6327751	0.8783258	878	391	744	495	604	689	927	401
ENSECAG00000000586	0.210298182	0.6327859	0.8783258	14	23	11	26	7	27	26	23
ENSECAG00000008095	0.93107846	0.6327873	0.8783258	36	39	46	29	23	27	31	38
ENSECAG00000024639	4.174381931	0.6328617	0.8783258	429	307	317	436	327	161	420	331
ENSECAG00000024342	5.396721986	0.6329619	0.8783258	858	697	861	1066	1077	500	738	533

ENSECAG00000010266	6.004761264	0.632976	0.8783258	801	1504	918	1484	968	850	1868	1284
ENSECAG00000015999	7.194522495	0.6331028	0.8783944	2624	2205	2875	3145	3509	2628	2944	1925
ENSECAG00000024898	6.139135015	0.6332353	0.8784709	1031	1436	1285	1402	891	1775	976	1579
ENSECAG00000013447	5.811054375	0.6333663	0.8785453	1024	936	1079	1115	853	1256	933	1121
ENSECAG00000013588	5.478514722	0.633567	0.8787164	875	654	870	918	895	656	1007	810
ENSECAG00000016617	3.626887831	0.633679	0.8787645	210	211	255	347	231	139	252	214
ENSECAG00000006549	6.178446721	0.6337843	0.8788032	1256	1498	1415	1768	1403	1265	1448	785
ENSECAG00000024817	1.339985126	0.6339952	0.8789883	36	41	44	54	26	35	80	52
ENSECAG00000009146	4.544887966	0.6342236	0.8791398	494	353	488	590	430	323	470	365
ENSECAG00000021305	6.213524593	0.6343013	0.8791398	1269	1305	1362	1577	1342	1413	1452	1335
ENSECAG00000024218	2.12595554	0.6343366	0.8791398	84	60	82	140	92	87	63	33
ENSECAG00000023084	6.831560037	0.6345046	0.8791819	2347	1592	2104	2373	2949	1347	2930	1517
ENSECAG00000013636	9.467011131	0.6345218	0.8791819	12722	14029	15196	9022	17062	11177	16684	9121
ENSECAG00000015740	6.292829629	0.6348829	0.8795214	1718	1192	1452	1441	1836	1145	1548	1368
ENSECAG00000015497	5.085217833	0.6349217	0.8795214	601	619	488	798	395	601	734	847
ENSECAG00000008235	1.81440461	0.6353865	0.8799557	96	96	42	51	33	38	26	124
ENSECAG00000010390	1.936874389	0.6353901	0.8799557	58	62	73	78	75	55	98	61
ENSECAG00000009002	0.535577518	0.6357044	0.8802836	16	24	11	46	15	41	29	19
ENSECAG00000019458	8.239391697	0.6358328	0.8803044	5360	4928	6101	5847	6360	3459	8497	5071
ENSECAG00000014586	4.91519532	0.6358744	0.8803044	720	466	680	603	553	371	644	499
ENSECAG00000004251	2.072928047	0.6359617	0.8803179	107	27	191	25	74	29	122	48
ENSECAG00000011144	5.072579347	0.6361957	0.880348	941	411	656	784	792	420	502	544
ENSECAG00000000623	6.87577687	0.6362318	0.880348	2585	1952	2641	2452	2166	2037	1915	1793
ENSECAG00000012851	6.502791856	0.6362892	0.880348	2076	1559	1843	1929	1379	1236	2256	1418
ENSECAG00000000974	7.735202692	0.6362934	0.880348	4623	3961	3934	5017	3027	4233	3992	3106
ENSECAG00000009606	7.234034984	0.6364063	0.8803969	2778	2437	2727	3238	2715	2421	3459	2820
ENSECAG00000019554	2.441809544	0.6365411	0.8804431	102	93	114	137	69	66	104	122
ENSECAG00000015112	5.369913894	0.6365947	0.8804431	905	521	856	1161	821	466	918	636
ENSECAG00000015731	4.196941373	0.6367438	0.880456	387	333	359	430	224	183	505	359
ENSECAG00000014191	6.613858175	0.6368226	0.880456	1942	1803	1901	2376	1390	1351	1979	2030
ENSECAG00000014734	2.606086171	0.6369273	0.880456	74	97	99	166	64	50	156	208
ENSECAG00000014826	4.450748346	0.6369637	0.880456	366	334	449	467	398	425	470	346
ENSECAG00000018538	6.786453953	0.6370672	0.880456	2472	1623	2016	2012	2851	1467	2688	1414
ENSECAG00000021620	3.781703489	0.6372368	0.880456	275	184	276	272	327	199	299	215
ENSECAG00000003138	1.909808444	0.6372732	0.880456	37	88	61	75	44	92	80	64
ENSECAG00000015033	4.526425098	0.63741	0.880456	467	315	339	591	579	383	372	387
ENSECAG00000000797	9.052028869	0.6374415	0.880456	9150	7661	9798	12654	13633	5807	14998	6735
ENSECAG00000000001	5.63827679	0.6374485	0.880456	852	1043	735	1034	1001	1032	817	842
ENSECAG00000001013	3.995705035	0.6374782	0.880456	320	243	246	367	266	258	320	360
ENSECAG00000004124	3.71545106	0.637545	0.880456	225	150	288	304	183	174	331	323
ENSECAG00000021004	0.570602972	0.637682	0.880456	44	21	20	33	37	15	17	20
ENSECAG00000000716	5.382565838	0.6376892	0.880456	724	783	809	731	875	977	767	474
ENSECAG00000023160	2.827855003	0.6378109	0.8804661	151	169	137	118	83	65	154	176
ENSECAG00000005760	6.219068908	0.6378516	0.8804661	1284	1383	1464	2009	915	940	1681	1624
ENSECAG00000000861	5.827145156	0.6379673	0.8805189	1315	855	1302	1173	1167	759	1064	887
ENSECAG00000001336	1.434420342	0.6381391	0.880649	44	57	59	21	35	41	49	78
ENSECAG00000026981	7.984023067	0.6382958	0.8807044	4743	4603	4262	5008	5791	4782	4912	3505
ENSECAG00000012547	2.400428466	0.6383343	0.8807044	106	102	55	111	90	133	87	80
ENSECAG00000016250	1.656317814	0.6386096	0.8809772	45	62	86	60	38	39	66	63
ENSECAG00000010421	4.845101017	0.6388128	0.8810583	521	571	562	701	417	333	637	594
ENSECAG00000013617	3.462702858	0.6388663	0.8810583	224	162	282	241	232	105	244	165
ENSECAG00000012634	3.78560308	0.6389773	0.8810583	338	208	270	320	224	263	242	187
ENSECAG00000003330	0.608961247	0.6389973	0.8810583	33	26	29	32	10	25	21	37
ENSECAG00000019441	5.144954486	0.6390572	0.8810583	610	416	631	981	674	610	1129	316
ENSECAG00000018536	5.366742862	0.6391338	0.8810583	546	626	894	1015	667	764	782	880
ENSECAG00000015444	4.211407166	0.6392364	0.8810929	333	316	472	396	297	232	454	288
ENSECAG00000006403	5.58201419	0.6393524	0.8811073	857	814	785	1108	1107	794	1013	678
ENSECAG00000000588	0.820026563	0.6394833	0.8811073	31	39	28	42	13	31	40	26
ENSECAG00000021890	0.099691534	0.6395753	0.8811073	23	27	19	11	12	12	21	19
ENSECAG00000011562	4.877949846	0.639618	0.8811073	523	472	637	816	330	322	522	834
ENSECAG00000011953	5.35246643	0.6396347	0.8811073	903	590	865	1035	699	295	1123	734
ENSECAG00000017202	6.576262665	0.6398699	0.8812425	2204	1443	1898	2340	1244	1085	1695	2537
ENSECAG00000011664	4.868726173	0.6399482	0.8812425	839	445	298	551	543	349	697	661
ENSECAG00000016324	2.96547033	0.6400306	0.8812425	158	204	169	99	74	177	117	136
ENSECAG00000009733	1.743485301	0.6400799	0.8812425	58	49	78	48	84	57	71	37
ENSECAG00000021003	3.159537363	0.6401207	0.8812425	144	151	163	289	82	155	193	166
ENSECAG00000016211	2.34561376	0.6403804	0.881493	98	63	144	115	69	47	80	139
ENSECAG00000008079	0.119243811	0.6405457	0.8816138	21	21	25	16	5	9	22	29
ENSECAG00000019392	6.26897476	0.6406633	0.8816143	1428	1217	1676	1372	1397	1791	1350	1169
ENSECAG00000008241	2.131741458	0.6407013	0.8816143	79	70	85	132	90	53	122	22
ENSECAG00000013582	5.074581828	0.6408168	0.8816664	395	775	534	753	453	669	516	891
ENSECAG00000016265	8.505831474	0.6409319	0.881718	7197	6367	8192	8157	5962	5557	8272	5093
ENSECAG00000002390	1.827391229	0.6411045	0.8818486	68	69	56	50	88	97	30	42
ENSECAG00000014719	2.600468394	0.6411903	0.8818599	89	57	102	189	193	55	189	38
ENSECAG00000006600	2.963304513	0.6413286	0.8819275	151	124	110	178	135.007	207	103	128
ENSECAG00000006341	3.373245748	0.6413948	0.8819275	160	192	201	197	229	162	176	209
ENSECAG00000016500	7.358200398	0.641564	0.8820249	3127	3689	3214	3388	1698	3065	2425	3826
ENSECAG00000015666	6.072122992	0.6416257	0.8820249	1392	1009	1273	1929	1517	928	1496	641

ENSECAG00000010157	10.32444077	0.6416986	0.8820249	25274	18750	24920	25937	28764	22413	22258	22794
ENSECAG00000008405	3.953127226	0.6418251	0.8820921	224	220	179	720	235	341	289	142
ENSECAG00000013523	4.484347241	0.6419206	0.8821166	420	353	452	632	263	347	363	539
ENSECAG00000019476	8.482967188	0.6420201	0.8821466	7904	5646	7492	8706	7783	5024	8252	3244
ENSECAG00000017536	5.562703231	0.6423699	0.882367	942	708	840	1035	791	684	1282	847
ENSECAG00000007814	4.704891177	0.642418	0.882367	501	565	524	528	308	415	505	547
ENSECAG00000012713	6.069574247	0.6424674	0.882367	1124	1152	1196	1522	1664	1164	1139	1028
ENSECAG00000016136	4.259030875	0.6425628	0.882367	427	286	351	340	388	313	456	290
ENSECAG00000020081	3.311816008	0.6426034	0.882367	205	156	190	167	234	142	208	165
ENSECAG00000013772	4.252104517	0.6426466	0.882367	340	274	445	325	585	329	273	231
ENSECAG00000009836	8.466787404	0.6430115	0.8826823	7249	4759	6782	7253	10074	4217	9056	3878
ENSECAG00000012159	3.189936398	0.6430789	0.8826823	139	191	169	256	76	130	185	221
ENSECAG00000016734	6.84323387	0.6431314	0.8826823	1841	2023	2156	2460	1949	2784	2336	1487
ENSECAG00000022209	8.382052599	0.643187	0.8826823	6320	7013	6091	7945	5636	5735	5779	5390
ENSECAG00000026810	3.439014438	0.6433063	0.8827393	225	196	169	308	132	118	240	248
ENSECAG00000012556	5.060056508	0.6435069	0.8828263	523	632	677	603	517	888	609	454
ENSECAG00000016919	7.104707944	0.6435251	0.8828263	2396	2369	2353	4360	2240	2222	3067	1870
ENSECAG00000018238	6.212404852	0.643615	0.882843	1462	1165	1428	1434	1280	1747	1134	1307
ENSECAG00000003617	2.960204752	0.6436951	0.8828462	203	86	191	166	88	85	129	216
ENSECAG00000008029	7.897000465	0.6438429	0.8829423	5020	5061	4926	4411	2688	4486	4855	4145
ENSECAG00000002734	5.666199049	0.644044	0.883066	857	847	934	1153	924	929	957	975
ENSECAG00000024592	5.006776292	0.6442483	0.883066	614	468	550	768	567	474	734	660
ENSECAG00000010479	7.93330834	0.6442729	0.883066	4268	4621	4017	5074	3645	5920	4071	4543
ENSECAG00000007197	5.835248549	0.6442957	0.883066	1197	1121	1190	1120	812	937	969	1173
ENSECAG00000000711	5.049801388	0.6443218	0.883066	728	498	671	840	766	346	644	505
ENSECAG00000015584	4.422734251	0.644638	0.8832369	388	279	556	560	310	250	358	538
ENSECAG00000020627	4.018740053	0.6447224	0.8832369	333	224	336	455	274	194	245	379
ENSECAG00000008578	0.677933442	0.6447503	0.8832369	30	7	33	38	43	19	45	12
ENSECAG00000013161	3.195808136	0.6447575	0.8832369	219	128	168	139	225	83	290	117
ENSECAG00000001441	7.564293269	0.6450273	0.8835	3416	3614	4011	2757	3038	3346	3651	4253
ENSECAG00000009900	2.964060202	0.6454819	0.8838503	146	71	199	249	94	73	290	76
ENSECAG00000019231	3.850310474	0.6455192	0.8838503	295	205	365	143	73	231	199	617
ENSECAG00000017176	4.797812324	0.6455338	0.8838503	468	523	569	725	367	400	592	548
ENSECAG00000017991	8.67022636	0.6455943	0.8838503	7586	7892	8072	9973	5490	6255	9354	6894
ENSECAG00000005764	3.230650746	0.6457242	0.8839216	175	135	186	177	84	265	109	237
ENSECAG00000010159	5.257816314	0.6458226	0.8839239	800	619	745	659	646	634	770	825
ENSECAG00000017149	5.947162796	0.6458815	0.8839239	1371	1045	1159	1480	1266	734	1300	951
ENSECAG00000015757	10.58460897	0.6463827	0.8845032	32298	23032	29284	28518	28755	31076	22417	32311
ENSECAG00000016827	5.982931872	0.6464674	0.8845126	982	1168	957	1620	1041	1212	1276	1202
ENSECAG00000006343	5.393590133	0.647321	0.8855636	814	849	938	804	600	527	953	831
ENSECAG00000013755	6.381111527	0.6476842	0.8855636	1796	1277	1758	1305	1900	1160	2084	1191
ENSECAG00000007970	2.022784803	0.647828	0.8855636	76.0008	44	87	82.0002	60	37.0003	128	90
ENSECAG00000026952	1.985572647	0.6478287	0.8855636	68	65	54	143	73	73	64	43
ENSECAG00000014218	3.960036769	0.6478404	0.8855636	3	794	2	37	1	474	2	849
ENSECAG00000023774	6.6398765	0.6478528	0.8855636	1787	1609	1766	2281	1969	2035	1950	1467
ENSECAG00000023335	1.708827236	0.6478821	0.8855636	63	52	74	29	146	14	33	55
ENSECAG00000014559	3.727704905	0.647929	0.8855636	226	182	371	315	223	188	225	253
ENSECAG00000020397	8.148242708	0.6479489	0.8855636	6391	4570	6089	6358	6028	3415	6565	3458
ENSECAG00000026317	0.487282669	0.6480152	0.8855636	32	21	12	27	16	46	25	12
ENSECAG00000015438	3.252961973	0.6481038	0.8855765	176	131	175	217	208	153	198	157
ENSECAG00000017626	5.387926719	0.6481806	0.8855765	858	554	911	1147	580	554	1035	732
ENSECAG00000008585	8.547620988	0.6482677	0.885589	7112	6234	6001	11940	5548	6310	7445	6119
ENSECAG00000000898	2.094072136	0.6484032	0.8856645	97	65	57	135	82	50	99	50
ENSECAG00000005662	4.777266146	0.6484789	0.8856645	558	422	640	643	576	477	528	257
ENSECAG00000021168	0.307182129	0.6489388	0.8861861	23	10	26	41	40	11	7	13
ENSECAG00000014124	5.833610001	0.6491926	0.8862292	1392	814	1328	1158	1287	486	1394	777
ENSECAG00000017466	0.942983393	0.6493242	0.8862292	53	26	35	39	19	21	36	46
ENSECAG00000023152	8.07802297	0.6493421	0.8862292	4723	5713	5986	5622	4189	4757	5382	4032
ENSECAG00000009453	3.511123652	0.6495393	0.8862292	227	183	255	275	197	114	324	148
ENSECAG00000006951	4.05788207	0.6496746	0.8862292	369	252	352	228	484	278	288	196
ENSECAG00000006093	7.097541034	0.6497097	0.8862292	2231	2444	3038	2357	3154	2248	2718	2171
ENSECAG00000019778	4.44378678	0.6497188	0.8862292	422	407	412	344	406	295	417	527
ENSECAG00000018199	4.763094537	0.6497588	0.8862292	405	663	757	387	235	297	404	894
ENSECAG00000006454	5.804749169	0.6499195	0.8862292	676	1309	999	1105	706	1130	1130	1237
ENSECAG00000018495	6.437876576	0.6499258	0.8862292	1814	1294	1361	2022	1793	1584	1680	1409
ENSECAG00000000855	5.043689954	0.6500285	0.8862292	591	626	582	629	485	551	665	783
ENSECAG00000023073	5.245038781	0.6500772	0.8862292	884	711	668	835	786	354	705	759
ENSECAG00000000351	2.987010378	0.650193	0.8862292	187	133	157	172	116	80	215	130
ENSECAG00000021408	5.580840592	0.6501994	0.8862292	975	853	1264	788	634	656	910	1085
ENSECAG00000022059	7.165088337	0.6502786	0.8862292	3810	2309	2326	3436	1629	3114	1916	2877
ENSECAG00000010536	1.113707208	0.6502953	0.8862292	31	42	24	52	27	39	61	34
ENSECAG00000013455	4.015668186	0.6502967	0.8862292	304	286	350	387	354	183	284	273
ENSECAG00000016935	5.312380555	0.6505783	0.8865066	921	546	986	811	671	374	974	741
ENSECAG00000024554	4.480453564	0.6508158	0.8867238	308	580	318	636	174	429	406	484
ENSECAG00000000737	7.472471147	0.6509655	0.8867762	4674	2420	4187	3410	4114	1654	4443	1999
ENSECAG00000014542	5.55021256	0.6510104	0.8867762	494	488	638	2588	300	328	603	1886
ENSECAG00000008226	6.814613974	0.6511356	0.8868404	3347	1531	2106	2365	2675	1122	2929	989
ENSECAG00000008948	4.825894182	0.6512358	0.8868705	394	298	427	1025	693	362	749	378

ENSECAG00000009108	1.557931333	0.6514646	0.8869812	44	39	49	77	38	39	63	81
ENSECAG00000011778	6.359900703	0.6515214	0.8869812	1820	1274	1944	1671	1646	1046	1821	1149
ENSECAG00000020547	11.39605069	0.6515924	0.8869812	52773	41987.1	55701	47956	61506	48145	43592	48357
ENSECAG00000012358	2.048541718	0.6516346	0.8869812	54	94	86	102	52	83	85	48
ENSECAG00000024698	4.751384227	0.6517122	0.8869812	628	413	461	727	447	333	733	349
ENSECAG00000012218	3.454907164	0.6520051	0.8869812	161	180	216	246	235	142	252	203
ENSECAG00000009068	6.296862128	0.6520076	0.8869812	1475	1375	2029	1527	1554	881	1863	1148
ENSECAG00000020042	2.147768773	0.6520256	0.8869812	85	95	121	54	90	70	73	53
ENSECAG00000008658	1.525802803	0.652086	0.8869812	39	51	63	81	30	31	57	70
ENSECAG00000011385	5.827381321	0.6521622	0.8869812	1074	909	1179	1051	980	1180	1061	993
ENSECAG00000000026	3.310391608	0.6522103	0.8869812	238	93	158	222	58	371	97	204
ENSECAG00000014550	6.017486933	0.6524255	0.8869812	1176	1399	1281	1393	959.001	999.001	1140	1336
ENSECAG00000012257	6.714707867	0.6525091	0.8869812	2047	1749	2156	2705	2192	1284	2354	1404
ENSECAG00000016025	5.002460595	0.6525689	0.8869812	736	486	684	727	671	522	601	370
ENSECAG00000013870	1.246250829	0.6526571	0.8869812	49	65	32	40	33	39	35	43
ENSECAG00000009627	5.978299287	0.6527431	0.8869812	1440	904	1115	1231	1102	792	1627	1295
ENSECAG00000021573	3.776149579	0.652752	0.8869812	284	248	256	335	203	161	266	303
ENSECAG00000010807	3.032081943	0.6528078	0.8869812	102	126	93	279	227	74	195	134
ENSECAG00000019957	3.632719771	0.6529157	0.8869812	259	151	276	217	331	220	177	193
ENSECAG00000006675	6.360222583	0.652943	0.8869812	1671	1253	1541	1658	1460	1041	1995	1757
ENSECAG00000015717	1.189618747	0.6529569	0.8869812	37	39	41	68	16	30	65	37
ENSECAG00000021207	9.174470278	0.6531445	0.8870206	12206	9677	12880	12793	11398	6421	10577	11161
ENSECAG00000019601	7.433106805	0.6532417	0.8870206	3662	3022	3659	3816	3455	2476	3410	2461
ENSECAG00000021997	6.77271869	0.6532601	0.8870206	2526	1420	2333	2791	1960	1211	2839	1585
ENSECAG00000015402	3.250061394	0.6533528	0.8870206	144	186	166	192	124	209	179	198
ENSECAG00000022743	5.324011802	0.6534684	0.8870206	655	810	534	960	530	525	934	1080
ENSECAG00000000918	1.832328598	0.6535988	0.8870206	47	43	76	87	78	35	114	47
ENSECAG00000014065	5.663553803	0.6536791	0.8870206	1163	679	1045	1311	879	429	1253	973
ENSECAG00000019974	4.890663615	0.6537248	0.8870206	613	575	663	566	332	550	402	702
ENSECAG00000012495	6.266864136	0.6537578	0.8870206	1462	1243	1609	1354	2125	1088	1745	861
ENSECAG00000015106	8.024089145	0.6537667	0.8870206	5793	4232	4854	6664	3674	3108	7202	4159
ENSECAG00000013874	2.943425763	0.6540384	0.8870265	14	383	5	36	1	239	4	384
ENSECAG00000015817	6.006696638	0.6540754	0.8870265	1122	1197	1120	1279	740	1783	825	1380
ENSECAG00000012825	4.64311654	0.6543703	0.8870265	376	543	924	190	245	266	436	732
ENSECAG00000014967	3.958951297	0.6543853	0.8870265	389	214	293	396	290	123	444	217
ENSECAG00000014992	0.810741845	0.654582	0.8870265	10	82	27	15	20	19	30	39
ENSECAG00000019187	6.102802615	0.6546603	0.8870265	1313	1369	1467	858	1333	1124	1401	1309
ENSECAG00000016811	1.617508449	0.6547226	0.8870265	58	39	68	88	68	22	81	30
ENSECAG00000020281	7.1282956	0.6547281	0.8870265	3045	2130	3410	2932	2977	1344	3392	1964
ENSECAG00000023790	3.846440121	0.6548386	0.8870265	318	224	330	306	246	160	362	220
ENSECAG00000014803	7.462345358	0.6549876	0.8870265	3550	2840	3059	3627	2672	3964	3281	3224
ENSECAG00000012308	3.312324371	0.6550474	0.8870265	192	127	235	167	239	169	169	164
ENSECAG00000022752	6.832328733	0.655062	0.8870265	2514	1856	2221	2790	1932	1425	2929	1624
ENSECAG00000020638	2.663413414	0.6551049	0.8870265	99	177	120	112	77	80	132	135
ENSECAG00000021796	8.22998873	0.6551187	0.8870265	5904	7262	6963	4256	2989	3975	4442	8909
ENSECAG00000024149	9.014593073	0.6552858	0.8870265	9118	8217	10515	10400	12028	9710	9704	7265
ENSECAG00000019055	2.390174939	0.6553193	0.8870265	104	57	87	132	84	59	107	149
ENSECAG00000024284	7.42684753	0.655323	0.8870265	3955	2868	3927	3314	3320	2119	3858	2555
ENSECAG00000015016	2.396814769	0.6553655	0.8870265	20	73	141	247	167	109	0	19
ENSECAG00000005207	3.773281319	0.6554111	0.8870265	331	186	262	218	243	251	281	251
ENSECAG00000024672	5.844734332	0.6555038	0.8870265	1018	894	1125	1245	1398	780	1365	802
ENSECAG00000014429	9.464544418	0.6555392	0.8870265	12462	13772	10227	15740	11743	14699	14150	12415
ENSECAG00000024506	3.365008094	0.6555653	0.8870265	202	180	249	214	86	127	230	257
ENSECAG00000023366	4.669419602	0.6556564	0.8870265	556	390	563	575	340	372	561	475
ENSECAG00000010175	5.71107657	0.6556887	0.8870265	903	1117	1081	1177	721	1210	584	946
ENSECAG00000003569	6.317064638	0.6557233	0.8870265	1671	1228	2224	1459	855	514	1755	2414
ENSECAG00000013345	7.780490024	0.6558108	0.8870392	4210	4563	4323	4857	2855	2970	5164	4235
ENSECAG00000020969	7.049959364	0.6559382	0.887068	2908	2684	2194	1836	2357	2881	2940	1768
ENSECAG00000023266	5.77976832	0.6560528	0.887068	1301	840	1433	919	657	468	1390	1323
ENSECAG00000022172	6.547297929	0.6561445	0.887068	2251	2437	1168	1679	1535	1560	1989	1289
ENSECAG00000016402	3.321088104	0.6561502	0.887068	196	185	171	281	176	243	166	58
ENSECAG00000007750	8.542638095	0.6562226	0.887068	7625	7151	6040	9953	4957	6630	6948	6788
ENSECAG00000017598	1.656186191	0.6563084	0.8870784	37	54	82	30	2	168	9	50
ENSECAG00000017334	4.766606671	0.656396	0.887085	685	458	431	415	524	435	522	565
ENSECAG00000008746	9.004371702	0.6565095	0.887085	11512	7955	8969	9289	13333	7252	12511	6072
ENSECAG00000009633	5.374677813	0.6565899	0.887085	903	688	870	942	570	375	1086	874
ENSECAG00000014319	3.737976578	0.6566257	0.887085	324	251	272	235	231	245	234	179
ENSECAG00000019266	4.401738033	0.6569697	0.8874135	379	381	392	399	356	340	345	542
ENSECAG00000007397	10.40208367	0.657025	0.8874135	28246	19133	26613	26166	28392	27586	21593	23148
ENSECAG00000007007	6.80585713	0.6571557	0.8874362	2547	1455	2348	2946	1742	1158	3090	1828
ENSECAG00000023341	3.574423486	0.657276	0.8874362	253	143	204	274	294	131	354	140
ENSECAG00000023185	7.250104495	0.657367	0.8874362	2932	2633	3228	3710	2369	2713	2610	2633
ENSECAG00000005707	2.988054286	0.6574886	0.8874362	117	201	168	157	66	114	173	180
ENSECAG00000011098	4.278649962	0.6575432	0.8874362	268	370	307	487	228	411	390	427
ENSECAG00000020206	4.369289887	0.6575736	0.8874362	360	449	425	443	290	385	363	354
ENSECAG00000009936	7.23852402	0.6575888	0.8874362	2925	2967	2599	3904	2308	2280	2690	3041
ENSECAG00000012113	8.085582896	0.657667	0.8874363	4689	4264	5698	5495	6434	4507	4668	4710
ENSECAG00000023695	0.642979962	0.6578565	0.8875866	27	30	18	49	16	25	25	30

ENSECAG00000003667	1.55958837	0.657996	0.8876488	48	51	47	61	62	63	55	35
ENSECAG00000022282	6.265111531	0.6581038	0.8876488	1407	1346	1395	2182	1114	1312	1402	1427
ENSECAG00000009318	7.284046364	0.658137	0.8876488	3056	1941	2491	4243	3258	2472	3917	2200
ENSECAG00000023179	12.3012505	0.6583951	0.8878793	107160	71820	94930	100303	98123	95269	81802	102041
ENSECAG00000018097	4.623799659	0.6585781	0.8878793	432	435	329	605	256	255	402	993
ENSECAG00000012270	6.101169171	0.658588	0.8878793	1266	1214	1309	1314	1251	1077	1448	1376
ENSECAG00000007669	4.657240498	0.6586645	0.8878793	450	386	540	490	498	498	444	432
ENSECAG00000003427	3.89834599	0.6586987	0.8878793	244	204	380	411	257	261	318	165
ENSECAG00000021351	6.341834888	0.6593673	0.888675	1555	2076	1791	1110	766	1446	1110	2125
ENSECAG00000011291	8.370624575	0.6595529	0.8887197	8082	4934	7692	6451	5565	4110	7759	5427
ENSECAG00000008441	2.988219686	0.6595569	0.8887197	177	60	298	125	139	61	140	187
ENSECAG00000015202	3.364637286	0.6596916	0.8887601	186	192	184	181	108	183	216	273
ENSECAG00000021056	6.275804864	0.6597434	0.8887601	1377	1383	1038	2001	1068	1551	1730	1469
ENSECAG00000026969	2.214560089	0.6598952	0.8888592	61	87	90	83	18	73	63	202
ENSECAG00000003722	3.114916525	0.6601497	0.8890013	138	74	197	228	100	105	252	217
ENSECAG00000015812	7.478523231	0.6602128	0.8890013	3171	2739	2481	4846	2897	5452	3123	1695
ENSECAG00000012328	7.55999428	0.6602355	0.8890013	4121	3072	4150	4153	4136	2400	3791	2582
ENSECAG00000014040	3.556408282	0.6604207	0.8890596	178	233	224	206	169	87	226	431
ENSECAG00000004844	3.83354294	0.6604353	0.8890596	235	317	259	361	151	247	161	384
ENSECAG00000016767	4.394901953	0.6606214	0.8892047	458	404	404	447	284	375	515	261
ENSECAG00000019012	1.550197631	0.6609619	0.8895346	62	43	65	68	31	20	86	58
ENSECAG00000017713	7.010718332	0.6610231	0.8895346	2445	2589	2587	2886	1793	2196	2708	2145
ENSECAG00000020192	3.953155338	0.6613111	0.8898167	304	250	322	254	237	211	356	375
ENSECAG00000018694	3.323266712	0.6614902	0.8898533	160	126	174	282	214	124	233	190
ENSECAG00000013220	4.060976836	0.661559	0.8898533	325	254	342	306	265	341	321	316
ENSECAG00000023074	2.470180579	0.6615733	0.8898533	105	107	115	123	62	92	98	114
ENSECAG00000019611	3.868435728	0.6617571	0.8899191	355	201	270	239	348	167	330	266
ENSECAG00000011263	4.667962885	0.6617789	0.8899191	596	375	589	521	593	326	419	380
ENSECAG00000009415	5.693070894	0.6619143	0.8899958	1012	759	993	1107	987	1014	991	846
ENSECAG00000009943	6.142262288	0.6620554	0.8900802	1342	1275	1283	1335	1488	1387	1200	1147
ENSECAG00000019044	5.131579742	0.6621474	0.8900985	730	739	691	672	700	423	669	613
ENSECAG00000023146	3.583590551	0.6624565	0.8904086	280	168	298	234	237	140	211	221
ENSECAG00000026979	4.50110616	0.6627097	0.8906436	466	381	404	417	471	312	437	482
ENSECAG00000024863	4.986488731	0.662951	0.8908625	431	584	575	768	461	604	674	639
ENSECAG00000017092	2.573930753	0.6634023	0.8912315	69	207	46	69	34	189	20	203
ENSECAG00000002987	5.596121618	0.6634382	0.8912315	1102	693	828	1385	712	931	1083	568
ENSECAG00000020127	3.646326002	0.6635673	0.8912315	325	265	244	172	236	165	243	201
ENSECAG00000006959	6.430376553	0.6636034	0.8912315	1579	1629	1635	1541	1568	1613	1760	1490
ENSECAG00000015031	3.270550835	0.6636179	0.8912315	129	210	208	125	81	334	71	221
ENSECAG00000023470	4.766473879	0.6637187	0.8912614	523	452	400	653	446	485	557	551
ENSECAG00000014661	0.900274197	0.6640115	0.8914212	20	25	46	62	35	7	63	15
ENSECAG00000024681	7.918004804	0.6640969	0.8914212	4417	4184	4615	4670	5021	4851	4637	3524
ENSECAG00000017799	9.760501697	0.6641747	0.8914212	14704	16325	18464	21948	13193	16181	17393	12102
ENSECAG00000006591	3.37187294	0.6641935	0.8914212	119	152	173	313	176	222	339	60
ENSECAG00000015560	4.547862068	0.66423	0.8914212	501	419	405	381	606	259	555	360
ENSECAG00000017167	6.63762114	0.6645594	0.8916277	1895	1735	2314	2181	2062	1477	1994	1282
ENSECAG00000013660	1.332930072	0.6646413	0.8916277	98	67	13	21	28	36	52	43
ENSECAG00000012915	4.728259057	0.6646436	0.8916277	505	474	575	612	339	534	539	382
ENSECAG00000024661	6.104343833	0.6646979	0.8916277	1296	1469	1188	1645	918	1403	1169	1166
ENSECAG00000019639	5.386460535	0.6648293	0.8916987	969	619	945	883	862	633	767	586
ENSECAG00000022888	3.851138651	0.6653557	0.8921635	279	229	295	252	204	233	415	250
ENSECAG00000003211	1.704102561	0.6653719	0.8921635	76	46	62	83	18	66	53	71
ENSECAG00000019056	5.602984054	0.6654362	0.8921635	990	823	802	1006	946	648	1101	975
ENSECAG00000007770	5.140199235	0.6654901	0.8921635	751	539	714	598	574	437	770	901
ENSECAG00000009326	7.377674432	0.6656029	0.8922094	3683	2745	3099	4177	3432	1745	3709	2613
ENSECAG00000009946	6.725451819	0.6658471	0.8923989	2158	1865	2002	2637	1576	1867	1943	1838
ENSECAG00000009295	6.832204737	0.6662592	0.8923989	2193	1889	2020	2379	2478	1908	2401	1755
ENSECAG00000018952	3.873904752	0.6662892	0.8923989	306	281	310	291	262	193	292	251
ENSECAG00000012660	11.94449398	0.6662942	0.8923989	85075	74527	86252	37614	64816	59546	74993	101197
ENSECAG00000019992	2.885709206	0.666405	0.8923989	152	114	194	54	48	137	240	152
ENSECAG00000018866	7.804412354	0.666496	0.8923989	3538	4232	4060	4761	4072	3870	4818	4062
ENSECAG00000014508	5.307954397	0.6665402	0.8923989	705	834	746	930	638	627	777	673
ENSECAG00000004794	6.079847837	0.6665719	0.8923989	1326	1128	1235	1866	1179	948	1474	1076
ENSECAG00000004390	5.201120781	0.6666366	0.8923989	680	536	862	606	981	358	1185	338
ENSECAG00000002606	3.887073822	0.6666405	0.8923989	325	219	304	231	243	201	300	377
ENSECAG00000017022	6.652054281	0.6666911	0.8923989	2092	1793	2072	2231	1460	1360	2021	2119
ENSECAG00000011335	6.303786928	0.6667201	0.8923989	2079	749	1696	2072	1786	949	1770	904
ENSECAG00000014761	5.378348967	0.6669154	0.8923989	765	833	911	862	865	652	755	557
ENSECAG00000016647	7.947741951	0.6669593	0.8923989	4523	4214	4706	4855	3918	4721	4377	5445
ENSECAG00000017172	6.33179158	0.6669794	0.8923989	404	3782	630	479	1134	2253	861	1869
ENSECAG00000025040	4.865046116	0.6670012	0.8923989	532	439	594	603	588	431	631	542
ENSECAG00000013871	6.115152749	0.6671245	0.8924588	1313	1104	1455	1813	1089	1044	1234	1393
ENSECAG00000020041	9.057143782	0.6674447	0.892782	6361	3294	10437	19896	8529	10622	20614	2491
ENSECAG00000009033	6.58089246	0.6678195	0.8931781	1900	1520	2019	2413	1864	1157	2149	1470
ENSECAG00000009997	6.369500872	0.6680387	0.8932675	1605	1476	1685	1329	1358	1885	1611	1272
ENSECAG00000017326	6.556352299	0.6680812	0.8932675	1729	1657	1928	1673	1718	1700	1891	1708
ENSECAG00000010782	4.626845924	0.6681222	0.8932675	421	463	459	458	335	681	276	516
ENSECAG00000017089	1.236417807	0.6683172	0.8933772	26	25	69	39	14	20	18	127

ENSECAG00000022045	6.577927483	0.6683616	0.8933772	1982	1516	1800	1813	2135	1481	2143	1414
ENSECAG00000026824	2.279214395	0.6688001	0.8937821	103	84	71	87	78	88	62	131
ENSECAG00000022546	9.210735382	0.6688219	0.8937821	10054	10973	10004	12918	10603	12125	12069	9533
ENSECAG00000009554	1.816728772	0.6691856	0.894163	53	75	68	91	22	42	59	107
ENSECAG00000007495	5.098501898	0.6697562	0.8944218	723	577	738	758	614	679	533	473
ENSECAG00000015895	4.707215752	0.6697653	0.8944218	505	469	520	637	409	394	530	458
ENSECAG00000019546	7.291941843	0.6697763	0.8944218	2663	2430	2707	3970	2989	2789	3494	2504
ENSECAG00000012014	1.792830204	0.6697819	0.8944218	68	48	86	79	43	34	76	77
ENSECAG00000002410	1.055795126	0.6698483	0.8944218	39	35	37	55	16	32	54	32
ENSECAG00000012748	3.734330268	0.6698518	0.8944218	202	261	279	224	282	254	251	203
ENSECAG00000009563	3.972873848	0.6702224	0.8948115	497	265	284	227	250	180	267	366
ENSECAG00000005087	5.903592184	0.6705466	0.8951392	1063	1027	956	1440	861	1213	1252	1138
ENSECAG00000014752	5.339024897	0.6709222	0.8953318	669	692	802	841	640	988	719	635
ENSECAG00000023285	1.949482632	0.6709374	0.8953318	74	84	79	72	62	79	57	50
ENSECAG00000005805	6.978465813	0.6709437	0.8953318	3137	1704	2456	1952	3738	1412	2909	1557
ENSECAG00000015561	5.294681286	0.6710062	0.8953318	750	744	785	914	493	597	828	791
ENSECAG00000007323	5.538734952	0.6712041	0.8953991	887	703	887	999	953	535	1051	982
ENSECAG00000022614	4.281203254	0.6712772	0.8953991	372	303	395	359	432	432	288	276
ENSECAG00000018937	1.402980742	0.6712931	0.8953991	21	37	18	104	12	4	183	27
ENSECAG00000017803	5.116094163	0.6713991	0.8954352	710	487	647	745	683	417	864	674
ENSECAG00000024416	3.029515112	0.6716466	0.8956602	140	109	167	183	181	104	181	150
ENSECAG00000013003	3.658248599	0.671905	0.8957952	270	162	344	260	172	157	337	202
ENSECAG00000021299	7.871567856	0.6719812	0.8957952	4918	4107	4130	6173	5519	2859	4545	3078
ENSECAG00000019730	0.668198172	0.6719844	0.8957952	37	40	22	23	18	18	44	21
ENSECAG00000009343	5.2221154	0.6723612	0.8961923	664	807	570	702	509	711	717	854
ENSECAG00000014120	3.673232761	0.6726463	0.8964579	217	226	227	264	328	146	248	239
ENSECAG00000019547	3.564332766	0.6727183	0.8964579	251	200	296	211	202	159	210	229
ENSECAG00000014580	1.099668413	0.6729086	0.8965239	33	38	38	66	6	17	40	74
ENSECAG00000018636	3.822993179	0.6729257	0.8965239	266	323	250	305	218	229	256	253
ENSECAG00000020408	6.010632665	0.6733577	0.8969943	1408	838	1344	1733	1371	808	1260	993
ENSECAG00000003512	4.64144599	0.6736212	0.8972314	385	516	537	598	314	432	377	562
ENSECAG00000005951	0.787811495	0.6736937	0.8972314	30	25	21	42	18	54	27	23
ENSECAG00000018230	2.516237552	0.6739291	0.8974397	129	58	112	116	76	137	109	101
ENSECAG00000023244	4.293105076	0.6742571	0.8976753	422	304	327	398	400	252	416	411
ENSECAG00000000972	0.535097587	0.6742641	0.8976753	24	25	27	39	5	13	19	52
ENSECAG00000025017	2.166336805	0.6744133	0.8977527	80	63	71	111	74	93	103	63
ENSECAG00000008063	1.503927404	0.6745275	0.8977527	79	19	29	112	51	45.0027	60	24.001
ENSECAG00000018943	6.110280592	0.674629	0.8977527	1373	1210	1439	1617	1345	1255	1321	774
ENSECAG00000021379	6.071436121	0.6747666	0.8977527	1360	1141	1038	1497	1218	1035	1431	1361
ENSECAG00000001391	0.249600299	0.6747759	0.8977527	23	23	25	20	38	10	11	11
ENSECAG00000022092	6.303954122	0.6748244	0.8977527	1381	1281	1594	1579	818	2187	1002	1796
ENSECAG00000022893	6.497405785	0.6748755	0.8977527	1852	1119	1971	2572	1626	855	2515	1338
ENSECAG00000004706	1.64025477	0.6751717	0.8980416	88	10	36	87	57	23	106	58
ENSECAG00000020855	7.908825023	0.6753083	0.8981181	4353	5883	4652	4550	2843	3859	4210	5487
ENSECAG00000015321	7.143668978	0.6757361	0.8985397	3157	2677	2718	2932	2206	2158	2591	2746
ENSECAG00000019472	3.679822493	0.6757835	0.8985397	250	268	236	286	232	141	222	276
ENSECAG00000014597	3.282587288	0.6759303	0.8986298	226	212	121	235	118	134	152	248
ENSECAG00000006597	4.999675579	0.6762295	0.8989223	699	499	746	665	532	361	842	495
ENSECAG00000019610	4.734899009	0.6763785	0.8990142	596	368	605	378	754	361	532	356
ENSECAG00000009988	6.959311974	0.6765218	0.8990142	2416	2226	1975	2604	1267	2588	2696	2820
ENSECAG00000006301	2.030128189	0.6766145	0.8990142	86	57	64	132	32	38	97	106
ENSECAG00000026883	2.411378156	0.6766453	0.8990142	92	70	107	115	153	72	102	70
ENSECAG00000024080	3.211627616	0.6767003	0.8990142	151	164	149	212	108	127	266	211
ENSECAG00000022154	5.01521547	0.6767735	0.8990142	521	593	772	773	294	674	403	784
ENSECAG00000022536	6.21793085	0.6770177	0.8992334	1706	1321	1486	1544	1380	677	1895	1263
ENSECAG00000024367	4.133548952	0.6772977	0.8995001	79	706	147	210	23	251	109	1018
ENSECAG00000022567	10.65200148	0.6774656	0.8996179	33022	24074	31008	31421	31317	31621	25633	31043
ENSECAG00000008273	6.027194367	0.6776093	0.8997036	1121	1198	1161	1329	732	1846	834	1362
ENSECAG00000026808	5.188916992	0.6780515	0.8997319	654	616	773	652	648	798	506	728
ENSECAG00000023201	8.690271235	0.678052	0.8997319	7765	7582	9191	9243	6516	7862	7429	6188
ENSECAG00000006773	5.651883931	0.6781078	0.8997319	1098	910	1033	1031	862	949	898	708
ENSECAG00000003271	8.236176702	0.6781526	0.8997319	5924	5058	5554	5861	6023	4447	7088	5269
ENSECAG00000011968	9.286556158	0.6782012	0.8997319	11724	9947	9816	20988	10237	12175	14164	5658
ENSECAG00000015230	3.949947224	0.6782207	0.8997319	307	235	256	345	194	336	340	285
ENSECAG00000017430	4.496297972	0.678231	0.8997319	495	283	501	390	489	368	367	455
ENSECAG00000019229	5.83776242	0.6782643	0.8997319	1090	805	1299	1080	1004	881	1430	1002
ENSECAG00000014786	6.976454521	0.6784891	0.8997383	2665	2117	2716	2780	2501	1745	2648	1776
ENSECAG00000021916	6.449049956	0.6785687	0.8997383	1534	1511	1590	1903	1298	1689	1673	1840
ENSECAG00000016050	5.710439644	0.6786102	0.8997383	939	964	870	1141	919	822	1213	978
ENSECAG00000007822	6.54439604	0.678658	0.8997383	1894	1652	1999	2035	1670	1592	1471	1624
ENSECAG00000018735	0.749253885	0.6786945	0.8997383	23	28	26	38	19	19	37	48
ENSECAG00000018660	3.2628512	0.6787444	0.8997383	193	175	165	162	123	162	250	193
ENSECAG00000003017	2.187850038	0.6789676	0.8997431	66	89	85	133	69	39	78	117
ENSECAG00000010211	6.052559741	0.67903	0.8997431	1174	1206	1373	1667	1169	1087	1525	792
ENSECAG00000016441	0.727859364	0.6790978	0.8997431	30	25	29	29	33	33	26	25
ENSECAG00000017636	3.826068127	0.6791035	0.8997431	263	235	287	379	232	168	279	291
ENSECAG00000002233	5.615776261	0.6792142	0.8997431	901	960	1090	1042	626	477	871	1420
ENSECAG00000010812	7.403867615	0.6792232	0.8997431	2712	2928	2924	4126	3116	2794	3970	2890

ENSECAG00000019227	5.024826584	0.6793739	0.89976	742	590	833	475	457	233	820	764
ENSECAG00000011392	4.577965058	0.6793945	0.89976	298	255	356	1182	245	217	950	259
ENSECAG00000009270	4.693762375	0.6795961	0.8999221	514	428	491	700	259	312	583	639
ENSECAG00000022608	5.668826605	0.6798422	0.9001431	981	1001	946	1204	824	573	1241	921
ENSECAG00000006558	6.806575942	0.6800497	0.9002784	2437	1893	1976	1997	2154	1732	2712	1854
ENSECAG00000005312	2.431093525	0.6801029	0.9002784	180	43	98	131	75	19	136	133
ENSECAG00000022646	5.017003775	0.6802551	0.9003491	753	461	749	676	551	488	662	521
ENSECAG00000020591	4.688500032	0.68038	0.9003491	470.001	463	557.001	398.001	629.001	454.001	468.001	364
ENSECAG00000017757	5.576852526	0.6804839	0.9003491	935	830	979	1144	939	581	1055	736
ENSECAG00000003466	4.585902914	0.680504	0.9003491	843	292	637	215	354	127	1022	181
ENSECAG00000011387	5.78472222	0.6806583	0.9003491	1034	868	1059	1173	1052	877	1029	1147
ENSECAG00000004960	7.442298139	0.6806701	0.9003491	1505	5393	2259	2994	1467	4699	6772	746
ENSECAG00000020873	2.530270991	0.6807112	0.9003491	102	102	119	151	53	60	100	174
ENSECAG00000003570	3.877118291	0.6809775	0.9003903	253	307	308	324	200	286	302	203
ENSECAG00000000696	0.173848626	0.6810808	0.9003903	19	15	25	14	9	20	27	24
ENSECAG00000015767	5.767216452	0.6811103	0.9003903	1193	915	935	1013	1088	856	1159	969
ENSECAG00000020568	6.190945464	0.6811264	0.9003903	1290	1505	1433	1694	1033	1320	1335	1312
ENSECAG00000000331	6.050882358	0.6811386	0.9003903	1320	1582	1144	1277	1175	880	1145	1359
ENSECAG00000007650	0.249325309	0.6813533	0.9004681	32	17	23	2	34	20	23	8
ENSECAG00000011156	3.410729644	0.6813561	0.9004681	159	184	208	327	158	155	197	208
ENSECAG00000010964	6.59699305	0.6817564	0.9008486	1338	1847	1554	2485	1616	2426	2034	1096
ENSECAG00000007294	6.44297952	0.6818025	0.9008486	1470	1607	1570	1857	1581	1575	1772	1547
ENSECAG00000024002	3.802037158	0.6819671	0.9009254	167	343	297	329	124	260	338	223
ENSECAG00000008561	7.366838443	0.6820193	0.9009254	2819	3357	2915	4437	2377	3047	3240	2600
ENSECAG00000002413	7.373333893	0.6822589	0.9009453	3424	3313	3935	3058	718	1452	2711	6440
ENSECAG00000024078	5.059523977	0.6822605	0.9009453	680	501	606	702	459	611	708	719
ENSECAG00000012093	6.057234441	0.6822743	0.9009453	1124	1399	1060	1865	757	1118	1337	1370
ENSECAG00000014611	2.670282191	0.6824242	0.9009453	179	70	134	67	197	70	145	72
ENSECAG00000006925	3.633892695	0.6824309	0.9009453	229	153	259	281	231	231	267	197
ENSECAG00000019569	6.736682972	0.6826394	0.9010079	2111	1769	2289	1709	2543	1893	1668	1790
ENSECAG00000024956	8.833507205	0.6826403	0.9010079	5697	9950	9111	8757	6554	9662	9642	8384
ENSECAG00000023664	0.605256904	0.6827164	0.9010079	37	17	29	38	14	25	47	9
ENSECAG00000010580	4.259747575	0.6828315	0.9010552	401	258	347	421	419	340	329	331
ENSECAG00000013265	5.135758718	0.6830396	0.901225	744	549	626	695	833	621	581	564
ENSECAG00000010538	3.512193698	0.6832613	0.901333	243	185	208	197	242	138	241	240
ENSECAG00000020850	8.364260438	0.6832801	0.901333	6752	5326	6374	5987	6740	5023	7894	5276
ENSECAG00000000101	2.667464528	0.683791	0.9016823	94	143	110	106	97	104	116	158
ENSECAG00000010962	3.407235198	0.6838172	0.9016823	186	145	211	242	212	135	261	196
ENSECAG00000001359	6.410439818	0.6838426	0.9016823	1383	1700	1611	2239	1329	1306	1639	1590
ENSECAG00000010757	4.070894238	0.6838624	0.9016823	291	297	350	440	218	215	286	428
ENSECAG00000019382	8.091369801	0.6839721	0.9017223	4505	4510	4711	6680	3906	5585	4354	6455
ENSECAG00000005528	7.169337621	0.6841548	0.9018177	2957	2167	2511	3148	2670	2484	2582	2998
ENSECAG00000013968	5.669119772	0.6842815	0.9018177	893.001	719	1186	1006	1017	1043	889	803.024
ENSECAG00000022362	5.537502481	0.6842827	0.9018177	865	924	685	982	764	874	890	922
ENSECAG00000013086	1.673987909	0.6850819	0.9027663	46	43	58	116	40	37	74	60
ENSECAG00000012913	4.838914725	0.6853229	0.9029346	466	447	574	612	397	149	1016	718
ENSECAG00000021279	6.25506425	0.6853686	0.9029346	1481	1279	1588	1334	1484	1353	1604	1249
ENSECAG00000013583	4.055790666	0.6854782	0.9029658	305	223	286	421	433	354	277	162
ENSECAG00000016889	3.678881766	0.6855987	0.9029658	228	202	245	247	90	93	295	530
ENSECAG00000020412	1.98482842	0.6856308	0.9029658	91	41	83	67	67	40	120	76
ENSECAG00000003387	4.595194396	0.6861137	0.9030849	529	410	487	544	490	269	577	336
ENSECAG00000011148	4.689679117	0.6861543	0.9030849	399	523	470	511	519	522	481	387
ENSECAG00000019235	4.413715621	0.6861989	0.9030849	405.001	326	444.001	402.001	515.001	303.001	465.001	316
ENSECAG00000010564	3.315254946	0.6862758	0.9030849	159	169	151	256	175	126	230	223
ENSECAG00000012597	7.684636686	0.6862883	0.9030849	4964	2832	3714	3812	3546	2929	4720	4410
ENSECAG00000009527	5.139230904	0.6863336	0.9030849	871	507	734	780	830	300	842	469
ENSECAG00000016297	5.228536143	0.686345	0.9030849	957	767	560	740	691	514	732	640
ENSECAG00000007384	4.10712234	0.6863906	0.9030849	365	254	316	342	289	245	389	377
ENSECAG00000021103	8.206532798	0.6864367	0.9030849	6551	3171	4591	10720	3728	3556	6879	6291
ENSECAG00000013128	5.166426899	0.6865594	0.9031091	426	946	484	771	564	667	646	808
ENSECAG00000025091	8.199099099	0.6868545	0.9031091	4553	6045	4988	6074	3723	7329	4434	6241
ENSECAG00000004707	6.16434749	0.6868736	0.9031091	1865	1020	1415	1593	1526	1526	1024	691
ENSECAG00000019198	5.862136525	0.6868821	0.9031091	1117	941	1064	1237	919	1052	1069	1282
ENSECAG00000024491	4.14159724	0.6869211	0.9031091	338	296	261	417	267	335	335	377
ENSECAG00000024248	5.947362122	0.6871329	0.9031091	1252	1047	1325	1388	1147	756	1337	1051
ENSECAG00000000246	6.334562292	0.6871403	0.9031091	1387	1524	1434	1665	1628	1705	1284	1311
ENSECAG00000002024	0.63639114	0.6871579	0.9031091	27	15	36	28	27	26	29	29
ENSECAG00000000826	1.505464637	0.6871707	0.9031091	56	46	57	39	45	32	85	52
ENSECAG00000020235	3.03728134	0.6873178	0.9031979	180	83	200	132	151	215	110	124
ENSECAG00000020966	4.705390441	0.6877418	0.9035118	407	534	380	615	380	446	648	495
ENSECAG00000021777	7.230628691	0.6877651	0.9035118	2537	2760	2767	3094	3589	2110	3474	2159
ENSECAG00000023882	1.204500655	0.6879726	0.9035118	35	45	24	56	24	45	34	65
ENSECAG00000006626	5.118468531	0.6883867	0.9035118	667	617	671	893	388	360	811	868
ENSECAG00000004274	8.011694534	0.6884007	0.9035118	4769	4369	4297	5784	6394	3041	6652	3599
ENSECAG00000015198	5.684812856	0.6884336	0.9035118	1126	815	1128	670	1701	567	1196	447
ENSECAG00000019746	4.099524377	0.6884765	0.9035118	487	458	158	279	175	218	291	475
ENSECAG00000000421	2.17659599	0.6885872	0.9035118	86	61	108	113	84	60	79.0007	76
ENSECAG00000007483	6.526931189	0.6885901	0.9035118	1735	1823	1464	1838	1703	1762	1956	1438

ENSECAG00000001874	-0.28139151	0.6888316	0.9035118	19	25	2	0	4	22	2	29
ENSECAG00000008187	4.611682579	0.6888813	0.9035118	641	280	463	639	406	425	563	271
ENSECAG00000013139	3.530013624	0.6888923	0.9035118	217	196	238	193	227	187	247	203
ENSECAG00000018433	3.905555605	0.6888961	0.9035118	271	180	470	154	251	211	160	509
ENSECAG00000002855	5.116229757	0.6889031	0.9035118	452	1201	388	754	507	724	381	671
ENSECAG00000021856	3.44531986	0.6889041	0.9035118	133	337	170	87	72	62	66	662
ENSECAG00000024527	4.001834084	0.6889778	0.9035118	364	269	332	343	320	277	349	134
ENSECAG00000004043	5.238512954	0.6890829	0.9035118	752	655	690	711	612	571	884	775
ENSECAG00000013605	4.034081793	0.6891589	0.9035118	330	378	292	328	333	98	405	299
ENSECAG00000021821	4.928232094	0.6892386	0.9035118	584	502	594	583	596	562	530	564
ENSECAG00000017832	7.150347848	0.6892896	0.9035118	3145	2497	3031	2879	3034	2019	2877	1817
ENSECAG00000019041	4.911307299	0.6893997	0.9035118	578	444	622	596	403	485	618	757
ENSECAG00000021331	0.095411105	0.6894304	0.9035118	15	19	9	27	23	15	16	20
ENSECAG00000000036	6.477295756	0.6894693	0.9035118	1572	1511	1673	1919	1187	1738	1941	1788
ENSECAG00000015887	3.895440664	0.6894816	0.9035118	251	238	273	340	286	335	277	199
ENSECAG00000001628	3.512851376	0.6895452	0.9035118	212	196	236	186	180	282	167	207
ENSECAG00000010780	5.289192644	0.6897467	0.9036716	883	564	897	840	693	511	878	629
ENSECAG00000009644	5.844686392	0.6900152	0.9039192	886	1176	930	1277	788	1010	906	1580
ENSECAG00000004514	-0.014102738	0.6901391	0.9039771	7	19	24	25	8	16	26	9
ENSECAG00000009145	8.446017456	0.6903799	0.9041884	6644	6464	7900	7387	5673	6116	7102	4968
ENSECAG00000026984	6.512120853	0.6905888	0.9043469	1340	1434	1873	2159	1155	2836	1352	1306
ENSECAG00000021192	1.968955913	0.69068	0.9043469	59.9866	58.0005	65.001	143.985	45.0005	54.0007	140	23.2423
ENSECAG00000022023	6.188172548	0.6907885	0.9043469	1517	1112	1705	1599	1043	1283	1311	1362
ENSECAG00000007391	6.950869173	0.6908194	0.9043469	2216	1524	2223	3389	1532	1544	3294	3152
ENSECAG00000010070	3.410956811	0.6909974	0.9043469	210	182	185	198	269	138	273	125
ENSECAG00000007580	4.212292792	0.6910541	0.9043469	334	289	395	505	217	338	384	324
ENSECAG00000015243	4.451409066	0.6910583	0.9043469	462	404	449	455	385	380	349	366
ENSECAG00000012905	8.256839345	0.6912326	0.9044708	6174	4671	5766	6109	7864	4513	7344	3407
ENSECAG00000018688	6.611431113	0.691446	0.9046421	1797	2004	1808	2319	1330	1751	1846	1787
ENSECAG00000006356	2.215095566	0.6916069	0.9046421	87	76	82	89	78	60	103	107
ENSECAG00000006561	10.70432909	0.6916239	0.9046421	34091	19841	35714	51084	40813	22658	38875	10948
ENSECAG00000006975	7.579330473	0.691688	0.9046421	4486	2091	4265	3324	4866	2030	5512	2338
ENSECAG00000023693	5.01805434	0.6917617	0.9046421	579	499	642	699	811	501	628	472
ENSECAG00000022584	0.88940017	0.6919406	0.9047718	47	24	44	30	19	22	52	27
ENSECAG00000002783	4.107188163	0.6922106	0.9050207	404	297	296	405	262	232	389	305
ENSECAG00000024701	3.81604175	0.6923772	0.9050629	188	346	158	333	155	255	310	346
ENSECAG00000005996	0.380415581	0.6924612	0.9050629	12	30	21	23	17	24	28	23
ENSECAG00000010359	6.105455605	0.6925109	0.9050629	1374	1042	1500	1713	1155	1088	1035	1428
ENSECAG00000010373	5.821363126	0.6925616	0.9050629	1498	841	1119	1149	1044	513	1439	969
ENSECAG00000024677	5.795918437	0.6926758	0.9050978	1008	754	739	1711	1035	995	1600	584
ENSECAG00000024033	3.355930099	0.6927477	0.9050978	108	235	214	177	103	143	246	293
ENSECAG00000010526	2.904704164	0.6931279	0.9054904	208	108	119	104	120	134	131	173
ENSECAG00000026976	2.696144768	0.6936122	0.906019	131	109	120	107	74	107	149	158
ENSECAG00000026878	2.186391049	0.6938579	0.9060727	103	65	103	97	96	59	82	64
ENSECAG00000021617	7.18010773	0.6938736	0.9060727	2446	3424	3612	2160	2150	1570	3387	2999
ENSECAG00000017921	3.312511774	0.6938927	0.9060727	153	139	238	194	211	70	304	187
ENSECAG00000023897	2.626476983	0.6941194	0.9061789	105	108	109	184	97	91	91	131
ENSECAG00000022112	5.242921095	0.6942654	0.9061789	606	672	668	1171	514	602	825	673
ENSECAG00000006692	7.602539869	0.6942687	0.9061789	4187	3332	4284	3998	3342	3361	3305	3238
ENSECAG00000001582	7.157201666	0.6942931	0.9061789	3527	2332	3577	2138	3257	1349	2736	2488
ENSECAG00000017024	5.443179145	0.6943804	0.9061854	769	605	992	1214	769	639	938	658
ENSECAG00000000741	3.656717051	0.6944576	0.9061854	221	225	325	279	293	101	436	31
ENSECAG00000020443	3.282683937	0.6948657	0.9065958	238	118	187	265	211	198	152	75
ENSECAG00000018169	5.811067187	0.6949581	0.9065958	1120	1155	1318	908	901	1052	1118	766
ENSECAG00000004925	6.77049122	0.6951477	0.9065958	2060	1549	2147	2435	2699	1307	2608	1650
ENSECAG00000014848	7.001310791	0.6951546	0.9065958	2461	2590	2334	3022	1852	2272	2531	2139
ENSECAG00000004465	7.097044271	0.6951712	0.9065958	2537	2249	2477	2987	2765	1772	3474	2378
ENSECAG00000006796	1.965747089	0.6953302	0.9066991	62	50	95	113	82	47	64	63
ENSECAG00000012241	3.44741564	0.6954429	0.9067184	178	190	191	246	173	171	234	240
ENSECAG00000017412	3.585098456	0.6955188	0.9067184	212	234	231	298	200	152	218	248
ENSECAG00000003310	7.974816839	0.6956138	0.9067184	4763	4821	4742	6353	5216	4019	5925	2046
ENSECAG00000008893	1.327634739	0.6958128	0.9067184	41	55	56	46	37	30	31	63
ENSECAG00000019789	3.058271115	0.6958416	0.9067184	114	165	152	174	128	111	151	237
ENSECAG00000017858	6.110847265	0.6959451	0.9067184	1477	1006	1480	1677	1152	1118	1221	1257
ENSECAG00000017698	7.119484368	0.6959494	0.9067184	2309	2650	2240	3173	2093	3225	2633	2337
ENSECAG00000004005	3.716432871	0.6959945	0.9067184	251	212	376	228	328	159	222	175
ENSECAG00000017989	7.978022054	0.6960634	0.9067184	4781	3160	4718	6410	4378	3850	5480	5312
ENSECAG00000011570	1.159151695	0.6962803	0.906825	37	54	46	38	36	17	27	63
ENSECAG00000014132	1.88900436	0.6963049	0.906825	80	70	80	65	43	47	64	91
ENSECAG00000015020	4.202848969	0.6966036	0.9069535	400	235	369	367	301	324	354	393
ENSECAG00000015762	4.998801332	0.6966104	0.9069535	531	673	675	705	472	607	615	485
ENSECAG00000010641	3.705209951	0.6966624	0.9069535	257	214	258	225	203	302	305	160
ENSECAG00000020260	7.668372445	0.6967229	0.9069535	3173	4098	3587	4136	2533	5902	3085	3387
ENSECAG00000001043	6.449563209	0.6971006	0.9072648	1764	1449	1927	1955	1403	1286	1542	1800
ENSECAG00000022333	4.597977588	0.6971638	0.9072648	521	232	420	640	503	509	639	174
ENSECAG00000011134	5.604430812	0.6972017	0.9072648	934	932	957	1113	911	800	904	722
ENSECAG00000014046	3.559409515	0.6974473	0.9074804	211	208	275	267	167	106	344	205
ENSECAG00000015431	3.067944704	0.6976475	0.9075283	144	162	133	170	227	131	151	113

ENSECAG00000010288	7.285146158	0.6977292	0.9075283	3161	3046	3050	2064	1737	1640	3150	5500
ENSECAG00000000313	3.260347505	0.6977354	0.9075283	216	132	80	278	134	81	257	273
ENSECAG00000014148	3.161976378	0.6978037	0.9075283	169	163	153	246	114	148	172	170
ENSECAG00000019062	5.292414731	0.697953	0.9076186	871	614	693	732	979	424	963	605
ENSECAG00000023112	3.946467386	0.6981331	0.9077101	308	282	281	258	320	285.999	238	293
ENSECAG00000020523	10.55256594	0.6981958	0.9077101	26093	24953	29639	31417	23859	28889	28167	31158
ENSECAG00000014003	3.449575495	0.6983295	0.9077101	228	174	182	212	100	162	196	370
ENSECAG00000019355	8.289032293	0.6983431	0.9077101	4376	10372	6919	3265	2617	4535	6094	8084
ENSECAG00000001853	4.790252492	0.6986252	0.9077915	621	384	573	466	490	303	898	447
ENSECAG00000008887	6.6638129	0.6987055	0.9077915	2038	1511	1967	2072	2707	1418	1822	1603
ENSECAG00000013263	4.043470042	0.6987152	0.9077915	395	228	476	237	301	172	298	355
ENSECAG00000023172	7.551472337	0.6988613	0.9077915	2752	4949	2566	3257	1565	4850	2478	5058
ENSECAG00000010645	4.95462146	0.6988838	0.9077915	583	495	751	692	469	475	556	628
ENSECAG00000010231	4.265458945	0.6991221	0.9077915	215	473	238	487	238	383	428	397
ENSECAG00000009060	2.462014057	0.6993177	0.9077915	88	104	74	134	89	69	123	134
ENSECAG00000021171	5.018957929	0.6994518	0.9077915	562	546	631	680	488	696	597	613
ENSECAG00000011070	3.478974291	0.6994977	0.9077915	229	127	301	260	161	142	318	148
ENSECAG00000017707	6.287781633	0.699524	0.9077915	857	2055	1584	982	267	2576	626	2329
ENSECAG00000021626	3.495882842	0.6995454	0.9077915	224	171	195	244	199	154	303	199
ENSECAG00000016390	5.555788668	0.6995876	0.9077915	922	651	913	1435	816	390	1631	495
ENSECAG00000009363	3.916930678	0.6996275	0.9077915	82	579	97	236	17	217	46	917
ENSECAG00000016315	4.331138685	0.6996377	0.9077915	448	397	405	397	585	126	510	158
ENSECAG00000000883	6.21639312	0.6996793	0.9077915	1448	1233	1388	1486	1808	863	1510	1403
ENSECAG00000024800	1.670478153	0.6998106	0.9077915	53	34	51	87	118	15	90	21
ENSECAG00000020445	5.398354186	0.6998377	0.9077915	869	655	912	698	665	770	1058	671
ENSECAG00000023303	0.648158051	0.6999505	0.9077915	16	50	10	25	26	24	10	53
ENSECAG00000018203	6.355091795	0.7000499	0.9077915	1407	1298	1528	1943	1485	1658	1588	1315
ENSECAG00000000568	3.317637101	0.7001757	0.9077915	210	124	176	226	126	114	249	274
ENSECAG00000016644	1.117425713	0.7003033	0.9077915	16	63	29	30	0	16	32	122
ENSECAG00000023640	6.492000213	0.7003591	0.9077915	1696	1410	2156	2069	1311	1562	1443	1843
ENSECAG00000013823	6.975564922	0.7004802	0.9077915	1909	2461	2247	2768	2071	2754	2374	2110
ENSECAG00000006949	8.133374454	0.7006741	0.9077915	6170	5011	5127	6612	4868	3791	6833	4038
ENSECAG00000024532	6.82247559	0.7007385	0.9077915	1923	1849	2267	2444	1620	2023	2327	2499
ENSECAG00000022272	8.809211918	0.7007897	0.9077915	8514	7366	8175	9459	6924	6940	9287	10673
ENSECAG00000000279	4.638657793	0.7008123	0.9077915	513	471	417	436	505	457	419	459
ENSECAG00000023108	6.651206411	0.7008144	0.9077915	1835	1636	1605	2506	2170	1709	2174	1439
ENSECAG00000016150	8.000578145	0.700935	0.9077915	4658	4424	4548	5525	4334	4515	4782	5477
ENSECAG00000024526	0.044118617	0.7010059	0.9077915	11	11	19	27	22	18	7	23
ENSECAG00000000565	6.576886015	0.7012288	0.9077915	2113	1565	1852	2271	1537	790	2226	2161
ENSECAG00000020785	4.253842253	0.7013387	0.9077915	215	460	263	644	191	303	243	541
ENSECAG00000008406	2.110737507	0.7013689	0.9077915	79	56	83	95	62	76	78	103
ENSECAG00000007250	5.181877728	0.7013798	0.9077915	752	639	786	759	649	502	853	516
ENSECAG00000005161	6.163441436	0.7013969	0.9077915	887	1558	1343	1511	917	924	1333	2260
ENSECAG00000018815	4.928066448	0.7015654	0.9077915	636	509	737	576	477	481	584	549
ENSECAG00000003985	2.029335107	0.7015793	0.9077915	87	51	103	46	133	42	80	50
ENSECAG00000003051	4.965947517	0.7015927	0.9077915	571	585	643	491	415	822	422	625
ENSECAG00000012764	2.989787105	0.7016073	0.9077915	102	121	138	227	133	188	143	120
ENSECAG00000015276	4.659435667	0.7017299	0.9077915	505	356	479	743	385	402	570	383
ENSECAG00000011130	2.249601066	0.70177	0.9077915	107	67	86	128	79	43	125	76
ENSECAG00000001086	1.108978398	0.7018172	0.9077915	19	44	34	77	16	24	48	52
ENSECAG00000019527	6.933183272	0.7018421	0.9077915	2301	1927	2216	2683	2837	1946	3257	1199
ENSECAG00000008452	6.212108791	0.7022507	0.9082165	1468	1134	1565	1380	1600	1110	1351	1452
ENSECAG00000016077	7.78043784	0.7024174	0.9083286	4439	3308	4648	5661	3424	3798	4536	3351
ENSECAG00000020980	4.19418566	0.7026596	0.9084363	363	300	324	373	325	323	406	311
ENSECAG00000024265	5.059354409	0.7026957	0.9084363	595	489	658	764	611	676	620	553
ENSECAG00000012877	6.13276628	0.7028653	0.9084363	1013	1230	1981	944	1380	1158	1515	1204
ENSECAG00000024737	6.519589666	0.7029257	0.9084363	1565	1482	1750	2072	2419	1623	1832	935
ENSECAG00000004476	3.055611902	0.7030518	0.9084363	162	56	246	144	193	137	179	115
ENSECAG00000009909	6.677763154	0.7030788	0.9084363	2035	1999	1795	2507	1556	2292	1559	1484
ENSECAG00000024655	4.322907836	0.7031202	0.9084363	364	609	192	237	364	272	164	708
ENSECAG00000006587	4.708266424	0.7031404	0.9084363	552	397	451	553	570	416	465	488
ENSECAG00000008142	3.74433276	0.7033905	0.9085776	343	210	250	295	306	128	373	115
ENSECAG00000009846	2.407365431	0.7035401	0.9085776	93	51	59	252	39	74	150	93
ENSECAG00000011585	7.12400192	0.70368	0.9085776	2258	3249	2243	2440	1536	3160	1902	3744
ENSECAG00000019385	6.317629518	0.7036916	0.9085776	1690	1200	1515	1576	1712	1039	1922	1336
ENSECAG00000022763	6.872684576	0.7037488	0.9085776	2216	2138	2069	3164	1567	1756	2590	2226
ENSECAG00000014173	5.48757614	0.7038434	0.9085776	715	775	707	1167	386	939	984	1058
ENSECAG00000014264	4.111747528	0.7039174	0.9085776	358	277	332	444	241	247	377	327
ENSECAG00000001700	6.066332657	0.7040613	0.9085776	1941	913	1369	1234	1684	812	1278	815
ENSECAG00000015720	5.911745108	0.7041126	0.9085776	1047	1150	939	1370	774	977	1341	1435
ENSECAG00000008863	5.100756207	0.7041684	0.9085776	748	607	664	758	620	453	706	597
ENSECAG00000022421	3.922505433	0.7041973	0.9085776	297	277	289	370	279	231	332	194
ENSECAG00000017196	1.255437859	0.7042736	0.9085776	45	41	31	77	12	67	35	34
ENSECAG00000019865	4.38927883	0.7043402	0.9085776	422	341	387	403	381	227	548	441
ENSECAG00000012157	3.696120036	0.7044117	0.9085776	306	224	218	306	198	195	323	174
ENSECAG00000018375	5.503963108	0.7044496	0.9085776	850	988	921	876	742	718	851	816
ENSECAG00000011789	2.205617092	0.704541	0.9085924	69	94	87	125	39	71	68	126
ENSECAG00000012317	6.964757684	0.7046324	0.908607	2358	1931	2614	2421	2224	2893	1990	2058

ENSECAG00000007508	6.856525517	0.7047705	0.908663	2076	1760	2322	2478	1210	3332	2124	1861
ENSECAG00000018829	7.728795465	0.7049497	0.908663	4623	3347	5832	3378	3376	2688	4166	4441
ENSECAG00000010342	3.148006447	0.7049515	0.908663	143	191	158	228	91	145	172	191
ENSECAG00000020380	1.742880892	0.7050368	0.908663	77	50	48	62	94	39	78	38
ENSECAG00000011696	10.0076314	0.7051432	0.908663	18463	17432	20584	20116	22743	11603	25150	18898
ENSECAG00000011151	7.705721753	0.7053223	0.908663	4635	3018	3839	4135	4433	2912	4722	3662
ENSECAG00000014082	5.468514191	0.7053436	0.908663	729	1246	879	661	552	751	876	859
ENSECAG00000010270	2.714638977	0.70543	0.908663	65	95	160	159	102	63	134	201
ENSECAG00000003532	4.393899145	0.7055952	0.908663	368	377	478	484	262	385	368	415
ENSECAG00000013439	1.144202689	0.7056482	0.908663	18	63	10	56	6	89	13	50
ENSECAG00000013764	6.414757936	0.7056597	0.908663	1320	1747	2501	1288	1053	1500	1383	1881
ENSECAG00000026832	1.640893092	0.7057123	0.908663	62	32	60	69	77	43	81	31
ENSECAG00000023078	4.660842773	0.7057157	0.908663	413	408	551	701	420	369	582	377
ENSECAG00000007356	5.473503994	0.7059425	0.9088519	912	684	868	863	938	770	877	706
ENSECAG00000007525	4.213831205	0.7060252	0.9088554	314	353	340	506	322	322	318	296
ENSECAG00000015615	6.775170927	0.7061119	0.908864	2186	1808	1996	3006	1551	2184	1510	2159
ENSECAG00000025104	9.839462511	0.7062464	0.9089341	21476	15745	18529	18778	17749	13150	18929	13279
ENSECAG00000010059	2.532879369	0.7063395	0.908951	85	95	155	137	123	94	58	101
ENSECAG00000011899	7.319046157	0.7065477	0.9090479	2657	3363	2610	4463	1867	3505	3136	2330
ENSECAG00000005659	3.011023832	0.7067304	0.9090479	98	238	84	152	114	114	163	220
ENSECAG00000003090	4.832339661	0.7067483	0.9090479	890	317	572	294	765	480	548	339
ENSECAG00000019002	3.626987538	0.7067587	0.9090479	237	304	160	163	111	475	54	250
ENSECAG00000020144	5.947906953	0.7068308	0.9090479	2152	619	1217	439	1770	690	1920	430
ENSECAG00000016802	1.699910918	0.706895	0.9090479	58	97	73	26	48	23	116	32
ENSECAG00000012047	2.924781222	0.7072476	0.9092967	164	109	138	144	160	93	189	131
ENSECAG00000011711	5.856713471	0.7072486	0.9092967	1205	1120	1158	783	853	1000	1210	1271
ENSECAG00000011911	1.992263168	0.7079184	0.9100549	113	52	86	25	87	34	85	96
ENSECAG00000000860	7.155948275	0.7081701	0.9100718	506	7614	1132	2213	751	2245	1408	4869
ENSECAG00000018197	6.710275878	0.7082197	0.9100718	2010	1963	1795	2030	1977	2077	2314	1414
ENSECAG00000003863	1.427051167	0.7082281	0.9100718	28	67	51	33	10	30	26	138
ENSECAG00000016030	5.245728972	0.7082521	0.9100718	695	737	791	840	539	526	847	731
ENSECAG00000024437	4.861954381	0.7085283	0.9103238	617	453	485	618	431	398	688	682
ENSECAG00000021243	7.344780088	0.7087964	0.910469	3252	2681	3267	2876	3834	1917	3987	2588
ENSECAG00000016908	1.202183378	0.7089266	0.910469	70	43	49	17	27	51	32	34
ENSECAG00000024249	4.729224224	0.709002	0.910469	511	333	459	909	368	319	549	599
ENSECAG00000005653	8.124341046	0.709182	0.910469	6381	4173	5434	6878	4515	4473	4711	5419
ENSECAG00000001058	6.863758439	0.7093066	0.910469	1785.01	2984.01	2102.01	2579.01	865.001	1841.01	2035	3239.01
ENSECAG00000018237	6.47798609	0.7093906	0.910469	1845	1604	1894	1871	1182	1767	1295	1813
ENSECAG00000021288	5.310206324	0.70941	0.910469	700	781	597	881	589	768	1032	586
ENSECAG00000017665	4.920102787	0.7096	0.910469	859	422	556	380	739	477	727	336
ENSECAG00000016053	1.991609832	0.7096611	0.910469	51	61	73	103	54	98	55	81
ENSECAG00000018547	4.850844121	0.7096767	0.910469	594	493	541	723	356	488	529	606
ENSECAG00000016203	3.226345363	0.7097149	0.910469	179	139	262	179	100	166	180	185
ENSECAG00000023631	5.691701004	0.7097789	0.910469	1031	610	862	1409	1285	778	1353	485
ENSECAG00000018887	8.385524772	0.709849	0.910469	7166	5009	8512	6543	6071	5419	5835	5529
ENSECAG00000001258	1.035005409	0.7098881	0.910469	39	25	45	30	93	18	29	9
ENSECAG00000011768	8.767662098	0.7099055	0.910469	11426	7059	7125	6568	8891	7145	9822	6935
ENSECAG00000011292	0.487935355	0.7100553	0.910469	13	55	16	24	10	30	2	39
ENSECAG00000009970	7.16668229	0.7101094	0.910469	2582	2861	2248	4100	1504	2581	2638	3125
ENSECAG00000007655	4.804616092	0.7101897	0.910469	579	451	586	661	452	276	636	588
ENSECAG00000015533	3.922632289	0.7103118	0.910469	229	281	256	357	188	277	263	402
ENSECAG00000008139	4.612642045	0.710381	0.910469	533	420	495	535	317	299	570	520
ENSECAG00000024136	4.162545893	0.7104013	0.910469	318	274	360	382	343	210	452	352
ENSECAG00000023980	4.739455966	0.7104248	0.910469	359	525	436	674	350	576	511	543
ENSECAG00000015162	7.503622969	0.7105281	0.910469	3172	3282	3236	3870	4280	2982	3422	2807
ENSECAG00000021449	6.724953015	0.710565	0.910469	2100	1762	2012	2046	1892	1627	2518	1908
ENSECAG00000003600	4.728502317	0.7108723	0.91076	511	469	538	632	471	411	477	458
ENSECAG00000003469	1.622160983	0.7109707	0.9107833	27	88	79	48	24	47	59	71
ENSECAG00000006633	1.60671189	0.7111071	0.9108554	12	52	33	120	21	48	37	123
ENSECAG00000010037	5.315770169	0.711508	0.9110191	763	656	706	1135	613	672	841	621
ENSECAG00000021080	4.005354556	0.7115697	0.9110191	320	323	333	317	251	172	335	353
ENSECAG00000013214	5.523120279	0.7116339	0.9110191	878	841	891	1119	730	512	1055	929
ENSECAG00000018062	8.317417514	0.7117027	0.9110191	6324	5924	6668	6978	4559	4867	6750	5929
ENSECAG00000006481	6.85750003	0.7117139	0.9110191	2435	1687	2605	2750	2098	1706	2609	1623
ENSECAG00000016300	5.216862004	0.7117501	0.9110191	733	527	536	1025	556	672	789	757
ENSECAG00000019070	3.699796212	0.7118063	0.9110191	234	270	249	303	165	298	290	118
ENSECAG00000011376	6.94935137	0.7118765	0.9110191	2256	2247	2141	2610	2096	1807	2836	2563
ENSECAG00000008338	5.773404281	0.7120834	0.9111812	986	918	1049	1532	540	1081	1263	888
ENSECAG00000011341	3.037564974	0.7127427	0.9118906	149	100	175	257	161	71	196	134
ENSECAG00000024227	3.847288855	0.7129456	0.9118906	369	218	350	226	237	190	405	163
ENSECAG00000021415	4.400287577	0.7130502	0.9118906	325	499	552	311	241	286	428	499
ENSECAG00000019321	6.490689636	0.7130867	0.9118906	1916	1375	1768	2317	2269	975	1926	1052
ENSECAG00000018452	2.880375387	0.7133487	0.9118906	130	116	144	150	156	133	134	119
ENSECAG00000019898	1.081880672	0.713373	0.9118906	31	52	23	38	28	18	48	64
ENSECAG00000018090	6.828026723	0.7134273	0.9118906	2216	2261	1947	2798	1927	1795	2370	1760
ENSECAG00000023868	8.471074105	0.7135533	0.9118906	7860	5523	7531	8052	6603	5213	7413	5271
ENSECAG00000023612	2.938001425	0.7135874	0.9118906	158	138	155	168	134	75	135	177
ENSECAG00000000437	2.651213952	0.7135991	0.9118906	147	80	145	139	100	64.0003	165	101

ENSECAG00000006938	0.199239169	0.7136249	0.9118906	16	22	24	26	12	30	6	18
ENSECAG00000014759	5.82658367	0.7137192	0.9118906	1205	834	1417	1162	1335	589	1322	700
ENSECAG00000000012	6.638619333	0.7137261	0.9118906	2403	1152	2555	2090	2792	851	2019	1194
ENSECAG00000017956	5.125778106	0.7138394	0.9118906	535	621	742	681	398	333	763	1188
ENSECAG00000010986	7.354895845	0.7139917	0.9118906	3392	2586	3070	3209	3333	2316	3455	3175
ENSECAG00000003965	5.092204247	0.7140623	0.9118906	704	451	691	968	619	344	941	491
ENSECAG00000013227	4.919299877	0.7141028	0.9118906	660	438	603	558	612	446	698	511
ENSECAG00000022474	4.944814986	0.7142457	0.9118906	718	548	446	802	613	397	752	373
ENSECAG00000005412	4.751210107	0.7143173	0.9118906	569	424	585	609	530	389	560	375
ENSECAG00000007262	5.232551125	0.7143713	0.9118906	642	796	745	561	370	490	759	1244
ENSECAG00000020558	3.525614675	0.7145497	0.9118906	190	224	167	381	41	154	234	352
ENSECAG00000021619	4.637157491	0.7145701	0.9118906	466	499	432	620	335	441	541	394
ENSECAG00000018193	6.940559057	0.7147706	0.9118906	2548	1865	2469	2314	2462	2269	2408	1966
ENSECAG00000022640	7.580995643	0.7149128	0.9118906	4158	3181	4088	4130	3348	2797	3130	3881
ENSECAG00000000897	5.100935654	0.7149615	0.9118906	752	581	753	681	513	528	710	624
ENSECAG00000014405	1.975317692	0.715017	0.9118906	51	105	46	69	23	83	29	156
ENSECAG00000014972	4.603606773	0.7152499	0.9118906	569	419	439	543	462	289	568	370
ENSECAG00000008379	3.345306103	0.7152836	0.9118906	213	152	207	260	128	102	313	167
ENSECAG00000018200	4.71832848	0.7153296	0.9118906	521	518	488	610	667	260	550	337
ENSECAG00000013411	6.037809918	0.7153948	0.9118906	1070	1366	1161	1734	1036	1154	1052	1254
ENSECAG00000020472	3.82198805	0.7154046	0.9118906	266	238	225	320	181	190	337	364
ENSECAG00000010518	6.151714165	0.7154152	0.9118906	1373	1040	1458	1486	1400	1045	1798	1099
ENSECAG00000001299	3.754259709	0.7154847	0.9118906	336	129	304	347	238	115	291	286
ENSECAG00000011210	4.402204202	0.7157142	0.9118906	387	364	457	507	348	325	394	384
ENSECAG00000009115	5.842419579	0.7157273	0.9118906	1092	870	1073	1300	1050	939	1355	941
ENSECAG00000018572	6.404931862	0.7157616	0.9118906	1486	1417	1570	1916	1608	1624	1623	1397
ENSECAG00000011783	7.70839863	0.7157763	0.9118906	4622	3502	3863	5081	3043	2552	4841	4205
ENSECAG00000009886	3.885134393	0.7159787	0.9118906	355	202	326	322	332	138	291	253
ENSECAG00000016783	2.270622205	0.7160084	0.9118906	68	58	76	154	78	60	161	70
ENSECAG00000022750	5.100363118	0.7160331	0.9118906	617	609	712	855	552	729	468	557
ENSECAG00000023724	3.485404921	0.7160604	0.9118906	147	173	221	292	295	126	198	218
ENSECAG00000013973	6.291033918	0.7161174	0.9118906	1931	1607	1673	1030	1456	970	1598	1409
ENSECAG00000022843	6.679292412	0.7161324	0.9118906	2113	1557	2005	2701	1626	1413	2154	1945
ENSECAG00000009741	6.310868959	0.7161947	0.9118906	1395	1433	1308	1833	1776	986	1935	1283
ENSECAG00000019815	8.690344549	0.7162504	0.9118906	7677	6178	9232	7678	6014	10507	6766	7063
ENSECAG00000023825	5.583183631	0.7166685	0.9122026	937	825	969	1161	564	759	1176	850
ENSECAG00000007758	4.826168377	0.7167337	0.9122026	390	825	448	618	268	596	460	586
ENSECAG00000018179	6.604918775	0.7167364	0.9122026	1893	1683	2253	2039	1534	1610	1490	2042
ENSECAG00000010374	5.268991642	0.7169404	0.9122535	676	679	620	920	483	771	723	882
ENSECAG00000007881	2.687619457	0.7169791	0.9122535	99	200	81	133	91	100	158	85
ENSECAG00000017575	4.424973866	0.7170357	0.9122535	729	268	540	212	241	190	276	749
ENSECAG00000020201	1.935298401	0.7170976	0.9122535	103	91	70	40	61	29	153	17
ENSECAG00000001774	6.26927437	0.7172359	0.9123029	1178	1606	1503	1417	1154	2107	1185	1167
ENSECAG00000000371	5.909181268	0.717297	0.9123029	1436	919	1162	932	905	980	778	1801
ENSECAG00000021160	6.725060765	0.7174099	0.9123443	2168	1534	2326	2635	2170	1069	2518	1649
ENSECAG00000000370	11.7698464	0.7177334	0.9124673	69713	50881	68060	72825	68826	77739	52414	57486
ENSECAG00000012265	5.015937182	0.7177645	0.9124673	738	549	617	733	802	260	766	422
ENSECAG00000013284	1.053520798	0.717857	0.9124673	38	46	40	37	27	18	36	54
ENSECAG00000015988	6.301951748	0.7180991	0.9124673	1840	1308	1592	1629	1220	1181	1810	1294
ENSECAG00000016437	2.227399065	0.718122	0.9124673	90	83	84	123	37	56	101	122
ENSECAG00000002758	5.851400728	0.7181487	0.9124673	1167	889	1045	1237	1403	789	1377	760
ENSECAG00000013625	4.475095774	0.7182069	0.9124673	460	394	517	426	274	226	474	576
ENSECAG00000001807	2.979738218	0.7182644	0.9124673	166	102	200	173	154	101	144	134
ENSECAG00000013062	10.66822886	0.7183011	0.9124673	34668	25534	24111	37565	34282	35611	20671	28674
ENSECAG00000018847	3.020991706	0.7183667	0.9124673	149	91	191	168	133	130	229	121
ENSECAG00000021816	6.446104118	0.7184336	0.9124673	2009	1267	1578	1697	1815	1555	1477	1579
ENSECAG00000015843	8.176172068	0.7184706	0.9124673	5252	5710	5872	6632	4480	5149	5899	4367
ENSECAG00000026889	5.027535035	0.7186526	0.9124952	588	629	662	758	510	386	743	641
ENSECAG00000023517	7.50358051	0.7186532	0.9124952	3817	3344	4015	3473	2943	3291	3123	3046
ENSECAG00000007024	3.329193587	0.7187383	0.9125012	207.001	209	198	192	142.001	113	256	185.001
ENSECAG00000017555	5.372108226	0.7189569	0.9126276	731	527	830	1048	980	449	1006	696
ENSECAG00000008082	6.51141008	0.7190654	0.9126276	1886	1125	2142	1703	1191	1437	2358	1915
ENSECAG00000017694	6.54027016	0.7191153	0.9126276	2000	1868	1793	1829	1254	1003	2385	1935
ENSECAG00000022176	6.409868849	0.7191592	0.9126276	1786	1383	1573	1629	1297	1123	1989	2010
ENSECAG00000020011	3.421849027	0.7193328	0.9127459	182	217	238	136	258	155	260	132
ENSECAG00000010095	2.329638993	0.7196333	0.9129333	83	72	141	64	92	79	97	105
ENSECAG00000006687	5.474746632	0.7196413	0.9129333	853	737	1090	915	729	887	664	735
ENSECAG00000015595	5.732951036	0.7198463	0.9130915	1014	844	1144	1321	965	917	966	805
ENSECAG00000023460	7.473123338	0.7201659	0.9133949	3139	2914	3640	3571	2630	3946	2344	4128
ENSECAG00000024596	4.056044359	0.7203774	0.9134884	334	284	329	405	234	185	442	303
ENSECAG00000020569	7.519042768	0.720453	0.9134884	3019	4101	3097	4667	2740	2991	3409	3488
ENSECAG00000021629	9.108604243	0.7204809	0.9134884	11730	8517	10454	10560	11076	10453	9403	9908
ENSECAG00000023479	5.204432674	0.7206954	0.9136583	839	539	944	659	493	427	864	798
ENSECAG00000011751	8.478354886	0.7208634	0.9136987	5673	7621	5714	7527	6322	6384	8270	5754
ENSECAG00000011633	5.409583982	0.7208881	0.9136987	759	694	908	822	629	714	827	993
ENSECAG00000024069	4.265516927	0.7210779	0.9138358	428	306	504	315	320	155	405	457
ENSECAG00000012639	5.964261428	0.7211571	0.9138358	1207	1075	1128	1677	1278	1026	986	978
ENSECAG00000021318	9.828395889	0.7216679	0.9143023	17317	16484	18599	21917	20442	9890	21469	11437

ENSECAG00000021488	6.170559496	0.7216863	0.9143023	1059	1743	1487	1505	763	1534	1117	1471
ENSECAG00000004299	6.369588411	0.722273	0.9149435	1590	1609	1591	1877	1187	1303	1836	1438
ENSECAG00000011959	2.11592431	0.7225472	0.9151888	45	83	86	99	80	54	111	80
ENSECAG00000010814	4.82480852	0.7227363	0.915262	363	685	354	696	367	430	670	679
ENSECAG00000018215	6.608821973	0.7227824	0.915262	1744	1821	2181	2132	1721	1787	1697	1481
ENSECAG00000024143	1.459703231	0.7228895	0.915262	26	84	36	39	18	24	108	65
ENSECAG00000020378	5.127760084	0.7229273	0.915262	567	411	781	890	748	487	936	470
ENSECAG00000026858	3.898459369	0.7230819	0.9153557	264	259	314	263	314	274	303	212
ENSECAG00000024533	4.885454238	0.7232267	0.915356	469	629	531	766	397	534	598	501
ENSECAG00000024483	6.542932037	0.7232432	0.915356	2028	1622	1714	2200	1752	1016	2329	1464
ENSECAG00000022103	9.091328998	0.7235932	0.9156218	10398	10251	11286	12342	9365	8528	11490	8326
ENSECAG00000003621	4.084312015	0.7237235	0.9156218	400	231	399	350	396	177	373	222
ENSECAG00000016462	3.49311738	0.7237916	0.9156218	232	161	221	311	136	127	190	317
ENSECAG00000021561	10.01695966	0.7239444	0.9156218	20603	16100	19470	21500	18535	18289	16009	23985
ENSECAG00000023393	5.531663576	0.7240045	0.9156218	927	737	1041	1040	845	834	813	675
ENSECAG00000012073	3.239394413	0.7241192	0.9156218	162	183	169	168	109	282	81	210
ENSECAG00000024944	5.862701335	0.7242259	0.9156218	1095	864	1234	1191	1185	1071	1072	957
ENSECAG00000012787	7.097400157	0.7242335	0.9156218	2578	2417	2627	2569	1446	3202	2796	2719
ENSECAG00000023441	3.653016708	0.7243711	0.9156218	302	262	193	259	173	325	164	163
ENSECAG00000013727	4.722229513	0.7244467	0.9156218	487	405	469	628	466	454	510	526
ENSECAG00000004879	4.481990821	0.7244672	0.9156218	485	324	455	398	447	437	490	281
ENSECAG00000011247	4.097964905	0.7244678	0.9156218	291	187	353	464	285	214	449	352
ENSECAG00000013338	2.177009249	0.7245139	0.9156218	103	80	78	59	70	45	80	145
ENSECAG00000009680	8.716578	0.7248268	0.9156218	7285	8626	9443	8787	4508	8293	5760	9924
ENSECAG00000024001	5.960467909	0.724872	0.9156218	1364	1056	993	1661	747	1026	1291	1258
ENSECAG00000013606	4.937399439	0.724903	0.9156218	659	429	544	667	657	299	751	618
ENSECAG00000001385	7.427355381	0.7249109	0.9156218	4631	2448	3359	2279	4252	2423	3609	2634
ENSECAG00000012894	6.581365767	0.7249615	0.9156218	1987	1458	1744	2034	2119	1460	2057	1491
ENSECAG00000026879	3.474644188	0.725056	0.9156218	185	138	230	280	210	192	211	210
ENSECAG00000014564	3.774985753	0.7250654	0.9156218	268	212	282	251	254	168	326	284
ENSECAG00000022461	4.752689898	0.7252066	0.9156983	544	410	632	605	391	281	632	585
ENSECAG00000008772	6.2761223	0.7254065	0.9158489	1473	1256	1468	1646	1262	1417	1469	1582
ENSECAG00000011714	1.640183731	0.7255139	0.9158827	8.99956	70	97	80.9951	5	34	150.995	20
ENSECAG00000003237	6.296880268	0.7257336	0.9160004	1655	1179	1545	1537	1243	1134	1726	1791
ENSECAG00000017904	7.033988808	0.7258118	0.9160004	2506	2100	2598	2634	2758	2335	2334	2252
ENSECAG00000018674	0.549033492	0.7258714	0.9160004	19	22	38	20	28	25	18	31
ENSECAG00000010401	6.016060988	0.726002	0.9160004	1337	1235	1332	1292	999	850	1351	1323
ENSECAG00000014773	3.512692907	0.7260103	0.9160004	243	214	245	215	181	217	152	213
ENSECAG00000016317	0.405106236	0.7263925	0.9163808	30	14	22	22	26	8	50	14
ENSECAG00000007207	4.173257764	0.7264744	0.9163822	379	364	266	452	281	204	392	376
ENSECAG00000010336	7.554793554	0.7268904	0.916513	4400	2683	4124	4124	3812	2471	4225	2549
ENSECAG00000011362	7.019207784	0.7269677	0.916513	2826	2466	2035	3226	2566	1737	3159	1572
ENSECAG00000018873	3.331734686	0.7271433	0.916513	233	157	199	226	151	127	226	189
ENSECAG00000013803	6.56655438	0.7271445	0.916513	1791	1556	2014	1753	1892	1552	1831	1745
ENSECAG00000012575	3.531940949	0.7271518	0.916513	206	198	238	298	162	159	224	248
ENSECAG00000007156	8.063373813	0.727196	0.916513	5225	4439	5727	6418	5172	3133	6078	4294
ENSECAG00000019995	2.260770453	0.7272127	0.916513	117	62	149	55	53	82	94	89
ENSECAG00000019216	5.565463456	0.7273041	0.916513	845	908.002	905	1169	852.002	751	966	709
ENSECAG00000018501	1.348043235	0.7273042	0.916513	31	101	26	40	33	57	11	55
ENSECAG00000006211	4.887750832	0.7275484	0.91662	566	339	617	708	521	646	755	285
ENSECAG00000019220	3.32594928	0.7275505	0.91662	206	183	236	90	167	76	348	193
ENSECAG00000014904	7.306728068	0.727636	0.916626	2848	3179	2593	3191	2406	2579	3244	3618
ENSECAG00000007777	4.952934059	0.727836	0.9167202	595	551	562	813	583	305	677	599
ENSECAG00000018562	5.560553039	0.7278721	0.9167202	1439	614	1206	560	1009	474	1352	458
ENSECAG00000015443	2.152092629	0.7284761	0.9172419	98	66	97	95	78	65	86	68
ENSECAG00000015553	2.22386245	0.7284842	0.9172419	54	47	170	111	54	79	97	80
ENSECAG00000013422	4.993020893	0.7285286	0.9172419	720	523	701	626	610	321	672	618
ENSECAG00000016816	5.32198314	0.7286499	0.9172929	875	753	710	889	712	459	999	639
ENSECAG00000012925	3.024814594	0.7289154	0.9173084	171	120	140	168	184	78	201	152
ENSECAG00000020274	5.176568509	0.728955	0.9173084	911	563	728	465	572	439	978	767
ENSECAG00000020430	3.562352785	0.729018	0.9173084	200	173	242	263	191	290	211	171
ENSECAG00000001897	6.564393209	0.7290483	0.9173084	2110	1510	2070	1948	1784	1273	1837	1686
ENSECAG00000008251	4.324934661	0.7291352	0.9173084	425	296	436	478	304	178	586	347
ENSECAG00000018984	6.748928223	0.7291467	0.9173084	2050	1650	2097	2321	2111	1672	2014	2188
ENSECAG00000006801	5.772324328	0.7292297	0.9173112	1002	1086	994	1342	635	1070	872	1160
ENSECAG00000013019	2.666380575	0.7299117	0.917977	150	60	130	126	148	119	82	114
ENSECAG00000023467	3.195632282	0.7299888	0.917977	155	118	228	251	101	124	212	192
ENSECAG00000011574	3.653989381	0.7301737	0.917977	263	244	269	232	182	205	211	258
ENSECAG00000015051	5.424310666	0.730222	0.917977	893	727	863	1007	1033	629	876	409
ENSECAG00000010786	7.858791393	0.7302334	0.917977	4187	4140	4513	4518	4409	3544	4138	5196
ENSECAG00000016144	3.7147446	0.7302526	0.917977	296	201	233	345	337	110	255	197
ENSECAG00000019009	6.903072901	0.7303247	0.917977	2490	1859	2211	2442	2591	1851	2325	2125
ENSECAG00000017478	2.669709149	0.7305504	0.9180739	161	80	118	160	120	85	163	64
ENSECAG00000018174	0.701662789	0.7306172	0.9180739	38	27	30	31	19	9	26	50
ENSECAG00000000188	4.811431255	0.730695	0.9180739	704	477	510	575	586	324	542	490
ENSECAG00000008678	8.585024292	0.7307251	0.9180739	7769	5795	8075	7105	6379	5862	7347	9165
ENSECAG00000000965	7.283086362	0.7308728	0.9181579	2609	3985	2187	3815	1727	3137	3587	2259
ENSECAG00000010081	6.68984793	0.7310376	0.9182095	1983	1754	1678	2378	1855	1872	1866	2031

ENSECAG00000010744	6.96786184	0.7311487	0.9182095	2819	1883	2212	2475	3017	2236	2157	1814
ENSECAG00000011716	5.617692044	0.7311564	0.9182095	777	848	1030	1346	656	672	1207	913
ENSECAG00000006989	6.74719553	0.7316041	0.9182743	1714	1824	2090	2453	2094	2524	1720	1496
ENSECAG00000018130	5.949734452	0.7316541	0.9182743	1290	1135	1205	1344	1061	909	1378	965
ENSECAG00000013499	6.965528743	0.7317703	0.9182743	2425	2356	2444	2884	1788	2277	2376	2169
ENSECAG00000007910	3.724975301	0.731779	0.9182743	290	166	268	257	264	133	392	222
ENSECAG00000021719	0.603044055	0.7318796	0.9182743	26	14	35	30	37	20	26	24
ENSECAG00000017818	4.107154949	0.7318922	0.9182743	309	234	414	454	263	178	393	367
ENSECAG00000011752	3.166052864	0.7318989	0.9182743	169	149	212	195	162	88	216	154
ENSECAG00000008404	7.161124588	0.7320065	0.9182743	2655	2178	2733	3243	2892	2720	2652	2294
ENSECAG00000021787	1.849358212	0.7320075	0.9182743	64	44	69	85	46	55	83	83
ENSECAG00000017773	2.219282537	0.7320832	0.9182743	77	80	81	100	79	104	69	85
ENSECAG00000014898	5.86109662	0.7320972	0.9182743	1091	881	944	1490	1065	672	1754	943
ENSECAG00000000116	6.158877035	0.7321835	0.9182811	1087	1336	1191	1782	1025	1040	1555	1741
ENSECAG00000022613	7.029298151	0.732366	0.9184087	2811	2079	2332	2594	2449	2076	2585	2611
ENSECAG00000008732	4.934545996	0.7326119	0.9186156	411	724	461	662	353	712	469	715
ENSECAG00000009028	6.399194818	0.7328676	0.9188348	1483	1476	1494	1918	1169	1600	1883	1622
ENSECAG00000015119	6.085216712	0.7329951	0.9188932	2477	518	1552	363	1149	665	1841	1635
ENSECAG00000021899	6.75441237	0.7333585	0.9192353	2355	1796	2056	2551	2008	1887	1934	1583
ENSECAG00000016287	3.709132215	0.7335053	0.9192353	258	245	227	328	230	192	270	205
ENSECAG00000009977	1.17815069	0.7335108	0.9192353	46	33	42	61	9	33	25	77
ENSECAG00000013502	7.701003475	0.7335942	0.9192384	3776	3145	3234	7180	3652	3024	5684	2140
ENSECAG00000021836	6.141197905	0.7338334	0.9194367	1176	1508	1346	1647	1146	1245	1365	1116
ENSECAG00000024102	3.333244271	0.7340537	0.9195108	182	170	243	218	140	159	204	185
ENSECAG00000019969	3.885427198	0.7340544	0.9195108	161	330	228	376	201	300	334	266
ENSECAG00000017356	1.762440472	0.7341635	0.9195394	23	60	54	144	31	51	77	65
ENSECAG00000013923	8.470845344	0.734246	0.9195394	6848	6526	7203	8271	4350	4338	7758	8408
ENSECAG00000014742	3.903409199	0.7343201	0.9195394	265	251	259	339	235	170	304	419
ENSECAG00000012310	7.784158702	0.7347457	0.919971	4988	3681	4271	4964	3166	4245	3400	4220
ENSECAG00000006611	6.348231969	0.7348851	0.9200441	1576	1443	1348	1780	1420	1349	1755	1529
ENSECAG00000017882	1.313252337	0.7351384	0.9201595	38	40	56	40	46.0005	65.0013	45	21
ENSECAG00000010140	4.191022317	0.7352225	0.9201595	288	326	380	365	306	353	309	375
ENSECAG00000016438	5.025483137	0.7352684	0.9201595	507	645	592	682	741	642	640	374
ENSECAG00000000762	4.907033111	0.7353013	0.9201595	707	454	672	586	540	447	548	532
ENSECAG00000014994	7.353515153	0.7355047	0.9202157	2821	3100	2569	3774	2342	3932	2637	3070
ENSECAG00000015132	6.294798773	0.7355083	0.9202157	1464	1176	1238	2105	1113	1401	1810	1533
ENSECAG00000001235	5.093294072	0.7356401	0.9202512	646	503	699	719	769	640	534	555
ENSECAG00000022174	3.852681507	0.7356987	0.9202512	264	232	263	320	300	238	300	232
ENSECAG00000025004	4.901883275	0.7362325	0.9207969	707	488	578	640	518	365	647	555
ENSECAG00000004664	1.758625183	0.7362971	0.9207969	66	45	83	77	45	42	82	58
ENSECAG00000005263	4.345058233	0.7365833	0.9210148	362	413	280	456	457	270	513	288
ENSECAG00000020116	6.910693458	0.7367169	0.9210148	1881	2447	3065	2338	1083	2712	1651	2664
ENSECAG00000012624	5.406701251	0.7367524	0.9210148	901	675	821	773	1057	726	815	530
ENSECAG00000007431	5.483855907	0.7368119	0.9210148	928	750	911	755	749	613	1094	907
ENSECAG00000018745	7.694588651	0.7368767	0.9210148	3878	3518	3708	4489	3591	4045	4001	3677
ENSECAG00000013591	4.393955089	0.7370199	0.9210925	439	316	370	449	279	307	433	557
ENSECAG00000008438	0.676317588	0.7373657	0.9211846	24	20	31	52	20	11	65	9
ENSECAG00000022985	4.863001345	0.7373955	0.9211846	609	357	569	651	543	257	973	477
ENSECAG00000020508	4.833601415	0.7374474	0.9211846	574	505	723	250	228	878	344	632
ENSECAG00000022300	1.914218771	0.7375547	0.9211846	52	52	82	86	95	23	82	83
ENSECAG00000015122	6.587207946	0.7375659	0.9211846	1983	1707	1903	2170	1176	1445	2177	1940
ENSECAG00000021198	1.590618099	0.7375802	0.9211846	37	35	61	86	33	41	75	75
ENSECAG00000008657	0.287726326	0.7380018	0.9216098	13	22	29	17	22	30	17	14
ENSECAG00000013476	4.464788055	0.7380865	0.9216142	431	364	472	531	236	201	588	535
ENSECAG00000023111	6.329181894	0.7388339	0.9222865	1655	1018	1514	1923	1829	1111	1971	1116
ENSECAG00000013649	5.081357445	0.7388512	0.9222865	865	439	635	824	541	457	513	818
ENSECAG00000018855	0.555032775	0.7388685	0.9222865	25	24	30	20	30	24	32	18
ENSECAG00000000217	1.213967504	0.739045	0.9224054	30	56	44	29	35	42	32	58
ENSECAG00000013864	6.084116484	0.7391652	0.9224327	1464	982	1294	1375	1015	1487	1501	1004
ENSECAG00000009743	3.38436752	0.7392292	0.9224327	202	148	249	168	274	137	227	141
ENSECAG00000009976	5.545474416	0.7394405	0.922595	933	810	1046	967	910	714	969	645
ENSECAG00000006020	3.442823253	0.7396314	0.9227318	204	178	212	290	170	125	268	192
ENSECAG00000001037	5.237692463	0.7399604	0.9230408	453	485	555	1405	670	312	1134	803
ENSECAG00000021079	3.136138394	0.7401533	0.9231631	164	133	182	170	164	145	151	188
ENSECAG00000008886	0.890853089	0.7402718	0.9231631	20	27	32	52	22	36	33	41
ENSECAG00000026949	1.701205236	0.7404363	0.9231631	31	78	51	70	30	73	58	76
ENSECAG00000020152	8.40557165	0.7404642	0.9231631	7846	4063	7204	6367	7566	6362	6094	5030
ENSECAG00000018341	0.479477287	0.7404648	0.9231631	33	13	41	20	22	20	20	24
ENSECAG00000018250	6.053902785	0.7407073	0.9232957	1480	915	1423	1578	1258	839	1274	1257
ENSECAG00000009317	5.310971508	0.7407336	0.9232957	697	949	661	603	626	842	514	922
ENSECAG00000014738	5.695960375	0.7409678	0.9234863	828	1038	1092	1217	710	744	1006	1153
ENSECAG00000010511	5.417828483	0.7411914	0.9234983	640	829	770	977	671	632	1099	811
ENSECAG00000007976	8.756581229	0.741208	0.9234983	8670	6593	8943	8201	8583	9062	6571	7485
ENSECAG00000005600	2.868434076	0.7412214	0.9234983	114	92	152	246	127	79	109	178
ENSECAG00000020891	2.589415334	0.7414404	0.9235944	123	127	105	125	116	85	83	118
ENSECAG00000006583	0.839693835	0.741532	0.9235944	25	21	23	58	17	31	40	41
ENSECAG00000015022	3.532572464	0.7415425	0.9235944	220	172	246	219	267	132	283	188
ENSECAG00000022048	6.738670902	0.7416568	0.9236093	2018	1896	2310	2409	2224	1227	2287	1727

ENSECAG00000018553	7.692893954	0.7417243	0.9236093	4114	3000	3360	6626	2922	3351	4713	3399
ENSECAG00000017621	4.376792042	0.7417983	0.9236093	333	387	451	370	268	377	470	434
ENSECAG00000020504	6.483457755	0.7421197	0.9239081	1679	1630	1759	2159	1408	1252	1670	1906
ENSECAG00000008278	5.763823251	0.7425783	0.9243777	1068	1023	1119	1148	828	824	1147	994
ENSECAG00000015415	5.987877249	0.7428722	0.9244737	1094	1259	1207	1573	1310	674	1598	885
ENSECAG00000022895	4.004080872	0.7428966	0.9244737	296	294	346	357	309	283	264	232
ENSECAG00000013780	3.126215614	0.7429115	0.9244737	192	96	182	250.001	202	110	203	78
ENSECAG00000021302	4.506774044	0.7430307	0.9244737	592	286	405	576	400	469	383	283
ENSECAG00000007702	7.024011803	0.7431459	0.9244737	1965	2599	2207	3008	2123	2805	2451	2198
ENSECAG00000023543	3.590626788	0.7432274	0.9244737	229	202	222	241	265	206	239	178
ENSECAG00000008644	4.254692433	0.7432392	0.9244737	307	383	317	416	273	357	413	373
ENSECAG00000023340	4.637718309	0.7433065	0.9244737	450	433	451	531	580	391	481	387
ENSECAG00000010549	5.563819039	0.7434977	0.9246103	864	993	860	1091	617	541	946	1212
ENSECAG00000002798	4.144142486	0.7437209	0.9247866	334	265	385	456	259	199	475	309
ENSECAG00000006184	6.081852814	0.7443818	0.9254265	1209	1139	1308	1458	1194	1419	1251	1096
ENSECAG00000015326	4.832761992	0.7443984	0.9254265	658	498	620	506	499	454	521	487
ENSECAG00000010568	5.269572266	0.7446521	0.9256406	825	574	740	1000	482	559	862	795
ENSECAG00000009750	4.876477983	0.7451947	0.9259891	531	552	566.001	539	666	464	603	442
ENSECAG00000012844	5.033428249	0.7451987	0.9259891	621	517	651	669	748	353	804	562
ENSECAG00000007707	7.385928961	0.7453252	0.9259891	3461	2528	3598	4023	3061	2030	3557	3047
ENSECAG00000009063	5.629053067	0.7453912	0.9259891	1044	861	1028	1047	971	794	985	673
ENSECAG00000001560	2.599580736	0.7454205	0.9259891	72	96	99	183	156	69	124	102
ENSECAG00000020416	8.380816132	0.7454944	0.9259891	7509	5800	7146	6605	3469	8003	3975	6876
ENSECAG00000024188	3.788132465	0.7455301	0.9259891	280	238	339	255	184	286	333	138
ENSECAG00000021067	7.363545582	0.7455846	0.9259891	3687	2088	2844	3870	3895	2342	3659	2417
ENSECAG00000022905	5.929681756	0.7458166	0.9261033	1298	914	1130	1266	1175	1090	1279	957
ENSECAG00000011981	1.445499021	0.7458397	0.9261033	79	22	53	37	87	16	61	39
ENSECAG00000019628	4.466505834	0.7460568	0.9262716	514	330	457	485	406	358	410	344
ENSECAG00000018144	5.321651151	0.7465273	0.9267545	813	620	800	771	961	535	803	671
ENSECAG00000010897	9.581251008	0.7466446	0.9267988	17340	9290	17618	19258	21391	5469	19629	6859
ENSECAG00000010799	4.236358229	0.7468202	0.9269155	322	315	439	320	313	275	273	536
ENSECAG00000008453	5.05159736	0.7469066	0.9269215	688	610	627	748	679	363	766	514
ENSECAG00000015656	4.747341181	0.7470594	0.9269914	421	421	508	857	443	339	651	445
ENSECAG00000013737	4.785755827	0.7472031	0.9269914	607	445	578	597	554	488	433	402
ENSECAG00000009240	7.573092191	0.7472456	0.9269914	3430	3423	3923	4664	3278	3055	3610	3230
ENSECAG00000007150	7.020823928	0.7473153	0.9269914	2227	2708	2453	3083	2492	1943	2418	2133
ENSECAG00000014582	4.937462698	0.747371	0.9269914	714	451	575	542	757	307	864	401
ENSECAG00000021612	7.228540815	0.7475234	0.9270792	3240	2543	2819	3588	1551	2810	2723	3247
ENSECAG00000024958	6.218604407	0.7477093	0.9271777	939	1657	1247	1730	866	1579	1421	1642
ENSECAG00000018560	4.806687529	0.7479288	0.9271777	266	927	227	567	154	455	262	1254
ENSECAG00000021798	3.947331166	0.7480157	0.9271777	345	147	277	392	366	161	473	179
ENSECAG00000000455	1.840575267	0.7480612	0.9271777	59	50	53	101	55	60	75	72
ENSECAG00000022088	7.575076267	0.7480888	0.9271777	3095	3327	3889	5271	3137	3492	4201	2335
ENSECAG00000013644	3.845242392	0.748169	0.9271777	276	246	278	264	162	306	270	322
ENSECAG00000010787	5.734564625	0.7482302	0.9271777	1078	953	931	1347	702	656	1299	1104
ENSECAG00000010566	4.240479635	0.7482558	0.9271777	262	354	360	436	228	407	335	421
ENSECAG00000017207	5.020746017	0.7485643	0.9274588	768	524	596	530	746	436	733	509
ENSECAG00000000220	3.234815209	0.7487111	0.9275395	174	204	186	187	139	177	179	142
ENSECAG00000010957	3.521083069	0.748794	0.9275411	220	217	237	249	145	128	274	253
ENSECAG00000024911	2.976597813	0.7489127	0.9275721	210	109	156	159	130	91	177	143
ENSECAG00000008478	4.194567727	0.7492296	0.9275721	302	262	359	461	305	332	330	382
ENSECAG00000008212	7.076415012	0.749238	0.9275721	2808	1833	2698	2846	2187	3035	1853	2770
ENSECAG00000024606	6.164980738	0.7492802	0.9275721	1480	1145	1291	1913	1261	972	1589	1205
ENSECAG00000004444	6.24764366	0.7493774	0.9275721	2003	1334	1271	1005	1303	792	2380	1361
ENSECAG00000013570	3.795417895	0.7493991	0.9275721	247	292	197	389	180	210	266	298
ENSECAG00000014515	0.934399126	0.7494434	0.9275721	11	69	25	45	17	2	26	77
ENSECAG00000014883	5.926373024	0.7495302	0.9275721	1356	831	1227	1570	1079	422	1875	970
ENSECAG00000016947	5.630196009	0.7496332	0.9275721	916	903	711	1219	925	834	887	1004
ENSECAG00000022532	6.727239152	0.7496357	0.9275721	2141	2208	2427	1663	1381	1809	1998	2145
ENSECAG00000012553	3.742661596	0.7498337	0.9276623	270	269	271	261	191	161	364	221
ENSECAG00000016335	2.74167971	0.7498719	0.9276623	133	94	135	131	124	117	135	116
ENSECAG00000020494	3.855368606	0.7500094	0.9277313	200	122	362	375	41	104	110	872
ENSECAG00000000348	6.997420995	0.7502071	0.9277432	2275	2182	2286	2906	2712	1686	3207	2000
ENSECAG00000008514	7.124077991	0.7502576	0.9277432	2884	2303	2949	3152	2811	2167	2513	2134
ENSECAG00000027684	11.70078293	0.750264	0.9277432	60623	61878	53590	74026	65988	66314	62109	51029
ENSECAG00000011497	5.897270506	0.750407	0.9277766	973	1113	1200	1536	775	983	1111	1268
ENSECAG00000024689	5.23318898	0.7505058	0.9277766	709	619	861	849	804	411	791	621
ENSECAG00000009207	2.904351388	0.7507447	0.9277766	160	105	141	147	159	120	152	121
ENSECAG00000008676	9.961184519	0.7507735	0.9277766	24699	15302	20210	20614	20946	15322	16366	15745
ENSECAG00000009556	6.478103642	0.750796	0.9277766	2244	1091	939	3174	1332	988	2744	1249
ENSECAG00000022552	4.557353424	0.7508582	0.9277766	456	498	468	461	466	446	392	294
ENSECAG00000015742	7.606234003	0.7509062	0.9277766	3655	3164	3853	5264	2742	4241	3955	2392
ENSECAG00000018417	5.339474279	0.7509445	0.9277766	758	584	867	854	679	691	842	786
ENSECAG00000006923	5.317932203	0.7510694	0.92783	832	568	780	1073	745	734	727	527
ENSECAG00000007248	5.94958025	0.7512137	0.9278901	1214	1123	1133	1515	1090	945	1343	934
ENSECAG00000012588	4.864022265	0.7512814	0.9278901	533	492	571	590	426	547	624	559
ENSECAG00000014055	4.87512147	0.751567	0.928142	604	407	655	736	703	227	772	357
ENSECAG00000013048	3.441825064	0.7516667	0.9281642	230	194	201	250	185	120	241	207

ENSECAG00000010969	7.90655101	0.7519679	0.9283805	4963	4381	4424	5671	5302	3362	4481	3433
ENSECAG00000024388	2.369099241	0.7520054	0.9283805	204	4	204	19	215	29	41	36
ENSECAG00000005102	0.568333149	0.7522185	0.9285427	25	19	34	37	27	10	24	33
ENSECAG00000010240	6.037701509	0.7523488	0.9286027	1272	1112	1293	1647	1467	904	1370	823
ENSECAG00000021970	4.011815158	0.7525161	0.9286731	235	248	339	393	272	275	325	322
ENSECAG00000024705	3.873724706	0.7526536	0.9286731	12	821	10	53	8	387	12	743
ENSECAG00000016369	3.931438257	0.7527165	0.9286731	320	251	262	304	319	218	367	235
ENSECAG00000009667	5.222878915	0.7527329	0.9286731	770	510	865	660	754	527	948	570
ENSECAG00000020667	5.874852706	0.7528241	0.9286847	1061	1043	1156	1147	1153	1270	985	865
ENSECAG00000016192	7.565378096	0.7530963	0.9289196	3365	5085	3719	2876	2731	4414	2555	3019
ENSECAG00000015789	2.806186755	0.7531876	0.9289314	155	70	202	141	102	55	175	152
ENSECAG00000000753	6.743610198	0.7534886	0.9292017	2310	1702	2412	2221	2135	1256	2369	1753
ENSECAG00000023330	6.085491233	0.7536855	0.929292	1525	812	1363	1436	1731	727	1739	926
ENSECAG00000022271	2.409972645	0.7537254	0.929292	72	108	99	151	71	42	89	159
ENSECAG00000000031	5.733937063	0.7538117	0.9292974	1005	1005	890	1099	641	931	1330	1079
ENSECAG00000008867	5.303392247	0.7540996	0.9295223	722	700	759	781	650	887	609	726
ENSECAG00000023503	2.781060341	0.7542084	0.9295223	123	162	126	136	73	77	135	186
ENSECAG00000017322	2.489355927	0.7543173	0.9295223	86	90	81	157	65	90	81	179
ENSECAG00000007913	4.970018336	0.7543686	0.9295223	582	592	690	650	568	573	492	507
ENSECAG00000016031	5.413173777	0.7544032	0.9295223	994	557	730	952	644	654	972	913
ENSECAG00000022389	9.373983657	0.7547721	0.9298545	13036	11220	12528	13061	12000	12961	12895	11143
ENSECAG00000023041	4.965820884	0.7550007	0.9298545	531	534	585	894	451	419	642	669
ENSECAG00000002434	3.146408716	0.7550431	0.9298545	149	176	137	191	111	174	175	193
ENSECAG00000002165	2.495645136	0.7550755	0.9298545	146	99	153	53	19	133	64	149
ENSECAG00000015797	2.428010966	0.7550822	0.9298545	115	122	71	77	99	84	92	121
ENSECAG00000020695	4.635338661	0.7551699	0.9298595	744	267	567	440	447	255	730	317
ENSECAG00000006315	5.703113357	0.7552499	0.9298595	1218	868	1304	772	1212	625	1006	770
ENSECAG00000017604	6.775191664	0.7553532	0.9298859	2180	2073	1719	2266	1783	1863	2281	2203
ENSECAG00000010959	5.636016047	0.7555372	0.9300116	766	882	891	1237	850	813	1039	982
ENSECAG00000010148	2.670363637	0.7557647	0.9300987	99	119	136	160	62	139	86	134
ENSECAG00000009855	4.161591632	0.7557717	0.9300987	313	279	378	486	372	221	363	282
ENSECAG00000023946	2.34583982	0.7559418	0.9301104	87	146	90	75	126	46	76	92
ENSECAG00000026999	4.176100023	0.7559724	0.9301104	274	364	323	373	148	525	262	379
ENSECAG00000015449	6.776516677	0.7560641	0.9301104	2216	2220	2296	1345	2194	1190	2465	2427
ENSECAG00000010153	5.427548396	0.7561088	0.9301104	691	707	709	1063	92	1562	393	1085
ENSECAG00000009169	5.189984649	0.7562444	0.9301765	652	701	618	981	529	625	715	659
ENSECAG00000007536	6.432158274	0.7565348	0.9303663	2328	1977	1652	908	2433	986	1848	664
ENSECAG00000015364	0.580586559	0.7565816	0.9303663	18	17	37	46	40	10	30	14
ENSECAG00000017156	5.502189297	0.7567096	0.9303663	716	929	954	764	507	1085	725	996
ENSECAG00000006082	1.057180463	0.7567911	0.9303663	36	38	31	40	61	21	17	49
ENSECAG00000009427	0.446426645	0.7568082	0.9303663	23	14	34	21	7	36	25	27
ENSECAG00000011724	0.519528297	0.7570437	0.9304478	23	18	27	45	20	15	54	3
ENSECAG00000019570	5.249202711	0.7570627	0.9304478	804	637	878	748	453	927	455	715
ENSECAG00000024104	0.83894871	0.7571202	0.9304478	27	31	44	20	24	40	26	36
ENSECAG00000013929	6.576801324	0.7575204	0.9307012	1832	1739	2094	2002	1562	1828	1403	1742
ENSECAG00000014614	5.154736211	0.7575842	0.9307012	696	593	592	810	547	468	884	780
ENSECAG00000006219	4.737370474	0.7576399	0.9307012	565	465	621	487	568	268	568	458
ENSECAG00000010802	2.560259636	0.7576542	0.9307012	101	120	126	122	98	73	110	120
ENSECAG00000003181	5.732788996	0.7577384	0.930704	1241	594	1210	1322	772	441	1882	728
ENSECAG00000023883	4.787990018	0.7578923	0.9307923	579	518	579	534	468	471	434	520
ENSECAG00000006505	6.745924314	0.7582033	0.9310737	1871	1847	2154	2223	2039	2172	2037	1623
ENSECAG00000015324	5.664604982	0.758485	0.9313189	1135	666	908	1130	1121	1064	1019	514
ENSECAG00000005386	2.730500975	0.7586467	0.9313981	150	103	122	161	68	149	102	122
ENSECAG00000009628	8.059413477	0.7587135	0.9313981	5318	4516	4462	5850	4706	4764	5598	4716
ENSECAG00000000906	6.702068511	0.7589403	0.9315656	1669	2268	1750	2778	818	2017	1566	2721
ENSECAG00000021909	3.060820649	0.7590683	0.9315656	161	147	184	178	147	146	96	168
ENSECAG00000012054	2.252356293	0.759176	0.9315656	37	198	40	102	31	60	47	174
ENSECAG00000016897	4.147666171	0.759178	0.9315656	304	338	374	410	322	330	257	292
ENSECAG00000000946	1.403858381	0.759275	0.931584	5	82	27	65	13	8	48	136
ENSECAG00000003934	4.634140737	0.7594263	0.931669	384	340	464	705	584	313	521	432
ENSECAG00000004167	1.285270423	0.7596586	0.9317788	27	37	54	79	10	21	49	81
ENSECAG00000000796	4.916269236	0.7596799	0.9317788	577	480	575	646	532	561	533	589
ENSECAG00000015307	5.644664422	0.7599188	0.9319712	1097	960	959	980	890	895	935	732
ENSECAG00000020743	4.966986138	0.7600492	0.9320305	552	441	589	1007	630	595	710	198
ENSECAG00000021097	9.020936563	0.7604442	0.932263	9046	10943	8634	13471	7807	9927	9301	8634
ENSECAG00000021925	3.263580772	0.7604695	0.932263	170	192	190	218	103	161	205	190
ENSECAG00000000665	6.475806308	0.760489	0.932263	1634	1903	1712	1866	1506	1677	1735	1226
ENSECAG00000026974	3.595625691	0.7605671	0.932263	201	149	237	324	339	119	248	197
ENSECAG00000009278	6.646146623	0.7607468	0.9322878	2425	1342	2229	2223	3057	733	2485	689
ENSECAG00000014807	3.395673081	0.7607514	0.9322878	150	218	207	201	203	190	219	163
ENSECAG00000000580	7.35147833	0.7610004	0.9324923	2814	2834	3126	3559	2503	3076	3038	3438
ENSECAG00000002702	3.318963247	0.7611052	0.9325201	162	157	161	266	111	190	140	293
ENSECAG00000018965	5.944268659	0.7613337	0.9326994	1086	1141	1276	1448	1079	1032	1170	989
ENSECAG00000000642	8.079729788	0.7615869	0.932909	5789	4533	4412	5656	5048	3474	6660	5200
ENSECAG00000026307	1.042706035	0.7618533	0.9329171	138	23	1	5	3	33	2	83
ENSECAG00000015522	4.733020185	0.761872	0.9329171	518	469	563	597	403	647	423	316
ENSECAG00000018265	6.802431157	0.7619771	0.9329171	1678	2116	1800	2844	1374	2742	2014	2040
ENSECAG00000012083	10.06233538	0.7620017	0.9329171	21745	18167	22892	23699	21321	14751	21957	16372

ENSECAG00000014563	4.324623546	0.7620041	0.9329171	490	313	305	389	451	368	406	249
ENSECAG00000019687	4.171224967	0.7624442	0.9332899	365	409	273	265	135	368	321	516
ENSECAG00000009006	3.008549151	0.7624729	0.9332899	157	197	110	177	100	99	209	150
ENSECAG00000015074	2.9472523	0.7627022	0.9333305	105	162	123	174	76	200	92	191
ENSECAG00000012404	3.948121765	0.7628809	0.9333305	321	224	295	415	312	218	292	239
ENSECAG00000001583	1.892956018	0.7630207	0.9333305	61	75	77	52	45	65	88	75
ENSECAG00000016892	5.491018037	0.7630271	0.9333305	807	453	855	1351	898	756	1086	608
ENSECAG00000010688	0.710577837	0.7630364	0.9333305	15	22	36	41	21	15	51	33
ENSECAG00000007927	4.92697339	0.7631238	0.9333305	605	561	567	713	569	421	542	577
ENSECAG00000007966	5.963070956	0.7631726	0.9333305	1352	919	1133	1323	1307	854	1545	956
ENSECAG00000017072	2.828087787	0.763363	0.9333305	90	86	157	200	153	115	134	120
ENSECAG00000014391	5.982248303	0.7634353	0.9333305	1364	813	1218	1417	1124	1162	1480	915
ENSECAG00000014949	3.716589925	0.7634611	0.9333305	253	238	223	246	57	306	168	442
ENSECAG00000008555	7.191145025	0.7634745	0.9333305	2870	1534	2839	4994	3406	1744	3960	1075
ENSECAG00000009096	5.87307797	0.7634921	0.9333305	780	1004	760	1902	462	1916	755	1102
ENSECAG00000009500	3.152343361	0.7636434	0.933415	156	168	205	181	139	135	164	169
ENSECAG00000021401	6.686966784	0.7637855	0.9334882	2167	1870	2049	2182	1911	1564	2161	1550
ENSECAG00000009764	5.523513256	0.76395	0.9335888	913	805	987	986	883	722	984	612
ENSECAG00000017807	0.785821102	0.7641233	0.9337002	43	17	26	34	57	18	25	22
ENSECAG00000020931	4.768418478	0.7645373	0.9341056	525	469	542	663	529	408	440	499
ENSECAG00000023318	6.514676601	0.7647243	0.9341119	1380	2205	1642	1538	1611	858	2282	2198
ENSECAG00000016841	6.885949767	0.7647659	0.9341119	1776	2362	1900	2901	1762	2236	2661	2121
ENSECAG00000009102	8.04437275	0.7648541	0.9341119	4777	4639	4660	5843	3837	5814	4724	4999
ENSECAG00000011411	6.71219672	0.7650166	0.9341119	2057	1980	1662	2202	2167	1721	2171	1693
ENSECAG00000012309	4.774361364	0.7650452	0.9341119	541	560	455	641	395	522	460	498
ENSECAG00000013456	4.341222808	0.7651059	0.9341119	424	349	449	408	176	351	408	466
ENSECAG00000009156	4.974616995	0.7652339	0.9341119	702	380	611	686	752	371	785	452
ENSECAG00000018201	3.535147362	0.7652762	0.9341119	177	172	253	267	255	192	191	211
ENSECAG00000016819	4.791479985	0.7652842	0.9341119	551	472	553	658	600	353	518	448
ENSECAG00000013794	1.382813493	0.7653759	0.9341119	38	48	54	66	38	55	41	34
ENSECAG00000011795	2.682950549	0.7654923	0.9341119	108	127	137	142	71	53	164	159
ENSECAG00000021430	8.222563138	0.7655694	0.9341119	5317	6753	5623	4347	4834	3662	6183	7824
ENSECAG00000000282	3.417691343	0.7657003	0.9341119	168	165	213	331	102	149	168	311
ENSECAG00000020376	1.663075862	0.7657101	0.9341119	71	59	74	43	43	49	42	74
ENSECAG00000022122	5.258069329	0.7657761	0.9341119	622	697	761	800	783	759	720	536
ENSECAG00000024593	0.991978587	0.7659459	0.9342187	31	41	40	24	24	15	38	69
ENSECAG00000019388	5.134729786	0.7662346	0.9344067	771	493	706	883	768	439	752	489
ENSECAG00000019294	1.239334046	0.7662645	0.9344067	68	23	40	57	34	8	75	43
ENSECAG00000004119	3.327871843	0.766582	0.9345568	243	133	239	193	210	104	215	164
ENSECAG00000008163	7.057211832	0.766623	0.9345568	2399	2422	2405	2825	2363	2286	3048	2194
ENSECAG00000022756	4.984577308	0.7666374	0.9345568	620	585	789	542	356	823	489	459
ENSECAG00000001581	6.010059575	0.7667167	0.9345568	1103	1364	1233	1475	750	1296	951	1417
ENSECAG00000020879	6.405102313	0.7669262	0.9345929	1438	1525	1991	1858	1408	1042	2141	1407
ENSECAG00000019597	5.499697272	0.7670028	0.9345929	1035	669	954	984	752	619	928	869
ENSECAG00000004897	4.496866341	0.7674092	0.9345929	118	1006	218	218	453	406	408	427
ENSECAG00000022067	3.918160436	0.7675987	0.9345929	240	269	315	398	248	245	305	243
ENSECAG00000013563	5.743447534	0.7676769	0.9345929	972	975	1186	1159	948	781	1129	890
ENSECAG00000018921	2.298714289	0.7676962	0.9345929	97	71	102	88	75	45	107	142
ENSECAG00000024770	3.833852146	0.7678084	0.9345929	270	278	281	317	150	194	244	396
ENSECAG00000016589	5.340073441	0.7678305	0.9345929	657	828	730	816	687	941	823	509
ENSECAG00000020730	5.722739979	0.7679737	0.9345929	940	1168	1119	968	729	1024	964	926
ENSECAG00000023044	6.140870293	0.7679804	0.9345929	1634	1070	1296	1701	1304	947	1392	1292
ENSECAG00000021355	4.756510147	0.7680071	0.9345929	477	513	410	634	639	326	549	496
ENSECAG00000023271	5.662981373	0.7682254	0.9345929	858	867	1053	1317	856	896	956	799
ENSECAG00000005643	3.740864928	0.7682315	0.9345929	210	402	173	276	163	213	184	347
ENSECAG00000012312	7.523164686	0.7682959	0.9345929	3360	3289	3829	3318	2606	3442	3525	4034
ENSECAG00000018271	6.417781577	0.7683428	0.9345929	1630	1420	1616	1806	1787	1615	1573	1280
ENSECAG00000018059	3.828313284	0.7683883	0.9345929	434	176	256	284	322	169	339	151
ENSECAG00000018527	7.866208114	0.7684	0.9345929	4154	4028	4578	4840	4986	3425	5344	3644.02
ENSECAG00000024976	5.602387815	0.7684491	0.9345929	958	852	854	1264	643	651	963	1150
ENSECAG00000022061	6.504455822	0.7684588	0.9345929	2276	1269	1980	1813	2173	874	2137	1195
ENSECAG00000006117	5.791583913	0.7684713	0.9345929	846	1471	898	850	529	1064	1174	1363
ENSECAG00000001649	9.475599555	0.7684742	0.9345929	13900	15532	17555	9682	8352	10381	12461	18196
ENSECAG00000014234	5.580616247	0.7686713	0.9347326	907	636	936	1159	1086	528	1427	587
ENSECAG00000008131	7.608318765	0.768867	0.9348705	4285	2694	3799	3995	3584	2427	4543	4115
ENSECAG00000022635	7.92040275	0.7691991	0.9351383	4362	3926	4689	5350	3625	3038	5895	5740
ENSECAG00000000253	4.882572463	0.7692519	0.9351383	530	496	640	715	504	560	498	458
ENSECAG00000020276	6.30422957	0.7693944	0.9352114	1701	1335	1663	1635	1402	1086	1600	1448
ENSECAG00000026992	4.275468593	0.7699957	0.9357426	289	365	356	561	251	330	443	320
ENSECAG00000024129	5.906724642	0.7699961	0.9357426	1118	1135	1170	1390	1086	797	1241	1083
ENSECAG00000007689	5.911472363	0.7703482	0.9359754	996	947	1212	1430	1052	1121	1274	984
ENSECAG00000008915	6.645135895	0.770426	0.9359754	1738	1827	1856	2691	1214	1475	1889	2419
ENSECAG00000005101	3.067678316	0.7704349	0.9359754	156	100	178	192	193	144	186	95
ENSECAG00000020838	7.502312282	0.7705796	0.9360511	3320	3337	3011	4036	3129	2849	3840	3665
ENSECAG00000023870	6.071118542	0.770789	0.9361572	1383	950	1557	1542	1261	843	1355	1253
ENSECAG00000010711	6.954329844	0.770909	0.9361572	2336	2239	2072	2721	1987	2660	2188	2234
ENSECAG00000012166	5.637218554	0.7709546	0.9361572	13	2802	11	273	83	1191	46	2587
ENSECAG00000026989	2.782911447	0.7709966	0.9361572	163	78	133	187	99	63	189	128

ENSECAG00000026908	4.608129186	0.7711219	0.9362092	520	421	490	525	402	367	431	488
ENSECAG00000025164	3.494184243	0.7714685	0.9362785	224	193	209	211	170	168	286	218
ENSECAG00000005178	1.628026832	0.7715341	0.9362785	69	58	53	63	32	32	92	55
ENSECAG00000021383	1.796123266	0.771606	0.9362785	33	56	143	3	56	83	102	20
ENSECAG00000010726	1.271898665	0.7716475	0.9362785	30	53	52	32	15	30	66	69
ENSECAG00000017952	3.413674476	0.7716523	0.9362785	172	189	280	132	152	350	159	103
ENSECAG00000000967	2.093319109	0.7716735	0.9362785	74	60	90	121	41	104	72	63
ENSECAG00000011242	5.468331722	0.7717685	0.9362825	940	770	978	875	404	474	932	1313
ENSECAG00000014063	6.209639499	0.7718989	0.9362825	1375	1030	1588	1643	1456	1044	1797	1224
ENSECAG00000008277	1.26179231	0.7719241	0.9362825	44	41	64	37	23	35	68	34
ENSECAG00000010167	2.926450145	0.7724776	0.9366477	151	136	139	132	118	95	182	174
ENSECAG00000010902	4.75889598	0.7724978	0.9366477	441	473	577	534	282	675	449	568
ENSECAG00000000113	3.297548203	0.7725549	0.9366477	182	213	227	158	106	114	176	282
ENSECAG00000021937	4.174206491	0.7726731	0.9366477	274	263	350	483	390	205	450	310
ENSECAG00000010435	3.925487477	0.7727182	0.9366477	195	272	262	415	315	223	429	174
ENSECAG00000014846	6.079308106	0.7727199	0.9366477	1291	1086	1434	1297	1155	941	1281	1639
ENSECAG00000019532	5.069098218	0.7729796	0.9367614	580	593	673	684	598	606	679	586
ENSECAG00000018523	4.793158077	0.7730375	0.9367614	601	382	545	562	666	317	652	439
ENSECAG00000000298	6.702172499	0.7730611	0.9367614	1888	1795	1825	2384	1591	1269	2543	2442
ENSECAG00000020356	6.373937709	0.7731695	0.9367928	1771	1520	1708.98	1182	1976	1153	1667	1346.05
ENSECAG00000010400	3.139806598	0.7733511	0.9368223	192	138	168	214	112	81	209	213
ENSECAG00000023144	6.277330755	0.7733588	0.9368223	1479	1462	1259	2069	1029	1513	1308	1496
ENSECAG00000011931	4.600408193	0.7735868	0.9369986	328	637	340	482	258	460	423	656
ENSECAG00000017250	2.621372646	0.7738705	0.9371536	96	109	126	164	92	112	109	101
ENSECAG00000020403	4.939629779	0.7738798	0.9371536	653	576	563	664	551	410	623	559
ENSECAG00000006648	4.1054742	0.7742194	0.9372848	271	280	363	378	411	191	278	394
ENSECAG00000012176	5.057955156	0.7744594	0.9372848	573	524	601	834	406	602	694	766
ENSECAG00000008796	3.261633577	0.7744797	0.9372848	117	181	189	218	66	288	103	235
ENSECAG00000019794	3.072256873	0.7745236	0.9372848	148	169	148	209	127	126	202	125
ENSECAG00000000631	7.75296949	0.7745427	0.9372848	4094	4572	3264	5462	3710	2998	4927	3497
ENSECAG00000012943	4.650544917	0.7745971	0.9372848	504	400	439	553	504	302	504	559
ENSECAG00000011080	7.706863768	0.7746965	0.9372848	4130	4504	3894	4154	3064	3522	4309	3656
ENSECAG00000020524	3.00832421	0.7747731	0.9372848	142	146	191	164	147	137	97	158
ENSECAG00000000746	3.167407119	0.775022	0.9372848	212	80	171	208	262	118	218	72
ENSECAG00000015512	4.051360071	0.7751083	0.9372848	332	267	351	282	339	350	368	160
ENSECAG00000000629	4.082982668	0.7751258	0.9372848	325	274	332	339	405	220	358	273
ENSECAG00000012048	7.679968273	0.775148	0.9372848	3881	4105	4121	4436	2163	5389	2665	3552
ENSECAG00000017052	5.733989587	0.7753433	0.9372848	1032	1050	1015	1157	719	858	1262	902
ENSECAG00000012174	4.512585098	0.7753636	0.9372848	550	359	433	492	351	290	506	453
ENSECAG00000018888	4.481328359	0.7753772	0.9372848	329	569	479	411	132	603	239	504
ENSECAG00000023080	4.063467656	0.7754008	0.9372848	258	293	305	403	236	245	344	419
ENSECAG00000005243	4.820857274	0.7754503	0.9372848	563	538	510	657	412	437	519	594
ENSECAG00000014028	3.767083662	0.7754734	0.9372848	274	202	297	326	232	220	212	266
ENSECAG00000015197	3.442247767	0.7758412	0.9376296	179	228	201	192	166	253	171	198
ENSECAG00000010974	2.836157741	0.7760182	0.9376882	106	187	121	99	174	135	124	87
ENSECAG00000021507	7.854152375	0.7763033	0.9376882	4073	3963	4273	5212	3315	4725	4203	4768
ENSECAG00000019406	4.535218113	0.776339	0.9376882	447	410	413	472	493	396	461	353
ENSECAG00000014575	5.614714082	0.7763568	0.9376882	880	895	916	1008	665	913	1014	1022
ENSECAG00000000286	6.595709671	0.776387	0.9376882	1854	1656	1952	2331	1388	1802	1699	1806
ENSECAG00000024393	5.222370438	0.7763921	0.9376882	744	570	699	1019	500	528	669	906
ENSECAG00000010295	4.988750267	0.7764676	0.9376882	573	575	621	799	337	386	601	900
ENSECAG00000012019	8.89326392	0.7769976	0.9380443	10023	7153	7822	11045	12267	5381	10705	7290
ENSECAG00000002992	4.537494838	0.7771903	0.9380443	190	453	328	797	353	373	421	580
ENSECAG00000018754	6.137126055	0.7772526	0.9380443	1557	1088	1525	1487	1467	1163	1238	994
ENSECAG00000021885	7.203325384	0.7773058	0.9380443	2880	2522	2635	3108	2925	2331	3473	2232
ENSECAG00000018623	5.949211467	0.777519	0.9380443	1044	889	1011	1805	947	748	1519	1438
ENSECAG00000017951	6.088251642	0.7775668	0.9380443	1389	1150	1312	1286	1033	1279	1286	1398
ENSECAG00000006359	2.183669415	0.7776141	0.9380443	93	38	96	106	119	18	146	65
ENSECAG00000009482	6.945419306	0.7776555	0.9380443	2263	2293	2168	3245	2636	1751	2128	2027
ENSECAG00000007738	2.28999932	0.7777555	0.9380443	93	78	73	117	108	64	101	86
ENSECAG00000023319	0.913338184	0.7777778	0.9380443	25	40	32	33	21	20	30	65
ENSECAG00000005795	5.005764853	0.7777975	0.9380443	541	664	756	606	358	372	584	934
ENSECAG00000001324	4.739037963	0.7778062	0.9380443	650	628	478	158	651	471	622	248
ENSECAG00000024966	4.9580317	0.7778361	0.9380443	504	580	647	779	713	513	592	316
ENSECAG00000021502	2.881360755	0.7780988	0.9381435	131	102	130	189	150	108	150	135
ENSECAG00000022225	5.987957963	0.7781156	0.9381435	1112	1050	966	2070	1083	1081	1519	743
ENSECAG00000012628	0.198990628	0.778166	0.9381435	10	32	20	23	11	8	12	40
ENSECAG00000015142	6.411117806	0.7783776	0.938299	1865	1445	1612	1911	1509	1227	1568	1638
ENSECAG00000000469	4.654223001	0.7786205	0.9383292	541	414	578	483	313	372	479	589
ENSECAG00000000506	1.214304104	0.778745	0.9383292	31	33	68	51	22	53	24	48
ENSECAG00000011406	6.641288997	0.7788969	0.9383292	2270	1672	2427	1628	1450	760	2836	2112
ENSECAG00000017496	5.923769214	0.778974	0.9383292	1138	969	1312	1489	1075	554	1419	1261
ENSECAG00000016200	5.778101378	0.779088	0.9383292	867	1022	1179	1067	914	1113	1162	837
ENSECAG00000023311	6.242697304	0.7792216	0.9383292	1403	1014	1442	1908	981	627	1931	2253
ENSECAG00000019064	7.050000074	0.7792326	0.9383292	2765	2104	2895	2912	2409	2122	2658	2033
ENSECAG00000006983	6.654092909	0.7793057	0.9383292	2039	2145	1537	1798	1857.99	2250	2003	1248
ENSECAG00000026853	3.844924782	0.7793532	0.9383292	262	231	227	366	231	231	330	274
ENSECAG00000016016	3.271794303	0.7793685	0.9383292	176	153	191	267	101	62	245	272

ENSECAG00000010008	4.754063314	0.7794614	0.9383292	349	563	561	720	213	555	294	759
ENSECAG00000020781	6.023798979	0.7795321	0.9383292	1092	1242	1527	1335	1221	977	1286	1055
ENSECAG00000006545	5.770702337	0.7795325	0.9383292	1005	879	1046	1219	1144	972	1118	772
ENSECAG00000017053	4.960138978	0.7795592	0.9383292	483	653	457	754	474	501	750	594
ENSECAG00000021835	6.771728092	0.7796418	0.9383292	2082	1682	2136	2380	2812	1859	1992	1347
ENSECAG00000001515	0.801099272	0.779778	0.9383938	36	27	33	24	55	13	29	27
ENSECAG00000005273	1.273769338	0.7798965	0.938437	45	52	52	37	56	29	38	35
ENSECAG00000010792	7.165910269	0.7800325	0.9385012	2657	2991	2625	3241	2630	2525	2813	1974
ENSECAG00000023315	8.316413961	0.7801463	0.9385387	7067	5145	6528	7000	6334	4611	7218	4113
ENSECAG00000019517	2.992811419	0.7802439	0.9385566	120	154	138	178	160	148	133	138
ENSECAG00000020240	6.642611746	0.780471	0.9387305	2452	1699	2280	1553	2496	985	2264	1261
ENSECAG00000021086	2.735113993	0.7805944	0.93875	133	92	135	178	79	97	144	137
ENSECAG00000000678	6.228535963	0.7807384	0.93875	1376	1470	1452	1704	1262	1284	1538	1141
ENSECAG00000013694	3.852555223	0.7807516	0.93875	208	233	264	388	219	221	260	368
ENSECAG00000022460	6.961642304	0.7808179	0.93875	2405	2307	2308	3019	1935	1634	2545	2646
ENSECAG00000012499	6.806092559	0.781177	0.939071	2332	1689	2335	2093	2066	1234	2592	2521
ENSECAG00000022897	1.941233991	0.7813404	0.939071	80	48	104	73	62.0004	71.0001	55	65
ENSECAG00000024983	6.359297815	0.7814175	0.939071	1626	1301	1815	1860	1531	1177	1699	1345
ENSECAG00000019593	8.422834058	0.7816644	0.939071	8134	7106	6025	4062	6604	5943	6844	6030
ENSECAG00000022520	6.299783474	0.7817299	0.939071	1489	1251	1628	1583	1379	1913	958	1416
ENSECAG00000021963	5.391626992	0.7817663	0.939071	849	767	812	719	578	841	783	876
ENSECAG00000016209	5.503534522	0.7817699	0.939071	916	854	826	1035	861	743	841	703
ENSECAG00000011253	5.54638074	0.7818008	0.939071	724	719	852	1273	537	546	1142	1311
ENSECAG00000005017	1.059866421	0.7818289	0.939071	29	43	38	36	31	32	28	57
ENSECAG00000017124	4.774309916	0.7819255	0.9390878	491	396	522	672	546	312	705	493
ENSECAG00000007901	0.808920422	0.782059	0.9390941	30	14	35	45	32	11	36	48
ENSECAG00000004662	1.327693891	0.7821214	0.9390941	36	44	78	37	49	24	50	43
ENSECAG00000003681	0.686842438	0.7821788	0.9390941	21	29	29	33	27	36	24	24
ENSECAG00000011958	4.107005895	0.7822744	0.9391096	341	315	271	366	322	254	334	362
ENSECAG00000014983	5.21624291	0.7825928	0.9393	709	532	835	935	808	575	702	486
ENSECAG00000017635	5.12159483	0.7826651	0.9393	760	567	676	793	621	527	683	594
ENSECAG00000000698	2.161902432	0.7827566	0.9393	59	137	72	82	58	93	45	96
ENSECAG00000012525	5.977291459	0.7827638	0.9393	1072	988	1226	1511	1137	983	1848	759
ENSECAG00000024890	2.688935126	0.7828664	0.9393239	114	118	136	146	94	94	139	115
ENSECAG00000018698	6.073434505	0.7831458	0.9395599	1353	1217	1007	1518	1137	686	1502	1741
ENSECAG00000010532	8.308923756	0.7833693	0.939603	5993	5432	6933	7254	4553	6604	4876	5665
ENSECAG00000022693	8.068359151	0.783386	0.939603	6179	4140	5653	5729	6263	3099	6265	3188
ENSECAG00000005459	6.664333452	0.783545	0.939603	2200	1863	1995	2047	1651	1259	2227	2025
ENSECAG00000000104	3.459439308	0.7836029	0.939603	181	157	210	283	194	157	242	223
ENSECAG00000011223	2.560616666	0.7837513	0.939603	27	260	47	62	23	127	43	247
ENSECAG00000020322	4.346449517	0.7838608	0.939603	302	338	420	470	212	496	291	479
ENSECAG00000005065	2.798366591	0.78398	0.939603	118	82	120	205	119	124	174	97
ENSECAG00000013654	4.291592341	0.7843612	0.939603	361	316	405	391	304	409	362.002	354
ENSECAG00000005597	2.401147623	0.7843734	0.939603	145	69	94	115	113	69	112	64
ENSECAG00000017520	0.939838145	0.7843839	0.939603	3	86	11	23	10	24	20	88
ENSECAG00000009630	0.180559022	0.7846279	0.939603	14	13	21	39	9	8	23	31
ENSECAG00000001194	3.553781743	0.7846494	0.939603	262	268	204	193	144	221	194	241
ENSECAG00000023972	2.238110361	0.7847247	0.939603	102	73	85	84	110	40	111	90
ENSECAG00000021191	7.205940277	0.7847283	0.939603	3064	2590	2649	3616	2596	1657	3493	2703
ENSECAG00000006958	4.69047097	0.7849282	0.939603	446	475	485	536	430	536	400	510
ENSECAG00000015778	3.094021537	0.7851118	0.939603	252	138	151	78	166	164	154	141
ENSECAG00000017891	2.534929305	0.7851146	0.939603	78	68	122	165	109	55	203	75
ENSECAG00000013251	5.510994255	0.7851315	0.939603	815	921	915	985	818	715	759	871.999
ENSECAG00000016999	3.017017558	0.7851353	0.939603	146	115	159	239	77	133	150	193
ENSECAG00000006289	9.000443578	0.7851471	0.939603	9662	9258	10385	11999	9525	8099	10373	7644
ENSECAG00000018485	8.738193568	0.7852852	0.939603	8676	6977	8402	10732	10502	4686	10964	3815
ENSECAG00000000127	5.673621243	0.7852918	0.939603	1043	783	946	1112	952	920	954	909
ENSECAG00000013248	0.436405105	0.7854272	0.939603	36	22	24	19	18	13	43	14
ENSECAG00000009480	2.762719453	0.7854592	0.939603	134	87	147	136	116	81	143	164
ENSECAG00000008456	4.329831163	0.7855011	0.939603	444	280	388	523	345	149	543	392
ENSECAG00000004384	0.774788332	0.7855245	0.939603	36	14	39	45	43	8	34	26
ENSECAG00000008984	2.51188892	0.7855409	0.939603	72	97	100	154	85	114	169	55
ENSECAG00000013766	1.347825499	0.7855685	0.939603	58	36	41	47	32	40	71	43
ENSECAG00000000811	3.985623672	0.7855805	0.939603	302	205	343	345	265	369	303	215
ENSECAG00000013656	1.243477286	0.7857632	0.9396134	49	20	31	73	51	32	53	35
ENSECAG00000023021	8.271687811	0.7858232	0.9396134	6416	5443	6148	6910	4227	6145	4399	6396
ENSECAG00000004193	2.066936455	0.7858812	0.9396134	56	51	55	150	95	29	149	47
ENSECAG00000007945	8.17239965	0.7859201	0.9396134	5838	5861	6247	5089	3114	3687	6630	6961
ENSECAG00000019221	2.86612385	0.7860397	0.9396574	135	101	133	176	109	100	126	203
ENSECAG00000007415	5.010652228	0.7863005	0.9396684	499	778	689	398	395	516	614	874
ENSECAG00000002362	1.624523278	0.7863422	0.9396684	79	41	79	17	79	38	66	43
ENSECAG00000014990	6.38311117	0.7864908	0.9396684	1566	1980	1230	1447	1329	1373	1687	1785
ENSECAG00000010292	1.816232599	0.7865075	0.9396684	71	31	81	75	49	53	93	65
ENSECAG00000017195	5.891522776	0.7865154	0.9396684	1015	1103	1136	1237	1141	1131	1084	976
ENSECAG00000013761	4.208308315	0.7865452	0.9396684	327	354	321	492	183	456	333	281
ENSECAG00000022573	6.35808684	0.786686	0.9396742	1918	1311	1554	1399	1474	1000	1879	1781
ENSECAG00000013242	6.129800278	0.7867252	0.9396742	1223	1559	1385	1408	889	1052	1089	1840
ENSECAG00000017447	6.333379927	0.7867983	0.9396742	1400	1699	1335	1575	807	1719	813	2549

ENSECAG00000000236	8.598523723	0.7870011	0.9398177	7702	7256	7320	8982	4786	5330	6955	10042
ENSECAG00000014069	3.619894078	0.7872852	0.940026	271	256	162	218	196	184	182	343
ENSECAG00000017998	6.371351674	0.7874041	0.940026	1605	1108	1399	2246	2030	1166	1848	1092
ENSECAG00000007860	0.048449525	0.7876467	0.940026	18	8	23	20	25	6	27	14
ENSECAG00000010458	5.379668911	0.7877324	0.940026	717	704	783	954	707	460	856	1089
ENSECAG00000012559	7.272444517	0.7877339	0.940026	3156	2879	3264	3065	2536	2852	2937	2381
ENSECAG00000012042	6.109180488	0.7877531	0.940026	1186	1518	1077	1781	673	931	1167	2055
ENSECAG00000023715	7.619780243	0.7877724	0.940026	4049	3227	4538	3993	3974	3375	3112	3070
ENSECAG00000008240	4.152017229	0.7878436	0.940026	388	330	379	318	280	170	418	386
ENSECAG00000012732	7.839468556	0.7879204	0.940026	3976	5410	3565	5381	3536	3982	4507.99	3883.99
ENSECAG00000014842	6.507649396	0.7881228	0.9401687	1919	1552	1862	1966	1525	947	2226	1778
ENSECAG00000012674	5.467308547	0.7882863	0.940265	959	697	960	929	881	683	768	736
ENSECAG00000001767	4.312884559	0.7885522	0.9404132	395	202	438	471	520	237	558	181
ENSECAG00000017935	7.263724399	0.7885761	0.9404132	2957	2847	2828	2925	1958	3352	2672	3267
ENSECAG00000014107	1.735891634	0.7888716	0.9405368	81	56	61	63	56	41	86	43
ENSECAG00000024980	3.187781629	0.7890271	0.9405368	122	221	157	158	51	287	88	229
ENSECAG00000003091	6.050234141	0.789067	0.9405368	1167	1071	1299	1514	971	1207	1298	1395
ENSECAG00000020681	6.929645096	0.7891445	0.9405368	2331	1952	2419	2553	2439	2058	2397	2074
ENSECAG00000005487	8.071126496	0.7892475	0.9405368	986	68	1425	18372	401	617	22500	680
ENSECAG00000017121	3.870197297	0.7892495	0.9405368	304	214	284	296	351	171	273	286
ENSECAG00000019655	3.744424686	0.7893257	0.9405368	249	235	275	317	207	277	238	190
ENSECAG00000007644	9.157759541	0.7893421	0.9405368	9979	10552	9876	12832	9996	11089	9971	10818
ENSECAG00000016472	5.553292218	0.789744	0.940917	778	871	856	1282	849	802	947	667
ENSECAG00000015912	5.553690965	0.7898952	0.9409388	721	1169	833	746	439	967	838	1219
ENSECAG00000012163	6.878702877	0.7900335	0.9409388	2427	1900	2627	1890	1702	2297	2226	2428
ENSECAG00000009529	6.598810322	0.7900458	0.9409388	1926	1705	2079	2040	1817	1473	1915	1571
ENSECAG00000017834	0.713746324	0.7900937	0.9409388	35	19	31	42	16	31	24	33
ENSECAG00000013346	5.055329282	0.7902077	0.9409759	672	637	599	752	462	649	620	566
ENSECAG00000012000	0.213441938	0.7903555	0.9410533	17	6	19	39	17	25	26	11
ENSECAG00000022273	2.795499781	0.7905845	0.9412099	117	103	159	180	101	98	166	114
ENSECAG00000022499	1.185734891	0.7907369	0.9412099	28	46	44	59	34	29	51	37
ENSECAG00000023381	4.120520399	0.790835	0.9412099	357	258	368	420	277	184	360	399
ENSECAG00000007315	4.296395647	0.7908557	0.9412099	353	374	360	491	329	392	353	271
ENSECAG00000017711	7.982966517	0.7909014	0.9412099	3825	5827	4369	6315	2978	3620	5907	5337
ENSECAG00000015499	7.629713656	0.7911359	0.9413904	4255	3582	3411	3664	4064	3031	4375	3220
ENSECAG00000023376	4.345259052	0.7913008	0.9414289	439	347	320	422	294	360	326	510
ENSECAG00000006749	6.965520786	0.791334	0.9414289	2928	1124	2279	3254	3036	1430	3593	1404
ENSECAG00000014505	5.744197481	0.7915269	0.9415597	915	1022	845	1272	783	1476	801	790
ENSECAG00000024528	3.710836124	0.791839	0.9418324	193	182	251	371	215	189	277	290
ENSECAG00000006331	4.292595896	0.7919762	0.9418684	406	302	417	345	413	250	483	315
ENSECAG00000024130	6.396609552	0.7920352	0.9418684	1793	1384	1458	1749	2127	1100	1853	1163
ENSECAG00000008013	0.541030626	0.7922579	0.9420185	20	28	16	37	18	34	24	24
ENSECAG00000000721	0.616721761	0.7923768	0.9420185	22	22	33	41	27	21	31	19
ENSECAG00000020421	4.79217491	0.7924101	0.9420185	581	445	515	691	429	410	629	475
ENSECAG00000008301	6.815855333	0.7925571	0.9420947	2052	2074	1945	2462	1654	1527	2656	2600
ENSECAG00000015558	7.194363991	0.7926784	0.9421402	3431	2185	3026	2370	3606	2134	3106	1999
ENSECAG00000011133	3.61814269	0.7928111	0.9421994	171	303	252	166	126	298	166	302
ENSECAG00000025021	5.541628521	0.7931141	0.9423972	850	847	930	1123	499	646	760	1349
ENSECAG00000022322	6.798911508	0.7932449	0.9423972	2373	1772	2245	2022	2095	1997	1770	2273
ENSECAG00000018079	7.017188509	0.7932712	0.9423972	2673	2127	2516	3128	1981	1710	2768	2674
ENSECAG00000016333	6.080718443	0.7933107	0.9423972	1316	1373	1164	1238	1376	1052	1428	1134
ENSECAG00000012677	8.91371471	0.7934601	0.9423972	9622	9315	9003	10785	8602	8592	9112	7085
ENSECAG00000025100	7.473555193	0.7935308	0.9423972	3672	2924	3424	3409	4036	2308	3993	2894
ENSECAG00000021987	4.4551036	0.793604	0.9423972	441	394	449	354	379	430	374	415
ENSECAG00000012384	5.703276979	0.7936413	0.9423972	938	814	1058	1159	940	1059	883	902
ENSECAG00000023177	4.878135269	0.794052	0.9427618	733	462	580	578	544	332	683	514
ENSECAG00000021778	0.897842777	0.7941876	0.9427618	7	71	25	20	11	24	32	69
ENSECAG00000008144	2.619258284	0.7942181	0.9427618	105	115	147	120	84	68	168	108
ENSECAG00000017459	4.481004806	0.7942804	0.9427618	293	688	364	419	206	436	268	604
ENSECAG00000012746	0.758211138	0.7944463	0.9428603	29	39	19	42	21	29	35	24
ENSECAG00000008107	8.19021897	0.7947732	0.9430367	4730	5910	5881	6945	4666	5249	5649	4711
ENSECAG00000008859	1.778473867	0.7948588	0.9430367	66	47	65	72	95	49	40	61
ENSECAG00000023288	7.27108082	0.7948617	0.9430367	3221	2336	3086	3062	3647	2431	3039	2255
ENSECAG00000014129	1.336275504	0.7949447	0.9430367	42	38	49	53	50	51	40	37
ENSECAG00000005387	6.022093864	0.7950373	0.9430367	1385	885	1339	1344	1272	953	1488	1103
ENSECAG00000019336	7.488929089	0.7951792	0.9430367	4051	2704	3827	3879	3456	2606	3425	3047
ENSECAG00000023254	6.819282177	0.7952131	0.9430367	2443	1943	2417	2226	2025	1474	2569	1897
ENSECAG00000024821	0.415695748	0.7952591	0.9430367	20	19.9967	18.997	32.9923	22.9999	23.999	33.9996	11.9999
ENSECAG00000013036	1.109663972	0.7954966	0.9431605	56	20	31	48	48	28	29	48
ENSECAG00000003357	5.999663481	0.7956522	0.9431605	1096	1341	1146	1232	1042	1287	1140	1196
ENSECAG00000016464	10.77066812	0.7956852	0.9431605	29018	34807	31423	36477	27068	35515	35245	30701
ENSECAG00000021317	2.907204794	0.7956957	0.9431605	164	121	134	182	98	139	126	146
ENSECAG00000009694	6.456671787	0.7958222	0.943212	1423	1334	1507	2494	1587	1346	1363	2154
ENSECAG00000010514	1.826375372	0.7959961	0.9433197	51	74	60	69	26	35	85	120
ENSECAG00000017715	2.412942472	0.7964177	0.9436957	91	85	94	121	95	28	203	85
ENSECAG00000011190	2.207788493	0.7964795	0.9436957	63	106	101.001	61	69	125	69	66
ENSECAG00000021345	2.605935022	0.796571	0.9437057	138	90	154	100	108	103	114	87
ENSECAG00000013791	5.922186849	0.7968724	0.9438671	1096	910	1173	1458	816	1272	1214	1136

ENSECAG00000011936	0.182891285	0.7968735	0.9438671	27	8	11	31	15	31	30	2
ENSECAG00000019146	3.266564431	0.7970458	0.9438864	160	173	208	230	135	128	173	228
ENSECAG00000027628	1.528193555	0.7970634	0.9438864	86	67	26	20	44	84	43	33
ENSECAG00000016944	2.64134005	0.797139	0.9438864	81	105	122	157	90	140	112	109
ENSECAG00000019218	4.729601025	0.7972743	0.9439482	414	429	565	605	506	320	581	568
ENSECAG00000007657	7.262953005	0.7975344	0.9441577	2617	2904	2531	3613	2512	3035	3198	2541
ENSECAG00000020268	3.109158556	0.797853	0.9443536	205	75	135	238	196	170	181	83.9174
ENSECAG00000006431	4.15301726	0.7978662	0.9443536	238	284	321	515	390	283	318	312
ENSECAG00000018023	0.605563377	0.798095	0.9443831	37	13	23	33	31	7	40	32
ENSECAG00000022486	4.160028119	0.7981404	0.9443831	329	284	361	374	428	264	338	281
ENSECAG00000017247	3.724644323	0.7981714	0.9443831	304	227	241	284	236	192	310	182
ENSECAG00000015050	0.261340994	0.7982236	0.9443831	29	19	24	17	32	15	22	5
ENSECAG00000001218	8.674361915	0.7985008	0.9446017	8150	6727	7444	8673	7329	8147	7031	7339
ENSECAG00000016603	8.556542151	0.7986255	0.9446017	6034	7428	6295	8751	5225	7923	8369	6253
ENSECAG00000019052	6.760160007	0.7986579	0.9446017	3126	1226	1068	3569	1606	795	2591	2708
ENSECAG00000019202	0.664126514	0.7988266	0.9446544	28	41	21	17	15	25	30	42
ENSECAG00000007272	7.22484874	0.7988688	0.9446544	2479	3171	2987	3331	3155	2446	2925	1850
ENSECAG00000012796	0.671100314	0.7995155	0.9452324	21	30	40	30	25	24	23	29
ENSECAG00000017913	1.079383005	0.7996112	0.9452324	15	39	48	64	25	40	31	40
ENSECAG00000013518	5.407373884	0.7997881	0.9452324	748	760	652	1079	721	663	872	875
ENSECAG00000006365	7.016347667	0.799809	0.9452324	2462	2252	2428	3295	1810	2161	2798	2310
ENSECAG00000013565	6.042542598	0.7998151	0.9452324	1336	1119	1031	1541	1387	999	1253	1202
ENSECAG00000015226	7.334649514	0.7998568	0.9452324	3089	2884	2959	4090	1983	2977	3099	3176
ENSECAG00000004993	4.311483433	0.7999786	0.9452358	366	446	355	407	402	280	380	316
ENSECAG00000022296	5.432010216	0.8000262	0.9452358	720	764	703	1108	790	882	769	697
ENSECAG00000015116	4.219027539	0.8002043	0.945348	324	344	306	425	194	276	314	598
ENSECAG00000010683	4.295492531	0.8003418	0.9453547	338	326	371	448	220	290	390	560
ENSECAG00000020503	4.952969089	0.8003765	0.9453547	392	166	702	1136	581	716	905	119
ENSECAG00000019248	4.881180732	0.8007896	0.9456615	594	505	545	581	571	413	565	624
ENSECAG00000022303	4.932837429	0.8008395	0.9456615	756	492	518	533	497	475	658	630
ENSECAG00000007648	3.636041192	0.8009214	0.9456615	270	210	255	186	142	217	236	322
ENSECAG00000016769	4.635442251	0.8009692	0.9456615	567	348	570	510	540	277	446	469
ENSECAG00000006208	0.654432534	0.8010945	0.9457111	14	22	47	39	22	20	16	41
ENSECAG00000024110	1.515418486	0.8012556	0.945803	140	4	92	0	80	1	103	0
ENSECAG00000017560	5.593398179	0.8014583	0.9458417	995	877	872	899	667	717	1070	1133
ENSECAG00000014556	5.796335288	0.8014666	0.9458417	1212	907	1134	1194	811	714	1437	995
ENSECAG00000023202	5.466836094	0.8017383	0.9458417	820	794	714	1039	799	798	856	782
ENSECAG00000000460	2.113628025	0.8018104	0.9458417	94	61	91	99	47	45	67	135
ENSECAG00000016068	5.434265977	0.8018233	0.9458417	936	719	754	1075	1115	527	760	601
ENSECAG00000015926	1.366817561	0.8018836	0.9458417	61	29	50	46	48	32	52	54
ENSECAG00000011603	7.684542912	0.8018841	0.9458417	4652	3281	4167	4463	4975	2819	3993	2521
ENSECAG00000016937	6.781646447	0.8019545	0.9458417	2309	1746	2381	2394	1833	2012	1836	1932
ENSECAG00000016883	8.137557639	0.8021871	0.9460178	4610	5569	5209	5858	4646	6515	5058	4264
ENSECAG00000023071	2.454177513	0.8023742	0.9460557	128	67	121	124	89	38	176	82
ENSECAG00000015409	5.328698632	0.8024753	0.9460557	597	798	815	816	650	970	620	657
ENSECAG00000012230	6.285577995	0.8024757	0.9460557	1783	1249	1653	1547	1448	1066	1846	1149
ENSECAG00000010673	3.306175041	0.8025524	0.9460557	168	177	178	218	193	159	195	177
ENSECAG00000011140	3.858382577	0.802659	0.9460831	305	203	291	372	233	210	233	325
ENSECAG00000015124	4.549433296	0.8027765	0.9460914	450	378	412	652	429	311	479	419
ENSECAG00000017363	6.870091517	0.8028326	0.9460914	1918	2324	2205	2391	1888	1915	2223	2598
ENSECAG00000012279	7.233601892	0.8029218	0.9460984	2290	3249	2925	2834	3290	1897	3148	2848
ENSECAG00000013308	5.281157347	0.8033592	0.946366	710	731	623	876	387	743	741	995
ENSECAG00000022411	4.36701967	0.8033736	0.946366	398	403	320	431	335	319	416	452
ENSECAG00000019039	5.338851259	0.8034051	0.946366	787	665	1003	776	848	434	897	676
ENSECAG00000015084	6.481395689	0.8035645	0.946366	1813	1573	1749	2027	2090	1004	1798	1387
ENSECAG00000008184	6.636105253	0.8035655	0.946366	1511	1512	1984	2636	1644	1398	1940	2386
ENSECAG00000018272	7.748551138	0.803681	0.9463832	4389	3925	4191	3613	6241	2514	4131	3033
ENSECAG00000012436	3.558432954	0.8037591	0.9463832	208	206	194	275	131	134	272	349
ENSECAG00000000422	0.708677202	0.80383	0.9463832	15	28	22	64	21	24	51	10
ENSECAG00000008327	5.342738351	0.8040127	0.9465002	824	766	907	724	837	426	808	785
ENSECAG00000012124	5.581981494	0.8041736	0.9465182	1081	682	1087	992	975	641	1137	620
ENSECAG00000014547	6.469008337	0.8041996	0.9465182	1720	1622	1462	2334	1163	1049	1877	2192
ENSECAG00000015883	3.416736696	0.804315	0.9465182	219	203	208	157	254	119	172	241
ENSECAG00000019947	7.838898876	0.8043734	0.9465182	4490	3884	4253	4654	6463	2613	5512	2517
ENSECAG00000016283	6.265098185	0.8044825	0.9465182	1135	1512	1343	1864	1286	1369	1296	1671
ENSECAG00000016426	5.086586466	0.8045279	0.9465182	666	521	664	731	567	401	840	736
ENSECAG00000017758	8.027379965	0.804706	0.9465409	4874	4016	4749	6281	4785	5542	5429	3340
ENSECAG00000021564	5.600463206	0.8047218	0.9465409	991	829	914	944	866	766	1092	857
ENSECAG00000020821	5.668741609	0.8048772	0.9465409	1221	673	1123	1074	1163	643	1100	660
ENSECAG00000014142	4.422943695	0.8048806	0.9465409	482	320	546	360	348	259	489	414
ENSECAG00000020889	6.791542412	0.8053651	0.9469846	2267	1770	2077	2819	1712	1790	2269	1994
ENSECAG00000009695	7.031826776	0.8055091	0.9469846	2994	2248	2696	2505	1805	1990	3001	2436
ENSECAG00000020711	5.290104728	0.8056721	0.9469846	699	476	997	810	526	580	993	826
ENSECAG00000009805	4.024969811	0.8056888	0.9469846	239	334	343	285	176.001	456.001	119	412
ENSECAG00000012874	5.739859724	0.8057333	0.9469846	916	865	997	1314	890	776	1310	996
ENSECAG00000012132	4.062118861	0.8058685	0.9469846	274	290	306	493	109	180	273	602
ENSECAG00000005828	4.969230267	0.8059135	0.9469846	694	408	717	702	586	474	544	571
ENSECAG00000011360	4.00464519	0.8060074	0.9469846	244	295	317	353	312	275	352	240

ENSECAG00000018673	9.780941006	0.8060718	0.9469846	18400	12129	15980	20601	20610	15272	19914	9190
ENSECAG00000011978	2.710350231	0.8060916	0.9469846	150	80	117	182	124	77	156	95
ENSECAG00000020521	7.756290857	0.8061945	0.9470076	4433	3623	4116	5261	4787	2576	4447	3374
ENSECAG00000000675	6.106384949	0.8065746	0.9473561	1259	1375	1322	1540	1029	1366	1047	1308
ENSECAG00000017734	4.443393398	0.806836	0.9475147	363	305	418	578	523	330	349	385
ENSECAG00000013955	6.705546289	0.8068764	0.9475147	1611	2177	1831	2816	924	2503	1373	2296
ENSECAG00000007148	3.056568915	0.8071739	0.947766	179	124	201	158	102	141	149	178
ENSECAG00000019720	2.397753025	0.8074332	0.9479725	97	58	116	157	100	34	107	122
ENSECAG00000018149	6.73782651	0.8076495	0.9480428	2073	1903	1954	2641	1875	1644	2315	1665
ENSECAG00000026983	2.849289049	0.8076958	0.9480428	118	121	158	136	203	125	115	77
ENSECAG00000013324	6.90778535	0.8077435	0.9480428	2759	2104	2453	2269	1744	1692	1831	3107
ENSECAG00000013380	2.7045771	0.8080656	0.9483161	113	142	98	165	76	113	134	123
ENSECAG00000019684	3.277720466	0.8081433	0.9483161	193	123	214	194	291	90	252	92
ENSECAG00000020351	4.732499467	0.8082416	0.9483335	459	405	491	678	448	406	604	509
ENSECAG00000014275	4.294292801	0.8088619	0.9489633	278	339	320	664	321	265	531	266
ENSECAG00000017489	3.353453116	0.8090617	0.9490997	178	147	178	272	177	164	182	225
ENSECAG00000013898	3.291922938	0.8091532	0.949109	192	138	199	206	183	123	289	139
ENSECAG00000014755	4.261673038	0.809331	0.9492195	434	296	304	409	526	169	537	213
ENSECAG00000017645	8.348845603	0.8094689	0.9492832	7515	4999	6980	6677	6407	5159	6003	5109
ENSECAG00000000854	0.596894681	0.8095703	0.9493042	26	33	17	39	9	24	40	25
ENSECAG00000015108	3.358326872	0.8098986	0.9495162	188	175	161	247	181	122	290	176
ENSECAG00000007577	4.223844248	0.8099183	0.9495162	349.001	336	347.001	467	340.001	186.001	379	409.024
ENSECAG00000001316	3.86039688	0.8100468	0.9495688	257	271	248	394	156	168	295	400
ENSECAG00000014942	4.860770181	0.8101485	0.94959	610	453	635	629	608	347	615	470
ENSECAG00000017286	6.145852005	0.810334	0.9496496	1578	857	1532	1448	1266	1058	1564	1350
ENSECAG00000021790	6.685798008	0.8104604	0.9496496	2338	1404	2195	2401	2209	925	3150	1088
ENSECAG00000009193	5.606052353	0.8105066	0.9496496	884.001	914	1029	1087	397.001	1173	532	1195.02
ENSECAG00000018676	5.614921145	0.8105419	0.9496496	975	810	852	1314	651	838	825	1098
ENSECAG00000010103	8.345468111	0.8106174	0.9496496	5296	5988	5771	7715	5577	5938	7091	5372
ENSECAG00000009253	1.567943402	0.8110131	0.9498803	42	36	46	95	58	25	97	41
ENSECAG00000016054	3.254245919	0.8110345	0.9498803	121	232	194	147	88	138	133	348
ENSECAG00000015408	3.357352373	0.8110652	0.9498803	132	156	171	320	153	104	192	316
ENSECAG00000015145	3.680551503	0.8111899	0.9499284	325	235	215	245	172	318	221	154
ENSECAG00000012070	6.866689512	0.8115687	0.950272	2154	2290	2615	2250	1334	2617	1557	2464
ENSECAG00000013788	10.35176127	0.811736	0.950272	24330	20473	29262	31489	25619	21419	26675	17336
ENSECAG00000018617	7.204676949	0.8117866	0.950272	2787	2499	2527	3432	3107	1661	3345	2896
ENSECAG00000010193	4.423005529	0.8118179	0.950272	347	393	480	495	429	380	354	311
ENSECAG00000003001	1.80218819	0.8120559	0.9503704	44	56	86	68	76	42	84	53
ENSECAG00000010693	5.259911833	0.8121656	0.9503704	710	585	815	798	985	501	898	453
ENSECAG00000009014	2.9587828	0.8123535	0.9503704	149	99	205	121	206	72	222	84
ENSECAG00000008782	1.671606155	0.8123944	0.9503704	72	38	61	60	91	60	57	19
ENSECAG00000022821	3.147192327	0.8124085	0.9503704	197.001	82	171	220	156	75.0003	247	192
ENSECAG00000001958	3.385566883	0.8124606	0.9503704	151	251	223	213	28	99	152	442
ENSECAG00000024831	5.988598832	0.8124876	0.9503704	1868	920	1256	1024	1529	1006	1252	606
ENSECAG00000006737	7.223867388	0.8126542	0.9504673	3079	2222	2866	3233	3266	2672	2681	2291
ENSECAG00000010098	3.456687402	0.8129076	0.9506659	163	273	167	208	224	168	239	177
ENSECAG00000011218	5.773093962	0.8132186	0.9509316	1248	825	1037	1282	1047	615	1327	897
ENSECAG00000010162	2.361190432	0.8134408	0.9510936	45	119	70	145	67	125	81	96
ENSECAG00000013639	2.731973369	0.8140052	0.9516556	41	134	170	144	78	153	90	159
ENSECAG00000019910	6.988968422	0.814136	0.9516951	2382	1935	2304	3080	2366	3246	2059	1466
ENSECAG00000015620	3.84614301	0.8142066	0.9516951	264	213	322	358	243	154	315	299
ENSECAG00000020908	2.257855181	0.8144346	0.9518637	85	61	99	111	71	63	108	110
ENSECAG00000008645	2.758822729	0.8146924	0.9520586	127	99	102	181	158	121	134	78
ENSECAG00000021896	2.578183444	0.8148286	0.9520586	61	91	139	155	70	79	155	142
ENSECAG00000026867	3.046088199	0.8148529	0.9520586	158	176	136	186	87	163	106	202
ENSECAG00000018007	1.098469178	0.8150353	0.9521549	24	31	49	50	45	27	47	34
ENSECAG00000015110	1.34944442	0.8152738	0.9521549	40	59	35	64	38	19	75	42
ENSECAG00000000106	7.018343186	0.8153687	0.9521549	1513	892	2743	4979	1860	2447	4887	731
ENSECAG00000024369	2.900883575	0.8154863	0.9521549	218	85	133	166	231	61	122	94
ENSECAG00000020989	6.184250028	0.815488	0.9521549	1473	1268	1558	1494	1420	1229	1324	1088
ENSECAG00000018881	0.163157977	0.8155041	0.9521549	18	19	14	25	23	12	28	14
ENSECAG00000013408	3.62243328	0.8156783	0.9521549	273	186	269	256	135	155	332	249
ENSECAG00000007374	8.417052985	0.8158979	0.9521549	6558	5616	6707	8861	4767	9357	5624	3348
ENSECAG00000005564	2.990626448	0.8159199	0.9521549	169	124	129	218	108	76	245	134
ENSECAG00000014089	7.058949058	0.8159911	0.9521549	2703	1962	2676	2813	2364	1332	3847	2558
ENSECAG00000002116	2.667608728	0.8160033	0.9521549	163	82	126	98	141	81	170	79
ENSECAG00000023542	6.257942009	0.8160674	0.9521549	1196	1588	1268	1759	1075	1529	1431	1554
ENSECAG00000010350	2.727877502	0.8161649	0.9521549	144	107	159	113	106	130	117	95
ENSECAG00000019447	3.787459318	0.8161837	0.9521549	197	319	279	298	234	211	295	220
ENSECAG00000006540	5.839087892	0.8161926	0.9521549	1309	936	1110	1219	1023	677	1530	854
ENSECAG00000019667	5.860059358	0.8163267	0.9522134	1174	1000	1142	1335	1317	979	1034	691
ENSECAG00000018117	2.937957758	0.8167575	0.9526182	118	117	147	196	133	135	160	131
ENSECAG00000026987	5.864677141	0.8170249	0.9528322	1223	906	1219	1319	1121	966	899	1054
ENSECAG00000008395	6.424263863	0.8173146	0.9529967	1804	1402	1564	2135	1159	1043	1917	1990
ENSECAG00000000406	6.993382241	0.8173374	0.9529967	2627	2163	2578	2277	2466	1913	2608	2408
ENSECAG00000000194	4.530998329	0.8174176	0.9529967	395	450	303	605	232	382	481	620
ENSECAG00000010818	5.89082058	0.8176405	0.9531089	1688	683	1691	243	292	398	2841	1258
ENSECAG00000000393	7.565462221	0.8177006	0.9531089	3095	3405	3093	4902	3134	3836	3855	3039

ENSECAG00000023221	7.671518696	0.8177656	0.9531089	4073	3375	4163	3835	4165	3967	3178	3483
ENSECAG00000023670	5.89681308	0.8179546	0.9531938	1111	1056	969	1419	1085	888	1285	1135
ENSECAG00000015025	0.244738604	0.8182022	0.9531938	18	32	24	12	29	12	23	10
ENSECAG00000014812	1.322125191	0.8182291	0.9531938	47	35	55	43	40	38	52	49
ENSECAG00000013187	7.228584333	0.8183485	0.9531938	4232	2502	3100	2093	1451	3653	2219	2895
ENSECAG00000015076	4.752452035	0.8184286	0.9531938	513	476	620	402	720	253	698	349
ENSECAG00000000594	4.030762309	0.8184626	0.9531938	268	329	308	321	321	291	301	277
ENSECAG00000002911	2.461547349	0.8184742	0.9531938	62	57	94	208	71	87	102	144
ENSECAG00000024390	7.311380105	0.8185097	0.9531938	2541	3000	3327	3151	3333	3059	2500	2632
ENSECAG00000019630	5.023793911	0.8187427	0.953291	709	455	716	729	507	474	672	633
ENSECAG00000004691	6.964599308	0.8187611	0.953291	2362	2596	2194	2800	1966	1640	2780	2462
ENSECAG00000019093	5.965281638	0.8189344	0.9533951	928	1240	1311	1541	566	1581	862	1241
ENSECAG00000009181	6.451545707	0.8190925	0.9533959	1627	1220	1565	2338	1939	1054	2068	1445
ENSECAG00000015610	6.020940661	0.8191794	0.9533959	1374	1142	1460	1180	1143	1329	1007	987
ENSECAG00000003551	2.566650233	0.8192042	0.9533959	82	122	137	127	58	121	115	108
ENSECAG00000010107	9.579810806	0.8193546	0.9533959	15327	12900	15704	13746	10237	18884	9242	16795
ENSECAG00000018768	4.75327226	0.8193547	0.9533959	555	341	551	738	431	377	495	583
ENSECAG00000024368	7.39582709	0.8196666	0.9536611	3591	3941	2874	2881	3104	3118	3156	2291
ENSECAG00000011487	6.68853846	0.8197704	0.9536842	1800	1880	1895	2281	1878	2040	2033	1567
ENSECAG00000021699	2.366666384	0.8199838	0.9537903	106	88	116	96	72	51	100	133
ENSECAG00000016526	1.896424464	0.8200296	0.9537903	84	65	79	62	75	40	85	54
ENSECAG00000009496	5.461974659	0.8202228	0.9539174	912	812	816	781	520	719	837	1176
ENSECAG00000010954	6.010547245	0.8203219	0.9539349	1261	1160	1476	1218	1235	881	1293	1128
ENSECAG00000000010	3.826910725	0.8206241	0.9541146	340	248	272	268	278	109	371	249
ENSECAG00000022675	7.140261688	0.8206444	0.9541146	2704	2464	2624	3545	2072	2532	2816	2442
ENSECAG00000011346	5.728514344	0.8209391	0.9542326	895	837	1098	1229	936	1069	906	930
ENSECAG00000019980	3.825632308	0.820955	0.9542326	261	229	246	338	242	214	410	191
ENSECAG00000016920	4.504171965	0.8211176	0.9542326	439	366	439	579	347	260	532	470
ENSECAG00000022295	7.018833152	0.8211518	0.9542326	2449	2515	2203	2656	2234	2720	2124	2323
ENSECAG00000018249	6.01648504	0.8212501	0.9542326	1225	1229	1184	1530	1053	1248	1053	1128
ENSECAG00000009307	7.390318206	0.8213679	0.9542326	3597	2318	2926	4032	3445	2103	4152	2819
ENSECAG00000023522	3.208217243	0.8214283	0.9542326	118	213	135	276	86	202	157	182
ENSECAG00000015929	9.578500001	0.8214526	0.9542326	15240	13511	15560	16992	13918	8516	18298	13640
ENSECAG00000007266	3.115321892	0.8215019	0.9542326	196	166	164	108	94	130	135	279
ENSECAG00000009404	3.061697944	0.821793	0.9544634	145	79	221	181	174	132	272	48
ENSECAG00000019775	7.360828414	0.8218687	0.9544634	3097	3052	3005	4039	1948	2479	3776	3437.02
ENSECAG00000025119	7.798446372	0.8219918	0.9545088	3826	3822	3900	5496	3650	3244	5113	4536
ENSECAG00000023903	6.471274189	0.8221098	0.9545482	1535	1627	1480	2147	1620	1435	1961	1514
ENSECAG00000016532	5.070651448	0.8223274	0.9546234	864	434	698	706	797	325	843	413
ENSECAG00000019843	6.372333041	0.8223426	0.9546234	1629	1445	1573	1641	1804	909	2190	1295
ENSECAG00000023516	4.33319611	0.8225085	0.9546269	499	280	465	362	421	330	349	290
ENSECAG00000011798	1.329139417	0.8229242	0.9546269	43	36	53	65	34	33	43	57
ENSECAG00000002155	6.451421748	0.8229378	0.9546269	1633	1562	1941	1835	1598	1192	2148	1272
ENSECAG00000008649	5.24401336	0.8229712	0.9546269	803	668	794	743	675	620	738	615
ENSECAG00000019269	4.116700025	0.8230305	0.9546269	318	155	390	472	424	244	324	280
ENSECAG00000010187	4.200348602	0.8232038	0.9546269	273	301	363	553	437	285	344	201
ENSECAG00000005853	3.670580347	0.8232083	0.9546269	189	217	209	353	174	160	304	312
ENSECAG00000006878	4.36387324	0.8232579	0.9546269	386	300	367	521	411	358	474	268
ENSECAG00000015891	6.403349315	0.8233917	0.9546269	1917	1167	1653	1730	1891	1116	1884	1366.02
ENSECAG00000008822	4.863279063	0.8234437	0.9546269	619	529	517	656	605	389	606	441
ENSECAG00000006699	3.091918381	0.8234528	0.9546269	213	131	169	163	159	134	128	161
ENSECAG00000000327	5.712136077	0.8237437	0.9546269	931	834	1143	1298	786	965	1015	894
ENSECAG00000002317	2.989132665	0.8238215	0.9546269	95	95	217	244	35	209	134	152
ENSECAG00000000619	5.246863813	0.8238404	0.9546269	836	558	712	785	708	414	1027	699
ENSECAG00000024469	5.717589586	0.8239124	0.9546269	952	952	932	1169	1232	993	808	764
ENSECAG00000011258	2.086563034	0.8239597	0.9546269	84	72	59	123	55	54	89	93
ENSECAG00000010871	1.543373238	0.8240022	0.9546269	59	44	46	82	46	13	64	76
ENSECAG00000012623	2.802607316	0.8240024	0.9546269	100	106	154	164	127	114	135	132
ENSECAG00000022717	6.950298836	0.8240923	0.9546269	2323	1635	2532	3572	2356	1833	2933	1592
ENSECAG00000000485	6.358379833	0.824211	0.9546269	1468	1419	1579	2122	1057	1276	1850	1625
ENSECAG00000019742	1.871981443	0.8242402	0.9546269	63	50	63	94	41	31	64	134
ENSECAG00000021905	2.263968492	0.8242659	0.9546269	60	117	46	129	98	34	86	138
ENSECAG00000011232	3.670297314	0.8243554	0.9546269	232	195	228	373	211	163	256	259
ENSECAG00000014704	4.439880357	0.8243627	0.9546269	396	342	298	631	322	352	454	471
ENSECAG00000012044	0.627339381	0.824589	0.9547916	25	32	37	21	36	19	26	18
ENSECAG00000018293	4.474287655	0.8249135	0.9549022	435	386	454	498	315	328	596	336
ENSECAG00000010304	5.316185054	0.8250918	0.9549022	871	570	775	816	670	445	1252	646
ENSECAG00000021898	7.3312994	0.8251166	0.9549022	3343	2529	3126	3978	2811	1710	4227	2772
ENSECAG00000010659	4.829151055	0.8251539	0.9549022	591	439	562	688	580	401	517	485
ENSECAG00000021882	8.945952603	0.8252189	0.9549022	11723	7280	10563	7663	10492	6003	11128	9128
ENSECAG00000023647	6.857075414	0.8253449	0.9549022	1721	2323	2045	3255	1304	2188	2063	2514
ENSECAG00000016772	5.600331811	0.8253554	0.9549022	870	920	975	1102	818	1011	751	758
ENSECAG00000000566	3.387872115	0.8253569	0.9549022	179	154	289	214	195	139	286	113
ENSECAG00000014753	7.248655211	0.8255079	0.9549302	3215	2428	3616	2177	2306	1673	3974	3520
ENSECAG00000009435	3.300283287	0.8255493	0.9549302	230	149	167	244	213	125	233	114
ENSECAG00000017242	5.411532708	0.8257546	0.9550704	952	659	918	865	858	743	711	635
ENSECAG00000017044	6.389105325	0.8258506	0.9550842	1935	1178	2203	939	2792	1129	1246	946
ENSECAG00000018860	8.055014671	0.8264702	0.9556308	4478	6897	4429	3830	5019	6618	5591	2130

ENSECAG00000001400	4.253710864	0.8265652	0.9556308	350	341	399	431	309	396	353	254
ENSECAG00000019560	5.385086146	0.826646	0.9556308	931	609	813	1016	1004	371	1111	488
ENSECAG00000011666	0.867309592	0.8266597	0.9556308	47	16	41	37	48	6	45	22
ENSECAG00000000411	2.959337361	0.8267954	0.9556326	158	106	150	170	154	70	164	189
ENSECAG00000003959	1.978651305	0.8268296	0.9556326	81	65	101	60	63	53	80	72
ENSECAG00000024529	1.8107881	0.827249	0.9557537	70	67	43	97	64	58	79	35
ENSECAG00000008521	2.002180329	0.8273092	0.9557537	65	78	66	85	89	63	65	70
ENSECAG00000024762	6.12694526	0.8274799	0.9557537	1340	1017	1274	2031	906	951	1799	1332
ENSECAG00000012875	6.482725748	0.8275884	0.9557537	1674	1687	1696	1723	1614	1888	1702	1281
ENSECAG00000008623	0.494741267	0.8276434	0.9557537	9	58	11	26	7	12	22	49
ENSECAG00000012810	6.819043835	0.8277313	0.9557537	2612	1676	2429	2323	2162	1182	2775	1931
ENSECAG00000008997	6.43689815	0.8277564	0.9557537	1735	1300	1744	1853	1703	1487	1723	1417
ENSECAG00000015950	2.925784682	0.8277874	0.9557537	198	85	135	197	215	64	179	67
ENSECAG00000019963	4.496476697	0.8278241	0.9557537	415	427	403	556	365	351	422	438
ENSECAG00000023131	5.026466647	0.827954	0.9557537	638	533	668	629	768	644	497	438
ENSECAG00000014268	7.14570027	0.8279934	0.9557537	2870	2596	2905	2915	1626	2402	2801	3118
ENSECAG00000024212	6.747642884	0.8280234	0.9557537	2101	2174	1652	2205	1263	2184	2009	2405
ENSECAG00000020796	2.674757758	0.8280282	0.9557537	110	119	125	152	106	76	149	113
ENSECAG00000005903	2.32143928	0.8283846	0.9559413	108	73	107	79	138	77	86	59
ENSECAG00000017775	3.016087096	0.828393	0.9559413	129	137	152	232	158	72	156	177
ENSECAG00000015244	5.404550858	0.8284432	0.9559413	935	509	960	819	986	511	1232	446
ENSECAG00000024679	6.448166015	0.8286163	0.9560439	1813	1440	1664	2068	1577	1300	1838	1441
ENSECAG00000009401	1.683568941	0.8288355	0.956158	79	43	68	61	52	66	49	45
ENSECAG00000011384	6.299807524	0.8288835	0.956158	1614	1185	1605	1618	1430	1045	1747	1609
ENSECAG00000020384	6.725045859	0.8291852	0.9562465	1764	1346	3146	2336	2971	514	1352	2533
ENSECAG00000010077	2.705007983	0.8291919	0.9562465	142	73	103	174	107	71	219	95
ENSECAG00000011764	7.282683048	0.8292128	0.9562465	3071	2455	3103	3911	2610	1719	3720	3058
ENSECAG00000024092	6.440417286	0.8293655	0.9563255	1663	1464	1650	2220	1215	2312	1431	951
ENSECAG00000004593	3.850785995	0.8296525	0.956396	240	209	191	472	254	238	339	232
ENSECAG00000013420	6.038934098	0.8297357	0.956396	1105	1228	1352	1305	1094	1458	1214	990
ENSECAG00000020374	2.242601404	0.8297891	0.956396	201	80	68	19	119	38	135	32
ENSECAG00000022237	3.975646856	0.8297892	0.956396	190	294.002	315	394	235.002	330	318	262
ENSECAG00000008287	3.765472725	0.8298476	0.956396	264	163	341	330	354	158	292	140
ENSECAG00000013393	5.716722565	0.8300534	0.9564498	972	1494	705	706	827	889	891	1248
ENSECAG00000015712	4.146479056	0.8300627	0.9564498	293	273	282	508	177	317	531	299
ENSECAG00000014128	2.63497218	0.8306883	0.9570736	115	131	106	137	101	88	109	128
ENSECAG00000021865	0.766219706	0.8308011	0.9571064	11	63	17	36	18	22	37	34
ENSECAG00000020539	5.443359584	0.8309897	0.957195	741	787	925	1019	775	811	717	711
ENSECAG00000011619	5.271989119	0.8311237	0.957195	928	581	856	712	468	560	654	1027
ENSECAG00000017780	6.825933039	0.8311308	0.957195	2131	2188	1752	2569	1672	1693	2664	2396
ENSECAG00000016187	4.593760394	0.831297	0.957211	520	364	385	577	439	377	507	450
ENSECAG00000011079	3.942141101	0.8313132	0.957211	293	251	300	387	308	202	270	289
ENSECAG00000020120	5.429576999	0.8314465	0.9572404	852	761	759	1075	670	733	724	878
ENSECAG00000006415	1.028319126	0.8316128	0.9572404	19	37	41	49	25	39	31	48
ENSECAG00000023993	2.045810781	0.8316607	0.9572404	92	55	89	65	138	33	87	44
ENSECAG00000012902	5.190451153	0.8316758	0.9572404	784	624	789	702	611	745	650	517
ENSECAG00000019013	5.593137982	0.8323155	0.9575965	885	899	1000	1054	757	957	861	776
ENSECAG00000021238	2.714207847	0.8323158	0.9575965	96	190	101	84	57	136	87	196
ENSECAG00000010803	1.442993495	0.8323166	0.9575965	59	24	105	23	71	24	46	39
ENSECAG00000004024	6.778017425	0.8323224	0.9575965	1852	2321	1873	2715	1390	1944	2102	2252
ENSECAG00000024832	7.067094686	0.8324623	0.9576604	3138	2409	2315	2818	2211	1838	2826	2606
ENSECAG00000008577	6.008314904	0.8325758	0.9576941	1333	1039	1285	1488	1004	829	1374	1362
ENSECAG00000009755	4.11583054	0.8327011	0.9577412	383	405	510	51	378	236	444	142
ENSECAG00000015523	1.074482409	0.8329179	0.9578936	31	40	51	39	38	43	33	22
ENSECAG00000008331	5.055733887	0.8330224	0.9579168	570	635	615	703	579	700	583	542
ENSECAG00000016924	3.795656812	0.8332615	0.9580947	187	365	183	367	219	207	277	263
ENSECAG00000007051	5.941749929	0.8335899	0.9583474	1069	1064	1164	1640	797	950	1146	1433
ENSECAG00000026814	3.7425956	0.8337214	0.9583474	239	197	268	311	255	115	360	276
ENSECAG00000014186	5.685490996	0.8337344	0.9583474	718	1056	1402	664	1114	597	953	1138
ENSECAG00000021924	2.199077012	0.8340146	0.9584575	75	64	103	125	42	71	95	106
ENSECAG00000021076	4.885789169	0.8340644	0.9584575	502	581	629	507	360	344	684	826
ENSECAG00000008754	5.212217347	0.8343818	0.9584575	832	570	817	734	473	448	861	859
ENSECAG00000002645	2.181792599	0.8343981	0.9584575	77	120	59	99	29	122	82	68
ENSECAG00000010656	2.318940487	0.8344848	0.9584575	115	120	84	34	37	96	52	177
ENSECAG00000024881	2.413183539	0.8344983	0.9584575	97	96	82	117	33	51	109	206
ENSECAG00000021022	2.579846227	0.8345125	0.9584575	100	83	133	130	55	98	205	90
ENSECAG00000017291	4.083855561	0.8347002	0.9584575	306	300.999	356.999	386	211.999	292	346.999	332.999
ENSECAG00000018321	3.360318886	0.8347912	0.9584575	213	148	179	237	208	152	267	131
ENSECAG00000008809	3.738406789	0.8349158	0.9584575	174	523	173	152	202	200	312	214
ENSECAG00000010781	3.769150045	0.8350493	0.9584575	235	331	244	260	207	185	285	278
ENSECAG00000000728	3.339691751	0.8350792	0.9584575	206	131	156	279	130	137	221	262
ENSECAG00000014541	1.976246967	0.8350993	0.9584575	82	43	79	89	69.0004	55.0001	90	72
ENSECAG00000017791	6.913459978	0.8352497	0.9584575	2515	1907	2509	2717	1774	1671	2774	2345
ENSECAG00000018425	5.342105859	0.8352817	0.9584575	535	953	641	915	305	1113	483	1014
ENSECAG00000024994	7.448813854	0.8352874	0.9584575	2976	3588	3393	3957	2468	3015	3399	3368
ENSECAG00000018414	1.333260113	0.8353497	0.9584575	39	75	38	22	22	37	43	80
ENSECAG00000020149	4.565980992	0.8354447	0.9584575	448	455	470	507	438	255	473	506
ENSECAG00000025082	2.42362244	0.8354497	0.9584575	78	99	90	133	113	99	101	73

ENSECAG00000018114	5.844289828	0.8355178	0.9584575	986	970	1002	1457	918	746	1291	1306
ENSECAG00000000259	0.776303915	0.8357384	0.9586138	23	32	39	37	12	37	16	43
ENSECAG00000022058	1.318771371	0.8359951	0.9588114	39	58	34	62	22	42	40	61
ENSECAG00000009757	4.987312718	0.8361143	0.9588363	829	543	788	332	632	620	529	394
ENSECAG00000023534	8.200441119	0.8361856	0.9588363	5083	7489	6744	3659	5251	4792	5010	5407
ENSECAG00000012886	5.556369295	0.8363979	0.9589829	720	699	1093	1096	981	807	1124	544
ENSECAG00000027695	9.77250285	0.8365007	0.959004	17626	15499	16577	16656	19064	14690	16131	14029
ENSECAG00000022844	4.385055212	0.8367982	0.9592482	353	361	370	597	363	347	475	277
ENSECAG00000011592	2.117735065	0.8369037	0.9592723	1	237	5	35	4	40	4	285
ENSECAG00000016219	1.489015235	0.8370529	0.9592726	64	28	60	70	51	37	82	20
ENSECAG00000008724	4.415501976	0.8371518	0.9592726	331	459	400	401	172	705	285	353
ENSECAG00000000276	0.529517582	0.8371586	0.9592726	26	28	23	31	11	15	38	31
ENSECAG00000022003	7.966038349	0.8372417	0.9592726	5020	4241	5142	5598	4357	3130	4982	5216
ENSECAG00000021271	3.30147548	0.8374112	0.9593701	238	126	245	174	199	127	218	142
ENSECAG00000024549	5.864489236	0.8376686	0.959398	1114	959	1139	1471	833	488	1244	1604
ENSECAG00000008151	2.715712278	0.8376967	0.959398	106	103	152	162	143	102	107	95
ENSECAG00000020467	5.042419861	0.8377624	0.959398	599	615	689	715	662	397	718	553
ENSECAG00000008525	5.527632532	0.83783	0.959398	723	725	877	1232	806	909	978	667
ENSECAG00000007560	7.096708959	0.8379407	0.959398	2707	2863	2308	2995	2177	1978	2378	3081
ENSECAG00000005729	6.455979215	0.8379773	0.959398	1523	1315	2001	2242	2196	1191	1941	832
ENSECAG00000024838	3.573336087	0.8380269	0.959398	203	185.002	248.009	266.004	251.002	208.002	196.008	205.004
ENSECAG00000006181	4.243978873	0.838359	0.9596815	380	346	334	370	335	345	463	247
ENSECAG00000016281	7.417995455	0.8387102	0.9598794	3618	2578	3468	4073	3458	2157	3734	2751
ENSECAG00000010820	4.043092373	0.8387902	0.9598794	282	263	391	382	309	327	199	286
ENSECAG00000015049	3.666489764	0.8388146	0.9598794	242	182	261	277	304	150	310	175
ENSECAG00000011706	4.974059322	0.838972	0.9598794	686	529	658	621	436	465	680	639
ENSECAG00000014890	4.300494876	0.8391084	0.9598794	337	266	352	651	378	227	517	275
ENSECAG00000000489	9.836659015	0.8391165	0.9598794	16590	17149	16915	18765	14090	21303	15048	15630
ENSECAG00000018627	4.648869655	0.8391234	0.9598794	549	369	454	540	387	567	446	410
ENSECAG00000013047	1.27505762	0.8393443	0.9600354	42	46	38	47	22	19	83	56
ENSECAG00000009236	6.895934187	0.8396018	0.9601304	2029	1988	2046	3583	1889	2176	2843	1461
ENSECAG00000017054	0.280029266	0.8396107	0.9601304	16	30	11	33	17	11	30	20
ENSECAG00000015556	0.462225233	0.8397741	0.9601304	15	31	27	19	0	28	14	54
ENSECAG00000009826	4.305573833	0.839813	0.9601304	350	345	405	478	270	379	348	373
ENSECAG00000010824	6.079075944	0.83985	0.9601304	1414	1208	1292	1209	1052	808	1389	1767
ENSECAG00000016482	2.196839872	0.8400251	0.960234	141	132	33	11	75	37	96	136
ENSECAG00000012500	5.666786275	0.8401459	0.9602547	964	1054	1022	969	901	1024	880	711
ENSECAG00000019290	5.14849669	0.8402147	0.9602547	777	591	807	648	1019	304	796	380
ENSECAG00000016755	7.200398625	0.8403227	0.9602547	2333	3140	2382	3325	2690	2730	3144	2201
ENSECAG00000018048	0.889245541	0.8403814	0.9602547	19	23	26	67	23	31	50	28
ENSECAG00000024646	3.787904262	0.8406071	0.9603722	224	186	374	260	231	301	221	241
ENSECAG00000016977	5.555951463	0.8407271	0.9603722	1037	648	1132	747	1190	495	1068	735
ENSECAG00000000547	6.240822755	0.8407379	0.9603722	1404	1445	1448	1721	1490	812	1718	1369
ENSECAG00000010335	5.92290762	0.8408616	0.960417	1221	972	1188	1256	1346	890	1195	1006
ENSECAG00000022238	0.701246257	0.840994	0.9604415	36	24	39	24	23	41	15	22
ENSECAG00000016805	5.400390729	0.8410522	0.9604415	533	1106	869	597	286	967	381	1429
ENSECAG00000000387	7.760442218	0.8414044	0.9607471	3250	5897	4287	3613	2892	1848	3840	6812
ENSECAG00000009566	5.635486363	0.841573	0.9608431	943	773	1041	1228	765	547	1060	1170
ENSECAG00000019850	6.117739838	0.8418855	0.9611033	1542	1114	1381	1493	1252	894	1538	1248
ENSECAG00000010842	6.021638683	0.8422919	0.9614705	1147	1040	1291	1504	1085	1494	1036	1051
ENSECAG00000021637	6.997634391	0.8423999	0.9614972	2657	2168	2439	2460	2518	2486	2171	2078
ENSECAG00000008410	2.581243262	0.8427079	0.9617522	71	130	66	181	62	141	106	122
ENSECAG00000020556	0.593417061	0.8430592	0.9618105	22	32	21	30	26	36	20	20
ENSECAG00000024941	3.917070773	0.8430738	0.9618105	244	240	274	396	210	195	355	362
ENSECAG00000024751	4.211461972	0.8431485	0.9618105	381	298	409	384	367	257	293	371
ENSECAG00000014412	5.896150999	0.8431499	0.9618105	1336	797	1303	1342	1463	675	1287	779
ENSECAG00000020246	9.18395315	0.8432527	0.9618105	11910	8421	11776	12300	15256	9206	12537	5700
ENSECAG00000022331	1.97038418	0.843267	0.9618105	57	72	69	90	74	24	98	95
ENSECAG00000010516	5.448128285	0.8434311	0.9618881	925	724	846	823	984	702	932	573
ENSECAG00000010877	5.393072535	0.8435044	0.9618881	743	855	732	858	602	646	1021	840
ENSECAG00000011805	6.464286644	0.8435986	0.9618893	1701	1492	1716	1838	1804	1646	1643	1310
ENSECAG00000011087	6.714149372	0.8436749	0.9618893	2563	1599	1922	2302	1724	1427	2251	2042
ENSECAG00000023673	5.648165944	0.8438841	0.9618901	986	799	891	1358	554	876	914	1173
ENSECAG00000020447	7.96132011	0.8441231	0.9618901	4247	3976	4107	6838	2384	3405	4606	8176
ENSECAG00000013845	2.108091272	0.8441311	0.9618901	79	64	83	116	65	65	85	79
ENSECAG00000005750	3.414602517	0.8441887	0.9618901	259	126	175	301	194	145	274	135
ENSECAG00000015578	4.393456667	0.8444935	0.9618901	444	333	455	441	315	263	624	302
ENSECAG00000018835	2.006523365	0.8446675	0.9618901	64	29	92	144	139	28	76	27
ENSECAG00000021844	2.95245059	0.8447424	0.9618901	100	156	116	211	110	117	125	212
ENSECAG00000008444	6.160890596	0.8447917	0.9618901	1547	1232	1466	1428	1384	958	1450	1266
ENSECAG00000020822	8.569508277	0.8448565	0.9618901	7249	6433	7483	7818	7487	5741	8695	6122
ENSECAG00000002715	6.517124095	0.8448665	0.9618901	1798	1620	1767	2117	1341	1880	1569	1573
ENSECAG00000004762	5.414064235	0.8450835	0.9618901	994	1258	578	468	644	394	994	1009
ENSECAG00000014281	6.242845903	0.8451637	0.9618901	1689	1037	1568	1783	1760	1061	1447	1041
ENSECAG00000018672	2.186388311	0.8451952	0.9618901	47	55	124	140	67	43	110	96
ENSECAG00000014598	2.581869307	0.8452406	0.9618901	121	90	103	135	121	90	135	90
ENSECAG00000022937	6.317376297	0.8452578	0.9618901	1466	1560	1247	1812	1008	1177	1776	1946
ENSECAG00000016365	4.012281891	0.8454023	0.9618901	344	241	425	267	333	194	378	231

ENSECAG00000008394	6.999886465	0.8455171	0.9618901	2561	2009	2991	2164	2060	2136	2945	2293
ENSECAG00000018548	3.735149143	0.8457278	0.9618901	235	278	280	196	213	327	214	199
ENSECAG00000013557	5.841686153	0.845773	0.9618901	1283	854	1188	1042	1024	646	1603	1023
ENSECAG00000021951	5.398693873	0.8458054	0.9618901	437	1169	691	835	245	1188	611	1001
ENSECAG00000019709	4.962548539	0.8458152	0.9618901	604	452	634	817	604	381	770	460
ENSECAG00000015214	5.197905808	0.8458153	0.9618901	788	563	863	704	837	549	749	432
ENSECAG00000011656	3.452924504	0.8458247	0.9618901	225	99	276	285	175	138	267	190
ENSECAG00000023052	3.819450067	0.8458274	0.9618901	223	211	230	415	95	388	214	318
ENSECAG00000023652	4.721316262	0.8458781	0.9618901	551	387	499	579	552	332	624	442
ENSECAG00000008751	6.434612351	0.8459582	0.9618901	1566	1124	1469	2558	1115	2253	1243	1582
ENSECAG00000017941	5.761296972	0.845962	0.9618901	922	835	983	1438	951	1332	1063	560
ENSECAG00000023851	7.961266534	0.8461703	0.9619598	5518	4496	4577	5241	4014	3520	5053	5032
ENSECAG00000012444	3.210078273	0.8462366	0.9619598	170	164	191	208	130	153	149	206
ENSECAG00000009331	4.24322651	0.8462773	0.9619598	382	311	386	429	308	249	328	442
ENSECAG00000013894	3.557053264	0.8466438	0.9620423	247	178	249	212	204	139	267	261
ENSECAG00000011635	1.834445745	0.8466715	0.9620423	53	43	69	102	73	39	87	61
ENSECAG00000027669	11.971823	0.8466976	0.9620423	75302	87285	73422	82353	79134	64936	71791	64605
ENSECAG00000011639	7.324772278	0.8467879	0.9620423	2930	3076	2789	4020	2790	2214	3688	2687
ENSECAG00000013930	7.350441987	0.8468368	0.9620423	3347	2752	2806	3579	2786	2799	3971	2475
ENSECAG00000014343	1.480391661	0.8469436	0.9620423	35	56	63	47	31	45	55	69
ENSECAG00000014071	3.805162913	0.8470222	0.9620423	291	156	292	392	207	178	394	214
ENSECAG00000020337	8.86509001	0.8470939	0.9620423	10297	7294	9024	8907	9328	5788	11715	7921
ENSECAG00000017293	4.709206396	0.847142	0.9620423	575	398	496	623	466	302	683	415
ENSECAG00000001414	5.102413081	0.8472576	0.9620423	695	587	667.001	793	819	385	817	411
ENSECAG00000021485	1.350880887	0.8472816	0.9620423	16	39	60	69	1	45	38	101
ENSECAG00000015123	7.090877517	0.847478	0.9621157	2871	1942	2721	2925	2169	2494	2440	2832
ENSECAG00000012744	4.182609414	0.8475156	0.9621157	276	162	301	677	656	182	343	154
ENSECAG00000022845	0.528403265	0.8477665	0.9621386	25	21	57	5	5	50	8	23
ENSECAG00000009315	0.598681856	0.8478616	0.9621386	39	20	26	29	36	10	33	20
ENSECAG00000023057	6.679214232	0.8478702	0.9621386	2057	1829	2136	2114	1750	1213	2295	2050
ENSECAG00000020852	5.89985426	0.8478837	0.9621386	1363	895	1287	1203	1068	986	1045	1078
ENSECAG00000007221	3.742297993	0.8479797	0.9621386	266	191	378	163	202	277	331	170
ENSECAG00000019132	6.248551575	0.848044	0.9621386	1744	1269	1544	1469	1099	1208	1633	1439
ENSECAG00000009133	2.217022058	0.8482745	0.9622921	12	235	17	46	9	83	8	243
ENSECAG00000014115	0.378052984	0.8484149	0.9622921	24	28	13	24	12.0005	29.0013	21	26
ENSECAG00000008524	2.213990266	0.8484774	0.9622921	114	53	108	92	97	36	104	84
ENSECAG00000023953	7.448533264	0.8486912	0.9622921	3364	3194	3460	3898	3124	3125	3401	2565
ENSECAG00000017544	0.690759041	0.8487865	0.9622921	18	31	22	43	16	37	13	44
ENSECAG00000009424	3.605521501	0.848902	0.9622921	230	217	267	250	156	142	238	320
ENSECAG00000020631	3.419622085	0.8489026	0.9622921	179	176	149	313	112	180	239	257
ENSECAG00000007598	5.18955589	0.8489113	0.9622921	978	333	637	1028	702	248	924	742
ENSECAG00000015847	2.695794099	0.8489417	0.9622921	143	86	94	200	122	61	212	63
ENSECAG00000007124	5.840306993	0.8491216	0.9624	1060	910	980	1461	950	586	1672	1105
ENSECAG00000020196	2.130798518	0.8492442	0.9624428	70	66	146	61	32	59	59	147
ENSECAG00000022953	2.594139603	0.849509	0.9626469	116	98	119	117	135	74	141	92
ENSECAG00000007116	7.285402895	0.8496746	0.9627385	2629	2775	3171	3912	2566	2400	3367	2693
ENSECAG00000011680	1.187319996	0.8498479	0.962779	51	37	46	27	36	13	41	75
ENSECAG00000022001	4.306500432	0.8501609	0.962779	437	245	419	410	372	244	469	380
ENSECAG00000008690	0.901794467	0.8501833	0.962779	23	32	40	48	19	35	43	26
ENSECAG00000017041	2.758544743	0.85038	0.962779	111	100	102	235	85	71	192	130
ENSECAG00000007897	5.344225404	0.85039	0.962779	878	647	718	1008	928	459	757	718
ENSECAG00000021859	1.724039621	0.8504208	0.962779	24	126	40	62	37	19	51	118
ENSECAG00000009987	6.552138721	0.8504461	0.962779	2005	1449	1870	1856	1986	1432	1905	1539
ENSECAG00000013445	5.053479792	0.8504493	0.962779	650	551	625	709	698	592	653	468
ENSECAG00000023269	7.911177903	0.8504731	0.962779	5189	4026	4575	4541	4781	3933	4898	4018
ENSECAG00000013756	2.659039001	0.850567	0.9627893	140	90	137	102	142	139	80	86
ENSECAG00000015269	7.577980389	0.8507503	0.9628082	3599	2833	4189	3998	4249	2412	5165	2414
ENSECAG00000015840	1.120086788	0.8507532	0.9628082	26	33	22	79	23	46	42	42
ENSECAG00000009141	3.871783051	0.8509512	0.9628921	308	252	271	273	201	211	336	333
ENSECAG00000016635	4.709457377	0.8510217	0.9628921	485	448	548	502	393	621	366	491
ENSECAG00000013505	3.690772518	0.8510816	0.9628921	291	189	321	220	264	160	301	182
ENSECAG00000000474	7.073406321	0.8512136	0.9629454	3142	1770	2761	2628	2876	2181	3307	1562
ENSECAG00000024938	4.609580506	0.85156	0.9631192	348	539	347	722	294	396	404	609
ENSECAG00000015131	0.110635879	0.8516225	0.9631192	40	0	30	0	31	22	13	7
ENSECAG00000022628	5.858425533	0.8516438	0.9631192	1052	890	1017	1524	987	1192	1102	918
ENSECAG00000012562	4.182420572	0.8517551	0.9631192	375	329	335	331	288	252	367	428
ENSECAG00000016486	5.355151792	0.8519505	0.9631192	777	670	861	814	905	624	888	578
ENSECAG00000024928	5.910243469	0.8521374	0.9631192	866.001	1390	965.001	1332	635.001	1176	953	1616.02
ENSECAG00000023457	6.749586966	0.8521811	0.9631192	2293	928	2613	2374	2941	323	4246	885
ENSECAG00000000375	7.367123274	0.8522617	0.9631192	3649	2469	3918	2453	3652	2570	4200	1785
ENSECAG00000011732	1.428471932	0.8522962	0.9631192	31	44	68	67	42	50	46	40
ENSECAG00000021514	3.863316767	0.8523031	0.9631192	283	283	299	281	267	207	279	264
ENSECAG00000013309	8.02024254	0.8523078	0.9631192	4870	4785	4955	6108	4683	4173	5522	3910
ENSECAG00000020981	3.4220686	0.8524871	0.9631192	185	187	226	253	141	184	183	234
ENSECAG00000000844	0.570701245	0.852561	0.9631192	16	25	39	23	13	27	18	44
ENSECAG00000013687	3.303966933	0.852598	0.9631192	159	175	183	229	137	128	216	248
ENSECAG00000018978	6.382221577	0.85266	0.9631192	1406	1474	1451	2081	1192	1886	1410	1523
ENSECAG00000003230	0.97209108	0.8527238	0.9631192	27	27	30	70	28	13	60	32

ENSECAG00000015487	8.872955514	0.8529115	0.9631404	8751	8491	10515	9550	8946	9276	7391	6775
ENSECAG00000020490	1.462183598	0.852973	0.9631404	33	12	58	105	39	45	85	32
ENSECAG00000022687	7.209484069	0.852997	0.9631404	2635	2038	2753	4043	3084	1633	3561	2741
ENSECAG00000007070	4.133288638	0.853239	0.963318	317	295	326	399	233	305	377	370
ENSECAG00000002156	0.779904911	0.8533465	0.9633435	32	24	27	49	21	30	27	33
ENSECAG00000013073	6.519749034	0.8536306	0.9634919	2803	1761	1572	652	1551	1423	2247	1594
ENSECAG00000017018	5.508623579	0.8536476	0.9634919	878	669	1004	937	904	823	938	645
ENSECAG00000011320	2.730593799	0.8537474	0.9635089	147	54	184	148	113	51	221	87
ENSECAG00000011476	2.645889606	0.8539344	0.9636241	118	112	139	122	78	84	136	138
ENSECAG00000006216	4.183498092	0.8540676	0.9636788	404	289	306	382	348	222	493	288
ENSECAG00000002955	5.051819094	0.8543777	0.963878	662	479	676	715	927	304	840	397
ENSECAG00000013120	7.474667441	0.8544139	0.963878	3302	3402	3187	3598	1834	5049	2647	3192
ENSECAG00000004226	1.624635579	0.8546133	0.9639464	61	35	70	76	40	51	59	58
ENSECAG00000017006	5.755255621	0.8546442	0.9639464	1156	877	1041	1230	763	1084	834	1068
ENSECAG00000000187	5.408268531	0.8549585	0.9641127	872	715	855	933	680	838	804	638
ENSECAG00000009442	5.977470874	0.8551829	0.9641127	1072	1053	1259	1667	804	1244	1054	1289
ENSECAG00000020997	4.315603448	0.855203	0.9641127	394	293	450	376	304	412	342	379
ENSECAG00000016337	6.531037502	0.8552999	0.9641127	2095	1138	1891	2323	2153	1305	1716	1310
ENSECAG00000017524	3.604899759	0.8553264	0.9641127	200	225	305	230	187	262	158	220
ENSECAG00000011527	4.934301158	0.8554148	0.9641127	682	506	512	627	428	335	726	793
ENSECAG00000014716	2.15301452	0.8554158	0.9641127	90	66	89	106	45	67	66	125
ENSECAG00000015339	2.346007442	0.8554708	0.9641127	43	52	53	248	49	123	107	88
ENSECAG00000000692	8.043672482	0.855625	0.9641909	5164	4862	5067	5962	6319	3451	5878	2969
ENSECAG00000001333	0.69870707	0.855726	0.964209	32	19	37	35	26	19	49	14
ENSECAG00000022240	0.661291107	0.8561113	0.9644873	17.0001	29.0001	31.0001	34.0001	11	37.0001	28	33.0001
ENSECAG00000019444	1.162985863	0.8561427	0.9644873	21	35	31	93	2	17	83	52
ENSECAG00000024984	5.758807153	0.8562724	0.9645057	876	1154	931	1349	494	1051	982	1262
ENSECAG00000007715	6.805945628	0.8563289	0.9645057	2004	1873	1962	2770	1388	1708	2477	2704
ENSECAG00000019840	6.742859914	0.856468	0.9645667	1938	2184	1799	2592	1808	1672	2126	1948
ENSECAG00000008473	3.006480409	0.8566117	0.964633	121.001	131	194.001	193.001	95.0009	165.001	115.001	172
ENSECAG00000013800	3.537892696	0.8567329	0.9646738	235	225	206	202	310	107	230	207
ENSECAG00000008613	5.093605006	0.8569091	0.9646951	581	637	649	725	332	884	474	755
ENSECAG00000008835	7.536709982	0.8569217	0.9646951	3691	2903	4220	4028	3125	1990	5525	2772
ENSECAG00000007888	3.023642337	0.8570861	0.9647359	158	136	170	179	124	97	175	174
ENSECAG00000014250	1.647862732	0.8571533	0.9647359	25	109	39	47	28	44	47	108
ENSECAG00000008957	6.141870136	0.8572128	0.9647359	1251	1466	1570	944	326	2309	470	1899
ENSECAG00000026852	3.073468715	0.8573238	0.9647654	197	112	171	152	199	118	197	102
ENSECAG00000019089	6.374686139	0.8578927	0.9648023	1399	1568	1692	1948	911	1361	1457	2115
ENSECAG00000011249	3.8376001	0.8579928	0.9648023	186	409	189	269	109	164	228	564
ENSECAG00000010272	5.444663258	0.8579941	0.9648023	818	717	799	1008	1036	579	893	680
ENSECAG00000024806	0.549636076	0.85815	0.9648023	22	7	25	52	31	21	38	12
ENSECAG00000017676	2.140455268	0.8581566	0.9648023	27	58	128	115	78	34	188	37
ENSECAG00000021815	3.285939373	0.8583524	0.9648023	150	137	201	257	190	189	138	181
ENSECAG00000015786	3.34021268	0.858426	0.9648023	160	166	253	224	198	165	172	163
ENSECAG00000020154	4.690461707	0.8585135	0.9648023	500	450	453	568	451	372	611	468
ENSECAG00000012399	7.991517738	0.8585195	0.9648023	6276	3226	5246	4663	6832	3766	5782	2333
ENSECAG00000009349	4.161420704	0.858571	0.9648023	319	294	369	448	316	410	260	233
ENSECAG00000019261	2.416563525	0.858953	0.9648023	93	100	103	100	91	106	98	87
ENSECAG00000009199	0.865275273	0.8590493	0.9648023	30	40	30	37	25	30	29	35
ENSECAG00000011042	1.736888958	0.8590722	0.9648023	71	84	30	54	54	70	20	89
ENSECAG00000019014	3.042248515	0.8591451	0.9648023	146	150	146	213	154	104	233	89
ENSECAG00000013692	8.330296655	0.8591874	0.9648023	4159	4121	7400	9227	4305	6039	11724	2236
ENSECAG00000011163	5.979787641	0.8591986	0.9648023	1354	1138	1143	1167	1283	902	1510	952
ENSECAG00000022594	2.051387436	0.8592602	0.9648023	49	87	107	79	75	45	86	79
ENSECAG00000023235	6.558906961	0.8592971	0.9648023	2047	1512	1781	2175	1537	1195	2294	1723
ENSECAG00000011727	3.23283109	0.8593088	0.9648023	17	463	38	131	42	144	59	454
ENSECAG00000013885	5.466473504	0.859318	0.9648023	777	797	879	1065	747	807	790	744
ENSECAG00000000051	4.659896204	0.8593823	0.9648023	47	1813	29	12	62	120	41	1468
ENSECAG00000016244	5.480094536	0.8594237	0.9648023	739	780	844	1208	721	631	1039	784
ENSECAG00000007177	3.644370393	0.8594642	0.9648023	265	171	272	236	350	150	309	112
ENSECAG00000001080	3.553126794	0.8596748	0.9648023	170	197	273	296	181	164	334	153
ENSECAG00000011754	5.57116592	0.8597587	0.9648023	1199	645	1092	848	1403	503	895	529
ENSECAG00000009291	1.540229608	0.8598084	0.9648023	51	45	51	66	38	71	27	66
ENSECAG00000008929	4.864847867	0.8598145	0.9648023	592	483	688	541	611	440	580	411
ENSECAG00000021498	5.353696951	0.8599863	0.9648023	910	788	737	644	400	577	990	1080
ENSECAG00000013037	5.102691311	0.860018	0.9648023	746	454	820	751	966	582	713	101
ENSECAG00000017349	5.536717474	0.8600225	0.9648023	922	884	922	780	466	847	853	1230
ENSECAG00000012013	6.335730707	0.860053	0.9648023	1645	1265	1711	1557	1514	952	1886	1632
ENSECAG00000003775	7.529826798	0.8600747	0.9648023	3615	3342	3858	3870	3857	2290	4431	2575
ENSECAG00000008786	6.091065073	0.8601832	0.9648288	1406	998	1358	1700	1405	733	1584	1146
ENSECAG00000024824	3.989462482	0.8603748	0.9649484	287	384	173	342	401	120	524	158
ENSECAG00000010309	4.018728375	0.8605138	0.9649808	349	195	266	439	235	186	370	413
ENSECAG00000024461	8.998453377	0.8605866	0.9649808	9999	8915	10445	11431	9394	9249	10334	6833
ENSECAG00000006859	7.319251552	0.8606585	0.9649808	3390	2800	3594	2839	2696	2426	3367	2805
ENSECAG00000009597	3.339181065	0.8608531	0.9650288	154	195	176	238	83	246	180	220
ENSECAG00000023114	0.328598743	0.8608713	0.9650288	20	27	21	24	22	13	18	27
ENSECAG00000025107	5.932646123	0.8613354	0.9653965	1336	1203	1191	858	778	599	1580	1640
ENSECAG00000024304	4.343978562	0.8614	0.9653965	359	292	436	469	436	383	353	291

ENSECAG00000024440	4.976457804	0.8614543	0.9653965	602	471	667	671	693	401	673	545
ENSECAG00000013355	4.206485159	0.8617237	0.9655345	376	301	352	373	213	315	331	489
ENSECAG00000010629	6.119684306	0.8619154	0.9655345	1413	1583	653	1628	1076	939	1004	2080
ENSECAG00000007813	6.545631688	0.8620452	0.9655345	1429	2824	1564	1468	706	2015	961	2720
ENSECAG00000002400	3.595675497	0.862069	0.9655345	218	219	271	249	285	102	353	121
ENSECAG00000018168	1.066219756	0.8622233	0.9655345	32	40	46	31	49	30	33	34
ENSECAG00000019917	2.576256564	0.8622423	0.9655345	112	106	109	117	105	105	168	58
ENSECAG00000023654	2.102420152	0.8623098	0.9655345	71	75	66	109	76	65	94	75
ENSECAG00000006063	2.555123471	0.8623232	0.9655345	94	76	138	161	121	79	116	89
ENSECAG00000020436	4.373843606	0.8623933	0.9655345	344	406	330	501	305	415	408	375
ENSECAG00000012267	4.645801995	0.8624677	0.9655345	562	369	553	505	311	319	520	627
ENSECAG00000020723	2.420511185	0.8625124	0.9655345	89	125	115	88	23	91	72	178
ENSECAG00000019040	4.589784965	0.8626479	0.965591	594	323	430	490	683	283	398	389
ENSECAG00000019876	2.055611565	0.8630049	0.9658557	95	76	73	79	63	74	57	86
ENSECAG00000016930	0.696915204	0.8630544	0.9658557	27	21	31	45	41	12	41	13
ENSECAG00000024056	5.169744344	0.8637094	0.9663927	717	558	791	682	563	613	700	748
ENSECAG00000009639	1.676298249	0.8638408	0.9663927	36	37	71	113	82	28	97	12
ENSECAG00000008498	4.733998508	0.863964	0.9663927	501	595	420	487	477	561	363	504
ENSECAG00000007830	4.64862205	0.8639767	0.9663927	553	439	373	637	425	240	698	442
ENSECAG00000012635	5.055514987	0.8640213	0.9663927	634	590	562	757	650	715	575	447
ENSECAG00000018273	2.55633776	0.8641167	0.9663927	106	115	88	130	86	130	85	117
ENSECAG00000014795	3.83882592	0.8641299	0.9663927	319	111	247	434	237	137	384	315
ENSECAG00000002570	3.105764078	0.8642553	0.9664379	92	232	100	211	24	124	137	355
ENSECAG00000021734	2.539545156	0.8644996	0.9666159	93	103	102	139	85	112	95	124
ENSECAG00000019607	4.009717949	0.8647915	0.9668471	363	231	391	294	498	199	250	162
ENSECAG00000010678	3.671966884	0.8649352	0.9668525	181	231	196	366	142	331	187	249
ENSECAG00000010203	0.671323133	0.8650002	0.9668525	24	11	32	48	39	27	33	11
ENSECAG00000019549	3.861413828	0.8652797	0.9668525	398	107	342	326	322	193	386	120
ENSECAG00000019484	6.595764424	0.865325	0.9668525	1753	1476	1849	2394	1281	2089	1744	1878
ENSECAG00000000464	6.39578984	0.8653331	0.9668525	1732	1241	1813	1948	1977	754	2011	1283
ENSECAG00000008944	4.234962624	0.8653452	0.9668525	348	475	250	404	363	257	313	382
ENSECAG00000010099	2.316353428	0.8654008	0.9668525	81	101	75	136	42	104	84	108
ENSECAG00000018344	4.347201335	0.8654773	0.9668525	338	311	398	598	495	278	533	125
ENSECAG00000023777	6.575228658	0.8656409	0.9668913	1982	1625	1730	1960	2287	1590	1665	1355
ENSECAG00000024021	0.170404736	0.8656822	0.9668913	7	27	8	35	7	22	7	39
ENSECAG00000011412	4.191236027	0.8660564	0.9671636	155	306	462	471	143	242	474	512
ENSECAG00000015910	7.201157837	0.8661313	0.9671636	2985	2700	2540	3012	2865	2070	2672	3161
ENSECAG00000009267	5.118995658	0.8661815	0.9671636	642	607	586	943	495	619	688	639
ENSECAG00000019032	6.785388901	0.8663123	0.9672146	2264	1738	2132	2329	2266	1444	2774	1679
ENSECAG00000014668	4.546820989	0.8665315	0.9673643	681	189	568	328	510	161	858	262
ENSECAG00000006944	2.850591764	0.8667185	0.9674115	166	134	141	123	184	85	156	75
ENSECAG00000004011	1.35448283	0.8667441	0.9674115	48	48	30	60	22	44	48	68
ENSECAG00000009961	3.819623666	0.8668488	0.9674333	285	214	294	333	209	325	156	267
ENSECAG00000021834	5.551311814	0.8671858	0.9675401	903	969	788	900	610	933	964	904
ENSECAG00000010852	11.53960539	0.8671996	0.9675401	64839	44770	58101	60429	54037	56418	46759	58187
ENSECAG00000018147	4.516439035	0.8672195	0.9675401	478	429	396	512	401	200	709	346
ENSECAG00000017394	7.859535506	0.8672852	0.9675401	4715	3851	3481	5829	5974	2853	5429	2892
ENSECAG00000022610	7.691877674	0.8674187	0.9675939	3970	3534	4434	4507	3367	2981	4758	3612
ENSECAG00000011267	7.400152768	0.8675637	0.9676607	3377	3408	2843	3757	3053	2576	3688	2641
ENSECAG00000015504	6.014695433	0.8677315	0.9677528	1355	1212	1233	1304	1167	952	1061	1366
ENSECAG00000019482	3.399246871	0.867927	0.9678758	162	202	166	312	175	162	196	201
ENSECAG00000009310	2.990073402	0.8683118	0.9682056	150	100	181	171	131	133	126	183
ENSECAG00000024260	1.768941397	0.8683932	0.9682056	35	63	80	73	75	70	43	50
ENSECAG00000023757	0.897090432	0.8686347	0.9682356	25	23	46	40	43	22	42	24
ENSECAG00000022878	5.915314329	0.8686406	0.9682356	1002	869	991	2063	1410	981	1444	398
ENSECAG00000016420	5.256577551	0.8686758	0.9682356	759	561	785	830	873	431	895	623
ENSECAG00000017682	2.47563496	0.8689284	0.968422	45	233	49	100	27	121	38	182
ENSECAG00000004829	5.180963337	0.8691063	0.9684977	650	714	593	931	356	626	863	730
ENSECAG00000018758	5.168367075	0.8692059	0.9684977	660	656	623	928	471	432	857	817
ENSECAG00000018027	6.539906242	0.8692521	0.9684977	1996	1506	1766	2160	2316	1238	2150	874
ENSECAG00000012970	6.265667024	0.8694745	0.9686505	1536	1325	1218	1842	1496	1557	1411	1082
ENSECAG00000000346	5.890707048	0.8697623	0.9688761	1065	997	939	1771	751	856	1284	1333
ENSECAG00000018606	4.783875846	0.8699515	0.9689445	521	486	432	675	496	374	607	548
ENSECAG00000016301	5.720003988	0.8699942	0.9689445	1089	830	1074	1047	948	935	990	948
ENSECAG00000016867	4.227568268	0.8702073	0.9690011	135	177	211	1075	251	363	369	309
ENSECAG00000011553	10.50951869	0.8702421	0.9690011	30272	18634	29974	33751	29721	24842	31346	20708
ENSECAG00000015455	4.77945278	0.8704435	0.9690011	679	246	682	494	406	275	814	576
ENSECAG00000002810	-0.028930069	0.8704459	0.9690011	19	17	13	16	12	24	23	6
ENSECAG00000014950	2.711894325	0.8705573	0.9690011	113	120	136	120	113	131	110	114
ENSECAG00000023879	10.26150867	0.8707272	0.9690011	25015	22677	25696	23750	22050	19604	25402	19571
ENSECAG00000009898	2.709627354	0.8708327	0.9690011	108	129	131	146	78	56	183	149
ENSECAG00000020871	6.510179109	0.8709427	0.9690011	1824	1605	1909	1875	1840	1531	1713	1312
ENSECAG00000016127	6.152253804	0.8709559	0.9690011	1601	1010	1357	1505	1314	889	1818	1251
ENSECAG00000007125	8.091789749	0.8709601	0.9690011	5319	4956	5408	5983	4438	4375	6117	4410
ENSECAG00000024729	8.497681124	0.8710462	0.9690011	8060	5308	8107	7382	8807	3581	9036	4392
ENSECAG00000023697	6.184813912	0.8710894	0.9690011	1438	1443	1356	1501	1237	983	1831	1156
ENSECAG00000007521	6.009316291	0.8711642	0.9690011	1053	1365	1212	1269	1016	1286	1163	1190
ENSECAG00000023225	5.63163944	0.8712672	0.9690011	1067	698	1066	967	1287	729	948	638

ENSECAG00000024844	8.229845874	0.8713247	0.9690011	5661	5594	5432	6222	3769	7184	4891	5755
ENSECAG00000005798	5.981961732	0.8714746	0.9690117	1535	994	1217	1264	1378	698	1436	995
ENSECAG00000022849	7.155181709	0.8717439	0.9690117	2669	2570	2621	3060	2067	2593	2824	2912
ENSECAG00000002550	1.920730857	0.8717606	0.9690117	68	56	72	86	80	69	46	71
ENSECAG00000016641	5.814375496	0.8717929	0.9690117	955	967	1174	1221	1105	660	1447	961
ENSECAG00000015154	5.011480428	0.8718277	0.9690117	538	648	467	820	497	514	774	587
ENSECAG00000016553	3.208166832	0.8719203	0.9690117	155	128	188	234	160	118	140	254
ENSECAG00000013920	5.15055134	0.8719471	0.9690117	688	578	560	915	673	467	842	636
ENSECAG00000014571	2.340371519	0.8720167	0.9690117	87	57	106	132	156	24	116	77
ENSECAG00000026822	3.690744605	0.8724907	0.969307	264	226	283	243	287	145	257	218
ENSECAG00000014044	5.23650056	0.872494	0.969307	808	515	761	815	564	445	893	896
ENSECAG00000011865	5.719336636	0.8728144	0.969307	1000	982	991	1049	1129	656	1200	891
ENSECAG00000013768	6.230443381	0.8728575	0.969307	1451	1231	1428	1666	1409	1417	1433	1172
ENSECAG00000023450	2.764390914	0.8728714	0.969307	131	128	145	100	132	75	152	139
ENSECAG00000016632	5.472328689	0.872896	0.969307	960	705	876	984	742	539	998	893
ENSECAG00000019428	4.427139463	0.8731309	0.969307	449	331	463	464	459	308	455	291
ENSECAG00000017960	4.58490734	0.8731625	0.969307	414	496	389	527	443	331	517	469
ENSECAG00000009622	3.718375451	0.8732635	0.969307	392	183	233	233	292	102	354	191
ENSECAG00000012056	7.632065702	0.8732769	0.969307	3013	4351	3634	4090	3294	3641	3277	4169
ENSECAG00000006391	2.88359185	0.873296	0.969307	94	231	98	115	96	131	132	176
ENSECAG00000018661	5.284124131	0.8733902	0.969307	809	619	774	782	753	531	809	761
ENSECAG00000007943	0.393934094	0.8733917	0.969307	21	20	29	27	23	8	38	18
ENSECAG00000003229	9.002375109	0.8736977	0.9695493	11156	8355	10092	11233	8491	8870	10538	8237
ENSECAG00000002460	3.42538789	0.8737808	0.9695493	291	139	213	209	272	151	232	91
ENSECAG00000014160	5.857926005	0.873988	0.9695906	1070	878	1038	1509	1183	896	1276	883
ENSECAG00000024306	8.487810309	0.8742214	0.9695906	7721	7211	7333	5973	5070	4788	6139	9407
ENSECAG00000000648	5.210456649	0.8743053	0.9695906	543	623	658	1042	627	416	898	808
ENSECAG00000000462	2.4210701	0.8743205	0.9695906	49	263	19	36	44	122	62	158
ENSECAG00000002635	5.19254979	0.8743272	0.9695906	571	587	759	1017	403	552	729	908
ENSECAG00000000328	2.042588046	0.8743302	0.9695906	105	64	96	53	82	81	73	41
ENSECAG00000020354	3.94281523	0.8747089	0.9698642	243	254	275	409	199	254	356	319
ENSECAG00000022196	4.583333985	0.874773	0.9698642	458	386	481	507	573	270	494	421
ENSECAG00000021839	3.596361632	0.874833	0.9698642	219	247	206	283	162	173	174	333
ENSECAG00000000395	4.564700324	0.8752142	0.9701921	585	299	411	519	610	406	481	217
ENSECAG00000018788	6.698102898	0.8753812	0.9702378	2141	1746	1756	2646	1517	1895	2040	1857
ENSECAG00000012718	4.732727244	0.8754263	0.9702378	506	609	482	482	331	484	493	555
ENSECAG00000004353	0.419230811	0.8760027	0.9707819	15	13	30	37	30	15	30	17
ENSECAG00000014382	2.842054553	0.876389	0.9710606	139	112	120	171	194	68	171	93
ENSECAG00000000053	3.494339444	0.8765292	0.9710606	223	161	217	302	351	101	179	150
ENSECAG00000014969	3.288500946	0.8765412	0.9710606	178	257	131	199	133	282	64	165
ENSECAG00000010652	0.806753444	0.876671	0.9710606	28	27	36	42	37	22	37	19
ENSECAG00000009458	7.687855793	0.8766871	0.9710606	4414	4757	3320	3675	4153	2146	4658	3787
ENSECAG00000009067	5.78974669	0.8767671	0.9710606	1459	792	1550	546	1396	604	1495	441
ENSECAG00000009235	3.698839641	0.8769501	0.9710959	241	196	270	328	146	240	234	284
ENSECAG00000015150	9.112863431	0.877009	0.9710959	11848	9611	10616	11905	10358	9049	9344	10062
ENSECAG00000008257	3.266258326	0.8770688	0.9710959	219	113	202	196	255	80	226	149
ENSECAG00000023010	5.001872312	0.8771744	0.9710959	677	521	601	652	475	654	665	527
ENSECAG00000023501	8.129597244	0.8772264	0.9710959	5921	3797	6721	5955	5191	2551	8369	4207
ENSECAG00000013966	5.327509592	0.877523	0.9713295	623	640	602	1265	533	655	919	845
ENSECAG00000015564	3.190124433	0.8777377	0.9713907	142	158	209	178	96	210	152	196
ENSECAG00000022725	2.31919888	0.8778901	0.9713907	86	98	76	132	56	82	97	109
ENSECAG00000008186	7.660000386	0.8779875	0.9713907	4293	3213	4238	4349	4097	2232	4938	3227
ENSECAG00000014324	3.480559857	0.8780505	0.9713907	212	141	222	281	120	224	192	270
ENSECAG00000014872	3.388604657	0.8782034	0.9713907	181	161	204	253	165	187	165	237
ENSECAG00000008102	7.207267141	0.8782547	0.9713907	2451	3101	2911	3217	2672	2405	3469	1928
ENSECAG00000006228	3.275893237	0.8782563	0.9713907	217	146	189	216	193	149	267	71
ENSECAG00000001645	5.376002878	0.8784194	0.9713907	1199	607	746	734	752	531	1268	436
ENSECAG00000008349	2.768690516	0.8784308	0.9713907	140	65	179	131	153	107	161	74
ENSECAG00000024996	5.60523308	0.8784521	0.9713907	581	1240	1171	613	302	601	812	1912
ENSECAG00000007581	3.73111958	0.878519	0.9713907	295	173	265	280	288	166	362	165
ENSECAG00000014242	5.316875611	0.878737	0.9715373	746	748	867	779	883	631	686	587
ENSECAG00000001851	5.660520679	0.8788645	0.9715837	847	668	1322	1042	1574	566	1131	443
ENSECAG00000018454	4.24260393	0.8792927	0.9719431	325	455	317	384	320	314	281	401
ENSECAG00000021329	0.750849228	0.8793608	0.9719431	33	22	28	45	19	16	54	25
ENSECAG00000015177	5.186077955	0.8794738	0.9719735	806	578	718	785	818	415	753	597
ENSECAG00000002222	3.865506938	0.8796085	0.9720277	299	243	289	272	299	165	450	173
ENSECAG00000017344	4.652366916	0.8797605	0.9720633	434	470	442	653	484	384	436	458
ENSECAG00000013049	5.694971816	0.8798289	0.9720633	1013	777	1090	1107	1143	630	1122	907
ENSECAG00000008605	5.602468798	0.8798974	0.9720633	901	798	885	1158	522	702	1130	1241
ENSECAG00000022177	4.169710556	0.880193	0.9722953	350	253	377	399	415	178	380	351
ENSECAG00000008250	6.050478462	0.8803827	0.9724024	1280	1140	1458	1362	1311	1120	985	1210
ENSECAG00000022015	1.071830591	0.8804637	0.9724024	22	29	17	101	6	41	30	60
ENSECAG00000006455	5.366487287	0.8806604	0.9724024	1067	524	687	902	599	587	858	988
ENSECAG00000014534	0.709663433	0.8807121	0.9724024	29	8	31	60	48	9	31	19
ENSECAG00000012489	7.104846575	0.8808576	0.9724024	2855	1993	3123	2997	2998	2311	2222	2073
ENSECAG00000017002	8.164944926	0.8811024	0.9724024	6071	4892	5419	6446	6441	3465	5450	4966
ENSECAG00000004882	6.813926895	0.8811654	0.9724024	2250	1776	2162	2477	2197	1542	2636	1907
ENSECAG00000021521	4.506964339	0.881239	0.9724024	368	524	444	443	296	362	541	415

ENSECAG00000005394	4.775552794	0.8813244	0.9724024	463	513	499	618	359	481	412	731
ENSECAG00000008168	5.196942366	0.8813782	0.9724024	760	671	608	765	685	440	827	751
ENSECAG00000013572	0.696663953	0.8814036	0.9724024	21	30	43	27	17	36	15	35
ENSECAG00000014168	7.168098723	0.881464	0.9724024	3187	2241	3169	2802	2753	2728	2296	2230
ENSECAG00000000789	4.333018414	0.8814812	0.9724024	421	273	390	528	323	282	449	375
ENSECAG00000011135	4.116638556	0.8815608	0.9724024	361.001	186	409.001	376	485.001	189.001	324	267.024
ENSECAG00000027056	2.039588054	0.8816856	0.9724024	61	104	78	55	69	74	51	97
ENSECAG00000015730	7.905606802	0.8817694	0.9724024	4906	4701	5222	3109	1196	4388	3108	8894
ENSECAG00000020939	3.791306843	0.8818151	0.9724024	217	330	207	287	214	338	197	234
ENSECAG00000010433	4.668149455	0.8818995	0.9724024	567	480	344	547	491	333	677	379
ENSECAG00000010437	6.126755259	0.8819165	0.9724024	1582	1125	1807	966	937	1222	1359	1410
ENSECAG00000022667	2.364321587	0.8820437	0.9724189	91	59	92	174	101	113	110	23
ENSECAG00000014800	7.340843473	0.8821027	0.9724189	3542	2840	3967	2387	2239	3331	3627	2198
ENSECAG00000021124	4.252005378	0.8821962	0.9724277	445	268	383	356	481	208	354	348
ENSECAG00000008028	2.15574642	0.8824773	0.9726431	73	65	99	115	53	116	58	69
ENSECAG00000022385	5.5037342	0.8826774	0.9727692	763	875	1021	915	729	952	786	690
ENSECAG00000010426	3.965286135	0.8828077	0.9728184	255	254	264	429	380	161	355	253
ENSECAG00000014977	4.600655347	0.8829392	0.9728689	402	455	470	524	351	557	371	454
ENSECAG00000016198	2.080148085	0.8830959	0.9728983	40	52	84	167	81	24	162	34
ENSECAG00000017872	5.023214091	0.8831371	0.9728983	571	588	669	750	409	644	484	725
ENSECAG00000004246	4.02497455	0.8832378	0.9729148	290	266	388	342	309	269	298	260
ENSECAG00000003565	4.880347134	0.8833488	0.9729428	589	410	568	807	222	223	783	913
ENSECAG00000001462	4.551340579	0.8837667	0.973296	393	395	596	468	522	396	418	296
ENSECAG00000021725	5.722770956	0.8839211	0.973296	1284	706	932	1152	991	1094	953	757
ENSECAG00000013471	3.293920633	0.8839265	0.973296	156	169	197	223	173	148	155	232
ENSECAG00000023753	4.463676602	0.8841071	0.9734005	481	581	339	312	178	576	214	526
ENSECAG00000025056	6.865564982	0.8844897	0.9736714	2287	1918	2283	2805	2125	1705	3017	1486
ENSECAG00000013395	7.677694412	0.8846699	0.9736714	3671	3569	3443	5134	3960	3664	3732	3472
ENSECAG00000008229	0.216940156	0.8846928	0.9736714	14	27	17	21	11	14	31	24
ENSECAG00000016728	4.364496886	0.884696	0.9736714	403	355	423	446	377	273	390	414
ENSECAG00000007791	2.951283895	0.8849635	0.9738151	191	73	177	144	160	65	247	107
ENSECAG00000012891	7.848069898	0.8850878	0.9738151	4801	3681	4810	5017	3848	3809	4289	4299
ENSECAG00000008941	7.184215721	0.8851686	0.9738151	3204	2314	2719	2928	2627	2677	3158	2122
ENSECAG00000008176	5.741219856	0.8853328	0.9738151	1000	947	988	1176	1041	929	1115	796
ENSECAG00000001415	7.101697593	0.8853369	0.9738151	2575	1858	2469	3803	2945	1793	3268	2115
ENSECAG00000017076	3.609540815	0.885341	0.9738151	232	210	258	263	148	222	190	287
ENSECAG00000019360	5.185512376	0.8855629	0.9738454	648	615	646	898	686	607	579	754
ENSECAG00000008680	8.22498016	0.8856792	0.9738454	5169	6129	5212	7230	5640	4646	6544	4372
ENSECAG00000009197	5.217885546	0.8857971	0.9738454	977	660	684	597	809	475	888	475
ENSECAG00000026945	1.623865524	0.8858326	0.9738454	81	63	52	24	39	36	34	111
ENSECAG00000013057	4.512095274	0.8859031	0.9738454	540	454	533	245	318	293	314	675
ENSECAG00000018720	0.569313059	0.8859274	0.9738454	25	28	35	21	19	16	22	40
ENSECAG00000013117	4.837726411	0.886062	0.9738454	456	599	545	658	309	534	500	661
ENSECAG00000015555	4.342724392	0.8860919	0.9738454	346	384	401	413	481	265	382	347
ENSECAG00000018723	5.582372144	0.8862102	0.9738454	774	852	802	1276	821	617	772	1287
ENSECAG00000011831	1.461894519	0.8863299	0.9738454	59	44	54	55	32	16	44	97
ENSECAG00000023721	9.840845747	0.8863952	0.9738454	12262	23588	11706	21987	6696	25338	16611	17441
ENSECAG00000022660	8.14288464	0.8863973	0.9738454	5059	4898	5225	7404	4644	4655	6483	4287
ENSECAG00000008628	0.620104522	0.8865816	0.9739537	38	17	31	29	27	15	28	31
ENSECAG00000007848	2.187920411	0.8867142	0.9740051	92	55	84	131	33	68	115	102
ENSECAG00000012409	4.867198034	0.8868495	0.9740065	759	403	613	536	649	260	802	388
ENSECAG00000022785	5.111155304	0.8871174	0.9740065	745	571	774	529	890	564	727	326
ENSECAG00000009409	4.189439734	0.8871283	0.9740065	378	344	332	326	345	163	442	405
ENSECAG00000021002	7.800930679	0.8873106	0.9740065	4904	3802	4074	4913	3464	3552	4782	4038
ENSECAG00000010522	5.948239802	0.887402	0.9740065	1403	1236	1239	980	2400	549	842	483
ENSECAG00000015982	6.207476711	0.8875482	0.9740065	1552	1140	1567	1613	1795	673	1571	1239
ENSECAG00000022306	4.24398944	0.8876221	0.9740065	330	384	368	353	279	350	458	295
ENSECAG00000023923	1.551946331	0.8877404	0.9740065	29	86	46	62	33	66	48	48
ENSECAG00000020298	8.71190431	0.8877463	0.9740065	8048	6572	7993	9775	8371	7005	8438	6711
ENSECAG00000024610	1.827797415	0.887797	0.9740065	47	71	108	48	5	49	39	147
ENSECAG00000016697	3.986538152	0.8878157	0.9740065	355	229	316	309	285	255	406	215
ENSECAG00000008458	8.417677416	0.8878261	0.9740065	6843	5517	6511	8430	6701	4143	7325	6224
ENSECAG00000007605	7.494775884	0.8878741	0.9740065	2933	3546	2989	4409	2783	3824	3425	2994
ENSECAG00000022484	7.011748208	0.8879421	0.9740065	2446	2165	2148	3572	1910	2184	2360	2648
ENSECAG00000011975	6.240889169	0.8880017	0.9740065	1435	1322	1479	1570	1301	1535	1259	1336
ENSECAG00000007859	0.275562512	0.8883204	0.9741323	22	20	25	21	9	14	30	26
ENSECAG00000015427	4.318497233	0.8883424	0.9741323	299	406	389	483	237	364	364	433
ENSECAG00000017886	4.878745024	0.8883737	0.9741323	582	498	549	706	623	377	617	468
ENSECAG00000007490	3.562582836	0.8884633	0.9741366	180	250	200	265	158	231	211	251
ENSECAG00000016949	1.454573175	0.8888798	0.9743171	46	51	59	54	20	30	75	65
ENSECAG00000010554	4.548965525	0.8889721	0.9743171	593	245	493	541	374	353	624	317
ENSECAG00000011075	4.282906828	0.8891971	0.9743171	377	387	387	375	290	407	366	291
ENSECAG00000003998	4.617114153	0.889256	0.9743171	576	481	444	344	448	228	513	627
ENSECAG00000021853	2.945676651	0.8892929	0.9743171	139	125	178	137	169	83	205	110
ENSECAG00000022626	4.805966686	0.8892999	0.9743171	499	531	455	660	512	349	700	504
ENSECAG00000007757	2.822135562	0.8893048	0.9743171	155	98	148	158	70	67	154	210
ENSECAG00000007173	4.62257942	0.8893576	0.9743171	583	339	270	805	608	206	668	282
ENSECAG00000020143	4.553339103	0.8894577	0.9743171	515	409	478	374	590	297	593	246

ENSECAG00000026980	6.641189662	0.8895994	0.9743171	1796	2068	1384	2425	1627	1716	2017	1906
ENSECAG00000007236	5.345683603	0.8896489	0.9743171	617	947	728	905	472	844	693	830
ENSECAG00000020841	5.011982125	0.8899283	0.9743171	638	539	682	693	552	600	452	638
ENSECAG00000020561	1.223903922	0.8899553	0.9743171	42	26	34	71	52	27	71	18
ENSECAG00000023940	4.009261962	0.8899894	0.9743171	283	298.002	303	390	253.002	339	267	254
ENSECAG00000009893	3.836406857	0.8901209	0.9743171	283	236	294	271	214	136	397	320
ENSECAG00000016906	2.598812165	0.890222	0.9743171	124	94	130	105	111	74	80	171
ENSECAG00000022645	4.66390998	0.8902386	0.9743171	413	608	507	446	370	508	427	459
ENSECAG00000014741	4.620894537	0.8905102	0.9743171	789	230	417	536	263	666	461	298
ENSECAG00000019081	3.686207633	0.8905352	0.9743171	187	222	236	341	105	259	246	324
ENSECAG00000019384	3.245478643	0.8905888	0.9743171	142	171	177	263	172	203	146	127
ENSECAG00000024675	2.65475842	0.8906344	0.9743171	74	110	146	168	87	118	107	120
ENSECAG00000017011	1.07086183	0.8908974	0.9743171	35	49	43	20	12	28	31	78
ENSECAG00000026834	2.367012994	0.89091	0.9743171	101	80	104	119	84	68	117	91
ENSECAG00000000229	1.00654257	0.8909139	0.9743171	4	102	23	15	23	15	56	43
ENSECAG00000018521	6.328349477	0.8909746	0.9743171	1557	1428	1464	1730	1683	1188	1618	1360
ENSECAG00000000628	0.81754235	0.8910113	0.9743171	36	14	19	59	0	19	81	31
ENSECAG00000020917	7.059722638	0.8911232	0.9743171	2862	1932	2724	2762	2089	1874	3532	2389
ENSECAG00000000350	4.389713018	0.8911808	0.9743171	357	360	442	444	295	394	356	467
ENSECAG00000025068	7.773140239	0.8912139	0.9743171	4026	3844	4356	5191	2051	3699	4142	5611
ENSECAG00000010046	5.075210366	0.8913141	0.9743171	771	545	659	687	655	405	762	584
ENSECAG00000021754	6.90225644	0.8913435	0.9743171	1755	2089	2109	3327	1329	2508	2424	2429
ENSECAG00000012979	7.213876083	0.8913728	0.9743171	2797	2810	2683	3074	2232	2919	2097	3429
ENSECAG00000021154	2.437650517	0.8916706	0.9743571	122	69	88	130	66	33	158	149
ENSECAG00000010885	6.457803942	0.8917639	0.9743571	1643	1374	1935	1818	1368	1211	2240	1677
ENSECAG00000007838	7.078265185	0.8918817	0.9743571	2718	2263	3061	2277	2571	1894	3024	2432
ENSECAG00000015059	1.114384036	0.8920096	0.9743571	33	33	32	60	49	30	69	7
ENSECAG00000020650	6.038141122	0.8921174	0.9743571	1306	1165	1063	1692	925	1053	1229	1447
ENSECAG00000014336	5.308555763	0.8922571	0.9743571	546	527	801	1349	816	673	997	311
ENSECAG00000015322	7.353274503	0.8923169	0.9743571	3394	2868	3322	3337	2778	2416	3913	2572
ENSECAG00000006724	3.58902609	0.8923609	0.9743571	224	199	208	326	200	114	270	274
ENSECAG00000000019	6.079449497	0.892405	0.9743571	1479	1001	1471	1406	1354	1135	1301	970
ENSECAG00000011777	4.287291035	0.8924284	0.9743571	458	242	479	299	94	540	314	451
ENSECAG00000016141	4.017226816	0.8924816	0.9743571	383	103	310	465	397	141	506	170
ENSECAG00000020907	4.879859097	0.8926566	0.9743571	542	480	597	643	566	509	462	581
ENSECAG00000008557	0.305438318	0.8926907	0.9743571	17	22	19	34	13	30	20	14
ENSECAG00000001911	7.348694751	0.8926924	0.9743571	3329	2589	3014	3644	3174	2233	3732	2840
ENSECAG00000017319	7.110975131	0.892696	0.9743571	2661	1916	2761	3386	3072	2328	2977	1675
ENSECAG00000011972	5.500815119	0.8930555	0.9743838	967	700	978	935	639	629	1028	944
ENSECAG00000011021	2.775426033	0.8930641	0.9743838	130	112	155	139	111	96	177	98
ENSECAG00000025087	6.533443279	0.8932564	0.9743838	1894	1373	1867	2264	2565	1031	1875	1088
ENSECAG00000022420	0.567576263	0.8932577	0.9743838	25	39	28	16	11	30	23	31
ENSECAG00000017516	4.588253716	0.8933929	0.9743838	472	355	598	408	353	288	499	632
ENSECAG00000019702	5.919421537	0.8934129	0.9743838	1047	1050	905	1700	1104	1161	1211	897
ENSECAG00000000410	3.79243717	0.8935287	0.9743838	247	231	232	395	252	181	223	315
ENSECAG00000008733	5.831902494	0.8935781	0.9743838	956	1007	1115	1313	861	1086	1093	1079
ENSECAG00000006660	3.145413636	0.8936413	0.9743838	164	138	209	157	170	172	162	129
ENSECAG00000024012	6.261965751	0.8936923	0.9743838	809	2111	1127	2000	1009	1157	1767	1542
ENSECAG00000012977	4.635659614	0.8937378	0.9743838	559	350	475	525	442	241	607	555
ENSECAG00000011295	0.249022962	0.8937499	0.9743838	22	29	11	24	9	12	36	21
ENSECAG00000010363	9.393486716	0.8941283	0.9747028	12640	12199	13503	14972	11710	11931	13362	10398
ENSECAG00000020210	3.027742284	0.8944088	0.974915	31	225	259	77	120	206	166	93
ENSECAG00000009950	3.873220406	0.894755	0.9750925	307	239	269	345	187	288	239	304
ENSECAG00000022491	5.135316937	0.8948201	0.9750925	731	501	720	758	705	505	725	626
ENSECAG00000021407	4.731348682	0.8948292	0.9750925	483	580	436	587	468	463	477	453
ENSECAG00000008051	5.410438171	0.8950168	0.9752035	791	608	903	986	550	526	1121	965
ENSECAG00000012126	6.889443438	0.8951974	0.9753066	2403	2010	3096	1797	2076	1224	2630	2585
ENSECAG00000013544	3.725621261	0.895399	0.9753708	157	475	193	131	126	315	131	371
ENSECAG00000017095	5.890411389	0.8954866	0.9753708	1251	969	999	1359	1072	1011	1425	822
ENSECAG00000003013	3.577492325	0.8955322	0.9753708	263	153	276	254	273	100	306	173
ENSECAG00000000624	5.748863932	0.8956409	0.9753708	1049	955	1123	1110	951	790	977	1089
ENSECAG00000016089	4.011029014	0.8956856	0.9753708	380	179	383	291	351	243	354	225
ENSECAG00000018997	5.930101987	0.8959914	0.9755535	1110	1015	1393	1315	1558	606	1511	691
ENSECAG00000018379	7.072574003	0.8960251	0.9755535	2383	2645	1994	3354	1936	2636	2817	2388
ENSECAG00000016202	0.828233319	0.8961912	0.9756407	48	20	31	35	42	13	52	13
ENSECAG00000016246	7.486894866	0.8963004	0.9756661	3463	3529	3371	3322	2798	3720	3329	3092
ENSECAG00000013542	6.935712445	0.8964885	0.975696	2476	2299	2751	2076	2347	1963	2784	1592
ENSECAG00000008684	7.732496707	0.8966687	0.975696	3494	4479	3795	5054	3250	2739	4632	4609
ENSECAG00000020062	7.532510494	0.8966704	0.975696	3644	3592	3237	4179	4082	2382	4430	2322
ENSECAG00000016600	1.454312169	0.8966714	0.975696	47	34	78	52	30	44	47	64
ENSECAG00000018319	0.53973495	0.8968913	0.9758418	16	20	25	42	33	8	61	3
ENSECAG00000023761	5.878160566	0.8972128	0.9760323	1191	990	1166	1307	1134	747	1472	867
ENSECAG00000008889	2.414033974	0.8972788	0.9760323	115	67	135	100	87	112	107	58
ENSECAG00000015795	4.124470776	0.8973242	0.9760323	395	280	352	293	532	200	404	139
ENSECAG00000018511	1.307264899	0.8976756	0.9763192	46	30	38	69.9999	41	32	67	37
ENSECAG00000014298	2.647100311	0.8977598	0.9763192	120	122	111	136	65	81	122	171
ENSECAG00000010544	7.503124484	0.8980868	0.9764074	3612	2080	3054	5471	4400	2862	4662	1392
ENSECAG00000010835	2.695410788	0.898154	0.9764074	124	106	105	177	123	133	100	85

ENSECAG00000021950	8.535425925	0.8981904	0.9764074	9167	5167	7575	7564	7903	5563	6585	6083
ENSECAG00000003862	5.765252841	0.8982017	0.9764074	1137	963	948	1130	1099	748	1093	1024
ENSECAG00000003961	1.524999404	0.8982707	0.9764074	44	31	71	67	20	17	91	86
ENSECAG00000013959	2.74176014	0.8986839	0.976763	131	94	157	144	101	58	149	166
ENSECAG00000000420	4.004019806	0.8989349	0.9769423	283	273	331	382	237	297	356	238
ENSECAG00000010706	4.060271218	0.8991025	0.9770311	283	339	339	347	252.007	334	262	309
ENSECAG00000007477	8.162718556	0.8996992	0.9775859	5725	4027	5872	6569	6155	3349	7087	4591
ENSECAG00000008311	7.266123113	0.9002155	0.9780533	3027	2789	2964	3403	2229	3425	2387	2654
ENSECAG00000024939	3.198319972	0.9005375	0.97829	110	413	40	135	55	215	22	317
ENSECAG00000019987	4.41292296	0.9006752	0.97829	360	460	403	447	313	492	392	279
ENSECAG00000021146	6.959851237	0.9008263	0.97829	2868	1987	2459	2541	3009	1560	2879	1418
ENSECAG00000023326	4.558225004	0.9012076	0.97829	426	440	469	463	460	413	442	383
ENSECAG00000015692	2.854728761	0.9012979	0.97829	314	74	99	78	147	32	212	127
ENSECAG00000015514	8.954585897	0.9013146	0.97829	8389	11094	9058	10492	8833	8027	12440	6069
ENSECAG00000014570	4.858577094	0.9013181	0.97829	547	491	543	651	585	450	589	481
ENSECAG00000012505	2.753778755	0.9014527	0.97829	89	113	105	233	69	78	146	183
ENSECAG00000024696	7.218775562	0.9016865	0.97829	3456	2315	2696	3364	3372	1891	2893	2413
ENSECAG00000017219	5.449845466	0.9018555	0.97829	730	743	664	1265	706	831	1013	626
ENSECAG00000000771	2.774053657	0.9021585	0.97829	70	117	183	171	127	173	118	43
ENSECAG00000023789	6.026320308	0.9023401	0.97829	1101	1310	1197	1392	946	1153	1303	1330
ENSECAG00000022969	2.143981427	0.9023738	0.97829	240	27	60	15	129	26	132	21
ENSECAG00000010634	3.496647414	0.9024575	0.97829	168	205	179	313	106	318	185	190
ENSECAG00000023017	0.896431726	0.9024967	0.97829	41	26	11	58	76	19	20	12
ENSECAG00000015428	5.183090621	0.9025792	0.97829	870	580	729	586	809	272	982	649
ENSECAG00000015857	2.950445313	0.9026095	0.97829	221.001	212	55.0005	106	137.001	107	45	236.001
ENSECAG00000013333	4.876455625	0.9026569	0.97829	560	446	577	758	651	280	621	546
ENSECAG00000012815	6.786298476	0.9027349	0.97829	1993	2347	1964	2106	1343	2650	1855	2053
ENSECAG00000016707	5.726605921	0.902787	0.97829	950	856	1007	1288	1051	629	1331	894
ENSECAG00000016251	3.087727847	0.9030328	0.97829	136	171	206	150	84	74	132	310
ENSECAG00000009722	3.602821655	0.9030528	0.97829	272	206	225	217	156	286	173	247
ENSECAG00000010483	4.586715942	0.9031611	0.97829	460	450	449	534	333	505	412	424
ENSECAG00000014887	4.514874675	0.9032342	0.97829	423	460	465	441	374	445	360	412
ENSECAG00000015445	5.758505328	0.9032527	0.97829	1130	890	1065	1195	898	630	1178	1180
ENSECAG00000009190	10.31597117	0.9033112	0.97829	22011	23062	28436	24145	19000	19713	26350	28313
ENSECAG00000024050	1.934899772	0.903623	0.97829	80	74	54	75	50	56	64	103
ENSECAG00000010248	3.067294596	0.9036283	0.97829	123	93	151	285	128	72	191	229
ENSECAG00000000689	6.128479215	0.9036783	0.97829	1923	817	1241	1425	1487	660	1624	1406
ENSECAG00000000767	7.173272164	0.9036844	0.97829	3057	2347	2971	3056	1838	1596	3855	3194
ENSECAG00000017949	1.037453927	0.903774	0.97829	32	38	42	35	19	38	23	62
ENSECAG00000007352	3.089216793	0.9037803	0.97829	113	164	169	225	152	139	141	158
ENSECAG00000015489	4.701309327	0.9038363	0.97829	525	544	457	509	583	358	498	396
ENSECAG00000021030	3.417244904	0.9038583	0.97829	214	188	220	215	149	140	214	252
ENSECAG00000010776	6.038818592	0.9039159	0.97829	1516	846	1524	1161	1688	815	1718	620
ENSECAG00000008511	10.71933225	0.9039604	0.97829	41013	25019	33737	29491	39352	28099	26908	27128
ENSECAG00000018659	7.555450451	0.9039641	0.97829	3099	3519	3273	5104	2485	3932	3431	3341
ENSECAG00000011330	2.247748679	0.9039915	0.97829	40	40	66	227.001	137	54	121	33
ENSECAG00000018622	7.336575762	0.9040737	0.97829	3568	2690	3854	2574	3399	2461	2938	2601
ENSECAG00000010846	5.683413131	0.904158	0.97829	980	924	912	1257	651	598	1090	1356
ENSECAG00000011203	4.680606612	0.9041679	0.97829	459	391	515	617	513	432	424	475
ENSECAG00000010440	5.121608828	0.9042352	0.97829	625	519	713	840	579	596	802	558
ENSECAG00000016358	6.767679933	0.9042733	0.97829	2151	2242	2143	1997	1462	1908	1795	2513
ENSECAG00000009507	3.517277158	0.9043256	0.97829	204	244	206	240	157	163	262	230
ENSECAG00000012933	6.458660764	0.9043909	0.97829	1024	2500	2201	785	1180	1328	2116	1874
ENSECAG00000021423	5.921997199	0.9044051	0.97829	1246	902	1418	1088	694	1077	1551	1131
ENSECAG00000008288	5.008719948	0.9045363	0.97829	814	563	582	565	732	551	601	368
ENSECAG00000009477	8.058860227	0.9045705	0.97829	4794	4986	4604	6172	5021	4754	5515	4057
ENSECAG00000011992	6.413092166	0.9046534	0.97829	1641	1489	1764	1832	1675	1504	1533	1278
ENSECAG00000015019	5.933706873	0.9049592	0.9784792	1585	915	1181	1138	706	899	1654	1151
ENSECAG00000002357	3.931459509	0.9050006	0.9784792	293	240	333	299	265	319	286	222
ENSECAG00000018889	4.777018345	0.905202	0.9785399	478	424	531	755	367	391	616	584
ENSECAG00000008262	7.389947934	0.9052409	0.9785399	3236	2884	3110	4108	3027	3583	3230	1874
ENSECAG00000010941	4.998288984	0.9054163	0.9785399	694	795	487	499	317	576	720	656
ENSECAG00000020147	4.398308168	0.9055971	0.9785399	445	314	430	484	463	146	537	375
ENSECAG00000021380	7.962828229	0.9056135	0.9785399	4789	4260	4536	5681	4397	3564	4675	5567
ENSECAG00000001445	7.70918197	0.9057969	0.9785399	4480	3621	3943	4522	3137	5112	3401	2832
ENSECAG00000007001	8.815821273	0.9058005	0.9785399	7155	9558	7690	11293	6006	8284	9354	8267
ENSECAG00000018824	4.195960725	0.9058583	0.9785399	348	395	345	341	240	316	295	433
ENSECAG00000010730	0.358684946	0.9059066	0.9785399	36	20	19	13	26	31	10	17
ENSECAG00000010284	3.619910194	0.9059914	0.9785399	266	186	243	240	338	121	301	142
ENSECAG00000006875	6.01271031	0.9060044	0.9785399	1112	1295	1047	1514	1147	1394	956	1102
ENSECAG00000021471	6.965604486	0.9064309	0.9789074	2091	2296	2102	3495	1179	2808	1986	2747
ENSECAG00000011491	6.473049558	0.906525	0.978916	1616	1806	1567	2020	1134	1050	1492	2668
ENSECAG00000008884	6.61215362	0.9067231	0.9790369	1643	1140	1818	3403	2490	428	3413	846
ENSECAG00000022963	5.982277511	0.9071174	0.9793527	1260	972	1474	1291	1090	1345	801	1138
ENSECAG00000021659	2.445485062	0.9072257	0.9793527	83	108	94	125	75	115	105	92
ENSECAG00000015401	4.525499549	0.9072743	0.9793527	273	694	402	409	308	501	358	428
ENSECAG00000021121	0.136802973	0.9075822	0.9795339	21	22	22	13	10	14	27	20
ENSECAG00000003113	6.45477128	0.9076854	0.9795339	1967	1226	1681	1911	2344	1222	1704	1086

ENSECAG00000013813	3.51724391	0.9077008	0.9795339	240	150	238	250	225	177	215	209
ENSECAG00000020242	4.296909676	0.9079114	0.9795372	362	327	423	391	344	203	381	514
ENSECAG00000006784	7.537709966	0.9079207	0.9795372	3108	3323	3247	5152.01	3453	3250	3958	2479
ENSECAG00000020574	2.16994059	0.9079867	0.9795372	90	59	111	75	80	42	174	40
ENSECAG00000015376	3.345038474	0.9080684	0.9795372	171	202	166	262	122	239	137	197
ENSECAG00000010062	5.47666095	0.908135	0.9795372	1011	828	736	925	975	613	888	681
ENSECAG00000021972	3.019616366	0.9083876	0.9796952	149	152	193	136	197	104	155	109
ENSECAG00000022570	9.110417882	0.908454	0.9796952	9492	8882	11223	14577	9809	11517	10272	7043
ENSECAG00000023932	8.607856244	0.9088097	0.9799125	7795	6301	7294	8752	5144	6386	6897	9986
ENSECAG00000026848	6.281769086	0.9089768	0.9799125	1763	1218	1518	1481	1811	1441	1370	956
ENSECAG00000021661	5.318302865	0.9089968	0.9799125	711	681	768	1007	523	502	824	1010
ENSECAG00000014873	6.763140944	0.9090114	0.9799125	2271	1812	2304	2181	2391	1456	2464	1436
ENSECAG00000024489	2.881250408	0.9090869	0.9799125	174	150	119	105	120	107	102	200
ENSECAG00000024988	5.800026462	0.9092422	0.9799768	1224	858	933	1424	672	536	1435	1411
ENSECAG00000009323	5.406133197	0.9093621	0.9799768	855	627	867	1021	984	733	599	634
ENSECAG00000014051	4.042674101	0.9094054	0.9799768	313	245	379	325	359	257	344	232
ENSECAG00000014387	9.732043309	0.9095428	0.9799894	16247	15063	17712	18249	19853	11037	18269	11357
ENSECAG00000021812	2.741489961	0.9096912	0.9799894	86	137	119	164	83	157	118	116
ENSECAG00000011567	6.656853473	0.9097214	0.9799894	1987	1951	1959	2030	1324	1898	1694	2188
ENSECAG00000024341	6.84693574	0.9097622	0.9799894	2094	2092	2023	2677	2019	2390	2149	1711
ENSECAG00000011365	3.662047873	0.9100704	0.9801602	283	234	299	166	135	185	181	386
ENSECAG00000017893	4.916420108	0.9101509	0.9801602	538	513	671	662	410	605	428	660
ENSECAG00000006850	4.768802434	0.9102313	0.9801602	550	338	627	592	414	307	776	532
ENSECAG00000017669	5.12971133	0.9102859	0.9801602	619	598	594	901	720	531	828	474
ENSECAG00000021263	3.245401861	0.9103521	0.9801602	230	101	171	256	200	88	289	103
ENSECAG00000024671	1.577937132	0.9107203	0.9803904	59	61	53	44	32	74	32	68
ENSECAG00000020263	4.561163868	0.9107386	0.9803904	453	368	527	516	327	344	571	447
ENSECAG00000005884	4.357719741	0.9110271	0.9804068	417	418	399	364	449	344	301	332
ENSECAG00000009202	4.070563065	0.9110526	0.9804068	293	321	356	346	274	249	340	326
ENSECAG00000007009	3.581308539	0.9112697	0.9804068	213	223	261	241	122	231	139	335
ENSECAG00000021222	7.4491898	0.9114329	0.9804068	2953	3547	2692	4292	2628	3147	3578	3343
ENSECAG00000023803	6.354693605	0.9115047	0.9804068	1552	1209	1555	2223	1257	881	1943	1836
ENSECAG00000014866	4.449765553	0.9115198	0.9804068	392	410	395	527	263	355	489	448
ENSECAG00000016730	4.932053823	0.9115206	0.9804068	844	889.008	619.008	3	436.003	467.003	1270	1
ENSECAG00000010013	7.620305301	0.9115243	0.9804068	3891	3045	3828	4479	3692	3063	4198	3387
ENSECAG00000008989	4.57874571	0.9115306	0.9804068	433	379	625	445	258	668	329	377
ENSECAG00000011560	6.336384618	0.9117254	0.9805235	2065	1488	1052	1584	951	1638	1370	1866
ENSECAG00000015199	2.329942888	0.9119149	0.9805609	104	134	38	94	64	158	80	47
ENSECAG00000022720	2.51477387	0.9119329	0.9805609	136	74	102	138	117	49	161	79
ENSECAG00000008386	1.386393377	0.9120809	0.9806273	50	69	61	13	42	34	27	72
ENSECAG00000023223	5.448584406	0.9125684	0.9809845	720	847	828	961	644	913	678	887
ENSECAG00000023401	4.160755045	0.9126198	0.9809845	231	327	336	487	218	383	376	312
ENSECAG00000022357	3.107611717	0.9126723	0.9809845	237	228	68	136	22	281	23	238
ENSECAG00000020275	3.867496828	0.9131618	0.9813392	310	237	306	258	304	259	226	253
ENSECAG00000007688	0.301240064	0.9133648	0.9813392	26	17	23	24	21	15	29	15
ENSECAG00000011078	5.584853237	0.9134707	0.9813392	1101	932	911	700	909	780	939	847
ENSECAG00000022578	3.227268053	0.9135093	0.9813392	148	169	220	171	241	165	141	117
ENSECAG00000000759	4.316357462	0.9135614	0.9813392	362	384	392	380	266	399	464	314
ENSECAG00000022984	2.88775402	0.9135627	0.9813392	61	306	71	121	68	79	157	220
ENSECAG00000005354	3.891004664	0.913607	0.9813392	318	198	354	261	406	157	382	144
ENSECAG00000021929	5.852850107	0.9137584	0.981409	1239	1014	993	1203	1010	966	1372	863
ENSECAG00000000872	6.382423721	0.9138675	0.9814334	1744	1344	1657	1684	1873	1014	2016	1221
ENSECAG00000010094	5.684070431	0.9140642	0.9815519	1246	767	918	1031	1020	515	1215	1045
ENSECAG00000011632	3.709427313	0.9141786	0.9815819	283	327	192	208	128	224	178	382
ENSECAG00000006469	5.888560718	0.9146443	0.981884	1468	886	1200	990	1032	810	1314	1181
ENSECAG00000011272	4.522831899	0.914702	0.981884	431	418	358	565	499	359	424	375
ENSECAG00000020611	0.671797575	0.9149506	0.981884	26	43	20	22	21	30	32	26
ENSECAG00000023962	8.303924499	0.9149901	0.981884	5497	6001	5598	7286	5951	6158	5735	4885
ENSECAG00000005272	1.357218026	0.9149929	0.981884	34	35	65	55	35	33	64	51
ENSECAG00000022561	3.771175433	0.9151383	0.981884	242	225	242	342	233	197	383	190
ENSECAG00000011996	4.175622433	0.9151863	0.981884	280	314	227	586	174	410	409	313
ENSECAG00000021592	5.107634013	0.9153826	0.981884	592	602	579	973	397	565	630	852
ENSECAG00000000723	2.911713751	0.9153849	0.981884	168	95	151	180	115	155	136	113
ENSECAG00000001722	5.361383999	0.9154268	0.981884	798	834	706	804	656	876	610	787
ENSECAG00000009579	4.829581727	0.9155433	0.981884	623	458	538	629	772	234	627	399
ENSECAG00000002187	4.025009869	0.9155719	0.981884	372	275	331	256	410	251	349	166
ENSECAG00000023403	6.517564368	0.9155837	0.981884	1980	1611	1722	1718	1913	991	2227	1624
ENSECAG00000006659	5.011940116	0.9157077	0.9819243	584	661	601	684	713	557	594	398
ENSECAG00000010055	2.914742048	0.9158811	0.9819675	199	78	145	177	112	66	320	53
ENSECAG00000012578	2.342742495	0.9159873	0.9819675	67	71	76	177	57	106	153	51
ENSECAG00000017659	6.279346798	0.9160074	0.9819675	1600	1436	1236	1711	1851	1129	1696	969
ENSECAG00000001035	3.619395895	0.9163255	0.9820589	241	160	261	314	269	151	233	213
ENSECAG00000012781	5.360892148	0.9163371	0.9820589	639	788	747.001	1081	610	850	736	683
ENSECAG00000020320	1.060666602	0.916352	0.9820589	24	81	24	23	9	28	23	79
ENSECAG00000017586	3.707665337	0.9164409	0.9820615	254	255	240	272	215	223	244	234
ENSECAG00000017909	3.326704735	0.9165527	0.9820886	173	168	207	242	233	149	176	141
ENSECAG00000021369	7.20214161	0.9166904	0.9821436	2954	2467	2807	3140	2185	2471	3318	2774
ENSECAG00000012412	3.573181751	0.9168396	0.9822078	231	192	252	261	282	173	190	184

ENSECAG00000011669	2.304357044	0.9170503	0.9822078	106	46	94	147	87	65	147	49
ENSECAG00000018948	9.995721093	0.9170961	0.9822078	26530	13338	19720	19227	19907	14448	20198	19926
ENSECAG00000004314	5.064669807	0.9171274	0.9822078	648	466	696	856	528	348	964	594
ENSECAG00000017940	1.742541633	0.9171827	0.9822078	62	83	61	35	22	94	25	89
ENSECAG00000007727	3.315846894	0.9173202	0.9822624	194	153	223	212	151	109	282	174
ENSECAG00000012870	1.469738786	0.917676	0.982464	42	83	21	53	24	40	14	116
ENSECAG00000010283	7.531304138	0.9176814	0.982464	3645	2909	3773	3976	3147	2223	3984	4251
ENSECAG00000008284	2.221346445	0.9179766	0.9826874	75	94	73	121	59	93	88	80
ENSECAG00000011717	6.108202244	0.9182624	0.9828857	1333	972	1444	1616	1314	1140	1333	1199
ENSECAG00000015041	3.715326341	0.9183662	0.9828857	286	209	217	295	172	154	310	331
ENSECAG00000025006	5.031790374	0.9184462	0.9828857	827	437	705	540	678	536	611	534
ENSECAG00000008148	6.635269278	0.9185079	0.9828857	1862	1593	2121	2099	2075	883	2615	1812
ENSECAG00000000374	-0.187563291	0.9187526	0.9829787	25	10	21	5	0	7	26	23
ENSECAG00000013002	4.830564467	0.9188158	0.9829787	529	508	611	590	300	371	525	836
ENSECAG00000020401	7.360042492	0.9188544	0.9829787	2932	3494	2615	3912	1844	2784	3279	3791
ENSECAG00000019984	1.396774104	0.9189426	0.9829804	40	51	55	46	24	45	53	64
ENSECAG00000024795	7.104559694	0.919231	0.9831964	3480	3836	1025	1918	2815	1941	889	4229
ENSECAG00000009994	4.104930452	0.9194658	0.9832658	230	355	395	369	201	401	206	375
ENSECAG00000026929	2.161536805	0.9194691	0.9832658	32	86	126	105	18	50	131	119
ENSECAG00000020469	5.001347953	0.9201596	0.9834834	590	564	591	725	545	637	561	550
ENSECAG00000021188	6.801214169	0.9202017	0.9834834	1695	2012	2107	2846	1923	1827	2473	1896
ENSECAG00000019900	9.837598135	0.9203027	0.9834834	18333	14571	18944	18770	16160	20122	14624	14381
ENSECAG00000014373	5.785568473	0.9204273	0.9834834	1246	795	1293	1006	1187	987	847	834
ENSECAG00000022262	6.989677825	0.9204991	0.9834834	2442	2415	2252	2664	1363	2364	2770	2765
ENSECAG00000017870	4.891928758	0.9206405	0.9834834	481	376	549	923	375	236	704	901
ENSECAG00000003734	1.26350636	0.9208101	0.9834834	37	26	47	68	54	13	98	12
ENSECAG00000026897	4.36730896	0.9209348	0.9834834	467	310	440	405	418	342	394	297
ENSECAG00000022204	5.513233394	0.9209534	0.9834834	1012	680	939	973	748	623	983	916
ENSECAG00000015453	3.517959577	0.9209847	0.9834834	188	190	292	201	179	186	218	243
ENSECAG00000018247	1.095257361	0.921083	0.9834834	64	18	32	50	34	38	29	40
ENSECAG00000021258	3.124703428	0.9210969	0.9834834	164	160	195	138	168	158	126	167
ENSECAG00000014885	1.492947771	0.9212638	0.9834834	55	32	61	70	59	44	50	37
ENSECAG00000001651	6.108093969	0.9212692	0.9834834	1316	1136	1438	1436	1005	1042	1356	1619
ENSECAG00000014852	4.2328128	0.9212754	0.9834834	479	234	352	382	247	189	451	506
ENSECAG00000023410	4.988016445	0.9212806	0.9834834	594	632	575	688	469	397	723	692
ENSECAG00000023930	6.107073147	0.9212949	0.9834834	1672	1049	1417	1147	1706	816	1673	858
ENSECAG00000020498	3.418979645	0.9213066	0.9834834	212	127	200	318	344	105	211	89
ENSECAG00000008375	3.824635178	0.9213591	0.9834834	224	325	272	282	142	246	194	408
ENSECAG00000018678	3.156935324	0.9216141	0.9834834	193	102	259	146	267	93	205	61
ENSECAG00000013963	3.550105117	0.9216224	0.9834834	167	203	201	334	205	239	237	157
ENSECAG00000010010	2.098786901	0.9216904	0.9834834	73	81	107	55	71	62	81	92
ENSECAG00000009917	1.94911474	0.9217045	0.9834834	37	94	73	82	51	96	54	67
ENSECAG00000024379	4.050564658	0.9217505	0.9834834	332	330	276	324	245	343	298	299
ENSECAG00000023362	4.020075666	0.9220893	0.9837431	352	248	338	304	279	271	334	288
ENSECAG00000013910	3.862824081	0.9221672	0.9837431	255	248	315	327	133	270	282	340
ENSECAG00000020312	2.591754932	0.9224935	0.9839989	108	147	114	75	63	114	74	177
ENSECAG00000022748	1.556684359	0.9227866	0.9841456	57	18	64	83	24	21	122	53
ENSECAG00000009400	2.381531101	0.9228664	0.9841456	48	159	43	155	39	108	77	132
ENSECAG00000021426	3.822716431	0.9229284	0.9841456	293	218	303	298	233	160	327	293
ENSECAG00000016668	1.775883282	0.9229777	0.9841456	59	50	68	80	56	58	89	41
ENSECAG00000012196	2.787079431	0.9231781	0.9842469	186	116	80	158	151	74	190	75
ENSECAG00000013192	5.813223772	0.9232459	0.9842469	1149	982	1035	1265	930	777	1448	898
ENSECAG00000022307	6.018089337	0.9236319	0.9844977	1329	1002	1410	1252	1474	668	1759	891
ENSECAG00000024732	5.86681927	0.9236545	0.9844977	1298	967	1047	1189	1139	953	1191	938
ENSECAG00000014272	6.192649932	0.9238523	0.984508	1364	1169	1546	1707	1375	1264	1387	1144
ENSECAG00000016447	6.401754679	0.9238531	0.984508	1604	1576	1706	1751	1326	1261	1932	1544
ENSECAG00000023471	1.299653251	0.9239242	0.984508	36	40	51	54	32	35	62	46
ENSECAG00000004620	8.219696091	0.9241336	0.9846387	5652	5284	6135	5863	4942	6483	6095	3908
ENSECAG00000022438	8.817108784	0.9245159	0.9848639	6191	12523	7492	7671	2596	6634	4503	19179
ENSECAG00000005508	4.985580102	0.9245183	0.9848639	677	639	507	655	524	504	568	645
ENSECAG00000015589	5.156933123	0.9246304	0.9848909	601	609	737	811	592	579	726	688
ENSECAG00000024278	3.806939694	0.9248378	0.985009	257	262	271	307	280	122	368	239
ENSECAG00000014310	5.434974954	0.9249147	0.985009	927	672	640	1207	821	643	957	663
ENSECAG00000010512	1.578614968	0.9251272	0.9851429	41	24	54	120	29	57	66	51
ENSECAG00000012244	1.595626681	0.9255553	0.9853097	28	73	62	68	23	21	44	121
ENSECAG00000023747	4.584461314	0.925622	0.9853097	350	335	359	847	350	230	435	749
ENSECAG00000017750	5.104787035	0.9257777	0.9853097	851	555	693	592	633	505	709	601
ENSECAG00000022663	4.776095133	0.9258282	0.9853097	554	426	560	623	484	493	428	514
ENSECAG00000012731	4.094198035	0.9258447	0.9853097	277	277	315	455	242.007	322	314	349
ENSECAG00000012543	6.631282835	0.9259069	0.9853097	1877	2022	2100	1734	2356	1628	1822	1173
ENSECAG00000018583	5.05399388	0.9259405	0.9853097	643	547	620	759	451	563	673	722
ENSECAG00000023565	5.572279825	0.9259777	0.9853097	1006	744	1081	907	859	893	674	899
ENSECAG00000008214	4.382916747	0.9262045	0.985351	392	366	360	530	386	373	405	303
ENSECAG00000021393	6.073877797	0.9263299	0.985351	1938	618	1704	1068	1848	614	1552	811
ENSECAG00000017861	6.884510135	0.9263508	0.985351	2073	2240	2438	2330	2491	2003	2326	1706
ENSECAG00000021868	9.766328917	0.9265025	0.985351	20403	13470	15642	17782	19390	13482	16657	13498
ENSECAG00000018146	5.675137731	0.9265298	0.985351	1037	805	1068	1035	1058	604	1131	944
ENSECAG00000018765	5.575850913	0.9265371	0.985351	1002	873	833	1041	1053	728	970	630

ENSECAG00000020211	5.218247883	0.9266582	0.9853876	759	612	766	726	876	440	893	515
ENSECAG00000006022	3.776024761	0.9268426	0.98542	245	290	214	295	208	261	300	218
ENSECAG00000013136	8.790475699	0.9268621	0.98542	9784	6462	8669	9350	11951	5789	9651	4851
ENSECAG00000009022	6.280982426	0.9271829	0.9856582	1696	1207	1652	1572	1848	877	1774	1078
ENSECAG00000000901	4.884206596	0.9273948	0.9856582	575	521	406	787	843	442	452	373
ENSECAG00000023379	6.676401611	0.9274899	0.9856582	2227	1713	2021	1910	1930	1167	2482	1954
ENSECAG00000007763	6.341950322	0.9275449	0.9856582	1376	1417	1765	1707	1197	1171	1887	1687
ENSECAG00000021667	2.513392519	0.9275452	0.9856582	126	86	110	123	101	47	138	120
ENSECAG00000020636	5.184636141	0.9276068	0.9856582	747	564	732	834	338	509	682	1073
ENSECAG00000020357	4.339450881	0.9277517	0.9857199	347	281	415	529	142	328	440	573
ENSECAG00000019867	6.258126803	0.9279732	0.985863	1190	2497	896	1099	820	2178	389	1953
ENSECAG00000008210	9.113341681	0.9280708	0.9858745	12030	9897	9072	11713	9910	8842	14761	7135
ENSECAG00000022940	5.756789135	0.9283867	0.9860325	983	826	1005	1403	822	777	964	1363
ENSECAG00000015437	1.099340805	0.9283931	0.9860325	22	28	46	63	53	15	65	20
ENSECAG00000018641	1.123252631	0.9287348	0.9863032	31	28	44	58	12	28	42	72
ENSECAG00000003345	10.20176738	0.9290713	0.9864568	21402	21384	21869	26329	17833	25274	14795	25894
ENSECAG00000012879	6.91148488	0.9291428	0.9864568	1943	2113	2076	3471	1423	1523	2406	3307
ENSECAG00000022559	6.445534476	0.9291632	0.9864568	1612	1303	1951	1897	1493	1666	1508	1582
ENSECAG00000010911	6.169948862	0.9294924	0.9864568	1478	1213	1344	1644	1209	1333	1401	1149
ENSECAG00000024486	4.00706415	0.9295049	0.9864568	313	221	370	366	311	316	258	232
ENSECAG00000016370	4.274233454	0.9295215	0.9864568	378	324	567	232	527	322	237	249
ENSECAG00000019955	3.993134248	0.929582	0.9864568	249	395	278	311	234	242	347	309
ENSECAG00000007142	6.398103879	0.9296004	0.9864568	1657	1471	1579	1803	882	1187	1751	2368
ENSECAG00000022314	4.911073155	0.929661	0.9864568	702	442	644	517	869	330	707	290
ENSECAG00000012950	3.268790564	0.9298358	0.98655	162	181	234	150	201	183	187	116
ENSECAG00000024331	4.656359627	0.9300665	0.9866755	454	323	504	689	539	310	711	297
ENSECAG00000000471	3.602033914	0.9301539	0.9866755	214	194	226	302	181	129	311	274
ENSECAG00000015058	7.147257677	0.9302485	0.9866755	2811	2497	3018	2566	2224	2218	3624	2329
ENSECAG00000017193	5.766725767	0.9303812	0.9866755	1100	897	1148	1137	1167	734	916	1043
ENSECAG00000015821	6.892259998	0.9304179	0.9866755	2248	2249	2232	2615	2279	2045	2516	1589
ENSECAG00000009175	5.368134893	0.9304752	0.9866755	720	875	670	909	610	678	929	788
ENSECAG00000016731	6.331690554	0.9310187	0.9871196	1641	1368	1533	1683	1267	1374	1643	1548
ENSECAG00000021172	4.453921733	0.9311207	0.9871196	410	312	421	559	333	174	519	600
ENSECAG00000027060	0.743349633	0.9311548	0.9871196	20	35	13	54	24	50	15	21
ENSECAG00000022698	3.912761061	0.9313869	0.9872119	287	245	250	412	149	207	324	397
ENSECAG00000024560	6.139713978	0.9314156	0.9872119	1469	1115	1415	1455	1433	951	1575	1181
ENSECAG00000015490	3.084157132	0.9316062	0.987304	177	160	148	175	93	142	128	229
ENSECAG00000007302	6.390098665	0.9316904	0.987304	1612	1549	1601	1832	1240	974	1767	2068
ENSECAG00000003414	5.645818283	0.9318898	0.987304	1190	664	1166	957	1230	914	945	597
ENSECAG00000003312	5.198069681	0.9319167	0.987304	683	620	699	839	652	565	610	818
ENSECAG00000000423	6.147057261	0.9319371	0.987304	1578	1166	1222	1512	1514	684	1375	1613
ENSECAG00000007796	6.384276233	0.9321353	0.9874219	1757	1268	1536	1932	1554	937	1988	1666
ENSECAG00000010039	6.259390212	0.9325273	0.9874847	1415	1108	1802	1616	1655	1410	1227	1176
ENSECAG00000021820	7.548951968	0.9325799	0.9874847	5051	2738	3249	3750	5037	2620	3808	1793
ENSECAG00000007611	6.658018238	0.9326103	0.9874847	1719	2071	1640	2537	1990	1914	2212	1024
ENSECAG00000026963	0.673431939	0.9326229	0.9874847	35	8	33	41	30	46	23	6
ENSECAG00000009481	5.766913876	0.9326628	0.9874847	1088	865	1321	908	716	969	1086	1171
ENSECAG00000016214	6.360878948	0.9327567	0.9874847	1445	1510	1483	1931	1549	1201	1915	1328
ENSECAG00000013414	2.429560786	0.9328031	0.9874847	88	83	118	132	49	80	165	90
ENSECAG00000019788	4.088022872	0.9331523	0.9877623	295	338	352	341	267	345	279	295
ENSECAG00000016099	5.560863044	0.9335847	0.9880603	1179	706	1121	589	530	565	851	1517
ENSECAG00000000621	6.625247569	0.9336929	0.9880603	1674	2129	1842	2074	1545	1947	1641	1809
ENSECAG00000009463	3.809301413	0.9336948	0.9880603	224	322	244	300	178	258	308	247
ENSECAG00000019674	6.897996811	0.9338405	0.9881224	2090	2078	2713	2501	1979	2289	2065	2065
ENSECAG00000002386	3.052557881	0.9340538	0.988256	202	102	200	125	158	88	253	113
ENSECAG00000015601	4.747150332	0.9341742	0.9882914	531	471	512	552	534	333	633	463
ENSECAG00000000498	2.075738593	0.934416	0.9883	81	64	99	81	64	67	85	76
ENSECAG00000022869	5.482172945	0.9345331	0.9883	940	429	740	1431	922	909	963	415
ENSECAG00000013948	4.540249977	0.9345939	0.9883	510	355	443	485	526	388	549	219
ENSECAG00000000645	4.050644347	0.934604	0.9883	261	237	326	466	124	271	338	475
ENSECAG00000020904	3.357277316	0.9347463	0.9883	220	119	204	250	155	179	183	217
ENSECAG00000009120	1.375359546	0.9347641	0.9883	51	33	47	69	24	24	67	66
ENSECAG00000022158	5.974475388	0.9347914	0.9883	1346	1017	1335	1237	1129	859	1436	1092
ENSECAG00000024165	3.64871201	0.9349143	0.988338	194	269	263	224	206	243	165	275
ENSECAG00000005290	2.400476309	0.9351076	0.9884503	110	81	100	108	68	61	154	104
ENSECAG00000016468	2.45389872	0.9352016	0.9884577	104	82	98	133	72	43	141	147
ENSECAG00000013754	1.591078062	0.9354142	0.9884588	50	47	55	74	60	47	59	45
ENSECAG00000000755	6.826859374	0.9355675	0.9884588	2392	1424	1956	3145	2039	1136	2782	2439
ENSECAG00000023764	8.198599054	0.935624	0.9884588	6244	4360	7040	4987	6023	4220	5837	5253
ENSECAG00000026923	2.368534209	0.9356409	0.9884588	63	83	134	111	113	81	73	97
ENSECAG00000019636	6.02302536	0.9356646	0.9884588	1190	1052	1373	1424	906	1456	1167	1113
ENSECAG00000015422	5.284910487	0.9357248	0.9884588	761	613	761	950	848	343	901	724
ENSECAG00000007703	6.378557145	0.9358748	0.9885254	1485	1644	1485	1924	1624	1159	1881	1301
ENSECAG00000007908	4.745323363	0.9360603	0.9886118	636	417	548	499	478	418	456	543
ENSECAG00000008042	5.007624998	0.9361684	0.9886118	638	437	687	739	505	560	634	622
ENSECAG00000006256	2.405971799	0.9362177	0.9886118	102	72	132	106	130	66	88	83
ENSECAG00000012077	5.043464587	0.9364269	0.9886283	576	511	590	894	280	455	907	803
ENSECAG00000016850	4.76107642	0.9366383	0.9886283	585	396	446	727	416	332	698	514

ENSECAG00000018442	7.109360346	0.9366655	0.9886283	2703	2481	2994	2641	2648	2488	2163	2406
ENSECAG00000022873	5.485003265	0.9367681	0.9886283	884.996	575	1022	1076	584.999	408	1194	1088
ENSECAG00000019307	6.152246528	0.9368408	0.9886283	1375	1127	1546	1456	1088	1367	1204	1438
ENSECAG00000004224	6.705206293	0.9371117	0.9886283	2095	1801	1964	2210	2102	1611	2268	1588
ENSECAG00000006445	3.717664675	0.937113	0.9886283	191	286	314	199	346	189	346	75
ENSECAG00000013441	3.247233902	0.9371856	0.9886283	176	150	204	214	131	144	183	212
ENSECAG00000010012	5.512256081	0.9372455	0.9886283	804	617	878	1285	817	780	923	773
ENSECAG00000019354	4.442430379	0.9372738	0.9886283	339	366	385	602	453	361	490	266
ENSECAG00000008800	5.794527652	0.9373401	0.9886283	1287	747	995	1378	867	653	1427	1080
ENSECAG00000012040	2.977327068	0.9373686	0.9886283	116	223	129	116	75	128	165	202
ENSECAG00000023741	3.598993656	0.9374381	0.9886283	513	106	270	49	109	59	597	148
ENSECAG00000017721	1.761648193	0.9374518	0.9886283	73	40	66	85	77	65	44	41
ENSECAG00000011031	0.798726823	0.9378018	0.9887937	30	20	40	36	72	15	23	8
ENSECAG00000022936	4.994675799	0.9379507	0.9887937	595	470	584	890	669	559	695	331
ENSECAG00000014497	5.270628201	0.9380295	0.9887937	748	550	768	941	568	444	933	899
ENSECAG00000020890	1.544267656	0.9380554	0.9887937	48	42	60	68	25	49	69	64
ENSECAG00000011382	6.273719559	0.9383443	0.9887937	1489	1082	1807	1631	1402	1339	1845	1029
ENSECAG00000012788	4.281837923	0.9386679	0.9887937	432	263	485	344	443	216	331	389
ENSECAG00000009179	0.685576752	0.9386692	0.9887937	42	25	23	29	31	10	45	23
ENSECAG00000023455	9.021783676	0.9387869	0.9887937	9295	9214	10396	12137	8903	7870	12252	8291
ENSECAG00000009488	9.215296451	0.9387921	0.9887937	10943	11187	15098	7929	3967	14217	7053	17139
ENSECAG00000017476	1.037553496	0.9388287	0.9887937	24	20	41	75	51	31	37	17
ENSECAG00000006472	5.187314476	0.9389655	0.9887937	864	550	687	711	799	451	745	652
ENSECAG00000009159	4.98249571	0.9390644	0.9887937	158	1015	637	636	150	393	442	1267
ENSECAG00000009324	5.41382965	0.9391049	0.9887937	908	652	785	961	577	1082	690	665
ENSECAG00000001899	7.249838665	0.9391125	0.9887937	2831	2493	3074	3418	2792	2372	3269	2620
ENSECAG00000024709	5.479135718	0.9391403	0.9887937	952	805	677	1080	920	557	832	873
ENSECAG00000014356	7.186343046	0.9391895	0.9887937	2776	2443	2596	3515	2532	2250	3427	2413
ENSECAG00000000419	9.429486089	0.9392376	0.9887937	12434	7957	13441	20551	17582	7075	20906	5653
ENSECAG00000021658	7.383937356	0.9392595	0.9887937	3400	2492	3835	3447	3419	3062	2460	2761
ENSECAG00000018868	3.383784095	0.9392626	0.9887937	231	178	216	186	218	122	197	201
ENSECAG00000013454	5.523710448	0.9395402	0.98881	920	814	875	1004	944	886	720	668
ENSECAG00000025009	2.83403279	0.939563	0.98881	139	99	145	163	106	146	94	156
ENSECAG00000007952	4.67740502	0.9396706	0.98881	508	382	487	610	499	419	565	366
ENSECAG00000009502	4.39635191	0.9396814	0.98881	349	411	364	534	259	261	342	639
ENSECAG00000016263	5.847997789	0.9397579	0.98881	1182	957	1151	1160	1072	1099	1047	904
ENSECAG00000007288	4.151772274	0.9398468	0.98881	14	1089	24	185	67	185	43	937
ENSECAG00000002306	5.893459052	0.9398908	0.98881	1071	1149	1056	1399	827	1386	963	973
ENSECAG00000003453	0.507084017	0.940035	0.98881	18	19	38	30	5	11	37	43
ENSECAG00000000135	7.314163377	0.940133	0.98881	3218	2446	3044	3908	2914	2346	3855	2319
ENSECAG00000011526	3.659802747	0.9401486	0.98881	262	186	262	283	161	177	322	247
ENSECAG00000009877	1.275414467	0.9402403	0.988814	53	34	39	53	41	24	70	38
ENSECAG00000021680	2.459463235	0.9404007	0.988814	117	113	60	124	70	40	118	175
ENSECAG00000023532	7.884499669	0.9405518	0.988814	4096	4597	3911	6069	3487	4251	4466	4567
ENSECAG00000003368	8.032788881	0.9405736	0.988814	5934	4089	5601	4983	7643	2789	5323	2915
ENSECAG00000024638	7.310613388	0.9407015	0.988814	3059	2988	2224	4307	2596	2228	3559	3028
ENSECAG00000027691	8.409474157	0.9407722	0.988814	4900	7387	4500	9660	4874	5548	7040	7281
ENSECAG00000006379	5.297144837	0.9408	0.988814	833	735	725	724	841	558	727	711
ENSECAG00000018885	3.612240828	0.9409271	0.988814	209	195	250	286	74	140	211	471
ENSECAG00000009113	7.468640476	0.9409359	0.988814	3754	3159	2953	3837	3176	3380	3287	2870
ENSECAG00000012453	3.638652576	0.9412996	0.9890751	246	221	246	260	176	105	237	376
ENSECAG00000020426	3.704377697	0.9415315	0.9890751	243	191	284	287	249	185	310	202
ENSECAG00000017667	2.808075212	0.9415738	0.9890751	143	118	133	137	163	70	199	80
ENSECAG00000007781	6.636556563	0.9415803	0.9890751	1466	1967	1908	2366	1290	1798	2062	2056
ENSECAG00000009031	2.885082684	0.9416247	0.9890751	106	133	171	165	128	78	138	179
ENSECAG00000022215	6.579250691	0.9417068	0.9890751	2013	1520	1848	2034	1924	1521	2115	1367
ENSECAG00000018235	4.614338147	0.9419987	0.9891176	605	322	418	557	506	415	425	407
ENSECAG00000024753	3.311330794	0.942367	0.9891176	151	200	108	329	119	286	157	114
ENSECAG00000008252	6.008942212	0.9423709	0.9891176	1443	998	1113	1537	1524	889	1409	773
ENSECAG00000004150	0.150754595	0.9423858	0.9891176	17	16	27	20	7	21	14	28
ENSECAG00000015128	8.308000608	0.9424639	0.9891176	6093	5933	7203	5537	3851	7787	4219	6141
ENSECAG00000019018	6.320196561	0.9426188	0.9891176	1608	1179	1702	1719	1536	1507	1488	1192
ENSECAG00000018335	3.278327611	0.9426285	0.9891176	145	164	181	259	158	151	212	179
ENSECAG00000022386	8.129652904	0.9427354	0.9891176	4670	5124	4996	6989	4673	5873	5696	3860
ENSECAG00000003124	7.476153122	0.9428063	0.9891176	3188	2948	4136	3491	3489	2836	3434	3102
ENSECAG00000001389	5.967155641	0.9428119	0.9891176	1005	1104	1128	1723	997.001	832.001	1230	1427.02
ENSECAG00000011612	10.97763689	0.9428715	0.9891176	33784	38303	31266	53754	37332	33215	45846	30163
ENSECAG00000000626	4.748156425	0.9429506	0.9891176	553	394	585	545	449	374	697	448
ENSECAG00000023573	5.917286833	0.9431107	0.9891176	1139	1083	1257	1257	1109	807	1194	1216
ENSECAG00000022012	1.198548454	0.9432123	0.9891176	54	16	64	41	34	34	63	26
ENSECAG00000023878	3.780093705	0.9432336	0.9891176	259	239	249	310	267	222	295	205
ENSECAG00000013901	7.578883733	0.9432383	0.9891176	3416	2928	3283	5379	3431	3024	4259	3184
ENSECAG00000024094	2.606968865	0.9433621	0.9891176	132	78	138	115	72	98	119	148
ENSECAG00000021225	7.551574633	0.9434485	0.9891176	3629	3363	3014	4840	3513	3013	3213	3556
ENSECAG00000015120	2.522752517	0.943461	0.9891176	127	66	127	118	113	56	172	80
ENSECAG00000018687	5.614722071	0.9434932	0.9891176	992	605	1180	1030	1513	593	879	541
ENSECAG00000013226	4.504858309	0.9435758	0.9891176	452	340	370	642	440	267	527	397
ENSECAG00000015829	3.272005586	0.9437846	0.9892405	140	153	220	230	107	118	217	264

ENSECAG00000018290	5.333281119	0.9439268	0.9892405	816	678	1023	621	607	417	1094	822
ENSECAG00000010306	2.961990904	0.9440954	0.9892405	137	109	185	166	175	83	174	132
ENSECAG00000012184	4.305586599	0.9441456	0.9892405	403	321	409	388	273	391	352	398
ENSECAG00000008083	2.047011247	0.9441913	0.9892405	71.0006	76.0019	59.0011	115.004	56.0005	72.0008	85	73.0002
ENSECAG00000015938	3.043572013	0.9443143	0.9892405	160	150	167	148	153	152	160	122
ENSECAG00000006268	5.16046184	0.9443555	0.9892405	796	649	687	608	681	635	624	620
ENSECAG00000024783	3.976883151	0.9443898	0.9892405	381	211	335	281	428	170	385	164
ENSECAG00000012790	0.899041613	0.9446154	0.9893856	32	32	39	36	27	12	47	42
ENSECAG00000008477	4.821803155	0.9447195	0.9894034	523	634	530	505	432	378	376	816
ENSECAG00000009131	3.728939261	0.9450328	0.9896402	291	198	284	242	170	160	219	413
ENSECAG00000017019	1.657751934	0.9453765	0.9898551	56	52	43	87	71	61	61	26
ENSECAG00000011152	4.547916551	0.945595	0.9898551	795	256	386	354	465	179	975	159
ENSECAG00000021194	4.760736444	0.9456193	0.9898551	473	575	591	422	511	555	403	452
ENSECAG00000012394	1.727968145	0.9456509	0.9898551	44	76	60	72	62	55	77	34
ENSECAG00000008877	4.815227493	0.9457528	0.9898551	337	704	505	603	332	693	494	480
ENSECAG00000009059	7.848097062	0.9457609	0.9898551	4606	4158	4394	4633	4625	4100	3899	3881
ENSECAG00000010625	4.342843145	0.9459349	0.989946	388	341	425	436	308	366	371	387
ENSECAG00000018194	6.792213848	0.9461491	0.9899609	2085	2180	1628	2701	1377	2047	2286	2311
ENSECAG00000007021	5.305644085	0.9462082	0.9899609	774	884	908	473	932	483	950	472
ENSECAG00000000386	2.197988082	0.9462105	0.9899609	97	83	86	76	19	93	38	172
ENSECAG00000013256	6.208357523	0.9466166	0.990166	1332	1109	1237	2135	1229	1156	1789	1215
ENSECAG00000012255	3.427405377	0.9466298	0.990166	178	170	161	347	142	179	208	230
ENSECAG00000000078	5.978469665	0.9470068	0.990166	1442	1034	1514	911	1156	860	1469	1050
ENSECAG00000007427	3.893506658	0.9470239	0.990166	292	259	273	318	163	214	400	314
ENSECAG00000015382	3.993634848	0.9472304	0.990166	16	1059	6	71	60	231	69	739
ENSECAG00000010723	5.402282491	0.9473902	0.990166	832	738	710	1003	682	577	961	866
ENSECAG00000011822	2.051324108	0.9474075	0.990166	26	186	52	40	7	45	33	202
ENSECAG00000026973	4.34008756	0.94741	0.990166	387	297	375	516	431	326	555	161
ENSECAG00000019258	4.720161663	0.9474199	0.990166	507	439	534	555	551	501	500	329
ENSECAG00000010557	3.040362822	0.9475728	0.990166	210	146	171	104	131	112	178	162
ENSECAG00000023539	6.26728748	0.9476375	0.990166	1387	1380	1470	1735	1326	859	1952	1538
ENSECAG00000015965	7.319746797	0.9477232	0.990166	3945	2910	1995	3509	3102	1962	3428	3178
ENSECAG00000023718	2.727560419	0.9477786	0.990166	110	79	136	188	112	71	152	146
ENSECAG00000003103	6.910812611	0.9479207	0.990166	2520	1803	2505	2665	2345	1912	2362	1944
ENSECAG00000017504	6.701651042	0.9479443	0.990166	2196	1863	2033	1924	2245	1270	2501	1597
ENSECAG00000009412	3.823797079	0.9479894	0.990166	286	226	267	312	327	233	307	149
ENSECAG00000004675	5.402077451	0.9480219	0.990166	919	755	825	750	799	615	933	722
ENSECAG00000004470	6.129023135	0.9480296	0.990166	1466	1025	1295	1676	1297	990	1698	1122
ENSECAG00000008289	3.941153041	0.9481169	0.990166	249	278	275	408	178	216	360	349
ENSECAG00000011568	5.303046894	0.9482937	0.990166	1101	740	715	493	876	789	736	362
ENSECAG00000019661	5.946513829	0.9482985	0.990166	1263	929	1178	1434	1258	859	1440	931
ENSECAG00000010570	1.512614348	0.9483243	0.990166	70	42	53	46	62	31	56	52
ENSECAG00000023991	5.282159442	0.9485836	0.9902638	557	1258	552	539	509	598	689	1024
ENSECAG00000006965	5.277532067	0.9485947	0.9902638	692	675	661	992	713	712	697	653
ENSECAG00000019895	-0.010831379	0.9487112	0.9902638	33	12	20	4	30	8	21	4
ENSECAG00000025152	3.040015563	0.9488357	0.9902638	112	261	79	177	149	114	151	164
ENSECAG00000010321	2.82982951	0.9488539	0.9902638	154	187	93	106	143	81	112	163
ENSECAG00000013857	3.20498234	0.9490399	0.9902773	170	149	169	222	157	128	230	154
ENSECAG00000004387	4.933583829	0.94911	0.9902773	547	522	607	691	423	470	610	712
ENSECAG00000015079	8.198157998	0.9491283	0.9902773	5891	4417	6457	6024	7165	3375	6281	4580
ENSECAG00000014227	5.062924528	0.9494479	0.9904333	672	582	578	757	576	532	642	660
ENSECAG00000022557	5.28350756	0.9494523	0.9904333	962	635	711	737	793	474	925	613
ENSECAG00000022235	4.808876794	0.9495593	0.990454	612	405	613	535	444	421	594	574
ENSECAG00000021961	4.928194111	0.9499021	0.9906451	450	543	538	884	514	595	688	360
ENSECAG00000005670	0.387028769	0.9499169	0.9906451	35	17	31	11	21	14	28	23
ENSECAG00000020563	8.211425408	0.9503663	0.9907678	7410	6307	5945	2643	6827	7591	4185	2167
ENSECAG00000020565	3.45936426	0.9503749	0.9907678	198	144	262	262	193	149	179	255
ENSECAG00000009962	5.922899931	0.9503757	0.9907678	1144	1206	1074	1326	636	952	1028	1715
ENSECAG00000008651	4.928632594	0.9503835	0.9907678	555.999	563.999	614.999	609.999	585	440	716	471.998
ENSECAG00000019174	3.053972458	0.9508907	0.9910598	160	158	156	170	194	106	132	149
ENSECAG00000019169	4.852610032	0.9510731	0.9910598	735	416	546	568	479	469	632	481
ENSECAG00000000577	6.686624892	0.9511439	0.9910598	2039	1503	2047	2457	1740	1855	1937	1871
ENSECAG00000013560	3.205853184	0.9512369	0.9910598	181	122	167	245	105	90	246	240
ENSECAG00000010312	2.98928322	0.9512477	0.9910598	205	208	57	137	76	60	181	256
ENSECAG00000023633	3.215769706	0.9514584	0.9910598	141	138	196	244	189	105	211	169
ENSECAG00000024420	6.940462223	0.9515007	0.9910598	2278	2247	2305	2826	2287	2003	2421	2035
ENSECAG00000000162	8.501508306	0.9516104	0.9910598	6752	6723	6509	8555	4983	6622	7703	6559
ENSECAG00000013442	6.003926846	0.9516246	0.9910598	925	1362	1054	1634	892	1603	972	1061
ENSECAG00000014616	5.837271308	0.9516466	0.9910598	972	946	830	1822	473	873	1226	1547
ENSECAG00000017382	5.565132295	0.9516761	0.9910598	839	849	782	1273	517	920	909	1016
ENSECAG00000010086	6.5903003	0.9517106	0.9910598	1953	1552	2050	2014	1488	1574	1528	2248
ENSECAG00000007412	1.039015011	0.9518375	0.9911011	49	42	42	18	27	39	26	44
ENSECAG00000022160	6.719825369	0.9521396	0.9911242	1935	2065	2485	1701	1180	1897	1969	2463
ENSECAG00000019024	6.078553067	0.9521584	0.9911242	1390	1127	1521	1168	1200	1164	1130	1349
ENSECAG00000018080	6.151216026	0.9521864	0.9911242	1291	1376	1119	1733	1127	1159	1308	1531
ENSECAG00000012314	3.31774227	0.9522087	0.9911242	224	161	175	204	179	137	198	204
ENSECAG00000023171	4.235409943	0.9522975	0.9911259	372	334	363	403	316	322	439	264
ENSECAG00000014217	5.769907036	0.9524765	0.9911317	1044	834	1182	1240	740	729	1179	1288

ENSECAG00000015638	7.529521936	0.9525366	0.9911317	3301	3231	3388	4683	3321	2335	4300	3390
ENSECAG00000013449	4.63339091	0.9525649	0.9911317	442	338	485	678	410	224	775	441
ENSECAG00000007939	0.60561718	0.9526585	0.9911383	15	50	21	19	6	37	7	51
ENSECAG00000022976	5.181020413	0.9527938	0.9911882	748	544	806	709	731	507	762	627
ENSECAG00000003157	7.235327132	0.9531132	0.9913549	2987	2834	3077	2694	2644	2529	3035	2671
ENSECAG00000008774	5.071163041	0.9531285	0.9913549	655	509	639	814	799	258	813	604
ENSECAG00000019813	7.064743576	0.9532486	0.9913607	2553	2135	3324	2303	3866	1469	3053	1359
ENSECAG00000026972	0.627112677	0.9533086	0.9913607	26	33	21	33	23.0004	27.0001	30	22
ENSECAG00000007723	3.368493361	0.9534173	0.991383	206	127	229	252	131	118	283	215
ENSECAG00000010385	1.737209375	0.9535406	0.9914204	13	158	15	49	14	32	24	168
ENSECAG00000017137	2.845694121	0.9537472	0.9914807	94	159	188	110	46	121	79	254
ENSECAG00000020079	5.007014676	0.9537731	0.9914807	471	490	704	888	405	678	556	623
ENSECAG00000011107	8.085969549	0.9545568	0.9920305	4988	5287	5744	5185	4666	4211.01	5166	5355
ENSECAG00000013289	4.800962109	0.954607	0.9920305	415	638	530	585	250	308	747	733
ENSECAG00000015068	0.513918111	0.9547912	0.9920305	31	9	45	16	50	19	27	0
ENSECAG00000014531	5.23440517	0.9548159	0.9920305	688	592	737	957	690	574	736	683
ENSECAG00000007118	4.762846952	0.9548219	0.9920305	524	385	590	643	538	423	505	459
ENSECAG00000012609	5.221886789	0.954826	0.9920305	724	638	760	762	568	765	534	781
ENSECAG00000013281	1.961677841	0.9549857	0.9921056	105	57	81	46	77	60	94	46
ENSECAG00000014850	6.284545981	0.9551251	0.9921597	1887	1029	1727	1484	2009	1103	1334	1071
ENSECAG00000009379	6.320546521	0.9552453	0.9921938	1542	1566	1834	1146	490	2769	945	1334
ENSECAG00000002199	4.060223412	0.9556306	0.9924537	273	320	286	409	402	147	418	255
ENSECAG00000014652	8.752062716	0.9558285	0.9924537	8046	10122	8690	6410	5204	8894	5989	10208
ENSECAG00000014229	11.76071643	0.955835	0.9924537	55829	85323	75227	46456	55790	37352	65284	95712
ENSECAG00000003592	5.622694283	0.9560543	0.9924537	1110	755	895	1065	1229	489	1216	674
ENSECAG00000012224	4.964109976	0.9560668	0.9924537	766	432	577	682	628	425	798	396
ENSECAG00000012945	6.53860898	0.9560738	0.9924537	1806	1159	2146	2169	1469	1421	2322	1575
ENSECAG00000001674	1.450423701	0.9561071	0.9924537	44	27	60	75	29	15	108	50
ENSECAG00000017450	1.722804804	0.9562516	0.992513	63	36	58	101	50	57	80	41
ENSECAG00000001650	7.048602008	0.9564547	0.9925945	2747	2231	2572	2849	2759	2243	2741	1670
ENSECAG00000009889	9.351691581	0.9565049	0.9925945	12966	11128	12387	14265	9977	11034	14993	11469
ENSECAG00000008106	4.647225929	0.9566737	0.9926118	521	272	553	649	634	150	690	353
ENSECAG00000007748	4.427504888	0.9567787	0.9926118	356	394	384	559	281	361	454	439
ENSECAG00000023832	7.626433809	0.9568243	0.9926118	3448	3645	3710	4542	4396	2925	4123	2841
ENSECAG00000000497	4.825857752	0.9569715	0.9926118	719	372	576	559	595	401	576	449
ENSECAG00000000836	6.908877209	0.9570652	0.9926118	1828	3104	2481	1848	740	3245	1154	3151
ENSECAG00000008598	2.451357008	0.957109	0.9926118	130	65	92	142	157	74	103	45
ENSECAG00000009827	5.387053841	0.9572194	0.9926118	781	676	931	896	526	718	765	971
ENSECAG00000005421	3.528533602	0.9572206	0.9926118	274	146	264	218	261	122	324	124
ENSECAG00000015925	7.332043181	0.9573153	0.9926193	3343	2606	3416	3286	4028	2383	3052	1974
ENSECAG00000018093	9.177999681	0.9574161	0.9926332	11401	10299	10908	12288	9368	11105	8904	11982
ENSECAG00000012663	5.97583611	0.9575268	0.9926574	1497	839	1267	1282	2117	607	1377	465
ENSECAG00000009975	5.084517123	0.9576815	0.9926615	611	492	948	602	717	522	617	549
ENSECAG00000022381	3.926585996	0.9577396	0.9926615	291	255	270	379	350	138	279	319
ENSECAG00000001410	0.513636628	0.957793	0.9926615	13	12	44	38	10	27	24	32
ENSECAG00000021764	6.664657543	0.9583038	0.9930602	1744	2158	1687	2250	1824	1766	2014	1695
ENSECAG00000016102	8.467889548	0.9583525	0.9930602	6381	7024	6253	7747	6044	6455	6951	6020
ENSECAG00000018825	5.052749625	0.9584852	0.9931071	703	701	581	584	549	598	731	485
ENSECAG00000018958	6.610584304	0.9586269	0.9931633	2184	1403	2003	2106	2187	1626	1891	1226
ENSECAG00000018900	5.385391924	0.9588903	0.9933456	861	721	872	809	863	525	974	648
ENSECAG00000017105	6.432062264	0.9593562	0.9937217	2219	1024	1639	1859	1943	1109	2288	977
ENSECAG00000009329	3.266900472	0.9594283	0.9937217	178	159	176	229	175	174	178	157
ENSECAG00000017606	2.054613327	0.959768	0.9939829	118	60	70	70	60	37	64	130
ENSECAG00000014357	1.054679474	0.9598859	0.9940144	62	32	50	8	44	13	50	36
ENSECAG00000011861	3.330877525	0.96023	0.9942396	248	99	231	212	182	137	231	167
ENSECAG00000000042	8.315608908	0.9602785	0.9942396	5840	5432	5056	8636	4323	5632	6751	6362
ENSECAG00000009920	4.032695889	0.9606557	0.9944534	284	267	239	491	250	260	328	341
ENSECAG00000007386	1.194751542	0.96066	0.9944534	35	44	47	46	78	31	31	12
ENSECAG00000018600	10.48943863	0.9610886	0.9946085	28145	24751	25712	33332	19897	27605	26248	29516
ENSECAG00000016157	2.235577916	0.9611537	0.9946085	86	48	124	102	105	62	100	68
ENSECAG00000010581	5.434622705	0.9612006	0.9946085	981	671	830	870	879	663	929	651
ENSECAG00000026898	3.565672861	0.9612059	0.9946085	220	162	257	279	137	261	242	203
ENSECAG00000017916	7.600454711	0.9612477	0.9946085	3341	3944	3559	4349	2836	3853	3924	3174
ENSECAG00000020415	4.584823193	0.9615923	0.9947869	467	377	439	585	461	398	355	495
ENSECAG00000013335	3.558683473	0.9615952	0.9947869	216	136	187	390	306	152	306	89
ENSECAG00000007883	4.845707629	0.9616838	0.9947879	345	758	430	715	58	220	242	1545
ENSECAG00000018693	1.576655211	0.9619902	0.9949911	47	45	54	84	38	52	53	61
ENSECAG00000015304	1.938312512	0.9620554	0.9949911	64	61	101	67	67	79	74	42
ENSECAG00000007737	2.41543548	0.962177	0.9950263	50	65	143	163	81	56	151	94
ENSECAG00000007811	3.325745647	0.962352	0.9951167	178	160	206	229	134	194	162	221
ENSECAG00000019359	3.829893662	0.9627625	0.9954397	260	261	261	313	313	278	218	193
ENSECAG00000021782	3.652911424	0.9628648	0.9954397	305	184	275	213	333	135	293	137
ENSECAG00000004120	3.518698445	0.9629488	0.9954397	255	210	137	295	194	196	218	200
ENSECAG00000015471	4.376104546	0.9631577	0.9954397	488	363	378	385	302	429	387	344
ENSECAG00000009751	6.735006983	0.9633398	0.9954397	2133	1723	1880	2675	1799	1439	2529	1936
ENSECAG00000011195	3.314703763	0.9634882	0.9954397	191	151	181	247	254	119	211	131
ENSECAG00000022721	3.96869547	0.963517	0.9954397	290	218	331	379.001	380	205	346	198
ENSECAG00000007968	6.542492991	0.9635372	0.9954397	1884	1495	2002	1848	1811	1948	1435	1402

ENSECAG00000014284	1.945957949	0.9636816	0.9954397	68	54	97	71	107	44.0001	77	44
ENSECAG00000009178	5.870258317	0.9637515	0.9954397	1234	876	1144	1304	1223	740	1415	886
ENSECAG00000026826	1.921659952	0.9637866	0.9954397	58	45	112	71	52	90	50	69
ENSECAG00000021465	5.803082912	0.963802	0.9954397	1072	857	1229	1184	832	1085	1025	1052
ENSECAG00000008757	3.554678368	0.9638316	0.9954397	210	154	227	340	217	137	245	241
ENSECAG00000015035	4.368620203	0.9640418	0.9954397	435	302	427	437	408	229	515	358
ENSECAG00000013819	6.8873148	0.9641389	0.9954397	2176	1859	2182	3057	1895	1239	2573	2980
ENSECAG00000017457	8.184067955	0.9642655	0.9954397	6912	3812	6466	5692	5888	4300	7212	3545
ENSECAG00000016022	7.423560153	0.9644897	0.9954397	3281	2725	3273	4293	4025	1993	4168	2219
ENSECAG00000022178	5.006766677	0.9644905	0.9954397	713	446	610	738	686	473	658	499
ENSECAG00000008294	4.403036152	0.9646093	0.9954397	389	421	354	487	349	416	367	356
ENSECAG00000024626	8.682063861	0.9646377	0.9954397	6561	10316	5559	9152	4619	9218	7551	7973
ENSECAG00000019694	3.855894347	0.9647077	0.9954397	266	222	269	384	190	261	323	256
ENSECAG00000019949	3.739876012	0.9648375	0.9954397	177	224	176	473	68	570	81	177
ENSECAG00000020551	6.137248365	0.9648601	0.9954397	2306	816	1202	1192	1089	1625	975	1216
ENSECAG00000025131	5.792303438	0.9650513	0.9954397	877	1075	971	1441	494	1337	748	1293
ENSECAG00000012772	0.912082683	0.9650659	0.9954397	24	24	50	43	41	18	52	18
ENSECAG00000020122	9.542776401	0.9650886	0.9954397	14147	10517	12646	21491	6785	22951	8828	13368
ENSECAG00000015531	2.125629164	0.9653551	0.9954397	97	63	70	108	100	57	68	77
ENSECAG00000015346	5.453112125	0.9653857	0.9954397	853	21	1317	1403	1445	32	1475	277
ENSECAG00000024178	4.59141015	0.9653958	0.9954397	414	385	653	426	446	558	405	269
ENSECAG00000011944	7.306576149	0.9654862	0.9954397	3398	2692	3324	2948	3517	2549	2547	2585
ENSECAG00000012603	4.504523232	0.9657057	0.9954397	369	437	488	453	314	369	409	540
ENSECAG00000010670	3.043565443	0.9657566	0.9954397	158	141	135	200	156	81	198	165
ENSECAG00000013096	1.032404892	0.9659746	0.9954397	37	18	43	55	37	38	43	22
ENSECAG00000018445	4.681041157	0.9660651	0.9954397	547	372	500	577	459	355	543	500
ENSECAG00000010123	1.928306784	0.9661491	0.9954397	110	27	71	82	67	29	96	83
ENSECAG00000006358	3.219794773	0.9663309	0.9954397	162	151	127	297	107	80	191	294
ENSECAG00000003277	8.169682625	0.9663792	0.9954397	10786	5779	3312	1763	6548	2602	7164	4915
ENSECAG00000017675	3.309395381	0.9664122	0.9954397	176	139	220	232	222	117	180	192
ENSECAG00000005198	5.011651666	0.9664613	0.9954397	639	542	616	705	810	420	591	496
ENSECAG00000012980	4.047268026	0.9665049	0.9954397	263	269	283	494	284	279	318	290
ENSECAG00000012882	9.281453204	0.9665314	0.9954397	13564	9637	12260	12951	12395	8245	15210	9591
ENSECAG00000003448	6.022606598	0.966685	0.9954397	1115	1148	1303	1488	579	1263	1208	1629
ENSECAG00000002263	7.179740644	0.9666987	0.9954397	2559	2576	2720	3441	1773	2856	2622	3147
ENSECAG00000008716	4.184661919	0.9667122	0.9954397	437	252	313	411	328	225	434	337
ENSECAG00000023413	6.395676961	0.966783	0.9954397	1864	1174	1781	1735	2057	761	2208	1165
ENSECAG00000018212	8.256762199	0.9667901	0.9954397	5248	6108	5227	7175	5818	5038	6736	4513
ENSECAG00000008975	2.650372398	0.9668114	0.9954397	118	131	117	107	46	159	117	119
ENSECAG00000024587	6.64060323	0.9669018	0.9954397	2222	1863	1705	1994	2099	1080	1709	2272
ENSECAG00000011254	5.599646308	0.9671709	0.9954397	852	834	950	1180	671	388	1168	1328
ENSECAG00000011830	4.789040227	0.9671772	0.9954397	589	408	551	600	700	366	529	391
ENSECAG00000022038	7.036955394	0.9671903	0.9954397	2768	1982	2655	2934	2940	2225	1886	2195
ENSECAG00000005667	4.493145273	0.9672214	0.9954397	558	228	535	429	300	258	745	372
ENSECAG00000016061	5.268857699	0.9673702	0.9954884	683	819	721	739	464	666	719	928
ENSECAG00000020657	2.466384636	0.9675196	0.9954884	110	68	118	135	123	85	132	48
ENSECAG00000018381	8.402974691	0.9675317	0.9954884	6310	5354	6850	8206	6274	5315	7157	5504
ENSECAG00000010577	2.232753995	0.9676384	0.9955029	9	206	39	97	14	84	22	201
ENSECAG00000011743	6.059476158	0.967721	0.9955029	1149	1194	1194	1667	854	1094	1304	1568
ENSECAG00000020450	2.897156082	0.9680917	0.995794	127	123	140	192	100	85	204	148
ENSECAG00000012566	3.177317036	0.968297	0.9958561	204	157	177	160	168	97	224	161
ENSECAG00000022098	1.218707662	0.9683702	0.9958561	37	27	46	65	35	28	51	49
ENSECAG00000012896	6.719389732	0.9684151	0.9958561	2115	1671	2219	2187	2348	1862	1827	1474
ENSECAG00000004792	5.884319448	0.968529	0.9958831	1264	852	1157	1384	661	741	1443	1459
ENSECAG00000011011	2.570878718	0.9686924	0.995961	74	109	111	163	75	137	106	98
ENSECAG00000017968	4.687280929	0.9690684	0.9960496	566	350	483	634	451	282	735	412
ENSECAG00000011062	5.687348188	0.9693405	0.9960496	945	761	1213	1094	1043	856	892	892
ENSECAG00000002756	3.548451745	0.969421	0.9960496	252	156	235	263	227	141	359	131
ENSECAG00000009813	6.991313851	0.9695429	0.9960496	4481	2308	2397	347	2076	1447	4175	1793
ENSECAG00000024721	5.372296252	0.969603	0.9960496	929	705	768	800	943	513	953	598
ENSECAG00000013840	3.261043966	0.9696461	0.9960496	161	172	176	239	130	165	153	226
ENSECAG00000007043	2.348518982	0.9696563	0.9960496	95	85	101	106	61	110	91	95
ENSECAG00000015181	5.439113426	0.9696747	0.9960496	1653	790	610	253	578	808	1058	662
ENSECAG00000016258	2.504312851	0.9698032	0.9960496	75	141	83	136	63	81	82	172
ENSECAG00000020682	1.342606475	0.9699436	0.9960496	54	35	50	49	42	33	54	49
ENSECAG00000012554	1.251203027	0.9699439	0.9960496	34	23	56	67	39	31	51	45
ENSECAG00000021020	7.0436157	0.9700226	0.9960496	2323	2507	2308	3226	2450	2396	2802	1752
ENSECAG00000019782	8.641421036	0.9700427	0.9960496	7069	8305	6252	9684	4951	8589	8189	6637
ENSECAG00000002414	2.721482889	0.970101	0.9960496	105	151	130	117	96	84	139	150
ENSECAG00000022783	1.137948872	0.9702191	0.9960496	43	24	30	69	40	48	45	17
ENSECAG00000021671	1.499876365	0.9702837	0.9960496	20	83	90	14	17	122	16	25
ENSECAG00000006066	4.935679234	0.9703119	0.9960496	677	396	614	704	551	425	781	473
ENSECAG00000015352	4.891469456	0.9703784	0.9960496	517	532	549	709	400	550	492	678
ENSECAG00000009763	3.38423789	0.970506	0.9960496	203	176	243	186	130	184	127	289
ENSECAG00000022010	2.393808395	0.9705867	0.9960496	146	54	77	127	122	25	151	87
ENSECAG00000024276	4.737181485	0.97062	0.9960496	524	480	527	526	325	703	504	349
ENSECAG00000023304	6.638100532	0.9707114	0.9960534	2009	1732	1871	2193	1836	1208	2413	1770
ENSECAG00000021822	4.633389934	0.9709547	0.996213	541	418	388	581	285	409	504	595

ENSECAG00000024426	5.961230197	0.9711717	0.9963203	1359	947	1242	1341	1175	1182	1135	915
ENSECAG00000005128	4.226492132	0.9712347	0.9963203	371	290	325	489	188	233	417	517
ENSECAG00000016554	8.205145002	0.9715832	0.9963548	5038	5680	5365	6873	4215	5636	6086	5294
ENSECAG00000006877	4.995272096	0.9715964	0.9963548	328	1256	346	431	360	389	300	1247
ENSECAG00000012920	6.992811576	0.971633	0.9963548	2515	2171	2629	2568	2490	2167	2433	2045
ENSECAG00000023261	5.544425884	0.9716685	0.9963548	831	712	970	1169	678	938	720	953
ENSECAG00000013508	6.647312094	0.9717772	0.9963548	2123	1679	2202	1739	2227	1804	1769	1340
ENSECAG00000013168	5.92480833	0.9717945	0.9963548	828	1241	1130	1524	485	1499	812	1493
ENSECAG00000020451	2.339984858	0.9718986	0.9963715	122	95	82	82	136	82	70	64
ENSECAG00000011917	9.878580328	0.9724316	0.9967863	15879	18576	20756	18120	11560	20863	16190	17849
ENSECAG00000020679	7.956640458	0.9724787	0.9967863	4255	4836	3994	6297	4035	4498	5315	4050
ENSECAG00000008537	6.484102226	0.9730208	0.9970329	1435	1639	1659	2259	1155	1531	1693	2072
ENSECAG00000012454	4.604437411	0.9730293	0.9970329	439	434	474	538	414	394	459	478
ENSECAG00000014964	6.3618786	0.9730496	0.9970329	1877	1279	1471	1781	1498	1172	1598	1672
ENSECAG00000006891	3.813365978	0.9732926	0.9970329	258	208	208	442	134	243	292	334
ENSECAG00000000614	3.841351257	0.9732997	0.9970329	197	238	279	405	102	302	287	335
ENSECAG00000003238	5.997626808	0.9733827	0.9970329	1270	1035	1386	1307	1320	913	1219	1116
ENSECAG00000022765	2.094049047	0.9735127	0.9970329	96	68	62	98	52	56	99	99
ENSECAG00000020575	5.729530049	0.9735346	0.9970329	937	958	926	1352	741	1009	816	1186
ENSECAG00000004668	2.77117645	0.9737526	0.9970329	78	127	107	218	132	108	78	161
ENSECAG00000000712	4.200008414	0.973767	0.9970329	414	311	403	293	266	330	444	274
ENSECAG00000019810	6.468459484	0.973992	0.9970329	1985	1480	1492	1988	1832	1130	2050	1383
ENSECAG00000015036	8.415655383	0.9740343	0.9970329	6914	5780	7266	6737	5501	4322	8603	6431
ENSECAG00000022049	4.889797846	0.9740863	0.9970329	547	354	558	908	510	369	794	481
ENSECAG00000005594	1.975864219	0.974149	0.9970329	62	43	75	124	41	66	125	51
ENSECAG00000007497	3.181632682	0.9743066	0.9970329	320	35	109	262	355	79	171	31
ENSECAG00000024635	5.086093017	0.974325	0.9970329	715	592	695	618	619	658	461	662
ENSECAG00000012483	3.289142791	0.9743519	0.9970329	159	140	241	216	208	155	186	146
ENSECAG00000009030	6.220369344	0.9743597	0.9970329	1626	1212	1520	1467	1378	1340	1637	967
ENSECAG00000014482	4.069010429	0.974387	0.9970329	256	333	298	412	173	413	269	327
ENSECAG00000024720	4.09446692	0.9745997	0.9971338	275	335	308	403	274	387	311	234
ENSECAG00000005156	3.636058369	0.9746612	0.9971338	289	215	185	279	204	175	234	272
ENSECAG00000019857	5.212156798	0.9749891	0.9973362	670	741	704	768	636	723	587	665
ENSECAG00000008237	1.600824732	0.9753657	0.9973362	35	139	18	25	42	25	59	87
ENSECAG00000018792	1.982737673	0.9754333	0.9973362	41	65	95	100	24	67	97	94
ENSECAG00000011826	5.962827912	0.9755472	0.9973362	1118	1001	1344	1401	1303	1094	1061	984
ENSECAG00000004755	7.633641635	0.9756279	0.9973362	3380	3872	3786	4511	1892	4293	2843	4980
ENSECAG00000022850	2.006364415	0.9756545	0.9973362	62	51	66	136	35	50	143	62
ENSECAG00000008958	6.968369949	0.9756598	0.9973362	2447	2313	2703	2273	2305	2113	2279	2218
ENSECAG00000022323	3.454300888	0.9756875	0.9973362	195	185	167	306	103	260	170	238
ENSECAG00000002142	3.186828234	0.9757537	0.9973362	174	138	138	259	109	251	143	130
ENSECAG00000010774	8.088719534	0.9759341	0.9973362	5016	4925	5369	6012	4873	4421	5477	4713
ENSECAG00000022019	8.358666469	0.9760613	0.9973362	7030	5404	6091	7038	6355	5409	5479	6247
ENSECAG00000024913	4.491108199	0.9761312	0.9973362	553	270	385	554	416	418	417	350
ENSECAG00000017498	4.840104265	0.9761606	0.9973362	495	454	643	636	686	325	493	556
ENSECAG00000018186	5.924448436	0.9761711	0.9973362	1089	963	1142	1568	685	1276	1181	1203
ENSECAG00000024243	10.10959046	0.976176	0.9973362	21466	19679	21073	24364	20308	18992	21171	18300
ENSECAG00000017945	0.257106947	0.9762865	0.9973593	19	16	27	22	3	12	24	42
ENSECAG00000016672	4.867282851	0.9766972	0.9974651	596	606	554	478	528	394	643	550
ENSECAG00000017123	5.727650562	0.9768252	0.9974651	925	910	1120	1166	628	1082	699	1348
ENSECAG00000020567	5.695840801	0.9770219	0.9974651	858	1005	993	1196	705	1031	904	1033
ENSECAG00000005704	8.013140068	0.9770246	0.9974651	4595	4953	5362	5234	4763	3738	5744	4371
ENSECAG00000011767	6.072505881	0.9771347	0.9974651	1110	1233	1160	1798	1150	1280	1201	1138
ENSECAG00000011461	6.418273795	0.9772642	0.9974651	1913	1281	1657	1862	1465	1192	1670	1821
ENSECAG00000001256	0.516305931	0.9773282	0.9974651	16	39	19	27	17	13	33	35
ENSECAG00000023687	5.69433632	0.9773354	0.9974651	1107	806	1141	959	1108	979	797	780
ENSECAG00000019462	0.567462464	0.9774417	0.9974651	33	24	17	35	32	29	21	14
ENSECAG00000023232	4.803645818	0.9774419	0.9974651	597	456	526	604	554	459	556	418
ENSECAG00000009871	2.817429698	0.9775465	0.9974651	130	106	144	169	157	108	151	80
ENSECAG00000018245	2.90907813	0.9777673	0.9974651	138	134	136	168	108	129	141	156
ENSECAG00000015228	3.520991952	0.9779167	0.9974651	170	197	290	234	181	189	179	260
ENSECAG00000022310	3.442586865	0.9779261	0.9974651	136	192	233	291	137	194	169	265
ENSECAG00000022197	4.197703028	0.9779531	0.9974651	345	230	449	406	166	481	348	300
ENSECAG00000006910	3.96742322	0.9779687	0.9974651	305	191	397	334	475	137	331	176
ENSECAG00000018434	4.0510325	0.9780114	0.9974651	262	358	281	376	240	287	359	305
ENSECAG00000003158	3.599560107	0.9780147	0.9974651	213	223	255	240	189	156	274	259
ENSECAG00000012180	4.166542152	0.9780585	0.9974651	257	380	336	413	311	300	358	317
ENSECAG00000023827	0.597574787	0.9781792	0.9974987	27	29	25	27	42	13	33	15
ENSECAG00000020360	5.054180043	0.9783911	0.9975336	625	593	637	741	479	424	758	746
ENSECAG00000019952	4.731332362	0.9784265	0.9975336	410	934	220	425	430	354	384	740
ENSECAG00000018541	5.862070154	0.9785179	0.9975336	969	1197	889	1473	621	1379	1044	1084
ENSECAG00000006019	6.989367506	0.9785647	0.9975336	2451	2039	2818	2642	2331	2090	2659	2017
ENSECAG00000016381	3.112320395	0.9788107	0.9976711	192	154	186	124	239	85	185	112
ENSECAG00000021069	6.088230477	0.9788993	0.9976711	1386	1249	1555	1034	535.996	1997	771	1430
ENSECAG00000019914	6.824198655	0.9789769	0.9976711	2665	3991	1314	342.004	2765	2016	957	2219
ENSECAG00000018131	3.158226308	0.9791073	0.9976711	84	237	145	222	160	123	172	178
ENSECAG00000019028	3.586336037	0.9791388	0.9976711	179	208	211	338	226	120	280	248
ENSECAG00000008665	5.83475998	0.9793736	0.9978208	950	1130	1060	1291	985	729	1251	1184

ENSECAG00000010412	0.54907852	0.979624	0.997965	20	22	20	45	8	11	44	39
ENSECAG00000012032	1.488521126	0.9797115	0.997965	44	71	24	69	65	17	99	22
ENSECAG00000023592	8.658547795	0.9800035	0.997965	9026	6267	7962	8214	8408	7077	6346	6926
ENSECAG00000022961	3.985600082	0.9800064	0.997965	160	309	328	447	180	230	341	388
ENSECAG00000015658	4.483749113	0.980033	0.997965	484	294	583	382	673	202	413	310
ENSECAG00000006493	1.922658575	0.9800422	0.997965	76	58	92	62	69	72	62	57
ENSECAG00000019456	2.523432828	0.9803346	0.9981539	101	79	118	150	69	65	156	124
ENSECAG00000022603	5.868338441	0.9806804	0.9981539	968	1059	1015	1558	722	1305	1194	922
ENSECAG00000016499	5.158369388	0.980682	0.9981539	700	647	868	544	479	834	451	726
ENSECAG00000013553	5.469418697	0.9807764	0.9981539	709	763	897	1093	636	711	956	899
ENSECAG00000008041	4.0057202	0.9807948	0.9981539	313	294	349	276	237	365	249	278
ENSECAG00000012137	4.68401594	0.9809219	0.9981539	476	446	516	556	569	318	568	404
ENSECAG00000006526	4.027278258	0.9809337	0.9981539	346	226	292	410	349	216	395	219
ENSECAG00000009492	4.870388145	0.9812239	0.9981539	604	515	638	495	505	360	596	659
ENSECAG00000010839	0.841171396	0.9812989	0.9981539	43	18	38	35	25	30	18	45
ENSECAG00000017937	0.787754362	0.9813187	0.9981539	23	19	23	65	26	33	19	37
ENSECAG00000000809	9.349579303	0.9813912	0.9981539	12901	11613	12150	14426	10342	11588	11453	13090
ENSECAG00000009429	0.959623276	0.9814439	0.9981539	39	41	23	41	23	28	39	42
ENSECAG00000014270	3.553426732	0.9814559	0.9981539	233	202	210	262	181	138	260	272
ENSECAG00000010128	7.818924429	0.9815045	0.9981539	5103	2816	3999	5898	5660	2224	5331	3265
ENSECAG00000011192	8.271197749	0.9815458	0.9981539	7010	3901	6351	6977	7091	3459	8182	3891
ENSECAG00000018625	2.114731729	0.9817268	0.9981609	73	61	87	111	72	73	79	80
ENSECAG00000023095	4.717444634	0.9817354	0.9981609	462	444	472	678	433	347	449	665
ENSECAG00000020759	0.924451232	0.9818279	0.9981609	43	25	21	52	19	30	45	37
ENSECAG00000008949	5.213695627	0.9819603	0.9981609	738	634	716	812	714	621	901	426
ENSECAG00000008523	6.337692312	0.9820199	0.9981609	1242	1980	1503	1515	773	842	1429	2836
ENSECAG00000021698	2.443183053	0.9821616	0.9981609	55	109	47	214	98	69	114	107
ENSECAG00000009110	4.851092036	0.9824277	0.9981609	742	398	644	458	695	353	694	337
ENSECAG00000016925	7.667730301	0.9824597	0.9981609	4362	3106	3948	4471	3716	2996	4739	3302
ENSECAG00000021039	6.582586451	0.9824623	0.9981609	1677	2127	1722	1857	1091	1989	1362	2362
ENSECAG00000012027	0.56027177	0.9825403	0.9981609	49	17	10	33	50	4	27	17
ENSECAG00000004286	4.438554512	0.9825835	0.9981609	484	233	356	656	598	229	538	197
ENSECAG00000011942	6.210743863	0.9826072	0.9981609	1528	1328	1551	1347	1159	943	1353	1882
ENSECAG00000015289	5.959171307	0.9827272	0.9981935	1054	1304	807	1694	766	999	1224	1505
ENSECAG00000012325	1.003337733	0.9828772	0.9982283	26	39	31	54	47	31	39	18
ENSECAG00000015755	8.108033738	0.9830221	0.9982283	5394	4600	5259	6416	4586	3789	6240	5360
ENSECAG00000018213	5.850218946	0.9830251	0.9982283	875	1059	1060	1523	1109	1101	1117	784
ENSECAG00000021422	6.888012557	0.9831954	0.9982585	2335	1990	2088	2850	1312	2058	2484	2709
ENSECAG00000021027	7.942159213	0.9832305	0.9982585	4751	4205	4528	5731	3724	3690	5536	4910
ENSECAG00000015393	5.546174456	0.983403	0.9982632	1065	730	898	939	849	603	1132	818
ENSECAG00000015476	7.013593892	0.9837471	0.9982632	2267	2672	2140	3011	2115	2641	2674	1757
ENSECAG00000013314	5.679009152	0.9838209	0.9982632	1165	739	1069	1015	1074	863	1025	704
ENSECAG00000008882	7.052276823	0.9839397	0.9982632	2333	2535	2445	3071	1672	1947	2783	3193
ENSECAG00000005362	0.587982026	0.983994	0.9982632	11	53	19	22	14	51	5	26
ENSECAG00000018483	5.21987697	0.9840151	0.9982632	637	814	592	840	570	672	691	732
ENSECAG00000016874	6.338589127	0.9840195	0.9982632	1506	1402	1658	1733	1434	1398	1673	1306
ENSECAG00000012531	1.182975739	0.9840802	0.9982632	38	45	51	32	37	43	31	43
ENSECAG00000007192	11.22226202	0.9841456	0.9982632	48045	34077	48800	56606	48021	23368	71702	33749
ENSECAG00000020461	3.756781302	0.9842604	0.9982632	197	224	266	368	223	103	300	363
ENSECAG00000006964	3.773539304	0.9843066	0.9982632	259	200	288	316	104	388	157	299
ENSECAG00000017091	2.986264986	0.9844002	0.9982632	129	139	171	174	97	132	179	156
ENSECAG00000019774	1.202824664	0.9844535	0.9982632	49	27	32	66	37	32	35	54
ENSECAG00000006127	6.825212722	0.9844656	0.9982632	2310	1893	2034	2620	1552	1357	2671	2728
ENSECAG00000019645	3.923857429	0.984703	0.9982784	333	170	446	221	321	176	423	187
ENSECAG00000013118	6.81338957	0.9847968	0.9982784	1936	1983	2246	2615	1833	1929	2342	1989
ENSECAG00000022758	1.312784468	0.9848208	0.9982784	43	46	42	56	45	41	35	47
ENSECAG00000020480	6.284150644	0.9848321	0.9982784	1368	1364	1612	1728	1221	1004	2055	1429
ENSECAG00000011655	6.764822419	0.9850053	0.9983649	2202	1796	1746	2787	1730	1339	2764	2134
ENSECAG00000027692	11.34627561	0.9852341	0.9984295	52012	48781	43487	59497	41343	43943	49504	51588
ENSECAG00000018957	6.645376953	0.9853252	0.9984295	2200	1700	1808	2079	1914	1351	2398	1614
ENSECAG00000015209	5.104960105	0.9854779	0.9984295	646	563	704	768	667	656	615	505
ENSECAG00000014438	3.583770958	0.9855468	0.9984295	237	193	203	299	154	170	318	230
ENSECAG00000018651	2.460122448	0.9855484	0.9984295	99	94	110	117	124	72	100	93
ENSECAG00000022920	6.387889252	0.9855964	0.9984295	1949	1226	1383	2037	1409	1320	1904	1400
ENSECAG00000018817	5.521238369	0.9865196	0.9992755	780	908	855	1037	640	885	818	920
ENSECAG00000013071	5.103445505	0.9869726	0.9995124	541	467	706	1017	663	458	674	673
ENSECAG00000016012	3.66948517	0.9870044	0.9995124	169	260	256	303	185	236	274	209
ENSECAG00000022563	3.331978166	0.9870212	0.9995124	277	185	241.001	50	355	98	218	49
ENSECAG00000007371	7.899365395	0.9871963	0.9995124	4743	4690	4943	4109	5190	3892	3526	4302
ENSECAG00000011381	6.353961327	0.9872267	0.9995124	1250	2010	1194	1864	830	2236	1523	1174
ENSECAG00000020735	4.325661008	0.9872814	0.9995124	404	381	354	409	273	416	375	360
ENSECAG00000010461	3.736452932	0.9874469	0.999575	259	197	276	304	250	215	235	248
ENSECAG00000003561	3.323340916	0.9875192	0.999575	176	138	219	246	226	161	176	145
ENSECAG00000010489	5.970544087	0.9876413	0.9996095	1181	1092	1224	1407	1082	1272	1119	963
ENSECAG00000027675	11.79948283	0.9877622	0.9996428	59653	74157	57280	86762	53621	60800	78416	64629
ENSECAG00000007879	1.465116802	0.9880809	0.9997754	63	32	51	62	81	26	60	26
ENSECAG00000019823	5.178843265	0.9881053	0.9997754	700	701	600	822	550	526	729	801
ENSECAG00000013127	5.968676483	0.9882175	0.9997754	1391	955	1138	1411	1426	764	1448	903

ENSECAG00000000223	5.596609887	0.9882453	0.9997754	833	1434	854	511	288	1230	402	1461
ENSECAG00000007190	7.75977641	0.9883637	0.9998061	4303	3721	4046	4909	3444	4070	4420	3549
ENSECAG00000018861	3.349996756	0.9885262	0.9998815	242	151	201	191	267	135	212	113
ENSECAG00000013828	7.551551591	0.9888481	0.9999198	3438	3690	3194	4280	2506	2248	3335	5538
ENSECAG00000012353	4.580757072	0.9891	0.9999198	328	366	503	696	294	275	426	733
ENSECAG00000021396	6.190048464	0.9891724	0.9999198	1357	1297	1519	1520	956	1070	1532	1719
ENSECAG00000007672	3.755317218	0.9891876	0.9999198	292	195	234	332	255	173	161	370
ENSECAG00000018159	1.717798235	0.9892935	0.9999198	52	59	72	66	31	38	72	91
ENSECAG00000016302	2.914974258	0.9893139	0.9999198	127	112	169	175	141	84	190	130
ENSECAG00000017684	5.759807891	0.9894039	0.9999198	988	1082	1045	1091	666	1277	876	992
ENSECAG00000019669	7.048893614	0.989741	0.9999198	2716	2185	2587	2873	2305	1956	2994	2308
ENSECAG00000008480	5.334240145	0.9897493	0.9999198	873	696	736	838	707	545	779	870
ENSECAG00000016386	1.895643014	0.9897791	0.9999198	86	59	62	76	44	52	90	77
ENSECAG00000020181	7.805810499	0.9897942	0.9999198	4341	4043	4756	4270	2596	2396	5012	6380
ENSECAG00000021889	7.321862649	0.9900247	0.9999198	2797	3292	3002	3307	3444	2478	3620	1989
ENSECAG00000023356	4.759778578	0.9900638	0.9999198	495	542	522	539	483	341	644	494
ENSECAG00000013667	5.297692929	0.9900795	0.9999198	748	653	826	844	598	547	1000	713
ENSECAG00000012700	3.666499671	0.9901446	0.9999198	185	223	358	207	111	129	338	357
ENSECAG00000014853	5.472610541	0.990191	0.9999198	772	711	1141	821	688	949	678	819
ENSECAG00000025154	3.115473002	0.9904425	0.9999198	145	142	180	207	125	143	141	202
ENSECAG00000006043	5.918911924	0.9904494	0.9999198	1092	1137	1267	1192	843	731	1589	1278
ENSECAG00000013424	10.00545515	0.9904749	0.9999198	21124	16720	19780	22967	17842	17204	17183	21071
ENSECAG00000026970	2.010384335	0.9905175	0.9999198	105	55	77	67	61	66	85	72
ENSECAG00000022285	5.513881333	0.990597	0.9999198	876	890	853	918	613	741	1125	836
ENSECAG00000018964	1.426095145	0.9907844	0.9999198	75	23	51	53	43	17	130	8
ENSECAG00000021041	3.794460181	0.9908048	0.9999198	300	186	323	266	215	325	210	220
ENSECAG00000021753	2.065634287	0.990871	0.9999198	80	65	79	95	65	45	85	103
ENSECAG00000020564	7.169778135	0.9909193	0.9999198	2883	2460	2112	3862	2387	3922	2391	1356
ENSECAG00000024036	3.616796153	0.9910257	0.9999198	247	159	269	285	349	110	269	152
ENSECAG00000007949	2.853413223	0.9910275	0.9999198	85	150	224	85	121	189	111	78
ENSECAG00000020716	11.35153281	0.9910303	0.9999198	51780	41434	52056	59962	61653	35607	64762	26930
ENSECAG00000000329	7.890834974	0.991162	0.9999198	5035	4466	3753	5277	3012	4282	4654	5069
ENSECAG00000023572	3.23357142	0.9912049	0.9999198	179	122	215	218	151	118	245	165
ENSECAG00000024642	4.93961886	0.991464	1	648	438	658	657	595	405	738	481
ENSECAG00000011367	2.190202864	0.9915554	1	97	75	70	109	82	73	110	56
ENSECAG00000003601	7.135263257	0.9919002	1	2875	2496	2839	2730	3061	2690	2383	1797
ENSECAG00000023092	1.248279915	0.9920147	1	21	124.001	16	2	63	49	12	34
ENSECAG00000008310	6.213669171	0.9921219	1	2129	976	1417	1260	1822	636	2069	913
ENSECAG00000019387	3.39069619	0.9921691	1	186	150	228	252	159	229	147	198
ENSECAG00000017288	6.83474703	0.9922029	1	1858	2352	2094	2569	2006	1987	2385	1815
ENSECAG00000013900	2.299049567	0.9923815	1	69	78	108	123	84	107	89	62
ENSECAG00000010325	8.406906223	0.9924856	1	6550	5226	7398	7374	4679	8808	5396	4926
ENSECAG00000008939	7.025139903	0.9925758	1	2814	2114	2649	2559	2452	2711	1955	2066
ENSECAG00000007187	5.639012238	0.9925826	1	989	938	956	986	575	905	1094	1009
ENSECAG00000012963	2.253310996	0.992598	1	85	78	77	129	56	42	110	134
ENSECAG00000005326	3.607889817	0.9926927	1	253	157	254	287	245	185	273	170
ENSECAG00000022293	2.448643393	0.9928695	1	69	89	128	136	86	86	122	92
ENSECAG00000016427	4.920333766	0.9929142	1	706	404	458	818	333	428	612	821
ENSECAG00000022834	6.019548382	0.9929333	1	1180	1249	1111	1515	940	1335	1153	1182
ENSECAG00000022394	3.292365643	0.992943	1	156	192	258	142	130	227	85	233
ENSECAG00000017836	7.884669108	0.9930171	1	5203	3058	4854	5470	3991	4221	5300	3491
ENSECAG00000018517	4.818205203	0.9932116	1	569	546	465	606	401	490	687	458
ENSECAG00000021391	6.650471563	0.9932173	1	1938	1873	1939	2069	1675	1834	2074	1601
ENSECAG00000010759	7.484658613	0.9933328	1	3399	3084	3375	4178	4210	2566	3176	2839
ENSECAG00000013264	5.731428619	0.9933559	1	865	980	823	1509	901	693	1131	1125
ENSECAG00000009911	6.525182525	0.9935045	1	1552	2242	1543	1759	1364	1463	2179	1656
ENSECAG00000002904	5.460075348	0.9936132	1	1004	631	885	923	705	870	803	745
ENSECAG00000019538	2.52880256	0.9938727	1	103	114	116	105	57	151	80	110
ENSECAG00000008249	5.297163736	0.9938785	1	844	680	830	685	804	437	1057	577
ENSECAG00000007369	7.152896134	0.9939897	1	2580	2854	2623	3012	1781	2756	2727	2905
ENSECAG00000016149	5.233456569	0.9941315	1	632	766	670	853	592	648	631	812
ENSECAG00000020465	6.313208456	0.9941532	1	1593	990	1666	2041	1746	1069	2082	871
ENSECAG00000016822	5.461273549	0.9941882	1	914	753	700	1079	689	489	981	1054
ENSECAG00000020717	4.273711418	0.9943129	1	271	358	302	589	196	283	473	455
ENSECAG00000016794	5.543032413	0.9946436	1	1049	773	926	875	852	443	1013	1088
ENSECAG00000010878	4.77574665	0.9946549	1	617	474	465	570	622	386	548	407
ENSECAG00000022623	6.075687238	0.994712	1	1435	1065	1554	1187	1398	779	1463	1244
ENSECAG00000018106	5.649894635	0.994818	1	950	837	1144	969	835	633	1016	1152
ENSECAG00000024754	5.298169348	0.9949585	1	818	533	862	867	920	400	955	589
ENSECAG00000008572	3.070301254	0.995044	1	126	170	174	176	170	127	151	145
ENSECAG00000013770	6.304700292	0.9950537	1	1471	1361	1466	1898	786	1326	1548	2035
ENSECAG00000021007	6.419654722	0.9951986	1	1707	1712	1582	1630	1198	1892	1442	1516
ENSECAG00000009902	6.90895658	0.9952884	1	1476	3457	2013	2235	388	3258	1185	3571
ENSECAG00000000184	4.925200999	0.9954075	1	559	508	604	704	468	550	575	572
ENSECAG00000019073	7.200625953	0.99542	1	3246	2376	2892	2946	2453	3339	1879	2637
ENSECAG00000010618	2.877709389	0.9954364	1	108	130	109	226	43	141	156	184
ENSECAG00000017599	3.069113619	0.9955095	1	172	115	163	202	155	62	196	197
ENSECAG00000023865	5.965455936	0.9956828	1	1378	1120	1078	1279	770	1087	1086	1533

ENSECAG00000019921	3.237741971	0.995802	1	202	213	117	188	188	168	136	170
ENSECAG00000023042	3.365696649	0.9960351	1	189	169	197	247	129	125	210	278
ENSECAG00000022682	3.527935382	0.9962663	1	203	187	207	305	186	138	233	272
ENSECAG00000008876	7.065443114	0.9968392	1	2160	2995	2718	2451	1367	2711	3352	2242
ENSECAG00000017099	6.512235719	0.9968471	1	1666	1716	1734	1991	1573	1646	1647	1644
ENSECAG00000021202	7.952200724	0.9977477	1	4566	5035	6242	3132	3263	3052	4361	7276
ENSECAG00000007555	4.83595949	0.9985246	1	466	513	556	695	383	500	654	519
ENSECAG00000020077	4.882726379	0.9988141	1	528	329	744	727	568	408	643	506
ENSECAG00000007787	7.378823656	0.9989002	1	3536	2428	3614	3439	3017	2773	3627	2552
ENSECAG00000002428	4.958334247	0.9989841	1	839	455	662	444	890	279	863	234
ENSECAG00000022401	4.429483466	0.9990609	1	447	337	417	480	315	296	548	406
ENSECAG00000012998	6.192314312	0.999294	1	1319	1453	1440	1456	1157	1369	1259	1419
ENSECAG00000018805	6.06077763	0.999853	1	1207	1270	1109	1631	1198	1086	1442	1073
ENSECAG00000004362	6.208247593	0.9998586	1	1651	1205	1503	1388	1171	1217	1472	1450
ENSECAG00000011758	6.492623228	0.9998757	1	1720	1887	1647	1706	1428	1730	1549	1695
ENSECAG00000001472	0.784425262	1	1	46	21	51	6	37	12	51	19
ENSECAG00000008200	1.520132641	1	1	64	78	30	38	48	38	18	92
ENSECAG00000012717	0.527910192	1	1	20	18	26	43	46	5	35	11
ENSECAG00000012893	1.160402771	1	1	39	32	53	43	48	26	32	46
ENSECAG00000016658	1.286694346	1	1	52	42	51	36	87	13	46	22
ENSECAG00000024175	0.50971739	1	1	24	20	26	33	27	19	44	7
ENSECAG00000017907	0.3430505	1	1	16	30	22	21	9	11	28	38
ENSECAG00000010367	2.582789516	1	1	129	61	107	173	133	55	171	73
ENSECAG00000023189	1.755320712	1	1	37	56	70	96	22	101	39	65
ENSECAG00000007436	2.194890664	1	1	99	66	87	98	75	53	80	116
ENSECAG00000014363	0.563630536	1	1	27	24	29	26	17	32	23	26
ENSECAG00000017649	2.463671142	1	1	84	95	144	98	97	115	80	89
ENSECAG00000000508	2.318592575	1	1	88	89	103	100	103	41	119	95
ENSECAG00000023354	0.670546072	1	1	23	27	37	29	34	12	24	37
ENSECAG00000024553	2.412654353	1	1	103	65	122	120	33	57	147	151
ENSECAG00000011215	1.365954289	1	1	24	53	82	31	39	36	38	64
ENSECAG00000003947	2.035704543	1	1	69	83	66	94	88	69	71	56
ENSECAG00000022981	2.563342054	1	1	70	121	86	182	90	105	168	58
ENSECAG00000024859	0.906749073	1	1	41	27	45	24	36	12	57	26
ENSECAG00000014905	1.307296318	1	1	59	33	58	34	60	30	36	43
ENSECAG00000007136	2.972927318	1	1	153	110	171	175	76	114	180	194
ENSECAG00000017624	2.845422263	1	1	124	138	130	161	69	134	134	171
ENSECAG00000018145	3.22490382	1	1	215	105	172	241	315	94	157	94
ENSECAG00000016395	3.571033075	1	1	193	178	176	392	178	106	523	86
ENSECAG00000014009	1.344844523	1	1	31	61	54	41	24	46	27	76
ENSECAG00000007336	3.009782084	1	1	212	142	120	142	130	137	142	160
ENSECAG00000019190	2.438013072	1	1	66	114	94	142	79	78	144	87
ENSECAG00000020497	3.517299052	1	1	286	101	177	343	170	107	345	220
ENSECAG00000010257	3.285162264	1	1	146	145	235	233	227	162	144	150
ENSECAG00000008919	2.978617903	1	1	110	135	179.001	186	118	168	143	123
ENSECAG00000007086	3.533418061	1	1	203	140	231	340	227	260	72	235
ENSECAG00000014814	4.864377066	1	1	516	503	613	637	508	566	489	497
ENSECAG00000016657	3.900078517	1	1	323	291	265	269	296	198	368	215
ENSECAG00000010765	4.693465523	1	1	526	428	626	417	465	413	470	499
ENSECAG00000020016	1.864753926	1	1	55	100	37	81	22	106	48	71
ENSECAG00000008659	5.228033791	1	1	690	808	644	754	764	672	766	468
ENSECAG00000022246	5.503922945	1	1	958	690	914	984	1042	570	1069	601
ENSECAG00000024371	1.757146298	1	1	62	17	91	92	23	28	86	106
ENSECAG00000013824	5.419727113	1	1	918	755	738	922	762	790	793	700
