# Supplementary material for: Transcriptome analysis of immune genes in peripheral blood mononuclear cells of young foals and adult horses
Source: PLoS One. 2018 Sep 5;13(9):e0202646. doi: 10.1371/journal.pone.0202646 (PMC6124769; doi:10.1371/journal.pone.0202646)
Supplement: S3 Table — (PDF) [file pone.0202646.s003.pdf]

S3 Table. Day 1 versus adult PBMC RNA-Seq data

				Raw data (number of reads)							
Gene (Ensembl ID)	log fold change	p value (tag-wise dispersion)	adjusted p value (tag-wise dispersion)	day1foalA	day1foalB	day1foalC	day1foalD	mare1	mare2	mare3	mare4
ENSECAG00000027689	12.49877772	1.63E-66	1.85E-62	198914	110243	286985	212133	0	0	0	0
ENSECAG00000027671	11.87250314	7.38E-66	4.20E-62	137518	84132	158360	138085	0	0	0	0
ENSECAG00000027672	11.53018381	7.89E-61	3.00E-57	124700	64151	118245	90510	0	0	0	0
ENSECAG00000027679	8.260403457	2.66E-49	7.58E-46	11461	5470	15373	9851	0	0	0	0
ENSECAG00000009282	5.956606662	6.04E-39	1.38E-35	11	8	21	28	3532	2414	2662	1741
ENSECAG00000007258	8.105284773	1.32E-37	2.51E-34	1	1	6	7	22410	7892	9135	6466
ENSECAG00000009575	7.466077731	7.94E-35	1.29E-31	1	1	1	3	9479	2821	16138	2703
ENSECAG00000022666	5.451700351	1.20E-27	1.71E-24	7	13	12	19	1914	1396	3774	477
ENSECAG00000008721	6.354135097	1.61E-26	2.04E-23	5	3	32	23	4905	2148	4946	1929
ENSECAG00000024888	9.12124771	8.26E-26	9.41E-23	113	59	242	123	29386	19316	28624	16603
ENSECAG00000008322	7.386947503	1.43E-25	1.48E-22	24	10	25	13	8415	3934	14370	2470
ENSECAG00000009700	4.636418284	2.94E-25	2.79E-22	14	12	23	30	1242	570	1849	549
ENSECAG00000020640	4.231835379	4.46E-25	3.90E-22	0	1	1	1	1060	114	1630	489
ENSECAG00000016797	4.199267769	6.76E-23	5.49E-20	449	643	700	725	13	33	28	48
ENSECAG00000009556	5.189553868	9.26E-23	7.03E-20	0	1	19	2	1332	988	2744	1249
ENSECAG00000016605	3.402310397	7.47E-21	5.32E-18	2	11	11	13	564	392	582	197
ENSECAG00000001910	5.257890492	1.51E-20	1.01E-17	24	14	24	31	3250	1152	1366	479
ENSECAG00000009589	5.180877572	2.07E-20	1.31E-17	30	16	32	13	2609	1101	1728	547
ENSECAG00000017267	5.352175214	1.56E-19	9.32E-17	44	10	22	64	1445	1186	3134	1162
ENSECAG00000011113	2.42651029	2.11E-19	1.20E-16	81	125	223	378	1	0	3	6
ENSECAG00000006338	4.097352166	4.49E-19	2.43E-16	17	22	37	49	952	373	987	464
ENSECAG00000008606	5.37398048	5.21E-19	2.70E-16	37	9	30	14	2398	1106	2813	686
ENSECAG00000021413	6.137583474	6.30E-19	3.12E-16	77	84	81	166	3228	2076	5176	1222
ENSECAG00000016994	3.087610839	1.39E-18	6.59E-16	1	1	0	4	313	85	890	207
ENSECAG00000021710	7.624623824	1.52E-18	6.72E-16	222	278	296	315	9986	7353	10154	4645
ENSECAG00000024524	1.108750953	1.53E-18	6.72E-16	0	0	0	0	136	56	109	45
ENSECAG00000024763	2.196707629	1.63E-18	6.86E-16	0	0	1	4	87	149	181	339
ENSECAG00000023330	4.973165299	2.56E-18	1.04E-15	61	26	62	38	1731	727	1739	926
ENSECAG00000024870	3.053597476	6.70E-18	2.63E-15	9	2	15	8	444	225	331	343
ENSECAG00000010886	1.58296721	3.54E-17	1.34E-14	0	0	4	1	113	97	83	185
ENSECAG00000018564	4.424488348	7.92E-17	2.91E-14	60	79	69	49	801	639	940	938
ENSECAG00000015819	3.580684595	2.00E-16	7.12E-14	19	2	20	23	523	495	463	433
ENSECAG00000011874	2.438732579	2.43E-16	8.39E-14	1	0	1	9	303	175	115	278
ENSECAG00000020041	7.955118536	2.89E-16	9.68E-14	137	164	97	295	8529	10622	20614	2491
ENSECAG00000006176	2.235685193	3.79E-16	1.23E-13	0	1	2	1	424	138	135	59
ENSECAG00000020503	3.817503774	5.41E-16	1.71E-13	4	9	20	20	581	716	905	119
ENSECAG00000020435	0.638022316	6.95E-16	2.13E-13	41	32	63	75	0	1	2	0
ENSECAG00000003774	7.905811003	7.12E-16	2.13E-13	85	120	332	524	7373	10489	16197	6017
ENSECAG00000021052	3.925766579	4.93E-15	1.44E-12	29	37	45	37	536	299	1190	441
ENSECAG00000015834	0.549173932	7.02E-15	2.00E-12	0	0	0	0	28	43	44	113
ENSECAG00000011440	3.773890589	1.05E-14	2.92E-12	26	34	34	84	567	387	689	474
ENSECAG00000017316	0.165920185	1.82E-14	4.95E-12	0	0	0	0	36	52	24	55
ENSECAG00000019436	1.390608381	2.29E-14	6.07E-12	2	0	1	0	165	84	36	124
ENSECAG00000012681	2.414536261	3.25E-14	8.41E-12	100	152	277	205	12	3	19	8
ENSECAG00000001227	0.411174709	6.29E-14	1.59E-11	0	0	0	0	68	26	95	21
ENSECAG00000013979	2.451370534	6.55E-14	1.62E-11	5	9	1	4	91	136	337	342
ENSECAG00000009474	5.860732893	8.05E-14	1.95E-11	10	70	56	189	1755	2746	1292	3521
ENSECAG00000005719	0.895586307	1.01E-13	2.38E-11	0	0	0	3	35	102	113	44
ENSECAG00000024743	3.399931629	1.03E-13	2.38E-11	4	1	0	4	134	277	110	1223
ENSECAG00000015637	2.779524636	1.07E-13	2.44E-11	175	146	281	334	10	8	10	31
ENSECAG00000025070	1.69949044	1.14E-13	2.54E-11	4	2	6	4	115	118	224	63
ENSECAG00000017232	1.42086628	1.26E-13	2.76E-11	0	3	4	3	84	105	54	173
ENSECAG00000016880	1.664945128	1.45E-13	3.12E-11	3	3	2	4	93	54	270	107
ENSECAG00000024406	3.772450079	1.82E-13	3.84E-11	27	16	36	52	194	670	660	627
ENSECAG00000002389	3.047488559	3.16E-13	6.55E-11	7	4	4	11	94	484	131	609
ENSECAG00000016243	1.116419208	4.04E-13	8.22E-11	3	0	0	2	142	58	82	56
ENSECAG00000017525	1.070181265	4.12E-13	8.23E-11	3	1	0	0	101	68	128	36
ENSECAG00000019758	3.006792751	4.22E-13	8.28E-11	16	10	25	21	439	207	454	169
ENSECAG00000011051	2.327891026	4.64E-13	8.95E-11	1	7	7	15	240	197	287	78
ENSECAG00000011631	2.45628248	9.47E-13	1.79E-10	4	2	5	10	289	103	74	407
ENSECAG00000006647	4.100797215	9.61E-13	1.79E-10	23	58	59	48	849	441	1179	257
ENSECAG00000015927	2.226262266	9.89E-13	1.82E-10	59	163	110	347	5	7	9	15
ENSECAG00000017357	5.250612023	1.13E-12	2.01E-10	137	99	207	249	1627	956	1615	1454
ENSECAG00000025096	0.733418159	1.13E-12	2.01E-10	0	0	4	0	86	41	95	38
ENSECAG00000015147	6.020485674	1.20E-12	2.10E-10	164	198	97	309	2974	2414	3663	1086
ENSECAG00000019060	3.00297002	1.23E-12	2.12E-10	9	15	21	12	611	142	337	178
ENSECAG00000023062	5.493418341	1.42E-12	2.41E-10	10	8	5	77	428	987	1702	4508
ENSECAG00000017842	1.983495409	1.49E-12	2.49E-10	2	3	11	11	122	73	248	194
ENSECAG00000008726	3.443847797	1.82E-12	3.01E-10	3	8	37	21	614	333	640	176
ENSECAG00000013088	0.747764975	1.97E-12	3.20E-10	0	4	0	1	93	49	71	45
ENSECAG00000003313	1.344273062	2.87E-12	4.59E-10	80	21	73	180	1	3	4	1

ENSECAG00000002971	4.212343962	3.17E-12	5.01E-10	60	89	76	111	673	589	1060	443
ENSECAG00000012621	2.017142992	4.91E-12	7.66E-10	7	9	14	11	134	108	265	125
ENSECAG00000023601	3.652249414	1.12E-11	1.72E-09	11	14	12	72	221	448	545	796
ENSECAG00000004372	2.885337507	1.21E-11	1.84E-09	7	3	5	25	184	128	256	632
ENSECAG00000001010	1.273167146	2.42E-11	3.62E-09	1	1	3	12	117	57	111	89
ENSECAG00000005078	0.316931858	2.52E-11	3.73E-09	0	1	0	3	40	33	91	28
ENSECAG00000024394	1.714703943	2.76E-11	4.03E-09	4	1	4	1	213	83	202	30
ENSECAG00000008660	1.936686713	2.80E-11	4.04E-09	6	1	9	9	166	69	280	102
ENSECAG00000006060	2.193872504	3.66E-11	5.20E-09	4	5	0	9	307	83	290	65
ENSECAG00000019460	1.021175745	4.09E-11	5.74E-09	96	57	53	42	0	1	7	0
ENSECAG00000005328	1.040967298	4.23E-11	5.88E-09	2	2	1	8	57	72	143	49
ENSECAG00000009625	8.836603217	4.45E-11	6.10E-09	63	55	3	0	13136	25454	35817	4071
ENSECAG00000025076	1.606096683	4.69E-11	6.27E-09	9	5	10	8	127	104	138	88
ENSECAG00000010997	2.08681497	4.71E-11	6.27E-09	98	26	180	316	0	4	3	9
ENSECAG00000023509	2.209365968	4.73E-11	6.27E-09	8	10	9	12	242	81	320	92
ENSECAG00000005624	1.957127023	5.49E-11	7.18E-09	0	6	1	16	164	152	194	105
ENSECAG00000009450	1.530019286	5.62E-11	7.27E-09	84	28	225	48	0	2	2	5
ENSECAG00000022890	0.98492479	6.45E-11	8.25E-09	2	2	2	0	96	34	156	31
ENSECAG00000004839	0.118429018	8.30E-11	1.05E-08	0	1	0	2	70	30	23	35
ENSECAG00000025078	4.715959408	8.60E-11	1.08E-08	75	94	88	241	759	715	895	1514
ENSECAG00000008916	5.19809855	9.33E-11	1.15E-08	77	108	77	283	738	1155	1144	2561
ENSECAG00000010836	7.06803418	1.15E-10	1.41E-08	266	514	349	649	6195	1979	6354	6439
ENSECAG00000015014	0.980146368	1.37E-10	1.66E-08	1	4	3	8	40	50	110	103
ENSECAG00000024219	2.950424501	1.55E-10	1.85E-08	20	11	30	36	295	202	189	482
ENSECAG00000006722	1.28482209	2.08E-10	2.47E-08	3	4	0	4	61	28	149	153
ENSECAG00000006663	3.668569196	2.23E-10	2.62E-08	24	15	1	29	551	218	1078	282
ENSECAG00000022555	1.166233307	2.65E-10	3.08E-08	3.00713	1	4	8	128	71.0001	109	34
ENSECAG00000014203	1.761187814	3.28E-10	3.78E-08	1	1	2	7	35	45	130	343
ENSECAG00000008445	2.471395032	3.53E-10	4.02E-08	83	118	208	378	7	5	23	27
ENSECAG00000024691	3.199822024	3.92E-10	4.41E-08	36	17	37	34	266	278	254	580
ENSECAG00000012112	1.382471232	4.36E-10	4.86E-08	3	5	9	10	87	108	155	45
ENSECAG00000001064	3.671430796	7.52E-10	8.31E-08	3	6	5	46	88	501	422	1071
ENSECAG00000001621	3.561488861	8.15E-10	8.93E-08	15	25	17	23	463	84	1284	175
ENSECAG00000011559	2.867071103	8.84E-10	9.58E-08	105	309	235	286	30	32	58	28
ENSECAG00000016614	3.536140689	9.41E-10	1.01E-07	379	254	182	641	57	30	77	54
ENSECAG00000011413	2.524922039	1.07E-09	1.14E-07	14	26	20	31	125	250	320	163
ENSECAG00000023979	2.5120518	1.10E-09	1.16E-07	10	2	12	3	205	50	196	471
ENSECAG00000012441	6.167958931	1.14E-09	1.19E-07	140	220	343	331	4720	1411	3977	982
ENSECAG00000023912	0.495622635	1.20E-09	1.24E-07	18	37	76	55	1	2	4	6
ENSECAG00000017386	2.66514047	1.63E-09	1.68E-07	19	7	27	11	301	146	413	135
ENSECAG00000013057	3.457794756	1.74E-09	1.77E-07	37	45	41	84	318	293	314	675
ENSECAG00000022707	0.886930866	1.82E-09	1.84E-07	4	4	4	3	50	54	139	40
ENSECAG00000013094	2.284350264	1.95E-09	1.95E-07	12	13	19	23	298	115	209	105
ENSECAG00000009483	0.956692113	2.03E-09	2.01E-07	6	1	1	3	96	34	121	49
ENSECAG00000004258	1.090945317	2.57E-09	2.53E-07	3	6	5	2	153	34	67	65
ENSECAG00000007393	0.269230693	3.02E-09	2.94E-07	25	33	36	58	3	2	7	2
ENSECAG00000015657	0.483100496	3.59E-09	3.47E-07	0	3	0	5	36	59	26	84
ENSECAG00000016537	3.396952412	3.80E-09	3.63E-07	32	48	70	48	597	270	422	239
ENSECAG00000021378	3.129234823	5.23E-09	4.96E-07	23.0106	34.0001	29.0079	16.0001	555.001	151.001	506	143.024
ENSECAG00000017642	3.09814741	5.68E-09	5.34E-07	25	21	21	73	220	179	510	398
ENSECAG00000013869	2.51040019	5.73E-09	5.35E-07	4	10	17	26	257	346	208	52
ENSECAG00000023141	2.747445347	7.08E-09	6.56E-07	199	130	215	295	17	18	32	57
ENSECAG00000015574	2.510786127	8.30E-09	7.62E-07	14	6	19	23	401	123	265	84
ENSECAG00000008174	2.264113282	1.10E-08	1.00E-06	8	17	22	12	295	68	255	116
ENSECAG00000014137	0.808756912	1.30E-08	1.18E-06	3	3	8	8	74	36	95	51
ENSECAG00000022305	3.230061187	1.31E-08	1.18E-06	12	7	2	45	100	306	345	757
ENSECAG00000011848	1.90612	1.43E-08	1.27E-06	4	13	7	23	217	102	185	61
ENSECAG00000016513	0.705067183	1.71E-08	1.51E-06	0	2	9	5	77	38	79	47
ENSECAG00000023537	4.798833957	1.74E-08	1.52E-06	103	120	164	338	1221	643	1259	794
ENSECAG00000013661	1.905913723	1.75E-08	1.52E-06	9	1	14	7	255	71	180	73
ENSECAG00000010510	0.961042444	2.18E-08	1.88E-06	3	6	11	4	60	56	125	47
ENSECAG00000017696	1.774690693	2.27E-08	1.94E-06	71	70	134	149	20	17	16	21
ENSECAG00000024798	3.205324783	2.39E-08	2.03E-06	23	42	37	86	215	201	433	508
ENSECAG00000003105	2.207532748	2.43E-08	2.05E-06	2	1	15	7	288	50	372	49
ENSECAG00000022349	1.314879292	2.54E-08	2.13E-06	5	6	12	9	133	69	124	40
ENSECAG00000010937	0.435009068	2.61E-08	2.17E-06	3	3	5	5	38	31	71	54
ENSECAG00000004390	4.269322225	2.63E-08	2.17E-06	55	101	70	166	981	358	1185	338
ENSECAG00000011292	2.919979499	2.67E-08	2.18E-06	142	250	167	493	10	30	2	39
ENSECAG00000019505	2.078377639	3.12E-08	2.54E-06	14	16	25	22	190	104	210	107
ENSECAG00000008538	4.280120219	3.16E-08	2.55E-06	79	109	157	143	955	527	840	385
ENSECAG00000023015	1.015377993	3.68E-08	2.95E-06	27	49	79	106	11	5	12	8
ENSECAG00000000106	6.038020169	3.93E-08	3.13E-06	222	279	274	471	1860	2447	4887	731
ENSECAG00000000431	1.11287395	4.26E-08	3.37E-06	36	64	125	52	3	9	14	2
ENSECAG00000019940	1.616948696	4.46E-08	3.49E-06	30	83	72	259	2	11	3	14
ENSECAG00000023587	4.008660413	4.48E-08	3.49E-06	40	43	51	38	1318	153	888	165
ENSECAG00000014770	3.417649794	5.22E-08	4.04E-06	33	20	49	43	790	206	501	122

ENSECAG00000024310	2.047883115	5.25E-08	4.04E-06	7	12	6	18	282	119	195	33
ENSECAG00000010840	1.555879934	5.43E-08	4.15E-06	10	18	9	5	123	82	135	88
ENSECAG00000000022	5.247369046	5.54E-08	4.21E-06	109	110	186	454	1042	1231	902	2251
ENSECAG00000018160	6.294410528	5.64E-08	4.25E-06	163	243	113	835	1724	2282	3468	4476
ENSECAG00000008773	4.759927265	5.91E-08	4.43E-06	137	166	243	206	934	510	1178	1112
ENSECAG00000008355	2.633088654	6.07E-08	4.52E-06	15	11	31	28	341	124	407	80
ENSECAG00000017301	2.006137001	6.13E-08	4.53E-06	11	7	13	6	288	175	83	48
ENSECAG00000010486	0.454017394	6.50E-08	4.78E-06	18	37	33	96	1	8	2	3
ENSECAG00000019204	0.877983496	9.26E-08	6.76E-06	40	29	141	27	3	3	9	0
ENSECAG00000010888	2.656812202	9.52E-08	6.90E-06	30	35	26	37	239	174	349	145
ENSECAG00000012249	1.344621223	9.64E-08	6.95E-06	79	70	32	132	10	5	16	12
ENSECAG00000003385	1.309793003	1.03E-07	7.39E-06	27	37	171	116	0	5	0	11
ENSECAG00000018739	0.39993761	1.14E-07	8.11E-06	19	35	43	72	3	9	3	3
ENSECAG00000006803	4.999249174	1.22E-07	8.62E-06	150	119	192	334	1885	661	1031	878
ENSECAG00000016066	1.484001299	1.25E-07	8.75E-06	41	85	65	197	2	11	1	16
ENSECAG00000025109	3.716997391	1.26E-07	8.81E-06	125	390	757	476.001	117	44.0005	64.0006	24.0004
ENSECAG00000008710	3.970871127	1.28E-07	8.87E-06	60	67	105	108	190	443	715	930
ENSECAG00000026892	0.459364449	1.36E-07	9.35E-06	4	7	4	4	40	42	65	45
ENSECAG00000016422	3.055647109	1.39E-07	9.52E-06	24	39	60	82	310	182	370	310
ENSECAG00000014452	5.191610463	1.50E-07	1.02E-05	101	75	133	190	2525	391	2488	322
ENSECAG00000016953	2.752798485	1.55E-07	1.05E-05	15	6	22	22	558	72	347	75
ENSECAG00000022699	0.983502787	1.66E-07	1.12E-05	3	2	2	7	57	26	212	22
ENSECAG00000015692	1.723778872	1.76E-07	1.18E-05	7	0	13	11	147	32	212	127
ENSECAG00000008617	0.697488867	1.99E-07	1.33E-05	3	1	6	10	36	50	44	102
ENSECAG00000021075	1.65259383	2.01E-07	1.33E-05	48	60	90	220	17	12	8	21
ENSECAG00000012416	2.848720227	2.12E-07	1.39E-05	34	19	15	19	497	108	364	127
ENSECAG00000002920	4.014224158	2.21E-07	1.44E-05	59	111	139	121	535	268	869	602
ENSECAG00000019691	2.276620894	2.27E-07	1.48E-05	123	91	197	167	37	31	20	28
ENSECAG00000015663	1.549737723	2.31E-07	1.49E-05	4	4	17	9	135	49	226	42
ENSECAG00000016594	3.458878648	2.37E-07	1.52E-05	26	50	74	103	266	228	380	702
ENSECAG00000002739	5.007590491	2.40E-07	1.53E-05	125	133	85	277	958	277	2104	1557
ENSECAG00000000015	4.70973061	2.43E-07	1.55E-05	78	43	78	267	940	306	2008	811
ENSECAG00000017297	3.517304528	2.63E-07	1.66E-05	30	58	81	21	528	269	759	174
ENSECAG00000024523	3.812850215	2.75E-07	1.73E-05	62	41	19	21	868	170	987	202
ENSECAG00000001159	2.002833693	2.77E-07	1.73E-05	6	13	11	38	147	148	234	63
ENSECAG00000015055	3.882804709	2.97E-07	1.85E-05	366	445	344	531	83	68	201	123
ENSECAG00000010654	1.644874193	3.34E-07	2.07E-05	124	24	116	143	0	6	0	14
ENSECAG00000016033	5.533866049	3.39E-07	2.09E-05	198	272	248	428	2481	755	2493	878
ENSECAG00000023348	3.059443735	3.48E-07	2.13E-05	24	65	46	58	265	178	531	229
ENSECAG00000009884	0.781125945	3.60E-07	2.19E-05	7	1	7	7	36	60	54	94
ENSECAG00000001863	4.340201971	3.75E-07	2.27E-05	78	95	187	214	705	425	1169	529
ENSECAG00000008643	3.425696928	3.86E-07	2.32E-05	20	46	75	85	248	693	239	322
ENSECAG00000018716	1.898909016	3.87E-07	2.32E-05	11	12	9	5	266	86	34	163
ENSECAG00000008141	3.373797049	4.11E-07	2.45E-05	38	50	84	106	395	193	458	394
ENSECAG00000012830	4.040439102	4.16E-07	2.47E-05	91	59	98	107	896	272	899	309
ENSECAG00000020782	4.609516421	4.37E-07	2.58E-05	429	518	805	1121	132	193	228	295
ENSECAG00000000268	2.908572576	4.57E-07	2.68E-05	46	36	38	61	263	211	364	207
ENSECAG00000023251	3.048218818	4.75E-07	2.77E-05	27	70	35	51	149	228	432	381
ENSECAG00000016140	5.67842936	5.00E-07	2.90E-05	971	1491	1135	2705	226	158	471	643
ENSECAG00000014979	0.591484351	5.18E-07	2.99E-05	2	2	10	7	41	28	93	56
ENSECAG00000013462	1.315996919	5.68E-07	3.27E-05	3	13	7	20	42	82	117	118
ENSECAG00000017905	2.047939078	5.92E-07	3.39E-05	8	13	26	8	235	156	183	37
ENSECAG00000022653	3.834693981	6.05E-07	3.45E-05	43	69	118	97	598	184	956	352
ENSECAG00000012652	1.614730281	6.75E-07	3.82E-05	9	11	12	15	224	41	90	85
ENSECAG00000020490	0.515897067	6.82E-07	3.85E-05	5	6	3	7	39	45	85	32
ENSECAG00000005023	4.498428223	7.42E-07	4.15E-05	62	25	39	33	1501	60	2006	195
ENSECAG00000017003	1.946761106	7.43E-07	4.15E-05	86	99	131	145	18	18	16	43
ENSECAG00000023892	5.231468585	7.58E-07	4.21E-05	142	277	331	412	984	715	1699	1723
ENSECAG00000021073	-0.196411579	7.90E-07	4.37E-05	4	0	0	3	26	26	48	21
ENSECAG00000004042	4.210833023	8.12E-07	4.46E-05	110	612	357	1512	70	64	106	158
ENSECAG00000012913	3.947194062	9.60E-07	5.25E-05	75	83	83	94	397	149	1016	718
ENSECAG00000014430	2.435324262	1.09E-06	5.95E-05	14	13	42	40	178	94	370	158
ENSECAG00000019948	4.906339239	1.10E-06	5.98E-05	102	97	79	101	2586	333	1529	169
ENSECAG00000014755	3.342979533	1.15E-06	6.18E-05	53	38	63	79	526	169	537	213
ENSECAG00000000852	1.031976406	1.15E-06	6.19E-05	42	46	71	98	7	20	3	4
ENSECAG00000021044	4.509406212	1.29E-06	6.91E-05	664	980	261	875	110	217	31	68
ENSECAG00000010672	2.352423348	1.54E-06	8.21E-05	13	18	40	50	180	184	243	107
ENSECAG00000018841	4.356768114	1.58E-06	8.38E-05	66	2	82	14	1420	714	726	326
ENSECAG00000018258	1.202076935	1.59E-06	8.40E-05	8	11	7	21	47	79	85	106
ENSECAG00000016549	3.410129748	1.67E-06	8.75E-05	40	24	95	31	400	238	803	164
ENSECAG00000004705	0.851072204	1.72E-06	8.97E-05	3	6	10	1	83	32	128	26
ENSECAG00000009296	3.055474779	1.76E-06	9.16E-05	80	206	425	336	31	59	71	67
ENSECAG00000021383	0.881486139	1.86E-06	9.64E-05	6	8	7	6	56	83	102	20
ENSECAG00000002211	3.887753606	1.95E-06	0.000100342	52	87	82	186	350	344	428	917
ENSECAG00000019779	2.573310353	2.04E-06	0.000104743	32	10	41	35	122	167	242	323
ENSECAG00000010765	3.825905514	2.14E-06	0.000109182	82	86	112	181	465	413	470	499

ENSECAG00000009704	0.26827015	2.15E-06	0.000109182	1	4	10	3	34	52	34	42
ENSECAG00000020810	4.950889001	2.19E-06	0.00011066	150	123	121	172	2570	497	1094	326
ENSECAG00000000158	2.500021201	2.94E-06	0.000148067	16	30	51	42	147	106	251	290
ENSECAG00000017770	1.698848341	3.08E-06	0.00015423	13	12	15	25	160	97	157	42
ENSECAG00000023608	1.685200009	3.13E-06	0.00015611	22	108	48	271	8	21	12	12
ENSECAG00000019861	1.795154943	3.48E-06	0.000173091	14	10	34	20	113	83	181	108
ENSECAG00000003240	1.87430337	3.89E-06	0.000192802	16	3	18	10	198	41	244	74
ENSECAG00000015959	4.258129849	4.05E-06	0.000199565	105	188	143	232	521	580	634	710
ENSECAG00000017881	1.661141037	4.08E-06	0.000200166	12	11	16	32	72	65	133	172
ENSECAG00000008808	0.544746824	4.28E-06	0.000209313	0	9	6	7	76	36	67	25
ENSECAG00000000638	7.805769128	4.44E-06	0.000216232	975	1099	1532	2561	8135	5392	10152	7524
ENSECAG00000017239	1.005985278	4.52E-06	0.000219212	7	1	17	2	81	55	63	87
ENSECAG00000019868	4.433562447	4.57E-06	0.000220604	528	810	413	779	154	240	41	144
ENSECAG00000021419	3.123796662	4.64E-06	0.000222484	162	183	314	385	37	44	62	123
ENSECAG00000000771	1.744008764	4.65E-06	0.000222484	13	11	16	28	127	173	118	43
ENSECAG00000020578	1.27674865	4.70E-06	0.00022412	15	9	5	5	72	33	120	128
ENSECAG00000015723	4.139873226	4.88E-06	0.000231286	344	616	336	775	49	192	138	177
ENSECAG00000012987	3.441084339	5.00E-06	0.000236377	35	51	100	150	403	285	410	356
ENSECAG00000023561	1.761819434	5.54E-06	0.000260496	7	6	7	13	372	34	67	30
ENSECAG00000011770	6.051284774	5.77E-06	0.000270387	500	450	228	262	3659	1926	2559	1146
ENSECAG00000013977	0.273620926	5.86E-06	0.000273331	5	4	3	6	20	35	38	71
ENSECAG00000012506	5.773093904	6.26E-06	0.000290938	344	263	326	530	1952	548	3161	2081
ENSECAG00000011074	1.930342147	6.59E-06	0.00030479	88	37	192	141	19	18	32	28
ENSECAG00000012151	2.722140356	6.82E-06	0.000314502	288	229	13	317	1	22	0	15
ENSECAG00000024357	2.863051008	7.05E-06	0.000323739	31	47	49	69	260	184	437	127
ENSECAG00000021081	3.074489665	7.38E-06	0.000337481	7	28	25	93	129	112	473	575
ENSECAG00000014698	1.016174666	7.52E-06	0.000341701	7	2	4	16	41	22	107	128
ENSECAG00000010215	0.599231716	7.53E-06	0.000341701	1	5	7	12	36	36	118	28
ENSECAG00000011180	6.419820401	7.61E-06	0.000343037	361	468	827	834	3608	2176	4672	1352
ENSECAG00000017396	5.346482097	7.62E-06	0.000343037	266	288	382	511	1243	1181	1864	950
ENSECAG00000019884	2.49504666	7.81E-06	0.000349872	57	86	205	388	37	30	43	41
ENSECAG00000018420	3.996647626	7.86E-06	0.000350806	42	74	194	145	575	501	866	262
ENSECAG00000008096	2.428277538	8.41E-06	0.000374217	22	51	29	34	177	105	302	164
ENSECAG00000013978	3.046287998	8.55E-06	0.000378738	46	14	23	86	194	194	270	530
ENSECAG00000024630	2.63388745	9.33E-06	0.000411906	7	1	28	12	7	349	319	307
ENSECAG00000020859	3.057134302	9.43E-06	0.000414423	32	35	96	83	342	248	255	258
ENSECAG00000016938	2.533532182	1.01E-05	0.000440953	25	36	27	57	195	78	357	189
ENSECAG00000018476	3.52127084	1.02E-05	0.00044615	65	66	58	135	439	233	690	225
ENSECAG00000002307	0.004600932	1.05E-05	0.000457981	7	2	1	3	31	26	39	38
ENSECAG00000016056	0.264347132	1.09E-05	0.000470112	2	8	4	10	44	33	51	30
ENSECAG00000020162	1.593551112	1.15E-05	0.000496105	19	5	6	16	100	87	219	39
ENSECAG00000017185	5.568690176	1.20E-05	0.000514139	268	358	383	610	2335	1351	1389	1004
ENSECAG00000016882	1.255555077	1.20E-05	0.000515352	5	11	16	29	73	57	96	95
ENSECAG00000016405	2.549724722	1.25E-05	0.000533439	17	29	46	56	273	265	159	90
ENSECAG00000016384	6.177991963	1.26E-05	0.000533439	358	182	450	370	6195	1249	2278	720
ENSECAG00000017746	4.356818715	1.27E-05	0.000538488	97	122	150	270	366	515	595	1261
ENSECAG00000019369	2.116388345	1.29E-05	0.000541189	21	21	20	47	192	118	207	76
ENSECAG00000026891	1.684049318	1.29E-05	0.000541189	9	16	20	36	152	83	139	62
ENSECAG00000017304	1.77857439	1.46E-05	0.000609658	11	19	25	35	166	99	127	66
ENSECAG00000017483	1.911558841	1.47E-05	0.000614681	15	4	27	31	170	58	208	104
ENSECAG00000010028	1.165459836	1.50E-05	0.000624027	30	54	88	109	16	16	16	1
ENSECAG00000016361	2.590269943	1.53E-05	0.000631985	35	16	34	24	310	293	186	54
ENSECAG00000018615	0.110329824	1.53E-05	0.000632477	3	6	2	8	28	56	29	26
ENSECAG00000021024	0.409126548	1.63E-05	0.000670367	1	1	3	15	34	32	99	26
ENSECAG00000010675	1.471887242	1.69E-05	0.000692925	11	24	12	15	99	104	47	118
ENSECAG00000008283	1.81647692	1.73E-05	0.000707312	8	20	31	31	154	83	76	158
ENSECAG00000020495	3.371994511	1.80E-05	0.000730334	28	36	36	179	364	195	478	423
ENSECAG00000019160	3.444870206	1.87E-05	0.000758838	65	35	112	105	525	273	403	250
ENSECAG00000011694	3.516044943	1.93E-05	0.000780462	143	233	450	547	144	83	112	54
ENSECAG00000022353	3.609589313	1.97E-05	0.000791624	143	424	284	557	126	97	137	109
ENSECAG00000020949	4.505970438	1.98E-05	0.000794169	397	357	1034	737	265	212	219	208
ENSECAG00000000271	1.847859354	1.99E-05	0.000794848	9	8	43	21	109	71	209	122
ENSECAG00000016804	1.981096175	2.03E-05	0.000808246	10	43	20	20	84	113	134	211
ENSECAG00000020259	5.656944823	2.04E-05	0.000808246	251	163	486	686	2250	931	2460	1198
ENSECAG00000014020	1.723082107	2.04E-05	0.00080833	16	21	24	20	145	55	173	78
ENSECAG00000001555	2.077611049	2.06E-05	0.000812967	13	12	33	7	286	172	104	37
ENSECAG00000013954	0.620595632	2.15E-05	0.000845602	9	8	9	7	59	48	57	36
ENSECAG00000015318	2.646234871	2.23E-05	0.000873879	40	9	39	30	392	106	278	121
ENSECAG00000008178	3.058608981	2.34E-05	0.000910962	171	196	186	417	40	40	123	86
ENSECAG00000000134	5.84954876	2.41E-05	0.000934574	2645	2652	384	639	142	524	161	246
ENSECAG00000019137	4.081242016	2.53E-05	0.000978024	282	454	759	358	153	199	130	168
ENSECAG00000002575	6.420797395	2.59E-05	0.000999882	512	502	298	921	1936	1028	5187	4149
ENSECAG00000008270	6.362917707	2.61E-05	0.001005015	588	340	629	596	4648	2204	3369	1031
ENSECAG00000004442	2.339179952	2.77E-05	0.001060743	6	26	3	20	158	12	524	132
ENSECAG00000013680	4.711795278	2.79E-05	0.001064996	167	181	195	414	611	758	824	1132
ENSECAG00000022873	4.637297104	2.99E-05	0.001137313	183	192	189	276	584.999	408	1194	1088

ENSECAG00000008106	3.724194314	3.02E-05	0.001144607	93	53	101	108	634	150	690	353
ENSECAG00000016817	3.564754252	3.04E-05	0.001148811	79	124	109	69	408	302	412	400
ENSECAG00000018199	3.66608651	3.07E-05	0.001158258	21	25	60	196	235	297	404	894
ENSECAG00000007708	2.878006006	3.09E-05	0.001162798	39	58	60	46	280	143	439	145
ENSECAG00000016636	6.140205772	3.27E-05	0.001225904	384	120	703	362	3516	1520	4293	901
ENSECAG00000014135	4.498028262	3.32E-05	0.00123839	107	107	176	190	1710	356	775	265
ENSECAG00000020975	5.899066959	3.39E-05	0.001261878	303	341	296	557	4592	622	1823	1232
ENSECAG00000018979	0.874927335	3.47E-05	0.001288631	0	6	5	20	63	39	33	122
ENSECAG00000025265	6.303275737	3.50E-05	0.001293511	203	491	5409	5452	206	505	246	396
ENSECAG00000006853	5.575260789	3.60E-05	0.001325791	342	357	435	435	1509	654	2770	1440
ENSECAG00000021780	5.362778673	3.65E-05	0.001339355	211	262	368	346	2696	624	1647	596
ENSECAG00000018288	2.805278	3.74E-05	0.001369787	39	21	63	76	200	164	412	182
ENSECAG00000007545	3.567132614	3.81E-05	0.001391469	195	111	551	895	0	42	0	44
ENSECAG00000023266	4.815214736	3.84E-05	0.001397631	148	111	163	481	657	468	1390	1323
ENSECAG00000023589	2.750691124	3.89E-05	0.00141012	8	26	18	103	128	173	245	422
ENSECAG00000009935	2.539306921	4.07E-05	0.00147051	18.0001	51	34.0001	68.0006	180	167	328.001	112
ENSECAG00000004180	4.158510611	4.24E-05	0.001528456	58	168	159	196	503	288	509	1063
ENSECAG00000020971	2.505962237	4.34E-05	0.00155878	18	14	24	80	188	79	171	362
ENSECAG00000020074	2.235666058	4.52E-05	0.001619195	27	16	2	40	177	192	227	79
ENSECAG00000014030	2.490272864	4.71E-05	0.001681025	8	22	37	82	102	152	286	250
ENSECAG00000024622	4.553443294	4.94E-05	0.001756287	395	439	715	1074	146	312	321	205
ENSECAG00000012542	4.861506537	4.98E-05	0.001764994	160	238	246	381	1347	467	1353	612
ENSECAG00000014517	2.925531156	5.01E-05	0.001769569	31	44	55	110	242	293	334	131
ENSECAG00000001923	5.74681186	5.02E-05	0.001769569	399	247	689	562	1613	1879	1815	1489
ENSECAG00000006607	3.802854992	5.18E-05	0.001813846	101	80	83	170	554	265	782	271
ENSECAG00000011851	1.204138563	5.18E-05	0.001813846	9	1	17	16	132	86	60	36
ENSECAG00000020325	3.205048724	5.26E-05	0.00183819	255	320	129	361	85	105	39	19
ENSECAG00000011464	6.277953917	5.81E-05	0.002022386	619	536	621	933	3108	1675	3065	2110
ENSECAG00000014680	3.046540411	6.06E-05	0.002102515	49	31	83	73	449	175	321	157
ENSECAG00000012010	5.033441532	6.08E-05	0.002102515	212	428	135	283	1254	948	1407	656
ENSECAG00000015806	2.801038331	6.49E-05	0.002239216	44	38	43	74	214	257	370	94
ENSECAG00000019002	5.713885682	6.54E-05	0.002249752	3278	884	884	456	111	475	54	250
ENSECAG00000009746	2.027336291	6.62E-05	0.002270299	22	33	29	37	176	141	117	87
ENSECAG00000018532	3.297095682	6.79E-05	0.002321122	65	18	86	47	479	295	515	98
ENSECAG00000013169	1.232672268	6.81E-05	0.002321122	6	9	30	17	68	91	93	56
ENSECAG00000023703	2.853752916	7.02E-05	0.002379314	42	46	66	54	238	141	475	134
ENSECAG00000014487	0.220359016	7.02E-05	0.002379314	7	2	5	6	24	26	86	23
ENSECAG00000019790	3.771030924	7.16E-05	0.002418877	62	33	136	208	418	295	549	564
ENSECAG00000019453	5.096756251	7.27E-05	0.002449171	1146	1271	484	590	199	377	315	351
ENSECAG00000013692	7.407662709	7.30E-05	0.00245157	454	750	1236	2189	4305	6039	11724	2236
ENSECAG00000017906	2.225483909	7.44E-05	0.002489974	19	30	23	66	161	142	239	83
ENSECAG00000008118	2.986531925	7.51E-05	0.002504985	23	44	44	95	522	70	283	222
ENSECAG00000011973	0.393215931	7.52E-05	0.002504985	0	12	2	2	19	49	22	92
ENSECAG00000000596	4.645493996	7.79E-05	0.002584091	173	169	282	297	1034	402	834	886
ENSECAG00000001114	3.653897542	8.74E-05	0.002894013	90	77	143	132	247	498	454	387
ENSECAG00000021438	1.001108416	8.85E-05	0.002918969	8	7	16	18	47	40	51	126
ENSECAG00000011009	4.365313387	9.13E-05	0.003002766	143	123	264	262	746	454	706	652
ENSECAG00000024980	4.772567474	9.35E-05	0.003058442	380	1777	846	240	51	287	88	229
ENSECAG00000009125	0.377454981	9.35E-05	0.003058442	6	4	12	3	42	64	37	24
ENSECAG00000009918	3.611614175	9.47E-05	0.003089305	77	58	141	114	553	395	458	176
ENSECAG00000007415	4.241187581	9.62E-05	0.003130652	74	139	204	299	395	516	614	874
ENSECAG00000014530	2.780946049	9.71E-05	0.003148709	15	70	52	61	127	126	363	331
ENSECAG00000012730	4.487770168	9.97E-05	0.003225682	106	217	254	307	780	638	936	437
ENSECAG00000021301	5.476430464	0.000100021	0.003225891	300	241	387	638	1404	1974	1618	653
ENSECAG00000010624	2.250120094	0.000107042	0.003442566	12	27	30	33	389	124	96	42
ENSECAG00000003193	3.59790989	0.000115193	0.003694292	150	273	466	466	145	84	117	159
ENSECAG00000008785	3.864633433	0.000119648	0.003826388	96	69	95	211	516	407	821	194
ENSECAG00000004716	0.841323467	0.000121696	0.003880976	26	29	47	128	1	6	6	24
ENSECAG00000017181	6.974764998	0.000125516	0.003991611	6805	3336	1959	1716	93	941	105	907
ENSECAG00000022583	3.705049773	0.000127637	0.004047777	671	559	56	141	10	97	21	76
ENSECAG00000017903	1.262044128	0.000129859	0.004106777	54	42	50	129	17	22	28	8
ENSECAG00000001870	5.601476052	0.000130353	0.004110999	336	175	463	442	3328	828	1525	775
ENSECAG00000023766	2.704735874	0.000139099	0.004374687	46	135	665	47	3	17	1	47
ENSECAG00000012449	2.754457654	0.000140734	0.00441393	18.0014	55	43.0011	104.002	151.002	257.002	181.002	285
ENSECAG00000006678	0.462174044	0.00014207	0.004443601	20	26	44	81	2	9	0	16
ENSECAG00000017352	2.275801425	0.000142705	0.004451227	21	20	62	52	113	151	178	185
ENSECAG00000021182	1.912925065	0.000144465	0.004493802	131	259	33	9	5	27	6	11
ENSECAG00000003563	2.297776035	0.000151858	0.004710906	26	42	51	35	156	111	263	114
ENSECAG00000006253	4.378762315	0.000153177	0.004732304	146	64	236	111	975	409	1256	244
ENSECAG00000000494	2.215159764	0.000153379	0.004732304	11	27	17	64	68	126	110	333
ENSECAG00000016385	1.545270926	0.000154119	0.004742287	4	16	12	41	42	168	77	102
ENSECAG00000024438	6.036621092	0.000155423	0.004769511	370	459	628	1066	2151	1220	3358	1704
ENSECAG00000006916	2.40351485	0.000159544	0.004882834	59	30	581	28	8	14	18	38
ENSECAG00000016889	2.853246751	0.000168538	0.005144242	31	39	50	72	90	93	295	530
ENSECAG00000016251	2.147169837	0.000169478	0.005159099	30	19	28	36	84	74	132	310
ENSECAG00000020786	1.025317449	0.000172062	0.005223809	1	18	10	6	124	27	119	20

ENSECAG00000019677	3.406509982	0.000178544	0.005406172	123	251	276	694	28	49	55	189
ENSECAG00000019187	5.412901405	0.000179999	0.005435778	272	296	578	627	1333	1124	1401	1309
ENSECAG00000008799	3.274977909	0.000183678	0.005503343	74	35	60	87	432	141	645	132
ENSECAG00000016626	3.656656095	0.000183726	0.005503343	81	92	175	144	348	355	444	401
ENSECAG00000015612	1.089116652	0.00018373	0.005503343	21	114	30	86	11	17	8	21
ENSECAG00000021647	1.649629136	0.00018417	0.005503343	142	71	8	135	10	9	35	12
ENSECAG00000018307	6.573333243	0.000185031	0.005514603	594	493	1108	986	3896	1952	5162	1623
ENSECAG00000016417	5.624306084	0.000185915	0.00552649	383	235	516	457	2373	769	2627	778
ENSECAG00000012539	3.587321825	0.000191078	0.005665156	70	76	44	215	307	239	525	516
ENSECAG00000019229	5.159776949	0.000197294	0.005832672	225	365	395	548	1004	881	1430	1002
ENSECAG00000003573	1.748775765	0.000197752	0.005832672	66	239	42	43	6	35	8	19
ENSECAG00000000368	0.524510426	0.00020137	0.00590992	1	13	11	3	30	85	43	30
ENSECAG00000013053	4.293157613	0.00020141	0.00590992	180	100	96	218	691	370	1290	307
ENSECAG00000023566	3.99633805	0.000222516	0.006512448	558	1037	138	132	4	83	11	127
ENSECAG00000004645	0.651312126	0.000224466	0.006552681	6	2	18	10	27	32	70	80
ENSECAG00000021985	1.018936554	0.000229198	0.006673696	6	13	15	21	104	27	91	43
ENSECAG00000012498	7.338076683	0.000231361	0.006719502	758	741	1900	843	9852	3291	7495	1818
ENSECAG00000016219	0.552581245	0.000235927	0.006834677	7	6	8	16	51	37	82	20
ENSECAG00000006711	5.039159592	0.000236756	0.006841292	1506	2011	108	119	13	113	16	231
ENSECAG00000000521	5.062702403	0.000238414	0.006865182	641	734	708	1545	214	234	431	611
ENSECAG00000011776	4.336947789	0.000238789	0.006865182	106	221	215	222	480	365	1247	517
ENSECAG00000019318	5.166648377	0.000239789	0.006876565	221	526	121	162	1711	1260	742	954
ENSECAG00000016139	2.524062789	0.00024043	0.006877635	36	28	37	83	194	129	324	113
ENSECAG00000010876	2.745473313	0.000242729	0.006925984	14	37	58	107	242	224	315	108
ENSECAG00000021895	3.101504482	0.000247591	0.007047061	48	63	115	24	314	278	367	173
ENSECAG00000023280	1.911104599	0.000249622	0.00707579	86	97	68	156	41	21	31	48
ENSECAG00000019352	3.182506153	0.000249843	0.00707579	761	23	101	80	0	10	0	39
ENSECAG00000000942	3.054185969	0.000251862	0.007115259	45	21	108	37	465	174	389	113
ENSECAG00000009665	5.173464934	0.000257276	0.007250205	236	267	502	445	1354	617	1500	998
ENSECAG00000026945	2.876712618	0.00025844	0.007265044	87	86	213	560	39	36	34	111
ENSECAG00000016258	3.780682251	0.000260427	0.007302856	682	364	212	115	63	81	82	172
ENSECAG00000007470	3.077541245	0.000263153	0.007361172	47	70	64	138	213	234	378	237
ENSECAG00000010995	4.27164483	0.000269311	0.00751497	123	115	258	214	811	404	924	319
ENSECAG00000006002	0.838180809	0.000272552	0.00757713	7	5	21	19	57	49	71	49
ENSECAG00000010569	1.854462146	0.00027287	0.00757713	35	9	7	6	101	73	278	80
ENSECAG00000020022	3.664035519	0.000276786	0.00766717	81	89	129	147	499	237	706	202
ENSECAG00000021257	0.975074994	0.000287481	0.007944099	9	12	7	30	69	53	83	45
ENSECAG00000006248	2.909739204	0.000288275	0.00794676	99	223	140	411	82	89	41	65
ENSECAG00000014234	4.836395833	0.000289419	0.007959035	212	365	185	283	1086	528	1427	587
ENSECAG00000004592	2.111940697	0.000290258	0.007962859	20	3	39	26	254	80	242	41
ENSECAG00000008293	5.30685819	0.000296691	0.008119787	181	68	520	265	2469	604	2018	455
ENSECAG00000022085	6.248164334	0.000299738	0.008183498	6517	379	478	734	0	167	1	123
ENSECAG00000002429	3.430343281	0.00030138	0.008208639	244	305	202	342.999	121.997	111	144	117
ENSECAG00000008758	4.31759793	0.000308783	0.008390193	296	435	798	677	248	244	266	68
ENSECAG00000008570	3.441817301	0.000314544	0.008526394	157	262	264	574	48	56	200	132
ENSECAG00000008809	2.81230363	0.000316973	0.008571827	20	111	45	37	202	200	312	214
ENSECAG00000005266	5.493436915	0.000322751	0.008707405	2352	957	536	601	187	401	225	551
ENSECAG00000009352	0.740101123	0.000325098	0.008737701	21	46	62	56	10	10	28	9
ENSECAG00000018670	3.845953002	0.000325929	0.008737701	80	96	221	164	416	332	677	376
ENSECAG00000021082	2.166088304	0.000326177	0.008737701	16.0001	33	35.0001	51.0006	287	92.0005	118.001	83.0004
ENSECAG00000014384	1.943098762	0.00033032	0.008825661	18	30	17	53	98	130	216	62
ENSECAG00000015360	3.181612453	0.000331011	0.008825661	19	39	45	109	114	113	140	926
ENSECAG00000010656	3.923845181	0.000335054	0.008912596	602	91	872	123	37	96	52	177
ENSECAG00000012910	1.779692749	0.000339252	0.008998672	6	8	53	9	97	203	92	71
ENSECAG00000016469	6.587449317	0.000339871	0.008998672	324	500	428	1887	2561	1486	3291	5724
ENSECAG00000000281	2.53059673	0.00034492	0.009111165	41	43	27	69	214	146	310	88
ENSECAG00000019838	0.916069354	0.000349252	0.009204246	14	72	31	126	4	26	4	4
ENSECAG00000021198	0.817190587	0.000351599	0.0092447	9	8	20	13	33	41	75	75
ENSECAG00000014292	7.171905289	0.000354256	0.009272149	7526	3772	1024	2864	274	1639	484	971
ENSECAG00000022090	0.951209397	0.000354272	0.009272149	8	16	21	3	63	40	65	83
ENSECAG00000020592	5.596282771	0.000355613	0.009285893	605	1513	993	2263	368	292	907	635
ENSECAG00000019700	2.411432886	0.000365767	0.009525143	34	14	54	26	352	65	213	97
ENSECAG00000019668	1.487926419	0.000366448	0.009525143	30.0007	40	146.001	112.001	13.0009	23.001	32.0011	27
ENSECAG00000008140	4.790249313	0.000367587	0.009532989	201	178	255	228	1805	438	1076	305
ENSECAG00000024644	3.428770638	0.000372813	0.009646535	102	64	121	89	421	251	424	253
ENSECAG00000008390	5.631937883	0.000380238	0.00981635	951	1739	864	1552	404	429	596	881
ENSECAG00000021289	6.402770046	0.000382781	0.009859646	342	204	1050	589	4968	1871	4559	516
ENSECAG00000003546	0.261553152	0.000386272	0.009927109	4	3	15	9	40	42	33	32
ENSECAG00000012240	1.329481978	0.000390003	0.010000424	21	51	92	134	13	22	33	18
ENSECAG00000015341	1.315386631	0.000393338	0.010063259	84	99	30	48	17	13	8	33
ENSECAG00000000026	4.604968124	0.000394907	0.010080751	755	1106	323	428	58	371	97	204
ENSECAG00000009210	0.385887884	0.000397471	0.010101137	5	7	9	17	28	31	48	54
ENSECAG00000008137	1.675940976	0.00039748	0.010101137	17	12	25	30	136	43	217	42
ENSECAG00000000918	1.092567517	0.000401797	0.010166714	9	13	19	24	78	35	114	47
ENSECAG00000003996	1.436359303	0.000401846	0.010166714	22	6	15	17	117	72	156	24
ENSECAG00000024694	1.117924707	0.000405402	0.010233925	11	10	13	34	63	57	107	49

ENSECAG00000021484	6.862131937	0.000410176	0.010331536	707	1027	690	546	8965	2078	3305	1415
ENSECAG00000017321	2.44764317	0.000427752	0.010750454	64	47	268	310	27	19	28	85
ENSECAG00000027377	9.046064462	0.000428904	0.010755665	250	1900	908	87595	447	1381	1248	1319
ENSECAG00000023469	0.603677577	0.000432159	0.010803687	1	8	3	25	24	47	59	71
ENSECAG00000013783	3.047028349	0.000432717	0.010803687	90	19	39	11	283	264	464	161
ENSECAG00000003002	5.789492202	0.000435537	0.010850309	2228	2560	423	551	507	582	181	230
ENSECAG00000006522	6.521624985	0.00043774	0.010881381	491	394	95	1260	1617	956	6004	4974
ENSECAG00000020056	2.217435673	0.000449748	0.011155523	34	36	27	60	119	89	198	187
ENSECAG00000025034	5.06244257	0.000452477	0.011198815	249	244	412	544	944	768	1075	1199
ENSECAG00000008596	4.703398172	0.000459113	0.011338388	225	2138	272	410	95	230	148	274
ENSECAG00000017507	0.6151509	0.000460144	0.011339261	6	4	7	26	49	48	65	32
ENSECAG00000006595	5.340215303	0.000465761	0.011452887	1339	2865	202	158	45	298	105	313
ENSECAG00000023457	5.871133076	0.000469757	0.01151175	346	338	550	263	2941	323	4246	885
ENSECAG00000016860	1.892525782	0.000470177	0.01151175	25	30	32	34	90	144	166	67
ENSECAG00000021212	1.644387889	0.000474374	0.011589578	55	54	85	154	29	16	32	43
ENSECAG00000020188	3.608896287	0.000475959	0.011603406	178	467	214	482	121	49	202	146
ENSECAG00000013350	3.404535422	0.000480491	0.011688875	49	43	40	106	1044	74	165	178
ENSECAG00000000519	2.937960739	0.000483098	0.011719047	42	50	65	131	148	276	309	226
ENSECAG00000020612	2.008422168	0.00048379	0.011719047	3	12	21	69	67	69	229	207
ENSECAG00000023617	1.19831075	0.00048918	0.011786537	10	12	25	16	71	20	106	106
ENSECAG00000016054	2.439793236	0.000489517	0.011786537	32	53	25	52	88	138	133	348
ENSECAG00000013814	2.983115779	0.000489682	0.011786537	203	263	198	148	49	101	51	106
ENSECAG00000024512	4.593178938	0.000495787	0.01190831	157	112	291	289	1308	400	1041	357
ENSECAG00000023824	3.979185737	0.000499502	0.011972284	126	153	197	171	419	372	695	424
ENSECAG00000022498	8.096332893	0.00050256	0.012020258	2271	1307	2252	2809	13529	5246	11137	6164
ENSECAG00000005927	4.952355195	0.000516934	0.012338135	217	216	482	339	1304	809	977	644
ENSECAG00000012923	3.519970831	0.000519398	0.012371019	94	91	145	128	383	288	394	316
ENSECAG00000019455	2.213636117	0.000523065	0.012432345	23	46	46	48	137	114	238	99
ENSECAG00000019285	5.314166197	0.000539423	0.012794434	314	385	432	492	1626	710	1632	881
ENSECAG00000017406	3.549027601	0.000555167	0.013140489	531	357	103	176	113	122	88	47
ENSECAG00000003015	1.316574118	0.00056793	0.013414689	125	7	113	22	21	7	15	7
ENSECAG00000011579	3.541002909	0.000591764	0.013948732	61	99	136	175	476	323	267	332
ENSECAG00000005070	1.402947041	0.00059357	0.013962384	8	7	22	38	151	58	101	36
ENSECAG00000023989	2.033910472	0.000600946	0.014103396	19	36	47	49	146	91	147	120
ENSECAG00000001372	1.885389284	0.000602042	0.014103396	50	45	157	204	6	47	16	36
ENSECAG00000019241	2.441491551	0.000604885	0.014140893	34	19	59	49	79	152	132	347
ENSECAG00000006267	2.594500307	0.00060668	0.014153788	9	33	72	92	227	167	246	146
ENSECAG00000012359	4.623958977	0.000619667	0.014427227	1271	208	588	350	118	275	180	234
ENSECAG00000019130	5.412358438	0.000621416	0.01443842	1413	1903	955	490	61	678	46	181
ENSECAG00000002855	5.586688087	0.000625799	0.014510631	669	1323	1587	1425	507	724	381	671
ENSECAG00000014953	2.687428414	0.000633856	0.014667583	40	47	57	101	175	152	310	169
ENSECAG00000020957	6.31815347	0.000637863	0.014710757	6378	296	547	561	155	653	226	287
ENSECAG00000007805	1.125192809	0.000638306	0.014710757	5	12	5	29	32	17	166	95
ENSECAG00000024081	4.16340954	0.000646955	0.014854988	1023	616	63	139	22	182	63	97
ENSECAG00000009716	1.85527755	0.000647174	0.014854988	26	21	22	46	86	112	210	60
ENSECAG00000013268	1.265446061	0.000656324	0.015034703	20	6	23	15	119	52	92	46
ENSECAG00000015241	1.024005829	0.000662259	0.015127169	16	14	18	7	72	53	98	36
ENSECAG00000022423	3.964366126	0.000663018	0.015127169	75	42	76	281	178	283	568	1113
ENSECAG00000010127	2.333062115	0.000666347	0.01517271	111	59	310	101	25	26	12	81
ENSECAG00000004810	0.526441815	0.000668624	0.015194177	2	12	19	3	50	27	59	49
ENSECAG00000014711	3.84469333	0.000670724	0.015211543	399	340	341	405	185	133	235	42
ENSECAG00000023632	5.977345735	0.000683911	0.01545211	1133	1216	2538	1598	508	814	741	930
ENSECAG00000010108	6.097993784	0.000684046	0.01545211	925	3487	1139	1693	620	674	877	835
ENSECAG00000020868	3.543642556	0.000689473	0.015543577	151	186	421	519	150	119	123	138
ENSECAG00000021479	2.830819031	0.000690826	0.015543577	256	390	75	44	20	61	14	72
ENSECAG00000016443	3.355063889	0.000698779	0.015691514	61	13	124	22	496	137	724	131
ENSECAG00000009328	4.068777293	0.000726118	0.016273341	228	195	1028	510	214	195	132	45
ENSECAG00000015007	1.732892984	0.000732402	0.016381926	29	22	23	32	92	56	172	105
ENSECAG00000017605	1.103976082	0.000737503	0.01646366	18	62	39	137	11	19	13	27
ENSECAG00000008622	7.2229713	0.000741229	0.016514469	5272.01	6680	1732.01	1700	847.001	1804	998	1167.02
ENSECAG00000013077	4.525920317	0.000750043	0.016678211	119	178	179	481	464	471	863	1068
ENSECAG00000019780	2.133425069	0.000762871	0.01693038	18	23	29	75	134	89	281	78
ENSECAG00000018229	3.962242726	0.000771501	0.017088587	159	309	383	978	134	76	290	172
ENSECAG00000014186	4.871208636	0.000775779	0.017149979	73	89	559	321	1114	597	953	1138
ENSECAG00000000339	3.008955496	0.000780219	0.017214706	659	22	95	48	6	43	0	30
ENSECAG00000016503	0.226148557	0.000794852	0.017503653	3	4	12	15	37	27	28	49
ENSECAG00000019249	2.640211036	0.000821956	0.018065567	26	60	57	58	355	219	120	87
ENSECAG00000013261	4.664125449	0.000824569	0.018088082	160	365	209	246	1047	653	1053	369
ENSECAG00000011898	2.176221384	0.000834619	0.018273344	53	12	30	21	133	141	216	108
ENSECAG00000000968	4.083974549	0.000837702	0.018305647	174	1158	341	371	26	259	25	112
ENSECAG00000010595	0.449990783	0.000841342	0.018349958	14	5	2	10	25	43	59	47
ENSECAG00000023677	0.645986827	0.000846534	0.018427891	47	32	32	49	6	14	10	23
ENSECAG00000023970	4.638102666	0.000853471	0.018543446	497	532	793	1034	91	300	47	428
ENSECAG00000014225	2.202497992	0.000865825	0.01877604	14	29	49	77	167	130	179	101
ENSECAG00000016313	5.106235389	0.000868998	0.018809026	288	231	405	468	1171	649	1831	625
ENSECAG00000019027	2.032328013	0.000875442	0.018904909	51	122	128	158	15	29	41	72

ENSECAG00000018057	2.948214636	0.000876749	0.018904909	67	52	50	85	431	194	226	117
ENSECAG00000010728	1.568697153	0.000880952	0.018959609	84	94	25	131	19	14	5	46
ENSECAG00000021132	2.200507878	0.000883885	0.018986858	45	89	225	176	32	32	24	77
ENSECAG00000009288	2.83978903	0.000889396	0.019040562	24	73	35	122	142	242	178	344
ENSECAG00000017925	1.481796624	0.00088973	0.019040562	22	19	20	33	94	89	108	51
ENSECAG00000022150	1.961701767	0.000898744	0.019175098	63	72	157	133	37	23	74	25
ENSECAG00000007872	3.971704838	0.000899385	0.019175098	79	160	153	272	524	482	621	268
ENSECAG00000011658	1.78681601	0.000903738	0.019231873	25	9	47	19	119	58	147	126
ENSECAG00000011320	1.858572813	0.000907093	0.019267262	18	38	24	39	113	51	221	87
ENSECAG00000019760	4.495465958	0.00091573	0.019414497	1163	317	405	241	190	174	189	258
ENSECAG00000007681	7.649836675	0.000936439	0.019816654	1397	1413	1782	1268	10141	2300	12330	2817
ENSECAG00000018802	6.484569474	0.000945377	0.019958155	780	409	1127	509	3967	1504	4985	1445
ENSECAG00000010821	2.760746747	0.000946632	0.019958155	42	51	63	92	196	174	387	105
ENSECAG00000016123	3.673340377	0.000957352	0.020143541	97	85	191	131	454	300	542	264
ENSECAG00000009402	2.748716044	0.000958963	0.020143541	60	331	170	184	57	78	42	81
ENSECAG00000014059	2.460781449	0.000965057	0.020218245	28	27	39	101	224.999	122	258	100
ENSECAG00000016596	6.952351991	0.000966072	0.020218245	3616	5842	1760	2178	379	1856	368	1134
ENSECAG00000009568	1.141833555	0.000977606	0.020393495	10	14	17	36	65	48	103	57
ENSECAG00000017428	1.98770346	0.000978028	0.020393495	88	72	150	128	14	21	91	20
ENSECAG00000020412	1.234934228	0.000991893	0.020644794	13	13	8	40	67	40	120	76
ENSECAG00000019038	2.091108037	0.001011921	0.021023221	21	13	33	74	140	109	237	71
ENSECAG00000020920	2.42735437	0.001019925	0.021150913	20	39	14	102	100	120	296	204
ENSECAG00000003181	4.896378341	0.001030248	0.021326144	221	385	177	346	772	441	1882	728
ENSECAG00000000477	4.196173573	0.001058425	0.021869628	259	397	413	876	201	165	229	313
ENSECAG00000020711	4.616877884	0.001065064	0.021966937	163	197	348	387	526	580	993	826
ENSECAG00000017943	4.783589372	0.001067368	0.021974655	142	427	159	364	758	666	586	1347
ENSECAG00000012282	3.889527346	0.001083	0.022251703	97	103	175	170	866	199	520	271
ENSECAG00000016664	3.656173075	0.001084734	0.022251703	542	233	319	187	45	135	31	190
ENSECAG00000024613	5.173941732	0.001097291	0.022445875	1604	1131	464	279	240	475	286	261
ENSECAG00000007843	3.438640623	0.001098143	0.022445875	301	526	195	117	41	160	39	109
ENSECAG00000011152	3.721769076	0.001102827	0.02250123	75	147.999	101	106	465	179	975	159
ENSECAG00000020009	5.974801197	0.001109981	0.022606676	2346	2535	643	657	377	614	590	749
ENSECAG00000021264	0.188179367	0.001116985	0.022708702	5	8	13	7	28	31	52	26
ENSECAG00000019327	3.255369011	0.00111944	0.022718053	28	19	140	29	730	220	333	47
ENSECAG00000007656	4.571471303	0.001136323	0.023019636	159	213	274	300	1242	337	889	452
ENSECAG00000023779	1.16180199	0.00115407	0.023337627	11	10	26	29	83	69	82	39
ENSECAG00000000360	7.251629233	0.001163285	0.023482267	8078	3707	1918	1567	337	2069	624	1072
ENSECAG00000024909	1.095522906	0.001172194	0.023563432	6	14	15	38	75	44	58	86
ENSECAG00000000039	1.757660636	0.001172567	0.023563432	0	100	76	339	0	8	1	23
ENSECAG00000011229	1.389578313	0.001173515	0.023563432	41	45	35	183	22	19	34	21
ENSECAG00000011632	4.253809403	0.001179689	0.023626704	351	532	402	673	128	224	178	382
ENSECAG00000017862	0.007771904	0.001180816	0.023626704	1	5	6	16	30	42	20	27
ENSECAG00000019711	9.10445663	0.001186723	0.023680172	23685	27427	3759	4673	732	6098	867	3753
ENSECAG00000017800	5.478334704	0.001187648	0.023680172	184	261	351	900	643	1026	1120	2919
ENSECAG00000017773	3.177566935	0.001202545	0.023935281	299	373	92	136	79	104	69	85
ENSECAG00000002225	0.926714981	0.001209476	0.024031216	10	77	34	115	4	29	9	6
ENSECAG00000024810	5.939983482	0.001215913	0.024117018	2331	3363	358	657	39	676	146	510
ENSECAG00000013153	7.826708113	0.001245577	0.024662427	7675	3587	9175	3325	3138	2306	916	1424
ENSECAG00000003029	0.023000567	0.001262106	0.024946315	6	7	3	12	26	21	52	24
ENSECAG00000004817	2.796826106	0.001265402	0.024968112	43	66	58	95	267	192	301	99
ENSECAG00000016029	0.629978314	0.001268767	0.02499119	11	9	7	21	64	35	44	43
ENSECAG00000008859	2.652970458	0.001276381	0.025097746	44	183	260	233	95	49	40	61
ENSECAG00000014259	7.351377679	0.001278902	0.025103967	5109	1543	7283	5108	220	2302	264	1465
ENSECAG00000024992	1.580948829	0.001296448	0.025404574	20	16	24	31	157	50	141	36
ENSECAG00000023958	2.581569054	0.001309005	0.025606576	26	46	63	103	139	133	274	198
ENSECAG00000024200	1.902980917	0.001327424	0.025922338	12	39	50	38	114	96	151	99
ENSECAG00000010185	8.125769567	0.001350453	0.026326894	5051	25485	1891	1268	132	2497	321	1610
ENSECAG00000009400	3.320569786	0.001401909	0.027283298	109	657	175	160	39	108	77	132
ENSECAG00000022378	4.49450735	0.001406238	0.027320847	137	79	306	277	1428	306	729	407
ENSECAG00000020404	5.773403679	0.001409996	0.027347193	451.999	333	778.999	772.997	2158	1471	1569	1261
ENSECAG00000022571	3.971252794	0.00142034	0.027500961	886	539	103	137	2	127	15	122
ENSECAG00000010053	1.158472599	0.00142614	0.027566399	13	12	15	30	73	59	128	24
ENSECAG00000008363	1.705902815	0.001448135	0.027944094	1	3	5	58	60	42	87	283
ENSECAG00000014360	6.663076188	0.001478596	0.02848361	352	431	1174	1482	6531	1751	3952	1123
ENSECAG00000009896	9.219242624	0.001493041	0.028713298	42452	8187	3003	4021	1721	3225	1847	6071
ENSECAG00000026832	0.879004525	0.001510794	0.029005712	15	3	19	14	77	43	81	31
ENSECAG00000001214	2.525179121	0.001551376	0.029711942	38	69	47	54	131	99	193	293
ENSECAG00000019847	5.251111228	0.001552798	0.029711942	1614	1197	394	457	204	543	279	382
ENSECAG00000011454	5.542458897	0.001587395	0.030289484	397	332	537	580	2432	708	1725	839
ENSECAG00000023082	2.685860851	0.001589892	0.030289484	54	34	77	34	329	118	290	94
ENSECAG00000011689	6.320261821	0.001592137	0.030289484	609	481	1083	866	4029	1692	3045	1163
ENSECAG00000012371	8.021127145	0.001593623	0.030289484	20546	2840	1548	1923	16	1955	38	514
ENSECAG00000007351	3.996056553	0.001616149	0.030666425	418	1181	124	221	1	40	2	176
ENSECAG00000006485	4.940933039	0.001624538	0.030774325	1572	907	314	321	34	264	68	442
ENSECAG00000004960	6.6216194	0.001632203	0.030868153	216	1315	406	934	1467	4699	6772	746
ENSECAG00000021452	2.663841636	0.001637531	0.030917565	43	36	48	112	178	148	351	124

ENSECAG00000020792	3.088566254	0.001645487	0.031016331	36	53	58	159	412	92	429	175
ENSECAG00000024577	5.688667426	0.001663666	0.031307167	960	2067	764	1302	518	678	712	653
ENSECAG00000022064	3.824951377	0.001668389	0.03134423	137	111	168	177	387	302	653	359
ENSECAG00000013133	0.05333596	0.00167232	0.031366323	5	6	8	13	28	29	40	24
ENSECAG00000020198	1.189701002	0.001677933	0.031389255	38	53	93	73	8	43	15	4
ENSECAG00000001652	3.506688736	0.001679056	0.031389255	176	168	246	684	124	83	97	198
ENSECAG00000013625	3.666336399	0.001690409	0.031549689	73	115	114	236	274	226	474	576
ENSECAG00000013391	4.235964047	0.00170012	0.03166642	142	89	267	272	702	334	886	400
ENSECAG00000016867	4.709532606	0.001702227	0.03166642	344	1086	412	838	251	363	369	309
ENSECAG00000021555	3.91595846	0.001710645	0.031771109	211	262	484	613	162	182	176	243
ENSECAG00000007003	4.2877324	0.001718477	0.031864599	135	177	250	288	840	364	732	384
ENSECAG00000023006	3.597843026	0.001722493	0.031879656	400	507	312	107	15	166	10	108
ENSECAG00000010188	4.114764617	0.001727418	0.031879656	381	694	349	274	113	229	208	253
ENSECAG00000016515	0.181699256	0.00172769	0.031879656	5	1	13	14	29	28	42	38
ENSECAG00000000612	1.342619027	0.001733819	0.031940983	12	19	29	31	109	69	84	43
ENSECAG00000015981	5.173797742	0.001755474	0.03224138	876	1454	707	589	185	706	282	335
ENSECAG00000009277	1.520132667	0.001755789	0.03224138	1	19	16	35	74	18	259	64
ENSECAG00000001119	8.758290563	0.001774288	0.032528615	6542	5538	13534	24296	3932	3399	5708	5318
ENSECAG00000017676	1.312237321	0.001777278	0.032531038	4	9	27	33	78	34	188	37
ENSECAG00000008385	3.168316433	0.001791069	0.03272689	71	30	64	143	322	186	546	123
ENSECAG00000000972	1.401463196	0.001793727	0.03272689	29	58	93	130	5	13	19	52
ENSECAG00000024576	0.972424835	0.001803716	0.032856485	7	17	17	30	44	63	55	71
ENSECAG00000011202	1.809135647	0.001812776	0.032968777	15	22	34	32	260	53	101	36
ENSECAG00000017459	4.978938857	0.001816519	0.03298416	756	783	864	680	206	436	268	604
ENSECAG00000017103	2.93594047	0.001828287	0.033144986	62	67	54	124	220	209	335	164
ENSECAG00000000416	1.582580796	0.001832001	0.033159502	25	62	65	209	16	15	25	51
ENSECAG00000002513	1.119595341	0.001856767	0.033554433	6	27	12	18	57	30	164	38
ENSECAG00000007718	1.280185427	0.001872333	0.033771037	11	7	16	33	185	30	65	33
ENSECAG00000008818	2.027171886	0.001874686	0.033771037	15	1	34	15	199	15	392	21
ENSECAG00000007305	5.257440819	0.001883854	0.033882588	296	127	614	197	2272	785	1378	498
ENSECAG00000013839	7.209261823	0.001904663	0.034168242	2454	3983	3647	5721	977	1976	1476	2307
ENSECAG00000002313	1.319948115	0.001906884	0.034168242	16	24	23	31	83	57	84	71
ENSECAG00000008466	0.947774313	0.00190874	0.034168242	12	10	11	30	57	61	94	26
ENSECAG00000024074	4.898949102	0.001914193	0.034212071	162	336	330	535	943	526	1375	724
ENSECAG00000008745	4.425657494	0.00197178	0.035186072	735	1321	133	322	3	219	4	111
ENSECAG00000024489	2.130314984	0.001981709	0.03530792	30	31	61	39	120	107	102	200
ENSECAG00000016573	2.734100837	0.002000265	0.035582842	96	45	203	480	34	44	27	117
ENSECAG00000019220	2.559525691	0.002008788	0.035678706	23	18	43	122	167	76	348	193
ENSECAG00000006881	6.028567299	0.002049304	0.036341634	2241	2532	784	979	313	1200	345	516
ENSECAG00000021238	3.593549007	0.002064656	0.036556926	255	586	268	129	57	136	87	196
ENSECAG00000010191	3.720848691	0.002075546	0.036639253	561	666	82	139	8	190	9	37
ENSECAG00000021865	1.819632749	0.002075742	0.036639253	198	50	24	67	18	22	37	34
ENSECAG00000019617	4.629484046	0.002096131	0.036941878	1049	716	194	397	150	224	245	363
ENSECAG00000008897	4.387378958	0.002100733	0.036965752	69	148	167	496	354	768	540	903
ENSECAG00000012421	1.185295879	0.002130708	0.037435355	1	5	15	42	54	99	28	113
ENSECAG00000016076	5.149313865	0.00214159	0.037488995	209	254	571	555	1261	765	1543	674
ENSECAG00000023397	0.908161311	0.002142765	0.037488995	8	12	18	15	131	26	55	23
ENSECAG00000020343	7.513216314	0.00214364	0.037488995	9437	5948	1326	1380	442	1823	887	1982
ENSECAG00000022510	3.202285412	0.002160639	0.037728342	201	359	92	312	73	42	117	158
ENSECAG00000013746	1.5296184	0.00216816	0.037795636	40	25	168	116	5	11	10	56
ENSECAG00000021134	4.939696416	0.002171133	0.037795636	254	205	417	276	1702	632	1104	343
ENSECAG00000014330	7.487247744	0.002188397	0.038038013	1098	625	2678	1377	5061	2686	13578	3361
ENSECAG00000023527	5.189796346	0.002209847	0.038312239	201	235	474	702	1609	1005	1101	613
ENSECAG00000000141	2.153272108	0.002210904	0.038312239	42	176	109	185	11	62	24	61
ENSECAG00000014416	2.321732182	0.002237622	0.038716305	26	53	52	67	101	112	259	144
ENSECAG00000010860	6.978608629	0.002302791	0.039760273	12105	53	73	575	1	295	0	115
ENSECAG00000024430	2.161571766	0.002304943	0.039760273	14	39	31	82	90	87	146	240
ENSECAG00000013344	0.866283199	0.002330384	0.040097653	23	58	27	103	2	8	18	31
ENSECAG00000018272	7.006750657	0.002331546	0.040097653	986	739	1743	1450	6241	2514	4131	3033
ENSECAG00000019230	1.860779275	0.002342066	0.040217834	47	248	120	11	3	39	6	29
ENSECAG00000008485	8.65738152	0.002375722	0.040700534	3118	3457	4377	3774	15735	7025	20324	8604
ENSECAG00000011788	1.993521933	0.002377326	0.040700534	26	37	30	60	106	117	191	71
ENSECAG00000020494	2.996649895	0.002407077	0.041148013	11	76	37	65	41	104	110	872
ENSECAG00000013079	2.083280392	0.002427375	0.041432775	15	29	71	47	113	88	151	169
ENSECAG00000023010	5.496676159	0.002468089	0.042064655	1290	844	1178	894	475	654	665	527
ENSECAG00000019870	4.985836065	0.002480732	0.042156069	507	338	1164	1877	63	455	54	454
ENSECAG00000017295	8.551511112	0.002480858	0.042156069	24811	10017	774	1579	906	3853	906	1653
ENSECAG00000000546	3.688953663	0.00250187	0.042449762	55	83	199	237	368	348	393	422
ENSECAG00000023595	6.559125991	0.002518281	0.042664622	928	686	874	1264	4589	1832	2294	2611
ENSECAG00000027594	1.279785341	0.002549739	0.043133408	8	35	161	89	17	26	22	11
ENSECAG00000016395	2.715368955	0.00258067	0.043529608	55	64	22	54	178	106	523	86
ENSECAG00000021014	5.359125842	0.002580807	0.043529608	184	315	241	970	519	1157	1469	1984
ENSECAG00000014619	2.349731264	0.002587897	0.043584621	25	34	43	84	151	214	218	51
ENSECAG00000012252	5.367924601	0.002621905	0.044092151	188	269	351	1052	974	1146	1161	1682
ENSECAG00000025092	6.615012285	0.002626952	0.044111863	1660	2080	3194	3277	1213	1085	1951	545
ENSECAG00000008784	6.095064386	0.002651492	0.044458377	468	447	845	826	4254	990	2319	955

ENSECAG00000016962	7.63481429	0.00266692	0.044577652	7802	8223	2132	1501	1064	2455	1601	1705
ENSECAG00000012755	4.604832571	0.002668771	0.044577652	84.0007	252	381.001	312.001	507.001	595.001	508.001	1324
ENSECAG00000011854	2.603076666	0.002672589	0.044577652	55	20	84	40	252	227	171	102
ENSECAG00000020946	0.232945027	0.002679495	0.044577652	8	8	14	7	32	28	37	39
ENSECAG00000017337	4.101798108	0.002680605	0.044577652	108	112	296	227	510	561	691	279
ENSECAG00000017783	2.396602929	0.00268584	0.044577652	46	5	43	23	307	94	300	32
ENSECAG00000022739	0.444473862	0.002686014	0.044577652	33	33	53	32	0	13	0	19
ENSECAG00000023019	1.709420222	0.002705408	0.044834162	15	20	17	66	119	103	138	46
ENSECAG00000016362	4.979361896	0.002710468	0.044852738	1292	1255	261	247	290	432	166	215
ENSECAG00000003776	3.827400216	0.002715134	0.04486474	265	154	691	373	116	155	281	111
ENSECAG00000022891	4.189041773	0.002726436	0.044956595	136	136	250	267	663	244	823	470
ENSECAG00000019602	3.668297106	0.002728591	0.044956595	92	62	217	143	435	293	593	235
ENSECAG00000008465	5.423448605	0.002745632	0.045171998	649	899	891	1915	521	437	704	675
ENSECAG00000010859	2.29723694	0.002757457	0.045271904	37	41	46	70	146	117	240	94
ENSECAG00000020122	10.60041144	0.002759658	0.045271904	62405	55666	15126	22839	6785	22951	8828	13368
ENSECAG00000006191	2.055643344	0.002771108	0.045378	28	29	52	52	82	88	150	184
ENSECAG00000000107	2.654519157	0.002774096	0.045378	65	33	55	66	167	82	360	196
ENSECAG00000014784	7.755500288	0.002782323	0.045447266	3338	4655	5103	10957	2037	1010	4012	2795
ENSECAG00000022251	3.906598832	0.002786329	0.045447497	375	884	130	209	45	265	50	113
ENSECAG00000006964	4.464079131	0.002795346	0.045467112	445	997	323	482	104	388	157	299
ENSECAG00000003436	6.111951255	0.002795519	0.045467112	314	613	798	995	2722	932	4390	888
ENSECAG00000024640	9.406683181	0.002831758	0.045990824	15888	29484	26529	6519	405	6099	922	9484
ENSECAG00000013323	3.639895568	0.002850685	0.046232263	149	179	419	634	76	110	264	144
ENSECAG00000017374	2.433188694	0.002904494	0.047037934	225	162	38	102	55	66	43	28
ENSECAG00000006492	8.026749742	0.002915768	0.047153428	1724	1745	2866	3809	8305	3259	12241	9464
ENSECAG00000014315	2.999443749	0.002935461	0.047404573	73	90	20	28	442	251	281	72
ENSECAG00000001926	5.712219244	0.002943409	0.047444593	317	354	638	606	3619	748	1706	522
ENSECAG00000024845	3.281658409	0.002946274	0.047444593	282	721	42	105	1	87	1	52
ENSECAG00000018456	0.022259796	0.00296979	0.047755732	4	7	10	12	28	22	30	36
ENSECAG00000010089	6.386135478	0.002992282	0.048049551	3572	2222	1153	1550	205	754	176	1596
ENSECAG00000007432	4.868744401	0.003025561	0.048515515	149	183	371	319	1575	393	1696	178
ENSECAG00000009096	6.802669273	0.003035121	0.048600353	2723	5432	1773	1581	462	1916	755	1102
ENSECAG00000008748	3.371024558	0.003040584	0.048619445	58	84	71	207	514	174	401	182
ENSECAG00000001774	7.266163325	0.003050249	0.048705583	6335	5329	1542	1719	1154	2107	1185	1167
ENSECAG00000017019	0.883659547	0.003066088	0.048889928	11	9	15	29	71	61	61	26
ENSECAG00000008416	4.380971727	0.003075194	0.048966553	149	206	212	390	420	375	915	763
ENSECAG00000006095	7.150612386	0.003094165	0.049199815	558	1328	726	2346	912	3999	6783	7265
ENSECAG00000021241	7.958371858	0.003112648	0.049424689	12387	7612	1903	1970	1716	2835	1617	1946
ENSECAG00000016899	4.088281182	0.003140078	0.049790796	147	103	217	278	567	291	762	412
ENSECAG00000013698	3.939609995	0.00314562	0.049809296	201	313	621	456	295	97	199	168
ENSECAG00000004670	4.034252527	0.003161721	0.049948437	541	405	106	736	83	25	370	127
ENSECAG00000012207	1.533646321	0.003163182	0.049948437	23	17	26	33	116	43	157	46
ENSECAG00000017568	0.708474135	0.003221849	0.050804364	8	5	12	30	34	40	43	80
ENSECAG00000023901	4.695927925	0.003229945	0.050861581	225	288	272	287	1302	383	944	467
ENSECAG00000007582	7.326116399	0.00324793	0.051074142	1511	565	973	458	12237	803	8254	1484
ENSECAG00000022677	5.17083754	0.003256716	0.051141676	1563	880	294	620	234	394	353	528
ENSECAG00000009725	4.140013386	0.00326271	0.051165223	110	291	187	210	523	534	470	486
ENSECAG00000023483	4.229116797	0.003268232	0.051181317	142	126	289	219	749	685	544	232
ENSECAG00000000357	8.773080353	0.003279706	0.051287925	3036	2858	5137	6664	14333	8755	16768	14395
ENSECAG00000009649	5.744733636	0.003287448	0.051287925	2770	1875	469	352	38	720	80	433
ENSECAG00000018421	3.61377947	0.003288554	0.051287925	100	130	137	72	728	188	237	337
ENSECAG00000015424	5.598847319	0.003300532	0.051404324	236	453	619	1033	1550	1185	1642	1242
ENSECAG00000000426	0.46399895	0.003364398	0.052269785	4	15	13	17	37	43	43	36
ENSECAG00000007464	4.806090161	0.003365283	0.052269785	264	114	219	471	1403	625	1139	304
ENSECAG00000012560	2.01502064	0.003407627	0.052855353	71	20	112	359	0	23	0	42
ENSECAG00000019578	0.883619723	0.003417419	0.052935127	13	5	14	29	57	68	73	25
ENSECAG00000014320	2.887055442	0.003427429	0.053018046	36	50	115	58	181	167	94	480
ENSECAG00000021201	4.455423744	0.003433715	0.053043209	174	39	184	385	791	276	1379	454
ENSECAG00000022408	2.601377354	0.003442185	0.053102005	28	73	46	102	132	113	253	244
ENSECAG00000020393	5.502223015	0.003459707	0.053243056	520	962	787	2736	408	324	716.999	805
ENSECAG00000017346	5.498660302	0.003460682	0.053243056	331	465	499	688	2200	644	1619	901
ENSECAG00000006513	4.823716096	0.003466126	0.053254843	95	371	302	379	845	379	1969	451
ENSECAG00000010893	2.594178062	0.003473186	0.053291406	97	89	209	240	95	57	86	45
ENSECAG00000016641	5.179522335	0.00347952	0.053316731	284	366	469	617	1105	660	1447	961
ENSECAG00000013266	2.322998003	0.003501773	0.053585602	35	38	42	89	186	94	197	124
ENSECAG00000017893	5.338598351	0.003516231	0.053711349	879	1300	683	987	410	605	428	660
ENSECAG00000000701	6.811771924	0.003519426	0.053711349	866	461	13511	529	32	275	20	1240
ENSECAG00000023475	5.532844835	0.00355375	0.054144783	1934	1294	1125	270	111	740	127	503
ENSECAG00000022594	1.250026036	0.003557338	0.054144783	9	13	35	34	75	45	86	79
ENSECAG00000014710	0.815554628	0.003587074	0.054524481	11	11	18	25	40	40	79	49
ENSECAG00000002113	1.857651725	0.003620286	0.054955939	25	31	37	50	100	58	175	106
ENSECAG00000005870	4.683589116	0.00365737	0.055444953	133	338	247	493	723	677	1100	506
ENSECAG00000022218	1.551481002	0.00367875	0.055694911	18	9	29	39	23	86	96	166
ENSECAG00000017874	6.624115631	0.003697726	0.055907857	1824	6602	777	1229	394	1143	1079	1049
ENSECAG00000008827	1.629656262	0.003711871	0.055990996	27	23	16	43	97	43	168	78
ENSECAG00000003859	1.926161462	0.003714141	0.055990996	37	39	21	25	77	75	253	84

ENSECAG00000001166	5.274869429	0.003717979	0.055990996	720	687	1102	1281	674	508	589	319
ENSECAG00000020787	2.108163753	0.003744827	0.056320818	22	24	49	72	150	61	227	100
ENSECAG00000000196	2.975610561	0.003752912	0.056367943	7	38	20	183	87	232	238	510
ENSECAG00000016173	2.855003795	0.003758843	0.05638264	26	68	64	149	272	239	192	156
ENSECAG00000006979	5.189612988	0.0037929	0.056818633	353	824	685	2114	454	307	569	545
ENSECAG00000018188	4.575256376	0.003827022	0.057254459	983	847	139	310	118	326	211	253
ENSECAG00000012186	3.633421744	0.003927047	0.058673203	99	110	155	163	555	217	489	213
ENSECAG00000024287	3.219632479	0.003932161	0.058673203	179	328	286	183	34	157	36	143
ENSECAG00000009941	5.372908924	0.00394856	0.0588244	1395	1820	264	509	381	499	373	437
ENSECAG00000010682	5.352243305	0.003952874	0.0588244	324	301.996	560	736	1305	681	1458	1301
ENSECAG00000012061	2.006961093	0.003957795	0.0588244	288	78	25	16	1	47	1	5
ENSECAG00000016863	3.978956621	0.00398395	0.059070095	146	109	228	204	633	333	566	310
ENSECAG00000021773	5.998275586	0.003985483	0.059070095	2538	1850	642	879	551	613	734	919
ENSECAG00000009163	1.414148866	0.003989891	0.059070095	22	18	25	35	77	68	127	47
ENSECAG00000007737	1.620920944	0.003997061	0.059099405	11	14	30	64	81	56	151	94
ENSECAG00000008959	5.954244281	0.004016771	0.059313801	621	415	614	707	2232	646	3889	1143
ENSECAG00000024389	5.590607099	0.004023207	0.059331877	1668	1468	540	880	267	793	458	623
ENSECAG00000009302	2.017469099	0.00403292	0.059398177	24	21	71	36	153	80	171	91
ENSECAG00000013108	0.549635832	0.004050091	0.059574009	8	8	17	17	63	22	57	31
ENSECAG00000018137	2.849880919	0.00409383	0.060139683	45	63	89	95	334	112	295	132
ENSECAG00000020944	3.841082431	0.004103227	0.060200047	198	282	500	462	133	219	257	152
ENSECAG00000019873	6.371022205	0.004142731	0.0607014	4926	979	1702	970	30	829	32	846
ENSECAG00000007941	3.459714259	0.004167227	0.060981845	170	168	422	370	182	73	172	128
ENSECAG00000005083	6.225079009	0.004191515	0.061258528	5025	1322	435	337	177	1127	196	278
ENSECAG00000014741	5.116265432	0.004201887	0.06133139	1080	664	885	611	263	666	461	298
ENSECAG00000018353	6.372508261	0.004220968	0.061531008	491	697	987	1632	2988	1421	3373	2098
ENSECAG00000014016	6.586255005	0.004291838	0.062484118	439	589	669	2238	1328	2520	2782	5424
ENSECAG00000019746	3.290706559	0.004331394	0.062979458	57	119	93	157	175	218	291	475
ENSECAG00000012793	3.210042201	0.004342588	0.063061692	41	48	142	166	156	322	339	297
ENSECAG00000014908	4.983731127	0.004359173	0.063131552	257	207	442	378	1700	396	1274	486
ENSECAG00000021364	5.741212658	0.004360231	0.063131552	275	190	897	836	2930	1046	1549	1008
ENSECAG00000024519	0.64943549	0.004364034	0.063131552	7	15	12	24	36	41	72	35
ENSECAG00000008024	2.821884789	0.004380377	0.063287552	65	51	41	100	144	100	205	432
ENSECAG00000000237	3.408322575	0.0043932	0.063392369	300	793	65	93	1	124	1	31
ENSECAG00000025146	5.243033143	0.004423551	0.063749531	1279	2697	146	179	7	436	14	180
ENSECAG00000024553	1.632426847	0.004457483	0.064157321	15	24	33	41	33	57	147	151
ENSECAG00000005489	6.297759158	0.004464761	0.064180936	3224	3616	1321	523	19	789	16	711
ENSECAG00000013745	6.166614683	0.004520873	0.06481245	2752	4083	309	399	96	901	156	684
ENSECAG00000016449	7.298890179	0.004523334	0.06481245	7960	6817	805	1407	20	1523	70	1165
ENSECAG00000001165	0.842995208	0.004525771	0.06481245	49.0106	77.0001	41.0079	29.0001	0	11.0007	1.00012	31.0239
ENSECAG00000014232	8.540863267	0.004580511	0.065513969	1832	710	5562	4163	10265	3869	27122	10321
ENSECAG00000017937	1.52274843	0.004604541	0.065714453	39	23	124	132	26	33	19	37
ENSECAG00000006153	8.136316812	0.004609067	0.065714453	12009	12729	3720	1363	215	3498	270	2511
ENSECAG00000009121	0.353300483	0.004611844	0.065714453	12	9	2	12	30	21	77	31
ENSECAG00000007837	9.73703305	0.004626976	0.065847645	33497	39600	7686	9329	749	11337	1836	8682
ENSECAG00000000356	1.535784298	0.004660562	0.066176374	61	71	48	114	28	14	38	51
ENSECAG00000015782	9.41030748	0.004662354	0.066176374	8192	5469	3269	6113	13223	24772	28657	19522
ENSECAG00000014577	1.309754856	0.004667512	0.066176374	59	86	34	63	18	17	37	37
ENSECAG00000008637	4.703188162	0.00469007	0.066379815	224	331	222	411	1008	429	982	604
ENSECAG00000001312	4.414421721	0.004695914	0.066379815	93	87	362	204	1476	346	749	157
ENSECAG00000024218	2.615292512	0.004699353	0.066379815	65	127	226	246	92	87	63	33
ENSECAG00000019579	3.488651621	0.004725442	0.066571516	800	306	3	76	0	88	2	18
ENSECAG00000014229	10.96494195	0.004729312	0.066571516	9236	10704	36396	19358	55790	37352	65284	95712
ENSECAG00000008225	3.180546006	0.004730466	0.066571516	60	73	112	165	220	231	238	360
ENSECAG00000014369	2.968491798	0.004764385	0.066966073	46	105	64	108	229	100	393	237
ENSECAG00000018996	9.098681085	0.004834362	0.067773621	34251	7643	6848	2636	2093	5955	2480	5444
ENSECAG00000011008	3.299785147	0.004838954	0.067773621	45	73	135	191	327	161	343	339
ENSECAG00000018918	1.603165298	0.004839697	0.067773621	29	22	20	45	99	87	114	58
ENSECAG00000009934	1.839363178	0.004860489	0.067981168	37	52	162	137	50	38	44	25
ENSECAG00000023865	6.408293768	0.004883725	0.068222336	2000	2115	1785	2173	770	1087	1086	1533
ENSECAG00000017963	2.485284244	0.004925561	0.068561476	38	27	64	63	345	123	185	45
ENSECAG00000024574	5.084218526	0.004925851	0.068561476	201	174	591	356	1799	645	1478	304
ENSECAG00000019054	1.517079294	0.00492766	0.068561476	17	18	29	51	67	53	99	121
ENSECAG00000015053	6.848414244	0.004933977	0.068561476	5045	3237	1109	1422	635	1069	1411	1540
ENSECAG00000010552	6.06748406	0.004938112	0.068561476	872	795	1677	4069	899	470	977	976
ENSECAG00000001499	2.815329636	0.004949417	0.068634731	357	69	111	120	45	39	108	84
ENSECAG00000008912	6.010308795	0.004961817	0.068664625	3063	914	1044	801	850	677	585	639
ENSECAG00000015261	6.21899815	0.004980185	0.068664625	5294	1281	325	445	28	863	37	433
ENSECAG00000022400	4.209366569	0.004985626	0.068664625	350	361	391	694	176	240	274	332
ENSECAG00000007763	5.689251829	0.004986609	0.068664625	366	399	704	1047	1197	1171	1887	1687
ENSECAG00000009192	11.65704485	0.004989337	0.068664625	142427	128476	31200	16061	17569	41828	13733	27810
ENSECAG00000007623	3.734260685	0.00499057	0.068664625	115	69	159	152	696	146	645	186
ENSECAG00000015104	1.715860786	0.004993791	0.068664625	58	39	213	97	0	19	1	50
ENSECAG00000015835	6.000720562	0.005000086	0.068668255	1769	1835	1244	1614	253	1432	308	679
ENSECAG00000015208	0.184266399	0.005042686	0.069079651	11	4	5	15	22	27	43	41
ENSECAG00000021076	4.238019518	0.005046206	0.069079651	138	156	220	363	360	344	684	826

ENSECAG00000023922	4.395308084	0.005054007	0.069079651	502	807	239	479	137	361	256	263
ENSECAG00000015483	3.236991406	0.005054313	0.069079651	166	765	113	68	1	89	5	91
ENSECAG00000006205	5.239421902	0.005081926	0.069373776	234	252	768	497	1002	957	1620	853
ENSECAG00000020419	4.163964543	0.005091641	0.069423156	614	860	229	192	15	339	34	111
ENSECAG00000022447	0.880229431	0.005103649	0.06950364	26	33	39	109	14	2	36	18
ENSECAG00000014957	1.401773422	0.005138783	0.069898494	95	92	24	48	7	29	17	41
ENSECAG00000014582	4.304806523	0.005153167	0.070010507	176	146	286	272	757	307	864	401
ENSECAG00000027244	2.970400639	0.005177643	0.070259195	31	44	142	861	37	83	40	80
ENSECAG00000014775	1.280629809	0.005236339	0.070889428	16	16	36	11	91	22	107	83
ENSECAG00000014377	4.079013995	0.00523654	0.070889428	101	145	180	301	788	322	749	183
ENSECAG00000024981	2.983894241	0.005253948	0.070962182	80	46	89	109	188	135	298	327
ENSECAG00000012148	7.802980271	0.00525438	0.070962182	10202	10662	1624	914	123	3022	383	1447
ENSECAG00000016264	0.625778194	0.005280836	0.071234977	17	21	49	78	17	9	20	18
ENSECAG00000022172	7.164552106	0.005296623	0.071363372	2851	7235	2642	1611	1535	1560	1989	1289
ENSECAG00000019319	8.042027118	0.005336279	0.071812692	9071	13314	2734	1865	1139	4558	846	1680
ENSECAG00000002764	2.004824593	0.005378465	0.072294956	26	116	52	350	4	71	12	2
ENSECAG00000008906	4.835767487	0.005409342	0.072624248	303	327	204	381	826	355	1472	791
ENSECAG00000008718	5.250616133	0.005429964	0.072815237	366	366	404	607	1457	602	1583	789
ENSECAG00000009167	1.48591503	0.005450005	0.072998006	74	103	74	34	7	31	9	51
ENSECAG00000009363	5.780078667	0.005502541	0.073541062	1144	4439	468	311	17	217	46	917
ENSECAG00000020049	3.086061221	0.005503468	0.073541062	47	97	113	118	213	130	381	289
ENSECAG00000012899	2.422385836	0.005515629	0.073617152	29	37	42	99	85	66	301	231
ENSECAG00000005905	6.668720427	0.005565861	0.074200619	296	220	1242	1030	10017	1453	1925	335
ENSECAG00000010334	3.293911602	0.005592102	0.074463253	218	900	41	46	0	93	0	7
ENSECAG00000013390	2.934293607	0.005696748	0.075768075	47	49	92	155	180	170	290	263
ENSECAG00000019242	4.459151493	0.005727736	0.076091329	273	366	456	1269	170	211	337	422
ENSECAG00000013592	2.273140494	0.005743593	0.076213063	38	28	66	55	161	93	261	78
ENSECAG00000006290	0.777981279	0.005752194	0.07623834	31	70	33	37	8	31	16	12
ENSECAG00000023441	4.325305622	0.005788215	0.07662655	969	324	285	232	173	325	164	163
ENSECAG00000019977	4.078303978	0.005801207	0.076709337	658	573	291	262	9	287	18	154
ENSECAG00000015719	5.209923995	0.005838312	0.077110418	631	2161	395	646	235	641	155	467
ENSECAG00000001088	2.156274747	0.005963939	0.078678379	17	28	57	68	218	166	97	52
ENSECAG00000003418	5.798068878	0.0059766	0.078754153	723	4330	513	520	157	638	219	863
ENSECAG00000019150	0.303676586	0.006018364	0.079179578	9	26	27	79	6	5	5	25
ENSECAG00000002490	4.74483167	0.006022794	0.079179578	791	1509	218	223	91	483	83	238
ENSECAG00000021171	5.4569368	0.006033741	0.079232001	608	1677	845	1115	488	696	597	613
ENSECAG00000021651	6.456600407	0.006058496	0.079465414	536	640	1177	1540	1323	2339	2205	4595
ENSECAG00000020551	6.7338969	0.006110864	0.080060056	2851	4147	1480	1730	1089	1625	975	1216
ENSECAG00000018324	7.590895953	0.006129049	0.080182968	6842	5418	2812	3188	1185	2592	2578	2240
ENSECAG00000004433	6.898337189	0.006134332	0.080182968	3860	7913	739	465	59	1409	277	1077
ENSECAG00000008187	3.820261893	0.006160307	0.080333508	65	87	265	226	406	425	563	271
ENSECAG00000014949	4.381300195	0.006168349	0.080333508	355	520	588	710	57	306	168	442
ENSECAG00000018618	7.016664887	0.006172868	0.080333508	3027	10568	751	949	15	1255	32	951
ENSECAG00000019531	2.956981844	0.006174073	0.080333508	172	518	69	48	39	119	42	60
ENSECAG00000002675	0.290088932	0.00619229	0.080478566	3	10	6	24	29	29	57	29
ENSECAG00000014701	5.563033539	0.006200181	0.080489238	1351	1896	705	531	352	805	341	623
ENSECAG00000000949	2.727735229	0.006266725	0.081213542	184	206	157	227	4	54	0	105
ENSECAG00000016418	1.049214067	0.006270242	0.081213542	8	14	34	3	51	43	32	133
ENSECAG00000019918	5.724616009	0.006295575	0.081449006	1285	1465	1292	869	483	991	613	659
ENSECAG00000011174	6.455594927	0.006384876	0.082506686	349	447	796	2135	2210	1360	2136	5206
ENSECAG00000011733	8.453442499	0.006391822	0.082506686	8427	21174	5277	3846	317	2632	632	6523
ENSECAG00000009370	2.831898391	0.00640072	0.082527974	37	60	64	149	252	169	318	114
ENSECAG00000024082	8.391619373	0.006445335	0.083009208	13553	14496	2156	2974	1350	4517	1539	3833
ENSECAG00000019437	7.647142575	0.006464854	0.083130678	1239	1263	3605	1699	8260	5279	8743	2648
ENSECAG00000023758	3.043777925	0.00646937	0.083130678	208	404	85	121	37	128	68	103
ENSECAG00000017122	1.605909884	0.006526609	0.083771639	29	18	29	38	73	42	113	138
ENSECAG00000009990	0.523381866	0.006572351	0.084027666	7	16	11	14	19	56	26	65
ENSECAG00000021750	6.815453453	0.006576947	0.084027666	424	958	317	2817	1944	3923	4582	3877
ENSECAG00000023192	8.924129927	0.006584468	0.084027666	33614	9446	1004	2554	974	6811	1216	1603
ENSECAG00000006445	2.976531437	0.00658592	0.084027666	45	55	100	121	346	189	346	75
ENSECAG00000018519	3.406560989	0.006588981	0.084027666	68	108	182	80	188	311	590	201
ENSECAG00000010533	0.981324572	0.006590839	0.084027666	16	53	57	86	25	23	28	7
ENSECAG00000007365	6.190681941	0.00662824	0.084409963	708	836	939	827	1368	2075	2135	2784
ENSECAG00000004945	6.160099596	0.006636322	0.084418459	1279	1720	2066	1863	1336	547	1278	743
ENSECAG00000015382	5.077796452	0.006666544	0.084672964	405	619	2585	335	60	231	69	739
ENSECAG00000000396	5.824160501	0.006671203	0.084672964	564	578	725	398	2487	1160	2527	666
ENSECAG00000016070	3.593235919	0.006720864	0.085208276	66	140	110	232	199	251	392	583
ENSECAG00000013626	1.026052986	0.006820456	0.086374735	42	27	34	106	22	19	24	25
ENSECAG00000017109	4.112432688	0.006839478	0.086519395	405	936	400	220	14	214	3	211
ENSECAG00000000967	2.566901946	0.00688483	0.086996437	57	206	146	213	41	104	72	63
ENSECAG00000016842	0.361018497	0.006898899	0.087049471	7	7	8.00009	23	57.0021	37	21	30
ENSECAG00000020809	0.85780855	0.006904319	0.087049471	16	47	44	86	12	30	17	17
ENSECAG00000006929	0.338523426	0.006913178	0.087064744	38	44	21	19	5	24	5	8
ENSECAG00000013994	4.163628408	0.006921391	0.087071858	158	136	268	286	597	298	616	535
ENSECAG00000024552	2.57015137	0.007027055	0.088179522	51	37	88	60	227	110	275	104
ENSECAG00000010517	1.321809333	0.007027953	0.088179522	20	28	110	177	0	9	0	42

ENSECAG00000020753	3.386743198	0.007032675	0.088179522	146	108	547	316	141	64	214	76
ENSECAG00000021609	6.177322542	0.007068709	0.08853383	1506	6479	345	364	5	637	7	465
ENSECAG00000024376	5.634927377	0.007097142	0.088792271	356	449	550	1080	1196	831	1769	1939
ENSECAG00000008985	2.510141231	0.007114235	0.088908415	29	42	70	91	89	83	221	307
ENSECAG00000013569	5.04211123	0.007175845	0.089476904	1006	1039	540	456	322	635	260	349
ENSECAG00000010783	5.909496829	0.007185984	0.089476904	1410	3597	544	1097	61	1075	69	540
ENSECAG00000012104	4.143520154	0.007193836	0.089476904	385	446	961	410	0	107	2	236
ENSECAG00000002390	2.523165374	0.00719763	0.089476904	73	151	165	219	88	97	30	42
ENSECAG00000010736	9.556064928	0.007201248	0.089476904	14444	39280	9268	14734	5537	4446	15592	7535
ENSECAG00000023776	1.210610418	0.007218581	0.089476904	15	17	17	44	71	46	102	53
ENSECAG00000011862	1.123255577	0.007219359	0.089476904	18	18	24	23	41	59	51	99
ENSECAG00000013223	0.360162946	0.007222598	0.089476904	7	10	2	23	42	23	63	26
ENSECAG00000021813	3.936155426	0.007253206	0.08975843	200	152	79	127	618	512	536	172
ENSECAG00000008862	1.870305408	0.007273161	0.089907637	14	19	29	86	71	86	105	181
ENSECAG00000015375	6.589298687	0.00729055	0.090024847	3658	3793	1001	630	796	1380	794	862
ENSECAG00000023275	1.462882848	0.00734466	0.090573524	12	27	29	40	49	135	92	46
ENSECAG00000008109	7.002084912	0.007350895	0.090573524	828	1103	1148	2402	2038	2605	4072	6945
ENSECAG00000014897	0.909574104	0.00740137	0.091093011	21	12	69	130.008	1.00713	12	5	35
ENSECAG00000013777	4.546058731	0.007409058	0.091093011	239	102	130	357	1542	259	282	797
ENSECAG00000008285	5.514677539	0.007424176	0.091180414	606	2063	724	1188	357	820	393	701
ENSECAG00000020204	1.37239032	0.00747764	0.091630109	15	17	1	51	57	63	85	122
ENSECAG00000007197	5.126653437	0.007484927	0.091630109	211	356	526	686	812	937	969	1173
ENSECAG00000024961	3.118476749	0.007484936	0.091630109	48	97	113	156	222	241	292	232
ENSECAG00000024361	5.098406995	0.00750478	0.091774351	1072	932	564	674	171	467	219	742
ENSECAG00000019506	7.137464408	0.007528343	0.091963722	1996	10265	1576	1174	624	1877	976	1688
ENSECAG00000025062	3.682893567	0.007550452	0.09213494	154	277	290	642	68	146	188	253
ENSECAG00000024302	0.652784225	0.007562719	0.092185825	1	12	21	23	52	42	65	28
ENSECAG00000010778	6.491516651	0.007593582	0.09236746	1893	926	3851	6010	0	490	0	565
ENSECAG00000009247	6.838375808	0.007593847	0.09236746	653	529	1360	1703	5957	678	6176	2135
ENSECAG00000008020	2.317231805	0.00762445	0.092640731	142	162	81	84	34	76	60	59
ENSECAG00000013283	5.739078202	0.007652766	0.092885646	250	351	907	1065	2012	1087	1868	1256
ENSECAG00000018281	3.076920916	0.007669091	0.092984663	201	367	141.002	104.004	73.0019	144.002	67.0022	91.0022
ENSECAG00000019129	2.104267427	0.007697366	0.093228206	24	51	36	64	108	166	181	54
ENSECAG00000007974	4.118672868	0.007724585	0.093445292	498	710	225	175	200	170	209	249
ENSECAG00000023744	2.613755896	0.007735782	0.093445292	63	44	87	60	214	130	197	169
ENSECAG00000014753	6.567675862	0.007739913	0.093445292	695	574	754	2273	2306	1673	3974	3520
ENSECAG00000021069	7.064674658	0.00775901	0.093576614	4980	6194	813.996	1178	535.996	1997	771	1430
ENSECAG00000006930	5.674119279	0.007768268	0.093589136	390	384	782	854	2196	1367	957	1221
ENSECAG00000011797	8.6644787	0.007785161	0.093616761	22249	12480	3225	1556	1154	6693	899	2082
ENSECAG00000020052	5.110357928	0.007787007	0.093616761	140	278	740	390	787	817	695	1761
ENSECAG00000001308	5.993130041	0.007816754	0.093875261	4187	1119	345	435	65	1152	166	167
ENSECAG00000008527	4.609265014	0.007838691	0.094039511	226	195	265	441	631	315	815	1082
ENSECAG00000014273	5.831444644	0.007887176	0.094521582	177	527	981	1081	1552	1523	1851	1689
ENSECAG00000010881	8.678247883	0.007958806	0.095156906	22783	11213	3944	1981	1742	5401	318	3512
ENSECAG00000003033	1.719277517	0.007963718	0.095156906	27	22	186	168	12	5	22	69
ENSECAG00000006938	0.774149312	0.007965264	0.095156906	16	68	42	53	12	30	6	18
ENSECAG00000026882	2.726682152	0.007991352	0.095208571	80	267	111	210	60	88	105	79
ENSECAG00000002083	4.096078102	0.007992553	0.095208571	608	706	129	179	109	288	110	181
ENSECAG00000013822	2.17766762	0.007994677	0.095208571	29	35	40	89	147	75	215	104
ENSECAG00000000014	3.768686632	0.008127776	0.096692512	105	273	231	965	80	129	192	239
ENSECAG00000006973	0.588846802	0.008261645	0.098059276	4	6	29	12	47	61	25	41
ENSECAG00000015198	5.065271567	0.008266628	0.098059276	224	297	443	567	1701	567	1196	447
ENSECAG00000006658	2.44966865	0.008269679	0.098059276	29	66	51	79	81	243	230	95
ENSECAG00000024242	6.004623599	0.008277116	0.098059276	461	588	908	1240	1512	1321	2261	2210
ENSECAG00000018384	3.383850063	0.008285841	0.098060603	101	80	150	125	399	196	425	196
ENSECAG00000010200	6.92541216	0.008308097	0.0982219	2497	6601	1497	1570	765	1747	1099	1628
ENSECAG00000008535	2.067511021	0.008322907	0.098294907	15	26	75	57	111	71	135	186
ENSECAG00000000017	0.963166632	0.008368119	0.098726457	10	15	17	37	33	63	60	67
ENSECAG00000021462	5.527980606	0.008444058	0.099519257	282	239	402	1277	1314	876	1589	1679
ENSECAG00000011155	7.02782353	0.008531368	0.100444281	3550	4543	2475	2102	1019	2478	780	1509
ENSECAG00000019793	5.544985654	0.008546245	0.100515495	251	242	531	1236	1369	903	1494	1712
ENSECAG00000020937	5.327112122	0.008582255	0.100834855	312	243	622	595	1861	621	1750	556
ENSECAG00000023092	2.325584509	0.00859931	0.100931076	75	17.0004	408	78	63	49	12	34
ENSECAG00000020013	0.742557852	0.008684675	0.101731355	17	42	48	71	9	17	7	34
ENSECAG00000022970	5.331306909	0.008693156	0.101731355	924	860	1068	1009	419	782	211	624
ENSECAG00000006453	4.837472703	0.0086943	0.101731355	1275	511	382	369	262	475	255	300
ENSECAG00000020638	3.210708526	0.00878953	0.10274004	61	204	131	685	77	80	132	135
ENSECAG00000013529	6.773606952	0.008800635	0.102764334	968	571	1831	913	4950	2486	4549	1423
ENSECAG00000000103	4.496285511	0.008881741	0.10360514	137	209	352	435	764	651	641	453
ENSECAG00000012668	2.676935234	0.008894016	0.103642141	97	119	223	195	110	58	95	71
ENSECAG00000021586	1.618240736	0.008904742	0.103661028	13	2	54	16	89	44	258	32
ENSECAG00000019751	5.200888447	0.008914139	0.103664428	771	736	692	1244	683	381	578	475
ENSECAG00000017205	7.371412262	0.008943859	0.103903915	6125	6623	1523	1464	985	2352	1514	1807
ENSECAG00000022016	2.563308899	0.008989083	0.104251436	51	68	55	87	173	145	244	119
ENSECAG00000018888	4.939765977	0.008998057	0.104251436	638	1137	596	617	132	603	239	504
ENSECAG00000019529	4.620423332	0.009001244	0.104251436	184	199	344	444	994	335	966	550

ENSECAG00000005881	1.88823107	0.009125405	0.105582048	95	82	54	124	60	32	41	46
ENSECAG00000021987	5.097517817	0.009142966	0.105677834	1505	709	371	425	379	430	374	415
ENSECAG00000012742	1.550570383	0.009163205	0.105804349	156	69	51	23	0	49	2	11
ENSECAG00000017010	1.597374024	0.009187226	0.105917195	60	238	16	8	20	37	14	13
ENSECAG00000013700	1.909216852	0.009191584	0.105917195	125	121	37	72	20	59	45	36
ENSECAG00000004969	2.366647458	0.009205749	0.105944355	14	22	35	107	47	47	189	400
ENSECAG00000000795	2.235549031	0.009212553	0.105944355	16	40	78	74	126	110	193	126
ENSECAG00000016179	3.419885561	0.009262226	0.106408115	86	96	130	200	348	191	338	335
ENSECAG00000021011	5.872262405	0.009284692	0.106423346	793	1551	1828	1455	801	676	744	1103
ENSECAG00000024595	0.675459553	0.009290974	0.106423346	12	9	13	27	57	31	61	34
ENSECAG00000016138	2.625325923	0.009291595	0.106423346	144	197	118	120	46	87	91	87
ENSECAG00000017186	4.774123125	0.009323491	0.106654279	245	108	485	252	779	372	1826	476
ENSECAG00000017739	5.225313499	0.009330493	0.106654279	549	916	687	1619	749	236	443	600
ENSECAG00000018562	4.801067782	0.009363108	0.106919742	332	171	311	404	1009	474	1352	458
ENSECAG00000000516	3.299475361	0.009394685	0.107172833	22	76	132	214	349	263	393	175
ENSECAG00000000307	5.906037669	0.009432504	0.107463251	1979	2271	601	679	571	885	541	754
ENSECAG00000020573	3.463202893	0.009439021	0.107463251	122	102	125	132	291	256	551	192
ENSECAG00000014246	1.472213638	0.009454706	0.107465127	9	44	29	23	90	87	121	33
ENSECAG00000010118	3.05236998	0.009466783	0.107465127	27	37	197	58	309	130	424	185
ENSECAG00000008471	3.960827572	0.009467503	0.107465127	301	302	369	518	130	153	364	241
ENSECAG00000024499	6.15882656	0.009493369	0.107651397	2277	1484	1134	1548	1000	1012	851	894
ENSECAG00000018638	2.167075924	0.009525278	0.107905757	204	137	47	50	8	38	10	88
ENSECAG00000019083	10.5198322	0.009576718	0.108380652	56105	58760	14064	11972	11330	22290	9253	12730
ENSECAG00000019115	6.868737423	0.009629183	0.108752641	4288	4723	2034	1128	120	1565	106	1682
ENSECAG00000017223	5.426498109	0.009638228	0.108752641	2791	262	397	399	226	692	326	204
ENSECAG00000000138	2.478480991	0.009638245	0.108752641	35	40	73	74	340	109	93	112
ENSECAG00000005115	1.328085295	0.00966978	0.109000442	43	45	45	122	31	30	23	34
ENSECAG00000004240	4.765790215	0.009727823	0.109546251	656	1143	508	270	164	469	234	414
ENSECAG00000015521	1.067846575	0.009761712	0.109715019	28	31	55	105	14	15	33	35
ENSECAG00000002472	4.988195703	0.009762083	0.109715019	843	1825	302	163	120	491	309	328
ENSECAG00000002412	5.117777045	0.009790265	0.109923241	978	1801	448.004	247.004	108.001	700	125	343.001
ENSECAG00000016488	3.716705064	0.009803597	0.109941064	121	78	225	176	461	261	475	314
ENSECAG00000016185	1.477344677	0.009811166	0.109941064	10	29	34	41	79	95	117	35
ENSECAG00000014536	2.318512498	0.009843619	0.110099848	41	23	57	84	135	139	252	73
ENSECAG00000016829	5.758799913	0.009844677	0.110099848	1971	609	1026	1364	897	716	697	389
ENSECAG00000023399	1.994371639	0.009866387	0.110234366	13	56	366	51	28	63	9	10
ENSECAG00000004574	2.43944844	0.009900616	0.110308464	38	50	61	90	161	163	91	199
ENSECAG00000012880	5.701837649	0.009907795	0.110308464	1007	2113	970	833	735	947	467	500
ENSECAG00000003448	6.528338691	0.009923825	0.110308464	2445	3043	1592	1528	579	1263	1208	1629
ENSECAG00000015262	5.959459288	0.009929663	0.110308464	910	2487	1163	1435	523	961	843	1039
ENSECAG00000012502	7.036586043	0.009937775	0.110308464	4250	5730	1391	1332	751	2271	792	1449
ENSECAG00000011697	3.13930658	0.009938913	0.110308464	74	33	122	140	220	211	165	441
ENSECAG00000013205	0.436721121	0.009940842	0.110308464	7	12	19	14	42	36	31	40
ENSECAG00000019365	5.799201685	0.009986912	0.110711772	1116	3248	807	459	254	1142	327	503
ENSECAG00000003194	0.186718986	0.010009927	0.110858968	34	23	21	27	5	13	21	8
ENSECAG00000018686	6.606858133	0.010093726	0.111678393	871	737	1483	968	5474	1811	2470	1718
ENSECAG00000023588	1.224213036	0.010111077	0.111761761	11	14	39	32	41	57	100	75
ENSECAG00000012750	5.674462864	0.010132407	0.111888899	1280	2551	728	352	346	848	405	636
ENSECAG00000009285	5.521758674	0.010153082	0.112008562	2159	2030	281	148	41	795	79	278
ENSECAG00000007642	2.812585067	0.010172221	0.112111071	558	77	5	42	6	94	4	4
ENSECAG00000019488	3.412816294	0.010201977	0.112186973	157	177	311	463	108	78	87	263
ENSECAG00000010015	6.234402444	0.010202297	0.112186973	628	565	690	1269	4403	1287	2555	788
ENSECAG00000015687	8.24204082	0.010214873	0.112186973	8856	14252	3151	3035	2236	4203	2959	3385
ENSECAG00000024435	4.749958068	0.01022224	0.112186973	349	573	555	1216	318	313	328	559
ENSECAG00000024244	6.461993139	0.010233467	0.112186973	754	501	1454	921	4156	1179	4083	1349
ENSECAG00000026902	1.595554841	0.010246071	0.112186973	50	42	136	80	23	24	26	64
ENSECAG00000019990	4.862854694	0.010248085	0.112186973	305	580.997	753.998	1372	527	337	523.998	208
ENSECAG00000023860	8.116598657	0.010259409	0.112203045	22797	1632	819	484	209	3488	580	1008
ENSECAG00000010633	4.937269994	0.010292023	0.112451714	691	1029	412	697	391	517	295	439
ENSECAG00000024152	5.77041174	0.010309762	0.112537523	365	308	1041	911	2013	1096	1848	1317
ENSECAG00000022845	1.229587685	0.010373068	0.11312009	29	69	93	61	5	50	8	23
ENSECAG00000019596	9.55800814	0.010438122	0.113720589	44781	9103	13124	10862	154	5896	215	10426
ENSECAG00000011922	5.596736037	0.010449015	0.113730433	1201	2426	590	460	351	861	271	616
ENSECAG00000004869	0.812998711	0.010466851	0.11377215	16	36	50	83	11	11	29	28
ENSECAG00000011728	7.472484468	0.010472834	0.11377215	7091	5088	1864	2820	678	2867	1181	2502
ENSECAG00000023189	2.456324873	0.010508934	0.114055492	40	280	104	172	22	101	39	65
ENSECAG00000010437	5.474024983	0.010582475	0.114744265	412	393	778	611	937	1222	1359	1410
ENSECAG00000023498	2.45311001	0.010605756	0.114813741	36	62	60	88	188	81	198	166
ENSECAG00000004286	3.740959902	0.010609052	0.114813741	106	102	207	182	598	229	538	197
ENSECAG00000015095	5.84277098	0.010619403	0.114816623	2194	2677	261	471	206	1035	232	527
ENSECAG00000024716	6.848613936	0.010659076	0.11513622	1446	2307	2028	7063	954	625	1569	2440
ENSECAG00000025003	1.305285644	0.010765334	0.116173773	23	45	41	168	17	39	20	28
ENSECAG00000011978	1.975736662	0.010818512	0.116627884	26	35	58	55	124	77	156	95
ENSECAG00000016341	5.461234524	0.010836827	0.116627884	736	1591	1010	1236	111	898	222	736
ENSECAG00000003569	5.523451782	0.010838147	0.116627884	215	581	418	909	855	514	1755	2414
ENSECAG00000009027	1.160463785	0.010849657	0.1166415	8	11	24	41	19	76	56	116

ENSECAG00000009016	4.004872259	0.010886599	0.116806433	252	337	385	600	315	232	136	188
ENSECAG00000003772	5.746372048	0.010888561	0.116806433	986	2737	705	840	498	1100	374	506
ENSECAG00000017707	7.379507029	0.010895778	0.116806433	4929	8638	1494	2026	267	2576	626	2329
ENSECAG00000015853	5.56461266	0.010927749	0.117038967	1813	2045	229	339	484	634	383	396
ENSECAG00000011488	3.022101462	0.010960726	0.117281832	219	52	620	75	1	43	2	130
ENSECAG00000009647	7.767571861	0.010975087	0.117325228	14954	5480	419	1317	16	2894	62	339
ENSECAG00000009760	4.191551705	0.011041069	0.117919863	135	170	339	211	744	408	627	297
ENSECAG00000012954	6.843770343	0.01108908	0.118149199	2575	6316	1236	1392	679	2207	713	1086
ENSECAG00000023765	0.815619355	0.011096413	0.118149199	22	93	25	34	14	26	15	18
ENSECAG00000001069	8.635142774	0.011104	0.118149199	11894	20822	8418	2281	153	5807	367	4012
ENSECAG00000016693	6.049832682	0.011104053	0.118149199	1305	2529	1133	1208	614	786	978	1233
ENSECAG00000011660	1.896588091	0.011134489	0.118224957	83	113	42	157	4	37	36	72
ENSECAG00000013962	7.743249438	0.011137809	0.118224957	10052	7161	1378	1614	875	3742	1388	1558
ENSECAG00000027699	10.70885992	0.011142326	0.118224957	64868	17016	44770	42763	4512	15632	7451	37036
ENSECAG00000024727	5.959168769	0.011200688	0.118618722	1635	2407	785	1209	265	1406	495	638
ENSECAG00000007722	2.425628166	0.01120115	0.118618722	50	17	89	27	266	72	238	97
ENSECAG00000017976	3.409144387	0.011210693	0.118618722	53	84	116	238	162	215	342	537
ENSECAG00000024710	0.94003536	0.011232344	0.118704942	5	9	25	32	18	36	80	100
ENSECAG00000024558	1.138485542	0.011239695	0.118704942	14	9	20	46	60	46	108	46
ENSECAG00000007380	0.836059327	0.011298366	0.119183669	12	13	21	26	63	39	69	31
ENSECAG00000022930	2.252173699	0.011305961	0.119183669	10	47	43	105	214	102	125	123
ENSECAG00000016611	6.305358836	0.011351338	0.119466215	688	1315	781	792	1793	2771	2895	1583
ENSECAG00000022330	5.286352495	0.011358259	0.119466215	286	423	464	803	628	936	1494	1352
ENSECAG00000015126	5.051667264	0.011364243	0.119466215	466	628	697	1508	603	498	430	336
ENSECAG00000022899	3.71100051	0.011383013	0.119543904	183	561	295	223	143	197	120	215
ENSECAG00000015494	1.203270465	0.011399559	0.119543904	98	80	37	23	3	47	1	2
ENSECAG00000015947	0.31834401	0.011403134	0.119543904	4	21	99	28	0	3	0	23
ENSECAG00000008594	6.132786023	0.011417569	0.119585122	555	3210	1226	2239	887	840	1325	609
ENSECAG00000017998	5.768985284	0.011436907	0.119610148	385	751	494	1078	2030	1166	1848	1092
ENSECAG00000008441	2.140776468	0.011440971	0.119610148	55	43	34	28	139	61	140	187
ENSECAG00000020235	3.857577778	0.011491477	0.120019488	221	982	109	161	151	215	110	124
ENSECAG00000015109	8.364317704	0.011501209	0.120019488	2123	1155	4702	4314	5016	21268	4493	9187
ENSECAG00000006071	2.675500242	0.011537346	0.120286338	53	73	49	101	117	101	281	259
ENSECAG00000009685	3.864074312	0.01155353	0.120344863	103	188	143	297	385	310	526	410
ENSECAG00000025682	1.106590714	0.01159061	0.120620747	32	15	100	121	0	26	0	25
ENSECAG00000020341	4.28988706	0.011623069	0.12084807	555	934	434	220	3	343	18	166
ENSECAG00000017482	3.378596497	0.011693245	0.121466783	360	525	62	60	83	169	83	45
ENSECAG00000009811	3.122126893	0.011763612	0.122086345	90	29	105	7	497	141	452	75
ENSECAG00000011439	6.328325961	0.011808513	0.122440726	1143	4209	945	1723	526	1162	942	1302
ENSECAG00000015865	10.12963125	0.011876859	0.123037339	81289	4019	16577	4391	1587	16764	981	5671
ENSECAG00000009150	4.870347542	0.011906043	0.123189546	259	246	373	571	1067	463	1117	657
ENSECAG00000018064	5.459552249	0.011913192	0.123189546	336	698	566	556	1206	697	1480	1566
ENSECAG00000024127	3.558736715	0.011951159	0.123455708	84	105	114	238	248	244	724	198
ENSECAG00000004117	-0.283799299	0.011960619	0.123455708	1	7	6	15	20	23	24	22
ENSECAG00000001041	1.509897045	0.011993981	0.123687929	21	24	23	49	93	76	128	35
ENSECAG00000011185	0.837279208	0.012031831	0.123965967	12	16	22	14	101	34	45	25
ENSECAG00000007818	9.097515576	0.012051849	0.124027204	19453	21025	5524	6368	3650	10016	4314	4215
ENSECAG00000013414	1.70765753	0.012059562	0.124027204	19	37	42	40	49	80	165	90
ENSECAG00000013368	2.97786931	0.012071899	0.124042032	161	522	92	67	7	124	20	97
ENSECAG00000018785	0.596756515	0.012085158	0.124066293	19	37	67	27	10	26	11	16
ENSECAG00000023036	6.280224906	0.012097428	0.124080373	1510	3054	1309	1424	797	1109	852	1369
ENSECAG00000000438	5.883276809	0.012214631	0.125169738	1764	981	1259	1273	753	646	1159	761
ENSECAG00000012168	5.21665926	0.012293839	0.125868126	1368	475	654	785	562	373	575	471
ENSECAG00000005292	8.009251202	0.012417407	0.127019029	7965	12435	2681	2111	1409	4525	1385	2705
ENSECAG00000015951	4.611853629	0.012489659	0.127505131	168	310	338	457	559	539	863	737
ENSECAG00000024887	6.275413865	0.012494653	0.127505131	2887	2695	1436	933	85	1665	80	588
ENSECAG00000012183	0.825811358	0.012505079	0.127505131	10	15	16	26	73	69	41	17
ENSECAG00000015809	2.597916321	0.012514534	0.127505131	23	45	132	58	106	202	189	216
ENSECAG00000025028	4.865270021	0.012528944	0.127505131	538	682	548	1221	154	625	139	491
ENSECAG00000004925	6.193949915	0.012532125	0.127505131	639	841	887	1204	2699	1307	2608	1650
ENSECAG00000007880	2.005592015	0.012637426	0.128297689	24	23	24	92	81	47	204	165
ENSECAG00000000543	1.895766085	0.01264182	0.128297689	132	59	96	68	16	22	29	91
ENSECAG00000023440	3.419284917	0.012658737	0.128297689	137	237	218	488	94	157	139	193
ENSECAG00000000110	3.373699032	0.012658811	0.128297689	92	152	67	142	246	157	562	270
ENSECAG00000013476	3.72587882	0.012666368	0.128297689	145	96	129	220	236	201	588	535
ENSECAG00000023170	6.492345273	0.012677966	0.128301012	926	839	1249	981	3320	1767	4078	1378
ENSECAG00000010218	1.010529501	0.01273388	0.128752418	7	15	21	43	48	70	57	52
ENSECAG00000005547	1.272861382	0.012789367	0.129013717	12	25	19	40	115	66	74	24
ENSECAG00000022974	0.940362615	0.012789706	0.129013717	20	56	58	55	30	21	20	20
ENSECAG00000015728	1.213198567	0.012793719	0.129013717	40	52	56	74	26	17	37	38
ENSECAG00000008613	5.621573646	0.01285159	0.129482613	1344	1848	576	778	332	884	474	755
ENSECAG00000023798	5.220165189	0.012885961	0.129714112	677	999	644	1147	330	548	661	656
ENSECAG00000023216	8.906172853	0.012922583	0.129967847	19330	26513	2546	3272	97	4742	178	5583
ENSECAG00000017136	3.982100071	0.012960546	0.130234615	219	388	351	553	178	244	221	276
ENSECAG00000026456	0.933358371	0.013036888	0.130738982	7	21	22	195	7	22	6	24
ENSECAG00000016507	4.440683246	0.013042105	0.130738982	321	1443	230	263	84	484	116	164

ENSECAG00000014984	9.75561913	0.01304519	0.130738982	32151	29099	8769	11021	6455	13707	6699	9887
ENSECAG00000017405	5.478186713	0.013064757	0.130819927	929	2074	713	563	448	965	313	363
ENSECAG00000004897	5.064249524	0.013149447	0.131552246	1136	423	1119	327	453	406	408	427
ENSECAG00000018438	1.294040327	0.013164142	0.131583635	18	18	28	39	45	62	123	54
ENSECAG00000003473	5.305470345	0.013188329	0.131601961	1108	2414	284	266	95	850	94	241
ENSECAG00000022468	7.580953893	0.013189094	0.131601961	6166	2953	4426	4372	2330	3132	1732	2195
ENSECAG00000011392	3.771656251	0.013239283	0.131902643	97	183	160	156	245	217	950	259
ENSECAG00000020150	0.255468729	0.0132424	0.131902643	14	28	31	44	9	12	24	9
ENSECAG00000020576	3.697998864	0.013275327	0.132115031	114	119	171	199	408	201	667	237
ENSECAG00000019037	1.283365112	0.013290931	0.132130477	109	74	18	21	15	37	14	23
ENSECAG00000012550	1.221984177	0.01330009	0.132130477	13	70	25	156	29	22	25	21
ENSECAG00000006159	6.084266174	0.013352747	0.13253795	1388	2370	1465	1072	597	1203	787	1045
ENSECAG00000015498	6.241766201	0.013416254	0.1329428	1822	2101	1381	1591	735	1210	969	1270
ENSECAG00000018069	5.788011483	0.013416889	0.1329428	470	276	1104	752	1918	1212	2017	1195
ENSECAG00000018312	3.434862869	0.013457952	0.133233722	94	131	124	158	227	189	552	284
ENSECAG00000018230	1.919152081	0.0135195	0.133688603	25	50	47	45	76	137	109	101
ENSECAG00000000300	2.147186927	0.013527384	0.133688603	17	85	31	37	55	157	116	201
ENSECAG00000011982	1.957460971	0.013684179	0.135033995	27	44	42	65	100	112	135	88
ENSECAG00000022554	6.017414154	0.01368724	0.135033995	1083	2439	1093	1462	900	1059	684	929
ENSECAG00000009872	5.470245496	0.013716912	0.135209562	1111	1588	518	855	373	734	457	757
ENSECAG00000024637	6.224091823	0.013743739	0.135335753	878	281	838	1252	2158	1156	3474	2173
ENSECAG00000012073	3.819530671	0.013753488	0.135335753	289	562	249	254	109	282	81	210
ENSECAG00000006636	1.608083757	0.013801334	0.13568928	4	2	33	62	84	31	68	212
ENSECAG00000007476	3.371439872	0.013815695	0.135713276	505	169	101	157	115	184	77	48
ENSECAG00000014636	1.231288585	0.01388945	0.136320165	14	21	34	31	55	42	110	63
ENSECAG00000006886	5.550106106	0.013938642	0.136510599	2138	1319	272	440	336	667	475	549
ENSECAG00000007665	6.548944928	0.013944646	0.136510599	2703	3557	1136	1226	643	1290	1302	1405
ENSECAG00000022980	0.245821369	0.013944824	0.136510599	17	21	55	26	24	10	8	3
ENSECAG00000006471	2.99774831	0.013976295	0.13670113	198.001	360	75.001	117	68.0011	96.0005	119	101.001
ENSECAG00000015450	1.594037548	0.0140739	0.13744409	49	42	103	109	24	21	68	39
ENSECAG00000020698	1.830602066	0.014077995	0.13744409	34	19	49	37	140	63	168	52
ENSECAG00000020932	7.211581191	0.014088472	0.13744409	2533	8288	1822	2627	600	3069	916	1587
ENSECAG00000024701	3.207771383	0.014131704	0.13772404	48	78	89	230	155	255	310	346
ENSECAG00000000465	8.994808765	0.014141362	0.13772404	9663	26513	6053	9474	1942	6176	6033	8617
ENSECAG00000007321	5.752823808	0.014171907	0.13790356	868	1167	1152	2124	377	585	738	1330
ENSECAG00000012179	8.950143161	0.01419973	0.137992593	27184	16257	4046	1430	654	9033	386	1929
ENSECAG00000020992	0.214408673	0.014209236	0.137992593	5	11	18	10	36	27	35	29
ENSECAG00000002482	4.269512202	0.014217419	0.137992593	440	928	174	291	121	316	190	294
ENSECAG00000016312	1.689596477	0.014372489	0.139335378	29	23	33	51	105	61	153	56
ENSECAG00000014968	5.642423513	0.014389018	0.139335378	322	150	705	628	4363	510	851	480
ENSECAG00000005185	2.622941879	0.014392482	0.139335378	10	112	24	81	331	68	263	117
ENSECAG00000017682	3.086790083	0.014450282	0.139776091	138	395	140	200	27	121	38	182
ENSECAG00000022154	5.2956614	0.014475367	0.139899875	851	1178	707	892	294	674	403	784
ENSECAG00000021325	2.151570422	0.014515768	0.140171351	50	68	219	153	28	52	9	92
ENSECAG00000022985	4.277859745	0.014548857	0.140253982	228	180	238	241	543	257	973	477
ENSECAG00000016046	2.626508942	0.014548964	0.140253982	34	67	663	49	0	47	0	62
ENSECAG00000007779	4.581704154	0.014605025	0.140667626	198	279	402	320	602	513	1061	503
ENSECAG00000016581	2.23234625	0.014619951	0.140667626	163	148	26	107	31	76	42	52
ENSECAG00000004193	1.394959029	0.014638545	0.140667626	23	10	28	38	95	29	149	47
ENSECAG00000011613	4.509397079	0.014641352	0.140667626	570	1529	169	155	27	470	31	143
ENSECAG00000018757	2.82110142	0.01465365	0.140667626	54	71	97	96	243	104	339	139
ENSECAG00000015760	4.151161632	0.014712819	0.14084811	113.971	141	273	336.006	730	346.005	700	256.002
ENSECAG00000010091	0.638442099	0.014716596	0.14084811	11	12	24	7	74	45	23	33
ENSECAG00000009014	2.305115568	0.014716611	0.14084811	27	42	80	64	206	72	222	84
ENSECAG00000024054	1.043527324	0.014721937	0.14084811	19	16	26	25	51	62	69	46
ENSECAG00000016971	5.870247094	0.014769122	0.141180898	417	362	1214	794	2126	1288	2256	1055
ENSECAG00000013606	4.379823384	0.014841143	0.141698053	202	201	268	365	657	299	751	618
ENSECAG00000005912	1.499902472	0.014848114	0.141698053	7	35	37	44	89	51	111	77
ENSECAG00000010962	2.835002335	0.014870398	0.141791858	60	69	103	105	212	135	261	196
ENSECAG00000014524	7.612798827	0.014944302	0.142287948	7165	5343	3553	2223	1207	3982	1168	2062
ENSECAG00000020840	7.12904038	0.014947421	0.142287948	4036	7015	1142	1421	604	2473	1191	1498
ENSECAG00000019423	6.639631952	0.015037901	0.142961228	2449	5098	1029	986	731	1746	966	1037
ENSECAG00000005925	4.472158083	0.015048305	0.142961228	113	187	252	548	780	654	834	270
ENSECAG00000011006	1.743431384	0.01505582	0.142961228	159	148	4	28	2	54	5	18
ENSECAG00000023105	0.747748548	0.015086496	0.143133126	28	26	50	70	3	30	10	25
ENSECAG00000011492	4.517150401	0.01514647	0.1435176	401	394	524	859	453	229	423	241
ENSECAG00000017135	5.389945707	0.015152232	0.1435176	331	541	477	757	1700	1021	1206	687
ENSECAG00000016206	4.755186111	0.015193419	0.14378809	361	517	651	1067	431	417	386	371
ENSECAG00000013660	1.986020459	0.015240807	0.144116765	145	108	7	130	28	36	52	43
ENSECAG00000020645	2.639498987	0.015368854	0.145206973	42	27	34	158	161	101	383	132
ENSECAG00000020378	4.579148312	0.015426705	0.145627623	201	271	365	381	748	487	936	470
ENSECAG00000014332	3.764196596	0.015438958	0.145627623	193	209	322	876	13	131	53	337
ENSECAG00000020764	1.874457686	0.015503531	0.145977723	49	92	59	170	58	38	55	37
ENSECAG00000001488	3.986006851	0.015510486	0.145977723	140	51	258	106	536.001	133	1271	159
ENSECAG00000004162	8.719772496	0.015523691	0.145977723	20368	14169	2339	1882	2617	3505	4747	5139
ENSECAG00000006904	9.84785234	0.015527363	0.145977723	23712	23188	16277	24273	13092	12828	11371	10096

ENSECAG00000001853	4.234183715	0.015545641	0.145987293	143	240	293	253	490	303	898	447
ENSECAG00000004228	5.925945073	0.015562274	0.145987293	3115	2335	417	462	8	846	7	428
ENSECAG00000008975	3.160351025	0.015566849	0.145987293	187	345	204	109	46	159	117	119
ENSECAG00000008344	4.776386101	0.015581111	0.146000781	211	268	345	562	1023	700	923	393
ENSECAG00000016820	3.141025459	0.015625735	0.14629851	73	161	240	476	140	100	156	58
ENSECAG00000016436	4.820572042	0.015690301	0.146782318	366	545	540	1353	274	363	547	480
ENSECAG00000015333	2.526972769	0.01584042	0.148065014	155	187	62	136	58	80	43	97
ENSECAG00000013804	0.486203075	0.015958891	0.149012138	4	11	21	21	50	36	35	33
ENSECAG00000020078	1.522549569	0.015967923	0.149012138	17	28	36	41	65	41	73	151
ENSECAG00000024702	8.303021523	0.016001222	0.149200585	15396	7614	2660	3169	2008	6024	1947	2238
ENSECAG00000006995	7.686310041	0.016027947	0.149222869	6513	7261	2971	2259	1583	3023	2308	2833
ENSECAG00000000051	5.778301921	0.016029826	0.149222869	900	753	3982	833	62	120	41	1468
ENSECAG00000019147	5.154079615	0.016061134	0.149392165	916	807	804	654	379	549	333	726
ENSECAG00000009524	7.546098866	0.01609804	0.149613216	4085	8469	2502	2844	1842	3111	2228	1870
ENSECAG00000023084	6.256614164	0.016132582	0.14981195	694	528	1170	1276	2949	1347	2930	1517
ENSECAG00000014689	4.377860137	0.01625615	0.150836402	391	895	285	401	174	465	204	213
ENSECAG00000018046	8.80074119	0.016351581	0.151598326	13531	13655	7936	8023	1923	8611	2526	6518
ENSECAG00000018414	0.633123374	0.01639982	0.151832104	8	2	16	31	22	37	43	80
ENSECAG00000025141	5.296034285	0.016440336	0.151832104	886	1777	685	347	273	745	317	553
ENSECAG00000024784	4.368582107	0.016442053	0.151832104	332	624	394	564	200	239	370	419
ENSECAG00000020301	2.585022917	0.016442464	0.151832104	32	54	84	99	189	117	326	80
ENSECAG00000009758	4.56893815	0.016443477	0.151832104	146	333	292	317	1238	390	857	259
ENSECAG00000012615	1.961614614	0.016472621	0.151977951	53	39	152	254	0	53	1	44
ENSECAG00000000224	2.629964732	0.016568341	0.152737301	102	167	99	244	58	52	78	138
ENSECAG00000008730	7.750815544	0.016583851	0.152756587	6242	7016	3734	3359	1450	4417	1594	2550
ENSECAG00000005580	0.191473928	0.016657181	0.153308005	2	8	16	18	22	21	37	48
ENSECAG00000016966	3.516341783	0.016688095	0.153468465	82	82	43	289	220	227	255	667
ENSECAG00000014312	4.926737629	0.016711787	0.153501709	202	337	433	641	948	694	1071	620
ENSECAG00000007480	3.132017881	0.016718675	0.153501709	71	119	84	142	145	218	382	253
ENSECAG00000009136	4.454111988	0.016780428	0.153844669	159	253	326	380	916	455	570	451
ENSECAG00000024636	5.072632765	0.016783055	0.153844669	964	2170	382	109	16	605	19	234
ENSECAG00000024473	1.641687204	0.016839676	0.154239512	22	18	52	40	105	60	145	53
ENSECAG00000000505	5.41374314	0.016926933	0.15491409	3116	363	561	294	0	419	1	215
ENSECAG00000015074	3.460689347	0.016982955	0.155301965	224	421	193	198	76	200	92	191
ENSECAG00000007084	2.124307941	0.016997721	0.155312238	31	70	155	261	18	64	6	76
ENSECAG00000022923	5.350776435	0.017124832	0.156348203	334	309	634	710	741	1098	733	1989
ENSECAG00000013291	8.258856762	0.017146087	0.156416826	1725	842	6658	2328	14621	11158	8774	3180
ENSECAG00000019367	1.369233218	0.017169518	0.156425869	14	17	41	42	54	93	73	68
ENSECAG00000022399	0.019309161	0.017174557	0.156425869	16	2	6	2	33	30	26	27
ENSECAG00000003759	7.2938513	0.017276982	0.157117283	4326	6761	1998	1782	1124	2948	1313	1736
ENSECAG00000023904	1.714610093	0.017278071	0.157117283	99	183	21	16	14	54	26	23
ENSECAG00000017470	4.275892881	0.01746574	0.158697085	401	816	313	323	133	451	111	234
ENSECAG00000013988	4.66160221	0.017525818	0.159115979	920	172	1261	346	23	366	13	380
ENSECAG00000018101	2.13841024	0.017564123	0.159336686	10	12	112	19	108	220	161	58
ENSECAG00000013528	4.62647665	0.01761376	0.159659758	288	749	747	913	34	563	121	349
ENSECAG00000015381	2.788899296	0.017641734	0.159731502	33	40	75	143	118	99	129	489
ENSECAG00000022402	3.085099631	0.017649735	0.159731502	62	59	101	143	535	148	167	130
ENSECAG00000003916	4.283450521	0.017704295	0.160087649	182	213	304	256	754	364	692.001	348
ENSECAG00000005092	0.3905925	0.017726245	0.160087649	4	5	24	13	71	12	32	36
ENSECAG00000006192	0.781339928	0.017731271	0.160087649	13	7	15	33	31	43	93	33
ENSECAG00000006867	1.671957204	0.017845929	0.160919427	12	19	65	27	116	156	50	45
ENSECAG00000012873	1.900387324	0.017851668	0.160919427	71	288	24	48	0	58	0	18
ENSECAG00000013072	1.587069523	0.017884525	0.160963183	26	26	44	22	121	46	140	46
ENSECAG00000009314	4.427196234	0.017884798	0.160963183	155	196	435	306	691	648	555	444
ENSECAG00000007533	7.774099421	0.017940109	0.161333448	2351	332	2265	264	15483	2841	11263	720
ENSECAG00000000458	4.687844932	0.017977563	0.161542665	609	891	690	510	43	367	24	555
ENSECAG00000011792	2.060379499	0.018011705	0.161622718	52	105	106	161	33	41	33	101
ENSECAG00000011929	6.337837692	0.018037351	0.161622718	468	11	1250	475	3964	616	6183	793
ENSECAG00000016017	5.605731989	0.018038891	0.161622718	1913	2816	352	438	1	594	0	187
ENSECAG00000003398	5.928137374	0.018043256	0.161622718	3787	1052	506	321	98	1269	64	297
ENSECAG00000015547	7.955483498	0.018120136	0.16218376	8529	13721	1522	1457	490	5147	551	1507
ENSECAG00000010300	1.404826987	0.018169701	0.16243937	19	28	16	54	68	68	104	59
ENSECAG00000019157	5.992737662	0.018177229	0.16243937	1362	1735	1093	1599	545	868	1160	1165
ENSECAG00000007499	2.918222429	0.018199443	0.162510323	397	226	35	123	2	135	1	32
ENSECAG00000014898	5.312152503	0.018232285	0.162675993	345	446	621	618	1065	672	1754	943
ENSECAG00000017949	1.828881243	0.018259777	0.162774273	165	88	24	43	19	38	23	62
ENSECAG00000011711	6.201670915	0.018271895	0.162774273	1509	1092	2069	2155	853	1000	1210	1271
ENSECAG00000020103	3.89442056	0.018322103	0.163093938	196	148	555	598	179	162	295	202
ENSECAG00000023690	5.533911391	0.018467575	0.164260421	1341	1727	771	397	394	940	321	561
ENSECAG00000010577	3.076779661	0.018539543	0.164771819	112	509	107	180	14	84	22	201
ENSECAG00000000400	8.055984649	0.018574473	0.164904801	6538	17629	2535	1389	398	4307	398	3249
ENSECAG00000019671	8.868826802	0.018583475	0.164904801	16169	18622	3849	5143	3418	7959	4178	4622
ENSECAG00000018228	6.945868398	0.018657492	0.165432665	2521	8909	1637	880	14	1166	74	1906
ENSECAG00000006350	3.142068197	0.018717597	0.165793548	373	305	25	131	24	61	97	167
ENSECAG00000024301	5.254631102	0.018727317	0.165793548	470	647	1430	1119	459	579	503	707
ENSECAG00000020164	7.928211517	0.018775404	0.166011471	6358	13061	4071	1240	864	5217	647	1982

ENSECAG00000021110	6.904869574	0.018792718	0.166011471	3978	7015	878	678	62	1872	126	1330
ENSECAG00000003955	6.911556905	0.018795677	0.166011471	961	1170	1155	2433	3476	1915	5726	3050
ENSECAG00000014645	4.23202066	0.018854695	0.166403645	86	440	21	58	448	324	1320	426
ENSECAG00000022569	1.78361971	0.01889028	0.166588567	47	45	127	218	0	25	0	63
ENSECAG00000020081	3.661145175	0.019031814	0.167649377	202	247	319	419	234	142	208	165
ENSECAG00000023737	1.245959395	0.019040021	0.167649377	19	11	32	38	39	49	102	83
ENSECAG00000021899	7.074708457	0.019107635	0.168084519	3529	2814	2944	3026	2008	1887	1934	1583
ENSECAG00000005038	6.394624198	0.019118968	0.168084519	1919	2766	1390	1603	646	1437	1247	1315
ENSECAG00000015505	6.739095827	0.019178869	0.168471068	611	985	1801	1977	3114	2935	3079	2862
ENSECAG00000019705	2.676143754	0.019192532	0.168471068	45	42	60	146	176	199	283	84
ENSECAG00000021368	2.704933129	0.019225464	0.168529368	121	85	224	220	64	75	40	148
ENSECAG00000007062	1.399523115	0.019239457	0.168529368	28	83	72	77	16	30	58	31
ENSECAG00000022197	4.840285636	0.019243582	0.168529368	793	1450	271	167	166	481	348	300
ENSECAG00000023081	5.790430003	0.019266178	0.168597565	316	489	1046	1032	1910	1329	1681	1190
ENSECAG00000015869	4.178243069	0.019323843	0.168972317	469	594	309	222	251	218	255	278
ENSECAG00000010153	6.721925649	0.019359745	0.169156325	1149	9665	601	385	92	1562	393	1085
ENSECAG00000016428	6.267565833	0.019385166	0.169248553	541	419	1586	1095	1702	1285	4062	2094
ENSECAG00000015849	7.337709446	0.019463169	0.169718306	826	983	1315	4448	4053	2530	6631	6407
ENSECAG00000011902	6.421688907	0.0194858	0.169718306	3742	2056	1066	656	897	1481	743	766
ENSECAG00000004338	1.290130749	0.019493264	0.169718306	18	42	21	15	87	55	95	43
ENSECAG00000014903	4.41933544	0.019498599	0.169718306	247	219	331	170	802	476	842	303
ENSECAG00000014752	5.661649992	0.019550846	0.17004307	813	1595	1018	1213	640	988	719	635
ENSECAG00000000562	4.265028798	0.019628656	0.170581941	106	203	242	475	531	397	640	554
ENSECAG00000004055	7.063341661	0.019642769	0.170581941	1319	268	1761	1033	9642	1990	4778	708
ENSECAG00000017325	6.428099216	0.019697652	0.170908182	1946	3811	1136	1038	831	1273	1387	1007
ENSECAG00000020482	1.128263248	0.019714525	0.170908182	4	6	23	55	31	70	115	49
ENSECAG00000015394	1.414607986	0.019725371	0.170908182	9	28	17	61	43	49	137	88
ENSECAG00000019507	3.54808357	0.019763065	0.171104563	128	160	309	632	119	82	249	194
ENSECAG00000018694	2.749716475	0.019784519	0.171160145	44	60	65	159	214	124	233	190
ENSECAG00000018054	1.487158109	0.019834788	0.171464743	43	58	59	113	38	36	24	46
ENSECAG00000007646	6.732092594	0.01988164	0.171739353	881	702	1728	1628	4716	1853	3848	1893
ENSECAG00000012397	8.006399677	0.019937515	0.172091435	13279	6498	4368	1683	85	4293	98	2222
ENSECAG00000020763	5.495938437	0.020022251	0.172606644	2024	1295	869	538	6	514	7	652
ENSECAG00000007170	5.375990185	0.020027525	0.172606644	317	380	571	832	531	860	1698	1638
ENSECAG00000000258	5.351500694	0.020229867	0.174042095	516	2592	328	610	327	353	574	743
ENSECAG00000008154	2.278317964	0.020251724	0.174042095	297	37	39	65	32	92	27	12
ENSECAG00000007528	1.244675591	0.020254615	0.174042095	13	13	46	19	103	50	103	23
ENSECAG00000014883	5.22934656	0.020255228	0.174042095	461	336	375	577	1079	422	1875	970
ENSECAG00000019169	5.078442553	0.020455431	0.175629776	483	1142	673	779	479	469	632	481
ENSECAG00000003761	4.869383545	0.020499621	0.175876549	538	1317	480	462	164	662	203	411
ENSECAG00000013685	5.106763005	0.02053457	0.175942374	240	373	629	577	828	873	1391	689
ENSECAG00000020819	2.171451294	0.020538201	0.175942374	53	270	78	66	12	93	23	51
ENSECAG00000009453	2.767532176	0.020564943	0.176039001	60	56	106	92	197	114	324	148
ENSECAG00000017048	4.604697145	0.020612489	0.176226692	1245	449	218	199	98	347	221	387
ENSECAG00000007382	4.782848191	0.020624733	0.176226692	261	226	467	479	739	582	735	920
ENSECAG00000000721	0.947853174	0.020643801	0.176226692	21	38	57	73	27	21	31	19
ENSECAG00000014813	2.319710521	0.020663118	0.176226692	44	55	39	86	168	147	182	67
ENSECAG00000000199	6.653487926	0.020664263	0.176226692	1890	7115	628	821	289	2124	424	816
ENSECAG00000001099	2.06601385	0.020683101	0.176255321	38	30	53	52	208	92	137	45
ENSECAG00000009073	1.522594371	0.020759584	0.176730809	9	15	41	64	74	95	104	56
ENSECAG00000005838	2.626983934	0.020815226	0.176730809	357	179	49	48	0	94	0	25
ENSECAG00000004025	8.266909678	0.020817172	0.176730809	10885	8916	4908	3429	2198	5535	2500	4019
ENSECAG00000014874	4.836361777	0.020828814	0.176730809	216	240	363	683	976	454	900	825
ENSECAG00000014691	10.06485423	0.020841676	0.176730809	31104	50564	10074	12724	4881	23273	6552	8938
ENSECAG00000008655	1.068069666	0.020843934	0.176730809	14	22	10	41	31	53	106	53
ENSECAG00000024817	0.761321217	0.020848937	0.176730809	14	19	21	12	26	35	80	52
ENSECAG00000014175	6.835015418	0.020863084	0.176730809	3568	4572	1399	1344	323	2027	715	1857
ENSECAG00000014015	2.150079101	0.020987509	0.177534987	55	35	46	36	147	151	173	44
ENSECAG00000017042	6.752665784	0.020989204	0.177534987	5159	3641	1114	918	50	1610	37	1300
ENSECAG00000018412	4.428741194	0.021046246	0.177735078	246	245	322	230	705	350	824	508
ENSECAG00000016299	3.887133024	0.02105283	0.177735078	151	340	303	691	139	173	303	247
ENSECAG00000019148	3.399333883	0.021059694	0.177735078	53	106	180	164	376	309	404	112
ENSECAG00000001399	7.804613609	0.021086356	0.177828271	4255	12413	2120	3004	1605	2342	3852	2970
ENSECAG00000017253	2.151443836	0.021145478	0.17810504	18	27	56	105	110	79	127	198
ENSECAG00000021455	6.105177087	0.021150462	0.17810504	1352	1921	1385	1828	572	1404	523	1241
ENSECAG00000003748	2.051529263	0.021209321	0.178200638	18	53	63	50	69	163	82	151
ENSECAG00000021722	4.537501424	0.021235355	0.178200638	451	959	512	374	50	525	137	346
ENSECAG00000013718	5.158010978	0.021242652	0.178200638	295	267	635	618	1125	645	1576	667
ENSECAG00000024058	1.724023362	0.021246062	0.178200638	12	47	41	40	159	80	100	39
ENSECAG00000017469	0.517100424	0.021265734	0.178200638	6	14	17	22	61	37	32	25
ENSECAG00000021652	6.569657397	0.021267533	0.178200638	620	686	1235	2021	1457	2743	1819	4859
ENSECAG00000019432	6.156900489	0.021271381	0.178200638	1609	3468	897	1004	304	1616	308	908
ENSECAG00000023923	1.896455037	0.021395971	0.17910132	40	111	113	98	33	66	48	48
ENSECAG00000022648	1.231402574	0.021410355	0.17910132	10	21	43	22	36	44	65	126
ENSECAG00000009343	4.689620435	0.02143066	0.179139551	242	217	392	495	509	711	717	854
ENSECAG00000017388	1.643628075	0.021478254	0.17931778	24	23	19	72	94	102	104	53

ENSECAG00000014400	4.98214384	0.021483483	0.17931778	274	182	580	495	1309	606	1194	471
ENSECAG00000002449	1.51411048	0.021533598	0.179604408	25	21	44	24	96	56	150	33
ENSECAG00000020861	2.701340035	0.021575147	0.179819216	62	87	62	68	150	102	394	127
ENSECAG00000019708	6.701481182	0.021592969	0.179836102	2846	2979	1078	2720	832	1029	2033	1755
ENSECAG00000014216	7.325395498	0.021616254	0.179898426	3746	6009	2679	2452	1479	2176	1828	2855
ENSECAG00000004462	3.628079675	0.02163812	0.179948868	57	130	190	119	942	182	243	102
ENSECAG00000008273	6.494348326	0.021747086	0.180723048	2360	2579	1605	1657	732	1846	834	1362
ENSECAG00000009733	2.293384569	0.021782645	0.180886513	49	70	220	165	84	57	71	37
ENSECAG00000007383	1.955454209	0.021846409	0.181240455	16	39	48	78	114	66	182	86
ENSECAG00000020945	1.08046137	0.021859122	0.181240455	15	7	36	31	52	41	75	72
ENSECAG00000023946	1.582864789	0.021873025	0.181240455	24	31	14	59	126	46	76	92
ENSECAG00000020790	4.030906418	0.021903644	0.181249138	180	155	239	183	655	283	671	242
ENSECAG00000020491	4.450773579	0.021910921	0.181249138	173	307	340	273	488	298	910	761
ENSECAG00000011779	4.532700667	0.021930883	0.181249138	535	857	345	391	104	437	314	369
ENSECAG00000023177	4.241104199	0.021937752	0.181249138	157	186	249	404	544	332	683	514
ENSECAG00000011223	3.368556301	0.021960934	0.181277871	131	440	393	110	23	127	43	247
ENSECAG00000020407	1.705560199	0.021973075	0.181277871	11	21	29	79	57	56	221	67
ENSECAG00000022243	0.99775779	0.022013272	0.181477985	10	24	27	28	60	42	76	42
ENSECAG00000016595	1.604972494	0.02204199	0.181583255	35	68	86	111	25	27	54	56
ENSECAG00000022935	4.600533415	0.022064016	0.181633277	488	833	393	454	239	399	397	380
ENSECAG00000013263	3.316824853	0.022122257	0.181981142	67	53	168	202	301	172	298	355
ENSECAG00000023126	4.201544846	0.022272434	0.18308423	575	1026	53	122	107	336	165	106
ENSECAG00000021398	8.051457225	0.022325659	0.183389339	3720	11079	5390	5886	2176	4937	2868	3680
ENSECAG00000007403	0.563039903	0.022370956	0.183628935	27	81	11	25	4	25	8	19
ENSECAG00000016702	7.614317756	0.022422807	0.183921946	7917	8282	1767	1551	248	3895	198	1837
ENSECAG00000000462	3.057005907	0.022441672	0.183944161	153	437	120	94	44	122	62	158
ENSECAG00000015175	7.010567424	0.022473079	0.18402875	2275	5103	1614	3367	1123	1872	2124	1849
ENSECAG00000005263	4.771559459	0.022484321	0.18402875	448	1259	373	446	457	270	513	288
ENSECAG00000019659	3.662764015	0.022513759	0.184137314	445	769	108	96	0	188	0	47
ENSECAG00000016168	4.709980188	0.022570597	0.18443636	205	218	253	495	1165	285	1477	235
ENSECAG00000026816	7.298982132	0.022582722	0.18443636	5471	5767	1867	1337	1390	3045	1168	1418
ENSECAG00000023433	0.375656925	0.022620181	0.184517657	8	6	11	21	78	9	42	20
ENSECAG00000016553	2.601582426	0.02262509	0.184517657	50	64	81	93	160	118	140	254
ENSECAG00000020400	1.542986086	0.022659772	0.184668217	44	56	56	133	45	31	23	49
ENSECAG00000024360	6.128543596	0.022703633	0.184776248	2404	1917	1045	796	832	1339	622	723
ENSECAG00000000639	2.043041045	0.022705487	0.184776248	43	49	151	243	6	77	7	60
ENSECAG00000026853	3.277481091	0.022738423	0.184912107	68	87	152	192	231	231	330	274
ENSECAG00000021851	5.183026447	0.022772042	0.184940393	193	491	372	826	680	686	998	1707
ENSECAG00000006824	2.175604394	0.022790335	0.184940393	60	125	88	169	34	70	87	54
ENSECAG00000014580	1.644837055	0.022790634	0.184940393	32	65	60	193	6	17	40	74
ENSECAG00000000773	4.864978836	0.022863538	0.185399846	168	281	454	681	706	624	1039	828
ENSECAG00000022716	6.742684052	0.022898758	0.18555328	3920	5310	1198	1345	6	1594	10	820
ENSECAG00000010204	1.279663152	0.023042061	0.186490289	16	19	25	50	55	52	111	56
ENSECAG00000006429	6.668691196	0.023047153	0.186490289	3311	1738	2553	1443	1062	1250	1057	2018
ENSECAG00000024062	4.46136315	0.023089923	0.186703677	172	180	197	572	411	353	989	774
ENSECAG00000014372	5.832852791	0.023112205	0.186751208	1682	2385	535	545	749	640	1079	413
ENSECAG00000009170	6.692036854	0.023150126	0.186924957	1844	5570	1259	1462	530	2000	753	1528
ENSECAG00000004727	3.279882841	0.02319089	0.18712139	84	21	145	49	885	69	207	70
ENSECAG00000006856	2.968880601	0.023211986	0.187158968	448	123	127	115	0	129	0	33
ENSECAG00000021294	5.824970606	0.023276608	0.187457826	1542	1361	814	1260	591	687	1018	1031
ENSECAG00000020892	3.784742113	0.023281982	0.187457826	67	175	86	298	269	108	799	518
ENSECAG00000000583	3.16867717	0.023318842	0.187621919	397	445	83	45	0	141	0	25
ENSECAG00000022058	1.755756301	0.023359089	0.187813013	120	61	62	56	22	42	40	61
ENSECAG00000025086	5.941458695	0.023417596	0.188150554	931	1675	1283	1855	557	1187	996	913
ENSECAG00000015186	4.719273531	0.023786927	0.190983187	733	866	326	376	266	316	508	396
ENSECAG00000011344	2.953588849	0.023830061	0.19098581	12	111	151	63	203	163	366	200
ENSECAG00000003495	3.802878683	0.023830549	0.19098581	612	372	132	100	136	244	114	133
ENSECAG00000022694	5.053207621	0.023853959	0.19098581	944	1062	354	546	472	313	643	448
ENSECAG00000022249	5.628973383	0.023854354	0.19098581	649	514	398	373	3175	770	1388	480
ENSECAG00000015807	7.078412826	0.023895759	0.191182867	2495	6658	1748	2078	909	2372	2049	1442
ENSECAG00000020368	2.376399133	0.023943706	0.191424244	48	43	56	90	210	84	197	98
ENSECAG00000011535	3.699859784	0.023959556	0.191424244	125	175	140	183	351	168	636	327
ENSECAG00000012791	5.089385154	0.024017801	0.191755025	239	597	283	525	872	328	1320	1399
ENSECAG00000011247	3.544810706	0.02405381	0.191776196	88	105	137	273	285	214	449	352
ENSECAG00000010367	1.900719919	0.024057344	0.191776196	43	24	38	52	133	55	171	73
ENSECAG00000008634	3.321501544	0.02408479	0.191776196	82	75	144	188	308	214	459	152
ENSECAG00000007874	1.435453426	0.024087831	0.191776196	33	88	100	90	1	27	0	56
ENSECAG00000011590	4.489904182	0.024105847	0.191785516	577	1102	303	141	105	490	154	249
ENSECAG00000012050	4.535121845	0.024127689	0.191825236	221	214	401	347	845	404	791	493
ENSECAG00000010858	0.515515624	0.024234003	0.192455035	15	32	49	45	13	10	8	34
ENSECAG00000020767	2.083017439	0.024240713	0.192455035	12	28	37	113	121	46	157	183
ENSECAG00000012132	4.693107506	0.02430808	0.192855392	292	1803	186	368	109	180	273	602
ENSECAG00000017771	3.922320686	0.024335909	0.192917157	347	147	693	280	371	125	166	98
ENSECAG00000023365	4.165078114	0.024349755	0.192917157	108	47	279	364	787	145	450	760
ENSECAG00000014113	7.661533197	0.024412907	0.193179094	8834	4283	2424	2248	1754	3378	1762	2486
ENSECAG00000000735	6.959967206	0.024421055	0.193179094	2508	8089	1103	1021	300	2732	604	1095

ENSECAG00000020010	6.79900348	0.024433719	0.193179094	1518	2175	3657	3488	1888	1012	1836	1699
ENSECAG00000011262	6.470886139	0.02460665	0.194191456	607	566	1315	1428	6042	1503	1670	1081
ENSECAG00000021428	5.588377207	0.024624936	0.194191456	1478	1469	536	720	420	858	704	631
ENSECAG00000020904	3.724678493	0.024632617	0.194191456	400	345	168	236	155	179	183	217
ENSECAG00000009794	7.13675762	0.024642532	0.194191456	3424	5214	1811	2557	1124	2049	1842	2383
ENSECAG00000020213	6.474489136	0.024647049	0.194191456	2674	2376	1179	1749	939	1559	666.001	1506
ENSECAG00000012838	4.815233871	0.024719219	0.194518224	715	733	230	910	263	546	396	352
ENSECAG00000023512	3.1030897	0.024722694	0.194518224	169	273	141	250	24	116	123	180
ENSECAG00000010351	5.081042267	0.024750785	0.19460476	786	1512	504	255	325	420	497	548
ENSECAG00000021887	1.334375618	0.024910615	0.195668434	14	26	41	34	76	67	86	48
ENSECAG00000019932	10.02466972	0.024924708	0.195668434	45084	48783	4997	6027	830	21542	1037	5540
ENSECAG00000009258	4.314362989	0.024937628	0.195668434	282	237	474	1008	232	150	416	362
ENSECAG00000007459	3.052963651	0.0250263	0.196228945	120	102	57	66	236	151	311	255
ENSECAG00000008796	3.935712206	0.025130242	0.19690833	575	500	124	182	66	288	103	235
ENSECAG00000004931	2.799791907	0.025163706	0.197034937	119	69	257	242	106	55	145	73
ENSECAG00000011735	1.886330031	0.025200739	0.197081555	33	100	68	197	16	30	35	94
ENSECAG00000021359	5.712669723	0.025204281	0.197081555	1278	1518	979	793	729	827	688	806
ENSECAG00000015726	3.36020153	0.025263433	0.197408499	135	292	201	454	29	116	67	285
ENSECAG00000012655	6.486341973	0.025314779	0.197516385	4867	2127	456	622	303	1868	284	775
ENSECAG00000005957	4.856968705	0.025314832	0.197516385	366	692	718	1007	545	256	704	243
ENSECAG00000017242	5.577078471	0.025330195	0.197516385	889	1003	1034	1353	858	743	711	635
ENSECAG00000009131	3.107757891	0.025346635	0.197516385	64	107	96	153	170	160	219	413
ENSECAG00000016126	5.210435877	0.025395527	0.197762019	745	898	733	924	695	610	420	492
ENSECAG00000010751	3.812359154	0.025440308	0.197975328	42	123	139	380	284	266	303	783
ENSECAG00000013188	1.921156268	0.025461658	0.198006132	28	40	29	79	92	115	152	66
ENSECAG00000015678	6.702226961	0.025479298	0.19800806	2386	4363	1326	1459	1030	1574	1375	1530
ENSECAG00000008506	1.44728507	0.02555836	0.198486994	25	14	38	39	73	44	147	52
ENSECAG00000021809	1.737058618	0.025699796	0.199410576	45	32	12	29	151	92	115	33
ENSECAG00000006847	9.265134205	0.025712317	0.199410576	12950	22354	11436	11389	5597	11002	9559	5986
ENSECAG00000024918	2.647456758	0.025741099	0.199497901	126	141	193	120	77	131	61	63
ENSECAG00000003382	4.796483831	0.025783066	0.199687216	859	1227	299	292	76	655	67	319
ENSECAG00000023002	1.518907793	0.025810597	0.199764546	53	28	90	157	1	23	5	71
ENSECAG00000023828	4.053792986	0.025852042	0.199949385	715	697	97	89	45	370	18	108
ENSECAG00000009530	7.879815302	0.025877151	0.200007714	1970	1148	4167	2630	12138	5499	8026	2343
ENSECAG00000020007	0.41370166	0.025903149	0.200072827	37	21	14	83	0	0	0	31
ENSECAG00000016457	2.375964075	0.025937413	0.200201656	38	37	59	114	141	125	232	92
ENSECAG00000010506	0.110215093	0.026178807	0.201927996	2	20	1	15	38	27	34	22
ENSECAG00000015790	2.529142914	0.026268322	0.202481277	50	65	57	95	85	103	290	189
ENSECAG00000009090	6.282242466	0.026404327	0.20331812	1931	2541	1248	1128	992	1023	1282	1157
ENSECAG00000019821	5.401602341	0.026412604	0.20331812	1422	1469	431	412	387	769	325	604
ENSECAG00000011309	3.569170607	0.026513506	0.203708086	101	64	221	196	375	223	485	256
ENSECAG00000023888	4.896385268	0.026521695	0.203708086	950	1333	470	401	8	525	8	352
ENSECAG00000014475	5.067454275	0.026524059	0.203708086	398	309	557	305	1235	754	1308	454
ENSECAG00000018681	5.596554301	0.026534835	0.203708086	336	1454	1074	2056	394	573	763	1054
ENSECAG00000001697	4.495788325	0.026568543	0.20382942	471	792	462	374	144	507	136	380
ENSECAG00000024033	2.782095107	0.026617118	0.204064569	32	71	103	129	103	143	246	293
ENSECAG00000023009	10.99458561	0.026750193	0.204946799	94630	49602	16885	17913	25682	22779	24401	18770
ENSECAG00000001164	3.337314292	0.026777712	0.205019672	80	83	165	192	295	193	332	287
ENSECAG00000010546	3.167204517	0.0268397	0.205356173	336	129	171	156	193	60	112	92
ENSECAG00000001714	0.385888338	0.026883276	0.205453248	8	12	8	24	33	12	44	57
ENSECAG00000010425	5.737471256	0.02688848	0.205453248	1283	1926	858	796	392	1261	452	674
ENSECAG00000022885	3.220665893	0.02696028	0.205863707	74	85	126	169	396	140	345	166
ENSECAG00000020137	2.003557084	0.026987258	0.205921878	22	42	74	52	100	73	148	128
ENSECAG00000022507	6.438611723	0.0270107	0.205921878	1337	4325	1239	1454	469	1739	985	1233
ENSECAG00000007620	1.888040054	0.027022159	0.205921878	35	42	26	60	111	56	184	75
ENSECAG00000018574	4.016224682	0.02705212	0.206012302	74	225	110	421	521	287	456	554
ENSECAG00000010260	6.184686735	0.027072063	0.206026359	1742	2588	990	1155	866	1235	1186	796
ENSECAG00000018131	2.537438861	0.027092905	0.206042249	53	56	81	95	160	123	172	178
ENSECAG00000000906	7.066530313	0.027110346	0.206042249	1464	5293	2688	4000	818	2017	1566	2721
ENSECAG00000010087	6.163326437	0.027168692	0.206325866	2212	2074	986	920	1007	867	1141	971
ENSECAG00000002320	4.984502292	0.027183909	0.206325866	432	380	1147	1199	199	402	397	788
ENSECAG00000021405	1.675653057	0.027229402	0.206533469	151	120	22	35	0	66	1	5
ENSECAG00000024139	5.93982433	0.027322034	0.206973153	828	4897	640	290	42	1107	135	728
ENSECAG00000023625	5.770594556	0.027338349	0.206973153	1250	2235	843	602	504	1063	549	777
ENSECAG00000001640	2.917407653	0.027344318	0.206973153	141	427	133	104	2.00073	84	16.0001	163
ENSECAG00000004463	6.519611525	0.027360087	0.206973153	3393	2411	1113	1078	1144	1644	832	980
ENSECAG00000019698	5.646205338	0.027396346	0.207109829	271	508	827	1135	1317	1348	1683	1095
ENSECAG00000001441	6.993789259	0.027419747	0.207149182	834	1042	2027	2565	3038	3346	3651	4253
ENSECAG00000016330	6.535502425	0.027438741	0.207155218	2190	3593	1999	1015	565	2349	500	938
ENSECAG00000024158	3.822083621	0.027462876	0.207200032	60	110	377	140	381	380	460	378
ENSECAG00000004701	4.872266624	0.027483419	0.207217698	360	262	380	419	985	445	1340	518
ENSECAG00000000751	3.136973796	0.027527336	0.207385317	48	75	156	171	179	211	289	297
ENSECAG00000017209	5.397963924	0.027542082	0.207385317	325	813	443	564	1432	728	1431	1031
ENSECAG00000014615	3.291537777	0.027589759	0.207607009	461	170	143	106	26	183	36	158
ENSECAG00000008601	10.21909502	0.027625171	0.207665247	30188	51159	14554	17441	7985	24664	9580	13473
ENSECAG00000021499	6.026569907	0.027658394	0.207665247	2931	463	2220	1675	5	656	1	841

ENSECAG00000015061	7.843722745	0.027679342	0.207665247	5924	7273	3806	3828	2973	3743	2860	2710
ENSECAG00000019025	6.075122824	0.027691155	0.207665247	2404	3148	409	468	104	1411	439	657
ENSECAG00000006582	3.098298555	0.02769386	0.207665247	48	121	113.002	159	252	237	235	203
ENSECAG00000020292	6.279201381	0.02770694	0.207665247	417	905	1125	1732	2178	1920	2406	1999
ENSECAG00000022482	5.494769172	0.027787318	0.208130671	990	1426	807	707	566	679	768	685
ENSECAG00000009631	4.46868665	0.027992947	0.209477223	395	792	469	329	270	373	235	397
ENSECAG00000017025	3.228949321	0.028003894	0.209477223	298	146	183	190	102	136	120	161
ENSECAG00000017970	6.982252901	0.02809707	0.209959794	4031	6294	1895	821	53	2470	138	1318
ENSECAG00000004312	7.608115789	0.028123539	0.209959794	7956	6249	3418	1854	155	2697	114	3478
ENSECAG00000009629	4.860273626	0.028123732	0.209959794	318	438	1145	975	445	520	516	180
ENSECAG00000024169	4.627989504	0.028145463	0.209984338	209	146	433	488	824	412	913	596
ENSECAG00000009707	1.942481995	0.028185264	0.210143568	55	28	109	218	48	31	26	78
ENSECAG00000009535	10.14241588	0.028340522	0.211162854	43898	46060	11540	11816	675	22878	1568	11482
ENSECAG00000016133	2.156143125	0.028412798	0.211562919	97	286	48	44	1	97	6	28
ENSECAG00000021267	3.918212269	0.028557235	0.212499422	189	343	350	575	124	275	190	295
ENSECAG00000016495	2.741667179	0.02860233	0.212695967	73	59	57	103	244	121	341	74
ENSECAG00000018585	3.946294692	0.028687804	0.21319233	659	595	89	79	86	337	42	113
ENSECAG00000017865	3.556450986	0.028719874	0.213204722	124	94	218	115	350	189	542	256
ENSECAG00000023733	12.14398138	0.028726925	0.213204722	412179	7274	7069	7230	47	41309	16	8621
ENSECAG00000024354	6.802813449	0.028808259	0.213531332	2829	2433	2451	2503	1404	2442	1152	1033
ENSECAG00000000470	6.262211657	0.028808443	0.213531332	538	1808	1512	4796	431	508	579	2265
ENSECAG00000009100	2.119587524	0.028860298	0.21364054	301	23	27	29	12	43	11	74
ENSECAG00000022964	1.195783032	0.028860707	0.21364054	19	22	27	32	54	34	122	50
ENSECAG00000011141	2.629702093	0.028936214	0.213948917	67	127	122	312	72	80	69	117
ENSECAG00000007626	6.811187848	0.02893995	0.213948917	1340	6144	1703	1840	770	1385	1369	2226
ENSECAG00000014087	4.188849226	0.029150029	0.21521231	110	123	142	474	201	243	542	1193
ENSECAG00000002133	3.096344522	0.029156371	0.21521231	67	107	101	160	297	171	279	185
ENSECAG00000015871	2.081614151	0.029167553	0.21521231	34	22	47	92	134	121	164	60
ENSECAG00000014746	6.404957626	0.029323916	0.216225898	764	1558	1580	5169	722	755	1318	1891
ENSECAG00000022051	10.77711534	0.029398703	0.216637042	68350	29198	26631	33616	15301	31053	14675	24095
ENSECAG00000010196	7.848753364	0.029444222	0.216832127	3928	13860	2900	3359	438	5308	625	2562
ENSECAG00000012770	5.260556858	0.029508005	0.21716137	319	440	569	766	1213	883	929	1018
ENSECAG00000000572	2.809259434	0.029550949	0.217257615	31	50	95	173	237	121	283	160
ENSECAG00000016061	5.507590078	0.029579623	0.217257615	780	1441	826	1032	464	666	719	928
ENSECAG00000026927	4.847475776	0.029589165	0.217257615	675	1130	396	403	296	643	211	357
ENSECAG00000002866	6.054079854	0.029597414	0.217257615	774	667	767	927	2841	1006	2737	942
ENSECAG00000024198	2.536303907	0.029622958	0.21730501	45	47	82	103	187	76	264	134
ENSECAG00000023172	8.055381442	0.029651372	0.217373384	5521	7964	5527	6358	1565	4850	2478	5058
ENSECAG00000008545	3.100932563	0.029712741	0.217683115	174	81	279	448	7	102	8	199
ENSECAG00000018094	3.216763994	0.029739507	0.217738693	176	476	170	123	18	250	28	72
ENSECAG00000022162	7.972802135	0.029758578	0.217738693	3575	5437	4569	11746	2555	2643	5233	3859
ENSECAG00000016500	7.638715774	0.029821112	0.218056111	4328	5830	4180	4044	1698	3065	2425	3826
ENSECAG00000013081	9.401017942	0.029992669	0.219169789	28660	21346	9769	12101	58	11094	127	6421
ENSECAG00000019606	4.052145328	0.030062605	0.219539935	258	251	417	634	315	201	294	200
ENSECAG00000018696	1.85751671	0.030104161	0.219702484	28	37	44	56	144	110	100	44
ENSECAG00000024847	8.742168949	0.030228761	0.220347155	23899	14614	2752	1071	110	8124	130	1718
ENSECAG00000006022	3.999157113	0.030246613	0.220347155	301	410	355	341	208	261	300	218
ENSECAG00000014251	4.509497132	0.030250558	0.220347155	923	1001	158	242	5	449	9	191
ENSECAG00000012354	1.198570997	0.03028709	0.220472201	18	12	42	29	42	57	74	81
ENSECAG00000014198	5.569285099	0.030341965	0.220598792	869	1074	844	1547	508	578	706	1124
ENSECAG00000018970	5.834506769	0.030343233	0.220598792	343	601	754	1409	1149	1311	1723	2059
ENSECAG00000012018	1.051696591	0.030397245	0.22074399	12	9	35	34	52	41	108	36
ENSECAG00000019275	0.476199491	0.030411561	0.22074399	39	9	49	32	25	17	10	8
ENSECAG00000022676	5.423464729	0.030421372	0.22074399	1651	166	1315	735	516	613	640	386
ENSECAG00000011834	6.893767419	0.030532833	0.221411659	2935	5439	2029	1692	167	2310	196	1961
ENSECAG00000018788	6.979100587	0.030648026	0.22210552	1758	4221	3041	2840	1517	1895	2040	1857
ENSECAG00000023753	4.775805379	0.030731019	0.222565299	360	400	1114	837	178	576	214	526
ENSECAG00000013384	0.300947097	0.03076316	0.222656437	3	14	11	23	36	13	43	44
ENSECAG00000006674	1.273890544	0.03083191	0.223012262	41	16	125	58	33	38	19	21
ENSECAG00000018755	3.576891349	0.030921405	0.22346744	132	106	133	235	287	227	459	337
ENSECAG00000014929	2.410915317	0.030934096	0.22346744	23	63	76	99	212	97	131	148
ENSECAG00000015413	5.678051788	0.030973567	0.223610692	382	290	573	1294	2627	785	1661	756
ENSECAG00000024457	8.856676493	0.031005246	0.223697548	13595	20715	5082	5283	1811	11186	2038	4267
ENSECAG00000007961	5.139286389	0.031111531	0.224221711	207	364	543	739	481	730	1035	1673
ENSECAG00000010189	3.173797176	0.031125895	0.224221711	91	45	160	105	392	149	371	129
ENSECAG00000026935	5.897182011	0.031136981	0.224221711	2144	1811	650	650	442	1330	444	678
ENSECAG00000025155	1.335593235	0.03125306	0.224830466	13	12	30	60	97	67	45	69
ENSECAG00000022883	8.127630773	0.031261013	0.224830466	8755	10802	2772	3915	1277	6010	1551	3685
ENSECAG00000014364	6.043162947	0.031409218	0.225753757	2263	1289	1206	995	522	1502	584	855
ENSECAG00000020144	5.380136309	0.031451418	0.225914444	563	418	463	375	1770	690	1920	430
ENSECAG00000009639	0.910752334	0.03149578	0.226090454	16	16	13	27	82	28	97	12
ENSECAG00000013497	2.62013154	0.03155256	0.226355319	46	79	400	153	138	30	87	13
ENSECAG00000023782	0.253286604	0.031580743	0.226414836	5	10	1	29	16	35	41	43
ENSECAG00000011905	1.674585004	0.031720726	0.227275311	37	47	56	190	35	40	39	51
ENSECAG00000013504	4.506763658	0.031826093	0.227788503	302	213	239	252	925	282	1147	285
ENSECAG00000002700	2.610227054	0.031832368	0.227788503	38	33	136	63	284	121	216	83

ENSECAG00000013595	6.773340213	0.031864349	0.227874132	2384	5401	1348	1310	610	2639	937	938
ENSECAG00000013437	4.888140765	0.031886474	0.227889206	328	208	418	556	1109	442	1017	681
ENSECAG00000002984	1.372901949	0.03196407	0.228300461	23	16	43	20	91	26	46	133
ENSECAG00000008116	2.162618619	0.032142205	0.229428845	44	47	57	30	169	58	260	49
ENSECAG00000019484	6.955578507	0.03216852	0.229472806	2757	3972	2093	2419	1281	2089	1744	1878
ENSECAG00000015967	1.071421579	0.032214304	0.229655513	14	21	18	40	72	57	80	23
ENSECAG00000011493	5.802887017	0.032304578	0.230154957	1924	332	2764	386	1395	273	515	198
ENSECAG00000016196	2.514128365	0.032420411	0.23083576	58	92	195	256	35	51	45	157
ENSECAG00000014100	1.677725469	0.032515947	0.231371283	96	140	46	18	10	66	15	37
ENSECAG00000009653	3.679317946	0.032586739	0.231730183	208	351	370	324	55	262	65	272
ENSECAG00000016329	1.695237749	0.032719668	0.232530224	56	163	55	31	16	31	48	61
ENSECAG00000014074	5.357520596	0.032831591	0.233180074	238	486	138	1099	683	524	1898	1816
ENSECAG00000010483	4.845321298	0.032860189	0.233237687	820	762	420	444	333	505	412	424
ENSECAG00000006372	0.463480963	0.03290049	0.233378242	42	47	30	8	7	33	7	4
ENSECAG00000014962	3.183966033	0.03297088	0.233724767	51	86	183	118	334	289	283	104
ENSECAG00000009148	2.153375777	0.0329904	0.233724767	15	48	55	95	173	83	179	72
ENSECAG00000021542	4.396666633	0.033071427	0.234082574	482	638	439	337	107	325	236	505
ENSECAG00000011864	7.296621578	0.033082026	0.234082574	3531	7865	1675	2186	562	3710	1115	1690
ENSECAG00000008295	5.213046902	0.033118277	0.234193527	552	253	1825	2121	3	427	3	579
ENSECAG00000017598	2.793069151	0.033238455	0.234897464	174	339	113	99	2	168	9	50
ENSECAG00000011623	4.210403838	0.033272202	0.234901638	200	213	268	275	616	340	653	383
ENSECAG00000003025	3.654272635	0.033280311	0.234901638	221	477	375	206	20	306	35	180
ENSECAG00000004611	2.004612991	0.033318868	0.235028075	107	252	33	27	2	86	2	25
ENSECAG00000004669	2.603522051	0.033415533	0.235563988	107	45	8	24	281	136	232	101
ENSECAG00000014202	2.997170523	0.033501292	0.236022406	87	86	93	99.0001	297	180	335	87
ENSECAG00000009119	7.242389858	0.033674359	0.237094975	4291	6248	1355	1978	1050	2785	1568	2046
ENSECAG00000009996	0.17663595	0.033744621	0.237442836	7	7	10	23	32	42	25	19
ENSECAG00000009899	3.365895021	0.033772515	0.237492333	65	124	144	188	362	107	370	314
ENSECAG00000019730	1.017897774	0.033943922	0.238550342	21	36	26	128	18	18	44	21
ENSECAG00000022722	5.222358835	0.033994192	0.23871078	443	2378	432	484	156	914	228	401
ENSECAG00000023398	4.194684775	0.034008686	0.23871078	835	667	50	75	140	281	209	127
ENSECAG00000014055	4.206998432	0.034038108	0.238770091	207	192	208	303	703	227	772	357
ENSECAG00000010702	4.398748614	0.034128514	0.239116199	452	1693	143	133	0	194	1	264
ENSECAG00000024318	3.841924318	0.034136286	0.239116199	365	936	73	95	20	331	18	107
ENSECAG00000006850	4.21782625	0.034158208	0.239116199	175	256	225	323	414	307	776	532
ENSECAG00000018403	0.598520847	0.034171459	0.239116199	17	45	22	65	13	33	14	10
ENSECAG00000018443	3.232638081	0.034323091	0.240017415	84	80	106	218	255	230	322	215
ENSECAG00000017955	6.694592862	0.034342413	0.240017415	2804	2752	1649	2054	1056	2125	916	1550
ENSECAG00000024168	6.386809034	0.034373428	0.240086798	3121	2902	639	762	523	1765	686	939
ENSECAG00000017074	1.967694704	0.034421006	0.240188231	24	33	60	75	128	92	135	74
ENSECAG00000019394	4.578581045	0.034430144	0.240188231	243	243	441	288	833	391	875	505
ENSECAG00000026866	3.067545195	0.03447955	0.240212221	119	165	168	345	119	97	160	127
ENSECAG00000001037	5.589851446	0.034484215	0.240212221	814	1737	749	1089	670	312	1134	803
ENSECAG00000014764	1.034992993	0.03449688	0.240212221	21	15	22	26	82	17	78	54
ENSECAG00000015331	1.283259597	0.034569587	0.240571363	36	36	109	57	7	25	49	41
ENSECAG00000022696	0.662185448	0.034599314	0.240631146	51	18	38	41	12	16	2	36
ENSECAG00000006067	5.692156875	0.034640103	0.240637448	1710	1794	691	499	218	1374	327	470
ENSECAG00000001996	1.817497882	0.034657892	0.240637448	21	245	55	39	16	60	29	45
ENSECAG00000008705	10.03300338	0.034663629	0.240637448	33868	42296	11479	11150	3969	24733	5281	10753
ENSECAG00000022072	9.328793824	0.034720221	0.24071368	6797	5311	7788	7654	13587	9366	45257	11121
ENSECAG00000009290	7.671249926	0.034737667	0.24071368	5732	8725	2017	2455	864	3239	1941	3724
ENSECAG00000010080	5.051609376	0.034738039	0.24071368	765	984	460	656	452	554	526	475
ENSECAG00000017883	6.019530331	0.034774158	0.240817393	2094	1980	511	1016	864	790	1093	842
ENSECAG00000009572	3.581672273	0.034857248	0.241246059	267	427	167	211	108	246	127	177
ENSECAG00000016494	6.695759789	0.0349516	0.241556858	3466	3539	1048	1118	697	1843	1105	1560
ENSECAG00000007264	0.071658851	0.034965078	0.241556858	8	10	14	10	36	24	26	23
ENSECAG00000013151	7.232329119	0.034965806	0.241556858	3964	1034	7967	1531	2010	1873	1133	2206
ENSECAG00000009355	2.692774297	0.035062002	0.241705161	50	69	114	85	176	135	279	127
ENSECAG00000024290	7.302147872	0.035069261	0.241705161	3359	5955	2948	2708	945	3407	954	2576
ENSECAG00000019830	7.258005611	0.035073545	0.241705161	2767	11009	1413	1780	26	2002	82	2477
ENSECAG00000007977	0.9839543	0.035088334	0.241705161	23	80	56	29	14	34	12	33
ENSECAG00000023335	1.078293873	0.035093424	0.241705161	8	7	46	15	146	14	33	55
ENSECAG00000000487	1.883085137	0.035182597	0.242172836	32	57	29	23	102	204	81	30
ENSECAG00000006407	5.555956818	0.035271716	0.242593948	509	1206	2009	2010	12	680	6	796
ENSECAG00000017226	2.256839522	0.035300862	0.242593948	35	42	81	67	67	90	195	190
ENSECAG00000003121	10.71473235	0.035307701	0.242593948	47415	64165	25609	22395	7306	39561	8399	20345
ENSECAG00000000288	8.294701515	0.03540785	0.242973683	23947	2973	2360	2497	3	3997	0	228
ENSECAG00000008951	1.930711581	0.035414426	0.242973683	23	79	27	36	66	123	174	73
ENSECAG00000008319	7.294548148	0.035426993	0.242973683	1469	1129	2612	1954	6962	2510	6685	2128
ENSECAG00000021430	7.638262859	0.035473796	0.243052913	1837	2017	1892	4121	4834	3662	6183	7824
ENSECAG00000013655	4.435948499	0.035482849	0.243052913	232	287	219	290	225	483	1304	491
ENSECAG00000018613	4.138927636	0.035535254	0.243052913	286	298	418	612	194	233	346	337
ENSECAG00000016534	6.286226029	0.035537736	0.243052913	2546	3929	781	331	301	1956	121	465
ENSECAG00000001514	6.20192397	0.035545288	0.243052913	1702	4020	1081	1036	23	1896	85	416
ENSECAG00000024278	3.171531557	0.035611664	0.243360624	82	62	136	168	280	122	368	239
ENSECAG00000022776	6.068373659	0.035647811	0.243461508	1064	2572	2075	1088	202	1068	140	1663

ENSECAG00000001262	2.341660541	0.03569869	0.243569031	56	82	155	212	45	64	65	106
ENSECAG00000017213	1.972551118	0.03572274	0.243569031	16	23	78	65	162	54	147	86
ENSECAG00000009903	2.136591448	0.035727737	0.243569031	22	39	51	69	18	73	154	288
ENSECAG00000003315	7.213501848	0.035806691	0.243961205	3597	2133	3311	5200	790	2834	1600	2713
ENSECAG00000007739	3.256278373	0.035846349	0.244085337	83	102	122	127	412	142	472	82
ENSECAG00000009799	5.039650319	0.035919681	0.244438476	938	154	1302	668	415	542	264	485
ENSECAG00000010956	0.590467644	0.036137337	0.245566183	8	17	22	14	46	51	57	11
ENSECAG00000012142	5.48192165	0.036178079	0.245566183	967	1819	828	486	358	1018	252	664
ENSECAG00000015326	4.206534123	0.036200519	0.245566183	154	155	359	327	499	454	521	487
ENSECAG00000024047	8.196864364	0.036205405	0.245566183	10068	12545	2915	1952	1115	6807	1393	2806
ENSECAG00000020416	8.745147161	0.036208065	0.245566183	12005	12522	6485	8114	3469	8003	3975	6876
ENSECAG00000012634	3.94659463	0.036214811	0.245566183	198	599	297	312	224	263	242	187
ENSECAG00000007320	3.421446019	0.036253282	0.245680721	136	350	306	204	87	181	133	215
ENSECAG00000023134	1.939185093	0.036304035	0.245878308	18	44	51	63	152	60	185	44
ENSECAG00000018649	9.407145307	0.036398426	0.246371035	16574	30099	10509	5903	5910	11948	5880	9358
ENSECAG00000010808	4.161441818	0.036428248	0.246426383	297	171	826	427	276	111	427	216
ENSECAG00000019430	3.8522493	0.036480753	0.246635021	125	253	102	212	255	197	758	455
ENSECAG00000023555	0.791601347	0.036534951	0.246854849	13	22	16	26	53	49	53	29
ENSECAG00000024790	2.987789772	0.036623368	0.247161698	234	149	138	334	2	142	8	132
ENSECAG00000022118	6.329940057	0.036623784	0.247161698	660	1814	168	379	2758	1146	4852	1362
ENSECAG00000014952	6.588041917	0.036699385	0.247525178	2526	5296	884	638	234	2120	354	1211
ENSECAG00000018600	10.97234105	0.036808061	0.247956659	79734	30425	33813	31464	19897	27605	26248	29516
ENSECAG00000008083	2.312927662	0.036837746	0.247956659	70.0016	120.002	83	200.038	56.0005	72.0008	85	73.0002
ENSECAG00000016324	3.475375107	0.036844132	0.247956659	589	122	161	60	74	177	117	136
ENSECAG00000020336	5.979372318	0.036850476	0.247956659	1352	2844	756	730	624	1090	694	1005
ENSECAG00000005863	9.759494627	0.036886698	0.248053782	20163	28308	20953	15556	3254	19909	2945	13494
ENSECAG00000013139	3.844444362	0.036963401	0.248422854	347	398	222	269	227	187	247	203
ENSECAG00000006218	1.404406974	0.037005659	0.248560136	50	196	20	17	1	43	0	29
ENSECAG00000020227	8.139802677	0.037066484	0.248599005	6602	11130	4786	3669	2156	6199	2428	3389
ENSECAG00000007580	4.469115137	0.037067645	0.248599005	480	928	231	322	217	338	384	324
ENSECAG00000009472	2.725128222	0.037088961	0.248599005	68	45	70	127	265	132	259	88
ENSECAG00000024746	1.37386553	0.037098789	0.248599005	40	34	60	122	37	13	57	31
ENSECAG00000020037	2.452692018	0.037197281	0.249112381	64	38	55	88	123	61	273	178
ENSECAG00000019874	5.903165724	0.037235725	0.249140892	672	736	673	981	1281	920	2553	1863
ENSECAG00000018503	3.51591907	0.037245305	0.249140892	106	126	136	241	263	266	358	335
ENSECAG00000020111	0.639029456	0.037321789	0.249505915	20.0106	12.0001	8.00789	23.0001	49.0006	38.0007	39.0001	41.0239
ENSECAG00000003836	2.214668843	0.037513339	0.250441843	65	173	67	132	54	76	48	74
ENSECAG00000003632	7.27422983	0.037520532	0.250441843	1845	1028	1855	1882	9427	2706	3602	2068
ENSECAG00000010327	6.716231457	0.037565216	0.250441843	1076	731	1926	1102	4466	2035	4222	1335
ENSECAG00000021968	10.50591619	0.037574602	0.250441843	71917	33181	15931	8867	4723	30598	5942	16424
ENSECAG00000013248	0.737586122	0.037593795	0.250441843	25	27	55	49	18	13	43	14
ENSECAG00000006195	3.105740982	0.037620259	0.250441843	330	257	188	155	0	45	0	185
ENSECAG00000019946	6.034841424	0.03763069	0.250441843	1537	1812	1071	1350	606	1332	773	1123
ENSECAG00000014660	8.328406028	0.037637768	0.250441843	9081	16625	2580	2806	1830	6653	697	3881
ENSECAG00000012393	7.043437774	0.037663444	0.250466306	3927	5511	1451	984	1498	2264	1218	1466
ENSECAG00000022290	4.636666029	0.037722513	0.250620712	1257	513	174	167	188	540	178	191
ENSECAG00000007505	6.415272782	0.03773069	0.250620712	1035	1667	2422	3010	1023	1035	1974	1282
ENSECAG00000012627	1.051747736	0.037799304	0.250930075	3	30	23	31	35	78	25	93
ENSECAG00000015346	4.561366698	0.037834385	0.251016594	117	7	217	442	1445	32	1475	277
ENSECAG00000007487	1.297360406	0.037923454	0.251460991	20	19	24	52	52	55	113	52
ENSECAG00000013623	7.655014096	0.038034905	0.252053195	5708	4422	2411	5735	2328	1864	3691	3528
ENSECAG00000024723	9.714744895	0.038094433	0.252300826	27829	28068	11885	8359	6836	18761	6473	7546
ENSECAG00000018808	7.307015303	0.038194766	0.252818262	9059	4634	2051	272	17	2761	4	497
ENSECAG00000005770	6.824098736	0.038272735	0.253187151	3942	3315	1705	907	1205	2059	1334	1165
ENSECAG00000013486	9.496522764	0.038326997	0.253398874	36249	22990	4327	5884	340	14504	641	6888
ENSECAG00000011343	4.649596512	0.038359425	0.253466078	267	323	330	387	700	883	622	426
ENSECAG00000016722	6.216770308	0.038432887	0.253733616	1798	4617	700	286	160	1730	208	713
ENSECAG00000010339	8.797803627	0.038444487	0.253733616	21585	5794	8940	3412	993	7013	2790	8087
ENSECAG00000016244	4.899430837	0.03866062	0.254925573	208	339	540	582	721	631	1039	784
ENSECAG00000011078	5.783495108	0.038669869	0.254925573	688	942	1738	1750	909	780	939	847
ENSECAG00000005086	3.965540123	0.038727748	0.25515938	309	284	549	320	105	143	141	479
ENSECAG00000010251	4.045508706	0.038766437	0.255177294	248	521	297	491	90	253	195	411
ENSECAG00000018058	1.463448035	0.038775294	0.255177294	27	42	34	16	70	85	84	61
ENSECAG00000011637	6.204257615	0.039022006	0.256652537	322	532	1779	947	3839	1220	2182	1167
ENSECAG00000012006	6.798298229	0.039068177	0.256807852	1191	1677	4080	4289	1725	1074	2661	998
ENSECAG00000013689	4.53997616	0.039105322	0.256903688	193	283	381	430	527	479	838	625
ENSECAG00000021080	3.351613814	0.039135897	0.256956281	72	106	136	230	251	172	335	353
ENSECAG00000023882	1.639695109	0.039272147	0.257647558	77	57	68	77	24	45	34	65
ENSECAG00000003905	3.446687097	0.039286444	0.257647558	172	346	159	330	124	99	181	246
ENSECAG00000022232	0.003195903	0.039355892	0.257954419	27	13	27	21	9	21	9	5
ENSECAG00000007942	9.307002363	0.039425415	0.258199528	28421	19700	5943	3962	1824	15438	2341	4807
ENSECAG00000012059	4.933896709	0.039451994	0.258199528	97	386	404	784	770	899	1130	570
ENSECAG00000026969	2.921998667	0.039461324	0.258199528	55	101	203	507	18	73	63	202
ENSECAG00000013677	5.865946333	0.039663323	0.259372164	847	3155	1050	709	165	1299	275	980
ENSECAG00000015006	4.689699632	0.03978198	0.259998762	849	1440	206	217	7	475	2	242
ENSECAG00000018828	5.355238622	0.03983769	0.260213482	1311	2855	168	193	2	404	0	471

ENSECAG00000023628	3.22099255	0.03988883	0.260398127	304	294	77	148	100	201	89	78
ENSECAG00000013348	1.370885053	0.039917855	0.26043827	28	41	24	15	53	60	95	79
ENSECAG00000011718	0.319120562	0.040048833	0.261143164	9	15	14	15	44	23	40	24
ENSECAG00000021545	6.179624774	0.040075155	0.261165219	494	599	824	1884	1052	1219	3046	2946
ENSECAG00000018824	4.471867499	0.040132618	0.261335531	517	813	326	268	240	316	295	433
ENSECAG00000010678	3.987376214	0.040147198	0.261335531	212	643	311	292	142	331	187	249
ENSECAG00000012573	6.779119323	0.040295287	0.262149625	1439	1151	2367	6486	1338	1013	1910	1962
ENSECAG00000004973	7.750453362	0.040347794	0.262337259	621	2669	2097	5080	7309	4860	11006	2488
ENSECAG00000024816	0.923989881	0.040370213	0.262337259	23	14	21	14	70	23	99	26
ENSECAG00000003702	2.61914556	0.040438252	0.262463332	250	40	98	213	14	162	18	49
ENSECAG00000003137	3.058954229	0.040452382	0.262463332	65	91	125	160	219	176	256	241
ENSECAG00000019111	8.733863438	0.040458774	0.262463332	11338	2901	29883	7749	13	4465	2	4527
ENSECAG00000013322	3.088292065	0.040518087	0.26269842	131	293	208	192	36	142	46	211
ENSECAG00000004387	5.178243329	0.040571871	0.26280496	902	1007	605	561	423	470	610	712
ENSECAG00000024495	3.368663998	0.040580687	0.26280496	96	86	164	169	258	143	544	223
ENSECAG00000001058	7.135580004	0.040606408	0.262822033	3123	3762.01	2734.01	3309.01	865.001	1841.01	2035	3239.01
ENSECAG00000018769	4.009202176	0.040689836	0.263212374	138	212	227	306	383	365	555	399
ENSECAG00000012496	5.241650715	0.04074684	0.263431442	748	1420	584	605	318	549	478	868
ENSECAG00000023347	7.660955677	0.040891402	0.264216012	7830	9551	1407	1553	132	4934	108	990
ENSECAG00000021220	2.757683714	0.040978731	0.264630093	35	79	106	129	117	215	197	205
ENSECAG00000018548	3.989633193	0.04104221	0.264744156	207	366	564	317	213	327	214	199
ENSECAG00000018322	1.535704473	0.041060305	0.264744156	33	22	21	54	84	84	109	42
ENSECAG00000020631	3.683091134	0.041066155	0.264744156	185	396	257	330	112	180	239	257
ENSECAG00000005857	1.374116173	0.041096539	0.264790094	11	35	41	28	41	118	43	79
ENSECAG00000000313	2.72481579	0.041268103	0.265745108	56	65	74	129	134	81	257	273
ENSECAG00000002321	6.366284586	0.041329258	0.265988469	1144	689	1005	1013	3659	1267	2853	1431
ENSECAG00000023981	8.25897531	0.041417043	0.266377777	6727	12175	4639	4672	2686	6287	3475	3688
ENSECAG00000017012	7.274712899	0.041436543	0.266377777	4619	3844	2163	3219	1098	2902	1663	2682
ENSECAG00000011144	5.181833434	0.04153785	0.266878343	632	556	791	1351	792	420	502	544
ENSECAG00000001080	2.909832566	0.041585469	0.267033599	62	64	143	107	181	164	334	153
ENSECAG00000017647	1.685680398	0.041649494	0.267160835	30	36	27	60	83	62	139	70
ENSECAG00000017134	5.363206267	0.041652216	0.267160835	1316	1503	577	434	126	1157	122	396
ENSECAG00000026984	7.018185074	0.041703891	0.267341668	2223	6397	1969	1618	1155	2836	1352	1306
ENSECAG00000003599	7.227486637	0.041736988	0.267403269	5418	7270	923	1008	201	3637	242	1160
ENSECAG00000018086	5.251784879	0.041791962	0.267486119	595	1780	593	690	128	710	261	858
ENSECAG00000003387	3.957628122	0.041796909	0.267486119	177	196	223	198	490	269	577	336
ENSECAG00000009787	3.603463835	0.04183278	0.267565278	67	123	772	353	113	231	55	180
ENSECAG00000020311	2.719100157	0.0419696	0.268063819	440	134	10	13	5	121	7	46
ENSECAG00000021312	4.104454185	0.041981074	0.268063819	76	212	312	341	422	515	581	333
ENSECAG00000023478	6.647768464	0.04198136	0.268063819	2791	4600	1581	1253	58	2388	99	1071
ENSECAG00000000284	1.396720147	0.042076597	0.268451188	25	30	28	38	61	50	129	51
ENSECAG00000015438	2.720730859	0.042089185	0.268451188	33	62	123	124	208	153	198	157
ENSECAG00000004906	5.400789021	0.042215355	0.269105162	222	511	321	1187	560	845	1561	1814
ENSECAG00000011450	5.689961727	0.042312514	0.26957357	524	521	737	953	1865	885	1921	909
ENSECAG00000009293	4.55473934	0.042449661	0.27029608	205	276	330	492	776	476	666	546
ENSECAG00000007789	1.929047548	0.042489766	0.270400215	40	18	40	75	58	81	113	176
ENSECAG00000016002	2.608017496	0.042539791	0.27056733	59	40	90	93	268	95	246	80
ENSECAG00000009336	7.878422519	0.042681113	0.271274006	1396	1665	2829	6136	6057	3617	8751	8703
ENSECAG00000017465	4.319657912	0.042698552	0.271274006	187	258	349	265	698	371	619	427
ENSECAG00000009608	5.093987679	0.042740506	0.271389104	832	1194	381	526	493	406	578	559
ENSECAG00000005360	1.043804499	0.042764421	0.271389593	17	35	120	64	0	17	1	50
ENSECAG00000018147	3.890559639	0.042827201	0.271587377	174	143	139	289	401	200	709	346
ENSECAG00000020898	5.370538985	0.042846257	0.271587377	215	515	499	1055	1632	960	1043	798
ENSECAG00000016508	10.00363637	0.042884468	0.271587377	40652	32535	13124	16396	477	22214	698	11407
ENSECAG00000021518	3.64692194	0.042891006	0.271587377	280	290	283	249	87	226	185	244
ENSECAG00000016707	5.199225635	0.042973482	0.271958359	401	447	526	604	1051	629	1331	894
ENSECAG00000022135	6.749804743	0.043046152	0.272120431	1829	9175	1564	190	3	1277	0	757
ENSECAG00000016328	2.913771501	0.043046895	0.272120431	37	55	689	68	39	69	29	165
ENSECAG00000006476	6.661631598	0.04318689	0.272853906	1842	3093	1841	2333	1218	1065	2135	1797
ENSECAG00000004770	3.846832841	0.043294737	0.273383575	346	735	109	106	163	199	211	164
ENSECAG00000016662	3.663224246	0.043352093	0.273594004	164	241	356	413	156	225	181	218
ENSECAG00000022880	4.023859542	0.043384751	0.273625011	365	403	501	249	67	314	78	378
ENSECAG00000012768	4.936325615	0.043416685	0.273625011	1553	1289	41	101	2	563	67	207
ENSECAG00000023705	5.181781003	0.043429108	0.273625011	340	429	498	648	1338	452	1453	717
ENSECAG00000016273	5.506600748	0.043464405	0.273662565	880	971	1256	863	509.002	842	613	859
ENSECAG00000020784	3.304227327	0.043483143	0.273662565	138	310	210	245	146	123	242	91
ENSECAG00000016578	5.069410047	0.043750659	0.275194065	1316	919	567	409	41	614	21	628
ENSECAG00000012930	6.869952742	0.043825972	0.275515565	5164	5143	578	958	8	2566	75	534
ENSECAG00000013597	0.131295456	0.043883409	0.275724399	14	24	21	44	7	18	6	20
ENSECAG00000025099	5.523670326	0.043931051	0.275765385	579	447	2378	2565	0	585	5	613
ENSECAG00000001481	5.936077067	0.043938376	0.275765385	863	4210	285	635	330	498	1105	1058
ENSECAG00000018133	9.332798539	0.043996508	0.27584365	19343	26476	9659.01	6545.02	1037	15886	2722.01	7235.01
ENSECAG00000022842	6.435882415	0.044003971	0.27584365	1772	4136	1531	1165	178	2357	203	968
ENSECAG00000022471	5.08862659	0.044038406	0.27584365	706	661	763	830	698	396	690	383
ENSECAG00000022695	4.208429883	0.044047761	0.27584365	195.002	290	690	695	123.001	350.001	108	424
ENSECAG00000024492	2.399609991	0.044080016	0.275893888	37	61	82	82	120	100	119	234

ENSECAG00000013749	5.099971752	0.044134696	0.275957917	981	662	663	555	446	560	583	515
ENSECAG00000012789	6.219672603	0.044138723	0.275957917	2982	3776	427	435	5	1316	25	719
ENSECAG00000014934	2.50295194	0.044242674	0.276456004	45	65	79	99	132	108	150	221
ENSECAG00000013018	1.09963563	0.044349372	0.276725327	25	19	21	26	62	51	100	24
ENSECAG00000015699	3.872539049	0.044363111	0.276725327	599	703	114	146	0	259	0	75
ENSECAG00000014046	2.870482507	0.044391191	0.276725327	52	122	66	123	167	106	344	205
ENSECAG00000023066	3.289113821	0.044405695	0.276725327	103	69	121	196	276	118	413	285
ENSECAG00000013776	3.597522367	0.044407306	0.276725327	107	132	166	206	441	130	537	242
ENSECAG00000005764	3.683153867	0.044449587	0.276826177	357	374	192	206	84	265	109	237
ENSECAG00000020694	4.184414696	0.044472119	0.276826177	145	137	336	364	350	354	713	564
ENSECAG00000026951	3.49957409	0.044500216	0.276849706	54	115	148	302	310	307	361	245
ENSECAG00000013656	0.698566317	0.044674071	0.277666652	15	17	17	24	51	32	53	35
ENSECAG00000007222	7.940998562	0.044680308	0.277666652	6127	9060	4856	2997	1197	5594	1150	3882
ENSECAG00000014178	4.766443446	0.044737115	0.277868005	538	790	469	546	341	403	438	533
ENSECAG00000020795	2.476909575	0.04477162	0.277930696	110	365	50	94	0	118	1	32
ENSECAG00000022397	2.509560552	0.044800651	0.277939388	96	360	56	59	15	131	13	68
ENSECAG00000025138	4.007963362	0.044821846	0.277939388	139	362	395	657	244	195	240	311
ENSECAG00000013937	2.540591842	0.044948271	0.278571618	69	48	66	58	87	142	64	367
ENSECAG00000004845	3.595319131	0.044995381	0.278711867	80	75	219	273	235	312	393	376
ENSECAG00000022383	2.266394459	0.045029336	0.278714453	49	25	99	21	165	81	77	228
ENSECAG00000011704	0.83852538	0.045052062	0.278714453	16	78	43	42	3	14	12	50
ENSECAG00000017241	9.805309378	0.045069241	0.278714453	58747	2953	12128	2845	5093	16282	6688	4633
ENSECAG00000021570	3.3893573	0.045144869	0.279030586	47	78	144	297	256	228	398	280
ENSECAG00000007024	2.66231939	0.045179838	0.279095202	45.0011	58	111.001	111	142.001	113	256	185.001
ENSECAG00000021476	10.62446482	0.045205922	0.279098096	126308	13991	3793	10668	20	20170	1	3003
ENSECAG00000021580	6.969003805	0.045229336	0.279098096	5900	414	3655	4153	3	158	6	2781
ENSECAG00000006725	6.992995481	0.045276136	0.27923554	3264	5302	1651	1523	780	3030	805	1615
ENSECAG00000009417	3.676736793	0.045456024	0.280080249	162	306	259	458	153	195	203	245
ENSECAG00000003585	2.153987274	0.045462301	0.280080249	22	46	71	81	179	131	81	87
ENSECAG00000017369	6.104788502	0.045486911	0.280080303	873	668	850	807	2511	613	3437	1361
ENSECAG00000015249	3.350049694	0.045521439	0.280080692	67	122	166	190	278	268	380	170
ENSECAG00000017419	6.847720902	0.045554843	0.280080692	2839	2187	2724	2405	1668	1924	1430	1752
ENSECAG00000014969	3.583228015	0.045560777	0.280080692	294	324	247	206	133	282	64	165
ENSECAG00000025164	2.969746349	0.045612481	0.280177607	72	96	98	143	170	168	286	218
ENSECAG00000007742	4.765602309	0.045640993	0.280177607	334	603	521	1060	475	354	500	418
ENSECAG00000009540	5.770429622	0.04565037	0.280177607	443	523	835	1238	1607	1177	1948	1098
ENSECAG00000014699	9.406180736	0.045686728	0.280240375	41182	5947	9561	5409	456	15913	774	4687
ENSECAG00000012161	3.484868448	0.045709827	0.280240375	117	256	343	351	90	118	232	242
ENSECAG00000010020	4.922558315	0.045781396	0.280528093	36	8	782	10	178	2264	396	1166
ENSECAG00000015267	5.573798704	0.045822517	0.280629022	466	604	2116	1404	731	685	745	729
ENSECAG00000017754	4.005402976	0.046020561	0.281378641	250	512	267	400	191	203	305	311
ENSECAG00000011136	4.409944212	0.046039971	0.281378641	461	1125	197	212	109	547	116	227
ENSECAG00000022357	3.678473315	0.046041396	0.281378641	267	511	205	297	22	281	23	238
ENSECAG00000012688	3.230121278	0.046043802	0.281378641	180	359	123	180	90	98	144	209
ENSECAG00000015759	5.846446654	0.046068492	0.281378641	1289	2283	851	800	369	1509	474	678
ENSECAG00000024209	7.402704436	0.046199183	0.282025574	6442	7314	1064	1304	291	4437	392	1138
ENSECAG00000024002	3.093154418	0.046226827	0.282043102	40	116	86	204	124	260	338	223
ENSECAG00000004400	3.956592939	0.046466368	0.28335276	558	626	80	144	46	360	90	186
ENSECAG00000026920	4.399874927	0.046551036	0.283717101	595	582	396	316	100	517	119	359
ENSECAG00000002564	0.80146725	0.046620947	0.283991159	19	20	50	107	4	19	0	42
ENSECAG00000026932	5.733858729	0.046683649	0.284221039	592	393	916	728	2077	1077	2156	590
ENSECAG00000006398	0.748142083	0.046734375	0.284377799	7	14	33	24	37	50	42	48
ENSECAG00000021723	1.409317186	0.046790002	0.284564196	79	87	65	19	10	64	3	28
ENSECAG00000022504	3.672967898	0.046854357	0.284803444	228	697	155	198	25	242	23	257
ENSECAG00000011365	2.969231873	0.046926906	0.28509222	34	37	101	222	135	185	181	386
ENSECAG00000012725	8.807497651	0.046984168	0.28528787	12896	13587	5887	8035	3501	9087	3966	6828
ENSECAG00000024181	7.287505023	0.047067215	0.285620591	7697	5038	1570	1232	26	3166	17	1103
ENSECAG00000022363	2.554211109	0.047089139	0.285620591	157	320	59	48	4	150	13	44
ENSECAG00000020076	6.849958498	0.047204671	0.286168894	3292	4365	1344	1104	1257	1753	1543	1644
ENSECAG00000019928	8.507130945	0.047374684	0.287046715	397	6333	40	4884	1833	17061	18875	11544
ENSECAG00000013421	4.734004313	0.047450973	0.287356027	198	843	490	1070	361	519	296	398
ENSECAG00000012048	7.982665781	0.04748654	0.28741853	4841	9535	3573	5599	2163	5389	2665	3552
ENSECAG00000000419	8.782617471	0.04751794	0.287455763	4384	4078	6201	5652	17582	7075	20906	5653
ENSECAG00000012693	4.582356323	0.047693466	0.288364374	332	360	195	269	666	688	880	360
ENSECAG00000000193	6.081482578	0.047769288	0.288669504	833	1494	1781	2156	1262	1067	1099	827
ENSECAG00000017886	4.311138161	0.047920599	0.289347242	160	221	319	411	623	377	617	468
ENSECAG00000020570	6.039572533	0.04793227	0.289347242	599	502	1243	1226	1965	1349	2203	1534
ENSECAG00000019053	2.573885528	0.048019593	0.289584477	44	73	102	79	132	95	267	165
ENSECAG00000007058	0.48017734	0.048022441	0.289584477	9	10	17	26	52	23	23	48
ENSECAG00000010729	8.423691428	0.0480719	0.289729262	3311	6298	9797	15416	4526	4842	6364	4212
ENSECAG00000008679	7.031161478	0.048133938	0.289949674	4166	5736	1151	1075	616	3394	529	1143
ENSECAG00000010395	0.362875574	0.048182371	0.29008794	9	38	38	37	9	21	9	23
ENSECAG00000004314	4.495259379	0.048259159	0.290396682	263	285	279	361	528	348	964	594
ENSECAG00000014096	4.717970881	0.048428667	0.291262742	642	695	575	330	402	209	570	438
ENSECAG00000024536	6.698285626	0.048481366	0.291425738	2169	7229	1266	1280	0	1110	11	1400
ENSECAG00000006442	6.048722671	0.048613184	0.292063905	1841	1704	977	1332	398	1188	539	1559

ENSECAG00000013691	6.13833209	0.04867626	0.292288619	654	704	686	1301	3847	527	2670	1046
ENSECAG00000004216	11.47253737	0.04872693	0.292438642	73502	91206	70409	29759	14882	51497	14458	58233
ENSECAG00000025052	2.818557824	0.048776456	0.292567578	71	83	83	107	236	104	322	122
ENSECAG00000009191	2.283182774	0.04886203	0.292567578	95	97	128	120	53	108	53	60
ENSECAG00000014207	4.007610983	0.048870133	0.292567578	200	474	446	356	168	201	184	421
ENSECAG00000025068	8.158894004	0.048873682	0.292567578	6756	11542	3063	4658	2051	3699	4142	5611
ENSECAG00000023139	3.093524912	0.048876902	0.292567578	48	11	101	220	329	86	148	450
ENSECAG00000007129	2.234535275	0.048983356	0.293050715	76	66	173	127	37	67	56	104
ENSECAG00000020201	1.136334201	0.049249812	0.294431798	23	14	23	27	61	29	153	17
ENSECAG00000004523	6.163935249	0.049265927	0.294431798	779	712	856	1392	2570	1315	2771	1156
ENSECAG00000016367	2.365160498	0.049315888	0.294476746	86	131	192	65	52	112	69	45
ENSECAG00000026965	2.31226772	0.049325178	0.294476746	12	27	101	73	262	198	33	70
ENSECAG00000022664	7.953457568	0.049432859	0.294964938	8144	7151	3260	3083	2047	4606	2474	3890
ENSECAG00000015031	3.776439883	0.049621268	0.295912326	268	553	183	274	81	334	71	221
ENSECAG00000009907	5.587953536	0.049643614	0.295912326	326	403	370	1356	615	602	1595	2736
ENSECAG00000020003	2.61328512	0.049723249	0.296231915	76	26	333	224	137	69	18	50
ENSECAG00000011856	0.93060318	0.049849366	0.29679231	7	23	21	41	47	55	63	38
ENSECAG00000011072	4.408822366	0.04986945	0.29679231	189	412	530	917	383	330	297	305
ENSECAG00000016653	10.58456323	0.049933262	0.296824696	13133	14698	25771	23202	49267	41169	40853	37822
ENSECAG00000021303	4.574276587	0.049937831	0.296824696	1028	980	158	166	12	640	11	70
ENSECAG00000016667	6.386699146	0.049953106	0.296824696	2568	3229	455	1535	187	758	839	2281
ENSECAG00000019133	3.674894941	0.050000062	0.296942411	184	435	309	213	250	164	151	201
ENSECAG00000004913	2.652351294	0.050025081	0.296942411	77	237	172	121	28	121	41	134
ENSECAG00000018117	3.174606435	0.050082099	0.297125948	149	257	176	216	133	135	160	131
ENSECAG00000013653	4.128486251	0.050164036	0.297457054	320	410	479	328	165	239	251	429
ENSECAG00000001648	5.0055095	0.050228691	0.297685396	394	441	403	455	946	549	1185	731
ENSECAG00000013883	2.883352836	0.050345114	0.298092857	49	38	655	64	87	59	39	132
ENSECAG00000020973	2.751580037	0.050349808	0.298092857	141	279	108	80	37	87	87	150
ENSECAG00000024503	6.4413973	0.050391451	0.298135734	1631	2926	1582	1466	1240	1252	1346	1481
ENSECAG00000022190	6.563237822	0.050409424	0.298135734	2775	2397	1279	1579	1266	1416	1285	1584
ENSECAG00000007242	7.396486527	0.050531086	0.298630063	3288	5137	4621	2430	1448	3414	1376	2996
ENSECAG00000021765	8.253919444	0.050565418	0.298630063	8642	14928	3159	4042	370	8345	314	2141
ENSECAG00000022574	7.5222103	0.050587644	0.298630063	6200	5879	2384	1685	1592	4184	1636	1762
ENSECAG00000008991	5.922680915	0.050597926	0.298630063	787	2059	1035	1698	641	1043	730	1281
ENSECAG00000008268	2.583327316	0.050675664	0.298933904	55	50	94	89	183	109	306	77
ENSECAG00000017890	5.057861489	0.050702987	0.29894019	1631	1328	145	167	4	752	9	150
ENSECAG00000011528	6.579311154	0.05075425	0.299087547	3217	2870	1181	841	1486	1386	1417	929
ENSECAG00000000101	2.154081129	0.050887863	0.299719772	28	52	69	80	97	104	116	158
ENSECAG00000018631	3.086553184	0.050925256	0.299784921	109	624	86	77	4	195	17	89
ENSECAG00000016526	1.244516462	0.051081128	0.300466096	17	19	38	41	75	40	85	54
ENSECAG00000022897	1.270726151	0.051093752	0.300466096	22.0071	37	19	35	62.0004	71.0001	55	65
ENSECAG00000023154	2.6408064	0.05116843	0.300749909	96	107	210	303	0	68	10.0001	154
ENSECAG00000013849	3.132347519	0.051225431	0.300929581	78	71	105	198	273	105	341	250
ENSECAG00000024882	2.99164987	0.051281771	0.301020236	286	148	59	381	2	117	0	129
ENSECAG00000004675	5.574730969	0.051304066	0.301020236	1037	1169	905	925	799	615	933	722
ENSECAG00000007863	1.845497862	0.051320182	0.301020236	36	36	48	52	129	60	136	65
ENSECAG00000016204	3.062223866	0.051393187	0.301162571	333	239	95	46	69	170	67	94
ENSECAG00000008540	0.949293492	0.051422911	0.301162571	17	20	20	30	56	24	93	39
ENSECAG00000010107	9.996278994	0.051469885	0.301162571	18924	41052	18160	16535	10237	18884	9242	16795
ENSECAG00000021993	5.401742872	0.051488947	0.301162571	2280	648	327	325	157	1116	231	304
ENSECAG00000004844	3.972609636	0.051503892	0.301162571	205	510	310	418	151	247	161	384
ENSECAG00000019877	6.635210401	0.051508849	0.301162571	3969	4265	571	592	126	2641	196	655
ENSECAG00000016283	6.505192615	0.051551907	0.301162571	1083	2727	1665	2981	1286	1369	1296	1671
ENSECAG00000018159	2.051124316	0.051573182	0.301162571	40	69	115	192	31	38	72	91
ENSECAG00000021754	7.221837207	0.051582522	0.301162571	2506	5657	2693	2767	1329	2508	2424	2429
ENSECAG00000015839	6.973621892	0.051723599	0.30183146	3702	4878	1607	1108	928	3153	1086	1001
ENSECAG00000021543	4.624080371	0.051826523	0.302277134	151	96	510	540	874	484	957	437
ENSECAG00000008751	6.804660123	0.052101	0.30358789	2245	4635	1455	1774	1115	2253	1243	1582
ENSECAG00000005671	2.85646896	0.052104588	0.30358789	54	72	34	204	121	185	260	246
ENSECAG00000016857	3.756694681	0.052137007	0.303621395	69	152	181	354	407	293	416	330
ENSECAG00000008826	7.380395188	0.052194015	0.303797988	4370	5257	2898	2194	1443	2884	1714	3139
ENSECAG00000011705	5.65500227	0.052262416	0.304040676	993	1435	751	1176	724	935	788	728
ENSECAG00000014290	2.760797915	0.052399218	0.304676665	81	40	104	81	347	100	196	111
ENSECAG00000024698	4.116427758	0.052445586	0.304676665	142	171	277	369	447	333	733	349
ENSECAG00000021034	2.786075418	0.052452021	0.304676665	33	137	141	431	96	115	87	69
ENSECAG00000020098	4.006209401	0.052514021	0.304881251	172	157	200	326	353	232	654	505
ENSECAG00000016071	6.532158844	0.052655189	0.30554502	2908	1598	2058	1491	676	2353	1019	1030
ENSECAG00000012047	2.413736733	0.052757397	0.305982154	41	76	64	93	160	93	189	131
ENSECAG00000016912	7.829766387	0.052839099	0.306192943	9498	4147	2642	2843	2689	3802	2833	2600
ENSECAG00000016684	5.85228554	0.052867858	0.306192943	1583	1967	701	737	548	1258	560	846
ENSECAG00000006899	5.308062978	0.052874425	0.306192943	223	491	699.99	840	1186	837	1326	848
ENSECAG00000016672	4.338862643	0.05290354	0.306205795	164	236	310	440	528	394	643	550
ENSECAG00000020448	0.628822261	0.053031097	0.306788132	17	15	12	24	40	34	61	29
ENSECAG00000019684	2.7010565	0.053095246	0.30697571	72	76	84	66	291	90	252	92
ENSECAG00000018702	7.854801037	0.053150802	0.30697571	7904	9640	1890	1747	851	5671	1153	2502
ENSECAG00000025055	7.449753318	0.053200973	0.30697571	2831	5416	4027	3825	1783	3755	2290	2321

ENSECAG00000012514	2.392795001	0.053222715	0.30697571	29.0071	84	47	101	103	85.0001	279	128
ENSECAG00000003259	3.28691053	0.053222724	0.30697571	73	141	149	156	345	232	269	181
ENSECAG00000018202	6.129695325	0.053225301	0.30697571	619	1364	1671	3298	633	1039	1102	1483
ENSECAG00000023101	1.532039151	0.053280192	0.30698666	53	79	48	76	29	35	60	45
ENSECAG00000024327	6.84422418	0.053281128	0.30698666	3414	4589	1040	1180	656	2236	1176	1782
ENSECAG00000008274	5.41601844	0.053310224	0.306998939	1226	2996	216	224	4	574	0	470
ENSECAG00000012951	4.544569815	0.053351747	0.307082729	310	1015	364	438	184	334	313	551
ENSECAG00000015266	8.47719902	0.053479584	0.307662992	6652	14395	4116	7595	4295	5078	5148	5641
ENSECAG00000011996	4.380856669	0.053522771	0.307755932	226	610	548	525	174	410	409	313
ENSECAG00000024194	3.747285008	0.05358409	0.307800484	126	140	250	203	343	385	429	254
ENSECAG00000011686	5.277584205	0.05358459	0.307800484	1300	1308	358	424	232	937	220	553
ENSECAG00000004384	0.025173606	0.053623858	0.307870711	3	8	3	27	43	8	34	26
ENSECAG00000016811	0.856879643	0.053822475	0.308714745	7	14	18	45	68	22	81	30
ENSECAG00000023358	8.805024675	0.0538251	0.308714745	11097	19404	7517	3635	3512	11422	2343	3795
ENSECAG00000017926	1.409498488	0.053924407	0.309128589	13	42	5	60	55	76	88	79
ENSECAG00000019479	5.917028687	0.053956605	0.309157498	1053	1332	1128	1847	723	933	1327	941
ENSECAG00000020437	6.511267662	0.054076036	0.309628127	1770	904	3036	2795	1850	1490	1001	982
ENSECAG00000024475	5.851220539	0.054093135	0.309628127	502	480	880	1345	1422	1078	1536	2107
ENSECAG00000015676	7.302981435	0.054238396	0.310142509	3685	8104	2272	1441	337	4230	340	1642
ENSECAG00000007601	4.904774309	0.054263162	0.310142509	775	1034	354	386	380	599	439	325
ENSECAG00000001900	1.314995948	0.054264724	0.310142509	84	29	67	41	24	61	12	19
ENSECAG00000011924	4.187255951	0.054300779	0.31019286	201	180	306	278	534	259	653	502
ENSECAG00000011780	5.369966276	0.054342507	0.31027555	279	468	526	914	687	1041	2475	485
ENSECAG00000023941	4.747796265	0.054370499	0.310279765	817	933	235	313	195	431	334	526
ENSECAG00000017044	5.83616666	0.054402236	0.31030534	593	475	1023	819	2792	1129	1246	946
ENSECAG00000016460	1.894981548	0.054475613	0.310308822	9	0	94	3	25	244	70	135
ENSECAG00000001141	7.269112038	0.054521816	0.310308822	8130	4899	714	1227	29	3175	23	1145
ENSECAG00000000003	4.509883413	0.054527509	0.310308822	479	979	295	304	207	614	174	210
ENSECAG00000013071	5.23955853	0.054531662	0.310308822	570	1069	765	896	663	458	674	673
ENSECAG00000008884	5.933503581	0.054539126	0.310308822	750	673	672	644	2490	428	3413	846
ENSECAG00000000074	4.752576828	0.054608087	0.310545988	259	171	574	207	1298	587	1045	158
ENSECAG00000010055	2.215416211	0.054689485	0.310853615	43	36	82	40	112	66	320	53
ENSECAG00000014199	5.910208313	0.054724694	0.310898525	1401	1929	860	1008	514	1121	826	1122
ENSECAG00000011640	5.063416583	0.054790275	0.31111585	194	645	691	1913	528	479	445	532
ENSECAG00000019533	0.68603282	0.054843958	0.311265434	4	6	20	37	100	23	46	12
ENSECAG00000023071	1.780573046	0.054954578	0.311737855	25	45	44	50	89	38	176	82
ENSECAG00000024788	10.12733317	0.055018037	0.311792492	28843	36696	13738	20064	15180	14687	20013	13447
ENSECAG00000009173	3.702370282	0.055018983	0.311792492	59	37	242	265	607	146	713	104
ENSECAG00000020317	6.669466694	0.055301597	0.313138932	2553	3592	1310	1365	1145	1641	1395	1682
ENSECAG00000007228	6.732588128	0.055311585	0.313138932	815	575	2059	2117	3308	2388	2331	3580
ENSECAG00000012411	6.61523503	0.055358517	0.313248863	1835	4199	1880	1191	535.001	2441	662.001	1450
ENSECAG00000018141	7.032839545	0.055454497	0.313561435	2593	4819	2015	2328	1177	2091	1820	2499
ENSECAG00000019660	8.323900321	0.055504983	0.313561435	15169	8827	3719	2886	156	8173	186	2159
ENSECAG00000018512	1.76513284	0.055560098	0.313561435	159	55	82	16	1	73	9	40
ENSECAG00000009549	7.86236089	0.055569397	0.313561435	4244	10585	3271	3334	1984	4796	2699	3096
ENSECAG00000007176	1.745635805	0.055575042	0.313561435	138	111	32	38	1	51	2	64
ENSECAG00000021853	2.37081094	0.055621352	0.313561435	61	39	76	72	169	83	205	110
ENSECAG00000017005	5.80407398	0.055633455	0.313561435	1847	2440	803	315	37	1331	68	680
ENSECAG00000007453	4.434915841	0.055634089	0.313561435	149	300	442	310	604	585	528	521
ENSECAG00000009013	3.349402595	0.055670335	0.313610475	56	114	180	214	294	242	300	242
ENSECAG00000022912	0.642509446	0.055758141	0.313865306	12	74	43	31	2	27	1	30
ENSECAG00000009619	4.497314571	0.055770708	0.313865306	169	252	448	392	759	514	625	454
ENSECAG00000021551	4.356606927	0.055838155	0.314089623	417	299	582	490	290	311	248	437
ENSECAG00000025135	3.844792234	0.055897521	0.314268285	312	1009	65	135	4	357	7	81
ENSECAG00000012707	5.115448003	0.055947021	0.314301532	475	713	854	1062	510	674	492	565
ENSECAG00000024563	5.077143194	0.055958648	0.314301532	1821	589	405	511	2	721	3	237
ENSECAG00000023787	1.280844134	0.05599897	0.314372915	68	73	26	41	26	37	26	39
ENSECAG00000014600	5.152332292	0.056053847	0.314525897	255	292	682	786	858	928	816	1114
ENSECAG00000022743	4.855952914	0.056103343	0.314592993	253	314	434	622	530	525	934	1080
ENSECAG00000014537	5.399878559	0.05614196	0.314592993	392	369	764	782	1409	643	1570	902
ENSECAG00000023967	4.223964906	0.056148701	0.314592993	82	183	324	397	211	594	348	888
ENSECAG00000016336	1.174897579	0.056178473	0.314594882	11	28	22	51	55	40	84	64
ENSECAG00000018997	5.318074726	0.056233009	0.314594882	235	328	619	1022	1558	606	1511	691
ENSECAG00000018541	6.038125296	0.056238376	0.314594882	910	1764	1665	1572	621	1379	1044	1084
ENSECAG00000024800	1.085207105	0.056259568	0.314594882	22	23	22	13	118	15	90	21
ENSECAG00000009713	5.023827241	0.056406546	0.315261916	187	193	740	500	1558	599	1267	259
ENSECAG00000022772	1.34143073	0.056524783	0.315639565	39	21	18	30	82	60	52	77
ENSECAG00000024979	6.304295661	0.05654794	0.315639565	817	2033	2614	1982	554	1061	1617	1657
ENSECAG00000020366	6.145025657	0.056557287	0.315639565	2338	2306	888	577	639	1758	662	583
ENSECAG00000024959	0.25730969	0.056690746	0.316198371	8	10	11	24	18	24	55	30
ENSECAG00000016346	5.336747708	0.056712962	0.316198371	976	1203	624	623	416	772	541	722
ENSECAG00000018406	6.353764741	0.056781646	0.316426352	594	750	1731	1053	3856	1368	2859	990
ENSECAG00000003698	2.850742627	0.056850627	0.31659299	109	253	100	192	78	99	114	139
ENSECAG00000013090	4.157767377	0.056867164	0.31659299	204	164	266	327	470	279	647	503
ENSECAG00000008158	0.409471055	0.056915926	0.316709587	7	12	12	26	30	9	71	38
ENSECAG00000012156	2.831270026	0.057052842	0.317160451	46	213	113	388	24	139	62	136

ENSECAG00000014092	2.525971066	0.05707833	0.317160451	142	223	90	67	22	126	41	97
ENSECAG00000021756	2.192522249	0.057090648	0.317160451	56	34	34	88	112	117	221	62
ENSECAG00000013644	4.114257705	0.057108382	0.317160451	323	598	304	276	162	306	270	322
ENSECAG00000023697	5.608802621	0.057232362	0.317502752	437	356	866	1070	1237	983	1831	1156
ENSECAG00000014987	4.782293391	0.057248015	0.317502752	254	323	450	542	609	692	808	723
ENSECAG00000011822	2.743071777	0.057266584	0.317502752	25	189	397	130	7	45	33	202
ENSECAG00000015857	2.252271059	0.057281568	0.317502752	63.0011	42	55.001	41.0005	137.001	107	45	236.001
ENSECAG00000014757	4.778400146	0.057511999	0.318515362	364	584	589	930	512	434	447	398
ENSECAG00000018825	4.514940539	0.05753109	0.318515362	212	319	394	347	549	598	731	485
ENSECAG00000010439	0.051495253	0.057548186	0.318515362	3	2	21	16	19	25	17	49
ENSECAG00000021951	5.882322957	0.05767196	0.31904532	768	3449	480	967	245	1188	611	1001
ENSECAG00000007916	6.255731151	0.057768761	0.319320981	983	2923	1435	1629	709	1506	1548	897
ENSECAG00000012804	8.614788488	0.057805158	0.319320981	9783	13164	7521	4709	3949	8959	3575	4339
ENSECAG00000002370	2.252189922	0.057822343	0.319320981	57	385	15	29	9	42	30	107
ENSECAG00000006915	8.146084733	0.05783398	0.319320981	8789	10948	2010	3134	4126	3591	4603	2599
ENSECAG00000016069	2.699302289	0.057874114	0.31938768	44	75	114	103	89	182	218	212
ENSECAG00000018952	3.252113421	0.057985267	0.319774218	90	131	129	163	262	193	292	251
ENSECAG00000017366	0.566798883	0.05800033	0.319774218	12	8	28	18	30	47	46	32
ENSECAG00000019738	7.803086327	0.058058258	0.319938656	4510	7820	3705	3911	2694	3854	2952	3461
ENSECAG00000017172	5.773666403	0.058175082	0.320427337	77	1202	679	691	1134	2253	861	1869
ENSECAG00000019809	4.469874523	0.058233276	0.320592768	123	105	471	519	561	468	631	747
ENSECAG00000000734	3.235281739	0.058398361	0.3211445	105	149	314	317	150	151	147	129
ENSECAG00000014279	5.05741803	0.058431638	0.3211445	333	326	471	733	827	529	1127	1037
ENSECAG00000003158	3.030484103	0.058441084	0.3211445	57	70	134	182	189	156	274	259
ENSECAG00000012784	2.191099818	0.058446325	0.3211445	49	29	51	87	143	50	223	100
ENSECAG00000008108	5.593585289	0.058477522	0.321160922	1135	1396	746	799	583	952	738	712
ENSECAG00000000836	7.284345701	0.058562622	0.321339898	2879	5932	2163	3800	740	3245	1154	3151
ENSECAG00000015502	6.235124822	0.058589989	0.321339898	1741	2088	1374	1222	1111	1302	1415	822
ENSECAG00000017922	9.004684693	0.058594785	0.321339898	11643	24456	5495	5788	3897	11910	5433	4596
ENSECAG00000008446	8.085068585	0.05864685	0.321470579	11033	8829	2629	2035	541	6013	960	4083
ENSECAG00000024543	3.754503594	0.058747871	0.321760063	171	291	356	401	254	171	227	213
ENSECAG00000000715	5.118480109	0.058756185	0.321760063	363	296	510	690	1089	393	1499	809
ENSECAG00000023741	4.177247018	0.058824074	0.321976964	899	87	299	284	109	59	597	148
ENSECAG00000015412	3.677323666	0.058897353	0.322223146	130	138	186	243	376	267	426	273
ENSECAG00000011173	6.173342713	0.058958424	0.322402333	1400	1905	1314	1623	1168	962	1088	1376
ENSECAG00000014670	4.498526049	0.059181062	0.323464422	527	640	346	416	304	174	577	375
ENSECAG00000016131	4.908438655	0.059248902	0.32367982	90	191	538	819	521	590	868	1382
ENSECAG00000019995	1.588647173	0.059312027	0.32386927	24	37	50	37	53	82	94	89
ENSECAG00000019509	8.607578629	0.059393176	0.324155708	9169	10945	7563	6667	5386	6153	5956	5332
ENSECAG00000014795	3.311637702	0.059442092	0.324155708	88	107	134	203	237	137	384	315
ENSECAG00000012334	1.877084119	0.059449901	0.324155708	95	141	50	60	3	42	25	98
ENSECAG00000011021	2.168089168	0.059604375	0.324755393	46	45	59	77	111	96	177	98
ENSECAG00000016116	6.405581545	0.059649712	0.324755393	519	799	1124	1822	4949	911	2767	954
ENSECAG00000019268	1.718138802	0.059689962	0.324755393	26	53	42	37	46	84	127	99
ENSECAG00000016824	4.295784436	0.059696819	0.324755393	137	68	329	510	692	543	351	533
ENSECAG00000020933	6.78488946	0.059702507	0.324755393	1003	1748	2634	5933	1577	801	2204	1985
ENSECAG00000016694	4.315364679	0.059844665	0.325373212	350	876	199	371	133	220	331	479
ENSECAG00000008690	1.117863231	0.05990144	0.325442996	26	43	47	87	19	35	43	26
ENSECAG00000020451	2.622380671	0.05991467	0.325442996	76	82	209	227	136	82	70	64
ENSECAG00000004757	5.102467705	0.060014341	0.325828933	625	717	702	942	567	359	861	497
ENSECAG00000011068	3.209641849	0.060053808	0.325887802	472	330	43	71	1	208	0	29
ENSECAG00000026911	6.10753552	0.060099637	0.325981118	1795	1718	1109	1191	912	1296	953	1045
ENSECAG00000019613	3.903202679	0.06014478	0.326070631	91	291	190	229	292	469	575	264
ENSECAG00000016922	7.015630205	0.060223046	0.326224107	6254	1063	2347	1096	1029	2297	1447	1692
ENSECAG00000017166	5.711035044	0.060230397	0.326224107	1052	1000	1048	1442	612	717	931	1151
ENSECAG00000014498	7.266612978	0.060277504	0.326324005	3301	4743	2302	3358	2319	1289	3158	2538
ENSECAG00000002438	4.24441445	0.060345264	0.326535565	1020	456	237	138	2	374	0	145
ENSECAG00000005421	2.9293011	0.060390158	0.326583961	60	112	93	126	261	122	324	124
ENSECAG00000007194	1.485331847	0.06043583	0.326583961	8	18	56	39	154	33	120	18
ENSECAG00000023064	5.748148473	0.060440264	0.326583961	521	2338	805	1318	704	611	804	1166
ENSECAG00000012998	6.408317801	0.060472933	0.326605478	1537	3082	1314	1496	1157	1369	1259	1419
ENSECAG00000014994	7.669377468	0.060524011	0.326688048	5026	4658	3857	4336	2342	3932	2637	3070
ENSECAG00000012595	3.079489878	0.060550105	0.326688048	110	196	143	337	114	136	135	136
ENSECAG00000021278	1.160613582	0.060574306	0.326688048	49	11	138	17	44	21	11	22
ENSECAG00000018560	5.235762955	0.060614217	0.326748511	628	943	1057	938	154	455	262	1254
ENSECAG00000023355	2.032448774	0.06073064	0.327177516	24	28	71	89	102	115	141	82
ENSECAG00000000362	6.804647786	0.060751275	0.327177516	3633	3653	1104	1167	1509	2013	1425	1089
ENSECAG00000017373	3.529139437	0.060841291	0.327507372	73	141	170	239	358	387	329	146
ENSECAG00000018538	6.297345401	0.06103761	0.328408877	928	793	1214	1166	2851	1467	2688	1414
ENSECAG00000017516	4.06490565	0.061091544	0.328543803	159	161	231	374	353	288	499	632
ENSECAG00000020165	1.88857255	0.061614348	0.331044101	10	19	69	87	84	82	87	157
ENSECAG00000008327	4.779936622	0.061651422	0.331044101	263	391	394	486	837	426	808	785
ENSECAG00000001317	7.283164931	0.061708057	0.331044101	2435	8206	2639	1085	2131	2116	1939	2200
ENSECAG00000021118	3.253221651	0.061722772	0.331044101	74	26	135	211	421	73	167	430
ENSECAG00000018034	6.561520167	0.061735256	0.331044101	2505	2120	1856	1486	1193	1640	1487	1452
ENSECAG00000002905	3.998663475	0.061758471	0.331044101	180	633	399	311	94	471	129	168

ENSECAG00000000927	4.614228114	0.061760006	0.331044101	1148	488	533	47	49	671	44	173
ENSECAG00000011111	2.891703086	0.06194297	0.33186857	130	115	259	190	90	51	66	215
ENSECAG00000003056	4.689174079	0.062169834	0.332927358	380	881	520	488	251	526	392	443
ENSECAG00000014401	2.89401709	0.062268232	0.333297517	66	67	143	82	355	136	229	93
ENSECAG00000024319	0.97911067	0.06233156	0.333435869	13	47	73	58	45	20	24	13
ENSECAG00000024878	3.137200524	0.062352654	0.333435869	62	53	230	89	275	149	204	338
ENSECAG00000002716	6.089015991	0.0626028	0.334616374	1860	2032	910	934	686	1261	1167	953
ENSECAG00000022117	7.096212464	0.062655857	0.334742812	2062.02	3973	3106	3320	1591.99	2342	2907	1604.05
ENSECAG00000012878	5.226725847	0.062922035	0.33591464	356	310	773	560	1364	510	1401	762
ENSECAG00000011688	6.059152852	0.062972757	0.33591464	1129	3468.01	803.003	763.998	554.001	1769.99	543.008	605.004
ENSECAG00000005487	7.26200439	0.062990976	0.33591464	102	28	276	4183	401	617	22500	680
ENSECAG00000024436	0.749703705	0.062993215	0.33591464	35	38	23	53	21	21	12	35
ENSECAG00000013514	7.398906583	0.063030854	0.335957991	1841	413	3533	1834	6455	4474	5787	2634
ENSECAG00000024762	5.565441911	0.063135861	0.336208393	510	659	591	862	906	951	1799	1332
ENSECAG00000021389	1.775469256	0.063144764	0.336208393	39	98	54	133	32	57	40	65
ENSECAG00000018369	3.573200337	0.063180594	0.336208393	72	162	439	545	145	137	134	271
ENSECAG00000007123	7.028229878	0.063195956	0.336208393	3699	4884	1684	1351	901	3101	829	1826
ENSECAG00000022014	3.136102591	0.063280041	0.336394806	143	245	205	213	93	103	60	252
ENSECAG00000017338	5.08129206	0.06329009	0.336394806	1052	1081	426	400	141	862	215	535
ENSECAG00000023869	3.560536115	0.063421387	0.336935365	120	291	183	548	123	141	186	271
ENSECAG00000021627	4.441722422	0.063478556	0.336992642	250	231	346	344	632	295	773	601
ENSECAG00000005675	6.363649805	0.063491367	0.336992642	1648	3053	1692	747	1171	1240	1177	1307
ENSECAG00000017450	1.043844207	0.063548057	0.337136361	7	25	6	59	50	57	80	41
ENSECAG00000010526	3.243405301	0.063611692	0.337316775	311	144	175	155	120	134	131	173
ENSECAG00000013220	4.378237443	0.063913567	0.338439326	332	947	218	342	265	341	321	316
ENSECAG00000012903	3.795409026	0.06392311	0.338439326	119	143	161	337	306	198	589	416
ENSECAG00000011650	4.513505726	0.063932893	0.338439326	548	683	390	315	261	475	223	401
ENSECAG00000000097	2.016120468	0.063942292	0.338439326	48	357	8	44	0	95	3	3
ENSECAG00000025124	4.094405189	0.064063121	0.338921299	129	176	224	432	519	398	625	268
ENSECAG00000010932	6.579662588	0.06412416	0.339086653	2611	3690	1198	892	898	2361	809	971
ENSECAG00000007598	4.588721612	0.064211484	0.339251517	208	272	495	296	702	248	924	742
ENSECAG00000017581	3.396722378	0.06421532	0.339251517	90	132	181	159	357	266	317	164
ENSECAG00000001554	1.561457358	0.064308286	0.339251517	103	100	24	25.0001	24.0007	15	51.0001	59
ENSECAG00000008028	2.415143336	0.064318998	0.339251517	41	174	182	112	53	116	58	69
ENSECAG00000014620	7.167210654	0.064342456	0.339251517	1636	2938	3551	5723	1915	2157	1927	2746
ENSECAG00000007115	4.516489694	0.064377477	0.339251517	380	903	358	364	293	505	324	256
ENSECAG00000016531	5.059360784	0.064403759	0.339251517	484	684	832	1000	368	296	782	752
ENSECAG00000013944	5.90641175	0.064441798	0.339251517	819	2318	981	1210	846	1061	910	917
ENSECAG00000017059	4.683879062	0.064442782	0.339251517	1011	531	255	278	298	359	432	386
ENSECAG00000014514	8.805486822	0.064453318	0.339251517	7838	16696	8001	7938	6938	6622	6176	6322
ENSECAG00000017356	1.9688322	0.06456378	0.339675893	38	163	65	100	31	51	77	65
ENSECAG00000013577	5.30549971	0.064656424	0.340006184	704	787	996	896	615	554	787	680
ENSECAG00000010104	2.069228555	0.06502012	0.341760882	27	37	67	74	82	210	100	56
ENSECAG00000015522	4.82598455	0.065187207	0.342481013	438	853	530	668	403	647	423	316
ENSECAG00000004791	4.108472631	0.065367882	0.343271834	66	132	422	286	792	270	572	272
ENSECAG00000021672	10.80378923	0.065418959	0.343381671	29471	46756	62975	28635	14361	30235	15195	39421
ENSECAG00000016161	7.834570317	0.065464556	0.343462661	14792	3547	1503	2275	8	4572	4	837
ENSECAG00000010050	6.569777936	0.065538525	0.343692359	1083	4741	1203	1848	951	1833	1012	1596
ENSECAG00000014941	6.446913863	0.065612993	0.343911915	683	1133	993	1997	1416	1621	3507	2964
ENSECAG00000012085	5.622447022	0.065643576	0.343911915	1173	1536	626	847	399	847	882	915
ENSECAG00000019599	4.954805454	0.065690256	0.343911915	612	1186	381	532	208	594	468	588
ENSECAG00000014573	0.650543248	0.065701222	0.343911915	16	14	18	23	57	23	49	35
ENSECAG00000009297	3.233334863	0.065824691	0.344256446	134	58	180	65	227	261	285	249
ENSECAG00000009910	5.276715482	0.065827517	0.344256446	202	361	812	829	1318	687	1290	840
ENSECAG00000012102	4.541734851	0.065895812	0.344455379	261	591	406	873	343	373	403	396
ENSECAG00000006668	3.005473521	0.065980829	0.344584723	24	119	123	155	280	270	180	130
ENSECAG00000021055	5.498154367	0.066011131	0.344584723	478	1460	849	1322	542	579	861	952
ENSECAG00000010973	7.492900946	0.066011355	0.344584723	2072	969	3150	2074	6893	2202	9423	2529
ENSECAG00000009926	6.845845941	0.066071553	0.344617999	3335	2404	2338	1665	1461	2081	1542	1696
ENSECAG00000011798	1.548984685	0.066078269	0.344617999	22	51	100	110	34	33	43	57
ENSECAG00000001347	4.560143711	0.066140346	0.344770795	197	338	364	1601	251	278	203	606
ENSECAG00000016535	3.717371946	0.066206644	0.344770795	108	143	761	339	75	302	76	226
ENSECAG00000018373	2.587492417	0.066221368	0.344770795	48	98	85	66	266	99	147	130
ENSECAG00000020385	6.351572748	0.066275141	0.344770795	1665	3415	1409	927	502	2009	482	1341
ENSECAG00000011664	4.421488576	0.066287592	0.344770795	218	188	333	462	543	349	697	661
ENSECAG00000021224	1.725286983	0.066289264	0.344770795	124	86	61	38	2	70	3	54
ENSECAG00000005775	1.32378116	0.066353576	0.344858727	15	33	43	33	55	81	73	52
ENSECAG00000006364	5.285353959	0.066366752	0.344858727	831	1252	569	647	530	529	566	829
ENSECAG00000024413	8.249838966	0.066399354	0.344869094	6413	8361	6714	5543	3415	6083	4080	4327
ENSECAG00000023607	6.14952697	0.06642933	0.344869094	885	1484	399	454	2323	1327	2864	1468
ENSECAG00000013142	3.537147563	0.066660782	0.345772182	134	116	168	202	331	190	381	322
ENSECAG00000012342	4.131988514	0.066664026	0.345772182	367	503	317	303	182	231	286	408
ENSECAG00000022213	6.879474316	0.066767784	0.346152649	1880	4510	2027	2513	863	2811	988	1911
ENSECAG00000013328	1.804740396	0.06681758	0.346253138	29	21	30	97	75	71	110	123
ENSECAG00000009798	3.14040212	0.066931974	0.346688138	189	382	96	179	11	131	45	236
ENSECAG00000015544	7.375281038	0.066985096	0.346805509	4237	10115	1463	1054	57	4406	193	1142

ENSECAG00000014112	5.990732401	0.067109743	0.34717891	484	648	1123	1383	1833	1201	2050	1601
ENSECAG00000024869	6.235809127	0.067149931	0.34717891	909	2159	1604	2175	943	1491	1131	1213
ENSECAG00000022420	0.850744145	0.067159193	0.34717891	12	38	44	83	11	30	23	31
ENSECAG00000012045	5.017049695	0.067179195	0.34717891	660	1503	834	395	5	648	16	535
ENSECAG00000003345	10.51345151	0.067277603	0.347416434	38544	24609	34397	32493	17833	25274	14795	25894
ENSECAG00000019815	9.038782751	0.067286187	0.347416434	9012	18922	9327	10774	6014	10507	6766	7063
ENSECAG00000024196	4.21373096	0.067404183	0.347703156	129	213	159	543	477	517	674	312
ENSECAG00000017034	4.86572248	0.067410497	0.347703156	565	800	583	504	367	451	606	489
ENSECAG00000023874	1.447493949	0.067433339	0.347703156	26	45	63	136	42	21	69	22
ENSECAG00000000608	6.285992016	0.06762118	0.348513866	1471	3759	642	1318	582	1989	582	966
ENSECAG00000025091	8.564665581	0.067698767	0.348755868	8662	11704	6609	6602	3723	7329	4434	6241
ENSECAG00000003934	4.151241434	0.067761402	0.348920654	150	276	315	232	584	313	521	432
ENSECAG00000021617	6.528934616	0.067825366	0.349092129	530	757	2087	1699	2150	1570	3387	2999
ENSECAG00000024705	4.954264535	0.067865487	0.349140791	500	365	2186	389	8	387	12	743
ENSECAG00000000007	5.517531268	0.067965699	0.349498409	1398	1645	514	354	343	1052	415	649
ENSECAG00000012623	2.262893631	0.068003945	0.349537206	29	40	92	94	127	114	135	132
ENSECAG00000011257	8.245213268	0.068094141	0.349842867	12241	9660	4432	1263	633	8570	927	2300
ENSECAG00000018687	5.067929597	0.068147147	0.349848624	337	337	614	502	1513	593	879	541
ENSECAG00000025150	4.267993185	0.068198243	0.349848624	269	478	451	486	285	230	372	393
ENSECAG00000007021	4.745738403	0.068199556	0.349848624	238	516	286	405	932	483	950	472
ENSECAG00000004709	0.333904553	0.068218177	0.349848624	6	7	23	19	11	51	33	37
ENSECAG00000011019	2.226223902	0.068307471	0.350148833	26	30	86	102	77	147	153	128
ENSECAG00000023479	4.613301469	0.068381698	0.350307706	260	300	264	538	493	427	864	798
ENSECAG00000022568	3.995897389	0.068400003	0.350307706	480	588	154	141	89	249	208	328
ENSECAG00000000633	1.958546568	0.06851015	0.350528952	29	36	43	94	120	99	128	66
ENSECAG00000010419	0.483861147	0.068526165	0.350528952	7	11	31	6	38	40	71	7
ENSECAG00000001869	5.959994543	0.068535569	0.350528952	393	848	952	1374	1378	1778	1377	1921
ENSECAG00000012960	4.620489768	0.06857077	0.350551511	324	392	317	221	599	706	543	703
ENSECAG00000002238	0.941696966	0.06867558	0.350929746	21	17	16	31	77	21	81	30
ENSECAG00000009031	2.297707358	0.068779206	0.351277904	50	48	61	93	128	78	138	179
ENSECAG00000019917	2.842876714	0.068817551	0.351277904	109	101	247	205	105	105	168	58
ENSECAG00000005090	8.040364702	0.06887145	0.351277904	12665	10089	1299	781	227	7107	140	893
ENSECAG00000015151	4.863269612	0.068930905	0.351277904	433	796	595	774	383	738	428	265
ENSECAG00000008014	4.536433947	0.068932847	0.351277904	160	962	377	796	158	344	172	636
ENSECAG00000015608	5.354680594	0.068976135	0.351277904	671	512	995	1555	640	417	1029	598
ENSECAG00000016948	11.23467951	0.068982211	0.351277904	88921	37891	48762	29461	29241	46226	26123	28220
ENSECAG00000019631	6.483841238	0.068990548	0.351277904	987	800	1152	1355	4849	1169	3238	781
ENSECAG00000022780	1.040965285	0.06908397	0.351596334	14	27	10	46	35	37	58	92
ENSECAG00000006177	2.585753275	0.069120326	0.351624179	48	85	71	111	130	109	273	138
ENSECAG00000017715	1.841284562	0.06918579	0.351800011	21	25	63	63	95	28	203	85
ENSECAG00000007960	8.391951606	0.06946594	0.353064733	5389	12408	5552	7436	3122	6542	5010	4996
ENSECAG00000015902	3.558075823	0.069496536	0.353064733	130	132	198	175	333	288	345	252
ENSECAG00000019264	8.09727409	0.069601536	0.353430836	7927	10324	3110	2412	2731	4981	3190	3846
ENSECAG00000017744	6.672092703	0.069630686	0.353430836	1388	2385	2267	3484	843	1891	1285	2235
ENSECAG00000006359	1.62739509	0.06974938	0.353875531	25	36	40	40	119	18	146	65
ENSECAG00000014867	10.27701093	0.069855907	0.354258131	27695	50715	38420	14336	284	20366	421	23880
ENSECAG00000014905	0.720564263	0.069896722	0.354307291	15	15	23	24	60	30	36	43
ENSECAG00000005667	3.957562631	0.070048282	0.354917529	151	215	190	288	300	258	745	372
ENSECAG00000021244	0.790779336	0.070189905	0.355476899	20	36	53	52	12	18	15	47
ENSECAG00000009193	5.777458414	0.070317207	0.355640436	989.011	1729	860.008	1316	397.001	1173	532	1195.02
ENSECAG00000014340	2.604893501	0.070325489	0.355640436	64.0034	57	77	108	129.003	77.9924	305	161.998
ENSECAG00000015123	7.316404818	0.070336336	0.355640436	2863	3503	3538	4389	2169	2494	2440	2832
ENSECAG00000022905	6.226778262	0.070347146	0.355640436	2192	2143	980	892	1175	1090	1279	957
ENSECAG00000024185	5.319467655	0.070505966	0.355965104	672	440	1437	1932	19	586	27	1029
ENSECAG00000013228	4.204560879	0.070549562	0.355965104	570	554	315	209	78	429	42	359
ENSECAG00000015734	4.764444942	0.070551571	0.355965104	157	309	418	708	845	617	749	611
ENSECAG00000013059	3.221776649	0.070580128	0.355965104	123	240	222	268	59	159	182	178
ENSECAG00000000537	8.056711839	0.070580768	0.355965104	5637	11699	4241	2318	2004	4773	3137	4605
ENSECAG00000013000	2.202835366	0.070598964	0.355965104	51	56	41	70	133	76	246	59
ENSECAG00000022888	3.372857765	0.07064164	0.355975252	98	87	170	211	204	233	415	250
ENSECAG00000025059	6.258592086	0.070663615	0.355975252	973	1345	1923	2812	1032	1157	1322	1455
ENSECAG00000001583	1.362653522	0.070724654	0.355975252	24	16	30	58	45	65	88	75
ENSECAG00000011895	9.572812083	0.070726045	0.355975252	33149	38482	4781	3238	8	13230	9	4216
ENSECAG00000015569	0.039487283	0.070772982	0.356054089	3	4	23	12	41	8	25	35
ENSECAG00000001581	6.172130857	0.07086455	0.356357291	935	2910	1259	1369	750	1296	951	1417
ENSECAG00000007922	4.064928738	0.071015184	0.356957118	370	727	508	217	2	128	0	421
ENSECAG00000024627	5.935795661	0.071124024	0.357346429	1228	1843	1066	1144	716	1328	1182	587
ENSECAG00000008331	5.194979526	0.071174764	0.357441743	733	695	783	895	579	700	583	542
ENSECAG00000011196	7.00266203	0.071234252	0.357441743	3849	7882	961	1394	3	2545	1	592
ENSECAG00000003626	4.16842189	0.071245444	0.357441743	218	1704	58	58	2	320	0	137
ENSECAG00000007145	3.053673492	0.071268578	0.357441743	84	85	514	213	24	232	11	92
ENSECAG00000010727	7.695695333	0.07138477	0.357798863	6544	6448	2480	2328	1794	4554	2312	2566
ENSECAG00000011747	1.527668659	0.071402637	0.357798863	40	33	90	116	66	13	52	28
ENSECAG00000007134	6.759126807	0.071453639	0.357825439	1913	3513	2053	2198	1086	2540	1339	1481
ENSECAG00000011939	6.343451366	0.071470799	0.357825439	1313	2767	1612	1512	756	1814	957	1396
ENSECAG00000021678	6.697565573	0.071504219	0.357835397	2555	2886	1598	1813	1622	1684	1194	1767

ENSECAG00000021704	5.303505142	0.071542137	0.357867852	502	1000	752	1305	485	818	566	681
ENSECAG00000007534	6.39804396	0.071601768	0.358008841	1310	3425	1233	1850	556	2204	622	1317
ENSECAG00000011514	0.030678344	0.071682175	0.358253537	5	4	22	13	35	19	31	20
ENSECAG00000005199	7.539115728	0.071874315	0.359056199	6205	5302	2320	2991	591	3566	618	4316
ENSECAG00000015385	2.794041271	0.071918787	0.359120783	66	84	161	402	53	60	141	148
ENSECAG00000000732	2.046480934	0.072081272	0.35965825	3	36	63	103	111	115	163	80
ENSECAG00000009095	7.844499449	0.072089603	0.35965825	2841	6624	5011	6910	3517	3928	3618	3035
ENSECAG00000007250	4.59814015	0.072159908	0.359816944	258	428	308	324	649	502	853	516
ENSECAG00000018167	5.346408133	0.07218462	0.359816944	1661.01	1743	324	382	3.00043	1014	3	167
ENSECAG00000012116	3.044577966	0.072238082	0.359840724	26	42	80	290	115	269	290	256
ENSECAG00000008742	6.292718177	0.072282773	0.359840724	692	366	1703	1414	2636	1588	1806	2387
ENSECAG00000011496	8.438660571	0.072315148	0.359840724	5253	6706	13749	7086	2549	7137	3430	6610
ENSECAG00000024689	4.663413202	0.072315817	0.359840724	276	303	416	409	804	411	791	621
ENSECAG00000015058	6.578373056	0.07238344	0.360019864	1102	522	1941	1381	2224	2218	3624	2329
ENSECAG00000005194	2.824470867	0.072492055	0.360281641	112	118	210	295	42	9	288	39
ENSECAG00000000478	2.920359425	0.072499362	0.360281641	70	59	148	111	187	99	342	204
ENSECAG00000024000	6.432160928	0.072815399	0.36169429	584	1027	899	2252	1222	1876	2347	3926
ENSECAG00000000309	0.272174751	0.073105491	0.362976891	31	34	19	20	21	3	31	5
ENSECAG00000011954	7.854662733	0.073200817	0.36329176	4602	9203	3782	3262	1915	4869	2070	4014
ENSECAG00000008957	6.579455604	0.073346072	0.363751553	2268	3275	1467	1762	326	2309	470	1899
ENSECAG00000020958	0.467951657	0.073400647	0.363751553	12	8	16	27	46	15	54	31
ENSECAG00000013747	6.395597483	0.073445082	0.363751553	2232	2423	1686	1477	308	1753	155	1981
ENSECAG00000018521	6.591597921	0.073448785	0.363751553	3204	1436	1531	1823	1683	1188	1618	1360
ENSECAG00000013066	3.641634725	0.07348259	0.363751553	70	70	218	339	406	243	332	355
ENSECAG00000011545	5.201470298	0.073485162	0.363751553	843	1330	442	595	192	756	416	768
ENSECAG00000023570	0.937321449	0.073712385	0.364609757	23	119	27	18	5	54	4	13
ENSECAG00000021295	6.847475335	0.073778329	0.364609757	1862	2244	1810	4828	1931	1417	2262	1616
ENSECAG00000001414	4.516277359	0.073790095	0.364609757	124	270	345.002	574	819	385	817	411
ENSECAG00000020874	5.201132938	0.073796773	0.364609757	630	746	1366	920	77	645	77	1047
ENSECAG00000007007	6.191275315	0.073835201	0.364609757	693	926	1115	1309	1742	1158	3090	1828
ENSECAG00000019914	7.17702155	0.073850689	0.364609757	1293	1517	7292	4609.04	2765	2016	957	2219
ENSECAG00000010420	6.836286417	0.073915551	0.3647718	814	911	1402	2912	1454	1794	4395	5294
ENSECAG00000021631	6.009821039	0.073983932	0.364818029	1277	1935	1453	901	729	1525	653	977
ENSECAG00000013478	3.044354598	0.07400682	0.364818029	75	89	116	163	279	117	279	201
ENSECAG00000016916	7.966295092	0.074037468	0.364818029	2939	4539	6044	10773	2846	3123	4310	5081
ENSECAG00000013419	3.238573979	0.074107621	0.364818029	108	381	126	248	155	166	129	114
ENSECAG00000024460	4.285231076	0.074110052	0.364818029	302	585	409	371	275	282	409	322
ENSECAG00000013238	1.290940539	0.074117181	0.364818029	12	16	49	40	117	60	52	31
ENSECAG00000000398	4.767393452	0.074176136	0.364950436	366	449	848	765	381	440	390	572
ENSECAG00000009892	5.374618963	0.07423363	0.365075543	920	1306	570	675	554	592	894	625
ENSECAG00000004464	0.561352589	0.074281024	0.365150887	25.0001	19	40.0001	47.0006	17.0001	24.0005	16.0006	23.0004
ENSECAG00000009881	6.877945514	0.074325129	0.365210008	3120	6722	1482	655	18	2737	75	1239
ENSECAG00000016143	2.434667566	0.07436271	0.365237036	28	55	64	146	115	126	131	204
ENSECAG00000013474	1.08940674	0.074402484	0.365274811	10	28	35	32	76	39	73	36
ENSECAG00000007599	6.531274123	0.074492156	0.365557414	1438	3036	1732	1917	1109	1345	1560	1881
ENSECAG00000021721	0.659170693	0.074571887	0.365791011	8	27	9	31	46	35	45	38
ENSECAG00000018973	5.850445077	0.074771136	0.366610413	2511	2695	401	387	0	1024	1	382
ENSECAG00000014262	4.201890787	0.074823214	0.36670783	208	191	312	311	437	381	503	572
ENSECAG00000014923	5.660900974	0.075062922	0.367724342	350	548	810	1098	1337	1371	2265	492
ENSECAG00000024334	2.929250921	0.075163032	0.368056394	41	65	188	98	112	162	223	336
ENSECAG00000024053	2.889844148	0.075242274	0.368184035	95	52	123	69	308	102	329	92
ENSECAG00000009804	0.933471531	0.075281049	0.368184035	17	28	14	21	113	15	45	34
ENSECAG00000023093	3.174036522	0.075286116	0.368184035	94	296	157	286	107	102	155	198
ENSECAG00000012862	9.182010943	0.075483281	0.368741072	19256	19986	5276	6480	3355	13482	4033	8354
ENSECAG00000016382	0.786745832	0.075496853	0.368741072	25	8	19	22	26	50	57	50
ENSECAG00000000424	0.997691857	0.075497184	0.368741072	16	17	22	45	63	40	53	50
ENSECAG00000025139	2.256239863	0.075536618	0.368775469	43	106	89	228	53	60	66	102
ENSECAG00000017382	5.709032378	0.075602905	0.36894088	682	1709	776	1527	517	920	909	1016
ENSECAG00000017827	7.108304677	0.075650577	0.368968857	3481	5443	1762	1455	1229	3042	1085	2090
ENSECAG00000013998	4.155490623	0.075673455	0.368968857	166	225	184	421	634	266	505	458
ENSECAG00000008769	7.524339398	0.075715929	0.369017916	5273	4527	3519	2505	2043	4246	1866	2236
ENSECAG00000016998	5.809056103	0.075769345	0.369077423	622	1372	1096	2157	440	801	1032	1325
ENSECAG00000019742	2.260572677	0.075819537	0.369077423	70	192	62	120	41	31	64	134
ENSECAG00000022868	4.542427263	0.075825392	0.369077423	195	236	439	401	891	284	917	409
ENSECAG00000020563	8.530104667	0.075992622	0.369733333	5837	5337	13428	11007	6827	7591	4185	2167
ENSECAG00000017538	5.248337045	0.076074991	0.369803643	837	1700	605	226	248	837	278	673
ENSECAG00000022151	5.593349587	0.076120195	0.369803643	326	1134	1174	2202	521	823	311	1185
ENSECAG00000000395	4.760092661	0.076120641	0.369803643	460	362	878	724	610	406	481	217
ENSECAG00000017421	2.166855698	0.076137	0.369803643	18	67	63	93	129	104	137	105
ENSECAG00000023322	5.118380812	0.076175197	0.369831393	204	940	786	1455	784	383	555	439
ENSECAG00000017148	6.153712234	0.076215866	0.369871113	2345	2205	700	627	914	1130	1227	831
ENSECAG00000013304	5.805293307	0.076322067	0.370228688	1532	1625	707	751	688	952	865	932
ENSECAG00000017287	1.889459213	0.076440007	0.370642881	25	31	56	84	78	73	115	127
ENSECAG00000013757	6.393551501	0.076515427	0.370850634	2439	2307	1117	1090	1518	1155	1244	1166
ENSECAG00000011044	1.438831676	0.076702294	0.371598134	21	152	47	34	28	33	21	57
ENSECAG00000024092	6.679473723	0.076830109	0.371832869	3296	2346	1393	1558	1215	2312	1431	951

ENSECAG00000000190	1.720711216	0.076855354	0.371832869	19	27	57	71	62	81	113	93
ENSECAG00000008388	2.070056731	0.076871698	0.371832869	32	29	80	71	56	157	110	123
ENSECAG00000009003	6.44422017	0.076881386	0.371832869	1555	2332	1439	2320	870	1135	1783	1853
ENSECAG00000010772	4.750486335	0.077026232	0.372375223	372	488	628	908	527	359	537	376
ENSECAG00000001784	9.325858349	0.07708814	0.372516328	29422	7703	16380	4346	1007	14730	1094	9931
ENSECAG00000007444	6.238201504	0.077395304	0.373841974	1241	3101	1171	1091	1024	1454	971	1123
ENSECAG00000022323	3.646560167	0.077485841	0.373964804	169	413	283	249	103	260	170	238
ENSECAG00000022078	6.229514542	0.077517543	0.373964804	2098	3624	893	456	160	2164	117	758
ENSECAG00000001129	6.985839615	0.077519274	0.373964804	3375	2736	2579	2333	1425	3239	1333	1275
ENSECAG00000019803	3.575174755	0.077578218	0.374090644	110	378	229	387	148	233	154	203
ENSECAG00000020935	6.474025412	0.077630256	0.374183093	941	1042	1400	1442	2185	1301	3400	2626
ENSECAG00000018640	4.460368184	0.07766733	0.374203366	228	200	385	433	544	551	475	672
ENSECAG00000021961	4.368610119	0.077770857	0.374543656	114	252	292	546	514	595	688	360
ENSECAG00000004470	5.633084217	0.077890802	0.374587269	509	656	755	920	1297	990	1698	1122
ENSECAG00000021812	2.972813263	0.077897673	0.374587269	84	282	193	151	83	157	118	116
ENSECAG00000016238	8.919738436	0.077902236	0.374587269	11410	17170	6851	7246	4396	9706	6142	7257
ENSECAG00000020717	3.720361271	0.07791152	0.374587269	85	130	223	305	196	283	473	455
ENSECAG00000019232	3.674138614	0.077961405	0.374591703	169	137	133	120	751	86	467	146
ENSECAG00000009180	5.978444785	0.077978247	0.374591703	2483	1762	881	318	136	1397	344	1148
ENSECAG00000021932	3.858956742	0.078064859	0.374849607	136	263	144	228	203	361	647	347
ENSECAG00000018901	4.141281862	0.078159765	0.375070296	127	236	253	400	366	309	546	622
ENSECAG00000010270	2.170389189	0.07818545	0.375070296	10	43	72	108	102	63	134	201
ENSECAG00000011042	2.122693999	0.078209652	0.375070296	72	98	179	50	54	70	20	89
ENSECAG00000017628	3.521109251	0.078360918	0.375637497	401	346	79	197	41	330	40	135
ENSECAG00000024826	5.410290352	0.078477578	0.376038393	321	644	631	883	1003	1205	1016	1092
ENSECAG00000022712	5.697826702	0.078569769	0.376196408	593	1035	1141	1985	617	890	1010	920
ENSECAG00000002871	4.944107573	0.078618222	0.376196408	579	1456	318	347	267	677	465	368
ENSECAG00000026958	6.035981614	0.078629424	0.376196408	1645	2408	712	813	627	1318	754	1137
ENSECAG00000014479	3.931279325	0.078642727	0.376196408	384	587	232	94	226	207	148	283
ENSECAG00000026846	7.204150044	0.078892409	0.377156631	2668	5845	2529	2155	1675	3310	1426	2007
ENSECAG00000017969	5.33164401	0.078909714	0.377156631	1446	1239	951	338	9	756	6	690
ENSECAG00000008724	4.664968039	0.079015476	0.377398743	260	639	843	631	172	705	285	353
ENSECAG00000014145	1.232142263	0.079043459	0.377398743	18	17	42	38	85	44	86	34
ENSECAG00000003192	9.96773946	0.079059816	0.377398743	28942	41261	13142	11728	1861	28792	1841	10809
ENSECAG00000011679	9.428189382	0.079200222	0.377910529	15406	29441	9602	8147	4766	16895	4639	9585
ENSECAG00000006305	1.033737571	0.079233568	0.377911256	18	39	60	73	24	26	32	36
ENSECAG00000014825	6.314022958	0.079326419	0.378195679	766	1319	1066	1252	2344	1843	2190	1848
ENSECAG00000015874	0.808182683	0.079511505	0.378919418	18	14	28	20	72	29	49	32
ENSECAG00000015520	5.464682012	0.079669168	0.379511915	322	674	875	637	1341	902	1407	922
ENSECAG00000015994	3.408623686	0.079708836	0.379542073	298	406	161	90	15	259	83	164
ENSECAG00000020627	4.069284572	0.079936839	0.380468609	203	472	327	498	274	194	245	379
ENSECAG00000021453	1.61415127	0.08005601	0.380876586	18	29	17	93	94	85	96	53
ENSECAG00000017684	5.898809177	0.080127068	0.38091497	670	1559	1148	2109	666	1277	876	992
ENSECAG00000009841	5.591334832	0.080130993	0.38091497	626	779	1126	1873	1041	606	818	704
ENSECAG00000022082	3.532281093	0.080356917	0.38179568	114	183	169	155	336	189	434	259
ENSECAG00000002977	2.01189998	0.080383333	0.38179568	15	68	51	55	251	46	99	49
ENSECAG00000015485	3.775463765	0.080479902	0.382094949	155	281	314.004	500	234	232	225	195
ENSECAG00000017687	8.581387548	0.080702734	0.382965109	9530	11619	6091	6339	3063	8495	4817	5529
ENSECAG00000019885	7.462702403	0.080730458	0.382965109	2926	7947	2750	2195	2285	3037	1924	2881
ENSECAG00000022563	2.68100576	0.080849254	0.38336891	36	43	64.0016	176	355	98	218	49
ENSECAG00000009909	6.791228231	0.080925929	0.383518624	1330	2734	2444	3789	1556	2292	1559	1484
ENSECAG00000005250	5.883275695	0.0809482	0.383518624	1500	1510	1018	893	649	1246	724	1018
ENSECAG00000011878	1.369698535	0.080984658	0.38353175	17	29	13	69	72	64	108	36
ENSECAG00000009398	2.924030443	0.081211559	0.38437717	93	45	69	180	276	117	247	180
ENSECAG00000011276	5.297441893	0.081240526	0.38437717	932	903	690	712	639	609	802	532
ENSECAG00000000614	4.112748538	0.081264457	0.38437717	188	844	238	315	102	302	287	335
ENSECAG00000018832	4.909782681	0.081299544	0.384383431	135	393	636	577	972	585	808	766
ENSECAG00000006110	0.739679642	0.081467363	0.384958796	8	16	53	93	17	25	26	18
ENSECAG00000020193	8.917810921	0.081488862	0.384958796	6615	25527	10374	5586	1330	9752	920	10490
ENSECAG00000023599	1.247296976	0.081589745	0.385275506	27	13	12	59	52	38	103	68
ENSECAG00000003887	7.318620624	0.081685054	0.385511691	2684	5688	2925	2852	1865	3025	2469	2344
ENSECAG00000013026	3.495620861	0.081707484	0.385511691	115	186	341	409	133	172	254	168
ENSECAG00000015703	5.336219596	0.081765118	0.385623806	518	918	926	1227	510	493	770	939
ENSECAG00000010036	6.339091452	0.081909798	0.38614619	2473	2181	939	1371	373	1849	738	1558
ENSECAG00000011960	5.087794813	0.081959128	0.386167704	212	366	495	936	721	629	1057	1159
ENSECAG00000009834	2.987731089	0.081982199	0.386167704	75	205	107	393	60	151	105	149
ENSECAG00000010670	2.495498159	0.082133048	0.386718259	48	55	102	93	156	81	198	165
ENSECAG00000021538	3.593295012	0.082214536	0.386733911	130	70	252	176	396	174	461	272
ENSECAG00000009984	5.619346407	0.082224154	0.386733911	785	1452	885	1209	445	831	547	1290
ENSECAG00000009676	6.462654178	0.082239092	0.386733911	777	747	1724	1702	2437	1728	3066	2097
ENSECAG00000023736	0.033997552	0.082272247	0.386733911	15	8	12	4	31	27	25	21
ENSECAG00000013287	0.933958176	0.082663175	0.388411164	26	70	59	32	2	17	3	62
ENSECAG00000019161	1.538267208	0.082727688	0.388505803	17	29	38	71	64	71	102	67
ENSECAG00000016721	9.367325467	0.082751565	0.388505803	33411	15217	7738	2714	878	19020	655	4188
ENSECAG00000015652	6.114414059	0.082844792	0.388776981	744	1189	1883	2666	1288	1204	1152	798
ENSECAG00000021764	6.120350692	0.082877623	0.388776981	344	832	1038	1863	1824	1766	2014	1695

ENSECAG00000015022	3.035258149	0.083247571	0.390351561	104	123	80	107	267	132	283	188
ENSECAG00000012669	4.104405817	0.083414291	0.390822032	142	133	308	392	471	256	482	597
ENSECAG00000015236	5.193639614	0.08341656	0.390822032	227	182	837	518	2523	421	911	267
ENSECAG00000022988	0.467629348	0.083454763	0.39084018	35	6	48	41	4	20	3	34
ENSECAG00000011086	7.552700006	0.083651119	0.391598679	8716	6510	1454	771	106	5656	170	563
ENSECAG00000011249	4.10847993	0.083763455	0.391963392	330	408	404	387	109	164	228	564
ENSECAG00000014470	2.87634472	0.083894429	0.392414986	61	73	161	88	245	107	221	208
ENSECAG00000009486	4.498099805	0.084033871	0.392881713	190	294	348	495	473	550	654	621
ENSECAG00000016075	8.903672337	0.084063228	0.392881713	12539	24848	7114	1394	851	12868	1091	5004
ENSECAG00000012974	2.699452948	0.084099288	0.39288896	61	63	89	93.0001	336	90	258	41
ENSECAG00000022023	6.26499571	0.084161179	0.393016826	1134	2397	1671	1465	1043	1283	1311	1362
ENSECAG00000009520	4.295834334	0.084299146	0.393499705	182	292	303	346	494	502	633	372
ENSECAG00000021208	7.054118427	0.084351545	0.393582924	3278	4196	1911	1931	1627	2361	1933	2051
ENSECAG00000010400	3.33867143	0.084470125	0.393974755	231	364	109	162	112	81	209	213
ENSECAG00000021173	5.919840621	0.084534879	0.394039549	1062	2756	834	776	470	1536	426	943
ENSECAG00000008873	1.419344242	0.084599192	0.394039549	33	29	27	31	40	33	159	68
ENSECAG00000017077	8.642948703	0.084619519	0.394039549	9655	15263	4637	5398	4423	8049	5141	4996
ENSECAG00000008761	0.821528168	0.084622459	0.394039549	16	64	39	39	20	29	30	20
ENSECAG00000025131	5.976439597	0.084771813	0.394385606	1139	2132	1019	1199	494	1337	748	1293
ENSECAG00000011602	1.907665724	0.084790359	0.394385606	16	8	46	102	59	18	135	234
ENSECAG00000016134	7.891491439	0.0848007	0.394385606	4906	10182	3139	3158	1832	4501	1592	5061
ENSECAG00000013544	3.948974385	0.084908211	0.394517153	199	340	338	560	126	315	131	371
ENSECAG00000012297	1.988263154	0.084914408	0.394517153	38	54	111	195	17	51	59	96
ENSECAG00000007798	4.774977666	0.084932942	0.394517153	226	274	492	619	692	613	706	777
ENSECAG00000015268	3.967541989	0.085056464	0.394582122	157	209	200	313	315	268	502	542
ENSECAG00000023059	2.671111142	0.085103614	0.394582122	83	86	46	83	305	81	203	104
ENSECAG00000019000	5.282834157	0.085111344	0.394582122	848	1557	552	368	344	810	440	696
ENSECAG00000011364	2.970999319	0.085116386	0.394582122	69	104	67	163	207	67	392	208
ENSECAG00000002508	6.330488435	0.085129725	0.394582122	1122	2065	1631	2283	1131	1360	1476	1350
ENSECAG00000021876	5.866517557	0.085154877	0.394582122	986	3286	521	676	221	1676	317	698
ENSECAG00000012336	6.487417883	0.085222433	0.394681708	2620	3439	885	682	992	1988	882	1044
ENSECAG00000015628	1.789246247	0.085245702	0.394681708	40	29	48	52	73	52	170	81
ENSECAG00000010145	1.903288628	0.08534066	0.394960736	57	48	90	184	17	10	46	124
ENSECAG00000014742	3.410242885	0.085543657	0.395739347	71	89	212	220	235	170	304	419
ENSECAG00000016782	8.578122236	0.08561449	0.395782046	8684	15362	5813	3943	3257	8840	4545	4454
ENSECAG00000023155	4.778251759	0.085622414	0.395782046	329	274	321	610	858	410	934	660
ENSECAG00000014990	6.572114843	0.085681823	0.395823649	1136	3026	1968	2377	1329	1373	1687	1785
ENSECAG00000003853	5.011959823	0.085700948	0.395823649	750	1291	449	368	215	972	167	363
ENSECAG00000001445	7.913015885	0.085807109	0.396051275	6074	5386	4352	4982	3137	5112	3401	2832
ENSECAG00000014490	3.185960051	0.085819806	0.396051275	207	255	100	239	77	181	74	197
ENSECAG00000020196	1.439732574	0.085938808	0.396258859	12	35	14	71	32	59	59	147
ENSECAG00000010882	5.360676945	0.085968536	0.396258859	404	765	977	1663	656	692	808	562
ENSECAG00000017629	6.461710925	0.085969204	0.396258859	1626	2969	1470	1474	1680	1245	1474	1194
ENSECAG00000012567	3.051077057	0.086093753	0.396672348	90	43	113	182	320	110	302	168
ENSECAG00000020995	1.305670288	0.086208519	0.397040449	23	69	80	51	32	37	50	26
ENSECAG00000018528	2.56903246	0.086366571	0.397220722	43	60	123	93	192	127	175	126
ENSECAG00000024259	8.717800169	0.086385094	0.397220722	4551	3503	5900	7769	9358	6611	19708	11383
ENSECAG00000023356	4.246767764	0.086417532	0.397220722	249	248	224	319	483	341	644	494
ENSECAG00000026999	4.441436097	0.086434689	0.397220722	278	860	376	437	148	525	262	379
ENSECAG00000010242	6.057697274	0.086462886	0.397220722	1968	3317	517	304	135	1656	197	991
ENSECAG00000018834	1.887402252	0.086491612	0.397220722	32	47	26	74	82	35	221	81
ENSECAG00000008017	4.231598483	0.08649189	0.397220722	201	175	287	392	487	273	711	502
ENSECAG00000006558	6.34414345	0.086583663	0.397481855	803	851	1354	1602	2154	1732	2712	1854
ENSECAG00000026811	2.060654004	0.086635817	0.397560974	41	45	40	56	101	22	325	52
ENSECAG00000024590	4.589862329	0.086676405	0.397586977	171	316	429	467	834	606	659	368
ENSECAG00000024124	6.401533252	0.086873435	0.398235275	673	1371	1203	1441	2507	1353	3577	1599
ENSECAG00000023088	2.59432945	0.086906391	0.398235275	63	114	177	204	107	89	102	78
ENSECAG00000018018	7.668125534	0.086922675	0.398235275	1926	1803	3861	2851	8743	4001	6501	2860
ENSECAG00000017935	7.578661216	0.087013998	0.398352603	4932	6268	2232	2955	1958	3352	2672	3267
ENSECAG00000011885	2.12774516	0.087018263	0.398352603	20	28	76	91	128	75	268	32
ENSECAG00000023949	2.963513323	0.087190926	0.398982592	111	118	450	130	6	113	14	218
ENSECAG00000007767	7.798498239	0.087263418	0.399024501	7033	5530	3486	2598	3644	4012	2313	2744
ENSECAG00000012475	0.944615564	0.087278261	0.399024501	15	18	21	41	48	58	71	23
ENSECAG00000023706	5.480058616	0.087312724	0.399024501	1033	1367	552	816	589	1060	497	584
ENSECAG00000007668	6.672131044	0.087340277	0.399024501	545	1170	1912	2145	2777	1777	4214	2268
ENSECAG00000000901	5.091756083	0.087501765	0.399559182	441	367	1480	867	843	442	452	373
ENSECAG00000022092	6.568916031	0.087527501	0.399559182	1660	2829	1789	2120	818	2187	1002	1796
ENSECAG00000015712	3.625738982	0.087578559	0.399632021	117	245	104	181	177	317	531	299
ENSECAG00000010136	6.838158014	0.087858373	0.400748226	2622	3919	1519	1748	1605	1050	2215	2169
ENSECAG00000014181	6.93290189	0.087920818	0.400872454	1428	1324	1616	1889	3441	1509	6060	2568
ENSECAG00000020138	5.104004007	0.087958338	0.40088298	492	720	707	1201	241	733	470	753
ENSECAG00000006784	7.757813269	0.087998986	0.400907747	2704	9150	3585	4266	3453	3250	3958	2479
ENSECAG00000011117	3.891665141	0.088054435	0.400999897	399	894	103	79	12	351	2	169
ENSECAG00000018264	7.088801098	0.088111858	0.40110096	1270	1254	2056	2857	3986	2077	4841	3780
ENSECAG00000023942	0.742832856	0.088330115	0.401911461	4	25	26	26	25	64	45	39
ENSECAG00000016970	4.33686386	0.08837464	0.401911461	87	159	340	439	1361	168	569	171

ENSECAG00000021447	4.82811607	0.08839581	0.401911461	906	626	344	423	412	501	594	251
ENSECAG00000011752	2.550655335	0.088628222	0.40280731	45	66	89	119	162	88	216	154
ENSECAG00000000387	8.113034242	0.088715797	0.403044434	6720	11150	4969	1574	2892	1848	3840	6812
ENSECAG00000015553	2.35353688	0.089078171	0.404430301	47	78	128	242	54	79	97	80
ENSECAG00000009253	1.04655126	0.089091892	0.404430301	13	31	24	33	58	25	97	41
ENSECAG00000019499	5.448836117	0.089160351	0.404579753	929	2590	253	196	316	1059	313	473
ENSECAG00000007773	3.986230405	0.089256942	0.404856689	194	217	152	294	431	217	589	433
ENSECAG00000003597	1.825719493	0.089426658	0.40509006	25	47	42	72	93	77	151	55
ENSECAG00000021525	10.03690339	0.089462382	0.40509006	24912	32147	15035	20694	17587	22026	12119	8340
ENSECAG00000008829	2.880739981	0.089477771	0.40509006	32	59	211	68	103	160	227	326
ENSECAG00000024271	2.221619623	0.089482112	0.40509006	41	24	62	89	28	77	112	312
ENSECAG00000024236	4.101476035	0.089486298	0.40509006	206	326	330	719	205	205	332	389
ENSECAG00000017584	5.693102987	0.089585213	0.405256735	357	635	851	1159	1207	687	2086	1517
ENSECAG00000020169	0.202434145	0.089594308	0.405256735	12	12	15	11	24	43	24	23
ENSECAG00000026878	1.546969361	0.0896405	0.405304643	29	46	34	37	96	59	82	64
ENSECAG00000021451	6.098675342	0.089694693	0.405388677	1187	2821	794	1149	757	1289	1012	1161
ENSECAG00000016979	4.311948603	0.089731704	0.405395018	131	252	384	384	614	476	625	327
ENSECAG00000024171	5.258449145	0.089831785	0.405686187	950	716	708	775	544	793	599	549
ENSECAG00000002522	1.404146187	0.089975481	0.406174009	54	46	66	59	33	37	38	51
ENSECAG00000015666	6.148717059	0.090192624	0.406992877	952	1037	2232	2288	1517	928	1496	641
ENSECAG00000008811	7.275147782	0.090328668	0.407445278	7009	6667	1044	785	4	2964	3	1312
ENSECAG00000003532	4.443052845	0.09036914	0.407466401	309	715	380	467	262	385	368	415
ENSECAG00000017462	3.118201213	0.090568704	0.408183625	89	249	190	264	108	188	135	111
ENSECAG00000009966	6.863488122	0.090599914	0.408183625	6039	3465	337	1195	8	3236	65	332
ENSECAG00000008375	3.235879787	0.090641803	0.40821081	71	102	107	239	142	246	194	408
ENSECAG00000003981	7.424330594	0.090723913	0.408267226	1540	1017	3549	2150	8850	1438	6976	2371
ENSECAG00000002387	5.00081531	0.090752132	0.408267226	746	1440	407	333	79	181	232	1184
ENSECAG00000023717	7.059283875	0.09076191	0.408267226	3005	4977	1530	2003	2038	2459	1827	1549
ENSECAG00000013636	8.963470478	0.090897057	0.408608839	3795	8522	5216	8861	17062	11177	16684	9121
ENSECAG00000012455	5.675174226	0.090909635	0.408608839	893	1632	816	981	762	593	1110	939
ENSECAG00000003013	2.996995571	0.090990811	0.408697924	57	132	115	123	273	100	306	173
ENSECAG00000019905	0.993869818	0.091001251	0.408697924	8	17	19	50	19	64	21	109
ENSECAG00000010123	1.376847291	0.091148223	0.40907426	22	25	40	44	67	29	96	83
ENSECAG00000023926	5.943426304	0.091156908	0.40907426	1149	1564	1117	1363	970	906	1346	905
ENSECAG00000016546	3.196736956	0.091211834	0.409159468	73	105	198	129	192	231	284	244
ENSECAG00000017766	4.3370407	0.091264835	0.409235977	204	207	350	393	661	340	512	549
ENSECAG00000010208	1.150659823	0.091310345	0.409278851	35	60	47	52	26	41	16	43
ENSECAG00000009680	8.915716784	0.091508742	0.4100067	9963	16665	7655	8357	4508	8293	5760	9924
ENSECAG00000014760	6.265749832	0.091595908	0.410235806	553	1138	1493	1127	1455	2714	1931	1829
ENSECAG00000014658	0.531362028	0.091753068	0.410778088	11	15	24	17	36	15	65	36
ENSECAG00000013955	6.898622178	0.091836941	0.410900653	1511	4526	1769	3267	924	2503	1373	2296
ENSECAG00000008821	7.391193339	0.091852627	0.410900653	7494	7695	991	558	9	3418	3	1221
ENSECAG00000012900	9.858246822	0.092011289	0.411211922	23798	29054	12630	14148	15137	10527	18555	11489
ENSECAG00000011402	7.262390916	0.092060803	0.411211922	1234	1419	2198	3675	3805	2594	4540	5572
ENSECAG00000007445	4.214560303	0.092064378	0.411211922	161	226	353	330	343	445	628	490
ENSECAG00000010331	3.447205999	0.092066683	0.411211922	160	203	347	239	172	171	220	152
ENSECAG00000021270	4.832048571	0.092136171	0.411360907	495	648	702	601	278	488	344	739
ENSECAG00000024928	6.086609529	0.092292177	0.411895897	801.011	1867	1687.01	1758	635.001	1176	953	1616.02
ENSECAG00000021921	2.000666984	0.092360683	0.412040115	36	34	75	57	111	46	178	99
ENSECAG00000019765	5.717307228	0.092430279	0.412189081	826	3059	922	331	155	1730	87	357
ENSECAG00000017840	7.760617748	0.092512084	0.412392355	6200	9745	2489	2329	281	6024	179	2612
ENSECAG00000020924	4.406335013	0.092579683	0.412493927	123	333	347	432	355	648	708	477
ENSECAG00000017056	4.597126905	0.092621139	0.412493927	508	856	348	355	138	515	404	424
ENSECAG00000021269	8.907713747	0.092643564	0.412493927	25229	3726	10248	2616	961	14392	1067	3185
ENSECAG00000014493	2.066271813	0.092788732	0.412978779	76	287	33	27	3	103	1	44
ENSECAG00000019890	4.070910354	0.092902166	0.413322062	237	213	236	188	459	345	682	282
ENSECAG00000006998	0.658510782	0.092949991	0.413373298	19	22	86	21	8	29	6	33
ENSECAG00000015122	6.733531822	0.093079765	0.413759384	2593	2959	1396	2036	1176	1445	2177	1940
ENSECAG00000012077	5.290874592	0.09311319	0.413759384	606	1771	488	573	280	455	907	803
ENSECAG00000023420	11.30775061	0.093156838	0.413759384	32385	70707	89722	62178	10213	45892	5116	67697
ENSECAG00000013981	6.203825086	0.093182175	0.413759384	3090	3439	239	561	4	1780	5	407
ENSECAG00000020524	3.095182445	0.093292909	0.414089579	106	179	263	215	147	137	97	158
ENSECAG00000019201	3.056372801	0.093330647	0.414095641	107	93	88	148	183	135	347	221
ENSECAG00000005515	3.812803878	0.093538306	0.414753415	298	250	416	261	314	205	276	66
ENSECAG00000022565	2.364604833	0.093551758	0.414753415	39	437	65	16	0	96	3	70
ENSECAG00000010117	9.289501671	0.093588324	0.414754015	32705	13766	5341	12317	12	13469	2	2529
ENSECAG00000010613	7.745827078	0.093698256	0.41507963	7648	3814	6011	2730	216	5242	187	3651
ENSECAG00000010482	5.750308377	0.093770794	0.415239398	1206	1797	713	793	534	1117	764	938
ENSECAG00000011621	6.352446474	0.093823915	0.415313092	977	1375	1832	3423	1124	955	1530	1760
ENSECAG00000013045	4.151938301	0.093873877	0.415372752	494	375	268	310	180	291	306	359
ENSECAG00000004274	7.514389294	0.094035483	0.415926175	1866	1908	2765	3228	6394	3041	6652	3599
ENSECAG00000012884	6.923174339	0.094075675	0.415942354	1902	3150	2708	3069	1368	2402	1521	2315
ENSECAG00000019774	1.411456607	0.094139081	0.416061118	52	77	39	58	37	32	35	54
ENSECAG00000006234	7.49457654	0.094526211	0.417482277	6683	9116	1065	881	30	4685	34	1149
ENSECAG00000023673	5.713764371	0.094533975	0.417482277	716	1179	1148	1582	554	876	914	1173
ENSECAG00000009611	7.801753459	0.094573844	0.417496398	8803	4347	2827	2368	1745	4822	2226	3428

ENSECAG00000011890	10.63831678	0.094681406	0.417650803	34640	68048	20879	18634	14898	39669	15168	16990
ENSECAG00000012280	6.417321862	0.094682189	0.417650803	965	2964	1886	1933	1020	2156	960	1111
ENSECAG00000015743	7.277642433	0.094842949	0.418197898	1132	1419	2490	3417	7006	2975	3998	2619
ENSECAG00000007287	0.423560532	0.094920601	0.41837826	4	19	12	31	40	29	35	32
ENSECAG00000009757	5.086014033	0.094958763	0.418384488	372	413	1352	991	632	620	529	394
ENSECAG00000002612	0.586928204	0.095059555	0.418604706	46	23	51	7	16	35	8	12
ENSECAG00000000588	0.975788453	0.095082281	0.418604706	17	71	61	28	13	31	40	26
ENSECAG00000022470	1.896582009	0.09525758	0.419214359	52	51	121	122	97	44	37	36
ENSECAG00000015456	4.380609318	0.095410245	0.419449968	171	214	312	524	421	554	786	395
ENSECAG00000024474	5.016763392	0.095414738	0.419449968	347	477	512	521	759	622	1081	835
ENSECAG00000009654	3.706629017	0.095456394	0.419449968	152	164	185	205	450	178	433	300
ENSECAG00000009046	9.669610898	0.095458486	0.419449968	18978	29287	11821	10453	8779	15530	13017	10377
ENSECAG00000011226	1.665715818	0.095543823	0.419662974	33	29	40	60	77	44	132	84
ENSECAG00000018367	2.16452408	0.095616645	0.419820866	32	38	87	83	111	67	171	128
ENSECAG00000002142	3.344044897	0.095692921	0.419993794	206	241	185	237	109	251	143	130
ENSECAG00000026993	1.202107989	0.095958845	0.420998632	17	26	21	56	60	52	54	69
ENSECAG00000021042	3.601098875	0.09611339	0.421514232	128	181	263	588	125	174	198	273
ENSECAG00000019343	2.711778195	0.096214779	0.421796402	28	74	110	44	20	158	18	586
ENSECAG00000003049	1.740291563	0.096333575	0.422082805	37	56	159	53	41	53	42	57
ENSECAG00000023581	0.108056216	0.096354257	0.422082805	6	6	15	22	27	9	52	25
ENSECAG00000019837	7.71080433	0.09642612	0.422235145	2946	1154	3227	2902	8576	4978	6122	3067
ENSECAG00000001471	10.44571037	0.096581048	0.422750956	49898	42751	38510	13047	47	27418	53	16340
ENSECAG00000014268	7.278670887	0.096790578	0.423447977	2885	3449	3678	3577	1626	2402	2801	3118
ENSECAG00000013759	8.297467185	0.096854948	0.423447977	5209.02	10954	5433.02	6730	2148.99	5363.97	5137	6446.05
ENSECAG00000014963	4.483079499	0.096867203	0.423447977	171	341	322	457	359	422	994	580
ENSECAG00000019095	5.366979288	0.096908924	0.423447977	110	607	609	930	550	512	2627	1071
ENSECAG00000011638	7.794687966	0.096926256	0.423447977	4335	10750	2418	2183	2678	4591	2450	2664
ENSECAG00000014095	3.962807721	0.097000593	0.423493292	135	145	197	416	405	218	647	386
ENSECAG00000010268	7.540252475	0.097011024	0.423493292	3403	5052	3544	4245	2665	2556	3073	3574
ENSECAG00000016491	8.945789678	0.097100761	0.42356972	7379	22484	7835	7747	3586	11901	4173	7691
ENSECAG00000005480	5.412654634	0.09710294	0.42356972	246	470	472	1156	486	1131	2686	509
ENSECAG00000008310	5.685210879	0.097464876	0.424985679	490	705	691	996	1822	636	2069	913
ENSECAG00000011067	5.835409889	0.097660485	0.425521262	2927	2191	187	457	0	1141	0	239
ENSECAG00000008007	4.34555355	0.097671662	0.425521262	161	281	364	385	594	481	454	510
ENSECAG00000003343	3.445530601	0.097711169	0.425521262	415	277	193	111	8	337	12	102
ENSECAG00000009428	2.046528444	0.097771795	0.425521262	42	42	70	58	152	96	113	66
ENSECAG00000024964	5.131932897	0.097778622	0.425521262	1498	422	405	462	440	216	836	606
ENSECAG00000025004	4.354908597	0.097811958	0.425521262	202	248	369	364	518	365	647	555
ENSECAG00000017111	5.068282066	0.097890177	0.425669782	448	814	786	848	497	386	742	683
ENSECAG00000002456	1.676855386	0.097935857	0.425669782	29	33	39	54	43	28	161	124
ENSECAG00000012769	5.86092398	0.097958263	0.425669782	703	684	779	1143	1358	1230	1768	1583
ENSECAG00000023099	5.50274459	0.098070348	0.425780428	554	2029	591	862	468	1040	581	692
ENSECAG00000009921	8.197881868	0.098093651	0.425780428	16275	2988	2660	1940	1916	8034	1929	1652
ENSECAG00000017221	8.900583959	0.098095921	0.425780428	8127	20741	6659	7799	3369	10608	5184	7980
ENSECAG00000019390	1.705614379	0.098277935	0.426407884	42	23	55	22	151	56	124	27
ENSECAG00000023752	5.271490303	0.098478226	0.427114133	315	611	703	608	977	1148	849	893
ENSECAG00000016637	3.519719602	0.098573697	0.42725438	67	107	188	290	176	200	368	480
ENSECAG00000009327	9.288839303	0.098623127	0.42725438	13766	14239	12627	13637	10213	9521	9210	9876
ENSECAG00000000484	2.163261149	0.098623145	0.42725438	49	47	59	76	118	55	161	141
ENSECAG00000004437	6.317344196	0.098761641	0.427662358	1155	2081	1551	2160	1289	1379	1344	1284
ENSECAG00000024048	8.247351832	0.098870362	0.427662358	9390	4341	5994	5837	4583	5394	4315	3967
ENSECAG00000024658	9.386257896	0.098887307	0.427662358	23992	18208	5978	7321	10818	7006	13579	6849
ENSECAG00000019777	5.350257105	0.098909683	0.427662358	389	657	755	504	901	1150	1018	1050
ENSECAG00000006923	5.352158977	0.098939286	0.427662358	544	1159	930	876	745	734	727	527
ENSECAG00000020841	5.071331372	0.098942701	0.427662358	683	636	642	851	552	600	452	638
ENSECAG00000018634	4.780183008	0.098991503	0.427710917	251	146	542	613	675	409	816	1003
ENSECAG00000017004	2.129701131	0.099107003	0.427934714	63	72	100	171	48	52	42	119
ENSECAG00000009313	2.880831022	0.099118475	0.427934714	59	58	119	121	348	40	364	87
ENSECAG00000005704	7.447890219	0.099206575	0.428070678	1160	1756	2768	4244	4763	3738	5744	4371
ENSECAG00000012182	5.647650636	0.099225166	0.428070678	1644	1050	610	774	477	1220	540	759
ENSECAG00000010914	6.870898862	0.09929762	0.428220984	3828	3359	1153	1295	1806	1960	1441	1571
ENSECAG00000021699	2.518825327	0.099496914	0.428917975	69	76	187	202	72	51	100	133
ENSECAG00000020759	0.37500775	0.09968655	0.429572812	10	13	19	20	19	30	45	37
ENSECAG00000013451	3.743343396	0.099727778	0.429587876	221	177	439	367	155	359	153	153
ENSECAG00000008461	0.167859847	0.099956279	0.430288705	7	7	9	29	12	32	45	27
ENSECAG00000008164	9.410365975	0.099966063	0.430288705	6581	6391	9834	13046	26320	12610	24164	10900
ENSECAG00000014069	3.805943573	0.100018687	0.430352512	169	344	242	514	196	184	182	343
ENSECAG00000017324	7.773601045	0.100127401	0.430657521	3625	1183	2725	3090	5516	6106	6008	5948
ENSECAG00000012484	6.472281721	0.100197218	0.430795064	3216	1246	1896	782	1482	1391	1180	1211
ENSECAG00000007009	3.70997733	0.100290708	0.430877975	152	290	355	394	122	231	139	335
ENSECAG00000019841	6.538091809	0.100292194	0.430877975	2398	2556	1404	1452	970	1885	821	1932
ENSECAG00000015339	2.572838891	0.100635151	0.432188305	64	168	113	202	49	123	107	88
ENSECAG00000022853	6.727527237	0.100690912	0.432264719	1086	172	2460	974	5834	2058	3624	726
ENSECAG00000010325	8.659520683	0.100775433	0.43238484	9160	13462	6882	5454	4679	8808	5396	4926
ENSECAG00000011932	3.290652501	0.100821752	0.43238484	75	109	149	224	240	202	414	172
ENSECAG00000018425	5.557890516	0.10084677	0.43238484	647	1558	914	1092	305	1113	483	1014

ENSECAG00000003516	7.515818227	0.100870807	0.43238484	3186	3862	5425	3566	2953	2579	2709	3391
ENSECAG00000005968	2.858033342	0.10096907	0.432643156	37	80	118	168	266	132	262	110
ENSECAG00000011844	5.369746575	0.101041997	0.432659297	796	687	1020	1001	517	674	590	975
ENSECAG00000016769	4.0758493	0.101048842	0.432659297	166	153	229	424	540	277	446	469
ENSECAG00000015884	4.931741	0.101205537	0.433167306	199	324	603	672	855	437	1156	747
ENSECAG00000015514	9.15302195	0.101372404	0.433718459	10677	17265	12901	9016	8833	8027	12440	6069
ENSECAG00000018293	3.913863269	0.101422683	0.433770564	133	117	277	340	315	328	596	336
ENSECAG00000019900	10.09433062	0.101478014	0.433844231	32008	22875	16695	20822	16160	20122	14624	14381
ENSECAG00000014936	2.518825013	0.101635798	0.434355692	51	69	79	109	167	75	241	126
ENSECAG00000011261	3.445539761	0.101730437	0.434597009	32	354	51	53	468	227	385	162
ENSECAG00000009835	4.336938863	0.101822447	0.434826916	225	103	410	268	1036	308	690	172
ENSECAG00000004558	5.686693276	0.101955652	0.435232509	792	1014	1142	1485	805	950	1049	711
ENSECAG00000008517	0.483300836	0.102063649	0.435530228	20	17	7	3	90	7	28	29
ENSECAG00000021509	2.279459297	0.102114883	0.435585593	57	173	94	106	66	72	62	97
ENSECAG00000012215	4.762890476	0.102155327	0.435594906	1019	668	205	283	490	494	329	243
ENSECAG00000010834	2.139129651	0.102247161	0.435662366	35	42	79	56	180	56	37	198
ENSECAG00000010180	3.770326701	0.102264178	0.435662366	295	301	253	301	156	333	178	196
ENSECAG00000011667	6.358277638	0.102285947	0.435662366	1906	3332	958	731	1162	1560	962	1160
ENSECAG00000023870	5.529487275	0.102401027	0.435989416	450	462	742	1066	1261	843	1355	1253
ENSECAG00000001854	2.106742895	0.102504977	0.436146145	44	138	102	107	45	101	52	57
ENSECAG00000011218	5.237553766	0.10255225	0.436146145	516	448	517	609	1047	615	1327	897
ENSECAG00000001256	0.770711403	0.102581656	0.436146145	34	53	34	21	17	13	33	35
ENSECAG00000021167	6.223336454	0.102591074	0.436146145	2028	2166	1098	1150	475	2138	422	1144
ENSECAG00000020879	5.843525833	0.102645801	0.436215916	336	754	915	1422	1408	1042	2141	1407
ENSECAG00000024254	1.024754767	0.102728102	0.436332038	28	11	87	67	19	33	13	42
ENSECAG00000009865	6.16195857	0.102749775	0.436332038	1917	3318	905	561	101	2024	83	985
ENSECAG00000012796	0.051693133	0.103054139	0.436939424	8	7	13	21	25	24	23	29
ENSECAG00000008628	0.780551737	0.103079005	0.436939424	30	28	51	37	27	15	28	31
ENSECAG00000016638	4.534632369	0.103081874	0.436939424	223	1327	359	188	204	412	370	372
ENSECAG00000001194	3.605748492	0.103088898	0.436939424	202	305	270	256	144	221	194	241
ENSECAG00000019120	4.580859646	0.103108463	0.436939424	163	461	763	987	203	154	307	798
ENSECAG00000004259	1.000526236	0.103123077	0.436939424	32	6	19	25	49	93	20	47
ENSECAG00000008948	4.408345339	0.103221534	0.437103291	208	333	327	332	693	362	749	378
ENSECAG00000001584	2.403143179	0.103238538	0.437103291	34	113	62	51	77	107	309	98
ENSECAG00000000641	4.441139552	0.10331231	0.437253028	578	274	370	574	377	262	419	376
ENSECAG00000014169	5.287343412	0.103373528	0.437349543	416	277	837	668	1237	769	1264	773
ENSECAG00000015468	6.213120931	0.103448282	0.437358878	2377	2303	1011	544	430	2069	613	919
ENSECAG00000014368	6.28435807	0.103452566	0.437358878	591	922	1206	1683	3017	1672	2610	896
ENSECAG00000017445	1.017345917	0.103563758	0.437578224	24	23	45	100	33	40	22	17
ENSECAG00000012474	5.041237591	0.103612435	0.437578224	199	231	702	801	873	599	1325	692
ENSECAG00000005295	1.992022487	0.103619753	0.437578224	75	186	44	45	29	91	32	61
ENSECAG00000006093	6.636427966	0.103662668	0.437597135	739	1098	1658	2278	3154	2248	2718	2171
ENSECAG00000019222	1.334396853	0.103730399	0.437648748	39	59	41	98	10	23	21	84
ENSECAG00000008907	2.785788364	0.103751776	0.437648748	101	128	158	235	64	78	125	173
ENSECAG00000012257	6.126702268	0.103813363	0.437670664	734	721	1149	1373	2192	1284	2354	1404
ENSECAG00000004972	1.044854311	0.103861349	0.437670664	26	45	43	69	21	29	30	43
ENSECAG00000018627	4.773937536	0.1038723	0.437670664	514	763	488	506	387	567	446	410
ENSECAG00000018864	2.910129229	0.103992896	0.437924874	130	326	96	99	88	151	130	77
ENSECAG00000000760	3.234186986	0.104009562	0.437924874	217	128	251	214	93	114	302	97
ENSECAG00000001627	3.839715716	0.10419659	0.438550157	158	442	292	379	137	285	257	254
ENSECAG00000013947	4.195639734	0.104241048	0.438575142	102	235	276	477	307	451	586	553
ENSECAG00000020079	5.124419106	0.104442351	0.439259758	491	1320	455	749	405	678	556	623
ENSECAG00000012386	5.256882682	0.104619303	0.439471711	532	1331	944	484	404	702	518	825
ENSECAG00000018625	2.327554571	0.104631377	0.439471711	84	199	73	74	72	73	79	80
ENSECAG00000024298	6.037678878	0.10464466	0.439471711	1036	1057	460	756	1719	1080	2090	2077
ENSECAG00000000329	8.071968449	0.104647151	0.439471711	4985	7281	3839	7743	3012	4282	4654	5069
ENSECAG00000015794	8.232793542	0.104820192	0.440036094	8053	6554	6802	3938	3453	3688	4560	6739
ENSECAG00000015805	7.079972372	0.104922391	0.440212636	3257	4991	1430	1788	1532	2163	2296	2194
ENSECAG00000006800	6.013275121	0.104974363	0.440212636	1058	2264	1138	1059	784	1479	917	932
ENSECAG00000016445	5.283490474	0.104990031	0.440212636	909	1073	649	528	574	651	648	676
ENSECAG00000021185	3.145810366	0.10501691	0.440212636	104	80	134	161	225	150	372	186
ENSECAG00000011456	10.38211859	0.105293307	0.44116688	39216	36349	18469	18411	15685	27840	16962	17187
ENSECAG00000008015	2.225674651	0.1053532	0.44116688	39	41	94	66	188	138	70	85
ENSECAG00000006895	2.734015178	0.105384911	0.44116688	45	120	234	243	62	143	120	77
ENSECAG00000000264	0.435353554	0.105431399	0.44116688	8	8	34	12	41	22	54	24
ENSECAG00000023363	2.506810423	0.105438303	0.44116688	87	144	101	175	48	55	121	136
ENSECAG00000016150	8.228745172	0.105608823	0.441670854	6146	6599	6083	7180	4334	4515	4782	5477
ENSECAG00000017796	5.717961888	0.10563634	0.441670854	517	625	825	1101	1299	1221	1956	926
ENSECAG00000009181	5.977151034	0.105681928	0.441699247	739	926	664	1204	1939	1054	2068	1445
ENSECAG00000006450	1.681365521	0.105765184	0.441797104	32	26	48	62	101	50	99	82
ENSECAG00000018714	9.493742869	0.105782952	0.441797104	18609	33074	10682	7095	512	21188	963	8023
ENSECAG00000015128	8.50770167	0.105863083	0.441969639	8115	9862	5894	7788	3851	7787	4219	6141
ENSECAG00000005501	1.963649676	0.105943035	0.442141294	43	29	35	78	42	129	229	37
ENSECAG00000008277	0.621005347	0.106036159	0.442177119	16	20	22	12	23	35	68	34
ENSECAG00000008358	5.112616493	0.1060554	0.442177119	271	538	488	778	758	871	976	873
ENSECAG00000020508	5.13114203	0.106068135	0.442177119	985	980	442	506	228	878	344	632

ENSECAG00000012816	4.292648749	0.106143958	0.442331244	175	215	375	356	656	535	507	285
ENSECAG00000024352	4.846591231	0.106238751	0.442564282	393	588	368	1294	457	264	497	677
ENSECAG00000002017	6.251779234	0.106387236	0.44302073	1697	3355	823	1207	192	2724	349	476
ENSECAG00000017196	1.388945685	0.106448525	0.443113879	34	75	58	70	12	67	35	34
ENSECAG00000007609	2.234628587	0.10676479	0.444109319	48	49	89	48	109	68	252	86
ENSECAG00000019243	4.982960898	0.106765675	0.444109319	1159	1133	359	274	8	564	28	740
ENSECAG00000019626	6.205030654	0.106807505	0.444121052	260	310	1110	2468	1283	1124	2261	3603
ENSECAG00000012553	3.157461954	0.106900765	0.444138979	74	90	176	160	191	161	364	221
ENSECAG00000000647	1.28281285	0.106916905	0.444138979	29	29	26	29	54	68	117	21
ENSECAG00000016625	3.349618444	0.10694841	0.444138979	0	33	162	183	198	2	541	634
ENSECAG00000012705	3.862593323	0.10696786	0.444138979	107	95	397	187	554	302	463	221
ENSECAG00000020675	3.850242641	0.107036696	0.444262773	310	383	276	223	319	184	264	167
ENSECAG00000006117	6.090475759	0.107174262	0.444521802	807	3555	715	1053	529	1064	1174	1363
ENSECAG00000000259	0.959154706	0.107177193	0.444521802	22	60	46	46	12	37	16	43
ENSECAG00000017402	0.811353151	0.107274049	0.444761488	11	28.0004	25	20	50	55	59	16
ENSECAG00000023263	7.389481939	0.107350223	0.444915286	4205	4889	2612	2365	2570	3104	2244	2300
ENSECAG00000008973	3.816394001	0.107404442	0.444918995	120	76	365	188	537	394	334	210
ENSECAG00000011467	8.476929402	0.107429277	0.444918995	11471	19521	1540	2573	93	10030	102	2284
ENSECAG00000004355	2.736929105	0.107468382	0.444919103	42	120	88	121	147	133	229	185
ENSECAG00000020136	7.790813033	0.107518877	0.444966345	4467	9938	3660	2913	624	7802	725	1418
ENSECAG00000000455	2.136975582	0.107784995	0.445905585	82	179	61	52	55	60	75	72
ENSECAG00000004256	4.772147982	0.107832035	0.445938148	584	847	401	420	454	560	410	331
ENSECAG00000016768	0.352284866	0.108196761	0.447273552	6	4	29	20	17	29	29	57
ENSECAG00000010789	6.496416706	0.108233521	0.447273552	2599	1608	2045	1277	691	1989	887	1869
ENSECAG00000009494	3.762000015	0.108490585	0.448042715	173	208	509	318	154	181	239	314
ENSECAG00000026130	2.74085049	0.108498355	0.448042715	52	32	167	460	93	111	105	64
ENSECAG00000019661	5.464019768	0.10864866	0.448500722	462	476	790	844	1258	859	1440	931
ENSECAG00000003257	3.760034226	0.108701308	0.448506756	114	107	333	205	287	275	406	441
ENSECAG00000013456	4.407618605	0.108728911	0.448506756	355	668	393	379	176	351	408	466
ENSECAG00000016207	0.640025291	0.108794197	0.448613522	14	14	29	15	21	38	78	27
ENSECAG00000024982	5.82316077	0.108997949	0.449175783	1444	1719	900	644	514	1477	608	802
ENSECAG00000005467	7.338188573	0.109009459	0.449175783	2241	2595	4985	5340	3526	1239	3955	1507
ENSECAG00000020118	5.274049303	0.109239824	0.449962154	518	902	637	1434	329	569	603	1051
ENSECAG00000019975	8.210068992	0.109423875	0.450557257	5395	3684	11366	6900	6350	4499	5294	1452
ENSECAG00000023565	5.642251117	0.109510075	0.450620721	1092	829	1134	1073	859	893	674	899
ENSECAG00000016034	5.283877046	0.109548131	0.450620721	828	1107	636	611	691	653	680	555
ENSECAG00000017704	5.828300808	0.109558166	0.450620721	661	568	914	946	1905	549	2401	1220
ENSECAG00000019713	3.393825017	0.109597609	0.450620721	158	213	163	397	159	148	219	172
ENSECAG00000016519	3.721740152	0.109686252	0.450785888	247	314	256	292	148	166	277	293
ENSECAG00000007173	4.042561535	0.109716969	0.450785888	184	123	358	204	608	206	668	282
ENSECAG00000018940	5.092539131	0.110410371	0.453070794	248	575	538	651	659	1052	753	951
ENSECAG00000023790	3.242568942	0.110432592	0.453070794	116	93	168	132	246	160	362	220
ENSECAG00000024659	3.611814869	0.110481322	0.453070794	134	127	206	212	389	184	448	248
ENSECAG00000007879	0.867580634	0.110491355	0.453070794	28	9	23	18	81	26	60	26
ENSECAG00000011917	10.13480438	0.110502428	0.453070794	25395	42676	14671	14152	11560	20863	16190	17849
ENSECAG00000010847	10.53778111	0.110542249	0.453070794	58657	63483	14238	15125	43.0004	35047	18	8693
ENSECAG00000011753	3.83565357	0.110552866	0.453070794	111	271	158	87	88	1090	158	208
ENSECAG00000014885	0.925291091	0.110591457	0.453070794	18	28	20	29	59	44	50	37
ENSECAG00000008051	4.927562463	0.110723251	0.453447557	209	400	441	771	550	526	1121	965
ENSECAG00000004679	4.716972903	0.110802362	0.453607126	919	59	1455	482	0	68	0	709
ENSECAG00000010246	5.743039778	0.110841899	0.453607126	529	878	1313	2238	825	732	1267	799
ENSECAG00000017203	9.489673147	0.110936045	0.453763292	20426	16306	13614	10274	5906	11139	10436	15802
ENSECAG00000012569	4.88351538	0.110959772	0.453763292	444	652	603	804	424	479	577	565
ENSECAG00000006239	4.894587252	0.111027316	0.453876479	338	250	406	721	765	431	992	892
ENSECAG00000003051	5.238152163	0.111112053	0.454059844	1368	475	647	512	415	822	422	625
ENSECAG00000000602	1.499706215	0.111306651	0.454691861	33	22	43	33	91	41	155	24
ENSECAG00000022638	3.15567386	0.111393673	0.454884133	209	367	86	90	70	127	147	183
ENSECAG00000007835	4.459559996	0.111441071	0.45491452	169	313	344	473	400	591	464	757
ENSECAG00000015546	5.826051095	0.111660397	0.455334925	580	528	1144	844	2235	752	2207	829
ENSECAG00000014226	11.08688015	0.111661365	0.455334925	11269	24824	121029	65204	30128	37531	24055	31512
ENSECAG00000016815	3.783065166	0.111664041	0.455334925	384	621	247	53	27	374	3	165
ENSECAG00000016826	5.095352965	0.111765126	0.455583944	542	648	796	965	458	314	764	816
ENSECAG00000021841	6.742664939	0.111930524	0.456094852	3351	3757	1097	980	906	3279	564	821
ENSECAG00000000168	6.987790068	0.11200573	0.45611406	1849	2209	6721	2792	126	422	89	5058
ENSECAG00000012670	9.085490718	0.112015363	0.45611406	20097	10458	8154	5468	7596	8492	5676	9450
ENSECAG00000018578	1.030428975	0.112120479	0.456378853	12	25	16	42	11	27	55	131
ENSECAG00000010823	4.333617895	0.112340812	0.456919366	213	157	344	429	683	268	737	416
ENSECAG00000014572	5.068354939	0.112388587	0.456919366	572	691	660	915	374	554	690	693
ENSECAG00000022301	4.669730965	0.112461916	0.456919366	209	329	445	509	899	557	477	612
ENSECAG00000014973	5.293950564	0.11247325	0.456919366	643	290	525	456	1781	1062	929	352
ENSECAG00000005817	4.12494519	0.112485883	0.456919366	163	395	198	167	293	327	792	453
ENSECAG00000023911	3.806231466	0.11249407	0.456919366	202	302	302	398	206	236	248	255
ENSECAG00000012302	1.299361774	0.112558282	0.457017135	19	161	24	26	15	44	13	49
ENSECAG00000015913	1.204865979	0.112640084	0.457023582	15	37	38	24	59	86	35	52
ENSECAG00000015003	2.858534829	0.112640156	0.457023582	76	91	83	153	149	150	243	206
ENSECAG00000026857	4.152814035	0.112769248	0.457274601	152	208	374	300	459	389	615	350

ENSECAG00000010694	6.28304664	0.112782352	0.457274601	1031	2892	1489	1418	962	1907	975	1039
ENSECAG00000023460	7.697353403	0.113090183	0.458359465	3152	5802	4394	5111	2630	3946	2344	4128
ENSECAG00000013632	4.765367872	0.113184391	0.458578041	801	599	307	468	489	398	370	484
ENSECAG00000000214	6.513079606	0.113291077	0.458846996	3413	3636	430	315	415	2503	708	790
ENSECAG00000015583	1.068843226	0.113404578	0.459061732	59	56	48	38	0	40	0	44
ENSECAG00000006391	3.068996756	0.113424739	0.459061732	89	169	144	383	96	131	132	176
ENSECAG00000018002	9.252697405	0.113482564	0.459112629	6997.01	1117	8328	8581	2706	12823	4899	52237
ENSECAG00000011160	2.181649982	0.113517967	0.459112629	117	181	54	39	66	113	39	18
ENSECAG00000022390	2.371437629	0.113722041	0.459390156	8	152	46	26	171	117	253	55
ENSECAG00000003755	3.016474129	0.113776515	0.459390156	132	152	170	247	114	138	129	149
ENSECAG00000008989	4.680714029	0.113804736	0.459390156	486	709	570	390	258	668	329	377
ENSECAG00000020695	4.0640546	0.113810487	0.459390156	203	188	226	315	447	255	730	317
ENSECAG00000010554	3.980124867	0.113812985	0.459390156	292	176	137	173	374	353	624	317
ENSECAG00000014508	4.737034382	0.113828689	0.459390156	154	316	423	734	638	627	777	673
ENSECAG00000010842	6.185519983	0.114019529	0.459736324	706	2773	1334	1691	1085	1494	1036	1051
ENSECAG00000018575	3.204391787	0.114041915	0.459736324	70	131	163	139	293	110	410	170
ENSECAG00000021782	3.774052223	0.114096874	0.459736324	262	306	281	314	333	135	293	137
ENSECAG00000021143	0.655229274	0.114148981	0.459736324	24	12	22	6	66	25	51	24
ENSECAG00000007182	2.173636704	0.114159433	0.459736324	29	41	86	91	124	86	188	78
ENSECAG00000001688	0.688606088	0.11417861	0.459736324	21	22	33	83	2	17	10	51
ENSECAG00000009570	8.329047273	0.11419713	0.459736324	12657	14091	1199	1555	315	10096	383	1757
ENSECAG00000020614	1.94721459	0.114487674	0.460480093	43	66	80	168	63	52	53	70
ENSECAG00000000216	7.287653	0.114488738	0.460480093	3051	4486	2969	2897	2173	3232	1963	2416
ENSECAG00000009049	7.803543567	0.114515338	0.460480093	2344	1528	4539	2731	9674	3976	9120	1986
ENSECAG00000017252	3.985849218	0.114594857	0.460480093	189	437	236	557	172	203	309	371
ENSECAG00000018007	0.591842424	0.114598971	0.460480093	4	26	19	27	45	27	47	34
ENSECAG00000013319	5.862099624	0.114631542	0.460480093	720	2555	1049	994	328	1815	468	719
ENSECAG00000017024	4.90141645	0.114691427	0.460480093	357	354	508	508	769	639	938	658
ENSECAG00000012922	4.240563755	0.11470545	0.460480093	241	173	507	905	123	189	353	527
ENSECAG00000004928	0.803099765	0.114896978	0.461077072	14	19	24	30	36	19	69	57
ENSECAG00000013554	8.29180473	0.114938546	0.461077072	9484	8338	4673	3382	3794	5404	3796	5612
ENSECAG00000024043	1.521971374	0.115024761	0.461077072	134	63	15	22	33	64	6	30
ENSECAG00000000690	4.029229533	0.115057264	0.461077072	173	174	233	370	406	311	415	518
ENSECAG00000007644	9.388778066	0.115090241	0.461077072	16011	15541	11807	13723	9996	11089	9971	10818
ENSECAG00000007467	4.212293579	0.115097149	0.461077072	259	269	253	213	322	554	595	421
ENSECAG00000019644	1.394400883	0.115486604	0.462279286	25	84	60	66	29	32	32	64
ENSECAG00000014890	3.755758927	0.115509691	0.462279286	117	181	174	311	378	227	517	275
ENSECAG00000017476	0.417736869	0.115528659	0.462279286	8	4	23	29	51	31	37	17
ENSECAG00000007926	0.569162581	0.11558349	0.462279286	19	3	22	15	76	21	56	8
ENSECAG00000023236	7.48210113	0.115637985	0.462279286	2768	7230	2744	3468	1001	4104	1707	3613
ENSECAG00000008121	1.95525945	0.11569324	0.462279286	34	46	57	66	133	48	161	68
ENSECAG00000016393	4.634371937	0.115707578	0.462279286	260	707	584	623	314	515	447	391
ENSECAG00000014648	4.363773418	0.115722087	0.462279286	206	331	305	341	351	402	700	650
ENSECAG00000012821	5.350630016	0.115883124	0.462760213	609	942	835	1125	514	829	531	860
ENSECAG00000019687	3.676011952	0.115975088	0.46296507	91	100	257	268	135	368	321	516
ENSECAG00000020718	4.040403831	0.11618694	0.463468894	311	672	513	274	0	359	1	226
ENSECAG00000004556	7.987146531	0.116220281	0.463468894	5278	12965	2549	2178	2083	7728	1481	1667
ENSECAG00000011966	7.664684736	0.116223425	0.463468894	4988	6372	2224	3512	2532	2709	3613	3806
ENSECAG00000002676	8.825162567	0.116455675	0.464232445	6059	3635	7666	5288	18056	8698	14784	7466
ENSECAG00000010095	1.877457467	0.1166022	0.46465385	26	46	69	60	92	79	97	105
ENSECAG00000010523	5.678408543	0.116673568	0.464775569	1948	2088	355	447	7	1679	39	193
ENSECAG00000005682	3.980156447	0.116828944	0.464780955	63	442	236	928	95	68	281	472
ENSECAG00000000692	7.4617088	0.11683841	0.464780955	1507	2076	2668	3583	6319	3451	5878	2969
ENSECAG00000009271	8.687922818	0.116842776	0.464780955	23277	7723	3295	6889	2	9251	1	1399
ENSECAG00000024566	6.830799886	0.116853915	0.464780955	1346	1912	3754	3743	2706	2087	1393	728
ENSECAG00000007426	6.884635367	0.11687904	0.464780955	2556	2559	2035	3010	1150	1825	1813	2801
ENSECAG00000011272	4.616020932	0.116988232	0.464976614	251	438	698	810	499	359	424	375
ENSECAG00000003722	2.664869255	0.117009925	0.464976614	54	104	57	128	100	105	252	217
ENSECAG00000006641	0.740726076	0.117309217	0.466003292	15	10	26	32	47	48	26	45
ENSECAG00000006745	8.563824626	0.117528187	0.466533719	2098	5808	6284	6419	9855	10813	5188.01	13981
ENSECAG00000023076	9.277021434	0.117556739	0.466533719	18766	24019	5672	5750	3246	18314	3099	6863
ENSECAG00000014388	1.721263158	0.117567392	0.466533719	21	35	37	89	91	53	106	92
ENSECAG00000010521	3.149707566	0.117621226	0.466533719	287	226	202	117	11	101	2	284
ENSECAG00000010227	6.911471847	0.117647634	0.466533719	774	1697	2195	2184	2861	3805	2612	3122
ENSECAG00000019355	7.637623108	0.117759192	0.466813508	1915	1831	3039	4121	2617	4535	6094	8084
ENSECAG00000014117	3.512411265	0.118092069	0.467763569	354	341	97	137	128	90	296	189
ENSECAG00000019452	5.943440826	0.118098408	0.467763569	1026	1231	1442	1505	862	1111	1121	1110
ENSECAG00000025174	1.6553626	0.118160603	0.467763569	35	21	45	51	163	49	90	31
ENSECAG00000024339	5.639355852	0.118185986	0.467763569	412	450	945	1145	1596	760	1666	1117
ENSECAG00000006587	4.282107241	0.118233567	0.467763569	221	217	302	392	570	416	465	488
ENSECAG00000022947	3.779816393	0.118278275	0.467763569	133	128	232	307	360	307	441	289
ENSECAG00000000331	6.08180333	0.118286457	0.467763569	1287	1744	1113	1519	1175	880	1145	1359
ENSECAG00000020616	2.634268469	0.118404217	0.46806667	45	57	84	159	78	125	223	234
ENSECAG00000016438	5.160516818	0.118705323	0.468943203	420	986	663	1074	741	642	640	374
ENSECAG00000023646	3.051483735	0.118708328	0.468943203	138	355	110	115	69	116	103	212
ENSECAG00000018189	3.615538827	0.118751952	0.468952818	124	231	144	150	296	129	569	322

ENSECAG00000016862	5.953757655	0.118920443	0.469431462	1789	1758	992	602	697	1786	494	593
ENSECAG00000008399	6.88438977	0.118955623	0.469431462	2340	3998	1957	1713	1489	1720	2744	1642
ENSECAG00000021649	2.691273494	0.119118814	0.46987958	73	62	99	109	204	81	266	132
ENSECAG00000023930	5.616325894	0.119215629	0.46987958	558	427	893	911	1706	816	1673	858
ENSECAG00000019726	4.029962145	0.119227619	0.46987958	244	936	235	137	44	539	33	161
ENSECAG00000015245	7.72278282	0.119256634	0.46987958	11233	1920	1436	2242	1416	5559	1796	1848
ENSECAG00000013382	2.414504112	0.119275537	0.46987958	24	38	90	148	98	172	122	163
ENSECAG00000020212	1.70224896	0.119350639	0.46988491	26	46	46	57	85	83	107	56
ENSECAG00000020322	4.516182159	0.119359434	0.46988491	249	795	430	547	212	496	291	479
ENSECAG00000010877	4.933361392	0.11947129	0.47016268	212	376	587	674	602	646	1021	840
ENSECAG00000004942	8.316101571	0.119512744	0.4701633	3224	1603	5043	6349	7948	6644	3069	16963
ENSECAG00000021771	6.347095085	0.119595398	0.47019337	2346	1609	1137	1496	958	1530	1684	1103
ENSECAG00000017875	3.216654218	0.119658195	0.47019337	91	78	175	169	355	136	281	192
ENSECAG00000021409	5.056043218	0.119672373	0.47019337	330	417	535	689	864	965	773	690
ENSECAG00000018928	2.021036842	0.119685585	0.47019337	29	55	48	94	116	87	117	94
ENSECAG00000014123	4.736943227	0.119753432	0.470297626	217	268	572	506	945	444	740	584
ENSECAG00000018095	0.305135586	0.119842555	0.470485341	12	6	17	22	20	13	49	46
ENSECAG00000012002	4.998003998	0.119914333	0.470604854	409	340	827	1380	485	280	742	639
ENSECAG00000007726	5.489049483	0.119995462	0.470760968	432	499	697	1052	948	1115	1576	918
ENSECAG00000009393	1.246777049	0.120070602	0.470801681	26	54	86	45	44	13	39	45
ENSECAG00000008155	3.26008403	0.120088545	0.470801681	69	119	120	223	110	148	301	465
ENSECAG00000024380	1.362321523	0.120172512	0.470908972	14	49	4	39	60	28	214	10
ENSECAG00000004339	1.814400662	0.120220538	0.470908972	29	21	65	73	120	63	117	66
ENSECAG00000006600	3.182826731	0.120292176	0.470908972	94	188	221	329.008	135.007	207	103	128
ENSECAG00000015500	6.008933464	0.120305436	0.470908972	1087	4086	703	518	2	555	1114	1293
ENSECAG00000020436	3.906517853	0.120352309	0.470908972	122	191	235	345	305	415	408	375
ENSECAG00000011219	5.875258089	0.120364085	0.470908972	1275	2255	807	802	452	1945	352	520
ENSECAG00000009898	2.124770076	0.120530292	0.471397242	36	39	86	70	78	56	183	149
ENSECAG00000015344	0.484226363	0.120608961	0.471542934	10	16	16	28	25	21	46	49
ENSECAG00000018190	7.448219535	0.120723522	0.471730226	2517	1383	2590	1963	7432	3020	7059	1690
ENSECAG00000010099	2.486657563	0.120814965	0.471730226	34	193	160	135	42	104	84	108
ENSECAG00000000466	6.958250506	0.120834244	0.471730226	2841	5301	1472	1221	1276	3565	1080	935
ENSECAG00000015363	3.885280222	0.120846697	0.471730226	133	148	321	256	376	299	450	375
ENSECAG00000019802	6.620706829	0.120864038	0.471730226	764	1814	1799	669	4013	2258	3278	1008
ENSECAG00000003965	4.535530433	0.121070043	0.472372322	277	324	367	331	619	344	941	491
ENSECAG00000010800	3.873713768	0.12113784	0.472460174	221	341	252	469	179	155	241	403
ENSECAG00000016195	4.186613834	0.121213014	0.472460174	372	674	197	274	220	231	282	431
ENSECAG00000026818	0.212135546	0.121217055	0.472460174	8	14	7	26	40	30	14	29
ENSECAG00000020406	7.413788463	0.121328621	0.472646023	4270	5577	2048	2488	1307	3320	3292	2517
ENSECAG00000011892	2.022606629	0.121386265	0.472646023	35	72	30	69	103	36	167	133
ENSECAG00000015086	9.750035621	0.121389282	0.472646023	16751	37207	8685	13187	8369	19453	13220	9298
ENSECAG00000017202	5.994928102	0.121518077	0.472924351	579	761	898	1504	1244	1085	1695	2537
ENSECAG00000023847	5.810054755	0.121543843	0.472924351	693	571	957	982	1637	1090	1771	1204
ENSECAG00000014868	7.118094326	0.121707126	0.473269641	2314	6149	1871	2091	885	3587	1207	2291
ENSECAG00000010479	8.180141272	0.121715723	0.473269641	5669	8476	5539	5223	3645	5920	4071	4543
ENSECAG00000008833	8.993180881	0.121893593	0.47339945	8925	15264	8641	11750	7580	8621	7418	8574
ENSECAG00000013123	5.745395096	0.121895919	0.47339945	896	1539	1132	940	700	1248	940	662
ENSECAG00000011669	1.696376017	0.121909126	0.47339945	51	18	27	58	87	65	147	49
ENSECAG00000022585	4.174802397	0.121915431	0.47339945	325	234	494	559	142	121	536	400
ENSECAG00000007522	5.930682564	0.122222952	0.474431745	1076	2308	979	733	994	976	971	1003
ENSECAG00000007417	6.0470022	0.122376594	0.474788226	1706	3141	616	869	29	2072	68	690
ENSECAG00000016767	4.422020605	0.122427085	0.474788226	466	589	369	318	284	375	515	261
ENSECAG00000013289	4.262612628	0.122439897	0.474788226	113	231	275	510	250	308	747	733
ENSECAG00000007912	2.922641349	0.122542428	0.475024018	67	66	142	157	190	180	218	184
ENSECAG00000007179	5.204300738	0.122609111	0.475120738	545	397	505	508	1192	383	1525	799
ENSECAG00000024972	2.817910853	0.122678044	0.475226106	70	122	109	73	235	148	154	181
ENSECAG00000011828	1.273550241	0.122850489	0.475732252	18	26	51	30	74	51	45	73
ENSECAG00000013881	4.566928142	0.123108893	0.476279497	225	237	384	565	610	390	812	606
ENSECAG00000024202	1.409355105	0.123135233	0.476279497	19	89	70	60	36	45	39	39
ENSECAG00000015264	1.85440389	0.123222275	0.476279497	163	33	63	26	34	74	36	48
ENSECAG00000011558	1.743612485	0.123225738	0.476279497	24	49	88	165	72	39	35	50
ENSECAG00000022231	1.687760069	0.123231882	0.476279497	24	29	66	50	59	74	155	53
ENSECAG00000009456	4.469845746	0.123242811	0.476279497	825	602	148	195	180	487	421	198
ENSECAG00000018298	3.98893896	0.123331165	0.476306779	162	233	236	295	434	301	473	385
ENSECAG00000005015	2.508610002	0.123333543	0.476306779	45	148	128	274	10	103	13	162
ENSECAG00000016376	2.202440129	0.123949967	0.478380317	40	114	151	117	56	105	80	38
ENSECAG00000012941	9.338616565	0.123985756	0.478380317	18058	20394	7848	7894	7524	13785	8825	8064
ENSECAG00000024977	5.55720956	0.124030453	0.478380317	506	563	944	699	1442	932	1448	936
ENSECAG00000020463	6.720072345	0.124050954	0.478380317	1163	3492	2310	2830	785	2785	733	1982
ENSECAG00000010657	4.701399975	0.124130468	0.478380317	283	259	382	600	697	395	733	819
ENSECAG00000012607	8.598091685	0.12414695	0.478380317	8104	21437	3141	3683	1003	12655	1103	3377
ENSECAG00000009576	3.343761473	0.124164588	0.478380317	70	90	222	207	189	233	330	295
ENSECAG00000009276	0.952552637	0.1242903	0.478555011	13	32	47	104	14	48	4	30
ENSECAG00000024031	6.43617177	0.12432609	0.478555011	3175	1972	998	740	1074	1998	1107	886
ENSECAG00000010158	2.128552136	0.124364209	0.478555011	22	95	64	50	88	132	106	124
ENSECAG00000010611	3.381298745	0.124378066	0.478555011	299	137	260	144	158	174	190	142

ENSECAG00000016158	6.489356765	0.12448296	0.478643239	1640	4180	1311	987	484	3107	552	645
ENSECAG00000017075	5.016105609	0.124485079	0.478643239	1203	1024	195	220	304	906	221	296
ENSECAG00000020963	6.109524041	0.124735261	0.479443263	1007	1382	1557	2014	1210	941	1359	1243
ENSECAG00000006573	2.80547762	0.124820337	0.479527862	64	100	71	118	107	41	327	306
ENSECAG00000022756	5.10569029	0.124852757	0.479527862	745	1319	452	340	356	823	489	459
ENSECAG00000019899	5.196061676	0.124883628	0.479527862	824	689	518	1018	574	353	796	780
ENSECAG00000019128	6.524101548	0.124999113	0.479809476	2717	2364	1273	1046	1833	975	1789	1237
ENSECAG00000001012	2.169731131	0.125058104	0.479874121	25	54	68	98	185	128	71	74
ENSECAG00000000744	5.765757425	0.125562305	0.481526993	1247	1857	1304	742	58	1884	71	556
ENSECAG00000019269	3.661019867	0.125573442	0.481526993	117	151	231	232	424	244	324	280
ENSECAG00000020940	1.484441892	0.125723342	0.481875851	30	26	94	159	0	29	4	88
ENSECAG00000019913	7.049809187	0.125749069	0.481875851	2214	5162	2032	2041	2218	2488	2140	1412
ENSECAG00000024914	5.535995436	0.126183814	0.482877658	447	359	1070	718	1491	770	2012	635
ENSECAG00000008923	8.543928437	0.126184085	0.482877658	4125	3183	32640	551	798	2261	357	12328
ENSECAG00000017831	5.89540954	0.126198419	0.482877658	545	1033	817	1034	2113	1072	1702	1159
ENSECAG00000011680	0.675005334	0.126217114	0.482877658	7	21	24	27	36	13	41	75
ENSECAG00000017079	2.773826607	0.126241498	0.482877658	44	74	133	142	143	180	214	163
ENSECAG00000015552	2.281352411	0.126264979	0.482877658	50	51	80	64	156	52	255	67
ENSECAG00000022804	2.139277066	0.126483408	0.483089621	50	44	167	251	0	89	0	77
ENSECAG00000023413	5.891819407	0.126489944	0.483089621	677	584	1013	1065	2057	761	2208	1165
ENSECAG00000020473	2.332875664	0.126490338	0.483089621	35	32	111	94	196	123	128	75
ENSECAG00000007830	4.105778995	0.126513172	0.483089621	201	174	158	446	425	240	698	442
ENSECAG00000021665	0.373534534	0.126612078	0.483089621	31	38	21	16	34	15	13	6
ENSECAG00000008876	7.292822627	0.126619136	0.483089621	3672	5647	2285	1579	1367	2711	3352	2242
ENSECAG00000023070	6.732135192	0.126638919	0.483089621	2789	3200	1468	1365	1174	2653	1248	1340
ENSECAG00000024240	4.346923301	0.126659861	0.483089621	191	211	400	375	714	392	658	308
ENSECAG00000019716	7.195540771	0.126743548	0.483149193	871	1347	2532	3586	5846	2600	5069	2166
ENSECAG00000015508	5.654617508	0.126760355	0.483149193	651	1349	960	1351	693	836	1032	934
ENSECAG00000018963	1.714762135	0.126922166	0.483587776	28	75	163	80	2	39	1	102
ENSECAG00000016532	4.525911635	0.127002679	0.483587776	275	212	393	419	797	325	843	413
ENSECAG00000009595	5.675983256	0.12700285	0.483587776	955	1254	1091	901	906	739	1201	675
ENSECAG00000000984	5.355044936	0.127176223	0.484086024	769	547	1107	1078	643	470	1232	473
ENSECAG00000008479	3.851051769	0.127419919	0.484851531	190	142	209	275	361	272	464	368
ENSECAG00000011138	1.113569997	0.127480982	0.484921812	40	120	39	9	0	64	3	17
ENSECAG00000011029	4.428316846	0.127561742	0.485066947	210	255	273	536	362	394	732	718
ENSECAG00000004420	6.793187793	0.127674261	0.485311718	3292	2143	2247	1288	1817	2002	1642	1460
ENSECAG00000016053	2.240478543	0.127711366	0.485311718	38	147	99	150	54	98	55	81
ENSECAG00000008533	4.12971752	0.127818839	0.485351408	211	368	480	486	128	304	376	372
ENSECAG00000008887	6.21572362	0.127821974	0.485351408	650	784	1243	1693	2707	1418	1822	1603
ENSECAG00000016390	4.962456942	0.127849703	0.485351408	336	340	592	457	816	390	1631	495
ENSECAG00000001249	4.783485464	0.128079235	0.486060697	326	553	1233	601	180	1048	65	120
ENSECAG00000009744	6.065284521	0.128321425	0.486743626	535.001	1649	273.001	1041	1395	2297	1055	2273
ENSECAG00000012199	6.095496688	0.128344696	0.486743626	779	4553	638	619	100	1468	232	1570
ENSECAG00000017370	6.384455571	0.128470381	0.486944307	1593	1972	1966	1492	1061	1226	1028	2181
ENSECAG00000019915	5.018685685	0.128509836	0.486944307	594	755	565	776	547	427	486	759
ENSECAG00000007364	4.421395857	0.128525924	0.486944307	370	527	405	515	273	561	236	325
ENSECAG00000014602	2.480346712	0.128649475	0.487250256	52	39	122	76	160	136	245	55
ENSECAG00000016917	6.837039745	0.128855447	0.487868061	3454	3522	1104	1204	1893	1380	2070	1669
ENSECAG00000003998	4.137494038	0.128957203	0.488091011	127	197	248	477	448	228	513	627
ENSECAG00000018383	5.185079468	0.129024246	0.488182466	424	436	527	680	884	502	1740	689
ENSECAG00000022965	8.406375492	0.129152101	0.488503876	5830	11567	7419	4701	5682	7134	4452	3474
ENSECAG00000008899	3.99983184	0.129205669	0.488544187	134	239	169	390	270	323	391	652
ENSECAG00000024316	5.683169293	0.129456998	0.489308728	576	411	907	1111	1232	906	1813	1318
ENSECAG00000017400	5.278230176	0.129493825	0.489308728	516	457	1124	1353	562	588	893	625
ENSECAG00000013764	5.854959066	0.129602333	0.489535314	462.001	764	1060	1166	1053	1500	1383	1881
ENSECAG00000007945	7.578674439	0.129664686	0.489535314	2172	2149	2010	4147	3114	3687	6630	6961
ENSECAG00000018606	4.338235842	0.129724579	0.489535314	186	262	354	409	496	374	607	548
ENSECAG00000014505	5.88367077	0.129759292	0.489535314	649	1812	1284	1463	783	1476	801	790
ENSECAG00000020600	5.364270648	0.129768782	0.489535314	565	411	749	566	1256	794	1359	786
ENSECAG00000006079	6.806589941	0.129892024	0.489801235	894	1007	1772	2842	2115	2194	3786	3700
ENSECAG00000023910	3.008371505	0.129925317	0.489801235	49	248	176	297	53	100	120	223
ENSECAG00000008454	7.435051505	0.130089735	0.490258734	1320	2054	3335	3190	4481	4695	4218	4425
ENSECAG00000014278	5.222035971	0.130133462	0.490259283	759	2559	543	204	0	970	0	93
ENSECAG00000011673	5.766630226	0.130211994	0.490259283	1148	91	68	98	3692	2722	59	105
ENSECAG00000013105	2.407436317	0.130232413	0.490259283	88	85	85	227	41	69	72	146
ENSECAG00000014763	3.924110328	0.130262128	0.490259283	160	214	178	294	167	320	743	379
ENSECAG00000022360	1.633106853	0.130422981	0.490702458	34	209	24	47	0	67	3	55
ENSECAG00000013070	8.115755454	0.130567334	0.491083282	9070	12031	2092	2148	316	9366	440	1973
ENSECAG00000023459	8.634047355	0.130639712	0.491193235	11175	4905	10559	7311	4797	10008	3916	4782
ENSECAG00000022690	8.081104541	0.130723273	0.491345152	3655	1597	4516	4192	10674	4315	8714	5367
ENSECAG00000019741	2.711395556	0.130794469	0.49143955	72	79	104	100	154	91	256	182
ENSECAG00000008160	4.869439941	0.130834719	0.49143955	400	432	312	487	905	459	1000	617
ENSECAG00000020295	1.286279963	0.13093035	0.491508155	25	16	23	64	48	49	75	79
ENSECAG00000011777	4.544581871	0.130939327	0.491508155	305	1110	323	337	94	540	314	451
ENSECAG00000021079	3.288944031	0.131085091	0.491893131	148	283	210	180	164	145	151	188
ENSECAG00000019949	4.007970703	0.131159611	0.492010598	329	459	343	305	68	570	81	177

ENSECAG00000009618	2.744276483	0.131422205	0.492833267	43	57	167	95	98	149	345	133
ENSECAG00000000176	3.705263938	0.131479635	0.492886282	157	281	364	345	112	270	230	253
ENSECAG00000009855	3.604633277	0.131549538	0.492889638	71	162	214	278	372	221	363	282
ENSECAG00000011273	4.908329232	0.131567115	0.492889638	361	636	604	1027	408	517	498	646
ENSECAG00000008173	6.637686757	0.131674383	0.492996005	2049	3053	1836	1388	1307	884	1921	2399
ENSECAG00000022511	7.573160327	0.131682113	0.492996005	5172	10353	1249	1402	127	6569	202	884
ENSECAG00000019124	7.779348741	0.1317551	0.493029713	5305	9461	2071	1980	1825	4402	1842	4425
ENSECAG00000026880	3.05592065	0.131810905	0.493029713	159	317	119	128	61	249	63	103
ENSECAG00000014719	2.126874944	0.131821032	0.493029713	49	27	73	64	193	55	189	38
ENSECAG00000015406	5.76895127	0.13199311	0.493511186	857	678	2095	1188	663	1313	490	989
ENSECAG00000011750	1.478403731	0.132231579	0.493986696	37	177	42	11	1	79	10	29
ENSECAG00000007625	8.321671206	0.132305804	0.493986696	11580	12771	3476	3212	28	9955	64	1555
ENSECAG00000003736	4.491091254	0.132341105	0.493986696	168	178	507	448	915	451	635	307
ENSECAG00000017143	5.172988425	0.132388531	0.493986696	224	8	264	1091	1857	81	337	2083
ENSECAG00000010693	4.801584962	0.132425996	0.493986696	273	265	578	503	985	501	898	453
ENSECAG00000015327	4.656796069	0.132445176	0.493986696	288	386	294	476	878	384	775	519
ENSECAG00000019855	4.250814558	0.132461858	0.493986696	749	332	195	221	160	495	206	249
ENSECAG00000024868	0.446470554	0.132498838	0.493986696	21	7	8	21	63	17	51	12
ENSECAG00000019901	3.546369443	0.132537772	0.493986696	60	109	271	252	274	281	328	312
ENSECAG00000013808	2.712869514	0.132554181	0.493986696	56	71	87	160	158	106	192	218
ENSECAG00000020522	4.907667909	0.132694004	0.494345955	678	1110	497	287	141	883	199	491
ENSECAG00000008786	5.586282705	0.132773086	0.494478764	482	676	656	1035	1405	733	1584	1146
ENSECAG00000018529	7.000024278	0.132900345	0.494549422	1072	1428	1207	3416	3313	1122	4563	4948
ENSECAG00000020865	5.7014547	0.132913058	0.494549422	517	572	800	1183	1204	699	1915	1552
ENSECAG00000015106	8.123767117	0.132922374	0.494549422	2516	4929	6923	12567	3674	3108	7202	4159
ENSECAG00000019100	1.835459874	0.133049125	0.494751787	41	130	37	114	34	35	72	78
ENSECAG00000000437	2.053128886	0.133063678	0.494751787	35	58	54.0001	81	100	64.0003	165	101
ENSECAG00000000489	10.05356453	0.133216367	0.494914506	21325	33797	18637	17176	14090	21303	15048	15630
ENSECAG00000015306	4.720553737	0.133217297	0.494914506	396	921	346	597	259	597	349	507
ENSECAG00000012246	5.954485299	0.133262128	0.494914506	932	1335	1376	1620	979	1092	1022	1173
ENSECAG00000004874	2.947604551	0.133281324	0.494914506	77	69	119	171	124	161	279	235
ENSECAG00000000757	1.546246087	0.133503413	0.495577553	31	32	42	49	89	49	105	55
ENSECAG00000025060	2.237420548	0.133706581	0.496169957	53	102	173	94	82	66	116	31
ENSECAG00000005273	1.354502075	0.133856623	0.496564891	37	45	59	82	56	29	38	35
ENSECAG00000007392	6.950497197	0.134082407	0.497108248	3009	3968	1526	1803	1135	2478	1922	2159
ENSECAG00000011831	0.844770678	0.134128757	0.497108248	14	17	12	45	32	16	44	97
ENSECAG00000016548	5.822938149	0.134134083	0.497108248	964	1074	1178	1534	975	884	1145	941
ENSECAG00000007813	6.678435595	0.134182627	0.497126328	1427	3179	1986	2594	706	2015	961	2720
ENSECAG00000006763	0.648768806	0.134306532	0.497423511	13	48	22	65	17	26	3	35
ENSECAG00000009780	5.85751466	0.13440564	0.497628686	1163	1395	1013	1163	912	909	1085	1073
ENSECAG00000008473	3.083870013	0.134574569	0.497777488	130.001	235	120.001	251.001	95.0009	165.001	115.001	172
ENSECAG00000011120	4.937346452	0.134575716	0.497777488	254	343	599	616	898	540	1246	470
ENSECAG00000012877	6.386889463	0.134584656	0.497777488	1165	4146	856	1081	1380	1158	1515	1204
ENSECAG00000011465	6.251760843	0.134620719	0.497777488	2097	2011	1555	691	606	2223	517	1075
ENSECAG00000013308	5.40269422	0.134670035	0.497798165	560	1270	776	1031	387	743	741	995
ENSECAG00000021304	4.739893397	0.13487034	0.498376769	223	222	595	555	796	606	675	604
ENSECAG00000024991	9.595233654	0.134928319	0.498429238	15162	27671	10977	13121	8820	17196	9294	11652
ENSECAG00000016370	3.739212648	0.135152987	0.498985484	95	204	206	268	527	322	237	249
ENSECAG00000007934	1.976500901	0.135174693	0.498985484	51	43	53	46	174	43	131	66
ENSECAG00000017160	5.191142343	0.135238194	0.498985484	378	960	1456	695	100	614	160	1213
ENSECAG00000015920	6.385621447	0.135295678	0.498985484	576	907	1126	2400	2690	1315	1992	2611
ENSECAG00000015460	9.375679751	0.13529804	0.498985484	20615	16657	7855	9095	10118	10836	10736	8874
ENSECAG00000014855	5.459506955	0.135478667	0.49948984	379	2229	791	850	112	1579	208	415
ENSECAG00000006090	4.877625545	0.135543291	0.499566323	427	524	535	1057	519	413	613	508
ENSECAG00000005377	3.992660949	0.135946399	0.500831705	202	161	208	316	472	168	610	409
ENSECAG00000006457	1.340788675	0.135974598	0.500831705	13	20	46	59	54	88	69	45
ENSECAG00000024249	4.755105923	0.13606363	0.50099755	210	798	504	935	368	319	549	599
ENSECAG00000020262	5.867576162	0.136343513	0.501651744	1152	1963	929	837	533	1489	740	934
ENSECAG00000015230	4.152524256	0.136361076	0.501651744	378	621	226	227	194	336	340	285
ENSECAG00000008251	3.763597823	0.136373487	0.501651744	146	164	193	271	304	178	586	347
ENSECAG00000014875	7.744448708	0.136495703	0.501919759	1093	2652	2862	6003	6038	5530	4793	5957
ENSECAG00000007671	7.128658531	0.136534518	0.501919759	1492	1816	2061	2243	5966	1959	4009	2719
ENSECAG00000023991	5.373841727	0.136596277	0.501984704	913	879	842	727	509	598	689	1024
ENSECAG00000014666	0.067244532	0.136643038	0.50199451	9	4	20	14	46	23	23	11
ENSECAG00000021789	5.213333538	0.136805359	0.502428712	608	772	778	922	638	551	766	658
ENSECAG00000009911	5.999842304	0.137009075	0.503014613	310	570	1008	2046	1364	1463	2179	1656
ENSECAG00000015370	5.941082393	0.137224158	0.503641856	1125	1652	929	1814	298	1486	378	1536
ENSECAG00000003766	3.821767025	0.137361853	0.503984757	77	144	223	375	622	344	342	149
ENSECAG00000008774	4.580841707	0.137542404	0.504397216	257	207	433	474	799	258	813	604
ENSECAG00000020910	2.457309503	0.137562877	0.504397216	33	81	80	122	140	109	176	136
ENSECAG00000000449	3.239479636	0.137904909	0.505488534	99	280	210	253	82	122	148	263
ENSECAG00000016754	5.551073899	0.137960397	0.505529165	881	1334	758	871	642	803	726	1007
ENSECAG00000013773	1.341979893	0.138025979	0.505606747	45	45	50	82	56	30	54	13
ENSECAG00000009239	4.592483513	0.138085214	0.505612884	202	279	359	625	484	428	818	723
ENSECAG00000004867	5.670555434	0.138116475	0.505612884	547	623	774	1055	1580	728	1629	1225
ENSECAG00000023722	5.310943072	0.138165701	0.505630507	628	2328	382	306	127	933	112	889

ENSECAG00000023169	1.34698895	0.138283912	0.505900495	21	33	49	32	53	64	70	67
ENSECAG00000009430	6.691510231	0.13850251	0.50604146	1963	2937	1581	2211	1455	1875	1455	2007
ENSECAG00000011935	5.994835848	0.138503646	0.50604146	921	1638	1204	1670	787	1053	1121	1425
ENSECAG00000011090	7.79265466	0.13850941	0.50604146	3815	10801	2952	2589	1265	7026	951	2583
ENSECAG00000016808	6.194369519	0.138562953	0.50604146	893	648	2648	2525	1168	1147	1277	1239
ENSECAG00000010616	4.393535913	0.138585117	0.50604146	191	370	864	548	186	461	108	520
ENSECAG00000001389	6.016886379	0.138589133	0.50604146	927.011	1669	1167.01	1760	997.001	832.001	1230	1427.02
ENSECAG00000014118	2.643111774	0.138653228	0.506113177	65	173	116	222	33	77	133	153
ENSECAG00000022750	5.091041477	0.138811699	0.506456068	550	891	538	885	552	729	468	557
ENSECAG00000000253	4.87913232	0.138836134	0.506456068	506	780	507	628	504	560	498	458
ENSECAG00000011163	6.078195378	0.138915285	0.506458617	986	1104	1353	2517	1283	902	1510	952
ENSECAG00000011563	2.498693179	0.138925802	0.506458617	48	59	105	106	158	103	130	181
ENSECAG00000010928	7.065885392	0.139011719	0.506555409	4537	6039	898	1232	39	4449	48	699
ENSECAG00000002581	4.131778936	0.139100561	0.506555409	183	508	261	605	351	213	368	257
ENSECAG00000019247	5.838318142	0.139125882	0.506555409	1304	1602	865	949	416	1141	766	1369
ENSECAG00000024077	2.591061535	0.139130326	0.506555409	74	173	205	82	172	55	61	75
ENSECAG00000019812	5.521544946	0.139261399	0.506870532	392	458	691	1259	1301	823.997	1507	1032
ENSECAG00000018469	1.759352157	0.139345787	0.507015591	31	49	58	44	87	83	103	67
ENSECAG00000016946	6.03747854	0.139571978	0.50767635	912	1160	1648	1952	973	1229	1106	1213
ENSECAG00000011108	6.230749797	0.139987794	0.509026202	929	1162	988	1072	2151	1591	2938	1078
ENSECAG00000014970	2.238788592	0.140091059	0.509116908	54	82	54	59	129	95	157	101
ENSECAG00000017237	7.260996679	0.140102176	0.509116908	10540	1683	328	659	30	4187	7	716
ENSECAG00000000090	6.99919152	0.140156337	0.509151212	3023	2004	4143	1565	1697	2409	1581	2347
ENSECAG00000019543	1.244691205	0.140246837	0.509317462	73	94	23	27	0	70	2	25
ENSECAG00000014594	1.160143985	0.140328789	0.509441188	23	35	31	21	83	59	48	33
ENSECAG00000004417	3.676208067	0.14041135	0.509441188	85	193	198	286	381	261	415	237
ENSECAG00000014668	4.059754945	0.140415147	0.509441188	218	182	237	251	510	161	858	262
ENSECAG00000017567	1.262324579	0.140673505	0.510215945	41	125	16	23	21	40	30	41
ENSECAG00000019263	8.20858672	0.140910393	0.510912364	6284	10939	3458	4502	2998	6444	4457	4246
ENSECAG00000006428	3.09235771	0.141110423	0.511474744	88	250	161	262	105	187	103	152
ENSECAG00000016911	6.181083592	0.141222891	0.511719484	1805	2403	1184	564	783	1678	722	1232
ENSECAG00000018498	3.135918108	0.141311273	0.511876818	69	110	138	190	178	121	289	321
ENSECAG00000000125	6.528456007	0.141410878	0.511945663	1904	2018	1331	2509	1145	1702	1409	1900
ENSECAG00000018195	7.577298328	0.141420212	0.511945663	3288	6491	3437	3105	3363	3464	2467	2825
ENSECAG00000006162	8.372128415	0.141540674	0.512218874	9130	14741	2465	2612	1418	10317	1907	3408
ENSECAG00000020025	2.562584653	0.141624703	0.512360101	85	99	201	138	109	90	141	35
ENSECAG00000003831	2.060989802	0.14167527	0.512380226	21	45	78	89	155	57	137	84
ENSECAG00000022494	7.118078896	0.141819805	0.512740069	3863	4046	1761	1676	1434	2938	2143	2063
ENSECAG00000007629	3.885444255	0.141883303	0.512806796	176	382	436	263	221	254	270	258
ENSECAG00000020478	6.981162837	0.141978377	0.512918655	879	1766	1444	3120	3104	4977	1800	3007
ENSECAG00000005171	4.885331894	0.142004357	0.512918655	279	240	733	482	678	793	794	699
ENSECAG00000000147	1.3289722	0.142106879	0.51312617	54	72	49	32	17	57	35	39
ENSECAG00000021864	7.123981057	0.142201673	0.513191015	900	1776	2649	2778	3502	4181	4284	2437
ENSECAG00000009873	3.82356694	0.142254863	0.513191015	137	838	189	137	84	438	101	148
ENSECAG00000009533	9.642566255	0.142260065	0.513191015	23270	16122	13403	12635	11975	13615	12673	11847
ENSECAG00000016744	4.75455772	0.142346721	0.513340962	349	519	674	771	470	392	655	386
ENSECAG00000019099	1.963446863	0.142429165	0.513386267	37	49	158	117	61	53	97	31
ENSECAG00000013276	4.982070342	0.14244947	0.513386267	503	573	781	926	284	819	237	680
ENSECAG00000016036	2.144589311	0.142580791	0.513696931	40	30	94	78	131	97	143	82
ENSECAG00000018547	4.852324694	0.142839771	0.514306824	511	859	454	545	356	488	529	606
ENSECAG00000014998	3.569161669	0.14284042	0.514306824	213	145	369	289	189	129	328	159
ENSECAG00000020369	6.0504411	0.142913278	0.514370767	193	790	1132	1907	1585	2013	1478	1748
ENSECAG00000009961	3.87377402	0.142948538	0.514370767	188	453	250	377	209	325	156	267
ENSECAG00000008013	0.749933464	0.143045541	0.514557182	19	43	40	42	18	34	24	24
ENSECAG00000009379	6.566254049	0.1431412	0.514738647	2806	2561	878	1692	490	2769	945	1334
ENSECAG00000002156	0.865128126	0.143318755	0.515214407	22	31	46	59	21	30	27	33
ENSECAG00000014399	4.959879982	0.143383989	0.51528621	442	1495	830	183	46	1160	132	188
ENSECAG00000018344	3.779028387	0.143449595	0.515359306	142	126	249	242	495	278	533	125
ENSECAG00000013048	3.497250628	0.143624086	0.515823411	125	424	199	227	185	120	241	207
ENSECAG00000024161	6.858608926	0.143688488	0.515885803	703	1151	1859	3117	2389	2197	2762	4808
ENSECAG00000024038	8.077026282	0.143764328	0.515885803	4637	11266	4203	3596	2015	7476	3101	3343
ENSECAG00000009982	7.120016775	0.143777396	0.515885803	2730	3204	3083	2762	2902	2412	2287	1442
ENSECAG00000019547	3.550161441	0.143983812	0.516230914	221	229	256	259	202	159	210	229
ENSECAG00000022604	6.850428723	0.143996268	0.516230914	1631	2617	2696	3108	1665	2086	2145	1861
ENSECAG00000009902	7.214723107	0.144009608	0.516230914	1916	7056	1789	3079	388	3258	1185	3571
ENSECAG00000006801	5.826404286	0.144086843	0.516271805	1132	1893	813	815	635	1070	872	1160
ENSECAG00000013913	4.785100012	0.144111708	0.516271805	257	334	346	746	724	637	820	567
ENSECAG00000021855	7.212098257	0.14425746	0.516631387	3511	3610	2369	2769	2377	2712	2111	2364
ENSECAG00000020734	1.945997848	0.144455497	0.517177934	131	148	41	20	2	71	6	95
ENSECAG00000002798	3.60160797	0.144644893	0.517693213	112	166	211	217	259	199	475	309
ENSECAG00000008529	5.028305661	0.144707555	0.517754718	327	316	661	623	768	504	867	1158
ENSECAG00000006122	3.373079991	0.144808996	0.517954893	105	352	190	297	45	148	184	281
ENSECAG00000020669	8.403030202	0.144991639	0.518445292	9457	6724	6292	5296	6706	5178	4265	5073
ENSECAG00000015011	5.718414619	0.145093088	0.518625548	666	656	784	872	1571	744	2151	967
ENSECAG00000025107	5.465569998	0.145223395	0.518625548	365	416	738	1124	778	599	1580	1640
ENSECAG00000024392	0.899021819	0.145224366	0.518625548	18	20	28	29	47	27	78	38

ENSECAG00000020881	6.787963761	0.145255153	0.518625548	1153	1441	1549	2082	2504	1432	4768	3010
ENSECAG00000016226	2.65478756	0.145277398	0.518625548	151	380	58	25	0	179	4	56
ENSECAG00000016509	2.606933596	0.145315371	0.518625548	89	71	231	280	0	97	2	158
ENSECAG00000008486	3.503719524	0.145399721	0.51876397	117	464	210	197	246	189	143	143
ENSECAG00000000064	5.754190003	0.145743904	0.519758968	1504	1516	791	592	467	1609	495	672
ENSECAG00000005359	3.774059059	0.145769906	0.519758968	286	333	219	291	218	322	160	188
ENSECAG00000012566	2.661404605	0.145867499	0.519944108	70	87	108	78	168	97	224	161
ENSECAG00000023594	5.497054084	0.145992775	0.520227776	881	1544	644	607	615	847	826	723
ENSECAG00000001989	5.558495429	0.146062302	0.520312675	2008	951	545	321	173	1667	252	336
ENSECAG00000008937	4.374441571	0.146149128	0.520319398	486	1204	162	139	5	600	11	253
ENSECAG00000010918	8.402399665	0.14621958	0.520319398	14408	13909	1585	1844	25	8230	32	3868
ENSECAG00000019620	5.424055859	0.146222286	0.520319398	509	920	1418	890	554	1110	539	630
ENSECAG00000017041	2.187302466	0.146289514	0.520319398	58	74	47	54	85	71	192	130
ENSECAG00000023403	6.042945487	0.1462927	0.520319398	611	586	1137	1611	1913	991	2227	1624
ENSECAG00000006526	3.545344299	0.14678229	0.521897679	106	197	177	194	349	216	395	219
ENSECAG00000020561	0.714677042	0.147055751	0.522475005	15	17	28	20	52	27	71	18
ENSECAG00000025023	6.859494004	0.147093003	0.522475005	2814	4192	1395	1245	1632	2687	1421	1282
ENSECAG00000006141	3.772611283	0.147105953	0.522475005	130	255	161	228	185	446	401	348
ENSECAG00000020842	2.734742046	0.147128227	0.522475005	78	81	236	376	0	108	0	154
ENSECAG00000025069	0.701567785	0.14719911	0.522563724	31.0002	41.0006	46.0004	16.0007	18.0008	40	17.0003	16.0009
ENSECAG00000017348	4.890324506	0.147365215	0.522965834	680	731	492	451	456	465	588	523
ENSECAG00000020689	0.562187751	0.14743925	0.522965834	16	18	23	14	41	32	24	47
ENSECAG00000010007	8.26514928	0.147450183	0.522965834	7531	7776	4558	5747	3877	6116	4236	5257
ENSECAG00000012767	0.758771785	0.147501082	0.522983438	10	24	24	31	29	37	48	54
ENSECAG00000009805	4.209475743	0.147868752	0.523648991	200	363	501	636	176.001	456.001	119	412
ENSECAG00000012263	8.481152742	0.147926039	0.523648991	5958	10565	7799	6468	5718	7219	4558	5255
ENSECAG00000024686	6.190444822	0.147949353	0.523648991	1224	1713	1017	2376	816	829	1386	1932
ENSECAG00000015195	5.159760058	0.147974912	0.523648991	478	479	706	1555	601	361	871	645
ENSECAG00000013244	6.543899386	0.147998848	0.523648991	1834	2745	1659	1537	991	2223	1193	1626
ENSECAG00000012495	5.843708251	0.147999837	0.523648991	418	812	1055	1130	2125	1088	1745	861
ENSECAG00000021434	1.454041169	0.148010756	0.523648991	22	12	75	27	90	77	36	77
ENSECAG00000018112	1.187447993	0.148103372	0.523813885	18	9	51	40	66	70	56	38
ENSECAG00000014267	2.831027307	0.148191969	0.523964463	79	451.003	77.0018	62.0025	13	228	76.002	40
ENSECAG00000009778	1.847166622	0.148323213	0.524181818	30	95	79	119	57	38	79	58
ENSECAG00000019889	6.352641258	0.148345526	0.524181818	2220	1730	1540	991	1347	1108	1399	1553
ENSECAG00000000009	1.04046871	0.148601858	0.524924651	18	23	30	38	30	52	61	62
ENSECAG00000009751	6.248034325	0.14864835	0.524926012	786	900	1192	1639	1799	1439	2529	1936
ENSECAG00000022754	1.508792012	0.148723223	0.525027563	27	75	73	73	40	33	54	55
ENSECAG00000010441	0.235004384	0.148809313	0.525168639	4	15	20	21	27	23	37	28
ENSECAG00000018924	8.315961257	0.148867629	0.525169869	9364	5876	5948	5126	3532	8194	4338	3381
ENSECAG00000012348	0.463889273	0.148901918	0.525169869	8	6	25	26	46	10	80	12
ENSECAG00000018466	2.63821147	0.149099002	0.525597687	68	112	174	202	89	69	156	102
ENSECAG00000004306	2.632095398	0.149115746	0.525597687	89	134	99	234	52	99	188	67
ENSECAG00000009471	5.1583994	0.149161715	0.525597687	252	1077	558	1441	332	516	560	951
ENSECAG00000014409	5.380157695	0.149401644	0.526184591	719	557	1086	1175	538	506	879	984
ENSECAG00000020068	6.883042508	0.149422076	0.526184591	611	1297	2160	2843	4495	2703	3381	1714
ENSECAG00000016031	5.529018865	0.149466927	0.526184591	961	1270	715	773	644	654	972	913
ENSECAG00000010890	4.477655784	0.149637764	0.526545004	470	962	457	189	20	484	20	565
ENSECAG00000023800	5.052988896	0.149661803	0.526545004	605	824	534	764	414	529	609	754
ENSECAG00000021673	2.707261829	0.149721611	0.52659269	48	64	88	179	199	185	117	153
ENSECAG00000017156	5.625200546	0.149882884	0.526997109	1159	955	889	992	507	1085	725	996
ENSECAG00000022012	0.612125176	0.149975782	0.52702802	20	10	31	8	34	34	63	26
ENSECAG00000000650	7.009306817	0.149998409	0.52702802	2249	8823	1157	525	50	3797	55	1355
ENSECAG00000018173	5.632869554	0.15003055	0.52702802	953	1278	877	934	758	1087	750	772
ENSECAG00000010560	8.789183949	0.150083758	0.527052309	8875	8950	21663	6424	87	6497	61	12159
ENSECAG00000021917	3.068917187	0.150187466	0.52725387	102	97	144	110	211	171	373	118
ENSECAG00000000881	0.858177135	0.150243491	0.527287962	28	21	22	9	54	43	69	21
ENSECAG00000003775	6.990003631	0.150603232	0.528387609	1215	1793	1950	2309	3857	2290	4431	2575
ENSECAG00000008940	1.610924287	0.150793547	0.52889234	22	41	133	90	20	45	46	72
ENSECAG00000010872	3.944819399	0.151002098	0.529460697	277	420	293	297	142	403	197	260
ENSECAG00000011634	4.962091428	0.15124013	0.529632374	267	423	513	670	1001	879	717	473
ENSECAG00000014730	1.091421964	0.151318037	0.529632374	17	20	84	76	36	26	27	37
ENSECAG00000022310	3.634954947	0.151353771	0.529632374	88	131	733	218	137	194	169	265
ENSECAG00000013037	4.481843658	0.15135746	0.529632374	273	300	369	174	966	582	713	101
ENSECAG00000003968	3.790300165	0.1513621	0.529632374	159	160	214	275	392	253	400	336
ENSECAG00000009624	6.77254487	0.151369078	0.529632374	2023	2391	2640	2101	1821	1993	1656	1819
ENSECAG00000008029	7.936898458	0.151376701	0.529632374	4772	6116	4116	5852	2688	4486	4855	4145
ENSECAG00000001265	2.575415035	0.1514916	0.529790562	79	155	131	144	107	97	91	97
ENSECAG00000024210	4.281394444	0.151514982	0.529790562	215	459	381	641	380	328	280	343
ENSECAG00000020530	4.909144674	0.151648804	0.530095682	257	557	839	1048	545	583	472	444
ENSECAG00000022953	2.124569956	0.151764833	0.530338436	49	47	67	75	135	74	141	92
ENSECAG00000024771	7.615669998	0.151831598	0.530408941	1239	2055	3165	5187	4542	5152	4871	5669
ENSECAG00000018995	6.181804204	0.151938352	0.53061906	1489	2372	995	1067	926	1181	1252	1474
ENSECAG00000008185	0.64520331	0.152089274	0.530983253	33	38	14	53	16	4	60	9
ENSECAG00000024353	5.306258584	0.152140184	0.53099816	513	739	779	1424	645	448	957	744
ENSECAG00000000386	2.506255282	0.152208613	0.531074182	70	264	80	103	19	93	38	172

ENSECAG00000015822	1.612236861	0.15239629	0.531442826	20	6	254	52	2	45	2	78
ENSECAG00000024046	8.995826133	0.152407626	0.531442826	13893	18192	7041	5847	1605	17967	2182	4742
ENSECAG00000019762	7.368228825	0.152590149	0.531865937	2840	6116	2167	3362	1078	3816	1793	3436
ENSECAG00000014515	1.143205048	0.152632927	0.531865937	53	37	50	55	17	2	26	77
ENSECAG00000020116	6.965407674	0.152669116	0.531865937	1846	2894	2483	3887	1083	2712	1651	2664
ENSECAG00000019299	5.768893419	0.152813557	0.532206285	535	1174	1199	1940	989	983	865	906
ENSECAG00000014736	6.146642972	0.152862846	0.532215137	1389	918	1161	2647	904	1119	1489	1332
ENSECAG00000015919	5.124436745	0.152951694	0.532361673	521	962	544	892	458	569	656	751
ENSECAG00000014240	4.166203094	0.153097639	0.532528332	208	96	503	160	477	395	509	477
ENSECAG00000009279	3.139162233	0.153098208	0.532528332	54	100	149	217	128	187	281	311
ENSECAG00000018269	7.195421516	0.153141611	0.532528332	918	1831	2983	2889	3891	3652	4328	3206
ENSECAG00000006452	5.096604371	0.153186674	0.532528332	419	764	788	950	598	409	670	740
ENSECAG00000021859	1.887088058	0.153280629	0.532692295	96	57	63	98	37	19	51	118
ENSECAG00000017727	3.425431467	0.153425464	0.533017061	86	148	188	213	213	191	403	287
ENSECAG00000007966	6.080205444	0.153489478	0.533017061	1446	1552	1240	1252	1307	854	1545	956
ENSECAG00000015181	5.493381264	0.153514532	0.533017061	981	584	1243	876	578	808	1058	662
ENSECAG00000014499	1.356466784	0.153631247	0.53325968	29	24	36	41	87	21	98	60
ENSECAG00000013617	2.867183944	0.153693825	0.533314295	57	80	126	160	232	105	244	165
ENSECAG00000006474	7.144310557	0.1538012	0.533524274	3010	6009	1748	1194	1643	3398	1502	1848
ENSECAG00000019329	4.094879445	0.153997378	0.533942259	43	171	264	520	170	327	471	875
ENSECAG00000019300	4.028966228	0.154015492	0.533942259	251	697	304	299	23	451	20	340
ENSECAG00000017361	5.250455236	0.154237985	0.53424429	247	318	570	1179	707	500	1208	1563
ENSECAG00000022430	5.467410972	0.154240393	0.53424429	576	262	1037	571	1400	924	1396	816
ENSECAG00000006885	7.381693631	0.154243389	0.53424429	2589	3220	3489	5399	3137	2950	2404	2512
ENSECAG00000022444	3.006529714	0.154438971	0.534543565	97	157	176	282	88	103	193	161
ENSECAG00000020381	3.491509148	0.154498684	0.534543565	229	343	144	202	145	213	188	196
ENSECAG00000021745	4.692172293	0.15451646	0.534543565	241	294	515	488	729	797	552	459
ENSECAG00000021937	3.736503823	0.15470623	0.534543565	106	250	153	272	390	205	450	310
ENSECAG00000025116	1.76230465	0.154771676	0.534543565	53	187	41	30	6	115	13	29
ENSECAG00000018866	7.345564887	0.154825058	0.534543565	1061	1613	2782	4244	4072	3870	4818	4062
ENSECAG00000008085	3.544715072	0.154831756	0.534543565	228	295	417	98	21	245	85	309
ENSECAG00000007329	5.626467293	0.154855464	0.534543565	1186	2596	255	211	345	1376	334	594
ENSECAG00000023764	7.700181405	0.154858772	0.534543565	1930	2541	3271	4300	6023	4220	5837	5253
ENSECAG00000008387	6.589366249	0.154903627	0.534543565	1123	3527	1886	1777	1417	1985	1602	1352
ENSECAG00000011130	1.644492218	0.154920245	0.534543565	29	24	57	59	79	43	125	76
ENSECAG00000018791	3.08276737	0.1549448	0.534543565	100	197	206	233	90	164	174	142
ENSECAG00000019831	5.110969461	0.154959931	0.534543565	552	741	706	872	416	641	664	709
ENSECAG00000026992	4.265822527	0.155024313	0.534543565	229	328	601	500	251	330	443	320
ENSECAG00000004316	0.508150296	0.155034067	0.534543565	27	25	25	46	6	7	23	45
ENSECAG00000001394	5.928797084	0.155164831	0.534832456	934	773	2956	799	1062	259	912	1607
ENSECAG00000024674	2.537819038	0.155345169	0.535291993	32	48	94	119	60	18	423	183
ENSECAG00000016464	10.98712536	0.155461699	0.535503781	50064	58716	28139	31346	27068	35515	35245	30701
ENSECAG00000021679	6.093334094	0.155568078	0.535503781	868	949	716	1367	2285	1244	2106	1282
ENSECAG00000018957	6.162286884	0.155580038	0.535503781	788	623	1350	1451	1914	1351	2398	1614
ENSECAG00000023364	6.165456149	0.155594774	0.535503781	955	2144	1706	1474	507	1553	717	1844
ENSECAG00000019277	4.65817647	0.155935811	0.53631382	292	527	396	1028	412	412	397	511
ENSECAG00000002165	2.655517366	0.155953696	0.53631382	89	283	75	116	19	133	64	149
ENSECAG00000007910	3.279395671	0.155971459	0.53631382	150	109	89	170	264	133	392	222
ENSECAG00000017860	5.785491431	0.156197789	0.536893032	464.002	995.002	2042	1460.04	828	803.001	752	1331
ENSECAG00000019508	0.536553176	0.156234222	0.536893032	8	14	35	84	22	8	18	32
ENSECAG00000020309	6.847493067	0.156306174	0.536978212	1137	792	2379	2248	2550	3923	3077	2244
ENSECAG00000026825	2.417956028	0.156463682	0.537280179	21	124	84	54	121	110	259	83
ENSECAG00000016697	3.531528107	0.156488456	0.537280179	116	158	207	191	285	255	406	215
ENSECAG00000012139	3.34785397	0.156551267	0.537333789	88	93	178	244	278	206	362	188
ENSECAG00000003961	0.996940419	0.156704404	0.537697299	19	14	21	45	20	17	91	86
ENSECAG00000024138	3.963224045	0.156776137	0.537781355	213	659	461	211	5	198	14	537
ENSECAG00000021505	5.708932379	0.156883666	0.537861582	1238	1270	557	1212	1095	520	746	1130
ENSECAG00000021058	1.757465739	0.156894011	0.537861582	33	43	51	54	77	37	135	101
ENSECAG00000018392	3.1299767	0.156960686	0.53792818	129	133	324	170	130	88	138	221
ENSECAG00000019306	3.500912517	0.157325636	0.539016662	66	130	195	299	291	283	265	291
ENSECAG00000002992	4.775552204	0.157425308	0.539195888	83	1114	386	1057	353	373	421	580
ENSECAG00000004790	2.492474913	0.157647309	0.539477356	70	73	92	62	148	112	229	92
ENSECAG00000016705	5.364281419	0.157649244	0.539477356	796	1219	632	788	369	369	761	1273
ENSECAG00000024232	1.957693623	0.157723145	0.539477356	44	51	153	132	9	30	23	152
ENSECAG00000015898	1.583333343	0.157781651	0.539477356	81	23	126	53	0	36	7	97
ENSECAG00000017984	7.568370203	0.157795353	0.539477356	7031	6112	1416	707	3029	3066	3443	1206
ENSECAG00000017908	4.098258619	0.157854396	0.539477356	207	380	402	478	204	311	322	354
ENSECAG00000008472	3.993538423	0.157940773	0.539477356	252	442	360	314	86	374	182	379
ENSECAG00000020269	2.221960798	0.157977165	0.539477356	134	104	69	65	48	58	74	112
ENSECAG00000024441	1.548649253	0.158000479	0.539477356	28	38	39	52	66	41	134	61
ENSECAG00000021567	7.552919972	0.158006191	0.539477356	3486	6115	3082	3196	2400	4231	2916	2486
ENSECAG00000013303	2.447537813	0.15802872	0.539477356	30	106	138	257	69	106	43	110
ENSECAG00000019646	4.035016885	0.158303999	0.540255106	97	142	375	369	601	317.999	454	290
ENSECAG00000020312	2.774297827	0.15846266	0.540547559	63	122	129	337	63	114	74	177
ENSECAG00000017383	6.106501366	0.158484651	0.540547559	2181	649	1618	1017	934	1207	1185	1155
ENSECAG00000013393	5.794014295	0.15858105	0.540639226	745	1458	1283	1172	827	889	891	1248

ENSECAG00000015405	7.058600559	0.158606501	0.540639226	914	1265	1339	4016	1685	1456	4257	7230
ENSECAG00000010966	3.720014741	0.159089473	0.5420619	101	143	167	347	174	142	451	633
ENSECAG00000018736	2.978981673	0.159149783	0.5420619	204	126	155	149	89	152	151	126
ENSECAG00000020462	7.575517342	0.159166704	0.5420619	3751	6834	3067	2612	1836	5140	1960	2673
ENSECAG00000017207	5.131687472	0.159227049	0.542105249	790	634	825	552	746	436	733	509
ENSECAG00000014491	7.840400058	0.159343221	0.542273749	1875	3265	3772	4297	6675	5418	7660	3903
ENSECAG00000012834	4.858433785	0.159407441	0.542273749	252.011	1063	435.008	809	291.001	497.001	614	570.024
ENSECAG00000022473	4.370426161	0.159447175	0.542273749	145	202	442	469	630	445	664	343
ENSECAG00000007886	0.614136814	0.159477861	0.542273749	7	42	52	50	0	32	6	35
ENSECAG00000024875	1.284174752	0.159536486	0.542273749	39	94	18	60	17	19	43	66
ENSECAG00000012743	5.185658926	0.159562324	0.542273749	418	702	482	431	1110	468	1412	751
ENSECAG00000013298	3.076651198	0.159633231	0.542297566	95	81	153	137	297	235	209	106
ENSECAG00000008917	7.590068728	0.159664597	0.542297566	3696	4784	4364	3306	2605	3821	3406	2861
ENSECAG00000023879	10.42784967	0.160058907	0.543441841	39016	38479	18340	15976	22050	19604	25402	19571
ENSECAG00000010201	1.282922398	0.160126827	0.543441841	14	40	125	56	8	20	21	84
ENSECAG00000006245	2.427930984	0.160144697	0.543441841	68	77	261	131	12	64	4	193
ENSECAG00000019676	5.197292884	0.160220278	0.543444318	337	510	715	669	871	883	985	862
ENSECAG00000025037	4.906085421	0.160240894	0.543444318	304	996	547	763	264	412	701	694
ENSECAG00000023902	6.0066787	0.160311046	0.543520326	873	2160	941	1524	716	1469	935	1170
ENSECAG00000019424	3.067796225	0.160544685	0.544102467	89	79	184	120	214	152	290	198
ENSECAG00000015817	6.199768792	0.16062485	0.544102467	1910	1702	1043	1296	740	1783	825	1380
ENSECAG00000009719	5.945929902	0.160654377	0.544102467	828	1511	1265	1661	693	1102	1075	1400
ENSECAG00000020654	2.44282199	0.160673912	0.544102467	43	58	104	98	181	57	204	123
ENSECAG00000006038	1.421658523	0.160744242	0.544178767	21	66	16	29	56	44	133	52
ENSECAG00000010790	4.328583019	0.161033152	0.544994778	277	237	306	324	507	285	743	502
ENSECAG00000016155	1.469934688	0.161177783	0.545209669	28	32	32	59	62	55	107	56
ENSECAG00000021007	6.486502899	0.161192424	0.545209669	1062	2677	2093	1872	1198	1892	1442	1516
ENSECAG00000008212	7.224646001	0.161329693	0.545511897	2539	3331	3129	3856	2187	3035	1853	2770
ENSECAG00000022288	2.265942336	0.161633682	0.546307388	46	58	167	186	28	78	47	137
ENSECAG00000014410	2.894131372	0.161660922	0.546307388	54	35	163	155	359	130	207	83
ENSECAG00000007796	5.927497987	0.161808356	0.546619937	638	781	852	1374	1554	937	1988	1666
ENSECAG00000015187	1.207210927	0.161849434	0.546619937	30	19	92	64	36	27	27	49
ENSECAG00000023658	4.968157026	0.162036804	0.54683897	501	688	809	564	455	496	597	656
ENSECAG00000005312	1.758365714	0.162133829	0.54683897	42	34	32	61	75	19	136	133
ENSECAG00000007663	7.11970755	0.162142875	0.54683897	1368	1556	2748	1861	6886	2078	3576	1971
ENSECAG00000014869	6.077177265	0.162150531	0.54683897	713	1390	1871	1928	1187	1078	1106	1295
ENSECAG00000016162	4.831670939	0.162161054	0.54683897	484	327	321	415	984	465	835	589
ENSECAG00000019560	4.861312999	0.162202477	0.54683897	309	339	565	465	1004	371	1111	488
ENSECAG00000017054	0.386621424	0.162366084	0.547228497	9	40	26	38	17	11	30	20
ENSECAG00000008196	3.414754917	0.162461446	0.547387857	291	269	155	144	95	111	144	330
ENSECAG00000014083	6.446780811	0.162557595	0.547405974	1524	2349	1572	1776	1569	1612	1309	1428
ENSECAG00000020987	3.787816617	0.162629943	0.547405974	167	83	322	203	340	327	419	316
ENSECAG00000009992	4.078803841	0.162664425	0.547405974	304	456	296.001	323	291	358	251	243
ENSECAG00000021685	0.718400946	0.162694276	0.547405974	11	16	28	32	56	26	54	27
ENSECAG00000018624	7.181039978	0.16270723	0.547405974	2626	3992	2342	3272	2200	2127	3238	2242
ENSECAG00000017586	3.733138164	0.162868166	0.547785544	206	195	390	331	215	223	244	234
ENSECAG00000012325	0.463314093	0.162964449	0.547947506	7	11	23	31	47	31	39	18
ENSECAG00000015134	1.265151354	0.163199215	0.548574865	32	81	38	55	61	32	19	31
ENSECAG00000003357	6.076107214	0.163494151	0.54906571	752	1496	1599	2025	1042	1287	1140	1196
ENSECAG00000024224	1.165607404	0.163504875	0.54906571	20	32	22	48	54	61	52	52
ENSECAG00000002987	5.569060662	0.163530136	0.54906571	806	1313	938	827	712	931	1083	568
ENSECAG00000010263	7.416059105	0.163538149	0.54906571	2504	4793	3458	3916	2310	2577	3556	3111
ENSECAG00000014555	2.113259355	0.163638465	0.549240542	39	66	64	67	66	93	203	87
ENSECAG00000015745	5.720514885	0.163767606	0.549511994	462	517	588	1633	1105	752	1624	1948
ENSECAG00000008076	6.096197111	0.163821102	0.549529535	887	1482	1586	1938	849	1501	1202	1156
ENSECAG00000019458	7.80035517	0.163995619	0.54953665	2066	2459	4537	3574	6360	3459	8497	5071
ENSECAG00000018758	4.669376197	0.164032263	0.54953665	251	273	344	676	471	432	857	817
ENSECAG00000006341	2.941091665	0.164050193	0.54953665	32	93	153	179	229	162	176	209
ENSECAG00000017556	4.784735823	0.164066528	0.54953665	708	552	438	473	411	309	550	630
ENSECAG00000006486	4.250954524	0.164101661	0.54953665	254	267	509	636	341	268	514	204
ENSECAG00000007908	4.269465519	0.164131943	0.54953665	226	263	225	435	478	418	456	543
ENSECAG00000017390	4.41938591	0.164161102	0.54953665	210	254	474	306	774	488	518	340
ENSECAG00000021391	6.716510816	0.164280831	0.549775796	2017	2194	2303	2199	1675	1834	2074	1601
ENSECAG00000024527	3.415670136	0.164367893	0.549834158	96	101	236	179	320	277	349	134
ENSECAG00000015410	1.722256801	0.164406216	0.549834158	44	46	160	70	10	105	3	44
ENSECAG00000012389	7.253545953	0.164479756	0.549834158	3036	4557	2566	2742	1965	4056	1910	1683
ENSECAG00000021023	3.96440588	0.164538515	0.549834158	149	190	238	368	411	413	388	319
ENSECAG00000014575	5.716152109	0.164539743	0.549834158	1118	1427	765	907	665	913	1014	1022
ENSECAG00000008640	3.275379367	0.164792149	0.550436316	76	144	180	160	333	258	224	145
ENSECAG00000000186	7.675418524	0.164816636	0.550436316	4411	2753	5556	4687	1965	2563	4905	4250
ENSECAG00000000566	2.835125244	0.164887948	0.550512988	73	62	122	148	195	139	286	113
ENSECAG00000008242	3.137631639	0.165005519	0.550630321	94	356	306	108	17	108	4	311
ENSECAG00000024208	5.854983447	0.165031389	0.550630321	714	1054	1401	1865	938	703	1057	1361
ENSECAG00000014326	4.049246663	0.165068185	0.550630321	197	251	414	611	218	225	241	444
ENSECAG00000018243	8.359537858	0.165156547	0.550763705	5385	7552	6646	8889	3939	4402	7139	6679
ENSECAG00000002758	5.43363022	0.165212302	0.550788305	323	577	778	970	1403	789	1377	760

ENSECAG00000017331	6.694962447	0.165260917	0.550789091	2914	2496	1443	1374	1837	1890	1695	1291
ENSECAG00000014502	3.701655652	0.165327697	0.550850405	173	87	225	246	284	193	410	447
ENSECAG00000022786	6.480483992	0.165655813	0.551782162	3072	1800	1084	1140	733	2467	786	1386
ENSECAG00000016890	5.095345187	0.165844264	0.552097189	580	780	613	849	460	400	810	766
ENSECAG00000016884	6.113344019	0.165847377	0.552097189	1570	1431	1268	1276	1091	1181	1267	1223
ENSECAG00000010634	3.62075295	0.165909646	0.552143034	108	356	231	418	106	318	185	190
ENSECAG00000008677	1.051648485	0.165963784	0.552161799	7	27	36	42	28	34	73	78
ENSECAG00000012274	5.793310777	0.16613011	0.552553697	507	896	955	985	1408	1489	1343	1191
ENSECAG00000022108	4.05354004	0.166233463	0.552735974	117	187	325	395	386	422	441	393
ENSECAG00000012403	4.111865885	0.166316856	0.552851797	184	421	308	603	211	282	320	384
ENSECAG00000007020	2.616234707	0.166579472	0.553563133	39	33	159	115	156	105	244	139
ENSECAG00000017915	4.519413541	0.1666911	0.553772447	195	101	458	587	485	462	392	1013
ENSECAG00000020012	5.046826365	0.166968474	0.554464983	338	424	611	639	797	710	948	791
ENSECAG00000008879	4.635404482	0.166996963	0.554464983	378	581	444	699	280	266	628	584
ENSECAG00000014214	3.230255848	0.167054318	0.554488157	76	130	170	178	175	180	341	254
ENSECAG00000002428	4.430750662	0.16710135	0.554488157	327	187	369	241	890	279	863	234
ENSECAG00000020449	6.250057355	0.167228432	0.554748165	1612	1549	1193	1861	1168	1328	1556	1218
ENSECAG00000008974	7.626686162	0.167381986	0.55509581	2982	2559	5945	6015	3096	3181	3623	3318
ENSECAG00000023194	0.769237297	0.167485001	0.555275694	16	18	32	22	40	26	58	45
ENSECAG00000013452	1.301152887	0.167622844	0.555345743	17	19	60	37	74	40	79	56
ENSECAG00000009120	0.824717673	0.167646579	0.555345743	20	19	28	20	24	24	67	66
ENSECAG00000021999	9.167310686	0.167652465	0.555345743	11983	21609	6673	8251	7117	12343	7252	8839
ENSECAG00000001117	3.50884564	0.167959409	0.555578972	165	187	303	326	272	176	207	112
ENSECAG00000014153	7.613574293	0.167969972	0.555578972	5619	3544	4072	2857	1432	4813	2038	3776
ENSECAG00000017880	4.171566483	0.167985814	0.555578972	228	172	347	288	397	301	721	416
ENSECAG00000010440	5.170482515	0.167989664	0.555578972	565	1064	582	741	579	596	802	558
ENSECAG00000025129	4.044806396	0.167995767	0.555578972	183	644	421	187	215	313	109	394
ENSECAG00000014197	4.999185092	0.168128659	0.555578972	329	1394	402	665	431	791	344	484
ENSECAG00000002833	4.968126507	0.168137142	0.555578972	330	837	618	920	341	601	633	613
ENSECAG00000000541	1.362788685	0.168139208	0.555578972	26	66	45	89	26	47	51	39
ENSECAG00000003091	6.155967917	0.168162067	0.555578972	1514	1940	1004	1298	971	1207	1298	1395
ENSECAG00000013640	3.089075992	0.168340055	0.556005665	76	155	176	379	118	99	143	205
ENSECAG00000015458	3.065415789	0.168470369	0.556224513	171	247	116	149	131	88	213	131
ENSECAG00000012029	5.668001879	0.168504027	0.556224513	607	1602	798	1338	601	861	1280	822
ENSECAG00000015973	4.252926231	0.168739787	0.556841296	227	398	358	668	335	244	407	360
ENSECAG00000001439	5.389995917	0.168815877	0.556930966	756	888	630	1187	810	654	850	639
ENSECAG00000016830	6.744390659	0.169272719	0.558276334	1977	3596	1624	1657	1809	1607	2022	1729
ENSECAG00000012945	6.07602234	0.169496909	0.558853838	679	1264	877	1151	1469	1421	2322	1575
ENSECAG00000023534	7.618139895	0.169578462	0.558960855	925	2204	2101	6657	5251	4792	5010	5407
ENSECAG00000009129	2.248252826	0.169679915	0.559133381	34	28	132	20	163	232	111	9
ENSECAG00000023620	4.008346308	0.169805765	0.559231294	155	217	256	327	225	329	489	576
ENSECAG00000005718	6.060372115	0.169807869	0.559231294	619	472	1144	1774	1743	1030	1644	2379
ENSECAG00000013378	2.404589673	0.170091648	0.559939862	77	112	110	152	74	64	108	111
ENSECAG00000013285	6.513719143	0.170121386	0.559939862	810	961	1702	2038	1754	1877	2841	2789
ENSECAG00000016246	7.611329439	0.170303688	0.560377888	4011	5170	3533	3474	2798	3720	3329	3092
ENSECAG00000010289	4.182202061	0.170387179	0.560413084	89	283	394	310	703	335	535	268
ENSECAG00000007993	6.822215802	0.170452688	0.560413084	2212	4263	1534	1384	1420	2349	1913	1579
ENSECAG00000017579	6.954175712	0.170462056	0.560413084	3077	2914	2083	1915	1736	1870	2418	2254
ENSECAG00000005944	5.64329249	0.170599269	0.560702273	484	563	956	1058	1342	973	1444	1181
ENSECAG00000023029	4.405590576	0.170745883	0.560867487	243	499	477	616	189	321	428	540
ENSECAG00000003882	2.974437716	0.170787768	0.560867487	98	75	132	138	208	147	279	161
ENSECAG00000018111	5.393521427	0.170797328	0.560867487	566	1699	537	887	268	1095	324	925
ENSECAG00000016378	4.39546122	0.170867363	0.560935678	229	679	371	546	238	551	273	335
ENSECAG00000010768	6.48925383	0.171002441	0.561184338	1030	724	1574	1905	2667	1400	3256	1907
ENSECAG00000000373	2.083750449	0.171057042	0.561184338	52	63	56	227	59	71	40	90
ENSECAG00000021766	2.73915537	0.171132284	0.561184338	28	47	110	214	190	178	159	154
ENSECAG00000018114	5.41004817	0.171140274	0.561184338	353	445	767	1069	918	746	1291	1306
ENSECAG00000012559	7.36458878	0.171210524	0.561253041	4063	4784	2246	2262	2536	2852	2937	2381
ENSECAG00000022257	3.069367074	0.171374248	0.561550051	68	54	284	573	1	178	2	181
ENSECAG00000019530	6.488379896	0.171430974	0.561550051	2419	2095	1437	1291	863	2510	1089	1221
ENSECAG00000014546	5.314560958	0.171499457	0.561550051	741	555	1011	1166	220	396	677	1362
ENSECAG00000017870	4.419996976	0.171500621	0.561550051	166	359	228	513	375	236	704	901
ENSECAG00000016098	0.369780223	0.171677758	0.561550051	7	40	4	4	37	29	28	38
ENSECAG00000010434	4.96326323	0.171697093	0.561550051	293	253	581	790	882	669	852	690
ENSECAG00000021271	2.773722006	0.171725923	0.561550051	78	86	108	114	199	127	218	142
ENSECAG00000010407	4.879128388	0.171764706	0.561550051	306	395	532	479	1060	397	1073	456
ENSECAG00000005814	7.659539057	0.171788195	0.561550051	2972	2364	2722	2386	5464	2956	10552	2937
ENSECAG00000023809	3.786936472	0.171794363	0.561550051	141	208	196	269	377	229	497	284
ENSECAG00000012013	5.888219228	0.172040946	0.561606166	752	524	890	1321	1514	952	1886	1632
ENSECAG00000006315	5.686116114	0.17206784	0.561606166	687	940	1071	1752	1212	625	1006	770
ENSECAG00000014032	7.772515865	0.172103088	0.561606166	4137	5323	3821	5091	3167	3418	4568	3647
ENSECAG00000000341	2.114780743	0.172126926	0.561606166	18	39	72	84	60	334	34	22
ENSECAG00000018076	6.456663944	0.172163213	0.561606166	1060	1918	2013	2612	1199	1551	1768	1613
ENSECAG00000010249	5.864769785	0.172194733	0.561606166	912	1325	981	1668	871	916	1057	1253
ENSECAG00000005757	9.319280275	0.172224264	0.561606166	26149	10568	6416	6297	9713	8357	13503	7559
ENSECAG00000019004	2.796090922	0.172224812	0.561606166	161	160	104	135	95	93	115	157

ENSECAG00000012036	6.482756652	0.172282834	0.561606166	1759	2128	1687	1719	1129	1694	1804	1538
ENSECAG00000014563	3.887516853	0.172304817	0.561606166	144	116	319	295	451	368	406	249
ENSECAG00000016265	8.576112721	0.172430113	0.561607839	6965	14008	5377	5826	5962	5557	8272	5093
ENSECAG00000023522	3.285691132	0.172432771	0.561607839	57	372	213	240	86	202	157	182
ENSECAG00000008755	7.734013069	0.172453316	0.561607839	6368	6737	2164	2050	2100	5736	2047	2681
ENSECAG00000011928	6.849748974	0.172574761	0.561687084	1384	881	1845	2370	3675	1802	4808	1849
ENSECAG00000014635	8.49761432	0.172576321	0.561687084	4109	3679	6991	4705	13552	8170	11705	4347
ENSECAG00000017595	3.412910229	0.172642197	0.561740902	64	190	163	219	199	299	235	324
ENSECAG00000019280	5.855125767	0.172802652	0.562102342	497	871	966	1148	2067	1320	1368	956
ENSECAG00000024933	4.176636387	0.173227763	0.563271616	337	470	343	313	425	238	316	263
ENSECAG00000000640	3.441160133	0.173278062	0.563271616	98	158	164	205	258	160	548	167
ENSECAG00000021187	5.970856732	0.173310538	0.563271616	1002	1326	1240	1674	930	919	1072	1486
ENSECAG00000013054	5.289346164	0.173585773	0.563864385	1266	520	554	683	750	550	790	537
ENSECAG00000011585	7.309755952	0.173661877	0.563864385	1694	3339	4764	4577	1536	3160	1902	3744
ENSECAG00000015773	6.364935103	0.173665114	0.563864385	646	1202	1447	1702	2360	1299	2562	2088
ENSECAG00000017474	3.773246923	0.173691032	0.563864385	222	414	224	259	221	220	261	239
ENSECAG00000007741	5.398003447	0.173860105	0.564252366	453	679	642	693	769	708	1951	898
ENSECAG00000016792	1.251069505	0.173921183	0.564253473	75	118	18	7	0	59	0	33
ENSECAG00000020211	4.766540611	0.173959569	0.564253473	306	335	559	387	876	440	893	515
ENSECAG00000020672	7.504435066	0.174206992	0.56477278	2519	4713	3540	5005	2737	3356	3660	2510
ENSECAG00000011232	3.157751644	0.174218885	0.56477278	81	138	132	175	211	163	256	259
ENSECAG00000014662	5.236206183	0.174406825	0.565179878	506	981	695	973	510	563	940	688
ENSECAG00000010795	6.644362387	0.17444375	0.565179878	937	2088	2282	3911	1069	1617	1219	2730
ENSECAG00000023430	8.479465584	0.17458414	0.565450681	13231	14013	3453	2454	53	11420	48	2675
ENSECAG00000024908	4.532422374	0.174626666	0.565450681	354	466	567	520	437	380	355	454
ENSECAG00000010731	5.03591125	0.174799685	0.565849991	1313	732	211	343	110	950	255	536
ENSECAG00000017672	1.322917886	0.174883044	0.56592062	39	23	93	61	27	32	64	35
ENSECAG00000019103	3.238496894	0.174920919	0.56592062	245	148	129	241	118	133	168	213
ENSECAG00000020127	3.065630315	0.175059037	0.566206571	111	147	115	74	236	165	243	201
ENSECAG00000009143	5.266292198	0.175460539	0.567258529	834	895	593	738	509	615	818	763
ENSECAG00000009690	1.550718775	0.17548393	0.567258529	100	46	84	30	3	74	0	57
ENSECAG00000011361	5.184309136	0.175615763	0.56750104	478	1027	785	839	329	903	326	838
ENSECAG00000006612	2.901281549	0.175658644	0.56750104	81	233	146	184	80	134	150	137
ENSECAG00000000113	2.727725163	0.175816134	0.567658705	69	60	85	162	106	114	176	282
ENSECAG00000017120	4.659842047	0.175936573	0.567658705	375	584	681	436	404	375	447	550
ENSECAG00000020299	0.679287891	0.17596301	0.567658705	46	12	11	69	38	10	16	24
ENSECAG00000002706	3.010270181	0.175969035	0.567658705	58	148	133	129	218	129	252	213
ENSECAG00000011829	1.118123644	0.175971906	0.567658705	12	19	51	15	23	58	5	145
ENSECAG00000000781	2.519946224	0.176052714	0.567658705	38	113	108	288	70	106	68	117
ENSECAG00000012726	4.152034898	0.176068283	0.567658705	217	459	370	475	193	271	276	487
ENSECAG00000008817	3.273355673	0.176106328	0.567658705	700	80	3	28	5	285	0	20
ENSECAG00000022438	9.08324397	0.176300065	0.567796201	5927	19918	13866	10252	2596	6634	4503	19179
ENSECAG00000019982	2.533016583	0.176302004	0.567796201	44	41	125	124	158	174	136	110
ENSECAG00000009195	5.03374671	0.176328872	0.567796201	636	64	455	264	1021	274	2308	241
ENSECAG00000019439	4.898938048	0.17636621	0.567796201	333	848	562	831	294	411	566	809
ENSECAG00000015671	2.377473335	0.176402261	0.567796201	120	146	105	67	60	60	189	15
ENSECAG00000019132	6.277037634	0.176459481	0.567796201	1825	1486	1200	1724	1099	1208	1633	1439
ENSECAG00000019577	5.381124119	0.17649809	0.567796201	393	831	884	1640	416	683	787	1007
ENSECAG00000003872	4.580055267	0.176663822	0.567991772	191	401	384	491	667	493	732	467
ENSECAG00000000235	2.799869831	0.176694149	0.567991772	39	91	75	207	107	139	202	269
ENSECAG00000017560	5.669451578	0.176742598	0.567991772	1084	1122	886	973	667	717	1070	1133
ENSECAG00000020728	1.264542011	0.176758441	0.567991772	27	36	52	118	2	20	37	77
ENSECAG00000013627	3.546314508	0.17700496	0.568433819	239	260	242	203	121	165	246	271
ENSECAG00000022304	6.389248756	0.177030696	0.568433819	1637	1611	2133	1503	1093	1998	1344	1232
ENSECAG00000022933	5.900633317	0.17704579	0.568433819	1152	685	599	387	2766	1463	1473	530
ENSECAG00000020981	3.430104037	0.177326557	0.569174755	131	212	237	353	141	184	183	234
ENSECAG00000012612	7.863009467	0.177689598	0.56992332	6028	7012	2574	3325	2734	4505	3097	4423
ENSECAG00000021680	2.611494346	0.177707354	0.56992332	113	150	113	139	70	40	118	175
ENSECAG00000024844	8.367628337	0.177709951	0.56992332	6585	8340	5721	7169	3769	7184	4891	5755
ENSECAG00000020656	5.33385452	0.177770229	0.569956086	532	311	843	646	988	935	1346	776
ENSECAG00000017624	2.33874335	0.177864726	0.570033096	43	55	88	103	69	134	134	171
ENSECAG00000020431	8.83065565	0.177894386	0.570033096	8340	10150	10653	9795	5493	6785	5828	11758
ENSECAG00000007508	7.087604995	0.17814497	0.570675433	3358	4474	1750	1533	1210	3332	2124	1861
ENSECAG00000009157	6.533325272	0.178225284	0.570772112	1861	2905	1289.01	1502	1154	1991	1414	1602
ENSECAG00000017618	8.973851728	0.178608866	0.571550576	10392	13460	10044	8524	5092	10898	5215	10669
ENSECAG00000019748	4.176379146	0.178616881	0.571550576	159	166	419	342	377	310	646	496
ENSECAG00000018342	7.430369491	0.178618968	0.571550576	3777	4489	3117	2728	2907	3166	2443	2827
ENSECAG00000001410	0.644103925	0.178809412	0.5719992	23	49	35	21	10	27	24	32
ENSECAG00000018720	0.019700108	0.178920299	0.57214647	6	12	20	11	19	16	22	40
ENSECAG00000007419	3.32587214	0.178955958	0.57214647	93	120	123	265	202	180	300	326
ENSECAG00000011348	6.07370614	0.179230649	0.572822931	720	699	785	1798	2185	1053	2584	1090
ENSECAG00000018084	3.669102315	0.179275396	0.572822931	107	142	234	287	215	320	328	398
ENSECAG00000010545	5.618750025	0.179333732	0.572822931	498	473	980	1025	1344	899	1771	917
ENSECAG00000023740	4.714635501	0.179368797	0.572822931	301	313	416	547	723	659	757	436
ENSECAG00000010318	4.66704699	0.179423741	0.572837716	196	365	449	548	405	793	778	535
ENSECAG00000013768	6.326938678	0.17952464	0.572999167	2062	1781	1234	1250	1409	1417	1433	1172

ENSECAG00000010231	4.365251216	0.17960853	0.573106254	431	362	430	432	238	383	428	397
ENSECAG00000022404	0.633250818	0.180056277	0.574373975	15	20	64	37	24	27	31	9
ENSECAG00000018813	3.432984659	0.180212084	0.574709966	73	194	297	487	42	124	163	352
ENSECAG00000017336	6.03460408	0.180562205	0.575665278	1492	1759	1017	956	847	1491	982	1075
ENSECAG00000012738	8.715477453	0.180745633	0.575954081	9666	12967	5827	6471	6938	9692	4463	5093
ENSECAG00000018860	8.21414927	0.180753969	0.575954081	4944	5954	7408	7613	5019	6618	5591	2130
ENSECAG00000017887	7.380948536	0.180967575	0.576473375	3964	2110	4288	3501	2863	3449	2297	2256
ENSECAG00000017758	8.187898042	0.18106578	0.576624868	7228	6822	4253	5544	4785	5542	5429	3340
ENSECAG00000011250	1.816057552	0.181409097	0.577184756	50	72	80	97	44	39	94	59
ENSECAG00000021602	3.010842192	0.181416113	0.577184756	100	174	158	281	106	121	79	223
ENSECAG00000023965	5.008951527	0.181468943	0.577184756	467	927	627	612	496	598	633	545
ENSECAG00000014130	6.30482485	0.181515842	0.577184756	1468	2377	1259	1353	1035	1649	1164	1490
ENSECAG00000020115	8.063183882	0.181575542	0.577184756	2415	5657	5011	11959	4033	4397	5564	3833
ENSECAG00000009579	4.334431489	0.181629745	0.577184756	221	218	332	414	772	234	627	399
ENSECAG00000021929	5.415360588	0.181643279	0.577184756	483	474	795	827	1010	966	1372	863
ENSECAG00000019465	3.215464642	0.181681617	0.577184756	64	105	196	128	535	236	66	109
ENSECAG00000017644	7.157026986	0.181697863	0.577184756	2723	4580	2982	2866	432	4105	180	3017
ENSECAG00000021107	6.780115919	0.181818797	0.5772371	1467	3522	1958	2490	1194	1588	1949	2719
ENSECAG00000012955	1.542378841	0.181888194	0.5772371	22	50	194	23	5	75	2	55
ENSECAG00000022114	5.021138497	0.181968132	0.5772371	452	679	507	1122	565	476	659	613
ENSECAG00000010373	5.291937551	0.181985022	0.5772371	486	385	608	841	1044	513	1439	969
ENSECAG00000004183	1.386006241	0.182013481	0.5772371	40	64	57	55	37	54	46	30
ENSECAG00000015530	6.046731234	0.18201855	0.5772371	1566	1075	1313	1312	1218	910	1437	1061
ENSECAG00000021693	6.52484479	0.182493602	0.578582474	2385	2281	1177	1504	1379	813	2057	2003
ENSECAG00000023448	4.622165748	0.182606472	0.578779143	184	180	685	344	378	460	1490	340
ENSECAG00000013060	4.834778967	0.182732646	0.57901786	492	467	446	1162	157	254	428	1044
ENSECAG00000020664	0.589916692	0.182798252	0.579064579	5	19	28	28	22	27	40	60
ENSECAG00000021662	7.192655698	0.18290495	0.57922006	3749	2757	2822	2727	3390	2083	3127	923
ENSECAG00000023751	0.896182904	0.182949085	0.57922006	12	35	71	49	12	41	27	30
ENSECAG00000000325	9.278858927	0.183081135	0.579440589	2886	6979	8586	18296	13437	10391	13528	28249
ENSECAG00000009480	2.327669266	0.18312053	0.579440589	36	68	92	95	116	81	143	164
ENSECAG00000019396	2.250833092	0.183232635	0.579506783	34	70	85	84	97	75	177	135
ENSECAG00000007743	8.468721086	0.183243252	0.579506783	2533	3461	6391	8676	7852	6718	13313	9190
ENSECAG00000024191	6.391459938	0.183363295	0.579725384	1826	1309	1919	1756.99	1569	1445	1653.99	1137
ENSECAG00000002199	3.587576979	0.183881199	0.581201404	93	148	257	210	402	147	418	255
ENSECAG00000011486	6.909609447	0.184142603	0.581634803	708	1456	2345	1826	462	3761	1435	7293
ENSECAG00000021529	5.141188631	0.184182807	0.581634803	368	447	602	722	1073	792	1062	543
ENSECAG00000012382	1.861781215	0.184185452	0.581634803	38	66	79	152	14	71	68	74
ENSECAG00000018789	6.122441825	0.18422267	0.581634803	1278	1998	1067	1388	712	930	1399	1777
ENSECAG00000022429	3.644684227	0.184455958	0.582122407	336	263	214	163	229	238	217	146
ENSECAG00000017536	5.177500279	0.184530321	0.582122407	358	529	438	904	791	684	1282	847
ENSECAG00000008598	1.915727528	0.184563003	0.582122407	24	49	67	71	157	74	103	45
ENSECAG00000019813	6.572458	0.184581633	0.582122407	802	972	1568	2315	3866	1469	3053	1359
ENSECAG00000011967	5.553911823	0.18467018	0.582240376	536	508	917	829	1531	1026	1325	754
ENSECAG00000014783	3.863311667	0.184792489	0.582464697	40	107	154	566	332	131	855	309
ENSECAG00000024404	5.064914942	0.18513214	0.583131928	363	337	750	480	1340	539	1012	479
ENSECAG00000009229	2.664446396	0.185143811	0.583131928	73	83	88	110	153	74	220	200
ENSECAG00000012548	3.461048223	0.185157832	0.583131928	100	84	175	298	172	255	263	419
ENSECAG00000000269	3.75980767	0.185236307	0.583217742	136	171	267	230	382	253	431	278
ENSECAG00000025151	3.169695234	0.185366272	0.583465581	102	107	123	192	215	140	332	222
ENSECAG00000023040	4.865975823	0.185448823	0.583564083	462	626	615	683	513	582	461	501
ENSECAG00000011063	0.404164982	0.185697511	0.584185178	14	27	45	34	4	14	3	48
ENSECAG00000017436	8.79471016	0.18579555	0.584224699	13218	15045	12599	2638	31	10690	12	6817
ENSECAG00000001546	8.223633991	0.185865677	0.584224699	2490	3169	6063	5623	9294	7393	9463	4545
ENSECAG00000015742	7.637998711	0.185914748	0.584224699	3191	5586	4215	3865	2742	4241	3955	2392
ENSECAG00000012265	4.480203126	0.185915335	0.584224699	243	281	383	402	802	260	766	422
ENSECAG00000024610	1.146245974	0.186272318	0.58512542	14	4	36	52	5	49	39	147
ENSECAG00000025051	2.325922405	0.186304756	0.58512542	44	72	47	99	52	32	185	275
ENSECAG00000018273	2.656521345	0.186367817	0.585162051	76	156	129	182	86	130	85	117
ENSECAG00000016141	3.544987116	0.186623694	0.585803902	156	102	209	171	397	141	506	170
ENSECAG00000000173	6.867818147	0.186797646	0.585913508	2674	4828	1059	1086	1770	2381	951	1989
ENSECAG00000007791	2.482435939	0.186803156	0.585913508	65	76	83	79	160	65	247	107
ENSECAG00000002250	3.182934448	0.186813003	0.585913508	136	53	137	133	103	461	102	254
ENSECAG00000020708	2.247476248	0.18707809	0.586307328	45	52	72	105	97	85	172	125
ENSECAG00000009114	5.218433123	0.18710721	0.586307328	435	435	543	800	1393	492	975	839
ENSECAG00000018921	1.845416943	0.187136773	0.586307328	33	21	76	67	75	45	107	142
ENSECAG00000023217	5.523974423	0.187144561	0.586307328	307	506	966	1096	1001	1344	1346	862
ENSECAG00000017879	1.271196772	0.187336693	0.586747798	28	45	38	104	32	29	30	60
ENSECAG00000012839	3.845927748	0.187521562	0.587043106	284	899	130	57	6	501	5	105
ENSECAG00000010564	2.910984226	0.187534104	0.587043106	92	83	132	119	175	126	230	223
ENSECAG00000011134	5.096207731	0.187728315	0.587338329	480	463	549	498	911	800	904	722
ENSECAG00000010744	7.145154393	0.187808481	0.587338329	3410	4017	2139	1853	3017	2236	2157	1814
ENSECAG00000018467	7.12437331	0.187816968	0.587338329	3624	4281	1786	1457	2248	2462	2226	2052
ENSECAG00000024671	1.753641606	0.18783477	0.587338329	28	48	95	143	32	74	32	68
ENSECAG00000005169	6.100776151	0.188034314	0.587800841	495	1040	1633	3287	899	1234	1248	1276
ENSECAG00000013982	2.161079055	0.188148472	0.587869168	35	42	86	95	74	136	130	106

ENSECAG00000000348	6.56953537	0.188159442	0.587869168	929	949	1646	2204	2712	1686	3207	2000
ENSECAG00000006619	3.238305695	0.188418539	0.588361485	45	145	191	183	194	324	237	182
ENSECAG00000014688	6.722332017	0.188420375	0.588361485	1111	2107	2758	3439	1315	1872	1531	2456
ENSECAG00000017662	4.352220335	0.188554987	0.588476883	325	412	1171	107	13	225	8	747
ENSECAG00000012231	6.918283913	0.188560709	0.588476883	1503	1609	1861	1813	4088	1906	3996	2392
ENSECAG00000010304	4.88581955	0.188738532	0.588870426	447	279	440	522	670	445	1252	646
ENSECAG00000019527	7.167485123	0.188916898	0.589043986	3317	5026	2159	1246	2837	1946	3257	1199
ENSECAG00000014896	8.128378689	0.188936332	0.589043986	6130	7548	4180	5184	3195	5567	4782	4937
ENSECAG00000020011	3.004104264	0.188949375	0.589043986	57	99	161	158	258	155	260	132
ENSECAG00000015644	1.421052637	0.189140892	0.589479622	58	72	94	14	11	61	1	60
ENSECAG00000015887	4.005242308	0.189452021	0.58980577	150	269	400	619	286	335	277	199
ENSECAG00000026979	4.116966306	0.189478918	0.58980577	170	313	214	347	471	312	437	482
ENSECAG00000021260	2.977841915	0.189533607	0.58980577	74	223	102	307	124	99	163	144
ENSECAG00000004762	5.448591025	0.18956493	0.58980577	601	445	955	1933	644	394	994	1009
ENSECAG00000012890	5.63425689	0.189566302	0.58980577	536	1026	1020	1763	1137	867	700	739
ENSECAG00000010385	1.171421066	0.18963032	0.58980577	14	21	19	59	14	32	24	168
ENSECAG00000023795	1.836659084	0.189659366	0.58980577	61	53	93	95	38	47	51	96
ENSECAG00000023437	5.958093578	0.189687347	0.58980577	682	1571	1286	1810	849	1208	1006	1278
ENSECAG00000007757	2.285053081	0.189755453	0.58980577	44	57	77	95	70	67	154	210
ENSECAG00000002481	8.301190269	0.189807926	0.58980577	12828	5299	2926	3092	4574	3728	7300	4168
ENSECAG00000012220	6.569165172	0.189846899	0.58980577	1692	3144	1551	1455	957	1633	1326	2426
ENSECAG00000011358	0.14547795	0.189867207	0.58980577	8	38	22	25	9	7	25	24
ENSECAG00000008468	4.870473744	0.190005826	0.590075377	394	785	442	844	370	497	484	703
ENSECAG00000008568	1.698482956	0.190210183	0.590543087	33	38	57	51	63	53	94	114
ENSECAG00000008053	0.496839514	0.190260171	0.590543087	36	46	14	15	7	31	18	23
ENSECAG00000013149	8.701834743	0.190409142	0.590844396	9259	10648	6680	7648	7626	5107	8627	6520
ENSECAG00000016278	7.269224018	0.190524717	0.591041937	4574	3723	1818	2032	2542	2120	3090	2373
ENSECAG00000012926	0.392638995	0.190603952	0.591126666	8	17	20	23	22	17	54	37
ENSECAG00000007658	0.473507202	0.190658297	0.591134181	20	54	21	17	13	29	20	19
ENSECAG00000026976	2.876491525	0.190719797	0.591138697	123	261	90	134	74	107	149	158
ENSECAG00000025956	3.120257716	0.190763599	0.591138697	200	215	152	134	134	224	87	110
ENSECAG00000007221	3.305245251	0.190973379	0.591627734	106	165	139	162	202	277	331	170
ENSECAG00000006602	3.550505869	0.191051505	0.591708755	271	352	160	155	286	128	249	112
ENSECAG00000016016	2.698956975	0.191385899	0.591878089	48	87	97	138	101	62	245	272
ENSECAG00000023446	3.512319493	0.191432583	0.591878089	129	132	198	211	261	182	434	271
ENSECAG00000019747	7.100479636	0.191454695	0.591878089	1554	455	3266	1650	7833	1602	4428	1076
ENSECAG00000021229	2.752401197	0.191489589	0.591878089	64	151	150	231	138	94	94	124
ENSECAG00000022137	1.751438221	0.191630954	0.591878089	49	57	63	120	50	41	64	68
ENSECAG00000011157	3.788478416	0.191687676	0.591878089	152	152	201	329	287	282	435	366
ENSECAG00000005886	3.160706159	0.191718085	0.591878089	94	187	181	335	133	113	111	243
ENSECAG00000014611	2.254811445	0.191721551	0.591878089	61	53	81	57	197	70	145	72
ENSECAG00000009742	11.27039867	0.191780332	0.591878089	118348	44066	27664	56731	35	57581	0	20860
ENSECAG00000014144	7.423029085	0.191789143	0.591878089	1746	1887	2428	4059	3990	3026	6450	4244
ENSECAG00000000897	5.066961423	0.191812871	0.591878089	444	383.999	894	1188	513	528	710	624
ENSECAG00000016701	4.91532587	0.191868565	0.591878089	361	622	590	997	517	473	595	579
ENSECAG00000024663	6.190519835	0.192016591	0.591878089	1637	1074	1860	1305	1208	902	1831	1192
ENSECAG00000023994	4.850496878	0.192093371	0.591878089	330	404	380	613	702	354	895	933
ENSECAG00000016323	1.082953893	0.192137018	0.591878089	63	28	60	17	15	53	18	35
ENSECAG00000012914	3.551618549	0.192143106	0.591878089	124	117	170	284	403	154	354	266
ENSECAG00000018837	5.894421232	0.192165237	0.591878089	1088	1351	1377	1083	554	1098	758	1638
ENSECAG00000018458	1.454444764	0.192205604	0.591878089	17	43	47	45	31	75	100	69
ENSECAG00000007801	6.95621859	0.192207345	0.591878089	1454	4839	2031	2378	1748	2570	1839	2023
ENSECAG00000022506	6.558617245	0.192218044	0.591878089	1961	2280	2223	1348	964	2951	1120	981
ENSECAG00000012016	3.383406006	0.192272733	0.591878089	120	308	203	255	231	116	201	171
ENSECAG00000000384	5.717670579	0.192291661	0.591878089	673	981	1122	1766	688	1019	738	1224
ENSECAG00000009878	5.355708108	0.192366999	0.591878089	293	560	946	737	944	945	1189	954
ENSECAG00000020525	1.307290483	0.192377045	0.591878089	32	33	69	82	27	37	27	64
ENSECAG00000008754	4.71591913	0.192405868	0.591878089	254	253	396	717	473	448	861	859
ENSECAG00000019122	5.988837575	0.192587416	0.592276536	748	542	1500	797	2182	1419	2100	748
ENSECAG00000018130	5.951627817	0.192646245	0.592297461	1408	1572	963	934	1061	909	1378	965
ENSECAG00000024277	5.147258315	0.192886467	0.592875926	459	464	435	706	901	386	1468	865
ENSECAG00000013288	3.532476856	0.193036967	0.593118106	109	213	218	507	195	199	258	141
ENSECAG00000023254	6.312678084	0.193088982	0.593118106	1081	886	1407	1214	2025	1474	2569	1897
ENSECAG00000006957	6.458077138	0.193168771	0.593118106	372	1026	825	3164	1283	1502	2412	4070
ENSECAG00000010891	5.512651728	0.19318705	0.593118106	517	1576	984	849	381	1162	458	1000
ENSECAG00000016944	2.74554807	0.193292566	0.593118106	75	169	142	191	90	140	112	109
ENSECAG00000019190	1.947139586	0.193376598	0.593118106	26	55	50	94	79	78	144	87
ENSECAG00000012409	4.370542708	0.19337715	0.593118106	294	288	293	286	649	260	802	388
ENSECAG00000000759	4.344111145	0.193384382	0.593118106	314	431	521	393	266	399	464	314
ENSECAG00000008241	2.204680385	0.193455069	0.593118106	39	71	230	82	90	53	122	22
ENSECAG00000014791	3.414490431	0.193486223	0.593118106	148	60	392	376	245	98	248	104
ENSECAG00000018665	3.028697191	0.193541929	0.593129169	32	112	83	271	181	237	236	174
ENSECAG00000006134	7.002705373	0.193617967	0.593202518	1809	2806	2394	4103	2059	1796	2640	2392
ENSECAG00000004707	6.152654635	0.19410035	0.594372703	1127	804	1976	2295	1526	1526	1024	691
ENSECAG00000023761	5.406248936	0.194104322	0.594372703	397	461	744	1029	1134	747	1472	867
ENSECAG00000007337	7.412461731	0.194252458	0.594541099	3203	6304	2977	1960	1533	5100	1415	2322

ENSECAG00000010605	4.61996948	0.194360259	0.594541099	283	839	367	593	284	400	440	584
ENSECAG00000008237	1.711073039	0.19440981	0.594541099	70	73	62	60	42	25	59	87
ENSECAG00000017457	8.367192434	0.194429309	0.594541099	11470	7579	3447	3231	5888	4300	7212	3545
ENSECAG00000021272	1.294107501	0.194443406	0.594541099	38	20	40	24	57	83	57	44
ENSECAG00000006233	5.059360597	0.194472644	0.594541099	335	280	789	556	1156	572	1201	449
ENSECAG00000010659	4.792489101	0.194537381	0.594570675	337	539	673	789	580	401	517	485
ENSECAG00000012854	8.227644817	0.194586766	0.594570675	5781	11452	3937	4319	3087	9043	3041	3070
ENSECAG00000018823	9.133974806	0.194690835	0.594729048	12094	17205	7994	9263	6022	11002	7865	11452
ENSECAG00000016298	4.610841163	0.194958242	0.595366794	230	408	337	502	396	415	749	880
ENSECAG00000018231	5.429682628	0.195004196	0.595366794	672	865	931	1069	876	681	854	680
ENSECAG00000021781	6.992142828	0.19514087	0.595505666	1848	2470	2745	3935	2466	1956	2451	1904
ENSECAG00000000286	6.582841631	0.195154294	0.595505666	1318	2258	1980	2558	1388	1802	1699	1806
ENSECAG00000024958	6.325010221	0.195239698	0.595606635	645	2248	1838	2381	866	1579	1421	1642
ENSECAG00000007917	7.596519106	0.195418581	0.595992645	2191	1919	4137	2519	7248	3472	5974	3226
ENSECAG00000008534	3.53618213	0.195543898	0.596215126	119	138	228	180	500	174	289	194
ENSECAG00000017061	7.464516972	0.195881992	0.597086074	1452	1907	3236	4027	4436	4352	4811	4218
ENSECAG00000000031	5.827353112	0.196008049	0.597216445	947	1838	792	1060	641	931	1330	1079
ENSECAG00000000987	6.136912255	0.19609794	0.597216445	1399	1814	1155	1269	1296	1086	1415	1128
ENSECAG00000004119	2.789425176	0.196130971	0.597216445	87	71	114	118	210	104	215	164
ENSECAG00000019612	5.454261527	0.196175464	0.597216445	504	516	612	1006	1369	548	1437	1036
ENSECAG00000020506	6.183624045	0.196187045	0.597216445	915	904	1043	1309	2533	1199	2750	917
ENSECAG00000018030	4.108851141	0.196314291	0.597369324	195	345	330	645	186	235	327	460
ENSECAG00000007189	1.586081383	0.196549508	0.597369324	18	35	31	88	58	43	78	125
ENSECAG00000018153	3.731292662	0.196610293	0.597369324	185	414	225	278	178	297	229	204
ENSECAG00000008587	3.304868049	0.1966416	0.597369324	121	159	259	312	151	188	188	157
ENSECAG00000009306	1.368905174	0.196692676	0.597369324	107	38	43	30	7	103	3	4
ENSECAG00000013024	2.199798454	0.196705245	0.597369324	47	82	106	201	17	52	43	167
ENSECAG00000022837	2.843426373	0.196752232	0.597369324	164	113	137	166	87	95	153	155
ENSECAG00000017921	2.889186052	0.196795767	0.597369324	66	57	107	201	211	70	304	187
ENSECAG00000007374	8.472335393	0.196819747	0.597369324	4466	7884	9898	9328	4767	9357	5624	3348
ENSECAG00000012756	1.375425025	0.196827146	0.597369324	25	73	13	3	56	72	67	79
ENSECAG00000021039	6.639461587	0.196862733	0.597369324	1554	2296	1992	2609	1091	1989	1362	2362
ENSECAG00000006773	5.600090825	0.196866904	0.597369324	990	1001	953	908	862	949	898	708
ENSECAG00000002924	3.238176279	0.197021913	0.597680382	97	57	111	283	333	109	349	188
ENSECAG00000011075	4.259034513	0.197099423	0.597715447	272	424	449	432	290	407	366	291
ENSECAG00000016421	6.946714639	0.197138472	0.597715447	7836	2473	249	724	0	3336	0	190
ENSECAG00000021093	2.560189807	0.197264519	0.597843127	77	311	78	74	13	230	34	29
ENSECAG00000008967	0.218766234	0.197285606	0.597843127	10	5	19	25	26	38	32	15
ENSECAG00000008338	5.256114456	0.197472522	0.598250308	376	584	607	758	540	1081	1263	888
ENSECAG00000015496	1.312412746	0.197610479	0.598508993	40	40	53	77	21	28	57	56
ENSECAG00000010356	6.677391765	0.197702712	0.598521478	1001	1734	2840	3766	2716	882	1928	1407
ENSECAG00000022955	9.148023884	0.197719743	0.598521478	11159	21782	6484	8083	7680	13046	8232	6842
ENSECAG00000020277	1.771381786	0.197814149	0.598648082	8	39	51	102	61	86	90	110
ENSECAG00000017760	5.699514503	0.197921334	0.598668015	601	464	1011	1024	1736	763	1576	1113
ENSECAG00000021116	10.28333972	0.197984707	0.598668015	26022	30796	20565	26213	20649	23196	22223	16694
ENSECAG00000014561	7.476531791	0.198011285	0.598668015	1779	1878	3011	3863	3642	3964	4694	5729
ENSECAG00000014723	2.51442897	0.198054173	0.598668015	81	60	87	80	206	102	176	90
ENSECAG00000012055	6.691066689	0.198083655	0.598668015	2148	2970	1371	1868	1708	1853	1992	1486
ENSECAG00000017726	4.927466783	0.198192115	0.598733958	436	732	649	683	442	576	472	666
ENSECAG00000003954	4.389665374	0.198228359	0.598733958	643	380	406	224	277	654	198	166
ENSECAG00000006640	5.260985013	0.198263243	0.598733958	371	498	639	863	624	989	980	1153
ENSECAG00000012166	6.232518958	0.198371448	0.598860602	713	972	4240	1790	83	1191	46	2587
ENSECAG00000023992	11.33333943	0.198410381	0.598860602	95104	91230	34728	25780	94	80904	57	16594
ENSECAG00000012126	6.380704324	0.19856789	0.599124135	754	610	1237	2506	2076	1224	2630	2585
ENSECAG00000019093	5.974423049	0.198602941	0.599124135	727	1872	1430	1351	566	1581	862	1241
ENSECAG00000022601	6.154485966	0.198700456	0.599259521	738	1056	1037	1481	1910	1307	2460	1423
ENSECAG00000016779	3.385162087	0.198908505	0.599728106	146	314	146	269	197	122	209	195
ENSECAG00000024248	5.440179095	0.199028684	0.599931576	530	427	694	984	1147	756	1337	1051
ENSECAG00000021016	5.231221418	0.199156306	0.600157369	311	334	688	1000	1374	674	973	699
ENSECAG00000022099	4.325793769	0.199265874	0.600264997	233	203	372	385	652	260	571	507
ENSECAG00000003600	4.227917421	0.199312388	0.600264997	191	239	316	408	471	411	477	458
ENSECAG00000007581	3.290391889	0.199350193	0.600264997	101	115	154	209	288	166	362	165
ENSECAG00000021281	7.679978882	0.199514608	0.600594247	4098	5778	3097	3895	2911	3557	3232	4068
ENSECAG00000016749	2.382891892	0.199637508	0.600594247	46	57	75	120	163	161	142	53
ENSECAG00000009988	7.101473916	0.199663953	0.600594247	2514	3232	2433	3340	1267	2588	2696	2820
ENSECAG00000011301	3.462985682	0.199670551	0.600594247	99	221	263	401	131	168	166	288
ENSECAG00000013170	7.145839212	0.19979481	0.600663656	1017	1501	2826	3196	3659	2588	5463	2897
ENSECAG00000023898	1.075376629	0.199799145	0.600663656	39	61	27	55	14	32	5	68
ENSECAG00000019202	0.210473002	0.199874236	0.600730776	12	5	13	28	15	25	30	42
ENSECAG00000022644	0.560678282	0.199928085	0.600734031	33	22	23	45	11	9	15	51
ENSECAG00000017772	4.758290361	0.200026274	0.600748694	279	374	499	513	613	613	708	686
ENSECAG00000026940	2.691940569	0.200038498	0.600748694	76	163	128	187	97	88	174	87
ENSECAG00000008432	4.163857993	0.200157136	0.600872404	326	141.001	337	776	224	183	434	395
ENSECAG00000015948	1.046759478	0.200211497	0.600872404	19	32	27	35	46	45	69	41
ENSECAG00000026917	5.506376541	0.200238024	0.600872404	741	822	1278	888	856	968	619	714
ENSECAG00000011700	3.846447678	0.200330813	0.60099244	114	438	461	347	31	380	61	367

ENSECAG00000010696	2.293974075	0.200527734	0.601308778	66	29	95	73	115	111	201	76
ENSECAG00000018308	1.53911586	0.200541891	0.601308778	25	24	57	48	85	38	157	26
ENSECAG00000012531	0.688845583	0.200682162	0.601570936	15	16	33	20	37	43	31	43
ENSECAG00000026922	3.293889396	0.201061104	0.602548216	99	306	176	259	136	193	191	152
ENSECAG00000010990	8.033196506	0.201261068	0.602883078	4541	13843	3254	3165	193	8326	177	4025
ENSECAG00000013742	7.10524012	0.201278751	0.602883078	2939	6148	1078	1359	1690	3768	1768	1051
ENSECAG00000017747	6.302518233	0.201393823	0.602923997	1198	2003	1753	1830	634	2220	653	1628
ENSECAG00000005362	0.883774541	0.201404637	0.602923997	8	47	95	21	14	51	5	26
ENSECAG00000022698	3.41626797	0.201451285	0.602923997	79	123	178	268	149	207	324	397
ENSECAG00000021675	5.617931536	0.201804182	0.603821448	438	730	1660	1514	684	966	853	923
ENSECAG00000023035	4.601121874	0.2018992	0.60394703	237	342	397	519	697	445	765	473
ENSECAG00000004299	6.339593547	0.202035603	0.604196308	931	1900	1623	2512	1187	1303	1836	1438
ENSECAG00000012080	5.871681175	0.202143137	0.604359142	817	799	822	959	1432	903	2106	1433
ENSECAG00000011953	4.806075494	0.202269597	0.604476162	365	335	322	619	699	295	1123	734
ENSECAG00000004900	4.936333311	0.202288465	0.604476162	345	341	509	638	1000	490	1138	439
ENSECAG00000020001	6.756468497	0.202409809	0.60468005	1726	2607	2046	2682	1648	1312	2492	2159
ENSECAG00000024738	2.850897933	0.202673428	0.60502541	53	305	85	214	50	69	130	213
ENSECAG00000026502	3.220594727	0.202711288	0.60502541	3	61	91	250	3	399	4	708
ENSECAG00000008732	5.007945113	0.202754154	0.60502541	462	785	670	751	353	712	469	715
ENSECAG00000008317	4.363445578	0.202788147	0.60502541	294	203.979	318	393	413.99	375	624	624
ENSECAG00000007187	5.652015446	0.202791126	0.60502541	972	1179	851	1022	575	905	1094	1009
ENSECAG00000008088	1.719360797	0.202977396	0.605422491	42	38	55	38	114	41	109	68
ENSECAG00000013457	10.51250706	0.203079956	0.60556975	50994	69657	13628	7795	10	43113	23	8341
ENSECAG00000019570	5.267900938	0.203187265	0.605731085	490	1603	497	647	453	927	455	715
ENSECAG00000018292	0.985083065	0.203281983	0.605854811	12	46	23	22	59	37	74	28
ENSECAG00000015693	4.508088446	0.203468942	0.606253312	317	427	597	534	387	364	485	406
ENSECAG00000021603	8.625454071	0.203618211	0.606539332	6632	15820	5659	5032	4214	7186	7978	6449
ENSECAG00000021768	4.91525243	0.204041328	0.607640734	563	548	584	833	252	800	308	664
ENSECAG00000013038	7.504094135	0.204199823	0.607687764	3096	6912	3064	2957	703	5918	883	3082
ENSECAG00000017235	5.165396659	0.204220008	0.607687764	555	976	679	721	734	493	456	828
ENSECAG00000016715	0.730108599	0.204254702	0.607687764	14	44	8	13	35	52	26	50
ENSECAG00000017014	1.24070536	0.204334175	0.607687764	5	34	43	23	3	92	6	155
ENSECAG00000006965	5.300351707	0.204373814	0.607687764	333	869	859	1409	713	712	697	653
ENSECAG00000009219	8.305437912	0.204377378	0.607687764	9828	6071	3655	5523	5739	3109	6951	5113
ENSECAG00000010159	5.321730663	0.204446459	0.607734447	718	832	756	911	646	634	770	825
ENSECAG00000011761	6.518126891	0.204620632	0.607849387	742	766	1978	2119	2504	1450	3159	2203
ENSECAG00000024416	2.637894631	0.204641131	0.607849387	66	99	90	102	181	104	181	150
ENSECAG00000017686	1.040365545	0.204691	0.607849387	19	18	42	25	41	44	17	102
ENSECAG00000011304	7.345038912	0.204724105	0.607849387	3692	1000	5581	3689	2502	2891	1654	3319
ENSECAG00000007556	8.408245886	0.204752077	0.607849387	7887	9265	4819	6247	3563	9863	3734	4198
ENSECAG00000023975	3.441147589	0.204940567	0.60786344	110	179	137	221	199	220	300	353
ENSECAG00000013908	6.361329946	0.20495851	0.60786344	777	1236	1548	1369	2286	2305	2115	1355
ENSECAG00000018816	4.452674336	0.20496925	0.60786344	190	244	215	699	312	389	666	856
ENSECAG00000017119	3.053864006	0.204970377	0.60786344	180	128	144	225	148	96	205	127
ENSECAG00000002306	5.906845956	0.205194857	0.608292654	1113	1502	1209	1004	827	1386	963	973
ENSECAG00000005856	6.957585466	0.205221966	0.608292654	995	1562	1875	3040	2252	1794	4812	4048
ENSECAG00000011351	1.680119502	0.205324355	0.608437735	12	92	28	28	36	170	49	70
ENSECAG00000023914	4.219905039	0.205413226	0.608542694	184	242	302	419	527	298	556	449
ENSECAG00000015564	3.263969739	0.205595676	0.608772026	132	257	184	233	96	210	152	196
ENSECAG00000019131	8.862887628	0.205597579	0.608772026	8988	16783	4932	8876	4334	12877	5530	6333
ENSECAG00000014343	1.629342321	0.20572086	0.608978677	20	73	70	116	31	45	55	69
ENSECAG00000018135	0.002485881	0.206010895	0.609546121	2	11	10	30	27	27	24	16
ENSECAG00000017691	6.397716181	0.206047668	0.609546121	1105	1489	1872	2769	1490	1443	1566	1461
ENSECAG00000011419	4.568972651	0.206073168	0.609546121	284	230	372	527	765	356	898	368
ENSECAG00000013232	4.330494507	0.206208189	0.609710146	143	210	376	548	438	452	516	566
ENSECAG00000016250	1.064890239	0.206299002	0.609710146	10	29	33	47	38	39	66	63
ENSECAG00000011592	2.698229812	0.206342648	0.609710146	87	139	236	187	4	40	4	285
ENSECAG00000002362	1.187606432	0.206348144	0.609710146	10	49	37	27	79	38	66	43
ENSECAG00000009721	8.803668038	0.20639639	0.609710146	6942	8858	9053	13830	7183	6938	9013	7474
ENSECAG00000008683	11.34163615	0.206546538	0.609995418	60114	77680	49485	36233	11575	88967	12244	36538
ENSECAG00000019875	6.31078075	0.20670633	0.610242056	1563	2915	919	1082	789	1887	978	1525
ENSECAG00000018321	2.922489754	0.206737252	0.610242056	98	79	137	114	208	152	267	131
ENSECAG00000022971	7.313917492	0.206993823	0.610708606	3820	5171	1441	2527	1511	2651	2378	3796
ENSECAG00000016223	5.852835005	0.207002592	0.610708606	633	1113	1280	1985	785	898	1161	1287
ENSECAG00000019360	5.202400955	0.207099619	0.610836571	444	876	854	894	686	607	579	754
ENSECAG00000005816	0.451601579	0.207159262	0.610854233	3	19	29	21	33	26	21	52
ENSECAG00000020999	4.976298317	0.207240747	0.610930705	499	1005	487	610	231	547	525	863
ENSECAG00000016580	6.256185418	0.207303026	0.610930705	554	827	1284	2080	1397	1359	1842	3095
ENSECAG00000003124	7.545305399	0.207456244	0.610930705	2626	4826	3766	4694	3489	2836	3434	3102
ENSECAG00000024122	6.227813663	0.207463167	0.610930705	1563	2191	906	1327	1310	1264	1299	1288
ENSECAG00000024696	6.72401459	0.207472947	0.610930705	1152	1264	1410	2500	3372	1891	2893	2413
ENSECAG00000006041	0.138640606	0.207507162	0.610930705	20	34	29	7	5	30	2	19
ENSECAG00000017817	2.58548896	0.207661888	0.61122818	95	93	254	61	85	102	101	103
ENSECAG00000024245	1.682012796	0.20796125	0.611951107	94	96	21	50	31	104	29	16
ENSECAG00000011333	3.485887855	0.208187075	0.612457326	155	320	230	224	166	152	157	296
ENSECAG00000020998	5.925521778	0.208468117	0.613010274	1151	1804	1006	886	1011	898	1040	1287

ENSECAG00000006906	2.225688563	0.208482721	0.613010274	75	229	39	48	46	102	59	76
ENSECAG00000016300	5.281759008	0.208633913	0.613296436	406	1090	630	1197	556	672	789	757
ENSECAG00000020954	4.170033626	0.208933757	0.614019315	249	370	422	448	394	252	338	301
ENSECAG00000013878	0.878895359	0.209004216	0.614067871	25	105	17	11	6	55	11	23
ENSECAG00000005146	4.568544228	0.209196719	0.614474883	327	446	450	778	375	315	461	555
ENSECAG00000000023	5.237676776	0.209520424	0.615266965	332	383	803	801	1290	945	858	583
ENSECAG00000021263	2.718393451	0.209686289	0.615372585	47	77	77	189	200	88	289	103
ENSECAG00000017082	6.023613363	0.209721208	0.615372585	1762	1770	863	796	523	2104	701	759
ENSECAG00000022252	5.17750354	0.209765179	0.615372585	436	365	532	876	846	543	1133	1067
ENSECAG00000004389	7.113068374	0.209772596	0.615372585	2035	2379	3347	4229	1733	2388	2094	3267
ENSECAG00000021819	4.529620818	0.209952266	0.615740995	268	344	431	344	561	496	596	582
ENSECAG00000015455	4.360019989	0.210089419	0.615844118	263	303	248	392	406	275	814	576
ENSECAG00000022242	6.835375158	0.210095613	0.615844118	2794	2988	1785	1508	2415	1887	1919	1388
ENSECAG00000024971	2.962432289	0.210333749	0.616383457	145	354	93	75	27	239	43	136
ENSECAG00000020477	5.24824154	0.210553638	0.616550842	331	480	682	905	809	949	916	1003
ENSECAG00000014942	4.383489557	0.210569034	0.616550842	218	272	335	460	608	347	615	470
ENSECAG00000016838	5.60624649	0.210603615	0.616550842	517	607	908	972	1210	1077	1528	940
ENSECAG00000020430	3.643654748	0.210607486	0.616550842	213	257	297	250	191	290	211	171
ENSECAG00000010600	0.351453541	0.210662115	0.616552231	10	34	23	44	16	32	19	6
ENSECAG00000012963	1.755977828	0.210952199	0.617090561	29	36	54	72	56	42	110	134
ENSECAG00000021302	3.987844945	0.210999054	0.617090561	194	168	289	292	400	469	383	283
ENSECAG00000019628	3.970625039	0.211008656	0.617090561	159	197	298	305	406	358	410	344
ENSECAG00000020991	6.135419193	0.211232916	0.617587762	1299	617	338	1317	2980	1126	692	2424
ENSECAG00000021620	3.404538653	0.211305142	0.617640318	142	153	144	169	327	199	299	215
ENSECAG00000014151	5.864679	0.211417001	0.617766393	770	1269	1183	1666	857	1010	1054	1250
ENSECAG00000011220	6.767907392	0.211492852	0.617766393	2362	363	1447	647	5042	1579	4585	806
ENSECAG00000008716	3.739945992	0.211511058	0.617766393	143	144	282	229	328	225	434	337
ENSECAG00000004773	5.040641669	0.211789926	0.61842224	431	382	605	503	1263	583	742	627
ENSECAG00000024116	5.643371738	0.212024881	0.618949556	650.004	1259	1159.01	1087	798.008	1035.01	600	1057
ENSECAG00000022821	2.720787289	0.212101151	0.619013486	80.0001	95	90	104	156	75.0003	247	192
ENSECAG00000016746	6.660312399	0.212352644	0.61910576	754	2803	2277	3228	1240	1966	1589	2108
ENSECAG00000019173	2.426461411	0.212357852	0.61910576	113	98	231	47	9	155	4	108
ENSECAG00000021941	7.130772705	0.212381103	0.61910576	3141	3201	2628	2465	2579	1223	4182	1689
ENSECAG00000002155	5.97323486	0.212490694	0.61910576	649	819	1102	1284	1598	1192	2148	1272
ENSECAG00000015342	11.27720065	0.212521246	0.61910576	69808	86020	37796	46183	169	60293	249	48737
ENSECAG00000020179	4.427948164	0.212556049	0.61910576	390	608	407	312	391	469	285	331
ENSECAG00000008144	2.083564258	0.212561669	0.61910576	32	53	63	101	84	68	168	108
ENSECAG00000016337	6.540616745	0.212582334	0.61910576	1029	1487	2570	3112	2153	1305	1716	1310
ENSECAG00000009024	4.717284876	0.21262218	0.61910576	295	401	540	1120	248	311	580	715
ENSECAG00000017442	4.521291963	0.212879729	0.619600304	162	387	452	420	590	631	627	383
ENSECAG00000024065	9.581222094	0.212900869	0.619600304	37214	12831	5382	2579	9656	19472	8670	4569
ENSECAG00000017631	3.410944846	0.213053018	0.619884644	125	125	143	237	191	193	284	386
ENSECAG00000014974	6.660394537	0.21314718	0.619885651	2557	3333	1042	1124	1050	1926	1224	2343
ENSECAG00000019839	3.416328787	0.213188921	0.619885651	192	206	204	264	167	197	212	175
ENSECAG00000006723	0.840531275	0.213216707	0.619885651	13	26	25	28	44	27	14	91
ENSECAG00000019024	6.105662017	0.213509075	0.62039323	1204	1825	1189	1340	1200	1164	1130	1349
ENSECAG00000012660	11.52498852	0.213550559	0.62039323	31029	32120	50613	56743	64816	59546	74993	101197
ENSECAG00000012174	4.028411597	0.213554771	0.62039323	177	227	272	316	351	290	506	453
ENSECAG00000011125	8.295910512	0.213644918	0.620496784	6845	9300	4566	4933	3947	6993	4548	5121
ENSECAG00000016725	4.362954009	0.213732289	0.620551839	205	282	375	394.001	687	336	549	434
ENSECAG00000026898	3.602870489	0.213772886	0.620551839	255	256	215	242	137	261	242	203
ENSECAG00000024911	2.443838883	0.213861779	0.620651632	63	61	74	117	130	91	177	143
ENSECAG00000011696	9.571594078	0.21392029	0.620663228	8109	7829	11246	17153	22743	11603	25150	18898
ENSECAG00000014847	3.308775529	0.214009127	0.62076278	95	206	266	311	104	177	102	279
ENSECAG00000014002	8.494802951	0.214105434	0.620883945	6455	10434	6381	6637	4829	6623	6924	6076
ENSECAG00000017418	9.776404511	0.214313654	0.6213295	21763	31914	9339	9967	4727	19722	6119	22143
ENSECAG00000024635	5.091842675	0.214432024	0.621407711	363	675	728	1181	619	658	461	662
ENSECAG00000014912	4.113598858	0.214449793	0.621407711	211	182	276	370	479	241	578	420
ENSECAG00000020574	1.687440168	0.214520279	0.621431128	38	29	57	46	80	42	174	40
ENSECAG00000023517	7.550318478	0.214587575	0.621431128	5383	4237	2512	2546	2943	3291	3123	3046
ENSECAG00000013942	4.9745715	0.214621624	0.621431128	788	599	453	615	680	359	751	432
ENSECAG00000019923	6.481218478	0.214702939	0.621508506	1500	2031	1423	2580	813	1431	1451	2500
ENSECAG00000009110	4.394218836	0.214760091	0.621515922	278	251	359	342	695	353	694	337
ENSECAG00000005945	5.934447629	0.214818423	0.621517241	943	2329	915	796	841	1207	1254	896
ENSECAG00000017231	6.067774908	0.21488842	0.621517241	468	1079	1163	1501	1511	1651	1701	1696
ENSECAG00000018186	5.938835691	0.214943652	0.621517241	996	1460	1210	1350	685	1276	1181	1203
ENSECAG00000024365	4.934832796	0.214994908	0.621517241	273	371	486	781	496	553	864	1115
ENSECAG00000013885	5.430967334	0.215109014	0.621517241	458	841	1086	1296	747	807	790	744
ENSECAG00000021626	3.093946422	0.215118604	0.621517241	71	78	179	188	199	154	303	199
ENSECAG00000014142	4.401726652	0.215181823	0.621517241	284	258	444	855	348	259	489	414
ENSECAG00000013605	3.470290785	0.215197274	0.621517241	93	105	169	300	333	98	405	299
ENSECAG00000008186	7.152568883	0.215293932	0.621638705	1752	1672	2552	2169	4097	2232	4938	3227
ENSECAG00000016369	3.530811314	0.215527798	0.622156182	96	138	219	258	319	218	367	235
ENSECAG00000005829	2.832465282	0.215643021	0.622331	70	67	125	124	248	53	398	68
ENSECAG00000015275	3.043251189	0.215770378	0.622415095	55	92	237	86	273	195	128	222
ENSECAG00000016348	2.383065948	0.2157815	0.622415095	54	100	117	185	74	74	97	110

ENSECAG00000012815	6.884061346	0.215889029	0.622567526	2726	3042	1704	2030	1343	2650	1855	2053
ENSECAG00000011540	2.170527789	0.216058125	0.622803939	88	131	88	70	42	135	19	72
ENSECAG00000022059	7.171558542	0.216080418	0.622803939	2015	1503	3225	6440	1629	3114	1916	2877
ENSECAG00000021494	3.94586467	0.216146249	0.622836003	88	188.992	295	390	467	242.997	304	506.982
ENSECAG00000023667	8.127576421	0.216353393	0.623038195	9306	6123	4580	2266	3966	8502	1715	2251
ENSECAG00000022599	2.821838701	0.216418333	0.623038195	73	93	127	121	125	156	239	181
ENSECAG00000016933	6.197797681	0.216421889	0.623038195	1073	2952	889	1203	1333	1684	1101	745
ENSECAG00000009527	4.60899219	0.216435315	0.623038195	266	245	426	535	830	300	842	469
ENSECAG00000011384	6.406820631	0.216724339	0.623663495	1626	3036	1042	1112	1430	1045	1747	1609
ENSECAG00000009928	7.442612195	0.216798737	0.623663495	2956	5723	2909	2775	3430	2987	2951	2339
ENSECAG00000009893	3.386341699	0.216816874	0.623663495	136	83	130	259	214	136	397	320
ENSECAG00000022379	5.744987682	0.21688655	0.623706332	1769	927	765	566	936	939	993	741
ENSECAG00000011725	0.191575196	0.217085186	0.62411991	14	8	24	8	22	16	38	35
ENSECAG00000006991	6.209632674	0.217497469	0.625064678	1165	517	534	1804	908	827	2483	3659
ENSECAG00000005833	2.653472051	0.217523606	0.625064678	80	95	218	142	115	103	95	111
ENSECAG00000012844	4.645436581	0.217660487	0.625297138	312	371	375	430	748	353	804	562
ENSECAG00000024465	5.202777291	0.217714348	0.625297138	627	297	430	668	1246	318	1465	778
ENSECAG00000015000	2.266932954	0.217826277	0.625320441	44	59	194	140	88	108	77	30
ENSECAG00000014595	0.385405852	0.217832312	0.625320441	14	8	20	24	50	13	30	33
ENSECAG00000016834	3.94444648	0.217962349	0.625518595	336	482	186	203	285	208	290	274
ENSECAG00000015689	5.620043932	0.218011224	0.625518595	785	819	1011	1446	1044	733	881	882
ENSECAG00000016114	1.182782061	0.218077225	0.625550318	6	153	15	44	7	4	20	86
ENSECAG00000019180	7.144402953	0.218283988	0.625846836	3925	3073	2262	1838	2431	1668	3575	1950
ENSECAG00000021655	4.728721528	0.218290539	0.625846836	237	195	655	510	950	428	875	410
ENSECAG00000011085	3.717752124	0.218446815	0.626075712	98	262	171	250	164	230	415	522
ENSECAG00000006901	4.286188034	0.218480352	0.626075712	323	461	350	441	278	260	481	397
ENSECAG00000024201	2.935988436	0.21860094	0.626169294	46	122	90	188	73	158	163	388
ENSECAG00000003634	3.129831344	0.218704109	0.626169294	100	148	115	137	204	225	329	115
ENSECAG00000007914	5.79777851	0.21872953	0.626169294	569	1043	1144	2107	972	843	1054	1106
ENSECAG00000017989	8.115203418	0.218733007	0.626169294	4511	8503	4027	6200	4378	3850	5480	5312
ENSECAG00000019492	5.64901446	0.218850295	0.626305732	1252	1481	692	475	1015	790	1114	487
ENSECAG00000004471	3.670506139	0.218944935	0.626305732	441	347	97	83	198	242	246	115
ENSECAG00000015368	1.74165883	0.218970121	0.626305732	38	39	54	48	113	46	144	38
ENSECAG00000000366	3.038446208	0.219041607	0.626305732	57	68	215	152	210	221	196	183
ENSECAG00000017249	6.394070321	0.219055725	0.626305732	696	938	1896	1638	2574	1770	1849	2059
ENSECAG00000008630	3.588576878	0.219175752	0.626443199	107	215	245	524	216	152	144	305
ENSECAG00000018586	4.430257997	0.219300156	0.626443199	260	276	337	447	530	519	538	483
ENSECAG00000009142	5.219095152	0.219307058	0.626443199	228	387	333	1325	344	588	975	1991
ENSECAG00000000893	1.616409846	0.219323899	0.626443199	29	70	39	145	20	21	71	84
ENSECAG00000015512	3.636396825	0.219561598	0.62677809	144	181	213	185	339	350	368	160
ENSECAG00000020608	1.748075735	0.219644596	0.62677809	35	54	45	54	106	56	97	70
ENSECAG00000015619	1.740881067	0.21967306	0.62677809	57	117	54	47	32	62	25	88
ENSECAG00000008410	2.679270124	0.219700083	0.62677809	75	129	132	222	62	141	106	122
ENSECAG00000002540	5.284211503	0.219716413	0.62677809	306	552	434	1074	653	901	2100	406
ENSECAG00000013658	3.286275825	0.219847372	0.62699457	89	322	135	309	113	172	133	238
ENSECAG00000017091	2.498024795	0.219905553	0.627003437	42	47	106	145	97	132	179	156
ENSECAG00000005486	5.729094464	0.220116587	0.627448007	666	2021	912	781	651	1067	861	1074
ENSECAG00000023124	4.522819614	0.220220135	0.627465012	340	385	251	305	857	368	751	321
ENSECAG00000014724	8.033552543	0.220246605	0.627465012	3230	6143	5339.01	8111	3842	4479	5209	4604
ENSECAG00000010357	2.218801336	0.220287892	0.627465012	52	64	61	75	97	156	39	164
ENSECAG00000006213	5.226510544	0.220503562	0.627922225	387	1377	842	855	76	1304	139	661
ENSECAG00000024358	2.981033597	0.220986375	0.629139755	78	28	476	161	184	126	114	49
ENSECAG00000012952	6.215124549	0.221140052	0.629155277	1101	1602	1706	1704	1315	1090	1488	1446
ENSECAG00000010941	4.516974377	0.22132003	0.629155277	224	189	412	594	317	576	720	656
ENSECAG00000023121	2.439007315	0.221553387	0.629155277	70	66	60	91	286	45	127	93
ENSECAG00000013258	4.542566883	0.221560705	0.629155277	258	235	419	497	744	441	815	310
ENSECAG00000024175	0.643172071	0.221620739	0.629155277	17	26	50	40	27	19	44	7
ENSECAG00000014116	3.527015816	0.221636595	0.629155277	118	106	171	293	236	123	369	443
ENSECAG00000006306	3.876669544	0.2216616	0.629155277	120	163	179	464	346	377	342	368
ENSECAG00000014383	5.580638754	0.221675495	0.629155277	693	551	395	957	229	853	2166	1868
ENSECAG00000015010	8.73779357	0.221694878	0.629155277	12797	18698	3425	3846	120	16051	213	3036
ENSECAG00000010539	3.520376402	0.221713329	0.629155277	78	241	318	387	191	231	200	164
ENSECAG00000008757	3.089915835	0.221806067	0.629155277	79	131	108	195	217	137	245	241
ENSECAG00000009970	7.153469305	0.221823199	0.629155277	1832	3505	3450	3390	1504	2581	2638	3125
ENSECAG00000021433	4.339396467	0.221830788	0.629155277	145	292	313	533	548	556	440	405
ENSECAG00000012646	4.363307656	0.221857912	0.629155277	197	218	347	488	776	298	708	276
ENSECAG00000021144	0.181410156	0.221870902	0.629155277	12	24	29	29	10	18	8	30
ENSECAG00000017389	5.44918992	0.221907836	0.629155277	529	994	980	1128	779	768	856	758
ENSECAG00000012390	5.108408364	0.221931277	0.629155277	672	842	524	700	582	758	588	501
ENSECAG00000024824	4.094404027	0.222074029	0.62930982	286	330	420	393	401	120	524	158
ENSECAG00000014564	3.389680526	0.222133296	0.62930982	106	138	176	214	254	168	326	284
ENSECAG00000009496	5.52159812	0.222151617	0.62930982	868	1236	779	775	520	719	837	1176
ENSECAG00000015912	5.641337956	0.222280383	0.629376421	708	1706	710	1009	439	967	838	1219
ENSECAG00000023883	4.286971859	0.222316929	0.629376421	234	210	453	256	468	471	434	520
ENSECAG00000024193	6.926690423	0.222365325	0.629376421	1815	2681	2517	3276	1742	2483	2037	2160
ENSECAG00000000895	3.300661767	0.222407649	0.629376421	100	166	145	170	316	184	309	161

ENSECAG00000023171	4.221241522	0.222451534	0.629376421	217	358	541	447	316	322	439	264
ENSECAG00000004924	4.657033215	0.222610996	0.629581233	256	441	602	884	403	400	477	530
ENSECAG00000011040	0.312216024	0.222634523	0.629581233	10	30	26	41	8	8	19	39
ENSECAG00000014585	6.779054148	0.222920308	0.630160645	2426	735	576	1526	1556	2768	1841	5337
ENSECAG00000024355	6.619800524	0.222950117	0.630160645	923	891.045	2215	1766	4307	1916	1909	1645
ENSECAG00000013874	3.762697519	0.223154408	0.630528637	27	359	796	295	1	239	4	384
ENSECAG00000022063	6.179007598	0.223239942	0.630528637	1397	2348	1498	583	1620	1307	1149	747
ENSECAG00000018848	4.657156584	0.223246459	0.630528637	195	189	453	767	614	449	757	689
ENSECAG00000009943	6.264209504	0.223498319	0.631083424	1865	2096	1100	925	1488	1387	1200	1147
ENSECAG00000000485	6.342615166	0.223682134	0.631302223	1199	2209	1671	1568	1057	1276	1850	1625
ENSECAG00000024402	6.372556713	0.223715211	0.631302223	1474	2365	1165	1719	1049	1256	1638	1903
ENSECAG00000018347	-0.016487672	0.223742158	0.631302223	12	23	20	25	15	17	4	20
ENSECAG00000017292	5.147452683	0.22396667	0.63166661	538	775	639	956	762	661	544	557
ENSECAG00000025054	6.897084941	0.224051577	0.63166661	2424	3144	2047	2007	2336	2186	1987	1606
ENSECAG00000011357	6.173507692	0.224075087	0.63166661	1151	1449	1662	1633	1293	1080	1623	1218
ENSECAG00000014705	3.000731609	0.224093231	0.63166661	90	110	122	142	134	124	332	220
ENSECAG00000009464	1.418789906	0.22419388	0.63166854	49	92	40	37	21	80	35	26
ENSECAG00000018794	5.151358398	0.224248698	0.63166854	438	399	745	533	1027	779	1121	555
ENSECAG00000021898	6.822736279	0.224278168	0.63166854	1474	1616	1572	1834	2811	1710	4227	2772
ENSECAG00000021459	5.699499321	0.224315846	0.63166854	741	1178	1175	1142	921	909	965	957
ENSECAG00000023474	4.9745514	0.224458362	0.631694817	245	506	624	585	963	571	912	618
ENSECAG00000022656	0.367764986	0.224458395	0.631694817	13	24	15	10	33	48	26	15
ENSECAG00000014040	3.161223969	0.224491632	0.631694817	77	88	176	180	169	87	226	431
ENSECAG00000008488	4.09136674	0.224802841	0.63241422	239	265	361	575	259	312	278	361
ENSECAG00000000725	7.32885373	0.22500455	0.632825296	1753	1550	2999	3234	4458	2939	4875	3928
ENSECAG00000014686	5.405427085	0.225191772	0.632911733	468	591	721	843	924	870	1311	1014
ENSECAG00000018583	5.052492387	0.225199052	0.632911733	552	693	597	844	451	563	673	722
ENSECAG00000017191	3.406296122	0.225202058	0.632911733	242	289	242	77	214	111	79	275
ENSECAG00000008915	6.134630514	0.225339498	0.632993369	541	751	1089	2056	1214	1475	1889	2419
ENSECAG00000000928	3.0149094	0.225390428	0.632993369	115	73	120	149	109	165	298	244
ENSECAG00000010292	1.408124158	0.225397902	0.632993369	26	38	46	38	49	53	93	65
ENSECAG00000023920	2.882006317	0.225570182	0.633320967	64	128	108	134	239	126	235	130
ENSECAG00000022361	5.639515994	0.225687509	0.633390336	635	923	1016	1686	676	544	1146	1253
ENSECAG00000007736	2.600934384	0.225706156	0.633390336	66	134	187	145	42	164	58	123
ENSECAG00000005942	0.269377729	0.225770684	0.633415286	12	8	20	21	23	13	28	52
ENSECAG00000012020	7.352792179	0.225885284	0.633580674	1184	2604	1942	4233	2806	3532	4773	5482
ENSECAG00000008943	4.463751856	0.226006292	0.633697314	506	628	264	311	379	361	433	365
ENSECAG00000002544	8.516172953	0.22603819	0.633697314	8335	8230	6368	6643	6657	5821	7564	4910
ENSECAG00000007431	5.108013479	0.226172111	0.633916662	369	479	568	730	749	613	1094	907
ENSECAG00000008931	3.566386945	0.226248693	0.633975231	138	222	254	405	128	206	256	249
ENSECAG00000023260	3.94129747	0.226406484	0.63415649	218	228	372	487	243	165	471	232
ENSECAG00000013447	5.877645076	0.226424781	0.63415649	899	1439	1147	1319	853	1256	933	1121
ENSECAG00000024006	5.34472011	0.226887306	0.635222929	465	696	821	1528	706	666	699	839
ENSECAG00000019597	5.451782529	0.22696993	0.635222929	870	644	810	1207	752	619	928	869
ENSECAG00000002611	3.724267214	0.226972936	0.635222929	163	139	263	216	270	261	488	292
ENSECAG00000024825	0.739607922	0.22718392	0.635491484	12	14	32	33	55	47	37	20
ENSECAG00000020843	2.082140536	0.227425055	0.635491484	50	104	71	140	62	39	76	108
ENSECAG00000011335	6.27727904	0.227428978	0.635491484	1601	863	2352	1542	1786	949	1770	904
ENSECAG00000014304	5.663487438	0.227475715	0.635491484	826	726	1464	1212	1236	765	1139	431
ENSECAG00000002414	2.249652078	0.227733149	0.635491484	45	54	87	93	96	84	139	150
ENSECAG00000024331	4.22162589	0.22782595	0.635491484	149	249	303	463	539	310	711	297
ENSECAG00000013450	2.01126941	0.227872274	0.635491484	87	192	63	12	12	154	5	26
ENSECAG00000021142	3.876292793	0.227912126	0.635491484	200	199	204	262	334	239	508	369
ENSECAG00000006883	5.133691092	0.227913113	0.635491484	412	637	728	1254	454	371	899	831
ENSECAG00000016711	3.120233924	0.227939225	0.635491484	83	75	201	150	292	137	309	136
ENSECAG00000015795	3.649486893	0.227970793	0.635491484	163	72	253	213	532	200	404	139
ENSECAG00000017245	5.025237835	0.227987947	0.635491484	504	1120	413	649	317	920	495	470
ENSECAG00000017791	6.928363042	0.228008658	0.635491484	1832	3349	1821	3255	1774	1671	2774	2345
ENSECAG00000019451	7.371081707	0.228020307	0.635491484	4399	4423	1984	2280	3797	2028	3266	1943
ENSECAG00000001066	5.238850792	0.228038929	0.635491484	532	1676	557	371	1053	483	659	292
ENSECAG00000027060	0.955742855	0.228049821	0.635491484	7	27	99	46	24	50	15	21
ENSECAG00000010948	7.28726108	0.22806335	0.635491484	4335	3422	2451	2177	2248	4214	1723	1844
ENSECAG00000007495	5.027480604	0.228073623	0.635491484	725	513	647	683	614	679	533	473
ENSECAG00000024652	6.049386079	0.228268234	0.635878111	1185	1835	1246	1078	724	725	2001	1316
ENSECAG00000006854	4.6547302	0.228528343	0.636446963	158	354	517	546	416	584	500	967
ENSECAG00000002088	5.748553261	0.228720828	0.636491936	783	1119	934	1643	915	911	807	1208
ENSECAG00000021751	2.910115211	0.228751107	0.636491936	95	82	100	154	144	101	335	188
ENSECAG00000021792	4.659443582	0.228759485	0.636491936	329	339	397	425	804	482	858	345
ENSECAG00000006942	6.005913713	0.228770445	0.636491936	829	1471	1153	1934	1094	966	1384	1215
ENSECAG00000018434	3.612766391	0.228824022	0.636491936	100	198	201	258	240	287	359	305
ENSECAG00000011807	8.672549616	0.22909675	0.637088104	9881	13216	5244	4715	4907	11163	4675	4951
ENSECAG00000011353	3.993518978	0.229150267	0.637088104	152	478	395	362	117	399	146	390
ENSECAG00000021408	5.072920174	0.229239329	0.637180116	301	396	589	847	634	656	910	1085
ENSECAG00000015012	3.773309494	0.229541918	0.637865447	107	376	172	729	17	72	103	613
ENSECAG00000016420	4.851074742	0.229676627	0.63808404	358	368	446	581	873	431	895	623
ENSECAG00000015427	4.304692029	0.229762317	0.638166377	153	426	553	591	237	364	364	433

ENSECAG00000017872	4.995982879	0.230041407	0.638733869	349	866	600	875	409	644	484	725
ENSECAG00000012944	3.530126853	0.23007884	0.638733869	231	352	166	168	121	222	193	252
ENSECAG00000015791	3.243912309	0.230192857	0.638894608	113	101	153	196	257	143	294	241
ENSECAG00000020535	6.763179548	0.230382246	0.639179564	1332	3322	2919	2228	513	3783	418	1801
ENSECAG00000007355	5.501234635	0.230407811	0.639179564	575	469	1502	1464	1033	1078	494	409
ENSECAG00000002668	6.416830958	0.230608651	0.639580874	1072	1635	891	1100	697	1511	3399	3321
ENSECAG00000007349	5.55594276	0.230680808	0.639625182	801	980	809	1210	920	674	983	846
ENSECAG00000007904	1.699678987	0.230906962	0.640096364	52	55	84	75	44	65	59	49
ENSECAG00000013843	1.59908606	0.231296484	0.641020075	25	29	53	70	55	67	78	95
ENSECAG00000010571	4.763688666	0.231441796	0.641200359	343	291	472	521	541	345	920	903
ENSECAG00000022270	6.173662862	0.231474175	0.641200359	1010	1332	1535	2209	855	1153	1643	1565
ENSECAG00000011837	8.864165352	0.231556172	0.64127147	5683	11352	10222	13395	7319	9853	6745	7576
ENSECAG00000005303	3.151252546	0.231769705	0.641706734	100	126	134	169	229	165	288	189
ENSECAG00000012875	6.52494934	0.231988232	0.641848225	1193	2456	1884	2154	1614	1888	1702	1281
ENSECAG00000018884	5.418915043	0.232001957	0.641848225	578	977	779	1217	724	500	991	916
ENSECAG00000019206	1.055957738	0.232021244	0.641848225	20	18	39	35	33	23	80	73
ENSECAG00000007800	6.382030799	0.232046315	0.641848225	851	928	1299	1999	2958	1056	2686	1658
ENSECAG00000006101	3.626814483	0.232535364	0.642954487	165	116	187	262	385	228	381	221
ENSECAG00000013939	2.175416121	0.23257019	0.642954487	38	173	81	100	31	65	86	114
ENSECAG00000022436	5.880778044	0.232638412	0.642954487	819	1072	1218	1850	867	740	1371	1314
ENSECAG00000019908	5.757639936	0.232722926	0.642954487	616	1115	1088	1802	872	1128	850	999
ENSECAG00000013724	4.73045887	0.232744307	0.642954487	358	660	217	180	754	552	969	430
ENSECAG00000018883	3.214667	0.232785103	0.642954487	208	277	224	206	0	240	0	175
ENSECAG00000011639	6.827714304	0.232975547	0.643324426	1171	1178	2221	2279	2790	2214	3688	2687
ENSECAG00000000564	2.800911448	0.23308604	0.643473465	121	77	63	109	257	112	218	114
ENSECAG00000011122	4.652024396	0.233231518	0.643541072	406	261	332	428	786	307	980	462
ENSECAG00000020251	0.197056584	0.233253208	0.643541072	33	16	20	22	3	8	6	46
ENSECAG00000020504	5.99372881	0.233280106	0.643541072	520	820	991	1729	1408	1252	1670	1906
ENSECAG00000004908	6.134274607	0.233345402	0.643565263	1897	1496	1132	867	1460	997	1520	890
ENSECAG00000022388	8.494083041	0.233517171	0.643883019	6757	9294	7348	6185	5615	7618	5662	5463
ENSECAG00000005116	3.623673265	0.233582377	0.643906867	260	425	147	151	180	327	159	136
ENSECAG00000019640	6.832113896	0.233703938	0.644086017	1071	1115	2443	2238	3760	2376	2739	2421
ENSECAG00000023248	4.645239595	0.233969261	0.644435681	214	403	601	1014	374	554	391	428
ENSECAG00000023181	5.870112494	0.234002231	0.644435681	736	1440	1340	1332	767	1242	1129	1047
ENSECAG00000016651	5.395735184	0.234016012	0.644435681	483	398	818	902	1113	752	1224	1020
ENSECAG00000021824	4.279867431	0.234057228	0.644435681	383	286	445	436	274	328	355	435
ENSECAG00000012380	6.262942502	0.234263244	0.644819836	724.458	857.369	1422.03	1762.6	1757.64	1298.91	1931.14	2582.74
ENSECAG00000013162	5.691774749	0.23431895	0.644819836	453	521	1145	1090	1562	1051	1736	758
ENSECAG00000007856	4.138534438	0.234366665	0.644819836	256	643	236	294	382	263	330	250
ENSECAG00000015196	2.998132913	0.234627874	0.645382543	101	456	102	39	32	251	46	121
ENSECAG00000020880	1.93038781	0.234806421	0.645717657	46	93	76	105	48	52	77	83
ENSECAG00000008727	2.550782469	0.234923231	0.645747862	73	141	234	131	6	68	0	224
ENSECAG00000024650	2.00180356	0.234930843	0.645747862	65	80	70	119	96	60	43	65
ENSECAG00000008291	7.709225259	0.235224092	0.646256149	3067	3492	3806	7987	3558	2220	4569	4292
ENSECAG00000018835	1.380396653	0.235229291	0.646256149	5	72	31	24	139	28	76	27
ENSECAG00000005210	6.950998909	0.235452238	0.646712601	1357	1775	2231	1813	3980	2310	4348	1853
ENSECAG00000020930	0.61198877	0.235749513	0.647250136	34	38	23	26	11	47	16	15
ENSECAG00000011896	4.393952793	0.235761644	0.647250136	280	296	318	372	529	453	599	445
ENSECAG00000021248	6.139242765	0.235831444	0.647285678	1081	745	1340	608	3031	824	2407	974
ENSECAG00000011315	0.235849413	0.235914455	0.647357453	15	18	34	32	3	32	12	20
ENSECAG00000024173	5.969057212	0.236040885	0.647433976	596	595	1483	1041	2538	1521	1450	670
ENSECAG00000012982	4.281130059	0.236109943	0.647433976	185	171	403	452	562	374	606	363
ENSECAG00000024651	5.916865648	0.236121731	0.647433976	672	594	1334	1015	2039	918	1987	1070
ENSECAG00000016394	4.638642717	0.236169811	0.647433976	1305	139	278	188	84	904	43	264
ENSECAG00000005195	6.041702543	0.236506326	0.647977538	795	1211	1371	2291	877	872	1252	1737
ENSECAG00000017882	0.888123421	0.23654337	0.647977538	10	22	33	38	46.0005	65.0013	45	21
ENSECAG00000020740	8.049761869	0.236555776	0.647977538	7691	4739	4316	3975	3741	4652	6368	3207
ENSECAG00000023456	2.213671724	0.236595751	0.647977538	36	49	74	117	175	75	117	89
ENSECAG00000011756	6.058320721	0.23677194	0.648304121	1263	1253	1148	1725	846	1034	1452	1484
ENSECAG00000024775	6.243409958	0.236912768	0.64853375	997	2473	1072	1802	1113	1746	1556	854
ENSECAG00000011712	2.809483924	0.236972829	0.648542225	83	77	115	131	185	89	266	164
ENSECAG00000009427	0.66473068	0.237042435	0.648576813	23	59	27	19	7	36	25	27
ENSECAG00000021798	3.553900349	0.237117231	0.648625581	135	146	201	207	366	161	473	179
ENSECAG00000009105	2.72592541	0.237378185	0.649105341	56	59	137	147	115	152	200	187
ENSECAG00000021048	7.507167067	0.237406644	0.649105341	3679	5762	3455	3046	558	5905	421	3626
ENSECAG00000016144	3.185781438	0.237469677	0.649121795	85	139	143	173	337	110	255	197
ENSECAG00000008638	6.720692689	0.237747581	0.649625425	1648	1435	3117	2759	1969	1914	2450	1036
ENSECAG00000021265	6.326857839	0.237768041	0.649625425	899	1455	2958	1532	1240	1078	1231	2073
ENSECAG00000023208	3.607622762	0.238199409	0.650527328	136	166	170	256	307	144	496	278
ENSECAG00000008767	6.469239967	0.238212422	0.650527328	1686	1716	2132	1611	1134	1705	1224	2123
ENSECAG00000012216	3.850255386	0.2383258	0.650617352	181	386	230	403	268	267	261	226
ENSECAG00000021202	7.459948616	0.238383802	0.650617352	1204	2170	2193	5284	3263	3052	4361	7276
ENSECAG00000008082	6.636709839	0.238439304	0.650617352	1593	3865	1215	1459	1191	1437	2358	1915
ENSECAG00000019538	2.575731187	0.23848005	0.650617352	78	131	122	177	57	151	80	110
ENSECAG00000011057	7.586969902	0.238531122	0.650617352	1802	2149	2991	4796	5100	3763	6048	4408
ENSECAG00000017058	3.769421722	0.238590693	0.650623961	179	168	201	267	322	227	422	364

ENSECAG00000013126	6.689506897	0.238751723	0.650760392	2124	4186	2399	918	26	3588	40	1364
ENSECAG00000014299	2.436634048	0.238755043	0.650760392	62	79	124	216	40	80	123	125
ENSECAG00000010255	3.10394106	0.238813353	0.65076353	67	118	118	224	154	165	199	329
ENSECAG00000001318	6.379360789	0.239046526	0.650807511	1121	831	1591	1330	2558	1252	1961	2446
ENSECAG00000019336	7.485271848	0.239173505	0.650807511	2835	4488	3567	3983	3456	2606	3425	3047
ENSECAG00000005550	4.680854549	0.239237985	0.650807511	465	962	504	238	129	1008	144	246
ENSECAG00000003621	3.568570972	0.239265214	0.650807511	142	155	212	188	396	177	373	222
ENSECAG00000005447	1.015596804	0.23927292	0.650807511	13	35	73	55	25	24	50	32
ENSECAG00000000436	10.23201766	0.239295436	0.650807511	23203	20780	58930	22433	36	15411	20	37094
ENSECAG00000019290	4.640063277	0.239313865	0.650807511	283	185	480	536	1019	304	796	380
ENSECAG00000004412	0.494293969	0.239324146	0.650807511	14	38	36	29	9	38	18	19
ENSECAG00000010803	0.893876488	0.239343966	0.650807511	13	18	21	53	71	24	46	39
ENSECAG00000014422	4.720530767	0.23959606	0.651337426	192	322	352	836	565	517	900	623
ENSECAG00000008688	5.57066622	0.239807875	0.651368605	555	893	1440	1349	482	1134	178	1284
ENSECAG00000023408	4.529233916	0.239808204	0.651368605	347	219	481	261	492	306	719	796
ENSECAG00000020815	2.361335783	0.239822206	0.651368605	44	50	85	129	85	159	138	120
ENSECAG00000020673	0.553293456	0.239885661	0.651368605	34	25	29	25	31	22	27	11
ENSECAG00000012169	5.313314999	0.239893593	0.651368605	433	1114	725	1161	449	1097	353	788
ENSECAG00000018643	3.453887498	0.240021964	0.651419865	117	130	136	285	206	173	374	337
ENSECAG00000012105	0.736433366	0.240065391	0.651419865	7	12	44	29	36	24	45	58
ENSECAG00000006011	6.96178796	0.240125464	0.651419865	1120	1269	1215	3927	2037	1604	4070	5255
ENSECAG00000018646	3.453236568	0.240194229	0.651419865	100	104	224	247	245	192	367	278
ENSECAG00000024106	0.432302455	0.240198559	0.651419865	13	23	9	23	36	53	18	19
ENSECAG00000010547	4.44982977	0.240390063	0.651783965	184	478	313	352	372	424	592	754
ENSECAG00000010335	5.961500403	0.24062255	0.652236737	1407	1215	1122	1119	1346	890	1195	1006
ENSECAG00000015384	2.703151353	0.240671632	0.652236737	63	117	211	174	64	86	176	128
ENSECAG00000019348	0.93251726	0.240770835	0.652350299	26	51	26	55	15	22	43	44
ENSECAG00000017522	2.406589464	0.240996993	0.652638692	190	153	38	23	130	34	89	69
ENSECAG00000024703	5.96530508	0.241028422	0.652638692	1180	1999	802	1053	581	993	940	1799
ENSECAG00000009156	4.589563769	0.241049249	0.652638692	282	267	420	502	752	371	785	452
ENSECAG00000013326	3.670967092	0.2413264	0.653233729	158	202	322	405	146	200	232	325
ENSECAG00000011016	3.691785319	0.241494834	0.653380649	56	39	491	137	509	122	396	345
ENSECAG00000017861	6.925636514	0.241495456	0.653380649	2084	2198	2777	3028	2491	2003	2326	1706
ENSECAG00000007794	6.169883962	0.241780675	0.653909014	1140	1721	1369	1649	1194	1645	1053	1172
ENSECAG00000021362	4.024110779	0.241805617	0.653909014	274	328	325	376	297	253	330	299
ENSECAG00000004436	4.674065186	0.241925797	0.654042345	320	361	403	473	536	481	747	708
ENSECAG00000000680	3.425402853	0.241969816	0.654042345	138	118	183	178	392	154	383	145
ENSECAG00000011823	2.279844009	0.242304257	0.654790877	55	37	89	91	98	64	260	83
ENSECAG00000013993	4.375766265	0.242549527	0.655208886	235	428	407	693	426	405	341	306
ENSECAG00000010347	7.359355235	0.242574041	0.655208886	1558	785	3997	3347	4304	3544	5930	3117
ENSECAG00000010683	4.35189422	0.242771725	0.655508849	234	560	334	598	220	290	390	560
ENSECAG00000017723	3.652669207	0.242836616	0.655508849	138	99	254	264	419	188	403	237
ENSECAG00000022582	4.791343145	0.242871949	0.655508849	341	487	385	447	476	840	740	606
ENSECAG00000015935	11.11751085	0.242915401	0.655508849	82408	40237	22880	23573	40745	25175	54479	24535
ENSECAG00000014294	3.507504958	0.242982367	0.655511014	138	249	272	293	183	227	145	238
ENSECAG00000007694	8.139905365	0.243031356	0.655511014	4868	8402	4841	5200	3301	6565	4339	4833
ENSECAG00000021031	9.053502011	0.243195064	0.655797207	7206	7712	17781	13959	9492	8534	13769	4587
ENSECAG00000007730	0.987173695	0.243414855	0.656221174	27	72	27	37	9	38	29	47
ENSECAG00000018423	7.170270266	0.24349427	0.656221174	1917	4660	2919	2532	2120	2985	2157	2584
ENSECAG00000009767	2.98009659	0.243525205	0.656221174	77	129	165	352	53	80	157	244
ENSECAG00000012836	2.873225982	0.243757568	0.656591631	98	67	121	138	221	117	219	165
ENSECAG00000012601	5.701196526	0.243778026	0.656591631	616	391	1048	1110	1734	1049	1409	900
ENSECAG00000017856	3.754786267	0.243897603	0.656758327	119	89	301	324	387	308	385	247
ENSECAG00000026973	4.388650195	0.244012554	0.656912491	231	266	753	573	431	326	555	161
ENSECAG00000008722	4.880079742	0.244311806	0.657562627	551	927	520	336	352	674	341	640
ENSECAG00000024665	5.254310118	0.244386639	0.657608575	294	459	691	1023	900	849	1060	884
ENSECAG00000000020	2.92869673	0.244460885	0.657652923	88	220	142	186	133	123	111	159
ENSECAG00000021154	1.973789938	0.244667707	0.657746078	45	32	47	95	66	33	158	149
ENSECAG00000005661	5.362043669	0.244673967	0.657746078	807	1273	664	485	788	425	1062	655
ENSECAG00000024732	5.450995786	0.244751912	0.657746078	507	476	917	792	1139	953	1191	938
ENSECAG00000018994	4.895366747	0.244779962	0.657746078	375	574	494	1085	586	401	735	450
ENSECAG00000011396	1.007298485	0.244784377	0.657746078	16	15	51	28	45	55	70	26
ENSECAG00000002944	8.922993972	0.24502372	0.65823385	7359	14630	9299	9453	5429	10393	7747	9297
ENSECAG00000022940	5.760234109	0.245084325	0.658241341	680	1283	1012	1550	822	777	964	1363
ENSECAG00000018569	5.53572486	0.24528874	0.65843131	423	568	857	1108	1194	953	1332	1006
ENSECAG00000021139	1.108264395	0.245325608	0.65843131	11	36	39	32	18	82	72	40
ENSECAG00000014363	0.660169957	0.245405355	0.65843131	14	53	27	37	17	32	23	26
ENSECAG00000023195	4.472608611	0.245437922	0.65843131	223	348	299	505	337	408	655	777
ENSECAG00000014674	2.271513865	0.245444223	0.65843131	96	43	111	180	25	64	206	21
ENSECAG00000022924	6.412172679	0.24581251	0.659263939	797	872	1536	2112	2686	1404	2351	1948
ENSECAG00000011786	3.582411232	0.245951216	0.659480592	89	202	205	242	192	209	402	384
ENSECAG00000006063	2.059037819	0.24601292	0.65949072	55	45	61	76	121	79	116	89
ENSECAG00000010917	2.629827334	0.246158676	0.659726113	46	92	126	91	186	98	70	256
ENSECAG00000014310	4.996802092	0.246339901	0.660056431	349	593	407	617	821	643	957	663
ENSECAG00000022025	2.100244036	0.24650977	0.660356172	66	65	63	176	81	41	105	65
ENSECAG00000006943	5.551138393	0.246693469	0.660466166	691	546	678	852	1286	595	1665	1109

ENSECAG00000001336	1.065435954	0.246723119	0.660466166	16	14	35	55	35	41	49	78
ENSECAG00000011098	4.345129756	0.246724866	0.660466166	382	443	402	378	228	411	390	427
ENSECAG00000016003	3.493790469	0.246805614	0.660527014	143	142	160	211	220	120	465	337
ENSECAG00000016399	6.122384266	0.247309439	0.661619609	765	750	1250	1529	1587	1104	2505	1726
ENSECAG00000017833	0.665841206	0.247330088	0.661619609	27	26	44	29	14	21	44	24
ENSECAG00000015714	6.19815936	0.247479387	0.661794329	1522.01	2466	1106.01	748	836.001	2035	1023	976.024
ENSECAG00000012582	4.71730843	0.24751681	0.661794329	535	630	343	564	494	532	448	388
ENSECAG00000003138	1.528332428	0.247597732	0.661794329	14	48	51	55	44	92	80	64
ENSECAG00000014263	0.673659578	0.247627918	0.661794329	5	13	31	42	37	48	28	38
ENSECAG00000023978	2.110654401	0.247747863	0.661831362	39.9996	37	86	89.9991	82	71.9993	181.999	98
ENSECAG00000007411	3.685109309	0.247758038	0.661831362	171	197	187	193	374	176	435	285
ENSECAG00000016202	0.970983752	0.247919876	0.662108326	13	18	100	46	42	13	52	13
ENSECAG00000011981	1.03534039	0.248001325	0.662170516	28	15	28	35	87	16	61	39
ENSECAG00000014866	4.480908402	0.24806847	0.662194497	531	692	239	257	263	355	489	448
ENSECAG00000000645	3.603737133	0.24821378	0.66239456	103	158	209	269	124	271	338	475
ENSECAG00000023808	4.852375635	0.248259779	0.66239456	284	269	404	856	633	447	1305	533
ENSECAG00000016355	2.561805877	0.248375044	0.662546832	59	75	102	111	213	97	159	108
ENSECAG00000008080	3.090566629	0.248693626	0.663241258	100	189	147	291	123	105	203	175
ENSECAG00000017309	1.23101324	0.248798648	0.663365951	37	28	47	84	43	32	41	39
ENSECAG00000021916	6.506710543	0.24894148	0.663591372	1680	1884	1723	2025	1298	1689	1673	1840
ENSECAG00000022749	2.512486174	0.249053544	0.663605252	96	57	89	51	215	91	210	65
ENSECAG00000014314	2.068370564	0.249063262	0.663605252	21	68	30	131	90	70	185	85
ENSECAG00000009265	3.14543405	0.249214681	0.663853332	69.0071	117	181	160	225	285	274	86
ENSECAG00000013828	7.61758354	0.249410067	0.66407005	2423	4305	3353	7270	2506	2248	3335	5538
ENSECAG00000006166	3.181120076	0.249445066	0.66407005	143	218	170	210	149	87	264	151
ENSECAG00000001192	5.6175317	0.249471024	0.66407005	324	612	966	1222	1135	591	1395	1758
ENSECAG00000021973	2.83703138	0.249561594	0.664138306	70	85	99	179	155	115	207	227
ENSECAG00000009781	3.598617109	0.249613334	0.664138306	150	176	162	242	318	247	339	266
ENSECAG00000019876	1.552085171	0.249748587	0.664342913	22	34	51	66	63	74	57	86
ENSECAG00000012417	4.062236495	0.250181266	0.665338405	208	242	315	702	155	222	360	451
ENSECAG00000024338	1.459153888	0.250367722	0.665443286	11	47	31	67	24	91	42	114
ENSECAG00000010710	5.459628933	0.25044078	0.665443286	579	679	958	1500	647	654	911	1004
ENSECAG00000017667	2.348841248	0.25044867	0.665443286	54	46	102	90	163	70	199	80
ENSECAG00000010097	3.807832853	0.250461156	0.665443286	164	209	283	573	156	191	262	381
ENSECAG00000002830	6.376821122	0.250570557	0.665443286	1074	2179	1879	1850	810	2336	1202	1374
ENSECAG00000006911	4.391860143	0.250571398	0.665443286	243	160	446	440	550	373	616	512
ENSECAG00000008391	4.762528819	0.251133912	0.666077846	427	410	422	1028	352	430	526	650
ENSECAG00000023897	2.573823086	0.251202157	0.666077846	59	122	128	206	97	91	91	131
ENSECAG00000006958	4.71735091	0.251202851	0.666077846	337	754	524	525	430	536	400	510
ENSECAG00000012479	4.441853433	0.251263757	0.666077846	234	254	372	494	553	352	797	430
ENSECAG00000002437	8.257142384	0.251281836	0.666077846	11149	5420	4478	2214	5795	7071	3128	3044
ENSECAG00000010243	5.089271231	0.251417008	0.666077846	512	657	668	952	555	400	692	852
ENSECAG00000014366	5.433246983	0.251435214	0.666077846	615	643	595	652	1388	608	1666	648
ENSECAG00000022499	0.648090717	0.251509181	0.666077846	8	11	37	32	34	29	51	37
ENSECAG00000013247	3.846090458	0.251529417	0.666077846	105	196	291	297	521	350	310	206
ENSECAG00000017447	6.427398435	0.251536123	0.666077846	1310	1614	2282	2066	807	1719	813	2549
ENSECAG00000000861	5.328651662	0.251567668	0.666077846	553	501	530	861	1167	759	1064	887
ENSECAG00000023980	4.775510449	0.251598636	0.666077846	329	686	605	631	350	576	511	543
ENSECAG00000007336	2.550410196	0.251617989	0.666077846	60	50	108	133	130	137	142	160
ENSECAG00000021934	6.069597942	0.251712128	0.666077846	493	781	1342	1686	1624	1470	2016	1460
ENSECAG00000009368	9.2579287	0.251739708	0.666077846	11242	3244	42	11637	15502	28132	7453	16566
ENSECAG00000005419	1.12667737	0.251801311	0.666077846	27.0071	115	11	34	6.00043	63.0001	22	32
ENSECAG00000010336	7.034577651	0.251905871	0.666077846	1554	1884	2100	2190	3812	2471	4225	2549
ENSECAG00000016582	3.753917573	0.25192834	0.666077846	347	436	222	181	27	589	8	71
ENSECAG00000017011	0.605030628	0.251977325	0.666077846	12	27	14	25	12	28	31	78
ENSECAG00000009858	3.62602536	0.251980438	0.666077846	141	131	182	303	319	193	328	364
ENSECAG00000011772	0.379834386	0.252042049	0.666086057	12	59	12	25	10	30	8	26
ENSECAG00000012021	3.018666618	0.252152433	0.666223127	86	143	174	303	175	95	127	166
ENSECAG00000023264	1.586102895	0.252342698	0.666427197	19	51	19	79	54	26	174	63
ENSECAG00000016991	4.618805453	0.252346741	0.666427197	175	296	493	595	763	445	843	367
ENSECAG00000017913	1.079561515	0.252639033	0.666721475	15	50	44	74	25	40	31	40
ENSECAG00000015437	0.629283794	0.252688012	0.666721475	18	19	22	19	53	15	65	20
ENSECAG00000016259	5.476155713	0.252713135	0.666721475	433	269	876	1185	1658	895	1110	711
ENSECAG00000000902	0.50618737	0.252728605	0.666721475	8	10	31	30	38	25	41	31
ENSECAG00000020538	6.974352354	0.252750978	0.666721475	1794	2400	2683	3787	2423	2272	2220	1923
ENSECAG00000017106	0.59302816	0.252978204	0.667103377	32	13	42	32	19	24	20	31
ENSECAG00000011633	5.041883006	0.253012945	0.667103377	411	479	482	653	629	714	827	993
ENSECAG00000020442	3.116766096	0.253332701	0.667791804	74	130	122	210	223	157	283	186
ENSECAG00000010496	6.722527766	0.253439087	0.667856187	3366	5506	412	494	3	3512	5	822
ENSECAG00000016351	1.521666562	0.253474447	0.667856187	46	34	38	21	22	47	72	153
ENSECAG00000009183	7.366809697	0.253688796	0.668232385	3811	3263	2717	3375	2844	2151	4001	2640
ENSECAG00000017121	3.477949584	0.253827694	0.668232385	109	130	217	226	351	171	273	286
ENSECAG00000020032	3.916119493	0.254010745	0.668232385	200	213	315	565	198	202	302	382
ENSECAG00000015971	4.526082483	0.25403905	0.668232385	299	299	350	459	621	416	743	452
ENSECAG00000015558	6.778769968	0.254139845	0.668232385	1317	1339	2110	1709	3606	2134	3106	1999
ENSECAG00000008772	6.324780678	0.254264044	0.668232385	1550	1933	1344	1513	1262	1417	1469	1582

ENSECAG00000023476	6.472349731	0.254320715	0.668232385	1569	2886	1233	1549	917	2404	987	1654
ENSECAG00000024373	6.44697221	0.254327319	0.668232385	906	1317	1640	1422	2265	1315	3204	1894
ENSECAG00000012442	4.743681905	0.254349108	0.668232385	204	475	410	610	780	587	762	455
ENSECAG00000020895	4.694083153	0.254429253	0.668232385	249	310	414	660	504	375	844	827
ENSECAG00000003758	5.114180605	0.254474044	0.668232385	884	1295	470	341	48	1378	32	387
ENSECAG00000022958	5.788293013	0.254491222	0.668232385	784	754	884	821	1773	952	1817	885
ENSECAG00000017675	3.330350635	0.254535113	0.668232385	111	188	210	356	222	117	180	192
ENSECAG00000016089	3.58668695	0.254535813	0.668232385	185	195	136	165	351	243	354	225
ENSECAG00000020350	4.933684493	0.254695645	0.668232385	302	500	516	585	970	646	761	544
ENSECAG00000021161	3.348174585	0.2547124	0.668232385	153	129	142	165	236	178	336	249
ENSECAG00000021040	4.508366755	0.254773522	0.668232385	165	328	501	440	402	475	547	783
ENSECAG00000024620	4.322880446	0.254776253	0.668232385	192	253	1217	537	0	163	0	728
ENSECAG00000023524	6.713815739	0.254791013	0.668232385	1700	1854	2271	2894	2251	1816	1831	1527
ENSECAG00000011778	5.855527107	0.25479111	0.668232385	878	690	891	954	1646	1046	1821	1149
ENSECAG00000017092	2.915601644	0.255076837	0.66882768	237	106	67	220	34	189	20	203
ENSECAG00000007559	4.989346262	0.25528507	0.669089588	343	385	617	636	961	530	828	738
ENSECAG00000024623	8.863264484	0.255294262	0.669089588	4898	8165	7928	5837	12604	11574	10086	11915
ENSECAG00000015033	4.595577379	0.255414818	0.66922598	251	386	654	785	579	383	372	387
ENSECAG00000018783	2.419509157	0.255463866	0.66922598	46	66	103	107	102	133	159	126
ENSECAG00000019828	1.369325301	0.255610899	0.669457117	22	73	16.9999	17	23	69	92	80
ENSECAG00000024618	4.348965259	0.255685171	0.669497625	111	215	326	677	553	355	676	436
ENSECAG00000023596	7.575390588	0.25627894	0.670818721	4607	5500	2338	2642	4523	2835	3272	2181
ENSECAG00000015548	5.948092896	0.256380483	0.670818721	438	831	1294	1380	1458	1110	1930	1555
ENSECAG00000012584	6.999020418	0.25643316	0.670818721	2736	4125	1761	1667	1840	3548	1506	1458
ENSECAG00000025031	3.178584766	0.256453285	0.670818721	193	238	133	141	146	139	203	147
ENSECAG00000023145	6.574502935	0.256484312	0.670818721	720	1398	2045	1736	2933	2407	2282	1646
ENSECAG00000007379	2.29882102	0.256713035	0.671076853	45	172	111	109	23	97	24	159
ENSECAG00000022286	4.604755541	0.256729799	0.671076853	463	868	324	255	263	433	551	455
ENSECAG00000021219	7.751628935	0.256773634	0.671076853	5042	5359	3505	3139	4628	3477	4129	2504
ENSECAG00000018648	5.722467378	0.256891405	0.671076853	1287	1284	713	793	584	1220	768	1081
ENSECAG00000001856	3.578250052	0.256951918	0.671076853	111	178	160	266	174	128	372	533
ENSECAG00000014977	4.632109752	0.256983049	0.671076853	161	517	612	920	351	557	371	454
ENSECAG00000021807	4.751679453	0.256995615	0.671076853	244	319	561	582	617	712	553	681
ENSECAG00000011355	7.688397954	0.257246955	0.671579129	2842	5982	3284	5355	2083	3220	5190	4074
ENSECAG00000020240	6.133358362	0.257330585	0.671643446	902	527	1399	1333	2496	985	2264	1261
ENSECAG00000016668	1.311615319	0.257513999	0.671938199	11	21	57	57	56	58	89	41
ENSECAG00000017353	9.002201092	0.257598341	0.671938199	5148	6114	10773	8117	13975	9702	18848	9574
ENSECAG00000010205	5.640986456	0.257652329	0.671938199	727	1254	1019	1003	855	957	936	882
ENSECAG00000008042	4.596778548	0.257679594	0.671938199	228	286	421	612	505	560	634	622
ENSECAG00000017173	2.541578986	0.257822578	0.672106214	55	67	99	129	115	95	230	139
ENSECAG00000022039	4.206374148	0.257862094	0.672106214	229	130	339	428	554	316	535	401
ENSECAG00000017666	2.24873819	0.258473677	0.673546076	29	67	112	59	198	104	141	33
ENSECAG00000023220	4.404075115	0.258576773	0.673660541	102	238	257	793	426	437	640	602
ENSECAG00000016127	5.754048658	0.258928813	0.674423368	668	761	740	1155	1314	889	1818	1251
ENSECAG00000022545	6.424560807	0.259130993	0.674795597	1212	3180	1263	1502	1063	2623	926	1072
ENSECAG00000023375	5.025667455	0.259192112	0.674800411	383	402	557	694	755	629	1041	720
ENSECAG00000006293	6.142371994	0.2592547	0.674809045	903	1423	1530	2048	994	1384	1355	1380
ENSECAG00000024999	6.141553659	0.259344103	0.674887453	622	847	1304	1695	1357	1419	1808	2305
ENSECAG00000026955	6.022576708	0.259462005	0.675017861	1800	1007	1392	779	1191	1557	827	830
ENSECAG00000008764	6.116882897	0.259512796	0.675017861	2161	3353	731	280	2	2175	0	625
ENSECAG00000026994	5.480078605	0.259627313	0.675161479	675	1054	854	998	861	775	824	797
ENSECAG00000008262	7.402850605	0.259798019	0.675258191	2687	4342	3853	3056	3027	3583	3230	1874
ENSECAG00000007521	5.605839589	0.259837284	0.675258191	419	610	927	1170	1016	1286	1163	1190
ENSECAG00000014773	3.466246767	0.259842436	0.675258191	105	188	193	500	181	217	152	213
ENSECAG00000008850	0.54796181	0.259923133	0.675293232	30	45	43	3	1	47	15	13
ENSECAG00000019881	6.286299599	0.259974549	0.675293232	1102	2770	876	1689	891	1586	1455	1530
ENSECAG00000013206	5.37612501	0.260110197	0.675491467	498	750	695	1610	689	598	967	789
ENSECAG00000016028	4.029395536	0.260361375	0.675896187	195	242	269	289	331	325	419	495
ENSECAG00000024898	6.241978014	0.260402488	0.675896187	728	2584	1253	1903	891	1775	976	1579
ENSECAG00000022654	2.761490695	0.260444144	0.675896187	179	154	87	100	95	75	150	147
ENSECAG00000007051	5.900129116	0.260511964	0.675918119	614	1548	1289	1577	797	950	1146	1433
ENSECAG00000010650	4.078869227	0.260609293	0.676016588	283	194	475	428	319	255	347	298
ENSECAG00000013250	6.61031275	0.260856763	0.676449336	2034	2114	1775	1780	1942	1574	1810	1616
ENSECAG00000011553	10.61117108	0.260894953	0.676449336	34077	44522	23042	23748	29721	24842	31346	20708
ENSECAG00000008048	4.67222375	0.261098131	0.676822001	343	501	529	749	321	292	574	684
ENSECAG00000019651	3.886359768	0.261198137	0.67692711	173	550	232	263	218	358	254	190
ENSECAG00000013255	8.191740852	0.261322364	0.677094929	5216	6559	5999	6518	2742	7075	4172	5905
ENSECAG00000006288	7.711170181	0.261634341	0.677699066	4196	236	3974	1246	8235	4808	7607	2040
ENSECAG00000006898	0.371620811	0.26167458	0.677699066	9	6	34	20	38	24	30	30
ENSECAG00000010986	7.465023378	0.261787735	0.677837926	4622	3391	2623	3180	3333	2316	3455	3175
ENSECAG00000022185	4.174862398	0.261893237	0.677924185	490	635	180	195	55	769	51	135
ENSECAG00000012175	6.821801242	0.261945155	0.677924185	1726	2980	1584	3178	1614	1317	2588	2554
ENSECAG00000018858	5.571423254	0.262029165	0.677924185	538	927	1297	1232	567	1013	728	1100
ENSECAG00000013950	8.049533307	0.262059231	0.677924185	3113	3181	4299	5036	8390	6780	6970	4039
ENSECAG00000015579	3.10825854	0.262273202	0.678246161	157	218	160	145	152	125	157	174
ENSECAG00000000962	1.265606965	0.262302841	0.678246161	14	35	39	51	35	91	54	49

ENSECAG00000022295	7.076075462	0.262446909	0.678287979	1401	2596	3026	4853	2234	2720	2124	2323
ENSECAG00000012824	3.313861383	0.262462136	0.678287979	77	298	168	319	166	118	208	210
ENSECAG00000020889	6.766643557	0.262497746	0.678287979	1306	3171	1899	2769	1712	1790	2269	1994
ENSECAG00000000607	1.508300405	0.26259748	0.678391721	47	12	36	41	159	60	59	10
ENSECAG00000008284	2.211979837	0.262721937	0.678554196	61	101	112	107	59	93	88	80
ENSECAG00000014283	6.020508457	0.262779574	0.678554196	711	624	851	1841	1268	848	1779	2575
ENSECAG00000012054	2.280568048	0.262975099	0.678905104	39	65	169	174	31	60	47	174
ENSECAG00000005869	5.666300866	0.26314348	0.679185789	452	635	1002	1158	1306	1017	1543	1041
ENSECAG00000006890	8.863265418	0.263401498	0.679657972	6816	7718	10294	15318	6615	6917	10392	8916
ENSECAG00000021810	1.915979287	0.263445817	0.679657972	42	48	52	76	87	50	103	129
ENSECAG00000006184	6.142293591	0.263604565	0.679913451	1828	1405	1041	1126	1194	1419	1251	1096
ENSECAG00000009774	4.555771191	0.26431722	0.681493287	286	394	462	846	449	365	479	425
ENSECAG00000011060	2.023199547	0.264447102	0.681493287	31	59	67	88	95	81	133	86
ENSECAG00000017711	7.975976756	0.264478464	0.681493287	2643	6789	5764	6496	2978	3620	5907	5337
ENSECAG00000018870	10.42377174	0.264505921	0.681493287	41787	56068	9397	5766	5494	50494	4163	14537
ENSECAG00000011177	5.517584248	0.264533206	0.681493287	2301	353	255	386	609	1168	735	271
ENSECAG00000013756	2.722587689	0.264641112	0.681493287	84	113	185	175	142	139	80	86
ENSECAG00000020620	5.644366847	0.264647843	0.681493287	471	692	808	1225	888	1022	1606	1342
ENSECAG00000022849	7.227036119	0.264725715	0.681493287	3566	3732	2076	2370	2067	2593	2824	2912
ENSECAG00000017561	2.761814332	0.264755802	0.681493287	37	198	170	217	71	207	36	108
ENSECAG00000019125	7.304210249	0.264965039	0.681751898	1625	3397	3485	5297	2834	2137.99	3432	2836
ENSECAG00000008835	7.045132635	0.264976034	0.681751898	1607	2110	1822	2179	3125	1990	5525	2772
ENSECAG00000003363	5.504665595	0.265148153	0.682040606	500	1003	995	1293	940	817	807	736
ENSECAG00000008541	1.360372692	0.2652485	0.682140648	38	23	24	55	80	39	82	50
ENSECAG00000017281	5.132860063	0.265419554	0.682140648	246	317	699	968	1257	850	940	383
ENSECAG00000008536	5.125927446	0.265423443	0.682140648	267	392	674	933	856	753	1111	667
ENSECAG00000000421	1.646737162	0.265426708	0.682140648	32	47	49	53	84	60	79.0007	76
ENSECAG00000017854	0.84690543	0.265590836	0.682403591	7	8	55	29	58	26	66	29
ENSECAG00000009177	5.559624736	0.265648899	0.682403591	617	1079	999	1170	613	993	822	996
ENSECAG00000014528	4.829205253	0.266023493	0.6832117	467	530	274	316	854	450	950	559
ENSECAG00000016333	5.702752485	0.266100563	0.683255505	587	754	902	1006	1376	1052	1428	1134
ENSECAG00000014846	5.691268579	0.266235261	0.683447227	427	611	1277	938	1155	941	1281	1639
ENSECAG00000019642	4.654203349	0.266448179	0.683839613	128	407	523	538	864	578	652	351
ENSECAG00000008162	3.710502245	0.266527657	0.683889424	88	132	118	495	192	250	531	360
ENSECAG00000007524	6.362906007	0.266907094	0.684574112	1154	1524	1791	2415	1862	1623	1245	1108
ENSECAG00000021614	3.271419635	0.266946939	0.684574112	122	137	163	142	258	243	305	132
ENSECAG00000013889	4.847987676	0.266974884	0.684574112	301	435	441	593	433	407	809	1188
ENSECAG00000007177	3.207644546	0.267135719	0.684752835	113	67	190	164	350	150	309	112
ENSECAG00000005178	1.090388627	0.267234981	0.684752835	14	41	35	28	32	32	92	55
ENSECAG00000025163	5.035403327	0.267260041	0.684752835	416	461	592	526	935	642	992	588
ENSECAG00000025095	3.503314077	0.267306412	0.684752835	94	157	234	211	336	278	336	149
ENSECAG00000013488	1.745851629	0.26734531	0.684752835	42	71	33	32	109	64	103	54
ENSECAG00000000584	5.54144468	0.267506211	0.685010844	569	536	901	876	1242	1047	1066	1081
ENSECAG00000012759	1.563760044	0.267861539	0.685533014	30	73	22	33	75	99	63	48
ENSECAG00000010375	6.667319084	0.26804828	0.685533014	1865	2259	2071	1956	2142	1431	2430	1298
ENSECAG00000023331	4.423164616	0.268091491	0.685533014	260	243	349	475	424	319	743	616
ENSECAG00000013884	0.693124178	0.268195211	0.685533014	13	18	27	29	79	23	27	24
ENSECAG00000018530	0.793120231	0.268361273	0.685533014	18	17	20	37	14	20	80	61
ENSECAG00000016661	8.408527952	0.268404207	0.685533014	1978	1458	9553	6618	5405	3593	23931	5122
ENSECAG00000025154	2.65299125	0.268435553	0.685533014	39	76	110	167	125	143	141	202
ENSECAG00000023234	1.134264477	0.268443481	0.685533014	16	30	41	39	75	33	55	46
ENSECAG00000018900	4.962747811	0.268468444	0.685533014	393	481	432	610	863	525	974	648
ENSECAG00000009169	4.724255803	0.268492266	0.685533014	253	326	503	605	529	625	715	659
ENSECAG00000008523	5.849375726	0.268521014	0.685533014	254	615	831	1992	773	842	1429	2836
ENSECAG00000008844	4.273260326	0.268592463	0.685533014	353	333	198	152	513	283	623	513
ENSECAG00000012682	2.943749763	0.268596712	0.685533014	98	85	146	121	237	132	215	164
ENSECAG00000018150	3.911013109	0.268618726	0.685533014	260	203	380	377	253	174	403	272
ENSECAG00000023990	5.077531824	0.268643893	0.685533014	690	760	653	512	716	609	698.001	404
ENSECAG00000006869	0.152065111	0.268753913	0.685533014	1	20	1	36	15	28	34	32
ENSECAG00000016302	2.474753376	0.268782298	0.685533014	57	82	86	105	141	84	190	130
ENSECAG00000016242	9.489982414	0.268812713	0.685533014	13435	17643	13653	14772	8890	19785	8737	10532
ENSECAG00000017991	8.623704066	0.268900429	0.685533014	5771	6887	9515	11414	5490	6255	9354	6894
ENSECAG00000011841	1.907105015	0.268953792	0.685533014	47	51	55	51	61	34	173	116
ENSECAG00000007696	2.445127755	0.269013518	0.685533014	87	194	52	116	44	64	101	157
ENSECAG00000000095	4.75075829	0.269088348	0.685533014	315	360	434	566	623	410	906	679
ENSECAG00000017930	5.091631089	0.269095041	0.685533014	552	939	641	570	639	767	572	452
ENSECAG00000018511	0.884934802	0.269161115	0.685547941	18	20	34.9999	30	41	32	67	37
ENSECAG00000020181	7.330305452	0.26928062	0.685566188	1618	1444	2566	4150	2596	2396	5012	6380
ENSECAG00000000755	6.394538302	0.269337914	0.685566188	750	874	1171	2552	2039	1136	2782	2439
ENSECAG00000017559	1.976667606	0.269357234	0.685566188	51	86	87	103	89	54	89	38
ENSECAG00000024525	0.28798606	0.269420879	0.685566188	20	12	35	31	31	9	20	15
ENSECAG00000017734	4.468023493	0.269517201	0.685566188	275	326	452	837	523	330	349	385
ENSECAG00000015182	2.757155728	0.26954213	0.685566188	94	57	102	142	151	127	169	212
ENSECAG00000016082	7.825688285	0.269589796	0.685566188	4738	4807	4222	4380	4153	3399	5017	3465
ENSECAG00000008692	6.25607104	0.269667037	0.685609472	827	2030	1440	2157	848	1440	1709	1533
ENSECAG00000007385	6.671398317	0.269770536	0.685719481	5588	735	482	415	2075	1192	2079	797

ENSECAG00000009197	4.757849692	0.270155628	0.686438652	429	418	261	465	809	475	888	475
ENSECAG00000020104	5.759563225	0.270174054	0.686438652	392	972	947	1145	1121	1383	1192	1486
ENSECAG00000014732	2.386626571	0.27043037	0.686936581	29	60	128	97	104	68	162	189
ENSECAG00000023223	5.430810565	0.270491932	0.686939692	504	879	1048	1153	644	913	678	887
ENSECAG00000009340	3.887682278	0.270800012	0.687521695	167	337	279	457	239	287	235	300
ENSECAG00000006013	4.430741262	0.27084188	0.687521695	203	259	490	386	556	675	486	334
ENSECAG00000016654	5.455321531	0.271016347	0.687572226	347	863	508	1068	744	977	1261	1283
ENSECAG00000006779	5.137791736	0.271029778	0.687572226	372	464	649	725	1139	596	868	765
ENSECAG00000022948	4.441733163	0.271042964	0.687572226	298	487	465	503	380	410	378	418
ENSECAG00000012399	7.539221499	0.271147403	0.687626538	2605	1649	3651	2589	6832	3766	5782	2333
ENSECAG00000020587	8.039483056	0.271227324	0.687626538	2925	7265	9518	5091	421	6429	302	7921
ENSECAG00000019822	7.19901403	0.271245567	0.687626538	2687	3461	2670	3092	2262	2198	3988	2091
ENSECAG00000014679	2.703374647	0.271392485	0.687658491	73	109	73	122	139	73	281	165
ENSECAG00000019014	2.5514356	0.271529096	0.687658491	51	94	102	101	154	104	233	89
ENSECAG00000000223	5.64589525	0.271650195	0.687658491	702	1208	1033	1327	288	1230	402	1461
ENSECAG00000012932	8.508133251	0.271704752	0.687658491	3524	4945	5145	8567	9440	5874	11833	9443
ENSECAG00000022649	2.789598556	0.271767261	0.687658491	167	90	159	126	170	91	105	106
ENSECAG00000017794	8.78852159	0.271796521	0.687658491	3497	10227	15033	11801	3681	14216	2723	7296
ENSECAG00000017997	2.234753268	0.271817682	0.687658491	22	66	80	114	163	67	194	52
ENSECAG00000023648	5.066997694	0.271862529	0.687658491	411	479	515	674	860	562	1083	732
ENSECAG00000005394	4.809536016	0.271903608	0.687658491	223	494	524	1280	359	481	412	731
ENSECAG00000019984	0.955359763	0.271979703	0.687658491	16	18	29	48	24	45	53	64
ENSECAG00000022127	8.511452603	0.27198149	0.687658491	17755	2093	8492	1885	196	15007	113	1699
ENSECAG00000000544	1.685855526	0.272047679	0.687658491	37	136	55	39	16	70	45	73
ENSECAG00000003080	4.860581889	0.272081186	0.687658491	282	287	585	697	548	559	788	915
ENSECAG00000020820	6.305050415	0.272103777	0.687658491	937	1285	1174	1407	2423	1720	1944	1523
ENSECAG00000017646	6.578324058	0.272394401	0.68824018	709	1845	1441	1971	2396	2347	3027	1588
ENSECAG00000020408	5.523174104	0.272564347	0.688516773	653	742	594	762	1371	808	1260	993
ENSECAG00000010584	5.12138474	0.272727675	0.68867087	559	790	659	770	517	433	771	856
ENSECAG00000022626	4.414954283	0.272746329	0.68867087	267	264	338	452	512	349	700	504
ENSECAG00000004755	7.68209714	0.272857291	0.688695628	2486	6998	3626	4308	1892	4293	2843	4980
ENSECAG00000007518	5.893883489	0.272918268	0.688695628	1852	2819	269	153	46	2337	33	479
ENSECAG00000000475	4.804604338	0.272937608	0.688695628	265	328	684	473	683	679	797	522
ENSECAG00000018142	6.191915215	0.273056674	0.688747596	480	864	899	2425	1016	1278	1777	3261
ENSECAG00000022851	1.154828893	0.273079196	0.688747596	21	28	29	49	29	67	48	67
ENSECAG00000022918	7.333260105	0.273214864	0.688937148	2529	5089	2824	2736	3189	3254	2528	2130
ENSECAG00000022984	3.025121159	0.273526339	0.689569833	35	66	529	132	68	79	157	220
ENSECAG00000013810	1.302447477	0.273754473	0.689857017	13	61	135	32	18	96	3	0
ENSECAG00000019669	6.611211411	0.27379017	0.689857017	1005	1193	1973	1882	2305	1956	2994	2308
ENSECAG00000015966	6.39797664	0.273858871	0.689857017	1173	1071	1554	1096	2788	1550	2832	1167
ENSECAG00000010848	4.825104882	0.273964443	0.689857017	300	494	532	1111	397	527	604	542
ENSECAG00000020158	6.386229384	0.274005011	0.689857017	840	867	1791	1554	2777	1700	3067	804
ENSECAG00000009149	6.709218636	0.27403109	0.689857017	842	2459	1754	4913	444	1016	1015	4376
ENSECAG00000018000	6.160115078	0.274073431	0.689857017	1023	1333	1235	2370	1212	1265	1321	1395
ENSECAG00000009246	3.024090374	0.274125002	0.689857017	243	192	80	96	152	107	124	164
ENSECAG00000009796	1.42232236	0.27424515	0.68988415	25	16	67	44	66	46	61	87
ENSECAG00000010942	7.805198321	0.274256975	0.68988415	5809	4612	3454	3492	3883	3926	3763	3855
ENSECAG00000016736	4.655259438	0.274411634	0.689982444	292	257	459	572	721	523	654	511
ENSECAG00000001215	2.024478439	0.27441726	0.689982444	42	74	126	101	47	57	96	83
ENSECAG00000013898	2.895077424	0.274568378	0.690209975	69	124	120	134	183	123	289	139
ENSECAG00000023417	8.665824447	0.274769095	0.690441139	14274	9648	3741	3094	3098	9926	7206	5798
ENSECAG00000015379	4.910226665	0.274781625	0.690441139	1057	733	326	204	85	1090	208	414
ENSECAG00000019473	1.63142231	0.274942242	0.690692283	15	40	35	99	91	50	97	66
ENSECAG00000019359	3.864147564	0.275035302	0.690773641	90	335	240	657	313	278	218	193
ENSECAG00000002756	3.10623684	0.275134549	0.690870497	91	93	183	145	227	141	359	131
ENSECAG00000017694	6.054719448	0.275347878	0.691033215	872	773	982	1429	1254	1003	2385	1935
ENSECAG00000011550	5.397841067	0.275378736	0.691033215	657	1433	591	673	562	817	704	917
ENSECAG00000018395	9.435818079	0.27538144	0.691033215	15692	28570	11246	5581	650	26060	609	9162
ENSECAG00000007541	6.438683776	0.275511146	0.691206345	1470	1882	1135	2694	1160	1881	1150	1907
ENSECAG00000020598	8.068190853	0.275635727	0.691366546	4599	8965	3676	4671	2821	5455	4792	5389
ENSECAG00000013633	0.77502964	0.275763632	0.691535012	12	22	69	50	11	24	7	59
ENSECAG00000005768	4.059991295	0.275988174	0.691549541	199	192	280	353	410	210	580	437
ENSECAG00000013481	7.101523177	0.276144981	0.691549541	3862	4206	1240	1295	2228	1523	4090	1533
ENSECAG00000017217	3.523528329	0.276180853	0.691549541	671	127	15	29	47	368	30	144
ENSECAG00000004944	5.296771399	0.276191609	0.691549541	397	362	827	888	1158	702	1312	665
ENSECAG00000023813	3.546333022	0.276234832	0.691549541	180	214	441	251	11	259	9	382
ENSECAG00000000047	4.538745021	0.276323941	0.691549541	207	410	374	435	972	363	579	335
ENSECAG00000000420	3.967346617	0.276327302	0.691549541	173	474	233	418	237	297	356	238
ENSECAG00000021237	2.19483613	0.276429682	0.691549541	43	54	61	119	124	76	113	130
ENSECAG00000011549	3.307736938	0.276457303	0.691549541	139	240	208	213	167	201	175	162
ENSECAG00000020629	3.396333503	0.276491724	0.691549541	86	105	178	296	165	242	265	353
ENSECAG00000007822	6.461551079	0.276523043	0.691549541	1306	1992	1738	2145	1670	1592	1471	1624
ENSECAG00000002556	2.810690124	0.276583657	0.691549541	103	47	146	84	219	58	309	138
ENSECAG00000013120	7.542109414	0.276643899	0.691549541	2782	4684	4352	3733	1834	5049	2647	3192
ENSECAG00000006840	4.206662788	0.276664733	0.691549541	158	204	257	541	167	499	737	443
ENSECAG00000014958	5.222350085	0.276718839	0.691549541	429	1232	516	898	576	708	603	789

ENSECAG00000010620	0.254278777	0.276741301	0.691549541	37	20	25	12	2	23	0	37
ENSECAG00000022714	1.44777896	0.276835705	0.691633641	16	22	86	129	36	30	75	38
ENSECAG00000016486	5.380085366	0.277034385	0.691978165	377	1301	560	1318	905	624	888	578
ENSECAG00000013403	3.407937616	0.277137667	0.692084304	114	109	207	221	279	209	361	185
ENSECAG00000011310	3.532045609	0.277450374	0.692557206	146	130	181	235	318	174	470	185
ENSECAG00000003368	7.545609612	0.277492973	0.692557206	2542	2273	2798	3105	7643	2789	5323	2915
ENSECAG00000017929	7.401476598	0.277509528	0.692557206	3252	5163	2904	2147	3070	3241	3180	2179
ENSECAG00000011445	3.904307588	0.277718576	0.692792716	242	298	345	316	299	317	290	165
ENSECAG00000008684	7.256336186	0.2777256	0.692792716	1829	1564	2189	3707	3250	2739	4632	4609
ENSECAG00000000887	5.624594189	0.277814733	0.692863249	844	691	1405	1060	1280	801	933	518
ENSECAG00000011647	8.450042572	0.278121611	0.693215441	6522	14416	3612	4169	3649	10571	4243	3890
ENSECAG00000005196	1.012158749	0.278253597	0.693215441	7	43	110	34	2	48	1	52
ENSECAG00000000648	4.825548936	0.27826467	0.693215441	318	360	512	587	627	416	898	808
ENSECAG00000023793	5.071677374	0.278265164	0.693215441	403	424	511	772	723	537	1032	961
ENSECAG00000000826	1.136804677	0.278358245	0.693215441	23	20	37	45	45	32	85	52
ENSECAG00000017290	7.211551239	0.278388908	0.693215441	2135	3180	3076	4040	2656	3245	2593	1907
ENSECAG00000011084	1.850557746	0.27839254	0.693215441	35	33	57	88	59	55	101	139
ENSECAG00000015274	2.467508119	0.278472955	0.693215441	61	104	52	104	132	75	189	151
ENSECAG00000006577	4.601678759	0.278599345	0.693215441	311	294	351	539	619	316	738	690
ENSECAG00000020149	4.113279488	0.278614186	0.693215441	193	198	287	405	438	255	473	506
ENSECAG00000005320	7.135948822	0.278625723	0.693215441	2786	2977	2680	2832	2741	2771	2443	1906
ENSECAG00000010623	2.677236939	0.278899742	0.693367962	65	99	105	115	160	114	161	181
ENSECAG00000001181	4.550102318	0.278922828	0.693367962	145	233	596	520	785	482	650	362
ENSECAG00000008101	5.551092717	0.278922878	0.693367962	588	412	821	1075	1375	678	1617	934
ENSECAG00000016465	6.071207931	0.27895987	0.693367962	1613	1097	1105	1382	1319	1045	1328	1155
ENSECAG00000017912	2.781949172	0.278991536	0.693367962	80	69	204	254	85	79	99	207
ENSECAG00000005758	5.788569377	0.279065525	0.693400481	704	1201	1066	1598	718	1062	1122	1156
ENSECAG00000022758	0.848020957	0.279376054	0.693878732	13	16	35	40	45	41	35	47
ENSECAG00000004617	6.201597869	0.279379895	0.693878732	482	1024	1390	1841	1328	1812	2663	1443
ENSECAG00000009168	7.155278506	0.279562435	0.694178786	860	1522	6658	4496	1923	2504	640	4015
ENSECAG00000014859	3.271193026	0.279622654	0.694178786	135	123	132	173	216	125	415	209
ENSECAG00000009324	5.430379435	0.279758286	0.694307296	967	1138	665	541	577	1082	690	665
ENSECAG00000008193	7.326027446	0.279796388	0.694307296	10698	1301	399	580	6	5990	4	154
ENSECAG00000004107	5.403344366	0.279993227	0.694576368	298	684	1007	515	2154	755	849	403
ENSECAG00000019270	5.045321857	0.280026836	0.694576368	550	1211	419	471	353	978	398	504
ENSECAG00000021194	4.7821373	0.280135939	0.694685583	358	1081	412	361	511	555	403	452
ENSECAG00000016019	6.678610597	0.280197431	0.694685583	2530	2625	1477	1260	2173	1893	1849	1155
ENSECAG00000014533	5.440152452	0.28025392	0.694685583	620	1298	807	748	583	655	816	1104
ENSECAG00000015829	2.822202715	0.280345371	0.694761002	41	82	100	221	107	118	217	264
ENSECAG00000009317	5.335765116	0.280428247	0.694777557	543	835	976	947	626	842	514	922
ENSECAG00000013346	4.978677574	0.280483702	0.694777557	525	824	464	682	462	649	620	566
ENSECAG00000017555	5.012186114	0.280581616	0.694777557	335	370	624	704	980	449	1006	696
ENSECAG00000012825	4.089240446	0.280596153	0.694777557	258	259	213	244	245	266	436	732
ENSECAG00000005556	6.142651333	0.280792743	0.695113151	400	1118	1240	1813	1185	1803	1949	1951
ENSECAG00000022687	6.798493469	0.280946419	0.695118604	1253	1619	1606	2290	3084	1633	3561	2741
ENSECAG00000010784	4.305133721	0.280973092	0.695118604	190	344	389	792	268	325	407	438
ENSECAG00000024955	1.189672631	0.280978113	0.695118604	29	84	23	55	7	42	39	56
ENSECAG00000024941	3.527172455	0.281086139	0.695178796	124	152	216	214	210	195	355	362
ENSECAG00000012942	1.340770174	0.281124565	0.695178796	40	4.00004	60	1.00012	42	41	181	24
ENSECAG00000017731	4.628602254	0.281278423	0.695408218	282	752	404	590	460	325	500	522
ENSECAG00000024080	2.847836032	0.281366421	0.695445151	64	98	123	154	108	127	266	211
ENSECAG00000010919	9.608587588	0.28141553	0.695445151	15991	25035	11674	10209	10308	20662	12132	8731
ENSECAG00000009562	6.75382952	0.281503685	0.695512034	847	1221	4875	2775	1597	2463	1227	1962
ENSECAG00000019333	6.747858567	0.281761353	0.695845341	2673	2426	2074	1174	1896	2111	961	2271
ENSECAG00000011248	6.188594878	0.281810142	0.695845341	1402	1674	1308	1351	1251	1583	1114	1250
ENSECAG00000013545	5.035662196	0.281821947	0.695845341	381	392	639	650	794	702	860	763
ENSECAG00000005354	3.446494397	0.282163941	0.696359123	124	65	215	245	406	157	382	144
ENSECAG00000013239	5.515746676	0.282179955	0.696359123	1395	337	948	839	624	711	566	1245
ENSECAG00000020260	7.784744722	0.282289887	0.696359123	2843	7780	3613	4276	2533	5902	3085	3387
ENSECAG00000012222	6.13407237	0.282332847	0.696359123	1306	3722	566	540	89	2882	151	689
ENSECAG00000018934	0.430939754	0.282335855	0.696359123	12	9	35	77	0	24	1	42
ENSECAG00000006769	5.079326742	0.282491336	0.696591695	436	295	805	500	1085	579	905	705
ENSECAG00000011970	4.381557855	0.28255983	0.696609715	133	167	294	774	359	510	503	669
ENSECAG00000020595	4.042084702	0.28268267	0.696761679	252	401	307	351	310	299	332	259
ENSECAG00000006589	6.421085344	0.282865925	0.697062458	1319	2213	1408	2091	1022	1161	1349	2603
ENSECAG00000017216	3.063252604	0.282939102	0.697091902	97	161	225	221	106	91	157	240
ENSECAG00000009270	4.193251286	0.283002591	0.697097468	186	220	323	414	259	312	583	639
ENSECAG00000003235	4.576110203	0.283107623	0.69720534	202	380	373	572	372	510	768	660
ENSECAG00000017977	3.886166526	0.284207944	0.699763719	159.542	112.631	228.968	430.401	355.357	268.09	504.858	321.257
ENSECAG00000011330	1.797330219	0.284324345	0.699898956	21	34	62	89.0001	137	54	121	33
ENSECAG00000024957	4.63685428	0.284463775	0.699964259	129	488	373	604	606	777	445	541
ENSECAG00000020031	4.789839904	0.284483222	0.699964259	371	580	572	734	626	386	665	370
ENSECAG00000015449	6.397755598	0.284565756	0.699964259	719	1118	1262	2291	2194	1190	2465	2427
ENSECAG00000006366	7.671676898	0.284695718	0.699964259	2224	7188	3380	4460	2538	4434	3316	3812
ENSECAG00000012176	5.109112184	0.284818853	0.699964259	724	1047	403	486	406	602	694	766
ENSECAG00000018244	4.046121427	0.284822058	0.699964259	141	199	355	356	361	310	558	373

ENSECAG00000022106	5.366774718	0.284842478	0.699964259	373	354	1048	845	934	969	1130	946
ENSECAG00000020670	2.887725629	0.285056479	0.699964259	77	91	113	171	133	133	207	247
ENSECAG00000016402	3.264914934	0.285082592	0.699964259	91	152	394	199	176	243	166	58
ENSECAG00000011004	6.137670811	0.285218673	0.699964259	724	1459	1240	2659	1051	1438	1189	1376
ENSECAG00000007635	3.653132889	0.28525409	0.699964259	125	141	212	302	479	172	298	267
ENSECAG00000015034	8.063169752	0.285329009	0.699964259	4708	2779	2473	4975	5053	5020	9142	7902
ENSECAG00000015479	4.039206742	0.285388464	0.699964259	187	336	391	450	213	281	323	385
ENSECAG00000018523	4.423401339	0.285393903	0.699964259	288	243	325	459	666	317	652	439
ENSECAG00000010957	3.020114986	0.285757496	0.699964259	74	82	170	172	145	128	274	253
ENSECAG00000023909	5.651281503	0.28583781	0.699964259	539	459	982	1190	1359	719	1777	1077
ENSECAG00000016473	6.678535708	0.285877237	0.699964259	1162	2343	2068	3104	1340	1908	2249	1923
ENSECAG00000022087	2.174522871	0.285892499	0.699964259	26	71	37	133	66	108	51	221
ENSECAG00000009621	4.78624638	0.285982748	0.699964259	272	316	608	522	644	478	1195	413
ENSECAG00000006771	5.245902052	0.286042633	0.699964259	706	406	1165	819	969	614	807	294
ENSECAG00000009627	6.045023421	0.28607271	0.699964259	1380	1677	1142	892	1102	792	1627	1295
ENSECAG00000016645	5.034922603	0.286081908	0.699964259	316	670	897	890	420	768	450	705
ENSECAG00000022727	4.046560729	0.286100287	0.699964259	232	190	276	296	475	302	528	292
ENSECAG00000000354	6.792628825	0.286136642	0.699964259	632	1536	2160	2296	1091	3511	1001	5489
ENSECAG00000018650	4.799609768	0.286148928	0.699964259	345	348	228	785	697	308	1174	622
ENSECAG00000000591	5.692348757	0.286149842	0.699964259	1693	996	669	486	558	1388	546	939
ENSECAG00000023324	5.761658944	0.286291112	0.699964259	611	504	1181	1073	1733	985	1569	967
ENSECAG00000009435	2.810906556	0.286299555	0.699964259	59	84	128	161	213	125	233	114
ENSECAG00000001985	3.054055464	0.286305305	0.699964259	193	268	96	80	127	146	130	159
ENSECAG00000022080	6.604546116	0.286364687	0.699964259	1461	2351	2185	1971	1148	2347	1193	2122
ENSECAG00000017083	1.937064107	0.286365664	0.699964259	52	74	97	93	59	67	39	93
ENSECAG00000012316	3.632528401	0.286379489	0.699964259	237	257	250	239	102	255	214	307
ENSECAG00000021401	6.62839997	0.286379756	0.699964259	1765	1634	2353	2210	1911	1564	2161	1550
ENSECAG00000011354	4.29023391	0.286724156	0.700480323	251	524	369	488	226	638	222	253
ENSECAG00000023182	2.912805307	0.286751213	0.700480323	84	78	167	122	205	160	174	183
ENSECAG00000009325	4.401560162	0.286834385	0.700480323	208	292	347	498	533	310	586	604
ENSECAG00000012523	2.011426376	0.286837002	0.700480323	42	45	68	85	99	67	131	94
ENSECAG00000009116	5.850445356	0.287138137	0.700883325	891	1149	1177	1423	890	924	1218	1244
ENSECAG00000000547	5.792912237	0.287217588	0.700883325	620	594	977	1306	1490	812	1718	1369
ENSECAG00000021411	2.084348321	0.287243035	0.700883325	24	129	98	119	47	57	74	109
ENSECAG00000013495	6.506196467	0.287248274	0.700883325	1447	1101	2035	3053	1256	1243	2028	2092
ENSECAG00000023224	6.626350839	0.287344809	0.700887241	1077	1016	1702	2366	1985	1670	3336	2777
ENSECAG00000020781	5.573611925	0.287476431	0.700887241	524	715	953	779	1221	977	1286	1055
ENSECAG00000016810	0.644345833	0.287478752	0.700887241	9	28	17	25	102	9	35	8
ENSECAG00000023227	8.191561108	0.287496128	0.700887241	3990	9611	5517	5274	2975	7775	4810	4362
ENSECAG00000021568	0.747570101	0.287643743	0.700923589	32	28	10	6	48	47	31	36
ENSECAG00000020676	7.070277811	0.287872921	0.700923589	1770	3539	2512	3365	2273	1884	3309	2257
ENSECAG00000014466	6.025766184	0.287905498	0.700923589	1232	1275	1173	1446	1073	1234	1206	1247
ENSECAG00000021486	6.029802427	0.287915927	0.700923589	1236	932	1682	1381	1375	1476	935	838
ENSECAG00000019476	8.467526763	0.287953543	0.700923589	10147	5848	6411	5018	7783	5024	8252	3244
ENSECAG00000013581	8.930585776	0.287999919	0.700923589	7080	15596	7393	10431	6655	9380	9726	8302
ENSECAG00000016627	0.790563912	0.288026171	0.700923589	16	53	43	27	23	22	38	31
ENSECAG00000006633	1.839971878	0.288059169	0.700923589	18	72	92	150	21	48	37	123
ENSECAG00000021172	4.034597948	0.28807713	0.700923589	142	199	229	466	333	174	519	600
ENSECAG00000010522	5.993271885	0.288184844	0.700923589	941	568	1812	2336	2400	549	842	483
ENSECAG00000008252	5.570016009	0.288188258	0.700923589	449	515	1132	886	1524	889	1409	773
ENSECAG00000008738	7.668971534	0.288316469	0.70102507	5687	3122	4540	2267	2961	3403	3509	4152
ENSECAG00000010902	4.794673842	0.288353131	0.70102507	472	798	415	514	282	675	449	568
ENSECAG00000009490	5.663609612	0.288736806	0.701618085	661	1066	1004	1415	908	749	1010	1095
ENSECAG00000022491	4.749978495	0.288741189	0.701618085	317	342	471	563	705	505	725	626
ENSECAG00000012492	5.618904343	0.288792345	0.701618085	588	400	1164	822	1582	892	1714	648
ENSECAG00000016216	6.059904528	0.288843563	0.701618085	1515	1632	1178	768	995	1361	1276	1112
ENSECAG00000010532	8.279910877	0.289032817	0.701900778	4705	8227	5557	7252	4553	6604	4876	5665
ENSECAG00000026822	3.230559204	0.289130676	0.701900778	126	108	140	187	287	145	257	218
ENSECAG00000015319	2.318914363	0.289144897	0.701900778	54	82	70	85	66	107	186	132
ENSECAG00000020230	4.914041848	0.289328919	0.702119994	347	732	562	868	577	539	339	707
ENSECAG00000014571	1.904225831	0.289358543	0.702119994	31	50	52	83	156	24	116	77
ENSECAG00000023113	1.642712026	0.289473451	0.702249145	32	32	49	69	88	32	107	79
ENSECAG00000020432	8.382898497	0.289535501	0.702250039	8212	9154	5349	3320	6710	5014	7887	3274
ENSECAG00000000372	5.048357757	0.289907109	0.702754546	394	413	620	646	868	597	987	715
ENSECAG00000016067	4.65481107	0.289936803	0.702754546	388	256	412	440	613	368	1001	503
ENSECAG00000013901	7.606668437	0.290096873	0.702754546	3837	4563	2862	4387	3431	3024	4259	3184
ENSECAG00000001076	0.827068069	0.290097055	0.702754546	63	11	33	37	0	17	10	71
ENSECAG00000006588	5.403366007	0.290165305	0.702754546	729	657	780	1256	645	560	997	948
ENSECAG00000019816	4.084834985	0.29021654	0.702754546	340	347	220	427	291	333	287	310
ENSECAG00000019750	6.386102105	0.29023482	0.702754546	1718	1120	2189	1700	1721	2004	1378	716
ENSECAG00000016926	4.761394443	0.290257717	0.702754546	483	430	528	715	510	400	599	505
ENSECAG00000015324	5.691563696	0.29034125	0.702754546	995	1035	1043	991	1121	1064	1019	514
ENSECAG00000026814	3.346251756	0.290360771	0.702754546	95	105	215	211	255	115	360	276
ENSECAG00000010799	3.852338689	0.290453466	0.702829482	121	125	249	427	313	275	273	536
ENSECAG00000007112	4.168204775	0.290552332	0.702919315	140	134	332	539	677	445	361	249
ENSECAG00000013959	2.269732332	0.290625673	0.702947373	51	77	61	94	101	58	149	166

ENSECAG00000014222	4.330428845	0.29077953	0.703125974	182	887	297	352	166	666	167	310
ENSECAG00000011846	6.850354898	0.2908588	0.703125974	1078	883	2234	2893	2145	1684	2491	5247
ENSECAG00000003319	3.650632848	0.291053196	0.703125974	131	180	192	277	267	175	388	392
ENSECAG00000001822	0.551975784	0.291056115	0.703125974	6	22	28	26	52	35	30	19
ENSECAG00000011182	4.561679525	0.291059294	0.703125974	353	579	368	643	202	292	469	749
ENSECAG00000014036	4.160812675	0.291070067	0.703125974	354	616	208	217	193	397	244	397
ENSECAG00000015940	5.05757748	0.291187286	0.703195275	392	398	540	778	855	560	981	798
ENSECAG00000009622	3.244554575	0.291307901	0.703195275	100	107	187	177	292	102	354	191
ENSECAG00000024781	3.204786685	0.291360143	0.703195275	115	123	128	188	277	128	289	203
ENSECAG00000016396	6.050222224	0.291381276	0.703195275	697	824	1278	1394	1370	1312	2007	1707
ENSECAG00000021588	4.96172758	0.291407581	0.703195275	310	644	572	1172	364	382	543	970
ENSECAG00000014948	3.590836456	0.291488968	0.703242615	120	136	209	297	310	225	345	277
ENSECAG00000009675	4.978473927	0.291620322	0.70341046	558	394	797	780	463	466	862	557
ENSECAG00000022311	3.706517463	0.29169003	0.703429569	149	217	303	440	322	200	254	159
ENSECAG00000019542	4.382172863	0.291752319	0.703430783	279	231	582	662	367	316	319	502
ENSECAG00000007350	4.591358336	0.292403882	0.704481834	151	365	447	582	872	602	448	370
ENSECAG00000015820	5.19248365	0.29243527	0.704481834	506	470	1162	981	1085	329	929	252
ENSECAG00000020125	5.0169805	0.292455989	0.704481834	293	435	823	474	935	647	742	763
ENSECAG00000001649	8.944451047	0.292581754	0.704481834	3506	8054	5601	13409	8352	10381	12461	18196
ENSECAG00000018655	4.27447128	0.292652745	0.704481834	484	430	253	291	380	269	451	296
ENSECAG00000017313	2.966473226	0.292679765	0.704481834	141	151	111	281	59	34	61	355
ENSECAG00000023202	5.466652766	0.292747455	0.704481834	863	1030	728	795	799	798	856	782
ENSECAG00000012505	2.744236852	0.292771567	0.704481834	77	170	138	175	69	78	146	183
ENSECAG00000022850	1.522176091	0.292836948	0.704481834	30	55	21	53	35	50	143	62
ENSECAG00000007579	4.938871326	0.292904204	0.704481834	252	498	862	1042	463	656	562	537
ENSECAG00000009479	3.688559418	0.292922792	0.704481834	228	289	60	71	370	206	514	240
ENSECAG00000021595	3.448888773	0.292930786	0.704481834	110	138	175	255	332	145	363	225
ENSECAG00000002701	6.752205198	0.293008666	0.704520309	2467	1525	1925	2787	953	1357	2147	3202
ENSECAG00000007807	4.757942938	0.293202804	0.704838245	197	1165	433	490	510	625	329	387
ENSECAG00000008227	3.825158796	0.293536763	0.705347088	220	521	209	179	197	369	222	185
ENSECAG00000022018	0.336486414	0.293538384	0.705347088	30	12	31	23	17	28	14	18
ENSECAG00000024114	8.172321395	0.293768581	0.705751274	3589	3835	4567	5191	7267	5187	7357	8613
ENSECAG00000018032	5.322522236	0.293860357	0.705753002	670.007	926	660	953	423	928	607	918
ENSECAG00000024672	5.490153539	0.293893279	0.705753002	435	457	844	1144	1398	780	1365	802
ENSECAG00000024184	4.065749201	0.294126916	0.706165107	115	189	387	357	699	186	454	303
ENSECAG00000011962	2.168102752	0.294319789	0.706479189	93	145	57	55	49	95	59	95
ENSECAG00000011806	7.045752328	0.294502039	0.70671642	1591	1488	2271	2635	4251	2212	3957	2596
ENSECAG00000003789	0.718137192	0.294542768	0.70671642	21	49	37	28	9	56	9	23
ENSECAG00000009069	9.94873818	0.294741537	0.707044332	7820	20394	14006	17536	27389	22842	26870	20592
ENSECAG00000011403	6.504247508	0.294952799	0.707268127	1075	1597	2316	2748	1255	994	1601	2720
ENSECAG00000014365	2.675781408	0.294959075	0.707268127	82	79	104	108	168	80	236	146
ENSECAG00000018065	1.153251206	0.295128059	0.707496099	29	29	25	39	85	33	59	35
ENSECAG00000020042	1.592469642	0.295178434	0.707496099	35	25	60	54	90	70	73	53
ENSECAG00000005386	2.665557989	0.295298379	0.70761559	92	156	132	134	68	149	102	122
ENSECAG00000008311	7.246960825	0.295467007	0.70761559	2713	3278	2948	3363	2229	3425	2387	2654
ENSECAG00000016883	8.203399155	0.29554013	0.70761559	5478	8083	5202	4870	4646	6515	5058	4264
ENSECAG00000009949	3.512304806	0.295612218	0.70761559	102	148	188	278	303	141	419	259
ENSECAG00000012229	8.948713718	0.295806008	0.70761559	5017	2371	12976	8699	12685	7335	12405	17860
ENSECAG00000007347	1.155985029	0.295856216	0.70761559	12	34	35	46	19	28	88	88
ENSECAG00000024243	10.12170852	0.295897031	0.70761559	16146	25144	20857	31544	20308	18992	21171	18300
ENSECAG00000000870	2.459127625	0.295902775	0.70761559	69	65	102	83	178	79	162	115
ENSECAG00000017941	5.7868196	0.295915938	0.70761559	721	1903	934	911	951	1332	1063	560
ENSECAG00000019023	6.49540454	0.295921902	0.70761559	2186	2609	1045	1094	1227	2254	1288	1404
ENSECAG00000008094	5.001739309	0.295940967	0.70761559	262	522	470	1686	422	291	635	938
ENSECAG00000014601	9.767956187	0.295974127	0.70761559	25682	28302	16562	5300	445	34880	251	8392
ENSECAG00000008003	4.209687109	0.296048086	0.707643809	267	330	426	469	322	296	442	316
ENSECAG00000019662	5.719928617	0.296161868	0.707650587	841	1909	719	688	929	1077	849	867
ENSECAG00000015075	1.988225512	0.296233401	0.707650587	35	65	63	72	93	115	115	56
ENSECAG00000025328	2.454621937	0.296237391	0.707650587	17	85	284	118	103	121	56	66
ENSECAG00000026842	1.693860461	0.296488992	0.707884911	54	53	113	50	23	35	18	129
ENSECAG00000021290	6.567396909	0.296557517	0.707884911	1169	3802	1191	1922	577	3263	916	1414
ENSECAG00000022446	6.40328057	0.296583809	0.707884911	433	1137	1346	2542	1286	1575	1739	3789
ENSECAG00000005752	5.137284734	0.296615385	0.707884911	259	419	811	793	970	776	1008	619
ENSECAG00000016077	7.783403932	0.296676081	0.707884911	7501	2562	2759	3807	3424	3798	4536	3351
ENSECAG00000017593	4.676287289	0.296739413	0.707884911	309	492	581	717	426	467	481	515
ENSECAG00000014992	0.718209655	0.296813052	0.707884911	25	29	20	60	20	19	30	39
ENSECAG00000008005	6.894946283	0.2968329	0.707884911	1421	2127	1538	2039	2656	3207	2989	2685
ENSECAG00000012562	4.17784313	0.296896033	0.707887192	214	311	460	516	288	252	367	428
ENSECAG00000022769	3.679278296	0.297088062	0.707984213	135	125	168	371	461	159	374	259
ENSECAG00000004476	2.695736922	0.297108682	0.707984213	46	75	119	167	193	137	179	115
ENSECAG00000018531	2.857598881	0.297123282	0.707984213	78	53	340	160	110	142	97	136
ENSECAG00000013141	4.927434656	0.297270056	0.708185727	316	600	532	1146	481	663	551	517
ENSECAG00000017650	6.224151525	0.297388462	0.708319589	891	1902	1430	1960	982	1567	1409	1476
ENSECAG00000010916	2.603955266	0.297462672	0.708348153	64	122	150	186	79	71	223	62
ENSECAG00000011326	4.943802048	0.297605992	0.708541242	475	539	759	679	533	464	602	688
ENSECAG00000021133	4.510003933	0.297761011	0.708717477	344	331	238	447	592	290	738	604

ENSECAG00000004395	1.219465915	0.297820708	0.708717477	21	48	37	18	19	60	78	72
ENSECAG00000013659	4.355518949	0.297947736	0.708717477	476	418	391	297	269	632	236	263
ENSECAG00000010529	4.341630485	0.297958555	0.708717477	315	431	422	434	366	344	436	358
ENSECAG00000008235	1.178718103	0.298088035	0.708717477	19	14	55	36	33	38	26	124
ENSECAG00000006963	0.846259163	0.298120129	0.708717477	8	36	78	44	4	34	3	59
ENSECAG00000000517	5.841918567	0.298171555	0.708717477	769	1232	1216	1461	705	1213	1108	1185
ENSECAG00000023501	7.644970657	0.298201964	0.708717477	2053	3784	2357	3751	5191	2551	8369	4207
ENSECAG00000014738	5.618616189	0.298347414	0.708717477	466	855	981	1954	710	744	1006	1153
ENSECAG00000005643	3.238701544	0.298349935	0.708717477	105	92	171	209	163	213	184	347
ENSECAG00000024986	5.300525548	0.298406956	0.708717477	661	505	625	416	1561	835	1021	418
ENSECAG00000007804	7.030143957	0.298427017	0.708717477	3345	3562	1626	1629	1158	3675	1865	2042
ENSECAG00000015840	0.693717302	0.298537907	0.708718073	4	18	29	44	23	46	42	42
ENSECAG00000018429	5.064407094	0.298601418	0.708718073	501	604	661	934	570	472	779	688
ENSECAG00000024988	5.349175267	0.298614018	0.708718073	448	820	350	863	672	536	1435	1411
ENSECAG00000005213	6.337737683	0.299004526	0.709496983	1725	1597	1395	1579	1026	1487	1535	1815
ENSECAG00000002607	1.383135214	0.299164923	0.709714336	25	23	42	65	64	48	78	59
ENSECAG00000012813	2.779533404	0.299220801	0.709714336	101	95	125	258	108	82	142	157
ENSECAG00000024286	8.098964923	0.299344508	0.709859867	8037	8363	2682	2491	2082	10290	2307	2115
ENSECAG00000019292	3.843250193	0.29942267	0.709897356	193	446	259	256	168	271	227	358
ENSECAG00000001674	0.997245763	0.299516056	0.709970914	16	25	24	47	29	15	108	50
ENSECAG00000009610	5.9411573	0.299651947	0.710145175	476	484	1126	1919	1143	1098	1805	2002
ENSECAG00000000711	4.554874682	0.29977165	0.710281006	244	256	531	439	766	346	644	505
ENSECAG00000011523	2.283322014	0.299903204	0.710368719	61	102	83	178	32	60	53	180
ENSECAG00000022309	4.995557295	0.299933459	0.710368719	216	425	607	835	999	611	952	511
ENSECAG00000021163	4.56577216	0.300157548	0.710751599	173	253	619	479	566	487	607	611
ENSECAG00000011900	3.911942576	0.30028131	0.7108968	128	156	217	470	265	239	522	455
ENSECAG00000001898	5.155106815	0.300366779	0.710951306	437	719	582	324	704	1365	850	495
ENSECAG00000014832	4.278503769	0.300464863	0.711035641	202	602	134	146	389	443	652	456
ENSECAG00000022710	7.606937144	0.300575796	0.711150339	3085	6269	3144	3294	3332	4191	2964	3052
ENSECAG00000023153	4.633313104	0.300782663	0.711491921	237	387	359	614	558	612	557	612
ENSECAG00000014039	3.662435761	0.300891696	0.711540281	171	166	188	239	365	181	388	290
ENSECAG00000014535	3.847961426	0.301010466	0.711540281	137	478	208.004	383	188	319	234	278
ENSECAG00000011126	5.804352483	0.301042511	0.711540281	534	564	1532	645	2049	1253	1948	346
ENSECAG00000020304	7.740240059	0.301071614	0.711540281	4828	6565	2565	2748	4707	3576	3365	3003
ENSECAG00000019069	7.281520112	0.301146752	0.711540281	2036	2492	2433	2058	3534	4789	2451	4233
ENSECAG00000020484	4.263938926	0.301210518	0.711540281	147	310	425	315	664	474	443	249
ENSECAG00000012277	1.556927464	0.30130157	0.711540281	46	41	69	83	52	34	74	45
ENSECAG00000024967	8.337273025	0.301313369	0.711540281	3340	7743	10171	8778	698	6232	922	12126
ENSECAG00000007948	1.020800602	0.301365589	0.711540281	17	53	42	62	19	36	10	62
ENSECAG00000010090	7.802631267	0.301457532	0.711609787	1835	3998	4499	9929	2807	3744	5080	4206
ENSECAG00000003253	5.183611566	0.301524356	0.711619983	351	473	605	927	683	942	997	828
ENSECAG00000020513	1.878845581	0.301609482	0.711672367	40	73	36	62	54	95	93	111
ENSECAG00000011215	1.453277426	0.301671572	0.711672367	99	24	39	39	39	36	38	64
ENSECAG00000015783	3.986282023	0.30182518	0.711703333	164	192	274	378	281	378	410	439
ENSECAG00000010491	6.015127745	0.301870269	0.711703333	452	821	1202	1706	2089	1222	2143	863
ENSECAG00000011275	5.604689209	0.301909823	0.711703333	654	802	665	855	1409	863	1382	999
ENSECAG00000016373	1.803564303	0.301934747	0.711703333	22	56	37	99	90	54	110	85
ENSECAG00000013212	4.034368193	0.302412902	0.712682858	385	320	216	337	303	305	382	191
ENSECAG00000011037	8.913193576	0.302551855	0.712862762	6423	6966	5082	9579	16348	9974	15153	6591
ENSECAG00000010078	3.292391407	0.30286374	0.7133955	101	93	151	260	271	187	395	115
ENSECAG00000007882	5.417712331	0.302911608	0.7133955	494	604	694	910	1191	724	1153	1003
ENSECAG00000024150	1.756439569	0.302993906	0.7133955	49	50	49	34	87	40	140	69
ENSECAG00000005217	5.609031167	0.303028602	0.7133955	516	736	1286	1601	1022	916	940	689
ENSECAG00000022369	7.863373175	0.303112489	0.713445459	5244	5748	4484	2890	3045	6617	3453	2599
ENSECAG00000020210	2.529432109	0.303463101	0.714035917	12	62	220	33	120	206	166	93
ENSECAG00000015219	1.989762084	0.303520792	0.714035917	40	90	63	145	77	57	74	67
ENSECAG00000005708	4.299256423	0.303589031	0.714035917	250	157	400	406	634	313	509	437
ENSECAG00000008184	6.668437372	0.303614218	0.714035917	1048	2944	1831	2735	1644	1398	1940	2386
ENSECAG00000012589	3.988968163	0.303767717	0.714249371	300	355	307	265	308	225	380	255
ENSECAG00000019231	3.475954708	0.303936984	0.714322	90	85	171	329	73	231	199	617
ENSECAG00000010321	2.82831521	0.303984379	0.714322	150	93	174	143	143	81	112	163
ENSECAG00000015901	4.100520822	0.303986833	0.714322	196	191	250	444	365	239	555	510
ENSECAG00000021261	6.786294355	0.304178779	0.714625547	1085	1133	2021	2709	1987	2194	3414	3271
ENSECAG00000014556	5.355148785	0.304439036	0.715089422	460	706	597	803	811	714	1437	995
ENSECAG00000010324	5.887394343	0.304581037	0.715173026	789	603	1454	664	1679	989	1864	1260
ENSECAG00000007461	5.149054434	0.304605084	0.715173026	628	622	854	668	548	671	710	697
ENSECAG00000000728	2.938930211	0.304663081	0.715173026	78	103	73	219	130	137	221	262
ENSECAG00000009159	4.484708937	0.304798029	0.715202126	130	316	490	427	150	393	442	1267
ENSECAG00000016164	2.325914054	0.304831641	0.715202126	58	45	74	121	143	59	189	105
ENSECAG00000020621	3.799754166	0.30488464	0.715202126	265	90	255	143	403	337	483	160
ENSECAG00000003157	7.24724612	0.304926756	0.715202126	1841	2102	3677	5556	2644	2529	3035	2671
ENSECAG00000008341	5.477395232	0.305011271	0.715253001	601	454	790	869	1566	718	1391	645
ENSECAG00000012840	4.697058411	0.305125061	0.715353282	180.007	681	460	933	333	485	481	575
ENSECAG00000011197	0.53355122	0.305186854	0.715353282	18	21	16	20	33	23	67	17
ENSECAG00000019351	1.389980049	0.305250345	0.715353282	29	31	54	35	66	56	76	51
ENSECAG00000025049	3.851676499	0.305305367	0.715353282	161	289	212	208	444	225	432	291

ENSECAG00000021994	6.23283302	0.305519521	0.715660668	580	1312	1367	1525	1960	2087	1453	1626
ENSECAG00000001415	6.692554498	0.305640092	0.715660668	1054	1069	2028	2340	2945	1793	3268	2115
ENSECAG00000003550	3.090163207	0.305668257	0.715660668	62	212	151	332	92	133	98	262
ENSECAG00000022184	5.907395672	0.305724885	0.715660668	739	1058	930	2428	648	912	1554	1311
ENSECAG00000010267	9.953331469	0.305750856	0.715660668	18776	28341	22170	10448	13844	25109	10953	16456
ENSECAG00000019720	1.872260173	0.305946865	0.715972262	30	55	25	107	100	34	107	122
ENSECAG00000020147	3.949617537	0.306732897	0.717619583	186	172	233	357	463	146	537	375
ENSECAG00000009021	3.515696422	0.306776857	0.717619583	79	169	260	208	203	355	359	185
ENSECAG00000000763	9.488019678	0.30687318	0.717685249	17639	6198	15500	19614	13263	12868	6598	15553
ENSECAG00000010930	6.847677829	0.306988783	0.717685249	1876	2298	2017	3271	2251	1846	1696	2447
ENSECAG00000011952	5.041780953	0.307036878	0.717685249	417	635	661	987	661	606	629	542
ENSECAG00000014882	6.41906868	0.307057079	0.717685249	732	2016	2133	2559	678	1618	1255	2507
ENSECAG00000008150	4.44348505	0.307224683	0.717808857	241	204	432	495	445	327	840	519
ENSECAG00000011211	2.855832868	0.307236061	0.717808857	55	61	153	174	106	84	213	322
ENSECAG00000002936	3.155341321	0.307376375	0.717852757	80	145	143	182	197	111	275	286
ENSECAG00000020134	5.949493111	0.307380956	0.717852757	1171	1094	1535	1134	1495	778	1746	466
ENSECAG00000011080	7.666704048	0.307523385	0.718038092	4130	4725	3500	3675	3064	3522	4309	3656
ENSECAG00000021098	4.862383799	0.307636231	0.718154293	252	331	313	956	523	253	976	1175
ENSECAG00000017638	1.75240571	0.307751562	0.718276248	48	36	61	39	59	67	161	49
ENSECAG00000002400	3.108047081	0.307904691	0.718486352	91	74	180	172	285	102	353	121
ENSECAG00000012134	4.056704143	0.308288332	0.719234152	271	380	271	407	387	226	411	198
ENSECAG00000012483	3.272228916	0.308376088	0.719291489	148	255	173	190	208	155	186	146
ENSECAG00000008161	4.643467407	0.308584493	0.719630162	727	668	544	282	2	1084	6	140
ENSECAG00000008226	6.273234814	0.308771668	0.719818946	1090	611	1560	1294	2675	1122	2929	989
ENSECAG00000023748	4.475696196	0.308791896	0.719818946	238	243	414	528	611	394	659	461
ENSECAG00000016700	4.784217263	0.309033437	0.720234529	328	893	556	427	444	528	463	563
ENSECAG00000008074	4.610356142	0.309135094	0.720323995	367	283	393	401	693	247	959	519
ENSECAG00000022937	6.328556568	0.309270345	0.720491688	1400	1905	1115	2019	1008	1177	1776	1946
ENSECAG00000021159	6.351533316	0.309413562	0.720677865	1135	1241	2249	2108	1312	1309	1349	1987
ENSECAG00000004031	5.231013533	0.309494977	0.720720049	619	636.007	580	181	856.003	434	1838	745
ENSECAG00000007136	2.978713134	0.309836759	0.720977117	109	240	124	165	76	114	180	194
ENSECAG00000011433	6.054840305	0.309842424	0.720977117	912	846	1117	1130	2524	1250	1305	1254
ENSECAG00000013920	4.776700208	0.309906665	0.720977117	264	374	525	603	673	467	842	636
ENSECAG00000015422	4.863463009	0.310095288	0.720977117	364	365	434	652	848	343	901	724
ENSECAG00000017788	4.28613293	0.310100041	0.720977117	230	210	412	369	521	436	477	409
ENSECAG00000017721	1.756481538	0.310107548	0.720977117	47	55	106	67	77	65	44	41
ENSECAG00000014057	3.617239281	0.310126388	0.720977117	100	123	213	358	327	216	306	329
ENSECAG00000011280	3.766755073	0.310130936	0.720977117	77	410	250	454	303	159	241	262
ENSECAG00000015224	3.02287672	0.310206048	0.720977117	359	239	11	84	0	228	0	139
ENSECAG00000021714	3.558533855	0.310268335	0.720977117	116	138	343	421	223	184	249	193
ENSECAG00000006462	6.012098729	0.310301965	0.720977117	814	803	1370	921	2068	1149	2010	1022
ENSECAG00000014213	5.251279019	0.310415359	0.721089039	439	463	685	828	864	621	1335	857
ENSECAG00000017735	1.429153258	0.310493306	0.721089039	15	40	96	99	9	61	12	80
ENSECAG00000010922	5.716183155	0.310540146	0.721089039	578	733	824	1207	1008	878	1536	1641
ENSECAG00000018417	5.344864721	0.310729875	0.721184829	703	1102	611	746	679	691	842	786
ENSECAG00000014516	5.230892669	0.310751683	0.721184829	498	714	711	1166	595	480	763	963
ENSECAG00000023564	3.514466355	0.310822394	0.721184829	172	200	155	430	261	225	174	146
ENSECAG00000024178	4.551113017	0.310834779	0.721184829	248	324	616	821	446	558	405	269
ENSECAG00000018284	2.325213937	0.310941421	0.721223013	39	75	89	106	85	130	134	129
ENSECAG00000013004	3.395919339	0.310977933	0.721223013	360	351	105	50	6	420	5	113
ENSECAG00000014138	4.405111461	0.311208614	0.721519025	342	290	429	675	388	204	576	413
ENSECAG00000016085	3.295308873	0.311232317	0.721519025	345	339	77	22	8	356	23	142
ENSECAG00000023964	0.537455461	0.311331023	0.721600915	5.01056	18.0001	20.0079	42.0001	36.0006	24.0007	38.0001	38.0239
ENSECAG00000023952	5.795161652	0.311572204	0.721911961	939	1417	914	1079	881	977	1333	931
ENSECAG00000013541	4.80667176	0.31159204	0.721911961	185	784	438	1187	103	353	274	1149
ENSECAG00000018501	1.305308896	0.311695585	0.722004932	21	64	54	73	33	57	11	55
ENSECAG00000008097	4.813213412	0.311936119	0.722307966	597	531	461	577	330	445	615	690
ENSECAG00000000444	1.474968676	0.312086253	0.722307966	31	26	63	31	124	33	88	28
ENSECAG00000003104	6.05004575	0.312135809	0.722307966	1368	1385	898.004	1601	1012	1870	1060	726.001
ENSECAG00000014655	2.211924858	0.31214243	0.722307966	34	85	56	109	136	105	113	87
ENSECAG00000018106	5.246123524	0.312184377	0.722307966	311	668	541	943	835	633	1016	1152
ENSECAG00000000753	6.280199802	0.312207071	0.722307966	898	1102	1108	1771	2135	1256	2369	1753
ENSECAG00000016942	0.412722994	0.312364477	0.722525308	8	32	36	34	22	28	19	14
ENSECAG00000015690	5.775695706	0.312440011	0.722553225	717	697	801	1189	1072	896	1405	1900
ENSECAG00000024984	5.705988844	0.312532683	0.722620754	639	1510	725	1443	494	1051	982	1262
ENSECAG00000023055	5.466056134	0.312648296	0.722647948	504	800	618	828	826	681	1568	1222
ENSECAG00000018793	0.892914552	0.312671391	0.722647948	15	14	36	38	11	28	59	85
ENSECAG00000008200	1.033114033	0.312765319	0.722718319	26	15	48	17	48	38	18	92
ENSECAG00000002235	3.555944373	0.31287814	0.722832311	146	89	167	318	363	133	433	236
ENSECAG00000006219	4.27991784	0.313012908	0.722996948	246	287	281	387	568	268	568	458
ENSECAG00000018641	0.67963278	0.313383761	0.723183626	14	20	13	42	12	28	42	72
ENSECAG00000003460	4.287768102	0.313399	0.723183626	319	381	341	521	442	346	412	233
ENSECAG00000013509	5.206395452	0.313448286	0.723183626	765	592	682	823	788	567	848	534
ENSECAG00000011530	6.706745276	0.313474965	0.723183626	1105	1700	1732	1922	2417	2503	3358	1850
ENSECAG00000016482	2.408137913	0.313490162	0.723183626	46	20	327	86	75	37	96	136
ENSECAG00000014421	4.893274178	0.313523877	0.723183626	403	566	589	865	547	336	685	663

ENSECAG00000024411	6.269203248	0.313557395	0.723183626	1104	1915	1790	1338	1273	1286	1718	1417
ENSECAG00000015076	4.368700327	0.313628322	0.723183626	208	186	430	473	720	253	698	349
ENSECAG00000003001	1.404563857	0.313665415	0.723183626	19	19	57	64	76	42	84	53
ENSECAG00000022401	4.018900501	0.31374854	0.723228816	127	290	244	381	315	296	548	406
ENSECAG00000008287	3.281626367	0.313827891	0.723265291	112	87	209	178	354	158	292	140
ENSECAG00000008893	1.282818636	0.313950383	0.723401155	23	24	66	99	37	30	31	63
ENSECAG00000007778	4.766407577	0.314148681	0.723711601	165	610	404	563	853	447	738	578
ENSECAG00000020438	3.180002736	0.314384418	0.723976014	209	116	203	180	183	113	264	91
ENSECAG00000018803	4.728486379	0.314390638	0.723976014	323	419	489	409	683	602	699	518
ENSECAG00000023807	4.829804578	0.314454851	0.723977447	278	362	584	612	580	654	787	669
ENSECAG00000010473	4.618658933	0.314529788	0.724003565	329	277	436	472	708	474	786	387
ENSECAG00000015542	6.817274612	0.314847777	0.724564079	2643	2783	1469	1781	2031	1887	2357	1762
ENSECAG00000019483	1.642654611	0.314909069	0.724564079	40	34	42	65	80	85	84	46
ENSECAG00000018811	6.765955977	0.314992784	0.724564079	2372	3153	1469	1509	3065	1344	2035	1143
ENSECAG00000023622	4.183028236	0.315027861	0.724564079	181	287	298	389	435	289	496	512
ENSECAG00000016099	5.571738331	0.315300237	0.724901648	751	992	564	1646	530	565	851	1517
ENSECAG00000015243	3.970112214	0.315301973	0.724901648	150	158	350	341	385	380	349	366
ENSECAG00000018885	3.670407142	0.315429358	0.7250481	181	172	337	400	74	140	211	471
ENSECAG00000001174	3.018544133	0.315626451	0.725354691	89	86	155	133	83	163	86	469
ENSECAG00000011089	1.16903466	0.315748036	0.725487666	16	84	79	22	7	80	1	33
ENSECAG00000008977	5.760088244	0.315846354	0.725567138	1587	568	1080	949	1631	1073	486	439
ENSECAG00000000609	6.337784704	0.315917234	0.72558356	1183	1199	1238	1235	1673	1858	2325	1915
ENSECAG00000009488	9.302819505	0.315986462	0.725596181	12729	17877	6846	14543	3967	14217	7053	17139
ENSECAG00000013377	5.420114632	0.316370459	0.726195362	333	1070	726	1796	204	407	482	1840
ENSECAG00000015533	3.975218693	0.316609202	0.726195362	225	512	203	319	188	277	263	402
ENSECAG00000012031	4.265568708	0.316707592	0.726195362	221	296	337	339	286	402	500	649
ENSECAG00000005653	8.057798864	0.316782016	0.726195362	4626	5083	5543	6395	4515	4473	4711	5419
ENSECAG00000012286	3.637779274	0.316835882	0.726195362	131	141	209	297	398	140	378	292
ENSECAG00000020320	1.115922036	0.316877907	0.726195362	33	51	56	36	9	28	23	79
ENSECAG00000023178	6.275766334	0.316890142	0.726195362	2118	1608	1005	1066	1306	1680	1388	1097
ENSECAG00000011636	2.802175812	0.316972905	0.726195362	113	32	176	4	160	145	401	59
ENSECAG00000011751	8.090944255	0.317052046	0.726195362	2731	2950	5425	5827	6322	6384	8270	5754
ENSECAG00000012040	3.005080682	0.317101278	0.726195362	56	192	202	245	75	128	165	202
ENSECAG00000009836	8.08640751	0.317128003	0.726195362	2416	2265	5201	6978	10074	4217	9056	3878
ENSECAG00000006984	5.761290609	0.317133854	0.726195362	621	773	1076	908	1099	1679	1275	1056
ENSECAG00000006826	1.833488696	0.317164931	0.726195362	31	122	68	80	31	55	36	114
ENSECAG00000015453	3.50538037	0.317172507	0.726195362	122	194	357	267	179	186	218	243
ENSECAG00000017435	4.659665935	0.317204175	0.726195362	316	652	481	603	254	533	521	550
ENSECAG00000000855	5.060577105	0.317304534	0.726279075	537	803	587	692	485	551	665	783
ENSECAG00000013583	4.113377168	0.317410292	0.72637511	176	368	304	637	433	354	277	162
ENSECAG00000027628	1.659026231	0.317552459	0.72655441	33	25	66	161	44	84	43	33
ENSECAG00000017349	5.54757218	0.317632737	0.726572315	897	1275	675	785	466	847	853	1230
ENSECAG00000013798	6.443714358	0.317687921	0.726572315	1044	1338	2484	2455	2234	1240	1621	1216
ENSECAG00000018790	0.851219788	0.318017066	0.727141783	30	65	20	24	23	33	25	35
ENSECAG00000009626	8.853367661	0.318116937	0.727141783	3895	1764	27938	9112	10434	4905	5705	9060
ENSECAG00000016972	4.654694763	0.318155403	0.727141783	355	710	497	458	252	684	362	494
ENSECAG00000018035	8.591060405	0.318312176	0.727141783	8228	9311	5210	8584	2315	10236	5572	8050
ENSECAG00000009550	11.14151028	0.318445272	0.727141783	42744	66350	38077	33086	45013	32434	53079	28792
ENSECAG00000016674	6.356323902	0.318445471	0.727141783	1516	2233	1448	1161	1408	1190	1503	1856
ENSECAG00000021619	4.168211266	0.31851356	0.727141783	141	277	323	427	335	441	541	394
ENSECAG00000019956	0.186934346	0.318524786	0.727141783	0	9	20	33	21	13	12	63
ENSECAG00000013780	2.606558669	0.31856884	0.727141783	89	62	81	121	202	110	203	78
ENSECAG00000012680	5.023230609	0.318575601	0.727141783	477	344	1009	903	315	623	348	1011
ENSECAG00000019354	4.044942781	0.318639719	0.727142353	159	214	323	355	453	361	490	266
ENSECAG00000009741	5.943761356	0.318936186	0.727582433	533	564	1370	1506	1776	986	1935	1283
ENSECAG00000016844	0.076963175	0.31896038	0.727582433	8	8	17	25	24	25	26	20
ENSECAG00000011381	6.339012692	0.319045413	0.727630614	776	1842	2393	1826	830	2236	1523	1174
ENSECAG00000010775	2.116414609	0.319222562	0.727888819	36	61	85	80	141	95	112	64
ENSECAG00000010820	3.995870614	0.319537735	0.728409945	108	313	295	707	309	327	199	286
ENSECAG00000002452	0.713251369	0.319579067	0.728409945	12	55	25	44	16	14	33	44
ENSECAG00000019904	5.617788945	0.319729697	0.728607406	909	1456	950	639	415	1910	402	499
ENSECAG00000015018	5.62293355	0.319944879	0.72889453	676	1137	900	1240	740	930	1016	995
ENSECAG00000013791	5.929067708	0.319983738	0.72889453	907	1898	924	1102	816	1272	1214	1136
ENSECAG00000018311	2.892168143	0.320137092	0.729061838	64	109	120	168	194	95	278	160
ENSECAG00000001845	3.131536516	0.32018526	0.729061838	121	46	308	284	126	134	122	220
ENSECAG00000013973	5.822086142	0.320329121	0.72924356	599	567	946	1545	1456	970	1598	1409
ENSECAG00000011048	6.335794455	0.320428906	0.72932489	1126	428	2057	824	3706	1772	2284	389
ENSECAG00000020956	3.007084147	0.320666667	0.72950093	170	375	103	73	0	347	6	44
ENSECAG00000005271	5.044384188	0.320700409	0.72950093	364	483	638	612	789	685	927	716
ENSECAG00000014359	1.626045585	0.320743885	0.72950093	93	35	50	54	76	51	35	40
ENSECAG00000021507	7.893372843	0.320812519	0.72950093	2905	5933	4834	6230	3315	4725	4203	4768
ENSECAG00000020160	2.927723438	0.320847877	0.72950093	91	72	132	158	325	106	201	106
ENSECAG00000019256	7.144446442	0.320890703	0.72950093	2001	5847	1915	1719	1533	3370	2471	2356
ENSECAG00000007323	5.196277119	0.321034313	0.729504856	375	386	631	984	953	535	1051	982
ENSECAG00000013762	5.052227098	0.321080946	0.729504856	1023	1365	154	135	49	1349	67	365
ENSECAG00000000217	1.313195398	0.321093219	0.729504856	27	30	69	83	35	42	32	58

ENSECAG00000011107	8.072413133	0.321225411	0.729504856	3325.01	6885	5524	6683.01	4666	4211.01	5166	5355
ENSECAG00000012333	5.838055342	0.321225814	0.729504856	776	679	1125	911	1748	1269	1484	940
ENSECAG00000020425	8.3583233	0.321323623	0.729504856	6224	7835	5139	7120	4214	5251	6532	7518
ENSECAG00000020110	5.980378348	0.321340962	0.729504856	940	1519	1127	1491	668	981	1582	1479
ENSECAG00000015330	2.166764291	0.321553285	0.729841338	61	28	67	107	111	60	151	119
ENSECAG00000016629	2.547508147	0.321806235	0.730106783	63	109	167	150	59	123	91	136
ENSECAG00000011830	4.385965537	0.321824991	0.730106783	203	230	424	482	700	366	529	391
ENSECAG00000015601	4.360642193	0.321862621	0.730106783	273	246	325	438	534	333	633	463
ENSECAG00000017743	5.043088026	0.322015831	0.730286112	288	350	730	708	325	649	808	1443
ENSECAG00000018867	2.16803984	0.322069966	0.730286112	57	149	69	87	77	49	117	75
ENSECAG00000017587	7.198899158	0.322407645	0.730832384	2333	3737	2703	3083	3101	2407	2350	2656
ENSECAG00000023532	7.863439663	0.322439268	0.730832384	2632	5525	5145	6323	3487	4251	4466	4567
ENSECAG00000025152	3.013737683	0.322665747	0.731089882	79	239	199	143	149	114	151	164
ENSECAG00000026919	1.154293168	0.322774907	0.731089882	20	36	90	38	37	45	32	32
ENSECAG00000012255	3.398893828	0.322798074	0.731089882	84	325	256	211	142	179	208	230
ENSECAG00000002606	3.54881117	0.322809735	0.731089882	142	177	131	274	243	201	300	377
ENSECAG00000007653	3.799150954	0.322951325	0.731265083	147	384	259	355	302	165	315	241
ENSECAG00000015037	1.685212422	0.323100649	0.731457722	27	193	24	32	12	119	29	26
ENSECAG00000000467	2.362829408	0.323195612	0.731527245	41	124	103	177	50	121	73	108
ENSECAG00000019482	3.330713034	0.323321622	0.731652596	106	183	248	302	175	162	196	201
ENSECAG00000021724	2.607718383	0.323396187	0.731652596	21	74	75	223	96	122	187	200
ENSECAG00000009447	2.628710274	0.323536174	0.731652596	54	65	101	163	108	78	260	170
ENSECAG00000000770	3.578428057	0.32374108	0.731652596	109	287	316	281	278	204	215	163
ENSECAG00000017170	1.448681196	0.323743007	0.731652596	39	27	50	32	60	54	124	31
ENSECAG00000002904	5.395011456	0.32376272	0.731652596	522	831	1068	982	705	870	803	745
ENSECAG00000002988	8.87441471	0.323779211	0.731652596	8506	14327	8144	6672	5422	12198	6622	7894
ENSECAG00000009655	3.541818148	0.323878841	0.731652596	171	208	224	351	219	104	262	272
ENSECAG00000013751	6.873738184	0.323884863	0.731652596	1620	1541	1828	2051	3105	1940	3581	2833
ENSECAG00000019395	2.864738307	0.32390524	0.731652596	78	134	98	130	190	109	245	161
ENSECAG00000015199	1.873864267	0.323966084	0.731652596	30	71	26	87	64	158	80	47
ENSECAG00000017799	9.706663335	0.324022169	0.731652596	9090	29240	13308	18664	13193	16181	17393	12102
ENSECAG00000019005	-0.064655142	0.324310632	0.732158744	6.00713	6	19	21	20.0004	22.0001	28	16
ENSECAG00000014300	6.999226569	0.324450081	0.732328345	1341	1328	2184	3212	3559	2522	3414	2923
ENSECAG00000008257	2.866060555	0.324692107	0.732705646	73	86	135	150	255	80	226	149
ENSECAG00000026960	5.107835863	0.324753023	0.732705646	268	531	682	780	760	675	1007	836
ENSECAG00000008585	8.470715518	0.324855351	0.732705646	4457	4840	10504	10539	5548	6310	7445	6119
ENSECAG00000012235	4.571499361	0.324921693	0.732705646	572	779	296	233	88	784	128	511
ENSECAG00000008163	6.680647791	0.324941436	0.732705646	1039	1286	1710	2507	2363	2286	3048	2194
ENSECAG00000000823	4.568699532	0.325003383	0.732705646	266	441	390	937	265	309	433	729
ENSECAG00000018093	9.190841356	0.325133586	0.732854065	9952	11705	13658	12210	9368	11105	8904	11982
ENSECAG00000010780	5.170424005	0.325312858	0.732910756	423	903	684	907	693	511	878	629
ENSECAG00000008059	5.091563518	0.325364785	0.732910756	299	514	556	1687	440	509	515	987
ENSECAG00000021189	0.955420864	0.325443647	0.732910756	12	19	38	47	52	38	49	42
ENSECAG00000017597	0.176486335	0.325497079	0.732910756	7	12	23	20	21	29	42	12
ENSECAG00000019862	4.87302572	0.325508378	0.732910756	373	255	485	709	864	298	1075	644
ENSECAG00000010085	5.056279398	0.325596972	0.732910756	352	455	629	697	824	862	741	680
ENSECAG00000010961	6.279431523	0.325665828	0.732910756	1556	2218	1443	831	2099	1376	1311	632
ENSECAG00000022704	4.197871884	0.325788042	0.732910756	143	387	258	368	244	345	550	642
ENSECAG00000007322	7.607002879	0.325819992	0.732910756	2465	3778	4115	6089	3568	3185	3569	3759
ENSECAG00000021443	6.166483786	0.32585925	0.732910756	1005	1277	1223	2483	1015	902	1520	1887
ENSECAG00000021326	1.908562605	0.325881782	0.732910756	43	64	67	39	121	55	115	71
ENSECAG00000008487	2.345972129	0.325931239	0.732910756	63	78	144	137	126	70	69	89
ENSECAG00000023353	5.59385608	0.326123676	0.733198668	681	786	817	587	1301	1366	1294	630
ENSECAG00000003511	5.752665376	0.326554139	0.734021496	1043	1415	890	784	889	1117	1157	780
ENSECAG00000008175	1.410967266	0.326934534	0.734636024	24	55	36	39	63	56	76	56
ENSECAG00000023723	5.050052695	0.326956586	0.734636024	442	349	536	696	554	333	1481	950
ENSECAG00000017286	5.78546815	0.327066813	0.734647741	651	922	859	1041	1266	1058	1564	1350
ENSECAG00000023820	8.516460774	0.327180798	0.734647741	4956	5574	12670	7968	4078	7214	3583	10308
ENSECAG00000012777	5.821947254	0.32721945	0.734647741	586	1127	855	980	1248	972	1591	1602
ENSECAG00000018887	7.885155476	0.327245491	0.734647741	2182	3456	4221	4950	6071	5419	5835	5529
ENSECAG00000007043	2.343222983	0.327343324	0.734647741	66	121	86	142	61	110	91	95
ENSECAG00000023078	4.542207122	0.327348967	0.734647741	241	444	532	720	420	369	582	377
ENSECAG00000016921	5.467222748	0.327420392	0.734663217	1135	1038	576	532	849	805	773	747
ENSECAG00000023791	1.406407558	0.327599937	0.7347407	14	17	2	109	1	77	85	122
ENSECAG00000011785	2.047820278	0.327619887	0.7347407	60	53	118	107	74	65	103	50
ENSECAG00000000242	7.663771758	0.327695974	0.7347407	3208	4788	3756	4678	3400	3569	3723	3950
ENSECAG00000018765	5.163025882	0.327723725	0.7347407	414	562	657	615	1053	728	970	630
ENSECAG00000008929	4.433848393	0.327777603	0.7347407	257	230	378	512	611	440	580	411
ENSECAG00000002317	3.060952971	0.327843026	0.734741646	84	496	43	81	35	209	134	152
ENSECAG00000015088	3.625970507	0.327907098	0.734741646	130	197	211	224	453	205	289	224
ENSECAG00000013193	3.672362431	0.328086471	0.734824486	92	158	146	433	369	264	438	171
ENSECAG00000013357	0.267326919	0.32809531	0.734824486	20	11	4	25	27	14	34	39
ENSECAG00000020735	4.268165065	0.328137698	0.734824486	253	453	362	480	273	416	375	360
ENSECAG00000011206	3.729836379	0.328483514	0.73545424	104	466	155	407	149	264	243	285
ENSECAG00000009510	6.350149689	0.32866449	0.735714751	918	883	1813	1501	2117	1970	1893	1766
ENSECAG00000018207	2.689780467	0.328831645	0.735944227	62	231	102	151	28	103	156	161

ENSECAG00000024262	5.419533089	0.329252053	0.736163243	552	1469	652	745	542	728	754	1098
ENSECAG00000016088	2.402826532	0.329264026	0.736163243	72	39	87	116	156	93	142	117
ENSECAG00000010884	3.076415586	0.329370973	0.736163243	112	137	147	95	226	128	295	172
ENSECAG00000011861	2.899945762	0.329382533	0.736163243	74	77	127	192	182	137	231	167
ENSECAG00000001756	3.832883136	0.329483665	0.736163243	163	191	245	295	373	254	471	265
ENSECAG00000022333	4.659932298	0.329545959	0.736163243	329	329	862	585	503	509	639	174
ENSECAG00000008456	3.858840463	0.329580457	0.736163243	190	259	154	265	345	149	543	392
ENSECAG00000008214	3.957501647	0.329588479	0.736163243	170	167	351	289	386	373	405	303
ENSECAG00000013439	1.410177234	0.329661408	0.736163243	17	104	52	61	6	89	13	50
ENSECAG00000013642	0.936947551	0.32971301	0.736163243	15	44	15	32	42	17	57	69
ENSECAG00000008664	7.329207141	0.329803717	0.736163243	1160	2263	2800	4077	3443	2796	4089	5423
ENSECAG00000023961	4.338714112	0.329827437	0.736163243	268	196	724	486	287	413	417	355
ENSECAG00000015139	5.238458645	0.329902614	0.736163243	332	804	756	1304	687	676	815	627
ENSECAG00000022770	6.249503137	0.32994106	0.736163243	897	1494	1102	1177	1792	1761	2166	1546
ENSECAG00000015051	4.946427625	0.32999188	0.736163243	224	393	576	826	1033	629	876	409
ENSECAG00000006312	3.485383338	0.330013007	0.736163243	135	324	212	239	180	280	131	197
ENSECAG00000008658	0.979357215	0.330028739	0.736163243	12	24	25	57	30	31	57	70
ENSECAG00000010761	3.005283868	0.33015308	0.736199925	70	172	146	303	102	139	152	182
ENSECAG00000021606	4.991182108	0.330174512	0.736199925	294	672	607	1124	387	623	647	696
ENSECAG00000018889	4.724524737	0.33036776	0.736403118	409	830	345	492	367	391	616	584
ENSECAG00000022350	3.24941758	0.330395005	0.736403118	108	145	124	202	162	110	328	335
ENSECAG00000015797	2.507281281	0.330546938	0.736597552	57	71	102	273	99	84	92	121
ENSECAG00000007248	5.504032306	0.330695705	0.736784853	408	584	797	1195	1090	945	1343	934
ENSECAG00000010493	4.658342473	0.331117138	0.737348046	297	260	474	582	629	479	745	547
ENSECAG00000006741	4.745609512	0.331142342	0.737348046	348	505	437	336	683	499	821	557
ENSECAG00000024945	4.623431712	0.331142781	0.737348046	190	389	588	415	609	582	589	542
ENSECAG00000010871	1.037235695	0.33135704	0.737371077	19	30	26	41	46	13	64	76
ENSECAG00000019869	1.201252281	0.331370909	0.737371077	27	7	70	19	57	47	54	65
ENSECAG00000003274	1.261142117	0.331409825	0.737371077	42	24	31	31	60	35	74	61
ENSECAG00000020785	4.163761178	0.331469311	0.737371077	117	443	374	634	191	303	243	541
ENSECAG00000024306	8.01273831	0.331476959	0.737371077	2527	2697	3605	7475	5070	4788	6139	9407
ENSECAG00000024265	5.048939334	0.331718947	0.73776523	402	725	658	901	611	676	620	553
ENSECAG00000018683	8.883758834	0.331817779	0.737840901	6099	16757	7678	8873	4491	12307	5261	10035
ENSECAG00000002178	0.80736959	0.332218042	0.738586683	29	32	36	38	22	37	28	30
ENSECAG00000004975	7.211447605	0.332401995	0.738713043	2013	3135	3094	4034	2293	1977	3171	3421
ENSECAG00000024917	3.053158462	0.332404648	0.738713043	80	158	163	310	92	144	169	193
ENSECAG00000011360	3.638233332	0.332498253	0.738776856	109	189	192	313	312	275	352	240
ENSECAG00000020020	7.393617879	0.332784772	0.739086975	2593	3976	3121	3954	2896	3248	3011	2970
ENSECAG00000006736	2.821036045	0.332830928	0.739086975	93	114	173	199	107	107	152	148
ENSECAG00000010938	5.983332244	0.33283258	0.739086975	592	688	1180	1667	1732	1097	1781	1446
ENSECAG00000008194	5.250467693	0.332946014	0.739194689	433	1326	881	581	532	1481	225	213
ENSECAG00000009473	7.109115548	0.33309024	0.739370713	2565	3765	1822	2799	2673	2273	2009	2868
ENSECAG00000013050	9.45736959	0.333155966	0.739372451	18403	21799	8150	5291	9788	14941	13107	9884
ENSECAG00000020969	7.10757678	0.333367311	0.739597276	2313	1919	3315	3785	2357	2881	2940	1768
ENSECAG00000011852	4.014591063	0.333387196	0.739597276	177	501	296	330	248	327	312	294
ENSECAG00000009959	6.671670373	0.333500414	0.739704308	1109	1952	1426	1692	3826	1484	2950	1663
ENSECAG00000000733	3.305233845	0.333649046	0.739714624	129	136	146	195	218	203	323	200
ENSECAG00000015383	1.286945654	0.333694039	0.739714624	17	70	53	64	23	51	53	38
ENSECAG00000016935	4.83973353	0.333699983	0.739714624	354	374	447	632	671	374	974	741
ENSECAG00000010580	3.923112022	0.333819091	0.739834603	154	198	295	321	419	340	329	331
ENSECAG00000019344	6.658029129	0.33390932	0.739890544	1343	3609	1654	1754	949	3556	1014	1244
ENSECAG00000016468	2.033526579	0.334132659	0.740241356	38	47	57	106	72	43	141	147
ENSECAG00000024211	2.620339954	0.334595997	0.741032753	70	92	115	92	137	120	140	186
ENSECAG00000010520	3.549938582	0.334649858	0.741032753	129	320	218	286	223	217	226	188
ENSECAG00000006701	1.474797904	0.334685148	0.741032753	29	54	87	62	30	23	34	100
ENSECAG00000024935	6.163967164	0.334901823	0.741280076	1531	1534	1332	1115	909	1896	1158	1164
ENSECAG00000017635	4.687683501	0.334993997	0.741280076	266	333	512	553	621	527	683	594
ENSECAG00000024930	3.876338552	0.335100164	0.741280076	158	226	236	293	298	180	635	332
ENSECAG00000024753	3.29688323	0.335365414	0.741280076	107	341	224	129	119	286	157	114
ENSECAG00000013770	6.27756636	0.335376596	0.741280076	1195	2097	1209	1713	786	1326	1548	2035
ENSECAG00000010774	8.070009119	0.335439733	0.741280076	3486	6673	5243	6832	4873	4421	5477	4713
ENSECAG00000015443	1.654911647	0.335450454	0.741280076	27	34	67	63	78	65	86	68
ENSECAG00000012070	6.808562164	0.33545415	0.741280076	1588	3007	2233	2287	1334	2617	1557	2464
ENSECAG00000007901	0.919554894	0.335581459	0.741280076	24	27	61	41	32	11	36	48
ENSECAG00000023513	4.963433895	0.335609516	0.741280076	459	664	578	783	447	470	649	781
ENSECAG00000015209	4.708825225	0.335686285	0.741280076	307	416	479	436	667	656	615	505
ENSECAG00000011568	5.271927493	0.335707966	0.741280076	320	474	1423	1135	876	789	736	362
ENSECAG00000011093	6.255634535	0.335723904	0.741280076	1742	776	1897	1544	1714	1096	1672	1081
ENSECAG00000003790	6.487261431	0.335779861	0.741280076	867	2334	1636	2695	1441	1640	1996	1538
ENSECAG00000024642	4.535110123	0.335872466	0.741280076	244	216	443	597	595	405	738	481
ENSECAG00000008240	4.083427786	0.335876288	0.741280076	274	159	453	508	280	170	418	386
ENSECAG00000019967	4.199376607	0.335903725	0.741280076	177	389	364	611	264	378	362	350
ENSECAG00000026952	1.895821162	0.335977824	0.741299909	28	64	67	170	73	73	64	43
ENSECAG00000009091	5.666709896	0.336342132	0.741959925	437	580	1111	1193	1250	1213	1000	1306
ENSECAG00000015490	2.628926056	0.336488681	0.74213941	46	74	105	165	93	142	128	229
ENSECAG00000018981	1.438739314	0.336644209	0.742338624	22.6574	63	27.04	123.175	34.3279	42.5809	46.804	59

ENSECAG00000020882	5.621316094	0.336933936	0.742568995	436	893	812	1047	1289	1157	1262	916
ENSECAG00000015946	4.245248261	0.336977785	0.742568995	255	249	358	307	478	450	495	359
ENSECAG00000018187	6.768363499	0.337020629	0.742568995	1231	2598	2043	3285	1928	1369	1966	2702
ENSECAG00000018238	6.234559909	0.337088918	0.742568995	1072	1737	1700	1520	1280	1747	1134	1307
ENSECAG00000016885	4.138816897	0.337106986	0.742568995	154	273	459	621	276	252	362	412
ENSECAG00000014586	4.779541127	0.33714002	0.742568995	483	595	494	553	553	371	644	499
ENSECAG00000018623	5.58762041	0.337206247	0.742571204	451	479	838	1387	947	748	1519	1438
ENSECAG00000022784	7.211968998	0.337513362	0.743103777	2421	1458	2392	2216	5006	2179	5505	2112
ENSECAG00000016135	0.2958591	0.337817926	0.743536861	27	16	30	18	12	28	13	24
ENSECAG00000011178	7.695669921	0.337840684	0.743536861	2688.01	4927	5024.01	4402	3626	4448	3382	3370.02
ENSECAG00000005161	6.211566003	0.33801975	0.743787176	945	1815	1046	2380	917	924	1333	2260
ENSECAG00000004662	0.829496492	0.338193466	0.744025626	15	29	19	40	49	24	50	43
ENSECAG00000012082	7.856316918	0.338322411	0.744055059	2146	2123	4678	5564	7931	4773	6901	3136
ENSECAG00000013778	2.404188628	0.338337553	0.744055059	83	105	83	159	68	69	94	146
ENSECAG00000022968	7.663012201	0.338423103	0.744099464	5242	5660	2292	2205	4123	3804	3949	2167
ENSECAG00000000511	3.436818397	0.338800784	0.744575363	159	224	227	252	215	201	224	154
ENSECAG00000020567	5.628719268	0.338937166	0.744575363	552	1046	920	1579	705	1031	904	1033
ENSECAG00000018435	9.730293707	0.3389692	0.744575363	17596	20519	15021	13713	14338	19033	12744	13246
ENSECAG00000014044	4.865071626	0.339014821	0.744575363	238	453	466	755	564	445	893	896
ENSECAG00000024159	4.730066372	0.339158266	0.744575363	568	617	373	455	326	450	654	542
ENSECAG00000016805	4.980127641	0.339192863	0.744575363	144	519	516	833	286	967	381	1429
ENSECAG00000019052	6.253018957	0.33921773	0.744575363	1021	466	660	2555	1606	795	2591	2708
ENSECAG00000022122	4.907212065	0.339314349	0.744575363	341	428	546	597	783	759	720	536
ENSECAG00000010628	5.364646354	0.33938097	0.744575363	465	830	787	1364	826	956	495	698
ENSECAG00000011367	1.754465887	0.33939233	0.744575363	32	35	55	84	82	73	110	56
ENSECAG00000016103	3.648713438	0.339405191	0.744575363	123	250	327	344	148	223	259	292
ENSECAG00000003793	2.181153258	0.339424342	0.744575363	50	44	125	19	200	44	192	31
ENSECAG00000011102	5.151343757	0.33972415	0.745089471	541	721	744	790	652	515	847	681
ENSECAG00000008378	3.189979519	0.339815538	0.745146361	125	110	126	185	209	93	357	241
ENSECAG00000018200	4.240048213	0.340005046	0.745317602	258	184	308	415	667	260	550	337
ENSECAG00000006983	6.700555497	0.340031295	0.745317602	2998	1509	1514	1802	1857.99	2250	2003	1248
ENSECAG00000014244	7.080217413	0.340090026	0.745317602	1291	1266	3584	1844	6292	2092	4064	1078
ENSECAG00000008117	3.549219051	0.340226596	0.745347723	139	173	281	382	206	186	200	264
ENSECAG00000023886	5.592645548	0.340301998	0.745347723	812	709	645	626	1699	691	1637	671.999
ENSECAG00000016487	6.323915917	0.340330112	0.745347723	1989	1703	1163	1149	1537	1110	1846	1372
ENSECAG00000026901	1.379644011	0.340392531	0.745347723	26	22	54	50	48	30	107	69
ENSECAG00000020102	6.317469356	0.340431107	0.745347723	1395	1717	1359	1823	1425	1722	1222	1440
ENSECAG00000019630	4.930683375	0.340742569	0.745766435	498	801	523	537	507	474	672	633
ENSECAG00000021084	4.552516859	0.340924207	0.745766435	185	297	407	669	481	540	549	641
ENSECAG00000019192	7.397116469	0.340951173	0.745766435	2562	3065	3544	4675	3304	2417	3319	3269
ENSECAG00000017669	4.749817407	0.340960994	0.745766435	321	328	400	675	720	531	828	474
ENSECAG00000024121	2.753428213	0.340963981	0.745766435	172	128	114	96	93	187	84	95
ENSECAG00000011268	1.850146195	0.341015376	0.745766435	31	35	78	76	95	97	92	55
ENSECAG00000002024	0.297990743	0.341107868	0.745783211	9	12	21	28	27	26	29	29
ENSECAG00000023269	7.511909403	0.341229991	0.745783211	1981	2199	3500	3693	4781	3933	4898	4018
ENSECAG00000023727	3.528102811	0.34126219	0.745783211	68	19	968	138	6	5	12	583
ENSECAG00000008733	5.786475861	0.341314487	0.745783211	622	1215	1106	1585	861	1086	1093	1079
ENSECAG00000004503	3.143333767	0.341418157	0.745783211	71	89	174	232	207	237	269	131
ENSECAG00000012241	3.10134535	0.34148451	0.745783211	68	105	170	208	173	171	234	240
ENSECAG00000024023	6.282512709	0.341616324	0.745783211	1340	1769	1870	1089	1436	1231	2022	1083
ENSECAG00000022642	2.763367956	0.34176036	0.745783211	33	96	154	146	149	230	153	112
ENSECAG00000023836	4.600813288	0.341760558	0.745783211	196	319	363	723	404	448	646	827
ENSECAG00000008927	5.084957267	0.3417823	0.745783211	526	228	729	571	752	871	794	804
ENSECAG00000018247	0.61501158	0.341796642	0.745783211	26	9	24	21	34	38	29	40
ENSECAG00000008300	1.334052492	0.341890644	0.745783211	27	60	22	29	27	79	63	71
ENSECAG00000019363	6.978809165	0.341975097	0.745783211	1738	1635	1852	2418	3480	2060	3930	2823
ENSECAG00000019512	0.668418881	0.341981749	0.745783211	24	10	32	13	28	20	95	17
ENSECAG00000003310	7.454581476	0.342005634	0.745783211	1414	2473	3163	4003	5216	4019	5925	2046
ENSECAG00000016471	4.531463372	0.342290556	0.746227368	227	493	470	774	252	642	280	474
ENSECAG00000011873	5.985838347	0.342340408	0.746227368	948	1387	1169	1561	1019	1336	950	1353
ENSECAG00000008739	0.925073428	0.342434548	0.746289687	11	44	63	43	11	55	23	31
ENSECAG00000023654	2.121622824	0.342775726	0.746635574	39	100	118	102	76	65	94	75
ENSECAG00000009633	4.897837009	0.342811501	0.746635574	366	309	488	725	570	375	1086	874
ENSECAG00000017397	1.692475972	0.342813607	0.746635574	21	68	30	72	42	39	107	131
ENSECAG00000008478	4.191975615	0.34285558	0.746635574	296	469	267	397	305	332	330	382
ENSECAG00000007275	3.35534254	0.343026679	0.746781706	100	66	230	250	295	198	305	188
ENSECAG00000013284	0.535904664	0.343057211	0.746781706	19	16	15	29	27	18	36	54
ENSECAG00000026937	4.338073056	0.343119464	0.746781706	255	478	455	425	366	376	310	441
ENSECAG00000008981	4.026948065	0.343289214	0.747008353	147	265	269	360	230	355	388	577
ENSECAG00000011942	5.809431276	0.343797459	0.747595179	518	921	960	1217	1159	943	1353	1882
ENSECAG00000004349	3.189453766	0.343968743	0.747595179	51	251	135	114	137	221	341	198
ENSECAG00000020426	3.311245786	0.344029553	0.747595179	83	188	174	180	249	185	310	202
ENSECAG00000008665	5.781780112	0.344111534	0.747595179	801	827	1168	1681	985	729	1251	1184
ENSECAG00000018895	4.68294571	0.344176902	0.747595179	176	511	255	1468	185	224	513	888
ENSECAG00000007313	8.842699355	0.344192229	0.747595179	10380	10435	7933	7057	9553	10080	7061	5200
ENSECAG00000017006	5.670937428	0.344229347	0.747595179	747	1397	795	1090	763	1084	834	1068

ENSECAG00000010244	4.200131777	0.34423912	0.747595179	223	344	424	502	337	286	351	399
ENSECAG00000008401	7.517037711	0.344328327	0.747595179	2096	4857	4400	4083	2163	3898	1562	5092
ENSECAG00000008976	5.73843222	0.344333223	0.747595179	1115	1231	796	910	1023	782	1108	1051
ENSECAG00000013877	7.609487209	0.344350684	0.747595179	2891	3593	4109	5504	3840	3287	3392	3636
ENSECAG00000002233	5.526629637	0.344386662	0.747595179	552	592	885	1955	626	477	871	1420
ENSECAG00000016850	4.354008415	0.344412535	0.747595179	274	271	286	451	416	332	698	514
ENSECAG00000006379	5.242074713	0.344570039	0.747794491	620	668	798	891	841	558	727	711
ENSECAG00000019151	2.408346823	0.344759254	0.747994162	47	144	44	68	128	64	236	105
ENSECAG00000018428	5.492063429	0.34480984	0.747994162	460	827	1186	1356	559	456	630	1636
ENSECAG00000009146	4.062119844	0.344938298	0.747994162	125	317	199	449	430	323	470	365
ENSECAG00000011192	8.325172003	0.345165789	0.747994162	9468	6802	3311	4624	7091	3459	8182	3891
ENSECAG00000010178	2.253961821	0.345317408	0.747994162	39	90	81	77	132	123	142	57
ENSECAG00000009106	4.127679221	0.345362918	0.747994162	215	401	362	442	461	188	386	261
ENSECAG00000009612	4.504318712	0.345376514	0.747994162	395	389	567	428	534	448	446	231
ENSECAG00000013118	6.779995876	0.345376603	0.747994162	1233	2153	2698	3095	1833	1929	2342	1989
ENSECAG00000001495	3.243700422	0.345387167	0.747994162	86	268	133	316	241	119	140	173
ENSECAG00000000746	2.78756909	0.345431143	0.747994162	66	79	160	109	262	118	218	72
ENSECAG00000012781	5.285014673	0.345467078	0.747994162	353	884	717.002	1331	610	850	736	683
ENSECAG00000011805	6.085392116	0.345499155	0.747994162	622	964	1115	1724	1804	1646	1643	1310
ENSECAG00000005081	7.686797909	0.345516144	0.747994162	2767	2321	4067	2994	6586	4349	6961	2388
ENSECAG00000015020	3.861733095	0.345696142	0.748171347	135	155	271	386	301	324	354	393
ENSECAG00000010042	7.457356516	0.345729421	0.748171347	1550	3127	3347	2928	3878	4016	4721	4363
ENSECAG00000013574	3.842378449	0.346266128	0.749190397	216	469	220	220	155	324	293	264
ENSECAG00000019852	6.72153937	0.346422988	0.749348082	1350	1333	1565	2278	2297	1480	3353	3181
ENSECAG00000010333	6.99852605	0.346470646	0.749348082	1376	4915	2282	2116	1248	4014	1247	2117
ENSECAG00000008559	4.906425053	0.34662334	0.749425457	385	433	541	490	847	910	578	451
ENSECAG00000020509	0.350784688	0.346760021	0.749425457	8	21	12	32	26	30	25	34
ENSECAG00000024013	6.206704683	0.346763716	0.749425457	859	1033	1329	1454	1942	1563	1829	1639
ENSECAG00000004152	4.446296409	0.346769724	0.749425457	191	180	383	665	825	288	615	376
ENSECAG00000013965	2.175987074	0.346845705	0.749433446	13	54	150	56	139	83	122	98
ENSECAG00000014665	3.876694679	0.34692461	0.749433446	170	219	227	306	322	210	541	344
ENSECAG00000020263	4.502181826	0.3469709	0.749433446	230	306	835	513	327	344	571	447
ENSECAG00000011267	6.951935489	0.347260801	0.749917342	1582	1684	2204	2089	3053	2576	3688	2641
ENSECAG00000022573	6.002524114	0.347484083	0.750183122	751	965	863	1507	1474	1000	1879	1781
ENSECAG00000020143	4.17144184	0.347631667	0.750183122	216	226	305	379	590	297	593	246
ENSECAG00000000391	2.901951356	0.347646965	0.750183122	67	56	156	194	226	156	155	172
ENSECAG00000020556	0.183248781	0.347647444	0.750183122	6	28	14	12	26	36	20	20
ENSECAG00000022822	5.229921745	0.347770736	0.75030696	514.003	671	953	869.003	831	575.003	912	507.001
ENSECAG00000017393	6.86901194	0.347879727	0.750368396	1795	2207	2125	3534	2522	1068	3056	1987
ENSECAG00000013324	6.445971863	0.348001455	0.750368396	1496	1234	1067	1288	1744	1692	1831	3107
ENSECAG00000015565	1.553605266	0.348110411	0.750368396	52	38	23	47	77	72	60	67
ENSECAG00000008292	4.156351472	0.348129154	0.750368396	231	320	325	586	296	313	297	414
ENSECAG00000012999	4.710192543	0.348216917	0.750368396	548	581	403	461	286	416	617	631
ENSECAG00000007703	5.969284583	0.348314139	0.750368396	691	743	1217	1371	1624	1159	1881	1301
ENSECAG00000014462	1.524685235	0.34848354	0.750368396	52	72	50	51	28.9998	91	37	32.999
ENSECAG00000016296	5.399105158	0.348509745	0.750368396	681	1160	668	784	596	1018	863	627
ENSECAG00000007315	4.196318843	0.348605944	0.750368396	167	310	452	623	329	392	353	271
ENSECAG00000026972	0.18645993	0.348606805	0.750368396	6.00713	15	18	26	23.0004	27.0001	30	22
ENSECAG00000009074	5.330390887	0.348755577	0.750368396	460	368	1153	1458	650	546	869	915
ENSECAG00000011865	5.344343695	0.348760782	0.750368396	520	385	844	809	1129	656	1200	891
ENSECAG00000009946	6.609029518	0.348871023	0.750368396	1454	1935	2042	2426	1576	1867	1943	1838
ENSECAG00000006985	1.616209071	0.348891361	0.750368396	4	81	135	81	2	111	2	47
ENSECAG00000005203	5.761143087	0.348959655	0.750368396	881	1770	665	976	427	1164	913	1397
ENSECAG00000024140	0.766618759	0.349027085	0.750368396	15	21	21	42	23	58	42	33
ENSECAG00000015185	3.187011085	0.349032865	0.750368396	112	112	149	191	251	145	247	222
ENSECAG00000010514	1.93116976	0.349047013	0.750368396	61	111	64	68	26	35	85	120
ENSECAG00000010433	4.290847856	0.349059733	0.750368396	329	245	289	302	491	333	677	379
ENSECAG00000010757	3.590752194	0.349168824	0.750368396	126	149	190	302	218	215	286	428
ENSECAG00000014476	4.880826732	0.34918329	0.750368396	257	423	569	668	768	367	859	823
ENSECAG00000022461	4.296993429	0.349370411	0.750523263	164	291	330	500	391	281	632	585
ENSECAG00000016018	2.41612211	0.349582669	0.750523263	53	56	111	94	49	56	202	230
ENSECAG00000016045	1.620161293	0.349589027	0.750523263	18	46	53	73	80	78	85	46
ENSECAG00000017777	3.954500136	0.349589482	0.750523263	130	292	251	322	275	420	450	318
ENSECAG00000009492	4.475453642	0.349757567	0.750523263	199	225	404	640	505	360	596	659
ENSECAG00000016281	7.370361125	0.349983793	0.750523263	3246	3449	3006	3314	3458	2157	3734	2751
ENSECAG00000009695	6.965214003	0.350002328	0.750523263	1359	2860	2643	3615	1805	1990	3001	2436
ENSECAG00000016959	7.021310751	0.350060678	0.750523263	2338	3803	2290	1757	2398	1971	3581	1498
ENSECAG00000008366	6.323851345	0.350130611	0.750523263	1100	2489	1456	1329	1025	1949	1525	1314
ENSECAG00000021188	6.407981763	0.350277376	0.750523263	655	1144	1855	1891	1923	1827	2473	1896
ENSECAG00000010435	3.562221541	0.35033968	0.750523263	78	178	194	319	315	223	429	174
ENSECAG00000004492	3.000966445	0.350399872	0.750523263	101	157	140	264	182	140	147	107
ENSECAG00000024094	2.192008418	0.350415292	0.750523263	51	45	54	131	72	98	119	148
ENSECAG00000013524	1.636326991	0.350436007	0.750523263	21	24	88	55	65	92	65	70
ENSECAG00000017455	2.484919974	0.350454448	0.750523263	88	95	60	62	249	93	140	60
ENSECAG00000010621	4.286740534	0.350503543	0.750523263	244	291	309	369	579	292	685	316
ENSECAG00000023916	5.234890079	0.350515036	0.750523263	593	757	713	904	747	718	576	755

ENSECAG00000014965	5.24943558	0.350545152	0.750523263	337	440	742	997	795	811	1207	803
ENSECAG00000020642	5.697101255	0.350548038	0.750523263	716	1220	961	1232	842	1094	954	976
ENSECAG00000022049	4.494122564	0.350590625	0.750523263	240	410	332	451	510	369	794	481
ENSECAG00000018424	5.434692259	0.350639722	0.750523263	587	552	675	912	1179	761	1157	984
ENSECAG00000016770	6.04426084	0.350835271	0.750609628	704	1843	1534	1256	1075	1438	1177	1161
ENSECAG00000010581	5.39591581	0.350878092	0.750609628	891	1008	605	674	879	663	929	651
ENSECAG00000008104	6.285625968	0.350913626	0.750609628	946	1732	1814	1861	1133	1083	1634	1986
ENSECAG00000006611	6.002417059	0.35094379	0.750609628	779	1044	955	1280	1420	1349	1755	1529
ENSECAG00000009706	9.245507995	0.351702045	0.752090117	10431	16600	11117	10176	12803	8351	12438	10074
ENSECAG00000024607	6.818015263	0.351916094	0.752336407	1129	1373	2241	2359	3435	2634	3125	1648
ENSECAG00000009278	6.128146936	0.351950148	0.752336407	779	542	1475	1464	3057	733	2485	689
ENSECAG00000007378	4.974521777	0.352161698	0.752336407	391	698	620	826	558	673	475	621
ENSECAG00000024711	6.510831218	0.352307952	0.752336407	1368	1019	1524	1632	2643	1785	2674	1638
ENSECAG00000010144	3.384736681	0.352312806	0.752336407	81	132	223	233	314	203	327	154
ENSECAG00000023953	7.400563259	0.352322842	0.752336407	3731	3285	2909	3158	3124	3125	3401	2565
ENSECAG00000010075	5.232200765	0.352332283	0.752336407	363	479	727	891	788	666	1002	1100
ENSECAG00000013800	3.160741152	0.352345869	0.752336407	96	124	140	195	310	107	230	207
ENSECAG00000024367	4.435929985	0.352732688	0.752679004	293	760	387	438	23	251	109	1018
ENSECAG00000026858	3.549509419	0.352752393	0.752679004	96	147	277	230	314	274	303	212
ENSECAG00000024602	6.325914684	0.352752671	0.752679004	1097	2107	1244	2041	1125	1579	1227	1940
ENSECAG00000009444	3.238172006	0.352881193	0.752679004	90	91	304	372	36	133	102	367
ENSECAG00000000626	4.700336231	0.35303287	0.752679004	429	638	434	516	449	374	697	448
ENSECAG00000024544	8.252090154	0.353094337	0.752679004	3108	4566	5517	5653	9469	6193	8812	5115
ENSECAG00000011172	8.215982043	0.35309583	0.752679004	2962	3445	6031	6231	8040	5760	8420	6662
ENSECAG00000014391	5.635153789	0.353101871	0.752679004	597	696	761	1136	1124	1162	1480	915
ENSECAG00000014690	6.17342139	0.353211809	0.752679004	1082	1199	1347	2252	1415	959	1876	1189
ENSECAG00000017265	6.488964205	0.353294713	0.752679004	1192	1139	1493	4063	861	730	2220	2761
ENSECAG00000009079	3.818938354	0.353295612	0.752679004	341	775	67	47	3	613	2	77
ENSECAG00000002413	7.349849436	0.35335965	0.752679004	1311	3306	2758	7798	718	1452	2711	6440
ENSECAG00000014914	5.276209256	0.353365769	0.752679004	769	437	635	1337	488	244	1318	855
ENSECAG00000021033	5.247464596	0.353575415	0.75298468	593	922	737	766	313	1066	610	757
ENSECAG00000013930	7.364929548	0.353700154	0.753109455	3198	4118	2540	3042	2786	2799	3971	2475
ENSECAG00000007086	3.517480769	0.3539189	0.753434307	120	253	246	349	227	260	72	235
ENSECAG00000016198	1.574530171	0.354016229	0.753465758	20	25	62	70	81	24	162	34
ENSECAG00000021328	4.529655418	0.354066035	0.753465758	314	425	491	651	202	399	496	627
ENSECAG00000010409	4.765737225	0.354133589	0.753468681	185	383	510	727	698	771	470	591
ENSECAG00000016233	3.598722312	0.354475182	0.75405455	339	254	91	230	86	260	264	244
ENSECAG00000012352	3.419252531	0.354570409	0.754057831	155	245	187	270	118	158	232	283
ENSECAG00000018603	3.117457554	0.35460919	0.754057831	95	242	258	181	12	79	24	431
ENSECAG00000017677	7.989429484	0.354730016	0.754173899	1800	2991	4618	7077	6153	3842	8407	6702
ENSECAG00000015796	3.157251838	0.354844245	0.754275901	125	107	106	200	261	94	249	258
ENSECAG00000017501	3.022545559	0.354926773	0.754310494	89	339	84	150	112	200	123	125
ENSECAG00000022204	5.433971284	0.355193224	0.754735881	820	762	794	962	748	623	983	916
ENSECAG00000026929	1.672229319	0.355463436	0.755169102	22	58	41	66	18	50	131	119
ENSECAG00000013639	2.335879437	0.355975555	0.756115987	22	54	112	133	78	153	90	159
ENSECAG00000015474	6.05756379	0.356082347	0.756201739	836	699	1045	1660	1613	967	2108	1728
ENSECAG00000015821	6.842773538	0.356329794	0.756346626	1735	2659	2400	2402	2279	2045	2516	1589
ENSECAG00000019386	5.211389305	0.356381169	0.756346626	416	1424	521	610	698	785	794	410
ENSECAG00000017599	2.643589762	0.356413332	0.756346626	70	63	79	173	155	62	196	197
ENSECAG00000014694	5.12177965	0.356416307	0.756346626	375	567	593	709	913	674	946	729
ENSECAG00000010590	4.748561111	0.356553466	0.756496685	284	607	577	725	422	582	550	454
ENSECAG00000022236	3.079407369	0.356683623	0.756631833	104	176	224	210	253	48	247	50
ENSECAG00000008011	4.701567825	0.356822717	0.756647881	248	286	373	802	377	539	679	893
ENSECAG00000000971	6.732904008	0.356824108	0.756647881	1801	1024	1888	1475	3667	2091	2404	2054
ENSECAG00000018579	1.392170307	0.356988591	0.7568557	47	24	70	69	55	21	43	61
ENSECAG00000022992	9.835035765	0.357132535	0.757019906	11509	32368	17041	14712	6919	12187	15318	29898
ENSECAG00000000512	5.15164756	0.357732607	0.758051899	356	453	674	808	937	452	1393	664
ENSECAG00000002072	3.899862253	0.357765268	0.758051899	145	241	178	386	250	185	474	542
ENSECAG00000016660	5.936791767	0.357890314	0.758051899	1099	1577	1179	844	1235	1174	1077	1005
ENSECAG00000009109	4.725556138	0.357919777	0.758051899	314	238	753	934	514	459	522	452
ENSECAG00000019274	5.50708106	0.357994482	0.758051899	859	1029	736	852	853	657	985	926
ENSECAG00000012497	6.049843701	0.358036184	0.758051899	443	789	1415	1755	1350	1394	1417	2137
ENSECAG00000010254	4.791536083	0.358294688	0.758051899	196	394	554	697	486	757	672	682
ENSECAG00000009947	4.195587837	0.35837305	0.758051899	284	241	304	274	511	463	444	298
ENSECAG00000023553	3.629390954	0.35842161	0.758051899	86	142	251	330	404	242	341	190
ENSECAG00000010009	4.519843302	0.358452536	0.758051899	310	322	349	449	512	311	832	551
ENSECAG00000021794	1.11926154	0.358470205	0.758051899	27	45	53	48	23	51	26	46
ENSECAG00000012977	4.261389625	0.358499123	0.758051899	182	204	367	495	442	241	607	555
ENSECAG00000013728	2.244224703	0.358533897	0.758051899	103	71	92	120	30	167	20	85
ENSECAG00000009640	3.817516572	0.358551557	0.758051899	182	336	339	262	248	178	367	266
ENSECAG00000016424	8.253336346	0.358709715	0.758245471	7366	7024	5021	4300	2936	9936	3898	3939
ENSECAG00000014482	4.024583982	0.358802641	0.758301108	161	398	346	442	173	413	269	327
ENSECAG00000009359	0.33498457	0.358952798	0.758358482	3	28	17	23	29	34	19	32
ENSECAG00000021982	5.843853326	0.358963009	0.758358482	729	654	983	1259	1911	814	1362	1359
ENSECAG00000013197	7.294308731	0.35908493	0.758475311	3861	2925	2320	2902	1947	3104	3087	3178
ENSECAG00000007441	6.170967252	0.35927092	0.758727403	786	540	1318	1979	1676	904	2725	1788

ENSECAG00000012279	6.855724587	0.359350978	0.758755728	1652	1858	1712	1690	3290	1897	3148	2848
ENSECAG00000010801	7.00665985	0.359571211	0.75893955	1804	3345	2559	2650	1842	2522	2381	2638
ENSECAG00000016520	3.85236405	0.35957136	0.75893955	157	163	390	523	240	224	438	170
ENSECAG00000014336	4.865118615	0.359846853	0.759280595	268	305	729	555	816	673	997	311
ENSECAG00000021997	6.296055006	0.359904744	0.759280595	967	1248	1290	1382	1960	1211	2839	1585
ENSECAG00000012173	3.090179917	0.360036468	0.759280595	50	195	120	161	208	80	384	179
ENSECAG00000015777	3.951576241	0.360048985	0.759280595	111	282	278	314	551	236	507	195
ENSECAG00000016142	4.734999922	0.360066397	0.759280595	272	389	678	898	388	398	569	644
ENSECAG00000008882	7.022559162	0.360542994	0.760144812	1737	2586	2371	4137	1672	1947	2783	3193
ENSECAG00000008202	5.660433586	0.360708912	0.760353817	597	611	1140	844	926	1454	1172	1180
ENSECAG00000013256	6.168341065	0.361115736	0.761034568	641	1428	1480	2574	1229	1156	1789	1215
ENSECAG00000019895	0.082703529	0.361165549	0.761034568	15	3	45	23	30	8	21	4
ENSECAG00000022922	3.939464046	0.361632168	0.761805592	192	149	312	305	454	214	493	309
ENSECAG00000018080	5.767179785	0.361703976	0.761805592	513	658	1046	1370	1127	1159	1308	1531
ENSECAG00000008524	1.74990398	0.361732194	0.761805592	38	49	50	62	97	36	104	84
ENSECAG00000010776	5.651572603	0.361901553	0.761828345	636	582	1045	843	1688	815	1718	620
ENSECAG00000015895	4.574743941	0.361930473	0.761828345	403	593	347	499	409	394	530	458
ENSECAG00000015392	7.016409713	0.361943743	0.761828345	1089	1358	2827	3043	3052	2411	4804	2440
ENSECAG00000015409	5.304022866	0.362208928	0.762245591	366	797	1042	1080	650	970	620	657
ENSECAG00000013307	5.256984527	0.362285498	0.762265828	375	544	839	706	1116	792	991	685
ENSECAG00000009830	3.365488829	0.362487186	0.762349061	120	224	247	243	225	197	152	174
ENSECAG00000004865	2.934363793	0.362509904	0.762349061	56	99	125	217	193	171	208	151
ENSECAG00000024187	8.773395937	0.362525939	0.762349061	4856	4235	9874	7772	14833	8407	12553	6954
ENSECAG00000014323	2.534472754	0.362681988	0.762536368	52	41	103	173	124	138	148	141
ENSECAG00000023311	5.867020151	0.362749068	0.762536583	448	537	850	2018	981	627	1931	2253
ENSECAG00000009251	5.430510556	0.362829177	0.762564184	678	802	551	502	1205	1099	1209	581
ENSECAG00000025043	0.730148378	0.362991468	0.762764463	9	4	36	50	37	35	44	40
ENSECAG00000023461	6.162071353	0.36307245	0.762793844	1130	1450	1261	1893	1062	773	1859	1731
ENSECAG00000023133	0.909678827	0.363342891	0.763221183	19	41	15	30	38	27	63	50
ENSECAG00000021043	4.528223293	0.363497059	0.763345733	372	455	304	142	973	377	632	264
ENSECAG00000014390	1.050431667	0.363536281	0.763345733	6	12	134	42	12	21	3	83
ENSECAG00000011237	5.552562498	0.363672363	0.763465165	531	761	1100	1481	776	903	938	909
ENSECAG00000019086	3.882127535	0.363736409	0.763465165	165	192	191	400	320	224	459	406
ENSECAG00000007072	3.936496488	0.363794337	0.763465165	192	204	281	269	513	196	461	291
ENSECAG00000017052	5.299056867	0.36429774	0.764246945	459	408	677	1012	719	858	1262	902
ENSECAG00000025122	2.010033984	0.364301113	0.764246945	71	72	105	64	66	62	61	95
ENSECAG00000024350	4.416577725	0.364663504	0.764537236	291	237	274	519	449	178	796	679
ENSECAG00000011156	2.914247077	0.364695743	0.764537236	37	145	134	169	158	155	197	208
ENSECAG00000005795	4.548418715	0.364722529	0.764537236	199	243	473	614	358	372	584	934
ENSECAG00000024673	4.199869657	0.364729927	0.764537236	225	210	392	312	635	297	548	270
ENSECAG00000015035	3.980618023	0.364775254	0.764537236	244	196	259	256	408	229	515	358
ENSECAG00000016157	1.829201099	0.364947854	0.764725656	24	57	71	67	105	62	100	68
ENSECAG00000016479	6.094923014	0.364999492	0.764725656	974	875	1210	1160	1323	1288	2177	1755
ENSECAG00000009638	3.67177313	0.365078221	0.76474987	156	150	244	241	305	212	509	206
ENSECAG00000016601	5.045053038	0.365253262	0.764878809	461	438	547	605	1126	547	768	656
ENSECAG00000025072	7.81115544	0.365296958	0.764878809	3180	4313	4753	6216	4040	3791	3918	4653
ENSECAG00000005699	4.412064219	0.365341323	0.764878809	409	836	159	242	262	584	236	376
ENSECAG00000024722	4.945797426	0.365476799	0.764887191	463	662	388	291	885	547	801	702
ENSECAG00000016906	2.207676577	0.365479694	0.764887191	45	71	59	108	111	74	80	171
ENSECAG00000005017	0.673233009	0.365625616	0.764994857	5	14	23	55	31	32	28	57
ENSECAG00000012227	3.897056156	0.365665526	0.764994857	174	307	305	425	268	285	245	305
ENSECAG00000008261	4.958173498	0.365793665	0.765122336	404	403	632	507	850	630	916	531
ENSECAG00000007896	5.255205398	0.366204223	0.765732143	475	505	894	1281	666	591	833	794
ENSECAG00000006415	0.63762111	0.36621972	0.765732143	9	23	13	47	25	39	31	48
ENSECAG00000006749	7.042418729	0.36634485	0.765853125	4021	2010	1594	2248	3036	1430	3593	1404
ENSECAG00000016094	2.184866922	0.366453283	0.765939164	54	122	64	132	47	101	97	77
ENSECAG00000015812	7.555613321	0.366548862	0.765998309	4063	3694	3147	3944	2897	5452	3123	1695
ENSECAG00000018415	2.500091398	0.367070046	0.766835175	59	73	246	100	42	73	89	188
ENSECAG00000014427	0.67004205	0.367135551	0.766835175	13	15	25	38	47	38	55	9
ENSECAG00000007377	6.038504699	0.367151387	0.766835175	768	2486	947	1052	954	1541	1336	943
ENSECAG00000000520	7.871202925	0.36734037	0.767089162	3281	5066	2630	2465	5854	4031	8819	4542
ENSECAG00000011429	3.373901039	0.367518608	0.767117938	124	83	83	360	363	134	332	185
ENSECAG00000000595	4.60211486	0.367568797	0.767117938	314	337	378	515	501	445	661	677
ENSECAG00000022341	8.741486564	0.367580567	0.767117938	9827	9184	6693	7571	5689	11649	6308	6264
ENSECAG00000000594	3.997344927	0.367623669	0.767117938	194	369	305	414	321	291	301	277
ENSECAG00000000749	5.18843806	0.367702575	0.767141986	515	411	596	758	1114	503	1142	718
ENSECAG00000024479	2.633588488	0.367925748	0.767466955	93	98	109	211	98	51	135	167
ENSECAG00000011300	0.499075302	0.36804544	0.76757599	23	15	21	13	34	14	62	25
ENSECAG00000002810	0.118460043	0.36834106	0.768035471	1	37	28	25	12	24	23	6
ENSECAG00000019218	4.386271223	0.368400677	0.768035471	200	284	427	449	506	320	581	568
ENSECAG00000000642	7.711083212	0.368513293	0.768106401	2482	2998	3910	3497	5048	3474	6660	5200
ENSECAG00000020984	7.36595458	0.368569633	0.768106401	2397	3844	2215	5577	1015	2095	2646	5987
ENSECAG00000008518	1.097989591	0.368809967	0.768466595	15	43	33	104	22	7	107	8
ENSECAG00000012033	1.547463003	0.368919594	0.76855436	35	45	35	53	38	31	117	100
ENSECAG00000006851	0.266231103	0.369112879	0.768816343	6	19	24	18	19	20	20	50
ENSECAG00000025015	6.129960294	0.369227708	0.768888015	922	1350	1251	2193	1320	1224	1392	1308

ENSECAG00000020780	5.847884025	0.36928236	0.768888015	636	1079	1216	1828	1194	821	1294	1043
ENSECAG00000022206	5.229934587	0.369516261	0.769234345	374	589	655	835	931	715	1089	788
ENSECAG00000009207	2.550064922	0.369633593	0.769337925	82	54	94	125	159	120	152	121
ENSECAG00000023190	4.28559714	0.36974571	0.769430617	338	662	237	261	270	362	557	249
ENSECAG00000006951	3.731116617	0.370043903	0.769573014	98	188	261	313	484	278	288	196
ENSECAG00000015393	5.490745031	0.370129707	0.769573014	950	867	786	791	849	603	1132	818
ENSECAG00000009429	0.514777425	0.370133793	0.769573014	12	15	38	14	23	28	39	42
ENSECAG00000012328	7.068804412	0.370158592	0.769573014	1606	1798	2026	2964	4136	2400	3791	2582
ENSECAG00000019894	6.413502786	0.370174787	0.769573014	1916	1917	1655	931	1702	1470	2107	963
ENSECAG00000018335	2.889382638	0.37021971	0.769573014	66	95	142	171	158	151	212	179
ENSECAG00000015517	1.800943874	0.37041723	0.769843038	20	68	60	160	47	49	66	80
ENSECAG00000020392	5.333102899	0.370539648	0.769866548	618	419	735	711	1138	628	1087	966
ENSECAG00000009111	5.867588904	0.370577145	0.769866548	1001	1175	1009	1387	851	1106	1023	1379
ENSECAG00000010892	6.080319709	0.370674218	0.769866548	744	1153	1273	2529	1239	975	1486	1381
ENSECAG00000007907	3.588239139	0.370699026	0.769866548	140	295	311	298	46	334	30	371
ENSECAG00000009967	4.526152908	0.371127319	0.770615452	229	367	504	346	728	319	811	355
ENSECAG00000019296	3.61178594	0.371629205	0.771516867	71	138	239	361	193	260	258	455
ENSECAG00000002386	2.657137944	0.371876464	0.771733678	73	76	97	142	158	88	253	113
ENSECAG00000018942	4.947501994	0.371917009	0.771733678	329	328	556	816	713	555	921	727
ENSECAG00000002911	2.51832649	0.371936995	0.771733678	55	231	81	97	71	87	102	144
ENSECAG00000024783	3.576199514	0.372104471	0.771940489	177	130	214	190	428	170	385	164
ENSECAG00000017164	9.424144138	0.372417718	0.772436664	6118	8311	15225	13505	20022	17796	15175	12920
ENSECAG00000009221	3.996021419	0.37247934	0.772436664	146	185	260	449	597	246	330	328
ENSECAG00000002831	3.307869693	0.372582693	0.772488363	254	229.998	137	136.998	85	332	52.9993	184
ENSECAG00000011251	3.100972836	0.372696302	0.772488363	80	130	159	177	162	195	253	199
ENSECAG00000018661	5.231067123	0.372707824	0.772488363	525	592	849	1047	753	531	809	761
ENSECAG00000017573	4.834933452	0.372862086	0.772667427	434	569	558	700	666	320	794	391
ENSECAG00000020252	9.697796577	0.373218563	0.773265394	18708	15684	14524	15469	12203	15792	15695	16161
ENSECAG00000017190	2.893713469	0.373446541	0.773532442	84	77	169	128	144	154	170	234
ENSECAG00000008318	3.233041356	0.373596851	0.773532442	125	94	150	207	122	144	377	278
ENSECAG00000006082	1.155845018	0.373629568	0.773532442	31	32	68	48	61	21	17	49
ENSECAG00000010991	5.879792868	0.373680812	0.773532442	616	634	1849	1920	1476	998	1112	716
ENSECAG00000017124	4.431025514	0.373739936	0.773532442	177	286	386	583	546	312	705	493
ENSECAG00000015471	3.967408698	0.373874858	0.773532442	220	158	303	290	302	429	387	344
ENSECAG00000007848	1.719467542	0.373900178	0.773532442	21	36	61	87	33	68	115	102
ENSECAG00000008288	4.587742449	0.373928514	0.773532442	322	234	481	470	732	551	601	368
ENSECAG00000002263	7.165056399	0.373958943	0.773532442	2288	4404	2204	2455	1773	2856	2622	3147
ENSECAG00000016617	3.493051987	0.374045399	0.773570729	115	202	211	420	231	139	252	214
ENSECAG00000010162	2.432006022	0.374138688	0.773623131	26	97	93	277	67	125	81	96
ENSECAG00000024440	4.621167404	0.374225699	0.773662535	322	325	437	470	693	401	673	545
ENSECAG00000023244	3.968603606	0.374676254	0.774453368	211	156	313	298	400	252	416	411
ENSECAG00000023115	3.378949374	0.374888666	0.774751763	75	82	509	255	169	293	84	149
ENSECAG00000015467	6.893035245	0.37537643	0.775550797	2299	2124	2068	2877	1703	1781	2905	2465
ENSECAG00000012578	1.925512033	0.375411545	0.775550797	49	78	39	49	57	106	153	51
ENSECAG00000009487	6.070916224	0.375639211	0.775584304	987	1422	1251	1691	1089	1253	1118	1557
ENSECAG00000018028	7.604504608	0.375654037	0.775584304	4912	5564	3992	1515	199	7754	314	3226
ENSECAG00000009028	6.391740895	0.375676394	0.775584304	1687	1675	1521	1540	1169	1600	1883	1622
ENSECAG00000026849	5.932642001	0.375700258	0.775584304	824	1242	910	680	1409	1911	1344	1064
ENSECAG00000017490	5.263760114	0.376070848	0.776208593	529	815	825	857	622	627	936	754
ENSECAG00000018299	5.829220896	0.376392298	0.776731251	926	1039	1238	1241	841	961	1237	1272
ENSECAG00000004093	2.091131898	0.376808207	0.777344488	61	54	52	84	81	67	171	93
ENSECAG00000024379	4.000614326	0.376873787	0.777344488	172	232	435	487	245	343	298	299
ENSECAG00000015830	5.241543509	0.376924673	0.777344488	331	476	603	1127	905	736	1115	815
ENSECAG00000020242	3.922282034	0.376992448	0.777344488	119	219	255	403	344	203	381	514
ENSECAG00000013399	2.290347247	0.377082227	0.777344488	36	77	103	86	72	141	88	157
ENSECAG00000018363	5.129114812	0.377247229	0.777344488	799	449	701	708	463	514	699	943
ENSECAG00000011702	3.760216685	0.377322739	0.777344488	106.984	429	180.985	419.976	239.974	269.974	211.969	257
ENSECAG00000008125	3.457074965	0.377361772	0.777344488	154	174	141	198	209	177	440	236
ENSECAG00000012788	3.873604616	0.377369715	0.777344488	189	156	242	338	443	216	331	389
ENSECAG00000026959	4.574944301	0.377372243	0.777344488	272	430	540	710	532	420	544	293
ENSECAG00000022168	5.932311865	0.377593114	0.777658756	836	960	1000	2304	685	767	1689	1485
ENSECAG00000010029	4.779980175	0.377861305	0.778070349	479	536	581	511	435	575	502	547
ENSECAG00000024364	5.86486827	0.378245225	0.778720052	634	1215	1346	1552	1087	937	1375	1024
ENSECAG00000000580	7.358445671	0.378411175	0.77874637	2492	3694	3058	3960	2503	3076	3038	3438
ENSECAG00000020278	6.299345326	0.378454461	0.77874637	1191	2314	724	2078	1324	1491	1560	1416
ENSECAG00000013475	7.107312121	0.378488396	0.77874637	1249	1935	3040	2541	3676	2481	4037	3081
ENSECAG00000020244	0.781575001	0.378531613	0.77874637	9	33	67	33	14	27	39	35
ENSECAG00000021804	7.928854049	0.378647263	0.778812706	8596	1960	5711	1778	5959	4874	4153	1316
ENSECAG00000020982	3.765971344	0.378717449	0.778812706	246	269	241	292	261	191	303	266
ENSECAG00000018846	3.016429052	0.378825807	0.778812706	113	184	181	160	106	130	208	158
ENSECAG00000018545	2.743313638	0.378865914	0.778812706	74	116	102	119	177	97	198	164
ENSECAG00000016189	4.666498194	0.378905892	0.778812706	270	569	445	804	333	440	544	599
ENSECAG00000000834	6.302567513	0.379065178	0.778967217	1101	1255	1222	1215	2493	1034	2703	1397
ENSECAG00000017299	1.098508857	0.379117905	0.778967217	3	33	36	60	32	58	50	62
ENSECAG00000017845	1.174169948	0.379659883	0.779901027	24	20	38	54	50	42	62	55
ENSECAG00000024076	6.929765378	0.379751348	0.779901027	1682	2750	2244	3223	1930	2127	2445	2530

ENSECAG00000011135	3.740682343	0.379777891	0.779901027	106.011	204	184.008	379	485.001	189.001	324	267.024
ENSECAG00000008183	2.986941635	0.379937369	0.780087817	103	523	43	53	0	333	2	61
ENSECAG00000018731	7.975029928	0.380024714	0.780126464	4490	6664	4002	4602	3389	5107	5429	4434
ENSECAG00000006540	5.416401828	0.380203602	0.780262052	467	473	776	1071	1023	677	1530	854
ENSECAG00000023341	3.24986826	0.380227832	0.780262052	112	184	136	141	294	131	354	140
ENSECAG00000022603	5.798549042	0.380471488	0.780455251	1032	1319	905	1008	722	1305	1194	922
ENSECAG00000020274	4.850154747	0.380484099	0.780455251	368	547	327	566	572	439	978	767
ENSECAG00000021844	2.954445712	0.380560307	0.780455251	74	155	189	227	110	117	125	212
ENSECAG00000006244	5.522262763	0.380596184	0.780455251	929	800	1044	742	1250	761	899	472
ENSECAG00000010181	3.049226635	0.380855077	0.780615152	98	77	159	186	202	149	217	214
ENSECAG00000007619	2.923094455	0.380975778	0.780615152	47	103	155	181	272	138	105	196
ENSECAG00000003770	3.061179724	0.381022819	0.780615152	86	290	87	225	100	97	229	185
ENSECAG00000000369	4.220818277	0.381047343	0.780615152	162	770	348	244	149	284	287	612
ENSECAG00000010008	4.668075078	0.38110746	0.780615152	221	841	500	544	213	555	294	759
ENSECAG00000016083	7.145205085	0.381145168	0.780615152	2160	3670	2485	2964	3093	1882	2984	2493
ENSECAG00000012599	2.000209213	0.381207363	0.780615152	36	88	59	156	81	68	59	72
ENSECAG00000006365	6.929782422	0.38135371	0.780615152	1722	2428	2277	3527	1810	2161	2798	2310
ENSECAG00000019029	6.886853468	0.381371089	0.780615152	1548	1860	2812	3599	2481	2146	2293	1778
ENSECAG00000024363	5.363773712	0.381398442	0.780615152	603	778	734	1166	644	875	741	833
ENSECAG00000008099	3.658024301	0.38154026	0.780615152	147	272	252	349	242	191	269	245
ENSECAG00000008712	4.926579924	0.381593222	0.780615152	483	553	594	754	563	391	793	588
ENSECAG00000002323	3.170166555	0.381733581	0.780615152	101	80	190	193	233	147	333	154
ENSECAG00000011036	5.334473022	0.381783794	0.780615152	739	737	1019	588	786	778	911	544
ENSECAG00000015459	6.141510106	0.381870247	0.780615152	644	772	1544	1657	2008	1602	1284	1703
ENSECAG00000015899	6.816653395	0.381886376	0.780615152	1809	2986	1929	2202	1639	2880	1893	1733
ENSECAG00000020867	8.459491004	0.381905747	0.780615152	9971	6854	4277	5111	6492	5235	7560	6011
ENSECAG00000012321	5.759124357	0.381908335	0.780615152	618	957	938	917	899	1204	1306	1676
ENSECAG00000017891	2.165172524	0.38243869	0.781558874	50	47	114	51	109	55	203	75
ENSECAG00000012654	4.11024928	0.38260971	0.781768046	100	237	324	499	535	317	447	331
ENSECAG00000019425	3.451746093	0.38274209	0.781804515	142	176	212	378	170	118	178	338
ENSECAG00000016530	3.982753511	0.382764898	0.781804515	311	247	369	293	207	175	302	492
ENSECAG00000007723	2.954635492	0.382841728	0.781821179	83	139	108	150	131	118	283	215
ENSECAG00000012467	3.843876047	0.383080011	0.782110576	143	177	452	438	336	232	326	162
ENSECAG00000001299	3.283253843	0.383120833	0.782110576	114	171	144	171	238	115	291	286
ENSECAG00000016771	0.609982114	0.383358606	0.782116599	18	30	37	35	10	15	45	35
ENSECAG00000021148	3.195683921	0.383573167	0.782116599	78	152	143	210	106	177	303	296
ENSECAG00000019865	4.060854189	0.383668947	0.782116599	231	162	325	322	381	227	548	441
ENSECAG00000019980	3.465195055	0.38368083	0.782116599	127	108	192	278	242	214	410	191
ENSECAG00000011341	2.544383776	0.383684773	0.782116599	46	122	66	129	161	71	196	134
ENSECAG00000013398	6.144273989	0.383745109	0.782116599	541	1073	1565	1386	2724	1092	1814	1100
ENSECAG00000024217	5.215354215	0.383772417	0.782116599	293	480	727	949	1288	650	1131	463
ENSECAG00000009174	2.124463139	0.383812782	0.782116599	60	94	69	128	57	90	105	62
ENSECAG00000020816	2.301281965	0.383814347	0.782116599	54	45	117	212	121	47	75	102
ENSECAG00000022752	6.365566837	0.383826315	0.782116599	939	1020	1565	1761	1932	1425	2929	1624
ENSECAG00000009140	1.937053	0.383879452	0.782116599	22	81	65	171	44	50	58	113
ENSECAG00000021513	4.316494655	0.383974507	0.782127904	190	441	342	694	257	279	424	538
ENSECAG00000024447	3.382126691	0.384038199	0.782127904	98	266	159	362	165	73	367	175
ENSECAG00000007422	7.952715073	0.384091095	0.782127904	2565	5414	4484	8689	2869	4755	4978	5514
ENSECAG00000000550	4.802669999	0.384376368	0.782470495	268	385	598	570	488	555	685	888
ENSECAG00000024341	6.819338619	0.384396792	0.782470495	1165	3051	2375	2713	2019	2390	2149	1711
ENSECAG00000026970	1.600845592	0.384554913	0.782652428	48	35	52	37	61	66	85	72
ENSECAG00000021530	5.797676353	0.38475289	0.782915399	786	1022	1114	1515	1015	1077	1060	1045
ENSECAG00000015036	7.996087121	0.384867791	0.783009258	3201	3493	4044	5074	5501	4322	8603	6431
ENSECAG00000021752	6.93344441	0.385045256	0.783230345	1566	875	2984	1995	4450	2057	3361	1992
ENSECAG00000021698	2.02578001	0.385459291	0.78393248	32	31	111	78	98	69	114	107
ENSECAG00000012420	5.62565235	0.385667167	0.784079137	505	702	873	1170	1063	878	1375	1321
ENSECAG00000015391	5.810927781	0.385669141	0.784079137	497	667	1185	1349	1513	1498	1340	888
ENSECAG00000013178	5.189749211	0.386047056	0.784433576	331	512	715	856	889	898	716	865
ENSECAG00000011466	6.27697966	0.386048307	0.784433576	783	2512	1456	1592	958	2102	951	1547
ENSECAG00000002889	7.211126814	0.386100129	0.784433576	1702	1565	2638	3508	3346	2665	4870	3473
ENSECAG00000008960	6.108972613	0.386251875	0.784433576	894	1769	1030	1868	976	1043	1732	1484
ENSECAG00000012876	5.060335244	0.386344763	0.784433576	704	821	489	513	328	354	907	925
ENSECAG00000005021	1.961259658	0.38636016	0.784433576	27	31	92	94	136	52	109	74
ENSECAG00000013534	8.184734385	0.386443505	0.784433576	6235	9790	2908	3348	5365	3797	7294	4500
ENSECAG00000020354	3.912211993	0.386596309	0.784433576	341	220	262	314	199	254	356	319
ENSECAG00000019714	4.785064104	0.386621485	0.784433576	467	682	361	630	354	399	645	692
ENSECAG00000015025	0.227555534	0.386623313	0.784433576	11	24	47	9	29	12	23	10
ENSECAG00000020618	4.018347078	0.386629254	0.784433576	164	363	285	524	237	305	322	348
ENSECAG00000019121	5.11120131	0.386721001	0.784433576	423	622	697	1087	403	521	727	970
ENSECAG00000016585	6.520949884	0.386739189	0.784433576	1612	2531	1274	1803	989	2387	1049	2137
ENSECAG00000017045	0.350997323	0.387010252	0.784843556	22	12	56	21	0	0	73	2
ENSECAG00000009463	3.397368905	0.387095362	0.784876348	106	202	147	212	178	258	308	247
ENSECAG00000023146	3.459481623	0.387612677	0.785293398	87	185	244	429	237	140	211	221
ENSECAG00000007577	4.120760197	0.387647837	0.785293398	248.011	435	298.008	387	340.001	186.001	379	409.024
ENSECAG00000011253	5.199875791	0.387675765	0.785293398	319	476	607	1031	537	546	1142	1311
ENSECAG00000004651	8.530895217	0.387724564	0.785293398	10746	8036	3879	4728	7540	4998	8466	5466

ENSECAG00000019673	6.304439675	0.387773199	0.785293398	1058	1419	1408	2601	1037	1162	2026	1779
ENSECAG00000023016	8.385762902	0.387787249	0.785293398	5110	6686	6929	8312	5861	5424	7137	6294
ENSECAG00000023858	2.460456968	0.387844896	0.785293398	70	176	86	103	86	72	101	138
ENSECAG00000004780	4.591722127	0.387852857	0.785293398	180	295	499	640	571	593	696	403
ENSECAG00000011870	1.900176136	0.388323252	0.785748751	33	65	46	86	102	49	124	80
ENSECAG00000023094	7.495662056	0.388384836	0.785748751	2865	3146	3817	4734	3030	3264	3326	3682
ENSECAG00000008406	1.779533651	0.388425192	0.785748751	36	48	46	81	62	76	78	103
ENSECAG00000023998	6.033100398	0.388454914	0.785748751	1013	759	1197	1014	2226	1011	1786	1192
ENSECAG00000023477	4.233413524	0.388483687	0.785748751	225	187	456	322	431	451	534	349
ENSECAG00000015740	5.967177264	0.388491851	0.785748751	667	565	1356	1485	1836	1145	1548	1368
ENSECAG00000008877	4.751745901	0.388689539	0.785887449	300	680	515	682	332	693	494	480
ENSECAG00000024044	4.020140286	0.388735449	0.785887449	238	201	273	293	385	250	457	439
ENSECAG00000011933	4.708351999	0.388847854	0.785887449	550	441	393	610	469	316	644	557
ENSECAG00000012829	8.442645157	0.388850045	0.785887449	5231	8410	7348	6817	6439	5266	7483	6475
ENSECAG00000013103	0.716364553	0.388905567	0.785887449	13	22	20	43	26	33	48	44
ENSECAG00000017962	5.214302087	0.389267297	0.786478824	599	810	630	836	675	574	812	771
ENSECAG00000011818	5.340934536	0.389347006	0.786500295	439.441	792.609	936.259	1127.48	679.159	694.877	770	926.458
ENSECAG00000005741	3.687227057	0.389569733	0.786550436	125	132	194	385	176	197	383	488
ENSECAG00000001304	1.653147216	0.389647126	0.786550436	32	45	34.0016	82	54	76	78	85
ENSECAG00000000266	5.587715222	0.389765929	0.786550436	634	645	900	807	1773	750	1368	663
ENSECAG00000018193	6.597254031	0.389809963	0.786550436	859	1475	1726	2274	2462	2269	2408	1966
ENSECAG00000013922	9.182923855	0.389838938	0.786550436	8754	14566	11690	11632	8838	10032	14974	9063
ENSECAG00000008165	4.492484297	0.389916383	0.786550436	236	320	451	444	576	315	786	464
ENSECAG00000019699	7.956613091	0.389989166	0.786550436	4293	6174	4889	4121	5167	4770	4575	3533
ENSECAG00000017807	0.42906372	0.389994988	0.786550436	6	14	36	21	57	18	25	22
ENSECAG00000016696	5.395849528	0.390066607	0.786550436	613	447	808	683	1412	614	1644	423
ENSECAG00000023114	0.321397027	0.390083308	0.786550436	36	28	8	16	22	13	18	27
ENSECAG00000017334	4.760835407	0.390203528	0.786550436	331	376	779	709	524	435	522	565
ENSECAG00000009644	5.855461635	0.39028823	0.786550436	425	1212	1285	2036	788	1010	906	1580
ENSECAG00000021912	1.438137192	0.390306542	0.786550436	39	37	29	40	15	139	25	74
ENSECAG00000009886	3.407463494	0.390339039	0.786550436	135	75	238	208	332	138	291	253
ENSECAG00000005306	1.370720198	0.390448034	0.786630838	41	37	31	26	58	20	25	146
ENSECAG00000020428	6.416014073	0.39067274	0.78667287	1202	2500	1644	1396	1257	2253	1129	1529
ENSECAG00000013442	5.939779271	0.390775514	0.78667287	720	1259	1337	1694	892	1603	972	1061
ENSECAG00000015420	5.265530842	0.390782029	0.78667287	948	707	678	514	478	1055	725	542
ENSECAG00000012891	7.792200679	0.390818869	0.78667287	4424	5219	3487	3993	3848	3809	4289	4299
ENSECAG00000007211	1.256006005	0.390850871	0.78667287	9	28	71	36	76	36	77	38
ENSECAG00000007513	8.009494863	0.39088348	0.78667287	3194	2187	5841	4648	8003	4836	7448	4654
ENSECAG00000009160	5.809119222	0.390989805	0.786673196	1210	1156	900	937	791	1004	1208	1210
ENSECAG00000024559	5.780498947	0.391025333	0.786673196	1042	1103	1049	1003	1208	832	1402	719
ENSECAG00000009347	7.792433176	0.391170223	0.786673196	6816	4206	3827	1675	2545	7883	1881	2318
ENSECAG00000019722	4.374665224	0.391227893	0.786673196	216	157	512	460	445	371	613	542
ENSECAG00000012745	3.82309583	0.391329329	0.786673196	109	184	263	386	284	290	363	392
ENSECAG00000024751	3.780209117	0.391342875	0.786673196	96	177	225	422	367	257	293	371
ENSECAG00000012866	3.248524602	0.391367324	0.786673196	164	237	148	182	167	190	155	182
ENSECAG00000009776	2.36079565	0.391569724	0.786814006	44	76	89	108	60	91	114	226
ENSECAG00000011246	0.474068904	0.391575596	0.786814006	17	42	24	24	9	27	16	38
ENSECAG00000018678	2.703977788	0.391982558	0.787492751	65	111	93	126	267	93	205	61
ENSECAG00000023119	0.187029003	0.392148688	0.787658902	12	6	36	40	26	23	11	9
ENSECAG00000005329	4.361642936	0.39225654	0.787658902	201	367	416	731	436	312	377	417
ENSECAG00000021734	2.536389873	0.392272813	0.787658902	47	107	124	216	85	112	95	124
ENSECAG00000017914	0.949734776	0.392432318	0.787771019	16	17	45	35	26	29	44	82
ENSECAG00000016949	0.993806182	0.392467037	0.787771019	22	37	18	36	20	30	75	65
ENSECAG00000021177	3.037086598	0.392755342	0.788210747	63	131	157	174	196	153	315	123
ENSECAG00000009860	3.451253464	0.392890244	0.788342515	162	258	207	275	39	356	109	244
ENSECAG00000019619	6.242760373	0.393007383	0.788438601	1189	2354	1262	1227	655	2714	810	1074
ENSECAG00000022483	7.586643889	0.39318616	0.788612204	5798	2165	3457	2814	4013	3191	3852	2718
ENSECAG00000023166	4.09319185	0.393232453	0.788612204	220	316	281	234	376	349	594	298
ENSECAG00000015048	0.752969055	0.393926831	0.789453756	32	35	35	25	23	9	60	25
ENSECAG00000012787	7.140969076	0.394015435	0.789453756	3361	3516	1806	1975	1446	3202	2796	2719
ENSECAG00000026914	5.187872984	0.394069544	0.789453756	794	925	582	412	803	377	1001	554
ENSECAG00000021471	6.908629163	0.394085132	0.789453756	1518	3253	1902	3194	1179	2808	1986	2747
ENSECAG00000021733	0.494037632	0.394149013	0.789453756	18	33	37	19	11	25	27	31
ENSECAG00000019028	3.197862084	0.39416303	0.789453756	91	106	170	222	226	120	280	248
ENSECAG00000007975	4.887143092	0.394171796	0.789453756	298	691	562	871	433	641	474	661
ENSECAG00000002652	2.874769847	0.394206816	0.789453756	61	96	147	159	104	100	193	309
ENSECAG00000001440	1.726621852	0.394335033	0.789571641	16	47	52	95	98	98	52	57
ENSECAG00000002379	5.942774875	0.394451389	0.78966574	688	571	1115	1631	1478	850	2225	1381
ENSECAG00000013147	2.934863405	0.394697848	0.790020218	153	147	124	163	87	183	134	149
ENSECAG00000022936	4.912214996	0.394841742	0.790169315	347	430	953	726	669	559	695	331
ENSECAG00000000311	5.555237492	0.394976314	0.790179938	554	926	825	1542	701	686	1099	1109
ENSECAG00000009994	4.021904765	0.394985861	0.790179938	170	408	365	376	201	401	206	375
ENSECAG00000010233	2.402750105	0.395058646	0.790186698	43	87	100	104	121	108	153	114
ENSECAG00000012469	5.948091852	0.395148673	0.790227937	964	1382	1197	1269	1098	1307	968	1228
ENSECAG00000011508	6.255270691	0.395294171	0.790380072	960	1420	1694	2131	1041	1284	1533	1911
ENSECAG00000020441	2.674434756	0.395478216	0.790588114	80	83	48	175	146	53	285	152

ENSECAG00000003681	0.729058729	0.395588655	0.790588114	20	24	44	43	27	36	24	24
ENSECAG00000023185	7.125764799	0.395606542	0.790588114	2070	3004	2965	3118	2369	2713	2610	2633
ENSECAG00000012357	6.678510349	0.395758786	0.790753559	1224	2420	1901	2802	1572	1896	1954	2223
ENSECAG00000016022	7.004949694	0.395845995	0.790773493	1255	1645	2448	2869	4025	1993	4168	2219
ENSECAG00000009693	4.878164611	0.395907678	0.790773493	285	513	516	605	824	476	824	627
ENSECAG00000012978	5.134281629	0.396261835	0.790855832	563	661	895	594	655	767	738	477
ENSECAG00000019097	5.153743828	0.3962712	0.790855832	896	639	543	526	718	602	684	645
ENSECAG00000010885	6.09082157	0.396321974	0.790855832	784	1301	857	1395	1368	1211	2240	1677
ENSECAG00000008782	1.73108431	0.396356429	0.790855832	44	50	107	68	91	60	57	19
ENSECAG00000021998	2.160622557	0.396357361	0.790855832	24	70	117	67	100	127	117	76
ENSECAG00000015889	6.235185313	0.39638448	0.790855832	509	964	1987	1474	2198	2000	1562	1296
ENSECAG00000021957	5.332955516	0.396435154	0.790855832	493	641	960	1174	689	597	880	913
ENSECAG00000023539	5.893323674	0.396519304	0.790885121	685	693	1056	1422	1326	859	1952	1538
ENSECAG00000011263	4.20787602	0.396779125	0.790915274	263	255	287	347	593	326	419	380
ENSECAG00000010501	4.707613626	0.396886467	0.790915274	250	462	482	477	922	351	763	435
ENSECAG00000005108	3.203967148	0.396921354	0.790915274	86	168	120	217	247	196	243	175
ENSECAG00000019384	3.168391075	0.396941931	0.790915274	89	161	149	365	172	203	146	127
ENSECAG00000019849	6.920436926	0.3969458	0.790915274	1575	2113	1632	2130	2984	1991	3927	2779
ENSECAG00000016383	1.843047333	0.397019003	0.790915274	42	59	71	35	106	45	129	64
ENSECAG00000009519	2.316472245	0.397020711	0.790915274	76	97	131	94	73	139	32	95
ENSECAG00000019056	5.287954481	0.397189087	0.790940127	345	624	617	1018	946	648	1101	975
ENSECAG00000016136	4.266404746	0.397238215	0.790940127	428	360	280	371	388	313	456	290
ENSECAG00000012451	5.188100352	0.397453545	0.790940127	306	451	1105	1263	643	739	430	848
ENSECAG00000009848	1.528283476	0.397462709	0.790940127	25	32	38	85	67	47	54	100
ENSECAG00000006414	3.985043888	0.397563289	0.790940127	169	339	241	245	353	422	396	299
ENSECAG00000009099	6.388047482	0.397582887	0.790940127	1334	1730	1859	1622	1623	1092	2294	1376
ENSECAG00000013763	7.614758018	0.397661246	0.790940127	2737	5441	3707	3796	3698	4335	3465	2741
ENSECAG00000023664	0.490284035	0.397677214	0.790940127	24	26	26	31	14	25	47	9
ENSECAG00000015317	2.439414528	0.397681286	0.790940127	50	99	121	200	36	169	50	112
ENSECAG00000014919	2.493774708	0.397727908	0.790940127	65	103	116	178	73	75	146	123
ENSECAG00000000508	2.306087407	0.397848747	0.791023188	78	47	125	154	103	41	119	95
ENSECAG00000021498	5.345923408	0.397908634	0.791023188	987	891	495	649	400	577	990	1080
ENSECAG00000024136	3.832415873	0.398193045	0.791450388	110	156	291	390	343	210	452	352
ENSECAG00000007301	8.328363662	0.398299275	0.791523346	3123	8240	6414	9278	3879	6666	6554	6429
ENSECAG00000008644	3.918146662	0.398411246	0.791607685	131	182	366	309	273	357	413	373
ENSECAG00000008421	3.552537483	0.398672072	0.791970577	146	174	281	354	241	167	236	234
ENSECAG00000013895	4.865152979	0.398733012	0.791970577	243	300	632	763	1020	518	635	549
ENSECAG00000025009	2.805411256	0.398832207	0.792029422	63	168	152	195	106	146	94	156
ENSECAG00000004289	6.304877571	0.398950282	0.792125733	1015	1384	900	1600	1108	1238	2041	3228
ENSECAG00000009537	3.339311523	0.399119759	0.792311066	140	151	216	311	232	139	206	177
ENSECAG00000014248	5.935912043	0.399182808	0.792311066	556	1161	1174	2304	993	739	1468	1445
ENSECAG00000023120	0.950356371	0.399335843	0.792379919	26	54	24	47	20	43	24	43
ENSECAG00000009399	4.027466881	0.399356695	0.792379919	141	210	344	384	372	341	436	372
ENSECAG00000010295	4.876270126	0.399454918	0.792436704	331	627	528	928	337	386	601	900
ENSECAG00000019685	5.807758613	0.399551833	0.792490875	755	1008	1271	1430	886	928	1218	1246
ENSECAG00000001775	1.578114978	0.399623589	0.792495133	42	26	61	39	40	70	35	135
ENSECAG00000024795	6.68966323	0.399750241	0.792608237	1419	1120	2442	1055	2815	1941	889	4229
ENSECAG00000024429	7.309145704	0.399921603	0.792783311	2003	5940	1899	3186	1937	5345	1565	1999
ENSECAG00000024034	5.104643449	0.400043299	0.792783311	364	283	685	936	758	505	1167	862
ENSECAG00000022717	6.520064417	0.400087063	0.792783311	1129	1244	1409	2083	2356	1833	2933	1592
ENSECAG00000016193	6.24471274	0.400117076	0.792783311	1120	2472	809	1576	966	1552	1428	1647
ENSECAG00000017157	7.302586601	0.400343912	0.793094734	1269	5742	3811	3732	142	2491	130	7031
ENSECAG00000019284	4.408684632	0.400472345	0.793211142	157	595	480	529	231	457	341	536
ENSECAG00000027691	8.006498207	0.400619452	0.793321338	3776	2663	4630	4594	4874	5548	7040	7281
ENSECAG00000021688	2.332395528	0.400744601	0.793321338	70	53	105	71	139	85	155	98
ENSECAG00000023851	7.887632021	0.400783719	0.793321338	3953	4787	3948	6263	4014	3520	5053	5032
ENSECAG00000026908	4.480823404	0.400890917	0.793321338	299	584	330	545	402	367	431	488
ENSECAG00000016726	8.218788592	0.400953525	0.793321338	6075	8867	3317	4852	2644	5852	5506	7406
ENSECAG00000011765	6.985302562	0.40103819	0.793321338	2128	2006	2565	3472	2842	2255	2081	2130
ENSECAG00000015891	6.370970227	0.401078432	0.793321338	895.011	1571	1956.01	2391	1891	1116	1884	1366.02
ENSECAG00000019910	6.99401015	0.40108543	0.793321338	1277	4043	2173	3056	2366	3246	2059	1466
ENSECAG00000013192	5.424238075	0.401181	0.793372535	412	731	714	968	930	777	1448	898
ENSECAG00000018450	1.758063457	0.401433213	0.793410043	12	46	53	108	85	88	67	75
ENSECAG00000010589	6.250160498	0.401471441	0.793410043	664	1197	1185	2000	1998	1282	2170	1750
ENSECAG00000026850	8.735981099	0.401508444	0.793410043	3002	4925	9084	10742	11930	12260	8241	8059
ENSECAG00000023934	4.641606875	0.401540549	0.793410043	148	228	674	623	513	734	592	502
ENSECAG00000017215	3.350379569	0.401569255	0.793410043	99	137	136	280	169	106	466	265
ENSECAG00000016504	6.134318359	0.401618101	0.793410043	1384	1426	1317	1198	1366	1308	1500	1096
ENSECAG00000018775	7.394863998	0.401934334	0.793465327	2413	2241	2379	3294	4738	2943	5190	3314
ENSECAG00000002578	5.79915455	0.401945367	0.793465327	765	852	1148	1742	1220	806	1076	1115
ENSECAG00000011061	4.196318777	0.401962304	0.793465327	289	260	279	290	296	501	427	481
ENSECAG00000014653	6.946823814	0.401963265	0.793465327	2003	2216	2617	2999	2415	1677	3153	2039
ENSECAG00000018237	6.34534386	0.401994555	0.793465327	1174	1504	1851	1949	1182	1767	1295	1813
ENSECAG00000022006	6.171499742	0.40269102	0.794586549	1072	1505	883	793	1874	1324	2772	1017
ENSECAG00000015476	6.607012457	0.402749377	0.794586549	857	1222	1994	2332	2115	2641	2674	1757
ENSECAG00000009044	6.379107526	0.40280657	0.794586549	785	1158	2502	580	1634	2821	2579	995

ENSECAG00000016149	5.154310086	0.402841771	0.794586549	362	599	708	1301	592	648	631	812
ENSECAG00000012042	5.669235935	0.403109705	0.794863696	452	760	909	1213	673	931	1167	2055
ENSECAG00000013333	4.477783293	0.403157006	0.794863696	267	278	414	471	651	280	621	546
ENSECAG00000026883	2.083951588	0.403243485	0.794863696	40	92	59	63	153	72	102	70
ENSECAG00000010069	0.718035117	0.403261547	0.794863696	16	26	21	32	52	24	51	24
ENSECAG00000010867	6.066351977	0.403366258	0.794932465	795	969	976	1582	1384	987	2275	1759
ENSECAG00000012358	1.535159896	0.403561741	0.795180066	24	42	50	63	52	83	85	48
ENSECAG00000001211	3.787890211	0.403877836	0.795665194	140	621	123	244	83	240	244	413
ENSECAG00000019618	5.587431639	0.404316667	0.796194216	619	852	663	916	919	807	1555	1269
ENSECAG00000007962	4.36288806	0.404333946	0.796194216	303	398	271	268	382	296	589	693
ENSECAG00000011391	5.286623038	0.404379819	0.796194216	476	480	1066	1213	1081	379	875	597
ENSECAG00000011526	3.253510545	0.40450769	0.796194216	107	102	196	201	161	177	322	247
ENSECAG00000012019	8.525973056	0.404528004	0.796194216	4218	4563	5919	8482	12267	5381	10705	7290
ENSECAG00000022145	3.493448678	0.404565967	0.796194216	149	175	165	207	252	148	437	245
ENSECAG00000005156	3.568030453	0.404829944	0.796539633	170	151	215	436	204	175	234	272
ENSECAG00000022235	4.445926466	0.405071207	0.796539633	225	293	355	571	444	421	594	574
ENSECAG00000012760	5.993864187	0.405187127	0.796539633	657	934	1336	1177	826	1552	2038	1628
ENSECAG00000008062	5.923801928	0.405235365	0.796539633	789	931	1350	1858	1233	985	1308	1085
ENSECAG00000019591	6.41976937	0.405247875	0.796539633	1121	1147	2225	2553	1672	1063	1367	2285
ENSECAG00000010771	4.632663722	0.405269349	0.796539633	309	457	331	478	601	397	729	602
ENSECAG00000018345	7.566187827	0.405511498	0.796539633	3137	3838	5095	3161	1498	6078	2246	3443
ENSECAG00000015525	0.880448386	0.405534188	0.796539633	14	41	20	31	37	18	83	38
ENSECAG00000012233	1.657887015	0.405535977	0.796539633	26	40	32	101	73	90	83	47
ENSECAG00000010551	6.803639832	0.405547696	0.796539633	2221	2814	1661	1851	2320	1280	2782	1962
ENSECAG00000015753	1.839637064	0.405615708	0.796539633	63	18	72	43	35	69	114	131
ENSECAG00000017319	6.729078553	0.405637427	0.796539633	1202	1393	2285	1802	3072	2328	2977	1675
ENSECAG00000012338	6.450868958	0.405651014	0.796539633	985	1214	1741	3488	1972	1106	1540	1896
ENSECAG00000016105	4.459127893	0.405762505	0.796621162	239	218	551	412	490	570	558	426
ENSECAG00000004655	7.697323544	0.405895315	0.796744511	1962	4163	5299	6129	2969	4644	3012	4446
ENSECAG00000022179	8.015202051	0.406299784	0.797167752	4710	7173	3902	4368	4014	6408	4557	3663
ENSECAG00000014319	3.580874238	0.406395891	0.797167752	177	160	289	330	231	245	234	179
ENSECAG00000012394	1.704654516	0.406459025	0.797167752	29	29	125	92	62	55	77	34
ENSECAG00000020276	6.187850589	0.406474406	0.797167752	1308	1418	1470	1397	1402	1086	1600	1448
ENSECAG00000021375	3.983038494	0.406509836	0.797167752	200	216	224	373	346	240	486	420
ENSECAG00000018673	9.808696054	0.40655016	0.797167752	18054	20913	17294	13221	20610	15272	19914	9190
ENSECAG00000022895	3.874388166	0.406606786	0.797167752	158	377	217.004	441	309	283	264	232
ENSECAG00000013834	3.095499169	0.406671085	0.797167752	32	102	267	111	37	310	72	415
ENSECAG00000009722	3.584956557	0.406808392	0.797299628	213	116	422	193	156	286	173	247
ENSECAG00000017046	3.347652014	0.406960622	0.797332081	121	237	166	298	152	221	197	184
ENSECAG00000005126	5.840553743	0.407000367	0.797332081	600	1196	787	1065	998	889	2260	1378
ENSECAG00000013277	2.132230158	0.407035051	0.797332081	44	122	87	99	57	83	102	76
ENSECAG00000019601	7.314549398	0.407266062	0.797508952	2850	3573	3115	2835	3455	2476	3410	2461
ENSECAG00000010503	3.654970609	0.407296355	0.797508952	111	157	195	365	396	169	420	216
ENSECAG00000022480	6.47000225	0.40733549	0.797508952	748	1557	1431	2045	1278	2146	1878	3057
ENSECAG00000024246	6.611328671	0.407635575	0.797959254	1492.99	1972	1910.99	2370.99	1838	1318	2557	1736
ENSECAG00000010148	2.566071706	0.407753335	0.798052556	55	184	111	132	62	139	86	134
ENSECAG00000020625	7.214447786	0.407872397	0.798148374	2075	4949	1887	2972	1477	2917	3132	3356
ENSECAG00000021612	7.125411939	0.408000911	0.798262653	2492	2625	2396	3568	1551	2810	2723	3247
ENSECAG00000010819	7.89435088	0.408213779	0.798454631	3513	6655	4012	4792	3819	5912	3955	3531
ENSECAG00000009356	6.74751279	0.408271692	0.798454631	1446	3331	1970	1795	1392	2972	1579	1797
ENSECAG00000007479	5.477224596	0.408345407	0.798454631	522	706	804	847	1079	873	1195	992
ENSECAG00000023660	5.276385344	0.408379562	0.798454631	293	423	783	1125	831	578	1230	1053
ENSECAG00000007602	4.885534297	0.408692979	0.798930214	432	480	585	861	781	421	540	488
ENSECAG00000013375	6.987944313	0.408815877	0.798994665	1761	3635	2287	2380	2268	2505	2796	1831
ENSECAG00000003481	6.148197775	0.409013022	0.798994665	727	1822	1067	626	2519	996	2448	934
ENSECAG00000000578	6.909128633	0.409029061	0.798994665	2441	2746	1842	2172	1924	1645	2692	2713
ENSECAG00000009145	8.36216819	0.409065791	0.798994665	8572	7009	4666	4212	5673	6116	7102	4968
ENSECAG00000017022	6.519447494	0.409076847	0.798994665	1118	1629	1838	2954	1460	1360	2021	2119
ENSECAG00000005228	2.857449743	0.409197481	0.799016814	64	107	120	341	158	120	164	82
ENSECAG00000017540	4.199484244	0.40922855	0.799016814	160	685	257	408	138	445	281	459
ENSECAG00000000965	7.170402207	0.409431761	0.799188503	2268	3020	3496	2608	1727	3137	3587	2259
ENSECAG00000007920	7.157144203	0.409489517	0.799188503	2218	2558	2915	3759	2793	2190	3130	2575
ENSECAG00000013853	2.942955625	0.409560371	0.799188503	126	175	116	189	101	67	211	196
ENSECAG00000024025	4.084171913	0.40959727	0.799188503	207	300	268	299	423	274	490	400
ENSECAG00000002187	3.631680158	0.409771488	0.799391431	185	77	270	220	410	251	349	166
ENSECAG00000009354	4.994496966	0.409868501	0.799443702	322	212	859	648	613	1230	590	529
ENSECAG00000016773	5.738772932	0.410147921	0.799616803	1024	1652	601	754	1114	750	1419	720
ENSECAG00000014779	6.274764719	0.410225906	0.799616803	1088	1698	1540	1782	1502	1396	1592	1370
ENSECAG00000012744	3.810630461	0.410227414	0.799616803	102	179	311	307	656	182	343	154
ENSECAG00000022570	9.056188407	0.410267821	0.799616803	11215	10227	9085	10806	9809	11517	10272	7043
ENSECAG00000014967	3.471730696	0.41030842	0.799616803	166	119	163	231	290	123	444	217
ENSECAG00000020270	1.583744924	0.410541606	0.799880527	31	35	43	71	72	47	145	27
ENSECAG00000022298	1.80106162	0.41058426	0.799880527	27	30	43	118	30	60	80	166
ENSECAG00000011552	4.122899615	0.410797293	0.79998344	249	217	290	345	406	367	444	398
ENSECAG00000012339	7.470821876	0.41087547	0.79998344	1880	2009	3951	3440	4778	3526	5210	3456
ENSECAG00000025090	5.516405237	0.410943561	0.79998344	566	650	961	1595	1048	855	664	844

ENSECAG00000009944	1.653197362	0.410992333	0.79998344	14	38	48	100	33	44	94	133
ENSECAG00000012939	3.56346569	0.411046331	0.79998344	80	399	99	137	218	271	378	275
ENSECAG00000009418	4.377222144	0.411058684	0.79998344	255.001	555	287.001	576.001	303.001	561.001	301.001	358
ENSECAG00000017910	7.036132366	0.411140879	0.799991753	2219	2537	1936	3806	2494	2451	2297	2452
ENSECAG00000015358	5.912952258	0.411252754	0.799991753	1421	1276	935	869	607	1421	762	1577
ENSECAG00000010106	2.017765576	0.411287144	0.799991753	24	24	105	96	164	28	161	48
ENSECAG00000018820	5.964227704	0.411344025	0.799991753	568	1559	1323	1669	841	1320	1306	1239
ENSECAG00000007563	6.289943956	0.411492838	0.800113507	722	1333.99	1695	2882.99	1030	1385.99	1587.99	1877.99
ENSECAG00000009668	0.886931071	0.411547185	0.800113507	44	59	26	21	0	71	0	29
ENSECAG00000000122	3.851505674	0.411700777	0.800275455	66	91	592	157	192	352	396	473
ENSECAG00000012886	5.20322131	0.41182795	0.800386004	366	715	761	497	981	807	1124	544
ENSECAG00000009442	5.867194782	0.411983669	0.800551984	767	1416	971	1493	804	1244	1054	1289
ENSECAG00000011894	3.655641692	0.412109364	0.800582618	122	164	222	315	242	182	356	412
ENSECAG00000012264	1.325454582	0.412140072	0.800582618	43	49	61	37	24	40	50	63
ENSECAG00000014322	6.035009345	0.412240103	0.800640324	823	1754	1325	1258	1319	1306	1326	984
ENSECAG00000008477	4.427335313	0.41234981	0.800658897	232	353	313	492	432	378	376	816
ENSECAG00000006910	3.557826906	0.412390318	0.800658897	105	139	236	277	475	137	331	176
ENSECAG00000009308	1.751766976	0.412505779	0.800746512	28	47	61	73	102	36	108	72
ENSECAG00000020376	1.183182775	0.412780415	0.801016207	32	17	40	43	43	49	42	74
ENSECAG00000020197	4.904316086	0.412838564	0.801016207	408	901	422	603	511	394	808	582
ENSECAG00000017333	6.41563731	0.412855784	0.801016207	1305	1034	1591	1265	2741	1670	2452	1222
ENSECAG00000016892	5.147326401	0.413272524	0.80168814	348	363	1054	505	898	756	1086	608
ENSECAG00000016565	4.032688328	0.413474568	0.801943433	226	358	252	464	320	242	430	257
ENSECAG00000024863	4.661115777	0.4137943	0.802347484	145	315	471	806	461	604	674	639
ENSECAG00000007095	5.306837362	0.413888273	0.802347484	414	260	578	1392	861	621	1078	1223
ENSECAG00000010985	4.812649697	0.41396222	0.802347484	468	500	664	518	476	551	575	536
ENSECAG00000009977	1.099345653	0.413964789	0.802347484	22	52	34	69	9	33	25	77
ENSECAG00000020875	4.258633058	0.41415851	0.802419538	180	570	223	193	438	485	452	423
ENSECAG00000003883	3.896934221	0.414205158	0.802419538	183	382	246	389	119	257	325	415
ENSECAG00000013300	1.905996994	0.414213406	0.802419538	3	26	245	84	22	18	20	171
ENSECAG00000022089	1.405891999	0.414368846	0.802573939	29	41	26	63	35	40	89	87
ENSECAG00000024257	8.922149744	0.414456872	0.802573939	4743	4026	10994	11222	14364	12026	9110	10583
ENSECAG00000014592	5.907225612	0.414517775	0.802573939	1007	1010	1338	1295	972	1159	1262	1169
ENSECAG00000005595	4.18049762	0.414575084	0.802573939	262	215	359	280	544	247	618	316
ENSECAG00000008511	10.70789973	0.414832472	0.802924383	24841	23509	40246	49667	39352	28099	26908	27128
ENSECAG00000019632	5.010634461	0.414897158	0.802924383	246	495	604	829	733	822	769	652
ENSECAG00000016920	4.091665524	0.415014863	0.803015672	155	274	263	435	347	260	532	470
ENSECAG00000018635	2.885673678	0.41519725	0.803021136	88	71	136	172	185	115	227	172
ENSECAG00000025184	1.498667721	0.415201729	0.803021136	25	40	80	91	36	72	29	56
ENSECAG00000019404	1.759568177	0.415229287	0.803021136	173	39	21	22	0	148	0	23
ENSECAG00000019843	6.02732777	0.415499633	0.803407494	878	947	1105	1177	1804	909	2190	1295
ENSECAG00000021657	11.33254383	0.415616755	0.803497496	36231	63692	47747	61489	32340	51596	47936	57002
ENSECAG00000024225	0.284338884	0.415689235	0.803501179	12	13	7	38	21	16	36	38
ENSECAG00000015132	6.263473019	0.415867536	0.803521565	1215	1602	1593	1558	1113	1401	1810	1533
ENSECAG00000013523	4.022131574	0.415887305	0.803521565	170	298	252	323	263	347	363	539
ENSECAG00000010103	8.342268228	0.415911514	0.803521565	6909	4687	6194	7484	5577	5938	7091	5372
ENSECAG00000009112	4.114695408	0.416230985	0.803985983	202	337	294	258	472	320	452	366
ENSECAG00000001958	3.383176138	0.416293137	0.803985983	110	482	154	111	28	99	152	442
ENSECAG00000000741	3.086391952	0.416614003	0.803999894	78	133	201	80	293	101	436	31
ENSECAG00000007352	2.67419739	0.416658577	0.803999894	79	85	106	128	152	139	141	158
ENSECAG00000006936	6.653048282	0.416696566	0.803999894	794	1117	2083	2736	2269	2043	3080	2190
ENSECAG00000005995	6.413581683	0.4167171	0.803999894	728	1445	2393	2638	1808	847	2154	1651
ENSECAG00000006575	5.531463741	0.416726635	0.803999894	660	683	843	1574	913	489	955	1167
ENSECAG00000009788	4.168395523	0.416742742	0.803999894	174	245	303	445	214	312	413	767
ENSECAG00000025056	6.451798257	0.416794675	0.803999894	1072	973	1845	1692	2125	1705	3017	1486
ENSECAG00000004150	0.125823749	0.416988064	0.804155037	10	21	33	20	7	21	14	28
ENSECAG00000016542	3.422529004	0.417016367	0.804155037	86	143	192	287	288	144	302	278
ENSECAG00000002816	4.936129165	0.417227239	0.804425422	287	397	626	733	635	632	731	838
ENSECAG00000011096	1.6293779	0.417400734	0.804623663	43	65	53	82	49	39	84	54
ENSECAG00000011014	3.683003566	0.417510687	0.804633156	104	213	203	330	331	310	278	265
ENSECAG00000017909	3.240919076	0.417547008	0.804633156	98	215	213	239	233	149	176	141
ENSECAG00000014460	8.282484248	0.417932946	0.805107881	7901	9172	3128	3247	3951	10106	3704	3489
ENSECAG00000016304	5.194333914	0.417954049	0.805107881	476	494	680	690	948	535	1168	795
ENSECAG00000010238	3.359833631	0.418058628	0.805107881	91	117	166	535	185	125	180	258
ENSECAG00000018474	5.348847566	0.418076223	0.805107881	290	459	565	1420	395	563	1330	1709
ENSECAG00000002856	5.685757915	0.41837764	0.805552078	824	710	1236	1297	1108	529	1353	968
ENSECAG00000023878	3.719660778	0.418522167	0.805614131	124	206	365	395	267	222	295	205
ENSECAG00000013226	4.121109394	0.418598389	0.805614131	187	320	257	359	440	267	527	397
ENSECAG00000019165	1.297299624	0.418661777	0.805614131	33	24	34	53	45	31	114	46
ENSECAG00000012107	6.349085819	0.418692913	0.805614131	1086	2060	1286	2055	1232	1402	1565	1960
ENSECAG00000009055	4.128657898	0.419021901	0.806097927	219	211	291	407	436	260	574	379
ENSECAG00000009847	8.053440833	0.419085958	0.806097927	3330	5846	5918	6482	4557	4621	5549	5085
ENSECAG00000021720	4.760015918	0.419235574	0.806113442	332	533	487	844	443	335	497	786
ENSECAG00000003697	1.328368904	0.419300364	0.806113442	6	40	42	72	64	61	71	38
ENSECAG00000016379	6.46447043	0.419306439	0.806113442	743	1366	1760	1955	2222	2191	2354	1490
ENSECAG00000004618	1.429967233	0.419379843	0.806118438	34	31	89	73	22	75	5	70

ENSECAG00000009904	6.224090132	0.41970471	0.806391604	1129	1025	1318	1150	1832	1642	2047	1480
ENSECAG00000010792	7.058651635	0.419708635	0.806391604	1875	2938	2498	3392	2630	2525	2813	1974
ENSECAG00000010480	7.161147511	0.419778334	0.806391604	2289	2673	3155	3234	3216	2592	2599	2188
ENSECAG00000003414	5.633274728	0.419832842	0.806391604	520	526	1386	1760	1230	914	945	597
ENSECAG00000022901	4.244241348	0.419876103	0.806391604	230	400	226	333	475	469	525	293
ENSECAG00000019493	2.627146324	0.420178828	0.806723282	62	129	136	180	65	129	117	140
ENSECAG00000009514	6.623931524	0.420190519	0.806723282	1411	2663	1533	2188	1464	2120	1547	2176
ENSECAG00000018452	2.552222406	0.420280883	0.806760724	49	109	110	102	156	133	134	119
ENSECAG00000026885	5.787551556	0.420470712	0.806989052	627	765	699	1546	1051	1257	1143	1681
ENSECAG00000019574	3.57122126	0.420646906	0.807068742	170	256	219	304	145	121	216	406
ENSECAG00000019428	4.024233344	0.420654011	0.807068742	168	221	324	348	459	308	455	291
ENSECAG00000009968	0.436801046	0.420968624	0.807536273	34	12	25	31	6	26	7	46
ENSECAG00000021661	4.915972711	0.42113105	0.807688211	280	364	379	1012	523	502	824	1010
ENSECAG00000000078	5.594393789	0.421189715	0.807688211	708	684	933	682	1156	860	1469	1050
ENSECAG00000014591	1.096198001	0.421409822	0.807899825	33	38	32	67	13	21	49	67
ENSECAG00000015054	0.833401771	0.421480872	0.807899825	12	40	29	66	35	28	28	30
ENSECAG00000005990	7.005512391	0.421566154	0.807899825	2246	4376	1429	1957	1441	3523	1692	2457
ENSECAG00000021030	3.006669959	0.421583914	0.807899825	104	94	155	147	149	140	214	252
ENSECAG00000011608	5.562555634	0.421752031	0.808085976	524	855	1088	1364	837	1021	909	816
ENSECAG00000008949	4.835738481	0.421956085	0.808336305	338	469	511	513	714	621	901	426
ENSECAG00000010394	6.015072371	0.422050385	0.808336305	1798	1850	594	450	1827	812	1330	650
ENSECAG00000018566	5.390961918	0.422095681	0.808336305	388	592	738	1089	931	785	1460	780
ENSECAG00000011453	6.498901273	0.422354667	0.808604917	1045	2845	1835	1489	946	2213	1377	2087
ENSECAG00000011022	3.569227515	0.422396902	0.808604917	103	184	154	346	284	265	237	311
ENSECAG00000000343	2.315594537	0.422498929	0.808604917	73	119	155	78	5	79	6	222
ENSECAG00000012127	3.496321301	0.42252004	0.808604917	145	338	178	224	197	251	241	146
ENSECAG00000022174	3.527616796	0.422773608	0.808954207	123	134	220	274	300	238	300	232
ENSECAG00000014371	7.11711287	0.422946376	0.809148797	1620	4025	2379	3296	2253	3854	2062	1839
ENSECAG00000008260	5.691485995	0.423131494	0.80915057	621	803	1365	1390	922	618	1165	1265
ENSECAG00000018599	6.383007221	0.423204737	0.80915057	1525	1581	1521	1792	1834	1300	1543	1617
ENSECAG00000013270	3.99036087	0.42321382	0.80915057	221	471	241	305	225	184	485	324
ENSECAG00000017343	7.545863419	0.423231589	0.80915057	1528	2920	4628	2861	4230	4272	4468	4756
ENSECAG00000009060	2.139796458	0.423742326	0.809731935	44	56	52	128	89	69	123	134
ENSECAG00000014089	6.702992217	0.423756012	0.809731935	1082	1145	1820	2752	2364	1332	3847	2558
ENSECAG00000010175	5.55683047	0.423770567	0.809731935	393	1178	945	1392	721	1210	584	946
ENSECAG00000020931	4.641902449	0.423928021	0.809731935	223	413	491	980	529	408	440	499
ENSECAG00000007272	7.140294548	0.424056874	0.809731935	1131	2037	4152	4877	3155	2446	2925	1850
ENSECAG00000010726	1.34008037	0.42406838	0.809731935	38	38	39	90	15	30	66	69
ENSECAG00000018413	2.823152072	0.42415501	0.809731935	60	90	150	155	210	94	199	164
ENSECAG00000003211	1.592377486	0.424225988	0.809731935	48	98	23	67	18	66	53	71
ENSECAG00000004830	1.837772266	0.424415969	0.809731935	38	52	64	66	93	62	98	78
ENSECAG00000012224	4.572903119	0.424439107	0.809731935	374	299	459	323	628	425	798	396
ENSECAG00000014975	8.568634736	0.42452227	0.809731935	8287	13031	3743	3823	8896	7762	6764	3219
ENSECAG00000013041	7.036824586	0.424528867	0.809731935	3780	1410	2776	1697	2669	2017	3026	1896
ENSECAG00000012436	3.205826455	0.424561408	0.809731935	74	128	111	293	131	134	272	349
ENSECAG00000000381	4.017199397	0.424590172	0.809731935	156	242	346	269	179	223	324	827
ENSECAG00000004807	8.270036772	0.424784224	0.809731935	5345	6223	2562	2844	13692	6416	6881	2906
ENSECAG00000008356	3.297258486	0.424809827	0.809731935	36	111	360	91	63	405	392	115
ENSECAG00000006892	5.404454639	0.42496124	0.809731935	291	980	1152	1112	454	1007	916	797
ENSECAG00000009564	4.676563334	0.425003029	0.809731935	272	334	419	661	511	381	990	556
ENSECAG00000015658	4.08904966	0.425085371	0.809731935	182	197	283	442	673	202	413	310
ENSECAG00000015062	1.501996843	0.425116807	0.809731935	26	57	61	88	29	41	55	77
ENSECAG00000016881	5.346244391	0.425141276	0.809731935	435	458	997	699	1121	577	1754	491
ENSECAG00000003773	2.214842332	0.425143712	0.809731935	76	169	63	44	74	62	128	71
ENSECAG00000008661	6.268647091	0.425185839	0.809731935	1021	1505	1734	1902	1484	1657	1022	1570
ENSECAG00000009543	4.370995981	0.425242621	0.809731935	310	185	401	378	669	239	772	310
ENSECAG00000014019	4.259827413	0.425385674	0.809868879	360	428	302	352	286	323	393	452
ENSECAG00000019732	0.273007078	0.425535198	0.809992409	15	14	20	17	35	30	22	19
ENSECAG00000024104	0.479170504	0.425592849	0.809992409	5	14	46	15	24	40	26	36
ENSECAG00000014441	2.603370161	0.425710624	0.810081138	63	91	93	134	135	63	237	150
ENSECAG00000008022	3.086290011	0.425977113	0.810336285	83	127	197	303	163	108	214	152
ENSECAG00000016819	4.371568755	0.42606619	0.810336285	219	247	414	483	600	353	518	448
ENSECAG00000018354	4.374828346	0.426121833	0.810336285	252	519	300	178	325	508	564	561
ENSECAG00000011768	8.808624361	0.426208418	0.810336285	12088	6455	8065	6793	8891	7145	9822	6935
ENSECAG00000013269	5.167622158	0.426370379	0.810336285	566	622	800	789	714	586	837	632
ENSECAG00000013910	3.782592248	0.426441807	0.810336285	250	352	236	204	133	270	282	340
ENSECAG00000024036	3.214085976	0.426472917	0.810336285	92	93	176	235	349	110	269	152
ENSECAG00000023450	2.407106861	0.426607767	0.810336285	68	66	88	105	132	75	152	139
ENSECAG00000016038	2.106347644	0.426628395	0.810336285	50	58	129	118	29	95	64	116
ENSECAG00000016968	4.743225309	0.426755773	0.810336285	310	318	430	681	761	291	1022	494
ENSECAG00000007672	3.360913843	0.426808274	0.810336285	172	107	150	188	255	173	161	370
ENSECAG00000010610	8.894038028	0.426819559	0.810336285	5699	6576	8396	9197	9575	10983	14214	10740
ENSECAG00000005690	1.672039165	0.42690115	0.810336285	44	50	54	109	30	53	72	75
ENSECAG00000000632	3.0858276	0.426921771	0.810336285	149	276	151	66	91	162	157	202
ENSECAG00000007236	5.236892934	0.426948452	0.810336285	327	903	735	1145	472	844	693	830
ENSECAG00000018756	4.093394594	0.427055096	0.810336285	203	335	373	480	191	376	180	504

ENSECAG00000011522	4.274834803	0.427136673	0.810336285	190	313	321	452	311	375	610	519
ENSECAG00000007113	4.790598914	0.427202051	0.810336285	464	424	604	652	468	345	740	593
ENSECAG00000007589	3.214442763	0.427268237	0.810336285	115	203	236	216	27	215	98	308
ENSECAG00000009630	0.13055113	0.427329694	0.810336285	6	15	23	47	9	8	23	31
ENSECAG00000009267	4.724251258	0.427339399	0.810336285	323	365	468	563	495	619	688	639
ENSECAG00000018741	6.90194055	0.427663422	0.810463731	1341	2099	1756	2335	3883	1654	3773	2156
ENSECAG00000021528	5.66727919	0.427666023	0.810463731	698	806	1056	1505	992	733	1353	854
ENSECAG00000019825	4.456046365	0.427701182	0.810463731	649	495	156	310	403	142	890	199
ENSECAG00000011858	5.837210268	0.427853642	0.810463731	1060	1197	881	1206	1010	1009	1273	1075
ENSECAG00000016288	3.542383028	0.427982029	0.810463731	110	206	219	215	353	209	283	234
ENSECAG00000019670	5.564533976	0.428028338	0.810463731	876	1419	551	717	653	1053	1019	814
ENSECAG00000017980	6.668444794	0.428030029	0.810463731	1449	2148	1963	2520	1682	1844	1817	2312
ENSECAG00000015355	7.946556903	0.428092584	0.810463731	2985	4801	6063	6454	3234	4773	3550	6569
ENSECAG00000020871	6.400935203	0.42816872	0.810463731	1110	1677	1799	2158	1840	1531	1713	1312
ENSECAG00000004264	0.921507234	0.428201678	0.810463731	4	5	63	43	63	32	40	43
ENSECAG00000022254	6.647040661	0.428352459	0.810463731	1178	1061	2063	2122	3008	1505	2132	2792
ENSECAG00000019783	5.02776748	0.42838005	0.810463731	268	512	558	858	982	591	733	708
ENSECAG00000014275	4.170359672	0.428394873	0.810463731	207	442	447	322	321	265	531	266
ENSECAG00000022042	4.58038641	0.428421844	0.810463731	234	463	220	654	405	470	722	661
ENSECAG00000012463	11.41097735	0.428474414	0.810463731	24331	32764	57026	60819	63502	71744	44756	77274
ENSECAG00000000454	6.339087755	0.428787688	0.810873104	671	976	869	2914	1209	1265	3826	1712
ENSECAG00000021210	3.605839908	0.428833286	0.810873104	149	146	248	229	298	164	396	293
ENSECAG00000024522	4.158205207	0.428905212	0.810874434	193	276	253	448	479	262	535	396
ENSECAG00000000605	2.180952849	0.429034404	0.810984009	61	91	95	112	114	59	98	60
ENSECAG00000023253	6.457863264	0.429165484	0.811097117	1514	1124	1441	3045	2232	893	2197	1315
ENSECAG00000005098	4.564078841	0.429249181	0.811120652	463	676	256	381	287	417	443	619
ENSECAG00000024960	5.660907418	0.429436862	0.811260008	545	437	928	1473	1207	925	1314	1290
ENSECAG00000013325	4.834782458	0.429630715	0.811260008	327	473	572	950	492	462	635	605
ENSECAG00000001999	4.843001383	0.429667989	0.811260008	370	359	557	570	666	644	746	589
ENSECAG00000021671	1.525840399	0.429687664	0.811260008	24	94	90	30	17	122	16	25
ENSECAG00000019879	5.668463609	0.429727253	0.811260008	635	1185	1121	1081	985	1282	717	796
ENSECAG00000008584	5.769349564	0.42979852	0.811260008	707	1615	691	1330	689	788	925	1698
ENSECAG00000011604	3.21006589	0.429841374	0.811260008	86	177	243	259	108	257	142	164
ENSECAG00000017440	5.560457423	0.429892985	0.811260008	695	1187	1016	724	873	1038	948	701
ENSECAG00000020878	7.682596334	0.430014918	0.811355625	2372	4401	4665	5682	2038	1778	5732	5965
ENSECAG00000012699	5.065343064	0.430213089	0.811517984	255	539	809	615	567	844	954	755
ENSECAG00000008379	2.872019945	0.430243527	0.811517984	65	167	109	111	128	102	313	167
ENSECAG00000015040	5.659231401	0.430381802	0.811644329	554	631	1015	1128	1266	840	1584	1047
ENSECAG00000018398	2.851970239	0.430534706	0.811798215	139	323	62	35	26	230	62	152
ENSECAG00000015914	6.660297225	0.430627568	0.811838857	1230	1495	1641	2118	2373	1739	2923	2518
ENSECAG00000009397	0.280432957	0.430819034	0.811947608	26	11	37	15	20	12	39	10
ENSECAG00000001316	3.758810016	0.430827888	0.811947608	134	240	238	530	156	168	295	400
ENSECAG00000020046	5.208114524	0.431075426	0.812141692	530	550	557	714	956	677	1005	790
ENSECAG00000006875	5.943550072	0.431084189	0.812141692	674	1602	1204	1448	1147	1394	956	1102
ENSECAG00000001468	3.952538689	0.431179047	0.812141692	325	305	224	316	193	166	376	441
ENSECAG00000010909	4.222502233	0.431259818	0.812141692	163	199	618	598	352	291	284	457
ENSECAG00000008307	5.743929718	0.431348985	0.812141692	718	1004	1034	1510	790	891	1186	1259
ENSECAG00000024078	5.034409102	0.431531815	0.812141692	591	804	534	534	459	611	708	719
ENSECAG00000018379	7.024277776	0.431561175	0.812141692	1717	2616	2794	3377	1936	2636	2817	2388
ENSECAG00000021600	2.509145529	0.431564267	0.812141692	71	50	108	120	230	69	138	99
ENSECAG00000010970	5.831424852	0.431619462	0.812141692	440	936	1221	1177	1425	1597	1367	874
ENSECAG00000003847	2.384559095	0.431704244	0.812141692	80	52	41	138	105	81	77	232
ENSECAG00000023067	3.736336709	0.431798075	0.812141692	159	145	242	304	474	266	341	161
ENSECAG00000015009	3.425090384	0.431834376	0.812141692	122	160	163	242	219	156	430	221
ENSECAG00000009030	6.143456905	0.431858217	0.812141692	1514	1555	1067	1132	1378	1340	1637	967
ENSECAG00000010786	7.858974645	0.43199748	0.812164713	4202	4384	4564	4976	4409	3544	4138	5196
ENSECAG00000027677	8.792463242	0.432064952	0.812164713	6236	4806	9831	6033	10590	9623	13477	8922
ENSECAG00000001234	4.198354017	0.432084468	0.812164713	204	274	325	396	341	407	452	494
ENSECAG00000022455	5.724137248	0.432248357	0.81233865	532	776	1394	1660	1047	827	1143	1021
ENSECAG00000011951	5.833079167	0.432585139	0.812563987	879	569	1148	944	1684	1352	1327	926
ENSECAG00000008984	2.15558878	0.432811336	0.812563987	24	82	77	101	85	114	169	55
ENSECAG00000023374	5.559765539	0.432872593	0.812563987	532	700	676	1208	984	782	1409	1245
ENSECAG00000024049	0.92184323	0.432921859	0.812563987	15	37	55	46	45	41	24	17
ENSECAG00000021896	2.234921688	0.433003378	0.812563987	45	93	73	79	70	79	155	142
ENSECAG00000010645	4.793497115	0.433042537	0.812563987	336	541	513	830	469	475	556	628
ENSECAG00000023152	7.946765377	0.433070275	0.812563987	4030	6457	4195	4564	4189	4757	5382	4032
ENSECAG00000017222	4.611365639	0.433157103	0.812563987	194	335	470.002	652	694	464	663	450
ENSECAG00000015717	0.654172556	0.433179278	0.812563987	13	39	20	15	16	30	65	37
ENSECAG00000014843	6.073930926	0.433217923	0.812563987	567	880	1521	1526	1534	1667	1726	1335
ENSECAG00000022819	7.793804027	0.433333465	0.812563987	2779	4419	4192	6965	2494	3116	5571	5576
ENSECAG00000014393	4.959838012	0.433351423	0.812563987	380	566	660	886	431	571	620	763
ENSECAG00000022389	9.372044709	0.433397436	0.812563987	9844	14459	14537	13987	12000	12961	12895	11143
ENSECAG00000009890	6.401766976	0.433431785	0.812563987	880	1311	1650	1653	1957	2292	1838	1717
ENSECAG00000022075	8.019765686	0.433438831	0.812563987	6105	7841	2595	3294	2776	9621	2402	2772
ENSECAG00000011210	4.246027997	0.433649256	0.812824627	217	345	421	540	348	325	394	384
ENSECAG00000021790	6.627489597	0.433953416	0.813260846	2875	1790	1167	1385	2209	925	3150	1088

ENSECAG00000021659	2.414625447	0.434174938	0.813542078	37	111	144	152	75	115	105	92
ENSECAG00000008177	1.990482453	0.434416094	0.813838178	52	214	69	0	7	123	8	88
ENSECAG00000018533	0.941691495	0.434515505	0.813838178	10	22	34	54	34	50	29	60
ENSECAG00000012435	3.427697128	0.434547412	0.813838178	143	123	200	213	269	154	315	272
ENSECAG00000015287	3.501444049	0.434738381	0.814061919	190	292	200	177	205	213	171	249
ENSECAG00000016295	4.465619936	0.434832937	0.814105079	221	492	231	479	340	377	673	702
ENSECAG00000016502	2.441913242	0.435070076	0.814415129	64	57	104	236	79	55	137	129
ENSECAG00000018688	6.175148513	0.435148112	0.814427298	734	1105	1388	1518	1330	1751	1846	1787
ENSECAG00000019657	3.908197907	0.435229938	0.814446556	157	202	324	291	383	372	390	239
ENSECAG00000017819	6.382754436	0.435571712	0.814917561	1025	1181	1455	1680	2509	1237	2590	1549
ENSECAG00000009711	5.039838206	0.435633702	0.814917561	281	461	588	889	553	575	963	999
ENSECAG00000009102	8.033253503	0.435696372	0.814917561	4490	5049	5610	5405	3837	5814	4724	4999
ENSECAG00000009502	3.994358285	0.43589244	0.815150366	129	193	256	490	259	261	342	639
ENSECAG00000019123	2.139012354	0.435983482	0.815186721	52	52	81	88	119	64	135	94
ENSECAG00000005100	4.173779266	0.436160947	0.815384627	216	316	464	447	414	350	375	222
ENSECAG00000012452	4.135995446	0.436540867	0.815960889	207	318	363	532	276	227	384	461
ENSECAG00000020647	7.505524973	0.436676113	0.816006627	2270	3334	4701	4458	2759	3757	4073	3020
ENSECAG00000006859	7.233258738	0.436708685	0.816006627	3040	3136	2502	2790	2696	2426	3367	2805
ENSECAG00000018099	2.213341831	0.436846372	0.816129954	75	12	128	30	243	72	115	25
ENSECAG00000005253	1.772306797	0.437136423	0.816537847	25	82	69	107	47	35	88	79
ENSECAG00000011567	6.558402837	0.437369435	0.816839077	1128	2193	2050	2163	1324	1898	1694	2188
ENSECAG00000019655	3.608389707	0.43753518	0.816897025	124	281	279	295	207	277	238	190
ENSECAG00000019324	2.372539082	0.437580891	0.816897025	254	92	5	44	0	247	2	14
ENSECAG00000000619	4.917375315	0.437729587	0.816897025	411	407	501	617	708	414	1027	699
ENSECAG00000007124	5.495943819	0.437731314	0.816897025	538	442	881	1087	950	586	1672	1105
ENSECAG00000019307	6.073870182	0.437759223	0.816897025	980	1503	1048	1777	1088	1367	1204	1438
ENSECAG00000021822	4.554343738	0.437976465	0.816948935	428	518	407	420	285	409	504	595
ENSECAG00000005773	4.71960741	0.438102749	0.816948935	139	1156	427	437	495	429	635	391
ENSECAG00000015445	5.369029705	0.438157437	0.816948935	433	436	693	1209	898	630	1178	1180
ENSECAG00000007596	4.759354778	0.438201813	0.816948935	250	230	609	715	965	468	601	485
ENSECAG00000016566	0.840977872	0.438205242	0.816948935	28	21	23	26	19	29	37	81
ENSECAG00000016666	4.120544987	0.43821758	0.816948935	397	647.007	88	178	79.0032	440	293	403
ENSECAG00000005107	3.585587626	0.438415684	0.817099276	170	278	194	306	115	217	240	330
ENSECAG00000001625	4.191802335	0.438534618	0.817099276	172	201	432	401	534	410	368	366
ENSECAG00000022834	5.930413739	0.438568647	0.817099276	1158	1213	1020	1233	940	1335	1153	1182
ENSECAG00000008254	2.142573653	0.438585303	0.817099276	15	40	107	129	89	81	159	95
ENSECAG00000024767	4.340633306	0.438692474	0.817165219	248	584	300	467	259	491	336	426
ENSECAG00000023680	6.231536243	0.438887283	0.817394359	1505	2600	696	810	1117	1205	1855	1414
ENSECAG00000020184	5.159136185	0.43908394	0.817522469	375	296	811	882	1036	622	1092	608
ENSECAG00000021803	3.418838402	0.439099684	0.817522469	206	267	125	220	80	249	209	247
ENSECAG00000016977	5.210860734	0.439234513	0.817639786	554	739	434	534	1190	495	1068	735
ENSECAG00000014258	4.942363208	0.439671421	0.818259217	346	470	607	583	695	506	916	746
ENSECAG00000009383	4.577835154	0.439806086	0.818259217	234	317	504	530	559	536	488	604
ENSECAG00000021022	2.205119882	0.439807158	0.818259217	33	48	139	65	55	98	205	90
ENSECAG00000023894	4.915620679	0.439854757	0.818259217	324	311	825	476	1025	494	821	487
ENSECAG00000000669	8.210010222	0.440080871	0.818546106	5213	7036	5380	5431	4438	7316	4180	5607
ENSECAG00000014209	3.326033319	0.440217184	0.8186659	91	128	164	280	155	214	243	323
ENSECAG00000008531	5.885832758	0.440389311	0.818852246	890	1022	1255	1460	1109	1135	1155	1115
ENSECAG00000006729	0.79578662	0.440939193	0.819740809	28	19	41	47	21	11	56	35
ENSECAG00000004824	6.092400232	0.4412184	0.819991744	1231	1139	1452	1406	1457	1264	1419	1039
ENSECAG00000012675	2.417901447	0.441280685	0.819991744	59	65	78	138	92	101	139	167
ENSECAG00000012543	6.540823562	0.441368846	0.819991744	1308	2353	1610	2064	2356	1628	1822	1173
ENSECAG00000015810	2.640444022	0.441432637	0.819991744	88	92	130	192	97	117	76	163
ENSECAG00000020161	9.534450245	0.44143429	0.819991744	13632	16520	12848	14682	11751	14751	12667	15439
ENSECAG00000009337	1.714093939	0.44155018	0.820073214	34	47	90	95	56	61	63	56
ENSECAG00000014496	0.487200896	0.441866981	0.82052774	7	37	17	19	19	26	51	33
ENSECAG00000005033	2.563156606	0.442028604	0.820694007	111	144	56	148	50	89	106	184
ENSECAG00000000697	5.19038854	0.442223493	0.820847391	283	1039	886	852	340	1170	228	833
ENSECAG00000024677	5.479863816	0.442290298	0.820847391	475	451	1041	954	1035	995	1600	584
ENSECAG00000024105	7.402874842	0.442327514	0.820847391	1170	2822	2728	8606	1567	2248	3146	5425
ENSECAG00000015431	2.753883708	0.44241571	0.820877259	82	95	112	132	227	131	151	113
ENSECAG00000009749	0.554181803	0.442591355	0.820996875	8	38	21	57	7	34	10	42
ENSECAG00000012124	5.472968388	0.442624402	0.820996875	900	1183	603	607	975	641	1137	620
ENSECAG00000023373	5.946393628	0.443081267	0.821474617	919	1325	1122	1452	1148	1475	998	1008
ENSECAG00000006787	6.912934717	0.443145111	0.821474617	1871	2605	2249	2777	1749	2701	2101	2422
ENSECAG00000009617	3.372407994	0.443280576	0.821474617	116	152	164	236	184	165	299	323
ENSECAG00000010374	5.226108506	0.443293131	0.821474617	454	622	911	1012	483	771	723	882
ENSECAG00000021350	4.503375472	0.44333807	0.821474617	244	494	319	403	490	521	636	450
ENSECAG00000007191	2.906619316	0.443339638	0.821474617	71	169	124	256	126	99	135	196
ENSECAG00000009958	11.18761068	0.443473839	0.821474617	37530	98951	30451	24899	5961	95054	5538	40781
ENSECAG00000011347	2.892419992	0.443491097	0.821474617	74	110	166	118	210	121	153	204
ENSECAG00000007346	7.025236842	0.443531354	0.821474617	2576	2904	2252	2157	1912	2666	2605	2532
ENSECAG00000007885	3.240991848	0.443641374	0.821514181	107	177	133	182	173	163	308	245
ENSECAG00000011808	1.498973225	0.443697031	0.821514181	20.0007	34	54.0005	67.001	134.001	36.001	48.0011	42
ENSECAG00000018001	1.551145783	0.443801815	0.821538524	46	57	43	89	20	26	43	117
ENSECAG00000000453	3.606645136	0.443874339	0.821538524	136	112	266	278	272	198	377	298

ENSECAG00000012453	3.235840989	0.444078318	0.821538524	110	124	130	235	176	105	237	376
ENSECAG00000011582	5.682704925	0.444122878	0.821538524	501	836	822	1276	1178	924	1529	1153
ENSECAG00000015398	3.714975604	0.444134167	0.821538524	163	214	133	326	298	162	511	287
ENSECAG00000020151	0.745502883	0.444213372	0.821538524	7	21	46	27	25	46	42	39
ENSECAG00000014185	3.17291503	0.444257433	0.821538524	82	126	170	211	177	164	330	181
ENSECAG00000011062	5.590284989	0.444287456	0.821538524	710	938	1091	983	1043	856	892	892
ENSECAG00000005326	3.232254678	0.444596757	0.821826051	107	117	185	194	245	185	273	170
ENSECAG00000019405	2.97791201	0.44462072	0.821826051	135	151	156	171	94	109	104	271
ENSECAG00000002460	3.388229684	0.444712061	0.821826051	367	74	181	121	272	151	232	91
ENSECAG00000012921	6.220160754	0.444786862	0.821826051	844	942	2412	2006	1207	1663	2208	501
ENSECAG00000012128	3.905794652	0.444818289	0.821826051	248	268	411	246	166	270	117	532
ENSECAG00000027056	2.085521628	0.444876061	0.821826051	23	46	232	60	69	74	51	97
ENSECAG00000006243	4.533079872	0.444955686	0.821839792	325	292	455	395	638	354	648	514
ENSECAG00000013471	2.934109639	0.445056616	0.821848671	81	73	161	177	173	148	155	232
ENSECAG00000009981	4.414721169	0.445106584	0.821848671	250	327	399	410	574	399	538	450
ENSECAG00000022755	3.366704012	0.445188779	0.821848671	158	174	126	163	341	124	328	184
ENSECAG00000023687	5.600412456	0.445300132	0.821848671	527	887	1026	1508	1108	979	797	780
ENSECAG00000008911	6.809307133	0.445321427	0.821848671	1384	2002	1656	1982	3278	1704	3785	1948
ENSECAG00000017942	5.810680291	0.445504467	0.821973298	912	1207	1138	1027	1165	1060	1170	887
ENSECAG00000016389	5.183153254	0.445533353	0.821973298	688	649	682	719	761	477	800	761
ENSECAG00000022421	3.479365581	0.445613705	0.821988339	102	162	194	279	279	231	332	194
ENSECAG00000009071	4.689915098	0.445852152	0.822196235	414	502	492	574	570	405	467	530
ENSECAG00000020674	3.73587127	0.445870844	0.822196235	240	254	221	303	164	253	300	295
ENSECAG00000015531	1.725936594	0.445979564	0.822263536	35	49	44	78	100	57	68	77
ENSECAG00000012481	7.345493664	0.446279406	0.822632173	2918	2236	2865	850	8100	2755	3698	1232
ENSECAG00000004249	5.050707773	0.446378909	0.822632173	685	807	532	458	504	1069	479	345
ENSECAG00000020173	4.372820013	0.446396272	0.822632173	202	419	725	411	82	491	86	770
ENSECAG00000017486	2.335264378	0.446527066	0.822740031	55	176	121	61	19	201	24	83
ENSECAG00000011655	6.395382874	0.446810212	0.822968498	846	1283	1413	2021	1730	1339	2764	2134
ENSECAG00000008127	0.618885575	0.446923383	0.822968498	15	30	14	25	12	85	10	29
ENSECAG00000008649	5.10717434	0.446936528	0.822968498	523	830	556	733	675	620	738	615
ENSECAG00000020273	3.135878391	0.446940204	0.822968498	109	205	156	225	156	160	199	147
ENSECAG00000020480	5.915729848	0.447124606	0.823174909	790	1190	823	1000	1221	1004	2055	1429
ENSECAG00000013420	5.979849687	0.447487552	0.823638287	745	1812	1112	1302	1094	1458	1214	990
ENSECAG00000013074	3.191579629	0.447520988	0.823638287	101	165	181	299	150	179	137	211
ENSECAG00000020796	2.241578401	0.447685603	0.82366341	39	53	76	141	106	76	149	113
ENSECAG00000020696	2.276026224	0.447688121	0.82366341	59	141	111	78	59	177	27	61
ENSECAG00000013578	5.40389743	0.447874284	0.82366341	423	504	817	1100	805	768	1280	1106
ENSECAG00000018910	5.101439548	0.447908019	0.82366341	279	411	684	948	1142	555	970	540
ENSECAG00000005097	3.751476159	0.448008487	0.82366341	84	217	222	385	287	430	257	260
ENSECAG00000008616	3.644272652	0.448016384	0.82366341	171	90	280	241	392	197	405	190
ENSECAG00000022725	2.222125417	0.448041062	0.82366341	50	97	101	127	56	82	97	109
ENSECAG00000010766	6.010199928	0.448413354	0.82421473	653	1315	1230	2105	814	982	1477	1683
ENSECAG00000022314	4.542939257	0.448572503	0.824259714	311	272	509	362	869	330	707	290
ENSECAG00000009712	2.59654688	0.448677633	0.824259714	43	94	122	135	115	142	196	112
ENSECAG00000012664	3.854312215	0.448716508	0.824259714	199	265	290	382	255	249	320	284
ENSECAG00000013510	10.35247638	0.448727423	0.824259714	10639	20357	23692	30338	28965	21709	27813	46316
ENSECAG00000021363	2.145522595	0.449009179	0.824644216	43	51	203	65	75	43	78	120
ENSECAG00000010237	4.497967714	0.449087351	0.824654756	160	467	384	917	291	493	403	503
ENSECAG00000000194	4.487645784	0.449275238	0.824866728	216	451	500	669	232	382	481	620
ENSECAG00000020821	5.261612819	0.44950328	0.825152345	527	659	581	659	1163	643	1100	660
ENSECAG00000003554	3.846156756	0.449622694	0.825238493	160	208	456	336	180	300	374	238
ENSECAG00000019963	4.360754347	0.44998754	0.825775006	278	287	491	575	365	351	422	438
ENSECAG00000019688	2.487711475	0.450102239	0.82579167	31	73	137	125	143	158	123	93
ENSECAG00000019969	3.863254819	0.450311527	0.82579167	113	336	291	465	201	300	334	266
ENSECAG00000023125	6.670295263	0.450457976	0.82579167	1155	1304	2566	3491	2402	1077	2259	1971
ENSECAG00000016852	6.652497223	0.450552944	0.82579167	1836	1131	3381	1454	2088	1227	2443	1826
ENSECAG00000023304	6.261114288	0.450649479	0.82579167	869	939	1320	1931	1836	1208	2413	1770
ENSECAG00000017511	3.891430922	0.450651178	0.82579167	148	291	223	584	178	204	251	479
ENSECAG00000008032	0.446601751	0.450679466	0.82579167	23	16	34	29	17	13	33	31
ENSECAG00000011670	10.54309528	0.450679556	0.82579167	10931	19964	29373	38029	41720	41209	27142	29888
ENSECAG00000003592	5.262559929	0.450766883	0.82579167	538	568	604	717	1229	489	1216	674
ENSECAG00000001013	3.975589697	0.450793317	0.82579167	237	330	271	379	266	258	320	360
ENSECAG00000000703	2.617532876	0.450810229	0.82579167	56	136	70	121	134	69	242	145
ENSECAG00000024684	6.894129702	0.45086702	0.82579167	1860	2143	2770	2571	2120	1990	2295	2571
ENSECAG00000025695	1.127142163	0.451145327	0.826168497	19	31	40	98	21	31	10	83
ENSECAG00000026827	2.915996146	0.451285679	0.826292612	78	132	95	180	190	139	239	137
ENSECAG00000012929	6.216912674	0.451411105	0.826389359	1094	664	1462	1366	3096	1654	1420	761
ENSECAG00000012515	4.628176816	0.451495381	0.826410758	267	530	533	640	487	418	531	467
ENSECAG00000013857	2.838655319	0.45178002	0.826562524	72	125	109	152	157	128	230	154
ENSECAG00000022291	7.755321955	0.451806737	0.826562524	3234	5413	4494	3777	4534	4516	3772	3147
ENSECAG00000007107	3.287658016	0.45190307	0.826562524	109	187	131	406	71	77	209	365
ENSECAG00000018544	1.790448016	0.451912187	0.826562524	32	55	35	92	33	44	125	131
ENSECAG00000016042	5.392780637	0.451941301	0.826562524	625	659	694	631	1195	579	1469	728
ENSECAG00000018144	5.273183841	0.452332485	0.827081807	626	781	740	815	961	535	803	671
ENSECAG00000005457	5.754831525	0.452374414	0.827081807	616	880	1338	1561	1001	1458	803	756

ENSECAG00000013096	0.640502147	0.452484779	0.827081807	19	14	15	44	37	38	43	22
ENSECAG00000016319	5.349650959	0.452515817	0.827081807	424	505	739	1064	882	658	1322	960
ENSECAG00000020315	3.733639549	0.452624657	0.827147948	150	182	221	309	355	166	473	264
ENSECAG00000016375	2.61804301	0.45312048	0.827745746	57	148	67	110	165	86	165	160
ENSECAG00000025085	3.122135109	0.453186469	0.827745746	122	120	182	274	159	115	218	169
ENSECAG00000023344	4.694781032	0.453234343	0.827745746	290	511	496	793	428	322	640	620
ENSECAG00000020062	7.119733124	0.45331073	0.827745746	1525	1929	2634	2850	4082	2382	4430	2322
ENSECAG00000002702	3.349923457	0.453315303	0.827745746	76	108	171	562	111	190	140	293
ENSECAG00000008519	5.364384469	0.453409739	0.827785419	405	823	867	1252	769	767	818	804
ENSECAG00000026775	0.489825908	0.453696291	0.827821343	7	26	18	32	18	32	15	60
ENSECAG00000018626	7.63594431	0.453779492	0.827821343	3036	4774	3737	4020	3580	3558	4217	3617
ENSECAG00000016254	5.924640491	0.453793996	0.827821343	968	1325	1102	1268	1311	1069	1239	1025
ENSECAG00000008904	1.39947707	0.453921204	0.827821343	31	37	43	108	20	35	44	87
ENSECAG00000024369	2.442861108	0.454025903	0.827821343	55	58	109	119	231	61	122	94
ENSECAG00000021100	6.205218863	0.454121061	0.827821343	1028	1666	1487	1569	1147	1435	1525	1538
ENSECAG00000022426	6.263404107	0.454186625	0.827821343	1047	1229	1321	2668	1227	828	2274	1633
ENSECAG00000022160	6.635211358	0.454191778	0.827821343	1729	2355	1412	2179	1180	1897	1969	2463
ENSECAG00000006712	4.261685596	0.454193523	0.827821343	204	305	334	422	471	372	561	367
ENSECAG00000009651	6.336935912	0.45421517	0.827821343	1071	830	1275	2070	1306	1445	2644	2282
ENSECAG00000019018	5.955388894	0.454229243	0.827821343	498	849	1371	1465	1536	1507	1488	1192
ENSECAG00000002197	0.534319555	0.454410038	0.827895816	8	59	27	19	10	18	27	42
ENSECAG00000008836	2.000471604	0.454415543	0.827895816	47	41	33	234	68	81	107	23
ENSECAG00000002942	6.089876078	0.454623074	0.828141392	1139	1244	1414	1449	1068	1379	1143	1576
ENSECAG00000013499	6.832972714	0.454706642	0.828161113	1620	2726	2136	2487	1788	2277	2376	2169
ENSECAG00000006023	5.451678284	0.455134613	0.828624967	782	1263	623	625	614	1182	633	802
ENSECAG00000022682	3.149363371	0.455173439	0.828624967	101	104	130	242	186	138	233	272
ENSECAG00000008891	6.173256414	0.455214411	0.828624967	704	1082	1876	2317	1561	1250	1498	1176
ENSECAG00000022245	1.113770533	0.455305599	0.828624967	14	20	51	49	77	39	57	25
ENSECAG00000014983	4.80752928	0.455350808	0.828624967	262	551	482	549	808	575	702	486
ENSECAG00000010284	3.252222718	0.455517362	0.828624967	142	92	196	149	338	121	301	142
ENSECAG00000008746	8.678735628	0.455562669	0.828624967	5243	4992	8020	7267	13333	7252	12511	6072
ENSECAG00000010959	5.308574979	0.455641603	0.828624967	277	470	822	1164	850	813	1039	982
ENSECAG00000010134	4.143320126	0.455651701	0.828624967	142	213	386	452	497	354	450	330
ENSECAG00000017318	2.27135339	0.455804562	0.828624967	41	155	103	86	70	75	84	122
ENSECAG00000001628	3.199032818	0.455869743	0.828624967	91	144	162	196	180	282	167	207
ENSECAG00000009791	5.424646521	0.455891503	0.828624967	422	686	735	1027	862	906	1431	804
ENSECAG00000018570	5.854443799	0.455938869	0.828624967	661	653	1270	1196	1651	835	1807	1143
ENSECAG00000010739	0.407925441	0.455980274	0.828624967	14	8	20	35	56	14	23	25
ENSECAG00000012879	6.835769458	0.456454246	0.829122702	1468	2781	1820	3161	1423	1523	2406	3307
ENSECAG00000021291	5.498883576	0.456474761	0.829122702	717	559	723	820	1273	522	1547	944
ENSECAG00000008632	6.516866135	0.456576979	0.829122702	1290	2156	1936	1733	1885	1653	1880	1547
ENSECAG00000011727	3.331122498	0.456615466	0.829122702	91	265	344	140	42	144	59	454
ENSECAG00000007853	1.631460677	0.456662121	0.829122702	25	51	48	67	35	36	91	132
ENSECAG00000019214	4.030989697	0.456691126	0.829122702	230	206	357	210	522	333	506	172
ENSECAG00000015752	4.688521079	0.456800657	0.82918933	380	489	570	549	559	383	564	485
ENSECAG00000024396	3.96937308	0.456916479	0.829267354	184	149	315	364	460	162	560	310
ENSECAG00000014838	3.408221123	0.457387003	0.829989006	86	212	218	371	140	194	179	279
ENSECAG00000026034	0.940039298	0.457598672	0.830030101	14	12	54	35	29	78	31	34
ENSECAG00000015238	0.23270033	0.45760733	0.830030101	7	25	20	42	16	20	14	27
ENSECAG00000007165	5.421281208	0.457648654	0.830030101	603	490	702	981	863	780	1437	934
ENSECAG00000013894	3.509448966	0.457773919	0.830030101	137	174	279	326	204	139	267	261
ENSECAG00000010174	3.931368471	0.457774177	0.830030101	211	368	185	154	313	413	371	317
ENSECAG00000006987	5.015556006	0.458347565	0.830881745	462	480	535	569	963	628	953	455
ENSECAG00000010203	0.311653686	0.458389832	0.830881745	16	8	18	28	39	27	33	11
ENSECAG00000022495	3.48986672	0.458750012	0.830911562	142	170	260	328	260	178	190	217
ENSECAG00000010236	5.296605291	0.458935445	0.830911562	398	765	952	1051	563	704	857	910
ENSECAG00000018978	6.324183788	0.459047573	0.830911562	790	2380	1464	1793	1192	1886	1410	1523
ENSECAG00000003466	4.056059374	0.459167136	0.830911562	262	165	219	355	354	127	1022	181
ENSECAG00000019555	3.915780672	0.45917322	0.830911562	232	363	289	278	182	389	192	352
ENSECAG00000017351	2.410127732	0.459298477	0.830911562	31	84	154	184	106	95	88	95
ENSECAG00000005594	1.964190028	0.459303362	0.830911562	39	162	53	52	41	66	125	51
ENSECAG00000015233	3.780485028	0.459305577	0.830911562	157	123	347	521	285	234	276	234
ENSECAG00000017463	5.506926659	0.459305887	0.830911562	779	540	1191	1009	742	721	1294	774
ENSECAG00000020833	2.090191602	0.459311451	0.830911562	50	60	66	91	80	94	129	90
ENSECAG00000012773	3.178211601	0.459392044	0.830911562	88	137	194	160	256	176	294	120
ENSECAG00000021086	2.265526642	0.459435491	0.830911562	32	54	38	196	79	97	144	137
ENSECAG00000012143	4.490147691	0.459503203	0.830911562	285	372	663	526	118	310	236	977
ENSECAG00000021441	2.564415773	0.459566805	0.830911562	72	70	109	118	166	82	221	91
ENSECAG00000015993	2.893968706	0.459714252	0.830911562	95	127	88	156	157	126	177	230
ENSECAG00000020279	0.532748794	0.459727339	0.830911562	13	43	19	38	19	40	23	14
ENSECAG00000022587	6.366471848	0.459732791	0.830911562	1121	1650	1684	2046	1597	1493	1541	1670
ENSECAG00000017430	4.201196444	0.459873207	0.830911562	179	279	320	448	489	368	367	455
ENSECAG00000019832	3.67387295	0.459885072	0.830911562	117	77	345	301	295	247	408	256
ENSECAG00000016091	5.827803358	0.459929794	0.830911562	547	771	1107	1391	1383	1039	1639	1216
ENSECAG00000022956	8.735455424	0.460113732	0.830911562	4465	8111	9960	12927	7264	8266	10033	6415
ENSECAG00000013345	7.649193637	0.460140882	0.830911562	3108	5821	3284	3355	2855	2970	5164	4235

ENSECAG00000019135	0.314336845	0.460262821	0.830911562	23	12	32	24	20	18	22	24
ENSECAG00000010500	7.913819081	0.460313549	0.830911562	2953	4353	3026	4760	6233	2961	9446	4796
ENSECAG00000016586	4.352987571	0.460379919	0.830911562	290	247	354	415	542	311	669	388
ENSECAG00000001653	5.429687223	0.460424814	0.830911562	432	531	849	1080	1183	724	1272	828
ENSECAG00000008831	5.839041399	0.460439838	0.830911562	833	722	1084	967	1911	877	1447	1085
ENSECAG00000008503	13.22780693	0.460449806	0.830911562	64769	118060	173852	287520	200019	220884	212054	277932
ENSECAG00000024970	2.699518325	0.460580573	0.830954402	38	99	383	63	3	149	17	238
ENSECAG00000010086	6.4911223	0.46061952	0.830954402	1384	1780	1796	2050	1488	1574	1528	2248
ENSECAG00000019554	1.959588724	0.46083718	0.831195299	44	55	49	96	69	66	104	122
ENSECAG00000009137	4.103558463	0.460899071	0.831195299	429	544	186	153	40	733	64	276
ENSECAG00000011724	0.427259352	0.461147704	0.831243419	18	36	27	21	20	15	54	3
ENSECAG00000006896	2.304616552	0.461210344	0.831243419	17	122	157	138	43	169	22	92
ENSECAG00000015492	3.235306999	0.461229441	0.831243419	151	81	172	157	307	140	387	76
ENSECAG00000015428	5.15066166	0.461233421	0.831243419	867	750	386	606	809	272	982	649
ENSECAG00000020275	3.789704252	0.461290814	0.831243419	185	286	316	289	304	259	226	253
ENSECAG00000022212	5.84157789	0.461465178	0.831426024	997	1171	1162	1001	1186	1100	1148	946
ENSECAG00000020584	3.243881848	0.461746682	0.831801578	83	175	234	285	190	166	168	185
ENSECAG00000017123	5.638962829	0.461900066	0.831818908	504	1141	1086	1293	628	1082	699	1348
ENSECAG00000007120	1.989881348	0.461902427	0.831818908	36	85	83	32	91	56	84	141
ENSECAG00000010445	6.462407485	0.462110662	0.831889519	1035	1192	1157	2415	1777	1273	3012	2329
ENSECAG00000007525	4.051894472	0.462165229	0.831889519	174	240	390	554	322	322	318	296
ENSECAG00000021028	3.696820738	0.462183958	0.831889519	139	316	195	158	353	286	305	252
ENSECAG00000018525	3.496194075	0.462331303	0.831889519	126	135	226	248	302	229	300	209
ENSECAG00000018506	3.458547347	0.462417756	0.831889519	113	79	174	361	372	135	321	212
ENSECAG00000022247	2.285183135	0.46248137	0.831889519	64	118	126	125	0	63	4	233
ENSECAG00000009295	6.817345557	0.462646215	0.831889519	1505	1964	2473	3136	2478	1908	2401	1755
ENSECAG00000001899	7.204585144	0.462664971	0.831889519	2986	3955	1940	2196	2792	2372	3269	2620
ENSECAG00000000785	2.069735739	0.462691473	0.831889519	57	25	49	127	147	37	132	85
ENSECAG00000013850	3.519631225	0.462745063	0.831889519	115	316	193	300	141	136	270	326
ENSECAG00000024008	1.937023446	0.462745395	0.831889519	44	33	58	104	66	43	93	159
ENSECAG00000009068	6.136931687	0.46293273	0.832094906	1074	1399	1173	1876	1554	881	1863	1148
ENSECAG00000018149	6.332091347	0.463185115	0.832417132	1082	1332	1378	1320	1875	1644	2315	1665
ENSECAG00000014076	4.106604373	0.463451944	0.832765211	164	231	207	541	183	284	452	718
ENSECAG00000026837	5.245634179	0.463842876	0.833336143	438	651	843	1111	833	556	863	687
ENSECAG00000024946	3.022846823	0.464004306	0.833393691	137	329	109	51	66	323	68	85
ENSECAG00000013359	4.906967148	0.464238579	0.833393691	301	408	1038	717	968	310	587	385
ENSECAG00000013870	1.118659965	0.464375308	0.833393691	17	38	94	23	33	39	35	43
ENSECAG00000019272	-0.203510944	0.464393733	0.833393691	8	23	8	28	3	2	26	25
ENSECAG00000017563	6.181662361	0.46442485	0.833393691	829	932	1127	1915	1220	1233	2145	2235
ENSECAG00000007911	3.8705546	0.464439386	0.833393691	177	271	288	463	118	322	162	472
ENSECAG00000006525	4.573521801	0.464686922	0.833393691	209	513	351	495	420	707	511	536
ENSECAG00000011079	3.539181277	0.464763846	0.833393691	113	169	232	250	308	202	270	289
ENSECAG00000015638	7.134746989	0.464811771	0.833393691	1284	1759	2578	3732	3321	2335	4300	3390
ENSECAG00000024737	6.200567582	0.4648346	0.833393691	864	1075	1482	1292	2419	1623	1832	935
ENSECAG00000016931	8.80410053	0.464874082	0.833393691	5736	6979	6677	8765	9969	10471	10902	10685
ENSECAG00000024968	4.13454639	0.464878462	0.833393691	149	480	220	606	341	412	258	287
ENSECAG00000010969	7.785756437	0.464879229	0.833393691	3278	3418	5545	5401	5302	3362	4481	3433
ENSECAG00000014289	4.810086917	0.464899722	0.833393691	248	322	690	630	634	571	690	688
ENSECAG00000011783	7.581458777	0.465160941	0.833429057	2881	3061	3981	5467	3043	2552	4841	4205
ENSECAG00000016497	5.875319237	0.465222226	0.833429057	575	1149	1208	1875	1022	1181	984	1273
ENSECAG00000005597	2.270847948	0.465306239	0.833429057	65	94	99	121	113	69	112	64
ENSECAG00000012858	2.111405399	0.46533759	0.833429057	64	11	122	51	159	67	111	75
ENSECAG00000017767	1.561899514	0.465367286	0.833429057	31	35	28	90	107	34	56	75
ENSECAG00000015269	7.214415669	0.465416259	0.833429057	1265	1560	3525	3439	4249	2412	5165	2414
ENSECAG00000011491	6.379261555	0.465446951	0.833429057	1130	1702	1343	2526	1134	1050	1492	2668
ENSECAG00000005374	7.975384436	0.465505083	0.833429057	4430	4968	4528	5632	4762	4341	4763	5048
ENSECAG00000019609	4.202905879	0.465627533	0.833517212	218	184	380	414	534	235	667	307
ENSECAG00000019571	6.269301002	0.465941264	0.833947695	1008	3002	1037	988	868	2581	1198	881
ENSECAG00000008863	4.687245739	0.466095786	0.833990752	255	470	494	478	620	453	706	597
ENSECAG00000008493	5.073998104	0.466111827	0.833990752	285	200	1489	1202	26	269	52	1851
ENSECAG00000022060	3.137120094	0.466204431	0.834025369	99	91	168	208	252	97	309	176
ENSECAG00000015237	3.148495067	0.466534257	0.834334737	116	138	158.999	306.999	98.9998	150.999	167	247.999
ENSECAG00000007820	2.027617696	0.466561419	0.834334737	31	55	50	128	56	56	108	165
ENSECAG00000010518	5.841990849	0.466665227	0.834334737	652	765	1217	1130	1400	1045	1798	1099
ENSECAG00000013601	4.156329424	0.466670497	0.834334737	221	234	332	372	439	294	583	344
ENSECAG00000021564	5.279052615	0.466897168	0.834608926	490	483	741	836	866	766	1092	857
ENSECAG00000017803	4.824978418	0.4672867	0.834829374	345	384	424	721	683	417	864	674
ENSECAG00000024142	4.586131141	0.467352577	0.834829374	252	403	623	658	573	328	495	449
ENSECAG00000015595	5.568851092	0.467382522	0.834829374	651	989	815	1260	965	917	966	805
ENSECAG00000006443	4.450365884	0.467545371	0.834829374	433	538	352	287	468	294	571	344
ENSECAG00000021592	4.987473797	0.46757598	0.834829374	372	602	612	961	397	565	630	852
ENSECAG00000019406	4.21375634	0.467667222	0.834829374	235	262	415	269	493	396	461	353
ENSECAG00000011569	5.705991618	0.467750339	0.834829374	554.011	1067	718.008	1010	441.001	1272	1670	1559.02
ENSECAG00000024883	6.733907478	0.467769814	0.834829374	1433	2744	1916	2263	1783	2199	1871	2165
ENSECAG00000006649	7.551614145	0.467892774	0.834829374	3584	4862	2602	3189	2836	3352	4181	3747
ENSECAG00000013334	7.626040044	0.467894454	0.834829374	3986	582	8458	3004	5660	3830	3039	1137

ENSECAG00000013713	5.104127467	0.467906742	0.834829374	568	791	661	576	590	431	986	683
ENSECAG00000019957	3.334680805	0.467914175	0.834829374	74	141	188	272	331	220	177	193
ENSECAG00000019322	6.89211809	0.467973743	0.834829374	1026	1896	2443	4895	2868	1446	3276	1376
ENSECAG00000009333	5.657722663	0.46826154	0.835088577	629	951	663	1029	1210	965	1522	983
ENSECAG00000017013	2.981905441	0.468283706	0.835088577	50	108	163	203	268	186	163	109
ENSECAG00000024564	5.86740012	0.468339092	0.835088577	620	841	944	1500	1319	1179	1303	1568
ENSECAG00000020891	2.447617157	0.468439047	0.835136008	62	124	106	141	116	85	83	118
ENSECAG00000009061	3.720506201	0.468579228	0.83520473	145	228	205	528	151	141	229	468
ENSECAG00000017550	7.355382628	0.468634065	0.83520473	2227	3162	2729	5161	2859	2368	3435	3768
ENSECAG00000011899	6.856932115	0.468840438	0.83520473	1351	1320	2282	2545	1867	3505	3136	2330
ENSECAG00000022192	3.96683826	0.468853278	0.83520473	209	224	337	490	429	154	359	250
ENSECAG00000013073	6.151026589	0.468886848	0.83520473	869	231	1816	1649	1551	1423	2247	1594
ENSECAG00000010122	2.134157544	0.468940177	0.83520473	30	64	79	113	72	136	125	71
ENSECAG00000022684	7.1734827	0.469055732	0.83520473	1560	2746	1838	3157	3729	2350	4219	3362
ENSECAG00000011911	1.684151722	0.469064474	0.83520473	25	16	69	96	87	34	85	96
ENSECAG00000010711	6.915142282	0.469367947	0.835614399	1841	2460	2310	2879	1987	2660	2188	2234
ENSECAG00000010597	4.830659838	0.469461823	0.835634476	326	440	653	878	403	457	602	739
ENSECAG00000021184	6.990369629	0.46952602	0.835634476	1106	1571	3115	2475	3788	2832	4058	1388
ENSECAG00000011505	6.495395564	0.469726194	0.835729454	998	1004	1724	2240	1794	1426	2464	2800
ENSECAG00000000096	2.672029868	0.469726199	0.835729454	67	221	117	94	75	153	104	130
ENSECAG00000015108	3.031569724	0.470036703	0.836098825	100	110	160	147	181	122	290	176
ENSECAG00000015215	4.985629005	0.470208538	0.836098825	360	428	636	668	880	606	856	569
ENSECAG00000022266	1.657262591	0.470303421	0.836098825	35	83	55	76	27	45	98	63
ENSECAG00000002186	6.641814748	0.470394702	0.836098825	1239	2206	2477	2430	432	3104	415	2962
ENSECAG00000013335	3.503120587	0.470423631	0.836098825	188	166	275	258	306	152	306	89
ENSECAG00000002091	3.546850061	0.470431354	0.836098825	116	193	192	266	338	176	298	267
ENSECAG00000012438	3.687297331	0.470447877	0.836098825	144	165	228	308	292	186	423	305
ENSECAG00000018522	3.119122107	0.470881116	0.836455679	83	103	204	175	201	136	298	181
ENSECAG00000022458	4.75633331	0.470891175	0.836455679	337	592	511	682	377	571	537	593
ENSECAG00000007776	1.983113874	0.47092613	0.836455679	39	76	45	87	107	60	75	121
ENSECAG00000007758	4.682084257	0.470942547	0.836455679	196	969	339	576	268	596	460	586
ENSECAG00000014082	5.038827862	0.471251042	0.836764775	458	522	664	424	552	751	876	859
ENSECAG00000000350	4.035407306	0.471263569	0.836764775	98	220	343	464	295	394	356	467
ENSECAG00000000916	4.953273862	0.471339119	0.836768419	212	583	590	713	759	518	1010	606
ENSECAG00000022492	5.925785595	0.471423937	0.836768636	1034	815	850	1016	1659	699	2403	1068
ENSECAG00000018297	4.708555281	0.471636212	0.836768636	263	400	452	633	455	454	780	743
ENSECAG00000017805	5.528379539	0.471652289	0.836768636	647	609	754	957	990	583	1462	1304
ENSECAG00000020908	1.926790282	0.471665546	0.836768636	32	60	76	73	71	63	108	110
ENSECAG00000017804	3.505457344	0.471736445	0.836768636	197	388	165	107	108	308	123	271
ENSECAG00000015206	5.232001019	0.471780226	0.836768636	323	893	594	658	940	734	930	857
ENSECAG00000011027	4.087853713	0.472045116	0.837107823	148	343	267	348	545	164	593	310
ENSECAG00000003535	6.379362555	0.472118518	0.837107823	906	3996	2153	470	3	2753	2	1912
ENSECAG00000020965	3.374228189	0.472251201	0.837161948	144	161	289	221	110	206	183	279
ENSECAG00000010573	1.982222158	0.472329808	0.837161948	13	18	125	94	181	39	132	32
ENSECAG00000022442	7.939934833	0.472461008	0.837161948	2486.01	3200	3810	6711	5003	6087	4953	6871
ENSECAG00000025074	4.886150867	0.472470254	0.837161948	298	395	577	704	555	478	658	1045
ENSECAG00000005649	2.208032185	0.472516704	0.837161948	36	111	100	131	48	69	112	114
ENSECAG00000006072	6.091972076	0.472638169	0.837178625	628	1335	1165	1377	1525	1806	1613	1315
ENSECAG00000000294	5.639100262	0.472708003	0.837178625	613	841	1083	1467	633	632	1216	1404
ENSECAG00000020218	4.031921012	0.472746718	0.837178625	220	214	332	264	574	240	447	258
ENSECAG00000018333	9.249152531	0.473254883	0.837758434	9913	11652	7065	6685	23552	11308	15639	7561
ENSECAG00000021840	6.993960561	0.473315813	0.837758434	1773	1882	2946	3673	2526	1972	3023	2207
ENSECAG00000018843	5.202134742	0.473354231	0.837758434	776	667	519	775	764	496	816	764
ENSECAG00000024706	6.690731582	0.473368468	0.837758434	1258	1967	2551	2537	2937	1267	2220	1408
ENSECAG00000011236	4.144181666	0.473512474	0.837883046	206	225	364	360	418	395	307	486
ENSECAG00000006321	7.370465651	0.47360846	0.83792266	3354	3433	3394	2278	4076	2551	3519	2232
ENSECAG00000026874	6.071624099	0.473887433	0.838277954	939	984	1731	1671	1737	1036	1166	1174
ENSECAG00000018048	0.921272497	0.474075857	0.838277954	8	21	58	74	23	31	50	28
ENSECAG00000018277	4.906119195	0.474124991	0.838277954	303	531	511	635	862	721	734	420
ENSECAG00000020564	7.120589025	0.474176968	0.838277954	2127	1220	5192	2724	2387	3922	2391	1356
ENSECAG00000018943	5.969599814	0.474177429	0.838277954	1080	2022	856	736	1345	1255	1321	774
ENSECAG00000015465	5.455474141	0.474413677	0.838367551	496	831	617	895	1260	512	1618	765
ENSECAG00000022285	5.404848832	0.47443653	0.838367551	626	734	874	1058	613	741	1125	836
ENSECAG00000013119	4.348670502	0.474449024	0.838367551	186	207	435	550	502	292	546	556
ENSECAG00000014943	4.536393533	0.474527373	0.838375876	254	464	373	414	391	601	484	640
ENSECAG00000011091	3.70325877	0.474740632	0.838379913	279	289	201	184	263	182	252	287
ENSECAG00000018174	0.199930463	0.474793463	0.838379913	16	7	25	13	19	9	26	50
ENSECAG00000015855	2.347276288	0.474886482	0.838379913	46	155	92	113	66	91	88	128
ENSECAG00000018148	5.330781194	0.474927472	0.838379913	390	597	755	971	903	942	1016	820
ENSECAG00000024860	7.300781079	0.474970503	0.838379913	1990	3178	2693	1840	4201	3484	4449	2711
ENSECAG00000026223	0.682210774	0.475005948	0.838379913	4	13	21	51	0	45	1	110
ENSECAG00000021482	3.127191977	0.475045131	0.838379913	74	222	185	224	143	127	197	196
ENSECAG00000022451	0.784477113	0.475250876	0.838483892	10	21	53	18	62	37	24	30
ENSECAG00000012154	5.856858866	0.475251344	0.838483892	694	1409	1137	1326	943	1422	898	1116
ENSECAG00000007008	2.917583167	0.475464253	0.838486286	87	202	147	156	108	123	143	193
ENSECAG00000000464	6.001669523	0.475501662	0.838486286	716	862	1354	1243	1977	754	2011	1283

ENSECAG00000021235	1.718559352	0.475620855	0.838486286	44	40	52	62	106	49	114	38
ENSECAG00000023743	2.571112752	0.475658509	0.838486286	76	87	94	115	180	112	128	123
ENSECAG00000022801	4.817337866	0.475659536	0.838486286	464	366	497	418	604	689	927	401
ENSECAG00000005906	5.901694106	0.475694591	0.838486286	670	1059	1381	1720	818	980	1131	1667
ENSECAG00000008405	3.440193339	0.475886179	0.838602929	87	79	298	252	235	341	289	142
ENSECAG00000019681	5.336148067	0.47595185	0.838602929	615	320	829	775	1383	489	1288	658
ENSECAG00000020154	4.363064956	0.476041604	0.838602929	267	325	403	323	451	372	611	468
ENSECAG00000002016	4.770035958	0.476097019	0.838602929	375	409	483	477	748	433	869	485
ENSECAG00000021826	6.383662075	0.476198619	0.838602929	872	958	1517	2238	1799	1222	2753	2117
ENSECAG00000002281	5.066583487	0.476202717	0.838602929	430	574	516	650	576	598	1080	867
ENSECAG00000009682	3.678344667	0.476318472	0.838635697	117	176	323	221	213	336	324	305
ENSECAG00000026897	3.986650916	0.476385997	0.838635697	190	204	358	272	418	342	394	297
ENSECAG00000014406	2.29677502	0.476442309	0.838635697	108	210	40	17	5	151	39	120
ENSECAG00000008041	3.918078948	0.4768502	0.839135487	135	472	268	330	237	365	249	278
ENSECAG00000009673	7.13243462	0.476873658	0.839135487	1549	2370	3279	4385	2854	1619	3791	2505
ENSECAG00000024506	2.887467297	0.476957281	0.839152934	70	125	89	195	86	127	230	257
ENSECAG00000022700	5.424048038	0.477105666	0.839208717	576	694	833	1327	618	538	1003	1186
ENSECAG00000016316	6.64104183	0.47713641	0.839208717	1520	2610	1767	1758	1821	1921	1982	1857
ENSECAG00000018806	4.813776686	0.477383482	0.839360838	318	308	486	765	453	420	965	814
ENSECAG00000008147	4.944902669	0.477416314	0.839360838	348	372	662	658	707	579	1000	572
ENSECAG00000021160	6.298883244	0.477444074	0.839360838	990	1433	1271	1317	2170	1069	2518	1649
ENSECAG00000013191	7.820443285	0.477564054	0.839400493	3745	4614	4398	4907	3865	5696	3237	3889
ENSECAG00000013161	2.903638794	0.477614088	0.839400493	98	63	136	173	225	83	290	117
ENSECAG00000025119	7.454016505	0.477708202	0.839436314	1922	2697	2923	3860	3650	3244	5113	4536
ENSECAG00000010283	7.168015788	0.477839627	0.839483762	1495	1800	2644	3526	3147	2223	3984	4251
ENSECAG00000010769	6.220437227	0.477882676	0.839483762	1014	1238	1150	1355	2160	1487	1689	1519
ENSECAG00000015121	4.410283331	0.478006088	0.839494237	249	402	298	436	399	359	653	564
ENSECAG00000010315	4.229146529	0.478036112	0.839494237	265	301	408	502	248	247	588	389
ENSECAG00000016358	6.65833355	0.478127005	0.839524361	1693	1632	2197	2324	1462	1908	1795	2513
ENSECAG00000009920	3.680605687	0.47837156	0.839735761	138	178	265	259	250	260	328	341
ENSECAG00000023237	8.137204986	0.478394918	0.839735761	4193	3261	5375	4541	8273	5549	8417	4420
ENSECAG00000022010	2.005626231	0.4784778	0.839751773	58	40	71	69	122	25	151	87
ENSECAG00000017363	6.537327959	0.478738614	0.839986287	755	1510	1516	2512	1888	1915	2223	2598
ENSECAG00000009602	6.241305482	0.478815241	0.839986287	866	1002	1556	1530	1915	1159	2409	1607
ENSECAG00000022070	6.197865463	0.478859541	0.839986287	899	2792	1082	908	1577	1407	1716	797
ENSECAG00000021598	2.585419411	0.478906543	0.839986287	98	57	225	86	134	78	101	125
ENSECAG00000023327	0.712619814	0.479452438	0.840711424	19	52	19	36	13	14	35	52
ENSECAG00000009022	5.903501149	0.479467657	0.840711424	781	938	944	1191	1848	877	1774	1078
ENSECAG00000007536	5.982002446	0.47957915	0.840731585	900	1090	962	952	2433	986	1848	664
ENSECAG00000008919	2.88880804	0.479626846	0.840731585	109	181	130.002	148	118	168	143	123
ENSECAG00000020988	2.941789974	0.480075371	0.841347166	116	169	152	152	112	118	150	200
ENSECAG00000023186	4.195625603	0.480125827	0.841347166	241	169	433	333	556	314	430	388
ENSECAG00000017149	5.515946158	0.48030395	0.841529774	525	434	1028	1047	1266	734	1300	951
ENSECAG00000020779	2.58463974	0.480495512	0.841735867	34	79	196	71	145	188	139	83
ENSECAG00000023640	6.331482749	0.480587266	0.841760252	989	1679	1485	2271	1311	1562	1443	1843
ENSECAG00000004362	6.109102568	0.48069028	0.841760252	1222	1581	1111	1286	1171	1217	1472	1450
ENSECAG00000024886	4.398016259	0.480759395	0.841760252	231	552	523	680	0	693	2	533
ENSECAG00000023095	4.62266443	0.480958152	0.841760252	285	643	403	609	433	347	449	665
ENSECAG00000000708	6.164191465	0.480990943	0.841760252	1068	1317	1796	1345	1342	1442	1470	1244
ENSECAG00000014787	1.657368995	0.48099141	0.841760252	24	84	38	46	75	42	76	99
ENSECAG00000014063	6.150488048	0.481070857	0.841760252	914	1631	1424	1584	1456	1044	1797	1224
ENSECAG00000005459	6.529309535	0.481154085	0.841760252	1203	1860	1628	2674	1651	1259	2227	2025
ENSECAG00000019341	0.98101264	0.481225795	0.841760252	20	15	27	57	13	24	88	64
ENSECAG00000016591	2.302957863	0.481248791	0.841760252	48	68	95	104	158	107	104	80
ENSECAG00000006455	5.028514272	0.481546944	0.842100387	220	452	723	848	599	587	858	988
ENSECAG00000010019	0.766029643	0.481591183	0.842100387	24	40	29	34	18	25	40	38
ENSECAG00000021012	3.049441785	0.481770752	0.842167896	53	166	122	388	77.0021	217	68	222
ENSECAG00000019212	3.742215257	0.481777735	0.842167896	148	216	282	445	354	158	301	207
ENSECAG00000010370	5.014598536	0.481856458	0.842176201	276	392	717	819	867	633	735	723
ENSECAG00000017271	4.011670744	0.481941736	0.842195957	224	150	298	669	262	130	446	400
ENSECAG00000023201	8.544079939	0.482296801	0.842570892	5617	8829	6652	8233	6516	7862	7429	6188
ENSECAG00000018699	1.530231954	0.482337697	0.842570892	17	49	39.9705	80	76	61	93	36
ENSECAG00000014072	4.817666628	0.482378311	0.842570892	262	321	618	711	583	464	885	694
ENSECAG00000024086	6.046082393	0.482540325	0.842646621	1183	868	891	1090	2024	1043	2159	991
ENSECAG00000017404	7.610347383	0.482677214	0.842646621	1628	2669	4411	4202	4650	5521	3765	4138
ENSECAG00000012848	5.126870853	0.482707176	0.842646621	581	640	506	977	515	453	934	836
ENSECAG00000007688	0.236050859	0.482717722	0.842646621	8	19	26	40	21	15	29	15
ENSECAG00000023585	8.036129016	0.482926666	0.842882123	3453	2112	6383	3906	13943	3325	5847	2035
ENSECAG00000011371	4.617654168	0.483169173	0.842894352	339	341	439	467	591	448	635	577
ENSECAG00000012271	3.625331005	0.483197099	0.842894352	127	425	158	270	148	192	198	384
ENSECAG00000011988	3.208181527	0.483265661	0.842894352	77	206	90	205	59	138	197	496
ENSECAG00000000382	4.644806478	0.483295099	0.842894352	531	373	301	226	466	785	649	423
ENSECAG00000017444	4.884677824	0.48330385	0.842894352	507	521	547	663	590	466	646	595
ENSECAG00000018969	0.285002891	0.483718375	0.843325725	19	14	15	17	49	9	30	21
ENSECAG00000011234	3.293724676	0.48382051	0.843325725	70	149	268	149	136	313	208	244
ENSECAG00000000077	1.604026776	0.483871875	0.843325725	21	72	43	52	42	77	57	101

ENSECAG00000006302	4.820757821	0.483882518	0.843325725	199	600	485	618	624	752	516	672
ENSECAG00000011011	2.515891918	0.48392156	0.843325725	33	107	118	232	75	137	106	98
ENSECAG00000011995	5.659678314	0.484011488	0.84335335	623	976	1116	1272	1298	761	1026	815
ENSECAG00000019388	4.729583348	0.484191902	0.843390763	392	408	390	498	768	439	752	489
ENSECAG00000017451	1.06532181	0.48426493	0.843390763	6	52	58	60	41	13	71	23
ENSECAG00000013582	4.782689909	0.484382964	0.843390763	210	685	488	407	453	669	516	891
ENSECAG00000010359	5.938081727	0.484393746	0.843390763	784	1143	1398	1503	1155	1088	1035	1428
ENSECAG00000016731	6.239163249	0.484598452	0.843390763	1290	1461	1350	1662	1267	1374	1643	1548
ENSECAG00000019087	5.062588368	0.484628486	0.843390763	324	504	688	726	624	866	793	760
ENSECAG00000021103	7.729411843	0.484684033	0.843390763	2016	1491	3648	7115	3728	3556	6879	6291
ENSECAG00000024938	4.47428738	0.484764661	0.843390763	229	356	467	769	294	396	404	609
ENSECAG00000014704	4.121298418	0.484798009	0.843390763	171	247	368	374	322	352	454	471
ENSECAG00000007689	5.844297389	0.484815073	0.843390763	835	1434	1103	1006	1052	1121	1274	984
ENSECAG00000014357	0.616756038	0.48484783	0.843390763	17	2	36	33	44	13	50	36
ENSECAG00000018233	8.363244764	0.484944787	0.843430553	6204	6837	5508	6665	5197	6090	6833	6743
ENSECAG00000008878	5.586239755	0.485131316	0.843626093	857	613	986	1264	1108	475	1262	895
ENSECAG00000017018	5.418994604	0.485611402	0.844243075	568	843	809	1130	904	823	938	645
ENSECAG00000012764	2.97297904	0.485634423	0.844243075	79	192	153	203	133	188	143	120
ENSECAG00000013492	6.365225658	0.485808296	0.844307515	1702	1943	1110	1368	1341	2109	1320.01	1384
ENSECAG00000007152	7.432628688	0.485819809	0.844307515	1591	4085	2697	2649	3588	3996	5315	3404
ENSECAG00000021928	5.892064927	0.485997225	0.844365665	862	1255	926	1599	1068	784	1283	1475
ENSECAG00000016442	8.060070076	0.486001599	0.844365665	2772	2019	7062	5488	5633	5789	6085	7651
ENSECAG00000000188	4.401455817	0.486095144	0.844399331	272	275	356	482	586	324	542	490
ENSECAG00000007205	5.142463571	0.486227931	0.844501143	441	557	633	672	737	640	1224	683
ENSECAG00000023118	0.793275818	0.486353945	0.844543508	8	49	14	33	39	32	54	33
ENSECAG00000007375	0.668285911	0.486455138	0.844543508	17	12	44	19	39	24	48	33
ENSECAG00000006403	5.537315654	0.486474864	0.844543508	640	829	984	1176	1107	794	1013	678
ENSECAG00000018653	4.757422931	0.486591389	0.844566824	402	367	434	523	842	409	647	586
ENSECAG00000009750	4.564526961	0.486748226	0.844566824	191	296	405.002	740	666	464	603	442
ENSECAG00000012847	3.888612481	0.486768589	0.844566824	219	203	202	315	337	173	463	416
ENSECAG00000019857	5.09574457	0.486835457	0.844566824	318	548	883	1041	636	723	587	665
ENSECAG00000007965	6.721774541	0.486947772	0.844566824	1373	2653	2088	2211	1322	2872	1606	2061
ENSECAG00000018815	4.510426506	0.486984885	0.844566824	203	332	341	686	477	481	584	549
ENSECAG00000008289	3.566521219	0.487007572	0.844566824	121	166	230	263	178	216	360	349
ENSECAG00000012208	4.435074747	0.487141091	0.844669711	248	493	385	588	427	521	381	312
ENSECAG00000005267	5.248735483	0.487265276	0.844700398	388	571	1004	1148	575	903	1040	401
ENSECAG00000023542	6.192940684	0.487307177	0.844700398	668	1211	1740	2435	1075	1529	1431	1554
ENSECAG00000008386	1.301704537	0.487579296	0.845007671	34	42	67	46	42	34	27	72
ENSECAG00000025134	5.474759694	0.487820103	0.845007671	542	961	834	1198	433	805	911	1282
ENSECAG00000026943	3.348214036	0.487888115	0.845007671	112	216	229	249	144	157	233	248
ENSECAG00000024596	3.63370186	0.487947644	0.845007671	126	192	240	256	234	185	442	303
ENSECAG00000006518	4.99209358	0.487963278	0.845007671	344	543	553	1186	353	494	579	1013
ENSECAG00000005510	2.412158781	0.488090577	0.845007671	18	74	158	226	52	131	50	137
ENSECAG00000008557	0.208577244	0.488100029	0.845007671	22	20	23	17	13	30	20	14
ENSECAG00000007747	6.050870597	0.488156558	0.845007671	812	900	1209	1413	1337	962	2543	1431
ENSECAG00000022513	1.649522568	0.488190894	0.845007671	9	92	125	58	1	95	1	90
ENSECAG00000005750	3.006158809	0.488287389	0.845007671	94	99	161	162	194	145	274	135
ENSECAG00000022015	0.582934126	0.488507475	0.845007671	6	15	11	61	6	41	30	60
ENSECAG00000011738	5.804454818	0.488568614	0.845007671	970	1176	1029	1037	984	1323	1065	892
ENSECAG00000020822	8.537574736	0.488603912	0.845007671	6921	9444	6329	5442	7487	5741	8695	6122
ENSECAG00000016543	7.933586914	0.488618802	0.845007671	3637	3322	3950	4317	6421	3910	7822	5048
ENSECAG00000015792	5.401845792	0.488641731	0.845007671	623	510	628	900	690	478	2328	662
ENSECAG00000021147	8.38023617	0.488733338	0.845007671	5113	3189	5400	6862	8239	3372	14831	6322
ENSECAG00000021493	5.837134425	0.488750794	0.845007671	1064	1327	825	1049	1139	849	1360	1082
ENSECAG00000016214	6.01489072	0.488820424	0.845007671	551	965	1250	1635	1549	1201	1915	1328
ENSECAG00000018662	5.529054338	0.48902404	0.845032936	395	834	551	1346	630	951	1138	1580
ENSECAG00000024501	6.782944859	0.489219518	0.845032936	1468	2506	2101	2594	1975	2135	2464	1850
ENSECAG00000008612	3.636364075	0.489231841	0.845032936	145	173	288	188	337	170	392	261
ENSECAG00000024655	4.364618665	0.48923607	0.845032936	237	283	848	290	364	272	164	708
ENSECAG00000016183	1.410784128	0.489310918	0.845032936	24	47	42	51	28	64	98	56
ENSECAG00000021181	2.378043356	0.489448035	0.845032936	70	90	84	173	81	49	109	150
ENSECAG00000017076	3.488601858	0.489475894	0.845032936	152	298	181	236	148	222	190	287
ENSECAG00000018119	6.095583647	0.489500768	0.845032936	1055	1401	1116	1726	998	1131	1718	1482
ENSECAG00000010984	3.156073262	0.489516343	0.845032936	70	123	241	312	159	121	217	180
ENSECAG00000013694	3.812380438	0.489638156	0.845032936	102	332	291	432	219	221	260	368
ENSECAG00000012979	7.153335714	0.489651496	0.845032936	1970	2308	3645	3433	2232	2919	2097	3429
ENSECAG00000012089	10.12572278	0.489814421	0.845185994	13423	25056	21797	30704	14837	23644	16632	27928
ENSECAG00000004706	1.36316838	0.489919148	0.845238597	15	34	30	87	57	23	106	58
ENSECAG00000010464	5.312679153	0.490063666	0.845302233	721	1054	556	632	437	758	879	978
ENSECAG00000011405	4.269847465	0.490112725	0.845302233	274	356	294	590	403	326	382	375
ENSECAG00000011101	4.216335781	0.490299226	0.845302233	127	253	311	592	341	413	338	614
ENSECAG00000019325	3.617028887	0.490308622	0.845302233	119	164	193	340	190	179	309	469
ENSECAG00000019955	3.615182082	0.490327268	0.845302233	98	232	255	224	234	242	347	309
ENSECAG00000007198	8.356690962	0.490754766	0.845752488	2535	4422	4631	10612	5897	6543	7453	11100
ENSECAG00000024954	2.405130414	0.49079405	0.845752488	54	100	118	154	76	72	136	117
ENSECAG00000003330	0.097155687	0.490811304	0.845752488	7	14	11	31	10	25	21	37

ENSECAG00000002173	6.550930556	0.4909871	0.84592738	910	1318	1713	2315	1356	1712	2558	3175
ENSECAG00000009171	8.393657645	0.491493912	0.846672445	2757	3963	6273	9630	9025	5878	10918	6188
ENSECAG00000013086	1.20225323	0.491636193	0.84669614	19	51	28	43	40	37	74	60
ENSECAG00000013477	2.779379626	0.491702713	0.84669614	77	343	104	25	26	303	48	65
ENSECAG00000015586	0.422989021	0.491735638	0.84669614	23	31	19	24	17	20	22	33
ENSECAG00000014833	4.323514116	0.491805145	0.84669614	241	315	409	331	536	303	575	433
ENSECAG00000024299	4.030337296	0.49202486	0.846880235	208	236	402	447	263	331	350	313
ENSECAG00000017188	4.50445523	0.492060848	0.846880235	388	436	401	489	325	433	504	499
ENSECAG00000027002	4.511565955	0.492197787	0.846923863	332	382	455	604	342	481	434	501
ENSECAG00000000879	1.792144241	0.492300562	0.846923863	30	62	47	83	75	57	95	90
ENSECAG00000016013	5.426270179	0.492309365	0.846923863	633	782	819	1107	994	834	918	568
ENSECAG00000011693	3.03645233	0.492571009	0.84702906	38	192	141	339	161	175	102	155
ENSECAG00000010011	3.731142614	0.49262456	0.84702906	231	232	395	163	251	448	126	115
ENSECAG00000010001	6.087472427	0.492660023	0.84702906	995	1621	1246	1371	1118	1470	1024	1552
ENSECAG00000024709	5.118229026	0.492688022	0.84702906	441	459	703	669	920	557	832	873
ENSECAG00000022541	4.391439783	0.492742509	0.84702906	249	239	433	463	456	310	523	649
ENSECAG00000013521	2.969872498	0.493002824	0.847348604	73	54	210	175	202	168	238	122
ENSECAG00000024384	0.587396273	0.493190847	0.847543818	10	19	23	39	15	24	25	71
ENSECAG00000010489	5.855096826	0.493557651	0.847951352	831	1377	1174	1039	1082	1272	1119	963
ENSECAG00000021308	2.554823985	0.493576953	0.847951352	30	148	108	212	107	136	86	95
ENSECAG00000014324	3.133318857	0.493807732	0.848219829	71	149	137	225	120	224	192	270
ENSECAG00000015883	3.092544887	0.493925904	0.848294828	101	87	198	158	254	119	172	241
ENSECAG00000012312	7.504475733	0.494211718	0.848529727	4012	4503	2272	2648	2606	3442	3525	4034
ENSECAG00000023931	8.173934876	0.494211736	0.848529727	3449	6206	5517	8222	4219	4589	6932	6377
ENSECAG00000005590	3.043264741	0.494340364	0.848622594	67	155	125	193	260	91	236	182
ENSECAG00000004439	1.482399239	0.494810249	0.848979899	34	64	38	83	56	39	44	63
ENSECAG00000012113	7.770307784	0.494917248	0.848979899	1688	2922	3750	6443	6434	4507	4668	4710
ENSECAG00000023488	8.777298086	0.494924716	0.848979899	10041	9691	6539	6206	7529	9456	8119	7442
ENSECAG00000017141	4.575483595	0.494995379	0.848979899	231	307	359	731	506	430	538	716
ENSECAG00000025403	0.169815653	0.49505486	0.848979899	8	14	21	23	19	28	34	17
ENSECAG00000018804	4.06376163	0.495154084	0.848979899	155	143	402	423	527	256	463	306
ENSECAG00000022083	7.395846069	0.495160925	0.848979899	3308	3481	2803	3123	2976	3419	3149	3150
ENSECAG00000019198	5.806956475	0.495235427	0.848979899	916	1203	895	1248	919	1052	1069	1282
ENSECAG00000023338	4.729260371	0.495292365	0.848979899	311	298	539	621	603	516	727	588
ENSECAG00000024165	3.285718825	0.495294202	0.848979899	62	121	234	241	206	243	165	275
ENSECAG00000021352	5.707402181	0.495650963	0.849348983	592	1055	1226	1246	878	1059	1023	1080
ENSECAG00000014569	4.795179376	0.49565873	0.849348983	310	578	469	859	519	579	526	513
ENSECAG00000025153	6.353479043	0.495781382	0.849431306	916	1357	1480	1609	2212	1898	1752	1598
ENSECAG00000017542	2.090546669	0.495862686	0.849442774	64	64	116	99	19	35	49	200
ENSECAG00000013635	6.59303408	0.496017718	0.849540544	1126	1595	2125	2997	1866	1927	1353	2144
ENSECAG00000016097	5.55093523	0.496130136	0.849540544	681	1060	760	1116	884	556	1133	1100
ENSECAG00000002520	5.004362305	0.496143617	0.849540544	472	1213	371	373	764	743	474	378
ENSECAG00000018370	5.719159818	0.496391218	0.849614272	611	709	1032	1137	1388	841	1632	1022
ENSECAG00000005548	4.512223935	0.49650296	0.849614272	238	346	478	442	276	568	694	573
ENSECAG00000014495	6.786408082	0.496559598	0.849614272	1273	2249	2272	3106	1777	2544	2047	1994
ENSECAG00000021856	3.148080447	0.496605285	0.849614272	82	102	133	233	72	62	66	662
ENSECAG00000015656	4.341962711	0.496669652	0.849614272	175	346	372	477	443	339	651	445
ENSECAG00000000428	5.302496369	0.496717135	0.849614272	776	472	772	968	776	652	848	778
ENSECAG00000015581	7.606443141	0.496783425	0.849614272	3100	4175	3446	4444	3407	4028	3573	3714
ENSECAG00000005988	4.873517816	0.496783681	0.849614272	421	673	552	600	355	556	578	778
ENSECAG00000020532	6.432387957	0.497012707	0.849742754	1251	1448	2036	2006	1672	1866	1540	1511
ENSECAG00000018339	7.950313428	0.497031101	0.849742754	3598	4417	5571	6003	5403	3391	6129	4033
ENSECAG00000003616	6.773594322	0.497082718	0.849742754	1776	2407	1964	2281	2528	2020	2335	1434
ENSECAG00000017954	7.129158204	0.497510716	0.850205339	3675	3222	1784	1374	3035	2067	3392	1986
ENSECAG00000013055	5.284255174	0.497525835	0.850205339	461.992	1054.99	698	831.027	402.997	987.994	771	812.999
ENSECAG00000004843	5.633125634	0.497608367	0.850205339	866	928	776	1202	852	726	1041	1248
ENSECAG00000000529	6.501998976	0.497652031	0.850205339	900	2671	1406	2357	1093	2671	1235	1706
ENSECAG00000013963	3.475058248	0.497788993	0.850311731	77	213	252	386	205	239	237	157
ENSECAG00000025030	5.988211305	0.497953571	0.850465257	671	1015	1458	2020	1206	1087	1036	1539
ENSECAG00000010748	3.966961232	0.498142332	0.850660035	225	504	278	168	270	327	344	241
ENSECAG00000012663	5.602608487	0.498410322	0.850984668	820	535	761	856	2117	607	1377	465
ENSECAG00000009840	3.51361946	0.498493938	0.850984668	144	183	163	247	242	168	304	342
ENSECAG00000022043	5.709734585	0.498556674	0.850984668	944	719	1268	1028	840	1101	1065	1026
ENSECAG00000000099	1.684638874	0.498797029	0.851267302	44	49	58	104	71	39	96	32
ENSECAG00000016937	6.6427965	0.498925478	0.851358898	1027	1724	2238	3196	1833	2012	1836	1932
ENSECAG00000022396	6.920784199	0.499075183	0.851486732	1574	1500	2162	2597	2739	2190	3730	2719
ENSECAG00000014425	4.054431493	0.499186129	0.851548409	219	238	301	316	334	258	532	418
ENSECAG00000006950	6.807348003	0.499333009	0.851671358	1526	3053	1904	2223	1759	1820	2395	2610
ENSECAG00000016309	4.308223201	0.499662967	0.852106482	289	306	582	348	463	418	364	257
ENSECAG00000010463	6.027720377	0.499741106	0.8521121	828	1601	1226	1401	1106	1243	1413	1299
ENSECAG00000015730	7.905778981	0.499870344	0.852204832	2712	3348	6748	7456	1196	4388	3108	8894
ENSECAG00000018471	7.751317678	0.500260291	0.852523163	3010	5055	4766	3995	2854	4736	4511	4227
ENSECAG00000016403	6.149721839	0.500325576	0.852523163	797	1374	1374	2195	1033	1715	1047	1582
ENSECAG00000021641	0.673700266	0.500354236	0.852523163	6	40	34	50	26	30	21	31
ENSECAG00000019466	5.721177728	0.50035659	0.852523163	697	1017	736	939	1512	911	1325	1094
ENSECAG00000007334	4.419743456	0.500496331	0.852633657	191	348	572	628	463	358	416	410

ENSECAG00000016315	3.856179665	0.500727782	0.852900328	219	197	174	303	585	126	510	158
ENSECAG00000022219	5.122169726	0.500856643	0.852992203	529	942	584	571	586	852	644	572
ENSECAG00000010450	4.47791899	0.500993519	0.853097699	260	243	582	358	652	501	595	298
ENSECAG00000009724	4.471344983	0.501076509	0.853111419	346	312	293	463	636	349	485	558
ENSECAG00000019309	5.522988526	0.501243583	0.853130157	542	894	630	932	729	781	1201	1554
ENSECAG00000010415	2.810131451	0.501290044	0.853130157	55	151	128	118	108	186	172	177
ENSECAG00000024482	3.673962301	0.50141057	0.853130157	199	254	300	325	11	230	19	596
ENSECAG00000007498	3.768714279	0.501431231	0.853130157	125	165	224	408	314	179	322	449
ENSECAG00000000496	7.26591432	0.501462188	0.853130157	1757	3636	3297	3650	2295	4035	2630	2554
ENSECAG00000011965	1.464360702	0.501562975	0.853174133	52	156	3	5	3	74	4	83
ENSECAG00000018810	2.9330743	0.501749147	0.853363316	67	108	119	213	103	126	257	232
ENSECAG00000013823	6.946499956	0.501834501	0.853380446	1062	2449	2651	4122	2071	2754	2374	2110
ENSECAG00000009951	2.478281094	0.501909132	0.853380446	34	60	95	190	133	114	168	102
ENSECAG00000015596	2.332434598	0.50202669	0.853452869	59	96	94	151	92	69	97	120
ENSECAG00000011764	7.168165351	0.502272904	0.853721562	2487	3038	2164	3430	2610	1719	3720	3058
ENSECAG00000024766	9.098780498	0.502423325	0.853721562	9475	10033	10876	12230	11329	10782	10948	8211
ENSECAG00000000860	6.993588819	0.502452205	0.853721562	964	2710	4937	2043	751	2245	1408	4869
ENSECAG00000015983	4.491775038	0.502517046	0.853721562	292	266	459	451	445	465	427	710
ENSECAG00000021329	0.330079997	0.502695491	0.853721562	13	13	27	19	19	16	54	25
ENSECAG00000024143	1.147653212	0.502702011	0.853721562	46	21	29	21	18	24	108	65
ENSECAG00000015288	6.318995511	0.502777935	0.853721562	1608	2025	1029	1219	1531	1238	1787	1575
ENSECAG00000003017	2.035030011	0.502784635	0.853721562	36	89	69	142	69	39	78	117
ENSECAG00000023061	10.05916789	0.502920095	0.853824229	24586	28447	14229	10955	18380	20986	20012	19508
ENSECAG00000024974	6.836206572	0.503131332	0.853946139	2923	1125	1114	1058	3682	2191	2683	2268
ENSECAG00000014794	4.657150413	0.503141915	0.853946139	245	734	472	540	412	663	436	396
ENSECAG00000022306	4.149138904	0.503366719	0.854200343	259	296	363	456	279	350	458	295
ENSECAG00000009291	1.181378709	0.5035222	0.85431445	9	24	59	53	38	71	27	66
ENSECAG00000019769	1.72347901	0.503584038	0.85431445	25	36	78	140	60	46	85	51
ENSECAG00000008092	1.743243752	0.503703354	0.854389553	47	29	59	71	104	67	66	65
ENSECAG00000021869	5.243182512	0.503781206	0.854394313	620	730	800	708	613	833	648	817
ENSECAG00000019472	3.507180155	0.504043469	0.854686337	132	173	245	366	232	141	222	276
ENSECAG00000005458	1.950385348	0.5041405	0.854686337	23	30	139	51	41	112	125	85
ENSECAG00000020583	5.725181068	0.504178607	0.854686337	1013	440	1581	1116	2117	491	737	531
ENSECAG00000003822	1.02068353	0.50430307	0.854770054	15	57	17	29	49	10	69	63
ENSECAG00000010211	5.884266122	0.504761138	0.855385203	1031	1401	1042	939	1169	1087	1525	792
ENSECAG00000019228	5.245099125	0.504816265	0.855385203	820	593	628	748	745	567	899	742
ENSECAG00000001749	2.763472094	0.504972298	0.855485866	102	84	118	117	183	126	194	122
ENSECAG00000012586	4.096594037	0.505025956	0.855485866	193	265	345	321	354	391	503	317
ENSECAG00000011033	4.107120833	0.505193664	0.855642645	204	219	491	460	281	251	425	384
ENSECAG00000016381	2.735827477	0.505308578	0.855709975	77	50	161	135	239	85	185	112
ENSECAG00000022748	1.186171461	0.505689567	0.856108233	17	50	24	46	24	21	122	53
ENSECAG00000008635	4.569980127	0.505740533	0.856108233	227	318	394	684	481	385	707	625
ENSECAG00000008326	3.463384981	0.505769343	0.856108233	98	173	169.004	304	268	188	290	267
ENSECAG00000014780	7.288174381	0.505859382	0.856133353	1820	2021	3449	2802	3188	4330	4005	2960
ENSECAG00000017625	4.063725305	0.505954365	0.856156702	247	118	199	508	653	147	491	291
ENSECAG00000022386	8.052949079	0.506023579	0.856156702	3223	6347	5375	6214	4673	5873	5696	3860
ENSECAG00000002531	2.553389836	0.506212096	0.856348397	53	80	137	110	150	103	187	103
ENSECAG00000005604	1.270324367	0.506601387	0.856615885	19	46	46	38	49	63	67	38
ENSECAG00000014435	8.296563814	0.506638793	0.856615885	6040	8144	4513	5092	3494	4541	6864	8950
ENSECAG00000011626	7.543945109	0.506723493	0.856615885	4438	2059	1777	1848	7729	2390	6565	1509
ENSECAG00000017145	8.032291207	0.506735907	0.856615885	3859	4854	5228	6730	5063	4436	5294	5175
ENSECAG00000015900	3.35589211	0.506746419	0.856615885	105	145	215	209	267	231	226	203
ENSECAG00000003238	5.645336218	0.506935891	0.856808955	634	634	938	1109	1320	913	1219	1116
ENSECAG00000011235	5.656047937	0.507153535	0.856834858	816	957	844	1243	795	749	1068	1329
ENSECAG00000020997	3.989932231	0.507175045	0.856834858	203	206	286	346	304	412	342	379
ENSECAG00000019366	6.751260889	0.507176997	0.856834858	1312	1156	1904	2747	2035	2109	3085	2839
ENSECAG00000009141	3.545598667	0.507268893	0.856862959	88	184	209	312	201	211	336	333
ENSECAG00000005633	3.557701704	0.507441354	0.857027119	100	247	220	204	313	283	230	239
ENSECAG00000013793	4.35634705	0.50782633	0.857447162	212	247	345	583	321	370	541	661
ENSECAG00000010965	8.243820137	0.507964051	0.857447162	3880	8568	4831	6924	3440	8194	3528	7059
ENSECAG00000015120	2.162376439	0.507977879	0.857447162	49	39	80	120	113	56	172	80
ENSECAG00000000556	4.504184351	0.508048242	0.857447162	280	555	395	526	422	400	456	483
ENSECAG00000025008	5.644688656	0.508066628	0.857447162	375	842	1058	1134	1437	851	1431	894
ENSECAG00000021708	2.904632232	0.508296767	0.857668787	101	198	107	179	128	110	267	69
ENSECAG00000021573	3.338606192	0.508348614	0.857668787	126	139	145	250	203	161	266	303
ENSECAG00000010391	4.839395845	0.508885814	0.858392695	265	414	638	1049	399	467	628	729
ENSECAG00000015352	4.782353896	0.508928475	0.858392695	496	584	426	549	400	550	492	678
ENSECAG00000021839	3.199702078	0.509063397	0.858490942	111	115	141	231	162	173	174	333
ENSECAG00000021106	5.068464533	0.509137535	0.858490942	515	838	547	631	565	740	628	650
ENSECAG00000000326	2.778258594	0.50932866	0.858686036	45	100	146	166	231	125	157	115
ENSECAG00000003471	7.140450137	0.5094866	0.858825132	2278	3730	2521	2215	2442	3183	2962	2074
ENSECAG00000002267	6.768344064	0.509629246	0.85893841	1806	3010	1478	1983	1619	2052	2383	2283
ENSECAG00000021258	2.771064183	0.509932268	0.859226517	48	86	155	168	168	158	126	167
ENSECAG00000014438	3.223001364	0.509951126	0.859226517	70	122	182	259	154	170	318	230
ENSECAG00000008495	5.964079115	0.510186184	0.859468315	632	1037	1273	2159	1048	1190	1274	1316
ENSECAG00000012064	0.330066759	0.510245616	0.859468315	6	22	20	28	22	36	30	21

ENSECAG00000000700	7.432381326	0.510405393	0.859610266	2329	2129	2920	3749	4471	2974	4892	3832
ENSECAG00000002972	4.363225484	0.510565866	0.859753347	272	283	333	454	375	277	583	676
ENSECAG00000010316	6.410435402	0.510874624	0.860146051	1366	1503	1856	1798	1910	1716	1465	1404
ENSECAG00000013046	7.188303929	0.511137593	0.860366526	1233	912	4995	5551	2157	3137	2178	3247
ENSECAG00000011763	6.319864909	0.511216536	0.860366526	881	1958	1512	1964	1306	1412	1647	1822
ENSECAG00000018487	3.709420293	0.511249306	0.860366526	172	222	183	257	371	135	399	315
ENSECAG00000011408	0.27455602	0.511338987	0.860366526	16	21	26	29	29	35	8	5
ENSECAG00000009286	7.448501287	0.51140803	0.860366526	2944	4077	3276	3007	3207	3751	3252	2985
ENSECAG00000008964	6.018767789	0.511503882	0.860366526	1061	1453	1064	1322	1086	1241	1287	1408
ENSECAG00000019114	6.174599504	0.511544335	0.860366526	1083	1000	1414	2204	1545	1309	1606	1126
ENSECAG00000012712	6.429011976	0.511610134	0.860366526	644	1688	2676	2051	1321	1954	1502	1758
ENSECAG00000011614	5.951631555	0.511852159	0.860533616	895	1298	996	1614	875	1089	1395	1470
ENSECAG00000010971	2.229174941	0.511867336	0.860533616	39	52	101	205	80	73	110	85
ENSECAG00000002892	4.309642765	0.511972192	0.860533616	212	334	259	527	403	431	618	370
ENSECAG00000017776	7.461461078	0.512086873	0.860533616	1507	3523	3137	3427	3912	4473	4854	3147
ENSECAG00000010874	7.867194568	0.512087418	0.860533616	3579	5716	4307	4495	3640	6216	4360	3251
ENSECAG00000010777	6.295650783	0.512220866	0.860630838	2193	1388	872	1138	1784	1184	2109	906
ENSECAG00000013158	2.298559977	0.512407692	0.860816194	72	124	99	76	98	103	95	68
ENSECAG00000007895	4.273162536	0.512675086	0.860816194	186	487	275	622	234	301	324	619
ENSECAG00000000796	4.615118826	0.512726111	0.860816194	293	382	484	452	532	561	533	589
ENSECAG00000020500	6.709647592	0.51273054	0.860816194	1810	2447	1551	2130	1845	1786	2517	1938
ENSECAG00000018059	3.389239887	0.51274779	0.860816194	158	243	96	132	322	169	339	151
ENSECAG00000004024	6.637309617	0.512881938	0.860816194	1342	1989	1942	2554	1390	1944	2102	2252
ENSECAG00000005065	2.479072523	0.512911612	0.860816194	45	62	119	144	119	124	174	97
ENSECAG00000014904	7.26876362	0.512936061	0.860816194	2331	3249	2816	3622	2406	2579	3244	3618
ENSECAG00000014989	5.635842813	0.513206895	0.861029711	797	1047	917	999	704	779	1174	1248
ENSECAG00000014597	3.101811084	0.513214547	0.861029711	144	142	202	163	118	134	152	248
ENSECAG00000008892	2.111575131	0.513655132	0.86157397	48	82	46	95	66	60	129	148
ENSECAG00000012818	3.72858187	0.513690304	0.86157397	204	320	234	249	246	345	258	149
ENSECAG00000022067	3.751104831	0.513920256	0.861832687	145	290	262	368	248	245	305	243
ENSECAG00000004168	1.9533303	0.514287034	0.862194864	43	77	62	56	51	85	99	120
ENSECAG00000004461	1.31034498	0.514287688	0.862194864	45	53	33	53	26	32	49	74
ENSECAG00000007978	7.18955232	0.51442811	0.862303304	1573	1835	2703	3574	3471	2400	4539	3306
ENSECAG00000020351	4.427450461	0.514710116	0.862649003	227	310	403	511	448	406	604	509
ENSECAG00000023144	6.119457116	0.51512632	0.862813438	799	1696	1164	1841	1029	1513	1308	1496
ENSECAG00000022617	5.387504963	0.515137912	0.862813438	699	1239	623	537	830	722	979	701
ENSECAG00000014125	4.796329791	0.515168666	0.862813438	189	740	325	1100	291	303	659	884
ENSECAG00000011695	6.144263354	0.515183719	0.862813438	647	1088	1724	2372	1305	1539	1298	1266
ENSECAG00000000513	4.695418774	0.515187154	0.862813438	270	395	534	530	754	444	623	537
ENSECAG00000021930	7.593264289	0.515501243	0.86321248	3244	4368	3056	4164	3549	2911	4373	4004
ENSECAG00000023468	6.190207927	0.515597224	0.863246234	1693	1580	941	1066	1193	1047	1971	1457
ENSECAG00000015694	3.319064633	0.51568793	0.863271149	98	210	133	205	131	146	372	294
ENSECAG00000012679	5.523015157	0.515956576	0.863593887	506	641	613	1317	758	466	1600	1544
ENSECAG00000013673	6.046307929	0.516076988	0.863668457	658	1219	1416	2055	1013	1297	1519	1315
ENSECAG00000011773	3.237598959	0.516428591	0.863889036	174	122	115	144	317	69	371	148
ENSECAG00000013983	7.343103466	0.516446011	0.863889036	2356	3920	2991	3309	2965	2781	3697	3033
ENSECAG00000021907	6.889262978	0.51646064	0.863889036	2854	2723	1644	1358	2666	1982	2781	1514
ENSECAG00000016757	4.02033665	0.516512312	0.863889036	124	629	163	387	315	349	308	241
ENSECAG00000016568	6.528577037	0.516810127	0.864260179	1182	1722	2059	2303	1527	1509	1882	2249
ENSECAG00000024632	0.287168755	0.516954571	0.86434751	19	17	15	16	24	33	32	17
ENSECAG00000024996	5.254148312	0.517014189	0.86434751	239	418	1039	864	302	601	812	1912
ENSECAG00000013254	2.396633764	0.517137879	0.86442736	57	101	84	186	58	70	105	162
ENSECAG00000006865	4.325801591	0.517359633	0.864638879	228	558	306	472	289	330	396	537
ENSECAG00000017131	4.770929226	0.517416309	0.864638879	421	339	518	469	638	526	780	557
ENSECAG00000022657	4.065842055	0.517545445	0.86472775	227	251	319	255	601	222	555	186
ENSECAG00000007716	0.627325061	0.517655023	0.864783924	15	30	38	36	13	16	50	32
ENSECAG00000014342	3.274100613	0.517795006	0.864842189	89	147	141	263	170	85	363	304
ENSECAG00000024978	1.862888973	0.517906008	0.864842189	58	54	55	117	77	36	59	96
ENSECAG00000015589	4.825719305	0.51791779	0.864842189	337	443	642	430	592	579	726	688
ENSECAG00000010387	5.418432343	0.518050661	0.864937202	450	508	959	968	749	906	1241	1036
ENSECAG00000011124	6.354208313	0.518153512	0.864982073	997	2081	1186	2222	1517	1991	1021	1620
ENSECAG00000019321	6.067744082	0.518594683	0.865591624	637	869	1525	1457	2269	975	1926	1052
ENSECAG00000004829	5.051327448	0.519279162	0.866607045	472	1045	418	582	356	626	863	730
ENSECAG00000020016	1.799602196	0.519451722	0.866653495	41	86	84	60	22	106	48	71
ENSECAG00000019410	7.2297466	0.51945924	0.866653495	1267	2653	2941	3101	3529	4028	2669	3506
ENSECAG00000009606	6.939693871	0.51975949	0.866754354	1314	1674	2383	2770	2715	2421	3459	2820
ENSECAG00000000910	6.124030511	0.519823871	0.866754354	955	1271	1474	1814	1962	1245	1529	584
ENSECAG00000022115	2.918555319	0.519837186	0.866754354	133	104	90	130	226	91	234	154
ENSECAG00000007359	8.180603851	0.519873995	0.866754354	6517	6390	4290	4179	4825	7001	5146	4647
ENSECAG00000016480	4.88243571	0.51990035	0.866754354	372	402	625	535	479	627	780	812
ENSECAG00000022999	1.405575545	0.519977763	0.86675649	23	95	24	68	26	30	54	81
ENSECAG00000000767	6.77778733	0.52010307	0.86683845	1063	975	1801	3635	1838	1596	3855	3194
ENSECAG00000024850	0.402802648	0.52035104	0.867073392	19	19	27	36	14	5	16	55
ENSECAG00000007727	2.928588949	0.520474845	0.867073392	94	63	159	176	151	109	282	174
ENSECAG00000024675	2.532998772	0.520509541	0.867073392	44	103	149	178	87	118	107	120
ENSECAG00000016117	2.632313401	0.520548672	0.867073392	102	185	68	116	38	129	162	134

ENSECAG00000008210	8.745037077	0.520952713	0.867468528	4671	9830	5997	6637	9910	8842	14761	7135
ENSECAG00000003276	6.398363687	0.520975749	0.867468528	2717	1648	675	802	1825	754	2581	1243
ENSECAG00000011128	5.652725048	0.521085746	0.867468528	648	1067	1238	923	1006	1202	986	669
ENSECAG00000005331	3.265394044	0.521191674	0.867468528	76	131	151	291	108	203	209	370
ENSECAG00000000287	7.19790775	0.521240465	0.867468528	1515	3069	3150	4160	2658	2682	3201	2733
ENSECAG00000013506	4.283168498	0.521578752	0.867468528	225	409	359	538	327	372	356	452
ENSECAG00000020851	6.245265124	0.521594705	0.867468528	867	1040	2011	2182	1396	1468	1509	1483
ENSECAG00000010224	7.129058751	0.521649279	0.867468528	2376	2297	2854	3276	2798	2423	2784	2722
ENSECAG00000001964	5.132941999	0.521660255	0.867468528	493	509	570	707	991	477	1042	725
ENSECAG00000022581	2.910436846	0.521661006	0.867468528	128	108	155	185	116	96	234	137
ENSECAG00000023044	5.755101881	0.521699394	0.867468528	662	785	960	1183	1304	947	1392	1292
ENSECAG00000010361	2.682771321	0.521700221	0.867468528	79	109	150	169	66	145	157	118
ENSECAG00000011605	4.298089132	0.522069669	0.867956073	217	283	333	491	406	342	555	499
ENSECAG00000010939	0.48368984	0.522367088	0.86832374	17	4	21	41	16	22	47	42
ENSECAG00000017105	6.079606371	0.522632632	0.868582494	1071	740	1203	1276	1943	1109	2288	977
ENSECAG00000012196	2.384359263	0.52271836	0.868582494	74	45	124	76	151	74	190	75
ENSECAG00000017659	5.940096397	0.522831722	0.868582494	761	721	1332	1218	1851	1129	1696	969
ENSECAG00000001035	3.500956049	0.52289099	0.868582494	87	237	237	367	269	151	233	213
ENSECAG00000017940	1.718757261	0.522970634	0.868582494	40	71	81	67	22	94	25	89
ENSECAG00000001426	8.668983707	0.522987418	0.868582494	5482	8096	8791	9978	5871	9635	6888	8256
ENSECAG00000020077	4.54091226	0.523056792	0.868582494	296	312	409	527	568	408	643	506
ENSECAG00000008489	6.087197029	0.523189225	0.868615077	798	830	1191	1725	1504	917	2231	1706
ENSECAG00000013579	4.663441152	0.523278528	0.868615077	313	294	513	562	566	402	622	730
ENSECAG00000017477	4.696142523	0.523400833	0.868615077	623	519	365	358	282	389	464	846
ENSECAG00000015289	5.847346059	0.523456205	0.868615077	723	866	1325	1661	766	999	1224	1505
ENSECAG00000013897	6.791045374	0.523587069	0.868615077	1640	2051	2261	2739	2917	1326	2879	1437
ENSECAG00000009189	1.160033041	0.523603885	0.868615077	22	50	38	65	29	46	34	51
ENSECAG00000017225	2.538349421	0.523610476	0.868615077	73	84	131	173	105	90	167	82
ENSECAG00000020180	-0.008217053	0.52390354	0.868974621	12	29	25	9	0	30	2	27
ENSECAG00000005549	8.29623573	0.52412901	0.86920938	3419	5483	5814	5647	7322	5704	9323	7119
ENSECAG00000010655	4.173884736	0.524341509	0.86920938	224	407	405	357	243	357	305	483
ENSECAG00000012193	4.180450873	0.52442942	0.86920938	250	511	242	378	347	318	421	319
ENSECAG00000023546	5.542986127	0.524471707	0.86920938	1361	986	451	432	875	899	722	984
ENSECAG00000016539	2.104341415	0.524505654	0.86920938	37	68	84	90	87	79	140	89
ENSECAG00000019795	7.381606606	0.524573756	0.86920938	3099	2845	2487	4561	1961	4779	2218	3396
ENSECAG00000007814	4.277155825	0.524579504	0.86920938	171	379	272	490	308	415	505	547
ENSECAG00000014692	7.997517644	0.524955788	0.869551063	3271	4108	4861	3999	6888	4895	7224	4830
ENSECAG00000017956	4.83328365	0.52498388	0.869551063	227	327	605	788	398	333	763	1188
ENSECAG00000022240	0.657637233	0.525058602	0.869551063	13	21.0001	34.0001	59.0001	11	37.0001	28	33.0001
ENSECAG00000024189	6.516470274	0.525146367	0.869551063	724	809	1371	3502	1468	1736	2233	3143
ENSECAG00000006065	7.637360547	0.525167598	0.869551063	1283	2877	3636	5917	4633	5255	4126	4344
ENSECAG00000024018	4.617055244	0.525354634	0.86973426	382	150	924	470	496	313	612	463
ENSECAG00000010461	3.624213892	0.525475829	0.869808419	168	289	243	239	250	215	235	248
ENSECAG00000022110	7.946326933	0.525567401	0.869833532	5142	4695	4121	4508	4302	6014	4743	3488
ENSECAG00000017546	5.300911638	0.525760569	0.86992608	232	568	825	1136	703	1071	969	858
ENSECAG00000017347	3.134298746	0.52577614	0.86992608	125	91	122	191	31	142	107	560
ENSECAG00000020989	6.027218151	0.525968045	0.870117145	1034	1061	1212	1718	1420	1229	1324	1088
ENSECAG00000006748	5.802486522	0.526335013	0.870476787	1106	1036	912	1065	1069	1058	1184	1022
ENSECAG00000010343	7.553067588	0.526451636	0.870476787	1883	6671	2761	3619	2275	4648	3092	3987
ENSECAG00000020517	3.415336382	0.526477934	0.870476787	128	263	212	219	197	202	202	216
ENSECAG00000020421	4.406790991	0.526491274	0.870476787	284	282	452	361	429	410	629	475
ENSECAG00000011652	7.740334177	0.526714565	0.870709667	2698	4206	4324	5861	3345	4974	4002	3923
ENSECAG00000007371	7.523454015	0.526832181	0.870709667	1858	2316	4089	3912	5190	3892	3526	4302
ENSECAG00000011743	5.949021815	0.526861563	0.870709667	649	1350	1298	1608	854	1094	1304	1568
ENSECAG00000024528	3.676480046	0.526974801	0.870770408	110	512	147	235	215	189	277	290
ENSECAG00000002691	6.153644269	0.527111094	0.870869222	856	1357	979	1487	1663	1280	1925	1675
ENSECAG00000025087	6.144080485	0.527546954	0.871223396	1272	851	1181	1023	2565	1031	1875	1088
ENSECAG00000022048	6.335823953	0.527574221	0.871223396	841	1180	1356	2073	2224	1227	2287	1727
ENSECAG00000017857	7.042480258	0.527643801	0.871223396	1880	2016	3589	2860	2909	2439	2846	1890
ENSECAG00000015081	1.057163843	0.527721231	0.871223396	14	20	33	67	46	68	22	46
ENSECAG00000008695	5.666542718	0.527750504	0.871223396	1107	802	990	794	965	766	1321	927
ENSECAG00000022412	4.114748068	0.527784608	0.871223396	177	357	294	567	453	254	301	321
ENSECAG00000016738	4.183168111	0.527920737	0.87131372	188	160	501	336	765	283	404	219
ENSECAG00000011923	6.111267295	0.527992389	0.87131372	715	700	1700	1485	2474	1035	1825	1063
ENSECAG00000020499	2.977837711	0.528138624	0.871428729	92	88	199	257	128	134	179	160
ENSECAG00000023859	4.288831591	0.528370542	0.871685063	140	136	715	313	344	488	515	467
ENSECAG00000026852	2.734943542	0.528505203	0.871780894	79	49	175	121	199	118	197	102
ENSECAG00000017839	3.210095615	0.528640885	0.871815248	222	141	65	69	79	185	424	217
ENSECAG00000000877	3.012950578	0.52872038	0.871815248	92	161	111	153	239	121	224	157
ENSECAG00000021299	7.710445732	0.528894977	0.871815248	2530	3116	5719	5554	5519	2859	4545	3078
ENSECAG00000018553	7.537264974	0.52898164	0.871815248	2771	3384	4041	4297	2922	3351	4713	3399
ENSECAG00000023862	7.176417515	0.528983736	0.871815248	2053	2850	2882	3581	1935	2720	2648	3776
ENSECAG00000020745	3.592545601	0.528985484	0.871815248	58	197	249	331	297	271	315	222
ENSECAG00000014306	4.997540869	0.529223125	0.872003043	482	451	503	605	986	371	1038	574
ENSECAG00000007483	6.231201501	0.529298108	0.872003043	752	1306	1546	1393	1703	1762	1956	1438
ENSECAG00000012851	6.079790307	0.529387797	0.872003043	984	731	1182	1516	1379	1236	2256	1418

ENSECAG00000020630	4.837691539	0.5294058	0.872003043	259	380	435	894	473	364	1018	835
ENSECAG00000015321	6.97542707	0.529601865	0.872046878	2062	2196	2700	2716	2206	2158	2591	2746
ENSECAG00000023164	3.278476506	0.529644321	0.872046878	97	137	153	261	183	128	351	246
ENSECAG00000027692	10.9550278	0.529662202	0.872046878	36050	18905	37317	27460	41343	43943	49504	51588
ENSECAG00000007592	2.642621696	0.529827115	0.872192266	56	127	143	175	113	141	101	111
ENSECAG00000014339	3.561091695	0.530041476	0.872324042	74	242	162	325	189	193	319	395
ENSECAG00000018949	5.523153624	0.530060406	0.872324042	546	902	916	1258	764	917	731	1145
ENSECAG00000006833	1.567167923	0.530648458	0.873165586	47	74	55	43	48	74	39	52
ENSECAG00000010813	6.955377507	0.530780826	0.873257183	1636	2632	1522	2052	2703	2840	2490	3380
ENSECAG00000009597	3.284725024	0.530935246	0.873260486	87	304	137	253	83	246	180	220
ENSECAG00000017656	5.056556357	0.530936239	0.873260486	372	723	622	916	667	842	466	556
ENSECAG00000000299	-0.130959574	0.53121817	0.873533108	5	24	14	26	24	10	20	6
ENSECAG00000023197	5.404043296	0.531343243	0.873533108	394	872	793	1371	693	989	696	873
ENSECAG00000024721	5.031534812	0.531370268	0.873533108	517	482	587	477	943	513	953	598
ENSECAG00000022702	4.767835702	0.531408898	0.873533108	376	272	581	1000	299	341	368	1064
ENSECAG00000012108	5.105249152	0.531520562	0.873557033	379	721	510	664	815	543	1046	761
ENSECAG00000017449	5.078131285	0.53157691	0.873557033	279	363	864	820	732	741	936	682
ENSECAG00000020471	2.000487667	0.531666775	0.873564	44	68	63	79	76	62	115	113
ENSECAG00000024700	6.74283333	0.531795781	0.873564	1614	1185	2760	2972	3102	1274	2153	1647
ENSECAG00000011151	7.399172514	0.531869202	0.873564	2094	1885	3171	3933	4433	2912	4722	3662
ENSECAG00000019768	5.673598929	0.531899586	0.873564	665	548	1134	1012	1072	922	1784	961
ENSECAG00000023326	4.233808312	0.531964797	0.873564	208	305	387	345	460	413	442	383
ENSECAG00000021313	2.272403931	0.532257374	0.873918402	67	65	95	72	115	80	141	107
ENSECAG00000023757	0.870625103	0.532671057	0.874431674	17	34	33	60	43	22	42	24
ENSECAG00000000775	5.232128607	0.532846376	0.874431674	462	932	746	748	536	607	867	946
ENSECAG00000016230	2.572640224	0.532869252	0.874431674	67	113	69	133	149	82	174	143
ENSECAG00000008572	2.972544894	0.532891815	0.874431674	53	203	153	230	170	127	151	145
ENSECAG00000015813	4.488545942	0.532954008	0.874431674	241	452	674	381	373	556	384	401
ENSECAG00000001048	2.673398156	0.533279275	0.874685269	72	55	149	139	133	146	136	164
ENSECAG00000015161	5.97573994	0.533404377	0.874685269	924	1032	1347	1558	1043	1280	1212	1359
ENSECAG00000009033	6.414072132	0.53341026	0.874685269	1844	1568	1317	1527	1864	1157	2149	1470
ENSECAG00000027676	10.44025683	0.533415882	0.874685269	31656	14115	36720	29727	1994	31195	2830	58741
ENSECAG00000013923	8.329198143	0.533679467	0.874890573	4419	7604	4696	8810	4350	4338	7758	8408
ENSECAG00000004860	3.681789063	0.533694777	0.874890573	123	291	357	280	69	402	67	375
ENSECAG00000023277	2.557052434	0.534202017	0.875506524	82	119	124	128	55	99	135	159
ENSECAG00000007391	6.657858453	0.534224314	0.875506524	1299	2068	1287	1788	1532	1544	3294	3152
ENSECAG00000006301	1.548803817	0.534349277	0.875585279	23	64	29	68	32	38	97	106
ENSECAG00000016435	3.854832499	0.534451285	0.875626404	103	551	197	299	211	261	347	288
ENSECAG00000015402	3.222319673	0.534648878	0.875824097	90	186	176	301	124	209	179	198
ENSECAG00000019833	4.402941897	0.534857761	0.876005939	299	420	227	394	415	212	737	629
ENSECAG00000019989	5.859402899	0.534913772	0.876005939	850	893	922	1072	1718	1096	1526	961
ENSECAG00000008002	3.389161792	0.535228816	0.876395811	179	367	89	144	139	124	234	298
ENSECAG00000007477	8.094372544	0.535362655	0.876488903	3813	5668	5518	6482	6155	3349	7087	4591
ENSECAG00000018565	3.258174074	0.535614776	0.876675947	89	248	116	323	143	121	221	250
ENSECAG00000013413	5.16176627	0.535630908	0.876675947	281	803	599	733	963	823	830	623
ENSECAG00000014250	1.631042017	0.535797381	0.876822363	50	58	47	82	28	44	47	108
ENSECAG00000020678	7.535955706	0.535955116	0.876834048	3425	3982	3393	3141	3941	3646	3339	3161
ENSECAG00000010446	4.159079808	0.535982384	0.876834048	201	219	367	410	317	412	551	354
ENSECAG00000015439	2.546106658	0.536111118	0.876834048	104	105	63	68	130	52	201	170
ENSECAG00000009000	4.403496491	0.536125968	0.876834048	371	543	347	293	464	301	508	375
ENSECAG00000024598	3.983769702	0.536189604	0.876834048	194	271	272	295	380	219	472	384
ENSECAG00000020885	9.532218633	0.536280487	0.87685672	13800	17641	12603	11547	14084	13235	15241	13730
ENSECAG00000011457	6.58572491	0.53640784	0.876939009	1710	1594	2121	1822	2242	1395	2286	1547
ENSECAG00000015506	4.785170727	0.536907215	0.877491123	355	464	562	391	717	737	667	370
ENSECAG00000024998	2.808579526	0.536946128	0.877491123	73	140	116	114	223	72	248	113
ENSECAG00000016847	5.613677954	0.537008926	0.877491123	610	708	667	1295	870	822	1274	1535
ENSECAG00000008537	6.373560738	0.537053856	0.877491123	1010	1872	1392	2258	1155	1531	1693	2072
ENSECAG00000018378	6.644187843	0.537242167	0.877672848	1830	2381	1775	1418	2018	1737	2176	1784
ENSECAG00000013985	6.841484133	0.537518329	0.877961891	1488	3188	1707	2618	1132	2101	2120	3412
ENSECAG00000016812	4.795687231	0.537573328	0.877961891	301	390	654	503	605	519	939	495
ENSECAG00000013272	4.012096602	0.537898489	0.877966166	210	213	296	343	389	288	383	403
ENSECAG00000007777	4.77535524	0.537908982	0.877966166	329	554	575	677	583	305	677	599
ENSECAG00000015448	6.458354402	0.537934326	0.877966166	1049	2499	1402	1878	1831	1304	2237	1498
ENSECAG00000017095	5.567560312	0.537996889	0.877966166	494	750	922	1033	1072	1011	1425	822
ENSECAG00000000497	4.475622585	0.538042495	0.877966166	328	302	412	394	595	401	576	449
ENSECAG00000012180	3.820350097	0.538093591	0.877966166	101	203	281	392	311	300	358	317
ENSECAG00000015768	6.056101836	0.538190477	0.877966166	1718	600	1256	1267	1556	958	1667	949
ENSECAG00000007864	8.142826901	0.538192874	0.877966166	4078	7128.97	5015.97	5657.99	4380.98	6626	6029.94	4480.97
ENSECAG00000007751	8.336963259	0.538330669	0.87804366	5844	7548	4854	6405	3626	6442	5181	9175
ENSECAG00000001851	5.323671452	0.538394623	0.87804366	295	814	497	1110	1574	566	1131	443
ENSECAG00000024871	1.587275078	0.538710874	0.878165652	30	62	61	83	37	59	52	73
ENSECAG00000000691	7.077547977	0.538973	0.878165652	1866	2459	2106	2116	3651	2401	3743	2737
ENSECAG00000007952	4.560889356	0.539036089	0.878165652	395	404	467	509	499	419	565	366
ENSECAG00000010232	6.945156485	0.539116206	0.878165652	1490	2987	1689	1578	3164	2690	3102	2438
ENSECAG00000024786	6.594340753	0.53924342	0.878165652	1171	1671	1629	1840	2483	2186	2647	1541
ENSECAG00000022050	6.386945498	0.539246964	0.878165652	766	1729	1100	3415	1297	761	2242	2243

ENSECAG00000004593	3.781481828	0.539258359	0.878165652	117	410	307	242	254	238	339	232
ENSECAG00000024314	3.735537766	0.539386188	0.878165652	109	173	382	195	436	407	302	75
ENSECAG00000014873	6.656704753	0.539580696	0.878165652	2298	2367	1243	1396	2391	1456	2464	1436
ENSECAG00000012684	1.746485661	0.539594046	0.878165652	17	62	45	97	112	37	69	88
ENSECAG00000015229	4.038788724	0.539634434	0.878165652	84	255	440	295	97	162	360	966
ENSECAG00000009511	4.737999478	0.539679133	0.878165652	261	393	500	659	614	486	949	416
ENSECAG00000017272	6.05658681	0.539692191	0.878165652	750	746	1322	1688	1451	1208	1884	1577
ENSECAG00000001359	6.230319035	0.539823775	0.878165652	1032	1845	1212	1675	1329	1306	1639	1590
ENSECAG00000018265	6.746149362	0.539981543	0.878165652	1147	2946	1893	2526	1374	2742	2014	2040
ENSECAG00000019607	3.608115723	0.540000248	0.878165652	124	141	201	351	498	199	250	162
ENSECAG00000009392	2.099610722	0.540019836	0.878165652	44	85	78	138	38	100	59	117
ENSECAG00000024215	0.533263393	0.540156848	0.878165652	20	9	21	35	45	38	32	11
ENSECAG00000009999	5.093598424	0.54016371	0.878165652	539	1058	500	482	231	1153	554	597
ENSECAG00000013631	4.012682854	0.540338535	0.878165652	303	264	264	374	313	247	349	349
ENSECAG00000009552	8.760454967	0.540343107	0.878165652	6688	9351	7946	9636	6418	7678	10580	8811
ENSECAG00000002408	5.354910158	0.540679721	0.878165652	481	522	1129	1131	1077	620	883	598
ENSECAG00000024349	9.096075151	0.54076097	0.878165652	8302	6847	9011	10314	15864	8745	16345	10436
ENSECAG00000014766	4.700490703	0.541054205	0.878165652	212	371	550	659	441	489	818	648
ENSECAG00000001949	6.980484731	0.541105784	0.878165652	5508	2302	1466	154	2	5782	3	1288
ENSECAG00000015826	4.319180223	0.541123971	0.878165652	231	501	495	311	596	286	415	234
ENSECAG00000012292	8.209421142	0.541158723	0.878165652	4876	5857	5854	6131	5230	6327	5683	5330
ENSECAG00000010846	5.320038102	0.54122029	0.878165652	484	540	585	1071	651	598	1090	1356
ENSECAG00000011860	4.55380281	0.541300313	0.878165652	211	611	321	384	583	768	445	315
ENSECAG00000014830	7.708854087	0.54131642	0.878165652	2079	2454	4467	5038	4982	3609	5512	5362
ENSECAG00000017769	5.495415159	0.541348093	0.878165652	570	774	733	1502	875	728	964	971
ENSECAG00000009897	3.440187003	0.541436679	0.878165652	99	150	212	273	280	171	320	224
ENSECAG00000010835	2.566924114	0.541437977	0.878165652	58	95	158	164	123	133	100	85
ENSECAG00000017344	4.500049671	0.541499689	0.878165652	219	442	526	609	484	384	436	458
ENSECAG00000015049	3.353734189	0.54153153	0.878165652	100	150	201	229	304	150	310	175
ENSECAG00000024397	8.987384492	0.541553926	0.878165652	7004	12463	8078	12367	8952	6330	13391	10706
ENSECAG00000016866	6.210987486	0.541849833	0.878165652	1548	901	1958	1073	1262	1829	1148	1364
ENSECAG00000012137	4.345338205	0.541858761	0.878165652	239	222	444	452	569	318	568	404
ENSECAG00000019377	5.037911955	0.54186677	0.878165652	341	489	682	690.001	1023	693	809.001	449
ENSECAG00000017994	3.616868371	0.541953329	0.878165652	162	176	213	240	203	151	450	346
ENSECAG00000018844	5.137067165	0.542049304	0.878165652	431	513	590	817	869	513	1112	743
ENSECAG00000009975	4.730863217	0.542087515	0.878165652	198	513	441	676	717	522	617	549
ENSECAG00000023048	5.975704944	0.54215929	0.878165652	728	1036	1035	1397	1660	1151	1883	1077
ENSECAG00000012701	8.067394454	0.542387721	0.878165652	5038	3167	6178	6285	4243	5589	4428	6065
ENSECAG00000007001	8.699214769	0.542401642	0.878165652	5511	10180	7859	8986	6006	8284	9354	8267
ENSECAG00000021615	5.998990785	0.542431158	0.878165652	1080	1273	997	1477	1177	1012	1413	1419
ENSECAG00000014448	2.13844045	0.542444988	0.878165652	58	71	71	149	82	42	108	102
ENSECAG00000014807	3.338493657	0.542504617	0.878165652	81	181	281	274	203	190	219	163
ENSECAG00000015276	4.253216273	0.542535978	0.878165652	217	227	353	493	385	402	570	383
ENSECAG00000021778	0.940882535	0.54263757	0.878165652	43	18	46	33	11	24	32	69
ENSECAG00000017554	6.174155292	0.54264889	0.878165652	1110	1556	1356	1459	1575	1542	1314	1132
ENSECAG00000005805	6.692928337	0.54274356	0.878165652	1468	1599	1882	1576	3738	1412	2909	1557
ENSECAG00000019266	4.126639598	0.542756533	0.878165652	170	256	251	518	356	340	345	542
ENSECAG00000022176	6.117395142	0.542779152	0.878165652	856	916	1165	1697	1297	1123	1989	2010
ENSECAG00000015585	3.677474045	0.542806623	0.878165652	124	210	247	271	270	191	485	245
ENSECAG00000008861	4.830059734	0.542896219	0.878165652	354	310	488	762	827	448	791	535
ENSECAG00000021305	6.157766224	0.542950834	0.878165652	1317	991	1307	1799	1342	1413	1452	1335
ENSECAG00000014960	3.718707247	0.543004266	0.878165652	192	220	311	284	313	251	235	210
ENSECAG00000018401	7.44376023	0.543020307	0.878165652	2046	2414	3227	3784	3971	3435	4564	4130
ENSECAG00000017168	2.087540448	0.543183629	0.87821337	39	93	151	80	3	128	2	140
ENSECAG00000015293	4.046140935	0.543204089	0.87821337	165	323	268	337	532	353	380	227
ENSECAG00000015244	5.089293579	0.543384764	0.878380739	408	306	683	882	986	511	1232	446
ENSECAG00000010897	9.119918715	0.543694548	0.878756733	7643	5126	10625	11822	21391	5469	19629	6859
ENSECAG00000016419	5.851821709	0.543945467	0.878994575	823	1182	863	774	1692	884	1741	1018
ENSECAG00000023493	5.896844003	0.544072602	0.878994575	1198	1278	768	1145	1452	962	1318	884
ENSECAG00000024095	10.44333959	0.544073322	0.878994575	13756	17133	30963	30504	34448	32862	28335	32682
ENSECAG00000004232	3.862322053	0.544173344	0.87903143	189	182	297	507	324	113	477	228
ENSECAG00000022113	4.63002711	0.54452214	0.879463881	288	307	439	640	493	442	610	710
ENSECAG00000024214	4.143267291	0.544712668	0.879463881	148	230	357	486	361	398	507	344
ENSECAG00000020481	6.736656915	0.544741058	0.879463881	2130	2719	1412	1568	2206	1958	2203	1794
ENSECAG00000022790	6.593296259	0.544750048	0.879463881	1322	774	2246	1906	3158	2030	2351	1341
ENSECAG00000015808	0.737987716	0.544939204	0.879633276	27	51	26	20	2	51	14	43
ENSECAG00000007291	0.555513881	0.545009497	0.879633276	37	42	24	7	0	76	0	7
ENSECAG00000014821	6.442938442	0.545111048	0.879672471	1132	1921	1810	1851	1766	1533	2016	1486
ENSECAG00000003619	6.626155077	0.545257347	0.879763859	1220	1257	2170	1842	2273	1873	2582	2351
ENSECAG00000021458	3.702610236	0.545322227	0.879763859	161	211	184	291	286	121	414	395
ENSECAG00000021422	6.792758116	0.54544904	0.879805868	1851	2910	1481	2156	1312	2058	2484	2709
ENSECAG00000012864	2.442560406	0.545502822	0.879805868	76	70	80	115	110	56	234	114
ENSECAG00000021318	9.390276773	0.54571429	0.880022265	9541	8850	11483	12662	20442	9890	21469	11437
ENSECAG00000000545	5.504622962	0.545858099	0.88012075	684	759	818	1232	975	742	1032	824
ENSECAG00000008819	7.647423754	0.545929972	0.88012075	2647	4332	3277	5654	2597	3218	4696	5052
ENSECAG00000009563	3.54366847	0.546135869	0.88032803	125	175	196	282	250	180	267	366

ENSECAG00000015945	3.323784591	0.546223185	0.880344134	85	166	175	245	173	115	316	330
ENSECAG00000017064	7.282358567	0.54670947	0.881003158	1910	3360	3466	3545	2077	3908	2832	3035
ENSECAG00000017425	7.936716873	0.546849432	0.881103989	3716	7747	4154	3015	4087	7039	4255	2781
ENSECAG00000014696	5.552402153	0.547027691	0.881266487	760	723	1036	1075	511	621	1628	1000
ENSECAG00000009158	2.145790664	0.547160017	0.881354951	42	71	126	116	98	67	129	39
ENSECAG00000000371	5.866729372	0.547654904	0.881898012	1312	898	1065	985	905	980	778	1801
ENSECAG00000023297	0.55207171	0.547746114	0.881898012	11	51	12	6	16	58	20	35
ENSECAG00000008563	6.735179467	0.547805708	0.881898012	1394	2910	1801	2182	1673	3245	1579	1469
ENSECAG00000002061	0.181604912	0.547830255	0.881898012	9	19	5	34	9	26	25	39
ENSECAG00000023974	5.839372102	0.547884465	0.881898012	764	1157	1220	1277	1025	1479	923	968
ENSECAG00000012022	7.391542705	0.548192162	0.882200841	1598	2598	2831	4301	3649	4229	4125	3414
ENSECAG00000016919	6.917219059	0.548247644	0.882200841	1658	1979	2642	3258	2240	2222	3067	1870
ENSECAG00000021114	5.965736416	0.548305064	0.882200841	668	1210	1292	1810	902	932	1561	1547
ENSECAG00000020755	9.030755677	0.548571093	0.882448519	6422	5242	12614	9468	15651	12291	11783	8562
ENSECAG00000012319	2.535843995	0.54861402	0.882448519	42	105	123	105	222	61	184	72
ENSECAG00000010845	5.456880554	0.548981638	0.882840854	828	1084	582	727	1104	653	907	743
ENSECAG00000008588	3.525413056	0.549013021	0.882840854	126	299	201	270	185	159	309	249
ENSECAG00000010668	5.856424786	0.549132442	0.882908185	592	852	1063	1425	853	1027	1774	1706
ENSECAG00000020055	6.560771131	0.549565187	0.88319712	900	1696	1684	2080	2234	1757	2755	1942
ENSECAG00000009768	2.217458936	0.549593116	0.88319712	43	124	129	116	0	143	0	143
ENSECAG00000015015	3.821616031	0.549635225	0.88319712	195	274	263	354	252	279	312	258
ENSECAG00000017653	2.239250733	0.549690863	0.88319712	49	78	73	105	76	68	152	140
ENSECAG00000019245	6.161635067	0.549866967	0.88319712	840	1291	1689	1803	1234	1423	1624	1326
ENSECAG00000016217	8.199350016	0.549909496	0.88319712	3461	3609	4503	7901	7375	3725	10496	6286
ENSECAG00000013696	6.410554149	0.549912932	0.88319712	1001	1943	1334	1179	1901	2235	2187	1440
ENSECAG00000013366	10.02605663	0.549932752	0.88319712	10294	12815	17765	29389	21149	16697	35334	25530
ENSECAG00000013688	4.071354982	0.550082852	0.883313578	283	220	267	269	521	143	642	280
ENSECAG00000007066	1.976684955	0.550229194	0.88342397	37	102	89	96	14	35	18	209
ENSECAG00000006357	3.953523499	0.550349434	0.883492429	164	294	424	332	191	366	320	319
ENSECAG00000007184	0.80378061	0.550567104	0.883540733	13	34	32	28	48	33	39	33
ENSECAG00000007542	0.99847133	0.550649824	0.883540733	19	28	37	36	12	33	29	107
ENSECAG00000019715	3.158217503	0.550661198	0.883540733	121	133	231	209	240	163	122	146
ENSECAG00000015228	3.163667712	0.550890408	0.883540733	83	142	138	241	181	189	179	260
ENSECAG00000008944	4.096998537	0.550910394	0.883540733	124	518	420	286	363	257	313	382
ENSECAG00000015004	2.409132951	0.550921818	0.883540733	77	88	120	121	112	76	139	80
ENSECAG00000017604	6.705653413	0.550922764	0.883540733	1430	1648	2055	3120	1783	1863	2281	2203
ENSECAG00000007816	5.249628684	0.551237438	0.883607526	476	602	871	513	1164	693	987	619
ENSECAG00000007150	6.619565621	0.551310679	0.883607526	1020	1316	1912	2408	2492	1943	2418	2133
ENSECAG00000010010	1.765202761	0.551317806	0.883607526	46	44	68	52	71	62	81	92
ENSECAG00000017101	2.80878565	0.551318008	0.883607526	92	77	145	131	211	78	239	124
ENSECAG00000020976	6.065868568	0.55135247	0.883607526	679	1236	1249	2287	898	793	2049	1589
ENSECAG00000023096	2.854758723	0.551684089	0.883913571	81	78	179	130	208	106	229	124
ENSECAG00000004992	5.145685707	0.551698712	0.883913571	311	493	733	912	825	691	805	879
ENSECAG00000014276	5.199387134	0.551855246	0.883955704	475	475	616	900	759	760	986	829
ENSECAG00000022057	2.682460033	0.551922138	0.883955704	69	61	155	135	140	106	161	180
ENSECAG00000013796	6.005541949	0.551957936	0.883955704	908	1597	1057	1323	1216	1438	1294	1035
ENSECAG00000021406	3.290254009	0.552083897	0.884027696	114	207	192	252	138	164	168	282
ENSECAG00000013754	1.239734765	0.552158186	0.884027696	12	62	28	48	60	47	59	45
ENSECAG00000015307	5.258110761	0.552326343	0.884172583	373	507	772	979	890	895	935	732
ENSECAG00000006549	5.97595523	0.55260562	0.884437346	701	1140	1527	1607	1403	1265	1448	785
ENSECAG00000023593	6.634363443	0.552647104	0.884437346	1060	1529	1804	2284	1973	2123	2905	2133
ENSECAG00000022610	7.305579789	0.552962003	0.884816923	1359	3184	2862	3140	3367	2981	4758	3612
ENSECAG00000020832	5.19956113	0.55314808	0.884913987	190	778	570	1033	974	709	869	799
ENSECAG00000019546	6.999397599	0.553178115	0.884913987	1344	1844	2587	2759	2989	2789	3494	2504
ENSECAG00000000327	5.546277755	0.553286315	0.884962728	686	1130	839	855	786	965	1015	894
ENSECAG00000007244	3.547467699	0.553371416	0.884974516	147	106	178	350	344	163	283	280
ENSECAG00000019226	1.810871498	0.55374866	0.885135648	42	47	81	107	84	48	90	41
ENSECAG00000011129	7.245334647	0.553871233	0.885135648	2557	4734	1956	2099	2313	2397	4442	2625
ENSECAG00000011444	6.83212997	0.554019171	0.885135648	1482	2077	2412	3000	1879	1931	2627	2456
ENSECAG00000015836	4.829452463	0.554043336	0.885135648	398	457	660	663	589	452	661	544
ENSECAG00000017182	7.601469886	0.554068556	0.885135648	2247	2121	3020	5572	3469	2344	6472	6178
ENSECAG00000016887	3.374002774	0.554113379	0.885135648	152	219	138	132	252	136	331	242
ENSECAG00000000974	7.550156469	0.554140454	0.885135648	3282	4241	3397	3170	3027	4233	3992	3106
ENSECAG00000021355	4.468267359	0.554189335	0.885135648	293	377	309	475	639	326	549	496
ENSECAG00000019474	9.315970144	0.554221049	0.885135648	20698	135	130	8213	22661	10471	16576	15670
ENSECAG00000001767	4.009132428	0.554308029	0.885135648	132	271	400	261	520	237	558	181
ENSECAG00000022843	6.282309611	0.554419842	0.885135648	789	1361	1268	1848	1626	1413	2154	1945
ENSECAG00000017575	4.271506198	0.554429588	0.885135648	114	258	297	1047	241	190	276	749
ENSECAG00000018880	7.273936045	0.554534318	0.885135648	1993	2605	3842	3767	3134	2586	3052	3182
ENSECAG00000018394	4.976258198	0.554657285	0.885135648	289	494	901	838	669	543	536	695
ENSECAG00000010812	7.110368615	0.554807938	0.885135648	1321	2515	2502	2880	3116	2794	3970	2890
ENSECAG00000010098	3.383654297	0.554892581	0.885135648	92	228	240	269	224	168	239	177
ENSECAG00000021336	1.551593551	0.554927021	0.885135648	37	66	71	50	40	100	39	28
ENSECAG00000006356	2.195466806	0.555137603	0.885135648	90	87	69	90	78	60	103	107
ENSECAG00000021636	7.190354952	0.55515009	0.885135648	1586	2749	2178	3132	2873	2832	4218	3622
ENSECAG00000020246	8.844216598	0.555160783	0.885135648	5475	6648	9562	7837	15256	9206	12537	5700

ENSECAG00000012723	6.232262145	0.555284333	0.885135648	901	1108	1264	1762	1639	1282	2119	1859
ENSECAG00000021283	5.496995204	0.555285859	0.885135648	683	986	737	1007	915	733	1053	864
ENSECAG00000018628	7.296640518	0.555321986	0.885135648	2845	3783	2778	2302	2766	3058	4082	2242
ENSECAG00000019200	4.879963394	0.555338071	0.885135648	412	382	660	842	670	521	606	504
ENSECAG00000000273	5.674046395	0.555824285	0.885786153	446	550	1342	1172	1136	887	1413	1248
ENSECAG00000014852	3.909800097	0.555921426	0.885786153	185	217	240	350	247	189	451	506
ENSECAG00000007289	3.616716704	0.555979609	0.885786153	115	194	241	465	150	227	142	406
ENSECAG00000013938	3.575937049	0.556122835	0.885862357	164	185	159	263	144	232	336	386
ENSECAG00000024852	4.958633139	0.556183059	0.885862357	226	566	294	1045	255	667	569	1400
ENSECAG00000015953	7.71500555	0.55626868	0.885874797	4056	2464	3670	2191	8495	3129	5451	2601
ENSECAG00000000696	0.199737122	0.556487808	0.886099818	12	18	23	33	9	20	27	24
ENSECAG00000020565	3.098588389	0.556778633	0.886427223	88	110	201	166	193	149	179	255
ENSECAG00000021974	2.254585453	0.556862531	0.886427223	50	69	68	206	90	52	121	99
ENSECAG00000006230	2.340861071	0.556935172	0.886427223	39	62	102	137	162	78	120	99
ENSECAG00000005938	3.893413955	0.557107759	0.886427223	137.011	284	236.008	347	362.001	348.001	358	269.024
ENSECAG00000022088	7.156562378	0.557168169	0.886427223	1524	2861	2251	2710	3137	3492	4201	2335
ENSECAG00000010929	2.65984005	0.557233377	0.886427223	67	77	117	158	122	111	161	181
ENSECAG00000021905	2.249545099	0.557308142	0.886427223	38	107	78	167	98	34	86	138
ENSECAG00000013136	8.43464221	0.557316299	0.886427223	5153	4112	6653	5873	11951	5789	9651	4851
ENSECAG00000014543	3.566492323	0.557767335	0.886677916	104	186	215	301	287	174	357	268
ENSECAG00000010431	6.116642967	0.557848202	0.886677916	967	983	1091	1519	1364	1195	2285	1572
ENSECAG00000014140	2.857509908	0.557921574	0.886677916	99	133	130	202	81	106	186	189
ENSECAG00000001003	3.118838289	0.557940448	0.886677916	102	113	186	296	195	170	167	129
ENSECAG00000005330	5.206066022	0.557949125	0.886677916	412	1023	610	806	572	605	764	957
ENSECAG00000022485	4.291084394	0.558040967	0.886677916	227	406	409	479	271	380	396	485
ENSECAG00000012986	5.371345451	0.558095805	0.886677916	605	376	944	1355	790	636	920	896
ENSECAG00000007319	3.405142417	0.558096965	0.886677916	100	210	200	345	182	251	138	228
ENSECAG00000013260	8.839453785	0.558580333	0.887054366	6941	10095	8917	9369	8102	10310	9096	7444
ENSECAG00000004371	2.636336167	0.558595284	0.887054366	53	118	185	139	78	156	121	112
ENSECAG00000000042	8.219191042	0.558624805	0.887054366	3453	6790	5883	7537	4323	5632	6751	6362
ENSECAG00000006203	0.74286824	0.558711859	0.887054366	23	17	29	31	52	25	35	34
ENSECAG00000000540	6.138980275	0.558726964	0.887054366	1185	1220	1134	802	1820	1916	1543	1106
ENSECAG00000018029	2.376877686	0.558801397	0.887054366	61	62	121	172	59	90	128	119
ENSECAG00000024362	4.409208645	0.55893705	0.887146007	251	354	428	641	426	312	554	391
ENSECAG00000015452	1.891469227	0.55901893	0.887152288	31	48	57	111	57	69	90	120
ENSECAG00000022373	4.44023577	0.559391556	0.887539653	268	463	412	530	433	330	454	492
ENSECAG00000019522	4.076486944	0.559418933	0.887539653	300	302	239	175	474	266	621	214
ENSECAG00000025040	4.598026389	0.559632788	0.887601924	308	381	384	540	588	431	631	542
ENSECAG00000013557	5.758162714	0.55967202	0.887601924	1140	1052	790	959	1024	646	1603	1023
ENSECAG00000012242	1.757955538	0.55969207	0.887601924	57	62	36	44	81	68	114	46
ENSECAG00000005600	2.70754472	0.559775573	0.887610711	49	171	97	215	127	79	109	178
ENSECAG00000013455	3.818646027	0.559886439	0.887662876	182	186	298	453	354	183	284	273
ENSECAG00000004444	6.190357747	0.560371217	0.888011978	1205	1125	1557	1695	1303	792	2380	1361
ENSECAG00000018388	0.370130139	0.560637505	0.888011978	4	18	14	45	7	36	20	50
ENSECAG00000011836	4.329091123	0.560643589	0.888011978	320	313	300	616	358	292	435	495
ENSECAG00000024924	3.310971784	0.560672924	0.888011978	142	272	126	214	146	117	231	277
ENSECAG00000013603	4.532415411	0.560688328	0.888011978	246	334	666	586	545	334	567	372
ENSECAG00000018882	4.300914636	0.560726607	0.888011978	222	343	438	548	285	340	412	511
ENSECAG00000009876	4.498906837	0.560761554	0.888011978	315	330	558	536	498	410	509	355
ENSECAG00000021909	2.622934084	0.56080936	0.888011978	23	120	80	201	147	146	96	168
ENSECAG00000024469	5.623461971	0.56087682	0.888011978	432	895	1088	1587	1232	993	808	764
ENSECAG00000018129	4.0657897	0.56089666	0.888011978	336	301	308	274	278	222	436	384
ENSECAG00000024129	5.733856575	0.560964616	0.888011978	892	978	1053	1054	1086	797	1241	1083
ENSECAG00000011819	7.128824722	0.561321176	0.888452883	2435	2826	2570	2726	2143	2584	2940	3181
ENSECAG00000010849	6.528432165	0.561404358	0.888461025	1141	1107	2052	1806	2492	1579	2428	1958
ENSECAG00000017293	4.549390745	0.561712711	0.888825464	325	427	403	657	466	302	683	415
ENSECAG00000009404	2.737665572	0.561804026	0.88884642	80	75	170	92	174	132	272	48
ENSECAG00000017201	6.255342247	0.561962726	0.888862931	740	960	1820	1654	2340	1213	1932	1501
ENSECAG00000008043	5.083896807	0.561970609	0.888862931	271	598	742	1167	740	649	817	442
ENSECAG00000024329	4.335894551	0.562173431	0.889060219	148	254	521	472	513	418	575	333
ENSECAG00000006917	0.978446702	0.562318933	0.889100785	20	34	76	20	19	63	23	31
ENSECAG00000027675	11.42730001	0.56235527	0.889100785	44036	27333	58963	41455	53621	60800	78416	64629
ENSECAG00000005166	7.012740545	0.562435106	0.889103538	1262	1514	3104	2780	3735	3068	2611	2339
ENSECAG00000008715	4.588082694	0.562660332	0.889116653	413	678	378	289	395	585	424	436
ENSECAG00000017792	4.899608264	0.56271474	0.889116653	437	343	574	595	641	384	899	827
ENSECAG00000016657	3.550503095	0.562825126	0.889116653	138	99	260	284	296	198	368	215
ENSECAG00000016557	3.241630476	0.562945408	0.889116653	165	143	197	210	158	84	315	190
ENSECAG00000017713	6.592179151	0.563041395	0.889116653	1176	1907	1465	1756	1793	2196	2708	2145
ENSECAG00000024069	4.089456212	0.563047653	0.889116653	290	406	282	284	320	155	405	457
ENSECAG00000021234	4.743386788	0.563265012	0.889116653	345	583	439	725	387	286	502	924
ENSECAG00000019978	3.317871936	0.563329464	0.889116653	86	91	225	277	354	172	216	162
ENSECAG00000021432	4.641994842	0.56342912	0.889116653	498	307	287	454	509	571	641	550
ENSECAG00000024088	0.042939571	0.563483877	0.889116653	8	16	25	29	20	26	15	7
ENSECAG00000006066	4.605990978	0.563625075	0.889116653	211	372	538	547	551	425	781	473
ENSECAG00000018375	5.308731346	0.563670315	0.889116653	555	668	850	965	742	718	851	816
ENSECAG00000022942	8.270513831	0.563819346	0.889116653	4423	6782	5755	6974	5210	5955	6550	6134

ENSECAG00000023238	7.184589387	0.563860639	0.889116653	1918	3568	2621	3180	2727	3320	2786	2314
ENSECAG00000001042	7.646366674	0.56392625	0.889116653	1359	4991	3528	3384	3959	6049	4713	3660
ENSECAG00000011687	4.592902167	0.563952623	0.889116653	301	360	433	837	304	411	402	757
ENSECAG00000022962	4.059634124	0.563971712	0.889116653	186	247	323	585	403	267	396	234
ENSECAG00000009389	10.84866346	0.564016482	0.889116653	16398	23311	38194	46709	48583	46827	36933	36577
ENSECAG00000015824	0.374349552	0.564025094	0.889116653	10	21	33	35	12	23	36	21
ENSECAG00000017291	3.91915343	0.564044328	0.889116653	215	365.999	265	292.999	211.999	292	346.999	332.999
ENSECAG00000014814	4.724419816	0.564083407	0.889116653	341	521	456	731	508	566	489	497
ENSECAG00000007240	7.262230861	0.56435336	0.889377161	2082	2966	2092	2690	3198	3190	4335	3463
ENSECAG00000009846	1.94010384	0.564446713	0.889377161	33	68	57	91	39	74	150	93
ENSECAG00000026921	4.681171849	0.564541865	0.889377161	333	549	449	642	505	360	686	490
ENSECAG00000009590	4.350177914	0.56458445	0.889377161	296	358	358	554	465	340	474	324
ENSECAG00000010618	2.510478739	0.564639273	0.889377161	53	85	99	136	43	141	156	184
ENSECAG00000004434	5.801820857	0.564873204	0.889622551	790	734	1042	1082	1526	781	1699	1122
ENSECAG00000014672	0.640038653	0.564960488	0.889636952	14	25	16	70	20	14	33	44
ENSECAG00000015177	4.828975937	0.565440066	0.890079112	359	363	494	692	818	415	753	597
ENSECAG00000010212	7.646737818	0.565544337	0.890079112	3249	3692	2201	3156	8184	2789	4344	3224
ENSECAG00000024391	4.559551026	0.565563793	0.890079112	464	484	211	236	521	343	704	627
ENSECAG00000023200	3.387005165	0.565637179	0.890079112	97	142	270	185	282	184	331	162
ENSECAG00000013416	4.500864253	0.565658318	0.890079112	173	356	434	612	452	561	438	578
ENSECAG00000001897	6.176188021	0.56571036	0.890079112	936	1119	1231	1501	1784	1273	1837	1686
ENSECAG00000020720	1.814080182	0.565905483	0.890263082	42	41	36	107	62	45	148	74
ENSECAG00000023543	3.2940566	0.566053145	0.890372348	91	92	244	236	265	206	239	178
ENSECAG00000025123	5.749074939	0.566242873	0.890414454	747	872	755	1173	1369	955	1331	1219
ENSECAG00000013186	3.057203636	0.566248309	0.890414454	142	117	219	145	204	138	160	133
ENSECAG00000013892	1.862032854	0.566314542	0.890414454	42	50	96	98	39	67	60	104
ENSECAG00000021072	2.659365251	0.566422638	0.8904315	95	171	75	135	88	111	181	107
ENSECAG00000009754	2.454768193	0.566610663	0.8904315	52	108	90	191	77	96	136	111
ENSECAG00000002919	6.909347387	0.566902288	0.8904315	2239	3327	2028	1233	2693	1637	3227	1743
ENSECAG00000012376	1.826093694	0.566991692	0.8904315	56.0071	78	29	16	2.00043	35.0001	8	304
ENSECAG00000007088	1.411281169	0.567016638	0.8904315	26	28	52	61	59	14	122	56
ENSECAG00000008321	2.471027009	0.567053209	0.8904315	45	55	133	139	132	81	129	163
ENSECAG00000022136	3.504120339	0.567115442	0.8904315	131	145	233	246	198	283	305	239
ENSECAG00000012956	5.126895491	0.567126159	0.8904315	363	619	668	708	743	638	790	982
ENSECAG00000013502	7.539223841	0.567331562	0.8904315	2764	5194	2749	3524	3652	3024	5684	2140
ENSECAG00000014271	6.929024669	0.567459158	0.8904315	1349	1836	2102	2866	2885	2146	3623	2611
ENSECAG00000000500	3.761825916	0.567523849	0.8904315	78	404	251	375	138	282	222	388
ENSECAG00000013668	7.010591016	0.5675591	0.8904315	1066	2850	2488	4222	1840	3489	2206	2240
ENSECAG00000006400	7.190159654	0.567573883	0.8904315	4162	2095	2204	1820	3519	2504	3234	1832
ENSECAG00000017066	5.774265057	0.567635891	0.8904315	788	660	938	1210	1591	691	1628	1125
ENSECAG00000003862	5.452547536	0.567774005	0.8904315	473	805	702	977	1099	748	1093	1024
ENSECAG00000004465	6.806815931	0.567829698	0.8904315	1707	1635	1642	2194	2765	1772	3474	2378
ENSECAG00000007328	7.650052978	0.567852146	0.8904315	1451	2737	4446	8615	4288	2536	5515	3239
ENSECAG00000022705	7.883000854	0.567922478	0.8904315	2498	6518	5298	4241	4737	5487	5181	2586
ENSECAG00000021232	2.650787584	0.567969102	0.8904315	77	79	77	179	122	86	147	220
ENSECAG00000011500	4.400083541	0.5679733	0.8904315	239	335	342	506	411	310	737	489
ENSECAG00000018485	8.314348885	0.568083059	0.8904315	4247	5468	5517	4843	10502	4686	10964	3815
ENSECAG00000018072	5.647854876	0.568107994	0.8904315	707	964	1166	961	1041	1027	1092	773
ENSECAG00000007683	5.192548796	0.568124235	0.8904315	361	602	593	1441	663	415	870	940
ENSECAG00000022164	6.283610329	0.568234689	0.890475263	1073	1047	2109	1839	1360	1064	2335	1440
ENSECAG00000007384	3.835495119	0.568308587	0.890475263	129	208	264	379	289	245	389	377
ENSECAG00000013622	4.189552912	0.568470255	0.890532759	269	283	261	366	400	238	677	379
ENSECAG00000017269	6.003028597	0.568532876	0.890532759	712	622	1292	2898	227	839	805	2935
ENSECAG00000017566	2.730395346	0.568579941	0.890532759	40	210	107	59	166	95	255	110
ENSECAG00000023724	3.443329084	0.568724418	0.890636519	102	199	237	337	295	126	198	218
ENSECAG00000003388	6.850937003	0.568908946	0.890708358	1690	2523	2121	2526	2523	2329	2051	1977
ENSECAG00000022374	2.328315046	0.568926762	0.890708358	52	246	52	42	28	232	24	50
ENSECAG00000009007	5.070638728	0.569194227	0.890836995	486	482	730	898	859	463	778	549
ENSECAG00000010340	1.88726213	0.569216962	0.890836995	43.0071	83	77	80	92.0004	58.0001	83	45
ENSECAG00000018608	4.900510193	0.569243666	0.890836995	439	474	675	684	478	512	742	642
ENSECAG00000016734	6.805466429	0.569606381	0.891002776	1731	3085	1852	1753	1949	2784	2336	1487
ENSECAG00000015762	4.797036198	0.569637776	0.891002776	253	554	577	836	472	607	615	485
ENSECAG00000008470	4.264408227	0.569848624	0.891002776	204	252	379	473	419.001	492.001	371	431
ENSECAG00000021942	3.758914595	0.569968449	0.891002776	143	227	200	309	84	151	421	633
ENSECAG00000009901	11.11804771	0.569976436	0.891002776	54983	35362	39835	30508	37739	39552	41391	50351
ENSECAG00000008548	11.12167668	0.569983825	0.891002776	20628	29220	48364	51367	57467	55878	42975	47381
ENSECAG00000010559	3.23169538	0.570014591	0.891002776	92	110	158	275	118	142	353	267
ENSECAG00000012094	3.561986606	0.570025354	0.891002776	88	220	235	261	281	248	311	228
ENSECAG00000021950	8.151929828	0.57005395	0.891002776	3716	2991	5613	6353	7903	5563	6585	6083
ENSECAG00000017572	3.868688159	0.570190857	0.891094427	239	211	317	338	347.999	227	338.998	227.999
ENSECAG00000022011	1.170730797	0.570315044	0.891166179	32	22	66	51	27	23	63	55
ENSECAG00000016783	1.996174619	0.570460671	0.891201652	28	64	85	86	78	60	161	70
ENSECAG00000017053	4.663973071	0.5705029	0.891201652	183	319	624	643	474	501	750	594
ENSECAG00000008939	6.90788785	0.570572582	0.891201652	1856	2070	2605	2714	2452	2711	1955	2066
ENSECAG00000015042	6.826777883	0.571057242	0.891580503	1403	1860	1789	2382	3441	1455	3465	2186
ENSECAG00000011617	2.535638855	0.571069644	0.891580503	47	67	127	148	161	112	184	73

ENSECAG00000020533	5.223148926	0.571114825	0.891580503	446	1061	502	867	747	807	577	750
ENSECAG00000009827	5.247618151	0.571210423	0.891580503	551	980	663	649	526	718	765	971
ENSECAG00000013560	2.870736322	0.571270678	0.891580503	73	132	112	163	105	90	246	240
ENSECAG00000014548	4.862881448	0.571285004	0.891580503	325	305	617	728	931	479	785	451
ENSECAG00000021601	5.428044498	0.571391233	0.891599333	512	811	716	1353	916	808	908	749
ENSECAG00000020493	6.066758289	0.571453696	0.891599333	914	1088	854	2507	1388	1400	1557	864
ENSECAG00000002732	4.419286048	0.57203857	0.892389575	236	388	408	395	475	438	633	392
ENSECAG00000000053	3.106420249	0.572292573	0.89254799	102	126	145	186	351	101	179	150
ENSECAG00000011342	8.320918887	0.572296911	0.89254799	5059	6350	5515	7811	7042	7132	5867	4318
ENSECAG00000008349	2.448104039	0.572379403	0.892554376	63	73	116	99	153	107	161	74
ENSECAG00000019987	4.256797604	0.572731528	0.892841312	334	415	357	299	313	492	392	279
ENSECAG00000000370	11.73040129	0.572822864	0.892841312	38714	49217	91714	96084	68826	77739	52414	57486
ENSECAG00000022215	6.248621474	0.572993264	0.892841312	1070	1278	1316	1258	1924	1521	2115	1367
ENSECAG00000015499	7.315289713	0.573079801	0.892841312	1498	1850	3428	4084	4064	3031	4375	3220
ENSECAG00000011363	7.231233954	0.573094783	0.892841312	1577	3235	2997	4234	2391	3606	3220	2358
ENSECAG00000010572	6.44613878	0.57314764	0.892841312	1020	1389	1482	1947	2246	1507	2519	1706
ENSECAG00000009202	3.923993391	0.573172874	0.892841312	144	279	267	534	274	249	340	326
ENSECAG00000011243	7.686787304	0.573190791	0.892841312	2894	4131	3735	5297	3838	3721	4449	4005
ENSECAG00000020128	4.789826429	0.573495499	0.893083703	263	531	607	449	420	627	762	704
ENSECAG00000023516	3.948629548	0.573640731	0.893083703	172	145	382	338	421	330	349	290
ENSECAG00000015214	4.828088213	0.57372326	0.893083703	402	355	574	528	837	549	749	432
ENSECAG00000023318	6.21877833	0.573763526	0.893083703	748	1886	1165	1030	1611	858	2282	2198
ENSECAG00000024661	5.900758428	0.573800083	0.893083703	653	1410	1200	1390	918	1403	1169	1166
ENSECAG00000008521	1.942970445	0.573817065	0.893083703	47	57	109	83	89	63	65	70
ENSECAG00000008851	9.709907196	0.57401162	0.893152207	15418	14300	20179	14397	6696	27558	7289	19571
ENSECAG00000016291	6.470719454	0.574029496	0.893152207	1054	1209	1739	1932	1514	1618	2617	2415
ENSECAG00000011509	5.474556432	0.574256335	0.893152207	800	1006	642	824	823	602	1194	918
ENSECAG00000020684	4.089075764	0.574375627	0.893152207	184	298	430	422	341	397	273	299
ENSECAG00000022561	3.450798129	0.5743855	0.893152207	131	164	215	208	233	197	383	190
ENSECAG00000005573	2.120709994	0.574385966	0.893152207	86	80	87	66	52	146	54	62
ENSECAG00000010068	7.99648467	0.574571045	0.893152207	4356	4549	4939	5616	5360	5046	4953	4295
ENSECAG00000004260	4.052178415	0.574579851	0.893152207	157	274	230	479	280	302	460	472
ENSECAG00000007059	3.471348731	0.57462887	0.893152207	28	232	489	240	13	472	13	229
ENSECAG00000025118	4.046979705	0.574645579	0.893152207	230	272	291	494	340	174	377	412
ENSECAG00000000565	6.168934771	0.574736577	0.893171708	822	880	947	2260	1537	790	2226	2161
ENSECAG00000010978	5.445534411	0.574937693	0.893346789	993	666	777	698	1018	677	1176	552
ENSECAG00000023074	2.0246055	0.575093259	0.893346789	50	50	83	76	62	92	98	114
ENSECAG00000023789	5.910797454	0.575172618	0.893346789	647	948	1298	1922	946	1153	1303	1330
ENSECAG00000019081	3.358276744	0.575299629	0.893346789	127	194	123	220	105	259	246	324
ENSECAG00000022893	6.086565003	0.575387652	0.893346789	718	1015	1293	1561	1626	855	2515	1338
ENSECAG00000018459	6.162069018	0.575407285	0.893346789	1025	784	1344	1564	1985	1067	1938	1565
ENSECAG00000012014	1.342611136	0.575447891	0.893346789	29	51	25	52	43	34	76	77
ENSECAG00000023502	5.347726927	0.575476974	0.893346789	516	540	738	948	947	800	1108	828
ENSECAG00000024024	2.756434763	0.575669589	0.893378967	58	67	168	161	184	116	205	113
ENSECAG00000011374	7.501121884	0.575730664	0.893378967	3270	4251	2905	3094	2828	4482	3183	3317
ENSECAG00000009882	3.335560122	0.575733661	0.893378967	147	153	153	195	298	140	265	214
ENSECAG00000023598	4.230229769	0.575811582	0.893378967	138	266	345	557	363	192	668	533
ENSECAG00000023784	5.342991438	0.575955756	0.893480894	479	489	747	1050	717	762	1200	1027
ENSECAG00000023203	4.857990862	0.576100592	0.89358382	355	419	507	668	622	568	991	469
ENSECAG00000008142	3.310444853	0.576249249	0.893686302	122	111	182	236	306	128	373	115
ENSECAG00000004868	5.986217208	0.576323656	0.893686302	895	1562	1074	1255	1005	1232	1431	1327
ENSECAG00000023376	4.2562395	0.576435553	0.893738086	255	435	311	460	294	360	326	510
ENSECAG00000012724	7.000188234	0.576629137	0.893916493	2117	3078	2133	2361	1363	3721	2289	2347
ENSECAG00000003034	1.292050892	0.576867755	0.894164655	37	37	21	52	45	19	86	76
ENSECAG00000010563	2.081210359	0.577114224	0.894369821	48	70	78	74	96	60	130	99
ENSECAG00000000939	1.207568819	0.577157231	0.894369821	22	45	44	68	32	34	61	46
ENSECAG00000018203	6.067100629	0.577354324	0.894488543	670	1476	1070	1262	1485	1658	1588	1315
ENSECAG00000010084	5.083149122	0.57739098	0.894488543	551	487	427	702	722	324	1405	746
ENSECAG00000013225	3.054258509	0.577533606	0.894587769	40	192	348	110	31	149	60	357
ENSECAG00000008462	4.370117002	0.577690132	0.894680508	248	260	566	555	541	317	334	409
ENSECAG00000024549	5.728958631	0.577764241	0.894680508	566	504	1167	2177	833	488	1244	1604
ENSECAG00000006181	4.154047479	0.577958108	0.894680508	375	389	249	264	335	345	463	247
ENSECAG00000022645	4.497968988	0.578016586	0.894680508	245	384	525	623	370	508	427	459
ENSECAG00000026834	2.228077161	0.578031025	0.894680508	74	101	77	97	84	68	117	91
ENSECAG00000019110	6.977283943	0.57810172	0.894680508	2117	2567	1959	2927	2652	1917	2790	2475
ENSECAG00000024056	4.865402057	0.578143566	0.894680508	363	375	615	601	563	613	700	748
ENSECAG00000014168	7.022517463	0.578246187	0.894717701	1732	2730	2466	3195	2753	2728	2296	2230
ENSECAG00000008653	4.920187035	0.578457587	0.894923172	427	747	494	605	539	559	689	606
ENSECAG00000020424	7.194674749	0.57860494	0.894923385	1301	2605	3369	4747	2972	2747	3741	1927
ENSECAG00000015602	5.071889325	0.578715741	0.894923385	278	1045	598	731	388	762	500	935
ENSECAG00000012172	8.077178745	0.57874613	0.894923385	9638	2654	4954	1715	1242	13265	1119	2527
ENSECAG00000016898	6.263654748	0.578794295	0.894923385	847	1447	982	1913	1669	1526	2008	1789
ENSECAG00000026989	2.35490383	0.578850751	0.894923385	32	156	53	87	99	63	189	128
ENSECAG00000019911	4.451535198	0.579090414	0.895172351	363	397	426	442	367	328	569	479
ENSECAG00000015563	6.430136068	0.579237101	0.895201483	1181	1146	1260	2131	1995	1244	3083	1697
ENSECAG00000025094	3.331578422	0.579301927	0.895201483	89	131	160	312	151	162	295	319

ENSECAG00000009763	3.258949321	0.579345149	0.895201483	117	162	212	260	130	184	127	289
ENSECAG00000017146	6.586371242	0.579508089	0.895331739	989	1115	3321	2289	1987	1702	2354	1448
ENSECAG00000017951	5.99246439	0.57978294	0.89563484	1098	1276	1202	1135	1033	1279	1286	1398
ENSECAG00000004604	7.071805948	0.580189866	0.896141857	1944	2290	1894	2509	3608	2558	3917	2306
ENSECAG00000006210	6.268599499	0.580284155	0.896165912	1075	1529	1513	1785	1579	1184	1617	1699
ENSECAG00000016995	7.107633572	0.580459259	0.896260402	4181	2598	1341	1423	3531	1495	3827	1710
ENSECAG00000000393	7.244705766	0.580599922	0.896260402	1327	2141	3544	3275	3134	3836	3855	3039
ENSECAG00000015974	4.527099141	0.580621746	0.896260402	171	406	490	528	602	433	539	501
ENSECAG00000008852	6.815512427	0.580660778	0.896260402	1428	1653	2904	3091	1286	739	2429	4325
ENSECAG00000019624	7.773191826	0.580738953	0.896260402	3504	3753	3421	2926	6291	4133	5811	3924
ENSECAG00000006808	5.948330091	0.580956342	0.896317953	883	1218	1328	1248	978	1145	1367	1391
ENSECAG00000023089	4.050014927	0.580971378	0.896317953	187	191	372	364	430	266	515	301
ENSECAG00000010502	7.241663369	0.581012428	0.896317953	1215	2703	3217	3086	2776	4296	4052	2725
ENSECAG00000010190	1.605157015	0.581099654	0.89633106	33	77	37	39	55	44	100	79
ENSECAG00000004668	2.672940584	0.581266002	0.896351101	55	145	107	206	132	108	78	161
ENSECAG00000013733	7.615440592	0.581272394	0.896351101	3005	2374	3384	3787	5830	3370	5855	3104
ENSECAG00000014429	9.174342956	0.581348839	0.896351101	5722	7770	12291	13007	11743	14699	14150	12415
ENSECAG00000010110	8.078766572	0.581604593	0.896624007	3560	5106	5708	6822	5369	5031	5988	4618
ENSECAG00000023209	3.239234303	0.581753915	0.896646221	86	108	200	252	174	192	276	217
ENSECAG00000012948	7.73728466	0.58179412	0.896646221	2997	5780	3586	3956	3334	5477	3963	3521
ENSECAG00000017458	3.826619543	0.581871941	0.896646221	147	179	305	324	436	208	443	211
ENSECAG00000011769	0.790196596	0.582023501	0.896646221	13	31	30	63	29	23	50	24
ENSECAG00000014261	5.917084899	0.582027515	0.896646221	660	816	1439	1926	1100	1071	1489	1116
ENSECAG00000011103	3.944863641	0.582122377	0.896646221	258	346	219	325	230	183	435	376
ENSECAG00000000789	4.178158528	0.582234854	0.896646221	216	509	264	393	323	282	449	375
ENSECAG00000008502	8.497727886	0.582318519	0.896646221	4735	6113	8653	8940	6295	6510	7745	7525
ENSECAG00000018906	6.929925631	0.582421776	0.896646221	1265	1765	1961	3304	2404	2153	3528	3183
ENSECAG00000021742	6.507666581	0.58251213	0.896646221	1491	1644	1494	2289	1512	1597	1862	2176
ENSECAG00000000383	6.587083078	0.582570174	0.896646221	1942	1613	1826	1664	1948	1561	2388	1662
ENSECAG00000011050	4.492873822	0.582606416	0.896646221	189	329.003	517.003	507.002	675.002	552.002	497.008	291.004
ENSECAG00000024529	1.397983657	0.582642841	0.896646221	16	26	61	72	64	58	79	35
ENSECAG00000018657	5.833213332	0.582767163	0.896660812	601	870	1188	1195	1266	1624	1033	1150
ENSECAG00000009766	5.422600449	0.582834126	0.896660812	537	398	1152	1339	551	737	1167	944
ENSECAG00000022969	2.094934037	0.58290869	0.896660812	86	7	196	41	129	26	132	21
ENSECAG00000017534	4.847522194	0.582994022	0.896660812	479	437	532	726	587	406	565	715
ENSECAG00000010851	6.670461309	0.583091759	0.896660812	1456	2004	1885	2492	2162	1892	2169	1741
ENSECAG00000019667	5.484367716	0.583124871	0.896660812	482	547	1035	972	1317	979	1034	691
ENSECAG00000020469	4.871462589	0.583342872	0.896874895	309	610	588	782	545	637	561	550
ENSECAG00000025039	3.515568136	0.583812817	0.897476225	239	162	284	150	316	248	202	93
ENSECAG00000019250	2.52649212	0.583905246	0.897497128	38	60	140	155	108	141	193	85
ENSECAG00000016647	7.899350261	0.584202968	0.897713263	3421	5099	4704	5158	3918	4721	4377	5445
ENSECAG00000018221	3.543866986	0.584203563	0.897713263	113	378	137	76	181	506	302	81
ENSECAG00000004044	1.614239639	0.584445551	0.897859162	35	34	37	92	54	32	118	79
ENSECAG00000012554	0.914220065	0.584502023	0.897859162	14	33	30	43	39	31	51	45
ENSECAG00000019842	5.237586306	0.584598324	0.897859162	472	435	670	982	657	637	1019	1128
ENSECAG00000005865	2.817873411	0.584631781	0.897859162	75	223	99	145	105	100	182	159
ENSECAG00000021762	5.35258132	0.584704975	0.897859162	466	579	1007	632	1594	605	934	571
ENSECAG00000021209	5.515248792	0.584868655	0.897859162	589	571	776	1124	1345	1019	1287	495
ENSECAG00000009789	6.661317869	0.58497939	0.897859162	1270	2866	1562	2062	1728	2155	2113	1874
ENSECAG00000006495	5.104962321	0.585005255	0.897859162	382	501	590	856	406	548	985	1234
ENSECAG00000011007	3.579737531	0.585078588	0.897859162	189	303	173	224	177	292	232	219
ENSECAG00000011541	4.123262154	0.585087143	0.897859162	211	199	277	494	420	407	523	230
ENSECAG00000018348	11.94941875	0.58523093	0.897895198	66794	72773	81664	80855	68533	91886	57934	81754
ENSECAG00000011721	5.313529461	0.585325919	0.897895198	479	547	786	873	980	771	1020	811
ENSECAG00000013042	2.977524981	0.58540751	0.897895198	85	136	170	230	157	127	154	170
ENSECAG00000018445	4.361467868	0.585426092	0.897895198	290	319	354	385	459	355	543	500
ENSECAG00000015881	5.212005502	0.585613578	0.898061771	464	584	891	936	919	631	776	590
ENSECAG00000017571	5.581401728	0.58578477	0.89809425	884	729	691	688	996	967	1334	1060
ENSECAG00000024884	4.738800598	0.585792525	0.89809425	223	469	345	810	362	368	729	1012
ENSECAG00000013321	3.150587044	0.586019772	0.898277319	100	119	156	217	119	161	308	230
ENSECAG00000000332	5.952513021	0.586069734	0.898277319	820	941	1242	1066	1518	1542	1212	1260
ENSECAG00000002222	3.547893402	0.586416216	0.898456553	185	108	209	248	299	165	450	173
ENSECAG00000010404	6.939462005	0.586583084	0.898456553	2151	2014	2200	2963	1858	2171	2505	3044
ENSECAG00000019276	5.909578692	0.586593768	0.898456553	849	871	1073	1135	1699	985	1938	890
ENSECAG00000023256	7.56908787	0.586653917	0.898456553	1837	3387	3756	3612	4773	3079	7357	2634
ENSECAG00000020450	2.539838526	0.586695923	0.898456553	66	67	83	171	100	85	204	148
ENSECAG00000016872	2.640322753	0.586800573	0.898456553	68	230	86	87	135	125	109	98
ENSECAG00000014387	9.351741201	0.586814514	0.898456553	7846	7601	14239	13265	19853	11037	18269	11357
ENSECAG00000023559	5.65643296	0.586818	0.898456553	598	761	926	1126	1272	1047	1139	1059
ENSECAG00000000124	7.916471299	0.587035556	0.898587301	3668	4343	5556	4953	4794	4034	6164	3929
ENSECAG00000021388	3.309812234	0.587061251	0.898587301	70	285	238	191	115	245	165	227
ENSECAG00000020539	5.265071003	0.587413493	0.898874933	416	823	775	979	775	811	717	711
ENSECAG00000013449	4.302986837	0.587538803	0.898874933	156	535	263	356	410	224	775	441
ENSECAG00000027694	10.77982187	0.587571506	0.898874933	23482	18403	44548	26415	40012	37673	49517	35888
ENSECAG00000004794	5.877208042	0.587641912	0.898874933	746	1165	1198	1417	1179	948	1474	1076
ENSECAG00000023944	2.525815537	0.587643928	0.898874933	63	60	142	112	129	92	172	132

ENSECAG00000000133	1.604299436	0.587826145	0.899032866	28	34	63	72	94	71	81	25
ENSECAG00000012624	5.121138462	0.587939396	0.899085296	567	579	554	506	1057	726	815	530
ENSECAG00000000933	5.292740886	0.588077318	0.899175432	586	638	832	919	715	834	695	828
ENSECAG00000014621	5.578954757	0.588544084	0.899768281	519	635	994	1121	1188	1019	1201	889
ENSECAG00000000590	5.135739061	0.588671083	0.899792956	432	524	847	935	654	646	622	841
ENSECAG00000009891	3.658198769	0.58871829	0.899792956	106	208	206	353	274	243	318	304
ENSECAG00000008148	6.315610535	0.589000479	0.900075093	1116	1203	1263	1627	2075	883	2615	1812
ENSECAG00000018419	4.150061826	0.589226846	0.900075093	253	141	708	274	536	237	256	315
ENSECAG00000023577	4.648709998	0.589291042	0.900075093	377	370	551	609	491	396	657	455
ENSECAG00000015832	11.48438981	0.589310869	0.900075093	23209	36595	63900	71843	76874	75005	54638	54299
ENSECAG00000011150	5.171413849	0.589361834	0.900075093	302	576	668	971	641	720	929	971
ENSECAG00000007905	4.910461374	0.589377235	0.900075093	330	604	793	590	704	492	723	459
ENSECAG00000003845	8.834756426	0.589599463	0.900293707	3298	15722	11701	5531	6335	11138	11476	5806
ENSECAG00000015926	1.078578796	0.589698976	0.900324909	21	33	38	41	48	32	52	54
ENSECAG00000024666	4.694939505	0.589833648	0.900365351	317	620	358	356	264	890	489	686
ENSECAG00000018929	5.285948719	0.589968178	0.900365351	679	932	672	581	506	1126	662	705
ENSECAG00000016681	6.691421539	0.589994648	0.900365351	1132	1717	1959	2122	2401	1882	2828	2328
ENSECAG00000018326	1.391367291	0.590041799	0.900365351	30	43	27	67	41	51	58	83
ENSECAG00000022394	2.920480911	0.590155763	0.900394319	46	114	121	241	130	227	85	233
ENSECAG00000007013	2.193205171	0.590309232	0.900394319	49	80	75	159	80	89	95	83
ENSECAG00000003512	4.429020965	0.590316275	0.900394319	217	492	450	520	314	432	377	562
ENSECAG00000024179	4.436992303	0.590402785	0.900394319	274	427	391	578	516	355	386	441
ENSECAG00000019587	1.215423837	0.590456213	0.900394319	37	48	35	50	44	33	42	53
ENSECAG00000020928	1.17048457	0.590725756	0.900684711	40	27	35	66	44	50	45	25
ENSECAG00000023388	2.085586867	0.590858822	0.900695498	36	60	191	51	50	69	30	157
ENSECAG00000015149	7.399796619	0.590891056	0.900695498	2215	3040	3429	4578	3798	2545	3700	3155
ENSECAG00000011931	4.522873087	0.591519059	0.90153206	274	512	577	391	258	460	423	656
ENSECAG00000007744	1.572494579	0.591648954	0.901609336	19	68	45	60	32	75	105	58
ENSECAG00000019403	4.422505662	0.591800891	0.901720174	251	205	463	542	568	388	547	432
ENSECAG00000022613	6.957028598	0.592029494	0.901947784	1754	2518	2428	2856	2449	2076	2585	2611
ENSECAG00000015480	3.237396203	0.592262408	0.902083034	88	113	215	356	174	125	220	215
ENSECAG00000025048	5.843026521	0.592341741	0.902083034	794	834	919	2004	822	631	1800	1362
ENSECAG00000014047	7.376118196	0.592355974	0.902083034	2361	4519	2422	3358	3229	2750	3522	3434
ENSECAG00000005951	0.800514943	0.592526742	0.90216681	31	46	22	25	18	54	27	23
ENSECAG00000011469	2.616210139	0.592684201	0.90216681	41.0007	144	92.0005	130.001	79.0009	162.001	134.001	178
ENSECAG00000009525	3.603119202	0.592909727	0.90216681	133	160	241	287	282	187	336	297
ENSECAG00000012270	5.830049986	0.592953604	0.90216681	917	691	1039	1042	1251	1077	1448	1376
ENSECAG00000011789	2.017795194	0.592961066	0.90216681	51	110	71	74	39	71	68	126
ENSECAG00000017413	5.392377965	0.593025194	0.90216681	355	793	835	1411	717	852	987	760
ENSECAG00000013093	2.545718814	0.593108163	0.90216681	71	74	171	138	80	103	140	127
ENSECAG00000009632	5.233768733	0.593136465	0.90216681	744	764	596	619	715	475	1028	786
ENSECAG00000018903	5.791007352	0.593224571	0.90216681	1121	1256	797	807	1063	838	1492	999
ENSECAG00000004121	4.082214541	0.593257051	0.90216681	215	214	391	299	398	254	455	430
ENSECAG00000024760	6.140347855	0.593282645	0.90216681	602	822	1764	1618	2777	961	1988	766
ENSECAG00000015144	3.077994808	0.59347034	0.902331708	185	127	215	86	239	48	114	233
ENSECAG00000024708	3.843635498	0.593666898	0.902510033	147	218	230	389	341	294	352	301
ENSECAG00000026974	3.315218992	0.593890915	0.902600895	84	199	176	207	339	119	248	197
ENSECAG00000016415	5.841542889	0.594014754	0.902600895	495	953	871	1684	1159	853	1582	1655
ENSECAG00000012630	6.840392229	0.594103542	0.902600895	1697	2739	2151	2127	1757	3364	1694	1882
ENSECAG00000010980	4.380463325	0.594108382	0.902600895	235	284	373	521	422	249	735	518
ENSECAG00000019610	4.474551786	0.594123066	0.902600895	272	248	498	468	754	361	532	356
ENSECAG00000019392	6.19922368	0.594541672	0.903053008	856	1558	1296	2048	1397	1791	1350	1169
ENSECAG00000014808	7.262540617	0.594579302	0.903053008	1937	1983	2894	3384	3221	2784	4207	3905
ENSECAG00000009518	1.427201642	0.594797162	0.903185708	58	54	45	32	59	59	54	25
ENSECAG00000021300	7.162334944	0.594825335	0.903185708	2331	3970	2161	2210	3359	2538	2766	2383
ENSECAG00000012314	2.987426993	0.594927755	0.903220762	61	95	152	242	179	137	198	204
ENSECAG00000002746	4.586533734	0.595097933	0.903358662	316	432	367	472	404	548	537	665
ENSECAG00000006043	5.58546928	0.595179879	0.903362608	485	451	866	1558	843	731	1589	1278
ENSECAG00000013610	6.07632919	0.595287045	0.903404827	1006	923	1148	1311	2029	780	1997	1396
ENSECAG00000012083	9.654097074	0.595621957	0.90360175	6736	13413	12483	22662	21321	14751	21957	16372
ENSECAG00000013971	4.468707807	0.595715292	0.90360175	329	394	331	380	404	400	672	538
ENSECAG00000016406	0.491264578	0.595732291	0.90360175	19	9	39	13	23	33	11	54
ENSECAG00000001057	1.464221151	0.595734276	0.90360175	18	65	49	91	28	22	63	93
ENSECAG00000000352	7.076793773	0.596009526	0.90380065	1322	2404	3527	3569	2121	2430	3880	2228
ENSECAG00000008443	3.39056923	0.596024179	0.90380065	119	270	135	294	141	274	215	175
ENSECAG00000021311	7.078776166	0.59611251	0.903814213	1802	2955	2687	2971	2791	2607	2974	2176
ENSECAG00000020044	3.868541654	0.596395982	0.904111871	200	140	346	266	462	166	373	331
ENSECAG00000006021	5.65463079	0.596467656	0.904111871	517.001	525.001	1174	1248	1589	899.002	1200	856.001
ENSECAG00000000891	5.882445958	0.596586234	0.904169979	718	1441	1019	1350	1253	802	1303	1320
ENSECAG00000010381	4.832789766	0.596664827	0.904169979	307	664	547	683	538	580	665	472
ENSECAG00000019039	4.973499505	0.596777349	0.904220138	388	443	485	815	848	434	897	676
ENSECAG00000018499	4.775902863	0.596920521	0.904316718	314	469	523	537	548	497	789	644
ENSECAG00000002350	8.064531481	0.597039236	0.904376225	2486	4159	5732	5723	6173	6221	6603	5421
ENSECAG00000024608	8.062573452	0.597370443	0.904456511	5307	5296	3436	5894	5694	5499	5405	3985
ENSECAG00000016387	3.818207351	0.597386325	0.904456511	157	228	258	477	248	231	330	303
ENSECAG00000011555	4.232038677	0.597386506	0.904456511	256	293	416	477	351	268	527	357

ENSECAG00000013281	1.61620763	0.597463378	0.904456511	30	16	76	78	77	60	94	46
ENSECAG00000014862	5.041736784	0.59753657	0.904456511	277	1054	518	227	586	1101	393	880
ENSECAG00000022368	7.757783346	0.597595412	0.904456511	5077	4372	2594	3552	4625	4498	4025	3400
ENSECAG00000000392	6.54474963	0.597648338	0.904456511	1100	1070	1737	2447	1912	1501	2852	2324
ENSECAG00000015838	6.803484929	0.597925642	0.904669211	1567	2238	2075	2720	2296	1748	2409	2327
ENSECAG00000014129	1.277360502	0.597963852	0.904669211	29	52	35	68	50	51	40	37
ENSECAG00000010745	2.732958045	0.598184427	0.904669211	56	50	136	214	128	82	173	229
ENSECAG00000013355	4.100183639	0.598195963	0.904669211	229	294	375	419	213	315	331	489
ENSECAG00000008993	9.130362071	0.598244396	0.904669211	7016	15047	7559	14391	7477	12437	9580	13436
ENSECAG00000007780	3.290327612	0.598351329	0.904669211	284	141	150	95	111	240	200	190
ENSECAG00000012095	1.479560798	0.59838768	0.904669211	27	52	34	112	42	34	34	94
ENSECAG00000023983	0.219575542	0.598433901	0.904669211	2	31	12	26	12	34	18	35
ENSECAG00000015929	9.189158799	0.59850404	0.904669211	6089	9524	10487	12896	13918	8516	18298	13640
ENSECAG00000013915	10.64475694	0.598597626	0.904690558	15488	21432	31288	40359	43614	37217	30524	34103
ENSECAG00000008822	4.499243783	0.598967909	0.905088624	227	271	463	604	605	389	606	441
ENSECAG00000013904	5.515364458	0.599020008	0.905088624	656	816	668	849	953	644	1428	1165
ENSECAG00000006920	6.337749893	0.599376644	0.905507311	1132	1008	1546	1654	1870	1233	2733	1618
ENSECAG00000008093	6.339822358	0.599742813	0.905940284	852.99	1258	1316	2118	1531.99	1221	2387	2301
ENSECAG00000022228	6.090321465	0.599915419	0.906052099	960	2004	1113	1018	914	1827	1224	1290
ENSECAG00000020293	6.589576716	0.600053829	0.906052099	995	2532	1852	2123	1608	2203	1797	1880
ENSECAG00000006717	5.299791419	0.600055584	0.906052099	248	1167	1071	708	1246	1080	331	207
ENSECAG00000017167	6.235435582	0.600159493	0.906063899	849	1293	1523	1365	2062	1477	1994	1282
ENSECAG00000018659	7.418587057	0.600222567	0.906063899	2119	5440	2497	3041	2485	3932	3431	3341
ENSECAG00000016947	5.531781634	0.600415511	0.906106167	727	975	899	828	925	834	887	1004
ENSECAG00000024151	6.31113019	0.600416666	0.906106167	1269	1726	1484	1458	1389	2101	1234	1393
ENSECAG00000014380	5.293430852	0.600489331	0.906106167	292	482	737	1285	816	1021	965	725
ENSECAG00000012657	6.448776067	0.600590451	0.906138655	1123	1503	1428	1731	1456	1719	2470	2327
ENSECAG00000013435	4.653861541	0.600671917	0.906141484	391	589	297	281	440	737	809	313
ENSECAG00000011277	4.744605686	0.600767539	0.906165664	381	434	515	410	798	452	694	472
ENSECAG00000024987	3.412689208	0.600859103	0.906183718	92	204	151	275	147	254	186	369
ENSECAG00000025173	4.223040008	0.601015154	0.90629901	262	304	358	506	362	373	379	355
ENSECAG00000019106	2.689873798	0.601164162	0.906403653	67	129	107	214	69	65	254	123
ENSECAG00000021787	1.81040575	0.601657188	0.906728271	45	98	62	55	46	55	83	83
ENSECAG00000018215	6.418970823	0.60167159	0.906728271	1226	2166	1302	1765	1721	1787	1697	1481
ENSECAG00000006648	3.818949905	0.601724412	0.906728271	153	141	241	434	411	191	278	394
ENSECAG00000015847	2.283159008	0.601773683	0.906728271	104	40	73	66	122	61	212	63
ENSECAG00000012571	6.246871859	0.601851124	0.906728271	615	2348	1409	1610	1253	1999	1157	1411
ENSECAG00000002193	5.179841658	0.601857316	0.906728271	462	684	767	858	738	642	880	630
ENSECAG00000013945	3.70950389	0.602117554	0.906919616	157	257	170	277	221	170	390	421
ENSECAG00000016401	6.062452162	0.60214818	0.906919616	1032.01	1140	1504.01	1347	1217	1263	1507	1307.02
ENSECAG00000006670	4.90168066	0.602223303	0.906919616	350	924	465	543	488	952	355	461
ENSECAG00000012266	5.418845957	0.602538696	0.907078356	535	491	949	892	1427	651	958	815
ENSECAG00000014767	6.251949376	0.60276549	0.907078356	545	1062	1808	1913	1403	1810	2511	1262
ENSECAG00000013775	5.002436718	0.602804409	0.907078356	335	605	643	939	591	702	745	495
ENSECAG00000014950	2.62587622	0.602807636	0.907078356	43	135	107	217	113	131	110	114
ENSECAG00000019897	3.918521976	0.602813018	0.907078356	140	260	300	517	398	222	303	257
ENSECAG00000013485	4.773996117	0.602869112	0.907078356	402	504	423	414	972	280	842	419
ENSECAG00000004167	0.825469155	0.602886422	0.907078356	13	34	17	46	10	21	49	81
ENSECAG00000015008	3.69956748	0.602998805	0.907127562	116	167	208	412	393	217	378	192
ENSECAG00000021548	5.699147086	0.603189625	0.90717785	581	684	1157	1101	1442	824	1557	902
ENSECAG00000019199	4.541107429	0.603191597	0.90717785	244	303	545	493	416	403	589	699
ENSECAG00000007504	2.916378626	0.603579532	0.9075576	150	114	149	141	222	144	117	86
ENSECAG00000020569	7.336619125	0.603658658	0.9075576	1717	3895	2740	4449	2740	2991	3409	3488
ENSECAG00000005393	4.838176735	0.603683242	0.9075576	493	537	395	706	505	524	603	632
ENSECAG00000006561	10.24871603	0.6039191	0.907792309	15614	15812	26849	19839	40813	22658	38875	10948
ENSECAG00000000800	3.396581212	0.604043098	0.907858835	136	217	245	204	233	88	291	221
ENSECAG00000006876	5.698662772	0.604235711	0.907936352	695	694	748	1374	1078	815	1416	1414
ENSECAG00000023323	0.375130537	0.604254171	0.907936352	2	5	41	35	16	27	46	26
ENSECAG00000021330	6.839267802	0.60460897	0.908349581	1903	1528	2155	1595	3771	2341	2691	1607
ENSECAG00000000116	5.88012901	0.604747717	0.908434437	561	1078	874	1547	1025	1040	1555	1741
ENSECAG00000020623	5.412485059	0.604825036	0.908434437	567	646.999	939	1130	723.999	713	999	965
ENSECAG00000000794	6.749575045	0.605249183	0.908802224	2429	3022	1212	976	1191	2603	1902	2489
ENSECAG00000021393	5.718235294	0.605332736	0.908802224	633	562	955	1424	1848	614	1552	811
ENSECAG00000022486	3.873980104	0.605360509	0.908802224	172	215	245	358	428	264	338	281
ENSECAG00000022877	2.158797301	0.605389202	0.908802224	53	103	56	69	65	95	112	130
ENSECAG00000010322	1.446952185	0.605489324	0.90883269	6	37	55	148	4	34	24	127
ENSECAG00000002814	6.991993815	0.605616348	0.908889302	1486	1838	3082	1967	3159	2857	3188	2368
ENSECAG00000021791	3.174503489	0.605766183	0.908889302	114	157	142	173	201	108	356	172
ENSECAG00000018855	0.268832125	0.605865748	0.908889302	0	21	19	37	30	24	32	18
ENSECAG00000020502	3.246871133	0.605960493	0.908889302	117	103	206	197	287	81	284	221
ENSECAG00000011543	5.261909121	0.605986752	0.908889302	417	485	1027	639	1287	700	874	587
ENSECAG00000020298	8.617107784	0.606006033	0.908889302	6101	9198	6660	7808	8371	7005	8438	6711
ENSECAG00000002621	5.87863937	0.606092431	0.908899147	1085	1159	1016	1046	1121	1321	1261	912
ENSECAG00000010755	3.44395458	0.60666432	0.909537633	128	210	242	266	192	275	102	253
ENSECAG00000010663	6.206647512	0.60683258	0.909537633	1143	532	1729	1308	3303	835	1757	893
ENSECAG00000017606	1.691049118	0.606894119	0.909537633	41	36	36	95	60	37	64	130

ENSECAG00000000066	8.761187896	0.606903455	0.909537633	4870	4865	9194	10262	8799	9555	9467	11800
ENSECAG00000012155	3.99753522	0.606917646	0.909537633	231	412	339	196	376	246	387	245
ENSECAG00000014110	1.60691898	0.607177392	0.909802178	40	44	51	55	52	39	127	62
ENSECAG00000014806	3.962275408	0.607253996	0.909802178	124	336	216	395	276	283	376	475
ENSECAG00000022296	5.319778119	0.607633756	0.909992228	414	846	857	991	790	882	769	697
ENSECAG00000020797	5.147933742	0.607716098	0.909992228	491	744	689	746	547	623	797	861
ENSECAG00000015778	3.023088587	0.607789238	0.909992228	119	94	216	193	166	164	154	141
ENSECAG00000017017	4.55887093	0.607798652	0.909992228	208	378	373	1024	177	277	337	1026
ENSECAG00000024834	6.75490919	0.607814169	0.909992228	1702	2281	1945	2218	1522	2533	1992	2352
ENSECAG00000005917	3.110561793	0.607874114	0.909992228	73	123	172	224	167	156	190	264
ENSECAG00000008070	5.117181757	0.60794035	0.909992228	453	899	536	728	715	630	782	624
ENSECAG00000014894	2.353260798	0.608280536	0.910201681	52	85	91	182	87	43	131	135
ENSECAG00000016510	2.141335803	0.608437604	0.910201681	96	30	42	95	111	71	142	81
ENSECAG00000017756	8.035975857	0.608617242	0.910201681	2850	3930	5375	5419	5171	6713	6101	5834
ENSECAG00000018980	8.405630138	0.608661257	0.910201681	3565	6693	7062	9830	5087	6052	7498	7913
ENSECAG00000013227	4.651878949	0.608696119	0.910201681	258	325	521	632	612	446	698	511
ENSECAG00000021275	5.469906244	0.608881449	0.910201681	673	565	828	845	1207	843	1064	861
ENSECAG00000004620	8.112663133	0.608997795	0.910201681	3329	7246	4447	6550	4942	6483	6095	3908
ENSECAG00000009454	2.566102987	0.609004862	0.910201681	62	171	122	99	47	148	87	162
ENSECAG00000018205	1.873631644	0.609107221	0.910201681	55	36	70	68	81	41	125	88
ENSECAG00000000713	5.890876049	0.609129842	0.910201681	1366	2223	312	409	212	2849	366	677
ENSECAG00000013542	6.789755259	0.609141092	0.910201681	1780	2753	1993	1691	2347	1963	2784	1592
ENSECAG00000014848	6.805621367	0.609268546	0.910201681	1662	2311	2042	2492	1852	2272	2531	2139
ENSECAG00000012317	6.893019681	0.609382513	0.910201681	1689	2581	2282	2527	2224	2893	1990	2058
ENSECAG00000022071	2.266272142	0.609412887	0.910201681	51	126	89	26	75	151	86	119
ENSECAG00000007369	7.02709056	0.609431958	0.910201681	1595	3697	2097	2631	1781	2756	2727	2905
ENSECAG00000015587	6.76591012	0.609524732	0.910201681	1300	2092	2397	2704	2288	2245	2022	1924
ENSECAG00000010505	2.962653374	0.609529623	0.910201681	98	102	209	196	198	129	153	120
ENSECAG00000001358	6.634889288	0.609548779	0.910201681	1727	1443	2007	2305	1708	1726	2310	2122
ENSECAG00000009529	6.421297995	0.609599281	0.910201681	918	1644	1666	2549	1817	1473	1915	1571
ENSECAG00000011230	5.631488266	0.609895227	0.910468506	858	959	972	849	1110	1043	922	810
ENSECAG00000007573	6.09807497	0.609937927	0.910468506	1027	1102	1441	1639	1454	1249	1599	1127
ENSECAG00000006975	7.304489884	0.61011602	0.910594362	2033	3291	1944	2936	4866	2030	5512	2338
ENSECAG00000013570	3.601265464	0.610182203	0.910594362	182	295	205	218	180	210	266	298
ENSECAG00000007824	6.476056622	0.610422766	0.91083397	1053.01	1438	1709	2870	2142	1808	1570	1405
ENSECAG00000000232	3.934276121	0.610826695	0.910914548	184	457	241	268	255	280	291	369
ENSECAG00000003667	1.531789149	0.610829137	0.910914548	34	50	61	76	62	63	55	35
ENSECAG00000020268	3.042577412	0.610976297	0.910914548	114	93	238	191.628	196	170	181	83.9174
ENSECAG00000023167	4.188983672	0.610988409	0.910914548	227	299	382	502	349	332	367	392
ENSECAG00000011366	7.411625192	0.610996198	0.910914548	2079	2999	3937	4363	3808	3576	3061	2725
ENSECAG00000008623	0.36949167	0.611002537	0.910914548	10	16	23	54	7	12	22	49
ENSECAG00000010062	5.320564911	0.611036838	0.910914548	492	442	995	1229	975	613	888	681
ENSECAG00000011732	1.278416693	0.611205366	0.910949604	22	86	30	44	42	50	46	40
ENSECAG00000005626	3.117855941	0.611306349	0.910949604	89	136	177	179	233	167	234	145
ENSECAG00000010332	4.012245315	0.611362528	0.910949604	241	216	281	313	492	208	435	326
ENSECAG00000008105	3.878917381	0.611380406	0.910949604	179	307	233	418	271	230	379	290
ENSECAG00000015180	0.713295999	0.611478678	0.910976806	22	48	26	22	23	27	15	51
ENSECAG00000004124	3.470185974	0.611761014	0.911246806	98	161	215	288	183	174	331	323
ENSECAG00000017183	0.770168547	0.61181999	0.911246806	21	24	17	44	33	29	38	48
ENSECAG00000015373	3.439707427	0.612106424	0.911433474	143	102	271	191	260	121	401	224
ENSECAG00000011181	5.977571069	0.612122703	0.911433474	472	754	1262	2001	1066	1232	1776	1679
ENSECAG00000023411	3.832058613	0.612185487	0.911433474	176.996	469.997	234.988	182.98	290	378.99	181.995	223.992
ENSECAG00000017285	3.192878942	0.612451076	0.91166318	112	158	136	314	145	153	178	236
ENSECAG00000019558	4.747542384	0.612499927	0.91166318	300	446	529	537	636	382	897	536
ENSECAG00000015226	7.163801725	0.612690562	0.911827719	1789	3329	2811	3144	1983	2977	3099	3176
ENSECAG00000016932	4.891086907	0.61283814	0.911928143	188	412	668	846	620	556	736	768
ENSECAG00000025168	4.916728495	0.613034623	0.912074168	336	536	588	904	616	545	711	533
ENSECAG00000009813	6.639456103	0.61316308	0.912074168	1534	336	2208	2409	2076	1447	4175	1793
ENSECAG00000013585	4.187550282	0.613176608	0.912074168	265	321	258	344	273	396	535	445
ENSECAG00000018725	2.652582117	0.613280593	0.912109674	67	81	127	145	149	151	130	127
ENSECAG00000024204	2.806051737	0.613444008	0.912233546	82	75	151	155	131	203	173	117
ENSECAG00000013727	4.455021434	0.613739205	0.912303827	235	353	422	488	466	454	510	526
ENSECAG00000013422	4.802364058	0.613804605	0.912303827	432	277	622	826	610	321	672	618
ENSECAG00000009108	1.541345634	0.613825401	0.912303827	33	72	46	69	38	39	63	81
ENSECAG00000019195	1.533506577	0.613856445	0.912303827	41	51	73	53	26	46	27	113
ENSECAG00000000375	7.0523558	0.613915125	0.912303827	1805	1617	2895	2341	3652	2570	4200	1785
ENSECAG00000007200	6.15979816	0.613972061	0.912303827	953	994	1328	1487	1805	971	2194	1570
ENSECAG00000017695	4.19632291	0.614190119	0.912430616	198	262	430	344	307	353	660	357
ENSECAG00000024547	5.646897612	0.614217676	0.912430616	456	853	1148	976	919	1160	1534	920
ENSECAG00000011754	5.199754813	0.614714528	0.913049563	415	320	861	903	1403	503	895	529
ENSECAG00000016930	0.567039211	0.614971207	0.913311661	23	27	23	35	41	12	41	13
ENSECAG00000016981	2.399361251	0.615131081	0.913356882	69	75	120	143	87	65	125	133
ENSECAG00000006613	1.52169366	0.615252571	0.913356882	24	42	68	95	78	26	70	42
ENSECAG00000015504	5.846271055	0.615295967	0.913356882	1000	1090	958	1207	1167	952	1061	1366
ENSECAG00000019235	4.339540839	0.615323819	0.913356882	224.001	542	357.001	422.001	515.001	303.001	465.001	316
ENSECAG00000018675	1.431545598	0.615725926	0.913356882	12	68	47	41	12	83	18	128

ENSECAG00000006687	5.26562726	0.615986231	0.913356882	399	842	761	991	729	887	664	735
ENSECAG00000021051	7.431449031	0.615990627	0.913356882	2254	3856	2693	4669	4395	2112	4283	2799
ENSECAG00000025102	5.576034734	0.616044132	0.913356882	904	1032	802	726	709	1139	578	1252
ENSECAG00000017698	7.049794703	0.616045336	0.913356882	1339	3166	2780	3138	2093	3225	2633	2337
ENSECAG00000012069	7.663082071	0.616161331	0.913356882	3350	5670	3313	2638	3543	4759	4364	2990
ENSECAG00000011198	5.448831363	0.616220127	0.913356882	469	314	1241	951	1245	713	1329	709
ENSECAG00000007105	7.566540474	0.616294339	0.913356882	2393	2531	4761	5311	3628	3117	4069	4057
ENSECAG00000024195	2.613516429	0.616321866	0.913356882	93	114	66	108	178	50	179	155
ENSECAG00000011972	5.152042315	0.616388867	0.913356882	641	394	429	841	639	629	1028	944
ENSECAG00000010625	4.180880683	0.616512258	0.913356882	242	356	395	375	308	366	371	387
ENSECAG00000008626	5.365944165	0.616584674	0.913356882	552	483	990	1189	868	543	1089	803
ENSECAG00000001722	5.046352247	0.616675637	0.913356882	352	459	826	599	656	876	610	787
ENSECAG00000009667	4.942380205	0.616686264	0.913356882	532	524	518	352	754	527	948	570
ENSECAG00000019044	4.926366442	0.616700395	0.913356882	228	448	790	1012	700	423	669	613
ENSECAG00000022579	5.9076721	0.616715613	0.913356882	845	823	986	1348	1558	895	1707	1292
ENSECAG00000012337	6.766846238	0.616730246	0.913356882	1188	3090	1683	2482	1922	2071	2129	2395
ENSECAG00000006268	4.838222281	0.616837916	0.913356882	324	223	695	724	681	635	624	620
ENSECAG00000013200	4.970293427	0.616846822	0.913356882	331	405	588	852	751	504	774	791
ENSECAG00000022334	6.953265567	0.616994867	0.913371417	1851	2573	2614	2325	1913	2846	2410	2487
ENSECAG00000016788	4.551999714	0.617156555	0.913371417	192	451	570	369	298	708	494	597
ENSECAG00000018676	5.430128311	0.617172284	0.913371417	566	607	1040	1117	651	838	825	1098
ENSECAG00000018377	4.49268479	0.617177542	0.913371417	387	255	404	384	735	238	747	351
ENSECAG00000021900	0.704564633	0.617343767	0.913431509	5	46	26	23	29	64	38	10
ENSECAG00000000423	5.836353635	0.617585957	0.913431509	684	722	1054	1430	1514	684	1375	1613
ENSECAG00000018754	5.768379819	0.617622808	0.913431509	720	766	1010	1161	1467	1163	1238	994
ENSECAG00000020813	7.213823314	0.617899835	0.913431509	2474	2592	2961	3158	2624	2579	3486	3023
ENSECAG00000000817	5.855674653	0.617940462	0.913431509	817	897	891	1231	1438	774	1910	1182
ENSECAG00000016918	6.772124823	0.617975758	0.913431509	1762	2130	2158	2142	2125	2195	2437	1832
ENSECAG00000002380	5.462743512	0.618097789	0.913431509	577	933	760	561	1649	677	1117	548
ENSECAG00000016785	6.124960233	0.618189503	0.913431509	980	948	1252	1451	1124	1257	2085	1908
ENSECAG00000014880	2.929575726	0.618214368	0.913431509	96	160	131	98	187	189	171	131
ENSECAG00000022405	7.09211605	0.618240995	0.913431509	1672	2576	2232	2426	4177	3179	3545	1486
ENSECAG00000010383	2.90776622	0.618261336	0.913431509	81	116	164	230	140	151	138	150
ENSECAG00000024311	7.13044937	0.618270816	0.913431509	2076	2934	2063	3689	2805	2125	3153	2962
ENSECAG00000006955	4.881833804	0.618306192	0.913431509	400	494	422	636	938	319	811	610
ENSECAG00000008928	10.46513449	0.618438036	0.913431509	14718	16602	32084	32096	35100	33694	30873	28730
ENSECAG00000010261	4.768114076	0.618495756	0.913431509	257	426	457	765	538	587	746	576
ENSECAG00000013080	6.273763185	0.618501845	0.913431509	966	1022	1463	1765	1825	1244	2658	1372
ENSECAG00000023688	2.362030357	0.618818321	0.913609495	51	65	89	137	43	97	181	153
ENSECAG00000012171	7.05734304	0.618852162	0.913609495	1815	2778	2714	2901	2210	3063	2486	2608
ENSECAG00000025029	3.812901055	0.618863103	0.913609495	96	372	291	360	285	345	238	211
ENSECAG00000000675	5.919421832	0.618965355	0.913641976	823	974	1252	1614	1029	1366	1047	1308
ENSECAG00000017714	2.626163255	0.619321688	0.914048707	66	76	165	95	192	86	155	124
ENSECAG00000017510	3.835253665	0.61946028	0.914048707	177	242	215	318	197	291	396	407
ENSECAG00000006331	4.012285547	0.619559477	0.914048707	208	189	301	389	413	250	483	315
ENSECAG00000023826	7.91860743	0.619562044	0.914048707	1412	6546	3494	4561	1190	9042	4367	7637
ENSECAG00000009937	3.579479858	0.619924289	0.914347156	132	189	169	323	323	273	210	250
ENSECAG00000019019	6.452783364	0.619924963	0.914347156	1274	1451	1291	1723	2264	1274	2622	1841
ENSECAG00000005451	3.746306864	0.620156356	0.914402724	156	237	398	233	253	302	272	218
ENSECAG00000016078	5.827370139	0.620190275	0.914402724	996	1089	773	624	653	832	1310	2452
ENSECAG00000007061	5.553430819	0.620203587	0.914402724	389	973	706	1791	397	829	876	1579
ENSECAG00000012470	3.06191178	0.620323032	0.914460406	119	159	161	79	189	270	164	118
ENSECAG00000000220	3.037211908	0.620428713	0.914497785	77	139	188	247	139	177	179	142
ENSECAG00000022273	2.403722608	0.620562545	0.914576644	76	66	110	84	101	98	166	114
ENSECAG00000023573	5.762023343	0.620842456	0.914676056	735	878	886	1720	1109	807	1194	1216
ENSECAG00000020917	6.958283252	0.62095093	0.914676056	2249	2923	1816	2171	2089	1874	3532	2389
ENSECAG00000015818	6.135652141	0.620994913	0.914676056	998	1377	1466	1476	1413	1637	1289	1164
ENSECAG00000021223	9.357241776	0.62114023	0.914676056	8916	12844	11159	18884	6992	12366	9332	21798
ENSECAG00000013154	2.916642928	0.621184189	0.914676056	70	160	142	232	91	85	331	98
ENSECAG00000022315	4.076726314	0.621275925	0.914676056	325	394	201	296	331	294	355	346
ENSECAG00000009481	5.44862929	0.621375411	0.914676056	384	444	985	1269	716	969	1086	1171
ENSECAG00000014542	5.045193982	0.621426459	0.914676056	171	266	378	1581	300	328	603	1886
ENSECAG00000016965	0.316120556	0.621562801	0.914676056	11	17	18	28	2	34	45	29
ENSECAG00000012304	3.534363361	0.62161849	0.914676056	187	164	155	240	203	159	392	310
ENSECAG00000022422	4.831203056	0.621783312	0.914676056	371	505	370	666	493	519	822	741
ENSECAG00000010247	4.930350608	0.621816253	0.914676056	316	862	503	654	488	625	602	691
ENSECAG00000022785	4.803422054	0.621834717	0.914676056	427	358	560	465	890	564	727	326
ENSECAG00000000621	6.46864542	0.621924127	0.914676056	889	1889	1611	2622	1545	1947	1641	1809
ENSECAG00000015844	0.581878119	0.621931967	0.914676056	11	14	53	38	25	20	22	39
ENSECAG00000000102	6.880575083	0.622203283	0.914676056	1963	1522	1723	2343	3546	1472	3444	2390
ENSECAG00000018799	6.854076163	0.622223351	0.914676056	1641	2169	2386	2654	2425	2179	2380	2096
ENSECAG00000024877	5.859762285	0.622244505	0.914676056	642	970	1205	1091	1822	943	1547	927
ENSECAG00000014382	2.536399151	0.622272916	0.914676056	57	84	92	156	194	68	171	93
ENSECAG00000019497	8.208200673	0.622348306	0.914676056	4357	5579	4642	8395	4336	4441	7050	7543
ENSECAG00000018890	5.351180529	0.622360637	0.914676056	412	871	753	1161	675	807	845	913
ENSECAG00000016571	4.216061945	0.622397488	0.914676056	145	394	335	403	462	371	552	291

ENSECAG00000019289	2.373052784	0.62288021	0.915250667	41	77	104	129	78	111	115	159
ENSECAG00000012837	5.179060938	0.622949268	0.915250667	402	666	549	843	1183	603	871	587
ENSECAG00000000306	5.525824707	0.62315709	0.915437867	674	821	952	1017	905	736	1097	955
ENSECAG00000017007	3.721546295	0.6233056	0.915537899	155	203	338	329	186	163	436	277
ENSECAG00000011390	6.553110932	0.62364153	0.91591316	987	1781	2357	2219	1744	2142	1824	1646
ENSECAG00000023466	5.769263555	0.623784791	0.916005398	856	1266	865	1051	1010	794	1322	1242
ENSECAG00000022527	10.19218147	0.623881245	0.916028884	18220	39472	16418	12820	22614	30780	21870	13266
ENSECAG00000008550	7.450029117	0.623997326	0.916081181	2056	1640	4249	3781	4920	4388	3621	2833
ENSECAG00000014013	2.381700174	0.624213837	0.916280884	61	83	110	82	136	86	160	88
ENSECAG00000010873	3.376374166	0.624389619	0.9162972	115	204	180	188	163	280	226	254
ENSECAG00000007666	0.669466338	0.624424462	0.9162972	17	28	25	50	30	16	38	33
ENSECAG00000015116	4.137526692	0.62453639	0.9162972	153	304	479	467	194	276	314	598
ENSECAG00000012700	3.565640718	0.624620053	0.9162972	70	206	222	500	111	129	338	357
ENSECAG00000022623	5.752028248	0.624627367	0.9162972	532	699	945	1601	1398	779	1463	1244
ENSECAG00000022326	6.854578456	0.624820452	0.916462361	2384	2789	1474	1707	1393	2585	2427	2599
ENSECAG00000009536	6.523202274	0.625015644	0.916494701	1359	1729	1675	2244	1230	1440	2134	2533
ENSECAG00000019528	4.050747435	0.625081007	0.916494701	194	281	361	454	218	279	304	506
ENSECAG00000022061	6.122460875	0.625084001	0.916494701	822	793	1640	1434	2173	874	2137	1195
ENSECAG00000011881	2.972256229	0.625255836	0.916615824	90	143	151	128	107	170	195	234
ENSECAG00000011947	4.833280114	0.625538114	0.916615824	321	617	451	518	675	511	868	520
ENSECAG00000022424	1.816449904	0.625591201	0.916615824	112	28	45	56	67	101	33	51
ENSECAG00000022449	3.800764373	0.62571939	0.916615824	184	247	260	215	332	192	494	258
ENSECAG00000009412	3.696282523	0.625731631	0.916615824	198	266	247	255	327	233	307	149
ENSECAG00000005884	4.009321424	0.62576876	0.916615824	223	239	347	240	449	344	301	332
ENSECAG00000015950	2.52316301	0.625809535	0.916615824	57	55	136	133	215	64	179	67
ENSECAG00000022744	2.900043385	0.625810698	0.916615824	38	126	169	290	120	152	153	147
ENSECAG00000010141	4.134650925	0.626089003	0.916806999	233	277	279	583	395	413	281	271
ENSECAG00000017115	2.155159792	0.62615626	0.916806999	82	31	117	105	120	51	106	62
ENSECAG00000014022	0.612495819	0.626306873	0.916806999	13	36	40	25	11	18	26	55
ENSECAG00000005344	3.831959743	0.626357835	0.916806999	97	151	287	478	406	290	422	173
ENSECAG00000013049	5.39763241	0.626503493	0.916806999	465	524	913	995	1143	630	1122	907
ENSECAG00000020190	6.721239286	0.626543285	0.916806999	1341	1635	2269	3013	2035	1959	2070	2239
ENSECAG00000017987	2.521403176	0.626581526	0.916806999	56	116	94	113	117	87	188	129
ENSECAG00000009917	1.852001116	0.626585442	0.916806999	35	86	97	56	51	96	54	67
ENSECAG00000016699	6.226868218	0.626778631	0.916959353	648	1223	1453	1885	1136	1492	1957	2214
ENSECAG00000017953	6.685108433	0.627058848	0.916959353	1176	1565	2145	2039	1818	2498	2373	2572
ENSECAG00000000248	5.372627536	0.627247402	0.916959353	540	970	486	647	551	1654	371	1077
ENSECAG00000017685	3.337157079	0.627293015	0.916959353	102	171	197	212	233	254	211	195
ENSECAG00000010640	6.488741713	0.627304168	0.916959353	1483	1948	1626	1577	2101	1485	1849	1650
ENSECAG00000026317	0.507832974	0.627569159	0.916959353	18	29	35	21	16	46	25	12
ENSECAG00000023379	6.557072232	0.627609599	0.916959353	1690	1683	1407	2247	1930	1167	2482	1954
ENSECAG00000008542	11.43227059	0.627657087	0.916959353	22570	37648	64332	65109	64274	77507	51656	55759
ENSECAG00000011994	9.384552948	0.627725636	0.916959353	9414	12533	9395	11715	16124	10742	20076	14775
ENSECAG00000006436	5.369223232	0.627832433	0.916959353	683	375	810	830	825	844	1459	652
ENSECAG00000017806	4.655519226	0.627875363	0.916959353	351	547	451	546	356	365	698	614
ENSECAG00000011055	6.30339502	0.627966062	0.916959353	844	736	2057	1767	1958	1130	2369	1778
ENSECAG00000011975	6.111863478	0.627995605	0.916959353	730	1174	1588	1958	1301	1535	1259	1336
ENSECAG00000012639	5.760693233	0.628002658	0.916959353	637	767	1394	1430	1278	1026	986	978
ENSECAG00000013484	1.127226576	0.628025879	0.916959353	9	24	60	43	3	48	7	140
ENSECAG00000005949	4.919823435	0.628156666	0.916959353	320	484	547	729	594	412	1008	750
ENSECAG00000026890	3.343084314	0.628178959	0.916959353	48	336	132	309	217	135	221	212
ENSECAG00000019947	7.539335919	0.628523968	0.916959353	2446	2862	3541	3265	6463	2613	5512	2517
ENSECAG00000022325	6.509075272	0.628527056	0.916959353	1681	1208	1693	2249	2237	1070	2112	1813
ENSECAG00000009561	2.897153095	0.628639574	0.916959353	114	122	105	230	138	102	126	211
ENSECAG00000024420	6.796454696	0.628656463	0.916959353	1084	1743	2505	3632	2287	2003	2421	2035
ENSECAG00000019373	6.858184401	0.628670062	0.916959353	1545	1645	1648	2938	2762	2122	3445	2270
ENSECAG00000014550	5.79893848	0.6286732	0.916959353	838.001	968	1184	1186	959.001	999.001	1140	1336
ENSECAG00000019188	4.121153994	0.628736138	0.916959353	234	163	345	433	339	295	501	441
ENSECAG00000022627	9.122966192	0.628836094	0.916959353	8512	9693	11272	13297	11853	9123	11845	10871
ENSECAG00000017035	1.650351347	0.628932865	0.916959353	16	55	70	69	58	55	115	56
ENSECAG00000013312	3.354569885	0.628967144	0.916959353	179	163	189	228	214	118	315	169
ENSECAG00000008704	5.159032113	0.628991546	0.916959353	293	700	599	1300	526	671	806	836
ENSECAG00000019590	1.42088747	0.629233175	0.916959353	26	48	49	85	29	56	53	63
ENSECAG00000006081	6.308419775	0.629284225	0.916959353	935	1332	1639	1398	1901	1760	1929	1512
ENSECAG00000014405	1.997593733	0.629372457	0.916959353	38	47	145	89	23	83	29	156
ENSECAG00000021561	9.728609017	0.629470005	0.916959353	10044	11371	17008	18240	18535	18289	16009	23985
ENSECAG00000010051	8.945902368	0.629491195	0.916959353	5293	5521	11460	11020	12665	9335	15734	7772
ENSECAG00000008558	4.447469585	0.629505579	0.916959353	336	467	326	500	321	315	646	480
ENSECAG00000012583	3.830186221	0.629707654	0.916959353	126	257	315	270	346	238	348	343
ENSECAG00000014743	4.740922493	0.629760992	0.916959353	341	554	391	486	669	560	648	506
ENSECAG00000014661	0.412212956	0.629795173	0.916959353	18	14	23	22	35	7	63	15
ENSECAG00000011681	5.636239588	0.629801781	0.916959353	656	921	996	1212	966	1003	1037	951
ENSECAG00000021821	4.830645295	0.629904655	0.916959353	412	496	590	640	596	562	530	564
ENSECAG00000024419	5.029130046	0.629911208	0.916959353	347	420	682	814	727	609	881	714
ENSECAG00000022076	5.066649274	0.630182355	0.917157238	243	1067	499	827	683	526	754	690
ENSECAG00000020691	4.479368526	0.630208263	0.917157238	213	490	330	484	308	582	621	493

ENSECAG00000018492	5.673376135	0.630531106	0.917411032	566	913	713	1287	740	732	1407	1787
ENSECAG00000023644	3.823179976	0.630594775	0.917411032	198	150	303	460	205	141	436	354
ENSECAG00000019797	5.67097128	0.630624395	0.917411032	778	543	789	1256	1605	441	1750	915
ENSECAG00000013761	4.016766107	0.630709042	0.917416947	113	252	413	548	183	456	333	281
ENSECAG00000023042	3.027938351	0.631026988	0.917663069	74	76	188	222	129	125	210	278
ENSECAG00000019712	3.853914291	0.631082829	0.917663069	141	292	295	237	335	403	352	195
ENSECAG00000005903	2.021950317	0.631120055	0.917663069	61	36	82	77	138	77	86	59
ENSECAG00000020069	4.284437859	0.631538591	0.918154368	201	399	528	378	232	607	344	321
ENSECAG00000011265	0.38700214	0.631953305	0.918271566	8	9	25	42	17	24	46	27
ENSECAG00000015936	5.890731503	0.631998049	0.918271566	392	946	1229	1651	1397	1373	1455	1096
ENSECAG00000023612	2.748145611	0.632013132	0.918271566	68	97	114	264	134	75	135	177
ENSECAG00000000036	6.387124505	0.632128463	0.918271566	1192	1857	1512	1739	1187	1738	1941	1788
ENSECAG00000001596	7.098084943	0.632162278	0.918271566	1417	1910	3205	2746	3165	3343	3097	2725
ENSECAG00000014716	1.770023157	0.632164088	0.918271566	33	45	60	87	45	67	66	125
ENSECAG00000024754	4.981477049	0.632183797	0.918271566	410	524	506	674	920	400	955	589
ENSECAG00000022209	8.172848534	0.632317127	0.918341992	3088	4800	6354	8863	5636	5735	5779	5390
ENSECAG00000000946	1.471461626	0.632546878	0.918341992	38	39	44	100	13	8	48	136
ENSECAG00000010209	5.114110195	0.632673606	0.918341992	664	551	640	662	703	490	914	677
ENSECAG00000012736	6.814168475	0.632707024	0.918341992	1256	824	3269	3860	2726	1999	2700	1329
ENSECAG00000022536	6.013194364	0.632714776	0.918341992	1111	1226	1075	1381	1380	677	1895	1263
ENSECAG00000013130	2.223232911	0.632779088	0.918341992	115	54	32	64	122	97	72	127
ENSECAG00000009727	7.769316816	0.63279692	0.918341992	4209	4702	3825	3231	3038	5482	2857	5311
ENSECAG00000008131	7.529491356	0.632958393	0.918394542	3083	2607	3871	4506	3584	2427	4543	4115
ENSECAG00000000507	5.909661158	0.633120909	0.918394542	752	829	1190	1961	953	903	1573	1403
ENSECAG00000013505	3.328194935	0.633162094	0.918394542	153	121	165	217	264	160	301	182
ENSECAG00000018636	3.598548758	0.633280599	0.918394542	181	215	230	282	218	229	256	253
ENSECAG00000016512	3.536354501	0.633432897	0.918394542	161	144	209	249	380	220	312	129
ENSECAG00000001757	6.246995342	0.633514035	0.918394542	1259	1247	1694	1466	1289	2066	1169	1365
ENSECAG00000007233	5.953422867	0.633585798	0.918394542	773	1061	1368	1576	1108	1250	1053	1478
ENSECAG00000018407	5.060651492	0.633728821	0.918394542	446	456	597	740	1018	396	901	701
ENSECAG00000024774	6.248071415	0.633859102	0.918394542	739	1514	1083	2833	859	925	1911	2379
ENSECAG00000003179	3.471775012	0.63392145	0.918394542	116	197	180	388	170	176	258	273
ENSECAG00000009628	7.973373434	0.63397252	0.918394542	3647	4865	4653	6090	4706	4764	5598	4716
ENSECAG00000006405	6.021261386	0.634019801	0.918394542	723	1131	1479	1725	1075	1082	1691	1377
ENSECAG00000013025	0.669106101	0.634107799	0.918394542	9	13	44	62	25	16	55	21
ENSECAG00000023340	4.370263916	0.634252504	0.918394542	231	312	445	415	580	391	481	387
ENSECAG00000017126	3.539691878	0.634267261	0.918394542	83	171	173	399	229	187	324	315
ENSECAG00000017219	5.143529826	0.634290505	0.918394542	324	444	920	763	706	831	1013	626
ENSECAG00000019261	2.101080214	0.634354012	0.918394542	85	76	42	50	91	106	98	87
ENSECAG00000010671	2.970477493	0.634370298	0.918394542	95	158	124	228	176	122	175	140
ENSECAG00000003669	7.840668449	0.634403836	0.918394542	1941	3306	4272	6395	3515	4919	4379	8025
ENSECAG00000008278	5.564936262	0.634446471	0.918394542	672	583	1136	1236	828	824	1147	994
ENSECAG00000008404	7.076910083	0.634570035	0.918456629	2255	2737	2307	2792	2892	2720	2652	2294
ENSECAG00000011023	3.964223862	0.634716479	0.918488326	143	286	343	465	183	351	393	309
ENSECAG00000017900	6.75273338	0.634753285	0.918488326	1191	2985	1765	2395	1967	2814	1875	1685
ENSECAG00000015030	7.783707287	0.635010534	0.918660541	3175	1277	7247	2227	6011	4374	7418	2768
ENSECAG00000022196	4.290933545	0.635086917	0.918660541	214	255	358	523	573	270	494	421
ENSECAG00000011648	6.95287994	0.635169086	0.918660541	1805	2636	2550	2356	3035	2124	2565	1999
ENSECAG00000015801	6.030987174	0.6351983	0.918660541	707	955	1366	2169	1249	1081	1430	1452
ENSECAG00000005071	2.835846313	0.635275752	0.918660541	58	265	125	97	173	72	177	131
ENSECAG00000008249	4.976236878	0.63536566	0.918673868	565	430	517	488	804	437	1057	577
ENSECAG00000000295	4.665406971	0.635851207	0.919105874	310	339	444	926	396	272	712	665
ENSECAG00000009585	7.790825476	0.635908191	0.919105874	1743	2606	5149	6004	5245	5200	4454	5070
ENSECAG00000017193	5.434965088	0.635944017	0.919105874	439	586	799	1198	1167	734	916	1043
ENSECAG00000002018	5.286801691	0.635987358	0.919105874	557	854	825	659	834	706	941	635
ENSECAG00000021913	5.518874429	0.636336825	0.919494194	441	1126	1003	982	625	1022	687	1257
ENSECAG00000026905	11.06407958	0.636580244	0.919655415	18506	28206	48037	52621	52124	53670	39595	47580
ENSECAG00000000127	5.39212466	0.636609954	0.919655415	472	527	833	1075	952	920	954	909
ENSECAG00000021041	3.644694265	0.63681318	0.919832283	192	212	247	287	215	325	210	220
ENSECAG00000019518	3.909865542	0.637000745	0.919979891	130	258	292	533	256	162	414	372
ENSECAG00000007288	3.726712121	0.637076984	0.919979891	122	146	275	321	67	185	43	937
ENSECAG00000021354	3.522823218	0.637605153	0.92062583	143	191	239	317	355	165	183	188
ENSECAG00000015670	7.053740136	0.637818487	0.920750795	1306	2103	2755	4587	2302	2393	2845	2930
ENSECAG00000015464	9.308349805	0.637874959	0.920750795	6742	7697	13352	15327	15050	15838	11666	14480
ENSECAG00000018849	6.963490813	0.638028187	0.920750795	1726	1678	2937	3364	2276	2631	2440	2433
ENSECAG00000013553	5.319854326	0.638062937	0.920750795	418	793	699	1223	636	711	956	899
ENSECAG00000010338	2.154722505	0.638171736	0.920750795	68	62	93	49.0001	170	50	126	57
ENSECAG00000024238	7.265946138	0.638222911	0.920750795	2618	4134	2420	2121	3915	2497	3457	2160
ENSECAG00000014132	1.469543789	0.638288888	0.920750795	25	34	53	71	43	47	64	91
ENSECAG00000021071	5.458844169	0.638413242	0.920750795	409	738	910	995	855	1042	1105	920
ENSECAG00000020980	4.106862953	0.638437804	0.920750795	281	440	261	270	325	323	406	311
ENSECAG00000021719	0.340473266	0.638500441	0.920750795	14	20	11	32	37	20	26	24
ENSECAG00000010706	3.905086128	0.638610579	0.920792989	72	290	277	617.008	252.007	334	262	309
ENSECAG00000019694	3.699039987	0.638753006	0.920881724	123	238	296	363	190	261	323	256
ENSECAG00000009554	1.603505558	0.638839256	0.920889457	41	82	41	62	22	42	59	107
ENSECAG00000023774	6.376756394	0.639172803	0.921115735	755	1247	1626	2175	1969	2035	1950	1467

ENSECAG00000016695	4.491008278	0.63926573	0.921115735	174	388	389	639	438	434	627	523
ENSECAG00000024895	0.012346593	0.639308882	0.921115735	11	10	15	24	25	9	28	23
ENSECAG00000005272	1.049709757	0.639343412	0.921115735	32	31	30	32	35	33	64	51
ENSECAG00000000001	5.37737599	0.639497284	0.921115735	322	424	1004	1211	1001	1032	817	842
ENSECAG00000018052	1.098564297	0.639584002	0.921115735	19	31	39	49	28	30	51	80
ENSECAG00000009577	1.200700356	0.639614122	0.921115735	18	29	41	64	62	31	66	44
ENSECAG00000008301	6.53292813	0.639643479	0.921115735	1031	1556	1409	2341	1654	1527	2656	2600
ENSECAG00000007231	3.94029607	0.639931039	0.921284438	206	199	224	401	259	169	541	447
ENSECAG00000006597	4.615051006	0.640034952	0.921284438	329	398	392	519	532	361	842	495
ENSECAG00000006855	5.209680778	0.640177389	0.921284438	614	718	725	654	697	791	868	590
ENSECAG00000005248	4.497598399	0.640497297	0.921284438	294	335	351	540	378	277	640	764
ENSECAG00000009190	10.21506102	0.640515456	0.921284438	17910	31989	15660	24235	19000	19713	26350	28313
ENSECAG00000014560	7.114545875	0.640535069	0.921284438	1527	2630	2122	3052	3430	2137	4278	2854
ENSECAG00000018555	6.894236075	0.640560246	0.921284438	1596	1912	2656	3035	2394	2169	2360	2446
ENSECAG00000007148	2.882667024	0.640624255	0.921284438	184	113	115	101	102	141	149	178
ENSECAG00000021253	3.114102117	0.64064664	0.921284438	96	171	200	197	170	160	235	118
ENSECAG00000019823	4.860291014	0.640676711	0.921284438	289	375	571	800	550	526	729	801
ENSECAG00000019622	3.03792516	0.64070286	0.921284438	83	80	237	256	155	145	181	158
ENSECAG00000017645	8.178183411	0.640786408	0.921284438	5432	5602	5113	5171	6407	5159	6003	5109
ENSECAG00000008264	7.783466739	0.64084222	0.921284438	2591	4137	3750	4156	5036	5471	4667	4605
ENSECAG00000006481	6.658679976	0.641032424	0.921284438	1635	2449	1717	1641	2098	1706	2609	1623
ENSECAG00000012110	8.488854872	0.641043643	0.921284438	4206	5345	6354	8269	9109	5520	8530	9585
ENSECAG00000016677	5.255632643	0.641055364	0.921284438	221	524	889	1104	679	1151	702	855
ENSECAG00000007859	0.139377022	0.641299129	0.921293427	14	16	22	27	9	14	30	26
ENSECAG00000013563	5.548640732	0.641478896	0.921293427	404	845	1103	1362	948	781	1129	890
ENSECAG00000019217	6.799820888	0.641510569	0.921293427	1548	2012	2473	2474	1811	2634	2016	2256
ENSECAG00000006280	6.874289544	0.641701599	0.921293427	1445	2080	2025	2258	2920	2467	2721	2442
ENSECAG00000011487	6.57286398	0.641744887	0.921293427	1182	2174	1932	1954	1878	2040	2033	1567
ENSECAG00000016733	3.943105819	0.64184207	0.921293427	190	305	257	265	241	296	427	421
ENSECAG00000020888	8.675752863	0.641971583	0.921293427	5708	7574	9943	8014	8068	6697	7902	9418
ENSECAG00000015910	7.094874424	0.641982804	0.921293427	2476	2015	2979	2695	2865	2070	2672	3161
ENSECAG00000006707	3.057223423	0.642084163	0.921293427	122	188	101	112	208	116	268	166
ENSECAG00000009845	3.896010785	0.642223785	0.921293427	196	277	224	290	347	278	408	299
ENSECAG00000018042	5.707585915	0.642236844	0.921293427	883	916	901	589	1053	849	2030	892
ENSECAG00000023837	6.104866324	0.6422846	0.921293427	759	1356	1700	1514	1100	2007	1135	1101
ENSECAG00000012731	3.959964392	0.642333351	0.921293427	148	317	259	507.008	242.007	322	314	349
ENSECAG00000012555	3.758022234	0.642384183	0.921293427	104	252	255	325	342	347	332	181
ENSECAG00000010566	3.954596861	0.642446808	0.921293427	86	238	202	596	228	407	335	421
ENSECAG00000015615	6.563887605	0.642491617	0.921293427	1086	1666	2222	2397	1551	2184	1510	2159
ENSECAG00000013340	3.531917649	0.642510662	0.921293427	172	136	241	203	330	161	351	205
ENSECAG00000022111	1.79661834	0.642518209	0.921293427	38	76	69	81	58	36	81	93
ENSECAG00000014759	5.437936556	0.642801106	0.921554425	513	363	967	1135	1335	589	1322	700
ENSECAG00000000623	6.666812514	0.642862121	0.921554425	655	1858	1694	4311	2166	2037	1915	1793
ENSECAG00000000498	1.718898832	0.643009931	0.921650267	32	29	52	110	64	67	85	76
ENSECAG00000024770	3.4494516	0.643150829	0.921673372	95	197	216	235	150	194	244	396
ENSECAG00000019775	6.990936206	0.643243955	0.921673372	1320.01	2035	2386.01	2932	1948	2479	3776	3437.02
ENSECAG00000019032	6.672696722	0.643298961	0.921673372	1616	1446	1976	2755	2266	1444	2774	1679
ENSECAG00000009449	4.264494342	0.643395644	0.921673372	232	168	409	500	689	272	509	263
ENSECAG00000011460	2.619762609	0.643430827	0.921673372	67	78	88	185	110	119	197	129
ENSECAG00000015767	5.669108281	0.643775799	0.921757611	808	794	1166	1012	1088	856	1159	969
ENSECAG00000016914	2.158093522	0.643793842	0.921757611	47	97	73	127	74	81	100	90
ENSECAG00000024483	6.168831995	0.64381806	0.921757611	758	1092	1379	1723	1752	1016	2329	1464
ENSECAG00000009240	7.382582846	0.643844515	0.921757611	2319	3272	3564	3554	3278	3055	3610	3230
ENSECAG00000018172	2.627872732	0.643896165	0.921757611	73	107	83	147	121	108	155	169
ENSECAG00000007427	3.583212926	0.644074924	0.921757611	169	172	196	254	163	214	400	314
ENSECAG00000022255	8.483937929	0.644138396	0.921757611	8503	6516	5946	4427	8062	6831	8670	4300
ENSECAG00000008525	5.233611962	0.644200864	0.921757611	287	527	797	1067	806	909	978	667
ENSECAG00000020658	3.353135575	0.644218297	0.921757611	183	162	196	208	196	130	265	223
ENSECAG00000014051	3.742004464	0.644306019	0.921767282	156	239	234	268	359	257	344	232
ENSECAG00000012525	5.69538529	0.644652207	0.922146673	402	760	1111	1387	1137	983	1848	759
ENSECAG00000022134	5.661066636	0.644843138	0.922222888	485	636	888	1575	932	1155	1110	1316
ENSECAG00000014440	3.051745981	0.644924597	0.922222888	102	95	122	244	165	153	196	228
ENSECAG00000008645	2.471403297	0.644948497	0.922222888	60	87	114	104	158	121	134	78
ENSECAG00000015867	2.331085833	0.645202635	0.922443256	45	86	76	133	78	81	128	165
ENSECAG00000018826	1.655328902	0.645300039	0.922443256	49	102	32	47	31	120	32	42
ENSECAG00000021436	8.330953352	0.645345677	0.922443256	2674	4037	7924	7649	8467	8006	6565	5914
ENSECAG00000023718	2.414313322	0.645692391	0.92257321	53	72	77	161	112	71	152	146
ENSECAG00000020774	3.141841668	0.645754482	0.92257321	89	97	257	258	151	113	251	185
ENSECAG00000009312	4.127871487	0.64577177	0.92257321	205	330	289	351	437	252	435	442
ENSECAG00000007465	5.821726186	0.645877351	0.92257321	616	1354	983	1405	838	1105	1161	1401
ENSECAG00000025125	5.734305046	0.646017323	0.92257321	764	686	1118	948	1622	1132	1270	722
ENSECAG00000007682	6.740847283	0.64603949	0.92257321	1916	2358	1591	1991	2663	1706	2380	1690
ENSECAG00000011195	3.192112919	0.646069378	0.92257321	85	169	167	308	254	119	211	131
ENSECAG00000014831	7.850121672	0.646186962	0.92257321	4809	5827	2770	3432	1385	7547	2650	5710
ENSECAG00000009669	4.539659841	0.64619665	0.92257321	403	403	422	478	463	375	639	395
ENSECAG00000020854	5.940913502	0.646246934	0.92257321	1344	764	981	1364	1343	886	1465	1206

ENSECAG00000013890	6.569844982	0.646714638	0.923125144	805	1606	1480	2780	1513	1667	2427	3036
ENSECAG00000013084	4.774011533	0.646878908	0.923184214	430	461	582	556	647	389	563	585
ENSECAG00000012267	4.478603554	0.646918196	0.923184214	341	668	287	332	311	319	520	627
ENSECAG00000024966	4.5837014	0.647338567	0.923540298	217	406	387	664	713	513	592	316
ENSECAG00000016353	4.869817177	0.647437052	0.923540298	389	389	576	621	578	547	698	786
ENSECAG00000019259	2.02701355	0.647480363	0.923540298	43	24	99	106	60	105	81	115
ENSECAG00000024729	8.1275708	0.647492197	0.923540298	3269	3475	6470	5443	8807	3581	9036	4392
ENSECAG00000018542	6.587625578	0.648042729	0.924060864	1280	1852	1585	1620	1832	2064	2492	2275
ENSECAG00000013019	2.396362726	0.648044459	0.924060864	89	46	85	114	148	119	82	114
ENSECAG00000021500	2.945432635	0.648100659	0.924060864	31	113	199	191	83	191	94	323
ENSECAG00000019104	3.467982467	0.648252532	0.924161222	100	131	244	294	169	279	300	242
ENSECAG00000013600	7.267946217	0.648333393	0.924161222	1180	2452	3142	3980	2427	4013	2989	4434
ENSECAG00000023271	5.303764666	0.648950852	0.924862962	422	678	749	866	856	896	956	799
ENSECAG00000009318	7.224213656	0.649008919	0.924862962	2337	2197	4252	2461	3258	2472	3917	2200
ENSECAG00000020146	2.658527615	0.649069395	0.924862962	69	79	92	189	136	75	190	172
ENSECAG00000009015	3.84655529	0.649242108	0.924993292	125.014	308	317	385	167.001	432	228	282
ENSECAG00000015831	6.387145433	0.649514143	0.92507625	1262	1367	1590	1187	2333	1436	2626	1213
ENSECAG00000005662	4.3704725	0.64956008	0.92507625	220	406	374	395	576	477	528	257
ENSECAG00000009976	5.339347786	0.649600057	0.92507625	639	867	673	807	910	714	969	645
ENSECAG00000008582	5.354174629	0.649681331	0.92507625	648	762	697	514	913	987	1029	719
ENSECAG00000007811	3.201853379	0.649706605	0.92507625	64	121	204	372	134	194	162	221
ENSECAG00000025208	1.586949755	0.649835937	0.925086566	24	36	77	106	30	92	27	68
ENSECAG00000008167	5.887516461	0.650076369	0.925086566	995	878	805	1155	1538	643	1617	1592
ENSECAG00000006839	6.802407506	0.650208431	0.925086566	1400	1823	2945	2431	1671	2188	2984	2066
ENSECAG00000000010	3.658493486	0.650315308	0.925086566	175	137	181	514	278	109	371	249
ENSECAG00000000878	2.077628893	0.650391952	0.925086566	87	77	68	69	54	110	54	98
ENSECAG00000008063	1.04162735	0.650429858	0.925086566	19.0027	12	51.0001	52.0029	51	45.0027	60	24.001
ENSECAG00000024994	7.274241799	0.650498079	0.925086566	1872	3348	2729	4065	2468	3015	3399	3368
ENSECAG00000023548	0.513782849	0.650560409	0.925086566	12	17	50	29	10	31	14	45
ENSECAG00000015440	6.427169098	0.65058369	0.925086566	2178	1612	1296	877	1387	1922	1023	2277
ENSECAG00000009583	8.566076676	0.650634599	0.925086566	5705	6322	8689	8171	6834	8805	6291	7495
ENSECAG00000022668	5.482059036	0.650675341	0.925086566	312	575	740	2182	492	679	704	1620
ENSECAG00000020589	5.627055813	0.650688908	0.925086566	616	965	998	1178	872	904	1153	1046
ENSECAG00000020655	4.982718411	0.650970785	0.925232349	480	707	526	618	608	529	708	690
ENSECAG00000000762	4.701443969	0.651061315	0.925232349	460	301	406	798	540	447	548	532
ENSECAG00000001560	2.336561926	0.651104353	0.925232349	34	100	82	128	156	69	124	102
ENSECAG00000018612	1.112441032	0.65111652	0.925232349	31	49	40	36	24	28	56	56
ENSECAG00000016704	4.72737735	0.651796298	0.926079775	306	614	413	429	563	539	763	511
ENSECAG00000011958	3.834636113	0.651912694	0.926079775	129	199	299	368	322	254	334	362
ENSECAG00000006431	4.048921368	0.652029405	0.926079775	126	409	317	453	390	283	318	312
ENSECAG00000018343	8.416055053	0.652122096	0.926079775	4793	6896	4791	5229	4871	14631	7025	4030
ENSECAG00000012706	5.712725726	0.65229497	0.926079775	539	973	1245	1315	1053	1075	982	1050
ENSECAG00000023627	2.679525012	0.652382933	0.926079775	28	113	128	178	143	211	94	113
ENSECAG00000021276	6.10885481	0.652403374	0.926079775	790	1297	1594	1632	1374	1332	1654	1156
ENSECAG00000020582	2.002786163	0.652584459	0.926079775	26	77	159	51	51	88	47	111
ENSECAG00000014375	2.79352108	0.652668971	0.926079775	66	144	113	227	98	74	146	225
ENSECAG00000008294	4.075341549	0.652727954	0.926079775	158	269	346	395	349	416	367	356
ENSECAG00000025075	4.995846006	0.652783539	0.926079775	487	482	772	638	689	641	771	444
ENSECAG00000007762	4.777675858	0.652796285	0.926079775	341	280	926	564	549	567	632	422
ENSECAG00000021736	4.342298384	0.652896648	0.926079775	196	380	487	515	339	474	448	347
ENSECAG00000023151	4.782309633	0.652993424	0.926079775	326	597	565	593	521	524	633	527
ENSECAG00000007497	2.787921543	0.653127658	0.926079775	184	35	83	79	355	79	171	31
ENSECAG00000018208	5.943479167	0.653135203	0.926079775	695	1441	998	958	1471	1401	1413	1195
ENSECAG00000025083	5.153275403	0.653297717	0.926079775	249	661	667	1333	980	585	672	561
ENSECAG00000014918	4.54457444	0.653328309	0.926079775	250	295	363	726	495	300	708	617
ENSECAG00000003503	4.802525404	0.653360427	0.926079775	386	439	565	734	720	391	647	477
ENSECAG00000012666	6.905966816	0.653614748	0.926079775	1657	1984	2299	1848	3348	1673	4329	1724
ENSECAG00000010810	6.757076529	0.653654499	0.926079775	1553	1354	1758	2516	3880	1585	2681	1634
ENSECAG00000009809	2.253335732	0.653826006	0.926079775	34	113	76	158	62	122	74	102
ENSECAG00000024231	2.775394213	0.653843465	0.926079775	85	108	125	213	104	125	111	191
ENSECAG00000006124	4.401298736	0.653944683	0.926079775	291	236	443	672	521	317	534	314
ENSECAG00000026810	3.025318672	0.654143011	0.926079775	97	126	109	216	132	118	240	248
ENSECAG00000013629	8.493282529	0.654201113	0.926079775	6939	4278	4792	6479	9035	3856	13791	7136
ENSECAG00000010534	6.729476597	0.654203442	0.926079775	1439	2023	2281	2318	1960	1850	2490	2169
ENSECAG00000012406	5.117186873	0.654203443	0.926079775	344	718	509	822	598	667	864	979
ENSECAG00000023011	4.743152231	0.654208277	0.926079775	304	377	464	707	512	468	583	827
ENSECAG00000013331	6.986130096	0.654313574	0.926079775	1345	3626	2287	2492	2016	2236	2111	3580
ENSECAG00000003146	3.285481669	0.654352154	0.926079775	141	242	164	172	130	178	201	259
ENSECAG00000007795	7.719817817	0.654352648	0.926079775	2349	3998	4163	3493	5929	3688	6141	3439
ENSECAG00000012845	5.680029118	0.654445327	0.926079775	1018	1062	743	863	1292	720	1497	601
ENSECAG00000021026	5.421552781	0.654478513	0.926079775	462	294	1188	1024	952	887	1165	859
ENSECAG00000016372	4.268099429	0.654617622	0.926118842	332	218	463	424	388	287	638	241
ENSECAG00000021731	3.196893637	0.654668813	0.926118842	132	101	224	243	175	137	240	176
ENSECAG00000026027	1.341292042	0.65476974	0.926145498	16	35	58	95	53	62	33	37
ENSECAG00000000548	11.10061127	0.654850352	0.926145498	14121	28875	31904	80016	14524	49102	123508	26191
ENSECAG00000006721	7.015441574	0.654992713	0.926231777	3323	2343	1899	1426	2410	3448	2188	1866

ENSECAG00000026934	3.224838118	0.655143185	0.92624938	49	241	184	280	154	203	171	195
ENSECAG00000015080	2.631711245	0.655348306	0.92624938	54	77	107	191	98	137	220	106
ENSECAG00000019938	4.153272032	0.655389655	0.92624938	210	295	391	480	236	226	545	438
ENSECAG00000013624	2.794517609	0.655393341	0.92624938	96	107	137	188	150	135	109	142
ENSECAG00000017040	5.489374823	0.655411946	0.92624938	554	901	851	1121	931	665	911	1094
ENSECAG00000010467	1.122759507	0.655818819	0.926709352	18	36	36	52	49	36	74	33
ENSECAG00000008668	3.004942528	0.655984609	0.926734955	45	49	468	97	121	296	80	79
ENSECAG00000022646	4.637573318	0.655999737	0.926734955	213	402	516	616	551	488	662	521
ENSECAG00000021222	7.324970101	0.656180378	0.926875138	1684	2735	3672	4610	2628	3147	3578	3343
ENSECAG00000014281	6.06062177	0.656346315	0.926994516	918	955	1555	1683	1760	1061	1447	1041
ENSECAG00000014124	5.436736195	0.656572877	0.927118971	568	549	691	1141	1287	486	1394	777
ENSECAG00000018805	5.747580216	0.6565973	0.927118971	645	910	881	1236	1198	1086	1442	1073
ENSECAG00000023693	4.765583176	0.656781424	0.927177381	284	425	589	569	811	501	628	472
ENSECAG00000021426	3.487842256	0.656843378	0.927177381	137	153	166	308	233	160	327	293
ENSECAG00000007600	6.700409281	0.656882982	0.927177381	1208	2585	1973	2190	1772	2637	1839	1882
ENSECAG00000015124	4.19814563	0.657129133	0.927391101	208	296	326	427	429	311	479	419
ENSECAG00000022038	6.888050121	0.657197313	0.927391101	1800	1762	2778	2661	2940	2225	1886	2195
ENSECAG00000012197	1.119957799	0.657309482	0.927434435	6	35	52	52	82	37	44	26
ENSECAG00000009063	5.419706549	0.657448132	0.927515115	611	848	907	818	971	794	985	673
ENSECAG00000024797	7.417892755	0.657687221	0.927737456	2321	3426	4205	2987	3690	3479	2828	3352
ENSECAG00000011827	6.000870461	0.657811363	0.927762707	1413	1730	589	789	1557	1789	850	656
ENSECAG00000021502	2.792550978	0.657868102	0.927762707	88	109	181	146	150	108	150	135
ENSECAG00000013712	6.105254027	0.65797997	0.927805543	1402	2561	369	1177	2	2485	1	1990
ENSECAG00000003816	9.376855715	0.658325069	0.927909586	2756	26193	13022	12161	5556	22150	4263	16872
ENSECAG00000014518	6.880619281	0.658329201	0.927909586	533	4270	1423	1432	603	5850	2489	1759
ENSECAG00000004034	4.500263975	0.658353104	0.927909586	541	282	259	240	614	180	1037	311
ENSECAG00000003601	6.797991737	0.658395872	0.927909586	1720	1483	2230	1783	3061	2690	2383	1797
ENSECAG00000011539	3.157510362	0.658615071	0.927909586	86	168	155	297	135	116	228	231
ENSECAG00000023292	5.857148424	0.658616994	0.927909586	596	1250	1039	1626	1046	1143	1318	1131
ENSECAG00000021989	4.88631907	0.658624274	0.927909586	378	1079	304	420	536	411	711	696
ENSECAG00000020805	5.154575398	0.658871144	0.928024946	409	760	694	854	737	715	653	726
ENSECAG00000000809	9.210144453	0.659070688	0.928024946	6710	9583	15762	14471	10342	11588	11453	13090
ENSECAG00000010652	0.428732832	0.659090711	0.928024946	7	11	26	44	37	22	37	19
ENSECAG00000010360	6.237720572	0.659337724	0.928024946	843	267	2856	2164	1853	1357	1538	1088
ENSECAG00000014473	7.634597674	0.659376499	0.928024946	2708	4815	2585	2469	7772	2498	4870	2986
ENSECAG00000008113	3.513314611	0.659420566	0.928024946	149	144	201	265	118	160	276	486
ENSECAG00000001496	5.642624359	0.659474432	0.928024946	854	810	902	1122	872	1103	1038	968
ENSECAG00000019625	3.898783821	0.659529261	0.928024946	157	261	307	288	373	252	388	317
ENSECAG00000020182	6.674668455	0.659641201	0.928024946	1242	1449	1946	3463	1914	1167	2852	2307
ENSECAG00000010536	0.876102638	0.659706839	0.928024946	14	10	51	45	27	39	61	34
ENSECAG00000019475	6.915239254	0.659747045	0.928024946	1162	1321	4181	2997	2769	2535	1891	2148
ENSECAG00000022469	10.64692711	0.659983309	0.928024946	15529	21776	32797	40350	37650	39687	30832	35653
ENSECAG00000015618	5.490049069	0.660004131	0.928024946	681	779	811	644	1383	813	1181	644
ENSECAG00000013864	5.993087075	0.660058782	0.928024946	1768	946	813	821	1015	1487	1501	1004
ENSECAG00000017197	5.733074249	0.660067291	0.928024946	565	919	964	1221	1248	1240	1267	959
ENSECAG00000016493	7.711867545	0.66008577	0.928024946	2122	3658	3734	4801	3744	5604	4175	5226
ENSECAG00000015087	2.603628731	0.660100353	0.928024946	66	67	132	144	118	91	230	115
ENSECAG00000004993	3.951952676	0.660218726	0.928024946	175	165	346	379	402	280	380	316
ENSECAG00000015135	8.912107751	0.660302821	0.928024946	6104	6395	10197	9389	11213	11571	9335	11064
ENSECAG00000007826	2.700967701	0.660503157	0.928024946	84	125	127	155	141	110	163	97
ENSECAG00000010149	6.958229545	0.660536647	0.928024946	1680	3012	1893	2877	1714	2716	2374	2984
ENSECAG00000001515	0.540483937	0.660549492	0.928024946	16	15	27	32	55	13	29	27
ENSECAG00000024304	4.217259979	0.660587996	0.928024946	271	474	281	345	436	383	353	291
ENSECAG00000005659	2.952986789	0.660662467	0.928024946	102	131	187	164	114	114	163	220
ENSECAG00000008889	2.046270701	0.661090637	0.928511831	38	47	108	82	87	112	107	58
ENSECAG00000022747	0.275482834	0.661482026	0.928832667	7	18	25	43	24	27	16	17
ENSECAG00000022163	4.853051846	0.661513614	0.928832667	484	629	400	609	360	492	689	783
ENSECAG00000018309	6.439615364	0.661744491	0.928832667	1896	1181	1115	1089	2292	1545	2721	1361
ENSECAG00000013840	3.106949116	0.661777789	0.928832667	109	197	131	216	130	165	153	226
ENSECAG00000003230	0.588482306	0.66177938	0.928832667	19	24	19	29	28	13	60	32
ENSECAG00000022534	6.873027563	0.661820834	0.928832667	2025	1684	2646	2385	2855	2031	2589	1788
ENSECAG00000005853	3.567599187	0.661976756	0.928832667	118	274	203	325	174	160	304	312
ENSECAG00000009331	4.05754174	0.662001353	0.928832667	214	290	272	506	308	249	328	442
ENSECAG00000010549	5.196454367	0.662070557	0.928832667	364	777	450	937	617	541	946	1212
ENSECAG00000001294	3.215139364	0.662338243	0.928832667	71	134	217	217	361	112	217	141
ENSECAG00000023207	0.26091078	0.662458678	0.928832667	18	10	18	25	29	19	29	24
ENSECAG00000011518	5.596949181	0.662480622	0.928832667	643	896	954	709	944	1163	1216	981
ENSECAG00000014227	4.759317345	0.66260617	0.928832667	285	324	681	582	576	532	642	660
ENSECAG00000007149	7.931013462	0.662618038	0.928832667	3191	4136	5630	5886	5032	4581	6323	3450
ENSECAG00000015691	3.884421453	0.662716935	0.928832667	179	143	394	442	371	251	328	214
ENSECAG00000013771	7.03728051	0.662723666	0.928832667	1784	463	4578	1471	6191	1831	3584	613
ENSECAG00000001651	5.966676471	0.662784242	0.928832667	1011	986	1220	1450	1005	1042	1356	1619
ENSECAG00000021097	8.826689894	0.662787579	0.928832667	6370	9212	9218	9771	7807	9927	9301	8634
ENSECAG00000012512	7.282842465	0.663076022	0.92899409	1884	3139	2999	4069	3199	3746	2610	2577
ENSECAG00000012486	6.55714621	0.663173444	0.92899409	936	1450	2112	1965	2441	1626	3081	1409
ENSECAG00000011306	1.024248203	0.663186657	0.92899409	19	32	48	56	45	30	58	20

ENSECAG00000000844	0.272153264	0.663258444	0.92899409	0	18	22	39	13	27	18	44
ENSECAG00000021485	1.032803662	0.663339	0.92899409	5	28	27	76	1	45	38	101
ENSECAG00000014231	5.576410288	0.663392354	0.92899409	385	604	1011	1416	1102	1010	1192	951
ENSECAG00000000637	3.963598777	0.66361595	0.929192915	127	188	391	391	298	330	461	311
ENSECAG00000020716	11.22518093	0.664009111	0.929374784	42093	49342	47185	38558	61653	35607	64762	26930
ENSECAG00000019055	2.153306706	0.664082612	0.929374784	33	51	72	154	84	59	107	149
ENSECAG00000012518	5.775932063	0.664158703	0.929374784	529	650	877	1837	1077	652	1508	1756
ENSECAG00000006704	2.946621425	0.664216268	0.929374784	122	111	150	196	149	78	225	164
ENSECAG00000006289	8.816646652	0.664365572	0.929374784	7091	8867	8741	9176	9525	8099	10373	7644
ENSECAG00000023239	6.875144155	0.66436635	0.929374784	1379	1633	2462	2493	2524	2346	2848	2842
ENSECAG00000002021	3.606275177	0.664385475	0.929374784	192	217	210	287	208	187	384	204
ENSECAG00000000035	5.299338561	0.66439889	0.929374784	529	756	604	1116	675	577	911	1014
ENSECAG00000020238	6.955919898	0.664962032	0.929892497	1464	6035	1189	511	1440	3802	2074	1987
ENSECAG00000017712	5.700353901	0.665013004	0.929892497	557	796	1169	1031	1584	657	1478	970
ENSECAG00000022979	4.580993934	0.665170481	0.929892497	190	550	415	746	427	614	450	387
ENSECAG00000018988	6.632600909	0.665274922	0.929892497	1382	1124	2049	2093	2859	1550	2464	2058
ENSECAG00000004246	3.683818224	0.665295599	0.929892497	165	182	259	256	309	269	298	260
ENSECAG00000003982	2.886589341	0.665302569	0.929892497	78	146	46	223	79	69	309	240
ENSECAG00000005996	0.343958097	0.665416241	0.929892497	13	22	26	31	17	24	28	23
ENSECAG00000014810	7.787075969	0.665489346	0.929892497	3489	4720	3640	4704	3032	4637	4646	5142
ENSECAG00000009053	4.207550277	0.665539949	0.929892497	205	114	412	553	614	251	506	299
ENSECAG00000018063	3.68042553	0.665585767	0.929892497	71	197	264	386	227	291	261	361
ENSECAG00000016518	7.166316659	0.665741008	0.929995262	2049	2504	3185	3177	2359	3332	2975	2660
ENSECAG00000007692	0.037369639	0.665977984	0.930212164	2	30	25	19	9	21	22	19
ENSECAG00000014614	4.893264412	0.666359782	0.930406954	376	441	454	767	547	468	884	780
ENSECAG00000018075	5.969441101	0.666397139	0.930406954	724	975	1100	1507	1630	1545	1184	1153
ENSECAG00000014071	3.450005937	0.666411755	0.930406954	171	177	170	188	207	178	394	214
ENSECAG00000011148	4.599928991	0.666444331	0.930406954	159	491	457	861	519	522	481	387
ENSECAG00000014836	2.976322999	0.66656005	0.93045441	103	186	158	138	119	101	210	197
ENSECAG00000014669	3.627625146	0.66665905	0.930465031	142	196	262	358	178	233	256	314
ENSECAG00000002434	3.064658946	0.666756292	0.930465031	54	162	155	310	111	174	175	193
ENSECAG00000024437	4.612511129	0.666812841	0.930465031	332	383	412	518	431	398	688	682
ENSECAG00000003514	0.086391016	0.667016075	0.930537487	15	11	25	9	25	23	20	20
ENSECAG00000011254	5.452171877	0.66716678	0.930537487	576	865	645	1291	671	388	1168	1328
ENSECAG00000000737	7.045771348	0.667217338	0.930537487	2699	2105	1624	1600	4114	1654	4443	1999
ENSECAG00000011959	2.07148102	0.667270005	0.930537487	23	73	65	187	80	54	111	80
ENSECAG00000017834	0.31065497	0.667326967	0.930537487	6	14	29	30	16	31	24	33
ENSECAG00000021397	4.101937525	0.667419106	0.930537487	228	258	335	328	475	292	424	334
ENSECAG00000006857	4.739601989	0.667436901	0.930537487	355	660	423	553	490	528	494	612
ENSECAG00000015868	6.889086067	0.667721294	0.930723712	1257	1720	2380	2784	3101	2036	3408	2191
ENSECAG00000018617	7.104672725	0.667733973	0.930723712	1735	2064	2984	3982	3107	1661	3345	2896
ENSECAG00000005229	6.484557932	0.668430857	0.931411214	1317	1775	1293	1461	1978	1517	2581	2020
ENSECAG00000018607	0.572686474	0.668458196	0.931411214	25	42	10	27	13	36	30	28
ENSECAG00000007133	3.459643741	0.668472642	0.931411214	72	299	373	138	39	174	35	565
ENSECAG00000010150	6.197480393	0.668715659	0.931608064	895.003	829	1727	1556	1916	1315	1852	1489
ENSECAG00000001645	5.204229677	0.66894109	0.931608064	418	361	1122	1002	752	531	1268	436
ENSECAG00000006660	2.846078111	0.669123123	0.931608064	74	103	128	186	170	172	162	129
ENSECAG00000022578	2.916971599	0.66913163	0.931608064	59	153	88	222	241	165	141	117
ENSECAG00000023150	8.117560228	0.669219251	0.931608064	2436	3430	6716	6736	5780	7578	5415	6030
ENSECAG00000009087	4.089532449	0.669220514	0.931608064	194	340	312	470	200	302	292	555
ENSECAG00000010557	2.705012878	0.669243308	0.931608064	60	74	136	181	131	112	178	162
ENSECAG00000015600	4.933676338	0.669268543	0.931608064	281	553	529	775	725	603	926	482
ENSECAG00000021489	4.304815463	0.669551834	0.931853801	251	569	318	327	240	361	442	535
ENSECAG00000023506	4.080168268	0.669608778	0.931853801	126	243	421	400	422.002	395	396	283
ENSECAG00000024554	4.063765868	0.669697408	0.931863235	151	295	311	399	174	429	406	484
ENSECAG00000015720	5.656473599	0.669894398	0.932005207	554	1048	831	996	774	977	1341	1435
ENSECAG00000022815	6.548894421	0.670074085	0.932005207	953	1100	2121	2357	1695	1987	2673	2089
ENSECAG00000008909	6.747871466	0.67007636	0.932005207	1609	1432	2136	1877	3166	1653	2674	2190
ENSECAG00000009215	6.057494171	0.670232316	0.932005207	1684	59	2185	994	2208	685	1635	539
ENSECAG00000008342	5.522650846	0.670309339	0.932005207	369	842	994	1008	1019	984	1459	671
ENSECAG00000020060	2.990746981	0.670429978	0.932005207	49	103	219	181	133	248	113	202
ENSECAG00000015739	6.487028029	0.670464716	0.932005207	1192	875	1845	2205	2156	1521	2614	1820
ENSECAG00000017933	5.959026254	0.670477153	0.932005207	613	1736	940	817	1061	2071	1400	989
ENSECAG00000007845	6.221954596	0.670564971	0.932005207	1260	1163	1054	1371	1688	1217	2314	1544
ENSECAG00000014347	5.699063218	0.670618064	0.932005207	527	1106	1041	1356	667	1151	1007	1314
ENSECAG00000018217	7.74115775	0.670950641	0.932117314	3241	3444	4116	5532	4331	2715	5400	4710
ENSECAG00000026829	2.428620375	0.67097369	0.932117314	63	66	105	125	170	72	121	114
ENSECAG00000008151	2.350068472	0.671020401	0.932117314	51	98	71	122	143	102	107	95
ENSECAG00000007621	7.597342138	0.671026219	0.932117314	1565	3506	1831	6683	2769	2088	4804	8328
ENSECAG00000022826	3.818106192	0.671279791	0.932355792	159	215	241	350	234	198	506	348
ENSECAG00000010579	0.76490795	0.671508972	0.93244106	18	38	16	33	24	39	38	45
ENSECAG00000000665	6.272007557	0.671577385	0.93244106	662	1379	1830	2273	1506	1677	1735	1226
ENSECAG00000022413	9.343916436	0.671586886	0.93244106	6588	7934	16048	13839	17621	14468	13624	12604
ENSECAG00000010197	3.080787312	0.671975579	0.932824497	83	105	242	140	197	148	249	165
ENSECAG00000008071	2.418167215	0.672055057	0.932824497	118	35	78	92	95	80	133	175
ENSECAG00000024134	4.477411961	0.672204286	0.932824497	292	380	429	381	688	431	530	322

ENSECAG00000017332	3.119586751	0.672383238	0.932824497	42	164	152	260	188	222	167	189
ENSECAG00000024309	4.084890927	0.672521157	0.932824497	77	295	355	490	315	546	257	370
ENSECAG00000017524	3.425565306	0.67255611	0.932824497	65	244	165	403	187	262	158	220
ENSECAG00000023731	4.229085603	0.672576375	0.932824497	229	234	335	493	438	261	516	468
ENSECAG00000024822	5.988791796	0.67267371	0.932824497	956	1460	1145	1144	698	1222	1069	2049
ENSECAG00000013180	2.621396466	0.672684742	0.932824497	35	66	217	100	197	63	188	112
ENSECAG00000013845	1.735839095	0.6728016	0.932824497	64	33	51	56	65	65	85	79
ENSECAG00000024780	4.709002723	0.672850655	0.932824497	299	567	432	451	621	346	970	449
ENSECAG00000007634	3.640734822	0.672921509	0.932824497	168	295	184	289	269	130	278	319
ENSECAG00000009877	0.965975179	0.672948946	0.932824497	28	16	28	51	41	24	70	38
ENSECAG00000023406	5.132307136	0.673010138	0.932824497	393	574	736	1015	717	696	726	666
ENSECAG00000015083	2.136470657	0.673183883	0.932844167	39	100	33	114	96	66	238	15
ENSECAG00000004120	3.35450236	0.673188202	0.932844167	139	127	260	251	194	196	218	200
ENSECAG00000009153	7.456905669	0.67346459	0.932919717	2567	4763	2099	3752	2778	3048	4757	3465
ENSECAG00000000314	5.435704064	0.673471989	0.932919717	444	701	859	999	1111	647	1260	868
ENSECAG00000007356	5.363410624	0.673488551	0.932919717	616	829	854	731	938	770	877	706
ENSECAG00000009133	2.254862922	0.67366837	0.933055278	38	111	93	152	9	83	8	243
ENSECAG00000000716	5.294549134	0.673854153	0.933199067	538	873	766	748	875	977	767	474
ENSECAG00000011238	2.367917787	0.674099631	0.933241957	42	58	136	117	117	121	125	92
ENSECAG00000021938	0.651557208	0.674176078	0.933241957	14	23	34	28	22	40	44	28
ENSECAG00000008403	10.65653079	0.674185122	0.933241957	11973	22059	34492	45300	41547	40542	28573	33549
ENSECAG00000006116	2.594957447	0.674213008	0.933241957	34	123	125	134	148	145	122	116
ENSECAG00000014264	3.889537672	0.674509563	0.933476002	153	255	301	447	241	247	377	327
ENSECAG00000020171	3.457746943	0.674546076	0.933476002	101	145	269	205	37	246	65	650
ENSECAG00000022359	3.553324191	0.674872795	0.933795568	131	192	231	357	205	210	276	246
ENSECAG00000024839	4.002777292	0.674941039	0.933795568	171	231	290	415	322	184	587	372
ENSECAG00000020054	1.017169527	0.675166008	0.933913579	23	26	40	64	46	31	36	38
ENSECAG00000007549	5.42617255	0.675190397	0.933913579	636	727	655	844	916	747	1092	1082
ENSECAG00000022302	3.368174286	0.675658478	0.934354254	170	194	123	164	293	114	412	131
ENSECAG00000024730	2.407031945	0.675675854	0.934354254	47	97	83	134	121	74	143	135
ENSECAG00000000263	3.376078067	0.675785605	0.934354254	129	206	217	229	196	191	211	225
ENSECAG00000023300	6.806546744	0.675837267	0.934354254	1694	2756	1482	2441	1736	1989	1983	3147
ENSECAG00000000187	5.217709352	0.676078748	0.934574617	793	539	627	693	680	838	804	638
ENSECAG00000011478	2.216401106	0.676176172	0.934595814	57	75	63	167	63	64	107	129
ENSECAG00000018267	5.303905739	0.676335386	0.934672577	582	934	571	848	569	546	1367	759
ENSECAG00000017545	6.557347185	0.676431193	0.934672577	1008	1344	2140	1963	2830	2078	1877	1548
ENSECAG00000009309	6.247991874	0.676478001	0.934672577	763	1310	1497	1681	1358	1969	1547	1848
ENSECAG00000000576	2.206105656	0.676923379	0.935174453	26	100	91	97	93	127	109	76
ENSECAG00000026889	4.809021896	0.677018944	0.935192997	342	465	631	700	510	386	743	641
ENSECAG00000013040	4.60131726	0.677109896	0.935205163	414	323	425	411	529	327	865	489
ENSECAG00000010946	3.493986636	0.677653938	0.935758091	76	197	283	227	197	264	277	263
ENSECAG00000009307	7.105857596	0.67778426	0.935758091	1416	2471	2470	3073	3445	2103	4152	2819
ENSECAG00000004664	1.545122731	0.677799179	0.935758091	44	57	39	74	45	42	82	58
ENSECAG00000017104	2.84988992	0.677838996	0.935758091	57	52	274	93	303	62	233	65
ENSECAG00000022502	6.282100344	0.678089237	0.935931709	897	1278	1415	2525	1335	931	2230	1837
ENSECAG00000008107	7.998312551	0.678253787	0.935931709	2995	5034	4836	7054	4666	5249	5649	4711
ENSECAG00000000040	3.214350896	0.678326769	0.935931709	154	124	188	225	162	120	304	163
ENSECAG00000020187	5.130862047	0.678356606	0.935931709	456	521	848	833	804	612	766	630
ENSECAG00000011565	5.346704048	0.678375798	0.935931709	415	553	845	1040	751	1019	914	909
ENSECAG00000017489	3.080847922	0.67881494	0.936239776	79	130	139	240	177	164	182	225
ENSECAG00000011742	4.816607019	0.67883838	0.936239776	364	536	485	499	683	686	707	412
ENSECAG00000024592	4.7719761	0.678855765	0.936239776	321	451	491	613	567	474	734	660
ENSECAG00000011826	5.65739251	0.678928027	0.936239776	596	826	904	1141	1303	1094	1061	984
ENSECAG00000001700	5.68293306	0.679327288	0.936298301	747	538	1214	918	1684	812	1278	815
ENSECAG00000018483	5.060893144	0.679366936	0.936298301	254	793	612	975	570	672	691	732
ENSECAG00000021243	7.274648572	0.679469991	0.936298301	3403	3226	1783	2634	3834	1917	3987	2588
ENSECAG00000011499	3.45091738	0.67957506	0.936298301	107	228	177	229	277	131	363	219
ENSECAG00000016128	11.1461848	0.679590353	0.936298301	25563	27593	47311	54380	61151	47619	44540	49754
ENSECAG00000001532	2.121103628	0.679629677	0.936298301	32	80	51	136	65	55	139	135
ENSECAG00000013736	6.697682214	0.679639592	0.936298301	1183	1857	2437	2552	2468	2082	1993	1663
ENSECAG00000003967	6.640382879	0.679688217	0.936298301	1566	2045	1858	1911	2259	1967	2104	1594
ENSECAG00000000252	4.595957755	0.679788639	0.936298301	223	491	572	587	481	516	393	522
ENSECAG00000024055	9.268822033	0.679852733	0.936298301	3449	15078	9023	13976	1557	26033	5317	22816
ENSECAG00000008790	4.24279663	0.679990882	0.936298301	130	293	200	735	191	239	404	900
ENSECAG00000007404	5.183812937	0.680073065	0.936298301	336	610	680	1264	730	645	878	672
ENSECAG00000014053	8.50055652	0.680138877	0.936298301	5350	6338	6211	9871	7949	4650	9232	7171
ENSECAG00000000012	6.237016326	0.680189019	0.936298301	882	449	1642	2262	2792	851	2019	1194
ENSECAG00000018454	3.90217634	0.680204062	0.936298301	172	290	310	225	320	314	281	401
ENSECAG00000008760	0.872756029	0.680633694	0.936684616	21	27	16	53	25	35	76	26
ENSECAG00000003947	1.707665294	0.680720684	0.936684616	26	39	65	93	88	69	71	56
ENSECAG00000009407	2.786354717	0.680791125	0.936684616	22	217	121	42	0	335	0	314
ENSECAG00000017904	6.937193007	0.680813808	0.936684616	1318	1896	2752	3722	2758	2335	2334	2252
ENSECAG00000011782	10.00693174	0.681064624	0.936865783	9035	16312	19608	26258	31214	29244	15915	14666
ENSECAG00000009178	5.717684648	0.681110065	0.936865783	818	1075	888	1107	1223	740	1415	886
ENSECAG00000000483	6.028538135	0.681276791	0.936914198	517	598	2034	2237	832	608	2902	1037
ENSECAG00000011424	4.568572651	0.681384269	0.936914198	217	341	518	594	496	520	640	451

ENSECAG00000000303	4.474135204	0.681392144	0.936914198	163	146	892	607	467	388	379	502
ENSECAG00000010666	2.264127639	0.681506033	0.936914339	49	50	109	118	91	95	141	100
ENSECAG00000019952	4.417957811	0.68183493	0.936914339	207	203	693	359	430	354	384	740
ENSECAG00000023057	6.339467418	0.68186558	0.936914339	930	1187	1312	2192	1750	1213	2295	2050
ENSECAG00000014270	3.4063781	0.681879829	0.936914339	157	209	201	217	181	138	260	272
ENSECAG00000000678	6.02190166	0.68191076	0.936914339	779	1469	1199	1471	1262	1284	1538	1141
ENSECAG00000011651	2.044823583	0.68196635	0.936914339	68	106	43	24	76	164	87	36
ENSECAG00000020036	5.834540703	0.681993911	0.936914339	833	741	1395	1336	1148	1109	1380	949
ENSECAG00000007738	2.02369449	0.682195437	0.936914339	42	58	51	125	108	64	101	86
ENSECAG00000017836	7.558911334	0.682205908	0.936914339	2551	3538	3119	3214	3991	4221	5300	3491
ENSECAG00000020148	2.341819649	0.682267182	0.936914339	80	21	165	122	112	60	160	63
ENSECAG00000023831	2.957727709	0.682332559	0.936914339	68	178	147	214	182	176	170	75
ENSECAG00000024530	5.816493205	0.682393473	0.936914339	546	714	1733	1451	1119	1504	1060	738
ENSECAG00000001462	4.209107813	0.682536188	0.936914339	139	351	436	358	522	396	418	296
ENSECAG00000023235	6.37966472	0.682544359	0.936914339	1242	2047	1262	1601	1537	1195	2294	1723
ENSECAG00000019051	5.016785955	0.682775079	0.936919778	469	420	621	656	773	362	822	954
ENSECAG00000000592	3.765280417	0.68280433	0.936919778	224	292	237	235	336	277	271	186
ENSECAG00000018517	4.656903346	0.682948074	0.936919778	400	607	408	415	401	490	687	458
ENSECAG00000024346	4.014132647	0.682954647	0.936919778	155	174	251	572	294	221	475	474
ENSECAG00000000811	3.713763702	0.683054719	0.936919778	133	241	241	282	265	369	303	215
ENSECAG00000024085	2.120998927	0.683166397	0.936919778	49	51	103	134	83	66	89	100
ENSECAG00000005707	2.568272473	0.683185299	0.936919778	67	117	79	130	66	114	173	180
ENSECAG00000011412	4.104541038	0.683206675	0.936919778	82	170	574	620	143	242	474	512
ENSECAG00000013265	5.031737025	0.683684448	0.937218928	463	544	739	699	833	621	581	564
ENSECAG00000021307	2.56184864	0.683703933	0.937218928	87	139	109	98	48	121	118	173
ENSECAG00000015556	0.48146069	0.683734239	0.937218928	9	19	24	61	0	28	14	54
ENSECAG00000022608	5.299731596	0.683824161	0.937218928	420	461	876	997	824	573	1241	921
ENSECAG00000022524	5.581546472	0.68383642	0.937218928	492	1060	764.004	1415	836	1254	691	977
ENSECAG00000018567	5.145086133	0.684023574	0.93736259	420	629	666	697	765	731	989	660
ENSECAG00000016412	7.48258816	0.68413281	0.937381674	2821	3115	2858	4766	2982	2556	4275	4544
ENSECAG00000018276	5.82005171	0.684244174	0.937381674	936	999	987	1238	964	1007	1494	1116
ENSECAG00000006802	0.426498616	0.684298556	0.937381674	8	12	32	34	8	33	55	21
ENSECAG00000012136	7.138077207	0.68436684	0.937381674	2045	2552	2755	3313	2336	2069	3771	3197
ENSECAG00000007103	5.564200504	0.684590687	0.937575481	592	979	930	1063	1044	856	943	951
ENSECAG00000018622	7.165737964	0.684699926	0.9376123	1974	2957	2766	3162	3399	2461	2938	2601
ENSECAG00000004270	3.755430415	0.684928101	0.937811958	261	241	259	205	173	192	496	240
ENSECAG00000017398	4.804732981	0.6853728	0.938069055	271	553	217	858	130	244	647	1594
ENSECAG00000012309	4.556065637	0.685480408	0.938069055	274	356	487	704	395	522	460	498
ENSECAG00000010818	5.856007333	0.685504771	0.938069055	1549	678	943	915	292	398	2841	1258
ENSECAG00000009214	5.822447526	0.685505881	0.938069055	745	1380	732	860	972	1497	1321	1231
ENSECAG00000003565	4.525980426	0.685527847	0.938069055	235	357	246	768	222	223	783	913
ENSECAG00000023299	5.193765345	0.68563919	0.938108663	394	662	738	1037	765	542	869	787
ENSECAG00000007175	3.409597295	0.685754066	0.938153094	164	128	266	237	269	125	240	211
ENSECAG00000008411	4.017455801	0.685868451	0.938196842	71	328	401	328	138	652	280	358
ENSECAG00000015197	3.337709088	0.686141587	0.938280426	83	217	187	310	166	253	171	198
ENSECAG00000020581	1.420448597	0.686173423	0.938280426	44	40	38	44	50	61	65	56
ENSECAG00000016476	4.002369224	0.686257582	0.938280426	146	294	393	414	393	291	337	252
ENSECAG00000012882	8.961093763	0.686314209	0.938280426	7185	6211	10128	9396	12395	8245	15210	9591
ENSECAG00000023180	7.357177886	0.686434413	0.938280426	1708	2213	3720	3641	3795	4194	3273	3292
ENSECAG00000009175	5.061283272	0.686480822	0.938280426	187	351	721	1204	610	678	929	788
ENSECAG00000001187	5.945978608	0.686506452	0.938280426	637.011	1413	1098.01	1602	1121	1212	1275	1331.02
ENSECAG00000014503	5.129370206	0.686801143	0.938377897	633	767	509	601	616	569	901	748
ENSECAG00000024130	6.285862042	0.686822423	0.938377897	986	914	2106	2039	2127	1100	1853	1163
ENSECAG00000003494	2.864475781	0.686897265	0.938377897	77	64	200	147	111	257	78	187
ENSECAG00000016814	7.31049024	0.68693057	0.938377897	2013	3194	2691	2591	4499	3158	3886	2678
ENSECAG00000014049	5.300603249	0.687055807	0.938377897	540	676	741	1023	976	704	808	667
ENSECAG00000014842	6.313347086	0.687148	0.938377897	1314	1345	1436	1808	1525	947	2226	1778
ENSECAG00000011422	4.721386751	0.687279744	0.938377897	390	829	511	165	632	540	493	394
ENSECAG00000011889	4.73474133	0.687307631	0.938377897	362	673	370	574	444	398	624	685
ENSECAG00000015119	6.039945538	0.687319568	0.938377897	805	315	1503	2755	1149	665	1841	1635
ENSECAG00000001101	5.694117376	0.687576111	0.938512258	486	884	1059	1765	213	841	900	2136
ENSECAG00000026844	4.604003985	0.68758285	0.938512258	333	325	739	421	486	496	515	441
ENSECAG00000023122	1.358331795	0.687936696	0.938708399	4	73	38	98	32	94	22.9999	31
ENSECAG00000008394	6.701230166	0.687936752	0.938708399	1644	2690	949	1277	2060	2136	2945	2293
ENSECAG00000023319	0.653107693	0.688043905	0.938708399	3	17	24	64	21	20	30	65
ENSECAG00000018110	4.696424959	0.688056354	0.938708399	271	382	578	796	524	448	627	491
ENSECAG00000020641	3.341965388	0.688240262	0.93883179	28	126	92	513	358	114	246	204
ENSECAG00000007486	2.911086002	0.688327592	0.93883179	103	121	127	141	144	79	237	222
ENSECAG00000019532	4.941648716	0.688394184	0.93883179	337	707	652	622	598	606	679	586
ENSECAG00000009344	8.514143217	0.688541799	0.938920634	5846	6933	7055	7438	6262	6479	8544	7906
ENSECAG00000013490	3.881695131	0.688624566	0.93892104	182	311	209	284	401	270	353	279
ENSECAG00000010272	5.163217644	0.688817457	0.939058537	364	399	745	1027	1036	579	893	680
ENSECAG00000025016	5.097744717	0.688890373	0.939058537	356	550	522	983	632	716	785	899
ENSECAG00000023583	6.063397392	0.689045863	0.939081398	1404	1640	707	973	1681	770	2032	906
ENSECAG00000022098	0.91281818	0.689072112	0.939081398	15	35	25	48	35	28	51	49
ENSECAG00000022303	4.667292013	0.689389305	0.939310142	324	329	570	506	497	475	658	630

ENSECAG00000000712	4.028652601	0.689404967	0.939310142	245	241	399	325	266	330	444	274
ENSECAG00000020649	4.757165502	0.689511714	0.939343169	306	406	575	573	577	387	754	704
ENSECAG00000022721	3.836420678	0.689824155	0.939569316	100	172	266	653	380	205	346	198
ENSECAG00000001893	2.61271056	0.689842768	0.939569316	82	124	54	140	148	146	154	92
ENSECAG00000009287	1.439220815	0.69019733	0.939935577	28	42	67	68	53	25	66	66
ENSECAG00000020948	4.966543676	0.690341169	0.939935577	526	626	489	635	665	487	675	692
ENSECAG00000012427	1.961629479	0.69050091	0.939935577	52	69	71	100	46	46	112	104
ENSECAG00000014769	6.946056578	0.690523317	0.939935577	1489	2013	3158	2853	2853	2309	3254	1425
ENSECAG00000001807	2.5906786	0.690580582	0.939935577	66	70	85	195	154	101	144	134
ENSECAG00000000390	6.017268116	0.690718431	0.939935577	610	838	1350	1796	1163	1459	1425	1697
ENSECAG00000021317	2.530988445	0.690742394	0.939935577	47	81	165	98	98	139	126	146
ENSECAG00000017466	0.710941226	0.690843809	0.939935577	33	16	28	38	19	21	36	46
ENSECAG00000008886	0.64324129	0.690867875	0.939935577	9	24	20	51	22	36	33	41
ENSECAG00000021995	4.48767021	0.690937272	0.939935577	256	383	458	428	526	255	668	572
ENSECAG00000008798	5.307269752	0.691056026	0.939968153	362.011	589	810.008	1042	826.001	889.001	1088	714.024
ENSECAG00000012970	6.123109505	0.691126342	0.939968153	1091	1192	1373	1522	1496	1557	1411	1082
ENSECAG00000013816	3.958419311	0.691339064	0.940145156	259	356	183	346	157	187	445	471
ENSECAG00000009409	3.901750944	0.691550417	0.940320255	296	224	211	199	345	163	442	405
ENSECAG00000011227	3.374271406	0.691761026	0.940494301	49	30	363	289	429	131	335	61
ENSECAG00000013215	0.856650597	0.692005444	0.940640172	27	22	33	50	31	36	29	38
ENSECAG00000023415	6.489827805	0.692138053	0.940640172	1338	1542	1985	1840	2110	1813	1561	1638
ENSECAG00000000551	5.04441353	0.69215359	0.940640172	274	344	612	1514	503	339	838	983
ENSECAG00000009762	4.476596866	0.692198802	0.940640172	66.8056	443.991	152.822	888.985	22.999	110	151.997	1841.4
ENSECAG00000003539	3.300354005	0.692412731	0.940818588	145	151	162	287	160	139	267	227
ENSECAG00000017251	6.08651547	0.692659123	0.940835168	996	1096	1321	1070	1864	1303	1750	1133
ENSECAG00000021399	2.902263185	0.692738798	0.940835168	59	208	105	213	134	198	110	131
ENSECAG00000024815	1.475992854	0.692812231	0.940835168	39	39	59	69	62	37	56	58
ENSECAG00000012933	6.160930457	0.692836119	0.940835168	296	1534	1354	1977	1180	1328	2116	1874
ENSECAG00000023369	7.240133547	0.692908279	0.940835168	2490	2403	2732	2077	3909	2930	3903	2845
ENSECAG00000018441	5.376761	0.692920763	0.940835168	387	608	912	1023	975	907	935	845
ENSECAG00000017585	4.577925951	0.693080503	0.940939843	240	497	428	692	453	255	551	674
ENSECAG00000018679	1.019967956	0.69317699	0.940958631	24	17	71	39	46	29	32	43
ENSECAG00000008987	5.016828481	0.693404356	0.941155054	339	599	640	930	586	707	560	726
ENSECAG00000005632	0.821418525	0.693592939	0.941156855	8	22	32	58	37	31	37	46
ENSECAG00000008231	2.982737699	0.693617278	0.941156855	38	220	148	129	188	271	129	101
ENSECAG00000010187	3.842373005	0.693653682	0.941156855	154	319	177	323	437	285	344	201
ENSECAG00000001043	6.082617081	0.69389504	0.941319699	637	896	1590	1646	1403	1286	1542	1800
ENSECAG00000019387	3.235912012	0.694001069	0.941319699	73	187	221	260	159	229	147	198
ENSECAG00000014026	3.468895174	0.69410324	0.941319699	79	256	135	298	143	131	298	436
ENSECAG00000008955	4.053218253	0.694104424	0.941319699	232	320	269	424	403	243	350	334
ENSECAG00000022300	1.8725507	0.694343204	0.941531369	39	52	54	145	95	23	82	83
ENSECAG00000017127	1.283070257	0.694546322	0.941694638	32	22	34	71	58	47	59	47
ENSECAG00000024426	5.783797291	0.694682967	0.941703143	793	1037	1133	1127	1175	1182	1135	915
ENSECAG00000011564	5.515545192	0.694786518	0.941703143	993	791	726	698	950	794	1091	844
ENSECAG00000010574	4.432317747	0.694871698	0.941703143	274	428	440	475	397	389	440	510
ENSECAG00000020605	2.767144425	0.694923602	0.941703143	72	70	68	256	56	57	110	401
ENSECAG00000020722	3.831886093	0.694966166	0.941703143	196	255	294	319	359	212	304	263
ENSECAG00000006686	4.70187054	0.695155909	0.9418231	231	272	407	1246	546	418	697	425
ENSECAG00000010981	6.871306234	0.695220144	0.9418231	1447	1830	2495	2097	3193	2396	2848	2007
ENSECAG00000000816	4.903336469	0.695327685	0.941856716	320	313	404	1458	233	224	590	1335
ENSECAG00000014189	4.769897864	0.695413806	0.941861311	291	428	639	504	500	924	597	365
ENSECAG00000020617	0.6478069	0.695690284	0.942123693	16	22	33	27	43	30	41	19
ENSECAG00000018796	8.031394468	0.695903313	0.942300098	2802	6176	5010	6239	5192	6060	5320	4067
ENSECAG00000020445	5.149711499	0.696301154	0.942614923	484	694	598	589	665	770	1058	671
ENSECAG00000016554	8.056610436	0.696301406	0.942614923	3353	5651	5028	6316	4215	5636	6086	5294
ENSECAG00000014338	11.63125339	0.696758838	0.943122028	91382	53565	35910	47179	1298	52633	11954	167738
ENSECAG00000014712	4.264527812	0.697028415	0.943167647	184	247	363	568	286	347	515	572
ENSECAG00000023968	6.222443008	0.697118064	0.943167647	880	789	1537	1996	2135	1389	1596	1495
ENSECAG00000010723	5.112001115	0.697222137	0.943167647	397	504	709	785	682	577	961	866
ENSECAG00000024907	5.824717581	0.697391139	0.943167647	635	872	1256	1127	1618	874	1587	986
ENSECAG00000004547	4.046073242	0.697426483	0.943167647	266	243	279	290	522	315	401	221
ENSECAG00000020091	6.088102005	0.697459817	0.943167647	1103	1071	1410	1458	1785	869	1711	1131
ENSECAG00000016589	5.221794	0.697697342	0.943167647	266	1052	651	952	687	941	823	509
ENSECAG00000012993	4.199782221	0.697728681	0.943167647	193	92	736	202	381	611	306	324
ENSECAG00000009310	2.873965907	0.697757231	0.943167647	156	89	140	137	131	133	126	183
ENSECAG00000007083	4.297698471	0.697771113	0.943167647	145	315	321	638	401	456	503	381
ENSECAG00000011683	6.377663849	0.697780857	0.943167647	1131	1817	1504	1763	1602	1865	1719	1451
ENSECAG00000016600	1.285735526	0.697786657	0.943167647	19	57	54	54	30	44	47	64
ENSECAG00000024439	4.296861847	0.697885149	0.943188797	288	306	421	448	416	337	387	436
ENSECAG00000011118	3.542597725	0.698070215	0.943219238	135	188	218	253	246	257	238	280
ENSECAG00000022742	7.244715584	0.698094115	0.943219238	2684	3698	2390	2181	3417	1919	3692	3129
ENSECAG00000019408	7.006473457	0.698156216	0.943219238	1315	2591	3103	2966	2182	1669	2872	3622
ENSECAG00000023160	2.595327857	0.698324982	0.943335302	43	96	91	266	83	65	154	176
ENSECAG00000010977	6.904606099	0.698409124	0.943337036	996	3410	2155	2784	2373	2950	2428	1670
ENSECAG00000014678	5.262946549	0.698762466	0.943492957	611	770	733	680	763	772	912	648
ENSECAG00000017464	6.428546213	0.698824006	0.943492957	1165	1490	1338	1822	1711	1704	2328	1953

ENSECAG00000022120	8.447459661	0.698887329	0.943492957	4007	5495	6249	8132	8239	7080	8829	7181
ENSECAG00000017376	5.992141543	0.699197444	0.943492957	649	1080	1099	2241	642	918	1418	2162
ENSECAG00000018645	6.991984541	0.699303261	0.943492957	1587	2915	2521	2602	2174	2898	2306	2693
ENSECAG00000013925	7.022869093	0.699358002	0.943492957	1702	1662	2579	2880	2340	2387	3673	3347
ENSECAG00000011340	3.569664378	0.699384639	0.943492957	124.011	244	155.008	405	229.001	183.001	248	286.024
ENSECAG00000021435	4.350419444	0.699458873	0.943492957	212	366	468	348	471	376	501	456
ENSECAG00000003116	2.826796695	0.69948641	0.943492957	65	101	144	254	144	95	201	126
ENSECAG00000019491	2.872015844	0.699538858	0.943492957	104	141	125	184	137	32	174	239
ENSECAG00000002087	5.677332328	0.6996166	0.943492957	697	887	968	1305	892	970	1142	1134
ENSECAG00000013408	3.266424146	0.699738519	0.943492957	127	169	120	228	135	155	332	249
ENSECAG00000016426	4.966214115	0.699801209	0.943492957	645	546	505	514	567	401	840	736
ENSECAG00000023592	8.511303993	0.6998061	0.943492957	4703	6007	7442	10111	8408	7077	6346	6926
ENSECAG00000024819	4.43553595	0.699848505	0.943492957	205	451	315	735	292	293	548	622
ENSECAG00000009956	0.786380454	0.699850507	0.943492957	2	36	31	46	10	42	34	63
ENSECAG00000014887	4.183987351	0.700312749	0.944004339	159	283	343	503	374	445	360	412
ENSECAG00000018674	0.306774907	0.700445023	0.944011279	14	10	22	31	28	25	18	31
ENSECAG00000020667	5.747849541	0.700483732	0.944011279	699	1025	1101	1219	1153	1270	985	865
ENSECAG00000010680	4.622955855	0.70067075	0.944082522	293	414	575	588	427	467	526	565
ENSECAG00000007955	9.289564363	0.700931568	0.944082522	9425	11089	10546	9556	12899	15750	14587	12734
ENSECAG00000008340	4.411081059	0.700993147	0.944082522	256	395	546	409	286	508	259	617
ENSECAG00000000963	4.124608462	0.701061625	0.944082522	199	277	473	377	359	406	307	307
ENSECAG00000013127	5.664457253	0.701076739	0.944082522	839	762	965	731	1426	764	1448	903
ENSECAG00000019411	6.227367232	0.701195942	0.944082522	406	4333	593	398	496	2247	727	2121
ENSECAG00000011758	6.326971176	0.70124146	0.944082522	1151	2009	1319	1430	1428	1730	1549	1695
ENSECAG00000016841	6.772607878	0.70139015	0.944082522	1182	2586	2200	2388	1762	2236	2661	2121
ENSECAG00000000629	3.82297509	0.701465843	0.944082522	177	150	315	325	405	220	358	273
ENSECAG00000015154	4.725838241	0.701466877	0.944082522	138	507	539	726	497	514	774	587
ENSECAG00000009182	4.085353004	0.701583238	0.944082522	219	164	379	401	406	414	363	305
ENSECAG00000005351	3.378508377	0.701703323	0.944082522	56	89	353	364	176	150	176	311
ENSECAG00000015977	6.484082394	0.701733181	0.944082522	1101	903	2822	2103	2271	1510	2191	1182
ENSECAG00000015965	7.014812744	0.701787794	0.944082522	1948	1382	2369	2974	3102	1962	3428	3178
ENSECAG00000009504	6.928678172	0.701819604	0.944082522	1423	1649	2488	2800	3452	2172	3145	2138
ENSECAG00000015193	7.178886483	0.701890989	0.944082522	1816	2567	3142	3593	3091	2085	3949	2588
ENSECAG00000025161	3.158769008	0.701946293	0.944082522	58	104	140	348	166	224	232	170
ENSECAG00000009499	3.240300905	0.702330495	0.944210583	91	114	238	209	211	221	269	136
ENSECAG00000012032	1.386842102	0.702464372	0.944210583	48	21	59	64	65	17	99	22
ENSECAG00000021884	3.95951833	0.702469052	0.944210583	143	278	248	424	354	346	285	375
ENSECAG00000014906	5.329831787	0.702530174	0.944210583	408	878	663	811.008	648.007	1194	913	778
ENSECAG00000011203	4.529606818	0.702556035	0.944210583	232	548	480	492	513	432	424	475
ENSECAG00000000471	3.310557404	0.702670993	0.944210583	90	193	146	258	181	129	311	274
ENSECAG00000023054	5.149298335	0.702679358	0.944210583	287	567	713	967	812	886	825	596
ENSECAG00000006511	0.5763193	0.702704987	0.944210583	17	4	56	13	29	30	44	26
ENSECAG00000022189	3.304219092	0.70292517	0.944314926	160	131	194	248	161	171	235	224
ENSECAG00000020387	1.683830545	0.703134143	0.944314926	56	55	33	55	69	58	105	52
ENSECAG00000010199	4.331867313	0.703522902	0.944314926	314	386	288	328	378	322	517	579
ENSECAG00000024532	6.719542938	0.703663557	0.944314926	1223	2000	2052	2857	1620	2023	2327	2499
ENSECAG00000016984	2.276840493	0.703823555	0.944314926	131	70	51	86	38	220	26	64
ENSECAG00000004212	4.389126214	0.703876333	0.944314926	247	315	436	619	549	272	509	361
ENSECAG00000012046	10.6322893	0.703974495	0.944314926	11506	22170	38891	39509	38059	41358	31787	30070
ENSECAG00000009759	7.360178395	0.704038271	0.944314926	2287	4016	3217	2657	3090	3191	3854	2997
ENSECAG00000011882	5.19308035	0.704056242	0.944314926	358	569	650	1002	1198	692	668	644
ENSECAG00000022500	9.626152972	0.704120994	0.944314926	10008	11398	15702	16149	20107	14059	22390	14855
ENSECAG00000011574	3.427570912	0.704147566	0.944314926	111	205	243	264	182	205	211	258
ENSECAG00000018146	5.389557591	0.70420139	0.944314926	568	644	700	943	1058	604	1131	944
ENSECAG00000017543	4.73563995	0.704351987	0.944314926	347	391	673	595	453	468	654	586
ENSECAG00000013933	0.36627819	0.704431335	0.944314926	5	36	20	19	15	43	24	25
ENSECAG00000016280	4.330831158	0.704467182	0.944314926	130	545	382	529	248	265	461	648
ENSECAG00000023381	3.916497129	0.704470519	0.944314926	130	297	361	386	277	184	360	399
ENSECAG00000017033	7.139166419	0.704573727	0.944314926	1359	2361	2835	3225	2852	3770	3186	2692
ENSECAG00000011804	0.888988536	0.704667986	0.944314926	7	34	14	69	9	12	35	109
ENSECAG00000017531	7.578409459	0.704682265	0.944314926	3226	2942	3986	4042	3344	4252	4766	2901
ENSECAG00000006430	10.7489005	0.704686468	0.944314926	15202	24091	36723	44579	42269	43383	31522	35520
ENSECAG00000009275	6.180973522	0.704695191	0.944314926	927	1171	1656	1755	1513	1232	1735	1390
ENSECAG00000017830	5.666746474	0.704807698	0.944314926	498	714	1278	1500	770	922	1024	1374
ENSECAG00000001300	1.899164408	0.704819514	0.944314926	29	71	51	145	59	83	70	74
ENSECAG00000011056	2.021025298	0.704841013	0.944314926	36	88	87	99	61	55	126	80
ENSECAG00000019674	6.723147704	0.704878379	0.944314926	975	1679	2442	3298	1979	2289	2065	2065
ENSECAG00000010781	3.568567288	0.70498947	0.944314926	177	250	211	231	207	185	285	278
ENSECAG00000026861	3.845297147	0.705060557	0.944314926	158	217	280	338	326	295	275	362
ENSECAG00000007142	6.102030946	0.705105067	0.944314926	701	1214	992	1895	882	1187	1751	2368
ENSECAG00000001072	4.66041029	0.70522863	0.944369319	302	558	321	533	485	686	522	520
ENSECAG00000010167	2.673102755	0.705361329	0.944422695	73	127	97	128	118	95	182	174
ENSECAG00000024809	3.865535027	0.705434396	0.944422695	146	209	307	355	334	246	329	378
ENSECAG00000019520	6.59309924	0.705765108	0.944754351	1315	1400	1983	1780	2784	1687	2499	1641
ENSECAG00000019772	6.690129183	0.706133731	0.945008013	1933	1648	1314	1680	3118	1981	2368	1722
ENSECAG00000015700	5.238887824	0.706151822	0.945008013	410	607	677	944	960	586	1012	799

ENSECAG00000021195	6.806266572	0.706234207	0.945008013	1541	1493	1873	2673	3006	1382	3373	2383
ENSECAG00000021191	6.849717206	0.706300405	0.945008013	1728	1631	1860	2479	2596	1657	3493	2703
ENSECAG00000019719	9.892186646	0.706369626	0.945008013	9628	12500	21143	22575	22445	22651	17794	21501
ENSECAG00000000302	6.586888188	0.706570853	0.945136481	740	1408	2653	2910	1594	2138	2075	1859
ENSECAG00000008680	7.879662694	0.706834728	0.945136481	1939	3178	4975	6651	5640	4646	6544	4372
ENSECAG00000015376	2.996774969	0.706878929	0.945136481	57	129	180	191	122	239	137	197
ENSECAG00000011200	5.513653492	0.706983242	0.945136481	540	586	927	1108	749	555	1734	1142
ENSECAG00000020653	5.417955743	0.706983983	0.945136481	407	466	1098	1055	904	857	991	1033
ENSECAG00000017322	2.421155793	0.706997264	0.945136481	35	109	111	172	65	90	81	179
ENSECAG00000012218	3.228157375	0.707139835	0.945136481	94	135	187	233	235	142	252	203
ENSECAG00000003090	4.586473621	0.707298987	0.945136481	234	147	775	515	765	480	548	339
ENSECAG00000014700	10.39742156	0.707343933	0.945136481	22059	26540	26783	24205	26947	25075	27670	27451
ENSECAG00000020357	4.041759088	0.707371342	0.945136481	143	249	271	502	142	328	440	573
ENSECAG00000024654	7.662311793	0.707416001	0.945136481	1984	5364	2318	4019	4654	4706	5761	3105
ENSECAG00000026983	2.57710399	0.707461844	0.945136481	48	125	88	147	203	125	115	77
ENSECAG00000020237	6.180964675	0.707758006	0.945348679	797	1090	1389	1780	1588	1098	2076	1747
ENSECAG00000019540	3.2677169	0.707786749	0.945348679	73	115	222	275	163	194	253	246
ENSECAG00000013755	6.289049705	0.708123778	0.945581633	1300	1158	1574	1758	1900	1160	2084	1191
ENSECAG00000012598	4.670980924	0.708191598	0.945581633	328	395	528	688	703	420	457	452
ENSECAG00000002550	1.636964812	0.708276593	0.945581633	22	57	56	76	80	69	46	71
ENSECAG00000013711	6.01214204	0.708347241	0.945581633	797	1004	1028.01	2214	1903	1190	1089	922
ENSECAG00000021716	6.085178255	0.708376438	0.945581633	591	1569	1105	1454	1551	1273	1604	1601
ENSECAG00000020374	1.824125265	0.708662829	0.945716777	29	16	156	26	119	38	135	32
ENSECAG00000016706	4.344180258	0.708798841	0.945716777	275	107	570	399	702	170	824	186
ENSECAG00000019637	4.549825305	0.708847683	0.945716777	205	322	244	930	512	334	594	661
ENSECAG00000012353	4.269375542	0.708853921	0.945716777	139	323	313	609	294	275	426	733
ENSECAG00000016447	6.232465643	0.708898441	0.945716777	1428	1996	792	1115	1326	1261	1932	1544
ENSECAG00000025132	0.815860554	0.709020823	0.945716777	21	45	13	29	39	26	50	37
ENSECAG00000021279	6.14563164	0.709059149	0.945716777	1289	853	1473	1581	1484	1353	1604	1249
ENSECAG00000021753	1.927659416	0.7092293	0.945816351	65	82	60	64	65	45	85	103
ENSECAG00000021923	4.36853733	0.709299957	0.945816351	341	301	408	471	308	310	577	492
ENSECAG00000021199	4.462737751	0.709492499	0.945858253	256	438	518	444	470	488	427	374
ENSECAG00000025100	7.201338117	0.709497539	0.945858253	2160	2234	2498	2890	4036	2308	3993	2894
ENSECAG00000015515	4.335515074	0.709596335	0.945879203	234	308	421	592	332	312	448	542
ENSECAG00000005666	6.131499273	0.709993378	0.94621152	917	985	1268	1631	1581	993	2072	1657
ENSECAG00000007172	1.385972245	0.710125567	0.94621152	7	18	94	61	33	55	78	66
ENSECAG00000008250	5.855348608	0.71014643	0.94621152	765	1027	1107	1478	1311	1120	985	1210
ENSECAG00000022532	6.50863956	0.710208297	0.94621152	996	2014	1607	2376	1381	1809	1998	2145
ENSECAG00000010024	2.395239241	0.710395195	0.94621152	106	65	120	84	133	128	57	80
ENSECAG00000008865	3.791145698	0.710504531	0.94621152	113	279	225	355	240	325	298	354
ENSECAG00000012163	6.759491657	0.710582738	0.94621152	1829	2305	1492	2332	1702	2297	2226	2428
ENSECAG00000008577	5.811099447	0.710747316	0.94621152	953	1114	859	1162	1004	829	1374	1362
ENSECAG00000010128	7.680216957	0.710762542	0.94621152	3466	2964	3012	6141	5660	2224	5331	3265
ENSECAG00000013380	2.498359081	0.710808901	0.94621152	63	83	149	135	76	113	134	123
ENSECAG00000023647	6.660880173	0.710832574	0.94621152	774	2325	1601	3408	1304	2188	2063	2514
ENSECAG00000014481	5.4701446	0.710842963	0.94621152	470	764	901	1298	698	851	1081	969
ENSECAG00000018946	4.139034801	0.711009692	0.946275824	146.007	280	363	616	321	416	305	351
ENSECAG00000006861	4.516518095	0.71107477	0.946275824	462	441	350	358	342	418	615	481
ENSECAG00000016030	5.01848398	0.711140619	0.946275824	438	413	683	939	539	526	847	731
ENSECAG00000020558	3.296615625	0.711237242	0.946293795	114	185	143	330	41	154	234	352
ENSECAG00000023262	5.465193482	0.711328762	0.946304973	560.011	724	657.008	1109	952.001	946.001	1001	987.024
ENSECAG00000020637	9.527126799	0.711711538	0.946476338	8813	15694	15172	16836	16488	14547	15695	11857
ENSECAG00000018017	3.759210133	0.71185294	0.946476338	166	133	328	295	415	176	426	199
ENSECAG00000015016	1.767313632	0.711869422	0.946476338	6	40	69	118	167	109	0	19
ENSECAG00000022003	7.617608649	0.711872391	0.946476338	2894	3311	2474	4418	4357	3130	4982	5216
ENSECAG00000012093	5.823445393	0.711900262	0.946476338	509	1031	1146	1778	757	1118	1337	1370
ENSECAG00000008895	5.451902058	0.711981701	0.946476338	770	613	1157	621	1010	968	738	749
ENSECAG00000011593	6.872985602	0.712039511	0.946476338	2388	2074	1445	2563	2129	2563	2210	2387
ENSECAG00000020120	5.223063018	0.71237479	0.946811461	604	741	626	750	670	733	724	878
ENSECAG00000006358	2.898116724	0.712625498	0.947034119	82	102	114	213	107	80	191	294
ENSECAG00000024719	8.611186591	0.712857896	0.947126551	8808	11705	3952	2681	5541	4993	17335	3959
ENSECAG00000023316	6.564467626	0.712874917	0.947126551	1236	1803	1612	2552	2265	1659	1946	1719
ENSECAG00000010312	2.850197674	0.712944624	0.947126551	50	117	104	326	76	60	181	256
ENSECAG00000022346	4.321449994	0.713223505	0.94738649	235	87	517	539	741	286	612	170
ENSECAG00000017895	7.629952075	0.713756957	0.947766695	3147	3516	3008	5242	3073	2887	4852	5202
ENSECAG00000008126	6.526538876	0.713798156	0.947766695	1231	1346	1785	1868	2658	1319	2907	1421
ENSECAG00000019840	6.409064361	0.713888929	0.947766695	845	1176	1708	2280	1808	1672	2126	1948
ENSECAG00000020652	3.738711307	0.713952703	0.947766695	176	163	237	337	334	180	353	321
ENSECAG00000021111	6.386916408	0.714034979	0.947766695	1201	1338	1453	1620	2289	1565	1948	1601
ENSECAG00000020575	5.54998342	0.71405643	0.947766695	531	813	1171	1048	741	1009	816	1186
ENSECAG00000023041	4.748701018	0.7141318	0.947766695	221	638	478	758	451	419	642	669
ENSECAG00000015150	8.934296371	0.714295449	0.947766695	5409	6955	12753	13233	10358	9049	9344	10062
ENSECAG00000023954	6.629210963	0.714319085	0.947766695	923	1865	2038	2980	1260	1561	2261	2926
ENSECAG00000013036	0.852558101	0.714342206	0.947766695	18	13	58	26	48	28	29	48
ENSECAG00000009401	1.484925044	0.714571387	0.947960293	57	48	46	43	52	66	49	45
ENSECAG00000006724	3.408426387	0.714667684	0.947977582	120	161	221	319	200	114	270	274

ENSECAG00000006424	3.462960014	0.714911289	0.948156192	114	132	281	233	284	252	242	187
ENSECAG00000026807	3.224146609	0.714968898	0.948156192	115	189	188	207	153	199	196	195
ENSECAG00000000376	1.945856775	0.71521628	0.948166888	52	52	72	76	78	82	98	78
ENSECAG00000012988	4.302087709	0.715220744	0.948166888	355	363	358	335	413	294	509	385
ENSECAG00000021117	2.425244917	0.715296841	0.948166888	43.0106	100	133.008	141	90.0006	125.001	116	88.0239
ENSECAG00000016974	1.573140631	0.715436532	0.948166888	34	113	28	40	35	43	100	54
ENSECAG00000022703	7.232643659	0.715447362	0.948166888	3000	2234	2197	3607	2907	1357	5029	3018
ENSECAG00000020397	7.924977111	0.715476656	0.948166888	5650	4713	3161	3476	6028	3415	6565	3458
ENSECAG00000010077	2.436526206	0.715650528	0.948210819	69	58	140	88	107	71	219	95
ENSECAG00000022177	4.035574573	0.715676379	0.948210819	255	335	265	348	415	178	380	351
ENSECAG00000013515	3.202480997	0.715790415	0.948251557	125	359	99	86	71	369	96	149
ENSECAG00000010788	5.370675389	0.715987477	0.94840226	317	848	946	1134	867	734	831	896
ENSECAG00000019556	4.302486163	0.716302677	0.948706732	243	453	229	582	241	266	480	614
ENSECAG00000024639	3.926263527	0.716383994	0.948706732	220	255	298	360	327	161	420	331
ENSECAG00000015188	5.160392205	0.716503184	0.948720189	925	642	312	561	802	619	866	576
ENSECAG00000013091	7.842599232	0.716560817	0.948720189	1884	2331	3026	9570	4681	3722	5994	6434
ENSECAG00000023400	2.063700837	0.716645356	0.948721788	28	88	91	120	74	69	66	114
ENSECAG00000024040	5.641237185	0.716787302	0.948799376	700	1142	667	788	1053	774	1512	1140
ENSECAG00000005760	5.978735616	0.716907374	0.948807966	853	1401	1056	1393	915	940	1681	1624
ENSECAG00000017775	2.662641862	0.717001687	0.948807966	65	63	123	189	158	72	156	177
ENSECAG00000003277	7.864528042	0.717043807	0.948807966	3002	2395	5233	5222	6548	2602	7164	4915
ENSECAG00000016499	4.97777633	0.717199943	0.948900702	436	728	510	660	479	834	451	726
ENSECAG00000009113	7.320576718	0.717280583	0.948900702	3156	2734	2825	2771	3176	3380	3287	2870
ENSECAG00000018596	1.08758762	0.717682311	0.949221839	18	34	35	54	26	32	81	48
ENSECAG00000013740	8.379107286	0.717707632	0.949221839	3322.01	4577	8276.01	6819	7067	8426	6643	7360.02
ENSECAG00000005293	5.229573882	0.717777339	0.949221839	566	700	615	895	649	629	885	891
ENSECAG00000016728	4.172350323	0.71787116	0.949221839	207	374	264	535	377	273	390	414
ENSECAG00000014115	0.308256984	0.717940207	0.949221839	7	17	37	32	12.0005	29.0013	21	26
ENSECAG00000019650	4.810526942	0.718108148	0.949333635	420	586	464	593	537	328	721	709
ENSECAG00000016854	7.033355881	0.718387552	0.949592741	3982	1550	811	860	2668	4162	2897	2050
ENSECAG00000010934	2.730314339	0.718925604	0.949855015	83	53	210	84.0001	178	78	306	52
ENSECAG00000007234	5.075059228	0.71893568	0.949855015	324	253	1114	648	1224	582	775	410
ENSECAG00000024263	2.023249581	0.718953012	0.949855015	28	111	62	114	49	70	88	110
ENSECAG00000013992	3.000200721	0.71897686	0.949855015	113	92	154	263	62	58	180	338
ENSECAG00000001385	7.333447199	0.71900312	0.949855015	2465	2425	3218	4071	4252	2423	3609	2634
ENSECAG00000018318	9.554736918	0.719326524	0.950172001	14320	13011	13573	13793	15901	18490	12557	11960
ENSECAG00000020665	11.96726308	0.719607037	0.950330046	40119	47777	92804	98086	102738	103807	72406	74900
ENSECAG00000001608	1.203071686	0.719680959	0.950330046	8	47	37	66	65	46	47	39
ENSECAG00000014093	4.095581497	0.719708878	0.950330046	192	342	357	402	273	363	332	402
ENSECAG00000016632	5.151469058	0.71978006	0.950330046	527	412	701	769	742	539	998	893
ENSECAG00000010430	6.459590865	0.719928853	0.95041628	1115	1548	1538	1784	2357	1623	2201	1619
ENSECAG00000024789	5.464439754	0.720138315	0.950487835	771	1048	617	726	1090	811	1090	562
ENSECAG00000005528	7.071158874	0.720191371	0.950487835	1708	1680	3538	3432	2670	2484	2582	2998
ENSECAG00000007412	0.873931565	0.720502603	0.950487835	38	17	29	44	27	39	26	44
ENSECAG00000015633	2.333431482	0.720587456	0.950487835	39	88	47	179	61	120	84	177
ENSECAG00000013819	6.741311763	0.720657899	0.950487835	1726	2043	1476	2741	1895	1239	2573	2980
ENSECAG00000011726	2.496361147	0.720909263	0.950487835	87	109	66	99	96	68	207	140
ENSECAG00000002783	3.738430276	0.720909508	0.950487835	133	169	286	350	262	232	389	305
ENSECAG00000025106	5.947473687	0.72092079	0.950487835	847	1102	897	1319	782	1605	1298	1760
ENSECAG00000012746	0.560419536	0.721027321	0.950487835	13	22	24	52	21	29	35	24
ENSECAG00000022411	4.108250367	0.721104659	0.950487835	230	222	353	371	335	319	416	452
ENSECAG00000020852	5.698970481	0.721152803	0.950487835	909	580	952	1418	1068	986	1045	1078
ENSECAG00000019611	3.641258468	0.721168571	0.950487835	133	121	347	259	348	167	330	266
ENSECAG00000015415	5.632806456	0.72125061	0.950487835	753	1098	689	690	1310	674	1598	885
ENSECAG00000023073	5.00284319	0.721265007	0.950487835	416	588	599	816	786	354	705	759
ENSECAG00000008516	4.58915939	0.721343163	0.950487835	260	356	659	568	613	445	474	393
ENSECAG00000009658	7.018567762	0.721394436	0.950487835	1434	3256	2453	2737	1793	3890	2247	2248
ENSECAG00000008129	7.826975895	0.721406948	0.950487835	2586	3941	4669	4250	4921	4385	5883	5134
ENSECAG00000021193	6.35530236	0.721485803	0.950487835	1061	1376	2059	1662	1511	2110	1425	1451
ENSECAG00000000279	4.523242181	0.721718166	0.950683943	303	306	529	604	505	457	419	459
ENSECAG00000024424	6.379379273	0.721938662	0.950774655	1304	1645	1480	1684	1317	1426	2219	1829
ENSECAG00000022271	2.205869817	0.721954053	0.950774655	37	115	53	158	71	42	89	159
ENSECAG00000014136	3.654134022	0.722161159	0.950937404	188	260	209	267	228	248	206	318
ENSECAG00000007936	1.284907837	0.72232059	0.950957033	29	66	24	32	51	84	38	33
ENSECAG00000021027	7.788036168	0.722357091	0.950957033	3954	4014	3662	4528	3724	3690	5536	4910
ENSECAG00000000407	5.062886871	0.722491746	0.950957033	519	624	555	768	522	411	870	938
ENSECAG00000018536	5.128379143	0.722510174	0.950957033	183	504	930	929	667	764	782	880
ENSECAG00000008938	5.253334509	0.722751582	0.950985121	307	738	862	774	851	931	781	770
ENSECAG00000003760	0.965701548	0.722851247	0.950985121	26	47	23	22	36	19	83	36
ENSECAG00000020139	7.953598499	0.722896668	0.950985121	4828	4277	4475	4272	5312	5071	5734	3661
ENSECAG00000020723	2.23791113	0.722908651	0.950985121	50	78	120	115	23	91	72	178
ENSECAG00000010954	5.680806269	0.723083289	0.950985121	515	902	1005	1172	1235	881	1293	1128
ENSECAG00000009482	6.740591522	0.723086119	0.950985121	925	1785	2009	3873	2636	1751	2128	2027
ENSECAG00000004257	3.946901588	0.723177212	0.950985121	153	215	286	437	380	280	386	313
ENSECAG00000010999	2.808842129	0.723199752	0.950985121	90	71	199	184	81	115	37	301
ENSECAG00000013562	5.599229044	0.723426616	0.950993234	653	854	782	1007	902	861	1426	1132

ENSECAG00000010862	8.096819722	0.723591232	0.950993234	3902	7506	4484	4242	6424	5072	6081	4235
ENSECAG00000009953	4.194816204	0.723619118	0.950993234	183	291	304	509	196	390	514	535
ENSECAG00000018457	2.657428388	0.72366693	0.950993234	76	115	127	160	53	104	144	201
ENSECAG00000017751	5.837149893	0.72375938	0.950993234	487	887	1154	1559	1600	1108	1305	1020
ENSECAG00000019064	6.841190025	0.723856871	0.950993234	1769	2374	2185	2098	2409	2122	2658	2033
ENSECAG00000012790	0.566701398	0.723873137	0.950993234	13	17	11	57	27	12	47	42
ENSECAG00000016587	3.686070538	0.723874164	0.950993234	130	260	163	338	187	184	439	354
ENSECAG00000016957	2.953524851	0.724129618	0.951055812	96	166	168	144	204	137	121	145
ENSECAG00000017729	5.17476202	0.724197956	0.951055812	455	515	656	870	738	470	1167	873
ENSECAG00000007078	3.162389582	0.72426503	0.951055812	192	155	117	166	118	166	273	164
ENSECAG00000000016	5.018269053	0.724419533	0.951055812	502	492	495	689	590	656	841	778
ENSECAG00000002176	4.843489068	0.724640597	0.951055812	306	1085	395	326	349	363	1047	601
ENSECAG00000000603	3.307214937	0.724786182	0.951055812	108	156	170	339	111	189	201	288
ENSECAG00000006493	1.759874323	0.724955759	0.951055812	44	36	81	94	69	72	62	57
ENSECAG00000012841	1.048729	0.724960148	0.951055812	12	41	39	69	22	18	66	54
ENSECAG00000024555	5.152166595	0.72498412	0.951055812	606	614	576	794	771	541	870	714
ENSECAG00000000212	4.610857763	0.724987482	0.951055812	278	431	541	608	377	398	745	488
ENSECAG00000024758	4.722673918	0.7250143	0.951055812	591	336	407	539	507	224	824	613
ENSECAG00000018336	7.150722086	0.725029481	0.951055812	1936	1871	2606	4603	2842	2284	3162	3148
ENSECAG00000017916	7.427227894	0.725063734	0.951055812	2137	3927	2943	4043	2836	3853	3924	3174
ENSECAG00000015145	3.306065148	0.725131775	0.951055812	183	192	99	146	172	318	221	154
ENSECAG00000020855	7.68336947	0.725174835	0.951055812	2405	6854	2554	3551	2843	3859	4210	5487
ENSECAG00000016107	2.802850983	0.725457314	0.951269582	82	168	102	176	49	159	143	198
ENSECAG00000013646	-0.03329572	0.725519016	0.951269582	1	23	9	29	17	33	21	8
ENSECAG00000013433	5.380103892	0.725588498	0.951269582	239	485	1013	1326	707	1163	882	911
ENSECAG00000015859	0.79985283	0.7257493	0.951273649	19	18	33	42	18	22	68	45
ENSECAG00000010798	5.413116776	0.725845566	0.951273649	497	1029	778	881	749	680	1202	863
ENSECAG00000021885	6.936202435	0.725856427	0.951273649	1298	1692	2804	2705	2925	2331	3473	2232
ENSECAG00000009054	4.688741029	0.72592582	0.951273649	361	550	503	530	258	709	105	901
ENSECAG00000003570	3.494185811	0.726021635	0.951281381	110	180	267	219	200	286	302	203
ENSECAG00000019985	1.378143446	0.726336686	0.951281381	31	49	41	44	47	14	100	73
ENSECAG00000021972	2.686622734	0.726433563	0.951281381	71	71	153	144	197	104	155	109
ENSECAG00000015816	11.9102434	0.726629598	0.951281381	44746	50779	102547	102818	94524	76549	58433	72856
ENSECAG00000022803	2.279171148	0.726675043	0.951281381	70	43	126	128	79	53	194	66
ENSECAG00000020066	6.270900255	0.726802434	0.951281381	828	1743	1347	1333	2003	1851	1380	1509
ENSECAG00000009117	7.758422289	0.72680676	0.951281381	3169	3717	4056	5395	5286	3333	4795	3979
ENSECAG00000008458	8.083543627	0.726827104	0.951281381	3401	4743	3956	6389	6701	4143	7325	6224
ENSECAG00000017417	5.033503706	0.726864404	0.951281381	306	570	908	736	476	837	701	612
ENSECAG00000013443	8.047049681	0.726892043	0.951281381	2792	7843	3785	5750	5342	6312	5851	3454
ENSECAG00000006470	1.490162473	0.726958916	0.951281381	15	100	15	90	27	50	52	83
ENSECAG00000014042	2.127290184	0.726984843	0.951281381	67	24	131	54	124	63	166	42
ENSECAG00000007999	4.360806751	0.727236266	0.951281381	232	286	470	406	873	354	453	131
ENSECAG00000019563	6.369587857	0.72729862	0.951281381	828	1646	1629	2306	1372	2036	1593	1608
ENSECAG00000021667	2.346218855	0.727321028	0.951281381	67	58	113	149	101	47	138	120
ENSECAG00000012210	8.408572736	0.727475029	0.951281381	3834	6586	5820	6883	7244	9034	7611	6180
ENSECAG00000006925	3.398645149	0.727488199	0.951281381	98	207	232	185	231	231	267	197
ENSECAG00000008834	4.842541151	0.727506955	0.951281381	114	819	988	426	24	538	63	1513
ENSECAG00000005889	2.73050409	0.727519279	0.951281381	45	132	93	273	46	86	97	288
ENSECAG00000013128	5.056648041	0.727700713	0.95140935	377	599	628	944	564	667	646	808
ENSECAG00000014999	1.842484525	0.727996742	0.951477787	57	51	49	109	55	54	67	103
ENSECAG00000012659	5.401165227	0.728029582	0.951477787	510	1133	815	661	546	1114	857	861
ENSECAG00000003561	3.16610468	0.728084582	0.951477787	74	244	152	219	226	161	176	145
ENSECAG00000019856	1.03024037	0.72808735	0.951477787	20	23	33	59	16	31	71	62
ENSECAG00000016833	3.450642277	0.728620446	0.951848552	92	195	198	384	127	194	194	354
ENSECAG00000022709	2.733320951	0.728675002	0.951848552	114	92	122	95	127	76	213	182
ENSECAG00000020415	4.294231949	0.728724538	0.951848552	200	309	397	468	461	398	355	495
ENSECAG00000021946	7.075138946	0.72877096	0.951848552	1640	3068	1924	3725	1630	2588	2305	4236
ENSECAG00000003837	10.06751878	0.728867631	0.951848552	17593	13683	25404	11023	28743	14335	51543	6274
ENSECAG00000021777	7.137701627	0.728872699	0.951848552	2569	2758	2511	2315	3589	2110	3474	2159
ENSECAG00000019159	6.010023082	0.728998554	0.95188594	729	1292	1290	1588	980	1314	1419	1509
ENSECAG00000010259	5.879869334	0.729068546	0.95188594	635	838	1348	1757	929	1165	1506	1190
ENSECAG00000008526	2.436881275	0.729183031	0.951919396	75	86	140	96	128	85	114	100
ENSECAG00000008678	8.328511791	0.729270823	0.951919396	3816	4665	5182	8608	6379	5862	7347	9165
ENSECAG00000016434	6.091928082	0.729345006	0.951919396	1355	1385	1154	895	1313	1286	1358	1519
ENSECAG00000002086	3.772034312	0.729662841	0.95197777	213	178	238	278	391	169	421	240
ENSECAG00000021488	5.796094583	0.729737052	0.95197777	360	1150	1026	1443	763	1534	1117	1471
ENSECAG00000007156	7.847697309	0.729743427	0.95197777	2625	4026	4515	6757	5172	3133	6078	4294
ENSECAG00000026371	0.573791998	0.729848759	0.95197777	13	21	33	44	26	36	32	15
ENSECAG00000017946	7.913233212	0.730234032	0.95197777	2632	4760	5284	5917	5182	3049	7164	4264
ENSECAG00000007386	1.054621411	0.73027733	0.95197777	19	41	69	24	78	31	31	12
ENSECAG00000023986	7.590605394	0.730335861	0.95197777	3487	6072	492	1087	7219	7716	1267	768
ENSECAG00000012593	3.412648751	0.730426517	0.95197777	100	134	268	237	265	270	292	110
ENSECAG00000017248	4.345493307	0.730442085	0.95197777	215	199	377	655	270	275	579	710
ENSECAG00000017665	4.818834904	0.730688462	0.95197777	453	318	704	616	739	477	727	336
ENSECAG00000016068	5.095579932	0.730823693	0.95197777	423	539	666	717	1115	527	760	601
ENSECAG00000020633	7.323745385	0.730862875	0.95197777	2411.01	2865	2866.01	3854	3729	2608	3332	3193.02

ENSECAG00000000854	0.221616717	0.730934825	0.95197777	6	16	18	36	9	24	40	25
ENSECAG00000012538	2.296209387	0.731105745	0.95197777	25	97	84	140	82	153	98	92
ENSECAG00000022100	4.606255134	0.731132349	0.95197777	320	412	489	608	478	334	618	562
ENSECAG00000008103	4.58332881	0.731258766	0.95197777	409	467	308	359	475	247	847	611
ENSECAG00000014734	2.403402912	0.73130733	0.95197777	44	80	109	130	64	50	156	208
ENSECAG00000014104	2.537403478	0.731398911	0.95197777	59	236	63	71	69	218	41	101
ENSECAG00000021891	5.524401301	0.731428979	0.95197777	534	593	932	1171	923	947	1282	922
ENSECAG00000016556	2.128960593	0.731501297	0.95197777	46	40	136	70	93	85	113	93
ENSECAG00000010079	5.071290953	0.731627084	0.95197777	460	469	599	776	983	620	735	605
ENSECAG00000016063	5.117017004	0.73165899	0.95197777	312	512	722	934	579	808	905	769
ENSECAG00000002867	1.362485635	0.731719079	0.95197777	43	6	79	22	147	23	59	1
ENSECAG00000000631	7.395382951	0.731750005	0.95197777	2021	2010	3908	3641	3710	2998	4927	3497
ENSECAG00000021085	5.901728095	0.731753969	0.95197777	797	996	1098	1160	1009	891	1545	1899
ENSECAG00000020925	4.602755255	0.731920636	0.95197777	179	409	413	761	547	509	555	523
ENSECAG00000008662	5.856468792	0.732027055	0.95197777	724	561	1642	1028	1195	723	1733	1577
ENSECAG00000007797	5.485917188	0.732098115	0.95197777	670	657	876	1143	1009	974	803	802
ENSECAG00000020540	3.496907613	0.732165829	0.95197777	141	247	221	227	219	183	277	233
ENSECAG00000011376	6.840177334	0.732252187	0.95197777	1735	2413	2017	2283	2096	1807	2836	2563
ENSECAG00000019536	4.88285832	0.732297918	0.95197777	404	579	605	590	687	597	666	430
ENSECAG00000009413	7.64798228	0.732477451	0.95197777	1672	4805	3532	3803	4010	6077	4011	3539
ENSECAG00000023505	8.309123041	0.732494818	0.95197777	5188	5958	5229	7114	5134	6005	6950	7403
ENSECAG00000014802	0.757739882	0.732545513	0.95197777	13	32	32	50	30	22	46	30
ENSECAG00000018773	10.1505192	0.732563445	0.95197777	13437	13023	25442	26443	27490	25220	22455	25536
ENSECAG00000008567	5.973508478	0.732655173	0.95197777	1070	1007	1181	1343	1767	832	1632	871
ENSECAG00000014133	6.089566415	0.732821939	0.95197777	898	1454	1418	1286	986	1825	1271	1352
ENSECAG00000023692	8.154989891	0.732839388	0.95197777	3984	3533	6127	5546	6738	5854	7141	5674
ENSECAG00000013314	5.505636846	0.732913014	0.95197777	593	739	1257	786	1074	863	1025	704
ENSECAG00000022027	3.69380757	0.733045383	0.95197777	126	277	183	312	258	283	341	257
ENSECAG00000012454	4.311346571	0.733075771	0.95197777	155	308	389	579	414	394	459	478
ENSECAG00000018515	5.463372605	0.733124526	0.95197777	569	765	999	964	893	820	911	949
ENSECAG00000016802	1.456736059	0.733196426	0.95197777	28	54	51	74	48	23	116	32
ENSECAG00000008659	5.048329996	0.733219432	0.95197777	424	737	515	798	764	672	766	468
ENSECAG00000004752	0.241015639	0.73334968	0.95197777	10	20	18	26	24	30	29	14
ENSECAG00000018551	5.179846583	0.733356943	0.95197777	416	557	682	874	618	581	1011	1019
ENSECAG00000009232	6.450199099	0.733400434	0.95197777	822	1734	1831	2421	1417	2371	1631	1538
ENSECAG00000007306	0.66376458	0.733403339	0.95197777	22	21	50	19	34	18	59	9
ENSECAG00000020913	6.293444933	0.733507902	0.952004955	1045	919	1524	2583	1576	1093	2157	1586
ENSECAG00000005572	7.48186986	0.733660337	0.95209426	2654	3686	3285	3629	3918	3193	4387	2913
ENSECAG00000019415	5.969214045	0.733849114	0.952139712	707	937	1194	1545	1055	1243	1500	1746
ENSECAG00000026924	3.872094105	0.733862624	0.952139712	168	256	279	298	199	276	361	461
ENSECAG00000015930	4.867566599	0.734047477	0.952247922	446	452	595	668	805	409	755	403
ENSECAG00000022516	4.43739775	0.734166159	0.952247922	432	204	497	428	477	267	674	354
ENSECAG00000016501	1.825221234	0.734284292	0.952247922	44	44	58	91	72	59	124	58
ENSECAG00000025597	1.244168834	0.734391059	0.952247922	8	13	29	163	2	80	4	76
ENSECAG00000012867	4.137141266	0.734528804	0.952247922	161	260	393	431	351	356	432	408
ENSECAG00000002378	1.198844039	0.734707227	0.952247922	30	43	29	67	41	38	52	45
ENSECAG00000004996	1.372711094	0.734730499	0.952247922	19	97	16	63	61	55	42	35
ENSECAG00000015578	4.060178108	0.735035655	0.952247922	216	185	351	395	315	263	624	302
ENSECAG00000024638	6.992248288	0.735183542	0.952247922	1530	1642	2537	3115	2596	2228	3559	3028
ENSECAG00000000985	4.089745801	0.735269523	0.952247922	239	266	422	343	525	217	482	149
ENSECAG00000018251	4.944009877	0.735275127	0.952247922	407	608	616	649	562	529	747	675
ENSECAG00000023962	8.155442061	0.735282467	0.952247922	4000	5884	5821	5542	5951	6158	5735	4885
ENSECAG00000009294	2.471104506	0.735338016	0.952247922	56	62	169	142	179	71	90	90
ENSECAG00000023657	1.320435399	0.735358663	0.952247922	49	27	27	79	50	75	25	33
ENSECAG00000013174	8.117607835	0.735632656	0.952247922	3067	4372	6294	7936	6768	3989	6768	4887
ENSECAG00000025149	6.584116411	0.735688859	0.952247922	1693	2700	1279	1129	2319	1766	2511	1106
ENSECAG00000018509	6.069264987	0.735701195	0.952247922	825	1039	1592	1639	1372	1372	1439	1237
ENSECAG00000012925	2.789559915	0.735776866	0.952247922	80	89	136	167	184	78	201	152
ENSECAG00000021868	9.466817355	0.735796407	0.952247922	10168	9157	13463	14750	19390	13482	16657	13498
ENSECAG00000008450	7.525259287	0.735825612	0.952247922	2521	3358	2937	3529	3964	4377	3579	4309
ENSECAG00000022559	6.28101505	0.735879335	0.952247922	885	1308	1503	2304	1493	1666	1508	1582
ENSECAG00000010824	5.814844909	0.735907397	0.952247922	672	748	1106	1399	1052	808	1389	1767
ENSECAG00000002541	6.876583086	0.735946976	0.952247922	2012	2650	1786	2043	1980	2107	2811	2603
ENSECAG00000004870	3.582950075	0.736005202	0.952247922	119	294	208	288	229	162	308	273
ENSECAG00000013468	2.643039775	0.736037041	0.952247922	86	126	93	160	92	124	97	177
ENSECAG00000012570	4.30432855	0.736514629	0.952757534	164	499	323.011	383.002	526	383	439	383.002
ENSECAG00000019294	0.846362208	0.737014348	0.953059139	9	24	34	54	34	8	75	43
ENSECAG00000021835	6.647540582	0.737161005	0.953059139	1231	1901	2103	2373	2812	1859	1992	1347
ENSECAG00000000346	5.690083157	0.737312461	0.953059139	541	1191	913	1284	751	856	1284	1333
ENSECAG00000013516	3.464085642	0.737341392	0.953059139	89	158	200	443	141	225	204	306
ENSECAG00000020310	3.564681268	0.737436189	0.953059139	202	193	232	126	288	281	312	160
ENSECAG00000008036	5.732721172	0.737439523	0.953059139	458	1057	930.001	1319	873	1540	1130	1101
ENSECAG00000019332	5.353071954	0.737479728	0.953059139	572	687	737	1064	764	599	1052	938
ENSECAG00000020996	4.302270955	0.737494377	0.953059139	137	501	314.004	597	326	400	485	382
ENSECAG00000021514	3.529818399	0.737501187	0.953059139	131	119	208	356	267	207	279	264
ENSECAG00000013820	3.062652765	0.737642899	0.953134083	54	154	245	204	111	220	107	210

ENSECAG00000018014	3.027154829	0.73772896	0.953137109	148	107	141	122	203	87	274	170
ENSECAG00000006497	1.206978932	0.737964066	0.953247147	42	51	41	24	64	33	42	36
ENSECAG00000024267	4.924926722	0.738258873	0.953247147	385	292	612	836	769	383	816	738
ENSECAG00000008902	4.831889011	0.738331442	0.953247147	470	571	508	498	568	477	732	548
ENSECAG00000003103	6.730822544	0.738351852	0.953247147	1830	1991	1827	2069	2345	1912	2362	1944
ENSECAG00000022544	8.33124722	0.738492813	0.953247147	3751	5054	6021	7339	6730	4858	9706	7805
ENSECAG00000020929	7.504809608	0.738505	0.953247147	2552	3668	4646	2534	4079	4033	3985	2365
ENSECAG00000015158	2.163856228	0.738511723	0.953247147	83	67	101	69	120	127	37	51
ENSECAG00000013676	8.236279043	0.738517475	0.953247147	5484	6866	5308	3859	6850	4662	6861	5843
ENSECAG00000023021	8.059679571	0.738581981	0.953247147	2761	3957	6781	7411	4227	6145	4399	6396
ENSECAG00000023410	4.677779314	0.738716332	0.953247147	335	276	498	670	469	397	723	692
ENSECAG00000021686	7.217696057	0.738839274	0.953247147	2728	2385	1874	2565	4226	1647	4919	2747
ENSECAG00000021629	9.005368759	0.738969688	0.953247147	6233	7010	13552	12874	11076	10453	9403	9908
ENSECAG00000024417	4.522401874	0.739030163	0.953247147	276	471	326	496	381	372	457	819
ENSECAG00000011661	6.545249668	0.739031575	0.953247147	1035	1131	1882	2544	2234	1818	2493	1741
ENSECAG00000018090	6.46986087	0.739070054	0.953247147	1080	1315	1856	1858	1927	1795	2370	1760
ENSECAG00000010996	4.80256283	0.739181233	0.95326974	232	451	572	745	600	668	597	568
ENSECAG00000000361	4.213525361	0.739255032	0.95326974	256	291	364	340	438	300	548	359
ENSECAG00000020838	7.239918598	0.739491564	0.953393629	1981	1789	3091	3519	3129	2849	3840	3665
ENSECAG00000018693	1.247477969	0.739518589	0.953393629	37	18	30	69	38	52	53	61
ENSECAG00000004792	5.709334275	0.739642791	0.953404769	683	1259	732	1234	661	741	1443	1459
ENSECAG00000014786	6.607543325	0.739805966	0.953404769	854	1775	1776	2509	2501	1745	2648	1776
ENSECAG00000020610	6.134585214	0.739818082	0.953404769	961	1483	1389	1358	1199	1656	1330	1453
ENSECAG00000014964	6.070336731	0.739862199	0.953404769	919	857	1270	1577	1498	1172	1598	1672
ENSECAG00000006638	4.823674057	0.739956526	0.953418407	251	502	402	874	506	395	714	909
ENSECAG00000018513	0.873689927	0.740070067	0.95343267	15	32	21	73	23	41	48	26
ENSECAG00000022005	8.501366868	0.740306361	0.95343267	4767	7831	6179	8614	6793	4787	9398	8503
ENSECAG00000009098	0.416956654	0.740395205	0.95343267	2	13	38	36	53	28	21	8
ENSECAG00000011279	3.956876123	0.740466873	0.95343267	177	222	221	471	292	192	485	425
ENSECAG00000009020	2.659329685	0.740593461	0.95343267	50	132	164	138	74	112	177	144
ENSECAG00000005265	6.425460889	0.740600102	0.95343267	1118	1745	790	2218	1272	1714	1890	2770
ENSECAG00000022331	1.876064467	0.740601665	0.95343267	58	50	75	86	74	24	98	95
ENSECAG00000012023	6.320517445	0.740637552	0.95343267	1054	1536	1565	1844	1731	2033	1563	1045
ENSECAG00000006109	1.54613168	0.741199484	0.954048178	80	19	34	23	12	198	5	32
ENSECAG00000004262	5.234568081	0.741367998	0.954157208	472	982	550	791	569	1049	632	743
ENSECAG00000016924	3.580681558	0.741478322	0.95419133	133	182	306	287	219	207	277	263
ENSECAG00000010673	3.050070958	0.74182956	0.954495738	102	113	159	192	193	159	195	177
ENSECAG00000022508	6.631847848	0.741882546	0.954495738	1172	2140	1935	2249	1887	2458	1535	1994
ENSECAG00000021207	8.787811973	0.742068025	0.95452237	6236	6526	7273	9795	11398	6421	10577	11161
ENSECAG00000021815	3.14970138	0.742193013	0.95452237	75	146	205	268	190	189	138	181
ENSECAG00000015463	5.73720821	0.742315451	0.95452237	382	603	1261	1651	706	1142	1352	1549
ENSECAG00000013788	10.15506248	0.742321871	0.95452237	25135	22906	14815	17178	25619	21419	26675	17336
ENSECAG00000021725	5.569238372	0.742322447	0.95452237	731	389	1333	1109	991	1094	953	757
ENSECAG00000012464	6.172968267	0.742413992	0.954532276	892	1001	1380	1733	1573	970	1914	1982
ENSECAG00000013295	4.187760004	0.742928505	0.954857913	191	169	457	471	412	350	404	434
ENSECAG00000008999	4.145209429	0.742941408	0.954857913	202	309	239	485	377	268	397	518
ENSECAG00000012606	3.808411369	0.742983795	0.954857913	159	227	353	319	272	231	341	292
ENSECAG00000009826	4.090386567	0.743002745	0.954857913	168	331	369	434	270	379	348	373
ENSECAG00000011968	9.042932256	0.743182567	0.954981211	6858	6909	15197	11343	10237	12175	14164	5658
ENSECAG00000015176	2.682503696	0.743383506	0.955044874	74	112	122	125	130	101	160	173
ENSECAG00000018478	5.742833729	0.743443574	0.955044874	676	596	1449	1367	1278	1096	876	1025
ENSECAG00000017741	7.331339221	0.743740993	0.955044874	2752	3390	2542	3004	3156	2603	4111	3200
ENSECAG00000019070	3.310839551	0.743800593	0.955044874	110	177	186	202	165	298	290	118
ENSECAG00000016210	5.255850983	0.743964235	0.955044874	339	597	1086	954	744	773	1234	367
ENSECAG00000010349	2.84452204	0.744093017	0.955044874	86	110	162	189	130	229	87	105
ENSECAG00000007104	7.896083819	0.744107816	0.955044874	2592	5685	5128	4637	3677	5698	4812	4841
ENSECAG00000000002	4.567460536	0.744109314	0.955044874	285	498	455	537	324	391	557	664
ENSECAG00000019146	2.915551669	0.744110752	0.955044874	62	114	135	224	135	128	173	228
ENSECAG00000018113	10.18001803	0.744371489	0.955044874	10831	28770	37938	16474	147	26853	306	56873
ENSECAG00000019326	6.774558252	0.744414272	0.955044874	1439	2903	1542	2250	2487	2351	2018	1867
ENSECAG00000019902	5.466130519	0.744586203	0.955044874	586	707	886	830	735	1193	930	995
ENSECAG00000024190	3.729201518	0.744666934	0.955044874	191	271	247	260	237	211	298	328
ENSECAG00000016212	5.16135682	0.744689079	0.955044874	588	378	769	928	690	412	1014	830
ENSECAG00000010193	4.202014424	0.74484052	0.955044874	183	324	416	490	429	380	354	311
ENSECAG00000019040	4.325764079	0.744847416	0.955044874	239	296	337	523	683	283	398	389
ENSECAG00000014291	7.048204949	0.744906627	0.955044874	1890	4024	1732	1984	2060	2688	2907	2971
ENSECAG00000000402	4.423221367	0.744975447	0.955044874	150	413	450	689	388	573	363	376
ENSECAG00000021124	3.984500356	0.745106338	0.955044874	209	215	287	374	481	208	354	348
ENSECAG00000016939	10.36936608	0.745116928	0.955044874	15315	15958	28997	31342	30075	31823	24984	29755
ENSECAG00000025005	5.0785454	0.745192837	0.955044874	341	518	860	879	645	718	710	658
ENSECAG00000012801	0.714097671	0.745373958	0.955044874	24	24	24	29	28	20	49	44
ENSECAG00000019470	0.607907339	0.745419337	0.955044874	17	33	18	43	17	17	31	49
ENSECAG00000019416	6.277935442	0.745527137	0.955044874	1035	1084	1592	2200	1138	1259	2060	1907
ENSECAG00000013580	5.332231214	0.745559526	0.955044874	854	571	764	643	1176	735	750	553
ENSECAG00000010839	0.681815853	0.745575891	0.955044874	23	27	34	29	25	30	18	45
ENSECAG00000014812	1.0649285	0.745976507	0.955044874	21	20	45	53	40	38	52	49

ENSECAG00000016039	4.2232179	0.745981066	0.955044874	114	337	455	430	515	382	471	273
ENSECAG00000005084	5.239010677	0.746213836	0.955044874	477	362	888	900	1031	709	1011	582
ENSECAG00000007700	4.406925984	0.746334077	0.955044874	213	552	303	560	718	451	309	179
ENSECAG00000008857	4.238322783	0.746405173	0.955044874	314	324	279	319	419	254	649	372
ENSECAG00000021473	4.292477405	0.746411063	0.955044874	183	518	306	486	336	243	511	514
ENSECAG00000011676	2.473211418	0.746489136	0.955044874	53	93	93	145	107	105	147	128
ENSECAG00000009424	3.404232051	0.746600471	0.955044874	144	126	221	314	156	142	238	320
ENSECAG00000017818	3.749533838	0.746613693	0.955044874	107	312	222	298	263	178	393	367
ENSECAG00000016552	5.608742009	0.746667009	0.955044874	490	706	1078	1181	852	896	1377	1206
ENSECAG00000018611	5.455104831	0.746706262	0.955044874	471	596	1066	927	834	1343	994	648
ENSECAG00000003079	8.530578769	0.746707616	0.955044874	4735	5281	7292	7931	8834	5836	9178	9210
ENSECAG00000016807	6.612909372	0.746724503	0.955044874	1222	1918	1987	2235	1900	1988	2004	2004
ENSECAG00000016287	3.350026854	0.746819325	0.955044874	102	124	256	229	230	192	270	205
ENSECAG00000012217	5.586690615	0.746825146	0.955044874	187	2638	11	35	1630	2322	99	236
ENSECAG00000010787	5.509245677	0.746831035	0.955044874	661	707	826	1215	702	656	1299	1104
ENSECAG00000014532	6.02256104	0.74683922	0.955044874	892	1131	1213	1633	1459	1006	1775	1089
ENSECAG00000026875	2.324492321	0.746928859	0.95505223	50	69	81	194	71	99	144	84
ENSECAG00000017473	4.599947574	0.747190157	0.955279049	300	449	332	595	518	386	684	561
ENSECAG00000021091	5.086511709	0.747324616	0.955343673	428	530	624	769	864	658	729	713
ENSECAG00000008513	2.636484936	0.747475503	0.95542928	85	111	83	135	137	91	157	162
ENSECAG00000005075	4.259021911	0.747565391	0.955436908	214	319	384	410	386	413	353.001	512
ENSECAG00000023213	6.011102355	0.747781239	0.955605501	665	1619	1376	1230	618	2430	1022	969
ENSECAG00000020058	3.868609339	0.748313994	0.956079394	205	227	195	371	257	270	389	374
ENSECAG00000023676	2.955679527	0.748320025	0.956079394	84	94	197	157	135	154	196	197
ENSECAG00000008848	6.841402109	0.748408319	0.956084909	1469	1940	1849	2588	2343	2193	3835	1932
ENSECAG00000004006	4.258484431	0.748624413	0.956253667	301	613	114	155	285	707	358	325
ENSECAG00000017710	3.087445864	0.749068987	0.956318306	100	108	229	135	228	196	200	117
ENSECAG00000011476	2.308021376	0.74907763	0.956318306	42	87	88	126	78	84	136	138
ENSECAG00000007968	6.367538048	0.749429974	0.956318306	961	1563	1820	1905	1811	1948	1435	1402
ENSECAG00000019449	4.558540686	0.749626305	0.956318306	371	497	395	257	487	484	835	304
ENSECAG00000000411	2.702173077	0.749918219	0.956318306	78	157	75	120	154	70	164	189
ENSECAG00000017368	4.572008315	0.749997515	0.956318306	281	371	439	561	529	295	613	673
ENSECAG00000020611	0.393430121	0.750097799	0.956318306	12	22	27	21	21	30	32	26
ENSECAG00000009513	7.47351201	0.750105881	0.956318306	1994	2714	3169	4531	4022	2880	4838	4152
ENSECAG00000022735	7.098673554	0.750130256	0.956318306	1922	3375	1974	2901	1994	2306	2610	4138
ENSECAG00000024188	3.550355828	0.750178577	0.956318306	213	123	268	248	184	286	333	138
ENSECAG00000005198	4.844595661	0.750270552	0.956318306	558	546	435	489	810	420	591	496
ENSECAG00000001324	4.488820458	0.750496708	0.956318306	193	778	268	251	651	471	622	248
ENSECAG00000017116	6.398894157	0.750729214	0.956318306	799	1101	1683	2470	1641	1835	1506	2411
ENSECAG00000016673	10.09915827	0.750750699	0.956318306	14889	18430	20673	29573	23794	19300	22769	22260
ENSECAG00000022720	2.332738553	0.750802485	0.956318306	68	94	96	114	117	49	161	79
ENSECAG00000000004	3.311299398	0.750978152	0.956318306	124	156	202	269	120	151	276	262
ENSECAG00000010826	4.407349254	0.751008859	0.956318306	175	386	325	636	353	286	541	711
ENSECAG00000014996	6.772009422	0.751113365	0.956318306	3470	1321	1411	938	2667	2716	2053	1094
ENSECAG00000015597	8.970647469	0.751114392	0.956318306	6675	3236	14209	9974	13954	11619	10235	8596
ENSECAG00000015605	3.735518952	0.751160401	0.956318306	146	316	200	346	203	203	257	411
ENSECAG00000022180	2.066115649	0.751164891	0.956318306	24	108	75	120	49	112	53	106
ENSECAG00000011706	4.759230226	0.751211817	0.956318306	359	474	519	670	436	465	680	639
ENSECAG00000012244	1.440080831	0.751293122	0.956318306	22	38	57	96	23	21	44	121
ENSECAG00000002265	3.556464498	0.751324006	0.956318306	160	224	207	281	252	184	310	210
ENSECAG00000018250	5.713052748	0.751344684	0.956318306	722	643	1102	1137	1258	839	1274	1257
ENSECAG00000017911	5.276013975	0.75157095	0.956318306	674	732	594	791	748	538	835	1041
ENSECAG00000025158	2.868101017	0.751614879	0.956318306	66	157	120	159	171	152	209	109
ENSECAG00000011760	0.337293751	0.751652443	0.956318306	4	8	62	22	18	33	22	16
ENSECAG00000017478	2.291206655	0.751726966	0.956318306	61	75	98	91	120	85	163	64
ENSECAG00000010288	7.210303683	0.751732928	0.956318306	2478	3198	1928	3304	1737	1640	3150	5500
ENSECAG00000023250	2.903075892	0.751760365	0.956318306	71	116	183	208	190	162	155	82
ENSECAG00000005483	4.829752368	0.751945765	0.956318306	266	595	583	738	518	741	484	527
ENSECAG00000017504	6.541352184	0.751967063	0.956318306	1654	1373	1750	2008	2245	1270	2501	1597
ENSECAG00000022539	3.554906705	0.752128224	0.956318306	122	195	214	290	199	222	336	281
ENSECAG00000023698	4.340746259	0.752252791	0.956318306	269	321	518	394	314	500	346	460
ENSECAG00000009782	2.552530305	0.752256253	0.956318306	92	131	100	96	91	135	144	96
ENSECAG00000001815	2.466111189	0.752282626	0.956318306	56	69	110	148	141	134	119	83
ENSECAG00000022855	5.937422711	0.752303652	0.956318306	1220	1122	1110	841	1148	1307	1247	1234
ENSECAG00000006410	1.26940643	0.752328037	0.956318306	20	27	45	73	40	29	79	63
ENSECAG00000024378	5.237908903	0.752354412	0.956318306	264	296	1049	1162	823	869	821	802
ENSECAG00000018327	2.068902657	0.752390341	0.956318306	47	87	76	103	53	78.3333	110	92
ENSECAG00000021003	2.904223717	0.752415083	0.956318306	57	263	79	171	82	155	193	166
ENSECAG00000024646	3.523077231	0.752634541	0.956318306	118	217	213	246	231	301	221	241
ENSECAG00000006078	5.533387647	0.75264609	0.956318306	565.001	875.001	812.001	905.003	1726	616.002	1098	639.001
ENSECAG00000010039	5.974256555	0.752653063	0.956318306	678	997	1173	1578	1655	1410	1227	1176
ENSECAG00000011001	0.412109243	0.752654391	0.956318306	7	31	16	32	32	16	39	25
ENSECAG00000020568	5.945330514	0.752665431	0.956318306	769	1262	1166	1411	1033	1320	1335	1312
ENSECAG00000021522	2.354741473	0.752753385	0.956318306	34	117	121	123	95	127	66	106
ENSECAG00000014218	4.203398051	0.752790923	0.956318306	188	280	636	411	1	474	2	849
ENSECAG00000013903	5.161262157	0.753094194	0.956596832	303	468	893	1168	619	711	689	869

ENSECAG00000023140	6.68487114	0.753249531	0.956687406	1397	2952	1401	1792	1413	2294	1956	2590
ENSECAG00000010070	3.179168443	0.753337194	0.956692019	96	73	277	176	269	138	273	125
ENSECAG00000024838	3.431891873	0.753430277	0.956703514	107	205.003	208.003	308.002	251.002	208.002	196.008	205.004
ENSECAG00000020316	11.47117222	0.753905064	0.957015864	29105	38592	65944	64274	66177	70505	49413	63181
ENSECAG00000023976	6.88761082	0.754007773	0.957015864	1372	1776	2561	3443	2433	2114	2776	2270
ENSECAG00000007554	3.141710904	0.754015503	0.957015864	118	121	189	235	144	167	254	150
ENSECAG00000007446	5.189487762	0.754127763	0.957015864	611	581	542	685	833	491	1143	792
ENSECAG00000013667	5.115802839	0.754137036	0.957015864	333	798	596	925	598	547	1000	713
ENSECAG00000021501	5.59490813	0.754180618	0.957015864	737	825	860	1145	770	632	1262	1323
ENSECAG00000023354	0.362302984	0.754390237	0.957051025	6	20	18	41	34	12	24	37
ENSECAG00000023651	2.763383264	0.754475679	0.957051025	71	122	229	90	40	106	76	307
ENSECAG00000006568	4.69201871	0.754539871	0.957051025	251	403	492	696	531	467	721	563
ENSECAG00000000577	6.514306214	0.754544576	0.957051025	1225	1714	1795	2088	1740	1855	1937	1871
ENSECAG00000008402	1.417793711	0.754746339	0.957200297	35	35	62	38	95	38	78	21
ENSECAG00000006228	2.933313019	0.755076506	0.957367012	93	134	178	98	193	149	267	71
ENSECAG00000012905	8.167550123	0.755223972	0.957367012	5374	6677	4310	4110	7864	4513	7344	3407
ENSECAG00000007605	7.34353106	0.75532929	0.957367012	1575	3752	3067	4125	2783	3824	3425	2994
ENSECAG00000000738	3.222817428	0.75534954	0.957367012	154	108	139	222	176	175	272	202
ENSECAG00000009395	3.436760615	0.755470604	0.957367012	137	154	174	281	247	147	351	220
ENSECAG00000014827	3.066443666	0.755522679	0.957367012	69	172	163	245	172	165	171	159
ENSECAG00000024801	6.313400956	0.755546116	0.957367012	1592	1557	1259	1183	2024	1744	1540	1037
ENSECAG00000007207	3.820236436	0.755610051	0.957367012	147	181	313	352	281	204	392	376
ENSECAG00000012383	4.558285217	0.755634604	0.957367012	322	297	443	562	543	360	745	447
ENSECAG00000011717	5.939846403	0.755971377	0.957589737	666	1092	1369	1551	1314	1140	1333	1199
ENSECAG00000013404	7.418376486	0.755978617	0.957589737	1973	1397	4902	3592	4350	3347	4314	3208
ENSECAG00000012459	6.593324188	0.756298635	0.957805383	1041	1460	1958	2318	1857	1904	2060	2684
ENSECAG00000009573	11.69290624	0.756317119	0.957805383	36206	41282	78266	74385	74312	75665	63678	78292
ENSECAG00000000780	5.230398164	0.756460107	0.957879915	397	430	852	1263	754	593	695	996
ENSECAG00000005508	4.787539133	0.756687705	0.95797763	367	328	650	742	524	504	568	645
ENSECAG00000016569	2.299624196	0.756705563	0.95797763	42	60	134	103	138	74	144	77
ENSECAG00000018278	1.291093294	0.756897736	0.958028078	22	56	29	53	31	41	31	107
ENSECAG00000012075	0.993437724	0.756913707	0.958028078	33	56	24	23	51	42	38	18
ENSECAG00000014215	5.950216425	0.757018937	0.958054757	1366	992	967	979	1544	787	1710	1015
ENSECAG00000004043	5.115296824	0.757342752	0.958255947	452	397	821	963	612	571	884	775
ENSECAG00000010177	3.253515506	0.757346246	0.958255947	83	161	146	289	171	189	236	241
ENSECAG00000001650	6.86742008	0.757520916	0.958370444	1665	2802	2101	1983	2759	2243	2741	1670
ENSECAG00000016020	3.280459764	0.757695422	0.958484709	94	79	290	213	320	178	253	104
ENSECAG00000014139	3.922347313	0.757814662	0.958529044	183	208	328	436	363	266	308	284
ENSECAG00000024759	2.393758689	0.758094498	0.95859968	37	86	140	99	76.9997	120	79.9999	178
ENSECAG00000012574	4.545716791	0.758146227	0.95859968	299	376	437	653	472	283	577	585
ENSECAG00000023053	2.364497624	0.758314628	0.95859968	64	74	101	106	147	77	158	71
ENSECAG00000013117	4.640158858	0.758388549	0.95859968	140	488	423	976	309	534	500	661
ENSECAG00000017858	5.868928509	0.758452944	0.95859968	806	1076	1029	1441	1152	1118	1221	1257
ENSECAG00000014616	5.528706114	0.758478213	0.95859968	520	632	754	1362	473	873	1226	1547
ENSECAG00000001222	2.489543058	0.75850508	0.95859968	34	141	106	156	87	132	105	116
ENSECAG00000009005	6.343433766	0.758544095	0.95859968	1521	1095	2032	1150	2204	1545	1668	1091
ENSECAG00000023572	2.928748757	0.758710115	0.958703069	93	207	72	129	151	118	245	165
ENSECAG00000012025	1.847854536	0.758900221	0.958835723	50	29	73	87	80	44	133	63
ENSECAG00000018179	6.36855194	0.758983534	0.958835723	1048	1471	1645	2062	1534	1610	1490	2042
ENSECAG00000024919	3.016263971	0.759458966	0.959303514	123	82	147	192	179	125	261	154
ENSECAG00000024434	4.708362055	0.759540863	0.959303514	294	588	417	493	446	639	635	566
ENSECAG00000023131	4.877254077	0.759606603	0.959303514	304	603	514	830	768	644	497	438
ENSECAG00000010458	5.248988629	0.759872597	0.959456348	721	635	487	896	707	460	856	1089
ENSECAG00000011715	5.982246058	0.759979863	0.959456348	930	738	1231	1885	1414	874	1578	1310
ENSECAG00000022391	5.212271504	0.760115223	0.959456348	395	434	797	1016	923	837	806	661
ENSECAG00000013231	7.088703102	0.760141715	0.959456348	1597	2578	2230	2945	4074	2193	3553	2261
ENSECAG00000019549	3.516405945	0.760148991	0.959456348	193	95	264	198	322	193	386	120
ENSECAG00000023853	5.287920777	0.760293057	0.95953181	370	557	765	1112	593	682	1093	1104
ENSECAG00000019950	7.418706518	0.760829459	0.960040131	3416	3388	2592	2730	4057	3179	4732	1863
ENSECAG00000013309	7.817744806	0.76086448	0.960040131	2906	4931	4439	4564	4683	4173	5522	3910
ENSECAG00000024443	4.453853621	0.761065066	0.960107765	157	409	271	763	375	382	464	712
ENSECAG00000007206	5.391662912	0.761164008	0.960107765	480	627	846	1276	847	545	1268	815
ENSECAG00000024015	3.851403271	0.761186632	0.960107765	206	238	291	331	240	230	297	404
ENSECAG00000022859	5.443398907	0.761274001	0.960107765	444	1090	669	1096	786	607	1182	1011
ENSECAG00000016432	6.977006151	0.761492859	0.960107765	1128	2376	2357	3029	3125	2987	2589	2327
ENSECAG00000000246	6.093276436	0.761500444	0.960107765	576	1387	1355	1507	1628	1705	1284	1311
ENSECAG00000005530	4.825803106	0.761790737	0.960107765	368	452	542	772	620	448	750	510
ENSECAG00000019598	5.756949711	0.76182084	0.960107765	741	731.989	1072.98	1170.98	1472.99	928	1394	961.993
ENSECAG00000019557	5.601389241	0.761855528	0.960107765	1058	491	1333	509	1296	1128	561	816
ENSECAG00000023362	3.744254116	0.761969573	0.960107765	153	267	208	295	279	271	334	288
ENSECAG00000024442	3.747572963	0.762015802	0.960107765	124	173	457	301	162	410	33	411
ENSECAG00000024428	1.199701255	0.762034847	0.960107765	32	25	60	51	13	34	40	89
ENSECAG00000018249	5.686149639	0.762481397	0.960107765	421	768	1056	1489	1053	1248	1053	1128
ENSECAG00000020071	4.99367498	0.762576904	0.960107765	540	737	432	567	606	372	960	698
ENSECAG00000017498	4.556487788	0.762799148	0.960107765	346	296	451	508	686	325	493	556
ENSECAG00000023633	2.93358631	0.762986494	0.960107765	63	158	123	190	189	105	211	169

ENSECAG00000019749	3.327232024	0.763243416	0.960107765	156	167	161	178	248	130	301	211
ENSECAG00000014446	9.575086719	0.763246664	0.960107765	11265	7164	19662	20183	19906	8750	20580	12702
ENSECAG00000017154	11.89737745	0.763293416	0.960107765	40249	50993	97036	110807	74991	74717	65918	89008
ENSECAG00000007330	0.804930232	0.763341072	0.960107765	15	31	40	43	38	26	50	19
ENSECAG00000001582	6.787879515	0.763376347	0.960107765	1740	1667	1750	2224	3257	1349	2736	2488
ENSECAG00000020265	3.062293767	0.763385118	0.960107765	82	139	168	192	204	157	184	181
ENSECAG00000023803	6.165709382	0.763450255	0.960107765	1318	1427	1151	1210	1257	881	1943	1836
ENSECAG00000004691	6.634190398	0.763766181	0.960107765	1547	1373	1756	2011	1966	1640	2780	2462
ENSECAG00000019645	3.757727422	0.763826277	0.960107765	199	166	363	271	321	176	423	187
ENSECAG00000000555	5.775062303	0.763842031	0.960107765	750	790	887	1355	1131	705	1885	1199
ENSECAG00000024284	7.051332803	0.763850515	0.960107765	2013	1586	1999	3463	3320	2119	3858	2555
ENSECAG00000014091	2.267110512	0.763851221	0.960107765	42	80	65	148	46	77	98	203
ENSECAG00000001258	0.827945319	0.763861648	0.960107765	20	24	29	40	93	18	29	9
ENSECAG00000015572	5.08189259	0.763969244	0.960107765	350	539	673	833	889	762	695	589
ENSECAG00000010092	6.074805072	0.763980577	0.960107765	904	903	1228	2111	1356	1030	1755	1391
ENSECAG00000023567	8.093595267	0.764156744	0.960107765	3228	4146	6205	7454	6794	4299	5210	5671
ENSECAG00000012372	6.252283331	0.76416055	0.960107765	751	1349	1619	2186	1056	1892	1497	1699
ENSECAG00000010252	6.713618156	0.764174948	0.960107765	1189	2022	1823	2216	2377	2492	2629	1726
ENSECAG00000020591	4.46424807	0.76418143	0.960107765	227.001	348	446.001	535.001	629.001	454.001	468.001	364
ENSECAG00000016895	4.167202804	0.764189717	0.960107765	158.007	302	408	528	277	383	344	444
ENSECAG00000015278	4.688175146	0.764237414	0.960107765	282	440	446	646	539	443	709	581
ENSECAG00000021650	3.985424262	0.76424318	0.960107765	224	293	254	404	265	197	465	380
ENSECAG00000014211	5.056487901	0.764319557	0.960107765	554	410	641	615	813	568	1032	538
ENSECAG00000017917	7.593599259	0.764418879	0.960107765	1851	2768	3417	5672	2309	2649	3995	8425
ENSECAG00000004513	6.257037245	0.764421459	0.960107765	844	1351	1123	2060	1054	1242	2360	2207
ENSECAG00000004050	1.435526601	0.764520331	0.960107765	33	27	45	75	35	27	45	128
ENSECAG00000004595	4.418640913	0.764614375	0.960107765	212	367	461	446	773	391	520	187
ENSECAG00000017978	3.577043108	0.764628643	0.960107765	133	277	191	208	210	212	301	327
ENSECAG00000023762	7.234302525	0.764729842	0.960128943	1858	2558	3204	3826	3318	2605	3311	2961
ENSECAG00000011448	5.115089028	0.764921617	0.960171989	562	604	711	624	638	507	1108	622
ENSECAG00000013936	7.755951579	0.764932801	0.960171989	2255	3221	5025	6365	3495	4887	4997	4069
ENSECAG00000016467	6.57550922	0.765141847	0.960220361	1061	2036	1622	1844	1820	2261	2046	2222
ENSECAG00000010414	0.86714164	0.765340708	0.960220361	24	45	40	19	14	29	14	78
ENSECAG00000012228	5.812767879	0.765493126	0.960220361	664	1553	819	1121	1168	996	1332	1087
ENSECAG00000019853	2.65539017	0.765576444	0.960220361	136	23	120	192	271	24	172	23
ENSECAG00000022437	6.165653673	0.765585093	0.960220361	1541	1227	1046	1191	1368	1473	1622	1353
ENSECAG00000014028	3.422021484	0.765585401	0.960220361	137	203	138	254	232	220	212	266
ENSECAG00000011337	5.217016633	0.765648071	0.960220361	483	711	757	806	730	614	1116	613
ENSECAG00000005290	2.122127643	0.765732776	0.960220361	51	70	72	100	68	61	154	104
ENSECAG00000011527	4.67328686	0.765837867	0.960220361	263	285	392	907	428	335	726	793
ENSECAG00000004932	5.377333249	0.765869307	0.960220361	541	722	857	712	754	832	1015	1048
ENSECAG00000000260	5.416194029	0.765916314	0.960220361	709	584	819	731	916	813	999	1016
ENSECAG00000002401	4.014043803	0.765983427	0.960220361	169	285	366	408	235	350	328	392
ENSECAG00000004011	1.09123308	0.766171968	0.960317504	28	35	44	27	22	44	48	68
ENSECAG00000026906	1.723446278	0.766229618	0.960317504	31	49	58	88	80	39	92	77
ENSECAG00000015725	6.248293944	0.766325213	0.960331597	1056	1347	1260	1505	1714	1330	1999	1670
ENSECAG00000014208	4.166113755	0.766683145	0.960674401	119	322	343.002	651	313	386	359	385
ENSECAG00000007006	7.330373928	0.766902524	0.960695052	1824	2270	3297	5167	3069	2079	4404	3653
ENSECAG00000006197	6.377825558	0.766943161	0.960695052	1135	1240	1654	1654	1754	1381	1983	2223
ENSECAG00000010911	5.968788441	0.766952773	0.960695052	714	1095	1366	1571	1209	1333	1401	1149
ENSECAG00000013909	5.523318148	0.767166934	0.960857595	451	908	813	1377	871	609	1287	1035
ENSECAG00000007894	6.866460917	0.767309981	0.960869515	2051	2265	1660	1515	3004	2886	2487	1847
ENSECAG00000024965	8.066967468	0.767403749	0.960869515	4121	4555	5231	5957	6156	4315	6043	5220
ENSECAG00000012221	6.704527657	0.767448426	0.960869515	1106	1585	2172	2461	2783	2361	2227	1740
ENSECAG00000013454	5.322545967	0.767514043	0.960869515	450	775	877	908	944	886	720	668
ENSECAG00000011547	10.9247678	0.767774084	0.961089384	21171	23257	46979	44292	42694	46936	37897	43371
ENSECAG00000007680	4.380830158	0.767926782	0.961161228	341	334	341	509	507	275	626	306
ENSECAG00000010378	5.499371473	0.768000325	0.961161228	547	776	781	1047	1014	760	1107	1092
ENSECAG00000015395	3.952369468	0.768153499	0.961247262	58	678	138	162	135	267	457	551
ENSECAG00000003160	1.212992885	0.768573615	0.961274662	32	46	39	28	43	40	86	33
ENSECAG00000005827	5.004665564	0.768641216	0.961274662	506	431	664	759	536	566	899	645
ENSECAG00000015682	4.423300485	0.76879548	0.961274662	218	367	418	650	342	365	440	600
ENSECAG00000013274	2.689213032	0.768829452	0.961274662	61	83	144	162	157	158	166	79
ENSECAG00000017761	2.845785003	0.768958391	0.961274662	52	113	145	264	81	105	139	249
ENSECAG00000019205	5.786953451	0.769022308	0.961274662	953	1459	834	627	1213	1130	1152	956
ENSECAG00000015493	6.877443777	0.769047168	0.961274662	896	1399	3582	3530	1738	2233	2018	3445
ENSECAG00000010277	9.151276412	0.769093517	0.961274662	6901	7145	17559	12070	4995	13874	7318	18734
ENSECAG00000005179	0.736340798	0.769099465	0.961274662	4	28	23	80	10	47	27	37
ENSECAG00000011930	2.553995014	0.769190672	0.961274662	84	42	105	147	19	13	22	483
ENSECAG00000022861	5.248010073	0.769239896	0.961274662	536	1062	587	543	707	793	808	770
ENSECAG00000022869	5.193391716	0.769305817	0.961274662	413	365	1132	618	922	909	963	415
ENSECAG00000012364	1.730675952	0.769403246	0.961274662	20	48	67	99	82	79	59	62
ENSECAG00000008201	7.050292406	0.769407507	0.961274662	2144	2404	2481	2648	2905	2253	3153	2464
ENSECAG00000012677	8.694956787	0.769518002	0.961274662	5667	6865	9222	9376	8602	8592	9112	7085
ENSECAG00000017195	5.74232357	0.769561599	0.961274662	524	875	1099	1658	1141	1131	1084	976
ENSECAG00000023520	4.3766572	0.769610764	0.961274662	219	371	381	620	426	343	520	411

ENSECAG00000007927	4.59417265	0.769976836	0.961626402	257	374	489	584	569	421	542	577
ENSECAG00000007190	7.454105038	0.770266419	0.961882547	2426	3126	3219	3015	3444	4070	4420	3549
ENSECAG00000006019	6.688042068	0.770581185	0.962110614	1239	2334	1594	1843	2331	2090	2659	2017
ENSECAG00000007707	7.161738291	0.770618067	0.962110614	1942	1901	3103	3947	3061	2030	3557	3047
ENSECAG00000012618	4.05651075	0.770993093	0.962232439	158.001	524	273.001	287.001	158.001	395.001	234.001	534
ENSECAG00000024491	4.022792353	0.771073214	0.962232439	151	402	292	385	267	335	335	377
ENSECAG00000020501	7.586303179	0.771129223	0.962232439	2841	3478	4031	3827	3970	3575	4454	3589
ENSECAG00000011059	4.4387746	0.77132326	0.962232439	228	475	249	559	305	264	574	796
ENSECAG00000013423	9.050748606	0.771398298	0.962232439	5512	7349	13534	15072	8686	10627	8562	14550
ENSECAG00000009381	5.440510522	0.771507015	0.962232439	905	670	717	751	1128	844	877	651
ENSECAG00000012902	4.965055866	0.771558816	0.962232439	350	442	728	867	611	745	650	517
ENSECAG00000011095	3.112061805	0.771679198	0.962232439	116	105	192	237	198	132	195	171
ENSECAG00000020746	6.407348113	0.77188618	0.962232439	924	1815	1477	2253	1109	1306	2302	2288
ENSECAG00000019399	4.751605568	0.771905498	0.962232439	392	425	364	664	512	369	719	788
ENSECAG00000025017	1.945510526	0.772115858	0.962232439	30	42	81	119	74	93	103	63
ENSECAG00000024764	3.336426103	0.772122701	0.962232439	151	148	200	244	206	183	220	206
ENSECAG00000008056	4.879142764	0.772190965	0.962232439	252	606	596	829	738	373	981	339
ENSECAG00000005570	5.730359454	0.772263703	0.962232439	795	890	1089	758	741	1537	1198	1154
ENSECAG00000021706	5.20793836	0.772265418	0.962232439	368	607	729	913	563	603	788	1303
ENSECAG00000023825	5.344963879	0.772287443	0.962232439	698	931	539	728	564	759	1176	850
ENSECAG00000015336	8.307223934	0.772505234	0.962232439	4601	6218	4751	5492	9566	6311	6534	5405
ENSECAG00000002284	3.77421188	0.772539441	0.962232439	145	259	238	402	273	236	296	304
ENSECAG00000016010	7.427429695	0.772556958	0.962232439	2375	2761	3064	3477	4930	2544	4678	3158
ENSECAG00000016332	6.603355081	0.772659885	0.962232439	773	1229	2161	2921	1624	2203	2319	2427
ENSECAG00000007829	5.097013396	0.772734068	0.962232439	384	381	898	736	840	427	1024	755
ENSECAG00000020289	7.149689728	0.772817855	0.962232439	1857	1946	2904	4189	1912	1781	3972	4047
ENSECAG00000012703	5.49674272	0.77285709	0.962232439	520	673	947	1027	980	964	943	1039
ENSECAG00000006458	2.596539455	0.772909789	0.962232439	79	111	128	79	90	135	186	121
ENSECAG00000003668	4.707441768	0.772914082	0.962232439	276	681	323	695	475	376	617	671
ENSECAG00000012260	4.96156724	0.772980465	0.962232439	290	1259	278	477	323	705	733	751
ENSECAG00000014531	4.940215799	0.772997619	0.962232439	338	546	653	599	690	574	736	683
ENSECAG00000009743	3.152533975	0.773593767	0.962681842	70	111	222	230	274	137	227	141
ENSECAG00000014389	2.576949959	0.773725648	0.962681842	89	58	135	163	78	85	160	159
ENSECAG00000017256	9.285110821	0.773784777	0.962681842	3900	9369	13593	18589	14739	18007	9654	11898
ENSECAG00000017626	5.02892639	0.773799891	0.962681842	377	723	507	639	580	554	1035	732
ENSECAG00000022864	4.751552102	0.773808187	0.962681842	392	452	497	644	603	489	644	464
ENSECAG00000019788	3.888263218	0.773865983	0.962681842	116	283	267	497	267	345	279	295
ENSECAG00000010012	5.334219969	0.774012873	0.962719572	474	631	781	1189	817	780	923	773
ENSECAG00000015142	6.185800286	0.7741233	0.962719572	947	1128	1555	1843	1509	1227	1568	1638
ENSECAG00000016772	5.272273563	0.774149994	0.962719572	430	785	577	907	818	1011	751	758
ENSECAG00000003462	4.872762283	0.774335038	0.962844518	375	373	922	504	634	611	572	546
ENSECAG00000017621	4.139421563	0.774631902	0.963037862	200	204	426	405	268	377	470	434
ENSECAG00000014796	5.689024885	0.774659705	0.963037862	364	930	1246	1521	981	1140	974	1068
ENSECAG00000026930	5.157439159	0.774800796	0.963108097	304	710	848	880	669	842	599	759
ENSECAG00000010953	0.653966787	0.774958343	0.96319877	13	20	53	29	36	13	39	31
ENSECAG00000012556	4.945331412	0.775179029	0.963219057	353	788	470	685	517	888	609	454
ENSECAG00000022552	4.312465114	0.775207309	0.963219057	248	315	479	440	466	446	392	294
ENSECAG00000000723	2.588123955	0.775228478	0.963219057	61	78	129	150	115	155	136	113
ENSECAG00000023451	5.007826813	0.775365581	0.963284279	376	371	661	868	975	437	800	616
ENSECAG00000010807	2.977755499	0.775469396	0.963296852	40	278	133	153	227	74	195	134
ENSECAG00000024811	5.071031983	0.775563743	0.963296852	343	286	1066	673	938	607	764	636
ENSECAG00000011132	11.1061478	0.775629534	0.963296852	45757	40575	30783	39068	28698	42164	46070	61003
ENSECAG00000020824	7.885640664	0.775733255	0.963320583	4608	4680	2917	4672	3754	4128	5574	5782
ENSECAG00000022269	7.097500075	0.775838304	0.963345958	1320	3225	2893	3002	2045	3547	2486	2884
ENSECAG00000015256	6.138731164	0.776072793	0.963410145	716	1665	1238	1244	1387	1554	1564	1659
ENSECAG00000023993	1.958418425	0.776227621	0.963410145	35	26	148	92	138	33	87	44
ENSECAG00000009415	5.464203542	0.776459085	0.963410145	637	603	924	846	987	1014	991	846
ENSECAG00000015551	5.808423272	0.77646897	0.963410145	611	1417	1046	1097	1213	1239	1234	854
ENSECAG00000010629	5.859561947	0.776571137	0.963410145	764	887	675	1737	1076	939	1004	2080
ENSECAG00000008866	5.892443272	0.776574915	0.963410145	672	1292	1258	1239	984	1901	711	1083
ENSECAG00000012206	1.480389538	0.776620983	0.963410145	30	50	27	81	63	28	79	73
ENSECAG00000026848	6.114323025	0.776687779	0.963410145	996	946	1656	1554	1811	1441	1370	956
ENSECAG00000011043	4.76260916	0.776742163	0.963410145	257	340	582	958	451	454	598	713
ENSECAG00000013844	3.550280194	0.776820301	0.963410145	103	215	203	392	198	206	262	283
ENSECAG00000006182	2.479196046	0.776939916	0.963410145	74	85	84	132	110	51	170	165
ENSECAG00000009052	2.99497026	0.77695451	0.963410145	93	86	190	182	189	158	203	145
ENSECAG00000008409	4.018084888	0.777011305	0.963410145	114	242	607	295	149	282	288	583
ENSECAG00000017576	2.895004203	0.777074692	0.963410145	67	131	135	189	142	162	209	137
ENSECAG00000019639	5.030584956	0.777353156	0.963555026	429	404	667	776	862	633	767	586
ENSECAG00000001903	4.720822182	0.777398376	0.963555026	295	362	564	639	505	510	789	521
ENSECAG00000003312	5.022106389	0.777445452	0.963555026	406	642	592	780	652	565	610	818
ENSECAG00000017934	4.501713001	0.777622168	0.963624248	284	455	361	599	265	479	427	665
ENSECAG00000008176	5.474317468	0.777670583	0.963624248	581	716	814	962	1041	929	1115	796
ENSECAG00000012485	6.113339424	0.777921423	0.963830167	833	2040	1073	1126	1762	1005	1854	1043
ENSECAG00000015091	5.079241715	0.778032036	0.963862321	297	763	685	843	662	906	475	648
ENSECAG00000017703	3.720424195	0.778495384	0.964143232	169	301	269	222	329	197	333	213

ENSECAG00000016577	3.860069409	0.778540361	0.964143232	125	265	241	402	527	251	295	186
ENSECAG00000008574	2.620036621	0.778542933	0.964143232	72	101	111	135	141	114	121	155
ENSECAG00000012898	0.719363563	0.778627175	0.964143232	16	14	49	27	16	57	29	34
ENSECAG00000022660	7.943075981	0.77882257	0.964143232	3197	6037	3817	5235	4644	4655	6483	4287
ENSECAG00000016120	5.628310724	0.778832081	0.964143232	616	1105	799	1156	758	906	1082	1306
ENSECAG00000013487	1.631724714	0.778860104	0.964143232	20	39	69	90	66	72	66	60
ENSECAG00000008867	5.157728976	0.778936272	0.964143232	407	684	607	1011	650	887	609	726
ENSECAG00000015282	3.799111299	0.779301494	0.964378952	143	187	286	467	181	231	415	318
ENSECAG00000005234	2.331845694	0.779487311	0.964378952	53	60	123	109	127	110	76	117
ENSECAG00000015322	7.15392979	0.779493272	0.964378952	2170	2596	2724	2977	2778	2416	3913	2572
ENSECAG00000021450	0.301081594	0.779596308	0.964378952	9	10	22	41	18	23	38	23
ENSECAG00000000934	7.128003945	0.77966562	0.964378952	1919	2769	2424	2274	3090	2947	3177	3080
ENSECAG00000016809	4.487213409	0.779736109	0.964378952	176	315	425	728	443	462	531	524
ENSECAG00000004023	3.302241835	0.779800518	0.964378952	144	158	145	219	201	166	294	208
ENSECAG00000021889	7.023571178	0.779804359	0.964378952	1189	3271	2255	2254	3444	2478	3620	1989
ENSECAG00000007717	6.001410113	0.779944664	0.964447703	864	1177	1270	1432	1546	1075	1435	1177
ENSECAG00000012159	2.788258208	0.780358448	0.964662548	30	117	129	228	76	130	185	221
ENSECAG00000000743	4.128883582	0.780408549	0.964662548	218	414	298	358	249	256	571	377
ENSECAG00000015518	5.629032382	0.780525304	0.964662548	577	761	1015	1412	987	910	1150	1008
ENSECAG00000017211	4.323921604	0.780584826	0.964662548	324	325	438	353	335	377	400	516
ENSECAG00000011657	6.455036403	0.780604442	0.964662548	794	2132	2269	1435	853	1816	1516	2896
ENSECAG00000021648	4.401568652	0.780769408	0.964662548	157	210	530	648	719	423	424	268
ENSECAG00000009828	9.017465124	0.780854646	0.964662548	8335	17698	6397	4206	9887	13762	12502	5019
ENSECAG00000014431	5.920212208	0.780869476	0.964662548	870	1050	1116	1441	1160	871	1601	1372
ENSECAG00000000291	8.441580375	0.780941854	0.964662548	4566	6699	4363	10984	4820	5790	8922	8912
ENSECAG00000016459	11.11820298	0.781048935	0.964662548	23025	26428	53380	53967	54808	47759	46459	46496
ENSECAG00000016874	6.05264361	0.78105045	0.964662548	776	999	1390	1483	1434	1398	1673	1306
ENSECAG00000016489	10.93420827	0.781291651	0.964855782	22402	21909	47883	44298	48963	42814	36079	43647
ENSECAG00000005737	0.818311481	0.781486563	0.9648883	6	15	39	64	32	47	15	51
ENSECAG00000010476	5.453820913	0.781541431	0.9648883	583	556	822	1094	1101	615	1380	796
ENSECAG00000024012	5.939428818	0.781670753	0.9648883	251	999	1109	2269	1009	1157	1767	1542
ENSECAG00000010756	4.66564933	0.781681288	0.9648883	431	373	453	423	565	408	815	463
ENSECAG00000018671	2.135126936	0.781741737	0.9648883	49	163	55	52	13	126	42	152
ENSECAG00000015380	3.773572522	0.782165948	0.965033028	160	185	275	337	245	265	348	339
ENSECAG00000011291	8.119917305	0.782177173	0.965033028	4099	4675	5121	6860	5565	4110	7759	5427
ENSECAG00000012896	6.539097714	0.782235686	0.965033028	1166	1838	1788	2164	2348	1862	1827	1474
ENSECAG00000020939	3.635095762	0.78237871	0.965033028	90	180	338	370	214	338	197	234
ENSECAG00000018573	3.082340877	0.782389265	0.965033028	74	258	133	100	133	277	150	168
ENSECAG00000016599	4.544951245	0.782403156	0.965033028	298	307	411	623	528	460	540	504
ENSECAG00000011767	5.875844496	0.782497893	0.965033028	830	923	1270	1319	1150	1280	1201	1138
ENSECAG00000019810	6.176807733	0.78262785	0.965033028	921	996	1464	1653	1832	1130	2050	1383
ENSECAG00000017829	3.402900486	0.782629109	0.965033028	103	201	198	235	164	192	233	336
ENSECAG00000018723	5.422895567	0.78270663	0.965033028	466	846	665	1309	821	617	772	1287
ENSECAG00000000586	0.194684482	0.783100039	0.965370565	4	19	30	32	7	27	26	23
ENSECAG00000006472	5.013590394	0.783149982	0.965370565	470	433	667	831	799	451	745	652
ENSECAG00000018468	5.157151482	0.783267956	0.965411463	399.996	478.998	1007.99	814.996	877.992	770.995	657	566.997
ENSECAG00000013766	1.113171072	0.783539923	0.965550482	26	26	37	54	32	40	71	43
ENSECAG00000012908	3.97567927	0.783626022	0.965550482	179	251	302	468	439	222	282	326
ENSECAG00000015110	0.998866805	0.783719918	0.965550482	23	35	43	26	38	19	75	42
ENSECAG00000021356	2.584939021	0.783719983	0.965550482	58	165	94	130	72	122	130	155
ENSECAG00000011147	8.081083904	0.783815074	0.965563148	5444	4478	4422	4859	4801	4303	6967	6089
ENSECAG00000010779	4.876694592	0.784167039	0.965710672	431	472	585	670	710	586	616	473
ENSECAG00000014344	2.262987965	0.784178814	0.965710672	56	72	94	141	59	57	115	155
ENSECAG00000007343	3.367040629	0.784189298	0.965710672	60	254	212	281	166	313	144	184
ENSECAG00000010368	3.550329945	0.78451932	0.966004195	135	207	187	285	199	305	285	227
ENSECAG00000017508	3.362391342	0.784597347	0.966004195	103	136	218	269	231	137	369	180
ENSECAG00000007373	1.566465654	0.784896945	0.966090671	44	43	51	51	31	31	51	145
ENSECAG00000017527	2.456996182	0.784986443	0.966090671	50	97	115	116	178	92	121	83
ENSECAG00000015528	5.41865773	0.78499357	0.966090671	674	948	696	754	544	1059	859	1005
ENSECAG00000019888	4.106115105	0.785023073	0.966090671	205	312	360	408	366	317	397	324
ENSECAG00000024125	4.87539643	0.785135652	0.966090671	382	315	614	747	510	518	755	800
ENSECAG00000014325	2.895625504	0.785248153	0.966090671	105	95	176	175	138	83	262	130
ENSECAG00000009086	4.456417587	0.785412802	0.966090671	233	331	565	390	714	378	629	209
ENSECAG00000008221	9.548410427	0.785561078	0.966090671	6149	14739	18108	13008	9951	22006	8462	24773
ENSECAG00000016800	8.835787518	0.785656631	0.966090671	5498	7003	7643	11735	10156	6768	14033	10009
ENSECAG00000022878	5.579695038	0.7857401	0.966090671	529	405	1548	837	1410	981	1444	398
ENSECAG00000021036	4.416842696	0.786092863	0.966090671	312	302	414	428	402	460	496	500
ENSECAG00000001830	2.194490429	0.786173238	0.966090671	23	116	91	127	74	121	47	109
ENSECAG00000010593	5.449471831	0.786186216	0.966090671	355	909	870	1257.01	970.007	1053	701	778
ENSECAG00000023225	5.471308858	0.786206844	0.966090671	505	751	1166	903	1287	729	948	638
ENSECAG00000014402	3.982057597	0.786208621	0.966090671	195	206	328	371	329	222	515	337
ENSECAG00000011663	1.985363985	0.786232808	0.966090671	45	45	123	82	89	70	112	43
ENSECAG00000010342	2.892053547	0.786421078	0.966090671	84	132	172	166	91	145	172	191
ENSECAG00000008963	6.21542734	0.786448934	0.966090671	1121	1003	1574	1806	1459	1372	1996	1282
ENSECAG00000013418	3.822190924	0.786525718	0.966090671	187	281	254	319	304	260	315	270
ENSECAG00000012444	2.99604408	0.786622698	0.966090671	61	143	148	274	130	153	149	206

ENSECAG00000010644	8.205626999	0.786732055	0.966090671	4701	6142	5029	5581	5731	5341	7560	5473
ENSECAG00000018122	1.163275782	0.786755507	0.966090671	16	44	37	73	33	34	43	63
ENSECAG00000004370	3.169182027	0.78677261	0.966090671	147	136	99	219	154	262	188	171
ENSECAG00000022987	3.730262986	0.786774226	0.966090671	114	403	172	215	181	224	486	301
ENSECAG00000023579	3.242726272	0.786788995	0.966090671	144	115	250	184	252	214	187	97
ENSECAG00000017952	3.160205332	0.787287061	0.966561532	69	275	110	153	152	350	159	103
ENSECAG00000007748	4.13247706	0.787483357	0.966561532	212	248	341	426	281	361	454	439
ENSECAG00000021331	0.05261986	0.787637157	0.966561532	8	14	27	25	23	15	16	20
ENSECAG00000016517	5.376137916	0.787648493	0.966561532	526	635	858	860	732	985	874	1013
ENSECAG00000000518	7.542884629	0.787660055	0.966561532	1833	1717	4061	5877	5234	3050	4514	3698
ENSECAG00000001039	4.252333206	0.787681853	0.966561532	265	326	351	353	426	389	500	347
ENSECAG00000012720	4.337348651	0.787888646	0.966711094	135	490	381	432	459	469	502	330
ENSECAG00000009730	6.191056008	0.788142342	0.966883304	1379	807	1631	1424	1584	1407	1623	1329
ENSECAG00000008058	1.807608473	0.788248049	0.966883304	46	41	62	86	92	48	102	62
ENSECAG00000015487	8.661608419	0.788283779	0.966883304	5906	10131	6833	6577	8946	9276	7391	6775
ENSECAG00000006052	2.307269527	0.788462605	0.966940651	48	78	112	138	89	68	120	121
ENSECAG00000021846	3.474229058	0.788507949	0.966940651	177	116	180	279	211	117	384	285
ENSECAG00000020333	4.762500897	0.788585327	0.966940651	286	423	631	570	610	534	652	568
ENSECAG00000020261	7.605431279	0.788697035	0.966972868	2521	3746	3682	4674	5375	3167	4206	3021
ENSECAG00000020559	2.421614689	0.788789801	0.966972868	69	70	142	115	83	110	156	82
ENSECAG00000012141	5.820560131	0.788973872	0.966972868	479	1309	772.999	1469	1038	1539	1065	1247
ENSECAG00000022272	8.696050192	0.788980139	0.966972868	6026	7381	6781	10860	6924	6940	9287	10673
ENSECAG00000003209	1.852905958	0.789036271	0.966972868	33	74	34	108	68	51	143	58
ENSECAG00000011964	7.014016837	0.789156671	0.967016327	885	2222	2454	3867.02	3563.01	2691	3254	1864
ENSECAG00000017137	2.521779338	0.789324391	0.967073412	36	118	126	118	46	121	79	254
ENSECAG00000014740	6.749520726	0.789373142	0.967073412	1395	1641	1846	2581	2918	1916	2923	1731
ENSECAG00000022077	6.821197406	0.789658573	0.967077858	1475	1813	2597	2585	2465	2276	2347	2074
ENSECAG00000014173	5.259734805	0.789795398	0.967077858	549	631	671	752	386	939	984	1058
ENSECAG00000014633	5.902545826	0.789798798	0.967077858	459	869	1066	2421	1061	992	1420	1427
ENSECAG00000012315	7.404964055	0.789806818	0.967077858	2222	2028	4087	3233	4625	2844	6216	1572
ENSECAG00000013539	4.688526807	0.789834779	0.967077858	378	342	476	572	548	363	779	593
ENSECAG00000011296	5.264749668	0.78988643	0.967077858	718	700	648	639	877	611	912	752
ENSECAG00000012966	4.608265212	0.790078186	0.967133542	241	490	390	752	386	566	523	506
ENSECAG00000015580	6.964374152	0.790238389	0.967133542	1863	2117	2470	2763	2673	1887	3251	2446
ENSECAG00000015005	7.745741925	0.790284198	0.967133542	2951	4197	3668	3500	3033	8176	2501	4711
ENSECAG00000016109	3.485162047	0.790316057	0.967133542	138	275	134	205	200	125	379	299
ENSECAG00000022811	4.248669285	0.790666997	0.967133542	233	249	328	530	301	299	520	559
ENSECAG00000022278	3.785490527	0.79078861	0.967133542	155	138	170	533	256	190	555	244
ENSECAG00000024995	4.814407322	0.790813258	0.967133542	243	463	604	884	383	687	548	660
ENSECAG00000004313	7.338884382	0.791075549	0.967133542	2430	2906	2323	3245	3144	3577	4180	3370
ENSECAG00000024390	7.164377618	0.79114827	0.967133542	1532	2917	2793	3703	3333	3059	2500	2632
ENSECAG00000000482	5.661368559	0.791185967	0.967133542	397	1025	957	1254	1018	1404	797	1128
ENSECAG00000012545	4.308223746	0.791408723	0.967133542	146	397	336	679	431	391	395	385
ENSECAG00000017230	3.734070101	0.79150342	0.967133542	129	148	346	322	237	348	287	279
ENSECAG00000024587	6.349530413	0.791548343	0.967133542	905	1332	1411	2115	2099	1080	1709	2272
ENSECAG00000020497	3.23249947	0.791553415	0.967133542	96	168	197	188	170	107	345	220
ENSECAG00000010855	6.43930811	0.791560703	0.967133542	1074	1920	1440	2030	1449	1698	1847	2094
ENSECAG00000013032	3.457106598	0.791639714	0.967133542	137	243	144	218	231	101	359	291
ENSECAG00000020475	6.801642114	0.791641509	0.967133542	2027	2555	1714	1563	2871	2159	2154	1793
ENSECAG00000009254	1.083089915	0.791759087	0.967133542	17	72	27	37	61	28	57	17
ENSECAG00000015649	4.104551932	0.791903584	0.967133542	162	321	328	407	339	385	337	425
ENSECAG00000006884	3.232692339	0.792031933	0.967133542	90	144	196	233	211	148	194	264
ENSECAG00000016252	6.133023389	0.792239804	0.967133542	1014	1585	1023	1055	1667	1360	1885	1258
ENSECAG00000005781	4.888073212	0.792392432	0.967133542	340	537	588	774	411	533	435	1020
ENSECAG00000008215	10.34646997	0.792477767	0.967133542	17449	35036	23629	19181	15359	29775	18099	40626
ENSECAG00000007702	6.889898115	0.792643463	0.967133542	1048	2536	2172	3453	2123	2805	2451	2198
ENSECAG00000000722	2.938157145	0.792695474	0.967133542	113	64	189	149	208	115	225	126
ENSECAG00000015523	0.724711144	0.792740884	0.967133542	12	23	43	31	38	43	33	22
ENSECAG00000019067	5.961423415	0.792797699	0.967133542	475	1095	1203	2161	976	1195	1317	1598
ENSECAG00000014107	1.514236345	0.79281355	0.967133542	22	38	95	60	56	41	86	43
ENSECAG00000005413	2.700384723	0.792847419	0.967133542	57	123	119	156	126	138	132	164
ENSECAG00000006208	0.430635457	0.793175234	0.967133542	13	21	24	40	22	20	16	41
ENSECAG00000005876	3.616446459	0.793223862	0.967133542	140	151	250	322	373	202	263	225
ENSECAG00000019791	3.681638328	0.79330181	0.967133542	176	248	180	347	241	192	286	328
ENSECAG00000019207	8.255681348	0.793337312	0.967133542	4450	3943	6032	8669	6753	4754	7727	5766
ENSECAG00000022225	5.750835767	0.793440283	0.967133542	459	901	1315	1503	1083	1081	1519	743
ENSECAG00000011599	6.225990808	0.793568662	0.967133542	816	1411	1652	1774	1803	1083	1828	1439
ENSECAG00000018062	8.076877002	0.793597292	0.967133542	3612	6233	4184	5994	4559	4867	6750	5929
ENSECAG00000009913	3.836720026	0.793623635	0.967133542	205	235	302	303	272	352	295	232
ENSECAG00000025021	5.31389625	0.793627933	0.967133542	410	767	663	1220	499	646	760	1349
ENSECAG00000016794	5.25838702	0.793677353	0.967133542	427	394	616	1337	852	443	1013	1088
ENSECAG00000016462	3.24829749	0.793765822	0.967133542	86	181	164	305	136	127	190	317
ENSECAG00000021996	6.960711309	0.793788872	0.967133542	1682	1575	2766	2532	2616	2334	4113	2046
ENSECAG00000007964	4.562665622	0.79387359	0.967133542	202	216	415	921	450	333	528	774
ENSECAG00000014664	4.649190986	0.794017689	0.967133542	230	473	714	477	635	419	414	558
ENSECAG00000015165	5.619473254	0.79422285	0.967133542	431	943	1077	1333	1275	884	897	930

ENSECAG00000022614	4.061814128	0.794230952	0.967133542	200	302	312	337	432	432	288	276
ENSECAG00000019789	2.961209622	0.794322318	0.967133542	65	125	133	290	128	111	151	237
ENSECAG00000014065	5.306383507	0.794329032	0.967133542	560	884	526	686	879	429	1253	973
ENSECAG00000007881	2.453379377	0.794356357	0.967133542	190	99	21	28	91	100	158	85
ENSECAG00000009972	2.472559985	0.794364583	0.967133542	65	98	104	147	100	101	124	120
ENSECAG00000013948	4.266333062	0.794707775	0.967133542	227	277	415	421	526	388	549	219
ENSECAG00000004091	3.099906224	0.794781739	0.967133542	66	73	213	272	209	153	182	201
ENSECAG00000024939	2.996841261	0.794788372	0.967133542	63	120	260	182	55	215	22	317
ENSECAG00000013789	0.964398426	0.794788852	0.967133542	12	23	38	62	14	36	66	52
ENSECAG00000015938	2.875532829	0.79481912	0.967133542	54	131	155	232	153	152	160	122
ENSECAG00000016050	5.573776704	0.79485282	0.967133542	642	857	923	1094	919	822	1213	978
ENSECAG00000017355	1.847786058	0.794911836	0.967133542	33	57	52	109	77	93	70	65
ENSECAG00000016209	5.269894887	0.794930633	0.967133542	364	652	749	1223	861	743	841	703
ENSECAG00000010544	7.226467863	0.794948083	0.967133542	1982	2118	3861	2119	4400	2862	4662	1392
ENSECAG00000024276	4.549477461	0.79496958	0.967133542	499	322	378	439	325	703	504	349
ENSECAG00000016343	6.375255118	0.795079062	0.967133542	763	1493	1688	2490	1550	1614	1876	1748
ENSECAG00000010140	3.956602016	0.795113742	0.967133542	103	288	316	419	306	353	309	375
ENSECAG00000019848	4.219812551	0.795232892	0.967175139	294	277	294	511	260	208	644	448
ENSECAG00000018672	1.981984731	0.795472096	0.967183871	16	61	116	120	67	43	110	96
ENSECAG00000024905	6.125122347	0.795522538	0.967183871	919	934.001	1389	1611	1832	1112	2100	1126
ENSECAG00000003237	6.064280718	0.79556521	0.967183871	871	1290	1131	1298	1243	1134	1726	1791
ENSECAG00000012511	4.381197426	0.795589459	0.967183871	238	259	485	486	449	337	629	422
ENSECAG00000017588	4.934600683	0.795664834	0.967183871	318	452	636	961	361	395	699	1068
ENSECAG00000009936	6.874333961	0.796049418	0.967299653	1201	1468	2277	3494	2308	2280	2690	3041
ENSECAG00000010601	3.67576237	0.796173922	0.967299653	127	202	266	306	203	378	245	271
ENSECAG00000018303	7.667366878	0.796245392	0.967299653	2785	4943	3221	3964	3727	3945	4456	4413
ENSECAG00000023393	5.193798799	0.796279375	0.967299653	399	559	714	929	845	834	813	675
ENSECAG00000024126	6.095385506	0.796298582	0.967299653	666	877	1241	2185	1421	945	1956	1751
ENSECAG00000011591	7.296933472	0.796691229	0.967299653	1562	2278	3173	4226	3211	3228	4131	3298
ENSECAG00000024704	4.209375012	0.796754213	0.967299653	306	277	278	374	315	186	614	544
ENSECAG00000026980	6.374276849	0.79676409	0.967299653	874	1073	1970	1982	1627	1716	2017	1906
ENSECAG00000022693	7.845211248	0.796774969	0.967299653	4467	4033	3884	3927	6263	3099	6265	3188
ENSECAG00000018313	2.368627202	0.796864437	0.967299653	64	87	124	103	93	90	133	100
ENSECAG00000023875	0.690229538	0.796937656	0.967299653	20	15	30	40	29	32	46	28
ENSECAG00000011164	3.158199009	0.797028864	0.967299653	107	191	134	235	89	209	187	233
ENSECAG00000013411	5.694474025	0.797091788	0.967299653	384	805	1188	1401	1036	1154	1052	1254
ENSECAG00000010426	3.81214148	0.797094791	0.967299653	138	230	327	379	380	161	355	253
ENSECAG00000011938	6.091820495	0.797190462	0.967299653	866	1107	1299	1460	1589	1563	1612	1166
ENSECAG00000003229	8.678767179	0.79720272	0.967299653	5310	7852	6713	8090	8491	8870	10538	8237
ENSECAG00000004756	4.163277633	0.797265149	0.967299653	232	244	358	412	354	354	420	430
ENSECAG00000022567	10.54100478	0.797354117	0.967299653	16954	20733	37671	39168	31317	31621	25633	31043
ENSECAG00000003510	5.510687639	0.797455851	0.967299653	814	543	1040	877	892	738	1389	762
ENSECAG00000006249	4.537495456	0.797459336	0.967299653	229	247	510	694	378	398	575	691
ENSECAG00000000021	3.104749061	0.797635595	0.967343506	132	62	161	231	167	78	300	228
ENSECAG00000022112	4.897288438	0.797665423	0.967343506	266	471	665	739	514	602	825	673
ENSECAG00000019778	4.237574242	0.797811531	0.967417646	235	277	359	445	406	295	417	527
ENSECAG00000018873	2.979755755	0.798058711	0.967552929	96	83	178	190	151	127	226	189
ENSECAG00000024681	7.802320275	0.798144655	0.967552929	2957	4669	4412	4478	5021	4851	4637	3524
ENSECAG00000006696	4.310756765	0.798178051	0.967552929	132	346	325	660	368	302	511	568
ENSECAG00000024418	4.586893886	0.798474919	0.967573893	392	304	459	459	677	268	706	475
ENSECAG00000016338	3.723470301	0.798494134	0.967573893	209	289	176	191	247	328	269	297
ENSECAG00000021727	7.019543975	0.798516345	0.967573893	1680	641	3461	4405	3341	1193	2656	3330
ENSECAG00000020192	3.844454159	0.798535292	0.967573893	264	214	189	368	237	211	356	375
ENSECAG00000015559	6.585830759	0.799056655	0.968078297	1163	2565	1104	2321	1555	1836	2251	2220
ENSECAG00000016813	4.568337475	0.799121637	0.968078297	355	398	421	563	449	328	718	479
ENSECAG00000008412	3.741054905	0.799217901	0.968091904	163	226	224	315	261	202	320	387
ENSECAG00000023549	4.996715509	0.79952316	0.968126546	467	535	654	480	958	666	895	274
ENSECAG00000000682	4.975629677	0.799562521	0.968126546	332	534	658	870	547	537	655	842
ENSECAG00000013540	6.217971602	0.799656405	0.968126546	688	796	2070	2283	1739	1438	1453	1399
ENSECAG00000021867	4.680125544	0.799699181	0.968126546	433	400	444	578	786	407	512	372
ENSECAG00000007125	7.881841261	0.799713468	0.968126546	3220	3743	4817	5904	4438	4375	6117	4410
ENSECAG00000012927	5.967731019	0.799756712	0.968126546	510	1139	1230	2032	713	1168	1579	1698
ENSECAG00000020403	4.61322104	0.799855522	0.968133672	188	386	494	720	551	410	623	559
ENSECAG00000024956	8.604086917	0.799943805	0.968133672	3575	8136	7929	7571	6554	9662	9642	8384
ENSECAG00000017521	4.674001711	0.800077714	0.968133672	478	438	440	277	713	484	775	277
ENSECAG00000020284	5.123024918	0.800143337	0.968133672	365	611	896	747	656	852	665	649
ENSECAG00000013654	4.139562971	0.800257851	0.968133672	172	317.001	395.002	455	304	409	362.002	354
ENSECAG00000012713	5.852093059	0.800274036	0.968133672	523	1131	1027	1447	1664	1164	1139	1028
ENSECAG00000016635	4.543604768	0.80035785	0.968133672	256	528	387	577	393	621	366	491
ENSECAG00000009822	7.236230154	0.800449836	0.968142079	1516	1733	3829	3642	4749	2561	3320	2582
ENSECAG00000013445	4.886533101	0.800658515	0.968147334	300	527	699	711	698	592	653	468
ENSECAG00000001360	4.492965213	0.800882906	0.968147334	270	402	391	508	417	418	629	509
ENSECAG00000014598	2.333058097	0.800896774	0.968147334	48	83	100	119	121	90	135	90
ENSECAG00000016753	3.57158504	0.800916722	0.968147334	120	199	189	337	166	300	315	254
ENSECAG00000021492	6.184841852	0.800964816	0.968147334	1204	982	1249	1468	1910	1063	1819	1602
ENSECAG00000024091	9.492156303	0.801029646	0.968147334	7897	8982	17542	16169	17621	16108	13600	15486

ENSECAG00000009659	5.337682114	0.801207057	0.968147334	562	710	770	935	649	694	1012	989
ENSECAG00000007576	4.84611563	0.801243785	0.968147334	394	238	625	948	754	287	907	435
ENSECAG00000018817	5.2314541	0.801449329	0.968147334	289	606	756	1096	640	885	818	920
ENSECAG00000020401	7.049992774	0.80149949	0.968147334	1619	2183	2141	3312	1844	2784	3279	3791
ENSECAG00000020775	0.340478663	0.80153333	0.968147334	7	39	15	31	13	5	33	44
ENSECAG00000024333	6.07755125	0.801771947	0.968147334	966	897	1111	1717	1218	1088	1917	1753
ENSECAG00000006004	4.636303009	0.801806175	0.968147334	299	570	467	492	564	542	473	434
ENSECAG00000022741	5.378269292	0.801842465	0.968147334	506	1890	310	234	1113	727	1007	490
ENSECAG00000026808	5.06024192	0.801951941	0.968147334	264	733	540	1061	648	798	506	728
ENSECAG00000018012	1.881602559	0.802028892	0.968147334	4	53	159	36	21	139	14	140
ENSECAG00000022972	2.111293472	0.802119955	0.968147334	24	77	65	145	69	83	77	143
ENSECAG00000002931	4.050986294	0.802146543	0.968147334	189	252	366	444	358	319	357	315
ENSECAG00000007147	7.853709242	0.802270317	0.968147334	3285	4649	4143	4969	4271	3896	5726	5097
ENSECAG00000011186	0.366843586	0.802313979	0.968147334	11	29	24	28	20	2	48	29
ENSECAG00000021820	7.35347419	0.802324682	0.968147334	2306	2637	3245	4052	5037	2620	3808	1793
ENSECAG00000002455	0.896481191	0.802324997	0.968147334	15	41	18	50	23	38	63	35
ENSECAG00000011287	4.832432233	0.802501268	0.968257411	182	414	312	1220	224	310	470	1545
ENSECAG00000000473	3.946978693	0.802658445	0.968344431	212	258	201	390	327	145	505	400
ENSECAG00000016289	5.909953205	0.802808945	0.968356655	814	1083	1072	1488	1258	1007	1449	1239
ENSECAG00000012308	3.109454627	0.802883448	0.968356655	93	150	155	203	239	169	169	164
ENSECAG00000016440	5.610115389	0.802982654	0.968356655	481	1031	828	1384	972	1012	980	1019
ENSECAG00000009365	2.848204817	0.803008799	0.968356655	95	95	139	161	94	86	200	258
ENSECAG00000001130	5.141100994	0.803146993	0.968420728	443	619	648	927	724	537	981	686
ENSECAG00000020668	0.94214568	0.803409006	0.968634071	18	14	56	56	38	18	61	33
ENSECAG00000018168	0.94407021	0.803521079	0.968666612	16	25	34	71	49	30	33	34
ENSECAG00000008021	2.508406422	0.80385008	0.968956591	85.0017	76	125	129	124.001	90.0013	180	68.0004
ENSECAG00000012822	9.85509099	0.804183614	0.968956591	9043	12657	21963	22096	20493	23824	16346	19766
ENSECAG00000026909	6.860806179	0.804271758	0.968956591	1744	1334	2710	2042	3572	1703	3964	1154
ENSECAG00000012674	5.147207147	0.804272632	0.968956591	455	619	654	725	881	683	768	736
ENSECAG00000002462	2.402499151	0.804275955	0.968956591	53	130	62	156	40	80	108	195
ENSECAG00000000698	1.915084922	0.804307158	0.968956591	35	71	67	113	58	93	45	96
ENSECAG00000014995	2.741488119	0.804450522	0.968956591	92	102	109	194	125	97	157	163
ENSECAG00000009870	1.786571151	0.804457274	0.968956591	20	49	75	102	89	73	72	59
ENSECAG00000015301	5.307091906	0.804561432	0.968956591	449	143	1554	921	1004	632	1165	423
ENSECAG00000010817	0.714825697	0.804671531	0.968956591	11	24	40	33	11	21	31	76
ENSECAG00000011472	2.94367071	0.804709463	0.968956591	103	159	141	156	136	139	219	134
ENSECAG00000023695	0.364617877	0.805150419	0.968956591	11	32	16	32	16	25	25	30
ENSECAG00000008098	7.163604409	0.805184358	0.968956591	1889	1492	3523	2941	4721	2575	4491	958
ENSECAG00000011380	7.166805901	0.805301751	0.968956591	3012	3382	1906	1566	3367	3304	3315	1575
ENSECAG00000018393	4.972859555	0.805327854	0.968956591	420	596	518	620	724	561	963	512
ENSECAG00000018495	6.21620487	0.805411336	0.968956591	862	916	1885	1559	1793	1584	1680	1409
ENSECAG00000008439	8.340359682	0.805464625	0.968956591	5142	6381	6437	5471	8358	5768	7141	5031
ENSECAG00000013251	5.188221672	0.805471551	0.968956591	291.999	513	730.998	1146	818	715	759	871.999
ENSECAG00000000162	8.30704998	0.805515609	0.968956591	5494	6138	5481	5578	4983	6622	7703	6559
ENSECAG00000017967	3.534284519	0.805561784	0.968956591	130	140	209	345	243	142	347	292
ENSECAG00000019997	5.295444724	0.805600765	0.968956591	598	471	732	895	979	491	1279	739
ENSECAG00000013460	1.964194819	0.805634	0.968956591	28	48	90	110	141	55	64	72
ENSECAG00000014721	0.90987935	0.805815916	0.968993044	14	35	34	43	37	33	59	30
ENSECAG00000022606	3.29912535	0.805909963	0.968993044	117	139	187	244	224	129	261	248
ENSECAG00000014260	11.81191176	0.806375995	0.968993044	35356	123923	74946	37507	4681	138257	6181	121223
ENSECAG00000015620	3.522485571	0.806413212	0.968993044	134	124	221	336	243	154	315	299
ENSECAG00000001598	5.090177105	0.806520713	0.968993044	534	787	449	671	658	731	768	626
ENSECAG00000000397	7.151996946	0.806858549	0.968993044	1866	2200	2713	3037	3658	2139	3764	2998
ENSECAG00000020281	6.87480511	0.806888598	0.968993044	2444	2416	1590	1685	2977	1344	3392	1964
ENSECAG00000007660	6.246824794	0.806907228	0.968993044	1117	883	1528	1708	1409	1153	2288	1892
ENSECAG00000015107	5.261492973	0.807012775	0.968993044	596	741	607	829	731	726	841	850
ENSECAG00000012024	5.29363187	0.807020325	0.968993044	313	954	920	530	327	788	1028	1337
ENSECAG00000010166	4.802095725	0.807039493	0.968993044	339	404	579	802	268	463	405	1130
ENSECAG00000015162	7.378925109	0.807082605	0.968993044	1649	2327	3882	5119	4280	2982	3422	2807
ENSECAG00000013630	3.423263287	0.807117404	0.968993044	142	147	172	351	183	132	345	229
ENSECAG00000008514	6.78507261	0.807163375	0.968993044	1271	1659	2032	2831	2811	2167	2513	2134
ENSECAG00000025866	0.709251627	0.807232181	0.968993044	5	27	34	47	25	49	32	28
ENSECAG00000021990	3.829864842	0.807254651	0.968993044	141	195	276	409	326	260	306	340
ENSECAG00000013687	3.156574867	0.807260721	0.968993044	127	156	127	249	137	128	216	248
ENSECAG00000023261	5.341346224	0.807285195	0.968993044	351	789	888	1084	678	938	720	953
ENSECAG00000016326	6.066675686	0.807384774	0.968993044	918	1110	1361	1196	1421	1255	1962	1259
ENSECAG00000000869	4.099925119	0.807397522	0.968993044	247	264	391	351	207	273	461	478
ENSECAG00000008605	5.437564846	0.807519045	0.968993044	489	980	651	1137	522	702	1130	1241
ENSECAG00000013424	9.822602634	0.807536759	0.968993044	10387	10832	25385	22836	17842	17204	17183	21071
ENSECAG00000001110	1.154060387	0.80781478	0.969220187	29	17	75	21	56	40	57	36
ENSECAG00000020873	2.270528559	0.807921136	0.969220187	59	127	54	114	53	60	100	174
ENSECAG00000018271	6.171762874	0.807981449	0.969220187	541	1173	1401	2175	1787	1615	1573	1280
ENSECAG00000010306	2.692755972	0.808126939	0.969292583	61	82	132	184	175	83	174	132
ENSECAG00000026888	2.447519063	0.808417577	0.969539041	60	112	92	143	83	95	100	158
ENSECAG00000015096	4.474428584	0.808751317	0.969837133	229	375	370	742	345	360	375	731
ENSECAG00000009080	1.431353211	0.808841882	0.969843583	24	55	51	71	72	17	57	66

ENSECAG00000021297	9.539272661	0.809084379	0.969949927	12054	10403	14723	12307	19142	10657	24567	12273
ENSECAG00000015984	8.044942385	0.809100962	0.969949927	3806	5997	3658	5992	6771	3123	7277	4591
ENSECAG00000012842	0.962069182	0.809320951	0.970001998	13	20	42	60	38	47	30	46
ENSECAG00000021784	2.956309871	0.809369326	0.970001998	87	109	132	218	161	177	145	182
ENSECAG00000015338	5.657629867	0.809478373	0.970001998	551	949	940	1102	1375	1302	979	675
ENSECAG00000022701	5.11186715	0.809564674	0.970001998	445	621	599	910	587	756	680	800
ENSECAG00000016106	2.589613402	0.809596209	0.970001998	93	74	101	177	87	67	152	184
ENSECAG00000014128	2.306434915	0.809655598	0.970001998	45	68	88	150	101	88	109	128
ENSECAG00000023887	1.329031999	0.809801729	0.970040255	7	57	52	59	30	65	54	64
ENSECAG00000000606	2.585738355	0.810008143	0.970040255	143	78	71	73	139	98	197	96
ENSECAG00000017282	3.580090431	0.810159117	0.970040255	150	167	255	247	134	208	154	544
ENSECAG00000005101	2.834616091	0.810170404	0.970040255	71	195	99	117	193	144	186	95
ENSECAG00000011003	8.405618586	0.810315609	0.970040255	5865	3495	6352	6856	11451	6188	9951	2465
ENSECAG00000023709	6.295563105	0.810384435	0.970040255	1154	620	1896	1765	1775	1619	2080	1416
ENSECAG00000010037	5.061235495	0.810404748	0.970040255	314	508	757	995	613	672	841	621
ENSECAG00000010047	5.775701563	0.810410718	0.970040255	638	942	1005	1272	1097	1053	1464	1178
ENSECAG00000026939	1.511945324	0.810454361	0.970040255	30	59	32	71	63	44	79	59
ENSECAG00000024456	5.606639675	0.810864349	0.970306294	530	774	903	1268	1255	875	1245	872
ENSECAG00000018169	5.548900694	0.811143195	0.970306294	610	961	937	917	901	1052	1118	766
ENSECAG00000020966	4.57878801	0.811144818	0.970306294	173	370	454	905	380	446	648	495
ENSECAG00000011331	8.148605865	0.811157094	0.970306294	3027	5034	5694	6127	4722	6164	5963	8011
ENSECAG00000024820	5.295443739	0.811220962	0.970306294	479	431	945	911	1162	617	940	704
ENSECAG00000018037	5.098599482	0.811307293	0.970306294	540	365	537	928	794	408	834	979
ENSECAG00000010635	5.641438863	0.811364322	0.970306294	577	1039	1073	1007	1079	1205	977	788
ENSECAG00000012184	4.033209252	0.81138831	0.970306294	175	244	369	366	273	391	352	398
ENSECAG00000007770	5.020178259	0.81151761	0.970306294	592	453	427	879	574	437	770	901
ENSECAG00000002048	5.075293848	0.8115289	0.970306294	347	851	529	792	570	575	831	809
ENSECAG00000007788	5.875576343	0.811648868	0.970313999	748	1318	1107	1138	811	1698	940	1272
ENSECAG00000020914	4.851122384	0.8117058	0.970313999	248	518	643	812	603	636	544	560
ENSECAG00000012779	7.304553564	0.811795077	0.97031884	2541	2689	2955	2279	4110	2829	4491	2529
ENSECAG00000014920	1.732775516	0.812060231	0.970395096	43	53	81	39	58	85	76	64
ENSECAG00000008647	4.661207345	0.812120334	0.970395096	372	509	359	464	406	348	1008	512
ENSECAG00000017165	7.218350799	0.812140125	0.970395096	2250	2198	2761	2881	3856	3549	3055	2452
ENSECAG00000023470	4.636404743	0.8122738	0.970395096	277	350	648	598	446	485	557	551
ENSECAG00000000328	1.826394084	0.812285048	0.970395096	37	37	93	101	82	81	73	41
ENSECAG00000016955	7.065261736	0.812384364	0.970411917	1892	2384	2398	3255	2299	2932	2767	2905
ENSECAG00000026813	10.72440698	0.812564114	0.970524805	18232	20257	39137	42439	40284	38082	31989	36995
ENSECAG00000006422	2.142062449	0.8126976	0.970551102	35	97	76	129	42	74	87	149
ENSECAG00000009497	5.429706407	0.812857616	0.970551102	413	424	1440	1044	818	1063	672	910
ENSECAG00000023985	3.649134303	0.8129027	0.970551102	160	198	296	272	325	192	246	255
ENSECAG00000026987	5.631894098	0.812927124	0.970551102	632	751	966	1374	1121	966	899	1054
ENSECAG00000021925	3.018088391	0.813035213	0.970578368	78	129	166	248	103	161	205	190
ENSECAG00000008348	4.741083943	0.813154244	0.97061869	257	518	549	708	488	393	651	680
ENSECAG00000018577	1.674087591	0.813383818	0.970725028	37	52	38	91	85	46	90	53
ENSECAG00000008651	4.660424914	0.813413858	0.970725028	377.999	444	446.999	439.998	585	440	716	471.998
ENSECAG00000017692	5.632062017	0.813647064	0.970780569	646	624	1215	1225	662	926	1348	1179
ENSECAG00000019486	4.660420448	0.813689178	0.970780569	369	318	537	513	568	461	675	503
ENSECAG00000002715	6.288437348	0.813716203	0.970780569	1053	1430	1464	1834	1341	1880	1569	1573
ENSECAG00000021056	6.145330135	0.813874451	0.970783218	857	1040	1872	1518	1068	1551	1730	1469
ENSECAG00000023972	2.01211316	0.814108495	0.970783218	54	34	80	107	110	40	111	90
ENSECAG00000006527	2.383529302	0.814116581	0.970783218	49	71	115	131	137	112	113	84
ENSECAG00000026820	4.631123857	0.814122294	0.970783218	276	111	994	286	63	415	161	1568
ENSECAG00000013847	5.670717954	0.814144766	0.970783218	637	871	852	1208	938	821	1575	1166
ENSECAG00000011336	3.596497522	0.81423848	0.970793287	203	164	254	248	280	174	312	226
ENSECAG00000015662	1.820923695	0.814343796	0.970817184	65	43	66	50	90	67	84	61
ENSECAG00000011076	7.354952457	0.814444782	0.970825983	2053	2357	2915	4157	4085	2627	4587	3164
ENSECAG00000001393	5.038332496	0.814521721	0.970825983	579	470	543	597	951	589	747	562
ENSECAG00000020900	4.871488872	0.814756668	0.971004361	348	435	652	766	409	546	780	691
ENSECAG00000007939	0.486749831	0.814947482	0.971015567	14	15	46	26	6	37	7	51
ENSECAG00000008396	7.623739344	0.815128813	0.971015567	3570	4014	2993	2033	5459	2835	6273	3037
ENSECAG00000011707	4.530501261	0.815241129	0.971015567	181	405	457	762	492	457	504	433
ENSECAG00000013871	5.855863688	0.815322965	0.971015567	702	1282	968	1337	1089	1044	1234	1393
ENSECAG00000023563	6.272927216	0.815362027	0.971015567	740	1947	1482	1635	1384	1997	1560	1327
ENSECAG00000026867	2.704066162	0.815476164	0.971015567	47	105	127	189	87	163	106	202
ENSECAG00000011387	5.564238744	0.815537619	0.971015567	605	692	948	1067	1052	877	1029	1147
ENSECAG00000020380	1.542981235	0.815597429	0.971015567	27	43	73	54	94	39	78	38
ENSECAG00000021483	7.527417975	0.81560351	0.971015567	2231	4211	3201	4089	3030	3288	4986	3928
ENSECAG00000023455	8.82388865	0.815663059	0.971015567	5617	8412	9615	10295	8903	7870	12252	8291
ENSECAG00000010759	7.296872907	0.815734911	0.971015567	1823	3260	2641	4159	4210	2566	3176	2839
ENSECAG00000011012	7.779488974	0.815789538	0.971015567	2416	3350	4879	4756	6065	4808	5927	2465
ENSECAG00000006077	4.402777831	0.815915347	0.971063792	165	354	360	791	407	445	507	369
ENSECAG00000022081	5.319791946	0.816079819	0.971119067	510	644	643	1007	703	639	1170	1009
ENSECAG00000023814	4.818800142	0.816279128	0.971119067	469	285	503	667	652	292	968	607
ENSECAG00000009185	4.681731936	0.816466959	0.971119067	282	544	500	583	517	492	584	514
ENSECAG00000018279	3.411048632	0.816686117	0.971119067	60	207	252	249	207	219	258	240
ENSECAG00000008427	2.515896668	0.816758904	0.971119067	49	96	62	204	103	131	184	81

ENSECAG00000018209	2.964841128	0.816839295	0.971119067	96	104	162	177	169	89	230	199
ENSECAG00000007943	0.066145019	0.816862406	0.971119067	18	12	23	7	23	8	38	18
ENSECAG00000026826	1.640099857	0.8168643	0.971119067	33	51	57	70	52	90	50	69
ENSECAG00000007070	3.883951349	0.816882748	0.971119067	171	239	291	332	233	305	377	370
ENSECAG00000017484	4.035553687	0.816972684	0.971119067	222	264	341	294	290	309	452	382
ENSECAG00000009688	6.59897905	0.817098591	0.971119067	1152	2078	1989	1962	1594	2227	1791	2268
ENSECAG00000012776	7.180706291	0.817171259	0.971119067	2088	2524	3126	2907	2809	3329	2436	3140
ENSECAG00000014058	5.777985838	0.817309096	0.971119067	474	853	1207	1744	1433	1059	1042	923
ENSECAG00000016335	2.51667025	0.817320658	0.971119067	55	82	101	168	124	117	135	116
ENSECAG00000017129	3.619188387	0.81741373	0.971119067	177	174	215	275	200	172	380	334
ENSECAG00000001892	7.197586752	0.817457572	0.971119067	1326	2840	2562	3760	2051	3583	2994	4183
ENSECAG00000003474	5.777416884	0.817498914	0.971119067	1328	603	716	1138	1044	857	1855	815
ENSECAG00000000506	0.845487758	0.817534788	0.971119067	12	27	31	53	22	53	24	48
ENSECAG00000001533	0.905987916	0.817582455	0.971119067	24	40	26	28	24	25	71	42
ENSECAG00000022472	5.360535727	0.81769895	0.971156118	387	570	1010	985	1039	956	829	711
ENSECAG00000012194	4.774001282	0.81787587	0.971245915	262	472	564	799	748	455	597	436
ENSECAG00000009236	6.574462432	0.817945176	0.971245915	898	1673	1807	2457	1889	2176	2843	1461
ENSECAG00000019312	6.523117486	0.818053856	0.971273663	1321	2028	1436	1905	1870	1536	2332	1858
ENSECAG00000008711	4.259893069	0.818157977	0.971293514	233	282	372	459	304	416	470	474
ENSECAG00000010494	1.363439886	0.818282876	0.971293514	56	37	36	25	62	31	82	48
ENSECAG00000014003	3.346739311	0.818326516	0.971293514	130	219	212	180	100	162	196	370
ENSECAG00000011411	6.562035922	0.818454127	0.971343713	1078	1736	1888	2418	2167	1721	2171	1693
ENSECAG00000020166	3.554575492	0.818630337	0.971356589	168	220	222	241	230	182	255	294
ENSECAG00000010132	9.110992951	0.818635614	0.971356589	10235	11202	8158	5526	14624	14722	13461	5546
ENSECAG00000006505	6.594438787	0.818750926	0.971392174	1151	1882	1842	2357	2039	2172	2037	1623
ENSECAG00000021449	6.585750919	0.818893975	0.971429038	1511	1654	1748	2061	1892	1627	2518	1908
ENSECAG00000024383	5.236322829	0.81903896	0.971429038	537	579	558	944	836	471	1086	933
ENSECAG00000007212	2.314497535	0.819059655	0.971429038	38	68	145	89	22	163	17	221
ENSECAG00000007883	4.681308411	0.819123299	0.971429038	286	555	622	476	58	220	242	1545
ENSECAG00000026949	1.48811937	0.819228206	0.971435033	26	55	35	76	30	73	58	76
ENSECAG00000015673	3.416600168	0.819299006	0.971435033	107	148	207	363	153	163	265	296
ENSECAG00000016297	4.888013537	0.819741043	0.971677029	310	403	576	847	691	514	732	640
ENSECAG00000019013	5.360533808	0.81979865	0.971677029	343	716	1048	1046	757	957	861	776
ENSECAG00000012041	4.749800289	0.820133231	0.971677029	304	368	788	396	861	548	668	258
ENSECAG00000020657	2.168094674	0.82019249	0.971677029	45	56	98	114	123	85	132	48
ENSECAG00000017496	5.605845727	0.820203556	0.971677029	593	670	920	1266	1075	554	1419	1261
ENSECAG00000020814	5.206480872	0.820352653	0.971677029	491	628	675	942	850	551	929	727
ENSECAG00000017541	6.744036438	0.820377472	0.971677029	1083	2173	1995	2922	1813	2388	2454	2115
ENSECAG00000011242	5.229165876	0.820432387	0.971677029	511	590	685	1015	404	474	932	1313
ENSECAG00000017176	4.446556764	0.820467126	0.971677029	321	318	424	428	367	400	592	548
ENSECAG00000008941	7.016570423	0.820629106	0.971677029	2468	2518	2151	1926	2627	2677	3158	2122
ENSECAG00000017529	0.263076711	0.820759511	0.971677029	12	15	19	31	27	11	23	37
ENSECAG00000016263	5.577866734	0.820760216	0.971677029	537	652	1092	1110	1072	1099	1047	904
ENSECAG00000011992	6.196684561	0.820770067	0.971677029	1035	1496	1294	1521	1675	1504	1533	1278
ENSECAG00000002353	1.886276043	0.820819615	0.971677029	14	84	56	110	33	111	51	118
ENSECAG00000014876	4.49590311	0.820825172	0.971677029	352	336	306	548	280	303	760	672
ENSECAG00000023656	3.199783262	0.820868658	0.971677029	99	150	119	283	145	97	301	275
ENSECAG00000018660	3.054473793	0.821078529	0.971824415	64	115	176	247	123	162	250	193
ENSECAG00000021521	4.204064806	0.821252629	0.971929437	196	322	346	440	296	362	541	415
ENSECAG00000007490	3.396717523	0.821423709	0.971951363	94	142	212	375	158	231	211	251
ENSECAG00000019139	4.696166082	0.821672205	0.971951363	263	387	447	911	445	551	589	539
ENSECAG00000016027	3.314750695	0.821677982	0.971951363	71	169	199	283	163	181	284	243
ENSECAG00000009329	2.987030827	0.821690718	0.971951363	43	150	111	281	175	174	178	157
ENSECAG00000012516	10.20325458	0.821698012	0.971951363	19207	21005	23330	21978	15977	32677	15328	30086
ENSECAG00000018948	9.725246875	0.821868343	0.971980285	13350	10309	16558	17200	19907	14448	20198	19926
ENSECAG00000023713	4.948527145	0.821940758	0.971980285	383	749	497	631	382	682	662	806
ENSECAG00000024292	6.353124501	0.821978584	0.971980285	1097	1244	1697	2043	1501	1552	1994	1698
ENSECAG00000015755	7.819856528	0.822490914	0.972430284	2874	3515	3403	6089	4586	3789	6240	5360
ENSECAG00000000479	5.996003093	0.822529963	0.972430284	768	1171	1272	1547	1272	1010	1498	1500
ENSECAG00000006252	2.668171311	0.822743067	0.972519623	75	143	102	108	135	70	187	166
ENSECAG00000009997	6.234331151	0.822776373	0.972519623	1210	1420	1669	1054	1358	1885	1611	1272
ENSECAG00000000285	3.286274189	0.823084468	0.972782796	104	231	162	226	201	216	196	174
ENSECAG00000021637	6.833502825	0.823618111	0.973312455	1664	1681	2579	2470	2518	2486	2171	2078
ENSECAG00000006959	6.293343289	0.82382062	0.973328503	1160	1652	1512	1311	1568	1613	1760	1490
ENSECAG00000021059	4.058669435	0.823870427	0.973328503	129	268	388	511	366	343	395	255
ENSECAG00000017080	1.661856093	0.82394791	0.973328503	25	51	68	76	60	62	74	73
ENSECAG00000023732	4.347433167	0.824041159	0.973328503	195	427	428	379	391	472	500	398
ENSECAG00000017894	1.960982497	0.824072706	0.973328503	14	46	121	130	49	88	117	56
ENSECAG00000023288	7.02756677	0.824146934	0.973328503	1451	2083	2541	3147	3647	2431	3039	2255
ENSECAG00000020679	7.674420155	0.824230136	0.973328503	2452	3815	3903	3976	4035	4498	5315	4050
ENSECAG00000003367	1.541585622	0.824589349	0.973609213	33	40	51	93	53	46	54	76
ENSECAG00000022282	5.998336403	0.824706235	0.973609213	688	1391	1195	1506	1114	1312	1402	1427
ENSECAG00000011158	4.961790519	0.824730741	0.973609213	425	491	606	779	667	625	686	579
ENSECAG00000015944	4.512872214	0.824921996	0.973609213	169	364	385	772	384	484	451	657
ENSECAG00000009859	4.734165425	0.82496981	0.973609213	254	543	557	654	472	453	729	556
ENSECAG00000000539	6.999622097	0.824980947	0.973609213	2581	2199	2342	1778	2783	1844	4154	1860

ENSECAG00000012780	5.672650695	0.825349082	0.973900869	822	751	925	956	1164	766	1462	1090
ENSECAG00000010568	4.943034619	0.825399164	0.973900869	430	531	439	732	482	559	862	795
ENSECAG00000023312	4.302612284	0.825596155	0.973972924	327	520	232	309	507	219	650	265
ENSECAG00000010248	2.814531174	0.82563133	0.973972924	53	131	90	234	128	72	191	229
ENSECAG00000009459	5.431262574	0.825908963	0.974199496	364	661	947	1173	999	751	1145	873
ENSECAG00000009349	3.926170242	0.826042001	0.974230962	143	315	308	383	316	410	260	233
ENSECAG00000021478	9.260674444	0.826142021	0.974230962	7989	7305	15889	11081	16245	13307	12708	11101
ENSECAG00000002357	3.754651621	0.826192353	0.974230962	110	361	254	288	265	319	286	222
ENSECAG00000022381	3.733013614	0.826405176	0.974293	118	182	262	480	350	138	279	319
ENSECAG00000018726	5.024017586	0.826416118	0.974293	280	516	437	1137	214	343	573	1768
ENSECAG00000021095	2.80332316	0.826822598	0.974671286	68	116	130	220	117	77	155	220
ENSECAG00000018235	4.425749999	0.827058813	0.97469165	333	413	333	469	506	415	425	407
ENSECAG00000006080	3.328577169	0.827101799	0.97469165	163	180	169	148	229	113	371	181
ENSECAG00000005087	5.761430946	0.827248461	0.97469165	712	899	1191	1197	861	1213	1252	1138
ENSECAG00000019974	4.610131551	0.827264924	0.97469165	318	437	491	554	332	550	402	702
ENSECAG00000011876	2.467005866	0.827347355	0.97469165	58	107	97	118	63	125	128	161
ENSECAG00000018637	3.969948554	0.827494488	0.97469165	251	159	400	239	259	323	254	516
ENSECAG00000015348	5.538360442	0.827508705	0.97469165	610	890	691	1272	567	613	1317	1396
ENSECAG00000020597	6.26187253	0.827642437	0.97469165	1156	1251	1307	1526	1741	1286	1951	1732
ENSECAG00000018986	8.027274271	0.827737905	0.97469165	3076	6056	5622	4471	5248	6241	5184	4460
ENSECAG00000010511	5.192710537	0.827775257	0.97469165	408	530	807	853	671	632	1099	811
ENSECAG00000023052	3.66766288	0.827794095	0.97469165	90	271	263	362	95	388	214	318
ENSECAG00000021146	6.754023863	0.827867216	0.97469165	2401	1610	1994	1392	3009	1560	2879	1418
ENSECAG00000013047	1.169738611	0.828029047	0.974781378	16	23	58	76	22	19	83	56
ENSECAG00000018958	6.319712182	0.828299918	0.9749966	1152	1462	1573	1208	2187	1626	1891	1226
ENSECAG00000024653	7.353959797	0.828646358	0.9749966	2365	3716	3202	2494	2831	3843	3548	3105
ENSECAG00000007532	5.76240261	0.828755171	0.9749966	791	727	1085	1416	1205	633	1574	1125
ENSECAG00000023756	3.059376033	0.828804261	0.9749966	98	167	141	211	176	141	184	177
ENSECAG00000017169	4.523860549	0.828886886	0.9749966	330	251	449	576	420	377	646	574
ENSECAG00000000463	2.984366733	0.828921421	0.9749966	56	170	129	259	156	119	178	190
ENSECAG00000017960	4.41216903	0.828933038	0.9749966	329	384	374	445	443	331	517	469
ENSECAG00000015906	2.671394994	0.829180281	0.9749966	57	102	109	186	105	97	199	158
ENSECAG00000015905	6.624152648	0.829259421	0.9749966	1071	1191	2509	2240	2763	1654	1883	2253
ENSECAG00000017234	6.829632563	0.82932374	0.9749966	1002	2292	1623	4002	1454	1510	2968	3519
ENSECAG00000017250	2.278335291	0.829326116	0.9749966	47	88	84	119	92	112	109	101
ENSECAG00000021192	1.692958257	0.829326262	0.9749966	22.5565	49.0001	94.0079	81.0001	45.0005	54.0007	140	23.2423
ENSECAG00000024029	6.550929423	0.829559412	0.9749966	1162	1402	1863	2661.99	2705	1625.99	1958	1359.99
ENSECAG00000020643	5.654697173	0.829665882	0.9749966	540	802	974	1530	1206	704	1080	1165
ENSECAG00000003551	2.224222643	0.829670266	0.9749966	25	60	159	87	58	121	115	108
ENSECAG00000000104	3.317914833	0.829797277	0.9749966	111	105	239	306	194	157	242	223
ENSECAG00000002063	0.39018109	0.829802089	0.9749966	18	23	12	29	22	18	12	54
ENSECAG00000012384	5.539077079	0.829942051	0.9749966	380	732	929	1604	940	1059	883	902
ENSECAG00000022335	4.380843924	0.830067918	0.9749966	210	282	502	601	249	428	459	575
ENSECAG00000019621	5.729287357	0.830100183	0.9749966	701	835	918	1249	992	605	1843	1294
ENSECAG00000014412	5.586943236	0.830128712	0.9749966	839	832	742	770	1463	675	1287	779
ENSECAG00000011332	2.326714908	0.830150598	0.9749966	44	57	115	174	46	97	105	154
ENSECAG00000024750	2.545002241	0.830243721	0.9749966	111	54	156	89	158	54	202	64
ENSECAG00000012588	4.711990681	0.83028242	0.9749966	357	659	459	399	426	547	624	559
ENSECAG00000012708	6.082722649	0.830393974	0.9749966	1061	943	1368	1226	1899	1166	1781	1068
ENSECAG00000022670	4.643309339	0.830438474	0.9749966	299	465	431	552	584	335	763	516
ENSECAG00000015084	6.250015769	0.830759904	0.975178973	842	1238	1608	2087	2090	1004	1798	1387
ENSECAG00000011221	1.849333092	0.830845527	0.975178973	107	58	19	14	51	153	64	37
ENSECAG00000006981	8.70182063	0.831003289	0.975178973	8419	7982	4225	8836	4730	6601	8709	14137
ENSECAG00000023935	5.85854994	0.831154242	0.975178973	877	826	824	1496	1219	722	1801	1399
ENSECAG00000022459	5.316199294	0.831165831	0.975178973	422	696	822	887	927	717	929	881
ENSECAG00000007500	9.692771949	0.831176734	0.975178973	10196	11418	19782	21266	17290	18425	14813	16427
ENSECAG00000008102	6.896311562	0.83123102	0.975178973	905	2550	2350	2753	2672	2405	3469	1928
ENSECAG00000015924	7.073343811	0.831390681	0.975178973	1739	2385	2417	2776	2801	2410	4171	2518
ENSECAG00000019385	6.097824222	0.831422331	0.975178973	937	1328	1143	1286	1712	1039	1922	1336
ENSECAG00000013402	4.201482005	0.83154301	0.975178973	278	210	461	406	599	294	508	97
ENSECAG00000005797	7.18224232	0.831664799	0.975178973	2622	2803	2361	2478	3281	2663	3278	2671
ENSECAG00000024212	6.499171026	0.831829795	0.975178973	1188	1663	1421	2003	1263	2184	2009	2405
ENSECAG00000016199	4.062282711	0.831842991	0.975178973	178	274	444	349	330	284	328	425
ENSECAG00000016996	8.225086759	0.831879446	0.975178973	3644	4221	6530	8332	5958	5027	7835	5894
ENSECAG00000004224	6.43910611	0.831944167	0.975178973	981	1486	1791	1850	2102	1611	2268	1588
ENSECAG00000012698	5.534895519	0.831964284	0.975178973	748	732	796	1079	746	898	1113	1079
ENSECAG00000019233	7.53864475	0.832338477	0.975517146	2926	4149	2860	3413	3582	3258	4615	3871
ENSECAG00000013002	4.61917628	0.832692347	0.975768212	323	498	395	595	300	371	525	836
ENSECAG00000017640	6.742715736	0.83276664	0.975768212	1051	1315	2398	2994	2716	1820	2873	1983
ENSECAG00000000460	1.772252037	0.832809813	0.975768212	50	76	42	52	47	45	67	135
ENSECAG00000017630	5.730325857	0.833003134	0.975777398	1172	1208	621	570	1337	1198	1004	747
ENSECAG00000008452	5.989626742	0.833071842	0.975777398	1212	1013	868	1087	1600	1110	1351	1452
ENSECAG00000006714	3.447659376	0.833074774	0.975777398	61	52	529	223	305	277	84	177
ENSECAG00000001599	11.55126628	0.833491551	0.976165138	27966	37557	66637	85380	74282	68034	52019	65341
ENSECAG00000007118	4.552465649	0.833692343	0.976226629	231	520	416	596	538	423	505	459
ENSECAG00000000129	7.016640662	0.833843153	0.976226629	1503	1695	2727	3266	1830	2836	3840	2915

ENSECAG00000024913	4.292669921	0.833924871	0.976226629	303	278	358	490	416	418	417	350
ENSECAG00000022211	4.18789303	0.833946292	0.976226629	233	254	443	420	384	462	322	303
ENSECAG00000008428	4.791237571	0.833972789	0.976226629	292	401	575	708	330	531	469	1072
ENSECAG00000015610	5.704197453	0.834149029	0.976332548	715	719	979	1232	1143	1329	1007	987
ENSECAG00000000008	8.084071704	0.834265113	0.976368042	2001	4019	5459	8540	6365	7081	4294	5593
ENSECAG00000025080	10.97163842	0.83445243	0.976486887	21349	24973	47202	49606	49522	46529	36727	40986
ENSECAG00000017565	6.303103619	0.83457161	0.97652598	833	1205	1475	2569	1196	1319	1888	2142
ENSECAG00000012792	5.696403874	0.834862492	0.976738535	268	1147	1069	1321	1337	1471	847	766
ENSECAG00000004219	6.381468654	0.834959982	0.976738535	648	887	1385	3375	2218	1459	2319	1322
ENSECAG00000019186	5.726011546	0.835010642	0.976738535	695	716	1007	1310	1120	876	1647	1012
ENSECAG00000007423	4.964567648	0.835100758	0.976743593	306	546	892	587	661	595	695	615
ENSECAG00000005616	3.745958938	0.83526163	0.976807218	157	313	210	308	310	163	351	285
ENSECAG00000010852	11.37577473	0.835385894	0.976807218	31963	38616	60608	70496	54037	56418	46759	58187
ENSECAG00000006216	4.020469452	0.835451136	0.976807218	271	306	243	336	348	222	493	288
ENSECAG00000013760	3.895342375	0.835498347	0.976807218	186	202	220	527	290	168	342	428
ENSECAG00000005400	2.423412248	0.835634449	0.976807268	75	92	141	78	85	42	45	254
ENSECAG00000007949	2.661406747	0.835748578	0.976807268	83	170	112	84	121	189	111	78
ENSECAG00000017558	4.185565123	0.835821367	0.976807268	169	263	285	712	308	208	568	438
ENSECAG00000003332	4.046961649	0.835918222	0.976807268	190	246	386	333	419	411	404	187
ENSECAG00000011346	5.554734871	0.835952887	0.976807268	567	761	1037	1136	936	1069	906	930
ENSECAG00000012377	1.110455675	0.836013177	0.976807268	12	38	42	58	31	62	24	60
ENSECAG00000010363	9.177433094	0.836172596	0.976825658	7897	9542	11103	14629	11710	11931	13362	10398
ENSECAG00000023631	5.561382272	0.836200515	0.976825658	657	530	1301	1002	1285	778	1353	485
ENSECAG00000013194	0.201803725	0.836477227	0.976925887	7	21	14	34	25	19	21	27
ENSECAG00000016147	3.590594074	0.836504398	0.976925887	176	172	349	169	211	247	136	370
ENSECAG00000023777	6.40387951	0.836607534	0.976925887	1118	872	2207	2163	2287	1590	1665	1355
ENSECAG00000016816	4.99682189	0.836707711	0.976925887	451	436	646	680	712	459	999	639
ENSECAG00000010392	4.852332964	0.836875497	0.976925887	452	549	471	600	616	450	706	622
ENSECAG00000016122	3.521451207	0.836889311	0.976925887	128	137	260	289	272	161	304	264
ENSECAG00000017300	3.603315869	0.837020781	0.976925887	113	326	165	240	280	143	424	230
ENSECAG00000015099	7.090332992	0.837022946	0.976925887	2087	2195	2584	2345	3094	2078	3981	2879
ENSECAG00000025104	9.585447103	0.83710861	0.976925887	14517	12667	13389	13822	17749	13150	18929	13279
ENSECAG00000013565	5.804924877	0.837144396	0.976925887	552	927	1169	1366	1387	999	1253	1202
ENSECAG00000007294	6.222480978	0.837392951	0.977115788	835	1145	1428	1927	1581	1575	1772	1547
ENSECAG00000022130	5.601383435	0.837484947	0.977122989	906	832	1028	602	867	1149	1108	853
ENSECAG00000004882	6.642406798	0.837601166	0.977158446	1495	2283	1758	1606	2197	1542	2636	1907
ENSECAG00000021593	5.292047202	0.83773318	0.977212321	448	755	765	776	718	695	1035	971
ENSECAG00000026946	0.737816868	0.838046061	0.977243729	17	27	26.0036	40	46	49	21	18
ENSECAG00000024664	3.52564933	0.838185496	0.977243729	90	309	249	146	230	334	267	157
ENSECAG00000012499	6.660939392	0.838247993	0.977243729	1973	1748	1482	1903	2066	1234	2592	2521
ENSECAG00000023551	3.041721999	0.838311904	0.977243729	79	113	177	218	174	108	269	173
ENSECAG00000000227	6.180400883	0.838358522	0.977243729	799	1257	1384	1711	1364	1440	1981	1549
ENSECAG00000008134	1.254885028	0.838380736	0.977243729	20	52	51	51	67	33	57	30
ENSECAG00000015133	5.265044702	0.838473312	0.977243729	485	646	673	1072	918	554	1209	533
ENSECAG00000005517	7.180971334	0.838541433	0.977243729	2684	2843	1950	2777	2981	2366	3656	2983
ENSECAG00000009761	8.589262846	0.838590117	0.977243729	7176.99	6353	6970.98	6804	10409	5407	10271	5665
ENSECAG00000026941	3.660384455	0.838703913	0.977243729	158	278	208	283	321	148	406	176
ENSECAG00000022914	1.987626249	0.838718687	0.977243729	20	67	52	152	89	63	105	85
ENSECAG00000024260	1.622096889	0.839021285	0.977243729	18	62	58	98	75	70	43	50
ENSECAG00000008434	0.888672644	0.839064451	0.977243729	15	21	43	46	10	22	58	69
ENSECAG00000019517	2.855007334	0.839094468	0.977243729	87	233	77	121	160	148	133	138
ENSECAG00000005815	5.469924884	0.839129558	0.977243729	628	873	890	815	842	1344	785	601
ENSECAG00000016982	7.078294448	0.839133482	0.977243729	1450	2323	2929	3553	2715	2451	2696	3215
ENSECAG00000009691	5.611594797	0.839248482	0.977277689	675	644	929	1178	1343	905	1098	875
ENSECAG00000015019	5.71531563	0.839384952	0.977336641	844	1031	808	1083	706	899	1654	1151
ENSECAG00000016203	2.869948794	0.839510002	0.977348387	85	91	127	214	100	166	180	185
ENSECAG00000025860	0.786530156	0.839709474	0.977348387	27	14	43	24	33	59	35	14
ENSECAG00000009993	5.394693433	0.839738054	0.977348387	411.011	549	847.008	1266	976.001	804.001	1055	816.024
ENSECAG00000006542	5.494932664	0.839738421	0.977348387	561	743	879	1193	374	924	1143	1302
ENSECAG00000020379	3.358607196	0.839879653	0.977412843	114	344	133	75	406	281	101	76
ENSECAG00000000319	6.58673267	0.840208964	0.977696143	1721	1307	1926	1409	2162	2165	2430	1600
ENSECAG00000018745	7.462561589	0.840469709	0.977821157	2025	2989	3298	4132	3591	4045	4001	3677
ENSECAG00000012953	4.789463667	0.840636589	0.977821157	178	436	411	1229	192	290	499	1294
ENSECAG00000011809	6.608030873	0.8406489	0.977821157	871	1283	2629	2783	2776	1805	1898	1455
ENSECAG00000017367	0.997043986	0.840816969	0.977821157	20	26	22	69	17	28	76	51
ENSECAG00000022178	4.74221254	0.840841911	0.977821157	305	526	484	562	686	473	658	499
ENSECAG00000012973	7.658119904	0.840903751	0.977821157	2010	4632	3290	5406	3409	4780	4681	3663
ENSECAG00000022650	4.173369206	0.840917606	0.977821157	270	334	326	286	356	304	472	439
ENSECAG00000014311	10.26516491	0.841213295	0.977949575	14164	15434	28051	29522	28605	29215	21273	27086
ENSECAG00000000409	2.000889445	0.841228942	0.977949575	60	73	80	69	62	78	107	76
ENSECAG00000010114	8.173568001	0.841327214	0.977949575	3653	3007	5293	8657	7302	3552	8211	6515
ENSECAG00000016472	5.308822664	0.841373643	0.977949575	402	994	699	844	849	802	947	667
ENSECAG00000022763	6.535849432	0.841457535	0.977949575	1147	1782	1457	2106	1567	1756	2590	2226
ENSECAG00000000204	6.692798609	0.841940318	0.978209826	906	1921	2034	3132	1884	1965	2537	2160
ENSECAG00000012635	4.877663477	0.842019687	0.978209826	241	522	500	1032	650	715	575	447
ENSECAG00000019020	2.139627435	0.84221995	0.978209826	39	62	121	110	79	41	108	130

ENSECAG00000006127	6.554314319	0.842295011	0.978209826	1422	1626	1223	2185	1552	1357	2671	2728
ENSECAG00000001342	3.8144753	0.842336388	0.978209826	66	295	265	412	332	432	177	246
ENSECAG00000017479	6.145686574	0.842385998	0.978209826	584	997	1225	2461	1331	1401	1808	1639
ENSECAG00000017868	4.625285985	0.842432779	0.978209826	263	477	455	550	461	504	759	431
ENSECAG00000014872	3.139156897	0.842619797	0.978209826	77	179	130	244	165	187	165	237
ENSECAG00000004879	4.2622164	0.842698842	0.978209826	233	353	435	299	447	437	490	281
ENSECAG00000017326	6.414747586	0.842713295	0.978209826	1041	2155	1449	1571	1718	1700	1891	1708
ENSECAG00000018925	5.302207545	0.842788521	0.978209826	459	1106	535	778	746	706	788	1004
ENSECAG00000020651	7.477465802	0.84280441	0.978209826	1925	3284	2993	4455	3508	3047	4913	4216
ENSECAG00000024079	10.94861783	0.842816246	0.978209826	20441	23601	47511	50156	46857	45737	36132	42121
ENSECAG00000015125	0.219012586	0.842885779	0.978209826	8	14	24	39	15	30	22	18
ENSECAG00000010540	8.396033974	0.843004968	0.978209826	3555	5340	6433	8753	7147	5497	9535	7534
ENSECAG00000019935	0.532102191	0.843056198	0.978209826	4	30	35	28	19	38	40	22
ENSECAG00000022158	5.766217664	0.843198282	0.978274986	1072	1233	609	831	1129	859	1436	1092
ENSECAG00000024648	7.743926722	0.84330602	0.97830029	2913	3938	4115	4843	4131	4972	3904	4423
ENSECAG00000014428	4.370655023	0.843467216	0.978387596	241	395	320	498	336	249	591	648
ENSECAG00000013517	4.681295631	0.843733459	0.978413237	464	302	429	531	623	363	769	496
ENSECAG00000010350	2.393848132	0.843792543	0.978413237	38	63	151.001	123	106	130	117	95
ENSECAG00000026860	5.081093553	0.843820802	0.978413237	453	510	622	780	763	382	1095	752
ENSECAG00000007888	2.715822361	0.843905366	0.978413237	70	112	113	166	124	97	175	174
ENSECAG00000011497	5.573973679	0.843919015	0.978413237	411	1104	726	1182	775	983	1111	1268
ENSECAG00000010421	4.571767618	0.844202167	0.978641856	349	445	256	703	417	333	637	594
ENSECAG00000019154	6.658236763	0.84437883	0.978734499	1529	3920	685	925	1065	4367	1176	1248
ENSECAG00000023915	3.924686256	0.844454017	0.978734499	187	190	330	433	339	226	376	313
ENSECAG00000010684	6.926443355	0.844672323	0.978793005	1612	1970	1874	3066	2109	2150	3415	3034
ENSECAG00000002635	4.890703334	0.844694718	0.978793005	240	350	729	866	403	552	729	908
ENSECAG00000027681	11.49960568	0.844874793	0.978793005	46268	33269	72027	42721	67550	74746	57792	50241
ENSECAG00000014782	4.741370409	0.844905411	0.978793005	361	455	539	489	568	427	726	611
ENSECAG00000000660	1.802838251	0.844999281	0.978793005	82	71	33	18	78	79	118	28
ENSECAG00000010947	4.860769925	0.845020329	0.978793005	675	424	454	230	951	996	345	159
ENSECAG00000015858	8.223783803	0.845109802	0.978797059	6824	3931	5198	2649	9686	4988	8076	3542
ENSECAG00000022663	4.554131288	0.845385605	0.979016897	279	425	438	605	484	493	428	514
ENSECAG00000008744	5.982924428	0.845600643	0.979166327	1200	1540	591	1062	1508	691	1974	1119
ENSECAG00000018197	6.487726462	0.845966432	0.979230754	1053	1413	2058	1774	1977	2077	2314	1414
ENSECAG00000009012	6.659686245	0.846026173	0.979230754	956	2799	1451	2425.01	1370.01	3445	937	2245
ENSECAG00000012306	7.501592822	0.846163278	0.979230754	2008	3626	3725	4238	3536	4987	3129	2972
ENSECAG00000023716	5.008541985	0.846248032	0.979230754	449	491	581	855	822	513	781	546
ENSECAG00000022275	4.691354054	0.846260788	0.979230754	344	296	462	733	519	402	833	519
ENSECAG00000024268	10.35404515	0.846367038	0.979230754	13049	16474	31677	32840	32042	31211	24082	25573
ENSECAG00000011133	3.476192864	0.84638981	0.979230754	244	211	149	148	126	298	166	302
ENSECAG00000024001	5.701326481	0.846542803	0.979230754	629	1116	928	1157	747	1026	1291	1258
ENSECAG00000021858	0.052442606	0.846609567	0.979230754	8	22	23	18	21	19	23	13
ENSECAG00000010033	7.199888557	0.846659108	0.979230754	1813	2195	3153	3130	4271	2230	3317	2984
ENSECAG00000014981	5.153192537	0.84668157	0.979230754	420	381	798	948	1051	461	1008	593
ENSECAG00000023179	12.18050262	0.846912005	0.979230754	56103	58372	111559	126852	98123	95269	81802	102041
ENSECAG00000006437	4.417782328	0.846950043	0.979230754	259	329	434	483	449	344	640	435
ENSECAG00000024197	5.440423248	0.847054751	0.979230754	618	597	834	1160	809	741	908	1131
ENSECAG00000016755	7.032378661	0.847063912	0.979230754	1333	2461	2922	3179	2690	2730	3144	2201
ENSECAG00000021369	7.019307052	0.84720038	0.979230754	1656	2348	2505	3124	2185	2471	3318	2774
ENSECAG00000023794	2.712949371	0.84720761	0.979230754	96	203	66	94.9998	48	134	212	144
ENSECAG00000013242	5.810252058	0.847262856	0.979230754	434	770	1400	1518	889	1052	1089	1840
ENSECAG00000006543	3.252158265	0.847290484	0.979230754	146	141	170	236	258	119	285	124
ENSECAG00000001086	0.845099093	0.847405895	0.979264729	9	47	18	61	16	24	48	52
ENSECAG00000017657	7.285248335	0.847615617	0.979407673	4101	2011	2495	635	5566	2903	4192	1068
ENSECAG00000010933	3.829893792	0.847769747	0.979486358	135	210	203	492	201	235	354	452
ENSECAG00000019221	2.643022174	0.848397688	0.980112397	70	65	129	178	109	100	126	203
ENSECAG00000016570	0.878274016	0.848729503	0.980212063	24	30	39	36	48	37	15	38
ENSECAG00000016012	3.384501054	0.848732603	0.980212063	51	196	196	333	185	236	274	209
ENSECAG00000012056	7.46217438	0.84874225	0.980212063	1648	2138	5596	4148	3294	3641	3277	4169
ENSECAG00000012365	6.116143971	0.848914697	0.98022882	1071	970	1752	1215	2029	1370	1508	752
ENSECAG00000014404	4.187799923	0.848928956	0.98022882	141	281.999	376.998	540	362	412	456.998	342
ENSECAG00000000029	4.393210952	0.849081496	0.980247818	334	381	326	377	500	375	532	412
ENSECAG00000021622	3.138207744	0.849117609	0.980247818	82	123	215	251	172	112	202	237
ENSECAG00000009962	5.713174178	0.849252121	0.980303701	603	808	878	1715	636	952	1028	1715
ENSECAG00000009950	3.644260183	0.849346238	0.980312948	155	218	275	269	187	288	239	304
ENSECAG00000003502	2.725179285	0.849666986	0.980337653	67	153	122	146	141	66	197	142
ENSECAG00000017288	6.632725127	0.849770649	0.980337653	1134	1795	2148	2331	2006	1987	2385	1815
ENSECAG00000017636	3.554709277	0.849932327	0.980337653	145	214	203	307	232	168	279	291
ENSECAG00000017412	3.320002947	0.849995604	0.980337653	84	178	186	320	200	152	218	248
ENSECAG00000010538	3.313252842	0.850013074	0.980337653	122	128	185	266	242	138	241	240
ENSECAG00000024461	8.770488836	0.85002421	0.980337653	6799	8234	8433	7999	9394	9249	10334	6833
ENSECAG00000013190	4.611210534	0.85010204	0.980337653	205	367	580	734	463	501	511	531
ENSECAG00000019867	6.082511853	0.850130973	0.980337653	1175	1724	1002	836	820	2178	389	1953
ENSECAG00000005538	5.565492723	0.850148908	0.980337653	527	822	698	1357	864	771	1358	1157
ENSECAG00000009438	3.463623126	0.850311543	0.980337653	178	226	178	199	310	118	324	161
ENSECAG00000005608	8.000926637	0.850314828	0.980337653	4061	4707	4429	5302	4869	4216	6836	5360

ENSECAG00000026944	2.948442582	0.850434526	0.980376375	129	180	115	112	126	171	153	174
ENSECAG00000009059	7.577957042	0.850640243	0.980444523	2390	2841	3992	4167	4625	4100	3899	3881
ENSECAG00000017730	2.907108892	0.850668552	0.980444523	69	122	136	211	153	158	179	153
ENSECAG00000014009	1.171566333	0.850751993	0.980444523	11	33	56	73	24	46	27	76
ENSECAG00000013649	4.822451312	0.8509402	0.980534892	404	386	560	734	541	457	513	818
ENSECAG00000022488	5.503124001	0.851002659	0.980534892	629	788	1091	766	1137	885	892	801
ENSECAG00000007715	6.560721777	0.851342476	0.980700645	1162	1963	1491	1952	1388	1708	2477	2704
ENSECAG00000021882	8.796611878	0.851476463	0.980700645	9548	6026	9005	6086	10492	6003	11128	9128
ENSECAG00000020803	3.842875301	0.851534324	0.980700645	202	182	320	353	441	151	374	218
ENSECAG00000012894	6.357459923	0.851664229	0.980700645	1308	1640	1324	1210	2119	1460	2057	1491
ENSECAG00000010081	6.46415674	0.851675206	0.980700645	1162	1979	1292	1632	1855	1872	1866	2031
ENSECAG00000010303	3.083865992	0.851855103	0.980700645	82	130	151	245	149	174	141	260
ENSECAG00000022001	4.071047553	0.852021691	0.980700645	207	277	289	410	372	244	469	380
ENSECAG00000016357	6.000600165	0.852238296	0.980700645	798	1014	1236	1468	1398	1142	1659	1368
ENSECAG00000002890	3.630595765	0.852402816	0.980700645	145	231	248	292	366	141	298	214
ENSECAG00000011168	7.436806368	0.852455979	0.980700645	1616.08	2534.59	3420.8	5955.13	3350.95	3210.58	4290.05	3461.37
ENSECAG00000018128	0.545230129	0.85259877	0.980700645	9	26	29	43	7	16	38	52
ENSECAG00000008620	4.653612598	0.852686072	0.980700645	197	435	508	816	678	522	474	372
ENSECAG00000019652	3.584676871	0.852701019	0.980700645	145	126	418	194	354	166	240	215
ENSECAG00000010894	2.033758225	0.852943288	0.980700645	39	90	84	87	68	75	107	81
ENSECAG00000005798	5.688526731	0.853035074	0.980700645	811	765	937	1011	1378	698	1436	995
ENSECAG00000017258	7.038321678	0.853096688	0.980700645	2054	3569	1844	1858	1494	5439	1734	1690
ENSECAG00000009862	6.115308322	0.853144235	0.980700645	1607	1719	639	630	1413	1876	1594	734
ENSECAG00000004636	5.579936	0.853148091	0.980700645	650	635	1028	1028	753	1225	841	1275
ENSECAG00000008855	7.911108633	0.853193918	0.980700645	3503	3980	4561	5639	3922	4177	4922	6825
ENSECAG00000021818	7.686296584	0.853244346	0.980700645	3407	4032	3023	3231	5180	2986	6640	3428
ENSECAG00000009323	5.181669015	0.853281061	0.980700645	381	483	842	1061	984	733	599	634
ENSECAG00000021020	6.75754568	0.853315314	0.980700645	911	2165	1934	2828	2450	2396	2802	1752
ENSECAG00000021477	5.650412564	0.853410309	0.980700645	494	753	1037	1570	1011	1071	940	1094
ENSECAG00000014853	5.197239108	0.853492291	0.980700645	311	566	821	982	688	949	678	819
ENSECAG00000019283	5.66531579	0.853507377	0.980700645	511	1026	1054	1001	1273	1128	1122	835
ENSECAG00000010129	2.986765245	0.853577746	0.980700645	73	146	172	206	129	209	155	145
ENSECAG00000017247	3.476507349	0.853601445	0.980700645	141	169	246	263	236	192	310	182
ENSECAG00000007787	7.09904291	0.853614586	0.980700645	2008	2990	2394	1781	3017	2773	3627	2552
ENSECAG00000021403	7.077650447	0.853644567	0.980700645	1834	2661	2328	2432	2862	3417	3095	2290
ENSECAG00000010814	4.619013798	0.853913817	0.980807184	335	332	501	548	367	430	670	679
ENSECAG00000004253	5.86402693	0.854058542	0.980807184	768	1467	781	934	2056	814	1356	823
ENSECAG00000000568	3.202118035	0.854125134	0.980807184	188	131	122	201	126	114	249	274
ENSECAG00000001455	4.784876064	0.854259899	0.980807184	307	374	691	583	726	620	461	538
ENSECAG00000015489	4.411931911	0.854290665	0.980807184	221	336	497	460	583	358	498	396
ENSECAG00000014119	9.769506799	0.85429309	0.980807184	13178	15110	17324	17923	16315	22274	20767	11987
ENSECAG00000003783	0.55483432	0.854340346	0.980807184	26	26	12	26	34	18	33	36
ENSECAG00000014879	4.695492091	0.854519943	0.980868038	401	531	402	401	730	270	834	450
ENSECAG00000012637	4.849569128	0.854565662	0.980868038	319	749	502	534	627	706	550	464
ENSECAG00000015112	5.025283944	0.855064053	0.981098457	336	499	634	883	821	466	918	636
ENSECAG00000009115	5.686580135	0.855074894	0.981098457	658	1156	872	1062	1050	939	1355	941
ENSECAG00000022464	4.247402707	0.855255398	0.981098457	260	301	359	397	360	311	516	466
ENSECAG00000006944	2.527465511	0.855301513	0.981098457	132	30	127	71	184	85	156	75
ENSECAG00000023652	4.485275628	0.855349485	0.981098457	280	476	352	444	552	332	624	442
ENSECAG00000011179	3.488492606	0.855423635	0.981098457	176	151	220	264	231	174	261	258
ENSECAG00000020384	6.493453797	0.855507053	0.981098457	1163	1265	1757	2629	2971	514	1352	2533
ENSECAG00000011322	6.060996059	0.855602745	0.981098457	1434	1047	1091	995	1716	911	1748	1170
ENSECAG00000008046	8.396317356	0.85574978	0.981098457	4061	5120	7407	8778	7971	5359	7567	6881
ENSECAG00000021658	7.168855886	0.855826891	0.981098457	1676	2552	2596	4034	3419	3062	2460	2761
ENSECAG00000022028	4.924576834	0.855919133	0.981098457	722	585	345	364	483	653	680	679
ENSECAG00000008100	7.338455285	0.855927424	0.981098457	2493	2533	2716	4125	3280	2486	4052	3623
ENSECAG00000018364	3.416960409	0.855945751	0.981098457	117	191	181	313	217	208	271	181
ENSECAG00000019979	6.823729179	0.855972857	0.981098457	1446	1591	2642	2770	2547	1956	2944	1941
ENSECAG00000018727	1.677819941	0.856141056	0.981159183	35	56	61	82	56	58	60	80
ENSECAG00000024744	6.563061261	0.856198198	0.981159183	1219	1641	1989	2120	2174	1591	2439	1648
ENSECAG00000015041	3.541234402	0.856440577	0.981289652	129	124	268	367	172	154	310	331
ENSECAG00000000898	1.800057786	0.856484433	0.981289652	43	67	85	52	82	50	99	50
ENSECAG00000016958	4.328485536	0.85658402	0.981304998	154	419	302	688	354	195	463	659
ENSECAG00000023366	4.392947193	0.85694644	0.98158647	340	469	323	344	340	372	561	475
ENSECAG00000018851	6.188704697	0.857118674	0.98158647	681	1354	1373	2173	1488	1580	1495	1426
ENSECAG00000025162	7.923456189	0.857262046	0.98158647	2634	4112	5057	6628	3678	4929	4411	6883
ENSECAG00000018768	4.512455873	0.857278026	0.98158647	220	408	347	778	431	377	495	583
ENSECAG00000006287	4.063709483	0.85733985	0.98158647	276	282	266	300	368	232	476	384
ENSECAG00000009292	4.499114569	0.857347023	0.98158647	331	549	340	289	494	411	595	463
ENSECAG00000009844	5.667563352	0.857690975	0.981807523	958	945	827	800	1076	937	1602	630
ENSECAG00000019174	2.843366098	0.857843806	0.981807523	83	123	147	177	194	106	132	149
ENSECAG00000013966	5.083673053	0.857855653	0.981807523	331	600	695	811	533	655	919	845
ENSECAG00000022981	2.280403957	0.857964453	0.981807523	45	47	127	126	90	105	168	58
ENSECAG00000018009	3.864427246	0.857971282	0.981807523	177	280	260	358	343	317	375	159
ENSECAG00000012412	3.354034765	0.858446952	0.982202978	115	128	256	268	282	173	190	184
ENSECAG00000009660	2.629526247	0.858489401	0.982202978	36	53	82	342	118	78	84	214

ENSECAG00000015349	3.822220651	0.85867949	0.982276696	202	388	209	196	269	216	344	340
ENSECAG00000012037	3.794416617	0.858750756	0.982276696	157	341	240	273	224	273	351	298
ENSECAG00000010151	6.682039299	0.858839899	0.982276696	1521	2726	1082	1511	1702	2720	1683	2707
ENSECAG00000010701	5.303984713	0.858898947	0.982276696	469	759	679	875	1012	689	932	777
ENSECAG00000006680	4.170344543	0.859088562	0.982287577	165	427	361	391	217	501	265	471
ENSECAG00000021981	4.601250073	0.859105458	0.982287577	313	424	491	552	647	478	451	406
ENSECAG00000019350	4.640257079	0.859274021	0.982287577	310	433	517	577	629	312	556	570
ENSECAG00000022385	5.269127131	0.859337525	0.982287577	327	725	777	1126	729	952	786	690
ENSECAG00000018489	7.318653694	0.859445395	0.982287577	3189	1290	2605	3599	3150	2772	3658	4376
ENSECAG00000010665	3.310826719	0.85946955	0.982287577	80	160	211	268	172	167	203	313
ENSECAG00000012976	5.975355655	0.859548148	0.982287577	528	1185	1150.01	1732	1503	1324	1517	1087
ENSECAG00000013436	5.937399672	0.859647434	0.982287577	1102	910	1024	1059	1536	873	1706	1244
ENSECAG00000018868	3.097707795	0.859684973	0.982287577	104	81	214	203	218	122	197	201
ENSECAG00000019447	3.541243715	0.859801929	0.982288155	128	215	240	280	234	211	295	220
ENSECAG00000013168	5.723490866	0.859975349	0.982288155	477	1201	1043	1270	485	1499	812	1493
ENSECAG00000022576	6.313724633	0.860025229	0.982288155	1030	1237	1393	2289	1333	1213	1994	2103
ENSECAG00000022237	3.805294016	0.860030596	0.982288155	134	265	298	353	235.002	330	318	262
ENSECAG00000018829	7.46667851	0.860127753	0.982300578	2044	4673	2565	3740	3376	2688	4166	4441
ENSECAG00000007555	4.629203853	0.86046335	0.982437625	203	470	539	677	383	500	654	519
ENSECAG00000020681	6.765854243	0.86050485	0.982437625	1205	2008	2317	2613	2439	2058	2397	2074
ENSECAG00000004287	5.361087559	0.860506631	0.982437625	407	955	641	1081	658	663	1098	1019
ENSECAG00000021396	5.918594603	0.860784629	0.982572884	716	1345	992	1159	956	1070	1532	1719
ENSECAG00000001100	3.153000743	0.860797711	0.982572884	127	169	185	153	172	176	204	175
ENSECAG00000007897	5.048916048	0.861197684	0.98293089	372	512	656	826	928	459	757	718
ENSECAG00000014613	5.586356236	0.861423948	0.98300452	511	842	1314	887	889	1100	1185	796
ENSECAG00000022392	4.5803421	0.861434879	0.98300452	187	338	546	694	369	572	623	505
ENSECAG00000020905	5.878174708	0.861624361	0.983060655	650	1252	1172	1277	957	1410	1088	1365
ENSECAG00000018875	1.6546814	0.861656765	0.983060655	17	47	96	61	78	46	113	35
ENSECAG00000002876	4.702680547	0.862172299	0.98350868	300	387	500	776	614	368	616	564
ENSECAG00000010014	0.299299811	0.862222234	0.98350868	26	9	31	3	23	63	5	5
ENSECAG00000015828	5.196481463	0.862676471	0.983687356	399	571	680	993	904	619	921	729
ENSECAG00000022139	2.085111632	0.862726345	0.983687356	28	79	132	54	49	103	75	133
ENSECAG00000006343	5.118102167	0.862734913	0.983687356	496	751	553	695	600	527	953	831
ENSECAG00000008095	0.638382147	0.862825046	0.983687356	10	20	25	62	23	27	31	38
ENSECAG00000019709	4.726683525	0.862887002	0.983687356	415	472	508	491	604	381	770	460
ENSECAG00000013294	6.393955765	0.862897288	0.983687356	1130	1738	1494	1406	1946	1934	1632	1693
ENSECAG00000012603	4.242505921	0.863158947	0.983887125	186	384	282	507	314	369	409	540
ENSECAG00000000557	3.961480678	0.863323191	0.983927636	137	238	342	478	323	276	356	329
ENSECAG00000020472	3.618968745	0.863577051	0.983927636	140	239	189	296	181	190	337	364
ENSECAG00000020730	5.465266287	0.863609196	0.983927636	462	984	827	1005	729	1024	964	926
ENSECAG00000004005	3.365741836	0.86382611	0.983927636	68	163	289	226	328	159	222	175
ENSECAG00000023123	5.364808254	0.863834194	0.983927636	544	872	633	799	623	721	1151	1107
ENSECAG00000008840	7.311956796	0.863837859	0.983927636	2408	2339	3174	3694	3980	2660	4108	2413
ENSECAG00000020604	1.629622716	0.863929843	0.983927636	24	65	44	100	62	47	53	83
ENSECAG00000018050	5.481054683	0.863933446	0.983927636	786	656	922	605	1200	864	1033	751
ENSECAG00000021402	1.05919579	0.863972295	0.983927636	22	47	20	62	27	33	62	43
ENSECAG00000019219	4.004117384	0.864114734	0.983991423	187	161	477	375	445	268	354	247
ENSECAG00000007654	2.9832343	0.864263197	0.984005849	108	146	130	191	92	101	242	225
ENSECAG00000014148	2.812942528	0.864300263	0.984005849	45	124	195	139	114	148	172	170
ENSECAG00000021817	5.037101849	0.864491182	0.984031777	336	745	587	771	716	587	786	634
ENSECAG00000020596	4.256690706	0.864495901	0.984031777	184	140	649	398	603	380	473	192
ENSECAG00000017553	6.352579202	0.864614394	0.984068267	1561	1762	1166	1138	2041	978	2187	1612
ENSECAG00000024336	10.34578106	0.86485906	0.984087418	13588	16504	33404	29548	31931	32860	22887	23869
ENSECAG00000006339	4.828886244	0.864982537	0.984087418	322	565	518	704	725	356	750	541
ENSECAG00000000982	4.037402777	0.865207431	0.984087418	133	222.999	426	495.999	555	176.999	538.999	88.9999
ENSECAG00000024629	6.625054804	0.865241596	0.984087418	1022	2362	1475	2099	1386	1869	2694	2700
ENSECAG00000017544	0.564581274	0.865349453	0.984087418	12	31	19	45	16	37	13	44
ENSECAG00000015148	5.933183096	0.865643673	0.984087418	691.999	884.996	1246	1802.99	1181	789.997	1584	1564
ENSECAG00000000643	5.312056911	0.86584934	0.984087418	446	659	713	1026	609	761	845	1219
ENSECAG00000020590	6.056810388	0.866009695	0.984087418	677	948	1640	1791	1234	980	2184	1220
ENSECAG00000021472	5.128139998	0.86602613	0.984087418	414	611	528	946	682	689	849	800
ENSECAG00000013794	1.127081841	0.866128086	0.984087418	10	61	27	66	38	55	41	34
ENSECAG00000013222	3.99833187	0.866330074	0.984087418	189	230	319	459	276	246	422	388
ENSECAG00000006626	4.78385463	0.866348719	0.984087418	291	461	399	845	388	360	811	868
ENSECAG00000019782	8.428619664	0.86641722	0.984087418	4796	5801	7240	7428	4951	8589	8189	6637
ENSECAG00000017437	1.240752929	0.866458827	0.984087418	34	35	35	52	43	46	59	50
ENSECAG00000021927	10.59119641	0.866569157	0.984087418	14950	21311	36879	38089	42939	36773	25821	26478
ENSECAG00000011656	3.224617644	0.866578406	0.984087418	306	75	95	112	175	138	267	190
ENSECAG00000022566	6.546888015	0.866639103	0.984087418	1210	2013	1577	2033	1917	2283	1698	1735
ENSECAG00000012872	2.859191243	0.866700204	0.984087418	87	95	169	154	177	105	223	125
ENSECAG00000000469	4.340224945	0.866727589	0.984087418	258	298	380	494	313	372	479	589
ENSECAG00000021066	5.316705677	0.866886927	0.984087418	484	676	885	895	563	650	1263	888
ENSECAG00000014676	9.319284896	0.867032178	0.984087418	9743	16990	7316	8139	13186	17228	12968	11654
ENSECAG00000020692	6.158706655	0.867090062	0.984087418	863	991	1215	2053	1146	698	2657	1898
ENSECAG00000011630	2.948481736	0.867326752	0.984087418	67	60	142	345	172	117	185	156
ENSECAG00000021200	5.460220394	0.867403255	0.984087418	813	740	769	748	984	779	835	1028

ENSECAG00000024605	7.62529334	0.867460129	0.984087418	3203	3436	3923	3545	4670	3658	5210	2812
ENSECAG00000020521	7.516875326	0.867481218	0.984087418	2538	3887	3327	3524	4787	2576	4447	3374
ENSECAG00000017787	3.982557632	0.867492997	0.984087418	189	425	182	288	398	242	443	291
ENSECAG00000012943	4.492355398	0.867636382	0.984087418	227	407	451	609	504	302	504	559
ENSECAG00000002171	4.540791507	0.867783634	0.984087418	289	219	797	293	417	320	185	1081
ENSECAG00000013671	7.618102808	0.867806271	0.984087418	2858	2923	3843	4829	5101	3090	4654	3402
ENSECAG00000002521	6.466893124	0.867912141	0.984087418	1360	1527	1363	1816	2286	1641	2284	1455
ENSECAG00000000060	4.343521624	0.867960491	0.984087418	240	303	497	383	554	427	498	263
ENSECAG00000010746	3.705350042	0.868001026	0.984087418	179	141	340	297	302	228	347	200
ENSECAG00000014000	4.078064986	0.868084352	0.984087418	154	319	286	464	393	257	427	385
ENSECAG00000013604	7.084153061	0.868095613	0.984087418	2180	2900	1857	2758	2446	3053	2921	2735
ENSECAG00000012086	3.11244113	0.868172316	0.984087418	72	182	187	170	202	208	218	111
ENSECAG00000010266	5.880640341	0.868267575	0.984087418	888	1529	791	956	968	850	1868	1284
ENSECAG00000019850	5.822715239	0.868279262	0.984087418	716	654	1147	1536	1252	894	1538	1248
ENSECAG00000020479	4.856478819	0.868352358	0.984087418	342	376	490	888	666	423	687	734
ENSECAG00000019736	6.267789705	0.868380653	0.984087418	1203	1808	974	1174	1380	1872	1694	1691
ENSECAG00000021351	6.054072477	0.868430623	0.984087418	414	1330	1282	2198	766	1446	1110	2125
ENSECAG00000018245	2.642629484	0.868535231	0.984087418	42	89	134	193	108	129	141	156
ENSECAG00000014217	5.496166858	0.868905784	0.984087418	548	713	753	1210	740	729	1179	1288
ENSECAG00000024916	5.424852652	0.869030594	0.984087418	564	624	820	1027	863	733	1127	1007
ENSECAG00000000135	7.029073098	0.869050342	0.984087418	1730	1936	3051	2341	2914	2346	3855	2319
ENSECAG00000003118	3.443936361	0.869152864	0.984087418	111	172	199	299	276	147	294	226
ENSECAG00000010754	5.694938425	0.869308585	0.984087418	502	1296	908	1138	1089	1050	1118	1019
ENSECAG00000014480	3.465628452	0.869330398	0.984087418	60	224	119	421	81	92	262	545
ENSECAG00000000226	4.502829229	0.869368505	0.984087418	197	519	448	536	524	467	445	422
ENSECAG00000009478	5.59010638	0.869369212	0.984087418	638	565	881	1353	700	700	1279	1544
ENSECAG00000016340	10.85155501	0.869380715	0.984087418	15718	25370	40462	54317	43846	43838	35218	36022
ENSECAG00000020519	8.334602241	0.869399797	0.984087418	8505	7263	2578	2721	2297	13902	2948	6082
ENSECAG00000010206	2.930750121	0.869439052	0.984087418	81	170	112	202	100	142	173	210
ENSECAG00000021345	2.350368175	0.869452684	0.984087418	90	91	88	83	108	103	114	87
ENSECAG00000015172	9.973211474	0.869488906	0.984087418	19411	15757	18778	10776	28014	34725	8052	13526
ENSECAG00000015444	3.864290812	0.86959061	0.984087418	138	201	345	368	297	232	454	288
ENSECAG00000023135	0.57986202	0.869605489	0.984087418	15	28	36	25	19	23	44	30
ENSECAG00000020255	4.314821035	0.8696719	0.984087418	338	420	375	244	413	371	488	374
ENSECAG00000009940	1.780171875	0.869731014	0.984087418	47	37	89	56	48	124	53	59
ENSECAG00000009648	7.67228291	0.86998252	0.984093219	5146	4766	1828	1538	5102	4086	4514	2935
ENSECAG00000000229	0.789599051	0.870326941	0.984093219	12	20	63	30	23	15	56	43
ENSECAG00000023395	0.311704429	0.870412018	0.984093219	5	13	26	41	24	9	21	47
ENSECAG00000000872	6.196646505	0.870468624	0.984093219	1276	1515	1102	1249	1873	1014	2016	1221
ENSECAG00000018926	5.90524487	0.870490269	0.984093219	835	891	1131	1331	1686	1090	1105	1247
ENSECAG00000021249	6.258581562	0.870500432	0.984093219	1278	1616	953	1589	1740	1112	2157	1398
ENSECAG00000007875	2.937362851	0.870542803	0.984093219	143	106	126	127	150	102	272	149
ENSECAG00000024925	2.797793097	0.870597076	0.984093219	84	61	291	69	152	212	81	99
ENSECAG00000023204	4.276502094	0.870663723	0.984093219	199	510	317	320	467	331	536	346
ENSECAG00000019381	4.517593218	0.870769913	0.984093219	274	505	367	449	394	600	561	409
ENSECAG00000024102	2.998420424	0.870849206	0.984093219	61	119	139	270	140	159	204	185
ENSECAG00000013772	4.13611646	0.870885486	0.984093219	286	211	501	249	585	329	273	231
ENSECAG00000021816	6.29039075	0.87108359	0.984093219	946	963	2048	1888	1815	1555	1477	1579
ENSECAG00000000792	6.151021399	0.871113562	0.984093219	752	1355	1764	1390	1371	1741	1393	1319
ENSECAG00000021707	5.57678157	0.871195549	0.984093219	339.999	751.999	1077	1560	949.998	895.988	1240	884
ENSECAG00000022246	5.298220818	0.871216171	0.984093219	641	659	698	810	1042	570	1069	601
ENSECAG00000018240	6.697888398	0.871258031	0.984093219	1763	1597	1785	2291	1989	1858	2644	2152
ENSECAG00000025018	6.418010639	0.87135435	0.984093219	709	2087	1530	1797	1775	1410	2031	2216
ENSECAG00000020866	4.342101655	0.871412704	0.984093219	284	280	457	383	451	416	558	326
ENSECAG00000006877	4.733533226	0.871506891	0.984093219	310	261	776	528	360	389	300	1247
ENSECAG00000015560	4.356653295	0.871554155	0.984093219	319	254	491	328	606	259	555	360
ENSECAG00000009243	0.613757971	0.871704794	0.984093219	9	20	31	55	10	32	28	46
ENSECAG00000019035	2.173913656	0.871724208	0.984093219	53	74	100	101	76	79	101	110
ENSECAG00000000030	3.952619024	0.871886491	0.984095723	173	250	265	419	226	258	364	494
ENSECAG00000020064	6.216838034	0.871899302	0.984095723	1094	1236	1566	1470	1578	1504	1826	1259
ENSECAG00000016190	11.38021427	0.872119433	0.984246605	29885	34515	56277	70841	64730	59486	48637	56235
ENSECAG00000019776	4.819714125	0.872315265	0.98432291	486	632	461	379	683	409	940	339
ENSECAG00000011998	2.648326355	0.872463721	0.98432291	38	165	75	173	118	191	111	107
ENSECAG00000008611	4.650406113	0.872479178	0.98432291	446	322	503	507	593	382	617	495
ENSECAG00000017514	10.68735675	0.872599201	0.98432291	21648	20397	40592	40441	36391	34604	29256	34377
ENSECAG00000020291	4.682847752	0.872619335	0.98432291	206	462	713	460	523	708	467	482
ENSECAG00000002745	5.10120092	0.872845583	0.984354034	607	837	376	421	814	635	788	726
ENSECAG00000000689	5.948565877	0.872981187	0.984354034	962	1305	978	1149	1487	660	1624	1406
ENSECAG00000019462	0.277271018	0.873001458	0.984354034	18	12	20	26	32	29	21	14
ENSECAG00000005163	3.024956709	0.873106325	0.984354034	130	130	171	146	89	117	146	315
ENSECAG00000018098	9.98499245	0.873189126	0.984354034	14024	16767	19184	24806	24162	14962	25441	19657
ENSECAG00000020856	6.739155704	0.873205212	0.984354034	2227	1941	1439	1734	1630	2678	1446	2909
ENSECAG00000024649	4.261031938	0.873333427	0.984354034	244	298	445	347	474	329	532	324
ENSECAG00000012622	6.314177479	0.873441604	0.984354034	1128	1133	1745	1814	1820	1372	2104	1335
ENSECAG00000026017	1.929297559	0.87344789	0.984354034	34	106	41	76	16	198	24	73
ENSECAG00000012810	6.582182108	0.873536232	0.984354034	1634	1241	1706	2336	2162	1182	2775	1931

ENSECAG00000003122	4.679341984	0.873597994	0.984354034	392	629	382	396	485	444	680	527
ENSECAG00000007745	5.815401122	0.873771612	0.984439971	685	740	1206	1594	1153	898	1328	1313
ENSECAG00000010782	4.492535646	0.873854423	0.984439971	150	483	513	581	335	681	276	516
ENSECAG00000000536	5.665870148	0.87397942	0.984439971	610	1183	777	1150	763	777	1226	1483
ENSECAG00000008395	6.185005375	0.874300671	0.984439971	948	968	1401	2130	1159	1043	1917	1990
ENSECAG00000007704	4.447971768	0.874307779	0.984439971	374	287	295	628	652	322	583	250
ENSECAG00000020699	10.85107033	0.874408414	0.984439971	21200	26851	38882	44678	35731	33408	38583	52756
ENSECAG00000022920	6.177566769	0.8745633	0.984439971	928	745	1931	1792	1409	1320	1904	1400
ENSECAG00000021836	5.821566187	0.874591771	0.984439971	538	1266	904	1351	1146	1245	1365	1116
ENSECAG00000020206	4.024221685	0.874636995	0.984439971	156	243	359	419	290	385	363	354
ENSECAG00000018145	3.029754936	0.874734718	0.984439971	134	98	162	194	315	94	157	94
ENSECAG00000014301	5.063980993	0.874885303	0.984439971	412	487	796	779	928	393	875	597
ENSECAG00000012331	4.593992145	0.874972441	0.984439971	628	338	160	375	518	341	847	432
ENSECAG00000023086	4.787808196	0.87499874	0.984439971	456	362	481	596	665	462	776	493
ENSECAG00000012035	6.135319431	0.875079838	0.984439971	520	735	1597	2456	1759	1417	1619	1268
ENSECAG00000018411	4.668232416	0.875258962	0.984439971	455	582.999	401	304	470	530	574	523.999
ENSECAG00000017863	7.02608526	0.875367437	0.984439971	1136	2078	2759	4135	4011	2037	2903	1772
ENSECAG00000003030	7.581035047	0.875446444	0.984439971	2230	3681	3553	4005	4474	3511	4882	3775
ENSECAG00000020123	3.22371525	0.875477252	0.984439971	89	151	211	214	309	176	174	132
ENSECAG00000017928	6.086782148	0.875493953	0.984439971	602	1085	1421	1871	1674	1371	1377	1406
ENSECAG00000020085	4.34545471	0.875676998	0.984439971	189	612	349	245	256	738	403	327
ENSECAG00000010837	3.790611273	0.875747347	0.984439971	95	150	271	531	217	201	390	403
ENSECAG00000010516	5.267210392	0.875768537	0.984439971	474	625	920	807	984	702	932	573
ENSECAG00000006710	5.596572246	0.875791099	0.984439971	1004	711	908	682	1134	901	1033	925
ENSECAG00000005495	2.684837866	0.875816453	0.984439971	115	82	123	104	152	93	181	129
ENSECAG00000016806	4.040240807	0.875835965	0.984439971	219	307	378	278	365	301	475	227
ENSECAG00000013518	5.233514142	0.875945438	0.984465825	404	624	769	1028	721	663	872	875
ENSECAG00000018213	5.582670073	0.876107733	0.984474906	359	878	1067	1212	1109	1101	1117	784
ENSECAG00000013860	2.057868718	0.87612646	0.984474906	63	87	56	87	66	63	121	91
ENSECAG00000020326	3.030063302	0.876281123	0.984551523	144	94	160	188	312	48	272	42
ENSECAG00000006601	3.534788258	0.876489191	0.984571704	136	172	200	317	252	155	407	203
ENSECAG00000009702	7.541211612	0.876695637	0.984571704	2556	3150.01	3398	4622	3508	2520	5442	4196.02
ENSECAG00000025140	4.60178029	0.876801788	0.984571704	350	559	301	443	450	677	538	406
ENSECAG00000010401	5.701946933	0.876862065	0.984571704	693	872	872	1227	999	850	1351	1323
ENSECAG00000005540	8.353057094	0.87693491	0.984571704	3396	5191	4801	10434	4639	5778	6377	11677
ENSECAG00000008935	4.938146827	0.876945978	0.984571704	486	717	419	423	575	591	718	763
ENSECAG00000019092	2.680568821	0.877023067	0.984571704	95	107	107	124	157	82	151	161
ENSECAG00000017461	6.594516022	0.877116922	0.984571704	759	2263	1679	2688	1672	2482	1674	2058
ENSECAG00000022307	5.767821599	0.877150183	0.984571704	1053	1036	742	719	1474	668	1759	891
ENSECAG00000013062	10.45127416	0.877163882	0.984571704	16240	20005	23510	41045	34282	35611	20671	28674
ENSECAG00000013165	2.432366421	0.877293973	0.984588554	77	85	107	94	65	104	105	187
ENSECAG00000003055	4.037185886	0.877420622	0.984588554	183	272	375	387	365	386	268	315
ENSECAG00000009566	5.34370054	0.877549585	0.984588554	505	642	690	1055	765	547	1060	1170
ENSECAG00000009465	2.09875939	0.877668835	0.984588554	25	98	64	142	68	81	69	124
ENSECAG00000001472	0.512575759	0.877691905	0.984588554	25	14	22	28	37	12	51	19
ENSECAG00000011795	2.428363614	0.877697781	0.984588554	56	63	136	149	71	53	164	159
ENSECAG00000022963	5.747667529	0.877856237	0.984590925	591	940	981	1530	1090	1345	801	1138
ENSECAG00000014150	5.223747691	0.87789575	0.984590925	427	435	850	1120	997	423	896	799
ENSECAG00000009985	4.746002042	0.878060888	0.984590925	299	301	604	740	535	484	580	711
ENSECAG00000013501	3.920250148	0.878099315	0.984590925	105	360	296	330	350	405	271	254
ENSECAG00000000474	6.831548393	0.878132301	0.984590925	1876	1622	2577	1571	2876	2181	3307	1562
ENSECAG00000023445	1.948872285	0.878410534	0.984798362	66	52	66	87	72	44	117	84
ENSECAG00000000526	6.083818661	0.878490309	0.984798362	770	1124	1561	1589	1333	946	1699	1704
ENSECAG00000021497	2.811231935	0.878744618	0.984934745	57	91	342	34	14	112	8	415
ENSECAG00000013216	5.94790854	0.878818823	0.984934745	861	1080	1030	1546	1217	798	1685	1494
ENSECAG00000012920	6.79023212	0.87889384	0.984934745	1700	2241	1443	2608	2490	2167	2433	2045
ENSECAG00000006934	6.610257201	0.878985773	0.984934745	1493	3287	1065	952	965	4529	914	1227
ENSECAG00000003314	1.21175158	0.879214462	0.984934745	17	36	71	33	57	37	66	35
ENSECAG00000009756	2.707217686	0.879263827	0.984934745	93	101	133	143	124	164	153	88
ENSECAG00000005731	6.7131848	0.879314786	0.984934745	1604	1613	2035	2349	2570	1759	2467	1909
ENSECAG00000024606	5.854748491	0.879494231	0.984934745	642	1040	1189	1244	1261	972	1589	1205
ENSECAG00000025006	4.835549509	0.879565524	0.984934745	425	502	553	564	678	536	611	534
ENSECAG00000017002	7.940073107	0.879646372	0.984934745	2539	2712	6130	7531	6441	3465	5450	4966
ENSECAG00000007044	0.939483114	0.879688628	0.984934745	15	24	4	108	11	14	69	59
ENSECAG00000009396	7.195426182	0.879712006	0.984934745	1513	1901	2974	4306	2753	3680	2882	3256
ENSECAG00000013725	5.054236562	0.87973662	0.984934745	460	420	631	952	553	448	899	895
ENSECAG00000022045	6.431434203	0.879904403	0.984951601	1313	1171	1912	1856	2135	1481	2143	1414
ENSECAG00000010622	10.47241884	0.879957468	0.984951601	15274	17243	32211	37967	31904	35273	23501	30764
ENSECAG00000016822	5.202693733	0.880029241	0.984951601	493	630	591	874	689	489	981	1054
ENSECAG00000019062	5.092357977	0.880097728	0.984951601	530	490	800	494	979	424	963	605
ENSECAG00000022831	2.838759254	0.880336219	0.985094689	108	104	110	198	99	70	232	199
ENSECAG00000008453	4.792525814	0.880398635	0.985094689	364	378	610	691	679	363	766	514
ENSECAG00000011097	4.11421314	0.880561101	0.985179652	102.011	206	727.008	304	319.001	319.001	48.0001	688.024
ENSECAG00000014737	6.036464812	0.880717863	0.985258217	841	966	976	1919	996	914	2233	1663
ENSECAG00000012138	3.572431044	0.880813574	0.985268475	90	235	194	353	260	223	301	239
ENSECAG00000020724	8.00423076	0.881077101	0.985457651	3648	4237	4936	4908	5449	4405	7634	5004

ENSECAG00000013765	4.88955083	0.881194743	0.985457651	354	368	490	941	713	412	802	649
ENSECAG00000026871	3.060244876	0.881242367	0.985457651	141	165	125	118	149	161	270	144
ENSECAG00000016433	8.086222036	0.881460768	0.985522492	5456	4321	4493	4606	8626	3564	6723	3576
ENSECAG00000016392	7.465623048	0.881473477	0.985522492	2858	3376	3024	2574	4945	2481	5611	2500
ENSECAG00000021466	7.973379316	0.881750374	0.985626139	2220	3542	5326	7422	5142	4109	7214	5564
ENSECAG00000020083	4.918746035	0.88192645	0.985626139	417	718	475	548	613	478	781	657
ENSECAG00000009032	3.664030246	0.882030215	0.985626139	105	320	188	343	115	209	336	397
ENSECAG00000008775	1.388517401	0.882045312	0.985626139	25	84	39	35	59	54	52	41
ENSECAG00000006931	8.077947335	0.882058331	0.985626139	1629	4464	5987	8088	5896	8813	2820	5207
ENSECAG00000006020	3.11837842	0.882085615	0.985626139	121	136	138	207	170	125	268	192
ENSECAG00000025022	9.365537708	0.88243405	0.98583508	7693	7830	15442	16256	13828	15091	11835	15859
ENSECAG00000010135	3.203683112	0.882637353	0.98583508	94	196	170	221	219	200	141	184
ENSECAG00000013588	5.335022665	0.88266599	0.98583508	402	944	746	896	895	656	1007	810
ENSECAG00000021834	5.362173551	0.882692095	0.98583508	371	1070	686	934	610	933	964	904
ENSECAG00000008222	1.041115075	0.882814732	0.98583508	24	35	20	60	39	17	76	44
ENSECAG00000010198	4.587950719	0.882955154	0.98583508	339	550	325	428	541	328	768	468
ENSECAG00000023484	0.364718014	0.882968002	0.98583508	36	14	17	11	42	24	20	10
ENSECAG00000016200	5.602678096	0.883194285	0.98583508	441	839	1067	1344	914	1113	1162	837
ENSECAG00000002580	5.664501562	0.883234142	0.98583508	453	1330	937	1039	950	1165	1155	923
ENSECAG00000022635	7.701763102	0.883389235	0.98583508	2247	3756	3457	5493	3625	3038	5895	5740
ENSECAG00000011714	1.215834941	0.883443446	0.98583508	1	40.9997	86	33.999	5	34	150.995	20
ENSECAG00000017416	1.063232497	0.883565091	0.98583508	37	27	26	54	38	18	44	66
ENSECAG00000021005	5.463417252	0.883593527	0.98583508	576	682	671	1213	603	691	1374	1208
ENSECAG00000017063	3.147822182	0.883659891	0.98583508	122	141	156	227	157	165	136	263
ENSECAG00000021487	2.133143845	0.883674572	0.98583508	57	54	72	141	66	70	109	113
ENSECAG00000012489	6.8708096	0.883725638	0.98583508	1718	1730	2608	2441	2998	2311	2222	2073
ENSECAG00000000399	5.979859829	0.883744648	0.98583508	910	1479	816	1333	2118	698	1382	1027
ENSECAG00000021610	7.067159833	0.883852968	0.985859316	2449	2733	1808	2373	2591	2899	2396	3112
ENSECAG00000022195	3.879536194	0.884055926	0.985989099	149	268	286	402	437	320	243	191
ENSECAG00000022600	2.788489484	0.884374911	0.986185365	72	118	168	123	206	134	145	100
ENSECAG00000015982	5.98195142	0.884405145	0.986185365	1209	1163	933	1073	1795	673	1571	1239
ENSECAG00000023584	7.362535557	0.884901461	0.986609241	2586	2895	2235	4257	2175	2272	4178	5193
ENSECAG00000006692	7.328375884	0.884958592	0.986609241	2390	3110	2665	3432	3342	3361	3305	3238
ENSECAG00000020548	2.824844475	0.885252721	0.986702259	59	148	92	212	111	87	273	154
ENSECAG00000000880	0.434431703	0.885308995	0.986702259	22	19	21	22	32	20	17	39
ENSECAG00000001444	1.623011501	0.88572909	0.986702259	32	52	61	80	39	91	41	70
ENSECAG00000000091	0.909079881	0.885787036	0.986702259	16	46	27	36	19	57	44	34
ENSECAG00000008346	6.028145108	0.885795521	0.986702259	641.003	1121	1586	1555	1199	881.003	2407	1076
ENSECAG00000014497	5.076149376	0.885836668	0.986702259	381	498	626	1042	568	444	933	899
ENSECAG00000013970	4.865526659	0.88592587	0.986702259	336	431	626	690	447	470	929	707
ENSECAG00000023303	0.505635423	0.885949126	0.986702259	12	14	19	53	26	24	10	53
ENSECAG00000000057	4.182537043	0.88597457	0.986702259	249	239	387	456	345	279	523	376
ENSECAG00000018079	6.711417293	0.886055771	0.986702259	1159	2023	1789	2504	1981	1710	2768	2674
ENSECAG00000012575	3.204004159	0.886058718	0.986702259	77	199	164	220	162	159	224	248
ENSECAG00000018350	5.542834286	0.886082028	0.986702259	620	864	899	1006	1245	646	1255	756
ENSECAG00000022022	6.028867307	0.886495548	0.986832576	594	1259	1592	1451	906	1564	1595	1359
ENSECAG00000004450	3.917433113	0.886753031	0.986832576	242	167	403	207	419	189	437	269
ENSECAG00000009900	2.577456353	0.886773423	0.986832576	83	67	160	94	94	73	290	76
ENSECAG00000007276	4.891722364	0.88685754	0.986832576	363	280	857	596	792	394	1210	238
ENSECAG00000024660	2.513717683	0.887017172	0.986832576	57	65	92	226	151	68	178	74
ENSECAG00000020454	0.984085282	0.887087826	0.986832576	20	32	28	64	38	28	29	59
ENSECAG00000022640	7.314947999	0.887094709	0.986832576	1957	1909	4078	3959	3348	2797	3130	3881
ENSECAG00000019763	6.224798717	0.88715804	0.986832576	769	1313	1455	2108	1299	1537	1586	1779
ENSECAG00000015731	3.90693731	0.887278963	0.986832576	255	225	236	353	224	183	505	359
ENSECAG00000017655	3.935319497	0.887328904	0.986832576	337	154	131	455	257	217	416	387
ENSECAG00000023932	8.421874924	0.887438204	0.986832576	3509	6021	6534	10029	5144	6386	6897	9986
ENSECAG00000022847	5.100914109	0.887611229	0.986832576	579	624	516	591	945	396	1103	562
ENSECAG00000013508	6.383785723	0.887725662	0.986832576	1192	1168	1908	1520	2227	1804	1769	1340
ENSECAG00000019481	5.903407097	0.887757939	0.986832576	835	881	1120	1370	1259	1147	1478	1279
ENSECAG00000023499	5.082899916	0.8877745	0.986832576	301	604	762	900	660.002	884	566	656
ENSECAG00000023674	6.714772496	0.887782339	0.986832576	1181	1887	2096	2676	2321	2302	2147	1880
ENSECAG00000023282	-0.176570255	0.88788917	0.986832576	1	25	23	6	10	22	22	15
ENSECAG00000023503	2.515059888	0.887902214	0.986832576	85	94	92	139	73	77	135	186
ENSECAG00000022844	4.13578369	0.887996711	0.986832576	214	268	373	443	363	347	475	277
ENSECAG00000017200	3.655455111	0.888014347	0.986832576	103	206	159	471	204	200	325	364
ENSECAG00000024216	0.574057427	0.888080368	0.986832576	9	11	33	61	1	19	24	69
ENSECAG00000002847	3.264474045	0.888209786	0.986832576	119	153	221	209	290	113	162	219
ENSECAG00000002280	6.196467621	0.888246527	0.986832576	894	915	1817	1836	1577	1267	1774	1495
ENSECAG00000020858	4.139425458	0.888310348	0.986832576	199	596	263	188	440	482	287	209
ENSECAG00000014617	7.701295769	0.888410009	0.986832576	2688	3219	4432	4264	5025	3805	4785	4365
ENSECAG00000004549	5.955270061	0.888452693	0.986832576	952	763	1495	1272	1837	851	1579	901
ENSECAG00000004586	5.376895878	0.888548189	0.98684133	159	459	633	2048	898	766	827	1093
ENSECAG00000010913	7.529814599	0.888633932	0.98684133	1674	2247	5576	4653	4550	2054	4422	4396
ENSECAG00000019248	4.657608163	0.88883742	0.986897822	257	254	615	723	571	413	565	624
ENSECAG00000022375	8.282775236	0.88885817	0.986897822	4205	4965	6624	7069	6356	6437	7090	5887
ENSECAG00000013722	6.650423355	0.889080194	0.986950236	712	2113	3166	1562	1437	3117	1412	2150

ENSECAG00000005915	2.376455724	0.889132853	0.986950236	41.0071	117	109	118	75.0004	115	94	135
ENSECAG00000006796	1.612913474	0.889165444	0.986950236	50	39	59	53	82	47	64	63
ENSECAG00000017243	1.075491964	0.889323082	0.986972549	25	27	28	74	15	29	62	63
ENSECAG00000010512	1.280345181	0.889362896	0.986972549	22	42	43	61	29	57	66	51
ENSECAG00000024699	7.806400205	0.889598835	0.986972549	3686	4917	3457	2774	6787	3857	5424	3295
ENSECAG00000006675	6.216143943	0.889746777	0.986972549	1075	1080	1265	2045	1460	1041	1995	1757
ENSECAG00000005412	4.478262582	0.889844531	0.986972549	296	392	355	591	530	389	560	375
ENSECAG00000010712	5.137910429	0.889918637	0.986972549	574	750	616	516	736	852	761	549
ENSECAG00000000450	5.479069978	0.890212551	0.986972549	649	1035	697	783	990	653	994	1082
ENSECAG00000021002	7.570253963	0.890258853	0.986972549	3307	3982	2524	3545	3464	3552	4782	4038
ENSECAG00000016978	2.058395209	0.890283565	0.986972549	12	31	177	112	67	5	267	17
ENSECAG00000011406	6.389303886	0.890595568	0.986972549	1209	1232	1442	2290	1450	760	2836	2112
ENSECAG00000026975	2.592921941	0.890621065	0.986972549	89	113	99	127	98	100	152	147
ENSECAG00000023868	8.201348041	0.890765746	0.986972549	4610	4884	5287	6438	6603	5213	7413	5271
ENSECAG00000026981	7.789031827	0.890843981	0.986972549	2468	3331	4979	5024	5791	4782	4912	3505
ENSECAG00000019073	6.925923716	0.890977344	0.986972549	1438	1804	2859	2505	2453	3339	1879	2637
ENSECAG00000003959	1.736706677	0.89122083	0.986972549	23	29	71	137	63	53	80	72
ENSECAG00000012445	8.240654568	0.891256303	0.986972549	3891	6000	4817	7594	6419	4739	6794	7216
ENSECAG00000011432	4.641048322	0.891396564	0.986972549	297	459	459	545	305	456	511	880
ENSECAG00000021415	4.122214434	0.891501222	0.986972549	187	461	237	389	241	286	428	499
ENSECAG00000022619	2.874636979	0.891530056	0.986972549	52	142	120	222	152	200	100	161
ENSECAG00000006545	5.595035035	0.891793104	0.986972549	859	815	799	915	1144	972	1118	772
ENSECAG00000000686	10.59223268	0.891879514	0.986972549	22791	31527	24727	26104	39648	26906	37868	29444
ENSECAG00000023670	5.668584275	0.891901358	0.986972549	774	870	825	1045	1085	888	1285	1135
ENSECAG00000007970	1.860798785	0.891969625	0.986972549	38.0001	52	77	86.0003	60	37.0003	128	90
ENSECAG00000007267	3.914425956	0.89199538	0.986972549	265	222	312	254	282	330	404	234
ENSECAG00000007963	6.121904193	0.892103466	0.986972549	1554	901	1176	832	1630	926	2674	948
ENSECAG00000024976	5.296723056	0.892108209	0.986972549	579	792	625	696	643	651	963	1150
ENSECAG00000017850	5.057057269	0.892135112	0.986972549	418	403	612	968	562	678	694	922
ENSECAG00000018433	3.73062309	0.892151333	0.986972549	81	194	385	316	251	211	160	509
ENSECAG00000019349	1.092927294	0.89221352	0.986972549	39	35	28	32	34	38	59	48
ENSECAG00000005471	4.197615559	0.892226597	0.986972549	263	305	423	260	507	309	439	319
ENSECAG00000009039	7.430636947	0.89227964	0.986972549	3497	3063	1423	3366	4454	1995	5853	2964
ENSECAG00000020447	7.799053943	0.892283421	0.986972549	1884	5621	3177	6284	2384	3405	4606	8176
ENSECAG00000022460	6.655531053	0.8922841	0.986972549	1350	1919	1732	2021	1935	1634	2545	2646
ENSECAG00000010570	1.264146978	0.892294513	0.986972549	18	29	62	59	62	31	56	52
ENSECAG00000016301	5.526874547	0.892337658	0.986972549	493	695	1225	1013	948	935	990	948
ENSECAG00000000308	6.391850356	0.892442491	0.986972549	1626	1677	1204	1250	1548	1841	1951	1621
ENSECAG00000018283	3.939238968	0.892501323	0.986972549	73	322	290	463	223	247	432	435
ENSECAG00000004819	3.23105958	0.892532301	0.986972549	127	154	132	245	167	204	228	203
ENSECAG00000011239	6.045568565	0.892622715	0.986972549	552	515	1898	2214	1764	1123	1144	1391
ENSECAG00000019089	6.135101116	0.892716915	0.986972549	610	1093	1453	2269	911	1361	1457	2115
ENSECAG00000008807	3.919051581	0.893007424	0.986972549	175	216	292	402	351	252	400	300
ENSECAG00000005717	4.721478064	0.893021397	0.986972549	390	364	527	632	528	447	564	654
ENSECAG00000009686	4.797181036	0.893152798	0.986972549	476	694	274	488	550	529	863	388
ENSECAG00000015957	4.378937069	0.893254394	0.986972549	251	207	464	579	318	323	595	578
ENSECAG00000024983	6.056370086	0.89331445	0.986972549	582	1225	1069	2035	1531	1177	1699	1345
ENSECAG00000020897	4.208359764	0.893496632	0.986972549	182	300	477	419	364	468	328	349
ENSECAG00000024591	3.381350901	0.893530521	0.986972549	169	258	102	160	131	191	311	274
ENSECAG00000008170	3.389655945	0.893562645	0.986972549	97	70	253	356	290	194	306	114
ENSECAG00000013187	6.971303412	0.893848362	0.986972549	2860	2702	788	2113	1451	3653	2219	2895
ENSECAG00000016839	1.081018904	0.89388359	0.986972549	37	49	10	48	13	70	27	52
ENSECAG00000023230	3.927822626	0.894065589	0.986972549	132	306	314	343	234	414	321	324
ENSECAG00000011077	3.087432703	0.894228386	0.986972549	132	143	138	193	200	147	230	125
ENSECAG00000015073	2.560174557	0.894308812	0.986972549	54	69	178	117	180	122	134	62
ENSECAG00000026824	2.164165755	0.894309203	0.986972549	54	65	90	119	78	88	62	131
ENSECAG00000024902	9.721661623	0.894348866	0.986972549	7799	11996	18532	23574	19426	19949	15276	17498
ENSECAG00000007526	5.702443867	0.894354885	0.986972549	497	973	1077	1216	1007	1275	1278	902
ENSECAG00000012404	3.683266366	0.894555692	0.986972549	130	224	257	350	312	218	292	239
ENSECAG00000024693	5.114470919	0.894692144	0.986972549	558	559	759	567	909	555	852	569
ENSECAG00000014926	3.560151769	0.894742701	0.986972549	150	188	206	287	319	194	272	223
ENSECAG00000011570	0.800970574	0.894788958	0.986972549	19	26	26	46	36	17	27	63
ENSECAG00000022675	6.891501948	0.894917129	0.986972549	1196	1757	2969	3050	2072	2532	2816	2442
ENSECAG00000023602	7.166438973	0.895131405	0.986972549	1263	1930	2721	4788	3123	1840	3945	3672
ENSECAG00000019654	2.841455453	0.895187351	0.986972549	49	122	115	273	182	147	116	132
ENSECAG00000014559	3.43212738	0.895215874	0.986972549	149	250	175	191	223	188	225	253
ENSECAG00000008220	2.498269264	0.895431854	0.986972549	38	106	109	157	115	151	79	126
ENSECAG00000017938	5.760568109	0.895628942	0.986972549	670	943	665	1624	942	1007	1423	1325
ENSECAG00000008723	5.904012828	0.895781415	0.986972549	758	839	1022	1686	1241	1122	1305	1477
ENSECAG00000016730	4.707671186	0.895794082	0.986972549	663	343	723	1	436.003	467.003	1270	1
ENSECAG00000012289	3.775035757	0.895915479	0.986972549	124.002	247	213	465	147.001	183.001	314	499
ENSECAG00000018902	1.106875503	0.895916094	0.986972549	19	23	69	35	26	26	39	89
ENSECAG00000020453	5.659831538	0.895941103	0.986972549	602	711	1042	1277	1174	983	988	1171
ENSECAG00000008926	5.588629908	0.895947356	0.986972549	752	694	863	1164	902	697	1286	1168
ENSECAG00000025567	1.998515036	0.896071698	0.986972549	41	51	108	78	70	127	64	69
ENSECAG00000015765	0.561332224	0.89609774	0.986972549	19	37	26	16	29	23	45	18

ENSECAG00000007050	5.553065501	0.896165922	0.986972549	803	541	773	1275	992	613	1395	966
ENSECAG00000017453	5.502508178	0.896178181	0.986972549	505	577	791	1590	948	712	1066	1061
ENSECAG00000011662	3.645605444	0.896269609	0.986972549	158	275	178	297	262	200	309	269
ENSECAG00000023259	1.584717946	0.896311821	0.986972549	22	86	43	68	21	90	52	73
ENSECAG00000002759	3.009659228	0.89638463	0.986972549	95	138	154	204	143	119	191	214
ENSECAG00000010468	4.391602716	0.896543107	0.986972549	268	328	464	412	499	474	456	356
ENSECAG00000012620	5.449996158	0.896651051	0.986972549	677	759	701	999	1053	609	1166	842
ENSECAG00000007262	5.093862473	0.896703749	0.986972549	512	722	368	864	370	490	759	1244
ENSECAG00000022765	1.84135478	0.896751878	0.986972549	34	45	76	99	52	56	99	99
ENSECAG00000017372	6.306114698	0.8967823	0.986972549	850	1422	1852	1493	1709	2034	1477	1499
ENSECAG00000026845	4.97715366	0.896889858	0.986972549	170	739	585	843	629	905	567	562
ENSECAG00000022976	4.931203277	0.897121657	0.986972549	342	412	637	818	731	507	762	627
ENSECAG00000023315	8.059940674	0.897144602	0.986972549	4280	3923	6120	4719	6334	4611	7218	4113
ENSECAG00000012800	6.044612121	0.897261193	0.986972549	953	1095	1330	1380	1267	1294	1390	1534
ENSECAG00000014355	2.447141866	0.897324882	0.986972549	64	53	135	132	148	62	137	120
ENSECAG00000000359	3.789064019	0.897388742	0.986972549	112.014	224	396	308	268.981	296	285	286
ENSECAG00000016416	6.77054025	0.89741001	0.986972549	1278	1707	2317	2843	1873	2096	2499	2628
ENSECAG00000000429	1.590207225	0.897410814	0.986972549	23	48	38	121	54	78	51	54
ENSECAG00000017795	9.146472124	0.897489434	0.986972549	4762	6744	17312	14882	13878	14679	9674	7789
ENSECAG00000009764	5.216471552	0.89751168	0.986972549	616	670	628	598	883	722	984	612
ENSECAG00000014345	5.008259024	0.89770356	0.986972549	249	595	758	749	900	719	554	548
ENSECAG00000017899	8.445315379	0.897729562	0.986972549	4611	5868	5577	8558	6627	5778	9239	8746
ENSECAG00000011435	4.565973844	0.897810687	0.986972549	304	236	548	680	591	323	606	459
ENSECAG00000011907	2.493675004	0.897871344	0.986972549	42	123	58	187	110	178	90	90
ENSECAG00000007781	6.381543877	0.897910332	0.986972549	594	1444	1595	2605	1290	1798	2062	2056
ENSECAG00000021880	7.963351281	0.898026868	0.986972549	2609	4934	4153	6232	5077	3934	5798	6853
ENSECAG00000003236	4.288435285	0.898146998	0.986972549	266	431	279	371	398	269	500	525
ENSECAG00000011944	7.032667843	0.898161322	0.986972549	1495	1967	2451	3531	3517	2549	2547	2585
ENSECAG00000024920	6.017199326	0.898178567	0.986972549	616	1170	1070	1889	1163	911	1837	1746
ENSECAG00000012398	2.914906826	0.898201369	0.986972549	67	142	180	171	181	110	179	151
ENSECAG00000021705	10.49406718	0.898860977	0.987573231	16430	19768	35720	36354	29147	30076	27729	31909
ENSECAG00000021288	5.149465432	0.899011901	0.987573231	528	513	852	646	589	768	1032	586
ENSECAG00000016359	4.559563744	0.899097805	0.987573231	226	394	522	561	667	283	785	325
ENSECAG00000007803	4.548082173	0.899154003	0.987573231	311	308	473	567	530	414	533	528
ENSECAG00000022238	0.454641247	0.899230029	0.987573231	5	25	23	49	23	41	15	22
ENSECAG00000016188	6.764278789	0.899268484	0.987573231	1235	1656	2359	2537	1888	2381	2392	2686
ENSECAG00000016437	1.883231309	0.899524046	0.987664557	28	51	77	110	37	56	101	122
ENSECAG00000000410	3.557851166	0.89959619	0.987664557	144	170	252	302	252	181	223	315
ENSECAG00000012611	5.926527825	0.89964802	0.987664557	894	1330	1001	1094	1329	1035	1711	1032
ENSECAG00000019339	1.163979205	0.89969865	0.987664557	25	41	41	43	54	39	74	21
ENSECAG00000020850	8.164560984	0.900033685	0.987893058	4146	4153	5361	6394	6740	5023	7894	5276
ENSECAG00000024022	2.274000545	0.900153944	0.987893058	69	85	104	64	104	112	146	50
ENSECAG00000003995	3.221534587	0.900167113	0.987893058	133	146	187	204	218	143	246	167
ENSECAG00000007363	5.281787302	0.90038455	0.987971548	426	542	887	954	688	699	861	1099
ENSECAG00000024832	6.771158533	0.900422865	0.987971548	1565	1275	2367	2475	2211	1838	2826	2606
ENSECAG00000000685	11.73033246	0.90050198	0.987971548	48674	89548	48312	41327	79302	62832	81139	71031
ENSECAG00000008069	3.33583	0.900644315	0.987971548	140	190	196	194	200	214	228	189
ENSECAG00000014253	7.020327429	0.900849664	0.987971548	1703	1907	2777	2758	3016	2146	3108	2955
ENSECAG00000010156	5.175163719	0.900908629	0.987971548	454	574	689	847	1135	442	775	749
ENSECAG00000009436	3.735528965	0.900921565	0.987971548	154	201	291	339	284	209	349	269
ENSECAG00000012843	7.090401043	0.901086262	0.987971548	2804	2329	1660	2074	4828	1533	4280	1298
ENSECAG00000001409	0.41638669	0.901107402	0.987971548	15	26	18	33	21	28	33	20
ENSECAG00000013737	4.513445932	0.901174411	0.987971548	271	374	382	675	554	488	433	402
ENSECAG00000011434	5.913042138	0.901193196	0.987971548	565	1273	1141	1538	1159	967	1739	1216
ENSECAG00000014809	6.55986637	0.901304109	0.987988183	1013	1882	1750	2378	1979	2173	2065	1585
ENSECAG00000003291	0.835810269	0.901441327	0.987988183	12	18	40	64	33	19	63	27
ENSECAG00000009458	7.455284492	0.901468709	0.987988183	3151	2958	2924	3296	4153	2146	4658	3787
ENSECAG00000014762	5.616349936	0.901556616	0.987989418	654	734	760	1353	1436	711	1343	751
ENSECAG00000004218	4.062850806	0.901656517	0.988003796	251	353	320	248	358	390	388	241
ENSECAG00000026879	3.273309343	0.902062243	0.988140151	119	122	146	309	210	192	211	210
ENSECAG00000018442	6.882683003	0.902084183	0.988140151	1003	1863	2139	4173	2648	2488	2163	2406
ENSECAG00000008583	6.723609626	0.902116142	0.988140151	1150	1614	2050	2848	2112	2185	2195	2572
ENSECAG00000019845	6.284106769	0.902128128	0.988140151	1150	1619	1341	1444	1198	2339	1496	1351
ENSECAG00000012980	3.823383172	0.902246185	0.988174393	142	309	275	316	284	279	318	290
ENSECAG00000007022	2.635191762	0.902646888	0.988327669	83	77	97	181	144	94	174	121
ENSECAG00000016963	2.987637456	0.902755824	0.988327669	100	136	167	145	177	99	241	170
ENSECAG00000015963	2.516914638	0.902867512	0.988327669	44	85	120	189	67	151	121	125
ENSECAG00000010715	4.476856457	0.902931215	0.988327669	367	434	327	438	543	313	543	461
ENSECAG00000020761	3.86461894	0.903099279	0.988327669	131	248	274	454	274	280	375	283
ENSECAG00000009755	3.785859634	0.903197612	0.988327669	128	43	312	559	378	236	444	142
ENSECAG00000009231	4.861901571	0.903248264	0.988327669	327	439	633	766	568	452	751	671
ENSECAG00000011969	6.394906062	0.903282733	0.988327669	1452	1374	1615	1466	2252	1313	1953	1491
ENSECAG00000007913	4.700382347	0.90337707	0.988327669	242	424	601	693	568	573	492	507
ENSECAG00000010646	4.792149872	0.903397164	0.988327669	380	492	440	705	503	425	796	614
ENSECAG00000018554	9.513422246	0.903455751	0.988327669	7534	10506	15306	19847	16626	16954	12288	16391
ENSECAG00000019293	10.78740736	0.903466957	0.988327669	18758	23375	39259	46979	37869	41597	31759	39682

ENSECAG00000013493	5.35577787	0.903514658	0.988327669	407	479	856	1409	622	633	980	1202
ENSECAG00000015789	2.535406264	0.903815684	0.988561971	107	94	90	109	102	55	175	152
ENSECAG00000012881	2.973107161	0.90391822	0.988579148	86	146	120	206	134	146	186	205
ENSECAG00000016758	9.585449078	0.904285815	0.988886179	9699	10182	18776	18346	15048	16868	12264	18847
ENSECAG00000019041	4.75933481	0.904402524	0.988918747	445	458	357	676	403	485	618	757
ENSECAG00000016843	8.082538931	0.904586968	0.988918747	3083	4465	5366	7400	4965	4955	6918	5848
ENSECAG00000025036	6.128588435	0.904697342	0.988918747	812	1007	1749	1618	2009	1172	1460	1153
ENSECAG00000010257	3.020641878	0.904870747	0.988918747	58	167	148	218	227	162	144	150
ENSECAG00000023232	4.538133123	0.904909085	0.988918747	221	324	513	646	554	459	556	418
ENSECAG00000013900	2.094215012	0.904978642	0.988918747	25	96	63	142	84	107	89	62
ENSECAG00000011087	6.424371259	0.905032325	0.988918747	934	1176	1655	2513	1724	1427	2251	2042
ENSECAG00000019916	8.290412578	0.905283779	0.988918747	4086	4553	6025	8605	7339	4669	7896	6299
ENSECAG00000012500	5.413769276	0.905316866	0.988918747	366	808	902	1150	901	1024	880	711
ENSECAG00000023913	4.153782501	0.905331144	0.988918747	157	273	282	611	255	304	378	595
ENSECAG00000020847	5.35078067	0.905464561	0.988918747	395	697	821	1058	1100	709	645	1009
ENSECAG00000015527	1.384064044	0.90548348	0.988918747	14	96	42	34	30	38	66	76
ENSECAG00000000025	2.125706337	0.905608571	0.988918747	39	46	138	102	118	70	100	65
ENSECAG00000019992	2.785292238	0.905729703	0.988918747	102	85	78	245	48	137	240	152
ENSECAG00000020319	1.541421991	0.905915185	0.988918747	47	26	83	45	47	32	76	82
ENSECAG00000012351	5.684119953	0.905950748	0.988918747	577	750	1031	1502	1078	881	1250	1095
ENSECAG00000010964	6.393420734	0.905960065	0.988918747	742	1950	1482	1885	1616	2426	2034	1096
ENSECAG00000000160	5.590333322	0.90616248	0.988918747	583	757	981	1242	1100	959	884	1039
ENSECAG00000011619	4.9754276	0.906167012	0.988918747	276	321	714	1045	468	560	654	1027
ENSECAG00000010758	0.927616196	0.906269164	0.988918747	22	61	19	27	43	40	40	25
ENSECAG00000000048	6.44357918	0.906353878	0.988918747	1328	895	1964	1898	2335	1150	2521	1571
ENSECAG00000004590	2.796433211	0.906371658	0.988918747	98	84	134	190	172	123	131	140
ENSECAG00000008992	6.369882275	0.906570807	0.988918747	1024	1857	1277	1872	1666	2220	1241	1621
ENSECAG00000015202	3.234622446	0.906577294	0.988918747	135	173	182	179	108	183	216	273
ENSECAG00000014411	3.812930529	0.906632565	0.988918747	200	269	254	240	307	211	321	367
ENSECAG00000009093	6.173816412	0.906751401	0.988918747	1287	1077	1317	1398	1883	802	2333	1103
ENSECAG00000011145	7.300781953	0.906796678	0.988918747	1266	4155	3575	2698	3427	4093	3372	2055
ENSECAG00000016354	5.982818429	0.906821869	0.988918747	706	924	1358	1582	1367	1254	1437	1362
ENSECAG00000002212	6.765855249	0.906834583	0.988918747	1211	1026	2211	3990	4750	988	2347	874
ENSECAG00000013159	5.133909968	0.907029012	0.989035031	311	527	783	1060	872	685	757	594
ENSECAG00000017780	6.599017653	0.907238589	0.989035031	1068	1281	2111	2583	1672	1693	2664	2396
ENSECAG00000014541	1.75296761	0.907271918	0.989035031	20.0071	119	27	67	69.0004	55.0001	90	72
ENSECAG00000017838	5.730305823	0.907288701	0.989035031	494	1881	723	734	1247	1483	1338	284
ENSECAG00000011835	5.716648411	0.907528187	0.989115237	897	989	798	1003	1097	944	1336	1027
ENSECAG00000009689	0.88537818	0.907627403	0.989115237	13	50	36	32	16	57	7	59
ENSECAG00000010753	4.779816855	0.907705658	0.989115237	333	571	431	675	370	554	657	716
ENSECAG00000005437	5.701673803	0.907736377	0.989115237	800	1056	873	950	1186	1047	1191	903
ENSECAG00000020660	10.07881432	0.907796672	0.989115237	9234	14433	27410	28645	22694	27777	20755	20994
ENSECAG00000008498	4.541090599	0.907995149	0.98923682	275	396	499	539	477	561	363	504
ENSECAG00000011612	10.72396768	0.908387901	0.989422545	20640	29187	31567	38636	37332	33215	45846	30163
ENSECAG00000006878	4.139710845	0.908515187	0.989422545	157	275	340	527	411	358	474	268
ENSECAG00000013110	8.150781783	0.908534156	0.989422545	3448	5298	4080	8410	5475	3601	8035	6910
ENSECAG00000020253	11.23721914	0.908601367	0.989422545	27191	33582	59105	61223	52570	55036	40726	49622
ENSECAG00000014176	3.287166333	0.908800171	0.989422545	147	109	161	297	207	153	275	179
ENSECAG00000008958	6.70099082	0.908827686	0.989422545	1132	1865	1844	2665	2305	2113	2279	2218
ENSECAG00000007192	11.02291055	0.9088413	0.989422545	42562	34926	35060	29771	48021	23368	71702	33749
ENSECAG00000014090	11.40340637	0.908931905	0.989422545	26853	34139	63345	74023	62219	64483	47890	56122
ENSECAG00000022322	6.621432588	0.908947773	0.989422545	1271	1659	1718	2613	2095	1997	1770	2273
ENSECAG00000018402	4.517148305	0.90914749	0.989486802	207	466	336	730	301	492	473	634
ENSECAG00000019829	5.779874752	0.909180627	0.989486802	882	1139	786	921	1192	872	1411	1274
ENSECAG00000024371	1.49043081	0.90933517	0.989506799	46	75	17	40	23	28	86	106
ENSECAG00000010542	5.731073742	0.909406588	0.989506799	445	914	1082	1482	944	1337	1025	1207
ENSECAG00000013865	3.805233161	0.909459741	0.989506799	211	178	240	388	274	174	402	327
ENSECAG00000015069	6.852626258	0.909644829	0.989613605	951	489	5051	1914	4248	1720	2840	1184
ENSECAG00000012298	4.905333786	0.909785179	0.989671724	297	373	565	988	465	527	682	908
ENSECAG00000012718	4.485814428	0.910009196	0.989685523	318	452	375	455	331	484	493	555
ENSECAG00000022169	1.241768071	0.910011014	0.989685523	31	41	39	55	52	38	50	48
ENSECAG00000014459	1.521200955	0.910133244	0.989685523	30	37	72	59	40	38	66	98
ENSECAG00000015497	4.943266342	0.91014558	0.989685523	337	501	624	833	395	601	734	847
ENSECAG00000008481	2.797323828	0.910279651	0.98973678	92	107	118	191	108	59	217	201
ENSECAG00000010802	2.240016566	0.910409509	0.989783447	55	38	90	157	98	73	110	120
ENSECAG00000011175	3.570808721	0.910556913	0.98984918	126	215	236	270	259	248	241	259
ENSECAG00000013929	6.265286026	0.910730369	0.989943217	676	1041	1857	2112	1562	1828	1403	1742
ENSECAG00000023494	5.375719242	0.911037106	0.989976473	347	546	1281	976	1021	1098	645	614
ENSECAG00000021475	4.41520675	0.911105891	0.989976473	453	475	220	274	358	414	605	410
ENSECAG00000008072	5.82246198	0.911197806	0.989976473	683	660	1259	1488	1247	880	1666	1122
ENSECAG00000010046	4.798646302	0.911253353	0.989976473	359	629	498	435	655	405	762	584
ENSECAG00000021954	4.234463529	0.911288471	0.989976473	217	305	375	448	326	262	574	477
ENSECAG00000022866	1.830158203	0.911548043	0.989976473	25.0008	79.0015	56	107.004	28.0022	108.004	27.0022	115.003
ENSECAG00000014570	4.62047413	0.911559858	0.989976473	323	452	507	430	585	450	589	481
ENSECAG00000014761	5.051264219	0.911653757	0.989976473	262	590	672	930	865	652	755	557
ENSECAG00000014272	5.918388741	0.911682073	0.989976473	773	1012	1271	1207	1375	1264	1387	1144

ENSECAG00000013738	2.250194399	0.911709196	0.989976473	61	54	98	120	120	78	94	108
ENSECAG00000022848	5.088834767	0.911723371	0.989976473	431	583	575	916	561	478	941	900
ENSECAG00000008614	5.013379111	0.911938697	0.989976473	289	839.002	571	686.002	413	845.003	717.004	701.003
ENSECAG00000015576	2.940179139	0.91196617	0.989976473	51	202	126	200	241	44	282	78
ENSECAG00000018023	0.422101229	0.912053034	0.989976473	17	16	30	23	31	7	40	32
ENSECAG00000015561	5.011780906	0.912156111	0.989976473	565	629	495	549	493	597	828	791
ENSECAG00000017673	8.257314473	0.912171229	0.989976473	4282	5767	6287	5732	3081	6200	4253	11712
ENSECAG00000024569	6.249835036	0.912365429	0.989976473	970	1203	1547	1872	1590	1215	2028	1573
ENSECAG00000015223	1.081996339	0.912414338	0.989976473	0	74	40	46	42	56	1	58
ENSECAG00000019340	5.108401445	0.912478455	0.989976473	437	450	716	868	732	464	985	815
ENSECAG00000013990	4.595096955	0.912531406	0.989976473	236	494	448	563	524	469	518	550
ENSECAG00000017832	6.822002208	0.912587007	0.989976473	1778	1717	2351	1903	3034	2019	2877	1817
ENSECAG00000001218	8.448313446	0.912865265	0.989985003	3679	4380	9134	8083	7329	8147	7031	7339
ENSECAG00000021105	0.494941818	0.912894522	0.989985003	9	30	18	39	33	15	43	24
ENSECAG00000011017	4.547082705	0.912938789	0.989985003	212	370	461	740	633	401	599	314
ENSECAG00000019065	5.527663281	0.912977913	0.989985003	614	562	1340	834	1436	437	1362	655
ENSECAG00000024511	4.350127131	0.913029647	0.989985003	219	363	242	662	212	265	438	856
ENSECAG00000022718	6.015090747	0.913142567	0.990013154	760	894	1384	1627	1447	1152	1448	1499
ENSECAG00000021642	2.438189833	0.913254986	0.990040756	67	32	124	185	181	42	106	112
ENSECAG00000000282	3.093939301	0.913468005	0.990171282	78	129	153	264	102	149	168	311
ENSECAG00000019179	2.625797331	0.913652362	0.990171282	87	128	87	136	166	91	126	122
ENSECAG00000018490	5.946828122	0.913908251	0.990171282	888	1297	767	1497	1692	774	1534	1174
ENSECAG00000010390	1.828781817	0.913930215	0.990171282	17	75	54	127	75	55	98	61
ENSECAG00000000298	6.491114873	0.913950977	0.990171282	1168	1463	1377	2423	1591	1269	2543	2442
ENSECAG00000020872	6.033813204	0.913992798	0.990171282	1074	942	1406	1041	1912	861	1888	1021
ENSECAG00000021306	5.03741021	0.914057938	0.990171282	481	494	614	707	612	575	881	766
ENSECAG00000007420	0.935508723	0.91419023	0.990171282	16	20	55	48	31	26	15	76
ENSECAG00000013112	2.650742422	0.914250079	0.990171282	87	196	58	95	41	89	157	235
ENSECAG00000009315	0.301252419	0.914271147	0.990171282	5	11	17	55	36	10	33	20
ENSECAG00000017228	8.433917058	0.914459563	0.990171282	5748	5042	5755	7219	9415	5590	8359	6469
ENSECAG00000020688	4.975852832	0.914482351	0.990171282	951	297	321	327	837	509	883	499
ENSECAG00000025110	8.125735085	0.914506019	0.990171282	4205	4784	3886	6593	3840	4567	8899	7238
ENSECAG00000000828	4.414757146	0.914804387	0.990341051	240	217	510	585	417	314	470	633
ENSECAG00000023523	7.853929177	0.914836788	0.990341051	3474	5184	3810	3890	4418	4739	5619	4523
ENSECAG00000018242	7.749011006	0.915096717	0.990484812	2793	3584	4696	4631	4892	4285	4919	3792
ENSECAG00000007838	6.840780504	0.915143587	0.990484812	1237	1604	2397	3111	2571	1894	3024	2432
ENSECAG00000008334	7.456059886	0.915301163	0.990561193	1641	2630	4001	4599	4208	3610	4173	3082
ENSECAG00000015928	3.920574513	0.915581668	0.990688863	129	250	293	483	289	329	331	303
ENSECAG00000013964	3.6679563	0.915639046	0.990688863	166	150	219	377	214	179	381	330
ENSECAG00000016427	4.666712807	0.915680185	0.990688863	267	445	454	673	333	428	612	821
ENSECAG00000016220	4.14806324	0.915913579	0.990766293	243	373	330	314	446	226	484	333
ENSECAG00000012605	4.448670717	0.9159258	0.990766293	492	254	356	373	462	325	666	389
ENSECAG00000015861	4.611917511	0.916142558	0.990821158	360	360	486	554	557	337	611	548
ENSECAG00000021732	4.885166881	0.916150578	0.990821158	523	511	496	524	672	614	636	528
ENSECAG00000005685	3.259527246	0.916320282	0.990910563	112	175	186	200	126	219	280	197
ENSECAG00000014298	2.361321116	0.916533878	0.990948002	41	95	107	125	65	81	122	171
ENSECAG00000004311	10.20978972	0.916555105	0.990948002	10552	17955	27103	35231	26471	29867	18486	21910
ENSECAG00000023132	2.589030327	0.916616022	0.990948002	60	185	87	96	106	112	146	131
ENSECAG00000013803	6.396438193	0.916742514	0.990990649	1159	1570	1759	1551	1892	1552	1831	1745
ENSECAG00000026833	6.264509828	0.916857439	0.991020786	881	1687	1189	1917	487	1809	1876	2266
ENSECAG00000024656	1.694278299	0.917192881	0.99119526	47	32	61	81	93	33	115	36
ENSECAG00000025013	5.292678655	0.91739984	0.99119526	399	601	895	953	844	830	873	795
ENSECAG00000010974	2.631629548	0.91742117	0.99119526	73	35	140	201	174	135	124	87
ENSECAG00000024128	1.040883441	0.917572097	0.99119526	18	34	44	45	45	50	38	34
ENSECAG00000023285	1.591292753	0.917589272	0.99119526	16	40	84	78	62	79	57	50
ENSECAG00000019925	4.632309303	0.917599466	0.99119526	241	405	561	593	637	403	631	457
ENSECAG00000017649	2.195959926	0.917718562	0.99119526	38	93	77	119	97	115	80	89
ENSECAG00000003925	7.675949967	0.917797501	0.99119526	2379	6025	2590	3844	1823	9748	1188	3303
ENSECAG00000003985	1.863314549	0.917974961	0.99119526	47	88	60	46	133	42	80	50
ENSECAG00000013217	5.308533413	0.918083424	0.99119526	686	881	563	497	572	1332	634	788
ENSECAG00000020050	4.697721471	0.918100898	0.99119526	366	465	466	508	416	443	657	722
ENSECAG00000015212	0.963250435	0.918204308	0.99119526	24	24	74	11	25	41	8	75
ENSECAG00000004911	5.84377888	0.91824303	0.99119526	895	1131	886	1020	1183	836	1681	1292
ENSECAG00000025088	5.763636571	0.918461017	0.99119526	415	943	1141	1681	975	1025	1940	677
ENSECAG00000010422	3.15633625	0.918615777	0.99119526	79	82	172	335	157	136	193	277
ENSECAG00000004846	0.064674753	0.918634071	0.99119526	22	16	5	23	12	17	24	26
ENSECAG00000003271	8.041603212	0.918659934	0.99119526	4302	4933	3808	4932	6023	4447	7088	5269
ENSECAG00000023977	1.638063426	0.918662564	0.99119526	45	37	47	95	62	27	89	77
ENSECAG00000017520	0.864935912	0.918787262	0.99119526	14	22	59	36	10	24	20	88
ENSECAG00000021956	9.652566078	0.918813799	0.99119526	6797	13676	18323	20288	18399	19830	14645	15487
ENSECAG00000021833	4.957671067	0.918847148	0.99119526	325	486	603	853	586	698	716	650
ENSECAG00000010355	5.215706885	0.919017627	0.99121784	489	380	689	1139	839	690	834	815
ENSECAG00000010689	5.581636525	0.91921446	0.99121784	452	708	912	1591	742	873	1290	1132
ENSECAG00000003427	3.551735031	0.919250829	0.99121784	83	177	255	359	257	261	318	165
ENSECAG00000019346	7.261310623	0.919257348	0.99121784	2147	2108	3322	3203	3710	2498	3990	3062
ENSECAG00000016724	6.51054448	0.919303396	0.99121784	1576	1622	1681	1475	2601	1354	2293	1381

ENSECAG00000019898	0.976182165	0.919630296	0.99132537	27	32	38	38	28	18	48	64
ENSECAG00000000684	3.743104769	0.919945975	0.99132537	159	118	274	463	352	187	299	273
ENSECAG00000006913	10.66305266	0.919978215	0.99132537	23402	25832	36357	26166	29635	48099	19025	39484
ENSECAG00000013489	4.954888473	0.919982422	0.99132537	395	600	545	714	554	393	631	1024
ENSECAG00000000940	4.329208413	0.920010137	0.99132537	182	659	340	263	666	329	463	201
ENSECAG00000005828	4.708853403	0.920073409	0.99132537	317	423	578	594	586	474	544	571
ENSECAG00000007976	8.592091938	0.920144231	0.99132537	3919	5788	8906	10547	8583	9062	6571	7485
ENSECAG00000007750	8.246668175	0.920199155	0.99132537	3622	5318	5539	8068	4957	6630	6948	6788
ENSECAG00000018984	6.542617571	0.920296395	0.99132537	1378	1569	1527	2036	2111	1672	2014	2188
ENSECAG00000021690	4.424199085	0.920354917	0.99132537	204	373	358	642	522	556	333	387
ENSECAG00000005765	3.449063604	0.920401546	0.99132537	126	204	186	286	267	226	223	181
ENSECAG00000016102	8.213318954	0.920448	0.99132537	3836	4817	6317	5943	6044	6455	6951	6020
ENSECAG00000011635	1.672485564	0.921010142	0.991781708	40	29	98	60	73	39	87	61
ENSECAG00000014191	6.288330513	0.921060582	0.991781708	973	1485	1235	1869	1390	1351	1979	2030
ENSECAG00000013320	6.574081857	0.921218115	0.991781708	1417	1168	1609	2812	1990	1266	2205	2550
ENSECAG00000014850	6.01764451	0.921220163	0.991781708	1024	739	1414	1329	2009	1103	1334	1071
ENSECAG00000019988	2.82789474	0.9214304	0.991907767	52	167	129	183	143	148	188	106
ENSECAG00000023805	5.845798057	0.92155522	0.991907767	874	1353	753	928	1474	1607	780	946
ENSECAG00000010596	6.362661567	0.921598626	0.991907767	605	1488	2194	1752	1744	1918	1803	1549
ENSECAG00000012727	3.361264034	0.922011173	0.992087875	90	148	269	239	241	182	252	201
ENSECAG00000019260	4.004091682	0.922017955	0.992087875	158	223	352	478	421	254	343	313
ENSECAG00000017179	6.380741382	0.922027388	0.992087875	930	1519	1581	1958	1776	1740	1833	1767
ENSECAG00000021924	1.890814174	0.922309334	0.992297464	29	56	60	125	42	71	95	106
ENSECAG00000015334	3.593056429	0.922415919	0.992318364	127	240	234	286	256	271	335	140
ENSECAG00000018610	1.478918523	0.922694173	0.992523919	31	29	55	90	79	57	59	26
ENSECAG00000021748	1.797068796	0.922888811	0.992527768	27	52	87	82	73	84	57	72
ENSECAG00000017968	4.429172727	0.922940925	0.992527768	254	343	444	498	451	282	735	412
ENSECAG00000016908	0.839253517	0.923042311	0.992527768	11	9	49	59	27	51	32	34
ENSECAG00000022293	2.188920583	0.923173364	0.992527768	38	73	82	138	86	86	122	92
ENSECAG00000012732	7.575137491	0.923193445	0.992527768	1568	3347	3152	6705	3536	3982	4507.99	3883.99
ENSECAG00000012758	3.354813417	0.923220822	0.992527768	90	125	250	287	171	190	259	256
ENSECAG00000007657	7.081109677	0.923457966	0.992653047	1413	2624	2696	3338	2512	3035	3198	2541
ENSECAG00000010639	8.06980449	0.923511732	0.992653047	2947	5270	4424	7411	5086	4622	6355	6467
ENSECAG00000006211	4.686149145	0.923751222	0.992816734	248	717	442	399	521	646	755	285
ENSECAG00000020523	10.35199059	0.924003602	0.992994243	12610	16470	31721	35377	23859	28889	28167	31158
ENSECAG00000022348	0.253625056	0.924410537	0.993265894	13	9	18	45	21	14	32	24
ENSECAG00000024113	6.514541243	0.924443805	0.993265894	1448	911	2110	1891	2606	1055	3475	915
ENSECAG00000005000	3.666026377	0.924560543	0.993265894	100	235	236	363	174	136	384	415
ENSECAG00000009500	2.882200456	0.924689186	0.993265894	59	132	105	269	139	135	164	169
ENSECAG00000017485	0.281170722	0.924860231	0.993265894	14	25	21	16	13	19	47	21
ENSECAG00000017296	3.06946126	0.924879003	0.993265894	157	155	99	143	114	115	300	205
ENSECAG00000001235	4.88833385	0.924900199	0.993265894	310	520	588	724	769	640	534	555
ENSECAG00000009485	5.434952836	0.924958421	0.993265894	1208	1037	298	190	20	2651	31	585
ENSECAG00000008447	0.700755079	0.92513009	0.993265894	15	14	34	49	47	20	43	23
ENSECAG00000015634	6.397710066	0.925191965	0.993265894	978	1085	1757	2316	1903	1300	2033	2026
ENSECAG00000002645	1.85783823	0.925230645	0.993265894	47	68	44	90	29	122	82	68
ENSECAG00000015089	11.30860377	0.925335753	0.993265894	26270	32978	57187	69332	62171	57179	44384	51596
ENSECAG00000018362	3.399300917	0.925576748	0.993265894	139	143	195	271	163	139	322	292
ENSECAG00000026923	2.176612838	0.925590887	0.993265894	20	76	111	141	113	81	73	97
ENSECAG00000010265	6.009963468	0.925659021	0.993265894	901	1229	1131	1366	1555	1069	1329	1424
ENSECAG00000005256	2.1698673	0.925681156	0.993265894	53	81	58	124	62	67	117	138
ENSECAG00000022797	6.212997342	0.925817147	0.993265894	920	1320	1590	1403	1657	1878	1619	1139
ENSECAG00000007164	6.045181756	0.925826761	0.993265894	761	1625	1211	1006	1690	1416	1732	814
ENSECAG00000000909	4.449850743	0.9262327	0.993471612	274	485	318	456	298	485	478	604
ENSECAG00000011562	4.5783891	0.926308856	0.993471612	238	267	449	887	330	322	522	834
ENSECAG00000015070	4.706140605	0.926354435	0.993471612	326	469	459	655	338	387	717	771
ENSECAG00000009670	3.54251571	0.926776436	0.993471612	113	239	176	313	295	91	388	243
ENSECAG00000008555	6.867098976	0.926808787	0.993471612	1277	3186	1373	2381	3406	1744	3960	1075
ENSECAG00000021962	4.997154352	0.926819534	0.993471612	426	381	887	626	835	594	1053	205
ENSECAG00000013253	6.80153831	0.92689904	0.993471612	1248	1497	2817	2748	2257	2524	2019	2419
ENSECAG00000016043	5.878467892	0.926927341	0.993471612	511	841	1385	1623	1594	1104	1475	862
ENSECAG00000019258	4.472692167	0.926947644	0.993471612	270	318	485	514	551	501	500	329
ENSECAG00000007274	4.193193172	0.927004889	0.993471612	267	156	559	286	511	359	475	217
ENSECAG00000017246	5.255422093	0.927030436	0.993471612	714	615	633	675	830	655	992	733
ENSECAG00000024859	0.707875045	0.927065648	0.993471612	29	27	21	31	36	12	57	26
ENSECAG00000011611	4.622955378	0.927171188	0.99347405	282	411	359	740	477	348	656	648
ENSECAG00000011370	6.879366662	0.927302732	0.99347405	1354	3021	1693	2190	2085	1958	3311	2873
ENSECAG00000017720	5.740699157	0.927329709	0.99347405	491	893	1353	1269	1359	1294	1138	623
ENSECAG00000023575	7.153763817	0.927481394	0.993543063	3760	1735	2294	1442	3935	2779	3874	1270
ENSECAG00000023969	1.16202565	0.927573461	0.993548203	18	32	45	70	52	45	54	26
ENSECAG00000001568	5.034633267	0.928223431	0.994150871	351	497	795	719	455	821	948	592
ENSECAG00000019459	4.789005058	0.928467591	0.994318834	369	463	486	638	554	379	850	618
ENSECAG00000011722	8.39201647	0.928742536	0.994512433	3400	5518	7753	7651	7321	8331	5850	6936
ENSECAG00000016276	6.662847429	0.928823074	0.994512433	1189	2427	1687	1804	2331	1724	2594	2078
ENSECAG00000021445	1.582462718	0.92906761	0.994559195	27	83	43	61	23	30	52	138
ENSECAG00000018310	1.71476683	0.929124138	0.994559195	29	43	77	94	74	62	87	43

ENSECAG00000017892	6.248755872	0.929128818	0.994559195	1147	1593	1373	1242	1480	2335	1582	850
ENSECAG00000019227	4.694934473	0.929486	0.994627881	213	334	362	1068	457	233	820	764
ENSECAG00000024388	1.949068173	0.929554085	0.994627881	67	3	171	5	215	29	41	36
ENSECAG00000007847	7.539070371	0.929563039	0.994627881	3748	3913	2316	2081	5593	4144	3898	2171
ENSECAG00000011385	5.632902016	0.929585762	0.994627881	426.007	730	1150	1377	980	1180	1061	993
ENSECAG00000006737	7.002087382	0.929694008	0.994627881	1649	2401	2374	2606	3266	2672	2681	2291
ENSECAG00000021875	5.03580564	0.929717164	0.994627881	291	578	773	752	689	668	842	608
ENSECAG00000008209	6.635675808	0.930007728	0.994845249	1353	654	2918	2170	2281	1963	2608	1703
ENSECAG00000024180	4.878810894	0.930254479	0.994929077	424	403	566	683	660	428	749	694
ENSECAG00000010152	2.293885408	0.930260872	0.994929077	50	82	73	160	97	64	114	129
ENSECAG00000018341	0.145390905	0.930631177	0.994999603	3	4	27	45	22	20	20	24
ENSECAG00000009432	3.701736025	0.930643877	0.994999603	99	361	179	331	201	340	197	324
ENSECAG00000020152	8.201291337	0.930668495	0.994999603	3746	3490	7502	6236	7566	6362	6094	5030
ENSECAG00000023721	9.599249592	0.930679443	0.994999603	11942	17643	10398	12726	6696	25338	16611	17441
ENSECAG00000016227	4.241554955	0.930763792	0.994999603	143	338	343	587	228	404	426	560
ENSECAG00000022265	5.801569382	0.931034082	0.995170701	770	687	1035	1505	1303	728	1718	1100
ENSECAG00000016891	0.952709424	0.931098666	0.995170701	19	27	45	47	49	34	43	26
ENSECAG00000019675	9.109462977	0.931431129	0.995359327	8932	9141	9954	11222	14004	7726	15239	9621
ENSECAG00000009051	2.879546894	0.931630931	0.995359327	58	114	178	205	145	161	162	136
ENSECAG00000011946	2.055521106	0.931694567	0.995359327	52	53	87	98	102	67	95	85
ENSECAG00000024205	10.2917451	0.931729324	0.995359327	14921	20389	25035	32790	17140	30815	14423	40388
ENSECAG00000010878	4.555417126	0.931873241	0.995359327	293	412	399	618	622	386	548	407
ENSECAG00000019253	2.244287912	0.932031075	0.995359327	19	83	120	129	138	52	92	117
ENSECAG00000022762	3.165818273	0.932108134	0.995359327	110	130	182	215	190	123	271	189
ENSECAG00000007669	4.469307927	0.932128964	0.995359327	197	394	387	659	498	498	444	432
ENSECAG00000001079	0.693171948	0.932187667	0.995359327	10	33	46	17	0	29	1	101
ENSECAG00000010240	5.735193686	0.932216488	0.995359327	516	922	1219	1213	1467	904	1370	823
ENSECAG00000017001	4.776665127	0.932236847	0.995359327	362	367	542	741	649	391	636	624
ENSECAG00000016603	8.375037787	0.932527588	0.995426436	3688	6440	7236	7021	5225	7923	8369	6253
ENSECAG00000006392	7.097685317	0.932529172	0.995426436	1948	1768	3539	2617	3265	2683	3036	2424
ENSECAG00000015988	6.025669626	0.932562	0.995426436	887	1092	1236	1502	1220	1181	1810	1294
ENSECAG00000000019	5.803801905	0.932761653	0.99554621	608	861	1162	1431	1354	1135	1301	970
ENSECAG00000022557	5.053527967	0.933115792	0.995590561	554	457	700	626	793	474	925	613
ENSECAG00000015911	4.939158686	0.933249204	0.995590561	422	650	490	627	491	597	793	701
ENSECAG00000023027	8.188969257	0.933324237	0.995590561	5911	1032	7343	6330	5639	4001	11817	3608
ENSECAG00000011139	2.076005166	0.933470896	0.995590561	37	71	86	107	40	56	133	134
ENSECAG00000014334	11.35098368	0.933707326	0.995590561	28970	36906	63924	65120	58290	61303	45359	50288
ENSECAG00000011281	4.053409447	0.933823417	0.995590561	185	261	486	270	380	317	365	317
ENSECAG00000008522	5.891551573	0.933856951	0.995590561	665	1006	1188	1537	1274	1173	1465	1064
ENSECAG00000027669	11.67796123	0.933953331	0.995590561	56739	33983	85160	46671	79134	64936	71791	64605
ENSECAG00000024614	2.453487175	0.934040785	0.995590561	70	50	151	126	84	83	127	159
ENSECAG00000018097	4.4659061	0.934061132	0.995590561	196	388	321	737	256	255	402	993
ENSECAG00000000037	5.5955722	0.934203668	0.995590561	495	777	1041	1211	1005	1276	865	922
ENSECAG00000024944	5.687236455	0.934288852	0.995590561	474	873	997	1559	1185	1071	1072	957
ENSECAG00000021423	5.683957842	0.934370916	0.995590561	537	439	1060	1821	694	1077	1551	1131
ENSECAG00000024929	1.028076654	0.934498541	0.995590561	20	30	33	64	43	29	59	33
ENSECAG00000014928	10.44630972	0.93451556	0.995590561	14177	18781	33722	35370	29946	32874	26249	29420
ENSECAG00000022434	5.926776366	0.934610096	0.995590561	1252	903	783	1112	1295	814	1840	1343
ENSECAG00000002257	2.62869882	0.934629732	0.995590561	24	156	125	170	21	38	98	356
ENSECAG00000006138	5.277131961	0.934669381	0.995590561	511	610	841	776	882	781	712	907
ENSECAG00000021970	3.810654912	0.934697018	0.995590561	163	286	228	319	272	275	325	322
ENSECAG00000018845	7.387778322	0.934705189	0.995590561	1892	2784	3079	4373	4408	2704	4562	2769
ENSECAG00000015408	3.158512356	0.934746304	0.995590561	86	211	154	181	153	104	192	316
ENSECAG00000023030	4.246825222	0.935040789	0.995590561	316	326	318	369	319	274	591	430
ENSECAG00000013214	5.219408803	0.93504621	0.995590561	446	661	677	884	730	512	1055	929
ENSECAG00000000889	3.272491937	0.935058854	0.995590561	135	245	117	182	166	111	281	258
ENSECAG00000008800	5.533062202	0.935127286	0.995590561	435	947	769	1236	867	653	1427	1080
ENSECAG00000024560	5.900583743	0.935183163	0.995590561	821	721	1356	1349	1433	951	1575	1181
ENSECAG00000017901	6.545138901	0.935353717	0.995590561	1607	2080	1222	1549	1622	1426	2619	2255
ENSECAG00000007760	7.45680328	0.935369949	0.995590561	2804	2364	3277	3778	4831	2455	4983	2937
ENSECAG00000018405	6.719382507	0.935508947	0.995590561	1077	1661	2500	2630	2505	1881	2647	1808
ENSECAG00000017757	5.264662433	0.935525214	0.995590561	389	471	855	1127	939	581	1055	736
ENSECAG00000023818	6.022258848	0.935631152	0.995590561	743	1421	870	1629	1094	1002	1668	1854
ENSECAG00000024227	3.555927676	0.935660801	0.995590561	116	301	237	193	237	190	405	163
ENSECAG00000004567	4.781361044	0.935688977	0.995590561	282	301	742	742	796	461	584	444
ENSECAG00000009646	4.230338451	0.936172954	0.995941185	112	323	431	541	472	95	768	330
ENSECAG00000021948	2.669990529	0.936382566	0.995941185	49	124	86	210	165	119	153	100
ENSECAG00000011430	6.12782095	0.936414676	0.995941185	959	760	1473	1829	1314	1283	1654	1749
ENSECAG00000011974	7.615622733	0.93643111	0.995941185	1824	3250	4213	5124	3578	4711	4419	4026
ENSECAG00000022262	6.782114052	0.936679635	0.995941185	1534	1727	1948	2812	1363	2364	2770	2765
ENSECAG00000014982	2.92286003	0.936693505	0.995941185	85	230	97	124	96	133	150	248
ENSECAG00000011270	3.734038882	0.936759552	0.995941185	159	227	238	326	279	260	341	254
ENSECAG00000005014	7.927500535	0.936880931	0.995941185	3032	4157	5060	5555	5061	4843	6084	4427
ENSECAG00000010059	2.189374929	0.937080162	0.995941185	37	55	101	141	123	94	58	101
ENSECAG00000016187	4.403536094	0.937169322	0.995941185	302	423	388	383	439	377	507	450
ENSECAG00000016279	2.599439905	0.937336563	0.995941185	108	105	109	76	59	158	133	161

ENSECAG00000011855	3.655553738	0.937355502	0.995941185	121	234	228	357	246	261	264	273
ENSECAG00000023108	6.453586371	0.937372861	0.995941185	1069	1588	1694	1890	2170	1709	2174	1439
ENSECAG00000007611	6.417576106	0.937523927	0.995941185	747	1167	2156	2442	1990	1914	2212	1024
ENSECAG00000001400	3.987618718	0.937543313	0.995941185	153	314	268	448	309	396	353	254
ENSECAG00000008627	4.704666257	0.937561643	0.995941185	437	523	385	467	514	382	682	624
ENSECAG00000002115	6.966573338	0.937587334	0.995941185	1719	2153	2676	2445	2657	3297	2623	1763
ENSECAG00000024896	6.473334485	0.937642372	0.995941185	1134	2194	1278	1760	1496	1530	1967	2475
ENSECAG00000000624	5.479012113	0.937680594	0.995941185	680	721	832	866	951	790	977	1089
ENSECAG00000024773	5.982088669	0.938001619	0.996021961	939	1390	1013	1111	1462	1736	1222	781
ENSECAG00000018500	4.886356445	0.938227259	0.996021961	305	484	610	755	437	630	612	839
ENSECAG00000016084	3.137571625	0.938365293	0.996021961	85	114	197	266	163	177	147	235
ENSECAG00000018290	5.106469909	0.938397761	0.996021961	620	1015	291	388	607	417	1094	822
ENSECAG00000006583	0.667098845	0.938457674	0.996021961	18	22	26	41	17	31	40	41
ENSECAG00000013916	5.756948051	0.938468309	0.996021961	795	918	894	1184	1153	826	1393	1283
ENSECAG00000016798	3.629974444	0.93850203	0.996021961	107	216	294	281	419	298	156	151
ENSECAG00000020760	6.174921476	0.938535732	0.996021961	1017	1190	1269	1752	1264	1162	1874	1814
ENSECAG00000014477	7.817323312	0.938632405	0.996021961	3055	3272	4647	5023	4796	3767	6851	4158
ENSECAG00000020356	6.196698767	0.938661837	0.996021961	697.021	1359	1723.02	1671	1976	1153	1667	1346.05
ENSECAG00000003113	6.251607677	0.938718985	0.996021961	1008	1043	1682	1837	2344	1222	1704	1086
ENSECAG00000011258	1.828376965	0.938929001	0.996151959	17	67	66	121	55	54	89	93
ENSECAG00000019323	5.292920549	0.939262115	0.996331497	471	605	874	856	833	709	805	990
ENSECAG00000015543	5.879639882	0.939454216	0.996331497	836	817	1064	1472	1156	980	1661	1286
ENSECAG00000016644	1.090632579	0.939549056	0.996331497	13	38	63	41	0	16	32	122
ENSECAG00000021067	7.196672097	0.939560856	0.996331497	2409	2429	2154	3456	3895	2342	3659	2417
ENSECAG00000008603	5.752915333	0.939573933	0.996331497	486.007	854	1012	1762	1241	933	1270	1080
ENSECAG00000018893	3.029865983	0.939623301	0.996331497	105	128	170	169	125	182	231	158
ENSECAG00000011438	5.137945534	0.940025078	0.996574615	510	577	876	521	673	810	965	505
ENSECAG00000016192	7.239867751	0.940063942	0.996574615	1309	3744	2162	3798	2731	4414	2555	3019
ENSECAG00000024842	7.750217928	0.940193386	0.996574615	2175	3329	3303	7689	2605	4391	3332	7566
ENSECAG00000011537	3.796898697	0.940211503	0.996574615	155	388	209	239	238	180	455	310
ENSECAG00000013051	3.11197379	0.940406803	0.996574615	133	127	138	219	170	113	282	166
ENSECAG00000017992	4.156705098	0.940507008	0.996574615	174	234	518	363	498	464	384	152
ENSECAG00000010213	4.775581367	0.940510185	0.996574615	338	374	715	518	533	656	558	568
ENSECAG00000021136	10.78960272	0.940552854	0.996574615	18581	25169	41374	43923	38804	43171	30925	36775
ENSECAG00000007266	2.912012123	0.940665299	0.996601008	72	93	127	265	94	130	135	279
ENSECAG00000023747	4.356481748	0.940919227	0.99665952	183	208	438	714	350	230	435	749
ENSECAG00000008008	6.902324961	0.941092167	0.99665952	1681	2679	2121	1736	2552	2850	2749	2031
ENSECAG00000006989	6.540988846	0.941110363	0.99665952	794	1725	1695	2696	2094	2524	1720	1496
ENSECAG00000007094	5.25327255	0.941169031	0.99665952	303	643	945	925	809	1088	644	651
ENSECAG00000009635	4.735533321	0.94127941	0.99665952	363	460	409	658	429	438	770	665
ENSECAG00000007201	10.1351441	0.941296138	0.99665952	10654	15041	27652	29604	23853	24004	20993	26770
ENSECAG00000010105	5.880343107	0.941333318	0.99665952	541	998	1219	1586	1157	1084	1526	1291
ENSECAG00000016211	2.030820691	0.942007753	0.997141833	46	62	38	159	69	47	80	139
ENSECAG00000010405	5.994483469	0.942047997	0.997141833	733	494	1282	2375	1488	1137	1550	1171
ENSECAG00000022321	4.908847701	0.942111301	0.997141833	491	351	514	805	749	329	1030	460
ENSECAG00000008781	4.61800441	0.942162381	0.997141833	404	520	311	477	435	516	483	609
ENSECAG00000024063	6.669032606	0.942259196	0.997141833	1451	1190	2172	2424	2802	1475	2644	1831
ENSECAG00000020839	4.375552718	0.942314359	0.997141833	265	424	260	526	350	277	605	567
ENSECAG00000016742	0.644843119	0.942513719	0.997260102	25	17	29	30	35	19	18	53
ENSECAG00000018305	6.751557885	0.942659611	0.99732178	1164	1667	2179	2851	2937	1599	2964	1784
ENSECAG00000011503	4.593623696	0.942791074	0.997368182	355	386	440	546	543	404	604	478
ENSECAG00000001155	6.662608399	0.942888258	0.997378316	800	1583	1866	3609	1370	1199	2653	3410
ENSECAG00000014127	4.641570705	0.943083188	0.997491833	252	396	621	578	577	469	661	388
ENSECAG00000006539	4.470231941	0.943478424	0.997817172	356	333	419	421	535	394	600	367
ENSECAG00000016305	6.777737502	0.943569056	0.997820333	1245	1603	2290	2839	1902	2024	2747	2766
ENSECAG00000023306	6.394014758	0.943729161	0.997896953	1558	1888	1047	1065	2236	1322	2638	1085
ENSECAG00000024412	5.236583642	0.943831791	0.997900056	412	1291	511	357	649	1234	819	472
ENSECAG00000023715	7.348959341	0.943930616	0.997900056	1940	2657	3492	3901	3974	3375	3112	3070
ENSECAG00000008561	7.041511599	0.944039655	0.997900056	1331	1717	2859	3839	2377	3047	3240	2600
ENSECAG00000017443	2.133019433	0.944089689	0.997900056	49	98	76	80	92	60	124	96
ENSECAG00000018651	2.216133318	0.944257626	0.997900056	30	69	84	164	124	72	100	93
ENSECAG00000005128	4.000349673	0.944390633	0.997900056	218	225	267	461	188	233	417	517
ENSECAG00000020051	7.31752203	0.944528008	0.997900056	2738	4257	1007	3039	3444	2892	3739	3288
ENSECAG00000015897	6.450251375	0.944696796	0.997900056	854	1333	2125	2240	1427	2564	1298	1887
ENSECAG00000019559	3.348919432	0.944746772	0.997900056	116	365	87	116	86	557	32	143
ENSECAG00000010661	10.17893911	0.944758721	0.997900056	13062	15390	26945	28857	24871	27864	19985	25246
ENSECAG00000016439	7.471537661	0.944770215	0.997900056	3890	4199	1614	1552	7220	2583	3383	1871
ENSECAG00000000018	4.614187045	0.944830607	0.997900056	209	582	442	542	333	452	618	701
ENSECAG00000017701	4.374595788	0.945045862	0.997900056	181	243	511	611	484	357	730	226
ENSECAG00000006087	6.219180899	0.945123211	0.997900056	781	1277	1663	1673	1775	1692	1592	1250
ENSECAG00000018761	0.261928264	0.945148263	0.997900056	14	20	25	21	17	17	36	23
ENSECAG00000024526	-0.04983775	0.94517953	0.997900056	2	27	20	17	22	18	7	23
ENSECAG00000022320	5.492885545	0.945319262	0.997900056	416	855	1045	955	1017	923	924	953
ENSECAG00000013441	2.97959404	0.945346105	0.997900056	130	160	97	145	131	144	183	212
ENSECAG00000002377	3.376932801	0.945397454	0.997900056	127	251	141	205	181	128	230	350
ENSECAG00000000301	4.646532384	0.94561139	0.997956648	273	353	461	793	508	514	603	471

ENSECAG00000018798	2.525741183	0.94581658	0.997956648	84	68	128	130	131	89	94	157
ENSECAG00000019077	7.322202737	0.945833443	0.997956648	2696	3778	2228	1997	2883	3185	4656	3103
ENSECAG00000013896	8.126494105	0.945916967	0.997956648	2518	4075	5340	9558	3880	6520	4962	7866
ENSECAG00000018965	5.648459245	0.945941807	0.997956648	468	889	1053	1272	1079	1032	1170	989
ENSECAG00000022692	9.279004138	0.946053012	0.997956648	7631	7815	14015	15419	13742	12728	11926	14421
ENSECAG00000003393	3.224548501	0.946083433	0.997956648	166	183	121	172	160	129	250	247
ENSECAG00000022294	1.046850117	0.946218975	0.997956648	7	61	32	45	40	59	26	40
ENSECAG00000006468	4.487117562	0.946239966	0.997956648	259	375	517	482	530	420	500	422
ENSECAG00000020194	4.581555504	0.94639144	0.99798053	367	352	473	510	499	482	547	473
ENSECAG00000014256	1.52809248	0.946533841	0.99798053	14	46	84	71	48	63	48	71
ENSECAG00000023951	6.525972485	0.94662701	0.99798053	1059	1343	1807	2661	2087	1510	2289	1881
ENSECAG00000017724	7.998889614	0.94679253	0.99798053	6888	2217	3854	3139	7421	5161	6439	2851
ENSECAG00000011140	3.591327259	0.946997869	0.99798053	126	176	185	423	233	210	233	325
ENSECAG00000008168	4.973649512	0.947171268	0.99798053	483	426	610	682	685	440	827	751
ENSECAG00000009694	6.245716834	0.947181928	0.99798053	943	815	1424	2390	1587	1346	1363	2154
ENSECAG00000022913	7.403889591	0.947209378	0.99798053	2711	2253	4233	2741	4307	3546	3593	2638
ENSECAG00000000644	5.803765604	0.947293779	0.99798053	687	941	1090	1282	1296	771	1477	1266
ENSECAG00000023726	5.409161306	0.94730403	0.99798053	411	692	742	1307	1106	848	896	748
ENSECAG00000012310	7.498625425	0.947347619	0.99798053	2238	3237	3984	3657	3166	4245	3400	4220
ENSECAG00000015738	1.091101277	0.947369717	0.99798053	32	20	24	70	34	16	47	80
ENSECAG00000019317	4.649374043	0.947502859	0.99798053	294	342	665	534	780	408	637	273
ENSECAG00000017750	4.839196358	0.94753722	0.99798053	620	403	492	352	633	505	709	601
ENSECAG00000018847	2.869837948	0.947694352	0.99798053	87	197	113	117	133	130	229	121
ENSECAG00000024410	6.20348797	0.947818084	0.99798053	1036	1245	1607	1254	1616	1566	1678	1408
ENSECAG00000004251	1.708932098	0.947823459	0.99798053	38	66	92	29	74	29	122	48
ENSECAG00000009006	2.727591066	0.947918403	0.99798053	78	74	177	153	100	99	209	150
ENSECAG00000003617	2.640194586	0.948003475	0.99798053	103	62	130	145	88	85	129	216
ENSECAG00000014242	5.041728519	0.948113265	0.99798053	363	423	747	875	883	631	686	587
ENSECAG00000006300	2.624895343	0.948269403	0.99798053	49	127	129	152	65	76	185	194
ENSECAG00000024881	2.221730488	0.948295832	0.99798053	21	52	65	225	33	51	109	206
ENSECAG00000015168	4.70774049	0.94846669	0.99798053	283	511	400	741	661	469	596	458
ENSECAG00000012968	3.886235173	0.948598765	0.99798053	150	278	259	410	205	207	460	388
ENSECAG00000008052	5.169994074	0.948867284	0.99798053	445	671	858	607	650	610	1444	394
ENSECAG00000012609	4.976506462	0.949057505	0.99798053	422	458	741	600	568	765	534	781
ENSECAG00000010524	6.977171365	0.949345017	0.99798053	1874	1362	2810	2873	3595	2341	2758	2018
ENSECAG00000000261	5.640773895	0.949517138	0.99798053	603	755	1032	1206	1444	765	1167	877
ENSECAG00000015584	4.117392493	0.94980558	0.99798053	178	370	247	490	310	250	358	538
ENSECAG00000005207	3.599229112	0.949950363	0.99798053	162	180	206	315	243	251	281	251
ENSECAG00000024742	0.944002834	0.94995268	0.99798053	27	16	48	42	29	36	55	34
ENSECAG00000021963	5.203100503	0.950042516	0.99798053	381	546	883	927	578	841	783	876
ENSECAG00000000085	5.788286591	0.950100406	0.99798053	570	874	1219	1373	912	1127	1041	1613
ENSECAG00000007827	7.631507754	0.950182175	0.99798053	2071	3904	3745	4629	3423	5003	4405	4022
ENSECAG00000020978	10.43486429	0.950237543	0.99798053	16264	17724	35165	33273	31414	30502	25334	27548
ENSECAG00000018572	6.226535322	0.950261932	0.99798053	763	1500	1667	1562	1608	1624	1623	1397
ENSECAG00000019034	4.914421693	0.950334934	0.99798053	255	504	548	949	438	617	744	781
ENSECAG00000013245	5.640068509	0.950378553	0.99798053	608	1044	711	1275	608	776	1224	1625
ENSECAG00000015231	2.690863328	0.950472801	0.99798053	54	252	74	58	100	263	111	56
ENSECAG00000006378	1.391934958	0.950557611	0.99798053	21	44	47	75	31	39	82	69
ENSECAG00000008139	4.328184723	0.950626482	0.99798053	232	233	423	612	317	299	570	520
ENSECAG00000024534	5.256331025	0.95063306	0.99798053	455	639	717	997	916	512	1040	772
ENSECAG00000015057	6.370366167	0.950730854	0.99798053	690	1644	1624	2153	1591	1310	1860	2332
ENSECAG00000018381	8.143880872	0.951020251	0.99798053	3798	4339	5766	6188	6274	5315	7157	5504
ENSECAG00000006891	3.553983679	0.951038426	0.99798053	125	302	162	240	134	243	292	334
ENSECAG00000015203	6.038013014	0.951101308	0.99798053	719	1173	1444	1395	1365	1458	1543	1220
ENSECAG00000024890	2.417399163	0.951167919	0.99798053	28	103	102	176	94	94	139	115
ENSECAG00000012473	2.699021533	0.95118402	0.99798053	24	139	83	266	99	225	73	120
ENSECAG00000019391	3.187312377	0.951204912	0.99798053	57	393	64	119	78	134	397	196
ENSECAG00000003428	10.7598881	0.951223145	0.99798053	33930	25074	31522	28480	39181	40232	27658	36044
ENSECAG00000014803	7.302029453	0.951717674	0.99798053	1932	2546	3527	3517	2672	3964	3281	3224
ENSECAG00000009259	7.19546552	0.951841752	0.99798053	1599	2447	2576	4100	2088	3015	3581	3899
ENSECAG00000006129	3.508773518	0.952011005	0.99798053	81	286	182	303	347	127	240	233
ENSECAG00000010226	4.777876298	0.952037729	0.99798053	313	490	459	762	596	380	688	655
ENSECAG00000020358	8.472361258	0.952287964	0.99798053	2396	5830	8807	9651	8217	8136	4632	8832
ENSECAG00000010719	10.24287871	0.952352144	0.99798053	13410	15139	27828	32392	31354	26397	19587	24762
ENSECAG00000017582	2.006459187	0.952542714	0.99798053	66	71	44	90	75	40	140	90
ENSECAG00000022535	5.728964462	0.952600237	0.99798053	559	968	895	1529	1115	1114	1298	924
ENSECAG00000020347	4.000580056	0.952603331	0.99798053	170	275	324	411	330	225	432	366
ENSECAG00000021517	3.660439599	0.95260555	0.99798053	166	201	247	298	226	247	299	285
ENSECAG00000007400	4.595491824	0.952702897	0.99798053	290	461	563	419	513	425	687	418
ENSECAG00000019370	7.16571162	0.952726627	0.99798053	1893	2476	2822	3206	2723	3681	3375	2199
ENSECAG00000009987	6.352330744	0.952820845	0.99798053	1324	1401	1514	1489	1986	1432	1905	1539
ENSECAG00000027684	11.52609501	0.952915061	0.99798053	56662	35315	68320	42316	65988	66314	62109	51029
ENSECAG00000020459	5.517697954	0.953042721	0.99798053	683	833	775	901	1073	719	1291	856
ENSECAG00000019441	5.00270227	0.953053114	0.99798053	405	380	953	581	674	610	1129	316
ENSECAG00000022520	6.109675125	0.95308009	0.99798053	667	1314	1546	1586	1379	1913	958	1416
ENSECAG00000022620	5.736858951	0.95309758	0.99798053	762	863	1101	1082	1146	873	1246	1229

ENSECAG00000016453	3.108872888	0.953133602	0.99798053	47	178	131	296	204	162	180	180
ENSECAG00000013124	5.219249494	0.953138126	0.99798053	705	702	697	413	1067	1293	397	229
ENSECAG00000009708	5.973706876	0.953143115	0.99798053	819	634	1499	1681	1453	1155	1577	1102
ENSECAG00000014800	7.083449969	0.953266806	0.99798053	1595	3396	1862	2957	2239	3331	3627	2198
ENSECAG00000015925	7.071016974	0.953341264	0.99798053	1918	1785	3001	2790	4028	2383	3052	1974
ENSECAG00000002570	2.906075324	0.953344721	0.99798053	24	136	74	354	24	124	137	355
ENSECAG00000019921	2.988462242	0.95337991	0.99798053	90	147	115	217	188	168	136	170
ENSECAG00000007560	6.814262065	0.953534492	0.99798053	1412	1534	2159	3054	2177	1978	2378	3081
ENSECAG00000021796	7.916534803	0.953646686	0.99798053	2034	3867	4302	8420	2989	3975	4442	8909
ENSECAG00000021365	4.288809103	0.953674261	0.99798053	344	216	413	367	424	240	606	422
ENSECAG00000011678	6.315777573	0.953709548	0.99798053	1015	804	1700	2414	1683	1168	2115	1789
ENSECAG00000022024	6.518578469	0.953754312	0.99798053	1244	1769	1943	1561	2120	2105	1991	1422
ENSECAG00000009889	9.10772618	0.953929967	0.99798053	7936	10432	8907	11358	9977	11034	14993	11469
ENSECAG00000009773	4.551910808	0.95393708	0.99798053	169	253	871	437	216	435	483	875
ENSECAG00000023641	4.118616691	0.953962811	0.99798053	135	271	330	597	405	313	336	390
ENSECAG00000010094	5.457800968	0.954033622	0.99798053	567	636	749	1227	1020	515	1215	1045
ENSECAG00000023112	3.761078497	0.954080179	0.99798053	136	138	346.999	378	320	285.999	238	293
ENSECAG00000024843	7.062491854	0.95412936	0.99798053	1008	1606	2553	5344	2714	2821	3300	2380
ENSECAG00000005422	5.446180935	0.954198511	0.99798053	493	593	781	1405	796	626	1390	918
ENSECAG00000011099	7.044906831	0.95433019	0.99798053	2181	3260	1516	2163	2186	2732	3203	2989
ENSECAG00000005243	4.527062886	0.95441259	0.99798053	248	433	454	527	412	437	519	594
ENSECAG00000006469	5.678997959	0.954512622	0.99798053	726	606	1189	1184	1032	810	1314	1181
ENSECAG00000012859	5.634391109	0.954531445	0.99798053	743	1156	812	747	1445	810	1326	599
ENSECAG00000025082	2.24546123	0.954568607	0.99798053	22	87	85	172	113	99	101	73
ENSECAG00000015251	4.902030729	0.95478683	0.99798053	386	599	615	529	670	622	715	496
ENSECAG00000015469	4.733678867	0.955183113	0.99798053	155	201	1046	606	680	676	543	337
ENSECAG00000023832	7.384809785	0.955218536	0.99798053	2114	3356	2998	3355	4396	2925	4123	2841
ENSECAG00000007655	4.498327988	0.955473738	0.99798053	239	267	384	799	452	276	636	588
ENSECAG00000012378	3.726672456	0.955531225	0.99798053	159	310	216	260	286	211	309	303
ENSECAG00000021774	2.589600739	0.955600949	0.99798053	38	92	122	210	122	107	119	147
ENSECAG00000013572	0.404720316	0.955653736	0.99798053	9	16	36	29	17	36	15	35
ENSECAG00000011914	6.627637821	0.95573273	0.99798053	1798	2090	1214	1520	2320	876	3838	1684
ENSECAG00000008676	9.653131853	0.955737052	0.99798053	10128	9747	16915	21677	20946	15322	16366	15745
ENSECAG00000014265	4.949395809	0.955799731	0.99798053	539	742	373	450	556	582	944	540
ENSECAG00000004159	5.55565556	0.955818539	0.99798053	598	516	1010	1296	1405	704	1091	805
ENSECAG00000014558	3.932940602	0.955857187	0.99798053	139	212	321	463	267	289	299	438
ENSECAG00000024728	4.997450785	0.956022837	0.99798053	354	465	763	771	673	595	737	677
ENSECAG00000023672	3.99165004	0.956039507	0.99798053	181	214	271	526	245	132	385	590
ENSECAG00000006335	0.910672042	0.956039746	0.99798053	4	34	46	52	52	36	26	36
ENSECAG00000025223	1.139464497	0.956064386	0.99798053	18	42	33	67	34	37	36	68
ENSECAG00000024285	3.977513796	0.95632755	0.99798053	152	235	325	470	340	276	314	383
ENSECAG00000007311	6.117204969	0.956363444	0.99798053	612	1008	1698	1931	1849	1249	1620	1100
ENSECAG00000020360	4.804585955	0.956409341	0.99798053	287	339	563	891	479	424	758	746
ENSECAG00000011622	3.215332276	0.956506467	0.99798053	74	235	130	250	165	97	265	260
ENSECAG00000006377	7.525501275	0.956508055	0.99798053	2095	2636	4004	4962	4855	2342	5305	3157
ENSECAG00000000364	2.969148562	0.956547735	0.99798053	108	151	107	181	176	133	197	157
ENSECAG00000010222	7.749191488	0.956554509	0.99798053	3596	2508	5196	3509	6220	4052	4776	3231
ENSECAG00000010508	5.564671296	0.956779684	0.99798053	591	889	778	1187	987	642	1119	1258
ENSECAG00000021379	5.902451655	0.956799598	0.99798053	797	775	1258	1540	1218	1035	1431	1361
ENSECAG00000021247	4.555750716	0.956917531	0.99798053	253	402	313	798	470	377	482	643
ENSECAG00000008480	5.086505542	0.956959238	0.99798053	535	428	682	736	707	545	779	870
ENSECAG00000014356	6.948508129	0.956969775	0.99798053	1450	1839	2707	2936	2532	2250	3427	2413
ENSECAG00000023359	2.900334767	0.957065611	0.99798053	75	177	112	166	172	98	167	196
ENSECAG00000014160	5.633161486	0.957173593	0.99798053	475	825	1027	1333	1183	896	1276	883
ENSECAG00000016191	2.980320722	0.957203313	0.99798053	107	161	100	183	159	140	206	165
ENSECAG00000013999	6.922471421	0.957304053	0.99798053	1730	2064	2365	2519	2944	2141	2868	2243
ENSECAG00000021065	11.62243423	0.957325334	0.99798053	36759	38680	78322	75129	70221	72043	55208	68675
ENSECAG00000000377	4.775920494	0.95734806	0.99798053	286	282	754	726	527	565	685	524
ENSECAG00000010685	6.710699849	0.957395112	0.99798053	1830	1422	2154	1757	2109	2456	2512	1830
ENSECAG00000011843	3.347853077	0.957612114	0.998115345	167	150	198	195	220	166	267	201
ENSECAG00000014886	0.472887857	0.957873729	0.998168044	7	38	29	22	15	36	33	23
ENSECAG00000017394	7.665316121	0.95797959	0.998168044	2646	3627	4021	4379	5974	2853	5429	2892
ENSECAG00000008091	8.394786697	0.958248556	0.998168044	5825	4930	6186	6153	7860	7555	7332	5799
ENSECAG00000024679	6.169146791	0.958259765	0.998168044	1067	1407	1223	1299	1577	1300	1838	1441
ENSECAG00000014940	6.25506153	0.958262347	0.998168044	859	855	2057	1794	2076	1491	1813	1109
ENSECAG00000010550	6.791521608	0.958423991	0.998168044	895.922	1724.41	2105.2	3803.87	1983.05	2489.42	2660.95	2164.63
ENSECAG00000017144	5.581604531	0.958518714	0.998168044	594	712	1134	1038	1093	876	1136	922
ENSECAG00000008642	7.900875481	0.958519067	0.998168044	2973	3306	4553	6880	4171	3394	6054	6693
ENSECAG00000015843	7.886216274	0.958552041	0.998168044	2808	5442	3986	4845	4480	5149	5899	4367
ENSECAG00000006103	0.538367224	0.958597698	0.998168044	18	28	26	25	10	20	45	41
ENSECAG00000010456	4.380560378	0.958691875	0.998168044	239	497	342	418	500	341	550	364
ENSECAG00000014547	6.20555272	0.958714762	0.998168044	995	1196	1463	1676	1163	1049	1877	2192
ENSECAG00000018839	6.668071709	0.959370182	0.998340092	1041	2371	1801	2224	1744	2647	2144	1938
ENSECAG00000020461	3.52023718	0.959421608	0.998340092	178	128	150	362	223	103	300	363
ENSECAG00000009199	0.615001493	0.959573338	0.998340092	18	10	33	47	25	30	29	35
ENSECAG00000016999	2.709790637	0.959630149	0.998340092	50	163	99	161	77	133	150	193

ENSECAG00000019636	5.781070517	0.959679178	0.998340092	643	955	1045	1327	906	1456	1167	1113
ENSECAG00000001075	6.549031141	0.959795258	0.998340092	1214	1772	1908	1822	1939	2212	1859	1787
ENSECAG00000010844	5.223327662	0.959819974	0.998340092	260	700	848	984	727	825	710	891
ENSECAG00000000140	7.090130861	0.960021401	0.998340092	2718	2198	2320	1995	2945	2696	3610	2236
ENSECAG00000000859	7.264076099	0.960053072	0.998340092	1199	2842	3772	3547	3923	2583	3151	3416
ENSECAG00000018698	5.874639072	0.960067407	0.998340092	610	1123	810	1777	1137	686	1502	1741
ENSECAG00000013591	4.224177586	0.96014549	0.998340092	225	223	407	526	279	307	433	557
ENSECAG00000022217	4.676605596	0.960234448	0.998340092	369	339	559	567	682	310	717	460
ENSECAG00000026938	9.762443109	0.960259922	0.998340092	7575	11954	20811	24511	22347	24294	12021	13648
ENSECAG00000011716	5.337496103	0.960368263	0.998340092	328	750	802	1178	656	672	1207	913
ENSECAG00000009547	5.462426223	0.960400759	0.998340092	504	570	1076	1055	928	882	873	1048
ENSECAG00000011690	1.368779763	0.960554548	0.998340092	43	44	37	46	50	37	61	66
ENSECAG00000012658	3.947338547	0.960591807	0.998340092	88	294	242	555	370	426	205	275
ENSECAG00000025867	5.453929873	0.96070046	0.998340092	52	402	744	2548	62	1860	34	1518
ENSECAG00000016025	4.67706729	0.960719659	0.998340092	390	430	519	429	671	522	601	370
ENSECAG00000016925	7.444001787	0.960737236	0.998340092	2726	3013	3044	3529	3716	2996	4739	3302
ENSECAG00000011451	7.747511201	0.960833903	0.998340092	2937	3112	4254	5036	5061	4335	4606	4243
ENSECAG00000015555	4.140951746	0.961021073	0.998340092	158	290	388	485	481	265	382	347
ENSECAG00000006256	2.142183881	0.961057748	0.998340092	48	91	88	80	130	66	88	83
ENSECAG00000021785	8.902310351	0.961151977	0.998340092	397	9391	17047	10944	1602	14538	11204	12606
ENSECAG00000022484	6.739460669	0.961323095	0.998340092	1060	2236	1816	2683	1910	2184	2360	2648
ENSECAG00000015786	3.057354883	0.961432087	0.998340092	58	196	138	219	198	165	172	163
ENSECAG00000019456	2.297750431	0.961532428	0.998340092	73	126	75	59	69	65	156	124
ENSECAG00000016153	4.362260827	0.961783168	0.998340092	315	309	396	437	504	324	529	376
ENSECAG00000010402	1.913562189	0.961787645	0.998340092	40	79	62	82	35	44	191	60
ENSECAG00000000718	6.593470315	0.961828643	0.998340092	1268	1402	1825	2571	1883	1751	2121	2373
ENSECAG00000026954	5.811866433	0.961995976	0.998340092	569	796	1250	1532	1248	1029	1359	1153
ENSECAG00000011529	1.030561451	0.962206214	0.998340092	19	50	19	52	22	27	18	99
ENSECAG00000021465	5.575955679	0.962375121	0.998340092	585	857	841	1194	832	1085	1025	1052
ENSECAG00000006592	3.874421604	0.962433409	0.998340092	118	331	277	345	236	258	349	407
ENSECAG00000008435	4.764384319	0.962462303	0.998340092	181	458	426	1021	486	524	617	686
ENSECAG00000008759	0.98366647	0.962621651	0.998340092	12	48	52	22	21.9999	20.9999	19	98.9998
ENSECAG00000006603	5.707061713	0.962673785	0.998340092	404	840	793	2020	806	614	1364	1677
ENSECAG00000014373	5.519592282	0.962718935	0.998340092	545	584	1104	1092	1187	987	847	834
ENSECAG00000009636	7.430201712	0.962753492	0.998340092	2782	2618	3464	3059	5190	3273	3728	2413
ENSECAG00000014972	4.298266614	0.962778297	0.998340092	384	322	303	303	462	289	568	370
ENSECAG00000003931	6.585695357	0.962950116	0.998340092	1802	1629	1530	1502	2539	1262	2757	1722
ENSECAG00000015757	10.43097337	0.963320847	0.998340092	16293	18871	32219	34548	28755	31076	22417	32311
ENSECAG00000016058	4.227445337	0.963330667	0.998340092	231	275	286	594	330	307	522	429
ENSECAG00000008913	1.315849257	0.963381154	0.998340092	28	22	70	58	8	45	20	125
ENSECAG00000011362	6.713081928	0.963529089	0.998340092	1079	1530	2210	2926	2566	1737	3159	1572
ENSECAG00000010631	5.492337519	0.963591784	0.998340092	492	530	767	1579	1027	574	1061	1196
ENSECAG00000020743	4.671262007	0.963632109	0.998340092	363	380	680	373	630	595	710	198
ENSECAG00000005934	0.161485085	0.963747843	0.998340092	21	33	7	7	4	40	4	34
ENSECAG00000006921	3.810046608	0.963800342	0.998340092	177	200	284	360	311	231	309	323
ENSECAG00000018194	6.571254451	0.963903675	0.998340092	1208	1337	2319	2039	1377	2047	2286	2311
ENSECAG00000020636	4.942460373	0.963997564	0.998340092	246	629	523	927	338	509	682	1073
ENSECAG00000016215	6.360815169	0.964048166	0.998340092	920	1253	1808	1999	1548	2000	1801	1604
ENSECAG00000007906	4.086849486	0.964248236	0.998340092	219	244	351	401	404	249	426	370
ENSECAG00000022546	9.047929136	0.964268889	0.998340092	4817	10808	11839	11757	10603	12125	12069	9533
ENSECAG00000019569	6.551787805	0.964289893	0.998340092	1007	1332	2011	2549	2543	1893	1668	1790
ENSECAG00000026873	9.5952116	0.964441037	0.998340092	14850	11584	13795	11749	15233	18302	17804	14383
ENSECAG00000013824	5.186056583	0.964489539	0.998340092	453	804	676	661	762	790	793	700
ENSECAG00000005219	8.328644815	0.964721557	0.998340092	4220	4731	6642	7429	9215	7172	6121	4535
ENSECAG00000006659	4.761821693	0.964744285	0.998340092	369	519	566	465	713	557	594	398
ENSECAG00000009871	2.562494877	0.964751978	0.998340092	68	81	99	179	157	108	151	80
ENSECAG00000012230	6.002409609	0.964800815	0.998340092	755	711	1421	1824	1448	1066	1846	1149
ENSECAG00000009390	7.615325458	0.964924197	0.998340092	2594	3300	3548	4558	4561	4080	5278	2829
ENSECAG00000007436	1.979717754	0.965275894	0.998340092	41	62	70	115	75	53	80	116
ENSECAG00000008719	3.698281365	0.965287853	0.998340092	174	154	271	345	248	188	379	286
ENSECAG00000000797	8.893005407	0.96544394	0.998340092	5165	8448	10031	10778	13633	5807	14998	6735
ENSECAG00000013264	5.505032383	0.96550208	0.998340092	479	894	820	1150	901	693	1131	1125
ENSECAG00000014798	1.957941066	0.965551002	0.998340092	62	61	48	100	101	48	84	86
ENSECAG00000013968	5.494579447	0.965666544	0.998340092	475.011	790	839.008	1240	1017	1043	889	803.024
ENSECAG00000020337	8.652185929	0.9658011	0.998340092	7014	7169	6163	7117	9328	5788	11715	7921
ENSECAG00000023846	4.890646016	0.965854937	0.998340092	356	452	592	775	503	492	893	642
ENSECAG00000000365	2.388637078	0.965908732	0.998340092	52	61	126	140	62	48	140	199
ENSECAG00000002734	5.482557254	0.965929941	0.998340092	526	701	957	1047	924	929	957	975
ENSECAG00000015063	0.58567844	0.966055381	0.998340092	28	25	22	18	21	9	19	72
ENSECAG00000004544	1.979396743	0.966120006	0.998340092	48	165	29	11	43	110	103	74
ENSECAG00000016612	6.975864518	0.966132388	0.998340092	1659	1866	2313	3368	2941	2396	2952	2294
ENSECAG00000015242	9.492000484	0.966153408	0.998340092	8931	8739	17463	16943	13976	15378	12699	18844
ENSECAG00000018287	5.361713663	0.966217435	0.998340092	574	602	804	985	621	828	984	1038
ENSECAG00000024604	9.120653246	0.96622033	0.998340092	10819	5525	12115	10024	13241	10826	15097	7808
ENSECAG00000022783	0.899502941	0.966262829	0.998340092	28	24	37	34	40	48	45	17
ENSECAG00000000883	6.031881877	0.966466063	0.998340092	998	850	1194	1598	1808	863	1510	1403

ENSECAG00000007069	3.72011159	0.966524047	0.998340092	156.002	242	219	328	269.001	164.001	434	272
ENSECAG00000007492	1.328851016	0.966594412	0.998340092	27	52	34	60	14	33	66	98
ENSECAG00000001065	2.231002702	0.966635677	0.998340092	31	141	82	85	89	124	84	83
ENSECAG00000022641	4.395070117	0.966669989	0.998340092	219	341	311	688	375	661	420	299
ENSECAG00000020507	6.78226877	0.966754344	0.998340092	1351	1688	2445	2426	1985	2119	3217	2148
ENSECAG00000017661	1.283958857	0.966813455	0.998340092	15	49	52	62	19	48	25	101
ENSECAG00000016787	0.65159056	0.966867365	0.998340092	25	29	13	38	8	57	19	36
ENSECAG00000017289	1.88727833	0.966933439	0.998340092	20	64.0004	80	115	93	94	60	49
ENSECAG00000012038	6.189686798	0.967002422	0.998340092	795	1124	1445	2066	1206	1302	1898	1790
ENSECAG00000021579	6.699051929	0.967061546	0.998340092	1192	1522	2259	2701	2185	1981	2069	2474
ENSECAG00000023719	5.477217811	0.967121786	0.998340092	503	795	894	1067	910	781	944	1112
ENSECAG00000011983	3.781843236	0.96725133	0.998340092	163	232	257	335	200	137	404	451
ENSECAG00000003869	7.59447476	0.967264376	0.998340092	2663	3897	3304	3919	3509	4422	4099	4154
ENSECAG00000012915	4.404520772	0.967313715	0.998340092	234	376	432	486	339	534	539	382
ENSECAG00000027000	2.712173736	0.967380795	0.998340092	53	85	125	237	66	104	135	241
ENSECAG00000015430	0.836136263	0.967444125	0.998340092	31	23	16	51	58	17	35	31
ENSECAG00000019593	8.222480903	0.96753473	0.998340092	2604	4323	7879	7352	6604	5943	6844	6030
ENSECAG00000021986	5.67215034	0.967554891	0.998340092	693	911	946	1103	1269	792	1202	1039
ENSECAG00000022445	3.539810683	0.967699513	0.998340092	132	134	278	301	260	147	332	258
ENSECAG00000023940	3.749512868	0.967804557	0.998340092	131	225	254	392	253.002	339	267	254
ENSECAG00000008073	8.065609502	0.967836037	0.998340092	3658	4752	4053	6667	6717	4455	6193	5483
ENSECAG00000007410	6.480619197	0.967888762	0.998340092	1162	2089	893	2208	1539	1178	2642	2386
ENSECAG00000020465	6.066905964	0.968132388	0.998340092	882	1065	1200	1666	1746	1069	2082	871
ENSECAG00000022267	5.270763985	0.968514504	0.998340092	392	586	871	987	790	926	756	774
ENSECAG00000024846	5.049309857	0.968522695	0.998340092	281	614	716	866	770	580	773	663
ENSECAG00000016628	4.050494914	0.968778446	0.998340092	236	315	298	320	357	374	311	332
ENSECAG00000021380	7.753543516	0.968859835	0.998340092	2527	3287	4100	6071	4397	3564	4675	5567
ENSECAG00000016986	6.019699898	0.968925782	0.998340092	959	1382	1144	1050	1535	809	1894	1303
ENSECAG00000006591	3.242017883	0.968942862	0.998340092	57	165	277	212	176	222	339	60
ENSECAG00000023221	7.475200661	0.968948141	0.998340092	1712	3011	3522	5114	4165	3967	3178	3483
ENSECAG00000005387	5.815277342	0.969008143	0.998340092	637	849	1165	1460	1272	953	1488	1103
ENSECAG00000012735	3.157376427	0.969014489	0.998340092	70	166	116	328	152	83	179	336
ENSECAG00000009326	7.078447994	0.969110717	0.998340092	1789	1961	2455	3688	3432	1745	3709	2613
ENSECAG00000005335	1.101780192	0.969115901	0.998340092	18	52	39	41	36	30	66	43
ENSECAG00000010027	2.840974804	0.969178138	0.998340092	105	109	160	127	174	115	148	157
ENSECAG00000011190	2.01132954	0.969202335	0.998340092	27	58	78.0016	137	69	125	69	66
ENSECAG00000021823	6.682228307	0.969232026	0.998340092	1124	1820	2221	2366	2078	2200	2552	1819
ENSECAG00000012400	6.766617978	0.96936103	0.998340092	136	2309	3619	2469	343	4805	289	3252
ENSECAG00000022474	4.637144929	0.969455568	0.998340092	348	263	675	475	613	397	752	373
ENSECAG00000024408	2.300293553	0.969461208	0.998340092	20	77	95	185	79	90	110	134
ENSECAG00000019502	6.989707353	0.9695827	0.998340092	1811	2271	2040	2868	2616	2587	2977	2634
ENSECAG00000012794	3.17966112	0.969790164	0.998340092	108	187	151	204	196	145	225	193
ENSECAG00000000558	7.181310478	0.969819119	0.998340092	1837	2367	3039	3144	2796	2788	3409	3387
ENSECAG00000012487	2.944587145	0.970064289	0.998340092	90	171	120	170	72	117	195	265
ENSECAG00000008693	8.039883836	0.970130506	0.998340092	5553	6137	2843	2776	6438	5686	6134	3775
ENSECAG00000024342	5.087499975	0.970130988	0.998340092	312	593	597	1061	1077	500	738	533
ENSECAG00000009738	11.57581777	0.970136159	0.998340092	32178	41350	75122	75398	69145	71111	55403	61340
ENSECAG00000002116	2.473062641	0.970171866	0.998340092	51	94	79	184	141	81	170	79
ENSECAG00000000622	1.048314826	0.970191899	0.998340092	12	47	50	38	23	38	67	40
ENSECAG00000020072	8.29629208	0.970366338	0.99842935	3125	6500	5637	8017	5646	5580	7106	8228
ENSECAG00000017026	4.273804044	0.970567221	0.998511708	297	292	336	431	457	235	605	371
ENSECAG00000020682	1.142353543	0.970621789	0.998511708	21	26	32	83	42	33	54	49
ENSECAG00000020622	4.614746552	0.970816925	0.998562937	338	442	447	519	546	463	681	380
ENSECAG00000022628	5.636278467	0.970866074	0.998562937	661	1039	816	991	987	1192	1102	918
ENSECAG00000011589	2.948690097	0.970934713	0.998562937	54	152	114	267	90	125	175	257
ENSECAG00000022019	8.132147743	0.971171083	0.998715814	3642	3388	7041	6366	6355	5409	5479	6247
ENSECAG00000026942	2.985410236	0.971497496	0.998926088	84	144	162	185	122	198	180	156
ENSECAG00000021984	3.430317627	0.971748016	0.998926088	146	173	158	292	256	132	259	270
ENSECAG00000006519	6.529017232	0.971777579	0.998926088	1008	2024	1611	2002	2143	1657	2365	1717
ENSECAG00000019234	6.870603685	0.971859903	0.998926088	1546.99	2424.99	1876.99	2537	2070	2354	2738.99	2708.99
ENSECAG00000023738	3.993429051	0.971900427	0.998926088	150	216	398	424	351	294	379	312
ENSECAG00000014652	8.50646509	0.972101082	0.998926088	3694	5603	7333	10662	5204	8894	5989	10208
ENSECAG00000009507	3.251584787	0.97231881	0.998926088	117	194	212	139	157	163	262	230
ENSECAG00000001911	7.140625886	0.972514644	0.998926088	2272	2195	2763	2685	3174	2233	3732	2840
ENSECAG00000014420	3.353039038	0.97251764	0.998926088	104	191	186	269	186	189	270	214
ENSECAG00000024533	4.593042745	0.972565433	0.998926088	153	430	562	696	397	534	598	501
ENSECAG00000014501	1.228938999	0.972595231	0.998926088	33	91	19	8	44	92	21	23
ENSECAG00000021926	5.262887185	0.972628911	0.998926088	575	712	575	828	793	335	1237	978
ENSECAG00000022109	6.786283464	0.972866957	0.998926088	1630	2607	1567	1809	2112	1909	3093	2374
ENSECAG00000019986	4.975823348	0.972882431	0.998926088	424	493	612	738	653	464	870	691
ENSECAG00000010993	2.400331314	0.972882928	0.998926088	69	87	92	122	113	66	131	136
ENSECAG00000006544	7.62115559	0.973074419	0.998926088	2629	2642	3679	5480	4199	2833	4753	4922
ENSECAG00000023959	3.001660774	0.973101581	0.998926088	131	105	146	171	201	94	209	177
ENSECAG00000015043	4.935168245	0.973135943	0.998926088	423	507	496	756	809	436	684	670
ENSECAG00000013246	2.332608704	0.973151258	0.998926088	55	67	133	108	69	73	155	127
ENSECAG00000016855	6.883732619	0.973196343	0.998926088	1563	2125	1946	2807	2282	2724	2385	2582

ENSECAG00000000628	0.653908107	0.973241144	0.998926088	25	22	21	37	0	19	81	31
ENSECAG00000012028	4.766081452	0.97330585	0.998926088	326	407	536	727	348	553	775	637
ENSECAG00000008815	4.648160686	0.973615835	0.999154163	498	466	402	285	694	468	671	297
ENSECAG00000007640	7.395510751	0.973911011	0.999229702	2317	3720	3677	1870	2925	4273	3726	3320
ENSECAG00000008211	3.801744789	0.974046729	0.999229702	156	225	316	305	97	294	503	314
ENSECAG00000023080	3.883210107	0.974093329	0.999229702	124	214	312	468	236	245	344	419
ENSECAG00000016583	0.915697096	0.974206497	0.999229702	35	40	15	29	7	68	24	51
ENSECAG00000005769	4.535546016	0.974250419	0.999229702	224	382	497	603	357	466	453	685
ENSECAG00000012157	3.381569352	0.9743528	0.999229702	89	209	236	222	198	195	323	174
ENSECAG00000007808	0.214066582	0.974484338	0.999229702	13	14	24	25	12	22	14	42
ENSECAG00000004504	1.174002693	0.974512956	0.999229702	11	36	44	78	69	48	26	33
ENSECAG00000024020	6.75614545	0.974582069	0.999229702	1415	1623	2199	2612	1721	2086	2693	2662
ENSECAG00000010157	10.15340169	0.974588655	0.999229702	11285	14456	25750	33488	28764	22413	22258	22794
ENSECAG00000013735	3.452155528	0.974654883	0.999229702	182	239	126	195	149	147	377	276
ENSECAG00000000406	6.784275176	0.97492696	0.999418642	1795	2082	1517	2246	2466	1913	2608	2408
ENSECAG00000016365	3.734465863	0.975025572	0.999429742	158	304	265	203	333	194	378	231
ENSECAG00000011382	6.051479693	0.975139433	0.999456468	956	1777	1013	825	1402	1339	1845	1029
ENSECAG00000023467	2.882694619	0.97570547	0.999917006	89	121	107	214	101	124	212	192
ENSECAG00000020572	4.578559962	0.975764422	0.999917006	291	461	502	456	522	508	568	406
ENSECAG00000013825	3.606629579	0.975940842	1	106	173	369	252	91	300	150	464
ENSECAG00000023907	5.277987833	0.976367412	1	310	1000	533	1017	539	505	1392	954
ENSECAG00000025137	0.266748987	0.976598057	1	13	32	8	27	29	8	34	23
ENSECAG00000016864	5.718607584	0.976617747	1	467	613	1011	1928	1096	783	1328	1302
ENSECAG00000011269	3.336595528	0.976709231	1	89	171	236	251	195	180	250	223
ENSECAG00000011461	6.1830104	0.976844799	1	865	884	1649	1969	1465	1192	1670	1821
ENSECAG00000006337	5.326630303	0.976913283	1	554	966	840	380	755	976	967	697
ENSECAG00000027695	9.559623681	0.977092251	1	14342	8813	17615	10193	19064	14690	16131	14029
ENSECAG00000024026	1.572934412	0.977117891	1	28	40	73	70	61	37	76	74
ENSECAG00000014120	3.508530536	0.977129001	1	144	141	207	334	328	146	248	239
ENSECAG00000022103	8.799213772	0.977190094	1	4017	7530	9245	12748	9365	8528	11490	8326
ENSECAG00000018212	8.017516584	0.977356129	1	2920	4060	4746	7319	5818	5038	6736	4513
ENSECAG00000010293	7.405343338	0.977383261	1	2573	3886	2798	2494	4608	3048	3801	2786
ENSECAG00000013338	2.023876128	0.977550627	1	31	57	70	145	70	45	80	145
ENSECAG00000019702	5.692580449	0.977693252	1	642	874	1118	1065	1104	1161	1211	897
ENSECAG00000000782	9.072178567	0.977731466	1	9471	9438	7988	9996	11710	9952	13156	11095
ENSECAG00000005987	4.558069313	0.977835101	1	178.007	494	522	561	690	438	453	379
ENSECAG00000023391	0.306194341	0.977929086	1	10	44	14	13	12	32	30	21
ENSECAG00000015098	6.857811861	0.977949087	1	1955	2167	1868	2046	1591	2926	2786	2463
ENSECAG00000014554	1.49347545	0.978159574	1	29	84	42	34	40	40	81	75
ENSECAG00000008968	7.218279534	0.978216924	1	2036	2118	3073	3430	3946	2221	3480	3041
ENSECAG00000016827	5.803231684	0.978414091	1	614	1149	1007	1264	1041	1212	1276	1202
ENSECAG00000009512	8.548099307	0.978470193	1	5470	5082	8554	7622	10456	7471	8118	5293
ENSECAG00000015079	7.980387341	0.978590852	1	3929	4926	3700	5222	7165	3375	6281	4580
ENSECAG00000012273	5.095150782	0.97868109	1	534	549	528	814	590	395	1005	946
ENSECAG00000016897	3.859008883	0.978892237	1	119	249	264	465	322	330	257	292
ENSECAG00000017072	2.646321572	0.978910709	1	33	102	144	194	153	115	134	120
ENSECAG00000020848	5.289054429	0.979002418	1	559	243	1479	489	832	536	1128	841
ENSECAG00000009065	11.61748612	0.979216575	1	35679	43384	67754	86310	72820	73427	53863	61140
ENSECAG00000016386	1.667028009	0.979288746	1	51	30	70	68	44	52	90	77
ENSECAG00000021385	9.06553631	0.979315968	1	6215	9209	9903	14073	11266	7383	15919	11343
ENSECAG00000012870	1.288280673	0.979469881	1	21	31	71	52	24	40	14	116
ENSECAG00000015304	1.690813739	0.979485314	1	50	66	55	46	67	79	74	42
ENSECAG00000022667	2.089196391	0.979543398	1	36	80	98	95	101	113	110	23
ENSECAG00000024050	1.721278572	0.979576859	1	46	39	68	75	50	56	64	103
ENSECAG00000015850	3.930309408	0.979629718	1	171	293	223	420	417	179	320	375
ENSECAG00000012431	6.379352182	0.97966349	1	933	1112	1696	2407	1500	1586	1737	2242
ENSECAG00000013410	1.608213851	0.979685848	1	36	84	35	55	34	69	56	88
ENSECAG00000000404	2.509895216	0.979745238	1	38	267	61	18	38	293	63	62
ENSECAG00000021225	7.306863968	0.979764515	1	1304	1542	4021	5344	3513	3013	3213	3556
ENSECAG00000000442	2.411440917	0.979917222	1	82	108	82	88	104	118	108	112
ENSECAG00000005244	5.710153096	0.979997068	1	666.001	848	1100	1136	1246	859	1474	900.001
ENSECAG00000009477	7.842605822	0.980156773	1	2295	4184	4464	6103	5021	4754	5515	4057
ENSECAG00000015999	7.035711916	0.980477831	1	1349	2425	2599	3317	3509	2628	2944	1925
ENSECAG00000009188	10.42105414	0.980828281	1	17650	18775	33644	29221	27852	33453	22447	30232
ENSECAG00000009002	0.447615492	0.980843456	1	2	32	27	38	15	41	29	19
ENSECAG00000018257	5.337146269	0.980956948	1	551	818	644	864	643	913	863	977
ENSECAG00000018201	3.356047452	0.980978653	1	96	201	197	258	255	192	191	211
ENSECAG00000018358	5.250248744	0.981017791	1	425	663	890	762	1121	606	852	640
ENSECAG00000015423	5.311972534	0.981124055	1	371	823	659	1099	780	586	861	1135
ENSECAG00000015401	4.252916542	0.981280736	1	92	322	611	409	308	501	358	428
ENSECAG00000019257	5.265629539	0.98132676	1	743	646	587	633	702	756	1098	713
ENSECAG00000024510	4.073761826	0.981521536	1	154	70	571	484	417	151	436	427
ENSECAG00000022793	5.496116133	0.981617351	1	495	724	927	1156	869	863	1176	939
ENSECAG00000005469	4.958180107	0.98168047	1	473	652	529	504	753	575	740	549
ENSECAG00000023720	0.482209225	0.98181478	1	20	18	38	15	11	23	11	63
ENSECAG00000021515	7.624271077	0.981892313	1	1335	2888	2336	8951	2137	2323	5312	7363

ENSECAG00000016040	4.584195038	0.981937806	1	276	403	412	672	365	313	656	718
ENSECAG00000020650	5.775039265	0.98197367	1	572	856	962	1678	925	1053	1229	1447
ENSECAG00000011920	1.914651286	0.982068314	1	48	85	40	88	82	55	104	75
ENSECAG00000001626	5.320027986	0.982148692	1	451	509	815	1198	627	754	899	1097
ENSECAG00000019545	8.383831519	0.982191654	1	4254	5446	4436	10298	5783	3840	9655	9683
ENSECAG00000024393	4.935833111	0.982230231	1	268	461	549	1035	500	528	669	906
ENSECAG00000024452	1.9740554	0.982305215	1	49	71	71	82	96	45	107	82
ENSECAG00000016960	5.477228854	0.982395435	1	472	819	754	1249	1014	970	856	880
ENSECAG00000011329	4.253999624	0.982491524	1	181	363	278	606	229	251	434	709
ENSECAG00000016621	6.02500397	0.982575575	1	738	1199	1236	1550	1311	1325	1535	1353
ENSECAG00000009714	6.886743612	0.982710436	1	1472	3775	1455	1523	2093	3380	1973	2357
ENSECAG00000007116	7.021488871	0.983025636	1	1241	1666	3120	3756	2566	2400	3367	2693
ENSECAG00000024593	0.837930245	0.983330784	1	17	27	32	48	24	15	38	69
ENSECAG00000000446	5.637294487	0.983333449	1	587	581	935	1615	625	720	1429	1502
ENSECAG00000020739	4.651908115	0.983456648	1	209	541	519	590	474	482	494	659
ENSECAG00000015258	10.26656937	0.983525329	1	9357	17920	29046	37942	26358	32498	20777	22559
ENSECAG00000019162	7.113796336	0.983613297	1	1686	2709	2495	3124	3228	2441	3737	2353
ENSECAG00000003075	0.708112114	0.983626491	1	6	56	17	34	29	16	37	50
ENSECAG00000012893	0.938031132	0.983639444	1	19	32	20	66	48	26	32	46
ENSECAG00000020771	2.072214835	0.983690988	1	38	72	63	138	70	72	101	105
ENSECAG00000022522	3.85404956	0.983843329	1	170	144	425	314	419	304	294	182
ENSECAG00000024368	7.10979545	0.983893908	1	1601	2414	3395	2479	3104	3118	3156	2291
ENSECAG00000023111	6.139473742	0.983991846	1	858	835	1611	1850	1829	1111	1971	1116
ENSECAG00000011070	3.161481532	0.984180457	1	86	222	177	149	161	142	318	148
ENSECAG00000021001	6.461793445	0.984199604	1	1158	1241	1711	2268	1536	1753	2067	2140
ENSECAG00000017294	5.067359967	0.984293448	1	559	681	438	626	775	560	720	784
ENSECAG00000012600	6.321074436	0.984459243	1	915	1276	1777	1837	1576	1925	1722	1513
ENSECAG00000013813	3.306827402	0.984496522	1	84	193	170	290	225	177	215	209
ENSECAG00000006699	2.817983281	0.984498626	1	73	95	150	202	159	134	128	161
ENSECAG00000024149	8.852190543	0.984591826	1	6386	9171	8156	9109	12028	9710	9704	7265
ENSECAG00000018894	5.56252693	0.984739447	1	455	784	880	1422	742	972	878	1377
ENSECAG00000020174	1.102408917	0.98478607	1	26	19	55	48	38	51	41	43
ENSECAG00000022440	7.092332456	0.984833094	1	1131	1605	3277	4366	3203	2011	3969	2545
ENSECAG00000019246	6.759299859	0.984844482	1	1134	1363	2705	2803	2125	1898	2263	2912
ENSECAG00000013171	5.566077444	0.985061069	1	664	682	1017	1006	1119	875	1248	788
ENSECAG00000014822	6.386530247	0.985104957	1	978	1277	1446	2453	1504	1166	2129	2383
ENSECAG00000000412	4.33944132	0.985141881	1	382	300	349	339	485	444	443	328
ENSECAG00000009335	5.62881942	0.985241947	1	885	876	811	793	951	808	1358	1122
ENSECAG00000013815	7.401100406	0.985310292	1	2153	2177	3682	4351	3073	3581	3106	4416
ENSECAG00000020726	6.984240955	0.985473585	1	1770	1616	2830	2907	3456	2309	2959	1939
ENSECAG00000022362	5.358450536	0.985504036	1	422	882	805	873	764	874	890	922
ENSECAG00000021437	2.147835544	0.985511069	1	40	124	69	81	107	111	78	63
ENSECAG00000022950	5.048799222	0.985524712	1	403	349	850	824	595	598	871	747
ENSECAG00000024831	5.708801717	0.985594297	1	844	613	1149	1087	1529	1006	1252	606
ENSECAG00000013003	3.346112609	0.985802915	1	108	192	280	143	172	157	337	202
ENSECAG00000011560	6.108706353	0.986103945	1	1094	1325	662	1783	951	1638	1370	1866
ENSECAG00000013918	5.98156551	0.986461005	1	684	1353	986	1599	587	1294	1308	2159
ENSECAG00000022681	2.206035345	0.986546385	1	66	91	63	98	60	166	87	63
ENSECAG00000006819	4.425590977	0.986574816	1	328	271	419	513	557	247	601	426
ENSECAG00000011603	7.399079882	0.986674112	1	1790	2470	4041	4090	4975	2819	3993	2521
ENSECAG00000013213	7.660577178	0.986702021	1	2604	3757	4374	3605	3543	4681	5388	3608
ENSECAG00000015297	3.022459282	0.986765898	1	106	114	179	177	149	165	275	103
ENSECAG00000024626	8.441358649	0.987038228	1	3041	5677	7901	9327	4619	9218	7551	7973
ENSECAG00000007397	10.23797372	0.98711011	1	13067	15945	27662	33172	28392	27586	21593	23148
ENSECAG00000013618	3.444896789	0.987209247	1	237	183	130	163	253	235	251	174
ENSECAG00000009989	1.071612736	0.987225	1	45	39	13	38	62	48	40	16
ENSECAG00000020234	7.817073228	0.987466728	1	2810	4072	3499	6073	4680	3909	6147	4474
ENSECAG00000020443	2.956358543	0.987527835	1	101	104	216	127	211	198	152	75
ENSECAG00000008444	5.898953684	0.98766434	1	940	794	1217	1268	1384	958	1450	1266
ENSECAG00000010410	7.176273343	0.98770026	1	2213	1434	2881	3929	3170	1886	4318	3040
ENSECAG00000014461	4.449280041	0.987722743	1	155	452	333	722	320	317	469	746
ENSECAG00000018426	6.011078093	0.987798719	1	468	1019	2085	1233	1512	1606	1392	876
ENSECAG00000018222	7.334687248	0.988094978	1	2314	2969	3119	2997	3329	3021	4630	2774
ENSECAG00000006454	5.638770788	0.988229737	1	348	1433	782	1119	706	1130	1130	1237
ENSECAG00000022194	6.697079749	0.988427442	1	1987	1548	1646	1879	2393	1636	2567	2252
ENSECAG00000024486	3.751937651	0.98856765	1	153	209	235	394	311	316	258	232
ENSECAG00000016590	4.887906611	0.988616245	1	263	556	526	860	575	814	499	574
ENSECAG00000014826	4.280251781	0.988774839	1	168	322	446	505	398	425	470	346
ENSECAG00000013115	6.25489718	0.988899332	1	1059	1265	1486	1618	1357	1897	1564	1604
ENSECAG00000018274	4.557656233	0.989079749	1	488	262	416	429	479	333	738	468
ENSECAG00000017261	6.982602981	0.989095021	1	2501	2208	1591	2279	5045	2091	1917	1417
ENSECAG00000009128	1.449987685	0.989159772	1	49	18	42	77	65	30	89	45
ENSECAG00000008615	3.854424802	0.989188204	1	145	290	302	308	254	420	289	240
ENSECAG00000007302	6.140337088	0.989329831	1	769	1028	1221	2232	1240	974	1767	2068
ENSECAG00000018527	7.672442	0.989389667	1	3210.01	3369	3961.01	3672	4986	3425	5344	3644.02
ENSECAG00000012748	3.572169216	0.989539174	1	122	248	240	249	282	254	251	203

ENSECAG00000020168	6.502350439	0.989624081	1	985	1280	1586	2943	1357	1261	2357	2794
ENSECAG00000019794	2.784362414	0.989648831	1	94	72	160	167	127	126	202	125
ENSECAG00000006451	4.064047623	0.989912168	1	169	192	497	373	487	362	416	132
ENSECAG00000009067	5.517190287	0.990121589	1	1201	644	515	545	1396	604	1495	441
ENSECAG00000010983	3.838832482	0.990174496	1	161	237	278	368	281	261	330	332
ENSECAG00000021178	6.101113591	0.990192753	1	1031	1065	1554	1147	1460	1505	1564	1267
ENSECAG00000014284	1.721617279	0.99024946	1	31.0071	49	78	77	107	44.0001	77	44
ENSECAG00000011671	7.591164831	0.990297858	1	2115	5681	2701	3187	5307	4045	4029	2764
ENSECAG00000006949	7.828883932	0.990354634	1	3292	3557	4834	4370	4868	3791	6833	4038
ENSECAG00000019178	5.968868599	0.990413829	1	985	1002	1045	1370	1837	991	1533	950
ENSECAG00000009739	1.336443066	0.990429631	1	11	20	107	46	65	67	57	14
ENSECAG00000020977	3.371203498	0.990507369	1	67	119	342	253	181	258	289	139
ENSECAG00000013395	7.457150929	0.990588011	1	2726	2335	3547	3813	3960	3664	3732	3472
ENSECAG00000008451	5.392786288	0.990790011	1	490	713	913	930	896	1190	620	760
ENSECAG00000016675	7.83586244	0.990993851	1	5165	3852	3477	2415	8156	2744	6147	2346
ENSECAG00000016692	7.390232892	0.991005227	1	2170	2844	3336	3731	3926	3843	3623	2676
ENSECAG00000000655	3.043827767	0.991268169	1	129	174	118	142	218	117	208	153
ENSECAG00000016334	6.240642833	0.991560139	1	894	1607	1366	1547	1507	1421	1892	1613
ENSECAG00000007991	0.85492745	0.991644053	1	5	65	17	43	21	109	0	2
ENSECAG00000012597	7.510666547	0.991806408	1	3658	3128	2774	2630	3546	2929	4720	4410
ENSECAG00000004919	6.383451479	0.991847424	1	1276	1181	1846	1559	2537	1212	2130	1218
ENSECAG00000019382	7.9145765	0.991899926	1	2565	4908	5572	4313	3906	5585	4354	6455
ENSECAG00000021407	4.471868295	0.992043354	1	183	316	546	625	468	463	477	453
ENSECAG00000023903	6.266106043	0.992100016	1	986	1340	1386	1847	1620	1435	1961	1514
ENSECAG00000019817	4.990274882	0.992248562	1	516	448	588	681	756	612	732	576
ENSECAG00000005916	3.186734539	0.992432034	1	125	204	132	172	90	170	233	280
ENSECAG00000000084	5.706993178	0.992734172	1	607	865	1092	1240	1487	738	1169	1020
ENSECAG00000010329	3.785338824	0.992790102	1	105	420	230	240	159	302	319	381
ENSECAG00000008697	3.40006313	0.992821039	1	96	207	157	325	189	207	214	272
ENSECAG00000023401	3.940987981	0.992943224	1	56	349	213	583	218	383	376	312
ENSECAG00000022961	3.746983874	0.993035279	1	107	302	221	367	180	230	341	388
ENSECAG00000023996	2.061801967	0.993253358	1	45	129	71	37	75	67	166	48
ENSECAG00000007474	5.254386301	0.993405635	1	481	1064	458	670	671	1037	773	713
ENSECAG00000019009	6.718940768	0.993420159	1	1858	1979	1730	1627	2591	1851	2325	2125
ENSECAG00000010309	3.82105224	0.993426204	1	119	204	269	480	235	186	370	413
ENSECAG00000000925	3.578118862	0.993608064	1	98	218	183	402	192	229	348	244
ENSECAG00000009162	8.729934566	0.993680619	1	7408	7415	7373	6883	11673	7180	11198	6076
ENSECAG00000000265	4.350470979	0.993722092	1	403	337	273	360	529	246	638	338
ENSECAG00000011713	6.24497981	0.9937747	1	799	1122	1720	1966	2018	1215	1691	1482
ENSECAG00000019814	6.743871308	0.993889783	1	1118	1422	2681	2695	1624	2049	2813	2673
ENSECAG00000011409	4.614897311	0.994007362	1	285	365	506	631	587	530	502	434
ENSECAG00000019553	3.739319134	0.994020658	1	117	197	267	424	272	305	292	246
ENSECAG00000023666	2.551008889	0.994026496	1	94	67	106	144	139	78	158	117
ENSECAG00000011746	7.413780259	0.994153937	1	4311	1201	3956	1482	5196	3453	4546	1231
ENSECAG00000015733	5.792159891	0.994802112	1	631	1379	730	1247	722	831	1657	1564
ENSECAG00000018027	6.275416233	0.995204428	1	971	1214	1776	1617	2316	1238	2150	874
ENSECAG00000003636	7.041291291	0.995535006	1	2116	1365	2297	3690	2742	1149	3130	4276
ENSECAG00000017574	4.161398312	0.995553215	1	333	327	209	350.999	402.998	212	509	405
ENSECAG00000020907	4.660257585	0.995851826	1	327	455	446	582	566	509	462	581
ENSECAG00000009261	7.164594418	0.995964335	1	4185	1992	808	1997	1599	3360	1952	5088
ENSECAG00000005729	6.181343058	0.996093393	1	1068	1208	1545	1268	2196	1191	1941	832
ENSECAG00000018861	3.120309151	0.996148043	1	124	87	234	170	267	135	212	113
ENSECAG00000012391	5.653438399	0.996335928	1	381	859	1059	1510	1401	1129	889	774
ENSECAG00000006997	5.066769845	0.996445311	1	622	502	613	547	1016	533	742	529
ENSECAG00000000184	4.684530458	0.996514313	1	353	417	524	535	468	550	575	572
ENSECAG00000020498	3.165004514	0.9965993	1	84	113	250	213	344	105	211	89
ENSECAG00000023917	3.911951558	0.996717406	1	88	242	371	448	217	426	305	304
ENSECAG00000008950	4.99701008	0.996846756	1	336	968	431	526	744	629	898	437
ENSECAG00000017341	7.095267163	0.996853124	1	1804	2213	2672	3155	2796	2632	3240	2917
ENSECAG00000012118	6.486767873	0.996854011	1	1450	1890	1749	1021	2103	2470	2028	891
ENSECAG00000013531	7.111872454	0.996855937	1	1351	2251	3216	3402	2898	3280	2750	2678
ENSECAG00000015390	5.913558897	0.996971579	1	948	728	1281	1304	1640	709	1556	1242
ENSECAG00000000236	8.313630285	0.997044389	1	3449	4336	7308	8426	4786	5330	6955	10042
ENSECAG00000000413	6.119935071	0.997097304	1	1055	1078	1226	1573	1412	1008	1893	1638
ENSECAG00000012874	5.537018231	0.997172137	1	768	790	746	923	890	776	1310	996
ENSECAG00000019583	3.683864269	0.997213172	1	156	185	249	345	224	208	396	269
ENSECAG00000008997	6.227155702	0.99747913	1	979	1344	1466	1560	1703	1487	1723	1417
ENSECAG00000022787	6.533118558	0.997539524	1	1307	1692	1641	1929	2131	1918	2084	1672
ENSECAG00000020547	11.2323601	0.997602818	1	19932	34739	60630	66170	61506	48145	43592	48357
ENSECAG00000002955	4.8538988	0.997821503	1	362	257	758	740	927	304	840	397
ENSECAG00000013383	5.627732488	0.997944834	1	709	877	838	1081	1264	676	1397	891
ENSECAG00000008068	5.624538182	0.998006748	1	673	639	1189	1032	1339	664	1141	1039
ENSECAG00000023659	4.044094425	0.998010846	1	190	242	363	404	344	177	445	446
ENSECAG00000024657	5.629298603	0.998325651	1	653	928	962	973	1213	734	1378	902
ENSECAG00000013409	5.846282261	0.998375075	1	791	1309	712	1262	1119	530	2182	1195
ENSECAG00000018629	4.628261404	0.998538373	1	351	408	369	642	718	340	561	471

ENSECAG00000019216	5.275618041	0.998623127	1	352	606	824	1119	852.002	751	966	709
ENSECAG00000020467	4.774593664	0.998856834	1	353	341	564	734	662	397	718	553
ENSECAG00000010013	7.399474701	0.998895864	1	1791	2295	3731	4733	3692	3063	4198	3387
ENSECAG00000022013	3.583300634	0.999051986	1	118	168	345	242	234	291	249	224
ENSECAG00000022066	5.837087139	0.99912504	1	1136	985	728	1011	1158	831	1605	1303
ENSECAG00000020283	7.642870677	0.999200285	1	2093	3582	4375	4576	3703	5154	3904	3975
ENSECAG00000000367	4.533916642	0.999291062	1	293	406	360	617	455	420	574	515
ENSECAG00000024720	3.855171156	0.99976016	1	93	259	318	425	274	387	311	234
ENSECAG00000009350	3.85091387	0.999839878	1	157	133	336	449	270	290	332	323
ENSECAG00000001580	3.751850568	0.999869519	1	171	132	322	355	374	194	399	178
ENSECAG00000021155	1.23845017	1	1	17	90	18	36	33	69	61	28
ENSECAG00000016130	0.897841781	1	1	8	53	28	43	21	12	62	59
ENSECAG00000005102	0.279433531	1	1	12	24	4	44	27	10	24	33
ENSECAG00000007238	0.005328198	1	1	9	22	10	26	4	8	31	35
ENSECAG00000022525	0.023106338	1	1	28	1	26	2	33	7	40	1
ENSECAG00000023334	0.349162353	1	1	3	17	41	30	37	25	23	12
ENSECAG00000006609	0.437477349	1	1	7	25	26	38	41	14	37	14
ENSECAG00000008354	0.273815103	1	1	11	12	30	30	11	25	33	25
ENSECAG00000021560	2.476829497	1	1	71	66	142	115	111	97	112	143
ENSECAG00000011295	-0.020365661	1	1	12	6	22	24	9	12	36	21
ENSECAG00000014603	2.585932116	1	1	35	91	142	186	124	127	121	125
ENSECAG00000019108	0.516668165	1	1	14	19	27	39	41	20	41	11
ENSECAG00000013122	2.833053836	1	1	71	103	157	191	144	135	187	132
ENSECAG00000019012	1.208985332	1	1	24	39	47	50	31	20	86	58
ENSECAG00000020890	1.326605093	1	1	29	54	44	44	25	49	69	64
ENSECAG00000013905	2.104927017	1	1	32	73	95	117	50	107	93	104
ENSECAG00000014727	2.86377756	1	1	119	73	149	167	190	95	205	126
ENSECAG00000014236	2.148026249	1	1	59	88	57	106	68	53	123	130
ENSECAG00000005564	2.711994529	1	1	97	52	133	188	108	76	245	134
ENSECAG00000012547	2.255013774	1	1	34	54	163	99	90	133	87	80
ENSECAG00000014955	3.290530031	1	1	70	135	188	355	217	103	173	330
ENSECAG00000010641	3.531223969	1	1	119	280	211	214	203	302	305	160
ENSECAG00000016441	0.595861903	1	1	9	24	19	58	33	33	26	25
ENSECAG00000015387	0.079606915	1	1	14	8	25	21	24	13	33	13
ENSECAG00000018881	-0.011513933	1	1	11	7	22	25	23	12	28	14
ENSECAG00000023471	1.100262771	1	1	14	53	41	43	32	35	62	46
ENSECAG00000022584	0.566251481	1	1	10	15	10	74	19	22	52	27
ENSECAG00000014285	0.126741733	1	1	21	21	18	5	10	39	31	4
ENSECAG00000011597	2.067290121	1	1	24	105	83	94	44	36	113	161
ENSECAG00000024821	0.256615069	1	1	15	20.9995	19.9991	21.9997	22.9999	23.999	33.9996	11.9999
ENSECAG00000001463	1.592081894	1	1	35	62	72	36	63	70	59	53
ENSECAG00000008025	2.43731811	1	1	49	64	80	217	143	101	122	82
ENSECAG00000026947	0.938941707	1	1	19	7	67	42	29	31	38	56
ENSECAG00000009235	3.43104816	1	1	97	163	193	357	146	240	234	284
ENSECAG00000012871	0.68183616	1	1	23	29	28	25	17	61	32	15
ENSECAG00000007648	3.444762016	1	1	141	226	194	204	142	217	236	322
ENSECAG00000012526	3.887503193	1	1	257	206	335	211	251	263	470	282
ENSECAG00000024472	0.139812232	1	1	5	13	25	34	14	28	31	12
ENSECAG00000020512	2.933096044	1	1	111	100	125	208	145	111	219	173
ENSECAG00000009179	0.43857568	1	1	14	6	64	5	31	10	45	23
ENSECAG00000004226	1.35058894	1	1	19	48	44	73	40	51	59	58
ENSECAG00000023404	1.137576023	1	1	8	96	24	23	25	95	34	18
ENSECAG00000009594	2.226494956	1	1	57	59	86	135	52	73	161	112
ENSECAG00000026862	2.92773718	1	1	89	85	177	202	133	154	156	192
ENSECAG00000017375	1.955309175	1	1	28	55	82	123	69	80	124	51
ENSECAG00000000351	2.663677447	1	1	133	83	82	125	116	80	215	130
ENSECAG00000018792	1.756288984	1	1	33	49	72	89	24	67	97	94
ENSECAG00000003791	5.50106804	1	1	726	969	648	780	985	1001	1228	621
ENSECAG00000010638	1.07532598	1	1	27	42	31	43	30	33	56	53
ENSECAG00000012950	3.046170208	1	1	137	108	143	187	201	183	187	116
ENSECAG00000011531	5.862255075	1	1	432	1320	1083	1508	1232	1541	1120	958
ENSECAG00000017099	6.271952369	1	1	747	1311	1549	2140	1573	1646	1647	1644
ENSECAG00000009932	2.596735946	1	1	45	90	145	170	87	110	141	169
ENSECAG00000020825	5.204784372	1	1	459	622	766	805	686	674	893	877
ENSECAG00000010537	0.886625476	1	1	16	40	43	27	22	45	14	64
ENSECAG00000018697	5.131821955	1	1	386	778	599.967	764.994	769.999	606.999	1008	604.995
