# Supplementary material for: Transcriptome analysis of immune genes in peripheral blood mononuclear cells of young foals and adult horses
Source: PLoS One. 2018 Sep 5;13(9):e0202646. doi: 10.1371/journal.pone.0202646 (PMC6124769; doi:10.1371/journal.pone.0202646)
Supplement: S1 Fig — Histograms were constructed to assess the distribution of p-values after differential gene expression analysis. For each comparison between age groups, the distribution of p-values greater than 0.05 was uniform. Each comparison results in more than 1,500 differentially expressed genes with p-values < 0.05. (PDF) [file pone.0202646.s004.pdf]

Transcriptome analysis of immune genes in peripheral blood mononuclear cells of young foals and adult horses

Supporting information

Distribution of p-values from RNA-Seq data sets.

Histograms were constructed to assess the distribution of adjusted p-values after differential gene expression analysis (supplemental figure 1). For each comparison between age groups, the distribution of adjusted p-values greater than 0.05 is uniform. Each comparison results in more than 1,500 differentially expressed genes with adjusted p-values < 0.05.

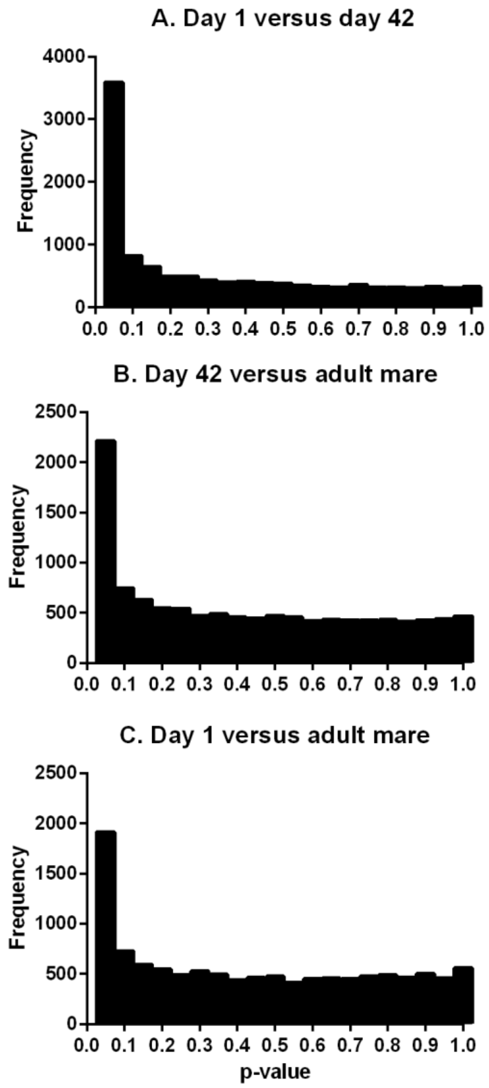

S1 Fig. Distribution of RNA-Seq differential gene expression adjusted p-values. The frequency of p-values is plotted from zero to one. Each comparison is plotted separately: A) day 1 versus day 42, B) day 42 versus adult mare, and C) day 1 versus adult mare.
